# Tobacco-induced hyperglycemia promotes lung cancer progression via cancer cell-macrophage interaction through paracrine IGF2/IR/NPM1-driven PD-L1 expression

**DOI:** 10.1038/s41467-024-49199-9

**Published:** 2024-06-08

**Authors:** Hyun-Ji Jang, Hye-Young Min, Yun Pyo Kang, Hye-Jin Boo, Jisung Kim, Jee Hwan Ahn, Seung Ho Oh, Jin Hwa Jung, Choon-Sik Park, Jong-Sook Park, Seog-Young Kim, Ho-Young Lee

**Affiliations:** 1 Creative Research Initiative Center for concurrent control of emphysema and lung cancer, College of Pharmacy, Seoul National University, Seoul, 08826 Republic of Korea; 2 Natural Products Research Institute, College of Pharmacy, Seoul National University, Seoul, 08826 Republic of Korea; 3 College of Pharmacy and Research Institute of Pharmaceutical Sciences, Seoul National University, Seoul, 08826 Republic of Korea; 4 Department of Histology, College of Medicine, Jeju National University, Jeju, 63243 Republic of Korea; 5 Department of Molecular Medicine and Biopharmaceutical Sciences, Graduate School of Convergence Science and Technology and College of Pharmacy, Seoul National University, Seoul, 08826 Republic of Korea; 6 PET core, Convergence Medicine Research Center, Asan Medical Center, Seoul, 05505 Republic of Korea; 7 Soonchunhyang University Bucheon Hospital, Bucheon-si, Gyeonggi-do 14584 Republic of Korea; 8 Department of Convergence Medicine, University of Ulsan College of Medicine, Seoul, 05505 Republic of Korea

**Keywords:** Cancer metabolism, Cancer microenvironment, Growth factor signalling, Non-small-cell lung cancer

## Abstract

Tobacco smoking (TS) is implicated in lung cancer (LC) progression through the development of metabolic syndrome. However, direct evidence linking metabolic syndrome to TS-mediated LC progression remains to be established. Our findings demonstrate that 4-(methylnitrosamino)−1-(3-pyridyl)−1-butanone and benzo[a]pyrene (NNK and BaP; NB), components of tobacco smoke, induce metabolic syndrome characteristics, particularly hyperglycemia, promoting lung cancer progression in male C57BL/6 J mice. NB enhances glucose uptake in tumor-associated macrophages by increasing the expression and surface localization of glucose transporter (GLUT) 1 and 3, thereby leading to transcriptional upregulation of insulin-like growth factor 2 (IGF2), which subsequently activates insulin receptor (IR) in LC cells in a paracrine manner, promoting its nuclear import. Nuclear IR binds to nucleophosmin (NPM1), resulting in IR/NPM1-mediated activation of the *CD274* promoter and expression of programmed death ligand-1 (PD-L1). Restricting glycolysis, depleting macrophages, or blocking PD-L1 inhibits NB-mediated LC progression. Analysis of patient tissues and public databases reveals elevated levels of IGF2 and GLUT1 in tumor-associated macrophages, as well as tumoral PD-L1 and phosphorylated insulin-like growth factor 1 receptor/insulin receptor (pIGF-1R/IR) expression, suggesting potential poor prognostic biomarkers for LC patients. Our data indicate that paracrine IGF2/IR/NPM1/PD-L1 signaling, facilitated by NB-induced dysregulation of glucose levels and metabolic reprogramming of macrophages, contributes to TS-mediated LC progression.

## Abstract (web summary)

Tobacco smoking is an important risk factor for lung cancer. Here the authors report that tobacco carcinogens promote metabolic syndrome and metabolic reprogramming of macrophages, associated with lung cancer progression in preclinical models.

## Introduction

Lung cancer (LC) remains the primary cause of cancer-related deaths globally^1^. Despite the development of numerous promising medications, resistance inevitably arises, leading to common tumor relapses^2^. Therefore, a deeper understanding of LC pathophysiology is imperative for developing treatment strategies. Tobacco smoking (TS) is by far the most significant risk factor for LC^3^. Two representative tobacco carcinogens (TCs), 4-(methylnitrosamino)−1-(3-pyridyl)−1-butanone (NNK) and benzo[a]pyrene (BaP), have been shown to induce LC development through genetic/epigenetic modifications of proto-oncogenes and tumor suppressor genes^4^, as well as stimulation of oncogenic signaling networks^5^, contributing to tumor activities in LC cells. However, the mechanisms driving TS-mediated LC progression remain elusive.

Cancer progression is a complex process governed by intricate cancer-stroma communications in the tumor microenvironment (TMiE) and host factors in the tumor macroenvironment (TMaE)^6,7^. Various environmental factors, including biological hazards and dietary factors, impact stromal cells in the TMiE and host systems in the TMaE, thereby promoting tumor initiation and progression^6,7^. TS has been linked to the emergence of metabolic syndrome, also known as insulin resistance syndrome^8^, which consists of at least three medical conditions, including high blood glucose, elevated serum triglyceride (TG) levels, low high-density lipoprotein (HDL), high blood pressure, and central adiposity^8,9^. Different metabolic syndrome components, particularly hyperglycemia and dyslipidemia, have been implicated in the etiology of tumor formation and progression for certain malignancies, including LC^10,11^. Changes in glucose and lipid metabolism affect tumor-associated macrophages (TAMs)^12^, one of the main types of stromal cells in the TMiE^13^. TAMs play a crucial role in cancer progression through various mechanisms, including the secretion of proteases, inflammatory mediators, and growth factors, and the disruption of anti-tumor immunity^14^. Consequently, it is hypothesized that elevated glucose and lipid circulation in patients with metabolic syndrome may contribute to LC progression by modifying the immunomodulatory function of TAMs rather than solely being responsible for the hypermetabolism of LC cells^14^. However, the precise effects and underlying mechanisms of TS-mediated metabolic syndrome on LC progression remain largely unknown.

Tumor cells and their neighboring stromal cells are implicated in the expression of immune checkpoint proteins to deactivate the host antitumor immune system^15^. The interaction between the programmed death 1 receptor (PD-1) and its ligand programmed death ligand 1 (PD-L1) primarily affects T-cell activity in TMiE^16^. By inducing cancer stem-like cell (CSC) properties^17^ and immune evasion^18^, PD-L1 promotes cancer progression. Multiple cytokines and growth factors, including interferon-gamma (IFN-γ), epidermal growth factor (EGF), interleukins (ILs), tumor necrosis factor-alpha (TNF-α), and insulin-like growth factor 2 (IGF2), control the transcription of PD-L1^19,20^. Recent studies have demonstrated that nucleophosmin (also known as NPM1 or B23) is a transcriptional activator of PD-L1^21^. However, little is known about its oncogenic function and the underlying mechanism of action in LC.

This study aims to determine the development of metabolic syndrome during TS-induced LC progression, elucidate the mechanistic link between TS-induced metabolic syndrome and LC progression, and identify potential molecular and cellular targets to inhibit TS-mediated LC progression. Here, we show that TS-induced hyperglycemia in TMaE and altered glucose metabolism in TAMs cooperate to promote LC progression through paracrine activation of the IGF2/IR/NPM1/PD-L1 signaling cascade in LC cells.

## Results

### Metabolic syndrome occurs in mice in which exposure to NNK and BaP, the primary TS components, induces lung cancer progression

**Fig. 1:**
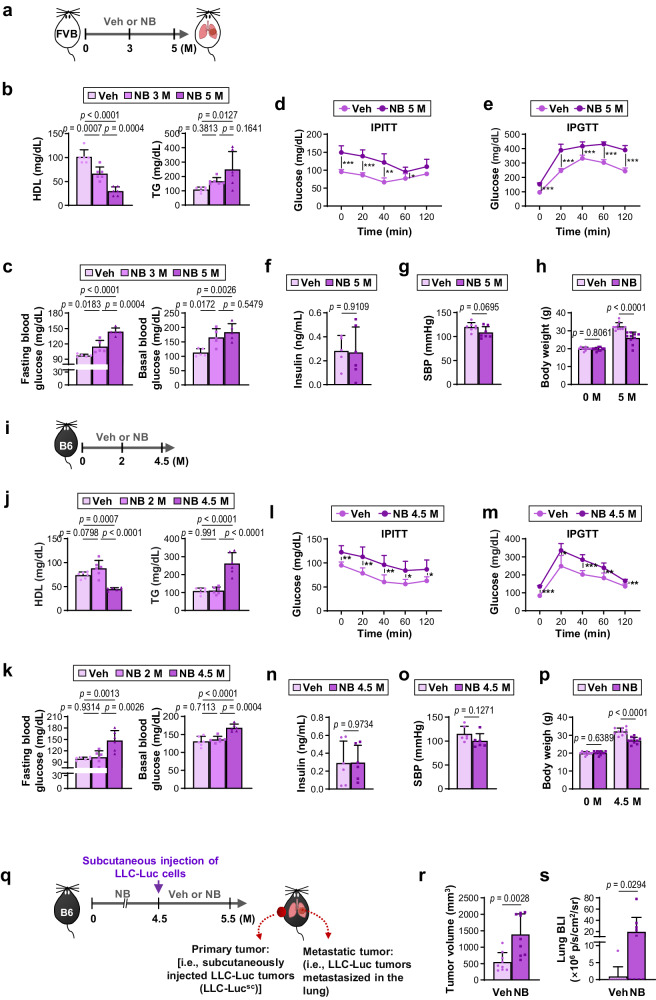
Chronic exposure to NB causes metabolic syndrome in mice. **a**–**p** Two-month-old male FVB/N mice (FVB, **a**–**h**) and C57BL/6 J mice (B6, **i**–**p**) were given vehicle (Veh) or NB (NNK/BaP). The experimental schedule is summarized in (**a**) and (**i**). **b**, **j** Blood high-density lipoprotein (HDL) and triglyceride (TG) levels in mice (*n* = 6/group). **c**, **k** Fasting and basal blood glucose levels in mice (*n* = 5/group for FVB; *n* = 6/group for B6). **d**, **l** Changes in blood glucose concentration determined by intraperitoneal insulin tolerance (IPITT) test (*n* = 5/group). *p*-values of Veh vs NB (FVB): 0 min, *p* = 0.0002; 20 min, *p* = 0.0002; 40 min, *p* = 0.0015; 60 min, *p* = 0.0387; 120 min, *p* = 0.0621. *p*-values of Veh vs NB (B6): 0 min, *p* = 0.0027; 20 min, *p* = 0.0092; 40 min, *p* = 0.0075; 60 min, *p* = 0.0167; 120 min, *p* = 0.0349. **e**, **m** Changes in blood glucose concentration determined by intraperitoneal glucose tolerance (IPGTT) test (*n* = 5/group). *p*-values of Veh vs NB (FVB): 0 min, *p* < 0.0001; 20 min, *p* = 0.0001; 40 min, *p* = 0.0004; 60 min, *p* < 0.0001; 120 min, *p* < 0.0001. *p*-values of Veh vs NB (B6): 0 min, *p* < 0.0001; 20 min, *p* = 0.0123; 40 min, *p* = 0.0005; 60 min, *p* = 0.007; 120 min, *p* = 0.0014. **f**, **n** Serum insulin levels in mice (*n* = 5/group for the Veh group of FVB; *n* = 6/group for other groups of FVB and B6). **g**, **o** Systolic blood pressure (SBP) of mice (FVB: *n* = 7/group; B6: *n* = 6/group). **h**, **p** Body weight of mice (*n* = 10/group). **q** Schematic diagram of the experimental schedule. **r** The tumor volume of subcutaneous LLC tumors (LLC-Luc^sc^) (*n* = 9/group). **s** Changes in lung tumor formation determined by ex vivo bioluminescence imaging (BLI) (*n* = 9/group). The data are presented as the mean ± SD. *p < 0.05, **p < 0.01, and ***p < 0.001, determined by a two-tailed Student’s *t*-test (**d, e, l, m**). *p*-values were determined by using one-way ANOVA with Tukey’s post-hoc test (**b**, **c**, **j**, **k**), a two-tailed Student’s *t*-test (**f**–**h, n**–**p**), or two-tailed Mann–Whitney test (**r, s**). Source data are provided as a Source Data file.

To assess whether metabolic syndrome occurs during TS-induced lung carcinogenesis, we examined metabolic syndrome characteristics in FVB/N (FVB) mice exposed to mixtures of NNK and BaP (NB) for five months following a well-established protocol for NB-induced LC development (3 μmol each, twice weekly via oral gavage)^22^ (Fig. 1a). Metabolic syndrome diagnosis typically involves the presence of high blood glucose ( ≥ 100 mg/dL fasting plasma glucose), elevated triglyceride (TG) ( ≥ 150 mg/dL), low high-density lipoprotein (HDL) ( < 40 mg/dL for men and <50 mg/dL for women), high blood pressure ( ≥ 130 mmHg systolic or ≥85 mmHg diastolic), and central adiposity^9,23^. Periodic blood sample analysis during NB exposure revealed time-dependent changes in HDL, TG, and glucose levels (fasting and basal) (Fig. 1b, c). We observed that exposure to NB for more than five months altered HDL-cholesterol, TG, and glucose concentrations to levels within the metabolic syndrome threshold (Fig. 1b, c). Furthermore, prolonged NB exposure increased insulin resistance (Fig. 1d) and reduced glucose tolerance (Fig. 1e) without significant changes in serum insulin levels (Fig. 1f). Consistent with findings in TS-exposed humans and mice^24,25^, NB-exposed mice exhibited lower systolic blood pressure (SBP) (Fig. 1g) and body weight (BW) (Fig. 1h) compared to control mice. As expected, all NB-exposed FVB mice developed lung tumors (Supplementary Fig. 1a), with 100% immunohistochemistry (IHC) positivity against thyroid transcription factor 1 (TTF1), a highly specific marker for lung adenocarcinoma^26^ (Supplementary Fig. 1b). Notably, 70-100% of NB-exposed animals exhibited tumor nodules in the liver, stomach, kidney, spleen, or colon, which were negative for TTF1 (Supplementary Fig. 1a, b). Humans and mice with the carcinogen-susceptible FVB or A/J background have displayed tumor nodules in various organs, including the colon, kidney, liver, and stomach, in addition to the lung after exposure to TS or NB^27–29^. Therefore, chronic NB exposure likely led to primary tumor development in multiple organs of tumor-prone mice.

We then explored whether the observed metabolic syndrome in NB-exposed FVB mice resulted from concurrent tumor development or prolonged NB exposure. To address this, we examined the effects of NB exposure on mice with a C57BL/6 J (B6) background, which are resistant to chemically-induced LC formation^30^. The components of NB-induced metabolic syndrome and changes in SBP and BW observed in FVB mice (presented in Fig. 1a–h) were also evident in B6 mice after exposure to NB for 4.5 months (Fig. 1i–p). As anticipated, B6 mice exposed to NB for 4.5 months showed no discernible tumor nodules in any of the organ sites (Supplementary Fig. 1c). Therefore, chronic NB exposure induces metabolic syndrome in rodents.

We then investigated whether the elevations of blood glucose or TG induced by NB exposure promote LC progression. Given that chronic NB exposure resulted in primary tumors in multiple organs of cancer-permissive FVB mice, which could lead to false positives when assessing lung cancer progression, we employed the well-defined Lewis lung carcinoma (LLC) mouse model^31^. B6 mice with induced metabolic syndrome due to NB exposure for 4.5 months were subcutaneously injected with luciferase-labeled LLC cells (LLC-Luc) and then exposed to vehicle (Veh) or NB for 1 month (Fig. 1q). We found that the growth of subcutaneously injected LLC-Luc tumors (LLC-Luc^sc^) was significantly greater in NB-exposed mice than in Veh-exposed mice (Fig. 1r). The NB-exposed mice also showed significantly greater bioluminescent intensity (BLI) in the lung compared with Veh-exposed mice (Fig. 1s), suggesting that NB exposure promotes LC progression. Several organs, including the pancreas that plays a key role in regulating glucose and lipid metabolism, are pathologically affected by tobacco use^32^. Therefore, we investigated whether NB-induced impairment of glucose homeostasis played a major role in NB-mediated LC progression. To this end, B6 mice carrying metabolic syndrome due to exposure to NB for 4.5 months were subcutaneously injected with LLC-Luc cells. Mice with palpable LLC-Luc^sc^ tumors were exposed to Veh or NB, either alone or in combination with daily administration of exendin-4 (EX4) or metformin (Met) (Supplementary Fig. 1d). We found that treatment with EX4 or Met significantly suppressed the NB-induced growth of LLC-Luc^sc^ tumors and BLI in the lung (Supplementary Fig. 1d). These results suggest that NB-induced metabolic alterations, particularly hyperglycemia, could exacerbate LC progression.

### NB promotes LC progression in a glucose-rich environment

**Fig. 2:**
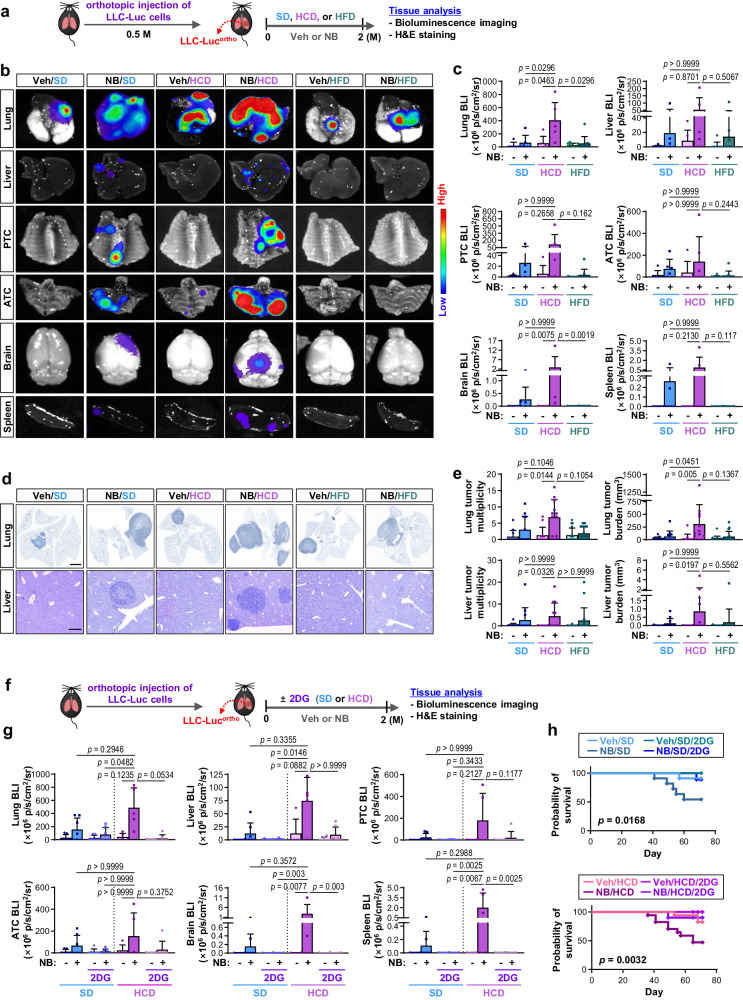
Chronic exposure to NB promotes lung cancer progression in high-carbohydrate diet (HCD)-fed mice. **a**–**e** Two-month-old male C57BL/6 J (B6) mice were inoculated orthotopically with LLC-Luc cells. After lung tumor formation was confirmed, mice with LLC-Luc orthotopic tumors (LLC-Luc^ortho^) were exposed to Veh or NB under standard diet (SD), high-carbohydrate diet (HCD), or high-fat diet (HFD) conditions for two months. The data is representative of two independent experiments with similar results. **a** Schematic diagram of the experimental schedule. **b** Representative ex vivo bioluminescence images of the lung, liver, posterior thoracic cage (PTC), anterior thoracic cage (ATC), brain, and spleen. **c** Quantitative analyzes of bioluminescence intensity (BLI) of analyzed organs (*n* = 6/group for Veh/SD, NB/SD, Veh/HCD, and NB/HCD groups; *n* = 10/group for Veh/HFD and NB/HFD groups). **d** Representative photographs of the H&E-stained sections of the lungs and liver. Scale bars: 2.5 μm (lung image); 50 μm (liver image). **e** Microscopic evaluation of H&E-stained lung and liver tissues for tumor multiplicity and burden (*n* = 12/group for Veh/SD, NB/SD, Veh/HCD, and NB/HCD groups; *n* = 17/group for Veh/HFD and NB/HFD groups). **f**–**h** B6 mice with LLC-Luc^ortho^ were exposed to Veh or NB under SD or HCD conditions, either alone or together with 2-deoxy-D-glucose (2DG, 500 mg/kg) for 2 months. **f** Schematic diagram of the experimental schedule. **g** Quantitative analyzes of BLI of analyzed organs (*n* = 5/group for Veh/HCD and NB/HCD groups; *n* = 7/group for other groups). **h** Kaplan–Meier survival curve of the mice in each group (*n* = 11 for Veh/SD and NB/SD groups; *n* = 9 for Veh/SD/2DG and NB/SD/2DG groups; *n* = 17 for Veh/HCD and NB/HCD groups; *n* = 10 for Veh/HCD/2DG and NB/HCD/2DG groups). The data are presented as the mean ± SD. *p*-values were determined by using Kruskal–Wallis test with Dunn’s post-hoc test (**c**, **e**, **g**) or a log-rank test (**h**). Source data are provided as a Source Data file.

Because exposure to NB induces genetic and epigenetic modifications, oncogenic signal transductions, and immune function disruptions^4,5^, which can impact cancer progression, we next aimed to separate the NB-induced tumor activities from metabolic syndrome. For this purpose, B6 mice carrying orthotopic tumors of LLC-Luc cells (LLC-Luc^ortho^) were exposed to NB for two months, which had no discernible effects on blood glucose or TG levels (Fig. 1j, k). During NB exposure, mice were fed either a high-carbohydrate diet (HCD) or high-fat diet (HFD) to increase circulating glucose or TG concentrations, respectively (Fig. 2a, Supplementary Fig. 2a, b). Mice subjected to HCD or HFD feeding for 2 months showed significantly elevated levels of blood glucose or TG, respectively (Supplementary Fig. 2c, d), akin to levels observed in B6 mice with NB-induced metabolic syndrome (Fig. 1j, k). Studies have shown that prolonged high-fat intake can elevate insulin production and induce diabetes (glucose intolerance) and hepatic insulin resistance (elevated gluconeogenesis)^33–35^. HCD, comprising more than 70% of calorie intake, has also been associated with an increased risk of diabetes^36^. Indeed, B6 mice placed on a HCD or HFD for 14 weeks, particularly those on the HFD, exhibited glucose intolerance (Supplementary Fig. 2e). Thus, long-term HCD or HFD feeding may upregulate glucose or triglyceride (TG) levels by causing metabolic alterations. We then assessed the impact of the diets on the physiological regulation of their metabolic status by conducting metabolic phenotyping of mice maintained on a HCD or HFD for two months without NB exposure. We observed that HFD-fed mice had marginal changes in food intake and significant increases in BW and the masses of inguinal and epididymal white adipose tissue (iWAT and eWAT, respectively) compared to standard diet (SD)-fed control mice (Supplementary Fig. 2f–h). Dual-energy X-ray absorptiometry (DEXA) analysis revealed that HFD-fed mice displayed significantly lower lean contents and increased fat mass compared to SD-fed control mice (Supplementary Fig. 2i). In contrast, HCD-fed mice showed no significant differences in diet intake, BW, and iWAT and eWAT masses compared to SD-fed control mice (Supplementary Fig. 2f–h). Both HCD-fed and HFD-fed mice showed no detectable difference in bone mineral content (BMC) and bone mineral density (BMD) compared to control mice (Supplementary Fig. 2j). To comprehensively assess energy regulation, we measured the respiratory exchange ratio (RER) and energy expenditure (EE) using a comprehensive laboratory animal monitoring system (CLAMS). An RER value near 1.0 indicates that carbohydrates are the main energy source, while an RER value of 0.7 indicates that fat is the predominant fuel source^37^. Values between 0.7 and 1.0 indicate a mix of both carbohydrates and fats as the energy source^37^. The RER values of mice fed SD, HCD, or HFD in the light phase were 0.76 ± 0.02, 0.80 ± 0.02, and 0.73 ± 0.01, respectively, and those in the dark phase were 0.82 ± 0.03, 0.87 ± 0.02, and 0.74 ± 0.02, respectively (Supplementary Fig. 2k). These findings indicate a mixed use of carbohydrates and fats in both the light and dark phases, with carbohydrates being the relatively more predominant energy source in the dark phase. Additionally, fat and carbohydrate are relatively predominant energy sources for HFD-fed and HCD-fed mice, respectively, compared to SD-fed control mice, as expected. Moreover, consistent with the previous finding^38^, mice fed HCD had significantly greater EE than mice fed SD in both the light and dark phases (*p* = 0.0177 in the light phase and *p* = 0.0379 in the dark phase) (Supplementary Fig. 2l). In contrast, there was no significant difference in EE between mice fed HFD and those fed SD (Supplementary Fig. 2l). We also noted that HCD-fed and HFD-fed mice displayed no significant differences in glucose clearance, insulin sensitivity, insulin production, and the levels of fasting blood glucose compared with SD-fed control mice (Supplementary Fig. 2m–p). Hence, it is probable that the diet-induced excessive intake of nutrients, but not metabolic alterations, elevates blood glucose or TG levels in mice fed HCD or HFD for 2 months.

We then performed bioluminescence imaging (BLI)-based detection of primary and metastatic tumor growth in mice carrying orthotopic LLC-Luc tumors (LLC-Luc^ortho^) that had been exposed to Veh or NB for two months under SD, HCD, or HFD conditions (LLC-Luc^ortho^-Veh/SD, LLC-Luc^ortho^-NB/SD, LLC-Luc^ortho^-Veh/HCD, LLC-Luc^ortho^-NB/HCD, LLC-Luc^ortho^-Veh/HFD, or LLC-Luc^ortho^-NB/HFD, respectively). We found that the LLC-Luc^ortho^-NB/SD and LLC-Luc^ortho^-NB/HCD groups exhibited higher signals in the primary (lung) and distant organ sites, including the liver, posterior and anterior thoracic cage, brain, and spleen, compared with the LLC-Luc^ortho^-Veh/SD and LLC-Luc^ortho^-Veh/HCD groups, respectively (Fig. 2b, c). Histological analysis also revealed greater tumor burden and multiplicity in the lungs and livers of the LLC-Luc^ortho^-NB/SD and LLC-Luc^ortho^-NB/HCD groups compared with those in their control groups (Fig. 2d, e). Conversely, LLC-Luc^ortho^-carrying mice exposed to Veh or NB under HFD conditions (LLC-Luc^ortho^-Veh/HFD or LLC-Luc^ortho^-NB/HFD) showed similar levels of tumor growth (Fig. 2b–e). These findings suggest the impact of high glucose intake on NB-induced LC progression.

To test this hypothesis, we evaluated the effects of 2-deoxy-D-glucose (2DG), a glycolysis inhibitor, on the growth and metastasis of Luc^ortho^ in mice exposed to Veh or NB for two months under SD or HCD conditions (i.e., LLC-Luc^ortho^-Veh/SD, LLC-Luc^ortho^-NB/SD, LLC-Luc^ortho^-Veh/HCD, or LLC-Luc^ortho^-NB/HCD, respectively) (Fig. 2f). 2DG treatment suppressed primary and metastatic tumor growth in the LLC-Luc^ortho^-NB/SD and LLC-Luc^ortho^-NB/HCD groups as measured by BLI (Fig. 2g, Supplementary Fig. 3a–c) and histology-based analyzes (Supplementary Fig. 3d). Survival of the LLC-Luc^ortho^-NB/SD and LLC-Luc^ortho^-NB/HCD groups was also significantly improved by 2DG treatment (Fig. 2h). We observed that NB exposure and HCD feeding had additive impacts on tumor growth in primary and distant organs. Hence, it is probable that high glucose intake and NB exposure cooperate to promote LC progression.

### TAMs exposed to NB in a glucose-rich environment promote LC progression

**Fig. 3:**
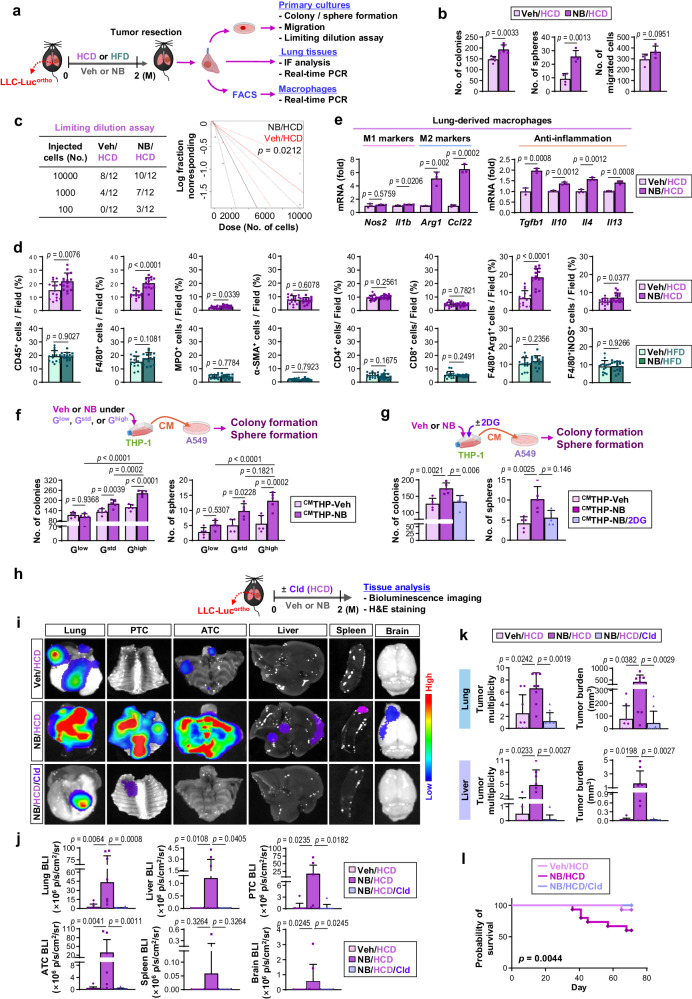
M2-like macrophages recruited and preconditioned by NB-induced metabolic reprogramming promote lung cancer progression. **a**–**e** C57BL/6 J (B6) mice with LLC-Luc^ortho^ were exposed to Veh or NB under HCD or HFD conditions for 2 months. **a** Schematic diagram of the experimental schedule. **b** Anchorage-independent (AID) colony formation (left, *n* = 5/group), sphere formation (middle, *n* = 4/group), and migration (right, *n* = 4/group) of LLC-Luc cells isolated from mice. **c** Tumorigenicity (*n* = 12/group) of LLC-Luc cells isolated from mice. The log-fraction plot was produced using online Extreme limiting dilution analysis (ELDA) software (right). **d** Immunofluorescence (IF) analysis of the indicated immune cells in lung tumors (*n* = 15/group). **e** Real-time PCR analysis of indicated marker expression in lung-derived primary macrophages (the CD45^+^F4/80^+^ population, *n* = 3/group). **f**–**g** The AID colony formation and sphere formation capacities of A549 cells exposed to THP-1-derived conditioned medium (CM) that was produced under the indicated conditions (*n* = 5/group). **h**–**l** B6 mice carrying LLC-Luc^ortho^ were exposed to Veh or NB under HCD conditions. Clodronate liposomes (Cld) were administered as summarized in (**h**). **i**–**j** Ex vivo bioluminescence images (**i**) and quantitative analyzes of bioluminescence intensity (BLI) (**j**) of analyzed organs (*n* = 8 for the NB/HCD group; *n* = 10 for Veh/HCD and NB/HCD/Cld groups). **k** Quantitative analyzes of tumor multiplicity and burden using H&E-stained lung and liver tissues (*n* = 8 for Veh/HCD and NB/HCD groups; *n* = 10 for the NB/HCD/Cld group). **l** Kaplan–Meier survival curve of mice in each group (*n* = 14 for the Veh/HCD group; *n* = 15 for NB/HCD and NB/HCD/Cld groups). The data are presented as the mean ± SD. *p*-values were determined by using a two-tailed Student’s t-test (**b**, **d**, **e**), two-tailed Mann–Whitney test (**d**), one-way ANOVA with Tukey’s post-hoc test (**f**, **g**), Kruskal–Wallis test with Dunn’s post-hoc test (**j**, **k**), or a log-rank test (**l**). The data shown in (**g**) are representative of two independent experiments with similar results, and the data shown in **b**, **e**, and **f** are representative of three independent experiments with similar results. Source data are provided as a Source Data file.

To investigate the mechanisms by which high glucose intake promotes NB-induced LC progression, we analyzed lung tissues isolated from the LLC-Luc^ortho^-Veh/HCD, LLC-Luc^ortho^-NB/HCD, LLC-Luc^ortho^-Veh/HFD, and LLC-Luc^ortho^-NB/HFD groups. We assessed the tumor activities of LC cells derived from primary cultures of LLC-Luc^ortho^ by performing a series of experiments described in Fig. 3a. Compared to LC cells from LLC^ortho^-Veh/HCD, those from LLC^ortho^-NB/HCD were significantly more capable of colony and sphere formation without significant differences in migration (Fig. 3b). The limiting dilution assay revealed that LLC-Luc^ortho^-NB/HCD-derived LC cells demonstrated significantly greater tumorigenic potential than LLC-Luc^ortho^-Veh/HCD-derived LC cells (Fig. 3c). Then, we evaluated the direct effects of NB exposure on LC cells. Two different LC cells (A549 and LLC) exposed to Veh or NB for two months exhibited no significant differences in their capacities for colony formation, sphere formation, and migration (Supplementary Fig. 4a). Therefore, it is plausible that NB exposure promotes LC progression by modulating specific stromal cells, rather than having direct impacts on LC cells.

We then performed immunofluorescence (IF) analysis of CD45^+^ leukocytes, F4/80^+^ macrophages, MPO^+^ neutrophils, α-SMA^+^ cancer-associated fibroblasts, and CD4^+^ or CD8^+^ T lymphocytes in LLC-Luc^ortho^ to investigate the potential stromal cells responsible for NB-mediated LC progression in HCD-fed mice. Compared to the LLC-Luc^ortho^-Veh/HCD group, the LLC-Luc^ortho^-NB/HCD group had higher levels of CD45^+^ leukocytes, particularly F4/80^+^ macrophages (Fig. 3d, Supplementary Fig. 4b). We also found significantly higher levels of arginase 1 (Arg1)^+^ M2-type macrophages (Arg1^+^F4/80^+^), rather than inducible nitric oxide synthase (iNOS)^+^ M1-type macrophages (iNOS^+^F4/80^+^), in the LLC-Luc^ortho^-NB/HCD group compared to those in the LLC-Luc^ortho^-Veh/HCD group (Fig. 3d, Supplementary Fig. 4b). We then analyzed macrophages and LC cells isolated from the tumors by fluorescence-activated cell sorting (FACS) (Supplementary Fig. 4c). We observed transcriptional upregulation of genes encoding M2 markers and anti-inflammatory mediators in LLC-Luc^ortho^-NB/HCD-derived pulmonary macrophages compared to those in LLC-Luc^ortho^-Veh/HCD-derived control macrophages (Fig. 3e). We further confirmed that mouse bone marrow-derived monocytes (BMDMs) and THP-1 human monocytes (THP-1s) had increased expression of M2-specific markers [i.e., mannose receptor (CD206) and Arg1], but not M1-specific marker (i.e., iNOS), following in vitro exposure to NB compared to their corresponding control cells exposed to Veh for the identical duration (Supplementary Fig. 4d). These NB-induced alterations were not observed in the LLC-Luc^ortho^-NB/HFD group when compared to the LLC-Luc^ortho^-Veh/HFD group (Fig. 3d, Supplementary Fig. 4b). Therefore, it is probable that NB-induced tumor infiltration of macrophages and their acquisition of M2-like pro-tumoral phenotypes, particularly under a glucose-rich environment, contribute to LC progression.

We investigated the impact of exposure to NB under a glucose-rich environment on the pro-tumoral function of macrophages. We conducted in vitro investigations on the effects of NB in the absence and presence of glucose supplementation on THP-1s and BMDMs. Typical in vitro cultures of various cell types are carried out in culture media that contain 25 mM glucose^39^, which is higher than normal physiological glucose (5–7 mM)^40^. We cultured THP-1s and BMDMs in media supplemented with varying concentrations of glucose. The standard culture media for THP-1s and BMDMs contain 2 g/L glucose (11 mM; G^std^), which is within the hyperglycemic range^41^ and represents blood glucose levels in FVB and B6 mice with NB-induced metabolic syndrome (as shown in Fig. 1). Because survival of THP-1s and BMDMs in glucose-free culture medium was found to be limited to one week, the cells were cultivated in glucose-free media supplemented with glucose at concentrations of 0.8 g/L (4.4 mM, G^low^), which approximately corresponds to suboptimal blood sugar levels, or 10 g/L (G^high^; 55 mM), which is analogous to diabetic levels^41^. We also established the physiologically relevant concentrations of NNK and BaP for in vitro studies based on previously published research on blood NNK or BaP levels in smokers. The average steady-state serum concentration of NNK in active smokers was reported to be 2 × 10^−4^ μM, rising abruptly from 10–100 μM in serum or to 1 mM at the mucosal surface shortly after smoking^42^. Additionally, human serum contained a maximum concentration of 177.5 ng/mL (0.7 μM) BaP^43^. Given BaP’s rapid turnover and elimination^44^, a relatively high concentration may accurately represent the physiological concentration of BaP in the distal region of smokers, such as the lungs^43,45^. Based on these parameters, we chose a concentration of 1 μM for both NNK and BaP for in vitro investigations on NB.

We collected conditioned media (CM) from THP-1s (^CM^THP) that had Veh or NB exposure under G^low^ (^CM^THP-Veh/G^low^ or ^CM^THP-NB/G^low^), G^std^ (^CM^THP-Veh or ^CM^THP-NB), or G^high^ (^CM^THP-Veh/G^high^ or ^CM^THP-NB/G^high^) conditions and added them to A549 cells. The colony-forming and sphere-forming capacities of A549 cells incubated with ^CM^THP-NB/G^high^, ^CM^THP-NB, or THP-NB/G^low^ were greater than those incubated with ^CM^THP-Veh/G^high^, ^CM^THP-Veh, or THP-Veh/G^low^, respectively (Fig. 3f). The tumor activities of A549 cells were greatest when incubated with ^CM^THP-NB/G^high^ compared to when incubated with ^CM^THP-NB or THP-NB/G^low^. We next collected CM from BMDMs that had Veh or NB exposure under the G^low^ (^CM^BMDM-Veh/G^low^ or ^CM^BMDM-NB/G^low^), G^std^ (^CM^BMDM-Veh or ^CM^BMDM-NB), or G^high^ (^CM^BMDM-Veh/G^high^ or ^CM^BMDM-NB/G^high^) conditions and added them to LLC cells. The aforementioned tumor activities of LLC cells were greatest when incubated with ^CM^BMDM-NB/G^high^ compared to when incubated with the other five BMDM-derived CM (Supplementary Fig. 5a). Additionally, these tumor activities of A549 and LLC cells were considerably diminished when incubated with CM from THP-1 or BMDM that had been exposed to NB in the presence of 2DG (^CM^THP-NB/2DG or ^CM^BMDM-NB/2DG), respectively, compared to when incubated with ^CM^THP-NB or ^CM^BMDM-NB, respectively (Fig. 3g, Supplementary Fig. 5b).

We conducted an animal experiment wherein LLC-Luc^ortho^-bearing mice were exposed to Veh or NB under HCD conditions (i.e., LLC-Luc^ortho^-Veh/HCD or LLC-Luc^ortho^-NB/HCD, respectively), either alone or in conjunction with clodronate (Cld), a liposomal reagent used to deplete macrophages^46^ (Fig. 3h). BLI-based analysis (Fig. 3i, j) and histology-based tumor quantification (Fig. 3k, Supplementary Fig. 5c) demonstrated that administration of clodronate significantly inhibited the tumor growth and metastasis in LLC-Luc^ortho^-NB/HCD group. The clodronate-treated LLC-Luc^ortho^-NB/HCD group also showed significantly higher overall survival than the Veh-treated LLC-Luc^ortho^-NB/HCD group (Fig. 3l). These results suggest that TAMs, particularly those exposed to NB under a glucose-rich TMiE, are programmed to promote LC progression.

### NB increases the expression and membrane location of GLUT1 and GLUT3, resulting in metabolic pre-programming of macrophages to acquire pro-tumor phenotypes

To investigate the mechanism by which NB exposure induces LC progression in a glucose-rich TMiE, we analyzed the effects of NB on glucose uptake in LLC-Luc^ortho^-carrying mice that had been exposed to Veh or NB. Positron emission tomography/magnetic resonance imaging (PET/MRI) analysis with 18F-fluorodeoxyglucose (18F-FDG) uptake and associated quantification by standardized uptake value ratio (SUVR) revealed significantly increased pulmonary uptake of 18F-FDG in mice that had NB exposure for one month compared to Veh-exposed control mice (Supplementary Fig. 6a). To assess how NB increases glucose uptake in lung tumors, we conducted a series of experiments outlined in Supplementary Fig. 6b. Real-time PCR analysis of the effects of NB exposure on the pulmonary expression of glucose transporters (GLUTs) revealed significantly elevated levels of *Slc2a1* and *Slc2a3* mRNA and a greater number of GLUT1^high^ and GLUT3^high^ macrophages (GLUT1^high^F4/80^+^ and GLUT3^high^ F4/80^+^, respectively) in the lungs of LLC-Luc^ortho^-carrying mice with 2-month exposure to NB compared with mice with 2-month exposure to Veh (Supplementary Fig. 6c, d). Consistently, LLC-Luc^ortho^-derived macrophages from NB-exposed mice exhibited higher levels of *Slc2a1* and *Slc2a3* expression than those from Veh-exposed mice (Supplementary Fig. 6e; left). Comparatively, LLC-Luc^ortho^-derived LC cells from NB-exposed mice and those from Veh-exposed mice exhibited similar levels of *Slc2a1* and *Slc2a3* expression (Supplementary Fig. 6f; left). In line with these findings, LLC-Luc^ortho^-derived macrophages from NB-exposed mice showed greater levels of 2-NBDG uptake compared with those from Veh-exposed mice (Supplementary Fig. 6e, f; right).

**Fig. 4:**
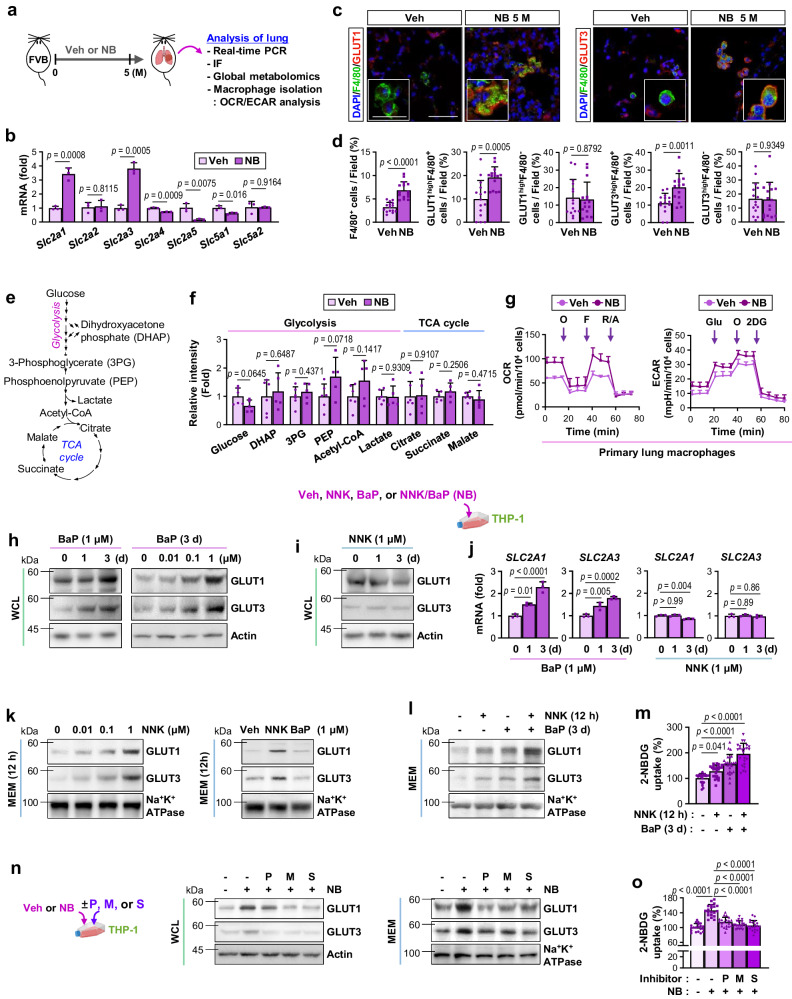
NB increases glucose consumption in macrophages through elevated expression and membranous localization of GLUT1 and GLUT3. **a**–**f** Lungs isolated from Veh- or NB-treated FVB mice for 5 months were subjected to various biological analyzes summarized in (**a**). **b** Real-time PCR analysis of the indicated GLUTs in the lungs (*n* = 3/group). **c**, **d** IF staining (**c**) and quantitative analysis (**d**) of GLUT1 and GLUT3 expression in the lungs of Veh- or NB-treated mice (*n* = 15/group). Scale bar: 50 μm. Scale bar (inset): 25 μm. **e**, **f** Schematic diagram of key glycolysis-TCA cycle metabolites (**e**) and these metabolites in the lungs (**f**) determined by LC/MS-based metabolomics (*n* = 6/group for the Veh group; *n* = 5/group for the NB group). **g** Seahorse analysis of oxygen consumption rate (OCR) (left) and extracellular acidification rate (ECAR) (right) in lung macrophages isolated from Veh- or NB-treated mice (*n* = 5/group). O: oligomycin; F: carbonyl cyanide p-trifluoromethoxy-phenylhydrazone (FCCP); R/A: rotenone/antimycin; 2DG: 2-deoxy-D-glucose. **h**, **i** Western blot (WB) analysis of THP-1 whole cell lysates (WCLs). **j** Real-time PCR analysis of *SLC2A1* (encoding GLUT1) and *SLC2A3* (encoding GLUT3) mRNA expression in THP-1 cells (*n* = 3/group). **k**, **l** WB analysis of the membrane fraction (MEM) of THP-1 cells. **m** Operetta high content imaging analysis of 2-NBDG fluorescent tracer uptake (*n* = 24/group) **n**, **o** THP-1 cells exposed to Veh or NB for 2 months were harvested. Vehicle (Veh) or the indicated inhibitors (P: 10 μM propranolol; M: 10 μM mecamylamine; and S: 1 μM SR1) were added to the cells 3 days before harvesting. **n** WB of GLUT1 and GLUT3 expression in WCL and MEM fraction. **o** Operetta high content imaging analysis of 2-NBDG fluorescent tracer uptake (*n* = 20/group). The data are presented as the mean ± SD. *p*-values were determined by using a two-tailed Student’s t-test (**b**, **d**, **f**), one-way ANOVA with Dunnett’s post-hoc test (**j**, **o**), or Kruskal–Wallis test with Dunn’s post-hoc test (**m**). The data are representative of two (data in **b**, **g**–**l**, **n**) or three (data in **m**, **o**) independent experiments with similar results. Source data are provided as a Source Data file.

We then conducted additional experiments using lung tissues from FVB mice, in which NB exposure for 5 months induced metabolic syndrome and tumor formation (presented in Fig. 1 and Supplementary Fig. 1). Similar to the observations in LLC-Luc^ortho^-carrying mice, NB exposure induced transcriptional increases of *Slc2a1* and *Slc2a3* mRNA in the lungs of FVB compared with Veh-exposed control mice (Fig. 4b). Immunofluorescence (IF) staining (Fig. 4c) and associated statistical analysis (Fig. 4d) further showed a greater number of pulmonary macrophages, particularly those with high levels of GLUT1 or GLUT3 (GLUT1^high^F4/80^+^ and GLUT3^high^F4/80^+^, respectively), in the lung tissues of NB-exposed mice compared with Veh-exposed mice. Contrarily, non-macrophage cell populations with elevated levels of GLUT1 or GLUT3 (GLUT1^high^F4/80^-^ and GLUT3^high^ F4/80^-^, respectively) in NB-exposed mice did not significantly differ from those in control mice. We then determined if exposure to NB affected glucose metabolism in TAMs. LC/MS-based global metabolomics on lung tissues from FVB mice revealed that none of the glycolysis and TCA cycle metabolites (Fig. 4e) were significantly altered in the lungs of NB-exposed mice compared with control mice (Fig. 4f). In contrast, a Seahorse-based investigation of glycolytic and oxidative fluxes revealed that pulmonary macrophages from NB-exposed mice had significantly greater oxygen consumption rates (OCR) and extracellular acidification rates (ECAR) compared with those from control mice (Fig. 4g). We further confirmed that THP-1s had significantly increased 2-NBDG uptake, OCR, ECAR, and ATP production after direct exposure to NB (Supplementary Fig. 7a–c). In contrast, these metabolic parameters remained unchanged in LC cells (A549 and H226Br) after NB exposure (Supplementary Fig. 7d–f). These results suggest that NB exposure increases glucose utilization by pulmonary cell populations, particularly by TAMs.

To investigate the mechanism underlying NB-induced glucose utilization in TAMs, we analyzed the relative contribution of each NNK and BaP exposure to GLUT expression in macrophages. THP-1s exhibited time-dependent increases in GLUT1 and GLUT3 protein expression after exposure to BaP at concentrations as low as 0.01 μM for 3 days, with the maximal increase occurring at 1 μM (Fig. 4h). In contrast, GLUT1 and GLUT3 expression remained unchanged after 3-day exposure to NNK (1 μM) (Fig. 4i). Time-dependent increases in *SLC2A1* and *SLC2A3* expression were also found in THP-1s exposed to BaP (1 μM), but not in those exposed to NNK (1 μM) (Fig. 4j). Notably, GLUT1 and GLUT3 protein levels in the THP-1 membrane (MEM) fraction were significantly elevated after 12 h of exposure to NNK, but not BaP (Fig. 4k). When compared to single exposure, the combined exposure to NNK and BaP (NB; 1 μM each) had additive effects on the glucose transporter levels on the MEM fraction (Fig. 4l) and 2-NBDG uptake (Fig. 4m). NNK is a ligand for both the β-adrenergic receptor (β-AR) and the nicotinic acetylcholine receptor (nAChR), whereas BaP is a ligand for the aryl hydrocarbon receptor (AhR)^47^. Indeed, pretreatment of THP-1s with β-AR (propranolol; P), nAChR (mecamylamine; M), or AhR (SR1; S) inhibitors reduced the NB-induced increase in GLUT1 and GLUT3 expression in whole-cell lysate (WCL) and MEM fraction (Fig. 4n) and glucose uptake (Fig. 4o). Together, these results suggest that BaP/AhR- and NNK/nAchR-mediated signaling pathways cooperate to facilitate glucose uptake in macrophages through transcriptional upregulation and membrane translocation of GLUT1 and GLUT3, respectively, thereby conferring pro-tumorigenic characteristics.

### NB-primed macrophages promote LC progression through paracrine activation of insulin receptor in LC cells

**Fig. 5:**
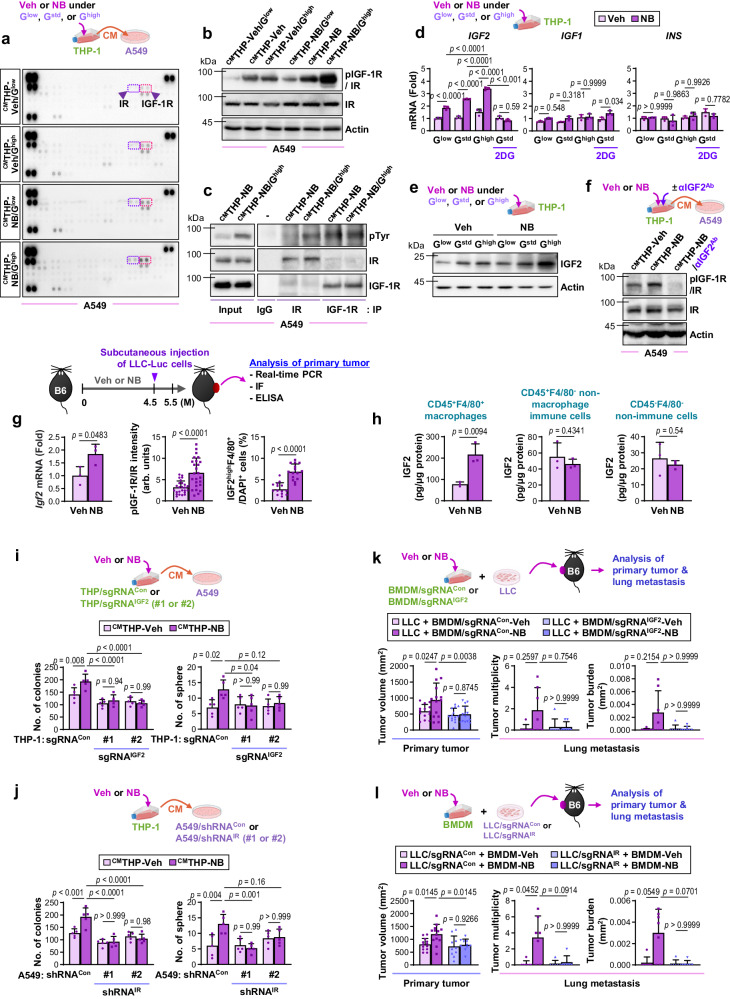
NB-primed macrophages under glucose-supplemented conditions upregulate IGF2 expression and activate IR in tumor cells in a paracrine manner. **a** A phospho-receptor tyrosine kinase (RTK) array with lysates of the indicated CM-treated A549 cells for 30 min. **b**, **f** Western blot (WB) analysis of phosphorylated IGF-1R/IR (Y1135/36 for IGF-1R, Y1150/51 for IR) and IR levels in the indicated CM-treated A549 cells for 30 min. αIGF2^Ab^: IGF2 neutralizing antibody (5 μg/mL) **c** Immunoprecipitation (IP) analysis of IGF-1R and IR phosphorylation in the indicated CM-treated A549 cells for 30 min. **d** Real-time PCR analysis of *IGF2*, *IGF1*, or *INS* expression in THP-1 cells (*n* = 3 biologically independent replicates per group). 2DG: 5 mM 2-deoxy-D-glucose. **e** WB analysis of IGF2 expression in THP-1 cells. **g** Real-time PCR analysis of *Igf2* mRNA (*n* = 3/group), immunohistochemistry (IHC) analysis of pIGF-1R/IR (Y1131 for IGF-1R, Y1146 for IR) expression (*n* = 25/group), and IF analysis of IGF2-expressing macrophages (IGF2^+^F4/80^+^) (*n* = 15/group) in LLC-Luc^sc^ tumors. arb. units.: arbitrary units. **h** IGF2 ELISA using CM from CD45^-^F4/80^-^ non-immune cells, CD45^+^F4/80^-^ non-macrophage immune cells, and CD45^+^F4/80^+^ macrophages isolated from LLC-Luc^sc^ tumors (*n* = 3/group). **i, j** Anchorage-independent colony formation and sphere formation of the indicated A549 cells treated with the CM from the indicated THP-1 cells (*n* = 5 biologically independent replicates per group). **k** The tumor volume of primary tumors of LLC cells co-injected with the indicated BMDMs (*n* = 14/group) and microscopic evaluation H&E-stained lung tissues (*n* = 7/group). **l** The tumor volume of primary tumors (*n* = 13 for LLC/sgRNA^Con^ groups and *n* = 10 for LLC/sgRNA^IR^ groups) and microscopic evaluation H&E-stained lung tissues (*n* = 7 for LLC/sgRNA^Con^ groups and *n* = 6 for LLC/sgRNA^IR^ groups). The data are presented as the mean ± SD. *p*-values were determined by using one-way ANOVA with Tukey’s post-hoc test (**d**, **i**, **j**), a two-tailed Student’s *t*-test (**g**, **h**), or Kruskal–Wallis test with Dunn’s post-hoc test (**k**, **l**). The data shown in **b**–**f**, **i**, **j** are representative of two independent experiments with similar results. Source data are provided as a Source Data file.

To understand how NB-enhanced glucose uptake contributes to the pro-tumoral function of macrophages, we examined the role of growth factors and cytokines in cancer-stroma communication^48^ utilizing THP-1s that had Veh or NB exposure under G^low^, G^std^, or G^high^ conditions and A549 cells exposed to CM from these THP-1s (i.e., ^CM^THP-Veh/G^low^, ^CM^THP-Veh, ^CM^THP-Veh/G^high^, ^CM^THP-NB/G^low^, ^CM^THP-NB, or ^CM^THP-NB/G^high^, respectively). We analyzed A549 cells for the activation profiles of receptor tyrosine kinases (RTKs) involved in various cancer cell activities^49^. A549 cells showed the most prominent phosphorylation (activation) of the insulin-like growth factor receptor/insulin receptor (IGF-1R/IR) when incubated with ^CM^THP-NB/G^high^ compared to when incubated with ^CM^THP-Veh/G^low^, ^CM^THP-Veh, ^CM^THP-Veh/G^high^, ^CM^THP-NB/G^low^, or ^CM^THP-NB (Fig. 5a). Western blot analysis confirmed greater IGF-1R/IR phosphorylation in A549 cells exposed to ^CM^THP-NB/G^high^, ^CM^THP-NB, or ^CM^THP-NB/G^low^ compared to those exposed to ^CM^THP-Veh/G^high^, ^CM^THP-Veh, or ^CM^THP-Veh/G^low^, respectively (Fig. 5b). We also analyzed LLC cells that were incubated with CM from BMDMs that had Veh or NB exposure under G^low^, G^std^, or G^high^ conditions (i.e., ^CM^THP-Veh/G^low^, ^CM^THP-Veh, ^CM^THP-Veh/G^high^, ^CM^THP-NB/G^low^, ^CM^THP-NB, or ^CM^THP-NB/G^high^, respectively). LLC cells incubated with CM from these BMDMs yielded highly similar results (Supplementary Fig. 8a). Notably, immunoprecipitation (IP) analysis showed greater IR phosphorylation rather than IGF-1R phosphorylation in A549 cells exposed to ^CM^THP-NB/G^high^ (Fig. 5c). These findings suggest that macrophages exposed to NB and glucose supply exert predominant effects on IR activation in LC cells.

Next, we assessed the expression of insulin-like growth factor 1 receptor/insulin receptor (IGF-1R/IR) ligands (IGF1, IGF2, and insulin [Ins])^50^ in NB-exposed macrophages. Real-time PCR analysis revealed a substantial upregulation of *IGF2* mRNA in NB-exposed THP-1s (Fig. 5d). Western blot and IF analyzes validated the elevated expression of IGF2 protein in NB-exposed THP-1s and BMDMs (Fig. 5e, Supplementary Fig. 8b, c). The NB-induced transcriptional increases in IGF2 expression were more pronounced under G^high^ condition than under G^std^ or G^low^ conditions, but 2DG treatment attenuated these effects (Fig. 5d, e). We also noted that CM from NB-exposed THP-1s in the presence of an IGF2-neutralizing antibody (^CM^THP-NB/αIGF2^Ab^) did not induce IGF-1R/IR phosphorylation in A549 cells (Fig. 5f), suggesting that IGF2 produced by NB-primed macrophages activated IGF-1R/IR in LC cells through a paracrine mechanism. The LLC-Luc^ortho^-NB/HCD mice presented in Fig. 2 also showed significant elevations in both the number of IGF2-expressing macrophages (IGF2^+^F4/80^+^) and tumoral phosphorylated IGF-1R/IR expression compared to the LLC-Luc^ortho^-Veh/HCD and the LLC-Luc^ortho^-NB/HCD treated with 2DG (Supplementary Fig. 8d, e). The majority of IGF2 expression in the LLC-Luc^ortho^-NB/HCD was detected in F4/80^+^ macrophages. Significant increases in IGF2 transcripts, IGF-1R/IR phosphorylation, and IGF2-expressing macrophage quantities were also observed in the lung tumors of FVB mice (Supplementary Fig. 9a–d) and LLC-Luc^sc^ tumors of B6 mice (Fig. 5g, Supplementary Fig. 9e, f) wherein chronic exposure to NB induced metabolic syndrome (as shown in Fig. 1). To investigate whether TAMs in mice with NB-induced metabolic syndrome played a major role in IGF2 delivery to LC cells, LLC-Luc^sc^ tumors of B6 mice showing NB-induced metabolic syndrome signatures were analyzed. ELISA of the CM from LLC-Luc^sc^-derived cells revealed that macrophages (CD45^+^F4/80^+^) in the NB-exposed tumors produced significantly more IGF2 than those in the Veh-exposed tumors (Fig. 5h). In contrast, non-immune cells (CD45^-^F4/80^-^) and F4/80^-^ immune cells (CD45^+^F4/80^-^) collected from NB-exposed LLC-Luc^sc^ tumors showed no significant difference in IGF2 production compared to their corresponding control cells collected from Veh-exposed tumors. Therefore, it is probable that NB-enhanced glucose metabolism endowed macrophages with transcription-dependent IGF2 production, leading to paracrine IGF-1R/IR activation in LC cells.

To investigate direct evidence supporting the role of paracrine IGF2 signaling in tumorigenic activities in LC cells, we generated THP-1s and BMDMs in which IGF2 expression was either intact or deleted using the CRISPR/Cas9 system (THP/sgRNA^con^ or THP/sgRNA^IGF2^, respectively). Subsequently, CM were collected from THP-1s and their IGF2-deficient sublines that were exposed to Veh or NB (^CM^THP/sgRNA^con^-Veh, ^CM^THP/sgRNA^con^-NB, ^CM^THP/sgRNA^IGF2^-Veh, and ^CM^THP/sgRNA^IGF2^-NB, respectively). A549 cells exposed to ^CM^THP/sgRNA^con^-NB exhibited significant increases in colony-forming and sphere-forming capabilities compared to those exposed to ^CM^THP/sgRNA^con^-Veh (Fig. 5i). In contrast, these tumorigenic activities of A549 cells exposed to ^CM^THP/sgRNA^IGF2^-NB were not significantly different from those of cells exposed to ^CM^THP/sgRNA^IGF2^-Veh. Subsequently, we collected CM from NB-exposed BMDMs, in which IGF2 expression was intact or deleted using the CRISPR/Cas9 system (i.e., ^CM^BMDM/sgRNA^con^-Veh, ^CM^BMDM/sgRNA^con^-NB, ^CM^BMDM/sgRNA^IGF2^-Veh, and ^CM^BMDM/sgRNA^IGF2^-NB). LLC cells exposed to ^CM^BMDM/sgRNA^con^-NB exhibited enhanced capabilities for forming colonies and spheres compared to those exposed to ^CM^BMDM/sgRNA^con^-Veh (Supplementary Fig. 9g). Contrarily, these tumorigenic activities of LLC cells exposed to ^CM^BMDM/sgRNA^IGF2^-NB and those of cells exposed to ^CM^BMDM/sgRNA^IGF2^-Veh did not differ significantly. Then, CM from THP-1s exposed to Veh or NB (^CM^THP-Veh or, ^CM^THP-NB) was added to A549 cells, in which IR expression was either unaltered or eliminated using IR-specific shRNA. In contrast to control cells transfected with control shRNA (A549/shRNA^con^), A549 cells transfected with IR-specific shRNA (A549/shRNA^IR^) showed no detectable increases in colony-forming and sphere-forming capabilities in response to ^CM^THP-NB (Fig. 5j). Similar results were observed when LLC cells, in which IR expression was intact or knocked out by the CRISPR/Cas9 method (LLC/sgRNA^Con^ or LLC/sgRNA^IR^), were incubated with ^CM^BMDM-Veh or ^CM^BMDM-NB (Supplementary Fig. 9h).

We subsequently conducted animal experiments. For the initial experiment, LLC cells were injected into mice along with BMDM/sgRNA^con^ or BMDM/sgRNA^IGF2^ that had been exposed to Veh or NB (i.e., BMDM/sgRNA^con^-Veh, BMDM/sgRNA^con^-NB, BMDM/sgRNA^IGF2^-Veh, or BMDM/sgRNA^IGF2^-NB). The tumor growth and lung metastasis in mice co-injected with LLC plus BMDM/sgRNA^Con^-NB were found to be significantly greater compared to those in mice co-injected with LLC plus BMDM/sgRNA^Con^-Veh, LLC plus BMDM/sgRNA^IGF2^-NB, or LLC plus BMDM/sgRNA^IGF2^-Veh (Fig. 5k). For the second set of experiments, LLC/sgRNA^Con^ or LLC/sgRNA^IR^ was co-injected with BMDMs that had been exposed to Veh or NB (i.e., BMDM-Veh or BMDM-NB). As shown in Fig. 5l, the tumor growth and lung metastasis in mice co-injected with LLC/sgRNA^Con^ plus BMDM-NB were significantly greater compared to those in mice co-injected with LLC/sgRNA^Con^ plus BMDM-Veh. In contrast, tumor growth and metastasis in mice co-injected with LLC/sgRNA^IR^ plus BMDM-NB were not significantly different from those in mice co-injected with LLC/sgRNA^IR^ plus BMDM-Veh. These findings underscore the role of macrophage-derived IGF2 in NB-induced LC progression.

### IGF2 induces activation and nuclear translocation of IR and its interaction with NPM1 in LC cells

**Fig. 6:**
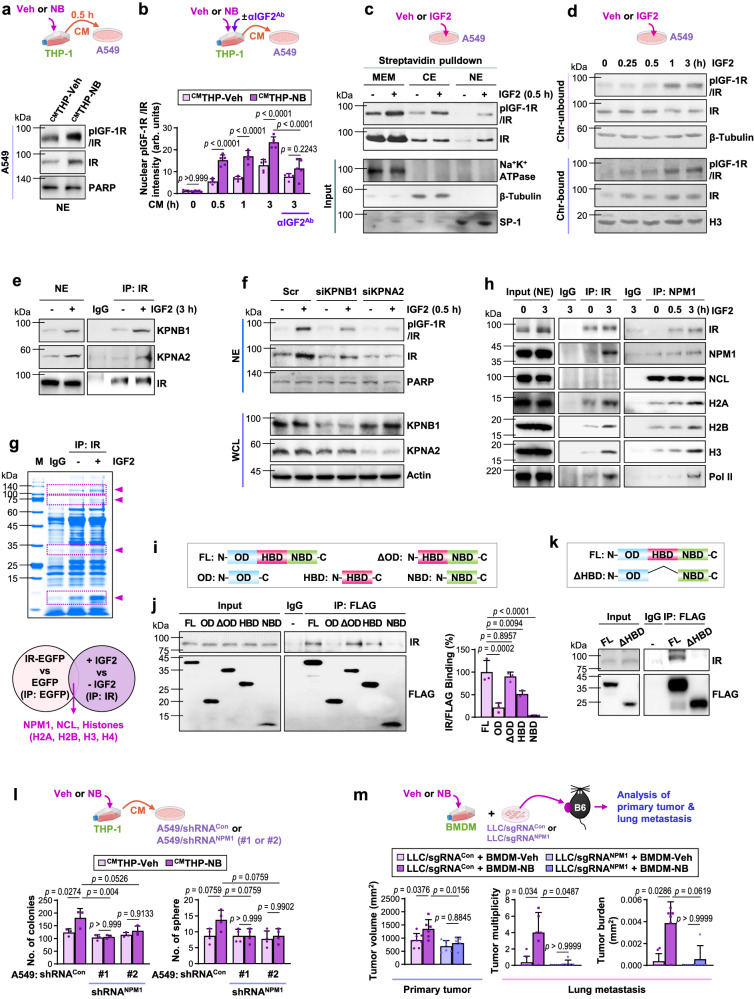
IGF2 induces nuclear translocation of IR and its association with NPM1. **a** Western blot (WB) analysis using the nuclear extract (NE) of CM-treated A549 cells. **b** Quantification of the immunofluorescence staining of nuclear phosphorylated IGF-1R/IR (pIGF-1R/IR, [Y1131 for IGF-1R, Y1146 for IR]) (*n* = 5 biologically independent replicates/group). arb. units.: arbitrary units. **c** Streptavidin pulldown analysis on membrane fractions (MEM), cytosol extracts (CE), and NE of IGF2 (50 ng/mL)-stimulated A549 cells. **d** WB analysis of non-chromatin (Chr-unbound) and chromatin (Chr-bound) fractions. **e** Immunoprecipitation (IP) analysis of the association of IR with KPNB1 and KPNA2 in IGF2-stimulated A549 cells. **f** WB analysis of nuclear pIGF-1R/IR (Y1135/36 for IGF-1R, Y1150/51 for IR) and IR levels in A549 cells. WCL: whole-cell lysates. **g** A representative image of Coomassie blue-stained gel on resolved anti-IR immunoprecipitants and common nuclear IR-associated proteins identified by LC-MS/MS analysis. NCL: nucleolin. **h** IP of the association of IR with NPM1, histones, and RNA polymerase II (Pol II) in A549 cells. **i** Schematic diagram depicting full-length (FL) and truncation mutants of NPM1. OD: oligomerization domain. HBD: histone binding domain. NBD: nucleic acid-binding domain. **j** Representative WB images of IP analysis and quantitative analysis (*n* = 3 biologically independent replicates/group) for the association of IR with NPM1 (FL or truncation mutants) in IGF2-stimulated H226Br cells for 3 h. **k** The association of IR with FL or HBD deletion mutant (ΔHBD) of NPM1 in IGF2-stimulated H226Br cells for 3 h. **l** Anchorage-independent colony formation (*n* = 3 biologically independent replicates/group) and sphere formation (*n* = 4 biologically independent replicates/group) capacities of the indicated A549 cells. **m** Schematic diagrams of experiments and quantitative analyzes of primary and metastatic lung tumor growth (LLC/sgRNA^Con^ + BMDM-Veh: *n* = 8; LLC/sgRNA^Con^ + BMDM-NB and LLC/sgRNA^NPM1^ + BMDM-Veh: *n* = 6; LLC/sgRNA^NPM1^ + BMDM-NB: *n* = 5). The data are presented as the mean ± SD. *p*-values were determined by using one-way ANOVA with Tukey’s post-hoc test (**b**, **j**, **l**) or Kruskal-Wallis test with Dunn’s post-hoc test (**m**). The data are representative of two (data shown in **a**–**f**, **h**, **k**, **l**) or three (data shown in **j**) independent experiments with similar results. Source data are provided as a Source Data file.

We determined the mechanism by which macrophage-driven IGF2 promoted LC progression. Notably, LY294002 (a PI3K inhibitor) or U0126 (a MEK inhibitor) had marginal effects on ^CM^THP-NB-induced colony and sphere formation in LC cells (Supplementary Fig. 10a). Hence, macrophage-driven IGF2 seemed to exert pro-tumor effects in LC cells via mechanisms distinct from canonical IGF-1R/IR signaling. Recent studies have demonstrated that IR translocates into the nucleus and forms complexes with RNA polymerase II and transcription regulators^51^. Western blot (Fig. 6a) and IF analyzes (Fig. 6b, Supplementary Fig. 10b) revealed that the CM from THP-1s that were exposed to NB (i.e., ^CM^THP-NB) induced obvious increases in nuclear pIGF-1R/IR levels in A549 cells as early as 30 minutes after the incubation. In contrast, the CM from THP-1s that had been exposed to NB in the presence of an IGF2-neutralizing antibody (^CM^THP-NB/αIGF2^Ab^) failed to increase nuclear pIGF-1R/IR levels. We then analyzed LLC-Luc^ortho^-carrying mice illustrated in Fig. 2. Notably, the LLC-Luc^ortho^-NB/HCD group exhibited significantly elevated levels of nuclear pIGF-1R/IR in comparison to the LLC-Luc^ortho^-Veh/HCD, LLC-Luc^ortho^-Veh/SD, LLC-Luc^ortho^-NB/SD, LLC-Luc^ortho^-Veh/HFD, LLC-Luc^ortho^-NB/HFD, LLC-Luc^ortho^-Veh/SD/2DG, LLC-Luc^ortho^-NB/SD/2DG, LLC-Luc^ortho^-Veh/HCD/2DG, and LLC-Luc^ortho^-NB/HCD/2DG groups (Supplementary Fig. 10c). We then evaluated the effects of recombinant IGF2 on LC cells. Within three minutes, IGF2 treatment increased the nuclear pIGF-1R/IR levels in LC cells (A549 and H226Br) (Supplementary Fig. 10d-e). Upon stimulation with IGF2, IGF-1R/IR was translocated to the cytosol and nucleus, as measured by streptavidin pulldown analysis of A549 cells that had undergone biotinylation of cell surface proteins (Fig. 6c). The association between nuclear pIGF-1R/IR and chromatin was observed upon analysis of the non-chromatin and chromatin fractions of A549 cells treated with IGF2 (Fig. 6d). Hence, it is plausible that IGF2 stimulated activation, nuclear translocation, and chromatin binding of IGF-1R/IR.

Despite the presence of a possible nuclear localization sequence (NLS)^52^, nuclear translocation of IR requires additional proteins, including importin-1 (karyopherin subunit beta 1; KPNB1) and importin-2 (karyopherin subunit alpha 2; KPNA2), an adapter protein involved in importin-mediated nuclear import of proteins^51^. Notably, IGF2 induced complex formation between IR, KPNB1, and KPNA2 in A549 cells (Fig. 6e). When KPNA2 or KPNB1 was silenced by siRNA transfection, the IGF2-induced increase in nuclear pIGF-1R/IR levels was significantly reduced (Fig. 6f). Moreover, the ^CM^THP-NB-induced sphere- and colony-forming capacities were significantly abolished in two distinct A549 subclones that were subjected to shRNA-mediated KPNB1 ablation (Supplementary Fig. 10f). Hence, it is likely that macrophage-derived IGF2 promotes aggressive phenotypes in LCs through the complementary action of nuclear IGF-1R/IR.

We then performed LC-MS/MS analysis of nuclear proteins associated with IR. The results of two independent LC-MS/MS analyzes of cells with induced overexpression of IR (IR-EGFP) and those with IGF2 stimulation revealed that IGF2 induces nuclear IR association with nucleophosmin (NPM1), nucleolin (NCL), and histones (Fig. 6g). Based on previous findings showing that: 1) NPM1 mainly localizes in the nucleoli with NCL, and shuttles through the nucleoli, nucleoplasm, and cytoplasm^53^; 2) NPM1 plays a role in chromatin remodeling and interacts with core histones^53^; and 3) NPM1 acts as a transcriptional activator of PD-L1^21^, we hypothesized that NPM1 functions as an IR-associated nuclear protein that regulates the transcriptional machinery responsible for NB-induced LC progression. Notably, a publicly available Gene Expression Omnibus (GEO) dataset (GSE30219) and nine prognosis-associated markers (KIAA0101, CDKN3, TPX2, MCM2, UBE2C, RRM2, PRC1, FGFR2, and GPC3) that have demonstrated prognostic significance in both lung adenocarcinoma and squamous cell carcinoma^54^ revealed a correlation between the expression levels of NPM1 and six markers associated with prognosis (KIAA0101, CDKN3, MCM2, RRM2, PRC1, and FGFR2) (Supplementary Fig. 11a). Gene set enrichment analysis (GSEA) also revealed that several gene sets associated with invasiveness and metastasis were significantly enriched in the NPM1^high^IGF2^high^ population compared with the NPM1^low^IGF2^low^ population (normalized enrichment score [NES] > 1.2, false discovery rate [FDR] <0.25), suggesting a possible association between NPM1 and IGF2 with protumor function in lung cancer (Supplementary Fig. 11b). Moreover, a co-immunoprecipitation study demonstrated the formation of an IGF2-induced complex among nuclear IR, NPM1, RNA polymerase II (Pol II), and core histones (Fig. 6h, Supplementary Fig. 11c). The association between IR and NPM1 was also detected in the primary tumors of FVB and B6 mice illustrated in Fig. 1 (Supplementary Fig. 11d), in which long-term exposure to NB resulted in a substantial rise in blood glucose levels, satisfying the metabolic syndrome criteria.

We determined the IR binding domain of NPM1 by generating mammalian expression vectors with FLAG-tagged full-length (FL), oligomerization domain (OD; N-terminal), FL without OD (ΔOD), histone binding domain (HBD; middle), or nucleic acid-binding domain (NBD) of NPM (Fig. 6i, top). The IP analysis and associated statistics of IGF2-stimulated LC cells (H226Br) using an anti-FLAG antibody revealed that FL, ΔOD, and HBD NPM1 mutants were associated with IR (Fig. 6j). In contrast, truncation mutants lacking HBD (OD and NBD) were incapable of interacting with IR. To validate the involvement of NPM1’s HBD in IR interaction, we established the H226Br subline in which NPM1 was knocked down by shRNA transfection and then either an NPM1 FL or a deletion mutant lacking HBD (ΔHBD) was reintroduced (H226Br/NPM1^FL^ or H226Br/NPM1^ΔHBD^, respectively) (Fig. 6k, top). Compared to H226Br/NPM1^FL^ cells, H226Br/NPM1^ΔHBD^ cells exhibited a significantly diminished IR-NPM1 interaction in response to IGF2 (Fig. 6k, bottom). These results suggest that NPM1 interacts with nuclear IR through the HBD.

We evaluated the role of the IR/NPM1 complex in the induction of aggressive phenotypes in LC cells by NB-primed macrophages. A549 cells (A549/shRNA^NPM1^), in which NPM1 expression was stably knocked down by shRNA transfection, were incubated with ^CM^THP-Veh or ^CM^THP-NB. Compared to control A549 cells (A549/shRNA^con^), two distinct A549/shRNA^NPM1^ subclones exhibited significantly diminished sphere- and colony-forming abilities in response to ^CM^THP-NB (Fig. 6l). These ^CM^THP-NB-induced tumor activities were similarly less in H226Br/NPM1^ΔHBD^ cells than in H226Br/NPM1^FL^ cells (Supplementary Fig. 11e). Then, we monitored tumors inm mice that were subcutaneously co-injected with NB-primed BMDMs and LLC cells in which NPM1 expression was either unaffected (LLC/sgRNA^Con^) or eliminated by the CRISPR/Cas9 system (LLC/sgRNA^NPM1^). Mice co-injected with LLC/sgRNA^Con^ plus NB-exposed BMDM exhibited significantly greater tumor growth and lung metastasis compared to mice co-injected with LLC/sgRNA^Con^ plus Veh-exposed BMDM, LLC/sgRNA^NPM1^ plus NB-exposed BMDM, or LLC/sgRNA^NPM1^ plus Veh-exposed BMDM combinations (Fig. 6m). These findings suggest that IGF2 produced by macrophages induces NPM1-mediated interactions between IR, Pol II, and histones on chromatin, resulting in aggressive LC cell behavior.

### IGF2-induced nuclear IR/NPM1 binding to the CD274 promoter stimulates PD-L1 expression, which is essential for NB-driven LC progression

**Fig. 7:**
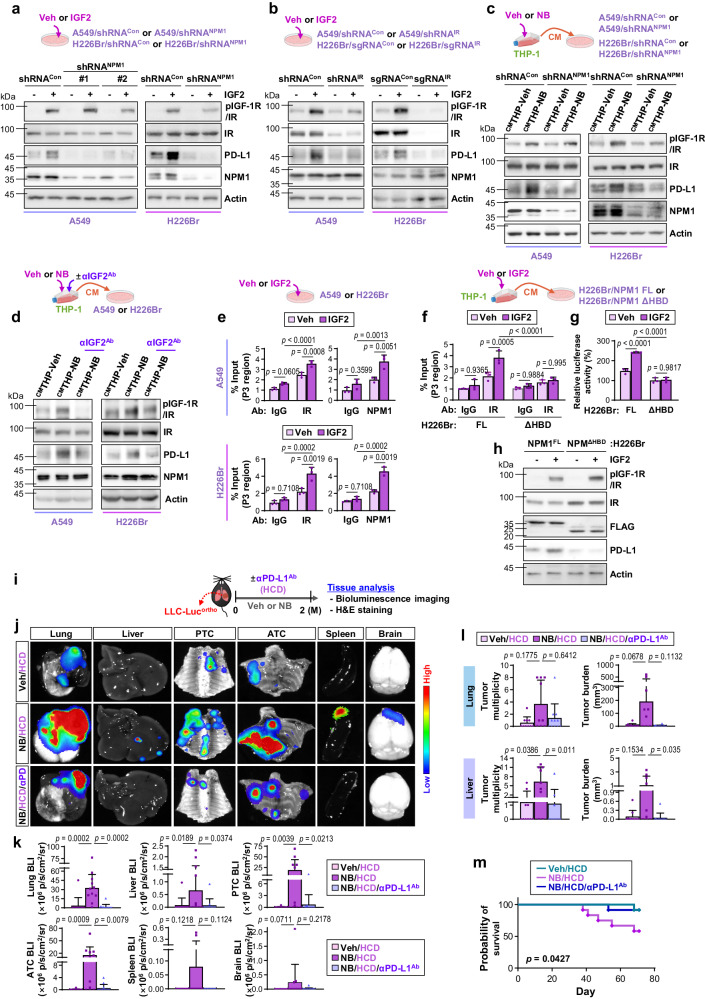
IGF2-induced association between IR and NPM1 in the nucleus increases PD-L1 expression, promoting LC progression. **a**–**d** Western blot (WB) analysis of the indicated protein expression in A549 and H226Br cells and their subclones. Cells were stimulated with IGF2 (50 ng/mL) for one day (**a**, **b**) or incubated with ^CM^THP-Veh or ^CM^THP-NB for one day (**c**, **d**). **e**–**g** Indicated LC cells were exposed to IGF2 (50 ng/mL) for 3 h (for chromatin immunoprecipitation [ChIP] assay) (**e**, **f**) or 24 h (for luciferase reporter assay) (**g**). **e**, **f** ChIP assay of IR (**e**, **f**) or NPM1 (**e**) binding to the P3 region of the *CD274* promoter (*n* = 3 biologically independent replicates/group). **g** Luciferase reporter assay of activation of the *CD274* promoter (*n* = 3 biologically independent replicates/group). **h** WB analysis of the effect of IGF2 stimulation (50 ng/mL for 24 h) on the indicated protein expression in H226Br cells. **i**–**m** B6 mice carrying LLC-Luc^ortho^ were exposed to Veh or NB under HCD condition, either alone or together with intraperitoneal injection of anti-PD-L1 antibody (αPD-L1^Ab^, 100 μg in 100 μL/mouse, twice a week). The data is representative of two independent experiments. **i** Schematic diagram of the experimental schedule. **j** Representative ex vivo bioluminescence images of analyzed organs **k** Quantitative analyzes of bioluminescence intensity (BLI) of analyzed organs (*n* = 12/group for the Veh/HCD group; *n* = 11/group for the NB/HCD group; *n* = 13/group for the NB/HCD/αPD-L1^Ab^ group). **l** Microscopic evaluation of H&E-stained lung and liver tissues for tumor multiplicity and burden (*n* = 10/group for the Veh/HCD group; *n* = 9/group for the NB/HCD group; *n* = 11/group for the NB/HCD/αPD-L1^Ab^ group). **m** Kaplan–Meier survival curve of the mice in each group (*n* = 12/group). The data are presented as the mean ± SD. *p*-values were determined by using one-way ANOVA with Tukey’s post-hoc test (**e**–**g**), Kruskal–Wallis test with Dunn’s post-hoc test (**k**, **l**), or a log-rank test (**m**). The data shown in **a**–**h** are representative of two independent experiments with similar results. Source data are provided as a Source Data file.

To determine the putative cellular targets of the NPM1/nuclear IR complex, gene expression profiles of NPM1^high^ vs. NPM1^low^ groups in lung cancer were compared using three GEO datasets (GSE37745, GSE50081, and GSE77803). Twenty genes were commonly upregulated in the NPM1^high^ group relative to the NPM1^low^ group in these three datasets (fold change ≥ 1.5; *p* < 0.05, *q* < 0.01) (Supplementary Fig. 12a, left). STRING protein interaction analysis^55^ using 20 common upregulated genes revealed that NPM1 is functionally associated with six markers (MAD2L1, ANP32E, SRSF3, EEF1A1, TFRC, and CD274) (Supplementary Fig. 12a, right). Four of these six genes (*ANP32E*, *EEF1A1*, *TFRC*, and *CD274*) were consistently downregulated in two distinct A549/shRNA^NPM1^ compared to A549/shRNA^Con^ cells (Supplementary Fig. 12b). Among the four genes, we focused on *CD274*, which encodes PD-L1, because ^CM^THP-NB (Supplementary Fig. 12c) and IGF2 (Supplementary Fig. 12d) consistently increased *CD274* transcription. Furthermore, IGF2-induced transcriptional upregulation of PD-L1 expression was markedly reduced in LC cells (A549 or H226Br) stably transfected with shRNA specifically targeting NPM1, IR, or KPNB1 (Fig. 7a, b, Supplementary Fig. 12d, e). ^CM^THP-NB or ^CM^BMDM-NB also upregulated PD-L1 expression in control LC cells, but not in those with IR or NPM1 deletion (Fig. 7c**;** Supplementary Fig. 12f, g). CM from THP-1s that had been exposed to NB in the presence of an IGF2-neutralizing antibody (^CM^THP-NB/αIGF2^Ab^) did not increase pIGF-1R/IR and PD-L1 expression in LC cells (Fig. 7d). Upregulation of PD-L1 expression was also seen in the primary tumors of FVB and B6 mice (Supplementary Fig. 12h–j), in which prolonged exposure to NB significantly increased blood glucose to meet metabolic syndrome criteria (presented in Fig. 1), and in LLC-Luc^ortho^-NB/HCD mice (presented in Fig. 2). PD-L1 mRNA and protein expression in LC cells, on the other hand, remained unchanged following in vitro exposure to NB for 2 months (Supplementary Fig. 12k). These results suggest that nuclear IR/NPM1 complex mediated by macrophage-derived IGF2 induces transcriptional upregulation of PD-L1 expression in LC cells.

The PD-L1 promoter possesses three potential NPM1-binding regions (P1-P3)^21^. NPM1 has a NLS sequence in its HBD^56^ and is predominantly bound to the 469–690 bp region of the PD-L1 promoter via its NBD^21^. Chromatin precipitation (ChIP) demonstrated that IR was mostly bound to the P3 region of the *CD274* promoter (Supplementary Fig. 12l). Additionally, IGF2 increased IR and NPM1 binding to the P3 region in two different LC cells (Fig. 7e). IGF2-induced IR binding to the P3 region (Fig. 7f) and activation of *CD274* promoter **(**Fig. 7g) were significantly lower in H226Br/NPM1^ΔHBD^ cells than in H226Br/NPM1^FL^ cells. Furthermore, IGF2-induced pIGF-1R/IR and PD-L1 expression was lower in H226Br/NPM1^ΔHBD^ cells than in H226Br/NPM1^FL^ cells (Fig. 7h). In response to ^CM^THP-NB, H226Br/NPM1^FL^ cells, but not H226Br/NPM1^ΔHBD^ cells, displayed significantly increased IR binding to the P3 region and *CD274* promoter activity (Supplementary Fig. 12m). Therefore, macrophage-derived IGF2 mediates the formation of nuclear IR/NPM1 complexes via NPM’s HBD domain, resulting in transcription-dependent PD-L1 expression.

We assessed the functional role of macrophage-mediated PD-L1 expression in the progression of NB-induced LC. Mice harboring LLC-Luc^ortho^ were exposed to NB under HCD conditions, either alone or in combination with a PD-L1-neutralizing antibody (αPD-L1^Ab^) (Fig. 7i). BLI-based analysis (Fig. 7j-k) and histology-based tumor quantification (Fig. 7l) revealed that administration of αPD-L1^Ab^ significantly inhibited the growth and metastasis of LLC-Luc^orto^-NB/HCD. In addition, the αPD-L1^Ab^-treated LLC-Luc^orto^-NB/HCD group demonstrated substantially improved survival compared to the LLC-Luc^orto^-NB/HCD group (Fig. 7m). These data suggest that PD-L1 expression elevated by macrophage-derived IGF2 plays a critical role in NB-induced LC progression.

### Expression of IGF2 and GLUT1 in macrophages correlates with the severity and metastatic status of lung cancer

**Fig. 8:**
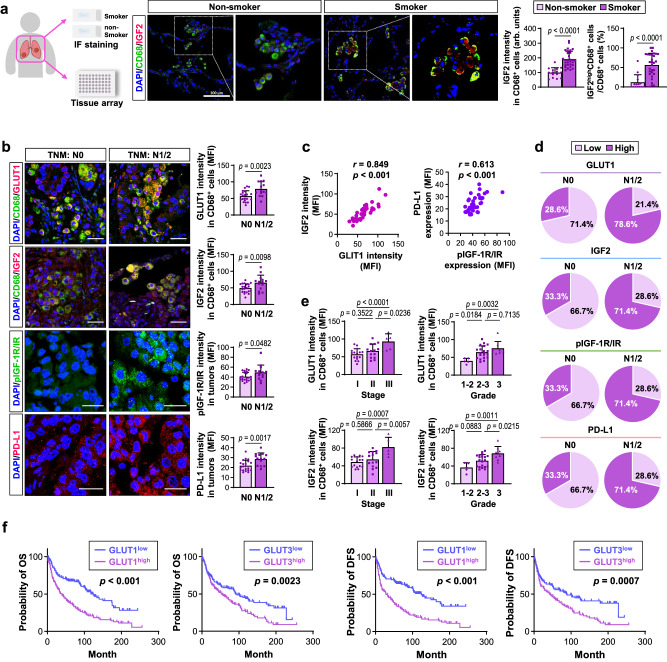
IGF2 and GLUT1 expression levels in macrophages and pIGF-1R/IR and PD-L1 levels in tumors are associated with the metastatic status of lung cancer patients. **a** Representative immunofluorescence (IF) images and quantitative analyzes of the indicated markers in patient-derived lung tissues from non-smokers (*n* = 3/group, 5 fields/slide [*n* = 15/group]) and smokers (*n* = 5/group, 6 fields/slide [*n* = 30/group]). Scale bar: 100 μm. **b** Representative IF staining images of the indicated markers using a tissue microarray (TMA) (*n* = 21 for the TNM N0 group; *n* = 14 for the TNM N1/2 group). Scale bars: 20 μm. **c**, left Correlation analysis for the Pearson correlation coefficient between IGF2 and GLUT1 levels in macrophages (*n* = 35/group). **c**, right Correlation analysis for the Spearman rank correlation coefficient between nuclear pIGF-1R/IR (Y1131 for IGF-1R, Y1146 for IR) and PD-L1 in tumor cells (*n* = 35/group). **d** Pie charts showing the levels of indicated markers in tumor tissues from patients with or without lymph node metastasis (N0 or N1/2, respectively; *n* = 21 for the TNM N0 group; *n* = 14 for the TNM N1/2 group). **e** Association of the expression level of the indicated markers with cancer stage (left, *n* = 16 for the stage I group; *n* = 14 for the stage II group; *n* = 5 for the stage III group) and tumor grade (right, *n* = 5 for the grade 1–2 group; *n* = 20 for the grade 2-3 group; *n* = 8 for the grade 3 group). **f** Analysis of a publicly available dataset (GSE30219) to determine the association of GLUT1 or GLUT3 levels with overall and disease-free survival (OS [*n* = 136/group] and DFS [*n* = 129/group], respectively) in patients with NSCLC. The data are presented as the mean ± SD. *p*-values were determined by using a two-tailed Student’s *t*-test (**a**, **b**), two-tailed Mann-Whitney test (**a**), one-way ANOVA with Tukey’s post-hoc test (**e**), Kruskal–Wallis test with Dunn’s post-hoc test (**e**), or a log-rank test (**f**). Source data are provided as a Source Data file.

Previous research has indicated that IGF2, GLUT1, and GLUT3 may serve as prognostic biomarkers for lung cancer^57–59^. To confirm the clinical relevance of our findings, we examined IGF2 expression in lung macrophages from lung cancer patients with a smoking history. IF analysis revealed increased IGF2 expression in CD68^+^ macrophages and a higher number of IGF2-expressing macrophages (IGF2^+^CD68^+^) in the lung tissues of smokers compared to non-smokers (Fig. 8a). In addition, we analyzed GLUT1 and IGF2 levels in macrophages as well as IR and PD-L1 expression in a tissue microarray containing 70 lung cancer tissues derived from 35 patients with lung cancer and clinicopathological characteristics, including TNM staging, lymph node metastasis, cancer stage, and tumor grade. The expression levels of GLUT1 and IGF2 in tumor-associated macrophages as well as tumor pIGF-1R/IR and PD-L1 were significantly higher in tumors from lung cancer patients with positive lymph node metastasis (N1/2) than in tumors from patients with negative lymph node metastasis (N0) (Fig. 8b). IGF2 expression was positively associated with GLUT1 expression in CD68^+^macrophages (Fig. 8c). The correlation between pIGF-1R/IR and PD-L1 expression in tumors was also substantial (Fig. 8c). Patients with lymph node metastasis were more likely than those without lymph node metastasis to exhibit high levels of GLUT1 or IGF2 expression in CD68^+^ macrophages and pIGF-1R/IR and PD-L1 expression in tumors [GLUT1:11 of 17 (64.7%), *p* = 0.0059; IGF2:10 of 17 (58.8%), *p* = 0.0409; pIGF-1R/IR:10 of 17 (58.8%), *p* = 0.0409; PD-L1:10 of 17 (58.8%), *p* = 0.0409] (Fig. 8d, Supplementary Table 1, 2).

The correlation between the expression of these markers and other clinicopathological characteristics was then evaluated. GLUT1 and IGF2 expression in CD68^+^ macrophages were higher in advanced-stage (stage III) or high-grade (grade 2–3 or 3) lung cancer tumors than in early-stage (stage I) or low-grade tumors (Fig. 8e). The expression of GLUT1 and IGF2 in CD68^+^ macrophages was positively correlated with lymphatic metastasis, advanced TNM stage, and lower grade (Supplementary Table 1). There were no significant associations between tumoral pIGF-1R/IR or PD-L1 expression and cancer stage or grade (Supplementary Table 2). According to an analysis of the GEO dataset (GSE30219), lung cancer patients with high GLUT1 or GLUT3 expression had a shorter lifespan and reduced disease-free survival than those with low GLUT1 or GLUT3 expression (Fig. 8f). These findings imply that GLUT1/IGF2 expression in TAMs and tumoral pIGF-1R/IR and PD-L1 expression serve as biomarkers for LC progression and therapeutic targets for LC treatment.

## Discussion

**Fig. 9:**
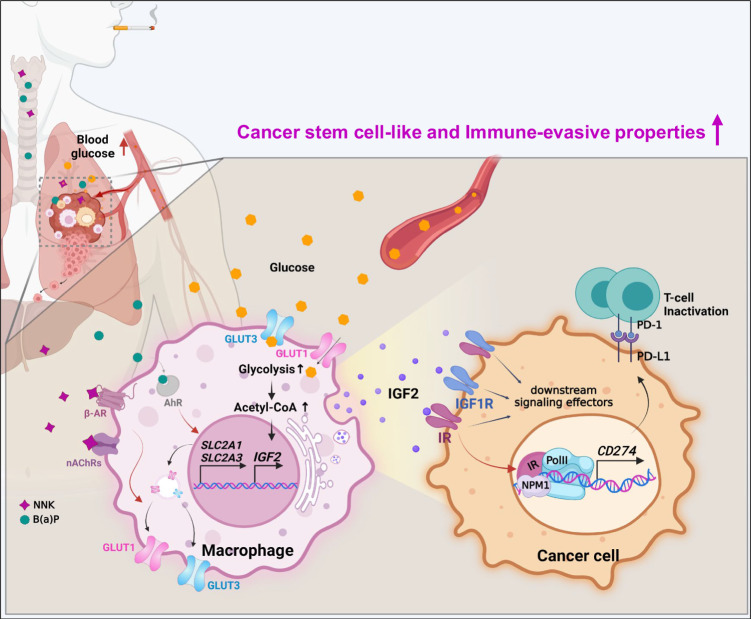
A proposed model illustrating NB-induced lung cancer progression mediated by the tumor-prone microenvironment with metabolically reprogrammed macrophages. In a glucose-rich microenvironment caused by systemic NB-induced hyperglycemia, NB increases glucose utilization in macrophages through GLUT1 and GLUT3 transcription and membranous localization upregulation, thereby inducing *IGF2* transcription in macrophages. IGF2 stimulates IR in tumor cells in a paracrine manner, resulting in PD-L1 expression upregulation through IR nuclear translocation and a complex formation with NPM1 and Pol II. These events lead to the acquisition of cancer stem cell-like and immune-evasive properties in tumor cells, ultimately mediating metastatic tumor formation.

Epidemiological, clinical, and experimental studies support the etiological role of TS-mediated metabolic syndrome in LC progression, although the underlying mechanism is unknown^60^. Here, we showed that NBs are important TS components that induce metabolic syndrome, especially hyperglycemia and dyslipidemia. NB induces transcriptional upregulation of genes implicated in the infiltration of monocytes/macrophages in TMiE and glucose uptake in TAMs by upregulating GLUT1 and GLUT3 expression and their membrane location. NB-primed macrophages produce IGF2, which induces activation and nuclear translocation of IR in LC cells. Nuclear IR forms a complex with NPM1, histones, and Pol II on the *CD274* promoter to induce PD-L1 expression, providing LC cells CSC and immune-evasive traits for rapid growth and metastasis. Disruption of the intercellular IGF2/IR/NPM/PD-L1 signaling cascade by genetic ablation of IGF2, IR, or NPM, pharmacological suppression of macrophages or glycolysis, and targeting of the PD-1/PD-L1 axis inhibited NB-induced LC progression (Fig. 9). Our findings highlight a unique model in which NB-induced metabolic disorders in TMaE, specifically hyperglycemia, are co-opted to create a tumor niche in TMiE, where monocytes/macrophages are recruited and trained to induce PD-L1 expression in LC cells, promoting LC progression.

Several metabolic syndrome components are currently considered to be additional risk factors for LC development and progression^10,11^; nevertheless, the function and mechanism of metabolic syndrome signature in TS-induced LC progression have long been unknown. Our findings supported the role of NB-induced hyperglycemia in LC progression. Glycolysis is typically hyperactive in cancer cells to generate enough energy for fast multiplication^61^. Elevated glucose levels cause DNA mutations and transcriptional, translational, and post-translational regulation of multiple oncogenic pathways in cancer cells^62^. Our findings show that NB exposure enhances glucose consumption in monocytes/macrophages via a two-step pathway: BaP/AhR-mediated transcriptional upregulation of GLUT1 and GLUT3 and NNK/nAChR-triggered membrane translocation of the transporters, leading to upregulation of genes involved in tumor infiltration and pro-tumoral activities. Therefore, although the involvement of additional mechanisms cannot be ruled out, it is probable that NB exposure promotes LC progression by inducing hyperglycemia in TMaE, which preprogram monocytes/macrophages in TMiE to acquire protumoral phenotypes.

Proinflammatory mediators and inducible nitric oxide synthase from M1 macrophages induce tissue injury^63^. M2 macrophages suppress the immune system and contribute to tissue remodeling/repair, angiogenesis, and tumor promotion by secreting various anti-inflammatory mediators and expressing Arg1, CD206, and scavenger receptors^63^. The significance of glucose metabolism in macrophage immunomodulation has been established^12^. M1 macrophages predominantly perform glycolysis^64^, whereas M2 macrophages have a high mitochondrial OCR^65^. M2 macrophages are commonly believed to be TAMs, which are significant glucose consumers^66^. TAMs exhibit M1 and M2 phenotypes dependent on TMiE and are associated with a specific pathogenic state^63^. TAM subsets, including interferon-primed, immune regulatory, inflammatory cytokine-enriched, lipid-associated, pro-angiogenic, resident-tissue macrophage-like, and proliferating TAMs, were further described utilizing a single cell-based technique^67^. Therefore, TAMs, despite their similarities, are not just a subset of M2 macrophages. Our findings indicate that NB-primed macrophages undergo metabolic reprogramming to perform enhanced glucose metabolism and acquire M2-type phenotypes, creating immunosuppressive TMiE.

Another essential question is how macrophages trained by NB-induced metabolic preprogramming confer aggressive characteristics on LC cells. Kinase profiling data revealed that NB-primed macrophages undergo transcriptional upregulation of IGF2 via increased glycolysis, which results in paracrine activation of IR signaling in LC cells. The IGF axis consists of IGF1, IGF2, their receptors, and IGF binding proteins^50^. Despite IGFs frequently exhibiting similar physiological and cellular functions, such as fetal growth, development, tumor progression, and metabolism^50^, they have distinct functions in regulating glucose metabolism in monocytes. IGF1-mediated IGF-1R activation causes monocytes to execute aerobic glycolysis and pro-inflammatory activities^68^. In contrast, IGF2 pre-programs mature macrophages to engage in anti-inflammatory activities^20^. The ability of macrophage-derived IGF2 to stimulate the aggressiveness of LC cells via transcriptional upregulation of PD-L1 is a remarkable aspect of our findings. A traditional paradigm for IGF-1R/IR signaling involves ligand binding at the cell surface, autophosphorylation, cytosolic protein docking, and biological output^50^. Notably, macrophage-derived IGF2 induced PD-L1 expression via KPNB1- and KPNA2-mediated nuclear localization of IR. IR lacks apparent nucleic acid-binding domains^51^. Hence, we postulated that a nuclear partner may enable IR binding to the PD-L1 promoter. Our discovery that IGF2 induces complex formation between IR, histones, and Pol II through the action of NPM1 supports this hypothesis. NPM1 binds to the PD-L1 promoter by interacting with various transcription factors, such as c-Myc or NF-κB, facilitating transcription^21^. Therefore, NPM1 may function as a transcriptional coactivator of IR to promote PD-L1 expression. IR has IR-A and IR-B isoforms^69^. IGF2-induced PD-L1 expression may be mediated by IR-A owing to its high IR-A affinity^70^ and high IR-A:IR-B ratio in cancer cells^69^. Further studies are needed to determine how IR isoforms regulate IGF2-induced PD-L1 expression and cancer progression. In our study, HFD did not substantially contribute to NB-induced LC progression. Previous research has shown that HFD-fed mice are more susceptible to urethane-induced lung tumorigenesis or metastasis^71^. This discrepancy may be attributed to experimental variables, such as HFD feeding time and the mouse model’s genetic origin. Additional research is also required to resolve this issue.

The results of our study carry translational implications. Our findings explain how hyperglycemia and diabetes mellitus act as important risk factors for the development and progression of some cancers, including LC^72^; and how patients’ smoking status affects the efficacy of anti-PD-1/PD-L1 immunotherapy^73^. Our mechanistic insights into the NB-induced metabolic complications in TMaE and TMiE further point to macrophage-associated GLUT1 and GLUT3 and IGF2/IR/NPM1/PD-L1 signaling components as prognostic biomarkers and therapeutic targets for patients with LC and persistent TS.

Our findings suggest that TS promotes the progression of LC through a key mechanism involving cancer-stroma communication facilitated by NB-mediated systemic and local complications in glucose metabolism. The hypothetical mechanisms derived from LLC tumors may not be applicable to the progression of LC induced by TS, considering the intricate nature of molecular and cellular compartments in TMiE and TMaE. Single-cell-based multiomics analyzes utilizing LC tissues from rodents exposed to NB and patients with a history of TS are necessary to determine whether our findings cast light on the pathogenesis of TS-induced LC progression. In particular, research examining the correlation between GLUT1, GLUT3, and IGF2 expression in pulmonary macrophages, growth and metastasis of lung tumors, and metabolic and smoking status of patients with LC may provide further substantial insights.

## Methods

The experiments complied with all relevant ethical regulations. All mouse experiments complied with the Seoul National University’s Institutional Animal Care and Use Committee authorized protocols (approval no. SNU-190628, SNU-200721, and SNU-231212-2-2) and the Institutional Animal Care and Use Committee of Asan Institute for Life Sciences (approval no. 2023-40-308). Experiments using patient-derived tissues from the Biobank of Soonchunhyang University Bucheon Hospital (schbc-biobank-2016-011-01), a member of the Korea Biobank Network, were conducted using the protocol approved by Seoul National University Institutional review board (IRB; approval No. E1608/001-001), ensuring compliance with the ethical guidelines of the Declaration of Helsinki.

### Reagents

Supplementary Table 3 lists the manufacturer, catalog number, application, and dilution ratio of the antibodies used for western blot analysis, IHC, and immunofluorescence analysis. Reagents for cell culture (fetal bovine serum [FBS, cat. No. S001-07], culture media [RPMI 1640: cat. no. LM011-01; DMEM: cat. no. LM001-05], phosphate-buffered saline [PBS, cat. no. LB001-02], antibiotics [cat. no. LS203-01], and trypsin-EDTA [cat. no. LS015-02]) were bought from Welgene (Gyeongsan-si, Republic of Korea). We purchased an IGF2 neutralizing antibody (cat. no. AF-292-NA), anti-mouse PD-L1 antibody for in vivo experiments (cat. no. BE0101), control and clodronate liposomes (cat. no. F70101C-NC), and SR-1 (cat. no. HY-15001) from R&D Systems (Minneapolis, MN, USA), BioXCell (Lebanon, NH, USA), FormuMax Scientific Inc. (Sunnyvale, CA, USA), and MedChemExpress LLC (Monmouth Junction, NJ, USA), respectively. NNK (cat. no. M325750) was acquired from Toronto Research Chemicals (Toronto, Ontario, Canada), and propranolol (cat. no. 0624) and mecamylamine (cat. no. 2843) were purchased from Tocris Bioscience (Bristol, UK). Exendin-4 (cat. no. GC13391) was acquired from GlpBio (Montclair, CA, USA). 2-NBDG (cat. no. 72987), 2DG (cat. no. D6134), metformin (cat. no. D150959), and BaP (cat. no. B1760), and other chemicals, unless otherwise specified, were purchased from Sigma-Aldrich (St. Louis, MO, USA). Small molecule inhibitors, NNK, and BaP were dissolved in dimethyl sulfoxide (DMSO) unless otherwise specified.

### Cell culture

A549 (cat. no. CCL-185) and Lewis lung cancer (LLC, cat. no. CRL-1642) cells were obtained from the American Type Culture Collection (ATCC, Manassas, VA, USA). Drs. John V. Heymach (University of Texas M.D. Anderson Cancer Center, Houston, TX, USA) and Kyu-Won Kim (Seoul National University, Seoul, Republic of Korea) kindly provided the H226Br and THP-1 cells, respectively. L929 cells (cat. no. 10001) were acquired from the Korean Cell Line Bank (Seoul, Republic of Korea). A549, H226Br, L929, and THP-1 cells were grown in a RPMI 1640 medium supplemented with 10% FBS and antibiotics at 37 °C in a humidified environment with 5% CO_2_. LLC cells were maintained in DMEM supplemented with 10% FBS and antibiotics. The glucose concentrations of RPMI 1640 and DMEM were 2 g/L (11 mM) and 4.5 g/L (25 mM), respectively. Unless otherwise specified, we diluted the vehicle (DMSO), NB, or other inhibitors in the medium containing 2 g/L glucose (the G^std^ condition). Authentication and verification of human cancer cell lines were performed using the AmplFLSTR identifier PCR Amplification Kit (Applied Biosystems, Foster, CA; cat. no. 4322288). We utilized mycoplasma-free cells that had been maintained for less than 2 months after recovery of validated cells.

### Mouse experiments

C57BL/6 J (B6) mice were purchased from DBL (Chungcheongbuk-do, Republic of Korea) and FVB/N mice from Japan SLC, Inc. (Hamamatsu, Japan). Mice were cared for according to the Association for Assessment and Accreditation of Laboratory Animal Care Standards and the US Public Health Service Policy on Human Care and Use of Laboratory Animals guidelines. Mice were maintained in a pathogen-free environment, freely accessed food and water, and housed at 22 ± 2 °C with a 12:12-h light:dark cycle. In all in vivo experiments, mice were randomly grouped. Monitoring tumor growth and metastatic tumor formation was performed in a blinded fashion. We did not statistically determine the sample size for animal experiments.

For animal experiments, NNK and BaP were dissolved in DMSO and then diluted in corn oil (final dose: 3 μmol NNK and 3 μmol BaP; treatment volume: 100 μL/mouse). 2DG were dissolved in PBS (final dose: 500 mg/kg body weight [BW]; treatment volume: 100–150 μL/mouse according to BW). Clodronate liposome was diluted in control neutral liposome solution (final dose: 0.7 mg/mouse; treatment volume: 100 μL/mouse). Anti-PD-L1 antibody was diluted in PBS (final dose: 100 μg/mouse; treatment volume: 100 μL/mouse).

To monitor NB-induced tumor development and metabolic alterations, 2-month-old FVB/N and B6 male mice were administered vehicle (Veh, 6% DMSO in corn oil) or NB via oral gavage twice a week for up to 5 months.

For subcutaneous tumor models, 2-month-old B6 male mice were administered Veh or NB twice a week for 4.5 months before being subcutaneously inoculated with LLC-Luc cells (1 × 10^6^ cells per mouse in 100 μL of PBS containing 25% Matrigel). These mice were then given Veh or NB for 1 additional month. When necessary, after forming palpable tumors (approximately 1 week after cell inoculation, tumor volume ≥ 50 mm^3^), mice were subjected to the daily administration of exendin-4 (EX4, 20 μg/kg) or metformin (Met, 50 mg/kg) by intraperitoneal injection for 3 weeks. We complied with the ethical guideline that subcutaneous tumors should not exceed 15 to 17 mm in diameter or be below 10% of the mouse body weight (approximately less than 2,000 mm^3^ in volume).

For orthotopic tumor models, LLC cells (1 × 10^5^ cells per mouse in 100 μL PBS) were injected intratracheally into 2-month-old B6 male mice. After lung tumor formation was confirmed at 0.5 months by bioluminescence imaging of two representative mice each group, mice were fed with a standard, high-fat, and high-carbohydrate diet (SD [cat. no. 38057, Purina Co., Seoul, Republic of Korea], HFD [cat. no. TD.06414, Envigo Teklad diets, Madison, WI, USA], and HCD [cat. no. TD.98090, Envigo Teklad diets], respectively) for 2 months, with oral gavage administration of Veh or NB twice a week, either alone or in combination with the indicated inhibitors (2DG: oral gavage, five times a week; clodronate liposome: intraperitoneal injection once a week combined with intratracheal administration once a week, a total of twice a week; anti-PD-L1 antibody: intraperitoneal administration, twice a week).

For subcutaneous tumor models employing co-injection of LLC cells and BMDMs, LLC cells and BMDMs (1 × 10^6^ LLC cells and 2 × 10^5^ BMDMs per mouse in 200 μL of PBS containing 25% Matrigel) were co-injected into the right flank of the 2-month-old B6 male mice. Prior to the co-injection, BMDMs and their sublines had Veh or NB exposure (1 μM NNK and 1 μM BaP in combination) for 10 days.

Tumor volume was calculated by measuring the short and long diameters of the tumors using a caliper and by using the formula: tumor volume (V) (mm^3^) = (long diameter x short diameter^2^)/2, as described in our previous report^74^. The size of the tumor was measured in a blinded manner, and its volume was assessed every 2 days. Tumor burden was calculated using the formula: tumor burden = mean tumor number (N) x mean tumor volume (V). as described previously^74^. Briefly, the number and size of tumors in five sections uniformly distributed throughout each lung were calculated to determine tumor multiplicity and burden. Mice were euthanized 1 month after cell inoculation or when tumors reached 10% of the body weight, the tumor size exceeded 2,000 mm^3^, or the mice were moribund.

We used IVIS-Spectrum microCT and Living Image software (ver. 4.2) (PerkinElmer, Waltham, MA, USA), in compliance with the manufacturer’s specifications, to monitor primary tumor development and metastatic tumor formation in multiple organs. Ex vivo imaging analysis was conducted to identify photons generated by metastatic lung cancers. Prior to anesthesia, mice were injected with luciferin (150 mg/kg) and were euthanized after 10–15 min. The lung, liver, thoracic cage, spleen, and brain tissues were removed and placed in a 100 mm dish. The tissues were photographed immediately after a 30 s exposure. Using the Living Image program (PerkinElmer), the luminescence levels of the regions of interest (ROI) in the presented images were quantified as radiance (photons/sec/cm^2^/sr). The experimental repeats and total animal numbers for each in vivo experiment are summarized in Supplementary Table 4.

### Determination of blood lipid levels and blood pressure in mice

Intraperitoneal insulin tolerance and glucose tolerance tests were performed as described previously^75^. In brief, after 4 h or overnight (10–12 h) fasting, mice were intraperitoneally injected with 0.5 U/kg BW insulin or 2 g/kg BW glucose for insulin tolerance or glucose tolerance tests, respectively. Glucose levels were monitored by collecting blood from a tail tip and using a blood glucose meter (Roche Diagnostics, Basel, Switzerland). Fasting glucose level was determined after overnight fasting^76^, and basal and fasting glucose levels were determined using a blood glucose meter. Triglyceride and high-density lipoprotein concentrations in blood were determined using a veterinary hematology analyzer (Fuji DRI-Chem 3500 s; Fujifilm, Tokyo, Japan) in accordance with the manufacturer’s instructions. Blood pressure in mice was measured by the noninvasive tail-cuff technique using the BP-2000 Blood Pressure Analysis System (Visitech Systems, Apex, NC, USA).

### Determination of serum insulin levels

Serum insulin was measured using the Ultra-Sensitive Mouse Insulin ELISA Kit (cat. no. 90080, Crystal Chem, IL, USA) according to the manufacturer’s instructions.

### Whole-body metabolic analysis

To assess changes in the respiratory exchange ratio and energy expenditure, we utilized the Comprehensive Laboratory Animal Monitoring System (Columbus Instruments, Columbus, OH, USA). Mice were individually acclimatized in a CLAMS cage and allow to freely access food and water. Mice were housed for 3 days. Average data for drawing bar graphs of RER (light and dark phases), hourly plots of RER and EE, regression plots, and statistical analysis results, as shown by group effect and mass effect, were obtained using the CalR software (https://calrapp.org/)^77^.

### Body composition analysis

The bone mineral content (BMC), bone mass density (BMD), lean mass, and fat mass were measured by the dual-energy X-ray absorptiometry (DEXA) using the Inalyzer DEXA system (Medikors, Seongnam-si, Gyeonggi-do, Republic of Korea). The mice were anesthetized using isoflurane during the body scan.

### Measurement of in vivo glucose uptake using 18F-FDG PET/MRI scanning

We measured glucose uptake in murine lung tissues, we utilized PET/MRI hybrid imaging using a nanoScan PET/MRI system (1 T, Mediso, Hungary). Prior to imaging, mice were anesthetized and maintained under 1 ~ 1.5% isoflurane to minimize motion artifacts with consistent internal body temperature of 33-34 °C by a Multicell heating system (Mediso). As detailed in previous study^78^, static PET imaging was performed after administering average 9.5 ± 0.8 MBq of FDG in 0.2 ml intravenously via the tail vein. T1-weighted MR images were acquired using a 3D gradient echo sequence (TR = 25 ms. TE_eff = 3.4 ms, FOV = 64 mm, matrix = 128 × 128). PET images were reconstructed using the Tera-Tomo 3D engine (Mediso) in full detector mode with all corrections enabled and employing high regularization through eight iterations at a voxel size of 0.5 mm. MR- derived anatomical contours were used to establish three-dimensional volume of interest (VOI) for organs and tumors using the InterView Fusion software (version 3.03, Mediso). Standard uptake values (SUV) were calculated for each defined VOI, with VOIs fixed at a diameter of 2 mm spheres drawn for tumor and muscle sites. Quantification of data was carried out by determination of the standard uptake value ratio (SUVR), calculated as the SUV of VOI in the tumor region of the lungs divided by the SUV of VOIs in the muscle region.

### Limiting dilution assay

Live LLC primary culture cells, verified via trypan blue exclusion assay, were mixed with Matrigel at a 1:1 ratio. The cells were inoculated into the flanks of C57BL/6 J mice. The number of tumors formed were counted manually, and the number of tumor-initiating cells was calculated using the Extreme Limiting Dilution Analysis (ELDA) online software (http://bioinf.wehi.edu.au/software/elda/)^79^.

### Isolating BMDMs

BMDMs were isolated from the bone marrow of femurs and tibias of 2-month-old C57BL/6 J mice. After lysing red blood cells (RBCs) using an RBC lysis buffer (cat. no. 64010-00, BioGems, Westlake Village, CA, USA), the remaining bone marrow cells were cultured in RPMI 1640 containing 10% heat-inactivated FBS, antibiotics, and a 10% L929 conditioned medium for differentiation. During maturation, BMDMs were treated with NNK (1 μM) and BaP (1 μM) every 2 days for 10 days.

### Primary culture of LLC cells and isolation of lung-derived macrophages

Tumorous lungs were excised from C57BL/6 J mice and cut into tiny pieces using surgical scissors to isolate LLC cells from orthotopic allograft tumors. Lung tumor pieces were further dissociated into single cells using the mouse tumor dissociation kit (cat. no. 130-096-730, Miltenyi Biotec, Bergisch Gladbach, Germany) according to the procedure recommended by the manufacturer’s instructions. Isolated cells were subjected to RNA preparation or were grown in RPMI 1640 medium supplemented with 10% FBS and antibiotics for subsequent experiments.

The remaining non-tumor regions of the lung were dissociated into single cells using the mouse lung dissociation kit (cat. no. 130-095-927, Miltenyi Biotec) according to the manufacturer’s instructions. RBCs were lysed by incubating cell pellets with RBC lysis buffer (BIogems). Isolated cells were incubated on ice for 15 min with TruStain fcX^TM^ (cat. no. 101320, BioLegend, San Diego, CA, USA) diluted in FACS buffer (PBS containing 1% BSA, 2 mM EDTA, and 0.05% sodium azide) at a 1:50 ratio. The cells were then stained for 30 min on ice with allophycocyanin (APC)/cyanine7 (Cy7)-conjugated anti-mouse CD45 (cat. no. 103116, BioLegend, 1:100 ratio) and phycoerythrin (PE)/Cy7-conjugated anti-mouse F4/80 (cat. no. 123114, BioLegend, 1:100 ratio) antibodies. After being washed twice with FACS buffer, cells were sorted using a FACS Aria III flow cytometer (BD Biosciences, San Jose, CA, USA). Isolated cells were subjected to RNA preparation or CM preparation for IGF2 ELISA. The gating strategy is shown in Supplementary Fig. 4c.

### Immunohistochemistry analysis

Immunohistochemical analysis was performed as described previously^74^. Antigen retrieval was performed using a citrate-based antigen unmasking solution (Vector Laboratories, Burlingame, CA, USA) on formalin-fixed, paraffin-embedded (FFPE) tissue specimens that had been deparaffinized and rehydrated. The slides were permeabilized using 0.3% Triton X-100 solution diluted in TBST (Tris-buffered saline containing 0.1% Tween-20), then blocked using blocking solution (5% normal serum in TBST) for 1 h at room temperature (RT). The slides were incubated overnight at 4 °C with 1:100-diluted primary antibodies (anti-PD-L1 [BioLegend], anti-pIGF-1R/IR (Y1131 for IGF-1R, Y1146 for IR, Cell Signaling Technology, Danvers, MA, USA], and anti-TTF1 [Santa Cruz Biotechnology, Dallas, TX, USA] antibodies) diluted in TBST containing 3 % BSA, followed by three washes with TBST. The secondary antibody (Vector Laboratories) was diluted in TBST containing 3 % BSA (1:500) and incubated with the slides for 1 h at RT after which the slides were treated with 0.3 % hydrogen peroxide solution. Solutions A (ABC-Elite, Vector Laboratories) and B (ABC-Elite, Vector Laboratories) were applied concurrently for 30 min, and the signals were detected using a 3,3’-diaminobenzidine (DAB) substrate kit (Vector Laboratories). Hematoxylin was used to counterstain the slides.

### Immunofluorescence staining

Immunofluorescence staining was performed as described previously^74^. Deparaffinization, rehydration, and antigen retrieval from FFPE tissues were performed as described above. After treatment with 0.3% hydrogen peroxide solution, the slides were incubated for 1 h at RT with a blocking solution (5% normal serum in TBS containing 0.025% Triton X-100). The primary antibodies (1:100 dilution) were incubated overnight at 4 °C on the slides. The slides were rinsed several times with a wash buffer (TBS with 0.01 % Tween-20), incubated for 1 h at RT with fluorochrome-labeled secondary antibodies (Thermo Fisher Scientific, Waltham, MA, USA), and washed several times with a wash buffer. The slides were counterstained using 4′,6-diamidino-2-phenylindole (DAPI) and were examined under a fluorescence microscope (Zeiss Axio Observer Z1, Carl Zeiss AG, Oberkochen, Germany) or Leica TCS SP8 (Leica Microsystems, Wetzlar, Germany) laser scanning confocal microscope. When sections of OCT-embedded tissues were used, the slides were fixed in 4% paraformaldehyde for 15 min at RT, then permeabilized before immunofluorescence staining. Quantification of fluorescent images was performed using photographs obtained from three to six independent fields of slide from at least three biological replicates per group.

### CM preparation

THP-1 cells and BMDMs were treated with vehicle (Veh, DMSO) or 1 μM NNK and 1 μM BaP in combination every other day for 2 months or 8 days, respectively, in culture media containing 2 g/L glucose (G^std^ condition). When necessary, these cells were further incubated for additional 2 days under low-glucose (G^low^, 0.8 g/L glucose, ^CM^THP-Veh/G^low^ or ^CM^THP-NB/G^low^), medium-glucose (G^std^, 2 g/L glucose, ^CM^THP-Veh or ^CM^THP-NB), or high-glucose (G^high^, 10 g/L glucose, ^CM^THP-Veh/G^high^ or ^CM^THP-NB/G^high^) culture conditions in the absence or presence of the indicated inhibitors (5 mM 2DG or 5 μg/mL IGF2 neutralizing antibody [αIGF2^Ab^]). Unless otherwise noted, the level of glucose in the medium was 2 g/L (the G^std^ condition). After incubating these THP-1 cells or BMDMs in fresh medium for one day, CM was harvested. Serum-free fresh medium was used for CM preparation for a RTK array, and fresh growth medium containing FBS was used for CM preparation for other experiments.

### Anchorage-independent colony formation and sphere formation assays

Anchorage-independent (AID) colony and sphere formation assays were performed, as described previously^74^. A549, H226Br, and LLC cells were exposed to the indicated CM for one week. CM was diluted with fresh growth medium at a 1:2 ratio, and the CM-containing medium was replaced every 2 or 3 days (three times in total). Thereafter, these CM-treated cells were subjected to colony and sphere formation assays to examine their AID colony formation and sphere formation capacities for 2 and 3 weeks, respectively.

### Real-time PCR

Real-time PCR analysis was performed as described previously^74^. In brief, total RNA was extracted using an easy-BLUE Total RNA Extraction Kit (Intron Biotechnology, Seongnam-si, Gyeonggi-do, Republic of Korea) according to the manufacturer’s instructions. All real-time PCR experiments were performed on an Applied Biosystems 7300 Real-Time PCR System or QuantStudio^TM^ 5 Real-Time PCR System (Thermo Fisher Scientific) using a real-time PCR master mix solution containing SYBR Green (Enzynomics, Daejeon, Republic of Korea) and gene-specific primers. The following thermocycler settings were used: 15 min pre-incubation at 95 °C; 40–45 cycles at 95 °C for 10 s, 60 °C for 15 s, and 72 °C for 30 s; and a final melt-curve analysis to assess the reaction specificity. The comparative cycle threshold (CT) approach was used to quantify mRNA expression. Supplementary Table 5 lists the primer sequences used in the PCR experiments.

### Determining glucose uptake

The cells were seeded onto black 96-well plates overnight. Cells were incubated with 2-NBDG (100 μg/mL) in a glucose-free media for 1 h and then removed from the 2-NBDG staining solution. After washing the cells using PBS, glucose-free media was added, and the fluorescence intensity was analyzed using an Operetta high-content imaging system (PerkinElmer).

### Energy metabolism measurements

Mitochondrial OCRs and ECARs in cells were measured using an XF Extracellular Flux Analyzer (Seahorse Bioscience, Agilent, Santa Clara, CA, USA), according to the manufacturer’s instructions.

### ATP production determination

Intracellular ATP levels were assessed according to the manufacturer’s instructions using an ATPlite^TM^ system (PerkinElmer). Cells (1×10^4^ cells/well) were seeded into a 96-well plate and ATP production was determined after 24 h. Luminescence was measured using a SpectraMax M5 Multi-Mode Microplate Reader (Molecular Devices, San Jose, CA, USA).

### Lung tissue global metabolomics

The sample preparation and instrumental analysis methods were slightly modified from previous study^80^. The lung tissue was pulverized using a pre-chilled BioPulverizer (59012MS, BioSpec). The tissue metabolite was extracted with 80% MeOH at a final tissue concentration of 25 mg/mL for 24 h at –80 °C. After centrifugation (17,000 × *g*, 20 min, 4 °C), the supernatant was analyzed by Vanquish UPLC system coupled to a Q Exactive Plus mass spectrometer (Thermo Fisher Scientific). The column was a SeQuant Zic-pHILIC guard column, 20 ×4.6 mm (MilliporeSigma, Burlington, MA, USA). The mobile phase A was 10 mM ammonium carbonate and 0.05% ammonium hydroxide. The mobile phase B was 100% acetonitrile. The column temperature was 30 °C. The gradient condition was as follows: 0 ~ 13 min: 80% to 20% of mobile phase B, 13–14 min: 20% of mobile phase B. The ESI mode was negative. The MS scan range was set to 60 to 900 m/z. The mass resolution was set to 70,000 and the AGC target was 1 ×10^6^. The 5 μL of the sample was loaded. The metabolite peaks were extracted by EL-Maven^81^ matching with a standard-based in-house metabolite library. After normalization of the median value of the entire metabolite peak area, further statistical analysis was conducted.

### IGF2 ELISA

The isolation of mouse-derived primary cells was carried out as described above. Isolated cells were cultured in serum-free RPMI 1640 medium containing antibiotics at 37 °C in a humidified environment with 5% CO_2_ for a day. CM was prepared by collecting cell culture medium. The level of IGF2 in CM was determined by an IGF2 ELISA kit (cat. no. MG200, R&D Systems, Minneapolis, MN, USA) according to the manufacturer’s instructions and normalized by the protein concentration of attached cells remaining after CM collection.

### Subcellular fractionation, immunoprecipitation (IP), and western blot analysis

Nuclear, cytoplasmic, and plasma membrane fractions of the cellular extracts were produced using a Subcellular Protein Fractionation Kit (cat. no. 78840, Thermo Fisher Scientific) per the manufacturer’s instructions. We employed a previously reported approach to isolate chromatin-bound proteins using the Triton extract buffer^82^. IP and western blot analyzes were performed as previously described^74^. For IP, immunoprecipitants were prepared from 500 μg of nuclear extract using IR- or NPM1-targeting antibodies.

### Analyzing biotinylated cell surface protein translocation

The cells were incubated with 5 mL PBS containing 1 mM sulfo-NHS-biotin (cat. no. 21217, Thermo Fisher Scientific) for 30 min at RT. The reactions were quenched for 10 min with 10 mL PBS containing 100 mM glycine. Cells were stimulated with IGF2 for 30 min, and the subcellular fractions were isolated. The IP assay used streptavidin agarose beads. The subcellular extracts were incubated with streptavidin agarose beads overnight at 4 °C and then subjected to western blot analysis using IR- and pIGF1R/IR-targeting antibodies.

### Determining receptor tyrosine kinase activation profiles

Activation levels of a panel of receptor tyrosine kinases were analyzed using a Phospho-RTK Array Kit (cat. no. ARY001B, R&D Systems), per the manufacturer’s instructions. The kinase activation levels were quantified by densitometric analysis using ImageJ software.

### Liquid chromatography-tandem mass spectrometry (LC-MS/MS)

#### Sample preparation

H226Br cells were stably transfected with control vector (EGFP) or expression vector for IR (pEGFP-IR, IR-EGFP), and A549 cells were stimulated with IGF2 (50 ng/mL) for 3 h. Nuclear extracts were prepared from these cells and then subjected to immunoprecipitation with anti-IR antibodies. Anti-IR immunoprecipitants were electrophoresed on a 13% SDS-PAGE gel. Bands that were differentially greater in the IR-EGFP overexpressing group or the IGF2-stimulated group were collected and further analyzed by LC-MS/MS.

#### In-gel tryptic digestion

The gel spots of interest for analysis were excised from the preparative gel, and the spots were transferred into each 1.5 mL tube. The band was washed with 100 μL of distilled water; then, 100 μL of 50 mM NH_4_HCO_3_ (pH 7.8) and acetonitrile (6:4) were added to the band and shook for in 10 min. This process was repeated at least three times until the Coomassie brilliant blue G250 dye disappeared. The supernatant was decanted, and the band was dried in speed vacuum concentrator (LaBoGeneAps, Lynge, Denmark) for 10 min. The solution was decanted and then digested with sequence-grade modified trypsin (Promega Co., Madison, WI, USA) (enzyme to substrate ratio = 1:50) at 37 °C with shaking for 16 h.

#### LC-MS/MS for peptides analysis

Nano LC–MS/MS analysis was performed with a nano HPLC system (Agilent, Wilmington, DE). The nano chip column (Agilent, Wilmington, DE, 150 mm × 0.075 mm) was used for peptide separation. The mobile phase A for LC separation was 0.1% formic acid in deionized water and the mobile phase B was 0.1% formic acid in acetonitrile. The chromatography gradient was designed for a linear increase from 3% B to 45% B in 30 min, 45% B to 95% B in 1 min, 95% B in 4 min, and 3% B in 10 min. The flow rate was maintained at 300 nL/min. Product ion spectra were collected in the information-dependent acquisition (IDA) mode and were analyzed by Agilent 6530 Accurate-Mass Q-TOF using continuous cycles of one full scan TOF MS from 300-2000 *m*/*z* (1.0 s) plus three product ion scans from 150–2000 *m*/*z* (1.5 s each). Precursor *m*/*z* values were selected starting with the most intense ion, using a selection quadrupole resolution of 3 Da. The rolling collision energy feature was used, which determines collision energy based on the precursor value and charge state. The dynamic exclusion time for precursor ion *m*/*z* values was 60 s.

#### Database searching

The mascot server 2.6 (Matrixscience, USA) was used to identify peptide sequences present in a protein sequence database. Database search criteria were, database name; NCBI Human_20160921_curated (1042220 sequences; 354829666 residues), fixed modification; carbamidomethylated at cysteine residues; variable modification; oxidized at methionine residues, maximum allowed missed cleavage; 2, MS tolerance; 10 ppm, MS/MS tolerance; 0.8 Da. The peptides were filtered with a significance threshold of *p* < 0.05.

### In vitro electroporation

BMDM cells (1 × 10^6^ cells/mL), differentiated for 4 days in RPMI 1640 medium containing L929 cells, were suspended in 100 μL Opti-MEM medium, incubated with 10 μg EV vector (pCAG-SpCas9-GFP-U6, Addgene plasmid #79144) or pCAG-SpCas9-GFP-U6-sgIGF2, and electroporated with the Super Electroporator NEPA 21 (NEPA GENE Co. Ltd., Chiba, Japan) under the following conditions: (A) pulse voltage, 100 V; pulse interval, 50 ms; pulse length, two ms; and pulse number, 2; or (B) pulse voltage, 125 V; pulse interval, 50 ms; pulse length, 5 ms; and pulse number, 2. The EV vector (pCAG-SpCas9-GFP-U6) or pCAG-SpCas9-GFP-U6-sgIGF2 (10 μg DNA) was electroporated into 1 × 10^6^ THP-1 cells in 100 μL Opti-MEM under the following conditions: (A) pulse voltage, 150 V; pulse interval, 50 ms; pulse length, 5 ms; and pulse number, 2; or (B) pulse voltage, 175 V; pulse interval, 50 ms; pulse length, 8 ms; and pulse number, 2; 24 h after transfection, GFP-positive cells were sorted using FACS Aria (BD Biosciences, San Jose, CA, USA).

### Establishment of stable cell lines using lentiviral transduction, the CRISPR/Cas9 system, and transfection with an expression vector

For stable knockdown cell line establishment, shRNA bacterial glycerol stock complementary to each human gene-coding sequence (INSR, NM_000208; NPM1, NM_002520; KPNB1, NM_001276453.2) was purchased from Sigma. The DNA construct (in the pLKO.1 lentiviral vector backbone) was isolated from the bacterial culture. The plasmids were transfected into HEK293T cells, and the supernatant containing the lentiviral particles was collected 48 h after transfection. Cancer cells (A549 and H226Br cells) were transduced with lentiviral particles and polybrene (cat # sc-134220, Santa Cruz Biotechnology). The infected cells were selected using puromycin for at least 1 week.

H226Br cells were transfected with the EV vector (pSpCas9[BB)]−2A-Puro [PX459] V2.0, Addgene plasmid # 62988) or pSpCas9[BB]−2A-Puro-sgIR using the jetPRIME® transfection reagent (cat. no. 101000046, Polyplus transfection, Illkirch-Graffenstaden, France). The transfected cells were selected using puromycin for at least 1 week.

LLC cells were transfected with a vector (pCAG-SpCas9-GFP-U6, Addgene plasmid # 79144) or pCAG-SpCas9-GFP-U6-sgIR using jetPRIME® transfection reagent (Polyplus transfection). After 24 h transfection, GFP-positive cells were sorted using FACS Aria (BD Biosciences).

H226Br cells with deleted NPM1 expression by stable NPM1 shRNA transfection were additionally transfected with empty (EV, pcDNA3.1-Flag) or NPM1 expression vectors (pcDNA3.1-Flag-NPM1 full-length [FL] or truncation mutants) using jetPRIME® transfection reagent (Polyplus transfection) and then selected with G418 for at least 1 week.

### Transwell migration assay

For the Transwell (8.0 μm pore size, Corning, Corning, NY, USA) migration assay, the outer membrane was coated with 0.05% gelatin. A549 cells exposed to NB, either alone or combined with D-glucose, were seeded in the lower compartments (2 × 10^5^ cells/well) in RPMI 1640 medium with 10% heat-inactivated FBS and 1% penicillin-streptomycin (cat. no. LS202-02, Welgene). After 1 day, THP-1 cells that were exposed to NB, either alone or combined with D-glucose were seeded onto upper wells (1 × 10^5^ cells/well) with RPMI 1640 medium without serum, and then the plates were further incubated for 18 h at 37 °C in a 5% CO_2_ incubator. After incubation, the membrane was stained using hematoxylin solution and mounted onto a glass slide. The number of stained cells per field were counted using a Nuance microscope (PerkinElmer).

### Dual luciferase reporter assay

pGL3 2 kb prom.CD274 was a gift from Julian Downward (Addgene plasmid # 107003; http://n2t.net/addgene:107003; RRID: Addgene_107003). Cells were seeded into 24 wells and then transfected with 0.5 μg/well reporter vectors using jetPRIME® transfection reagent (Polyplus transfection). Then, 10 ng/well pRL-Tk vectors were co-transfected to normalize the transfection efficiency. After 12 h of transfection, the media was replaced with serum-free media for 12 h. Transfected cells were incubated with IGF2 or CM from macrophages for 24 h and luciferase activity was measured using the Dual-Glo Luciferase Assay System (cat # E1910; Promega, Madison, WI, USA) according to the manufacturer’s instructions.

### Chromatin immunoprecipitation (ChIP) assay

A ChIP assay was conducted using a SimpleChIP Enzymatic Chromatin IP kit (cat. no. 9002S, Cell Signaling Technology) per the manufacturer’s instructions. IP was performed using IR- (Santa Cruz Biotechnology) and NPM1- (Santa Cruz Biotechnology) targeting antibodies and a negative control. Cross-linked chromatin was treated with a normal mouse IgG antibody (Santa Cruz Biotechnology) overnight at 4 °C with agitation. Real-time PCR was used to assess the enrichment of precipitated DNA and normalize it to 2% of the total input.

### Analyzing patient-derived tissue samples

The Biobank of Soonchunhyang University Bucheon Hospital (schbc-biobank-2016-011-01), a member of the Korea Biobank Network, provided human lung tissue samples with a history of smoking. The informed consent requirement was waived by our IRB protocol. A human lung carcinoma tissue microarray (TMA) was purchased from US Biomax (Rockville, MD, USA; cat. no. LC821). The TMA comprised of 80 cores from 40 patients (70 lung cancer tissues and 10 normal lung tissues) with a smoking history (2015 WHO classification) and other clinicopathological features, including pathology grade, TNM, and clinical stage according to the 8th UICC/AJCC TNM edition for non-small cell lung cancer staging^83^. Immunofluorescence staining was performed using antibodies targeting GLUT1, IGF2, CD68, pIGF-1R/IR (Y1131 for IGF-1R, Y1146 for IR), and PD-L1. GLUT1 and IGF2 expression in tumor-infiltrated CD68^+^ macrophages and pIGF-1R/IR (Y1131 for IGF-1R, Y1146 for IR) and PD-L1 expression in tumors were quantified using LAS X (Leica Application Suite X) software. The fluorescence intensity within the whole tissue area per field was measured under five randomly selected microscopic fields using LAS X software. The 50th percentile was used to define high and low levels of each marker. The association between protein expression and clinicopathological features, including TNM, tumor stage, and grade, was analyzed.

### In silico analysis

We utilized datasets available in the Gene Expression Omnibus (GEO) (National Center for Biotechnology Information, Bethesda, MD, USA) by manually downloading gene expression values and clinical information. Three datasets (GSE37745, GSE50081, and GSE77803) were used to identify PD-L1 as a marker functionally associated with NPM1. GSE37745 contains 196 non-small cell lung cancer (NSCLC) specimens, GSE50081 includes of 181 NSCLC cases, and GSE77803 contains 156 NSCLC specimens. STRING protein interaction analysis^55^ of genes that were significantly upregulated in the NPM1^high^ group (fold change ≥ 2; *p* < 0.05 and *q* < 0.05) was performed to determine the NPM1-associated network. A GSE30219 dataset, which comprises the mRNA profiles of 272 NSCLC samples, 21 small cell lung cancer samples, and 14 non-tumoral lung tissue samples, was used for survival analysis, the correlation of NPM1 with prognosis-associated genes, and GSEA. 272 NSCLC cases were used for analysis. The prognosis-associated genes were defined as the genes that were overlapped in at least two prognostic gene signatures for both lung adenocarcinoma and squamous cell carcinoma^54^.

The survival rates of patients with lung cancer were analyzed using Kaplan–Meier curves. The median value of the data was used to define the GLUT1^high^ versus GLUT1^low^ and GLUT3^high^ versus GLUT3^low^ groups for survival analysis. The significance of the results was determined using a log-rank test. GSEA was performed using the GSEA software (ver. 4.3.2) according to the GSEA user guide. 37 gene sets related to invasiveness and metastasis were obtained from the Molecular Signature Database (MSigDB) available on the GSEA homepage (www.gsea-msigdb.org). The NPM1^high^IGF2^high^ versus NPM1^low^IGF2^low^ groups were defined by sorting the data according to the NPM1 and IGF2 expression values. The following probes were used to obtain gene expression levels: NPM1: 221691_x_at; SLC2A1: 201250_s_at; IGF2: 202409_at; SLC2A3: 202499_s_at; KIAA0101: 202503_s_at; CDKN3: 1555758_a_at; MCM2: 202107_s_at; RRM2: 201890_at; PRC1: 218009_s_at; FGFR2: 208229_at; TPX2: 210052_s_at; UBE2C: 202954_at; GPC3: 243243_at.

### Statistical analyses

Data are presented as the mean ± standard deviation. All in vitro experiments were performed at least twice independently and a representative result is shown. Graphs show the results of multiple biological replicates in a representative experiment. The sample sizes for the in vitro and in vivo tests were not statistically determined. Statistical significance was evaluated using a two-tailed Student’s t-test, two-tailed Mann–Whitney test, one-way ANOVA, or Kruskal–Wallis test using GraphPad Prism software (version 10, GraphPad Software Inc., La Jolla, CA, USA). A Shapiro–Wilk test was used to determine whether the in vitro or in vivo data were normally distributed. To check whether the variances of the two test groups were equivalent, an F-test for variance equality was conducted. The Brown–Forsythe test for variance equality was used to ensure that more than three experimental groups had the same variance. *p* < 0.05 was considered statistically significant. The quantified TMA data were analyzed using a two-sided Fisher’s exact test.

### Reporting summary

Further information on research design is available in the Nature Portfolio Reporting Summary linked to this article.

### Supplementary information

Supplementary Information

Description of Additional Supplementary Files

Supplementary Data 1

Reporting Summary

### Source data

Source Data

## Data Availability

A set of mass spectrometry proteomics data (the ‘+IGF2 vs. -IGF2’ group) has been deposited to the ProteomeXchange Consortium via the PRIDE^84^ partner repository with the dataset identifier PXD051519. Another set of mass spectrometry proteomics data (the ‘IR-EGFP vs. EGFP’ group) could not be deposited to the ProteomeXchange Consortium due to the unavailability of raw LC-MS/MS data; the lack of raw LC-MS/MS data was caused by the complete removal of raw data from the service system of Yonsei Proteome Research Center (YPRC) due to the closure of YPRC. The processed data (results of a peptide sequence search on the Mascot server) are available in Supplementary Data 1. The publicly available human lung cancer data used in this study are available in the GEO database under accession code GSE30219, GSE37745, GSE50081, and GSE77803. The remaining data are available within the Article, Supplementary Information, or Source Data file. Source data are provided with this paper.
