# Supplementary material for: Tobacco-induced hyperglycemia promotes lung cancer progression via cancer cell-macrophage interaction through paracrine IGF2/IR/NPM1-driven PD-L1 expression
Source: Nat Commun. 2024 Jun 8;15:4909. doi: 10.1038/s41467-024-49199-9 (PMC11162468; doi:10.1038/s41467-024-49199-9)

Supplementary Information

Tobacco-induced hyperglycemia promotes lung cancer progression via cancer cell-macrophage interaction through paracrine IGF2/IR/NPM1-driven PD-L1 expression

Hyun-Ji Jang, Hye-Young Min, Yun Pyo Kang, Hye-Jin Boo, Jisung Kim, Jee Hwan Ahn, Seung Ho Oh, Jin Hwa Jung, Choon-Sik Park, Jong-Sook Park, Seog-Young Kim, Ho-Young Lee

Supplementary Table 1. Association between GLUT1 or IGF2 expression in macrophages and various clinicopathological factors of lung cancer patients.

p-values were determined by using a two-sided Fisher's exact test.

**Variables**		**Expression intensity**					
		**GLUT1 in CD68$^+$ macrophages**			**IGF2 in CD68$^+$ macrophages**		
		**Low (*n* = 18)**	**High (*n* = 17)**	***p* value**	**Low (*n* = 18)**	**High (*n* = 17)**	***p* value**
**Sex**	Female	2	0	0.4857	2	0	0.4857
	Male	16	17		16	17	
**Age**	< 65	5	12	**0.0184**	7	10	0.3175
	≥ 65	13	5		11	7	
**Smoking history (year)**	≤ 30	7	10	0.3175	9	8	>0.9999
	> 30	11	7		9	9	
**Dosage/day (PCS)**	≤ 20	11	10	>0.9999	11	10	>0.9999
	> 20	7	7		7	7	
**TNM (T)**	T1	1	2	0.3399	1	2	0.3399
	T2, T2a, T2b	15	10		15	10	
	T3, T4	2	5		2	5	
**TNM (N)**	N0	15	6	**0.0059**	14	7	**0.0409**
	N1/2	3	11		4	10	
**Grade**	G1, G1~2	5	0	**0.0364**	5	0	**0.0364**
	G2, G2~3	11	9		11	9	
	G3	2	6		2	6	
**Stage**	I, IA, IB	12	4	**0.0069**	11	5	**0.0283**
	IIA, IIB	6	8		7	7	
	IIIA	0	5		0	5	

Supplementary Table 2. Association between pIGF-1R (Y1131)/IR (Y1146) or PD-L1 expression in tumor cells and various clinicopathological factors of lung cancer patients.

p-values were determined by using a two-sided Fisher's exact test.

Variables		Expression intensity					
		pIGF-1R (Y1131)/IR (Y1146) in tumors			PD-L1 in tumors		
		Low (*n* = 18)	High (*n* = 17)	*p* value	Low (*n* = 18)	High (*n* = 17)	*p* value
**Sex**	Female	0	2	0.2286	0	2	0.2286
	Male	18	15		18	15	
**Age**	< 65	4	13	**0.0022**	7	10	0.3175
	≥ 65	14	4		11	7	
**Smoking history (year)**	≤ 30	6	11	0.0943	6	11	0.0943
	> 30	12	6		12	6	
**Dosage/day (PCS)**	≤ 20	9	12	0.3053	10	11	0.7332
	> 20	9	5		8	6	
**TNM (T)**	T1	2	1	>0.9999	2	1	>0.9999
	T2, T2a, T2b	13	12		13	12	
	T3, T4	3	4		3	4	
**TNM (N)**	N0	14	7	**0.0409**	14	7	**0.0409**
	N1/2	4	10		4	10	
**Grade**	G1, G1~2	4	1	0.5432	4	1	0.2649
	G2, G2~3	10	10		8	12	
	G3	4	4		5	3	
**Stage**	I, IA, IB	11	5	0.1343	10	6	0.2817
	IIA, IIB	6	8		7	7	
	IIIA	1	4		1	4	

Supplementary Table 3. Antibodies used in this study

Target	Vendor	Catalogue	Clone	Dilution ratio	Application
CD45	BD Bioscience	550539	30-F11	1:200	IF
F4/80	Bio-Rad	MCA497		1:200	IF
MPO	Abcam	208670		1:200	IF
α-SMA	Sigma Aldrich	A2547		1:200	IF
CD4	Bio-Rad	MCA1767T		1:200	IF
CD8	Abcam	22378		1:200	IF
CD206	Proteintech	18704-1-AP		1:1000	WB
Arginase 1	Cell signaling	93668	D4E3M	1:200 (IF)<br>1:1000 (WB)	IF, WB
iNOS	Abcam	ab178945		1:200	IF
Granzyme B	R&D	AF-1865		1:200	IF
GLUT1	Novus Biologicals	NB110-39113		1:500 (IF)<br>1:1000 (WB)	IF, WB
GLUT3	Proteintech	20403-1-AP		1:500 (IF)<br>1:1000 (WB)	IF, WB
NA+K+ATPase	Cell signaling	3010		1:1000	WB
pIGF-1R (Y1131) /IR (Y1146)	Cell signaling	3021		1:100 (IHC)<br>1:200 (IF)	IF, IHC
pIGF-1R (Y1135/36) /IR (Y1150/1151)	Cell signaling	3024	19H7	1:1000	WB
IR	Santa Cruz	sc-711	C-19	1:200	CHIP
IR	Santa Cruz	sc-57342	CT-3	1:200 (IP)<br>1:1000 (WB)	IP, WB
IR	Cell signaling	3020	L55B10	1:1000	WB
IGF-1R	Cell signaling	3027		1:1000	WB
IGF2	Abcam	ab9574		1:1000 (WB)<br>1:200 (IF)	WB<br>IF
NPM1	Abcam	10530		1:1000	WB
NPM1	Santa Cruz	sc-32256		1:200	IP, CHIP
PD-L1	Cell signaling	13684	E1L3N	1:1000	WB
OCT-A	Santa Cruz	sc-166355	H-5	1:1000 (WB)<br>1:200 (IP)	WB, IP
OCT-A	Santa Cruz	807	D-8	1:1000 (WB)	WB
human CD274	BioLegend	329402	29E.2A3	1:100	IF
mouse CD274	BioLegend	124302	10F.9G2	1:100	IHC

mouse PD-L1	BioXCell	BE0101	10F.9G2	8 μg/mL (WB)<br>10 μg/mL (blockade)	WB, Blockade
TTF1	Santa Cruz	sc-13040		1:100	IHC
APC/Cy7 anti-mouse CD45	BioLegend	103116	30-F11	1:100	FACS
PE/Cy7 anti-mouse F4/80	BioLegend	123114	BM8	1:100	FACS

[1)]Application - WB: Western blot analysis; IF: Immunofluorescence staining; IHC: Immunohistochemistry; IP: Immunoprecipitation; FACS: Fluorescence-Activated Cell Sorting,

Supplementary Table 4. The experimental repeats and total animal numbers for each *in vivo* experiment

Figure	Group	Number of mice		Total number of mice per group
		Experiment 1	Experiment 2	
Fig. 1a-h, Supplementary Fig. 1a-b	Veh	6	5	11
	NB 3 months	6		6
	NB 5 months	6	5	11
Fig. 1i-p, Supplementary Fig. 1c	Veh	10	3	13
	NB 2.5 months	7		7
	NB 4.5 months	10	3	13
Fig. 1q-s	Veh	11	3	14
	NB	11	3	14
Supplementary Fig. 1d	Veh	7		7
	NB	21		21
Fig. 2a-h, Supplementary Fig. 2c-d, f-g, o-p, Supplementary Fig. 3a-d	Veh/SD	10	11	21
	NB/SD	10	11	21
	Veh/HCD	10	17	27
	NB/HCD	10	17	27
	Veh/HFD	10	10	20
	NB/HFD	10	10	20
	Veh/SD/2DG		9	9
	NB/SD/2DG		9	9
	Veh/HCD/2DG		10	10
	NB/HCD/2DG		10	10
Fig. 3h-l	Veh/HCD	5	15	20
	NB/HCD	5	15	20
	NB/HCD/Cld	5	15	20
Fig. 5k	LLC+BMDM-sgRNA^{Con}/Veh	14		14
	LLC+BMDM-sgRNA^{Con}/NB	14		14
	LLC+BMDM-sgRNA^{IGF2}/Veh	14		14
	LLC+BMDM-sgRNA^{IGF2}/NB	14		14
Fig. 5l	LLC-sgRNA^{Con} + BMDM/Veh	13		13
	LLC-sgRNA^{Con} + BMDM/NB	13		13
	LLC-sgRNA^{IR} + BMDM/Veh	10		10
	LLC-sgRNA^{IR} + BMDM/NB	10		10
Fig. 6m	LLC+BMDM-sgRNA^{Con}/Veh	8		8
	LLC+BMDM-sgRNA^{Con}/NB	6		6
	LLC+BMDM-sgRNA^{NPM1}/Veh	6		6
	LLC+BMDM-sgRNA^{NPM1}/NB	5		5
Fig. 7i-m	Veh/HCD	5	13	18
	NB/HCD	5	13	18
	NB/HCD/αPD-L1^{Ab}	5	13	18
Supplementary Fig. 2e	SD	8		8
	HCD	6		6
	HFD	11		11
Supplementary Fig. 2h-n	SD	10		10
	HCD	10		10
	HFD	10		10
Supplementary Fig. 6a	Veh	4		4
	NB	4		4

Supplementary Table 5. Primer sequences used in this study

Gene	Forward sequence (5´-3´)	Reverse sequence (5´-3´)	Species
SLC2A1	ATT GGC TCC GGT ATC GTC AAC	GCT CAG ATA GGA CAT CCA GGG TA	Human
SLC2A3	GGT GGC TGC TTT ATG GGA CT	GTA AAA CCC AGT AGC AGC GG	Human
ARG1	GTGGAAACTTGCATGGACAAC	AATCCTGGCACATCGGGAATC	Human
CCL22	ATC GCC TAC AGA CTG CAC TC	GAC GGT AAC GGA CGT AAT CAC	Human
IL4	CGG CAA CTT TGT CCA CGG A	TCT GTT ACG GTC AAC TCG GTG	Human
IL13	AAC ATC ACC CAG AAC CAG AAG	CAG AAT CCG CTC AGC ATC C	Human
IL1B	ATG ATG GCT TAT TAC AGT GGC AA	GTC GGA GAT TCG TAG CTG GA	Human
NOS2	TTC AGT ATC ACA ACC TCA GCA AG	TGG ACC TGC AAG TTA AAA TCC C	Human
IGF1	GCTCTTCAGTTCGTGTGTGGA	GCCTCCTTAGATCACAGCTCC	Human
IGF2	CTT GGA CTT TGA GTC AAA TTG G	GGT CGT GCC AAT TAC ATT TCA	Human
INS	GCA GCC TTT GTG AAC CAA CAC	CCC CGC ACA CTA GGT AGA GA	Human
CCR2	CAG GTG ACA GAG ACT CTT GGG A	GGC AAT CCT ACA GCC AAG AGC T	Human
CCR5	TTC TGG GCT CCC TAC AAC ATT	TTG GTC CAA CCT GTT AGA GCT A	Human
CD274	GCT GCA CTA ATT GTC TAT TGG GA	AAT TCG CTT GTA GTC GGC ACC	Human
CCL2	CAG CCA GAT GCA ATC AAT GCC	TGG AAT CCT GAA CCC ACT TCT	Human
CCL7	ACA GAA GGA CCA CCA GTA GCC A	GGT GCT TCA TAA AGT CCT GGA CC	Human
KPNB1	CCA CTT TCC TTG TGG AAC TGT	CTC TGC TGA TAT TGT GCC TTG A	Human
KPNA2	CTGTTGGCTCTCCTTGCAGTTC	GCAGGATTCTTGTTGCGGCAAAG	Human
ANP32E	TGC CTG TGT GTC AAT GGG G	GCA GAG CTT CTA CTG TAC TGA GA	Human
MAD2L1	GTT CTT CTC ATT CGG CAT CAA CA	GAG TCC GTA TTT CTG CAC TCG	Human
EEF1A1	TGT CGT CAT TGG ACA CGT AGA	ACG CTC AGC TTT CAG TTT ATC C	Human
SRSF3	TGG CAA CAA GAC GGA ATT GGA	CAA AGC CGG GTG GGT TTC TA	Human
TFRC	GGC TAC TTG GGC TAT TGT AAA GG	CAG TTT CTC CGA CAA CTT TCT CT	Human
ACTB	CATGTACGTTGCTATCCAGGC	CTCCTTAATGTCACGCACGAT	Human
Slc2a1	CTC TGT CGG CCT CTT TGT TAA T	CCA GTT TGG AGA AGC CCA TAA G	Mouse
Slc2a2	TCA GAA GAC AAG ATC ACC GGA	GCT GGT GTG ACT GTA AGT GGG	Mouse
Slc2a3	ATG GGG ACA ACG AAG GTG AC	GTC TCA GGT GCA TTG ATG ACT C	Mouse
Slc2a4	GTG ACT GGA ACA CTG GTC CTA	CCA GCC ACG TTG CAT TGT AG	Mouse
Slc2a5	CCA ATA TGG GTA CAA CGT AGC TG	GCG TCA AGG TGA AGG ACT CAA TA	Mouse

Sglt1	CAC CGA GGG CTG ACT CAT TC	TGA TCC GTA CAC CAG TAC CAC	Mouse
Sglt2	ATG GAG CAA CAC GTA GAG GC	ATG ACC AGC AGG AAA TAG GCA	Mouse
Ccl2	GCT ACA AGA GGA TCA CCA GCA G	GTC TGG ACC CAT TCC TTC TTG G	Mouse
Ccl4	TTC CTG CTG TTT CTC TTA CAC CT	CTG TCT GCC TCT TTT GGT CAG	Mouse
Ccl5	CCTGCTGCTTTGCCTACCTCTC	ACACACTTGGCGGTTCCTTCGA	Mouse
Ccl7	CAG AAG GAT CAC CAG TAG TCG G	ATA GCC TCC TCG ACC CAC TTC T	Mouse
Ccl17	TAC CAT GAG GTC ACT TCA GAT GC	GCA CTC TCG GCC TAC ATT GG	Mouse
Ccl20	GTG GGT TTC ACA AGA CAG ATG GC	CCA GTT CTG CTT TGG ATC AGC G	Mouse
Ccl22	AGG TCC CTA TGG TGC CAA TGT	CGG CAG GAT TTT GAG GTC CA	Mouse
Cxcl2	CATCCAGAGCTTGAGTGTGACG	GGCTTCAGGGTCAAGGCAAACT	Mouse
Cxcl5	CCGCTGGCATTTCTGTTGCTGT	CAGGGATCACCTCCAAATTAGCG	Mouse
Cxcl9	TCCTTTTGGGCATCATCTTC	TTCCCCCTCTTTTGCTTTTT	Mouse
Cxcl10	ATC ATC CCT GCG AGC CTA TCC T	GAC CTT TTT TGG CTA AAC GCT TTC	Mouse
Cxcl11	GGC TTC CTT ATG TTC AAA CAG GG	GCC GTT ACT CGG GTA AAT TAC A	Mouse
Cxcl12	GGA GGA TAG ATG TGC TCT GGA AC	AGT GAG GAT GGA GAC CGT GGT G	Mouse
Cxcl16	GGCTTTGGACCCTTGTCTCTTG	TTGCGCTCAAAGCAGTCCACT	Mouse
Cxcr2	CTC TAT TCT GCC AGA TGC TGT CC	ACA AGG CTC AGC AGA GTC ACC A	Mouse
Cxcr3	TAC GAT CAG CGC CTC AAT GCC A	AGC AGG AAA CCA GCC ACT AGC T	Mouse
Cxcr4	GAC TGG CAT AGT CGG CAA TGG A	CAA AGA GGA GGT CAG CCA CTG A	Mouse
Ccr2	GCT GTG TTT GCC TCT CTA CCA G	CAA GTA GAG GCA GGA TCA GGC T	Mouse
Ccr4	GGACTAGGTCTGTGCAAGATCG	TGCCTTCAAGGAGAATACCGCG	Mouse
Ccr5	GTC TAC TTT CTC TTC TGG ACT CC	CCA AGA GTC TCT GTT GCC TGC A	Mouse
Ccr6	CCT GGG CAA CAT TAT GGT GGT	CAG AAC GGT AGG GTG AGG ACA	Mouse
Tnf	CAGCCGATGGGTTGTACCTT	TGTGGGTGAGGAGCACGTAGT	Mouse
Nos2	CAGCTGGGCTGTACAAACCTT	CATTGGAAGTGAAGCGTTTCG	Mouse
Ifng	ATG AAC GCT ACA CAC TGC ATC	CCATCCTTTTGCCAGTTCCTC	Mouse
Il1b	AACCTGCTGGTGTGTGACGTTC	CAGCACGAGGCTTTTTTGTTGT	Mouse
Tgfb1	CTCCCGTGGCTTCTAGTGC	GCCTTAGTTTGGACAGGATCTG	Mouse
Il10	ATGCTGCCTGCTCTTACTGACTG	CCCAAGTAACCCTTAAAGTCCTGC	Mouse
Il4	ACAGGAGAAGGGACGCCAT	ACCTTGGAAGCCCTACAGA	Mouse
Il13	CCTGGCTCTTGCTTGCCTT	GGTCTTGTGTGATGTTGCTCA	Mouse

Igf1	CTGGACCAGAGACCCTTTGC	GGACGGGGACTTCTGAGTCTT	Mouse
Igf2	TGGCCCTCCTGGAGACATACTGTGC	TTGGAAGAACTTGCCCACGGGGTATC	Mouse
Igf2	GCGGCTTCTACTTCAGCAG	CAGGTGTCATATTGGAAGAAC	Mouse
Ins	CACTTCCTACCCCTGCTGG	ACCACAAAGATGCTGTTTGACA	Mouse
Actb	TGTCCACCTTCCAGCAGATGT	AGCTCAGTAACAGTCCGCCTAG	Mouse

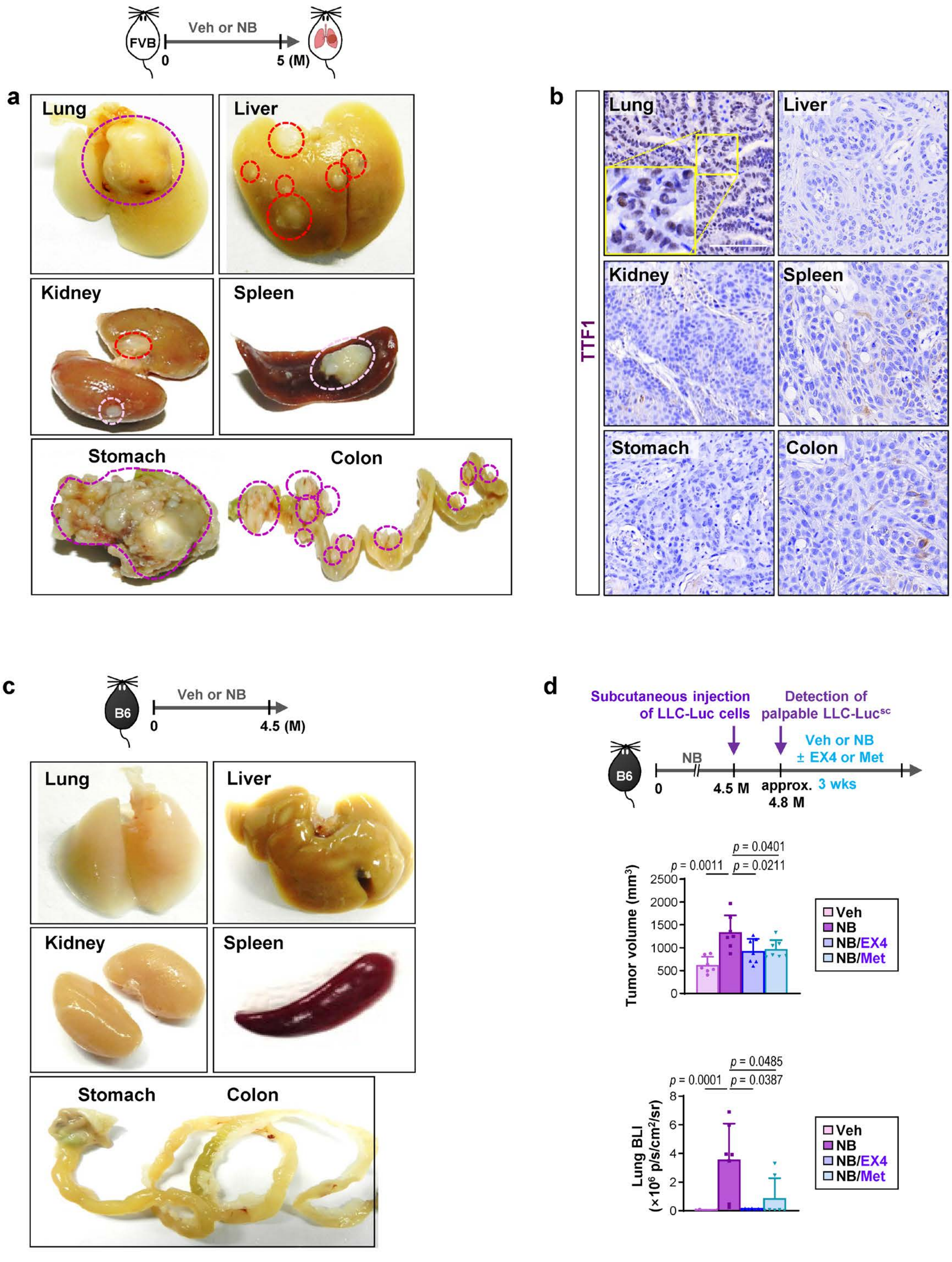

Supplementary Fig. 1. The effects of chronic NB exposure on tumor formation and progression in FVB/N mice and C57BL/6J mice.

a-c. Two-month-old FVB/N (FVB) (**a, b**) and C57BL/6J (B6) (**c**) mice were given vehicle (Veh) or NB (NNK/BaP, 3 μmol each in 100 μL corn oil) for the indicated times (twice a week by oral gavage).

a. Representative images of tumor nodules in the indicated organs in NB-treated FVB mice.

b. Immunohistochemical staining of TTF1 within the tumors in the indicated organs of FVB mice. Scale bar: 100 μm.

c. Gross evaluation of various organs in NB-treated B6 mice.

d. LLC-Luc cells were subcutaneously inoculated with B6 mice given Veh or NB (NNK/BaP, 3 μmol each diluted in corn oil, 100 μL/mouse, twice a week by oral gavage) for 4.5 months, and these mice were continuously given Veh or NB. NB-treated mice were additionally administered vehicle (PBS), exendin-4 (EX4, 20 μg/kg) or metformin (Met, 50 mg/kg) for 3 weeks.
Top: Schematic diagram of the experimental schedule.
Bottom: The tumor volume of subcutaneous LLC tumors (LLC-Luc^{sc}) (*n* = 7/group) and the level of metastatic tumor formation in the lung, determined by bioluminescence imaging (*n* = 7/group). The data are presented as the mean ± SD. *p*-values were determined by using one-way ANOVA with Dunnett's post-hoc test by comparison with the NB group. Source data are provided as a Source Data file.

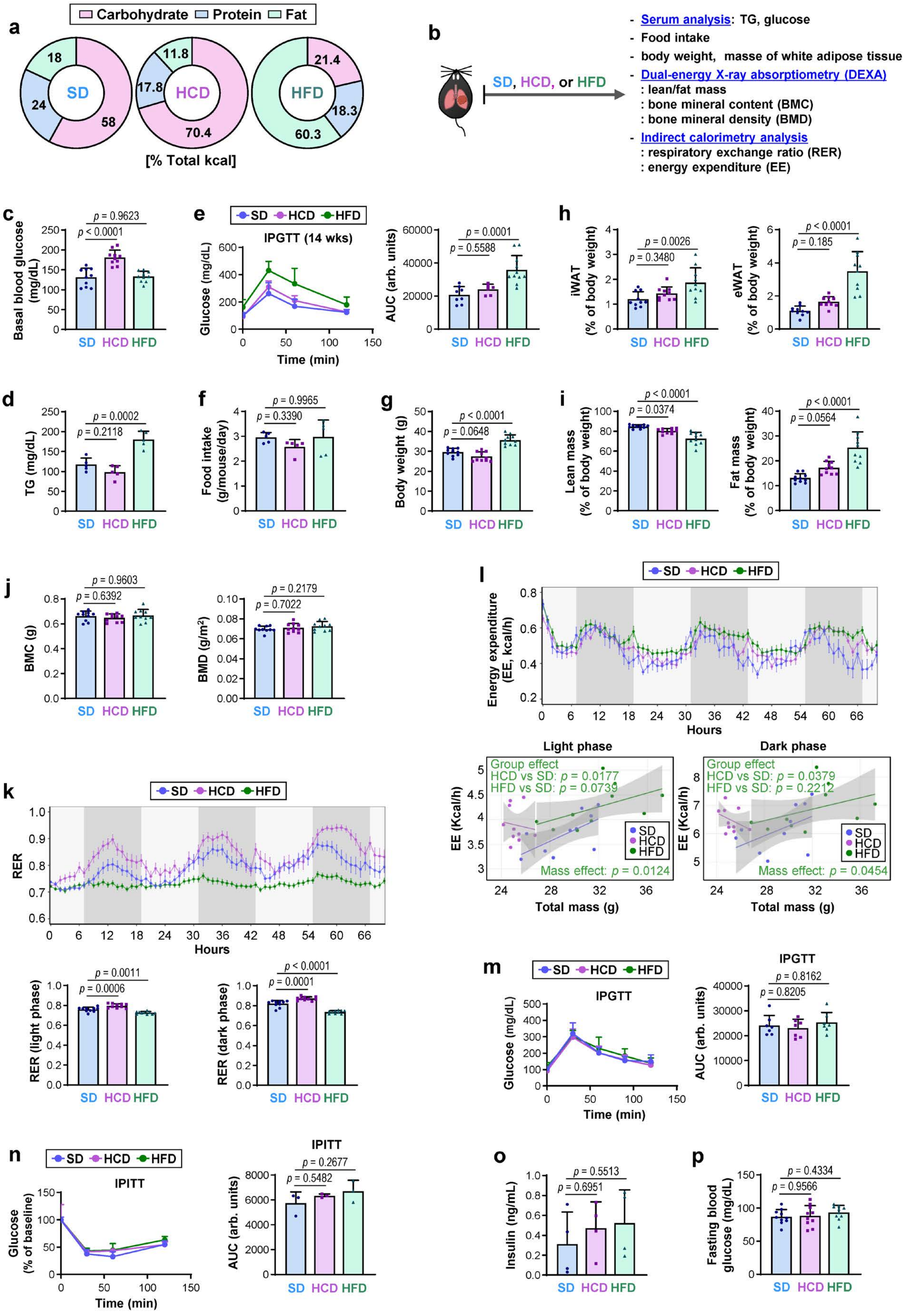

Supplementary Fig. 2. Dietary effects on metabolic changes in C57BL/6J mice.

a. Standard diet (SD), high-carbohydrate diet (HCD), and high-fat diet (HFD) composition.
b. Schematic diagram of the experimental schedule.
c-d. Changes in basal blood glucose (*n* = 10/group) (**c**) and blood triglyceride (TG) (*n* = 5/group) (**d**) in C57BL/6J (B6) mice that fed SD, HCD, or HFD for 2 months.
e. Changes in blood glucose concentration determined by intraperitoneal glucose tolerance (IPGTT) test in B6 mice fed SD (*n* = 8/group), HCD (*n* = 6/group), or HFD (*n* = 11/group) for 14 weeks. The bar graph in the right panel shows the area under the curve (AUC) of GTT in each group.
f-h. Changes in food intake (*n* = 5/group) (**f**), body weight (*n* = 10/group) (**g**), and the weight of inguinal and epididymal white adipose tissues (iWAT and eWAT, *n* = 10/group) (**h**) in B6 mice that fed SD, HCD, or HFD for 2 months.
i-j. The dual-energy X-ray absorptiometry (DEXA) analysis for changes in lean mass (**i**), fat mass (**i**), bone mineral content (BMC) (**j**), and bone mineral density (BMD) (**j**) in B6 mice that fed SD, HCD, or HFD for two months (*n* = 10/group).
k-l. Evaluation of respiratory exchange ratio (RER) and energy expenditure (EE) in B6 mice that fed SD, HCD, or HFD for 2 months using a comprehensive laboratory animal monitoring system (CLAMS) (*n* = 10/group). The regression plots and analysis results, obtained by using the CalR software, in **k** show the relationship between EE and total body mass.
m. Changes in blood glucose concentration determined by IPGTT test in B6 mice fed SD, HCD, or HFD for 2 months (*n* = 7/group). Right: AUC of GTT in each group.
n. Changes in blood glucose concentration determined by insulin tolerance (IPITT) test in B6 mice fed SD, HCD, or HFD for 2 months (*n* = 3/group). Right: AUC of ITT in each group.
o-p. Blood insulin (*n* = 4/group) (**o**) and fasting blood glucose (*n* = 10/group) (**p**) levels in B6 mice that fed SD, HCD, or HFD for 2 months.
The data are presented as the mean ± SD. *p*-values were determined by using one-way ANOVA with Dunnett's post-hoc test by comparison with the SD group (**c-k, m-p**). Source data are provided as a Source Data file.

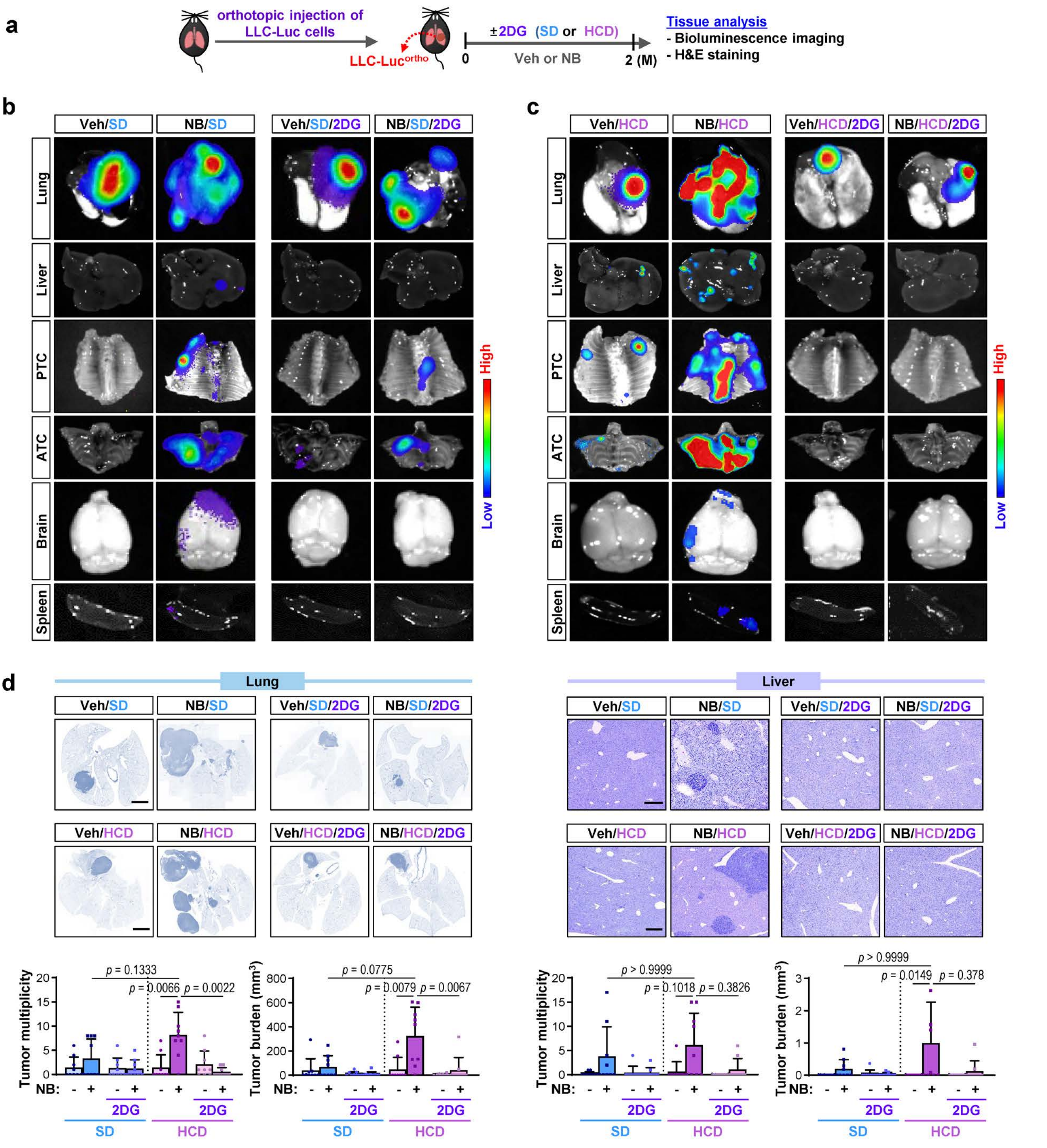

Supplementary Fig. 3. NB promotes lung cancer progression in a glucose-rich environment.
a. Schematic diagram of the experimental schedule. C57BL/6J mice carrying LLC-Luc^{ortho} were exposed to vehicle (Veh) or NB (NNK/BaP, 3 μmol each in 100 μL corn oil) for 2 months (twice a week by oral gavage) under standard diet (SD) or high-carbohydrate diet (HCD) conditions, either alone or together with 2-deoxy-D-glucose (2DG, 500 mg/kg, five times a week by oral gavage).
b, c. Representative ex vivo bioluminescence images of the lung, liver, posterior thoracic cage (PTC), anterior thoracic cage (ATC), brain, and spleen. Quantitative analyses are shown in **Fig. 2g**.
d. Top: Representative photographs of the H&E-stained sections of the lungs and liver. Scale bars: 200 μm. Bottom: Microscopic evaluation of H&E-stained lung and liver tissues for tumor multiplicity and burden (n = 9/group). The data are presented as the mean ± SD. p-values were determined by using Kruskal–Wallis test with Dunn's post-hoc test. Source data are provided as a Source Data file.

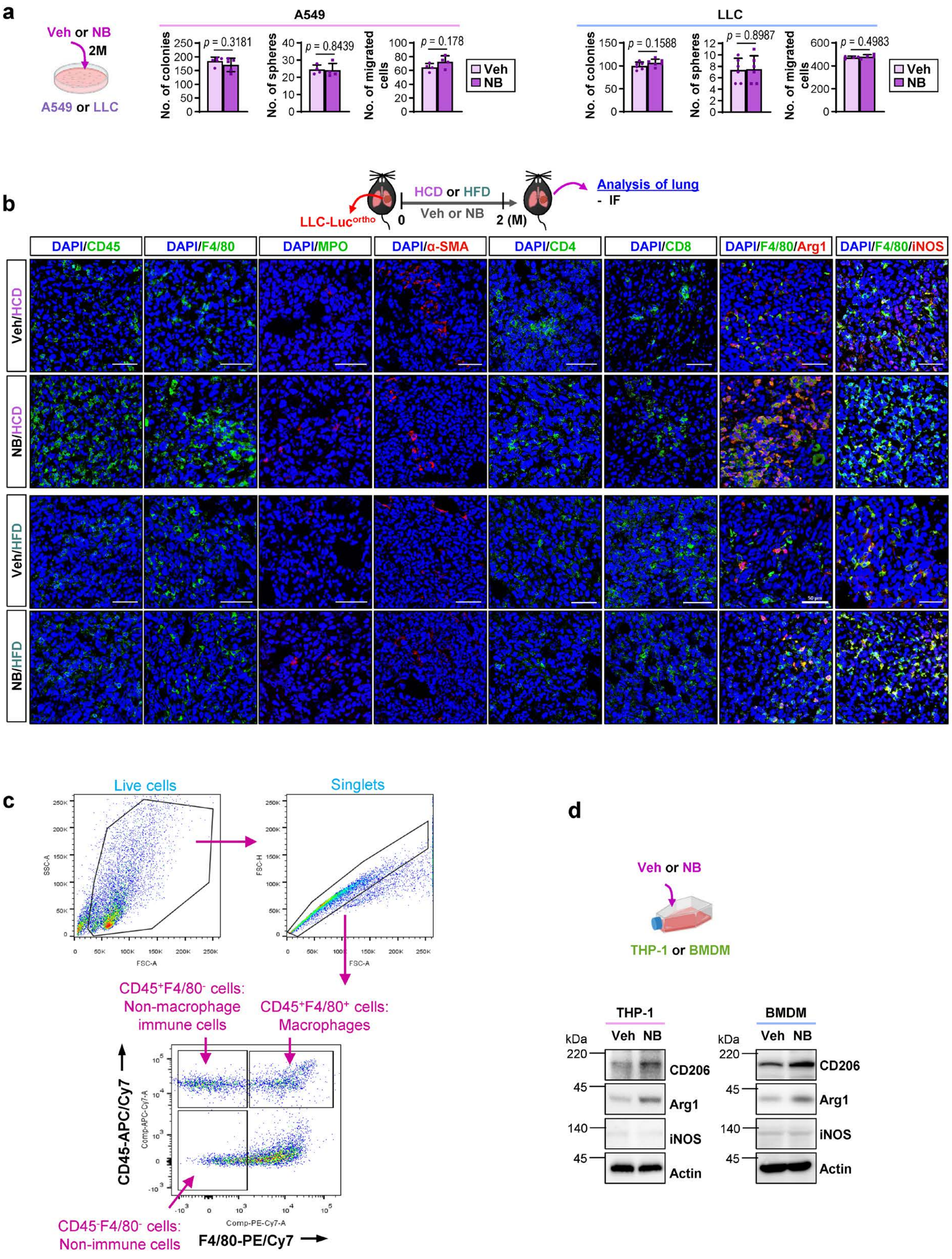

Supplementary Fig. 4. Chronic NB exposure promotes lung cancer progression by recruiting macrophages.

a. Changes in anchorage-independent colony formation, migration, and sphere formation in A549 and LLC cells treated with vehicle (Veh, DMSO) or NB (1 μM NNK and 1 μM BaP in combination)

every other day for 2 months (n = 5 biologically independent replicates/group for the colony formation assay in A549 cells; n = 4 biologically independent replicates/group for sphere formation and migration assays in A549 cells and the migration assay in LLC cells; n = 6 biologically independent replicates/group for colony formation and sphere formation assays in LLC cells). The data are presented as the mean ± SD. p-values were determined by using a two-tailed Student's t-test.

b. C57BL/6J (B6) mice harboring LLC-Luc^{ortho} were exposed to Veh or NB for 2 months (twice a week by oral gavage) under HCD or HFD conditions. Representative immunofluorescence images showing the infiltration of indicated immune cells into the lung. Scale bars: 50 μm. Quantitative analyses are shown in **Fig. 3d**.

c. Gating strategies for isolating lung-derived macrophages ($CD45^+F4/80^+$ cells), non-macrophage immune cells ($CD45^+F4/80^-$ cells), and non-immune cells ($CD45^-F4/80^-$ cells).

d. Western blot analysis for indicated markers in THP-1 cells treated with Veh or NB (1 μM NNK and 1 μM BaP in combination) every other day for 2 months and BMDMs treated with Veh or NB every other day for 10 days. The in vitro data, including WB image results, is representative of at least two independent experiments with similar results. Source data are provided as a Source Data file. Uncropped western blot images are included at the end of the Supplementary Information.

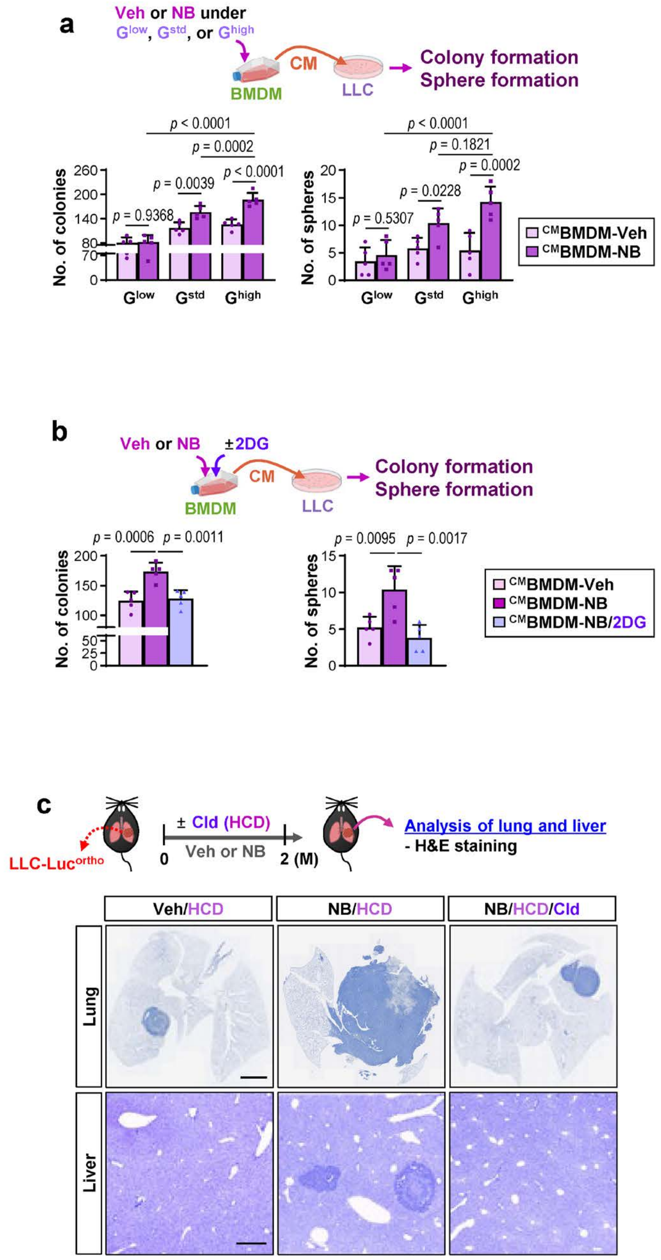

Supplementary Fig. 5. Chronic NB exposure-promoted lung cancer progression is facilitated by macrophage-mediated tumor-promoting activities.

a-b. BMDMs exposed to Veh or NB for 8 days under G^{std} culture conditions were subjected to an additional 2 days of NB exposure under glucose supplementation at concentrations of 0.8 g/L (G^{low}), 2 g/L (G^{std}), or 10 g/L (G^{high}) in the absence or presence of 2DG (5 mM). Conditioned media (CM) were collected after incubating BMDMs in fresh medium for 1 day. LLC cells incubated with BMDM-derived CM for one week were analyzed for the capacities of anchorage-independent colony formation and sphere formation (n = 5 biologically independent replicates/group). The data are presented as the mean ± SD. p-values were determined by using one-way ANOVA with Tukey's post-hoc test.

c. B6 mice harboring LLC-Luc^{ortho} were exposed to Veh or NB (NNK/BaP, 3 μmol each in 100 μL corn oil) under HCD conditions in the absence or presence of clodronate liposomes (Cld; 0.7 mg in 100

μL, once a week by intraperitoneal injection combined with once a week by intratracheal instillation, twice a week in total). Representative H&E images of lung and liver (*n* = 8 for Veh/HCD and NB/HCD groups; *n* = 10 for the NB/HCD/Cld group). Scale bars: 2.5 μm. The in vitro data is representative of at least two independent experiments with similar results. Source data are provided as a Source Data file.

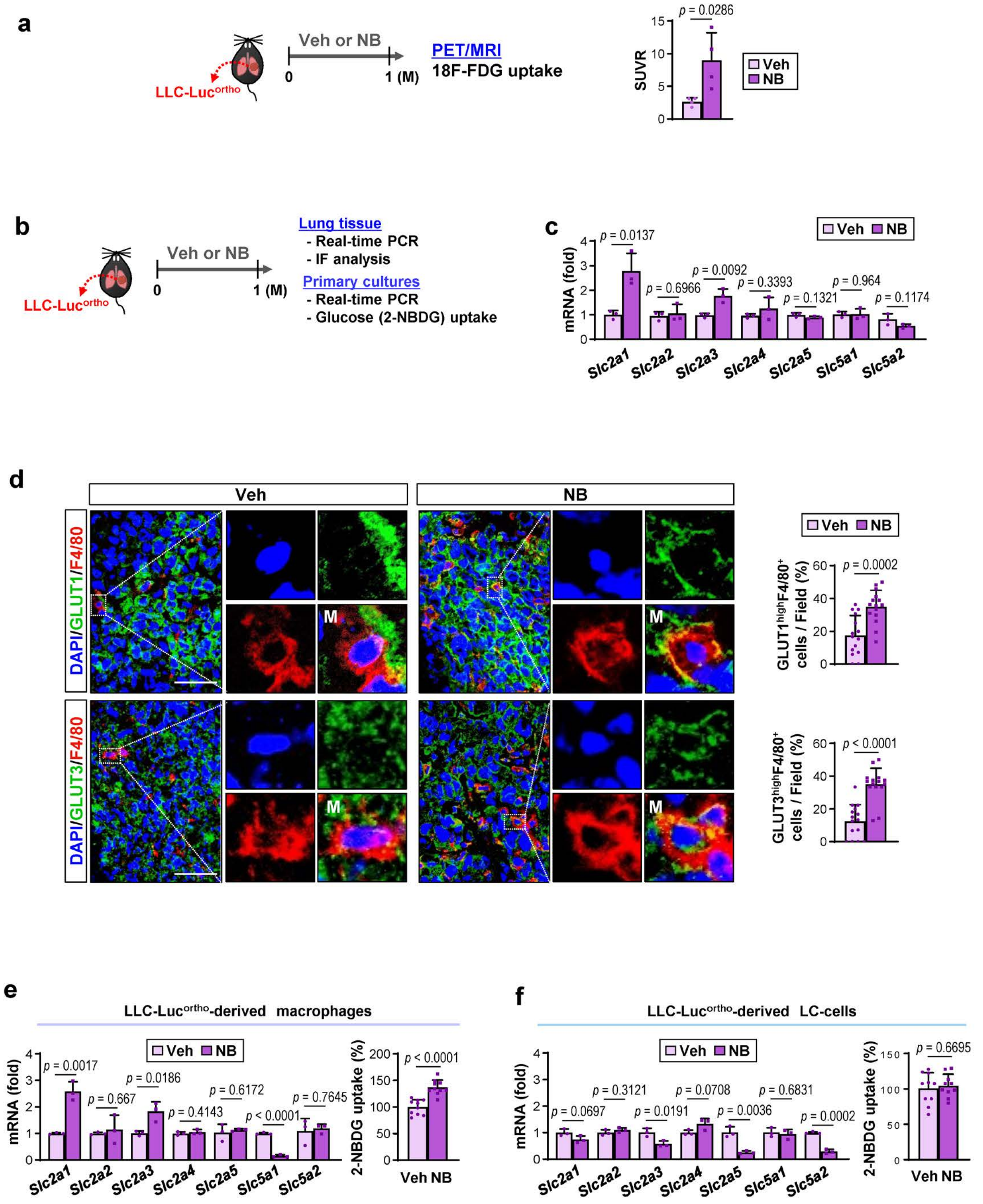

Supplementary Fig. 6. Modulation of GLUT expression and glucose uptake in the lung macrophages in orthotopic LLC-Luc-bearing C57BL/6J mice that were exposed to NB.

a-f. C57BL/6J (B6) mice harboring LLC-Luc^{ortho} were exposed to Veh or NB (NNK/BaP, 3 μmol each) for two months (twice a week by oral gavage).

a. Changes in glucose uptake in Veh- or NB-treated mice for 1 month, as determined by standard uptake value ratio (SUVR) analysis using 18F-FDG PET/MRI scanning images (*n* = 4/group).

b. Schematic diagram of the experiments.

c. Expression of genes encoding GLUTs in the lungs measured by real-time PCR analysis (*n* = 3/group).

d. Representative immunofluorescence images (left) and quantitative analysis (right, *n* = 3/group, 5 fields/slide [*n* = 15/group]) of GLUT1 and GLUT3 expression in the F4/80^{+} macrophages of the lungs.

Scale bars: 50 μm. M: merge.

e-f. Expression of genes encoding GLUTs measured by real-time PCR analyses of (*n* = 3/group) and 2-NBDG fluorescent tracer uptake glucose uptake measured by Operetta high content imaging analysis (*n* = 10/group) in the isolated lung macrophages (**e**) and LLC-Luc cells (**f**) from LLC-Luc^{ortho}-Veh or LLC-Luc^{ortho}-NB groups.

The data are presented as the mean ± SD. *p*-values were as determined by using two-tailed Mann-Whitney test (**a, d**) or a two-tailed Student's *t*-test (**c-f**). The in vitro data is representative of at least two independent experiments with similar results. Source data are provided as a Source Data file.

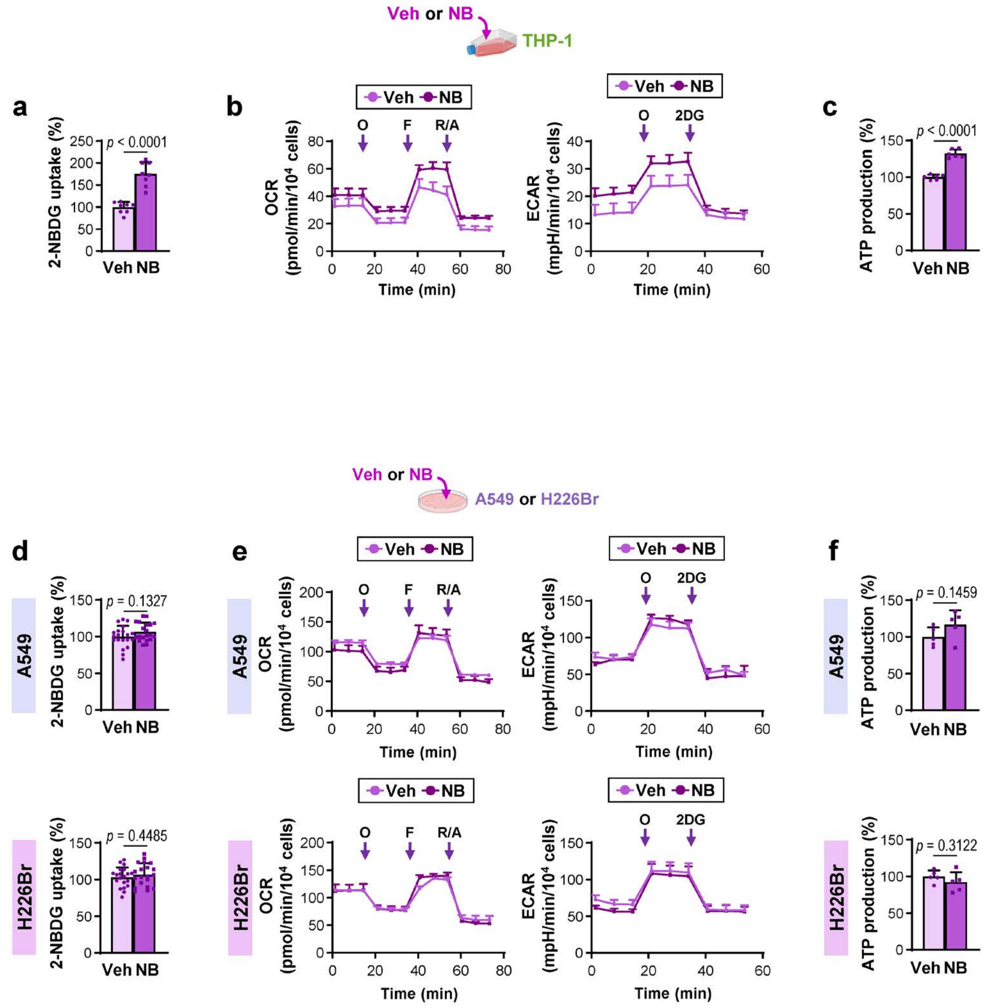

Supplementary Fig. 7. Modulation of glucose uptake, GLUT expression, glycolysis, and ATP production in macrophages, but not in lung cancer cells, by treatment with NB.

a-f. THP-1 cells (**a-c**) and lung cancer cells (**d-f**) were exposed to vehicle (Veh) or a mixture of 1 μM NNK and 1 μM BaP (NB) for 2 months.

a, d. 2-NBDG fluorescent tracer uptake glucose uptake measured by Operetta high content imaging analysis (*n* = 8/group for THP-1 cells; *n* = 20/group for A549 and H226Br cells)

b, e. Seahorse analysis of OCR and ECAR (*n* = 3/group for OCR analysis in THP-1 cells; *n* = 4/group for ECAR analysis in THP-1 cells; *n* = 3/group for OCR and ECAR analysis in A549 and H226Br cells). O: oligomycin; F: carbonyl cyanide p-trifluoromethoxy-phenylhydrazone (FCCP); R/A: rotenone/antimycin; 2DG: 2-deoxy-D-glucose.

c, f. Luminescent analysis of ATP production (*n* = 6/group for THP-1 cells; *n* = 5/group for A549 and H226Br cells).

The data are presented as the mean ± SD. *p*-values were determined by using a two-tailed Student's *t*-test. The in vitro data is representative of at least two independent experiments with similar results. Source data are provided as a Source Data file.

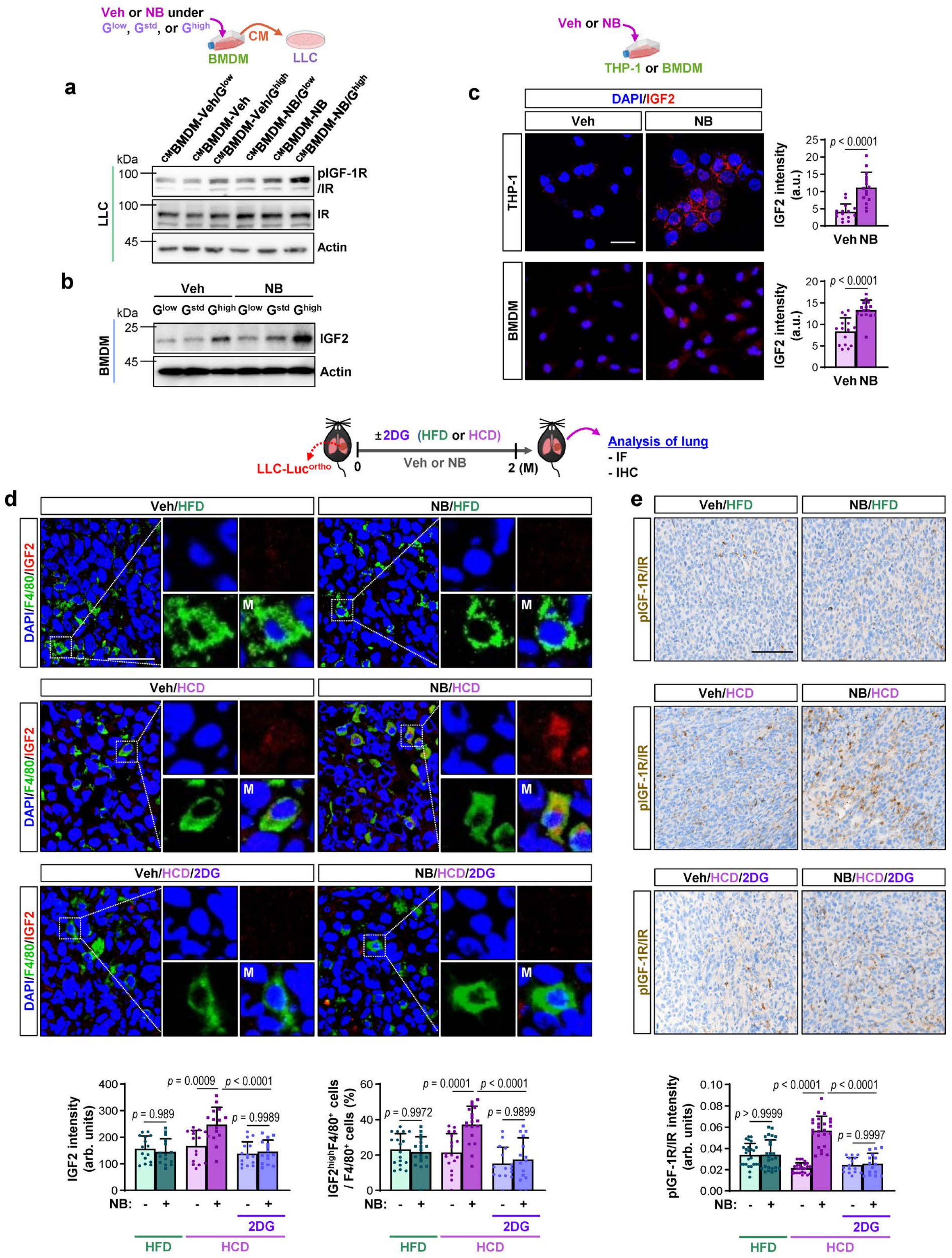

Supplementary Fig. 8. Paracrine IR activation in lung cancer cells by IGF2 from NB-primed macrophages under glucose-supplemented conditions.

a-c. BMDMs had vehicle (Veh) or NB (1 μM NNK and 1 μM BaP in combination) exposure for eight days and additional two days of Veh or NB exposure under low-glucose (G^{low}; 0.8 g/L glucose), standard-glucose (G^{std}; 2 g/L glucose), or high-glucose (G^{high}; 10 g/L glucose) culture conditions (**a-**

c). THP-1 cells had Veh or NB exposure for two months (**c**). Conditioned medium (CM) were collected after incubating BMDMs and THP-1 cells in fresh medium for one day.

a. LLC-Luc cells were treated with the BMDM-derived CMs for 30 min. Western blot (WB) analysis of pIGF-1R/IR (Y1135/36 for IGF-1R, Y1150/51 for IR) and IR levels in whole-cell lysates of LLC Cells.

b. WB analysis of IGF2 levels in whole-cell lysates of BMDMs.

c. Immunofluorescence (IF) analysis of IGF2 expression in THP-1 cells and BMDMs (*n* = 3/group, 5 fields/slide [*n* = 15/group]). Scale bar: 50 μm.

d-e. C57BL/6J mice harboring LLC-Luc^{ortho} were exposed to Veh or NB (NNK/BaP, 3 μmol each) in 100 μL corn oil for 2 months (twice a week by oral gavage) under HFD or HCD conditions, either alone or together with 2-deoxy-D-glucose (2DG, 500 mg/kg, five times a week by oral gavage).

d. IF analysis of IGF2 expression in F4/80$^+$ macrophages in the lungs of mice and statistical analysis of the IF results (*n* = 3/group, 4–6 fields/slide; IGF2 intensity: *n* = 12/group; IGF2^{high}F4/80$^+$ cells/F4/80$^+$ cells (%): *n* = 18/group of HFD and HCD groups, *n* = 15/group of the HCD+2DG group). Scale bar: 50 μm. M: merge.

e. Immunohistochemical analysis pIGF-1R/IR (Y1131 for IGF-1R, Y1146 for IR) expression in the lungs of mice and quantitative analysis (*n* = 5/group, 3 or 5 fields/slide; *n* = 25/group of HFD and HCD groups, *n* = 15/group of the HCD+2DG group). Scale bar: 100 μm.

The data are presented as the mean ± SD. *p*-values were determined by using a two-tailed Student's t-test with Welch's correction (**c**), a two-tailed Student's t-test (**c**) or one-way ANOVA with Tukey's post-hoc test (**d, e**). The in vitro data, including WB image results, is representative of at least two independent experiments with similar results. Source data are provided as a Source Data file. Uncropped western blot images are included at the end of the Supplementary Information.

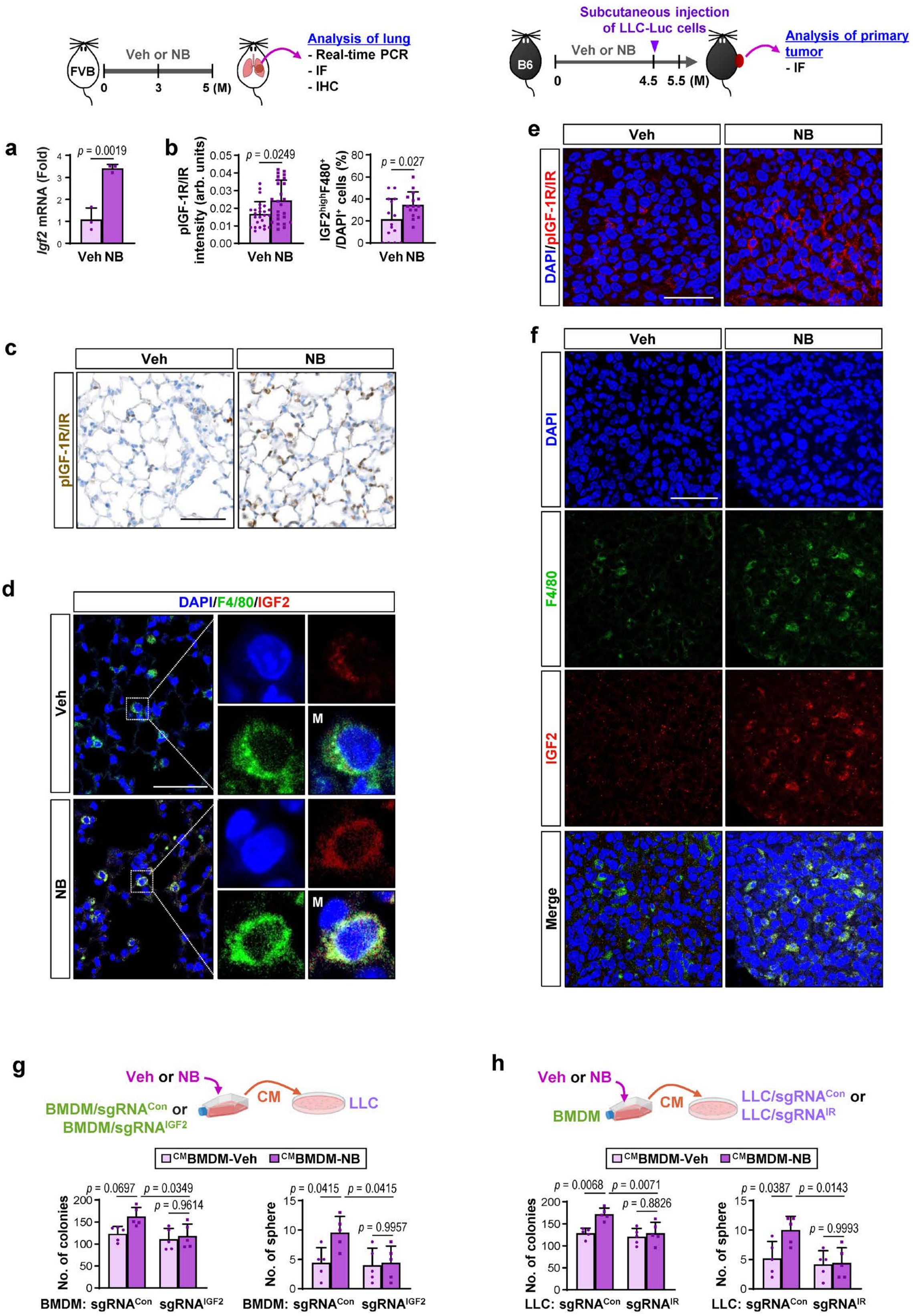

Supplementary Fig. 9. Paracrine IR activation in lung cancer cells and enhancement of their colony- and sphere-forming activities by NB-primed macrophages under glucose-supplemented conditions.

a-d. Two-month-old FVB mice were given vehicle (Veh) or NB (NNK/BaP, 3 μmol each in 100 μL corn oil) for 3 or 5 months (twice a week by oral gavage).
a. Real-time PCR analysis of *Igf2* mRNA expression in the lung tissues from Veh- or NB-treated FVB mice (*n* = 3 biologically independent replicates/group)
b-d. Immunohistochemistry (IHC) analysis of pIGF-1R/IR (Y1131 for IGF-1R, Y1146 for IR) expression and immunofluorescence (IF) analysis of IGF2-expressing macrophages ($IGF2^{+}F4/80^{+}$) in the lung tissues from Veh- or NB-treated FVB mice.
b. Quantification results of IHC and IF images (*n* = 5/group, 5 fields/slide for IHC analysis [*n* = 25/group]; *n* = 3/group, 5 fields/slide for IF analysis [*n* = 15/group]).
c-d. Representative images of IHC (**c**) and IF (**d**) analyses. Scale bars: 50 μm (IF image); 100 μm (IHC image). M: merge.
e-f. Two-month-old B6 mice given Veh or NB as described above for 4.5 months were inoculated subcutaneously with LLC-Luc cells and then given Veh or NB for additional 1 month. $LLC\text{-}Luc^{sc}$ tumors were dissected and subjected to IF analysis of tumoral pIGF-1R/IR (Y1131 for IGF-1R, Y1146 for IR) expression (**e**) and IGF2 expression in $F4/80^{+}$ macrophages (**f**). Representative images are shown (*n* = 5/group). Scale bars: 50 μm.
g-h. BMDM sublines, in which IGF2 expression was intact or eliminated using the CRISPR/Cas9 system (**g**), were treated with Veh or NB (a mixture of 1 μM NNK and 1 μM BaP) for ten days. CM were collected from BMDMs after incubating them in fresh growth medium for one day. LLC-Luc cells, either untransfected (**g**) or stably transfected with control or IR-specific sgRNAs ($LLC/sgRNA^{con}$ or $LLC/sgRNA^{IR}$, respectively) (**h**), were incubated with the BMDM-derived CM for one week and then subjected to anchorage-independent colony formation and sphere formation assays (*n* = 5 biologically independent replicates/group).
The data are presented as the mean ± SD. *p*-values were determined by using a two-tailed Student's *t*-test (**a**, **b**), two-tailed Mann-Whitney test (**b**), or one-way ANOVA with Tukey's post-hoc test (**g**, **h**). The in vitro data is representative of at least two independent experiments with similar results. Source data are provided as a Source Data file.

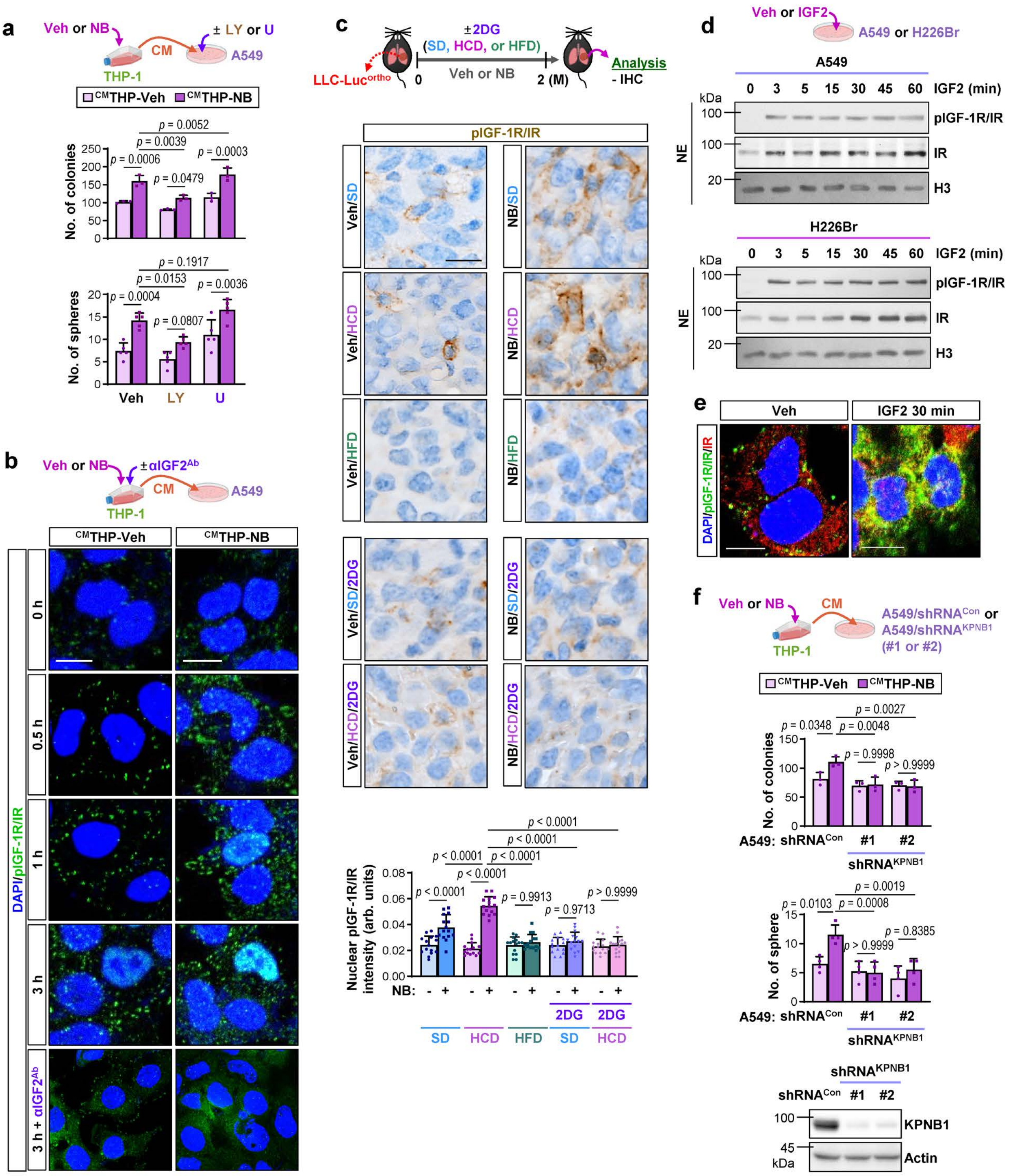

Supplementary Fig. 10. Activation and nuclear localization of IR in lung cancer cells by IGF2 from NB-primed macrophages.

a-b. THP-1 cells exposed to vehicle (Veh) or NB (1 μM NNK and 1 μM BaP in combination) for two months had additional two days of NB exposure in the absence or presence of αIGF2^{Ab} (5 μg/mL) (**b**). CMs (^{CM}THP-Veh and ^{CM}THP-NB) were collected from Veh- or NB-exposed THP-1 cells after incubating them in fresh growth medium for one day.

a. The anchorage-independent colony formation (*n* = 3 biologically independent replicates/group) and sphere formation (*n* = 5 biologically independent replicates/group) capacities of A549 cells incubated with THP-1-derived CMs in the absence or presence of LY (LY294001, 5 μM) or U (U0126,

5 μM) for 1 week.

b. Representative immunofluorescence (IF) images of phosphorylated IGF-1R/IR (Y1131 for IGF-1R, Y1146 for IR) nuclear translocation in A549 cells after incubation with THP-1-derived CM for the indicated periods (*n* = 5 biologically independent replicates/group). **Fig. 6b** depicts the quantitative analysis of the IF results. Scale bars: 50 μm.

c. C57BL/6 mice harboring LLC-Luc^{ortho} were exposed to vehicle (Veh) or NB (NNK/BaP, 3 μmol each in 100 μL corn oil) for 2 months (twice a week by oral gavage) under SD, HFD or HCD conditions, either alone or together with 2-deoxy-D-glucose (2DG, 500 mg/kg, five times a week by oral gavage). Representative immunohistochemistry images (top) and quantitative analysis (bottom, *n* = 3/group, 5 fields/slide [*n* = 15/group]) of pIGF-1R/IR (Y1131 for IGF-1R, Y1146 for IR) in mouse lung tumors. Scale bar: 100 μm.

d. A549 and H226Br cells were stimulated with IGF2 (50 ng/mL) for the indicated time periods. Western blot (WB) analysis of total and pIGF-1R/IR (Y1135/36 for IGF-1R, Y1150/51 for IR) using nuclear extract (NE). The images are is representative of at least two independent experiments with similar results.

e. IF images of total and pIGF-1R/IR (Y1131 for IGF-1R, Y1146 for IR) in A549 cells stimulated with IGF2 (50 ng/mL) for 30 min. Scale bars: 50 μm. The images are is representative of at least two independent experiments with similar results.

f. A549 cells stably transfected with control or either one of two different KPNB1 shRNAs were subjected to the analyses of anchorage-independent colony formation (*n* = 3 biological independent replicates/group) and sphere formation (*n* = 4 biological independent replicates /group) in response to ^{CM}THP-Veh or ^{CM}THP-NB (top) and expression the indicated proteins by WB analysis (bottom).

The data are presented as the mean ± SD. *p*-values were determined by using or one-way ANOVA with Tukey's post-hoc test (**a, c, f**). The in vitro data, including WB image results, is representative of at least two independent experiments with similar results. Source data are provided as a Source Data file. Uncropped western blot images are included at the end of the Supplementary Information.

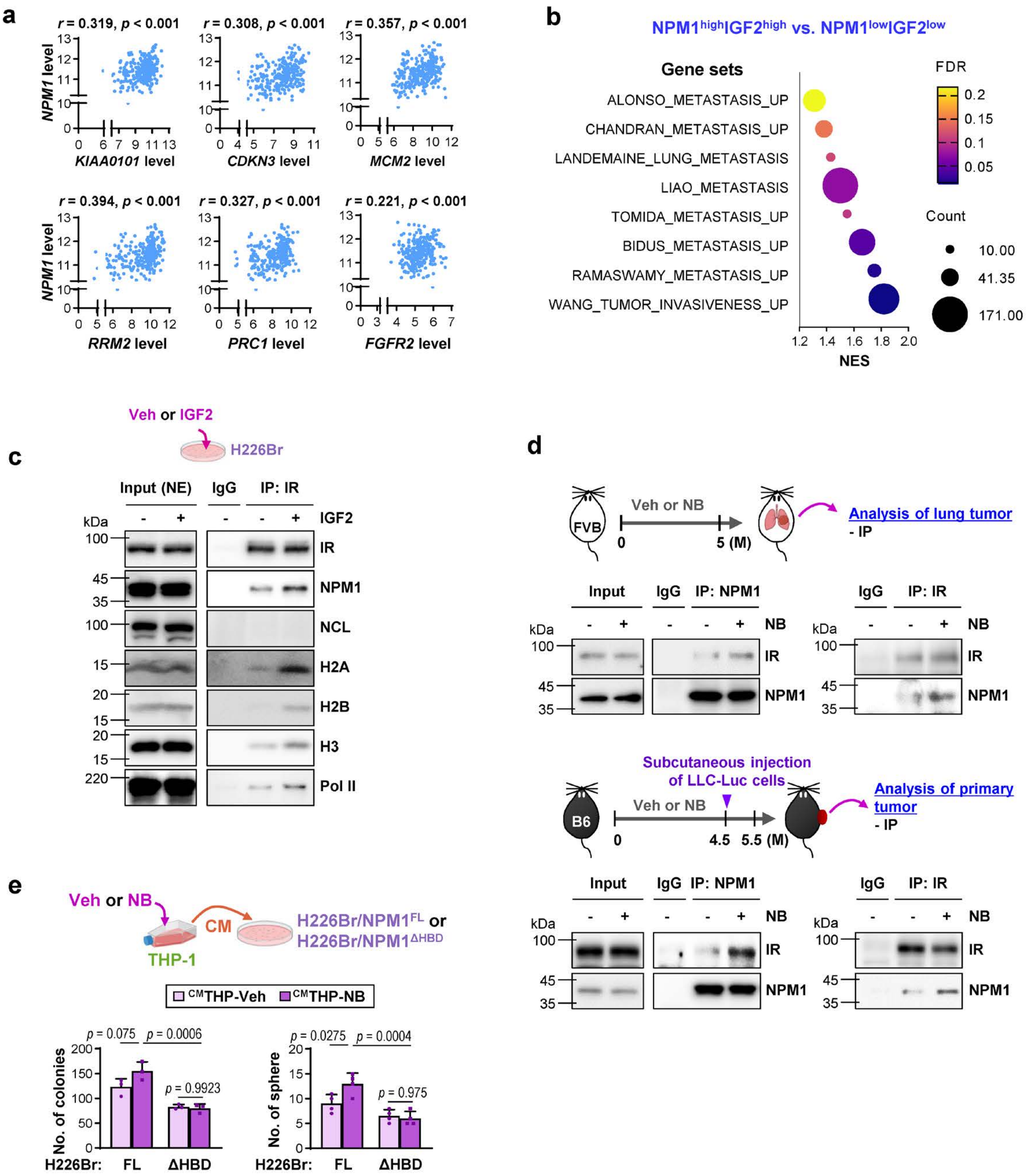

Supplementary Fig. 11. Association of nucleophosmin 1 (NPM1) with the prognosis of lung cancer patients and increased interaction between IR and NPM1 by IGF2 in lung cancer cells and in the lungs of NB-treated mice.

a. Analysis of the GSE30219 dataset using Spearman rank correlation to determine the association of NPM1 with prognosis-associated genes in NSCLC (n = 272).

b. A bubble chart depicting gene sets that are significantly enriched in the $NPM1^{high}IGF2^{high}$ population (n = 68) compared with the $NPM1^{low}IGF2^{low}$ population (n = 68) of the GSE30219 dataset (false discovery rate [FDR] < 0.25). Gene sets with a normalized enrichment score [NES] above 1.2 were shown. The Count indicates the number of genes that have the largest impact on the enrichment result.

c. Immunoprecipitation (IP) analysis of the association of IR with NPM1, histones, and RNA

polymerase II (RNA Pol II) in H226Br cells stimulated with IGF2 (50 ng/mL) for 3 h.

d. IP analyses of the association of IR with NPM1 in the tumors from the indicated mouse models. Detailed information on the indicated mouse models is shown in **Fig. 1**.

e. Anchorage-independent colony formation (n = 3 biologically independent replicates/group) and sphere formation (n = 4 biologically independent replicates/group) capacities of H226Br/NPM1^{FL} and H226Br/NPM1$^{\Delta HBD}$ cells in response to ^{CM}THP-Veh or ^{CM}THP-NB for 1 week. CM collection was performed as described in **Supplementary Fig. 10a**.

The data are presented as the mean ± SD. p-values were determined by using one-way ANOVA with Tukey's post-hoc test. The in vitro data, including WB image results, is representative of at least two independent experiments with similar results. Source data are provided as a Source Data file. Uncropped western blot images are included at the end of the Supplementary Information.

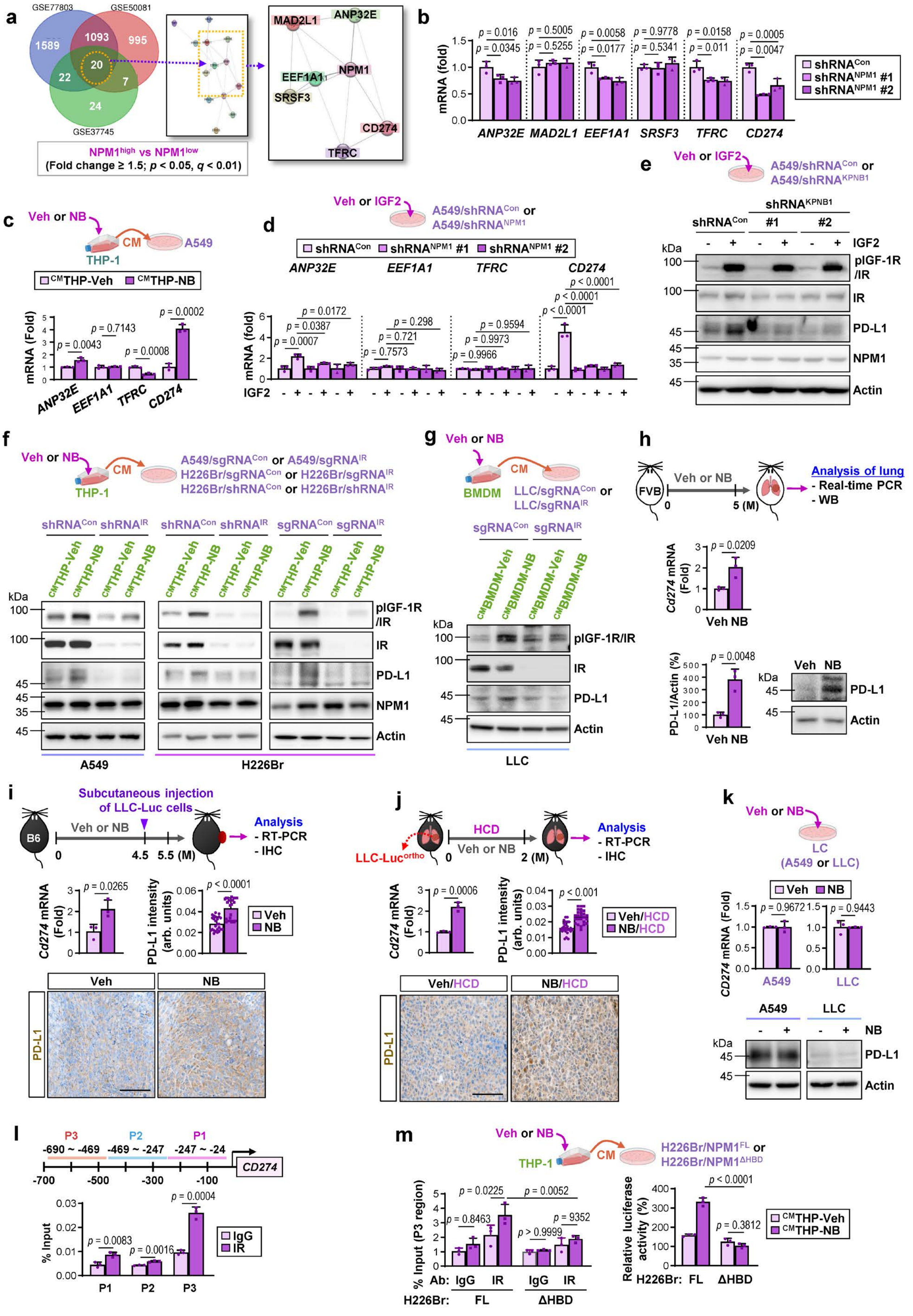

Supplementary Fig. 12. Complex formation of nuclear IR and NPM1 through macrophage-

derived IGF2 induces transcriptional upregulation of PD-L1 expression in lung cancer cells, resulting in lung cancer progression.

a. STRING protein interaction analysis of the common markers that were significantly upregulated in the NPM1^{high} group in three GEO datasets (GSE37745, GSE50081, and GSE77803) (Fold change ≥ 1.5, p < 0.05, and q < 0.01).

b. Real-time PCR analyses of the indicated gene expression in A549 cells stably transfected with control or NPM1 shRNAs (A549/shRNA^{Con} and two subclones of A549/shRNA^{NPM1}) (n = 3 biologically independent replicates/group).

c-d. A549 cells and their subclones, in which NPM1 expression was intact or ablated by shRNA transfection, were incubated with ^{CM}THP-Veh or ^{CM}THP-NB for one day (**c**) or stimulated with IGF2 (50 ng/mL) for 1 day (**d**). CM collection was performed under the conditions same as in **Fig. 5a-f**. Real-time PCR analyses of the indicated gene expression were performed (n = 3 biologically independent replicates/group).

e. Western blot (WB) analysis of the effects of one-day exposure to IGF2 (50 ng/mL) on the indicated protein expression in A549 cells in which KPNB1 expression was ablated by shRNA transfection.

f-g. THP-1 and BMDM cells were exposed to Veh or NB (1 μM NNK and 1 μM BaP in combination) for 2 months or 10 days, respectively. CM were collected after incubating these cells in fresh medium for 1 day. WB analysis of the effects of 1-day exposure to THP-1-derived conditioned medium (CM; ^{CM}THP-Veh or ^{CM}THP-NB)(**f**)or BMDM-derived CM (^{CM}BMDM-Veh or ^{CM}BMDM-NB)(**g**) on the indicated protein expression in A549, H226Br, and LLC cells in which IR expression was intact or eliminated by shRNA transfection or by the CRISPR/Cas9 system.

h. Real-time PCR of *Cd274* (top, n = 3 biologically independent replicates/group) and WB analyses of PD-L1 expression (bottom; quantification [n = 3/group]) in the lungs from FVB/N mice treated with vehicle (Veh) or NB (3 μmol NNK and 3 μmol BaP in combination in 100 μL corn oil, twice a week by oral gavage) for 5 months.

i-j. C57BL/6J (B6) mice were given Veh or NB (3 μmol NNK and 3 μmol BaP in combination in 100 μL corn oil, twice a week by oral gavage) for 4.5 months, subcutaneously inoculated with LLC-Luc cells (LLC-Luc^{sc}), and then exposed to Veh or NB for an additional 1 month (**i**). B6 mice carrying LLC-Luc^{ortho} were given Veh or NB under high-carbohydrate diet (HCD) conditions, either alone or together with 2-deoxy-D-glucose (2DG, 500 mg/kg, five times a week by oral gavage) for 2 months (**j**). Real-time PCR (left, n = 3/group) of *Cd274* expression and immunohistochemistry (IHC) analysis of PD-L1 expression in the lungs (n = 5/group, 5 fields/slide [n = 25/group]) were performed. Representative IHC images are shown. Scale bars: 100 μm. arb. units: arbitrary units.

k. Real-time PCR (top, n = 3 biologically independent replicates/group) and WB (bottom) analyses of PD-L1 expression in A549 and LLC cells exposed to Veh or NB (1 μM NNK and 1 μM BaP in combination) for 2 months.

l. Chromatin immunoprecipitation (ChIP) assay of IR binding to the three regions (P1, P2, and P3) of the *CD274* promoter in A549 cells (n = 3 biologically independent replicates/group).

m. H226Br cells, in which NPM1 expression was ablated by shRNA transfection and then the NPM1 FL or ΔHBD was reintroduced (H226Br/NPM1^{FL} or H226Br/NPM1$^{\Delta HBD}$, respectively), were exposed to ^{CM}THP-Veh/G^{std} or ^{CM}THP-NB/G^{std} (top) or to Veh or IGF2 (50 ng/mL) for 3 h (for ChIP assay) or 24 h (for luciferase reporter assay). ChIP assay of IR binding to the P3 region of the *CD274* promoter (n = 3 biologically independent replicates/group) (left) and luciferase reporter assay of activation of the *CD274* promoter (n = 3 biologically independent replicates/group) (right) were performed.

The data are presented as the mean ± SD. p-values were determined by one-way ANOVA with Dunnett's post-hoc test (**b**), one-way ANOVA with Tukey's post-hoc test (**d, m**), a two-tailed Student's

t-test (**c, h-l**), or two-tailed Mann-Whitney test (**i**). The in vitro data, including WB image results, is representative of at least two independent experiments with similar results. Source data are provided as a Source Data file. Uncropped western blot images are included at the end of the Supplementary Information.

Uncropped images of blots that are presented in supplementary figures

Supplementary Fig. 4d

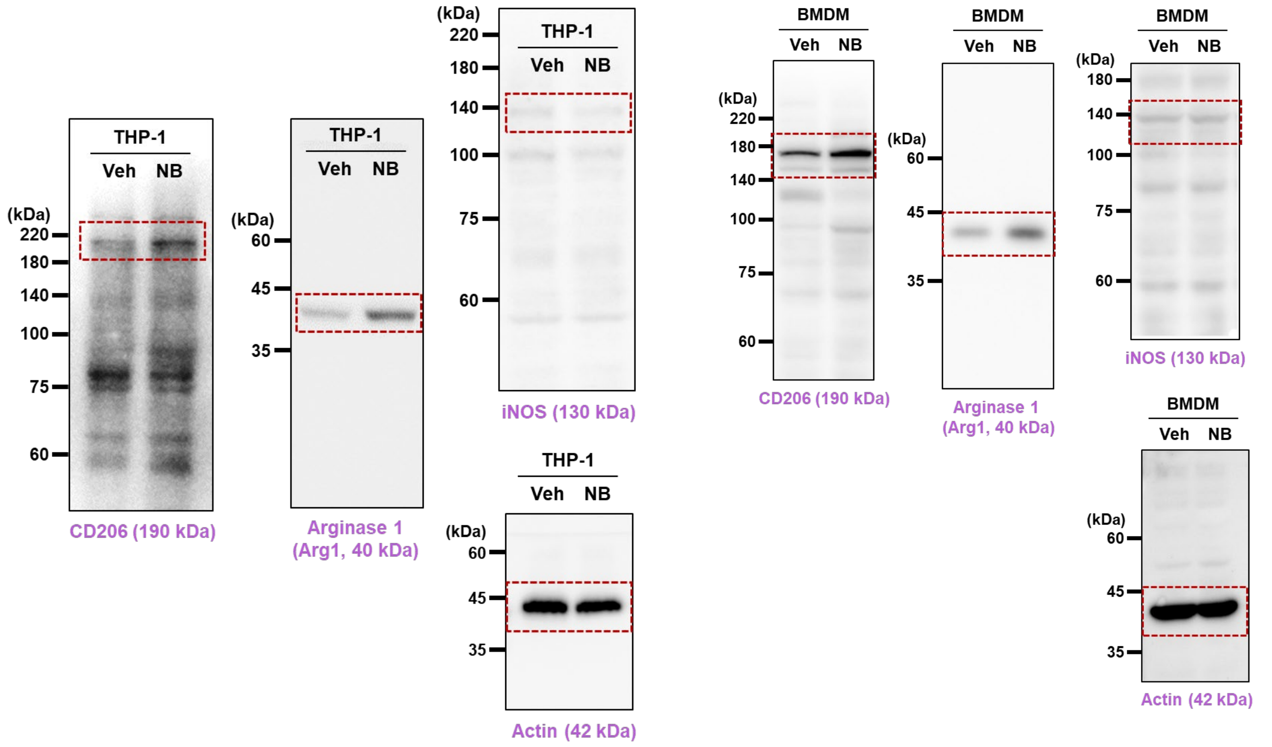

Supplementary Fig. 8a

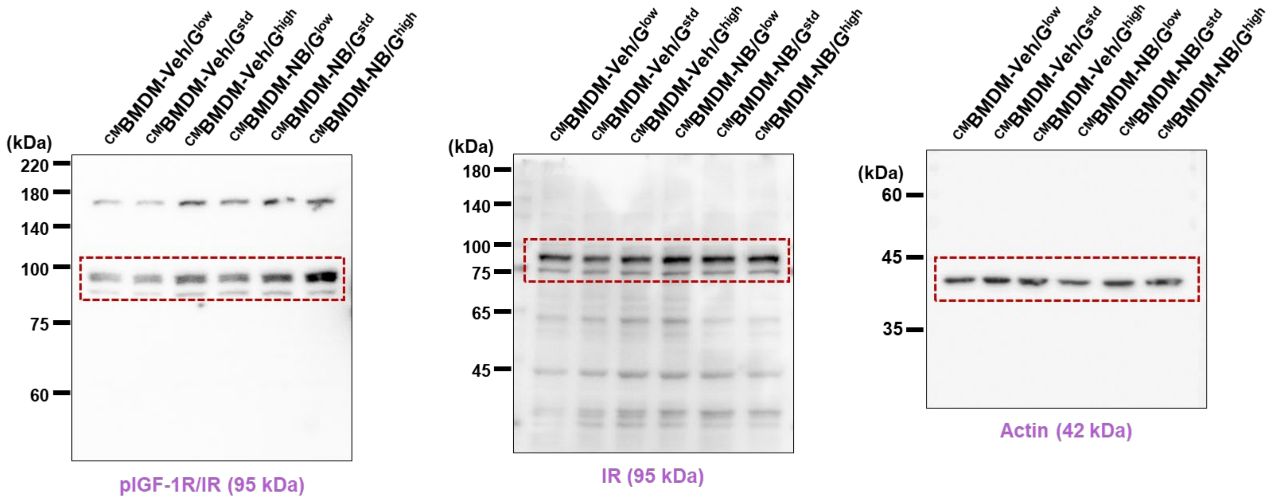

Supplementary Fig. 8b

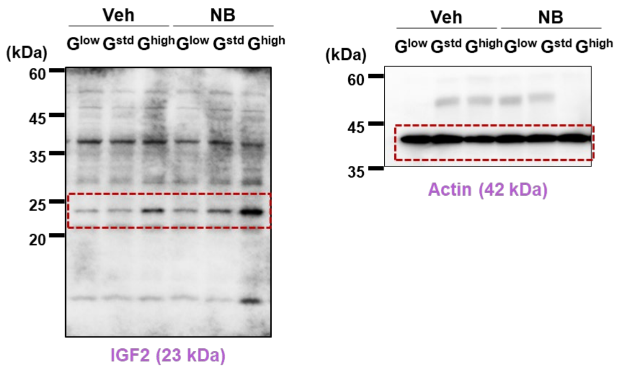

Supplementary Fig. 10d

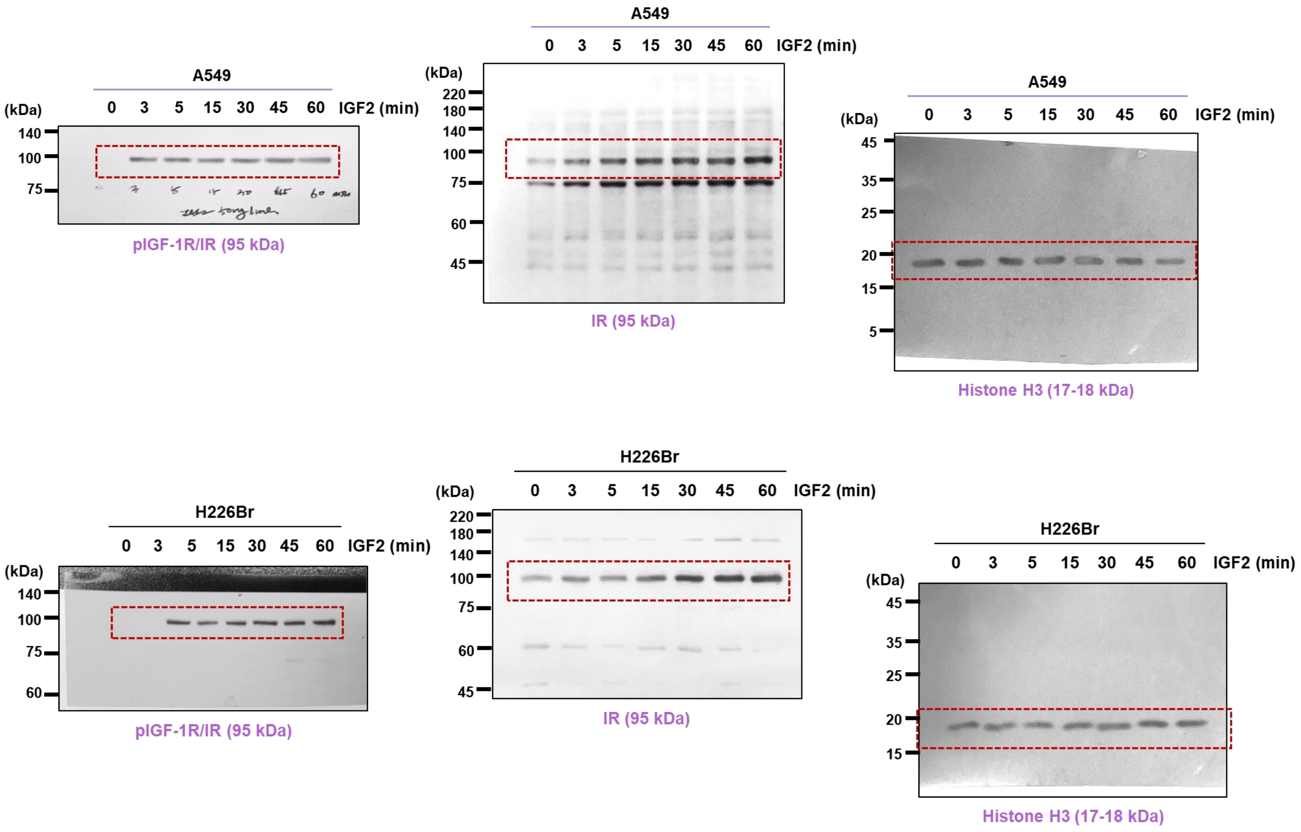

Supplementary Fig. 10f

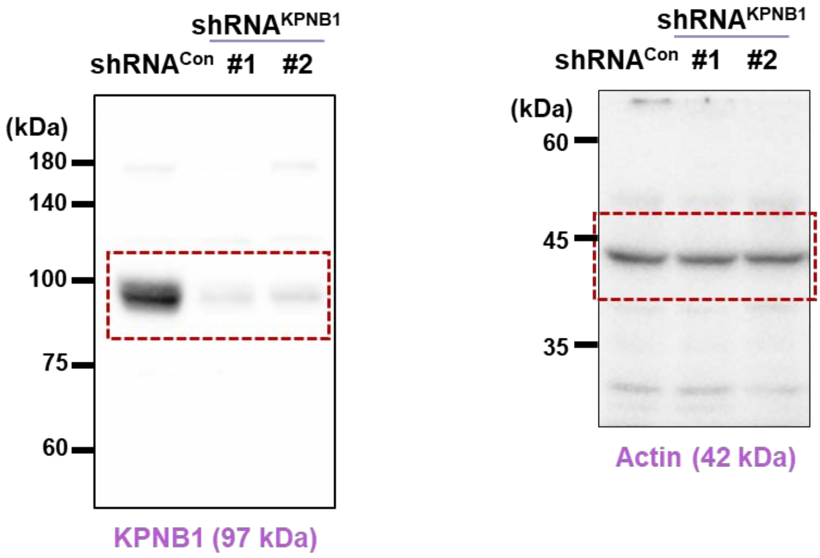

Supplementary Fig. 11c

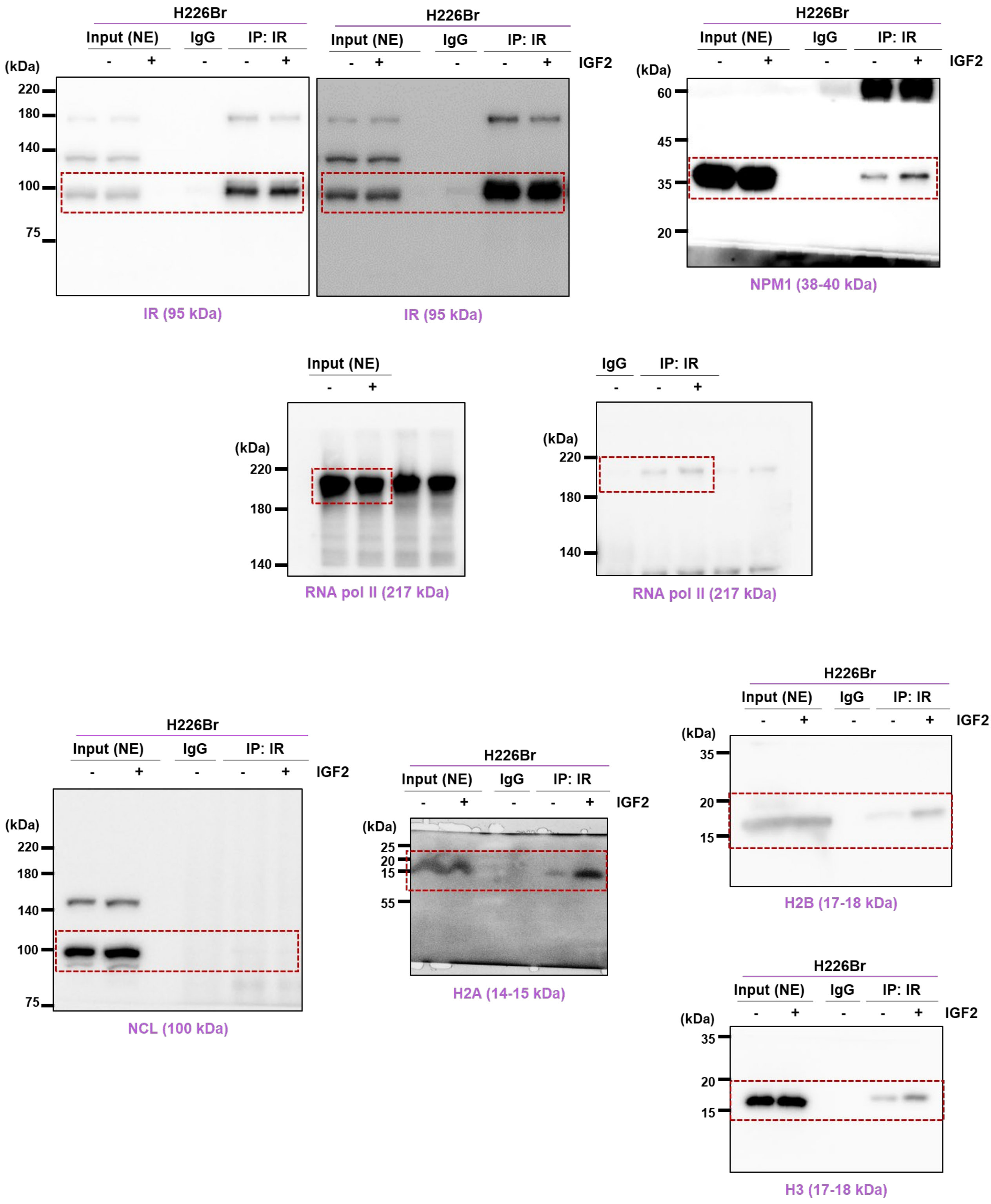

Supplementary Fig. 11d

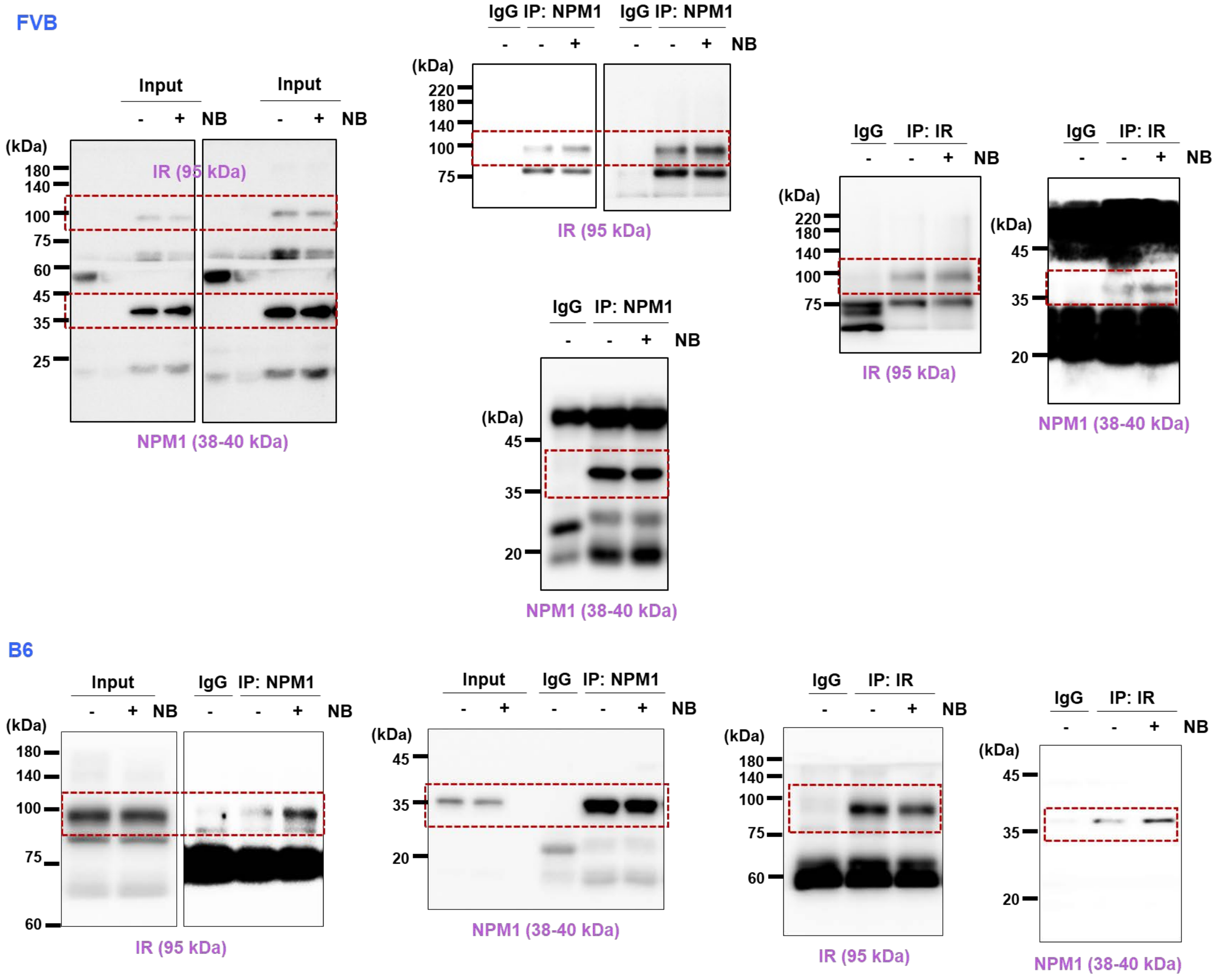

Supplementary Fig. 12e

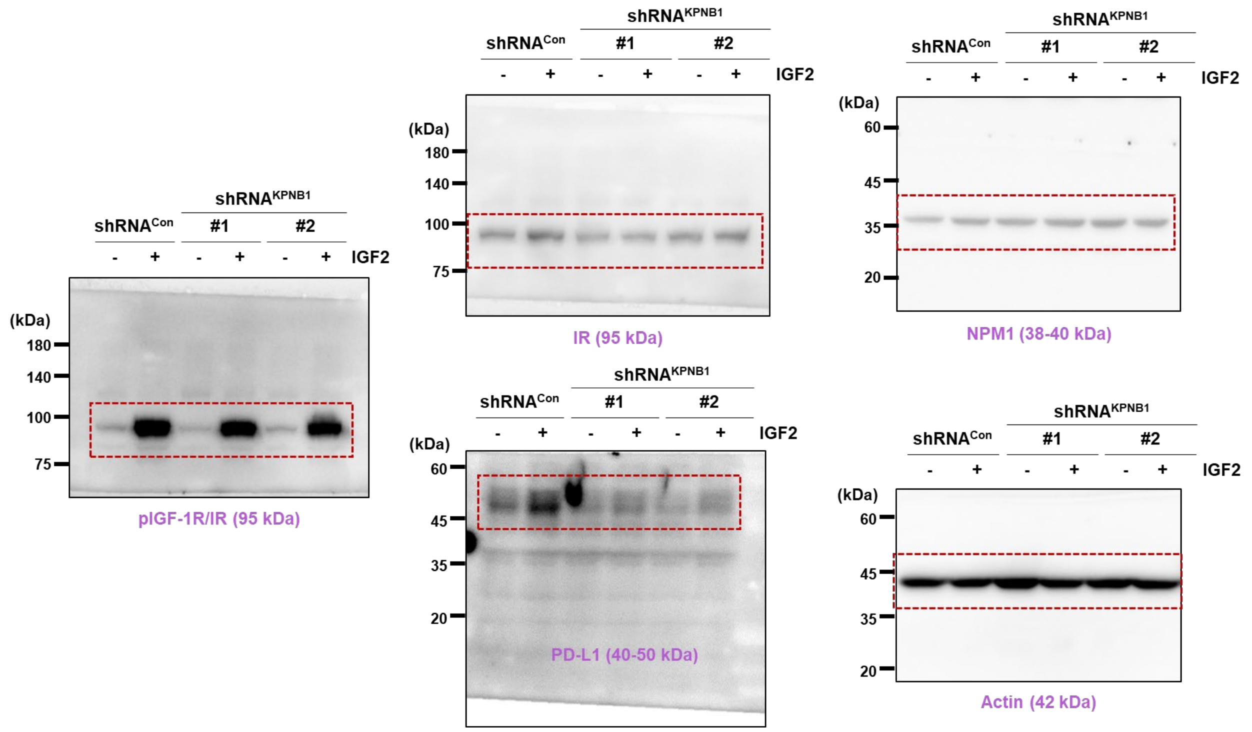

Supplementary Fig. 12f

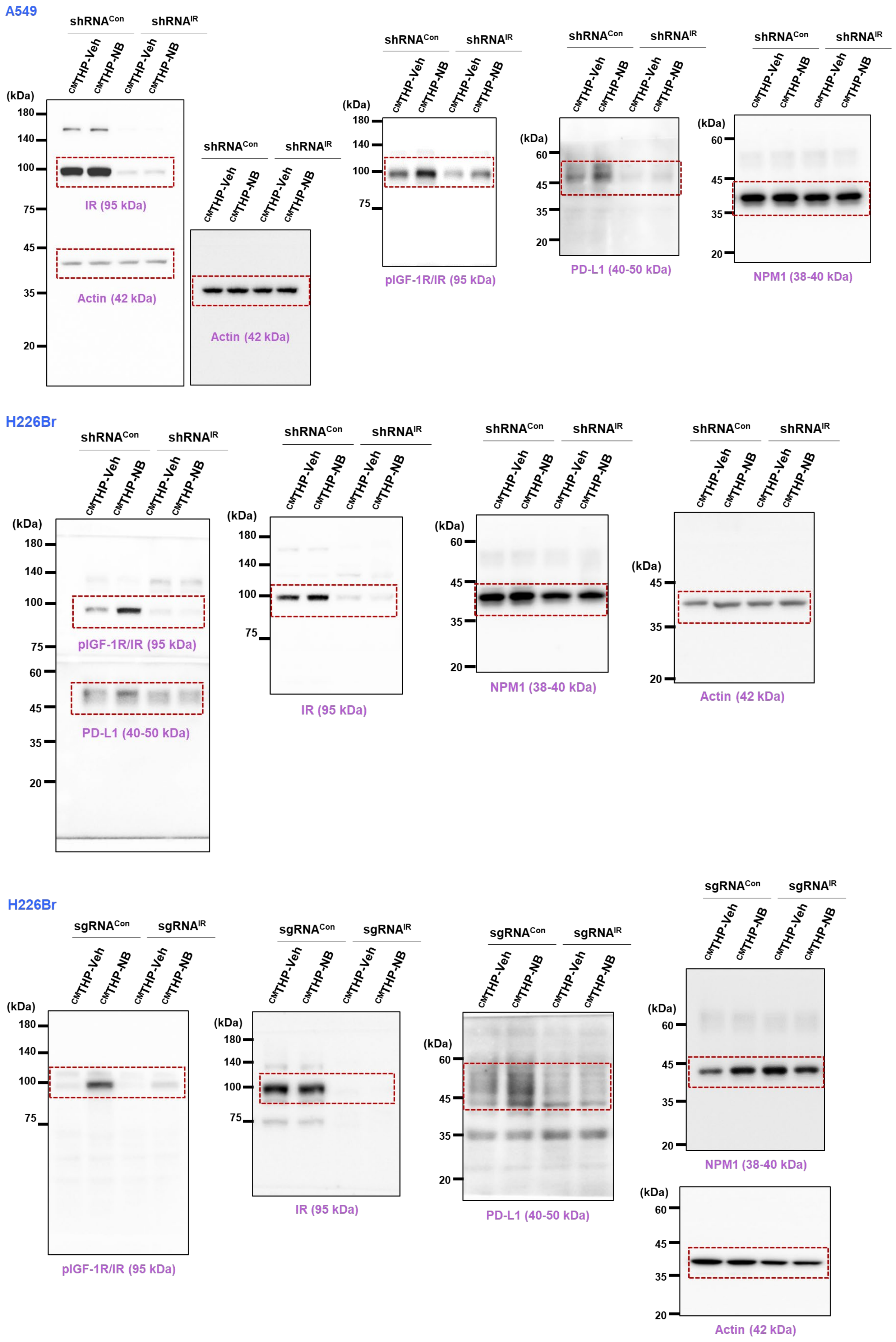

Supplementary Fig. 12g

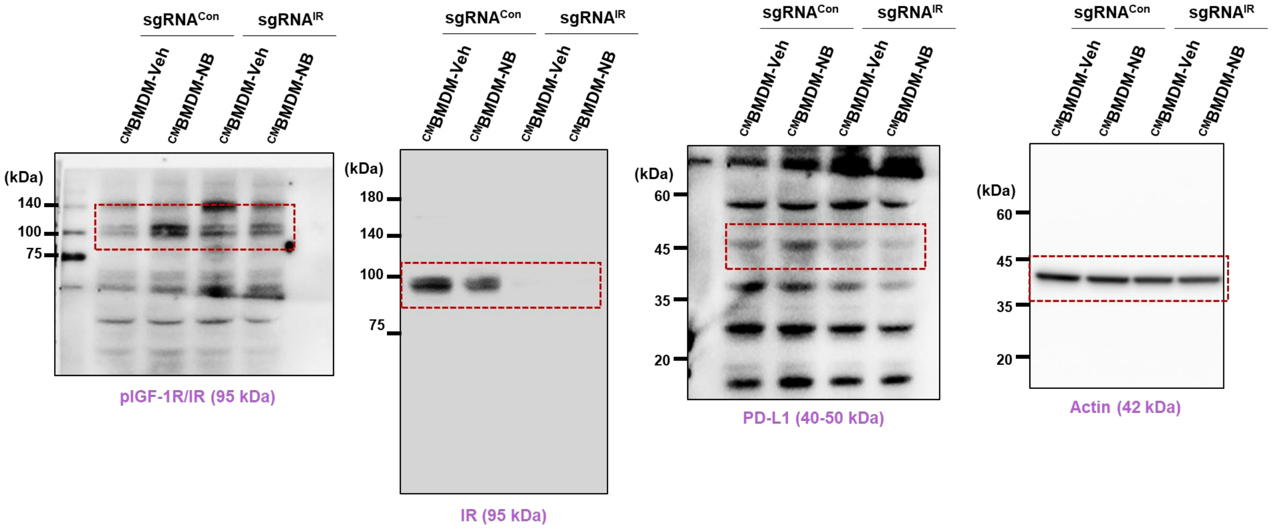

Supplementary Fig. 12h

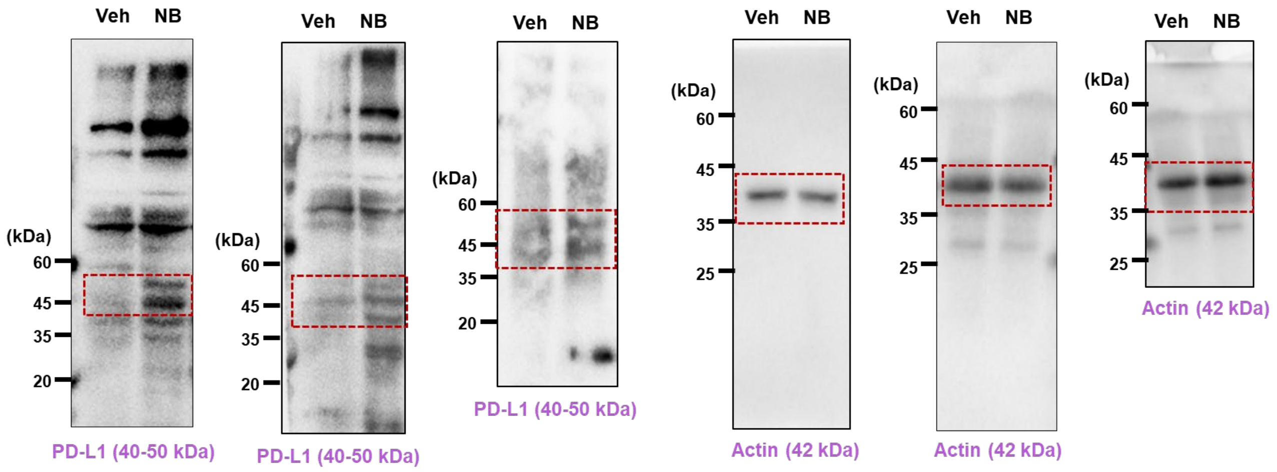

Supplementary Fig. 12k

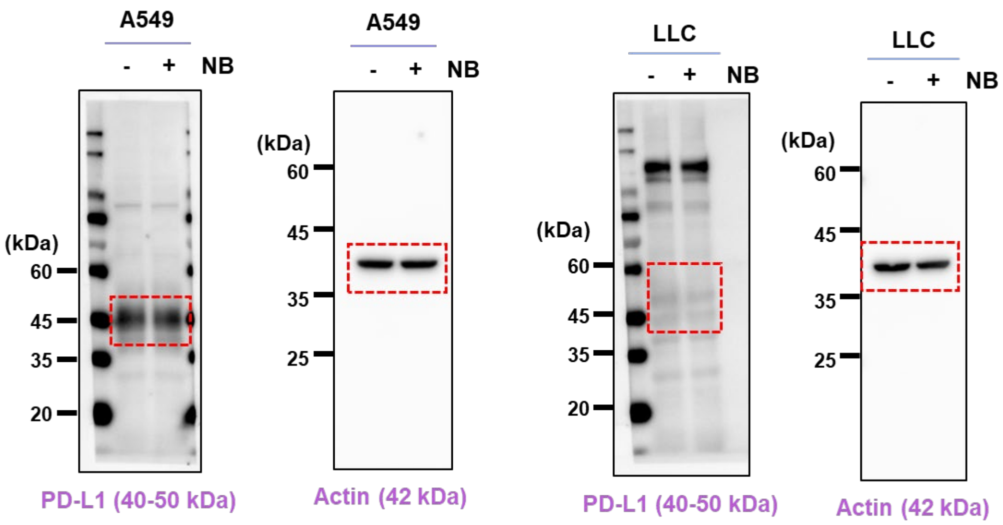

Supplement: Supplementary file 1 — Supplementary Information [file 41467_2024_49199_MOESM1_ESM.pdf]
