# Supplementary material for: Tobacco-induced hyperglycemia promotes lung cancer progression via cancer cell-macrophage interaction through paracrine IGF2/IR/NPM1-driven PD-L1 expression
Source: Nat Commun. 2024 Jun 8;15:4909. doi: 10.1038/s41467-024-49199-9 (PMC11162468; doi:10.1038/s41467-024-49199-9)
Supplement: Supplementary file 2 — Description of Additional Supplementary Files [file 41467_2024_49199_MOESM2_ESM.pdf]

Description of Additional Supplementary Files

File Name: Supplementary Data 1

Description: Processed results of protein LC-MS/MS analysis. Proteins that were differentially expressed in the IR-EGFP group compared with those in the EGFP group were analyzed by LC-MS/MS. These HTML files are the results of a peptide sequence search on the Mascot server using LC-MS/MS data.
