# Supplementary material for: Tobacco-induced hyperglycemia promotes lung cancer progression via cancer cell-macrophage interaction through paracrine IGF2/IR/NPM1-driven PD-L1 expression
Source: Nat Commun. 2024 Jun 8;15:4909. doi: 10.1038/s41467-024-49199-9 (PMC11162468; doi:10.1038/s41467-024-49199-9)
Supplement: Supplementary file 3 — Supplementary Data 1 [file 41467_2024_49199_MOESM3_ESM.zip › Supplementary Data 1/1.htm]

Peptide Summary Report (../data/20120627/F011166.dat)


# Mascot Search Results

```
User            : yprc
Email           : info
Search title    : 
MS data file    : 1.xml
Database        : NCBInr 110704 (14481393 sequences; 4958963357 residues)
Taxonomy        : Homo sapiens (human) (217342 sequences)
Timestamp       : 27 Jun 2012 at 01:32:28 GMT

|  |  |  |
| --- | --- | --- |
| Protein hits    : | gi|47115317 | VIM [Homo sapiens] |
|  | gi|193787214 | unnamed protein product [Homo sapiens] |
|  | gi|5030431 | vimentin [Homo sapiens] |
|  | gi|34782901 | ATP5A1 protein [Homo sapiens] |
|  | gi|13129104 | coiled-coil domain-containing protein 86 [Homo sapiens] |
|  | gi|73760405 | thymopoietin isoform beta [Homo sapiens] |
|  | gi|885683 | thymopoietin alpha [Homo sapiens] |
|  | gi|18088719 | Tubulin, beta [Homo sapiens] |
|  | gi|17066105 | Titin [Homo sapiens] |
|  | gi|42558279 | tubulin beta-8 chain isoform 1 [Homo sapiens] |
|  | gi|7657381 | pre-mRNA-processing factor 19 [Homo sapiens] |
|  | gi|225131084 | titin [Homo sapiens] |
|  | gi|119631909 | nebulin, isoform CRA_f [Homo sapiens] |
|  | gi|307358 | peripherin [Homo sapiens] |
|  | gi|119631418 | titin, isoform CRA_a [Homo sapiens] |
|  | gi|103472005 | antigen KI-67 isoform 1 [Homo sapiens] |
|  | gi|415819 | antigen of the monoclonal antibody Ki-67 [Homo sapiens] |
|  | gi|119631910 | nebulin, isoform CRA_g [Homo sapiens] |
|  | gi|356168 | histone H1b |
|  | gi|33694244 | nucleophosmin [Homo sapiens] |
```

### Probability Based Mowse Score

Ions score is -10\*Log(P), where P is the
probability that the observed match is a random event.  
Individual ions scores
> 47 indicate identity or extensive homology (p<0.05).  
Protein scores
are derived from ions scores as a non-probabilistic basis for ranking protein
hits.

### Peptide Summary Report

|  |  |  |  |
| --- | --- | --- | --- |
|  | Peptide Summary Select Summary (protein hits) Select Summary (unassigned) Export Search Results |  | Help |
|  | Significance threshold p< | Max. number of hits |  |
|  | Standard scoring  MudPIT scoring | Ions score or expect cut-off | Show sub-sets |
|  | Show pop-ups  Suppress pop-ups | Sort unassigned  Decreasing Score Increasing query / Mr Decreasing Intensity | Require bold red |

  
 
                
             


  


  
     **Error tolerant**    

|  |  |
| --- | --- |
| **1.** | gi|47115317    **Mass:** 53579    **Score:** 1340   **Queries matched:** 99   **emPAI:** 4.63 |
|  | VIM [Homo sapiens] |

|  |  |
| --- | --- |
|  | Check to include this hit in error tolerant search or archive report |
|  |  |

|  |  |  |  |  |  |  |  |  |  |  |
| --- | --- | --- | --- | --- | --- | --- | --- | --- | --- | --- |
|  | **Query** | **Observed** | **Mr(expt)** | **Mr(calc)** | **Delta** | **Miss** | **Score** | **Expect** | **Rank** | **Peptide** |
|  | 1279 | **458.2179** | **914.4209** | **913.9716** | **0.4493** | **0** | **24** | **12** | **1** | **R.SYVTTSTR.T** |
|  | 1565 | **485.9523** | **969.8899** | **970.1276** | **-0.2377** | **1** | **32** | **1.4** | **1** | **R.LRSSVPGVR.L** |
|  | 2229 | **587.3734** | **1172.7319** | **1173.4028** | **-0.6709** | **1** | **51** | **0.021** | **1** | **R.TLLIKTVETR.D** |
|  | 2231 | **587.3816** | **1172.7484** | **1173.4028** | **-0.6544** | **1** | **(12)** | **1.6e+02** | **1** | **R.TLLIKTVETR.D** |
|  | 440 | **392.2943** | **1173.8609** | **1173.4028** | **0.4580** | **1** | **(31)** | **1.7** | **1** | **R.TLLIKTVETR.D** |
|  | 442 | **392.3950** | **1174.1627** | **1173.4028** | **0.7599** | **1** | **(21)** | **21** | **1** | **R.TLLIKTVETR.D** |
|  | 2691 | **655.6322** | **1309.2496** | **1309.3800** | **-0.1304** | **0** | **31** | **1.7** | **1** | **K.NLQEAEEWYK.S** |
|  | 1158 | **444.5180** | **1330.5318** | **1329.5885** | **0.9433** | **2** | **16** | **84** | **2** | **K.RTLLIKTVETR.D** |
|  | 1381 | **465.8632** | **1394.5675** | **1393.5016** | **1.0658** | **1** | **25** | **9** | **1** | **R.DVRQQYESVAAK.N** |
|  | 3000 | **714.6857** | **1427.3566** | **1428.5456** | **-1.1891** | **0** | **(11)** | **1.9e+02** | **1** | **R.SLYASSPGGVYATR.S** |
|  | 3001 | **714.9583** | **1427.9018** | **1428.5456** | **-0.6438** | **0** | **(20)** | **22** | **1** | **R.SLYASSPGGVYATR.S** |
|  | 3003 | **715.1034** | **1428.1920** | **1428.5456** | **-0.3536** | **0** | **(37)** | **0.48** | **1** | **R.SLYASSPGGVYATR.S** |
|  | 3005 | **715.1392** | **1428.2635** | **1428.5456** | **-0.2821** | **0** | **(65)** | **0.00081** | **1** | **R.SLYASSPGGVYATR.S** |
|  | 3006 | **715.2098** | **1428.4049** | **1428.5456** | **-0.1407** | **0** | **(56)** | **0.0068** | **1** | **R.SLYASSPGGVYATR.S** |
|  | 3007 | **715.2242** | **1428.4337** | **1428.5456** | **-0.1119** | **0** | **79** | **3.6e-05** | **1** | **R.SLYASSPGGVYATR.S** |
|  | 3008 | **715.2937** | **1428.5726** | **1428.5456** | **0.0270** | **0** | **(37)** | **0.49** | **1** | **R.SLYASSPGGVYATR.S** |
|  | 3010 | **715.4422** | **1428.8696** | **1428.5456** | **0.3240** | **0** | **(24)** | **11** | **1** | **R.SLYASSPGGVYATR.S** |
|  | 3011 | **715.5910** | **1429.1672** | **1428.5456** | **0.6216** | **0** | **(21)** | **20** | **1** | **R.SLYASSPGGVYATR.S** |
|  | 3012 | **715.6136** | **1429.2125** | **1428.5456** | **0.6669** | **0** | **(49)** | **0.029** | **1** | **R.SLYASSPGGVYATR.S** |
|  | 3014 | **715.7111** | **1429.4073** | **1428.5456** | **0.8617** | **0** | **(61)** | **0.0022** | **1** | **R.SLYASSPGGVYATR.S** |
|  | 3015 | **715.8400** | **1429.6653** | **1428.5456** | **1.1196** | **0** | **(77)** | **6e-05** | **1** | **R.SLYASSPGGVYATR.S** |
|  | 1613 | **491.1848** | **1470.5323** | **1470.6769** | **-0.1446** | **2** | **(27)** | **4.9** | **1** | **R.SSAVRLRSSVPGVR.L** |
|  | 3061 | **736.2912** | **1470.5676** | **1470.6769** | **-0.1092** | **2** | **(12)** | **1.6e+02** | **2** | **R.SSAVRLRSSVPGVR.L** |
|  | 1615 | **491.2809** | **1470.8207** | **1470.6769** | **0.1438** | **2** | **28** | **3.7** | **2** | **R.SSAVRLRSSVPGVR.L** |
|  | 1617 | **491.5699** | **1471.6877** | **1470.6769** | **1.0108** | **2** | **(28)** | **5.1** | **1** | **R.SSAVRLRSSVPGVR.L** |
|  | 1658 | **499.2558** | **1494.7451** | **1494.6320** | **0.1132** | **0** | **(24)** | **11** | **1** | **R.MFGGPGTASRPSSSR.S** |
|  | 1659 | **499.2778** | **1494.8113** | **1494.6320** | **0.1794** | **0** | **(27)** | **4.6** | **1** | **R.MFGGPGTASRPSSSR.S** |
|  | 1660 | **499.4523** | **1495.3347** | **1495.6365** | **-0.3018** | **0** | **(2)** | **1.3e+03** | **8** | **R.TYSLGSALRPSTSR.S** |
|  | 3174 | **748.7148** | **1495.4148** | **1494.6320** | **0.7828** | **0** | **(30)** | **2.4** | **1** | **R.MFGGPGTASRPSSSR.S** |
|  | 3175 | **748.7802** | **1495.5455** | **1495.6365** | **-0.0910** | **0** | **(20)** | **28** | **1** | **R.TYSLGSALRPSTSR.S** |
|  | 3176 | **748.8155** | **1495.6162** | **1495.6365** | **-0.0203** | **0** | **(41)** | **0.23** | **1** | **R.TYSLGSALRPSTSR.S** |
|  | 3177 | **748.8173** | **1495.6199** | **1495.6365** | **-0.0167** | **0** | **41** | **0.22** | **1** | **R.TYSLGSALRPSTSR.S** |
|  | 1664 | **499.9474** | **1496.8202** | **1495.6365** | **1.1836** | **0** | **(11)** | **2.1e+02** | **2** | **R.TYSLGSALRPSTSR.S** |
|  | 1688 | **504.1835** | **1509.5282** | **1510.6314** | **-1.1031** | **0** | **51** | **0.021** | **1** | **R.MFGGPGTASRPSSSR.S + Oxidation (M)** |
|  | 3223 | **756.2562** | **1510.4977** | **1510.6314** | **-0.1337** | **0** | **(33)** | **1.3** | **1** | **R.MFGGPGTASRPSSSR.S + Oxidation (M)** |
|  | 3224 | **756.2725** | **1510.5303** | **1510.6314** | **-0.1011** | **0** | **(18)** | **36** | **1** | **R.MFGGPGTASRPSSSR.S + Oxidation (M)** |
|  | 1690 | **504.6530** | **1510.9370** | **1510.6314** | **0.3056** | **0** | **(34)** | **1.2** | **1** | **R.MFGGPGTASRPSSSR.S + Oxidation (M)** |
|  | 1693 | **504.7305** | **1511.1693** | **1510.6314** | **0.5379** | **0** | **(38)** | **0.37** | **1** | **R.MFGGPGTASRPSSSR.S + Oxidation (M)** |
|  | 1694 | **504.7505** | **1511.2293** | **1510.6314** | **0.5979** | **0** | **(15)** | **62** | **1** | **R.MFGGPGTASRPSSSR.S + Oxidation (M)** |
|  | 1695 | **504.7949** | **1511.3624** | **1510.6314** | **0.7310** | **0** | **(46)** | **0.053** | **1** | **R.MFGGPGTASRPSSSR.S + Oxidation (M)** |
|  | 3225 | **756.7136** | **1511.4123** | **1510.6314** | **0.7810** | **0** | **(27)** | **4** | **1** | **R.MFGGPGTASRPSSSR.S + Oxidation (M)** |
|  | 1732 | **509.3900** | **1525.1477** | **1524.6296** | **0.5181** | **1** | **(30)** | **2.2** | **1** | **K.NLQEAEEWYKSK.F** |
|  | 3262 | **763.6478** | **1525.2809** | **1524.6296** | **0.6512** | **1** | **60** | **0.0022** | **1** | **K.NLQEAEEWYKSK.F** |
|  | 3273 | **767.4732** | **1532.9316** | **1533.7640** | **-0.8324** | **1** | **56** | **0.006** | **1** | **R.KVESLQEEIAFLK.K** |
|  | 1744 | **512.5246** | **1534.5516** | **1533.7640** | **0.7876** | **1** | **(44)** | **0.11** | **1** | **R.KVESLQEEIAFLK.K** |
|  | 1885 | **530.5061** | **1588.4961** | **1587.6872** | **0.8089** | **1** | **34** | **1.2** | **1** | **R.TNEKVELQELNDR.F** |
|  | 2036 | **551.4794** | **1651.4159** | **1650.8177** | **0.5983** | **1** | **(22)** | **14** | **1** | **R.RMFGGPGTASRPSSSR.S** |
|  | 2059 | **555.1577** | **1662.4510** | **1661.9363** | **0.5146** | **2** | **52** | **0.016** | **1** | **R.KVESLQEEIAFLKK.L** |
|  | 3468 | **834.2979** | **1666.5809** | **1666.8171** | **-0.2362** | **1** | **26** | **7** | **1** | **R.RMFGGPGTASRPSSSR.S + Oxidation (M)** |
|  | 2070 | **556.7693** | **1667.2857** | **1666.8171** | **0.4686** | **1** | **(6)** | **6e+02** | **5** | **R.RMFGGPGTASRPSSSR.S + Oxidation (M)** |
|  | 3471 | **835.1633** | **1668.3119** | **1668.8229** | **-0.5111** | **1** | **(12)** | **1.2e+02** | **1** | **R.LGDLYEEEMRELR.R + Oxidation (M)** |
|  | 2074 | **557.2157** | **1668.6249** | **1668.8229** | **-0.1980** | **1** | **(33)** | **1.4** | **1** | **R.LGDLYEEEMRELR.R + Oxidation (M)** |
|  | 3472 | **835.6997** | **1669.3846** | **1668.8229** | **0.5617** | **1** | **(5)** | **6.1e+02** | **2** | **R.LGDLYEEEMRELR.R + Oxidation (M)** |
|  | 2076 | **557.5576** | **1669.6507** | **1668.8229** | **0.8277** | **1** | **(30)** | **2.5** | **1** | **R.LGDLYEEEMRELR.R + Oxidation (M)** |
|  | 2077 | **557.6202** | **1669.8384** | **1668.8229** | **1.0154** | **1** | **46** | **0.085** | **1** | **R.LGDLYEEEMRELR.R + Oxidation (M)** |
|  | 2143 | **569.3221** | **1704.9443** | **1704.8569** | **0.0874** | **1** | **(45)** | **0.093** | **1** | **R.VEVERDNLAEDIMR.L + Oxidation (M)** |
|  | 2145 | **569.4583** | **1705.3526** | **1704.8569** | **0.4957** | **1** | **55** | **0.0077** | **1** | **R.VEVERDNLAEDIMR.L + Oxidation (M)** |
|  | 3530 | **889.3395** | **1776.6642** | **1776.8612** | **-0.1971** | **1** | **(48)** | **0.038** | **1** | **K.FADLSEAANRNNDALR.Q** |
|  | 2281 | **593.5367** | **1777.5881** | **1776.8612** | **0.7268** | **1** | **(52)** | **0.014** | **1** | **K.FADLSEAANRNNDALR.Q** |
|  | 2282 | **593.5908** | **1777.7503** | **1776.8612** | **0.8891** | **1** | **56** | **0.0065** | **1** | **K.FADLSEAANRNNDALR.Q** |
|  | 2397 | **609.3042** | **1824.8904** | **1825.0086** | **-0.1182** | **2** | **(43)** | **0.15** | **1** | **R.LGDLYEEEMRELRR.Q + Oxidation (M)** |
|  | 2398 | **609.3533** | **1825.0376** | **1825.0086** | **0.0290** | **2** | **56** | **0.0076** | **1** | **R.LGDLYEEEMRELRR.Q + Oxidation (M)** |
|  | 2400 | **609.5724** | **1825.6950** | **1824.9854** | **0.7095** | **1** | **33** | **1.3** | **1** | **R.ETNLDSLPLVDTHSKR.T** |
|  | 2401 | **609.6833** | **1826.0277** | **1825.0086** | **1.0191** | **2** | **(44)** | **0.13** | **1** | **R.LGDLYEEEMRELRR.Q + Oxidation (M)** |
|  | 2402 | **609.6973** | **1826.0698** | **1825.0086** | **1.0612** | **2** | **(27)** | **7.3** | **1** | **R.LGDLYEEEMRELRR.Q + Oxidation (M)** |
|  | 2444 | **613.2791** | **1836.8152** | **1836.8238** | **-0.0087** | **0** | **(25)** | **8.6** | **1** | **R.DGQVINETSQHHDDLE.-** |
|  | 3547 | **919.6611** | **1837.3075** | **1836.8238** | **0.4836** | **0** | **66** | **0.00064** | **1** | **R.DGQVINETSQHHDDLE.-** |
|  | 3548 | **919.6991** | **1837.3834** | **1836.8238** | **0.5596** | **0** | **(49)** | **0.034** | **1** | **R.DGQVINETSQHHDDLE.-** |
|  | 2450 | **613.6031** | **1837.7873** | **1836.8238** | **0.9634** | **0** | **(24)** | **10** | **1** | **R.DGQVINETSQHHDDLE.-** |
|  | 3559 | **965.3582** | **1928.7015** | **1929.0949** | **-0.3934** | **1** | **43** | **0.11** | **1** | **R.SLYASSPGGVYATRSSAVR.L** |
|  | 2626 | **644.2406** | **1929.6996** | **1929.0949** | **0.6047** | **1** | **(15)** | **85** | **1** | **R.SLYASSPGGVYATRSSAVR.L** |
|  | 2627 | **644.3286** | **1929.9637** | **1929.0949** | **0.8687** | **1** | **(24)** | **12** | **1** | **R.SLYASSPGGVYATRSSAVR.L** |
|  | 2731 | **664.9528** | **1991.8361** | **1992.1108** | **-0.2747** | **2** | **20** | **20** | **1** | **K.SKFADLSEAANRNNDALR.Q** |
|  | 2733 | **665.1976** | **1992.5705** | **1992.1108** | **0.4597** | **2** | **(18)** | **40** | **1** | **K.SKFADLSEAANRNNDALR.Q** |
|  | 2972 | **702.1501** | **2103.4283** | **2104.2406** | **-0.8124** | **2** | **(70)** | **0.00024** | **1** | **K.FADLSEAANRNNDALRQAK.Q** |
|  | 2975 | **702.6874** | **2105.0401** | **2104.2406** | **0.7995** | **2** | **83** | **1.2e-05** | **1** | **K.FADLSEAANRNNDALRQAK.Q** |
|  | 3055 | **733.6553** | **2197.9436** | **2198.4382** | **-0.4946** | **2** | **(13)** | **1.1e+02** | **1** | **R.SLYASSPGGVYATRSSAVRLR.S** |
|  | 3057 | **734.0897** | **2199.2470** | **2198.4382** | **0.8088** | **2** | **15** | **67** | **1** | **R.SLYASSPGGVYATRSSAVRLR.S** |
|  | 3346 | **793.2315** | **2376.6723** | **2377.5844** | **-0.9121** | **1** | **(41)** | **0.21** | **1** | **R.QVQSLTCEVDALKGTNESLER.Q + Carbamidomethyl (C)** |
|  | 3347 | **793.6980** | **2378.0718** | **2377.5844** | **0.4874** | **1** | **(68)** | **0.00031** | **1** | **R.QVQSLTCEVDALKGTNESLER.Q + Carbamidomethyl (C)** |
|  | 3348 | **793.7485** | **2378.2232** | **2377.5844** | **0.6388** | **1** | **79** | **2.6e-05** | **1** | **R.QVQSLTCEVDALKGTNESLER.Q + Carbamidomethyl (C)** |
|  | 3349 | **793.7788** | **2378.3143** | **2377.5844** | **0.7298** | **1** | **(52)** | **0.014** | **1** | **R.QVQSLTCEVDALKGTNESLER.Q + Carbamidomethyl (C)** |
|  | 3603 | **1190.3260** | **2378.6373** | **2377.5844** | **1.0529** | **1** | **(73)** | **0.00013** | **1** | **R.QVQSLTCEVDALKGTNESLER.Q + Carbamidomethyl (C)** |
|  | 3361 | **798.1281** | **2391.3620** | **2391.5929** | **-0.2309** | **1** | **(34)** | **0.9** | **1** | **R.SYVTTSTRTYSLGSALRPSTSR.S** |
|  | 3604 | **1197.0317** | **2392.0487** | **2391.5929** | **0.4558** | **1** | **(9)** | **2.2e+02** | **1** | **R.SYVTTSTRTYSLGSALRPSTSR.S** |
|  | 3365 | **798.3941** | **2392.1601** | **2391.5929** | **0.5672** | **1** | **(14)** | **1.1e+02** | **1** | **R.SYVTTSTRTYSLGSALRPSTSR.S** |
|  | 3366 | **798.4783** | **2392.4128** | **2391.5929** | **0.8199** | **1** | **38** | **0.4** | **1** | **R.SYVTTSTRTYSLGSALRPSTSR.S** |
|  | 3367 | **798.4875** | **2392.4403** | **2391.5929** | **0.8474** | **1** | **(31)** | **2.1** | **1** | **R.SYVTTSTRTYSLGSALRPSTSR.S** |
|  | 3368 | **798.4979** | **2392.4714** | **2391.5929** | **0.8785** | **1** | **(26)** | **5.8** | **1** | **R.SYVTTSTRTYSLGSALRPSTSR.S** |
|  | 3405 | **808.7103** | **2423.1088** | **2423.4624** | **-0.3536** | **1** | **52** | **0.014** | **1** | **K.TVETRDGQVINETSQHHDDLE.-** |
|  | 3407 | **808.8766** | **2423.6078** | **2423.4624** | **0.1453** | **1** | **(45)** | **0.088** | **1** | **K.TVETRDGQVINETSQHHDDLE.-** |
|  | 3408 | **809.0497** | **2424.1269** | **2423.4624** | **0.6644** | **1** | **(46)** | **0.055** | **1** | **K.TVETRDGQVINETSQHHDDLE.-** |
|  | 3409 | **809.2024** | **2424.5850** | **2423.4624** | **1.1226** | **1** | **(44)** | **0.091** | **1** | **K.TVETRDGQVINETSQHHDDLE.-** |
|  | 3521 | **879.1796** | **2634.5165** | **2634.8094** | **-0.2929** | **1** | **18** | **33** | **1** | **R.QMREMEENFAVEAANYQDTIGR.L + 2 Oxidation (M)** |
|  | 3561 | **969.5146** | **2905.5218** | **2906.1669** | **-0.6451** | **1** | **(25)** | **8** | **1** | **R.TYSLGSALRPSTSRSLYASSPGGVYATR.S** |
|  | 3563 | **969.8363** | **2906.4867** | **2906.1669** | **0.3198** | **1** | **29** | **2.6** | **1** | **R.TYSLGSALRPSTSRSLYASSPGGVYATR.S** |
|  | 3573 | **998.1837** | **2991.5290** | **2992.2114** | **-0.6824** | **2** | **(60)** | **0.0028** | **1** | **R.TLLIKTVETRDGQVINETSQHHDDLE.-** |
|  | 3574 | **998.2974** | **2991.8699** | **2992.2114** | **-0.3415** | **2** | **(61)** | **0.0015** | **1** | **R.TLLIKTVETRDGQVINETSQHHDDLE.-** |
|  | 3575 | **998.5247** | **2992.5518** | **2992.2114** | **0.3404** | **2** | **61** | **0.0017** | **1** | **R.TLLIKTVETRDGQVINETSQHHDDLE.-** |

  

|  |  |
| --- | --- |
|  | |
|  | **Proteins matching the same set of peptides:** |

|  |  |
| --- | --- |
|  | gi|62414289    **Mass:** 53651    **Score:** 1338   **Queries matched:** 99 |
|  | vimentin [Homo sapiens] |

---

|  |  |
| --- | --- |
| **2.** | gi|193787214    **Mass:** 46976    **Score:** 1079   **Queries matched:** 81   **emPAI:** 4.10 |
|  | unnamed protein product [Homo sapiens] |

|  |  |
| --- | --- |
|  | Check to include this hit in error tolerant search or archive report |
|  |  |

|  |  |  |  |  |  |  |  |  |  |  |
| --- | --- | --- | --- | --- | --- | --- | --- | --- | --- | --- |
|  | **Query** | **Observed** | **Mr(expt)** | **Mr(calc)** | **Delta** | **Miss** | **Score** | **Expect** | **Rank** | **Peptide** |
|  | 1279 | 458.2179 | 914.4209 | 913.9716 | 0.4493 | 0 | 24 | 12 | 1 | R.SYVTTSTR.T |
|  | 1565 | 485.9523 | 969.8899 | 970.1276 | -0.2377 | 1 | 32 | 1.4 | 1 | R.LRSSVPGVR.L |
|  | 2691 | 655.6322 | 1309.2496 | 1309.3800 | -0.1304 | 0 | 31 | 1.7 | 1 | K.NLQEAEEWYK.S |
|  | 2872 | **682.6369** | **1363.2590** | **1363.4359** | **-0.1768** | **0** | **(25)** | **6.6** | **1** | **M.FGGPGTASRPSSSR.S** |
|  | 1252 | **455.7674** | **1364.2801** | **1363.4359** | **0.8442** | **0** | **40** | **0.23** | **1** | **M.FGGPGTASRPSSSR.S** |
|  | 1381 | 465.8632 | 1394.5675 | 1393.5016 | 1.0658 | 1 | 25 | 9 | 1 | R.DVRQQYESVAAK.N |
|  | 3000 | 714.6857 | 1427.3566 | 1428.5456 | -1.1891 | 0 | (11) | 1.9e+02 | 1 | R.SLYASSPGGVYATR.S |
|  | 3001 | 714.9583 | 1427.9018 | 1428.5456 | -0.6438 | 0 | (20) | 22 | 1 | R.SLYASSPGGVYATR.S |
|  | 3003 | 715.1034 | 1428.1920 | 1428.5456 | -0.3536 | 0 | (37) | 0.48 | 1 | R.SLYASSPGGVYATR.S |
|  | 3005 | 715.1392 | 1428.2635 | 1428.5456 | -0.2821 | 0 | (65) | 0.00081 | 1 | R.SLYASSPGGVYATR.S |
|  | 3006 | 715.2098 | 1428.4049 | 1428.5456 | -0.1407 | 0 | (56) | 0.0068 | 1 | R.SLYASSPGGVYATR.S |
|  | 3007 | 715.2242 | 1428.4337 | 1428.5456 | -0.1119 | 0 | 79 | 3.6e-05 | 1 | R.SLYASSPGGVYATR.S |
|  | 3008 | 715.2937 | 1428.5726 | 1428.5456 | 0.0270 | 0 | (37) | 0.49 | 1 | R.SLYASSPGGVYATR.S |
|  | 3010 | 715.4422 | 1428.8696 | 1428.5456 | 0.3240 | 0 | (24) | 11 | 1 | R.SLYASSPGGVYATR.S |
|  | 3011 | 715.5910 | 1429.1672 | 1428.5456 | 0.6216 | 0 | (21) | 20 | 1 | R.SLYASSPGGVYATR.S |
|  | 3012 | 715.6136 | 1429.2125 | 1428.5456 | 0.6669 | 0 | (49) | 0.029 | 1 | R.SLYASSPGGVYATR.S |
|  | 3014 | 715.7111 | 1429.4073 | 1428.5456 | 0.8617 | 0 | (61) | 0.0022 | 1 | R.SLYASSPGGVYATR.S |
|  | 3015 | 715.8400 | 1429.6653 | 1428.5456 | 1.1196 | 0 | (77) | 6e-05 | 1 | R.SLYASSPGGVYATR.S |
|  | 1613 | 491.1848 | 1470.5323 | 1470.6769 | -0.1446 | 2 | (27) | 4.9 | 1 | R.SSAVRLRSSVPGVR.L |
|  | 3061 | 736.2912 | 1470.5676 | 1470.6769 | -0.1092 | 2 | (12) | 1.6e+02 | 2 | R.SSAVRLRSSVPGVR.L |
|  | 1615 | 491.2809 | 1470.8207 | 1470.6769 | 0.1438 | 2 | 28 | 3.7 | 2 | R.SSAVRLRSSVPGVR.L |
|  | 1617 | 491.5699 | 1471.6877 | 1470.6769 | 1.0108 | 2 | (28) | 5.1 | 1 | R.SSAVRLRSSVPGVR.L |
|  | 1658 | 499.2558 | 1494.7451 | 1494.6320 | 0.1132 | 0 | (24) | 11 | 1 | -.MFGGPGTASRPSSSR.S |
|  | 1659 | 499.2778 | 1494.8113 | 1494.6320 | 0.1794 | 0 | (27) | 4.6 | 1 | -.MFGGPGTASRPSSSR.S |
|  | 1660 | 499.4523 | 1495.3347 | 1495.6365 | -0.3018 | 0 | (2) | 1.3e+03 | 8 | R.TYSLGSALRPSTSR.S |
|  | 3174 | 748.7148 | 1495.4148 | 1494.6320 | 0.7828 | 0 | (30) | 2.4 | 1 | -.MFGGPGTASRPSSSR.S |
|  | 3175 | 748.7802 | 1495.5455 | 1495.6365 | -0.0910 | 0 | (20) | 28 | 1 | R.TYSLGSALRPSTSR.S |
|  | 3176 | 748.8155 | 1495.6162 | 1495.6365 | -0.0203 | 0 | (41) | 0.23 | 1 | R.TYSLGSALRPSTSR.S |
|  | 3177 | 748.8173 | 1495.6199 | 1495.6365 | -0.0167 | 0 | 41 | 0.22 | 1 | R.TYSLGSALRPSTSR.S |
|  | 1664 | 499.9474 | 1496.8202 | 1495.6365 | 1.1836 | 0 | (11) | 2.1e+02 | 2 | R.TYSLGSALRPSTSR.S |
|  | 1688 | 504.1835 | 1509.5282 | 1510.6314 | -1.1031 | 0 | 51 | 0.021 | 1 | -.MFGGPGTASRPSSSR.S + Oxidation (M) |
|  | 3223 | 756.2562 | 1510.4977 | 1510.6314 | -0.1337 | 0 | (33) | 1.3 | 1 | -.MFGGPGTASRPSSSR.S + Oxidation (M) |
|  | 3224 | 756.2725 | 1510.5303 | 1510.6314 | -0.1011 | 0 | (18) | 36 | 1 | -.MFGGPGTASRPSSSR.S + Oxidation (M) |
|  | 1690 | 504.6530 | 1510.9370 | 1510.6314 | 0.3056 | 0 | (34) | 1.2 | 1 | -.MFGGPGTASRPSSSR.S + Oxidation (M) |
|  | 1693 | 504.7305 | 1511.1693 | 1510.6314 | 0.5379 | 0 | (38) | 0.37 | 1 | -.MFGGPGTASRPSSSR.S + Oxidation (M) |
|  | 1694 | 504.7505 | 1511.2293 | 1510.6314 | 0.5979 | 0 | (15) | 62 | 1 | -.MFGGPGTASRPSSSR.S + Oxidation (M) |
|  | 1695 | 504.7949 | 1511.3624 | 1510.6314 | 0.7310 | 0 | (46) | 0.053 | 1 | -.MFGGPGTASRPSSSR.S + Oxidation (M) |
|  | 3225 | 756.7136 | 1511.4123 | 1510.6314 | 0.7810 | 0 | (27) | 4 | 1 | -.MFGGPGTASRPSSSR.S + Oxidation (M) |
|  | 1732 | 509.3900 | 1525.1477 | 1524.6296 | 0.5181 | 1 | (30) | 2.2 | 1 | K.NLQEAEEWYKSK.F |
|  | 3262 | 763.6478 | 1525.2809 | 1524.6296 | 0.6512 | 1 | 60 | 0.0022 | 1 | K.NLQEAEEWYKSK.F |
|  | 3273 | 767.4732 | 1532.9316 | 1533.7640 | -0.8324 | 1 | 56 | 0.006 | 1 | R.KVESLQEEIAFLK.K |
|  | 1744 | 512.5246 | 1534.5516 | 1533.7640 | 0.7876 | 1 | (44) | 0.11 | 1 | R.KVESLQEEIAFLK.K |
|  | 1885 | 530.5061 | 1588.4961 | 1587.6872 | 0.8089 | 1 | 34 | 1.2 | 1 | R.TNEKVELQELNDR.F |
|  | 2059 | 555.1577 | 1662.4510 | 1661.9363 | 0.5146 | 2 | 52 | 0.016 | 1 | R.KVESLQEEIAFLKK.L |
|  | 3471 | 835.1633 | 1668.3119 | 1668.8229 | -0.5111 | 1 | (12) | 1.2e+02 | 1 | R.LGDLYEEEMRELR.R + Oxidation (M) |
|  | 2074 | 557.2157 | 1668.6249 | 1668.8229 | -0.1980 | 1 | (33) | 1.4 | 1 | R.LGDLYEEEMRELR.R + Oxidation (M) |
|  | 3472 | 835.6997 | 1669.3846 | 1668.8229 | 0.5617 | 1 | (5) | 6.1e+02 | 2 | R.LGDLYEEEMRELR.R + Oxidation (M) |
|  | 2076 | 557.5576 | 1669.6507 | 1668.8229 | 0.8277 | 1 | (30) | 2.5 | 1 | R.LGDLYEEEMRELR.R + Oxidation (M) |
|  | 2077 | 557.6202 | 1669.8384 | 1668.8229 | 1.0154 | 1 | 46 | 0.085 | 1 | R.LGDLYEEEMRELR.R + Oxidation (M) |
|  | 2143 | 569.3221 | 1704.9443 | 1704.8569 | 0.0874 | 1 | (45) | 0.093 | 1 | R.VEVERDNLAEDIMR.L + Oxidation (M) |
|  | 2145 | 569.4583 | 1705.3526 | 1704.8569 | 0.4957 | 1 | 55 | 0.0077 | 1 | R.VEVERDNLAEDIMR.L + Oxidation (M) |
|  | 3530 | 889.3395 | 1776.6642 | 1776.8612 | -0.1971 | 1 | (48) | 0.038 | 1 | K.FADLSEAANRNNDALR.Q |
|  | 2281 | 593.5367 | 1777.5881 | 1776.8612 | 0.7268 | 1 | (52) | 0.014 | 1 | K.FADLSEAANRNNDALR.Q |
|  | 2282 | 593.5908 | 1777.7503 | 1776.8612 | 0.8891 | 1 | 56 | 0.0065 | 1 | K.FADLSEAANRNNDALR.Q |
|  | 2397 | 609.3042 | 1824.8904 | 1825.0086 | -0.1182 | 2 | (43) | 0.15 | 1 | R.LGDLYEEEMRELRR.Q + Oxidation (M) |
|  | 2398 | 609.3533 | 1825.0376 | 1825.0086 | 0.0290 | 2 | 56 | 0.0076 | 1 | R.LGDLYEEEMRELRR.Q + Oxidation (M) |
|  | 2401 | 609.6833 | 1826.0277 | 1825.0086 | 1.0191 | 2 | (44) | 0.13 | 1 | R.LGDLYEEEMRELRR.Q + Oxidation (M) |
|  | 2402 | 609.6973 | 1826.0698 | 1825.0086 | 1.0612 | 2 | (27) | 7.3 | 1 | R.LGDLYEEEMRELRR.Q + Oxidation (M) |
|  | 3559 | 965.3582 | 1928.7015 | 1929.0949 | -0.3934 | 1 | 43 | 0.11 | 1 | R.SLYASSPGGVYATRSSAVR.L |
|  | 2626 | 644.2406 | 1929.6996 | 1929.0949 | 0.6047 | 1 | (15) | 85 | 1 | R.SLYASSPGGVYATRSSAVR.L |
|  | 2627 | 644.3286 | 1929.9637 | 1929.0949 | 0.8687 | 1 | (24) | 12 | 1 | R.SLYASSPGGVYATRSSAVR.L |
|  | 2731 | 664.9528 | 1991.8361 | 1992.1108 | -0.2747 | 2 | 20 | 20 | 1 | K.SKFADLSEAANRNNDALR.Q |
|  | 2733 | 665.1976 | 1992.5705 | 1992.1108 | 0.4597 | 2 | (18) | 40 | 1 | K.SKFADLSEAANRNNDALR.Q |
|  | 2972 | 702.1501 | 2103.4283 | 2104.2406 | -0.8124 | 2 | (70) | 0.00024 | 1 | K.FADLSEAANRNNDALRQAK.Q |
|  | 2975 | 702.6874 | 2105.0401 | 2104.2406 | 0.7995 | 2 | 83 | 1.2e-05 | 1 | K.FADLSEAANRNNDALRQAK.Q |
|  | 3055 | 733.6553 | 2197.9436 | 2198.4382 | -0.4946 | 2 | (13) | 1.1e+02 | 1 | R.SLYASSPGGVYATRSSAVRLR.S |
|  | 3057 | 734.0897 | 2199.2470 | 2198.4382 | 0.8088 | 2 | 15 | 67 | 1 | R.SLYASSPGGVYATRSSAVRLR.S |
|  | 3346 | 793.2315 | 2376.6723 | 2377.5844 | -0.9121 | 1 | (41) | 0.21 | 1 | R.QVQSLTCEVDALKGTNESLER.Q + Carbamidomethyl (C) |
|  | 3347 | 793.6980 | 2378.0718 | 2377.5844 | 0.4874 | 1 | (68) | 0.00031 | 1 | R.QVQSLTCEVDALKGTNESLER.Q + Carbamidomethyl (C) |
|  | 3348 | 793.7485 | 2378.2232 | 2377.5844 | 0.6388 | 1 | 79 | 2.6e-05 | 1 | R.QVQSLTCEVDALKGTNESLER.Q + Carbamidomethyl (C) |
|  | 3349 | 793.7788 | 2378.3143 | 2377.5844 | 0.7298 | 1 | (52) | 0.014 | 1 | R.QVQSLTCEVDALKGTNESLER.Q + Carbamidomethyl (C) |
|  | 3603 | 1190.3260 | 2378.6373 | 2377.5844 | 1.0529 | 1 | (73) | 0.00013 | 1 | R.QVQSLTCEVDALKGTNESLER.Q + Carbamidomethyl (C) |
|  | 3361 | 798.1281 | 2391.3620 | 2391.5929 | -0.2309 | 1 | (34) | 0.9 | 1 | R.SYVTTSTRTYSLGSALRPSTSR.S |
|  | 3604 | 1197.0317 | 2392.0487 | 2391.5929 | 0.4558 | 1 | (9) | 2.2e+02 | 1 | R.SYVTTSTRTYSLGSALRPSTSR.S |
|  | 3365 | 798.3941 | 2392.1601 | 2391.5929 | 0.5672 | 1 | (14) | 1.1e+02 | 1 | R.SYVTTSTRTYSLGSALRPSTSR.S |
|  | 3366 | 798.4783 | 2392.4128 | 2391.5929 | 0.8199 | 1 | 38 | 0.4 | 1 | R.SYVTTSTRTYSLGSALRPSTSR.S |
|  | 3367 | 798.4875 | 2392.4403 | 2391.5929 | 0.8474 | 1 | (31) | 2.1 | 1 | R.SYVTTSTRTYSLGSALRPSTSR.S |
|  | 3368 | 798.4979 | 2392.4714 | 2391.5929 | 0.8785 | 1 | (26) | 5.8 | 1 | R.SYVTTSTRTYSLGSALRPSTSR.S |
|  | 3521 | 879.1796 | 2634.5165 | 2634.8094 | -0.2929 | 1 | 18 | 33 | 1 | R.QMREMEENFAVEAANYQDTIGR.L + 2 Oxidation (M) |
|  | 3561 | 969.5146 | 2905.5218 | 2906.1669 | -0.6451 | 1 | (25) | 8 | 1 | R.TYSLGSALRPSTSRSLYASSPGGVYATR.S |
|  | 3563 | 969.8363 | 2906.4867 | 2906.1669 | 0.3198 | 1 | 29 | 2.6 | 1 | R.TYSLGSALRPSTSRSLYASSPGGVYATR.S |

  


---

|  |  |
| --- | --- |
| **3.** | gi|5030431    **Mass:** 41562    **Score:** 792    **Queries matched:** 42   **emPAI:** 2.68 |
|  | vimentin [Homo sapiens] |

|  |  |
| --- | --- |
|  | Check to include this hit in error tolerant search or archive report |
|  |  |

|  |  |  |  |  |  |  |  |  |  |  |
| --- | --- | --- | --- | --- | --- | --- | --- | --- | --- | --- |
|  | **Query** | **Observed** | **Mr(expt)** | **Mr(calc)** | **Delta** | **Miss** | **Score** | **Expect** | **Rank** | **Peptide** |
|  | 2229 | 587.3734 | 1172.7319 | 1173.4028 | -0.6709 | 1 | 51 | 0.021 | 1 | R.TLLIKTVETR.D |
|  | 2231 | 587.3816 | 1172.7484 | 1173.4028 | -0.6544 | 1 | (12) | 1.6e+02 | 1 | R.TLLIKTVETR.D |
|  | 440 | 392.2943 | 1173.8609 | 1173.4028 | 0.4580 | 1 | (31) | 1.7 | 1 | R.TLLIKTVETR.D |
|  | 442 | 392.3950 | 1174.1627 | 1173.4028 | 0.7599 | 1 | (21) | 21 | 1 | R.TLLIKTVETR.D |
|  | 961 | **428.2896** | **1281.8465** | **1281.4628** | **0.3837** | **2** | **9** | **2.1e+02** | **2** | **-.RFANYIDKVR.F** |
|  | 2691 | 655.6322 | 1309.2496 | 1309.3800 | -0.1304 | 0 | 31 | 1.7 | 1 | K.NLQEAEEWYK.S |
|  | 1158 | 444.5180 | 1330.5318 | 1329.5885 | 0.9433 | 2 | 16 | 84 | 2 | K.RTLLIKTVETR.D |
|  | 1732 | 509.3900 | 1525.1477 | 1524.6296 | 0.5181 | 1 | (30) | 2.2 | 1 | K.NLQEAEEWYKSK.F |
|  | 3262 | 763.6478 | 1525.2809 | 1524.6296 | 0.6512 | 1 | 60 | 0.0022 | 1 | K.NLQEAEEWYKSK.F |
|  | 3273 | 767.4732 | 1532.9316 | 1533.7640 | -0.8324 | 1 | 56 | 0.006 | 1 | R.KVESLQEEIAFLK.K |
|  | 1744 | 512.5246 | 1534.5516 | 1533.7640 | 0.7876 | 1 | (44) | 0.11 | 1 | R.KVESLQEEIAFLK.K |
|  | 2059 | 555.1577 | 1662.4510 | 1661.9363 | 0.5146 | 2 | 52 | 0.016 | 1 | R.KVESLQEEIAFLKK.L |
|  | 3471 | 835.1633 | 1668.3119 | 1668.8229 | -0.5111 | 1 | (12) | 1.2e+02 | 1 | R.LGDLYEEEMRELR.R + Oxidation (M) |
|  | 2074 | 557.2157 | 1668.6249 | 1668.8229 | -0.1980 | 1 | (33) | 1.4 | 1 | R.LGDLYEEEMRELR.R + Oxidation (M) |
|  | 3472 | 835.6997 | 1669.3846 | 1668.8229 | 0.5617 | 1 | (5) | 6.1e+02 | 2 | R.LGDLYEEEMRELR.R + Oxidation (M) |
|  | 2076 | 557.5576 | 1669.6507 | 1668.8229 | 0.8277 | 1 | (30) | 2.5 | 1 | R.LGDLYEEEMRELR.R + Oxidation (M) |
|  | 2077 | 557.6202 | 1669.8384 | 1668.8229 | 1.0154 | 1 | 46 | 0.085 | 1 | R.LGDLYEEEMRELR.R + Oxidation (M) |
|  | 2143 | 569.3221 | 1704.9443 | 1704.8569 | 0.0874 | 1 | (45) | 0.093 | 1 | R.VEVERDNLAEDIMR.L + Oxidation (M) |
|  | 2145 | 569.4583 | 1705.3526 | 1704.8569 | 0.4957 | 1 | 55 | 0.0077 | 1 | R.VEVERDNLAEDIMR.L + Oxidation (M) |
|  | 3530 | 889.3395 | 1776.6642 | 1776.8612 | -0.1971 | 1 | (48) | 0.038 | 1 | K.FADLSEAANRNNDALR.Q |
|  | 2281 | 593.5367 | 1777.5881 | 1776.8612 | 0.7268 | 1 | (52) | 0.014 | 1 | K.FADLSEAANRNNDALR.Q |
|  | 2282 | 593.5908 | 1777.7503 | 1776.8612 | 0.8891 | 1 | 56 | 0.0065 | 1 | K.FADLSEAANRNNDALR.Q |
|  | 2397 | 609.3042 | 1824.8904 | 1825.0086 | -0.1182 | 2 | (43) | 0.15 | 1 | R.LGDLYEEEMRELRR.Q + Oxidation (M) |
|  | 2398 | 609.3533 | 1825.0376 | 1825.0086 | 0.0290 | 2 | 56 | 0.0076 | 1 | R.LGDLYEEEMRELRR.Q + Oxidation (M) |
|  | 2400 | 609.5724 | 1825.6950 | 1824.9854 | 0.7095 | 1 | 33 | 1.3 | 1 | R.ETNLDSLPLVDTHSKR.T |
|  | 2401 | 609.6833 | 1826.0277 | 1825.0086 | 1.0191 | 2 | (44) | 0.13 | 1 | R.LGDLYEEEMRELRR.Q + Oxidation (M) |
|  | 2402 | 609.6973 | 1826.0698 | 1825.0086 | 1.0612 | 2 | (27) | 7.3 | 1 | R.LGDLYEEEMRELRR.Q + Oxidation (M) |
|  | 2444 | 613.2791 | 1836.8152 | 1836.8238 | -0.0087 | 0 | (25) | 8.6 | 1 | R.DGQVINETSQHHDDLE.- |
|  | 3547 | 919.6611 | 1837.3075 | 1836.8238 | 0.4836 | 0 | 66 | 0.00064 | 1 | R.DGQVINETSQHHDDLE.- |
|  | 3548 | 919.6991 | 1837.3834 | 1836.8238 | 0.5596 | 0 | (49) | 0.034 | 1 | R.DGQVINETSQHHDDLE.- |
|  | 2450 | 613.6031 | 1837.7873 | 1836.8238 | 0.9634 | 0 | (24) | 10 | 1 | R.DGQVINETSQHHDDLE.- |
|  | 2731 | 664.9528 | 1991.8361 | 1992.1108 | -0.2747 | 2 | 20 | 20 | 1 | K.SKFADLSEAANRNNDALR.Q |
|  | 2733 | 665.1976 | 1992.5705 | 1992.1108 | 0.4597 | 2 | (18) | 40 | 1 | K.SKFADLSEAANRNNDALR.Q |
|  | 2972 | 702.1501 | 2103.4283 | 2104.2406 | -0.8124 | 2 | (70) | 0.00024 | 1 | K.FADLSEAANRNNDALRQAK.Q |
|  | 2975 | 702.6874 | 2105.0401 | 2104.2406 | 0.7995 | 2 | 83 | 1.2e-05 | 1 | K.FADLSEAANRNNDALRQAK.Q |
|  | 3405 | 808.7103 | 2423.1088 | 2423.4624 | -0.3536 | 1 | 52 | 0.014 | 1 | K.TVETRDGQVINETSQHHDDLE.- |
|  | 3407 | 808.8766 | 2423.6078 | 2423.4624 | 0.1453 | 1 | (45) | 0.088 | 1 | K.TVETRDGQVINETSQHHDDLE.- |
|  | 3408 | 809.0497 | 2424.1269 | 2423.4624 | 0.6644 | 1 | (46) | 0.055 | 1 | K.TVETRDGQVINETSQHHDDLE.- |
|  | 3409 | 809.2024 | 2424.5850 | 2423.4624 | 1.1226 | 1 | (44) | 0.091 | 1 | K.TVETRDGQVINETSQHHDDLE.- |
|  | 3573 | 998.1837 | 2991.5290 | 2992.2114 | -0.6824 | 2 | (60) | 0.0028 | 1 | R.TLLIKTVETRDGQVINETSQHHDDLE.- |
|  | 3574 | 998.2974 | 2991.8699 | 2992.2114 | -0.3415 | 2 | (61) | 0.0015 | 1 | R.TLLIKTVETRDGQVINETSQHHDDLE.- |
|  | 3575 | 998.5247 | 2992.5518 | 2992.2114 | 0.3404 | 2 | 61 | 0.0017 | 1 | R.TLLIKTVETRDGQVINETSQHHDDLE.- |

  


---

|  |  |
| --- | --- |
| **4.** | gi|34782901    **Mass:** 48795    **Score:** 546    **Queries matched:** 31   **emPAI:** 0.80 |
|  | ATP5A1 protein [Homo sapiens] |

|  |  |
| --- | --- |
|  | Check to include this hit in error tolerant search or archive report |
|  |  |

|  |  |  |  |  |  |  |  |  |  |  |
| --- | --- | --- | --- | --- | --- | --- | --- | --- | --- | --- |
|  | **Query** | **Observed** | **Mr(expt)** | **Mr(calc)** | **Delta** | **Miss** | **Score** | **Expect** | **Rank** | **Peptide** |
|  | 29 | **362.3192** | **722.6236** | **722.8757** | **-0.2522** | **0** | **45** | **0.066** | **1** | **K.APGIIPR.I** |
|  | 731 | **408.3027** | **814.5907** | **814.9264** | **-0.3358** | **0** | **32** | **1.4** | **1** | **R.ELIIGDR.Q** |
|  | 1748 | **513.8945** | **1025.7742** | **1026.1875** | **-0.4133** | **0** | **77** | **5.2e-05** | **1** | **K.AVDSLVPIGR.G** |
|  | 181 | **374.1740** | **1119.5000** | **1120.3880** | **-0.8881** | **1** | **(24)** | **12** | **1** | **R.VGLKAPGIIPR.I** |
|  | 182 | **374.2171** | **1119.6291** | **1120.3880** | **-0.7589** | **1** | **(4)** | **1.1e+03** | **4** | **R.VGLKAPGIIPR.I** |
|  | 183 | **374.2230** | **1119.6469** | **1120.3880** | **-0.7412** | **1** | **(11)** | **1.9e+02** | **3** | **R.VGLKAPGIIPR.I** |
|  | 184 | **374.2428** | **1119.7062** | **1120.3880** | **-0.6818** | **1** | **(9)** | **3.1e+02** | **2** | **R.VGLKAPGIIPR.I** |
|  | 185 | **374.2470** | **1119.7188** | **1120.3880** | **-0.6693** | **1** | **(14)** | **1e+02** | **1** | **R.VGLKAPGIIPR.I** |
|  | 2092 | **561.2475** | **1120.4802** | **1120.3880** | **0.0922** | **1** | **(43)** | **0.13** | **1** | **R.VGLKAPGIIPR.I** |
|  | 2093 | **561.3041** | **1120.5935** | **1120.3880** | **0.2054** | **1** | **(42)** | **0.19** | **1** | **R.VGLKAPGIIPR.I** |
|  | 192 | **374.6886** | **1121.0436** | **1120.3880** | **0.6556** | **1** | **(54)** | **0.0095** | **1** | **R.VGLKAPGIIPR.I** |
|  | 194 | **374.7598** | **1121.2572** | **1120.3880** | **0.8692** | **1** | **55** | **0.01** | **1** | **R.VGLKAPGIIPR.I** |
|  | 431 | **391.1487** | **1170.4239** | **1171.3008** | **-0.8769** | **0** | **10** | **2.2e+02** | **1** | **R.VVDALGNAIDGK.G** |
|  | 436 | **391.2175** | **1170.6304** | **1170.4021** | **0.2283** | **2** | **33** | **0.97** | **1** | **K.LIKEGDIVKR.T** |
|  | 763 | **410.8920** | **1229.6538** | **1229.3831** | **0.2707** | **1** | **15** | **91** | **1** | **R.ELIIGDRQTGK.T** |
|  | 764 | **410.9585** | **1229.8533** | **1229.3831** | **0.4702** | **1** | **(14)** | **1e+02** | **1** | **R.ELIIGDRQTGK.T** |
|  | 939 | **426.7690** | **1277.2850** | **1276.5737** | **0.7112** | **2** | **46** | **0.058** | **1** | **R.RVGLKAPGIIPR.I** |
|  | 940 | **426.7752** | **1277.3035** | **1276.5737** | **0.7298** | **2** | **(23)** | **14** | **1** | **R.RVGLKAPGIIPR.I** |
|  | 941 | **426.7785** | **1277.3132** | **1276.5737** | **0.7395** | **2** | **(36)** | **0.63** | **1** | **R.RVGLKAPGIIPR.I** |
|  | 942 | **426.8435** | **1277.5083** | **1276.5737** | **0.9346** | **2** | **(43)** | **0.13** | **1** | **R.RVGLKAPGIIPR.I** |
|  | 2891 | **684.5450** | **1367.0753** | **1367.5537** | **-0.4783** | **1** | **31** | **1.7** | **1** | **K.AVDSLVPIGRGQR.E** |
|  | 1302 | **459.1536** | **1374.4385** | **1374.6060** | **-0.1674** | **1** | **(14)** | **1.2e+02** | **8** | **R.ISVREPMQTGIK.A + Oxidation (M)** |
|  | 2934 | **688.7366** | **1375.4584** | **1374.6060** | **0.8524** | **1** | **32** | **2.1** | **1** | **R.ISVREPMQTGIK.A + Oxidation (M)** |
|  | 2935 | **688.7412** | **1375.4676** | **1374.6060** | **0.8617** | **1** | **(25)** | **9.3** | **2** | **R.ISVREPMQTGIK.A + Oxidation (M)** |
|  | 3297 | **777.3478** | **1552.6808** | **1553.6725** | **-0.9918** | **0** | **11** | **2e+02** | **3** | **R.EAYPGDVFYLHSR.L** |
|  | 1845 | **524.7727** | **1571.2959** | **1570.7493** | **0.5466** | **2** | **19** | **31** | **1** | **R.GQRELIIGDRQTGK.T** |
|  | 3421 | **813.1962** | **1624.3776** | **1624.8333** | **-0.4558** | **0** | **67** | **0.00043** | **1** | **R.TGAIVDVPVGEELLGR.V** |
|  | 3422 | **813.2565** | **1624.4982** | **1624.8333** | **-0.3352** | **0** | **(47)** | **0.056** | **1** | **R.TGAIVDVPVGEELLGR.V** |
|  | 2156 | **571.5284** | **1711.5632** | **1710.9258** | **0.6374** | **1** | **26** | **5** | **1** | **R.VVDALGNAIDGKGPIGSK.T** |
|  | 2295 | **594.8775** | **1781.6103** | **1781.0190** | **0.5913** | **1** | **10** | **2.5e+02** | **2** | **K.RTGAIVDVPVGEELLGR.V** |
|  | 3353 | **795.1943** | **2382.5607** | **2382.7782** | **-0.2175** | **2** | **47** | **0.055** | **1** | **R.ISVREPMQTGIKAVDSLVPIGR.G + Oxidation (M)** |

  

|  |  |
| --- | --- |
|  | |
|  | **Proteins matching the same set of peptides:** |

|  |  |
| --- | --- |
|  | gi|158259937    **Mass:** 54493    **Score:** 546    **Queries matched:** 31 |
|  | unnamed protein product [Homo sapiens] |

|  |  |
| --- | --- |
|  | gi|4757810    **Mass:** 59750    **Score:** 544    **Queries matched:** 31 |
|  | ATP synthase subunit alpha, mitochondrial precursor [Homo sapiens] |

|  |  |
| --- | --- |
|  | gi|15030240    **Mass:** 59808    **Score:** 544    **Queries matched:** 31 |
|  | ATP synthase, H+ transporting, mitochondrial F1 complex, alpha subunit 1, cardiac muscle [Homo sapiens] |

|  |  |
| --- | --- |
|  | gi|127798841    **Mass:** 59707    **Score:** 544    **Queries matched:** 31 |
|  | ATP synthase, H+ transporting, mitochondrial F1 complex, alpha subunit 1, cardiac muscle [Homo sapiens] |

---

|  |  |
| --- | --- |
| **5.** | gi|13129104    **Mass:** 40235    **Score:** 401    **Queries matched:** 15   **emPAI:** 0.61 |
|  | coiled-coil domain-containing protein 86 [Homo sapiens] |

|  |  |
| --- | --- |
|  | Check to include this hit in error tolerant search or archive report |
|  |  |

|  |  |  |  |  |  |  |  |  |  |  |
| --- | --- | --- | --- | --- | --- | --- | --- | --- | --- | --- |
|  | **Query** | **Observed** | **Mr(expt)** | **Mr(calc)** | **Delta** | **Miss** | **Score** | **Expect** | **Rank** | **Peptide** |
|  | 1274 | **458.1040** | **914.1932** | **913.0730** | **1.1202** | **0** | **22** | **19** | **1** | **K.AEVVQVIR.N** |
|  | 1811 | **521.7531** | **1041.4913** | **1041.2453** | **0.2461** | **1** | **59** | **0.0026** | **1** | **R.KAEVVQVIR.N** |
|  | 1962 | **539.3251** | **1076.6354** | **1077.2341** | **-0.5988** | **1** | **(13)** | **1.5e+02** | **2** | **K.QPPQQPAAKI.-** |
|  | 1964 | **539.7473** | **1077.4798** | **1077.2341** | **0.2457** | **1** | **13** | **1.1e+02** | **2** | **K.QPPQQPAAKI.-** |
|  | 534 | **402.3078** | **1203.9013** | **1203.3063** | **0.5951** | **2** | **11** | **2.2e+02** | **10** | **K.RKGSSSQAPASK.K** |
|  | 2937 | **690.2652** | **1378.5156** | **1378.5962** | **-0.0805** | **0** | **(14)** | **1e+02** | **1** | **R.FSQMLQDKPLR.T + Oxidation (M)** |
|  | 1327 | **460.7045** | **1379.0912** | **1378.5962** | **0.4951** | **0** | **33** | **1** | **1** | **R.FSQMLQDKPLR.T + Oxidation (M)** |
|  | 1426 | **470.5605** | **1408.6594** | **1407.6971** | **0.9623** | **2** | **40** | **0.28** | **1** | **K.KLNKEELPVIPK.G** |
|  | 1557 | **485.1796** | **1452.5167** | **1451.7133** | **0.8034** | **2** | **23** | **13** | **1** | **R.KAEVVQVIRNPAK.L** |
|  | 2644 | **647.8005** | **1940.3794** | **1941.1503** | **-0.7709** | **1** | **45** | **0.098** | **1** | **R.RLGGLRPESPESLTSVSR.T** |
|  | 2648 | **648.1024** | **1941.2851** | **1941.1503** | **0.1347** | **1** | **(14)** | **1e+02** | **1** | **R.RLGGLRPESPESLTSVSR.T** |
|  | 2679 | **654.1571** | **1959.4491** | **1960.2776** | **-0.8285** | **2** | **(43)** | **0.13** | **1** | **R.DTLALLQKQPPQQPAAKI.-** |
|  | 2683 | **654.5872** | **1960.7393** | **1960.2776** | **0.4617** | **2** | **71** | **0.00016** | **1** | **R.DTLALLQKQPPQQPAAKI.-** |
|  | 3497 | **852.6745** | **2555.0013** | **2555.7080** | **-0.7066** | **1** | **(66)** | **0.0006** | **1** | **R.ALVEFESNPEETREPGSPPSVQR.A** |
|  | 3498 | **852.9993** | **2555.9756** | **2555.7080** | **0.2677** | **1** | **84** | **1.1e-05** | **1** | **R.ALVEFESNPEETREPGSPPSVQR.A** |

  


---

|  |  |
| --- | --- |
| **6.** | gi|73760405    **Mass:** 50670    **Score:** 370    **Queries matched:** 10   **emPAI:** 0.37 |
|  | thymopoietin isoform beta [Homo sapiens] |

|  |  |
| --- | --- |
|  | Check to include this hit in error tolerant search or archive report |
|  |  |

|  |  |  |  |  |  |  |  |  |  |  |
| --- | --- | --- | --- | --- | --- | --- | --- | --- | --- | --- |
|  | **Query** | **Observed** | **Mr(expt)** | **Mr(calc)** | **Delta** | **Miss** | **Score** | **Expect** | **Rank** | **Peptide** |
|  | 1265 | **457.5388** | **913.0629** | **912.0449** | **1.0180** | **0** | **20** | **35** | **1** | **K.GGPLQALTR.E** |
|  | 259 | **381.7391** | **1142.1951** | **1141.3643** | **0.8308** | **2** | **36** | **0.82** | **1** | **R.AKTPVTLKQR.R** |
|  | 2933 | **688.7117** | **1375.4086** | **1374.5348** | **0.8738** | **0** | **55** | **0.0097** | **1** | **M.PEFLEDPSVLTK.D** |
|  | 1363 | **463.1016** | **1386.2825** | **1385.5258** | **0.7567** | **1** | **24** | **9** | **1** | **K.GGPLQALTRESTR.G** |
|  | 1553 | **483.3265** | **1446.9574** | **1447.6088** | **-0.6514** | **1** | **9** | **2.7e+02** | **6** | **R.MEESFSSKYVPK.Y + Oxidation (M)** |
|  | 1970 | **540.4410** | **1618.3009** | **1617.7945** | **0.5065** | **1** | **38** | **0.37** | **1** | **M.PEFLEDPSVLTKDK.L** |
|  | 3457 | **824.7311** | **1647.4475** | **1647.7392** | **-0.2917** | **0** | **92** | **1.4e-06** | **1** | **R.SSTPLPTISSSAENTR.Q** |
|  | 2330 | **598.6791** | **1793.0151** | **1793.0279** | **-0.0129** | **0** | **6** | **7.8e+02** | **5** | **K.HASPILPITEFSDIPR.R** |
|  | 3503 | **857.8336** | **2570.4787** | **2570.6795** | **-0.2008** | **1** | **(87)** | **3.8e-06** | **1** | **K.GPPDFSSDEEREPTPVLGSGAAAAGR.S** |
|  | 3505 | **858.0713** | **2571.1917** | **2570.6795** | **0.5122** | **1** | **92** | **1.6e-06** | **1** | **K.GPPDFSSDEEREPTPVLGSGAAAAGR.S** |

  


---

|  |  |
| --- | --- |
| **7.** | gi|885683    **Mass:** 75492    **Score:** 278    **Queries matched:** 6   **emPAI:** 0.19 |
|  | thymopoietin alpha [Homo sapiens] |

|  |  |
| --- | --- |
|  | Check to include this hit in error tolerant search or archive report |
|  |  |

|  |  |  |  |  |  |  |  |  |  |  |
| --- | --- | --- | --- | --- | --- | --- | --- | --- | --- | --- |
|  | **Query** | **Observed** | **Mr(expt)** | **Mr(calc)** | **Delta** | **Miss** | **Score** | **Expect** | **Rank** | **Peptide** |
|  | 2031 | **550.9249** | **1099.8351** | **1099.2612** | **0.5738** | **0** | **2** | **1.5e+03** | **7** | **K.SGIQPLCPER.S** |
|  | 2933 | 688.7117 | 1375.4086 | 1374.5348 | 0.8738 | 0 | 55 | 0.0097 | 1 | M.PEFLEDPSVLTK.D |
|  | 1970 | 540.4410 | 1618.3009 | 1617.7945 | 0.5065 | 1 | 38 | 0.37 | 1 | M.PEFLEDPSVLTKDK.L |
|  | 3457 | 824.7311 | 1647.4475 | 1647.7392 | -0.2917 | 0 | 92 | 1.4e-06 | 1 | R.SSTPLPTISSSAENTR.Q |
|  | 3503 | 857.8336 | 2570.4787 | 2570.6795 | -0.2008 | 1 | (87) | 3.8e-06 | 1 | K.GPPDFSSDEEREPTPVLGSGAAAAGR.S |
|  | 3505 | 858.0713 | 2571.1917 | 2570.6795 | 0.5122 | 1 | 92 | 1.6e-06 | 1 | K.GPPDFSSDEEREPTPVLGSGAAAAGR.S |

  

|  |  |
| --- | --- |
|  | |
|  | **Proteins matching the same set of peptides:** |

|  |  |
| --- | --- |
|  | gi|913174    **Mass:** 57648    **Score:** 278    **Queries matched:** 6 |
|  | TRPP [Homo sapiens] |

|  |  |
| --- | --- |
|  | gi|4507555    **Mass:** 75491    **Score:** 278    **Queries matched:** 6 |
|  | thymopoietin isoform alpha [Homo sapiens] |

---

|  |  |
| --- | --- |
| **8.** | gi|18088719    **Mass:** 49671    **Score:** 155    **Queries matched:** 6   **emPAI:** 0.21 |
|  | Tubulin, beta [Homo sapiens] |

|  |  |
| --- | --- |
|  | Check to include this hit in error tolerant search or archive report |
|  |  |

|  |  |  |  |  |  |  |  |  |  |  |
| --- | --- | --- | --- | --- | --- | --- | --- | --- | --- | --- |
|  | **Query** | **Observed** | **Mr(expt)** | **Mr(calc)** | **Delta** | **Miss** | **Score** | **Expect** | **Rank** | **Peptide** |
|  | 1805 | **521.1210** | **1040.2272** | **1041.1807** | **-0.9535** | **0** | **11** | **2e+02** | **7** | **K.DMMAACDPR.H + 2 Oxidation (M)** |
|  | 2200 | **580.7714** | **1159.5281** | **1159.4011** | **0.1270** | **0** | **53** | **0.011** | **1** | **K.LAVNMVPFPR.L + Oxidation (M)** |
|  | 3432 | **816.6194** | **1631.2241** | **1631.8459** | **-0.6217** | **0** | **(38)** | **0.38** | **1** | **R.AILVDLEPGTMDSVR.S + Oxidation (M)** |
|  | 3433 | **816.7033** | **1631.3918** | **1631.8459** | **-0.4540** | **0** | **47** | **0.047** | **1** | **R.AILVDLEPGTMDSVR.S + Oxidation (M)** |
|  | 2007 | **546.7794** | **1637.3161** | **1636.9135** | **0.4026** | **0** | **46** | **0.053** | **1** | **R.LHFFMPGFAPLTSR.G + Oxidation (M)** |
|  | 2008 | **546.8675** | **1637.5803** | **1636.9135** | **0.6668** | **0** | **(28)** | **3.6** | **1** | **R.LHFFMPGFAPLTSR.G + Oxidation (M)** |

  


---

|  |  |
| --- | --- |
| **9.** | gi|17066105    **Mass:** 3816172  **Score:** 139    **Queries matched:** 61 |
|  | Titin [Homo sapiens] |

|  |  |
| --- | --- |
|  | Check to include this hit in error tolerant search or archive report |
|  |  |

|  |  |  |  |  |  |  |  |  |  |  |
| --- | --- | --- | --- | --- | --- | --- | --- | --- | --- | --- |
|  | **Query** | **Observed** | **Mr(expt)** | **Mr(calc)** | **Delta** | **Miss** | **Score** | **Expect** | **Rank** | **Peptide** |
|  | 145 | **370.6359** | **739.2570** | **738.8753** | **0.3817** | **1** | **6** | **4.1e+02** | **2** | **K.LKEPPR.F** |
|  | 150 | **371.1405** | **740.2662** | **739.8601** | **0.4061** | **1** | **17** | **43** | **2** | **K.KEAPPAK.V** |
|  | 152 | **371.1479** | **740.2811** | **739.8601** | **0.4210** | **1** | **(16)** | **57** | **2** | **K.KEAPPAK.V** |
|  | 156 | **371.2527** | **740.4906** | **739.8601** | **0.6305** | **1** | **(8)** | **2.7e+02** | **6** | **K.KEAPPAK.V** |
|  | 217 | **377.1982** | **752.3817** | **752.9019** | **-0.5202** | **0** | **12** | **1.3e+02** | **6** | **K.IHVAVSK.R** |
|  | 251 | **379.9627** | **757.9107** | **758.9064** | **-0.9957** | **1** | **13** | **1.6e+02** | **1** | **R.VKGLTNK.K** |
|  | 253 | **380.0245** | **758.0342** | **758.8402** | **-0.8060** | **0** | **7** | **6.2e+02** | **8** | **K.CSSTSFK.L** |
|  | 295 | **385.2104** | **768.4061** | **768.9875** | **-0.5814** | **1** | **15** | **56** | **1** | **K.KVVAKPK.E** |
|  | 374 | **388.1563** | **774.2979** | **774.9041** | **-0.6062** | **0** | **16** | **80** | **1** | **K.EPAAFLK.R** |
|  | 377 | **388.1808** | **774.3468** | **774.9041** | **-0.5573** | **0** | **(15)** | **86** | **2** | **K.EPAAFLK.R** |
|  | 656 | **407.0448** | **812.0749** | **812.9571** | **-0.8822** | **0** | **5** | **7.5e+02** | **7** | **R.VNSRPIK.D** |
|  | 810 | **414.8762** | **827.7376** | **827.9254** | **-0.1878** | **1** | **5** | **7.5e+02** | **10** | **K.KELPEGR.W** |
|  | 884 | **419.2829** | **836.5510** | **835.9904** | **0.5606** | **1** | **8** | **3.7e+02** | **9** | **K.YKVTGLR.D** |
|  | 895 | **419.9751** | **837.9354** | **838.9912** | **-1.0558** | **1** | **7** | **4.5e+02** | **4** | **K.KVEAPPAK.V** |
|  | 1000 | **430.8234** | **859.6319** | **860.0334** | **-0.4014** | **0** | **14** | **1.3e+02** | **5** | **K.IAGSLPMR.V + Oxidation (M)** |
|  | 1111 | **438.0734** | **874.1321** | **873.9971** | **0.1350** | **2** | **18** | **50** | **4** | **K.SNKERIK.D** |
|  | 1265 | 457.5388 | 913.0629 | 912.0451 | 1.0179 | 0 | 6 | 7.6e+02 | 7 | R.NAVGVSLPR.E |
|  | 1276 | **458.1125** | **914.2102** | **913.0696** | **1.1406** | **0** | **8** | **3.8e+02** | **4** | **R.ITIENVPK.K** |
|  | 1607 | **489.7600** | **977.5053** | **978.1264** | **-0.6211** | **1** | **3** | **9.9e+02** | **8** | **K.WSECARVK.S** |
|  | 1628 | **494.1905** | **986.3663** | **985.1803** | **1.1860** | **1** | **15** | **94** | **3** | **K.VKEPPIFR.K** |
|  | 1935 | **536.4972** | **1070.9796** | **1070.1770** | **0.8026** | **1** | **6** | **5.7e+02** | **10** | **K.LDDGTYRCK.V** |
|  | 53 | **364.1732** | **1089.4973** | **1090.1851** | **-0.6878** | **1** | **11** | **1.8e+02** | **5** | **K.NGVEIKSTDK.C** |
|  | 82 | **367.8962** | **1100.6665** | **1101.3170** | **-0.6505** | **0** | **11** | **2.3e+02** | **3** | **K.DVLPGSAVCLK.S** |
|  | 2038 | **551.6353** | **1101.2558** | **1100.2296** | **1.0263** | **2** | **7** | **7e+02** | **6** | **R.GARELVKGDR.C** |
|  | 205 | **377.0463** | **1128.1166** | **1128.2793** | **-0.1627** | **1** | **12** | **1.5e+02** | **4** | **K.LTSGEAPGIRK.E** |
|  | 284 | **385.0680** | **1152.1818** | **1151.3591** | **0.8227** | **1** | **14** | **88** | **4** | **K.VHKLTIADVR.A** |
|  | 2270 | **592.9449** | **1183.8751** | **1184.3078** | **-0.4326** | **0** | **8** | **4.3e+02** | **5** | **K.GRPQATVNWR.K** |
|  | 2364 | **604.0964** | **1206.1781** | **1205.4249** | **0.7532** | **0** | **5** | **9.2e+02** | **5** | **K.LTCVVESSVLR.A** |
|  | 675 | **407.3267** | **1218.9580** | **1219.4315** | **-0.4735** | **0** | **13** | **1e+02** | **1** | **K.VLGSSIHMECK.V + Oxidation (M)** |
|  | 683 | **407.5414** | **1219.6022** | **1218.4482** | **1.1539** | **2** | **12** | **1.9e+02** | **6** | **K.GRYKIVLQNK.H** |
|  | 741 | **408.9569** | **1223.8486** | **1224.3422** | **-0.4935** | **1** | **7** | **5.8e+02** | **7** | **R.EVKSQMTETR.E + Oxidation (M)** |
|  | 803 | **413.9915** | **1238.9522** | **1238.3257** | **0.6265** | **0** | **17** | **44** | **1** | **K.SSCTAVVDVSDR.A** |
|  | 2586 | **637.1836** | **1272.3524** | **1271.3819** | **0.9706** | **1** | **9** | **3.7e+02** | **4** | **R.EERFEVLHGR.E** |
|  | 2606 | **642.1375** | **1282.2601** | **1281.4530** | **0.8072** | **0** | **3** | **1.2e+03** | **3** | **K.VLEADPYFTVK.L** |
|  | 2654 | **649.8001** | **1297.5854** | **1298.4238** | **-0.8383** | **0** | **9** | **4.1e+02** | **8** | **R.LHEGMEYTFR.V + Oxidation (M)** |
|  | 2755 | **666.7589** | **1331.5030** | **1331.4720** | **0.0310** | **0** | **5** | **1e+03** | **6** | **R.ETVTAVTVQDLR.V** |
|  | 2967 | **700.1125** | **1398.2103** | **1397.4836** | **0.7267** | **0** | **2** | **1.5e+03** | **4** | **K.DDTSTSLELFAAK.A** |
|  | 1411 | **468.4284** | **1402.2631** | **1402.5928** | **-0.3297** | **1** | **7** | **5e+02** | **5** | **K.AQVKELSSTAQLK.V** |
|  | 3198 | **749.3215** | **1496.6282** | **1496.6660** | **-0.0378** | **1** | **2** | **1.7e+03** | **6** | **K.GNEYIFRVTGVNK.Y** |
|  | 3218 | **753.0555** | **1504.0963** | **1504.6865** | **-0.5902** | **1** | **5** | **6.7e+02** | **3** | **R.ASNVAGSKSFPVNVK.V** |
|  | 1682 | **503.5047** | **1507.4918** | **1507.7333** | **-0.2415** | **1** | **8** | **4.8e+02** | **4** | **R.SPTPPSIAAKAQLAR.Q** |
|  | 1692 | **504.7005** | **1511.0793** | **1511.8049** | **-0.7255** | **1** | **3** | **1e+03** | **2** | **R.CNMKLVPELTYK.V + Carbamidomethyl (C); Oxidation (M)** |
|  | 1742 | **512.3596** | **1534.0567** | **1534.6710** | **-0.6143** | **2** | **6** | **5.7e+02** | **4** | **K.ESFPVQWKRDDK.T** |
|  | 2091 | **560.6112** | **1678.8114** | **1679.8738** | **-1.0623** | **1** | **5** | **1.2e+03** | **9** | **R.NVDSVVNGTCRLDCK.I + Carbamidomethyl (C)** |
|  | 3494 | **849.0225** | **1696.0303** | **1694.9300** | **1.1003** | **2** | **1** | **2.3e+03** | **7** | **K.DKPAVAPATKKAAVDGR.L** |
|  | 2176 | **575.9305** | **1724.7693** | **1723.9214** | **0.8479** | **0** | **6** | **6.6e+02** | **4** | **R.AGSDLVLDAAVGGKPEPK.I** |
|  | 2244 | **590.4874** | **1768.4399** | **1768.0170** | **0.4230** | **1** | **14** | **84** | **2** | **K.NEKGLSDPVTIGPITVK.E** |
|  | 2276 | **593.1105** | **1776.3094** | **1775.9547** | **0.3548** | **1** | **10** | **2.7e+02** | **10** | **K.ATNEVGSDTCSCSVKFK.E** |
|  | 2419 | **611.7848** | **1832.3322** | **1833.0060** | **-0.6738** | **1** | **(8)** | **4.8e+02** | **4** | **K.ATNEVGSDTCSCSVKFK.E + Carbamidomethyl (C)** |
|  | 2422 | **611.8551** | **1832.5431** | **1831.9980** | **0.5451** | **1** | **13** | **1.1e+02** | **1** | **R.MAHEGALTGVTTDQKEK.Q + Oxidation (M)** |
|  | 2491 | **620.3240** | **1857.9498** | **1859.1275** | **-1.1778** | **2** | **3** | **1.4e+03** | **8** | **R.IEEGKSLRFPLALEEK.Q** |
|  | 2625 | **644.1377** | **1929.3909** | **1929.1148** | **0.2761** | **1** | **4** | **9.4e+02** | **6** | **R.VCAENAAGPGKFSPPSDPK.T + Carbamidomethyl (C)** |
|  | 2670 | **652.6118** | **1954.8133** | **1954.3164** | **0.4969** | **2** | **1** | **1.7e+03** | **7** | **K.VGLKGVEFNVPRLLEGVK.Y** |
|  | 2894 | **685.0886** | **2052.2437** | **2052.2855** | **-0.0418** | **2** | **15** | **85** | **1** | **K.TIDTTAEQTSFRILEAKK.G** |
|  | 3187 | **749.1390** | **2244.3949** | **2245.5560** | **-1.1611** | **2** | **0** | **2.2e+03** | **10** | **R.VLAKNAAGVISKGSESTGPVTCR.D** |
|  | 3257 | **763.4155** | **2287.2244** | **2286.6294** | **0.5950** | **2** | **4** | **1.1e+03** | **1** | **R.VRAENRYGVSQPLVSSIIVAK.H** |
|  | 3270 | **766.5801** | **2296.7181** | **2295.5714** | **1.1466** | **2** | **9** | **2.9e+02** | **3** | **K.DCIRTDGGQYILKLSNVGGTK.S + Carbamidomethyl (C)** |
|  | 3299 | **777.4265** | **2329.2574** | **2329.6706** | **-0.4133** | **1** | **4** | **1e+03** | **6** | **K.IRIYAMNSEGLGEPALVPGTPK.A + Oxidation (M)** |
|  | 3303 | **777.7145** | **2330.1213** | **2329.5845** | **0.5367** | **1** | **5** | **6.2e+02** | **9** | **K.SSGLTDGIAYEFRVIAENMAGK.S** |
|  | 3438 | **817.2358** | **2448.6853** | **2447.8036** | **0.8818** | **2** | **2** | **1.7e+03** | **3** | **R.EPPSFVKKVDPSYLMLPGESAR.L** |
|  | 3474 | **839.8387** | **2516.4941** | **2515.7929** | **0.7011** | **1** | **15** | **67** | **2** | **R.IYAMNSEGLGEPALVPGTPKAEDR.M** |

  

|  |  |
| --- | --- |
|  | |
|  | **Proteins matching the same set of peptides:** |

|  |  |
| --- | --- |
|  | gi|108861911    **Mass:** 3816142  **Score:** 139    **Queries matched:** 61 |
|  | RecName: Full=Titin; AltName: Full=Connectin; AltName: Full=Rhabdomyosarcoma antigen MU-RMS-40.14 |

---

|  |  |
| --- | --- |
| **10.** | gi|42558279    **Mass:** 49775    **Score:** 137    **Queries matched:** 5   **emPAI:** 0.14 |
|  | tubulin beta-8 chain isoform 1 [Homo sapiens] |

|  |  |
| --- | --- |
|  | Check to include this hit in error tolerant search or archive report |
|  |  |

|  |  |  |  |  |  |  |  |  |  |  |
| --- | --- | --- | --- | --- | --- | --- | --- | --- | --- | --- |
|  | **Query** | **Observed** | **Mr(expt)** | **Mr(calc)** | **Delta** | **Miss** | **Score** | **Expect** | **Rank** | **Peptide** |
|  | 2200 | 580.7714 | 1159.5281 | 1159.4011 | 0.1270 | 0 | 53 | 0.011 | 1 | K.LAVNMVPFPR.L + Oxidation (M) |
|  | 803 | 413.9915 | 1238.9522 | 1238.4379 | 0.5143 | 1 | 16 | 61 | 2 | R.YLTAAAIFRGR.M |
|  | 3411 | **809.3981** | **1616.7815** | **1617.8194** | **-1.0379** | **0** | **22** | **17** | **1** | **R.AVLVDLEPGTMDSVR.S + Oxidation (M)** |
|  | 2007 | 546.7794 | 1637.3161 | 1636.9135 | 0.4026 | 0 | 46 | 0.053 | 1 | R.LHFFMPGFAPLTSR.G + Oxidation (M) |
|  | 2008 | 546.8675 | 1637.5803 | 1636.9135 | 0.6668 | 0 | (28) | 3.6 | 1 | R.LHFFMPGFAPLTSR.G + Oxidation (M) |

  

|  |  |
| --- | --- |
|  | |
|  | **Proteins matching the same set of peptides:** |

|  |  |
| --- | --- |
|  | gi|55962054    **Mass:** 48725    **Score:** 137    **Queries matched:** 5 |
|  | novel protein similar to beta-tubulin 4Q (TUBB4Q) (LOC253936) [Homo sapiens] |

|  |  |
| --- | --- |
|  | gi|73487292    **Mass:** 49359    **Score:** 137    **Queries matched:** 5 |
|  | TUBB8 protein [Homo sapiens] |

---

|  |  |
| --- | --- |
| **11.** | gi|7657381    **Mass:** 55180    **Score:** 134    **Queries matched:** 8   **emPAI:** 0.12 |
|  | pre-mRNA-processing factor 19 [Homo sapiens] |

|  |  |
| --- | --- |
|  | Check to include this hit in error tolerant search or archive report |
|  |  |

|  |  |  |  |  |  |  |  |  |  |  |
| --- | --- | --- | --- | --- | --- | --- | --- | --- | --- | --- |
|  | **Query** | **Observed** | **Mr(expt)** | **Mr(calc)** | **Delta** | **Miss** | **Score** | **Expect** | **Rank** | **Peptide** |
|  | 985 | **429.3219** | **856.6291** | **857.0045** | **-0.3755** | **1** | **28** | **3.2** | **1** | **R.SLKFYSL.-** |
|  | 1726 | **507.8262** | **1013.6377** | **1013.1258** | **0.5119** | **0** | **13** | **1.3e+02** | **3** | **K.FIASTGMDR.S + Oxidation (M)** |
|  | 1302 | 459.1536 | 1374.4385 | 1374.5397 | -0.1012 | 1 | (20) | 28 | 1 | K.LQDKATVLTTER.K |
|  | 2934 | 688.7366 | 1375.4584 | 1374.5397 | 0.9186 | 1 | (22) | 20 | 3 | K.LQDKATVLTTER.K |
|  | 2935 | 688.7412 | 1375.4676 | 1374.5397 | 0.9279 | 1 | 35 | 0.94 | 1 | K.LQDKATVLTTER.K |
|  | 2298 | **595.4871** | **1783.4392** | **1783.0285** | **0.4107** | **1** | **33** | **1.1** | **1** | **R.GKTVPEELVKPEELSK.Y** |
|  | 2596 | **640.1578** | **1917.4513** | **1917.1638** | **0.2875** | **1** | **(24)** | **10** | **1** | **K.TVPEELVKPEELSKYR.Q** |
|  | 2598 | **640.4520** | **1918.3339** | **1917.1638** | **1.1701** | **1** | **25** | **8.3** | **1** | **K.TVPEELVKPEELSKYR.Q** |

  

|  |  |
| --- | --- |
|  | |
|  | **Proteins matching the same set of peptides:** |

|  |  |
| --- | --- |
|  | gi|119594310    **Mass:** 45730    **Score:** 134    **Queries matched:** 8 |
|  | PRP19/PSO4 pre-mRNA processing factor 19 homolog (S. cerevisiae), isoform CRA\_a [Homo sapiens] |

---

|  |  |
| --- | --- |
| **12.** | gi|225131084    **Mass:** 3713667  **Score:** 133    **Queries matched:** 60 |
|  | titin [Homo sapiens] |

|  |  |
| --- | --- |
|  | Check to include this hit in error tolerant search or archive report |
|  |  |

|  |  |  |  |  |  |  |  |  |  |  |
| --- | --- | --- | --- | --- | --- | --- | --- | --- | --- | --- |
|  | **Query** | **Observed** | **Mr(expt)** | **Mr(calc)** | **Delta** | **Miss** | **Score** | **Expect** | **Rank** | **Peptide** |
|  | 145 | 370.6359 | 739.2570 | 738.8753 | 0.3817 | 1 | 6 | 4.1e+02 | 2 | K.LKEPPR.F |
|  | 150 | 371.1405 | 740.2662 | 739.8601 | 0.4061 | 1 | 17 | 43 | 2 | K.KEAPPAK.V |
|  | 152 | 371.1479 | 740.2811 | 739.8601 | 0.4210 | 1 | (16) | 57 | 2 | K.KEAPPAK.V |
|  | 156 | 371.2527 | 740.4906 | 739.8601 | 0.6305 | 1 | (8) | 2.7e+02 | 6 | K.KEAPPAK.V |
|  | 251 | 379.9627 | 757.9107 | 757.9198 | -0.0092 | 0 | 13 | 1.6e+02 | 1 | R.FLTLHK.V |
|  | 253 | 380.0245 | 758.0342 | 758.8402 | -0.8060 | 0 | 7 | 6.2e+02 | 8 | K.CSSTSFK.L |
|  | 295 | 385.2104 | 768.4061 | 768.9875 | -0.5814 | 1 | 15 | 56 | 1 | K.KVVAKPK.E |
|  | 374 | 388.1563 | 774.2979 | 774.9041 | -0.6062 | 0 | 16 | 80 | 1 | K.EPAAFLK.R |
|  | 377 | 388.1808 | 774.3468 | 774.9041 | -0.5573 | 0 | (15) | 86 | 2 | K.EPAAFLK.R |
|  | 656 | 407.0448 | 812.0749 | 812.9571 | -0.8822 | 0 | 5 | 7.5e+02 | 7 | R.VNSRPIK.D |
|  | 810 | 414.8762 | 827.7376 | 827.9254 | -0.1878 | 1 | 5 | 7.5e+02 | 10 | K.KELPEGR.W |
|  | 884 | 419.2829 | 836.5510 | 835.9904 | 0.5606 | 1 | 8 | 3.7e+02 | 9 | K.YKVTGLR.D |
|  | 895 | 419.9751 | 837.9354 | 838.9912 | -1.0558 | 1 | 7 | 4.5e+02 | 4 | K.KVEAPPAK.V |
|  | 1000 | 430.8234 | 859.6319 | 860.0334 | -0.4014 | 0 | 14 | 1.3e+02 | 5 | K.IAGSLPMR.V + Oxidation (M) |
|  | 1111 | 438.0734 | 874.1321 | 873.9971 | 0.1350 | 2 | 18 | 50 | 4 | K.SNKERIK.D |
|  | 1265 | 457.5388 | 913.0629 | 912.0451 | 1.0179 | 0 | 6 | 7.6e+02 | 7 | R.NAVGVSLPR.E |
|  | 1276 | 458.1125 | 914.2102 | 913.0696 | 1.1406 | 0 | 8 | 3.8e+02 | 4 | R.ITIENVPK.K |
|  | 1479 | **475.4606** | **948.9065** | **949.0836** | **-0.1771** | **1** | **8** | **4.1e+02** | **5** | **K.EGSRLEMK.V** |
|  | 1607 | 489.7600 | 977.5053 | 978.1264 | -0.6211 | 1 | 3 | 9.9e+02 | 8 | K.WSECARVK.S |
|  | 1628 | 494.1905 | 986.3663 | 985.1803 | 1.1860 | 1 | 15 | 94 | 3 | K.VKEPPIFR.K |
|  | 1935 | 536.4972 | 1070.9796 | 1070.1770 | 0.8026 | 1 | 6 | 5.7e+02 | 10 | K.LDDGTYRCK.V |
|  | 53 | 364.1732 | 1089.4973 | 1090.1851 | -0.6878 | 1 | 11 | 1.8e+02 | 5 | K.NGVEIKSTDK.C |
|  | 82 | 367.8962 | 1100.6665 | 1101.3170 | -0.6505 | 0 | 11 | 2.3e+02 | 3 | K.DVLPGSAVCLK.S |
|  | 2038 | 551.6353 | 1101.2558 | 1100.2296 | 1.0263 | 2 | 7 | 7e+02 | 6 | R.GARELVKGDR.C |
|  | 205 | 377.0463 | 1128.1166 | 1128.2793 | -0.1627 | 1 | 12 | 1.5e+02 | 4 | K.LTSGEAPGIRK.E |
|  | 284 | 385.0680 | 1152.1818 | 1151.3591 | 0.8227 | 1 | 14 | 88 | 4 | K.VHKLTIADVR.A |
|  | 2270 | 592.9449 | 1183.8751 | 1184.3078 | -0.4326 | 0 | 8 | 4.3e+02 | 5 | K.GRPQATVNWR.K |
|  | 2364 | 604.0964 | 1206.1781 | 1205.4249 | 0.7532 | 0 | 5 | 9.2e+02 | 5 | K.LTCVVESSVLR.A |
|  | 675 | 407.3267 | 1218.9580 | 1219.4315 | -0.4735 | 0 | 13 | 1e+02 | 1 | K.VLGSSIHMECK.V + Oxidation (M) |
|  | 683 | 407.5414 | 1219.6022 | 1218.4482 | 1.1539 | 2 | 12 | 1.9e+02 | 6 | K.GRYKIVLQNK.H |
|  | 741 | 408.9569 | 1223.8486 | 1224.3422 | -0.4935 | 1 | 7 | 5.8e+02 | 7 | R.EVKSQMTETR.E + Oxidation (M) |
|  | 803 | 413.9915 | 1238.9522 | 1238.3257 | 0.6265 | 0 | 17 | 44 | 1 | K.SSCTAVVDVSDR.A |
|  | 2586 | 637.1836 | 1272.3524 | 1271.3819 | 0.9706 | 1 | 9 | 3.7e+02 | 4 | R.EERFEVLHGR.E |
|  | 2606 | 642.1375 | 1282.2601 | 1281.4530 | 0.8072 | 0 | 3 | 1.2e+03 | 3 | K.VLEADPYFTVK.L |
|  | 2654 | 649.8001 | 1297.5854 | 1298.4238 | -0.8383 | 0 | 9 | 4.1e+02 | 8 | R.LHEGMEYTFR.V + Oxidation (M) |
|  | 2755 | 666.7589 | 1331.5030 | 1331.4720 | 0.0310 | 0 | 5 | 1e+03 | 6 | R.ETVTAVTVQDLR.V |
|  | 2967 | 700.1125 | 1398.2103 | 1397.4836 | 0.7267 | 0 | 2 | 1.5e+03 | 4 | K.DDTSTSLELFAAK.A |
|  | 1411 | 468.4284 | 1402.2631 | 1402.5928 | -0.3297 | 1 | 7 | 5e+02 | 5 | K.AQVKELSSTAQLK.V |
|  | 3198 | 749.3215 | 1496.6282 | 1496.6660 | -0.0378 | 1 | 2 | 1.7e+03 | 6 | K.GNEYIFRVTGVNK.Y |
|  | 3218 | 753.0555 | 1504.0963 | 1504.6865 | -0.5902 | 1 | 5 | 6.7e+02 | 3 | R.ASNVAGSKSFPVNVK.V |
|  | 1682 | 503.5047 | 1507.4918 | 1507.7333 | -0.2415 | 1 | 8 | 4.8e+02 | 4 | R.SPTPPSIAAKAQLAR.Q |
|  | 1692 | 504.7005 | 1511.0793 | 1511.8049 | -0.7255 | 1 | 3 | 1e+03 | 2 | R.CNMKLVPELTYK.V + Carbamidomethyl (C); Oxidation (M) |
|  | 1742 | 512.3596 | 1534.0567 | 1534.6710 | -0.6143 | 2 | 6 | 5.7e+02 | 4 | K.ESFPVQWKRDDK.T |
|  | 2091 | 560.6112 | 1678.8114 | 1679.8738 | -1.0623 | 1 | 5 | 1.2e+03 | 9 | R.NVDSVVNGTCRLDCK.I + Carbamidomethyl (C) |
|  | 3494 | 849.0225 | 1696.0303 | 1694.9300 | 1.1003 | 2 | 1 | 2.3e+03 | 7 | K.DKPAVAPATKKAAVDGR.L |
|  | 2176 | 575.9305 | 1724.7693 | 1723.9214 | 0.8479 | 0 | 6 | 6.6e+02 | 4 | R.AGSDLVLDAAVGGKPEPK.I |
|  | 2244 | 590.4874 | 1768.4399 | 1768.0170 | 0.4230 | 1 | 14 | 84 | 2 | K.NEKGLSDPVTIGPITVK.E |
|  | 2276 | 593.1105 | 1776.3094 | 1775.9547 | 0.3548 | 1 | 10 | 2.7e+02 | 10 | K.ATNEVGSDTCSCSVKFK.E |
|  | 2419 | 611.7848 | 1832.3322 | 1833.0060 | -0.6738 | 1 | (8) | 4.8e+02 | 4 | K.ATNEVGSDTCSCSVKFK.E + Carbamidomethyl (C) |
|  | 2422 | 611.8551 | 1832.5431 | 1831.9980 | 0.5451 | 1 | 13 | 1.1e+02 | 1 | R.MAHEGALTGVTTDQKEK.Q + Oxidation (M) |
|  | 2625 | 644.1377 | 1929.3909 | 1929.1148 | 0.2761 | 1 | 4 | 9.4e+02 | 6 | R.VCAENAAGPGKFSPPSDPK.T + Carbamidomethyl (C) |
|  | 2670 | 652.6118 | 1954.8133 | 1954.3164 | 0.4969 | 2 | 1 | 1.7e+03 | 7 | K.VGLKGVEFNVPRLLEGVK.Y |
|  | 2894 | 685.0886 | 2052.2437 | 2052.2855 | -0.0418 | 2 | 15 | 85 | 1 | K.TIDTTAEQTSFRILEAKK.G |
|  | 3187 | 749.1390 | 2244.3949 | 2245.5560 | -1.1611 | 2 | 0 | 2.2e+03 | 10 | R.VLAKNAAGVISKGSESTGPVTCR.D |
|  | 3257 | 763.4155 | 2287.2244 | 2286.6294 | 0.5950 | 2 | 4 | 1.1e+03 | 1 | R.VRAENRYGVSQPLVSSIIVAK.H |
|  | 3270 | 766.5801 | 2296.7181 | 2295.5714 | 1.1466 | 2 | 9 | 2.9e+02 | 3 | K.DCIRTDGGQYILKLSNVGGTK.S + Carbamidomethyl (C) |
|  | 3299 | 777.4265 | 2329.2574 | 2329.6706 | -0.4133 | 1 | 4 | 1e+03 | 6 | K.IRIYAMNSEGLGEPALVPGTPK.A + Oxidation (M) |
|  | 3303 | 777.7145 | 2330.1213 | 2329.5845 | 0.5367 | 1 | 5 | 6.2e+02 | 9 | K.SSGLTDGIAYEFRVIAENMAGK.S |
|  | 3438 | 817.2358 | 2448.6853 | 2447.8036 | 0.8818 | 2 | 2 | 1.7e+03 | 3 | R.EPPSFVKKVDPSYLMLPGESAR.L |
|  | 3474 | 839.8387 | 2516.4941 | 2515.7929 | 0.7011 | 1 | 15 | 67 | 2 | R.IYAMNSEGLGEPALVPGTPKAEDR.M |

  


---

|  |  |
| --- | --- |
| **13.** | gi|119631909    **Mass:** 777920   **Score:** 132    **Queries matched:** 30 |
|  | nebulin, isoform CRA\_f [Homo sapiens] |

|  |  |
| --- | --- |
|  | Check to include this hit in error tolerant search or archive report |
|  |  |

|  |  |  |  |  |  |  |  |  |  |  |
| --- | --- | --- | --- | --- | --- | --- | --- | --- | --- | --- |
|  | **Query** | **Observed** | **Mr(expt)** | **Mr(calc)** | **Delta** | **Miss** | **Score** | **Expect** | **Rank** | **Peptide** |
|  | 169 | **372.6250** | **743.2352** | **743.8918** | **-0.6566** | **1** | **22** | **16** | **6** | **R.KVQELK.T** |
|  | 517 | **401.7036** | **801.3925** | **800.9432** | **0.4493** | **2** | **13** | **1.2e+02** | **6** | **R.KKVDPSK.F** |
|  | 973 | **429.0564** | **856.0979** | **856.0480** | **0.0499** | **1** | **13** | **1.4e+02** | **9** | **K.AHMLKTR.N** |
|  | 975 | **429.0998** | **856.1849** | **856.0480** | **0.1368** | **1** | **(5)** | **7.5e+02** | **10** | **K.AHMLKTR.N** |
|  | 1055 | **434.1796** | **866.3445** | **865.9700** | **0.3744** | **0** | **12** | **1.7e+02** | **3** | **K.ALSDVAYK.K** |
|  | 1080 | **435.9347** | **869.8546** | **870.9930** | **-1.1384** | **0** | **17** | **48** | **3** | **R.QGLTLSPR.L** |
|  | 1084 | **436.1782** | **870.3417** | **870.9931** | **-0.6515** | **0** | **6** | **7.2e+02** | **5** | **R.QRPETLK.F** |
|  | 1399 | **467.0483** | **932.0818** | **931.0882** | **0.9936** | **1** | **8** | **4.5e+02** | **6** | **K.AAKLSSQVK.Y** |
|  | 1888 | **530.6460** | **1059.2772** | **1059.2605** | **0.0168** | **2** | **12** | **2.2e+02** | **1** | **K.KAAKLSSQVK.Y** |
|  | 1916 | **534.7700** | **1067.5253** | **1068.2905** | **-0.7652** | **0** | **10** | **2.3e+02** | **7** | **K.LHKPVTDMK.E** |
|  | 1923 | **536.0461** | **1070.0774** | **1070.1770** | **-0.0997** | **0** | **6** | **6.4e+02** | **5** | **R.TTENLPCHV.- + Carbamidomethyl (C)** |
|  | 88 | **368.1640** | **1101.4697** | **1102.2818** | **-0.8121** | **2** | **(7)** | **5.4e+02** | **8** | **K.KAGEILSEKK.Y** |
|  | 92 | **368.2580** | **1101.7519** | **1102.2818** | **-0.5299** | **2** | **13** | **1.1e+02** | **1** | **K.KAGEILSEKK.Y** |
|  | 635 | **406.2022** | **1215.5845** | **1216.3413** | **-0.7569** | **1** | **(10)** | **2.1e+02** | **6** | **R.QLKAAGDALSDK.L** |
|  | 640 | **406.3466** | **1216.0175** | **1216.3413** | **-0.3238** | **1** | **14** | **87** | **8** | **R.QLKAAGDALSDK.L** |
|  | 926 | **423.2154** | **1266.6240** | **1267.3452** | **-0.7211** | **1** | **10** | **2.9e+02** | **6** | **K.VTAQNSDKNYK.A** |
|  | 950 | **427.8523** | **1280.5348** | **1280.4234** | **0.1114** | **0** | **8** | **4.5e+02** | **4** | **K.VTDQISDIVYK.D** |
|  | 1049 | **433.5409** | **1297.6005** | **1296.4889** | **1.1116** | **0** | **13** | **1.5e+02** | **6** | **K.CQILVSDIDYK.H** |
|  | 1095 | **437.0404** | **1308.0990** | **1308.4798** | **-0.3808** | **1** | **3** | **1.3e+03** | **9** | **R.ATEILSDKIYR.Q** |
|  | 1647 | **496.3236** | **1485.9487** | **1486.7324** | **-0.7837** | **1** | **10** | **2.5e+02** | **2** | **K.GKMIGVLSINDDPK.M** |
|  | 1819 | **523.0095** | **1566.0064** | **1566.7575** | **-0.7511** | **0** | **4** | **1.1e+03** | **8** | **R.GLNAMANETPDFMR.A** |
|  | 1936 | **536.5109** | **1606.5104** | **1605.9212** | **0.5893** | **1** | **11** | **2e+02** | **1** | **K.LVLSMNVAKMQSER.E** |
|  | 1981 | **541.9464** | **1622.8171** | **1621.9206** | **0.8965** | **1** | **(8)** | **4e+02** | **3** | **K.LVLSMNVAKMQSER.E + Oxidation (M)** |
|  | 2153 | **570.5983** | **1708.7728** | **1709.9611** | **-1.1882** | **2** | **4** | **1.2e+03** | **6** | **K.VMKDANNLASEVKYK.A** |
|  | 2304 | **595.9377** | **1784.7911** | **1783.9879** | **0.8031** | **2** | **3** | **1.3e+03** | **8** | **K.QLGHHIGARAIRDDPK.M** |
|  | 2383 | **607.6240** | **1819.8497** | **1820.0517** | **-0.2020** | **1** | **5** | **8.6e+02** | **3** | **K.EAIGQGTPIPDLPEVKR.V** |
|  | 2404 | **609.7632** | **1826.2676** | **1826.9980** | **-0.7305** | **0** | **5** | **9.5e+02** | **3** | **K.ENLGTGIPTTVTPEIER.V** |
|  | 2784 | **668.8446** | **2003.5116** | **2003.2615** | **0.2502** | **2** | **4** | **1e+03** | **8** | **K.ENMRKATPTPVTPEMER.A + Oxidation (M)** |
|  | 3313 | **778.1982** | **2331.5726** | **2332.7594** | **-1.1868** | **2** | **5** | **7.5e+02** | **2** | **K.MKIHIVPDMVEMVTAKDSQK.K + 2 Oxidation (M)** |
|  | 3467 | **833.4502** | **2497.3284** | **2497.8457** | **-0.5172** | **2** | **10** | **2.8e+02** | **4** | **K.DKTQVHIMPDTPEIMLARQNK.I + 2 Oxidation (M)** |

  


---

|  |  |
| --- | --- |
| **14.** | gi|307358    **Mass:** 53878    **Score:** 132    **Queries matched:** 6   **emPAI:** 0.13 |
|  | peripherin [Homo sapiens] |

|  |  |
| --- | --- |
|  | Check to include this hit in error tolerant search or archive report |
|  |  |

|  |  |  |  |  |  |  |  |  |  |  |
| --- | --- | --- | --- | --- | --- | --- | --- | --- | --- | --- |
|  | **Query** | **Observed** | **Mr(expt)** | **Mr(calc)** | **Delta** | **Miss** | **Score** | **Expect** | **Rank** | **Peptide** |
|  | 2129 | **566.0983** | **1130.1819** | **1131.1989** | **-1.0170** | **1** | **10** | **2.5e+02** | **1** | **R.AGFSSTSYRR.T** |
|  | 372 | **388.1103** | **1161.3087** | **1160.2782** | **1.0305** | **0** | **10** | **3.3e+02** | **4** | **R.LLGSASPSSSVR.L** |
|  | 751 | **409.1196** | **1224.3367** | **1223.2462** | **1.0905** | **1** | **23** | **15** | **1** | **R.SELDKSSAHSY.-** |
|  | 2691 | 655.6322 | 1309.2496 | 1309.3800 | -0.1304 | 0 | 31 | 1.7 | 1 | K.NLQEAEEWYK.S |
|  | 1732 | 509.3900 | 1525.1477 | 1524.6296 | 0.5181 | 1 | (30) | 2.2 | 1 | K.NLQEAEEWYKSK.Y |
|  | 3262 | 763.6478 | 1525.2809 | 1524.6296 | 0.6512 | 1 | 60 | 0.0022 | 1 | K.NLQEAEEWYKSK.Y |

  

|  |  |
| --- | --- |
|  | |
|  | **Proteins matching the same set of peptides:** |

|  |  |
| --- | --- |
|  | gi|21264345    **Mass:** 53650    **Score:** 132    **Queries matched:** 6 |
|  | peripherin [Homo sapiens] |

|  |  |
| --- | --- |
|  | gi|119578464    **Mass:** 53778    **Score:** 132    **Queries matched:** 6 |
|  | peripherin, isoform CRA\_a [Homo sapiens] |

|  |  |
| --- | --- |
|  | gi|193785065    **Mass:** 53680    **Score:** 132    **Queries matched:** 6 |
|  | unnamed protein product [Homo sapiens] |

---

|  |  |
| --- | --- |
| **15.** | gi|119631418    **Mass:** 3881215  **Score:** 132    **Queries matched:** 61 |
|  | titin, isoform CRA\_a [Homo sapiens] |

|  |  |
| --- | --- |
|  | Check to include this hit in error tolerant search or archive report |
|  |  |

|  |  |  |  |  |  |  |  |  |  |  |
| --- | --- | --- | --- | --- | --- | --- | --- | --- | --- | --- |
|  | **Query** | **Observed** | **Mr(expt)** | **Mr(calc)** | **Delta** | **Miss** | **Score** | **Expect** | **Rank** | **Peptide** |
|  | 145 | 370.6359 | 739.2570 | 738.8753 | 0.3817 | 1 | 6 | 4.1e+02 | 2 | K.LKEPPR.F |
|  | 150 | 371.1405 | 740.2662 | 739.8601 | 0.4061 | 1 | 17 | 43 | 2 | K.KEAPPAK.V |
|  | 152 | 371.1479 | 740.2811 | 739.8601 | 0.4210 | 1 | (16) | 57 | 2 | K.KEAPPAK.V |
|  | 156 | 371.2527 | 740.4906 | 739.8601 | 0.6305 | 1 | (8) | 2.7e+02 | 6 | K.KEAPPAK.V |
|  | 251 | 379.9627 | 757.9107 | 757.9198 | -0.0092 | 0 | 13 | 1.6e+02 | 1 | R.FLTLHK.V |
|  | 253 | 380.0245 | 758.0342 | 758.8402 | -0.8060 | 0 | 7 | 6.2e+02 | 8 | K.CSSTSFK.L |
|  | 295 | 385.2104 | 768.4061 | 768.9875 | -0.5814 | 1 | 15 | 56 | 1 | K.KVVAKPK.E |
|  | 374 | 388.1563 | 774.2979 | 774.9041 | -0.6062 | 0 | 16 | 80 | 1 | K.EPAAFLK.R |
|  | 377 | 388.1808 | 774.3468 | 774.9041 | -0.5573 | 0 | (15) | 86 | 2 | K.EPAAFLK.R |
|  | 656 | 407.0448 | 812.0749 | 812.9571 | -0.8822 | 0 | 5 | 7.5e+02 | 7 | R.VNSRPIK.D |
|  | 810 | 414.8762 | 827.7376 | 827.9254 | -0.1878 | 1 | 5 | 7.5e+02 | 10 | K.KELPEGR.W |
|  | 884 | 419.2829 | 836.5510 | 835.9904 | 0.5606 | 1 | 8 | 3.7e+02 | 9 | K.YKVTGLR.D |
|  | 895 | 419.9751 | 837.9354 | 838.9912 | -1.0558 | 1 | 7 | 4.5e+02 | 4 | K.KVEAPPAK.V |
|  | 1000 | 430.8234 | 859.6319 | 860.0334 | -0.4014 | 0 | 14 | 1.3e+02 | 5 | K.IAGSLPMR.V + Oxidation (M) |
|  | 1111 | 438.0734 | 874.1321 | 873.9971 | 0.1350 | 2 | 18 | 50 | 4 | K.SNKERIK.D |
|  | 1265 | 457.5388 | 913.0629 | 912.0451 | 1.0179 | 0 | 6 | 7.6e+02 | 7 | R.NAVGVSLPR.E |
|  | 1276 | 458.1125 | 914.2102 | 913.0696 | 1.1406 | 0 | 8 | 3.8e+02 | 4 | R.ITIENVPK.K |
|  | 1479 | 475.4606 | 948.9065 | 949.0836 | -0.1771 | 1 | 8 | 4.1e+02 | 5 | K.EGSRLEMK.V |
|  | 1607 | 489.7600 | 977.5053 | 978.1264 | -0.6211 | 1 | 3 | 9.9e+02 | 8 | K.WSECARVK.S |
|  | 1628 | 494.1905 | 986.3663 | 985.1803 | 1.1860 | 1 | 15 | 94 | 3 | K.VKEPPIFR.K |
|  | 1935 | 536.4972 | 1070.9796 | 1070.1770 | 0.8026 | 1 | 6 | 5.7e+02 | 10 | K.LDDGTYRCK.V |
|  | 53 | 364.1732 | 1089.4973 | 1090.1851 | -0.6878 | 1 | 11 | 1.8e+02 | 5 | K.NGVEIKSTDK.C |
|  | 82 | 367.8962 | 1100.6665 | 1101.3170 | -0.6505 | 0 | 11 | 2.3e+02 | 3 | K.DVLPGSAVCLK.S |
|  | 2038 | 551.6353 | 1101.2558 | 1100.2296 | 1.0263 | 2 | 7 | 7e+02 | 6 | R.GARELVKGDR.C |
|  | 205 | 377.0463 | 1128.1166 | 1128.2793 | -0.1627 | 1 | 12 | 1.5e+02 | 4 | K.LTSGEAPGIRK.E |
|  | 284 | 385.0680 | 1152.1818 | 1151.3591 | 0.8227 | 1 | 14 | 88 | 4 | K.VHKLTIADVR.A |
|  | 2270 | 592.9449 | 1183.8751 | 1184.3078 | -0.4326 | 0 | 8 | 4.3e+02 | 5 | K.GRPQATVNWR.K |
|  | 2364 | 604.0964 | 1206.1781 | 1205.4249 | 0.7532 | 0 | 5 | 9.2e+02 | 5 | K.LTCVVESSVLR.A |
|  | 675 | 407.3267 | 1218.9580 | 1219.4315 | -0.4735 | 0 | 13 | 1e+02 | 1 | K.VLGSSIHMECK.V + Oxidation (M) |
|  | 683 | 407.5414 | 1219.6022 | 1218.4482 | 1.1539 | 2 | 12 | 1.9e+02 | 6 | K.GRYKIVLQNK.H |
|  | 741 | 408.9569 | 1223.8486 | 1224.3422 | -0.4935 | 1 | 7 | 5.8e+02 | 7 | R.EVKSQMTETR.E + Oxidation (M) |
|  | 803 | 413.9915 | 1238.9522 | 1238.3257 | 0.6265 | 0 | 17 | 44 | 1 | K.SSCTAVVDVSDR.A |
|  | 2586 | 637.1836 | 1272.3524 | 1271.3819 | 0.9706 | 1 | 9 | 3.7e+02 | 4 | R.EERFEVLHGR.E |
|  | 2606 | 642.1375 | 1282.2601 | 1281.4530 | 0.8072 | 0 | 3 | 1.2e+03 | 3 | K.VLEADPYFTVK.L |
|  | 2654 | 649.8001 | 1297.5854 | 1298.4238 | -0.8383 | 0 | 9 | 4.1e+02 | 8 | R.LHEGMEYTFR.V + Oxidation (M) |
|  | 2755 | 666.7589 | 1331.5030 | 1331.4720 | 0.0310 | 0 | 5 | 1e+03 | 6 | R.ETVTAVTVQDLR.V |
|  | 2967 | 700.1125 | 1398.2103 | 1397.4836 | 0.7267 | 0 | 2 | 1.5e+03 | 4 | K.DDTSTSLELFAAK.A |
|  | 1411 | 468.4284 | 1402.2631 | 1402.5928 | -0.3297 | 1 | 7 | 5e+02 | 5 | K.AQVKELSSTAQLK.V |
|  | 3198 | 749.3215 | 1496.6282 | 1496.6660 | -0.0378 | 1 | 2 | 1.7e+03 | 6 | K.GNEYIFRVTGVNK.Y |
|  | 3218 | 753.0555 | 1504.0963 | 1504.6865 | -0.5902 | 1 | 5 | 6.7e+02 | 3 | R.ASNVAGSKSFPVNVK.V |
|  | 1682 | 503.5047 | 1507.4918 | 1507.7333 | -0.2415 | 1 | 8 | 4.8e+02 | 4 | R.SPTPPSIAAKAQLAR.Q |
|  | 1692 | 504.7005 | 1511.0793 | 1511.8049 | -0.7255 | 1 | 3 | 1e+03 | 2 | R.CNMKLVPELTYK.V + Carbamidomethyl (C); Oxidation (M) |
|  | 1742 | 512.3596 | 1534.0567 | 1534.6710 | -0.6143 | 2 | 6 | 5.7e+02 | 4 | K.ESFPVQWKRDDK.T |
|  | 2091 | 560.6112 | 1678.8114 | 1679.8738 | -1.0623 | 1 | 5 | 1.2e+03 | 9 | R.NVDSVVNGTCRLDCK.I + Carbamidomethyl (C) |
|  | 3494 | 849.0225 | 1696.0303 | 1694.9300 | 1.1003 | 2 | 1 | 2.3e+03 | 7 | K.DKPAVAPATKKAAVDGR.L |
|  | 2176 | 575.9305 | 1724.7693 | 1723.9214 | 0.8479 | 0 | 6 | 6.6e+02 | 4 | R.AGSDLVLDAAVGGKPEPK.I |
|  | 2244 | 590.4874 | 1768.4399 | 1768.0170 | 0.4230 | 1 | 14 | 84 | 2 | K.NEKGLSDPVTIGPITVK.E |
|  | 2276 | 593.1105 | 1776.3094 | 1775.9547 | 0.3548 | 1 | 10 | 2.7e+02 | 10 | K.ATNEVGSDTCSCSVKFK.E |
|  | 2419 | 611.7848 | 1832.3322 | 1833.0060 | -0.6738 | 1 | (8) | 4.8e+02 | 4 | K.ATNEVGSDTCSCSVKFK.E + Carbamidomethyl (C) |
|  | 2422 | 611.8551 | 1832.5431 | 1831.9980 | 0.5451 | 1 | 13 | 1.1e+02 | 1 | R.MAHEGALTGVTTDQKEK.Q + Oxidation (M) |
|  | 2491 | 620.3240 | 1857.9498 | 1859.1275 | -1.1778 | 2 | 3 | 1.4e+03 | 8 | R.IEEGKSLRFPLALEEK.Q |
|  | 2625 | 644.1377 | 1929.3909 | 1929.1148 | 0.2761 | 1 | 4 | 9.4e+02 | 6 | R.VCAENAAGPGKFSPPSDPK.T + Carbamidomethyl (C) |
|  | 2670 | 652.6118 | 1954.8133 | 1954.3164 | 0.4969 | 2 | 1 | 1.7e+03 | 7 | K.VGLKGVEFNVPRLLEGVK.Y |
|  | 2894 | 685.0886 | 2052.2437 | 2052.2855 | -0.0418 | 2 | 15 | 85 | 1 | K.TIDTTAEQTSFRILEAKK.G |
|  | 3187 | 749.1390 | 2244.3949 | 2245.5560 | -1.1611 | 2 | 0 | 2.2e+03 | 10 | R.VLAKNAAGVISKGSESTGPVTCR.D |
|  | 3257 | 763.4155 | 2287.2244 | 2286.6294 | 0.5950 | 2 | 4 | 1.1e+03 | 1 | R.VRAENRYGVSQPLVSSIIVAK.H |
|  | 3270 | 766.5801 | 2296.7181 | 2295.5714 | 1.1466 | 2 | 9 | 2.9e+02 | 3 | K.DCIRTDGGQYILKLSNVGGTK.S + Carbamidomethyl (C) |
|  | 3299 | 777.4265 | 2329.2574 | 2329.6706 | -0.4133 | 1 | 4 | 1e+03 | 6 | K.IRIYAMNSEGLGEPALVPGTPK.A + Oxidation (M) |
|  | 3303 | 777.7145 | 2330.1213 | 2329.5845 | 0.5367 | 1 | 5 | 6.2e+02 | 9 | K.SSGLTDGIAYEFRVIAENMAGK.S |
|  | 3438 | 817.2358 | 2448.6853 | 2447.8036 | 0.8818 | 2 | 2 | 1.7e+03 | 3 | R.EPPSFVKKVDPSYLMLPGESAR.L |
|  | 3474 | 839.8387 | 2516.4941 | 2515.7929 | 0.7011 | 1 | 15 | 67 | 2 | R.IYAMNSEGLGEPALVPGTPKAEDR.M |

  


---

|  |  |
| --- | --- |
| **16.** | gi|103472005    **Mass:** 358689   **Score:** 120    **Queries matched:** 17 |
|  | antigen KI-67 isoform 1 [Homo sapiens] |

|  |  |
| --- | --- |
|  | Check to include this hit in error tolerant search or archive report |
|  |  |

|  |  |  |  |  |  |  |  |  |  |  |
| --- | --- | --- | --- | --- | --- | --- | --- | --- | --- | --- |
|  | **Query** | **Observed** | **Mr(expt)** | **Mr(calc)** | **Delta** | **Miss** | **Score** | **Expect** | **Rank** | **Peptide** |
|  | 173 | **373.1169** | **744.2191** | **744.7474** | **-0.5283** | **0** | **15** | **1.1e+02** | **5** | **K.EPAGEDK.G** |
|  | 825 | **416.0808** | **830.1467** | **828.9563** | **1.1904** | **0** | **9** | **4.4e+02** | **5** | **K.ATLLQQR.R** |
|  | 944 | **427.1951** | **852.3754** | **853.0210** | **-0.6455** | **1** | **14** | **1e+02** | **1** | **R.KPIPRDK.V** |
|  | 985 | 429.3219 | 856.6291 | 857.0362 | -0.4071 | 1 | 11 | 1.6e+02 | 7 | R.CPKTRPR.K |
|  | 1072 | **435.6170** | **869.2192** | **869.9190** | **-0.6997** | **0** | **4** | **9.1e+02** | **9** | **K.EQPQPSGK.Q** |
|  | 2229 | 587.3734 | 1172.7319 | 1172.3783 | 0.3536 | 2 | 15 | 79 | 4 | K.TLTPRKLSTR.N |
|  | 2382 | **607.5442** | **1213.0736** | **1213.3808** | **-0.3072** | **1** | **6** | **5.6e+02** | **4** | **K.VQVKEEPSAVK.F** |
|  | 640 | 406.3466 | 1216.0175 | 1215.2308 | 0.7867 | 2 | 16 | 53 | 5 | R.TRSHRDSEDI.- |
|  | 685 | **407.5475** | **1219.6202** | **1219.3057** | **0.3145** | **2** | **11** | **2.1e+02** | **4** | **K.RGGGKDGSVTGTK.R** |
|  | 928 | **423.4051** | **1267.1932** | **1266.4463** | **0.7468** | **1** | **8** | **4.8e+02** | **7** | **K.LDLPGNLPGSKR.W** |
|  | 1210 | **451.0157** | **1350.0248** | **1350.5829** | **-0.5581** | **1** | **3** | **1.3e+03** | **10** | **K.DIKAFMGTPVQK.L + Oxidation (M)** |
|  | 1254 | **456.2148** | **1365.6224** | **1365.6837** | **-0.0613** | **1** | **10** | **2.6e+02** | **4** | **R.KSLVMHTPPVLK.K + Oxidation (M)** |
|  | 2494 | **620.5226** | **1858.5456** | **1859.0234** | **-0.4778** | **2** | **8** | **3.2e+02** | **2** | **K.VACKSSQPDPDKNPASSK.R** |
|  | 2658 | **650.5939** | **1948.7594** | **1949.1443** | **-0.3849** | **2** | **7** | **4.4e+02** | **4** | **K.IPCKSSPELEDTATSSKR.R** |
|  | 2715 | **659.6386** | **1975.8937** | **1975.2049** | **0.6888** | **1** | **8** | **4.3e+02** | **9** | **K.IACKSPPPESMDTPTSTR.R + Carbamidomethyl (C)** |
|  | 3286 | **775.7935** | **2324.3582** | **2323.4957** | **0.8625** | **2** | **1** | **2e+03** | **9** | **K.ELFQTRGHTEESMTNDKTAK.V** |
|  | 3444 | **819.1430** | **2454.4068** | **2453.7086** | **0.6983** | **2** | **12** | **1.2e+02** | **5** | **K.ESAKQMLDPANYGTGMERWPR.T + Oxidation (M)** |

  

|  |  |
| --- | --- |
|  | |
|  | **Proteins matching the same set of peptides:** |

|  |  |
| --- | --- |
|  | gi|119569563    **Mass:** 358608   **Score:** 120    **Queries matched:** 17 |
|  | antigen identified by monoclonal antibody Ki-67, isoform CRA\_b [Homo sapiens] |

---

|  |  |
| --- | --- |
| **17.** | gi|415819    **Mass:** 358741   **Score:** 119    **Queries matched:** 17 |
|  | antigen of the monoclonal antibody Ki-67 [Homo sapiens] |

|  |  |
| --- | --- |
|  | Check to include this hit in error tolerant search or archive report |
|  |  |

|  |  |  |  |  |  |  |  |  |  |  |
| --- | --- | --- | --- | --- | --- | --- | --- | --- | --- | --- |
|  | **Query** | **Observed** | **Mr(expt)** | **Mr(calc)** | **Delta** | **Miss** | **Score** | **Expect** | **Rank** | **Peptide** |
|  | 173 | 373.1169 | 744.2191 | 744.7474 | -0.5283 | 0 | 15 | 1.1e+02 | 5 | K.EPAGEDK.G |
|  | 825 | 416.0808 | 830.1467 | 828.9563 | 1.1904 | 0 | 9 | 4.4e+02 | 5 | K.ATLLQQR.R |
|  | 944 | 427.1951 | 852.3754 | 853.0210 | -0.6455 | 1 | 14 | 1e+02 | 1 | R.KPIPRDK.V |
|  | 985 | 429.3219 | 856.6291 | 857.0362 | -0.4071 | 1 | 11 | 1.6e+02 | 7 | R.CPKTRPR.K |
|  | 1072 | 435.6170 | 869.2192 | 869.9190 | -0.6997 | 0 | 4 | 9.1e+02 | 9 | K.EQPQPSGK.Q |
|  | 2229 | 587.3734 | 1172.7319 | 1172.3783 | 0.3536 | 2 | 15 | 79 | 4 | K.TLTPRKLSTR.N |
|  | 2382 | 607.5442 | 1213.0736 | 1213.3808 | -0.3072 | 1 | 6 | 5.6e+02 | 4 | K.VQVKEEPSAVK.F |
|  | 670 | **407.2534** | **1218.7380** | **1218.3837** | **0.3542** | **1** | **16** | **55** | **2** | **R.GGERVATCLQK.R + Carbamidomethyl (C)** |
|  | 685 | 407.5475 | 1219.6202 | 1219.3057 | 0.3145 | 2 | 11 | 2.1e+02 | 4 | K.RGGGKDGSVTGTK.R |
|  | 759 | **410.2754** | **1227.8040** | **1227.3655** | **0.4385** | **0** | **12** | **1.4e+02** | **2** | **K.AQPLEDLAGWK.E** |
|  | 928 | 423.4051 | 1267.1932 | 1266.4463 | 0.7468 | 1 | 8 | 4.8e+02 | 7 | K.LDLPGNLPGSKR.W |
|  | 1210 | 451.0157 | 1350.0248 | 1350.5829 | -0.5581 | 1 | 3 | 1.3e+03 | 10 | K.DIKAFMGTPVQK.L + Oxidation (M) |
|  | 1254 | 456.2148 | 1365.6224 | 1365.6837 | -0.0613 | 1 | 10 | 2.6e+02 | 4 | R.KSLVMHTPPVLK.K + Oxidation (M) |
|  | 2658 | 650.5939 | 1948.7594 | 1949.1443 | -0.3849 | 2 | 7 | 4.4e+02 | 4 | K.IPCKSSPELEDTATSSKR.R |
|  | 2715 | 659.6386 | 1975.8937 | 1975.2049 | 0.6888 | 1 | 8 | 4.3e+02 | 9 | K.IACKSPPPESMDTPTSTR.R + Carbamidomethyl (C) |
|  | 3286 | 775.7935 | 2324.3582 | 2323.4957 | 0.8625 | 2 | 1 | 2e+03 | 9 | K.ELFQTRGHTEESMTNDKTAK.V |
|  | 3444 | 819.1430 | 2454.4068 | 2453.7086 | 0.6983 | 2 | 12 | 1.2e+02 | 5 | K.ESAKQMLDPANYGTGMERWPR.T + Oxidation (M) |

  


---

|  |  |
| --- | --- |
| **18.** | gi|119631910    **Mass:** 772969   **Score:** 118    **Queries matched:** 29 |
|  | nebulin, isoform CRA\_g [Homo sapiens] |

|  |  |
| --- | --- |
|  | Check to include this hit in error tolerant search or archive report |
|  |  |

|  |  |  |  |  |  |  |  |  |  |  |
| --- | --- | --- | --- | --- | --- | --- | --- | --- | --- | --- |
|  | **Query** | **Observed** | **Mr(expt)** | **Mr(calc)** | **Delta** | **Miss** | **Score** | **Expect** | **Rank** | **Peptide** |
|  | 169 | 372.6250 | 743.2352 | 743.8918 | -0.6566 | 1 | 22 | 16 | 6 | R.KVQELK.T |
|  | 517 | 401.7036 | 801.3925 | 800.9432 | 0.4493 | 2 | 13 | 1.2e+02 | 6 | R.KKVDPSK.F |
|  | 973 | 429.0564 | 856.0979 | 856.0480 | 0.0499 | 1 | 13 | 1.4e+02 | 9 | K.AHMLKTR.N |
|  | 975 | 429.0998 | 856.1849 | 856.0480 | 0.1368 | 1 | (5) | 7.5e+02 | 10 | K.AHMLKTR.N |
|  | 1055 | 434.1796 | 866.3445 | 865.9700 | 0.3744 | 0 | 12 | 1.7e+02 | 3 | K.ALSDVAYK.K |
|  | 1084 | 436.1782 | 870.3417 | 870.9931 | -0.6515 | 0 | 6 | 7.2e+02 | 5 | R.QRPETLK.F |
|  | 1399 | 467.0483 | 932.0818 | 931.0882 | 0.9936 | 1 | 8 | 4.5e+02 | 6 | K.AAKLSSQVK.Y |
|  | 1888 | 530.6460 | 1059.2772 | 1059.2605 | 0.0168 | 2 | 12 | 2.2e+02 | 1 | K.KAAKLSSQVK.Y |
|  | 1916 | 534.7700 | 1067.5253 | 1068.2905 | -0.7652 | 0 | 10 | 2.3e+02 | 7 | K.LHKPVTDMK.E |
|  | 88 | 368.1640 | 1101.4697 | 1102.2818 | -0.8121 | 2 | (7) | 5.4e+02 | 8 | K.KAGEILSEKK.Y |
|  | 92 | 368.2580 | 1101.7519 | 1102.2818 | -0.5299 | 2 | 13 | 1.1e+02 | 1 | K.KAGEILSEKK.Y |
|  | 635 | 406.2022 | 1215.5845 | 1216.3413 | -0.7569 | 1 | (10) | 2.1e+02 | 6 | R.QLKAAGDALSDK.L |
|  | 640 | 406.3466 | 1216.0175 | 1216.3413 | -0.3238 | 1 | 14 | 87 | 8 | R.QLKAAGDALSDK.L |
|  | 926 | 423.2154 | 1266.6240 | 1267.3452 | -0.7211 | 1 | 10 | 2.9e+02 | 6 | K.VTAQNSDKNYK.A |
|  | 950 | 427.8523 | 1280.5348 | 1280.4234 | 0.1114 | 0 | 8 | 4.5e+02 | 4 | K.VTDQISDIVYK.D |
|  | 1049 | 433.5409 | 1297.6005 | 1296.4889 | 1.1116 | 0 | 13 | 1.5e+02 | 6 | K.CQILVSDIDYK.H |
|  | 1095 | 437.0404 | 1308.0990 | 1308.4798 | -0.3808 | 1 | 3 | 1.3e+03 | 9 | R.ATEILSDKIYR.Q |
|  | 1647 | 496.3236 | 1485.9487 | 1486.7324 | -0.7837 | 1 | 10 | 2.5e+02 | 2 | K.GKMIGVLSINDDPK.M |
|  | 1819 | 523.0095 | 1566.0064 | 1566.7575 | -0.7511 | 0 | 4 | 1.1e+03 | 8 | R.GLNAMANETPDFMR.A |
|  | 1936 | 536.5109 | 1606.5104 | 1605.9212 | 0.5893 | 1 | 11 | 2e+02 | 1 | K.LVLSMNVAKMQSER.E |
|  | 1981 | 541.9464 | 1622.8171 | 1621.9206 | 0.8965 | 1 | (8) | 4e+02 | 3 | K.LVLSMNVAKMQSER.E + Oxidation (M) |
|  | 2153 | 570.5983 | 1708.7728 | 1709.9611 | -1.1882 | 2 | 4 | 1.2e+03 | 6 | K.VMKDANNLASEVKYK.A |
|  | 2304 | 595.9377 | 1784.7911 | 1783.9879 | 0.8031 | 2 | 3 | 1.3e+03 | 8 | K.QLGHHIGARAIRDDPK.M |
|  | 2383 | 607.6240 | 1819.8497 | 1820.0517 | -0.2020 | 1 | 5 | 8.6e+02 | 3 | K.EAIGQGTPIPDLPEVKR.V |
|  | 2404 | 609.7632 | 1826.2676 | 1826.9980 | -0.7305 | 0 | 5 | 9.5e+02 | 3 | K.ENLGTGIPTTVTPEIER.V |
|  | 2784 | 668.8446 | 2003.5116 | 2003.2615 | 0.2502 | 2 | 4 | 1e+03 | 8 | K.ENMRKATPTPVTPEMER.A + Oxidation (M) |
|  | 3258 | **763.5494** | **2287.6261** | **2287.6788** | **-0.0526** | **1** | **1** | **2.2e+03** | **8** | **K.TQIHIMPDTPEIMLARMNK.I + 3 Oxidation (M)** |
|  | 3313 | 778.1982 | 2331.5726 | 2332.7594 | -1.1868 | 2 | 5 | 7.5e+02 | 2 | K.MKIHIVPDMVEMVTAKDSQK.K + 2 Oxidation (M) |
|  | 3467 | 833.4502 | 2497.3284 | 2497.8457 | -0.5172 | 2 | 10 | 2.8e+02 | 4 | K.DKTQVHIMPDTPEIMLARQNK.I + 2 Oxidation (M) |

  


---

|  |  |
| --- | --- |
| **19.** | gi|356168    **Mass:** 21734    **Score:** 116    **Queries matched:** 5   **emPAI:** 0.33 |
|  | histone H1b |

|  |  |
| --- | --- |
|  | Check to include this hit in error tolerant search or archive report |
|  |  |

|  |  |  |  |  |  |  |  |  |  |  |
| --- | --- | --- | --- | --- | --- | --- | --- | --- | --- | --- |
|  | **Query** | **Observed** | **Mr(expt)** | **Mr(calc)** | **Delta** | **Miss** | **Score** | **Expect** | **Rank** | **Peptide** |
|  | 2123 | **565.5554** | **1129.0959** | **1130.2953** | **-1.1994** | **2** | **0** | **2.6e+03** | **7** | **K.ATGAATPKKSAK.K** |
|  | 2544 | **630.6600** | **1259.3053** | **1260.4801** | **-1.1749** | **1** | **34** | **1.2** | **1** | **K.SLVSKGTLVQTK.G** |
|  | 2729 | **664.6874** | **1327.3600** | **1326.5383** | **0.8217** | **1** | **52** | **0.016** | **1** | **R.KASGPPVSELITK.A** |
|  | 1644 | **495.5614** | **1483.6619** | **1482.7240** | **0.9380** | **2** | **30** | **3.5** | **1** | **K.RKASGPPVSELITK.A** |
|  | 1862 | **527.0581** | **1578.1521** | **1578.6820** | **-0.5299** | **1** | **2** | **1.6e+03** | **6** | **K.ALAAAGYDVEKNNSR.I** |

  

|  |  |
| --- | --- |
|  | |
|  | **Proteins matching the same set of peptides:** |

|  |  |
| --- | --- |
|  | gi|4885379    **Mass:** 21865    **Score:** 116    **Queries matched:** 5 |
|  | histone H1.4 [Homo sapiens] |

|  |  |
| --- | --- |
|  | gi|66365795    **Mass:** 21893    **Score:** 116    **Queries matched:** 5 |
|  | Histone cluster 1, H1e [Homo sapiens] |

---

|  |  |
| --- | --- |
| **20.** | gi|33694244    **Mass:** 32603    **Score:** 106    **Queries matched:** 4   **emPAI:** 0.34 |
|  | nucleophosmin [Homo sapiens] |

|  |  |
| --- | --- |
|  | Check to include this hit in error tolerant search or archive report |
|  |  |

|  |  |  |  |  |  |  |  |  |  |  |
| --- | --- | --- | --- | --- | --- | --- | --- | --- | --- | --- |
|  | **Query** | **Observed** | **Mr(expt)** | **Mr(calc)** | **Delta** | **Miss** | **Score** | **Expect** | **Rank** | **Peptide** |
|  | 1378 | **465.2904** | **928.5660** | **928.0844** | **0.4817** | **2** | **1** | **1.9e+03** | **6** | **R.EKTPKTPK.G** |
|  | 2131 | **566.3429** | **1130.6710** | **1130.2490** | **0.4220** | **1** | **75** | **8.3e-05** | **1** | **K.GPSSVEDIKAK.M** |
|  | 225 | **377.9084** | **1130.7029** | **1130.2490** | **0.4539** | **1** | **(38)** | **0.37** | **1** | **K.GPSSVEDIKAK.M** |
|  | 2694 | **655.8890** | **1964.6449** | **1964.1619** | **0.4830** | **1** | **31** | **1.9** | **1** | **R.MTDQEAIQDLWQWRK.S + Oxidation (M)** |

  


---

**Peptide matches not assigned to protein hits:** (no details means no
match)  
  

|  |  |  |  |  |  |  |  |  |  |  |
| --- | --- | --- | --- | --- | --- | --- | --- | --- | --- | --- |
|  | **Query** | **Observed** | **Mr(expt)** | **Mr(calc)** | **Delta** | **Miss** | **Score** | **Expect** | **Rank** | **Peptide** |
|  | 1048 | **433.4252** | **1297.2533** | **1296.4326** | **0.8207** | **1** | **56** | **0.0076** | **1** | **AIESSRDLLHR** |
|  | 866 | **418.8493** | **835.6838** | **835.9044** | **-0.2206** | **0** | **55** | **0.0099** | **1** | **SPPADPPR** |
|  | 3287 | **775.8000** | **1549.5852** | **1549.8527** | **-0.2675** | **0** | **53** | **0.012** | **1** | **LLGASELPIVTPALR** |
|  | 3009 | **715.4397** | **2143.2969** | **2144.2151** | **-0.9182** | **1** | **53** | **0.013** | **1** | **EGQEDQGLTKDYGNSPLHR** |
|  | 2465 | **614.8793** | **1227.7438** | **1228.3571** | **-0.6133** | **1** | **51** | **0.018** | **1** | **AAPGAEFAPNKR** |
|  | 2097 | **562.1088** | **1122.2029** | **1121.1577** | **1.0452** | **0** | **51** | **0.022** | **1** | **AFEEDQVAGR** |
|  | 3535 | **894.8789** | **1787.7430** | **1787.0217** | **0.7213** | **0** | **51** | **0.019** | **1** | **VLAQQGEYSEAIPILR** |
|  | 3428 | **815.3929** | **1628.7711** | **1628.7424** | **0.0288** | **0** | **50** | **0.028** | **1** | **SSGPYGGGGQYFAKPR** |
|  | 1691 | **504.6577** | **1007.3006** | **1007.1411** | **0.1595** | **0** | **48** | **0.039** | **1** | **VSIVNQYGK** |
|  | 2475 | **616.6362** | **1231.2577** | **1231.4008** | **-0.1431** | **0** | **47** | **0.064** | **1** | **GIVEFSGKPAAR** |
|  | 2641 | **647.1118** | **1292.2088** | **1291.3697** | **0.8391** | **0** | **44** | **0.094** | **1** | **QNLSQFEAQAR** |
|  | 1839 | **524.2775** | **1046.5403** | **1046.2200** | **0.3202** | **0** | **44** | **0.12** | **1** | **SSLNPILFR** |
|  | 2931 | **688.3643** | **1374.7137** | **1374.4932** | **0.2205** | **0** | **43** | **0.12** | **1** | **SSSPSEILQELGK** |
|  | 1078 | **435.8250** | **869.6352** | **869.9190** | **-0.2838** | **0** | **42** | **0.16** | **1** | **ISSSSFSR** |
|  | 918 | **422.1837** | **842.3527** | **841.9949** | **0.3578** | **0** | **40** | **0.31** | **1** | **GITLSVRP** |
|  | 1183 | **447.9425** | **1340.8052** | **1340.5648** | **0.2404** | **1** | **39** | **0.29** | **1** | **KATGPPVSELITK** |
|  | 2848 | **679.8777** | **2036.6109** | **2036.2065** | **0.4044** | **1** | **38** | **0.42** | **1** | **SNMDNMFESYINNLRR + 2 Oxidation (M)** |
|  | 3183 | **749.0328** | **1496.0509** | **1495.6301** | **0.4208** | **0** | **38** | **0.36** | **1** | **AAGTVFTTVEDLGSK** |
|  | 921 | **422.2867** | **842.5585** | **841.9949** | **0.5636** | **0** | **38** | **0.41** | **1** | **GITLSVRP** |
|  | 1049 | 433.5409 | 1297.6005 | 1296.4326 | 1.1679 | 1 | 37 | 0.56 | 1 | AIESSRDLLHR |
|  | 1361 | **462.9572** | **923.8996** | **923.0248** | **0.8749** | **0** | **37** | **0.49** | **1** | **EPPVAGPTR** |
|  | 1432 | **471.0457** | **940.0766** | **940.0088** | **0.0678** | **0** | **36** | **0.53** | **1** | **STETALYR** |
|  | 2689 | **655.2574** | **1308.5001** | **1308.3938** | **0.1064** | **2** | **36** | **0.61** | **1** | **IALEFDKDRSD** |
|  | 778 | **411.6789** | **1232.0145** | **1231.4008** | **0.6138** | **0** | **35** | **0.69** | **1** | **GIVEFSGKPAAR** |
|  | 1628 | 494.1905 | 986.3663 | 986.2078 | 0.1584 | 0 | 34 | 0.99 | 1 | DLPLLLFR |
|  | 2192 | **579.3528** | **1735.0364** | **1734.9570** | **0.0793** | **2** | **34** | **0.97** | **1** | **AIRLELQGPRGSPNAR** |
|  | 3178 | **748.8282** | **1495.6416** | **1495.6301** | **0.0115** | **0** | **33** | **1.6** | **1** | **AAGTVFTTVEDLGSK** |
|  | 2760 | **667.0127** | **1998.0159** | **1997.9879** | **0.0281** | **2** | **32** | **1.3** | **1** | **NQGGYGGSSSSSSYGSGRRF** |
|  | 2883 | **684.0323** | **1366.0499** | **1365.5807** | **0.4692** | **1** | **32** | **1.5** | **1** | **GALVLGSSLKQHR** |
|  | 1073 | **435.6815** | **1304.0222** | **1303.5314** | **0.4908** | **2** | **32** | **1.4** | **1** | **AKLRDMLSDVR** |
|  | 926 | 423.2154 | 1266.6240 | 1266.4498 | 0.1742 | 0 | 31 | 2 | 1 | VARPAQLSAPTR |
|  | 923 | **422.2926** | **842.5705** | **841.9949** | **0.5755** | **0** | **30** | **2.2** | **1** | **GITLSVRP** |
|  | 998 | **430.7094** | **859.4041** | **858.9823** | **0.4218** | **0** | **30** | **2.4** | **1** | **SIALTQAR** |
|  | 2137 | **568.1998** | **1701.5771** | **1701.9803** | **-0.4031** | **1** | **30** | **2.7** | **1** | **KLEPIWNEVGLEMK + Oxidation (M)** |
|  | 455 | **394.8143** | **1181.4207** | **1182.2009** | **-0.7801** | **1** | **30** | **2.9** | **1** | **GRSDYDGIGSR** |
|  | 791 | **413.4742** | **824.9335** | **825.9954** | **-1.0619** | **0** | **30** | **3** | **1** | **QLSILPR** |
|  | 358 | **387.9106** | **1160.7097** | **1160.4138** | **0.2958** | **1** | **29** | **3.9** | **1** | **MALGPRCGAIR + Oxidation (M)** |
|  | 1615 | 491.2809 | 1470.8207 | 1469.7119 | 1.1088 | 2 | 29 | 2.9 | 1 | SCQRKGVPIQTPR |
|  | 2476 | **616.6735** | **1231.3321** | **1231.3989** | **-0.0668** | **0** | **29** | **4.2** | **1** | **SGISASLAISGLR** |
|  | 1480 | **475.5199** | **1423.5375** | **1422.6056** | **0.9319** | **0** | **29** | **4.5** | **1** | **GSCYPATADLLVGR** |
|  | 3236 | **758.6386** | **1515.2623** | **1514.8536** | **0.4088** | **0** | **29** | **2.9** | **1** | **CMSALSMPMLATSR + Oxidation (M)** |
|  | 169 | 372.6250 | 743.2352 | 742.7315 | 0.5037 | 0 | 29 | 3.3 | 1 | EEPAPSN |
|  | 1367 | **463.9114** | **925.8080** | **925.1300** | **0.6780** | **2** | **28** | **3.5** | **1** | **QIKVKGPR** |
|  | 1205 | **450.4680** | **898.9213** | **898.9203** | **0.0009** | **0** | **28** | **4.5** | **1** | **QQGAGEPGR** |
|  | 1400 | **467.1614** | **932.3080** | **933.0444** | **-0.7364** | **1** | **28** | **4.4** | **1** | **AAARDMNGK** |
|  | 403 | **389.1729** | **1164.4966** | **1164.3544** | **0.1421** | **0** | **28** | **4.2** | **1** | **LDLNLAAAVHK** |
|  | 2477 | **616.7761** | **1231.5375** | **1231.3989** | **0.1385** | **0** | **28** | **4.5** | **1** | **SGISASLAISGLR** |
|  | 1344 | **461.9337** | **921.8527** | **921.1428** | **0.7098** | **1** | **28** | **4** | **1** | **SCLACRLR** |
|  | 159 | **371.2751** | **1110.8031** | **1110.2642** | **0.5389** | **2** | **28** | **2.9** | **1** | **KEDPQLPRK** |
|  | 1331 | **460.8132** | **919.6117** | **919.0361** | **0.5756** | **0** | **27** | **5** | **1** | **FPVVSGASR** |
|  | 369 | **388.0398** | **774.0648** | **774.8660** | **-0.8012** | **1** | **27** | **6.2** | **1** | **TTELRR** |
|  | 1752 | **514.4221** | **1026.8295** | **1027.1391** | **-0.3096** | **2** | **27** | **3.9** | **1** | **LRGSEGPRR** |
|  | 163 | **372.2415** | **742.4683** | **742.9466** | **-0.4783** | **1** | **27** | **4.1** | **1** | **KELILK** |
|  | 2457 | **614.1031** | **1226.1915** | **1225.4375** | **0.7540** | **1** | **27** | **5.1** | **1** | **GNAIEKQKPLK** |
|  | 3274 | **767.8444** | **2300.5111** | **2299.6279** | **0.8831** | **0** | **27** | **6.2** | **1** | **SAEHCDCLQCFFIIHSMGGGK + Oxidation (M)** |
|  | 2458 | **614.1979** | **1226.3811** | **1225.5434** | **0.8377** | **0** | **27** | **5.2** | **1** | **LCPPTLSILLR** |
|  | 2147 | **570.1063** | **1138.1977** | **1137.3741** | **0.8237** | **2** | **27** | **5.5** | **1** | **MSKIEDMKR** |
|  | 800 | **413.7607** | **825.5066** | **825.9954** | **-0.4888** | **0** | **27** | **4.5** | **1** | **QLSILPR** |
|  | 3026 | **720.1871** | **2157.5392** | **2156.4032** | **1.1361** | **1** | **27** | **5.5** | **1** | **AGFAGDDAPRAVFPSIVGRPR** |
|  | 466 | **396.8748** | **791.7348** | **791.8334** | **-0.0987** | **0** | **27** | **6.2** | **1** | **GQSAGGCR + Carbamidomethyl (C)** |
|  | 573 | **404.1041** | **1209.2900** | **1208.1967** | **1.0934** | **0** | **26** | **6.1** | **1** | **ANDHGYDNFR** |
|  | 510 | **400.7414** | **799.4680** | **799.8689** | **-0.4008** | **0** | **26** | **5.9** | **1** | **AGPEELGK** |
|  | 771 | **411.3186** | **1230.9336** | **1231.4453** | **-0.5116** | **0** | **26** | **5.4** | **1** | **IFSMSWCWR + Oxidation (M)** |
|  | 15 | **361.9575** | **721.9002** | **721.8465** | **0.0537** | **0** | **26** | **6.2** | **1** | **MECPAR + Oxidation (M)** |
|  | 1202 | **450.2914** | **898.5681** | **899.0464** | **-0.4783** | **0** | **26** | **5.5** | **1** | **VQTVPLSR** |
|  | 385 | **388.9870** | **1163.9389** | **1163.3914** | **0.5476** | **0** | **26** | **7.9** | **1** | **CAHLCMDSVK + Carbamidomethyl (C)** |
|  | 1525 | **479.0975** | **956.1803** | **956.1010** | **0.0793** | **1** | **26** | **6.8** | **1** | **ARTVLSPGR** |
|  | 451 | **393.6914** | **1178.0520** | **1177.3203** | **0.7317** | **1** | **26** | **5.9** | **1** | **RPRERPGGPR** |
|  | 2287 | **593.9347** | **1778.7819** | **1779.1089** | **-0.3270** | **0** | **26** | **6.7** | **1** | **MALDNLLANLPPAKPGK + Oxidation (M)** |
|  | 535 | **402.3830** | **802.7512** | **802.8346** | **-0.0834** | **0** | **26** | **8.6** | **1** | **GYAEAHR** |
|  | 993 | **430.2329** | **1287.6767** | **1287.5020** | **0.1746** | **0** | **26** | **7.4** | **1** | **ISTEINSALVLK** |
|  | 394 | **389.1300** | **1164.3679** | **1164.4010** | **-0.0330** | **1** | **26** | **8.1** | **1** | **EMFPPLCRR + Oxidation (M)** |
|  | 309 | **386.0730** | **1155.1967** | **1154.2769** | **0.9198** | **0** | **25** | **6.9** | **1** | **MDGPGFGGMNR + Oxidation (M)** |
|  | 748 | **409.0331** | **1224.0771** | **1224.4693** | **-0.3922** | **0** | **25** | **8.7** | **1** | **SAAICYTAALLK** |
|  | 1065 | **435.0051** | **1301.9932** | **1301.5984** | **0.3948** | **2** | **25** | **7.1** | **1** | **KVLGEMVERLK** |
|  | 1993 | **543.8075** | **1085.6002** | **1086.3120** | **-0.7118** | **1** | **25** | **6.9** | **1** | **QLLCGLRAR + Carbamidomethyl (C)** |
|  | 2103 | **562.4869** | **1684.4386** | **1683.7963** | **0.6424** | **1** | **25** | **6.7** | **1** | **QSMSRIPASSNSTSSK + Oxidation (M)** |
|  | 2979 | **705.8861** | **1409.7574** | **1408.6352** | **1.1222** | **2** | **25** | **8.2** | **1** | **LAHQQCLQRRR** |
|  | 459 | **396.0140** | **790.0133** | **788.9554** | **1.0578** | **0** | **25** | **9.8** | **1** | **ALLDVCR** |
|  | 1578 | **487.7775** | **973.5402** | **973.2342** | **0.3060** | **2** | **25** | **8.2** | **1** | **KKAALSMPK** |
|  | 891 | **419.5810** | **1255.7209** | **1255.4007** | **0.3202** | **2** | **25** | **9.8** | **1** | **EKSFQCNKSGK** |
|  | 2115 | **563.8414** | **1688.5021** | **1688.8028** | **-0.3006** | **1** | **25** | **7.2** | **1** | **SGRPSGRFSAERPER** |
|  | 2690 | **655.2943** | **1308.5738** | **1308.5942** | **-0.0203** | **1** | **25** | **9.1** | **1** | **RQLPCMASQMK + Oxidation (M)** |
|  | 787 | **413.4178** | **824.8209** | **825.9954** | **-1.1745** | **0** | **25** | **8.4** | **1** | **QLSILPR** |
|  | 47 | **363.3268** | **1086.9583** | **1086.2809** | **0.6774** | **0** | **25** | **8.2** | **1** | **MLTSMLSGSK + 2 Oxidation (M)** |
|  | 1253 | **455.8362** | **909.6576** | **908.9550** | **0.7025** | **0** | **25** | **9** | **1** | **APSQYSTR** |
|  | 2535 | **629.6834** | **1886.0279** | **1886.1994** | **-0.1715** | **1** | **25** | **11** | **1** | **SPDEPLPVVRIPVALQR** |
|  | 3075 | **739.9250** | **1477.8352** | **1477.7920** | **0.0432** | **1** | **25** | **8.9** | **1** | **KMRPLSGLMETAK + Oxidation (M)** |
|  | 1453 | **472.6192** | **943.2236** | **943.1435** | **0.0801** | **0** | **25** | **9.9** | **1** | **LLGPPPPPR** |
|  | 615 | **406.0088** | **1215.0041** | **1215.4658** | **-0.4617** | **0** | **24** | **8.7** | **1** | **ICGGLAMYECR** |
|  | 1077 | **435.8242** | **869.6337** | **870.0283** | **-0.3946** | **0** | **24** | **9.3** | **1** | **TPCPSLPR** |
|  | 1149 | **443.9071** | **885.7994** | **886.9893** | **-1.1898** | **0** | **24** | **10** | **1** | **VPQTSDLK** |
|  | 2116 | **564.1200** | **1689.3378** | **1688.9700** | **0.3679** | **1** | **24** | **9.8** | **1** | **QLVRGEPNVSMICSR** |
|  | 2800 | **670.1854** | **2007.5341** | **2007.2547** | **0.2794** | **2** | **24** | **9.9** | **1** | **ECGRGFCDKSTLIVHER + Carbamidomethyl (C)** |
|  | 1071 | **435.1722** | **1302.4944** | **1303.6373** | **-1.1429** | **2** | **24** | **9.6** | **1** | **ECKIKGKPLCK + Carbamidomethyl (C)** |
|  | 909 | **421.7348** | **1262.1822** | **1262.4762** | **-0.2939** | **2** | **24** | **9.1** | **1** | **AEEEIMKKIR + Oxidation (M)** |
|  | 2117 | **564.1954** | **1689.5641** | **1688.9700** | **0.5942** | **1** | **24** | **10** | **1** | **QLVRGEPNVSMICSR** |
|  | 81 | **367.2994** | **1098.8761** | **1098.2963** | **0.5797** | **0** | **24** | **10** | **1** | **CCIAAAYDLR** |
|  | 1465 | **473.8347** | **1418.4820** | **1418.5062** | **-0.0242** | **0** | **24** | **11** | **1** | **DMMSEGGPPGAEPQ + Oxidation (M)** |
|  | 1 | **360.3220** | **1077.9439** | **1078.1942** | **-0.2503** | **0** | **24** | **12** | **1** | **LLDDAMAADK + Oxidation (M)** |
|  | 450 | **393.6326** | **1177.8756** | **1178.3415** | **-0.4658** | **1** | **24** | **8.7** | **1** | **RGETLPVPGPR** |
|  | 1172 | **445.8158** | **1334.4251** | **1334.5256** | **-0.1005** | **1** | **24** | **12** | **1** | **MARGDQAVMAPR + 2 Oxidation (M)** |
|  | 1424 | **470.2748** | **1407.8023** | **1408.6438** | **-0.8414** | **1** | **24** | **8.7** | **1** | **CKLVMDQISEAR + Oxidation (M)** |
|  | 1681 | **503.4756** | **1507.4047** | **1506.5550** | **0.8497** | **1** | **24** | **11** | **1** | **MGERAGSPGTDQER + Oxidation (M)** |
|  | 911 | **421.8853** | **841.7558** | **841.9949** | **-0.2392** | **0** | **24** | **11** | **1** | **GITLSVRP** |
|  | 1045 | **433.2023** | **1296.5848** | **1297.4574** | **-0.8725** | **0** | **24** | **11** | **1** | **TCDPVEMSYPR** |
|  | 77 | **367.0945** | **1098.2615** | **1099.2384** | **-0.9769** | **1** | **24** | **12** | **1** | **SEGVPVEKVR** |
|  | 2808 | **671.6097** | **1341.2047** | **1340.5648** | **0.6399** | **1** | **24** | **9.2** | **1** | **KATGPPVSELITK** |
|  | 2910 | **686.2354** | **1370.4560** | **1370.5972** | **-0.1412** | **0** | **24** | **11** | **1** | **ADPECMLGHLLR + Oxidation (M)** |
|  | 508 | **400.4803** | **1198.4187** | **1198.3295** | **0.0892** | **2** | **24** | **13** | **1** | **QSSKLNEHKK** |
|  | 494 | **399.4868** | **1195.4382** | **1196.4459** | **-1.0077** | **2** | **24** | **12** | **1** | **QRLRELLIR** |
|  | 2186 | **578.8562** | **1733.5464** | **1732.8718** | **0.6747** | **0** | **24** | **9.6** | **1** | **HPAKPDPSGECNPDLR** |
|  | 1010 | **431.1296** | **1290.3667** | **1290.5358** | **-0.1691** | **2** | **23** | **14** | **1** | **KMASATRLIQR + Oxidation (M)** |
|  | 1019 | **431.6852** | **1292.0334** | **1291.5023** | **0.5310** | **1** | **23** | **11** | **1** | **SAGVPSRVIHIR** |
|  | 392 | **389.1201** | **776.2255** | **776.8819** | **-0.6564** | **0** | **23** | **13** | **1** | **DPCCPSR** |
|  | 638 | **406.2567** | **1215.7479** | **1215.3797** | **0.3681** | **0** | **23** | **9.3** | **1** | **SEAACLAAGPGIR** |
|  | 1516 | **477.9274** | **1430.7600** | **1429.6017** | **1.1584** | **2** | **23** | **11** | **1** | **HSNPKDRMTSLK + Oxidation (M)** |
|  | 123 | **369.3056** | **1104.8946** | **1105.3089** | **-0.4142** | **0** | **23** | **11** | **1** | **AKPWAVCFPS** |
|  | 640 | 406.3466 | 1216.0175 | 1216.3249 | -0.3074 | 0 | 23 | 9.7 | 1 | MHSAGTPGLSSR + Oxidation (M) |
|  | 3142 | **742.6709** | **2224.9905** | **2224.5250** | **0.4655** | **2** | **23** | **9.5** | **1** | **MYNSQHRSAISCIRTVWR + Oxidation (M)** |
|  | 265 | **382.3479** | **1144.0216** | **1144.3037** | **-0.2820** | **1** | **23** | **14** | **1** | **MKGTDSGSCCR** |
|  | 270 | **384.1334** | **1149.3779** | **1148.2956** | **1.0823** | **2** | **23** | **12** | **1** | **RMEGGWREK** |
|  | 1158 | 444.5180 | 1330.5318 | 1329.3733 | 1.1586 | 1 | 23 | 17 | 1 | NSPSPGGKEAETR |
|  | 307 | **386.0608** | **1155.1602** | **1156.2632** | **-1.1030** | **0** | **23** | **12** | **1** | **TEEEVFSGMK** |
|  | 57 | **364.3400** | **1089.9978** | **1089.3129** | **0.6850** | **2** | **23** | **11** | **1** | **RMQRLIEK + Oxidation (M)** |
|  | 2081 | **558.2952** | **1114.5756** | **1115.2375** | **-0.6620** | **1** | **23** | **13** | **1** | **GELAVKDANAK** |
|  | 34 | **363.0817** | **1086.2228** | **1087.2273** | **-1.0045** | **0** | **23** | **13** | **1** | **LEQGTSALIR** |
|  | 93 | **368.2641** | **1101.7702** | **1101.1711** | **0.5991** | **1** | **23** | **11** | **1** | **DQRIGELDR** |
|  | 413 | **389.2405** | **1164.6994** | **1164.2935** | **0.4060** | **2** | **23** | **12** | **1** | **EDSARVMGRK + Oxidation (M)** |
|  | 524 | **401.9553** | **801.8957** | **801.8515** | **0.0442** | **1** | **23** | **15** | **1** | **RSGQQAR** |
|  | 509 | **400.5207** | **1198.5400** | **1198.4173** | **0.1227** | **0** | **23** | **14** | **1** | **AVFPSIVGRPR** |
|  | 684 | **407.5428** | **1219.6064** | **1219.5177** | **0.0887** | **0** | **23** | **14** | **1** | **MKPALLEVMR + 2 Oxidation (M)** |
|  | 635 | 406.2022 | 1215.5845 | 1215.2688 | 0.3156 | 1 | 23 | 12 | 1 | GKYENYNEAK |
|  | 1589 | **488.1449** | **1461.4126** | **1461.6798** | **-0.2672** | **0** | **23** | **15** | **1** | **IIPQGADSTMLATK + Oxidation (M)** |
|  | 560 | **403.9533** | **1208.8377** | **1208.3643** | **0.4734** | **0** | **23** | **14** | **1** | **AHNDVVEVVVK** |
|  | 406 | **389.1872** | **776.3595** | **776.9233** | **-0.5637** | **0** | **23** | **14** | **1** | **VVSGLFR** |
|  | 3157 | **744.1466** | **1486.2784** | **1486.6316** | **-0.3531** | **2** | **23** | **13** | **1** | **MQKSSSTRAGDMR + 2 Oxidation (M)** |
|  | 793 | **413.5331** | **825.0515** | **825.9954** | **-0.9439** | **0** | **23** | **14** | **1** | **QLSILPR** |
|  | 1947 | **538.0886** | **1074.1625** | **1075.2200** | **-1.0576** | **1** | **23** | **15** | **1** | **LIAASTSRTR** |
|  | 1314 | **459.7928** | **917.5708** | **917.1278** | **0.4430** | **0** | **23** | **14** | **1** | **ILNVVGMR + Oxidation (M)** |
|  | 1391 | **466.1626** | **1395.4655** | **1396.6561** | **-1.1905** | **1** | **23** | **15** | **1** | **MSILGGWTTKFR** |
|  | 486 | **399.2029** | **1194.5867** | **1195.3701** | **-0.7835** | **1** | **23** | **11** | **1** | **QSYRLIYPR** |
|  | 237 | **379.1541** | **1134.4403** | **1135.3184** | **-0.8781** | **0** | **23** | **14** | **1** | **MGAGVGVAGCTR + Carbamidomethyl (C)** |
|  | 2469 | **615.6973** | **1229.3799** | **1228.4001** | **0.9798** | **1** | **23** | **18** | **1** | **FESIRLPGGPR** |
|  | 371 | **388.0966** | **1161.2676** | **1162.2525** | **-0.9849** | **0** | **23** | **17** | **1** | **LTAHSNYTQK** |
|  | 1464 | **473.8249** | **945.6350** | **946.1060** | **-0.4711** | **0** | **23** | **15** | **1** | **ACHEMALR + Oxidation (M)** |
|  | 2585 | **637.0459** | **1908.1155** | **1907.1308** | **0.9848** | **2** | **23** | **15** | **1** | **GDSGAAPDVDDKLCLRMK + Oxidation (M)** |
|  | 53 | 364.1732 | 1089.4973 | 1088.3246 | 1.1727 | 1 | 22 | 11 | 1 | NGRALCILTK |
|  | 87 | **368.1331** | **1101.3773** | **1102.2255** | **-0.8482** | **1** | **22** | **16** | **1** | **MRSHGNSGLK + Oxidation (M)** |
|  | 444 | **392.4427** | **1174.3061** | **1174.3892** | **-0.0831** | **2** | **22** | **17** | **1** | **KLEDGPKFLK** |
|  | 3158 | **744.2850** | **1486.5552** | **1486.6316** | **-0.0764** | **2** | **22** | **15** | **1** | **MQKSSSTRAGDMR + 2 Oxidation (M)** |
|  | 3550 | **925.0199** | **1848.0250** | **1847.2064** | **0.8187** | **2** | **22** | **16** | **1** | **IKSEASPRPLKSVIPPK** |
|  | 1454 | **472.6742** | **1415.0004** | **1414.5209** | **0.4795** | **2** | **22** | **13** | **1** | **KSPESQKGVDSPR** |
|  | 168 | **372.5046** | **742.9944** | **743.8915** | **-0.8971** | **0** | **22** | **17** | **1** | **SNGLIIK** |
|  | 3124 | **741.6284** | **1481.2419** | **1480.8373** | **0.4047** | **2** | **22** | **12** | **1** | **KQVPCFASMLTKK** |
|  | 3439 | **817.3348** | **2448.9822** | **2448.7799** | **0.2023** | **1** | **22** | **15** | **1** | **SPASGQGPMCNVKAPLGSPSPRPR + Carbamidomethyl (C)** |
|  | 2473 | **616.5267** | **1231.0387** | **1230.3266** | **0.7121** | **0** | **22** | **13** | **1** | **VDGIAWSPETR** |
|  | 152 | 371.1479 | 740.2811 | 739.8235 | 0.4575 | 1 | 22 | 12 | 1 | TRQAHK |
|  | 1524 | **478.8722** | **1433.5944** | **1433.5605** | **0.0339** | **2** | **22** | **16** | **1** | **KEISEIDKEGTGK** |
|  | 1170 | **445.7171** | **1334.1290** | **1333.5621** | **0.5669** | **1** | **22** | **15** | **1** | **VQRAFMQGQLR** |
|  | 1513 | **477.7534** | **1430.2381** | **1430.6278** | **-0.3897** | **1** | **22** | **12** | **1** | **MIRYFTSNPASK + Oxidation (M)** |
|  | 410 | **389.2281** | **1164.6623** | **1164.2915** | **0.3707** | **0** | **22** | **15** | **1** | **IWATATCGER + Carbamidomethyl (C)** |
|  | 289 | **385.1022** | **1152.2845** | **1153.3085** | **-1.0240** | **0** | **22** | **15** | **1** | **LNNNYLMSGK** |
|  | 287 | **385.0858** | **1152.2353** | **1152.4118** | **-0.1765** | **1** | **22** | **15** | **1** | **MCLAGCTPRK + Carbamidomethyl (C); Oxidation (M)** |
|  | 8 | **360.4388** | **718.8628** | **719.8322** | **-0.9693** | **1** | **22** | **24** | **1** | **ERFIR** |
|  | 1329 | **460.7809** | **1379.3206** | **1378.5729** | **0.7476** | **0** | **22** | **16** | **1** | **AQSACTYGALCYK** |
|  | 2033 | **550.9758** | **1649.9053** | **1649.9404** | **-0.0351** | **1** | **22** | **18** | **1** | **CAAHGRVCTECCLAR + Carbamidomethyl (C)** |
|  | 1028 | **432.4003** | **862.7858** | **863.9791** | **-1.1933** | **0** | **22** | **17** | **1** | **MAATDIAR + Oxidation (M)** |
|  | 2232 | **587.4363** | **1759.2867** | **1760.0361** | **-0.7495** | **0** | **22** | **15** | **1** | **SLDSFLLSPEAAVGLLK** |
|  | 1377 | **465.0118** | **1392.0133** | **1392.6063** | **-0.5929** | **1** | **22** | **18** | **1** | **EPQHCCPPTPRK** |
|  | 1971 | **540.5052** | **1618.4934** | **1618.8123** | **-0.3189** | **0** | **22** | **16** | **1** | **FPAAGMSRPLDTSPR + Oxidation (M)** |
|  | 933 | **424.1316** | **846.2484** | **845.9406** | **0.3078** | **0** | **22** | **20** | **1** | **TATSTLPR** |
|  | 3410 | **809.2292** | **1616.4437** | **1616.7398** | **-0.2961** | **1** | **22** | **16** | **1** | **HGQRGHGQQLLETR** |
|  | 1447 | **472.3138** | **942.6128** | **943.0987** | **-0.4860** | **0** | **22** | **14** | **1** | **LLQVNTGAK** |
|  | 655 | **407.0380** | **1218.0919** | **1218.2811** | **-0.1892** | **2** | **22** | **16** | **1** | **SARERSSAAAGR** |
|  | 2632 | **645.1791** | **1288.3434** | **1288.4538** | **-0.1104** | **0** | **22** | **19** | **1** | **CPSVSGPGCPATR + Carbamidomethyl (C)** |
|  | 1539 | **480.6822** | **1439.0244** | **1438.5388** | **0.4856** | **0** | **22** | **17** | **1** | **EGNPAPEYTWFK** |
|  | 2675 | **653.7247** | **1958.1520** | **1958.2836** | **-0.1316** | **1** | **22** | **22** | **1** | **DSLRMAIMVQSPMFDGK + 2 Oxidation (M)** |
|  | 1016 | **431.2576** | **1290.7505** | **1291.4346** | **-0.6840** | **0** | **21** | **18** | **1** | **GVCQSSVVAGTAR + Carbamidomethyl (C)** |
|  | 1042 | **433.1375** | **1296.3904** | **1297.4688** | **-1.0784** | **2** | **21** | **19** | **1** | **GPWRRAGTVAAR** |
|  | 2483 | **618.3606** | **1234.7064** | **1234.3549** | **0.3515** | **1** | **21** | **19** | **1** | **KQEGTPEGLYL** |
|  | 3150 | **743.2783** | **1484.5419** | **1484.5293** | **0.0126** | **0** | **21** | **19** | **1** | **AWGAGPPEGGGGGSATR** |
|  | 304 | **386.0342** | **770.0536** | **768.8681** | **1.1856** | **2** | **21** | **17** | **1** | **AGPGRRR** |
|  | 1877 | **529.1118** | **1584.3133** | **1583.8280** | **0.4853** | **2** | **21** | **20** | **1** | **SDEKAAVAGKKPVVGK** |
|  | 3331 | **789.3461** | **1576.6775** | **1576.8185** | **-0.1411** | **1** | **21** | **19** | **1** | **ALIFGGEMVAGEARR** |
|  | 1017 | **431.2979** | **1290.8716** | **1291.5386** | **-0.6671** | **0** | **21** | **19** | **1** | **LEPGALAPLAALR** |
|  | 2603 | **641.4410** | **1921.3009** | **1922.3226** | **-1.0216** | **2** | **21** | **21** | **1** | **MRRCENDCICMMLSK + Carbamidomethyl (C); 2 Oxidation (M)** |
|  | 978 | **429.1210** | **856.2273** | **855.9786** | **0.2487** | **0** | **21** | **20** | **1** | **SPTLSVPR** |
|  | 1111 | 438.0734 | 874.1321 | 873.9937 | 0.1383 | 1 | 21 | 23 | 1 | SLELREK |
|  | 2963 | **698.3650** | **2092.0728** | **2091.3614** | **0.7114** | **0** | **21** | **20** | **1** | **DDYFQVLCMADVVISTAK + Carbamidomethyl (C); Oxidation (M)** |
|  | 1281 | **458.3438** | **1372.0093** | **1372.4825** | **-0.4732** | **0** | **21** | **17** | **1** | **FDNPAAVSPTPTR** |
|  | 924 | **422.5727** | **843.1306** | **842.8571** | **0.2735** | **0** | **21** | **22** | **1** | **GQSQGGGPR** |
|  | 1001 | **430.8831** | **859.7513** | **860.0367** | **-0.2853** | **1** | **21** | **25** | **1** | **INMAGAKR** |
|  | 883 | **419.2787** | **1254.8138** | **1255.3393** | **-0.5255** | **0** | **21** | **20** | **1** | **GSPGGPGAAGFPGAR** |
|  | 2144 | **569.3991** | **1705.1752** | **1704.8846** | **0.2905** | **2** | **21** | **19** | **1** | **HQKGTWKAGCDGSCL + 2 Carbamidomethyl (C)** |
|  | 625 | **406.1353** | **1215.3838** | **1216.3859** | **-1.0022** | **0** | **21** | **21** | **1** | **FDIPLGLQGTR** |
|  | 37 | **363.1129** | **724.2110** | **723.8407** | **0.3703** | **0** | **21** | **22** | **1** | **FLCER + Carbamidomethyl (C)** |
|  | 2540 | **630.2092** | **1258.4037** | **1258.3781** | **0.0255** | **0** | **21** | **22** | **1** | **TDQEVLGELVR** |
|  | 1689 | **504.5955** | **1510.7644** | **1509.6417** | **1.1227** | **1** | **21** | **27** | **1** | **TDRIVGQNSGTSMK + Oxidation (M)** |
|  | 3385 | **803.4600** | **1604.9053** | **1605.8996** | **-0.9943** | **2** | **21** | **21** | **1** | **LGKSLTPKAEFCVR + Carbamidomethyl (C)** |
|  | 1491 | **476.1722** | **1425.4945** | **1426.6225** | **-1.1280** | **1** | **21** | **22** | **1** | **DGGCNHMQCSKCK + Oxidation (M)** |
|  | 1796 | **520.2394** | **1557.6960** | **1556.7593** | **0.9366** | **2** | **21** | **22** | **1** | **ASEEKIKQLGAGTPK** |
|  | 3058 | **735.1896** | **2202.5466** | **2201.4571** | **1.0894** | **1** | **21** | **22** | **1** | **AARSAEPELDAAAATATCAAVIK** |
|  | 2969 | **700.2299** | **1398.4449** | **1397.5716** | **0.8734** | **1** | **21** | **21** | **1** | **EMQDKVLDMEK + 2 Oxidation (M)** |
|  | 3018 | **717.3156** | **1432.6163** | **1431.6140** | **1.0023** | **0** | **21** | **23** | **1** | **MVNALENLAGIDR + Oxidation (M)** |
|  | 2670 | 652.6118 | 1954.8133 | 1954.3660 | 0.4473 | 0 | 21 | 19 | 1 | NLPRPVLVRPGPLLVSAR |
|  | 974 | **429.0915** | **1284.2523** | **1284.4221** | **-0.1698** | **2** | **21** | **23** | **1** | **STQVAHGKSNKK** |
|  | 796 | **413.6053** | **825.1958** | **825.9954** | **-0.7996** | **0** | **21** | **16** | **1** | **QLSILPR** |
|  | 2246 | **590.9991** | **1179.9834** | **1179.3015** | **0.6819** | **1** | **21** | **23** | **1** | **VMKTGGENTDK** |
|  | 980 | **429.1487** | **856.2826** | **855.9785** | **0.3041** | **0** | **21** | **24** | **1** | **SPSSLLPR** |
|  | 2347 | **599.8343** | **1197.6538** | **1198.4173** | **-0.7635** | **0** | **21** | **20** | **1** | **AVFPSIVGRPR** |
|  | 1317 | **459.9282** | **1376.7623** | **1375.5691** | **1.1933** | **0** | **20** | **26** | **1** | **LCAVWMDSSYK + Carbamidomethyl (C); Oxidation (M)** |
|  | 663 | **407.1300** | **812.2452** | **811.8828** | **0.3623** | **0** | **20** | **23** | **1** | **HGGTAELK** |
|  | 3413 | **809.8699** | **1617.7250** | **1618.8504** | **-1.1255** | **2** | **20** | **26** | **1** | **MEQKTPEKTLAAQK + Oxidation (M)** |
|  | 1318 | **460.0101** | **1377.0081** | **1376.6849** | **0.3233** | **0** | **20** | **27** | **1** | **MCSVLPVAVTGVK + Carbamidomethyl (C); Oxidation (M)** |
|  | 1587 | **488.1293** | **1461.3656** | **1461.6039** | **-0.2382** | **2** | **20** | **27** | **1** | **DRDGVRVPMASSR + Oxidation (M)** |
|  | 613 | **405.9626** | **809.9105** | **808.9850** | **0.9255** | **0** | **20** | **22** | **1** | **FIGMVDK** |
|  | 2754 | **666.7515** | **1997.2322** | **1998.2943** | **-1.0621** | **2** | **20** | **29** | **1** | **RQDAPKPTPAACRCSGLAR** |
|  | 2004 | **546.3048** | **1635.8923** | **1634.8529** | **1.0394** | **0** | **20** | **26** | **1** | **QRPEMGVIAFDIWG + Oxidation (M)** |
|  | 804 | **414.1928** | **1239.5564** | **1238.5702** | **0.9861** | **2** | **20** | **21** | **1** | **LLWLLRLRR** |
|  | 377 | 388.1808 | 774.3468 | 774.7766 | -0.4298 | 0 | 20 | 27 | 1 | GAGAGGEEK |
|  | 1512 | **477.5336** | **1429.5786** | **1428.7164** | **0.8623** | **0** | **20** | **28** | **1** | **DLVPCVPATPAMAK + Oxidation (M)** |
|  | 557 | **403.8775** | **1208.6103** | **1209.3888** | **-0.7784** | **0** | **20** | **26** | **1** | **EEPVIVTPPTK** |
|  | 1879 | **529.3453** | **1585.0138** | **1585.7871** | **-0.7733** | **1** | **20** | **22** | **1** | **VLCIGNENNQRAVR** |
|  | 1152 | **443.9216** | **885.8285** | **885.9681** | **-0.1396** | **2** | **20** | **26** | **1** | **HSSSKRGK** |
|  | 2942 | **692.3730** | **2074.0970** | **2074.4499** | **-0.3529** | **0** | **20** | **24** | **1** | **AFLGSAGAAPPHPPVVLMWR** |
|  | 2038 | 551.6353 | 1101.2558 | 1100.2991 | 0.9568 | 2 | 20 | 32 | 1 | RLPGCVSGRR |
|  | 503 | **400.2079** | **1197.6014** | **1198.3030** | **-0.7016** | **0** | **20** | **19** | **1** | **NDTAFNEIMK + Oxidation (M)** |
|  | 593 | **405.1254** | **1212.3539** | **1211.5435** | **0.8105** | **0** | **20** | **24** | **1** | **CLWMPGMAMR + Oxidation (M)** |
|  | 44 | **363.2374** | **1086.6899** | **1086.1765** | **0.5135** | **0** | **20** | **21** | **1** | **GENPPTSCPK + Carbamidomethyl (C)** |
|  | 166 | **372.3310** | **1113.9709** | **1113.2016** | **0.7693** | **0** | **20** | **22** | **1** | **QYNCSGSLGK + Carbamidomethyl (C)** |
|  | 1530 | **479.9225** | **957.8302** | **957.1487** | **0.6815** | **0** | **20** | **27** | **1** | **VTAPCTPLR** |
|  | 334 | **386.9370** | **771.8593** | **772.9328** | **-1.0735** | **1** | **20** | **29** | **1** | **GGLKALSK** |
|  | 2602 | **641.2767** | **1920.8080** | **1921.0893** | **-0.2813** | **0** | **20** | **29** | **1** | **TCATLNPATFLPDNEEK + Carbamidomethyl (C)** |
|  | 3464 | **828.2546** | **2481.7417** | **2480.7722** | **0.9696** | **2** | **20** | **24** | **1** | **ESVGKQATGEVAGKGGPVGGKPTLQK** |
|  | 1730 | **509.1764** | **1524.5069** | **1523.7095** | **0.7974** | **0** | **20** | **29** | **1** | **SFAAVIQALDGEMR + Oxidation (M)** |
|  | 1603 | **489.3837** | **1465.1288** | **1465.5245** | **-0.3956** | **0** | **20** | **23** | **1** | **ENVGGPGAPEGTPAGR** |
|  | 1102 | **437.2315** | **1308.6724** | **1308.3755** | **0.2969** | **0** | **20** | **27** | **1** | **GGEEVCQLGSSSR** |
|  | 359 | **387.9139** | **1160.7196** | **1161.2926** | **-0.5730** | **1** | **20** | **33** | **1** | **RCLAGAAGGETR** |
|  | 671 | **407.2653** | **1218.7736** | **1218.4020** | **0.3716** | **2** | **20** | **20** | **1** | **TLTHYKSAVKA** |
|  | 600 | **405.2264** | **808.4381** | **807.8512** | **0.5869** | **1** | **20** | **21** | **1** | **FDRENK** |
|  | 1335 | **460.8940** | **1379.6599** | **1379.6060** | **0.0539** | **1** | **20** | **28** | **1** | **VCSDTPSVMRLR + Oxidation (M)** |
|  | 348 | **387.2142** | **1158.6204** | **1158.3055** | **0.3150** | **1** | **20** | **26** | **1** | **SGVSEPLSRVK** |
|  | 1251 | **455.6834** | **909.3520** | **908.9550** | **0.3970** | **0** | **20** | **21** | **1** | **QGDHLSPGV** |
|  | 1698 | **504.9194** | **1511.7360** | **1510.7637** | **0.9723** | **2** | **20** | **27** | **1** | **MAAAPQAPGRGSLRK** |
|  | 1537 | **480.2615** | **1437.7623** | **1437.6018** | **0.1605** | **0** | **20** | **27** | **1** | **NGHWTGLVGDLLR** |
|  | 3485 | **845.5312** | **2533.5716** | **2532.7920** | **0.7796** | **1** | **20** | **26** | **1** | **VNPFRPGDSEPPPAPGAQRAQMGR** |
|  | 2643 | **647.6890** | **1940.0447** | **1939.2036** | **0.8411** | **1** | **20** | **31** | **1** | **GWRCNFPLQGPAGLTHK + Carbamidomethyl (C)** |
|  | 335 | **386.9397** | **1157.7969** | **1158.2856** | **-0.4887** | **0** | **20** | **31** | **1** | **QPPVGGVSDMR + Oxidation (M)** |
|  | 2507 | **623.0469** | **1866.1185** | **1864.9832** | **1.1353** | **1** | **20** | **28** | **1** | **ETVNNQKYMSFTSGDK + Oxidation (M)** |
|  | 174 | **373.6862** | **745.3575** | **744.8366** | **0.5209** | **0** | **20** | **30** | **1** | **QLLSER** |
|  | 1456 | **472.7095** | **1415.1064** | **1415.6379** | **-0.5315** | **1** | **20** | **23** | **1** | **SMYLCASSLERR** |
|  | 2474 | **616.5347** | **1231.0547** | **1231.3989** | **-0.3443** | **0** | **20** | **24** | **1** | **SGISASLAISGLR** |
|  | 3162 | **744.8744** | **2231.6010** | **2231.5974** | **0.0036** | **2** | **20** | **32** | **1** | **RWRTFPVDCVAMCGDCVEK + Oxidation (M)** |
|  | 3537 | **897.7296** | **1793.4444** | **1793.1358** | **0.3087** | **2** | **20** | **25** | **1** | **ISMPDVDLHLKGPKVK + Oxidation (M)** |
|  | 2188 | **578.9500** | **1733.8277** | **1734.9704** | **-1.1427** | **0** | **20** | **27** | **1** | **AVYLALNQCSVSTTHK** |
|  | 3424 | **814.6194** | **2440.8362** | **2440.5723** | **0.2638** | **1** | **20** | **26** | **1** | **EEEDGEGAGPGEQGGGKLVLSSLPK** |
|  | 1093 | **436.9913** | **871.9678** | **872.0674** | **-0.0996** | **0** | **20** | **31** | **1** | **HPVSMACK** |
|  | 2890 | **684.2194** | **2049.6359** | **2050.2579** | **-0.6220** | **1** | **20** | **27** | **1** | **RSPSTGPWPCPQDPLGAAR + Carbamidomethyl (C)** |
|  | 1039 | **433.0899** | **1296.2474** | **1295.4168** | **0.8307** | **0** | **20** | **28** | **1** | **MSSSATAVETPAK + Oxidation (M)** |
|  | 2742 | **666.5956** | **1996.7646** | **1997.2964** | **-0.5319** | **1** | **20** | **23** | **1** | **QVQLLQSAAEVKKPGSSVK** |
|  | 2810 | **671.6907** | **2012.0498** | **2012.2779** | **-0.2280** | **2** | **20** | **29** | **1** | **RYACRGGGTCQMDAFMR + Carbamidomethyl (C); 2 Oxidation (M)** |
|  | 887 | **419.3192** | **1254.9353** | **1255.3393** | **-0.4040** | **0** | **20** | **28** | **1** | **GSPGGPGAAGFPGAR** |
|  | 2917 | **686.3114** | **2055.9120** | **2056.2144** | **-0.3024** | **0** | **20** | **28** | **1** | **LSGSCSAPSLAAPDGSAPSAPR + Carbamidomethyl (C)** |
|  | 1229 | **452.6674** | **903.3201** | **903.1660** | **0.1541** | **0** | **20** | **26** | **1** | **MVTMCMR + 2 Oxidation (M)** |
|  | 670 | 407.2534 | 1218.7380 | 1218.4465 | 0.2914 | 0 | 20 | 22 | 1 | GQLSGQPLCMK + Carbamidomethyl (C) |
|  | 1212 | **451.1673** | **1350.4797** | **1351.4400** | **-0.9603** | **0** | **20** | **32** | **1** | **DITEEIMSGGGSR** |
|  | 1706 | **505.3684** | **1008.7220** | **1009.1537** | **-0.4317** | **1** | **20** | **22** | **1** | **ETKDPPPLL** |
|  | 605 | **405.3117** | **1212.9129** | **1213.4268** | **-0.5139** | **1** | **20** | **22** | **1** | **KEFSACAIGCK + Carbamidomethyl (C)** |
|  | 1184 | **448.0027** | **1340.9860** | **1341.7335** | **-0.7475** | **2** | **20** | **28** | **1** | **MMMLVGMGKRR + 2 Oxidation (M)** |
|  | 21 | **362.1895** | **722.3641** | **722.8328** | **-0.4686** | **0** | **20** | **22** | **1** | **PSPPGLR** |
|  | 71 | **366.1306** | **730.2464** | **729.8255** | **0.4209** | **1** | **20** | **31** | **1** | **KSSPVGR** |
|  | 2736 | **666.4608** | **1330.9068** | **1330.5268** | **0.3800** | **0** | **20** | **28** | **1** | **LISPVSLQTSTGK** |
|  | 2783 | **668.7903** | **2003.3487** | **2002.4251** | **0.9236** | **0** | **20** | **35** | **1** | **EILPLACFLGLRPLYQR** |
|  | 2276 | **593.1105** | **1776.3094** | **1777.0784** | **-0.7689** | **1** | **20** | **30** | **1** | **FAKVALAAGSPARPPPAR** |
|  | 1599 | **488.5644** | **975.1140** | **976.1487** | **-1.0346** | **0** | **20** | **39** | **1** | **MALSPSATAK** |
|  | 1240 | **453.6650** | **1357.9729** | **1358.4758** | **-0.5029** | **1** | **19** | **26** | **1** | **FDKFGPEMSER + Oxidation (M)** |
|  | 761 | **410.6483** | **1228.9228** | **1228.3787** | **0.5441** | **0** | **19** | **26** | **1** | **QMSHDLVAVGR + Oxidation (M)** |
|  | 2774 | **668.6200** | **2002.8378** | **2003.3536** | **-0.5158** | **2** | **19** | **26** | **1** | **NCGKSGVLQALLGRNLMR + Carbamidomethyl (C); Oxidation (M)** |
|  | 1080 | 435.9347 | 869.8546 | 870.9965 | -1.1419 | 1 | 19 | 29 | 1 | AGSGKAKPR |
|  | 1106 | **437.3240** | **1308.9498** | **1308.5328** | **0.4170** | **1** | **19** | **28** | **1** | **MCPFGRAGLQGR + Oxidation (M)** |
|  | 1307 | **459.4002** | **1375.1785** | **1375.7048** | **-0.5264** | **1** | **19** | **28** | **1** | **LTQMLKCHVFR** |
|  | 3098 | **740.5709** | **2218.6906** | **2217.4961** | **1.1945** | **1** | **19** | **27** | **1** | **GVKLLAALMDDEVGSGEDLLR + Oxidation (M)** |
|  | 483 | **399.1535** | **1194.4383** | **1195.3669** | **-0.9285** | **0** | **19** | **27** | **1** | **TFFISPGSLAR** |
|  | 3082 | **740.3669** | **2218.0787** | **2218.6615** | **-0.5828** | **1** | **19** | **30** | **1** | **MVMSFRVSDLQMLLGFVGR + 2 Oxidation (M)** |
|  | 67 | **366.0216** | **1095.0427** | **1096.2043** | **-1.1615** | **2** | **19** | **36** | **1** | **PRGRGGGGGGLR** |
|  | 219 | **377.2461** | **1128.7161** | **1129.1880** | **-0.4718** | **2** | **19** | **23** | **1** | **SGRGGKSSTHR** |
|  | 1710 | **505.6498** | **1513.9273** | **1514.8500** | **-0.9227** | **1** | **19** | **30** | **1** | **VALLKLVSLTGSWK** |
|  | 3491 | **848.2390** | **1694.4632** | **1694.9231** | **-0.4599** | **1** | **19** | **28** | **1** | **SLPGLASSVKEPLPQAT** |
|  | 2260 | **592.7053** | **1775.0938** | **1775.9382** | **-0.8444** | **2** | **19** | **37** | **1** | **SVFMSSTTSASGTGRKR + Oxidation (M)** |
|  | 79 | **367.1750** | **732.3353** | **732.7400** | **-0.4047** | **0** | **19** | **29** | **1** | **ESAPSSR** |
|  | 132 | **369.3606** | **1105.0597** | **1106.2110** | **-1.1513** | **1** | **19** | **35** | **1** | **RMLPSDESR + Oxidation (M)** |
|  | 253 | 380.0245 | 758.0342 | 757.7925 | 0.2417 | 0 | 19 | 36 | 1 | HSGASATK |
|  | 820 | **415.9092** | **829.8037** | **828.9564** | **0.8473** | **1** | **19** | **36** | **1** | **AALAAERK** |
|  | 1299 | **458.9689** | **1373.8846** | **1374.6921** | **-0.8074** | **2** | **19** | **35** | **1** | **MKELCAMYGKK + Carbamidomethyl (C); Oxidation (M)** |
|  | 418 | **389.3656** | **1165.0746** | **1165.3922** | **-0.3176** | **2** | **19** | **34** | **1** | **LPGRGVAALRR** |
|  | 1064 | **434.9946** | **867.9744** | **867.0890** | **0.8855** | **0** | **19** | **28** | **1** | **ILMSTMR + Oxidation (M)** |
|  | 1747 | **513.8032** | **1538.3875** | **1537.8008** | **0.5867** | **0** | **19** | **25** | **1** | **MSEILLSQVTNMR + Oxidation (M)** |
|  | 981 | **429.1506** | **1284.4295** | **1283.4769** | **0.9526** | **1** | **19** | **32** | **1** | **DQVLLAARELR** |
|  | 425 | **389.9215** | **777.8282** | **776.9198** | **0.9084** | **0** | **19** | **33** | **1** | **AFLGELK** |
|  | 90 | **368.1872** | **1101.5393** | **1101.2971** | **0.2422** | **2** | **19** | **28** | **1** | **KKFFQEFK** |
|  | 2854 | **680.7676** | **2039.2806** | **2040.1969** | **-0.9163** | **2** | **19** | **38** | **1** | **RGFAEGQGGSGTESGKLVFR** |
|  | 205 | 377.0463 | 1128.1166 | 1127.4422 | 0.6745 | 2 | 19 | 31 | 1 | ETKCMIKMK + Oxidation (M) |
|  | 112 | **369.2688** | **1104.7841** | **1104.3011** | **0.4831** | **0** | **19** | **28** | **1** | **MHPSLATMGK + 2 Oxidation (M)** |
|  | 428 | **390.8988** | **1169.6742** | **1169.4373** | **0.2370** | **0** | **19** | **31** | **1** | **MSKPPDLLLR** |
|  | 1825 | **523.3350** | **1044.6553** | **1044.1631** | **0.4922** | **1** | **19** | **31** | **1** | **TPVAAAGSSKR** |
|  | 2813 | **672.2896** | **1342.5643** | **1341.5958** | **0.9685** | **1** | **19** | **32** | **1** | **LGTTLELTPLRK** |
|  | 3137 | **742.5419** | **1483.0690** | **1482.6611** | **0.4079** | **2** | **19** | **30** | **1** | **KKEQWSMTSVSR + Oxidation (M)** |
|  | 356 | **387.9095** | **773.8043** | **773.8780** | **-0.0736** | **2** | **19** | **40** | **1** | **GKQGEKK** |
|  | 995 | **430.5237** | **859.0327** | **859.9673** | **-0.9346** | **0** | **19** | **45** | **1** | **TTLSVPSR** |
|  | 1973 | **540.5595** | **1618.6564** | **1618.9461** | **-0.2898** | **1** | **19** | **36** | **1** | **VSNCICPLIGRACR + 2 Carbamidomethyl (C)** |
|  | 284 | 385.0680 | 1152.1818 | 1152.3008 | -0.1190 | 1 | 19 | 29 | 1 | VLHAEQEKAK |
|  | 1544 | **481.8336** | **1442.4787** | **1442.7295** | **-0.2508** | **1** | **19** | **33** | **1** | **KCCQAGMVLGGFR + Carbamidomethyl (C); Oxidation (M)** |
|  | 3169 | **747.3281** | **1492.6415** | **1492.7251** | **-0.0837** | **1** | **19** | **33** | **1** | **HYLFGPPKLHQR** |
|  | 1297 | **458.9430** | **1373.8068** | **1373.6461** | **0.1607** | **2** | **19** | **37** | **1** | **HMKKFHGDMVK + Oxidation (M)** |
|  | 1800 | **520.4955** | **1038.9763** | **1038.1517** | **0.8246** | **0** | **19** | **29** | **1** | **FIEIGSGTSK** |
|  | 1200 | **450.2248** | **898.4347** | **899.0495** | **-0.6148** | **1** | **19** | **33** | **1** | **LGRTSLPR** |
|  | 617 | **406.0181** | **1215.0322** | **1214.3719** | **0.6603** | **1** | **19** | **31** | **1** | **LQKADVVAQSR** |
|  | 596 | **405.1700** | **1212.4879** | **1211.3084** | **1.1795** | **1** | **19** | **31** | **1** | **MGFSEQGRQR + Oxidation (M)** |
|  | 2538 | **630.0299** | **1887.0675** | **1886.1694** | **0.8981** | **1** | **19** | **34** | **1** | **ETLEELKAFYELICK + Carbamidomethyl (C)** |
|  | 632 | **406.1783** | **1215.5126** | **1214.3950** | **1.1176** | **0** | **19** | **33** | **1** | **CFCSQPCGQNK** |
|  | 1390 | **466.1072** | **1395.2995** | **1395.5208** | **-0.2212** | **2** | **19** | **37** | **1** | **RNEIDAEPPAKR** |
|  | 361 | **387.9455** | **773.8761** | **772.8866** | **0.9896** | **0** | **19** | **43** | **1** | **VAILDDK** |
|  | 2133 | **566.7784** | **1131.5421** | **1132.2283** | **-0.6862** | **1** | **19** | **30** | **1** | **LSSQGNVSGKR** |
|  | 2187 | **578.8947** | **1155.7745** | **1156.3723** | **-0.5978** | **1** | **19** | **29** | **1** | **QVLVKAELEK** |
|  | 806 | **414.2184** | **826.4220** | **826.9373** | **-0.5153** | **0** | **19** | **27** | **1** | **GGLASTPPK** |
|  | 329 | **386.8570** | **1157.5488** | **1158.3931** | **-0.8442** | **0** | **19** | **39** | **1** | **LCPAMGYTFR** |
|  | 2032 | **550.9512** | **1649.8315** | **1649.9951** | **-0.1636** | **2** | **19** | **36** | **1** | **MTDLGRLGAFITKVK** |
|  | 31 | **362.9244** | **1085.7511** | **1085.1702** | **0.5809** | **0** | **19** | **35** | **1** | **GEEHLPTFR** |
|  | 603 | **405.2706** | **1212.7895** | **1212.3328** | **0.4567** | **0** | **19** | **27** | **1** | **AGHCAPSEAIEK** |
|  | 2199 | **580.1458** | **1737.4153** | **1736.8804** | **0.5349** | **1** | **19** | **38** | **1** | **VTNEPSVDNHLDLRK** |
|  | 2881 | **683.9673** | **1365.9198** | **1365.5807** | **0.3391** | **1** | **19** | **29** | **1** | **GALVLGSSLKQHR** |
|  | 1113 | **438.1845** | **1311.5313** | **1311.5352** | **-0.0039** | **2** | **19** | **39** | **1** | **HEKTLRPRFK** |
|  | 2238 | **589.1416** | **1764.4026** | **1765.1008** | **-0.6982** | **1** | **19** | **39** | **1** | **EEMTKNQVILTCLVK + Oxidation (M)** |
|  | 1234 | **453.0106** | **1356.0095** | **1355.6074** | **0.4021** | **1** | **19** | **39** | **1** | **GRYLTVACIFR + Carbamidomethyl (C)** |
|  | 1778 | **518.9227** | **1553.7460** | **1553.6943** | **0.0518** | **1** | **19** | **35** | **1** | **IRMDSIGSTVSSER + Oxidation (M)** |
|  | 1227 | **452.3015** | **1353.8823** | **1354.5995** | **-0.7173** | **2** | **19** | **34** | **1** | **KLADKFIHAGVR** |
|  | 2552 | **631.4498** | **1891.3273** | **1890.1679** | **1.1594** | **1** | **19** | **36** | **1** | **LTMWRLADIEQSTGIR** |
|  | 40 | **363.1520** | **1086.4339** | **1085.3704** | **1.0635** | **2** | **19** | **34** | **1** | **GAIRLLMRR** |
|  | 3578 | **1002.1842** | **2002.3536** | **2003.1486** | **-0.7949** | **0** | **19** | **37** | **1** | **YTPASPSSPDFTTWSCAK + Carbamidomethyl (C)** |
|  | 171 | **373.0315** | **744.0483** | **744.9427** | **-0.8944** | **0** | **19** | **46** | **1** | **LQMPLK + Oxidation (M)** |
|  | 3152 | **743.4597** | **2227.3568** | **2226.5591** | **0.7977** | **2** | **19** | **38** | **1** | **CFGTKCAGCAQGISPSDLVRR + Carbamidomethyl (C)** |
|  | 1033 | **432.9447** | **1295.8119** | **1295.5955** | **0.2165** | **2** | **19** | **37** | **1** | **RCAIEADMKMK** |
|  | 2513 | **623.8557** | **1868.5450** | **1868.0549** | **0.4900** | **1** | **19** | **32** | **1** | **CLNAGSGTETAERVQMK + Carbamidomethyl (C); Oxidation (M)** |
|  | 1340 | **461.8019** | **921.5891** | **921.9092** | **-0.3201** | **1** | **19** | **33** | **1** | **EKSSNSDR** |
|  | 1754 | **514.7753** | **1541.3036** | **1541.8375** | **-0.5338** | **1** | **19** | **29** | **1** | **VLVSGMFLSNRMR + 2 Oxidation (M)** |
|  | 1999 | **545.8856** | **1089.7563** | **1089.1985** | **0.5578** | **1** | **18** | **34** | **1** | **TWKESPSAIA** |
|  | 2049 | **553.0734** | **1656.1981** | **1657.0506** | **-0.8525** | **0** | **18** | **41** | **1** | **MPPVSLFLFCFSLR** |
|  | 2250 | **592.0627** | **1182.1107** | **1182.3732** | **-0.2624** | **2** | **18** | **36** | **1** | **LKGSSHAVQKK** |
|  | 2058 | **554.2453** | **1659.7137** | **1658.8680** | **0.8458** | **0** | **18** | **40** | **1** | **DPGETVGGDEIVMVIK** |
|  | 2884 | **684.0554** | **1366.0961** | **1365.4036** | **0.6924** | **0** | **18** | **35** | **1** | **YDSGSFATQAYR** |
|  | 3320 | **783.9234** | **1565.8320** | **1566.7957** | **-0.9637** | **1** | **18** | **43** | **1** | **FKGPFTDVVTTNLK** |
|  | 1727 | **508.2167** | **1014.4187** | **1014.1337** | **0.2849** | **1** | **18** | **40** | **1** | **HTAESLKTK** |
|  | 2471 | **616.3842** | **1230.7535** | **1230.3679** | **0.3856** | **0** | **18** | **39** | **1** | **IENDVATILSR** |
|  | 254 | **380.1120** | **1137.3138** | **1138.2973** | **-0.9835** | **1** | **18** | **44** | **1** | **CASLQKFGER** |
|  | 2204 | **581.0002** | **1739.9784** | **1740.0132** | **-0.0348** | **1** | **18** | **41** | **1** | **LLAARGATIQELNTIR** |
|  | 2889 | **684.2092** | **1366.4036** | **1365.5807** | **0.8228** | **1** | **18** | **38** | **1** | **GALVLGSSLKQHR** |
|  | 1379 | **465.3328** | **928.6508** | **928.0677** | **0.5831** | **0** | **18** | **32** | **1** | **CSVVPPNR + Carbamidomethyl (C)** |
|  | 1481 | **475.6085** | **1423.8032** | **1423.6135** | **0.1897** | **2** | **18** | **46** | **1** | **KLIDYGLSGSSRK** |
|  | 220 | **377.2478** | **1128.7212** | **1129.0952** | **-0.3739** | **0** | **18** | **30** | **1** | **GSGSSQSSGYGR** |
|  | 777 | **411.6432** | **1231.9074** | **1232.3853** | **-0.4779** | **0** | **18** | **33** | **1** | **NSAYLHLSSLK** |
|  | 1675 | **501.8502** | **1502.5283** | **1502.6308** | **-0.1024** | **1** | **18** | **39** | **1** | **HLEGTNKEAPPPGR** |
|  | 3289 | **776.0609** | **1550.1070** | **1549.7236** | **0.3834** | **1** | **18** | **32** | **1** | **SKWYNEYAVYVK** |
|  | 2128 | **566.0624** | **1695.1650** | **1694.0262** | **1.1388** | **2** | **18** | **41** | **1** | **FLYLYCSKEMPRK + Oxidation (M)** |
|  | 968 | **428.9134** | **1283.7180** | **1283.5200** | **0.1980** | **1** | **18** | **36** | **1** | **CFTCGKIVGNK + 2 Carbamidomethyl (C)** |
|  | 2210 | **582.6946** | **1745.0618** | **1745.0103** | **0.0515** | **2** | **18** | **49** | **1** | **SVSQKVAAAMPVRAADK + Oxidation (M)** |
|  | 2393 | **608.6837** | **1215.3527** | **1216.2619** | **-0.9093** | **1** | **18** | **50** | **1** | **RTGENPATASGR** |
|  | 2645 | **647.9260** | **1940.7557** | **1941.3687** | **-0.6130** | **1** | **18** | **32** | **1** | **CGGRGLLLGLAVAAAAVMAAR** |
|  | 951 | **427.9222** | **1280.7444** | **1280.4483** | **0.2960** | **1** | **18** | **38** | **1** | **GKAGTATLAMSEK + Oxidation (M)** |
|  | 1404 | **467.8656** | **1400.5747** | **1399.5739** | **1.0008** | **0** | **18** | **43** | **1** | **MCGSAAGATELCR + 2 Carbamidomethyl (C); Oxidation (M)** |
|  | 3339 | **791.7731** | **1581.5315** | **1580.6979** | **0.8335** | **1** | **18** | **33** | **1** | **QQEELNAQVEKHK** |
|  | 2560 | **632.4844** | **1262.9540** | **1262.4395** | **0.5144** | **1** | **18** | **36** | **1** | **VLGRCGSSQSIR** |
|  | 1304 | **459.2089** | **916.4029** | **917.0187** | **-0.6157** | **0** | **18** | **45** | **1** | **SSSVPSVVR** |
|  | 2771 | **667.9033** | **2000.6878** | **2000.1926** | **0.4952** | **1** | **18** | **35** | **1** | **NPQEAESYCAQYKVLTR** |
|  | 149 | **371.0728** | **1110.1962** | **1111.3384** | **-1.1422** | **2** | **18** | **34** | **1** | **KMKFPCER + Carbamidomethyl (C); Oxidation (M)** |
|  | 750 | **409.0689** | **1224.1846** | **1225.3515** | **-1.1669** | **1** | **18** | **46** | **1** | **YTTATGRNITK** |
|  | 802 | **413.8739** | **1238.5995** | **1238.4230** | **0.1765** | **1** | **18** | **35** | **1** | **VHAARCGLQGAR** |
|  | 439 | **391.3124** | **1170.9150** | **1171.2643** | **-0.3494** | **1** | **18** | **30** | **1** | **GAQKGAAPTGTGR** |
|  | 724 | **408.1218** | **1221.3431** | **1222.3906** | **-1.0475** | **0** | **18** | **44** | **1** | **TASFGGITVLTR** |
|  | 862 | **418.1558** | **1251.4453** | **1250.5548** | **0.8905** | **1** | **18** | **42** | **1** | **MGNPMAKNLMK + Oxidation (M)** |
|  | 1457 | **472.7649** | **1415.2724** | **1414.6084** | **0.6640** | **0** | **18** | **34** | **1** | **MCSGWSSSVIWR + Oxidation (M)** |
|  | 3558 | **964.2253** | **2889.6539** | **2890.3611** | **-0.7073** | **1** | **18** | **31** | **1** | **ALNPDSAPRPMPALEYLPHPIKLHPV + Oxidation (M)** |
|  | 965 | **428.5959** | **855.1769** | **854.9507** | **0.2263** | **0** | **18** | **33** | **1** | **NGVPEALR** |
|  | 2943 | **692.9177** | **2075.7310** | **2075.2823** | **0.4487** | **0** | **18** | **35** | **1** | **HSIIHTGEKPYECSECGK + Carbamidomethyl (C)** |
|  | 2640 | **646.9106** | **1291.8065** | **1291.3218** | **0.4847** | **0** | **18** | **33** | **1** | **INSDSEELTQR** |
|  | 1067 | **435.0881** | **868.1614** | **866.9648** | **1.1966** | **2** | **18** | **38** | **1** | **DPRSKHK** |
|  | 2136 | **567.9816** | **1700.9227** | **1699.7542** | **1.1685** | **0** | **18** | **43** | **1** | **MEAGSGPPGGPGSESPNR + Oxidation (M)** |
|  | 1151 | **443.9102** | **1328.7085** | **1329.4642** | **-0.7558** | **1** | **18** | **45** | **1** | **EGAFPAAQVQRR** |
|  | 1458 | **472.8332** | **1415.4773** | **1416.6426** | **-1.1653** | **0** | **18** | **41** | **1** | **LSFCDSPGQMMK + Carbamidomethyl (C); Oxidation (M)** |
|  | 2044 | **552.0586** | **1102.1024** | **1102.2456** | **-0.1432** | **2** | **18** | **44** | **1** | **RKTTASPSVR** |
|  | 569 | **404.0644** | **1209.1711** | **1208.4089** | **0.7622** | **1** | **18** | **43** | **1** | **MMIDPNAKTR + 2 Oxidation (M)** |
|  | 1223 | **452.0077** | **902.0006** | **902.0087** | **-0.0081** | **0** | **18** | **51** | **1** | **HQSIYVR** |
|  | 1298 | **458.9481** | **1373.8220** | **1373.4258** | **0.3963** | **0** | **18** | **46** | **1** | **TSGDPPSLASQSAR** |
|  | 2409 | **610.0858** | **1827.2351** | **1827.2231** | **0.0120** | **1** | **18** | **43** | **1** | **MGCLSPCTLRSWMLR + Carbamidomethyl (C); Oxidation (M)** |
|  | 173 | 373.1169 | 744.2191 | 743.8916 | 0.3275 | 1 | 18 | 51 | 1 | KLLEGGK |
|  | 242 | **379.3176** | **756.6205** | **756.8475** | **-0.2270** | **0** | **18** | **34** | **1** | **IPSVEGR** |
|  | 246 | **379.4885** | **1135.4434** | **1134.2593** | **1.1841** | **0** | **18** | **51** | **1** | **MTMETQMSQ + 3 Oxidation (M)** |
|  | 279 | **385.0058** | **1151.9952** | **1152.3636** | **-0.3684** | **0** | **18** | **37** | **1** | **VQSSIAFLCGK** |
|  | 499 | **400.1735** | **798.3322** | **797.9426** | **0.3896** | **1** | **18** | **37** | **1** | **TPVTPRK** |
|  | 3013 | **715.6323** | **2143.8746** | **2144.2964** | **-0.4217** | **0** | **18** | **35** | **1** | **LLPSVWSESEDGAGSPPPYR** |
|  | 516 | **401.6784** | **1202.0131** | **1201.2952** | **0.7179** | **2** | **18** | **40** | **1** | **RDKQYQAHR** |
|  | 263 | **382.1461** | **762.2775** | **761.9731** | **0.3044** | **1** | **18** | **50** | **1** | **KCILGTK** |
|  | 2896 | **685.1034** | **1368.1920** | **1367.5572** | **0.6348** | **2** | **18** | **44** | **1** | **TAAPSVRPEKRR** |
|  | 623 | **406.1033** | **810.1919** | **809.8737** | **0.3182** | **1** | **18** | **42** | **1** | **VEHNRR** |
|  | 627 | **406.1396** | **1215.3967** | **1214.2924** | **1.1042** | **2** | **18** | **42** | **1** | **LRSHSRSSER** |
|  | 1498 | **476.3650** | **950.7153** | **950.0022** | **0.7131** | **0** | **18** | **34** | **1** | **GTTVAVSSASA** |
|  | 3538 | **897.7386** | **1793.4625** | **1794.0208** | **-0.5583** | **2** | **18** | **38** | **1** | **FLRARDFDLDLAWR** |
|  | 18 | **362.1209** | **722.2270** | **722.7252** | **-0.4982** | **0** | **18** | **41** | **1** | **DSCQDR** |
|  | 32 | **362.9998** | **1085.9774** | **1086.2260** | **-0.2487** | **1** | **18** | **43** | **1** | **RLEACPQNR** |
|  | 2681 | **654.4448** | **1306.8749** | **1307.5579** | **-0.6831** | **1** | **18** | **41** | **1** | **ILPSDACKIYK + Carbamidomethyl (C)** |
|  | 706 | **407.8638** | **1220.5693** | **1221.4142** | **-0.8448** | **2** | **18** | **43** | **1** | **GSVNCRCAAKR + Carbamidomethyl (C)** |
|  | 1653 | **498.3605** | **1492.0592** | **1492.5233** | **-0.4640** | **0** | **18** | **34** | **1** | **MEGGFGSDFGGSGSGK + Oxidation (M)** |
|  | 1526 | **479.3470** | **1435.0188** | **1434.6230** | **0.3958** | **1** | **18** | **35** | **1** | **LRHLSFSGMSGAR + Oxidation (M)** |
|  | 1686 | **504.0283** | **1509.0626** | **1509.7925** | **-0.7299** | **2** | **18** | **47** | **1** | **QKFVSMKVDHMK + 2 Oxidation (M)** |
|  | 1919 | **535.1090** | **1602.3049** | **1601.9060** | **0.3989** | **2** | **18** | **47** | **1** | **LIPVSGKLEKNMEK + Oxidation (M)** |
|  | 2897 | **685.1193** | **1368.2237** | **1367.5572** | **0.6666** | **2** | **18** | **45** | **1** | **TAAPSVRPEKRR** |
|  | 645 | **406.6321** | **1216.8741** | **1217.3809** | **-0.5068** | **2** | **18** | **32** | **1** | **RRSPSPAPPPR** |
|  | 2638 | **646.2745** | **1290.5342** | **1289.4882** | **1.0460** | **2** | **18** | **47** | **1** | **MRNQDPGKMGR** |
|  | 3239 | **759.8193** | **1517.6239** | **1516.7450** | **0.8788** | **1** | **18** | **49** | **1** | **ALPATGSAGCARCPGK + Carbamidomethyl (C)** |
|  | 266 | **383.1229** | **1146.3465** | **1145.3994** | **0.9471** | **1** | **18** | **46** | **1** | **RCVPGTLPMR + Oxidation (M)** |
|  | 3148 | **742.9432** | **2225.8075** | **2226.4003** | **-0.5928** | **2** | **18** | **42** | **1** | **TLVVHEKABBLGKGGBZESTK** |
|  | 1751 | **514.3588** | **1540.0543** | **1539.6888** | **0.3655** | **0** | **18** | **35** | **1** | **LGGIWHTPAGPYDGV** |
|  | 2102 | **562.4425** | **1684.3053** | **1685.0408** | **-0.7355** | **2** | **18** | **36** | **1** | **GQKDVCIVLAKEIIR** |
|  | 762 | **410.6802** | **819.3456** | **818.7860** | **0.5596** | **0** | **18** | **39** | **1** | **AAAQDSEQ** |
|  | 1582 | **487.9575** | **973.9002** | **973.1250** | **0.7752** | **1** | **18** | **51** | **1** | **TGPVSVGTKK** |
|  | 1560 | **485.3319** | **1452.9736** | **1453.5965** | **-0.6230** | **0** | **18** | **33** | **1** | **AQQALSELHTVEK** |
|  | 482 | **399.1493** | **1194.4258** | **1195.3519** | **-0.9261** | **0** | **18** | **40** | **1** | **AGLGKPEACGHR** |
|  | 108 | **369.2556** | **1104.7446** | **1104.2746** | **0.4700** | **0** | **18** | **39** | **1** | **AIDPSLVDMK + Oxidation (M)** |
|  | 150 | 371.1405 | 740.2662 | 740.8051 | -0.5389 | 1 | 18 | 36 | 1 | EEKAHK |
|  | 176 | **373.9316** | **1118.7728** | **1119.2378** | **-0.4650** | **1** | **18** | **59** | **1** | **RPRQGPGSHK** |
|  | 548 | **403.3555** | **1207.0442** | **1206.3299** | **0.7143** | **1** | **18** | **43** | **1** | **ERELLSSGGCR** |
|  | 614 | **406.0016** | **1214.9828** | **1215.4461** | **-0.4633** | **0** | **18** | **42** | **1** | **MDANVLMHLR + Oxidation (M)** |
|  | 1289 | **458.7894** | **1373.3459** | **1373.6244** | **-0.2784** | **1** | **18** | **45** | **1** | **MGCNLCTFQKR + Carbamidomethyl (C); Oxidation (M)** |
|  | 2069 | **556.6918** | **1667.0531** | **1667.7354** | **-0.6823** | **1** | **18** | **52** | **1** | **EDKASGGGAGALSSAPHR** |
|  | 1137 | **443.1565** | **884.2982** | **885.0661** | **-0.7679** | **1** | **18** | **45** | **1** | **ALTRVVAR** |
|  | 544 | **403.0849** | **1206.2325** | **1205.3668** | **0.8658** | **1** | **18** | **52** | **1** | **GRAGPSPPTPIR** |
|  | 2114 | **563.7878** | **1688.3414** | **1687.9123** | **0.4290** | **0** | **18** | **38** | **1** | **SGAAQGLAEVMAGLGVEK** |
|  | 3350 | **794.1439** | **2379.4096** | **2378.6451** | **0.7645** | **1** | **18** | **37** | **1** | **GIHHGPSVAQPIHLDSTQLSRK** |
|  | 798 | **413.6722** | **825.3297** | **825.9160** | **-0.5863** | **1** | **18** | **31** | **1** | **GQARAAPR** |
|  | 1511 | **477.5272** | **953.0397** | **953.0540** | **-0.0143** | **0** | **18** | **51** | **1** | **GQASPAPAVR** |
|  | 2272 | **593.0021** | **1775.9842** | **1776.9294** | **-0.9451** | **2** | **18** | **48** | **1** | **SPGGSSRGSPSVSCSRLR** |
|  | 1159 | **444.7989** | **887.5830** | **887.0984** | **0.4845** | **1** | **18** | **53** | **1** | **IICEGPKK** |
|  | 1411 | 468.4284 | 1402.2631 | 1402.5132 | -0.2500 | 0 | 18 | 45 | 1 | SFNHFTSLGHQK |
|  | 3426 | **814.7451** | **2441.2132** | **2440.8142** | **0.3990** | **0** | **17** | **36** | **1** | **MPWSPALSCVSSSAQVGSLTLMR + 2 Oxidation (M)** |
|  | 95 | **368.2743** | **1101.8006** | **1101.2160** | **0.5847** | **1** | **17** | **41** | **1** | **KQVETGFHR** |
|  | 488 | **399.2174** | **1194.6300** | **1194.2729** | **0.3571** | **0** | **17** | **35** | **1** | **SLGEQMAAESR + Oxidation (M)** |
|  | 144 | **370.5162** | **739.0176** | **739.7803** | **-0.7628** | **0** | **17** | **38** | **1** | **GGIGSGHR** |
|  | 1160 | **444.8745** | **1331.6015** | **1330.5104** | **1.0911** | **0** | **17** | **56** | **1** | **TAQSPAMVGSPIR + Oxidation (M)** |
|  | 336 | **386.9408** | **1157.8002** | **1157.3205** | **0.4798** | **0** | **17** | **53** | **1** | **MLCGGGAFNSK + Carbamidomethyl (C); Oxidation (M)** |
|  | 1085 | **436.2003** | **870.3857** | **870.9932** | **-0.6074** | **1** | **17** | **48** | **1** | **KAVPGNSAK** |
|  | 2775 | **668.6396** | **1335.2645** | **1335.5270** | **-0.2624** | **1** | **17** | **42** | **1** | **MAKISSPTETVR + Oxidation (M)** |
|  | 3110 | **740.7582** | **1479.5016** | **1478.6888** | **0.8127** | **0** | **17** | **46** | **1** | **LLPLPGEERPETK** |
|  | 520 | **401.9088** | **801.8027** | **800.8586** | **0.9442** | **0** | **17** | **55** | **1** | **FFSASSR** |
|  | 1953 | **538.2538** | **1611.7392** | **1611.7466** | **-0.0074** | **0** | **17** | **50** | **1** | **ELGFGATVIENIEGY** |
|  | 473 | **398.8294** | **795.6440** | **795.9300** | **-0.2859** | **1** | **17** | **47** | **1** | **RVPATPR** |
|  | 4 | **360.3709** | **1078.0904** | **1077.1746** | **0.9158** | **1** | **17** | **64** | **1** | **RGEGWGEMR** |
|  | 1268 | **457.6053** | **1369.7937** | **1369.6293** | **0.1644** | **0** | **17** | **47** | **1** | **LNTPMGPGGTVVVK** |
|  | 165 | **372.2458** | **742.4769** | **742.9499** | **-0.4731** | **1** | **17** | **37** | **1** | **KIITIR** |
|  | 370 | **388.0681** | **774.1213** | **773.8399** | **0.2815** | **0** | **17** | **58** | **1** | **EHHVPR** |
|  | 381 | **388.2744** | **1161.8011** | **1162.2047** | **-0.4036** | **0** | **17** | **46** | **1** | **DSEDSQAVIAK** |
|  | 1824 | **523.3065** | **1044.5983** | **1045.1544** | **-0.5561** | **1** | **17** | **50** | **1** | **SISRPRSSR** |
|  | 2595 | **640.0638** | **1278.1129** | **1278.4324** | **-0.3195** | **0** | **17** | **49** | **1** | **TIAMDGTEGLVR + Oxidation (M)** |
|  | 703 | **407.8437** | **1220.5090** | **1219.4480** | **1.0610** | **0** | **17** | **49** | **1** | **IFEMGYVFTL** |
|  | 1508 | **477.2435** | **952.4722** | **953.0938** | **-0.6215** | **0** | **17** | **47** | **1** | **LTPTHVTGK** |
|  | 2542 | **630.6093** | **1259.2037** | **1259.4970** | **-0.2932** | **1** | **17** | **44** | **1** | **NGLDVLFKVVR** |
|  | 1225 | **452.1537** | **902.2927** | **902.0071** | **0.2855** | **1** | **17** | **60** | **1** | **SAGKAEIAR** |
|  | 785 | **413.2806** | **824.5464** | **823.9815** | **0.5649** | **0** | **17** | **34** | **1** | **SAMSAMAR** |
|  | 856 | **418.0440** | **1251.1098** | **1250.4072** | **0.7027** | **0** | **17** | **51** | **1** | **PSAGLCSCWGGR + Carbamidomethyl (C)** |
|  | 990 | **430.1931** | **858.3714** | **858.0176** | **0.3538** | **0** | **17** | **55** | **1** | **MLPSASPR** |
|  | 2793 | **669.0850** | **1336.1551** | **1335.4688** | **0.6864** | **2** | **17** | **49** | **1** | **NNVDKDKHLPR** |
|  | 137 | **369.4279** | **1105.2616** | **1104.3011** | **0.9605** | **0** | **17** | **62** | **1** | **MHPSLATMGK + 2 Oxidation (M)** |
|  | 1312 | **459.5889** | **1375.7444** | **1376.5391** | **-0.7946** | **2** | **17** | **61** | **1** | **AMKEAGKGGVADSR** |
|  | 487 | **399.2166** | **1194.6277** | **1195.3520** | **-0.7243** | **1** | **17** | **36** | **1** | **NDLCKGPHVR + Carbamidomethyl (C)** |
|  | 3431 | **816.5391** | **2446.5950** | **2446.1285** | **0.4665** | **0** | **17** | **49** | **1** | **IIIGCFVAITLMAAVMLVIFYK + Oxidation (M)** |
|  | 64 | **365.9009** | **729.7870** | **729.8252** | **-0.0382** | **0** | **17** | **58** | **1** | **GGASGLLR** |
|  | 495 | **400.0579** | **798.1010** | **798.9272** | **-0.8262** | **0** | **17** | **43** | **1** | **QSGVPLAK** |
|  | 3020 | **717.9168** | **2150.7283** | **2151.3553** | **-0.6271** | **1** | **17** | **47** | **1** | **TPSSSSLSQGVCSTLTKNTPR** |
|  | 1809 | **521.6117** | **1561.8129** | **1561.6566** | **0.1563** | **2** | **17** | **58** | **1** | **RSRSALSVAGTGDER** |
|  | 770 | **411.3148** | **1230.9224** | **1230.4159** | **0.5065** | **1** | **17** | **43** | **1** | **SIAYLHAATRK** |
|  | 2804 | **671.0663** | **1340.1179** | **1340.5713** | **-0.4534** | **2** | **17** | **46** | **1** | **KKKPGLLNSNNK** |
|  | 3109 | **740.7059** | **2219.0956** | **2218.5801** | **0.5155** | **1** | **17** | **41** | **1** | **LMAGCAAVGASLAAPGRLCEQR + Carbamidomethyl (C); Oxidation (M)** |
|  | 3444 | 819.1430 | 2454.4068 | 2454.7198 | -0.3130 | 2 | 17 | 38 | 1 | RCRCDIQMTQSPSSLSASVGDR + Carbamidomethyl (C) |
|  | 3455 | **823.9557** | **1645.8967** | **1644.8511** | **1.0456** | **2** | **17** | **57** | **1** | **AGILARLEDMRDER** |
|  | 120 | **369.2969** | **1104.8686** | **1105.2626** | **-0.3939** | **0** | **17** | **45** | **1** | **VIQMIDENK + Oxidation (M)** |
|  | 863 | **418.1750** | **1251.5030** | **1250.3577** | **1.1453** | **0** | **17** | **51** | **1** | **ETGFCGFGDSCK** |
|  | 2016 | **548.6082** | **1642.8025** | **1641.8189** | **0.9835** | **1** | **17** | **58** | **1** | **TIKFGTNIDLYDNK** |
|  | 102 | **369.1349** | **1104.3824** | **1105.3089** | **-0.9265** | **0** | **17** | **55** | **1** | **AKPWAVCFPS** |
|  | 124 | **369.3098** | **1104.9074** | **1104.3011** | **0.6063** | **0** | **17** | **47** | **1** | **MHPSLATMGK + 2 Oxidation (M)** |
|  | 1950 | **538.1840** | **1611.5299** | **1610.7521** | **0.7778** | **1** | **17** | **54** | **1** | **SRQGGSVCGCDPCER + Carbamidomethyl (C)** |
|  | 2424 | **612.0506** | **1833.1296** | **1834.0881** | **-0.9585** | **2** | **17** | **52** | **1** | **ALVAHTQAIKADNGRLR** |
|  | 2467 | **615.2278** | **1842.6612** | **1842.1003** | **0.5608** | **2** | **17** | **51** | **1** | **DKLAPPTPSIYLENKR** |
|  | 331 | **386.8906** | **771.7665** | **772.8898** | **-1.1234** | **1** | **17** | **57** | **1** | **KDLELR** |
|  | 664 | **407.1445** | **1218.4112** | **1217.3477** | **1.0636** | **0** | **17** | **50** | **1** | **MLENFFSEGK + Oxidation (M)** |
|  | 982 | **429.1565** | **1284.4473** | **1285.4864** | **-1.0391** | **1** | **17** | **52** | **1** | **TKEGSIVDPLVK** |
|  | 1294 | **458.8414** | **915.6680** | **915.1750** | **0.4930** | **2** | **17** | **56** | **1** | **AMKKMYK + Oxidation (M)** |
|  | 1336 | **461.4108** | **920.8068** | **919.8502** | **0.9567** | **0** | **17** | **44** | **1** | **DDNGGEASR** |
|  | 101 | **369.1257** | **1104.3550** | **1105.3089** | **-0.9538** | **0** | **17** | **57** | **1** | **AKPWAVCFPS** |
|  | 354 | **387.8402** | **773.6657** | **772.8466** | **0.8190** | **0** | **17** | **63** | **1** | **LLNNGDK** |
|  | 3171 | **748.0406** | **2241.0998** | **2241.5028** | **-0.4030** | **1** | **17** | **41** | **1** | **GTFSEVSLAQDPKPQQRKPK** |
|  | 3486 | **846.9883** | **2537.9427** | **2536.7656** | **1.1770** | **0** | **17** | **61** | **1** | **ISWEGLLALDNGEMEVLESTTGR + Oxidation (M)** |
|  | 1003 | **431.0163** | **1290.0267** | **1290.6433** | **-0.6166** | **2** | **17** | **64** | **1** | **RLKPLIPGLRK** |
|  | 765 | **411.0869** | **1230.2385** | **1229.4776** | **0.7609** | **1** | **17** | **57** | **1** | **LRCMCTCNR + 2 Carbamidomethyl (C); Oxidation (M)** |
|  | 823 | **415.9644** | **829.9141** | **828.9564** | **0.9576** | **1** | **17** | **62** | **1** | **AALAAERK** |
|  | 2539 | **630.1858** | **1258.3568** | **1258.3781** | **-0.0213** | **0** | **17** | **52** | **1** | **TDQEVLGELVR** |
|  | 223 | **377.5767** | **1129.7080** | **1130.4077** | **-0.6998** | **2** | **17** | **38** | **1** | **NALMKLLRR + Oxidation (M)** |
|  | 686 | **407.5494** | **1219.6261** | **1220.3533** | **-0.7272** | **1** | **17** | **52** | **1** | **ELEEKMSQAR** |
|  | 1094 | **437.0164** | **872.0180** | **872.9230** | **-0.9050** | **1** | **17** | **58** | **1** | **NPEEKTR** |
|  | 52 | **364.1213** | **1089.3417** | **1089.2549** | **0.0869** | **2** | **17** | **48** | **1** | **KLRGHVSHR** |
|  | 1043 | **433.1472** | **864.2795** | **864.9705** | **-0.6909** | **1** | **17** | **52** | **1** | **RTQAMSR + Oxidation (M)** |
|  | 1676 | **502.2515** | **1503.7322** | **1503.6237** | **0.1085** | **1** | **17** | **56** | **1** | **MAHQGCTAGDRQR + Carbamidomethyl (C); Oxidation (M)** |
|  | 2124 | **565.6063** | **1693.7966** | **1693.8174** | **-0.0208** | **0** | **17** | **65** | **1** | **ESEELQHVQWRPR** |
|  | 363 | **387.9844** | **773.9540** | **773.8778** | **0.0761** | **0** | **17** | **65** | **1** | **ISIQASR** |
|  | 768 | **411.2548** | **1230.7421** | **1230.4124** | **0.3297** | **0** | **17** | **45** | **1** | **INPIAGIGNYAK** |
|  | 2989 | **708.7170** | **2123.1288** | **2122.3701** | **0.7587** | **1** | **17** | **49** | **1** | **CIVGSRPGGGGDGPGWIPQRR** |
|  | 3481 | **843.4537** | **1684.8926** | **1683.9732** | **0.9193** | **2** | **17** | **49** | **1** | **CQRNASLERVLPGLK** |
|  | 3483 | **845.3490** | **1688.6832** | **1688.9950** | **-0.3118** | **1** | **17** | **50** | **1** | **GPRPPRVMAAPAPVTR + Oxidation (M)** |
|  | 3506 | **858.0875** | **1714.1601** | **1714.9824** | **-0.8223** | **1** | **17** | **44** | **1** | **LRSVTAANTAVFYCAK** |
|  | 3513 | **866.3346** | **1730.6544** | **1730.9651** | **-0.3107** | **2** | **17** | **52** | **1** | **KPAQEALINYSRRGK** |
|  | 3525 | **886.2155** | **1770.4161** | **1771.0655** | **-0.6493** | **0** | **17** | **42** | **1** | **DAIFPIPVACDAPCPK + 2 Carbamidomethyl (C)** |
|  | 2875 | **683.6898** | **2048.0473** | **2047.2457** | **0.8015** | **2** | **17** | **50** | **1** | **DSGVYYCATWKDYYKK + Carbamidomethyl (C)** |
|  | 3511 | **865.4041** | **1728.7933** | **1728.9525** | **-0.1592** | **1** | **17** | **50** | **1** | **CPEQPGLHRATCGFR + Carbamidomethyl (C)** |
|  | 1144 | **443.7863** | **1328.3366** | **1327.5759** | **0.7607** | **2** | **17** | **51** | **1** | **MNISARRFTLC + Oxidation (M)** |
|  | 1413 | **468.5051** | **1402.4930** | **1403.6471** | **-1.1541** | **1** | **17** | **66** | **1** | **EKVLITTNVCAR + Carbamidomethyl (C)** |
|  | 2745 | **666.6618** | **1996.9632** | **1997.2996** | **-0.3363** | **1** | **17** | **50** | **1** | **ALYWACMKVSHPVQNFS + Oxidation (M)** |
|  | 387 | **389.0399** | **1164.0975** | **1163.1960** | **0.9015** | **1** | **17** | **59** | **1** | **GNKQGSSDGVSK** |
|  | 694 | **407.7334** | **1220.1781** | **1220.3350** | **-0.1568** | **1** | **17** | **49** | **1** | **DVRQIFNNSK** |
|  | 1046 | **433.2601** | **864.5055** | **864.9672** | **-0.4617** | **1** | **17** | **44** | **1** | **MASAKDAR + Oxidation (M)** |
|  | 1763 | **516.6110** | **1546.8107** | **1545.7369** | **1.0739** | **0** | **17** | **69** | **1** | **TPLHCVMEVDTER + Oxidation (M)** |
|  | 2606 | 642.1375 | 1282.2601 | 1282.4558 | -0.1956 | 1 | 17 | 51 | 1 | MCHGTAGAHGRK + Carbamidomethyl (C) |
|  | 127 | **369.3376** | **1104.9905** | **1104.3011** | **0.6894** | **0** | **17** | **54** | **1** | **MHPSLATMGK + 2 Oxidation (M)** |
|  | 1096 | **437.0721** | **1308.1941** | **1308.5280** | **-0.3339** | **1** | **17** | **60** | **1** | **SLSGCPRATSAMK** |
|  | 2073 | **557.0370** | **1668.0890** | **1668.7897** | **-0.7008** | **2** | **17** | **51** | **1** | **RTYEGGNALDGGRMR + Oxidation (M)** |
|  | 2279 | **593.3588** | **1777.0543** | **1776.0704** | **0.9839** | **1** | **17** | **57** | **1** | **MAGFWVGTAPLVAAGRR + Oxidation (M)** |
|  | 3126 | **741.6811** | **2222.0211** | **2221.6037** | **0.4174** | **2** | **17** | **41** | **1** | **LFGLVRQGLKCDGCGLNYHK** |
|  | 3227 | **757.1972** | **1512.3796** | **1511.7085** | **0.6711** | **1** | **17** | **50** | **1** | **ELCFIHHWRDR** |
|  | 2940 | **691.2148** | **2070.6224** | **2070.2627** | **0.3597** | **0** | **17** | **53** | **1** | **DVYCLHPGETPAPPEECR + Carbamidomethyl (C)** |
|  | 3144 | **742.7537** | **1483.4925** | **1483.7532** | **-0.2607** | **1** | **17** | **48** | **1** | **QAQIAITVPKTWK** |
|  | 341 | **387.0003** | **771.9858** | **771.8620** | **0.1237** | **0** | **17** | **61** | **1** | **AQDVLAR** |
|  | 931 | **423.9007** | **1268.6800** | **1269.4106** | **-0.7306** | **1** | **17** | **62** | **1** | **QRSAPSGGAVALR** |
|  | 2394 | **608.8268** | **1215.6388** | **1216.1693** | **-0.5305** | **0** | **17** | **48** | **1** | **EEDQDGSPSPR** |
|  | 3553 | **938.1775** | **1874.3402** | **1873.3096** | **1.0306** | **1** | **17** | **43** | **1** | **AVFLALSAQLLQARLMK** |
|  | 1486 | **475.7856** | **949.5565** | **949.0869** | **0.4697** | **1** | **17** | **48** | **1** | **MEASSLRR** |
|  | 2526 | **628.3834** | **1882.1281** | **1881.1413** | **0.9868** | **1** | **17** | **55** | **1** | **NKLCVAALSVNNFCDNR** |
|  | 3068 | **738.2661** | **2211.7760** | **2210.6608** | **1.1151** | **0** | **17** | **54** | **1** | **MALAGLAMGCIDTVANMQLVR + 2 Oxidation (M)** |
|  | 2987 | **708.4067** | **2122.1980** | **2121.3344** | **0.8637** | **2** | **17** | **54** | **1** | **RAPAAEPSPAAAPAGREMENK** |
|  | 422 | **389.7898** | **777.5648** | **776.9017** | **0.6632** | **0** | **17** | **56** | **1** | **IGSNCVK + Carbamidomethyl (C)** |
|  | 585 | **404.9852** | **1211.9333** | **1211.3696** | **0.5637** | **0** | **17** | **51** | **1** | **TGQSMTLQCAR + Oxidation (M)** |
|  | 1774 | **518.8519** | **1035.6890** | **1036.1856** | **-0.4966** | **1** | **17** | **47** | **1** | **HVKNGGSIPK** |
|  | 3412 | **809.7092** | **1617.4037** | **1617.6472** | **-0.2435** | **0** | **17** | **43** | **1** | **DSDSVCDTGVETSFR** |
|  | 3572 | **993.9100** | **1985.8053** | **1985.2127** | **0.5926** | **2** | **17** | **40** | **1** | **HKPFAANGRSPSRFSIGR** |
|  | 366 | **388.0260** | **774.0372** | **772.8467** | **1.1904** | **0** | **17** | **70** | **1** | **EAGLDLR** |
|  | 2015 | **548.5798** | **1642.7171** | **1643.8598** | **-1.1426** | **1** | **17** | **61** | **1** | **LEGWCCQMDKETK + Carbamidomethyl (C); Oxidation (M)** |
|  | 390 | **389.0977** | **1164.2709** | **1163.2373** | **1.0336** | **0** | **17** | **62** | **1** | **YIGTGHADTTK** |
|  | 696 | **407.7526** | **1220.2356** | **1219.2394** | **0.9962** | **0** | **17** | **55** | **1** | **TMSSSSGQGHGSP** |
|  | 2765 | **667.6482** | **1999.9224** | **2000.0235** | **-0.1011** | **0** | **17** | **52** | **1** | **NNNYQACDHNTEFSNTK** |
|  | 36 | **363.0972** | **1086.2695** | **1086.3255** | **-0.0559** | **0** | **17** | **57** | **1** | **FIACLMSTK + Carbamidomethyl (C); Oxidation (M)** |
|  | 480 | **399.1265** | **796.2382** | **795.9019** | **0.3363** | **0** | **17** | **51** | **1** | **MNTVTSK + Oxidation (M)** |
|  | 1912 | **534.3881** | **1066.7613** | **1067.1796** | **-0.4183** | **0** | **17** | **50** | **1** | **GAEHCPGLQR** |
|  | 404 | **389.1734** | **1164.4979** | **1164.2900** | **0.2080** | **0** | **17** | **58** | **1** | **MSLGSQGVSGGGK** |
|  | 685 | 407.5475 | 1219.6202 | 1219.3651 | 0.2550 | 0 | 17 | 57 | 1 | QAMLENASDIK |
|  | 68 | **366.0235** | **1095.0482** | **1094.1820** | **0.8663** | **2** | **17** | **66** | **1** | **RSRNSQTVF** |
|  | 1068 | **435.1154** | **1302.3241** | **1302.5283** | **-0.2042** | **1** | **17** | **53** | **1** | **GWQAAAHMMRK + Oxidation (M)** |
|  | 1980 | **541.9387** | **1622.7938** | **1623.0425** | **-0.2487** | **2** | **17** | **56** | **1** | **IRHCLLIPVRATCK** |
|  | 384 | **388.6072** | **1162.7995** | **1162.2957** | **0.5038** | **1** | **17** | **53** | **1** | **QDAKGLFEVR** |
|  | 1765 | **517.7451** | **1550.2132** | **1549.6855** | **0.5277** | **0** | **17** | **50** | **1** | **TYWVPNSSGGLVNR** |
|  | 1974 | **540.6421** | **1079.2694** | **1078.1808** | **1.0886** | **0** | **17** | **68** | **1** | **LSAHCSCDSR** |
|  | 1308 | **459.4521** | **916.8895** | **917.9999** | **-1.1105** | **0** | **17** | **64** | **1** | **ELSLEAEK** |
|  | 658 | **407.0751** | **1218.2032** | **1217.2899** | **0.9134** | **2** | **17** | **55** | **1** | **DGTPREGTRTK** |
|  | 56 | **364.2924** | **1089.8552** | **1090.1685** | **-0.3133** | **0** | **17** | **42** | **1** | **GSGDGEMGKPR** |
|  | 3338 | **791.0368** | **2370.0882** | **2369.6287** | **0.4596** | **1** | **17** | **46** | **1** | **QMPGKGLEWMGVTFPSDSETR + Oxidation (M)** |
|  | 611 | **405.9174** | **809.8199** | **808.8823** | **0.9377** | **0** | **17** | **52** | **1** | **PSAQPPGR** |
|  | 138 | **369.4375** | **1105.2903** | **1104.2547** | **1.0357** | **0** | **17** | **72** | **1** | **LDTYCMSAK + Carbamidomethyl (C); Oxidation (M)** |
|  | 928 | 423.4051 | 1267.1932 | 1267.4776 | -0.2845 | 1 | 17 | 67 | 1 | GPLRVGAIVETR |
|  | 1600 | **488.9554** | **1463.8442** | **1463.6528** | **0.1914** | **0** | **17** | **61** | **1** | **SADDVVVLGMNLSK + Oxidation (M)** |
|  | 2185 | **578.8036** | **1733.3886** | **1733.0176** | **0.3710** | **1** | **17** | **49** | **1** | **CFKMQSAAFSIAADVK + Oxidation (M)** |
|  | 3515 | **869.0219** | **2604.0436** | **2605.0829** | **-1.0394** | **1** | **17** | **63** | **1** | **KIWTCFEFSIIQCPELMMDR + 2 Carbamidomethyl (C)** |
|  | 555 | **403.8483** | **805.6819** | **804.8968** | **0.7850** | **0** | **17** | **59** | **1** | **AATIHHR** |
|  | 3083 | **740.3845** | **1478.7543** | **1477.6013** | **1.1529** | **1** | **17** | **57** | **1** | **GPVQRYPGNQTTC + Carbamidomethyl (C)** |
|  | 3470 | **835.1447** | **1668.2745** | **1668.8878** | **-0.6132** | **1** | **17** | **43** | **1** | **VQEAMESMVKSLER + 2 Oxidation (M)** |
|  | 3584 | **1024.6412** | **2047.2677** | **2047.3597** | **-0.0921** | **0** | **17** | **52** | **1** | **CNLHLVDIWNMIEAFR + Carbamidomethyl (C); Oxidation (M)** |
|  | 350 | **387.4007** | **1159.1798** | **1160.3412** | **-1.1613** | **0** | **17** | **80** | **1** | **MAKPLTDQEK** |
|  | 1517 | **477.9383** | **953.8619** | **954.0618** | **-0.1999** | **0** | **17** | **54** | **1** | **LADGPGHCK + Carbamidomethyl (C)** |
|  | 517 | 401.7036 | 801.3925 | 801.9310 | -0.5385 | 1 | 17 | 56 | 1 | KSLADIR |
|  | 1153 | **443.9453** | **885.8758** | **885.0198** | **0.8561** | **1** | **17** | **63** | **1** | **VNPNVKSK** |
|  | 1674 | **501.3265** | **1000.6382** | **1000.1071** | **0.5311** | **1** | **17** | **51** | **1** | **AITPKDNNK** |
|  | 1552 | **483.1953** | **1446.5636** | **1445.6190** | **0.9445** | **0** | **17** | **62** | **1** | **GDVWLTSSAAGLLR** |
|  | 732 | **408.3471** | **814.6794** | **813.8988** | **0.7807** | **0** | **17** | **52** | **1** | **SPITPGSR** |
|  | 808 | **414.5756** | **1240.7047** | **1241.4617** | **-0.7571** | **0** | **17** | **49** | **1** | **ALQMSCCSLGR + Carbamidomethyl (C); Oxidation (M)** |
|  | 3113 | **740.8884** | **2219.6429** | **2219.4738** | **0.1692** | **0** | **17** | **65** | **1** | **YSYCNECMLDPDCGFCNK + 2 Carbamidomethyl (C)** |
|  | 3475 | **840.2996** | **1678.5843** | **1677.8976** | **0.6867** | **1** | **17** | **54** | **1** | **TEHICDSIMKISER + Oxidation (M)** |
|  | 1910 | **534.0930** | **1066.1713** | **1067.3022** | **-1.1310** | **2** | **17** | **63** | **1** | **KIICDKYK + Carbamidomethyl (C)** |
|  | 2109 | **562.7694** | **1123.5240** | **1124.2012** | **-0.6772** | **0** | **17** | **48** | **1** | **STIDQYELR** |
|  | 2697 | **656.1512** | **1310.2877** | **1309.3833** | **0.9044** | **0** | **17** | **56** | **1** | **GNFIPYANEER** |
|  | 996 | **430.5855** | **859.1562** | **859.9273** | **-0.7711** | **0** | **17** | **67** | **1** | **GAAGSSALAR** |
|  | 238 | **379.1844** | **756.3539** | **756.8044** | **-0.4504** | **0** | **17** | **50** | **1** | **GLPEGER** |
|  | 868 | **418.9319** | **1253.7737** | **1254.4787** | **-0.7051** | **1** | **17** | **69** | **1** | **TILKIPSAQQR** |
|  | 1148 | **443.9058** | **1328.6953** | **1328.5590** | **0.1363** | **1** | **17** | **63** | **1** | **NVVKIPDFGLAR** |
|  | 2417 | **610.5243** | **1828.5507** | **1829.1544** | **-0.6037** | **2** | **16** | **50** | **1** | **GQSPKILRLKPHGLER** |
|  | 1197 | **450.1343** | **898.2538** | **898.0567** | **0.1971** | **0** | **16** | **61** | **1** | **MGLEMSSK + Oxidation (M)** |
|  | 3127 | **741.6910** | **2222.0509** | **2221.5412** | **0.5098** | **2** | **16** | **46** | **1** | **MSHCARPLSSLEQSKKCSR + Carbamidomethyl (C); Oxidation (M)** |
|  | 559 | **403.9509** | **805.8871** | **805.8769** | **0.0102** | **1** | **16** | **62** | **1** | **VKESSTR** |
|  | 1005 | **431.0494** | **1290.1261** | **1290.3404** | **-0.2143** | **1** | **16** | **74** | **1** | **GNTSSERAALER** |
|  | 2699 | **656.7534** | **1311.4921** | **1312.5181** | **-1.0261** | **2** | **16** | **69** | **1** | **NIKQVAERINK** |
|  | 2767 | **667.7113** | **2000.1117** | **1999.4047** | **0.7070** | **2** | **16** | **71** | **1** | **LRLLAAGCGPGLLADAKMR + Carbamidomethyl (C); Oxidation (M)** |
|  | 2703 | **657.3367** | **1312.6586** | **1313.5260** | **-0.8675** | **1** | **16** | **58** | **1** | **KQLSNSAMGLHK** |
|  | 2410 | **610.0975** | **1218.1802** | **1219.3687** | **-1.1885** | **1** | **16** | **61** | **1** | **QRSMSPTSTPK** |
|  | 969 | **428.9463** | **855.8778** | **856.9236** | **-1.0458** | **0** | **16** | **57** | **1** | **GSPVVGEGR** |
|  | 224 | **377.9017** | **1130.6829** | **1131.3479** | **-0.6649** | **0** | **16** | **52** | **1** | **CVPPYVQIR + Carbamidomethyl (C)** |
|  | 713 | **407.9934** | **813.9720** | **812.9770** | **0.9951** | **2** | **16** | **62** | **1** | **SYKKCGK** |
|  | 126 | **369.3262** | **1104.9564** | **1104.2778** | **0.6787** | **0** | **16** | **58** | **1** | **VLMDLQNQK + Oxidation (M)** |
|  | 1874 | **528.9468** | **1583.8183** | **1584.7099** | **-0.8915** | **1** | **16** | **60** | **1** | **NGKTPAEMHAQSADK** |
|  | 518 | **401.8087** | **801.6026** | **800.9431** | **0.6595** | **0** | **16** | **71** | **1** | **VVGAQSLK** |
|  | 846 | **417.5184** | **833.0221** | **831.9155** | **1.1065** | **0** | **16** | **79** | **1** | **GAHGSYLK** |
|  | 1092 | **436.9868** | **1307.9381** | **1308.5543** | **-0.6162** | **2** | **16** | **68** | **1** | **LRRWMFGGGTK** |
|  | 2494 | 620.5226 | 1858.5456 | 1859.1739 | -0.6284 | 1 | 16 | 49 | 1 | CELEGKLCGASNCTVCTK |
|  | 311 | **386.0951** | **1155.2631** | **1154.3201** | **0.9431** | **1** | **16** | **57** | **1** | **ITSKPPEARR** |
|  | 973 | 429.0564 | 856.0979 | 854.9341 | 1.1639 | 0 | 16 | 58 | 1 | MNHGPQR + Oxidation (M) |
|  | 1150 | **443.9098** | **1328.7072** | **1329.4643** | **-0.7571** | **2** | **16** | **66** | **1** | **QYPPRSRNPSK** |
|  | 3117 | **741.2787** | **1480.5427** | **1481.6595** | **-1.1168** | **2** | **16** | **59** | **1** | **EAPQLLSQRQRR** |
|  | 1119 | **439.1035** | **1314.2883** | **1314.5737** | **-0.2855** | **1** | **16** | **66** | **1** | **LTKNMLCAGYK + Carbamidomethyl (C); Oxidation (M)** |
|  | 1385 | **466.0553** | **930.0958** | **929.1368** | **0.9590** | **0** | **16** | **66** | **1** | **FMLLFSR + Oxidation (M)** |
|  | 1614 | **491.2424** | **980.4700** | **980.2468** | **0.2232** | **1** | **16** | **60** | **1** | **ATGKVMVMK + Oxidation (M)** |
|  | 2781 | **668.7654** | **1335.5160** | **1334.6298** | **0.8862** | **1** | **16** | **76** | **1** | **SGPVLGHLKAMPK** |
|  | 587 | **405.0153** | **1212.0237** | **1211.4128** | **0.6109** | **0** | **16** | **57** | **1** | **GCMEMPGCPDR + Oxidation (M)** |
|  | 833 | **416.1977** | **1245.5708** | **1246.4552** | **-0.8844** | **0** | **16** | **70** | **1** | **MEGDCLSCMK + 2 Carbamidomethyl (C); Oxidation (M)** |
|  | 1611 | **490.3511** | **1468.0312** | **1467.6494** | **0.3818** | **2** | **16** | **50** | **1** | **DLSKGGCKNGYLR + Carbamidomethyl (C)** |
|  | 1820 | **523.0929** | **1044.1710** | **1045.1477** | **-0.9767** | **1** | **16** | **66** | **1** | **LTAQKAEER** |
|  | 929 | **423.4335** | **1267.2784** | **1267.3253** | **-0.0468** | **0** | **16** | **81** | **1** | **ETNPNGETFCR** |
|  | 154 | **371.2173** | **1110.6297** | **1111.2158** | **-0.5861** | **2** | **16** | **40** | **1** | **QEPVGGRGRR** |
|  | 252 | **380.0029** | **1136.9864** | **1137.2663** | **-0.2798** | **1** | **16** | **72** | **1** | **QWKGMENTK + Oxidation (M)** |
|  | 301 | **385.7021** | **769.3895** | **768.8812** | **0.5082** | **0** | **16** | **47** | **1** | **GTGICYR** |
|  | 1476 | **475.3868** | **948.7589** | **949.1051** | **-0.3462** | **2** | **16** | **55** | **1** | **EKAKQAFK** |
|  | 2239 | **589.6219** | **1765.8437** | **1766.1565** | **-0.3129** | **0** | **16** | **77** | **1** | **MSPSPTALFCLGLCLGR** |
|  | 2447 | **613.3704** | **1837.0889** | **1837.1087** | **-0.0198** | **2** | **16** | **62** | **1** | **LRSRVQIGVVGGSDYCK** |
|  | 2669 | **652.4763** | **1302.9377** | **1302.4109** | **0.5269** | **1** | **16** | **56** | **1** | **GEKNGMTFSSTK + Oxidation (M)** |
|  | 2436 | **612.5622** | **1834.6644** | **1834.2089** | **0.4555** | **0** | **16** | **52** | **1** | **LYLCPSFPHQSLMLK + Carbamidomethyl (C)** |
|  | 2898 | **685.1794** | **2052.5162** | **2053.3844** | **-0.8683** | **0** | **16** | **64** | **1** | **EICHFGQVLLTVPVTSPR + Carbamidomethyl (C)** |
|  | 652 | **406.9197** | **811.8247** | **811.9689** | **-0.1443** | **1** | **16** | **56** | **1** | **QKAPQIK** |
|  | 476 | **399.0641** | **796.1134** | **794.9419** | **1.1715** | **0** | **16** | **57** | **1** | **KPVQAPR** |
|  | 1979 | **541.7834** | **1622.3282** | **1622.9053** | **-0.5772** | **1** | **16** | **50** | **1** | **KQALVTVCESGMQTK** |
|  | 2680 | **654.3506** | **1960.0296** | **1960.1337** | **-0.1042** | **2** | **16** | **62** | **1** | **EEASRNLSTPPLRCSSR + Carbamidomethyl (C)** |
|  | 1029 | **432.4191** | **1294.2352** | **1294.4168** | **-0.1816** | **1** | **16** | **69** | **1** | **QEAEVHKLGQR** |
|  | 3463 | **828.1218** | **2481.3433** | **2480.7722** | **0.5711** | **2** | **16** | **47** | **1** | **ESVGKQATGEVAGKGGPVGGKPTLQK** |
|  | 3437 | **817.1455** | **2448.4144** | **2448.6406** | **-0.2263** | **2** | **16** | **51** | **1** | **GYHQYTYDGKDYIALKEDLR** |
|  | 3514 | **868.6955** | **1735.3762** | **1735.9552** | **-0.5790** | **1** | **16** | **56** | **1** | **APESLFLLEEMRER + Oxidation (M)** |
|  | 902 | **420.5973** | **1258.7696** | **1258.3616** | **0.4081** | **1** | **16** | **51** | **1** | **VTKQHNDECK + Carbamidomethyl (C)** |
|  | 1000 | 430.8234 | 859.6319 | 859.8844 | -0.2524 | 1 | 16 | 77 | 1 | KDDAQQR |
|  | 2834 | **678.2755** | **2031.8044** | **2031.3026** | **0.5017** | **2** | **16** | **65** | **1** | **ARHPQLAPTLQPRTECR + Carbamidomethyl (C)** |
|  | 2244 | 590.4874 | 1768.4399 | 1769.0991 | -0.6592 | 2 | 16 | 52 | 1 | FPAVNSLIQRINLRK |
|  | 976 | **429.1020** | **1284.2837** | **1284.4185** | **-0.1348** | **0** | **16** | **64** | **1** | **ELQQALEGQLR** |
|  | 2980 | **705.9058** | **1409.7967** | **1409.5638** | **0.2329** | **0** | **16** | **59** | **1** | **QPGDSATYLCAVK + Carbamidomethyl (C)** |
|  | 1805 | 521.1210 | 1040.2272 | 1041.2850 | -1.0578 | 1 | 16 | 62 | 1 | GLKEVVVGIK |
|  | 540 | **402.9091** | **803.8034** | **802.8363** | **0.9670** | **1** | **16** | **73** | **1** | **AEGSGARR** |
|  | 2600 | **641.2261** | **1920.6562** | **1920.1244** | **0.5318** | **0** | **16** | **71** | **1** | **LSEDVDAPPPLGGAELALR** |
|  | 320 | **386.1926** | **770.3704** | **770.8358** | **-0.4654** | **0** | **16** | **50** | **1** | **GPGAGAWR** |
|  | 1563 | **485.5353** | **969.0558** | **970.1674** | **-1.1116** | **2** | **16** | **71** | **1** | **KKIPETVR** |
|  | 2768 | **667.7476** | **2000.2207** | **2000.1578** | **0.0629** | **1** | **16** | **82** | **1** | **HDVTCTVSGGGRSAQAGAIR + Carbamidomethyl (C)** |
|  | 966 | **428.6993** | **855.3839** | **855.9785** | **-0.5946** | **0** | **16** | **46** | **1** | **SPSSLLPR** |
|  | 1399 | 467.0483 | 932.0818 | 931.1114 | 0.9704 | 1 | 16 | 70 | 1 | AKPACKADK |
|  | 2878 | **683.9329** | **2048.7764** | **2048.4028** | **0.3736** | **0** | **16** | **54** | **1** | **DLSLSEDVMVCFGNMFIK** |
|  | 601 | **405.2448** | **1212.7121** | **1213.2514** | **-0.5393** | **0** | **16** | **49** | **1** | **DPDIEAPSNQK** |
|  | 160 | **371.2976** | **1110.8706** | **1110.3502** | **0.5204** | **2** | **16** | **43** | **1** | **KEPRGALLVK** |
|  | 2887 | **684.1659** | **2049.4755** | **2049.3511** | **0.1245** | **2** | **16** | **64** | **1** | **DKAYEGGQLCAMCFSPKK + Carbamidomethyl (C); Oxidation (M)** |
|  | 3303 | 777.7145 | 2330.1213 | 2330.6833 | -0.5621 | 0 | 16 | 50 | 1 | WCPAPDCGYAVIAFGCASCPK + 3 Carbamidomethyl (C) |
|  | 855 | **418.0411** | **1251.1012** | **1251.4385** | **-0.3372** | **1** | **16** | **69** | **1** | **RSRPCTWSCV + Carbamidomethyl (C)** |
|  | 1808 | **521.4365** | **1040.8583** | **1040.1080** | **0.7503** | **0** | **16** | **50** | **1** | **SSSWMGGQGK + Oxidation (M)** |
|  | 2801 | **670.7598** | **2009.2571** | **2009.0883** | **0.1688** | **0** | **16** | **78** | **1** | **DYGGGFDYWGQGTLVTVSS** |
|  | 481 | **399.1317** | **796.2486** | **795.9694** | **0.2792** | **0** | **16** | **59** | **1** | **GPQLLLR** |
|  | 2390 | **608.2833** | **1821.8278** | **1821.1654** | **0.6624** | **0** | **16** | **70** | **1** | **LPCGEYAMFIAWLYK + Oxidation (M)** |
|  | 3235 | **758.4001** | **2272.1783** | **2272.4672** | **-0.2889** | **1** | **16** | **64** | **1** | **KPYECSECGKSFAESSSFTK + Carbamidomethyl (C)** |
|  | 216 | **377.1817** | **752.3486** | **751.8526** | **0.4961** | **1** | **16** | **54** | **1** | **SSKGSMR** |
|  | 961 | 428.2896 | 1281.8465 | 1282.5320 | -0.6855 | 1 | 16 | 47 | 1 | CLCKPGYKGEGK |
|  | 1568 | **486.3479** | **1456.0215** | **1456.6848** | **-0.6633** | **1** | **16** | **52** | **1** | **KVWALAESGAISPK** |
|  | 1718 | **506.2275** | **1515.6603** | **1516.7385** | **-1.0782** | **0** | **16** | **64** | **1** | **QLVNMCINPDPEK + Oxidation (M)** |
|  | 2172 | **575.2626** | **1722.7655** | **1722.9695** | **-0.2040** | **1** | **16** | **70** | **1** | **CHGVSGSCTLRTCWR + Carbamidomethyl (C)** |
|  | 3104 | **740.6241** | **1479.2335** | **1479.6820** | **-0.4485** | **2** | **16** | **53** | **1** | **MKETPLSNCERR + Oxidation (M)** |
|  | 3229 | **757.6285** | **2269.8633** | **2269.5842** | **0.2791** | **2** | **16** | **52** | **1** | **TLERRPQGGRSMPTTPVLTR + Oxidation (M)** |
|  | 977 | **429.1148** | **856.2148** | **856.0214** | **0.1934** | **0** | **16** | **68** | **1** | **ALLSPSLR** |
|  | 2807 | **671.4360** | **1340.8572** | **1340.4838** | **0.3734** | **1** | **16** | **65** | **1** | **KNVEAMSGMEGR + 2 Oxidation (M)** |
|  | 2300 | **595.5191** | **1189.0234** | **1188.3727** | **0.6508** | **0** | **16** | **60** | **1** | **IKPGFVQISAE** |
|  | 3221 | **754.7658** | **1507.5168** | **1506.7536** | **0.7633** | **0** | **16** | **65** | **1** | **SCICHVCSTHMNR + Oxidation (M)** |
|  | 247 | **379.8217** | **1136.4431** | **1137.4402** | **-0.9971** | **1** | **16** | **75** | **1** | **MMNCPKILR + 2 Oxidation (M)** |
|  | 1022 | **432.0377** | **1293.0908** | **1292.4376** | **0.6532** | **0** | **16** | **74** | **1** | **CCESASEDCMAK + Oxidation (M)** |
|  | 1777 | **518.9116** | **1035.8085** | **1035.1148** | **0.6937** | **1** | **16** | **66** | **1** | **ARFGVASGDR** |
|  | 2795 | **669.7633** | **2006.2677** | **2007.2099** | **-0.9421** | **1** | **16** | **83** | **1** | **SPDLWWRNPCDCATTK + 2 Carbamidomethyl (C)** |
|  | 1157 | **444.3445** | **886.6743** | **886.9495** | **-0.2752** | **1** | **16** | **61** | **1** | **GGGTPKNEK** |
|  | 1651 | **496.6250** | **1486.8529** | **1486.6099** | **0.2431** | **1** | **16** | **82** | **1** | **AGSDGARGMPGQTGPK** |
|  | 310 | **386.0856** | **1155.2345** | **1154.3597** | **0.8749** | **0** | **16** | **63** | **1** | **LFCMGDVNAK + Carbamidomethyl (C)** |
|  | 490 | **399.2537** | **796.4926** | **796.9561** | **-0.4635** | **0** | **16** | **47** | **1** | **MSQMLR + 2 Oxidation (M)** |
|  | 1103 | **437.2402** | **1308.6985** | **1308.4647** | **0.2338** | **0** | **16** | **68** | **1** | **ICQAGNGFHAYK** |
|  | 1244 | **454.2792** | **1359.8154** | **1359.5319** | **0.2835** | **2** | **16** | **61** | **1** | **KPRATTEVSSRK** |
|  | 1992 | **543.0469** | **1084.0790** | **1084.3177** | **-0.2388** | **2** | **16** | **65** | **1** | **WKLNVIRR** |
|  | 2886 | **684.1285** | **2049.3633** | **2050.2794** | **-0.9162** | **1** | **16** | **67** | **1** | **KAIMHHEGHMDDGISLSR + Oxidation (M)** |
|  | 3226 | **757.0461** | **2268.1161** | **2267.7121** | **0.4039** | **0** | **16** | **52** | **1** | **MALAGLAMGCIDTVANMQLVR + Carbamidomethyl (C); 2 Oxidation (M)** |
|  | 221 | **377.3282** | **1128.9625** | **1128.2777** | **0.6848** | **0** | **16** | **54** | **1** | **NFQAVPLDPK** |
|  | 3579 | **1007.6444** | **3019.9111** | **3020.3522** | **-0.4412** | **1** | **16** | **61** | **1** | **ISRVEAEDVGIYYCMQGTHWPPYTF + Carbamidomethyl (C)** |
|  | 291 | **385.1247** | **1152.3519** | **1153.2905** | **-0.9385** | **1** | **16** | **60** | **1** | **QCRSSGIDCK + Carbamidomethyl (C)** |
|  | 360 | **387.9258** | **773.8367** | **773.8349** | **0.0019** | **0** | **16** | **85** | **1** | **EIVNGSR** |
|  | 665 | **407.1536** | **1218.4385** | **1219.3983** | **-0.9597** | **2** | **16** | **67** | **1** | **QNPCGSKACRR** |
|  | 522 | **401.9176** | **1202.7306** | **1203.4554** | **-0.7247** | **2** | **16** | **80** | **1** | **KGKPASMVKNK + Oxidation (M)** |
|  | 2785 | **668.8900** | **2003.6479** | **2003.2214** | **0.4265** | **0** | **16** | **60** | **1** | **QPPGGQGFPASAAPAQVPAVR** |
|  | 2140 | **568.9974** | **1703.9701** | **1702.9947** | **0.9754** | **1** | **16** | **74** | **1** | **GSVVPKQQLLQQHIK** |
|  | 2291 | **594.7747** | **1187.5345** | **1187.2604** | **0.2742** | **0** | **16** | **70** | **1** | **CCESGDAFNNK** |
|  | 3325 | **786.5374** | **2356.5899** | **2355.7096** | **0.8803** | **1** | **16** | **69** | **1** | **IQRMPSESAAQSLAVALPLQTK + Oxidation (M)** |
|  | 1605 | **489.5912** | **977.1676** | **978.0783** | **-0.9107** | **0** | **16** | **86** | **1** | **LLESEECR** |
|  | 3096 | **740.5500** | **2218.6278** | **2219.8198** | **-1.1920** | **1** | **16** | **64** | **1** | **MLLVLIPLLGIHFVLRTVR + Oxidation (M)** |
|  | 3539 | **898.8209** | **1795.6271** | **1795.0405** | **0.5865** | **1** | **16** | **51** | **1** | **FTRFIIEEVLDDLGK** |
|  | 2055 | **553.9888** | **1105.9628** | **1105.1647** | **0.7980** | **1** | **16** | **74** | **1** | **QQWGRTSSR** |
|  | 3147 | **742.9334** | **2225.7781** | **2225.3725** | **0.4055** | **2** | **16** | **69** | **1** | **LTELHGESSGSEKATGHKTGAK** |
|  | 901 | **420.5198** | **1258.5372** | **1257.4828** | **1.0544** | **1** | **16** | **76** | **1** | **CLMSKFTAQR + Carbamidomethyl (C); Oxidation (M)** |
|  | 1323 | **460.3646** | **1378.0716** | **1377.6543** | **0.4173** | **0** | **16** | **61** | **1** | **PIPLNQGCLAPVR** |
|  | 82 | 367.8962 | 1100.6665 | 1101.2557 | -0.5892 | 0 | 16 | 78 | 1 | YHQCMEFK + Oxidation (M) |
|  | 3130 | **741.8004** | **2222.3789** | **2222.4166** | **-0.0377** | **1** | **16** | **75** | **1** | **DMRMGGGGAMNMGDPYGSGGQK + 3 Oxidation (M)** |
|  | 2654 | 649.8001 | 1297.5854 | 1296.4326 | 1.1528 | 1 | 16 | 77 | 1 | AIESSRDLLHR |
|  | 3474 | 839.8387 | 2516.4941 | 2516.7645 | -0.2704 | 1 | 16 | 59 | 1 | LMENMRNDIASHPPVEGSYAPR + 2 Oxidation (M) |
|  | 1169 | **445.4724** | **1333.3952** | **1333.5326** | **-0.1374** | **2** | **16** | **97** | **1** | **FVVEKAEQQKK** |
|  | 317 | **386.1778** | **1155.5112** | **1154.4724** | **1.0388** | **2** | **16** | **59** | **1** | **RKMKPLLPR + Oxidation (M)** |
|  | 492 | **399.3214** | **1194.9420** | **1195.2610** | **-0.3190** | **1** | **16** | **50** | **1** | **ISSKDSCQDR + Carbamidomethyl (C)** |
|  | 1116 | **438.3557** | **874.6966** | **874.0154** | **0.6813** | **0** | **16** | **65** | **1** | **ECTCTSCK** |
|  | 3237 | **758.7812** | **2273.3214** | **2272.6428** | **0.6786** | **2** | **16** | **68** | **1** | **MKVEGMTCHSCTSTIEGKIGK + 2 Oxidation (M)** |
|  | 228 | **378.1386** | **754.2623** | **753.8916** | **0.3707** | **1** | **16** | **63** | **1** | **MSCGRK + Carbamidomethyl (C); Oxidation (M)** |
|  | 250 | **379.9448** | **757.8747** | **756.9154** | **0.9594** | **0** | **16** | **81** | **1** | **MGMCSR + Carbamidomethyl (C); Oxidation (M)** |
|  | 465 | **396.2090** | **1185.6047** | **1185.5478** | **0.0569** | **1** | **16** | **69** | **1** | **MMMLVGMGKR + 2 Oxidation (M)** |
|  | 1291 | **458.8017** | **915.5886** | **915.0025** | **0.5860** | **0** | **16** | **72** | **1** | **EIQLQER** |
|  | 2000 | **546.2114** | **1090.4079** | **1089.3096** | **1.0984** | **1** | **16** | **74** | **1** | **LQGVERIMK + Oxidation (M)** |
|  | 3276 | **769.7592** | **1537.5035** | **1536.6966** | **0.8070** | **1** | **16** | **60** | **1** | **RPPSGIWGGPGRGSR** |
|  | 2510 | **623.5680** | **1245.1212** | **1244.3945** | **0.7267** | **0** | **16** | **59** | **1** | **DLGLSVLDVSAR** |
|  | 3108 | **740.7009** | **2219.0806** | **2218.3880** | **0.6927** | **2** | **16** | **58** | **1** | **NRHSQNPNLHKNIPFTSVD** |
|  | 3308 | **778.0020** | **2330.9839** | **2331.6352** | **-0.6513** | **1** | **16** | **59** | **1** | **NMSGQVSMGPAFIHHHPPKSR + Oxidation (M)** |
|  | 84 | **367.9708** | **1100.8902** | **1100.1897** | **0.7005** | **2** | **16** | **79** | **1** | **DLRQGEARR** |
|  | 2584 | **636.8407** | **1271.6666** | **1270.5379** | **1.1288** | **0** | **16** | **64** | **1** | **MLLTEVPLNPK + Oxidation (M)** |
|  | 1146 | **443.8409** | **1328.5007** | **1328.5441** | **-0.0434** | **0** | **16** | **74** | **1** | **CMNMAHPPAAGR + Carbamidomethyl (C); Oxidation (M)** |
|  | 740 | **408.9470** | **815.8792** | **815.7888** | **0.0904** | **0** | **16** | **80** | **1** | **GHSADSSR** |
|  | 897 | **420.1733** | **1257.4977** | **1257.3768** | **0.1209** | **0** | **16** | **69** | **1** | **DMAAAGLHSNVR + Oxidation (M)** |
|  | 3090 | **740.5024** | **2218.4850** | **2219.6691** | **-1.1842** | **0** | **16** | **69** | **1** | **QPPPSLCGPALVALVLACGLSR + Carbamidomethyl (C)** |
|  | 405 | **389.1750** | **1164.5028** | **1163.3717** | **1.1311** | **2** | **16** | **73** | **1** | **MPPRDKTMR + 2 Oxidation (M)** |
|  | 1324 | **460.4061** | **918.7974** | **918.0298** | **0.7677** | **1** | **16** | **66** | **1** | **ACREPSQK** |
|  | 907 | **421.1776** | **1260.5108** | **1260.4370** | **0.0737** | **2** | **16** | **72** | **1** | **KSEALESIKQK** |
|  | 2312 | **596.8010** | **1191.5873** | **1191.3203** | **0.2670** | **0** | **16** | **72** | **1** | **SYCPARPGQR + Carbamidomethyl (C)** |
|  | 2321 | **597.2287** | **1788.6639** | **1788.0560** | **0.6079** | **0** | **16** | **80** | **1** | **LSLPGQLGALTSQPLHR** |
|  | 1156 | **444.2189** | **1329.6346** | **1329.5074** | **0.1273** | **1** | **16** | **79** | **1** | **HHTSSAIPVPKR** |
|  | 1726 | 507.8262 | 1013.6377 | 1013.1043 | 0.5334 | 0 | 16 | 62 | 1 | DAMCSSSANK |
|  | 807 | **414.3339** | **826.6530** | **825.8896** | **0.7634** | **0** | **16** | **52** | **1** | **ENGFCTR** |
|  | 1120 | **439.3421** | **876.6695** | **876.0759** | **0.5936** | **2** | **16** | **63** | **1** | **KIMGDGKK** |
|  | 2072 | **556.9048** | **1111.7948** | **1111.2953** | **0.4995** | **2** | **16** | **68** | **1** | **VQKARGLDPK** |
|  | 445 | **393.0358** | **784.0569** | **783.9357** | **0.1212** | **0** | **16** | **67** | **1** | **GVGLGPGPM** |
|  | 913 | **421.9163** | **1262.7268** | **1263.3417** | **-0.6149** | **2** | **16** | **70** | **1** | **MSAGGRDEERR** |
|  | 1638 | **494.7211** | **1481.1410** | **1481.6330** | **-0.4920** | **1** | **16** | **64** | **1** | **ISVRALCNGDYDR** |
|  | 167 | **372.4532** | **1114.3375** | **1114.2576** | **0.0798** | **1** | **16** | **91** | **1** | **IHRLFEGSR** |
|  | 650 | **406.7909** | **1217.3506** | **1218.4285** | **-1.0779** | **2** | **16** | **70** | **1** | **KMKYNIHER** |
|  | 829 | **416.1583** | **1245.4527** | **1244.3150** | **1.1378** | **0** | **16** | **87** | **1** | **GTGHPNSYHFK** |
|  | 3596 | **1123.1611** | **2244.3075** | **2245.4929** | **-1.1855** | **2** | **16** | **54** | **1** | **LRRSSSLDTEGCFQTGLCSK + Carbamidomethyl (C)** |
|  | 1647 | 496.3236 | 1485.9487 | 1485.6418 | 0.3069 | 1 | 16 | 67 | 1 | RASAGTPSLSAGVSPK |
|  | 2236 | **588.1880** | **1761.5418** | **1761.9726** | **-0.4309** | **2** | **16** | **79** | **1** | **IKSKGDDGTAVYAAPLR** |
|  | 2985 | **708.0272** | **1414.0397** | **1413.4746** | **0.5651** | **0** | **16** | **59** | **1** | **EMQEGHGGWNPR + Oxidation (M)** |
|  | 3205 | **749.4278** | **1496.8408** | **1495.8351** | **1.0057** | **2** | **16** | **71** | **1** | **RRLIPEALLAGMR** |
|  | 3305 | **777.8201** | **1553.6254** | **1552.6382** | **0.9872** | **0** | **16** | **74** | **1** | **NFGDLVSIQSESEK** |
|  | 229 | **378.1713** | **1131.4917** | **1132.2366** | **-0.7448** | **2** | **16** | **59** | **1** | **GSHRSHPRAK** |
|  | 489 | **399.2204** | **796.4261** | **795.8802** | **0.5459** | **0** | **16** | **53** | **1** | **SLFSTNK** |
|  | 2816 | **672.7391** | **2015.1952** | **2015.2074** | **-0.0121** | **0** | **16** | **85** | **1** | **CNIQMTQSPSAMSASVGDR + 2 Oxidation (M)** |
|  | 2829 | **675.3838** | **1348.7528** | **1349.5765** | **-0.8237** | **0** | **16** | **77** | **1** | **MGLSSNVLCAGPK + Carbamidomethyl (C); Oxidation (M)** |
|  | 827 | **416.1344** | **1245.3811** | **1245.3695** | **0.0116** | **1** | **16** | **88** | **1** | **DRDGRPQVMR + Oxidation (M)** |
|  | 1122 | **440.2501** | **1317.7282** | **1318.3010** | **-0.5728** | **0** | **16** | **75** | **1** | **SSDGEDEQQVPK** |
|  | 1637 | **494.6785** | **1481.0134** | **1480.5608** | **0.4527** | **2** | **16** | **72** | **1** | **SKSEDQRAMAEGR + Oxidation (M)** |
|  | 2127 | **566.0155** | **1695.0243** | **1693.9650** | **1.0594** | **2** | **16** | **79** | **1** | **SRTTPFMGIIDKTAR** |
|  | 2173 | **575.5151** | **1723.5232** | **1723.0059** | **0.5173** | **1** | **16** | **63** | **1** | **EEIQKLMGQIHQLR** |
|  | 2901 | **685.8496** | **2054.5267** | **2054.1967** | **0.3299** | **0** | **16** | **84** | **1** | **LDNSYCEQFITNPNNPK + Carbamidomethyl (C)** |
|  | 599 | **405.2204** | **1212.6390** | **1213.2514** | **-0.6123** | **0** | **16** | **59** | **1** | **DPDIEAPSNQK** |
|  | 1536 | **480.2367** | **1437.6880** | **1436.6769** | **1.0111** | **0** | **16** | **77** | **1** | **MFWPSVNSALLR + Oxidation (M)** |
|  | 2025 | **550.1627** | **1647.4660** | **1647.8732** | **-0.4072** | **0** | **16** | **74** | **1** | **NSPSLFPCAPLCER + 2 Carbamidomethyl (C)** |
|  | 938 | **426.1829** | **1275.5265** | **1275.3689** | **0.1576** | **2** | **15** | **77** | **1** | **SRESLEEAAKR** |
|  | 388 | **389.0765** | **1164.2073** | **1163.4111** | **0.7961** | **0** | **15** | **85** | **1** | **MSLCSLLSPR + Carbamidomethyl (C)** |
|  | 543 | **403.0384** | **1206.0931** | **1206.3300** | **-0.2370** | **1** | **15** | **84** | **1** | **ANSEVAQCRTK** |
|  | 1117 | **438.4395** | **874.8643** | **875.0712** | **-0.2068** | **0** | **15** | **89** | **1** | **LCLSHMR + Oxidation (M)** |
|  | 2594 | **639.4340** | **1276.8533** | **1276.3983** | **0.4549** | **1** | **15** | **76** | **1** | **LRVTEGGEPYR** |
|  | 779 | **411.8383** | **1232.4926** | **1231.3429** | **1.1497** | **2** | **15** | **83** | **1** | **RHQSGSMREK + Oxidation (M)** |
|  | 2042 | **551.9193** | **1652.7356** | **1651.9449** | **0.7907** | **1** | **15** | **75** | **1** | **MFPIGEMEIVKEGR + Oxidation (M)** |
|  | 2099 | **562.3381** | **1683.9920** | **1683.9470** | **0.0451** | **2** | **15** | **73** | **1** | **KEGIMNPEVGMKYR + 2 Oxidation (M)** |
|  | 217 | 377.1982 | 752.3817 | 752.8389 | -0.4572 | 0 | 15 | 59 | 1 | GVFDCR + Carbamidomethyl (C) |
|  | 1650 | **496.5576** | **1486.6505** | **1486.7623** | **-0.1118** | **1** | **15** | **98** | **1** | **AVMQSQKPPKNCR** |
|  | 2956 | **696.5305** | **2086.5694** | **2087.2914** | **-0.7220** | **1** | **15** | **68** | **1** | **HGTKIGTLGSATTELLSGSTR** |
|  | 452 | **394.0651** | **1179.1732** | **1179.4108** | **-0.2376** | **0** | **15** | **82** | **1** | **KPGSSVMVSCK + Carbamidomethyl (C)** |
|  | 2155 | **570.9384** | **1709.7929** | **1710.8427** | **-1.0499** | **0** | **15** | **69** | **1** | **DFLGTNWSSSAAWLR** |
|  | 207 | **377.1139** | **1128.3196** | **1128.3059** | **0.0137** | **1** | **15** | **72** | **1** | **RLVPCSGSPR + Carbamidomethyl (C)** |
|  | 612 | **405.9553** | **1214.8438** | **1214.3686** | **0.4752** | **0** | **15** | **70** | **1** | **EAGFDMCALGK + Carbamidomethyl (C); Oxidation (M)** |
|  | 1585 | **488.0756** | **1461.2045** | **1460.6801** | **0.5244** | **0** | **15** | **86** | **1** | **QACTSMLCCGQSR + Carbamidomethyl (C); Oxidation (M)** |
|  | 2459 | **614.2628** | **1226.5107** | **1225.4375** | **1.0732** | **1** | **15** | **73** | **1** | **GNAIEKQKPLK** |
|  | 979 | **429.1326** | **1284.3755** | **1284.5082** | **-0.1326** | **1** | **15** | **77** | **1** | **CSPKMPPAPSGR + Carbamidomethyl (C)** |
|  | 1334 | **460.8752** | **1379.6034** | **1378.4225** | **1.1809** | **1** | **15** | **80** | **1** | **SPDPDKSTGTDCR** |
|  | 1448 | **472.3464** | **1414.0170** | **1414.6117** | **-0.5947** | **0** | **15** | **60** | **1** | **VVEALHQNIVHR** |
|  | 2340 | **599.5749** | **1795.7025** | **1796.0518** | **-0.3494** | **0** | **15** | **64** | **1** | **CAEMIISMDSSQIHSK + Oxidation (M)** |
|  | 3234 | **758.2166** | **2271.6275** | **2272.7037** | **-1.0762** | **1** | **15** | **73** | **1** | **EWVLKSSILIAMAVYTYLR + Oxidation (M)** |
|  | 375 | **388.1642** | **1161.4703** | **1162.2973** | **-0.8270** | **0** | **15** | **87** | **1** | **IFYVDHVNR** |
|  | 262 | **382.1079** | **1143.3016** | **1144.2787** | **-0.9772** | **1** | **15** | **90** | **1** | **KTALAEDQLR** |
|  | 575 | **404.1123** | **1209.3146** | **1210.3864** | **-1.0718** | **2** | **15** | **77** | **1** | **SLLGSKQGHKR** |
|  | 992 | **430.2136** | **1287.6187** | **1286.4742** | **1.1445** | **1** | **15** | **82** | **1** | **IDIINAVESGKK** |
|  | 1988 | **542.6576** | **1083.3004** | **1082.3236** | **0.9768** | **2** | **15** | **89** | **1** | **NCPLPRKVR** |
|  | 2660 | **651.1227** | **1300.2306** | **1300.5656** | **-0.3350** | **0** | **15** | **74** | **1** | **MSSSMEICVIK + Carbamidomethyl (C); Oxidation (M)** |
|  | 1893 | **531.7304** | **1592.1691** | **1592.7981** | **-0.6290** | **0** | **15** | **70** | **1** | **HHMFSLISGSGTMR + 2 Oxidation (M)** |
|  | 2068 | **556.4080** | **1666.2019** | **1666.9211** | **-0.7192** | **0** | **15** | **67** | **1** | **HGEGPLLLHLASPVAR** |
|  | 1634 | **494.4271** | **1480.2592** | **1480.6283** | **-0.3691** | **1** | **15** | **66** | **1** | **RGSHSGAQGLLAAQK** |
|  | 463 | **396.1081** | **790.2015** | **790.9249** | **-0.7235** | **0** | **15** | **88** | **1** | **LTEAMNL** |
|  | 1081 | **436.0000** | **1304.9779** | **1304.4679** | **0.5100** | **0** | **15** | **75** | **1** | **TSFLASDLCYK + Carbamidomethyl (C)** |
|  | 3560 | **966.3674** | **1930.7201** | **1930.1626** | **0.5575** | **2** | **15** | **61** | **1** | **KVQPSELVGSVWTKEDK** |
|  | 526 | **401.9982** | **801.9817** | **800.8636** | **1.1181** | **1** | **15** | **91** | **1** | **AEVRGGGR** |
|  | 3214 | **751.4147** | **2251.2218** | **2250.4268** | **0.7951** | **1** | **15** | **74** | **1** | **GARCDIQMTQSPSSPSASVGDR** |
|  | 395 | **389.1303** | **776.2458** | **775.9153** | **0.3306** | **0** | **15** | **86** | **1** | **AWGMAPK + Oxidation (M)** |
|  | 1035 | **432.9832** | **863.9516** | **863.0123** | **0.9393** | **0** | **15** | **79** | **1** | **GFNLTLAK** |
|  | 970 | **428.9888** | **1283.9443** | **1284.5082** | **-0.5639** | **1** | **15** | **74** | **1** | **CSPKMPPAPSGR + Carbamidomethyl (C)** |
|  | 2266 | **592.8271** | **1775.4593** | **1776.1119** | **-0.6526** | **1** | **15** | **66** | **1** | **FCRMDVAVLSYCVR + 2 Carbamidomethyl (C)** |
|  | 529 | **402.0766** | **1203.2076** | **1204.3060** | **-1.0983** | **0** | **15** | **93** | **1** | **VGFTEECEYK** |
|  | 588 | **405.0425** | **1212.1055** | **1211.4059** | **0.6995** | **0** | **15** | **72** | **1** | **LFGSFSLEALK** |
|  | 2752 | **666.6989** | **1997.0744** | **1997.2136** | **-0.1392** | **2** | **15** | **86** | **1** | **MDRGAKSCPANFLAAADDK + Oxidation (M)** |
|  | 2941 | **692.3563** | **2074.0468** | **2074.4117** | **-0.3649** | **1** | **15** | **75** | **1** | **DMIARPRQPVAQWHQLK** |
|  | 30 | **362.5065** | **1084.4972** | **1085.1718** | **-0.6746** | **0** | **15** | **74** | **1** | **QVQEQVAQR** |
|  | 285 | **385.0759** | **1152.2055** | **1152.3241** | **-0.1186** | **1** | **15** | **69** | **1** | **GPMFKGVASSR + Oxidation (M)** |
|  | 880 | **419.2646** | **1254.7716** | **1255.3393** | **-0.5677** | **0** | **15** | **76** | **1** | **GSPGGPGAAGFPGAR** |
|  | 1427 | **470.7110** | **1409.1108** | **1408.7281** | **0.3827** | **2** | **15** | **57** | **1** | **DLVRKLIVLDPK** |
|  | 2484 | **619.0829** | **1236.1510** | **1235.5052** | **0.6457** | **1** | **15** | **79** | **1** | **CPLLPARRPR + Carbamidomethyl (C)** |
|  | 3129 | **741.7288** | **1481.4427** | **1480.7708** | **0.6719** | **0** | **15** | **65** | **1** | **LESWEPAVCCLCK** |
|  | 186 | **374.2574** | **1119.7499** | **1119.3288** | **0.4210** | **0** | **15** | **77** | **1** | **LLEVLSGEML + Oxidation (M)** |
|  | 519 | **401.8957** | **801.7766** | **800.8602** | **0.9164** | **0** | **15** | **90** | **1** | **APTAQASR** |
|  | 845 | **417.3510** | **832.6872** | **832.9004** | **-0.2131** | **0** | **15** | **72** | **1** | **HSDSLFK** |
|  | 2666 | **651.6442** | **1951.9105** | **1951.2311** | **0.6794** | **0** | **15** | **71** | **1** | **SIAQAGMCSGVISAHYSLR** |
|  | 318 | **386.1795** | **1155.5164** | **1156.2465** | **-0.7300** | **0** | **15** | **65** | **1** | **QAQPSVASSPGK** |
|  | 1501 | **476.5319** | **1426.5735** | **1425.5898** | **0.9837** | **1** | **15** | **94** | **1** | **EECWNSMAVKGR + Oxidation (M)** |
|  | 408 | **389.2130** | **776.4113** | **776.9198** | **-0.5085** | **0** | **15** | **72** | **1** | **EALGFLK** |
|  | 2799 | **670.0833** | **2007.2276** | **2006.4172** | **0.8104** | **1** | **15** | **79** | **1** | **TTQHPLALLARVWVLCK + Carbamidomethyl (C)** |
|  | 69 | **366.0605** | **1095.1594** | **1095.2727** | **-0.1133** | **1** | **15** | **89** | **1** | **ALMAEFNRK + Oxidation (M)** |
|  | 710 | **407.9247** | **1220.7520** | **1221.5085** | **-0.7566** | **1** | **15** | **80** | **1** | **ALKEELLFMK** |
|  | 3545 | **911.7598** | **2732.2571** | **2733.1890** | **-0.9319** | **2** | **15** | **68** | **1** | **FRNYLIPKGTAIMALLTSVLHDDK + Oxidation (M)** |
|  | 33 | **363.0052** | **1085.9935** | **1085.2546** | **0.7389** | **0** | **15** | **77** | **1** | **EMGNSLGCFK** |
|  | 618 | **406.0405** | **1215.0994** | **1214.5211** | **0.5783** | **1** | **15** | **73** | **1** | **LKMKPDVVLR + Oxidation (M)** |
|  | 1233 | **452.8168** | **1355.4283** | **1356.5226** | **-1.0943** | **1** | **15** | **85** | **1** | **GKIYSLNEAYAK** |
|  | 1639 | **494.9111** | **1481.7112** | **1482.5948** | **-0.8835** | **2** | **15** | **84** | **1** | **DDPKALGEEPKQR** |
|  | 822 | **415.9637** | **829.9126** | **828.9564** | **0.9562** | **1** | **15** | **95** | **1** | **AALAAERK** |
|  | 1072 | 435.6170 | 869.2192 | 870.0697 | -0.8504 | 0 | 15 | 66 | 1 | YFGMKPK |
|  | 2487 | **619.3334** | **1236.6520** | **1235.5052** | **1.1467** | **1** | **15** | **79** | **1** | **CPLLPARRPR + Carbamidomethyl (C)** |
|  | 2559 | **632.4420** | **1894.3039** | **1894.9427** | **-0.6388** | **0** | **15** | **79** | **1** | **VWGPPGGEGTGDLDEFDF** |
|  | 2009 | **547.4049** | **1092.7950** | **1092.2059** | **0.5892** | **1** | **15** | **65** | **1** | **GERGVPGPPGAV** |
|  | 1406 | **468.0234** | **1401.0480** | **1400.4975** | **0.5505** | **0** | **15** | **88** | **1** | **DHCCQGTGHSVEK** |
|  | 2080 | **558.1805** | **1114.3463** | **1113.2215** | **1.1248** | **0** | **15** | **82** | **1** | **HGASASLTISGL** |
|  | 1180 | **447.5606** | **893.1064** | **892.0323** | **1.0742** | **1** | **15** | **97** | **1** | **KAQAAEMK + Oxidation (M)** |
|  | 1760 | **516.0120** | **1030.0091** | **1029.1484** | **0.8608** | **1** | **15** | **86** | **1** | **SERGSLGVPK** |
|  | 313 | **386.1078** | **1155.3013** | **1155.2849** | **0.0164** | **0** | **15** | **75** | **1** | **LHVEGPTCSR + Carbamidomethyl (C)** |
|  | 2493 | **620.3735** | **1858.0984** | **1859.0482** | **-0.9497** | **0** | **15** | **80** | **1** | **GPEQNPAEHKPSVIVTR** |
|  | 2721 | **663.1818** | **1324.3487** | **1325.4228** | **-1.0740** | **1** | **15** | **79** | **1** | **GKMEDSAVPDTC + Carbamidomethyl (C); Oxidation (M)** |
|  | 2778 | **668.7549** | **1335.4951** | **1335.5713** | **-0.0762** | **0** | **15** | **1e+02** | **1** | **AQSSLGYIPLMR** |
|  | 3423 | **814.2593** | **1626.5039** | **1626.8954** | **-0.3915** | **2** | **15** | **76** | **1** | **LNSDIIRTTGLPKAK** |
|  | 3546 | **917.8745** | **1833.7342** | **1832.9725** | **0.7617** | **0** | **15** | **61** | **1** | **VTAACGGNHGCSQSSALR + 2 Carbamidomethyl (C)** |
|  | 958 | **428.2430** | **1281.7068** | **1282.6000** | **-0.8932** | **1** | **15** | **62** | **1** | **RTLMLMAQMR + 2 Oxidation (M)** |
|  | 1209 | **451.0101** | **1350.0082** | **1350.6475** | **-0.6392** | **1** | **15** | **88** | **1** | **CTVSMSLPKQLK + Oxidation (M)** |
|  | 199 | **376.1014** | **750.1880** | **749.8118** | **0.3763** | **0** | **15** | **77** | **1** | **SNTGFPK** |
|  | 3088 | **740.4714** | **2218.3920** | **2217.4812** | **0.9107** | **2** | **15** | **81** | **1** | **TPAGIRYRIAVIADLDTESR** |
|  | 264 | **382.2369** | **1143.6885** | **1143.2080** | **0.4805** | **2** | **15** | **79** | **1** | **KEQEPNKDR** |
|  | 1823 | **523.1920** | **1044.3691** | **1044.1432** | **0.2260** | **0** | **15** | **88** | **1** | **GEHVVCGSTR** |
|  | 5 | **360.3732** | **1078.0974** | **1079.1425** | **-1.0450** | **0** | **15** | **1.1e+02** | **1** | **NPMNTSSQGK + Oxidation (M)** |
|  | 558 | **403.9478** | **805.8808** | **805.9431** | **-0.0623** | **1** | **15** | **85** | **1** | **QMRVEK + Oxidation (M)** |
|  | 2460 | **614.3517** | **1840.0329** | **1839.1458** | **0.8870** | **1** | **15** | **82** | **1** | **LWLRLFEAVFAGNFR** |
|  | 1538 | **480.6743** | **1439.0006** | **1439.8514** | **-0.8508** | **2** | **15** | **76** | **1** | **MITILQLPKVRK** |
|  | 3103 | **740.6077** | **2218.8010** | **2219.6424** | **-0.8414** | **0** | **15** | **67** | **1** | **LLPIGLNICAPGDQELIALAK + Carbamidomethyl (C)** |
|  | 767 | **411.1852** | **1230.5333** | **1231.5068** | **-0.9735** | **1** | **15** | **86** | **1** | **VLHSSLVCKVF** |
|  | 3044 | **729.7650** | **1457.5152** | **1456.6717** | **0.8436** | **2** | **15** | **87** | **1** | **SPRRMSFSGIFR + Oxidation (M)** |
|  | 151 | **371.1413** | **740.2677** | **740.8911** | **-0.6233** | **1** | **15** | **66** | **1** | **KILDPR** |
|  | 249 | **379.9402** | **757.8655** | **757.8736** | **-0.0080** | **0** | **15** | **95** | **1** | **FTIYSK** |
|  | 3492 | **848.8214** | **2543.4421** | **2543.8526** | **-0.4106** | **2** | **15** | **65** | **1** | **FPPTANMLLPTGEGQSGRAALRDK + Oxidation (M)** |
|  | 339 | **386.9869** | **771.9591** | **771.8191** | **0.1400** | **0** | **15** | **93** | **1** | **DVGPTQR** |
|  | 145 | 370.6359 | 739.2570 | 738.9131 | 0.3439 | 0 | 15 | 55 | 1 | LLSFFI |
|  | 885 | **419.2996** | **1254.8765** | **1255.4487** | **-0.5722** | **1** | **15** | **81** | **1** | **MMCAGDLRGGR + Carbamidomethyl (C); 2 Oxidation (M)** |
|  | 3036 | **727.0312** | **2178.0714** | **2177.5199** | **0.5515** | **1** | **15** | **67** | **1** | **IQPAKLEDSAVYLCASSLLR** |
|  | 500 | **400.1815** | **1197.5223** | **1198.4340** | **-0.9117** | **0** | **15** | **71** | **1** | **QAVMEMMSQK + Oxidation (M)** |
|  | 1277 | **458.1331** | **914.2515** | **914.0361** | **0.2154** | **0** | **15** | **88** | **1** | **MSFWSEK** |
|  | 3222 | **755.8243** | **2264.4509** | **2264.4468** | **0.0041** | **1** | **15** | **97** | **1** | **SEDTAVYYCASRVPDGMDVW** |
|  | 795 | **413.5914** | **1237.7521** | **1238.4761** | **-0.7240** | **2** | **15** | **61** | **1** | **AVIKILHSKET** |
|  | 2338 | **599.3898** | **1795.1471** | **1794.9642** | **0.1829** | **0** | **15** | **79** | **1** | **SAPLPSAAAHQQQLYGR** |
|  | 2466 | **614.9573** | **1841.8498** | **1841.0246** | **0.8252** | **1** | **15** | **72** | **1** | **HSVETPDIKDLASTLSK** |
|  | 3128 | **741.6938** | **2222.0594** | **2221.4830** | **0.5764** | **2** | **15** | **65** | **1** | **SPSQGRQPAFRPPAWNRLR** |
|  | 741 | 408.9569 | 1223.8486 | 1224.4081 | -0.5595 | 0 | 15 | 94 | 1 | IDTSCVCALSR + Carbamidomethyl (C) |
|  | 3319 | **781.5591** | **1561.1034** | **1561.6929** | **-0.5896** | **1** | **15** | **80** | **1** | **GSSGSSGIKEVNQALK** |
|  | 58 | **364.8220** | **727.6292** | **726.8845** | **0.7447** | **1** | **15** | **81** | **1** | **AFKSMK + Oxidation (M)** |
|  | 234 | **378.2679** | **1131.7816** | **1131.2867** | **0.4949** | **2** | **15** | **59** | **1** | **TIRSAGSVKGR** |
|  | 1523 | **478.8314** | **955.6481** | **955.1128** | **0.5352** | **1** | **15** | **79** | **1** | **LAAAPTGAKR** |
|  | 3156 | **744.0732** | **2229.1976** | **2228.3962** | **0.8013** | **1** | **15** | **68** | **1** | **TQQEDSAVYLCASSLGTKGGR + Carbamidomethyl (C)** |
|  | 191 | **374.4978** | **1120.4711** | **1119.3354** | **1.1357** | **1** | **15** | **1.1e+02** | **1** | **KLLQDIMSR + Oxidation (M)** |
|  | 2396 | **609.2285** | **1824.6634** | **1825.0102** | **-0.3468** | **1** | **15** | **90** | **1** | **HNMDIGTWDNKGPVPK + Oxidation (M)** |
|  | 3594 | **1090.7844** | **2179.5541** | **2178.5577** | **0.9964** | **1** | **15** | **74** | **1** | **GCFHCEVCKMALNMNNYK + Carbamidomethyl (C); Oxidation (M)** |
|  | 164 | **372.2442** | **742.4736** | **741.8790** | **0.5946** | **0** | **15** | **66** | **1** | **QLGGIVR** |
|  | 344 | **387.0486** | **1158.1237** | **1158.2422** | **-0.1186** | **0** | **15** | **99** | **1** | **DHSGGIGDICK + Carbamidomethyl (C)** |
|  | 760 | **410.4583** | **1228.3528** | **1229.4066** | **-1.0538** | **1** | **15** | **1.1e+02** | **1** | **VKHETELAMR + Oxidation (M)** |
|  | 133 | **369.3619** | **1105.0636** | **1104.3028** | **0.7608** | **0** | **15** | **96** | **1** | **ASVPPVPGKPR** |
|  | 2903 | **686.0167** | **1370.0185** | **1370.4848** | **-0.4662** | **0** | **15** | **70** | **1** | **VSLDVDNGNMYK + Oxidation (M)** |
|  | 1201 | **450.2853** | **1347.8338** | **1347.4780** | **0.3559** | **1** | **15** | **70** | **1** | **AVREGMCDSDHK** |
|  | 2021 | **549.7293** | **1646.1658** | **1646.8571** | **-0.6914** | **0** | **15** | **77** | **1** | **EPESILQVLSQMEK + Oxidation (M)** |
|  | 60 | **365.0992** | **1092.2753** | **1093.2302** | **-0.9549** | **0** | **15** | **91** | **1** | **EELTGYLLR** |
|  | 1547 | **482.3243** | **962.6338** | **962.0177** | **0.6161** | **0** | **15** | **75** | **1** | **QQHSTSFK** |
|  | 1657 | **498.9865** | **1493.9373** | **1494.8008** | **-0.8636** | **2** | **15** | **81** | **1** | **TYSRNVLAMALKK** |
|  | 243 | **379.3304** | **756.6460** | **757.7940** | **-1.1479** | **0** | **15** | **74** | **1** | **GGWPGER** |
|  | 1492 | **476.2108** | **1425.6103** | **1424.5354** | **1.0748** | **0** | **15** | **86** | **1** | **ADSQLFCVAEER + Carbamidomethyl (C)** |
|  | 2715 | 659.6386 | 1975.8937 | 1975.3633 | 0.5303 | 1 | 15 | 82 | 1 | ALCLICRGCIPSSLAQR + 3 Carbamidomethyl (C) |
|  | 589 | **405.0428** | **808.0708** | **807.8578** | **0.2130** | **1** | **15** | **80** | **1** | **AGDPHRR** |
|  | 2486 | **619.2815** | **1236.5482** | **1237.3653** | **-0.8171** | **0** | **15** | **85** | **1** | **GTNIQHIALDR** |
|  | 534 | **402.3078** | **1203.9013** | **1203.4585** | **0.4429** | **2** | **15** | **82** | **1** | **GRLLSLGMKGR + Oxidation (M)** |
|  | 2216 | **585.0637** | **1168.1127** | **1169.2651** | **-1.1524** | **0** | **15** | **87** | **1** | **SSALDMENFR** |
|  | 756 | **409.4179** | **1225.2315** | **1225.4375** | **-0.2060** | **0** | **15** | **1.1e+02** | **1** | **TPLPFHSWIK** |
|  | 1193 | **449.9140** | **1346.7198** | **1347.4315** | **-0.7117** | **0** | **15** | **84** | **1** | **GSTAPAASAATSSLR** |
|  | 3569 | **974.3284** | **2919.9629** | **2920.0661** | **-0.1031** | **2** | **15** | **67** | **1** | **DTFMYNQEQQENSAESMQPKRGER + Oxidation (M)** |
|  | 274 | **384.9003** | **767.7858** | **768.9245** | **-1.1387** | **1** | **15** | **74** | **1** | **FMAKTR + Oxidation (M)** |
|  | 6 | **360.3786** | **718.7424** | **719.8289** | **-1.0865** | **0** | **15** | **1.2e+02** | **1** | **FVADLR** |
|  | 1250 | **455.2580** | **1362.7518** | **1362.4909** | **0.2610** | **1** | **15** | **76** | **1** | **RESVVNLENFR** |
|  | 1604 | **489.5802** | **1465.7183** | **1464.7070** | **1.0114** | **0** | **15** | **1.1e+02** | **1** | **NLLTMGVDIDMAR + Oxidation (M)** |
|  | 2365 | **604.1018** | **1809.2832** | **1810.2950** | **-1.0118** | **1** | **15** | **91** | **1** | **MGPKLWIHLLIFLTK** |
|  | 3592 | **1075.3088** | **2148.6029** | **2149.4552** | **-0.8523** | **2** | **15** | **73** | **1** | **SYEDCCGSRCCVRALSIQR** |
|  | 812 | **415.0777** | **828.1407** | **827.0036** | **1.1371** | **0** | **15** | **90** | **1** | **MPPDVIR** |
|  | 1685 | **504.0276** | **1509.0606** | **1508.6371** | **0.4235** | **1** | **15** | **94** | **1** | **MAAAEAGGDDARCVR + Oxidation (M)** |
|  | 1946 | **537.6820** | **1073.3492** | **1072.2144** | **1.1349** | **0** | **15** | **1e+02** | **1** | **NLSGFPAAAPK** |
|  | 2288 | **594.2708** | **1779.7901** | **1780.0542** | **-0.2641** | **1** | **15** | **96** | **1** | **KNMPVEETAWSCCPK + Carbamidomethyl (C)** |
|  | 3232 | **757.9431** | **2270.8072** | **2270.5355** | **0.2717** | **2** | **15** | **83** | **1** | **KDGLKFYTDPSYFFDLWK** |
|  | 903 | **420.6888** | **839.3628** | **839.9410** | **-0.5782** | **0** | **15** | **64** | **1** | **GHPFVQR** |
|  | 1014 | **431.2109** | **860.4070** | **861.0613** | **-0.6542** | **0** | **15** | **99** | **1** | **MASVLNVK** |
|  | 3022 | **718.9546** | **2153.8416** | **2153.6243** | **0.2173** | **0** | **15** | **74** | **1** | **VIIIFLLPPYVFISEMSR + Oxidation (M)** |
|  | 3155 | **743.6904** | **2228.0491** | **2227.6103** | **0.4388** | **2** | **15** | **69** | **1** | **MPSETHAMLATLARVAALRR + 2 Oxidation (M)** |
|  | 2310 | **596.6785** | **1787.0132** | **1787.0071** | **0.0062** | **0** | **15** | **1.2e+02** | **1** | **SHMPYTDAVVHFVQR** |
|  | 3241 | **760.1992** | **2277.5753** | **2277.5379** | **0.0374** | **1** | **15** | **85** | **1** | **LLIYGNSNRPSGVPQRFSGSK** |
|  | 3379 | **802.2369** | **1602.4591** | **1601.8065** | **0.6526** | **1** | **15** | **85** | **1** | **YKPSHIPGNSAKFR** |
|  | 890 | **419.5642** | **837.1136** | **836.9106** | **0.2031** | **0** | **15** | **1e+02** | **1** | **ICSSDAGK + Carbamidomethyl (C)** |
|  | 1190 | **449.2987** | **896.5826** | **897.0602** | **-0.4776** | **1** | **15** | **67** | **1** | **NLVCHRR** |
|  | 1789 | **519.2758** | **1036.5367** | **1036.1244** | **0.4124** | **1** | **15** | **86** | **1** | **MRNTGAGGTR + Oxidation (M)** |
|  | 1053 | **434.1274** | **1299.3600** | **1300.5504** | **-1.1905** | **0** | **15** | **86** | **1** | **IQEVSCCLLHR** |
|  | 1588 | **488.1338** | **974.2528** | **974.0235** | **0.2293** | **0** | **15** | **98** | **1** | **SEQPEGSLK** |
|  | 851 | **417.9453** | **1250.8136** | **1250.5099** | **0.3036** | **1** | **15** | **94** | **1** | **LCLAVKDYIR + Carbamidomethyl (C)** |
|  | 2499 | **621.4167** | **1861.2281** | **1862.1330** | **-0.9050** | **1** | **15** | **82** | **1** | **ELVFWSDVTLDRILR** |
|  | 3324 | **786.3796** | **2356.1167** | **2356.6561** | **-0.5394** | **0** | **15** | **87** | **1** | **HEYLYGSYPLCQFQVQKPR** |
|  | 117 | **369.2848** | **736.5547** | **736.8793** | **-0.3245** | **0** | **15** | **78** | **1** | **AFQMPK + Oxidation (M)** |
|  | 1929 | **536.2355** | **1605.6842** | **1604.8484** | **0.8358** | **1** | **15** | **93** | **1** | **LGTAALLLTFRSDAR** |
|  | 493 | **399.4040** | **796.7932** | **796.8717** | **-0.0785** | **0** | **15** | **85** | **1** | **HSVEVAR** |
|  | 1044 | **433.1627** | **1296.4659** | **1296.4445** | **0.0214** | **1** | **15** | **89** | **1** | **EEIDTLKMSSK + Oxidation (M)** |
|  | 1181 | **447.7828** | **1340.3263** | **1341.5163** | **-1.1900** | **2** | **15** | **79** | **1** | **LQRGELSPKASR** |
|  | 1737 | **510.6449** | **1528.9126** | **1527.7212** | **1.1914** | **0** | **15** | **1.1e+02** | **1** | **ATQDQILQTLIQR** |
|  | 1742 | 512.3596 | 1534.0567 | 1534.6479 | -0.5912 | 2 | 15 | 72 | 1 | EKGDPVKMSNGNDK + Oxidation (M) |
|  | 2966 | **699.5922** | **2095.7543** | **2095.3633** | **0.3911** | **2** | **15** | **70** | **1** | **SEQVLPCSVCQETFRRR + Carbamidomethyl (C)** |
|  | 1793 | **519.9814** | **1556.9222** | **1557.6943** | **-0.7722** | **2** | **15** | **87** | **1** | **ETRHCISRQQSR + Carbamidomethyl (C)** |
|  | 2998 | **714.5399** | **2140.5976** | **2140.4418** | **0.1557** | **2** | **15** | **82** | **1** | **WRPQALDTVELEKLASRK** |
|  | 682 | **407.5041** | **1219.4901** | **1218.3803** | **1.1098** | **0** | **15** | **1e+02** | **1** | **CEVNINGATLK + Carbamidomethyl (C)** |
|  | 1231 | **452.7860** | **903.5571** | **904.1106** | **-0.5535** | **1** | **15** | **89** | **1** | **ILSRLFR** |
|  | 1968 | **540.1750** | **1078.3353** | **1079.2334** | **-0.8980** | **1** | **15** | **93** | **1** | **NAINKNFCR** |
|  | 2750 | **666.6801** | **1331.3454** | **1331.6060** | **-0.2605** | **2** | **15** | **91** | **1** | **QKHGVPPLKLSK** |
|  | 1755 | **515.4427** | **1028.8707** | **1029.1053** | **-0.2345** | **1** | **15** | **74** | **1** | **IGESDRQPK** |
|  | 3355 | **795.7496** | **2384.2267** | **2384.7441** | **-0.5174** | **0** | **15** | **75** | **1** | **NVILEVNEDMLSEALIQNLVK** |
|  | 1945 | **537.5259** | **1609.5555** | **1609.9943** | **-0.4388** | **2** | **15** | **97** | **1** | **LPVTKMKYSGNLMK** |
|  | 2085 | **559.5040** | **1675.4897** | **1675.1138** | **0.3759** | **2** | **15** | **79** | **1** | **MLLRASMIFAHLKK + Oxidation (M)** |
|  | 2900 | **685.8474** | **2054.5201** | **2054.3079** | **0.2122** | **2** | **15** | **1e+02** | **1** | **MPLAEDIKGSCFQSGNKR + Carbamidomethyl (C); Oxidation (M)** |
|  | 852 | **417.9510** | **833.8872** | **833.9529** | **-0.0657** | **0** | **15** | **96** | **1** | **CLNSLER** |
|  | 869 | **419.0181** | **836.0213** | **834.8981** | **1.1233** | **0** | **15** | **1.1e+02** | **1** | **ANTSCSPR** |
|  | 1008 | **431.0871** | **1290.2391** | **1291.3452** | **-1.1061** | **0** | **15** | **1.1e+02** | **1** | **MDATANDVPSDR** |
|  | 584 | **404.9612** | **1211.8614** | **1211.2869** | **0.5745** | **1** | **15** | **85** | **1** | **MERDGCAGGGSR + Oxidation (M)** |
|  | 654 | **407.0158** | **1218.0252** | **1217.4817** | **0.5434** | **2** | **15** | **82** | **1** | **ITLQDLKRCK** |
|  | 3097 | **740.5557** | **2218.6450** | **2219.2503** | **-0.6053** | **1** | **15** | **83** | **1** | **DTKDNYCIISSSEESELDN + Carbamidomethyl (C)** |
|  | 545 | **403.1058** | **804.1969** | **803.9039** | **0.2929** | **0** | **15** | **1e+02** | **1** | **SQITVTR** |
|  | 1550 | **483.1532** | **964.2917** | **964.0600** | **0.2317** | **1** | **15** | **97** | **1** | **CPGRGDFR + Carbamidomethyl (C)** |
|  | 397 | **389.1600** | **1164.4579** | **1163.4542** | **1.0037** | **0** | **15** | **98** | **1** | **CLAAVLTLMR + Carbamidomethyl (C); Oxidation (M)** |
|  | 604 | **405.2799** | **1212.8175** | **1212.3626** | **0.4549** | **2** | **15** | **69** | **1** | **RRQVQADALR** |
|  | 811 | **414.9128** | **827.8107** | **826.8976** | **0.9132** | **1** | **15** | **91** | **1** | **PGGSPKER** |
|  | 2209 | **582.6667** | **1744.9779** | **1745.0347** | **-0.0568** | **2** | **15** | **1.1e+02** | **1** | **NLSRLRYLGVTLSPR** |
|  | 2518 | **627.3768** | **1879.1083** | **1878.2234** | **0.8849** | **2** | **15** | **89** | **1** | **LGERRLLILGELRPDK** |
|  | 2809 | **671.6765** | **1341.3382** | **1340.5053** | **0.8330** | **2** | **15** | **86** | **1** | **MERDAAFTQKK + Oxidation (M)** |
|  | 3114 | **741.0655** | **2220.1743** | **2219.5831** | **0.5912** | **2** | **15** | **72** | **1** | **DHADMLKQYATCLSRLLPK + Oxidation (M)** |
|  | 1207 | **450.7379** | **899.4611** | **900.0376** | **-0.5765** | **2** | **15** | **77** | **1** | **GLRAAREK** |
|  | 1276 | 458.1125 | 914.2102 | 913.9781 | 0.2320 | 1 | 15 | 94 | 1 | AEGAPGRTR |
|  | 1697 | **504.8426** | **1511.5055** | **1510.6696** | **0.8360** | **2** | **15** | **81** | **1** | **SDKETRLATSMAGK + Oxidation (M)** |
|  | 2041 | **551.8302** | **1101.6456** | **1101.1680** | **0.4777** | **0** | **15** | **79** | **1** | **SLPAVQSDER** |
|  | 2932 | **688.5442** | **2062.6104** | **2062.4144** | **0.1960** | **0** | **15** | **79** | **1** | **FALGPSCVSSTMTASLQCCR** |
|  | 1147 | **443.8817** | **885.7487** | **886.0473** | **-0.2986** | **0** | **15** | **95** | **1** | **LLQGLTNK** |
|  | 511 | **401.1092** | **800.2035** | **800.8569** | **-0.6533** | **0** | **15** | **1e+02** | **1** | **NLGPGDTK** |
|  | 1635 | **494.4652** | **986.9156** | **987.1516** | **-0.2360** | **2** | **15** | **90** | **1** | **GKVEAEKVK** |
|  | 1853 | **526.0956** | **1050.1765** | **1049.2044** | **0.9721** | **1** | **15** | **98** | **1** | **MDVYPPRR + Oxidation (M)** |
|  | 595 | **405.1635** | **1212.4685** | **1212.3858** | **0.0826** | **1** | **15** | **86** | **1** | **CAAGRAPRPTR + Carbamidomethyl (C)** |
|  | 98 | **368.6831** | **1103.0271** | **1102.2899** | **0.7372** | **1** | **15** | **94** | **1** | **RIGAGFVLNR** |
|  | 355 | **387.9054** | **773.7959** | **772.8467** | **0.9492** | **0** | **15** | **1.1e+02** | **1** | **AQEQLGK** |
|  | 372 | 388.1103 | 1161.3087 | 1162.2609 | -0.9522 | 1 | 15 | 1.1e+02 | 1 | RSCGEMGHGR + Carbamidomethyl (C); Oxidation (M) |
|  | 530 | **402.0898** | **802.1647** | **802.9158** | **-0.7511** | **1** | **15** | **1.1e+02** | **1** | **ASLEKAGK** |
|  | 565 | **403.9979** | **1208.9715** | **1208.3722** | **0.5993** | **1** | **15** | **96** | **1** | **ATAGLAFHRHK** |
|  | 1036 | **433.0084** | **1296.0029** | **1295.3983** | **0.6046** | **1** | **15** | **95** | **1** | **YSLGQVSKEER** |
|  | 1504 | **476.7888** | **951.5629** | **952.0692** | **-0.5062** | **1** | **15** | **76** | **1** | **KDNHALVR** |
|  | 292 | **385.1407** | **1152.4001** | **1151.3791** | **1.0210** | **2** | **15** | **82** | **1** | **EEAFLMRKK** |
|  | 539 | **402.7856** | **803.5564** | **803.8609** | **-0.3044** | **0** | **15** | **1e+02** | **1** | **SVTLDNR** |
|  | 1764 | **517.6727** | **1549.9958** | **1549.7103** | **0.2855** | **1** | **15** | **1e+02** | **1** | **QSGTRGCTWEVLR + Carbamidomethyl (C)** |
|  | 424 | **389.9048** | **1166.6922** | **1167.3584** | **-0.6663** | **1** | **15** | **95** | **1** | **APQQQVQKLK** |
|  | 1098 | **437.0940** | **1308.2598** | **1308.5659** | **-0.3061** | **1** | **15** | **1.1e+02** | **1** | **TEDKILHIVIK** |
|  | 1534 | **480.1731** | **1437.4971** | **1437.6453** | **-0.1481** | **2** | **15** | **98** | **1** | **MDAQCSAKVNARK + Oxidation (M)** |
|  | 2088 | **560.1383** | **1677.3927** | **1677.8114** | **-0.4187** | **1** | **15** | **1e+02** | **1** | **STGYCSVTGNLSGCKGS + Carbamidomethyl (C)** |
|  | 50 | **363.5140** | **725.0132** | **725.7440** | **-0.7309** | **0** | **14** | **85** | **1** | **YAESEK** |
|  | 753 | **409.2084** | **1224.6030** | **1223.4418** | **1.1613** | **1** | **14** | **90** | **1** | **MFGQGTKVEVK** |
|  | 1162 | **444.9069** | **1331.6984** | **1332.3788** | **-0.6803** | **0** | **14** | **1.1e+02** | **1** | **HGSFNTVQASER** |
|  | 3403 | **806.8653** | **2417.5737** | **2416.8111** | **0.7626** | **2** | **14** | **1.1e+02** | **1** | **MPKFSMPSLKGEGPEVDVNLPK + Oxidation (M)** |
|  | 399 | **389.1625** | **1164.4654** | **1164.3100** | **0.1555** | **0** | **14** | **1e+02** | **1** | **NNCVMPEDVK + Oxidation (M)** |
|  | 1586 | **488.1005** | **974.1861** | **973.0918** | **1.0944** | **2** | **14** | **1.1e+02** | **1** | **RSAVATRGR** |
|  | 3212 | **749.8928** | **2246.6563** | **2247.6863** | **-1.0300** | **2** | **14** | **1e+02** | **1** | **MKRHEMVAKPPAMCSHFAK + 3 Oxidation (M)** |
|  | 2619 | **643.6044** | **1927.7909** | **1927.2068** | **0.5842** | **2** | **14** | **78** | **1** | **CEFVMEVTNKTRADVK + Carbamidomethyl (C)** |
|  | 657 | **407.0743** | **812.1338** | **811.8863** | **0.2475** | **1** | **14** | **91** | **1** | **KHTGVDR** |
|  | 972 | **429.0280** | **1284.0620** | **1284.4883** | **-0.4263** | **1** | **14** | **89** | **1** | **CCVTHDRCYK + Carbamidomethyl (C)** |
|  | 1165 | **445.1022** | **1332.2843** | **1332.5031** | **-0.2187** | **1** | **14** | **1.1e+02** | **1** | **DNSTMGYMAAKK + Oxidation (M)** |
|  | 1285 | **458.5239** | **1372.5495** | **1373.4737** | **-0.9242** | **1** | **14** | **1.2e+02** | **1** | **SKGQDVVGAGWGGR** |
|  | 1540 | **480.7259** | **1439.1555** | **1439.5267** | **-0.3712** | **0** | **14** | **81** | **1** | **SPFNAFDIWGQGT** |
|  | 2430 | **612.2402** | **1833.6985** | **1834.0154** | **-0.3168** | **0** | **14** | **97** | **1** | **GMIDGSTPTITPNPNFR + Oxidation (M)** |
|  | 521 | **401.9129** | **801.8110** | **800.8601** | **0.9509** | **0** | **14** | **1.1e+02** | **1** | **AEQGLQR** |
|  | 847 | **417.6169** | **833.2191** | **833.9530** | **-0.7339** | **0** | **14** | **87** | **1** | **GSVEACIR** |
|  | 1781 | **518.9797** | **1553.9170** | **1554.8343** | **-0.9173** | **1** | **14** | **93** | **1** | **LPLTLFPSANLRGR** |
|  | 1030 | **432.5737** | **1294.6990** | **1295.5260** | **-0.8269** | **0** | **14** | **1e+02** | **1** | **VCLTDVTIMER + Oxidation (M)** |
|  | 1995 | **544.0175** | **1629.0302** | **1629.9161** | **-0.8859** | **0** | **14** | **1e+02** | **1** | **SMITFMDDLNMPAK + Oxidation (M)** |
|  | 2263 | **592.8124** | **1775.4150** | **1775.1418** | **0.2731** | **1** | **14** | **83** | **1** | **VYRPLGSFILKIPSGK** |
|  | 2501 | **621.4805** | **1861.4192** | **1862.1330** | **-0.7138** | **1** | **14** | **75** | **1** | **ELVFWSDVTLDRILR** |
|  | 306 | **386.0449** | **1155.1125** | **1154.4243** | **0.6882** | **1** | **14** | **84** | **1** | **KAFIGCPPPPK** |
|  | 3378 | **802.0924** | **1602.1700** | **1602.8924** | **-0.7223** | **2** | **14** | **75** | **1** | **SKFGDGYIVTMKIK + Oxidation (M)** |
|  | 2428 | **612.2239** | **1222.4330** | **1222.3940** | **0.0390** | **2** | **14** | **98** | **1** | **NRTKQLFSTK** |
|  | 1983 | **542.0139** | **1623.0194** | **1622.9515** | **0.0679** | **1** | **14** | **92** | **1** | **NGLKMCEPCGGLCPK + Carbamidomethyl (C); Oxidation (M)** |
|  | 1831 | **523.8159** | **1568.4254** | **1567.6793** | **0.7461** | **1** | **14** | **83** | **1** | **FEEKCGDNGSIVGR + Carbamidomethyl (C)** |
|  | 2583 | **636.2860** | **1270.5572** | **1269.4042** | **1.1531** | **1** | **14** | **95** | **1** | **GVDPEKAPSLTR** |
|  | 2620 | **643.6172** | **1927.8294** | **1928.2444** | **-0.4150** | **1** | **14** | **81** | **1** | **MLVAAAASVASCRPHSGKR + Oxidation (M)** |
|  | 1856 | **526.5240** | **1051.0332** | **1051.1573** | **-0.1241** | **2** | **14** | **98** | **1** | **GKFRTSAER** |
|  | 2269 | **592.9108** | **1775.7103** | **1774.9731** | **0.7373** | **1** | **14** | **82** | **1** | **GSDCTAKEPPLANVCR + 2 Carbamidomethyl (C)** |
|  | 340 | **386.9988** | **771.9829** | **770.9205** | **1.0624** | **1** | **14** | **1.1e+02** | **1** | **KVVVNGR** |
|  | 914 | **421.9285** | **841.8422** | **841.9798** | **-0.1377** | **0** | **14** | **95** | **1** | **WCIHQR** |
|  | 1243 | **454.2078** | **1359.6011** | **1360.4918** | **-0.8907** | **0** | **14** | **1e+02** | **1** | **TTSSMDPGDMMR + 2 Oxidation (M)** |
|  | 3300 | **777.5140** | **2329.5199** | **2330.2002** | **-0.6802** | **0** | **14** | **95** | **1** | **SIDDEDVDENEDDVYGNSSGR** |
|  | 505 | **400.2565** | **1197.7473** | **1197.4111** | **0.3362** | **1** | **14** | **69** | **1** | **NSPRPMKVPR + Oxidation (M)** |
|  | 582 | **404.7250** | **807.4352** | **806.9278** | **0.5074** | **0** | **14** | **80** | **1** | **MSSIVDR** |
|  | 986 | **429.3978** | **1285.1711** | **1285.4497** | **-0.2786** | **1** | **14** | **90** | **1** | **NRSLASPLQATK** |
|  | 2743 | **666.6112** | **1996.8114** | **1996.3778** | **0.4337** | **2** | **14** | **79** | **1** | **AFKAFVQMTGHFLNLKK + Oxidation (M)** |
|  | 195 | **375.0962** | **1122.2664** | **1123.2397** | **-0.9733** | **0** | **14** | **1.1e+02** | **1** | **DLAGWSTMAR + Oxidation (M)** |
|  | 504 | **400.2254** | **1197.6539** | **1198.4340** | **-0.7800** | **0** | **14** | **70** | **1** | **QAVMEMMSQK + Oxidation (M)** |
|  | 3192 | **749.2220** | **1496.4293** | **1495.5936** | **0.8358** | **1** | **14** | **94** | **1** | **DGEAGRAGAAPVAPEK** |
|  | 135 | **369.4065** | **1105.1972** | **1104.2812** | **0.9161** | **1** | **14** | **1.2e+02** | **1** | **VNLAMEGRAK + Oxidation (M)** |
|  | 1040 | **433.1176** | **1296.3308** | **1297.4574** | **-1.1266** | **0** | **14** | **98** | **1** | **TCDPVEMSYPR** |
|  | 1553 | 483.3265 | 1446.9574 | 1446.6519 | 0.3055 | 1 | 14 | 84 | 1 | EFAHYCNFCKK + Carbamidomethyl (C) |
|  | 1871 | **528.3862** | **1582.1365** | **1581.7722** | **0.3644** | **2** | **14** | **76** | **1** | **RAGDLLEDSPKRPK** |
|  | 202 | **376.2531** | **1125.7373** | **1125.3186** | **0.4187** | **2** | **14** | **75** | **1** | **GPASPLEAKKK** |
|  | 63 | **365.8908** | **1094.6502** | **1094.1506** | **0.4996** | **0** | **14** | **1.1e+02** | **1** | **EGNVSMDTEL** |
|  | 477 | **399.1083** | **796.2018** | **796.8716** | **-0.6697** | **0** | **14** | **88** | **1** | **QAVSHQK** |
|  | 1937 | **536.5621** | **1606.6642** | **1606.7865** | **-0.1223** | **1** | **14** | **1.2e+02** | **1** | **HDRYAPPRPGTLAR** |
|  | 315 | **386.1273** | **770.2397** | **769.8893** | **0.3505** | **1** | **14** | **90** | **1** | **NPVGQKK** |
|  | 1129 | **441.4304** | **1321.2689** | **1321.5465** | **-0.2776** | **0** | **14** | **95** | **1** | **HFQQVFQMLK + Oxidation (M)** |
|  | 1167 | **445.3009** | **888.5870** | **889.0085** | **-0.4215** | **1** | **14** | **93** | **1** | **SAQAAKVSK** |
|  | 1996 | **544.3636** | **1630.0688** | **1630.8676** | **-0.7989** | **2** | **14** | **97** | **1** | **REAEQLRMELAIR + Oxidation (M)** |
|  | 2258 | **592.6693** | **1183.3238** | **1182.2905** | **1.0334** | **2** | **14** | **1.2e+02** | **1** | **TKDPHTRATR** |
|  | 111 | **369.2672** | **1104.7794** | **1104.3277** | **0.4517** | **2** | **14** | **86** | **1** | **RVAAMSVAKR + Oxidation (M)** |
|  | 1449 | **472.3841** | **942.7534** | **943.0758** | **-0.3224** | **0** | **14** | **78** | **1** | **MTQPPPEK + Oxidation (M)** |
|  | 2054 | **553.9608** | **1658.8601** | **1659.0035** | **-0.1434** | **2** | **14** | **1e+02** | **1** | **KAQLAILGMTSLKER** |
|  | 2148 | **570.1277** | **1707.3609** | **1707.9053** | **-0.5444** | **0** | **14** | **1e+02** | **1** | **GPQLPGCSASSPLSPPR + Carbamidomethyl (C)** |
|  | 1325 | **460.5003** | **918.9858** | **918.1125** | **0.8733** | **1** | **14** | **1.3e+02** | **1** | **IEEIMKR** |
|  | 1975 | **540.6770** | **1079.3392** | **1080.4056** | **-1.0664** | **1** | **14** | **1.1e+02** | **1** | **IVIVSKMMK + 2 Oxidation (M)** |
|  | 1228 | **452.4288** | **902.8429** | **902.0468** | **0.7961** | **0** | **14** | **1.1e+02** | **1** | **QATTIIAGK** |
|  | 1230 | **452.7249** | **1355.1526** | **1355.6654** | **-0.5129** | **1** | **14** | **89** | **1** | **CYMSLILKAEGK** |
|  | 2002 | **546.2449** | **1635.7124** | **1635.8113** | **-0.0989** | **0** | **14** | **1.1e+02** | **1** | **AEAYAFLTPVEEAPK** |
|  | 2139 | **568.5415** | **1135.0682** | **1134.3120** | **0.7563** | **1** | **14** | **92** | **1** | **TGRCPFQGLR** |
|  | 99 | **368.9323** | **1103.7749** | **1103.2501** | **0.5248** | **0** | **14** | **1.1e+02** | **1** | **IMENTAPGVR + Oxidation (M)** |
|  | 949 | **427.8102** | **1280.4086** | **1279.3592** | **1.0494** | **0** | **14** | **1e+02** | **1** | **GEVGPPGPAGSAGAR** |
|  | 2180 | **577.2487** | **1152.4825** | **1151.3113** | **1.1713** | **0** | **14** | **98** | **1** | **EGEMEVAMQK** |
|  | 1607 | 489.7600 | 977.5053 | 978.0818 | -0.5765 | 1 | 14 | 83 | 1 | SEGEVARCK |
|  | 3249 | **762.1664** | **2283.4772** | **2284.2593** | **-0.7822** | **1** | **14** | **96** | **1** | **LSSTDNEDEELGTEGSTSEKR** |
|  | 3091 | **740.5162** | **1479.0177** | **1478.6887** | **0.3290** | **1** | **14** | **96** | **1** | **QLTLLESDLYRK** |
|  | 256 | **380.2907** | **758.5667** | **758.9694** | **-0.4027** | **0** | **14** | **94** | **1** | **MVAIPTK** |
|  | 1522 | **478.4254** | **1432.2539** | **1432.4930** | **-0.2391** | **1** | **14** | **80** | **1** | **DGSGVSSLGSKSSHK** |
|  | 1597 | **488.4394** | **1462.2959** | **1462.7458** | **-0.4498** | **2** | **14** | **93** | **1** | **RLRVLGDRPKPR** |
|  | 1066 | **435.0379** | **1302.0916** | **1301.5764** | **0.5152** | **1** | **14** | **89** | **1** | **GLQLGGKAFGLLK** |
|  | 2383 | 607.6240 | 1819.8497 | 1818.9444 | 0.9053 | 1 | 14 | 1.1e+02 | 1 | ASDTAMYYCARHSNR + Carbamidomethyl (C); Oxidation (M) |
|  | 3390 | **804.2239** | **2409.6495** | **2409.7865** | **-0.1371** | **1** | **14** | **98** | **1** | **SGASVGIHLLCSIFPPHRWACT + Carbamidomethyl (C)** |
|  | 1759 | **515.9702** | **1544.8883** | **1545.8228** | **-0.9345** | **0** | **14** | **1.1e+02** | **1** | **LLSMTLSPDLHMR + 2 Oxidation (M)** |
|  | 1792 | **519.9766** | **1556.9075** | **1556.7477** | **0.1599** | **0** | **14** | **97** | **1** | **CHFPAGTSRPGTGLR** |
|  | 2770 | **667.8464** | **1333.6781** | **1333.4067** | **0.2714** | **1** | **14** | **1.1e+02** | **1** | **ERPGRYPSEDK** |
|  | 1812 | **521.8617** | **1041.7086** | **1041.1590** | **0.5496** | **0** | **14** | **89** | **1** | **GPGGSCEFCGK** |
|  | 277 | **384.9515** | **1151.8323** | **1152.3734** | **-0.5412** | **1** | **14** | **87** | **1** | **HGILGACGRLR** |
|  | 1612 | **490.4572** | **978.8995** | **979.1756** | **-0.2761** | **0** | **14** | **90** | **1** | **ICLGEAMAR + Oxidation (M)** |
|  | 2353 | **601.0131** | **1200.0115** | **1200.4149** | **-0.4034** | **1** | **14** | **1e+02** | **1** | **MRPRLGGLER + Oxidation (M)** |
|  | 2462 | **614.4283** | **1840.2629** | **1841.0708** | **-0.8080** | **0** | **14** | **92** | **1** | **GNVSLSTAIVADLGSPLAR** |
|  | 3063 | **737.2258** | **1472.4369** | **1472.6644** | **-0.2276** | **0** | **14** | **96** | **1** | **AEMTLATNFFATR** |
|  | 873 | **419.1988** | **1254.5742** | **1255.4471** | **-0.8729** | **1** | **14** | **1.1e+02** | **1** | **HDKVNMQVLR + Oxidation (M)** |
|  | 1242 | **454.1877** | **906.3605** | **906.0556** | **0.3050** | **0** | **14** | **1.1e+02** | **1** | **MVLSESPK + Oxidation (M)** |
|  | 1433 | **471.1141** | **1410.3201** | **1409.5886** | **0.7314** | **1** | **14** | **91** | **1** | **CTGNERSLADCIK** |
|  | 2305 | **596.0265** | **1785.0573** | **1784.9877** | **0.0695** | **2** | **14** | **1.1e+02** | **1** | **YQQLQKDLSKSMSGR + Oxidation (M)** |
|  | 2378 | **606.7999** | **1211.5849** | **1212.4620** | **-0.8770** | **0** | **14** | **88** | **1** | **SCFHIMDICK + Oxidation (M)** |
|  | 598 | **405.2003** | **1212.5786** | **1213.3886** | **-0.8100** | **0** | **14** | **86** | **1** | **LNSCHFAYCR** |
|  | 1803 | **521.0024** | **1039.9901** | **1041.0762** | **-1.0861** | **0** | **14** | **96** | **1** | **GEAQGSTVHR** |
|  | 3070 | **738.5080** | **2212.5018** | **2211.4965** | **1.0053** | **2** | **14** | **1e+02** | **1** | **TWYVGKINRTQAEEMLSGK** |
|  | 324 | **386.2164** | **1155.6270** | **1156.3060** | **-0.6790** | **0** | **14** | **74** | **1** | **EIDMTYQLK + Oxidation (M)** |
|  | 805 | **414.2152** | **826.4156** | **825.9129** | **0.5028** | **1** | **14** | **80** | **1** | **VHTGEKR** |
|  | 984 | **429.3099** | **1284.9075** | **1285.4730** | **-0.5655** | **2** | **14** | **83** | **1** | **KKSCPNPGEIR + Carbamidomethyl (C)** |
|  | 2443 | **613.2115** | **1836.6125** | **1836.1405** | **0.4720** | **0** | **14** | **1e+02** | **1** | **MAFFTGLWGPFTCVSR + Oxidation (M)** |
|  | 1584 | **488.0169** | **974.0190** | **973.1693** | **0.8497** | **0** | **14** | **1.2e+02** | **1** | **LLIFGASPR** |
|  | 2343 | **599.7326** | **1197.4504** | **1197.3813** | **0.0692** | **1** | **14** | **1.2e+02** | **1** | **DVRLPLEVEK** |
|  | 2370 | **605.7609** | **1814.2606** | **1814.0687** | **0.1919** | **0** | **14** | **1.1e+02** | **1** | **NLFPCGDSMVCIIDDR + Oxidation (M)** |
|  | 1295 | **458.8636** | **915.7124** | **915.0226** | **0.6898** | **1** | **14** | **1.1e+02** | **1** | **YEGTCSKK** |
|  | 2257 | **592.6669** | **1774.9784** | **1775.0144** | **-0.0360** | **1** | **14** | **1.2e+02** | **1** | **IGFDVVTLSGTRGPLSR** |
|  | 2702 | **657.2747** | **1968.8018** | **1968.3526** | **0.4492** | **0** | **14** | **99** | **1** | **MQCVHLHPLLQVSLHR + Carbamidomethyl (C)** |
|  | 3266 | **765.1752** | **2292.5033** | **2291.5413** | **0.9621** | **1** | **14** | **95** | **1** | **DHLSFTMVRGGPLNWFDPW + Oxidation (M)** |
|  | 2533 | **629.0798** | **1256.1449** | **1255.5513** | **0.5936** | **2** | **14** | **1e+02** | **1** | **KKIVVLGANWK** |
|  | 3143 | **742.6777** | **2225.0110** | **2224.5135** | **0.4975** | **0** | **14** | **78** | **1** | **SVLSVCFPGCLLTSGEAEQQR** |
|  | 597 | **405.1786** | **808.3425** | **807.9590** | **0.3835** | **1** | **14** | **93** | **1** | **STAVKMR + Oxidation (M)** |
|  | 261 | **382.1014** | **1143.2820** | **1144.2787** | **-0.9968** | **1** | **14** | **1.2e+02** | **1** | **KTALAEDQLR** |
|  | 420 | **389.4022** | **776.7897** | **775.8509** | **0.9388** | **0** | **14** | **1.3e+02** | **1** | **SGVVTASR** |
|  | 583 | **404.9146** | **1211.7217** | **1211.4079** | **0.3138** | **1** | **14** | **95** | **1** | **IEVVLPEKER** |
|  | 712 | **407.9753** | **1220.9037** | **1221.3811** | **-0.4775** | **0** | **14** | **1e+02** | **1** | **LMSPETTLGTR + Oxidation (M)** |
|  | 954 | **428.1294** | **1281.3660** | **1280.4963** | **0.8698** | **2** | **14** | **1.1e+02** | **1** | **DSKGKAMGALFR** |
|  | 561 | **403.9599** | **1208.8574** | **1208.3640** | **0.4934** | **2** | **14** | **1.1e+02** | **1** | **EKGYIDLSKR** |
|  | 1625 | **493.6846** | **1478.0317** | **1477.7139** | **0.3178** | **2** | **14** | **91** | **1** | **CRCTPAWATRAK + 2 Carbamidomethyl (C)** |
|  | 824 | **415.9697** | **829.9247** | **828.9149** | **1.0097** | **0** | **14** | **1.2e+02** | **1** | **APASAAWR** |
|  | 1004 | **431.0444** | **1290.1109** | **1290.5143** | **-0.4033** | **2** | **14** | **1.3e+02** | **1** | **ASVFSNLRIRK** |
|  | 1894 | **532.0663** | **1062.1178** | **1061.2763** | **0.8415** | **0** | **14** | **1.1e+02** | **1** | **MHVICSDLK + Oxidation (M)** |
|  | 737 | **408.8892** | **1223.6455** | **1223.4217** | **0.2238** | **2** | **14** | **1.1e+02** | **1** | **KAASSLIYSRK** |
|  | 816 | **415.8847** | **829.7546** | **828.9084** | **0.8462** | **0** | **14** | **1.2e+02** | **1** | **YYADAVK** |
|  | 1911 | **534.1188** | **1066.2228** | **1066.2281** | **-0.0053** | **0** | **14** | **1.1e+02** | **1** | **SMQLFDNVI** |
|  | 2079 | **557.7867** | **1670.3381** | **1670.7161** | **-0.3780** | **0** | **14** | **86** | **1** | **ASNQPPGGGGGTGGDCPGGK** |
|  | 426 | **390.0041** | **1166.9900** | **1167.2724** | **-0.2824** | **1** | **14** | **1.1e+02** | **1** | **AEPEAIPERR** |
|  | 1795 | **520.1995** | **1038.3841** | **1037.2199** | **1.1642** | **2** | **14** | **1e+02** | **1** | **ALKLRSGHR** |
|  | 417 | **389.2772** | **1164.8095** | **1164.2503** | **0.5592** | **1** | **14** | **93** | **1** | **NLENMRSER + Oxidation (M)** |
|  | 1576 | **487.2019** | **972.3891** | **973.1296** | **-0.7406** | **1** | **14** | **1.1e+02** | **1** | **RWTELIR** |
|  | 1907 | **533.8713** | **1065.7279** | **1066.1040** | **-0.3761** | **1** | **14** | **90** | **1** | **DANCGSSREK** |
|  | 42 | **363.2004** | **1086.5791** | **1087.3401** | **-0.7609** | **2** | **14** | **85** | **1** | **INVAAKRGMK** |
|  | 2171 | **574.0435** | **1719.1082** | **1718.9712** | **0.1370** | **1** | **14** | **1.1e+02** | **1** | **TAPLRMPEGPEPAAPGK** |
|  | 649 | **406.7889** | **811.5631** | **812.0122** | **-0.4491** | **0** | **14** | **1e+02** | **1** | **GVVVAIVR** |
|  | 893 | **419.7704** | **1256.2891** | **1256.4928** | **-0.2038** | **0** | **14** | **97** | **1** | **LLNSGVYLIHK** |
|  | 1012 | **431.1585** | **1290.4534** | **1290.5143** | **-0.0608** | **2** | **14** | **1.2e+02** | **1** | **ASVFSNLRIRK** |
|  | 1216 | **451.7621** | **1352.2643** | **1351.5146** | **0.7497** | **2** | **14** | **1.1e+02** | **1** | **GRDSAPGPALRVR** |
|  | 1332 | **460.8205** | **919.6261** | **918.9516** | **0.6746** | **1** | **14** | **1.1e+02** | **1** | **RQSSGEQK** |
|  | 1592 | **488.2603** | **1461.7586** | **1461.6616** | **0.0970** | **0** | **14** | **1.1e+02** | **1** | **NSGVLKPFSGSIQK** |
|  | 22 | **362.1936** | **722.3724** | **722.8295** | **-0.4571** | **0** | **14** | **81** | **1** | **LFGTGTK** |
|  | 994 | **430.2514** | **858.4881** | **858.0788** | **0.4093** | **0** | **14** | **1e+02** | **1** | **AFLPAVIK** |
|  | 2150 | **570.4561** | **1138.8973** | **1138.1700** | **0.7274** | **1** | **14** | **85** | **1** | **RDMASNEGSR + Oxidation (M)** |
|  | 367 | **388.0343** | **1161.0808** | **1160.3395** | **0.7414** | **0** | **14** | **1.3e+02** | **1** | **MVTDYGAFIK + Oxidation (M)** |
|  | 2333 | **599.1114** | **1196.2081** | **1196.3765** | **-0.1684** | **1** | **14** | **1e+02** | **1** | **LTYSSRCPAAK** |
|  | 2912 | **686.2628** | **2055.7663** | **2055.4252** | **0.3410** | **1** | **14** | **1.1e+02** | **1** | **MPSETHAMLATLARVAALR + Oxidation (M)** |
|  | 1608 | **489.9512** | **977.8877** | **977.0753** | **0.8123** | **1** | **14** | **1.1e+02** | **1** | **TQATSWKR** |
|  | 619 | **406.0661** | **1215.1762** | **1214.3439** | **0.8324** | **0** | **14** | **1e+02** | **1** | **FPSTGVDTMPY** |
|  | 997 | **430.6326** | **859.2504** | **858.0605** | **1.1899** | **0** | **14** | **1e+02** | **1** | **LAAMPALR + Oxidation (M)** |
|  | 2709 | **659.1834** | **1316.3519** | **1316.5448** | **-0.1929** | **0** | **14** | **1.1e+02** | **1** | **SLLQAVANLPYK** |
|  | 2951 | **695.3190** | **2082.9347** | **2082.4636** | **0.4711** | **1** | **14** | **1.1e+02** | **1** | **MEIRADGLGIPQLLEAVLK + Oxidation (M)** |
|  | 1134 | **442.6903** | **1325.0488** | **1324.3633** | **0.6856** | **1** | **14** | **83** | **1** | **DKPRGSGSGGGGHR** |
|  | 1218 | **451.8220** | **901.6291** | **901.0656** | **0.5636** | **2** | **14** | **1.3e+02** | **1** | **KPKGSTKR** |
|  | 1488 | **475.8956** | **1424.6647** | **1423.4678** | **1.1969** | **0** | **14** | **1.1e+02** | **1** | **AFSDSSSCAQHQR** |
|  | 1593 | **488.2621** | **974.5095** | **974.0732** | **0.4364** | **1** | **14** | **1.1e+02** | **1** | **GDRVTITGR** |
|  | 2911 | **686.2432** | **1370.4717** | **1371.5389** | **-1.0673** | **0** | **14** | **1.1e+02** | **1** | **STAPLNTQISALR** |
|  | 621 | **406.0963** | **810.1777** | **809.9085** | **0.2693** | **0** | **14** | **1e+02** | **1** | **CMNEEK + Carbamidomethyl (C)** |
|  | 1236 | **453.1941** | **1356.5601** | **1357.6447** | **-1.0846** | **0** | **14** | **1.2e+02** | **1** | **CLALLTLNHMGR + Oxidation (M)** |
|  | 1259 | **457.1382** | **912.2615** | **912.0466** | **0.2149** | **1** | **14** | **1.1e+02** | **1** | **RYFTLGR** |
|  | 1895 | **532.1431** | **1062.2713** | **1061.1702** | **1.1011** | **0** | **14** | **1.1e+02** | **1** | **QLQEEMQR** |
|  | 11 | **360.4926** | **1078.4557** | **1079.2320** | **-0.7763** | **2** | **14** | **1.3e+02** | **1** | **KRTQSMQGK + Oxidation (M)** |
|  | 323 | **386.2145** | **1155.6214** | **1156.1604** | **-0.5389** | **1** | **14** | **79** | **1** | **RGPGEAAPTEGD** |
|  | 491 | **399.2725** | **796.5301** | **795.8419** | **0.6882** | **0** | **14** | **75** | **1** | **GSQYWR** |
|  | 2290 | **594.6428** | **1187.2707** | **1188.3944** | **-1.1236** | **0** | **14** | **1.3e+02** | **1** | **IVPVVSQSECK** |
|  | 758 | **410.1753** | **818.3358** | **817.8907** | **0.4451** | **1** | **14** | **1.2e+02** | **1** | **QSAAGTKR** |
|  | 781 | **413.0305** | **1236.0695** | **1235.4985** | **0.5709** | **1** | **14** | **95** | **1** | **RLDLFSCLLR** |
|  | 134 | **369.3758** | **1105.1053** | **1105.3089** | **-0.2036** | **0** | **14** | **1.3e+02** | **1** | **AKPWAVCFPS** |
|  | 656 | 407.0448 | 812.0749 | 811.9244 | 0.1505 | 0 | 14 | 98 | 1 | EMGNSMK + Oxidation (M) |
|  | 1020 | **431.7111** | **1292.1112** | **1292.4422** | **-0.3311** | **0** | **14** | **97** | **1** | **LSGNPLTCDCR + 2 Carbamidomethyl (C)** |
|  | 681 | **407.4953** | **1219.4636** | **1219.2625** | **0.2011** | **1** | **14** | **1.3e+02** | **1** | **EIQGRTESGSR** |
|  | 1401 | **467.1721** | **932.3293** | **931.9935** | **0.3359** | **1** | **14** | **1.2e+02** | **1** | **AVASRSADR** |
|  | 736 | **408.8849** | **1223.6324** | **1224.4362** | **-0.8039** | **1** | **14** | **1.2e+02** | **1** | **GACRAAPPGGVLR** |
|  | 1182 | **447.8560** | **893.6971** | **892.9556** | **0.7415** | **0** | **14** | **1.1e+02** | **1** | **VEYAQQR** |
|  | 122 | **369.3018** | **1104.8831** | **1104.3011** | **0.5820** | **1** | **14** | **98** | **1** | **HAMKECLEK + Oxidation (M)** |
|  | 272 | **384.7704** | **1151.2889** | **1151.3625** | **-0.0736** | **2** | **14** | **98** | **1** | **RKKPLPQER** |
|  | 1409 | **468.2783** | **934.5417** | **933.9398** | **0.6020** | **0** | **14** | **1e+02** | **1** | **DCEESSHK** |
|  | 1580 | **487.8821** | **1460.6243** | **1461.5838** | **-0.9595** | **1** | **14** | **1.2e+02** | **1** | **GCLQGMEDSHRR + Carbamidomethyl (C); Oxidation (M)** |
|  | 3220 | **753.5140** | **2257.5199** | **2256.4643** | **1.0556** | **1** | **14** | **1.1e+02** | **1** | **KDYTQVDYLINGMYADSEM** |
|  | 14 | **361.1682** | **1080.4823** | **1081.2660** | **-0.7837** | **2** | **14** | **93** | **1** | **QEKKLGPGPK** |
|  | 484 | **399.1583** | **1194.4528** | **1194.4435** | **0.0094** | **1** | **14** | **97** | **1** | **MLAKISTTWK + Oxidation (M)** |
|  | 874 | **419.2191** | **1254.6350** | **1253.5108** | **1.1242** | **0** | **14** | **1.2e+02** | **1** | **TPPVGMSPQVLK** |
|  | 1292 | **458.8196** | **915.6244** | **915.0126** | **0.6119** | **2** | **14** | **1.2e+02** | **1** | **GRVGAGSRR** |
|  | 3322 | **784.7501** | **1567.4853** | **1567.8930** | **-0.4076** | **1** | **14** | **87** | **1** | **DICLKMSMPIASSR + Oxidation (M)** |
|  | 3 | **360.3381** | **718.6614** | **717.7682** | **0.8931** | **0** | **14** | **1.3e+02** | **1** | **LDNSAAK** |
|  | 1998 | **544.4463** | **1630.3167** | **1630.8444** | **-0.5277** | **1** | **14** | **1e+02** | **1** | **TRSLGGLISAVGVGSTR** |
|  | 532 | **402.1802** | **1203.5184** | **1202.3578** | **1.1605** | **1** | **14** | **1.2e+02** | **1** | **LPSQSLSSKQK** |
|  | 1403 | **467.5523** | **933.0898** | **932.0298** | **1.0601** | **0** | **14** | **1.4e+02** | **1** | **FDFGVGYK** |
|  | 1483 | **475.7087** | **1424.1039** | **1424.6515** | **-0.5476** | **2** | **14** | **96** | **1** | **RSAKPSEKPRLR** |
|  | 85 | **368.0289** | **1101.0646** | **1100.2260** | **0.8386** | **0** | **14** | **1.2e+02** | **1** | **LAQGISQLDR** |
|  | 469 | **398.0797** | **1191.2168** | **1191.3999** | **-0.1831** | **2** | **14** | **1.2e+02** | **1** | **HKTLMFKTEG** |
|  | 875 | **419.2245** | **836.4341** | **836.0748** | **0.3593** | **0** | **14** | **1.1e+02** | **1** | **ALLCSVCK** |
|  | 3360 | **798.0888** | **2391.2442** | **2390.6824** | **0.5618** | **1** | **14** | **86** | **1** | **LLSELEYEAWMEEFKNDML** |
|  | 76 | **366.2620** | **1095.7638** | **1095.2345** | **0.5293** | **1** | **14** | **1e+02** | **1** | **GMNPKFQHH** |
|  | 148 | **371.0710** | **1110.1909** | **1111.2125** | **-1.0216** | **2** | **14** | **92** | **1** | **RSHPSGSGAKK** |
|  | 190 | **374.4751** | **746.9354** | **746.8295** | **0.1059** | **0** | **14** | **1.6e+02** | **1** | **MEPENK** |
|  | 746 | **409.0048** | **815.9947** | **815.9145** | **0.0802** | **0** | **14** | **1.2e+02** | **1** | **LAELAGSR** |
|  | 1027 | **432.3952** | **862.7756** | **862.0276** | **0.7480** | **0** | **14** | **1.1e+02** | **1** | **VGGLLTFR** |
|  | 2753 | **666.7402** | **1997.1983** | **1997.2998** | **-0.1014** | **2** | **14** | **1.3e+02** | **1** | **LDEPVIRMGCVYSSSKR + Carbamidomethyl (C)** |
|  | 2495 | **620.6874** | **1859.0401** | **1860.0179** | **-0.9778** | **2** | **14** | **1.3e+02** | **1** | **KCTGQARNASAGTPPTSR + Carbamidomethyl (C)** |
|  | 550 | **403.6845** | **1208.0314** | **1207.3877** | **0.6438** | **2** | **14** | **95** | **1** | **RCVRNMAER + Carbamidomethyl (C); Oxidation (M)** |
|  | 1301 | **459.1213** | **1374.3418** | **1373.5517** | **0.7902** | **2** | **14** | **1.3e+02** | **1** | **SEIQELKGDVKK** |
|  | 2727 | **664.1798** | **1326.3448** | **1326.4951** | **-0.1503** | **1** | **14** | **1.1e+02** | **1** | **LIKEPVQEDQK** |
|  | 128 | **369.3404** | **1104.9990** | **1105.3089** | **-0.3099** | **0** | **14** | **1.1e+02** | **1** | **AKPWAVCFPS** |
|  | 383 | **388.4387** | **774.8626** | **773.9442** | **0.9184** | **1** | **14** | **1.6e+02** | **1** | **IPGCTKR** |
|  | 1272 | **457.8908** | **1370.6503** | **1371.5852** | **-0.9349** | **1** | **14** | **1.1e+02** | **1** | **LYARTGFLFQR** |
|  | 1842 | **524.3213** | **1569.9417** | **1569.7862** | **0.1555** | **1** | **14** | **1.1e+02** | **1** | **RLPGVNMFEQGHGK** |
|  | 801 | **413.8186** | **1238.4335** | **1238.4977** | **-0.0641** | **1** | **14** | **97** | **1** | **MSPKLYVWAK + Oxidation (M)** |
|  | 971 | **428.9985** | **855.9822** | **855.9985** | **-0.0162** | **1** | **14** | **1.1e+02** | **1** | **GVDYMKK + Oxidation (M)** |
|  | 35 | **363.0941** | **1086.2601** | **1087.2772** | **-1.0171** | **1** | **14** | **1.1e+02** | **1** | **CDQCRHVVK** |
|  | 125 | **369.3188** | **1104.9343** | **1105.3089** | **-0.3746** | **0** | **14** | **1.1e+02** | **1** | **AKPWAVCFPS** |
|  | 130 | **369.3514** | **1105.0321** | **1105.3089** | **-0.2767** | **0** | **14** | **1.2e+02** | **1** | **AKPWAVCFPS** |
|  | 828 | **416.1545** | **1245.4415** | **1244.3548** | **1.0866** | **0** | **14** | **1.3e+02** | **1** | **AGATNGTISTPVR** |
|  | 10 | **360.4785** | **718.9422** | **718.8673** | **0.0749** | **0** | **14** | **1.4e+02** | **1** | **LHMFR + Oxidation (M)** |
|  | 332 | **386.8975** | **1157.6703** | **1158.2624** | **-0.5921** | **0** | **14** | **1.2e+02** | **1** | **TQVTNNPGTVK** |
|  | 412 | **389.2351** | **776.4555** | **776.8372** | **-0.3817** | **0** | **14** | **1e+02** | **1** | **TGHTSFK** |
|  | 699 | **407.7765** | **1220.3074** | **1220.3979** | **-0.0906** | **0** | **14** | **1.1e+02** | **1** | **TSMALLFEHR + Oxidation (M)** |
|  | 1985 | **542.1971** | **1082.3795** | **1081.1832** | **1.1964** | **1** | **14** | **1.1e+02** | **1** | **TITHNRDPK** |
|  | 2905 | **686.0873** | **2055.2398** | **2054.3459** | **0.8939** | **1** | **14** | **1.1e+02** | **1** | **NILVESEAHVKIADFGLAK** |
|  | 1452 | **472.4656** | **1414.3747** | **1413.6221** | **0.7527** | **2** | **14** | **1.2e+02** | **1** | **NEKNRPSLKSLK** |
|  | 1494 | **476.2484** | **1425.7229** | **1426.7254** | **-1.0025** | **2** | **14** | **1.1e+02** | **1** | **MEAMEKLKSGMR + Oxidation (M)** |
|  | 2947 | **694.6626** | **1387.3104** | **1386.3417** | **0.9688** | **1** | **14** | **92** | **1** | **GSSSRSGGGGYGDSR** |
|  | 215 | **377.1781** | **752.3414** | **752.8157** | **-0.4743** | **0** | **14** | **93** | **1** | **AGGQVPGAP** |
|  | 1261 | **457.1802** | **1368.5184** | **1368.5168** | **0.0016** | **0** | **14** | **1.2e+02** | **1** | **TFIQCSHFTER** |
|  | 738 | **408.8926** | **1223.6557** | **1224.3252** | **-0.6695** | **1** | **14** | **1.2e+02** | **1** | **YAEWSSGLRR** |
|  | 2633 | **645.2096** | **1288.4044** | **1287.4507** | **0.9537** | **1** | **14** | **1.2e+02** | **1** | **MAGVGRSGGPWGR** |
|  | 888 | **419.3427** | **836.6705** | **836.9138** | **-0.2433** | **0** | **14** | **1.1e+02** | **1** | **GSGLCSASR** |
|  | 2111 | **562.8665** | **1685.5774** | **1684.8736** | **0.7038** | **0** | **14** | **89** | **1** | **DQMSHLFNVAHTLR + Oxidation (M)** |
|  | 129 | **369.3434** | **1105.0081** | **1105.3089** | **-0.3008** | **0** | **14** | **1.2e+02** | **1** | **AKPWAVCFPS** |
|  | 953 | **428.0871** | **854.1594** | **852.9778** | **1.1815** | **0** | **14** | **1.1e+02** | **1** | **AAPAAPLSR** |
|  | 109 | **369.2625** | **1104.7653** | **1104.2879** | **0.4774** | **2** | **14** | **99** | **1** | **RSAVVMGRGR + Oxidation (M)** |
|  | 1100 | **437.2069** | **872.3990** | **871.9811** | **0.4179** | **1** | **14** | **1.2e+02** | **1** | **KIAEQQR** |
|  | 1320 | **460.1328** | **918.2508** | **919.1007** | **-0.8498** | **1** | **14** | **1.3e+02** | **1** | **MAGGKKPSK + Oxidation (M)** |
|  | 3425 | **814.6589** | **2440.9546** | **2439.7763** | **1.1783** | **0** | **14** | **94** | **1** | **NLEALALDLMEPEQAVDLTLPK + Oxidation (M)** |
|  | 203 | **377.0250** | **1128.0528** | **1127.1590** | **0.8939** | **0** | **14** | **1.1e+02** | **1** | **GVDASGETTYK** |
|  | 538 | **402.7717** | **803.5286** | **802.9158** | **0.6128** | **1** | **14** | **1.3e+02** | **1** | **IKSGAEAK** |
|  | 772 | **411.3213** | **1230.9417** | **1230.4077** | **0.5340** | **0** | **14** | **98** | **1** | **AFDLYFVLDK** |
|  | 2158 | **571.6327** | **1711.8759** | **1712.0893** | **-0.2134** | **2** | **14** | **1.4e+02** | **1** | **CPICEKVIQGAGKLPR** |
|  | 1287 | **458.7048** | **1373.0923** | **1372.5087** | **0.5837** | **0** | **14** | **1e+02** | **1** | **GSWLQGPGGMGPGR + Oxidation (M)** |
|  | 2607 | **642.1733** | **1923.4978** | **1924.1911** | **-0.6933** | **2** | **14** | **1.1e+02** | **1** | **TAPTCSGAPRTRCTACTAR** |
|  | 2738 | **666.5018** | **1996.4833** | **1996.3298** | **0.1535** | **0** | **14** | **99** | **1** | **ITATAFFVELLQMEQVR** |
|  | 3531 | **890.2390** | **2667.6949** | **2667.1310** | **0.5639** | **0** | **14** | **94** | **1** | **CMQVGGNASALWLDSCVVPPLSPPPK** |
|  | 553 | **403.8243** | **1208.4508** | **1207.4008** | **1.0500** | **0** | **14** | **1.2e+02** | **1** | **SCMCTAGYSLR + Oxidation (M)** |
|  | 662 | **407.1193** | **1218.3357** | **1217.3972** | **0.9385** | **2** | **14** | **1.1e+02** | **1** | **CLGSARWKEPA** |
|  | 2039 | **551.6914** | **1101.3680** | **1100.2908** | **1.0773** | **0** | **14** | **1.4e+02** | **1** | **EGWIPMPVR + Oxidation (M)** |
|  | 118 | **369.2890** | **1104.8449** | **1105.3089** | **-0.4639** | **0** | **14** | **1e+02** | **1** | **AKPWAVCFPS** |
|  | 365 | **387.9946** | **773.9745** | **772.8966** | **1.0779** | **2** | **14** | **1.4e+02** | **1** | **RGRVSAK** |
|  | 105 | **369.2484** | **1104.7231** | **1105.3089** | **-0.5858** | **0** | **14** | **1e+02** | **1** | **AKPWAVCFPS** |
|  | 458 | **395.9943** | **1184.9606** | **1184.3044** | **0.6562** | **0** | **14** | **1.3e+02** | **1** | **ATPHFSGLAAGR** |
|  | 818 | **415.9042** | **1244.6904** | **1244.4442** | **0.2461** | **0** | **14** | **1.3e+02** | **1** | **HDACSALCVVAR** |
|  | 1309 | **459.4801** | **1375.4181** | **1374.6934** | **0.7247** | **0** | **14** | **1.5e+02** | **1** | **ILGPIPQHMIQK** |
|  | 2880 | **683.9546** | **2048.8416** | **2048.2801** | **0.5615** | **2** | **14** | **93** | **1** | **MYAKEGLKQQSHEIQNK + Oxidation (M)** |
|  | 2961 | **696.7728** | **1391.5309** | **1390.8008** | **0.7301** | **2** | **14** | **1.3e+02** | **1** | **VLKLLKMAVGMR + 2 Oxidation (M)** |
|  | 3295 | **777.2273** | **2328.6597** | **2328.8166** | **-0.1569** | **2** | **14** | **1.1e+02** | **1** | **TLMLLAQMRGISLFSCLKDR + 2 Oxidation (M)** |
|  | 567 | **404.0448** | **1209.1122** | **1209.3951** | **-0.2829** | **2** | **14** | **1.2e+02** | **1** | **ALNHEIEKKK** |
|  | 636 | **406.2029** | **1215.5866** | **1215.3566** | **0.2300** | **0** | **14** | **99** | **1** | **FWAELAPEPR** |
|  | 3037 | **727.2976** | **1452.5804** | **1451.6023** | **0.9781** | **1** | **14** | **1.1e+02** | **1** | **LCTSATESEVARGK** |
|  | 17 | **362.1195** | **1083.3363** | **1084.2746** | **-0.9383** | **1** | **14** | **1.1e+02** | **1** | **AAAALLRWGR** |
|  | 121 | **369.2975** | **1104.8705** | **1105.3089** | **-0.4384** | **0** | **14** | **1e+02** | **1** | **AKPWAVCFPS** |
|  | 975 | **429.0998** | **856.1849** | **856.9003** | **-0.7154** | **0** | **14** | **1.2e+02** | **1** | **QSYCGGDK** |
|  | 3296 | **777.2942** | **1552.5736** | **1551.7844** | **0.7892** | **0** | **14** | **1.1e+02** | **1** | **MTLDRPGEGATMLK + 2 Oxidation (M)** |
|  | 114 | **369.2734** | **1104.7980** | **1105.3089** | **-0.5109** | **0** | **14** | **1e+02** | **1** | **AKPWAVCFPS** |
|  | 629 | **406.1571** | **810.2995** | **810.8519** | **-0.5524** | **0** | **14** | **1.1e+02** | **1** | **EYAVSSR** |
|  | 910 | **421.7933** | **841.5719** | **841.9949** | **-0.4231** | **0** | **14** | **1.2e+02** | **1** | **GITLSVRP** |
|  | 496 | **400.1274** | **1197.3601** | **1197.2585** | **0.1015** | **1** | **14** | **1e+02** | **1** | **RQDSQGIEHK** |
|  | 835 | **416.2976** | **830.5804** | **830.8896** | **-0.3092** | **1** | **14** | **1.1e+02** | **1** | **ADGTVGRR** |
|  | 1918 | **534.8654** | **1067.7159** | **1068.3352** | **-0.6192** | **1** | **14** | **1.1e+02** | **1** | **DLCMKVMR + Carbamidomethyl (C); Oxidation (M)** |
|  | 106 | **369.2516** | **1104.7327** | **1105.3089** | **-0.5762** | **0** | **14** | **1e+02** | **1** | **AKPWAVCFPS** |
|  | 1024 | **432.1158** | **1293.3251** | **1292.4373** | **0.8877** | **1** | **14** | **1.3e+02** | **1** | **LNGEVFGGGTKLT** |
|  | 1652 | **496.8615** | **1487.5625** | **1488.5444** | **-0.9820** | **0** | **14** | **1.2e+02** | **1** | **HGASCQNTHGTYR + Carbamidomethyl (C)** |
|  | 1807 | **521.3400** | **1040.6653** | **1040.1311** | **0.5341** | **0** | **14** | **96** | **1** | **LGNSAPGPATR** |
|  | 2267 | **592.8384** | **1775.4930** | **1776.0554** | **-0.5625** | **2** | **14** | **98** | **1** | **RGRGCTLQYQHAMVR** |
|  | 136 | **369.4224** | **1105.2451** | **1105.3089** | **-0.0638** | **0** | **14** | **1.5e+02** | **1** | **AKPWAVCFPS** |
|  | 556 | **403.8735** | **805.7323** | **805.9430** | **-0.2107** | **1** | **14** | **1.2e+02** | **1** | **GAGMAQKK + Oxidation (M)** |
|  | 925 | **423.1309** | **1266.3706** | **1266.6402** | **-0.2697** | **2** | **14** | **1.3e+02** | **1** | **MLTCRKIACVI + Oxidation (M)** |
|  | 2710 | **659.2059** | **1316.3971** | **1315.4695** | **0.9276** | **2** | **14** | **1.2e+02** | **1** | **EEPKEEVTVKK** |
|  | 89 | **368.1718** | **1101.4934** | **1100.3091** | **1.1843** | **1** | **14** | **1.1e+02** | **1** | **VISLKEANVK** |
|  | 819 | **415.9044** | **829.7939** | **830.9689** | **-1.1750** | **0** | **14** | **1.4e+02** | **1** | **LTLSELR** |
|  | 1629 | **494.2088** | **1479.6042** | **1478.6789** | **0.9254** | **1** | **14** | **1.2e+02** | **1** | **QHPAVRGSLGPGMR + Oxidation (M)** |
|  | 2515 | **624.3857** | **1870.1351** | **1870.1602** | **-0.0251** | **1** | **14** | **1.2e+02** | **1** | **NQVAFTEVMGMLWRR + 2 Oxidation (M)** |
|  | 680 | **407.4886** | **1219.4438** | **1220.3134** | **-0.8697** | **1** | **14** | **1.4e+02** | **1** | **ANLGSGCEEKR + Carbamidomethyl (C)** |
|  | 1041 | **433.1225** | **1296.3452** | **1297.4804** | **-1.1351** | **0** | **14** | **1.2e+02** | **1** | **NIVLSGGSTMFR + Oxidation (M)** |
|  | 1131 | **442.2451** | **1323.7132** | **1324.4808** | **-0.7677** | **0** | **14** | **99** | **1** | **CMDLEDLGLSR + Carbamidomethyl (C); Oxidation (M)** |
|  | 3092 | **740.5219** | **2218.5434** | **2218.5751** | **-0.0318** | **1** | **14** | **1.1e+02** | **1** | **IPSLGSGLEQAHAMPSPRILK + Oxidation (M)** |
|  | 267 | **383.3430** | **764.6711** | **764.9309** | **-0.2597** | **0** | **14** | **1.1e+02** | **1** | **MASLTVK + Oxidation (M)** |
|  | 2464 | **614.6461** | **1840.9162** | **1840.1258** | **0.7904** | **0** | **14** | **1.3e+02** | **1** | **WTGPYLVIYSTLTAVR** |
|  | 107 | **369.2551** | **1104.7430** | **1105.3089** | **-0.5658** | **0** | **14** | **1e+02** | **1** | **AKPWAVCFPS** |
|  | 607 | **405.3347** | **1212.9819** | **1213.3440** | **-0.3622** | **1** | **14** | **90** | **1** | **DAPRNLSALTR** |
|  | 1873 | **528.9308** | **1055.8468** | **1055.2072** | **0.6396** | **1** | **14** | **1.2e+02** | **1** | **AKSDPCAMNC + Oxidation (M)** |
|  | 2623 | **643.7023** | **1285.3899** | **1286.5023** | **-1.1125** | **0** | **14** | **1.4e+02** | **1** | **MALPQGHLTFR + Oxidation (M)** |
|  | 13 | **361.0682** | **1080.1825** | **1079.2751** | **0.9075** | **2** | **14** | **1.2e+02** | **1** | **RSVSLSMRK + Oxidation (M)** |
|  | 183 | 374.2230 | 1119.6469 | 1119.3288 | 0.3181 | 0 | 14 | 1.2e+02 | 1 | LLEVLSGEML + Oxidation (M) |
|  | 865 | **418.6328** | **1252.8761** | **1252.5890** | **0.2871** | **2** | **14** | **1.2e+02** | **1** | **IKPIPKKTVTK** |
|  | 872 | **419.1947** | **836.3746** | **836.9322** | **-0.5576** | **0** | **14** | **1.3e+02** | **1** | **QDPPVGPK** |
|  | 1112 | **438.1216** | **1311.3427** | **1312.4455** | **-1.1028** | **0** | **14** | **1.3e+02** | **1** | **VVEMYNIDEGK + Oxidation (M)** |
|  | 1114 | **438.2011** | **1311.5810** | **1312.5347** | **-0.9538** | **1** | **14** | **1.3e+02** | **1** | **NTAYLQMKSLK + Oxidation (M)** |
|  | 1328 | **460.7465** | **1379.2173** | **1379.4967** | **-0.2794** | **1** | **14** | **1e+02** | **1** | **ENPMTNRSTVSK + Oxidation (M)** |
|  | 1571 | **486.7817** | **1457.3230** | **1457.6743** | **-0.3513** | **0** | **14** | **99** | **1** | **GLEASWIWVQLR** |
|  | 115 | **369.2787** | **1104.8140** | **1105.2229** | **-0.4089** | **1** | **13** | **1e+02** | **1** | **EKSPQMGANK + Oxidation (M)** |
|  | 1330 | **460.7904** | **1379.3491** | **1379.7233** | **-0.3741** | **1** | **13** | **1.1e+02** | **1** | **CCCCCRVCCR + 4 Carbamidomethyl (C)** |
|  | 1398 | **466.9384** | **1397.7929** | **1397.5827** | **0.2102** | **2** | **13** | **1.2e+02** | **1** | **ANINANLQGKARK** |
|  | 1662 | **499.6318** | **1495.8732** | **1496.6460** | **-0.7728** | **1** | **13** | **1.2e+02** | **1** | **YCLRHVENDGYK** |
|  | 2261 | **592.7849** | **1183.5549** | **1184.2598** | **-0.7049** | **1** | **13** | **1.1e+02** | **1** | **GAEGPEAERLR** |
|  | 3054 | **733.6473** | **1465.2798** | **1465.7200** | **-0.4402** | **2** | **13** | **92** | **1** | **EMVKLNYVAARR + Oxidation (M)** |
|  | 3489 | **847.4603** | **2539.3588** | **2538.8608** | **0.4980** | **1** | **13** | **1.2e+02** | **1** | **RPLSKCGMGPGGGDASLTLHGLQNR + Oxidation (M)** |
|  | 1423 | **470.1094** | **938.2040** | **937.0545** | **1.1494** | **1** | **13** | **1.1e+02** | **1** | **GTEHRIPK** |
|  | 1756 | **515.5055** | **1543.4943** | **1542.7790** | **0.7153** | **2** | **13** | **1.2e+02** | **1** | **ARTKGLQSGVDIGLK** |
|  | 964 | **428.4389** | **1282.2945** | **1282.5320** | **-0.2374** | **1** | **13** | **1.2e+02** | **1** | **FMEKLDACIR + Carbamidomethyl (C)** |
|  | 1123 | **440.2739** | **878.5331** | **878.0057** | **0.5274** | **1** | **13** | **1.1e+02** | **1** | **IAKDMER + Oxidation (M)** |
|  | 2320 | **597.0964** | **1788.2670** | **1789.0993** | **-0.8323** | **1** | **13** | **1.3e+02** | **1** | **MTLSSAIDSVDKVPVVK** |
|  | 842 | **417.1037** | **832.1926** | **833.0079** | **-0.8153** | **0** | **13** | **1.4e+02** | **1** | **NTLACLAK** |
|  | 97 | **368.2891** | **1101.8450** | **1101.3201** | **0.5249** | **0** | **13** | **1.1e+02** | **1** | **QLLMVGGLDR** |
|  | 956 | **428.2114** | **1281.6119** | **1281.4332** | **0.1788** | **0** | **13** | **1e+02** | **1** | **EMTSSVLTVNGK + Oxidation (M)** |
|  | 1984 | **542.0914** | **1623.2519** | **1623.8768** | **-0.6248** | **1** | **13** | **1.2e+02** | **1** | **VTMRQFDHPHIVK + Oxidation (M)** |
|  | 2089 | **560.2873** | **1118.5598** | **1117.3625** | **1.1973** | **1** | **13** | **1.3e+02** | **1** | **RLSALLTGICA** |
|  | 523 | **401.9309** | **801.8470** | **800.9663** | **0.8808** | **0** | **13** | **1.4e+02** | **1** | **MVHTGLK + Oxidation (M)** |
|  | 592 | **405.1225** | **1212.3454** | **1212.4420** | **-0.0966** | **1** | **13** | **1.1e+02** | **1** | **KQLAALTQAIR** |
|  | 1319 | **460.0457** | **1377.1149** | **1377.5850** | **-0.4701** | **0** | **13** | **1.3e+02** | **1** | **CYLMPFGTGTSK + Carbamidomethyl (C); Oxidation (M)** |
|  | 1962 | 539.3251 | 1076.6354 | 1076.2661 | 0.3693 | 0 | 13 | 1.2e+02 | 1 | EMAPSWVLK + Oxidation (M) |
|  | 1013 | **431.1824** | **860.3500** | **861.0198** | **-0.6698** | **0** | **13** | **1.4e+02** | **1** | **GPHYLMK + Oxidation (M)** |
|  | 78 | **367.1581** | **1098.4521** | **1099.1967** | **-0.7446** | **0** | **13** | **1.2e+02** | **1** | **AGPGVEDLWR** |
|  | 200 | **376.1728** | **1125.4961** | **1125.2787** | **0.2174** | **1** | **13** | **1e+02** | **1** | **VKLAGDAAAGPR** |
|  | 1835 | **524.0582** | **1046.1017** | **1045.1046** | **0.9970** | **0** | **13** | **1.3e+02** | **1** | **AGPSAASSAAAGK** |
|  | 2285 | **593.7865** | **1185.5582** | **1185.2942** | **0.2640** | **2** | **13** | **1.2e+02** | **1** | **TPGALGRDRSR** |
|  | 2916 | **686.2982** | **2055.8723** | **2055.5523** | **0.3200** | **1** | **13** | **1.2e+02** | **1** | **GSRLLCWVLLCLLGAGPVK + Carbamidomethyl (C)** |
|  | 400 | **389.1649** | **776.3150** | **775.8906** | **0.4244** | **0** | **13** | **1.3e+02** | **1** | **MECYSK + Oxidation (M)** |
|  | 211 | **377.1529** | **1128.4364** | **1128.3456** | **0.0909** | **1** | **13** | **1.1e+02** | **1** | **MLLSGHTKNK** |
|  | 288 | **385.0979** | **1152.2716** | **1153.3551** | **-1.0835** | **1** | **13** | **1.1e+02** | **1** | **AFTCITSVRR** |
|  | 3414 | **809.9419** | **2426.8035** | **2426.7263** | **0.0772** | **1** | **13** | **1.4e+02** | **1** | **NGVNCEHSPVDLSKVDLHFMK + Carbamidomethyl (C)** |
|  | 2503 | **621.5584** | **1861.6529** | **1862.1330** | **-0.4802** | **1** | **13** | **95** | **1** | **ELVFWSDVTLDRILR** |
|  | 3238 | **759.0616** | **2274.1628** | **2274.4693** | **-0.3066** | **1** | **13** | **96** | **1** | **ALLGHCSNSAGSTNSWELKNK + Carbamidomethyl (C)** |
|  | 73 | **366.1732** | **1095.4973** | **1095.2328** | **0.2645** | **0** | **13** | **1.2e+02** | **1** | **TCSHHAAALK + Carbamidomethyl (C)** |
|  | 245 | **379.4044** | **1135.1909** | **1135.3134** | **-0.1225** | **1** | **13** | **1.5e+02** | **1** | **TGSDVLFIRK** |
|  | 357 | **387.9101** | **773.8054** | **773.9211** | **-0.1156** | **1** | **13** | **1.5e+02** | **1** | **RVISTAK** |
|  | 1572 | **486.8171** | **971.6195** | **972.0541** | **-0.4346** | **0** | **13** | **1.1e+02** | **1** | **VVSTQAPDR** |
|  | 1921 | **535.8840** | **1069.7533** | **1069.1891** | **0.5642** | **0** | **13** | **1.1e+02** | **1** | **GMAFTLEER + Oxidation (M)** |
|  | 728 | **408.2068** | **1221.5981** | **1222.2169** | **-0.6189** | **1** | **13** | **1.1e+02** | **1** | **RTTTEDENEK** |
|  | 839 | **416.5720** | **1246.6938** | **1247.4400** | **-0.7462** | **1** | **13** | **1.4e+02** | **1** | **EVVNKVFNIVS** |
|  | 1007 | **431.0859** | **860.1570** | **859.0949** | **1.0620** | **2** | **13** | **1.5e+02** | **1** | **ACLRIRK** |
|  | 1851 | **525.3754** | **1048.7361** | **1049.3051** | **-0.5691** | **0** | **13** | **1.1e+02** | **1** | **IIPFTIAFK** |
|  | 2419 | 611.7848 | 1832.3322 | 1833.0950 | -0.7628 | 1 | 13 | 1.2e+02 | 1 | DVVRWASGGLALQGIYK |
|  | 2749 | **666.6774** | **1997.0099** | **1996.3264** | **0.6835** | **0** | **13** | **1.2e+02** | **1** | **GLYLTEMEWMSLVMYN + Oxidation (M)** |
|  | 430 | **391.1484** | **1170.4230** | **1169.2915** | **1.1315** | **1** | **13** | **1.2e+02** | **1** | **PAQTDRVNLR** |
|  | 644 | **406.6098** | **811.2048** | **811.9028** | **-0.6980** | **1** | **13** | **92** | **1** | **QMKENY** |
|  | 2678 | **654.1227** | **1959.3459** | **1958.1924** | **1.1534** | **1** | **13** | **1.2e+02** | **1** | **AGETMYLYEKANTPELK** |
|  | 3120 | **741.5571** | **2221.6490** | **2221.4749** | **0.1741** | **0** | **13** | **1.1e+02** | **1** | **YTAVVMPVHYQHGTGQSSCR** |
|  | 161 | **372.1920** | **1113.5539** | **1113.2480** | **0.3058** | **0** | **13** | **98** | **1** | **LPGGCGAPGSAAR** |
|  | 314 | **386.1195** | **1155.3363** | **1154.2769** | **1.0595** | **0** | **13** | **1.1e+02** | **1** | **CHGTSGSCQFK** |
|  | 393 | **389.1244** | **776.2340** | **775.9352** | **0.2988** | **0** | **13** | **1.4e+02** | **1** | **LFKPSGK** |
|  | 502 | **400.2040** | **1197.5898** | **1198.4355** | **-0.8457** | **1** | **13** | **95** | **1** | **MIHIVQAKDK + Oxidation (M)** |
|  | 936 | **425.3893** | **848.7638** | **849.8034** | **-1.0396** | **0** | **13** | **1.2e+02** | **1** | **NSESGNSR** |
|  | 1711 | **505.8603** | **1514.5587** | **1513.5426** | **1.0161** | **0** | **13** | **1.1e+02** | **1** | **EEASADMQADFQR + Oxidation (M)** |
|  | 1127 | **441.1373** | **1320.3898** | **1319.4694** | **0.9205** | **1** | **13** | **1.2e+02** | **1** | **ASSWHVCPRSCS** |
|  | 1671 | **500.4375** | **1498.2903** | **1497.6788** | **0.6115** | **1** | **13** | **96** | **1** | **KSSCQGGLAPSVAHR** |
|  | 3132 | **741.8611** | **1481.7074** | **1481.6911** | **0.0162** | **1** | **13** | **1.4e+02** | **1** | **ANLIKEAEEMCSK + Oxidation (M)** |
|  | 236 | **378.4220** | **1132.2437** | **1132.2944** | **-0.0507** | **1** | **13** | **1.3e+02** | **1** | **VGGAKINTNCR** |
|  | 1083 | **436.1106** | **1305.3096** | **1306.3810** | **-1.0714** | **0** | **13** | **1.2e+02** | **1** | **YLSLSGNHSSNK** |
|  | 282 | **385.0421** | **1152.1041** | **1152.3636** | **-0.2596** | **1** | **13** | **1.1e+02** | **1** | **GFQCLKIESK** |
|  | 319 | **386.1877** | **1155.5411** | **1156.2265** | **-0.6854** | **0** | **13** | **98** | **1** | **CAVNSGGSNYK + Carbamidomethyl (C)** |
|  | 1964 | 539.7473 | 1077.4798 | 1076.2893 | 1.1906 | 2 | 13 | 1.1e+02 | 1 | FKDVAKQLK |
|  | 2740 | **666.5624** | **1331.1100** | **1330.4472** | **0.6627** | **0** | **13** | **1e+02** | **1** | **SECSAQCGLGYR + Carbamidomethyl (C)** |
|  | 3033 | **725.8749** | **1449.7350** | **1450.7284** | **-0.9934** | **0** | **13** | **1.4e+02** | **1** | **LPDKPLCQHMPR + Oxidation (M)** |
|  | 3240 | **760.1815** | **1518.3481** | **1518.7611** | **-0.4129** | **1** | **13** | **1.2e+02** | **1** | **TRGTQGLPMTMGPR + Oxidation (M)** |
|  | 1940 | **537.1464** | **1072.2781** | **1073.1579** | **-0.8798** | **0** | **13** | **1.4e+02** | **1** | **QLTGGVDVER** |
|  | 457 | **395.7832** | **1184.3274** | **1183.2984** | **1.0291** | **1** | **13** | **1.4e+02** | **1** | **ESCPEHAVRR** |
|  | 634 | **406.1937** | **1215.5589** | **1216.5138** | **-0.9549** | **2** | **13** | **1.1e+02** | **1** | **MSFAMTLKKK + 2 Oxidation (M)** |
|  | 2237 | **588.4556** | **1762.3445** | **1762.0740** | **0.2706** | **2** | **13** | **1.1e+02** | **1** | **ALEVLEEVKVTKEMK + Oxidation (M)** |
|  | 2256 | **592.6343** | **1774.8807** | **1773.8839** | **0.9968** | **2** | **13** | **1.5e+02** | **1** | **GGRGSCGGSKGDCGSCGGSK + Carbamidomethyl (C)** |
|  | 2497 | **621.0614** | **1860.1620** | **1861.0228** | **-0.8607** | **2** | **13** | **1.2e+02** | **1** | **TTTLSPGRSTTPSVSGRR** |
|  | 86 | **368.1041** | **1101.2902** | **1101.2556** | **0.0346** | **1** | **13** | **1.4e+02** | **1** | **THLFAKQEK** |
|  | 110 | **369.2645** | **1104.7714** | **1105.3089** | **-0.5375** | **0** | **13** | **1.1e+02** | **1** | **AKPWAVCFPS** |
|  | 1890 | **530.7900** | **1589.3479** | **1589.9168** | **-0.5688** | **1** | **13** | **1.1e+02** | **1** | **KLTLGMDYMFQVK + Oxidation (M)** |
|  | 572 | **404.0782** | **806.1416** | **805.9446** | **0.1970** | **0** | **13** | **1.3e+02** | **1** | **WVAGAMR + Oxidation (M)** |
|  | 1466 | **473.8829** | **1418.6266** | **1419.6496** | **-1.0230** | **0** | **13** | **1.4e+02** | **1** | **YVAICHPLQYR + Carbamidomethyl (C)** |
|  | 2295 | 594.8775 | 1781.6103 | 1780.8996 | 0.7107 | 2 | 13 | 1.1e+02 | 1 | GSGGAAGAPGERGRTGPLGR |
|  | 2407 | **610.0005** | **1826.9795** | **1827.0459** | **-0.0665** | **1** | **13** | **1.3e+02** | **1** | **CYSSCGKVQDAFISYR** |
|  | 2909 | **686.2014** | **2055.5821** | **2056.3070** | **-0.7250** | **1** | **13** | **1.2e+02** | **1** | **MYRFAGLAFASHIGAQSGR + Oxidation (M)** |
|  | 3321 | **784.6218** | **2350.8431** | **2351.5784** | **-0.7353** | **2** | **13** | **1.1e+02** | **1** | **AGLQRGQQMDPDRAFICGESR + Oxidation (M)** |
|  | 447 | **393.4798** | **1177.4173** | **1177.4380** | **-0.0207** | **1** | **13** | **1.5e+02** | **1** | **AKVMMEVGQGK** |
|  | 905 | **421.1463** | **1260.4167** | **1259.4787** | **0.9380** | **2** | **13** | **1.3e+02** | **1** | **AEVRKALANCK + Carbamidomethyl (C)** |
|  | 919 | **422.2063** | **1263.5966** | **1262.4429** | **1.1537** | **2** | **13** | **1.3e+02** | **1** | **GTLGRRMAQTR + Oxidation (M)** |
|  | 734 | **408.4920** | **1222.4539** | **1221.4241** | **1.0298** | **0** | **13** | **1.6e+02** | **1** | **TAILVGGMSTQK + Oxidation (M)** |
|  | 1124 | **440.4228** | **1318.2462** | **1318.5012** | **-0.2550** | **0** | **13** | **1.4e+02** | **1** | **KPDLPQTNFCR** |
|  | 2135 | **567.5264** | **1699.5569** | **1698.8938** | **0.6632** | **0** | **13** | **1.1e+02** | **1** | **LSVMEEDQACAMESR** |
|  | 2757 | **666.8460** | **1997.5159** | **1998.1536** | **-0.6377** | **1** | **13** | **1.3e+02** | **1** | **ASGVPDRFSGSGSGTDFILK** |
|  | 2780 | **668.7620** | **2003.2637** | **2004.3137** | **-1.0499** | **0** | **13** | **1.6e+02** | **1** | **LPQDPCGPDCAPPAPCLPR + Carbamidomethyl (C)** |
|  | 80 | **367.2446** | **732.4744** | **732.7829** | **-0.3084** | **0** | **13** | **1.1e+02** | **1** | **NLSAGSGK** |
|  | 867 | **418.8820** | **1253.6237** | **1254.4407** | **-0.8170** | **2** | **13** | **1.5e+02** | **1** | **SKKGHGAIPGFR** |
|  | 1750 | **514.1900** | **1539.5478** | **1538.7921** | **0.7557** | **1** | **13** | **1.2e+02** | **1** | **VSVDWGKCMNPFR** |
|  | 2389 | **608.2747** | **1214.5345** | **1213.3443** | **1.1903** | **1** | **13** | **1.3e+02** | **1** | **TVRGSPVVGEGR** |
|  | 1079 | **435.9030** | **1304.6867** | **1304.4530** | **0.2336** | **1** | **13** | **1.2e+02** | **1** | **ETRWPYLSPR** |
|  | 1570 | **486.6983** | **971.3819** | **972.0143** | **-0.6324** | **1** | **13** | **1.1e+02** | **1** | **DRASGVPDR** |
|  | 1963 | **539.5840** | **1077.1532** | **1076.2048** | **0.9484** | **1** | **13** | **1.6e+02** | **1** | **VSRYGYFGK** |
|  | 3404 | **806.8689** | **2417.5845** | **2416.7563** | **0.8282** | **2** | **13** | **1.4e+02** | **1** | **SPFTKVALSSAGARSTGGTCAMCR + Carbamidomethyl (C)** |
|  | 1310 | **459.5547** | **917.0945** | **916.0769** | **1.0177** | **2** | **13** | **1.7e+02** | **1** | **KAAGGGKVTK** |
|  | 2734 | **665.5814** | **1329.1481** | **1328.5161** | **0.6320** | **1** | **13** | **1e+02** | **1** | **SQLSTTMSRCAK + Oxidation (M)** |
|  | 3541 | **901.4642** | **1800.9137** | **1801.9751** | **-1.0614** | **0** | **13** | **1.2e+02** | **1** | **APQTEVGCQANQLISSR** |
|  | 162 | **372.2334** | **1113.6781** | **1114.3406** | **-0.6625** | **1** | **13** | **98** | **1** | **MGHFMKGFK + 2 Oxidation (M)** |
|  | 345 | **387.1073** | **1158.2998** | **1157.3604** | **0.9395** | **1** | **13** | **1.5e+02** | **1** | **KSGLVVLGQEK** |
|  | 716 | **408.0251** | **1221.0531** | **1221.4291** | **-0.3760** | **0** | **13** | **1.3e+02** | **1** | **VPLPSGPMNPGR** |
|  | 2882 | **684.0126** | **1366.0104** | **1365.4700** | **0.5404** | **1** | **13** | **1e+02** | **1** | **AAMDSRASDAINK + Oxidation (M)** |
|  | 3583 | **1019.4988** | **2036.9828** | **2036.4252** | **0.5576** | **2** | **13** | **1.1e+02** | **1** | **MRRCENDCICMMLSK + 3 Carbamidomethyl (C); 2 Oxidation (M)** |
|  | 208 | **377.1335** | **1128.3785** | **1128.2792** | **0.0992** | **0** | **13** | **1.2e+02** | **1** | **ATANIIVNGQK** |
|  | 700 | **407.7776** | **1220.3105** | **1219.3684** | **0.9421** | **0** | **13** | **1.3e+02** | **1** | **DCIATGIQSVR + Carbamidomethyl (C)** |
|  | 943 | **427.1593** | **1278.4557** | **1279.5050** | **-1.0492** | **1** | **13** | **1.2e+02** | **1** | **IDMKVIEPYR + Oxidation (M)** |
|  | 983 | **429.2459** | **1284.7154** | **1284.5130** | **0.2023** | **2** | **13** | **1.1e+02** | **1** | **FLRRAAVAPQR** |
|  | 1047 | **433.2841** | **1296.8303** | **1296.5438** | **0.2865** | **1** | **13** | **1e+02** | **1** | **TVCRMGAAVCR + 2 Carbamidomethyl (C); Oxidation (M)** |
|  | 2242 | **590.1521** | **1178.2894** | **1177.3583** | **0.9312** | **1** | **13** | **1.3e+02** | **1** | **GGGVGCKMAAGGR + Carbamidomethyl (C)** |
|  | 2537 | **629.8924** | **1886.6550** | **1886.2238** | **0.4312** | **0** | **13** | **1e+02** | **1** | **WVQVLHAYCIISAGIR + Carbamidomethyl (C)** |
|  | 1372 | **464.1641** | **1389.4702** | **1389.6899** | **-0.2197** | **1** | **13** | **1.3e+02** | **1** | **MAQAAQCRLLLR + Oxidation (M)** |
|  | 1702 | **505.0739** | **1512.1997** | **1512.7451** | **-0.5454** | **1** | **13** | **1.2e+02** | **1** | **MLKAEMEDLVSSK + 2 Oxidation (M)** |
|  | 2164 | **572.7576** | **1715.2505** | **1715.9951** | **-0.7446** | **1** | **13** | **1.3e+02** | **1** | **RILFCTDCCPDCGNR** |
|  | 1125 | **441.0233** | **880.0318** | **878.9688** | **1.0631** | **0** | **13** | **1.3e+02** | **1** | **GLVDSFNK** |
|  | 1196 | **450.0468** | **898.0787** | **898.0200** | **0.0587** | **0** | **13** | **1.2e+02** | **1** | **QLTHFPR** |
|  | 2698 | **656.4946** | **1310.9745** | **1311.5962** | **-0.6218** | **1** | **13** | **1.1e+02** | **1** | **NLCKQPILQVR** |
|  | 3089 | **740.4930** | **2218.4570** | **2218.5157** | **-0.0587** | **2** | **13** | **1.2e+02** | **1** | **NTIDWMKKPRCGVPDQTR + Carbamidomethyl (C); Oxidation (M)** |
|  | 744 | **408.9949** | **1223.9626** | **1223.3755** | **0.5872** | **0** | **13** | **1.4e+02** | **1** | **LPTPAGGPVGTEK** |
|  | 1463 | **473.8001** | **1418.3782** | **1417.6340** | **0.7442** | **2** | **13** | **1.2e+02** | **1** | **ARMEQTIADVKR** |
|  | 3146 | **742.9034** | **2225.6880** | **2225.4882** | **0.1998** | **2** | **13** | **1.4e+02** | **1** | **RTCGQLTLQQGSGPKSLTHR + Carbamidomethyl (C)** |
|  | 3245 | **761.1925** | **2280.5553** | **2279.4876** | **1.0677** | **1** | **13** | **1.3e+02** | **1** | **AFYITGGARACDGSTGPELQHK** |
|  | 639 | **406.2737** | **1215.7991** | **1215.3037** | **0.4954** | **2** | **13** | **98** | **1** | **TMDHNRNRR + Oxidation (M)** |
|  | 676 | **407.4158** | **812.8168** | **812.9371** | **-0.1203** | **0** | **13** | **1.4e+02** | **1** | **EGHLVCR** |
|  | 1025 | **432.1279** | **1293.3615** | **1292.4592** | **0.9024** | **0** | **13** | **1.4e+02** | **1** | **MQATAVVETASGK** |
|  | 1531 | **480.0943** | **1437.2607** | **1436.7002** | **0.5605** | **0** | **13** | **1.4e+02** | **1** | **VPPCPANFCVFSR** |
|  | 1577 | **487.2343** | **1458.6809** | **1459.5877** | **-0.9069** | **1** | **13** | **1.4e+02** | **1** | **CTARGGSLAQPADR + Carbamidomethyl (C)** |
|  | 2433 | **612.2772** | **1222.5397** | **1221.4556** | **1.0841** | **2** | **13** | **1.3e+02** | **1** | **LKDRPPLRAR** |
|  | 2688 | **655.0689** | **1962.1846** | **1962.2540** | **-0.0694** | **1** | **13** | **1.3e+02** | **1** | **THSNEKPYVCKIPGCTK + Carbamidomethyl (C)** |
|  | 3275 | **767.9243** | **2300.7508** | **2299.5621** | **1.1887** | **2** | **13** | **1.4e+02** | **1** | **TSPSPSPSPSPRTPMSWSRIK + Oxidation (M)** |
|  | 1645 | **496.2581** | **990.5013** | **990.1535** | **0.3478** | **0** | **13** | **1.4e+02** | **1** | **LLATNPSFK** |
|  | 1728 | **508.5117** | **1015.0086** | **1016.1960** | **-1.1874** | **2** | **13** | **1.4e+02** | **1** | **KLKQGVTSR** |
|  | 2431 | **612.2444** | **1222.4741** | **1222.3773** | **0.0968** | **1** | **13** | **1.3e+02** | **1** | **EHAKQHGMIR + Oxidation (M)** |
|  | 113 | **369.2704** | **1104.7890** | **1105.3089** | **-0.5199** | **0** | **13** | **1.1e+02** | **1** | **AKPWAVCFPS** |
|  | 832 | **416.1959** | **1245.5655** | **1244.4822** | **1.0833** | **0** | **13** | **1.5e+02** | **1** | **LDCSTLCCACV + 2 Carbamidomethyl (C)** |
|  | 1606 | **489.6043** | **1465.7908** | **1465.5245** | **0.2664** | **0** | **13** | **1.6e+02** | **1** | **ENVGGPGAPEGTPAGR** |
|  | 1636 | **494.4788** | **1480.4142** | **1479.6371** | **0.7771** | **1** | **13** | **1.4e+02** | **1** | **CTFEGCTKAYSR + 2 Carbamidomethyl (C)** |
|  | 1677 | **502.4598** | **1002.9049** | **1002.1893** | **0.7156** | **1** | **13** | **1.2e+02** | **1** | **CLDKAPTVR** |
|  | 2423 | **611.9591** | **1832.8551** | **1833.1780** | **-0.3229** | **2** | **13** | **1.2e+02** | **1** | **EKITEMPSIIKDLCR + Carbamidomethyl (C)** |
|  | 2662 | **651.1919** | **1950.5535** | **1951.2529** | **-0.6994** | **0** | **13** | **1.3e+02** | **1** | **TWVPIPPRPMASSGSVPR + Oxidation (M)** |
|  | 283 | **385.0537** | **768.0925** | **768.9442** | **-0.8517** | **0** | **13** | **1.1e+02** | **1** | **ITVALPR** |
|  | 1783 | **519.0179** | **1036.0211** | **1036.1244** | **-0.1033** | **1** | **13** | **1.3e+02** | **1** | **MRNTGAGGTR + Oxidation (M)** |
|  | 2293 | **594.8079** | **1781.4014** | **1781.9771** | **-0.5757** | **2** | **13** | **1.2e+02** | **1** | **LGRSCTTRHHCPSGGR + Carbamidomethyl (C)** |
|  | 2735 | **666.3978** | **1996.1713** | **1995.0916** | **1.0797** | **0** | **13** | **1.3e+02** | **1** | **QSGMHLTLPDAHDADSGSR** |
|  | 3115 | **741.2067** | **1480.3987** | **1480.7511** | **-0.3524** | **1** | **13** | **1.2e+02** | **1** | **MLDMGFEPQIRK + Oxidation (M)** |
|  | 547 | **403.2558** | **1206.7451** | **1206.4542** | **0.2910** | **0** | **13** | **1.2e+02** | **1** | **GQLLPMPAVPAP + Oxidation (M)** |
|  | 1450 | **472.4273** | **942.8398** | **943.0360** | **-0.1961** | **0** | **13** | **1.1e+02** | **1** | **ACEPGVDPR** |
|  | 2748 | **666.6737** | **1331.3326** | **1332.5294** | **-1.1968** | **2** | **13** | **1.3e+02** | **1** | **KQLQDEMLRR + Oxidation (M)** |
|  | 74 | **366.1799** | **1095.5176** | **1096.1498** | **-0.6321** | **0** | **13** | **1.2e+02** | **1** | **YNFPSSEPR** |
|  | 248 | **379.8250** | **1136.4530** | **1135.2538** | **1.1992** | **1** | **13** | **1.4e+02** | **1** | **MAHGKDFASR + Oxidation (M)** |
|  | 1203 | **450.3247** | **1347.9520** | **1347.6021** | **0.3500** | **0** | **13** | **1.1e+02** | **1** | **VMGQYLVQYCK + Oxidation (M)** |
|  | 2303 | **595.9300** | **1784.7678** | **1785.0324** | **-0.2646** | **1** | **13** | **1.2e+02** | **1** | **MLENYARFITAASAAR** |
|  | 2179 | **577.0133** | **1152.0118** | **1152.3438** | **-0.3320** | **1** | **13** | **1.3e+02** | **1** | **HLLGAVGTKEK** |
|  | 2960 | **696.7629** | **2087.2666** | **2088.3871** | **-1.1204** | **1** | **13** | **1.5e+02** | **1** | **LLGPKAPGPSTGDLTGPGPCPR** |
|  | 920 | **422.2084** | **842.4021** | **841.9519** | **0.4502** | **1** | **13** | **1.4e+02** | **1** | **GIPGDKGAK** |
|  | 1479 | 475.4606 | 948.9065 | 950.0685 | -1.1620 | 0 | 13 | 1.4e+02 | 1 | MTVEEAVR + Oxidation (M) |
|  | 2461 | **614.3710** | **1840.0907** | **1840.1258** | **-0.0351** | **0** | **13** | **1.3e+02** | **1** | **WTGPYLVIYSTLTAVR** |
|  | 333 | **386.9219** | **771.8291** | **772.9362** | **-1.1072** | **2** | **13** | **1.5e+02** | **1** | **LRASKAK** |
|  | 501 | **400.1976** | **1197.5705** | **1197.3862** | **0.1844** | **2** | **13** | **1e+02** | **1** | **TLCSSDRGCKK** |
|  | 719 | **408.0494** | **1221.1260** | **1221.3893** | **-0.2633** | **1** | **13** | **1.3e+02** | **1** | **FQLGRSMNPR + Oxidation (M)** |
|  | 1055 | 434.1796 | 866.3445 | 865.8938 | 0.4507 | 0 | 13 | 1.3e+02 | 1 | HAGGGAGSPR |
|  | 3100 | **740.5892** | **1479.1636** | **1479.5989** | **-0.4354** | **2** | **13** | **1.1e+02** | **1** | **QRNYAWETRQK** |
|  | 3165 | **745.3747** | **1488.7346** | **1489.6501** | **-0.9155** | **0** | **13** | **1.3e+02** | **1** | **TFSTYGSHLAFVC + Carbamidomethyl (C)** |
|  | 316 | **386.1607** | **1155.4599** | **1156.2265** | **-0.7665** | **0** | **13** | **1.2e+02** | **1** | **CAVNSGGSNYK + Carbamidomethyl (C)** |
|  | 571 | **404.0760** | **1209.2058** | **1208.4302** | **0.7756** | **1** | **13** | **1.3e+02** | **1** | **IPTQLSYCRK** |
|  | 809 | **414.7260** | **1241.1559** | **1240.4158** | **0.7401** | **1** | **13** | **1.1e+02** | **1** | **RLPHSCPDCGR** |
|  | 661 | **407.1107** | **1218.3098** | **1218.3010** | **0.0088** | **1** | **13** | **1.3e+02** | **1** | **RSTPGCSPGGSR + Carbamidomethyl (C)** |
|  | 729 | **408.2692** | **814.5235** | **813.8987** | **0.6248** | **0** | **13** | **1.1e+02** | **1** | **ATPAGLER** |
|  | 1089 | **436.7207** | **871.4266** | **870.9932** | **0.4335** | **1** | **13** | **1.1e+02** | **1** | **KAVPGNSAK** |
|  | 2646 | **648.0015** | **1940.9824** | **1940.0729** | **0.9095** | **1** | **13** | **1.2e+02** | **1** | **IKSTVDGATTDYAGTVQGR** |
|  | 609 | **405.7842** | **1214.3305** | **1213.4568** | **0.8738** | **2** | **13** | **1.3e+02** | **1** | **RVSPAGVCLRR** |
|  | 1836 | **524.1148** | **1046.2148** | **1045.1958** | **1.0191** | **1** | **13** | **1.4e+02** | **1** | **RKPFDVQR** |
|  | 2296 | **594.9456** | **1781.8145** | **1781.7902** | **0.0243** | **0** | **13** | **1.3e+02** | **1** | **FCSDSSSDCGSSSGSVR + 2 Carbamidomethyl (C)** |
|  | 2240 | **589.6364** | **1177.2580** | **1177.3053** | **-0.0473** | **0** | **13** | **1.7e+02** | **1** | **STNATSAILTAK** |
|  | 2415 | **610.2877** | **1218.5605** | **1219.4545** | **-0.8940** | **1** | **13** | **1.4e+02** | **1** | **RSVIGSSCLIK + Carbamidomethyl (C)** |
|  | 347 | **387.1996** | **1158.5765** | **1158.4114** | **0.1651** | **0** | **13** | **1.3e+02** | **1** | **KPNIAIVEMK + Oxidation (M)** |
|  | 156 | 371.2527 | 740.4906 | 739.7820 | 0.7085 | 1 | 13 | 86 | 1 | QYRHH |
|  | 528 | **402.0524** | **802.0901** | **800.9431** | **1.1470** | **0** | **13** | **1.6e+02** | **1** | **AVVISGAGK** |
|  | 648 | **406.7567** | **1217.2479** | **1216.3048** | **0.9430** | **1** | **13** | **1.2e+02** | **1** | **RLQQGLDSGSR** |
|  | 715 | **408.0004** | **1220.9789** | **1220.4043** | **0.5746** | **0** | **13** | **1.4e+02** | **1** | **ILINHCHTNR** |
|  | 1266 | **457.5488** | **1369.6242** | **1368.6211** | **1.0031** | **1** | **13** | **1.6e+02** | **1** | **KVNGTAIIQLPSK** |
|  | 1408 | **468.2698** | **1401.7872** | **1402.5945** | **-0.8073** | **1** | **13** | **1.3e+02** | **1** | **FSTLKIQPSEPR** |
|  | 1500 | **476.5116** | **1426.5127** | **1426.5792** | **-0.0665** | **0** | **13** | **1.6e+02** | **1** | **ALSALGAAHFGAGAGR** |
|  | 3131 | **741.8083** | **2222.4027** | **2221.7083** | **0.6944** | **1** | **13** | **1.5e+02** | **1** | **AMEDAAKGMPLICCLWMHK + Carbamidomethyl (C); Oxidation (M)** |
|  | 922 | **422.2889** | **1263.8446** | **1264.3629** | **-0.5183** | **0** | **13** | **1.2e+02** | **1** | **EKPSNCAASTEK** |
|  | 1762 | **516.5964** | **1546.7671** | **1546.8805** | **-0.1133** | **2** | **13** | **1.7e+02** | **1** | **CCNPSVPKQRVMK + Carbamidomethyl (C)** |
|  | 2376 | **606.6505** | **1816.9294** | **1815.9388** | **0.9906** | **0** | **13** | **1.5e+02** | **1** | **DSCQGDSGGPLVCGDHLR** |
|  | 342 | **387.0023** | **771.9899** | **772.8899** | **-0.9000** | **1** | **13** | **1.5e+02** | **1** | **KGSGLPSK** |
|  | 1916 | 534.7700 | 1067.5253 | 1067.2412 | 0.2841 | 0 | 13 | 1.2e+02 | 1 | GCMANVTVTR + Oxidation (M) |
|  | 1358 | **462.2549** | **1383.7425** | **1383.6028** | **0.1397** | **1** | **13** | **1.2e+02** | **1** | **MRHGCHTVIASR + Oxidation (M)** |
|  | 2327 | **598.2245** | **1791.6513** | **1791.9142** | **-0.2629** | **1** | **13** | **1.3e+02** | **1** | **SSVPSQKTYQGSYGFR** |
|  | 2637 | **645.6776** | **1289.3404** | **1288.4801** | **0.8603** | **2** | **13** | **1.6e+02** | **1** | **ANKALGMGGTRGR** |
|  | 3017 | **716.4498** | **2146.3271** | **2147.3515** | **-1.0244** | **2** | **13** | **1.3e+02** | **1** | **REGLVLGSARNSSISGPFGSR** |
|  | 96 | **368.2867** | **734.5586** | **734.8616** | **-0.3030** | **0** | **13** | **1.2e+02** | **1** | **GQLASMI + Oxidation (M)** |
|  | 210 | **377.1480** | **752.2813** | **752.8556** | **-0.5743** | **1** | **13** | **1.2e+02** | **1** | **EKTFTK** |
|  | 213 | **377.1591** | **1128.4550** | **1129.3719** | **-0.9169** | **0** | **13** | **1.2e+02** | **1** | **LGAMTSMVCK + Carbamidomethyl (C); 2 Oxidation (M)** |
|  | 222 | **377.3798** | **752.7449** | **751.8741** | **0.8708** | **1** | **13** | **1.4e+02** | **1** | **APVPVRGG** |
|  | 239 | **379.2256** | **1134.6548** | **1135.1526** | **-0.4979** | **1** | **13** | **1.1e+02** | **1** | **HYDDHRHR** |
|  | 1264 | **457.3605** | **1369.0594** | **1368.6211** | **0.4383** | **1** | **13** | **1.1e+02** | **1** | **KVNGTAIIQLPSK** |
|  | 1288 | **458.7828** | **1373.3262** | **1374.4153** | **-1.0892** | **0** | **13** | **1.3e+02** | **1** | **THNSASWSDTLR** |
|  | 2113 | **563.7632** | **1688.2674** | **1687.9586** | **0.3088** | **1** | **13** | **1.2e+02** | **1** | **VASIALLDLTGARCER** |
|  | 3327 | **787.6721** | **2359.9940** | **2360.3587** | **-0.3647** | **1** | **13** | **1.1e+02** | **1** | **EGTENQGVEPQDEVDGGDTQKK** |
|  | 3595 | **1113.4956** | **2224.9764** | **2224.3396** | **0.6368** | **0** | **13** | **94** | **1** | **GLEWVSGISGSGSSTHYADSVK** |
|  | 788 | **413.4254** | **824.8361** | **825.9095** | **-1.0733** | **0** | **13** | **1.3e+02** | **1** | **LSGPAEPR** |
|  | 1442 | **471.8535** | **1412.5385** | **1412.7814** | **-0.2429** | **2** | **13** | **1.3e+02** | **1** | **KMLKFISGIFTK** |
|  | 1678 | **502.6213** | **1504.8418** | **1504.6268** | **0.2151** | **1** | **13** | **1.7e+02** | **1** | **SFQPCESAKHSAR + Carbamidomethyl (C)** |
|  | 2527 | **628.4481** | **1882.3222** | **1881.1347** | **1.1875** | **1** | **13** | **1.2e+02** | **1** | **LSENGAPEKDVLVAIGLR** |
|  | 2586 | 637.1836 | 1272.3524 | 1273.4373 | -1.0849 | 1 | 13 | 1.4e+02 | 1 | LLDYSLHKER |
|  | 3476 | **840.4569** | **1678.8990** | **1677.8826** | **1.0164** | **1** | **13** | **1.3e+02** | **1** | **NMIDHIVLHREER + Oxidation (M)** |
|  | 1548 | **482.3578** | **1444.0514** | **1444.6096** | **-0.5582** | **1** | **13** | **1.2e+02** | **1** | **IDCQGIPPSSKDK + Carbamidomethyl (C)** |
|  | 1556 | **485.0444** | **968.0741** | **969.1395** | **-1.0654** | **0** | **13** | **1.2e+02** | **1** | **MSCVPGFR + Carbamidomethyl (C); Oxidation (M)** |
|  | 3125 | **741.6387** | **2221.8940** | **2221.5958** | **0.2983** | **1** | **13** | **1e+02** | **1** | **MSVDHLYCYLPNVTFFKK + Oxidation (M)** |
|  | 240 | **379.2551** | **756.4954** | **755.9886** | **0.5068** | **0** | **13** | **1.1e+02** | **1** | **CLVFMK + Oxidation (M)** |
|  | 709 | **407.8901** | **1220.6482** | **1219.4513** | **1.1969** | **0** | **13** | **1.3e+02** | **1** | **LTVASLCSQVAK** |
|  | 1070 | **435.1509** | **1302.4306** | **1301.5155** | **0.9152** | **1** | **13** | **1.3e+02** | **1** | **HTLMRVLSSGGK + Oxidation (M)** |
|  | 1132 | **442.2492** | **882.4837** | **882.9791** | **-0.4954** | **0** | **13** | **1.1e+02** | **1** | **LSQSSMSK + Oxidation (M)** |
|  | 1616 | **491.4182** | **1471.2323** | **1471.6812** | **-0.4490** | **0** | **13** | **1.1e+02** | **1** | **SAQCGSSMIYHCK + Carbamidomethyl (C)** |
|  | 2051 | **553.8080** | **1105.6012** | **1105.2263** | **0.3749** | **2** | **13** | **1.2e+02** | **1** | **KMEPEGRSR + Oxidation (M)** |
|  | 2432 | **612.2498** | **1222.4847** | **1221.4556** | **1.0292** | **2** | **13** | **1.4e+02** | **1** | **LKDRPPLRAR** |
|  | 3203 | **749.4105** | **1496.8063** | **1496.7105** | **0.0957** | **1** | **13** | **1.3e+02** | **1** | **LGRLDQAVLDLQR** |
|  | 790 | **413.4701** | **824.9253** | **823.9335** | **0.9919** | **0** | **13** | **1.4e+02** | **1** | **TAAVSTFK** |
|  | 2228 | **587.2092** | **1758.6055** | **1759.0516** | **-0.4460** | **0** | **13** | **1.4e+02** | **1** | **SMIVGVITTSYGMDVW** |
|  | 2245 | **590.7180** | **1179.4211** | **1178.3580** | **1.0631** | **1** | **13** | **1.6e+02** | **1** | **YSTKMTYLR + Oxidation (M)** |
|  | 2976 | **703.0461** | **1404.0774** | **1404.6318** | **-0.5545** | **2** | **13** | **1.2e+02** | **1** | **EKADLATKCLQGK** |
|  | 3028 | **721.7732** | **2162.2974** | **2162.3846** | **-0.0872** | **2** | **13** | **1.5e+02** | **1** | **ERNSRPSSKNLTNSSIPMK + Oxidation (M)** |
|  | 1118 | **439.0855** | **876.1563** | **875.0482** | **1.1081** | **2** | **13** | **1.5e+02** | **1** | **KRAPMEK + Oxidation (M)** |
|  | 1032 | **432.8326** | **1295.4756** | **1295.5110** | **-0.0354** | **1** | **13** | **1.4e+02** | **1** | **MVGGVRASALYR + Oxidation (M)** |
|  | 1405 | **467.9917** | **1400.9529** | **1401.5006** | **-0.5476** | **0** | **13** | **1.5e+02** | **1** | **MECTEADGDAMR + Carbamidomethyl (C); Oxidation (M)** |
|  | 1490 | **476.1268** | **950.2388** | **950.1777** | **0.0611** | **1** | **13** | **1.5e+02** | **1** | **GKTTMPGMK** |
|  | 2230 | **587.3759** | **1172.7369** | **1173.2969** | **-0.5599** | **1** | **13** | **1.4e+02** | **1** | **KEPSQPAECK + Carbamidomethyl (C)** |
|  | 2990 | **709.2165** | **1416.4182** | **1417.5877** | **-1.1695** | **1** | **13** | **1.3e+02** | **1** | **RAAVPVLDCGSSDK** |
|  | 201 | **376.1895** | **1125.5462** | **1126.3298** | **-0.7836** | **0** | **13** | **1.1e+02** | **1** | **AVGSPLCVPAR + Carbamidomethyl (C)** |
|  | 401 | **389.1653** | **1164.4736** | **1165.4503** | **-0.9767** | **0** | **13** | **1.5e+02** | **1** | **MVNMLGCLPR + 2 Oxidation (M)** |
|  | 1913 | **534.6005** | **1067.1861** | **1066.2564** | **0.9298** | **1** | **13** | **1.7e+02** | **1** | **TRMLDCVR + Carbamidomethyl (C); Oxidation (M)** |
|  | 2067 | **556.3128** | **1110.6108** | **1110.3336** | **0.2772** | **1** | **13** | **1.5e+02** | **1** | **IPSRHTMLR** |
|  | 3154 | **743.6039** | **2227.7897** | **2228.5753** | **-0.7856** | **2** | **13** | **1.1e+02** | **1** | **MGRAPEVCSQQPSRVMPPGK + Carbamidomethyl (C); Oxidation (M)** |
|  | 3270 | 766.5801 | 2296.7181 | 2295.7903 | 0.9278 | 1 | 13 | 1.2e+02 | 1 | LPILGSRTMRPMTIFSMATR + Oxidation (M) |
|  | 1260 | **457.1724** | **912.3300** | **912.0416** | **0.2884** | **0** | **13** | **1.4e+02** | **1** | **LDPILADR** |
|  | 100 | **369.0150** | **1104.0227** | **1104.3011** | **-0.2784** | **1** | **13** | **1.6e+02** | **1** | **CMAAAPGVKGPS + Oxidation (M)** |
|  | 679 | **407.4734** | **1219.3979** | **1219.2642** | **0.1337** | **0** | **13** | **1.6e+02** | **1** | **HSSPPQEPGAGR** |
|  | 1262 | **457.2914** | **1368.8520** | **1368.5781** | **0.2738** | **1** | **13** | **1.2e+02** | **1** | **TDHIVGSKALLSK** |
|  | 2325 | **597.8778** | **1790.6113** | **1790.9772** | **-0.3660** | **0** | **13** | **1.1e+02** | **1** | **TARPGGGTPLLNGAGPGAAR** |
|  | 3078 | **740.1687** | **2217.4839** | **2217.5644** | **-0.0805** | **2** | **13** | **1.3e+02** | **1** | **SVVCKMNPMTDAASCGSEVKK + 2 Oxidation (M)** |
|  | 1736 | **510.5388** | **1528.5942** | **1527.6817** | **0.9124** | **1** | **13** | **1.7e+02** | **1** | **EANLGTDAGVAVRVR** |
|  | 1863 | **527.0974** | **1578.2701** | **1577.6709** | **0.5992** | **0** | **13** | **1.4e+02** | **1** | **YGISSGDAGSTFMER** |
|  | 3281 | **771.5571** | **2311.6492** | **2312.5854** | **-0.9362** | **2** | **13** | **1.3e+02** | **1** | **LQQENMNLLSDARSARMYR + Oxidation (M)** |
|  | 70 | **366.0881** | **1095.2420** | **1096.3038** | **-1.0617** | **0** | **13** | **1.5e+02** | **1** | **ACFHCEVCK + Carbamidomethyl (C)** |
|  | 380 | **388.2502** | **774.4856** | **773.7488** | **0.7369** | **0** | **13** | **1.3e+02** | **1** | **ASNDPDR** |
|  | 721 | **408.0938** | **1221.2593** | **1221.4060** | **-0.1467** | **1** | **13** | **1.5e+02** | **1** | **AMFCSSFREK + Oxidation (M)** |
|  | 1948 | **538.1725** | **1611.4953** | **1610.8381** | **0.6572** | **2** | **13** | **1.5e+02** | **1** | **DKGNCGVGKSCLCNR + Carbamidomethyl (C)** |
|  | 755 | **409.3615** | **1225.0624** | **1224.3635** | **0.6989** | **0** | **13** | **1.4e+02** | **1** | **APMECQESWK + Oxidation (M)** |
|  | 1101 | **437.2080** | **1308.6018** | **1307.5630** | **1.0388** | **1** | **13** | **1.5e+02** | **1** | **KIVQYGCPFPR** |
|  | 2921 | **686.7173** | **1371.4198** | **1371.4562** | **-0.0364** | **1** | **13** | **1.4e+02** | **1** | **RAQEAEEAGAALR** |
|  | 49 | **363.3664** | **724.7181** | **724.7842** | **-0.0661** | **0** | **13** | **1.5e+02** | **1** | **ASSGMTR + Oxidation (M)** |
|  | 1797 | **520.2789** | **1557.8146** | **1558.8233** | **-1.0087** | **0** | **13** | **1.4e+02** | **1** | **NTEMCNVMMQLR + Carbamidomethyl (C); 2 Oxidation (M)** |
|  | 3335 | **789.8881** | **2366.6422** | **2367.7170** | **-1.0748** | **2** | **13** | **1.6e+02** | **1** | **AGLSAAMSAKAISEQTGKELLYK** |
|  | 175 | **373.6958** | **745.3768** | **745.9306** | **-0.5538** | **0** | **13** | **1.6e+02** | **1** | **IVNCLK + Carbamidomethyl (C)** |
|  | 182 | 374.2171 | 1119.6291 | 1119.2775 | 0.3517 | 2 | 13 | 1.4e+02 | 1 | RAAGFLRSNK |
|  | 378 | **388.1926** | **1161.5557** | **1161.3557** | **0.2000** | **1** | **13** | **1.5e+02** | **1** | **KDNLTMHMR + Oxidation (M)** |
|  | 438 | **391.3074** | **1170.9000** | **1171.3768** | **-0.4768** | **1** | **13** | **1e+02** | **1** | **LCRGRPGLSR + Carbamidomethyl (C)** |
|  | 1666 | **499.9885** | **1496.9433** | **1496.6494** | **0.2939** | **1** | **13** | **1.3e+02** | **1** | **TYSHGTYRAGPMR** |
|  | 2426 | **612.2119** | **1833.6136** | **1832.9863** | **0.6273** | **1** | **13** | **1.4e+02** | **1** | **RTTSSAVTETGPPEMPR + Oxidation (M)** |
|  | 2706 | **658.8589** | **1973.5545** | **1973.3707** | **0.1838** | **2** | **13** | **1.3e+02** | **1** | **MAVCGLGSRLGLGSRLGLR + Carbamidomethyl (C)** |
|  | 1475 | **475.2337** | **948.4526** | **948.0127** | **0.4400** | **0** | **13** | **1.5e+02** | **1** | **TCEGQGTPR** |
|  | 1745 | **513.5483** | **1537.6227** | **1537.8289** | **-0.2062** | **2** | **13** | **1.6e+02** | **1** | **QRDPGLPKMQVIR** |
|  | 1121 | **439.6993** | **1316.0758** | **1315.4743** | **0.6016** | **0** | **13** | **1.2e+02** | **1** | **LMSQSMSSVSSR + Oxidation (M)** |
|  | 2913 | **686.2657** | **2055.7751** | **2055.4384** | **0.3366** | **2** | **13** | **1.4e+02** | **1** | **MEDRKELLTEIPVAGIIK** |
|  | 2456 | **613.9733** | **1838.8978** | **1839.0499** | **-0.1521** | **1** | **13** | **1.3e+02** | **1** | **LKDSGPLFNTDYDILK** |
|  | 3228 | **757.2781** | **2268.8121** | **2269.3983** | **-0.5863** | **0** | **13** | **1.3e+02** | **1** | **EESGGENETDGQINLGCLLYK** |
|  | 3551 | **927.1960** | **1852.3773** | **1853.1033** | **-0.7260** | **0** | **13** | **1.1e+02** | **1** | **GGMTPGLMTPGTGELDMR + 2 Oxidation (M)** |
|  | 2791 | **669.0315** | **2004.0723** | **2003.0197** | **1.0527** | **1** | **13** | **1.3e+02** | **1** | **MDEDEETEHETKAGPER** |
|  | 2907 | **686.1664** | **2055.4770** | **2054.3079** | **1.1691** | **2** | **13** | **1.4e+02** | **1** | **MPLAEDIKGSCFQSGNKR + Carbamidomethyl (C); Oxidation (M)** |
|  | 3190 | **749.1555** | **2244.4444** | **2245.5030** | **-1.0586** | **0** | **13** | **1.3e+02** | **1** | **LTIITDESTTTAFMELSSLR + Oxidation (M)** |
|  | 3283 | **772.3557** | **2314.0450** | **2313.5040** | **0.5410** | **1** | **13** | **1.3e+02** | **1** | **CDRLPEQGNPDTLCMDYNR + Carbamidomethyl (C); Oxidation (M)** |
|  | 116 | **369.2817** | **1104.8230** | **1104.2991** | **0.5238** | **0** | **13** | **1.2e+02** | **1** | **QQLLIGAYAK** |
|  | 1819 | 523.0095 | 1566.0064 | 1564.8297 | 1.1767 | 2 | 13 | 1.5e+02 | 1 | SRTMPRTVPGSTMK + Oxidation (M) |
|  | 2970 | **700.6450** | **1399.2753** | **1399.5325** | **-0.2573** | **1** | **13** | **1.1e+02** | **1** | **AECTAKLAGDGHAR** |
|  | 3039 | **727.8553** | **1453.6959** | **1452.6995** | **0.9964** | **1** | **13** | **1.6e+02** | **1** | **ILVCGTCGTRSSQK** |
|  | 1254 | 456.2148 | 1365.6224 | 1364.6525 | 0.9699 | 2 | 13 | 1.4e+02 | 1 | NFTELKIKMPK + Oxidation (M) |
|  | 2386 | **607.7914** | **1820.3520** | **1820.0880** | **0.2640** | **0** | **13** | **1.4e+02** | **1** | **GPPCACCHLNGAPRPR + 3 Carbamidomethyl (C)** |
|  | 3139 | **742.6425** | **1483.2701** | **1483.6568** | **-0.3867** | **0** | **13** | **1.1e+02** | **1** | **WWHQHNLCFR + Carbamidomethyl (C)** |
|  | 1374 | **464.1950** | **1389.5628** | **1388.5894** | **0.9734** | **1** | **13** | **1.4e+02** | **1** | **DAIAQVEMDLKR** |
|  | 1407 | **468.1256** | **1401.3547** | **1400.4808** | **0.8738** | **2** | **13** | **1.6e+02** | **1** | **NKNSHCAGERER** |
|  | 3376 | **800.9679** | **2399.8815** | **2399.7671** | **0.1145** | **0** | **13** | **1.5e+02** | **1** | **ATVLQQLGWMHHNMDLVMDK + 2 Oxidation (M)** |
|  | 146 | **370.9024** | **1109.6849** | **1110.3057** | **-0.6207** | **1** | **13** | **1.1e+02** | **1** | **EMKAEIMAR + 2 Oxidation (M)** |
|  | 1326 | **460.5305** | **1378.5692** | **1378.5814** | **-0.0122** | **2** | **13** | **1.9e+02** | **1** | **KSAACGQPRMSSR** |
|  | 2028 | **550.6435** | **1099.2722** | **1098.2518** | **1.0205** | **1** | **13** | **1.8e+02** | **1** | **FGLTTFKER** |
|  | 1023 | **432.0502** | **1293.1285** | **1293.4255** | **-0.2970** | **0** | **13** | **1.6e+02** | **1** | **AGATYGTISTPVR** |
|  | 1849 | **525.0837** | **1048.1527** | **1049.2207** | **-1.0680** | **1** | **13** | **1.5e+02** | **1** | **KELIDYLR** |
|  | 3002 | **715.0123** | **2142.0146** | **2142.5240** | **-0.5093** | **1** | **13** | **1.2e+02** | **1** | **FCQFPQEIVLQMVERCR + Oxidation (M)** |
|  | 759 | 410.2754 | 1227.8040 | 1228.3937 | -0.5897 | 0 | 13 | 1.3e+02 | 1 | MSACSSSEISVK |
|  | 766 | **411.1657** | **1230.4751** | **1231.3344** | **-0.8594** | **0** | **13** | **1.5e+02** | **1** | **ADDTGLYFCAR** |
|  | 1296 | **458.9279** | **1373.7614** | **1373.5283** | **0.2331** | **0** | **13** | **1.6e+02** | **1** | **MAYYAYYLGDK + Oxidation (M)** |
|  | 1468 | **474.3661** | **946.7174** | **945.9803** | **0.7371** | **2** | **13** | **1.3e+02** | **1** | **GRRAEDSR** |
|  | 1602 | **489.3455** | **1465.0143** | **1465.7382** | **-0.7239** | **1** | **13** | **1.2e+02** | **1** | **MLRAMVASGSELGK + Oxidation (M)** |
|  | 2037 | **551.5867** | **1101.1587** | **1100.2692** | **0.8895** | **0** | **13** | **1.8e+02** | **1** | **FLAFSGPHPK** |
|  | 2183 | **578.0588** | **1154.1028** | **1154.2570** | **-0.1543** | **0** | **13** | **1.4e+02** | **1** | **HLTSEMHGAR + Oxidation (M)** |
|  | 2315 | **596.9402** | **1191.8657** | **1192.4110** | **-0.5453** | **1** | **13** | **1.4e+02** | **1** | **KEALHQIVVR** |
|  | 2449 | **613.4637** | **1837.3691** | **1838.0867** | **-0.7177** | **0** | **13** | **1.2e+02** | **1** | **EGGLYTCIATNLVGADLK** |
|  | 3416 | **812.3792** | **2434.1155** | **2433.7901** | **0.3254** | **2** | **13** | **1.4e+02** | **1** | **GSPGPPYRPPSQRLREAVPCLR** |
|  | 196 | **375.1825** | **748.3501** | **747.9252** | **0.4250** | **1** | **13** | **1.4e+02** | **1** | **TKKPFK** |
|  | 1315 | **459.8604** | **917.7059** | **917.9602** | **-0.2542** | **0** | **13** | **1.6e+02** | **1** | **NLVDENSK** |
|  | 1905 | **533.4559** | **1064.8971** | **1064.2785** | **0.6185** | **1** | **13** | **1.1e+02** | **1** | **LSVSYLRVK** |
|  | 2822 | **673.7612** | **1345.5077** | **1344.4755** | **1.0321** | **1** | **13** | **1.8e+02** | **1** | **GTRATWTELPGR** |
|  | 3041 | **727.9060** | **1453.7972** | **1454.7785** | **-0.9812** | **2** | **13** | **1.4e+02** | **1** | **KLCVKEMNSGMAK + Oxidation (M)** |
|  | 693 | **407.7121** | **813.4094** | **813.8591** | **-0.4496** | **1** | **13** | **1.2e+02** | **1** | **AGREPER** |
|  | 695 | **407.7393** | **1220.1956** | **1220.3813** | **-0.1857** | **2** | **13** | **1.4e+02** | **1** | **EHVPRRGGSLL** |
|  | 1901 | **532.7540** | **1595.2397** | **1594.7906** | **0.4491** | **0** | **13** | **1.3e+02** | **1** | **NGSIENCLHKPTPK + Carbamidomethyl (C)** |
|  | 2782 | **668.7715** | **2003.2925** | **2003.1719** | **0.1206** | **0** | **13** | **1.8e+02** | **1** | **FSDSSSAGGMGQKPVEMSGK + Oxidation (M)** |
|  | 2728 | **664.2883** | **1989.8428** | **1990.2573** | **-0.4145** | **1** | **13** | **1.4e+02** | **1** | **EQEIVASSPSLSGLKLGFK** |
|  | 2927 | **687.1816** | **2058.5226** | **2059.3463** | **-0.8237** | **1** | **13** | **1.4e+02** | **1** | **SMMSPMAERSMMSAYER + 4 Oxidation (M)** |
|  | 241 | **379.3042** | **1134.8905** | **1134.3467** | **0.5438** | **0** | **13** | **1.2e+02** | **1** | **SQAGIMASLLK + Oxidation (M)** |
|  | 281 | **385.0375** | **1152.0903** | **1152.1716** | **-0.0813** | **0** | **13** | **1.3e+02** | **1** | **DSGDGGLVNYR** |
|  | 338 | **386.9510** | **771.8873** | **771.8622** | **0.0251** | **1** | **13** | **1.6e+02** | **1** | **APTAEKR** |
|  | 1140 | **443.4624** | **1327.3651** | **1327.5329** | **-0.1677** | **2** | **13** | **1.7e+02** | **1** | **CSECGKVFNRK + Carbamidomethyl (C)** |
|  | 2385 | **607.6891** | **1820.0451** | **1819.1086** | **0.9365** | **1** | **13** | **1.7e+02** | **1** | **QLLEEKAAMTDAMVPR + Oxidation (M)** |
|  | 3093 | **740.5255** | **2218.5544** | **2219.4262** | **-0.8718** | **0** | **13** | **1.4e+02** | **1** | **PGPTSSATNVSMVVSAGPLSSEK + Oxidation (M)** |
|  | 38 | **363.1316** | **1086.3725** | **1087.1646** | **-0.7921** | **0** | **13** | **1.5e+02** | **1** | **EPNEDCKPR** |
|  | 884 | 419.2829 | 836.5510 | 835.9919 | 0.5591 | 0 | 13 | 1.4e+02 | 1 | CIQCQK + 2 Carbamidomethyl (C) |
|  | 1269 | **457.7246** | **1370.1517** | **1369.4899** | **0.6617** | **2** | **13** | **1.1e+02** | **1** | **QGQSLGPRAGRSR** |
|  | 1740 | **511.7071** | **1021.3995** | **1020.9986** | **0.4009** | **0** | **13** | **1.3e+02** | **1** | **SNSAAAWSNN** |
|  | 2551 | **631.4246** | **1260.8343** | **1260.3542** | **0.4801** | **1** | **13** | **1.5e+02** | **1** | **EGNSKVTGAELR** |
|  | 1429 | **470.7555** | **939.4962** | **940.0750** | **-0.5788** | **0** | **13** | **1.1e+02** | **1** | **ALVDTYCR** |
|  | 1716 | **506.1823** | **1010.3499** | **1010.1451** | **0.2048** | **2** | **13** | **1.4e+02** | **1** | **TKKFSQGSK** |
|  | 1915 | **534.6954** | **1067.3760** | **1068.2771** | **-0.9011** | **2** | **13** | **1.6e+02** | **1** | **CAQRFCKR + Carbamidomethyl (C)** |
|  | 515 | **401.5778** | **1201.7113** | **1201.2838** | **0.4275** | **0** | **13** | **1.5e+02** | **1** | **QTPGSALEPSSK** |
|  | 1917 | **534.7706** | **1601.2897** | **1601.6808** | **-0.3910** | **2** | **13** | **1.3e+02** | **1** | **SLASSHSRSRSPSSR** |
|  | 1930 | **536.2572** | **1070.4996** | **1071.1006** | **-0.6009** | **0** | **13** | **1.5e+02** | **1** | **FDEHDGPVR** |
|  | 2671 | **652.8982** | **1955.6724** | **1955.1667** | **0.5058** | **1** | **13** | **1.3e+02** | **1** | **RPCWSGGAGRGGGPAWAVR + Carbamidomethyl (C)** |
|  | 3462 | **828.0002** | **1653.9857** | **1653.8830** | **0.1027** | **2** | **13** | **1.6e+02** | **1** | **TSAASRQASMMKNVR + Oxidation (M)** |
|  | 1217 | **451.8054** | **1352.3939** | **1351.4961** | **0.8978** | **1** | **13** | **1.7e+02** | **1** | **SHCQWLRHER** |
|  | 3102 | **740.6069** | **2218.7984** | **2219.4624** | **-0.6639** | **1** | **13** | **1.2e+02** | **1** | **ASVCSCHPRSAGHTCSVGSVC + 3 Carbamidomethyl (C)** |
|  | 351 | **387.7652** | **1160.2735** | **1160.3012** | **-0.0277** | **0** | **13** | **1.8e+02** | **1** | **LALAAQECER + Carbamidomethyl (C)** |
|  | 1944 | **537.4393** | **1609.2958** | **1608.6980** | **0.5979** | **2** | **13** | **1.3e+02** | **1** | **ARWRGSCSSGNSQR + Carbamidomethyl (C)** |
|  | 298 | **385.3280** | **1152.9618** | **1153.3536** | **-0.3917** | **0** | **13** | **1.1e+02** | **1** | **QMMTMHNTK + 2 Oxidation (M)** |
|  | 1654 | **498.6770** | **995.3393** | **995.1304** | **0.2088** | **1** | **13** | **1.3e+02** | **1** | **IRTDTYVK** |
|  | 2241 | **589.9015** | **1177.7882** | **1177.2887** | **0.4995** | **2** | **13** | **1.3e+02** | **1** | **GLRDCTLRED** |
|  | 513 | **401.3287** | **1200.9639** | **1200.3420** | **0.6219** | **1** | **12** | **1.3e+02** | **1** | **EEAALAAQAKAK** |
|  | 537 | **402.5668** | **1204.6783** | **1204.3291** | **0.3493** | **0** | **12** | **1.6e+02** | **1** | **ANPTVTLFPASS** |
|  | 991 | **430.2001** | **1287.5782** | **1288.5613** | **-0.9830** | **2** | **12** | **1.7e+02** | **1** | **GLGNKMKFLHK + Oxidation (M)** |
|  | 1097 | **437.0736** | **872.1324** | **871.0578** | **1.0746** | **1** | **12** | **1.7e+02** | **1** | **MCKECGK + Carbamidomethyl (C); Oxidation (M)** |
|  | 1461 | **473.5024** | **944.9900** | **945.9736** | **-0.9835** | **0** | **12** | **1.9e+02** | **1** | **SPSGAGSGAQK** |
|  | 1590 | **488.1814** | **1461.5221** | **1462.4775** | **-0.9554** | **0** | **12** | **1.6e+02** | **1** | **SPQDPGDSVQYNR** |
|  | 1868 | **527.4006** | **1052.7864** | **1053.3021** | **-0.5158** | **1** | **12** | **1.1e+02** | **1** | **VLLVQRLGR** |
|  | 1987 | **542.3631** | **1624.0671** | **1624.8796** | **-0.8125** | **0** | **12** | **1.3e+02** | **1** | **ETTALGIMAMTWQR + Oxidation (M)** |
|  | 2720 | **662.2854** | **1322.5560** | **1323.4150** | **-0.8589** | **0** | **12** | **1.5e+02** | **1** | **NGLPGSRPGSPER** |
|  | 2899 | **685.3025** | **2052.8853** | **2053.2633** | **-0.3780** | **2** | **12** | **1.6e+02** | **1** | **GRQCGLAGPDRGDQGLIPSR** |
|  | 1333 | **460.8364** | **919.6580** | **919.0176** | **0.6404** | **0** | **12** | **1.6e+02** | **1** | **GSAACGALGR + Carbamidomethyl (C)** |
|  | 462 | **396.0939** | **790.1731** | **789.9006** | **0.2725** | **0** | **12** | **1.7e+02** | **1** | **MGTAPAAR + Oxidation (M)** |
|  | 1815 | **522.3029** | **1563.8864** | **1564.5661** | **-0.6798** | **0** | **12** | **1.5e+02** | **1** | **ESSPAEPSQAGAYDR** |
|  | 1880 | **529.3998** | **1585.1773** | **1583.9833** | **1.1941** | **2** | **12** | **1.2e+02** | **1** | **LFFRFMNVLIKR** |
|  | 2277 | **593.1820** | **1776.5238** | **1777.1362** | **-0.6124** | **1** | **12** | **1.6e+02** | **1** | **CELDIMNKCTSLCCK + Carbamidomethyl (C); Oxidation (M)** |
|  | 2776 | **668.6519** | **1335.2889** | **1334.5650** | **0.7239** | **0** | **12** | **1.4e+02** | **1** | **CLGELLAAACASR + Carbamidomethyl (C)** |
|  | 3372 | **799.5177** | **2395.5309** | **2396.6852** | **-1.1543** | **2** | **12** | **1.4e+02** | **1** | **NRTNCDINIHYKSGVCNTMR + Carbamidomethyl (C)** |
|  | 227 | **378.1224** | **1131.3451** | **1131.3277** | **0.0174** | **0** | **12** | **1.4e+02** | **1** | **LYFHWPLR** |
|  | 303 | **386.0083** | **1155.0027** | **1154.3033** | **0.6994** | **0** | **12** | **1.3e+02** | **1** | **SCSAAGLHRPR** |
|  | 414 | **389.2409** | **1164.7005** | **1165.3908** | **-0.6903** | **2** | **12** | **1.3e+02** | **1** | **MRGVGMRQAK + 2 Oxidation (M)** |
|  | 776 | **411.5973** | **821.1798** | **820.9080** | **0.2718** | **0** | **12** | **1.3e+02** | **1** | **GEMVLEQ + Oxidation (M)** |
|  | 900 | **420.3570** | **838.6992** | **838.9530** | **-0.2537** | **0** | **12** | **1.2e+02** | **1** | **VSNTCCR + Carbamidomethyl (C)** |
|  | 1772 | **518.8292** | **1035.6437** | **1035.1346** | **0.5091** | **0** | **12** | **1.2e+02** | **1** | **MGGAAFGPDGR** |
|  | 2208 | **582.4823** | **1162.9498** | **1163.4545** | **-0.5047** | **1** | **12** | **1.3e+02** | **1** | **KPGAMVKVSCK + Oxidation (M)** |
|  | 2871 | **682.6332** | **2044.8774** | **2045.3029** | **-0.4255** | **1** | **12** | **1.2e+02** | **1** | **REHCPPESPACPSTVHLK + Carbamidomethyl (C)** |
|  | 3517 | **871.7938** | **1741.5729** | **1740.9997** | **0.5731** | **2** | **12** | **1.2e+02** | **1** | **LRQQALETKHFIEK** |
|  | 858 | **418.0688** | **834.1228** | **834.8732** | **-0.7504** | **0** | **12** | **1.6e+02** | **1** | **QAEWSSK** |
|  | 2492 | **620.3518** | **1858.0332** | **1858.0419** | **-0.0086** | **2** | **12** | **1.5e+02** | **1** | **NPPCSGSSIATASPERRK** |
|  | 235 | **378.3636** | **754.7125** | **755.8627** | **-1.1501** | **0** | **12** | **1.3e+02** | **1** | **GGVGTLPR** |
|  | 577 | **404.1700** | **1209.4879** | **1210.3833** | **-0.8954** | **1** | **12** | **1.5e+02** | **1** | **GRSIPGAAEKPK** |
|  | 987 | **429.5538** | **1285.6393** | **1285.3125** | **0.3269** | **0** | **12** | **1.8e+02** | **1** | **EVDDFFEQEK** |
|  | 1163 | **444.9478** | **887.8808** | **889.0748** | **-1.1939** | **0** | **12** | **1.8e+02** | **1** | **MAAVVAATR** |
|  | 1661 | **499.5250** | **1495.5527** | **1496.6015** | **-1.0487** | **1** | **12** | **1.7e+02** | **1** | **DSSLEHKYSQMR + Oxidation (M)** |
|  | 19 | **362.1417** | **1083.4028** | **1083.1346** | **0.2682** | **1** | **12** | **1.4e+02** | **1** | **DSRSGSTMAR + Oxidation (M)** |
|  | 2925 | **687.1028** | **2058.2862** | **2059.1554** | **-0.8693** | **0** | **12** | **1.5e+02** | **1** | **MLDAQPDSEDHGCGQTQAR** |
|  | 416 | **389.2553** | **1164.7439** | **1165.2150** | **-0.4711** | **0** | **12** | **1.4e+02** | **1** | **AFNHSSNFNK** |
|  | 882 | **419.2710** | **1254.7908** | **1255.2914** | **-0.5006** | **0** | **12** | **1.5e+02** | **1** | **GVPEASGPQNDGK** |
|  | 947 | **427.6129** | **1279.8165** | **1280.5342** | **-0.7178** | **0** | **12** | **1.3e+02** | **1** | **VWSALPYTICK** |
|  | 391 | **389.1047** | **1164.2919** | **1164.3729** | **-0.0810** | **0** | **12** | **1.7e+02** | **1** | **EPVVYPWMK + Oxidation (M)** |
|  | 1938 | **536.5687** | **1071.1227** | **1071.1900** | **-0.0673** | **2** | **12** | **1.9e+02** | **1** | **SSGFRKSFR** |
|  | 2018 | **548.9941** | **1095.9735** | **1095.2082** | **0.7653** | **1** | **12** | **1.5e+02** | **1** | **MEADCKAGDR** |
|  | 2149 | **570.1860** | **1707.5359** | **1707.8871** | **-0.3512** | **1** | **12** | **1.5e+02** | **1** | **EMQQQELAQMRQR + 2 Oxidation (M)** |
|  | 2570 | **634.5131** | **1900.5170** | **1900.1198** | **0.3973** | **1** | **12** | **1.2e+02** | **1** | **LAGMTGREGGFSAFNQLK + Oxidation (M)** |
|  | 3297 | 777.3478 | 1552.6808 | 1553.7917 | -1.1109 | 2 | 12 | 1.5e+02 | 1 | TAACRTLGARHAGLR |
|  | 3523 | **880.2842** | **1758.5536** | **1757.8977** | **0.6558** | **1** | **12** | **1.4e+02** | **1** | **EVGWEEGQKLALENR** |
|  | 1753 | **514.4383** | **1026.8618** | **1027.1293** | **-0.2675** | **0** | **12** | **1.2e+02** | **1** | **GSDPAVASVPK** |
|  | 65 | **365.9780** | **1094.9119** | **1094.2483** | **0.6636** | **1** | **12** | **1.8e+02** | **1** | **VEHARMHAK + Oxidation (M)** |
|  | 435 | **391.2046** | **1170.5917** | **1170.3987** | **0.1930** | **1** | **12** | **1.2e+02** | **1** | **LLAEELNKLK** |
|  | 364 | **387.9871** | **1160.9391** | **1160.3676** | **0.5715** | **0** | **12** | **1.9e+02** | **1** | **GMMPPLNTQR + Oxidation (M)** |
|  | 1054 | **434.1748** | **1299.5023** | **1299.4597** | **0.0426** | **2** | **12** | **1.5e+02** | **1** | **NINKHTERCK + Carbamidomethyl (C)** |
|  | 2289 | **594.4470** | **1186.8791** | **1186.2757** | **0.6035** | **1** | **12** | **1.4e+02** | **1** | **LSAKNGTPQDR** |
|  | 155 | **371.2421** | **1110.7042** | **1111.2372** | **-0.5329** | **1** | **12** | **99** | **1** | **QCCCDRGR + 3 Carbamidomethyl (C)** |
|  | 1829 | **523.5846** | **1567.7316** | **1566.7775** | **0.9542** | **0** | **12** | **2e+02** | **1** | **TQAMALETPTPGPPR** |
|  | 1981 | 541.9464 | 1622.8171 | 1623.8981 | -1.0811 | 0 | 12 | 1.5e+02 | 1 | MAPQPMIHTQQGLR + Oxidation (M) |
|  | 2700 | **656.8842** | **1967.6303** | **1967.3134** | **0.3168** | **1** | **12** | **1.3e+02** | **1** | **SIQKSIFTVCLDATMPR + Carbamidomethyl (C)** |
|  | 3081 | **740.3526** | **2218.0356** | **2217.5473** | **0.4883** | **2** | **12** | **1.5e+02** | **1** | **GSGYKFATYAIGWVRQMPGK** |
|  | 3168 | **746.8892** | **2237.6453** | **2237.6662** | **-0.0208** | **1** | **12** | **1.7e+02** | **1** | **QLKQMYCPPCNCGWFSTCK** |
|  | 570 | **404.0719** | **1209.1936** | **1209.4679** | **-0.2743** | **2** | **12** | **1.6e+02** | **1** | **CLRVLKGHQR** |
|  | 673 | **407.2750** | **1218.8027** | **1218.5561** | **0.2467** | **1** | **12** | **1.2e+02** | **1** | **APACVCVRVGIM** |
|  | 1091 | **436.9493** | **1307.8257** | **1308.5047** | **-0.6790** | **0** | **12** | **1.7e+02** | **1** | **MGHNFSLPVYK + Oxidation (M)** |
|  | 1701 | **505.0470** | **1008.0792** | **1008.1475** | **-0.0683** | **1** | **12** | **1.5e+02** | **1** | **GELDTMSKK** |
|  | 1827 | **523.4749** | **1044.9350** | **1044.1446** | **0.7905** | **0** | **12** | **1.4e+02** | **1** | **WGGPGGPGCTR** |
|  | 2391 | **608.4551** | **1822.3431** | **1823.0854** | **-0.7424** | **1** | **12** | **1.3e+02** | **1** | **DARVVGLLAGTCGVAQHR** |
|  | 616 | **406.0169** | **810.0190** | **808.9850** | **1.0340** | **1** | **12** | **1.4e+02** | **1** | **FIPKAMS + Oxidation (M)** |
|  | 1313 | **459.7272** | **917.4396** | **917.9636** | **-0.5240** | **0** | **12** | **1.4e+02** | **1** | **VSADNTVGR** |
|  | 2223 | **586.4490** | **1756.3248** | **1755.1639** | **1.1609** | **1** | **12** | **1.3e+02** | **1** | **MPCPRPVVFHFLRR** |
|  | 2271 | **593.0018** | **1775.9831** | **1774.9318** | **1.0514** | **1** | **12** | **1.6e+02** | **1** | **GDSSHVVSEGVPRIHAK** |
|  | 2328 | **598.2877** | **1194.5605** | **1195.3486** | **-0.7880** | **0** | **12** | **1.5e+02** | **1** | **GNGLPPLDCPR + Carbamidomethyl (C)** |
|  | 2593 | **638.8772** | **1913.6094** | **1914.2160** | **-0.6065** | **2** | **12** | **1.3e+02** | **1** | **ALDMPFSFSRLCRASR + Carbamidomethyl (C)** |
|  | 2723 | **663.5256** | **1987.5547** | **1987.3474** | **0.2073** | **1** | **12** | **1.3e+02** | **1** | **YLINNRLITAQQWLIK** |
|  | 3272 | **766.7288** | **2297.1641** | **2296.6226** | **0.5415** | **2** | **12** | **1.2e+02** | **1** | **GCSLGAPGTMVKLGNNFAEKGTK + Oxidation (M)** |
|  | 3460 | **827.6628** | **2479.9664** | **2480.9556** | **-0.9893** | **1** | **12** | **1.4e+02** | **1** | **HLCLCQACTEILMRHPVYHR + Carbamidomethyl (C)** |
|  | 3496 | **852.3104** | **1702.6061** | **1702.7995** | **-0.1934** | **0** | **12** | **1.5e+02** | **1** | **TMPGGNDHEIFTDPR + Oxidation (M)** |
|  | 704 | **407.8510** | **1220.5308** | **1220.4046** | **0.1262** | **2** | **12** | **1.6e+02** | **1** | **NAFRRMGQPK + Oxidation (M)** |
|  | 1082 | **436.1062** | **1305.2964** | **1305.5222** | **-0.2258** | **1** | **12** | **1.6e+02** | **1** | **LLKLANDVTYR** |
|  | 2345 | **599.7795** | **1197.5443** | **1198.4340** | **-0.8897** | **1** | **12** | **1.5e+02** | **1** | **MDQMMPGKTK + 2 Oxidation (M)** |
|  | 2686 | **655.0155** | **1962.0243** | **1961.3537** | **0.6707** | **2** | **12** | **1.4e+02** | **1** | **MAAKCADLRMCLDVFK + 2 Carbamidomethyl (C); 2 Oxidation (M)** |
|  | 2856 | **681.2451** | **2040.7130** | **2041.4447** | **-0.7318** | **1** | **12** | **1.6e+02** | **1** | **CVLHFKPCGLFGKELHR + Carbamidomethyl (C)** |
|  | 2860 | **681.6377** | **1361.2606** | **1360.5363** | **0.7244** | **0** | **12** | **1.3e+02** | **1** | **MSTAVLENPGLGR + Oxidation (M)** |
|  | 687 | **407.5752** | **1219.7034** | **1220.3533** | **-0.6498** | **0** | **12** | **1.4e+02** | **1** | **DNTIGVTVNCK + Carbamidomethyl (C)** |
|  | 988 | **429.6423** | **1285.9046** | **1285.4497** | **0.4549** | **1** | **12** | **1.4e+02** | **1** | **NRSLASPLQATK** |
|  | 143 | **370.2415** | **1107.7023** | **1107.3924** | **0.3099** | **0** | **12** | **1.1e+02** | **1** | **GLLLGLLRPR** |
|  | 3386 | **803.8405** | **2408.4992** | **2409.6824** | **-1.1832** | **1** | **12** | **1.6e+02** | **1** | **RMLAQHCVDANNTFCFDNPR + Carbamidomethyl (C)** |
|  | 343 | **387.0052** | **1157.9935** | **1157.3635** | **0.6299** | **1** | **12** | **1.7e+02** | **1** | **QYKCTICNK + Carbamidomethyl (C)** |
|  | 817 | **415.8901** | **829.7655** | **828.9564** | **0.8091** | **1** | **12** | **1.8e+02** | **1** | **AALAAERK** |
|  | 1392 | **466.1848** | **1395.5321** | **1395.5620** | **-0.0299** | **1** | **12** | **1.7e+02** | **1** | **RDCLTQACSALTG + Carbamidomethyl (C)** |
|  | 2673 | **653.4316** | **1957.2728** | **1957.2389** | **0.0338** | **2** | **12** | **1.6e+02** | **1** | **SDRHNLLQACKMQDIK + Carbamidomethyl (C)** |
|  | 3061 | 736.2912 | 1470.5676 | 1471.6483 | -1.0806 | 1 | 12 | 1.5e+02 | 1 | GTRPRGCATGSRPR |
|  | 1384 | **466.0305** | **1395.0692** | **1394.6783** | **0.3909** | **1** | **12** | **1.7e+02** | **1** | **MDGYILSLVQKK** |
|  | 2334 | **599.1351** | **1794.3832** | **1793.2250** | **1.1582** | **2** | **12** | **1.5e+02** | **1** | **LVKLTCVLLRDPIPR + Carbamidomethyl (C)** |
|  | 3048 | **731.8726** | **1461.7303** | **1461.6699** | **0.0605** | **2** | **12** | **1.8e+02** | **1** | **EVIRVWFSNRR** |
|  | 3522 | **880.2655** | **1758.5162** | **1757.9028** | **0.6134** | **2** | **12** | **1.4e+02** | **1** | **QELAASRTARDAALER** |
|  | 889 | **419.4233** | **836.8318** | **835.8564** | **0.9755** | **0** | **12** | **2e+02** | **1** | **ESGSELSK** |
|  | 1854 | **526.1221** | **1575.3442** | **1574.8839** | **0.4603** | **2** | **12** | **1.7e+02** | **1** | **AALAKALKMTDAQVK + Oxidation (M)** |
|  | 2395 | **608.9064** | **1215.7980** | **1216.3844** | **-0.5865** | **1** | **12** | **1.3e+02** | **1** | **LSKDGVDLITR** |
|  | 214 | **377.1664** | **752.3181** | **751.8924** | **0.4257** | **1** | **12** | **1.4e+02** | **1** | **ATSAMKK + Oxidation (M)** |
|  | 894 | **419.9012** | **837.7876** | **836.8709** | **0.9167** | **1** | **12** | **1.4e+02** | **1** | **CRGDDSK + Carbamidomethyl (C)** |
|  | 1816 | **522.7583** | **1043.5018** | **1043.1351** | **0.3667** | **1** | **12** | **1.3e+02** | **1** | **SRDTAPQLR** |
|  | 2581 | **636.0104** | **1905.0090** | **1904.3688** | **0.6402** | **2** | **12** | **1.5e+02** | **1** | **TVAMMVPDRQIIMRVK + Oxidation (M)** |
|  | 2763 | **667.1331** | **1332.2513** | **1331.5398** | **0.7116** | **2** | **12** | **1.6e+02** | **1** | **KTGYSFVNCKK + Carbamidomethyl (C)** |
|  | 688 | **407.6219** | **1219.8435** | **1219.3486** | **0.4949** | **1** | **12** | **1.3e+02** | **1** | **GPYPYNQPKR** |
|  | 726 | **408.1854** | **814.3561** | **813.8987** | **0.4573** | **0** | **12** | **1.6e+02** | **1** | **LTSHTQK** |
|  | 1359 | **462.4969** | **1384.4685** | **1383.4207** | **1.0478** | **1** | **12** | **1.7e+02** | **1** | **NSDSGSSKTFPTR** |
|  | 1532 | **480.1411** | **958.2673** | **958.1116** | **0.1557** | **0** | **12** | **1.7e+02** | **1** | **VWQDGILK** |
|  | 2412 | **610.1682** | **1827.4823** | **1827.9431** | **-0.4608** | **1** | **12** | **1.7e+02** | **1** | **QYKDMMSEGGSPGAEPQ + Oxidation (M)** |
|  | 48 | **363.3497** | **724.6846** | **724.7195** | **-0.0349** | **0** | **12** | **1.6e+02** | **1** | **DYTGNR** |
|  | 432 | **391.1581** | **1170.4520** | **1171.3306** | **-0.8787** | **1** | **12** | **1.5e+02** | **1** | **RGTTVHLECR** |
|  | 2939 | **691.1187** | **2070.3338** | **2071.3981** | **-1.0644** | **1** | **12** | **1.6e+02** | **1** | **VVEILKPLCRSSQSTEPK + Carbamidomethyl (C)** |
|  | 3317 | **781.0540** | **2340.1397** | **2339.4287** | **0.7110** | **0** | **12** | **1.3e+02** | **1** | **QEASDGLITITPTTGSDGHPDAR** |
|  | 1090 | **436.8880** | **871.7612** | **870.9899** | **0.7714** | **0** | **12** | **1.7e+02** | **1** | **VIEAPGASK** |
|  | 1270 | **457.7631** | **1370.2670** | **1369.5891** | **0.6779** | **0** | **12** | **1.3e+02** | **1** | **NFHIFYQICK + Carbamidomethyl (C)** |
|  | 1867 | **527.2617** | **1578.7630** | **1577.8298** | **0.9331** | **2** | **12** | **1.5e+02** | **1** | **VHISRDKITRPGAK** |
|  | 325 | **386.2620** | **1155.7637** | **1155.3526** | **0.4112** | **0** | **12** | **1.1e+02** | **1** | **CFLGCHHPK + 2 Carbamidomethyl (C)** |
|  | 1445 | **472.1855** | **942.3561** | **943.0374** | **-0.6813** | **0** | **12** | **1.6e+02** | **1** | **QPYGCYR + Carbamidomethyl (C)** |
|  | 1646 | **496.2911** | **990.5675** | **991.1848** | **-0.6173** | **1** | **12** | **1.6e+02** | **1** | **VALVNGKYK** |
|  | 2020 | **549.5306** | **1645.5696** | **1644.9175** | **0.6521** | **2** | **12** | **1.4e+02** | **1** | **KMQVNNAKAMSAHAK + Oxidation (M)** |
|  | 3571 | **991.5245** | **2971.5514** | **2972.3942** | **-0.8428** | **1** | **12** | **1.4e+02** | **1** | **VTRAAAAAAAATMALAAPSSPTPESPTMLTK + Oxidation (M)** |
|  | 153 | **371.1512** | **1110.4316** | **1109.2330** | **1.1985** | **1** | **12** | **1.2e+02** | **1** | **TIYADSVKGR** |
|  | 479 | **399.1233** | **796.2319** | **796.8749** | **-0.6430** | **0** | **12** | **1.4e+02** | **1** | **AGSGPRPR** |
|  | 1546 | **482.2434** | **1443.7080** | **1444.6525** | **-0.9446** | **0** | **12** | **1.7e+02** | **1** | **MAGPELLLDSNIR + Oxidation (M)** |
|  | 2157 | **571.6193** | **1711.8358** | **1712.0231** | **-0.1873** | **2** | **12** | **1.9e+02** | **1** | **QEAICLVKGSHKTLK + Carbamidomethyl (C)** |
|  | 2591 | **637.9275** | **1273.8402** | **1272.6430** | **1.1972** | **2** | **12** | **1.4e+02** | **1** | **LGLGKMLTALKK** |
|  | 328 | **386.8123** | **771.6099** | **770.9635** | **0.6465** | **1** | **12** | **1.7e+02** | **1** | **MKACYR** |
|  | 337 | **386.9504** | **1157.8291** | **1157.3239** | **0.5053** | **2** | **12** | **1.8e+02** | **1** | **KEYGCKNCGR** |
|  | 411 | **389.2310** | **1164.6709** | **1165.2995** | **-0.6287** | **2** | **12** | **1.4e+02** | **1** | **IKRTGEGNYK** |
|  | 2066 | **556.2744** | **1110.5340** | **1111.3150** | **-0.7810** | **0** | **12** | **1.7e+02** | **1** | **SHLMLPLER + Oxidation (M)** |
|  | 2377 | **606.7722** | **1817.2945** | **1816.9666** | **0.3278** | **0** | **12** | **1.6e+02** | **1** | **EFVYQEAAGPHQTLAR** |
|  | 2730 | **664.8916** | **1991.6526** | **1991.1215** | **0.5312** | **2** | **12** | **1.4e+02** | **1** | **EYERMKTEAESNSNMR + Oxidation (M)** |
|  | 104 | **369.2250** | **736.4352** | **736.8793** | **-0.4440** | **0** | **12** | **1.4e+02** | **1** | **AFQMPK + Oxidation (M)** |
|  | 574 | **404.1086** | **1209.3035** | **1209.2627** | **0.0407** | **1** | **12** | **1.6e+02** | **1** | **KIEAYAEDDR** |
|  | 1649 | **496.4526** | **990.8905** | **990.0294** | **0.8610** | **2** | **12** | **1.5e+02** | **1** | **RGGTRDELS** |
|  | 1830 | **523.6301** | **1567.8682** | **1567.8053** | **0.0629** | **2** | **12** | **2e+02** | **1** | **TAPSGQKSMLEKFK + Oxidation (M)** |
|  | 2270 | 592.9449 | 1183.8751 | 1183.4027 | 0.4724 | 0 | 12 | 1.5e+02 | 1 | MEPVGCCGECR |
|  | 2863 | **681.8694** | **2042.5860** | **2043.3020** | **-0.7160** | **2** | **12** | **1.6e+02** | **1** | **TPSKLCSGSKSHDVQEVLK** |
|  | 497 | **400.1279** | **1197.3614** | **1198.4340** | **-1.0725** | **0** | **12** | **1.5e+02** | **1** | **QAVMEMMSQK + Oxidation (M)** |
|  | 708 | **407.8719** | **1220.5937** | **1221.4954** | **-0.9017** | **2** | **12** | **1.6e+02** | **1** | **LDMHKKMFR + Oxidation (M)** |
|  | 780 | **412.8622** | **823.7095** | **823.0114** | **0.6981** | **0** | **12** | **1.5e+02** | **1** | **NICVVLY** |
|  | 2550 | **631.2865** | **1260.5582** | **1259.4787** | **1.0795** | **1** | **12** | **1.7e+02** | **1** | **RACQDISVVIR** |
|  | 398 | **389.1602** | **776.3056** | **775.8905** | **0.4150** | **1** | **12** | **1.7e+02** | **1** | **KELSATK** |
|  | 932 | **424.0039** | **845.9931** | **845.9672** | **0.0259** | **1** | **12** | **1.9e+02** | **1** | **MVRGDPR + Oxidation (M)** |
|  | 2789 | **668.9463** | **2003.8167** | **2004.3153** | **-0.4986** | **1** | **12** | **1.3e+02** | **1** | **MQFGKLWCGCSGEFPTR + Carbamidomethyl (C)** |
|  | 620 | **406.0890** | **810.1632** | **809.0133** | **1.1499** | **1** | **12** | **1.6e+02** | **1** | **RMTMVR + Oxidation (M)** |
|  | 683 | 407.5414 | 1219.6022 | 1218.4899 | 1.1123 | 2 | 12 | 1.7e+02 | 1 | MVECKKAQPK + Carbamidomethyl (C) |
|  | 1194 | **450.0180** | **1347.0318** | **1347.4762** | **-0.4443** | **0** | **12** | **1.6e+02** | **1** | **CAMGSYQPDFR + Carbamidomethyl (C); Oxidation (M)** |
|  | 1221 | **451.9131** | **1352.7171** | **1352.6249** | **0.0921** | **1** | **12** | **1.9e+02** | **1** | **GFKNCLEILCR + Carbamidomethyl (C)** |
|  | 2175 | **575.8373** | **1724.4897** | **1724.8728** | **-0.3832** | **1** | **12** | **1.3e+02** | **1** | **DGYISHRELGDCMR + Carbamidomethyl (C); Oxidation (M)** |
|  | 188 | **374.3524** | **746.6901** | **745.9309** | **0.7592** | **1** | **12** | **2e+02** | **1** | **KGVPMAK + Oxidation (M)** |
|  | 453 | **394.0696** | **1179.1868** | **1180.3772** | **-1.1904** | **1** | **12** | **1.8e+02** | **1** | **STMGKPQRLY** |
|  | 836 | **416.4870** | **1246.4388** | **1246.3676** | **0.0713** | **1** | **12** | **2.3e+02** | **1** | **DVTDDVKSIVR** |
|  | 1105 | **437.2917** | **1308.8528** | **1309.4529** | **-0.6001** | **0** | **12** | **1.5e+02** | **1** | **TCQQAECPGGCR + Carbamidomethyl (C)** |
|  | 1257 | **456.4494** | **910.8841** | **911.0538** | **-0.1697** | **0** | **12** | **1.5e+02** | **1** | **IPATVPEGK** |
|  | 2184 | **578.5586** | **1732.6536** | **1733.0655** | **-0.4119** | **2** | **12** | **1.5e+02** | **1** | **INFKDFCRGVFAMK + Carbamidomethyl (C)** |
|  | 379 | **388.2231** | **1161.6473** | **1161.2032** | **0.4440** | **0** | **12** | **1.6e+02** | **1** | **QHNSCTTENK** |
|  | 1166 | **445.1979** | **888.3811** | **889.0745** | **-0.6934** | **1** | **12** | **1.9e+02** | **1** | **LLAKECR + Carbamidomethyl (C)** |
|  | 1282 | **458.4545** | **1372.3412** | **1371.6482** | **0.6930** | **0** | **12** | **1.8e+02** | **1** | **ILVNMAAGQVSIR** |
|  | 1373 | **464.1642** | **1389.4706** | **1390.6052** | **-1.1347** | **1** | **12** | **1.6e+02** | **1** | **YYAVCDPLRYK** |
|  | 2013 | **548.3324** | **1641.9750** | **1640.9502** | **1.0249** | **1** | **12** | **1.6e+02** | **1** | **MGFPYPCPRCALR + 2 Carbamidomethyl (C); Oxidation (M)** |
|  | 2548 | **630.9924** | **1889.9551** | **1890.2540** | **-0.2989** | **1** | **12** | **1.5e+02** | **1** | **MENLKHIITLGQVIHK + Oxidation (M)** |
|  | 1704 | **505.2041** | **1512.5902** | **1513.7180** | **-1.1278** | **2** | **12** | **1.6e+02** | **1** | **EHRGEMEQKIIK + Oxidation (M)** |
|  | 641 | **406.4085** | **1216.2034** | **1216.3246** | **-0.1213** | **1** | **12** | **1.7e+02** | **1** | **NILTGGRGPDGCG** |
|  | 892 | **419.7095** | **1256.1062** | **1256.3259** | **-0.2196** | **1** | **12** | **1.3e+02** | **1** | **APQTGPGTSRER** |
|  | 1360 | **462.6354** | **1384.8840** | **1384.4087** | **0.4753** | **0** | **12** | **1.4e+02** | **1** | **SSAAENPSAGGSPPR** |
|  | 1735 | **510.4586** | **1528.3535** | **1528.8205** | **-0.4670** | **2** | **12** | **1.5e+02** | **1** | **MMGFTCFYKRR + Carbamidomethyl (C); 2 Oxidation (M)** |
|  | 2381 | **607.5114** | **1213.0079** | **1213.4899** | **-0.4820** | **0** | **12** | **1.3e+02** | **1** | **CMVLVDMWK + Carbamidomethyl (C); 2 Oxidation (M)** |
|  | 2971 | **701.9529** | **1401.8910** | **1402.5101** | **-0.6191** | **2** | **12** | **1.3e+02** | **1** | **KKDGVANAEATNGK** |
|  | 41 | **363.1779** | **724.3411** | **723.8624** | **0.4787** | **0** | **12** | **1.4e+02** | **1** | **ACMEVR + Oxidation (M)** |
|  | 826 | **416.1100** | **1245.3077** | **1246.4815** | **-1.1738** | **0** | **12** | **2e+02** | **1** | **CPCSFCLFNR + Carbamidomethyl (C)** |
|  | 1749 | **513.9306** | **1538.7696** | **1539.7769** | **-1.0073** | **2** | **12** | **1.6e+02** | **1** | **ALATRMAQDTCKSK + Oxidation (M)** |
|  | 1957 | **538.4537** | **1074.8926** | **1074.2286** | **0.6639** | **1** | **12** | **1.4e+02** | **1** | **LIKSLETDR** |
|  | 2100 | **562.3462** | **1122.6776** | **1122.4025** | **0.2751** | **2** | **12** | **1.6e+02** | **1** | **KEMAMLQKK + Oxidation (M)** |
|  | 327 | **386.3494** | **770.6841** | **769.9126** | **0.7715** | **0** | **12** | **1.4e+02** | **1** | **HVMEVR** |
|  | 989 | **430.1569** | **1287.4484** | **1287.4458** | **0.0027** | **1** | **12** | **1.9e+02** | **1** | **RLLPDSSSGCPR** |
|  | 2944 | **693.3105** | **2076.9093** | **2077.3298** | **-0.4205** | **2** | **12** | **1.6e+02** | **1** | **MPGRCQSDAAMRVNGPASR + Carbamidomethyl (C); Oxidation (M)** |
|  | 1386 | **466.0611** | **930.1074** | **930.9588** | **-0.8514** | **0** | **12** | **1.8e+02** | **1** | **DNLNEAQK** |
|  | 1696 | **504.8363** | **1511.4868** | **1511.7023** | **-0.2154** | **1** | **12** | **1.4e+02** | **1** | **VHIEMGPDGRVTGK + Oxidation (M)** |
|  | 2335 | **599.1916** | **1196.3684** | **1195.4943** | **0.8741** | **1** | **12** | **1.6e+02** | **1** | **IAAAILKIPSAK** |
|  | 2411 | **610.0980** | **1827.2717** | **1826.0810** | **1.1907** | **0** | **12** | **1.7e+02** | **1** | **IHNNMFDPALIGDKPK + Oxidation (M)** |
|  | 2705 | **658.4299** | **1314.8451** | **1314.3916** | **0.4535** | **1** | **12** | **1.6e+02** | **1** | **GGRMSGQNSGAHR** |
|  | 3172 | **748.2015** | **2241.5824** | **2240.6452** | **0.9372** | **2** | **12** | **1.6e+02** | **1** | **CLGLLEPYFWKKGEACAVR + Carbamidomethyl (C)** |
|  | 1267 | **457.6006** | **1369.7798** | **1370.5543** | **-0.7746** | **2** | **12** | **1.6e+02** | **1** | **EVEKEGAALRLR** |
|  | 2446 | **613.3544** | **1837.0409** | **1837.1040** | **-0.0630** | **0** | **12** | **1.6e+02** | **1** | **MASSASLETMVPPACPR + Carbamidomethyl (C); 2 Oxidation (M)** |
|  | 536 | **402.4863** | **1204.4366** | **1205.3204** | **-0.8838** | **1** | **12** | **2.3e+02** | **1** | **ERDALEAVFR** |
|  | 1720 | **506.9255** | **1517.7543** | **1518.6966** | **-0.9423** | **2** | **12** | **1.7e+02** | **1** | **RRTEMPFPEPSR + Oxidation (M)** |
|  | 678 | **407.4621** | **1219.3642** | **1220.4393** | **-1.0751** | **1** | **12** | **2e+02** | **1** | **ATVLLSCSKGGK + Carbamidomethyl (C)** |
|  | 1076 | **435.7167** | **1304.1280** | **1304.4563** | **-0.3282** | **1** | **12** | **1.3e+02** | **1** | **GYGFRGPGPQLR** |
|  | 2520 | **627.4735** | **1879.3984** | **1880.1531** | **-0.7548** | **2** | **12** | **1.4e+02** | **1** | **LRGNSIGPQGAKALADALK** |
|  | 674 | **407.3216** | **1218.9427** | **1219.4116** | **-0.4689** | **1** | **12** | **1.3e+02** | **1** | **GQVSANMKIQK + Oxidation (M)** |
|  | 3427 | **815.2633** | **1628.5118** | **1628.7788** | **-0.2670** | **1** | **12** | **1.6e+02** | **1** | **NELLSPLKDSDEIR** |
|  | 1668 | **500.0831** | **1497.2270** | **1496.7274** | **0.4997** | **1** | **12** | **1.6e+02** | **1** | **EDTGKTAFVMLLR + Oxidation (M)** |
|  | 1761 | **516.0714** | **1545.1921** | **1544.6261** | **0.5660** | **2** | **12** | **1.8e+02** | **1** | **SRDKGQEGLGSPSAR** |
|  | 2382 | 607.5442 | 1213.0736 | 1212.3363 | 0.7373 | 0 | 12 | 1.3e+02 | 1 | EPVQVSTCGHR |
|  | 2574 | **635.6646** | **1269.3143** | **1269.3195** | **-0.0052** | **0** | **12** | **1.8e+02** | **1** | **GADTQYFGPGTR** |
|  | 23 | **362.2297** | **1083.6669** | **1084.2730** | **-0.6061** | **1** | **12** | **1.3e+02** | **1** | **QLRLAGTIGR** |
|  | 1670 | **500.3549** | **998.6951** | **998.1806** | **0.5144** | **1** | **12** | **1.3e+02** | **1** | **YCRMANVAA** |
|  | 2725 | **663.6249** | **1325.2350** | **1325.5831** | **-0.3482** | **1** | **12** | **1.4e+02** | **1** | **LAAVRCLHSLSR** |
|  | 141 | **370.1639** | **1107.4696** | **1106.3153** | **1.1543** | **1** | **12** | **1.4e+02** | **1** | **EKFAFCSMK + Oxidation (M)** |
|  | 1884 | **530.3521** | **1588.0340** | **1588.8078** | **-0.7738** | **0** | **12** | **1.7e+02** | **1** | **MSVDCLGQHAVLSGR + Oxidation (M)** |
|  | 2075 | **557.5433** | **1669.6076** | **1668.8925** | **0.7152** | **0** | **12** | **1.5e+02** | **1** | **GPMNQCLVATDTHGPK** |
|  | 630 | **406.1711** | **1215.4912** | **1214.3306** | **1.1606** | **1** | **12** | **1.6e+02** | **1** | **ATFYASSVRGR** |
|  | 837 | **416.5379** | **831.0610** | **829.9875** | **1.0734** | **2** | **12** | **2.3e+02** | **1** | **IKSAAGRK** |
|  | 1293 | **458.8314** | **1373.4719** | **1373.5979** | **-0.1259** | **0** | **12** | **1.9e+02** | **1** | **SIFQVFTHLPGK** |
|  | 1471 | **474.7097** | **947.4047** | **947.1506** | **0.2541** | **1** | **12** | **1.5e+02** | **1** | **VVEIEKCK** |
|  | 2247 | **591.5671** | **1181.1195** | **1180.3936** | **0.7258** | **0** | **12** | **1.5e+02** | **1** | **MYYSLGLSCK + Oxidation (M)** |
|  | 551 | **403.7937** | **1208.3589** | **1208.3873** | **-0.0283** | **0** | **12** | **1.8e+02** | **1** | **MAPIPQDPAPR + Oxidation (M)** |
|  | 1640 | **495.0888** | **1482.2442** | **1482.5814** | **-0.3371** | **1** | **12** | **1.8e+02** | **1** | **PRGCDPNNPGGVSGR** |
|  | 3430 | **816.4575** | **2446.3504** | **2445.7708** | **0.5795** | **2** | **12** | **1.7e+02** | **1** | **APKLLISGASTLDSWVPSRFSGR** |
|  | 512 | **401.3071** | **800.5994** | **800.9033** | **-0.3039** | **1** | **12** | **1.5e+02** | **1** | **KSPLSNR** |
|  | 1352 | **462.1270** | **1383.3589** | **1383.6357** | **-0.2769** | **2** | **12** | **1.6e+02** | **1** | **NLIRKIPDSISK** |
|  | 1520 | **478.2623** | **1431.7646** | **1430.6506** | **1.1140** | **1** | **12** | **1.5e+02** | **1** | **LLNHLNSEKLIH** |
|  | 789 | **413.4679** | **1237.3815** | **1238.5025** | **-1.1210** | **1** | **12** | **1.8e+02** | **1** | **QLMHEALKLR** |
|  | 843 | **417.1864** | **1248.5371** | **1247.5476** | **0.9895** | **1** | **12** | **1.9e+02** | **1** | **CLKMLDMSFK + 2 Oxidation (M)** |
|  | 1110 | **437.5712** | **1309.6915** | **1309.4299** | **0.2617** | **1** | **12** | **2.1e+02** | **1** | **GQCPSESSCRQK** |
|  | 1982 | **541.9488** | **1622.8242** | **1621.7513** | **1.0729** | **0** | **12** | **1.7e+02** | **1** | **YVGQNLSIHSGQYR** |
|  | 2915 | **686.2972** | **2055.8695** | **2056.2576** | **-0.3880** | **0** | **12** | **1.7e+02** | **1** | **GVQCQEQLVESGGGVVQPGK + Carbamidomethyl (C)** |
|  | 2658 | 650.5939 | 1948.7594 | 1948.3597 | 0.3998 | 0 | 12 | 1.4e+02 | 1 | VGPLRPLPLGMGAQGCLPR + Oxidation (M) |
|  | 1469 | **474.3819** | **1420.1235** | **1420.5948** | **-0.4713** | **1** | **12** | **1.5e+02** | **1** | **TGLGVGGSTMAAARR + Oxidation (M)** |
|  | 2374 | **606.4437** | **1210.8727** | **1211.3282** | **-0.4555** | **0** | **12** | **1.4e+02** | **1** | **DQNIVGNARPK** |
|  | 2572 | **634.6831** | **1901.0271** | **1901.2719** | **-0.2447** | **1** | **12** | **1.9e+02** | **1** | **LGVLNKITLGDSIMKPVS + Oxidation (M)** |
|  | 2836 | **678.6077** | **1355.2006** | **1355.4700** | **-0.2695** | **0** | **12** | **1.4e+02** | **1** | **LAYESDGIVASCQ** |
|  | 131 | **369.3596** | **1105.0568** | **1106.2422** | **-1.1855** | **2** | **12** | **1.9e+02** | **1** | **SLPRGRAHGR** |
|  | 952 | **428.0544** | **1281.1409** | **1281.3949** | **-0.2540** | **1** | **12** | **1.7e+02** | **1** | **GATISWDTRQM + Oxidation (M)** |
|  | 1591 | **488.1849** | **1461.5325** | **1461.6201** | **-0.0876** | **0** | **12** | **1.9e+02** | **1** | **AGHSLDDMLLDCR + Oxidation (M)** |
|  | 2052 | **553.8219** | **1658.4435** | **1658.9473** | **-0.5037** | **0** | **12** | **1.5e+02** | **1** | **HVIPGHMACSMACGGR + 2 Oxidation (M)** |
|  | 2259 | **592.6735** | **1183.3321** | **1182.2887** | **1.0435** | **0** | **12** | **2.1e+02** | **1** | **MNCNTQSQTR** |
|  | 1619 | **491.8445** | **981.6743** | **982.1549** | **-0.4806** | **0** | **12** | **1.5e+02** | **1** | **MQQSVYVK** |
|  | 2278 | **593.2550** | **1776.7428** | **1777.2039** | **-0.4611** | **2** | **12** | **1.8e+02** | **1** | **MKIWQVLCLARWLT + Oxidation (M)** |
|  | 2828 | **675.2644** | **2022.7710** | **2022.3441** | **0.4270** | **2** | **12** | **1.8e+02** | **1** | **ILQPMLDSSCSETPKTKK + Oxidation (M)** |
|  | 906 | **421.1654** | **1260.4740** | **1261.4283** | **-0.9544** | **0** | **12** | **1.7e+02** | **1** | **LECSGAVSAHCK + Carbamidomethyl (C)** |
|  | 3567 | **972.4545** | **2914.3412** | **2913.2206** | **1.1206** | **1** | **12** | **1.5e+02** | **1** | **MASLLQDLSISDVETIRNGHDSELLR** |
|  | 433 | **391.1689** | **780.3229** | **779.8377** | **0.4853** | **0** | **12** | **1.6e+02** | **1** | **DSGGGFIK** |
|  | 677 | **407.4366** | **1219.2876** | **1220.4196** | **-1.1320** | **0** | **12** | **2e+02** | **1** | **TCPSRPSCLEK** |
|  | 701 | **407.7795** | **1220.3165** | **1221.3363** | **-1.0199** | **0** | **12** | **1.8e+02** | **1** | **SSYYMIGEQK + Oxidation (M)** |
|  | 1219 | **451.8419** | **1352.5036** | **1351.4617** | **1.0419** | **0** | **12** | **2e+02** | **1** | **AVSANYSTGSPAVK** |
|  | 2078 | **557.6306** | **1113.2464** | **1114.2132** | **-0.9667** | **1** | **12** | **2.1e+02** | **1** | **AEATGEKRPR** |
|  | 2299 | **595.5109** | **1783.5106** | **1782.9685** | **0.5421** | **1** | **12** | **1.5e+02** | **1** | **DDAKNTLYLEMNSLR** |
|  | 119 | **369.2930** | **1104.8567** | **1104.2680** | **0.5888** | **2** | **12** | **1.5e+02** | **1** | **HRSDRMMR + Oxidation (M)** |
|  | 1142 | **443.5067** | **1327.4981** | **1326.4586** | **1.0395** | **0** | **12** | **2.1e+02** | **1** | **CDVCGQLFNDR + Carbamidomethyl (C)** |
|  | 2610 | **642.5396** | **1924.5967** | **1925.1214** | **-0.5247** | **1** | **12** | **1.3e+02** | **1** | **ENEKAPGPTTAVSYMSVK + Oxidation (M)** |
|  | 525 | **401.9716** | **1202.8928** | **1203.3938** | **-0.5011** | **1** | **12** | **2e+02** | **1** | **RLLALSHEHK** |
|  | 1757 | **515.6115** | **1029.2082** | **1029.1286** | **0.0797** | **0** | **12** | **2.2e+02** | **1** | **MPVSTEGHR + Oxidation (M)** |
|  | 12 | **360.5217** | **719.0287** | **719.7858** | **-0.7571** | **0** | **12** | **1.7e+02** | **1** | **NPFNTK** |
|  | 1145 | **443.8002** | **1328.3783** | **1327.4417** | **0.9366** | **1** | **12** | **1.7e+02** | **1** | **ESLFQYERQK** |
|  | 1969 | **540.3662** | **1078.7176** | **1078.2802** | **0.4374** | **0** | **12** | **1.5e+02** | **1** | **LITMTINEK + Oxidation (M)** |
|  | 2060 | **555.2238** | **1662.6491** | **1661.8139** | **0.8352** | **0** | **12** | **1.8e+02** | **1** | **TPGNPATAVSGTPAPPAR** |
|  | 2314 | **596.8705** | **1191.7262** | **1191.2740** | **0.4522** | **0** | **12** | **1.6e+02** | **1** | **QEGHSCVEFR** |
|  | 2441 | **613.1400** | **1836.3979** | **1837.0621** | **-0.6642** | **0** | **12** | **1.7e+02** | **1** | **LTESYIQLGLQCAGGAGR** |
|  | 3053 | **733.3972** | **2197.1695** | **2197.5927** | **-0.4232** | **0** | **12** | **1.7e+02** | **1** | **MYICPFMGAVSGTLTVTDFK + Oxidation (M)** |
|  | 697 | **407.7701** | **1220.2882** | **1220.3847** | **-0.0965** | **1** | **12** | **1.8e+02** | **1** | **GTLGRPPPGRGR** |
|  | 1063 | **434.9324** | **867.8500** | **867.0029** | **0.8472** | **0** | **12** | **1.5e+02** | **1** | **CQVADMK + Carbamidomethyl (C); Oxidation (M)** |
|  | 1667 | **500.0642** | **1497.1705** | **1497.8477** | **-0.6773** | **1** | **12** | **1.7e+02** | **1** | **NVCRALVILLEVR** |
|  | 2168 | **573.1156** | **1716.3246** | **1716.8326** | **-0.5079** | **2** | **12** | **1.8e+02** | **1** | **GGRGSCGGSKGDCGSCGGSK** |
|  | 2177 | **576.1252** | **1150.2357** | **1151.1900** | **-0.9543** | **1** | **12** | **1.8e+02** | **1** | **GGIGGRGSGYDR** |
|  | 2992 | **710.7471** | **1419.4794** | **1420.5849** | **-1.1056** | **0** | **12** | **1.9e+02** | **1** | **AVSDLGTVEIECK + Carbamidomethyl (C)** |
|  | 3442 | **817.7729** | **1633.5311** | **1633.8962** | **-0.3651** | **2** | **12** | **1.4e+02** | **1** | **GQVLNIQARRTLHK** |
|  | 232 | **378.2168** | **1131.6283** | **1132.2715** | **-0.6432** | **0** | **12** | **1.2e+02** | **1** | **MPGESTVCHR + Oxidation (M)** |
|  | 1460 | **473.4241** | **1417.2502** | **1417.5526** | **-0.3024** | **1** | **12** | **1.7e+02** | **1** | **HYNGGVGRCAQAK + Carbamidomethyl (C)** |
|  | 2479 | **617.2583** | **1232.5018** | **1231.3559** | **1.1459** | **0** | **12** | **1.9e+02** | **1** | **NELSGALTGLTR** |
|  | 769 | **411.3066** | **1230.8977** | **1230.4624** | **0.4353** | **1** | **12** | **1.5e+02** | **1** | **AAKCSCCMGAR + 2 Carbamidomethyl (C); Oxidation (M)** |
|  | 916 | **422.0743** | **842.1339** | **841.0316** | **1.1023** | **0** | **12** | **1.9e+02** | **1** | **CCTLGCNK** |
|  | 1368 | **463.9795** | **925.9442** | **926.0669** | **-0.1227** | **0** | **12** | **1.6e+02** | **1** | **AMVDMSEK + Oxidation (M)** |
|  | 1248 | **455.2015** | **1362.5823** | **1361.4978** | **1.0844** | **0** | **12** | **1.7e+02** | **1** | **STVTVNTIDLGNK** |
|  | 2167 | **573.0419** | **1716.1034** | **1715.8463** | **0.2572** | **2** | **12** | **1.9e+02** | **1** | **GRAGSDGARGMPGQTGPK + Oxidation (M)** |
|  | 2332 | **598.8726** | **1793.5957** | **1793.0697** | **0.5260** | **0** | **12** | **1.4e+02** | **1** | **VATTGWTMDPQEMVVK** |
|  | 2435 | **612.5488** | **1223.0829** | **1222.3276** | **0.7553** | **0** | **12** | **1.4e+02** | **1** | **FLPCENGGGSGGK** |
|  | 2739 | **666.5337** | **1996.5789** | **1997.2599** | **-0.6810** | **0** | **12** | **1.4e+02** | **1** | **YKPVCNQVECHPYFNR** |
|  | 3278 | **770.4568** | **2308.3482** | **2309.5336** | **-1.1854** | **2** | **12** | **1.7e+02** | **1** | **GLEWVSSIVGSGGRKNYADSVK** |
|  | 1380 | **465.4702** | **928.9256** | **929.1419** | **-0.2162** | **1** | **12** | **2e+02** | **1** | **MPVLSARR** |
|  | 2868 | **682.4083** | **2044.2028** | **2044.1178** | **0.0851** | **2** | **12** | **1.7e+02** | **1** | **TKSWGEQCSETSGTDSGRK** |
|  | 3542 | **909.0052** | **2723.9936** | **2724.1192** | **-0.1256** | **1** | **12** | **1.9e+02** | **1** | **WVDPTALCEELLLPLENPCQGRAR** |
|  | 3554 | **946.6649** | **2836.9726** | **2836.2243** | **0.7482** | **1** | **12** | **1.6e+02** | **1** | **QEVYCNIPDATSWSFPNGVLIKVVR** |
|  | 478 | **399.1162** | **796.2176** | **795.8867** | **0.3308** | **0** | **12** | **1.6e+02** | **1** | **SHGAGLVR** |
|  | 691 | **407.6647** | **1219.9719** | **1220.4012** | **-0.4292** | **1** | **12** | **1.4e+02** | **1** | **MDSLALGRWR + Oxidation (M)** |
|  | 821 | **415.9107** | **829.8066** | **829.9444** | **-0.1378** | **1** | **12** | **2.1e+02** | **1** | **NKTALQR** |
|  | 1664 | 499.9474 | 1496.8202 | 1495.6383 | 1.1819 | 0 | 12 | 1.7e+02 | 1 | VTAVTGPDFPAGAHR |
|  | 2892 | **684.9299** | **1367.8450** | **1367.6564** | **0.1886** | **1** | **12** | **1.6e+02** | **1** | **VKLSFSMSLLSR** |
|  | 373 | **388.1338** | **1161.3792** | **1160.3478** | **1.0314** | **2** | **12** | **2.1e+02** | **1** | **RVNECKVQK + Carbamidomethyl (C)** |
|  | 382 | **388.2809** | **774.5471** | **774.9721** | **-0.4250** | **1** | **12** | **1.7e+02** | **1** | **AKVACVK + Carbamidomethyl (C)** |
|  | 2169 | **573.6082** | **1717.8023** | **1717.9018** | **-0.0995** | **0** | **12** | **2.2e+02** | **1** | **CPPSPAGGPVPGPWADGR** |
|  | 2251 | **592.2869** | **1773.8384** | **1773.0301** | **0.8084** | **1** | **12** | **1.8e+02** | **1** | **AMVGHPGAQSRVGCCAR + Carbamidomethyl (C); Oxidation (M)** |
|  | 2480 | **617.4906** | **1849.4496** | **1849.9998** | **-0.5502** | **2** | **12** | **1.6e+02** | **1** | **KYHQPQRASGSSYGGVK** |
|  | 2827 | **675.1849** | **1348.3551** | **1347.5343** | **0.8208** | **1** | **12** | **1.8e+02** | **1** | **KEDVGMVVGIDLG + Oxidation (M)** |
|  | 170 | **373.0018** | **743.9888** | **742.9499** | **1.0388** | **1** | **12** | **2.2e+02** | **1** | **LITIRK** |
|  | 419 | **389.3921** | **1165.1540** | **1165.6136** | **-0.4596** | **0** | **12** | **2.2e+02** | **1** | **DAXXXXXYDK** |
|  | 1087 | **436.3384** | **1305.9930** | **1305.5040** | **0.4889** | **0** | **12** | **1.4e+02** | **1** | **SCMCTAGYSLR + 2 Carbamidomethyl (C)** |
|  | 2040 | **551.8152** | **1652.4234** | **1652.7176** | **-0.2942** | **0** | **12** | **1.5e+02** | **1** | **QNSDPTSENPPLPTR** |
|  | 2071 | **556.8385** | **1667.4933** | **1668.0368** | **-0.5434** | **2** | **12** | **1.5e+02** | **1** | **MRHFLWMLKDFK + Oxidation (M)** |
|  | 2962 | **698.2089** | **1394.4029** | **1393.6338** | **0.7691** | **1** | **12** | **1.7e+02** | **1** | **CYSCGKLGHIQK + Carbamidomethyl (C)** |
|  | 2220 | **585.6703** | **1169.3258** | **1169.3313** | **-0.0055** | **1** | **12** | **2.1e+02** | **1** | **GGSASRSLPLPK** |
|  | 850 | **417.9384** | **1250.7932** | **1250.5101** | **0.2831** | **0** | **12** | **1.9e+02** | **1** | **CPMSCYYIVR + Oxidation (M)** |
|  | 1128 | **441.2032** | **1320.5874** | **1321.4605** | **-0.8731** | **2** | **12** | **1.8e+02** | **1** | **RQKGGESIMDGK + Oxidation (M)** |
|  | 2307 | **596.2891** | **1190.5633** | **1189.3840** | **1.1794** | **1** | **12** | **1.9e+02** | **1** | **SMFQRITYK + Oxidation (M)** |
|  | 2751 | **666.6895** | **1997.0462** | **1996.1560** | **0.8902** | **1** | **12** | **1.9e+02** | **1** | **DLAPKCDVSFLQSEDGSGK** |
|  | 3313 | 778.1982 | 2331.5726 | 2330.5480 | 1.0245 | 2 | 12 | 1.7e+02 | 1 | EEISAETPLKTARVEATLSER |
|  | 1739 | **511.3462** | **1531.0164** | **1530.7916** | **0.2248** | **1** | **12** | **1.5e+02** | **1** | **LPCRCCVPSEGGPV + 2 Carbamidomethyl (C)** |
|  | 2161 | **571.9511** | **1141.8873** | **1142.2662** | **-0.3788** | **2** | **12** | **1.8e+02** | **1** | **KNGAAKQSNPK** |
|  | 2790 | **669.0258** | **2004.0551** | **2004.3782** | **-0.3231** | **0** | **12** | **1.6e+02** | **1** | **YVAICSPLHYPVIMNQR** |
|  | 3029 | **723.1508** | **2166.4301** | **2167.4639** | **-1.0338** | **2** | **12** | **1.8e+02** | **1** | **ESSALTKHKIIHTGEKPYK** |
|  | 1075 | **435.7103** | **1304.1088** | **1304.4301** | **-0.3212** | **1** | **12** | **1.4e+02** | **1** | **AESCGHATVSSKK** |
|  | 1660 | 499.4523 | 1495.3347 | 1495.7392 | -0.4045 | 0 | 12 | 1.4e+02 | 1 | TTDPMLALSVYLR + Oxidation (M) |
|  | 1733 | **509.4613** | **1525.3617** | **1525.7055** | **-0.3438** | **1** | **12** | **1.7e+02** | **1** | **LRLEASTSDPLPAR** |
|  | 1935 | **536.4972** | **1070.9796** | **1071.1437** | **-0.1641** | **0** | **12** | **1.6e+02** | **1** | **GSMGTSGEACR + Oxidation (M)** |
|  | 3373 | **800.5090** | **2398.5047** | **2397.7178** | **0.7869** | **1** | **12** | **1.7e+02** | **1** | **NEGCLHMTCAKCNHGFCWR + 3 Carbamidomethyl (C); Oxidation (M)** |
|  | 290 | **385.1059** | **768.1970** | **767.8337** | **0.3633** | **1** | **12** | **1.6e+02** | **1** | **RGPPGER** |
|  | 1430 | **470.7804** | **1409.3191** | **1409.4762** | **-0.1571** | **0** | **12** | **1.4e+02** | **1** | **LEMDNDSTVNQK + Oxidation (M)** |
|  | 2225 | **586.5385** | **1756.5932** | **1756.8719** | **-0.2787** | **2** | **12** | **1.5e+02** | **1** | **TEPSGRRGSDELTVPR** |
|  | 2668 | **652.2624** | **1953.7650** | **1953.0747** | **0.6903** | **1** | **12** | **1.8e+02** | **1** | **DQPLSESLNHSSQIRNK** |
|  | 2684 | **654.6845** | **1961.0314** | **1960.3025** | **0.7288** | **0** | **12** | **2e+02** | **1** | **CVEAAVMTGLALNCHINK + Carbamidomethyl (C); Oxidation (M)** |
|  | 3298 | **777.3555** | **1552.6962** | **1552.6894** | **0.0067** | **1** | **12** | **1.8e+02** | **1** | **RFSLAEFAQEQAR** |
|  | 774 | **411.4215** | **1231.2424** | **1231.3344** | **-0.0921** | **0** | **12** | **2.2e+02** | **1** | **ADDTGLYFCAR** |
|  | 1785 | **519.0766** | **1554.2076** | **1554.8560** | **-0.6484** | **1** | **12** | **1.8e+02** | **1** | **CYVHIMKEGLCSR + Oxidation (M)** |
|  | 1900 | **532.6760** | **1595.0057** | **1594.8704** | **0.1353** | **0** | **12** | **2.1e+02** | **1** | **LMNVDPMYSMYAK + 2 Oxidation (M)** |
|  | 2888 | **684.1941** | **2049.5601** | **2049.3310** | **0.2291** | **0** | **12** | **1.8e+02** | **1** | **LCPDGFYGLSCQAPCTCDR** |
|  | 39 | **363.1470** | **1086.4187** | **1086.3255** | **0.0933** | **0** | **12** | **1.7e+02** | **1** | **FIACLMSTK + Carbamidomethyl (C); Oxidation (M)** |
|  | 461 | **396.0630** | **790.1113** | **788.9554** | **1.1559** | **0** | **12** | **2.1e+02** | **1** | **ALLDVCR** |
|  | 1062 | **434.9290** | **1301.7649** | **1302.4771** | **-0.7122** | **1** | **12** | **1.6e+02** | **1** | **SMGGFMEDLRK + 2 Oxidation (M)** |
|  | 2802 | **670.7800** | **2009.3179** | **2010.4142** | **-1.0963** | **2** | **12** | **2.1e+02** | **1** | **HHCRACGKVFCASCCSLK + Carbamidomethyl (C)** |
|  | 197 | **375.1976** | **1122.5705** | **1123.4301** | **-0.8596** | **1** | **12** | **1.7e+02** | **1** | **LSKCALLMTK + Oxidation (M)** |
|  | 1224 | **452.0253** | **1353.0536** | **1353.5138** | **-0.4601** | **2** | **12** | **2.2e+02** | **1** | **GGAGPNARGPGRMR** |
|  | 1439 | **471.7175** | **941.4202** | **941.0631** | **0.3571** | **0** | **12** | **1.4e+02** | **1** | **SMSFQGIR + Oxidation (M)** |
|  | 1700 | **504.9538** | **1511.8392** | **1511.7652** | **0.0741** | **1** | **12** | **1.8e+02** | **1** | **KPIEDVLLSSVRR** |
|  | 2505 | **622.8173** | **1865.4298** | **1865.1154** | **0.3143** | **0** | **12** | **1.7e+02** | **1** | **HGAGVAMDGQDVQLPLLK + Oxidation (M)** |
|  | 1633 | **494.3896** | **986.7644** | **986.0423** | **0.7221** | **0** | **12** | **1.5e+02** | **1** | **NGGAHLDFR** |
|  | 1743 | **512.4066** | **1534.1975** | **1534.6447** | **-0.4472** | **1** | **12** | **1.4e+02** | **1** | **AEPSEVDMNSPKSK + Oxidation (M)** |
|  | 1168 | **445.4281** | **888.8414** | **887.9806** | **0.8608** | **1** | **12** | **2.1e+02** | **1** | **RSSEGCCF** |
|  | 1583 | **488.0159** | **974.0171** | **975.0413** | **-1.0242** | **0** | **12** | **2.1e+02** | **1** | **QRPNSCDR** |
|  | 2674 | **653.4742** | **1957.4006** | **1957.1526** | **0.2479** | **1** | **12** | **1.7e+02** | **1** | **ASQGIGNSLAWFQQKHGK** |
|  | 3107 | **740.6857** | **1479.3567** | **1478.5994** | **0.7572** | **0** | **12** | **1.5e+02** | **1** | **NSETFPTILEEAK** |
|  | 546 | **403.1293** | **1206.3657** | **1205.3900** | **0.9758** | **1** | **12** | **2e+02** | **1** | **MRHQSIYVR + Oxidation (M)** |
|  | 580 | **404.6911** | **1211.0510** | **1210.4445** | **0.6065** | **0** | **12** | **1.4e+02** | **1** | **VIAYSVHYMK** |
|  | 3484 | **845.4498** | **1688.8848** | **1689.8836** | **-0.9988** | **0** | **12** | **1.8e+02** | **1** | **VMFQSTHILPDEEK + Oxidation (M)** |
|  | 276 | **384.9496** | **1151.8266** | **1152.3208** | **-0.4942** | **1** | **12** | **1.6e+02** | **1** | **ENMTSPAKFK** |
|  | 725 | **408.1775** | **1221.5103** | **1221.4291** | **0.0812** | **0** | **12** | **1.9e+02** | **1** | **VPLPSGPMNPGR** |
|  | 848 | **417.6711** | **1249.9913** | **1250.5531** | **-0.5618** | **1** | **12** | **1.6e+02** | **1** | **GLMEPLLPPKR** |
|  | 1154 | **443.9549** | **1328.8425** | **1329.4545** | **-0.6120** | **0** | **12** | **2e+02** | **1** | **EDGNSISSMMVK + 2 Oxidation (M)** |
|  | 1455 | **472.7035** | **943.3922** | **943.1435** | **0.2487** | **0** | **12** | **1.5e+02** | **1** | **LLGPPPPPR** |
|  | 1927 | **536.1879** | **1070.3610** | **1070.2897** | **0.0713** | **0** | **12** | **1.9e+02** | **1** | **IVCCVPGHSR** |
|  | 1997 | **544.4381** | **1086.8614** | **1086.2426** | **0.6188** | **1** | **12** | **1.7e+02** | **1** | **EGALKLAATGR** |
|  | 2618 | **643.5354** | **1927.5840** | **1928.3237** | **-0.7397** | **2** | **12** | **1.5e+02** | **1** | **EKLSAMQAHLAGKMALTK** |
|  | 2814 | **672.2979** | **1342.5809** | **1341.4699** | **1.1110** | **0** | **12** | **1.8e+02** | **1** | **TAEGNPTGGLVGLR** |
|  | 782 | **413.1389** | **1236.3946** | **1236.3993** | **-0.0047** | **1** | **12** | **1.7e+02** | **1** | **VSTVMDTVGRR + Oxidation (M)** |
|  | 915 | **421.9666** | **841.9184** | **841.9122** | **0.0062** | **0** | **12** | **1.9e+02** | **1** | **SGAAAGVGPR** |
|  | 3329 | **788.7491** | **2363.2251** | **2362.4951** | **0.7299** | **1** | **12** | **1.4e+02** | **1** | **SAAGGRAAPDLTPGPPDSGCGTGHR + Carbamidomethyl (C)** |
|  | 3508 | **859.1458** | **2574.4153** | **2573.8985** | **0.5168** | **2** | **12** | **1.4e+02** | **1** | **GQMLKFSSMAPDLDRLNELGYR + 2 Oxidation (M)** |
|  | 1238 | **453.6621** | **1357.9642** | **1357.6646** | **0.2995** | **2** | **12** | **1.6e+02** | **1** | **RADIICGKLLAK + Carbamidomethyl (C)** |
|  | 2294 | **594.8457** | **1781.5149** | **1781.1681** | **0.3469** | **2** | **12** | **1.6e+02** | **1** | **RKTLVSMPPLPGLDLK + Oxidation (M)** |
|  | 2862 | **681.8348** | **2042.4823** | **2043.3763** | **-0.8939** | **2** | **12** | **2.1e+02** | **1** | **KGTCAMASHPAGGLLPFRR + Carbamidomethyl (C); Oxidation (M)** |
|  | 16 | **361.9766** | **1082.9076** | **1082.2160** | **0.6916** | **2** | **12** | **1.8e+02** | **1** | **GKERGCCSSR** |
|  | 714 | **407.9981** | **813.9814** | **812.9538** | **1.0277** | **0** | **12** | **1.9e+02** | **1** | **IIPTVDR** |
|  | 1966 | **540.1243** | **1078.2338** | **1078.2404** | **-0.0067** | **0** | **12** | **1.9e+02** | **1** | **AQTMGNSLLK + Oxidation (M)** |
|  | 669 | **407.2060** | **1218.5959** | **1218.3176** | **0.2783** | **1** | **12** | **1.6e+02** | **1** | **TLVEQRSGGSGK** |
|  | 896 | **420.0833** | **838.1518** | **838.9299** | **-0.7780** | **1** | **12** | **1.8e+02** | **1** | **RSSATCSK** |
|  | 1908 | **533.9427** | **1598.8061** | **1597.8064** | **0.9997** | **0** | **12** | **1.8e+02** | **1** | **STENVEVVFGLYLK** |
|  | 3466 | **828.8051** | **2483.3932** | **2483.6895** | **-0.2963** | **0** | **12** | **1.5e+02** | **1** | **ACLNSTYEYIFNNCHDLYSR + Carbamidomethyl (C)** |
|  | 1581 | **487.9384** | **1460.7932** | **1460.6984** | **0.0947** | **1** | **12** | **2.2e+02** | **1** | **TIEALMDRGAIVR + Oxidation (M)** |
|  | 3352 | **794.2810** | **1586.5472** | **1585.8105** | **0.7368** | **1** | **12** | **1.8e+02** | **1** | **QMGGHLRMVEANSR** |
|  | 3375 | **800.8300** | **1599.6451** | **1600.7706** | **-1.1254** | **1** | **12** | **1.8e+02** | **1** | **NCKGITSDGTATMTGK + Oxidation (M)** |
|  | 368 | **388.0348** | **774.0549** | **772.9529** | **1.1020** | **0** | **12** | **2.3e+02** | **1** | **AVPDMLK** |
|  | 434 | **391.1923** | **1170.5546** | **1169.4207** | **1.1339** | **2** | **12** | **1.5e+02** | **1** | **TKIASIRKPR** |
|  | 1876 | **529.0791** | **1584.2151** | **1584.7909** | **-0.5757** | **0** | **12** | **1.9e+02** | **1** | **MILEVGENNGFFSK** |
|  | 3161 | **744.7667** | **1487.5185** | **1487.7008** | **-0.1822** | **1** | **12** | **1.9e+02** | **1** | **STVPSVSSISRVLR** |
|  | 300 | **385.5251** | **1153.5531** | **1154.3863** | **-0.8332** | **0** | **11** | **1.6e+02** | **1** | **MGVAARPPALR + Oxidation (M)** |
|  | 531 | **402.1179** | **1203.3315** | **1202.2470** | **1.0845** | **0** | **11** | **2.2e+02** | **1** | **WDAMEYDEK + Oxidation (M)** |
|  | 3311 | **778.1420** | **2331.4039** | **2331.5096** | **-0.1057** | **0** | **11** | **1.5e+02** | **1** | **GESLMGEVATDWMDTECPEAK + 2 Oxidation (M)** |
|  | 1174 | **446.1733** | **1335.4978** | **1336.5165** | **-1.0186** | **0** | **11** | **2.1e+02** | **1** | **PHSPLGSMPEIR + Oxidation (M)** |
|  | 1199 | **450.2228** | **1347.6463** | **1347.6466** | **-0.0003** | **0** | **11** | **1.9e+02** | **1** | **AAALALGAAAGCMLK + Oxidation (M)** |
|  | 1451 | **472.4429** | **942.8711** | **942.0760** | **0.7951** | **1** | **11** | **1.7e+02** | **1** | **AVPWTRGR** |
|  | 1487 | **475.8215** | **1424.4423** | **1423.6765** | **0.7658** | **1** | **11** | **1.9e+02** | **1** | **MEIAEKHLDIPK** |
|  | 1682 | 503.5047 | 1507.4918 | 1507.7086 | -0.2168 | 2 | 11 | 2.2e+02 | 1 | EAVNAKCSSLEKTK |
|  | 2906 | **686.1128** | **2055.3162** | **2055.2792** | **0.0370** | **2** | **11** | **1.8e+02** | **1** | **QSRKAANVLGGACGLQPGDR + Carbamidomethyl (C)** |
|  | 602 | **405.2630** | **1212.7669** | **1213.4731** | **-0.7062** | **0** | **11** | **1.4e+02** | **1** | **LQCPVGPIQCR** |
|  | 930 | **423.6150** | **1267.8227** | **1268.4556** | **-0.6329** | **0** | **11** | **1.9e+02** | **1** | **VDLLVNLPLDTG** |
|  | 1192 | **449.7277** | **1346.1610** | **1345.4986** | **0.6624** | **0** | **11** | **1.5e+02** | **1** | **AGVNTVTTLVENK** |
|  | 1869 | **527.4259** | **1052.8370** | **1053.2177** | **-0.3806** | **1** | **11** | **1.4e+02** | **1** | **AFAKNPHLR** |
|  | 330 | **386.8665** | **1157.5774** | **1157.3206** | **0.2568** | **1** | **11** | **2.1e+02** | **1** | **DLNSRNVLVK** |
|  | 810 | **414.8762** | **827.7376** | **828.9367** | **-1.1991** | **1** | **11** | **1.8e+02** | **1** | **EHEMKR** |
|  | 1303 | **459.1713** | **1374.4918** | **1373.6474** | **0.8444** | **1** | **11** | **2.1e+02** | **1** | **LHPFHVIRINK** |
|  | 1709 | **505.6202** | **1513.8385** | **1514.7623** | **-0.9237** | **2** | **11** | **2.1e+02** | **1** | **FFFSKIKEAGLID** |
|  | 2796 | **669.8796** | **1337.7444** | **1338.5322** | **-0.7879** | **0** | **11** | **1.7e+02** | **1** | **QGLLQTGYVTCR** |
|  | 2981 | **705.9443** | **1409.8738** | **1409.5077** | **0.3661** | **2** | **11** | **1.6e+02** | **1** | **HPSSRASAKPRSE** |
|  | 3582 | **1017.7354** | **3050.1839** | **3049.4780** | **0.7059** | **2** | **11** | **1.6e+02** | **1** | **VLLDKSNEPSVRQQLLQGYDMLMNPK + 2 Oxidation (M)** |
|  | 1164 | **445.0364** | **1332.0870** | **1331.5116** | **0.5754** | **1** | **11** | **2.3e+02** | **1** | **EISETEAKALLK** |
|  | 2048 | **552.9952** | **1103.9757** | **1104.3457** | **-0.3700** | **1** | **11** | **2.1e+02** | **1** | **AGLVLPPPKGR** |
|  | 3415 | **810.6616** | **1619.3085** | **1618.8554** | **0.4531** | **2** | **11** | **1.6e+02** | **1** | **NDVMIRKEAYVHK + Oxidation (M)** |
|  | 626 | **406.1390** | **1215.3949** | **1214.3303** | **1.0645** | **1** | **11** | **1.8e+02** | **1** | **LGSRHWSLTTG** |
|  | 2045 | **552.1560** | **1102.2972** | **1102.2456** | **0.0517** | **2** | **11** | **2.1e+02** | **1** | **RKTTASPSVR** |
|  | 2311 | **596.6923** | **1787.0548** | **1786.1893** | **0.8654** | **2** | **11** | **2.5e+02** | **1** | **MKVVNLKQAILQAWK + Oxidation (M)** |
|  | 506 | **400.3220** | **1197.9439** | **1198.4554** | **-0.5115** | **2** | **11** | **1.4e+02** | **1** | **VRKDDLVLLK** |
|  | 1135 | **443.0781** | **1326.2122** | **1325.4374** | **0.7748** | **2** | **11** | **1.8e+02** | **1** | **SAAARRAGPGGGAAR** |
|  | 2034 | **551.3740** | **1651.0999** | **1651.7991** | **-0.6992** | **1** | **11** | **1.7e+02** | **1** | **MATGSAQGNFTGHTKK + Oxidation (M)** |
|  | 3179 | **748.9023** | **1495.7898** | **1494.6552** | **1.1346** | **1** | **11** | **2.1e+02** | **1** | **RLAQDGAHVVVSSR** |
|  | 2323 | **597.7812** | **1193.5477** | **1194.3176** | **-0.7698** | **0** | **11** | **1.8e+02** | **1** | **SICPGTAPAPSH + Carbamidomethyl (C)** |
|  | 386 | **389.0193** | **1164.0357** | **1164.4605** | **-0.4249** | **0** | **11** | **2.1e+02** | **1** | **VIIVGYMTIR** |
|  | 2615 | **643.1819** | **1926.5235** | **1927.2313** | **-0.7079** | **1** | **11** | **1.8e+02** | **1** | **LIEAVETAVMPQWQRR** |
|  | 775 | **411.5247** | **1231.5519** | **1231.3114** | **0.2405** | **0** | **11** | **2.3e+02** | **1** | **GSGGSTFYAASVK** |
|  | 1038 | **433.0411** | **864.0675** | **864.9901** | **-0.9226** | **0** | **11** | **1.9e+02** | **1** | **CCEAGLR + 2 Carbamidomethyl (C)** |
|  | 2991 | **710.4475** | **2128.3204** | **2127.3570** | **0.9634** | **1** | **11** | **2e+02** | **1** | **NMAFQKYSNMEQSLFTR + 2 Oxidation (M)** |
|  | 198 | **375.8845** | **749.7543** | **750.8215** | **-1.0672** | **0** | **11** | **1.9e+02** | **1** | **SMTGSPR + Oxidation (M)** |
|  | 1290 | **458.7916** | **1373.3525** | **1374.5431** | **-1.1905** | **0** | **11** | **1.9e+02** | **1** | **HCSIETMEVPR + Carbamidomethyl (C); Oxidation (M)** |
|  | 1787 | **519.1708** | **1554.4902** | **1553.8034** | **0.6867** | **1** | **11** | **1.9e+02** | **1** | **TTCSCSLAVQLSKGR** |
|  | 1791 | **519.9302** | **1037.8456** | **1037.1490** | **0.6966** | **1** | **11** | **1.9e+02** | **1** | **SPTSAGRMSK + Oxidation (M)** |
|  | 2375 | **606.4504** | **1210.8861** | **1210.3402** | **0.5459** | **0** | **11** | **1.5e+02** | **1** | **MFGCDVGPDGR + Carbamidomethyl (C)** |
|  | 3099 | **740.5740** | **2218.6998** | **2219.5849** | **-0.8851** | **1** | **11** | **1.7e+02** | **1** | **CTRFCSASLEMPMWHPYK + 2 Oxidation (M)** |
|  | 1057 | **434.2713** | **866.5278** | **866.9580** | **-0.4302** | **1** | **11** | **1.5e+02** | **1** | **EYLRLSS** |
|  | 1099 | **437.1827** | **872.3507** | **872.0011** | **0.3496** | **1** | **11** | **2.1e+02** | **1** | **CDIEKHK** |
|  | 1626 | **493.7867** | **1478.3379** | **1479.5246** | **-1.1867** | **0** | **11** | **1.6e+02** | **1** | **QDWVDSGCPEESK** |
|  | 1806 | **521.2344** | **1560.6810** | **1561.7178** | **-1.0368** | **0** | **11** | **1.9e+02** | **1** | **MSHPALHDALNDPK + Oxidation (M)** |
|  | 1896 | **532.3453** | **1594.0138** | **1592.8394** | **1.1744** | **0** | **11** | **1.8e+02** | **1** | **MLQLAGVSNSTCGGVR** |
|  | 3294 | **777.1530** | **1552.2911** | **1552.7094** | **-0.4182** | **1** | **11** | **1.7e+02** | **1** | **VTISCSGSRSNIGSK + Carbamidomethyl (C)** |
|  | 1031 | **432.7179** | **1295.1315** | **1294.4750** | **0.6566** | **1** | **11** | **1.6e+02** | **1** | **TITVDDKMSLR + Oxidation (M)** |
|  | 2252 | **592.3828** | **1774.1261** | **1772.9736** | **1.1524** | **1** | **11** | **1.9e+02** | **1** | **NPPISDTLGSDKQLCK + Carbamidomethyl (C)** |
|  | 962 | **428.3030** | **1281.8869** | **1281.5043** | **0.3827** | **2** | **11** | **1.4e+02** | **1** | **VVPKGKGEQIAR** |
|  | 1495 | **476.2506** | **950.4864** | **949.9624** | **0.5241** | **1** | **11** | **1.9e+02** | **1** | **SGVGESTRAS** |
|  | 2746 | **666.6669** | **1996.9784** | **1997.2168** | **-0.2383** | **1** | **11** | **1.9e+02** | **1** | **NFQTACGMSLPRGISQDR + Oxidation (M)** |
|  | 3121 | **741.5897** | **2221.7468** | **2221.5244** | **0.2224** | **0** | **11** | **1.6e+02** | **1** | **LESLDISNTSITDITALLACK** |
|  | 3590 | **1052.7892** | **2103.5636** | **2104.4925** | **-0.9290** | **2** | **11** | **1.6e+02** | **1** | **HIIQMTGFKMEEKEALAK** |
|  | 908 | **421.5322** | **841.0495** | **841.9518** | **-0.9023** | **0** | **11** | **2.3e+02** | **1** | **GLEISAPR** |
|  | 1139 | **443.2122** | **1326.6145** | **1325.5584** | **1.0561** | **1** | **11** | **1.8e+02** | **1** | **FLVGPDGVPLRR** |
|  | 2445 | **613.2900** | **1836.8479** | **1837.9247** | **-1.0767** | **0** | **11** | **1.9e+02** | **1** | **SQASTHEMGHGGPEAAVR + Oxidation (M)** |
|  | 3400 | **806.5906** | **2416.7496** | **2415.6156** | **1.1339** | **1** | **11** | **1.9e+02** | **1** | **QLFHPEQLITGKEDAANNYAR** |
|  | 2695 | **656.0891** | **1965.2452** | **1966.2637** | **-1.0186** | **0** | **11** | **1.9e+02** | **1** | **YFCALGVSGFLGGYWGIR** |
|  | 586 | **405.0135** | **1212.0182** | **1212.4207** | **-0.4025** | **1** | **11** | **1.8e+02** | **1** | **KTCTPACSGCK + 2 Carbamidomethyl (C)** |
|  | 1006 | **431.0525** | **1290.1352** | **1290.5143** | **-0.3790** | **2** | **11** | **2.4e+02** | **1** | **ASVFSNLRIRK** |
|  | 1535 | **480.2022** | **1437.5845** | **1437.5742** | **0.0103** | **1** | **11** | **2e+02** | **1** | **AMVVDKDFPEDR + Oxidation (M)** |
|  | 2043 | **551.9717** | **1652.8930** | **1653.8597** | **-0.9666** | **1** | **11** | **2.1e+02** | **1** | **MENCGALQRMPTDR + 2 Oxidation (M)** |
|  | 2182 | **577.9521** | **1153.8895** | **1153.2473** | **0.6422** | **0** | **11** | **1.9e+02** | **1** | **AFNVGQGYAAR** |
|  | 2762 | **667.0876** | **1998.2408** | **1999.2278** | **-0.9870** | **2** | **11** | **2e+02** | **1** | **CMQPKDFNFKTPENDK + Carbamidomethyl (C)** |
|  | 3580 | **1017.3326** | **2032.6505** | **2032.4496** | **0.2009** | **2** | **11** | **1.4e+02** | **1** | **LTLLQRKLQPAAMYEIK + Oxidation (M)** |
|  | 698 | **407.7744** | **1220.3010** | **1220.4362** | **-0.1352** | **0** | **11** | **2e+02** | **1** | **VIVMSATLDAGK + Oxidation (M)** |
|  | 273 | **384.8231** | **767.6315** | **766.9483** | **0.6832** | **0** | **11** | **1.8e+02** | **1** | **TLGCFVK** |
|  | 542 | **402.9342** | **1205.7804** | **1206.3497** | **-0.5693** | **0** | **11** | **2.2e+02** | **1** | **HIDGAYIYVR** |
|  | 834 | **416.2659** | **830.5170** | **830.8862** | **-0.3691** | **1** | **11** | **1.9e+02** | **1** | **KGAAANDGK** |
|  | 1155 | **444.1126** | **886.2104** | **885.0659** | **1.1445** | **1** | **11** | **2.3e+02** | **1** | **LLLSGARR** |
|  | 2203 | **580.9620** | **1739.8639** | **1738.8961** | **0.9678** | **1** | **11** | **2e+02** | **1** | **IALITVTDKDADHNGR** |
|  | 3269 | **765.6808** | **1529.3468** | **1529.7390** | **-0.3922** | **1** | **11** | **1.5e+02** | **1** | **SAHFLGVDKVITSR** |
|  | 257 | **380.3304** | **1137.9690** | **1137.2251** | **0.7439** | **1** | **11** | **2e+02** | **1** | **SRTATECDVR** |
|  | 2047 | **552.9383** | **1655.7927** | **1656.8835** | **-1.0908** | **2** | **11** | **2.1e+02** | **1** | **KIERFNPPSNVTVR** |
|  | 2354 | **601.1785** | **1200.3423** | **1199.3190** | **1.0232** | **1** | **11** | **2.1e+02** | **1** | **TLWHSAGKSGR** |
|  | 2434 | **612.5372** | **1834.5895** | **1833.9956** | **0.5940** | **1** | **11** | **1.6e+02** | **1** | **VIPEDGPAAQNPENVKR** |
|  | 2920 | **686.5125** | **2056.5152** | **2056.3303** | **0.1849** | **2** | **11** | **1.8e+02** | **1** | **WMASAWKGSSRTVWCQR + Oxidation (M)** |
|  | 3279 | **771.1544** | **1540.2941** | **1540.8327** | **-0.5387** | **2** | **11** | **1.8e+02** | **1** | **SSPGLGCRAKPLARK** |
|  | 2895 | **685.0935** | **1368.1722** | **1367.4509** | **0.7214** | **2** | **11** | **2e+02** | **1** | **RSGGRGGGGDCGFK + Carbamidomethyl (C)** |
|  | 3516 | **870.7345** | **2609.1813** | **2609.9573** | **-0.7759** | **2** | **11** | **1.7e+02** | **1** | **GARAPSPTMAYSEEHKGMPCGFIR + Oxidation (M)** |
|  | 180 | **374.1633** | **1119.4676** | **1119.3356** | **0.1321** | **2** | **11** | **2.2e+02** | **1** | **RLMDEIKAK + Oxidation (M)** |
|  | 554 | **403.8279** | **1208.4616** | **1209.4380** | **-0.9765** | **1** | **11** | **2.1e+02** | **1** | **LLLRGANPDLK** |
|  | 1543 | **481.3981** | **1441.1721** | **1441.5214** | **-0.3492** | **2** | **11** | **1.7e+02** | **1** | **GSSGSSGKCETKEK + Carbamidomethyl (C)** |
|  | 1802 | **520.9800** | **1559.9180** | **1558.8479** | **1.0700** | **2** | **11** | **1.9e+02** | **1** | **IQGTCYRGAAKCCK + Carbamidomethyl (C)** |
|  | 1898 | **532.5743** | **1063.1338** | **1064.1924** | **-1.0586** | **0** | **11** | **2.5e+02** | **1** | **YQVINNVSK** |
|  | 293 | **385.1459** | **1152.4155** | **1153.2839** | **-0.8683** | **0** | **11** | **1.7e+02** | **1** | **FYSSQLSPPK** |
|  | 1506 | **477.1146** | **1428.3215** | **1428.6515** | **-0.3299** | **0** | **11** | **1.9e+02** | **1** | **GFENLFMLSGGLK + Oxidation (M)** |
|  | 2023 | **550.0939** | **1647.2594** | **1648.0042** | **-0.7447** | **2** | **11** | **2e+02** | **1** | **CMRYSCSLPCVKK + 2 Carbamidomethyl (C); Oxidation (M)** |
|  | 2575 | **635.7191** | **1269.4235** | **1268.4028** | **1.0207** | **1** | **11** | **2.3e+02** | **1** | **KDQTHACPNVR** |
|  | 3562 | **969.6317** | **2905.8730** | **2905.1771** | **0.6959** | **2** | **11** | **1.8e+02** | **1** | **SHQADLELEVKNSLDTIHRLESELK** |
|  | 271 | **384.2865** | **766.5582** | **766.8885** | **-0.3304** | **0** | **11** | **1.5e+02** | **1** | **ILTGAHR** |
|  | 2649 | **648.1361** | **1941.3862** | **1942.1664** | **-0.7802** | **2** | **11** | **2e+02** | **1** | **CRHGYFGANCDTKCPR + 2 Carbamidomethyl (C)** |
|  | 3004 | **715.1086** | **2142.3038** | **2143.4674** | **-1.1636** | **1** | **11** | **1.9e+02** | **1** | **MDPNCSCATGGSCSCASSCKCK** |
|  | 3358 | **797.0751** | **2388.2032** | **2387.5763** | **0.6270** | **1** | **11** | **1.6e+02** | **1** | **MNEPVSGQEPTKVSQVESPEAK + Oxidation (M)** |
|  | 3479 | **841.5239** | **2521.5494** | **2520.8789** | **0.6705** | **2** | **11** | **1.9e+02** | **1** | **KSLSLLMNSLRAEDTAVYYCAR + Oxidation (M)** |
|  | 1496 | **476.2776** | **950.5403** | **951.0182** | **-0.4778** | **0** | **11** | **1.8e+02** | **1** | **MYNSQHR + Oxidation (M)** |
|  | 1656 | **498.8001** | **995.5854** | **996.1217** | **-0.5362** | **1** | **11** | **1.6e+02** | **1** | **NNSGLKVHK** |
|  | 1738 | **510.8458** | **1529.5151** | **1528.7973** | **0.7179** | **0** | **11** | **1.9e+02** | **1** | **SAPCRPMLGVYYR + Oxidation (M)** |
|  | 1818 | **522.9344** | **1565.7810** | **1565.8590** | **-0.0780** | **2** | **11** | **2e+02** | **1** | **MHMRETSGFTLKK** |
|  | 2936 | **689.5198** | **2065.5372** | **2065.3618** | **0.1753** | **2** | **11** | **1.8e+02** | **1** | **SIRWLDRCIAAHQRPDK** |
|  | 633 | **406.1792** | **810.3436** | **809.9118** | **0.4318** | **0** | **11** | **1.9e+02** | **1** | **MGPCSGSR + Oxidation (M)** |
|  | 1210 | **451.0157** | **1350.0248** | **1349.5551** | **0.4697** | **1** | **11** | **2.2e+02** | **1** | **NKEIMFHVSTK + Oxidation (M)** |
|  | 1715 | **506.1568** | **1010.2988** | **1009.1206** | **1.1782** | **1** | **11** | **1.8e+02** | **1** | **TQPPPGTRR** |
|  | 389 | **389.0961** | **776.1774** | **775.9137** | **0.2638** | **0** | **11** | **2.2e+02** | **1** | **MVNIDGK** |
|  | 2406 | **609.9290** | **1826.7647** | **1827.2229** | **-0.4582** | **2** | **11** | **1.7e+02** | **1** | **NLCKQPILQVRNLCK + Carbamidomethyl (C)** |
|  | 3377 | **801.5092** | **1601.0035** | **1599.9315** | **1.0721** | **2** | **11** | **2.1e+02** | **1** | **MKTFFEKSLIDIK** |
|  | 3520 | **879.0017** | **1755.9886** | **1756.1037** | **-0.1151** | **0** | **11** | **2.2e+02** | **1** | **CSPCCSLHCSPCCSLR + Carbamidomethyl (C)** |
|  | 305 | **386.0405** | **1155.0994** | **1156.2976** | **-1.1982** | **2** | **11** | **1.8e+02** | **1** | **AHSQARLRAF** |
|  | 622 | **406.0984** | **1215.2731** | **1216.3232** | **-1.0501** | **0** | **11** | **2e+02** | **1** | **NGDTAVYFCAR** |
|  | 702 | **407.8163** | **813.6178** | **813.9220** | **-0.3042** | **0** | **11** | **2.1e+02** | **1** | **MEQPGPR** |
|  | 2221 | **586.1006** | **1170.1864** | **1170.2728** | **-0.0865** | **0** | **11** | **2e+02** | **1** | **DINGGGATLPQK** |
|  | 2292 | **594.8011** | **1781.3811** | **1780.8684** | **0.5127** | **2** | **11** | **1.8e+02** | **1** | **SSMEDPRGINGQSKSAT + Oxidation (M)** |
|  | 1034 | **432.9465** | **1295.8172** | **1295.4214** | **0.3958** | **1** | **11** | **2e+02** | **1** | **LCSEYPENKR + Carbamidomethyl (C)** |
|  | 2030 | **550.8483** | **1649.5226** | **1648.7701** | **0.7525** | **0** | **11** | **1.7e+02** | **1** | **GDVLPNADETWYLR** |
|  | 2624 | **643.9316** | **1928.7728** | **1928.1897** | **0.5831** | **0** | **11** | **1.6e+02** | **1** | **YIFSMISDSSGVMVYGR + Oxidation (M)** |
|  | 2625 | 644.1377 | 1929.3909 | 1930.2998 | -0.9089 | 2 | 11 | 2e+02 | 1 | LKRMTASLCPRPAALQT + Carbamidomethyl (C); Oxidation (M) |
|  | 346 | **387.1440** | **1158.4097** | **1157.3452** | **1.0644** | **0** | **11** | **2.3e+02** | **1** | **FCIGLHSAPR + Carbamidomethyl (C)** |
|  | 2522 | **628.1006** | **1881.2798** | **1881.0322** | **0.2475** | **2** | **11** | **2e+02** | **1** | **MAREAEFEAEQERIR + Oxidation (M)** |
|  | 231 | **378.1833** | **1131.5278** | **1131.3446** | **0.1832** | **0** | **11** | **1.5e+02** | **1** | **YSCPPPALVK + Carbamidomethyl (C)** |
|  | 1354 | **462.1796** | **922.3445** | **922.0583** | **0.2862** | **2** | **11** | **1.9e+02** | **1** | **AMDKDAKK + Oxidation (M)** |
|  | 1942 | **537.1977** | **1072.3806** | **1071.3140** | **1.0666** | **1** | **11** | **2.3e+02** | **1** | **ILKLAASISR** |
|  | 2243 | **590.3876** | **1178.7605** | **1179.3923** | **-0.6319** | **1** | **11** | **2e+02** | **1** | **SYQPLMRLR + Oxidation (M)** |
|  | 2608 | **642.1783** | **1282.3419** | **1283.4406** | **-1.0986** | **2** | **11** | **2e+02** | **1** | **VDRLGVAGAGGRR** |
|  | 1519 | **478.0119** | **1431.0136** | **1430.6078** | **0.4058** | **1** | **11** | **1.9e+02** | **1** | **TRIHEGYISQVK** |
|  | 1834 | **523.9415** | **1568.8024** | **1567.8118** | **0.9906** | **0** | **11** | **2.2e+02** | **1** | **FGMGSAQACPCQVPR + Oxidation (M)** |
|  | 1052 | **434.0477** | **1299.1210** | **1299.3968** | **-0.2758** | **2** | **11** | **1.9e+02** | **1** | **LGQGERAADARR** |
|  | 2617 | **643.3278** | **1926.9611** | **1927.2047** | **-0.2436** | **0** | **11** | **2e+02** | **1** | **LAVLGSCHMFSDQYLDK** |
|  | 2893 | **685.0858** | **2052.2353** | **2051.3502** | **0.8850** | **1** | **11** | **2.1e+02** | **1** | **ASGLVASNLNLKPGEGLRVR** |
|  | 1311 | **459.5814** | **1375.7221** | **1376.6187** | **-0.8966** | **2** | **11** | **2.6e+02** | **1** | **MEVEQLKKEVK + Oxidation (M)** |
|  | 2273 | **593.0065** | **1775.9972** | **1775.8651** | **0.1321** | **0** | **11** | **2.1e+02** | **1** | **SAEPGTETSQVNLSDLK** |
|  | 2867 | **682.3773** | **1362.7399** | **1362.5555** | **0.1844** | **0** | **11** | **2e+02** | **1** | **CASQSGMTAYGMR** |
|  | 3056 | **734.0416** | **2199.1027** | **2198.4184** | **0.6843** | **1** | **11** | **1.6e+02** | **1** | **MAARAPPAAPAAEEPGNPGGPPR + Oxidation (M)** |
|  | 2317 | **597.0462** | **1788.1164** | **1786.9375** | **1.1790** | **0** | **11** | **2.3e+02** | **1** | **NTIYCNVEPTESNMR + Oxidation (M)** |
|  | 3052 | **733.1688** | **1464.3229** | **1463.4639** | **0.8590** | **1** | **11** | **2e+02** | **1** | **EDSNIRENSSGAGK** |
|  | 653 | **407.0028** | **1217.9862** | **1217.3742** | **0.6120** | **0** | **11** | **1.9e+02** | **1** | **MGSCCSCPDK + 3 Carbamidomethyl (C); Oxidation (M)** |
|  | 1350 | **462.1182** | **1383.3325** | **1383.5363** | **-0.2038** | **1** | **11** | **2e+02** | **1** | **SRNHLALMGGDGR** |
|  | 1621 | **492.1525** | **982.2902** | **982.1051** | **0.1851** | **2** | **11** | **1.9e+02** | **1** | **AGRGRRPGR** |
|  | 2046 | **552.2159** | **1653.6257** | **1652.8436** | **0.7821** | **1** | **11** | **2.3e+02** | **1** | **SKGAPVVEEPASPGSIK** |
|  | 2667 | **652.2289** | **1953.6645** | **1954.2269** | **-0.5625** | **0** | **11** | **2.1e+02** | **1** | **VAPEEHPVLLTEAPLNPK** |
|  | 859 | **418.0848** | **1251.2323** | **1250.4882** | **0.7441** | **1** | **11** | **2.2e+02** | **1** | **LLARGPPLDIW** |
|  | 1133 | **442.3075** | **882.6003** | **881.9742** | **0.6260** | **1** | **11** | **1.5e+02** | **1** | **YSGRWIT** |
|  | 1256 | **456.4280** | **910.8412** | **911.0636** | **-0.2224** | **2** | **11** | **1.7e+02** | **1** | **LKGNPARR** |
|  | 1850 | **525.1819** | **1572.5235** | **1572.7206** | **-0.1971** | **1** | **11** | **2.2e+02** | **1** | **GHDVTFYKTWYR** |
|  | 2302 | **595.7527** | **1189.4906** | **1189.4254** | **0.0652** | **1** | **11** | **2.3e+02** | **1** | **MLLVGKDGNVK + Oxidation (M)** |
|  | 1746 | **513.7845** | **1025.5542** | **1025.0520** | **0.5021** | **0** | **11** | **1.6e+02** | **1** | **DQSSCTTAR + Carbamidomethyl (C)** |
|  | 2612 | **642.6588** | **1924.9543** | **1924.1148** | **0.8394** | **2** | **11** | **2e+02** | **1** | **LCEEKCETDEIQKGGK + 2 Carbamidomethyl (C)** |
|  | 2509 | **623.5532** | **1245.0917** | **1244.3317** | **0.7600** | **0** | **11** | **1.7e+02** | **1** | **LSCEASGSTFSR** |
|  | 2563 | **632.6608** | **1263.3068** | **1263.5750** | **-0.2682** | **2** | **11** | **2.5e+02** | **1** | **QCKLMRLPFA + Carbamidomethyl (C)** |
|  | 2798 | **670.0491** | **2007.1250** | **2006.2832** | **0.8418** | **2** | **11** | **2e+02** | **1** | **VSTEHNKECLINISKYK** |
|  | 1126 | **441.1217** | **1320.3430** | **1319.4429** | **0.9002** | **1** | **11** | **2.1e+02** | **1** | **ELFHNKDCEK + Carbamidomethyl (C)** |
|  | 2308 | **596.3726** | **1786.0955** | **1786.9822** | **-0.8867** | **2** | **11** | **2.2e+02** | **1** | **DWSNKMPDMAYERK + Oxidation (M)** |
|  | 3218 | 753.0555 | 1504.0963 | 1503.7596 | 0.3367 | 1 | 11 | 1.6e+02 | 1 | VKLTLEEEACLQK |
|  | 1232 | **452.8051** | **1355.3933** | **1355.6091** | **-0.2158** | **1** | **11** | **2.2e+02** | **1** | **IFRAWFNMVR + Oxidation (M)** |
|  | 1575 | **487.1072** | **972.1997** | **972.0209** | **0.1788** | **2** | **11** | **2.2e+02** | **1** | **SSRRHSSR** |
|  | 2082 | **558.3749** | **1114.7351** | **1115.2374** | **-0.5023** | **1** | **11** | **2e+02** | **1** | **DIQNQLKEK** |
|  | 2556 | **632.2498** | **1262.4847** | **1263.5750** | **-1.0902** | **2** | **11** | **2.3e+02** | **1** | **QCKLMRLPFA + Carbamidomethyl (C)** |
|  | 757 | **410.0157** | **1227.0248** | **1227.4632** | **-0.4384** | **1** | **11** | **2.4e+02** | **1** | **CGHGRVLLCR + 2 Carbamidomethyl (C)** |
|  | 1305 | **459.2765** | **916.5383** | **916.0303** | **0.5080** | **0** | **11** | **2e+02** | **1** | **TSLPGGGLSK** |
|  | 643 | **406.5797** | **1216.7170** | **1216.4588** | **0.2581** | **2** | **11** | **1.7e+02** | **1** | **KCRLIYHQR** |
|  | 660 | **407.1064** | **1218.2971** | **1217.4040** | **0.8931** | **2** | **11** | **2.1e+02** | **1** | **VWRTCRSGPR** |
|  | 2214 | **584.5580** | **1167.1013** | **1166.3272** | **0.7741** | **0** | **11** | **1.8e+02** | **1** | **LDAGGIPSLAPR** |
|  | 2543 | **630.6576** | **1888.9506** | **1889.1353** | **-0.1847** | **1** | **11** | **2.3e+02** | **1** | **EDAMVASRLTPGSQGLLK + Oxidation (M)** |
|  | 2588 | **637.5133** | **1909.5177** | **1910.1393** | **-0.6216** | **1** | **11** | **1.8e+02** | **1** | **CNECGKVFNQLSNLAR + 2 Carbamidomethyl (C)** |
|  | 1926 | **536.1212** | **1070.2277** | **1070.2415** | **-0.0139** | **0** | **11** | **2.1e+02** | **1** | **FLGALPPAER** |
|  | 1009 | **431.0994** | **860.1840** | **860.9520** | **-0.7679** | **0** | **11** | **2.5e+02** | **1** | **TNESALVK** |
|  | 2379 | **606.9974** | **1211.9800** | **1211.3248** | **0.6551** | **1** | **11** | **2e+02** | **1** | **GTTSLPSHLKNG** |
|  | 2485 | **619.1041** | **1854.2902** | **1854.0680** | **0.2222** | **0** | **11** | **2.1e+02** | **1** | **TDQALAGSLVSSTCGVCGK + Carbamidomethyl (C)** |
|  | 2592 | **637.9581** | **1910.8522** | **1910.1744** | **0.6779** | **2** | **11** | **1.9e+02** | **1** | **DVPPDILLDFPERKQK** |
|  | 2843 | **679.0404** | **1356.0660** | **1356.6154** | **-0.5493** | **1** | **11** | **1.9e+02** | **1** | **QMPGCFNFLRK + Oxidation (M)** |
|  | 590 | **405.0646** | **808.1145** | **808.9452** | **-0.8307** | **1** | **11** | **1.9e+02** | **1** | **MGSGWKK + Oxidation (M)** |
|  | 1446 | **472.2625** | **1413.7652** | **1413.6916** | **0.0736** | **2** | **11** | **2e+02** | **1** | **LFSCRCGSPKCR + Carbamidomethyl (C)** |
|  | 1618 | **491.7885** | **1472.3432** | **1471.7276** | **0.6155** | **2** | **11** | **1.6e+02** | **1** | **MRLLLPGEARGSR + Oxidation (M)** |
|  | 1665 | **499.9681** | **997.9214** | **998.1575** | **-0.2361** | **0** | **11** | **2e+02** | **1** | **VHNGISMPK + Oxidation (M)** |
|  | 2708 | **658.9783** | **1973.9128** | **1972.9337** | **0.9791** | **0** | **11** | **1.8e+02** | **1** | **SGHTSTGDGLNGPSDASEQR** |
|  | 2818 | **673.2052** | **1344.3956** | **1343.5075** | **0.8882** | **0** | **11** | **2.1e+02** | **1** | **AMGAMEGSAASCGK + Carbamidomethyl (C); Oxidation (M)** |
|  | 792 | **413.4916** | **824.9685** | **823.9815** | **0.9870** | **0** | **11** | **2.2e+02** | **1** | **MQGVSMR + Oxidation (M)** |
|  | 2195 | **579.8198** | **1157.6249** | **1158.3120** | **-0.6872** | **2** | **11** | **1.8e+02** | **1** | **SAVTAQAARRK** |
|  | 2268 | **592.8498** | **1183.6848** | **1183.3546** | **0.3302** | **0** | **11** | **1.8e+02** | **1** | **KPLSEGQPSLK** |
|  | 747 | **409.0096** | **816.0045** | **814.9481** | **1.0564** | **1** | **11** | **2.4e+02** | **1** | **FFPSSKC** |
|  | 912 | **421.9068** | **1262.6981** | **1262.5057** | **0.1924** | **1** | **11** | **2.1e+02** | **1** | **RLCAGSALCVR + 2 Carbamidomethyl (C)** |
|  | 1051 | **434.0429** | **1299.1066** | **1300.2923** | **-1.1857** | **1** | **11** | **2e+02** | **1** | **DAGGAGPADREER** |
|  | 2534 | **629.2238** | **1256.4327** | **1256.4732** | **-0.0404** | **1** | **11** | **2.2e+02** | **1** | **GLMPGTAYKFR + Oxidation (M)** |
|  | 2974 | **702.5815** | **1403.1483** | **1402.5729** | **0.5754** | **0** | **11** | **1.7e+02** | **1** | **LMTGDTITAHAGAK + Oxidation (M)** |
|  | 3467 | 833.4502 | 2497.3284 | 2497.0265 | 0.3020 | 1 | 11 | 2.1e+02 | 1 | VCLKWPGGSCMAALTVTLMVLSSP + 2 Oxidation (M) |
|  | 2275 | **593.0593** | **1776.1558** | **1777.0485** | **-0.8927** | **2** | **11** | **2.2e+02** | **1** | **LESLQAEIKMLSDRK + Oxidation (M)** |
|  | 3598 | **1162.4548** | **3484.3423** | **3484.9903** | **-0.6480** | **2** | **11** | **1.5e+02** | **1** | **GVSTFMAEMLETASILRSATKDSLIIIDELGR + Oxidation (M)** |
|  | 61 | **365.4678** | **1093.3811** | **1093.3415** | **0.0397** | **2** | **11** | **2.6e+02** | **1** | **KTMTTRVIK + Oxidation (M)** |
|  | 280 | **385.0262** | **768.0376** | **768.8565** | **-0.8188** | **1** | **11** | **1.9e+02** | **1** | **QKFSPY** |
|  | 1143 | **443.5368** | **1327.5883** | **1326.5217** | **1.0667** | **1** | **11** | **2.5e+02** | **1** | **CMECGKAYSYR + Oxidation (M)** |
|  | 3065 | **737.5555** | **2209.6445** | **2210.4671** | **-0.8226** | **1** | **11** | **2e+02** | **1** | **MARSNMDNMFESYINNLK + 2 Oxidation (M)** |
|  | 730 | **408.3024** | **1221.8849** | **1222.3308** | **-0.4459** | **0** | **11** | **1.8e+02** | **1** | **CQSSGWQITR + Carbamidomethyl (C)** |
|  | 1351 | **462.1217** | **1383.3428** | **1383.4789** | **-0.1360** | **0** | **11** | **2.1e+02** | **1** | **VTETEDTQMVSK + Oxidation (M)** |
|  | 2954 | **696.3489** | **2086.0246** | **2085.3219** | **0.7028** | **0** | **11** | **2.2e+02** | **1** | **DAAHFGVCMSPPSWQPNPK + Oxidation (M)** |
|  | 3396 | **805.3039** | **2412.8895** | **2413.7952** | **-0.9057** | **1** | **11** | **2.1e+02** | **1** | **CQCHLRVVQCSDLELGTNPLK + Carbamidomethyl (C)** |
|  | 783 | **413.2221** | **824.4294** | **824.9279** | **-0.4985** | **2** | **11** | **1.6e+02** | **1** | **SHGQKRL** |
|  | 841 | **416.6321** | **1246.8741** | **1246.3242** | **0.5498** | **0** | **11** | **2e+02** | **1** | **SNNDGTVGELLK** |
|  | 1002 | **430.9626** | **859.9105** | **858.9790** | **0.9315** | **0** | **11** | **2.6e+02** | **1** | **LAASQIEK** |
|  | 1247 | **454.5551** | **1360.6430** | **1359.6108** | **1.0321** | **0** | **11** | **2.7e+02** | **1** | **IPNILSAFPFLQ** |
|  | 3209 | **749.5284** | **1497.0421** | **1496.7272** | **0.3149** | **1** | **11** | **2.1e+02** | **1** | **LKLPGDSSLMTYR + Oxidation (M)** |
|  | 1109 | **437.5321** | **1309.5741** | **1309.4680** | **0.1062** | **2** | **11** | **2.9e+02** | **1** | **KSIGAKEGSGFTK** |
|  | 2959 | **696.6867** | **1391.3586** | **1390.7146** | **0.6441** | **1** | **11** | **1.9e+02** | **1** | **GMLVGIVGKVGCGK + Carbamidomethyl (C); Oxidation (M)** |
|  | 3292 | **776.6400** | **1551.2652** | **1550.7168** | **0.5485** | **0** | **11** | **1.8e+02** | **1** | **MNSQQSVPATMNAR + Oxidation (M)** |
|  | 1189 | **449.1973** | **1344.5696** | **1343.5969** | **0.9728** | **2** | **11** | **2e+02** | **1** | **KVEMHWTIRK + Oxidation (M)** |
|  | 2766 | **667.6925** | **2000.0553** | **2000.2242** | **-0.1688** | **2** | **11** | **2.4e+02** | **1** | **SGDPRPSCAPKSPACRTR + 2 Carbamidomethyl (C)** |
|  | 935 | **424.4186** | **846.8225** | **847.9863** | **-1.1638** | **2** | **11** | **2.5e+02** | **1** | **GAKGRGMR + Oxidation (M)** |
|  | 1037 | **433.0180** | **1296.0318** | **1295.3967** | **0.6352** | **0** | **11** | **2.2e+02** | **1** | **AGDPLCESGSECK** |
|  | 1389 | **466.1007** | **1395.2800** | **1394.4650** | **0.8150** | **1** | **11** | **2.3e+02** | **1** | **ENSVASERDMEK** |
|  | 1518 | **477.9511** | **1430.8312** | **1431.6140** | **-0.7828** | **0** | **11** | **2e+02** | **1** | **CALGQVGELTGQQK** |
|  | 2914 | **686.2886** | **1370.5624** | **1369.4007** | **1.1617** | **1** | **11** | **2.2e+02** | **1** | **VQGNDHSATRER** |
|  | 879 | **419.2599** | **1254.7575** | **1254.3927** | **0.3647** | **1** | **11** | **2.1e+02** | **1** | **LNQPGTPTRTAV** |
|  | 2367 | **604.4209** | **1206.8270** | **1206.3085** | **0.5186** | **2** | **11** | **2e+02** | **1** | **DKDAEFLRGR** |
|  | 172 | **373.1151** | **744.2153** | **744.8334** | **-0.6181** | **0** | **11** | **2.7e+02** | **1** | **TVAAPSSL** |
|  | 1435 | **471.3612** | **1411.0614** | **1411.7102** | **-0.6489** | **0** | **11** | **1.6e+02** | **1** | **IWIPGPGAILCSK + Carbamidomethyl (C)** |
|  | 1573 | **487.0841** | **1458.2301** | **1457.8285** | **0.4015** | **2** | **11** | **2.3e+02** | **1** | **HLMLRVRLYLK + Oxidation (M)** |
|  | 1866 | **527.2302** | **1052.4457** | **1052.1685** | **0.2772** | **1** | **11** | **2.1e+02** | **1** | **MHDVKNHR + Oxidation (M)** |
|  | 2083 | **558.5950** | **1672.7629** | **1671.7639** | **0.9990** | **1** | **11** | **2.7e+02** | **1** | **KEAGAGAQDAGAAEGAAVK** |
|  | 752 | **409.2000** | **1224.5777** | **1223.3821** | **1.1956** | **1** | **11** | **2.2e+02** | **1** | **VGPGPVTGDRLR** |
|  | 1503 | **476.7700** | **1427.2879** | **1427.7315** | **-0.4436** | **1** | **11** | **1.7e+02** | **1** | **IYVMSIVCKNNK + Oxidation (M)** |
|  | 722 | **408.0950** | **1221.2628** | **1222.4353** | **-1.1724** | **1** | **11** | **2.4e+02** | **1** | **DGLLKANCGMK + Carbamidomethyl (C); Oxidation (M)** |
|  | 1258 | **456.9050** | **1367.6929** | **1368.5187** | **-0.8257** | **1** | **11** | **2.2e+02** | **1** | **NPMTNYTTVRR + Oxidation (M)** |
|  | 2024 | **550.1034** | **1647.2880** | **1647.8701** | **-0.5820** | **0** | **11** | **2.2e+02** | **1** | **VTVIFAGSPNITVSSR** |
|  | 3151 | **743.3411** | **2227.0010** | **2227.4643** | **-0.4633** | **2** | **11** | **2.3e+02** | **1** | **YNPQENRWHTIAPMGTRR** |
|  | 362 | **387.9531** | **773.8914** | **772.8302** | **1.0613** | **0** | **11** | **2.7e+02** | **1** | **NHCSGAGK** |
|  | 2235 | **588.0907** | **1761.2499** | **1761.8732** | **-0.6233** | **1** | **11** | **2.3e+02** | **1** | **HIHPSRTDACDPEAR + Carbamidomethyl (C)** |
|  | 2747 | **666.6729** | **1331.3309** | **1331.5345** | **-0.2036** | **0** | **11** | **2.2e+02** | **1** | **IDNLLQEMLLGG + Oxidation (M)** |
|  | 3251 | **762.5557** | **2284.6450** | **2284.5423** | **0.1027** | **0** | **11** | **2.1e+02** | **1** | **MQQQGIILEAETGPSPDTKPK + Oxidation (M)** |
|  | 189 | **374.3705** | **1120.0892** | **1119.3619** | **0.7272** | **1** | **11** | **2.9e+02** | **1** | **RICLGEAMAR** |
|  | 1822 | **523.1870** | **1566.5389** | **1565.8107** | **0.7281** | **0** | **11** | **2.3e+02** | **1** | **ESPPLVVLELWQR** |
|  | 2865 | **682.0833** | **2043.2276** | **2042.2327** | **0.9949** | **2** | **11** | **2.1e+02** | **1** | **DTEPVELNCNFSFSRKR** |
|  | 2371 | **606.2745** | **1210.5343** | **1211.3729** | **-0.8387** | **1** | **11** | **2.1e+02** | **1** | **SSAASACSCRLR** |
|  | 2821 | **673.6433** | **1345.2718** | **1344.4277** | **0.8442** | **1** | **11** | **1.9e+02** | **1** | **ISTEKNSSHVDK** |
|  | 3069 | **738.2906** | **2211.8496** | **2211.4567** | **0.3929** | **1** | **11** | **2.2e+02** | **1** | **CQCRLGSLGAACEQTQTEGAK + Carbamidomethyl (C)** |
|  | 157 | **371.2530** | **1110.7367** | **1110.3734** | **0.3634** | **1** | **11** | **1.4e+02** | **1** | **SHLPMKLLR + Oxidation (M)** |
|  | 1436 | **471.4347** | **940.8546** | **941.1722** | **-0.3176** | **1** | **11** | **1.7e+02** | **1** | **LLLLGRTR** |
|  | 1928 | **536.2272** | **1070.4396** | **1071.2162** | **-0.7767** | **1** | **11** | **2.3e+02** | **1** | **SRHIGWCR + Carbamidomethyl (C)** |
|  | 2022 | **549.7566** | **1646.2476** | **1646.8056** | **-0.5580** | **1** | **11** | **1.9e+02** | **1** | **SHECHECGKLFSR + 2 Carbamidomethyl (C)** |
|  | 258 | **381.2904** | **1140.8490** | **1140.3068** | **0.5423** | **0** | **11** | **2.1e+02** | **1** | **LPEPCPSTVTP** |
|  | 456 | **395.6424** | **1183.9050** | **1184.2665** | **-0.3615** | **2** | **11** | **2e+02** | **1** | **QSPRNSPRSR** |
|  | 3504 | **857.8842** | **1713.7535** | **1712.9896** | **0.7639** | **2** | **11** | **2e+02** | **1** | **QRLEWVGLVESKIR** |
|  | 1478 | **475.4527** | **1423.3359** | **1424.5155** | **-1.1796** | **0** | **11** | **2.3e+02** | **1** | **DPGGGAGAITVASHSK** |
|  | 2498 | **621.3681** | **1240.7214** | **1241.3607** | **-0.6393** | **2** | **11** | **2.2e+02** | **1** | **LQGAERGAARGR** |
|  | 427 | **390.8431** | **779.6714** | **779.8875** | **-0.2160** | **2** | **11** | **2.1e+02** | **1** | **AYRKSR** |
|  | 2438 | **612.8014** | **1835.3820** | **1835.3498** | **0.0322** | **2** | **11** | **2.1e+02** | **1** | **MVMTVFACLMGKGMKR + 2 Oxidation (M)** |
|  | 2481 | **617.9598** | **1850.8573** | **1850.3029** | **0.5544** | **2** | **11** | **2.1e+02** | **1** | **MLPVRCVPAPATLRLR + Carbamidomethyl (C)** |
|  | 2926 | **687.1049** | **2058.2926** | **2057.3168** | **0.9757** | **2** | **11** | **2.2e+02** | **1** | **TGPPSAPQGALAAPRSPAVRR** |
|  | 2604 | **641.7904** | **1281.5660** | **1282.3728** | **-0.8068** | **1** | **11** | **2.6e+02** | **1** | **RCNNHDQHCR** |
|  | 2722 | **663.5100** | **1325.0052** | **1324.3931** | **0.6121** | **0** | **11** | **1.9e+02** | **1** | **ALSSADTQAADFK** |
|  | 3291 | **776.5057** | **2326.4950** | **2325.6222** | **0.8728** | **2** | **11** | **2.2e+02** | **1** | **ACLEGECVEWLRRYLENGK + Carbamidomethyl (C)** |
|  | 3301 | **777.5809** | **2329.7204** | **2330.5927** | **-0.8722** | **1** | **11** | **2.1e+02** | **1** | **VSASPDPRPLKEEEEAPLLPR** |
|  | 3371 | **798.8156** | **2393.4247** | **2393.6913** | **-0.2667** | **2** | **11** | **2e+02** | **1** | **KNGQCSLKTSMSGYIPSYLDK + Carbamidomethyl (C); Oxidation (M)** |
|  | 1474 | **474.9196** | **1421.7368** | **1420.6792** | **1.0576** | **1** | **11** | **2.4e+02** | **1** | **KCGFGVCLDCYR + Carbamidomethyl (C)** |
|  | 2946 | **694.5071** | **2080.4991** | **2080.3620** | **0.1371** | **2** | **11** | **2e+02** | **1** | **SPLSVPRSKSEMSYIDGVK** |
|  | 1375 | **464.2144** | **1389.6210** | **1389.5127** | **0.1082** | **0** | **11** | **2.2e+02** | **1** | **TFNQSSILTNHK** |
|  | 1699 | **504.9526** | **1511.8356** | **1511.7023** | **0.1333** | **1** | **11** | **2.2e+02** | **1** | **VHIEMGPDGRVTGK + Oxidation (M)** |
|  | 2330 | 598.6791 | 1793.0151 | 1792.9900 | 0.0251 | 2 | 11 | 2.6e+02 | 1 | RKGSDGASPPASPSIIPR |
|  | 566 | **404.0114** | **806.0081** | **805.8833** | **0.1249** | **0** | **11** | **2.3e+02** | **1** | **HCSGGSCR** |
|  | 1462 | **473.6662** | **945.3176** | **945.0948** | **0.2228** | **0** | **11** | **2.2e+02** | **1** | **MATPAGLER** |
|  | 1220 | **451.9050** | **1352.6927** | **1353.5070** | **-0.8143** | **0** | **11** | **2.7e+02** | **1** | **MQSSGIPNGGHIR** |
|  | 3456 | **824.3649** | **1646.7150** | **1647.0426** | **-0.3276** | **2** | **11** | **2.2e+02** | **1** | **CIRKLMHLVQMSR + 2 Oxidation (M)** |
|  | 421 | **389.4066** | **776.7983** | **776.7957** | **0.0026** | **0** | **11** | **2.8e+02** | **1** | **TQSGAGTR** |
|  | 552 | **403.8105** | **805.6063** | **804.8490** | **0.7574** | **1** | **11** | **2.3e+02** | **1** | **RSAAGSEK** |
|  | 849 | **417.8361** | **833.6574** | **832.8988** | **0.7585** | **0** | **11** | **2.4e+02** | **1** | **VLSTEER** |
|  | 1339 | **461.8004** | **921.5860** | **921.0734** | **0.5127** | **1** | **11** | **2e+02** | **1** | **LKMQNGSK + Oxidation (M)** |
|  | 2530 | **628.6143** | **1255.2137** | **1255.4637** | **-0.2499** | **0** | **11** | **2.1e+02** | **1** | **QVDVLGTVIGVR** |
|  | 1891 | **530.9426** | **1589.8055** | **1588.6969** | **1.1087** | **1** | **11** | **2.5e+02** | **1** | **MNREELEDSFFR + Oxidation (M)** |
|  | 2190 | **579.0045** | **1733.9914** | **1733.7438** | **0.2476** | **0** | **11** | **2.3e+02** | **1** | **EGGWSVSFDYWGQDT** |
|  | 3555 | **948.8242** | **2843.4505** | **2842.3184** | **1.1320** | **1** | **11** | **1.8e+02** | **1** | **RTLTTLCPLVLCGGVMAPGPAVDQEAR + Carbamidomethyl (C); Oxidation (M)** |
|  | 353 | **387.8268** | **1160.4584** | **1161.4153** | **-0.9569** | **1** | **11** | **2.8e+02** | **1** | **AKLGMLNTVSK** |
|  | 1622 | **492.2357** | **1473.6850** | **1473.6891** | **-0.0041** | **1** | **11** | **2.2e+02** | **1** | **MELFVKESFEAK + Oxidation (M)** |
|  | 1108 | **437.4364** | **1309.2870** | **1310.4528** | **-1.1657** | **0** | **11** | **2.8e+02** | **1** | **ADSTVQLAPSPPK** |
|  | 1414 | **468.5351** | **1402.5832** | **1401.6280** | **0.9552** | **1** | **11** | **2.9e+02** | **1** | **MLDKEIPPSISR + Oxidation (M)** |
|  | 666 | **407.1805** | **1218.5193** | **1217.3327** | **1.1865** | **1** | **11** | **2.2e+02** | **1** | **VAQNKTLGSGSR** |
|  | 3557 | **951.2827** | **1900.5506** | **1899.8831** | **0.6675** | **1** | **11** | **1.7e+02** | **1** | **SQDLDSRQHPEESSER** |
|  | 1178 | **447.0643** | **892.1138** | **893.0432** | **-0.9295** | **0** | **11** | **2.5e+02** | **1** | **ACLNGCAK + 2 Carbamidomethyl (C)** |
|  | 51 | **364.0444** | **1089.1109** | **1090.1652** | **-1.0542** | **0** | **11** | **2.2e+02** | **1** | **GSCPSPSSSPK + Carbamidomethyl (C)** |
|  | 2105 | **562.6173** | **1684.8298** | **1685.9859** | **-1.1561** | **1** | **11** | **2.7e+02** | **1** | **MGLLQEKMGFCEQR + Oxidation (M)** |
|  | 2108 | **562.7339** | **1685.1795** | **1684.8322** | **0.3473** | **2** | **11** | **2.2e+02** | **1** | **IPARENPCEGGSKNR + Carbamidomethyl (C)** |
|  | 2687 | **655.0264** | **1308.0380** | **1308.3938** | **-0.3558** | **2** | **11** | **2.1e+02** | **1** | **IALEFDKDRSD** |
|  | 2995 | **711.5046** | **2131.4917** | **2132.4926** | **-1.0008** | **0** | **11** | **2.2e+02** | **1** | **CHYCQSIMHMVANCPHK + 2 Carbamidomethyl (C); Oxidation (M)** |
|  | 1949 | **538.1766** | **1074.3384** | **1075.2863** | **-0.9479** | **2** | **11** | **2.4e+02** | **1** | **DGKMSLLRR** |
|  | 2929 | **687.4373** | **2059.2896** | **2058.3662** | **0.9234** | **2** | **11** | **2.3e+02** | **1** | **CRSPWRPGSSKVWTPVSK** |
|  | 646 | **406.6864** | **811.3581** | **812.0088** | **-0.6507** | **1** | **11** | **1.7e+02** | **1** | **VKDPLIK** |
|  | 1050 | **434.0413** | **866.0678** | **864.9868** | **1.0810** | **1** | **11** | **2.2e+02** | **1** | **AKNSFIW** |
|  | 1485 | **475.7705** | **949.5262** | **950.1579** | **-0.6316** | **2** | **11** | **2e+02** | **1** | **KAVTMTRK + Oxidation (M)** |
|  | 1967 | **540.1535** | **1617.4383** | **1617.7744** | **-0.3361** | **1** | **11** | **2.4e+02** | **1** | **DYIECAEKLYDAK + Carbamidomethyl (C)** |
|  | 25 | **362.2470** | **1083.7189** | **1083.2421** | **0.4768** | **1** | **11** | **1.8e+02** | **1** | **GIEPGSLRVR** |
|  | 689 | **407.6415** | **813.2683** | **812.8311** | **0.4372** | **0** | **11** | **1.9e+02** | **1** | **AHAASSGGR** |
|  | 838 | **416.5396** | **1246.5966** | **1247.3537** | **-0.7572** | **1** | **11** | **3.1e+02** | **1** | **EERDWTIVLS** |
|  | 2096 | **561.7128** | **1121.4108** | **1122.1888** | **-0.7780** | **0** | **11** | **2.5e+02** | **1** | **QVSSPSFTNR** |
|  | 75 | **366.1928** | **1095.5562** | **1095.2096** | **0.3465** | **0** | **11** | **2.1e+02** | **1** | **LSTPGGLHTGR** |
|  | 1173 | **446.1111** | **1335.3111** | **1334.4773** | **0.8338** | **1** | **11** | **2.6e+02** | **1** | **LYNGRAAEAELK** |
|  | 1564 | **485.6316** | **1453.8726** | **1454.6657** | **-0.7931** | **0** | **11** | **2.2e+02** | **1** | **EIVTQFTTQFLK** |
|  | 1601 | **489.2206** | **1464.6396** | **1464.7765** | **-0.1369** | **2** | **11** | **2.5e+02** | **1** | **KHFMALIFSAKR + Oxidation (M)** |
|  | 1724 | **507.3900** | **1519.1479** | **1519.7406** | **-0.5928** | **1** | **11** | **1.8e+02** | **1** | **ISNVNKALDFIASK** |
|  | 1860 | **526.9901** | **1051.9653** | **1051.1573** | **0.8081** | **2** | **11** | **2.3e+02** | **1** | **GKFRTSAER** |
|  | 2091 | 560.6112 | 1678.8114 | 1678.9040 | -0.0925 | 1 | 11 | 2.9e+02 | 1 | MSPDEIKIPPEPPGR + Oxidation (M) |
|  | 3282 | **771.6265** | **2311.8574** | **2311.5759** | **0.2816** | **1** | **11** | **1.9e+02** | **1** | **RLSCAASGCTFSNYEMNWVR + Oxidation (M)** |
|  | 899 | **420.3172** | **838.6196** | **837.9021** | **0.7175** | **1** | **11** | **1.8e+02** | **1** | **SRTGMDR + Oxidation (M)** |
|  | 1420 | **469.4988** | **1405.4743** | **1406.6046** | **-1.1303** | **1** | **11** | **2.6e+02** | **1** | **TRSLISMDEINK** |
|  | 1641 | **495.1113** | **988.2079** | **987.1546** | **1.0532** | **1** | **11** | **2.5e+02** | **1** | **ESLRTLLR** |
|  | 2364 | 604.0964 | 1206.1781 | 1205.3418 | 0.8363 | 0 | 11 | 2.4e+02 | 1 | SQAEQCGTLIR |
|  | 825 | 416.0808 | 830.1467 | 829.9411 | 0.2056 | 0 | 11 | 2.9e+02 | 1 | LLASPSSR |
|  | 1505 | **476.8614** | **1427.5622** | **1428.6963** | **-1.1342** | **2** | **11** | **2.3e+02** | **1** | **LSKGEMKELLHK + Oxidation (M)** |
|  | 1844 | **524.7100** | **1571.1077** | **1570.8357** | **0.2720** | **1** | **11** | **2.2e+02** | **1** | **RLMSGPVPPSACSPR + Oxidation (M)** |
|  | 2361 | **603.1132** | **1806.3173** | **1805.1546** | **1.1627** | **2** | **11** | **2.4e+02** | **1** | **CIMQATDIMRKQGPR + Carbamidomethyl (C)** |
|  | 2938 | **690.8513** | **1379.6877** | **1378.5748** | **1.1130** | **1** | **11** | **2.6e+02** | **1** | **SVYPVAGGPTFKR** |
|  | 299 | **385.3942** | **1153.1605** | **1152.4516** | **0.7089** | **1** | **11** | **2.3e+02** | **1** | **TCLGPKSMMK + Carbamidomethyl (C)** |
|  | 1056 | **434.2637** | **866.5127** | **866.1010** | **0.4117** | **1** | **11** | **1.8e+02** | **1** | **MKEMLAK + Oxidation (M)** |
|  | 1989 | **542.9527** | **1083.8906** | **1084.2700** | **-0.3793** | **2** | **11** | **2.3e+02** | **1** | **APSGKLVERK** |
|  | 2107 | **562.7118** | **1685.1132** | **1684.8008** | **0.3124** | **2** | **11** | **2.5e+02** | **1** | **ALKEGREPDYSEYK** |
|  | 2724 | **663.5677** | **1325.1206** | **1324.6085** | **0.5121** | **0** | **11** | **1.8e+02** | **1** | **LFVINEVCEMK** |
|  | 1943 | **537.2435** | **1072.4723** | **1071.4021** | **1.0702** | **1** | **11** | **2.7e+02** | **1** | **VACNMMMKK + Oxidation (M)** |
|  | 1440 | **471.7710** | **941.5272** | **941.0847** | **0.4425** | **0** | **11** | **1.8e+02** | **1** | **MGMSNLTR + 2 Oxidation (M)** |
|  | 3079 | **740.3132** | **1478.6116** | **1479.6985** | **-1.0869** | **1** | **11** | **2.3e+02** | **1** | **MQSAKYFNYTVK** |
|  | 3219 | **753.0916** | **2256.2525** | **2257.3531** | **-1.1006** | **2** | **11** | **1.9e+02** | **1** | **AEDTAVYYCARGDSSSSRYR** |
|  | 3280 | **771.3793** | **2311.1156** | **2310.6492** | **0.4665** | **2** | **11** | **2.2e+02** | **1** | **GFPSVFRGGKYAATSQVLLPSK** |
|  | 960 | **428.2609** | **1281.7605** | **1281.3783** | **0.3822** | **0** | **11** | **1.7e+02** | **1** | **EAAGARPAGAGPTR** |
|  | 2571 | **634.6168** | **1900.8283** | **1901.0632** | **-0.2350** | **0** | **11** | **2e+02** | **1** | **HIQMTQSPSSLSASVGDR** |
|  | 864 | **418.1894** | **1251.5461** | **1252.3537** | **-0.8076** | **0** | **10** | **2.4e+02** | **1** | **MENNLAEFER** |
|  | 1136 | **443.1310** | **1326.3708** | **1326.3243** | **0.0464** | **0** | **10** | **2.3e+02** | **1** | **DGNGTTIDYWGQ** |
|  | 735 | **408.8115** | **1223.4124** | **1224.4530** | **-1.0406** | **2** | **10** | **2.5e+02** | **1** | **AGPLTKEKPKR** |
|  | 2031 | 550.9249 | 1099.8351 | 1099.2231 | 0.6119 | 0 | 10 | 2.4e+02 | 1 | HCTPAWATR + Carbamidomethyl (C) |
|  | 2249 | **591.6366** | **1771.8876** | **1772.9375** | **-1.0498** | **1** | **10** | **2.7e+02** | **1** | **EVSRTLGSGEGMAVSHR** |
|  | 2777 | **668.6580** | **2002.9517** | **2003.1950** | **-0.2433** | **0** | **10** | **2.2e+02** | **1** | **GDSGGPLVCGGVLEGVVTSGSR** |
|  | 1086 | **436.2290** | **1305.6647** | **1304.4946** | **1.1702** | **2** | **10** | **2.2e+02** | **1** | **YPLPTKKGSASR** |
|  | 1397 | **466.8989** | **931.7830** | **932.0347** | **-0.2516** | **1** | **10** | **2.5e+02** | **1** | **WKNSELR** |
|  | 1717 | **506.2060** | **1515.5958** | **1514.5968** | **0.9990** | **1** | **10** | **2.3e+02** | **1** | **RHTQAGEGTSLETK** |
|  | 1723 | **507.3886** | **1012.7623** | **1012.2273** | **0.5350** | **0** | **10** | **1.9e+02** | **1** | **VPPMVNVTR** |
|  | 3047 | **731.6851** | **1461.3553** | **1460.5939** | **0.7614** | **0** | **10** | **2e+02** | **1** | **QAQHILSSAHPSGK** |
|  | 308 | **386.0681** | **1155.1823** | **1155.3014** | **-0.1192** | **0** | **10** | **2.2e+02** | **1** | **IEPEAVLQTR** |
|  | 322 | **386.1988** | **1155.5743** | **1155.3247** | **0.2496** | **2** | **10** | **1.8e+02** | **1** | **EKYGDKMLR + Oxidation (M)** |
|  | 647 | **406.6915** | **1217.0524** | **1217.3276** | **-0.2752** | **0** | **10** | **1.8e+02** | **1** | **LDGWVLAGSTQA** |
|  | 1058 | **434.4162** | **1300.2265** | **1300.4182** | **-0.1916** | **0** | **10** | **2.2e+02** | **1** | **EVQLVQSGGEVR** |
|  | 1402 | **467.4028** | **932.7908** | **933.9663** | **-1.1754** | **1** | **10** | **2.1e+02** | **1** | **GSRSPSTSR** |
|  | 2576 | **635.7505** | **1904.2295** | **1904.1566** | **0.0728** | **2** | **10** | **2.8e+02** | **1** | **ALSLERARGTGASMAVAAR + Oxidation (M)** |
|  | 3210 | **749.7512** | **1497.4875** | **1498.6405** | **-1.1530** | **2** | **10** | **2.1e+02** | **1** | **TPQLTPGRKTEDR** |
|  | 1284 | **458.5205** | **1372.5394** | **1372.4842** | **0.0553** | **1** | **10** | **3.1e+02** | **1** | **VKSVSPQGNSVDR** |
|  | 1848 | **525.0297** | **1572.0670** | **1572.6279** | **-0.5609** | **0** | **10** | **2.5e+02** | **1** | **EFTETAAIFEDGSR** |
|  | 2234 | **587.8779** | **1760.6114** | **1760.1288** | **0.4826** | **0** | **10** | **2e+02** | **1** | **MFLAPCSIHLLNTVK + Carbamidomethyl (C); Oxidation (M)** |
|  | 498 | **400.1587** | **1197.4540** | **1197.4093** | **0.0447** | **1** | **10** | **2.1e+02** | **1** | **MAACGGTCKNK + 2 Carbamidomethyl (C)** |
|  | 1415 | **469.0297** | **936.0447** | **937.0083** | **-0.9636** | **0** | **10** | **2.5e+02** | **1** | **VPPRPGEDA** |
|  | 1434 | **471.3191** | **1410.9350** | **1410.4890** | **0.4461** | **1** | **10** | **1.8e+02** | **1** | **AGGGKGELDADPAPR** |
|  | 1687 | **504.1335** | **1006.2523** | **1005.1930** | **1.0593** | **1** | **10** | **2.6e+02** | **1** | **ISQRLCTGK** |
|  | 2716 | **659.7415** | **1976.2022** | **1975.2346** | **0.9676** | **2** | **10** | **3e+02** | **1** | **STPAAGSCCVRACPSAPRSGP** |
|  | 260 | **381.8125** | **1142.4153** | **1142.3540** | **0.0614** | **2** | **10** | **2.8e+02** | **1** | **KWLTSPVRR** |
|  | 1011 | **431.1575** | **860.3003** | **859.9308** | **0.3695** | **2** | **10** | **2.9e+02** | **1** | **ADRSRQK** |
|  | 1832 | **523.8352** | **1568.4834** | **1567.6346** | **0.8489** | **1** | **10** | **2.2e+02** | **1** | **DKAAAGTHDSFDMW + Oxidation (M)** |
|  | 2062 | **555.3552** | **1663.0433** | **1663.9158** | **-0.8725** | **0** | **10** | **2.3e+02** | **1** | **SPMNVMNVENPFIR + Oxidation (M)** |
|  | 2253 | **592.4631** | **1774.3670** | **1773.9222** | **0.4449** | **1** | **10** | **1.9e+02** | **1** | **GTEALRGGAMSEPAGDVR** |
[truncated: 3,419,005 more chars]
