# Supplementary material for: Tobacco-induced hyperglycemia promotes lung cancer progression via cancer cell-macrophage interaction through paracrine IGF2/IR/NPM1-driven PD-L1 expression
Source: Nat Commun. 2024 Jun 8;15:4909. doi: 10.1038/s41467-024-49199-9 (PMC11162468; doi:10.1038/s41467-024-49199-9)
Supplement: Supplementary file 3 — Supplementary Data 1 [file 41467_2024_49199_MOESM3_ESM.zip › Supplementary Data 1/2.htm]

Peptide Summary Report (../data/20120627/F011176.dat)


# Mascot Search Results

```
User            : yprc
Email           : info
Search title    : 
MS data file    : 2.xml
Database        : NCBInr 110704 (14481393 sequences; 4958963357 residues)
Taxonomy        : Homo sapiens (human) (217342 sequences)
Timestamp       : 27 Jun 2012 at 03:58:11 GMT

|  |  |  |
| --- | --- | --- |
| Protein hits    : | gi|62414289 | vimentin [Homo sapiens] |
|  | gi|193787214 | unnamed protein product [Homo sapiens] |
|  | gi|4757810 | ATP synthase subunit alpha, mitochondrial precursor [Homo sapiens] |
|  | gi|73760405 | thymopoietin isoform beta [Homo sapiens] |
|  | gi|5174735 | tubulin beta-2C chain [Homo sapiens] |
|  | gi|193785596 | unnamed protein product [Homo sapiens] |
|  | gi|7106439 | tubulin beta-5 chain [Mus musculus] |
|  | gi|4507729 | tubulin beta-2A chain [Homo sapiens] |
|  | gi|9755133 | XPMC2 protein [Homo sapiens] |
|  | gi|119589485 | tubulin, beta 4, isoform CRA_b [Homo sapiens] |
|  | gi|1297274 | beta-tubulin [Homo sapiens] |
|  | gi|10433717 | unnamed protein product [Homo sapiens] |
|  | gi|13129104 | coiled-coil domain-containing protein 86 [Homo sapiens] |
|  | gi|38014544 | Tubb5 protein [Rattus norvegicus] |
|  | gi|7657381 | pre-mRNA-processing factor 19 [Homo sapiens] |
|  | gi|119631904 | nebulin, isoform CRA_a [Homo sapiens] |
|  | gi|806562 | nebulin [Homo sapiens] |
|  | gi|119631909 | nebulin, isoform CRA_f [Homo sapiens] |
|  | gi|32189394 | ATP synthase subunit beta, mitochondrial precursor [Homo sapiens] |
|  | gi|28940 | unnamed protein product [Homo sapiens] |
```

### Probability Based Mowse Score

Ions score is -10\*Log(P), where P is the
probability that the observed match is a random event.  
Individual ions scores
> 47 indicate identity or extensive homology (p<0.05).  
Protein scores
are derived from ions scores as a non-probabilistic basis for ranking protein
hits.

### Peptide Summary Report

|  |  |  |  |
| --- | --- | --- | --- |
|  | Peptide Summary Select Summary (protein hits) Select Summary (unassigned) Export Search Results |  | Help |
|  | Significance threshold p< | Max. number of hits |  |
|  | Standard scoring  MudPIT scoring | Ions score or expect cut-off | Show sub-sets |
|  | Show pop-ups  Suppress pop-ups | Sort unassigned  Decreasing Score Increasing query / Mr Decreasing Intensity | Require bold red |

  
 
                
             


  


  
     **Error tolerant**    

|  |  |
| --- | --- |
| **1.** | gi|62414289    **Mass:** 53651    **Score:** 2151   **Queries matched:** 207   **emPAI:** 21.20 |
|  | vimentin [Homo sapiens] |

|  |  |
| --- | --- |
|  | Check to include this hit in error tolerant search or archive report |
|  |  |

|  |  |  |  |  |  |  |  |  |  |  |
| --- | --- | --- | --- | --- | --- | --- | --- | --- | --- | --- |
|  | **Query** | **Observed** | **Mr(expt)** | **Mr(calc)** | **Delta** | **Miss** | **Score** | **Expect** | **Rank** | **Peptide** |
|  | 1086 | **458.1244** | **914.2340** | **913.9716** | **0.2623** | **0** | **21** | **24** | **1** | **R.SYVTTSTR.T** |
|  | 1329 | **485.9291** | **969.8435** | **970.1276** | **-0.2841** | **1** | **(29)** | **3.4** | **1** | **R.LRSSVPGVR.L** |
|  | 1333 | **486.1971** | **970.3793** | **970.1276** | **0.2518** | **1** | **31** | **1.9** | **1** | **R.LRSSVPGVR.L** |
|  | 1334 | **486.2640** | **970.5132** | **970.1276** | **0.3856** | **1** | **(15)** | **85** | **2** | **R.LRSSVPGVR.L** |
|  | 1335 | **486.2930** | **970.5712** | **970.1276** | **0.4436** | **1** | **(28)** | **3.6** | **1** | **R.LRSSVPGVR.L** |
|  | 1723 | **532.1644** | **1062.3141** | **1062.1980** | **0.1160** | **0** | **15** | **98** | **5** | **K.LQEEMLQR.E + Oxidation (M)** |
|  | 1827 | **545.1134** | **1088.2120** | **1088.1293** | **0.0827** | **0** | **64** | **0.0011** | **1** | **R.QDVDNASLAR.L** |
|  | 1841 | **547.3596** | **1092.7045** | **1093.1474** | **-0.4430** | **0** | **71** | **0.00021** | **1** | **K.FADLSEAANR.N** |
|  | 1844 | **547.6176** | **1093.2203** | **1093.1474** | **0.0729** | **0** | **(56)** | **0.008** | **1** | **K.FADLSEAANR.N** |
|  | 1913 | **561.6705** | **1121.3263** | **1121.2403** | **0.0859** | **0** | **27** | **6.1** | **1** | **R.EYQDLLNVK.M** |
|  | 2043 | **585.6261** | **1169.2374** | **1169.4106** | **-0.1732** | **0** | **55** | **0.0093** | **1** | **K.ILLAELEQLK.G** |
|  | 2054 | **587.4657** | **1172.9166** | **1173.4028** | **-0.4862** | **1** | **(46)** | **0.057** | **1** | **R.TLLIKTVETR.D** |
|  | 2058 | **587.5499** | **1173.0851** | **1173.4028** | **-0.3178** | **1** | **69** | **0.0003** | **1** | **R.TLLIKTVETR.D** |
|  | 375 | **392.4074** | **1174.2001** | **1173.4028** | **0.7973** | **1** | **(29)** | **3.9** | **1** | **R.TLLIKTVETR.D** |
|  | 377 | **392.4977** | **1174.4709** | **1173.4028** | **1.0680** | **1** | **(36)** | **0.7** | **1** | **R.TLLIKTVETR.D** |
|  | 2534 | **655.4051** | **1308.7954** | **1309.3800** | **-0.5846** | **0** | **(32)** | **1.6** | **1** | **K.NLQEAEEWYK.S** |
|  | 2537 | **655.6068** | **1309.1988** | **1309.3800** | **-0.1812** | **0** | **(8)** | **3.2e+02** | **6** | **K.NLQEAEEWYK.S** |
|  | 2540 | **656.1443** | **1310.2738** | **1309.3800** | **0.8938** | **0** | **36** | **0.59** | **1** | **K.NLQEAEEWYK.S** |
|  | 979 | **444.2782** | **1329.8124** | **1329.5885** | **0.2239** | **2** | **27** | **4.9** | **1** | **K.RTLLIKTVETR.D** |
|  | 1177 | **465.4945** | **1393.4613** | **1393.5016** | **-0.0404** | **1** | **(36)** | **0.72** | **1** | **R.DVRQQYESVAAK.N** |
|  | 2789 | **697.7607** | **1393.5067** | **1393.5016** | **0.0051** | **1** | **45** | **0.1** | **1** | **R.DVRQQYESVAAK.N** |
|  | 2827 | **714.7654** | **1427.5160** | **1428.5456** | **-1.0297** | **0** | **(8)** | **4.8e+02** | **2** | **R.SLYASSPGGVYATR.S** |
|  | 2829 | **714.7822** | **1427.5497** | **1428.5456** | **-0.9960** | **0** | **(15)** | **99** | **1** | **R.SLYASSPGGVYATR.S** |
|  | 2830 | **714.8660** | **1427.7173** | **1428.5456** | **-0.8284** | **0** | **(10)** | **3.2e+02** | **1** | **R.SLYASSPGGVYATR.S** |
|  | 2831 | **714.9754** | **1427.9360** | **1428.5456** | **-0.6096** | **0** | **(66)** | **0.0005** | **1** | **R.SLYASSPGGVYATR.S** |
|  | 2832 | **715.0773** | **1428.1398** | **1428.5456** | **-0.4059** | **0** | **(49)** | **0.027** | **1** | **R.SLYASSPGGVYATR.S** |
|  | 2833 | **715.1013** | **1428.1877** | **1428.5456** | **-0.3579** | **0** | **(44)** | **0.1** | **1** | **R.SLYASSPGGVYATR.S** |
|  | 2834 | **715.1086** | **1428.2025** | **1428.5456** | **-0.3431** | **0** | **(52)** | **0.017** | **1** | **R.SLYASSPGGVYATR.S** |
|  | 2835 | **715.1232** | **1428.2316** | **1428.5456** | **-0.3141** | **0** | **(57)** | **0.0058** | **1** | **R.SLYASSPGGVYATR.S** |
|  | 2836 | **715.1440** | **1428.2733** | **1428.5456** | **-0.2723** | **0** | **(52)** | **0.017** | **1** | **R.SLYASSPGGVYATR.S** |
|  | 2837 | **715.1512** | **1428.2877** | **1428.5456** | **-0.2579** | **0** | **(58)** | **0.0038** | **1** | **R.SLYASSPGGVYATR.S** |
|  | 2838 | **715.1655** | **1428.3163** | **1428.5456** | **-0.2294** | **0** | **(71)** | **0.00021** | **1** | **R.SLYASSPGGVYATR.S** |
|  | 2839 | **715.1826** | **1428.3503** | **1428.5456** | **-0.1953** | **0** | **(59)** | **0.0032** | **1** | **R.SLYASSPGGVYATR.S** |
|  | 2840 | **715.2111** | **1428.4073** | **1428.5456** | **-0.1383** | **0** | **(52)** | **0.018** | **1** | **R.SLYASSPGGVYATR.S** |
|  | 2841 | **715.2139** | **1428.4131** | **1428.5456** | **-0.1326** | **0** | **(56)** | **0.0073** | **1** | **R.SLYASSPGGVYATR.S** |
|  | 2842 | **715.2271** | **1428.4393** | **1428.5456** | **-0.1063** | **0** | **(64)** | **0.00094** | **1** | **R.SLYASSPGGVYATR.S** |
|  | 2843 | **715.3545** | **1428.6942** | **1428.5456** | **0.1486** | **0** | **(55)** | **0.0085** | **1** | **R.SLYASSPGGVYATR.S** |
|  | 2844 | **715.4484** | **1428.8819** | **1428.5456** | **0.3363** | **0** | **(15)** | **80** | **1** | **R.SLYASSPGGVYATR.S** |
|  | 2845 | **715.5173** | **1429.0199** | **1428.5456** | **0.4742** | **0** | **82** | **1.6e-05** | **1** | **R.SLYASSPGGVYATR.S** |
|  | 2846 | **715.6626** | **1429.3104** | **1428.5456** | **0.7648** | **0** | **(57)** | **0.0043** | **1** | **R.SLYASSPGGVYATR.S** |
|  | 2847 | **715.7421** | **1429.4693** | **1428.5456** | **0.9237** | **0** | **(80)** | **2.9e-05** | **1** | **R.SLYASSPGGVYATR.S** |
|  | 2849 | **715.7794** | **1429.5441** | **1428.5456** | **0.9984** | **0** | **(39)** | **0.37** | **1** | **R.SLYASSPGGVYATR.S** |
|  | 1314 | **482.2364** | **1443.6870** | **1443.5652** | **0.1217** | **2** | **57** | **0.0054** | **1** | **R.RQVDQLTNDKAR.V** |
|  | 1381 | **491.5553** | **1471.6438** | **1470.6769** | **0.9669** | **2** | **29** | **4** | **1** | **R.SSAVRLRSSVPGVR.L** |
|  | 1382 | **491.5972** | **1471.7694** | **1470.6769** | **1.0926** | **2** | **(26)** | **7.6** | **1** | **R.SSAVRLRSSVPGVR.L** |
|  | 1429 | **498.8465** | **1493.5173** | **1494.6320** | **-1.1147** | **0** | **(18)** | **40** | **1** | **R.MFGGPGTASRPSSSR.S** |
|  | 2967 | **747.9111** | **1493.8075** | **1494.6320** | **-0.8245** | **0** | **(32)** | **1.8** | **1** | **R.MFGGPGTASRPSSSR.S** |
|  | 1431 | **499.1139** | **1494.3196** | **1494.6320** | **-0.3124** | **0** | **(20)** | **23** | **1** | **R.MFGGPGTASRPSSSR.S** |
|  | 1432 | **499.2308** | **1494.6701** | **1495.6365** | **-0.9664** | **0** | **(15)** | **83** | **1** | **R.TYSLGSALRPSTSR.S** |
|  | 2970 | **748.4442** | **1494.8735** | **1495.6365** | **-0.7630** | **0** | **(35)** | **0.85** | **1** | **R.TYSLGSALRPSTSR.S** |
|  | 1433 | **499.3059** | **1494.8956** | **1495.6365** | **-0.7410** | **0** | **(5)** | **7.5e+02** | **2** | **R.TYSLGSALRPSTSR.S** |
|  | 1434 | **499.3626** | **1495.0657** | **1495.6365** | **-0.5709** | **0** | **(16)** | **48** | **1** | **R.TYSLGSALRPSTSR.S** |
|  | 2971 | **748.5568** | **1495.0987** | **1495.6365** | **-0.5378** | **0** | **(34)** | **1** | **1** | **R.TYSLGSALRPSTSR.S** |
|  | 2972 | **748.6536** | **1495.2925** | **1494.6320** | **0.6605** | **0** | **(21)** | **16** | **1** | **R.MFGGPGTASRPSSSR.S** |
|  | 1435 | **499.4423** | **1495.3046** | **1495.6365** | **-0.3319** | **0** | **(24)** | **7.6** | **1** | **R.TYSLGSALRPSTSR.S** |
|  | 1437 | **499.4917** | **1495.4529** | **1495.6365** | **-0.1836** | **0** | **(26)** | **6.3** | **1** | **R.TYSLGSALRPSTSR.S** |
|  | 2974 | **748.7348** | **1495.4548** | **1495.6365** | **-0.1817** | **0** | **(3)** | **1e+03** | **3** | **R.TYSLGSALRPSTSR.S** |
|  | 1438 | **499.4945** | **1495.4613** | **1495.6365** | **-0.1752** | **0** | **(2)** | **1.4e+03** | **7** | **R.TYSLGSALRPSTSR.S** |
|  | 2976 | **748.7833** | **1495.5517** | **1495.6365** | **-0.0848** | **0** | **(31)** | **2.4** | **1** | **R.TYSLGSALRPSTSR.S** |
|  | 2977 | **748.7871** | **1495.5594** | **1495.6365** | **-0.0771** | **0** | **(8)** | **4.1e+02** | **2** | **R.TYSLGSALRPSTSR.S** |
|  | 2978 | **748.7964** | **1495.5780** | **1495.6365** | **-0.0585** | **0** | **(38)** | **0.41** | **1** | **R.TYSLGSALRPSTSR.S** |
|  | 2984 | **748.8477** | **1495.6807** | **1495.6365** | **0.0441** | **0** | **(42)** | **0.2** | **1** | **R.TYSLGSALRPSTSR.S** |
|  | 1440 | **499.6003** | **1495.7788** | **1495.6365** | **0.1422** | **0** | **(17)** | **55** | **1** | **R.TYSLGSALRPSTSR.S** |
|  | 1441 | **499.6466** | **1495.9176** | **1495.6365** | **0.2811** | **0** | **(23)** | **14** | **1** | **R.TYSLGSALRPSTSR.S** |
|  | 1442 | **499.6580** | **1495.9519** | **1495.6365** | **0.3154** | **0** | **(17)** | **47** | **2** | **R.TYSLGSALRPSTSR.S** |
|  | 1443 | **499.6650** | **1495.9729** | **1495.6365** | **0.3364** | **0** | **(19)** | **34** | **2** | **R.TYSLGSALRPSTSR.S** |
|  | 1445 | **499.6939** | **1496.0595** | **1495.6365** | **0.4229** | **0** | **(24)** | **7.9** | **1** | **R.TYSLGSALRPSTSR.S** |
|  | 2992 | **749.0385** | **1496.0622** | **1495.6365** | **0.4257** | **0** | **(2)** | **1.4e+03** | **4** | **R.TYSLGSALRPSTSR.S** |
|  | 1446 | **499.7245** | **1496.1515** | **1495.6365** | **0.5149** | **0** | **(25)** | **6.8** | **1** | **R.TYSLGSALRPSTSR.S** |
|  | 1447 | **499.7292** | **1496.1653** | **1495.6365** | **0.5288** | **0** | **(4)** | **8.4e+02** | **3** | **R.TYSLGSALRPSTSR.S** |
|  | 1449 | **499.7497** | **1496.2269** | **1495.6365** | **0.5904** | **0** | **(12)** | **1.3e+02** | **3** | **R.TYSLGSALRPSTSR.S** |
|  | 1450 | **499.7522** | **1496.2344** | **1495.6365** | **0.5979** | **0** | **(16)** | **46** | **2** | **R.TYSLGSALRPSTSR.S** |
|  | 1451 | **499.8096** | **1496.4066** | **1495.6365** | **0.7701** | **0** | **(15)** | **67** | **2** | **R.TYSLGSALRPSTSR.S** |
|  | 1452 | **499.8214** | **1496.4422** | **1495.6365** | **0.8056** | **0** | **(15)** | **70** | **2** | **R.TYSLGSALRPSTSR.S** |
|  | 1453 | **499.8350** | **1496.4827** | **1495.6365** | **0.8462** | **0** | **(20)** | **23** | **1** | **R.TYSLGSALRPSTSR.S** |
|  | 1454 | **499.8409** | **1496.5005** | **1495.6365** | **0.8639** | **0** | **(38)** | **0.34** | **1** | **R.TYSLGSALRPSTSR.S** |
|  | 1455 | **499.8478** | **1496.5213** | **1495.6365** | **0.8848** | **0** | **(18)** | **41** | **1** | **R.TYSLGSALRPSTSR.S** |
|  | 1456 | **499.8617** | **1496.5628** | **1495.6365** | **0.9263** | **0** | **46** | **0.066** | **1** | **R.TYSLGSALRPSTSR.S** |
|  | 3009 | **749.3035** | **1496.5922** | **1495.6365** | **0.9556** | **0** | **(11)** | **2e+02** | **1** | **R.TYSLGSALRPSTSR.S** |
|  | 1457 | **499.9070** | **1496.6990** | **1495.6365** | **1.0624** | **0** | **(42)** | **0.18** | **1** | **R.TYSLGSALRPSTSR.S** |
|  | 1458 | **499.9319** | **1496.7736** | **1495.6365** | **1.1370** | **0** | **(28)** | **4.3** | **1** | **R.TYSLGSALRPSTSR.S** |
|  | 1488 | **504.2891** | **1509.8450** | **1510.6314** | **-0.7864** | **0** | **(10)** | **2.9e+02** | **1** | **R.MFGGPGTASRPSSSR.S + Oxidation (M)** |
|  | 1490 | **504.4336** | **1510.2785** | **1510.6314** | **-0.3529** | **0** | **(33)** | **1.2** | **1** | **R.MFGGPGTASRPSSSR.S + Oxidation (M)** |
|  | 1491 | **504.4456** | **1510.3148** | **1510.6314** | **-0.3166** | **0** | **(45)** | **0.076** | **1** | **R.MFGGPGTASRPSSSR.S + Oxidation (M)** |
|  | 3030 | **756.1921** | **1510.3695** | **1510.6314** | **-0.2619** | **0** | **(29)** | **3** | **1** | **R.MFGGPGTASRPSSSR.S + Oxidation (M)** |
|  | 3031 | **756.2419** | **1510.4691** | **1510.6314** | **-0.1623** | **0** | **50** | **0.025** | **1** | **R.MFGGPGTASRPSSSR.S + Oxidation (M)** |
|  | 1492 | **504.5174** | **1510.5300** | **1510.6314** | **-0.1014** | **0** | **(44)** | **0.13** | **1** | **R.MFGGPGTASRPSSSR.S + Oxidation (M)** |
|  | 1493 | **504.5997** | **1510.7768** | **1510.6314** | **0.1455** | **0** | **(12)** | **2e+02** | **1** | **R.MFGGPGTASRPSSSR.S + Oxidation (M)** |
|  | 1496 | **504.6768** | **1511.0081** | **1510.6314** | **0.3767** | **0** | **(48)** | **0.041** | **1** | **R.MFGGPGTASRPSSSR.S + Oxidation (M)** |
|  | 1497 | **504.7342** | **1511.1803** | **1510.6314** | **0.5489** | **0** | **(32)** | **1.4** | **1** | **R.MFGGPGTASRPSSSR.S + Oxidation (M)** |
|  | 1498 | **504.7369** | **1511.1886** | **1510.6314** | **0.5573** | **0** | **(47)** | **0.047** | **1** | **R.MFGGPGTASRPSSSR.S + Oxidation (M)** |
|  | 3032 | **756.7118** | **1511.4088** | **1510.6314** | **0.7774** | **0** | **(28)** | **2.9** | **1** | **R.MFGGPGTASRPSSSR.S + Oxidation (M)** |
|  | 3033 | **756.7314** | **1511.4481** | **1510.6314** | **0.8167** | **0** | **(43)** | **0.097** | **1** | **R.MFGGPGTASRPSSSR.S + Oxidation (M)** |
|  | 1500 | **504.9255** | **1511.7543** | **1510.6314** | **1.1229** | **0** | **(27)** | **5** | **1** | **R.MFGGPGTASRPSSSR.S + Oxidation (M)** |
|  | 1538 | **508.9904** | **1523.9490** | **1524.6296** | **-0.6807** | **1** | **(37)** | **0.52** | **1** | **K.NLQEAEEWYKSK.F** |
|  | 3054 | **763.1932** | **1524.3716** | **1524.6296** | **-0.2581** | **1** | **(62)** | **0.0016** | **1** | **K.NLQEAEEWYKSK.F** |
|  | 3055 | **763.2119** | **1524.4089** | **1524.6296** | **-0.2207** | **1** | **67** | **0.00054** | **1** | **K.NLQEAEEWYKSK.F** |
|  | 1541 | **509.3192** | **1524.9356** | **1524.6296** | **0.3059** | **1** | **(31)** | **1.9** | **1** | **K.NLQEAEEWYKSK.F** |
|  | 1543 | **510.5560** | **1528.6457** | **1527.7229** | **0.9228** | **1** | **52** | **0.023** | **1** | **R.HLREYQDLLNVK.M** |
|  | 3077 | **767.7169** | **1533.4191** | **1533.7640** | **-0.3450** | **1** | **(64)** | **0.00081** | **1** | **R.KVESLQEEIAFLK.K** |
|  | 3078 | **767.7756** | **1533.5365** | **1533.7640** | **-0.2276** | **1** | **78** | **4.2e-05** | **1** | **R.KVESLQEEIAFLK.K** |
|  | 3079 | **767.7770** | **1533.5392** | **1533.7640** | **-0.2249** | **1** | **(35)** | **0.87** | **1** | **R.KVESLQEEIAFLK.K** |
|  | 1555 | **512.4220** | **1534.2438** | **1533.7640** | **0.4798** | **1** | **(58)** | **0.0031** | **1** | **R.KVESLQEEIAFLK.K** |
|  | 1556 | **512.4808** | **1534.4203** | **1533.7640** | **0.6563** | **1** | **(71)** | **0.00019** | **1** | **R.KVESLQEEIAFLK.K** |
|  | 1566 | **514.4370** | **1540.2889** | **1539.8147** | **0.4742** | **1** | **(17)** | **39** | **1** | **K.ILLAELEQLKGQGK.S** |
|  | 3083 | **771.2303** | **1540.4459** | **1539.8147** | **0.6312** | **1** | **60** | **0.0024** | **1** | **K.ILLAELEQLKGQGK.S** |
|  | 1568 | **514.5745** | **1540.7012** | **1539.8147** | **0.8865** | **1** | **(40)** | **0.3** | **1** | **K.ILLAELEQLKGQGK.S** |
|  | 1569 | **514.6674** | **1540.9799** | **1539.8147** | **1.1652** | **1** | **(42)** | **0.16** | **1** | **K.ILLAELEQLKGQGK.S** |
|  | 1716 | **530.4187** | **1588.2339** | **1587.6872** | **0.5467** | **1** | **40** | **0.22** | **1** | **R.TNEKVELQELNDR.F** |
|  | 1863 | **551.6482** | **1651.9224** | **1650.8177** | **1.1047** | **1** | **44** | **0.14** | **1** | **R.RMFGGPGTASRPSSSR.S** |
|  | 1864 | **551.6760** | **1652.0057** | **1650.8177** | **1.1880** | **1** | **(36)** | **0.76** | **1** | **R.RMFGGPGTASRPSSSR.S** |
|  | 1867 | **552.1337** | **1653.3788** | **1652.8235** | **0.5553** | **1** | **(42)** | **0.19** | **1** | **R.LGDLYEEEMRELR.R** |
|  | 1882 | **555.1788** | **1662.5143** | **1661.9363** | **0.5780** | **2** | **(42)** | **0.15** | **1** | **R.KVESLQEEIAFLKK.L** |
|  | 1883 | **555.2501** | **1662.7282** | **1661.9363** | **0.7919** | **2** | **58** | **0.004** | **1** | **R.KVESLQEEIAFLKK.L** |
|  | 3295 | **834.2674** | **1666.5200** | **1666.8171** | **-0.2971** | **1** | **(35)** | **0.87** | **1** | **R.RMFGGPGTASRPSSSR.S + Oxidation (M)** |
|  | 1893 | **556.8126** | **1667.4157** | **1666.8171** | **0.5986** | **1** | **(36)** | **0.51** | **1** | **R.RMFGGPGTASRPSSSR.S + Oxidation (M)** |
|  | 3296 | **835.2340** | **1668.4532** | **1668.8229** | **-0.3697** | **1** | **(29)** | **3** | **1** | **R.LGDLYEEEMRELR.R + Oxidation (M)** |
|  | 1898 | **557.4318** | **1669.2731** | **1668.8229** | **0.4502** | **1** | **46** | **0.049** | **1** | **R.LGDLYEEEMRELR.R + Oxidation (M)** |
|  | 1899 | **557.6702** | **1669.9885** | **1668.8229** | **1.1656** | **1** | **(31)** | **2.7** | **1** | **R.LGDLYEEEMRELR.R + Oxidation (M)** |
|  | 3324 | **853.1700** | **1704.3253** | **1704.8569** | **-0.5316** | **1** | **43** | **0.095** | **1** | **R.VEVERDNLAEDIMR.L + Oxidation (M)** |
|  | 2072 | **590.0087** | **1767.0040** | **1766.9478** | **0.0563** | **1** | **(25)** | **8.2** | **1** | **R.LQDEIQNMKEEMAR.H + 2 Oxidation (M)** |
|  | 3347 | **884.7201** | **1767.4254** | **1766.9478** | **0.4777** | **1** | **(40)** | **0.22** | **1** | **R.LQDEIQNMKEEMAR.H + 2 Oxidation (M)** |
|  | 2073 | **590.1520** | **1767.4339** | **1766.9478** | **0.4862** | **1** | **42** | **0.19** | **1** | **R.LQDEIQNMKEEMAR.H + 2 Oxidation (M)** |
|  | 2074 | **590.2034** | **1767.5881** | **1766.9478** | **0.6404** | **1** | **(37)** | **0.57** | **1** | **R.LQDEIQNMKEEMAR.H + 2 Oxidation (M)** |
|  | 2099 | **592.9519** | **1775.8335** | **1776.8612** | **-1.0277** | **1** | **(7)** | **4.8e+02** | **3** | **K.FADLSEAANRNNDALR.Q** |
|  | 2100 | **593.0111** | **1776.0112** | **1776.8612** | **-0.8501** | **1** | **(9)** | **3.3e+02** | **5** | **K.FADLSEAANRNNDALR.Q** |
|  | 3350 | **889.2459** | **1776.4769** | **1776.8612** | **-0.3843** | **1** | **(53)** | **0.01** | **1** | **K.FADLSEAANRNNDALR.Q** |
|  | 3352 | **889.2820** | **1776.5492** | **1776.8612** | **-0.3121** | **1** | **(49)** | **0.027** | **1** | **K.FADLSEAANRNNDALR.Q** |
|  | 2103 | **593.2668** | **1776.7782** | **1776.8612** | **-0.0831** | **1** | **(56)** | **0.0068** | **1** | **K.FADLSEAANRNNDALR.Q** |
|  | 2106 | **593.3073** | **1776.8998** | **1776.8612** | **0.0385** | **1** | **(9)** | **3.6e+02** | **3** | **K.FADLSEAANRNNDALR.Q** |
|  | 2107 | **593.3945** | **1777.1614** | **1776.8612** | **0.3002** | **1** | **(11)** | **1.9e+02** | **1** | **K.FADLSEAANRNNDALR.Q** |
|  | 2108 | **593.5016** | **1777.4826** | **1776.8612** | **0.6214** | **1** | **(30)** | **2.4** | **1** | **K.FADLSEAANRNNDALR.Q** |
|  | 2110 | **593.5886** | **1777.7437** | **1776.8612** | **0.8825** | **1** | **(6)** | **6.1e+02** | **4** | **K.FADLSEAANRNNDALR.Q** |
|  | 2112 | **593.6707** | **1777.9898** | **1776.8612** | **1.1286** | **1** | **(21)** | **28** | **1** | **K.FADLSEAANRNNDALR.Q** |
|  | 2113 | **593.6731** | **1777.9971** | **1776.8612** | **1.1359** | **1** | **67** | **0.00065** | **1** | **K.FADLSEAANRNNDALR.Q** |
|  | 2114 | **593.6738** | **1777.9993** | **1776.8612** | **1.1381** | **1** | **(63)** | **0.0017** | **1** | **K.FADLSEAANRNNDALR.Q** |
|  | 2131 | **595.4662** | **1783.3764** | **1783.0777** | **0.2987** | **2** | **(49)** | **0.027** | **1** | **K.ILLAELEQLKGQGKSR.L** |
|  | 2132 | **595.4887** | **1783.4440** | **1783.0777** | **0.3662** | **2** | **65** | **0.00082** | **1** | **K.ILLAELEQLKGQGKSR.L** |
|  | 2220 | **603.9775** | **1808.9103** | **1809.0092** | **-0.0990** | **2** | **45** | **0.084** | **1** | **R.LGDLYEEEMRELRR.Q** |
|  | 3368 | **913.2670** | **1824.5192** | **1825.0086** | **-0.4895** | **2** | **(18)** | **34** | **1** | **R.LGDLYEEEMRELRR.Q + Oxidation (M)** |
|  | 2255 | **609.2184** | **1824.6330** | **1825.0086** | **-0.3757** | **2** | **(34)** | **1.2** | **1** | **R.LGDLYEEEMRELRR.Q + Oxidation (M)** |
|  | 2257 | **609.3021** | **1824.8842** | **1825.0086** | **-0.1244** | **2** | **(17)** | **51** | **1** | **R.LGDLYEEEMRELRR.Q + Oxidation (M)** |
|  | 3369 | **913.5269** | **1825.0389** | **1824.9854** | **0.0535** | **1** | **62** | **0.0017** | **1** | **R.ETNLDSLPLVDTHSKR.T** |
|  | 2260 | **609.5756** | **1825.7045** | **1824.9854** | **0.7191** | **1** | **(34)** | **1** | **1** | **R.ETNLDSLPLVDTHSKR.T** |
|  | 2261 | **609.6250** | **1825.8528** | **1825.0086** | **0.8442** | **2** | **(32)** | **1.6** | **1** | **R.LGDLYEEEMRELRR.Q + Oxidation (M)** |
|  | 2262 | **609.6416** | **1825.9026** | **1825.0086** | **0.8940** | **2** | **(43)** | **0.15** | **1** | **R.LGDLYEEEMRELRR.Q + Oxidation (M)** |
|  | 2307 | **613.0337** | **1836.0789** | **1836.8238** | **-0.7449** | **0** | **(7)** | **6e+02** | **7** | **R.DGQVINETSQHHDDLE.-** |
|  | 3373 | **919.2212** | **1836.4276** | **1836.8238** | **-0.3962** | **0** | **(47)** | **0.042** | **1** | **R.DGQVINETSQHHDDLE.-** |
|  | 3374 | **919.2697** | **1836.5245** | **1836.8238** | **-0.2993** | **0** | **50** | **0.023** | **1** | **R.DGQVINETSQHHDDLE.-** |
|  | 2317 | **613.5677** | **1837.6811** | **1836.8238** | **0.8572** | **0** | **(18)** | **33** | **1** | **R.DGQVINETSQHHDDLE.-** |
|  | 3382 | **965.3291** | **1928.6434** | **1929.0949** | **-0.4515** | **1** | **42** | **0.12** | **1** | **R.SLYASSPGGVYATRSSAVR.L** |
|  | 3383 | **965.3950** | **1928.7753** | **1929.0949** | **-0.3197** | **1** | **(39)** | **0.31** | **1** | **R.SLYASSPGGVYATRSSAVR.L** |
|  | 2478 | **644.3561** | **1930.0461** | **1929.0949** | **0.9511** | **1** | **(20)** | **25** | **1** | **R.SLYASSPGGVYATRSSAVR.L** |
|  | 2479 | **644.3863** | **1930.1367** | **1929.0949** | **1.0418** | **1** | **(21)** | **23** | **1** | **R.SLYASSPGGVYATRSSAVR.L** |
|  | 2518 | **653.7000** | **1958.0777** | **1958.2007** | **-0.1231** | **2** | **11** | **2.6e+02** | **8** | **R.VEVERDNLAEDIMRLR.E** |
|  | 2520 | **654.0589** | **1959.1545** | **1958.2007** | **0.9538** | **2** | **(5)** | **8.2e+02** | **3** | **R.VEVERDNLAEDIMRLR.E** |
|  | 2579 | **665.3218** | **1992.9432** | **1992.1108** | **0.8323** | **2** | **40** | **0.27** | **1** | **K.SKFADLSEAANRNNDALR.Q** |
|  | 2801 | **702.3351** | **2103.9831** | **2104.2406** | **-0.2576** | **2** | **68** | **0.00045** | **1** | **K.FADLSEAANRNNDALRQAK.Q** |
|  | 2802 | **702.5337** | **2104.5789** | **2104.2406** | **0.3383** | **2** | **(62)** | **0.0014** | **1** | **K.FADLSEAANRNNDALRQAK.Q** |
|  | 2890 | **734.0176** | **2199.0306** | **2198.4382** | **0.5923** | **2** | **(9)** | **2.4e+02** | **1** | **R.SLYASSPGGVYATRSSAVRLR.S** |
|  | 2891 | **734.1108** | **2199.3103** | **2198.4382** | **0.8721** | **2** | **14** | **90** | **1** | **R.SLYASSPGGVYATRSSAVRLR.S** |
|  | 3134 | **789.9568** | **2366.8482** | **2367.5481** | **-0.6999** | **1** | **17** | **59** | **1** | **K.LQEEMLQREEAENTLQSFR.Q + Oxidation (M)** |
|  | 3419 | **1189.4766** | **2376.9383** | **2377.5844** | **-0.6461** | **1** | **(65)** | **0.00053** | **1** | **R.QVQSLTCEVDALKGTNESLER.Q + Carbamidomethyl (C)** |
|  | 3144 | **793.3457** | **2377.0149** | **2377.5844** | **-0.5695** | **1** | **(38)** | **0.39** | **1** | **R.QVQSLTCEVDALKGTNESLER.Q + Carbamidomethyl (C)** |
|  | 3145 | **793.3658** | **2377.0754** | **2377.5844** | **-0.5091** | **1** | **(68)** | **0.00042** | **1** | **R.QVQSLTCEVDALKGTNESLER.Q + Carbamidomethyl (C)** |
|  | 3146 | **793.5016** | **2377.4828** | **2377.5844** | **-0.1017** | **1** | **(42)** | **0.17** | **1** | **R.QVQSLTCEVDALKGTNESLER.Q + Carbamidomethyl (C)** |
|  | 3147 | **793.6010** | **2377.7809** | **2377.5844** | **0.1964** | **1** | **(22)** | **16** | **1** | **R.QVQSLTCEVDALKGTNESLER.Q + Carbamidomethyl (C)** |
|  | 3148 | **793.6812** | **2378.0213** | **2377.5844** | **0.4369** | **1** | **(53)** | **0.011** | **1** | **R.QVQSLTCEVDALKGTNESLER.Q + Carbamidomethyl (C)** |
|  | 3150 | **793.7402** | **2378.1985** | **2377.5844** | **0.6141** | **1** | **(24)** | **7.6** | **1** | **R.QVQSLTCEVDALKGTNESLER.Q + Carbamidomethyl (C)** |
|  | 3151 | **793.7443** | **2378.2108** | **2377.5844** | **0.6264** | **1** | **(36)** | **0.47** | **1** | **R.QVQSLTCEVDALKGTNESLER.Q + Carbamidomethyl (C)** |
|  | 3152 | **793.7446** | **2378.2117** | **2377.5844** | **0.6273** | **1** | **(29)** | **2.5** | **1** | **R.QVQSLTCEVDALKGTNESLER.Q + Carbamidomethyl (C)** |
|  | 3153 | **793.7689** | **2378.2846** | **2377.5844** | **0.7002** | **1** | **(62)** | **0.0013** | **1** | **R.QVQSLTCEVDALKGTNESLER.Q + Carbamidomethyl (C)** |
|  | 3154 | **793.7913** | **2378.3516** | **2377.5844** | **0.7672** | **1** | **73** | **0.00012** | **1** | **R.QVQSLTCEVDALKGTNESLER.Q + Carbamidomethyl (C)** |
|  | 3155 | **793.8789** | **2378.6145** | **2377.5844** | **1.0301** | **1** | **(27)** | **6.1** | **1** | **R.QVQSLTCEVDALKGTNESLER.Q + Carbamidomethyl (C)** |
|  | 3156 | **793.8878** | **2378.6411** | **2377.5844** | **1.0567** | **1** | **(66)** | **0.00083** | **1** | **R.QVQSLTCEVDALKGTNESLER.Q + Carbamidomethyl (C)** |
|  | 3163 | **797.9430** | **2390.8068** | **2391.5929** | **-0.7861** | **1** | **(17)** | **64** | **1** | **R.SYVTTSTRTYSLGSALRPSTSR.S** |
|  | 3164 | **797.9841** | **2390.9302** | **2390.5884** | **0.3419** | **1** | **(11)** | **2.3e+02** | **1** | **R.MFGGPGTASRPSSSRSYVTTSTR.T** |
|  | 3165 | **798.0231** | **2391.0470** | **2391.5929** | **-0.5459** | **1** | **(14)** | **86** | **1** | **R.SYVTTSTRTYSLGSALRPSTSR.S** |
|  | 3166 | **798.0303** | **2391.0688** | **2391.5929** | **-0.5241** | **1** | **(23)** | **10** | **1** | **R.SYVTTSTRTYSLGSALRPSTSR.S** |
|  | 3167 | **798.0769** | **2391.2085** | **2391.5929** | **-0.3844** | **1** | **(8)** | **3.6e+02** | **1** | **R.SYVTTSTRTYSLGSALRPSTSR.S** |
|  | 3169 | **798.2038** | **2391.5892** | **2391.5929** | **-0.0037** | **1** | **(7)** | **5.1e+02** | **2** | **R.SYVTTSTRTYSLGSALRPSTSR.S** |
|  | 3171 | **798.2360** | **2391.6859** | **2391.5929** | **0.0930** | **1** | **(17)** | **50** | **1** | **R.SYVTTSTRTYSLGSALRPSTSR.S** |
|  | 3173 | **798.2703** | **2391.7886** | **2391.5929** | **0.1957** | **1** | **(4)** | **9.2e+02** | **1** | **R.SYVTTSTRTYSLGSALRPSTSR.S** |
|  | 3174 | **798.2715** | **2391.7925** | **2391.5929** | **0.1995** | **1** | **(1)** | **2e+03** | **4** | **R.SYVTTSTRTYSLGSALRPSTSR.S** |
|  | 3175 | **798.3038** | **2391.8891** | **2391.5929** | **0.2962** | **1** | **(6)** | **6e+02** | **1** | **R.SYVTTSTRTYSLGSALRPSTSR.S** |
|  | 3176 | **798.3472** | **2392.0193** | **2391.5929** | **0.4264** | **1** | **(23)** | **13** | **1** | **R.SYVTTSTRTYSLGSALRPSTSR.S** |
|  | 3177 | **798.4191** | **2392.2352** | **2391.5929** | **0.6423** | **1** | **45** | **0.082** | **1** | **R.SYVTTSTRTYSLGSALRPSTSR.S** |
|  | 3178 | **798.4388** | **2392.2944** | **2391.5929** | **0.7014** | **1** | **(18)** | **36** | **1** | **R.SYVTTSTRTYSLGSALRPSTSR.S** |
|  | 3179 | **798.4550** | **2392.3427** | **2391.5929** | **0.7498** | **1** | **(4)** | **9.3e+02** | **1** | **R.SYVTTSTRTYSLGSALRPSTSR.S** |
|  | 3181 | **798.4991** | **2392.4751** | **2391.5929** | **0.8822** | **1** | **(24)** | **9.8** | **1** | **R.SYVTTSTRTYSLGSALRPSTSR.S** |
|  | 3182 | **798.5099** | **2392.5075** | **2391.5929** | **0.9146** | **1** | **(2)** | **1.7e+03** | **5** | **R.SYVTTSTRTYSLGSALRPSTSR.S** |
|  | 3184 | **798.5586** | **2392.6536** | **2391.5929** | **1.0607** | **1** | **(13)** | **1.3e+02** | **1** | **R.SYVTTSTRTYSLGSALRPSTSR.S** |
|  | 3187 | **798.5953** | **2392.7638** | **2391.5929** | **1.1709** | **1** | **(4)** | **1.1e+03** | **4** | **R.SYVTTSTRTYSLGSALRPSTSR.S** |
|  | 3191 | **798.7459** | **2393.2154** | **2393.7344** | **-0.5190** | **2** | **8** | **3.3e+02** | **2** | **R.ETNLDSLPLVDTHSKRTLLIK.T** |
|  | 3202 | **803.0779** | **2406.2115** | **2406.5878** | **-0.3763** | **1** | **30** | **2.1** | **1** | **R.MFGGPGTASRPSSSRSYVTTSTR.T + Oxidation (M)** |
|  | 3204 | **803.4690** | **2407.3848** | **2406.5878** | **0.7970** | **1** | **(29)** | **3.1** | **1** | **R.MFGGPGTASRPSSSRSYVTTSTR.T + Oxidation (M)** |
|  | 3225 | **808.7864** | **2423.3370** | **2423.4624** | **-0.1255** | **1** | **(45)** | **0.07** | **1** | **K.TVETRDGQVINETSQHHDDLE.-** |
|  | 3226 | **808.9152** | **2423.7233** | **2423.4624** | **0.2609** | **1** | **(40)** | **0.29** | **1** | **K.TVETRDGQVINETSQHHDDLE.-** |
|  | 3227 | **809.1433** | **2424.4078** | **2423.4624** | **0.9453** | **1** | **46** | **0.046** | **1** | **K.TVETRDGQVINETSQHHDDLE.-** |
|  | 3228 | **809.1620** | **2424.4638** | **2423.4624** | **1.0014** | **1** | **(42)** | **0.14** | **1** | **K.TVETRDGQVINETSQHHDDLE.-** |
|  | 3309 | **845.6527** | **2533.9360** | **2533.7701** | **0.1658** | **2** | **73** | **0.00011** | **1** | **R.RQVQSLTCEVDALKGTNESLER.Q + Carbamidomethyl (C)** |
|  | 3341 | **879.3679** | **2635.0816** | **2634.8094** | **0.2721** | **1** | **47** | **0.056** | **1** | **R.QMREMEENFAVEAANYQDTIGR.L + 2 Oxidation (M)** |
|  | 3384 | **969.5157** | **2905.5251** | **2906.1669** | **-0.6418** | **1** | **50** | **0.022** | **1** | **R.TYSLGSALRPSTSRSLYASSPGGVYATR.S** |
|  | 3385 | **970.0675** | **2907.1803** | **2906.1669** | **1.0134** | **1** | **(27)** | **5** | **1** | **R.TYSLGSALRPSTSRSLYASSPGGVYATR.S** |
|  | 3394 | **998.3890** | **2992.1449** | **2992.2114** | **-0.0665** | **2** | **(61)** | **0.0015** | **1** | **R.TLLIKTVETRDGQVINETSQHHDDLE.-** |
|  | 3395 | **998.5768** | **2992.7082** | **2992.2114** | **0.4968** | **2** | **63** | **0.0012** | **1** | **R.TLLIKTVETRDGQVINETSQHHDDLE.-** |
|  | 3396 | **998.5785** | **2992.7133** | **2992.2114** | **0.5019** | **2** | **(54)** | **0.0088** | **1** | **R.TLLIKTVETRDGQVINETSQHHDDLE.-** |

  


---

|  |  |
| --- | --- |
| **2.** | gi|193787214    **Mass:** 46976    **Score:** 1843   **Queries matched:** 186   **emPAI:** 17.55 |
|  | unnamed protein product [Homo sapiens] |

|  |  |
| --- | --- |
|  | Check to include this hit in error tolerant search or archive report |
|  |  |

|  |  |  |  |  |  |  |  |  |  |  |
| --- | --- | --- | --- | --- | --- | --- | --- | --- | --- | --- |
|  | **Query** | **Observed** | **Mr(expt)** | **Mr(calc)** | **Delta** | **Miss** | **Score** | **Expect** | **Rank** | **Peptide** |
|  | 1086 | 458.1244 | 914.2340 | 913.9716 | 0.2623 | 0 | 21 | 24 | 1 | R.SYVTTSTR.T |
|  | 1329 | 485.9291 | 969.8435 | 970.1276 | -0.2841 | 1 | (29) | 3.4 | 1 | R.LRSSVPGVR.L |
|  | 1333 | 486.1971 | 970.3793 | 970.1276 | 0.2518 | 1 | 31 | 1.9 | 1 | R.LRSSVPGVR.L |
|  | 1334 | 486.2640 | 970.5132 | 970.1276 | 0.3856 | 1 | (15) | 85 | 2 | R.LRSSVPGVR.L |
|  | 1335 | 486.2930 | 970.5712 | 970.1276 | 0.4436 | 1 | (28) | 3.6 | 1 | R.LRSSVPGVR.L |
|  | 1723 | 532.1644 | 1062.3141 | 1062.1980 | 0.1160 | 0 | 15 | 98 | 5 | K.LQEEMLQR.E + Oxidation (M) |
|  | 1827 | 545.1134 | 1088.2120 | 1088.1293 | 0.0827 | 0 | 64 | 0.0011 | 1 | R.QDVDNASLAR.L |
|  | 1841 | 547.3596 | 1092.7045 | 1093.1474 | -0.4430 | 0 | 71 | 0.00021 | 1 | K.FADLSEAANR.N |
|  | 1844 | 547.6176 | 1093.2203 | 1093.1474 | 0.0729 | 0 | (56) | 0.008 | 1 | K.FADLSEAANR.N |
|  | 1913 | 561.6705 | 1121.3263 | 1121.2403 | 0.0859 | 0 | 27 | 6.1 | 1 | R.EYQDLLNVK.M |
|  | 2043 | 585.6261 | 1169.2374 | 1169.4106 | -0.1732 | 0 | 55 | 0.0093 | 1 | K.ILLAELEQLK.G |
|  | 2534 | 655.4051 | 1308.7954 | 1309.3800 | -0.5846 | 0 | (32) | 1.6 | 1 | K.NLQEAEEWYK.S |
|  | 2537 | 655.6068 | 1309.1988 | 1309.3800 | -0.1812 | 0 | (8) | 3.2e+02 | 6 | K.NLQEAEEWYK.S |
|  | 2540 | 656.1443 | 1310.2738 | 1309.3800 | 0.8938 | 0 | 36 | 0.59 | 1 | K.NLQEAEEWYK.S |
|  | 2704 | **682.5181** | **1363.0213** | **1363.4359** | **-0.4145** | **0** | **(28)** | **3.3** | **1** | **M.FGGPGTASRPSSSR.S** |
|  | 1057 | **455.8164** | **1364.4270** | **1363.4359** | **0.9912** | **0** | **52** | **0.016** | **1** | **M.FGGPGTASRPSSSR.S** |
|  | 1177 | 465.4945 | 1393.4613 | 1393.5016 | -0.0404 | 1 | (36) | 0.72 | 1 | R.DVRQQYESVAAK.N |
|  | 2789 | 697.7607 | 1393.5067 | 1393.5016 | 0.0051 | 1 | 45 | 0.1 | 1 | R.DVRQQYESVAAK.N |
|  | 2827 | 714.7654 | 1427.5160 | 1428.5456 | -1.0297 | 0 | (8) | 4.8e+02 | 2 | R.SLYASSPGGVYATR.S |
|  | 2829 | 714.7822 | 1427.5497 | 1428.5456 | -0.9960 | 0 | (15) | 99 | 1 | R.SLYASSPGGVYATR.S |
|  | 2830 | 714.8660 | 1427.7173 | 1428.5456 | -0.8284 | 0 | (10) | 3.2e+02 | 1 | R.SLYASSPGGVYATR.S |
|  | 2831 | 714.9754 | 1427.9360 | 1428.5456 | -0.6096 | 0 | (66) | 0.0005 | 1 | R.SLYASSPGGVYATR.S |
|  | 2832 | 715.0773 | 1428.1398 | 1428.5456 | -0.4059 | 0 | (49) | 0.027 | 1 | R.SLYASSPGGVYATR.S |
|  | 2833 | 715.1013 | 1428.1877 | 1428.5456 | -0.3579 | 0 | (44) | 0.1 | 1 | R.SLYASSPGGVYATR.S |
|  | 2834 | 715.1086 | 1428.2025 | 1428.5456 | -0.3431 | 0 | (52) | 0.017 | 1 | R.SLYASSPGGVYATR.S |
|  | 2835 | 715.1232 | 1428.2316 | 1428.5456 | -0.3141 | 0 | (57) | 0.0058 | 1 | R.SLYASSPGGVYATR.S |
|  | 2836 | 715.1440 | 1428.2733 | 1428.5456 | -0.2723 | 0 | (52) | 0.017 | 1 | R.SLYASSPGGVYATR.S |
|  | 2837 | 715.1512 | 1428.2877 | 1428.5456 | -0.2579 | 0 | (58) | 0.0038 | 1 | R.SLYASSPGGVYATR.S |
|  | 2838 | 715.1655 | 1428.3163 | 1428.5456 | -0.2294 | 0 | (71) | 0.00021 | 1 | R.SLYASSPGGVYATR.S |
|  | 2839 | 715.1826 | 1428.3503 | 1428.5456 | -0.1953 | 0 | (59) | 0.0032 | 1 | R.SLYASSPGGVYATR.S |
|  | 2840 | 715.2111 | 1428.4073 | 1428.5456 | -0.1383 | 0 | (52) | 0.018 | 1 | R.SLYASSPGGVYATR.S |
|  | 2841 | 715.2139 | 1428.4131 | 1428.5456 | -0.1326 | 0 | (56) | 0.0073 | 1 | R.SLYASSPGGVYATR.S |
|  | 2842 | 715.2271 | 1428.4393 | 1428.5456 | -0.1063 | 0 | (64) | 0.00094 | 1 | R.SLYASSPGGVYATR.S |
|  | 2843 | 715.3545 | 1428.6942 | 1428.5456 | 0.1486 | 0 | (55) | 0.0085 | 1 | R.SLYASSPGGVYATR.S |
|  | 2844 | 715.4484 | 1428.8819 | 1428.5456 | 0.3363 | 0 | (15) | 80 | 1 | R.SLYASSPGGVYATR.S |
|  | 2845 | 715.5173 | 1429.0199 | 1428.5456 | 0.4742 | 0 | 82 | 1.6e-05 | 1 | R.SLYASSPGGVYATR.S |
|  | 2846 | 715.6626 | 1429.3104 | 1428.5456 | 0.7648 | 0 | (57) | 0.0043 | 1 | R.SLYASSPGGVYATR.S |
|  | 2847 | 715.7421 | 1429.4693 | 1428.5456 | 0.9237 | 0 | (80) | 2.9e-05 | 1 | R.SLYASSPGGVYATR.S |
|  | 2849 | 715.7794 | 1429.5441 | 1428.5456 | 0.9984 | 0 | (39) | 0.37 | 1 | R.SLYASSPGGVYATR.S |
|  | 1314 | 482.2364 | 1443.6870 | 1443.5652 | 0.1217 | 2 | 57 | 0.0054 | 1 | R.RQVDQLTNDKAR.V |
|  | 1381 | 491.5553 | 1471.6438 | 1470.6769 | 0.9669 | 2 | 29 | 4 | 1 | R.SSAVRLRSSVPGVR.L |
|  | 1382 | 491.5972 | 1471.7694 | 1470.6769 | 1.0926 | 2 | (26) | 7.6 | 1 | R.SSAVRLRSSVPGVR.L |
|  | 1429 | 498.8465 | 1493.5173 | 1494.6320 | -1.1147 | 0 | (18) | 40 | 1 | -.MFGGPGTASRPSSSR.S |
|  | 2967 | 747.9111 | 1493.8075 | 1494.6320 | -0.8245 | 0 | (32) | 1.8 | 1 | -.MFGGPGTASRPSSSR.S |
|  | 1431 | 499.1139 | 1494.3196 | 1494.6320 | -0.3124 | 0 | (20) | 23 | 1 | -.MFGGPGTASRPSSSR.S |
|  | 1432 | 499.2308 | 1494.6701 | 1495.6365 | -0.9664 | 0 | (15) | 83 | 1 | R.TYSLGSALRPSTSR.S |
|  | 2970 | 748.4442 | 1494.8735 | 1495.6365 | -0.7630 | 0 | (35) | 0.85 | 1 | R.TYSLGSALRPSTSR.S |
|  | 1433 | 499.3059 | 1494.8956 | 1495.6365 | -0.7410 | 0 | (5) | 7.5e+02 | 2 | R.TYSLGSALRPSTSR.S |
|  | 1434 | 499.3626 | 1495.0657 | 1495.6365 | -0.5709 | 0 | (16) | 48 | 1 | R.TYSLGSALRPSTSR.S |
|  | 2971 | 748.5568 | 1495.0987 | 1495.6365 | -0.5378 | 0 | (34) | 1 | 1 | R.TYSLGSALRPSTSR.S |
|  | 2972 | 748.6536 | 1495.2925 | 1494.6320 | 0.6605 | 0 | (21) | 16 | 1 | -.MFGGPGTASRPSSSR.S |
|  | 1435 | 499.4423 | 1495.3046 | 1495.6365 | -0.3319 | 0 | (24) | 7.6 | 1 | R.TYSLGSALRPSTSR.S |
|  | 1437 | 499.4917 | 1495.4529 | 1495.6365 | -0.1836 | 0 | (26) | 6.3 | 1 | R.TYSLGSALRPSTSR.S |
|  | 2974 | 748.7348 | 1495.4548 | 1495.6365 | -0.1817 | 0 | (3) | 1e+03 | 3 | R.TYSLGSALRPSTSR.S |
|  | 1438 | 499.4945 | 1495.4613 | 1495.6365 | -0.1752 | 0 | (2) | 1.4e+03 | 7 | R.TYSLGSALRPSTSR.S |
|  | 2976 | 748.7833 | 1495.5517 | 1495.6365 | -0.0848 | 0 | (31) | 2.4 | 1 | R.TYSLGSALRPSTSR.S |
|  | 2977 | 748.7871 | 1495.5594 | 1495.6365 | -0.0771 | 0 | (8) | 4.1e+02 | 2 | R.TYSLGSALRPSTSR.S |
|  | 2978 | 748.7964 | 1495.5780 | 1495.6365 | -0.0585 | 0 | (38) | 0.41 | 1 | R.TYSLGSALRPSTSR.S |
|  | 2984 | 748.8477 | 1495.6807 | 1495.6365 | 0.0441 | 0 | (42) | 0.2 | 1 | R.TYSLGSALRPSTSR.S |
|  | 1440 | 499.6003 | 1495.7788 | 1495.6365 | 0.1422 | 0 | (17) | 55 | 1 | R.TYSLGSALRPSTSR.S |
|  | 1441 | 499.6466 | 1495.9176 | 1495.6365 | 0.2811 | 0 | (23) | 14 | 1 | R.TYSLGSALRPSTSR.S |
|  | 1442 | 499.6580 | 1495.9519 | 1495.6365 | 0.3154 | 0 | (17) | 47 | 2 | R.TYSLGSALRPSTSR.S |
|  | 1443 | 499.6650 | 1495.9729 | 1495.6365 | 0.3364 | 0 | (19) | 34 | 2 | R.TYSLGSALRPSTSR.S |
|  | 1445 | 499.6939 | 1496.0595 | 1495.6365 | 0.4229 | 0 | (24) | 7.9 | 1 | R.TYSLGSALRPSTSR.S |
|  | 2992 | 749.0385 | 1496.0622 | 1495.6365 | 0.4257 | 0 | (2) | 1.4e+03 | 4 | R.TYSLGSALRPSTSR.S |
|  | 1446 | 499.7245 | 1496.1515 | 1495.6365 | 0.5149 | 0 | (25) | 6.8 | 1 | R.TYSLGSALRPSTSR.S |
|  | 1447 | 499.7292 | 1496.1653 | 1495.6365 | 0.5288 | 0 | (4) | 8.4e+02 | 3 | R.TYSLGSALRPSTSR.S |
|  | 1449 | 499.7497 | 1496.2269 | 1495.6365 | 0.5904 | 0 | (12) | 1.3e+02 | 3 | R.TYSLGSALRPSTSR.S |
|  | 1450 | 499.7522 | 1496.2344 | 1495.6365 | 0.5979 | 0 | (16) | 46 | 2 | R.TYSLGSALRPSTSR.S |
|  | 1451 | 499.8096 | 1496.4066 | 1495.6365 | 0.7701 | 0 | (15) | 67 | 2 | R.TYSLGSALRPSTSR.S |
|  | 1452 | 499.8214 | 1496.4422 | 1495.6365 | 0.8056 | 0 | (15) | 70 | 2 | R.TYSLGSALRPSTSR.S |
|  | 1453 | 499.8350 | 1496.4827 | 1495.6365 | 0.8462 | 0 | (20) | 23 | 1 | R.TYSLGSALRPSTSR.S |
|  | 1454 | 499.8409 | 1496.5005 | 1495.6365 | 0.8639 | 0 | (38) | 0.34 | 1 | R.TYSLGSALRPSTSR.S |
|  | 1455 | 499.8478 | 1496.5213 | 1495.6365 | 0.8848 | 0 | (18) | 41 | 1 | R.TYSLGSALRPSTSR.S |
|  | 1456 | 499.8617 | 1496.5628 | 1495.6365 | 0.9263 | 0 | 46 | 0.066 | 1 | R.TYSLGSALRPSTSR.S |
|  | 3009 | 749.3035 | 1496.5922 | 1495.6365 | 0.9556 | 0 | (11) | 2e+02 | 1 | R.TYSLGSALRPSTSR.S |
|  | 1457 | 499.9070 | 1496.6990 | 1495.6365 | 1.0624 | 0 | (42) | 0.18 | 1 | R.TYSLGSALRPSTSR.S |
|  | 1458 | 499.9319 | 1496.7736 | 1495.6365 | 1.1370 | 0 | (28) | 4.3 | 1 | R.TYSLGSALRPSTSR.S |
|  | 1488 | 504.2891 | 1509.8450 | 1510.6314 | -0.7864 | 0 | (10) | 2.9e+02 | 1 | -.MFGGPGTASRPSSSR.S + Oxidation (M) |
|  | 1490 | 504.4336 | 1510.2785 | 1510.6314 | -0.3529 | 0 | (33) | 1.2 | 1 | -.MFGGPGTASRPSSSR.S + Oxidation (M) |
|  | 1491 | 504.4456 | 1510.3148 | 1510.6314 | -0.3166 | 0 | (45) | 0.076 | 1 | -.MFGGPGTASRPSSSR.S + Oxidation (M) |
|  | 3030 | 756.1921 | 1510.3695 | 1510.6314 | -0.2619 | 0 | (29) | 3 | 1 | -.MFGGPGTASRPSSSR.S + Oxidation (M) |
|  | 3031 | 756.2419 | 1510.4691 | 1510.6314 | -0.1623 | 0 | 50 | 0.025 | 1 | -.MFGGPGTASRPSSSR.S + Oxidation (M) |
|  | 1492 | 504.5174 | 1510.5300 | 1510.6314 | -0.1014 | 0 | (44) | 0.13 | 1 | -.MFGGPGTASRPSSSR.S + Oxidation (M) |
|  | 1493 | 504.5997 | 1510.7768 | 1510.6314 | 0.1455 | 0 | (12) | 2e+02 | 1 | -.MFGGPGTASRPSSSR.S + Oxidation (M) |
|  | 1496 | 504.6768 | 1511.0081 | 1510.6314 | 0.3767 | 0 | (48) | 0.041 | 1 | -.MFGGPGTASRPSSSR.S + Oxidation (M) |
|  | 1497 | 504.7342 | 1511.1803 | 1510.6314 | 0.5489 | 0 | (32) | 1.4 | 1 | -.MFGGPGTASRPSSSR.S + Oxidation (M) |
|  | 1498 | 504.7369 | 1511.1886 | 1510.6314 | 0.5573 | 0 | (47) | 0.047 | 1 | -.MFGGPGTASRPSSSR.S + Oxidation (M) |
|  | 3032 | 756.7118 | 1511.4088 | 1510.6314 | 0.7774 | 0 | (28) | 2.9 | 1 | -.MFGGPGTASRPSSSR.S + Oxidation (M) |
|  | 3033 | 756.7314 | 1511.4481 | 1510.6314 | 0.8167 | 0 | (43) | 0.097 | 1 | -.MFGGPGTASRPSSSR.S + Oxidation (M) |
|  | 1500 | 504.9255 | 1511.7543 | 1510.6314 | 1.1229 | 0 | (27) | 5 | 1 | -.MFGGPGTASRPSSSR.S + Oxidation (M) |
|  | 1538 | 508.9904 | 1523.9490 | 1524.6296 | -0.6807 | 1 | (37) | 0.52 | 1 | K.NLQEAEEWYKSK.F |
|  | 3054 | 763.1932 | 1524.3716 | 1524.6296 | -0.2581 | 1 | (62) | 0.0016 | 1 | K.NLQEAEEWYKSK.F |
|  | 3055 | 763.2119 | 1524.4089 | 1524.6296 | -0.2207 | 1 | 67 | 0.00054 | 1 | K.NLQEAEEWYKSK.F |
|  | 1541 | 509.3192 | 1524.9356 | 1524.6296 | 0.3059 | 1 | (31) | 1.9 | 1 | K.NLQEAEEWYKSK.F |
|  | 1543 | 510.5560 | 1528.6457 | 1527.7229 | 0.9228 | 1 | 52 | 0.023 | 1 | R.HLREYQDLLNVK.M |
|  | 3077 | 767.7169 | 1533.4191 | 1533.7640 | -0.3450 | 1 | (64) | 0.00081 | 1 | R.KVESLQEEIAFLK.K |
|  | 3078 | 767.7756 | 1533.5365 | 1533.7640 | -0.2276 | 1 | 78 | 4.2e-05 | 1 | R.KVESLQEEIAFLK.K |
|  | 3079 | 767.7770 | 1533.5392 | 1533.7640 | -0.2249 | 1 | (35) | 0.87 | 1 | R.KVESLQEEIAFLK.K |
|  | 1555 | 512.4220 | 1534.2438 | 1533.7640 | 0.4798 | 1 | (58) | 0.0031 | 1 | R.KVESLQEEIAFLK.K |
|  | 1556 | 512.4808 | 1534.4203 | 1533.7640 | 0.6563 | 1 | (71) | 0.00019 | 1 | R.KVESLQEEIAFLK.K |
|  | 1566 | 514.4370 | 1540.2889 | 1539.8147 | 0.4742 | 1 | (17) | 39 | 1 | K.ILLAELEQLKGQGK.S |
|  | 3083 | 771.2303 | 1540.4459 | 1539.8147 | 0.6312 | 1 | 60 | 0.0024 | 1 | K.ILLAELEQLKGQGK.S |
|  | 1568 | 514.5745 | 1540.7012 | 1539.8147 | 0.8865 | 1 | (40) | 0.3 | 1 | K.ILLAELEQLKGQGK.S |
|  | 1569 | 514.6674 | 1540.9799 | 1539.8147 | 1.1652 | 1 | (42) | 0.16 | 1 | K.ILLAELEQLKGQGK.S |
|  | 1716 | 530.4187 | 1588.2339 | 1587.6872 | 0.5467 | 1 | 40 | 0.22 | 1 | R.TNEKVELQELNDR.F |
|  | 1867 | 552.1337 | 1653.3788 | 1652.8235 | 0.5553 | 1 | (42) | 0.19 | 1 | R.LGDLYEEEMRELR.R |
|  | 1882 | 555.1788 | 1662.5143 | 1661.9363 | 0.5780 | 2 | (42) | 0.15 | 1 | R.KVESLQEEIAFLKK.L |
|  | 1883 | 555.2501 | 1662.7282 | 1661.9363 | 0.7919 | 2 | 58 | 0.004 | 1 | R.KVESLQEEIAFLKK.L |
|  | 3296 | 835.2340 | 1668.4532 | 1668.8229 | -0.3697 | 1 | (29) | 3 | 1 | R.LGDLYEEEMRELR.R + Oxidation (M) |
|  | 1898 | 557.4318 | 1669.2731 | 1668.8229 | 0.4502 | 1 | 46 | 0.049 | 1 | R.LGDLYEEEMRELR.R + Oxidation (M) |
|  | 1899 | 557.6702 | 1669.9885 | 1668.8229 | 1.1656 | 1 | (31) | 2.7 | 1 | R.LGDLYEEEMRELR.R + Oxidation (M) |
|  | 3324 | 853.1700 | 1704.3253 | 1704.8569 | -0.5316 | 1 | 43 | 0.095 | 1 | R.VEVERDNLAEDIMR.L + Oxidation (M) |
|  | 2072 | 590.0087 | 1767.0040 | 1766.9478 | 0.0563 | 1 | (25) | 8.2 | 1 | R.LQDEIQNMKEEMAR.H + 2 Oxidation (M) |
|  | 3347 | 884.7201 | 1767.4254 | 1766.9478 | 0.4777 | 1 | (40) | 0.22 | 1 | R.LQDEIQNMKEEMAR.H + 2 Oxidation (M) |
|  | 2073 | 590.1520 | 1767.4339 | 1766.9478 | 0.4862 | 1 | 42 | 0.19 | 1 | R.LQDEIQNMKEEMAR.H + 2 Oxidation (M) |
|  | 2074 | 590.2034 | 1767.5881 | 1766.9478 | 0.6404 | 1 | (37) | 0.57 | 1 | R.LQDEIQNMKEEMAR.H + 2 Oxidation (M) |
|  | 2099 | 592.9519 | 1775.8335 | 1776.8612 | -1.0277 | 1 | (7) | 4.8e+02 | 3 | K.FADLSEAANRNNDALR.Q |
|  | 2100 | 593.0111 | 1776.0112 | 1776.8612 | -0.8501 | 1 | (9) | 3.3e+02 | 5 | K.FADLSEAANRNNDALR.Q |
|  | 3350 | 889.2459 | 1776.4769 | 1776.8612 | -0.3843 | 1 | (53) | 0.01 | 1 | K.FADLSEAANRNNDALR.Q |
|  | 3352 | 889.2820 | 1776.5492 | 1776.8612 | -0.3121 | 1 | (49) | 0.027 | 1 | K.FADLSEAANRNNDALR.Q |
|  | 2103 | 593.2668 | 1776.7782 | 1776.8612 | -0.0831 | 1 | (56) | 0.0068 | 1 | K.FADLSEAANRNNDALR.Q |
|  | 2106 | 593.3073 | 1776.8998 | 1776.8612 | 0.0385 | 1 | (9) | 3.6e+02 | 3 | K.FADLSEAANRNNDALR.Q |
|  | 2107 | 593.3945 | 1777.1614 | 1776.8612 | 0.3002 | 1 | (11) | 1.9e+02 | 1 | K.FADLSEAANRNNDALR.Q |
|  | 2108 | 593.5016 | 1777.4826 | 1776.8612 | 0.6214 | 1 | (30) | 2.4 | 1 | K.FADLSEAANRNNDALR.Q |
|  | 2110 | 593.5886 | 1777.7437 | 1776.8612 | 0.8825 | 1 | (6) | 6.1e+02 | 4 | K.FADLSEAANRNNDALR.Q |
|  | 2112 | 593.6707 | 1777.9898 | 1776.8612 | 1.1286 | 1 | (21) | 28 | 1 | K.FADLSEAANRNNDALR.Q |
|  | 2113 | 593.6731 | 1777.9971 | 1776.8612 | 1.1359 | 1 | 67 | 0.00065 | 1 | K.FADLSEAANRNNDALR.Q |
|  | 2114 | 593.6738 | 1777.9993 | 1776.8612 | 1.1381 | 1 | (63) | 0.0017 | 1 | K.FADLSEAANRNNDALR.Q |
|  | 2131 | 595.4662 | 1783.3764 | 1783.0777 | 0.2987 | 2 | (49) | 0.027 | 1 | K.ILLAELEQLKGQGKSR.L |
|  | 2132 | 595.4887 | 1783.4440 | 1783.0777 | 0.3662 | 2 | 65 | 0.00082 | 1 | K.ILLAELEQLKGQGKSR.L |
|  | 2220 | 603.9775 | 1808.9103 | 1809.0092 | -0.0990 | 2 | 45 | 0.084 | 1 | R.LGDLYEEEMRELRR.Q |
|  | 3368 | 913.2670 | 1824.5192 | 1825.0086 | -0.4895 | 2 | (18) | 34 | 1 | R.LGDLYEEEMRELRR.Q + Oxidation (M) |
|  | 2255 | 609.2184 | 1824.6330 | 1825.0086 | -0.3757 | 2 | (34) | 1.2 | 1 | R.LGDLYEEEMRELRR.Q + Oxidation (M) |
|  | 2257 | 609.3021 | 1824.8842 | 1825.0086 | -0.1244 | 2 | (17) | 51 | 1 | R.LGDLYEEEMRELRR.Q + Oxidation (M) |
|  | 2261 | 609.6250 | 1825.8528 | 1825.0086 | 0.8442 | 2 | (32) | 1.6 | 1 | R.LGDLYEEEMRELRR.Q + Oxidation (M) |
|  | 2262 | 609.6416 | 1825.9026 | 1825.0086 | 0.8940 | 2 | (43) | 0.15 | 1 | R.LGDLYEEEMRELRR.Q + Oxidation (M) |
|  | 3382 | 965.3291 | 1928.6434 | 1929.0949 | -0.4515 | 1 | 42 | 0.12 | 1 | R.SLYASSPGGVYATRSSAVR.L |
|  | 3383 | 965.3950 | 1928.7753 | 1929.0949 | -0.3197 | 1 | (39) | 0.31 | 1 | R.SLYASSPGGVYATRSSAVR.L |
|  | 2478 | 644.3561 | 1930.0461 | 1929.0949 | 0.9511 | 1 | (20) | 25 | 1 | R.SLYASSPGGVYATRSSAVR.L |
|  | 2479 | 644.3863 | 1930.1367 | 1929.0949 | 1.0418 | 1 | (21) | 23 | 1 | R.SLYASSPGGVYATRSSAVR.L |
|  | 2518 | 653.7000 | 1958.0777 | 1958.2007 | -0.1231 | 2 | 11 | 2.6e+02 | 8 | R.VEVERDNLAEDIMRLR.E |
|  | 2520 | 654.0589 | 1959.1545 | 1958.2007 | 0.9538 | 2 | (5) | 8.2e+02 | 3 | R.VEVERDNLAEDIMRLR.E |
|  | 2579 | 665.3218 | 1992.9432 | 1992.1108 | 0.8323 | 2 | 40 | 0.27 | 1 | K.SKFADLSEAANRNNDALR.Q |
|  | 2801 | 702.3351 | 2103.9831 | 2104.2406 | -0.2576 | 2 | 68 | 0.00045 | 1 | K.FADLSEAANRNNDALRQAK.Q |
|  | 2802 | 702.5337 | 2104.5789 | 2104.2406 | 0.3383 | 2 | (62) | 0.0014 | 1 | K.FADLSEAANRNNDALRQAK.Q |
|  | 2890 | 734.0176 | 2199.0306 | 2198.4382 | 0.5923 | 2 | (9) | 2.4e+02 | 1 | R.SLYASSPGGVYATRSSAVRLR.S |
|  | 2891 | 734.1108 | 2199.3103 | 2198.4382 | 0.8721 | 2 | 14 | 90 | 1 | R.SLYASSPGGVYATRSSAVRLR.S |
|  | 3134 | 789.9568 | 2366.8482 | 2367.5481 | -0.6999 | 1 | 17 | 59 | 1 | K.LQEEMLQREEAENTLQSFR.Q + Oxidation (M) |
|  | 3419 | 1189.4766 | 2376.9383 | 2377.5844 | -0.6461 | 1 | (65) | 0.00053 | 1 | R.QVQSLTCEVDALKGTNESLER.Q + Carbamidomethyl (C) |
|  | 3144 | 793.3457 | 2377.0149 | 2377.5844 | -0.5695 | 1 | (38) | 0.39 | 1 | R.QVQSLTCEVDALKGTNESLER.Q + Carbamidomethyl (C) |
|  | 3145 | 793.3658 | 2377.0754 | 2377.5844 | -0.5091 | 1 | (68) | 0.00042 | 1 | R.QVQSLTCEVDALKGTNESLER.Q + Carbamidomethyl (C) |
|  | 3146 | 793.5016 | 2377.4828 | 2377.5844 | -0.1017 | 1 | (42) | 0.17 | 1 | R.QVQSLTCEVDALKGTNESLER.Q + Carbamidomethyl (C) |
|  | 3147 | 793.6010 | 2377.7809 | 2377.5844 | 0.1964 | 1 | (22) | 16 | 1 | R.QVQSLTCEVDALKGTNESLER.Q + Carbamidomethyl (C) |
|  | 3148 | 793.6812 | 2378.0213 | 2377.5844 | 0.4369 | 1 | (53) | 0.011 | 1 | R.QVQSLTCEVDALKGTNESLER.Q + Carbamidomethyl (C) |
|  | 3150 | 793.7402 | 2378.1985 | 2377.5844 | 0.6141 | 1 | (24) | 7.6 | 1 | R.QVQSLTCEVDALKGTNESLER.Q + Carbamidomethyl (C) |
|  | 3151 | 793.7443 | 2378.2108 | 2377.5844 | 0.6264 | 1 | (36) | 0.47 | 1 | R.QVQSLTCEVDALKGTNESLER.Q + Carbamidomethyl (C) |
|  | 3152 | 793.7446 | 2378.2117 | 2377.5844 | 0.6273 | 1 | (29) | 2.5 | 1 | R.QVQSLTCEVDALKGTNESLER.Q + Carbamidomethyl (C) |
|  | 3153 | 793.7689 | 2378.2846 | 2377.5844 | 0.7002 | 1 | (62) | 0.0013 | 1 | R.QVQSLTCEVDALKGTNESLER.Q + Carbamidomethyl (C) |
|  | 3154 | 793.7913 | 2378.3516 | 2377.5844 | 0.7672 | 1 | 73 | 0.00012 | 1 | R.QVQSLTCEVDALKGTNESLER.Q + Carbamidomethyl (C) |
|  | 3155 | 793.8789 | 2378.6145 | 2377.5844 | 1.0301 | 1 | (27) | 6.1 | 1 | R.QVQSLTCEVDALKGTNESLER.Q + Carbamidomethyl (C) |
|  | 3156 | 793.8878 | 2378.6411 | 2377.5844 | 1.0567 | 1 | (66) | 0.00083 | 1 | R.QVQSLTCEVDALKGTNESLER.Q + Carbamidomethyl (C) |
|  | 3163 | 797.9430 | 2390.8068 | 2391.5929 | -0.7861 | 1 | (17) | 64 | 1 | R.SYVTTSTRTYSLGSALRPSTSR.S |
|  | 3164 | 797.9841 | 2390.9302 | 2390.5884 | 0.3419 | 1 | (11) | 2.3e+02 | 1 | -.MFGGPGTASRPSSSRSYVTTSTR.T |
|  | 3165 | 798.0231 | 2391.0470 | 2391.5929 | -0.5459 | 1 | (14) | 86 | 1 | R.SYVTTSTRTYSLGSALRPSTSR.S |
|  | 3166 | 798.0303 | 2391.0688 | 2391.5929 | -0.5241 | 1 | (23) | 10 | 1 | R.SYVTTSTRTYSLGSALRPSTSR.S |
|  | 3167 | 798.0769 | 2391.2085 | 2391.5929 | -0.3844 | 1 | (8) | 3.6e+02 | 1 | R.SYVTTSTRTYSLGSALRPSTSR.S |
|  | 3169 | 798.2038 | 2391.5892 | 2391.5929 | -0.0037 | 1 | (7) | 5.1e+02 | 2 | R.SYVTTSTRTYSLGSALRPSTSR.S |
|  | 3171 | 798.2360 | 2391.6859 | 2391.5929 | 0.0930 | 1 | (17) | 50 | 1 | R.SYVTTSTRTYSLGSALRPSTSR.S |
|  | 3173 | 798.2703 | 2391.7886 | 2391.5929 | 0.1957 | 1 | (4) | 9.2e+02 | 1 | R.SYVTTSTRTYSLGSALRPSTSR.S |
|  | 3174 | 798.2715 | 2391.7925 | 2391.5929 | 0.1995 | 1 | (1) | 2e+03 | 4 | R.SYVTTSTRTYSLGSALRPSTSR.S |
|  | 3175 | 798.3038 | 2391.8891 | 2391.5929 | 0.2962 | 1 | (6) | 6e+02 | 1 | R.SYVTTSTRTYSLGSALRPSTSR.S |
|  | 3176 | 798.3472 | 2392.0193 | 2391.5929 | 0.4264 | 1 | (23) | 13 | 1 | R.SYVTTSTRTYSLGSALRPSTSR.S |
|  | 3177 | 798.4191 | 2392.2352 | 2391.5929 | 0.6423 | 1 | 45 | 0.082 | 1 | R.SYVTTSTRTYSLGSALRPSTSR.S |
|  | 3178 | 798.4388 | 2392.2944 | 2391.5929 | 0.7014 | 1 | (18) | 36 | 1 | R.SYVTTSTRTYSLGSALRPSTSR.S |
|  | 3179 | 798.4550 | 2392.3427 | 2391.5929 | 0.7498 | 1 | (4) | 9.3e+02 | 1 | R.SYVTTSTRTYSLGSALRPSTSR.S |
|  | 3181 | 798.4991 | 2392.4751 | 2391.5929 | 0.8822 | 1 | (24) | 9.8 | 1 | R.SYVTTSTRTYSLGSALRPSTSR.S |
|  | 3182 | 798.5099 | 2392.5075 | 2391.5929 | 0.9146 | 1 | (2) | 1.7e+03 | 5 | R.SYVTTSTRTYSLGSALRPSTSR.S |
|  | 3184 | 798.5586 | 2392.6536 | 2391.5929 | 1.0607 | 1 | (13) | 1.3e+02 | 1 | R.SYVTTSTRTYSLGSALRPSTSR.S |
|  | 3187 | 798.5953 | 2392.7638 | 2391.5929 | 1.1709 | 1 | (4) | 1.1e+03 | 4 | R.SYVTTSTRTYSLGSALRPSTSR.S |
|  | 3202 | 803.0779 | 2406.2115 | 2406.5878 | -0.3763 | 1 | 30 | 2.1 | 1 | -.MFGGPGTASRPSSSRSYVTTSTR.T + Oxidation (M) |
|  | 3204 | 803.4690 | 2407.3848 | 2406.5878 | 0.7970 | 1 | (29) | 3.1 | 1 | -.MFGGPGTASRPSSSRSYVTTSTR.T + Oxidation (M) |
|  | 3309 | 845.6527 | 2533.9360 | 2533.7701 | 0.1658 | 2 | 73 | 0.00011 | 1 | R.RQVQSLTCEVDALKGTNESLER.Q + Carbamidomethyl (C) |
|  | 3341 | 879.3679 | 2635.0816 | 2634.8094 | 0.2721 | 1 | 47 | 0.056 | 1 | R.QMREMEENFAVEAANYQDTIGR.L + 2 Oxidation (M) |
|  | 3384 | 969.5157 | 2905.5251 | 2906.1669 | -0.6418 | 1 | 50 | 0.022 | 1 | R.TYSLGSALRPSTSRSLYASSPGGVYATR.S |
|  | 3385 | 970.0675 | 2907.1803 | 2906.1669 | 1.0134 | 1 | (27) | 5 | 1 | R.TYSLGSALRPSTSRSLYASSPGGVYATR.S |

  


---

|  |  |
| --- | --- |
| **3.** | gi|4757810    **Mass:** 59750    **Score:** 890    **Queries matched:** 54   **emPAI:** 1.36 |
|  | ATP synthase subunit alpha, mitochondrial precursor [Homo sapiens] |

|  |  |
| --- | --- |
|  | Check to include this hit in error tolerant search or archive report |
|  |  |

|  |  |  |  |  |  |  |  |  |  |  |
| --- | --- | --- | --- | --- | --- | --- | --- | --- | --- | --- |
|  | **Query** | **Observed** | **Mr(expt)** | **Mr(calc)** | **Delta** | **Miss** | **Score** | **Expect** | **Rank** | **Peptide** |
|  | 19 | **362.2872** | **722.5596** | **722.8757** | **-0.3161** | **0** | **46** | **0.055** | **1** | **K.APGIIPR.I** |
|  | 22 | **362.4806** | **722.9463** | **722.8757** | **0.0706** | **0** | **(31)** | **2** | **1** | **K.APGIIPR.I** |
|  | 627 | **408.7139** | **815.4130** | **814.9264** | **0.4866** | **0** | **35** | **0.72** | **1** | **R.ELIIGDR.Q** |
|  | 777 | **423.7170** | **845.4193** | **844.9957** | **0.4236** | **0** | **37** | **0.46** | **1** | **R.STVAQLVK.R** |
|  | 1471 | **501.6938** | **1001.3729** | **1001.1814** | **0.1916** | **1** | **57** | **0.0056** | **1** | **R.STVAQLVKR.L** |
|  | 1558 | **513.7992** | **1025.5836** | **1026.1875** | **-0.6038** | **0** | **77** | **4.2e-05** | **1** | **K.AVDSLVPIGR.G** |
|  | 1559 | **513.8011** | **1025.5875** | **1026.1875** | **-0.5999** | **0** | **(44)** | **0.074** | **1** | **K.AVDSLVPIGR.G** |
|  | 1574 | **515.1913** | **1028.3678** | **1028.2463** | **0.1214** | **1** | **24** | **10** | **7** | **R.GVRLTELLK.Q** |
|  | 160 | **374.0737** | **1119.1988** | **1120.3880** | **-1.1892** | **1** | **(10)** | **3.4e+02** | **6** | **R.VGLKAPGIIPR.I** |
|  | 161 | **374.1620** | **1119.4640** | **1120.3880** | **-0.9241** | **1** | **(8)** | **4.7e+02** | **5** | **R.VGLKAPGIIPR.I** |
|  | 162 | **374.1621** | **1119.4642** | **1120.3880** | **-0.9239** | **1** | **(10)** | **3.2e+02** | **1** | **R.VGLKAPGIIPR.I** |
|  | 163 | **374.1872** | **1119.5393** | **1120.3880** | **-0.8487** | **1** | **(21)** | **22** | **1** | **R.VGLKAPGIIPR.I** |
|  | 164 | **374.2019** | **1119.5835** | **1120.3880** | **-0.8045** | **1** | **(10)** | **2.3e+02** | **2** | **R.VGLKAPGIIPR.I** |
|  | 167 | **374.2353** | **1119.6838** | **1120.3880** | **-0.7043** | **1** | **(14)** | **1.1e+02** | **4** | **R.VGLKAPGIIPR.I** |
|  | 168 | **374.2372** | **1119.6894** | **1120.3880** | **-0.6987** | **1** | **(9)** | **3.1e+02** | **5** | **R.VGLKAPGIIPR.I** |
|  | 170 | **374.3008** | **1119.8803** | **1120.3880** | **-0.5078** | **1** | **(6)** | **6.3e+02** | **4** | **R.VGLKAPGIIPR.I** |
|  | 171 | **374.3029** | **1119.8864** | **1120.3880** | **-0.5017** | **1** | **(22)** | **18** | **1** | **R.VGLKAPGIIPR.I** |
|  | 172 | **374.3263** | **1119.9566** | **1120.3880** | **-0.4314** | **1** | **61** | **0.0024** | **1** | **R.VGLKAPGIIPR.I** |
|  | 175 | **374.3442** | **1120.0105** | **1120.3880** | **-0.3775** | **1** | **(6)** | **7.9e+02** | **9** | **R.VGLKAPGIIPR.I** |
|  | 176 | **374.6440** | **1120.9100** | **1120.3880** | **0.5219** | **1** | **(38)** | **0.37** | **1** | **R.VGLKAPGIIPR.I** |
|  | 177 | **374.7294** | **1121.1661** | **1120.3880** | **0.7781** | **1** | **(17)** | **63** | **1** | **R.VGLKAPGIIPR.I** |
|  | 178 | **374.7747** | **1121.3021** | **1120.3880** | **0.9140** | **1** | **(53)** | **0.015** | **1** | **R.VGLKAPGIIPR.I** |
|  | 1914 | **561.6744** | **1121.3341** | **1120.3880** | **0.9460** | **1** | **(57)** | **0.0059** | **1** | **R.VGLKAPGIIPR.I** |
|  | 1916 | **561.7734** | **1121.5321** | **1120.3880** | **1.1440** | **1** | **(60)** | **0.0021** | **1** | **R.VGLKAPGIIPR.I** |
|  | 292 | **386.5860** | **1156.7358** | **1156.2926** | **0.4432** | **1** | **27** | **4.9** | **1** | **R.GQRELIIGDR.Q** |
|  | 372 | **391.2711** | **1170.7912** | **1170.4021** | **0.3890** | **2** | **42** | **0.12** | **1** | **K.LIKEGDIVKR.T** |
|  | 2332 | **615.4514** | **1228.8881** | **1229.3831** | **-0.4951** | **1** | **45** | **0.07** | **1** | **R.ELIIGDRQTGK.T** |
|  | 649 | **410.9734** | **1229.8979** | **1229.3831** | **0.5148** | **1** | **(19)** | **37** | **1** | **R.ELIIGDRQTGK.T** |
|  | 650 | **411.0255** | **1230.0544** | **1229.3831** | **0.6712** | **1** | **(17)** | **52** | **1** | **R.ELIIGDRQTGK.T** |
|  | 2457 | **639.3472** | **1276.6797** | **1276.5737** | **0.1059** | **2** | **62** | **0.0019** | **1** | **R.RVGLKAPGIIPR.I** |
|  | 786 | **426.6718** | **1276.9931** | **1276.5737** | **0.4193** | **2** | **(53)** | **0.0095** | **1** | **R.RVGLKAPGIIPR.I** |
|  | 787 | **426.7507** | **1277.2298** | **1276.5737** | **0.6561** | **2** | **(43)** | **0.11** | **1** | **R.RVGLKAPGIIPR.I** |
|  | 788 | **426.8029** | **1277.3864** | **1276.5737** | **0.8126** | **2** | **(45)** | **0.08** | **1** | **R.RVGLKAPGIIPR.I** |
|  | 2552 | **659.2164** | **1316.4181** | **1316.5001** | **-0.0820** | **0** | **18** | **45** | **1** | **K.TSIAIDTIINQK.R** |
|  | 2731 | **684.5195** | **1367.0242** | **1367.5537** | **-0.5295** | **1** | **(25)** | **6.5** | **1** | **K.AVDSLVPIGRGQR.E** |
|  | 2732 | **684.8766** | **1367.7384** | **1367.5537** | **0.1847** | **1** | **30** | **2.5** | **1** | **K.AVDSLVPIGRGQR.E** |
|  | 2764 | **688.2203** | **1374.4259** | **1374.6060** | **-0.1801** | **1** | **(22)** | **15** | **2** | **R.ISVREPMQTGIK.A + Oxidation (M)** |
|  | 2767 | **688.6713** | **1375.3279** | **1374.6060** | **0.7219** | **1** | **33** | **1.1** | **1** | **R.ISVREPMQTGIK.A + Oxidation (M)** |
|  | 1110 | **459.4835** | **1375.4284** | **1374.6060** | **0.8224** | **1** | **(18)** | **60** | **1** | **R.ISVREPMQTGIK.A + Oxidation (M)** |
|  | 1111 | **459.5121** | **1375.5140** | **1374.6060** | **0.9080** | **1** | **(12)** | **2.3e+02** | **9** | **R.ISVREPMQTGIK.A + Oxidation (M)** |
|  | 2897 | **736.8651** | **1471.7154** | **1472.6858** | **-0.9704** | **1** | **47** | **0.054** | **1** | **K.TSIAIDTIINQKR.F** |
|  | 1385 | **492.0894** | **1473.2461** | **1472.6858** | **0.5603** | **1** | **(44)** | **0.089** | **1** | **K.TSIAIDTIINQKR.F** |
|  | 1387 | **492.1713** | **1473.4917** | **1472.6858** | **0.8059** | **1** | **(43)** | **0.12** | **1** | **K.TSIAIDTIINQKR.F** |
|  | 3100 | **777.7083** | **1553.4017** | **1553.6725** | **-0.2708** | **0** | **(25)** | **5.9** | **1** | **R.EAYPGDVFYLHSR.L** |
|  | 3113 | **778.1588** | **1554.3028** | **1553.6725** | **0.6303** | **0** | **49** | **0.029** | **1** | **R.EAYPGDVFYLHSR.L** |
|  | 1670 | **524.7755** | **1571.3042** | **1570.7493** | **0.5549** | **2** | **33** | **1.2** | **1** | **R.GQRELIIGDRQTGK.T** |
|  | 3240 | **812.8740** | **1623.7331** | **1624.8333** | **-1.1002** | **0** | **(58)** | **0.0045** | **1** | **R.TGAIVDVPVGEELLGR.V** |
|  | 3242 | **813.7031** | **1625.3915** | **1624.8333** | **0.5581** | **0** | **61** | **0.0018** | **1** | **R.TGAIVDVPVGEELLGR.V** |
|  | 1970 | **571.6774** | **1712.0101** | **1710.9258** | **1.0844** | **1** | **26** | **7.8** | **1** | **R.VVDALGNAIDGKGPIGSK.T** |
|  | 2122 | **594.6106** | **1780.8096** | **1781.0190** | **-0.2094** | **1** | **3** | **1.5e+03** | **5** | **K.RTGAIVDVPVGEELLGR.V** |
|  | 2547 | **657.6730** | **1969.9969** | **1970.3174** | **-0.3205** | **2** | **33** | **1.4** | **1** | **R.AMKQVAGTMKLELAQYR.E + 2 Oxidation (M)** |
|  | 3091 | **776.5295** | **2326.5663** | **2325.5959** | **0.9703** | **0** | **11** | **2.3e+02** | **9** | **K.QGQYSPMAIEEQVAVIYAGVR.G + Oxidation (M)** |
|  | 3157 | **795.4194** | **2383.2359** | **2382.7782** | **0.4578** | **2** | **47** | **0.048** | **1** | **R.ISVREPMQTGIKAVDSLVPIGR.G + Oxidation (M)** |
|  | 3158 | **795.5605** | **2383.6595** | **2382.7782** | **0.8813** | **2** | **(46)** | **0.067** | **1** | **R.ISVREPMQTGIKAVDSLVPIGR.G + Oxidation (M)** |

  

|  |  |
| --- | --- |
|  | |
|  | **Proteins matching the same set of peptides:** |

|  |  |
| --- | --- |
|  | gi|15030240    **Mass:** 59808    **Score:** 890    **Queries matched:** 54 |
|  | ATP synthase, H+ transporting, mitochondrial F1 complex, alpha subunit 1, cardiac muscle [Homo sapiens] |

|  |  |
| --- | --- |
|  | gi|34782901    **Mass:** 48795    **Score:** 890    **Queries matched:** 54 |
|  | ATP5A1 protein [Homo sapiens] |

|  |  |
| --- | --- |
|  | gi|127798841    **Mass:** 59707    **Score:** 890    **Queries matched:** 54 |
|  | ATP synthase, H+ transporting, mitochondrial F1 complex, alpha subunit 1, cardiac muscle [Homo sapiens] |

|  |  |
| --- | --- |
|  | gi|158259937    **Mass:** 54493    **Score:** 890    **Queries matched:** 54 |
|  | unnamed protein product [Homo sapiens] |

---

|  |  |
| --- | --- |
| **4.** | gi|73760405    **Mass:** 50670    **Score:** 434    **Queries matched:** 11   **emPAI:** 0.37 |
|  | thymopoietin isoform beta [Homo sapiens] |

|  |  |
| --- | --- |
|  | Check to include this hit in error tolerant search or archive report |
|  |  |

|  |  |  |  |  |  |  |  |  |  |  |
| --- | --- | --- | --- | --- | --- | --- | --- | --- | --- | --- |
|  | **Query** | **Observed** | **Mr(expt)** | **Mr(calc)** | **Delta** | **Miss** | **Score** | **Expect** | **Rank** | **Peptide** |
|  | 1071 | **457.5471** | **913.0793** | **912.0449** | **1.0344** | **0** | **25** | **11** | **1** | **K.GGPLQALTR.E** |
|  | 713 | **417.9412** | **1250.8015** | **1251.3921** | **-0.5906** | **0** | **10** | **2.7e+02** | **7** | **R.NRPPLPAGTNSK.G** |
|  | 2768 | **688.8065** | **1375.5983** | **1374.5348** | **1.0635** | **0** | **46** | **0.074** | **1** | **M.PEFLEDPSVLTK.D** |
|  | 1804 | **540.3986** | **1618.1735** | **1617.7945** | **0.3790** | **1** | **53** | **0.01** | **1** | **M.PEFLEDPSVLTKDK.L** |
|  | 3278 | **825.2048** | **1648.3948** | **1647.7392** | **0.6555** | **0** | **82** | **1.5e-05** | **1** | **R.SSTPLPTISSSAENTR.Q** |
|  | 2029 | **582.2886** | **1743.8435** | **1742.9281** | **0.9154** | **2** | **58** | **0.0047** | **1** | **K.KPLTRAEVGEKTEER.R** |
|  | 2163 | **598.6844** | **1793.0312** | **1793.0279** | **0.0032** | **0** | **(15)** | **1.1e+02** | **1** | **K.HASPILPITEFSDIPR.R** |
|  | 2168 | **598.9750** | **1793.9028** | **1793.0279** | **0.8748** | **0** | **33** | **1.3** | **1** | **K.HASPILPITEFSDIPR.R** |
|  | 2358 | **620.7269** | **1859.1584** | **1859.1244** | **0.0341** | **2** | **20** | **29** | **1** | **M.PEFLEDPSVLTKDKLK.S** |
|  | 3331 | **857.8328** | **2570.4763** | **2570.6795** | **-0.2032** | **1** | **(87)** | **3.9e-06** | **1** | **K.GPPDFSSDEEREPTPVLGSGAAAAGR.S** |
|  | 3332 | **858.1165** | **2571.3272** | **2570.6795** | **0.6477** | **1** | **107** | **4e-08** | **1** | **K.GPPDFSSDEEREPTPVLGSGAAAAGR.S** |

  


---

|  |  |
| --- | --- |
| **5.** | gi|5174735    **Mass:** 49830    **Score:** 390    **Queries matched:** 17   **emPAI:** 0.57 |
|  | tubulin beta-2C chain [Homo sapiens] |

|  |  |
| --- | --- |
|  | Check to include this hit in error tolerant search or archive report |
|  |  |

|  |  |  |  |  |  |  |  |  |  |  |
| --- | --- | --- | --- | --- | --- | --- | --- | --- | --- | --- |
|  | **Query** | **Observed** | **Mr(expt)** | **Mr(calc)** | **Delta** | **Miss** | **Score** | **Expect** | **Rank** | **Peptide** |
|  | 2019 | **580.1808** | **1158.3468** | **1159.4011** | **-1.0543** | **0** | **(41)** | **0.2** | **1** | **K.LAVNMVPFPR.L + Oxidation (M)** |
|  | 2024 | **581.2592** | **1160.5035** | **1159.4011** | **1.1025** | **0** | **60** | **0.003** | **1** | **K.LAVNMVPFPR.L + Oxidation (M)** |
|  | 2025 | **581.2952** | **1160.5756** | **1159.4011** | **1.1745** | **0** | **(41)** | **0.23** | **1** | **K.LAVNMVPFPR.L + Oxidation (M)** |
|  | 2482 | **644.6786** | **1287.3424** | **1287.5734** | **-0.2310** | **1** | **3** | **1.5e+03** | **10** | **R.KLAVNMVPFPR.L + Oxidation (M)** |
|  | 1204 | **468.2981** | **1401.8721** | **1401.5899** | **0.2823** | **1** | **5** | **7.8e+02** | **9** | **K.RISEQFTAMFR.R + Oxidation (M)** |
|  | 3231 | **809.6979** | **1617.3811** | **1617.8194** | **-0.4383** | **0** | **(28)** | **3** | **1** | **R.AVLVDLEPGTMDSVR.S + Oxidation (M)** |
|  | 3232 | **810.1809** | **1618.3470** | **1617.8194** | **0.5277** | **0** | **78** | **3.1e-05** | **1** | **R.AVLVDLEPGTMDSVR.S + Oxidation (M)** |
|  | 3265 | **819.3607** | **1636.7065** | **1636.9135** | **-0.2069** | **0** | **63** | **0.0012** | **1** | **R.LHFFMPGFAPLTSR.G + Oxidation (M)** |
|  | 1835 | **546.6984** | **1637.0729** | **1636.9135** | **0.1595** | **0** | **(13)** | **1.6e+02** | **1** | **R.LHFFMPGFAPLTSR.G + Oxidation (M)** |
|  | 3266 | **819.6091** | **1637.2035** | **1636.9135** | **0.2900** | **0** | **(45)** | **0.086** | **1** | **R.LHFFMPGFAPLTSR.G + Oxidation (M)** |
|  | 1837 | **546.8776** | **1637.6105** | **1636.9135** | **0.6970** | **0** | **(43)** | **0.11** | **1** | **R.LHFFMPGFAPLTSR.G + Oxidation (M)** |
|  | 3317 | **849.1711** | **1696.3275** | **1696.8561** | **-0.5286** | **0** | **22** | **14** | **1** | **K.NSSYFVEWIPNNVK.T** |
|  | 3327 | **854.6531** | **1707.2915** | **1707.9418** | **-0.6504** | **0** | **23** | **13** | **1** | **R.ALTVPELTQQMFDAK.N + Oxidation (M)** |
|  | 2334 | **615.8024** | **1844.3851** | **1844.0317** | **0.3534** | **1** | **31** | **2.3** | **1** | **R.INVYYNEATGGKYVPR.A** |
|  | 2663 | **677.7338** | **2030.1791** | **2030.3046** | **-0.1255** | **1** | **35** | **1.2** | **1** | **K.MSATFIGNSTAIQELFKR.I + Oxidation (M)** |
|  | 2821 | **710.1707** | **2127.4898** | **2126.4200** | **1.0698** | **1** | **43** | **0.12** | **1** | **-.MREIVHLQAGQCGNQIGAK.F + Carbamidomethyl (C); Oxidation (M)** |
|  | 3193 | **799.0768** | **2394.2084** | **2394.7012** | **-0.4928** | **2** | **30** | **2.3** | **1** | **K.IREEYPDRIMNTFSVVPSPK.V + Oxidation (M)** |

  

|  |  |
| --- | --- |
|  | |
|  | **Proteins matching the same set of peptides:** |

|  |  |
| --- | --- |
|  | gi|20809886    **Mass:** 49807    **Score:** 390    **Queries matched:** 17 |
|  | Tubulin, beta 2C [Homo sapiens] |

|  |  |
| --- | --- |
|  | gi|23958133    **Mass:** 49839    **Score:** 390    **Queries matched:** 17 |
|  | Tubulin, beta 2C [Homo sapiens] |

|  |  |
| --- | --- |
|  | gi|119608775    **Mass:** 48824    **Score:** 390    **Queries matched:** 17 |
|  | tubulin, beta 2C, isoform CRA\_b [Homo sapiens] |

---

|  |  |
| --- | --- |
| **6.** | gi|193785596    **Mass:** 44602    **Score:** 372    **Queries matched:** 17   **emPAI:** 0.53 |
|  | unnamed protein product [Homo sapiens] |

|  |  |
| --- | --- |
|  | Check to include this hit in error tolerant search or archive report |
|  |  |

|  |  |  |  |  |  |  |  |  |  |  |
| --- | --- | --- | --- | --- | --- | --- | --- | --- | --- | --- |
|  | **Query** | **Observed** | **Mr(expt)** | **Mr(calc)** | **Delta** | **Miss** | **Score** | **Expect** | **Rank** | **Peptide** |
|  | 2019 | 580.1808 | 1158.3468 | 1159.4011 | -1.0543 | 0 | (41) | 0.2 | 1 | K.LAVNMVPFPR.L + Oxidation (M) |
|  | 2024 | 581.2592 | 1160.5035 | 1159.4011 | 1.1025 | 0 | 60 | 0.003 | 1 | K.LAVNMVPFPR.L + Oxidation (M) |
|  | 2025 | 581.2952 | 1160.5756 | 1159.4011 | 1.1745 | 0 | (41) | 0.23 | 1 | K.LAVNMVPFPR.L + Oxidation (M) |
|  | 2482 | 644.6786 | 1287.3424 | 1287.5734 | -0.2310 | 1 | 3 | 1.5e+03 | 10 | R.KLAVNMVPFPR.L + Oxidation (M) |
|  | 1204 | 468.2981 | 1401.8721 | 1401.5899 | 0.2823 | 1 | 5 | 7.8e+02 | 9 | K.RISEQFTAMFR.R + Oxidation (M) |
|  | 3231 | 809.6979 | 1617.3811 | 1617.8194 | -0.4383 | 0 | (28) | 3 | 1 | R.AVLVDLEPGTMDSVR.S + Oxidation (M) |
|  | 3232 | 810.1809 | 1618.3470 | 1617.8194 | 0.5277 | 0 | 78 | 3.1e-05 | 1 | R.AVLVDLEPGTMDSVR.S + Oxidation (M) |
|  | 3265 | 819.3607 | 1636.7065 | 1636.9135 | -0.2069 | 0 | 63 | 0.0012 | 1 | R.LHFFMPGFAPLTSR.G + Oxidation (M) |
|  | 1835 | 546.6984 | 1637.0729 | 1636.9135 | 0.1595 | 0 | (13) | 1.6e+02 | 1 | R.LHFFMPGFAPLTSR.G + Oxidation (M) |
|  | 3266 | 819.6091 | 1637.2035 | 1636.9135 | 0.2900 | 0 | (45) | 0.086 | 1 | R.LHFFMPGFAPLTSR.G + Oxidation (M) |
|  | 1837 | 546.8776 | 1637.6105 | 1636.9135 | 0.6970 | 0 | (43) | 0.11 | 1 | R.LHFFMPGFAPLTSR.G + Oxidation (M) |
|  | 3317 | 849.1711 | 1696.3275 | 1696.8561 | -0.5286 | 0 | 22 | 14 | 1 | K.NSSYFVEWIPNNVK.T |
|  | 3327 | 854.6531 | 1707.2915 | 1707.9418 | -0.6504 | 0 | 23 | 13 | 1 | R.ALTVPELTQQMFDAK.N + Oxidation (M) |
|  | 2334 | 615.8024 | 1844.3851 | 1844.0317 | 0.3534 | 1 | 31 | 2.3 | 1 | R.INVYYNEATGGKYVPR.A |
|  | 2663 | 677.7338 | 2030.1791 | 2030.3046 | -0.1255 | 1 | 35 | 1.2 | 1 | K.MSATFIGNSTAIQELFKR.I + Oxidation (M) |
|  | 2821 | 710.1707 | 2127.4898 | 2127.3650 | 0.1248 | 1 | 26 | 7.2 | 3 | -.MRENVHLQAGQCGNQIGAK.F + Carbamidomethyl (C); Oxidation (M) |
|  | 3193 | 799.0768 | 2394.2084 | 2394.7012 | -0.4928 | 2 | 30 | 2.3 | 1 | K.IREEYPDRIMNTFSVVPSPK.V + Oxidation (M) |

  


---

|  |  |
| --- | --- |
| **7.** | gi|7106439    **Mass:** 49670    **Score:** 334    **Queries matched:** 18   **emPAI:** 0.57 |
|  | tubulin beta-5 chain [Mus musculus] |

|  |  |
| --- | --- |
|  | Check to include this hit in error tolerant search or archive report |
|  |  |

|  |  |  |  |  |  |  |  |  |  |  |
| --- | --- | --- | --- | --- | --- | --- | --- | --- | --- | --- |
|  | **Query** | **Observed** | **Mr(expt)** | **Mr(calc)** | **Delta** | **Miss** | **Score** | **Expect** | **Rank** | **Peptide** |
|  | 2019 | 580.1808 | 1158.3468 | 1159.4011 | -1.0543 | 0 | (41) | 0.2 | 1 | K.LAVNMVPFPR.L + Oxidation (M) |
|  | 2024 | 581.2592 | 1160.5035 | 1159.4011 | 1.1025 | 0 | 60 | 0.003 | 1 | K.LAVNMVPFPR.L + Oxidation (M) |
|  | 2025 | 581.2952 | 1160.5756 | 1159.4011 | 1.1745 | 0 | (41) | 0.23 | 1 | K.LAVNMVPFPR.L + Oxidation (M) |
|  | 2482 | 644.6786 | 1287.3424 | 1287.5734 | -0.2310 | 1 | 3 | 1.5e+03 | 10 | R.KLAVNMVPFPR.L + Oxidation (M) |
|  | 1204 | 468.2981 | 1401.8721 | 1401.5899 | 0.2823 | 1 | 5 | 7.8e+02 | 9 | K.RISEQFTAMFR.R + Oxidation (M) |
|  | 3250 | **816.6980** | **1631.3812** | **1631.8459** | **-0.4646** | **0** | **(59)** | **0.0028** | **1** | **R.AILVDLEPGTMDSVR.S + Oxidation (M)** |
|  | 3253 | **816.9507** | **1631.8866** | **1631.8459** | **0.0407** | **0** | **75** | **8.7e-05** | **1** | **R.AILVDLEPGTMDSVR.S + Oxidation (M)** |
|  | 3265 | 819.3607 | 1636.7065 | 1636.9135 | -0.2069 | 0 | 63 | 0.0012 | 1 | R.LHFFMPGFAPLTSR.G + Oxidation (M) |
|  | 1835 | 546.6984 | 1637.0729 | 1636.9135 | 0.1595 | 0 | (13) | 1.6e+02 | 1 | R.LHFFMPGFAPLTSR.G + Oxidation (M) |
|  | 3266 | 819.6091 | 1637.2035 | 1636.9135 | 0.2900 | 0 | (45) | 0.086 | 1 | R.LHFFMPGFAPLTSR.G + Oxidation (M) |
|  | 1837 | 546.8776 | 1637.6105 | 1636.9135 | 0.6970 | 0 | (43) | 0.11 | 1 | R.LHFFMPGFAPLTSR.G + Oxidation (M) |
|  | 1877 | **554.0420** | **1659.1038** | **1659.8774** | **-0.7736** | **0** | **(7)** | **5.4e+02** | **5** | **R.ALTVPELTQQVFDAK.N** |
|  | 3289 | **830.6453** | **1659.2759** | **1659.8774** | **-0.6016** | **0** | **(21)** | **18** | **2** | **R.ALTVPELTQQVFDAK.N** |
|  | 3290 | **830.7469** | **1659.4790** | **1659.8774** | **-0.3984** | **0** | **36** | **0.54** | **1** | **R.ALTVPELTQQVFDAK.N** |
|  | 1879 | **554.3004** | **1659.8791** | **1659.8774** | **0.0016** | **0** | **(10)** | **2.8e+02** | **3** | **R.ALTVPELTQQVFDAK.N** |
|  | 3317 | 849.1711 | 1696.3275 | 1696.8561 | -0.5286 | 0 | 22 | 14 | 1 | K.NSSYFVEWIPNNVK.T |
|  | 2821 | 710.1707 | 2127.4898 | 2126.4200 | 1.0698 | 1 | 43 | 0.12 | 1 | -.MREIVHIQAGQCGNQIGAK.F + Carbamidomethyl (C); Oxidation (M) |
|  | 3193 | 799.0768 | 2394.2084 | 2394.7012 | -0.4928 | 2 | 30 | 2.3 | 1 | K.IREEYPDRIMNTFSVVPSPK.V + Oxidation (M) |

  

|  |  |
| --- | --- |
|  | |
|  | **Proteins matching the same set of peptides:** |

|  |  |
| --- | --- |
|  | gi|18088719    **Mass:** 49671    **Score:** 334    **Queries matched:** 18 |
|  | Tubulin, beta [Homo sapiens] |

---

|  |  |
| --- | --- |
| **8.** | gi|4507729    **Mass:** 49906    **Score:** 318    **Queries matched:** 16   **emPAI:** 0.38 |
|  | tubulin beta-2A chain [Homo sapiens] |

|  |  |
| --- | --- |
|  | Check to include this hit in error tolerant search or archive report |
|  |  |

|  |  |  |  |  |  |  |  |  |  |  |
| --- | --- | --- | --- | --- | --- | --- | --- | --- | --- | --- |
|  | **Query** | **Observed** | **Mr(expt)** | **Mr(calc)** | **Delta** | **Miss** | **Score** | **Expect** | **Rank** | **Peptide** |
|  | 2019 | 580.1808 | 1158.3468 | 1159.4011 | -1.0543 | 0 | (41) | 0.2 | 1 | K.LAVNMVPFPR.L + Oxidation (M) |
|  | 2024 | 581.2592 | 1160.5035 | 1159.4011 | 1.1025 | 0 | 60 | 0.003 | 1 | K.LAVNMVPFPR.L + Oxidation (M) |
|  | 2025 | 581.2952 | 1160.5756 | 1159.4011 | 1.1745 | 0 | (41) | 0.23 | 1 | K.LAVNMVPFPR.L + Oxidation (M) |
|  | 2482 | 644.6786 | 1287.3424 | 1287.5734 | -0.2310 | 1 | 3 | 1.5e+03 | 10 | R.KLAVNMVPFPR.L + Oxidation (M) |
|  | 1041 | **452.4392** | **1354.2955** | **1355.4517** | **-1.1562** | **0** | **3** | **1.6e+03** | **8** | **R.INVYYNEAAGNK.Y** |
|  | 1204 | 468.2981 | 1401.8721 | 1401.5899 | 0.2823 | 1 | 5 | 7.8e+02 | 9 | K.RISEQFTAMFR.R + Oxidation (M) |
|  | 3250 | 816.6980 | 1631.3812 | 1631.8459 | -0.4646 | 0 | (59) | 0.0028 | 1 | R.AILVDLEPGTMDSVR.S + Oxidation (M) |
|  | 3253 | 816.9507 | 1631.8866 | 1631.8459 | 0.0407 | 0 | 75 | 8.7e-05 | 1 | R.AILVDLEPGTMDSVR.S + Oxidation (M) |
|  | 3265 | 819.3607 | 1636.7065 | 1636.9135 | -0.2069 | 0 | 63 | 0.0012 | 1 | R.LHFFMPGFAPLTSR.G + Oxidation (M) |
|  | 1835 | 546.6984 | 1637.0729 | 1636.9135 | 0.1595 | 0 | (13) | 1.6e+02 | 1 | R.LHFFMPGFAPLTSR.G + Oxidation (M) |
|  | 3266 | 819.6091 | 1637.2035 | 1636.9135 | 0.2900 | 0 | (45) | 0.086 | 1 | R.LHFFMPGFAPLTSR.G + Oxidation (M) |
|  | 1837 | 546.8776 | 1637.6105 | 1636.9135 | 0.6970 | 0 | (43) | 0.11 | 1 | R.LHFFMPGFAPLTSR.G + Oxidation (M) |
|  | 3317 | 849.1711 | 1696.3275 | 1696.8561 | -0.5286 | 0 | 22 | 14 | 1 | K.NSSYFVEWIPNNVK.T |
|  | 3327 | 854.6531 | 1707.2915 | 1707.9418 | -0.6504 | 0 | 12 | 1.6e+02 | 5 | R.ALTVPELTQQMFDSK.N |
|  | 2663 | 677.7338 | 2030.1791 | 2030.3046 | -0.1255 | 1 | 35 | 1.2 | 1 | K.MSATFIGNSTAIQELFKR.I + Oxidation (M) |
|  | 2821 | 710.1707 | 2127.4898 | 2126.4200 | 1.0698 | 1 | 43 | 0.12 | 1 | -.MREIVHIQAGQCGNQIGAK.F + Carbamidomethyl (C); Oxidation (M) |

  

|  |  |
| --- | --- |
|  | |
|  | **Proteins matching the same set of peptides:** |

|  |  |
| --- | --- |
|  | gi|27227551    **Mass:** 49915    **Score:** 318    **Queries matched:** 16 |
|  | class II beta tubulin isotype [Homo sapiens] |

---

|  |  |
| --- | --- |
| **9.** | gi|9755133    **Mass:** 46697    **Score:** 266    **Queries matched:** 7   **emPAI:** 0.41 |
|  | XPMC2 protein [Homo sapiens] |

|  |  |
| --- | --- |
|  | Check to include this hit in error tolerant search or archive report |
|  |  |

|  |  |  |  |  |  |  |  |  |  |  |
| --- | --- | --- | --- | --- | --- | --- | --- | --- | --- | --- |
|  | **Query** | **Observed** | **Mr(expt)** | **Mr(calc)** | **Delta** | **Miss** | **Score** | **Expect** | **Rank** | **Peptide** |
|  | 1468 | **501.1743** | **1000.3338** | **1000.1913** | **0.1425** | **0** | **36** | **0.66** | **1** | **K.ALQEWLLK.Q** |
|  | 1489 | **504.3960** | **1006.7773** | **1007.1411** | **-0.3638** | **0** | **49** | **0.028** | **1** | **R.VSIVNQYGK.C** |
|  | 2471 | **643.2952** | **1284.5756** | **1284.4617** | **0.1138** | **0** | **54** | **0.01** | **1** | **R.TAVSGIRPENLK.Q** |
|  | 1172 | **464.5963** | **1390.7668** | **1390.6303** | **0.1365** | **1** | **(33)** | **1.5** | **1** | **K.RAPSSPVAKPGPVK.T** |
|  | 1173 | **464.8682** | **1391.5825** | **1390.6303** | **0.9522** | **1** | **56** | **0.0066** | **1** | **K.RAPSSPVAKPGPVK.T** |
|  | 2879 | **730.2889** | **2187.8446** | **2188.4585** | **-0.6138** | **2** | **24** | **9.8** | **1** | **K.QGEELEVVQKEVAEMLKGR.I + Oxidation (M)** |
|  | 3088 | **775.4839** | **2323.4295** | **2323.6233** | **-0.1938** | **1** | **51** | **0.022** | **1** | **R.ALALDCEMVGVGPKGEESMAAR.V + Carbamidomethyl (C); 2 Oxidation (M)** |

  

|  |  |
| --- | --- |
|  | |
|  | **Proteins matching the same set of peptides:** |

|  |  |
| --- | --- |
|  | gi|76781492    **Mass:** 46671    **Score:** 266    **Queries matched:** 7 |
|  | RNA exonuclease 4 [Homo sapiens] |

---

|  |  |
| --- | --- |
| **10.** | gi|119589485    **Mass:** 54467    **Score:** 255    **Queries matched:** 14   **emPAI:** 0.26 |
|  | tubulin, beta 4, isoform CRA\_b [Homo sapiens] |

|  |  |
| --- | --- |
|  | Check to include this hit in error tolerant search or archive report |
|  |  |

|  |  |  |  |  |  |  |  |  |  |  |
| --- | --- | --- | --- | --- | --- | --- | --- | --- | --- | --- |
|  | **Query** | **Observed** | **Mr(expt)** | **Mr(calc)** | **Delta** | **Miss** | **Score** | **Expect** | **Rank** | **Peptide** |
|  | 2019 | 580.1808 | 1158.3468 | 1159.4011 | -1.0543 | 0 | (41) | 0.2 | 1 | K.LAVNMVPFPR.L + Oxidation (M) |
|  | 2024 | 581.2592 | 1160.5035 | 1159.4011 | 1.1025 | 0 | 60 | 0.003 | 1 | K.LAVNMVPFPR.L + Oxidation (M) |
|  | 2025 | 581.2952 | 1160.5756 | 1159.4011 | 1.1745 | 0 | (41) | 0.23 | 1 | K.LAVNMVPFPR.L + Oxidation (M) |
|  | 2482 | 644.6786 | 1287.3424 | 1287.5734 | -0.2310 | 1 | 3 | 1.5e+03 | 10 | R.KLAVNMVPFPR.L + Oxidation (M) |
|  | 1204 | 468.2981 | 1401.8721 | 1401.5899 | 0.2823 | 1 | 5 | 7.8e+02 | 9 | K.RISEQFTAMFR.R + Oxidation (M) |
|  | 3231 | 809.6979 | 1617.3811 | 1617.8194 | -0.4383 | 0 | (28) | 3 | 1 | R.AVLVDLEPGTMDSVR.S + Oxidation (M) |
|  | 3232 | 810.1809 | 1618.3470 | 1617.8194 | 0.5277 | 0 | 78 | 3.1e-05 | 1 | R.AVLVDLEPGTMDSVR.S + Oxidation (M) |
|  | 3265 | 819.3607 | 1636.7065 | 1636.9135 | -0.2069 | 0 | 63 | 0.0012 | 1 | R.LHFFMPGFAPLTSR.G + Oxidation (M) |
|  | 1835 | 546.6984 | 1637.0729 | 1636.9135 | 0.1595 | 0 | (13) | 1.6e+02 | 1 | R.LHFFMPGFAPLTSR.G + Oxidation (M) |
|  | 3266 | 819.6091 | 1637.2035 | 1636.9135 | 0.2900 | 0 | (45) | 0.086 | 1 | R.LHFFMPGFAPLTSR.G + Oxidation (M) |
|  | 1837 | 546.8776 | 1637.6105 | 1636.9135 | 0.6970 | 0 | (43) | 0.11 | 1 | R.LHFFMPGFAPLTSR.G + Oxidation (M) |
|  | 3317 | 849.1711 | 1696.3275 | 1696.8561 | -0.5286 | 0 | 22 | 14 | 1 | K.NSSYFVEWIPNNVK.T |
|  | 3327 | 854.6531 | 1707.2915 | 1707.9418 | -0.6504 | 0 | 23 | 13 | 1 | R.ALTVPELTQQMFDAK.N + Oxidation (M) |
|  | 2949 | **742.7908** | **2225.3501** | **2225.4817** | **-0.1316** | **0** | **5** | **8.7e+02** | **8** | **R.LPGAQGPCSAVSAASSTLAAAAAPR.A** |

  


---

|  |  |
| --- | --- |
| **11.** | gi|1297274    **Mass:** 50517    **Score:** 250    **Queries matched:** 11   **emPAI:** 0.21 |
|  | beta-tubulin [Homo sapiens] |

|  |  |
| --- | --- |
|  | Check to include this hit in error tolerant search or archive report |
|  |  |

|  |  |  |  |  |  |  |  |  |  |  |
| --- | --- | --- | --- | --- | --- | --- | --- | --- | --- | --- |
|  | **Query** | **Observed** | **Mr(expt)** | **Mr(calc)** | **Delta** | **Miss** | **Score** | **Expect** | **Rank** | **Peptide** |
|  | 2019 | 580.1808 | 1158.3468 | 1159.4011 | -1.0543 | 0 | (41) | 0.2 | 1 | K.LAVNMVPFPR.L + Oxidation (M) |
|  | 2024 | 581.2592 | 1160.5035 | 1159.4011 | 1.1025 | 0 | 60 | 0.003 | 1 | K.LAVNMVPFPR.L + Oxidation (M) |
|  | 2025 | 581.2952 | 1160.5756 | 1159.4011 | 1.1745 | 0 | (41) | 0.23 | 1 | K.LAVNMVPFPR.L + Oxidation (M) |
|  | 2482 | 644.6786 | 1287.3424 | 1287.5734 | -0.2310 | 1 | 3 | 1.5e+03 | 10 | R.KLAVNMVPFPR.L + Oxidation (M) |
|  | 1204 | 468.2981 | 1401.8721 | 1401.5899 | 0.2823 | 1 | 5 | 7.8e+02 | 9 | K.RISEQFTAMFR.R + Oxidation (M) |
|  | 3250 | 816.6980 | 1631.3812 | 1631.8459 | -0.4646 | 0 | (59) | 0.0028 | 1 | R.AILVDLEPGTMDSVR.S + Oxidation (M) |
|  | 3253 | 816.9507 | 1631.8866 | 1631.8459 | 0.0407 | 0 | 75 | 8.7e-05 | 1 | R.AILVDLEPGTMDSVR.S + Oxidation (M) |
|  | 3317 | 849.1711 | 1696.3275 | 1696.8561 | -0.5286 | 0 | 22 | 14 | 1 | K.NSSYFVEWIPNNVK.V |
|  | 3327 | 854.6531 | 1707.2915 | 1707.9418 | -0.6504 | 0 | 23 | 13 | 1 | R.ALTVPELTQQMFDAK.N + Oxidation (M) |
|  | 2663 | 677.7338 | 2030.1791 | 2030.3046 | -0.1255 | 1 | 23 | 15 | 2 | K.MSSTFIGNSTAIQELFKR.I |
|  | 2821 | 710.1707 | 2127.4898 | 2126.4200 | 1.0698 | 1 | 43 | 0.12 | 1 | -.MREIVHIQAGQCGNQIGAK.F + Carbamidomethyl (C); Oxidation (M) |

  

|  |  |
| --- | --- |
|  | |
|  | **Proteins matching the same set of peptides:** |

|  |  |
| --- | --- |
|  | gi|50592996    **Mass:** 50432    **Score:** 250    **Queries matched:** 11 |
|  | tubulin beta-3 chain isoform 1 [Homo sapiens] |

|  |  |
| --- | --- |
|  | gi|189054409    **Mass:** 50404    **Score:** 250    **Queries matched:** 11 |
|  | unnamed protein product [Homo sapiens] |

---

|  |  |
| --- | --- |
| **12.** | gi|10433717    **Mass:** 49843    **Score:** 213    **Queries matched:** 12   **emPAI:** 0.29 |
|  | unnamed protein product [Homo sapiens] |

|  |  |
| --- | --- |
|  | Check to include this hit in error tolerant search or archive report |
|  |  |

|  |  |  |  |  |  |  |  |  |  |  |
| --- | --- | --- | --- | --- | --- | --- | --- | --- | --- | --- |
|  | **Query** | **Observed** | **Mr(expt)** | **Mr(calc)** | **Delta** | **Miss** | **Score** | **Expect** | **Rank** | **Peptide** |
|  | 2019 | 580.1808 | 1158.3468 | 1159.4011 | -1.0543 | 0 | (41) | 0.2 | 1 | K.LAVNMVPFPR.L + Oxidation (M) |
|  | 2024 | 581.2592 | 1160.5035 | 1159.4011 | 1.1025 | 0 | 60 | 0.003 | 1 | K.LAVNMVPFPR.L + Oxidation (M) |
|  | 2025 | 581.2952 | 1160.5756 | 1159.4011 | 1.1745 | 0 | (41) | 0.23 | 1 | K.LAVNMVPFPR.L + Oxidation (M) |
|  | 2482 | 644.6786 | 1287.3424 | 1287.5734 | -0.2310 | 1 | 3 | 1.5e+03 | 10 | R.KLAVNMVPFPR.L + Oxidation (M) |
|  | 3265 | 819.3607 | 1636.7065 | 1636.9135 | -0.2069 | 0 | 63 | 0.0012 | 1 | R.LHFFMPGFAPLTSR.G + Oxidation (M) |
|  | 1835 | 546.6984 | 1637.0729 | 1636.9135 | 0.1595 | 0 | (13) | 1.6e+02 | 1 | R.LHFFMPGFAPLTSR.G + Oxidation (M) |
|  | 3266 | 819.6091 | 1637.2035 | 1636.9135 | 0.2900 | 0 | (45) | 0.086 | 1 | R.LHFFMPGFAPLTSR.G + Oxidation (M) |
|  | 1837 | 546.8776 | 1637.6105 | 1636.9135 | 0.6970 | 0 | (43) | 0.11 | 1 | R.LHFFMPGFAPLTSR.G + Oxidation (M) |
|  | 3317 | 849.1711 | 1696.3275 | 1696.8561 | -0.5286 | 0 | 22 | 14 | 1 | K.NSSYFVEWIPNNVK.V |
|  | 3337 | **868.6511** | **1735.2875** | **1735.9552** | **-0.6678** | **0** | **37** | **0.52** | **1** | **R.ALTVPELTQQMFDAR.N + Oxidation (M)** |
|  | 2632 | **670.3589** | **2008.0545** | **2009.2505** | **-1.1960** | **1** | **6** | **6.9e+02** | **10** | **M.REIVHIQAGQCGNQIGTK.F + Carbamidomethyl (C)** |
|  | 2663 | 677.7338 | 2030.1791 | 2030.3046 | -0.1255 | 1 | 23 | 15 | 2 | K.MASTFIGNSTAIQELFKR.I + Oxidation (M) |

  

|  |  |
| --- | --- |
|  | |
|  | **Proteins matching the same set of peptides:** |

|  |  |
| --- | --- |
|  | gi|14210536    **Mass:** 49857    **Score:** 213    **Queries matched:** 12 |
|  | tubulin beta-6 chain [Homo sapiens] |

|  |  |
| --- | --- |
|  | gi|194389900    **Mass:** 46701    **Score:** 213    **Queries matched:** 12 |
|  | unnamed protein product [Homo sapiens] |

---

|  |  |
| --- | --- |
| **13.** | gi|13129104    **Mass:** 40235    **Score:** 207    **Queries matched:** 8   **emPAI:** 0.27 |
|  | coiled-coil domain-containing protein 86 [Homo sapiens] |

|  |  |
| --- | --- |
|  | Check to include this hit in error tolerant search or archive report |
|  |  |

|  |  |  |  |  |  |  |  |  |  |  |
| --- | --- | --- | --- | --- | --- | --- | --- | --- | --- | --- |
|  | **Query** | **Observed** | **Mr(expt)** | **Mr(calc)** | **Delta** | **Miss** | **Score** | **Expect** | **Rank** | **Peptide** |
|  | 1218 | **470.2166** | **1407.6276** | **1407.6971** | **-0.0695** | **2** | **(37)** | **0.53** | **1** | **K.KLNKEELPVIPK.G** |
|  | 1219 | **470.5601** | **1408.6580** | **1407.6971** | **0.9610** | **2** | **38** | **0.48** | **1** | **K.KLNKEELPVIPK.G** |
|  | 1326 | **485.1013** | **1452.2817** | **1451.7133** | **0.5684** | **2** | **20** | **25** | **1** | **R.KAEVVQVIRNPAK.L** |
|  | 1881 | **555.0421** | **1662.1042** | **1662.9542** | **-0.8500** | **2** | **4** | **1.1e+03** | **10** | **K.KRFSQMLQDKPLR.T + Oxidation (M)** |
|  | 2524 | **654.4044** | **1960.1911** | **1960.2776** | **-0.0865** | **2** | **59** | **0.0034** | **1** | **R.DTLALLQKQPPQQPAAKI.-** |
|  | 2525 | **654.4287** | **1960.2640** | **1960.2776** | **-0.0137** | **2** | **(45)** | **0.085** | **1** | **R.DTLALLQKQPPQQPAAKI.-** |
|  | 2528 | **654.6758** | **1961.0052** | **1960.2776** | **0.7276** | **2** | **(17)** | **59** | **1** | **R.DTLALLQKQPPQQPAAKI.-** |
|  | 3325 | **853.2067** | **2556.5980** | **2555.7080** | **0.8900** | **1** | **86** | **5e-06** | **1** | **R.ALVEFESNPEETREPGSPPSVQR.A** |

  


---

|  |  |
| --- | --- |
| **14.** | gi|38014544    **Mass:** 24271    **Score:** 188    **Queries matched:** 15   **emPAI:** 0.67 |
|  | Tubb5 protein [Rattus norvegicus] |

|  |  |
| --- | --- |
|  | Check to include this hit in error tolerant search or archive report |
|  |  |

|  |  |  |  |  |  |  |  |  |  |  |
| --- | --- | --- | --- | --- | --- | --- | --- | --- | --- | --- |
|  | **Query** | **Observed** | **Mr(expt)** | **Mr(calc)** | **Delta** | **Miss** | **Score** | **Expect** | **Rank** | **Peptide** |
|  | 2019 | 580.1808 | 1158.3468 | 1159.4011 | -1.0543 | 0 | (41) | 0.2 | 1 | K.LAVNMVPFPR.L + Oxidation (M) |
|  | 2024 | 581.2592 | 1160.5035 | 1159.4011 | 1.1025 | 0 | 60 | 0.003 | 1 | K.LAVNMVPFPR.L + Oxidation (M) |
|  | 2025 | 581.2952 | 1160.5756 | 1159.4011 | 1.1745 | 0 | (41) | 0.23 | 1 | K.LAVNMVPFPR.L + Oxidation (M) |
|  | 2482 | 644.6786 | 1287.3424 | 1287.5734 | -0.2310 | 1 | 3 | 1.5e+03 | 10 | R.KLAVNMVPFPR.L + Oxidation (M) |
|  | 1204 | 468.2981 | 1401.8721 | 1401.5899 | 0.2823 | 1 | 5 | 7.8e+02 | 9 | K.RISEQFTAMFR.R + Oxidation (M) |
|  | 3265 | 819.3607 | 1636.7065 | 1636.9135 | -0.2069 | 0 | 63 | 0.0012 | 1 | R.LHFFMPGFAPLTSR.G + Oxidation (M) |
|  | 1835 | 546.6984 | 1637.0729 | 1636.9135 | 0.1595 | 0 | (13) | 1.6e+02 | 1 | R.LHFFMPGFAPLTSR.G + Oxidation (M) |
|  | 3266 | 819.6091 | 1637.2035 | 1636.9135 | 0.2900 | 0 | (45) | 0.086 | 1 | R.LHFFMPGFAPLTSR.G + Oxidation (M) |
|  | 1837 | 546.8776 | 1637.6105 | 1636.9135 | 0.6970 | 0 | (43) | 0.11 | 1 | R.LHFFMPGFAPLTSR.G + Oxidation (M) |
|  | 1877 | 554.0420 | 1659.1038 | 1659.8774 | -0.7736 | 0 | (7) | 5.4e+02 | 5 | R.ALTVPELTQQVFDAK.N |
|  | 3289 | 830.6453 | 1659.2759 | 1659.8774 | -0.6016 | 0 | (21) | 18 | 2 | R.ALTVPELTQQVFDAK.N |
|  | 3290 | 830.7469 | 1659.4790 | 1659.8774 | -0.3984 | 0 | 36 | 0.54 | 1 | R.ALTVPELTQQVFDAK.N |
|  | 1879 | 554.3004 | 1659.8791 | 1659.8774 | 0.0016 | 0 | (10) | 2.8e+02 | 3 | R.ALTVPELTQQVFDAK.N |
|  | 3317 | 849.1711 | 1696.3275 | 1696.8561 | -0.5286 | 0 | 22 | 14 | 1 | K.NSSYFVEWIPNNVK.T |
|  | 2626 | **669.2922** | **2004.8544** | **2005.2586** | **-0.4043** | **1** | **2** | **1.7e+03** | **9** | **M.SGVTTCLRFPGQLNADLR.K + Carbamidomethyl (C)** |

  


---

|  |  |
| --- | --- |
| **15.** | gi|7657381    **Mass:** 55180    **Score:** 185    **Queries matched:** 10   **emPAI:** 0.26 |
|  | pre-mRNA-processing factor 19 [Homo sapiens] |

|  |  |
| --- | --- |
|  | Check to include this hit in error tolerant search or archive report |
|  |  |

|  |  |  |  |  |  |  |  |  |  |  |
| --- | --- | --- | --- | --- | --- | --- | --- | --- | --- | --- |
|  | **Query** | **Observed** | **Mr(expt)** | **Mr(calc)** | **Delta** | **Miss** | **Score** | **Expect** | **Rank** | **Peptide** |
|  | 825 | **429.5084** | **857.0021** | **857.0045** | **-0.0025** | **1** | **31** | **2.8** | **1** | **R.SLKFYSL.-** |
|  | 827 | **429.5429** | **857.0710** | **857.0045** | **0.0665** | **1** | **(22)** | **22** | **1** | **R.SLKFYSL.-** |
|  | 1528 | **507.5145** | **1013.0143** | **1013.1258** | **-0.1115** | **0** | **27** | **5.5** | **1** | **K.FIASTGMDR.S + Oxidation (M)** |
|  | 1098 | **458.8220** | **1373.4437** | **1374.5397** | **-1.0960** | **1** | **(13)** | **1.5e+02** | **2** | **K.LQDKATVLTTER.K** |
|  | 2764 | 688.2203 | 1374.4259 | 1374.5397 | -0.1138 | 1 | 39 | 0.33 | 1 | K.LQDKATVLTTER.K |
|  | 2767 | 688.6713 | 1375.3279 | 1374.5397 | 0.7881 | 1 | (27) | 5 | 2 | K.LQDKATVLTTER.K |
|  | 1110 | 459.4835 | 1375.4284 | 1374.5397 | 0.8887 | 1 | (10) | 3.1e+02 | 10 | K.LQDKATVLTTER.K |
|  | 2130 | **595.3647** | **1783.0719** | **1783.0285** | **0.0434** | **1** | **32** | **1.9** | **1** | **R.GKTVPEELVKPEELSK.Y** |
|  | 2460 | **640.2979** | **1917.8714** | **1917.1638** | **0.7075** | **1** | **57** | **0.0057** | **1** | **K.TVPEELVKPEELSKYR.Q** |
|  | 2461 | **640.3253** | **1917.9538** | **1917.1638** | **0.7899** | **1** | **(29)** | **3.2** | **1** | **K.TVPEELVKPEELSKYR.Q** |

  

|  |  |
| --- | --- |
|  | |
|  | **Proteins matching the same set of peptides:** |

|  |  |
| --- | --- |
|  | gi|119594310    **Mass:** 45730    **Score:** 185    **Queries matched:** 10 |
|  | PRP19/PSO4 pre-mRNA processing factor 19 homolog (S. cerevisiae), isoform CRA\_a [Homo sapiens] |

---

|  |  |
| --- | --- |
| **16.** | gi|119631904    **Mass:** 740442   **Score:** 174    **Queries matched:** 31 |
|  | nebulin, isoform CRA\_a [Homo sapiens] |

|  |  |
| --- | --- |
|  | Check to include this hit in error tolerant search or archive report |
|  |  |

|  |  |  |  |  |  |  |  |  |  |  |
| --- | --- | --- | --- | --- | --- | --- | --- | --- | --- | --- |
|  | **Query** | **Observed** | **Mr(expt)** | **Mr(calc)** | **Delta** | **Miss** | **Score** | **Expect** | **Rank** | **Peptide** |
|  | 24 | **363.0088** | **724.0029** | **723.7929** | **0.2100** | **0** | **14** | **1e+02** | **5** | **K.DMVSEK.K + Oxidation (M)** |
|  | 148 | **372.5496** | **743.0843** | **743.9149** | **-0.8305** | **0** | **33** | **1.1** | **10** | **K.MLHSLK.V + Oxidation (M)** |
|  | 149 | **372.6229** | **743.2311** | **743.8918** | **-0.6607** | **1** | **(24)** | **10** | **1** | **R.KVQELK.T** |
|  | 150 | **372.6581** | **743.3014** | **743.8918** | **-0.5903** | **1** | **24** | **10** | **3** | **R.KVQELK.T** |
|  | 186 | **375.5199** | **749.0250** | **749.8333** | **-0.8083** | **0** | **0** | **2.6e+03** | **10** | **R.NIASDCK.Y** |
|  | 818 | **429.0431** | **856.0714** | **856.0480** | **0.0233** | **1** | **13** | **1.2e+02** | **1** | **K.AHMLKTR.N** |
|  | 820 | **429.1022** | **856.1896** | **856.0480** | **0.1416** | **1** | **(13)** | **1.4e+02** | **1** | **K.AHMLKTR.N** |
|  | 883 | **434.1199** | **866.2251** | **865.0284** | **1.1967** | **2** | **5** | **8.5e+02** | **5** | **K.YKADLKK.L** |
|  | 909 | **435.9534** | **869.8921** | **870.9930** | **-1.1010** | **0** | **18** | **43** | **1** | **R.QGLTLSPR.L** |
|  | 914 | **436.0844** | **870.1540** | **870.9930** | **-0.8390** | **0** | **(15)** | **82** | **4** | **R.QGLTLSPR.L** |
|  | 1717 | **530.6490** | **1059.2833** | **1059.2605** | **0.0229** | **2** | **(8)** | **6e+02** | **4** | **K.KAAKLSSQVK.Y** |
|  | 1718 | **530.6578** | **1059.3009** | **1059.2605** | **0.0404** | **2** | **13** | **1.6e+02** | **1** | **K.KAAKLSSQVK.Y** |
|  | 1777 | **538.0971** | **1074.1794** | **1074.1905** | **-0.0111** | **0** | **7** | **5e+02** | **7** | **K.GHYVGTLTAR.D** |
|  | 56 | **366.1620** | **1095.4639** | **1095.2495** | **0.2144** | **2** | **14** | **1e+02** | **3** | **K.NLSSQKKYK.E** |
|  | 57 | **366.2478** | **1095.7211** | **1095.2495** | **0.4717** | **2** | **(5)** | **7.1e+02** | **5** | **K.NLSSQKKYK.E** |
|  | 1939 | **564.1071** | **1126.1993** | **1127.3114** | **-1.1120** | **2** | **0** | **2.5e+03** | **9** | **K.DMVSEKKYK.I** |
|  | 260 | **385.0608** | **1152.1601** | **1152.4102** | **-0.2501** | **0** | **9** | **2.7e+02** | **2** | **K.MMWSMHVAK.I + 2 Oxidation (M)** |
|  | 551 | **406.4843** | **1216.4307** | **1216.2968** | **0.1339** | **2** | **7** | **5.7e+02** | **7** | **K.YKEDYEKNK.G** |
|  | 2263 | **609.6999** | **1217.3850** | **1216.3413** | **1.0437** | **1** | **13** | **1.8e+02** | **3** | **R.QLKAAGDALSDK.L** |
|  | 861 | **432.4976** | **1294.4705** | **1293.4685** | **1.0020** | **2** | **6** | **8.2e+02** | **9** | **K.AGEILSEKKYR.Q** |
|  | 877 | **433.3978** | **1297.1713** | **1296.4889** | **0.6824** | **0** | **11** | **1.8e+02** | **8** | **K.CQILVSDIDYK.H** |
|  | 893 | **434.8575** | **1301.5505** | **1300.4991** | **1.0513** | **0** | **16** | **69** | **1** | **K.GYDLPVDAIPIK.A** |
|  | 945 | **440.2520** | **1317.7339** | **1317.4687** | **0.2652** | **0** | **11** | **1.9e+02** | **3** | **K.ATATPVTPEMQR.V + Oxidation (M)** |
|  | 1200 | **468.1680** | **1401.4818** | **1402.5562** | **-1.0744** | **2** | **14** | **1.3e+02** | **2** | **K.KQYEANKAHWK.W** |
|  | 1204 | 468.2981 | 1401.8721 | 1402.5562 | -0.6841 | 2 | (10) | 2.3e+02 | 6 | K.KQYEANKAHWK.W |
|  | 1986 | **574.3182** | **1719.9325** | **1718.9680** | **0.9646** | **0** | **10** | **2.6e+02** | **2** | **K.TSFHTPVDMLSVVAAK.K + Oxidation (M)** |
|  | 2429 | **632.6989** | **1895.0744** | **1895.2094** | **-0.1350** | **2** | **6** | **8.8e+02** | **8** | **K.GCGWVPFGSLEMEKAKR.A** |
|  | 2437 | **634.8785** | **1901.6133** | **1902.1542** | **-0.5409** | **1** | **4** | **8.2e+02** | **7** | **K.ENMGKGTPLPVTPEMER.V + Oxidation (M)** |
|  | 2526 | **654.5477** | **1960.6210** | **1960.1934** | **0.4277** | **0** | **7** | **3.8e+02** | **7** | **K.EGSHGLSMLGRPDIEMAK.K + 2 Oxidation (M)** |
|  | 2603 | **667.2744** | **1998.8009** | **1999.3551** | **-0.5543** | **1** | **4** | **1e+03** | **4** | **K.MIGALSINDDPKMLHSLK.T + Oxidation (M)** |
|  | 3286 | **828.2256** | **2481.6546** | **2481.8446** | **-0.1900** | **2** | **4** | **1e+03** | **7** | **R.QHPSNFQFKKLTDSMDMVLAK.Q + Oxidation (M)** |

  


---

|  |  |
| --- | --- |
| **17.** | gi|806562    **Mass:** 773204   **Score:** 173    **Queries matched:** 32 |
|  | nebulin [Homo sapiens] |

|  |  |
| --- | --- |
|  | Check to include this hit in error tolerant search or archive report |
|  |  |

|  |  |  |  |  |  |  |  |  |  |  |
| --- | --- | --- | --- | --- | --- | --- | --- | --- | --- | --- |
|  | **Query** | **Observed** | **Mr(expt)** | **Mr(calc)** | **Delta** | **Miss** | **Score** | **Expect** | **Rank** | **Peptide** |
|  | 24 | 363.0088 | 724.0029 | 723.7929 | 0.2100 | 0 | 14 | 1e+02 | 5 | K.DMVSEK.K + Oxidation (M) |
|  | 148 | 372.5496 | 743.0843 | 743.9149 | -0.8305 | 0 | 33 | 1.1 | 10 | K.MLHSLK.V + Oxidation (M) |
|  | 149 | 372.6229 | 743.2311 | 743.8918 | -0.6607 | 1 | (24) | 10 | 1 | R.KVQELK.T |
|  | 150 | 372.6581 | 743.3014 | 743.8918 | -0.5903 | 1 | 24 | 10 | 3 | R.KVQELK.T |
|  | 186 | 375.5199 | 749.0250 | 749.8333 | -0.8083 | 0 | 0 | 2.6e+03 | 10 | R.NIASDCK.Y |
|  | 446 | **401.9682** | **801.9217** | **800.9432** | **0.9785** | **2** | **15** | **1e+02** | **3** | **R.KKVDPSK.F** |
|  | 818 | 429.0431 | 856.0714 | 856.0480 | 0.0233 | 1 | 13 | 1.2e+02 | 1 | K.AHMLKTR.N |
|  | 820 | 429.1022 | 856.1896 | 856.0480 | 0.1416 | 1 | (13) | 1.4e+02 | 1 | K.AHMLKTR.N |
|  | 883 | 434.1199 | 866.2251 | 865.0284 | 1.1967 | 2 | 5 | 8.5e+02 | 5 | K.YKADLKK.L |
|  | 1118 | **460.0421** | **918.0694** | **918.0031** | **0.0663** | **0** | **12** | **1.7e+02** | **3** | **R.SDQISQIK.Y** |
|  | 1717 | 530.6490 | 1059.2833 | 1059.2605 | 0.0229 | 2 | (8) | 6e+02 | 4 | K.KAAKLSSQVK.Y |
|  | 1718 | 530.6578 | 1059.3009 | 1059.2605 | 0.0404 | 2 | 13 | 1.6e+02 | 1 | K.KAAKLSSQVK.Y |
|  | 1777 | 538.0971 | 1074.1794 | 1074.1905 | -0.0111 | 0 | 7 | 5e+02 | 7 | K.GHYVGTLTAR.D |
|  | 56 | 366.1620 | 1095.4639 | 1095.2495 | 0.2144 | 2 | 14 | 1e+02 | 3 | K.NLSSQKKYK.E |
|  | 57 | 366.2478 | 1095.7211 | 1095.2495 | 0.4717 | 2 | (5) | 7.1e+02 | 5 | K.NLSSQKKYK.E |
|  | 1939 | 564.1071 | 1126.1993 | 1127.3114 | -1.1120 | 2 | 0 | 2.5e+03 | 9 | K.DMVSEKKYK.I |
|  | 260 | 385.0608 | 1152.1601 | 1152.4102 | -0.2501 | 0 | 9 | 2.7e+02 | 2 | K.MMWSMHVAK.I + 2 Oxidation (M) |
|  | 551 | 406.4843 | 1216.4307 | 1216.2968 | 0.1339 | 2 | 7 | 5.7e+02 | 7 | K.YKEDYEKNK.G |
|  | 2263 | 609.6999 | 1217.3850 | 1216.3413 | 1.0437 | 1 | 13 | 1.8e+02 | 3 | R.QLKAAGDALSDK.L |
|  | 861 | 432.4976 | 1294.4705 | 1293.4685 | 1.0020 | 2 | 6 | 8.2e+02 | 9 | K.AGEILSEKKYR.Q |
|  | 877 | 433.3978 | 1297.1713 | 1296.4889 | 0.6824 | 0 | 11 | 1.8e+02 | 8 | K.CQILVSDIDYK.H |
|  | 893 | 434.8575 | 1301.5505 | 1300.4991 | 1.0513 | 0 | 16 | 69 | 1 | K.GYDLPVDAIPIK.A |
|  | 945 | 440.2520 | 1317.7339 | 1317.4687 | 0.2652 | 0 | 11 | 1.9e+02 | 3 | K.ATATPVTPEMQR.V + Oxidation (M) |
|  | 1200 | 468.1680 | 1401.4818 | 1402.5562 | -1.0744 | 2 | 14 | 1.3e+02 | 2 | K.KQYEANKAHWK.W |
|  | 1204 | 468.2981 | 1401.8721 | 1402.5562 | -0.6841 | 2 | (10) | 2.3e+02 | 6 | K.KQYEANKAHWK.W |
|  | 1986 | 574.3182 | 1719.9325 | 1718.9680 | 0.9646 | 0 | 10 | 2.6e+02 | 2 | K.TSFHTPVDMLSVVAAK.K + Oxidation (M) |
|  | 2429 | 632.6989 | 1895.0744 | 1895.2094 | -0.1350 | 2 | 6 | 8.8e+02 | 8 | K.GCGWVPFGSLEMEKAKR.A |
|  | 2430 | **632.7946** | **1895.3615** | **1896.1875** | **-0.8261** | **1** | **5** | **9.7e+02** | **7** | **K.DKYLLPPDAPELVQAVK.N** |
|  | 2437 | 634.8785 | 1901.6133 | 1902.1542 | -0.5409 | 1 | 4 | 8.2e+02 | 7 | K.ENMGKGTPLPVTPEMER.V + Oxidation (M) |
|  | 2526 | 654.5477 | 1960.6210 | 1960.1934 | 0.4277 | 0 | 7 | 3.8e+02 | 7 | K.EGSHGLSMLGRPDIEMAK.K + 2 Oxidation (M) |
|  | 2603 | 667.2744 | 1998.8009 | 1999.3551 | -0.5543 | 1 | 4 | 1e+03 | 4 | K.MIGALSINDDPKMLHSLK.T + Oxidation (M) |
|  | 3286 | 828.2256 | 2481.6546 | 2481.8446 | -0.1900 | 2 | 4 | 1e+03 | 7 | R.QHPSNFQFKKLTDSMDMVLAK.Q + Oxidation (M) |

  


---

|  |  |
| --- | --- |
| **18.** | gi|119631909    **Mass:** 777920   **Score:** 173    **Queries matched:** 31 |
|  | nebulin, isoform CRA\_f [Homo sapiens] |

|  |  |
| --- | --- |
|  | Check to include this hit in error tolerant search or archive report |
|  |  |

|  |  |  |  |  |  |  |  |  |  |  |
| --- | --- | --- | --- | --- | --- | --- | --- | --- | --- | --- |
|  | **Query** | **Observed** | **Mr(expt)** | **Mr(calc)** | **Delta** | **Miss** | **Score** | **Expect** | **Rank** | **Peptide** |
|  | 24 | 363.0088 | 724.0029 | 723.7929 | 0.2100 | 0 | 14 | 1e+02 | 5 | K.DMVSEK.K + Oxidation (M) |
|  | 148 | 372.5496 | 743.0843 | 743.9149 | -0.8305 | 0 | 33 | 1.1 | 10 | K.MLHSLK.V + Oxidation (M) |
|  | 149 | 372.6229 | 743.2311 | 743.8918 | -0.6607 | 1 | (24) | 10 | 1 | R.KVQELK.T |
|  | 150 | 372.6581 | 743.3014 | 743.8918 | -0.5903 | 1 | 24 | 10 | 3 | R.KVQELK.T |
|  | 186 | 375.5199 | 749.0250 | 749.8333 | -0.8083 | 0 | 0 | 2.6e+03 | 10 | R.NIASDCK.Y |
|  | 446 | 401.9682 | 801.9217 | 800.9432 | 0.9785 | 2 | 15 | 1e+02 | 3 | R.KKVDPSK.F |
|  | 818 | 429.0431 | 856.0714 | 856.0480 | 0.0233 | 1 | 13 | 1.2e+02 | 1 | K.AHMLKTR.N |
|  | 820 | 429.1022 | 856.1896 | 856.0480 | 0.1416 | 1 | (13) | 1.4e+02 | 1 | K.AHMLKTR.N |
|  | 883 | 434.1199 | 866.2251 | 865.0284 | 1.1967 | 2 | 5 | 8.5e+02 | 5 | K.YKADLKK.L |
|  | 909 | 435.9534 | 869.8921 | 870.9930 | -1.1010 | 0 | 18 | 43 | 1 | R.QGLTLSPR.L |
|  | 914 | 436.0844 | 870.1540 | 870.9930 | -0.8390 | 0 | (15) | 82 | 4 | R.QGLTLSPR.L |
|  | 1717 | 530.6490 | 1059.2833 | 1059.2605 | 0.0229 | 2 | (8) | 6e+02 | 4 | K.KAAKLSSQVK.Y |
|  | 1718 | 530.6578 | 1059.3009 | 1059.2605 | 0.0404 | 2 | 13 | 1.6e+02 | 1 | K.KAAKLSSQVK.Y |
|  | 1777 | 538.0971 | 1074.1794 | 1074.1905 | -0.0111 | 0 | 7 | 5e+02 | 7 | K.GHYVGTLTAR.D |
|  | 1939 | 564.1071 | 1126.1993 | 1127.3114 | -1.1120 | 2 | 0 | 2.5e+03 | 9 | K.DMVSEKKYK.I |
|  | 260 | 385.0608 | 1152.1601 | 1152.4102 | -0.2501 | 0 | 9 | 2.7e+02 | 2 | K.MMWSMHVAK.I + 2 Oxidation (M) |
|  | 551 | 406.4843 | 1216.4307 | 1216.2968 | 0.1339 | 2 | 7 | 5.7e+02 | 7 | K.YKEDYEKNK.G |
|  | 2263 | 609.6999 | 1217.3850 | 1216.3413 | 1.0437 | 1 | 13 | 1.8e+02 | 3 | R.QLKAAGDALSDK.L |
|  | 861 | 432.4976 | 1294.4705 | 1293.4685 | 1.0020 | 2 | 6 | 8.2e+02 | 9 | K.AGEILSEKKYR.Q |
|  | 877 | 433.3978 | 1297.1713 | 1296.4889 | 0.6824 | 0 | 11 | 1.8e+02 | 8 | K.CQILVSDIDYK.H |
|  | 893 | 434.8575 | 1301.5505 | 1300.4991 | 1.0513 | 0 | 16 | 69 | 1 | K.GYDLPVDAIPIK.A |
|  | 945 | 440.2520 | 1317.7339 | 1317.4687 | 0.2652 | 0 | 11 | 1.9e+02 | 3 | K.ATATPVTPEMQR.V + Oxidation (M) |
|  | 1200 | 468.1680 | 1401.4818 | 1402.5562 | -1.0744 | 2 | 14 | 1.3e+02 | 2 | K.KQYEANKAHWK.W |
|  | 1204 | 468.2981 | 1401.8721 | 1402.5562 | -0.6841 | 2 | (10) | 2.3e+02 | 6 | K.KQYEANKAHWK.W |
|  | 1986 | 574.3182 | 1719.9325 | 1718.9680 | 0.9646 | 0 | 10 | 2.6e+02 | 2 | K.TSFHTPVDMLSVVAAK.K + Oxidation (M) |
|  | 2429 | 632.6989 | 1895.0744 | 1895.2094 | -0.1350 | 2 | 6 | 8.8e+02 | 8 | K.GCGWVPFGSLEMEKAKR.A |
|  | 2430 | 632.7946 | 1895.3615 | 1896.1875 | -0.8261 | 1 | 5 | 9.7e+02 | 7 | K.DKYLLPPDAPELVQAVK.N |
|  | 2437 | 634.8785 | 1901.6133 | 1902.1542 | -0.5409 | 1 | 4 | 8.2e+02 | 7 | K.ENMGKGTPLPVTPEMER.V + Oxidation (M) |
|  | 2526 | 654.5477 | 1960.6210 | 1960.1934 | 0.4277 | 0 | 7 | 3.8e+02 | 7 | K.EGSHGLSMLGRPDIEMAK.K + 2 Oxidation (M) |
|  | 2603 | 667.2744 | 1998.8009 | 1999.3551 | -0.5543 | 1 | 4 | 1e+03 | 4 | K.MIGALSINDDPKMLHSLK.T + Oxidation (M) |
|  | 3286 | 828.2256 | 2481.6546 | 2481.8446 | -0.1900 | 2 | 4 | 1e+03 | 7 | R.QHPSNFQFKKLTDSMDMVLAK.Q + Oxidation (M) |

  


---

|  |  |
| --- | --- |
| **19.** | gi|32189394    **Mass:** 56559    **Score:** 171    **Queries matched:** 7   **emPAI:** 0.06 |
|  | ATP synthase subunit beta, mitochondrial precursor [Homo sapiens] |

|  |  |
| --- | --- |
|  | Check to include this hit in error tolerant search or archive report |
|  |  |

|  |  |  |  |  |  |  |  |  |  |  |
| --- | --- | --- | --- | --- | --- | --- | --- | --- | --- | --- |
|  | **Query** | **Observed** | **Mr(expt)** | **Mr(calc)** | **Delta** | **Miss** | **Score** | **Expect** | **Rank** | **Peptide** |
|  | 2459 | **640.2028** | **1278.3907** | **1278.4324** | **-0.0417** | **0** | **27** | **4.9** | **1** | **R.TIAMDGTEGLVR.G + Oxidation (M)** |
|  | 1721 | **531.8220** | **1592.4439** | **1591.7852** | **0.6586** | **1** | **16** | **51** | **1** | **R.TIAMDGTEGLVRGQK.V + Oxidation (M)** |
|  | 3232 | 810.1809 | 1618.3470 | 1617.8242 | 0.5229 | 0 | 16 | 60 | 2 | K.VALVYGQMNEPPGAR.A + Oxidation (M) |
|  | 2462 | **640.8226** | **1919.4455** | **1919.2258** | **0.2197** | **1** | **(41)** | **0.21** | **1** | **K.VLDSGAPIKIPVGPETLGR.I** |
|  | 2463 | **640.9565** | **1919.8475** | **1919.2258** | **0.6216** | **1** | **86** | **6.6e-06** | **1** | **K.VLDSGAPIKIPVGPETLGR.I** |
|  | 3392 | **994.7258** | **1987.4369** | **1988.1984** | **-0.7615** | **0** | **7** | **4.6e+02** | **3** | **R.AIAELGIYPAVDPLDSTSR.I** |
|  | 2662 | **676.7817** | **2027.3230** | **2026.3575** | **0.9656** | **2** | **19** | **46** | **1** | **R.IMNVIGEPIDERGPIKTK.Q + Oxidation (M)** |

  

|  |  |
| --- | --- |
|  | |
|  | **Proteins matching the same set of peptides:** |

|  |  |
| --- | --- |
|  | gi|89574029    **Mass:** 48113    **Score:** 171    **Queries matched:** 7 |
|  | mitochondrial ATP synthase, H+ transporting F1 complex beta subunit [Homo sapiens] |

---

|  |  |
| --- | --- |
| **20.** | gi|28940    **Mass:** 57955    **Score:** 168    **Queries matched:** 7   **emPAI:** 0.06 |
|  | unnamed protein product [Homo sapiens] |

|  |  |
| --- | --- |
|  | Check to include this hit in error tolerant search or archive report |
|  |  |

|  |  |  |  |  |  |  |  |  |  |  |
| --- | --- | --- | --- | --- | --- | --- | --- | --- | --- | --- |
|  | **Query** | **Observed** | **Mr(expt)** | **Mr(calc)** | **Delta** | **Miss** | **Score** | **Expect** | **Rank** | **Peptide** |
|  | 2459 | 640.2028 | 1278.3907 | 1278.4324 | -0.0417 | 0 | 27 | 4.9 | 1 | R.TIAMDGTEGLVR.G + Oxidation (M) |
|  | 1721 | 531.8220 | 1592.4439 | 1591.7852 | 0.6586 | 1 | 16 | 51 | 1 | R.TIAMDGTEGLVRGQK.V + Oxidation (M) |
|  | 3231 | 809.6979 | 1617.3811 | 1616.8394 | 0.5417 | 0 | 13 | 98 | 6 | K.VALVYGQMNQPPGAR.A + Oxidation (M) |
|  | 2462 | 640.8226 | 1919.4455 | 1919.2258 | 0.2197 | 1 | (41) | 0.21 | 1 | K.VLDSGAPIKIPVGPETLGR.I |
|  | 2463 | 640.9565 | 1919.8475 | 1919.2258 | 0.6216 | 1 | 86 | 6.6e-06 | 1 | K.VLDSGAPIKIPVGPETLGR.I |
|  | 3392 | 994.7258 | 1987.4369 | 1988.1984 | -0.7615 | 0 | 7 | 4.6e+02 | 3 | R.AIAELGIYPAVDPLDSTSR.I |
|  | 2662 | 676.7817 | 2027.3230 | 2026.3575 | 0.9656 | 2 | 19 | 46 | 1 | R.IMNVIGEPIDERGPIKTK.Q + Oxidation (M) |

  


---

**Peptide matches not assigned to protein hits:** (no details means no
match)  
  

|  |  |  |  |  |  |  |  |  |  |  |
| --- | --- | --- | --- | --- | --- | --- | --- | --- | --- | --- |
|  | **Query** | **Observed** | **Mr(expt)** | **Mr(calc)** | **Delta** | **Miss** | **Score** | **Expect** | **Rank** | **Peptide** |
|  | 2850 | **715.7855** | **2144.3344** | **2144.2151** | **0.1193** | **1** | **74** | **0.00012** | **1** | **EGQEDQGLTKDYGNSPLHR** |
|  | 3359 | **896.7288** | **1791.4427** | **1790.9243** | **0.5184** | **0** | **74** | **9.8e-05** | **1** | **SYELPDGQVITIGNER** |
|  | 1635 | **521.3996** | **1561.1766** | **1560.6469** | **0.5298** | **0** | **54** | **0.0072** | **1** | **SGCNHPDLDVQYR + Carbamidomethyl (C)** |
|  | 2412 | **631.1270** | **1260.2391** | **1260.4801** | **-0.2410** | **1** | **54** | **0.01** | **1** | **SLVSKGTLVQTK** |
|  | 877 | 433.3978 | 1297.1713 | 1296.4326 | 0.7387 | 1 | 54 | 0.0096 | 1 | AIESSRDLLHR |
|  | 3351 | **889.2740** | **1776.5333** | **1775.9992** | **0.5341** | **0** | **52** | **0.015** | **1** | **AMGIMNSFVNDIFER + 2 Oxidation (M)** |
|  | 1946 | **565.8566** | **1129.6985** | **1130.2490** | **-0.5505** | **1** | **50** | **0.025** | **1** | **GPSSVEDIKAK** |
|  | 3213 | **806.0843** | **2415.2307** | **2415.6156** | **-0.3849** | **1** | **49** | **0.028** | **1** | **QLFHPEQLITGKEDAANNYAR** |
|  | 1018 | **449.2912** | **1344.8515** | **1344.4722** | **0.3793** | **1** | **49** | **0.027** | **1** | **TKDDIIEFAHR** |
|  | 2531 | **655.1063** | **1308.1979** | **1308.3938** | **-0.1959** | **2** | **48** | **0.04** | **1** | **IALEFDKDRSD** |
|  | 876 | **433.3928** | **1297.1563** | **1296.4326** | **0.7237** | **1** | **45** | **0.078** | **1** | **AIESSRDLLHR** |
|  | 1821 | **543.9626** | **1628.8658** | **1628.7424** | **0.1234** | **0** | **44** | **0.11** | **1** | **SSGPYGGGGQYFAKPR** |
|  | 1416 | **495.4630** | **1483.3668** | **1482.7240** | **0.6428** | **2** | **44** | **0.11** | **1** | **RKASGPPVSELITK** |
|  | 660 | **411.6490** | **1231.9248** | **1231.4008** | **0.5240** | **0** | **43** | **0.12** | **1** | **GIVEFSGKPAAR** |
|  | 2975 | **748.7531** | **1495.4914** | **1495.6301** | **-0.1386** | **0** | **43** | **0.14** | **1** | **AAGTVFTTVEDLGSK** |
|  | 768 | **422.2341** | **842.4534** | **841.9949** | **0.4585** | **0** | **42** | **0.15** | **1** | **GITLSVRP** |
|  | 2514 | **652.4581** | **1954.3522** | **1954.2269** | **0.1253** | **0** | **42** | **0.16** | **1** | **VAPEEHPVLLTEAPLNPK** |
|  | 212 | **377.9369** | **1130.7886** | **1130.2490** | **0.5396** | **1** | **41** | **0.16** | **1** | **GPSSVEDIKAK** |
|  | 1918 | **562.1081** | **1122.2014** | **1121.1577** | **1.0437** | **0** | **41** | **0.22** | **1** | **AFEEDQVAGR** |
|  | 1954 | **568.1805** | **1701.5193** | **1701.9803** | **-0.4610** | **1** | **40** | **0.28** | **1** | **KLEPIWNEVGLEMK + Oxidation (M)** |
|  | 1574 | 515.1913 | 1028.3678 | 1027.2582 | 1.1096 | 1 | 40 | 0.31 | 1 | KLAALAEALK |
|  | 1820 | **543.8131** | **1628.4172** | **1628.7424** | **-0.3252** | **0** | **38** | **0.37** | **1** | **SSGPYGGGGQYFAKPR** |
|  | 769 | **422.2438** | **842.4728** | **841.9949** | **0.4778** | **0** | **37** | **0.46** | **1** | **GITLSVRP** |
|  | 1399 | **493.7925** | **985.5703** | **986.2078** | **-0.6375** | **0** | **35** | **0.7** | **1** | **DLPLLLFR** |
|  | 770 | **422.2585** | **842.5022** | **841.9949** | **0.5072** | **0** | **34** | **0.93** | **1** | **GITLSVRP** |
|  | 148 | **372.5496** | **743.0843** | **742.9035** | **0.1808** | **0** | **34** | **1.1** | **1** | **AGLELLK** |
|  | 1567 | **514.4943** | **1026.9739** | **1027.1391** | **-0.1652** | **2** | **33** | **1.1** | **1** | **LRGSEGPRR** |
|  | 3356 | **894.4332** | **1786.8517** | **1787.0217** | **-0.1700** | **0** | **33** | **1.2** | **1** | **VLAQQGEYSEAIPILR** |
|  | 675 | **413.5580** | **825.1012** | **825.9954** | **-0.8943** | **0** | **33** | **1.1** | **1** | **QLSILPR** |
|  | 1887 | **555.8925** | **1664.6552** | **1664.7962** | **-0.1411** | **1** | **32** | **1.3** | **1** | **ALSRQEMQEVQSSR + Oxidation (M)** |
|  | 1248 | **473.0659** | **944.1169** | **944.0885** | **0.0285** | **0** | **32** | **1.8** | **1** | **AGLQFPVGR** |
|  | 1349 | **487.1404** | **972.2660** | **972.0538** | **0.2121** | **0** | **31** | **2.1** | **1** | **DIDNLVQR** |
|  | 849 | **431.6741** | **1292.0000** | **1291.5023** | **0.4977** | **1** | **31** | **1.9** | **1** | **SAGVPSRVIHIR** |
|  | 812 | **428.8852** | **855.7557** | **855.9389** | **-0.1832** | **1** | **31** | **1.9** | **1** | **ERVAEPR** |
|  | 3040 | **758.5914** | **1515.1681** | **1514.8536** | **0.3145** | **0** | **31** | **1.9** | **1** | **CMSALSMPMLATSR + Oxidation (M)** |
|  | 1012 | **448.2709** | **1341.7904** | **1341.5129** | **0.2775** | **1** | **31** | **1.8** | **1** | **LQAEIEGLKGQR** |
|  | 674 | **413.5270** | **1237.5589** | **1237.3656** | **0.1934** | **1** | **30** | **2.3** | **1** | **LSPESAPGPARR** |
|  | 2961 | **744.8025** | **1487.5902** | **1486.6316** | **0.9586** | **2** | **30** | **2.7** | **1** | **MQKSSSTRAGDMR + 2 Oxidation (M)** |
|  | 386 | **394.3414** | **786.6680** | **785.8886** | **0.7795** | **0** | **30** | **2.6** | **1** | **NSSILPR** |
|  | 409 | **399.7001** | **1196.0781** | **1196.3103** | **-0.2322** | **1** | **30** | **1.9** | **1** | **KGIVEHEEQK** |
|  | 1006 | **447.9402** | **1340.7986** | **1341.7335** | **-0.9349** | **2** | **30** | **2.7** | **1** | **MMMLVGMGKRR + 2 Oxidation (M)** |
|  | 3089 | **775.5065** | **1548.9982** | **1549.8527** | **-0.8546** | **0** | **29** | **3.2** | **1** | **LLGASELPIVTPALR** |
|  | 425 | **400.7465** | **1199.2172** | **1198.4173** | **0.7999** | **0** | **29** | **3.2** | **1** | **AVFPSIVGRPR** |
|  | 1005 | **447.8869** | **1340.6385** | **1340.5648** | **0.0737** | **1** | **29** | **3.3** | **1** | **KATGPPVSELITK** |
|  | 759 | **421.9408** | **1262.8002** | **1263.4887** | **-0.6885** | **1** | **29** | **3.6** | **1** | **IHLEIKQLNR** |
|  | 3004 | **749.2533** | **1496.4918** | **1495.6301** | **0.8618** | **0** | **29** | **3.5** | **1** | **AAGTVFTTVEDLGSK** |
|  | 1222 | **471.0300** | **940.0453** | **940.0088** | **0.0364** | **0** | **28** | **3.4** | **1** | **STETALYR** |
|  | 1938 | **563.9309** | **1688.7706** | **1688.8028** | **-0.0322** | **1** | **28** | **3.6** | **1** | **SGRPSGRFSAERPER** |
|  | 2322 | **614.0098** | **1226.0049** | **1225.3466** | **0.6583** | **0** | **28** | **4.3** | **1** | **IENNMTIEMD + Oxidation (M)** |
|  | 37 | **363.3080** | **1086.9017** | **1086.2029** | **0.6987** | **1** | **28** | **3.9** | **1** | **LQRAQVSER** |
|  | 1575 | **515.2686** | **1542.7837** | **1543.6962** | **-0.9125** | **0** | **28** | **4.7** | **1** | **EDMEGMLGEDVMR + 2 Oxidation (M)** |
|  | 676 | **413.7123** | **825.4098** | **825.9954** | **-0.5857** | **0** | **27** | **3.3** | **1** | **QLSILPR** |
|  | 136 | **371.2560** | **1110.7458** | **1111.2490** | **-0.5032** | **1** | **27** | **3.1** | **1** | **KEPEKPIDR** |
|  | 704 | **416.6494** | **831.2841** | **830.9689** | **0.3152** | **0** | **27** | **4.8** | **1** | **QITTLQK** |
|  | 35 | **363.2137** | **1086.6189** | **1087.2935** | **-0.6747** | **0** | **27** | **4.2** | **1** | **CIGNNLAVVK + Carbamidomethyl (C)** |
|  | 131 | **371.1935** | **1110.5583** | **1111.2490** | **-0.6907** | **1** | **27** | **3.6** | **1** | **KEPEKPIDR** |
|  | 274 | **385.4752** | **768.9357** | **768.8582** | **0.0775** | **0** | **27** | **5.7** | **1** | **EALSPPR** |
|  | 1015 | **448.7475** | **1343.2203** | **1342.5011** | **0.7192** | **2** | **27** | **4.3** | **1** | **LKEDVLEQRGR** |
|  | 1933 | **563.5487** | **1125.0826** | **1125.1878** | **-0.1051** | **0** | **27** | **5.2** | **1** | **DMSDEVGCVN + Carbamidomethyl (C)** |
|  | 21 | **362.3615** | **1084.0625** | **1083.2817** | **0.7807** | **1** | **27** | **6.2** | **1** | **ILKSPEIQR** |
|  | 928 | **437.0386** | **872.0625** | **872.0639** | **-0.0014** | **1** | **26** | **6.7** | **1** | **LLLTKER** |
|  | 497 | **404.1815** | **1209.5225** | **1209.2247** | **0.2978** | **2** | **26** | **5.9** | **1** | **NGSSGKKSDSSR** |
|  | 2959 | **744.6332** | **1487.2516** | **1486.6316** | **0.6200** | **2** | **26** | **5.1** | **1** | **MQKSSSTRAGDMR + 2 Oxidation (M)** |
|  | 815 | **429.0046** | **855.9945** | **855.9786** | **0.0159** | **0** | **26** | **5.9** | **1** | **SPTLSVPR** |
|  | 1562 | **514.1005** | **1539.2792** | **1538.6860** | **0.5933** | **0** | **26** | **6.2** | **1** | **GLTGGFGSHSVCGGFR** |
|  | 2676 | **679.4977** | **2035.4711** | **2036.2065** | **-0.7354** | **1** | **26** | **6.1** | **1** | **SNMDNMFESYINNLRR + 2 Oxidation (M)** |
|  | 3358 | **896.2214** | **1790.4281** | **1790.9243** | **-0.4962** | **0** | **26** | **5.1** | **1** | **SYELPDGQVITIGNER** |
|  | 1460 | **500.0394** | **1497.0959** | **1496.7505** | **0.3454** | **2** | **26** | **6.6** | **1** | **RKATGPPVSELITK** |
|  | 34 | **363.1876** | **724.3605** | **724.8088** | **-0.4484** | **0** | **26** | **5.7** | **1** | **LHQTAR** |
|  | 1561 | **514.0977** | **1539.2708** | **1539.7104** | **-0.4397** | **0** | **26** | **6.8** | **1** | **IIPGFECQGGDFTR** |
|  | 2013 | **579.0322** | **1734.0743** | **1734.8065** | **-0.7321** | **2** | **26** | **7.1** | **1** | **MGRANSANGKNGDSAER** |
|  | 1571 | **514.8109** | **1027.6069** | **1027.1391** | **0.4679** | **1** | **26** | **5.7** | **1** | **LGRTGDRPR** |
|  | 960 | **443.4217** | **1327.2428** | **1326.5383** | **0.7046** | **1** | **25** | **6.9** | **1** | **KASGPPVSELITK** |
|  | 2341 | **617.3189** | **1232.6230** | **1232.3740** | **0.2491** | **2** | **25** | **8.3** | **1** | **MAGSPSRAAGRR + Oxidation (M)** |
|  | 3280 | **827.5645** | **2479.6712** | **2478.6285** | **1.0426** | **1** | **25** | **7.7** | **1** | **GLEWVSAISGSGHSTNYADSVKGR** |
|  | 894 | **434.8777** | **1301.6110** | **1301.6613** | **-0.0503** | **2** | **25** | **7.5** | **1** | **MMKSKILPPEK** |
|  | 2206 | **600.7717** | **1199.5286** | **1198.3660** | **1.1626** | **0** | **25** | **8** | **1** | **ASGPPVSELITK** |
|  | 120 | **370.1347** | **738.2546** | **737.8109** | **0.4436** | **2** | **25** | **7.1** | **1** | **RAAQRH** |
|  | 60 | **367.1375** | **1098.3903** | **1097.2902** | **1.1001** | **0** | **25** | **8.7** | **1** | **ECMMQLGGR + Carbamidomethyl (C); Oxidation (M)** |
|  | 2252 | **608.8005** | **1823.3793** | **1823.0988** | **0.2805** | **1** | **25** | **8.1** | **1** | **EAITVQQKQMPFCDK + Carbamidomethyl (C)** |
|  | 240 | **382.1918** | **1143.5531** | **1144.3449** | **-0.7918** | **0** | **25** | **8.7** | **1** | **CLPSVAAASIR + Carbamidomethyl (C)** |
|  | 540 | **406.0725** | **810.1302** | **809.8456** | **0.2846** | **0** | **25** | **8.4** | **1** | **MQGDTSR + Oxidation (M)** |
|  | 1829 | **545.7988** | **1634.3743** | **1634.7869** | **-0.4126** | **0** | **25** | **7.6** | **1** | **VNDVNEFAPVFVER** |
|  | 378 | **393.2778** | **1176.8111** | **1176.3521** | **0.4591** | **1** | **25** | **7.1** | **1** | **MSRPRFNPR + Oxidation (M)** |
|  | 403 | **399.0956** | **796.1765** | **796.9577** | **-0.7812** | **1** | **25** | **8.4** | **1** | **KGLVPQR** |
|  | 1327 | **485.4022** | **968.7897** | **969.0565** | **-0.2669** | **1** | **24** | **6.9** | **1** | **NQADPRLR** |
|  | 150 | 372.6581 | 743.3014 | 743.8883 | -0.5869 | 0 | 24 | 8.7 | 1 | EEIIIK |
|  | 1565 | **514.3769** | **1540.1085** | **1540.6804** | **-0.5719** | **2** | **24** | **7.5** | **1** | **EDAGRELLAPGTRR** |
|  | 1996 | **576.6724** | **1151.3301** | **1152.3689** | **-1.0388** | **0** | **24** | **11** | **1** | **MHVMAASMAR + 3 Oxidation (M)** |
|  | 1182 | **466.0502** | **1395.1283** | **1394.6567** | **0.4716** | **1** | **24** | **11** | **1** | **TWLTFALLSKSK** |
|  | 1028 | **450.5233** | **899.0318** | **899.0495** | **-0.0178** | **1** | **24** | **12** | **1** | **LGRTSLPR** |
|  | 1352 | **487.8887** | **973.7627** | **973.0453** | **0.7174** | **2** | **24** | **11** | **1** | **DNKQANKR** |
|  | 564 | **406.9748** | **811.9347** | **810.8765** | **1.0583** | **0** | **24** | **9.2** | **1** | **SGFGHFC + Carbamidomethyl (C)** |
|  | 1032 | **450.7937** | **899.5726** | **899.0099** | **0.5627** | **2** | **24** | **10** | **1** | **RDRSLPR** |
|  | 988 | **445.2173** | **888.4199** | **888.0104** | **0.4095** | **1** | **24** | **12** | **1** | **RRPGCSR + Carbamidomethyl (C)** |
|  | 2346 | **618.2671** | **1851.7791** | **1852.1033** | **-0.3242** | **2** | **24** | **11** | **1** | **LAQELRDVRGNIQALR** |
|  | 1232 | **471.8926** | **1412.6556** | **1413.5990** | **-0.9434** | **1** | **24** | **10** | **1** | **KTLLEGEMSHPR + Oxidation (M)** |
|  | 1373 | **489.2100** | **976.4052** | **976.1368** | **0.2684** | **1** | **24** | **12** | **1** | **LRLHGPQR** |
|  | 475 | **403.7709** | **1208.2906** | **1209.4350** | **-1.1443** | **0** | **24** | **11** | **1** | **QKPSIPPTLTK** |
|  | 495 | **404.1643** | **806.3138** | **806.9923** | **-0.6785** | **2** | **24** | **11** | **1** | **FLSGKKK** |
|  | 227 | **379.3172** | **756.6196** | **756.8937** | **-0.2742** | **1** | **24** | **8.8** | **1** | **LLARER** |
|  | 382 | **393.5954** | **1177.7639** | **1178.3415** | **-0.5775** | **1** | **24** | **9.1** | **1** | **RGETLPVPGPR** |
|  | 3194 | **799.1622** | **1596.3097** | **1595.8399** | **0.4698** | **0** | **24** | **9** | **1** | **LGLLAFAQHQAEVAK** |
|  | 830 | **429.8852** | **857.7556** | **858.0177** | **-0.2621** | **1** | **24** | **13** | **1** | **EHVAKMK + Oxidation (M)** |
|  | 3408 | **1085.9124** | **3254.7149** | **3253.6848** | **1.0301** | **1** | **24** | **9** | **1** | **YLASDPDCRWCPAPDCGYAVIAYGCASCPK + Carbamidomethyl (C)** |
|  | 294 | **386.7847** | **1157.3320** | **1156.3971** | **0.9349** | **0** | **24** | **12** | **1** | **LCMGSIMNSK + Carbamidomethyl (C); Oxidation (M)** |
|  | 2446 | **636.0242** | **1905.0503** | **1905.1396** | **-0.0893** | **0** | **24** | **11** | **1** | **VLSRPNAQELPSMYQR + Oxidation (M)** |
|  | 774 | **422.4959** | **842.9770** | **841.9964** | **0.9806** | **0** | **24** | **15** | **1** | **ALSLWPR** |
|  | 760 | **422.0099** | **842.0050** | **841.0100** | **0.9950** | **0** | **24** | **12** | **1** | **AALLNAIR** |
|  | 200 | **377.1145** | **1128.3213** | **1128.2429** | **0.0784** | **2** | **24** | **11** | **1** | **IARQEAERR** |
|  | 1163 | **463.9205** | **925.8263** | **926.0683** | **-0.2421** | **0** | **24** | **11** | **1** | **LEELAVPR** |
|  | 381 | **393.4902** | **1177.4483** | **1177.4380** | **0.0104** | **1** | **24** | **14** | **1** | **AKVMMEVGQGK** |
|  | 1472 | **501.7256** | **1001.4365** | **1000.2561** | **1.1804** | **1** | **24** | **10** | **1** | **VLTVPCQKI** |
|  | 829 | **429.8295** | **1286.4664** | **1287.4492** | **-0.9827** | **2** | **23** | **13** | **1** | **GKARESGCPAVR + Carbamidomethyl (C)** |
|  | 155 | **373.8077** | **1118.4010** | **1119.2675** | **-0.8665** | **0** | **23** | **15** | **1** | **GIISPVGDAYK** |
|  | 1132 | **460.7923** | **919.5698** | **919.9827** | **-0.4129** | **1** | **23** | **11** | **1** | **ASATSRATR** |
|  | 1192 | **467.6492** | **933.2836** | **933.0180** | **0.2656** | **1** | **23** | **11** | **1** | **ESVASKGQK** |
|  | 2953 | **743.4625** | **1484.9103** | **1484.5293** | **0.3810** | **0** | **23** | **13** | **1** | **AWGAGPPEGGGGGSATR** |
|  | 2603 | 667.2744 | 1998.8009 | 1997.9879 | 0.8130 | 2 | 23 | 13 | 1 | NQGGYGGSSSSSSYGSGRRF |
|  | 1367 | **488.7274** | **975.4399** | **975.0182** | **0.4218** | **1** | **23** | **11** | **1** | **SSRAPAGSSR** |
|  | 1554 | **512.3717** | **1022.7286** | **1023.1902** | **-0.4615** | **2** | **23** | **10** | **1** | **QHRKVLDK** |
|  | 1713 | **530.0803** | **1058.1459** | **1059.2372** | **-1.0913** | **0** | **23** | **14** | **1** | **MSVPLGQSQL** |
|  | 1046 | **453.5634** | **905.1120** | **904.9911** | **0.1209** | **0** | **23** | **16** | **1** | **MAGAGAGAGAR + Oxidation (M)** |
|  | 3289 | 830.6453 | 1659.2759 | 1658.8098 | 0.4661 | 0 | 23 | 12 | 1 | NQVALNPQNTVFDAK |
|  | 1112 | **459.5202** | **1375.5383** | **1376.5355** | **-0.9972** | **0** | **23** | **18** | **1** | **BLZZGIZTLMGR + Oxidation (M)** |
|  | 1276 | **476.5982** | **951.1816** | **952.0327** | **-0.8511** | **2** | **23** | **15** | **1** | **ERAHRQR** |
|  | 479 | **403.8527** | **1208.5360** | **1208.4733** | **0.0626** | **1** | **23** | **14** | **1** | **KMYLQAAIVR + Oxidation (M)** |
|  | 45 | **364.4124** | **1090.2149** | **1089.3128** | **0.9021** | **2** | **23** | **15** | **1** | **CELARTLKR** |
|  | 921 | **436.6385** | **871.2621** | **870.9998** | **0.2624** | **2** | **23** | **12** | **1** | **LSPRRSR** |
|  | 1291 | **478.6694** | **1432.9859** | **1433.5605** | **-0.5746** | **2** | **23** | **12** | **1** | **KEISEIDKEGTGK** |
|  | 416 | **400.0923** | **1197.2546** | **1198.4340** | **-1.1794** | **0** | **23** | **12** | **1** | **QAVMEMMSQK + Oxidation (M)** |
|  | 891 | **434.3834** | **1300.1281** | **1300.5869** | **-0.4588** | **1** | **23** | **11** | **1** | **LTVTIIKASNLK** |
|  | 2793 | **699.5944** | **2095.7611** | **2096.3873** | **-0.6263** | **0** | **23** | **11** | **1** | **LSCAASGFIFGSYGMNWIR + Oxidation (M)** |
|  | 61 | **367.1508** | **1098.4302** | **1098.2833** | **0.1469** | **2** | **23** | **14** | **1** | **KQMQRHVR + Oxidation (M)** |
|  | 2680 | **679.8610** | **1357.7071** | **1358.3746** | **-0.6675** | **1** | **23** | **14** | **1** | **KQHSEATNSSNR** |
|  | 865 | **432.9545** | **1295.8414** | **1296.5319** | **-0.6905** | **1** | **23** | **14** | **1** | **QYESLKILICS** |
|  | 1252 | **473.7829** | **945.5510** | **945.1412** | **0.4098** | **0** | **23** | **13** | **1** | **QMMGCFR + Carbamidomethyl (C); Oxidation (M)** |
|  | 2338 | **616.7656** | **1231.5165** | **1231.3989** | **0.1175** | **0** | **23** | **16** | **1** | **SGISASLAISGLR** |
|  | 1401 | **494.1328** | **986.2509** | **986.2078** | **0.0431** | **0** | **23** | **15** | **1** | **DLPLLLFR** |
|  | 384 | **393.7366** | **1178.1877** | **1178.3445** | **-0.1568** | **1** | **23** | **14** | **1** | **QQRLLNGPPR** |
|  | 594 | **407.7838** | **1220.3291** | **1219.4812** | **0.8479** | **2** | **23** | **15** | **1** | **MMKLSNRAPR + Oxidation (M)** |
|  | 914 | 436.0844 | 870.1540 | 870.0283 | 0.1258 | 0 | 23 | 15 | 1 | TPCPSLPR |
|  | 1771 | **537.5132** | **1609.5176** | **1608.8786** | **0.6389** | **2** | **23** | **15** | **1** | **YKPLADTFLREKK** |
|  | 2270 | **610.1060** | **1218.1971** | **1218.4747** | **-0.2776** | **1** | **23** | **15** | **1** | **CHAVLLARAHK** |
|  | 740 | **419.2617** | **1254.7629** | **1255.4886** | **-0.7257** | **1** | **22** | **14** | **1** | **DAMMAMNGKVR + 2 Oxidation (M)** |
|  | 945 | 440.2520 | 1317.7339 | 1317.5345 | 0.1993 | 0 | 22 | 15 | 1 | QCIPISSCPQGK + Carbamidomethyl (C) |
|  | 394 | **398.0066** | **1190.9975** | **1190.3272** | **0.6703** | **0** | **22** | **15** | **1** | **DALCLEGGVSAR** |
|  | 793 | **427.7205** | **1280.1394** | **1279.3808** | **0.7586** | **0** | **22** | **12** | **1** | **GSSGSSGMNAAVVR** |
|  | 776 | **423.2019** | **1266.5835** | **1266.4498** | **0.1337** | **0** | **22** | **16** | **1** | **VARPAQLSAPTR** |
|  | 435 | **401.4071** | **800.7994** | **801.8448** | **-1.0455** | **0** | **22** | **19** | **1** | **DGNIDIR** |
|  | 2060 | **587.6171** | **1759.8290** | **1761.0229** | **-1.1938** | **1** | **22** | **18** | **1** | **VIDDSLVVGVKTTSSLK** |
|  | 962 | **443.4902** | **1327.4484** | **1326.5446** | **0.9038** | **2** | **22** | **18** | **1** | **NAGAVIGKGGKNIK** |
|  | 2825 | **714.6373** | **1427.2599** | **1427.6405** | **-0.3806** | **0** | **22** | **13** | **1** | **SSMLSAILGEMEK + 2 Oxidation (M)** |
|  | 408 | **399.6658** | **1195.9753** | **1196.3103** | **-0.3350** | **1** | **22** | **11** | **1** | **KGIVEHEEQK** |
|  | 977 | **443.9565** | **1328.8475** | **1328.4330** | **0.4144** | **1** | **22** | **18** | **1** | **RHEGFAAALGDGK** |
|  | 1936 | **563.7764** | **1688.3069** | **1688.9700** | **-0.6630** | **1** | **22** | **14** | **1** | **QLVRGEPNVSMICSR** |
|  | 1620 | **520.0123** | **1557.0148** | **1557.7043** | **-0.6895** | **2** | **22** | **16** | **1** | **ASEEKNKQLGAGTPK** |
|  | 1513 | **505.4274** | **1008.8401** | **1008.2816** | **0.5585** | **1** | **22** | **13** | **1** | **MVPALPPKR** |
|  | 542 | **406.0862** | **1215.2365** | **1215.4875** | **-0.2510** | **1** | **22** | **17** | **1** | **LVVLFGVGKQR** |
|  | 2570 | **663.6649** | **1987.9726** | **1988.5013** | **-0.5288** | **2** | **22** | **16** | **1** | **VPPLKIQLSKIGPPFIIK** |
|  | 78 | **369.1162** | **1104.3265** | **1104.2879** | **0.0386** | **2** | **22** | **20** | **1** | **RSAVVMGRGR + Oxidation (M)** |
|  | 523 | **405.4398** | **1213.2974** | **1213.4268** | **-0.1295** | **1** | **22** | **20** | **1** | **KEFSACAIGCK + Carbamidomethyl (C)** |
|  | 2906 | **740.0570** | **2217.1488** | **2216.5198** | **0.6291** | **1** | **22** | **14** | **1** | **TWPMASPRASVTTCTCAFR + 2 Carbamidomethyl (C); Oxidation (M)** |
|  | 653 | **411.1089** | **1230.3045** | **1230.5421** | **-0.2376** | **1** | **22** | **19** | **1** | **MVTMKMCGPK + Carbamidomethyl (C); 3 Oxidation (M)** |
|  | 113 | **369.3538** | **1105.0391** | **1105.2443** | **-0.2052** | **1** | **22** | **20** | **1** | **TIHTGGKTYK** |
|  | 3121 | **784.6663** | **1567.3177** | **1566.7957** | **0.5221** | **1** | **22** | **15** | **1** | **FKGPFTDVVTTNLK** |
|  | 1096 | **458.7098** | **915.4049** | **914.9695** | **0.4354** | **2** | **22** | **17** | **1** | **DGGARARGR** |
|  | 503 | **404.9426** | **1211.8055** | **1212.2749** | **-0.4693** | **1** | **22** | **17** | **1** | **HDSSFAKHQR** |
|  | 655 | **411.1963** | **1230.5667** | **1231.3991** | **-0.8324** | **1** | **22** | **19** | **1** | **LVTSDTAAGKIR** |
|  | 840 | **430.9637** | **859.9125** | **860.8690** | **-0.9565** | **0** | **21** | **23** | **1** | **DLNSQER** |
|  | 180 | **375.0243** | **1122.0508** | **1121.3794** | **0.6714** | **1** | **21** | **21** | **1** | **TRRPLLLPR** |
|  | 2606 | **667.7689** | **2000.2846** | **1999.4032** | **0.8814** | **1** | **21** | **24** | **1** | **GLHGAMAFILVMLPDRNK + Oxidation (M)** |
|  | 1484 | **504.0196** | **1006.0243** | **1005.2363** | **0.7881** | **2** | **21** | **20** | **1** | **GVKAMSLKR + Oxidation (M)** |
|  | 835 | **430.4872** | **858.9597** | **859.0255** | **-0.0657** | **1** | **21** | **26** | **1** | **SVRSGLLK** |
|  | 896 | **434.9264** | **867.8380** | **866.8967** | **0.9414** | **0** | **21** | **17** | **1** | **SHCDYWG** |
|  | 1213 | **469.8420** | **1406.5040** | **1405.5405** | **0.9635** | **2** | **21** | **18** | **1** | **EMGQRRAGNGVSK + Oxidation (M)** |
|  | 307 | **386.9987** | **1157.9740** | **1157.2792** | **0.6948** | **2** | **21** | **22** | **1** | **FFKGGGSSKSR** |
|  | 3044 | **759.2888** | **2274.8441** | **2273.7631** | **1.0810** | **1** | **21** | **19** | **1** | **MAVALICMSMEFGRAVWLR + Carbamidomethyl (C); 2 Oxidation (M)** |
|  | 1443 | 499.6650 | 1495.9729 | 1495.7273 | 0.2456 | 0 | 21 | 18 | 1 | HQLSHPGLPGALLR |
|  | 2666 | **678.0145** | **2031.0214** | **2031.3026** | **-0.2812** | **2** | **21** | **17** | **1** | **ARHPQLAPTLQPRTECR + Carbamidomethyl (C)** |
|  | 443 | **401.9072** | **1202.6994** | **1203.3925** | **-0.6931** | **0** | **21** | **23** | **1** | **ERPAEAMMPR + Oxidation (M)** |
|  | 1357 | **488.1117** | **1461.3129** | **1460.8244** | **0.4886** | **2** | **21** | **22** | **1** | **FCITMCKKLTEK + Oxidation (M)** |
|  | 1365 | **488.5385** | **975.0621** | **973.9870** | **1.0752** | **1** | **21** | **26** | **1** | **GQKGDQGER** |
|  | 2684 | **680.0295** | **2037.0665** | **2037.2297** | **-0.1632** | **0** | **21** | **18** | **1** | **STCPSAAPSASAPAMTTVENK + Oxidation (M)** |
|  | 2500 | **649.3671** | **1296.7193** | **1296.4326** | **0.2867** | **1** | **21** | **19** | **1** | **AIESSRDLLHR** |
|  | 1865 | **551.9332** | **1652.7775** | **1653.9042** | **-1.1267** | **1** | **21** | **21** | **1** | **AASPCLGPVAAAGSGLRR** |
|  | 412 | **399.9403** | **797.8658** | **796.9178** | **0.9480** | **1** | **21** | **17** | **1** | **QGLPRAR** |
|  | 897 | **435.1233** | **1302.3477** | **1303.3360** | **-0.9883** | **1** | **21** | **20** | **1** | **GPGTSAGSEQKER** |
|  | 710 | **417.4870** | **1249.4388** | **1249.4194** | **0.0195** | **1** | **21** | **27** | **1** | **VTQSSPPHLKR** |
|  | 1283 | **477.3948** | **952.7748** | **952.0809** | **0.6939** | **0** | **21** | **16** | **1** | **LSEESMLK + Oxidation (M)** |
|  | 1410 | **494.5048** | **986.9948** | **986.2078** | **0.7870** | **0** | **21** | **24** | **1** | **DLPLLLFR** |
|  | 583 | **407.4279** | **1219.2616** | **1218.3871** | **0.8744** | **1** | **21** | **24** | **1** | **ARGTGASMAVAAR** |
|  | 939 | **437.7435** | **873.4722** | **872.9675** | **0.5046** | **0** | **21** | **20** | **1** | **DVAHLYR** |
|  | 415 | **400.0758** | **1197.2052** | **1198.3758** | **-1.1706** | **1** | **21** | **19** | **1** | **LNRCGMADFR + Oxidation (M)** |
|  | 27 | **363.0705** | **724.1263** | **724.7809** | **-0.6546** | **0** | **21** | **22** | **1** | **MSEQSK + Oxidation (M)** |
|  | 1589 | **518.1199** | **1034.2250** | **1033.2081** | **1.0169** | **1** | **21** | **23** | **1** | **LCNWVSRR** |
|  | 1894 | **557.0634** | **1112.1120** | **1111.0833** | **1.0288** | **0** | **21** | **21** | **1** | **ASHGHSADSSR** |
|  | 426 | **400.8346** | **1199.4817** | **1198.4173** | **1.0644** | **0** | **21** | **22** | **1** | **AVFPSIVGRPR** |
|  | 317 | **387.3258** | **1158.9553** | **1159.3794** | **-0.4240** | **0** | **21** | **22** | **1** | **NFWIVKPGAK** |
|  | 167 | 374.2353 | 1119.6838 | 1119.3588 | 0.3250 | 1 | 21 | 22 | 1 | VGKACCVPTK + 2 Carbamidomethyl (C) |
|  | 405 | **399.1898** | **1194.5473** | **1194.4055** | **0.1418** | **2** | **21** | **18** | **1** | **LRKSLSSMTR + Oxidation (M)** |
|  | 1334 | 486.2640 | 970.5132 | 971.1156 | -0.6024 | 2 | 21 | 21 | 1 | LSGAKSPRR |
|  | 2895 | **734.9855** | **2201.9342** | **2201.4571** | **0.4771** | **1** | **21** | **18** | **1** | **AARSAEPELDAAAATATCAAVIK** |
|  | 2145 | **596.9989** | **1787.9745** | **1788.9369** | **-0.9623** | **1** | **21** | **25** | **1** | **EVSRTLGSGEGMAVSHR + Oxidation (M)** |
|  | 533 | **405.9885** | **1214.9432** | **1214.3091** | **0.6342** | **1** | **21** | **21** | **1** | **YEMRSQGATR + Oxidation (M)** |
|  | 648 | **410.9487** | **1229.8238** | **1229.4776** | **0.3463** | **1** | **21** | **24** | **1** | **LRCMCTCNR + 2 Carbamidomethyl (C); Oxidation (M)** |
|  | 2710 | **683.8600** | **1365.7052** | **1365.5807** | **0.1245** | **1** | **21** | **23** | **1** | **GALVLGSSLKQHR** |
|  | 1269 | **476.0718** | **950.1288** | **949.0836** | **1.0452** | **2** | **21** | **24** | **1** | **ECENAKKK** |
|  | 1303 | **480.4163** | **1438.2266** | **1437.5941** | **0.6325** | **2** | **21** | **20** | **1** | **EKSSPSATEKVFK** |
|  | 1892 | **556.6121** | **1666.8140** | **1667.7305** | **-0.9164** | **0** | **21** | **28** | **1** | **WVVIGDENYGEGSSR** |
|  | 1487 | **504.1822** | **1006.3497** | **1005.1501** | **1.1996** | **1** | **21** | **25** | **1** | **ALRGEAGMGK + Oxidation (M)** |
|  | 14 | **362.0524** | **1083.1349** | **1084.2303** | **-1.0953** | **2** | **21** | **23** | **1** | **KVSEARAAPR** |
|  | 1734 | **533.5737** | **1597.6990** | **1597.7020** | **-0.0030** | **0** | **20** | **27** | **1** | **APDITCTSEFQETR** |
|  | 858 | **432.2486** | **1293.7235** | **1294.4567** | **-0.7331** | **2** | **20** | **22** | **1** | **KSGPKGAPAAEPGK** |
|  | 2957 | **743.9318** | **2228.7733** | **2227.7243** | **1.0490** | **0** | **20** | **24** | **1** | **LSDVMILTVFCLSVFALIGL + Carbamidomethyl (C); Oxidation (M)** |
|  | 1299 | **480.0107** | **958.0065** | **957.0824** | **0.9242** | **0** | **20** | **25** | **1** | **TIGISVDPR** |
|  | 713 | 417.9412 | 1250.8015 | 1250.4072 | 0.3943 | 0 | 20 | 25 | 1 | PSAGLCSCWGGR + Carbamidomethyl (C) |
|  | 13 | **362.0084** | **722.0019** | **721.8448** | **0.1572** | **1** | **20** | **23** | **1** | **KSAAFAK** |
|  | 1813 | **542.0247** | **1623.0520** | **1623.8518** | **-0.7999** | **1** | **20** | **23** | **1** | **GDLIGVVEALTRQPR** |
|  | 261 | **385.0641** | **1152.1701** | **1153.3486** | **-1.1785** | **0** | **20** | **22** | **1** | **ETPPLPPACTK** |
|  | 2893 | **734.5312** | **2200.5716** | **2200.5531** | **0.0184** | **0** | **20** | **23** | **1** | **FGDCFLADPWYLVLLATAK + Carbamidomethyl (C)** |
|  | 862 | **432.6211** | **1294.8411** | **1295.5358** | **-0.6947** | **2** | **20** | **22** | **1** | **RRLEPPFRPK** |
|  | 981 | **444.9182** | **1331.7326** | **1332.4800** | **-0.7474** | **1** | **20** | **29** | **1** | **EKVPGEMEIER + Oxidation (M)** |
|  | 1228 | **471.7134** | **1412.1180** | **1411.4306** | **0.6873** | **0** | **20** | **18** | **1** | **EDGAELPADPAGGGR** |
|  | 146 | **372.3457** | **742.6766** | **743.8488** | **-1.1721** | **2** | **20** | **24** | **1** | **KEPDKK** |
|  | 1979 | **573.3721** | **1144.7294** | **1144.1927** | **0.5367** | **0** | **20** | **24** | **1** | **ASNPADVSDLR** |
|  | 1744 | **534.8141** | **1601.4201** | **1600.7804** | **0.6396** | **2** | **20** | **22** | **1** | **EQFRVASLHTRTR** |
|  | 3297 | **836.6261** | **1671.2374** | **1670.8634** | **0.3740** | **0** | **20** | **23** | **1** | **AANNGALPPDLSYIVR** |
|  | 2880 | **730.5291** | **2188.5652** | **2187.5115** | **1.0537** | **2** | **20** | **24** | **1** | **TMSMWAGPDHWRFRPRR** |
|  | 315 | **387.1402** | **772.2656** | **772.8898** | **-0.6243** | **1** | **20** | **29** | **1** | **EGAQLKK** |
|  | 609 | **408.0101** | **1221.0081** | **1220.5204** | **0.4877** | **0** | **20** | **26** | **1** | **TELLMIFPLK + Oxidation (M)** |
|  | 1485 | **504.0718** | **1509.1933** | **1509.7925** | **-0.5992** | **2** | **20** | **27** | **1** | **QKFVSMKVDHMK + 2 Oxidation (M)** |
|  | 1304 | **480.5198** | **959.0249** | **959.1214** | **-0.0965** | **0** | **20** | **32** | **1** | **DSCVIPLR + Carbamidomethyl (C)** |
|  | 965 | **443.8094** | **885.6040** | **885.0661** | **0.5379** | **2** | **20** | **27** | **1** | **VRKGLQGK** |
|  | 1197 | **467.7835** | **1400.3284** | **1400.6499** | **-0.3214** | **1** | **20** | **24** | **1** | **RPEMSRGLLAVR + Oxidation (M)** |
|  | 1366 | **488.6210** | **1462.8408** | **1462.6678** | **0.1730** | **1** | **20** | **32** | **1** | **LDKDLTIGQMQGK + Oxidation (M)** |
|  | 3360 | **897.9792** | **1793.9437** | **1795.1084** | **-1.1647** | **0** | **20** | **28** | **1** | **ICVITLAESHPVLQSGK** |
|  | 1203 | **468.2932** | **934.5715** | **935.1016** | **-0.5300** | **0** | **20** | **25** | **1** | **APAMFNIR + Oxidation (M)** |
|  | 1662 | **524.0674** | **1569.1802** | **1568.7717** | **0.4085** | **0** | **20** | **29** | **1** | **TSISQVLPPSFQHK** |
|  | 438 | **401.7416** | **801.4684** | **800.9033** | **0.5652** | **1** | **20** | **29** | **1** | **CRFSEC + Carbamidomethyl (C)** |
|  | 1987 | **574.4675** | **1720.3802** | **1720.9570** | **-0.5768** | **1** | **20** | **23** | **1** | **KLLEDIIAEFTTNLT** |
|  | 2405 | **630.1887** | **1258.3627** | **1258.3781** | **-0.0155** | **0** | **20** | **27** | **1** | **TDQEVLGELVR** |
|  | 2576 | **664.8456** | **1991.5146** | **1991.2073** | **0.3072** | **2** | **20** | **28** | **1** | **AGSIYYADSVKGRFTISR** |
|  | 2964 | **746.9243** | **2237.7506** | **2237.6265** | **0.1241** | **1** | **20** | **28** | **1** | **DCAAAEKCCINVCGLHSCVAAR** |
|  | 505 | **404.9697** | **807.9246** | **807.8545** | **0.0701** | **1** | **20** | **26** | **1** | **NRDTFR** |
|  | 1370 | **488.9094** | **975.8039** | **976.0856** | **-0.2817** | **0** | **20** | **29** | **1** | **SSVAGSLSLR** |
|  | 2251 | **608.7742** | **1215.5337** | **1216.3049** | **-0.7712** | **2** | **20** | **30** | **1** | **KKAGAGNANSNGK** |
|  | 2947 | **742.7335** | **2225.1782** | **2225.4667** | **-0.2885** | **1** | **20** | **23** | **1** | **TLLHGTRGTHQHQVELIER** |
|  | 2125 | **594.9326** | **1187.8503** | **1187.3004** | **0.5500** | **0** | **20** | **27** | **1** | **EKPSVAPSSTGK** |
|  | 302 | **386.9518** | **1157.8334** | **1158.2656** | **-0.4323** | **2** | **20** | **32** | **1** | **KEREAELGAR** |
|  | 963 | **443.5872** | **1327.7396** | **1327.5113** | **0.2283** | **1** | **20** | **30** | **1** | **CGVCGGNGSTCKK + 2 Carbamidomethyl (C)** |
|  | 1083 | **457.7784** | **1370.3132** | **1369.5513** | **0.7619** | **0** | **20** | **24** | **1** | **MRPEGAAQAWPR** |
|  | 1242 | **472.3613** | **942.7078** | **943.1022** | **-0.3945** | **2** | **20** | **23** | **1** | **ATKATKAPR** |
|  | 1307 | **481.2512** | **1440.7315** | **1441.5892** | **-0.8578** | **1** | **20** | **31** | **1** | **EVRSTPLTATPGGR** |
|  | 422 | **400.3568** | **798.6989** | **798.8941** | **-0.1952** | **2** | **20** | **23** | **1** | **RGPARSR** |
|  | 1572 | **514.8294** | **1027.6440** | **1027.0894** | **0.5546** | **0** | **20** | **24** | **1** | **VGAQAEEAPR** |
|  | 2939 | **741.8342** | **2222.4803** | **2222.4960** | **-0.0156** | **2** | **19** | **34** | **1** | **EKFNPEWYKGEPIWVTAK** |
|  | 280 | **386.0544** | **770.0940** | **768.9310** | **1.1631** | **1** | **19** | **28** | **1** | **MLRGHR** |
|  | 16 | **362.2003** | **722.3859** | **722.7865** | **-0.4006** | **0** | **19** | **23** | **1** | **FSVGDAK** |
|  | 982 | **444.9682** | **1331.8824** | **1331.3627** | **0.5198** | **1** | **19** | **36** | **1** | **DQDMYSDKSDK** |
|  | 46 | **365.0041** | **1091.9900** | **1091.3487** | **0.6413** | **2** | **19** | **31** | **1** | **TCPVCKQKK + Carbamidomethyl (C)** |
|  | 402 | **399.0737** | **1194.1990** | **1193.3311** | **0.8679** | **0** | **19** | **28** | **1** | **MFDWISHNK + Oxidation (M)** |
|  | 1201 | **468.2209** | **934.4271** | **933.9628** | **0.4642** | **0** | **19** | **33** | **1** | **TENQSSIR** |
|  | 304 | **386.9789** | **771.9430** | **772.8965** | **-0.9534** | **2** | **19** | **34** | **1** | **KGLSRGR** |
|  | 3119 | **781.2775** | **2340.8102** | **2340.5047** | **0.3056** | **0** | **19** | **30** | **1** | **SMAAFAGMCDGGSTEDGCVAASR + 2 Carbamidomethyl (C); 2 Oxidation (M)** |
|  | 1372 | **489.1997** | **1464.5770** | **1465.6735** | **-1.0964** | **2** | **19** | **33** | **1** | **CYKCDVCGKEF + 3 Carbamidomethyl (C)** |
|  | 2335 | **616.2957** | **1230.5765** | **1230.4556** | **0.1209** | **1** | **19** | **33** | **1** | **AADKFLSLIPR** |
|  | 1148 | **461.8906** | **1382.6495** | **1383.5284** | **-0.8789** | **0** | **19** | **31** | **1** | **MVVDCCTDPDGR + Carbamidomethyl (C); Oxidation (M)** |
|  | 351 | **389.1252** | **776.2356** | **775.8076** | **0.4279** | **0** | **19** | **34** | **1** | **ADTGSLGR** |
|  | 1479 | **503.0978** | **1506.2712** | **1505.6994** | **0.5718** | **0** | **19** | **36** | **1** | **GSHMMHSALDMTR + 2 Oxidation (M)** |
|  | 2865 | **722.0372** | **2163.0895** | **2162.5121** | **0.5775** | **1** | **19** | **24** | **1** | **TTLVCPYLVDTGMFRGCR + 2 Carbamidomethyl (C); Oxidation (M)** |
|  | 753 | **420.5871** | **839.1594** | **838.9497** | **0.2098** | **0** | **19** | **27** | **1** | **ECLGDMR + Oxidation (M)** |
|  | 989 | **445.2493** | **1332.7257** | **1333.6849** | **-0.9592** | **1** | **19** | **33** | **1** | **AVLCPPPVKKPGK** |
|  | 2152 | **597.8105** | **1193.6063** | **1193.2881** | **0.3182** | **0** | **19** | **27** | **1** | **MEQALAGASSGR + Oxidation (M)** |
|  | 2935 | **741.7776** | **2222.3106** | **2223.5040** | **-1.1935** | **0** | **19** | **32** | **1** | **MVNGQEELHCISMETTSIGK + Oxidation (M)** |
|  | 235 | **380.2879** | **1137.8414** | **1138.3802** | **-0.5388** | **2** | **19** | **30** | **1** | **CKKLEAVYK + Carbamidomethyl (C)** |
|  | 1285 | **477.4865** | **952.9583** | **953.1763** | **-0.2180** | **0** | **19** | **32** | **1** | **LLLPGELAK** |
|  | 2608 | **667.9149** | **2000.7226** | **2000.1974** | **0.5251** | **0** | **19** | **28** | **1** | **CWAEDPQERPPFQQIR** |
|  | 1675 | **525.3436** | **1573.0087** | **1573.8128** | **-0.8041** | **1** | **19** | **30** | **1** | **RELASLCLQDGGALK** |
|  | 2679 | **679.6180** | **2035.8318** | **2035.3060** | **0.5257** | **0** | **19** | **27** | **1** | **QVFLCAYMAAFNSINGNR + Oxidation (M)** |
|  | 1451 | 499.8096 | 1496.4066 | 1495.7273 | 0.6793 | 0 | 19 | 26 | 1 | HQLSHPGLPGALLR |
|  | 1578 | **515.8293** | **1029.6438** | **1029.1549** | **0.4889** | **2** | **19** | **29** | **1** | **TADRAARLR** |
|  | 2693 | **680.6669** | **2038.9784** | **2039.2734** | **-0.2950** | **2** | **19** | **29** | **1** | **EAREKGHLEPTELLMNR + Oxidation (M)** |
|  | 9 | **360.5078** | **719.0007** | **718.8624** | **0.1384** | **0** | **19** | **36** | **1** | **MNDIVK** |
|  | 98 | **369.2914** | **1104.8520** | **1104.2991** | **0.5529** | **0** | **19** | **30** | **1** | **QQLLIGAYAK** |
|  | 291 | **386.2469** | **1155.7187** | **1155.3247** | **0.3940** | **2** | **19** | **24** | **1** | **KGYKTVGDCK + Carbamidomethyl (C)** |
|  | 1200 | 468.1680 | 1401.4818 | 1402.6590 | -1.1772 | 2 | 19 | 37 | 1 | EALRIAKECIEK |
|  | 1125 | **460.4062** | **1378.1965** | **1378.5960** | **-0.3995** | **0** | **19** | **31** | **1** | **IFIQTLEANACR** |
|  | 1442 | 499.6580 | 1495.9519 | 1495.7273 | 0.2246 | 0 | 19 | 32 | 1 | HQLSHPGLPGALLR |
|  | 2518 | 653.7000 | 1958.0777 | 1957.2275 | 0.8501 | 0 | 19 | 39 | 1 | LVYSQPLDLPESVEVIR |
|  | 837 | **430.9001** | **859.7854** | **859.9273** | **-0.1419** | **0** | **19** | **40** | **1** | **GAAGSSALAR** |
|  | 532 | **405.9525** | **1214.8352** | **1215.3979** | **-0.5628** | **0** | **19** | **31** | **1** | **VVENGALLSWK** |
|  | 38 | **363.4684** | **1087.3832** | **1088.2801** | **-0.8969** | **0** | **19** | **38** | **1** | **EHCLNFVVK** |
|  | 450 | **402.0049** | **801.9951** | **800.8602** | **1.1349** | **0** | **19** | **40** | **1** | **QAAAVEGR** |
|  | 312 | **387.0627** | **772.1106** | **770.9567** | **1.1538** | **0** | **19** | **41** | **1** | **LTPSILK** |
|  | 2011 | **578.9449** | **1155.8751** | **1156.3327** | **-0.4575** | **1** | **19** | **33** | **1** | **KVVPIGETGTR** |
|  | 2455 | **638.6605** | **1275.3063** | **1274.3808** | **0.9254** | **1** | **19** | **37** | **1** | **GAEATETQAKLR** |
|  | 88 | **369.2626** | **736.5105** | **736.9009** | **-0.3904** | **0** | **19** | **30** | **1** | **MMNTPK + Oxidation (M)** |
|  | 681 | **414.9076** | **1241.7006** | **1242.4499** | **-0.7493** | **0** | **19** | **34** | **1** | **MAPGWAGVGAAVR** |
|  | 666 | **412.6497** | **1234.9270** | **1234.4906** | **0.4364** | **1** | **19** | **26** | **1** | **GLLEAPRILPR** |
|  | 483 | **403.9433** | **805.8719** | **806.9675** | **-1.0956** | **0** | **19** | **36** | **1** | **SIMTDIK** |
|  | 796 | **427.8808** | **1280.6202** | **1279.4405** | **1.1797** | **1** | **19** | **34** | **1** | **MACKSEDSPSPK** |
|  | 833 | **430.3734** | **858.7320** | **857.9776** | **0.7544** | **0** | **19** | **35** | **1** | **GGPGCAALR + Carbamidomethyl (C)** |
|  | 1839 | **547.0472** | **1638.1195** | **1636.9368** | **1.1828** | **2** | **19** | **36** | **1** | **KALMLAMGYHEKGR + 2 Oxidation (M)** |
|  | 516 | **405.2342** | **1212.6804** | **1213.4020** | **-0.7216** | **0** | **19** | **27** | **1** | **SWDFSALLMK + Oxidation (M)** |
|  | 543 | **406.1152** | **1215.3233** | **1215.5307** | **-0.2073** | **1** | **19** | **34** | **1** | **MVKAGTCCATCK** |
|  | 1452 | 499.8214 | 1496.4422 | 1495.7273 | 0.7148 | 0 | 19 | 29 | 1 | HQLSHPGLPGALLR |
|  | 1974 | **572.1449** | **1142.2750** | **1141.2384** | **1.0366** | **1** | **19** | **36** | **1** | **SPVAGAADAARR** |
|  | 399 | **398.9714** | **795.9280** | **795.8867** | **0.0413** | **0** | **19** | **31** | **1** | **SHGAGLVR** |
|  | 937 | **437.5338** | **873.0528** | **872.0675** | **0.9854** | **2** | **19** | **48** | **1** | **KVAKTAVR** |
|  | 1098 | 458.8220 | 1373.4437 | 1373.5584 | -0.1146 | 1 | 19 | 38 | 1 | LSTRPVTKTSQR |
|  | 2318 | **613.6910** | **1838.0508** | **1837.9427** | **0.1080** | **2** | **19** | **42** | **1** | **DPSGASREQYFGPGTRL** |
|  | 2223 | **604.2386** | **1206.4625** | **1205.4281** | **1.0345** | **2** | **19** | **38** | **1** | **MKTILGDQRK + Oxidation (M)** |
|  | 604 | **407.9490** | **1220.8248** | **1220.3351** | **0.4897** | **0** | **19** | **37** | **1** | **GRPPPPDDTLR** |
|  | 3271 | **820.7502** | **2459.2286** | **2458.8524** | **0.3761** | **1** | **19** | **27** | **1** | **CELQIRGLVAEDAGEYLCMCGK + Carbamidomethyl (C)** |
|  | 32 | **363.1410** | **1086.4008** | **1087.2109** | **-0.8101** | **0** | **19** | **35** | **1** | **NLVSMAGHSR + Oxidation (M)** |
|  | 1636 | **521.4733** | **1561.3976** | **1560.6469** | **0.7508** | **0** | **19** | **29** | **1** | **SGCNHPDLDVQYR + Carbamidomethyl (C)** |
|  | 1909 | **559.4783** | **1116.9419** | **1116.1825** | **0.7593** | **0** | **19** | **31** | **1** | **SAPAATLGSADR** |
|  | 51 | **366.0186** | **1095.0338** | **1095.1170** | **-0.0832** | **1** | **19** | **42** | **1** | **DSISSSGKWE** |
|  | 561 | **406.8940** | **1217.6599** | **1217.3295** | **0.3304** | **2** | **19** | **32** | **1** | **ANNSAKDEKIK** |
|  | 828 | **429.6180** | **857.2213** | **857.9497** | **-0.7284** | **0** | **19** | **35** | **1** | **DFPASPPK** |
|  | 571 | **407.1713** | **1218.4917** | **1219.3686** | **-0.8768** | **2** | **19** | **35** | **1** | **QEGKMQEGKGK** |
|  | 993 | **447.0638** | **892.1129** | **891.0838** | **1.0291** | **0** | **19** | **40** | **1** | **MLEEILK + Oxidation (M)** |
|  | 1347 | **487.1137** | **1458.3189** | **1458.7256** | **-0.4068** | **1** | **19** | **39** | **1** | **LPMTLSRQEVIR + Oxidation (M)** |
|  | 2057 | **587.5413** | **1173.0677** | **1173.1827** | **-0.1149** | **0** | **19** | **33** | **1** | **DVYEEFDEK** |
|  | 1139 | **461.2195** | **1380.6363** | **1381.5620** | **-0.9257** | **1** | **18** | **38** | **1** | **GICDAGRCVCDR + 2 Carbamidomethyl (C)** |
|  | 2067 | **589.5975** | **1177.1803** | **1176.3469** | **0.8333** | **1** | **18** | **41** | **1** | **TILNNGKTCR + Carbamidomethyl (C)** |
|  | 629 | **408.8337** | **1223.4790** | **1224.3252** | **-0.8462** | **1** | **18** | **40** | **1** | **YAEWSSGLRR** |
|  | 1250 | **473.3177** | **1416.9311** | **1417.6306** | **-0.6996** | **2** | **18** | **34** | **1** | **ELECIREIKER** |
|  | 714 | **417.9690** | **833.9232** | **833.8008** | **0.1224** | **0** | **18** | **40** | **1** | **SSDGTPDR** |
|  | 1765 | **537.1893** | **1608.5456** | **1608.8391** | **-0.2934** | **2** | **18** | **43** | **1** | **TIRDSSRTMDVCPK** |
|  | 270 | **385.2082** | **768.4015** | **767.9115** | **0.4901** | **0** | **18** | **27** | **1** | **LFVFDK** |
|  | 126 | **371.0213** | **1110.0416** | **1110.2841** | **-0.2424** | **1** | **18** | **30** | **1** | **ATTKCFTGPK + Carbamidomethyl (C)** |
|  | 1819 | **543.7716** | **1628.2926** | **1628.8668** | **-0.5741** | **1** | **18** | **34** | **1** | **LNTMSKTSLGQSMSK + Oxidation (M)** |
|  | 510 | **405.0300** | **1212.0678** | **1211.3084** | **0.7595** | **1** | **18** | **36** | **1** | **MGFSEQGRQR + Oxidation (M)** |
|  | 1449 | 499.7497 | 1496.2269 | 1495.7273 | 0.4996 | 0 | 18 | 30 | 1 | HQLSHPGLPGALLR |
|  | 1064 | **456.7570** | **1367.2487** | **1367.6363** | **-0.3876** | **1** | **18** | **28** | **1** | **LGGLSISPAGIVKR** |
|  | 1257 | **474.3051** | **1419.8930** | **1420.7171** | **-0.8241** | **0** | **18** | **35** | **1** | **IHCLDILFAFTK** |
|  | 1516 | **505.5871** | **1513.7391** | **1514.8500** | **-1.1109** | **1** | **18** | **44** | **1** | **VALLKLVSLTGSWK** |
|  | 2177 | **599.2082** | **1196.4016** | **1195.3687** | **1.0329** | **1** | **18** | **38** | **1** | **KQKPAQDVPGK** |
|  | 3046 | **759.6059** | **2275.7955** | **2275.5387** | **0.2568** | **1** | **18** | **34** | **1** | **NSLFKFESSSHAISMSAYLR** |
|  | 1428 | **498.7729** | **1493.2967** | **1493.7349** | **-0.4382** | **1** | **18** | **31** | **1** | **RFHFFCSMSCR + Carbamidomethyl (C); Oxidation (M)** |
|  | 805 | **428.2651** | **854.5155** | **855.0367** | **-0.5213** | **1** | **18** | **28** | **1** | **GLRAPTLK** |
|  | 1831 | **546.0900** | **1090.1653** | **1090.2944** | **-0.1291** | **1** | **18** | **42** | **1** | **CYEVGMMKGG + Oxidation (M)** |
|  | 846 | **431.0418** | **1290.1032** | **1289.4896** | **0.6135** | **1** | **18** | **49** | **1** | **LAKPGRLHGAGGR** |
|  | 1450 | 499.7522 | 1496.2344 | 1495.7273 | 0.5071 | 0 | 18 | 30 | 1 | HQLSHPGLPGALLR |
|  | 1723 | 532.1644 | 1062.3141 | 1061.1702 | 1.1438 | 0 | 18 | 41 | 1 | QLQEEMQR |
|  | 3123 | **784.7642** | **1567.5135** | **1567.8930** | **-0.3794** | **1** | **18** | **32** | **1** | **DICLKMSMPIASSR + Oxidation (M)** |
|  | 1024 | **450.0999** | **898.1850** | **898.0583** | **0.1268** | **0** | **18** | **39** | **1** | **GAVSLPVAGK** |
|  | 1732 | **533.3090** | **1596.9047** | **1595.9029** | **1.0018** | **1** | **18** | **39** | **1** | **IISAVIESMKYWR** |
|  | 2886 | **732.1788** | **1462.3429** | **1461.6136** | **0.7292** | **0** | **18** | **39** | **1** | **ACDTLGDLMYTDK + Oxidation (M)** |
|  | 1114 | **459.6407** | **917.2667** | **918.0726** | **-0.8059** | **0** | **18** | **39** | **1** | **GLCVSGGLR + Carbamidomethyl (C)** |
|  | 2299 | **612.2361** | **1222.4574** | **1223.2495** | **-0.7921** | **0** | **18** | **41** | **1** | **LENQGSTGFDR** |
|  | 3244 | **814.2911** | **1626.5674** | **1625.8295** | **0.7378** | **2** | **18** | **38** | **1** | **LHLYERTRDLPGR** |
|  | 1069 | **457.3699** | **912.7250** | **913.0727** | **-0.3477** | **0** | **18** | **33** | **1** | **TLSPGILGR** |
|  | 1544 | **510.5806** | **1528.7195** | **1528.6664** | **0.0531** | **2** | **18** | **52** | **1** | **QALSPAEAEKRSGGK** |
|  | 2075 | **590.2068** | **1178.3989** | **1177.3318** | **1.0671** | **1** | **18** | **42** | **1** | **NAEMLSTQKR** |
|  | 2820 | **709.5328** | **2125.5762** | **2126.5640** | **-0.9879** | **2** | **18** | **37** | **1** | **VIRGLMLLISGAKGQWSPSL** |
|  | 2246 | **608.1254** | **1821.3541** | **1821.1687** | **0.1854** | **0** | **18** | **42** | **1** | **STLMGHMLYLLGNINK + Oxidation (M)** |
|  | 621 | **408.2578** | **814.5007** | **813.7729** | **0.7279** | **0** | **18** | **34** | **1** | **DDHGNTR** |
|  | 1256 | **474.1812** | **946.3475** | **946.1725** | **0.1751** | **2** | **18** | **45** | **1** | **MARKVVSR** |
|  | 1371 | **489.1013** | **1464.2816** | **1464.7119** | **-0.4303** | **2** | **18** | **45** | **1** | **INVKVGGNHKGTLK** |
|  | 141 | **371.3995** | **740.7842** | **740.8481** | **-0.0638** | **0** | **18** | **41** | **1** | **SVISAHK** |
|  | 875 | **433.3896** | **1297.1468** | **1296.5587** | **0.5881** | **2** | **18** | **36** | **1** | **KPFKCKECEK + Carbamidomethyl (C)** |
|  | 697 | **416.2223** | **1245.6446** | **1245.4555** | **0.1891** | **2** | **18** | **43** | **1** | **MRIQKAGGNVR + Oxidation (M)** |
|  | 720 | **418.0918** | **1251.2533** | **1251.4170** | **-0.1637** | **1** | **18** | **44** | **1** | **AHPFSMYSRR** |
|  | 3069 | **764.9721** | **1527.9294** | **1527.7494** | **0.1801** | **0** | **18** | **37** | **1** | **FHCNSCPFLCSR + 2 Carbamidomethyl (C)** |
|  | 1087 | **458.2038** | **914.3929** | **915.0886** | **-0.6958** | **0** | **18** | **43** | **1** | **LSVLASGLR** |
|  | 83 | **369.2442** | **1104.7103** | **1105.0072** | **-0.2968** | **1** | **18** | **37** | **1** | **XDRAGLVXPR** |
|  | 96 | **369.2854** | **736.5560** | **735.8930** | **0.6631** | **1** | **18** | **37** | **1** | **AMATKAK + Oxidation (M)** |
|  | 845 | **431.0378** | **1290.0913** | **1289.4434** | **0.6478** | **2** | **18** | **53** | **1** | **DATRARLPAYR** |
|  | 1703 | **528.8989** | **1055.7831** | **1056.3028** | **-0.5198** | **2** | **18** | **40** | **1** | **AKTLLGKAVR** |
|  | 1830 | **545.9191** | **1634.7352** | **1634.7935** | **-0.0583** | **1** | **18** | **43** | **1** | **SRADAGHAGVSANMMK + 2 Oxidation (M)** |
|  | 1104 | **459.0344** | **1374.0810** | **1374.6487** | **-0.5677** | **0** | **18** | **49** | **1** | **ISAMGALSLGLGLR + Oxidation (M)** |
|  | 1523 | **506.7506** | **1517.2297** | **1516.5298** | **0.6998** | **1** | **18** | **34** | **1** | **GGEGSTGTERAGQGPR** |
|  | 2615 | **668.6074** | **2002.8001** | **2003.1950** | **-0.3949** | **0** | **18** | **36** | **1** | **GDSGGPLVCGGVLEGVVTSGSR** |
|  | 458 | **402.3145** | **1203.9214** | **1204.4201** | **-0.4987** | **0** | **18** | **41** | **1** | **MAFNGCCPDCK + Oxidation (M)** |
|  | 1762 | **536.5863** | **1071.1578** | **1070.3047** | **0.8532** | **1** | **18** | **55** | **1** | **DMKCLMEK + Carbamidomethyl (C); Oxidation (M)** |
|  | 2736 | **685.1443** | **1368.2738** | **1367.5572** | **0.7166** | **2** | **18** | **45** | **1** | **TAAPSVRPEKRR** |
|  | 40 | **364.1029** | **726.1910** | **725.8365** | **0.3544** | **1** | **18** | **41** | **1** | **NPILRN** |
|  | 135 | **371.2479** | **740.4809** | **740.8912** | **-0.4102** | **0** | **18** | **28** | **1** | **KPELVR** |
|  | 2536 | **655.5001** | **1963.4780** | **1964.1619** | **-0.6839** | **1** | **18** | **38** | **1** | **MTDQEAIQDLWQWRK + Oxidation (M)** |
|  | 1144 | **461.8324** | **921.6500** | **921.1445** | **0.5055** | **2** | **18** | **42** | **1** | **HKVLLRR** |
|  | 2282 | **611.1119** | **1830.3136** | **1830.0084** | **0.3052** | **2** | **18** | **45** | **1** | **QRSQDIGRSGAALETLK** |
|  | 232 | **380.0988** | **758.1828** | **758.8036** | **-0.6209** | **0** | **18** | **50** | **1** | **HTGCGER** |
|  | 272 | **385.2540** | **1152.7397** | **1153.3286** | **-0.5888** | **0** | **18** | **30** | **1** | **WVCEDMGLK + Carbamidomethyl (C); Oxidation (M)** |
|  | 185 | **375.2177** | **748.4205** | **748.8255** | **-0.4049** | **2** | **18** | **40** | **1** | **KKSSGDK** |
|  | 2813 | **706.7305** | **1411.4462** | **1410.7107** | **0.7354** | **1** | **18** | **43** | **1** | **ALGCPRIHLMAGR + Oxidation (M)** |
|  | 3258 | **817.2782** | **1632.5416** | **1631.8094** | **0.7322** | **0** | **18** | **43** | **1** | **VFPTHPDCPQFSTR** |
|  | 3230 | **809.4957** | **1616.9766** | **1616.7398** | **0.2367** | **1** | **18** | **43** | **1** | **HGQRGHGQQLLETR** |
|  | 129 | **371.1341** | **1110.3802** | **1111.3613** | **-0.9811** | **2** | **18** | **37** | **1** | **IKLCHGQKK + Carbamidomethyl (C)** |
|  | 2822 | **710.5012** | **2128.4813** | **2128.4943** | **-0.0130** | **1** | **18** | **44** | **1** | **EDMPSVWVVTIRLAVAVAR + Oxidation (M)** |
|  | 857 | **432.2350** | **1293.6830** | **1294.4830** | **-0.8001** | **1** | **18** | **42** | **1** | **LHAMRLDSHAK + Oxidation (M)** |
|  | 2735 | **685.1234** | **1368.2320** | **1367.4690** | **0.7630** | **0** | **18** | **47** | **1** | **WPGSHLATSQQR** |
|  | 2254 | **609.2048** | **1824.5923** | **1824.0404** | **0.5520** | **1** | **18** | **48** | **1** | **VEDAVEAIGFGKFQWK** |
|  | 2620 | **668.7681** | **2003.2820** | **2004.2275** | **-0.9455** | **1** | **18** | **56** | **1** | **EKALAELSNQHLQMGYR + Oxidation (M)** |
|  | 712 | **417.8162** | **1250.4263** | **1249.3365** | **1.0899** | **2** | **18** | **49** | **1** | **SPARHPGAGTGKN** |
|  | 2587 | **666.5078** | **1331.0008** | **1331.6209** | **-0.6200** | **0** | **18** | **39** | **1** | **YLYAFSIVPMK** |
|  | 2733 | **685.0383** | **1368.0619** | **1367.5572** | **0.5047** | **2** | **18** | **42** | **1** | **TAAPSVRPEKRR** |
|  | 1825 | **544.3160** | **1629.9259** | **1629.8597** | **0.0663** | **1** | **18** | **51** | **1** | **LRSSAPGECHQIMK + Carbamidomethyl (C); Oxidation (M)** |
|  | 2372 | **623.1560** | **1244.2972** | **1245.4075** | **-1.1102** | **0** | **18** | **47** | **1** | **VCADPQATWVR** |
|  | 199 | **377.0972** | **1128.2693** | **1128.2397** | **0.0297** | **1** | **18** | **44** | **1** | **VRSAQGQPASK** |
|  | 340 | **388.6658** | **1162.9751** | **1162.3999** | **0.5752** | **0** | **18** | **43** | **1** | **AMLALTSTLNK** |
|  | 2632 | **670.3589** | **2008.0545** | **2007.1071** | **0.9473** | **1** | **18** | **46** | **1** | **NFHNISNRCSYADHSNK** |
|  | 2594 | **666.6323** | **1996.8748** | **1996.2305** | **0.6443** | **2** | **18** | **39** | **1** | **RSSVNSSAMAETAANRICK** |
|  | 3259 | **817.4316** | **2449.2728** | **2449.7363** | **-0.4636** | **1** | **18** | **45** | **1** | **EGLLSCGISLDWDEKRPEFVR** |
|  | 24 | 363.0088 | 724.0029 | 723.7713 | 0.2316 | 0 | 18 | 44 | 1 | DEVFSK |
|  | 2565 | **663.1902** | **1324.3656** | **1323.6004** | **0.7651** | **1** | **18** | **45** | **1** | **KLMAFTLQDLK + Oxidation (M)** |
|  | 1560 | **513.9489** | **1538.8244** | **1538.7921** | **0.0323** | **1** | **18** | **45** | **1** | **VSVDWGKCMNPFR** |
|  | 2456 | **638.7506** | **1913.2297** | **1913.2262** | **0.0034** | **1** | **18** | **56** | **1** | **NHEKLACILDSVVNCVR** |
|  | 1494 | **504.6374** | **1510.8899** | **1511.7023** | **-0.8124** | **1** | **18** | **52** | **1** | **VHIEMGPDGRVTGK + Oxidation (M)** |
|  | 39 | **363.5142** | **1087.5205** | **1088.1691** | **-0.6487** | **0** | **18** | **42** | **1** | **SQLANTEPTK** |
|  | 775 | **422.9882** | **843.9616** | **842.9798** | **0.9818** | **0** | **18** | **54** | **1** | **GPEMYCK + Oxidation (M)** |
|  | 942 | **438.1875** | **1311.5403** | **1310.5621** | **0.9783** | **1** | **18** | **52** | **1** | **LGSAGSKVVAFMK + Oxidation (M)** |
|  | 1279 | **476.9260** | **951.8371** | **951.0843** | **0.7528** | **1** | **17** | **47** | **1** | **ALHGRELR** |
|  | 2355 | **619.8878** | **1237.7609** | **1237.3653** | **0.3955** | **0** | **17** | **36** | **1** | **GTNIQHIALDR** |
|  | 1311 | **481.8796** | **961.7445** | **961.8869** | **-0.1424** | **0** | **17** | **50** | **1** | **GENDGGEER** |
|  | 2142 | **596.6693** | **1786.9858** | **1787.0946** | **-0.1089** | **1** | **17** | **63** | **1** | **HALRCYCQAMQVYK + Carbamidomethyl (C); Oxidation (M)** |
|  | 1212 | **469.6783** | **1406.0126** | **1406.5633** | **-0.5507** | **2** | **17** | **37** | **1** | **HYDKKGDGMIDK** |
|  | 1960 | **569.3644** | **1136.7141** | **1136.2337** | **0.4804** | **0** | **17** | **48** | **1** | **QKPSGSEMEK + Oxidation (M)** |
|  | 2264 | **609.7620** | **1826.2637** | **1825.1377** | **1.1260** | **2** | **17** | **54** | **1** | **IGRLLMDSKYSTLTAR** |
|  | 256 | **384.9902** | **767.9656** | **766.8091** | **1.1566** | **1** | **17** | **41** | **1** | **GGRGGAHR** |
|  | 2675 | **679.1141** | **1356.2135** | **1355.5395** | **0.6740** | **0** | **17** | **47** | **1** | **GLNLAANAAVTAAAK** |
|  | 2870 | **725.3961** | **1448.7773** | **1447.5955** | **1.1819** | **2** | **17** | **48** | **1** | **ARTSPYPGSKVER** |
|  | 1917 | **562.0883** | **1683.2426** | **1683.9287** | **-0.6861** | **1** | **17** | **48** | **1** | **LRTYSTHAVVMYAR + Oxidation (M)** |
|  | 137 | **371.2625** | **740.5101** | **740.8912** | **-0.3810** | **0** | **17** | **31** | **1** | **KPELVR** |
|  | 456 | **402.2475** | **1203.7202** | **1204.3107** | **-0.5905** | **0** | **17** | **46** | **1** | **ADAEAECNILR** |
|  | 1287 | **478.1572** | **1431.4493** | **1432.4930** | **-1.0436** | **1** | **17** | **46** | **1** | **DGSGVSSLGSKSSHK** |
|  | 2048 | **586.0081** | **1170.0013** | **1170.4053** | **-0.4040** | **2** | **17** | **49** | **1** | **LIDLLRRSGK** |
|  | 407 | **399.5599** | **797.1050** | **797.9854** | **-0.8804** | **0** | **17** | **38** | **1** | **QVVALIR** |
|  | 1124 | **460.3030** | **1377.8869** | **1378.5068** | **-0.6198** | **0** | **17** | **44** | **1** | **DSWDMLGVKPGAS + Oxidation (M)** |
|  | 1586 | **517.7574** | **1033.5000** | **1034.2143** | **-0.7144** | **0** | **17** | **43** | **1** | **AANALCAGMR + Carbamidomethyl (C)** |
|  | 2448 | **636.2066** | **1270.3984** | **1269.3875** | **1.0110** | **0** | **17** | **48** | **1** | **MAQAAGPAGGGEPR** |
|  | 2940 | **741.8738** | **2222.5992** | **2223.5985** | **-0.9993** | **2** | **17** | **54** | **1** | **VMRLNPMAIDSPAMDFSRR + Oxidation (M)** |
|  | 2069 | **589.7644** | **1177.5140** | **1178.3613** | **-0.8472** | **0** | **17** | **53** | **1** | **CPSPPQPSLPR** |
|  | 388 | **395.1968** | **1182.5682** | **1181.4265** | **1.1417** | **1** | **17** | **48** | **1** | **RTLFLFGVTK** |
|  | 1521 | **506.1646** | **1010.3144** | **1009.2348** | **1.0797** | **2** | **17** | **45** | **1** | **MNRCARCR** |
|  | 1300 | **480.1948** | **958.3748** | **957.1918** | **1.1830** | **0** | **17** | **51** | **1** | **MKPDVVLR** |
|  | 3003 | **749.2357** | **1496.4565** | **1495.7525** | **0.7041** | **1** | **17** | **48** | **1** | **VGMGARTPRPGAGLR** |
|  | 6 | **360.3846** | **718.7543** | **718.8655** | **-0.1112** | **0** | **17** | **68** | **1** | **IALCSR + Carbamidomethyl (C)** |
|  | 1677 | **526.0178** | **1575.0311** | **1574.8425** | **0.1887** | **2** | **17** | **53** | **1** | **KISAYMKSSQFLR + Oxidation (M)** |
|  | 2361 | **621.3865** | **1861.1374** | **1862.0289** | **-0.8914** | **1** | **17** | **48** | **1** | **EEGAGPTRTVEWLNMR + Oxidation (M)** |
|  | 2739 | **685.2605** | **1368.5062** | **1367.5572** | **0.9491** | **2** | **17** | **52** | **1** | **TAAPSVRPEKRR** |
|  | 3276 | **822.5103** | **2464.5088** | **2463.7048** | **0.8039** | **2** | **17** | **49** | **1** | **YPSNLQLFSRSRLPGPWDSSR** |
|  | 1869 | **552.3307** | **1102.6466** | **1102.2374** | **0.4092** | **0** | **17** | **54** | **1** | **MMSSVSTESK + Oxidation (M)** |
|  | 2104 | **593.2837** | **1184.5526** | **1184.3228** | **0.2298** | **0** | **17** | **55** | **1** | **LSCVASGFTSR + Carbamidomethyl (C)** |
|  | 512 | **405.0656** | **808.1165** | **807.8761** | **0.2404** | **1** | **17** | **46** | **1** | **SRNMER + Oxidation (M)** |
|  | 84 | **369.2480** | **1104.7219** | **1105.3089** | **-0.5870** | **0** | **17** | **44** | **1** | **AKPWAVCFPS** |
|  | 2753 | **686.1225** | **2055.3453** | **2055.3786** | **-0.0333** | **1** | **17** | **49** | **1** | **SLLNQEIRNMQMISFAK + 2 Oxidation (M)** |
|  | 2611 | **668.0320** | **2001.0740** | **2002.2468** | **-1.1729** | **1** | **17** | **48** | **1** | **WTTATTMKVLSNTTTTTK + Oxidation (M)** |
|  | 2094 | **592.7626** | **1183.5104** | **1183.2701** | **0.2403** | **0** | **17** | **52** | **1** | **AAELFFGEGSR** |
|  | 1247 | **472.9574** | **943.9000** | **943.0624** | **0.8376** | **1** | **17** | **53** | **1** | **RAVTAQAAR** |
|  | 97 | **369.2913** | **1104.8517** | **1105.3089** | **-0.4572** | **0** | **17** | **45** | **1** | **AKPWAVCFPS** |
|  | 103 | **369.3084** | **1104.9029** | **1105.3089** | **-0.4060** | **0** | **17** | **47** | **1** | **AKPWAVCFPS** |
|  | 1414 | **494.9204** | **987.8260** | **987.1084** | **0.7176** | **1** | **17** | **54** | **1** | **DLVRDLEK** |
|  | 1476 | **502.5257** | **1504.5548** | **1505.6927** | **-1.1378** | **0** | **17** | **63** | **1** | **MASGNGLPSSSALVAK + Oxidation (M)** |
|  | 2328 | **614.8434** | **1227.6720** | **1228.4182** | **-0.7463** | **0** | **17** | **42** | **1** | **SASPLCQGAPLK + Carbamidomethyl (C)** |
|  | 2856 | **717.2877** | **1432.5607** | **1431.6140** | **0.9466** | **0** | **17** | **51** | **1** | **MVNALENLAGIDR + Oxidation (M)** |
|  | 2115 | **593.7823** | **1778.3247** | **1777.9122** | **0.4124** | **0** | **17** | **50** | **1** | **AFSESGSNLHALMNQR + Oxidation (M)** |
|  | 1838 | **547.0468** | **1638.1183** | **1636.9368** | **1.1815** | **2** | **17** | **53** | **1** | **KALMLAMGYHEKGR + 2 Oxidation (M)** |
|  | 296 | **386.8740** | **1157.5997** | **1158.2622** | **-0.6625** | **1** | **17** | **57** | **1** | **EILKEQENR** |
|  | 154 | **373.5341** | **1117.5802** | **1117.2087** | **0.3715** | **0** | **17** | **60** | **1** | **EGADGSPPFIK** |
|  | 1047 | **453.6024** | **905.1901** | **906.0222** | **-0.8321** | **1** | **17** | **56** | **1** | **GSSQCRLR** |
|  | 2687 | **680.3816** | **1358.7484** | **1359.6175** | **-0.8691** | **0** | **17** | **52** | **1** | **SPNVLLQNLCCR** |
|  | 3035 | **757.0935** | **2268.2583** | **2268.6620** | **-0.4036** | **2** | **17** | **40** | **1** | **MHWVRQGPGKGLEWVCGISK** |
|  | 1596 | **518.6688** | **1035.3229** | **1036.1212** | **-0.7983** | **1** | **17** | **56** | **1** | **SRTMDGPTR + Oxidation (M)** |
|  | 1992 | **576.0034** | **1724.9881** | **1723.9874** | **1.0006** | **1** | **17** | **53** | **1** | **SLFGSIGDIESCKLVR** |
|  | 2406 | **630.2446** | **1887.7117** | **1887.0643** | **0.6474** | **0** | **17** | **53** | **1** | **AEWFQHQHWLYWR** |
|  | 2092 | **592.7577** | **1775.2509** | **1774.9945** | **0.2564** | **0** | **17** | **55** | **1** | **ASGFIFSTYGMHWVR + Oxidation (M)** |
|  | 3037 | **757.7966** | **1513.5785** | **1514.6995** | **-1.1210** | **0** | **17** | **52** | **1** | **ADVNLPQDLGDVMK** |
|  | 1007 | **448.1136** | **894.2125** | **895.0345** | **-0.8220** | **0** | **17** | **52** | **1** | **IEVFCER** |
|  | 2776 | **691.0134** | **1380.0121** | **1379.5182** | **0.4939** | **0** | **17** | **44** | **1** | **GDGAPVTTVPVPNR** |
|  | 1106 | **459.1095** | **1374.3063** | **1373.5733** | **0.7331** | **0** | **17** | **60** | **1** | **VAYPMVAAYSASK + Oxidation (M)** |
|  | 1415 | **495.2157** | **1482.6249** | **1483.6425** | **-1.0175** | **1** | **17** | **56** | **1** | **YAMDVENKIQEK + Oxidation (M)** |
|  | 92 | **369.2684** | **1104.7830** | **1105.3089** | **-0.5258** | **0** | **17** | **46** | **1** | **AKPWAVCFPS** |
|  | 1530 | **507.6403** | **1519.8988** | **1519.7905** | **0.1082** | **1** | **17** | **59** | **1** | **IVTLTATVRGVHPR** |
|  | 2313 | **613.2420** | **1836.7038** | **1836.1405** | **0.5634** | **0** | **17** | **52** | **1** | **MAFFTGLWGPFTCVSR + Oxidation (M)** |
|  | 642 | **409.9019** | **1226.6836** | **1226.4042** | **0.2794** | **2** | **17** | **60** | **1** | **KDAMYWEKR** |
|  | 685 | **415.1451** | **1242.4131** | **1242.5311** | **-0.1180** | **1** | **17** | **56** | **1** | **NVLSMPIVNKK** |
|  | 145 | **372.2989** | **1113.8746** | **1113.2234** | **0.6512** | **1** | **17** | **42** | **1** | **TYRAGPSFSK** |
|  | 1903 | **558.3736** | **1114.7324** | **1114.1649** | **0.5675** | **0** | **17** | **51** | **1** | **TPGTSLTWHD** |
|  | 446 | 401.9682 | 801.9217 | 802.9192 | -0.9975 | 2 | 17 | 63 | 1 | KKGSQQK |
|  | 477 | **403.8229** | **1208.4465** | **1208.3444** | **0.1022** | **1** | **17** | **56** | **1** | **VTMTADVYRH + Oxidation (M)** |
|  | 2339 | **616.7789** | **1231.5431** | **1230.4738** | **1.0692** | **0** | **17** | **58** | **1** | **QISDLMGLIPK + Oxidation (M)** |
|  | 1511 | **505.1961** | **1512.5661** | **1513.7377** | **-1.1717** | **0** | **17** | **53** | **1** | **EMNPALGIDCLHK + Carbamidomethyl (C); Oxidation (M)** |
|  | 62 | **367.1643** | **1098.4707** | **1098.2502** | **0.2206** | **0** | **17** | **52** | **1** | **VLVGSQPSSPK** |
|  | 1563 | **514.2203** | **1026.4258** | **1026.2073** | **0.2185** | **0** | **17** | **54** | **1** | **LSAFLSNMK + Oxidation (M)** |
|  | 3366 | **910.2731** | **1818.5314** | **1818.0473** | **0.4840** | **2** | **17** | **42** | **1** | **EGLRSSRAGAPACLCGR + 2 Carbamidomethyl (C)** |
|  | 439 | **401.8075** | **801.6001** | **802.7867** | **-1.1865** | **0** | **17** | **64** | **1** | **NPTSGPSSG** |
|  | 1109 | **459.4283** | **1375.2627** | **1374.4933** | **0.7693** | **1** | **17** | **55** | **1** | **ISALGEEKSSPEK** |
|  | 3068 | **764.7978** | **1527.5808** | **1527.6419** | **-0.0611** | **2** | **17** | **54** | **1** | **RRAQEAEEAGAALR** |
|  | 124 | **370.7166** | **1109.1278** | **1108.2516** | **0.8762** | **0** | **17** | **44** | **1** | **AVQAPRPQNK** |
|  | 590 | **407.7326** | **1220.1757** | **1219.3436** | **0.8321** | **0** | **17** | **50** | **1** | **AWGQGTLVTVST** |
|  | 2294 | **612.1496** | **1833.4266** | **1834.0188** | **-0.5921** | **1** | **17** | **56** | **1** | **TGAAMSSSPWEPATLRR + Oxidation (M)** |
|  | 2401 | **629.7088** | **1257.4028** | **1257.4150** | **-0.0121** | **1** | **17** | **66** | **1** | **ETPCSKCCDSK + Carbamidomethyl (C)** |
|  | 109 | **369.3432** | **1105.0073** | **1104.3011** | **0.7063** | **0** | **17** | **56** | **1** | **MHPSLATMGK + 2 Oxidation (M)** |
|  | 2575 | **664.6877** | **1327.3606** | **1327.5477** | **-0.1872** | **0** | **17** | **58** | **1** | **YYAPLLDAMVR + Oxidation (M)** |
|  | 2773 | **689.8964** | **1377.7779** | **1376.6614** | **1.1165** | **1** | **17** | **49** | **1** | **LDQKTAMSLLLK + Oxidation (M)** |
|  | 1102 | **458.9907** | **915.9666** | **915.0491** | **0.9175** | **2** | **17** | **63** | **1** | **RVAGGAKEK** |
|  | 2 | **360.3274** | **718.6399** | **718.8457** | **-0.2057** | **0** | **17** | **62** | **1** | **LFFHR** |
|  | 1599 | **518.8922** | **1553.6545** | **1554.6807** | **-1.0262** | **2** | **17** | **53** | **1** | **DQQEKEEFMRSK** |
|  | 520 | **405.3235** | **1212.9484** | **1213.4700** | **-0.5217** | **2** | **17** | **42** | **1** | **RDVAVAIKTLK** |
|  | 630 | **408.8801** | **815.7454** | **815.9577** | **-0.2122** | **2** | **17** | **61** | **1** | **GADLKKGK** |
|  | 1672 | **525.1140** | **1572.3199** | **1572.8050** | **-0.4852** | **2** | **17** | **60** | **1** | **IKKGTQQNIAVSSAK** |
|  | 166 | **374.2281** | **746.4415** | **746.8758** | **-0.4342** | **0** | **17** | **55** | **1** | **MIPTNR + Oxidation (M)** |
|  | 824 | **429.3312** | **1284.9714** | **1285.4085** | **-0.4370** | **2** | **17** | **46** | **1** | **HKGYSPPESRK** |
|  | 887 | **434.2761** | **1299.8060** | **1300.4398** | **-0.6337** | **0** | **17** | **43** | **1** | **MTSSCMHTAATGS + Oxidation (M)** |
|  | 964 | **443.7944** | **1328.3610** | **1327.4879** | **0.8731** | **0** | **17** | **55** | **1** | **QINQVALEAWR** |
|  | 127 | **371.0887** | **1110.2441** | **1110.3934** | **-0.1493** | **2** | **17** | **47** | **1** | **KLLKVKPER** |
|  | 2259 | **609.3507** | **1825.0300** | **1824.1714** | **0.8586** | **1** | **17** | **59** | **1** | **KIGVVVVGFPATPLAEAR** |
|  | 471 | **403.1888** | **1206.5444** | **1206.4176** | **0.1267** | **1** | **17** | **58** | **1** | **LYAGLPRCTR + Carbamidomethyl (C)** |
|  | 999 | **447.1663** | **892.3177** | **892.0290** | **0.2888** | **1** | **17** | **59** | **1** | **MEIEDKK** |
|  | 1542 | **509.6713** | **1525.9916** | **1525.9369** | **0.0547** | **0** | **17** | **61** | **1** | **LFLLCFGIIFLIG + Carbamidomethyl (C)** |
|  | 2643 | **672.1693** | **1342.3237** | **1341.5179** | **0.8058** | **1** | **17** | **54** | **1** | **YLASRAGHTLPR** |
|  | 93 | **369.2793** | **736.5438** | **736.9009** | **-0.3571** | **0** | **17** | **49** | **1** | **SCCVPTK** |
|  | 250 | **384.8378** | **1151.4913** | **1152.3043** | **-0.8129** | **1** | **17** | **49** | **1** | **RAPPTTAVPSR** |
|  | 94 | **369.2821** | **1104.8241** | **1104.3011** | **0.5231** | **0** | **17** | **50** | **1** | **MHPSLATMGK + 2 Oxidation (M)** |
|  | 2265 | **609.8976** | **1826.6706** | **1826.1490** | **0.5215** | **2** | **17** | **47** | **1** | **VRVRGAETGLYICMNK + Oxidation (M)** |
|  | 358 | **389.2289** | **1164.6646** | **1165.2150** | **-0.5504** | **0** | **17** | **51** | **1** | **AFNHSSNFNK** |
|  | 967 | **443.8503** | **1328.5289** | **1329.4957** | **-0.9669** | **0** | **17** | **59** | **1** | **SLPAALSATEIEK** |
|  | 236 | **381.0778** | **1140.2112** | **1141.3195** | **-1.1083** | **1** | **17** | **66** | **1** | **VWIPDDLRK** |
|  | 631 | **408.9015** | **1223.6823** | **1224.2805** | **-0.5983** | **0** | **17** | **63** | **1** | **LGQGSTFSGSQR** |
|  | 1760 | **536.5110** | **1606.5108** | **1606.8246** | **-0.3138** | **0** | **17** | **53** | **1** | **SHAIGCVNQFIMDR + Oxidation (M)** |
|  | 310 | **387.0075** | **772.0002** | **771.9083** | **0.0919** | **1** | **17** | **64** | **1** | **GSALRLR** |
|  | 2714 | **684.0129** | **1366.0110** | **1366.4516** | **-0.4406** | **2** | **17** | **48** | **1** | **KETSECPSDKDK** |
|  | 2583 | **666.3569** | **1330.6991** | **1330.4241** | **0.2749** | **0** | **17** | **58** | **1** | **MATSLGSNTYNR + Oxidation (M)** |
|  | 1546 | **510.6588** | **1528.9543** | **1528.7723** | **0.1819** | **0** | **17** | **65** | **1** | **SILLNCATPDAVVR + Carbamidomethyl (C)** |
|  | 2066 | **589.2632** | **1764.7674** | **1764.1590** | **0.6084** | **1** | **17** | **64** | **1** | **KAAMTSLMDLTLLLAR + Oxidation (M)** |
|  | 2295 | **612.1718** | **1833.4931** | **1833.9987** | **-0.5057** | **1** | **17** | **60** | **1** | **HCDSPAPSGGGKYCLGER** |
|  | 2203 | **600.1797** | **1797.5169** | **1797.9435** | **-0.4266** | **0** | **17** | **63** | **1** | **LTPSCPNPASATGGGPAGSR** |
|  | 77 | **369.0621** | **1104.1640** | **1104.2991** | **-0.1351** | **0** | **17** | **67** | **1** | **QQLLIGAYAK** |
|  | 3129 | **789.4635** | **1576.9122** | **1575.9100** | **1.0023** | **0** | **17** | **56** | **1** | **MEWLLELMGYMK + 2 Oxidation (M)** |
|  | 65 | **367.4048** | **1099.1921** | **1100.2197** | **-1.0276** | **1** | **17** | **78** | **1** | **IDEPIEEKK** |
|  | 3270 | **820.5200** | **1639.0251** | **1638.8662** | **0.1589** | **0** | **17** | **56** | **1** | **MDGIFAGIICNQANR + Oxidation (M)** |
|  | 238 | **382.1522** | **1143.4344** | **1142.3504** | **1.0840** | **0** | **16** | **68** | **1** | **LLALGSGAWVR** |
|  | 801 | **428.0239** | **1281.0496** | **1281.4577** | **-0.4081** | **0** | **16** | **58** | **1** | **RPPLGEDSIGLK** |
|  | 2669 | **678.2052** | **2031.5934** | **2031.3026** | **0.2908** | **2** | **16** | **59** | **1** | **ARHPQLAPTLQPRTECR + Carbamidomethyl (C)** |
|  | 485 | **404.0202** | **1209.0384** | **1208.1967** | **0.8418** | **0** | **16** | **60** | **1** | **ANDHGYDNFR** |
|  | 1146 | **461.8666** | **1382.5777** | **1382.5218** | **0.0558** | **0** | **16** | **59** | **1** | **GHSLVNSLIETGR** |
|  | 1998 | **576.7164** | **1151.4181** | **1150.3463** | **1.0718** | **1** | **16** | **64** | **1** | **MNISKSEITK** |
|  | 114 | **369.3676** | **1105.0807** | **1106.2556** | **-1.1749** | **1** | **16** | **69** | **1** | **RGPEVGGFCK + Carbamidomethyl (C)** |
|  | 350 | **389.1198** | **1164.3373** | **1163.2838** | **1.0535** | **2** | **16** | **66** | **1** | **TGETASKRWK** |
|  | 1117 | **460.0386** | **1377.0935** | **1376.5388** | **0.5547** | **1** | **16** | **67** | **1** | **LRDGTGNQMLQK + Oxidation (M)** |
|  | 2668 | **678.1137** | **2031.3190** | **2031.3026** | **0.0163** | **2** | **16** | **59** | **1** | **ARHPQLAPTLQPRTECR + Carbamidomethyl (C)** |
|  | 2012 | **578.9653** | **1733.8738** | **1733.9559** | **-0.0821** | **1** | **16** | **60** | **1** | **QVEEEILALKASFEK** |
|  | 1570 | **514.7301** | **1027.4454** | **1028.1204** | **-0.6750** | **0** | **16** | **49** | **1** | **QEALGQGVAR** |
|  | 871 | **433.1439** | **1296.4096** | **1296.5438** | **-0.1342** | **1** | **16** | **60** | **1** | **TVCRMGAAVCR + 2 Carbamidomethyl (C); Oxidation (M)** |
|  | 1369 | **488.8437** | **975.6726** | **975.0165** | **0.6561** | **0** | **16** | **60** | **1** | **SSFPAHSSR** |
|  | 1603 | **518.9272** | **1553.7594** | **1552.6449** | **1.1145** | **1** | **16** | **59** | **1** | **VLDSPSRLDEEHR** |
|  | 313 | **387.0836** | **772.1523** | **771.8886** | **0.2638** | **1** | **16** | **71** | **1** | **CRASAHK** |
|  | 1580 | **516.0682** | **1545.1825** | **1544.6261** | **0.5565** | **2** | **16** | **66** | **1** | **SRDKGQEGLGSPSAR** |
|  | 298 | **386.8931** | **771.7713** | **770.9171** | **0.8543** | **0** | **16** | **67** | **1** | **SIVPSLR** |
|  | 941 | **438.0381** | **874.0615** | **873.0487** | **1.0128** | **1** | **16** | **71** | **1** | **KTELALAK** |
|  | 2090 | **592.6584** | **1774.9532** | **1774.9202** | **0.0329** | **0** | **16** | **74** | **1** | **ASIELVEAEVSELETR** |
|  | 432 | **401.3099** | **1200.9075** | **1200.3089** | **0.5987** | **1** | **16** | **54** | **1** | **HSRFVHDFR** |
|  | 68 | **368.1329** | **1101.3766** | **1102.3116** | **-0.9349** | **0** | **16** | **66** | **1** | **MLCHHCSSK + Carbamidomethyl (C)** |
|  | 822 | **429.2363** | **1284.6866** | **1285.4497** | **-0.7631** | **1** | **16** | **54** | **1** | **NRSLASPLQATK** |
|  | 254 | **384.9612** | **767.9075** | **768.9013** | **-0.9937** | **0** | **16** | **53** | **1** | **APSAKPAK** |
|  | 76 | **368.9313** | **735.8479** | **736.9025** | **-1.0546** | **0** | **16** | **70** | **1** | **VIPPGVR** |
|  | 283 | **386.1000** | **1155.2779** | **1154.2784** | **0.9995** | **2** | **16** | **57** | **1** | **EWAKRNHTL** |
|  | 1295 | **478.9100** | **1433.7078** | **1432.5990** | **1.1089** | **1** | **16** | **61** | **1** | **ERLDMAPDTLQK + Oxidation (M)** |
|  | 2070 | **589.7802** | **1177.5456** | **1177.3203** | **0.2254** | **2** | **16** | **62** | **1** | **RRPRGEPGPR** |
|  | 3091 | 776.5295 | 2326.5663 | 2327.6118 | -1.0455 | 1 | 16 | 61 | 1 | MGLAPKDPALPGSSAPQPPEPAQA |
|  | 2742 | **685.4493** | **2053.3259** | **2054.3277** | **-1.0019** | **1** | **16** | **64** | **1** | **LRALAPSAECPIAEESLAR + Carbamidomethyl (C)** |
|  | 659 | **411.4527** | **1231.3358** | **1231.4453** | **-0.1094** | **0** | **16** | **78** | **1** | **IFSMSWCWR + Oxidation (M)** |
|  | 1105 | **459.0472** | **1374.1195** | **1374.5430** | **-0.4234** | **0** | **16** | **71** | **1** | **LEVSSSCGPQCHK** |
|  | 1573 | **515.1398** | **1028.2648** | **1028.2694** | **-0.0047** | **0** | **16** | **63** | **1** | **MAPIIISQR** |
|  | 3076 | **766.8728** | **1531.7308** | **1530.8063** | **0.9245** | **0** | **16** | **71** | **1** | **YSVQLLTPANLLAK** |
|  | 5 | **360.3821** | **1078.1242** | **1077.1945** | **0.9297** | **1** | **16** | **86** | **1** | **AGRDGSFLVR** |
|  | 2022 | **580.8397** | **1739.4970** | **1739.0268** | **0.4702** | **2** | **16** | **53** | **1** | **HGKKQLDPLTIYGIR** |
|  | 2044 | **585.6478** | **1753.9213** | **1754.0134** | **-0.0921** | **1** | **16** | **73** | **1** | **EESLGKCLLASTYLAR** |
|  | 459 | **402.4755** | **1204.4043** | **1204.3291** | **0.0753** | **0** | **16** | **88** | **1** | **ANPTVTLFPASS** |
|  | 504 | **404.9437** | **807.8725** | **808.9254** | **-1.0528** | **2** | **16** | **58** | **1** | **GYKGKTR** |
|  | 1111 | 459.5121 | 1375.5140 | 1375.5938 | -0.0798 | 1 | 16 | 84 | 1 | LEGATKLTCLGGGR |
|  | 434 | **401.3571** | **1201.0493** | **1201.3550** | **-0.3057** | **1** | **16** | **64** | **1** | **MKGTDSGSCCR + Carbamidomethyl (C)** |
|  | 1272 | **476.2978** | **1425.8713** | **1425.5036** | **0.3676** | **0** | **16** | **53** | **1** | **GAASGGAAQSPEPVAR** |
|  | 2381 | **625.8926** | **1874.6556** | **1875.3472** | **-0.6916** | **2** | **16** | **51** | **1** | **SMAFLTCCLLKCVKK + 3 Carbamidomethyl (C); Oxidation (M)** |
|  | 2008 | **578.5002** | **1732.4784** | **1731.9235** | **0.5549** | **0** | **16** | **51** | **1** | **LASVTAADTAVYYCAR + Carbamidomethyl (C)** |
|  | 539 | **406.0385** | **1215.0935** | **1214.3685** | **0.7249** | **0** | **16** | **58** | **1** | **LLESGAVVNQGK** |
|  | 761 | **422.0414** | **842.0681** | **840.8860** | **1.1821** | **0** | **16** | **64** | **1** | **HAHFSSR** |
|  | 1660 | **524.0002** | **1045.9857** | **1045.2305** | **0.7552** | **1** | **16** | **68** | **1** | **SVTLLEAKGK** |
|  | 1501 | **504.9563** | **1511.8467** | **1512.7332** | **-0.8865** | **1** | **16** | **63** | **1** | **LPGRGAGAEMPATLR + Oxidation (M)** |
|  | 882 | **434.0975** | **866.1802** | **865.0348** | **1.1453** | **1** | **16** | **62** | **1** | **LKAHQLR** |
|  | 521 | **405.3347** | **1212.9819** | **1212.3625** | **0.6193** | **1** | **16** | **49** | **1** | **NCSHLKDHCR** |
|  | 651 | **411.0532** | **1230.1375** | **1230.3912** | **-0.2537** | **0** | **16** | **68** | **1** | **MLEGLGSPASPR + Oxidation (M)** |
|  | 719 | **418.0758** | **1251.2052** | **1250.3842** | **0.8211** | **2** | **16** | **67** | **1** | **KFGDMRSHSIG + Oxidation (M)** |
|  | 1426 | **496.6820** | **1487.0238** | **1487.6393** | **-0.6155** | **1** | **16** | **62** | **1** | **AGDTAVYYCARAAR** |
|  | 354 | **389.1654** | **1164.4740** | **1165.3029** | **-0.8290** | **2** | **16** | **68** | **1** | **LRRTSTFER** |
|  | 1685 | **527.0355** | **1578.0844** | **1577.7652** | **0.3191** | **2** | **16** | **62** | **1** | **NLSSASQATRQKMR** |
|  | 243 | **384.1952** | **766.3755** | **765.8328** | **0.5428** | **0** | **16** | **51** | **1** | **CSTGSPSK** |
|  | 3199 | **801.2012** | **2400.5813** | **2400.8446** | **-0.2632** | **1** | **16** | **63** | **1** | **MGGSRGVAALCIGGGMGIAMCVQR + Carbamidomethyl (C); 3 Oxidation (M)** |
|  | 334 | **388.2594** | **774.5040** | **774.8263** | **-0.3222** | **1** | **16** | **62** | **1** | **NVRGSSR** |
|  | 71 | **368.2263** | **1101.6569** | **1101.2141** | **0.4427** | **0** | **16** | **56** | **1** | **YCGSQCQQK + Carbamidomethyl (C)** |
|  | 417 | **400.1086** | **1197.3035** | **1197.4011** | **-0.0976** | **1** | **16** | **58** | **1** | **MIGFLTEDKK + Oxidation (M)** |
|  | 1591 | **518.2987** | **1551.8739** | **1552.7673** | **-0.8934** | **2** | **16** | **65** | **1** | **AKDSYSLALSKLEK** |
|  | 184 | **375.1970** | **748.3791** | **747.7115** | **0.6677** | **0** | **16** | **60** | **1** | **DQADGSR** |
|  | 322 | **387.8793** | **773.7438** | **772.8568** | **0.8871** | **2** | **16** | **78** | **1** | **RRGQTR** |
|  | 478 | **403.8471** | **1208.5191** | **1209.3935** | **-0.8743** | **1** | **16** | **66** | **1** | **ITSVKNSWFK** |
|  | 1312 | **482.0018** | **961.9888** | **961.1109** | **0.8779** | **0** | **16** | **69** | **1** | **VTISVDLSK** |
|  | 1990 | **575.3783** | **1148.7418** | **1148.2294** | **0.5125** | **1** | **16** | **63** | **1** | **RTEYGVGNPR** |
|  | 66 | **367.4168** | **1099.2283** | **1098.2946** | **0.9336** | **0** | **16** | **86** | **1** | **WTPELAILR** |
|  | 287 | **386.1476** | **1155.4207** | **1156.2844** | **-0.8638** | **0** | **16** | **59** | **1** | **TLFEDAGYLK** |
|  | 1244 | **472.5308** | **1414.5703** | **1414.6084** | **-0.0381** | **0** | **16** | **79** | **1** | **MCSGWSSSVIWR + Oxidation (M)** |
|  | 1356 | **488.1090** | **1461.3049** | **1461.7941** | **-0.4892** | **2** | **16** | **73** | **1** | **VKAAPGPKAALAVIR** |
|  | 756 | **421.0886** | **1260.2435** | **1259.4787** | **0.7648** | **2** | **16** | **64** | **1** | **AEVRKALANCK + Carbamidomethyl (C)** |
|  | 2263 | 609.6999 | 1217.3850 | 1216.3065 | 1.0784 | 1 | 16 | 81 | 1 | ELFRSTGHGGR |
|  | 2548 | **657.9232** | **1970.7475** | **1971.2409** | **-0.4934** | **1** | **16** | **51** | **1** | **MHVLDLGGSFPGTEGAKVR** |
|  | 2599 | **666.7784** | **1997.3132** | **1998.2928** | **-0.9796** | **2** | **16** | **79** | **1** | **MREMHRAATFIQSTFR + Oxidation (M)** |
|  | 2719 | **684.1079** | **2049.3016** | **2049.4204** | **-0.1189** | **1** | **16** | **64** | **1** | **YLCPFACLQKCSVSCGR + 3 Carbamidomethyl (C)** |
|  | 337 | **388.3436** | **1162.0085** | **1162.3419** | **-0.3334** | **1** | **16** | **70** | **1** | **THLKIHTGQK** |
|  | 658 | **411.2745** | **1230.8013** | **1231.5714** | **-0.7701** | **0** | **16** | **56** | **1** | **LVMALMPVGLR + 2 Oxidation (M)** |
|  | 1375 | **489.5945** | **977.1742** | **977.0324** | **0.1418** | **0** | **16** | **81** | **1** | **ESPSAPPHR** |
|  | 1337 | **486.4106** | **1456.2096** | **1455.6190** | **0.5905** | **2** | **16** | **52** | **1** | **RTSEQITHRLSK** |
|  | 404 | **399.1608** | **1194.4603** | **1193.4256** | **1.0348** | **2** | **16** | **58** | **1** | **KGLVPQRCHR** |
|  | 615 | **408.1555** | **1221.4444** | **1221.2569** | **0.1875** | **0** | **16** | **69** | **1** | **HFGDEDQAMR + Oxidation (M)** |
|  | 2248 | **608.6850** | **1823.0328** | **1822.8835** | **0.1494** | **2** | **16** | **83** | **1** | **WEEGDSLDARGGKTSSK** |
|  | 700 | **416.3806** | **830.7464** | **831.0187** | **-0.2722** | **0** | **16** | **74** | **1** | **CCQPKPR** |
|  | 800 | **427.9991** | **1280.9753** | **1280.4930** | **0.4823** | **1** | **16** | **65** | **1** | **MFDDLVQLRK + Oxidation (M)** |
|  | 1292 | **478.8551** | **1433.5431** | **1434.6063** | **-1.0632** | **2** | **16** | **65** | **1** | **ALQRLQAREGHR** |
|  | 956 | **443.2494** | **1326.7260** | **1325.6214** | **1.1046** | **1** | **16** | **57** | **1** | **CLGEMKCTLVR + Carbamidomethyl (C); Oxidation (M)** |
|  | 1448 | **499.7480** | **1496.2220** | **1495.7275** | **0.4945** | **1** | **16** | **51** | **1** | **MMITRGWEGWGR + Oxidation (M)** |
|  | 1852 | **549.4512** | **1096.8877** | **1097.3712** | **-0.4836** | **1** | **16** | **52** | **1** | **PCGKIPILEK** |
|  | 2354 | **619.8818** | **1237.7489** | **1237.3653** | **0.3836** | **0** | **16** | **50** | **1** | **GTNIQHIALDR** |
|  | 813 | **428.9880** | **1283.9419** | **1284.3358** | **-0.3938** | **0** | **16** | **63** | **1** | **QGGGGGGGSVPGIER** |
|  | 203 | **377.1468** | **752.2787** | **752.9433** | **-0.6645** | **0** | **16** | **62** | **1** | **CTMAALK + Oxidation (M)** |
|  | 1650 | **523.1609** | **1044.3071** | **1043.2643** | **1.0428** | **2** | **16** | **71** | **1** | **KITLSRAVR** |
|  | 2559 | **659.5579** | **1975.6514** | **1976.1512** | **-0.4998** | **1** | **16** | **55** | **1** | **MAYCDQIFQDETGKNR + Carbamidomethyl (C)** |
|  | 3326 | **854.2004** | **1706.3861** | **1705.9127** | **0.4734** | **0** | **16** | **54** | **1** | **PMGPGGMNQSGPPPPPR + 2 Oxidation (M)** |
|  | 1459 | **499.9594** | **1496.8561** | **1495.7273** | **1.1287** | **0** | **16** | **64** | **1** | **HQLSHPGLPGALLR** |
|  | 3264 | **818.6077** | **1635.2006** | **1635.0019** | **0.1986** | **2** | **16** | **62** | **1** | **KGLKDGTILCTLMNK** |
|  | 2038 | **584.4228** | **1166.8308** | **1166.3076** | **0.5232** | **1** | **16** | **56** | **1** | **YGAKMEAPQR + Oxidation (M)** |
|  | 449 | **402.0041** | **801.9933** | **800.8835** | **1.1099** | **0** | **16** | **79** | **1** | **MAEAHAR + Oxidation (M)** |
|  | 2728 | **684.2795** | **1366.5442** | **1367.4690** | **-0.9249** | **0** | **16** | **66** | **1** | **WPGSHLATSQQR** |
|  | 654 | **411.1429** | **1230.4064** | **1230.3497** | **0.0567** | **0** | **16** | **72** | **1** | **LSCAASGFDFGR** |
|  | 1667 | **524.3802** | **1046.7457** | **1047.2613** | **-0.5155** | **2** | **16** | **59** | **1** | **LPRRRPPR** |
|  | 189 | **376.0650** | **1125.1727** | **1126.2899** | **-1.1172** | **0** | **16** | **64** | **1** | **TGVLQGHCVR + Carbamidomethyl (C)** |
|  | 2777 | **692.1267** | **2073.3580** | **2073.4864** | **-0.1285** | **2** | **16** | **64** | **1** | **INASGGLILRMGAINRCLK + Carbamidomethyl (C); Oxidation (M)** |
|  | 724 | **418.7446** | **835.4745** | **835.9491** | **-0.4746** | **0** | **16** | **73** | **1** | **CGECTAPR** |
|  | 934 | **437.2739** | **872.5329** | **872.9229** | **-0.3899** | **1** | **16** | **63** | **1** | **ENNPSGKK** |
|  | 2388 | **627.7515** | **1880.2322** | **1879.0975** | **1.1347** | **1** | **16** | **76** | **1** | **EAVARTSVNTVSTQSLLC** |
|  | 2887 | **732.4512** | **1462.8876** | **1462.6714** | **0.2162** | **2** | **16** | **67** | **1** | **AADEQMALSKVRK + Oxidation (M)** |
|  | 2546 | **657.5303** | **1969.5686** | **1969.9877** | **-0.4191** | **1** | **16** | **54** | **1** | **AGHGHSAESSRQSGTHHAR** |
|  | 116 | **369.4211** | **1105.2413** | **1106.2540** | **-1.0127** | **1** | **16** | **85** | **1** | **RLGMGEGGVSK + Oxidation (M)** |
|  | 2415 | **631.4547** | **1260.8946** | **1260.4667** | **0.4279** | **1** | **16** | **67** | **1** | **TGSCQGLRLAVR** |
|  | 2784 | **696.4464** | **1390.8780** | **1390.5869** | **0.2911** | **1** | **16** | **72** | **1** | **NLLSLGFDRVTR** |
|  | 325 | **387.9154** | **1160.7240** | **1160.4968** | **0.2272** | **1** | **16** | **85** | **1** | **VIRMLIMPR + 2 Oxidation (M)** |
|  | 2741 | **685.3663** | **1368.7179** | **1367.5602** | **1.1576** | **2** | **16** | **73** | **1** | **QAAGIRSQKPRR** |
|  | 647 | **410.9209** | **1229.7404** | **1230.2802** | **-0.5398** | **0** | **16** | **74** | **1** | **YSAADQTSPYK** |
|  | 2812 | **706.1277** | **1410.2407** | **1409.6501** | **0.5907** | **1** | **16** | **67** | **1** | **YLSVKSLPEAMR + Oxidation (M)** |
|  | 156 | **373.8982** | **745.7817** | **745.8495** | **-0.0678** | **0** | **16** | **90** | **1** | **WPAGCR + Carbamidomethyl (C)** |
|  | 357 | **389.1970** | **776.3793** | **775.8773** | **0.5020** | **1** | **16** | **66** | **1** | **NAMRER** |
|  | 1278 | **476.6441** | **1426.9102** | **1427.7365** | **-0.8262** | **1** | **16** | **64** | **1** | **AVRMGMGAVFFNK** |
|  | 1743 | **534.8041** | **1067.5934** | **1067.2211** | **0.3723** | **1** | **16** | **61** | **1** | **KFQEACWR** |
|  | 2154 | **597.9291** | **1790.7651** | **1791.0354** | **-0.2704** | **2** | **16** | **58** | **1** | **LRASVSTKCNLEDQVK** |
|  | 2848 | **715.7717** | **2144.2928** | **2144.4503** | **-0.1575** | **1** | **16** | **80** | **1** | **QNMGQHLDVKLVPSSSYIK** |
|  | 299 | **386.8937** | **1157.6590** | **1158.3750** | **-0.7159** | **2** | **16** | **77** | **1** | **AMAAPVKGNRK + Oxidation (M)** |
|  | 947 | **441.1650** | **880.3153** | **880.0065** | **0.3088** | **1** | **16** | **70** | **1** | **GGAPGPLRR** |
|  | 1350 | **487.2152** | **1458.6234** | **1457.6316** | **0.9917** | **2** | **16** | **76** | **1** | **RTLKSGLTPEEAR** |
|  | 414 | **400.0659** | **798.1169** | **797.9424** | **0.1746** | **1** | **16** | **61** | **1** | **KGLLPDR** |
|  | 2465 | **642.2989** | **1923.8747** | **1923.1332** | **0.7415** | **1** | **16** | **67** | **1** | **AKEWCPGNMGDWSALGK + Carbamidomethyl (C); Oxidation (M)** |
|  | 869 | **433.1154** | **864.2159** | **863.9641** | **0.2519** | **0** | **16** | **71** | **1** | **QPPRPGGR** |
|  | 1920 | **562.1747** | **1122.3346** | **1121.3330** | **1.0015** | **1** | **16** | **71** | **1** | **ELLVHLNKR** |
|  | 2619 | **668.7477** | **1335.4806** | **1336.5116** | **-1.0310** | **0** | **16** | **86** | **1** | **SLTGESLNGMVTK** |
|  | 706 | **417.0386** | **1248.0935** | **1247.4680** | **0.6255** | **2** | **16** | **81** | **1** | **DMKTAIRQLR + Oxidation (M)** |
|  | 1097 | **458.7830** | **1373.3267** | **1372.4558** | **0.8710** | **2** | **16** | **68** | **1** | **RRGSGAQHHGGPR** |
|  | 20 | **362.3159** | **1083.9255** | **1084.2466** | **-0.3212** | **0** | **16** | **58** | **1** | **MAELEHLGGK** |
|  | 326 | **387.9925** | **773.9703** | **773.8548** | **0.1155** | **0** | **16** | **87** | **1** | **DPACSGPK** |
|  | 453 | **402.0303** | **802.0459** | **801.9974** | **0.0485** | **0** | **16** | **83** | **1** | **KPTGMIR** |
|  | 2640 | **671.9170** | **2012.7288** | **2012.1703** | **0.5585** | **1** | **16** | **57** | **1** | **GSGSHMASMTGGQQMGRGSR + 2 Oxidation (M)** |
|  | 3316 | **849.0591** | **1696.1035** | **1696.8596** | **-0.7561** | **1** | **16** | **63** | **1** | **DRQVMNMWSEQEK + Oxidation (M)** |
|  | 2912 | **740.5836** | **2218.7285** | **2217.5506** | **1.1779** | **0** | **16** | **61** | **1** | **QMQALGIAMCSVHGWVQER + Carbamidomethyl (C); Oxidation (M)** |
|  | 519 | **405.3226** | **1212.9455** | **1213.4268** | **-0.4813** | **1** | **16** | **54** | **1** | **KEFSACAIGCK + Carbamidomethyl (C)** |
|  | 1259 | **474.7100** | **1421.1077** | **1421.6671** | **-0.5594** | **0** | **16** | **63** | **1** | **MGNVLLQALGPHR + Oxidation (M)** |
|  | 509 | **405.0190** | **808.0231** | **808.9883** | **-0.9651** | **0** | **16** | **66** | **1** | **CVALTFR** |
|  | 3125 | **786.0489** | **2355.1247** | **2354.5375** | **0.5871** | **1** | **16** | **57** | **1** | **ASGYSFSSYGIHWVRQAPGQR** |
|  | 535 | **406.0199** | **810.0250** | **809.9365** | **0.0884** | **1** | **16** | **66** | **1** | **CYRAAAR** |
|  | 2726 | **684.2623** | **1366.5099** | **1365.4948** | **1.0151** | **1** | **16** | **71** | **1** | **RPSGISSRFSGSK** |
|  | 1661 | **524.0466** | **1569.1175** | **1568.7403** | **0.3773** | **2** | **16** | **77** | **1** | **HTGGVRHECGECRK** |
|  | 842 | **430.9667** | **1289.8779** | **1290.4298** | **-0.5518** | **1** | **16** | **89** | **1** | **RGAAFGFPGASPR** |
|  | 85 | **369.2482** | **1104.7225** | **1105.2227** | **-0.5002** | **0** | **16** | **63** | **1** | **QDGALMGEIR + Oxidation (M)** |
|  | 482 | **403.9417** | **1208.8029** | **1209.3570** | **-0.5542** | **2** | **16** | **75** | **1** | **SYRSYLRHK** |
|  | 743 | **419.3916** | **836.7684** | **835.9259** | **0.8426** | **0** | **16** | **79** | **1** | **TMDALNR + Oxidation (M)** |
|  | 973 | **443.8845** | **885.7541** | **884.9368** | **0.8173** | **0** | **16** | **76** | **1** | **THNALSSR** |
|  | 1116 | **459.7632** | **1376.2676** | **1375.4398** | **0.8277** | **0** | **16** | **66** | **1** | **DLTFLHEGNDSK** |
|  | 3118 | **780.1166** | **2337.3276** | **2336.4463** | **0.8812** | **0** | **16** | **57** | **1** | **IQNDSVAIETQAITDNCSEER** |
|  | 255 | **384.9797** | **767.9446** | **766.8854** | **1.0592** | **0** | **16** | **63** | **1** | **SLPTPPR** |
|  | 1126 | **460.4992** | **1378.4754** | **1378.5762** | **-0.1008** | **1** | **16** | **92** | **1** | **EAILAIHKEAQR** |
|  | 1268 | **475.9484** | **949.8821** | **951.0381** | **-1.1560** | **0** | **16** | **76** | **1** | **QVDSIPHR** |
|  | 1533 | **508.0005** | **1520.9792** | **1519.8319** | **1.1473** | **1** | **16** | **74** | **1** | **CLKVMGSYCPSCR + Carbamidomethyl (C); Oxidation (M)** |
|  | 2661 | **676.7401** | **1351.4654** | **1352.5985** | **-1.1331** | **0** | **16** | **92** | **1** | **AMTSLYGQLAGLK** |
|  | 2716 | **684.0482** | **2049.1225** | **2050.1901** | **-1.0676** | **2** | **16** | **66** | **1** | **SLSRWMSSSRWSESSCT + Carbamidomethyl (C); Oxidation (M)** |
|  | 567 | **407.0347** | **1218.0818** | **1217.4122** | **0.6696** | **1** | **16** | **66** | **1** | **SQTKLTIGLEK** |
|  | 808 | **428.2885** | **1281.8434** | **1281.4611** | **0.3822** | **0** | **16** | **51** | **1** | **GAASAAAPGALVAVR** |
|  | 1402 | **494.1545** | **986.2942** | **985.1156** | **1.1785** | **1** | **16** | **77** | **1** | **GYTGQKTCK** |
|  | 1901 | **558.0338** | **1671.0793** | **1671.9395** | **-0.8603** | **1** | **16** | **74** | **1** | **LEACPGAVAMTPEARR** |
|  | 1080 | **457.6313** | **913.2477** | **913.0297** | **0.2180** | **0** | **16** | **63** | **1** | **ASLAADIPR** |
|  | 1131 | **460.6828** | **1379.0263** | **1378.4919** | **0.5344** | **2** | **16** | **63** | **1** | **SKRSSNTSTLAAR** |
|  | 1394 | **492.8871** | **983.7595** | **983.1658** | **0.5937** | **0** | **16** | **75** | **1** | **LAILQVGNR** |
|  | 1302 | **480.2716** | **1437.7926** | **1436.6969** | **1.0958** | **1** | **16** | **71** | **1** | **ELTLQNMKMANK + Oxidation (M)** |
|  | 2359 | **621.2321** | **1860.6740** | **1860.1188** | **0.5552** | **1** | **16** | **71** | **1** | **WSSLKASDTAMYICAR + Carbamidomethyl (C)** |
|  | 308 | **387.0026** | **771.9903** | **772.7672** | **-0.7768** | **0** | **16** | **82** | **1** | **GGAGGGAGGGR** |
|  | 534 | **405.9908** | **1214.9501** | **1215.3964** | **-0.4464** | **2** | **16** | **67** | **1** | **LQEAEIKKEK** |
|  | 1165 | **464.0941** | **926.1733** | **927.0397** | **-0.8664** | **0** | **16** | **69** | **1** | **EMLAHGGGR** |
|  | 1204 | 468.2981 | 1401.8721 | 1401.7173 | 0.1548 | 1 | 16 | 67 | 1 | MQVRVSLLALAGK + Oxidation (M) |
|  | 2416 | **631.8042** | **1892.3904** | **1893.2366** | **-0.8461** | **0** | **16** | **80** | **1** | **AAAIGWMPVASGPMPAPPR + Oxidation (M)** |
|  | 581 | **407.4204** | **812.8260** | **813.9816** | **-1.1556** | **0** | **16** | **82** | **1** | **LIVTSPGK** |
|  | 755 | **421.0261** | **840.0373** | **839.9129** | **0.1245** | **0** | **16** | **66** | **1** | **FCAGEEK + Carbamidomethyl (C)** |
|  | 1871 | **552.6788** | **1655.0143** | **1653.9159** | **1.0984** | **2** | **16** | **91** | **1** | **QGFLVKYIATKEEK** |
|  | 867 | **433.0753** | **864.1357** | **864.9422** | **-0.8064** | **0** | **16** | **74** | **1** | **DLDSIFR** |
|  | 157 | **374.0247** | **1119.0518** | **1119.3158** | **-0.2640** | **1** | **16** | **93** | **1** | **VGDTKHGMMK + Oxidation (M)** |
|  | 2781 | **696.0879** | **2085.2415** | **2086.4127** | **-1.1712** | **1** | **16** | **75** | **1** | **QFPKQSLMLMATSNEGCK + Carbamidomethyl (C); Oxidation (M)** |
|  | 234 | **380.1456** | **1137.4146** | **1138.3604** | **-0.9457** | **1** | **16** | **82** | **1** | **EKVLQGLVPR** |
|  | 562 | **406.9399** | **1217.7975** | **1218.2794** | **-0.4820** | **2** | **16** | **67** | **1** | **EGRTWERER** |
|  | 794 | **427.8270** | **1280.4589** | **1279.3789** | **1.0800** | **0** | **16** | **76** | **1** | **FLPCENGGGSGGK + Carbamidomethyl (C)** |
|  | 1149 | **461.9125** | **1382.7153** | **1382.4788** | **0.2365** | **0** | **16** | **73** | **1** | **TAGYCSGGSCYTGR** |
|  | 1792 | **539.2694** | **1614.7861** | **1615.8744** | **-1.0884** | **1** | **16** | **79** | **1** | **AIVVHGAHLKDIQSK** |
|  | 2245 | **607.8523** | **1820.5347** | **1820.1894** | **0.3453** | **2** | **16** | **61** | **1** | **AVLRGVTVMMQSRSLR + Oxidation (M)** |
|  | 2311 | **613.1436** | **1224.2723** | **1224.3204** | **-0.0481** | **2** | **16** | **72** | **1** | **RDKLSGEYEK** |
|  | 905 | **435.7425** | **869.4703** | **870.0282** | **-0.5580** | **0** | **15** | **60** | **1** | **HVAEAICK** |
|  | 193 | **376.2089** | **1125.6046** | **1125.1943** | **0.4103** | **0** | **15** | **58** | **1** | **GYPSPGAHSPR** |
|  | 751 | **420.3242** | **838.6336** | **838.9942** | **-0.3607** | **0** | **15** | **57** | **1** | **LNPLVQR** |
|  | 839 | **430.9247** | **1289.7521** | **1289.3821** | **0.3699** | **2** | **15** | **90** | **1** | **AEGGEGRCRAAR + Carbamidomethyl (C)** |
|  | 1952 | **566.7838** | **1131.5528** | **1132.2283** | **-0.6754** | **1** | **15** | **64** | **1** | **LSSQGNVSGKR** |
|  | 2116 | **593.8386** | **1778.4937** | **1778.8972** | **-0.4035** | **2** | **15** | **66** | **1** | **GPDDRNPISFRMDDK + Oxidation (M)** |
|  | 3080 | **768.8050** | **1535.5952** | **1534.8003** | **0.7949** | **1** | **15** | **76** | **1** | **EVQSNMVPRSMLK + Oxidation (M)** |
|  | 2093 | **592.7579** | **1775.2516** | **1775.9596** | **-0.7079** | **0** | **15** | **78** | **1** | **EQSRPFVMGYMNER + 2 Oxidation (M)** |
|  | 359 | **389.2625** | **776.5101** | **776.8620** | **-0.3518** | **1** | **15** | **67** | **1** | **CRDAAGK + Carbamidomethyl (C)** |
|  | 526 | **405.7636** | **809.5125** | **809.8703** | **-0.3578** | **0** | **15** | **71** | **1** | **DHNGLVR** |
|  | 2946 | **742.7054** | **2225.0940** | **2225.4584** | **-0.3645** | **1** | **15** | **59** | **1** | **GFRGNGIDCEPITSCLEQTGK** |
|  | 259 | **385.0356** | **1152.0847** | **1151.2980** | **0.7867** | **2** | **15** | **63** | **1** | **MRKAASAASSR + Oxidation (M)** |
|  | 898 | **435.1920** | **1302.5537** | **1302.5828** | **-0.0291** | **0** | **15** | **71** | **1** | **SCLLLGATIGVAK + Carbamidomethyl (C)** |
|  | 1380 | **491.4435** | **1471.3085** | **1471.5257** | **-0.2173** | **1** | **15** | **61** | **1** | **EPREGGGAIEEEAK** |
|  | 2429 | 632.6989 | 1895.0744 | 1894.0952 | 0.9792 | 1 | 15 | 97 | 1 | LATNLSFQSLNPRYNR |
|  | 451 | **402.0117** | **802.0087** | **801.9312** | **0.0775** | **2** | **15** | **89** | **1** | **KAVGKGDK** |
|  | 2376 | **623.7538** | **1868.2394** | **1869.1935** | **-0.9542** | **1** | **15** | **90** | **1** | **QKMTSLSSCFAQLCHK + Carbamidomethyl (C)** |
|  | 473 | **403.3596** | **1207.0567** | **1207.2287** | **-0.1720** | **0** | **15** | **75** | **1** | **VSSNQDPDSCR** |
|  | 689 | **415.8194** | **829.6240** | **829.0194** | **0.6046** | **0** | **15** | **87** | **1** | **LLMPSPR + Oxidation (M)** |
|  | 3217 | **806.5008** | **2416.4802** | **2415.5910** | **0.8892** | **2** | **15** | **71** | **1** | **AASDKGSSCSSSRQNLTLSLSSTK** |
|  | 1534 | **508.1772** | **1521.5096** | **1521.7782** | **-0.2686** | **0** | **15** | **80** | **1** | **ITEMCPPLNCSEK + Carbamidomethyl (C)** |
|  | 1904 | **558.4426** | **1672.3057** | **1671.8069** | **0.4988** | **0** | **15** | **65** | **1** | **LPVGSGSAGEAAASLGTAR** |
|  | 3381 | **963.9043** | **1925.7938** | **1926.1936** | **-0.3998** | **0** | **15** | **54** | **1** | **MYPELTLPLFSEVSQR + Oxidation (M)** |
|  | 48 | **365.5098** | **1093.5072** | **1094.2697** | **-0.7624** | **1** | **15** | **82** | **1** | **GGRMTLGSCR + Carbamidomethyl (C)** |
|  | 2867 | **724.2532** | **1446.4916** | **1446.7795** | **-0.2879** | **0** | **15** | **75** | **1** | **MPGIITVGCFPVR + Carbamidomethyl (C)** |
|  | 249 | **384.7273** | **1151.1598** | **1150.2651** | **0.8947** | **0** | **15** | **65** | **1** | **TTMPTSGGGWR** |
|  | 601 | **407.9059** | **1220.6954** | **1221.4904** | **-0.7950** | **0** | **15** | **77** | **1** | **VLMLNSNMVGK + Oxidation (M)** |
|  | 1003 | **447.3518** | **892.6888** | **892.9508** | **-0.2620** | **0** | **15** | **65** | **1** | **LSVDSTGSK** |
|  | 2035 | **584.0535** | **1166.0922** | **1165.4520** | **0.6402** | **1** | **15** | **76** | **1** | **HAMMMKSWK + Oxidation (M)** |
|  | 2754 | **686.1461** | **2055.4162** | **2055.3853** | **0.0308** | **1** | **15** | **75** | **1** | **MCRPQPALQSLPLESRR + Carbamidomethyl (C); Oxidation (M)** |
|  | 696 | **416.2085** | **1245.6032** | **1245.3429** | **0.2603** | **1** | **15** | **83** | **1** | **IVRASQESEAR** |
|  | 1947 | **565.9037** | **1694.6889** | **1694.8123** | **-0.1235** | **1** | **15** | **72** | **1** | **DIDSEKEAAMEAEIK + Oxidation (M)** |
|  | 2932 | **741.7258** | **2222.1551** | **2221.5345** | **0.6207** | **0** | **15** | **63** | **1** | **SIMGQFDHPNVIHLEGVVTK** |
|  | 3307 | **845.5230** | **1689.0312** | **1688.8391** | **0.1922** | **1** | **15** | **75** | **1** | **SSGKHYGQFTCEGCK + Carbamidomethyl (C)** |
|  | 41 | **364.2495** | **1089.7262** | **1090.4862** | **-0.7601** | **1** | **15** | **55** | **1** | **LMKIILLCK + Oxidation (M)** |
|  | 814 | **428.9897** | **1283.9471** | **1283.5647** | **0.3824** | **0** | **15** | **73** | **1** | **MNGLCCMALDR + Carbamidomethyl (C)** |
|  | 173 | **374.3292** | **1119.9654** | **1120.3979** | **-0.4325** | **1** | **15** | **85** | **1** | **RCCGCNCCCR** |
|  | 442 | **401.9015** | **801.7882** | **801.8484** | **-0.0602** | **1** | **15** | **90** | **1** | **ASRSPER** |
|  | 2010 | **578.8766** | **1155.7385** | **1156.3755** | **-0.6370** | **0** | **15** | **64** | **1** | **LGLLQVTGVTR** |
|  | 2779 | **693.0681** | **2076.1822** | **2075.4544** | **0.7277** | **1** | **15** | **69** | **1** | **KCLLQSGAPAVPQLLHMPD + Carbamidomethyl (C)** |
|  | 1216 | **470.1053** | **938.1957** | **937.0544** | **1.1413** | **0** | **15** | **71** | **1** | **QELIAHAR** |
|  | 2581 | **665.8381** | **1994.4922** | **1995.1976** | **-0.7054** | **1** | **15** | **80** | **1** | **VAGALVQNTEKGPNAEQLR** |
|  | 2766 | **688.3921** | **2062.1541** | **2062.2737** | **-0.1196** | **2** | **15** | **80** | **1** | **SPGREPGRDHLNGVAMNVR** |
|  | 427 | **401.0919** | **800.1691** | **798.9704** | **1.1987** | **1** | **15** | **88** | **1** | **EKPAKVK** |
|  | 762 | **422.0940** | **842.1732** | **841.9949** | **0.1783** | **1** | **15** | **86** | **1** | **SLPKIER** |
|  | 328 | **388.0869** | **1161.2384** | **1160.3642** | **0.8742** | **0** | **15** | **94** | **1** | **WVLTPAAFAGK** |
|  | 379 | **393.3053** | **1176.8938** | **1176.3718** | **0.5220** | **1** | **15** | **60** | **1** | **RRPHELLQK** |
|  | 1767 | **537.2475** | **1608.7203** | **1609.7770** | **-1.0567** | **0** | **15** | **89** | **1** | **DLLAYAFALAGNQDK** |
|  | 445 | **401.9213** | **801.8278** | **800.9429** | **0.8849** | **0** | **15** | **91** | **1** | **ALESILR** |
|  | 2926 | **740.8816** | **1479.7484** | **1480.7079** | **-0.9595** | **1** | **15** | **88** | **1** | **EASIDILHSIVKR** |
|  | 3122 | **784.7288** | **1567.4427** | **1567.8930** | **-0.4502** | **1** | **15** | **61** | **1** | **DICLKMSMPIASSR + Oxidation (M)** |
|  | 1127 | **460.5056** | **918.9964** | **920.0638** | **-1.0674** | **1** | **15** | **1e+02** | **1** | **IPSAGGKYK** |
|  | 2360 | **621.3234** | **1860.9481** | **1861.2113** | **-0.2632** | **2** | **15** | **77** | **1** | **IKTVDISMILSEAIRR + Oxidation (M)** |
|  | 559 | **406.8028** | **1217.3863** | **1216.3843** | **1.0020** | **1** | **15** | **75** | **1** | **ISKLAANISGDK** |
|  | 1085 | **458.1156** | **914.2164** | **913.9749** | **0.2416** | **1** | **15** | **81** | **1** | **GKGDGVPER** |
|  | 2508 | **651.3387** | **1950.9941** | **1950.1837** | **0.8104** | **2** | **15** | **78** | **1** | **SSRGRGSFGMQVVSVGGPGK** |
|  | 2068 | **589.6041** | **1177.1935** | **1177.3948** | **-0.2013** | **1** | **15** | **91** | **1** | **KPLKCEDCGK + Carbamidomethyl (C)** |
|  | 1294 | **478.8809** | **955.7470** | **955.1525** | **0.5945** | **1** | **15** | **78** | **1** | **KILLSPER** |
|  | 2310 | **613.0830** | **1836.2269** | **1836.0264** | **0.2004** | **2** | **15** | **79** | **1** | **SALDEIMEIEEEKKR + Oxidation (M)** |
|  | 2672 | **678.9077** | **1355.8006** | **1355.6425** | **0.1582** | **0** | **15** | **65** | **1** | **MPPPVSSAVILTK + Oxidation (M)** |
|  | 481 | **403.8992** | **805.7836** | **806.8448** | **-1.0613** | **0** | **15** | **80** | **1** | **ANSEACR + Carbamidomethyl (C)** |
|  | 579 | **407.3022** | **1218.8844** | **1218.4019** | **0.4824** | **1** | **15** | **60** | **1** | **EAARIIAEAFK** |
|  | 636 | **408.9922** | **815.9697** | **816.8994** | **-0.9296** | **0** | **15** | **89** | **1** | **TVNIADGK** |
|  | 803 | **428.2513** | **1281.7319** | **1281.4214** | **0.3104** | **0** | **15** | **58** | **1** | **RPVAAAAAGSASPR** |
|  | 2127 | **595.0910** | **1782.2508** | **1781.0852** | **1.1657** | **0** | **15** | **86** | **1** | **QIPCSSPGCCLSFPSVR** |
|  | 2287 | **611.3793** | **1220.7438** | **1221.3925** | **-0.6488** | **1** | **15** | **81** | **1** | **THCGRGLGGVHK** |
|  | 3355 | **893.7461** | **1785.4774** | **1784.8803** | **0.5971** | **1** | **15** | **68** | **1** | **EMEAQSCEQETTARR + Oxidation (M)** |
|  | 371 | **391.2628** | **1170.7661** | **1171.3471** | **-0.5810** | **1** | **15** | **58** | **1** | **QAVELLGKASR** |
|  | 554 | **406.6588** | **1216.9541** | **1216.4306** | **0.5234** | **1** | **15** | **59** | **1** | **GHTFKLWLSK** |
|  | 1624 | **520.1852** | **1557.5334** | **1557.6199** | **-0.0865** | **0** | **15** | **76** | **1** | **DGGPPPPGPGPAEEGAR** |
|  | 2586 | **666.5050** | **1330.9952** | **1330.5568** | **0.4385** | **1** | **15** | **69** | **1** | **LAVNRSPVAMTR + Oxidation (M)** |
|  | 201 | **377.1156** | **1128.3245** | **1127.2547** | **1.0698** | **1** | **15** | **76** | **1** | **LPSNRGNTLR** |
|  | 284 | **386.1076** | **770.2004** | **769.9307** | **0.2697** | **2** | **15** | **74** | **1** | **KKFGYK** |
|  | 610 | **408.0326** | **1221.0756** | **1220.3136** | **0.7621** | **1** | **15** | **82** | **1** | **MRAQEDLEGR + Oxidation (M)** |
|  | 3107 | **777.9208** | **1553.8268** | **1552.6382** | **1.1886** | **0** | **15** | **87** | **1** | **NFGDLVSIQSESEK** |
|  | 2213 | **601.7776** | **1802.3108** | **1802.1457** | **0.1650** | **2** | **15** | **81** | **1** | **CPWREVLSKTQTLLK** |
|  | 2300 | **612.3740** | **1222.7333** | **1223.3192** | **-0.5859** | **1** | **15** | **81** | **1** | **EHSSAYSMRR** |
|  | 2593 | **666.6038** | **1996.7891** | **1996.2255** | **0.5636** | **1** | **15** | **66** | **1** | **RSCDMVFGPANLGEDAIK + Carbamidomethyl (C); Oxidation (M)** |
|  | 2649 | **672.8063** | **2015.3966** | **2016.2433** | **-0.8466** | **1** | **15** | **92** | **1** | **GDNVSWRHPTMGSVFIGR** |
|  | 506 | **404.9871** | **807.9595** | **809.0099** | **-1.0504** | **0** | **15** | **75** | **1** | **TVLMACR + Oxidation (M)** |
|  | 1862 | **551.6378** | **1101.2607** | **1101.0851** | **0.1757** | **0** | **15** | **1e+02** | **1** | **GDPGSQGSPGSR** |
|  | 2617 | **668.6744** | **2003.0011** | **2003.3106** | **-0.3095** | **2** | **15** | **80** | **1** | **HRHMACDYRFISLAPL + Carbamidomethyl (C); Oxidation (M)** |
|  | 56 | 366.1620 | 1095.4639 | 1096.2043 | -0.7404 | 2 | 15 | 80 | 1 | PRGRGGGGGGLR |
|  | 462 | **402.9255** | **1205.7543** | **1205.3900** | **0.3643** | **2** | **15** | **92** | **1** | **RMWNDTVRK** |
|  | 1707 | **529.0942** | **1056.1736** | **1056.2135** | **-0.0399** | **1** | **15** | **81** | **1** | **LTNPQVKEK** |
|  | 82 | **369.2198** | **736.4247** | **736.7949** | **-0.3701** | **0** | **15** | **71** | **1** | **EAGSSMR** |
|  | 855 | **432.0109** | **1293.0104** | **1292.5435** | **0.4670** | **1** | **15** | **90** | **1** | **LCDVTLKSFAVP** |
|  | 1089 | **458.2336** | **914.4523** | **914.0361** | **0.4162** | **0** | **15** | **82** | **1** | **MSFWSEK** |
|  | 595 | **407.7883** | **1220.3428** | **1220.3567** | **-0.0138** | **1** | **15** | **83** | **1** | **VDQVGRCATSK + Carbamidomethyl (C)** |
|  | 86 | **369.2560** | **736.4971** | **735.8499** | **0.6473** | **1** | **15** | **71** | **1** | **NMEAKK + Oxidation (M)** |
|  | 225 | **379.2023** | **1134.5847** | **1134.2406** | **0.3441** | **0** | **15** | **66** | **1** | **YIHPQYNSI** |
|  | 44 | **364.3753** | **1090.1038** | **1090.0624** | **0.0415** | **0** | **15** | **89** | **1** | **VNGEGGSGGNSR** |
|  | 2817 | **707.9092** | **1413.8036** | **1413.5127** | **0.2908** | **0** | **15** | **75** | **1** | **TNASFAGCSLTER + Carbamidomethyl (C)** |
|  | 197 | **377.0698** | **752.1247** | **751.8062** | **0.3186** | **0** | **15** | **79** | **1** | **EAASSCK + Carbamidomethyl (C)** |
|  | 366 | **390.0003** | **777.9858** | **778.8316** | **-0.8457** | **0** | **15** | **84** | **1** | **DACDTVR** |
|  | 607 | **407.9596** | **1220.8566** | **1221.4705** | **-0.6138** | **0** | **15** | **84** | **1** | **IMSCGMDHSLK** |
|  | 683 | **414.9216** | **1241.7427** | **1242.4879** | **-0.7452** | **0** | **15** | **81** | **1** | **MAATLILEPAGR** |
|  | 603 | **407.9489** | **1220.8247** | **1221.3446** | **-0.5199** | **0** | **15** | **84** | **1** | **GEGPCACPDCGR + Carbamidomethyl (C)** |
|  | 2585 | **666.4377** | **1330.8607** | **1330.6178** | **0.2429** | **2** | **15** | **81** | **1** | **ILIPKGSYGRVK** |
|  | 160 | 374.0737 | 1119.1988 | 1119.2214 | -0.0225 | 0 | 15 | 1e+02 | 1 | EEEIVFPEK |
|  | 1179 | **465.8698** | **929.7248** | **928.9895** | **0.7354** | **1** | **15** | **86** | **1** | **KAQPQDSR** |
|  | 3252 | **816.8687** | **1631.7226** | **1631.8522** | **-0.1295** | **1** | **15** | **89** | **1** | **NVKIWGLDFGDCHK** |
|  | 2212 | **601.6698** | **1201.3248** | **1202.4009** | **-1.0761** | **1** | **15** | **1e+02** | **1** | **QRTLTTLLEK** |
|  | 749 | **420.2012** | **1257.5814** | **1256.4549** | **1.1266** | **1** | **15** | **76** | **1** | **MLCGGGAFNSKR + Oxidation (M)** |
|  | 747 | **419.9379** | **1256.7915** | **1257.3107** | **-0.5192** | **2** | **15** | **77** | **1** | **DPDEVRKGDAR** |
|  | 391 | **396.3523** | **1186.0346** | **1186.3221** | **-0.2874** | **2** | **15** | **84** | **1** | **CEDCGRRYK + Carbamidomethyl (C)** |
|  | 625 | **408.5773** | **1222.7096** | **1223.5078** | **-0.7982** | **0** | **15** | **84** | **1** | **MLPGYALAMTR** |
|  | 628 | **408.7957** | **815.5765** | **815.8781** | **-0.3016** | **1** | **15** | **88** | **1** | **QTAGGRAR** |
|  | 1189 | **467.1981** | **1398.5722** | **1398.5593** | **0.0129** | **0** | **15** | **88** | **1** | **ASLGGLQPYTTYK** |
|  | 2966 | **747.8448** | **2240.5122** | **2239.6819** | **0.8303** | **1** | **15** | **96** | **1** | **VLQVGGRYLCISLAQAHILK + Carbamidomethyl (C)** |
|  | 3117 | **778.5411** | **2332.6012** | **2333.6643** | **-1.0631** | **1** | **15** | **78** | **1** | **VLPVMHESHHAQSEYIKSLK** |
|  | 1436 | **499.4699** | **1495.3876** | **1495.5868** | **-0.1992** | **1** | **15** | **70** | **1** | **TERELLESYIDGA** |
|  | 1595 | **518.5565** | **1552.6474** | **1553.7025** | **-1.0551** | **1** | **15** | **1e+02** | **1** | **QVQDRMVPSAHNR + Oxidation (M)** |
|  | 2402 | **629.7441** | **1886.2103** | **1886.1992** | **0.0110** | **1** | **15** | **1e+02** | **1** | **AGVYKLTGAIMHYGNMK + 2 Oxidation (M)** |
|  | 3376 | **927.1056** | **1852.1964** | **1852.0619** | **0.1345** | **2** | **15** | **84** | **1** | **AHSQTDREWLRIALR** |
|  | 153 | **373.1965** | **744.3783** | **744.7938** | **-0.4155** | **1** | **15** | **86** | **1** | **ESPERK** |
|  | 670 | **413.3177** | **824.6207** | **825.8201** | **-1.1993** | **0** | **15** | **58** | **1** | **QDGESYK** |
|  | 691 | **416.0000** | **829.9852** | **830.0504** | **-0.0652** | **1** | **15** | **1e+02** | **1** | **GCVRALVL** |
|  | 1398 | **493.7101** | **1478.1082** | **1477.6889** | **0.4192** | **0** | **15** | **70** | **1** | **IGCNHPLPGPGPYR** |
|  | 3338 | **869.2563** | **1736.4978** | **1736.9435** | **-0.4457** | **1** | **15** | **73** | **1** | **KVASMAPVTAEGFQER + Oxidation (M)** |
|  | 1631 | **521.1151** | **1560.3232** | **1560.6668** | **-0.3436** | **0** | **15** | **81** | **1** | **YTSWSPMGCDANGR + Oxidation (M)** |
|  | 2158 | **598.4042** | **1792.1904** | **1791.9555** | **0.2349** | **1** | **15** | **79** | **1** | **KINNITVSFEGNPSGSK** |
|  | 2236 | **606.7511** | **1817.2311** | **1816.0814** | **1.1498** | **1** | **15** | **90** | **1** | **YLYEPVYPKGSPQMK + Oxidation (M)** |
|  | 323 | **387.8924** | **1160.6549** | **1160.3031** | **0.3519** | **1** | **15** | **1e+02** | **1** | **MKGTDSGSCCR + Oxidation (M)** |
|  | 866 | **432.9940** | **1295.9598** | **1296.5319** | **-0.5721** | **1** | **15** | **86** | **1** | **QYESLKILICS** |
|  | 1503 | **505.0889** | **1512.2446** | **1511.6344** | **0.6103** | **2** | **15** | **82** | **1** | **NVDPKEYEKYAR** |
|  | 222 | **378.3344** | **1131.9811** | **1132.2879** | **-0.3069** | **0** | **15** | **64** | **1** | **TTEICGAPGVGK** |
|  | 589 | **407.6615** | **1219.9622** | **1220.4194** | **-0.4572** | **0** | **15** | **68** | **1** | **TLCATDALCPR + Carbamidomethyl (C)** |
|  | 585 | **407.4918** | **1219.4531** | **1220.3965** | **-0.9434** | **1** | **15** | **99** | **1** | **SRVTMSLDPSK** |
|  | 2900 | **738.2776** | **2211.8106** | **2211.4965** | **0.3141** | **2** | **15** | **85** | **1** | **TWYVGKINRTQAEEMLSGK** |
|  | 278 | **386.0228** | **1155.0461** | **1154.3183** | **0.7279** | **0** | **15** | **73** | **1** | **MSLNSSLSCR + Carbamidomethyl (C)** |
|  | 2111 | **593.6339** | **1185.2529** | **1185.3076** | **-0.0546** | **0** | **15** | **1e+02** | **1** | **GHEITEQPMK + Oxidation (M)** |
|  | 2230 | **606.3411** | **1816.0012** | **1815.1078** | **0.8934** | **0** | **15** | **83** | **1** | **WCLILCATCGSHGTHR + Carbamidomethyl (C)** |
|  | 2315 | **613.3765** | **1837.1074** | **1838.0272** | **-0.9198** | **2** | **15** | **84** | **1** | **ISRRVLNEDGYSSTIK** |
|  | 2305 | **612.8391** | **1835.4950** | **1836.2099** | **-0.7150** | **1** | **15** | **72** | **1** | **RILALCMGNHELYMR + Oxidation (M)** |
|  | 1029 | **450.5837** | **1348.7290** | **1348.5223** | **0.2067** | **0** | **15** | **95** | **1** | **TNGLGLDTSPVMK + Oxidation (M)** |
|  | 1066 | **457.2050** | **1368.5929** | **1368.5830** | **0.0098** | **2** | **15** | **88** | **1** | **VSLRNLRGYYK** |
|  | 2303 | **612.6466** | **1834.9176** | **1834.2089** | **0.7087** | **0** | **15** | **96** | **1** | **LYLCPSFPHQSLMLK + Carbamidomethyl (C)** |
|  | 3038 | **757.8342** | **2270.4803** | **2270.7193** | **-0.2390** | **1** | **15** | **95** | **1** | **AARMAFAECIAPACVMSWLR + Carbamidomethyl (C); Oxidation (M)** |
|  | 901 | **435.4325** | **1303.2754** | **1304.3837** | **-1.1082** | **0** | **15** | **85** | **1** | **EEAMTDLPAGDR** |
|  | 125 | **371.0146** | **1110.0216** | **1109.1967** | **0.8249** | **2** | **15** | **66** | **1** | **FRTRTADSR** |
|  | 938 | **437.5506** | **873.0864** | **871.9777** | **1.1087** | **0** | **15** | **1.1e+02** | **1** | **LGQEQGLK** |
|  | 3192 | **799.0049** | **1595.9950** | **1595.7772** | **0.2178** | **0** | **15** | **78** | **1** | **TTGSNIPMDQPCCR + Carbamidomethyl (C); Oxidation (M)** |
|  | 1588 | **518.0685** | **1034.1223** | **1033.2496** | **0.8727** | **2** | **15** | **93** | **1** | **RKMTSAIAR** |
|  | 2101 | **593.1946** | **1776.5616** | **1775.9661** | **0.5955** | **2** | **15** | **92** | **1** | **RFPPYHVGQTFDRR** |
|  | 424 | **400.4175** | **1198.2303** | **1197.4491** | **0.7812** | **0** | **15** | **96** | **1** | **CIMQATDIMR + Oxidation (M)** |
|  | 1063 | **456.6840** | **1367.0299** | **1367.5456** | **-0.5157** | **0** | **15** | **63** | **1** | **NMEIEVSVAECK + Oxidation (M)** |
|  | 669 | **413.2556** | **1236.7446** | **1237.4913** | **-0.7468** | **0** | **15** | **61** | **1** | **QVLAALLPVTGR** |
|  | 2065 | **589.1063** | **1764.2968** | **1764.9500** | **-0.6532** | **0** | **15** | **93** | **1** | **MDAELEFAIQPNTTGK** |
|  | 3137 | **791.4942** | **2371.4604** | **2370.8931** | **0.5673** | **1** | **15** | **87** | **1** | **SCRLLLYVSLCLVETALMNTK** |
|  | 319 | **387.8203** | **1160.4386** | **1159.2735** | **1.1650** | **0** | **15** | **1.1e+02** | **1** | **MSPAAAAAGAGER** |
|  | 690 | **415.9111** | **829.8075** | **828.9564** | **0.8510** | **1** | **15** | **1e+02** | **1** | **AALAAERK** |
|  | 1822 | **544.0024** | **1628.9850** | **1627.8438** | **1.1412** | **1** | **15** | **98** | **1** | **DSVADPHNLKICCR + Carbamidomethyl (C)** |
|  | 1659 | **523.8909** | **1568.6504** | **1567.8118** | **0.8387** | **0** | **15** | **91** | **1** | **FGMGSAQACPCQVPR + Oxidation (M)** |
|  | 1780 | **538.2450** | **1074.4752** | **1074.2155** | **0.2598** | **1** | **15** | **92** | **1** | **ARSSGPAAMAR** |
|  | 2126 | **594.9650** | **1187.9152** | **1188.4374** | **-0.5222** | **0** | **15** | **92** | **1** | **DVVAIIMAVNK + Oxidation (M)** |
|  | 2517 | **653.5480** | **1957.6217** | **1958.2504** | **-0.6286** | **1** | **15** | **74** | **1** | **MPRPGPFSVDPRHHLSK** |
|  | 2409 | **630.5792** | **1259.1437** | **1258.4212** | **0.7224** | **2** | **15** | **73** | **1** | **EQLGAVKEEKK** |
|  | 3319 | **849.2814** | **1696.5480** | **1696.9654** | **-0.4175** | **0** | **15** | **80** | **1** | **GPLPSLSPTMQAGTIAR** |
|  | 3024 | **753.0551** | **1504.0954** | **1503.8324** | **0.2630** | **1** | **15** | **69** | **1** | **MAVRLCDVASLLR + Carbamidomethyl (C)** |
|  | 556 | **406.7393** | **1217.1956** | **1216.4541** | **0.7415** | **2** | **15** | **76** | **1** | **MKKSQGVGPIR + Oxidation (M)** |
|  | 286 | **386.1271** | **770.2394** | **769.9539** | **0.2856** | **0** | **15** | **82** | **1** | **MCASACK + Carbamidomethyl (C)** |
|  | 2521 | **654.1460** | **1306.2772** | **1305.5222** | **0.7550** | **1** | **15** | **87** | **1** | **LLKLANDVTYR** |
|  | 3361 | **898.2389** | **1794.4630** | **1793.9283** | **0.5347** | **0** | **15** | **68** | **1** | **TSGYTFTSFDINWVR** |
|  | 30 | **363.1360** | **724.2573** | **723.8392** | **0.4181** | **1** | **15** | **88** | **1** | **SMNTKK + Oxidation (M)** |
|  | 2362 | **621.4274** | **1861.2599** | **1862.1330** | **-0.8731** | **1** | **15** | **81** | **1** | **ELVFWSDVTLDRILR** |
|  | 2551 | **659.0673** | **1974.1796** | **1974.2660** | **-0.0864** | **0** | **15** | **89** | **1** | **TWLPGEGAVQNHVLALLR** |
|  | 915 | **436.1468** | **870.2787** | **870.0118** | **0.2670** | **2** | **15** | **92** | **1** | **KSRAVPGR** |
|  | 2685 | **680.0680** | **2037.1818** | **2037.2756** | **-0.0938** | **1** | **15** | **85** | **1** | **AEAQEYGNKLVFGAGTILR** |
|  | 1807 | **540.7795** | **1619.3163** | **1618.8353** | **0.4809** | **2** | **15** | **73** | **1** | **LHEDHARTKTLLGK** |
|  | 2397 | **629.4206** | **1256.8264** | **1256.4482** | **0.3782** | **0** | **15** | **88** | **1** | **VASELINILER** |
|  | 2453 | **637.4741** | **1909.4002** | **1909.1234** | **0.2768** | **0** | **15** | **83** | **1** | **VSCLCLEENVENDPCK + 2 Carbamidomethyl (C)** |
|  | 1048 | **454.3486** | **1360.0236** | **1359.6379** | **0.3858** | **1** | **15** | **77** | **1** | **EMARVVVTVISR** |
|  | 33 | **363.1577** | **1086.4511** | **1085.2975** | **1.1535** | **2** | **15** | **83** | **1** | **ILNGGKDIKK** |
|  | 264 | **385.1193** | **768.2237** | **767.8718** | **0.3520** | **0** | **15** | **80** | **1** | **MCDQQK + Oxidation (M)** |
|  | 1731 | **533.0461** | **1064.0775** | **1063.2292** | **0.8483** | **1** | **15** | **92** | **1** | **RSLMDGLGAK + Oxidation (M)** |
|  | 2637 | **671.4554** | **1340.8961** | **1340.5284** | **0.3678** | **2** | **15** | **87** | **1** | **QPVQESLAKGRK** |
|  | 468 | **403.0959** | **1206.2655** | **1205.2592** | **1.0063** | **0** | **15** | **1e+02** | **1** | **GQSMNHTSATR + Oxidation (M)** |
|  | 1681 | **526.6515** | **1576.9323** | **1577.8499** | **-0.9176** | **2** | **15** | **1e+02** | **1** | **MAGGSPAAKRVVVYR + Oxidation (M)** |
|  | 74 | **368.3841** | **1102.1302** | **1103.1886** | **-1.0584** | **1** | **15** | **1.2e+02** | **1** | **RATADAWGQK** |
|  | 1464 | **500.4611** | **1498.3610** | **1497.6209** | **0.7402** | **2** | **15** | **75** | **1** | **AGHQAHGPRGPEKR** |
|  | 437 | **401.7274** | **801.4401** | **801.9312** | **-0.4911** | **2** | **15** | **94** | **1** | **AKNKDVK** |
|  | 599 | **407.8600** | **1220.5578** | **1219.3901** | **1.1677** | **0** | **15** | **91** | **1** | **MDPFHNMAPK + 2 Oxidation (M)** |
|  | 1151 | **462.0283** | **1383.0626** | **1382.5666** | **0.4960** | **2** | **15** | **88** | **1** | **HKTGLFEHKSAK** |
|  | 2100 | 593.0111 | 1776.0112 | 1776.0205 | -0.0094 | 1 | 15 | 94 | 1 | LYCKQDYQQLFAAK + Carbamidomethyl (C) |
|  | 782 | **425.3688** | **1273.0843** | **1272.3385** | **0.7459** | **0** | **15** | **83** | **1** | **LAEMYGGGESDK + Oxidation (M)** |
|  | 1873 | **553.2088** | **1656.6042** | **1655.9035** | **0.7008** | **1** | **15** | **98** | **1** | **RWCGMTGGACSCPR + 3 Carbamidomethyl (C)** |
|  | 2244 | **607.5964** | **1819.7671** | **1820.9389** | **-1.1717** | **2** | **15** | **84** | **1** | **SNMDRRQAEIGEGSVR + Oxidation (M)** |
|  | 3314 | **848.4366** | **1694.8585** | **1694.0576** | **0.8009** | **2** | **15** | **89** | **1** | **VARPPALMLRAQKSR** |
|  | 530 | **405.9506** | **809.8865** | **808.9007** | **0.9858** | **0** | **15** | **84** | **1** | **MVDATTR + Oxidation (M)** |
|  | 1658 | **523.8037** | **1045.5926** | **1045.2786** | **0.3141** | **0** | **15** | **80** | **1** | **MCMECLSR + Carbamidomethyl (C); Oxidation (M)** |
|  | 1842 | **547.3922** | **1639.1545** | **1639.9769** | **-0.8225** | **0** | **15** | **75** | **1** | **MIINLALFGMTQSGK + Oxidation (M)** |
|  | 1878 | **554.1224** | **1106.2301** | **1107.3331** | **-1.1030** | **1** | **15** | **97** | **1** | **MVRAPQLHR** |
|  | 518 | **405.2726** | **1212.7957** | **1213.2116** | **-0.4160** | **0** | **15** | **69** | **1** | **DAQEESIHER** |
|  | 725 | **418.9739** | **1253.8995** | **1253.2723** | **0.6272** | **0** | **15** | **1.1e+02** | **1** | **ESPSSTGSSTWK** |
|  | 3200 | **801.2818** | **2400.8232** | **2400.8396** | **-0.0163** | **0** | **15** | **93** | **1** | **MNCDVCGLSCISFNVLMVHK + 3 Carbamidomethyl (C); Oxidation (M)** |
|  | 91 | **369.2674** | **736.5201** | **736.8793** | **-0.3592** | **0** | **15** | **80** | **1** | **AFQMPK + Oxidation (M)** |
|  | 613 | **408.0548** | **1221.1423** | **1220.3714** | **0.7708** | **1** | **15** | **95** | **1** | **LQEALKDFEK** |
|  | 799 | **427.9966** | **1280.9675** | **1281.3718** | **-0.4043** | **1** | **15** | **90** | **1** | **YVLSGSGREEGK** |
|  | 1750 | **536.1215** | **1070.2281** | **1071.1916** | **-0.9635** | **2** | **15** | **93** | **1** | **SSSLTRHKR** |
|  | 2724 | **684.2172** | **1366.4195** | **1365.5545** | **0.8651** | **0** | **15** | **89** | **1** | **RPSSSLYCPVEK** |
|  | 3298 | **836.9926** | **1671.9704** | **1670.8701** | **1.1004** | **1** | **15** | **1e+02** | **1** | **WVHRPGKGSQGAFFP** |
|  | 1305 | **480.5384** | **959.0619** | **959.0553** | **0.0067** | **0** | **15** | **1.2e+02** | **1** | **TADVVQQAK** |
|  | 1397 | **493.5385** | **1477.5934** | **1477.6460** | **-0.0526** | **2** | **15** | **1.1e+02** | **1** | **WAGNEKNMVRAW + Oxidation (M)** |
|  | 2944 | **742.6671** | **2224.9792** | **2224.4739** | **0.5053** | **1** | **15** | **70** | **1** | **VSCKASGYTFSDAYMHWVR + Oxidation (M)** |
|  | 461 | **402.8485** | **1205.5234** | **1206.3945** | **-0.8712** | **2** | **15** | **1e+02** | **1** | **LKSGGYQAVKR** |
|  | 557 | **406.7875** | **811.5602** | **810.9379** | **0.6223** | **0** | **15** | **88** | **1** | **KPDPNIK** |
|  | 663 | **412.2730** | **822.5313** | **822.9934** | **-0.4621** | **0** | **15** | **77** | **1** | **TMPLSCR + Oxidation (M)** |
|  | 731 | **419.1667** | **1254.4780** | **1253.3833** | **1.0947** | **0** | **15** | **1.1e+02** | **1** | **MTAIVSSTGGTGR + Oxidation (M)** |
|  | 2629 | **669.7627** | **2006.2659** | **2006.2152** | **0.0507** | **0** | **15** | **1.1e+02** | **1** | **GAPGALLEYWGQGTLVTVSS** |
|  | 811 | **428.7765** | **855.5383** | **855.8990** | **-0.3606** | **1** | **15** | **79** | **1** | **RGSGHQSK** |
|  | 1738 | **534.0562** | **1066.0975** | **1065.1360** | **0.9616** | **2** | **15** | **99** | **1** | **SSTKETERK** |
|  | 89 | **369.2656** | **736.5163** | **736.8793** | **-0.3629** | **0** | **15** | **81** | **1** | **AFQMPK + Oxidation (M)** |
|  | 338 | **388.4183** | **774.8218** | **773.8349** | **0.9869** | **1** | **15** | **1.3e+02** | **1** | **RAAEEAK** |
|  | 611 | **408.0429** | **814.0711** | **812.9569** | **1.1142** | **0** | **15** | **95** | **1** | **AAGALAALR** |
|  | 961 | **443.4477** | **1327.3208** | **1328.4713** | **-1.1505** | **0** | **15** | **1e+02** | **1** | **VQVPGATESATIR** |
|  | 2670 | **678.7443** | **1355.4739** | **1356.6074** | **-1.1335** | **1** | **15** | **1.1e+02** | **1** | **SIVEVKVLDVQK** |
|  | 1629 | **521.0021** | **1559.9841** | **1559.7484** | **0.2357** | **2** | **15** | **87** | **1** | **KGMPFRQAHEASGK + Oxidation (M)** |
|  | 2734 | **685.0945** | **2052.2615** | **2051.3934** | **0.8681** | **0** | **15** | **96** | **1** | **NMQCPKPDCMYLHELR + Carbamidomethyl (C); Oxidation (M)** |
|  | 1826 | **545.0339** | **1632.0796** | **1632.8519** | **-0.7722** | **2** | **15** | **1e+02** | **1** | **NFRAKHHCNSCCR + Carbamidomethyl (C)** |
|  | 3387 | **971.2314** | **1940.4481** | **1940.2698** | **0.1783** | **2** | **15** | **72** | **1** | **LASRLCFLQEERTFVK** |
|  | 804 | **428.2624** | **1281.7650** | **1281.4413** | **0.3237** | **1** | **15** | **67** | **1** | **GPEPCLPEGRAR** |
|  | 2289 | **612.0535** | **1222.0922** | **1221.3199** | **0.7723** | **1** | **15** | **96** | **1** | **EPERVVHNEI** |
|  | 1258 | **474.5699** | **947.1250** | **946.1060** | **1.0190** | **0** | **15** | **1.2e+02** | **1** | **QPCACPATR** |
|  | 1406 | **494.2441** | **986.4734** | **987.1084** | **-0.6350** | **1** | **15** | **97** | **1** | **DLVRDLEK** |
|  | 2171 | **599.0917** | **1794.2529** | **1795.0042** | **-0.7514** | **0** | **15** | **89** | **1** | **QSATAGTPAMAGGAMALTR + 2 Oxidation (M)** |
|  | 541 | **406.0837** | **1215.2289** | **1216.3018** | **-1.0730** | **2** | **14** | **91** | **1** | **AARREVEEEK** |
|  | 885 | **434.2457** | **1299.7150** | **1299.4398** | **0.2752** | **0** | **14** | **76** | **1** | **TSCNLHNHAMR + Oxidation (M)** |
|  | 1737 | **534.0141** | **1599.0201** | **1599.8055** | **-0.7854** | **0** | **14** | **1e+02** | **1** | **AEETMEQPLWIPR** |
|  | 2796 | **700.4537** | **2098.3390** | **2097.3742** | **0.9649** | **0** | **14** | **90** | **1** | **MAAVHDLEMESMNLNMGR + 3 Oxidation (M)** |
|  | 2738 | **685.2599** | **2052.7575** | **2052.2953** | **0.4622** | **1** | **14** | **99** | **1** | **EAFQLQGMPNSSGLMNRR + Oxidation (M)** |
|  | 734 | **419.1969** | **836.3790** | **835.9889** | **0.3901** | **0** | **14** | **1.1e+02** | **1** | **MCSPASPK + Oxidation (M)** |
|  | 843 | **430.9867** | **859.9585** | **860.8690** | **-0.9105** | **0** | **14** | **1.2e+02** | **1** | **DLNSQER** |
|  | 991 | **445.6724** | **1333.9949** | **1333.4909** | **0.5040** | **1** | **14** | **89** | **1** | **GILGTLNKSTSSR** |
|  | 1306 | **480.7455** | **1439.2144** | **1438.6035** | **0.6109** | **1** | **14** | **82** | **1** | **FDIFSRYESMK + Oxidation (M)** |
|  | 1467 | **500.9018** | **999.7888** | **1000.2363** | **-0.4474** | **2** | **14** | **94** | **1** | **KASIVKNLK** |
|  | 2026 | **581.3085** | **1740.9032** | **1741.0609** | **-0.1577** | **0** | **14** | **1e+02** | **1** | **ALPGMGTTIDVILINGR** |
|  | 2443 | **635.6971** | **1269.3795** | **1270.3870** | **-1.0075** | **0** | **14** | **1.1e+02** | **1** | **SQEFYELLNK** |
|  | 3235 | **811.5292** | **2431.5655** | **2430.6953** | **0.8703** | **2** | **14** | **92** | **1** | **MEYEDKAGRPSKPPSPKQNVR + Oxidation (M)** |
|  | 281 | **386.0576** | **770.1005** | **770.0152** | **0.0854** | **1** | **14** | **87** | **1** | **IVKVALK** |
|  | 1214 | **469.8799** | **1406.6175** | **1406.5616** | **0.0559** | **1** | **14** | **90** | **1** | **QGKEGSMIDANLK + Oxidation (M)** |
|  | 3066 | **764.5875** | **1527.1601** | **1526.6722** | **0.4880** | **1** | **14** | **83** | **1** | **RMISTAGLTSGSSSR + Oxidation (M)** |
|  | 2105 | **593.2863** | **1776.8366** | **1777.9771** | **-1.1405** | **2** | **14** | **1e+02** | **1** | **GLKAEMEDMRGQQER** |
|  | 2061 | **587.6772** | **1760.0096** | **1761.0229** | **-1.0133** | **1** | **14** | **1.2e+02** | **1** | **VIDDSLVVGVKTTSSLK** |
|  | 1052 | **454.8925** | **907.7703** | **908.0099** | **-0.2397** | **0** | **14** | **1e+02** | **1** | **EDLPGHLK** |
|  | 1263 | **475.6602** | **1423.9586** | **1424.5205** | **-0.5619** | **1** | **14** | **90** | **1** | **SPRTPEGWGGQPR** |
|  | 2747 | **686.0323** | **1370.0498** | **1369.5246** | **0.5252** | **1** | **14** | **81** | **1** | **KESLGHWSQGLK** |
|  | 911 | **436.0272** | **1305.0595** | **1305.4348** | **-0.3753** | **2** | **14** | **93** | **1** | **SGAEVKKEGSSVK** |
|  | 2795 | **700.3823** | **1398.7499** | **1397.6625** | **1.0874** | **0** | **14** | **91** | **1** | **GEMGVAGPMGLPGPK** |
|  | 2954 | **743.5334** | **2227.5782** | **2226.4899** | **1.0883** | **1** | **14** | **96** | **1** | **VINKHSTEVTVGPEGDMPCR + Carbamidomethyl (C)** |
|  | 370 | **391.2277** | **1170.6609** | **1171.2710** | **-0.6100** | **2** | **14** | **71** | **1** | **NRTAAGGNRVR** |
|  | 2182 | **599.5520** | **1795.6338** | **1796.0518** | **-0.4180** | **0** | **14** | **77** | **1** | **CAEMIISMDSSQIHSK + Oxidation (M)** |
|  | 3074 | **766.2984** | **2295.8730** | **2296.5894** | **-0.7164** | **0** | **14** | **90** | **1** | **VELSMEDIETILNTLIYDGK** |
|  | 916 | **436.1569** | **870.2989** | **870.9534** | **-0.6544** | **1** | **14** | **99** | **1** | **QDLPRSR** |
|  | 1246 | **472.8797** | **1415.6168** | **1415.6379** | **-0.0211** | **1** | **14** | **99** | **1** | **SMYLCASSLERR** |
|  | 2539 | **655.7405** | **1964.1993** | **1965.1536** | **-0.9543** | **1** | **14** | **1.2e+02** | **1** | **DPRFCAFVQEAESRPR + Carbamidomethyl (C)** |
|  | 2651 | **673.4476** | **2017.3207** | **2016.3010** | **1.0197** | **0** | **14** | **96** | **1** | **VCLLGCGFSTGYGAAINNAK + Carbamidomethyl (C)** |
|  | 646 | **410.5243** | **1228.5507** | **1229.5173** | **-0.9666** | **2** | **14** | **1.2e+02** | **1** | **LAAGVKFRVLR** |
|  | 2336 | **616.5723** | **1231.1298** | **1231.4222** | **-0.2925** | **0** | **14** | **84** | **1** | **VTLGQPASISCR** |
|  | 223 | **378.4267** | **1132.2578** | **1132.3393** | **-0.0814** | **2** | **14** | **1e+02** | **1** | **HMEFRKLR + Oxidation (M)** |
|  | 958 | **443.3191** | **884.6234** | **884.8906** | **-0.2672** | **0** | **14** | **74** | **1** | **GPQTDDPR** |
|  | 2472 | **643.4103** | **1927.2087** | **1927.2068** | **0.0019** | **2** | **14** | **94** | **1** | **CEFVMEVTNKTRADVK + Carbamidomethyl (C)** |
|  | 2851 | **716.1868** | **1430.3588** | **1429.6196** | **0.7391** | **0** | **14** | **98** | **1** | **IHLASASGATINFK** |
|  | 352 | **389.1326** | **1164.3757** | **1164.1313** | **0.2445** | **0** | **14** | **1.1e+02** | **1** | **EDTVEEEEGK** |
|  | 568 | **407.0712** | **1218.1915** | **1219.3901** | **-1.1986** | **0** | **14** | **93** | **1** | **MDPFHNMAPK + 2 Oxidation (M)** |
|  | 1368 | **488.7845** | **975.5542** | **975.1306** | **0.4235** | **2** | **14** | **84** | **1** | **ACSLGRRR + Carbamidomethyl (C)** |
|  | 764 | **422.0979** | **842.1810** | **842.0612** | **0.1198** | **1** | **14** | **1.1e+02** | **1** | **GIHIMKK + Oxidation (M)** |
|  | 2172 | **599.1024** | **1794.2851** | **1795.0042** | **-0.7192** | **0** | **14** | **93** | **1** | **QSATAGTPAMAGGAMALTR + 2 Oxidation (M)** |
|  | 226 | **379.2935** | **1134.8582** | **1134.2226** | **0.6356** | **0** | **14** | **77** | **1** | **VCPNGYDPGGR** |
|  | 1529 | **507.5549** | **1013.0950** | **1012.1311** | **0.9639** | **2** | **14** | **1.2e+02** | **1** | **RRPTGGRGR** |
|  | 1818 | **543.2599** | **1084.5050** | **1084.2269** | **0.2781** | **0** | **14** | **99** | **1** | **ESLVIDRPR** |
|  | 2121 | **594.4376** | **1186.8603** | **1187.2189** | **-0.3586** | **0** | **14** | **88** | **1** | **DLDWSHASTR** |
|  | 2580 | **665.5931** | **1993.7572** | **1994.3453** | **-0.5881** | **2** | **14** | **79** | **1** | **RGCQVVGLYRLCGSAAVK + 2 Carbamidomethyl (C)** |
|  | 3136 | **790.3585** | **2368.0534** | **2366.8861** | **1.1673** | **2** | **14** | **94** | **1** | **NLKLVPLQVLICSYKCFTQR** |
|  | 1999 | **576.8689** | **1151.7230** | **1152.3636** | **-0.6406** | **0** | **14** | **77** | **1** | **AFNLCSVLTK + Carbamidomethyl (C)** |
|  | 2495 | **648.3701** | **1942.0882** | **1941.1585** | **0.9296** | **2** | **14** | **97** | **1** | **CRGGGAMCAGEEERCAQGR** |
|  | 263 | **385.0843** | **1152.2308** | **1151.3540** | **0.8767** | **0** | **14** | **87** | **1** | **GICGMELLDGK + Oxidation (M)** |
|  | 367 | **390.5670** | **779.1193** | **779.8857** | **-0.7664** | **0** | **14** | **83** | **1** | **QPPWPR** |
|  | 850 | **431.7286** | **1292.1636** | **1291.4577** | **0.7059** | **1** | **14** | **89** | **1** | **DFRNKPYPVR** |
|  | 1976 | **572.5434** | **1143.0720** | **1144.2391** | **-1.1670** | **1** | **14** | **90** | **1** | **ENKPSGRQTK** |
|  | 1421 | **496.3625** | **1486.0652** | **1486.7126** | **-0.6474** | **1** | **14** | **86** | **1** | **FYFMSPCEKYR + Oxidation (M)** |
|  | 2083 | **591.5040** | **1771.4897** | **1771.1336** | **0.3561** | **1** | **14** | **78** | **1** | **MWTMSSWTMKMMR + 4 Oxidation (M)** |
|  | 847 | **431.1044** | **860.1940** | **859.0949** | **1.0990** | **2** | **14** | **1.2e+02** | **1** | **ACLRIRK** |
|  | 2148 | **597.3116** | **1788.9128** | **1789.0993** | **-0.1865** | **1** | **14** | **1.1e+02** | **1** | **MTLSSAIDSVDKVPVVK** |
|  | 560 | **406.8767** | **811.7386** | **812.8710** | **-1.1323** | **0** | **14** | **88** | **1** | **GPRPSGDK** |
|  | 1423 | **496.5419** | **1486.6035** | **1486.7623** | **-0.1587** | **1** | **14** | **1.3e+02** | **1** | **AVMQSQKPPKNCR** |
|  | 80 | **369.1759** | **1104.5054** | **1105.2543** | **-0.7488** | **1** | **14** | **94** | **1** | **AGRAPGPRPAR** |
|  | 452 | **402.0175** | **802.0203** | **802.9391** | **-0.9188** | **0** | **14** | **1.2e+02** | **1** | **IPAEMAR + Oxidation (M)** |
|  | 1067 | **457.2537** | **1368.7389** | **1368.3759** | **0.3631** | **1** | **14** | **94** | **1** | **GGGGGGGGGGGGRGPQAR** |
|  | 1420 | **496.2447** | **1485.7119** | **1485.6930** | **0.0189** | **2** | **14** | **1.1e+02** | **1** | **HCGKAFSRSSFCR** |
|  | 2398 | **629.6251** | **1257.2355** | **1257.5293** | **-0.2938** | **2** | **14** | **95** | **1** | **KSGKMVCHPVR + Oxidation (M)** |
|  | 1583 | **517.1100** | **1032.2053** | **1031.1180** | **1.0874** | **1** | **14** | **1.1e+02** | **1** | **EAKVAAADEK** |
|  | 1133 | **460.7967** | **1379.3678** | **1378.5300** | **0.8379** | **0** | **14** | **94** | **1** | **TSPSSLVAFINSR** |
|  | 1801 | **540.2928** | **1617.8564** | **1616.8362** | **1.0202** | **1** | **14** | **1e+02** | **1** | **IFKANHPMDAEVTK + Oxidation (M)** |
|  | 2096 | **592.8246** | **1183.6344** | **1183.2934** | **0.3410** | **2** | **14** | **85** | **1** | **KNSSSSSKACK + Carbamidomethyl (C)** |
|  | 2711 | **683.9558** | **2048.8453** | **2049.4204** | **-0.5752** | **1** | **14** | **80** | **1** | **YLCPFACLQKCSVSCGR + 3 Carbamidomethyl (C)** |
|  | 2032 | **583.2736** | **1164.5323** | **1164.2072** | **0.3252** | **0** | **14** | **1.1e+02** | **1** | **SPASQGACSGSR + Carbamidomethyl (C)** |
|  | 3127 | **788.5585** | **1575.1022** | **1574.6669** | **0.4352** | **0** | **14** | **94** | **1** | **DEAIHCPPYSEEK + Carbamidomethyl (C)** |
|  | 216 | **378.1575** | **1131.4502** | **1132.2515** | **-0.8012** | **1** | **14** | **86** | **1** | **LDRMGSNPAR + Oxidation (M)** |
|  | 2723 | **684.2056** | **1366.3963** | **1365.4948** | **0.9016** | **1** | **14** | **96** | **1** | **GLTRGPTQGPPER** |
|  | 3198 | **800.8057** | **1599.5965** | **1599.8473** | **-0.2507** | **2** | **14** | **86** | **1** | **KVATSKSVQVDYMK + Oxidation (M)** |
|  | 321 | **387.8716** | **773.7284** | **772.8468** | **0.8817** | **0** | **14** | **1.2e+02** | **1** | **GPLGSGASK** |
|  | 2366 | **621.8490** | **1862.5248** | **1862.1330** | **0.3918** | **1** | **14** | **85** | **1** | **ELVFWSDVTLDRILR** |
|  | 2530 | **654.9846** | **1307.9545** | **1308.4847** | **-0.5302** | **0** | **14** | **82** | **1** | **GVGAEPLLPWNR** |
|  | 2602 | **666.8549** | **1997.5426** | **1998.2859** | **-0.7433** | **2** | **14** | **97** | **1** | **ICQRYDQLMEAWEKK + Carbamidomethyl (C)** |
|  | 342 | **389.0210** | **1164.0408** | **1164.3728** | **-0.3320** | **1** | **14** | **1.1e+02** | **1** | **INKTIMEATK + Oxidation (M)** |
|  | 1418 | **495.5855** | **1483.7343** | **1483.6487** | **0.0855** | **1** | **14** | **1.3e+02** | **1** | **DILSGLCERHGIN + Carbamidomethyl (C)** |
|  | 2002 | **577.1194** | **1152.2241** | **1152.2841** | **-0.0600** | **1** | **14** | **99** | **1** | **RLCFEGSQR + Carbamidomethyl (C)** |
|  | 1008 | **448.1822** | **1341.5244** | **1340.4655** | **1.0589** | **2** | **14** | **1e+02** | **1** | **MNFAEREGSKR + Oxidation (M)** |
|  | 2752 | **686.1162** | **2055.3265** | **2054.4205** | **0.9060** | **1** | **14** | **99** | **1** | **MMKNRPFMGSISQQNIR + Oxidation (M)** |
|  | 606 | **407.9574** | **1220.8499** | **1221.4043** | **-0.5543** | **0** | **14** | **1e+02** | **1** | **ELMDLPTCGAR + Oxidation (M)** |
|  | 1042 | **452.4606** | **1354.3595** | **1353.5621** | **0.7975** | **2** | **14** | **1.2e+02** | **1** | **EDDKMEALKMK + Oxidation (M)** |
|  | 2853 | **716.2598** | **1430.5048** | **1429.3848** | **1.1200** | **0** | **14** | **99** | **1** | **SSSSSSSSSCSHSR + Carbamidomethyl (C)** |
|  | 101 | **369.2946** | **1104.8615** | **1104.2812** | **0.5803** | **1** | **14** | **90** | **1** | **VNLAMEGRAK + Oxidation (M)** |
|  | 645 | **410.3181** | **1227.9320** | **1228.3157** | **-0.3838** | **1** | **14** | **91** | **1** | **AAPGRSSTPTQR** |
|  | 736 | **419.2103** | **1254.6088** | **1255.3393** | **-0.7305** | **0** | **14** | **1.1e+02** | **1** | **GSPGGPGAAGFPGAR** |
|  | 1729 | **532.7155** | **1063.4161** | **1063.2904** | **0.1257** | **1** | **14** | **99** | **1** | **VITKAYIQK** |
|  | 2921 | **740.7280** | **2219.1619** | **2219.5601** | **-0.3982** | **2** | **14** | **88** | **1** | **CLEMKYGNEIMNKDPVFR + 2 Oxidation (M)** |
|  | 3097 | **777.5707** | **2329.6900** | **2329.6923** | **-0.0022** | **2** | **14** | **97** | **1** | **KVTGASFVVFNGALKTSSGFLAK** |
|  | 2727 | **684.2710** | **2049.7908** | **2050.4665** | **-0.6757** | **2** | **14** | **1e+02** | **1** | **MYGKIIFVLLLSDTHKR + Oxidation (M)** |
|  | 2755 | **686.1749** | **1370.3351** | **1369.5296** | **0.8055** | **1** | **14** | **99** | **1** | **GLGATARQANALAR** |
|  | 265 | **385.1492** | **1152.4253** | **1151.2565** | **1.1689** | **0** | **14** | **89** | **1** | **GASVEHVCHR + Carbamidomethyl (C)** |
|  | 3370 | **917.8522** | **1833.6896** | **1833.9875** | **-0.2979** | **1** | **14** | **76** | **1** | **KENSETVVTGSLDDLVK** |
|  | 597 | **407.7975** | **1220.3703** | **1220.5021** | **-0.1319** | **0** | **14** | **1.1e+02** | **1** | **ELLIMINACK + Carbamidomethyl (C); Oxidation (M)** |
|  | 954 | **443.1208** | **884.2268** | **883.0470** | **1.1798** | **0** | **14** | **1e+02** | **1** | **ATVPLQVR** |
|  | 1152 | **462.0690** | **922.1232** | **921.9720** | **0.1512** | **0** | **14** | **98** | **1** | **GDECLGSGK + Carbamidomethyl (C)** |
|  | 1481 | **503.2457** | **1004.4766** | **1005.1898** | **-0.7133** | **0** | **14** | **1.1e+02** | **1** | **METAATLLR** |
|  | 2808 | **704.1067** | **2109.2979** | **2108.3403** | **0.9575** | **1** | **14** | **97** | **1** | **AMGEGAGQVGERGGSMQGLCGR** |
|  | 2945 | **742.6918** | **1483.3688** | **1483.7120** | **-0.3432** | **0** | **14** | **79** | **1** | **MPHSPLGSMPEIR + 2 Oxidation (M)** |
|  | 841 | **430.9657** | **1289.8749** | **1290.3800** | **-0.5051** | **0** | **14** | **1.3e+02** | **1** | **EYQWLHTGEK** |
|  | 1288 | **478.2252** | **1431.6534** | **1430.5613** | **1.0921** | **0** | **14** | **98** | **1** | **LEALGTGWEELGR** |
|  | 208 | **377.2990** | **752.5832** | **752.8786** | **-0.2953** | **0** | **14** | **79** | **1** | **IFQCDK** |
|  | 395 | **398.3216** | **1191.9426** | **1191.3369** | **0.6058** | **1** | **14** | **86** | **1** | **SLPRTFGQGTK** |
|  | 2320 | **613.7329** | **1838.1766** | **1838.9754** | **-0.7989** | **1** | **14** | **1.2e+02** | **1** | **GTRPHSLSLNGGSRETGL** |
|  | 672 | **413.3420** | **1237.0039** | **1236.3957** | **0.6081** | **1** | **14** | **72** | **1** | **SGLCSEAAAAKTK** |
|  | 1223 | **471.2460** | **1410.7159** | **1409.5968** | **1.1191** | **2** | **14** | **93** | **1** | **RRGGGLVQPGGSLR** |
|  | 2137 | **595.8011** | **1189.5875** | **1189.2977** | **0.2898** | **0** | **14** | **95** | **1** | **ANFTIYDCSR** |
|  | 1051 | **454.6881** | **907.3614** | **907.0287** | **0.3328** | **0** | **14** | **87** | **1** | **RPPPPTSR** |
|  | 1969 | **571.4341** | **1711.2801** | **1711.9139** | **-0.6339** | **2** | **14** | **82** | **1** | **ATEKQHITSALEKQK** |
|  | 1861 | **551.6028** | **1101.1908** | **1100.2693** | **0.9214** | **2** | **14** | **1.3e+02** | **1** | **VRKQLEAEK** |
|  | 2247 | **608.6751** | **1823.0032** | **1823.9838** | **-0.9807** | **0** | **14** | **1.3e+02** | **1** | **NEDQIQGLHQACQLAR** |
|  | 207 | **377.2359** | **752.4570** | **752.8604** | **-0.4033** | **0** | **14** | **78** | **1** | **SVWHPK** |
|  | 2050 | **586.2960** | **1755.8657** | **1756.8567** | **-0.9910** | **1** | **14** | **1.1e+02** | **1** | **NGHDGSMDVQQRAWR** |
|  | 374 | **391.3995** | **1171.1763** | **1170.3590** | **0.8173** | **1** | **14** | **1.1e+02** | **1** | **NITAQLPTKGK** |
|  | 1654 | **523.3381** | **1044.6615** | **1044.1664** | **0.4951** | **2** | **14** | **98** | **1** | **SKEGKSRPR** |
|  | 2337 | **616.7529** | **1231.4911** | **1232.3740** | **-0.8829** | **2** | **14** | **1.2e+02** | **1** | **MAGSPSRAAGRR + Oxidation (M)** |
|  | 638 | **409.0174** | **1224.0301** | **1224.4561** | **-0.4259** | **0** | **14** | **1.2e+02** | **1** | **MGLPQPGHCLR + Oxidation (M)** |
|  | 1084 | **458.0672** | **1371.1794** | **1370.5575** | **0.6219** | **2** | **14** | **1.1e+02** | **1** | **RSRESPLSLGLR** |
|  | 2239 | **607.0112** | **1818.0113** | **1819.0059** | **-0.9945** | **2** | **14** | **1e+02** | **1** | **DASQVSAPGTRRIMEGK + Oxidation (M)** |
|  | 1730 | **532.9293** | **1595.7658** | **1595.9112** | **-0.1454** | **0** | **14** | **1.1e+02** | **1** | **MACEVACGVLHLHR + Carbamidomethyl (C)** |
|  | 729 | **419.1252** | **1254.3533** | **1253.4760** | **0.8773** | **1** | **14** | **1.2e+02** | **1** | **MRGCPMAGGSVR + 2 Oxidation (M)** |
|  | 765 | **422.1083** | **1263.3027** | **1264.3629** | **-1.0601** | **0** | **14** | **1.2e+02** | **1** | **MSLETTGPQER + Oxidation (M)** |
|  | 994 | **447.0775** | **892.1401** | **891.9229** | **0.2172** | **1** | **14** | **1.1e+02** | **1** | **TELGRTSE** |
|  | 2496 | **648.5967** | **1295.1786** | **1294.5161** | **0.6624** | **0** | **14** | **84** | **1** | **SLSELLGPYGMK** |
|  | 2908 | **740.4633** | **2218.3676** | **2217.4016** | **0.9660** | **2** | **14** | **1e+02** | **1** | **RWPTASEELSFTWRHTGR** |
|  | 785 | **426.3812** | **850.7477** | **850.9801** | **-0.2325** | **0** | **14** | **92** | **1** | **LLSSGSCK + Carbamidomethyl (C)** |
|  | 267 | **385.1552** | **768.2956** | **767.7906** | **0.5050** | **0** | **14** | **90** | **1** | **SGAPSGHR** |
|  | 492 | **404.1113** | **806.2078** | **805.8600** | **0.3477** | **0** | **14** | **1.1e+02** | **1** | **ACNGSAAR + Carbamidomethyl (C)** |
|  | 990 | **445.6324** | **889.2499** | **889.1176** | **0.1323** | **1** | **14** | **1e+02** | **1** | **LLVTKCGR** |
|  | 1764 | **537.1654** | **1072.3160** | **1073.2041** | **-0.8881** | **1** | **14** | **1.2e+02** | **1** | **AALTQAKSQR** |
|  | 2268 | **610.0189** | **1827.0346** | **1826.1488** | **0.8858** | **0** | **14** | **1.1e+02** | **1** | **GHCGGTSLLALQTLCPVR** |
|  | 2399 | **629.6331** | **1257.2515** | **1258.3416** | **-1.0902** | **1** | **14** | **1.1e+02** | **1** | **DRGIDATAQVGR** |
|  | 81 | **369.1859** | **1104.5355** | **1104.2991** | **0.2364** | **0** | **14** | **98** | **1** | **QQLLIGAYAK** |
|  | 1971 | **571.8625** | **1712.5655** | **1712.8159** | **-0.2505** | **0** | **14** | **86** | **1** | **QAVVTQEPSASGTPGQR** |
|  | 268 | **385.1754** | **1152.5039** | **1153.2423** | **-0.7385** | **0** | **14** | **85** | **1** | **SLISDSQPIHG** |
|  | 423 | **400.4041** | **798.7933** | **798.8840** | **-0.0907** | **0** | **14** | **1.1e+02** | **1** | **GLPLDER** |
|  | 972 | **443.8820** | **885.7493** | **884.9402** | **0.8092** | **1** | **14** | **1.1e+02** | **1** | **GRAGAGSPGR** |
|  | 1982 | **573.5279** | **1717.5615** | **1717.8371** | **-0.2755** | **0** | **14** | **94** | **1** | **VQDLDAHGWTWFSR** |
|  | 1991 | **575.6752** | **1724.0033** | **1724.9126** | **-0.9093** | **1** | **14** | **1.3e+02** | **1** | **KHNVELGITISSPSSR** |
|  | 2279 | **610.7817** | **1829.3230** | **1828.1416** | **1.1814** | **2** | **14** | **1.1e+02** | **1** | **GAPERVLERCSSILIK + Carbamidomethyl (C)** |
|  | 2393 | **628.5762** | **1882.7063** | **1882.2087** | **0.4976** | **2** | **14** | **87** | **1** | **INEILSNALKRGEIIAK** |
|  | 592 | **407.7703** | **1220.2887** | **1219.3685** | **0.9202** | **0** | **14** | **1.1e+02** | **1** | **GLSAVVSQAECR** |
|  | 1140 | **461.4071** | **1381.1991** | **1381.6216** | **-0.4225** | **2** | **14** | **91** | **1** | **RYLRVETIFGK** |
|  | 318 | **387.7136** | **1160.1187** | **1160.2650** | **-0.1463** | **2** | **14** | **1.2e+02** | **1** | **RRGSMGDPER** |
|  | 1023 | **449.9638** | **897.9129** | **897.0370** | **0.8758** | **1** | **14** | **1e+02** | **1** | **AAGRPRAAK** |
|  | 1724 | **532.4286** | **1594.2638** | **1593.9153** | **0.3485** | **2** | **14** | **90** | **1** | **RSAFPKACIAGLMR + Carbamidomethyl (C); Oxidation (M)** |
|  | 2136 | **595.5991** | **1783.7752** | **1784.9431** | **-1.1679** | **0** | **14** | **1.2e+02** | **1** | **FSSCVSQSTLTEPGWR** |
|  | 2493 | **648.2925** | **1941.8553** | **1942.0657** | **-0.2104** | **0** | **14** | **1e+02** | **1** | **DIEMTQSPSFVSASVGDR + Oxidation (M)** |
|  | 102 | **369.3050** | **1104.8927** | **1105.3089** | **-0.4162** | **0** | **14** | **96** | **1** | **AKPWAVCFPS** |
|  | 198 | **377.0829** | **752.1511** | **752.8619** | **-0.7108** | **0** | **14** | **1e+02** | **1** | **SGILHAR** |
|  | 784 | **426.3652** | **1276.0735** | **1275.4136** | **0.6600** | **1** | **14** | **90** | **1** | **VANPRLDTFSR** |
|  | 3343 | **879.9937** | **2636.9588** | **2637.1284** | **-0.1696** | **1** | **14** | **1.2e+02** | **1** | **LPSVPFETIQALDVVMRHLPSMR** |
|  | 1477 | **502.5649** | **1504.6726** | **1503.7445** | **0.9281** | **1** | **14** | **1.4e+02** | **1** | **SNFFFINFIAKR** |
|  | 2193 | **599.8676** | **1796.5805** | **1796.9770** | **-0.3965** | **1** | **14** | **92** | **1** | **KIHVSAVNPSYNGGEPK** |
|  | 2775 | **690.7257** | **2069.1550** | **2068.2864** | **0.8685** | **2** | **14** | **1.2e+02** | **1** | **KQHKIPTNDELLYDPEK** |
|  | 3305 | **844.4516** | **1686.8884** | **1686.9741** | **-0.0857** | **2** | **14** | **1e+02** | **1** | **EKYSIMKSMNMHR + 2 Oxidation (M)** |
|  | 900 | **435.3582** | **868.7017** | **869.0069** | **-0.3052** | **1** | **14** | **80** | **1** | **LAGGHCRR** |
|  | 930 | **437.1027** | **1308.2860** | **1308.4585** | **-0.1725** | **2** | **14** | **1.2e+02** | **1** | **LASMKKQDEDK + Oxidation (M)** |
|  | 2274 | **610.1176** | **1218.2203** | **1217.2451** | **0.9753** | **1** | **14** | **1.1e+02** | **1** | **RTPSFDDDHK** |
|  | 2440 | **635.3395** | **1268.6642** | **1268.4192** | **0.2449** | **1** | **14** | **1.1e+02** | **1** | **AEGLLQGKHSTK** |
|  | 43 | **364.2923** | **1089.8546** | **1089.2448** | **0.6098** | **2** | **14** | **79** | **1** | **KAWTKQNTL** |
|  | 1041 | 452.4392 | 1354.2955 | 1354.5531 | -0.2576 | 0 | 14 | 1.2e+02 | 1 | VIPAASLGGPGPYR |
|  | 1021 | **449.8928** | **1346.6563** | **1346.4684** | **0.1879** | **1** | **14** | **1.1e+02** | **1** | **VDDTGRYYCVR** |
|  | 2759 | **686.4659** | **2056.3757** | **2055.2379** | **1.1377** | **2** | **14** | **1.1e+02** | **1** | **GRHHLEEIMYNENTRR** |
|  | 1388 | **492.1839** | **1473.5295** | **1473.6575** | **-0.1279** | **2** | **14** | **1.1e+02** | **1** | **VGQSRTGPKDCLR + Carbamidomethyl (C)** |
|  | 1419 | **496.1699** | **1485.4874** | **1485.7094** | **-0.2220** | **2** | **14** | **1.2e+02** | **1** | **SALFLGVGRYRSC + Carbamidomethyl (C)** |
|  | 2099 | 592.9519 | 1775.8335 | 1775.1187 | 0.7148 | 1 | 14 | 1e+02 | 1 | YGFLLLMKHPEVEAK |
|  | 29 | **363.1277** | **1086.3611** | **1085.2132** | **1.1479** | **1** | **14** | **1.1e+02** | **1** | **GKNPPPNFSK** |
|  | 1980 | **573.4040** | **1144.7932** | **1144.3616** | **0.4316** | **0** | **14** | **98** | **1** | **VVALFYFASK** |
|  | 1592 | **518.3367** | **1551.9878** | **1551.7428** | **0.2451** | **0** | **14** | **1e+02** | **1** | **QCQDLGAVVFGMPN + Carbamidomethyl (C); Oxidation (M)** |
|  | 2604 | **667.5040** | **1999.4899** | **2000.4129** | **-0.9229** | **2** | **14** | **98** | **1** | **RSEHMPLNGMLIMKQAK + Oxidation (M)** |
|  | 797 | **427.9120** | **853.8093** | **854.0505** | **-0.2412** | **1** | **14** | **1.1e+02** | **1** | **KSMGMIR + 2 Oxidation (M)** |
|  | 356 | **389.1757** | **1164.5050** | **1163.3068** | **1.1981** | **1** | **14** | **1.1e+02** | **1** | **GTHKGYCLER** |
|  | 494 | **404.1614** | **1209.4620** | **1209.4812** | **-0.0192** | **1** | **14** | **1.1e+02** | **1** | **IAMLWGSGKCK + Oxidation (M)** |
|  | 1616 | **519.1441** | **1554.4101** | **1553.7637** | **0.6465** | **1** | **14** | **1.1e+02** | **1** | **VPFHCSECGKSFR + Carbamidomethyl (C)** |
|  | 15 | **362.1923** | **722.3697** | **722.7896** | **-0.4199** | **0** | **14** | **85** | **1** | **AGLPAHSA** |
|  | 316 | **387.2177** | **1158.6310** | **1158.3500** | **0.2810** | **1** | **14** | **1.1e+02** | **1** | **LPGPGSSLRFK** |
|  | 2045 | **585.6859** | **1754.0356** | **1753.0487** | **0.9868** | **1** | **14** | **1.3e+02** | **1** | **LRTDAVLPLTVAEVQK** |
|  | 2433 | **633.3080** | **1264.6012** | **1265.3988** | **-0.7977** | **1** | **14** | **1.2e+02** | **1** | **GYTGVRCEQPR** |
|  | 47 | **365.4101** | **1093.2081** | **1093.2768** | **-0.0687** | **1** | **14** | **1.4e+02** | **1** | **KASAVPAPPQK** |
|  | 139 | **371.2895** | **1110.8463** | **1110.1781** | **0.6682** | **1** | **14** | **72** | **1** | **TYNADSVKGR** |
|  | 588 | **407.6446** | **813.2745** | **813.9683** | **-0.6938** | **2** | **14** | **89** | **1** | **KCHGKNK** |
|  | 231 | **380.0878** | **1137.2411** | **1137.3522** | **-0.1111** | **1** | **14** | **1.3e+02** | **1** | **CYLLDGGKIR** |
|  | 324 | **387.9080** | **1160.7017** | **1161.3969** | **-0.6952** | **2** | **14** | **1.4e+02** | **1** | **ARRLIELYK** |
|  | 2955 | **743.7177** | **2228.1310** | **2228.4608** | **-0.3299** | **2** | **14** | **91** | **1** | **LKKELVHYQQSPGEDTSLR** |
|  | 361 | **389.4034** | **1165.1881** | **1165.2749** | **-0.0868** | **0** | **14** | **1.4e+02** | **1** | **LQESVMEASR + Oxidation (M)** |
|  | 3034 | **756.8216** | **2267.4426** | **2267.6308** | **-0.1882** | **2** | **14** | **1.2e+02** | **1** | **GCWLGEASKTCLHMAKGCGK + 3 Carbamidomethyl (C); Oxidation (M)** |
|  | 52 | **366.0566** | **1095.1478** | **1096.2145** | **-1.0667** | **1** | **14** | **1.3e+02** | **1** | **RSMDPSYPK + Oxidation (M)** |
|  | 108 | **369.3394** | **1104.9959** | **1104.2991** | **0.6968** | **0** | **14** | **1.1e+02** | **1** | **QQLLIGAYAK** |
|  | 623 | **408.3461** | **1222.0162** | **1221.2766** | **0.7396** | **0** | **14** | **99** | **1** | **GEEAGGLASQFR** |
|  | 2909 | **740.4640** | **2218.3698** | **2218.3766** | **-0.0068** | **0** | **14** | **1.1e+02** | **1** | **MDLASEITSATQTSSLCSSGR + Carbamidomethyl (C); Oxidation (M)** |
|  | 3300 | **840.1932** | **1678.3716** | **1677.8976** | **0.4739** | **1** | **14** | **89** | **1** | **TEHICDSIMKISER + Oxidation (M)** |
|  | 517 | **405.2713** | **1212.7917** | **1213.3393** | **-0.5475** | **1** | **14** | **84** | **1** | **CNVSDEMKGSK + Oxidation (M)** |
|  | 1043 | **452.7120** | **1355.1138** | **1355.5829** | **-0.4691** | **1** | **14** | **1e+02** | **1** | **VTPVRQVASASIK** |
|  | 1963 | **569.9867** | **1137.9586** | **1137.2944** | **0.6642** | **1** | **14** | **1.2e+02** | **1** | **IMSQRCNNR + Oxidation (M)** |
|  | 241 | **382.2362** | **762.4576** | **761.9087** | **0.5489** | **0** | **14** | **1.1e+02** | **1** | **KPGFSVK** |
|  | 598 | **407.8101** | **1220.4080** | **1221.3844** | **-0.9764** | **0** | **14** | **1.2e+02** | **1** | **DPCAGPVCDCK + 2 Carbamidomethyl (C)** |
|  | 678 | **414.0448** | **826.0749** | **825.9127** | **0.1621** | **0** | **14** | **96** | **1** | **SGSHLVAR** |
|  | 429 | **401.2552** | **1200.7434** | **1200.3022** | **0.4411** | **1** | **14** | **1e+02** | **1** | **EVGNGAKQEIR** |
|  | 528 | **405.8762** | **1214.6063** | **1214.4350** | **0.1714** | **0** | **14** | **1e+02** | **1** | **MIVAAEAVAAPR + Oxidation (M)** |
|  | 237 | **382.1513** | **1143.4318** | **1144.3217** | **-0.8899** | **1** | **14** | **1.3e+02** | **1** | **DIRINLVSSK** |
|  | 1430 | **498.8769** | **1493.6085** | **1493.7133** | **-0.1048** | **1** | **14** | **1.1e+02** | **1** | **VSGRPSLGAPQRLR** |
|  | 1856 | **549.8132** | **1097.6116** | **1098.3164** | **-0.7048** | **0** | **14** | **89** | **1** | **MTPLALSPPR + Oxidation (M)** |
|  | 2920 | **740.7073** | **2219.0997** | **2219.5369** | **-0.4373** | **2** | **14** | **93** | **1** | **RGEKLLVITVATAETEGYLR** |
|  | 1486 | **504.1199** | **1509.3376** | **1509.8124** | **-0.4747** | **2** | **14** | **1.2e+02** | **1** | **YEVAVRKSAIMVK + Oxidation (M)** |
|  | 948 | **441.3693** | **1321.0858** | **1320.4691** | **0.6167** | **0** | **14** | **91** | **1** | **SPESSILDGMIR + Oxidation (M)** |
|  | 1522 | **506.5519** | **1011.0891** | **1012.2487** | **-1.1596** | **1** | **14** | **1.3e+02** | **1** | **FPVPKGVLR** |
|  | 2124 | **594.8738** | **1781.5993** | **1782.0452** | **-0.4458** | **0** | **14** | **98** | **1** | **LTVKPNIQVPDPAVYQ** |
|  | 396 | **398.9077** | **1193.7010** | **1194.5379** | **-0.8369** | **2** | **14** | **99** | **1** | **LMRQIRMCK + Oxidation (M)** |
|  | 792 | **427.4656** | **852.9164** | **853.0607** | **-0.1443** | **0** | **14** | **1.4e+02** | **1** | **ALKPPSIK** |
|  | 1717 | 530.6490 | 1059.2833 | 1060.1639 | -0.8806 | 0 | 14 | 1.5e+02 | 1 | AVQYSIHSR |
|  | 104 | **369.3119** | **1104.9136** | **1105.3089** | **-0.3953** | **0** | **14** | **1.1e+02** | **1** | **AKPWAVCFPS** |
|  | 783 | **425.7530** | **1274.2369** | **1273.4855** | **0.7514** | **0** | **14** | **1.1e+02** | **1** | **MMGSYECHCR + Carbamidomethyl (C)** |
|  | 1118 | 460.0421 | 918.0694 | 918.1987 | -0.1292 | 2 | 14 | 1.3e+02 | 1 | KMKISALK |
|  | 2647 | **672.6457** | **2014.9149** | **2015.3116** | **-0.3967** | **1** | **14** | **98** | **1** | **CLQNKSILDTVTPSVHME** |
|  | 3301 | **840.2810** | **1678.5472** | **1677.9025** | **0.6448** | **1** | **14** | **1.1e+02** | **1** | **MNGAGFLKHCLEER + Carbamidomethyl (C); Oxidation (M)** |
|  | 1050 | **454.5339** | **1360.5795** | **1361.5246** | **-0.9451** | **1** | **14** | **1.4e+02** | **1** | **MVVVTGREPDSR + Oxidation (M)** |
|  | 1107 | **459.1193** | **1374.3358** | **1373.5548** | **0.7810** | **0** | **14** | **1.3e+02** | **1** | **GDSAMYLCASSLR** |
|  | 1745 | **534.9648** | **1067.9149** | **1067.2594** | **0.6555** | **0** | **14** | **1.3e+02** | **1** | **MVESAVIFR + Oxidation (M)** |
|  | 2470 | **643.0323** | **1926.0749** | **1926.0727** | **0.0022** | **0** | **14** | **1e+02** | **1** | **DLLSPPTDNRPGQMDNR** |
|  | 662 | **412.1670** | **822.3192** | **822.0301** | **0.2891** | **1** | **14** | **1.2e+02** | **1** | **LVRFCK + Carbamidomethyl (C)** |
|  | 852 | **431.9432** | **1292.8074** | **1293.4554** | **-0.6479** | **1** | **14** | **1.3e+02** | **1** | **MTSPPRGPGTHR** |
|  | 2751 | **686.0999** | **2055.2774** | **2056.4528** | **-1.1754** | **2** | **14** | **1.1e+02** | **1** | **MGHSKQIRILLLNEMEK + Oxidation (M)** |
|  | 380 | **393.4684** | **1177.3832** | **1177.3718** | **0.0114** | **1** | **14** | **1.4e+02** | **1** | **LSMRDVTVEK** |
|  | 1123 | **460.2049** | **1377.5926** | **1378.5068** | **-0.9142** | **0** | **14** | **1.3e+02** | **1** | **DSWDMLGVKPGAS + Oxidation (M)** |
|  | 563 | **406.9650** | **1217.8729** | **1217.4386** | **0.4343** | **1** | **14** | **1e+02** | **1** | **KNVGIGSCLAAK + Carbamidomethyl (C)** |
|  | 1615 | **519.1259** | **1036.2369** | **1036.1179** | **0.1191** | **1** | **14** | **1.1e+02** | **1** | **DEKENVMR + Oxidation (M)** |
|  | 1640 | **521.6102** | **1561.8083** | **1561.7426** | **0.0658** | **1** | **14** | **1.4e+02** | **1** | **HFECSFCGKSFR + 2 Carbamidomethyl (C)** |
|  | 111 | **369.3465** | **1105.0173** | **1105.2111** | **-0.1938** | **1** | **14** | **1.2e+02** | **1** | **HQSTLARHR** |
|  | 159 | **374.0721** | **1119.1940** | **1119.2477** | **-0.0537** | **0** | **14** | **1.4e+02** | **1** | **IYAMGGGSYGK + Oxidation (M)** |
|  | 251 | **384.8710** | **767.7273** | **767.8535** | **-0.1262** | **0** | **14** | **99** | **1** | **SFQCAR + Carbamidomethyl (C)** |
|  | 2147 | **597.0291** | **1192.0434** | **1191.3088** | **0.7347** | **0** | **14** | **1.3e+02** | **1** | **LQDDSEMLPK + Oxidation (M)** |
|  | 2256 | **609.2439** | **1824.7095** | **1824.1695** | **0.5400** | **1** | **14** | **1.2e+02** | **1** | **LPVELGSVTTLKALNLR** |
|  | 90 | **369.2656** | **1104.7747** | **1105.3089** | **-0.5342** | **0** | **14** | **1e+02** | **1** | **AKPWAVCFPS** |
|  | 1535 | **508.2538** | **1521.7392** | **1520.6047** | **1.1345** | **1** | **14** | **1.2e+02** | **1** | **HAEGTFTSDFPRR** |
|  | 2916 | **740.6425** | **2218.9052** | **2218.3517** | **0.5534** | **0** | **14** | **92** | **1** | **GDMTLDPDTANPELILSEDR + Oxidation (M)** |
|  | 789 | **426.8395** | **1277.4963** | **1278.3712** | **-0.8749** | **0** | **14** | **1.2e+02** | **1** | **DPPVHSDLGSVR** |
|  | 955 | **443.1917** | **1326.5530** | **1327.4615** | **-0.9086** | **1** | **14** | **1.1e+02** | **1** | **EYWDCLEGKK + Carbamidomethyl (C)** |
|  | 1788 | **538.7112** | **1613.1114** | **1612.7896** | **0.3218** | **1** | **14** | **1.2e+02** | **1** | **HMPPPNMTTNERR + 2 Oxidation (M)** |
|  | 778 | **423.7243** | **845.4338** | **845.9852** | **-0.5514** | **0** | **14** | **1.1e+02** | **1** | **VIYGPAAR** |
|  | 2569 | **663.5955** | **1325.1761** | **1325.4687** | **-0.2926** | **0** | **14** | **93** | **1** | **NDYIITGGGFLR** |
|  | 2691 | **680.5723** | **2038.6946** | **2039.3380** | **-0.6434** | **1** | **14** | **93** | **1** | **LNPGDRGVMMDISAPFFR + Oxidation (M)** |
|  | 1524 | **506.7741** | **1517.3002** | **1517.8376** | **-0.5373** | **0** | **14** | **93** | **1** | **MNECCLCGTSVAMR** |
|  | 2600 | **666.8029** | **1997.3864** | **1997.1523** | **0.2341** | **1** | **14** | **1.4e+02** | **1** | **ALPQNDDHYVMQEHRK + Oxidation (M)** |
|  | 2903 | **738.7967** | **2213.3679** | **2214.3498** | **-0.9819** | **1** | **14** | **1.4e+02** | **1** | **QPSGDSGLAAETSAISQVPRSR** |
|  | 8 | **360.4774** | **1078.4102** | **1079.2269** | **-0.8167** | **1** | **14** | **1.5e+02** | **1** | **MKADLGDWK + Oxidation (M)** |
|  | 831 | **430.1745** | **858.3342** | **858.0177** | **0.3165** | **1** | **13** | **1.3e+02** | **1** | **EHVAKMK + Oxidation (M)** |
|  | 2628 | **669.6073** | **1337.1998** | **1336.5380** | **0.6618** | **1** | **13** | **95** | **1** | **AGMTEEAQRLCK** |
|  | 890 | **434.3752** | **1300.1035** | **1299.4398** | **0.6636** | **1** | **13** | **92** | **1** | **CSSCGQAFGQRR** |
|  | 1128 | **460.5561** | **1378.6461** | **1378.4701** | **0.1760** | **0** | **13** | **1.5e+02** | **1** | **GAWGNNMNSGLNK + Oxidation (M)** |
|  | 1164 | **463.9890** | **1388.9449** | **1388.3558** | **0.5891** | **0** | **13** | **1.1e+02** | **1** | **DNHLGGEDFDNR** |
|  | 2713 | **684.0010** | **1365.9872** | **1365.5807** | **0.4064** | **1** | **13** | **97** | **1** | **GALVLGSSLKQHR** |
|  | 2898 | **736.9540** | **2207.8398** | **2207.5492** | **0.2905** | **2** | **13** | **1e+02** | **1** | **GKNVCQLSITLEDLYKGVAR** |
|  | 276 | **385.9876** | **769.9604** | **770.9171** | **-0.9567** | **1** | **13** | **1e+02** | **1** | **KTNLAPK** |
|  | 823 | **429.2497** | **1284.7268** | **1285.5558** | **-0.8289** | **0** | **13** | **1e+02** | **1** | **MAVVSGILGIPGR + Oxidation (M)** |
|  | 1403 | **494.1626** | **1479.4657** | **1479.6587** | **-0.1930** | **2** | **13** | **1.3e+02** | **1** | **MSRQLNIKSSGDK + Oxidation (M)** |
|  | 112 | **369.3486** | **1105.0236** | **1105.3089** | **-0.2852** | **0** | **13** | **1.2e+02** | **1** | **AKPWAVCFPS** |
|  | 705 | **416.6535** | **1246.9382** | **1246.4569** | **0.4814** | **1** | **13** | **1.1e+02** | **1** | **KSFAMLSTCSR + Oxidation (M)** |
|  | 1235 | **472.1004** | **942.1860** | **943.1435** | **-0.9575** | **0** | **13** | **1.1e+02** | **1** | **LLGPPPPPR** |
|  | 3057 | **763.4216** | **2287.2427** | **2287.5496** | **-0.3069** | **2** | **13** | **1.2e+02** | **1** | **DLQSNVEHLTEKMKTTIQR + Oxidation (M)** |
|  | 290 | **386.2239** | **770.4330** | **769.8694** | **0.5636** | **0** | **13** | **86** | **1** | **GSPPCPGR** |
|  | 2292 | **612.1183** | **1833.3327** | **1833.0309** | **0.3018** | **1** | **13** | **1.2e+02** | **1** | **EVSVSEGRAHAVVFDCK** |
|  | 498 | **404.2253** | **1209.6537** | **1209.2727** | **0.3810** | **2** | **13** | **1e+02** | **1** | **RGSSKGHDTHK** |
|  | 1474 | **502.1997** | **1503.5769** | **1504.6664** | **-1.0895** | **0** | **13** | **1.3e+02** | **1** | **EGCQTEFCYHCK + Carbamidomethyl (C)** |
|  | 1799 | **540.2429** | **1617.7064** | **1618.7957** | **-1.0893** | **2** | **13** | **1.2e+02** | **1** | **CGRMFSDPSSFRR + Carbamidomethyl (C); Oxidation (M)** |
|  | 95 | **369.2843** | **1104.8306** | **1105.3089** | **-0.4782** | **0** | **13** | **1.1e+02** | **1** | **AKPWAVCFPS** |
|  | 204 | **377.1866** | **1128.5376** | **1129.2724** | **-0.7348** | **2** | **13** | **97** | **1** | **SRFLRGHEK** |
|  | 2197 | **599.9349** | **1796.7825** | **1796.0566** | **0.7258** | **0** | **13** | **1.1e+02** | **1** | **VLCIINPGNPTGQVQSR** |
|  | 2607 | **667.8811** | **1333.7474** | **1334.5437** | **-0.7962** | **0** | **13** | **1.1e+02** | **1** | **MPGHPGAAEQLVK** |
|  | 1251 | **473.6424** | **945.2699** | **945.0302** | **0.2398** | **0** | **13** | **1.3e+02** | **1** | **IHASFENK** |
|  | 1301 | **480.2671** | **958.5194** | **959.0950** | **-0.5756** | **0** | **13** | **1.2e+02** | **1** | **VIGDLDTVK** |
|  | 767 | **422.1488** | **842.2829** | **841.9948** | **0.2880** | **0** | **13** | **1.3e+02** | **1** | **SLSLPGLR** |
|  | 211 | **377.5097** | **1129.5069** | **1130.2967** | **-0.7898** | **0** | **13** | **1.1e+02** | **1** | **LHYCSYCGK + Carbamidomethyl (C)** |
|  | 239 | **382.1538** | **1143.4392** | **1143.2543** | **0.1848** | **2** | **13** | **1.4e+02** | **1** | **ARAPDTKAGTR** |
|  | 1354 | **488.0316** | **1461.0727** | **1461.6667** | **-0.5940** | **1** | **13** | **1.4e+02** | **1** | **DSLEVRVCACPGR + Carbamidomethyl (C)** |
|  | 1557 | **513.3452** | **1537.0135** | **1536.7777** | **0.2357** | **1** | **13** | **97** | **1** | **VQIGGGLVQPGRSLR** |
|  | 1795 | **539.9592** | **1077.9037** | **1078.3049** | **-0.4013** | **0** | **13** | **1.3e+02** | **1** | **LLLGPAGGGVPK** |
|  | 87 | **369.2587** | **1104.7540** | **1105.3089** | **-0.5549** | **0** | **13** | **1.1e+02** | **1** | **AKPWAVCFPS** |
|  | 147 | **372.3465** | **1114.0173** | **1114.1237** | **-0.1063** | **0** | **13** | **1.2e+02** | **1** | **DTAPGGDQTPR** |
|  | 758 | **421.8466** | **1262.5177** | **1263.5271** | **-1.0094** | **1** | **13** | **1.3e+02** | **1** | **QKFPFLVTVGK** |
|  | 1220 | **470.7339** | **939.4530** | **938.9811** | **0.4719** | **0** | **13** | **88** | **1** | **AEGPPEPSR** |
|  | 2535 | **655.4572** | **1963.3493** | **1964.1619** | **-0.8127** | **1** | **13** | **1.2e+02** | **1** | **MTDQEAIQDLWQWRK + Oxidation (M)** |
|  | 3072 | **765.7106** | **1529.4064** | **1529.7390** | **-0.3326** | **1** | **13** | **96** | **1** | **SAHFLGVDKVITSR** |
|  | 3 | **360.3643** | **1078.0707** | **1079.2286** | **-1.1579** | **0** | **13** | **1.6e+02** | **1** | **MYAYGFVGR + Oxidation (M)** |
|  | 69 | **368.1539** | **1101.4396** | **1101.2539** | **0.1857** | **1** | **13** | **1.2e+02** | **1** | **NAIAITNKEK** |
|  | 2380 | **625.3628** | **1873.0662** | **1872.1311** | **0.9351** | **0** | **13** | **1.2e+02** | **1** | **IPECLNCGMNDCPSYR + Carbamidomethyl (C)** |
|  | 100 | **369.2945** | **1104.8612** | **1105.3089** | **-0.4476** | **0** | **13** | **1.1e+02** | **1** | **AKPWAVCFPS** |
|  | 2140 | **596.1696** | **1190.3243** | **1189.3674** | **0.9570** | **2** | **13** | **1.3e+02** | **1** | **DKVRAGLFQR** |
|  | 544 | **406.1216** | **1215.3426** | **1214.4232** | **0.9194** | **2** | **13** | **1.2e+02** | **1** | **RPQNKFLRR** |
|  | 1001 | **447.2022** | **1338.5845** | **1338.4695** | **0.1150** | **1** | **13** | **1.3e+02** | **1** | **GAPGVTGPKGDVGAR** |
|  | 1180 | **465.8842** | **1394.6304** | **1394.5757** | **0.0547** | **1** | **13** | **1.3e+02** | **1** | **NHSGKVLGSSALPK** |
|  | 3313 | **847.4543** | **2539.3409** | **2540.1570** | **-0.8162** | **2** | **13** | **1.2e+02** | **1** | **AYRLTVAKLEPPLIPFMPLLIK + Oxidation (M)** |
|  | 1058 | **455.9452** | **1364.8133** | **1365.6455** | **-0.8322** | **1** | **13** | **1.1e+02** | **1** | **RQLPCMASQMK + Carbamidomethyl (C); Oxidation (M)** |
|  | 2084 | **591.5316** | **1181.0485** | **1181.3371** | **-0.2886** | **0** | **13** | **1e+02** | **1** | **FNFIADVVEK** |
|  | 110 | **369.3438** | **1105.0093** | **1105.3089** | **-0.2996** | **0** | **13** | **1.3e+02** | **1** | **AKPWAVCFPS** |
|  | 490 | **404.0763** | **806.1378** | **804.9765** | **1.1614** | **0** | **13** | **1.3e+02** | **1** | **AVTIVFR** |
|  | 10 | **360.5304** | **1078.5690** | **1079.2336** | **-0.6646** | **1** | **13** | **1.2e+02** | **1** | **RVGAADMFGR** |
|  | 63 | **367.1820** | **1098.5238** | **1099.2365** | **-0.7127** | **1** | **13** | **1.1e+02** | **1** | **YSDFAKLQK** |
|  | 547 | **406.2537** | **810.4926** | **809.8239** | **0.6687** | **0** | **13** | **94** | **1** | **AQGEYSR** |
|  | 695 | **416.0808** | **1245.2204** | **1246.3938** | **-1.1735** | **1** | **13** | **1.5e+02** | **1** | **NMAALDAADRAK** |
|  | 1796 | **540.0842** | **1078.1537** | **1078.2638** | **-0.1101** | **1** | **13** | **1.3e+02** | **1** | **GGSDGCKVPMK** |
|  | 2526 | 654.5477 | 1960.6210 | 1961.2922 | -0.6712 | 1 | 13 | 97 | 1 | CIVLGIAAHRSPTPVQGNK |
|  | 257 | **384.9910** | **1151.9508** | **1151.2696** | **0.6812** | **0** | **13** | **1.1e+02** | **1** | **ALQATVGNSYK** |
|  | 2250 | **608.7469** | **1823.2187** | **1824.1926** | **-0.9740** | **1** | **13** | **1.5e+02** | **1** | **GKNMCGLAACASYPIPLV + Oxidation (M)** |
|  | 3087 | **774.6819** | **1547.3490** | **1546.8936** | **0.4553** | **1** | **13** | **97** | **1** | **MQKEIAALAPSMLK + Oxidation (M)** |
|  | 552 | **406.5519** | **811.0890** | **809.9300** | **1.1591** | **0** | **13** | **1.1e+02** | **1** | **MDIFER** |
|  | 1881 | **555.0421** | **1662.1042** | **1661.9795** | **0.1247** | **2** | **13** | **1.2e+02** | **1** | **MRPRPRSAPGKPRR** |
|  | 3059 | **763.6176** | **1525.2204** | **1524.7604** | **0.4600** | **0** | **13** | **1.1e+02** | **1** | **EQGLEAQLLGIVVR** |
|  | 79 | **369.1642** | **1104.4706** | **1103.2766** | **1.1940** | **1** | **13** | **1.2e+02** | **1** | **MRVPGCGDPR + Oxidation (M)** |
|  | 99 | **369.2923** | **1104.8548** | **1104.2879** | **0.5669** | **2** | **13** | **1.1e+02** | **1** | **RSAVVMGRGR + Oxidation (M)** |
|  | 795 | **427.8748** | **1280.6021** | **1280.3621** | **0.2400** | **0** | **13** | **1.2e+02** | **1** | **YMNTEAFFGQG + Oxidation (M)** |
|  | 529 | **405.8834** | **1214.6280** | **1215.3930** | **-0.7650** | **0** | **13** | **1.2e+02** | **1** | **EELAALISELK** |
|  | 3251 | **816.6992** | **1631.3835** | **1631.7909** | **-0.4074** | **1** | **13** | **1e+02** | **1** | **GSPSSHLLGADHGLRK** |
|  | 904 | **435.6904** | **1304.0491** | **1304.4533** | **-0.4042** | **0** | **13** | **97** | **1** | **SPHSGMTTPMSR + Oxidation (M)** |
|  | 1260 | **475.3188** | **948.6229** | **948.0325** | **0.5905** | **1** | **13** | **1.1e+02** | **1** | **GNSKSGALSK** |
|  | 2550 | **658.1589** | **1314.3031** | **1314.4479** | **-0.1448** | **1** | **13** | **1.2e+02** | **1** | **LAAGSKAPASAQSR** |
|  | 2588 | **666.5107** | **1996.5101** | **1995.4093** | **1.1007** | **0** | **13** | **1.1e+02** | **1** | **FLLGIYCSLHHAMTIFI + Oxidation (M)** |
|  | 1241 | **472.3515** | **1414.0322** | **1414.5176** | **-0.4853** | **0** | **13** | **99** | **1** | **GGPASVPSSSPGTSVK** |
|  | 652 | **411.0963** | **1230.2667** | **1229.3848** | **0.8818** | **1** | **13** | **1.4e+02** | **1** | **LEHLFESRAK** |
|  | 1171 | **464.4355** | **1390.2842** | **1390.5489** | **-0.2647** | **2** | **13** | **1.1e+02** | **1** | **GSGPGRFPFKQGR** |
|  | 1537 | **508.9776** | **1015.9404** | **1016.1546** | **-0.2142** | **0** | **13** | **1.4e+02** | **1** | **VHPGPVSPAR** |
|  | 2494 | **648.3531** | **1942.0373** | **1941.0592** | **0.9780** | **1** | **13** | **1.2e+02** | **1** | **TDAEELVSRSYWDTLR** |
|  | 2706 | **682.6536** | **2044.9385** | **2044.3395** | **0.5991** | **2** | **13** | **1e+02** | **1** | **HGGDLYVVGGSIPRRMWK + Oxidation (M)** |
|  | 2353 | **619.8112** | **1237.6075** | **1237.4084** | **0.1991** | **1** | **13** | **1.1e+02** | **1** | **RGLNLAPSPANK** |
|  | 2701 | **682.3070** | **2043.8988** | **2044.1837** | **-0.2849** | **1** | **13** | **1.3e+02** | **1** | **HFQGTLELEVGDWKDNR** |
|  | 2748 | **686.0396** | **2055.0965** | **2054.2433** | **0.8532** | **0** | **13** | **1.1e+02** | **1** | **FDPNCSCAAGDSCTCAGSCK + 2 Carbamidomethyl (C)** |
|  | 3001 | **749.2180** | **1496.4213** | **1495.6763** | **0.7450** | **1** | **13** | **1.2e+02** | **1** | **ALLPDRSLPPSDSK** |
|  | 285 | **386.1216** | **1155.3428** | **1156.3755** | **-1.0328** | **0** | **13** | **1.2e+02** | **1** | **MQQAGMALYK + Oxidation (M)** |
|  | 555 | **406.6641** | **1216.9702** | **1216.3084** | **0.6618** | **2** | **13** | **93** | **1** | **RERKPSGGSSR** |
|  | 844 | **431.0254** | **860.0361** | **860.0732** | **-0.0371** | **0** | **13** | **1.6e+02** | **1** | **MLDAAVIK** |
|  | 863 | **432.8289** | **1295.4646** | **1296.5319** | **-1.0673** | **1** | **13** | **1.3e+02** | **1** | **QYESLKILICS** |
|  | 1092 | **458.5465** | **1372.6174** | **1373.6608** | **-1.0435** | **1** | **13** | **1.6e+02** | **1** | **KLQAMVIEISNK** |
|  | 1897 | **557.3401** | **1112.6655** | **1112.1971** | **0.4685** | **0** | **13** | **1.2e+02** | **1** | **GHCSGGSCYK + 2 Carbamidomethyl (C)** |
|  | 935 | **437.3988** | **872.7829** | **872.0905** | **0.6924** | **1** | **13** | **1.3e+02** | **1** | **MSLLPRR** |
|  | 2144 | **596.8486** | **1191.6825** | **1192.2787** | **-0.5962** | **1** | **13** | **1.2e+02** | **1** | **SYDKTTHNVK** |
|  | 2228 | **606.2308** | **1210.4468** | **1209.3553** | **1.0914** | **0** | **13** | **1.2e+02** | **1** | **AACCAGTSWSPR** |
|  | 2491 | **647.9606** | **1940.8595** | **1941.1470** | **-0.2875** | **0** | **13** | **1e+02** | **1** | **QSALTQDPAVSVALGQTVR** |
|  | 346 | **389.0725** | **1164.1953** | **1164.5051** | **-0.3099** | **1** | **13** | **1.4e+02** | **1** | **LCSCKCPLLGK** |
|  | 748 | **420.1968** | **1257.5682** | **1258.4905** | **-0.9224** | **1** | **13** | **1.2e+02** | **1** | **AILCNDGLVKR + Carbamidomethyl (C)** |
|  | 976 | **443.9098** | **1328.7072** | **1327.5724** | **1.1347** | **1** | **13** | **1.4e+02** | **1** | **AYPPPRISWLK** |
|  | 868 | **433.0883** | **1296.2426** | **1296.3433** | **-0.1007** | **1** | **13** | **1.3e+02** | **1** | **SKGFGSGSPGNSSK** |
|  | 2204 | **600.2896** | **1198.5643** | **1199.3606** | **-0.7962** | **2** | **13** | **1.4e+02** | **1** | **NQEVKNALKR** |
|  | 2584 | **666.4232** | **1996.2475** | **1995.1747** | **1.0728** | **1** | **13** | **1.3e+02** | **1** | **HYNSSLASRVTMSVDPSK + Oxidation (M)** |
|  | 2613 | **668.5609** | **2002.6604** | **2002.2763** | **0.3840** | **1** | **13** | **1.1e+02** | **1** | **ELRESSLLNQHACAVMK + Carbamidomethyl (C); Oxidation (M)** |
|  | 335 | **388.2937** | **774.5727** | **773.7488** | **0.8239** | **0** | **13** | **1.2e+02** | **1** | **ASNDPDR** |
|  | 2046 | **585.8064** | **1169.5980** | **1170.3192** | **-0.7212** | **0** | **13** | **1.1e+02** | **1** | **CEQCGTGFIGR** |
|  | 2387 | **627.7445** | **1253.4742** | **1254.4359** | **-0.9616** | **1** | **13** | **1.5e+02** | **1** | **RLQEVPQTVGK** |
|  | 2507 | **651.2798** | **1300.5448** | **1299.5394** | **1.0054** | **1** | **13** | **1.3e+02** | **1** | **NPAMEKLELVR** |
|  | 2703 | **682.4131** | **2044.2171** | **2043.2390** | **0.9781** | **2** | **13** | **1.2e+02** | **1** | **TTAQLQAVERELAEERAK** |
|  | 1727 | **532.6471** | **1594.9191** | **1594.8337** | **0.0854** | **1** | **13** | **1.6e+02** | **1** | **GMTFNLIGESTIRR** |
|  | 1930 | **563.2756** | **1686.8047** | **1687.9901** | **-1.1853** | **1** | **13** | **1.3e+02** | **1** | **VHCSCHLPNPRVLR + Carbamidomethyl (C)** |
|  | 856 | **432.0358** | **862.0568** | **860.9984** | **1.0585** | **1** | **13** | **1.4e+02** | **1** | **KSTTGLVR** |
|  | 922 | **436.7631** | **1307.2671** | **1307.4738** | **-0.2067** | **2** | **13** | **1.2e+02** | **1** | **EEAREKMLASK + Oxidation (M)** |
|  | 1035 | **451.6013** | **1351.7817** | **1351.5345** | **0.2472** | **1** | **13** | **1.6e+02** | **1** | **AASPPRMPGSQPR** |
|  | 1062 | **456.4859** | **1366.4355** | **1366.6567** | **-0.2211** | **1** | **13** | **1.4e+02** | **1** | **ICNAHGCKMAMR + 2 Oxidation (M)** |
|  | 2095 | **592.7994** | **1183.5840** | **1184.3261** | **-0.7421** | **1** | **13** | **1.2e+02** | **1** | **LGVHDMNDRK** |
|  | 2375 | **623.6840** | **1245.3531** | **1244.4013** | **0.9519** | **2** | **13** | **1.6e+02** | **1** | **VNNSSRSKKPK** |
|  | 1929 | **563.2442** | **1124.4736** | **1123.4170** | **1.0567** | **1** | **13** | **1.2e+02** | **1** | **AARLMMMGSR** |
|  | 2151 | **597.7965** | **1193.5782** | **1194.3407** | **-0.7624** | **0** | **13** | **1.1e+02** | **1** | **NGALPVPSGNIR** |
|  | 440 | **401.8386** | **1202.4937** | **1201.3964** | **1.0973** | **0** | **13** | **1.5e+02** | **1** | **MGMEMWAAAR + 3 Oxidation (M)** |
|  | 992 | **446.1595** | **890.3042** | **890.8520** | **-0.5478** | **0** | **13** | **1.5e+02** | **1** | **NSDQEGGGK** |
|  | 1768 | **537.2947** | **1072.5747** | **1072.1334** | **0.4413** | **1** | **13** | **1.5e+02** | **1** | **GGEGERSKPR** |
|  | 2269 | **610.0853** | **1827.2338** | **1827.2231** | **0.0108** | **1** | **13** | **1.3e+02** | **1** | **MGCLSPCTLRSWMLR + Carbamidomethyl (C); Oxidation (M)** |
|  | 2394 | **628.5975** | **1882.7704** | **1882.0635** | **0.7070** | **2** | **13** | **1.1e+02** | **1** | **MPPRTRTSFSVSTADGR + Oxidation (M)** |
|  | 214 | **378.1346** | **1131.3815** | **1132.2449** | **-0.8634** | **0** | **13** | **1.2e+02** | **1** | **DVMLADAQVGGG** |
|  | 1016 | **448.7780** | **1343.3119** | **1342.5243** | **0.7875** | **1** | **13** | **1.1e+02** | **1** | **CTHQEKVTIQR** |
|  | 1044 | **452.9337** | **903.8527** | **903.0152** | **0.8375** | **0** | **13** | **1.4e+02** | **1** | **NSVSPMPR + Oxidation (M)** |
|  | 2179 | **599.4314** | **1795.2720** | **1796.0518** | **-0.7798** | **0** | **13** | **1.1e+02** | **1** | **CAEMIISMDSSQIHSK + Oxidation (M)** |
|  | 2510 | **651.9419** | **1952.8035** | **1952.2543** | **0.5493** | **2** | **13** | **1e+02** | **1** | **MEALASTEKMLQDKVNK + Oxidation (M)** |
|  | 12 | **361.9187** | **721.8226** | **722.8113** | **-0.9887** | **0** | **13** | **1.3e+02** | **1** | **MSGSLGR + Oxidation (M)** |
|  | 1525 | **506.7901** | **1517.3480** | **1516.7367** | **0.6113** | **0** | **13** | **1e+02** | **1** | **IYCADMYLENPK + Carbamidomethyl (C)** |
|  | 2852 | **716.2279** | **1430.4410** | **1429.3848** | **1.0563** | **0** | **13** | **1.3e+02** | **1** | **SSSSSSSSSCSHSR + Carbamidomethyl (C)** |
|  | 602 | **407.9294** | **1220.7660** | **1221.2832** | **-0.5173** | **1** | **13** | **1.3e+02** | **1** | **ASAYNNRAQAR** |
|  | 1412 | **494.8557** | **987.6967** | **987.1381** | **0.5586** | **2** | **13** | **1.3e+02** | **1** | **NGGPQKRCK** |
|  | 1746 | **535.9221** | **1604.7440** | **1604.8552** | **-0.1112** | **1** | **13** | **1.3e+02** | **1** | **MSFCLHTGVPGRSR + Carbamidomethyl (C)** |
|  | 2327 | **614.3892** | **1840.1453** | **1839.0617** | **1.0836** | **1** | **13** | **1.3e+02** | **1** | **RASPPPPPPPGGAWEAVR** |
|  | 3085 | **771.6077** | **1541.2006** | **1540.8311** | **0.3695** | **1** | **13** | **1.1e+02** | **1** | **CGVISPRFHVQLK + Carbamidomethyl (C)** |
|  | 183 | **375.1806** | **1122.5196** | **1123.2796** | **-0.7599** | **0** | **13** | **1.3e+02** | **1** | **TPMAAAAFAEK + Oxidation (M)** |
|  | 1271 | **476.1951** | **950.3754** | **951.1441** | **-0.7686** | **0** | **13** | **1.3e+02** | **1** | **LVCVSFQR** |
|  | 2049 | **586.2070** | **1755.5987** | **1756.0824** | **-0.4836** | **2** | **13** | **1.3e+02** | **1** | **MRTGRKPDGCAICFK + Carbamidomethyl (C); Oxidation (M)** |
|  | 2925 | **740.8793** | **2219.6156** | **2219.4956** | **0.1201** | **2** | **13** | **1.5e+02** | **1** | **RSYNEPPLPTPVSPQPKSPK** |
|  | 210 | **377.4541** | **1129.3400** | **1128.3488** | **0.9912** | **2** | **13** | **1.4e+02** | **1** | **GAAGRACGPLKK** |
|  | 746 | **419.8985** | **1256.6734** | **1256.5610** | **0.1124** | **1** | **13** | **1.2e+02** | **1** | **ALKFMCSMPR + Carbamidomethyl (C); Oxidation (M)** |
|  | 2804 | **703.4880** | **2107.4418** | **2108.3501** | **-0.9084** | **0** | **13** | **1.3e+02** | **1** | **AVLLNLGTIELYGSNDPYR** |
|  | 196 | **377.0462** | **1128.1163** | **1127.3773** | **0.7390** | **1** | **13** | **1.3e+02** | **1** | **IGLKDVITLR** |
|  | 1341 | **486.8115** | **1457.4124** | **1456.6470** | **0.7654** | **2** | **13** | **1.2e+02** | **1** | **GVRSPTSSALRTPK** |
|  | 1267 | **475.7729** | **949.5310** | **949.0174** | **0.5136** | **1** | **13** | **1.2e+02** | **1** | **SGTSGEKVGK** |
|  | 1425 | **496.6663** | **991.3179** | **992.0852** | **-0.7673** | **2** | **13** | **1.4e+02** | **1** | **TKQDSASKK** |
|  | 2005 | **577.5866** | **1729.7377** | **1729.8893** | **-0.1516** | **1** | **13** | **1.4e+02** | **1** | **WLPPSEATSQGWKSR** |
|  | 496 | **404.1750** | **1209.5030** | **1210.3369** | **-0.8339** | **1** | **13** | **1.3e+02** | **1** | **EIEKNPPAQGK** |
|  | 959 | **443.3874** | **1327.1401** | **1326.5218** | **0.6183** | **2** | **13** | **1e+02** | **1** | **KVSSMGFGKDVR + Oxidation (M)** |
|  | 2787 | **696.6781** | **2087.0121** | **2087.4253** | **-0.4132** | **2** | **13** | **1.2e+02** | **1** | **LCRFQGCKYLSDINITR + Carbamidomethyl (C)** |
|  | 3067 | **764.7779** | **1527.5410** | **1527.7213** | **-0.1803** | **1** | **13** | **1.2e+02** | **1** | **EQQVADLQLKLSR** |
|  | 1233 | **471.9087** | **941.8026** | **942.1339** | **-0.3313** | **0** | **13** | **1.2e+02** | **1** | **EAITMLHK** |
|  | 262 | **385.0691** | **1152.1851** | **1151.3806** | **0.8045** | **0** | **13** | **1.2e+02** | **1** | **LGPGCGMMAGGK + Carbamidomethyl (C); Oxidation (M)** |
|  | 950 | **442.2689** | **1323.7844** | **1324.6183** | **-0.8339** | **2** | **13** | **1e+02** | **1** | **MRCLAARVNYK** |
|  | 1978 | **573.3364** | **1144.6581** | **1144.3021** | **0.3560** | **0** | **13** | **1.4e+02** | **1** | **AMAAVPQDVAR + Oxidation (M)** |
|  | 2983 | **748.8446** | **1495.6744** | **1495.7672** | **-0.0928** | **1** | **13** | **1.6e+02** | **1** | **VKLPYHATLGIQR** |
|  | 983 | **445.0252** | **888.0356** | **886.9959** | **1.0398** | **2** | **13** | **1.6e+02** | **1** | **RRIEGEK** |
|  | 1211 | **469.4047** | **936.7947** | **936.9835** | **-0.1888** | **0** | **13** | **1.1e+02** | **1** | **DPTMDNTK + Oxidation (M)** |
|  | 538 | **406.0319** | **1215.0734** | **1214.4513** | **0.6221** | **0** | **13** | **1.2e+02** | **1** | **SALILAVTLGEK** |
|  | 1130 | **460.6070** | **1378.7988** | **1377.6742** | **1.1246** | **1** | **13** | **1.5e+02** | **1** | **LLRAVILGPPGSGK** |
|  | 1353 | **487.9752** | **1460.9035** | **1460.7000** | **0.2035** | **2** | **13** | **1.5e+02** | **1** | **KKCTSPFSCCW + 3 Carbamidomethyl (C)** |
|  | 1805 | **540.4578** | **1618.3511** | **1618.8501** | **-0.4990** | **1** | **13** | **1.1e+02** | **1** | **FFCGEQYILEKGK + Carbamidomethyl (C)** |
|  | 3330 | **857.0308** | **1712.0467** | **1711.9006** | **0.1462** | **1** | **13** | **1.4e+02** | **1** | **RVNNCSDMCFHQK + 2 Carbamidomethyl (C); Oxidation (M)** |
|  | 1378 | **490.3179** | **1467.9316** | **1468.7634** | **-0.8317** | **0** | **13** | **1.1e+02** | **1** | **FPVFLIVCNGFR + Carbamidomethyl (C)** |
|  | 1411 | **494.8428** | **1481.5062** | **1481.6331** | **-0.1268** | **0** | **13** | **1.3e+02** | **1** | **HTGCKPFECNEC + 2 Carbamidomethyl (C)** |
|  | 975 | **443.9004** | **1328.6790** | **1327.5960** | **1.0830** | **0** | **13** | **1.4e+02** | **1** | **SYTVGVMMMHR + Oxidation (M)** |
|  | 1240 | **472.3080** | **1413.9020** | **1413.7280** | **0.1740** | **1** | **13** | **1.1e+02** | **1** | **GLGDMKLGKPLLR + Oxidation (M)** |
|  | 1321 | **483.6245** | **965.2342** | **966.1587** | **-0.9244** | **1** | **13** | **1.5e+02** | **1** | **ISGRGFCVK** |
|  | 1470 | **501.5571** | **1501.6491** | **1501.6377** | **0.0114** | **0** | **13** | **1.7e+02** | **1** | **TLTGAELELAEAQR** |
|  | 151 | **373.0330** | **1116.0767** | **1116.2058** | **-0.1290** | **1** | **13** | **1.7e+02** | **1** | **KEVHCSTQQ + Carbamidomethyl (C)** |
|  | 2549 | **658.0461** | **1971.1163** | **1970.2560** | **0.8602** | **0** | **13** | **1.3e+02** | **1** | **AAGPAHASVCPPTPSLLPAR + Carbamidomethyl (C)** |
|  | 688 | **415.7224** | **1244.1452** | **1243.4296** | **0.7155** | **1** | **13** | **1.3e+02** | **1** | **LSCKASGYSLSK** |
|  | 3292 | **831.5583** | **2491.6527** | **2492.8259** | **-1.1732** | **1** | **13** | **1.3e+02** | **1** | **DMVNVCETNLSKPNPPSLAKYR + Oxidation (M)** |
|  | 3308 | **845.6419** | **2533.9035** | **2533.0187** | **0.8848** | **2** | **13** | **1.3e+02** | **1** | **ELMVQLEGLMKLLKTQGGVSWR + Oxidation (M)** |
|  | 3390 | **981.4436** | **2941.3086** | **2940.2079** | **1.1007** | **2** | **13** | **1.1e+02** | **1** | **AYKRLDGSTECCNNHSLTDVCFSYR + Carbamidomethyl (C)** |
|  | 247 | **384.2513** | **1149.7318** | **1150.1575** | **-0.4258** | **1** | **13** | **1e+02** | **1** | **GTRAESEASSR** |
|  | 344 | **389.0540** | **776.0931** | **776.7940** | **-0.7008** | **0** | **13** | **1.5e+02** | **1** | **GGWGSDAK** |
|  | 1161 | **463.4017** | **924.7887** | **924.9295** | **-0.1409** | **0** | **13** | **1e+02** | **1** | **GDSGESSALC** |
|  | 2037 | **584.3655** | **1750.0743** | **1750.0251** | **0.0492** | **0** | **13** | **1.3e+02** | **1** | **VDGPVDPPAATVLPMVR + Oxidation (M)** |
|  | 491 | **404.0854** | **1209.2339** | **1210.4033** | **-1.1694** | **1** | **13** | **1.4e+02** | **1** | **VKEVFGTGAMR + Oxidation (M)** |
|  | 1202 | **468.2787** | **1401.8141** | **1401.4821** | **0.3319** | **1** | **13** | **1.3e+02** | **1** | **LEQGGTADGLRER** |
|  | 1628 | **520.9551** | **1559.8431** | **1558.6493** | **1.1937** | **1** | **13** | **1.3e+02** | **1** | **ARIASESAGSDPLER** |
|  | 105 | **369.3149** | **1104.9226** | **1104.2991** | **0.6235** | **0** | **13** | **1.3e+02** | **1** | **QQLLIGAYAK** |
|  | 1275 | **476.4611** | **950.9074** | **950.0285** | **0.8789** | **0** | **13** | **1.3e+02** | **1** | **GICNSSDVR** |
|  | 1363 | **488.4299** | **1462.2676** | **1462.5670** | **-0.2993** | **1** | **13** | **1.3e+02** | **1** | **FVSRAQSAEPSGAR** |
|  | 1824 | **544.2829** | **1086.5510** | **1086.2426** | **0.3084** | **1** | **13** | **1.5e+02** | **1** | **GAIVTGKGLDR** |
|  | 229 | **379.9742** | **1136.9005** | **1137.3094** | **-0.4089** | **0** | **13** | **1.6e+02** | **1** | **MVGGQIFVDR + Oxidation (M)** |
|  | 2770 | **688.9128** | **1375.8109** | **1375.3987** | **0.4123** | **1** | **13** | **1.2e+02** | **1** | **ERQVSEAEENGK** |
|  | 838 | **430.9124** | **1289.7149** | **1290.4863** | **-0.7714** | **1** | **13** | **1.6e+02** | **1** | **ESVTQIMKNPK + Oxidation (M)** |
|  | 1850 | **549.2292** | **1644.6654** | **1643.8848** | **0.7806** | **0** | **13** | **1.4e+02** | **1** | **GMSNCSMTPMTSAPR + Carbamidomethyl (C); Oxidation (M)** |
|  | 2928 | **741.3372** | **2220.9893** | **2221.5610** | **-0.5717** | **1** | **13** | **1.3e+02** | **1** | **APRSCPGAPAPEGLTWMGPAVR** |
|  | 1647 | **522.9263** | **1043.8378** | **1043.1716** | **0.6661** | **0** | **13** | **1.4e+02** | **1** | **AETINATVPK** |
|  | 2086 | **591.8716** | **1181.7284** | **1181.2312** | **0.4972** | **1** | **13** | **1.1e+02** | **1** | **DCSNTAEKEK + Carbamidomethyl (C)** |
|  | 586 | **407.5322** | **1219.5745** | **1220.3284** | **-0.7539** | **0** | **13** | **1.5e+02** | **1** | **ISEDLPYEVR** |
|  | 699 | **416.3690** | **830.7233** | **829.8650** | **0.8583** | **2** | **13** | **1.5e+02** | **1** | **RGGGRGDR** |
|  | 985 | **445.1070** | **888.1992** | **887.1017** | **1.0975** | **2** | **13** | **1.6e+02** | **1** | **GTRIKLCP** |
|  | 1190 | **467.2277** | **1398.6608** | **1399.7624** | **-1.1016** | **0** | **13** | **1.4e+02** | **1** | **CPLIALWCPITL + Carbamidomethyl (C)** |
|  | 2866 | **722.2881** | **1442.5614** | **1443.5618** | **-1.0004** | **1** | **13** | **1.4e+02** | **1** | **GEDQALLRGLESR** |
|  | 2938 | **741.8269** | **2222.4585** | **2223.3784** | **-0.9198** | **2** | **13** | **1.5e+02** | **1** | **SSPSQIATVTGNMESKEERR + Oxidation (M)** |
|  | 279 | **386.0476** | **770.0805** | **768.9029** | **1.1776** | **0** | **13** | **1.2e+02** | **1** | **SSMCLR + Carbamidomethyl (C); Oxidation (M)** |
|  | 546 | **406.2229** | **1215.6465** | **1216.3430** | **-0.6964** | **0** | **13** | **1.1e+02** | **1** | **GQLVESVGWNK** |
|  | 1328 | **485.7178** | **969.4208** | **970.0448** | **-0.6240** | **2** | **13** | **1e+02** | **1** | **SRDPSPRR** |
|  | 1564 | **514.2363** | **1026.4579** | **1027.1756** | **-0.7177** | **1** | **13** | **1.4e+02** | **1** | **RLTAAPPSSK** |
|  | 1896 | **557.2709** | **1668.7904** | **1667.8811** | **0.9093** | **0** | **13** | **1.4e+02** | **1** | **EAMNHTVSLLNITQP** |
|  | 1332 | **486.0894** | **1455.2459** | **1455.5741** | **-0.3282** | **0** | **13** | **1.3e+02** | **1** | **VLANSGDPNYLHR** |
|  | 2824 | **714.5808** | **2140.7203** | **2140.3574** | **0.3629** | **0** | **13** | **1.1e+02** | **1** | **MDPNCSCATGGSCSCASSCK + 4 Carbamidomethyl (C)** |
|  | 421 | **400.3029** | **798.5910** | **798.9272** | **-0.3362** | **0** | **13** | **99** | **1** | **LVSQAPGK** |
|  | 195 | **376.9622** | **751.9097** | **751.8924** | **0.0173** | **1** | **13** | **1.3e+02** | **1** | **MKGTSTK** |
|  | 187 | **376.0078** | **750.0007** | **748.8486** | **1.1522** | **0** | **13** | **1.4e+02** | **1** | **GDVSLCR** |
|  | 480 | **403.8640** | **805.7132** | **804.9185** | **0.7947** | **1** | **13** | **1.4e+02** | **1** | **VRGGCTR + Carbamidomethyl (C)** |
|  | 2009 | **578.8549** | **1733.5424** | **1732.9828** | **0.5596** | **2** | **13** | **1.2e+02** | **1** | **MNPQQRRMAAIGTDK + Oxidation (M)** |
|  | 1270 | **476.1492** | **950.2836** | **949.0902** | **1.1935** | **2** | **13** | **1.4e+02** | **1** | **SRSCGKVR + Carbamidomethyl (C)** |
|  | 1280 | **477.1375** | **1428.3902** | **1429.5798** | **-1.1897** | **0** | **13** | **1.4e+02** | **1** | **GCSYNICHSFGK + 2 Carbamidomethyl (C)** |
|  | 1099 | **458.8459** | **1373.5156** | **1374.4619** | **-0.9463** | **2** | **13** | **1.6e+02** | **1** | **AEPEFAGGRRER** |
|  | 3287 | **828.3006** | **2481.8796** | **2480.7722** | **1.1075** | **2** | **13** | **1.3e+02** | **1** | **ESVGKQATGEVAGKGGPVGGKPTLQK** |
|  | 3323 | **851.8149** | **1701.6151** | **1700.9192** | **0.6958** | **2** | **13** | **1.1e+02** | **1** | **TRCCRCASWWPSTS + Carbamidomethyl (C)** |
|  | 807 | **428.2838** | **1281.8294** | **1281.4908** | **0.3385** | **2** | **13** | **99** | **1** | **CQLPRQAPRR + Carbamidomethyl (C)** |
|  | 1854 | **549.7141** | **1646.1200** | **1646.0233** | **0.0967** | **2** | **13** | **1.4e+02** | **1** | **KTLVLPKKPEVPPVT** |
|  | 2695 | **680.9460** | **1359.8773** | **1360.5645** | **-0.6872** | **0** | **13** | **1.2e+02** | **1** | **LRPPTPSSPRPR** |
|  | 121 | **370.1722** | **1107.4945** | **1107.2617** | **0.2328** | **1** | **13** | **1.1e+02** | **1** | **LWANSNKFK** |
|  | 174 | **374.3311** | **746.6475** | **745.8644** | **0.7830** | **0** | **13** | **1.6e+02** | **1** | **SLATLGGK** |
|  | 668 | **413.2477** | **824.4806** | **823.8290** | **0.6517** | **0** | **13** | **99** | **1** | **SNSSMGGQG** |
|  | 2146 | **597.0210** | **1192.0272** | **1192.4343** | **-0.4071** | **2** | **13** | **1.6e+02** | **1** | **MGNFKSRKPK** |
|  | 2330 | **615.2773** | **1842.8097** | **1843.1546** | **-0.3450** | **1** | **13** | **1.4e+02** | **1** | **VYHYQMKIHVFSYK** |
|  | 164 | 374.2019 | 1119.5835 | 1119.2726 | 0.3110 | 0 | 13 | 1.4e+02 | 1 | CHGLSGSCEVK |
|  | 525 | **405.7297** | **1214.1670** | **1214.3952** | **-0.2281** | **0** | **13** | **1.2e+02** | **1** | **MRPAVLGSPDR + Oxidation (M)** |
|  | 816 | **429.0053** | **855.9958** | **854.9705** | **1.0253** | **0** | **13** | **1.3e+02** | **1** | **AIECYTR** |
|  | 2191 | **599.8225** | **1796.4454** | **1797.0017** | **-0.5564** | **2** | **13** | **1.3e+02** | **1** | **EHKEKNIQLVADGCR + Carbamidomethyl (C)** |
|  | 2878 | **729.7615** | **1457.5083** | **1456.6717** | **0.8366** | **2** | **13** | **1.5e+02** | **1** | **SPRRMSFSGIFR + Oxidation (M)** |
|  | 3106 | **777.8558** | **2330.5451** | **2330.5927** | **-0.0475** | **1** | **13** | **1.6e+02** | **1** | **VSASPDPRPLKEEEEAPLLPR** |
|  | 353 | **389.1501** | **1164.4280** | **1164.2105** | **0.2175** | **1** | **13** | **1.5e+02** | **1** | **GGGRGGMGSAGER + Oxidation (M)** |
|  | 974 | **443.8853** | **1328.6338** | **1327.5559** | **1.0778** | **2** | **13** | **1.5e+02** | **1** | **RCDLRAIAPQK + Carbamidomethyl (C)** |
|  | 2467 | **642.3594** | **1282.7040** | **1282.4194** | **0.2846** | **0** | **13** | **1.4e+02** | **1** | **SFNDDAMLIEK** |
|  | 152 | **373.1459** | **744.2770** | **744.8366** | **-0.5597** | **0** | **13** | **1.7e+02** | **1** | **ADLGTLR** |
|  | 168 | 374.2372 | 1119.6894 | 1119.3554 | 0.3340 | 0 | 13 | 1.4e+02 | 1 | GMCTSPPLIK + Carbamidomethyl (C); Oxidation (M) |
|  | 1447 | 499.7292 | 1496.1653 | 1495.7273 | 0.4380 | 0 | 13 | 1.1e+02 | 1 | HQLSHPGLPGALLR |
|  | 1655 | **523.3464** | **1044.6781** | **1045.1476** | **-0.4696** | **0** | **13** | **1.3e+02** | **1** | **TQISDVLGGR** |
|  | 1907 | **559.3749** | **1675.1025** | **1675.0079** | **0.0946** | **1** | **13** | **1.3e+02** | **1** | **CPAGGNPMPTMRWLK + Oxidation (M)** |
|  | 2224 | **604.2817** | **1206.5487** | **1207.4191** | **-0.8704** | **2** | **13** | **1.5e+02** | **1** | **KKGISFASELK** |
|  | 2621 | **668.8485** | **2003.5234** | **2003.3438** | **0.1796** | **0** | **13** | **1.4e+02** | **1** | **SWLPMGANYIIMNYSVK + Oxidation (M)** |
|  | 3303 | **841.2039** | **1680.3929** | **1680.0411** | **0.3519** | **1** | **13** | **1.2e+02** | **1** | **LKQAMEMVGFLPATK + Oxidation (M)** |
|  | 3334 | **865.8550** | **2594.5428** | **2593.9558** | **0.5869** | **0** | **13** | **1.2e+02** | **1** | **CPSPPYINLISVGGQHQGVFGLPR + Carbamidomethyl (C)** |
|  | 936 | **437.4174** | **872.8201** | **871.9780** | **0.8422** | **0** | **13** | **1.6e+02** | **1** | **VEEGAVLR** |
|  | 1070 | **457.3705** | **1369.0895** | **1368.6177** | **0.4717** | **0** | **13** | **1.2e+02** | **1** | **LNIIIVAEGAIDK** |
|  | 1935 | **563.6763** | **1688.0066** | **1689.0991** | **-1.0924** | **2** | **13** | **1.6e+02** | **1** | **CQSILNMAMFRCKK + Oxidation (M)** |
|  | 2902 | **738.6790** | **2213.0149** | **2212.2182** | **0.7966** | **1** | **13** | **1.2e+02** | **1** | **MQEEEEQSWKEEEEEAR + Oxidation (M)** |
|  | 1088 | **458.2246** | **1371.6517** | **1372.4874** | **-0.8357** | **1** | **13** | **1.5e+02** | **1** | **SISSDGRPLERR** |
|  | 2345 | **618.2551** | **1851.7432** | **1851.1584** | **0.5848** | **2** | **13** | **1.5e+02** | **1** | **LGVGAELLVDVGQRLRR** |
|  | 2791 | **698.7698** | **2093.2873** | **2092.5596** | **0.7278** | **2** | **13** | **1.7e+02** | **1** | **MVRPGLARMSLPRCWFR + Oxidation (M)** |
|  | 2164 | **598.7620** | **1195.5093** | **1195.3455** | **0.1638** | **0** | **13** | **1.4e+02** | **1** | **VCGGVTPSSFGGK** |
|  | 2374 | **623.3352** | **1244.6556** | **1244.4458** | **0.2099** | **0** | **13** | **1.5e+02** | **1** | **SHQCPLCPFR + Carbamidomethyl (C)** |
|  | 311 | **387.0166** | **1158.0275** | **1158.3517** | **-0.3241** | **2** | **13** | **1.6e+02** | **1** | **CCRSLFKTSG + Carbamidomethyl (C)** |
|  | 1532 | **507.8341** | **1520.4801** | **1520.5995** | **-0.1194** | **0** | **13** | **1.3e+02** | **1** | **STGSIVGQQPFGGAGSA** |
|  | 1941 | **564.2953** | **1689.8637** | **1689.8056** | **0.0581** | **0** | **13** | **1.6e+02** | **1** | **GPCVWDTFTHQGGER** |
|  | 3081 | **769.7532** | **1537.4917** | **1536.6966** | **0.7951** | **1** | **13** | **1.2e+02** | **1** | **RPPSGIWGGPGRGSR** |
|  | 730 | **419.1357** | **1254.3850** | **1255.5099** | **-1.1249** | **0** | **13** | **1.7e+02** | **1** | **SPPCTWCCACK + Carbamidomethyl (C)** |
|  | 2215 | **602.5630** | **1804.6668** | **1804.8647** | **-0.1980** | **0** | **13** | **1.3e+02** | **1** | **SHGDYGVSSYYGLDVW** |
|  | 2277 | **610.2721** | **1827.7941** | **1828.0556** | **-0.2614** | **2** | **13** | **1.5e+02** | **1** | **LKLMDEPALRSPGGGDR + Oxidation (M)** |
|  | 2395 | **628.6664** | **1882.9770** | **1882.1229** | **0.8541** | **1** | **13** | **1.6e+02** | **1** | **DMPTREIFDVCINQK + Carbamidomethyl (C); Oxidation (M)** |
|  | 2639 | **671.4965** | **2011.4672** | **2012.1620** | **-0.6947** | **2** | **13** | **1.3e+02** | **1** | **DSRRTGDYYYMDVWGK** |
|  | 640 | **409.1303** | **816.2458** | **816.8564** | **-0.6105** | **0** | **13** | **1.6e+02** | **1** | **EDIEGVR** |
|  | 1995 | **576.6204** | **1151.2259** | **1150.4159** | **0.8101** | **1** | **13** | **1.6e+02** | **1** | **MVSKMLQAAR + Oxidation (M)** |
|  | 2041 | **585.2051** | **1752.5931** | **1753.0040** | **-0.4109** | **1** | **13** | **1.5e+02** | **1** | **QLDKMVLEDNCSLTK + Oxidation (M)** |
|  | 2273 | **610.1149** | **1827.3224** | **1827.8667** | **-0.5442** | **1** | **13** | **1.5e+02** | **1** | **EGPAGGTGGSGGPGGSLGSRGR** |
|  | 584 | **407.4865** | **1219.4374** | **1218.2711** | **1.1662** | **0** | **13** | **1.7e+02** | **1** | **SILEPDTSNSR** |
|  | 1325 | **484.5241** | **967.0334** | **968.1580** | **-1.1245** | **1** | **13** | **1.6e+02** | **1** | **MHCNHKAK** |
|  | 2712 | **683.9583** | **1365.9017** | **1366.5638** | **-0.6620** | **0** | **13** | **1.2e+02** | **1** | **IYNFFNGNCMK + Oxidation (M)** |
|  | 2762 | **687.3809** | **1372.7471** | **1371.5590** | **1.1881** | **0** | **13** | **1.4e+02** | **1** | **MPISSELAPSSPR** |
|  | 2774 | **690.5284** | **2068.5632** | **2069.3838** | **-0.8207** | **1** | **13** | **1.3e+02** | **1** | **SAAAVICAYLVEYSPSRIR** |
|  | 202 | **377.1463** | **752.2778** | **752.9434** | **-0.6656** | **0** | **13** | **1.3e+02** | **1** | **MVMAASK + Oxidation (M)** |
|  | 333 | **388.2466** | **774.4784** | **774.8593** | **-0.3809** | **0** | **13** | **1.4e+02** | **1** | **SGLELEK** |
|  | 1340 | **486.7274** | **971.4401** | **971.0228** | **0.4174** | **0** | **13** | **1.2e+02** | **1** | **NEANDPALK** |
|  | 2384 | **627.1147** | **1878.3221** | **1877.2636** | **1.0584** | **2** | **13** | **1.4e+02** | **1** | **CVCMLGDIRLRGQTGVR** |
|  | 727 | **419.0974** | **1254.2700** | **1254.3663** | **-0.0963** | **0** | **13** | **1.7e+02** | **1** | **MDVGGLSDPYGK + Oxidation (M)** |
|  | 1205 | **469.0811** | **936.1473** | **935.1180** | **1.0293** | **0** | **13** | **1.5e+02** | **1** | **LGLGLFSTK** |
|  | 1290 | **478.5645** | **1432.6714** | **1433.7590** | **-1.0877** | **0** | **13** | **1.7e+02** | **1** | **NHMLLCLIEGMK + 2 Oxidation (M)** |
|  | 2189 | **599.7912** | **1796.3514** | **1796.0700** | **0.2814** | **2** | **13** | **1.4e+02** | **1** | **KVIKAPIEDLDLEGQK** |
|  | 2391 | **628.4420** | **1254.8693** | **1255.4272** | **-0.5579** | **2** | **13** | **1.3e+02** | **1** | **AKSSGLGVRAGPR** |
|  | 2721 | **684.1738** | **1366.3329** | **1365.5098** | **0.8231** | **0** | **13** | **1.4e+02** | **1** | **QIVSPSSSMAQSK + Oxidation (M)** |
|  | 182 | **375.1762** | **1122.5065** | **1121.3746** | **1.1318** | **1** | **13** | **1.4e+02** | **1** | **MAVCIETAKR** |
|  | 1195 | **467.7092** | **1400.1055** | **1399.5327** | **0.5728** | **2** | **13** | **1.3e+02** | **1** | **DRDAMPGARGPEK** |
|  | 2014 | **579.0428** | **1734.1062** | **1734.8262** | **-0.7200** | **1** | **13** | **1.5e+02** | **1** | **AHLGPQHAYGSASGREP** |
|  | 2378 | **623.9199** | **1868.7376** | **1868.1427** | **0.5949** | **2** | **13** | **1.2e+02** | **1** | **NPQREKEMMQLYIR + 2 Oxidation (M)** |
|  | 2411 | **631.0793** | **1890.2159** | **1890.0820** | **0.1339** | **0** | **13** | **1.5e+02** | **1** | **PDPNCSCAAGDSCTCAGSCK** |
|  | 2683 | **679.9264** | **1357.8380** | **1358.6131** | **-0.7751** | **2** | **13** | **1.2e+02** | **1** | **RVGRVLLCETR + Carbamidomethyl (C)** |
|  | 3070 | **765.0355** | **2292.0842** | **2292.7515** | **-0.6672** | **2** | **13** | **1.1e+02** | **1** | **MGRTQPPCARTPCVCLCLR + 3 Carbamidomethyl (C); Oxidation (M)** |
|  | 1100 | **458.8710** | **1373.5907** | **1373.6211** | **-0.0304** | **2** | **13** | **1.6e+02** | **1** | **KNAVELELAKCR** |
|  | 1293 | **478.8649** | **1433.5726** | **1433.6267** | **-0.0541** | **0** | **13** | **1.4e+02** | **1** | **MDTPQLTLTELR + Oxidation (M)** |
|  | 1617 | **519.2405** | **1554.6993** | **1555.7995** | **-1.1002** | **2** | **13** | **1.4e+02** | **1** | **FKELAAEFSRMAR** |
|  | 632 | **408.9202** | **1223.7383** | **1224.3387** | **-0.6004** | **0** | **13** | **1.6e+02** | **1** | **ITSVTSVASACE + Carbamidomethyl (C)** |
|  | 23 | **362.9388** | **1085.7942** | **1085.2862** | **0.5080** | **2** | **13** | **1.4e+02** | **1** | **MTRCRGSCR + Oxidation (M)** |
|  | 336 | **388.3188** | **1161.9344** | **1162.4266** | **-0.4922** | **1** | **13** | **1.5e+02** | **1** | **MTRLLEMPR + Oxidation (M)** |
|  | 726 | **419.0044** | **835.9939** | **836.9140** | **-0.9200** | **1** | **13** | **1.7e+02** | **1** | **GKMSEGGR + Oxidation (M)** |
|  | 1611 | **519.0425** | **1036.0703** | **1036.1179** | **-0.0476** | **1** | **13** | **1.4e+02** | **1** | **DEKENVMR + Oxidation (M)** |
|  | 1942 | **564.3891** | **1126.7634** | **1127.4422** | **-0.6788** | **2** | **13** | **1.4e+02** | **1** | **ETKCMIKMK + Oxidation (M)** |
|  | 2657 | **674.9316** | **2021.7728** | **2021.2184** | **0.5543** | **0** | **13** | **1.2e+02** | **1** | **TPEPARPGAPAAGASAVGCAGGR** |
|  | 282 | **386.0903** | **1155.2486** | **1156.2730** | **-1.0243** | **2** | **13** | **1.4e+02** | **1** | **RDSNFMRSK + Oxidation (M)** |
|  | 476 | **403.7797** | **1208.3168** | **1208.2564** | **0.0605** | **0** | **13** | **1.5e+02** | **1** | **EMQNSLNNDK + Oxidation (M)** |
|  | 1053 | **455.0453** | **908.0758** | **907.0900** | **0.9858** | **2** | **13** | **1.5e+02** | **1** | **MEKISKR + Oxidation (M)** |
|  | 1808 | **540.8217** | **1619.4428** | **1618.8801** | **0.5626** | **2** | **13** | **1.2e+02** | **1** | **DMRMLEEAARLQR** |
|  | 2227 | **606.1439** | **1210.2731** | **1209.4185** | **0.8546** | **1** | **13** | **1.4e+02** | **1** | **RGFTVIVDMR + Oxidation (M)** |
|  | 206 | **377.1987** | **1128.5739** | **1128.3225** | **0.2514** | **0** | **13** | **1.2e+02** | **1** | **GPPNGMMPAPK + 2 Oxidation (M)** |
|  | 971 | **443.8812** | **1328.6215** | **1327.4615** | **1.1600** | **0** | **13** | **1.5e+02** | **1** | **DLGGMNTALFSW + Oxidation (M)** |
|  | 2481 | **644.6729** | **1930.9964** | **1930.2998** | **0.6965** | **2** | **13** | **1.6e+02** | **1** | **LKRMTASLCPRPAALQT + Carbamidomethyl (C); Oxidation (M)** |
|  | 1809 | **541.0150** | **1620.0229** | **1620.7652** | **-0.7423** | **1** | **13** | **1.5e+02** | **1** | **CRSDDTAVYFCAR + 2 Carbamidomethyl (C)** |
|  | 3293 | **831.6852** | **2492.0334** | **2492.8936** | **-0.8602** | **1** | **13** | **1.2e+02** | **1** | **GLMSAANMGNYFTKSLHSIFMR + Oxidation (M)** |
|  | 2941 | **741.9268** | **2222.7581** | **2223.6115** | **-0.8534** | **2** | **13** | **1.4e+02** | **1** | **MATCIGEKIEDFKVGNLLGK + Carbamidomethyl (C)** |
|  | 3149 | **793.7061** | **1585.3973** | **1584.6053** | **0.7920** | **0** | **12** | **1.1e+02** | **1** | **GPYESGSGHSSGLGHR** |
|  | 368 | **391.1481** | **1170.4221** | **1169.3313** | **1.0909** | **1** | **12** | **1.4e+02** | **1** | **SHSNLIKGVSK** |
|  | 886 | **434.2639** | **1299.7694** | **1300.4581** | **-0.6887** | **0** | **12** | **1.2e+02** | **1** | **VVQETVLVEER** |
|  | 1637 | **521.4760** | **1040.9372** | **1040.2571** | **0.6802** | **0** | **12** | **1.2e+02** | **1** | **SAMCAFPIK + Carbamidomethyl (C); Oxidation (M)** |
|  | 1699 | **528.7600** | **1055.5052** | **1056.1290** | **-0.6237** | **0** | **12** | **1.2e+02** | **1** | **MGNSEGCNTK + Oxidation (M)** |
|  | 3141 | **792.2289** | **1582.4430** | **1581.7605** | **0.6825** | **1** | **12** | **1.4e+02** | **1** | **AGRGPSHHPCPVGPR + Carbamidomethyl (C)** |
|  | 1072 | **457.5602** | **1369.6584** | **1370.4601** | **-0.8017** | **0** | **12** | **1.8e+02** | **1** | **LGAEDGEMEMEK + 2 Oxidation (M)** |
|  | 1281 | **477.2580** | **1428.7519** | **1429.5798** | **-0.8279** | **0** | **12** | **1.4e+02** | **1** | **GCSYNICHSFGK + 2 Carbamidomethyl (C)** |
|  | 2810 | **705.2860** | **1408.5572** | **1408.5229** | **0.0344** | **2** | **12** | **1.5e+02** | **1** | **RVHEGSQKSQPR** |
|  | 3363 | **908.5739** | **1815.1329** | **1814.1547** | **0.9782** | **1** | **12** | **1.4e+02** | **1** | **MELENIVANSLLLKAR** |
|  | 295 | **386.8165** | **771.6182** | **771.9052** | **-0.2869** | **1** | **12** | **1.6e+02** | **1** | **ALGKEVR** |
|  | 2078 | **590.9828** | **1769.9264** | **1769.9700** | **-0.0436** | **1** | **12** | **1.5e+02** | **1** | **ELRMSSSYPTGLADVK + Oxidation (M)** |
|  | 486 | **404.0263** | **1209.0568** | **1208.4122** | **0.6447** | **1** | **12** | **1.5e+02** | **1** | **NLGMSMRVER + Oxidation (M)** |
|  | 951 | **442.3452** | **1324.0136** | **1324.3633** | **-0.3497** | **1** | **12** | **1.1e+02** | **1** | **DKPRGSGSGGGGHR** |
|  | 1742 | **534.6721** | **1600.9942** | **1600.8812** | **0.1130** | **2** | **12** | **1.7e+02** | **1** | **GCLLQRIDQELKK + Carbamidomethyl (C)** |
|  | 258 | **384.9919** | **767.9689** | **766.9101** | **1.0589** | **1** | **12** | **1.3e+02** | **1** | **RWVCF + Carbamidomethyl (C)** |
|  | 1236 | **472.1942** | **1413.5605** | **1412.6342** | **0.9263** | **2** | **12** | **1.5e+02** | **1** | **LRSPSVLEVREK** |
|  | 1243 | **472.4225** | **942.8303** | **943.0374** | **-0.2071** | **0** | **12** | **1.3e+02** | **1** | **QPYGCYR + Carbamidomethyl (C)** |
|  | 1520 | **506.0841** | **1010.1534** | **1010.1468** | **0.0066** | **1** | **12** | **1.4e+02** | **1** | **MDAENMRK + Oxidation (M)** |
|  | 1777 | 538.0971 | 1074.1794 | 1074.1723 | 0.0071 | 1 | 12 | 1.6e+02 | 1 | APNTERCAR + Carbamidomethyl (C) |
|  | 1093 | **458.5646** | **1372.6716** | **1373.5348** | **-0.8632** | **0** | **12** | **1.9e+02** | **1** | **FYNAGLAYCHSK** |
|  | 1253 | **473.9709** | **945.9270** | **947.1105** | **-1.1835** | **0** | **12** | **1.6e+02** | **1** | **LQCASLQK + Carbamidomethyl (C)** |
|  | 2042 | **585.4969** | **1168.9791** | **1168.3665** | **0.6127** | **1** | **12** | **1.2e+02** | **1** | **AAKSMLDPAHK** |
|  | 2763 | **687.7384** | **1373.4620** | **1374.4387** | **-0.9766** | **0** | **12** | **1.7e+02** | **1** | **ESMETHFGSHGR** |
|  | 1074 | **457.5756** | **1369.7046** | **1369.7187** | **-0.0141** | **2** | **12** | **1.8e+02** | **1** | **YHKVCTMLKCK + Oxidation (M)** |
|  | 1803 | **540.3671** | **1078.7193** | **1078.3251** | **0.3943** | **1** | **12** | **1.3e+02** | **1** | **SPLEKFMVK** |
|  | 297 | **386.8807** | **1157.6199** | **1157.3024** | **0.3175** | **0** | **12** | **1.7e+02** | **1** | **AASGACMTCNR + Carbamidomethyl (C); Oxidation (M)** |
|  | 1475 | **502.2500** | **1503.7279** | **1502.6953** | **1.0326** | **1** | **12** | **1.6e+02** | **1** | **EIVCNVTLGGERR + Carbamidomethyl (C)** |
|  | 1812 | **541.8293** | **1081.6439** | **1081.2049** | **0.4390** | **1** | **12** | **1.2e+02** | **1** | **VTMTRNTSR + Oxidation (M)** |
|  | 1965 | **570.5605** | **1708.6595** | **1708.9119** | **-0.2524** | **2** | **12** | **1.4e+02** | **1** | **ERERGMEISPMVDK + 2 Oxidation (M)** |
|  | 3412 | **1169.5995** | **3505.7763** | **3504.9715** | **0.8048** | **2** | **12** | **1.1e+02** | **1** | **DVVLTAQPGAEQCAESSQHIRILQPQFRLIR** |
|  | 54 | **366.0988** | **1095.2742** | **1094.1620** | **1.1122** | **0** | **12** | **1.7e+02** | **1** | **GHCPGGEGPAGR** |
|  | 656 | **411.2574** | **1230.7499** | **1231.4469** | **-0.6971** | **0** | **12** | **1.3e+02** | **1** | **GHLGLPGGGVVLR** |
|  | 946 | **440.4001** | **1318.1780** | **1318.5012** | **-0.3232** | **0** | **12** | **1.6e+02** | **1** | **KPDLPQTNFCR** |
|  | 1129 | **460.5817** | **1378.7230** | **1377.6577** | **1.0653** | **1** | **12** | **1.9e+02** | **1** | **CPPAPVALLGQRR** |
|  | 1162 | **463.4876** | **1387.4406** | **1388.6358** | **-1.1953** | **2** | **12** | **1.6e+02** | **1** | **EIKEAMVRALGR + Oxidation (M)** |
|  | 472 | **403.2995** | **804.5843** | **804.9548** | **-0.3706** | **0** | **12** | **1.4e+02** | **1** | **AAGAGMISK** |
|  | 698 | **416.2837** | **830.5527** | **829.8997** | **0.6530** | **0** | **12** | **1.5e+02** | **1** | **LWPSGDR** |
|  | 1885 | **555.4246** | **1663.2517** | **1662.8481** | **0.4035** | **2** | **12** | **1.2e+02** | **1** | **ILRDASGTSRGVGFAR** |
|  | 2451 | **637.2826** | **1272.5504** | **1273.5234** | **-0.9730** | **2** | **12** | **1.5e+02** | **1** | **ILKDPYLQRK** |
|  | 2555 | **659.3485** | **1316.6822** | **1316.4871** | **0.1952** | **2** | **12** | **1.5e+02** | **1** | **SPARTQSPGKCGK** |
|  | 2965 | **747.2340** | **1492.4532** | **1491.8617** | **0.5916** | **2** | **12** | **1.5e+02** | **1** | **ATGKVLVMKELMR + Oxidation (M)** |
|  | 464 | **402.9616** | **1205.8627** | **1205.2807** | **0.5820** | **0** | **12** | **1.7e+02** | **1** | **NHVNVEGATHK** |
|  | 1181 | **465.9924** | **1394.9551** | **1395.5406** | **-0.5855** | **1** | **12** | **1.6e+02** | **1** | **TLSHKYTNEMR + Oxidation (M)** |
|  | 2757 | **686.2412** | **1370.4676** | **1369.6290** | **0.8386** | **0** | **12** | **1.5e+02** | **1** | **GAQPSPLLCSVLK + Carbamidomethyl (C)** |
|  | 551 | 406.4843 | 1216.4307 | 1215.2706 | 1.1601 | 1 | 12 | 1.7e+02 | 1 | SNEGADGPVKNK |
|  | 957 | **443.2518** | **1326.7331** | **1327.4929** | **-0.7598** | **2** | **12** | **1.3e+02** | **1** | **RGRGESGGLLGLR** |
|  | 3036 | **757.5692** | **2269.6855** | **2269.4960** | **0.1894** | **0** | **12** | **1.4e+02** | **1** | **FHYGTHYSNSAGVMHYLIR + Oxidation (M)** |
|  | 365 | **389.9934** | **777.9720** | **776.9449** | **1.0272** | **1** | **12** | **1.6e+02** | **1** | **KATAAMGK** |
|  | 715 | **417.9847** | **1250.9319** | **1250.5101** | **0.4218** | **0** | **12** | **1.6e+02** | **1** | **CPMSCYYIVR + Oxidation (M)** |
|  | 780 | **424.8291** | **1271.4652** | **1271.3340** | **0.1313** | **1** | **12** | **1.7e+02** | **1** | **KDDVTSSTGPHK** |
|  | 1274 | **476.4239** | **950.8331** | **950.9503** | **-0.1173** | **0** | **12** | **1.3e+02** | **1** | **QTSGTSNTR** |
|  | 314 | **387.1376** | **1158.3905** | **1158.3038** | **0.0868** | **2** | **12** | **1.8e+02** | **1** | **EGKYYSKGVK** |
|  | 1643 | **522.2905** | **1042.5662** | **1043.1781** | **-0.6120** | **1** | **12** | **1.5e+02** | **1** | **CNECGKAYR** |
|  | 2007 | **578.4510** | **1732.3308** | **1731.9151** | **0.4156** | **2** | **12** | **1.2e+02** | **1** | **AAQRCGGGARADAGPMAR + Oxidation (M)** |
|  | 2882 | **730.9723** | **2189.8947** | **2189.4926** | **0.4021** | **1** | **12** | **1.3e+02** | **1** | **NLPLPPPPPPRGGDLMAYDR + Oxidation (M)** |
|  | 1215 | **470.0208** | **1407.0404** | **1406.6064** | **0.4340** | **1** | **12** | **1.4e+02** | **1** | **MPRDFQQVELK + Oxidation (M)** |
|  | 115 | **369.4200** | **1105.2377** | **1106.2554** | **-1.0177** | **0** | **12** | **1.9e+02** | **1** | **WSAWFHACV** |
|  | 1040 | **452.1660** | **1353.4757** | **1353.4825** | **-0.0068** | **0** | **12** | **1.9e+02** | **1** | **GMAQDSAAAQTMR + Oxidation (M)** |
|  | 158 | **374.0448** | **1119.1121** | **1120.2556** | **-1.1435** | **0** | **12** | **2e+02** | **1** | **SVPASNAIAYK** |
|  | 320 | **387.8272** | **1160.4595** | **1160.3478** | **0.1118** | **1** | **12** | **1.9e+02** | **1** | **ENQVIVRMR + Oxidation (M)** |
|  | 406 | **399.2199** | **1194.6376** | **1195.3685** | **-0.7309** | **1** | **12** | **1.1e+02** | **1** | **QTLLKAPDPGR** |
|  | 1417 | **495.4899** | **1483.4475** | **1484.6372** | **-1.1896** | **2** | **12** | **1.6e+02** | **1** | **HTVPSCRGEDAKGK** |
|  | 1834 | **546.5441** | **1636.6100** | **1635.8427** | **0.7673** | **2** | **12** | **1.7e+02** | **1** | **EARLCRQEFEQVK** |
|  | 2175 | **599.1127** | **1794.3158** | **1794.0607** | **0.2551** | **0** | **12** | **1.5e+02** | **1** | **MGWAAVMGLGGGTTALER + Oxidation (M)** |
|  | 2631 | **670.0836** | **1338.1525** | **1337.4846** | **0.6678** | **1** | **12** | **1.6e+02** | **1** | **AHTSTPREIGLR** |
|  | 2783 | **696.4144** | **1390.8141** | **1391.5503** | **-0.7363** | **1** | **12** | **1.6e+02** | **1** | **VATQEGKEITCR + Carbamidomethyl (C)** |
|  | 1264 | **475.7281** | **949.4415** | **949.1498** | **0.2918** | **1** | **12** | **1.3e+02** | **1** | **GAMGKCPTK + Carbamidomethyl (C)** |
|  | 1466 | **500.7536** | **1499.2387** | **1499.5870** | **-0.3482** | **1** | **12** | **1.2e+02** | **1** | **QQGPKNSSWAPGSR** |
|  | 2030 | **582.3261** | **1743.9560** | **1743.9160** | **0.0400** | **1** | **12** | **1.6e+02** | **1** | **CDECGKSFAQSSGLVR + Carbamidomethyl (C)** |
|  | 2445 | **635.8457** | **1904.5149** | **1904.1104** | **0.4046** | **2** | **12** | **1.4e+02** | **1** | **VRSPTRSPSGSMLAQADK + Oxidation (M)** |
|  | 880 | **433.9659** | **1298.8754** | **1298.5363** | **0.3391** | **2** | **12** | **1.5e+02** | **1** | **IPINPASRKFR** |
|  | 1594 | **518.4923** | **1552.4548** | **1551.7610** | **0.6937** | **0** | **12** | **1.5e+02** | **1** | **ISGLVEGTMYYFR + Oxidation (M)** |
|  | 2135 | **595.5959** | **1783.7655** | **1784.0229** | **-0.2574** | **1** | **12** | **1.7e+02** | **1** | **MPNGMYDGSALIRASGK + Oxidation (M)** |
|  | 1344 | **487.0341** | **972.0535** | **972.0968** | **-0.0433** | **1** | **12** | **1.7e+02** | **1** | **LNLAGRDLT** |
|  | 2468 | **642.5341** | **1283.0533** | **1282.4859** | **0.5675** | **2** | **12** | **1.2e+02** | **1** | **EAPKPKVEEKK** |
|  | 2648 | **672.7839** | **1343.5531** | **1342.4383** | **1.1148** | **1** | **12** | **1.8e+02** | **1** | **FTVSRDNSMNR + Oxidation (M)** |
|  | 2722 | **684.1969** | **1366.3790** | **1366.6549** | **-0.2759** | **2** | **12** | **1.5e+02** | **1** | **IAGIRGIQGVVRK** |
|  | 2876 | **727.9451** | **2180.8130** | **2181.4689** | **-0.6559** | **2** | **12** | **1.3e+02** | **1** | **KMAAGKSGGSAGEITFLEALAR + Oxidation (M)** |
|  | 59 | **367.1007** | **1098.2800** | **1099.2199** | **-0.9398** | **0** | **12** | **1.7e+02** | **1** | **SSYGMNWVR** |
|  | 3284 | **828.0637** | **2481.1690** | **2480.7722** | **0.3968** | **2** | **12** | **1.3e+02** | **1** | **ESVGKQATGEVAGKGGPVGGKPTLQK** |
|  | 233 | **380.1403** | **1137.3987** | **1136.3029** | **1.0957** | **2** | **12** | **1.8e+02** | **1** | **QHLKKAPWE** |
|  | 2209 | **601.2303** | **1200.4459** | **1199.3572** | **1.0887** | **1** | **12** | **1.6e+02** | **1** | **QAELEAARLAK** |
|  | 3094 | **776.9448** | **1551.8749** | **1552.6431** | **-0.7683** | **0** | **12** | **1.7e+02** | **1** | **VLQYYSAATEHDR** |
|  | 3397 | **1002.0409** | **2002.0670** | **2001.2435** | **0.8235** | **1** | **12** | **1.4e+02** | **1** | **EAYRQLLQTENLPAQVK** |
|  | 118 | **370.0778** | **1107.2112** | **1106.2390** | **0.9721** | **2** | **12** | **1.5e+02** | **1** | **RARTVAGYGR** |
|  | 570 | **407.0971** | **1218.2692** | **1219.3320** | **-1.0628** | **1** | **12** | **1.6e+02** | **1** | **NGTAVCATNRR + Carbamidomethyl (C)** |
|  | 596 | **407.7914** | **813.5680** | **812.8909** | **0.6772** | **0** | **12** | **1.6e+02** | **1** | **GFCETTR** |
|  | 2563 | **662.7902** | **1985.3483** | **1985.1562** | **0.1921** | **1** | **12** | **1.8e+02** | **1** | **LLDINKDFQPYYGEGGR** |
|  | 2803 | **702.9980** | **2105.9718** | **2106.3643** | **-0.3925** | **1** | **12** | **1.3e+02** | **1** | **NQRITSPVHVSFYVCNGK + Carbamidomethyl (C)** |
|  | 194 | **376.2813** | **750.5478** | **750.8645** | **-0.3166** | **0** | **12** | **1.2e+02** | **1** | **TMSGAIR + Oxidation (M)** |
|  | 303 | **386.9618** | **771.9088** | **771.9083** | **0.0005** | **1** | **12** | **1.8e+02** | **1** | **IRGSLAR** |
|  | 1463 | **500.3565** | **1498.0473** | **1498.6669** | **-0.6196** | **1** | **12** | **1.2e+02** | **1** | **VTSAPSALRSGHCR + Carbamidomethyl (C)** |
|  | 75 | **368.4286** | **1102.2637** | **1103.2136** | **-0.9499** | **1** | **12** | **2.2e+02** | **1** | **RVPGAGSSGCR + Carbamidomethyl (C)** |
|  | 107 | **369.3370** | **736.6592** | **736.8660** | **-0.2068** | **1** | **12** | **1.6e+02** | **1** | **LGRHVR** |
|  | 1759 | **536.4219** | **1070.8291** | **1070.2648** | **0.5642** | **0** | **12** | **1.3e+02** | **1** | **AATGACMTCNK** |
|  | 2758 | **686.3734** | **2056.0981** | **2055.5523** | **0.5457** | **1** | **12** | **1.6e+02** | **1** | **GSRLLCWVLLCLLGAGPVK + Carbamidomethyl (C)** |
|  | 619 | **408.2038** | **1221.5892** | **1220.3931** | **1.1962** | **1** | **12** | **1.5e+02** | **1** | **EEKALNLEMK + Oxidation (M)** |
|  | 2325 | **614.1257** | **1839.3548** | **1839.3165** | **0.0383** | **1** | **12** | **1.5e+02** | **1** | **MDMRVPAQLLGLLLLR** |
|  | 2868 | **724.7854** | **2171.3340** | **2171.5404** | **-0.2064** | **2** | **12** | **1.8e+02** | **1** | **EKTERLLCQMIPPSVAEAR** |
|  | 3019 | **749.6092** | **2245.8054** | **2246.3751** | **-0.5697** | **2** | **12** | **1.3e+02** | **1** | **TALSRSEATEEGGNQQMHRK + Oxidation (M)** |
|  | 889 | **434.3419** | **1300.0036** | **1300.5141** | **-0.5105** | **1** | **12** | **1.2e+02** | **1** | **CQNMLRSHVR + Carbamidomethyl (C)** |
|  | 1526 | **506.9631** | **1011.9114** | **1012.1210** | **-0.2097** | **0** | **12** | **1.6e+02** | **1** | **QASLLHTSR** |
|  | 1553 | **512.3474** | **1022.6800** | **1022.0696** | **0.6105** | **0** | **12** | **1.3e+02** | **1** | **DIQHAGVPGE** |
|  | 2167 | **598.9205** | **1793.7392** | **1792.8558** | **0.8834** | **2** | **12** | **1.3e+02** | **1** | **SQKNEKSEDIASQSNK** |
|  | 2454 | **638.0541** | **1911.1401** | **1911.3148** | **-0.1747** | **2** | **12** | **1.7e+02** | **1** | **ASLVFIGKVLVSSKCFR + Carbamidomethyl (C)** |
|  | 550 | **406.4014** | **1216.1820** | **1216.3692** | **-0.1872** | **0** | **12** | **1.6e+02** | **1** | **GCGGWGSLPGLR + Carbamidomethyl (C)** |
|  | 1693 | **527.4503** | **1052.8859** | **1053.2110** | **-0.3252** | **0** | **12** | **1.2e+02** | **1** | **AFISLPSYR** |
|  | 1753 | **536.1754** | **1605.5041** | **1605.7274** | **-0.2234** | **1** | **12** | **1.6e+02** | **1** | **TEDTAVYYCARGTR** |
|  | 3216 | **806.4889** | **2416.4445** | **2415.6621** | **0.7824** | **1** | **12** | **1.5e+02** | **1** | **QPSPGPTPPPFPGNRGTALGGGSIR** |
|  | 387 | **394.8344** | **1181.4810** | **1182.4193** | **-0.9384** | **2** | **12** | **1.8e+02** | **1** | **IQLRLAGQKR** |
|  | 1376 | **489.9523** | **1466.8348** | **1466.6415** | **0.1933** | **0** | **12** | **1.7e+02** | **1** | **ELAGHTGYLSCCR + Carbamidomethyl (C)** |
|  | 2486 | **646.8723** | **1291.7298** | **1291.3697** | **0.3601** | **0** | **12** | **1.4e+02** | **1** | **QNLSQFEAQAR** |
|  | 2798 | **700.7092** | **2099.1055** | **2098.3122** | **0.7933** | **1** | **12** | **1.5e+02** | **1** | **HLDFLDILLGARDEDDIK** |
|  | 339 | **388.4292** | **774.8436** | **774.8826** | **-0.0390** | **0** | **12** | **2.3e+02** | **1** | **EICADPK** |
|  | 2309 | **613.0516** | **1836.1326** | **1837.0456** | **-0.9131** | **2** | **12** | **1.6e+02** | **1** | **YNIFTYLRAGSGRYR** |
|  | 2918 | **740.6761** | **2219.0061** | **2218.5088** | **0.4973** | **1** | **12** | **1.3e+02** | **1** | **LALEDLQAAHRQEIQELLK** |
|  | 502 | **404.9333** | **1211.7777** | **1212.3529** | **-0.5752** | **0** | **12** | **1.5e+02** | **1** | **MEMAFHEASK + 2 Oxidation (M)** |
|  | 2063 | **587.8863** | **1173.7578** | **1174.2880** | **-0.5302** | **0** | **12** | **1.4e+02** | **1** | **AAAPGGLASSCGR + Carbamidomethyl (C)** |
|  | 2761 | **687.3510** | **1372.6872** | **1373.4688** | **-0.7816** | **2** | **12** | **1.6e+02** | **1** | **QAEEENELRKK** |
|  | 3092 | **776.8481** | **1551.6815** | **1551.8137** | **-0.1322** | **1** | **12** | **1.8e+02** | **1** | **LKITCHLGNNPWR** |
|  | 779 | **424.3214** | **1269.9421** | **1269.3875** | **0.5546** | **0** | **12** | **1.5e+02** | **1** | **MAQAAGPAGGGEPR** |
|  | 1943 | **564.4315** | **1690.2722** | **1690.7637** | **-0.4915** | **0** | **12** | **1.4e+02** | **1** | **DWGQDFGVVFDYWG** |
|  | 1613 | **519.0499** | **1036.0851** | **1035.1761** | **0.9090** | **1** | **12** | **1.6e+02** | **1** | **KLMSDNGVR + Oxidation (M)** |
|  | 1866 | **552.0875** | **1102.1601** | **1101.3665** | **0.7936** | **1** | **12** | **1.8e+02** | **1** | **KQTFCCCLR** |
|  | 2192 | **599.8555** | **1197.6962** | **1197.3843** | **0.3118** | **0** | **12** | **1.4e+02** | **1** | **DAGVIAGLNVLR** |
|  | 2351 | **619.3812** | **1855.1215** | **1854.0711** | **1.0504** | **0** | **12** | **1.6e+02** | **1** | **EGLPFVLFQFGNSWGR** |
|  | 1880 | **554.8287** | **1661.4640** | **1660.8011** | **0.6630** | **0** | **12** | **1.3e+02** | **1** | **LVGGPMDASVEEEGVR + Oxidation (M)** |
|  | 1928 | **563.1251** | **1686.3532** | **1685.9079** | **0.4453** | **1** | **12** | **1.6e+02** | **1** | **GCDAPPCCRNPGPWR + Carbamidomethyl (C)** |
|  | 3344 | **880.7029** | **1759.3910** | **1758.9735** | **0.4174** | **0** | **12** | **1.5e+02** | **1** | **CNSADLVNMHILQER + Oxidation (M)** |
|  | 1193 | **467.6652** | **933.3157** | **933.0475** | **0.2681** | **2** | **12** | **1.5e+02** | **1** | **QGRRGGSCL** |
|  | 1673 | **525.2118** | **1048.4088** | **1047.2909** | **1.1179** | **0** | **12** | **1.7e+02** | **1** | **SALVLLYLR** |
|  | 1749 | **536.0994** | **1070.1841** | **1069.2983** | **0.8858** | **0** | **12** | **1.7e+02** | **1** | **FQMCVAGIK + Carbamidomethyl (C); Oxidation (M)** |
|  | 2021 | **580.4218** | **1738.2433** | **1739.0237** | **-0.7804** | **1** | **12** | **1.4e+02** | **1** | **KWPGIKPTEVTIQNK** |
|  | 2589 | **666.5239** | **1331.0331** | **1331.4520** | **-0.4189** | **1** | **12** | **1.4e+02** | **1** | **KGPDSLSDGPACK + Carbamidomethyl (C)** |
|  | 508 | **404.9979** | **1211.9714** | **1212.4222** | **-0.4507** | **1** | **12** | **1.5e+02** | **1** | **DLLGLHNMKR + Oxidation (M)** |
|  | 1870 | **552.5134** | **1103.0121** | **1102.3281** | **0.6839** | **1** | **12** | **1.5e+02** | **1** | **ELCHCKLTV + Carbamidomethyl (C)** |
|  | 2373 | **623.2588** | **1244.5028** | **1243.4495** | **1.0533** | **0** | **12** | **1.7e+02** | **1** | **ALSGSATLVSIPK** |
|  | 2558 | **659.5085** | **1975.5035** | **1976.2820** | **-0.7786** | **1** | **12** | **1.4e+02** | **1** | **LNNTMNACAAIAALERVK + Carbamidomethyl (C); Oxidation (M)** |
|  | 3299 | **839.7924** | **2516.3549** | **2517.1469** | **-0.7920** | **0** | **12** | **1.3e+02** | **1** | **IAPWIMTPNILPPVSVFVCCMK + Carbamidomethyl (C)** |
|  | 1045 | **453.0656** | **904.1164** | **903.1192** | **0.9972** | **0** | **12** | **1.8e+02** | **1** | **VLFLGNLK** |
|  | 18 | **362.2025** | **1083.5854** | **1084.2516** | **-0.6662** | **0** | **12** | **1.3e+02** | **1** | **SGGLCQMCR + 2 Carbamidomethyl (C); Oxidation (M)** |
|  | 600 | **407.8851** | **813.7554** | **814.8040** | **-1.0486** | **0** | **12** | **1.6e+02** | **1** | **GSSHGQSR** |
|  | 1393 | **492.4047** | **1474.1918** | **1473.6857** | **0.5062** | **2** | **12** | **1.3e+02** | **1** | **LARPSERHRVPR** |
|  | 2350 | **619.2316** | **1236.4484** | **1236.3361** | **0.1123** | **1** | **12** | **1.6e+02** | **1** | **DWDPRTPPPR** |
|  | 2740 | **685.2684** | **1368.5221** | **1369.4124** | **-0.8903** | **0** | **12** | **1.7e+02** | **1** | **GPVSSTSDSSTNCK** |
|  | 591 | **407.7343** | **1220.1809** | **1219.4296** | **0.7512** | **1** | **12** | **1.5e+02** | **1** | **AYIKLNQLEK** |
|  | 1519 | **505.9700** | **1009.9253** | **1010.1716** | **-0.2463** | **2** | **12** | **1.5e+02** | **1** | **KQMREFR + Oxidation (M)** |
|  | 1691 | **527.2872** | **1052.5597** | **1053.2110** | **-0.6514** | **0** | **12** | **1.6e+02** | **1** | **AFISLPSYR** |
|  | 64 | **367.2838** | **732.5528** | **732.8491** | **-0.2963** | **0** | **12** | **1.5e+02** | **1** | **ALAEACR** |
|  | 3039 | **758.1526** | **2271.4356** | **2271.6227** | **-0.1871** | **1** | **12** | **1.5e+02** | **1** | **CTGTLRFVHQSCLHQWIK + 2 Carbamidomethyl (C)** |
|  | 433 | **401.3101** | **1200.9081** | **1201.5039** | **-0.5958** | **1** | **12** | **1.5e+02** | **1** | **MAFFCILRK + Carbamidomethyl (C); Oxidation (M)** |
|  | 587 | **407.5328** | **1219.5762** | **1220.4444** | **-0.8682** | **1** | **12** | **1.8e+02** | **1** | **VSRIPVSFCR + Carbamidomethyl (C)** |
|  | 1601 | **518.9110** | **1553.7109** | **1554.8346** | **-1.1237** | **0** | **12** | **1.6e+02** | **1** | **QPLPPYTPAMMHR + Oxidation (M)** |
|  | 2243 | **607.5323** | **1213.0498** | **1212.3363** | **0.7135** | **0** | **12** | **1.3e+02** | **1** | **EPVQVSTCGHR** |
|  | 67 | **368.0277** | **1101.0609** | **1101.2372** | **-0.1764** | **0** | **12** | **1.8e+02** | **1** | **AGSCPILGGGGR + Carbamidomethyl (C)** |
|  | 2730 | **684.4426** | **2050.3057** | **2049.1374** | **1.1683** | **0** | **12** | **1.6e+02** | **1** | **IQVSSGENMAGTAEGEGQQR** |
|  | 2123 | **594.8289** | **1187.6429** | **1187.4409** | **0.2021** | **1** | **12** | **1.5e+02** | **1** | **CHRAPIFMR + Carbamidomethyl (C)** |
|  | 2407 | **630.2478** | **1887.7212** | **1887.1458** | **0.5754** | **1** | **12** | **1.7e+02** | **1** | **LLIYEVSNRFSGVPHR** |
|  | 332 | **388.2225** | **1161.6454** | **1162.4414** | **-0.7959** | **0** | **12** | **1.6e+02** | **1** | **VPLDMSLFLK** |
|  | 457 | **402.3102** | **802.6056** | **802.9424** | **-0.3367** | **0** | **12** | **1.6e+02** | **1** | **KPGTCAAR** |
|  | 580 | **407.3109** | **1218.9106** | **1219.3468** | **-0.4363** | **1** | **12** | **1.3e+02** | **1** | **SKINYGGDIPR** |
|  | 2937 | **741.7842** | **2222.3306** | **2221.5082** | **0.8224** | **0** | **12** | **1.7e+02** | **1** | **SFSANMMEESEMCAVPGGLAK + 2 Oxidation (M)** |
|  | 3028 | **753.6752** | **2258.0035** | **2257.6015** | **0.4020** | **1** | **12** | **1.3e+02** | **1** | **DEITLSMLKSMLLMEAEDR + 2 Oxidation (M)** |
|  | 752 | **420.4705** | **1258.3892** | **1259.5581** | **-1.1689** | **2** | **12** | **1.9e+02** | **1** | **LLNKIKMDTAL** |
|  | 2438 | **634.9646** | **1901.8716** | **1901.2155** | **0.6561** | **2** | **12** | **1.4e+02** | **1** | **RTQYSCYCCKLSMK + 3 Carbamidomethyl (C); Oxidation (M)** |
|  | 1075 | **457.5841** | **1369.7301** | **1369.4371** | **0.2931** | **1** | **12** | **1.9e+02** | **1** | **NDSTLPSARAPPSG** |
|  | 1855 | **549.8003** | **1646.3787** | **1646.8438** | **-0.4650** | **1** | **12** | **1.3e+02** | **1** | **LFGFVRLHEDGAGTK** |
|  | 1231 | **471.8923** | **1412.6547** | **1412.4220** | **0.2327** | **1** | **12** | **1.6e+02** | **1** | **DREAAEGLGSHDR** |
|  | 1908 | **559.4564** | **1116.8981** | **1117.2070** | **-0.3090** | **0** | **12** | **1.4e+02** | **1** | **DINTIEDAVK** |
|  | 1078 | **457.6149** | **1369.8224** | **1369.4371** | **0.3854** | **1** | **12** | **1.6e+02** | **1** | **NDSTLPSARAPPSG** |
|  | 1154 | **462.0811** | **922.1474** | **921.1131** | **1.0343** | **0** | **12** | **1.6e+02** | **1** | **IISMNSLK + Oxidation (M)** |
|  | 2413 | **631.1440** | **1890.4100** | **1891.1149** | **-0.7050** | **2** | **12** | **1.8e+02** | **1** | **TTPTLGRTSSPRMAAATR + Oxidation (M)** |
|  | 2623 | **668.9501** | **1335.8854** | **1335.5468** | **0.3386** | **1** | **12** | **1.4e+02** | **1** | **LAVSPVCMEDKQ + Oxidation (M)** |
|  | 1606 | **518.9481** | **1035.8815** | **1036.2269** | **-0.3455** | **0** | **12** | **1.7e+02** | **1** | **LQLEACGMR + Oxidation (M)** |
|  | 2641 | **671.9336** | **1341.8524** | **1342.6268** | **-0.7744** | **2** | **12** | **1.4e+02** | **1** | **LLIKALSGGKNTK** |
|  | 3052 | **762.1850** | **1522.3552** | **1522.6733** | **-0.3181** | **2** | **12** | **1.7e+02** | **1** | **ARRWVGHGSAAAGAR** |
|  | 499 | **404.3533** | **1210.0376** | **1209.3340** | **0.7037** | **1** | **12** | **1.5e+02** | **1** | **LRSGNASTMTR + Oxidation (M)** |
|  | 1945 | **565.6676** | **1693.9806** | **1692.8940** | **1.0867** | **2** | **12** | **2.2e+02** | **1** | **QIFPSAKDNQKCTR + Carbamidomethyl (C)** |
|  | 2138 | **595.8480** | **1784.5217** | **1784.9677** | **-0.4459** | **0** | **12** | **1.5e+02** | **1** | **IMNYDEFQHCWNK + Carbamidomethyl (C)** |
|  | 2590 | **666.5247** | **1996.5520** | **1995.4031** | **1.1489** | **2** | **12** | **1.4e+02** | **1** | **ARPGQRPPKMCHLMKR + Carbamidomethyl (C); 2 Oxidation (M)** |
|  | 410 | **399.7075** | **1196.1002** | **1195.3719** | **0.7283** | **2** | **12** | **1.2e+02** | **1** | **KSPGIPAGANRK** |
|  | 2364 | **621.5163** | **1861.5267** | **1862.1330** | **-0.6063** | **1** | **12** | **1.3e+02** | **1** | **ELVFWSDVTLDRILR** |
|  | 2533 | **655.3695** | **1963.0863** | **1964.1619** | **-1.0756** | **1** | **12** | **1.7e+02** | **1** | **MTDQEAIQDLWQWRK + Oxidation (M)** |
|  | 400 | **399.0263** | **796.0378** | **796.9377** | **-0.8999** | **1** | **12** | **1.5e+02** | **1** | **SYLRCR** |
|  | 874 | **433.3526** | **1297.0355** | **1297.5218** | **-0.4862** | **0** | **12** | **1.4e+02** | **1** | **FMNPYNAVLTK** |
|  | 832 | **430.3611** | **1288.0613** | **1288.3693** | **-0.3080** | **0** | **12** | **1.6e+02** | **1** | **SPSSSSAARPWR** |
|  | 802 | **428.1067** | **1281.2979** | **1282.4444** | **-1.1465** | **0** | **12** | **1.8e+02** | **1** | **TDMTQLEACVR + Oxidation (M)** |
|  | 1090 | **458.2697** | **1371.7869** | **1370.6205** | **1.1664** | **0** | **12** | **1.6e+02** | **1** | **ASASGVFCCPLCR + Carbamidomethyl (C)** |
|  | 1091 | **458.5434** | **1372.6080** | **1373.5549** | **-0.9469** | **1** | **12** | **2.2e+02** | **1** | **ETLIDVARTSLR** |
|  | 170 | 374.3008 | 1119.8803 | 1120.3088 | -0.4285 | 1 | 12 | 1.7e+02 | 1 | RPRAPVTPAR |
|  | 575 | **407.2225** | **812.4302** | **812.9571** | **-0.5269** | **1** | **12** | **1.4e+02** | **1** | **RGFCAEM** |
|  | 940 | **437.8804** | **1310.6190** | **1309.5175** | **1.1015** | **2** | **12** | **2e+02** | **1** | **KISNHGSLRVAK** |
|  | 1527 | **506.9878** | **1011.9609** | **1012.0978** | **-0.1370** | **0** | **12** | **1.7e+02** | **1** | **SYQQAQCK + Carbamidomethyl (C)** |
|  | 2573 | **664.2648** | **1326.5148** | **1326.5248** | **-0.0101** | **1** | **12** | **1.7e+02** | **1** | **KGAQSLPGPCAAAR** |
|  | 3041 | **758.8589** | **1515.7030** | **1515.7387** | **-0.0357** | **2** | **12** | **2e+02** | **1** | **LNMGKRDPWNLR + Oxidation (M)** |
|  | 1715 | **530.3875** | **1058.7601** | **1059.2404** | **-0.4803** | **1** | **12** | **1.6e+02** | **1** | **SGMFWLRF + Oxidation (M)** |
|  | 401 | **399.0509** | **1194.1305** | **1193.4142** | **0.7164** | **2** | **12** | **1.6e+02** | **1** | **MKSVIENTKK + Oxidation (M)** |
|  | 944 | **439.1590** | **876.3031** | **877.0175** | **-0.7144** | **0** | **12** | **1.9e+02** | **1** | **LITEMDR** |
|  | 1031 | **450.7200** | **899.4252** | **899.0049** | **0.4203** | **1** | **12** | **1.4e+02** | **1** | **WPSPERK** |
|  | 2799 | **701.7883** | **2102.3426** | **2102.4996** | **-0.1570** | **2** | **12** | **2e+02** | **1** | **LKGQLCDVLLIVGDQKFR + Carbamidomethyl (C)** |
|  | 2828 | **714.7720** | **2141.2939** | **2142.3253** | **-1.0314** | **1** | **12** | **2e+02** | **1** | **VTTGGSNTEAFFGQGTRLTVV** |
|  | 2855 | **716.8816** | **2147.6226** | **2146.5556** | **1.0670** | **2** | **12** | **1.8e+02** | **1** | **RSFNFVLFFLQLCREVK** |
|  | 138 | **371.2767** | **740.5386** | **739.8601** | **0.6786** | **1** | **12** | **1.1e+02** | **1** | **KEAPPAK** |
|  | 142 | **372.1574** | **742.3000** | **742.8176** | **-0.5175** | **0** | **12** | **1.6e+02** | **1** | **ALGEEPK** |
|  | 343 | **389.0360** | **776.0571** | **776.8802** | **-0.8230** | **0** | **12** | **2e+02** | **1** | **GPFTISR** |
|  | 1296 | **479.7091** | **1436.1051** | **1436.7368** | **-0.6317** | **2** | **12** | **1.5e+02** | **1** | **ESMPSLMEKKLK + Oxidation (M)** |
|  | 2023 | **580.9798** | **1159.9448** | **1160.2831** | **-0.3382** | **1** | **12** | **1.8e+02** | **1** | **NTATWKNAVR** |
|  | 58 | **367.0575** | **1098.1504** | **1097.1827** | **0.9677** | **1** | **12** | **1.9e+02** | **1** | **TRTGSSFSVR** |
|  | 205 | **377.1869** | **752.3589** | **752.9434** | **-0.5844** | **0** | **12** | **1.4e+02** | **1** | **MVMAASK + Oxidation (M)** |
|  | 639 | **409.0182** | **1224.0323** | **1224.4481** | **-0.4158** | **1** | **12** | **2e+02** | **1** | **TQMEMLTKDK** |
|  | 1392 | **492.3868** | **1474.1383** | **1473.6957** | **0.4427** | **1** | **12** | **1.4e+02** | **1** | **AQPKEVMFPPGTR + Oxidation (M)** |
|  | 1682 | **526.7167** | **1051.4186** | **1050.2553** | **1.1633** | **1** | **12** | **1.5e+02** | **1** | **VLKNPPAGVR** |
|  | 1879 | 554.3004 | 1659.8791 | 1660.7377 | -0.8587 | 0 | 12 | 1.8e+02 | 1 | WISTYNGDTNYAQK |
|  | 2568 | **663.5267** | **1987.5580** | **1988.2216** | **-0.6636** | **0** | **12** | **1.4e+02** | **1** | **ICSTAVSYLEVYNEQIR** |
|  | 3111 | **778.0311** | **2331.0712** | **2331.3239** | **-0.2526** | **0** | **12** | **1.4e+02** | **1** | **SGTSHPEDFAEDAAPWSGAENR** |
|  | 926 | **436.9798** | **1307.9172** | **1307.6010** | **0.3162** | **0** | **12** | **2e+02** | **1** | **MVIEYIGTIIR** |
|  | 1199 | **468.0541** | **1401.1402** | **1401.5616** | **-0.4214** | **1** | **12** | **1.9e+02** | **1** | **NLQEALIDTEKK** |
|  | 2930 | **741.7104** | **2222.1092** | **2221.3820** | **0.7271** | **2** | **12** | **1.4e+02** | **1** | **NSHLLDRIYEFEKDDAQK** |
|  | 1396 | **493.3700** | **1477.0878** | **1476.5950** | **0.4929** | **1** | **12** | **1.4e+02** | **1** | **VWSRSGSAGWLDR** |
|  | 2149 | **597.4463** | **1192.8778** | **1192.3647** | **0.5131** | **1** | **12** | **1.6e+02** | **1** | **SQVIKFSQQK** |
|  | 1619 | **519.8183** | **1556.4327** | **1556.8552** | **-0.4225** | **2** | **12** | **1.4e+02** | **1** | **ALCIHTGRELRCK + Carbamidomethyl (C)** |
|  | 2333 | **615.7435** | **1844.2082** | **1845.0646** | **-0.8563** | **0** | **12** | **2.1e+02** | **1** | **GNVIPHTYCDHMSVAK + Carbamidomethyl (C); Oxidation (M)** |
|  | 1208 | **469.2033** | **1404.5878** | **1404.5292** | **0.0585** | **0** | **12** | **1.7e+02** | **1** | **ETTATHCGATPCR + Carbamidomethyl (C)** |
|  | 1461 | **500.1275** | **1497.3603** | **1496.6494** | **0.7109** | **1** | **12** | **1.7e+02** | **1** | **TYSHGTYRAGPMR** |
|  | 1890 | **556.2598** | **1665.7571** | **1665.7991** | **-0.0419** | **0** | **12** | **1.9e+02** | **1** | **CASSMSGLNTEAFFGQ + Oxidation (M)** |
|  | 70 | **368.1861** | **734.3575** | **733.8771** | **0.4804** | **0** | **12** | **1.6e+02** | **1** | **MTTIPR + Oxidation (M)** |
|  | 2997 | **749.1345** | **2244.3814** | **2244.4816** | **-0.1003** | **2** | **12** | **1.6e+02** | **1** | **MSSNSNTGDLQKSLKHGLTPI + Oxidation (M)** |
|  | 3220 | **806.7327** | **2417.1760** | **2416.5157** | **0.6603** | **0** | **12** | **1.4e+02** | **1** | **GLEWIGQINDSGNTNSNPSLSGR** |
|  | 470 | **403.1727** | **1206.4958** | **1206.4178** | **0.0781** | **1** | **12** | **1.9e+02** | **1** | **RVYMASLPNR** |
|  | 558 | **406.7886** | **1217.3435** | **1218.3606** | **-1.0170** | **2** | **12** | **1.7e+02** | **1** | **LRSSGLSEGKGK** |
|  | 1142 | **461.7750** | **921.5351** | **921.0734** | **0.4618** | **1** | **12** | **1.5e+02** | **1** | **LKMQNGSK + Oxidation (M)** |
|  | 2157 | **598.2922** | **1791.8545** | **1792.8759** | **-1.0213** | **0** | **12** | **1.8e+02** | **1** | **EPSTPPSPISSSSSSCSR** |
|  | 3392 | 994.7258 | 1987.4369 | 1987.0297 | 0.4071 | 0 | 12 | 1.5e+02 | 1 | MQSHAADNENNIASNQSR |
|  | 418 | **400.1740** | **1197.5000** | **1198.3362** | **-0.8362** | **2** | **12** | **1.5e+02** | **1** | **KPRGTDNRVR** |
|  | 513 | **405.0792** | **1212.2155** | **1213.3606** | **-1.1451** | **0** | **12** | **1.7e+02** | **1** | **SISFMGGSNGLK + Oxidation (M)** |
|  | 2170 | **599.0863** | **1794.2367** | **1794.9595** | **-0.7228** | **1** | **12** | **1.7e+02** | **1** | **ISRTSGVLSTTGTPFDR** |
|  | 2304 | **612.7037** | **1223.3926** | **1223.3987** | **-0.0061** | **0** | **12** | **2.1e+02** | **1** | **MEPVVYGGISR + Oxidation (M)** |
|  | 3248 | **815.5031** | **2443.4872** | **2442.6892** | **0.7980** | **2** | **12** | **1.8e+02** | **1** | **MADTDAAAPFGKNGSNCLPRHPR + Oxidation (M)** |
|  | 511 | **405.0308** | **1212.0703** | **1212.4634** | **-0.3931** | **0** | **12** | **1.7e+02** | **1** | **CGIQFLHLPK + Carbamidomethyl (C)** |
|  | 1515 | **505.5503** | **1513.6287** | **1513.6353** | **-0.0065** | **1** | **12** | **2e+02** | **1** | **RMEAGEAAPPAGAGGR + Oxidation (M)** |
|  | 2097 | **592.8689** | **1775.5845** | **1775.9644** | **-0.3798** | **2** | **12** | **1.5e+02** | **1** | **TNWPAPGGHERKVAQK** |
|  | 2537 | 655.6068 | 1309.1988 | 1309.4663 | -0.2674 | 0 | 12 | 1.5e+02 | 1 | GFVQSWLTSVVS |
|  | 3386 | **970.2896** | **2907.8465** | **2908.3534** | **-0.5069** | **1** | **12** | **1.3e+02** | **1** | **DLKYQAQDNFMMMDDAVLCMCFSR + 2 Oxidation (M)** |
|  | 537 | **406.0317** | **1215.0729** | **1215.4198** | **-0.3469** | **0** | **12** | **1.6e+02** | **1** | **KPAPTMPSATSK** |
|  | 1967 | **571.3145** | **1710.9212** | **1711.0184** | **-0.0972** | **2** | **12** | **1.8e+02** | **1** | **AASRLELNLVRLLSR** |
|  | 2233 | **606.5359** | **1211.0571** | **1211.3051** | **-0.2480** | **0** | **12** | **1.4e+02** | **1** | **MHDLPPDSGAR + Oxidation (M)** |
|  | 2326 | **614.1261** | **1839.3561** | **1839.1263** | **0.2299** | **1** | **12** | **1.7e+02** | **1** | **VMSLGPCSASCGLGTARR + Carbamidomethyl (C); Oxidation (M)** |
|  | 360 | **389.2861** | **1164.8360** | **1165.2566** | **-0.4205** | **0** | **12** | **1.6e+02** | **1** | **GPAGEAGASPPVR** |
|  | 1937 | **563.7883** | **1125.5618** | **1125.1940** | **0.3677** | **0** | **12** | **1.5e+02** | **1** | **AGHLNANDWK** |
|  | 2542 | **656.5460** | **1311.0773** | **1310.6330** | **0.4442** | **0** | **12** | **1.4e+02** | **1** | **LICMVACNACR + 2 Carbamidomethyl (C)** |
|  | 31 | **363.1405** | **724.2662** | **723.7977** | **0.4685** | **0** | **12** | **1.8e+02** | **1** | **DSPFCR** |
|  | 1138 | **460.9838** | **1379.9293** | **1380.4416** | **-0.5123** | **0** | **12** | **1.9e+02** | **1** | **MSSGADGSGGAAVAAR + Oxidation (M)** |
|  | 2365 | **621.5369** | **1861.5884** | **1862.0088** | **-0.4203** | **0** | **12** | **1.4e+02** | **1** | **NANLAPCGADPDASWGMR + Oxidation (M)** |
|  | 870 | **433.1263** | **864.2379** | **863.9971** | **0.2408** | **0** | **12** | **1.8e+02** | **1** | **ALDFIASK** |
|  | 1678 | **526.1780** | **1050.3413** | **1051.1060** | **-0.7647** | **0** | **12** | **1.9e+02** | **1** | **TLPSTSSSGSK** |
|  | 25 | **363.0459** | **724.0770** | **723.8224** | **0.2545** | **0** | **12** | **1.8e+02** | **1** | **CASQCR + Carbamidomethyl (C)** |
|  | 143 | **372.1868** | **742.3588** | **742.9465** | **-0.5877** | **0** | **12** | **1.5e+02** | **1** | **SIIGLLK** |
|  | 349 | **389.1054** | **1164.2941** | **1164.2716** | **0.0224** | **0** | **12** | **2e+02** | **1** | **YQCNQCSYR** |
|  | 501 | **404.8273** | **807.6399** | **806.8598** | **0.7801** | **0** | **12** | **1.8e+02** | **1** | **APFALSSD** |
|  | 57 | 366.2478 | 1095.7211 | 1095.2960 | 0.4252 | 2 | 12 | 1.7e+02 | 1 | KPRRVETLP |
|  | 431 | **401.2882** | **1200.8424** | **1201.3746** | **-0.5323** | **1** | **12** | **1.6e+02** | **1** | **DPLGRDFLLR** |
|  | 1004 | **447.5881** | **1339.7421** | **1339.6083** | **0.1339** | **2** | **12** | **1.9e+02** | **1** | **MCGVGEQMRKK + Carbamidomethyl (C); Oxidation (M)** |
|  | 3282 | **827.8513** | **2480.5316** | **2480.7819** | **-0.2503** | **1** | **12** | **1.7e+02** | **1** | **TPMTFPPRVIWGCTAQHDHGGR + Oxidation (M)** |
|  | 411 | **399.8174** | **1196.4300** | **1197.3878** | **-0.9578** | **1** | **12** | **1.6e+02** | **1** | **CDVCGKVFNR + Carbamidomethyl (C)** |
|  | 1355 | **488.0331** | **1461.0770** | **1461.5755** | **-0.4985** | **0** | **12** | **2.1e+02** | **1** | **AGEPGLQGPAGPPGEK** |
|  | 2207 | **600.9672** | **1199.9197** | **1200.4545** | **-0.5349** | **0** | **12** | **1.7e+02** | **1** | **GCTLLLTARPR** |
|  | 3029 | **755.6979** | **1509.3810** | **1509.7028** | **-0.3219** | **0** | **12** | **1.5e+02** | **1** | **VALTHLTLDLEER** |
|  | 1630 | **521.0675** | **1040.1202** | **1040.1711** | **-0.0508** | **1** | **12** | **1.7e+02** | **1** | **SSSVTRLYK** |
|  | 3346 | **881.3336** | **1760.6525** | **1760.1090** | **0.5434** | **1** | **12** | **1.8e+02** | **1** | **EDPLKGHVPLALCVLR** |
|  | 190 | **376.1186** | **750.2224** | **750.6658** | **-0.4434** | **0** | **12** | **1.7e+02** | **1** | **SSPSGDDS** |
|  | 1113 | **459.5364** | **1375.5871** | **1375.4912** | **0.0960** | **0** | **12** | **2.4e+02** | **1** | **GGVPGGDAAGAACCR + 2 Carbamidomethyl (C)** |
|  | 2159 | **598.4587** | **1194.9027** | **1194.2580** | **0.6447** | **1** | **12** | **1.5e+02** | **1** | **GPPTRPDGGQRG** |
|  | 2226 | **605.0828** | **1812.2261** | **1811.0450** | **1.1812** | **0** | **12** | **1.9e+02** | **1** | **ENGTDTVQMLFLCPAR + Oxidation (M)** |
|  | 2506 | **651.2568** | **1950.7483** | **1950.1569** | **0.5914** | **1** | **12** | **1.8e+02** | **1** | **ELRDHADSNIVIGLVGNK** |
|  | 633 | **408.9211** | **815.8273** | **815.8715** | **-0.0441** | **0** | **12** | **2e+02** | **1** | **NSSIPNGK** |
|  | 927 | **437.0362** | **1308.0865** | **1308.4402** | **-0.3537** | **1** | **12** | **2e+02** | **1** | **KLNTETFGVSGR** |
|  | 2529 | **654.7972** | **1961.3695** | **1962.2107** | **-0.8412** | **2** | **12** | **2.1e+02** | **1** | **EAHKNNKLALPADSVNIK** |
|  | 2557 | **659.4819** | **1975.4236** | **1975.2311** | **0.1925** | **2** | **12** | **1.7e+02** | **1** | **CISRGEALAPTTQSKLSR + Carbamidomethyl (C)** |
|  | 2725 | **684.2604** | **2049.7590** | **2049.3395** | **0.4195** | **1** | **12** | **1.8e+02** | **1** | **MGSLAHTPSPSQGCCMRGR + Carbamidomethyl (C); Oxidation (M)** |
|  | 3082 | **770.1096** | **1538.2045** | **1537.6485** | **0.5560** | **1** | **12** | **1.5e+02** | **1** | **KLEMDNDSTVNQK + Oxidation (M)** |
|  | 3409 | **1096.9780** | **2191.9413** | **2191.2932** | **0.6481** | **1** | **12** | **1.4e+02** | **1** | **MNSISDFPSSGRYEWDSGR** |
|  | 441 | **401.8809** | **1202.6205** | **1202.4025** | **0.2180** | **1** | **12** | **2.1e+02** | **1** | **RIPADTFAALK** |
|  | 1011 | **448.2675** | **1341.7804** | **1341.4699** | **0.3104** | **1** | **12** | **1.5e+02** | **1** | **IEQLQKENSPR** |
|  | 1957 | **568.5543** | **1702.6408** | **1701.9173** | **0.7234** | **1** | **12** | **1.8e+02** | **1** | **RWQTLLSVDDLVEK** |
|  | 2418 | **632.0206** | **1262.0265** | **1261.4334** | **0.5931** | **1** | **12** | **1.9e+02** | **1** | **HAARLEGVVPGR** |
|  | 2927 | **741.0950** | **1480.1752** | **1479.7216** | **0.4536** | **2** | **12** | **1.5e+02** | **1** | **KLDKWVPHELSK** |
|  | 390 | **396.2093** | **1185.6057** | **1186.2757** | **-0.6700** | **1** | **12** | **1.8e+02** | **1** | **GDKLSTQPNAR** |
|  | 618 | **408.1974** | **814.3799** | **813.9417** | **0.4382** | **1** | **12** | **1.8e+02** | **1** | **RGLVDIAA** |
|  | 1627 | **520.9301** | **1039.8453** | **1039.2525** | **0.5929** | **1** | **12** | **1.8e+02** | **1** | **APLTHKMNK** |
|  | 1722 | **532.1272** | **1062.2396** | **1062.1551** | **0.0846** | **0** | **12** | **2e+02** | **1** | **MGPGATAGGAEK + Oxidation (M)** |
|  | 1747 | **535.9562** | **1069.8977** | **1069.2322** | **0.6655** | **0** | **12** | **1.9e+02** | **1** | **FEGIVTMTR + Oxidation (M)** |
|  | 2234 | **606.5767** | **1211.1385** | **1211.3282** | **-0.1896** | **0** | **12** | **1.5e+02** | **1** | **DQNIVGNARPK** |
|  | 2653 | **673.9970** | **1345.9792** | **1345.5250** | **0.4543** | **1** | **12** | **1.6e+02** | **1** | **SCDKTHTCPPCP + Carbamidomethyl (C)** |
|  | 3219 | **806.7308** | **2417.1703** | **2416.8024** | **0.3679** | **2** | **12** | **1.5e+02** | **1** | **ECILGGRSIDGHSGSVCLMRLR + Carbamidomethyl (C)** |
|  | 489 | **404.0613** | **806.1079** | **805.9000** | **0.2079** | **0** | **12** | **1.9e+02** | **1** | **SCEVGVR + Carbamidomethyl (C)** |
|  | 1009 | **448.1945** | **894.3741** | **894.1125** | **0.2616** | **1** | **12** | **1.8e+02** | **1** | **KLAQIPPK** |
|  | 1309 | **481.7816** | **1442.3227** | **1442.6616** | **-0.3389** | **0** | **12** | **1.6e+02** | **1** | **QCQPLPFSMYR + Carbamidomethyl (C); Oxidation (M)** |
|  | 1390 | **492.2622** | **982.5096** | **982.0901** | **0.4194** | **0** | **12** | **1.8e+02** | **1** | **SQLCECDK + Carbamidomethyl (C)** |
|  | 2118 | **593.8713** | **1185.7278** | **1185.3340** | **0.3937** | **1** | **12** | **1.6e+02** | **1** | **QQTVLSRTPR** |
|  | 878 | **433.8067** | **1298.3980** | **1298.5612** | **-0.1631** | **1** | **12** | **1.8e+02** | **1** | **MAGGVRPLRGLR + Oxidation (M)** |
|  | 1160 | **463.0692** | **1386.1853** | **1385.6749** | **0.5104** | **1** | **12** | **1.7e+02** | **1** | **LLRAVIMGAPGSGK + Oxidation (M)** |
|  | 1931 | **563.3362** | **1686.9864** | **1685.8995** | **1.0868** | **0** | **12** | **1.8e+02** | **1** | **WVSTAIGCCSGQLCS + 3 Carbamidomethyl (C)** |
|  | 2238 | **606.8999** | **1817.6775** | **1818.0606** | **-0.3831** | **2** | **12** | **1.4e+02** | **1** | **RLLALTSSDLGCQPSRT** |
|  | 1038 | **452.0302** | **1353.0683** | **1353.6066** | **-0.5384** | **2** | **11** | **2.3e+02** | **1** | **APKAEPLASKTLK** |
|  | 2003 | **577.3137** | **1728.9188** | **1730.0215** | **-1.1027** | **2** | **11** | **1.9e+02** | **1** | **EILRGFKLNWANLR** |
|  | 2141 | **596.6105** | **1191.2062** | **1190.3106** | **0.8956** | **0** | **11** | **2.2e+02** | **1** | **DNPHWVGIPR** |
|  | 1081 | **457.6642** | **1369.9706** | **1369.5479** | **0.4227** | **1** | **11** | **1.5e+02** | **1** | **WVGDLPNGRVCP + Carbamidomethyl (C)** |
|  | 175 | 374.3442 | 1120.0105 | 1121.1824 | -1.1718 | 0 | 11 | 2.2e+02 | 1 | LVDGNNSCSGR |
|  | 275 | **385.7900** | **1154.3479** | **1153.2211** | **1.1268** | **0** | **11** | **1.7e+02** | **1** | **SQGIDTMETR + Oxidation (M)** |
|  | 970 | **443.8802** | **1328.6186** | **1329.5900** | **-0.9714** | **0** | **11** | **1.9e+02** | **1** | **MAICQFFLQGR + Oxidation (M)** |
|  | 2488 | **647.5946** | **1293.1744** | **1292.3975** | **0.7769** | **1** | **11** | **1.6e+02** | **1** | **GRGLGEGLNYEK** |
|  | 362 | **389.4182** | **1165.2325** | **1166.3142** | **-1.0817** | **2** | **11** | **2.4e+02** | **1** | **RKNHEHMSK** |
|  | 968 | **443.8663** | **1328.5766** | **1328.4514** | **0.1252** | **0** | **11** | **2e+02** | **1** | **APDASAVTHCQTK** |
|  | 1911 | **561.3718** | **1681.0933** | **1679.9335** | **1.1598** | **2** | **11** | **1.8e+02** | **1** | **MTLSQSLKTKGPSSSK** |
|  | 2368 | **622.4493** | **1864.3258** | **1864.1755** | **0.1504** | **1** | **11** | **1.7e+02** | **1** | **MRGLNPGTVNSCCIPTK + Carbamidomethyl (C); Oxidation (M)** |
|  | 733 | **419.1796** | **1254.5165** | **1255.4222** | **-0.9056** | **1** | **11** | **2.2e+02** | **1** | **WSTLPKSSPPR** |
|  | 1649 | **522.9366** | **1565.7878** | **1565.7496** | **0.0381** | **1** | **11** | **1.9e+02** | **1** | **EARVLEAVCEFSR + Carbamidomethyl (C)** |
|  | 2936 | **741.7811** | **1481.5474** | **1481.7258** | **-0.1784** | **2** | **11** | **1.9e+02** | **1** | **SGRLRLVGGPGPCR + Carbamidomethyl (C)** |
|  | 2943 | **742.5676** | **2224.6805** | **2223.4957** | **1.1849** | **2** | **11** | **1.7e+02** | **1** | **RVHTGEKPYRCPWCDYR + Carbamidomethyl (C)** |
|  | 522 | **405.3469** | **1213.0186** | **1212.2668** | **0.7518** | **1** | **11** | **1.5e+02** | **1** | **HGEEGVEAEKK** |
|  | 1413 | **494.8803** | **1481.6189** | **1482.7669** | **-1.1481** | **2** | **11** | **2e+02** | **1** | **TVEKGIPKQITLR** |
|  | 2780 | **694.6936** | **1387.3724** | **1386.5488** | **0.8237** | **0** | **11** | **1.7e+02** | **1** | **GILQMASDSMSSK + 2 Oxidation (M)** |
|  | 1039 | **452.1388** | **1353.3942** | **1353.4856** | **-0.0914** | **0** | **11** | **2.3e+02** | **1** | **YIGHSAHVTNVR** |
|  | 853 | **431.9631** | **861.9115** | **861.9367** | **-0.0252** | **0** | **11** | **2.1e+02** | **1** | **TEEAAITK** |
|  | 1135 | **460.9283** | **1379.7627** | **1379.4370** | **0.3257** | **2** | **11** | **2e+02** | **1** | **KQTSGTSNTRGSR** |
|  | 3215 | **806.4381** | **2416.2922** | **2416.7265** | **-0.4344** | **2** | **11** | **1.8e+02** | **1** | **SSDTRCCELCKYEFIMETK + 2 Carbamidomethyl (C); Oxidation (M)** |
|  | 28 | **363.0740** | **1086.1999** | **1086.2625** | **-0.0626** | **0** | **11** | **2e+02** | **1** | **QTPAALPTCK + Carbamidomethyl (C)** |
|  | 624 | **408.3992** | **814.7836** | **815.9014** | **-1.1178** | **1** | **11** | **2.2e+02** | **1** | **MNRSHR + Oxidation (M)** |
|  | 920 | **436.3203** | **1305.9386** | **1306.4309** | **-0.4923** | **1** | **11** | **1.5e+02** | **1** | **AGGPRGVASAPPGGR** |
|  | 1095 | **458.6880** | **1373.0420** | **1373.5334** | **-0.4914** | **0** | **11** | **1.7e+02** | **1** | **ENLYFQGMTVR + Oxidation (M)** |
|  | 2458 | **639.9786** | **1916.9137** | **1917.1566** | **-0.2429** | **1** | **11** | **1.7e+02** | **1** | **HGYIWDRHYNICLAR** |
|  | 2819 | **708.8866** | **2123.6376** | **2123.3946** | **0.2430** | **2** | **11** | **2e+02** | **1** | **LFGRLVSEELRGGGYGCGPR** |
|  | 3268 | **819.7433** | **2456.2079** | **2455.7443** | **0.4636** | **0** | **11** | **1.5e+02** | **1** | **MAQFPTAMNGGPNMWAITSEER + Oxidation (M)** |
|  | 851 | **431.7826** | **1292.3255** | **1293.4471** | **-1.1215** | **1** | **11** | **2e+02** | **1** | **SKTLGSGACGEVK + Carbamidomethyl (C)** |
|  | 1034 | **451.3001** | **1350.8782** | **1350.6557** | **0.2225** | **1** | **11** | **1.7e+02** | **1** | **KHMISMHPITR** |
|  | 3143 | **792.8508** | **2375.5303** | **2376.7154** | **-1.1851** | **1** | **11** | **2.1e+02** | **1** | **RQMQALGIAMYSVHGWVQER + Oxidation (M)** |
|  | 133 | **371.2184** | **1110.6329** | **1111.2125** | **-0.5796** | **2** | **11** | **1.3e+02** | **1** | **RSHPSGSGAKK** |
|  | 1143 | **461.8030** | **921.5912** | **922.0150** | **-0.4239** | **0** | **11** | **1.7e+02** | **1** | **SCTLEGGAK + Carbamidomethyl (C)** |
|  | 1155 | **462.1776** | **922.3404** | **922.9849** | **-0.6444** | **0** | **11** | **1.9e+02** | **1** | **GSPAPANPGR** |
|  | 1339 | **486.5728** | **1456.6964** | **1456.7063** | **-0.0100** | **0** | **11** | **2.3e+02** | **1** | **MLTHLSLAENALK + Oxidation (M)** |
|  | 1710 | **529.5912** | **1057.1677** | **1056.2384** | **0.9293** | **0** | **11** | **2.5e+02** | **1** | **MMGNMTSPR + 2 Oxidation (M)** |
|  | 834 | **430.3888** | **1288.1443** | **1288.4769** | **-0.3326** | **1** | **11** | **2e+02** | **1** | **RAMGEQAVALAR + Oxidation (M)** |
|  | 1153 | **462.0720** | **1383.1938** | **1382.4325** | **0.7613** | **1** | **11** | **1.9e+02** | **1** | **DEEVQERLDHL** |
|  | 397 | **398.9456** | **795.8765** | **795.0015** | **0.8750** | **0** | **11** | **1.7e+02** | **1** | **MIAVSFK** |
|  | 2645 | **672.3606** | **1342.7064** | **1343.6777** | **-0.9713** | **2** | **11** | **1.9e+02** | **1** | **LGCIKIAASLKGI + Carbamidomethyl (C)** |
|  | 2973 | **748.6617** | **1495.3086** | **1495.7260** | **-0.4174** | **1** | **11** | **1.5e+02** | **1** | **RVAPVSVGGSGLLQR** |
|  | 3279 | **827.2651** | **2478.7732** | **2479.7776** | **-1.0043** | **2** | **11** | **1.9e+02** | **1** | **AQRPQSCTSVGRVTVHSTPVRR + Carbamidomethyl (C)** |
|  | 883 | 434.1199 | 866.2251 | 866.9582 | -0.7331 | 0 | 11 | 1.9e+02 | 1 | VNSSVAYK |
|  | 1985 | **573.7946** | **1718.3615** | **1717.9350** | **0.4265** | **1** | **11** | **1.7e+02** | **1** | **KEVQEEQEILECLK** |
|  | 2225 | **604.4412** | **1810.3013** | **1811.0267** | **-0.7254** | **0** | **11** | **1.7e+02** | **1** | **EWMAAATHALSAPAEVR** |
|  | 2363 | **621.4791** | **1861.4150** | **1861.0789** | **0.3361** | **0** | **11** | **1.5e+02** | **1** | **EIVMTASPGTLSLSPGER + Oxidation (M)** |
|  | 140 | **371.3121** | **740.6094** | **739.8601** | **0.7494** | **1** | **11** | **1.3e+02** | **1** | **KEAPPAK** |
|  | 1249 | **473.2509** | **1416.7304** | **1417.6687** | **-0.9383** | **0** | **11** | **2.1e+02** | **1** | **TMVLPLSSLSSWP** |
|  | 1391 | **492.3276** | **1473.9606** | **1473.7834** | **0.1772** | **2** | **11** | **1.6e+02** | **1** | **ISDKVVGILMRAR + Oxidation (M)** |
|  | 1973 | **572.1335** | **1713.3785** | **1713.0128** | **0.3656** | **2** | **11** | **2e+02** | **1** | **RLGEMRFTLGTFLR + Oxidation (M)** |
|  | 569 | **407.0780** | **812.1413** | **811.7537** | **0.3876** | **0** | **11** | **1.9e+02** | **1** | **SGDQDHPG** |
|  | 1022 | **449.9396** | **897.8645** | **898.9668** | **-1.1023** | **1** | **11** | **1.8e+02** | **1** | **AGPSRAQGR** |
|  | 1065 | **457.1680** | **1368.4817** | **1368.5419** | **-0.0601** | **2** | **11** | **2e+02** | **1** | **AAVPTSPGTRQRK** |
|  | 1225 | **471.4720** | **1411.3940** | **1410.6196** | **0.7743** | **1** | **11** | **1.9e+02** | **1** | **IRELAEPWLQR** |
|  | 2297 | **612.1951** | **1833.5630** | **1833.0123** | **0.5507** | **2** | **11** | **2e+02** | **1** | **SFIGKSPNEYHIERR** |
|  | 2956 | **743.7884** | **2228.3430** | **2229.5069** | **-1.1639** | **0** | **11** | **2.1e+02** | **1** | **IEEVSNTPLLLTVEVQECR + Carbamidomethyl (C)** |
|  | 1576 | **515.2706** | **1542.7895** | **1541.7250** | **1.0646** | **0** | **11** | **2e+02** | **1** | **VQSSFMVSLGVSER + Oxidation (M)** |
|  | 2087 | **592.4306** | **1774.2696** | **1774.0279** | **0.2417** | **0** | **11** | **1.6e+02** | **1** | **GCTGSPSSAGPPALCLISR** |
|  | 2386 | **627.6947** | **1880.0619** | **1880.0903** | **-0.0284** | **2** | **11** | **2.3e+02** | **1** | **LRDQLGTAKNANEMFR + Oxidation (M)** |
|  | 798 | **427.9425** | **853.8703** | **854.8829** | **-1.0126** | **0** | **11** | **1.9e+02** | **1** | **AGGMSDSSK + Oxidation (M)** |
|  | 1234 | **472.0685** | **1413.1835** | **1413.5408** | **-0.3574** | **1** | **11** | **1.9e+02** | **1** | **SFSRSSSLIHHR** |
|  | 1073 | **457.5744** | **1369.7011** | **1369.5927** | **0.1085** | **1** | **11** | **2.3e+02** | **1** | **GLVCGPLSAPRGSR** |
|  | 1552 | **512.3015** | **1533.8824** | **1533.5571** | **0.3253** | **1** | **11** | **1.9e+02** | **1** | **SSGADGGGGAAVAARSDK** |
|  | 2081 | **591.2474** | **1770.7200** | **1770.9547** | **-0.2347** | **0** | **11** | **1.9e+02** | **1** | **ECTFSCASGEYLEMK + Carbamidomethyl (C); Oxidation (M)** |
|  | 2646 | **672.6078** | **2014.8012** | **2015.2932** | **-0.4920** | **2** | **11** | **1.6e+02** | **1** | **WIEPKICREDLTDAIR + Carbamidomethyl (C)** |
|  | 3020 | **749.6248** | **2245.8521** | **2245.4053** | **0.4468** | **1** | **11** | **1.6e+02** | **1** | **VLETDPAFAREHGTASTLSSR** |
|  | 3418 | **1189.0813** | **3564.2217** | **3564.2230** | **-0.0013** | **2** | **11** | **1.4e+02** | **1** | **AVPARVSLGITTVLTMTTIITGVNASMPRVSYVK + Oxidation (M)** |
|  | 72 | **368.2462** | **1101.7165** | **1101.2424** | **0.4741** | **1** | **11** | **1.7e+02** | **1** | **SHWMNRVR + Oxidation (M)** |
|  | 493 | **404.1151** | **1209.3232** | **1209.4384** | **-0.1152** | **1** | **11** | **2e+02** | **1** | **QAAVPLVKVER** |
|  | 549 | **406.3743** | **1216.1008** | **1215.3533** | **0.7475** | **1** | **11** | **1.7e+02** | **1** | **QKIAQLEEEK** |
|  | 2240 | **607.1508** | **1212.2867** | **1213.3871** | **-1.1004** | **1** | **11** | **1.9e+02** | **1** | **SRTGATAPALLR** |
|  | 2403 | **630.0560** | **1887.1457** | **1887.0995** | **0.0462** | **1** | **11** | **2e+02** | **1** | **VQEGVRNISNQLSITTK** |
|  | 3212 | **805.4346** | **2413.2815** | **2412.7643** | **0.5172** | **1** | **11** | **2e+02** | **1** | **MIVDMQSHTRVISYEGCLTR + Carbamidomethyl (C); Oxidation (M)** |
|  | 11 | **360.5460** | **719.0771** | **718.8426** | **0.2346** | **2** | **11** | **1.9e+02** | **1** | **TKGTKGK** |
|  | 1473 | **502.0079** | **1503.0014** | **1502.7649** | **0.2366** | **2** | **11** | **2.1e+02** | **1** | **GPLGGGRAGKMVCAR + Carbamidomethyl (C); Oxidation (M)** |
|  | 2169 | **599.0198** | **1794.0372** | **1794.1435** | **-0.1064** | **2** | **11** | **2e+02** | **1** | **LLKADLHGAIISVTKSK** |
|  | 301 | **386.9482** | **1157.8225** | **1157.3671** | **0.4554** | **2** | **11** | **2.2e+02** | **1** | **AEYKMGRMR + Oxidation (M)** |
|  | 806 | **428.2686** | **1281.7835** | **1281.4809** | **0.3026** | **1** | **11** | **1.4e+02** | **1** | **ALLEKYNTACR** |
|  | 881 | **434.0743** | **1299.2007** | **1299.4565** | **-0.2558** | **1** | **11** | **1.9e+02** | **1** | **DHLKNAMTGNAK** |
|  | 1013 | **448.3694** | **894.7240** | **895.1204** | **-0.3965** | **1** | **11** | **1.6e+02** | **1** | **LYGIKCAK** |
|  | 1400 | **494.0767** | **986.1387** | **985.1786** | **0.9600** | **0** | **11** | **2.1e+02** | **1** | **AYAVVCECK** |
|  | 2765 | **688.3512** | **2062.0314** | **2061.4429** | **0.5885** | **2** | **11** | **2e+02** | **1** | **ITDMQLGGSVEISKLITKK** |
|  | 2982 | **748.8386** | **2243.4935** | **2242.4840** | **1.0095** | **1** | **11** | **2.3e+02** | **1** | **IIAEGANGPTTPEADKIFLER** |
|  | 3304 | **841.2811** | **2520.8210** | **2521.8255** | **-1.0044** | **0** | **11** | **1.9e+02** | **1** | **MWPNGSSLGPCFRPTNITLEER + Oxidation (M)** |
|  | 687 | **415.2747** | **1242.8020** | **1243.4364** | **-0.6344** | **2** | **11** | **1.7e+02** | **1** | **LARHSSMADKK** |
|  | 721 | **418.1462** | **1251.4165** | **1251.4746** | **-0.0581** | **0** | **11** | **2.1e+02** | **1** | **NALWLGMAISC + Carbamidomethyl (C); Oxidation (M)** |
|  | 2242 | **607.2710** | **1212.5272** | **1213.3674** | **-0.8402** | **0** | **11** | **2e+02** | **1** | **TVRPCSGSGPPR** |
|  | 1379 | **490.3769** | **1468.1085** | **1468.5699** | **-0.4614** | **0** | **11** | **1.6e+02** | **1** | **EGGAAMAAGESMAQR + 2 Oxidation (M)** |
|  | 3329 | **856.5717** | **2566.6930** | **2565.8565** | **0.8364** | **1** | **11** | **2e+02** | **1** | **LPVTPGEPASISCRSSQSLLHSNGK** |
|  | 454 | **402.0343** | **802.0539** | **801.9313** | **0.1226** | **1** | **11** | **2.4e+02** | **1** | **RVVAETK** |
|  | 1988 | **575.3333** | **1722.9776** | **1723.0029** | **-0.0253** | **2** | **11** | **2.1e+02** | **1** | **KFKQMTEAIGPSTIR + Oxidation (M)** |
|  | 2447 | **636.1102** | **1270.2057** | **1269.3195** | **0.8861** | **0** | **11** | **2e+02** | **1** | **GADTQYFGPGTR** |
|  | 872 | **433.2708** | **1296.7902** | **1297.4836** | **-0.6935** | **0** | **11** | **1.6e+02** | **1** | **HMLALAPNSTAR + Oxidation (M)** |
|  | 3345 | **880.8187** | **1759.6227** | **1760.0228** | **-0.4002** | **1** | **11** | **1.6e+02** | **1** | **WMERGISALLLSPDR + Oxidation (M)** |
|  | 466 | **403.0511** | **804.0875** | **802.9193** | **1.1682** | **2** | **11** | **2.2e+02** | **1** | **REVKGSK** |
|  | 836 | **430.6523** | **1288.9349** | **1288.3907** | **0.5441** | **0** | **11** | **1.9e+02** | **1** | **QGAMNTAQQPSR** |
|  | 854 | **431.9633** | **1292.8676** | **1293.4352** | **-0.5676** | **1** | **11** | **2.2e+02** | **1** | **AGRGLQQASHLR** |
|  | 2001 | **576.9241** | **1727.7502** | **1728.0934** | **-0.3432** | **1** | **11** | **1.8e+02** | **1** | **WLQKLCCPCCFGR + 3 Carbamidomethyl (C)** |
|  | 2331 | **615.3804** | **1843.1191** | **1843.9374** | **-0.8183** | **0** | **11** | **2e+02** | **1** | **SISSSSSSYIYYADSVK** |
|  | 2439 | **635.0842** | **1902.2305** | **1901.9754** | **0.2551** | **2** | **11** | **1.9e+02** | **1** | **ESEKSDGDPIVDPEKEK** |
|  | 1175 | **465.0586** | **928.1024** | **927.0596** | **1.0428** | **1** | **11** | **2e+02** | **1** | **GLRGPGAATK** |
|  | 2958 | **743.9512** | **2228.8313** | **2228.5484** | **0.2829** | **1** | **11** | **1.8e+02** | **1** | **EIITFWQVMLRNTTCHY + Carbamidomethyl (C); Oxidation (M)** |
|  | 3406 | **1055.8783** | **3164.6127** | **3165.6644** | **-1.0517** | **1** | **11** | **1.7e+02** | **1** | **AFSTCTSHMCAIVITYVAAFFTFFTRR + Carbamidomethyl (C); Oxidation (M)** |
|  | 1265 | **475.7400** | **1424.1977** | **1424.6083** | **-0.4106** | **2** | **11** | **1.8e+02** | **1** | **GQQKAAVATARAPR** |
|  | 2610 | **667.9874** | **2000.9401** | **2001.2652** | **-0.3250** | **1** | **11** | **1.7e+02** | **1** | **YHVLEMIGEGSFGRVYK + Oxidation (M)** |
|  | 3093 | **776.9286** | **1551.8424** | **1551.6651** | **0.1773** | **1** | **11** | **2.3e+02** | **1** | **VPSDFHARHAATSR** |
|  | 3222 | **807.6815** | **2420.0224** | **2419.8892** | **0.1332** | **2** | **11** | **1.6e+02** | **1** | **AVCAGLICTALARMALLRETNR + Carbamidomethyl (C); Oxidation (M)** |
|  | 26 | **363.0598** | **1086.1571** | **1085.2563** | **0.9007** | **1** | **11** | **2.1e+02** | **1** | **CCTSKISSTR** |
|  | 1019 | **449.4261** | **1345.2563** | **1345.4986** | **-0.2423** | **0** | **11** | **1.8e+02** | **1** | **AGVNTVTTLVENK** |
|  | 1239 | **472.2871** | **942.5594** | **943.0591** | **-0.4996** | **1** | **11** | **1.7e+02** | **1** | **TGSGIRAGPK** |
|  | 1506 | **505.1061** | **1512.2962** | **1511.6423** | **0.6538** | **1** | **11** | **2e+02** | **1** | **YNHKTDAALWHR** |
|  | 1 | **360.3063** | **718.5978** | **717.8544** | **0.7434** | **1** | **11** | **2.1e+02** | **1** | **KITAASK** |
|  | 892 | **434.8430** | **1301.5067** | **1301.5171** | **-0.0104** | **2** | **11** | **1.9e+02** | **1** | **NMSFRFIKSR + Oxidation (M)** |
|  | 1059 | **456.3861** | **1366.1362** | **1365.4483** | **0.6878** | **0** | **11** | **1.5e+02** | **1** | **NAVGSPEGQELHK** |
|  | 1427 | **497.1878** | **992.3608** | **992.0884** | **0.2725** | **1** | **11** | **2.2e+02** | **1** | **FFEEKHR** |
|  | 3075 | **766.7808** | **1531.5467** | **1531.6936** | **-0.1468** | **2** | **11** | **1.9e+02** | **1** | **KNGLMEASPEQRR + Oxidation (M)** |
|  | 1499 | **504.8877** | **1007.7605** | **1007.1508** | **0.6097** | **1** | **11** | **2e+02** | **1** | **GQLLRHQR** |
|  | 1651 | **523.1779** | **1044.3409** | **1044.3133** | **0.0276** | **0** | **11** | **2.2e+02** | **1** | **IAWLLCLR + Carbamidomethyl (C)** |
|  | 1698 | **528.3952** | **1582.1634** | **1582.7570** | **-0.5935** | **0** | **11** | **1.6e+02** | **1** | **QVQLVESGGGVVTPGR** |
|  | 2858 | **718.4034** | **2152.1882** | **2152.4278** | **-0.2397** | **2** | **11** | **2e+02** | **1** | **EETAAFLTALLQRKMEER + Oxidation (M)** |
|  | 933 | **437.2649** | **872.5150** | **872.9924** | **-0.4774** | **1** | **11** | **1.9e+02** | **1** | **GGPGNRGMK** |
|  | 1103 | **459.0105** | **1374.0092** | **1374.6076** | **-0.5983** | **1** | **11** | **2.3e+02** | **1** | **ISHTGEKPFKCK** |
|  | 1915 | **561.7544** | **1682.2410** | **1682.9768** | **-0.7358** | **0** | **11** | **1.9e+02** | **1** | **FEDVYVGICLNLLK + Carbamidomethyl (C)** |
|  | 3047 | **759.7689** | **1517.5231** | **1517.7913** | **-0.2682** | **1** | **11** | **1.9e+02** | **1** | **CAVVDVPFGGAKAGVK** |
|  | 2942 | **742.4518** | **2224.3334** | **2223.3345** | **0.9988** | **2** | **11** | **2e+02** | **1** | **LFGFVRLHXDGARTXTLLGK** |
|  | 1101 | **458.8977** | **915.7806** | **916.0352** | **-0.2546** | **0** | **11** | **2.4e+02** | **1** | **IWAVSQGR** |
|  | 2873 | **727.0944** | **2178.2611** | **2178.5265** | **-0.2654** | **2** | **11** | **1.8e+02** | **1** | **AEMEQMALDVGLPSSKLKSK + Oxidation (M)** |
|  | 531 | **405.9513** | **809.8877** | **808.9668** | **0.9210** | **1** | **11** | **1.9e+02** | **1** | **CEMKGGK + Carbamidomethyl (C)** |
|  | 986 | **445.1607** | **1332.4600** | **1333.4925** | **-1.0325** | **0** | **11** | **2.4e+02** | **1** | **AHSGEHLLLGATK** |
|  | 1669 | **524.7570** | **1047.4991** | **1047.1934** | **0.3058** | **2** | **11** | **1.8e+02** | **1** | **NNMAAKRSR** |
|  | 1683 | **526.7926** | **1051.5704** | **1051.1573** | **0.4132** | **2** | **11** | **1.6e+02** | **1** | **GKFRTSAER** |
|  | 1846 | **547.9508** | **1640.8302** | **1640.8162** | **0.0141** | **1** | **11** | **2e+02** | **1** | **CPVGGGGPNPSTSASPKK** |
|  | 2221 | **604.0196** | **1809.0366** | **1810.1464** | **-1.1098** | **2** | **11** | **2.2e+02** | **1** | **FIMSLRKAAMAEPNAK + 2 Oxidation (M)** |
|  | 3048 | **759.8987** | **2276.6741** | **2277.6904** | **-1.0163** | **2** | **11** | **2.4e+02** | **1** | **MPPPPLLSLRRLGGGWSAVTR + Oxidation (M)** |
|  | 1531 | **507.6985** | **1520.0733** | **1519.7374** | **0.3358** | **1** | **11** | **1.9e+02** | **1** | **EQKDLSLTPFTIK** |
|  | 2280 | **610.8940** | **1829.6600** | **1828.9576** | **0.7023** | **1** | **11** | **1.7e+02** | **1** | **EQPTRTISSPTSCEHR** |
|  | 524 | **405.4942** | **808.9737** | **809.7529** | **-0.7792** | **0** | **11** | **2.3e+02** | **1** | **EEECEGD** |
|  | 1577 | **515.6146** | **1029.2145** | **1029.1879** | **0.0265** | **0** | **11** | **2.6e+02** | **1** | **EIADSLILR** |
|  | 2080 | **591.2106** | **1180.4064** | **1181.3820** | **-0.9756** | **0** | **11** | **2.1e+02** | **1** | **VGPVSAVGVTAPK** |
|  | 1221 | **471.0109** | **940.0070** | **939.1169** | **0.8901** | **2** | **11** | **1.9e+02** | **1** | **KASRKPPR** |
|  | 1343 | **486.9647** | **971.9145** | **972.0804** | **-0.1658** | **0** | **11** | **2.2e+02** | **1** | **SGLMQHQR + Oxidation (M)** |
|  | 1752 | **536.1746** | **1605.5015** | **1604.9099** | **0.5917** | **2** | **11** | **2.1e+02** | **1** | **IKKQANDLVSTLMK + Oxidation (M)** |
|  | 1781 | **538.2627** | **1611.7659** | **1611.9106** | **-0.1447** | **0** | **11** | **2.2e+02** | **1** | **MACEVACGVLHLHR + Carbamidomethyl (C); Oxidation (M)** |
|  | 2128 | **595.1834** | **1188.3520** | **1188.3944** | **-0.0423** | **0** | **11** | **2.4e+02** | **1** | **IVPVVSQSECK** |
|  | 1986 | 574.3182 | 1719.9325 | 1720.8181 | -0.8855 | 0 | 11 | 2.2e+02 | 1 | PGTQTPAPAEDPHSGCR |
|  | 2616 | **668.6691** | **1335.3234** | **1334.4790** | **0.8444** | **0** | **11** | **2.1e+02** | **1** | **DWRPLTFGGGTK** |
|  | 2694 | **680.8782** | **1359.7416** | **1359.6162** | **0.1254** | **1** | **11** | **2e+02** | **1** | **VRNVVLGVTQFK** |
|  | 3206 | **804.1962** | **2409.5665** | **2409.8663** | **-0.2998** | **2** | **11** | **2e+02** | **1** | **MSAPFSFLSNVKYNFMRIIK + Oxidation (M)** |
|  | 3364 | **908.9894** | **2723.9460** | **2725.1076** | **-1.1616** | **1** | **11** | **2.2e+02** | **1** | **CLDSMCCFPEGEAACASVGRMLER + 2 Carbamidomethyl (C); 2 Oxidation (M)** |
|  | 1395 | **493.3316** | **1476.9727** | **1477.7155** | **-0.7428** | **2** | **11** | **1.8e+02** | **1** | **ALLVDRVRGHWR** |
|  | 2235 | **606.5934** | **1816.7580** | **1816.1341** | **0.6238** | **1** | **11** | **1.8e+02** | **1** | **MVEADCHGKLCIGGLNR** |
|  | 2285 | **611.1819** | **1220.3490** | **1221.2403** | **-0.8913** | **1** | **11** | **2.2e+02** | **1** | **ETKHGGHENGR** |
|  | 2560 | **659.7684** | **1976.2831** | **1976.2820** | **0.0011** | **1** | **11** | **2.7e+02** | **1** | **SLSPICPRSQIGLNTMSR + Oxidation (M)** |
|  | 49 | **365.5313** | **1093.5717** | **1093.3415** | **0.2302** | **2** | **11** | **2.1e+02** | **1** | **KTMTTRVIK + Oxidation (M)** |
|  | 622 | **408.3429** | **1222.0064** | **1221.4291** | **0.5773** | **0** | **11** | **1.8e+02** | **1** | **VPLPSGPMNPGR** |
|  | 1478 | **502.7229** | **1505.1465** | **1504.6913** | **0.4552** | **0** | **11** | **1.9e+02** | **1** | **VYPQADAVIVHHR** |
|  | 1593 | **518.4518** | **1552.3332** | **1552.7061** | **-0.3730** | **0** | **11** | **1.8e+02** | **1** | **SSLSASVGDGVTITCR** |
|  | 2017 | **579.6270** | **1735.8587** | **1736.9302** | **-1.0715** | **2** | **11** | **2.6e+02** | **1** | **SRPTPGGTQKAVEPRR** |
|  | 2188 | **599.7095** | **1796.1062** | **1797.2290** | **-1.1227** | **0** | **11** | **2.6e+02** | **1** | **MMLIPMASVMAVTEPK + 3 Oxidation (M)** |
|  | 2743 | **685.7032** | **2054.0876** | **2055.1832** | **-1.0957** | **1** | **11** | **2.2e+02** | **1** | **LRSDDTAVYYCASAGGYW + Carbamidomethyl (C)** |
|  | 707 | **417.1267** | **1248.3580** | **1249.3994** | **-1.0414** | **1** | **11** | **2.4e+02** | **1** | **QGVWGGQSMRK + Oxidation (M)** |
|  | 2079 | **591.1122** | **1770.3144** | **1770.0806** | **0.2338** | **2** | **11** | **2.1e+02** | **1** | **LIRLLLYHSKQEEK** |
|  | 2519 | **653.7955** | **1958.3644** | **1958.1990** | **0.1655** | **2** | **11** | **2.5e+02** | **1** | **MLGNEWSKLPPEEKQR + Oxidation (M)** |
|  | 355 | **389.1660** | **776.3172** | **775.8922** | **0.4250** | **1** | **11** | **2.2e+02** | **1** | **SKEVWK** |
|  | 447 | **401.9830** | **1202.9268** | **1203.4554** | **-0.5285** | **2** | **11** | **2.5e+02** | **1** | **KSSKPIMEKR** |
|  | 932 | **437.1684** | **1308.4831** | **1309.5390** | **-1.0559** | **1** | **11** | **2.4e+02** | **1** | **LTCPCCNTRK + 3 Carbamidomethyl (C)** |
|  | 1266 | **475.7643** | **1424.2707** | **1424.6665** | **-0.3958** | **0** | **11** | **1.9e+02** | **1** | **MGCSVTAVPPMSGR + 2 Oxidation (M)** |
|  | 1857 | **550.8760** | **1099.7372** | **1099.1122** | **0.6249** | **0** | **11** | **1.8e+02** | **1** | **LSSANGHEER** |
|  | 2155 | **597.9813** | **1790.9218** | **1789.9662** | **0.9555** | **0** | **11** | **2.1e+02** | **1** | **MGPEPAPAATHAAPQATR + Oxidation (M)** |
|  | 2737 | **685.2030** | **1368.3912** | **1368.4740** | **-0.0827** | **2** | **11** | **2.2e+02** | **1** | **ASKAASMSNRSDK + Oxidation (M)** |
|  | 3371 | **917.8995** | **1833.7843** | **1833.0750** | **0.7092** | **0** | **11** | **1.6e+02** | **1** | **NIMNDWHLSLFISSR** |
|  | 2441 | **635.6477** | **1269.2806** | **1268.4192** | **0.8614** | **1** | **11** | **2.1e+02** | **1** | **AEGLLQGKHSTK** |
|  | 1868 | **552.1899** | **1653.5476** | **1652.8085** | **0.7391** | **1** | **11** | **2.4e+02** | **1** | **VNSLAQDPTHTKWR** |
|  | 2452 | **637.3798** | **1909.1171** | **1909.1666** | **-0.0495** | **2** | **11** | **2.3e+02** | **1** | **TLKDESSVTSKLACTAVR** |
|  | 2564 | **663.1744** | **1324.3341** | **1323.5193** | **0.8148** | **1** | **11** | **2.1e+02** | **1** | **FASKVAGLSGCHF** |
|  | 2678 | **679.5959** | **1357.1770** | **1356.5278** | **0.6492** | **1** | **11** | **1.7e+02** | **1** | **MYGCDSVKQPR + Carbamidomethyl (C); Oxidation (M)** |
|  | 1324 | **484.2367** | **1449.6880** | **1448.5833** | **1.1047** | **1** | **11** | **2.1e+02** | **1** | **YQCDACGKGFSR + 2 Carbamidomethyl (C)** |
|  | 732 | **419.1779** | **1254.5114** | **1253.4045** | **1.1069** | **0** | **11** | **2.5e+02** | **1** | **EHLGSQLIAGTK** |
|  | 1422 | **496.3753** | **990.7358** | **990.1786** | **0.5573** | **1** | **11** | **1.9e+02** | **1** | **MLPTASSKR** |
|  | 1728 | **532.6614** | **1594.9620** | **1595.7108** | **-0.7489** | **0** | **11** | **2.6e+02** | **1** | **AGALQETQYFGPGTR** |
|  | 2816 | **707.2982** | **1412.5817** | **1411.5366** | **1.0450** | **0** | **11** | **2.1e+02** | **1** | **EQDGALYLMAER + Oxidation (M)** |
|  | 913 | **436.0731** | **870.1313** | **868.9973** | **1.1341** | **0** | **11** | **2.1e+02** | **1** | **TVYAMER** |
|  | 2071 | **589.9835** | **1177.9523** | **1177.3750** | **0.5773** | **2** | **11** | **2.3e+02** | **1** | **VTRKENSLCK** |
|  | 2915 | **740.6348** | **1479.2548** | **1478.7601** | **0.4947** | **2** | **11** | **1.7e+02** | **1** | **KPGHTKKMNFFK + Oxidation (M)** |
|  | 3420 | **1190.7140** | **2379.4132** | **2378.7233** | **0.6899** | **1** | **11** | **1.7e+02** | **1** | **IMPNTVFVGGIDVRMDESEIR** |
|  | 741 | **419.2879** | **1254.8415** | **1255.5316** | **-0.6900** | **1** | **11** | **2.1e+02** | **1** | **MAKTLQTCMR + Carbamidomethyl (C); Oxidation (M)** |
|  | 1036 | **451.6725** | **901.3301** | **900.1635** | **1.1667** | **1** | **11** | **2.1e+02** | **1** | **ILMKVGHC** |
|  | 1483 | **503.6063** | **1005.1977** | **1004.2052** | **0.9926** | **0** | **11** | **2.9e+02** | **1** | **MLAVAVASSR** |
|  | 1701 | **528.7986** | **1055.5824** | **1056.1340** | **-0.5516** | **2** | **11** | **1.7e+02** | **1** | **GGREEKAPGR** |
|  | 1921 | **562.6535** | **1684.9383** | **1685.8371** | **-0.8987** | **2** | **11** | **2.6e+02** | **1** | **EVLEGARASVDRVER** |
|  | 420 | **400.2611** | **1197.7612** | **1198.3709** | **-0.6096** | **0** | **11** | **1.5e+02** | **1** | **CASSLTSGMLR + Carbamidomethyl (C); Oxidation (M)** |
|  | 1706 | **529.0467** | **1056.0786** | **1056.2564** | **-0.1778** | **1** | **11** | **2.1e+02** | **1** | **ELTSPRLIK** |
|  | 1959 | **568.9640** | **1703.8698** | **1702.8872** | **0.9826** | **2** | **11** | **2.3e+02** | **1** | **EALDRVARGDLAECK + Carbamidomethyl (C)** |
|  | 500 | **404.7645** | **1211.2713** | **1212.3775** | **-1.1061** | **0** | **11** | **2.1e+02** | **1** | **MNCLDCLDR + 2 Carbamidomethyl (C); Oxidation (M)** |
|  | 1115 | **459.7586** | **1376.2537** | **1375.5924** | **0.6614** | **1** | **11** | **2e+02** | **1** | **FDCGKPQVEPKK** |
|  | 1407 | **494.4517** | **986.8887** | **987.1115** | **-0.2229** | **1** | **11** | **2e+02** | **1** | **SLKENIQR** |
|  | 2760 | **686.6597** | **2056.9568** | **2056.3867** | **0.5701** | **1** | **11** | **1.8e+02** | **1** | **IEHEHEGCLKMSPASLMK + Oxidation (M)** |
|  | 3415 | **1184.8983** | **3551.6728** | **3551.0830** | **0.5897** | **2** | **11** | **1.8e+02** | **1** | **FHKHLQDLFAPLVVRYVDLMESSIAQSIHR** |
|  | 966 | **443.8193** | **1328.4357** | **1327.4817** | **0.9540** | **1** | **11** | **2.2e+02** | **1** | **NVDTEISKMSMG + Oxidation (M)** |
|  | 1159 | **462.5417** | **1384.6031** | **1384.4087** | **0.1944** | **0** | **11** | **2.4e+02** | **1** | **SSAAENPSAGGSPPR** |
|  | 2109 | **593.5872** | **1777.7393** | **1777.0979** | **0.6414** | **1** | **11** | **2.2e+02** | **1** | **KDLCGQCSPLGCLIR + 3 Carbamidomethyl (C)** |
|  | 2682 | **679.9243** | **1357.8338** | **1356.6371** | **1.1968** | **2** | **11** | **1.8e+02** | **1** | **GLIPVKRMPSSR + Oxidation (M)** |
|  | 3025 | **753.1321** | **1504.2494** | **1504.6218** | **-0.3724** | **0** | **11** | **1.9e+02** | **1** | **SVSMLDLQGDGPGGR + Oxidation (M)** |
|  | 53 | **366.0611** | **730.1073** | **729.7857** | **0.3217** | **1** | **11** | **2.4e+02** | **1** | **GRGVGER** |
|  | 467 | **403.0784** | **804.1420** | **804.8936** | **-0.7516** | **1** | **11** | **2.4e+02** | **1** | **GSGFPGKR** |
|  | 484 | **403.9819** | **805.9490** | **805.8833** | **0.0658** | **0** | **11** | **2.3e+02** | **1** | **HCSGGSCR** |
|  | 692 | **416.0092** | **830.0036** | **830.0506** | **-0.0469** | **1** | **11** | **2.6e+02** | **1** | **LPRMAVK + Oxidation (M)** |
|  | 1330 | **485.9749** | **1454.9026** | **1454.5018** | **0.4008** | **1** | **11** | **2e+02** | **1** | **THKANASNSPEGNK** |
|  | 3073 | **766.1504** | **1530.2860** | **1529.8020** | **0.4840** | **1** | **11** | **1.9e+02** | **1** | **VPWLSVMETPARK + Oxidation (M)** |
|  | 269 | **385.2034** | **1152.5881** | **1151.4668** | **1.1214** | **0** | **11** | **1.6e+02** | **1** | **TNVALMCMLR** |
|  | 1789 | **538.7668** | **1075.5189** | **1076.2891** | **-0.7702** | **0** | **11** | **1.9e+02** | **1** | **AIATTALIFR** |
|  | 2962 | **744.8926** | **2231.6556** | **2232.5372** | **-0.8817** | **1** | **11** | **2.5e+02** | **1** | **QPMNAASGAAMSLAGAEKNGLVK + Oxidation (M)** |
|  | 165 | **374.2221** | **746.4294** | **745.9309** | **0.4986** | **0** | **11** | **2.2e+02** | **1** | **EVVLMR** |
|  | 701 | **416.3938** | **1246.1593** | **1246.3343** | **-0.1749** | **2** | **11** | **2.6e+02** | **1** | **LSSPRGGSSSRR** |
|  | 929 | **437.0502** | **872.0856** | **872.0656** | **0.0200** | **0** | **11** | **2.5e+02** | **1** | **VLLQPFR** |
|  | 1550 | **512.1596** | **1022.3044** | **1022.0681** | **0.2364** | **1** | **11** | **2.2e+02** | **1** | **GKSTQSTEGK** |
|  | 1769 | **537.3157** | **1072.6166** | **1072.2591** | **0.3574** | **2** | **11** | **2.4e+02** | **1** | **QIAAKQGTKK** |
|  | 144 | **372.2732** | **1113.7973** | **1114.4049** | **-0.6076** | **1** | **11** | **1.7e+02** | **1** | **LIQRLCQLK** |
|  | 469 | **403.1185** | **1206.3334** | **1206.3515** | **-0.0181** | **0** | **11** | **2.5e+02** | **1** | **AHVAFPDFFR** |
|  | 790 | **427.1055** | **1278.2944** | **1278.3266** | **-0.0321** | **0** | **11** | **2.1e+02** | **1** | **SQSDTAVNVTTR** |
|  | 1634 | **521.2474** | **1560.7201** | **1561.8436** | **-1.1234** | **0** | **11** | **2.1e+02** | **1** | **TMPSNLVNFILNAK** |
|  | 1714 | **530.1758** | **1058.3369** | **1057.1650** | **1.1719** | **2** | **11** | **2.5e+02** | **1** | **ELAGARRER** |
|  | 1748 | **536.0403** | **1605.0989** | **1604.6830** | **0.4159** | **1** | **11** | **2.2e+02** | **1** | **GEVERGPQSPQQHR** |
|  | 1912 | **561.4732** | **1681.3974** | **1681.8880** | **-0.4905** | **0** | **11** | **1.9e+02** | **1** | **VTEGAGCMSGELAVWR + Oxidation (M)** |
|  | 266 | **385.1545** | **1152.4414** | **1151.3573** | **1.0840** | **1** | **11** | **1.9e+02** | **1** | **IPYGSRFIAK** |
|  | 413 | **400.0564** | **798.0979** | **797.8994** | **0.1986** | **0** | **11** | **1.9e+02** | **1** | **EGLVPQR** |
|  | 738 | **419.2436** | **1254.7087** | **1254.5436** | **0.1651** | **1** | **11** | **2.2e+02** | **1** | **KKPGMTMSCAK + Carbamidomethyl (C); Oxidation (M)** |
|  | 772 | **422.2781** | **842.5414** | **841.9485** | **0.5929** | **0** | **11** | **2e+02** | **1** | **LENEIPK** |
|  | 2018 | **580.0398** | **1737.0972** | **1737.7359** | **-0.6386** | **0** | **11** | **2.3e+02** | **1** | **QEEFDVANNGSSQANK** |
|  | 428 | **401.1655** | **1200.4743** | **1199.3590** | **1.1154** | **0** | **11** | **2.4e+02** | **1** | **CHESCMECK + 2 Carbamidomethyl (C); Oxidation (M)** |
|  | 1310 | **481.8379** | **961.6609** | **962.0855** | **-0.4246** | **1** | **11** | **2.2e+02** | **1** | **QTLERCR + Carbamidomethyl (C)** |
|  | 1444 | **499.6723** | **1495.9947** | **1495.7393** | **0.2555** | **1** | **11** | **2e+02** | **1** | **SNSFISIPKMEVK + Oxidation (M)** |
|  | 1549 | **511.8349** | **1021.6550** | **1021.1463** | **0.5087** | **2** | **11** | **1.9e+02** | **1** | **DGKKEDCVK** |
|  | 3379 | **953.4049** | **2857.1925** | **2856.9621** | **0.2304** | **2** | **11** | **1.9e+02** | **1** | **EEEEGKDIEEGAIVNPGRDSATNQIR** |
|  | 1641 | **521.8974** | **1041.7800** | **1041.3081** | **0.4720** | **0** | **11** | **2.2e+02** | **1** | **AIMLGAKPPK + Oxidation (M)** |
|  | 2664 | **677.7341** | **2030.1802** | **2029.1940** | **0.9863** | **2** | **11** | **2.8e+02** | **1** | **APSQFMYYRGSGSYRWG + Oxidation (M)** |
|  | 1652 | **523.1951** | **1044.3754** | **1043.3056** | **1.0697** | **1** | **11** | **2.4e+02** | **1** | **CLKCCECK + 2 Carbamidomethyl (C)** |
|  | 2609 | **667.9833** | **2000.9277** | **2000.1725** | **0.7551** | **0** | **11** | **1.9e+02** | **1** | **DLSSHQLNEFLAQTLQR** |
|  | 1665 | **524.2219** | **1046.4291** | **1047.2613** | **-0.8322** | **2** | **11** | **2.5e+02** | **1** | **LPRRRPPR** |
|  | 2948 | **742.7452** | **1483.4756** | **1482.6644** | **0.8112** | **2** | **11** | **2e+02** | **1** | **MENFTSQSVKRR** |
|  | 2541 | **656.5161** | **1966.5262** | **1967.2076** | **-0.6814** | **2** | **11** | **1.8e+02** | **1** | **SRITMSVDTSKNHFSLK + Oxidation (M)** |
|  | 42 | **364.2812** | **1089.8215** | **1089.4103** | **0.4112** | **0** | **11** | **1.6e+02** | **1** | **LTPPLLLLLP** |
|  | 566 | **407.0031** | **1217.9870** | **1218.4203** | **-0.4333** | **1** | **11** | **2e+02** | **1** | **VKGDMDISLPK + Oxidation (M)** |
|  | 711 | **417.6945** | **833.3743** | **833.9133** | **-0.5390** | **1** | **11** | **2e+02** | **1** | **EAARECR** |
|  | 1797 | **540.1165** | **1078.2181** | **1077.2557** | **0.9624** | **0** | **11** | **2.4e+02** | **1** | **MQSTLISAAR** |
|  | 2396 | **628.9608** | **1883.8603** | **1883.1770** | **0.6832** | **1** | **11** | **1.9e+02** | **1** | **DLYSALANKCCHVGCTK + Carbamidomethyl (C)** |
|  | 2271 | **610.1067** | **1218.1986** | **1217.4605** | **0.7381** | **2** | **11** | **2.3e+02** | **1** | **KPKVRFTVDK** |
|  | 3291 | **831.3221** | **2490.9441** | **2491.9436** | **-0.9996** | **1** | **11** | **2.2e+02** | **1** | **VSLMCPSWSPELKQSTCLSLPK + Carbamidomethyl (C)** |
|  | 860 | **432.4224** | **1294.2451** | **1294.5028** | **-0.2577** | **1** | **11** | **2.5e+02** | **1** | **GADRGPVLLGALR** |
|  | 1229 | **471.7913** | **1412.3516** | **1412.5164** | **-0.1648** | **2** | **11** | **1.8e+02** | **1** | **RGGAGAHGFSPSRR** |
|  | 1254 | **474.0811** | **946.1475** | **945.1380** | **1.0095** | **1** | **11** | **2.5e+02** | **1** | **RTVAAPSML** |
|  | 817 | **429.0261** | **1284.0560** | **1283.4141** | **0.6420** | **1** | **11** | **2.2e+02** | **1** | **SSGISPCFSSRR** |
|  | 1697 | **528.2965** | **1581.8674** | **1581.8978** | **-0.0305** | **2** | **11** | **2.2e+02** | **1** | **ARQLDALLEALKLK** |
|  | 2143 | **596.6844** | **1787.0312** | **1787.1329** | **-0.1017** | **1** | **11** | **3e+02** | **1** | **FQMMVTPLCLSGSRK + Carbamidomethyl (C); 2 Oxidation (M)** |
|  | 2290 | **612.0720** | **1222.1291** | **1221.3198** | **0.8093** | **0** | **11** | **2.3e+02** | **1** | **SAGGTTAYAAPVR** |
|  | 242 | **383.0868** | **1146.2381** | **1146.2366** | **0.0016** | **0** | **11** | **2.4e+02** | **1** | **CQHSGSQPFR** |
|  | 253 | **384.9533** | **767.8918** | **768.9245** | **-1.0327** | **1** | **11** | **2e+02** | **1** | **FMAKTR + Oxidation (M)** |
|  | 548 | **406.3032** | **810.5915** | **809.9297** | **0.6618** | **0** | **11** | **1.7e+02** | **1** | **NCIFSLN** |
|  | 1735 | **533.9191** | **1598.7350** | **1599.8239** | **-1.0889** | **2** | **11** | **2.2e+02** | **1** | **KSIIKEPESAAEAVK** |
|  | 1922 | **562.6750** | **1685.0030** | **1685.7012** | **-0.6982** | **0** | **11** | **2.7e+02** | **1** | **DDAPASVHSEITDSNK** |
|  | 2681 | **679.8695** | **2036.5863** | **2037.3006** | **-0.7142** | **1** | **11** | **2.3e+02** | **1** | **VNWVVVAATRGGYGMDVWG** |
|  | 3405 | **1052.9689** | **2103.9230** | **2104.3632** | **-0.4402** | **2** | **11** | **1.6e+02** | **1** | **AELLSREQKYEDGIALLR** |
|  | 1548 | **511.2670** | **1020.5193** | **1021.2588** | **-0.7395** | **1** | **11** | **2.3e+02** | **1** | **CLRMGQVAK + Oxidation (M)** |
|  | 1851 | **549.2513** | **1644.7319** | **1645.9235** | **-1.1917** | **2** | **11** | **2.3e+02** | **1** | **LSDFGLCTGLKKAHR** |
|  | 1950 | **566.2364** | **1130.4581** | **1129.3073** | **1.1509** | **0** | **11** | **2.4e+02** | **1** | **VLSQQAASVVK** |
|  | 2718 | **684.1012** | **1366.1876** | **1365.5096** | **0.6781** | **0** | **11** | **2.3e+02** | **1** | **NIISLMDTSGNGK + Oxidation (M)** |
|  | 2809 | **705.1401** | **2112.3982** | **2112.5443** | **-0.1460** | **1** | **11** | **2.2e+02** | **1** | **IHFNPPLPMMRNQMITR + Oxidation (M)** |
|  | 919 | **436.2098** | **1305.6072** | **1305.5456** | **0.0616** | **1** | **11** | **2.3e+02** | **1** | **LSRMGATPTPFK** |
|  | 2634 | **670.4944** | **2008.4610** | **2008.3239** | **0.1371** | **1** | **11** | **2.1e+02** | **1** | **MSICCCFFFRDYGSSK + 2 Carbamidomethyl (C)** |
|  | 709 | **417.4595** | **1249.3564** | **1248.4064** | **0.9500** | **0** | **11** | **3e+02** | **1** | **GTGTISEAMPLR + Oxidation (M)** |
|  | 1712 | **529.8618** | **1057.7088** | **1058.2343** | **-0.5254** | **1** | **11** | **2.2e+02** | **1** | **KNPGFAAVVR** |
|  | 744 | **419.3963** | **836.7778** | **835.9258** | **0.8520** | **0** | **11** | **2.6e+02** | **1** | **CLGTETR + Carbamidomethyl (C)** |
|  | 1076 | **457.5863** | **1369.7366** | **1370.5742** | **-0.8376** | **1** | **11** | **2.6e+02** | **1** | **ETFSTMTLLRR + Oxidation (M)** |
|  | 1609 | **519.0004** | **1035.9861** | **1037.1239** | **-1.1379** | **2** | **11** | **2.2e+02** | **1** | **YGLDGKKDAA** |
|  | 2298 | **612.2033** | **1833.5878** | **1833.1465** | **0.4413** | **2** | **11** | **2.4e+02** | **1** | **RMNSLRLVAPMWNGR + 2 Oxidation (M)** |
|  | 2432 | **633.0837** | **1896.2290** | **1897.1738** | **-0.9448** | **1** | **11** | **2.5e+02** | **1** | **KTQTFLLETPEIYWK** |
|  | 2807 | **704.0208** | **2109.0401** | **2108.3120** | **0.7281** | **0** | **11** | **1.8e+02** | **1** | **ASQSISSYLHWYQQKPGK** |
|  | 465 | **403.0105** | **1206.0092** | **1206.2572** | **-0.2480** | **1** | **11** | **2.6e+02** | **1** | **GKSEEELEASK** |
|  | 617 | **408.1786** | **1221.5135** | **1220.5007** | **1.0128** | **1** | **11** | **2.4e+02** | **1** | **TFAKVITTVLK** |
|  | 1462 | **500.2009** | **1497.5804** | **1496.7684** | **0.8120** | **0** | **11** | **2.3e+02** | **1** | **NSSLGPAIALFLMF + Oxidation (M)** |
|  | 1509 | **505.1375** | **1512.3902** | **1511.5284** | **0.8618** | **1** | **11** | **2.2e+02** | **1** | **GSSGSSGDKMDGAPSR + Oxidation (M)** |
|  | 1644 | **522.4351** | **1042.8555** | **1043.2841** | **-0.4287** | **1** | **11** | **1.9e+02** | **1** | **CAVLSRLPK + Carbamidomethyl (C)** |
|  | 1695 | **528.2058** | **1581.5953** | **1581.0142** | **0.5811** | **0** | **11** | **2.2e+02** | **1** | **LSPPVIPFMPLLLK + Oxidation (M)** |
|  | 2612 | **668.2351** | **2001.6831** | **2001.3331** | **0.3501** | **2** | **11** | **2.4e+02** | **1** | **AKVFQVMNLRENGMYGK + Oxidation (M)** |
|  | 918 | **436.1976** | **870.3804** | **871.0545** | **-0.6741** | **0** | **11** | **2.3e+02** | **1** | **FSVFMPK + Oxidation (M)** |
|  | 1068 | **457.3114** | **912.6080** | **911.9988** | **0.6092** | **0** | **11** | **1.9e+02** | **1** | **VELPDSPR** |
|  | 2697 | **681.2012** | **2040.5813** | **2041.2013** | **-0.6200** | **1** | **11** | **2.3e+02** | **1** | **CDSEILYNNHKFTNASK + Carbamidomethyl (C)** |
|  | 2990 | **748.9482** | **1495.8817** | **1495.7358** | **0.1459** | **0** | **11** | **2.2e+02** | **1** | **DIVINEAIYMVSI + Oxidation (M)** |
|  | 3372 | **918.9381** | **1835.8614** | **1836.0810** | **-0.2195** | **1** | **11** | **2.1e+02** | **1** | **MTFGRGGAASVVLNVGGAR + Oxidation (M)** |
|  | 608 | **407.9779** | **813.9410** | **812.8909** | **1.0502** | **0** | **11** | **2.4e+02** | **1** | **YMQSER** |
|  | 634 | **408.9793** | **815.9438** | **815.8747** | **0.0691** | **0** | **11** | **2.6e+02** | **1** | **AINQAGSR** |
|  | 1657 | **523.7200** | **1045.4252** | **1045.2339** | **0.1913** | **0** | **11** | **2.2e+02** | **1** | **TGLMAAMYR + 2 Oxidation (M)** |
|  | 1787 | **538.6364** | **1612.8871** | **1612.8459** | **0.0412** | **1** | **11** | **2.9e+02** | **1** | **FSLRLSVMSAADTAK + Oxidation (M)** |
|  | 1876 | **553.6719** | **1105.3290** | **1105.3123** | **0.0167** | **2** | **11** | **2.9e+02** | **1** | **KSQVLKSCR + Carbamidomethyl (C)** |
|  | 3404 | **1052.9225** | **3155.7453** | **3154.6101** | **1.1352** | **2** | **11** | **1.7e+02** | **1** | **DGTKFICMALYSELLAVSSKGELYQWK + Carbamidomethyl (C); Oxidation (M)** |
|  | 430 | **401.2712** | **800.5276** | **799.9581** | **0.5694** | **0** | **11** | **2.1e+02** | **1** | **IATGGLLR** |
|  | 1054 | **455.0883** | **908.1619** | **907.0751** | **1.0868** | **1** | **11** | **2.3e+02** | **1** | **RVVVAAHR** |
|  | 3201 | **802.9692** | **2405.8855** | **2405.0053** | **0.8802** | **1** | **11** | **2.5e+02** | **1** | **MSVVGGTLLLGIAICCCCCCRR + 2 Carbamidomethyl (C); Oxidation (M)** |
|  | 1948 | **566.1068** | **1695.2983** | **1695.9010** | **-0.6028** | **0** | **11** | **2.5e+02** | **1** | **LPRPQALCGGSGGVGGGGR** |
|  | 398 | **398.9557** | **1193.8448** | **1194.2993** | **-0.4545** | **1** | **10** | **2.1e+02** | **1** | **EHIKNPDWR** |
|  | 1209 | **469.2145** | **1404.6213** | **1404.6982** | **-0.0769** | **2** | **10** | **2.3e+02** | **1** | **IPKMAAVKDSCGK + Carbamidomethyl (C)** |
|  | 2064 | **588.0868** | **1761.2382** | **1761.0343** | **0.2039** | **2** | **10** | **2.5e+02** | **1** | **MGAKDATPVPCGRWAK + Carbamidomethyl (C); Oxidation (M)** |
|  | 2673 | **678.9230** | **2033.7467** | **2034.5088** | **-0.7620** | **2** | **10** | **1.9e+02** | **1** | **MFSGKLKPTMAFMSGKLK + 2 Oxidation (M)** |
|  | 1262 | **475.6154** | **1423.8239** | **1423.7180** | **0.1059** | **1** | **10** | **2.7e+02** | **1** | **TMPPYYLGPLKK + Oxidation (M)** |
|  | 2106 | 593.3073 | 1776.8998 | 1776.9278 | -0.0280 | 2 | 10 | 2.6e+02 | 1 | SGDVQYRSSVRCYTR |
|  | 2597 | **666.7124** | **1997.1150** | **1996.2520** | **0.8631** | **1** | **10** | **2.7e+02** | **1** | **IHSREKPFGCDQCSMK + 2 Carbamidomethyl (C); Oxidation (M)** |
|  | 2605 | **667.6447** | **1333.2746** | **1332.5294** | **0.7452** | **2** | **10** | **2.2e+02** | **1** | **AMRNKLEGEIR + Oxidation (M)** |
|  | 2745 | **685.9852** | **2054.9335** | **2055.3358** | **-0.4022** | **1** | **10** | **1.9e+02** | **1** | **TSDCLCLQGKEPPSPAGPVR** |
|  | 1002 | **447.2703** | **1338.7886** | **1338.6416** | **0.1470** | **1** | **10** | **2.1e+02** | **1** | **RLMTPIMYAAR + Oxidation (M)** |
|  | 2883 | **731.0414** | **2190.1022** | **2190.3530** | **-0.2509** | **1** | **10** | **2e+02** | **1** | **FRGLEEGQAQAGQCPSLEGR + Carbamidomethyl (C)** |
|  | 917 | **436.1758** | **1305.5051** | **1306.5535** | **-1.0484** | **1** | **10** | **2.4e+02** | **1** | **KALASVPSPALPR** |
|  | 1145 | **461.8563** | **921.6977** | **921.9754** | **-0.2776** | **1** | **10** | **2.4e+02** | **1** | **ACKTDGDGR** |
|  | 1814 | **542.0692** | **1082.1235** | **1083.1279** | **-1.0043** | **0** | **10** | **2.3e+02** | **1** | **MSDSNLDSSK** |
|  | 181 | **375.1058** | **1122.2953** | **1121.2868** | **1.0086** | **0** | **10** | **2.7e+02** | **1** | **TGLAGAPGPPGVK** |
|  | 1014 | **448.4707** | **1342.3898** | **1341.6423** | **0.7476** | **2** | **10** | **2.6e+02** | **1** | **KFYKCDMCCK + Carbamidomethyl (C); Oxidation (M)** |
|  | 2176 | **599.1477** | **1794.4209** | **1794.0607** | **0.3602** | **0** | **10** | **2.3e+02** | **1** | **MGWAAVMGLGGGTTALER + Oxidation (M)** |
|  | 329 | **388.1821** | **1161.5242** | **1162.2776** | **-0.7533** | **1** | **10** | **2.6e+02** | **1** | **VCVSAERENR** |
|  | 1621 | **520.0695** | **1038.1241** | **1039.1530** | **-1.0289** | **2** | **10** | **2.3e+02** | **1** | **GHGLRTRSR** |
|  | 2082 | **591.3032** | **1770.8873** | **1770.9779** | **-0.0906** | **1** | **10** | **2.4e+02** | **1** | **SGNKAAVELEMDVGFGM + Oxidation (M)** |
|  | 2450 | **637.2434** | **1272.4720** | **1271.3721** | **1.1000** | **0** | **10** | **2.4e+02** | **1** | **DMELDASSMEK + Oxidation (M)** |
|  | 2746 | **686.0297** | **1370.0447** | **1370.5342** | **-0.4896** | **0** | **10** | **2e+02** | **1** | **MHNLNSALDALR + Oxidation (M)** |
|  | 2324 | **614.1003** | **1839.2787** | **1840.1292** | **-0.8506** | **1** | **10** | **2.3e+02** | **1** | **ENILVSLGAVGKSAVQVR** |
|  | 3130 | **789.5980** | **2365.7719** | **2364.6120** | **1.1598** | **0** | **10** | **2.1e+02** | **1** | **YHEVHYILLDPSCSGSGMPSR + Oxidation (M)** |
|  | 572 | **407.1756** | **1218.5045** | **1217.3557** | **1.1488** | **0** | **10** | **2.3e+02** | **1** | **NNLNAAMNIAR + Oxidation (M)** |
|  | 1645 | **522.8799** | **1565.6177** | **1565.7344** | **-0.1168** | **2** | **10** | **2.3e+02** | **1** | **IKERIHGHGNNYK** |
|  | 1816 | **543.0635** | **1084.1123** | **1083.1792** | **0.9331** | **0** | **10** | **2.3e+02** | **1** | **DGTVAPAGHCR** |
|  | 507 | **404.9911** | **1211.9510** | **1212.3530** | **-0.4019** | **2** | **10** | **2.2e+02** | **1** | **ERTYSTVKTK** |
|  | 1187 | **466.9467** | **931.8787** | **933.0427** | **-1.1640** | **0** | **10** | **2.5e+02** | **1** | **VFCEHGNK** |
|  | 1953 | **567.6910** | **1700.0509** | **1699.9978** | **0.0531** | **2** | **10** | **2.9e+02** | **1** | **MTGMPQMGRMRSPR + 4 Oxidation (M)** |
|  | 2173 | **599.1039** | **1794.2895** | **1793.1373** | **1.1521** | **1** | **10** | **2.3e+02** | **1** | **FALTAETKAWMVVIGR** |
|  | 2316 | **613.4023** | **1837.1849** | **1838.1083** | **-0.9235** | **1** | **10** | **2.3e+02** | **1** | **ASEDLVVGIYQQKFLK** |
|  | 2400 | **629.6456** | **1257.2765** | **1256.4516** | **0.8248** | **2** | **10** | **2.5e+02** | **1** | **EKAAALERQLK** |
|  | 3342 | **879.7023** | **2636.0846** | **2635.0295** | **1.0551** | **1** | **10** | **2.2e+02** | **1** | **ECMCQKCSLPVSVGSSAHLSQGLR + 2 Carbamidomethyl (C)** |
|  | 3365 | **909.5229** | **2725.5467** | **2726.0920** | **-0.5454** | **1** | **10** | **2.3e+02** | **1** | **AILLLDGTLNGAECFGSPEMAFQRR + Oxidation (M)** |
|  | 1639 | **521.5653** | **1561.6738** | **1560.7247** | **0.9491** | **0** | **10** | **2.8e+02** | **1** | **WPNDIYYSDLMK + Oxidation (M)** |
|  | 2618 | **668.6776** | **2003.0107** | **2003.2778** | **-0.2672** | **2** | **10** | **2.5e+02** | **1** | **IPADATALTAMAKADGDVKK + Oxidation (M)** |
|  | 2910 | **740.4917** | **2218.4529** | **2218.4016** | **0.0514** | **1** | **10** | **2.4e+02** | **1** | **GILKDNTSTTSSMVASAEHPR + Oxidation (M)** |
|  | 3311 | **847.0232** | **2538.0474** | **2538.7234** | **-0.6760** | **0** | **10** | **2.7e+02** | **1** | **DNPCISEHAGDFLTGQAVCGFGDK + Carbamidomethyl (C)** |
|  | 578 | **407.2856** | **812.5565** | **812.8445** | **-0.2880** | **0** | **10** | **1.8e+02** | **1** | **ESSPSFC + Carbamidomethyl (C)** |
|  | 742 | **419.3802** | **836.7457** | **837.0197** | **-0.2740** | **0** | **10** | **2.5e+02** | **1** | **CLCSLGK + 2 Carbamidomethyl (C)** |
|  | 3421 | **1196.5846** | **2391.1544** | **2391.7730** | **-0.6186** | **2** | **10** | **1.6e+02** | **1** | **RVYQRGCVNPMINIEQLWR + Oxidation (M)** |
|  | 1374 | **489.2595** | **1464.7562** | **1464.6806** | **0.0756** | **0** | **10** | **2.5e+02** | **1** | **EVMDYTFIGVFK + Oxidation (M)** |
|  | 2591 | **666.5431** | **1996.6071** | **1996.3347** | **0.2724** | **0** | **10** | **2e+02** | **1** | **MAAIGAHLGCTSACVAVYK + 2 Carbamidomethyl (C); Oxidation (M)** |
|  | 718 | **418.0507** | **834.0867** | **832.9684** | **1.1183** | **2** | **10** | **2.6e+02** | **1** | **DCKSPKR** |
|  | 2055 | **587.5140** | **1759.5199** | **1759.0150** | **0.5050** | **2** | **10** | **2.1e+02** | **1** | **GLRKMLDNFDCFGDK** |
|  | 2420 | **632.2222** | **1262.4296** | **1263.4491** | **-1.0195** | **1** | **10** | **2.7e+02** | **1** | **CHECGKLFNR + Carbamidomethyl (C)** |
|  | 2431 | **632.7975** | **1895.3703** | **1896.2173** | **-0.8471** | **1** | **10** | **2.8e+02** | **1** | **RLYPAPCLEGPTPTKPR** |
|  | 3170 | **798.2042** | **1594.3937** | **1593.9071** | **0.4866** | **0** | **10** | **2.2e+02** | **1** | **AYLMSICCMVNEK + Carbamidomethyl (C); 2 Oxidation (M)** |
|  | 908 | **435.8638** | **1304.5691** | **1305.5025** | **-0.9333** | **1** | **10** | **2.4e+02** | **1** | **KCIGVFEGHTSK** |
|  | 1386 | **492.1204** | **1473.3391** | **1472.5865** | **0.7525** | **1** | **10** | **2.3e+02** | **1** | **MAAAAAQGGGGGEPRR + Oxidation (M)** |
|  | 1739 | **534.3800** | **1600.1178** | **1600.7554** | **-0.6376** | **1** | **10** | **2.2e+02** | **1** | **TDGAGNGLNKTHLMR + Oxidation (M)** |
|  | 436 | **401.4412** | **800.8675** | **801.9312** | **-1.0636** | **1** | **10** | **3.3e+02** | **1** | **TTSLPKR** |
|  | 1188 | **467.1977** | **1398.5708** | **1399.6766** | **-1.1058** | **1** | **10** | **2.6e+02** | **1** | **VPKAEYIPTIIR** |
|  | 1282 | **477.2774** | **1428.8101** | **1427.6883** | **1.1218** | **2** | **10** | **2.1e+02** | **1** | **SALAPNLLTSGKKK** |
|  | 1775 | **538.0913** | **1074.1678** | **1073.2009** | **0.9669** | **1** | **10** | **2.6e+02** | **1** | **VRIAEQTEK** |
|  | 1968 | **571.4108** | **1711.2103** | **1711.9670** | **-0.7567** | **2** | **10** | **2e+02** | **1** | **AKATRHPGVMPSCGAR + Carbamidomethyl (C); Oxidation (M)** |
|  | 2098 | **592.9004** | **1775.6790** | **1776.1054** | **-0.4264** | **1** | **10** | **2.1e+02** | **1** | **KVMVLDFVTPSPLGTR + Oxidation (M)** |
|  | 1010 | **448.1953** | **894.3758** | **895.0163** | **-0.6404** | **0** | **10** | **2.4e+02** | **1** | **MPCTSGQR + Oxidation (M)** |
|  | 2385 | **627.1608** | **1252.3069** | **1252.4200** | **-0.1131** | **1** | **10** | **2.4e+02** | **1** | **RMCGFYSSASK + Oxidation (M)** |
|  | 2307 | 613.0337 | 1836.0789 | 1837.1040 | -1.0251 | 0 | 10 | 2.5e+02 | 1 | MASSASLETMVPPACPR + Carbamidomethyl (C); 2 Oxidation (M) |
|  | 2492 | **648.2636** | **1941.7685** | **1942.1119** | **-0.3434** | **0** | **10** | **2.4e+02** | **1** | **MNSLQTDDTGMYFCAR + Carbamidomethyl (C); 2 Oxidation (M)** |
|  | 848 | **431.2460** | **860.4772** | **859.8844** | **0.5929** | **0** | **10** | **2.5e+02** | **1** | **SGGASATGPR** |
|  | 1025 | **450.1511** | **898.2874** | **898.0185** | **0.2689** | **0** | **10** | **2.6e+02** | **1** | **NVRPDGLK** |
|  | 1158 | **462.2042** | **922.3935** | **921.9092** | **0.4844** | **1** | **10** | **2.3e+02** | **1** | **GSSDDGKTR** |
|  | 684 | **415.0138** | **828.0128** | **826.9404** | **1.0724** | **1** | **10** | **2.5e+02** | **1** | **GLRGPTLGG** |
|  | 2423 | **632.2991** | **1262.5834** | **1263.4491** | **-0.8657** | **1** | **10** | **2.7e+02** | **1** | **CHECGKLFNR + Carbamidomethyl (C)** |
|  | 750 | **420.2454** | **1257.7140** | **1258.4243** | **-0.7103** | **1** | **10** | **1.9e+02** | **1** | **ARINESLSQLK** |
|  | 1668 | **524.7043** | **1571.0909** | **1571.7689** | **-0.6781** | **0** | **10** | **2.4e+02** | **1** | **LYLVSDVLYNSSAK** |
|  | 1884 | **555.3558** | **1663.0453** | **1663.9554** | **-0.9101** | **2** | **10** | **2.4e+02** | **1** | **LLLTTKISKNFETR** |
|  | 2977 | 748.7871 | 1495.5594 | 1494.6552 | 0.9043 | 1 | 10 | 2.6e+02 | 1 | RLAQDGAHVVVSSR |
|  | 3015 | **749.4107** | **2245.2099** | **2244.5234** | **0.6866** | **2** | **10** | **2.4e+02** | **1** | **MQKTDKTMTELEIDMNQR + 2 Oxidation (M)** |
|  | 1345 | **487.0966** | **1458.2677** | **1457.7971** | **0.4706** | **2** | **10** | **2.6e+02** | **1** | **LLLTTESAIKKIK** |
|  | 130 | **371.1552** | **1110.4433** | **1110.3021** | **0.1411** | **0** | **10** | **1.9e+02** | **1** | **AGISYIVYPK** |
|  | 641 | **409.7046** | **817.3943** | **816.9259** | **0.4685** | **1** | **10** | **2.3e+02** | **1** | **SHQKCSK** |
|  | 2160 | **598.4767** | **1194.9386** | **1194.2579** | **0.6806** | **2** | **10** | **2e+02** | **1** | **GRGGAEKNSYR** |
|  | 635 | **408.9887** | **815.9626** | **816.9689** | **-1.0063** | **0** | **10** | **2.8e+02** | **1** | **GVVLGGCR + Carbamidomethyl (C)** |
|  | 1684 | **526.9041** | **1051.7933** | **1051.1573** | **0.6361** | **2** | **10** | **2.4e+02** | **1** | **GKFRTSAER** |
|  | 1773 | **537.9955** | **1073.9762** | **1073.1180** | **0.8582** | **0** | **10** | **2.6e+02** | **1** | **TGQGLPSSGNR** |
|  | 1794 | **539.9382** | **1077.8617** | **1078.1757** | **-0.3141** | **0** | **10** | **2.6e+02** | **1** | **NIALNGEYGK** |
|  | 2281 | **611.0584** | **1830.1529** | **1830.0038** | **0.1491** | **1** | **10** | **2.6e+02** | **1** | **GVERVDATVSVPESWAK** |
|  | 2790 | **697.9410** | **2090.8009** | **2090.3996** | **0.4013** | **1** | **10** | **2e+02** | **1** | **LESHLQNLSTLMAPTYKK + Oxidation (M)** |
|  | 1958 | **568.9061** | **1135.7975** | **1135.3148** | **0.4826** | **1** | **10** | **2.3e+02** | **1** | **RWLLDFASK** |
|  | 2371 | **623.1127** | **1244.2106** | **1245.3047** | **-1.0942** | **0** | **10** | **2.6e+02** | **1** | **EHGAQVPQHSR** |
|  | 2815 | **707.1783** | **1412.3418** | **1411.5368** | **0.8050** | **0** | **10** | **2.4e+02** | **1** | **TGPLTSEMQEFR + Oxidation (M)** |
|  | 907 | **435.8440** | **1304.5098** | **1303.5528** | **0.9571** | **0** | **10** | **2.5e+02** | **1** | **VLTCTGAPWPCR** |
|  | 1623 | **520.1049** | **1557.2924** | **1557.8003** | **-0.5079** | **1** | **10** | **2.4e+02** | **1** | **RWCGMTGGACSCPR + Carbamidomethyl (C); Oxidation (M)** |
|  | 2501 | **649.9178** | **1297.8209** | **1297.4806** | **0.3404** | **2** | **10** | **2e+02** | **1** | **MKETAAAKFER + Oxidation (M)** |
|  | 3263 | **818.3732** | **1634.7317** | **1633.8962** | **0.8355** | **2** | **10** | **2.3e+02** | **1** | **GQVLNIQARRTLHK** |
|  | 106 | **369.3311** | **1104.9712** | **1104.2680** | **0.7032** | **2** | **10** | **2.5e+02** | **1** | **HRSDRMMR + Oxidation (M)** |
|  | 931 | **437.1211** | **1308.3412** | **1308.5280** | **-0.1868** | **1** | **10** | **2.9e+02** | **1** | **NGSVCLMDVAKR + Oxidation (M)** |
|  | 1582 | **516.8565** | **1031.6982** | **1032.2154** | **-0.5171** | **0** | **10** | **2.4e+02** | **1** | **MTVVSVPQR + Oxidation (M)** |
|  | 1751 | **536.1726** | **1070.3304** | **1069.2105** | **1.1200** | **1** | **10** | **2.6e+02** | **1** | **DSFANKFIK** |
[truncated: 3,252,452 more chars]
