# Supplementary material for: Tobacco-induced hyperglycemia promotes lung cancer progression via cancer cell-macrophage interaction through paracrine IGF2/IR/NPM1-driven PD-L1 expression
Source: Nat Commun. 2024 Jun 8;15:4909. doi: 10.1038/s41467-024-49199-9 (PMC11162468; doi:10.1038/s41467-024-49199-9)
Supplement: Supplementary file 3 — Supplementary Data 1 [file 41467_2024_49199_MOESM3_ESM.zip › Supplementary Data 1/3.htm]

Peptide Summary Report (../data/20120627/F011177.dat)


# Mascot Search Results

```
User            : yprc
Email           : info
Search title    : 
MS data file    : 3.xml
Database        : NCBInr 110704 (14481393 sequences; 4958963357 residues)
Taxonomy        : Homo sapiens (human) (217342 sequences)
Timestamp       : 27 Jun 2012 at 03:55:58 GMT

|  |  |  |
| --- | --- | --- |
| Protein hits    : | gi|4885381 | histone H1.5 [Homo sapiens] |
|  | gi|66365795 | Histone cluster 1, H1e [Homo sapiens] |
|  | gi|4885377 | histone H1.3 [Homo sapiens] |
|  | gi|4885375 | histone H1.2 [Homo sapiens] |
|  | gi|4885373 | histone H1.1 [Homo sapiens] |
|  | gi|435476 | cytokeratin 9 [Homo sapiens] |
|  | gi|11935049 | keratin 1 [Homo sapiens] |
|  | gi|225131084 | titin [Homo sapiens] |
|  | gi|17066105 | Titin [Homo sapiens] |
|  | gi|119631418 | titin, isoform CRA_a [Homo sapiens] |
|  | gi|1017427 | elastic titin [Homo sapiens] |
|  | gi|46812692 | Keratin 6A [Homo sapiens] |
|  | gi|17066104 | N2B-Titin Isoform [Homo sapiens] |
|  | gi|1212992 | titin [Homo sapiens] |
|  | gi|119631904 | nebulin, isoform CRA_a [Homo sapiens] |
|  | gi|115527120 | nebulin isoform 3 [Homo sapiens] |
|  | gi|623409 | keratin 10 [Homo sapiens] |
|  | gi|806562 | nebulin [Homo sapiens] |
|  | gi|184084 | histone H1t [Homo sapiens] |
|  | gi|62088916 | Insulin-like growth factor 2 receptor variant [Homo sapiens] |
```

### Probability Based Mowse Score

Ions score is -10\*Log(P), where P is the
probability that the observed match is a random event.  
Individual ions scores
> 47 indicate identity or extensive homology (p<0.05).  
Protein scores
are derived from ions scores as a non-probabilistic basis for ranking protein
hits.

### Peptide Summary Report

|  |  |  |  |
| --- | --- | --- | --- |
|  | Peptide Summary Select Summary (protein hits) Select Summary (unassigned) Export Search Results |  | Help |
|  | Significance threshold p< | Max. number of hits |  |
|  | Standard scoring  MudPIT scoring | Ions score or expect cut-off | Show sub-sets |
|  | Show pop-ups  Suppress pop-ups | Sort unassigned  Decreasing Score Increasing query / Mr Decreasing Intensity | Require bold red |

  
 
                
             


  


  
     **Error tolerant**    

|  |  |
| --- | --- |
| **1.** | gi|4885381    **Mass:** 22580    **Score:** 532    **Queries matched:** 23   **emPAI:** 3.60 |
|  | histone H1.5 [Homo sapiens] |

|  |  |
| --- | --- |
|  | Check to include this hit in error tolerant search or archive report |
|  |  |

|  |  |  |  |  |  |  |  |  |  |  |
| --- | --- | --- | --- | --- | --- | --- | --- | --- | --- | --- |
|  | **Query** | **Observed** | **Mr(expt)** | **Mr(calc)** | **Delta** | **Miss** | **Score** | **Expect** | **Rank** | **Peptide** |
|  | 2389 | **547.1691** | **1092.3234** | **1093.1872** | **-0.8639** | **0** | **64** | **0.0011** | **1** | **K.ALAAGGYDVEK.N** |
|  | 2557 | **583.7935** | **1165.5721** | **1166.2843** | **-0.7121** | **1** | **61** | **0.002** | **1** | **K.GTGASGSFKLNK.K** |
|  | 538 | **389.7013** | **1166.0816** | **1166.2843** | **-0.2026** | **1** | **(9)** | **3e+02** | **1** | **K.GTGASGSFKLNK.K** |
|  | 2558 | **584.1606** | **1166.3064** | **1166.2843** | **0.0221** | **1** | **(25)** | **8.2** | **1** | **K.GTGASGSFKLNK.K** |
|  | 541 | **389.8387** | **1166.4938** | **1166.2843** | **0.2095** | **1** | **(16)** | **75** | **1** | **K.GTGASGSFKLNK.K** |
|  | 2559 | **584.3058** | **1166.5968** | **1166.2843** | **0.3125** | **1** | **(54)** | **0.012** | **1** | **K.GTGASGSFKLNK.K** |
|  | 546 | **389.9907** | **1166.9500** | **1166.2843** | **0.6657** | **1** | **(29)** | **3.1** | **1** | **K.GTGASGSFKLNK.K** |
|  | 2680 | **607.5792** | **1213.1437** | **1212.3925** | **0.7511** | **0** | **48** | **0.037** | **1** | **K.ATGPPVSELITK.A** |
|  | 2681 | **607.6364** | **1213.2579** | **1212.3925** | **0.8654** | **0** | **(41)** | **0.23** | **1** | **K.ATGPPVSELITK.A** |
|  | 2796 | **631.2382** | **1260.4617** | **1260.4801** | **-0.0185** | **1** | **(67)** | **0.00058** | **1** | **K.SLVSKGTLVQTK.G** |
|  | 2797 | **631.7916** | **1261.5683** | **1260.4801** | **1.0882** | **1** | **85** | **8.9e-06** | **1** | **K.SLVSKGTLVQTK.G** |
|  | 2829 | **648.2100** | **1294.4053** | **1294.4566** | **-0.0513** | **2** | **(27)** | **5.8** | **1** | **K.GTGASGSFKLNKK.A** |
|  | 2830 | **648.2167** | **1294.4186** | **1294.4566** | **-0.0380** | **2** | **49** | **0.031** | **1** | **K.GTGASGSFKLNKK.A** |
|  | 1313 | **432.7587** | **1295.2538** | **1294.4566** | **0.7973** | **2** | **(28)** | **3.7** | **1** | **K.GTGASGSFKLNKK.A** |
|  | 2964 | **671.2705** | **1340.5262** | **1340.5648** | **-0.0386** | **1** | **(61)** | **0.0022** | **1** | **R.KATGPPVSELITK.A** |
|  | 2965 | **671.3223** | **1340.6298** | **1340.5648** | **0.0649** | **1** | **74** | **0.00011** | **1** | **R.KATGPPVSELITK.A** |
|  | 1488 | **448.0380** | **1341.0917** | **1340.5648** | **0.5269** | **1** | **(52)** | **0.017** | **1** | **R.KATGPPVSELITK.A** |
|  | 1490 | **448.2213** | **1341.6418** | **1340.5648** | **1.0769** | **1** | **(15)** | **79** | **1** | **R.KATGPPVSELITK.A** |
|  | 3340 | **782.9611** | **1563.9073** | **1564.6554** | **-0.7481** | **1** | **98** | **3.9e-07** | **1** | **K.ALAAGGYDVEKNNSR.I** |
|  | 3341 | **783.2235** | **1564.4322** | **1564.6554** | **-0.2232** | **1** | **(94)** | **1e-06** | **1** | **K.ALAAGGYDVEKNNSR.I** |
|  | 2222 | **522.4912** | **1564.4515** | **1564.6554** | **-0.2040** | **1** | **(26)** | **6.8** | **1** | **K.ALAAGGYDVEKNNSR.I** |
|  | 2223 | **522.8212** | **1565.4415** | **1564.6554** | **0.7861** | **1** | **(40)** | **0.23** | **1** | **K.ALAAGGYDVEKNNSR.I** |
|  | 2473 | **565.5194** | **1693.5361** | **1692.8277** | **0.7083** | **2** | **55** | **0.0083** | **1** | **K.KALAAGGYDVEKNNSR.I** |

  


---

|  |  |
| --- | --- |
| **2.** | gi|66365795    **Mass:** 21893    **Score:** 488    **Queries matched:** 22   **emPAI:** 3.81 |
|  | Histone cluster 1, H1e [Homo sapiens] |

|  |  |
| --- | --- |
|  | Check to include this hit in error tolerant search or archive report |
|  |  |

|  |  |  |  |  |  |  |  |  |  |  |
| --- | --- | --- | --- | --- | --- | --- | --- | --- | --- | --- |
|  | **Query** | **Observed** | **Mr(expt)** | **Mr(calc)** | **Delta** | **Miss** | **Score** | **Expect** | **Rank** | **Peptide** |
|  | 1275 | **430.2518** | **858.4889** | **858.9826** | **-0.4937** | **1** | **10** | **2.4e+02** | **4** | **K.VVAASKER.S** |
|  | 2557 | 583.7935 | 1165.5721 | 1166.2843 | -0.7121 | 1 | 61 | 0.002 | 1 | K.GTGASGSFKLNK.K |
|  | 538 | 389.7013 | 1166.0816 | 1166.2843 | -0.2026 | 1 | (9) | 3e+02 | 1 | K.GTGASGSFKLNK.K |
|  | 2558 | 584.1606 | 1166.3064 | 1166.2843 | 0.0221 | 1 | (25) | 8.2 | 1 | K.GTGASGSFKLNK.K |
|  | 541 | 389.8387 | 1166.4938 | 1166.2843 | 0.2095 | 1 | (16) | 75 | 1 | K.GTGASGSFKLNK.K |
|  | 2559 | 584.3058 | 1166.5968 | 1166.2843 | 0.3125 | 1 | (54) | 0.012 | 1 | K.GTGASGSFKLNK.K |
|  | 546 | 389.9907 | 1166.9500 | 1166.2843 | 0.6657 | 1 | (29) | 3.1 | 1 | K.GTGASGSFKLNK.K |
|  | 2658 | **600.1294** | **1198.2440** | **1198.3660** | **-0.1219** | **0** | **(38)** | **0.45** | **1** | **K.ASGPPVSELITK.V** |
|  | 2659 | **600.2422** | **1198.4697** | **1198.3660** | **0.1038** | **0** | **(52)** | **0.019** | **1** | **K.ASGPPVSELITK.V** |
|  | 2662 | **600.7306** | **1199.4464** | **1198.3660** | **1.0804** | **0** | **56** | **0.0075** | **1** | **K.ASGPPVSELITK.V** |
|  | 2796 | 631.2382 | 1260.4617 | 1260.4801 | -0.0185 | 1 | (67) | 0.00058 | 1 | K.SLVSKGTLVQTK.G |
|  | 2797 | 631.7916 | 1261.5683 | 1260.4801 | 1.0882 | 1 | 85 | 8.9e-06 | 1 | K.SLVSKGTLVQTK.G |
|  | 2829 | 648.2100 | 1294.4053 | 1294.4566 | -0.0513 | 2 | (27) | 5.8 | 1 | K.GTGASGSFKLNKK.A |
|  | 2830 | 648.2167 | 1294.4186 | 1294.4566 | -0.0380 | 2 | 49 | 0.031 | 1 | K.GTGASGSFKLNKK.A |
|  | 1313 | 432.7587 | 1295.2538 | 1294.4566 | 0.7973 | 2 | (28) | 3.7 | 1 | K.GTGASGSFKLNKK.A |
|  | 2870 | **663.9126** | **1325.8104** | **1326.5383** | **-0.7278** | **1** | **69** | **0.0003** | **1** | **R.KASGPPVSELITK.V** |
|  | 2873 | **664.2123** | **1326.4098** | **1326.5383** | **-0.1285** | **1** | **(50)** | **0.029** | **1** | **R.KASGPPVSELITK.V** |
|  | 1441 | **443.5318** | **1327.5731** | **1326.5383** | **1.0349** | **1** | **(40)** | **0.34** | **1** | **R.KASGPPVSELITK.V** |
|  | 3211 | **740.6421** | **1479.2694** | **1478.6028** | **0.6666** | **0** | **36** | **0.5** | **1** | **M.SETAPAAPAAPAPAEK.T** |
|  | 1998 | **495.4649** | **1483.3726** | **1482.7240** | **0.6487** | **2** | **39** | **0.32** | **1** | **K.RKASGPPVSELITK.V** |
|  | 2262 | **527.2079** | **1578.6015** | **1578.6820** | **-0.0806** | **1** | **(33)** | **1.4** | **1** | **K.ALAAAGYDVEKNNSR.I** |
|  | 3364 | **790.4298** | **1578.8448** | **1578.6820** | **0.1628** | **1** | **86** | **6.6e-06** | **1** | **K.ALAAAGYDVEKNNSR.I** |

  


---

|  |  |
| --- | --- |
| **3.** | gi|4885377    **Mass:** 22350    **Score:** 450    **Queries matched:** 21   **emPAI:** 3.05 |
|  | histone H1.3 [Homo sapiens] |

|  |  |
| --- | --- |
|  | Check to include this hit in error tolerant search or archive report |
|  |  |

|  |  |  |  |  |  |  |  |  |  |  |
| --- | --- | --- | --- | --- | --- | --- | --- | --- | --- | --- |
|  | **Query** | **Observed** | **Mr(expt)** | **Mr(calc)** | **Delta** | **Miss** | **Score** | **Expect** | **Rank** | **Peptide** |
|  | 1168 | **422.2008** | **842.3868** | **841.9951** | **0.3918** | **1** | **12** | **1.7e+02** | **8** | **K.VAGAATPKK.S** |
|  | 2557 | 583.7935 | 1165.5721 | 1166.2843 | -0.7121 | 1 | 61 | 0.002 | 1 | K.GTGASGSFKLNK.K |
|  | 538 | 389.7013 | 1166.0816 | 1166.2843 | -0.2026 | 1 | (9) | 3e+02 | 1 | K.GTGASGSFKLNK.K |
|  | 2558 | 584.1606 | 1166.3064 | 1166.2843 | 0.0221 | 1 | (25) | 8.2 | 1 | K.GTGASGSFKLNK.K |
|  | 541 | 389.8387 | 1166.4938 | 1166.2843 | 0.2095 | 1 | (16) | 75 | 1 | K.GTGASGSFKLNK.K |
|  | 2559 | 584.3058 | 1166.5968 | 1166.2843 | 0.3125 | 1 | (54) | 0.012 | 1 | K.GTGASGSFKLNK.K |
|  | 546 | 389.9907 | 1166.9500 | 1166.2843 | 0.6657 | 1 | (29) | 3.1 | 1 | K.GTGASGSFKLNK.K |
|  | 2658 | 600.1294 | 1198.2440 | 1198.3660 | -0.1219 | 0 | (38) | 0.45 | 1 | K.ASGPPVSELITK.A |
|  | 2659 | 600.2422 | 1198.4697 | 1198.3660 | 0.1038 | 0 | (52) | 0.019 | 1 | K.ASGPPVSELITK.A |
|  | 2662 | 600.7306 | 1199.4464 | 1198.3660 | 1.0804 | 0 | 56 | 0.0075 | 1 | K.ASGPPVSELITK.A |
|  | 2796 | 631.2382 | 1260.4617 | 1260.4801 | -0.0185 | 1 | (67) | 0.00058 | 1 | K.SLVSKGTLVQTK.G |
|  | 2797 | 631.7916 | 1261.5683 | 1260.4801 | 1.0882 | 1 | 85 | 8.9e-06 | 1 | K.SLVSKGTLVQTK.G |
|  | 2829 | 648.2100 | 1294.4053 | 1294.4566 | -0.0513 | 2 | (27) | 5.8 | 1 | K.GTGASGSFKLNKK.A |
|  | 2830 | 648.2167 | 1294.4186 | 1294.4566 | -0.0380 | 2 | 49 | 0.031 | 1 | K.GTGASGSFKLNKK.A |
|  | 1313 | 432.7587 | 1295.2538 | 1294.4566 | 0.7973 | 2 | (28) | 3.7 | 1 | K.GTGASGSFKLNKK.A |
|  | 2870 | 663.9126 | 1325.8104 | 1326.5383 | -0.7278 | 1 | 69 | 0.0003 | 1 | R.KASGPPVSELITK.A |
|  | 2873 | 664.2123 | 1326.4098 | 1326.5383 | -0.1285 | 1 | (50) | 0.029 | 1 | R.KASGPPVSELITK.A |
|  | 1441 | 443.5318 | 1327.5731 | 1326.5383 | 1.0349 | 1 | (40) | 0.34 | 1 | R.KASGPPVSELITK.A |
|  | 1998 | 495.4649 | 1483.3726 | 1482.7240 | 0.6487 | 2 | 39 | 0.32 | 1 | K.RKASGPPVSELITK.A |
|  | 2262 | 527.2079 | 1578.6015 | 1578.6820 | -0.0806 | 1 | (33) | 1.4 | 1 | K.ALAAAGYDVEKNNSR.I |
|  | 3364 | 790.4298 | 1578.8448 | 1578.6820 | 0.1628 | 1 | 86 | 6.6e-06 | 1 | K.ALAAAGYDVEKNNSR.I |

  


---

|  |  |
| --- | --- |
| **4.** | gi|4885375    **Mass:** 21364    **Score:** 442    **Queries matched:** 20   **emPAI:** 3.32 |
|  | histone H1.2 [Homo sapiens] |

|  |  |
| --- | --- |
|  | Check to include this hit in error tolerant search or archive report |
|  |  |

|  |  |  |  |  |  |  |  |  |  |  |
| --- | --- | --- | --- | --- | --- | --- | --- | --- | --- | --- |
|  | **Query** | **Observed** | **Mr(expt)** | **Mr(calc)** | **Delta** | **Miss** | **Score** | **Expect** | **Rank** | **Peptide** |
|  | 2557 | 583.7935 | 1165.5721 | 1166.2843 | -0.7121 | 1 | 61 | 0.002 | 1 | K.GTGASGSFKLNK.K |
|  | 538 | 389.7013 | 1166.0816 | 1166.2843 | -0.2026 | 1 | (9) | 3e+02 | 1 | K.GTGASGSFKLNK.K |
|  | 2558 | 584.1606 | 1166.3064 | 1166.2843 | 0.0221 | 1 | (25) | 8.2 | 1 | K.GTGASGSFKLNK.K |
|  | 541 | 389.8387 | 1166.4938 | 1166.2843 | 0.2095 | 1 | (16) | 75 | 1 | K.GTGASGSFKLNK.K |
|  | 2559 | 584.3058 | 1166.5968 | 1166.2843 | 0.3125 | 1 | (54) | 0.012 | 1 | K.GTGASGSFKLNK.K |
|  | 546 | 389.9907 | 1166.9500 | 1166.2843 | 0.6657 | 1 | (29) | 3.1 | 1 | K.GTGASGSFKLNK.K |
|  | 2658 | 600.1294 | 1198.2440 | 1198.3660 | -0.1219 | 0 | (38) | 0.45 | 1 | K.ASGPPVSELITK.A |
|  | 2659 | 600.2422 | 1198.4697 | 1198.3660 | 0.1038 | 0 | (52) | 0.019 | 1 | K.ASGPPVSELITK.A |
|  | 2662 | 600.7306 | 1199.4464 | 1198.3660 | 1.0804 | 0 | 56 | 0.0075 | 1 | K.ASGPPVSELITK.A |
|  | 2796 | 631.2382 | 1260.4617 | 1260.4801 | -0.0185 | 1 | (67) | 0.00058 | 1 | K.SLVSKGTLVQTK.G |
|  | 2797 | 631.7916 | 1261.5683 | 1260.4801 | 1.0882 | 1 | 85 | 8.9e-06 | 1 | K.SLVSKGTLVQTK.G |
|  | 2829 | 648.2100 | 1294.4053 | 1294.4566 | -0.0513 | 2 | (27) | 5.8 | 1 | K.GTGASGSFKLNKK.A |
|  | 2830 | 648.2167 | 1294.4186 | 1294.4566 | -0.0380 | 2 | 49 | 0.031 | 1 | K.GTGASGSFKLNKK.A |
|  | 1313 | 432.7587 | 1295.2538 | 1294.4566 | 0.7973 | 2 | (28) | 3.7 | 1 | K.GTGASGSFKLNKK.A |
|  | 2870 | 663.9126 | 1325.8104 | 1326.5383 | -0.7278 | 1 | 69 | 0.0003 | 1 | R.KASGPPVSELITK.A |
|  | 2873 | 664.2123 | 1326.4098 | 1326.5383 | -0.1285 | 1 | (50) | 0.029 | 1 | R.KASGPPVSELITK.A |
|  | 1441 | 443.5318 | 1327.5731 | 1326.5383 | 1.0349 | 1 | (40) | 0.34 | 1 | R.KASGPPVSELITK.A |
|  | 3211 | 740.6421 | 1479.2694 | 1478.6028 | 0.6666 | 0 | 36 | 0.5 | 1 | M.SETAPAAPAAAPPAEK.A |
|  | 2262 | 527.2079 | 1578.6015 | 1578.6820 | -0.0806 | 1 | (33) | 1.4 | 1 | K.ALAAAGYDVEKNNSR.I |
|  | 3364 | 790.4298 | 1578.8448 | 1578.6820 | 0.1628 | 1 | 86 | 6.6e-06 | 1 | K.ALAAAGYDVEKNNSR.I |

  


---

|  |  |
| --- | --- |
| **5.** | gi|4885373    **Mass:** 21842    **Score:** 283    **Queries matched:** 14   **emPAI:** 1.35 |
|  | histone H1.1 [Homo sapiens] |

|  |  |
| --- | --- |
|  | Check to include this hit in error tolerant search or archive report |
|  |  |

|  |  |  |  |  |  |  |  |  |  |  |
| --- | --- | --- | --- | --- | --- | --- | --- | --- | --- | --- |
|  | **Query** | **Observed** | **Mr(expt)** | **Mr(calc)** | **Delta** | **Miss** | **Score** | **Expect** | **Rank** | **Peptide** |
|  | 2329 | **536.2662** | **1070.5176** | **1070.2865** | **0.2311** | **2** | **2** | **1.7e+03** | **9** | **K.KPAKAAAASKK.K** |
|  | 2557 | 583.7935 | 1165.5721 | 1166.2843 | -0.7121 | 1 | 61 | 0.002 | 1 | K.GTGASGSFKLNK.K |
|  | 538 | 389.7013 | 1166.0816 | 1166.2843 | -0.2026 | 1 | (9) | 3e+02 | 1 | K.GTGASGSFKLNK.K |
|  | 2558 | 584.1606 | 1166.3064 | 1166.2843 | 0.0221 | 1 | (25) | 8.2 | 1 | K.GTGASGSFKLNK.K |
|  | 541 | 389.8387 | 1166.4938 | 1166.2843 | 0.2095 | 1 | (16) | 75 | 1 | K.GTGASGSFKLNK.K |
|  | 2559 | 584.3058 | 1166.5968 | 1166.2843 | 0.3125 | 1 | (54) | 0.012 | 1 | K.GTGASGSFKLNK.K |
|  | 546 | 389.9907 | 1166.9500 | 1166.2843 | 0.6657 | 1 | (29) | 3.1 | 1 | K.GTGASGSFKLNK.K |
|  | 2796 | 631.2382 | 1260.4617 | 1260.4801 | -0.0185 | 1 | (67) | 0.00058 | 1 | K.SLVSKGTLVQTK.G |
|  | 2797 | 631.7916 | 1261.5683 | 1260.4801 | 1.0882 | 1 | 85 | 8.9e-06 | 1 | K.SLVSKGTLVQTK.G |
|  | 2829 | 648.2100 | 1294.4053 | 1294.4566 | -0.0513 | 2 | (27) | 5.8 | 1 | K.GTGASGSFKLNKK.A |
|  | 2830 | 648.2167 | 1294.4186 | 1294.4566 | -0.0380 | 2 | 49 | 0.031 | 1 | K.GTGASGSFKLNKK.A |
|  | 1313 | 432.7587 | 1295.2538 | 1294.4566 | 0.7973 | 2 | (28) | 3.7 | 1 | K.GTGASGSFKLNKK.A |
|  | 2262 | 527.2079 | 1578.6015 | 1578.6820 | -0.0806 | 1 | (33) | 1.4 | 1 | K.ALAAAGYDVEKNNSR.I |
|  | 3364 | 790.4298 | 1578.8448 | 1578.6820 | 0.1628 | 1 | 86 | 6.6e-06 | 1 | K.ALAAAGYDVEKNNSR.I |

  


---

|  |  |
| --- | --- |
| **6.** | gi|435476    **Mass:** 62129    **Score:** 270    **Queries matched:** 5   **emPAI:** 0.11 |
|  | cytokeratin 9 [Homo sapiens] |

|  |  |
| --- | --- |
|  | Check to include this hit in error tolerant search or archive report |
|  |  |

|  |  |  |  |  |  |  |  |  |  |  |
| --- | --- | --- | --- | --- | --- | --- | --- | --- | --- | --- |
|  | **Query** | **Observed** | **Mr(expt)** | **Mr(calc)** | **Delta** | **Miss** | **Score** | **Expect** | **Rank** | **Peptide** |
|  | 831 | **406.6886** | **811.3625** | **811.9689** | **-0.6065** | **1** | **13** | **1.1e+02** | **9** | **K.KGPAAIQK.N** |
|  | 866 | **407.2516** | **812.4885** | **811.9689** | **0.5196** | **1** | **(5)** | **5.7e+02** | **10** | **K.KGPAAIQK.N** |
|  | 2445 | **562.1126** | **1122.2104** | **1121.2403** | **0.9701** | **0** | **26** | **6** | **1** | **R.QEYEQLIAK.N** |
|  | 2758 | **617.0690** | **1232.1232** | **1232.2610** | **-0.1379** | **0** | **113** | **1.3e-08** | **1** | **R.SGGGGGGGLGSGGSIR.S** |
|  | 3500 | **897.0138** | **1792.0128** | **1791.7036** | **0.3093** | **0** | **118** | **5.1e-09** | **1** | **R.GGSGGSYGGGGSGGGYGGGSGSR.G** |

  

|  |  |
| --- | --- |
|  | |
|  | **Proteins matching the same set of peptides:** |

|  |  |
| --- | --- |
|  | gi|55956899    **Mass:** 62064    **Score:** 270    **Queries matched:** 5 |
|  | keratin, type I cytoskeletal 9 [Homo sapiens] |

---

|  |  |
| --- | --- |
| **7.** | gi|11935049    **Mass:** 66066    **Score:** 202    **Queries matched:** 6   **emPAI:** 0.16 |
|  | keratin 1 [Homo sapiens] |

|  |  |
| --- | --- |
|  | Check to include this hit in error tolerant search or archive report |
|  |  |

|  |  |  |  |  |  |  |  |  |  |  |
| --- | --- | --- | --- | --- | --- | --- | --- | --- | --- | --- |
|  | **Query** | **Observed** | **Mr(expt)** | **Mr(calc)** | **Delta** | **Miss** | **Score** | **Expect** | **Rank** | **Peptide** |
|  | 1408 | **438.0411** | **874.0674** | **873.9937** | **0.0737** | **0** | **18** | **49** | **1** | **R.SLVNLGGSK.S** |
|  | 2137 | **517.7321** | **1033.4493** | **1033.0906** | **0.3587** | **0** | **43** | **0.11** | **1** | **R.TLLEGEESR.M** |
|  | 2307 | **534.1687** | **1066.3226** | **1065.1340** | **1.1886** | **0** | **50** | **0.026** | **1** | **K.AQYEDIAQK.S** |
|  | 2456 | **563.6682** | **1125.3216** | **1125.1860** | **0.1356** | **0** | **57** | **0.0055** | **1** | **K.AEAESLYQSK.Y** |
|  | 2525 | **576.8057** | **1151.5965** | **1152.2113** | **-0.6148** | **1** | **35** | **0.73** | **1** | **R.NKYEDEINK.R** |
|  | 382 | **385.0221** | **1152.0440** | **1152.2113** | **-0.1673** | **1** | **(7)** | **4.7e+02** | **7** | **R.NKYEDEINK.R** |

  

|  |  |
| --- | --- |
|  | |
|  | **Proteins matching the same set of peptides:** |

|  |  |
| --- | --- |
|  | gi|119395750    **Mass:** 66038    **Score:** 202    **Queries matched:** 6 |
|  | keratin, type II cytoskeletal 1 [Homo sapiens] |

|  |  |
| --- | --- |
|  | gi|189054178    **Mass:** 66019    **Score:** 202    **Queries matched:** 6 |
|  | unnamed protein product [Homo sapiens] |

---

|  |  |
| --- | --- |
| **8.** | gi|225131084    **Mass:** 3713667  **Score:** 189    **Queries matched:** 83 |
|  | titin [Homo sapiens] |

|  |  |
| --- | --- |
|  | Check to include this hit in error tolerant search or archive report |
|  |  |

|  |  |  |  |  |  |  |  |  |  |  |
| --- | --- | --- | --- | --- | --- | --- | --- | --- | --- | --- |
|  | **Query** | **Observed** | **Mr(expt)** | **Mr(calc)** | **Delta** | **Miss** | **Score** | **Expect** | **Rank** | **Peptide** |
|  | 220 | **371.1613** | **740.3078** | **739.8601** | **0.4477** | **1** | **16** | **44** | **1** | **K.KEAPPAK.V** |
|  | 229 | **371.3508** | **740.6868** | **739.8601** | **0.8268** | **1** | **(7)** | **3.9e+02** | **3** | **K.KEAPPAK.V** |
|  | 232 | **372.2370** | **742.4593** | **742.9102** | **-0.4510** | **1** | **(10)** | **2.2e+02** | **8** | **K.LRLSVR.G** |
|  | 236 | **372.3603** | **742.7059** | **742.9102** | **-0.2044** | **1** | **10** | **2.6e+02** | **5** | **K.LRLSVR.G** |
|  | 253 | **374.1826** | **746.3503** | **745.8282** | **0.5222** | **2** | **8** | **4e+02** | **3** | **K.RDATKR.T** |
|  | 286 | **376.9611** | **751.9075** | **752.8341** | **-0.9266** | **0** | **(6)** | **6.3e+02** | **4** | **R.CVETSSK.K** |
|  | 302 | **377.5815** | **753.1481** | **752.8341** | **0.3141** | **0** | **7** | **3.8e+02** | **9** | **R.CVETSSK.K** |
|  | 337 | **380.2560** | **758.4972** | **758.7342** | **-0.2370** | **0** | **1** | **1.8e+03** | **10** | **R.SESDGHK.R** |
|  | 477 | **387.8971** | **773.7794** | **772.9295** | **0.8499** | **0** | **12** | **2e+02** | **4** | **R.LILTEGK.N** |
|  | 479 | **387.9558** | **773.8968** | **772.8931** | **1.0037** | **1** | **12** | **2.3e+02** | **4** | **R.RITDLR.L** |
|  | 525 | **389.1608** | **776.3068** | **775.9168** | **0.3900** | **0** | **17** | **60** | **1** | **R.ISCGGAIR.S** |
|  | 534 | **389.3109** | **776.6071** | **775.8044** | **0.8028** | **0** | **7** | **5e+02** | **5** | **K.EGQDISK.R** |
|  | 572 | **394.1502** | **786.2857** | **785.9716** | **0.3141** | **1** | **7** | **5.2e+02** | **7** | **K.LKVEAVK.I** |
|  | 575 | **395.1189** | **788.2231** | **787.9013** | **0.3218** | **0** | **11** | **2.6e+02** | **2** | **K.EIKPSSK.Y** |
|  | 690 | **402.9113** | **803.8078** | **802.8331** | **0.9747** | **1** | **9** | **3.5e+02** | **4** | **K.NEAGERK.K** |
|  | 823 | **406.3112** | **810.6075** | **809.8854** | **0.7222** | **0** | **(1)** | **1.7e+03** | **9** | **R.CVETSSK.K + Carbamidomethyl (C)** |
|  | 867 | **407.2631** | **812.5114** | **812.0089** | **0.5025** | **1** | **(2)** | **1.2e+03** | **5** | **R.VPEVIKK.A** |
|  | 883 | **407.5434** | **813.0721** | **812.0089** | **1.0632** | **1** | **5** | **8.5e+02** | **3** | **R.VPEVIKK.A** |
|  | 932 | **408.2015** | **814.3883** | **813.8988** | **0.4894** | **0** | **5** | **7.2e+02** | **9** | **R.HQVTTTK.Y** |
|  | 994 | **413.0560** | **824.0972** | **822.9089** | **1.1884** | **2** | **1** | **1.9e+03** | **10** | **K.GDKGRYK.I** |
|  | 997 | **413.2073** | **824.3998** | **825.0077** | **-0.6078** | **1** | **17** | **41** | **1** | **K.KPVPEKK.V** |
|  | 1055 | **418.0395** | **834.0641** | **832.9651** | **1.0991** | **0** | **8** | **4.9e+02** | **9** | **K.VNAEACVK.T** |
|  | 1304 | **432.1916** | **862.3685** | **863.0126** | **-0.6441** | **0** | **10** | **2.9e+02** | **5** | **K.KPEAPPPK.V** |
|  | 1321 | **433.0001** | **863.9855** | **863.0126** | **0.9729** | **0** | **(8)** | **3.8e+02** | **4** | **K.KPEAPPPK.V** |
|  | 1496 | **448.3535** | **894.6921** | **895.1405** | **-0.4483** | **0** | **4** | **7.7e+02** | **7** | **R.VLGVPVIAK.D** |
|  | 1597 | **458.0739** | **914.1330** | **913.0696** | **1.0634** | **0** | **11** | **2.2e+02** | **3** | **R.ITIENVPK.K** |
|  | 1629 | **459.0482** | **916.0817** | **915.0425** | **1.0392** | **0** | **9** | **4.2e+02** | **5** | **R.IDVTPVGSK.L** |
|  | 1750 | **467.3486** | **932.6824** | **932.0332** | **0.6492** | **1** | **8** | **3.6e+02** | **3** | **K.IVKAGDSSR.L** |
|  | 1912 | **483.7343** | **965.4538** | **966.1950** | **-0.7412** | **0** | **7** | **4.5e+02** | **8** | **K.IMGYIIEK.I** |
|  | 2024 | **501.3096** | **1000.6045** | **1000.1470** | **0.4575** | **0** | **8** | **4.4e+02** | **10** | **K.VGVGPTIETK.T** |
|  | 2068 | **505.5834** | **1009.1521** | **1008.0000** | **1.1521** | **0** | **8** | **4.3e+02** | **8** | **K.NDAGYSEPR.E** |
|  | 2108 | **511.7337** | **1021.4527** | **1021.1278** | **0.3248** | **1** | **4** | **8.6e+02** | **4** | **R.IAAENRYGK.S** |
|  | 2187 | **520.1142** | **1038.2136** | **1037.1074** | **1.1063** | **0** | **12** | **1.5e+02** | **1** | **K.TAGPDCNFR.V + Carbamidomethyl (C)** |
|  | 78 | **364.2352** | **1089.6834** | **1090.1851** | **-0.5016** | **1** | **13** | **87** | **2** | **K.NGVEIKSTDK.C** |
|  | 2390 | **547.2822** | **1092.5497** | **1093.2950** | **-0.7453** | **1** | **19** | **34** | **2** | **R.MEIKSTIQK.T + Oxidation (M)** |
|  | 212 | **371.0298** | **1110.0673** | **1109.2083** | **0.8590** | **0** | **5** | **7e+02** | **6** | **K.GDSCEVTGTIK.A** |
|  | 277 | **376.1724** | **1125.4950** | **1125.3187** | **0.1763** | **1** | **14** | **85** | **1** | **K.DKPAVAPATKK.A** |
|  | 278 | **376.1878** | **1125.5413** | **1125.3187** | **0.2227** | **1** | **(5)** | **6.4e+02** | **6** | **K.DKPAVAPATKK.A** |
|  | 280 | **376.2140** | **1125.6200** | **1125.3187** | **0.3013** | **1** | **(6)** | **4.9e+02** | **8** | **K.DKPAVAPATKK.A** |
|  | 410 | **386.0735** | **1155.1982** | **1154.3151** | **0.8831** | **0** | **13** | **1.2e+02** | **2** | **K.DMCSAQLSVK.E + Carbamidomethyl (C); Oxidation (M)** |
|  | 457 | **387.1488** | **1158.4243** | **1158.4113** | **0.0130** | **0** | **8** | **5.2e+02** | **5** | **K.ALVGGTAPMTIK.W** |
|  | 495 | **388.3174** | **1161.9301** | **1161.3308** | **0.5993** | **1** | **15** | **94** | **3** | **K.ELPEGRWMK.A + Oxidation (M)** |
|  | 663 | **401.8616** | **1202.5627** | **1201.3779** | **1.1848** | **2** | **10** | **2.8e+02** | **1** | **K.ERNSLLWKR.A** |
|  | 843 | **406.9774** | **1217.9102** | **1217.3892** | **0.5210** | **0** | **4** | **9.4e+02** | **5** | **K.IELSPSMEAPK.I + Oxidation (M)** |
|  | 864 | **407.2437** | **1218.7089** | **1219.4315** | **-0.7226** | **0** | **11** | **1.5e+02** | **1** | **K.VLGSSIHMECK.V + Oxidation (M)** |
|  | 874 | **407.3152** | **1218.9236** | **1219.4315** | **-0.5079** | **0** | **(3)** | **1e+03** | **10** | **K.VLGSSIHMECK.V + Oxidation (M)** |
|  | 910 | **407.8531** | **1220.5371** | **1219.4315** | **1.1056** | **0** | **(10)** | **2.6e+02** | **3** | **K.VLGSSIHMECK.V + Oxidation (M)** |
|  | 985 | **411.9931** | **1232.9572** | **1233.3702** | **-0.4130** | **0** | **3** | **1.3e+03** | **8** | **K.WISVTTEEIR.E** |
|  | 1004 | **413.7383** | **1238.1927** | **1238.3257** | **-0.1330** | **0** | **11** | **1.7e+02** | **5** | **K.SSCTAVVDVSDR.A** |
|  | 1078 | **419.1846** | **1254.5316** | **1255.4406** | **-0.9089** | **0** | **9** | **3.6e+02** | **5** | **R.MSFVESTAVLR.L + Oxidation (M)** |
|  | 1088 | **419.3385** | **1254.9934** | **1254.4373** | **0.5561** | **2** | **6** | **7.1e+02** | **9** | **K.IRNYYLEKR.E** |
|  | 2811 | **638.1871** | **1274.3595** | **1273.4738** | **0.8856** | **0** | **24** | **10** | **4** | **K.EIELDFAVPLK.D** |
|  | 1283 | **431.0033** | **1289.9878** | **1289.4568** | **0.5310** | **0** | **9** | **4.1e+02** | **10** | **K.WVACGEPVAETK.M** |
|  | 1366 | **435.7154** | **1304.1240** | **1303.3757** | **0.7483** | **0** | **3** | **9.8e+02** | **8** | **K.GEYVCDCGTDK.T + 2 Carbamidomethyl (C)** |
|  | 2857 | **659.3555** | **1316.6963** | **1315.5386** | **1.1576** | **1** | **11** | **2.3e+02** | **1** | **R.LECKIAGSPEIR.V** |
|  | 1423 | **441.1475** | **1320.4204** | **1321.4357** | **-1.0153** | **0** | **11** | **2.3e+02** | **4** | **R.DTIVVNAGETFR.L** |
|  | 1450 | **443.8993** | **1328.6756** | **1329.6332** | **-0.9576** | **1** | **5** | **8.7e+02** | **8** | **R.LPGPPGKPKVLAR.T** |
|  | 1478 | **446.2575** | **1335.7503** | **1334.6068** | **1.1436** | **0** | **9** | **3e+02** | **2** | **R.IPAVVTGRPVPTK.V** |
|  | 3033 | **684.1815** | **1366.3483** | **1365.4665** | **0.8817** | **0** | **(9)** | **3e+02** | **4** | **R.NDAGTASCSIELK.V + Carbamidomethyl (C)** |
|  | 3037 | **684.2953** | **1366.5758** | **1365.4665** | **1.1093** | **0** | **11** | **1.9e+02** | **3** | **R.NDAGTASCSIELK.V + Carbamidomethyl (C)** |
|  | 3085 | **687.9270** | **1373.8392** | **1374.4570** | **-0.6177** | **2** | **4** | **8.5e+02** | **10** | **K.DKDATDLTRSPR.V** |
|  | 1680 | **461.7959** | **1382.3655** | **1382.5815** | **-0.2160** | **0** | **7** | **4.7e+02** | **10** | **K.LVIHDCTPEDIK.T** |
|  | 1721 | **464.2229** | **1389.6466** | **1390.6086** | **-0.9620** | **1** | **8** | **4.1e+02** | **6** | **K.MHVVWFKNDAK.L + Oxidation (M)** |
|  | 1813 | **474.1762** | **1419.5065** | **1419.7591** | **-0.2527** | **2** | **7** | **5.8e+02** | **5** | **R.KVVTIRACCTLR.L + Carbamidomethyl (C)** |
|  | 1835 | **476.0251** | **1425.0531** | **1424.6828** | **0.3702** | **1** | **10** | **2.6e+02** | **5** | **K.LVVTGLKEGAFYK.F** |
|  | 1871 | **478.1414** | **1431.4019** | **1432.5808** | **-1.1789** | **1** | **9** | **2.9e+02** | **3** | **K.GDTKLRPTPEYR.T** |
|  | 2132 | **517.0354** | **1548.0840** | **1548.7805** | **-0.6964** | **1** | **5** | **8.6e+02** | **7** | **K.DMCSAQLSVKEPPK.F + Oxidation (M)** |
|  | 2310 | **534.2443** | **1599.7106** | **1600.8120** | **-1.1013** | **0** | **10** | **3.2e+02** | **1** | **K.ADNSVGAVASSAVLVIK.A** |
|  | 2347 | **538.8981** | **1613.6722** | **1614.7923** | **-1.1200** | **1** | **11** | **2.3e+02** | **3** | **K.APEIIDVSSKAEEVK.I** |
|  | 2365 | **540.4541** | **1618.3401** | **1617.8624** | **0.4778** | **1** | **6** | **4.9e+02** | **9** | **K.DVNVIEGTKAVLECK.V** |
|  | 2394 | **547.9403** | **1640.7988** | **1639.8015** | **0.9972** | **1** | **10** | **2.8e+02** | **8** | **K.SLSLQYSAKDLTEGK.E** |
|  | 2411 | **552.5649** | **1654.6725** | **1654.7564** | **-0.0840** | **0** | **17** | **64** | **1** | **K.GVNYTQLSIDNCDR.N + Carbamidomethyl (C)** |
|  | 2472 | **565.5018** | **1693.4831** | **1693.9585** | **-0.4753** | **1** | **4** | **8.6e+02** | **6** | **R.VLGYVVEMQPKGTEK.W + Oxidation (M)** |
|  | 2514 | **574.8275** | **1721.4604** | **1722.0117** | **-0.5513** | **0** | **7** | **4.3e+02** | **6** | **R.MPVDPPGKPEVIDVTK.S** |
|  | 3516 | **915.3739** | **1828.7330** | **1827.9876** | **0.7454** | **1** | **6** | **5.6e+02** | **7** | **R.GDSGRYFLTLENTAGVK.T** |
|  | 2716 | **611.3873** | **1831.1398** | **1831.9980** | **-0.8582** | **1** | **6** | **6.2e+02** | **7** | **R.MAHEGALTGVTTDQKEK.Q + Oxidation (M)** |
|  | 2717 | **611.4086** | **1831.2035** | **1831.9980** | **-0.7945** | **1** | **(4)** | **1.1e+03** | **9** | **R.MAHEGALTGVTTDQKEK.Q + Oxidation (M)** |
|  | 2742 | **613.3709** | **1837.0904** | **1837.1221** | **-0.0318** | **1** | **7** | **5.1e+02** | **10** | **K.AGTKIELPATVTGKPEPK.I** |
|  | 2809 | **637.0549** | **1908.1426** | **1907.1075** | **1.0351** | **1** | **5** | **8.1e+02** | **5** | **R.VSAINGAGKGDSCEVTGTIK.A** |
|  | 3058 | **686.0137** | **2055.0188** | **2056.1895** | **-1.1707** | **0** | **8** | **3.2e+02** | **4** | **K.EDAGNYSFTIPALGLSTSGR.V** |
|  | 3166 | **732.2034** | **2193.5879** | **2192.5197** | **1.0682** | **2** | **11** | **1.9e+02** | **1** | **K.DLNMVVSAARISCGGAIRSQK.G + Oxidation (M)** |
|  | 3327 | **772.5742** | **2314.7003** | **2314.7255** | **-0.0252** | **2** | **17** | **51** | **1** | **K.VDPSYLMLPGESARLHCKLK.G + Carbamidomethyl (C)** |
|  | 3552 | **1117.2664** | **3348.7769** | **3347.8177** | **0.9592** | **2** | **9** | **3.3e+02** | **1** | **K.NTGGVLGASCILECKVAGSSPISVAWFHEKTK.I + Carbamidomethyl (C)** |

  


---

|  |  |
| --- | --- |
| **9.** | gi|17066105    **Mass:** 3816172  **Score:** 188    **Queries matched:** 85 |
|  | Titin [Homo sapiens] |

|  |  |
| --- | --- |
|  | Check to include this hit in error tolerant search or archive report |
|  |  |

|  |  |  |  |  |  |  |  |  |  |  |
| --- | --- | --- | --- | --- | --- | --- | --- | --- | --- | --- |
|  | **Query** | **Observed** | **Mr(expt)** | **Mr(calc)** | **Delta** | **Miss** | **Score** | **Expect** | **Rank** | **Peptide** |
|  | 220 | 371.1613 | 740.3078 | 739.8601 | 0.4477 | 1 | 16 | 44 | 1 | K.KEAPPAK.V |
|  | 229 | 371.3508 | 740.6868 | 739.8601 | 0.8268 | 1 | (7) | 3.9e+02 | 3 | K.KEAPPAK.V |
|  | 232 | 372.2370 | 742.4593 | 742.9102 | -0.4510 | 1 | (10) | 2.2e+02 | 8 | K.LRLSVR.G |
|  | 236 | 372.3603 | 742.7059 | 742.9102 | -0.2044 | 1 | 10 | 2.6e+02 | 5 | K.LRLSVR.G |
|  | 253 | 374.1826 | 746.3503 | 745.8282 | 0.5222 | 2 | 8 | 4e+02 | 3 | K.RDATKR.T |
|  | 286 | 376.9611 | 751.9075 | 752.8341 | -0.9266 | 0 | (6) | 6.3e+02 | 4 | R.CVETSSK.K |
|  | 302 | 377.5815 | 753.1481 | 752.8341 | 0.3141 | 0 | 7 | 3.8e+02 | 9 | R.CVETSSK.K |
|  | 337 | 380.2560 | 758.4972 | 758.7342 | -0.2370 | 0 | 1 | 1.8e+03 | 10 | R.SESDGHK.R |
|  | 477 | 387.8971 | 773.7794 | 772.9295 | 0.8499 | 0 | 12 | 2e+02 | 4 | R.LILTEGK.N |
|  | 479 | 387.9558 | 773.8968 | 772.8931 | 1.0037 | 1 | 12 | 2.3e+02 | 4 | R.RITDLR.L |
|  | 525 | 389.1608 | 776.3068 | 775.9168 | 0.3900 | 0 | 17 | 60 | 1 | R.ISCGGAIR.S |
|  | 534 | 389.3109 | 776.6071 | 775.8044 | 0.8028 | 0 | 7 | 5e+02 | 5 | K.EGQDISK.R |
|  | 572 | 394.1502 | 786.2857 | 785.9716 | 0.3141 | 1 | 7 | 5.2e+02 | 7 | K.LKVEAVK.I |
|  | 575 | 395.1189 | 788.2231 | 787.9013 | 0.3218 | 0 | 11 | 2.6e+02 | 2 | K.EIKPSSK.Y |
|  | 690 | 402.9113 | 803.8078 | 802.8331 | 0.9747 | 1 | 9 | 3.5e+02 | 4 | K.NEAGERK.K |
|  | 823 | 406.3112 | 810.6075 | 809.8854 | 0.7222 | 0 | (1) | 1.7e+03 | 9 | R.CVETSSK.K + Carbamidomethyl (C) |
|  | 867 | 407.2631 | 812.5114 | 812.0089 | 0.5025 | 1 | (2) | 1.2e+03 | 5 | R.VPEVIKK.A |
|  | 883 | 407.5434 | 813.0721 | 812.0089 | 1.0632 | 1 | 5 | 8.5e+02 | 3 | R.VPEVIKK.A |
|  | 932 | 408.2015 | 814.3883 | 813.8988 | 0.4894 | 0 | 5 | 7.2e+02 | 9 | R.HQVTTTK.Y |
|  | 994 | 413.0560 | 824.0972 | 822.9089 | 1.1884 | 2 | 1 | 1.9e+03 | 10 | K.GDKGRYK.I |
|  | 997 | 413.2073 | 824.3998 | 825.0077 | -0.6078 | 1 | 17 | 41 | 1 | K.KPVPEKK.V |
|  | 1055 | 418.0395 | 834.0641 | 832.9651 | 1.0991 | 0 | 8 | 4.9e+02 | 9 | K.VNAEACVK.T |
|  | 1304 | 432.1916 | 862.3685 | 863.0126 | -0.6441 | 0 | 10 | 2.9e+02 | 5 | K.KPEAPPPK.V |
|  | 1321 | 433.0001 | 863.9855 | 863.0126 | 0.9729 | 0 | (8) | 3.8e+02 | 4 | K.KPEAPPPK.V |
|  | 1496 | 448.3535 | 894.6921 | 895.1405 | -0.4483 | 0 | 4 | 7.7e+02 | 7 | R.VLGVPVIAK.D |
|  | 1597 | 458.0739 | 914.1330 | 913.0696 | 1.0634 | 0 | 11 | 2.2e+02 | 3 | R.ITIENVPK.K |
|  | 1629 | 459.0482 | 916.0817 | 915.0425 | 1.0392 | 0 | 9 | 4.2e+02 | 5 | R.IDVTPVGSK.L |
|  | 1750 | 467.3486 | 932.6824 | 932.0332 | 0.6492 | 1 | 8 | 3.6e+02 | 3 | K.IVKAGDSSR.L |
|  | 1912 | 483.7343 | 965.4538 | 966.1950 | -0.7412 | 0 | 7 | 4.5e+02 | 8 | K.IMGYIIEK.I |
|  | 2024 | 501.3096 | 1000.6045 | 1000.1470 | 0.4575 | 0 | 8 | 4.4e+02 | 10 | K.VGVGPTIETK.T |
|  | 2068 | 505.5834 | 1009.1521 | 1008.0000 | 1.1521 | 0 | 8 | 4.3e+02 | 8 | K.NDAGYSEPR.E |
|  | 2108 | 511.7337 | 1021.4527 | 1021.1278 | 0.3248 | 1 | 4 | 8.6e+02 | 4 | R.IAAENRYGK.S |
|  | 2187 | 520.1142 | 1038.2136 | 1037.1074 | 1.1063 | 0 | 12 | 1.5e+02 | 1 | K.TAGPDCNFR.V + Carbamidomethyl (C) |
|  | 78 | 364.2352 | 1089.6834 | 1090.1851 | -0.5016 | 1 | 13 | 87 | 2 | K.NGVEIKSTDK.C |
|  | 2390 | 547.2822 | 1092.5497 | 1093.2950 | -0.7453 | 1 | 19 | 34 | 2 | R.MEIKSTIQK.T + Oxidation (M) |
|  | 212 | 371.0298 | 1110.0673 | 1109.2083 | 0.8590 | 0 | 5 | 7e+02 | 6 | K.GDSCEVTGTIK.A |
|  | 2442 | **560.1669** | **1118.3190** | **1118.3244** | **-0.0055** | **1** | **4** | **1.2e+03** | **5** | **K.TATSAKLTVVK.R** |
|  | 277 | 376.1724 | 1125.4950 | 1125.3187 | 0.1763 | 1 | 14 | 85 | 1 | K.DKPAVAPATKK.A |
|  | 278 | 376.1878 | 1125.5413 | 1125.3187 | 0.2227 | 1 | (5) | 6.4e+02 | 6 | K.DKPAVAPATKK.A |
|  | 280 | 376.2140 | 1125.6200 | 1125.3187 | 0.3013 | 1 | (6) | 4.9e+02 | 8 | K.DKPAVAPATKK.A |
|  | 410 | 386.0735 | 1155.1982 | 1154.3151 | 0.8831 | 0 | 13 | 1.2e+02 | 2 | K.DMCSAQLSVK.E + Carbamidomethyl (C); Oxidation (M) |
|  | 457 | 387.1488 | 1158.4243 | 1158.4113 | 0.0130 | 0 | 8 | 5.2e+02 | 5 | K.ALVGGTAPMTIK.W |
|  | 495 | 388.3174 | 1161.9301 | 1161.3308 | 0.5993 | 1 | 15 | 94 | 3 | K.ELPEGRWMK.A + Oxidation (M) |
|  | 663 | 401.8616 | 1202.5627 | 1201.3779 | 1.1848 | 2 | 10 | 2.8e+02 | 1 | K.ERNSLLWKR.A |
|  | 843 | 406.9774 | 1217.9102 | 1217.3892 | 0.5210 | 0 | 4 | 9.4e+02 | 5 | K.IELSPSMEAPK.I + Oxidation (M) |
|  | 864 | 407.2437 | 1218.7089 | 1219.4315 | -0.7226 | 0 | 11 | 1.5e+02 | 1 | K.VLGSSIHMECK.V + Oxidation (M) |
|  | 874 | 407.3152 | 1218.9236 | 1219.4315 | -0.5079 | 0 | (3) | 1e+03 | 10 | K.VLGSSIHMECK.V + Oxidation (M) |
|  | 910 | 407.8531 | 1220.5371 | 1219.4315 | 1.1056 | 0 | (10) | 2.6e+02 | 3 | K.VLGSSIHMECK.V + Oxidation (M) |
|  | 985 | 411.9931 | 1232.9572 | 1233.3702 | -0.4130 | 0 | 3 | 1.3e+03 | 8 | K.WISVTTEEIR.E |
|  | 1004 | 413.7383 | 1238.1927 | 1238.3257 | -0.1330 | 0 | 11 | 1.7e+02 | 5 | K.SSCTAVVDVSDR.A |
|  | 1078 | 419.1846 | 1254.5316 | 1255.4406 | -0.9089 | 0 | 9 | 3.6e+02 | 5 | R.MSFVESTAVLR.L + Oxidation (M) |
|  | 1088 | 419.3385 | 1254.9934 | 1254.4373 | 0.5561 | 2 | 6 | 7.1e+02 | 9 | K.IRNYYLEKR.E |
|  | 2811 | 638.1871 | 1274.3595 | 1273.4738 | 0.8856 | 0 | 24 | 10 | 4 | K.EIELDFAVPLK.D |
|  | 1283 | 431.0033 | 1289.9878 | 1289.4568 | 0.5310 | 0 | 9 | 4.1e+02 | 10 | K.WVACGEPVAETK.M |
|  | 1366 | 435.7154 | 1304.1240 | 1303.3757 | 0.7483 | 0 | 3 | 9.8e+02 | 8 | K.GEYVCDCGTDK.T + 2 Carbamidomethyl (C) |
|  | 2857 | 659.3555 | 1316.6963 | 1315.5386 | 1.1576 | 1 | 11 | 2.3e+02 | 1 | R.LECKIAGSPEIR.V |
|  | 1423 | 441.1475 | 1320.4204 | 1321.4357 | -1.0153 | 0 | 11 | 2.3e+02 | 4 | R.DTIVVNAGETFR.L |
|  | 1450 | 443.8993 | 1328.6756 | 1329.6332 | -0.9576 | 1 | 5 | 8.7e+02 | 8 | R.LPGPPGKPKVLAR.T |
|  | 1478 | 446.2575 | 1335.7503 | 1334.6068 | 1.1436 | 0 | 9 | 3e+02 | 2 | R.IPAVVTGRPVPTK.V |
|  | 3033 | 684.1815 | 1366.3483 | 1365.4665 | 0.8817 | 0 | (9) | 3e+02 | 4 | R.NDAGTASCSIELK.V + Carbamidomethyl (C) |
|  | 3037 | 684.2953 | 1366.5758 | 1365.4665 | 1.1093 | 0 | 11 | 1.9e+02 | 3 | R.NDAGTASCSIELK.V + Carbamidomethyl (C) |
|  | 3085 | 687.9270 | 1373.8392 | 1374.4570 | -0.6177 | 2 | 4 | 8.5e+02 | 10 | K.DKDATDLTRSPR.V |
|  | 1680 | 461.7959 | 1382.3655 | 1382.5815 | -0.2160 | 0 | 7 | 4.7e+02 | 10 | K.LVIHDCTPEDIK.T |
|  | 1721 | 464.2229 | 1389.6466 | 1390.6086 | -0.9620 | 1 | 8 | 4.1e+02 | 6 | K.MHVVWFKNDAK.L + Oxidation (M) |
|  | 1813 | 474.1762 | 1419.5065 | 1419.7591 | -0.2527 | 2 | 7 | 5.8e+02 | 5 | R.KVVTIRACCTLR.L + Carbamidomethyl (C) |
|  | 1835 | 476.0251 | 1425.0531 | 1424.6828 | 0.3702 | 1 | 10 | 2.6e+02 | 5 | K.LVVTGLKEGAFYK.F |
|  | 1871 | 478.1414 | 1431.4019 | 1432.5808 | -1.1789 | 1 | 9 | 2.9e+02 | 3 | K.GDTKLRPTPEYR.T |
|  | 2132 | 517.0354 | 1548.0840 | 1548.7805 | -0.6964 | 1 | 5 | 8.6e+02 | 7 | K.DMCSAQLSVKEPPK.F + Oxidation (M) |
|  | 2310 | 534.2443 | 1599.7106 | 1600.8120 | -1.1013 | 0 | 10 | 3.2e+02 | 1 | K.ADNSVGAVASSAVLVIK.E |
|  | 2347 | 538.8981 | 1613.6722 | 1614.7923 | -1.1200 | 1 | 11 | 2.3e+02 | 3 | K.APEIIDVSSKAEEVK.I |
|  | 2365 | 540.4541 | 1618.3401 | 1617.8624 | 0.4778 | 1 | 6 | 4.9e+02 | 9 | K.DVNVIEGTKAVLECK.V |
|  | 2394 | 547.9403 | 1640.7988 | 1639.8015 | 0.9972 | 1 | 10 | 2.8e+02 | 8 | K.SLSLQYSAKDLTEGK.E |
|  | 2411 | 552.5649 | 1654.6725 | 1654.7564 | -0.0840 | 0 | 17 | 64 | 1 | K.GVNYTQLSIDNCDR.N + Carbamidomethyl (C) |
|  | 2472 | 565.5018 | 1693.4831 | 1693.9585 | -0.4753 | 1 | 4 | 8.6e+02 | 6 | R.VLGYVVEMQPKGTEK.W + Oxidation (M) |
|  | 2514 | 574.8275 | 1721.4604 | 1722.0117 | -0.5513 | 0 | 7 | 4.3e+02 | 6 | R.MPVDPPGKPEVIDVTK.S |
|  | 3516 | 915.3739 | 1828.7330 | 1827.9876 | 0.7454 | 1 | 6 | 5.6e+02 | 7 | R.GDSGRYFLTLENTAGVK.T |
|  | 2716 | 611.3873 | 1831.1398 | 1831.9980 | -0.8582 | 1 | 6 | 6.2e+02 | 7 | R.MAHEGALTGVTTDQKEK.Q + Oxidation (M) |
|  | 2717 | 611.4086 | 1831.2035 | 1831.9980 | -0.7945 | 1 | (4) | 1.1e+03 | 9 | R.MAHEGALTGVTTDQKEK.Q + Oxidation (M) |
|  | 2742 | 613.3709 | 1837.0904 | 1837.1221 | -0.0318 | 1 | 7 | 5.1e+02 | 10 | K.AGTKIELPATVTGKPEPK.I |
|  | 2809 | 637.0549 | 1908.1426 | 1907.1075 | 1.0351 | 1 | 5 | 8.1e+02 | 5 | R.VSAINGAGKGDSCEVTGTIK.A |
|  | 2840 | **653.3883** | **1957.1427** | **1957.1911** | **-0.0483** | **1** | **5** | **8.8e+02** | **7** | **K.AAYARDPQYPPAPPAFPK.V** |
|  | 3058 | 686.0137 | 2055.0188 | 2056.1895 | -1.1707 | 0 | 8 | 3.2e+02 | 4 | K.EDAGNYSFTIPALGLSTSGR.V |
|  | 3166 | 732.2034 | 2193.5879 | 2192.5197 | 1.0682 | 2 | 11 | 1.9e+02 | 1 | K.DLNMVVSAARISCGGAIRSQK.G + Oxidation (M) |
|  | 3327 | 772.5742 | 2314.7003 | 2314.7255 | -0.0252 | 2 | 17 | 51 | 1 | K.VDPSYLMLPGESARLHCKLK.G + Carbamidomethyl (C) |
|  | 3552 | 1117.2664 | 3348.7769 | 3347.8177 | 0.9592 | 2 | 9 | 3.3e+02 | 1 | K.NTGGVLGASCILECKVAGSSPISVAWFHEKTK.I + Carbamidomethyl (C) |

  

|  |  |
| --- | --- |
|  | |
|  | **Proteins matching the same set of peptides:** |

|  |  |
| --- | --- |
|  | gi|108861911    **Mass:** 3816142  **Score:** 188    **Queries matched:** 85 |
|  | RecName: Full=Titin; AltName: Full=Connectin; AltName: Full=Rhabdomyosarcoma antigen MU-RMS-40.14 |

---

|  |  |
| --- | --- |
| **10.** | gi|119631418    **Mass:** 3881215  **Score:** 186    **Queries matched:** 86 |
|  | titin, isoform CRA\_a [Homo sapiens] |

|  |  |
| --- | --- |
|  | Check to include this hit in error tolerant search or archive report |
|  |  |

|  |  |  |  |  |  |  |  |  |  |  |
| --- | --- | --- | --- | --- | --- | --- | --- | --- | --- | --- |
|  | **Query** | **Observed** | **Mr(expt)** | **Mr(calc)** | **Delta** | **Miss** | **Score** | **Expect** | **Rank** | **Peptide** |
|  | 220 | 371.1613 | 740.3078 | 739.8601 | 0.4477 | 1 | 16 | 44 | 1 | K.KEAPPAK.V |
|  | 229 | 371.3508 | 740.6868 | 739.8601 | 0.8268 | 1 | (7) | 3.9e+02 | 3 | K.KEAPPAK.V |
|  | 232 | 372.2370 | 742.4593 | 742.9102 | -0.4510 | 1 | (10) | 2.2e+02 | 8 | K.LRLSVR.G |
|  | 236 | 372.3603 | 742.7059 | 742.9102 | -0.2044 | 1 | 10 | 2.6e+02 | 5 | K.LRLSVR.G |
|  | 253 | 374.1826 | 746.3503 | 745.8282 | 0.5222 | 2 | 8 | 4e+02 | 3 | K.RDATKR.T |
|  | 286 | 376.9611 | 751.9075 | 752.8341 | -0.9266 | 0 | (6) | 6.3e+02 | 4 | R.CVETSSK.K |
|  | 302 | 377.5815 | 753.1481 | 752.8341 | 0.3141 | 0 | 7 | 3.8e+02 | 9 | R.CVETSSK.K |
|  | 337 | 380.2560 | 758.4972 | 758.7342 | -0.2370 | 0 | 1 | 1.8e+03 | 10 | R.SESDGHK.R |
|  | 477 | 387.8971 | 773.7794 | 772.9295 | 0.8499 | 0 | 12 | 2e+02 | 4 | R.LILTEGK.N |
|  | 479 | 387.9558 | 773.8968 | 772.8931 | 1.0037 | 1 | 12 | 2.3e+02 | 4 | R.RITDLR.L |
|  | 525 | 389.1608 | 776.3068 | 775.9168 | 0.3900 | 0 | 17 | 60 | 1 | R.ISCGGAIR.S |
|  | 534 | 389.3109 | 776.6071 | 775.8044 | 0.8028 | 0 | 7 | 5e+02 | 5 | K.EGQDISK.R |
|  | 572 | 394.1502 | 786.2857 | 785.9716 | 0.3141 | 1 | 7 | 5.2e+02 | 7 | K.LKVEAVK.I |
|  | 575 | 395.1189 | 788.2231 | 787.9013 | 0.3218 | 0 | 11 | 2.6e+02 | 2 | K.EIKPSSK.Y |
|  | 690 | 402.9113 | 803.8078 | 802.8331 | 0.9747 | 1 | 9 | 3.5e+02 | 4 | K.NEAGERK.K |
|  | 823 | 406.3112 | 810.6075 | 809.8854 | 0.7222 | 0 | (1) | 1.7e+03 | 9 | R.CVETSSK.K + Carbamidomethyl (C) |
|  | 867 | 407.2631 | 812.5114 | 812.0089 | 0.5025 | 1 | (2) | 1.2e+03 | 5 | R.VPEVIKK.A |
|  | 883 | 407.5434 | 813.0721 | 812.0089 | 1.0632 | 1 | 5 | 8.5e+02 | 3 | R.VPEVIKK.A |
|  | 932 | 408.2015 | 814.3883 | 813.8988 | 0.4894 | 0 | 5 | 7.2e+02 | 9 | R.HQVTTTK.Y |
|  | 994 | 413.0560 | 824.0972 | 822.9918 | 1.1054 | 1 | 1 | 1.8e+03 | 9 | K.KVPSAPPK.K |
|  | 997 | 413.2073 | 824.3998 | 825.0077 | -0.6078 | 1 | 17 | 41 | 1 | K.KPVPEKK.V |
|  | 1055 | 418.0395 | 834.0641 | 832.9651 | 1.0991 | 0 | 8 | 4.9e+02 | 9 | K.VNAEACVK.T |
|  | 1119 | **421.0947** | **840.1747** | **841.0501** | **-0.8754** | **2** | **9** | **3.7e+02** | **2** | **K.VPEIKKK.V** |
|  | 1304 | 432.1916 | 862.3685 | 863.0126 | -0.6441 | 0 | 10 | 2.9e+02 | 5 | K.KPEAPPPK.V |
|  | 1321 | 433.0001 | 863.9855 | 863.0126 | 0.9729 | 0 | (8) | 3.8e+02 | 4 | K.KPEAPPPK.V |
|  | 1496 | 448.3535 | 894.6921 | 895.1405 | -0.4483 | 0 | 4 | 7.7e+02 | 7 | R.VLGVPVIAK.D |
|  | 1597 | 458.0739 | 914.1330 | 913.0696 | 1.0634 | 0 | 11 | 2.2e+02 | 3 | R.ITIENVPK.K |
|  | 1629 | 459.0482 | 916.0817 | 915.0425 | 1.0392 | 0 | 9 | 4.2e+02 | 5 | R.IDVTPVGSK.L |
|  | 1750 | 467.3486 | 932.6824 | 932.0332 | 0.6492 | 1 | 8 | 3.6e+02 | 3 | K.IVKAGDSSR.L |
|  | 1912 | 483.7343 | 965.4538 | 966.1950 | -0.7412 | 0 | 7 | 4.5e+02 | 8 | K.IMGYIIEK.I |
|  | 2024 | 501.3096 | 1000.6045 | 1000.1470 | 0.4575 | 0 | 8 | 4.4e+02 | 10 | K.VGVGPTIETK.T |
|  | 2068 | 505.5834 | 1009.1521 | 1008.0000 | 1.1521 | 0 | 8 | 4.3e+02 | 8 | K.NDAGYSEPR.E |
|  | 2108 | 511.7337 | 1021.4527 | 1021.1278 | 0.3248 | 1 | 4 | 8.6e+02 | 4 | R.IAAENRYGK.S |
|  | 2187 | 520.1142 | 1038.2136 | 1037.1074 | 1.1063 | 0 | 12 | 1.5e+02 | 1 | K.TAGPDCNFR.V + Carbamidomethyl (C) |
|  | 78 | 364.2352 | 1089.6834 | 1090.1851 | -0.5016 | 1 | 13 | 87 | 2 | K.NGVEIKSTDK.C |
|  | 2390 | 547.2822 | 1092.5497 | 1093.2950 | -0.7453 | 1 | 19 | 34 | 2 | R.MEIKSTIQK.T + Oxidation (M) |
|  | 212 | 371.0298 | 1110.0673 | 1109.2083 | 0.8590 | 0 | 5 | 7e+02 | 6 | K.GDSCEVTGTIK.A |
|  | 2442 | 560.1669 | 1118.3190 | 1118.3244 | -0.0055 | 1 | 4 | 1.2e+03 | 5 | K.TATSAKLTVVK.R |
|  | 277 | 376.1724 | 1125.4950 | 1125.3187 | 0.1763 | 1 | 14 | 85 | 1 | K.DKPAVAPATKK.A |
|  | 278 | 376.1878 | 1125.5413 | 1125.3187 | 0.2227 | 1 | (5) | 6.4e+02 | 6 | K.DKPAVAPATKK.A |
|  | 280 | 376.2140 | 1125.6200 | 1125.3187 | 0.3013 | 1 | (6) | 4.9e+02 | 8 | K.DKPAVAPATKK.A |
|  | 410 | 386.0735 | 1155.1982 | 1154.3151 | 0.8831 | 0 | 13 | 1.2e+02 | 2 | K.DMCSAQLSVK.E + Carbamidomethyl (C); Oxidation (M) |
|  | 457 | 387.1488 | 1158.4243 | 1158.4113 | 0.0130 | 0 | 8 | 5.2e+02 | 5 | K.ALVGGTAPMTIK.W |
|  | 495 | 388.3174 | 1161.9301 | 1161.3308 | 0.5993 | 1 | 15 | 94 | 3 | K.ELPEGRWMK.A + Oxidation (M) |
|  | 663 | 401.8616 | 1202.5627 | 1201.3779 | 1.1848 | 2 | 10 | 2.8e+02 | 1 | K.ERNSLLWKR.A |
|  | 843 | 406.9774 | 1217.9102 | 1217.3892 | 0.5210 | 0 | 4 | 9.4e+02 | 5 | K.IELSPSMEAPK.I + Oxidation (M) |
|  | 864 | 407.2437 | 1218.7089 | 1219.4315 | -0.7226 | 0 | 11 | 1.5e+02 | 1 | K.VLGSSIHMECK.V + Oxidation (M) |
|  | 874 | 407.3152 | 1218.9236 | 1219.4315 | -0.5079 | 0 | (3) | 1e+03 | 10 | K.VLGSSIHMECK.V + Oxidation (M) |
|  | 910 | 407.8531 | 1220.5371 | 1219.4315 | 1.1056 | 0 | (10) | 2.6e+02 | 3 | K.VLGSSIHMECK.V + Oxidation (M) |
|  | 985 | 411.9931 | 1232.9572 | 1233.3702 | -0.4130 | 0 | 3 | 1.3e+03 | 8 | K.WISVTTEEIR.E |
|  | 1004 | 413.7383 | 1238.1927 | 1238.3257 | -0.1330 | 0 | 11 | 1.7e+02 | 5 | K.SSCTAVVDVSDR.A |
|  | 1078 | 419.1846 | 1254.5316 | 1255.4406 | -0.9089 | 0 | 9 | 3.6e+02 | 5 | R.MSFVESTAVLR.L + Oxidation (M) |
|  | 1088 | 419.3385 | 1254.9934 | 1254.4373 | 0.5561 | 2 | 6 | 7.1e+02 | 9 | K.IRNYYLEKR.E |
|  | 2811 | 638.1871 | 1274.3595 | 1273.4738 | 0.8856 | 0 | 24 | 10 | 4 | K.EIELDFAVPLK.D |
|  | 1283 | 431.0033 | 1289.9878 | 1289.4568 | 0.5310 | 0 | 9 | 4.1e+02 | 10 | K.WVACGEPVAETK.M |
|  | 1366 | 435.7154 | 1304.1240 | 1303.3757 | 0.7483 | 0 | 3 | 9.8e+02 | 8 | K.GEYVCDCGTDK.T + 2 Carbamidomethyl (C) |
|  | 2857 | 659.3555 | 1316.6963 | 1315.5386 | 1.1576 | 1 | 11 | 2.3e+02 | 1 | R.LECKIAGSPEIR.V |
|  | 1423 | 441.1475 | 1320.4204 | 1321.4357 | -1.0153 | 0 | 11 | 2.3e+02 | 4 | R.DTIVVNAGETFR.L |
|  | 1450 | 443.8993 | 1328.6756 | 1329.6332 | -0.9576 | 1 | 5 | 8.7e+02 | 8 | R.LPGPPGKPKVLAR.T |
|  | 1478 | 446.2575 | 1335.7503 | 1334.6068 | 1.1436 | 0 | 9 | 3e+02 | 2 | R.IPAVVTGRPVPTK.V |
|  | 3033 | 684.1815 | 1366.3483 | 1365.4665 | 0.8817 | 0 | (9) | 3e+02 | 4 | R.NDAGTASCSIELK.V + Carbamidomethyl (C) |
|  | 3037 | 684.2953 | 1366.5758 | 1365.4665 | 1.1093 | 0 | 11 | 1.9e+02 | 3 | R.NDAGTASCSIELK.V + Carbamidomethyl (C) |
|  | 3085 | 687.9270 | 1373.8392 | 1374.4570 | -0.6177 | 2 | 4 | 8.5e+02 | 10 | K.DKDATDLTRSPR.V |
|  | 1680 | 461.7959 | 1382.3655 | 1382.5815 | -0.2160 | 0 | 7 | 4.7e+02 | 10 | K.LVIHDCTPEDIK.T |
|  | 1721 | 464.2229 | 1389.6466 | 1390.6086 | -0.9620 | 1 | 8 | 4.1e+02 | 6 | K.MHVVWFKNDAK.L + Oxidation (M) |
|  | 1813 | 474.1762 | 1419.5065 | 1419.7591 | -0.2527 | 2 | 7 | 5.8e+02 | 5 | R.KVVTIRACCTLR.L + Carbamidomethyl (C) |
|  | 1835 | 476.0251 | 1425.0531 | 1424.6828 | 0.3702 | 1 | 10 | 2.6e+02 | 5 | K.LVVTGLKEGAFYK.F |
|  | 1871 | 478.1414 | 1431.4019 | 1432.5808 | -1.1789 | 1 | 9 | 2.9e+02 | 3 | K.GDTKLRPTPEYR.T |
|  | 2132 | 517.0354 | 1548.0840 | 1548.7805 | -0.6964 | 1 | 5 | 8.6e+02 | 7 | K.DMCSAQLSVKEPPK.F + Oxidation (M) |
|  | 2310 | 534.2443 | 1599.7106 | 1600.8120 | -1.1013 | 0 | 10 | 3.2e+02 | 1 | K.ADNSVGAVASSAVLVIK.A |
|  | 2347 | 538.8981 | 1613.6722 | 1614.7923 | -1.1200 | 1 | 11 | 2.3e+02 | 3 | K.APEIIDVSSKAEEVK.I |
|  | 2365 | 540.4541 | 1618.3401 | 1617.8624 | 0.4778 | 1 | 6 | 4.9e+02 | 9 | K.DVNVIEGTKAVLECK.V |
|  | 2394 | 547.9403 | 1640.7988 | 1639.8015 | 0.9972 | 1 | 10 | 2.8e+02 | 8 | K.SLSLQYSAKDLTEGK.E |
|  | 2411 | 552.5649 | 1654.6725 | 1654.7564 | -0.0840 | 0 | 17 | 64 | 1 | K.GVNYTQLSIDNCDR.N + Carbamidomethyl (C) |
|  | 2472 | 565.5018 | 1693.4831 | 1693.9585 | -0.4753 | 1 | 4 | 8.6e+02 | 6 | R.VLGYVVEMQPKGTEK.W + Oxidation (M) |
|  | 2514 | 574.8275 | 1721.4604 | 1722.0117 | -0.5513 | 0 | 7 | 4.3e+02 | 6 | R.MPVDPPGKPEVIDVTK.S |
|  | 2584 | **591.5008** | **1771.4802** | **1772.2029** | **-0.7227** | **2** | **5** | **7.5e+02** | **2** | **K.KMPLAPPKKPEVPPVK.V + Oxidation (M)** |
|  | 3516 | 915.3739 | 1828.7330 | 1827.9876 | 0.7454 | 1 | 6 | 5.6e+02 | 7 | R.GDSGRYFLTLENTAGVK.T |
|  | 2716 | 611.3873 | 1831.1398 | 1831.9980 | -0.8582 | 1 | 6 | 6.2e+02 | 7 | R.MAHEGALTGVTTDQKEK.Q + Oxidation (M) |
|  | 2717 | 611.4086 | 1831.2035 | 1831.9980 | -0.7945 | 1 | (4) | 1.1e+03 | 9 | R.MAHEGALTGVTTDQKEK.Q + Oxidation (M) |
|  | 2742 | 613.3709 | 1837.0904 | 1837.1221 | -0.0318 | 1 | 7 | 5.1e+02 | 10 | K.AGTKIELPATVTGKPEPK.I |
|  | 2809 | 637.0549 | 1908.1426 | 1907.1075 | 1.0351 | 1 | 5 | 8.1e+02 | 5 | R.VSAINGAGKGDSCEVTGTIK.A |
|  | 3058 | 686.0137 | 2055.0188 | 2056.1895 | -1.1707 | 0 | 8 | 3.2e+02 | 4 | K.EDAGNYSFTIPALGLSTSGR.V |
|  | 3166 | 732.2034 | 2193.5879 | 2192.5197 | 1.0682 | 2 | 11 | 1.9e+02 | 1 | K.DLNMVVSAARISCGGAIRSQK.G + Oxidation (M) |
|  | 3327 | 772.5742 | 2314.7003 | 2314.7255 | -0.0252 | 2 | 17 | 51 | 1 | K.VDPSYLMLPGESARLHCKLK.G + Carbamidomethyl (C) |
|  | 3552 | 1117.2664 | 3348.7769 | 3347.8177 | 0.9592 | 2 | 9 | 3.3e+02 | 1 | K.NTGGVLGASCILECKVAGSSPISVAWFHEKTK.I + Carbamidomethyl (C) |

  


---

|  |  |
| --- | --- |
| **11.** | gi|1017427    **Mass:** 883014   **Score:** 152    **Queries matched:** 29 |
|  | elastic titin [Homo sapiens] |

|  |  |
| --- | --- |
|  | Check to include this hit in error tolerant search or archive report |
|  |  |

|  |  |  |  |  |  |  |  |  |  |  |
| --- | --- | --- | --- | --- | --- | --- | --- | --- | --- | --- |
|  | **Query** | **Observed** | **Mr(expt)** | **Mr(calc)** | **Delta** | **Miss** | **Score** | **Expect** | **Rank** | **Peptide** |
|  | 220 | 371.1613 | 740.3078 | 739.8601 | 0.4477 | 1 | 16 | 44 | 1 | K.KEAPPAK.V |
|  | 229 | 371.3508 | 740.6868 | 739.8601 | 0.8268 | 1 | (7) | 3.9e+02 | 3 | K.KEAPPAK.V |
|  | 477 | 387.8971 | 773.7794 | 772.9295 | 0.8499 | 0 | 12 | 2e+02 | 4 | R.LILTEGK.N |
|  | 867 | 407.2631 | 812.5114 | 812.0089 | 0.5025 | 1 | (2) | 1.2e+03 | 5 | R.VPEVIKK.A |
|  | 883 | 407.5434 | 813.0721 | 812.0089 | 1.0632 | 1 | 5 | 8.5e+02 | 3 | R.VPEVIKK.A |
|  | 997 | 413.2073 | 824.3998 | 825.0077 | -0.6078 | 1 | 17 | 41 | 1 | K.KPVPEKK.V |
|  | 1304 | 432.1916 | 862.3685 | 863.0126 | -0.6441 | 0 | 10 | 2.9e+02 | 5 | K.KPEAPPPK.V |
|  | 1321 | 433.0001 | 863.9855 | 863.0126 | 0.9729 | 0 | (8) | 3.8e+02 | 4 | K.KPEAPPPK.V |
|  | 1496 | 448.3535 | 894.6921 | 894.0697 | 0.6225 | 1 | 3 | 9.9e+02 | 10 | K.KEKPPPAK.V |
|  | 1750 | 467.3486 | 932.6824 | 932.0332 | 0.6492 | 1 | 8 | 3.6e+02 | 3 | K.IVKAGDSSR.L |
|  | 277 | 376.1724 | 1125.4950 | 1125.3187 | 0.1763 | 1 | 14 | 85 | 1 | K.DKPAVAPATKK.A |
|  | 278 | 376.1878 | 1125.5413 | 1125.3187 | 0.2227 | 1 | (5) | 6.4e+02 | 6 | K.DKPAVAPATKK.A |
|  | 280 | 376.2140 | 1125.6200 | 1125.3187 | 0.3013 | 1 | (6) | 4.9e+02 | 8 | K.DKPAVAPATKK.A |
|  | 410 | 386.0735 | 1155.1982 | 1154.3151 | 0.8831 | 0 | 13 | 1.2e+02 | 2 | K.DMCSAQLSVK.E + Carbamidomethyl (C); Oxidation (M) |
|  | 457 | 387.1488 | 1158.4243 | 1158.4113 | 0.0130 | 0 | 8 | 5.2e+02 | 5 | K.ALVGGTAPMTIK.W |
|  | 864 | 407.2437 | 1218.7089 | 1219.4315 | -0.7226 | 0 | 11 | 1.5e+02 | 1 | K.VLGSSIHMECK.V + Oxidation (M) |
|  | 874 | 407.3152 | 1218.9236 | 1219.4315 | -0.5079 | 0 | (3) | 1e+03 | 10 | K.VLGSSIHMECK.V + Oxidation (M) |
|  | 910 | 407.8531 | 1220.5371 | 1219.4315 | 1.1056 | 0 | (10) | 2.6e+02 | 3 | K.VLGSSIHMECK.V + Oxidation (M) |
|  | 1004 | 413.7383 | 1238.1927 | 1238.3257 | -0.1330 | 0 | 11 | 1.7e+02 | 5 | K.SSCTAVVDVSDR.A |
|  | 1078 | 419.1846 | 1254.5316 | 1255.4406 | -0.9089 | 0 | 9 | 3.6e+02 | 5 | R.MSFVESTAVLR.L + Oxidation (M) |
|  | 2857 | 659.3555 | 1316.6963 | 1315.5386 | 1.1576 | 1 | 11 | 2.3e+02 | 1 | R.LECKIAGSPEIR.V |
|  | 3033 | 684.1815 | 1366.3483 | 1365.4665 | 0.8817 | 0 | (9) | 3e+02 | 4 | R.NDAGTASCSIELK.V + Carbamidomethyl (C) |
|  | 3037 | 684.2953 | 1366.5758 | 1365.4665 | 1.1093 | 0 | 11 | 1.9e+02 | 3 | R.NDAGTASCSIELK.V + Carbamidomethyl (C) |
|  | 1871 | 478.1414 | 1431.4019 | 1432.5808 | -1.1789 | 1 | 9 | 2.9e+02 | 3 | K.GDTKLRPTPEYR.T |
|  | 2132 | 517.0354 | 1548.0840 | 1548.7805 | -0.6964 | 1 | 5 | 8.6e+02 | 7 | K.DMCSAQLSVKEPPK.F + Oxidation (M) |
|  | 2310 | 534.2443 | 1599.7106 | 1600.8120 | -1.1013 | 0 | 10 | 3.2e+02 | 1 | K.ADNSVGAVASSAVLVIK.A |
|  | 2347 | 538.8981 | 1613.6722 | 1614.7923 | -1.1200 | 1 | 11 | 2.3e+02 | 3 | K.APEIIDVSSKAEEVK.I |
|  | 3327 | 772.5742 | 2314.7003 | 2314.7255 | -0.0252 | 2 | 17 | 51 | 1 | K.VDPSYLMLPGESARLHCKLK.G + Carbamidomethyl (C) |
|  | 3552 | 1117.2664 | 3348.7769 | 3347.8177 | 0.9592 | 2 | 9 | 3.3e+02 | 1 | K.NTGGVLGASCILECKVAGSSPISVAWFHEKTK.I + Carbamidomethyl (C) |

  


---

|  |  |
| --- | --- |
| **12.** | gi|46812692    **Mass:** 60074    **Score:** 126    **Queries matched:** 8 |
|  | Keratin 6A [Homo sapiens] |

|  |  |
| --- | --- |
|  | Check to include this hit in error tolerant search or archive report |
|  |  |

|  |  |  |  |  |  |  |  |  |  |  |
| --- | --- | --- | --- | --- | --- | --- | --- | --- | --- | --- |
|  | **Query** | **Observed** | **Mr(expt)** | **Mr(calc)** | **Delta** | **Miss** | **Score** | **Expect** | **Rank** | **Peptide** |
|  | 2422 | **554.6749** | **1107.3349** | **1107.1740** | **0.1609** | **0** | **47** | **0.063** | **1** | **K.AQYEEIAQR.S** |
|  | 2525 | 576.8057 | 1151.5965 | 1152.2113 | -0.6148 | 1 | 35 | 0.73 | 1 | K.NKYEDEINK.R |
|  | 382 | 385.0221 | 1152.0440 | 1152.2113 | -0.1673 | 1 | (7) | 4.7e+02 | 7 | K.NKYEDEINK.R |
|  | 900 | **407.7890** | **1220.3449** | **1220.3964** | **-0.0514** | **2** | **12** | **1.6e+02** | **7** | **R.GEMALKDAKNK.L + Oxidation (M)** |
|  | 1105 | **420.1940** | **1257.5599** | **1257.3967** | **0.1632** | **2** | **4** | **9.1e+02** | **9** | **R.VRAEEREQIK.T** |
|  | 2977 | **676.7477** | **1351.4806** | **1350.5166** | **0.9639** | **1** | **15** | **1.1e+02** | **7** | **R.TAAENEFVTLKK.D** |
|  | 2985 | **679.2632** | **1356.5116** | **1357.5090** | **-0.9974** | **1** | **9** | **3.2e+02** | **3** | **K.NKLEGLEDALQK.A** |
|  | 3140 | **713.4896** | **2137.4467** | **2137.4543** | **-0.0076** | **1** | **7** | **4.9e+02** | **9** | **K.QDLAWLLKEYQELMNVK.L + Oxidation (M)** |

  


---

|  |  |
| --- | --- |
| **13.** | gi|17066104    **Mass:** 2993008  **Score:** 126    **Queries matched:** 58 |
|  | N2B-Titin Isoform [Homo sapiens] |

|  |  |
| --- | --- |
|  | Check to include this hit in error tolerant search or archive report |
|  |  |

|  |  |  |  |  |  |  |  |  |  |  |
| --- | --- | --- | --- | --- | --- | --- | --- | --- | --- | --- |
|  | **Query** | **Observed** | **Mr(expt)** | **Mr(calc)** | **Delta** | **Miss** | **Score** | **Expect** | **Rank** | **Peptide** |
|  | 232 | 372.2370 | 742.4593 | 742.9102 | -0.4510 | 1 | (10) | 2.2e+02 | 8 | K.LRLSVR.G |
|  | 236 | 372.3603 | 742.7059 | 742.9102 | -0.2044 | 1 | 10 | 2.6e+02 | 5 | K.LRLSVR.G |
|  | 253 | 374.1826 | 746.3503 | 745.8282 | 0.5222 | 2 | 8 | 4e+02 | 3 | K.RDATKR.T |
|  | 286 | 376.9611 | 751.9075 | 752.8341 | -0.9266 | 0 | (6) | 6.3e+02 | 4 | R.CVETSSK.K |
|  | 302 | 377.5815 | 753.1481 | 752.8341 | 0.3141 | 0 | 7 | 3.8e+02 | 9 | R.CVETSSK.K |
|  | 337 | 380.2560 | 758.4972 | 758.7342 | -0.2370 | 0 | 1 | 1.8e+03 | 10 | R.SESDGHK.R |
|  | 479 | 387.9558 | 773.8968 | 772.8931 | 1.0037 | 1 | 12 | 2.3e+02 | 4 | R.RITDLR.L |
|  | 525 | 389.1608 | 776.3068 | 775.9168 | 0.3900 | 0 | 17 | 60 | 1 | R.ISCGGAIR.S |
|  | 534 | 389.3109 | 776.6071 | 775.8044 | 0.8028 | 0 | 7 | 5e+02 | 5 | K.EGQDISK.R |
|  | 572 | 394.1502 | 786.2857 | 785.9716 | 0.3141 | 1 | 7 | 5.2e+02 | 7 | K.LKVEAVK.I |
|  | 575 | 395.1189 | 788.2231 | 787.9013 | 0.3218 | 0 | 11 | 2.6e+02 | 2 | K.EIKPSSK.Y |
|  | 690 | 402.9113 | 803.8078 | 802.8331 | 0.9747 | 1 | 9 | 3.5e+02 | 4 | K.NEAGERK.K |
|  | 823 | 406.3112 | 810.6075 | 809.8854 | 0.7222 | 0 | (1) | 1.7e+03 | 9 | R.CVETSSK.K + Carbamidomethyl (C) |
|  | 932 | 408.2015 | 814.3883 | 813.8988 | 0.4894 | 0 | 5 | 7.2e+02 | 9 | R.HQVTTTK.Y |
|  | 994 | 413.0560 | 824.0972 | 822.9089 | 1.1884 | 2 | 1 | 1.9e+03 | 10 | K.GDKGRYK.I |
|  | 1055 | 418.0395 | 834.0641 | 832.9651 | 1.0991 | 0 | 8 | 4.9e+02 | 9 | K.VNAEACVK.T |
|  | 1496 | 448.3535 | 894.6921 | 895.1405 | -0.4483 | 0 | 4 | 7.7e+02 | 7 | R.VLGVPVIAK.D |
|  | 1597 | 458.0739 | 914.1330 | 913.0696 | 1.0634 | 0 | 11 | 2.2e+02 | 3 | R.ITIENVPK.K |
|  | 1629 | 459.0482 | 916.0817 | 915.0425 | 1.0392 | 0 | 9 | 4.2e+02 | 5 | R.IDVTPVGSK.L |
|  | 1912 | 483.7343 | 965.4538 | 966.1950 | -0.7412 | 0 | 7 | 4.5e+02 | 8 | K.IMGYIIEK.I |
|  | 2024 | 501.3096 | 1000.6045 | 1000.1470 | 0.4575 | 0 | 8 | 4.4e+02 | 10 | K.VGVGPTIETK.T |
|  | 2068 | 505.5834 | 1009.1521 | 1008.0000 | 1.1521 | 0 | 8 | 4.3e+02 | 8 | K.NDAGYSEPR.E |
|  | 2108 | 511.7337 | 1021.4527 | 1021.1278 | 0.3248 | 1 | 4 | 8.6e+02 | 4 | R.IAAENRYGK.S |
|  | 2187 | 520.1142 | 1038.2136 | 1037.1074 | 1.1063 | 0 | 12 | 1.5e+02 | 1 | K.TAGPDCNFR.V + Carbamidomethyl (C) |
|  | 78 | 364.2352 | 1089.6834 | 1090.1851 | -0.5016 | 1 | 13 | 87 | 2 | K.NGVEIKSTDK.C |
|  | 2390 | 547.2822 | 1092.5497 | 1093.2950 | -0.7453 | 1 | 19 | 34 | 2 | R.MEIKSTIQK.T + Oxidation (M) |
|  | 212 | 371.0298 | 1110.0673 | 1109.2083 | 0.8590 | 0 | 5 | 7e+02 | 6 | K.GDSCEVTGTIK.A |
|  | 2442 | 560.1669 | 1118.3190 | 1118.3244 | -0.0055 | 1 | 4 | 1.2e+03 | 5 | K.TATSAKLTVVK.R |
|  | 495 | 388.3174 | 1161.9301 | 1161.3308 | 0.5993 | 1 | 15 | 94 | 3 | K.ELPEGRWMK.A + Oxidation (M) |
|  | 663 | 401.8616 | 1202.5627 | 1201.3779 | 1.1848 | 2 | 10 | 2.8e+02 | 1 | K.ERNSLLWKR.A |
|  | 843 | 406.9774 | 1217.9102 | 1217.3892 | 0.5210 | 0 | 4 | 9.4e+02 | 5 | K.IELSPSMEAPK.I + Oxidation (M) |
|  | 864 | 407.2437 | 1218.7089 | 1219.2576 | -0.5487 | 0 | 7 | 3.8e+02 | 5 | K.WEKPESDGGSK.I |
|  | 985 | 411.9931 | 1232.9572 | 1233.3702 | -0.4130 | 0 | 3 | 1.3e+03 | 8 | K.WISVTTEEIR.E |
|  | 1088 | 419.3385 | 1254.9934 | 1254.4373 | 0.5561 | 2 | 6 | 7.1e+02 | 9 | K.IRNYYLEKR.E |
|  | 2811 | 638.1871 | 1274.3595 | 1273.4738 | 0.8856 | 0 | 24 | 10 | 4 | K.EIELDFAVPLK.D |
|  | 1283 | 431.0033 | 1289.9878 | 1289.4568 | 0.5310 | 0 | 9 | 4.1e+02 | 10 | K.WVACGEPVAETK.M |
|  | 1366 | 435.7154 | 1304.1240 | 1303.3757 | 0.7483 | 0 | 3 | 9.8e+02 | 8 | K.GEYVCDCGTDK.T + 2 Carbamidomethyl (C) |
|  | 1423 | 441.1475 | 1320.4204 | 1321.4357 | -1.0153 | 0 | 11 | 2.3e+02 | 4 | R.DTIVVNAGETFR.L |
|  | 1450 | 443.8993 | 1328.6756 | 1329.6332 | -0.9576 | 1 | 5 | 8.7e+02 | 8 | R.LPGPPGKPKVLAR.T |
|  | 1478 | 446.2575 | 1335.7503 | 1334.6068 | 1.1436 | 0 | 9 | 3e+02 | 2 | R.IPAVVTGRPVPTK.V |
|  | 3085 | 687.9270 | 1373.8392 | 1374.4570 | -0.6177 | 2 | 4 | 8.5e+02 | 10 | K.DKDATDLTRSPR.V |
|  | 1680 | 461.7959 | 1382.3655 | 1382.5815 | -0.2160 | 0 | 7 | 4.7e+02 | 10 | K.LVIHDCTPEDIK.T |
|  | 1721 | 464.2229 | 1389.6466 | 1390.6086 | -0.9620 | 1 | 8 | 4.1e+02 | 6 | K.MHVVWFKNDAK.L + Oxidation (M) |
|  | 1813 | 474.1762 | 1419.5065 | 1419.7591 | -0.2527 | 2 | 7 | 5.8e+02 | 5 | R.KVVTIRACCTLR.L + Carbamidomethyl (C) |
|  | 1835 | 476.0251 | 1425.0531 | 1424.6828 | 0.3702 | 1 | 10 | 2.6e+02 | 5 | K.LVVTGLKEGAFYK.F |
|  | 2365 | 540.4541 | 1618.3401 | 1617.8624 | 0.4778 | 1 | 6 | 4.9e+02 | 9 | K.DVNVIEGTKAVLECK.V |
|  | 2394 | 547.9403 | 1640.7988 | 1639.8015 | 0.9972 | 1 | 10 | 2.8e+02 | 8 | K.SLSLQYSAKDLTEGK.E |
|  | 2411 | 552.5649 | 1654.6725 | 1654.7564 | -0.0840 | 0 | 17 | 64 | 1 | K.GVNYTQLSIDNCDR.N + Carbamidomethyl (C) |
|  | 2472 | 565.5018 | 1693.4831 | 1693.9585 | -0.4753 | 1 | 4 | 8.6e+02 | 6 | R.VLGYVVEMQPKGTEK.W + Oxidation (M) |
|  | 2514 | 574.8275 | 1721.4604 | 1722.0117 | -0.5513 | 0 | 7 | 4.3e+02 | 6 | R.MPVDPPGKPEVIDVTK.S |
|  | 3516 | 915.3739 | 1828.7330 | 1827.9876 | 0.7454 | 1 | 6 | 5.6e+02 | 7 | R.GDSGRYFLTLENTAGVK.T |
|  | 2716 | 611.3873 | 1831.1398 | 1831.9980 | -0.8582 | 1 | 6 | 6.2e+02 | 7 | R.MAHEGALTGVTTDQKEK.Q + Oxidation (M) |
|  | 2717 | 611.4086 | 1831.2035 | 1831.9980 | -0.7945 | 1 | (4) | 1.1e+03 | 9 | R.MAHEGALTGVTTDQKEK.Q + Oxidation (M) |
|  | 2742 | 613.3709 | 1837.0904 | 1837.1221 | -0.0318 | 1 | 7 | 5.1e+02 | 10 | K.AGTKIELPATVTGKPEPK.I |
|  | 2809 | 637.0549 | 1908.1426 | 1907.1075 | 1.0351 | 1 | 5 | 8.1e+02 | 5 | R.VSAINGAGKGDSCEVTGTIK.A |
|  | 2840 | 653.3883 | 1957.1427 | 1957.1911 | -0.0483 | 1 | 5 | 8.8e+02 | 7 | K.AAYARDPQYPPAPPAFPK.V |
|  | 3058 | 686.0137 | 2055.0188 | 2056.1895 | -1.1707 | 0 | 8 | 3.2e+02 | 4 | K.EDAGNYSFTIPALGLSTSGR.V |
|  | 3166 | 732.2034 | 2193.5879 | 2192.5197 | 1.0682 | 2 | 11 | 1.9e+02 | 1 | K.DLNMVVSAARISCGGAIRSQK.G + Oxidation (M) |

  


---

|  |  |
| --- | --- |
| **14.** | gi|1212992    **Mass:** 2993415  **Score:** 125    **Queries matched:** 60 |
|  | titin [Homo sapiens] |

|  |  |
| --- | --- |
|  | Check to include this hit in error tolerant search or archive report |
|  |  |

|  |  |  |  |  |  |  |  |  |  |  |
| --- | --- | --- | --- | --- | --- | --- | --- | --- | --- | --- |
|  | **Query** | **Observed** | **Mr(expt)** | **Mr(calc)** | **Delta** | **Miss** | **Score** | **Expect** | **Rank** | **Peptide** |
|  | 232 | 372.2370 | 742.4593 | 742.9102 | -0.4510 | 1 | (10) | 2.2e+02 | 8 | K.LRLSVR.G |
|  | 236 | 372.3603 | 742.7059 | 742.9102 | -0.2044 | 1 | 10 | 2.6e+02 | 5 | K.LRLSVR.G |
|  | 253 | 374.1826 | 746.3503 | 745.8282 | 0.5222 | 2 | 8 | 4e+02 | 3 | K.RDATKR.T |
|  | 286 | 376.9611 | 751.9075 | 752.8341 | -0.9266 | 0 | (6) | 6.3e+02 | 4 | R.CVETSSK.K |
|  | 302 | 377.5815 | 753.1481 | 752.8341 | 0.3141 | 0 | 7 | 3.8e+02 | 9 | R.CVETSSK.K |
|  | 337 | 380.2560 | 758.4972 | 758.7342 | -0.2370 | 0 | 1 | 1.8e+03 | 10 | R.SESDGHK.R |
|  | 479 | 387.9558 | 773.8968 | 772.8931 | 1.0037 | 1 | 12 | 2.3e+02 | 4 | R.RITDLR.L |
|  | 525 | 389.1608 | 776.3068 | 775.9168 | 0.3900 | 0 | 17 | 60 | 1 | R.ISCGGAIR.S |
|  | 534 | 389.3109 | 776.6071 | 775.8044 | 0.8028 | 0 | 7 | 5e+02 | 5 | K.EGQDISK.R |
|  | 572 | 394.1502 | 786.2857 | 785.9716 | 0.3141 | 1 | 7 | 5.2e+02 | 7 | K.LKVEAVK.I |
|  | 575 | 395.1189 | 788.2231 | 787.9013 | 0.3218 | 0 | 11 | 2.6e+02 | 2 | K.EIKPSSK.Y |
|  | 690 | 402.9113 | 803.8078 | 802.8331 | 0.9747 | 1 | 9 | 3.5e+02 | 4 | K.NEAGERK.K |
|  | 823 | 406.3112 | 810.6075 | 809.8854 | 0.7222 | 0 | (1) | 1.7e+03 | 9 | R.CVETSSK.K + Carbamidomethyl (C) |
|  | 932 | 408.2015 | 814.3883 | 813.8988 | 0.4894 | 0 | 5 | 7.2e+02 | 9 | R.HQVTTTK.Y |
|  | 994 | 413.0560 | 824.0972 | 822.9089 | 1.1884 | 2 | 1 | 1.9e+03 | 10 | K.GDKGRYK.I |
|  | 1055 | 418.0395 | 834.0641 | 832.9651 | 1.0991 | 0 | 8 | 4.9e+02 | 9 | K.VNAEACVK.T |
|  | 1496 | 448.3535 | 894.6921 | 895.1405 | -0.4483 | 0 | 4 | 7.7e+02 | 7 | R.VLGVPVIAK.D |
|  | 1597 | 458.0739 | 914.1330 | 913.0696 | 1.0634 | 0 | 11 | 2.2e+02 | 3 | R.ITIENVPK.K |
|  | 1629 | 459.0482 | 916.0817 | 915.0425 | 1.0392 | 0 | 9 | 4.2e+02 | 5 | R.IDVTPVGSK.L |
|  | 1912 | 483.7343 | 965.4538 | 966.1950 | -0.7412 | 0 | 7 | 4.5e+02 | 8 | K.IMGYIIEK.I |
|  | 2024 | 501.3096 | 1000.6045 | 1000.1470 | 0.4575 | 0 | 8 | 4.4e+02 | 10 | K.VGVGPTIETK.T |
|  | 2068 | 505.5834 | 1009.1521 | 1008.0000 | 1.1521 | 0 | 8 | 4.3e+02 | 8 | K.NDAGYSEPR.E |
|  | 2108 | 511.7337 | 1021.4527 | 1021.1278 | 0.3248 | 1 | 4 | 8.6e+02 | 4 | R.IAAENRYGK.S |
|  | 2187 | 520.1142 | 1038.2136 | 1037.1074 | 1.1063 | 0 | 12 | 1.5e+02 | 1 | K.TAGPDCNFR.V + Carbamidomethyl (C) |
|  | 78 | 364.2352 | 1089.6834 | 1090.1851 | -0.5016 | 1 | 13 | 87 | 2 | K.NGVEIKSTDK.C |
|  | 2390 | 547.2822 | 1092.5497 | 1093.2950 | -0.7453 | 1 | 19 | 34 | 2 | R.MEIKSTIQK.T + Oxidation (M) |
|  | 212 | 371.0298 | 1110.0673 | 1109.2083 | 0.8590 | 0 | 5 | 7e+02 | 6 | K.GDSCEVTGTIK.A |
|  | 2442 | 560.1669 | 1118.3190 | 1118.3244 | -0.0055 | 1 | 4 | 1.2e+03 | 5 | K.TATSAKLTVVK.R |
|  | 495 | 388.3174 | 1161.9301 | 1161.3308 | 0.5993 | 1 | 15 | 94 | 3 | K.ELPEGRWMK.A + Oxidation (M) |
|  | 663 | 401.8616 | 1202.5627 | 1201.3779 | 1.1848 | 2 | 10 | 2.8e+02 | 1 | K.ERNSLLWKR.A |
|  | 843 | 406.9774 | 1217.9102 | 1217.3892 | 0.5210 | 0 | 4 | 9.4e+02 | 5 | K.IELSPSMEAPK.I + Oxidation (M) |
|  | 864 | 407.2437 | 1218.7089 | 1219.2576 | -0.5487 | 0 | 7 | 3.8e+02 | 5 | K.WEKPESDGGSK.I |
|  | 985 | 411.9931 | 1232.9572 | 1233.3702 | -0.4130 | 0 | 3 | 1.3e+03 | 8 | K.WISVTTEEIR.E |
|  | 1088 | 419.3385 | 1254.9934 | 1254.4373 | 0.5561 | 2 | 6 | 7.1e+02 | 9 | K.IRNYYLEKR.E |
|  | 2811 | 638.1871 | 1274.3595 | 1273.4738 | 0.8856 | 0 | 24 | 10 | 4 | K.EIELDFAVPLK.D |
|  | 1283 | 431.0033 | 1289.9878 | 1289.4568 | 0.5310 | 0 | 9 | 4.1e+02 | 10 | K.WVACGEPVAETK.M |
|  | 1366 | 435.7154 | 1304.1240 | 1303.3757 | 0.7483 | 0 | 3 | 9.8e+02 | 8 | K.GEYVCDCGTDK.T + 2 Carbamidomethyl (C) |
|  | 1423 | 441.1475 | 1320.4204 | 1321.4357 | -1.0153 | 0 | 11 | 2.3e+02 | 4 | R.DTIVVNAGETFR.L |
|  | 1450 | 443.8993 | 1328.6756 | 1329.6332 | -0.9576 | 1 | 5 | 8.7e+02 | 8 | R.LPGPPGKPKVLAR.T |
|  | 1478 | 446.2575 | 1335.7503 | 1334.6068 | 1.1436 | 0 | 9 | 3e+02 | 2 | R.IPAVVTGRPVPTK.V |
|  | 3085 | 687.9270 | 1373.8392 | 1374.4570 | -0.6177 | 2 | 4 | 8.5e+02 | 10 | K.DKDATDLTRSPR.V |
|  | 1680 | 461.7959 | 1382.3655 | 1382.5815 | -0.2160 | 0 | 7 | 4.7e+02 | 10 | K.LVIHDCTPEDIK.T |
|  | 1721 | 464.2229 | 1389.6466 | 1390.6086 | -0.9620 | 1 | 8 | 4.1e+02 | 6 | K.MHVVWFKNDAK.L + Oxidation (M) |
|  | 1813 | 474.1762 | 1419.5065 | 1419.7591 | -0.2527 | 2 | 7 | 5.8e+02 | 5 | R.KVVTIRACCTLR.L + Carbamidomethyl (C) |
|  | 1835 | 476.0251 | 1425.0531 | 1424.6828 | 0.3702 | 1 | 10 | 2.6e+02 | 5 | K.LVVTGLKEGAFYK.F |
|  | 3142 | **715.2814** | **1428.5480** | **1429.6630** | **-1.1150** | **1** | **5** | **8.5e+02** | **9** | **R.EKVQHLPVSAPPK.I** |
|  | 2365 | 540.4541 | 1618.3401 | 1617.8624 | 0.4778 | 1 | 6 | 4.9e+02 | 9 | K.DVNVIEGTKAVLECK.V |
|  | 2394 | 547.9403 | 1640.7988 | 1639.8015 | 0.9972 | 1 | 10 | 2.8e+02 | 8 | K.SLSLQYSAKDLTEGK.E |
|  | 2411 | 552.5649 | 1654.6725 | 1654.7564 | -0.0840 | 0 | 17 | 64 | 1 | K.GVNYTQLSIDNCDR.N + Carbamidomethyl (C) |
|  | 2472 | 565.5018 | 1693.4831 | 1693.9585 | -0.4753 | 1 | 4 | 8.6e+02 | 6 | R.VLGYVVEMQPKGTEK.W + Oxidation (M) |
|  | 2514 | 574.8275 | 1721.4604 | 1722.0117 | -0.5513 | 0 | 7 | 4.3e+02 | 6 | R.MPVDPPGKPEVIDVTK.S |
|  | 3516 | 915.3739 | 1828.7330 | 1827.9876 | 0.7454 | 1 | 6 | 5.6e+02 | 7 | R.GDSGRYFLTLENTAGVK.T |
|  | 2716 | 611.3873 | 1831.1398 | 1831.9980 | -0.8582 | 1 | 6 | 6.2e+02 | 7 | R.MAHEGALTGVTTDQKEK.Q + Oxidation (M) |
|  | 2717 | 611.4086 | 1831.2035 | 1831.9980 | -0.7945 | 1 | (4) | 1.1e+03 | 9 | R.MAHEGALTGVTTDQKEK.Q + Oxidation (M) |
|  | 2742 | 613.3709 | 1837.0904 | 1837.1221 | -0.0318 | 1 | 7 | 5.1e+02 | 10 | K.AGTKIELPATVTGKPEPK.I |
|  | 2809 | 637.0549 | 1908.1426 | 1907.1075 | 1.0351 | 1 | 5 | 8.1e+02 | 5 | R.VSAINGAGKGDSCEVTGTIK.A |
|  | 2840 | 653.3883 | 1957.1427 | 1957.1911 | -0.0483 | 1 | 5 | 8.8e+02 | 7 | K.AAYARDPQYPPAPPAFPK.V |
|  | 3058 | 686.0137 | 2055.0188 | 2056.1895 | -1.1707 | 0 | 8 | 3.2e+02 | 4 | K.EDAGNYSFTIPALGLSTSGR.V |
|  | 3166 | 732.2034 | 2193.5879 | 2192.5197 | 1.0682 | 2 | 11 | 1.9e+02 | 1 | K.DLNMVVSAARISCGGAIRSQK.G + Oxidation (M) |
|  | 3224 | **740.7553** | **2219.2438** | **2220.4206** | **-1.1768** | **1** | **3** | **1.2e+03** | **7** | **R.SNFISCREPSYTPGPPSAPR.V + Carbamidomethyl (C)** |

  


---

|  |  |
| --- | --- |
| **15.** | gi|119631904    **Mass:** 740442   **Score:** 121    **Queries matched:** 20 |
|  | nebulin, isoform CRA\_a [Homo sapiens] |

|  |  |
| --- | --- |
|  | Check to include this hit in error tolerant search or archive report |
|  |  |

|  |  |  |  |  |  |  |  |  |  |  |
| --- | --- | --- | --- | --- | --- | --- | --- | --- | --- | --- |
|  | **Query** | **Observed** | **Mr(expt)** | **Mr(calc)** | **Delta** | **Miss** | **Score** | **Expect** | **Rank** | **Peptide** |
|  | 87 | **365.1487** | **728.2826** | **727.9155** | **0.3671** | **0** | **12** | **1.7e+02** | **1** | **K.MLHSLK.V** |
|  | 240 | **372.6154** | **743.2159** | **743.8918** | **-0.6758** | **1** | **24** | **9.7** | **1** | **R.KVQELK.T** |
|  | 760 | **405.0511** | **808.0873** | **808.8757** | **-0.7883** | **0** | **8** | **4e+02** | **2** | **R.DIASEFK.Y** |
|  | 811 | **406.1271** | **810.2395** | **810.8484** | **-0.6090** | **0** | **12** | **1.5e+02** | **2** | **R.DIASDYK.Y** |
|  | 888 | **407.6146** | **813.2143** | **813.9467** | **-0.7323** | **1** | **8** | **3.8e+02** | **5** | **K.GKHIGFR.S** |
|  | 1001 | **413.5569** | **825.0991** | **824.9645** | **0.1346** | **1** | **8** | **3.4e+02** | **10** | **R.QPPDKLK.F** |
|  | 1240 | **428.9006** | **855.7864** | **856.0480** | **-0.2616** | **1** | **13** | **1.3e+02** | **6** | **K.AHMLKTR.N** |
|  | 1374 | **436.1344** | **870.2541** | **870.9930** | **-0.7390** | **0** | **10** | **3e+02** | **8** | **R.QGLTLSPR.L** |
|  | 1386 | **436.7892** | **871.5636** | **870.9930** | **0.5705** | **0** | **(6)** | **6.7e+02** | **10** | **R.QGLTLSPR.L** |
|  | 669 | **401.9709** | **1202.8905** | **1203.2781** | **-0.3876** | **0** | **10** | **3.4e+02** | **2** | **K.YQEDFENMK.D** |
|  | 834 | **406.7885** | **1217.3434** | **1216.3413** | **1.0020** | **1** | **12** | **1.7e+02** | **4** | **R.QLKAAGDALSDK.L** |
|  | 1303 | **432.1838** | **1293.5293** | **1294.4101** | **-0.8808** | **0** | **4** | **1.1e+03** | **10** | **R.NQENISSVLYK.E** |
|  | 1345 | **434.7484** | **1301.2229** | **1300.4991** | **0.7237** | **0** | **9** | **2.4e+02** | **2** | **K.GYDLPVDAIPIK.A** |
|  | 1542 | **451.9525** | **1352.8353** | **1353.5007** | **-0.6654** | **0** | **4** | **1.2e+03** | **8** | **K.MAQDVATNVNYK.Q** |
|  | 2027 | **502.0656** | **1503.1745** | **1502.6689** | **0.5056** | **0** | **13** | **1.5e+02** | **1** | **K.NMMQIQSDNVYK.E + 2 Oxidation (M)** |
|  | 2174 | **519.1104** | **1554.3091** | **1554.8694** | **-0.5603** | **0** | **5** | **8e+02** | **5** | **K.FSSLMDSIPMVLAK.N + Oxidation (M)** |
|  | 2586 | **591.6392** | **1771.8953** | **1771.9244** | **-0.0291** | **0** | **13** | **1.6e+02** | **1** | **K.ENLHYTTVADRPDIK.K** |
|  | 2880 | **666.0048** | **1994.9921** | **1995.3038** | **-0.3117** | **0** | **6** | **6.2e+02** | **7** | **K.FTSVPDSMGMMLAQHNTK.Q** |
|  | 3169 | **732.9736** | **2195.8987** | **2196.4207** | **-0.5219** | **1** | **5** | **6.7e+02** | **2** | **R.CSLDFMDSSNPPASATRIAR.T + Carbamidomethyl (C)** |
|  | 3417 | **830.2797** | **2487.8168** | **2488.7806** | **-0.9638** | **2** | **8** | **3.5e+02** | **4** | **K.HEGEKFKCHIPADAPQFIQHR.V** |

  

|  |  |
| --- | --- |
|  | |
|  | **Proteins matching the same set of peptides:** |

|  |  |
| --- | --- |
|  | gi|119631909    **Mass:** 777920   **Score:** 120    **Queries matched:** 20 |
|  | nebulin, isoform CRA\_f [Homo sapiens] |

---

|  |  |
| --- | --- |
| **16.** | gi|115527120    **Mass:** 772892   **Score:** 113    **Queries matched:** 17 |
|  | nebulin isoform 3 [Homo sapiens] |

|  |  |
| --- | --- |
|  | Check to include this hit in error tolerant search or archive report |
|  |  |

|  |  |  |  |  |  |  |  |  |  |  |
| --- | --- | --- | --- | --- | --- | --- | --- | --- | --- | --- |
|  | **Query** | **Observed** | **Mr(expt)** | **Mr(calc)** | **Delta** | **Miss** | **Score** | **Expect** | **Rank** | **Peptide** |
|  | 87 | 365.1487 | 728.2826 | 727.9155 | 0.3671 | 0 | 12 | 1.7e+02 | 1 | K.MLHSLK.V |
|  | 240 | 372.6154 | 743.2159 | 743.8918 | -0.6758 | 1 | 24 | 9.7 | 1 | R.KVQELK.T |
|  | 760 | 405.0511 | 808.0873 | 808.8757 | -0.7883 | 0 | 8 | 4e+02 | 2 | R.DIASEFK.Y |
|  | 811 | 406.1271 | 810.2395 | 810.8484 | -0.6090 | 0 | 12 | 1.5e+02 | 2 | R.DIASDYK.Y |
|  | 888 | 407.6146 | 813.2143 | 813.9467 | -0.7323 | 1 | 8 | 3.8e+02 | 5 | K.GKHIGFR.S |
|  | 1001 | 413.5569 | 825.0991 | 824.9645 | 0.1346 | 1 | 8 | 3.4e+02 | 10 | R.QPPDKLK.F |
|  | 1240 | 428.9006 | 855.7864 | 856.0480 | -0.2616 | 1 | 13 | 1.3e+02 | 6 | K.AHMLKTR.N |
|  | 669 | 401.9709 | 1202.8905 | 1203.2781 | -0.3876 | 0 | 10 | 3.4e+02 | 2 | K.YQEDFENMK.D |
|  | 834 | 406.7885 | 1217.3434 | 1216.3413 | 1.0020 | 1 | 12 | 1.7e+02 | 4 | R.QLKAAGDALSDK.L |
|  | 1303 | 432.1838 | 1293.5293 | 1294.4101 | -0.8808 | 0 | 4 | 1.1e+03 | 10 | R.NQENISSVLYK.E |
|  | 1345 | 434.7484 | 1301.2229 | 1300.4991 | 0.7237 | 0 | 9 | 2.4e+02 | 2 | K.GYDLPVDAIPIK.A |
|  | 1542 | 451.9525 | 1352.8353 | 1353.5007 | -0.6654 | 0 | 4 | 1.2e+03 | 8 | K.MAQDVATNVNYK.Q |
|  | 2027 | 502.0656 | 1503.1745 | 1502.6689 | 0.5056 | 0 | 13 | 1.5e+02 | 1 | K.NMMQIQSDNVYK.E + 2 Oxidation (M) |
|  | 2174 | 519.1104 | 1554.3091 | 1554.8694 | -0.5603 | 0 | 5 | 8e+02 | 5 | K.FSSLMDSIPMVLAK.N + Oxidation (M) |
|  | 2586 | 591.6392 | 1771.8953 | 1771.9244 | -0.0291 | 0 | 13 | 1.6e+02 | 1 | K.ENLHYTTVADRPDIK.K |
|  | 2880 | 666.0048 | 1994.9921 | 1995.2376 | -0.2455 | 0 | 6 | 6.2e+02 | 7 | K.FTSVPDSMGMVLAQHNTK.Q + 2 Oxidation (M) |
|  | 3417 | 830.2797 | 2487.8168 | 2488.7806 | -0.9638 | 2 | 8 | 3.5e+02 | 4 | K.HEGEKFKCHIPADAPQFIQHR.V |

  


---

|  |  |
| --- | --- |
| **17.** | gi|623409    **Mass:** 57247    **Score:** 110    **Queries matched:** 4   **emPAI:** 0.12 |
|  | keratin 10 [Homo sapiens] |

|  |  |
| --- | --- |
|  | Check to include this hit in error tolerant search or archive report |
|  |  |

|  |  |  |  |  |  |  |  |  |  |  |
| --- | --- | --- | --- | --- | --- | --- | --- | --- | --- | --- |
|  | **Query** | **Observed** | **Mr(expt)** | **Mr(calc)** | **Delta** | **Miss** | **Score** | **Expect** | **Rank** | **Peptide** |
|  | 748 | **404.4764** | **806.9380** | **806.8631** | **0.0750** | **0** | **35** | **1** | **1** | **R.LAADDFR.L** |
|  | 7 | **360.4062** | **1078.1963** | **1079.1210** | **-0.9247** | **1** | **8** | **5.2e+02** | **3** | **R.QGEPRDYSK.Y** |
|  | 2554 | **583.3600** | **1164.7052** | **1165.2531** | **-0.5479** | **0** | **17** | **53** | **4** | **R.LENEIQTYR.S** |
|  | 2799 | **632.2011** | **1262.3873** | **1262.2838** | **0.1035** | **0** | **53** | **0.014** | **1** | **R.SLLEGEGSSGGGGR.G** |

  


---

|  |  |
| --- | --- |
| **18.** | gi|806562    **Mass:** 773204   **Score:** 108    **Queries matched:** 17 |
|  | nebulin [Homo sapiens] |

|  |  |
| --- | --- |
|  | Check to include this hit in error tolerant search or archive report |
|  |  |

|  |  |  |  |  |  |  |  |  |  |  |
| --- | --- | --- | --- | --- | --- | --- | --- | --- | --- | --- |
|  | **Query** | **Observed** | **Mr(expt)** | **Mr(calc)** | **Delta** | **Miss** | **Score** | **Expect** | **Rank** | **Peptide** |
|  | 87 | 365.1487 | 728.2826 | 727.9155 | 0.3671 | 0 | 12 | 1.7e+02 | 1 | K.MLHSLK.V |
|  | 240 | 372.6154 | 743.2159 | 743.8918 | -0.6758 | 1 | 24 | 9.7 | 1 | R.KVQELK.T |
|  | 760 | 405.0511 | 808.0873 | 808.8757 | -0.7883 | 0 | 8 | 4e+02 | 2 | R.DIASEFK.Y |
|  | 811 | 406.1271 | 810.2395 | 810.8484 | -0.6090 | 0 | 12 | 1.5e+02 | 2 | R.DIASDYK.Y |
|  | 888 | 407.6146 | 813.2143 | 813.9467 | -0.7323 | 1 | 8 | 3.8e+02 | 5 | K.GKHIGFR.S |
|  | 1001 | 413.5569 | 825.0991 | 824.9645 | 0.1346 | 1 | 8 | 3.4e+02 | 10 | R.QPPDKLK.F |
|  | 1240 | 428.9006 | 855.7864 | 856.0480 | -0.2616 | 1 | 13 | 1.3e+02 | 6 | K.AHMLKTR.N |
|  | 669 | 401.9709 | 1202.8905 | 1203.2781 | -0.3876 | 0 | 10 | 3.4e+02 | 2 | K.YQEDFENMK.D |
|  | 834 | 406.7885 | 1217.3434 | 1216.3413 | 1.0020 | 1 | 12 | 1.7e+02 | 4 | R.QLKAAGDALSDK.L |
|  | 1303 | 432.1838 | 1293.5293 | 1294.4101 | -0.8808 | 0 | 4 | 1.1e+03 | 10 | R.NQENISSVLYK.E |
|  | 1345 | 434.7484 | 1301.2229 | 1300.4991 | 0.7237 | 0 | 9 | 2.4e+02 | 2 | K.GYDLPVDAIPIK.A |
|  | 1542 | 451.9525 | 1352.8353 | 1353.5007 | -0.6654 | 0 | 4 | 1.2e+03 | 8 | K.MAQDVATNVNYK.Q |
|  | 2027 | 502.0656 | 1503.1745 | 1502.6689 | 0.5056 | 0 | 13 | 1.5e+02 | 1 | K.NMMQIQSDNVYK.E + 2 Oxidation (M) |
|  | 2174 | 519.1104 | 1554.3091 | 1554.8694 | -0.5603 | 0 | 5 | 8e+02 | 5 | K.FSSLMDSIPMVLAK.N + Oxidation (M) |
|  | 2586 | 591.6392 | 1771.8953 | 1771.9244 | -0.0291 | 0 | 13 | 1.6e+02 | 1 | K.ENLHYTTVADRPDIK.K |
|  | 2880 | 666.0048 | 1994.9921 | 1995.3038 | -0.3117 | 0 | 6 | 6.2e+02 | 7 | K.FTSVPDSMGMMLAQHNTK.Q |
|  | 3265 | **742.6465** | **2224.9175** | **2224.5215** | **0.3959** | **0** | **6** | **5.3e+02** | **1** | **K.HPLHECICLPDQNDIIHAR.K** |

  


---

|  |  |
| --- | --- |
| **19.** | gi|184084    **Mass:** 22019    **Score:** 106    **Queries matched:** 5   **emPAI:** 0.33 |
|  | histone H1t [Homo sapiens] |

|  |  |
| --- | --- |
|  | Check to include this hit in error tolerant search or archive report |
|  |  |

|  |  |  |  |  |  |  |  |  |  |  |
| --- | --- | --- | --- | --- | --- | --- | --- | --- | --- | --- |
|  | **Query** | **Observed** | **Mr(expt)** | **Mr(calc)** | **Delta** | **Miss** | **Score** | **Expect** | **Rank** | **Peptide** |
|  | 2098 | **509.1266** | **1016.2385** | **1015.2080** | **1.0305** | **2** | **4** | **1.2e+03** | **8** | **K.VIPKSTRSK.A** |
|  | 1097 | **419.4839** | **1255.4296** | **1254.4824** | **0.9472** | **2** | **10** | **3.6e+02** | **3** | **K.KPRATTPKTVR.S** |
|  | 2262 | 527.2079 | 1578.6015 | 1578.6820 | -0.0806 | 1 | (33) | 1.4 | 1 | K.ALAAAGYDVEKNNSR.I |
|  | 3364 | 790.4298 | 1578.8448 | 1578.6820 | 0.1628 | 1 | 86 | 6.6e-06 | 1 | K.ALAAAGYDVEKNNSR.I |
|  | 2440 | **559.8954** | **1676.6640** | **1676.8436** | **-0.1796** | **0** | **8** | **3.6e+02** | **5** | **M.SETVPAASASAGVAAMEK.L** |

  

|  |  |
| --- | --- |
|  | |
|  | **Proteins matching the same set of peptides:** |

|  |  |
| --- | --- |
|  | gi|20544168    **Mass:** 22019    **Score:** 106    **Queries matched:** 5 |
|  | histone H1t [Homo sapiens] |

---

|  |  |
| --- | --- |
| **20.** | gi|62088916    **Mass:** 266111   **Score:** 105    **Queries matched:** 13 |
|  | Insulin-like growth factor 2 receptor variant [Homo sapiens] |

|  |  |
| --- | --- |
|  | Check to include this hit in error tolerant search or archive report |
|  |  |

|  |  |  |  |  |  |  |  |  |  |  |
| --- | --- | --- | --- | --- | --- | --- | --- | --- | --- | --- |
|  | **Query** | **Observed** | **Mr(expt)** | **Mr(calc)** | **Delta** | **Miss** | **Score** | **Expect** | **Rank** | **Peptide** |
|  | 594 | **398.9171** | **795.8194** | **794.9354** | **0.8840** | **0** | **(14)** | **87** | **1** | **K.VVVTYSK.G** |
|  | 596 | **398.9796** | **795.9444** | **794.9354** | **1.0090** | **0** | **(12)** | **1.5e+02** | **2** | **K.VVVTYSK.G** |
|  | 597 | **399.0620** | **796.1092** | **794.9354** | **1.1738** | **0** | **16** | **65** | **1** | **K.VVVTYSK.G** |
|  | 2057 | **505.0375** | **1008.0602** | **1007.0152** | **1.0450** | **0** | **6** | **5.9e+02** | **3** | **R.GGTPYNNER.H** |
|  | 374 | **384.9563** | **1151.8467** | **1152.3636** | **-0.5169** | **0** | **12** | **1.6e+02** | **2** | **R.VQSSIAFLCGK.T** |
|  | 440 | **386.9122** | **1157.7143** | **1158.3947** | **-0.6804** | **0** | **10** | **2.9e+02** | **5** | **R.ACPPGTAACLVR.G** |
|  | 614 | **399.7847** | **1196.3319** | **1197.3646** | **-1.0326** | **1** | **16** | **54** | **3** | **K.GASFGRLQSMK.L + Oxidation (M)** |
|  | 1042 | **416.1843** | **1245.5306** | **1246.4072** | **-0.8766** | **0** | **11** | **2.3e+02** | **3** | **K.TASSVIELTPTK.T** |
|  | 1428 | **442.1930** | **1323.5568** | **1322.4435** | **1.1134** | **0** | **10** | **2.5e+02** | **4** | **K.YVDGDLCPDGIR.K** |
|  | 1704 | **463.1116** | **1386.3125** | **1387.4737** | **-1.1612** | **0** | **4** | **1.1e+03** | **7** | **K.LWCSTTADYDR.D + Carbamidomethyl (C)** |
|  | 1852 | **476.2814** | **1425.8221** | **1425.5633** | **0.2589** | **1** | **(10)** | **2.2e+02** | **6** | **K.DGSPCPSKSGLSYK.S** |
|  | 1853 | **476.3821** | **1426.1242** | **1425.5633** | **0.5609** | **1** | **12** | **1.3e+02** | **7** | **K.DGSPCPSKSGLSYK.S** |
|  | 2943 | **668.8804** | **2003.6189** | **2004.2462** | **-0.6272** | **1** | **22** | **16** | **1** | **R.TVEACPVVRVEGDNCEVK.D + Carbamidomethyl (C)** |

  


---

**Peptide matches not assigned to protein hits:** (no details means no
match)  
  

|  |  |  |  |  |  |  |  |  |  |  |
| --- | --- | --- | --- | --- | --- | --- | --- | --- | --- | --- |
|  | **Query** | **Observed** | **Mr(expt)** | **Mr(calc)** | **Delta** | **Miss** | **Score** | **Expect** | **Rank** | **Peptide** |
|  | 1164 | **422.1893** | **842.3638** | **841.9949** | **0.3688** | **0** | **47** | **0.052** | **1** | **GITLSVRP** |
|  | 1167 | **422.2000** | **842.3851** | **841.9949** | **0.3902** | **0** | **42** | **0.17** | **1** | **GITLSVRP** |
|  | 1132 | **421.7762** | **841.5375** | **841.9949** | **-0.4574** | **0** | **41** | **0.2** | **1** | **GITLSVRP** |
|  | 1160 | **422.1656** | **842.3164** | **841.9949** | **0.3214** | **0** | **40** | **0.26** | **1** | **GITLSVRP** |
|  | 1879 | **479.1712** | **956.3277** | **956.1010** | **0.2267** | **1** | **40** | **0.29** | **1** | **ARTVLSPGR** |
|  | 1173 | **422.2375** | **842.4602** | **841.9949** | **0.4652** | **0** | **40** | **0.26** | **1** | **GITLSVRP** |
|  | 1175 | **422.2582** | **842.5016** | **841.9949** | **0.5066** | **0** | **40** | **0.26** | **1** | **GITLSVRP** |
|  | 1136 | **421.8817** | **841.7486** | **841.9949** | **-0.2464** | **0** | **40** | **0.29** | **1** | **GITLSVRP** |
|  | 1135 | **421.8705** | **841.7263** | **841.9949** | **-0.2686** | **0** | **39** | **0.29** | **1** | **GITLSVRP** |
|  | 3075 | **687.1680** | **1372.3212** | **1373.4290** | **-1.1079** | **1** | **39** | **0.35** | **1** | **GVEGSAGAGKEAQGR** |
|  | 1172 | **422.2363** | **842.4579** | **841.9949** | **0.4629** | **0** | **38** | **0.43** | **1** | **GITLSVRP** |
|  | 1178 | **422.3024** | **842.5900** | **841.9949** | **0.5950** | **0** | **37** | **0.41** | **1** | **GITLSVRP** |
|  | 1145 | **422.0986** | **842.1824** | **841.9949** | **0.1874** | **0** | **37** | **0.53** | **1** | **GITLSVRP** |
|  | 1158 | **422.1588** | **842.3029** | **841.9949** | **0.3080** | **0** | **37** | **0.54** | **1** | **GITLSVRP** |
|  | 1139 | **421.9519** | **841.8890** | **841.9949** | **-0.1059** | **0** | **37** | **0.51** | **1** | **GITLSVRP** |
|  | 1168 | 422.2008 | 842.3868 | 841.9949 | 0.3919 | 0 | 37 | 0.56 | 1 | GITLSVRP |
|  | 1156 | **422.1449** | **842.2750** | **841.9949** | **0.2800** | **0** | **37** | **0.59** | **1** | **GITLSVRP** |
|  | 1146 | **422.1038** | **842.1929** | **841.9949** | **0.1979** | **0** | **37** | **0.61** | **1** | **GITLSVRP** |
|  | 1155 | **422.1342** | **842.2536** | **841.9949** | **0.2587** | **0** | **37** | **0.6** | **1** | **GITLSVRP** |
|  | 1166 | **422.1965** | **842.3783** | **841.9949** | **0.3833** | **0** | **37** | **0.58** | **1** | **GITLSVRP** |
|  | 1142 | **422.0622** | **842.1096** | **841.9949** | **0.1147** | **0** | **37** | **0.58** | **1** | **GITLSVRP** |
|  | 1180 | **422.5980** | **843.1811** | **841.9949** | **1.1862** | **0** | **36** | **0.61** | **1** | **GITLSVRP** |
|  | 2811 | 638.1871 | 1274.3595 | 1273.4771 | 0.8824 | 2 | 36 | 0.67 | 1 | DKIDKWDLIK |
|  | 2245 | **524.1689** | **1046.3231** | **1046.1291** | **0.1940** | **0** | **35** | **0.83** | **1** | **LSSPADITDK** |
|  | 3345 | **784.6369** | **1567.2590** | **1566.7957** | **0.4634** | **1** | **35** | **0.69** | **1** | **FKGPFTDVVTTNLK** |
|  | 2826 | **646.5312** | **1291.0477** | **1291.4313** | **-0.3835** | **2** | **35** | **0.73** | **1** | **MKEEEQAGKNK** |
|  | 1143 | **422.0691** | **842.1233** | **841.9949** | **0.1284** | **0** | **35** | **0.88** | **1** | **GITLSVRP** |
|  | 1162 | **422.1767** | **842.3386** | **841.9949** | **0.3436** | **0** | **35** | **0.97** | **1** | **GITLSVRP** |
|  | 1140 | **421.9811** | **841.9474** | **841.9949** | **-0.0476** | **0** | **35** | **0.92** | **1** | **GITLSVRP** |
|  | 2725 | **612.2011** | **1222.3874** | **1223.3358** | **-0.9483** | **1** | **34** | **0.98** | **1** | **EEKQPTAPPAR** |
|  | 3391 | **813.1583** | **1624.3017** | **1623.9147** | **0.3870** | **2** | **34** | **0.92** | **1** | **QYVLKGGGMSSLAGKK** |
|  | 1170 | **422.2146** | **842.4144** | **841.9949** | **0.4195** | **0** | **33** | **1.3** | **1** | **GITLSVRP** |
|  | 1144 | **422.0939** | **842.1731** | **841.9949** | **0.1781** | **0** | **33** | **1.5** | **1** | **GITLSVRP** |
|  | 3343 | **784.1279** | **1566.2410** | **1566.7957** | **-0.5547** | **1** | **32** | **1.2** | **1** | **FKGPFTDVVTTNLK** |
|  | 1362 | **435.6194** | **869.2239** | **870.0283** | **-0.8043** | **0** | **32** | **1.3** | **1** | **TPCPSLPR** |
|  | 1525 | **450.7743** | **899.5338** | **899.0099** | **0.5239** | **2** | **32** | **1.6** | **1** | **RDRSLPR** |
|  | 1148 | **422.1082** | **842.2017** | **841.9949** | **0.2068** | **0** | **32** | **2** | **1** | **GITLSVRP** |
|  | 2625 | **594.5573** | **1187.0997** | **1186.3124** | **0.7873** | **1** | **31** | **1.8** | **1** | **SPVSKSPVEEK** |
|  | 2311 | **534.3021** | **1066.5895** | **1066.2281** | **0.3614** | **0** | **31** | **2.2** | **1** | **SMQLFDNVI** |
|  | 1279 | **430.4234** | **858.8320** | **857.9944** | **0.8377** | **1** | **31** | **2.6** | **1** | **QDQKVLK** |
|  | 1141 | **422.0427** | **842.0706** | **841.9949** | **0.0757** | **0** | **31** | **2.2** | **1** | **GITLSVRP** |
|  | 1881 | **479.1880** | **956.3613** | **956.1010** | **0.2604** | **1** | **30** | **2.7** | **1** | **ARTVLSPGR** |
|  | 2479 | **565.9802** | **1129.9455** | **1129.3038** | **0.6417** | **1** | **30** | **2.9** | **1** | **EKLLAELEGK** |
|  | 1128 | **421.7096** | **841.4044** | **841.9949** | **-0.5905** | **0** | **30** | **2.4** | **1** | **GITLSVRP** |
|  | 1126 | **421.6773** | **841.3398** | **841.9949** | **-0.6552** | **0** | **29** | **2.5** | **1** | **GITLSVRP** |
|  | 2241 | **523.8885** | **1045.7622** | **1045.1923** | **0.5699** | **0** | **29** | **3.1** | **1** | **LSSAHVYLR** |
|  | 2427 | **556.2315** | **1110.4482** | **1109.2728** | **1.1755** | **0** | **29** | **3.4** | **1** | **SPLLQEPTPK** |
|  | 1518 | **450.3012** | **898.5876** | **899.0495** | **-0.4620** | **1** | **29** | **2.8** | **1** | **LGRTSLPR** |
|  | 1013 | **414.1924** | **1239.5551** | **1238.4828** | **1.0723** | **0** | **29** | **3** | **1** | **MQSPPHLAVCR** |
|  | 2859 | **659.7136** | **1317.4125** | **1317.4884** | **-0.0760** | **1** | **29** | **4.6** | **1** | **TRTEELIVQTK** |
|  | 420 | **386.1692** | **770.3237** | **769.8462** | **0.4776** | **0** | **28** | **3.6** | **1** | **DGNLVPR** |
|  | 1523 | **450.6857** | **899.3566** | **899.0497** | **0.3069** | **1** | **28** | **3.8** | **1** | **VATISPRR** |
|  | 733 | **404.0409** | **1209.1004** | **1208.4949** | **0.6055** | **2** | **27** | **5** | **1** | **MLGGGMKLKNK + 2 Oxidation (M)** |
|  | 1118 | **421.0736** | **1260.1986** | **1259.4787** | **0.7198** | **2** | **27** | **4.8** | **1** | **AEVRKALANCK + Carbamidomethyl (C)** |
|  | 1838 | **476.1326** | **1425.3755** | **1425.6725** | **-0.2969** | **0** | **27** | **5.3** | **1** | **FCPSTVNSLNCLK** |
|  | 1154 | **422.1307** | **842.2467** | **841.9949** | **0.2517** | **0** | **27** | **5.7** | **1** | **GITLSVRP** |
|  | 1266 | **430.0018** | **857.9888** | **858.9791** | **-0.9903** | **0** | **27** | **6** | **1** | **ILDSVQGK** |
|  | 3505 | **899.6390** | **2695.8949** | **2696.8971** | **-1.0021** | **1** | **27** | **6** | **1** | **GLEWVAVMSYDGSNEFYADSVKGR + Oxidation (M)** |
|  | 513 | **389.0854** | **1164.2341** | **1165.2947** | **-1.0606** | **0** | **26** | **6.9** | **1** | **LECGGPAMEDK + Oxidation (M)** |
|  | 1137 | **421.9175** | **841.8203** | **841.9949** | **-0.1746** | **0** | **26** | **5.9** | **1** | **GITLSVRP** |
|  | 1251 | **429.0937** | **856.1727** | **855.9785** | **0.1942** | **0** | **26** | **6.3** | **1** | **SPSSLLPR** |
|  | 127 | **367.1500** | **732.2851** | **731.8214** | **0.4638** | **0** | **26** | **6.4** | **1** | **NMAPQR + Oxidation (M)** |
|  | 1176 | **422.2704** | **842.5261** | **841.9949** | **0.5312** | **0** | **26** | **5.6** | **1** | **GITLSVRP** |
|  | 1369 | **435.8876** | **1304.6405** | **1304.4084** | **0.2322** | **0** | **26** | **6.3** | **1** | **GLEWVSAVSGSGR** |
|  | 111 | **366.2142** | **1095.6203** | **1096.3006** | **-0.6802** | **0** | **26** | **6.1** | **1** | **VIVVSMNYR + Oxidation (M)** |
|  | 249 | **374.0830** | **746.1513** | **745.8495** | **0.3018** | **0** | **26** | **8** | **1** | **WPAGCR + Carbamidomethyl (C)** |
|  | 618 | **399.9938** | **1196.9593** | **1197.3713** | **-0.4119** | **2** | **26** | **5.7** | **1** | **GAGAKRHMTPR + Oxidation (M)** |
|  | 1130 | **421.7666** | **841.5184** | **841.9949** | **-0.4765** | **0** | **26** | **6.8** | **1** | **GITLSVRP** |
|  | 3307 | **760.4003** | **1518.7859** | **1519.6198** | **-0.8340** | **1** | **26** | **6.9** | **1** | **QEAQGARDCPECR + Carbamidomethyl (C)** |
|  | 1579 | **456.2706** | **1365.7896** | **1366.5838** | **-0.7941** | **0** | **25** | **6.1** | **1** | **CLFASGSPFGPVK + Carbamidomethyl (C)** |
|  | 2366 | **540.6741** | **1079.3335** | **1078.2703** | **1.0632** | **1** | **25** | **8.7** | **1** | **HFLPPGVRR** |
|  | 2689 | **608.6323** | **1822.8748** | **1823.0985** | **-0.2237** | **0** | **25** | **8.7** | **1** | **NIIIQDCMWSGVSAAAK + Oxidation (M)** |
|  | 470 | **387.8373** | **1160.4898** | **1161.4567** | **-0.9669** | **1** | **25** | **9.6** | **1** | **MPAKTPIYLK** |
|  | 399 | **385.6113** | **769.2079** | **769.8725** | **-0.6647** | **0** | **25** | **5.7** | **1** | **NLACHR + Carbamidomethyl (C)** |
|  | 2122 | **515.5709** | **1543.6906** | **1543.7224** | **-0.0318** | **1** | **25** | **10** | **1** | **DVWSNEAVKNIIR** |
|  | 3346 | **784.6526** | **1567.2904** | **1566.7957** | **0.4947** | **1** | **25** | **6.6** | **1** | **FKGPFTDVVTTNLK** |
|  | 1413 | **438.3178** | **874.6209** | **875.0514** | **-0.4305** | **2** | **25** | **7.5** | **1** | **KDGRLMR** |
|  | 380 | **385.0139** | **1152.0197** | **1152.3472** | **-0.3276** | **2** | **25** | **7.3** | **1** | **HLTAKTPTKR** |
|  | 476 | **387.8938** | **773.7729** | **773.8779** | **-0.1051** | **1** | **25** | **10** | **1** | **KLAGTER** |
|  | 2105 | **511.1928** | **1530.5563** | **1529.8864** | **0.6699** | **1** | **25** | **9.1** | **1** | **MVCLKLPGGSYMAK + 2 Oxidation (M)** |
|  | 1675 | **460.7947** | **919.5747** | **919.0543** | **0.5204** | **0** | **25** | **8.3** | **1** | **MSSPLADAK** |
|  | 974 | **411.1360** | **1230.3859** | **1229.4776** | **0.9083** | **1** | **25** | **9.7** | **1** | **LRCMCTCNR + 2 Carbamidomethyl (C); Oxidation (M)** |
|  | 3422 | **832.9955** | **1663.9763** | **1662.8250** | **1.1514** | **1** | **25** | **10** | **1** | **MHRVGELSGDNYLR + Oxidation (M)** |
|  | 235 | **372.3531** | **742.6914** | **741.8625** | **0.8288** | **1** | **25** | **9.2** | **1** | **CGARAHK** |
|  | 3203 | **740.4943** | **1478.9737** | **1479.6568** | **-0.6831** | **0** | **24** | **9.2** | **1** | **ACNLDVILGFDGSR** |
|  | 1262 | **429.3008** | **856.5868** | **856.0017** | **0.5851** | **0** | **24** | **7.8** | **1** | **SPSCPLPR** |
|  | 574 | **394.2959** | **1179.8654** | **1180.4219** | **-0.5564** | **1** | **24** | **8.3** | **1** | **KMSCTICGHK + Carbamidomethyl (C); Oxidation (M)** |
|  | 2305 | **534.1470** | **1599.4187** | **1598.8241** | **0.5947** | **0** | **24** | **11** | **1** | **NFDVGHVPMAGLLGR + Oxidation (M)** |
|  | 1184 | **423.1309** | **844.2471** | **843.9678** | **0.2792** | **0** | **24** | **11** | **1** | **TLGTVTPR** |
|  | 1153 | **422.1295** | **842.2443** | **841.9949** | **0.2493** | **0** | **24** | **11** | **1** | **GITLSVRP** |
|  | 2477 | **565.7042** | **1129.3937** | **1129.3932** | **0.0004** | **2** | **24** | **12** | **1** | **KALKLTLSQK** |
|  | 1225 | **427.8576** | **1280.5506** | **1281.4346** | **-0.8840** | **0** | **24** | **10** | **1** | **EVYEIELCQR** |
|  | 141 | **368.0730** | **1101.1968** | **1100.2724** | **0.9244** | **2** | **24** | **12** | **1** | **AIIKKSGNNR** |
|  | 1944 | **488.0991** | **1461.2750** | **1461.7079** | **-0.4329** | **2** | **24** | **12** | **1** | **LRRVSLSEIGFGK** |
|  | 612 | **399.7137** | **1196.1189** | **1195.3288** | **0.7900** | **1** | **24** | **7.6** | **1** | **KQSEAVHLQR** |
|  | 6 | **360.4024** | **718.7901** | **717.8775** | **0.9125** | **0** | **24** | **15** | **1** | **LLDAMR** |
|  | 2239 | **523.5880** | **1567.7417** | **1568.9027** | **-1.1610** | **1** | **24** | **14** | **1** | **DLVPCVPATPAMAKR** |
|  | 464 | **387.3692** | **1159.0853** | **1159.2900** | **-0.2047** | **1** | **24** | **13** | **1** | **IEAKGTLNGEK** |
|  | 626 | **400.1922** | **1197.5545** | **1198.3525** | **-0.7980** | **0** | **24** | **8.9** | **1** | **LGPPAGAAGCTQR** |
|  | 454 | **387.0607** | **1158.1600** | **1159.2569** | **-1.0968** | **1** | **24** | **13** | **1** | **SCRGGNCYSR + Carbamidomethyl (C)** |
|  | 86 | **365.1224** | **728.2300** | **728.8341** | **-0.6040** | **0** | **24** | **12** | **1** | **LEGSPVK** |
|  | 3301 | **759.4387** | **2275.2940** | **2275.5170** | **-0.2230** | **1** | **24** | **11** | **1** | **QPWSDFAVLNGGKINSDIWK** |
|  | 2390 | 547.2822 | 1092.5497 | 1092.2241 | 0.3256 | 0 | 24 | 11 | 1 | MSSTSPNLQK |
|  | 1821 | **474.5218** | **1420.5431** | **1421.5381** | **-0.9950** | **1** | **24** | **15** | **1** | **KNWMVGGEGGASGR + Oxidation (M)** |
|  | 48 | **363.1239** | **1086.3494** | **1085.2580** | **1.0915** | **0** | **24** | **11** | **1** | **MGPPSAPPCR + Carbamidomethyl (C); Oxidation (M)** |
|  | 542 | **389.8792** | **1166.6155** | **1166.4153** | **0.2002** | **2** | **24** | **12** | **1** | **RLMMEQSKK + Oxidation (M)** |
|  | 2378 | **542.1719** | **1623.4936** | **1623.8154** | **-0.3217** | **2** | **24** | **11** | **1** | **NRMPSPCRSFGNNK + Oxidation (M)** |
|  | 2851 | **655.5471** | **1309.0793** | **1308.4633** | **0.6161** | **1** | **24** | **9.6** | **1** | **NFGIGEKMQER** |
|  | 3020 | **684.0077** | **1366.0006** | **1365.5807** | **0.4199** | **1** | **23** | **9.7** | **1** | **GALVLGSSLKQHR** |
|  | 3397 | **818.7158** | **2453.1253** | **2452.6326** | **0.4926** | **0** | **23** | **9.1** | **1** | **LDSGTLPPAVAAAGGTGGGGSGGSGAGKPK** |
|  | 361 | **384.2239** | **766.4330** | **765.9007** | **0.5324** | **1** | **23** | **9.2** | **1** | **TPAPPRK** |
|  | 1152 | **422.1176** | **842.2203** | **841.9949** | **0.2254** | **0** | **23** | **13** | **1** | **GITLSVRP** |
|  | 3248 | **741.9856** | **2222.9346** | **2223.5652** | **-0.6306** | **0** | **23** | **9.6** | **1** | **QVQLLESGPGLVKPSETLSLK** |
|  | 2010 | **497.5511** | **1489.6310** | **1489.5906** | **0.0404** | **1** | **23** | **15** | **1** | **AHARVYHTYDEK** |
|  | 3025 | **684.0312** | **1366.0477** | **1365.5807** | **0.4670** | **1** | **23** | **11** | **1** | **GALVLGSSLKQHR** |
|  | 2092 | **508.1853** | **1521.5337** | **1521.6787** | **-0.1450** | **2** | **23** | **13** | **1** | **QWASAHEKKTAHK** |
|  | 1078 | 419.1846 | 1254.5316 | 1253.3647 | 1.1669 | 0 | 23 | 14 | 1 | AQLSNSAQGLHK |
|  | 1149 | **422.1091** | **842.2035** | **841.9949** | **0.2085** | **0** | **23** | **14** | **1** | **GITLSVRP** |
|  | 1329 | **433.4335** | **1297.2782** | **1297.4836** | **-0.2054** | **0** | **23** | **14** | **1** | **HMLALAPNSTAR + Oxidation (M)** |
|  | 2942 | **668.8767** | **2003.6080** | **2003.2377** | **0.3702** | **1** | **23** | **12** | **1** | **RGSSALLPFCLPPWDDTA + Carbamidomethyl (C)** |
|  | 3029 | **684.1040** | **1366.1932** | **1365.5807** | **0.6125** | **1** | **23** | **13** | **1** | **GALVLGSSLKQHR** |
|  | 3143 | **715.9868** | **2144.9383** | **2144.3246** | **0.6137** | **1** | **23** | **11** | **1** | **QENDMVDSAPQWEAVLRR** |
|  | 1457 | **444.1909** | **1329.5507** | **1330.5733** | **-1.0226** | **0** | **23** | **15** | **1** | **ILSEGVDHCMVK** |
|  | 1325 | **433.2578** | **864.5007** | **865.0103** | **-0.5095** | **1** | **23** | **11** | **1** | **KVGTGTMR + Oxidation (M)** |
|  | 225 | **371.2701** | **1110.7883** | **1110.3534** | **0.4348** | **1** | **23** | **8.6** | **1** | **VRVALGLGLGR** |
|  | 2977 | 676.7477 | 1351.4806 | 1351.4648 | 0.0158 | 1 | 23 | 17 | 1 | KINNTFGSGTANK |
|  | 2791 | **629.0774** | **1256.1400** | **1256.4287** | **-0.2887** | **1** | **23** | **13** | **1** | **HPMDTEVTKAK** |
|  | 1284 | **431.0090** | **1290.0049** | **1290.5358** | **-0.5309** | **2** | **23** | **16** | **1** | **KMASATRLIQR + Oxidation (M)** |
|  | 900 | 407.7890 | 1220.3449 | 1220.5071 | -0.1622 | 2 | 23 | 14 | 1 | IREKHLAILK |
|  | 2513 | **574.5460** | **1147.0771** | **1147.4116** | **-0.3345** | **1** | **23** | **13** | **1** | **LARTLFLWK** |
|  | 2807 | **636.2803** | **1270.5458** | **1271.4711** | **-0.9254** | **2** | **23** | **14** | **1** | **YSRLPRGLPGR** |
|  | 3331 | **774.8768** | **2321.6083** | **2322.5986** | **-0.9902** | **2** | **23** | **16** | **1** | **CRFMAGAEETNNKSCFWAEK** |
|  | 2923 | **667.8361** | **1333.6573** | **1332.6600** | **0.9973** | **2** | **23** | **16** | **1** | **ILRRLFNLCK + Carbamidomethyl (C)** |
|  | 1270 | **430.0977** | **858.1807** | **857.9115** | **0.2692** | **1** | **23** | **17** | **1** | **NSANSPRL** |
|  | 1147 | **422.1070** | **1263.2988** | **1264.3695** | **-1.0707** | **1** | **23** | **16** | **1** | **SSSGMGGRAPVSR + Oxidation (M)** |
|  | 1189 | **423.2212** | **844.4276** | **843.9678** | **0.4597** | **0** | **23** | **15** | **1** | **TLGTVTPR** |
|  | 610 | **399.5890** | **1195.7450** | **1196.3103** | **-0.5654** | **1** | **23** | **10** | **1** | **KGIVEHEEQK** |
|  | 1540 | **451.8366** | **1352.4877** | **1353.5801** | **-1.0925** | **0** | **22** | **18** | **1** | **MEDLLTLEYVK** |
|  | 912 | **407.8730** | **1220.5968** | **1219.4148** | **1.1819** | **1** | **22** | **15** | **1** | **ARAISLMASSGR** |
|  | 3319 | **765.8353** | **1529.6559** | **1529.7141** | **-0.0582** | **0** | **22** | **17** | **1** | **VTPFYAVWCNDSK** |
|  | 52 | **363.1691** | **1086.4852** | **1086.1566** | **0.3287** | **0** | **22** | **13** | **1** | **QGVLGADDGVR** |
|  | 1159 | **422.1617** | **842.3085** | **841.9949** | **0.3136** | **0** | **22** | **17** | **1** | **GITLSVRP** |
|  | 2306 | **534.1628** | **1066.3109** | **1066.2281** | **0.0828** | **0** | **22** | **17** | **1** | **SMQLFDNVI** |
|  | 1845 | **476.1950** | **1425.5628** | **1424.5373** | **1.0256** | **2** | **22** | **16** | **1** | **MKESSPSGSKSQR + Oxidation (M)** |
|  | 1183 | **423.1233** | **844.2318** | **843.9678** | **0.2640** | **0** | **22** | **18** | **1** | **TLGTVTPR** |
|  | 1289 | **431.0430** | **1290.1067** | **1290.5358** | **-0.4291** | **2** | **22** | **20** | **1** | **KMASATRLIQR + Oxidation (M)** |
|  | 786 | **405.2661** | **1212.7763** | **1212.3164** | **0.4599** | **1** | **22** | **12** | **1** | **RQVGPQSGEVR** |
|  | 979 | **411.4236** | **1231.2487** | **1230.3497** | **0.8990** | **0** | **22** | **20** | **1** | **LSCAASGFDFGR** |
|  | 1398 | **437.1306** | **872.2464** | **871.8951** | **0.3514** | **0** | **22** | **18** | **1** | **ENGVNSPR** |
|  | 2140 | **518.6968** | **1035.3788** | **1035.2175** | **0.1613** | **0** | **22** | **15** | **1** | **MFTLSPAPR + Oxidation (M)** |
|  | 827 | **406.3909** | **1216.1506** | **1216.3050** | **-0.1544** | **2** | **22** | **15** | **1** | **EELARSEARR** |
|  | 2695 | **609.7770** | **1217.5392** | **1216.5187** | **1.0205** | **1** | **22** | **18** | **1** | **VVRVHICPYM** |
|  | 579 | **396.0440** | **1185.1098** | **1184.3044** | **0.8053** | **0** | **22** | **18** | **1** | **ATPHFSGLAAGR** |
|  | 1171 | **422.2167** | **842.4186** | **841.9949** | **0.4236** | **0** | **22** | **17** | **1** | **GITLSVRP** |
|  | 1839 | **476.1464** | **950.2781** | **949.1267** | **1.1514** | **1** | **22** | **17** | **1** | **AMASVAQKK + Oxidation (M)** |
|  | 3037 | 684.2953 | 1366.5758 | 1365.6224 | 0.9534 | 2 | 22 | 17 | 1 | MGGVSCGRELTKK |
|  | 3213 | **740.6533** | **2218.9378** | **2219.6707** | **-0.7329** | **2** | **22** | **14** | **1** | **ITMICLPQWLELFKGRDR** |
|  | 3459 | **855.2023** | **2562.5848** | **2562.8806** | **-0.2958** | **0** | **22** | **14** | **1** | **MIFAECSPNTCPCGEQCCNQR + 4 Carbamidomethyl (C)** |
|  | 241 | **372.9508** | **1115.8303** | **1116.2254** | **-0.3951** | **1** | **22** | **21** | **1** | **LNSGKADGNIK** |
|  | 1831 | **475.7769** | **1424.3084** | **1424.6217** | **-0.3133** | **0** | **22** | **15** | **1** | **AVGPSSTQLYMVR + Oxidation (M)** |
|  | 622 | **400.0976** | **1197.2707** | **1198.4340** | **-1.1633** | **0** | **22** | **16** | **1** | **QAVMEMMSQK + Oxidation (M)** |
|  | 1564 | **453.4406** | **904.8663** | **905.0126** | **-0.1463** | **0** | **22** | **19** | **1** | **AHLATHQK** |
|  | 3297 | **758.1479** | **2271.4215** | **2271.5319** | **-0.1104** | **2** | **22** | **16** | **1** | **DSGLEVLAVQRVSKTQAWQR** |
|  | 3558 | **1180.3551** | **3538.0431** | **3537.9008** | **0.1424** | **0** | **22** | **17** | **1** | **EQEVQPQTLSTQTILENSPCPMMDPFQVEK + Carbamidomethyl (C); 2 Oxidation (M)** |
|  | 504 | **388.6514** | **775.2880** | **774.9024** | **0.3857** | **0** | **22** | **17** | **1** | **IVYFSF** |
|  | 1314 | **432.8034** | **1295.3880** | **1295.4678** | **-0.0798** | **1** | **22** | **18** | **1** | **GFSCGSAIVGGGKR** |
|  | 1165 | **422.1944** | **842.3741** | **841.9949** | **0.3791** | **0** | **22** | **19** | **1** | **GITLSVRP** |
|  | 1608 | **458.5130** | **1372.5169** | **1373.6013** | **-1.0843** | **1** | **22** | **23** | **1** | **MVQTIQVNCKH + Carbamidomethyl (C); Oxidation (M)** |
|  | 2837 | **652.2405** | **1302.4662** | **1303.5544** | **-1.0883** | **1** | **22** | **18** | **1** | **ACPTARALCEIR** |
|  | 412 | **386.1057** | **1155.2950** | **1154.3994** | **0.8955** | **0** | **22** | **17** | **1** | **AIGTPSVGVLLK** |
|  | 1157 | **422.1456** | **842.2764** | **841.9949** | **0.2814** | **0** | **22** | **20** | **1** | **GITLSVRP** |
|  | 2394 | 547.9403 | 1640.7988 | 1639.9790 | 0.8198 | 2 | 22 | 18 | 1 | TVLKSTSKLMTQMR + Oxidation (M) |
|  | 3366 | **792.7578** | **2375.2513** | **2375.6828** | **-0.4315** | **2** | **22** | **15** | **1** | **NVKSEGSGQRGGSMAVLVWLHM + 2 Oxidation (M)** |
|  | 1550 | **452.1735** | **1353.4983** | **1354.5466** | **-1.0483** | **0** | **22** | **22** | **1** | **VPIYFSTGLTEK** |
|  | 3243 | **741.8576** | **2222.5506** | **2221.5311** | **1.0196** | **1** | **22** | **21** | **1** | **KPNCFELYIPDNKDQVIK + Carbamidomethyl (C)** |
|  | 2832 | **649.2256** | **1296.4365** | **1295.5903** | **0.8462** | **1** | **22** | **18** | **1** | **MQIFLKTLTGK + Oxidation (M)** |
|  | 1622 | **458.8814** | **915.7481** | **916.0305** | **-0.2824** | **0** | **21** | **21** | **1** | **KPIGSEASK** |
|  | 136 | **367.3756** | **1099.1046** | **1100.1781** | **-1.0736** | **0** | **21** | **24** | **1** | **YIEYQGAEK** |
|  | 903 | **407.7997** | **1220.3770** | **1219.3072** | **1.0698** | **2** | **21** | **19** | **1** | **AGWERSNKGSK** |
|  | 1037 | **416.0719** | **830.1291** | **828.9564** | **1.1726** | **1** | **21** | **23** | **1** | **AALAAERK** |
|  | 3129 | **707.5154** | **1413.0160** | **1412.6538** | **0.3621** | **2** | **21** | **18** | **1** | **KSRYSCIVLSIS + Carbamidomethyl (C)** |
|  | 495 | 388.3174 | 1161.9301 | 1162.3867 | -0.4566 | 1 | 21 | 19 | 1 | NCLLMRGPSR + Oxidation (M) |
|  | 1032 | **415.8540** | **829.6933** | **828.9564** | **0.7369** | **1** | **21** | **22** | **1** | **AALAAERK** |
|  | 1371 | **436.0318** | **1305.0732** | **1304.4084** | **0.6649** | **0** | **21** | **18** | **1** | **GLEWVSAVSGSGR** |
|  | 3167 | **732.4747** | **1462.9347** | **1462.7358** | **0.1988** | **2** | **21** | **19** | **1** | **KQHEAKLAVTPLK** |
|  | 2823 | **645.6587** | **1289.3026** | **1288.3643** | **0.9383** | **1** | **21** | **21** | **1** | **EALNVEGSDAKR** |
|  | 2171 | **519.0963** | **1036.1778** | **1035.1993** | **0.9786** | **1** | **21** | **19** | **1** | **MTSKSGGCHK** |
|  | 713 | **403.9296** | **1208.7665** | **1209.3721** | **-0.6055** | **1** | **21** | **20** | **1** | **IEPMKAFDSR + Oxidation (M)** |
|  | 259 | **374.3105** | **746.6063** | **746.8094** | **-0.2032** | **0** | **21** | **21** | **1** | **AAIGSNSK** |
|  | 652 | **401.3528** | **1201.0363** | **1201.4378** | **-0.4015** | **0** | **21** | **20** | **1** | **VPGDAVIMPFR** |
|  | 1092 | **419.4116** | **1255.2125** | **1255.4240** | **-0.2115** | **2** | **21** | **24** | **1** | **RPEAKKVDAGGK** |
|  | 1049 | **417.8198** | **1250.4371** | **1249.4341** | **1.0030** | **0** | **21** | **22** | **1** | **CIETLLQSASGK** |
|  | 1770 | **470.0732** | **938.1317** | **939.1731** | **-1.0414** | **0** | **21** | **18** | **1** | **LPSPGMIPK** |
|  | 2953 | **669.6929** | **2006.0564** | **2006.1888** | **-0.1323** | **2** | **21** | **22** | **1** | **QNDCRTMNCGRGDYCR + 2 Carbamidomethyl (C)** |
|  | 3229 | **740.9826** | **2219.9256** | **2220.5281** | **-0.6025** | **2** | **21** | **17** | **1** | **RLENPQVEFNSIARLECMA** |
|  | 515 | **389.1046** | **1164.2918** | **1164.3100** | **-0.0182** | **0** | **21** | **23** | **1** | **NNCVMPEDVK + Oxidation (M)** |
|  | 650 | **401.2250** | **800.4353** | **800.9862** | **-0.5509** | **2** | **21** | **19** | **1** | **AKVDKLK** |
|  | 1161 | **422.1678** | **842.3209** | **841.9949** | **0.3260** | **0** | **21** | **23** | **1** | **GITLSVRP** |
|  | 3527 | **964.1121** | **2889.3140** | **2889.1314** | **0.1826** | **2** | **21** | **21** | **1** | **DNSKSTLYLQMSSLRADDTAVYYCA + Carbamidomethyl (C); Oxidation (M)** |
|  | 3434 | **845.1552** | **1688.2956** | **1687.8925** | **0.4032** | **1** | **21** | **16** | **1** | **CKLFDSPSLCSSSTR + Carbamidomethyl (C)** |
|  | 123 | **367.0741** | **1098.2002** | **1097.2687** | **0.9315** | **1** | **21** | **23** | **1** | **NSLGVPAGVKR** |
|  | 1252 | **429.0973** | **856.1799** | **856.0017** | **0.1782** | **0** | **21** | **21** | **1** | **SPSCPLPR** |
|  | 1836 | **476.0859** | **1425.2355** | **1424.5553** | **0.6802** | **0** | **21** | **23** | **1** | **VGYCSGGTCYSEAK** |
|  | 236 | 372.3603 | 742.7059 | 742.8607 | -0.1548 | 0 | 21 | 23 | 1 | IVTVGSPA |
|  | 614 | 399.7847 | 1196.3319 | 1197.3614 | -1.0295 | 1 | 21 | 18 | 1 | TWAGVSSKSMK + Oxidation (M) |
|  | 1268 | **430.0787** | **858.1426** | **858.8102** | **-0.6676** | **0** | **21** | **25** | **1** | **EDNDPNR** |
|  | 3079 | **687.2810** | **1372.5472** | **1373.6245** | **-1.0772** | **2** | **21** | **22** | **1** | **RVSRCSPTGLIK + Carbamidomethyl (C)** |
|  | 471 | **387.8586** | **773.7024** | **773.9176** | **-0.2153** | **1** | **21** | **26** | **1** | **ILSGEKK** |
|  | 216 | **371.1088** | **1110.3043** | **1111.1660** | **-0.8617** | **0** | **21** | **18** | **1** | **AGAPGPGGGSDLR** |
|  | 1609 | **458.5700** | **915.1252** | **914.1669** | **0.9584** | **1** | **21** | **28** | **1** | **GPIKLGMAK** |
|  | 1952 | **488.2180** | **1461.6317** | **1461.4893** | **0.1423** | **0** | **21** | **24** | **1** | **SDPQPYEDNLAGR** |
|  | 952 | **409.1759** | **1224.5054** | **1225.4625** | **-0.9570** | **0** | **21** | **23** | **1** | **NSLCVMCVAR + 2 Carbamidomethyl (C); Oxidation (M)** |
|  | 2099 | **509.1343** | **1016.2539** | **1015.1019** | **1.1520** | **0** | **21** | **24** | **1** | **EALGHMDSR** |
|  | 1124 | **421.5594** | **841.1041** | **841.8690** | **-0.7649** | **0** | **21** | **23** | **1** | **AEGPGGAGAR** |
|  | 1942 | **488.0739** | **1461.1996** | **1461.5972** | **-0.3976** | **0** | **21** | **25** | **1** | **VAAALPGMESTQDR + Oxidation (M)** |
|  | 1224 | **427.7128** | **1280.1161** | **1280.5558** | **-0.4397** | **0** | **21** | **18** | **1** | **CGLPYVEVLCK + Carbamidomethyl (C)** |
|  | 2892 | **666.5699** | **1996.6875** | **1996.3511** | **0.3363** | **1** | **21** | **18** | **1** | **NDMRLDSEGIFLLLCLK + Oxidation (M)** |
|  | 3183 | **737.2671** | **2208.7791** | **2208.4362** | **0.3428** | **2** | **21** | **21** | **1** | **YVRGENGPGGHIVLKSASNPR** |
|  | 781 | **405.1776** | **1212.5107** | **1211.4078** | **1.1029** | **0** | **21** | **20** | **1** | **EEAMIPCFGAK + Oxidation (M)** |
|  | 1810 | **474.0196** | **1419.0365** | **1418.6631** | **0.3734** | **0** | **21** | **25** | **1** | **ALLNPGCHLPAQK + Carbamidomethyl (C)** |
|  | 3046 | **685.1852** | **1368.3556** | **1368.5847** | **-0.2292** | **1** | **21** | **23** | **1** | **LMPSRCNTQYR** |
|  | 841 | **406.9584** | **1217.8529** | **1217.3956** | **0.4573** | **1** | **21** | **21** | **1** | **LQEKCQALER** |
|  | 1527 | **451.0247** | **900.0346** | **900.0375** | **-0.0029** | **1** | **21** | **25** | **1** | **GLAARSGLR** |
|  | 2833 | **650.2752** | **1298.5356** | **1299.4729** | **-0.9373** | **1** | **21** | **23** | **1** | **NKSGIISEPLNK** |
|  | 3165 | **731.4337** | **2191.2788** | **2191.5250** | **-0.2462** | **0** | **21** | **23** | **1** | **SPISAQLALDGVGTMVNCTIK + Carbamidomethyl (C); Oxidation (M)** |
|  | 1805 | **473.4544** | **1417.3410** | **1417.5114** | **-0.1703** | **2** | **21** | **25** | **1** | **MADDAGAAGGRGRGR** |
|  | 1273 | **430.2146** | **858.4144** | **857.9082** | **0.5061** | **0** | **21** | **25** | **1** | **DEPSALAR** |
|  | 43 | **363.0863** | **1086.2368** | **1085.2546** | **0.9823** | **0** | **21** | **24** | **1** | **EMGNSLGCFK** |
|  | 2020 | **500.2538** | **998.4928** | **999.1620** | **-0.6693** | **0** | **21** | **23** | **1** | **LPVSLSQQK** |
|  | 1179 | **422.5686** | **843.1224** | **842.9829** | **0.1394** | **0** | **21** | **26** | **1** | **VGALSQLR** |
|  | 2299 | **533.3452** | **1597.0135** | **1597.9536** | **-0.9401** | **1** | **21** | **21** | **1** | **MGKCCHHCFPCCR + Carbamidomethyl (C); Oxidation (M)** |
|  | 3156 | **724.6710** | **2170.9909** | **2171.5157** | **-0.5248** | **2** | **20** | **19** | **1** | **ARVVYGDTDSMFVLLKGATK** |
|  | 2066 | **505.3775** | **1008.7402** | **1008.2999** | **0.4403** | **2** | **20** | **18** | **1** | **MVDMKKLK + Oxidation (M)** |
|  | 1402 | **437.2872** | **872.5596** | **871.9812** | **0.5784** | **0** | **20** | **22** | **1** | **QVGVSLNR** |
|  | 640 | **401.0804** | **800.1461** | **799.8723** | **0.2738** | **0** | **20** | **27** | **1** | **ADSPVGVR** |
|  | 1947 | **488.1631** | **1461.4672** | **1461.6650** | **-0.1978** | **1** | **20** | **27** | **1** | **VALNAKVVGGPHGDK** |
|  | 2699 | **610.0240** | **1827.0500** | **1826.1308** | **0.9192** | **2** | **20** | **25** | **1** | **YRQIFSVMVNQRIR + Oxidation (M)** |
|  | 2524 | **576.5423** | **1151.0698** | **1152.1237** | **-1.0539** | **0** | **20** | **20** | **1** | **QTDTETQDSK** |
|  | 1972 | **490.0788** | **978.1428** | **977.1335** | **1.0093** | **0** | **20** | **25** | **1** | **EVNEIMVK + Oxidation (M)** |
|  | 3172 | **733.7537** | **1465.4927** | **1464.4734** | **1.0193** | **0** | **20** | **23** | **1** | **GDMGDPGSQWNER + Oxidation (M)** |
|  | 1957 | **488.4037** | **974.7927** | **974.0070** | **0.7857** | **1** | **20** | **22** | **1** | **AMREEHSD** |
|  | 345 | **382.2568** | **762.4988** | **762.7659** | **-0.2671** | **0** | **20** | **24** | **1** | **ESAAETR** |
|  | 1671 | **460.5838** | **919.1528** | **920.1483** | **-0.9954** | **0** | **20** | **30** | **1** | **MTLCSPLV + Carbamidomethyl (C)** |
|  | 1151 | **422.1141** | **842.2135** | **841.9949** | **0.2185** | **0** | **20** | **28** | **1** | **GITLSVRP** |
|  | 1903 | **482.0840** | **962.1533** | **961.9764** | **0.1769** | **2** | **20** | **27** | **1** | **DRGRSEDK** |
|  | 1581 | **456.2872** | **1365.8393** | **1365.5642** | **0.2751** | **2** | **20** | **20** | **1** | **RGARSYQVICR + Carbamidomethyl (C)** |
|  | 2248 | **524.6780** | **1047.3413** | **1048.1500** | **-0.8087** | **0** | **20** | **27** | **1** | **DSSPAHLPPK** |
|  | 2867 | **663.6111** | **1325.2074** | **1325.5366** | **-0.3292** | **1** | **20** | **20** | **1** | **WIQYKGHCYK** |
|  | 372 | **384.9245** | **767.8342** | **767.8319** | **0.0023** | **0** | **20** | **22** | **1** | **HSWGPGK** |
|  | 685 | **402.4824** | **1204.4249** | **1205.5971** | **-1.1722** | **0** | **20** | **36** | **1** | **QMPVIMVIMK + Oxidation (M)** |
|  | 2257 | **525.7072** | **1049.3996** | **1048.2162** | **1.1834** | **2** | **20** | **24** | **1** | **CYTSYRKK** |
|  | 78 | 364.2352 | 1089.6834 | 1090.1685 | -0.4850 | 0 | 20 | 19 | 1 | GSGDGEMGKPR |
|  | 624 | **400.1174** | **1197.3299** | **1198.4340** | **-1.1040** | **0** | **20** | **24** | **1** | **QAVMEMMSQK + Oxidation (M)** |
|  | 1294 | **431.1080** | **860.2013** | **859.0255** | **1.1758** | **1** | **20** | **31** | **1** | **KGSLSVLR** |
|  | 2268 | **528.2761** | **1581.8060** | **1581.7291** | **0.0769** | **0** | **20** | **26** | **1** | **MHCLDGPSDSPPPR + Carbamidomethyl (C); Oxidation (M)** |
|  | 3463 | **856.7703** | **2567.2886** | **2566.8846** | **0.4041** | **1** | **20** | **21** | **1** | **NRVVLTMTNMDPLDTGTYYCAR + 2 Oxidation (M)** |
|  | 2554 | 583.3600 | 1164.7052 | 1165.3840 | -0.6788 | 1 | 20 | 28 | 1 | IKDPFAHLPK |
|  | 689 | **402.8405** | **1205.4995** | **1206.3712** | **-0.8718** | **0** | **20** | **30** | **1** | **SQWMLEQLR + Oxidation (M)** |
|  | 1009 | **414.0561** | **826.0974** | **825.9557** | **0.1416** | **1** | **20** | **23** | **1** | **GLPQKAGR** |
|  | 2126 | **515.9456** | **1544.8147** | **1545.8673** | **-1.0527** | **0** | **20** | **29** | **1** | **TIAQHLPFSMLCK + Carbamidomethyl (C)** |
|  | 299 | **377.2252** | **1128.6535** | **1129.2640** | **-0.6105** | **0** | **20** | **20** | **1** | **NLWFTFSSK** |
|  | 90 | **365.2100** | **1092.6077** | **1093.3645** | **-0.7567** | **2** | **20** | **23** | **1** | **DCLKAIMKR + Oxidation (M)** |
|  | 1703 | **463.1002** | **924.1857** | **923.0245** | **1.1611** | **0** | **20** | **25** | **1** | **LSSGALYGR** |
|  | 645 | **401.1251** | **1200.3531** | **1201.3300** | **-0.9769** | **0** | **20** | **30** | **1** | **TNPSVSSLLQR** |
|  | 737 | **404.0669** | **1209.1785** | **1209.3751** | **-0.1966** | **0** | **20** | **28** | **1** | **DEPCLIHNLR** |
|  | 3420 | **832.8444** | **1663.6739** | **1662.8481** | **0.8259** | **2** | **20** | **25** | **1** | **NSNVLRRNNSIFTK** |
|  | 681 | **402.3537** | **1204.0389** | **1203.3043** | **0.7346** | **0** | **20** | **28** | **1** | **NWTITDANIR** |
|  | 897 | **407.7665** | **1220.2773** | **1221.4073** | **-1.1301** | **0** | **20** | **28** | **1** | **SNGLICGGNGVCK** |
|  | 918 | **408.0314** | **814.0480** | **813.8987** | **0.1493** | **0** | **20** | **28** | **1** | **GAPGINGTK** |
|  | 1832 | **475.8385** | **1424.4932** | **1425.6727** | **-1.1794** | **1** | **20** | **28** | **1** | **VVLLGKSVEPQTR** |
|  | 758 | **405.0272** | **808.0397** | **806.9278** | **1.1119** | **0** | **20** | **26** | **1** | **AVTAMNGK + Oxidation (M)** |
|  | 850 | **407.0795** | **1218.2163** | **1219.4082** | **-1.1919** | **0** | **20** | **27** | **1** | **VITVDGNICTGK** |
|  | 2893 | **666.5714** | **1996.6921** | **1997.2547** | **-0.5626** | **0** | **20** | **22** | **1** | **SWLHPDSFPILGLPDFR** |
|  | 2939 | **668.7316** | **2003.1725** | **2003.3537** | **-0.1812** | **0** | **20** | **34** | **1** | **HGGGVGGPCAMATSVLCCLR + 2 Carbamidomethyl (C)** |
|  | 1888 | **480.1730** | **958.3312** | **959.1480** | **-0.8168** | **0** | **20** | **29** | **1** | **VGMTVHCR + Carbamidomethyl (C)** |
|  | 895 | **407.7490** | **813.4832** | **813.9848** | **-0.5016** | **1** | **20** | **27** | **1** | **DLVAKLR** |
|  | 2138 | **517.7835** | **1550.3284** | **1550.8061** | **-0.4777** | **1** | **20** | **25** | **1** | **LRPFPSARLGPPSR** |
|  | 2848 | **654.5912** | **1960.7514** | **1961.3387** | **-0.5873** | **0** | **20** | **23** | **1** | **LRPCGVVGAWVGMGVCQR + Carbamidomethyl (C); Oxidation (M)** |
|  | 1445 | **443.7878** | **1328.3412** | **1329.4658** | **-1.1246** | **0** | **20** | **28** | **1** | **SRPVWAASWGGR** |
|  | 3220 | **740.7194** | **2219.1361** | **2218.7043** | **0.4318** | **1** | **20** | **24** | **1** | **MTCLFPWFRISPVGIYCGK** |
|  | 1163 | **422.1836** | **842.3523** | **841.9949** | **0.3574** | **0** | **20** | **32** | **1** | **GITLSVRP** |
|  | 2634 | **595.8557** | **1189.6965** | **1190.3521** | **-0.6556** | **2** | **20** | **25** | **1** | **EKEKHAAHLK** |
|  | 2772 | **623.6852** | **1245.3556** | **1245.3428** | **0.0127** | **0** | **20** | **36** | **1** | **SVYNCSGEACR + Carbamidomethyl (C)** |
|  | 232 | 372.2370 | 742.4593 | 742.8607 | -0.4014 | 0 | 20 | 23 | 1 | IVTVGSPA |
|  | 3103 | **697.6089** | **2089.8045** | **2090.3845** | **-0.5800** | **0** | **20** | **23** | **1** | **LSCAASGLSISDAWMHWVR** |
|  | 965 | **410.1883** | **1227.5426** | **1228.3322** | **-0.7895** | **0** | **20** | **31** | **1** | **SNCSGLTFGGGTK** |
|  | 1741 | **466.1876** | **1395.5405** | **1396.6611** | **-1.1206** | **1** | **20** | **31** | **1** | **CTKPALAAPGGVRR** |
|  | 1493 | **448.2680** | **1341.7818** | **1342.5410** | **-0.7591** | **1** | **20** | **24** | **1** | **GSKPSPLSVKNTK** |
|  | 1924 | **486.3758** | **970.7369** | **971.1951** | **-0.4583** | **0** | **20** | **23** | **1** | **ATVIVIVTR** |
|  | 1725 | **464.2886** | **926.5625** | **927.0397** | **-0.4773** | **0** | **19** | **24** | **1** | **LSGAPCSHR** |
|  | 2308 | **534.1814** | **1066.3480** | **1066.1519** | **0.1961** | **1** | **19** | **32** | **1** | **GMDRGGFGGGR** |
|  | 3531 | **990.8923** | **1979.7699** | **1980.1118** | **-0.3420** | **2** | **19** | **23** | **1** | **RQHAARPREDPHAAPDR** |
|  | 2674 | **606.4319** | **1210.8491** | **1211.2357** | **-0.3865** | **0** | **19** | **26** | **1** | **GDEVFTTTEGR** |
|  | 92 | **365.2239** | **728.4330** | **728.8374** | **-0.4043** | **0** | **19** | **26** | **1** | **VTLSGPR** |
|  | 670 | **401.9965** | **801.9781** | **800.9066** | **1.0715** | **2** | **19** | **36** | **1** | **ARRAEAK** |
|  | 102 | **366.0646** | **1095.1716** | **1094.2183** | **0.9533** | **1** | **19** | **34** | **1** | **QISAEKQYK** |
|  | 1253 | **429.1210** | **856.2272** | **855.9786** | **0.2486** | **0** | **19** | **32** | **1** | **ADVPLSVR** |
|  | 227 | **371.3024** | **740.5900** | **740.8051** | **-0.2151** | **1** | **19** | **20** | **1** | **KEAHEK** |
|  | 810 | **406.1257** | **1215.3548** | **1216.4325** | **-1.0776** | **1** | **19** | **30** | **1** | **SFCAFSCPRAK** |
|  | 628 | **400.2146** | **1197.6216** | **1198.3941** | **-0.7724** | **1** | **19** | **23** | **1** | **WVQMRTAYK + Oxidation (M)** |
|  | 1826 | **475.1581** | **1422.4521** | **1421.5367** | **0.9154** | **1** | **19** | **35** | **1** | **MRASEAAVSGDGVR + Oxidation (M)** |
|  | 2915 | **667.4380** | **1999.2918** | **1999.2080** | **0.0838** | **0** | **19** | **31** | **1** | **CNIQMTQSPSAMSASVGDR + Oxidation (M)** |
|  | 1278 | **430.3331** | **1287.9770** | **1287.4856** | **0.4915** | **2** | **19** | **29** | **1** | **AKSKSCHDLSVL** |
|  | 169 | **369.2304** | **1104.6691** | **1104.3011** | **0.3680** | **0** | **19** | **27** | **1** | **MHPSLATMGK + 2 Oxidation (M)** |
|  | 1981 | **492.1642** | **982.3135** | **983.1860** | **-0.8724** | **2** | **19** | **30** | **1** | **KQAKSYMK** |
|  | 3338 | **777.9700** | **2330.8877** | **2331.6532** | **-0.7655** | **1** | **19** | **29** | **1** | **RNMGVSSGLELITLPHGHQLR + Oxidation (M)** |
|  | 401 | **385.9979** | **1154.9714** | **1154.2769** | **0.6945** | **0** | **19** | **27** | **1** | **MDGPGFGGMNR + Oxidation (M)** |
|  | 510 | **389.0739** | **776.1330** | **776.8188** | **-0.6859** | **0** | **19** | **35** | **1** | **NAAGGCER** |
|  | 71 | **364.0872** | **1089.2394** | **1089.1604** | **0.0789** | **1** | **19** | **30** | **1** | **LDLGGSERSR** |
|  | 427 | **386.2985** | **1155.8732** | **1156.2877** | **-0.4145** | **0** | **19** | **23** | **1** | **FGFAIGSQTTK** |
|  | 1544 | **451.9984** | **1352.9731** | **1353.5867** | **-0.6137** | **0** | **19** | **39** | **1** | **TCVPAAAHLITEK** |
|  | 661 | **401.8059** | **1202.3956** | **1203.4352** | **-1.0395** | **1** | **19** | **37** | **1** | **GRLGFQVWLK** |
|  | 214 | **371.0997** | **1110.2769** | **1111.1182** | **-0.8412** | **1** | **19** | **27** | **1** | **ASSSGSASKSDK** |
|  | 729 | **404.0158** | **1209.0252** | **1208.4949** | **0.5303** | **2** | **19** | **32** | **1** | **MLGGGMKLKNK + 2 Oxidation (M)** |
|  | 1930 | **487.1792** | **972.3437** | **972.2492** | **0.0944** | **1** | **19** | **35** | **1** | **MAAGRLIIK** |
|  | 1616 | **458.8112** | **1373.4116** | **1373.6440** | **-0.2325** | **1** | **19** | **34** | **1** | **LRLLSGYLWPR** |
|  | 3276 | **744.6707** | **1487.3265** | **1486.6879** | **0.6387** | **0** | **19** | **26** | **1** | **MAPSVPAAEPEYPK** |
|  | 2849 | **655.1360** | **1962.3858** | **1963.1956** | **-0.8099** | **0** | **19** | **32** | **1** | **MAAPIEETAAASPAPFCGR + Carbamidomethyl (C); Oxidation (M)** |
|  | 3542 | **1049.9502** | **3146.8284** | **3145.7525** | **1.0759** | **2** | **19** | **23** | **1** | **VAAHHKCMMCSLCHCPGATIGCDVKTCHR + 2 Oxidation (M)** |
|  | 1131 | **421.7757** | **841.5366** | **841.8692** | **-0.3325** | **1** | **19** | **33** | **1** | **ATDDKHR** |
|  | 1280 | **430.4274** | **1288.2599** | **1287.5899** | **0.6701** | **0** | **19** | **40** | **1** | **YLGPYVMMIGK + Oxidation (M)** |
|  | 1627 | **459.0458** | **916.0768** | **916.9357** | **-0.8590** | **1** | **19** | **37** | **1** | **GAEEAERR** |
|  | 2599 | **592.8599** | **1775.5576** | **1775.0757** | **0.4819** | **0** | **19** | **28** | **1** | **AGPSSMVLLASLSSVSLR** |
|  | 938 | **408.3511** | **814.6874** | **814.8471** | **-0.1597** | **1** | **19** | **30** | **1** | **RGSADGPR** |
|  | 620 | **400.0757** | **798.1366** | **797.9027** | **0.2339** | **2** | **19** | **30** | **1** | **KSHNGKK** |
|  | 909 | **407.8484** | **1220.5231** | **1220.4046** | **0.1185** | **1** | **19** | **34** | **1** | **QKPHKCGTHGK** |
|  | 1211 | **426.1781** | **850.3415** | **850.9156** | **-0.5742** | **0** | **19** | **34** | **1** | **EPLSYSR** |
|  | 723 | **403.9895** | **1208.9463** | **1208.1967** | **0.7497** | **0** | **19** | **35** | **1** | **ANDHGYDNFR** |
|  | 2528 | **578.3413** | **1732.0018** | **1732.0825** | **-0.0807** | **1** | **19** | **34** | **1** | **MPGLHPRCPLSGVVPR + Oxidation (M)** |
|  | 1423 | 441.1475 | 1320.4204 | 1320.5155 | -0.0952 | 1 | 19 | 34 | 1 | ERATASMAQVLK + Oxidation (M) |
|  | 267 | **375.2671** | **748.5195** | **748.7393** | **-0.2198** | **0** | **19** | **30** | **1** | **QNSSDAK** |
|  | 1646 | **459.8616** | **917.7084** | **917.0383** | **0.6701** | **0** | **19** | **37** | **1** | **ACEVAPLQS** |
|  | 3332 | **776.1812** | **2325.5215** | **2325.7433** | **-0.2218** | **0** | **19** | **33** | **1** | **MQCSSLFSCFTPDSITLMLGK + Oxidation (M)** |
|  | 1539 | **451.7997** | **1352.3769** | **1352.6003** | **-0.2234** | **0** | **19** | **39** | **1** | **MPIFVPLSNYR + Oxidation (M)** |
|  | 1858 | **476.7172** | **1427.1294** | **1427.6304** | **-0.5010** | **1** | **19** | **27** | **1** | **QSRACQAAGTMCK + Carbamidomethyl (C); Oxidation (M)** |
|  | 1913 | **483.9337** | **1448.7790** | **1449.5696** | **-0.7907** | **0** | **19** | **36** | **1** | **AFGSGYQLSVHQR** |
|  | 3390 | **813.1331** | **1624.2513** | **1623.9745** | **0.2769** | **1** | **19** | **27** | **1** | **IKTTMDLMEGIFPK** |
|  | 1370 | **436.0028** | **1304.9863** | **1304.6237** | **0.3627** | **0** | **19** | **34** | **1** | **IIVFCTVPCPR + Carbamidomethyl (C)** |
|  | 2749 | **614.1497** | **1226.2845** | **1226.3795** | **-0.0949** | **1** | **19** | **33** | **1** | **ATSPQKSPSVPK** |
|  | 448 | **386.9712** | **1157.8914** | **1158.2856** | **-0.3942** | **0** | **19** | **39** | **1** | **QPPVGGVSDMR + Oxidation (M)** |
|  | 1276 | **430.2687** | **1287.7840** | **1288.5961** | **-0.8121** | **1** | **19** | **32** | **1** | **ILLMKDESVLK** |
|  | 638 | **401.0352** | **800.0555** | **800.8172** | **-0.7616** | **0** | **19** | **38** | **1** | **HSTGGTNK** |
|  | 1806 | **473.5126** | **945.0104** | **945.1245** | **-0.1142** | **1** | **19** | **46** | **1** | **RTICHCR + Carbamidomethyl (C)** |
|  | 791 | **405.8763** | **1214.6069** | **1215.3979** | **-0.7911** | **0** | **19** | **33** | **1** | **VVENGALLSWK** |
|  | 2068 | 505.5834 | 1009.1521 | 1008.2351 | 0.9170 | 0 | 19 | 40 | 1 | MVIIGNSFK |
|  | 2386 | **546.5162** | **1636.5263** | **1636.8525** | **-0.3261** | **1** | **19** | **33** | **1** | **KPSESVAQRAMCSAR + Oxidation (M)** |
|  | 2624 | **593.9191** | **1778.7352** | **1779.0695** | **-0.3343** | **2** | **19** | **32** | **1** | **DRPKSTLMNFSKVQK** |
|  | 3023 | **684.0270** | **1366.0392** | **1365.4948** | **0.5444** | **1** | **19** | **30** | **1** | **RPSGISSRFSGSK** |
|  | 3254 | **742.2264** | **1482.4381** | **1481.6794** | **0.7587** | **1** | **19** | **33** | **1** | **ELAGAHRTNKPCGK** |
|  | 1511 | **450.0849** | **1347.2325** | **1346.6355** | **0.5970** | **1** | **19** | **34** | **1** | **TQVMGEIKIALK + Oxidation (M)** |
|  | 2899 | **666.6582** | **1331.3016** | **1330.4441** | **0.8576** | **1** | **19** | **33** | **1** | **GIGVSSNGAKPEKS** |
|  | 224 | **371.2360** | **740.4572** | **740.8912** | **-0.4340** | **0** | **19** | **23** | **1** | **KPELVR** |
|  | 2070 | **505.9687** | **1009.9225** | **1009.1322** | **0.7903** | **0** | **19** | **33** | **1** | **ETSTEALMK** |
|  | 459 | **387.1582** | **1158.4523** | **1157.2776** | **1.1748** | **2** | **19** | **41** | **1** | **KSPGVKNQGDK** |
|  | 844 | **407.0120** | **1218.0137** | **1218.3555** | **-0.3418** | **0** | **19** | **33** | **1** | **LAQGEYIAPEK** |
|  | 1567 | **455.1938** | **908.3727** | **909.0874** | **-0.7147** | **1** | **19** | **36** | **1** | **SPLLRAPR** |
|  | 1112 | **420.4612** | **838.9075** | **837.9202** | **0.9874** | **2** | **19** | **40** | **1** | **KAGKFSNS** |
|  | 107 | **366.1287** | **1095.3638** | **1094.2879** | **1.0760** | **2** | **19** | **40** | **1** | **NRQIKASFM** |
|  | 279 | **376.2001** | **1125.5781** | **1125.2820** | **0.2961** | **2** | **19** | **29** | **1** | **EKRLSHSLR** |
|  | 1301 | **432.1074** | **1293.3001** | **1292.3961** | **0.9039** | **2** | **19** | **40** | **1** | **STQEKLSSREK** |
|  | 3475 | **866.3247** | **2595.9519** | **2597.1206** | **-1.1687** | **2** | **19** | **37** | **1** | **APPCRSRPGLAMLRRPAPALAPAAR** |
|  | 768 | **405.1129** | **1212.3166** | **1211.2867** | **1.0299** | **0** | **19** | **35** | **1** | **NNPNGFQVHGK** |
|  | 96 | **365.2897** | **728.5646** | **728.7944** | **-0.2298** | **0** | **19** | **32** | **1** | **EGAPTVR** |
|  | 466 | **387.7393** | **1160.1958** | **1159.2504** | **0.9454** | **0** | **19** | **43** | **1** | **AASSLQSGVPSR** |
|  | 2199 | **520.6741** | **1039.3335** | **1038.1583** | **1.1752** | **0** | **19** | **37** | **1** | **AADLAHITAR** |
|  | 2521 | **576.0184** | **1725.0331** | **1726.0330** | **-0.9999** | **2** | **19** | **38** | **1** | **SYLLRMCNDLLRR + Carbamidomethyl (C); Oxidation (M)** |
|  | 3253 | **742.0961** | **1482.1774** | **1482.5933** | **-0.4159** | **1** | **19** | **30** | **1** | **EAMAQKEDMEER + Oxidation (M)** |
|  | 2783 | **627.7534** | **1253.4921** | **1252.4845** | **1.0076** | **1** | **18** | **42** | **1** | **GTFGQVVKCWK** |
|  | 2082 | **506.9705** | **1517.8894** | **1516.6920** | **1.1974** | **1** | **18** | **37** | **1** | **ENLKLSSENIELK** |
|  | 3257 | **742.3345** | **1482.6543** | **1482.6807** | **-0.0265** | **2** | **18** | **36** | **1** | **LKNLHKAIEDSSK** |
|  | 2500 | **571.5145** | **1711.5214** | **1710.9458** | **0.5756** | **2** | **18** | **30** | **1** | **KIGQDIFMTEEQKK + Oxidation (M)** |
|  | 1958 | **488.4748** | **974.9347** | **975.0610** | **-0.1262** | **0** | **18** | **41** | **1** | **AGWNPAGFR** |
|  | 2567 | **586.0544** | **1755.1412** | **1755.9579** | **-0.8168** | **1** | **18** | **38** | **1** | **RCHGLFAGAAVQGAAGGR + Carbamidomethyl (C)** |
|  | 1419 | **440.9784** | **1319.9131** | **1319.4512** | **0.4619** | **2** | **18** | **40** | **1** | **REGAGLRNSMGR + Oxidation (M)** |
|  | 693 | **402.9767** | **803.9387** | **803.9702** | **-0.0315** | **0** | **18** | **43** | **1** | **GNVIMVR + Oxidation (M)** |
|  | 795 | **405.9580** | **1214.8519** | **1215.3797** | **-0.5278** | **0** | **18** | **35** | **1** | **SEAACLAAGPGIR** |
|  | 1326 | **433.3093** | **1296.9058** | **1296.5321** | **0.3737** | **0** | **18** | **31** | **1** | **LAMLDFVSSLGK + Oxidation (M)** |
|  | 1654 | **460.1296** | **918.2444** | **917.1710** | **1.0734** | **1** | **18** | **42** | **1** | **VMLVVRGK + Oxidation (M)** |
|  | 1976 | **490.4754** | **978.9360** | **978.1860** | **0.7500** | **0** | **18** | **37** | **1** | **LVPTIPPNK** |
|  | 2798 | **631.9213** | **1892.7416** | **1892.2352** | **0.5065** | **2** | **18** | **33** | **1** | **SRRLQAVVPGIPPWCR + Carbamidomethyl (C)** |
|  | 201 | **369.3383** | **736.6618** | **736.7700** | **-0.1082** | **0** | **18** | **39** | **1** | **DPNTYK** |
|  | 2304 | **533.9337** | **1598.7790** | **1597.8362** | **0.9428** | **1** | **18** | **37** | **1** | **TVSGMDGMKCGPGLR + Carbamidomethyl (C); 2 Oxidation (M)** |
|  | 1672 | **460.6716** | **1378.9925** | **1378.5118** | **0.4808** | **1** | **18** | **34** | **1** | **RCDMCSNYEK + 2 Carbamidomethyl (C); Oxidation (M)** |
|  | 2902 | **666.6837** | **1331.3525** | **1331.4981** | **-0.1455** | **0** | **18** | **40** | **1** | **WGCWGPSPSLGK + Carbamidomethyl (C)** |
|  | 497 | **388.3558** | **1162.0451** | **1161.2845** | **0.7607** | **0** | **18** | **44** | **1** | **DEITAYFMR + Oxidation (M)** |
|  | 2972 | **675.0502** | **2022.1283** | **2021.3412** | **0.7872** | **1** | **18** | **37** | **1** | **FRPELKVLVASATMDTAR + Oxidation (M)** |
|  | 3021 | **684.0142** | **2049.0205** | **2049.4818** | **-0.4614** | **1** | **18** | **32** | **1** | **LLQVASPFLEPHLVRMAK** |
|  | 233 | **372.2795** | **742.5442** | **742.0049** | **0.5392** | **2** | **18** | **30** | **1** | **KILKLK** |
|  | 1795 | **472.2068** | **1413.5984** | **1413.5773** | **0.0210** | **1** | **18** | **39** | **1** | **SLEASGRIYVYR** |
|  | 2369 | **541.0842** | **1620.2305** | **1620.8280** | **-0.5975** | **1** | **18** | **39** | **1** | **LAVTAHSMAYGTNRI + Oxidation (M)** |
|  | 3110 | **698.7797** | **2093.3168** | **2094.3484** | **-1.0316** | **0** | **18** | **46** | **1** | **LSCAASGFAFSTYTLNWVR** |
|  | 360 | **384.2227** | **1149.6461** | **1149.2836** | **0.3625** | **0** | **18** | **30** | **1** | **GHSHLCPSVR + Carbamidomethyl (C)** |
|  | 1412 | **438.2236** | **1311.6487** | **1311.4439** | **0.2048** | **1** | **18** | **42** | **1** | **KQLSNSAEGLHK** |
|  | 1800 | **472.4644** | **1414.3709** | **1413.6883** | **0.6826** | **2** | **18** | **40** | **1** | **TAHKAARLGITMK + Oxidation (M)** |
|  | 1820 | **474.4791** | **946.9435** | **947.9431** | **-0.9996** | **0** | **18** | **46** | **1** | **SQDQDDIK** |
|  | 2435 | **558.5159** | **1672.5254** | **1671.9143** | **0.6111** | **0** | **18** | **35** | **1** | **MWQNDLQPLLIER + Oxidation (M)** |
|  | 1873 | **478.2217** | **954.4287** | **954.1083** | **0.3204** | **1** | **18** | **38** | **1** | **APPASPRCR** |
|  | 410 | 386.0735 | 1155.1982 | 1154.3168 | 0.8814 | 0 | 18 | 37 | 1 | KPPVASNGVTGK |
|  | 2359 | **540.0508** | **1617.1302** | **1617.8042** | **-0.6741** | **2** | **18** | **41** | **1** | **EEERGAAIIQKAFR** |
|  | 2954 | **669.6991** | **2006.0751** | **2006.3297** | **-0.2545** | **1** | **18** | **44** | **1** | **MPGSVIPPPLVRGGQQASSK** |
|  | 1847 | **476.2108** | **950.4067** | **949.9656** | **0.4412** | **1** | **18** | **40** | **1** | **STSSGGGNRK** |
|  | 272 | **376.0757** | **1125.2049** | **1125.3017** | **-0.0968** | **0** | **18** | **38** | **1** | **HICGGALIADR** |
|  | 463 | **387.2025** | **1158.5854** | **1158.2193** | **0.3661** | **0** | **18** | **39** | **1** | **NALSSVDPEAR** |
|  | 796 | **405.9905** | **809.9662** | **808.9087** | **1.0576** | **1** | **18** | **37** | **1** | **FGRCNR + Carbamidomethyl (C)** |
|  | 2991 | **680.1498** | **2037.4273** | **2037.3435** | **0.0838** | **1** | **18** | **40** | **1** | **DSRAALTLQPLCPSPALQR** |
|  | 747 | **404.4644** | **1210.3711** | **1211.4128** | **-1.0417** | **0** | **18** | **47** | **1** | **GCMEMPGCPDR + Oxidation (M)** |
|  | 1717 | **464.1102** | **1389.3083** | **1388.5065** | **0.8018** | **0** | **18** | **38** | **1** | **MADGAAAGAGGSPSLR** |
|  | 128 | **367.1628** | **1098.4663** | **1099.2844** | **-0.8182** | **1** | **18** | **39** | **1** | **QLASKLGVQR** |
|  | 3042 | **684.8894** | **1367.7640** | **1367.5569** | **0.2071** | **2** | **18** | **39** | **1** | **AGAAGALPAQRTKR** |
|  | 3359 | **789.2372** | **2364.6894** | **2364.6798** | **0.0096** | **1** | **18** | **37** | **1** | **YTCHVQHEGLPEPLTLRWK + Carbamidomethyl (C)** |
|  | 2152 | **518.8992** | **1553.6753** | **1553.8020** | **-0.1267** | **2** | **18** | **39** | **1** | **AKMTDSDHFKVMK + Oxidation (M)** |
|  | 3431 | **841.9619** | **2522.8636** | **2521.7015** | **1.1621** | **2** | **18** | **45** | **1** | **NDPFARMETRGPQGAANPMDSSR + Oxidation (M)** |
|  | 1332 | **434.0140** | **866.0133** | **864.9852** | **1.0281** | **0** | **18** | **38** | **1** | **GSGGSFLIK** |
|  | 261 | **374.3747** | **746.7345** | **745.9339** | **0.8006** | **1** | **18** | **54** | **1** | **ICRINK** |
|  | 1971 | **490.0662** | **978.1177** | **978.1646** | **-0.0469** | **1** | **18** | **42** | **1** | **MAASLDKVK + Oxidation (M)** |
|  | 2205 | **521.2335** | **1560.6784** | **1559.9586** | **0.7198** | **0** | **18** | **40** | **1** | **MMGICPSCALWGMK + 2 Oxidation (M)** |
|  | 3290 | **755.8943** | **1509.7738** | **1509.7310** | **0.0428** | **0** | **18** | **47** | **1** | **SPAPPWAVGAACAVR + Carbamidomethyl (C)** |
|  | 1866 | **477.8880** | **1430.6417** | **1429.7274** | **0.9144** | **0** | **18** | **38** | **1** | **MSLPCSSFICLR + Carbamidomethyl (C); Oxidation (M)** |
|  | 2345 | **538.4785** | **1612.4134** | **1611.9073** | **0.5060** | **2** | **18** | **35** | **1** | **HGCLEIVLKGTTGKR** |
|  | 1901 | **481.9898** | **1442.9472** | **1442.6416** | **0.3056** | **1** | **18** | **44** | **1** | **EVHSGQARWLML + Oxidation (M)** |
|  | 2055 | **505.0207** | **1008.0266** | **1007.0649** | **0.9617** | **2** | **18** | **40** | **1** | **SFGRDRNR** |
|  | 1593 | **457.5807** | **1369.7200** | **1369.5910** | **0.1289** | **1** | **18** | **47** | **1** | **LCDMRASALCDR + Oxidation (M)** |
|  | 1840 | **476.1613** | **1425.4618** | **1424.7075** | **0.7542** | **1** | **18** | **41** | **1** | **QSRGMLLLYSLK + Oxidation (M)** |
|  | 1849 | **476.2167** | **950.4186** | **951.0810** | **-0.6625** | **0** | **18** | **41** | **1** | **ASAISHPLR** |
|  | 2021 | **500.8122** | **1499.4144** | **1498.5759** | **0.8385** | **1** | **18** | **34** | **1** | **TSSATSGLSCRSER + Carbamidomethyl (C)** |
|  | 25 | **362.0193** | **722.0238** | **722.7864** | **-0.7626** | **0** | **18** | **40** | **1** | **NSLDFK** |
|  | 3082 | **687.3503** | **2059.0289** | **2058.2535** | **0.7753** | **1** | **18** | **42** | **1** | **NPALQTSLSSLSSSVSRAPR** |
|  | 1374 | 436.1344 | 870.2541 | 870.0283 | 0.2258 | 0 | 18 | 43 | 1 | TPCPSLPR |
|  | 2064 | **505.3060** | **1008.5973** | **1008.2332** | **0.3640** | **0** | **18** | **37** | **1** | **WCFLDVLL** |
|  | 4 | **360.3632** | **1078.0675** | **1079.2286** | **-1.1611** | **0** | **18** | **55** | **1** | **MYAYGFVGR + Oxidation (M)** |
|  | 1875 | **478.3728** | **954.7309** | **955.1110** | **-0.3802** | **0** | **18** | **32** | **1** | **LITSAHWK** |
|  | 450 | **386.9904** | **771.9661** | **770.9633** | **1.0027** | **1** | **18** | **47** | **1** | **IILRTR** |
|  | 1639 | **459.2358** | **916.4568** | **916.0320** | **0.4248** | **0** | **18** | **45** | **1** | **LVDLDWR** |
|  | 1392 | **437.0765** | **872.1382** | **872.0242** | **0.1140** | **2** | **18** | **48** | **1** | **KAAAAAGGKK** |
|  | 2383 | **544.7687** | **1087.5226** | **1088.1310** | **-0.6084** | **0** | **18** | **39** | **1** | **DYHNLSSPR** |
|  | 3132 | **709.8417** | **2126.5030** | **2125.4950** | **1.0080** | **1** | **18** | **49** | **1** | **GQAPLPMAHIEKLAADCMR + Carbamidomethyl (C); Oxidation (M)** |
|  | 9 | **360.4232** | **1078.2473** | **1079.1673** | **-0.9200** | **0** | **18** | **59** | **1** | **GGQHSQAAPVK** |
|  | 1087 | **419.3313** | **1254.9718** | **1255.4240** | **-0.4522** | **2** | **18** | **42** | **1** | **NGRKSDVPGVVK** |
|  | 1954 | **488.2580** | **974.5013** | **975.0611** | **-0.5599** | **1** | **18** | **46** | **1** | **TGQISRASR** |
|  | 2678 | **606.9104** | **1211.8060** | **1212.3546** | **-0.5485** | **1** | **18** | **33** | **1** | **EELMESRMR + 2 Oxidation (M)** |
|  | 3043 | **684.9337** | **1367.8527** | **1367.5572** | **0.2955** | **2** | **18** | **37** | **1** | **TAAPSVRPEKRR** |
|  | 3085 | **687.9270** | **1373.8392** | **1374.5461** | **-0.7068** | **0** | **18** | **36** | **1** | **SPLHPGGTLWPGR** |
|  | 1317 | **432.9345** | **1295.7814** | **1295.5293** | **0.2522** | **1** | **18** | **44** | **1** | **SVMRVMGALGDK + 2 Oxidation (M)** |
|  | 2081 | **506.6739** | **1011.3329** | **1010.1865** | **1.1465** | **0** | **18** | **40** | **1** | **MVDLCNSTK** |
|  | 3396 | **817.5602** | **1633.1057** | **1633.7869** | **-0.6812** | **2** | **18** | **42** | **1** | **FCSGQHLRDSISRQ** |
|  | 752 | **404.9337** | **807.8526** | **806.8880** | **0.9646** | **0** | **18** | **41** | **1** | **SAQAQMR + Oxidation (M)** |
|  | 1214 | **426.5239** | **851.0331** | **850.9124** | **0.1207** | **0** | **18** | **52** | **1** | **DVNLEVY** |
|  | 1471 | **445.6775** | **1334.0104** | **1334.4591** | **-0.4487** | **1** | **18** | **41** | **1** | **QQLDQASKTCR + Carbamidomethyl (C)** |
|  | 300 | **377.3680** | **1129.0819** | **1130.2521** | **-1.1703** | **0** | **18** | **42** | **1** | **LAQTLVDSGAR** |
|  | 3074 | **687.0658** | **1372.1168** | **1372.4839** | **-0.3671** | **0** | **18** | **41** | **1** | **SAIASTPGTLQGNR** |
|  | 1223 | **427.4547** | **1279.3420** | **1280.4531** | **-1.1112** | **0** | **18** | **52** | **1** | **IEIHGVPADCAR** |
|  | 2944 | **668.8892** | **2003.6455** | **2004.3369** | **-0.6915** | **2** | **18** | **38** | **1** | **TEEAFPGGPLGALRAMCKR** |
|  | 2975 | **675.4382** | **2023.2925** | **2022.2708** | **1.0217** | **2** | **18** | **45** | **1** | **ECGKAYGWCSELIRHR + 2 Carbamidomethyl (C)** |
|  | 2595 | **592.7688** | **1775.2842** | **1774.9946** | **0.2897** | **1** | **18** | **45** | **1** | **DHVKNSVYLQMDGLR** |
|  | 3373 | **801.5419** | **2401.6034** | **2402.6170** | **-1.0136** | **2** | **18** | **47** | **1** | **AHTGEKTFECGECGKTFWEK + 2 Carbamidomethyl (C)** |
|  | 1338 | **434.1738** | **1299.4991** | **1299.4135** | **0.0857** | **1** | **18** | **43** | **1** | **SGSANAYMQSKR** |
|  | 79 | **364.2357** | **1089.6848** | **1089.3129** | **0.3719** | **2** | **18** | **32** | **1** | **GLREMLKAR + Oxidation (M)** |
|  | 1566 | **454.5138** | **907.0129** | **907.0319** | **-0.0190** | **2** | **18** | **56** | **1** | **NKQKHPR** |
|  | 2855 | **656.2242** | **1310.4336** | **1309.3387** | **1.0949** | **0** | **18** | **44** | **1** | **ESTNLANFNDGK** |
|  | 756 | **405.0130** | **808.0112** | **806.8598** | **1.1515** | **0** | **18** | **42** | **1** | **APFALSSD** |
|  | 2139 | **518.6660** | **1035.3173** | **1034.2509** | **1.0664** | **0** | **18** | **49** | **1** | **AMVCLLGPGT + Carbamidomethyl (C); Oxidation (M)** |
|  | 876 | **407.3418** | **1219.0031** | **1219.3003** | **-0.2972** | **0** | **18** | **35** | **1** | **YFDYWGQGAL** |
|  | 1589 | **457.1716** | **1368.4925** | **1368.4058** | **0.0867** | **1** | **18** | **46** | **1** | **EKASIYQNQNSS** |
|  | 1794 | **472.1951** | **942.3754** | **942.0710** | **0.3044** | **0** | **18** | **45** | **1** | **QETLAKPR** |
|  | 3106 | **698.1870** | **2091.5389** | **2091.3728** | **0.1660** | **2** | **18** | **44** | **1** | **DGVELRAGKTMAIAAQGACR + Carbamidomethyl (C); Oxidation (M)** |
|  | 2615 | **593.6085** | **1777.8034** | **1778.1889** | **-0.3855** | **1** | **18** | **50** | **1** | **CPVFMSFLPLPNRLK + Oxidation (M)** |
|  | 1254 | **429.1231** | **1284.3472** | **1283.4305** | **0.9167** | **1** | **18** | **47** | **1** | **INEDPKDALLR** |
|  | 2839 | **652.8222** | **1955.4444** | **1956.2296** | **-0.7851** | **2** | **18** | **48** | **1** | **KPGASVKVSCKASGYNFR + Carbamidomethyl (C)** |
|  | 1597 | 458.0739 | 914.1330 | 914.0656 | 0.0673 | 2 | 18 | 46 | 1 | WGGRLRGI |
|  | 2979 | **677.1970** | **2028.5689** | **2029.2785** | **-0.7097** | **0** | **18** | **48** | **1** | **NTTAFSVLYAVFGQHSMR** |
|  | 452 | **387.0259** | **1158.0556** | **1157.4067** | **0.6489** | **0** | **18** | **53** | **1** | **MAHGPGALMLK + 2 Oxidation (M)** |
|  | 969 | **411.0084** | **820.0021** | **818.8772** | **1.1249** | **1** | **18** | **49** | **1** | **DPRFER** |
|  | 3281 | **746.0311** | **1490.0475** | **1489.7215** | **0.3260** | **1** | **18** | **37** | **1** | **VKLVHSGGGVVQPGR** |
|  | 3486 | **878.9380** | **2633.7918** | **2634.9697** | **-1.1779** | **2** | **18** | **47** | **1** | **AGSCWQDPLAVALSRGRQLAAPPGR + Carbamidomethyl (C)** |
|  | 3205 | **740.5139** | **2218.5196** | **2218.4859** | **0.0336** | **1** | **18** | **44** | **1** | **ALPTSPTEYQMALSPPASRGK + Oxidation (M)** |
|  | 735 | **404.0480** | **1209.1218** | **1208.4089** | **0.7130** | **0** | **18** | **47** | **1** | **VLQMECVGTR + Carbamidomethyl (C); Oxidation (M)** |
|  | 2945 | **668.8899** | **2003.6475** | **2004.2540** | **-0.6065** | **1** | **18** | **40** | **1** | **TFITVSALFSHNRAHFR** |
|  | 1440 | **443.4240** | **1327.2499** | **1326.5814** | **0.6685** | **0** | **18** | **43** | **1** | **LVVLSTVGVPGTGK** |
|  | 1984 | **492.3570** | **982.6992** | **982.1381** | **0.5611** | **1** | **18** | **37** | **1** | **ARLPQEIR** |
|  | 138 | **367.6595** | **1099.9563** | **1100.3553** | **-0.3990** | **2** | **18** | **41** | **1** | **TKRILTLQK** |
|  | 2996 | **680.8077** | **2039.4010** | **2038.2705** | **1.1306** | **2** | **18** | **55** | **1** | **CMVCGGDRSGCSKQSGSFR + Carbamidomethyl (C); Oxidation (M)** |
|  | 407 | **386.0588** | **770.1028** | **768.9441** | **1.1587** | **0** | **18** | **43** | **1** | **IASLPIR** |
|  | 199 | **369.3320** | **1104.9737** | **1105.2443** | **-0.2706** | **1** | **18** | **46** | **1** | **TIHTGGKTYK** |
|  | 917 | **408.0274** | **1221.0599** | **1220.3348** | **0.7251** | **1** | **18** | **48** | **1** | **GRGLGGGLNYEK** |
|  | 2048 | **504.2309** | **1509.6704** | **1509.6417** | **0.0288** | **1** | **17** | **50** | **1** | **TDRIVGQNSGTSMK + Oxidation (M)** |
|  | 3047 | **685.2596** | **1368.5045** | **1367.5572** | **0.9473** | **2** | **17** | **49** | **1** | **TAAPSVRPEKRR** |
|  | 1311 | **432.5240** | **863.0331** | **861.9202** | **1.1130** | **0** | **17** | **60** | **1** | **EPGMGSER** |
|  | 3482 | **873.9471** | **2618.8193** | **2617.9081** | **0.9112** | **1** | **17** | **51** | **1** | **MMNSHFLDGNLVPLEGKEVDESR** |
|  | 438 | **386.8940** | **1157.6597** | **1157.2377** | **0.4219** | **1** | **17** | **52** | **1** | **LSAPGSQREGR** |
|  | 1766 | **469.2896** | **1404.8467** | **1404.6135** | **0.2331** | **2** | **17** | **39** | **1** | **LRGFEGKLTAQGK** |
|  | 1994 | **494.9438** | **1481.8094** | **1482.6277** | **-0.8184** | **1** | **17** | **50** | **1** | **QTACQAVQHQRGR** |
|  | 906 | **407.8306** | **813.6463** | **813.9003** | **-0.2539** | **0** | **17** | **49** | **1** | **AAGAPGGWK** |
|  | 812 | **406.1442** | **1215.4105** | **1215.4460** | **-0.0355** | **2** | **17** | **46** | **1** | **RYKCLSCTK + 2 Carbamidomethyl (C)** |
|  | 3258 | **742.3504** | **2224.0290** | **2223.7840** | **0.2451** | **0** | **17** | **46** | **1** | **VAIYMPMIPELVVAMLACAR + 2 Oxidation (M)** |
|  | 84 | **364.8394** | **727.6640** | **727.8921** | **-0.2281** | **0** | **17** | **46** | **1** | **LGQLGLK** |
|  | 508 | **389.0484** | **776.0820** | **775.9998** | **0.0823** | **1** | **17** | **54** | **1** | **KMGALLK + Oxidation (M)** |
|  | 1000 | **413.5304** | **825.0461** | **823.9152** | **1.1309** | **2** | **17** | **47** | **1** | **KGKGNSSM + Oxidation (M)** |
|  | 1458 | **444.2455** | **886.4762** | **886.0077** | **0.4685** | **1** | **17** | **48** | **1** | **AELERLR** |
|  | 1731 | **465.9175** | **1394.7305** | **1394.5791** | **0.1514** | **2** | **17** | **51** | **1** | **DPVPLGRSAGAAKR** |
|  | 3214 | **740.6538** | **1479.2928** | **1478.8394** | **0.4534** | **1** | **17** | **38** | **1** | **EMPICCIIKIVNS + Oxidation (M)** |
|  | 3219 | **740.6921** | **1479.3694** | **1479.6832** | **-0.3139** | **1** | **17** | **38** | **1** | **CSCREGWIGNGIK + Carbamidomethyl (C)** |
|  | 436 | **386.8478** | **771.6808** | **770.8840** | **0.7969** | **2** | **17** | **51** | **1** | **ARGGRVR** |
|  | 1046 | **417.1368** | **1248.3882** | **1249.4807** | **-1.0925** | **2** | **17** | **54** | **1** | **GASKGCVTITKK + Carbamidomethyl (C)** |
|  | 2496 | **571.1040** | **1140.1932** | **1139.3847** | **0.8085** | **0** | **17** | **46** | **1** | **ELLPVLISAGK** |
|  | 1439 | **443.3566** | **1327.0477** | **1326.4769** | **0.5708** | **1** | **17** | **37** | **1** | **SCLNEFPDFRV** |
|  | 1982 | **492.2639** | **982.5129** | **983.1629** | **-0.6499** | **0** | **17** | **46** | **1** | **VGVTVVGPQK** |
|  | 1433 | **443.0037** | **1325.9888** | **1326.5050** | **-0.5162** | **2** | **17** | **46** | **1** | **QRVEEQLRLR** |
|  | 139 | **367.9109** | **733.8071** | **732.8259** | **0.9811** | **0** | **17** | **54** | **1** | **LSSGTLR** |
|  | 2511 | **574.1051** | **1146.1954** | **1145.2286** | **0.9668** | **1** | **17** | **51** | **1** | **ARAWAAGETGR** |
|  | 3015 | **683.8815** | **1365.7482** | **1364.6821** | **1.0660** | **2** | **17** | **45** | **1** | **LLLAPRRGLTVR** |
|  | 627 | **400.2138** | **1197.6192** | **1198.2862** | **-0.6670** | **0** | **17** | **37** | **1** | **SCCSYISHQN + Carbamidomethyl (C)** |
|  | 3120 | **701.7766** | **1401.5384** | **1401.6511** | **-0.1127** | **2** | **17** | **57** | **1** | **QRSSIKTVELIK** |
|  | 1103 | **420.1658** | **838.3168** | **837.8772** | **0.4397** | **0** | **17** | **48** | **1** | **SGFGVSER** |
|  | 183 | **369.2752** | **1104.8035** | **1104.2991** | **0.5044** | **0** | **17** | **43** | **1** | **QQLLIGAYAK** |
|  | 3234 | **741.4536** | **2221.3385** | **2222.4628** | **-1.1243** | **0** | **17** | **48** | **1** | **GGGLLVRPSPPNSGPLAAPGDHR** |
|  | 730 | **404.0238** | **1209.0491** | **1209.3969** | **-0.3477** | **1** | **17** | **50** | **1** | **AVKCNMDSLR + Carbamidomethyl (C); Oxidation (M)** |
|  | 1996 | **495.0724** | **988.1301** | **988.0963** | **0.0339** | **2** | **17** | **53** | **1** | **ILDSGNKKN** |
|  | 2000 | **496.2357** | **1485.6851** | **1485.6252** | **0.0599** | **1** | **17** | **53** | **1** | **RSGSPCAPQSAPATR** |
|  | 1199 | **424.0807** | **1269.2199** | **1269.4472** | **-0.2273** | **1** | **17** | **56** | **1** | **TKDPNVVGQLAK** |
|  | 2738 | **613.1986** | **1836.5737** | **1836.1405** | **0.4332** | **0** | **17** | **49** | **1** | **MAFFTGLWGPFTCVSR + Oxidation (M)** |
|  | 516 | **389.1133** | **1164.3177** | **1163.4061** | **0.9115** | **1** | **17** | **55** | **1** | **AKILIDSIYK** |
|  | 710 | **403.9204** | **1208.7392** | **1209.3936** | **-0.6544** | **0** | **17** | **51** | **1** | **NGSVCLMDVAK + Carbamidomethyl (C); Oxidation (M)** |
|  | 1937 | **487.9334** | **1460.7781** | **1461.5509** | **-0.7728** | **0** | **17** | **56** | **1** | **EDPSSMNDVQPVK + Oxidation (M)** |
|  | 1244 | **428.9888** | **855.9628** | **855.8907** | **0.0721** | **0** | **17** | **47** | **1** | **GYYDNPK** |
|  | 3299 | **758.2315** | **1514.4482** | **1514.5652** | **-0.1170** | **1** | **17** | **48** | **1** | **SHQHASQRAEHAR** |
|  | 180 | **369.2640** | **1104.7698** | **1105.3089** | **-0.5391** | **0** | **17** | **43** | **1** | **AKPWAVCFPS** |
|  | 2890 | **666.5253** | **1331.0358** | **1330.3976** | **0.6381** | **0** | **17** | **41** | **1** | **DELELELAENR** |
|  | 271 | **376.0755** | **1125.2043** | **1125.2853** | **-0.0810** | **1** | **17** | **47** | **1** | **RPASLAQRAR** |
|  | 453 | **387.0442** | **772.0737** | **770.8839** | **1.1897** | **2** | **17** | **59** | **1** | **AARAARR** |
|  | 1598 | **458.0912** | **1371.2516** | **1370.5378** | **0.7138** | **2** | **17** | **51** | **1** | **MEPRSGGLRSHK + Oxidation (M)** |
|  | 2482 | **567.6508** | **1699.9303** | **1700.9375** | **-1.0072** | **1** | **17** | **62** | **1** | **HVFGAGGFPSAGSILKR** |
|  | 1242 | **428.9506** | **855.8865** | **856.0181** | **-0.1317** | **0** | **17** | **47** | **1** | **ISELGLPK** |
|  | 1378 | **436.1822** | **1305.5245** | **1306.4906** | **-0.9661** | **2** | **17** | **51** | **1** | **KKDYHAELMR + Oxidation (M)** |
|  | 60 | **363.2780** | **1086.8117** | **1087.3102** | **-0.4986** | **1** | **17** | **41** | **1** | **IVSTKAILDK** |
|  | 667 | **401.9092** | **1202.7055** | **1203.4735** | **-0.7679** | **0** | **17** | **58** | **1** | **NQVPIVPVPLK** |
|  | 944 | **408.8784** | **1223.6129** | **1223.3969** | **0.2160** | **0** | **17** | **55** | **1** | **LSCAASGITVSSK** |
|  | 3210 | **740.6376** | **2218.8905** | **2218.5571** | **0.3335** | **2** | **17** | **40** | **1** | **CTKCGKSFGMISCLTEHSR + 2 Carbamidomethyl (C); Oxidation (M)** |
|  | 3291 | **755.9417** | **1509.8686** | **1509.6665** | **0.2022** | **2** | **17** | **51** | **1** | **KKEQLDSGHRPSK** |
|  | 3416 | **830.1715** | **1658.3282** | **1657.9062** | **0.4220** | **2** | **17** | **39** | **1** | **KEQLKLYCETCDK + Carbamidomethyl (C)** |
|  | 3419 | **831.7706** | **1661.5264** | **1660.8738** | **0.6526** | **2** | **17** | **39** | **1** | **DCPMPRNAARISEK + Carbamidomethyl (C); Oxidation (M)** |
|  | 715 | **403.9388** | **1208.7942** | **1209.3983** | **-0.6042** | **1** | **17** | **52** | **1** | **LSSKLIQHQR** |
|  | 1760 | **468.2628** | **934.5109** | **935.0734** | **-0.5625** | **0** | **17** | **51** | **1** | **LSLLSSTSK** |
|  | 77 | **364.2028** | **1089.5861** | **1090.2909** | **-0.7048** | **0** | **17** | **36** | **1** | **LEITMLSQGV** |
|  | 251 | **374.1572** | **1119.4493** | **1118.2614** | **1.1880** | **1** | **17** | **57** | **1** | **NKEMPIDQK + Oxidation (M)** |
|  | 3039 | **684.8578** | **1367.7008** | **1368.4904** | **-0.7896** | **0** | **17** | **54** | **1** | **VSAQQFDDAFLK** |
|  | 281 | **376.2207** | **750.4267** | **750.7998** | **-0.3731** | **0** | **17** | **39** | **1** | **NFISDR** |
|  | 1437 | **443.1973** | **1326.5698** | **1326.4603** | **0.1095** | **0** | **17** | **49** | **1** | **MYGCHVGSDWR + Oxidation (M)** |
|  | 2726 | **612.2628** | **1222.5107** | **1223.3820** | **-0.8712** | **2** | **17** | **52** | **1** | **SKGQIPNSKHK** |
|  | 313 | **378.5375** | **1132.5904** | **1133.4100** | **-0.8196** | **2** | **17** | **42** | **1** | **GPRYLMKLR** |
|  | 807 | **406.1158** | **1215.3253** | **1214.4977** | **0.8275** | **2** | **17** | **50** | **1** | **ISLQKASVKLK** |
|  | 1761 | **468.4377** | **934.8605** | **935.0734** | **-0.2129** | **0** | **17** | **50** | **1** | **LSLLSSTSK** |
|  | 44 | **363.0882** | **1086.2425** | **1087.2505** | **-1.0080** | **0** | **17** | **52** | **1** | **QYPQCCDCK** |
|  | 503 | **388.4626** | **1162.3656** | **1161.3573** | **1.0083** | **1** | **17** | **73** | **1** | **MWHREQMK + Oxidation (M)** |
|  | 172 | **369.2324** | **1104.6749** | **1104.3011** | **0.3738** | **0** | **17** | **45** | **1** | **MHPSLATMGK + 2 Oxidation (M)** |
|  | 260 | **374.3640** | **746.7133** | **746.8758** | **-0.1625** | **0** | **17** | **67** | **1** | **SPSGMLR** |
|  | 763 | **405.0895** | **1212.2464** | **1213.3871** | **-1.1407** | **1** | **17** | **48** | **1** | **RGQVSAVLDIR** |
|  | 831 | 406.6886 | 811.3625 | 811.8366 | -0.4741 | 0 | 17 | 38 | 1 | GFSDATSK |
|  | 2080 | **506.5987** | **1011.1826** | **1010.1865** | **0.9961** | **0** | **17** | **60** | **1** | **MVDLCNSTK** |
|  | 3513 | **913.7275** | **2738.1604** | **2739.0712** | **-0.9107** | **0** | **17** | **46** | **1** | **VGSDCTTIHYNYMCNSSCMGCMNR + 3 Oxidation (M)** |
|  | 186 | **369.2817** | **1104.8230** | **1104.3011** | **0.5219** | **0** | **17** | **45** | **1** | **MHPSLATMGK + 2 Oxidation (M)** |
|  | 248 | **373.9434** | **1118.8079** | **1119.3322** | **-0.5243** | **1** | **17** | **67** | **1** | **LIEAAEMAKK + Oxidation (M)** |
|  | 920 | **408.0909** | **1221.2504** | **1220.4261** | **0.8243** | **2** | **17** | **55** | **1** | **NKMAAQNCRK + Carbamidomethyl (C)** |
|  | 2276 | **529.5778** | **1057.1407** | **1058.2757** | **-1.1349** | **2** | **17** | **65** | **1** | **SGTKIIGKVR** |
|  | 231 | **372.0459** | **1113.1155** | **1112.3876** | **0.7279** | **1** | **17** | **50** | **1** | **KLTCLECMR + Oxidation (M)** |
|  | 569 | **393.5099** | **785.0049** | **784.9866** | **0.0183** | **1** | **17** | **58** | **1** | **GALKVLGK** |
|  | 3520 | **930.0671** | **2787.1792** | **2787.0907** | **0.0885** | **1** | **17** | **56** | **1** | **TCPMKEGNPFGPFWDQFHVSFNK + Carbamidomethyl (C); Oxidation (M)** |
|  | 477 | 387.8971 | 773.7794 | 772.8898 | 0.8896 | 1 | 17 | 64 | 1 | QLKADAK |
|  | 197 | **369.3263** | **1104.9566** | **1105.3089** | **-0.3523** | **0** | **17** | **50** | **1** | **AKPWAVCFPS** |
|  | 1689 | **461.9728** | **1382.8963** | **1382.4973** | **0.3991** | **0** | **17** | **50** | **1** | **MAENSVLTSTTGR + Oxidation (M)** |
|  | 3430 | **839.1434** | **1676.2721** | **1676.7853** | **-0.5133** | **0** | **17** | **40** | **1** | **HHAATVNNLNVTEEK** |
|  | 3441 | **848.4528** | **1694.8907** | **1694.0925** | **0.7983** | **2** | **17** | **51** | **1** | **LAAKCLVMKAEMNGSK** |
|  | 3480 | **873.6239** | **1745.2330** | **1744.9453** | **0.2877** | **1** | **17** | **50** | **1** | **HLPLDEPAELGLRER** |
|  | 140 | **367.9725** | **1100.8952** | **1101.2574** | **-0.3621** | **2** | **17** | **59** | **1** | **GTAKANVGAGKK** |
|  | 779 | **405.1682** | **1212.4823** | **1211.4078** | **1.0745** | **0** | **17** | **48** | **1** | **IYMETPGCPGK + Oxidation (M)** |
|  | 806 | **406.1134** | **1215.3179** | **1216.2953** | **-0.9773** | **1** | **17** | **51** | **1** | **AAEEPSKVEEK** |
|  | 3374 | **803.5756** | **2407.7045** | **2407.8736** | **-0.1691** | **0** | **17** | **53** | **1** | **CLLSPVPQTQPVTMVLDHLCR + Carbamidomethyl (C)** |
|  | 244 | **373.1552** | **1116.4433** | **1117.3626** | **-0.9194** | **0** | **17** | **60** | **1** | **LIIVASQAMR + Oxidation (M)** |
|  | 1980 | **492.1542** | **982.2937** | **981.1069** | **1.1867** | **0** | **17** | **50** | **1** | **LHVNGNSIK** |
|  | 726 | **404.0067** | **805.9986** | **804.9151** | **1.0835** | **1** | **17** | **55** | **1** | **NMIRQGA + Oxidation (M)** |
|  | 490 | **388.2158** | **774.4168** | **774.8196** | **-0.4028** | **0** | **17** | **52** | **1** | **DEAATLR** |
|  | 1306 | **432.2667** | **862.5187** | **862.0494** | **0.4693** | **2** | **17** | **46** | **1** | **KAMKEAGK** |
|  | 2163 | **519.0184** | **1036.0220** | **1035.1362** | **0.8857** | **1** | **17** | **52** | **1** | **SKNATAACGR + Carbamidomethyl (C)** |
|  | 3038 | **684.6626** | **2050.9656** | **2051.3670** | **-0.4014** | **2** | **17** | **44** | **1** | **RDVTSAKVMNASAALEFLK** |
|  | 332 | **380.1556** | **1137.4447** | **1137.3973** | **0.0474** | **0** | **17** | **58** | **1** | **VTPAMGMQMR + Oxidation (M)** |
|  | 3358 | **788.5718** | **1575.1288** | **1574.8441** | **0.2846** | **2** | **17** | **50** | **1** | **AESCKVAIIDRLTR** |
|  | 699 | **403.1390** | **1206.3948** | **1205.3966** | **0.9982** | **2** | **17** | **59** | **1** | **RFCRPSRAR + Carbamidomethyl (C)** |
|  | 1708 | **463.1526** | **1386.4356** | **1386.6364** | **-0.2009** | **2** | **17** | **51** | **1** | **ASIVKNLKDTLGK** |
|  | 1748 | **467.2465** | **1398.7172** | **1397.5530** | **1.1642** | **0** | **17** | **54** | **1** | **NTLYLQMDDLR + Oxidation (M)** |
|  | 2995 | **680.6332** | **2038.8774** | **2038.2241** | **0.6533** | **2** | **17** | **44** | **1** | **HSRKQSEPPANDLFNAVK** |
|  | 40 | **363.0416** | **1086.1026** | **1087.2077** | **-1.1051** | **0** | **17** | **55** | **1** | **MPASGAPTPSR + Oxidation (M)** |
|  | 475 | **387.8811** | **1160.6212** | **1160.3891** | **0.2322** | **1** | **17** | **65** | **1** | **FKMLEHALR + Oxidation (M)** |
|  | 1169 | **422.2111** | **842.4074** | **841.9949** | **0.4124** | **0** | **17** | **55** | **1** | **GITLSVRP** |
|  | 1577 | **456.2527** | **1365.7359** | **1366.5590** | **-0.8231** | **0** | **17** | **46** | **1** | **KPGPLPSSLDDLK** |
|  | 654 | **401.3594** | **1201.0560** | **1201.5039** | **-0.4479** | **1** | **17** | **55** | **1** | **MAFFCILRK + Carbamidomethyl (C); Oxidation (M)** |
|  | 3026 | **684.0331** | **2049.0771** | **2048.3648** | **0.7123** | **2** | **17** | **47** | **1** | **MQPCLEGEECKVLPDRK + Carbamidomethyl (C); Oxidation (M)** |
|  | 2516 | **575.1605** | **1722.4592** | **1722.8735** | **-0.4143** | **0** | **17** | **58** | **1** | **YLQGMLEAGEGGAPSSR** |
|  | 228 | **371.3076** | **740.6003** | **740.8051** | **-0.2047** | **1** | **17** | **36** | **1** | **EEKAHK** |
|  | 491 | **388.2219** | **1161.6434** | **1162.3620** | **-0.7186** | **0** | **17** | **53** | **1** | **QPPVPPQRPM + Oxidation (M)** |
|  | 3274 | **743.8314** | **2228.4721** | **2227.6679** | **0.8042** | **0** | **17** | **63** | **1** | **TLLLVAAFGLVLQGPCANTLR + Carbamidomethyl (C)** |
|  | 3497 | **893.9642** | **1785.9137** | **1787.0020** | **-1.0883** | **2** | **17** | **59** | **1** | **AEDTAVYYCAKVRAGI + Carbamidomethyl (C)** |
|  | 3510 | **907.1377** | **1812.2606** | **1812.9947** | **-0.7341** | **1** | **17** | **50** | **1** | **TPDSLEPSPLKESPCR + Carbamidomethyl (C)** |
|  | 3511 | **908.9911** | **1815.9674** | **1817.1190** | **-1.1516** | **2** | **17** | **57** | **1** | **AGKTLLPSSRQAMTALR + Oxidation (M)** |
|  | 39 | **363.0325** | **1086.0753** | **1085.2148** | **0.8606** | **1** | **17** | **55** | **1** | **NAVIGNNKQK** |
|  | 3421 | **832.9752** | **2495.9035** | **2495.8131** | **0.0903** | **1** | **17** | **63** | **1** | **APTGACPIPGPDACRAPTGACTTPGR + Carbamidomethyl (C)** |
|  | 617 | **399.9605** | **1196.8593** | **1196.3334** | **0.5259** | **0** | **17** | **47** | **1** | **GFSCGSAIVGGGK + Carbamidomethyl (C)** |
|  | 658 | **401.6858** | **801.3568** | **801.9277** | **-0.5709** | **0** | **17** | **52** | **1** | **GTVDGILK** |
|  | 2918 | **667.5636** | **1999.6686** | **1999.2924** | **0.3763** | **0** | **17** | **46** | **1** | **MPSSISAFEGTCVSIPCR + 2 Carbamidomethyl (C)** |
|  | 137 | **367.4240** | **1099.2499** | **1099.3044** | **-0.0546** | **1** | **17** | **73** | **1** | **EHKTAALVCK** |
|  | 1521 | **450.4692** | **898.9237** | **899.0065** | **-0.0829** | **1** | **17** | **64** | **1** | **RATGIPER** |
|  | 1941 | **488.0671** | **1461.1792** | **1460.6204** | **0.5587** | **1** | **17** | **62** | **1** | **NGVACYLTNHGRR** |
|  | 2517 | **575.1733** | **1148.3318** | **1149.3582** | **-1.0264** | **0** | **17** | **59** | **1** | **QSLLDMSLVK + Oxidation (M)** |
|  | 2874 | **664.2498** | **1326.4847** | **1325.6841** | **0.8007** | **0** | **17** | **54** | **1** | **ILFCKPCLFK + 2 Carbamidomethyl (C)** |
|  | 3371 | **798.1374** | **2391.3900** | **2390.7091** | **0.6809** | **0** | **17** | **44** | **1** | **VTVSCSGGMSLEGPSAFLCGSSLK + Carbamidomethyl (C); Oxidation (M)** |
|  | 1815 | **474.3909** | **946.7670** | **946.1029** | **0.6641** | **1** | **17** | **48** | **1** | **SVKASSVLR** |
|  | 2980 | **678.5735** | **1355.1322** | **1354.4669** | **0.6652** | **0** | **17** | **45** | **1** | **LPSLNPWDAGER** |
|  | 1891 | **480.2390** | **958.4631** | **959.1246** | **-0.6615** | **1** | **17** | **57** | **1** | **CLAPNRASK** |
|  | 2869 | **663.6617** | **1987.9631** | **1988.2447** | **-0.2817** | **2** | **17** | **51** | **1** | **TVAGGGLETISNLKAKWDK** |
|  | 890 | **407.6478** | **1219.9213** | **1219.3106** | **0.6107** | **0** | **17** | **45** | **1** | **DHQRPSGVPAR** |
|  | 785 | **405.2635** | **1212.7684** | **1212.2898** | **0.4786** | **0** | **17** | **42** | **1** | **DSQGENMFLR + Oxidation (M)** |
|  | 1460 | **444.8669** | **1331.5787** | **1331.3492** | **0.2294** | **0** | **17** | **65** | **1** | **HSSGISNTSTANR** |
|  | 3351 | **786.0197** | **1570.0246** | **1570.8970** | **-0.8723** | **1** | **17** | **47** | **1** | **LFKVSVPHVLEMR + Oxidation (M)** |
|  | 119 | **366.3256** | **1095.9546** | **1096.3038** | **-0.3492** | **1** | **17** | **59** | **1** | **LKCGHSQPVK** |
|  | 1045 | **417.1034** | **1248.2880** | **1248.3932** | **-0.1051** | **1** | **17** | **63** | **1** | **GCCEGRGPVTGR + Carbamidomethyl (C)** |
|  | 3077 | **687.2161** | **1372.4173** | **1371.5192** | **0.8982** | **0** | **17** | **54** | **1** | **APEACGVSSLQPR + Carbamidomethyl (C)** |
|  | 1897 | **481.2490** | **960.4831** | **960.9916** | **-0.5084** | **1** | **17** | **58** | **1** | **GSGVRGASDR** |
|  | 1077 | **419.1720** | **1254.4938** | **1253.5108** | **0.9830** | **0** | **17** | **65** | **1** | **TPPVGMSPQVLK** |
|  | 2294 | **532.5020** | **1062.9892** | **1062.0889** | **0.9004** | **0** | **17** | **52** | **1** | **SASPSTQQEK** |
|  | 346 | **382.2653** | **1143.7736** | **1144.3266** | **-0.5530** | **1** | **17** | **54** | **1** | **ATATRLLGWR** |
|  | 411 | **386.0755** | **1155.2042** | **1156.3971** | **-1.1928** | **0** | **17** | **53** | **1** | **LCMGSIMNSK + Carbamidomethyl (C); Oxidation (M)** |
|  | 1309 | **432.3617** | **1294.0628** | **1293.5150** | **0.5478** | **2** | **17** | **49** | **1** | **RNPPGKTVINVV** |
|  | 1787 | **471.1320** | **940.2491** | **939.1762** | **1.0729** | **0** | **17** | **51** | **1** | **LLMLHGQK** |
|  | 1067 | **419.0747** | **836.1346** | **834.9410** | **1.1935** | **0** | **17** | **66** | **1** | **CSAALASR + Carbamidomethyl (C)** |
|  | 3349 | **785.2566** | **2352.7476** | **2352.4971** | **0.2505** | **2** | **17** | **55** | **1** | **DRDQVENEAEKDLQCHAPVR** |
|  | 3429 | **837.9634** | **2510.8681** | **2511.8850** | **-1.0169** | **2** | **17** | **63** | **1** | **AGTRWVLGALLRGCGCNCSSCR + 4 Carbamidomethyl (C)** |
|  | 1357 | **435.2068** | **1302.5982** | **1303.5760** | **-0.9778** | **2** | **17** | **52** | **1** | **KFILERNPMR** |
|  | 1612 | **458.7495** | **1373.2265** | **1373.5069** | **-0.2804** | **0** | **17** | **51** | **1** | **ETLPSIWDSPTK** |
|  | 474 | **387.8770** | **1160.6090** | **1161.3158** | **-0.7069** | **1** | **17** | **68** | **1** | **AHAQKSCTCR + Carbamidomethyl (C)** |
|  | 196 | **369.3084** | **1104.9029** | **1104.2991** | **0.6038** | **0** | **17** | **52** | **1** | **QQLLIGAYAK** |
|  | 755 | **405.0041** | **807.9933** | **806.9725** | **1.0209** | **0** | **17** | **53** | **1** | **FAPAVMR + Oxidation (M)** |
|  | 1890 | **480.2307** | **958.4467** | **959.1017** | **-0.6550** | **2** | **17** | **59** | **1** | **SEAVAKKAR** |
|  | 3275 | **743.9617** | **2228.8630** | **2228.5351** | **0.3279** | **2** | **17** | **49** | **1** | **CGSQRRSCSWAGLGLGSMSLR + Oxidation (M)** |
|  | 942 | **408.8116** | **1223.4125** | **1223.3805** | **0.0320** | **0** | **17** | **60** | **1** | **SQPSVSQMGMR + Oxidation (M)** |
|  | 2677 | **606.7881** | **1211.5614** | **1211.2836** | **0.2778** | **2** | **17** | **52** | **1** | **CDGDRDCKDK + Carbamidomethyl (C)** |
|  | 3049 | **685.3469** | **1368.6791** | **1367.5271** | **1.1520** | **0** | **17** | **60** | **1** | **FSDYYMSWIR** |
|  | 1377 | **436.1784** | **870.3420** | **871.0360** | **-0.6940** | **1** | **17** | **58** | **1** | **KAQILNGK** |
|  | 3438 | **845.7541** | **2534.2403** | **2533.6114** | **0.6288** | **0** | **17** | **44** | **1** | **VTGTEGSSSTLVDYTSTSSTGGSPVR** |
|  | 1465 | **445.0843** | **888.1538** | **889.0052** | **-0.8514** | **0** | **17** | **68** | **1** | **LVTDQSVK** |
|  | 837 | **406.8710** | **1217.5907** | **1218.2316** | **-0.6408** | **1** | **17** | **51** | **1** | **AEPGSRESETR** |
|  | 921 | **408.0966** | **1221.2678** | **1221.4142** | **-0.1464** | **2** | **17** | **62** | **1** | **GSVNCRCAAKR + Carbamidomethyl (C)** |
|  | 3140 | 713.4896 | 2137.4467 | 2137.5042 | -0.0575 | 1 | 17 | 55 | 1 | KACAECFMAVSCATCQEIR + Carbamidomethyl (C); Oxidation (M) |
|  | 3508 | **905.9597** | **2714.8570** | **2713.9043** | **0.9526** | **1** | **17** | **57** | **1** | **LSGRDLGAPAGSMGAASCEDEELEFK + Carbamidomethyl (C); Oxidation (M)** |
|  | 3192 | **740.2491** | **2217.7251** | **2218.4659** | **-0.7408** | **1** | **17** | **55** | **1** | **CGETRLEASLCSCSDDCLQK + Carbamidomethyl (C)** |
|  | 2775 | **624.0795** | **1246.1442** | **1246.3723** | **-0.2281** | **0** | **17** | **59** | **1** | **AVNLFPAGTNSR** |
|  | 367 | **384.5500** | **1150.6277** | **1150.2634** | **0.3643** | **0** | **17** | **46** | **1** | **AANMLQQSGSK + Oxidation (M)** |
|  | 1026 | **415.1865** | **1242.5374** | **1241.5249** | **1.0125** | **1** | **17** | **59** | **1** | **CPKCVMAECK + 2 Carbamidomethyl (C); Oxidation (M)** |
|  | 2887 | **666.5035** | **1330.9923** | **1331.4948** | **-0.5025** | **0** | **17** | **50** | **1** | **DPSLSINACLNK + Carbamidomethyl (C)** |
|  | 2136 | **517.6329** | **1549.8764** | **1550.7579** | **-0.8815** | **1** | **17** | **73** | **1** | **GDGKYCDPINPCLR** |
|  | 754 | **404.9821** | **807.9495** | **807.9374** | **0.0121** | **0** | **17** | **54** | **1** | **HPVAVASK** |
|  | 1487 | **447.3292** | **892.6437** | **892.0356** | **0.6081** | **2** | **17** | **49** | **1** | **EAKTKCR + Carbamidomethyl (C)** |
|  | 3302 | **759.8895** | **2276.6464** | **2275.5637** | **1.0827** | **1** | **17** | **67** | **1** | **LRLSCAASGFSVSSNSMTWVR + Oxidation (M)** |
|  | 961 | **410.0633** | **1227.1677** | **1227.1951** | **-0.0274** | **0** | **17** | **63** | **1** | **TSEGAEGSAQGPH** |
|  | 1834 | **476.0099** | **950.0050** | **950.0898** | **-0.0848** | **1** | **17** | **62** | **1** | **FVSIDNKK** |
|  | 2181 | **520.0375** | **1038.0603** | **1038.1170** | **-0.0567** | **0** | **17** | **55** | **1** | **MAGQDAGCGR + Carbamidomethyl (C); Oxidation (M)** |
|  | 2762 | **620.7780** | **1859.3117** | **1859.1375** | **0.1742** | **1** | **17** | **61** | **1** | **FVSDQAPRGMAALCQHK** |
|  | 1287 | **431.0267** | **860.0387** | **860.9155** | **-0.8768** | **1** | **17** | **72** | **1** | **QGQTSRGK** |
|  | 1293 | **431.0927** | **860.1707** | **859.9655** | **0.2052** | **0** | **17** | **71** | **1** | **DWEALVK** |
|  | 2916 | **667.5515** | **1333.0882** | **1333.4893** | **-0.4011** | **0** | **17** | **49** | **1** | **APSPSLLPASEHK** |
|  | 604 | **399.2798** | **796.5448** | **796.9079** | **-0.3632** | **0** | **17** | **41** | **1** | **ISSLSYK** |
|  | 3028 | **684.1002** | **2049.2785** | **2048.4342** | **0.8442** | **1** | **17** | **57** | **1** | **NPEMCRVLLTHEIMCSR + Oxidation (M)** |
|  | 588 | **398.1001** | **1191.2782** | **1192.3482** | **-1.0699** | **1** | **17** | **61** | **1** | **FSSHRAAAMAK + Oxidation (M)** |
|  | 2052 | **504.9401** | **1511.7980** | **1510.7637** | **1.0343** | **2** | **17** | **58** | **1** | **MAAAPQAPGRGSLRK** |
|  | 213 | **371.0797** | **1110.2168** | **1111.2753** | **-1.0586** | **1** | **17** | **49** | **1** | **SHHTTCLKK + Carbamidomethyl (C)** |
|  | 385 | **385.0787** | **1152.2140** | **1152.3455** | **-0.1314** | **1** | **17** | **52** | **1** | **ISKAHVPSWK** |
|  | 2455 | **563.6423** | **1687.9048** | **1686.9340** | **0.9709** | **1** | **17** | **68** | **1** | **GWDLGALRVGPWGMR + Oxidation (M)** |
|  | 2198 | **520.4667** | **1038.9186** | **1038.1517** | **0.7668** | **0** | **17** | **47** | **1** | **GDQLYTTLK** |
|  | 2676 | **606.7147** | **1211.4145** | **1211.4095** | **0.0051** | **0** | **17** | **67** | **1** | **LDTTQMAMIR + 2 Oxidation (M)** |
|  | 3323 | **768.6727** | **1535.3306** | **1535.7702** | **-0.4396** | **1** | **17** | **46** | **1** | **DKPTVVCREVHPR** |
|  | 2480 | **566.2318** | **1130.4488** | **1129.3139** | **1.1350** | **2** | **17** | **62** | **1** | **GRTLRSSKPK** |
|  | 935 | **408.2498** | **814.4849** | **815.0127** | **-0.5278** | **1** | **16** | **50** | **1** | **VLSQKLK** |
|  | 1269 | **430.0832** | **1287.2274** | **1287.5020** | **-0.2746** | **0** | **16** | **69** | **1** | **ISTEINSALVLK** |
|  | 2289 | **532.2546** | **1593.7416** | **1592.8247** | **0.9169** | **2** | **16** | **63** | **1** | **GGHGCRSGVVSPVPRK** |
|  | 3185 | **738.0577** | **1474.1007** | **1473.5682** | **0.5325** | **0** | **16** | **49** | **1** | **EVSSSHMVSQGGPR + Oxidation (M)** |
|  | 3356 | **788.1168** | **2361.3283** | **2360.8188** | **0.5095** | **1** | **16** | **47** | **1** | **FIGDPVTCLKSGAICHPVFCPR** |
|  | 3436 | **845.6155** | **2533.8244** | **2534.9650** | **-1.1406** | **2** | **16** | **57** | **1** | **LTFAQLKEEVDKAASGLLSIGLCK** |
|  | 529 | **389.2266** | **776.4383** | **775.8506** | **0.5877** | **0** | **16** | **54** | **1** | **YGFYAR** |
|  | 943 | **408.8318** | **1223.4733** | **1223.3604** | **0.1129** | **1** | **16** | **63** | **1** | **VSWAGSMRQW + Oxidation (M)** |
|  | 1358 | **435.2727** | **1302.7959** | **1302.4771** | **0.3189** | **1** | **16** | **45** | **1** | **SMGGFMEDLRK + 2 Oxidation (M)** |
|  | 1449 | **443.8990** | **1328.6749** | **1328.4449** | **0.2300** | **0** | **16** | **61** | **1** | **EAPEPMELDGPK + Oxidation (M)** |
|  | 2930 | **668.5123** | **1335.0099** | **1334.5222** | **0.4877** | **0** | **16** | **53** | **1** | **ECSTMGANVLPR + Carbamidomethyl (C)** |
|  | 794 | **405.9524** | **809.8900** | **808.8393** | **1.0507** | **0** | **16** | **55** | **1** | **VPGHEDR** |
|  | 1829 | **475.6349** | **1423.8827** | **1423.6234** | **0.2592** | **2** | **16** | **63** | **1** | **AQTLPGLRGAGRAR** |
|  | 660 | **401.7421** | **1202.2040** | **1202.3197** | **-0.1157** | **0** | **16** | **65** | **1** | **HPTPLQQEPR** |
|  | 499 | **388.3846** | **774.7544** | **774.9935** | **-0.2391** | **1** | **16** | **77** | **1** | **IRFVIK** |
|  | 1101 | **420.0579** | **838.1011** | **836.9321** | **1.1690** | **0** | **16** | **55** | **1** | **GLTFGGGTK** |
|  | 2246 | **524.2883** | **1569.8426** | **1568.8012** | **1.0414** | **1** | **16** | **64** | **1** | **RLTWGCALDALHR + Carbamidomethyl (C)** |
|  | 613 | **399.7306** | **1196.1697** | **1197.3646** | **-1.1949** | **2** | **16** | **46** | **1** | **NQKCKSQSFK** |
|  | 110 | **366.2001** | **1095.5781** | **1096.1995** | **-0.6213** | **2** | **16** | **55** | **1** | **EARDFRFR** |
|  | 902 | **407.7994** | **1220.3761** | **1219.3436** | **1.0325** | **0** | **16** | **62** | **1** | **AWGQGTLVTVST** |
|  | 3539 | **1029.9253** | **3086.7537** | **3086.6966** | **0.0571** | **2** | **16** | **44** | **1** | **LAFTGSQPTWVLTQAPLLMLGLRFIRR** |
|  | 134 | **367.2788** | **1098.8143** | **1098.2930** | **0.5213** | **0** | **16** | **55** | **1** | **EASLLPLLSR** |
|  | 1291 | **431.0903** | **860.1658** | **859.0520** | **1.1138** | **2** | **16** | **73** | **1** | **SKCPRLR** |
|  | 1331 | **433.8798** | **865.7449** | **864.8594** | **0.8855** | **0** | **16** | **59** | **1** | **YNDGSPGR** |
|  | 1742 | **466.2095** | **930.4042** | **929.9807** | **0.4236** | **1** | **16** | **64** | **1** | **GSGGGGRGGLR** |
|  | 776 | **405.1555** | **1212.4444** | **1212.4356** | **0.0088** | **1** | **16** | **56** | **1** | **SLCMFEIPKE + Oxidation (M)** |
|  | 1234 | **428.4315** | **1282.2722** | **1283.4124** | **-1.1402** | **0** | **16** | **58** | **1** | **FHMGLYGETGR + Oxidation (M)** |
|  | 135 | **367.3051** | **1098.8933** | **1099.2614** | **-0.3681** | **0** | **16** | **58** | **1** | **MPPQLQETR** |
|  | 664 | **401.8679** | **801.7209** | **801.9310** | **-0.2101** | **1** | **16** | **71** | **1** | **KLGTAGQK** |
|  | 914 | **407.9648** | **1220.8724** | **1220.3134** | **0.5589** | **0** | **16** | **62** | **1** | **CLTAQQQSGER** |
|  | 3102 | **697.5986** | **1393.1825** | **1393.6507** | **-0.4682** | **1** | **16** | **47** | **1** | **GTLSLMPEFKVR + Oxidation (M)** |
|  | 687 | **402.6545** | **1204.9415** | **1205.3668** | **-0.4253** | **0** | **16** | **56** | **1** | **RPGAPSLSPAPR** |
|  | 761 | **405.0629** | **1212.1666** | **1211.3649** | **0.8017** | **2** | **16** | **56** | **1** | **SPPPKATEEKK** |
|  | 1186 | **423.1752** | **844.3357** | **844.9162** | **-0.5805** | **1** | **16** | **69** | **1** | **TARGTPSR** |
|  | 2292 | **532.4421** | **1062.8695** | **1063.2077** | **-0.3382** | **1** | **16** | **52** | **1** | **SLFGQKEVR** |
|  | 1967 | **489.7443** | **1466.2107** | **1466.6019** | **-0.3912** | **1** | **16** | **51** | **1** | **GHVTERQQIVSGR** |
|  | 3035 | **684.1997** | **2049.5769** | **2049.4204** | **0.1565** | **1** | **16** | **59** | **1** | **YLCPFACLQKCSVSCGR + 3 Carbamidomethyl (C)** |
|  | 704 | **403.2901** | **1206.8481** | **1207.4010** | **-0.5528** | **1** | **16** | **55** | **1** | **QGLSMTAKTVR + Oxidation (M)** |
|  | 2889 | **666.5251** | **1331.0354** | **1330.4275** | **0.6079** | **1** | **16** | **51** | **1** | **KCHSSGEVQNGK + Carbamidomethyl (C)** |
|  | 1221 | **427.4191** | **852.8234** | **853.1036** | **-0.2802** | **1** | **16** | **64** | **1** | **KLALAPIK** |
|  | 2347 | 538.8981 | 1613.6722 | 1613.8571 | -0.1848 | 1 | 16 | 61 | 1 | MIFRVSAEGSQACAK + Oxidation (M) |
|  | 2959 | **670.6179** | **1339.2211** | **1339.4940** | **-0.2729** | **2** | **16** | **51** | **1** | **ASDYSKKSNVLK** |
|  | 364 | **384.3519** | **766.6890** | **767.8734** | **-1.1844** | **0** | **16** | **54** | **1** | **QISGVHK** |
|  | 2234 | **523.3333** | **1566.9778** | **1566.8006** | **0.1772** | **2** | **16** | **59** | **1** | **CQKVPSGDNPYKCK** |
|  | 2606 | **593.0293** | **1776.0657** | **1775.9230** | **0.1427** | **2** | **16** | **64** | **1** | **SRLSGGTTHYAASVRGR** |
|  | 14 | **360.5024** | **1078.4850** | **1078.1578** | **0.3272** | **1** | **16** | **68** | **1** | **RGDMSSNPAK + Oxidation (M)** |
|  | 671 | **401.9994** | **1202.9759** | **1203.2399** | **-0.2640** | **0** | **16** | **73** | **1** | **NTTSCNSGTYR** |
|  | 836 | **406.8333** | **1217.4776** | **1218.4469** | **-0.9693** | **0** | **16** | **59** | **1** | **MMEVGAKPAPR + 2 Oxidation (M)** |
|  | 162 | **369.1877** | **1104.5411** | **1105.3089** | **-0.7678** | **1** | **16** | **55** | **1** | **MDLGIGLSKR + Oxidation (M)** |
|  | 1501 | **449.0333** | **1344.0778** | **1343.5703** | **0.5076** | **2** | **16** | **56** | **1** | **NLKKWTSPLEK** |
|  | 1652 | **460.0379** | **1377.0914** | **1377.5053** | **-0.4139** | **0** | **16** | **70** | **1** | **GTSLHLAGPGGTPGR** |
|  | 1811 | **474.1085** | **946.2023** | **946.1277** | **0.0746** | **0** | **16** | **69** | **1** | **MPPHVPPR + Oxidation (M)** |
|  | 1115 | **420.5471** | **839.0793** | **838.8718** | **0.2076** | **2** | **16** | **63** | **1** | **DHAGGRRA** |
|  | 1321 | 433.0001 | 863.9855 | 862.9694 | 1.0161 | 0 | 16 | 63 | 1 | GTPGLSFGK |
|  | 442 | **386.9214** | **1157.7421** | **1158.4529** | **-0.7108** | **0** | **16** | **70** | **1** | **IITVMSMGMK + 3 Oxidation (M)** |
|  | 1686 | **461.9203** | **1382.7388** | **1383.4223** | **-0.6835** | **0** | **16** | **61** | **1** | **GSEMDNNCSPTR + Carbamidomethyl (C); Oxidation (M)** |
|  | 1807 | **473.7118** | **945.4089** | **945.0302** | **0.3787** | **0** | **16** | **56** | **1** | **SSLWEAPR** |
|  | 987 | **412.1933** | **822.3718** | **822.9982** | **-0.6265** | **2** | **16** | **62** | **1** | **AHRAKLK** |
|  | 505 | **388.6748** | **775.3348** | **774.8163** | **0.5184** | **0** | **16** | **60** | **1** | **EIAGEEK** |
|  | 981 | **411.4612** | **1231.3613** | **1230.6031** | **0.7582** | **0** | **16** | **80** | **1** | **MMLSCLFLLK + 2 Oxidation (M)** |
|  | 1631 | **459.0862** | **1374.2366** | **1374.4999** | **-0.2633** | **0** | **16** | **72** | **1** | **ELSQGGCMSSFR + Carbamidomethyl (C); Oxidation (M)** |
|  | 3051 | **685.4255** | **1368.8363** | **1369.6357** | **-0.7994** | **1** | **16** | **67** | **1** | **QQSPLMAVLRAR** |
|  | 3173 | **734.5590** | **1467.1033** | **1466.5339** | **0.5693** | **1** | **16** | **57** | **1** | **YGGGGCYEEYRGR** |
|  | 93 | **365.2357** | **1092.6850** | **1092.1628** | **0.5222** | **0** | **16** | **53** | **1** | **RPFSDSGQAK** |
|  | 446 | **386.9486** | **1157.8236** | **1157.3205** | **0.5030** | **2** | **16** | **71** | **1** | **GEAKGIIKEGR** |
|  | 762 | **405.0717** | **1212.1929** | **1212.2452** | **-0.0523** | **0** | **16** | **59** | **1** | **GSSGSSGMGDGAVK + Oxidation (M)** |
|  | 3052 | **685.4399** | **2053.2976** | **2053.3250** | **-0.0273** | **2** | **16** | **67** | **1** | **RISVQPSSSLSARMMSGSR + Oxidation (M)** |
|  | 37 | **362.9778** | **1085.9111** | **1085.2531** | **0.6580** | **1** | **16** | **64** | **1** | **IDCSMKTSK + Carbamidomethyl (C); Oxidation (M)** |
|  | 381 | **385.0190** | **768.0231** | **767.9166** | **0.1066** | **1** | **16** | **55** | **1** | **GKVTVHK** |
|  | 1315 | **432.8358** | **1295.4854** | **1296.5419** | **-1.0565** | **2** | **16** | **66** | **1** | **CLNGNPPKRLK + Carbamidomethyl (C)** |
|  | 1324 | **433.1002** | **1296.2785** | **1297.4574** | **-1.1789** | **0** | **16** | **65** | **1** | **TCDPVEMSYPR** |
|  | 2908 | **666.8809** | **1997.6204** | **1997.4436** | **0.1768** | **0** | **16** | **57** | **1** | **LLISIIIMVSASSSSCMGGK** |
|  | 2958 | **670.4429** | **2008.3064** | **2007.1569** | **1.1495** | **0** | **16** | **64** | **1** | **DEWLIDTWGQGTLVTVSS** |
|  | 2582 | **591.4207** | **1771.2400** | **1770.0609** | **1.1791** | **2** | **16** | **55** | **1** | **IGKIGEGSYGVVFKCR + Carbamidomethyl (C)** |
|  | 3175 | **735.3777** | **2203.1109** | **2202.5941** | **0.5167** | **1** | **16** | **62** | **1** | **MGHAMATGLVLIGTGSSVKLDK + Oxidation (M)** |
|  | 426 | **386.2932** | **770.5716** | **770.9171** | **-0.3454** | **0** | **16** | **46** | **1** | **LPSSVIR** |
|  | 641 | **401.0829** | **1200.2266** | **1199.3140** | **0.9125** | **0** | **16** | **72** | **1** | **LENNEAQLLR** |
|  | 1296 | **431.1621** | **1290.4642** | **1290.3800** | **0.0841** | **0** | **16** | **75** | **1** | **EYQWLHTGEK** |
|  | 1545 | **452.0066** | **901.9984** | **901.1251** | **0.8733** | **0** | **16** | **78** | **1** | **MLSIVPGGK** |
|  | 3407 | **824.1194** | **1646.2240** | **1645.8806** | **0.3433** | **2** | **16** | **50** | **1** | **ERIHTGKKPYECK + Carbamidomethyl (C)** |
|  | 1961 | **489.2425** | **976.4701** | **976.0625** | **0.4077** | **0** | **16** | **68** | **1** | **DGLDSNPMK** |
|  | 2570 | **586.6667** | **1756.9781** | **1757.0603** | **-0.0823** | **1** | **16** | **78** | **1** | **TILKESFPCIWYTR** |
|  | 3284 | **748.7806** | **2243.3196** | **2243.4735** | **-0.1540** | **0** | **16** | **66** | **1** | **YIVIEDPFDLNHNLGAGLSR** |
|  | 1258 | **429.1723** | **856.3298** | **855.8907** | **0.4392** | **0** | **16** | **64** | **1** | **GYYDNPK** |
|  | 2704 | **610.1105** | **1827.3092** | **1827.8667** | **-0.5574** | **1** | **16** | **65** | **1** | **EGPAGGTGGSGGPGGSLGSRGR** |
|  | 2924 | **667.9185** | **2000.7332** | **2000.3633** | **0.3699** | **1** | **16** | **55** | **1** | **MSELTSFCPPASMIRLK + Carbamidomethyl (C); 2 Oxidation (M)** |
|  | 35 | **362.2233** | **722.4319** | **721.8018** | **0.6301** | **0** | **16** | **49** | **1** | **DFVSVR** |
|  | 1385 | **436.3432** | **870.6717** | **871.0131** | **-0.3414** | **0** | **16** | **50** | **1** | **TPTKPPCQ** |
|  | 1469 | **445.1671** | **888.3195** | **889.0085** | **-0.6889** | **1** | **16** | **74** | **1** | **RSTTLSPK** |
|  | 884 | **407.5677** | **1219.6811** | **1219.4083** | **0.2728** | **2** | **16** | **60** | **1** | **GKKEGGSSMLPL + Oxidation (M)** |
|  | 1042 | 416.1843 | 1245.5306 | 1246.4338 | -0.9032 | 1 | 16 | 76 | 1 | SPVLEKSSMPR + Oxidation (M) |
|  | 1513 | **450.1925** | **898.3703** | **898.0630** | **0.3072** | **1** | **16** | **67** | **1** | **KAQLWPR** |
|  | 1494 | **448.2906** | **1341.8496** | **1342.3440** | **-0.4944** | **1** | **16** | **51** | **1** | **EDGKESTSSDCK + Carbamidomethyl (C)** |
|  | 2396 | **549.5139** | **1097.0129** | **1096.2127** | **0.8003** | **0** | **16** | **54** | **1** | **AAPAYGMDVW + Oxidation (M)** |
|  | 2986 | **679.7776** | **2036.3106** | **2037.3040** | **-0.9934** | **1** | **16** | **78** | **1** | **TCEMESRCVPQAGVQWR + Carbamidomethyl (C)** |
|  | 977 | **411.2451** | **1230.7131** | **1230.4376** | **0.2755** | **0** | **16** | **57** | **1** | **CGFVVCAECSR + Carbamidomethyl (C)** |
|  | 2935 | **668.6770** | **2003.0088** | **2002.2283** | **0.7805** | **2** | **16** | **66** | **1** | **LIEDNEATAREGAKFPIK** |
|  | 561 | **392.2225** | **1173.6453** | **1174.3377** | **-0.6924** | **2** | **16** | **52** | **1** | **GRRGAAAGSLCR** |
|  | 595 | **398.9589** | **1193.8545** | **1193.2898** | **0.5647** | **1** | **16** | **57** | **1** | **CAERHYDTAK** |
|  | 692 | **402.9521** | **803.8894** | **803.9072** | **-0.0179** | **1** | **16** | **74** | **1** | **SLTATRR** |
|  | 2476 | **565.6909** | **1694.0504** | **1692.9617** | **1.0887** | **1** | **16** | **81** | **1** | **QPSRAFACTLPGCWR** |
|  | 2808 | **637.0049** | **1907.9927** | **1907.2648** | **0.7279** | **0** | **16** | **61** | **1** | **CHFCQSISHMVASCPLK + Oxidation (M)** |
|  | 11 | **360.4909** | **1078.4506** | **1078.1329** | **0.3178** | **0** | **16** | **79** | **1** | **DFPSSEIQR** |
|  | 1414 | **439.5015** | **1315.4823** | **1314.4861** | **0.9962** | **0** | **16** | **85** | **1** | **NFSGSIPAVTPPK** |
|  | 3285 | **751.8068** | **2252.3981** | **2252.5731** | **-0.1750** | **2** | **16** | **72** | **1** | **KSLKCEQHLGHNAMYWYK + Oxidation (M)** |
|  | 451 | **387.0070** | **1157.9987** | **1157.3008** | **0.6979** | **0** | **16** | **73** | **1** | **HQGVTVGMGQK + Oxidation (M)** |
|  | 1797 | **472.3074** | **1413.9001** | **1413.5560** | **0.3442** | **0** | **16** | **54** | **1** | **AESEGAVLMTHPR + Oxidation (M)** |
|  | 2062 | **505.2869** | **1512.8385** | **1512.7316** | **0.1069** | **0** | **16** | **61** | **1** | **MGAGLGFGSVAPHSPK** |
|  | 2319 | **536.0143** | **1605.0209** | **1605.9394** | **-0.9185** | **2** | **16** | **65** | **1** | **ALVMKFIQDTPSKK** |
|  | 3256 | **742.3048** | **2223.8923** | **2224.3081** | **-0.4159** | **1** | **16** | **63** | **1** | **AADSEAAAGGAGAGAAAGVGAGPQGRR** |
|  | 1993 | **494.8420** | **987.6693** | **987.1116** | **0.5577** | **1** | **16** | **65** | **1** | **QAVSGQAAKK** |
|  | 28 | **362.1636** | **1083.4687** | **1084.2236** | **-0.7548** | **1** | **16** | **54** | **1** | **SKQLPSAEPK** |
|  | 1575 | **456.2003** | **1365.5789** | **1366.5890** | **-1.0101** | **1** | **16** | **64** | **1** | **VPVPTGPRSCPTR** |
|  | 2803 | **634.5299** | **1900.5675** | **1900.1016** | **0.4659** | **2** | **16** | **51** | **1** | **RLSDGSLSSRHTTLLTR** |
|  | 1848 | **476.2118** | **1425.6131** | **1424.4408** | **1.1723** | **0** | **16** | **66** | **1** | **QDNHQHPQQHR** |
|  | 1307 | **432.2913** | **862.5679** | **861.9003** | **0.6676** | **1** | **16** | **57** | **1** | **QGASSERK** |
|  | 1628 | **459.0474** | **916.0799** | **917.0252** | **-0.9452** | **2** | **16** | **76** | **1** | **NKVGSTRR** |
|  | 2701 | **610.0689** | **1218.1230** | **1217.3295** | **0.7935** | **0** | **16** | **68** | **1** | **EANGIVTTTGVR** |
|  | 3250 | **741.9957** | **2222.9650** | **2223.4625** | **-0.4975** | **2** | **16** | **52** | **1** | **KERLPAFLESDCYFEYR + Carbamidomethyl (C)** |
|  | 174 | **369.2525** | **1104.7354** | **1104.2680** | **0.4675** | **2** | **16** | **58** | **1** | **HRSDRMMR + Oxidation (M)** |
|  | 657 | **401.6795** | **801.3442** | **801.9279** | **-0.5836** | **1** | **16** | **63** | **1** | **TPGKTSLV** |
|  | 2770 | **623.1976** | **1866.5707** | **1867.1565** | **-0.5857** | **2** | **16** | **70** | **1** | **VGSAAQTRAMKQVAGTMK + 2 Oxidation (M)** |
|  | 1174 | **422.2558** | **842.4968** | **841.9949** | **0.5019** | **0** | **16** | **60** | **1** | **GITLSVRP** |
|  | 2365 | 540.4541 | 1618.3401 | 1617.7762 | 0.5639 | 0 | 16 | 55 | 1 | DTSTSTVYMGLSSLR |
|  | 3326 | **771.8926** | **2312.6556** | **2311.5544** | **1.1011** | **2** | **16** | **76** | **1** | **KNYGCCDTSPTAPRLAATATR + 2 Carbamidomethyl (C)** |
|  | 1959 | **488.5687** | **975.1227** | **975.2069** | **-0.0842** | **0** | **16** | **88** | **1** | **CCMFSALK + Carbamidomethyl (C); Oxidation (M)** |
|  | 2967 | **672.6829** | **2015.0264** | **2015.3959** | **-0.3695** | **0** | **16** | **65** | **1** | **YIAICNPLLYSVVMSQK + Carbamidomethyl (C); Oxidation (M)** |
|  | 1749 | **467.2823** | **932.5498** | **933.0840** | **-0.5343** | **0** | **16** | **61** | **1** | **EMAQIGLR + Oxidation (M)** |
|  | 2601 | **592.9720** | **1775.8940** | **1775.0657** | **0.8282** | **1** | **16** | **67** | **1** | **HHEGACVSMCLCARK + 2 Carbamidomethyl (C); Oxidation (M)** |
|  | 1979 | **491.4904** | **1471.4490** | **1470.7080** | **0.7409** | **0** | **16** | **66** | **1** | **EAPLPFILLGGSEK** |
|  | 2543 | **581.1362** | **1740.3865** | **1739.9010** | **0.4855** | **0** | **16** | **73** | **1** | **MVNGVTPSEELGEHPK + Oxidation (M)** |
|  | 1060 | **418.2173** | **1251.6297** | **1252.3769** | **-0.7472** | **1** | **16** | **62** | **1** | **TPEPPNLSRNK** |
|  | 156 | **369.1602** | **1104.4586** | **1103.2899** | **1.1687** | **1** | **16** | **68** | **1** | **AEAMKSPELK** |
|  | 160 | **369.1826** | **1104.5257** | **1104.2680** | **0.2577** | **2** | **16** | **63** | **1** | **HRSDRMMR + Oxidation (M)** |
|  | 537 | **389.5336** | **1165.5787** | **1166.2164** | **-0.6377** | **0** | **16** | **71** | **1** | **QAEVLSDSCTG + Carbamidomethyl (C)** |
|  | 724 | **403.9970** | **805.9792** | **805.9397** | **0.0396** | **0** | **16** | **70** | **1** | **EVSACIK + Carbamidomethyl (C)** |
|  | 1354 | **435.1405** | **1302.3993** | **1301.3201** | **1.0792** | **0** | **16** | **65** | **1** | **SHDTVQEAATSR** |
|  | 1190 | **423.2426** | **1266.7056** | **1266.5193** | **0.1862** | **2** | **16** | **66** | **1** | **VRLGPAPCRAAR** |
|  | 1422 | **441.0901** | **1320.2481** | **1320.6427** | **-0.3946** | **0** | **16** | **72** | **1** | **CLLLLSPHSLPK** |
|  | 482 | **388.0715** | **1161.1923** | **1162.2525** | **-1.0602** | **0** | **16** | **83** | **1** | **LTAHSNYTQK** |
|  | 1203 | **424.4688** | **1270.3841** | **1271.5473** | **-1.1633** | **0** | **16** | **92** | **1** | **VTLTLILHFSK** |
|  | 1655 | **460.1434** | **1377.4081** | **1376.5821** | **0.8261** | **2** | **16** | **76** | **1** | **LRDMLSDVRQK + Oxidation (M)** |
|  | 1740 | **466.1776** | **930.3403** | **931.0500** | **-0.7096** | **1** | **16** | **73** | **1** | **IHSGKSFR** |
|  | 126 | **367.1369** | **1098.3885** | **1099.2398** | **-0.8512** | **0** | **16** | **71** | **1** | **SAPFLTPGPGR** |
|  | 1679 | **461.7773** | **921.5399** | **921.9968** | **-0.4569** | **1** | **16** | **57** | **1** | **EQRAQYK** |
|  | 1730 | **465.3901** | **1393.1482** | **1392.5565** | **0.5917** | **0** | **16** | **56** | **1** | **TPPQSQTPGALPAK** |
|  | 2017 | **499.6346** | **1495.8818** | **1494.7975** | **1.0842** | **0** | **16** | **72** | **1** | **VTAQATVLMYILR + Oxidation (M)** |
|  | 349 | **382.3117** | **1143.9130** | **1143.2973** | **0.6157** | **2** | **16** | **67** | **1** | **ERNVQRLTK** |
|  | 782 | **405.1792** | **1212.5155** | **1211.5038** | **1.0118** | **1** | **16** | **63** | **1** | **VMIRIHPCAR + Oxidation (M)** |
|  | 3277 | **744.7167** | **1487.4186** | **1487.8068** | **-0.3882** | **1** | **16** | **59** | **1** | **MKYLKPMEEMR + 2 Oxidation (M)** |
|  | 1590 | **457.2897** | **1368.8470** | **1368.4805** | **0.3666** | **1** | **16** | **59** | **1** | **HARESECPHFR** |
|  | 2113 | **513.4471** | **1537.3193** | **1537.8009** | **-0.4816** | **1** | **16** | **54** | **1** | **MILGTDRVEQMTK + Oxidation (M)** |
|  | 2270 | **528.8726** | **1583.5955** | **1584.7263** | **-1.1308** | **0** | **16** | **61** | **1** | **ILEVVNQIQDEER** |
|  | 2785 | **628.2081** | **1254.4015** | **1255.3361** | **-0.9346** | **0** | **16** | **68** | **1** | **GEAGNSDSMCLR + Oxidation (M)** |
|  | 1633 | **459.1268** | **1374.3582** | **1374.5014** | **-0.1432** | **0** | **16** | **80** | **1** | **AFNQSSILTTHR** |
|  | 1281 | **430.8886** | **859.7623** | **858.9792** | **0.7832** | **1** | **16** | **85** | **1** | **TLKSEPGK** |
|  | 1617 | **458.8209** | **915.6269** | **916.0338** | **-0.4068** | **2** | **16** | **75** | **1** | **KKAGAENAK** |
|  | 1912 | 483.7343 | 965.4538 | 964.9704 | 0.4835 | 0 | 16 | 58 | 1 | SSLVNESET |
|  | 2179 | **519.9297** | **1037.8447** | **1037.1736** | **0.6711** | **1** | **16** | **67** | **1** | **AHQKVAIDR** |
|  | 434 | **386.8028** | **1157.3862** | **1157.2991** | **0.0872** | **0** | **16** | **73** | **1** | **CGVQSFYTPR** |
|  | 457 | 387.1488 | 1158.4243 | 1157.3438 | 1.0806 | 0 | 16 | 80 | 1 | VLSGHLMQTR + Oxidation (M) |
|  | 502 | **388.4345** | **1162.2813** | **1161.3524** | **0.9289** | **0** | **16** | **98** | **1** | **VEITHCGQMK + Oxidation (M)** |
|  | 598 | **399.1104** | **1194.3090** | **1195.3671** | **-1.0581** | **1** | **16** | **63** | **1** | **GLKAEMEDMR + Oxidation (M)** |
|  | 1264 | **429.5118** | **1285.5133** | **1284.4384** | **1.0749** | **0** | **16** | **88** | **1** | **ALGIHIDGDDMK** |
|  | 3034 | **684.1957** | **1366.3766** | **1365.4948** | **0.8818** | **1** | **16** | **67** | **1** | **GLTRGPTQGPPER** |
|  | 3014 | **683.8594** | **2048.5560** | **2047.4428** | **1.1131** | **1** | **16** | **72** | **1** | **TYAYLENRVRPLVLVIK** |
|  | 433 | **386.7388** | **1157.1942** | **1156.2928** | **0.9015** | **1** | **16** | **72** | **1** | **CNECEKAFR + Carbamidomethyl (C)** |
|  | 501 | **388.4198** | **1162.2371** | **1163.3880** | **-1.1509** | **1** | **16** | **99** | **1** | **AGKLCLSSTVK + Carbamidomethyl (C)** |
|  | 740 | **404.1108** | **1209.3103** | **1209.2727** | **0.0377** | **2** | **16** | **71** | **1** | **RGRATDSFSGR** |
|  | 1353 | **435.1143** | **1302.3206** | **1302.5383** | **-0.2177** | **0** | **16** | **65** | **1** | **AAPSVTLFPPSLC** |
|  | 1555 | **452.3265** | **1353.9573** | **1354.5899** | **-0.6326** | **1** | **16** | **64** | **1** | **EDIMVMDTKLK + 2 Oxidation (M)** |
|  | 1265 | **429.8636** | **1286.5688** | **1285.6419** | **0.9269** | **0** | **16** | **78** | **1** | **MLPALGMACPPK + Carbamidomethyl (C)** |
|  | 2620 | **593.7734** | **1778.2981** | **1777.9489** | **0.3492** | **0** | **16** | **72** | **1** | **LSSVTAADTAVYYCASR** |
|  | 2691 | **608.7069** | **1215.3990** | **1216.4324** | **-1.0333** | **1** | **16** | **88** | **1** | **EFICKFCGR + 2 Carbamidomethyl (C)** |
|  | 3460 | **855.5193** | **1709.0238** | **1708.8439** | **0.1799** | **1** | **16** | **68** | **1** | **TRDMGGYSTTTDFIK + Oxidation (M)** |
|  | 1461 | **444.9281** | **887.8414** | **888.8791** | **-1.0377** | **0** | **16** | **83** | **1** | **ADGNEDLR** |
|  | 2232 | **523.2534** | **1566.7381** | **1565.7298** | **1.0083** | **1** | **16** | **75** | **1** | **GAASASVREPTPLPGR** |
|  | 2425 | **556.1051** | **1665.2931** | **1665.8903** | **-0.5971** | **2** | **16** | **71** | **1** | **LAFPKSPPSVSHKDR** |
|  | 1880 | **479.1799** | **956.3451** | **956.1024** | **0.2426** | **1** | **16** | **73** | **1** | **QRTIPWR** |
|  | 857 | **407.1386** | **1218.3935** | **1218.4086** | **-0.0151** | **0** | **16** | **69** | **1** | **RPGVIISPSHR** |
|  | 881 | **407.4729** | **1219.3965** | **1219.4082** | **-0.0117** | **1** | **16** | **83** | **1** | **CLKLELDTER** |
|  | 310 | **378.2902** | **1131.8483** | **1132.2713** | **-0.4229** | **1** | **16** | **50** | **1** | **KHFLYGDPR** |
|  | 2565 | **585.7172** | **1754.1293** | **1753.8215** | **0.3079** | **0** | **16** | **79** | **1** | **QESGTGPASPGQAPENVK** |
|  | 2763 | **621.1152** | **1860.3235** | **1861.3436** | **-1.0201** | **1** | **16** | **68** | **1** | **RVIQCGAALMLALGMIGK + Oxidation (M)** |
|  | 3293 | **756.9299** | **1511.8450** | **1512.7083** | **-0.8634** | **2** | **16** | **71** | **1** | **KGKDYYQTLGLAR** |
|  | 293 | **377.1272** | **1128.3594** | **1128.3009** | **0.0585** | **0** | **16** | **68** | **1** | **LPSPDCPFPR** |
|  | 1850 | **476.2170** | **1425.6288** | **1426.6225** | **-0.9937** | **1** | **16** | **71** | **1** | **DGGCNHMQCSKCK + Oxidation (M)** |
|  | 2302 | **533.7969** | **1598.3686** | **1597.8578** | **0.5109** | **2** | **16** | **56** | **1** | **EKPYKCVECGKTR + Carbamidomethyl (C)** |
|  | 520 | **389.1485** | **776.2822** | **775.9566** | **0.3256** | **0** | **16** | **78** | **1** | **ILAMANK + Oxidation (M)** |
|  | 1572 | **456.1826** | **910.3503** | **910.1121** | **0.2383** | **2** | **16** | **70** | **1** | **KALPPEKK** |
|  | 2087 | **507.2603** | **1012.5057** | **1012.2039** | **0.3018** | **1** | **16** | **72** | **1** | **EKAVPLISR** |
|  | 1382 | **436.2098** | **1305.6072** | **1306.3810** | **-0.7739** | **0** | **16** | **71** | **1** | **YLSLSGNHSSNK** |
|  | 2493 | **570.1716** | **1707.4925** | **1706.8278** | **0.6647** | **1** | **16** | **73** | **1** | **RLADMYGTGQESLYS + Oxidation (M)** |
|  | 2548 | **582.2543** | **1743.7406** | **1743.1865** | **0.5542** | **0** | **16** | **76** | **1** | **VLAVVVIGAVVAAVMCR + Carbamidomethyl (C); Oxidation (M)** |
|  | 329 | **380.1336** | **1137.3787** | **1137.2562** | **0.1225** | **2** | **16** | **81** | **1** | **HRGRTNIQR** |
|  | 409 | **386.0707** | **1155.1899** | **1154.1810** | **1.0088** | **1** | **16** | **67** | **1** | **EEKEEYEAK** |
|  | 2640 | **596.5386** | **1786.5937** | **1787.1411** | **-0.5473** | **0** | **16** | **63** | **1** | **MTHCCSPCCQPTCCR + 2 Carbamidomethyl (C)** |
|  | 3048 | **685.2687** | **1368.5226** | **1367.4690** | **1.0535** | **0** | **16** | **76** | **1** | **WPGSHLATSQQR** |
|  | 219 | **371.1611** | **740.3074** | **740.9342** | **-0.6267** | **2** | **16** | **54** | **1** | **KKIPQK** |
|  | 2735 | **612.9306** | **1835.7696** | **1836.1405** | **-0.3708** | **0** | **16** | **60** | **1** | **MAFFTGLWGPFTCVSR + Oxidation (M)** |
|  | 2862 | **662.3505** | **1984.0292** | **1984.5343** | **-0.5051** | **1** | **16** | **71** | **1** | **MALAAILVTLVILPVFRK + Oxidation (M)** |
|  | 155 | **369.1462** | **1104.4165** | **1104.0808** | **0.3358** | **0** | **16** | **77** | **1** | **EYSNFDSDK** |
|  | 719 | **403.9511** | **1208.8312** | **1208.2532** | **0.5781** | **0** | **16** | **75** | **1** | **DMDNAVDGLDK + Oxidation (M)** |
|  | 745 | **404.3145** | **806.6143** | **806.8632** | **-0.2489** | **0** | **16** | **59** | **1** | **DEPPPPR** |
|  | 397 | **385.4251** | **1153.2532** | **1152.1286** | **1.1246** | **0** | **16** | **75** | **1** | **GSGFPDGEGSSR** |
|  | 418 | **386.1618** | **770.3088** | **769.9291** | **0.3797** | **1** | **16** | **64** | **1** | **ELPGVKK** |
|  | 963 | **410.1396** | **1227.3966** | **1226.3394** | **1.0572** | **0** | **16** | **80** | **1** | **QLLAGTGPGGEAR** |
|  | 1328 | **433.3909** | **864.7670** | **865.0068** | **-0.2398** | **0** | **16** | **62** | **1** | **LLGSNSMK + Oxidation (M)** |
|  | 1452 | **443.9107** | **885.8066** | **885.0594** | **0.7472** | **0** | **16** | **78** | **1** | **ITTIPNVK** |
|  | 1384 | **436.3431** | **870.6714** | **870.9915** | **-0.3201** | **1** | **16** | **56** | **1** | **KAGASFYK** |
|  | 3067 | **686.1857** | **1370.3566** | **1371.4562** | **-1.0997** | **1** | **16** | **70** | **1** | **RAQEAEEAGAALR** |
|  | 1238 | **428.7525** | **855.4902** | **854.9905** | **0.4997** | **0** | **16** | **57** | **1** | **IGNVEVPK** |
|  | 1824 | **475.0258** | **1422.0553** | **1422.6270** | **-0.5717** | **0** | **16** | **82** | **1** | **NICSSCGLEILDR** |
|  | 2883 | **666.3992** | **1330.7836** | **1331.6226** | **-0.8390** | **0** | **16** | **73** | **1** | **TAPSLVMVLSWK** |
|  | 1720 | **464.1564** | **926.2980** | **925.1464** | **1.1516** | **1** | **16** | **72** | **1** | **YITCLKGK** |
|  | 3432 | **842.0365** | **1682.0582** | **1680.9013** | **1.1569** | **0** | **16** | **71** | **1** | **LHVNYLTSLHLQDK** |
|  | 1318 | **432.9463** | **1295.8169** | **1296.5319** | **-0.7150** | **1** | **16** | **74** | **1** | **QYESLKILICS** |
|  | 2714 | **611.1320** | **1830.3737** | **1831.0348** | **-0.6611** | **2** | **16** | **76** | **1** | **RKSSPETWASSISVPAK** |
|  | 150 | **369.0648** | **1104.1721** | **1104.2564** | **-0.0843** | **1** | **16** | **84** | **1** | **GEPPAPKSPPK** |
|  | 528 | **389.1980** | **776.3813** | **775.9998** | **0.3815** | **1** | **16** | **69** | **1** | **KMGALLK + Oxidation (M)** |
|  | 1125 | **421.5757** | **1261.7049** | **1261.3357** | **0.3691** | **0** | **16** | **71** | **1** | **DELTASLEEVR** |
|  | 1299 | **431.2466** | **1290.7175** | **1291.4742** | **-0.7567** | **1** | **16** | **74** | **1** | **NMDSKLALIDR + Oxidation (M)** |
|  | 2009 | **497.5317** | **1489.5729** | **1488.7334** | **0.8395** | **1** | **16** | **89** | **1** | **CQPGFGMKGPSHVK + Oxidation (M)** |
|  | 2914 | **667.3577** | **1999.0508** | **1999.0737** | **-0.0229** | **0** | **16** | **75** | **1** | **DSQCTSGPSSYAQSLEGPGK** |
|  | 1393 | **437.0999** | **872.1850** | **872.9826** | **-0.7975** | **0** | **16** | **85** | **1** | **LEMSYSK + Oxidation (M)** |
|  | 468 | **387.8130** | **773.6113** | **773.8745** | **-0.2632** | **0** | **16** | **88** | **1** | **ITIANDK** |
|  | 2351 | **539.2832** | **1614.8274** | **1615.6841** | **-0.8566** | **0** | **16** | **77** | **1** | **CDCDHSDGCDPVHGR** |
|  | 535 | **389.4221** | **1165.2440** | **1166.2662** | **-1.0222** | **2** | **16** | **95** | **1** | **QDMSKSNGKR + Oxidation (M)** |
|  | 780 | **405.1736** | **808.3325** | **808.8608** | **-0.5283** | **1** | **16** | **68** | **1** | **KDSGSCR + Carbamidomethyl (C)** |
|  | 814 | **406.2076** | **1215.6005** | **1215.4260** | **0.1745** | **1** | **16** | **63** | **1** | **RCALLAAQANK + Carbamidomethyl (C)** |
|  | 1133 | **421.7889** | **1262.3446** | **1261.4482** | **0.8964** | **1** | **16** | **77** | **1** | **AESCKVAIIDR + Carbamidomethyl (C)** |
|  | 387 | **385.1003** | **768.1858** | **767.9164** | **0.2695** | **0** | **16** | **65** | **1** | **GPLVINR** |
|  | 2562 | **585.1481** | **1168.2814** | **1167.3586** | **0.9228** | **2** | **16** | **75** | **1** | **KTLQRAEPPK** |
|  | 184 | **369.2776** | **1104.8108** | **1104.3011** | **0.5097** | **0** | **16** | **65** | **1** | **MHPSLATMGK + 2 Oxidation (M)** |
|  | 720 | **403.9537** | **805.8926** | **805.8371** | **0.0555** | **1** | **16** | **77** | **1** | **TRTADSR** |
|  | 3514 | **913.7535** | **1825.4923** | **1825.0384** | **0.4539** | **2** | **16** | **64** | **1** | **VQRAAAGVGGSLRAQVER** |
|  | 2608 | **593.0851** | **1184.1555** | **1184.4303** | **-0.2748** | **1** | **16** | **77** | **1** | **IIHTGKKPYK** |
|  | 3238 | **741.6748** | **1481.3348** | **1480.7313** | **0.6036** | **1** | **16** | **57** | **1** | **MLVTSGAVAFGKQR + Oxidation (M)** |
|  | 1072 | **419.1379** | **836.2611** | **836.9969** | **-0.7358** | **2** | **15** | **87** | **1** | **AKTMKDK + Oxidation (M)** |
|  | 2401 | **550.5153** | **1648.5236** | **1648.7784** | **-0.2548** | **1** | **15** | **65** | **1** | **KAGDGSAAPAAAGALGAHR** |
|  | 3117 | **701.2948** | **2100.8622** | **2101.4075** | **-0.5452** | **2** | **15** | **74** | **1** | **MLSPANSLDIAMEKHQKR + 2 Oxidation (M)** |
|  | 1034 | **415.9650** | **829.9152** | **828.9149** | **1.0003** | **0** | **15** | **89** | **1** | **APASAAWR** |
|  | 1406 | **437.6824** | **1310.0249** | **1309.4265** | **0.5985** | **0** | **15** | **69** | **1** | **FGLTFQSPADAR** |
|  | 2272 | **528.9995** | **1055.9842** | **1056.1983** | **-0.2141** | **0** | **15** | **74** | **1** | **DVAHWLGCR** |
|  | 3348 | **785.2076** | **1568.4005** | **1567.7654** | **0.6351** | **0** | **15** | **71** | **1** | **SAAQSCNSSFFMCK + Carbamidomethyl (C)** |
|  | 439 | **386.8969** | **771.7790** | **770.8790** | **0.9000** | **0** | **15** | **84** | **1** | **SHKPFR** |
|  | 440 | 386.9122 | 1157.7143 | 1157.4662 | 0.2481 | 1 | 15 | 84 | 1 | IKELGMLIPK + Oxidation (M) |
|  | 2115 | **513.8505** | **1538.5292** | **1537.7459** | **0.7833** | **2** | **15** | **65** | **1** | **IRGAGGISFRCSTGR** |
|  | 2591 | **592.5533** | **1183.0918** | **1182.4195** | **0.6723** | **1** | **15** | **62** | **1** | **MSGCRVFIGR + Carbamidomethyl (C)** |
|  | 2781 | **627.6106** | **1879.8096** | **1880.1085** | **-0.2989** | **1** | **15** | **65** | **1** | **WNSLKASDTAMYYCAR** |
|  | 623 | **400.1138** | **1197.3192** | **1198.2433** | **-0.9241** | **1** | **15** | **68** | **1** | **KHDSGAADLER** |
|  | 688 | **402.7892** | **803.5636** | **802.9588** | **0.6047** | **1** | **15** | **85** | **1** | **LQSSLKK** |
|  | 1004 | 413.7383 | 1238.1927 | 1237.3009 | 0.8918 | 0 | 15 | 57 | 1 | MTLNGGGSGAGGSR + Oxidation (M) |
|  | 1068 | **419.0947** | **1254.2619** | **1255.4601** | **-1.1982** | **0** | **15** | **89** | **1** | **EIQSILPGISAK** |
|  | 1764 | **469.1683** | **1404.4826** | **1403.7115** | **0.7711** | **0** | **15** | **75** | **1** | **AFPMLCSLALHK + Carbamidomethyl (C); Oxidation (M)** |
|  | 1350 | **435.0071** | **867.9994** | **869.0202** | **-1.0208** | **1** | **15** | **68** | **1** | **LLPGADRK** |
|  | 2298 | **533.2110** | **1596.6108** | **1596.6942** | **-0.0833** | **1** | **15** | **77** | **1** | **DPPTTSGPQTDQPKK** |
|  | 600 | **399.1855** | **1194.5344** | **1194.2944** | **0.2399** | **1** | **15** | **63** | **1** | **QELRAYADTK** |
|  | 1240 | 428.9006 | 855.7864 | 856.0632 | -0.2768 | 1 | 15 | 68 | 1 | VMTSKMK + 2 Oxidation (M) |
|  | 1814 | **474.3666** | **1420.0777** | **1420.6064** | **-0.5287** | **0** | **15** | **66** | **1** | **LAFVSAVQEELSK** |
|  | 2001 | **496.2804** | **990.5459** | **990.9727** | **-0.4267** | **0** | **15** | **79** | **1** | **EWNGSENR** |
|  | 2661 | **600.5004** | **1198.9860** | **1199.4003** | **-0.4144** | **2** | **15** | **63** | **1** | **LKEVLQREGK** |
|  | 205 | **369.3737** | **1105.0988** | **1105.2940** | **-0.1952** | **1** | **15** | **91** | **1** | **NCRHFMPK + Carbamidomethyl (C); Oxidation (M)** |
|  | 1734 | **466.1054** | **1395.2940** | **1396.4195** | **-1.1255** | **1** | **15** | **82** | **1** | **SGRAPPEAEDPDR** |
|  | 2395 | **548.5518** | **1642.6331** | **1642.8470** | **-0.2139** | **1** | **15** | **76** | **1** | **ATFTYKEVLEQVSK** |
|  | 621 | **400.0959** | **1197.2654** | **1198.4620** | **-1.1966** | **0** | **15** | **70** | **1** | **MPPLRPAASCR** |
|  | 1796 | **472.2226** | **942.4304** | **942.0676** | **0.3628** | **0** | **15** | **76** | **1** | **ANGTVSIPLA** |
|  | 2539 | **580.0375** | **1737.0903** | **1736.9830** | **0.1072** | **0** | **15** | **82** | **1** | **MAEELNAVIVSIEYR** |
|  | 2978 | **676.7592** | **1351.5035** | **1350.5465** | **0.9570** | **0** | **15** | **99** | **1** | **ATNSPKPHMVPR + Oxidation (M)** |
|  | 1259 | **429.1967** | **856.3785** | **856.8804** | **-0.5018** | **0** | **15** | **76** | **1** | **LNSDSGHK** |
|  | 3347 | **785.1726** | **1568.3304** | **1567.6793** | **0.6512** | **1** | **15** | **70** | **1** | **ESCFDPGSIKNGTR + Carbamidomethyl (C)** |
|  | 992 | **412.8449** | **1235.5125** | **1234.4759** | **1.0366** | **1** | **15** | **73** | **1** | **CSRIMHMQR + Carbamidomethyl (C); Oxidation (M)** |
|  | 1528 | **451.1063** | **900.1978** | **900.1007** | **0.0971** | **2** | **15** | **86** | **1** | **QTKHMKK** |
|  | 3337 | **777.4264** | **2329.2570** | **2328.5814** | **0.6756** | **2** | **15** | **77** | **1** | **EKLELAGSQGRSFFIQSQFR** |
|  | 7 | 360.4062 | 1078.1963 | 1079.2501 | -1.0538 | 1 | 15 | 1.1e+02 | 1 | RLLDSPGPPK |
|  | 41 | **363.0505** | **1086.1293** | **1087.1512** | **-1.0220** | **2** | **15** | **79** | **1** | **QEAAGSRGRR** |
|  | 556 | **391.1953** | **1170.5638** | **1170.3410** | **0.2228** | **1** | **15** | **62** | **1** | **MPDCTSKCR + 2 Carbamidomethyl (C); Oxidation (M)** |
|  | 1365 | **435.7071** | **1304.0991** | **1303.5991** | **0.5000** | **1** | **15** | **60** | **1** | **RIIMMAWNPR + Oxidation (M)** |
|  | 2287 | **531.4326** | **1060.8503** | **1060.2053** | **0.6450** | **1** | **15** | **67** | **1** | **LAQSLRGTSK** |
|  | 3208 | **740.5812** | **2218.7215** | **2219.4096** | **-0.6880** | **2** | **15** | **67** | **1** | **DRTKGPAEAELPEDPSMMGR + 2 Oxidation (M)** |
|  | 808 | **406.1193** | **1215.3358** | **1215.2970** | **0.0388** | **0** | **15** | **76** | **1** | **EMQNLSQHGR + Oxidation (M)** |
|  | 1261 | **429.2617** | **856.5087** | **856.0282** | **0.4806** | **2** | **15** | **64** | **1** | **IARRGGVK** |
|  | 2370 | **541.1915** | **1620.5524** | **1620.8544** | **-0.3020** | **1** | **15** | **77** | **1** | **RPELNQPARLTLGR** |
|  | 2668 | **604.1033** | **1809.2876** | **1809.9758** | **-0.6881** | **0** | **15** | **81** | **1** | **CEHGYASSFPSMPSPR + Carbamidomethyl (C)** |
|  | 414 | **386.1140** | **1155.3200** | **1154.2769** | **1.0431** | **0** | **15** | **72** | **1** | **MDGPGFGGMNR + Oxidation (M)** |
|  | 2 | **360.3445** | **1078.0112** | **1077.2110** | **0.8002** | **0** | **15** | **96** | **1** | **GSLHFIEQM + Oxidation (M)** |
|  | 734 | **404.0418** | **806.0688** | **805.8569** | **0.2119** | **0** | **15** | **79** | **1** | **DTPACSR + Carbamidomethyl (C)** |
|  | 1634 | **459.1470** | **916.2793** | **917.1013** | **-0.8221** | **1** | **15** | **89** | **1** | **LKSLVETK** |
|  | 2898 | **666.6516** | **1996.9327** | **1996.3098** | **0.6228** | **0** | **15** | **72** | **1** | **LLLTSLQSTMCSATAGNLR + Oxidation (M)** |
|  | 429 | **386.3387** | **770.6626** | **771.8455** | **-1.1828** | **0** | **15** | **61** | **1** | **MQGSGHR** |
|  | 494 | **388.3127** | **1161.9161** | **1161.2895** | **0.6266** | **1** | **15** | **78** | **1** | **GVVGGKMDENR** |
|  | 1837 | **476.1069** | **950.1991** | **951.0995** | **-0.9004** | **2** | **15** | **83** | **1** | **KMGSAKDSK** |
|  | 1991 | **494.1008** | **1479.2803** | **1479.7215** | **-0.4412** | **1** | **15** | **81** | **1** | **NALECDISARMLK + Oxidation (M)** |
|  | 2430 | **556.8870** | **1111.7591** | **1112.2401** | **-0.4810** | **1** | **15** | **70** | **1** | **AKGTGGGGLPAAR** |
|  | 2309 | **534.1909** | **1066.3671** | **1066.2281** | **0.1390** | **0** | **15** | **85** | **1** | **SMQLFDNVI** |
|  | 217 | **371.1299** | **1110.3676** | **1109.2116** | **1.1560** | **0** | **15** | **65** | **1** | **TVLSTGMDNR + Oxidation (M)** |
|  | 356 | **383.4403** | **1147.2988** | **1147.3424** | **-0.0436** | **0** | **15** | **95** | **1** | **MQSLLEPSVK + Oxidation (M)** |
|  | 858 | **407.1411** | **1218.4012** | **1219.3322** | **-0.9310** | **2** | **15** | **77** | **1** | **GAPEGSMSRRR + Oxidation (M)** |
|  | 1435 | **443.1103** | **1326.3086** | **1325.5153** | **0.7933** | **1** | **15** | **75** | **1** | **ITQFGTVTRFR** |
|  | 2242 | **523.9348** | **1045.8548** | **1045.2786** | **0.5763** | **2** | **15** | **85** | **1** | **LRGKPVFKT** |
|  | 328 | **380.1224** | **1137.3451** | **1137.3158** | **0.0293** | **1** | **15** | **90** | **1** | **CRHENLPLR** |
|  | 662 | **401.8446** | **1202.5117** | **1202.4075** | **0.1043** | **2** | **15** | **93** | **1** | **GLCKKGDHCK + 2 Carbamidomethyl (C)** |
|  | 3072 | **686.2522** | **1370.4896** | **1370.4651** | **0.0245** | **1** | **15** | **79** | **1** | **RSPSVSSPEPAEK** |
|  | 94 | **365.2395** | **728.4642** | **728.9200** | **-0.4558** | **0** | **15** | **67** | **1** | **ALTALLK** |
|  | 264 | **375.0175** | **748.0201** | **748.8917** | **-0.8715** | **0** | **15** | **90** | **1** | **MVGSALR + Oxidation (M)** |
|  | 1977 | **491.2548** | **980.4947** | **981.1701** | **-0.6753** | **0** | **15** | **77** | **1** | **NVLSVMFR + Oxidation (M)** |
|  | 3322 | **767.6699** | **2299.9874** | **2299.6710** | **0.3164** | **1** | **15** | **65** | **1** | **GGERQVLLCFFLQSTNAIMR + Oxidation (M)** |
|  | 478 | **387.9207** | **1160.7398** | **1160.4287** | **0.3111** | **0** | **15** | **99** | **1** | **FLCIGLVPNK + Carbamidomethyl (C)** |
|  | 583 | **397.1860** | **792.3572** | **791.8484** | **0.5088** | **0** | **15** | **80** | **1** | **LSWGSDK** |
|  | 384 | **385.0701** | **1152.1880** | **1152.4085** | **-0.2204** | **0** | **15** | **72** | **1** | **QLPCMASQMK + Oxidation (M)** |
|  | 469 | **387.8369** | **1160.4886** | **1161.3605** | **-0.8719** | **1** | **15** | **96** | **1** | **MSLCHRWR + Carbamidomethyl (C); Oxidation (M)** |
|  | 1271 | **430.1378** | **1287.3911** | **1288.3643** | **-0.9733** | **1** | **15** | **91** | **1** | **EDRDSLTLSPR** |
|  | 2949 | **668.9239** | **2003.7497** | **2004.5651** | **-0.8154** | **0** | **15** | **66** | **1** | **LLPLLGAALLLMLPLLGTR + Oxidation (M)** |
|  | 122 | **367.0290** | **1098.0647** | **1097.2240** | **0.8407** | **0** | **15** | **90** | **1** | **SNNSMAQAMK + Oxidation (M)** |
|  | 462 | **387.1956** | **1158.5648** | **1159.2933** | **-0.7286** | **1** | **15** | **81** | **1** | **TASAGKLLQDR** |
|  | 2155 | **518.9535** | **1553.8383** | **1554.8990** | **-1.0607** | **2** | **15** | **79** | **1** | **AIKIMPAKALASNAR** |
|  | 750 | **404.7502** | **1211.2285** | **1210.4247** | **0.8038** | **1** | **15** | **74** | **1** | **MLADSINRMK + 2 Oxidation (M)** |
|  | 1507 | **449.4443** | **1345.3108** | **1345.5880** | **-0.2771** | **0** | **15** | **78** | **1** | **ASVSGSMPMPLPR + Oxidation (M)** |
|  | 2449 | **562.5392** | **1684.5956** | **1685.0044** | **-0.4089** | **2** | **15** | **72** | **1** | **MAARRGALIVLEGVGR + Oxidation (M)** |
|  | 2518 | **575.3480** | **1723.0219** | **1722.9614** | **0.0605** | **0** | **15** | **83** | **1** | **DPGMGAMGGMGGGMGGGMF + 3 Oxidation (M)** |
|  | 637 | **401.0036** | **799.9925** | **798.8212** | **1.1713** | **0** | **15** | **86** | **1** | **FDCSDR + Carbamidomethyl (C)** |
|  | 3055 | **685.9185** | **2054.7332** | **2054.4817** | **0.2515** | **2** | **15** | **67** | **1** | **LLLTRFIFCSATMRTHK + Oxidation (M)** |
|  | 493 | **388.2944** | **1161.8610** | **1162.3222** | **-0.4611** | **1** | **15** | **78** | **1** | **FQMAPGPSRR + Oxidation (M)** |
|  | 395 | **385.2835** | **1152.8284** | **1153.4395** | **-0.6111** | **1** | **15** | **56** | **1** | **KALLMCEACR + Oxidation (M)** |
|  | 2713 | **610.5215** | **1828.5423** | **1827.9398** | **0.6024** | **1** | **15** | **68** | **1** | **GVYSSTNELTTDSTPKK** |
|  | 339 | **381.1479** | **1140.4217** | **1139.2990** | **1.1227** | **0** | **15** | **91** | **1** | **VTTPEPTAVPK** |
|  | 682 | **402.3801** | **1204.1180** | **1203.2996** | **0.8184** | **1** | **15** | **93** | **1** | **DTAEEKELLR** |
|  | 3180 | **736.9545** | **2207.8412** | **2206.6770** | **1.1642** | **2** | **15** | **68** | **1** | **GADASMACRKLAVAHPLLLLR** |
|  | 1426 | **441.3810** | **880.7473** | **881.0724** | **-0.3251** | **1** | **15** | **66** | **1** | **AFLAGKFK** |
|  | 1614 | **458.7672** | **915.5195** | **916.0139** | **-0.4943** | **0** | **15** | **75** | **1** | **APSAASCPR + Carbamidomethyl (C)** |
|  | 1288 | **431.0280** | **1290.0619** | **1290.3800** | **-0.3182** | **0** | **15** | **1e+02** | **1** | **EYQWLHTGEK** |
|  | 2692 | **608.7789** | **1823.3146** | **1824.0684** | **-0.7538** | **1** | **15** | **85** | **1** | **EQLQQCDCQNSLMKR** |
|  | 1702 | **463.0818** | **1386.2232** | **1385.6516** | **0.5716** | **2** | **15** | **75** | **1** | **QLKALTRLDLSK** |
|  | 1819 | **474.4612** | **946.9077** | **946.0432** | **0.8646** | **1** | **15** | **89** | **1** | **ASSHKSCAR** |
|  | 1453 | **443.9216** | **1328.7425** | **1329.5950** | **-0.8525** | **2** | **15** | **86** | **1** | **CHARGLKMLER + Oxidation (M)** |
|  | 2574 | **589.0037** | **1175.9925** | **1176.2742** | **-0.2817** | **1** | **15** | **88** | **1** | **NIKITSEEDK** |
|  | 2951 | **669.2211** | **2004.6410** | **2004.2525** | **0.3885** | **2** | **15** | **82** | **1** | **SLDTCGHRTLTGAKECVR + Carbamidomethyl (C)** |
|  | 1204 | **425.0909** | **1272.2507** | **1272.4359** | **-0.1853** | **1** | **15** | **91** | **1** | **QWIEPRNCAR** |
|  | 1206 | **425.2803** | **848.5458** | **847.9830** | **0.5628** | **1** | **15** | **73** | **1** | **VRNSMNK** |
|  | 1657 | **460.2412** | **1377.7015** | **1376.5590** | **1.1425** | **1** | **15** | **86** | **1** | **NTPGKPMREDTM** |
|  | 2065 | **505.3597** | **1008.7046** | **1008.2816** | **0.4230** | **1** | **15** | **64** | **1** | **MVPALPPKR** |
|  | 319 | **379.3719** | **756.7291** | **755.8197** | **0.9095** | **0** | **15** | **93** | **1** | **HAVSGGTK** |
|  | 375 | **384.9633** | **767.9117** | **767.8732** | **0.0385** | **0** | **15** | **72** | **1** | **HSNGLIK** |
|  | 1323 | **433.0782** | **1296.2125** | **1295.5538** | **0.6586** | **1** | **15** | **83** | **1** | **MNLKGIQVHGAK** |
|  | 1552 | **452.2085** | **902.4022** | **902.0700** | **0.3321** | **0** | **15** | **1e+02** | **1** | **AASAAPLACK** |
|  | 1687 | **461.9438** | **921.8727** | **921.0271** | **0.8457** | **0** | **15** | **80** | **1** | **TVIEDCNK** |
|  | 327 | **380.0980** | **1137.2717** | **1137.3111** | **-0.0393** | **1** | **15** | **94** | **1** | **MRLLTGTSSR + Oxidation (M)** |
|  | 1747 | **467.0864** | **1398.2370** | **1397.6160** | **0.6210** | **0** | **15** | **89** | **1** | **TPSLPLQVLTSNK** |
|  | 3060 | **686.0311** | **1370.0475** | **1369.5396** | **0.5078** | **1** | **15** | **70** | **1** | **LLSLQEKESYC + Carbamidomethyl (C)** |
|  | 101 | **366.0157** | **1095.0248** | **1095.2097** | **-0.1849** | **1** | **15** | **95** | **1** | **STTAGYLARR** |
|  | 855 | **407.1167** | **1218.3278** | **1219.4779** | **-1.1500** | **0** | **15** | **82** | **1** | **GTCMFPSMCAR + Oxidation (M)** |
|  | 2858 | **659.6526** | **1317.2904** | **1316.4656** | **0.8248** | **1** | **15** | **83** | **1** | **QAVKHPPAEPSR** |
|  | 1485 | **447.2597** | **1338.7569** | **1338.4893** | **0.2677** | **1** | **15** | **76** | **1** | **ARSMDIDDFIR** |
|  | 2961 | **670.8177** | **1339.6206** | **1340.6176** | **-0.9971** | **1** | **15** | **94** | **1** | **GHKPRLGCCVLT + Carbamidomethyl (C)** |
|  | 2534 | **578.8130** | **1155.6113** | **1155.3675** | **0.2438** | **0** | **15** | **70** | **1** | **GCPALLLSEPR** |
|  | 3269 | **742.9020** | **1483.7892** | **1484.6617** | **-0.8726** | **1** | **15** | **93** | **1** | **RTCCYGAPWTNR + Carbamidomethyl (C)** |
|  | 386 | **385.0894** | **1152.2461** | **1153.2657** | **-1.0196** | **1** | **15** | **74** | **1** | **CPSGDSAGKFK + Carbamidomethyl (C)** |
|  | 1096 | **419.4516** | **836.8884** | **836.0350** | **0.8533** | **1** | **15** | **1.1e+02** | **1** | **CLQKCGK + Carbamidomethyl (C)** |
|  | 2024 | **501.3096** | **1000.6045** | **1001.0522** | **-0.4477** | **1** | **15** | **78** | **1** | **REDTPLDR** |
|  | 1376 | **436.1781** | **1305.5121** | **1304.5608** | **0.9514** | **0** | **15** | **86** | **1** | **SSCCSCCPVGCAK + Carbamidomethyl (C)** |
|  | 1688 | **461.9705** | **921.9263** | **923.0925** | **-1.1662** | **0** | **15** | **80** | **1** | **CIPCDCNR** |
|  | 3308 | **760.5350** | **1519.0553** | **1519.6198** | **-0.5646** | **1** | **15** | **82** | **1** | **QEAQGARDCPECR + Carbamidomethyl (C)** |
|  | 3418 | **831.4166** | **2491.2277** | **2490.9836** | **0.2442** | **2** | **15** | **83** | **1** | **SHCIMTFYLFGIRSFPKLWK + Oxidation (M)** |
|  | 2896 | **666.6324** | **1331.2501** | **1330.5136** | **0.7365** | **1** | **15** | **72** | **1** | **ATAVDPTCARLR + Carbamidomethyl (C)** |
|  | 3329 | **773.2080** | **2316.6019** | **2317.6848** | **-1.0830** | **2** | **15** | **82** | **1** | **SCISRTAPRLLCTLEPGVDTK + Carbamidomethyl (C)** |
|  | 3317 | **764.6418** | **1527.2689** | **1527.7859** | **-0.5170** | **0** | **15** | **66** | **1** | **CMNSGGLLTMSLER + Oxidation (M)** |
|  | 1966 | **489.6947** | **977.3746** | **978.2126** | **-0.8380** | **1** | **15** | **74** | **1** | **MPPPGKVPR** |
|  | 1978 | **491.3975** | **980.7803** | **979.9054** | **0.8749** | **0** | **15** | **65** | **1** | **GGGGSSSGSGDR** |
|  | 3245 | **741.8729** | **2222.5964** | **2223.6314** | **-1.0350** | **2** | **15** | **94** | **1** | **IRIDLTSMQIKTTDLLFSK** |
|  | 15 | **360.5028** | **718.9908** | **718.9055** | **0.0853** | **0** | **15** | **94** | **1** | **ISVCAVK** |
|  | 49 | **363.1346** | **724.2544** | **723.8407** | **0.4137** | **0** | **15** | **84** | **1** | **LFCADR** |
|  | 57 | **363.2421** | **1086.7041** | **1086.2260** | **0.4781** | **0** | **15** | **69** | **1** | **CPGSWHFPR** |
|  | 246 | **373.2123** | **1116.6148** | **1116.3714** | **0.2435** | **0** | **15** | **86** | **1** | **LEELMKPLK + Oxidation (M)** |
|  | 707 | **403.7518** | **805.4888** | **804.9548** | **0.5339** | **0** | **15** | **85** | **1** | **AGVSACIK + Carbamidomethyl (C)** |
|  | 1033 | **415.9645** | **1244.8714** | **1244.4642** | **0.4072** | **1** | **15** | **1e+02** | **1** | **AVVEKVNGCLGR** |
|  | 1889 | **480.2140** | **1437.6198** | **1437.6404** | **-0.0206** | **0** | **15** | **89** | **1** | **VVQGSPEVPGITVR** |
|  | 2228 | **523.0718** | **1044.1288** | **1045.2786** | **-1.1498** | **0** | **15** | **90** | **1** | **MCMECLSR + Carbamidomethyl (C); Oxidation (M)** |
|  | 835 | **406.8120** | **1217.4140** | **1217.4204** | **-0.0065** | **2** | **15** | **82** | **1** | **GLQAARAQKFK** |
|  | 1275 | 430.2518 | 858.4889 | 857.9116 | 0.5773 | 1 | 15 | 81 | 1 | QSDPQKR |
|  | 1356 | **435.1501** | **1302.4281** | **1302.4540** | **-0.0259** | **1** | **15** | **82** | **1** | **IEEKGVPEDMR** |
|  | 2716 | 611.3873 | 1831.1398 | 1832.0689 | -0.9291 | 2 | 15 | 87 | 1 | EQSPPGLRGGQLHSLKK |
|  | 2955 | **669.7560** | **2006.2458** | **2007.2097** | **-0.9640** | **0** | **15** | **1e+02** | **1** | **ACVFWNQGLLMHENQGS + Carbamidomethyl (C); Oxidation (M)** |
|  | 383 | **385.0471** | **768.0795** | **768.9013** | **-0.8217** | **0** | **15** | **75** | **1** | **LVGPEVR** |
|  | 1036 | **416.0251** | **830.0354** | **829.9875** | **0.0478** | **2** | **15** | **1e+02** | **1** | **KKSAGLAR** |
|  | 3119 | **701.5508** | **2101.6302** | **2102.3044** | **-0.6743** | **0** | **15** | **73** | **1** | **EIQQALVDAGDKPATFVGSR** |
|  | 3455 | **853.0983** | **1704.1818** | **1703.9812** | **0.2005** | **1** | **15** | **72** | **1** | **AVSRNPLCTLCLEER** |
|  | 799 | **406.0294** | **1215.0659** | **1214.3056** | **0.7603** | **0** | **15** | **79** | **1** | **MGTGEASGGYIR + Oxidation (M)** |
|  | 1290 | **431.0528** | **1290.1363** | **1290.5724** | **-0.4361** | **1** | **15** | **1.1e+02** | **1** | **KCSFLCDVTMK + Oxidation (M)** |
|  | 774 | **405.1400** | **808.2653** | **807.9192** | **0.3461** | **1** | **15** | **81** | **1** | **SCSTVRR** |
|  | 1503 | **449.2970** | **896.5792** | **895.9348** | **0.6444** | **0** | **15** | **65** | **1** | **VCGDSSSGK + Carbamidomethyl (C)** |
|  | 218 | **371.1559** | **1110.4454** | **1110.1781** | **0.2673** | **1** | **15** | **65** | **1** | **TYNADSVKGR** |
|  | 1197 | **424.0453** | **1269.1136** | **1268.5948** | **0.5188** | **0** | **15** | **1e+02** | **1** | **CLWMPGMAMR + Carbamidomethyl (C); Oxidation (M)** |
|  | 784 | **405.2395** | **808.4643** | **808.8790** | **-0.4147** | **1** | **15** | **66** | **1** | **GKYGEGAK** |
|  | 1939 | **487.9697** | **1460.8868** | **1460.6969** | **0.1900** | **0** | **15** | **98** | **1** | **VTFHMGSSSLPAVK** |
|  | 551 | **390.0518** | **778.0889** | **776.9019** | **1.1870** | **1** | **15** | **89** | **1** | **QEMVRV + Oxidation (M)** |
|  | 2760 | **619.1417** | **1854.4030** | **1855.2529** | **-0.8499** | **1** | **15** | **86** | **1** | **IGANIIGMRSLGVVLIGR + Oxidation (M)** |
|  | 3361 | **789.8480** | **1577.6813** | **1578.8078** | **-1.1266** | **0** | **15** | **92** | **1** | **VYTIMYSCWHEF** |
|  | 3494 | **890.7227** | **2669.1458** | **2669.0076** | **0.1382** | **2** | **15** | **83** | **1** | **QRAGGVPQPANLGAGAEPAGAALVRLAR** |
|  | 50 | **363.1424** | **724.2700** | **724.7195** | **-0.4494** | **0** | **15** | **85** | **1** | **DYTGNR** |
|  | 222 | **371.2131** | **1110.6172** | **1111.3613** | **-0.7441** | **2** | **15** | **57** | **1** | **IKLCHGQKK + Carbamidomethyl (C)** |
|  | 31 | **362.1719** | **1083.4936** | **1084.1010** | **-0.6073** | **0** | **15** | **70** | **1** | **VQGNDHSATR** |
|  | 1110 | **420.3732** | **838.7316** | **838.9447** | **-0.2131** | **0** | **15** | **70** | **1** | **ITSVEYK** |
|  | 1256 | **429.1564** | **1284.4469** | **1285.4100** | **-0.9631** | **0** | **15** | **88** | **1** | **LSATGAGRPGVSGR** |
|  | 1604 | **458.2080** | **1371.6019** | **1371.5605** | **0.0415** | **2** | **15** | **91** | **1** | **FEKQLACKSNF + Carbamidomethyl (C)** |
|  | 2184 | **520.0979** | **1038.1810** | **1039.2725** | **-1.0914** | **0** | **15** | **84** | **1** | **TLRPAVVGVK** |
|  | 362 | **384.2338** | **766.4527** | **765.8145** | **0.6383** | **0** | **15** | **67** | **1** | **DPGPGAPR** |
|  | 1018 | **414.7310** | **827.4473** | **826.8744** | **0.5730** | **0** | **15** | **71** | **1** | **CFDETR + Carbamidomethyl (C)** |
|  | 2201 | **521.0854** | **1560.2342** | **1560.8224** | **-0.5883** | **2** | **15** | **85** | **1** | **QMYVALNGKGAPRR** |
|  | 2734 | **612.4553** | **1834.3436** | **1833.8815** | **0.4621** | **0** | **15** | **75** | **1** | **DESTDSGLSMSSYSVPR + Oxidation (M)** |
|  | 880 | **407.4339** | **1219.2795** | **1219.3437** | **-0.0641** | **0** | **15** | **1e+02** | **1** | **FQIPVASDSQK** |
|  | 765 | **405.1086** | **1212.3036** | **1213.4534** | **-1.1498** | **1** | **15** | **83** | **1** | **GPLSSRPLKCR** |
|  | 1533 | **451.2824** | **900.5501** | **901.1249** | **-0.5748** | **0** | **15** | **81** | **1** | **LLGCSPALK** |
|  | 2335 | **537.5869** | **1609.7386** | **1609.9943** | **-0.2557** | **2** | **15** | **1.2e+02** | **1** | **LPVTKMKYSGNLMK** |
|  | 2884 | **666.4135** | **1996.2184** | **1997.2816** | **-1.0632** | **1** | **15** | **88** | **1** | **AMMSEFCTQGAEMCRR + 2 Carbamidomethyl (C); 2 Oxidation (M)** |
|  | 3053 | **685.5393** | **1369.0638** | **1368.4754** | **0.5885** | **0** | **15** | **80** | **1** | **GNAGNMLTFGGGTR + Oxidation (M)** |
|  | 3530 | **987.1835** | **1972.3522** | **1971.9840** | **0.3682** | **1** | **15** | **81** | **1** | **NECISEDMGDEDKEER + Carbamidomethyl (C); Oxidation (M)** |
|  | 47 | **363.1220** | **1086.3439** | **1085.2546** | **1.0893** | **0** | **15** | **90** | **1** | **EMGNSLGCFK** |
|  | 1394 | **437.1057** | **1308.2948** | **1308.6073** | **-0.3126** | **0** | **15** | **1e+02** | **1** | **LSMSLMEQLLK + Oxidation (M)** |
|  | 3212 | **740.6421** | **2218.9041** | **2218.5788** | **0.3253** | **0** | **15** | **70** | **1** | **NQPPSPEFVARPVGVCSMMR + Oxidation (M)** |
|  | 275 | **376.1336** | **1125.3786** | **1125.2324** | **0.1462** | **0** | **15** | **83** | **1** | **ATEGAIHAVEK** |
|  | 1024 | **415.1378** | **1242.3911** | **1242.4068** | **-0.0157** | **0** | **15** | **94** | **1** | **HQMALDWVSR** |
|  | 3408 | **824.9788** | **1647.9427** | **1647.9162** | **0.0265** | **1** | **15** | **99** | **1** | **LPDLRLVIAAADPGAR** |
|  | 2742 | **613.3709** | **1837.0904** | **1837.2146** | **-0.1243** | **2** | **15** | **88** | **1** | **QRTPKINMMLANLYK + Oxidation (M)** |
|  | 3217 | **740.6752** | **1479.3356** | **1479.7880** | **-0.4524** | **0** | **15** | **71** | **1** | **VSMPICVGATAMQR + Oxidation (M)** |
|  | 492 | **388.2916** | **774.5683** | **775.0151** | **-0.4467** | **1** | **15** | **87** | **1** | **IRVIMK + Oxidation (M)** |
|  | 1756 | **468.0835** | **934.1522** | **934.1002** | **0.0520** | **2** | **15** | **98** | **1** | **LRAGPRHK** |
|  | 3030 | **684.1128** | **2049.3162** | **2049.2631** | **0.0531** | **1** | **15** | **89** | **1** | **RSIAAAGYYYYYGMDVW** |
|  | 398 | **385.5175** | **1153.5304** | **1154.3615** | **-0.8311** | **1** | **15** | **81** | **1** | **GIKCCTSVTSR** |
|  | 589 | **398.1118** | **1191.3132** | **1192.3864** | **-1.0732** | **1** | **15** | **94** | **1** | **VTGGIKTVDMR + Oxidation (M)** |
|  | 1714 | **463.8486** | **1388.5235** | **1387.5614** | **0.9622** | **0** | **15** | **86** | **1** | **GNPIGLNMLSNNK + Oxidation (M)** |
|  | 1532 | **451.2582** | **900.5016** | **899.9516** | **0.5500** | **0** | **15** | **88** | **1** | **RPGVGSGDR** |
|  | 265 | **375.0728** | **748.1307** | **747.7513** | **0.3795** | **1** | **15** | **1e+02** | **1** | **DAGDKDK** |
|  | 1917 | **485.3543** | **1453.0407** | **1453.6644** | **-0.6237** | **1** | **15** | **66** | **1** | **CLKNTPAFFAER + Carbamidomethyl (C)** |
|  | 2533 | **578.7071** | **1733.0991** | **1733.0474** | **0.0517** | **2** | **15** | **1.1e+02** | **1** | **AGHPPAVKAGGMRIVQK + Oxidation (M)** |
|  | 3081 | **687.2903** | **2058.8487** | **2059.4516** | **-0.6029** | **1** | **15** | **91** | **1** | **LLQQIQLKDTPTILIGHK** |
|  | 585 | **397.3494** | **792.6840** | **793.8198** | **-1.1358** | **0** | **15** | **84** | **1** | **GTTVSVSSG** |
|  | 1960 | **489.0782** | **976.1416** | **975.0577** | **1.0839** | **0** | **15** | **99** | **1** | **SSNTALLNR** |
|  | 81 | **364.2450** | **1089.7127** | **1090.2759** | **-0.5632** | **1** | **15** | **65** | **1** | **LTKGFNLAAR** |
|  | 416 | **386.1292** | **1155.3655** | **1155.3526** | **0.0129** | **0** | **15** | **83** | **1** | **CFLGCHHPK + 2 Carbamidomethyl (C)** |
|  | 655 | **401.4028** | **1201.1861** | **1200.3023** | **0.8838** | **0** | **15** | **1.1e+02** | **1** | **ERPETSAALAR** |
|  | 800 | **406.0395** | **1215.0963** | **1214.5639** | **0.5323** | **1** | **15** | **83** | **1** | **IMVIINKVLR + Oxidation (M)** |
|  | 941 | **408.8056** | **1223.3946** | **1222.3493** | **1.0454** | **1** | **15** | **96** | **1** | **FEPYANPTKR** |
|  | 1418 | **440.9765** | **1319.9074** | **1319.5289** | **0.3785** | **1** | **15** | **95** | **1** | **ECFLIQPKER + Carbamidomethyl (C)** |
|  | 1653 | **460.0815** | **918.1481** | **918.9483** | **-0.8001** | **0** | **15** | **1e+02** | **1** | **DAIDSVSGR** |
|  | 2621 | **593.8138** | **1185.6129** | **1185.3108** | **0.3021** | **1** | **15** | **83** | **1** | **AYAGCTQRTSK** |
|  | 959 | **409.3989** | **1225.1745** | **1224.3667** | **0.8079** | **1** | **15** | **1.1e+02** | **1** | **LQSPGDKLPGGR** |
|  | 2466 | **564.9052** | **1127.7955** | **1127.2532** | **0.5424** | **0** | **15** | **89** | **1** | **WRPEPSSLR** |
|  | 2909 | **666.9929** | **1997.9566** | **1997.4717** | **0.4848** | **2** | **15** | **77** | **1** | **NVENMKILRLMLLHIK + 2 Oxidation (M)** |
|  | 2929 | **668.4810** | **2002.4207** | **2002.2100** | **0.2107** | **2** | **15** | **88** | **1** | **LQDAEIARLCEDLDRNK** |
|  | 2937 | **668.7189** | **2003.1344** | **2004.3138** | **-1.1793** | **1** | **15** | **1.1e+02** | **1** | **LPSLSPVARSFSACSVGLGR** |
|  | 198 | **369.3304** | **1104.9692** | **1104.3011** | **0.6681** | **0** | **15** | **89** | **1** | **MHPSLATMGK + 2 Oxidation (M)** |
|  | 821 | **406.2751** | **1215.8032** | **1216.3878** | **-0.5846** | **1** | **15** | **70** | **1** | **MSYQQKQCK + Carbamidomethyl (C); Oxidation (M)** |
|  | 1559 | **453.0022** | **1355.9843** | **1356.5955** | **-0.6111** | **1** | **15** | **1e+02** | **1** | **DMWCTACRWK + Carbamidomethyl (C)** |
|  | 2219 | **522.2971** | **1563.8690** | **1562.7636** | **1.1054** | **1** | **15** | **91** | **1** | **HLDSYEFLKAILN** |
|  | 964 | **410.1407** | **1227.3999** | **1226.3841** | **1.0157** | **1** | **15** | **1e+02** | **1** | **AFNYSSLLRR** |
|  | 2969 | **673.2699** | **2016.7875** | **2017.3092** | **-0.5217** | **2** | **15** | **93** | **1** | **DWLFDMVTTWFGAKKR + Oxidation (M)** |
|  | 460 | **387.1631** | **772.3115** | **771.9481** | **0.3634** | **1** | **15** | **1e+02** | **1** | **ALKALTR** |
|  | 3022 | **684.0236** | **2049.0487** | **2048.4029** | **0.6458** | **2** | **15** | **77** | **1** | **DPKSVEQAIVLKEGFMIK + Oxidation (M)** |
|  | 894 | **407.7171** | **813.4195** | **813.8159** | **-0.3965** | **0** | **15** | **81** | **1** | **ESTQGHR** |
|  | 1063 | **418.7112** | **1253.1114** | **1253.5569** | **-0.4455** | **0** | **15** | **93** | **1** | **LPLNCCVFCK + 2 Carbamidomethyl (C)** |
|  | 142 | **368.1255** | **1101.3542** | **1101.1315** | **0.2227** | **1** | **15** | **99** | **1** | **AAGDSGSRAGPR** |
|  | 406 | **386.0521** | **1155.1341** | **1154.3365** | **0.7976** | **0** | **15** | **84** | **1** | **AYLLMSDVSR** |
|  | 993 | **412.9145** | **823.8142** | **824.9279** | **-1.1138** | **2** | **15** | **81** | **1** | **SHGQKRL** |
|  | 1892 | **480.4097** | **1438.2069** | **1438.6796** | **-0.4726** | **1** | **15** | **80** | **1** | **HPPGEMMRWAAR** |
|  | 3091 | **692.6232** | **2074.8473** | **2074.4070** | **0.4403** | **1** | **15** | **72** | **1** | **MAHRLTLLSPHVAEEVVR + Oxidation (M)** |
|  | 103 | **366.0666** | **1095.1775** | **1094.2267** | **0.9508** | **1** | **15** | **1e+02** | **1** | **MCRGVGDQR + Carbamidomethyl (C); Oxidation (M)** |
|  | 158 | **369.1736** | **736.3324** | **736.8793** | **-0.5468** | **0** | **15** | **88** | **1** | **AFQMPK + Oxidation (M)** |
|  | 182 | **369.2658** | **736.5168** | **736.8793** | **-0.3624** | **0** | **15** | **81** | **1** | **AFQMPK + Oxidation (M)** |
|  | 292 | **377.1115** | **1128.3125** | **1127.2482** | **1.0642** | **0** | **15** | **88** | **1** | **ADPVAGIAGSAAK** |
|  | 544 | **389.9836** | **777.9525** | **776.9019** | **1.0506** | **0** | **15** | **94** | **1** | **MVAAVDR + Oxidation (M)** |
|  | 581 | **396.2128** | **1185.6161** | **1186.3817** | **-0.7655** | **0** | **15** | **90** | **1** | **GEMNVTLGIPR** |
|  | 3255 | **742.3021** | **1482.5895** | **1483.6211** | **-1.0316** | **0** | **15** | **89** | **1** | **EGQMESVEAAMSSK** |
|  | 607 | **399.4494** | **1195.3260** | **1195.2875** | **0.0386** | **1** | **15** | **96** | **1** | **MERDGCAGGGSR** |
|  | 1237 | **428.7383** | **1283.1929** | **1283.4339** | **-0.2410** | **1** | **15** | **69** | **1** | **YAKCDQCGNPK + Carbamidomethyl (C)** |
|  | 1520 | **450.3590** | **898.7032** | **899.0694** | **-0.3663** | **0** | **15** | **75** | **1** | **QVTCHLAK** |
|  | 2820 | **643.8478** | **1928.5212** | **1928.1795** | **0.3417** | **1** | **15** | **84** | **1** | **NGLPCMSFSPGGRSWCR + Carbamidomethyl (C); Oxidation (M)** |
|  | 741 | **404.1263** | **1209.3567** | **1209.4798** | **-0.1231** | **1** | **15** | **94** | **1** | **MISTRVMDIK + Oxidation (M)** |
|  | 1997 | **495.3102** | **988.6057** | **988.1660** | **0.4397** | **1** | **15** | **86** | **1** | **DALCRVVGR** |
|  | 152 | **369.1127** | **1104.3160** | **1105.3089** | **-0.9929** | **1** | **15** | **1e+02** | **1** | **MLALTEARGK + Oxidation (M)** |
|  | 1229 | **428.2346** | **1281.6816** | **1281.2940** | **0.3876** | **2** | **15** | **72** | **1** | **GSSGSSGRSSRTR** |
|  | 1322 | **433.0648** | **1296.1723** | **1296.5319** | **-0.3596** | **1** | **15** | **94** | **1** | **QYESLKILICS** |
|  | 1835 | 476.0251 | 1425.0531 | 1424.5403 | 0.5128 | 0 | 15 | 99 | 1 | ANSVSCWSGPGFR + Carbamidomethyl (C) |
|  | 2141 | **518.7422** | **1553.2044** | **1553.7868** | **-0.5824** | **2** | **15** | **76** | **1** | **LDKCGVCGGKGNSCR + Carbamidomethyl (C)** |
|  | 291 | **377.0984** | **1128.2731** | **1128.2795** | **-0.0064** | **0** | **14** | **89** | **1** | **KPSVGSPSLTR** |
|  | 1094 | **419.4436** | **1255.3085** | **1254.5055** | **0.8031** | **2** | **14** | **1.2e+02** | **1** | **APKASRPPKMR + Oxidation (M)** |
|  | 1531 | **451.1641** | **1350.4700** | **1351.5443** | **-1.0743** | **1** | **14** | **1e+02** | **1** | **GTGFIKTELISVS** |
|  | 1642 | **459.5934** | **917.1719** | **915.9922** | **1.1797** | **0** | **14** | **1.2e+02** | **1** | **ALADSHFR** |
|  | 1853 | 476.3821 | 1426.1242 | 1425.5666 | 0.5576 | 2 | 14 | 75 | 1 | GRNCELKDSFEK |
|  | 1951 | **488.2106** | **974.4064** | **975.1026** | **-0.6962** | **1** | **14** | **1e+02** | **1** | **ERFGKPNK** |
|  | 3040 | **684.8693** | **2051.5856** | **2051.1765** | **0.4092** | **0** | **14** | **96** | **1** | **ASDTAMYYCARPGGDDWR + Oxidation (M)** |
|  | 3294 | **757.2733** | **1512.5317** | **1513.6751** | **-1.1433** | **2** | **14** | **87** | **1** | **ELGKGPEKSEMHR + Oxidation (M)** |
|  | 485 | **388.1078** | **1161.3012** | **1162.2974** | **-0.9962** | **1** | **14** | **1.1e+02** | **1** | **CAEKMQDHK + Carbamidomethyl (C); Oxidation (M)** |
|  | 1918 | **485.5523** | **1453.6347** | **1454.6940** | **-1.0592** | **0** | **14** | **1e+02** | **1** | **VMPGSCFHVSCSK + Carbamidomethyl (C); Oxidation (M)** |
|  | 2673 | **606.2325** | **1815.6755** | **1815.0553** | **0.6202** | **0** | **14** | **89** | **1** | **ITENIGCVMTGMTADSR + Oxidation (M)** |
|  | 2779 | **626.6843** | **1877.0306** | **1876.1184** | **0.9122** | **2** | **14** | **1.1e+02** | **1** | **KRSVLPPDGNGSPVLPDK** |
|  | 1481 | **447.1614** | **892.3081** | **892.0323** | **0.2758** | **0** | **14** | **1e+02** | **1** | **MLATTSPR + Oxidation (M)** |
|  | 2602 | **592.9972** | **1183.9796** | **1184.3690** | **-0.3894** | **1** | **14** | **97** | **1** | **AHSIGKAICER** |
|  | 2613 | **593.1729** | **1184.3309** | **1183.3130** | **1.0179** | **0** | **14** | **1e+02** | **1** | **QHTVLWDTAI** |
|  | 3405 | **822.2528** | **2463.7363** | **2464.5988** | **-0.8625** | **1** | **14** | **92** | **1** | **EEPKLPQQSHSAFGATSSSSGFGK** |
|  | 1862 | **477.5755** | **1429.7043** | **1429.5566** | **0.1477** | **0** | **14** | **1e+02** | **1** | **CEGINISGNFYR + Carbamidomethyl (C)** |
|  | 168 | **369.2251** | **736.4354** | **736.8793** | **-0.4439** | **0** | **14** | **83** | **1** | **AFQMPK + Oxidation (M)** |
|  | 632 | **400.2437** | **798.4726** | **797.9424** | **0.5303** | **1** | **14** | **68** | **1** | **KGLLPDR** |
|  | 1455 | **444.0685** | **886.1223** | **885.0660** | **1.0563** | **2** | **14** | **1e+02** | **1** | **RLEALRK** |
|  | 2490 | **569.4194** | **1705.2361** | **1705.9770** | **-0.7409** | **0** | **14** | **82** | **1** | **KPTLLYCSNGGHFLR** |
|  | 668 | **401.9232** | **801.8317** | **801.8450** | **-0.0133** | **0** | **14** | **1.1e+02** | **1** | **AEEQAVR** |
|  | 1200 | **424.1537** | **1269.4388** | **1270.4351** | **-0.9963** | **0** | **14** | **1.1e+02** | **1** | **LFEAVFAGNFR** |
|  | 1943 | **488.0790** | **1461.2150** | **1461.7113** | **-0.4963** | **1** | **14** | **1.1e+02** | **1** | **AEAGMGMWRLAPR + Oxidation (M)** |
|  | 954 | **409.2130** | **816.4112** | **816.9209** | **-0.5096** | **0** | **14** | **91** | **1** | **DMFNFK + Oxidation (M)** |
|  | 1343 | **434.4782** | **866.9417** | **866.0197** | **0.9220** | **1** | **14** | **1.1e+02** | **1** | **HQGKIGVK** |
|  | 171 | **369.2317** | **736.4487** | **736.8793** | **-0.4306** | **0** | **14** | **84** | **1** | **AFQMPK + Oxidation (M)** |
|  | 226 | **371.2785** | **740.5422** | **739.8203** | **0.7219** | **0** | **14** | **62** | **1** | **RPNPEK** |
|  | 326 | **380.0842** | **1137.2304** | **1138.3770** | **-1.1465** | **1** | **14** | **1.1e+02** | **1** | **KMPLTEYLK + Oxidation (M)** |
|  | 480 | **387.9656** | **1160.8746** | **1160.4039** | **0.4706** | **1** | **14** | **1.2e+02** | **1** | **FYISYLVKK** |
|  | 1065 | **419.0163** | **1254.0268** | **1254.4787** | **-0.4519** | **1** | **14** | **1.1e+02** | **1** | **KGPTSLVLNGIR** |
|  | 118 | **366.3168** | **1095.9281** | **1095.1899** | **0.7383** | **1** | **14** | **1e+02** | **1** | **MGAHAGKHSSP + Oxidation (M)** |
|  | 1645 | **459.8373** | **1376.4898** | **1375.7032** | **0.7866** | **1** | **14** | **1e+02** | **1** | **VLARMVDILACR + Oxidation (M)** |
|  | 2675 | **606.5114** | **1211.0079** | **1210.4263** | **0.5816** | **2** | **14** | **73** | **1** | **LTRTHDVLKK** |
|  | 506 | **389.0072** | **775.9996** | **775.7680** | **0.2316** | **1** | **14** | **1.1e+02** | **1** | **SRAGDDR** |
|  | 2264 | **527.7513** | **1053.4879** | **1053.1333** | **0.3546** | **1** | **14** | **76** | **1** | **GAPRAGAGSPGR** |
|  | 2320 | **536.0485** | **1605.1232** | **1604.7212** | **0.4021** | **1** | **14** | **96** | **1** | **AAMENQRSYSMER + 2 Oxidation (M)** |
|  | 389 | **385.1198** | **1152.3373** | **1152.3903** | **-0.0530** | **1** | **14** | **85** | **1** | **VAWMRSGVCK + Oxidation (M)** |
|  | 815 | **406.2203** | **1215.6388** | **1215.3797** | **0.2591** | **0** | **14** | **78** | **1** | **GEQGLTGMPGIR** |
|  | 3105 | **697.9951** | **2090.9632** | **2090.3401** | **0.6230** | **1** | **14** | **76** | **1** | **RLETAEAYVGPGGPECPMR + Carbamidomethyl (C)** |
|  | 287 | **377.0178** | **752.0207** | **750.9307** | **1.0901** | **1** | **14** | **90** | **1** | **CKMGQK + Carbamidomethyl (C)** |
|  | 698 | **403.0850** | **1206.2329** | **1206.3267** | **-0.0938** | **0** | **14** | **1.1e+02** | **1** | **VGGDSAGDLMIR + Oxidation (M)** |
|  | 859 | **407.1521** | **1218.4340** | **1217.4022** | **1.0318** | **2** | **14** | **95** | **1** | **LRTAGRDGLCR** |
|  | 1344 | **434.6977** | **867.3805** | **866.9613** | **0.4192** | **0** | **14** | **72** | **1** | **AGPHALSSK** |
|  | 1723 | **464.2469** | **1389.7187** | **1389.5228** | **0.1959** | **2** | **14** | **92** | **1** | **RGGGGSAPPRAPPGR** |
|  | 1792 | **471.8609** | **1412.5606** | **1411.5599** | **1.0008** | **0** | **14** | **92** | **1** | **KPASSSSAPQNIPK** |
|  | 2039 | **503.0770** | **1004.1391** | **1003.1342** | **1.0050** | **0** | **14** | **1.1e+02** | **1** | **MNSLGVNPR + Oxidation (M)** |
|  | 193 | **369.2931** | **1104.8572** | **1105.2110** | **-0.3539** | **1** | **14** | **87** | **1** | **RLGHNQPQR** |
|  | 335 | **380.2412** | **1137.7013** | **1138.3156** | **-0.6143** | **1** | **14** | **91** | **1** | **GEFFELIRK** |
|  | 549 | **390.0244** | **778.0340** | **776.8621** | **1.1720** | **1** | **14** | **98** | **1** | **REGMER** |
|  | 599 | **399.1850** | **796.3552** | **795.8867** | **0.4685** | **1** | **14** | **80** | **1** | **GAKLHDR** |
|  | 996 | **413.1263** | **824.2378** | **824.9660** | **-0.7283** | **0** | **14** | **89** | **1** | **SCQISCK + Carbamidomethyl (C)** |
|  | 1526 | **451.0097** | **900.0047** | **899.9862** | **0.0185** | **0** | **14** | **1.1e+02** | **1** | **ELANYYK** |
|  | 1859 | **477.2054** | **1428.5941** | **1427.6038** | **0.9902** | **0** | **14** | **95** | **1** | **VQPWLSLATQER** |
|  | 396 | **385.2974** | **1152.8700** | **1153.2492** | **-0.3792** | **1** | **14** | **68** | **1** | **DARPGARGPEK** |
|  | 3006 | **681.4451** | **2041.3132** | **2040.3970** | **0.9162** | **2** | **14** | **98** | **1** | **RLGPVPPGLCGRHWEPLR** |
|  | 3310 | **761.1410** | **2280.4008** | **2280.6829** | **-0.2821** | **0** | **14** | **89** | **1** | **IMMFIGGPATQGPGMVVGDELK + 2 Oxidation (M)** |
|  | 273 | **376.0875** | **750.1602** | **749.8548** | **0.3055** | **0** | **14** | **93** | **1** | **AFSINAK** |
|  | 486 | **388.1125** | **1161.3153** | **1162.2922** | **-0.9769** | **0** | **14** | **1.2e+02** | **1** | **YLIPNAGDATK** |
|  | 2003 | **496.5106** | **1486.5095** | **1486.8037** | **-0.2942** | **2** | **14** | **1.2e+02** | **1** | **MYLKTRAGMPFR + Oxidation (M)** |
|  | 2877 | **665.4719** | **1993.3936** | **1992.3031** | **1.0905** | **1** | **14** | **94** | **1** | **QCCPSAHGVPKSMDLFSK + Carbamidomethyl (C)** |
|  | 3176 | **735.4261** | **2203.2563** | **2203.4828** | **-0.2265** | **2** | **14** | **95** | **1** | **CTIEGCNMVFSSLRSRNR + 2 Carbamidomethyl (C); Oxidation (M)** |
|  | 465 | **387.4826** | **772.9504** | **772.8931** | **0.0573** | **1** | **14** | **1.4e+02** | **1** | **LTAKANR** |
|  | 690 | 402.9113 | 803.8078 | 802.9192 | 0.8886 | 1 | 14 | 1.1e+02 | 1 | NSRLVSK |
|  | 839 | **406.8989** | **1217.6745** | **1218.4052** | **-0.7307** | **1** | **14** | **87** | **1** | **GGTRAVLEFIR** |
|  | 2104 | **511.1419** | **1020.2691** | **1021.1975** | **-0.9284** | **0** | **14** | **1e+02** | **1** | **HHSLRPMK + Oxidation (M)** |
|  | 2936 | **668.7039** | **2003.0894** | **2003.2858** | **-0.1964** | **1** | **14** | **1.1e+02** | **1** | **TGQPLPQSIQQAMRYLR + Oxidation (M)** |
|  | 3456 | **853.6466** | **1705.2784** | **1705.0569** | **0.2216** | **1** | **14** | **91** | **1** | **FISCFIRCVWFQR** |
|  | 1745 | **466.2592** | **1395.7554** | **1396.6960** | **-0.9406** | **1** | **14** | **97** | **1** | **KMLDPGLMTCSK + Carbamidomethyl (C); Oxidation (M)** |
|  | 46 | **363.1192** | **1086.3355** | **1087.3333** | **-0.9978** | **0** | **14** | **99** | **1** | **ILTAALTCPK + Carbamidomethyl (C)** |
|  | 769 | **405.1136** | **808.2125** | **807.9125** | **0.3001** | **0** | **14** | **93** | **1** | **MIDTGQK + Oxidation (M)** |
|  | 828 | **406.3956** | **1216.1646** | **1216.3846** | **-0.2199** | **1** | **14** | **95** | **1** | **SRVTISIDTPK** |
|  | 1233 | **428.3694** | **1282.0862** | **1281.3751** | **0.7111** | **2** | **14** | **72** | **1** | **GRFTISRDDSK** |
|  | 1366 | 435.7154 | 1304.1240 | 1304.4117 | -0.2877 | 0 | 14 | 77 | 1 | CSCNNGEMCDR + Carbamidomethyl (C); Oxidation (M) |
|  | 1446 | **443.8018** | **885.5889** | **884.9337** | **0.6552** | **0** | **14** | **99** | **1** | **AEPGATPSR** |
|  | 1580 | **456.2828** | **1365.8262** | **1365.4534** | **0.3729** | **1** | **14** | **77** | **1** | **DPEHQGRVHYK** |
|  | 403 | **386.0087** | **770.0027** | **769.9555** | **0.0472** | **0** | **14** | **86** | **1** | **RPIPCK + Carbamidomethyl (C)** |
|  | 711 | **403.9210** | **805.8271** | **804.9548** | **0.8723** | **0** | **14** | **1e+02** | **1** | **AGVSACIK + Carbamidomethyl (C)** |
|  | 1541 | **451.8824** | **1352.6252** | **1351.5742** | **1.0509** | **0** | **14** | **1.2e+02** | **1** | **MVTHTAASPALPR** |
|  | 3001 | **681.1812** | **2040.5213** | **2041.4366** | **-0.9154** | **1** | **14** | **99** | **1** | **FVYLVMELMRGGELLDR** |
|  | 203 | **369.3478** | **736.6809** | **736.8793** | **-0.1984** | **0** | **14** | **1e+02** | **1** | **AFQMPK + Oxidation (M)** |
|  | 587 | **398.0526** | **794.0903** | **794.8588** | **-0.7685** | **0** | **14** | **1e+02** | **1** | **QHLGQGR** |
|  | 603 | **399.2592** | **1194.7553** | **1195.3057** | **-0.5504** | **0** | **14** | **69** | **1** | **MGQTNVTSWR + Oxidation (M)** |
|  | 1587 | **456.7613** | **1367.2616** | **1366.5904** | **0.6712** | **0** | **14** | **75** | **1** | **GCSKPPWRPPGGK** |
|  | 2207 | **521.2814** | **1560.8219** | **1560.7282** | **0.0938** | **0** | **14** | **96** | **1** | **SSSNNSAPPKPMSLK + Oxidation (M)** |
|  | 3227 | **740.8931** | **1479.7715** | **1478.7104** | **1.0611** | **0** | **14** | **1.1e+02** | **1** | **IAPPEAPTGYMFGK** |
|  | 3388 | **811.7051** | **1621.3954** | **1620.8494** | **0.5459** | **1** | **14** | **78** | **1** | **KLCLNICVGESGDR + 2 Carbamidomethyl (C)** |
|  | 3389 | **811.9165** | **2432.7273** | **2432.8183** | **-0.0910** | **2** | **14** | **1.1e+02** | **1** | **RLTLSEIYQFLQARFPFFR** |
|  | 124 | **367.1088** | **1098.3042** | **1099.2811** | **-0.9769** | **0** | **14** | **1.1e+02** | **1** | **IVNVSLADLR** |
|  | 1219 | **427.3208** | **1278.9403** | **1279.5296** | **-0.5893** | **1** | **14** | **73** | **1** | **TMLQCLKQNK + Carbamidomethyl (C); Oxidation (M)** |
|  | 1588 | **457.0712** | **1368.1913** | **1367.4428** | **0.7486** | **2** | **14** | **99** | **1** | **KSANGSDDKASCK + Carbamidomethyl (C)** |
|  | 1635 | **459.1476** | **1374.4206** | **1373.6609** | **0.7596** | **1** | **14** | **1.1e+02** | **1** | **MLKASAASPAVALK + Oxidation (M)** |
|  | 2173 | **519.0980** | **1036.1813** | **1036.2040** | **-0.0227** | **1** | **14** | **97** | **1** | **SRITMSVDK** |
|  | 2277 | **529.6048** | **1057.1948** | **1057.1552** | **0.0396** | **1** | **14** | **1.3e+02** | **1** | **KNDPIDEVK** |
|  | 548 | **389.9991** | **777.9834** | **778.9823** | **-0.9988** | **0** | **14** | **1e+02** | **1** | **VVPLVPR** |
|  | 691 | **402.9279** | **1205.7616** | **1206.3898** | **-0.6282** | **0** | **14** | **1.1e+02** | **1** | **ATMASDPMPAAK + Oxidation (M)** |
|  | 1257 | **429.1661** | **856.3174** | **856.0016** | **0.3157** | **0** | **14** | **1e+02** | **1** | **MAEALHGK** |
|  | 1682 | **461.8256** | **1382.4547** | **1381.4478** | **1.0069** | **1** | **14** | **96** | **1** | **GGNQSGDGVSTKFK** |
|  | 2128 | **516.1387** | **1545.3938** | **1545.7366** | **-0.3428** | **2** | **14** | **1.1e+02** | **1** | **SDLEKASALNTKIR** |
|  | 487 | **388.1138** | **774.2128** | **773.8781** | **0.3348** | **1** | **14** | **1.2e+02** | **1** | **KTSVSPR** |
|  | 1245 | **429.0043** | **855.9938** | **855.9370** | **0.0568** | **0** | **14** | **95** | **1** | **WPQSNPK** |
|  | 3031 | **684.1172** | **1366.2197** | **1365.5096** | **0.7102** | **0** | **14** | **99** | **1** | **NIISLMDTSGNGK + Oxidation (M)** |
|  | 3262 | **742.4941** | **1482.9735** | **1483.6226** | **-0.6491** | **1** | **14** | **97** | **1** | **ALAAPVAEEKEEAR** |
|  | 1463 | **444.9861** | **887.9573** | **887.0356** | **0.9218** | **2** | **14** | **1.2e+02** | **1** | **KALNKEGK** |
|  | 872 | **407.3077** | **1218.9010** | **1219.4826** | **-0.5817** | **2** | **14** | **76** | **1** | **GVLGHLKARIR** |
|  | 2153 | **518.9446** | **1035.8744** | **1035.1762** | **0.6982** | **1** | **14** | **99** | **1** | **ESLEVRMR + Oxidation (M)** |
|  | 2718 | **611.6069** | **1221.1991** | **1220.3980** | **0.8011** | **2** | **14** | **99** | **1** | **DKGKECVWAK + Carbamidomethyl (C)** |
|  | 878 | **407.3473** | **1219.0197** | **1219.4776** | **-0.4579** | **1** | **14** | **80** | **1** | **NAICVNCIKK + 2 Carbamidomethyl (C)** |
|  | 1459 | **444.8020** | **1331.3838** | **1332.4832** | **-1.0993** | **1** | **14** | **1.1e+02** | **1** | **SQPSAAQPSKMGK + Oxidation (M)** |
|  | 377 | **384.9758** | **1151.9053** | **1151.3607** | **0.5446** | **1** | **14** | **88** | **1** | **LAEMCRSLR + Carbamidomethyl (C); Oxidation (M)** |
|  | 840 | **406.9557** | **1217.8448** | **1218.4054** | **-0.5606** | **1** | **14** | **92** | **1** | **SRVVLGEFGVR** |
|  | 1651 | **459.9963** | **917.9779** | **917.0220** | **0.9559** | **1** | **14** | **1.1e+02** | **1** | **ARETSVVR** |
|  | 298 | **377.2021** | **1128.5843** | **1129.1980** | **-0.6137** | **1** | **14** | **78** | **1** | **KDDYCEAGTK** |
|  | 1208 | **425.4241** | **1273.2500** | **1273.4177** | **-0.1676** | **1** | **14** | **1.2e+02** | **1** | **MSREYGPYVR + Oxidation (M)** |
|  | 634 | **400.3788** | **1198.1142** | **1198.3709** | **-0.2567** | **0** | **14** | **90** | **1** | **CASSLTSGMLR + Carbamidomethyl (C); Oxidation (M)** |
|  | 978 | **411.2498** | **1230.7271** | **1231.2964** | **-0.5693** | **2** | **14** | **88** | **1** | **RAGPGGKGADGCAD** |
|  | 1661 | **460.2983** | **1377.8728** | **1376.7113** | **1.1616** | **1** | **14** | **92** | **1** | **LACPVCSKMVPR + Carbamidomethyl (C); Oxidation (M)** |
|  | 3295 | **757.9788** | **1513.9429** | **1514.7956** | **-0.8527** | **2** | **14** | **86** | **1** | **ARLRVLMVVDGNR + Oxidation (M)** |
|  | 3300 | **759.0367** | **2274.0881** | **2274.5027** | **-0.4146** | **1** | **14** | **81** | **1** | **NIISLMDTSGNGKLEFDEFK + Oxidation (M)** |
|  | 552 | **390.0730** | **1167.1968** | **1168.2654** | **-1.0685** | **0** | **14** | **1e+02** | **1** | **SFSPHAGGRPR** |
|  | 2535 | **579.0081** | **1734.0022** | **1733.8831** | **0.1191** | **1** | **14** | **1e+02** | **1** | **AAPSPRPDRSPAGEGLR** |
|  | 484 | **388.0924** | **774.1700** | **773.8812** | **0.2888** | **1** | **14** | **1.2e+02** | **1** | **IRAASTR** |
|  | 567 | **393.2025** | **1176.5854** | **1177.3982** | **-0.8128** | **1** | **14** | **84** | **1** | **AMACGVAKTGPR + Oxidation (M)** |
|  | 2236 | **523.3424** | **1567.0050** | **1567.7474** | **-0.7423** | **1** | **14** | **95** | **1** | **MTGMTADSRSQVQR** |
|  | 132 | **367.2554** | **1098.7441** | **1098.3376** | **0.4065** | **0** | **14** | **93** | **1** | **FSLLQLHLK** |
|  | 379 | **385.0106** | **1152.0096** | **1151.2267** | **0.7829** | **1** | **14** | **89** | **1** | **WTKTAGSASDK** |
|  | 2469 | **565.4375** | **1693.2903** | **1693.9882** | **-0.6978** | **1** | **14** | **89** | **1** | **ECAMVLADFGARVVR + Carbamidomethyl (C)** |
|  | 2618 | **593.6600** | **1777.9579** | **1779.0261** | **-1.0682** | **1** | **14** | **1.3e+02** | **1** | **NADLCIGSGTKVALFNR** |
|  | 2998 | **681.0878** | **1360.1608** | **1360.4684** | **-0.3076** | **0** | **14** | **1e+02** | **1** | **ADYCPECYPDK + Carbamidomethyl (C)** |
|  | 3017 | **683.9336** | **2048.7786** | **2048.1708** | **0.6078** | **0** | **14** | **85** | **1** | **VGNGFGGYCSSTSCYHFDF** |
|  | 388 | **385.1103** | **768.2058** | **767.9580** | **0.2478** | **1** | **14** | **91** | **1** | **VSCCKTK** |
|  | 1187 | **423.1865** | **1266.5372** | **1266.3671** | **0.1701** | **2** | **14** | **1.1e+02** | **1** | **VSQHRGPDKSR** |
|  | 1934 | **487.3784** | **972.7419** | **973.0852** | **-0.3432** | **2** | **14** | **92** | **1** | **SKSRSPSPK** |
|  | 2088 | **507.3115** | **1518.9124** | **1518.7129** | **0.1995** | **1** | **14** | **93** | **1** | **DILRVSEAEFLAR** |
|  | 2702 | **610.1075** | **1827.3005** | **1827.1072** | **0.1932** | **2** | **14** | **1.1e+02** | **1** | **CLDDIEKKLASLPEPR** |
|  | 3518 | **917.8549** | **2750.5424** | **2751.0916** | **-0.5492** | **2** | **14** | **77** | **1** | **QPGTAQRPACRHGKSLSHLPLHSSK + Carbamidomethyl (C)** |
|  | 343 | **381.2781** | **1140.8122** | **1141.2317** | **-0.4195** | **0** | **14** | **1e+02** | **1** | **DAETGALIHSK** |
|  | 1228 | **428.2074** | **1281.6000** | **1281.3734** | **0.2266** | **1** | **14** | **89** | **1** | **DFGSGFPVKDGR** |
|  | 1403 | **437.3715** | **872.7283** | **873.0056** | **-0.2774** | **0** | **14** | **99** | **1** | **EVILTGGGK** |
|  | 1542 | 451.9525 | 1352.8353 | 1352.5807 | 0.2546 | 1 | 14 | 1.2e+02 | 1 | IEESVRSMVMR + Oxidation (M) |
|  | 1953 | **488.2327** | **1461.6759** | **1460.7430** | **0.9329** | **2** | **14** | **1.1e+02** | **1** | **LLKTWREMDIR** |
|  | 2368 | **541.0692** | **1080.1236** | **1079.1906** | **0.9331** | **1** | **14** | **1e+02** | **1** | **DRMSYHVR + Oxidation (M)** |
|  | 2441 | **560.0367** | **1677.0881** | **1675.9265** | **1.1616** | **1** | **14** | **1.1e+02** | **1** | **VPPGKNYTYVWPVR** |
|  | 100 | **365.9615** | **729.9081** | **728.8405** | **1.0676** | **1** | **14** | **1.2e+02** | **1** | **AGAGKIGR** |
|  | 1771 | **470.1262** | **1407.3565** | **1406.7154** | **0.6411** | **0** | **14** | **94** | **1** | **ACLVVSLCISWR + Carbamidomethyl (C)** |
|  | 2364 | **540.4028** | **1618.1861** | **1618.8818** | **-0.6956** | **2** | **14** | **84** | **1** | **SHREGVFKCPLCSR** |
|  | 980 | **411.4339** | **1231.2795** | **1230.3284** | **0.9512** | **1** | **14** | **1.3e+02** | **1** | **SVVKGGAADVDGR** |
|  | 1659 | **460.2842** | **1377.8304** | **1376.6449** | **1.1855** | **1** | **14** | **95** | **1** | **FCFPCSREFIK** |
|  | 1828 | **475.4780** | **948.9413** | **949.1018** | **-0.1605** | **1** | **14** | **1.2e+02** | **1** | **KFTSPLEK** |
|  | 898 | **407.7693** | **1220.2857** | **1220.3566** | **-0.0709** | **1** | **14** | **1.1e+02** | **1** | **LSVGKGADCSQR** |
|  | 1777 | **470.3255** | **1407.9544** | **1408.6025** | **-0.6480** | **2** | **14** | **76** | **1** | **AREKEKPTGPPAK** |
|  | 2247 | **524.5607** | **1047.1066** | **1048.2361** | **-1.1295** | **1** | **14** | **1.3e+02** | **1** | **KSGVGNIFVK** |
|  | 3062 | **686.0480** | **2055.1219** | **2054.3841** | **0.7378** | **0** | **14** | **92** | **1** | **IELIQDFEMPTVCTTIK + Carbamidomethyl (C); Oxidation (M)** |
|  | 591 | **398.1736** | **1191.4988** | **1192.6444** | **-1.1457** | **2** | **14** | **1e+02** | **1** | **IKMCLIKLCK** |
|  | 1674 | **460.7448** | **1379.2121** | **1378.6609** | **0.5512** | **2** | **14** | **90** | **1** | **LRLCMEKVDQK + Oxidation (M)** |
|  | 2051 | **504.8232** | **1511.4476** | **1511.6806** | **-0.2330** | **1** | **14** | **87** | **1** | **QAPGKGPEWVSSIR** |
|  | 2572 | **588.0833** | **1174.1518** | **1173.3199** | **0.8319** | **0** | **14** | **1.1e+02** | **1** | **YSNCMGEITR** |
|  | 285 | **376.3890** | **1126.1449** | **1126.3015** | **-0.1565** | **0** | **14** | **1.1e+02** | **1** | **FFLSSGLIDK** |
|  | 1603 | **458.2073** | **1371.5997** | **1371.5872** | **0.0126** | **1** | **14** | **1.1e+02** | **1** | **YRERPVLPVSR** |
|  | 2648 | **597.5290** | **1193.0432** | **1192.4741** | **0.5691** | **1** | **14** | **92** | **1** | **AGRVLVFVMGK + Oxidation (M)** |
|  | 458 | **387.1521** | **772.2894** | **771.8024** | **0.4870** | **0** | **14** | **1.2e+02** | **1** | **ACGDGHR + Carbamidomethyl (C)** |
|  | 732 | **404.0356** | **1209.0848** | **1209.4811** | **-0.3964** | **1** | **14** | **1.1e+02** | **1** | **LPLLTRLDLR** |
|  | 1855 | **476.4523** | **950.8899** | **951.0531** | **-0.1632** | **0** | **14** | **97** | **1** | **TLEEQMGK + Oxidation (M)** |
|  | 441 | **386.9180** | **771.8212** | **771.9283** | **-0.1071** | **0** | **14** | **1.2e+02** | **1** | **AGLVCPR + Carbamidomethyl (C)** |
|  | 606 | **399.3383** | **1194.9927** | **1195.2213** | **-0.2286** | **1** | **14** | **77** | **1** | **CTDDGAQSRSR** |
|  | 1127 | **421.6924** | **1262.0552** | **1261.5177** | **0.5374** | **1** | **14** | **87** | **1** | **VCCQDIRVLGR** |
|  | 1729 | **465.2072** | **928.3996** | **928.1140** | **0.2857** | **1** | **14** | **1.1e+02** | **1** | **AGAMRLGPR** |
|  | 2358 | **540.0470** | **1078.0792** | **1079.1706** | **-1.0914** | **2** | **14** | **1.1e+02** | **1** | **GRGGSSGAKFR** |
|  | 2988 | **680.0172** | **2037.0295** | **2037.3154** | **-0.2860** | **0** | **14** | **90** | **1** | **LQPISSEQLCLSSQMTAK + Carbamidomethyl (C); Oxidation (M)** |
|  | 1694 | **462.2449** | **1383.7124** | **1384.6039** | **-0.8915** | **0** | **14** | **96** | **1** | **NGLITMQSHVIR + Oxidation (M)** |
|  | 1728 | **465.1898** | **1392.5474** | **1391.4676** | **1.0798** | **0** | **14** | **1.1e+02** | **1** | **TSGTVSHSGNTMGR** |
|  | 3147 | **718.6216** | **1435.2284** | **1435.6971** | **-0.4687** | **2** | **14** | **85** | **1** | **CPCCLWRTPRS + 2 Carbamidomethyl (C)** |
|  | 757 | **405.0155** | **808.0162** | **806.9940** | **1.0223** | **0** | **14** | **98** | **1** | **MQKPCK + Carbamidomethyl (C); Oxidation (M)** |
|  | 1117 | **421.0551** | **840.0954** | **839.9776** | **0.1178** | **0** | **14** | **97** | **1** | **MDSQMTK** |
|  | 1607 | **458.4243** | **914.8337** | **915.0058** | **-0.1721** | **0** | **14** | **1e+02** | **1** | **GLSGAAAAAAR** |
|  | 2283 | **530.2350** | **1587.6830** | **1587.8909** | **-0.2079** | **1** | **14** | **1.2e+02** | **1** | **VLVEKCGHHCFCR + Carbamidomethyl (C)** |
|  | 2684 | **607.7892** | **1820.3454** | **1820.0338** | **0.3116** | **2** | **14** | **1e+02** | **1** | **TNKPMAGSKKTSSPTER** |
|  | 2956 | **669.8655** | **2006.5743** | **2006.1839** | **0.3904** | **1** | **14** | **1e+02** | **1** | **GSHTLQRMFGCDVGPDGR + Carbamidomethyl (C); Oxidation (M)** |
|  | 2654 | **598.2770** | **1194.5392** | **1194.2481** | **0.2911** | **0** | **14** | **1.1e+02** | **1** | **DSNGLSDPYVK** |
|  | 3536 | **1009.1198** | **3024.3371** | **3025.4151** | **-1.0781** | **2** | **14** | **1.1e+02** | **1** | **TPVFPPQIEGGIVYCSSSASIENKGKCR + Carbamidomethyl (C)** |
|  | 596 | 398.9796 | 795.9444 | 795.8835 | 0.0609 | 0 | 14 | 93 | 1 | AGAPGAPGAK |
|  | 1390 | **437.0512** | **1308.1315** | **1308.5923** | **-0.4609** | **0** | **14** | **1.2e+02** | **1** | **IIVISHNIPMR + Oxidation (M)** |
|  | 109 | **366.1812** | **1095.5213** | **1096.2791** | **-0.7578** | **0** | **14** | **1e+02** | **1** | **EMQTLMGVR + 2 Oxidation (M)** |
|  | 257 | **374.2417** | **1119.7030** | **1120.3052** | **-0.6022** | **2** | **14** | **1e+02** | **1** | **RLIYKASNR** |
|  | 3222 | **740.7541** | **2219.2403** | **2219.4294** | **-0.1891** | **1** | **14** | **1e+02** | **1** | **EMEQFFSTFGELTVEPRR + Oxidation (M)** |
|  | 3403 | **821.1081** | **1640.2014** | **1639.0571** | **1.1443** | **2** | **14** | **79** | **1** | **MKMVVFFSFKCAK + Carbamidomethyl (C); Oxidation (M)** |
|  | 1648 | **459.8963** | **1376.6667** | **1377.5204** | **-0.8537** | **0** | **14** | **1.2e+02** | **1** | **ILNCSETVVEDR** |
|  | 2646 | **597.1801** | **1788.5180** | **1789.0624** | **-0.5444** | **1** | **14** | **1.2e+02** | **1** | **GMISNLLGKGAVDQLTR + Oxidation (M)** |
|  | 947 | **409.0146** | **816.0144** | **814.8869** | **1.1275** | **1** | **14** | **1.2e+02** | **1** | **QAPESRK** |
|  | 2437 | **559.1018** | **1674.2832** | **1674.8305** | **-0.5473** | **0** | **14** | **1.1e+02** | **1** | **DNGLCGAGAEALAGALSK + Carbamidomethyl (C)** |
|  | 175 | **369.2527** | **1104.7360** | **1105.2031** | **-0.4671** | **2** | **14** | **94** | **1** | **KTPSSSSRQK** |
|  | 1298 | **431.1752** | **1290.5035** | **1290.5143** | **-0.0107** | **2** | **14** | **1.3e+02** | **1** | **ASVFSNLRIRK** |
|  | 1928 | **487.1191** | **1458.3353** | **1457.6383** | **0.6970** | **2** | **14** | **1.1e+02** | **1** | **RGSCVSPYSSRCR** |
|  | 151 | **369.0662** | **1104.1765** | **1105.2908** | **-1.1143** | **1** | **14** | **1.2e+02** | **1** | **RAVLAVHPDK** |
|  | 250 | **374.1530** | **1119.4370** | **1119.3354** | **0.1015** | **1** | **14** | **1.2e+02** | **1** | **KLLQDIMSR + Oxidation (M)** |
|  | 798 | **406.0182** | **810.0216** | **808.9683** | **1.0533** | **1** | **14** | **99** | **1** | **HLTARVL** |
|  | 1003 | **413.6297** | **1237.8668** | **1237.4086** | **0.4583** | **0** | **14** | **73** | **1** | **GPCGPDCAPPAPR** |
|  | 1733 | **466.0569** | **1395.1485** | **1394.6783** | **0.4701** | **2** | **14** | **1.2e+02** | **1** | **YMDKEIINLKK** |
|  | 252 | **374.1794** | **1119.5161** | **1120.2953** | **-0.7793** | **0** | **14** | **1.1e+02** | **1** | **DAGIYEVILK** |
|  | 2774 | **623.7775** | **1245.5402** | **1246.4983** | **-0.9581** | **0** | **14** | **1.2e+02** | **1** | **MCSSCLVCESK + Carbamidomethyl (C)** |
|  | 3159 | **725.8512** | **2174.5314** | **2173.3344** | **1.1970** | **0** | **14** | **1.3e+02** | **1** | **SFFPTMEEMFGGGAADDYGK + Oxidation (M)** |
|  | 348 | **382.2834** | **1143.8280** | **1144.2820** | **-0.4541** | **2** | **14** | **1e+02** | **1** | **ILKRESGEGR** |
|  | 1031 | **415.6803** | **1244.0186** | **1244.4410** | **-0.4223** | **1** | **14** | **1e+02** | **1** | **LDSAFRCMSSK** |
|  | 1618 | **458.8293** | **1373.4659** | **1373.5963** | **-0.1304** | **0** | **14** | **1.2e+02** | **1** | **SSKPNLLGPAVYK** |
|  | 1680 | **461.7959** | **1382.3655** | **1381.6798** | **0.6858** | **1** | **14** | **95** | **1** | **FLMSVTKLVESK** |
|  | 3104 | **697.6865** | **2090.0374** | **2089.3784** | **0.6589** | **1** | **14** | **93** | **1** | **CESPHRGETIQMQCVWK + Carbamidomethyl (C)** |
|  | 3376 | **804.1034** | **1606.1920** | **1605.9457** | **0.2463** | **0** | **14** | **89** | **1** | **YLIPCNHMMLSQR** |
|  | 1584 | **456.3340** | **910.6533** | **910.0060** | **0.6474** | **0** | **14** | **81** | **1** | **IYCPDSR + Carbamidomethyl (C)** |
|  | 1620 | **458.8632** | **1373.5675** | **1374.5595** | **-0.9921** | **0** | **14** | **1.2e+02** | **1** | **ALEPLEGLETMR + Oxidation (M)** |
|  | 2497 | **571.2662** | **1710.7765** | **1710.9306** | **-0.1541** | **2** | **14** | **1.1e+02** | **1** | **SFEQYLLLGSRNRK** |
|  | 3032 | **684.1587** | **1366.3026** | **1365.7661** | **0.5365** | **0** | **14** | **1.1e+02** | **1** | **ILGLLAAMLPPLK + Oxidation (M)** |
|  | 163 | **369.1906** | **1104.5497** | **1105.3089** | **-0.7592** | **0** | **14** | **97** | **1** | **AKPWAVCFPS** |
|  | 1434 | **443.0504** | **884.0860** | **884.0118** | **0.0743** | **0** | **14** | **1e+02** | **1** | **MASLFSGR + Oxidation (M)** |
|  | 2696 | **609.9280** | **1217.8413** | **1217.4852** | **0.3561** | **2** | **14** | **92** | **1** | **KSCQACRFMK + Oxidation (M)** |
|  | 2919 | **667.6607** | **1333.3066** | **1333.3931** | **-0.0865** | **0** | **14** | **1.1e+02** | **1** | **GAGHHHSLDSCR + Carbamidomethyl (C)** |
|  | 3195 | **740.3466** | **1478.6785** | **1477.6443** | **1.0342** | **2** | **14** | **1.1e+02** | **1** | **NKQWGKDSLCNK + Carbamidomethyl (C)** |
|  | 708 | **403.8510** | **1208.5308** | **1207.4257** | **1.1051** | **2** | **14** | **1.1e+02** | **1** | **PLVKRNIDPR** |
|  | 1241 | **428.9232** | **1283.7475** | **1284.5049** | **-0.7574** | **2** | **14** | **99** | **1** | **KQTCPYCKEK + Carbamidomethyl (C)** |
|  | 1427 | **442.1773** | **882.3398** | **883.0867** | **-0.7468** | **1** | **14** | **1e+02** | **1** | **KVLAIDPK** |
|  | 1456 | **444.0914** | **1329.2519** | **1329.4840** | **-0.2321** | **2** | **14** | **1.2e+02** | **1** | **RFATGRYLADC + Carbamidomethyl (C)** |
|  | 2790 | **628.6809** | **1255.3470** | **1255.5499** | **-0.2028** | **2** | **14** | **1.3e+02** | **1** | **KEKMVGAAFMK + Oxidation (M)** |
|  | 74 | **364.1583** | **1089.4526** | **1090.1835** | **-0.7308** | **0** | **14** | **89** | **1** | **AAVTEFADPVA** |
|  | 157 | **369.1674** | **1104.4802** | **1105.3089** | **-0.8287** | **0** | **14** | **1.1e+02** | **1** | **AKPWAVCFPS** |
|  | 1207 | **425.4104** | **848.8060** | **847.9863** | **0.8198** | **2** | **14** | **1.2e+02** | **1** | **SRGRGGMK** |
|  | 2547 | **582.2152** | **1162.4156** | **1163.3698** | **-0.9541** | **2** | **14** | **1.2e+02** | **1** | **GGSLLKAFSRK** |
|  | 517 | **389.1259** | **776.2370** | **775.9171** | **0.3199** | **1** | **14** | **1.2e+02** | **1** | **MKAPGTR + Oxidation (M)** |
|  | 1975 | **490.3197** | **978.6246** | **979.0746** | **-0.4501** | **1** | **14** | **93** | **1** | **TYHSCRR + Carbamidomethyl (C)** |
|  | 284 | **376.3475** | **750.6802** | **750.8065** | **-0.1262** | **1** | **14** | **99** | **1** | **SSHHRK** |
|  | 1066 | **419.0698** | **1254.1871** | **1254.4588** | **-0.2717** | **1** | **14** | **1.3e+02** | **1** | **CLGSPQHLKSK + Carbamidomethyl (C)** |
|  | 3263 | **742.5995** | **2224.7763** | **2223.6743** | **1.1020** | **1** | **14** | **88** | **1** | **LPLLQLVPAKLLNSSCSLEK + Carbamidomethyl (C)** |
|  | 3382 | **808.4423** | **1614.8697** | **1613.8253** | **1.0444** | **1** | **14** | **1.1e+02** | **1** | **KCHLHGHSCPQGPR + Carbamidomethyl (C)** |
|  | 125 | **367.1133** | **1098.3179** | **1097.1992** | **1.1187** | **0** | **14** | **1.2e+02** | **1** | **YNASCSVPEK** |
|  | 524 | **389.1554** | **776.2959** | **775.9169** | **0.3790** | **0** | **14** | **1.2e+02** | **1** | **AMLAGSAR** |
|  | 536 | **389.4387** | **1165.2940** | **1165.2731** | **0.0209** | **0** | **14** | **1.4e+02** | **1** | **DFCVQAPESL + Carbamidomethyl (C)** |
|  | 838 | **406.8807** | **1217.6200** | **1218.4054** | **-0.7853** | **1** | **14** | **97** | **1** | **SRVVLGEFGVR** |
|  | 2488 | **569.0436** | **1704.1086** | **1705.0338** | **-0.9253** | **2** | **14** | **1.2e+02** | **1** | **VSVLKQLFADRCLR + Carbamidomethyl (C)** |
|  | 2514 | 574.8275 | 1721.4604 | 1722.1255 | -0.6652 | 1 | 14 | 94 | 1 | LAALLPDLLVFRKPR |
|  | 1076 | **419.1700** | **1254.4880** | **1253.2969** | **1.1911** | **0** | **14** | **1.3e+02** | **1** | **DSWGMDVWGQG + Oxidation (M)** |
|  | 1553 | **452.2278** | **1353.6611** | **1353.5751** | **0.0860** | **1** | **14** | **1.3e+02** | **1** | **CHSCSCRVCGGK + 2 Carbamidomethyl (C)** |
|  | 2269 | **528.8207** | **1055.6266** | **1055.2717** | **0.3548** | **2** | **14** | **87** | **1** | **ERLKATLPK** |
|  | 647 | **401.1692** | **1200.4856** | **1201.3582** | **-0.8726** | **1** | **14** | **1.2e+02** | **1** | **SPPTGACWRAR** |
|  | 736 | **404.0572** | **1209.1494** | **1208.4088** | **0.7406** | **0** | **14** | **1.1e+02** | **1** | **IGTNQMAVCAK + Carbamidomethyl (C); Oxidation (M)** |
|  | 200 | **369.3347** | **1104.9818** | **1104.2779** | **0.7039** | **1** | **14** | **1.1e+02** | **1** | **NGKLMESTPK** |
|  | 1409 | **438.1425** | **874.2703** | **873.9492** | **0.3211** | **0** | **14** | **1.2e+02** | **1** | **MDTSSSCK + Oxidation (M)** |
|  | 191 | **369.2862** | **1104.8363** | **1105.3089** | **-0.4726** | **0** | **14** | **97** | **1** | **AKPWAVCFPS** |
|  | 421 | **386.1736** | **1155.4988** | **1155.3212** | **0.1775** | **0** | **14** | **93** | **1** | **DFSAISLACTK** |
|  | 523 | **389.1553** | **1164.4438** | **1163.3268** | **1.1171** | **1** | **14** | **1.2e+02** | **1** | **CSECGKFFSR** |
|  | 616 | **399.9484** | **1196.8229** | **1196.3103** | **0.5126** | **1** | **14** | **94** | **1** | **KGIVEHEEQK** |
|  | 1405 | **437.5688** | **873.1227** | **873.9076** | **-0.7849** | **0** | **14** | **1.4e+02** | **1** | **EALAAEDR** |
|  | 1524 | **450.6901** | **899.3654** | **899.0694** | **0.2960** | **0** | **14** | **93** | **1** | **QVTCHLAK** |
|  | 3083 | **687.3848** | **2059.1321** | **2059.3395** | **-0.2073** | **2** | **14** | **1.1e+02** | **1** | **VTAASSAPQRRPPGVRRPR** |
|  | 351 | **382.3382** | **1143.9924** | **1144.2986** | **-0.3061** | **0** | **14** | **1.2e+02** | **1** | **IDCQGIPPSSK** |
|  | 371 | **384.9101** | **767.8054** | **768.8152** | **-1.0097** | **0** | **14** | **96** | **1** | **THTGEKP** |
|  | 305 | **377.8078** | **1130.4011** | **1131.3066** | **-0.9054** | **1** | **14** | **1.1e+02** | **1** | **MPNGSAGVKVR + Oxidation (M)** |
|  | 1116 | **421.0111** | **840.0075** | **840.9408** | **-0.9333** | **0** | **14** | **1e+02** | **1** | **MPSSSFSV** |
|  | 1239 | **428.8130** | **1283.4167** | **1283.4937** | **-0.0770** | **0** | **14** | **1e+02** | **1** | **MAEDPAALKPPK + Oxidation (M)** |
|  | 237 | **372.3751** | **1114.1031** | **1114.2542** | **-0.1511** | **0** | **14** | **1.3e+02** | **1** | **INFDHSLIR** |
|  | 990 | **412.2363** | **1233.6868** | **1234.4196** | **-0.7328** | **0** | **14** | **97** | **1** | **EATLEGLQMVK + Oxidation (M)** |
|  | 2580 | **590.3533** | **1768.0378** | **1768.9388** | **-0.9010** | **0** | **14** | **1.2e+02** | **1** | **SCAVDLTTAAVAFGDEAK** |
|  | 3267 | **742.6904** | **1483.3661** | **1482.7850** | **0.5810** | **0** | **14** | **86** | **1** | **SGMGLTFLLAPFTK** |
|  | 161 | **369.1862** | **1104.5365** | **1105.3089** | **-0.7724** | **0** | **14** | **1e+02** | **1** | **AKPWAVCFPS** |
|  | 1668 | **460.5143** | **1378.5207** | **1378.4900** | **0.0306** | **0** | **14** | **1.4e+02** | **1** | **NNTLSSHLHIDK** |
|  | 2194 | **520.2742** | **1557.8005** | **1558.8332** | **-1.0326** | **2** | **14** | **1.1e+02** | **1** | **GTLQPRPRPPRKR** |
|  | 1522 | **450.5916** | **899.1683** | **899.0694** | **0.0989** | **0** | **14** | **1.2e+02** | **1** | **QVTCHLAK** |
|  | 243 | **373.1257** | **744.2366** | **743.8122** | **0.4243** | **1** | **14** | **1.4e+02** | **1** | **EAAAARR** |
|  | 590 | **398.1548** | **1191.4423** | **1192.3678** | **-0.9256** | **0** | **14** | **1.2e+02** | **1** | **AILHQLVDQR** |
|  | 1305 | **432.2161** | **862.4175** | **862.0527** | **0.3648** | **2** | **14** | **1.1e+02** | **1** | **GMKVRQK + Oxidation (M)** |
|  | 1312 | **432.5979** | **863.1810** | **862.9330** | **0.2480** | **0** | **14** | **1.1e+02** | **1** | **HASAAPPGR** |
|  | 1955 | **488.3181** | **1461.9320** | **1462.6013** | **-0.6693** | **0** | **14** | **1e+02** | **1** | **GLELGGLDAFDIWG** |
|  | 1451 | **443.9084** | **885.8021** | **885.9184** | **-0.1162** | **0** | **14** | **1.2e+02** | **1** | **LPDAADER** |
|  | 3365 | **792.0099** | **2373.0077** | **2372.5828** | **0.4249** | **2** | **14** | **96** | **1** | **RWQGGERSMSGHSGPGHMMDR + Oxidation (M)** |
|  | 1277 | **430.3044** | **858.5941** | **857.9942** | **0.5999** | **0** | **14** | **1e+02** | **1** | **AQAASLLGK** |
|  | 1738 | **466.1328** | **1395.3761** | **1395.6302** | **-0.2541** | **2** | **14** | **1.2e+02** | **1** | **KHVMEVRQQPK + Oxidation (M)** |
|  | 2123 | **515.6356** | **1029.2563** | **1030.2223** | **-0.9660** | **0** | **14** | **1.4e+02** | **1** | **HLQCVSCAL + Carbamidomethyl (C)** |
|  | 3353 | **787.2421** | **2358.7040** | **2357.8217** | **0.8824** | **2** | **14** | **1.1e+02** | **1** | **RLPKPQTAMEMLMQGRPGKR + 2 Oxidation (M)** |
|  | 702 | **403.1666** | **804.3183** | **804.9548** | **-0.6365** | **1** | **14** | **1.2e+02** | **1** | **ICQEKK + Carbamidomethyl (C)** |
|  | 1310 | **432.4085** | **862.8022** | **862.9941** | **-0.1919** | **0** | **14** | **1.2e+02** | **1** | **SICSLGAR + Carbamidomethyl (C)** |
|  | 2709 | **610.2073** | **1218.3998** | **1217.4438** | **0.9560** | **2** | **14** | **1.2e+02** | **1** | **MHKIFRETR** |
|  | 117 | **366.2577** | **1095.7509** | **1096.2641** | **-0.5132** | **2** | **14** | **1e+02** | **1** | **ERGRLMYR + Oxidation (M)** |
|  | 2532 | **578.6816** | **1733.0228** | **1732.9577** | **0.0650** | **1** | **14** | **1.4e+02** | **1** | **ISCKAFGYSFPHYR + Carbamidomethyl (C)** |
|  | 3036 | **684.2378** | **2049.6912** | **2049.3511** | **0.3401** | **2** | **14** | **1.1e+02** | **1** | **DKAYEGGQLCAMCFSPKK + Carbamidomethyl (C); Oxidation (M)** |
|  | 317 | **379.2220** | **756.4293** | **756.8907** | **-0.4614** | **0** | **14** | **90** | **1** | **ADVVVVR** |
|  | 966 | **410.2425** | **1227.7053** | **1227.4188** | **0.2865** | **1** | **14** | **1e+02** | **1** | **FRPAGPRATVR** |
|  | 2049 | **504.3192** | **1509.9354** | **1509.6863** | **0.2490** | **1** | **14** | **1.1e+02** | **1** | **YTCVAVNAAGEKQR** |
|  | 2403 | **551.0681** | **1100.1214** | **1099.1738** | **0.9477** | **0** | **14** | **1.2e+02** | **1** | **SMSSTSASAVR + Oxidation (M)** |
|  | 1909 | **482.4860** | **1444.4357** | **1445.5759** | **-1.1402** | **0** | **14** | **1.3e+02** | **1** | **GLEWVSSIGGNGAAK** |
|  | 2566 | **586.0381** | **1170.0615** | **1169.3313** | **0.7302** | **1** | **14** | **1.2e+02** | **1** | **IEWMNMTRG + 2 Oxidation (M)** |
|  | 190 | **369.2857** | **1104.8349** | **1105.3089** | **-0.4740** | **0** | **14** | **1e+02** | **1** | **AKPWAVCFPS** |
|  | 2210 | **521.5085** | **1041.0023** | **1041.2916** | **-0.2893** | **1** | **14** | **1.1e+02** | **1** | **IPMMKEHR** |
|  | 2293 | **532.4921** | **1062.9693** | **1063.1830** | **-0.2136** | **1** | **14** | **1e+02** | **1** | **MVDANKEEK** |
|  | 2903 | **666.7506** | **1997.2297** | **1998.1902** | **-0.9606** | **0** | **14** | **1.4e+02** | **1** | **MTEGDMGVTLEASTEADLK** |
|  | 725 | **404.0048** | **805.9948** | **805.9197** | **0.0751** | **0** | **14** | **1.2e+02** | **1** | **NPFPFGK** |
|  | 2723 | **612.1172** | **1222.2196** | **1221.4489** | **0.7706** | **1** | **14** | **1.2e+02** | **1** | **APALASTPRLPK** |
|  | 296 | **377.1763** | **752.3378** | **752.7280** | **-0.3901** | **0** | **14** | **96** | **1** | **GSSGSSGSK** |
|  | 1864 | **477.6593** | **1429.9557** | **1430.5200** | **-0.5643** | **2** | **14** | **94** | **1** | **EEKLLRDAQQNS** |
|  | 2303 | **533.8936** | **1598.6585** | **1597.8376** | **0.8209** | **2** | **14** | **1.1e+02** | **1** | **LHEDGARCKTLLGK + Carbamidomethyl (C)** |
|  | 3241 | **741.7493** | **2222.2258** | **2221.4052** | **0.8206** | **1** | **14** | **1e+02** | **1** | **VQNEGTGKSSWWMLNPEGGK + Oxidation (M)** |
|  | 192 | **369.2907** | **1104.8501** | **1105.3089** | **-0.4588** | **0** | **14** | **1e+02** | **1** | **AKPWAVCFPS** |
|  | 718 | **403.9505** | **1208.8293** | **1208.3939** | **0.4355** | **2** | **14** | **1.2e+02** | **1** | **RRFAGAGCVSK + Carbamidomethyl (C)** |
|  | 1177 | **422.2901** | **842.5655** | **841.9949** | **0.5705** | **0** | **14** | **1e+02** | **1** | **GITLSVRP** |
|  | 2050 | **504.5847** | **1510.7320** | **1510.7769** | **-0.0450** | **0** | **14** | **1.4e+02** | **1** | **ETGLNVFLLDMCR** |
|  | 3503 | **898.0510** | **2691.1309** | **2691.9961** | **-0.8652** | **0** | **14** | **1.3e+02** | **1** | **SQWPQSAHILLGSHTLLGAVGEPHR** |
|  | 1876 | **478.6296** | **955.2444** | **954.1265** | **1.1179** | **1** | **14** | **1.1e+02** | **1** | **MGLSSCRK + Carbamidomethyl (C); Oxidation (M)** |
|  | 2444 | **561.2789** | **1680.8146** | **1679.8720** | **0.9426** | **1** | **14** | **1.2e+02** | **1** | **DAAARLTISSPLEAHK** |
|  | 2773 | **623.7124** | **1868.1150** | **1867.1826** | **0.9324** | **2** | **14** | **1.4e+02** | **1** | **MAAAPPLRDRLSFLHR + Oxidation (M)** |
|  | 3113 | **700.4631** | **1398.9115** | **1398.6107** | **0.3008** | **2** | **14** | **1.1e+02** | **1** | **EQLLSERRLVR** |
|  | 391 | **385.1668** | **768.3188** | **768.8997** | **-0.5809** | **0** | **14** | **95** | **1** | **MAMQEK + 2 Oxidation (M)** |
|  | 2400 | **550.3952** | **1098.7756** | **1099.2416** | **-0.4659** | **2** | **14** | **99** | **1** | **KAGVAHSKSSK** |
|  | 3242 | **741.7656** | **1481.5165** | **1480.6649** | **0.8515** | **1** | **14** | **1.1e+02** | **1** | **TQSVNLPKELHSK** |
|  | 189 | **369.2850** | **1104.8327** | **1105.3089** | **-0.4761** | **0** | **14** | **1e+02** | **1** | **AKPWAVCFPS** |
|  | 461 | **387.1675** | **772.3202** | **772.8931** | **-0.5729** | **1** | **14** | **1.3e+02** | **1** | **KLAGATGR** |
|  | 701 | **403.1572** | **1206.4494** | **1207.4688** | **-1.0193** | **1** | **14** | **1.3e+02** | **1** | **ITQPVLKRPR** |
|  | 1569 | **455.7545** | **1364.2413** | **1364.5912** | **-0.3499** | **0** | **14** | **92** | **1** | **LSCSVLNSSPAMR** |
|  | 1929 | **487.1660** | **972.3172** | **972.2889** | **0.0282** | **1** | **14** | **1.3e+02** | **1** | **GLGLKIMLK** |
|  | 2233 | **523.2655** | **1566.7743** | **1565.6653** | **1.1091** | **2** | **14** | **1.2e+02** | **1** | **TDCQESSKEAVRR + Carbamidomethyl (C)** |
|  | 3087 | **689.1022** | **1376.1896** | **1377.3665** | **-1.1769** | **0** | **14** | **1.2e+02** | **1** | **GSGDDTYYADSVK** |
|  | 1075 | **419.1698** | **1254.4872** | **1253.4760** | **1.0112** | **1** | **14** | **1.4e+02** | **1** | **MRGCPMAGGSVR + 2 Oxidation (M)** |
|  | 2204 | **521.2007** | **1560.5799** | **1559.6785** | **0.9014** | **0** | **14** | **1.2e+02** | **1** | **QNYIEDLQNGPIR** |
|  | 2795 | **629.9404** | **1886.7991** | **1887.2088** | **-0.4097** | **2** | **14** | **96** | **1** | **DMRLMQGDEICLRYK + Oxidation (M)** |
|  | 89 | **365.2047** | **1092.5920** | **1092.2242** | **0.3678** | **1** | **14** | **99** | **1** | **CLETDVEKR** |
|  | 178 | **369.2622** | **1104.7644** | **1104.2991** | **0.4653** | **0** | **14** | **1e+02** | **1** | **QQLLIGAYAK** |
|  | 206 | **369.4707** | **1105.3900** | **1105.3089** | **0.0812** | **0** | **14** | **1.4e+02** | **1** | **AKPWAVCFPS** |
|  | 1123 | **421.3331** | **1260.9770** | **1261.4516** | **-0.4745** | **1** | **14** | **95** | **1** | **IVGENISRMSR** |
|  | 1316 | **432.9133** | **1295.7176** | **1296.5319** | **-0.8143** | **1** | **14** | **1.2e+02** | **1** | **QYESLKILICS** |
|  | 1753 | **467.9209** | **1400.7406** | **1401.6311** | **-0.8905** | **1** | **14** | **1.3e+02** | **1** | **DPKQTTLLCLGR + Carbamidomethyl (C)** |
|  | 3496 | **891.8281** | **1781.6415** | **1782.1579** | **-0.5164** | **1** | **14** | **89** | **1** | **RLFETMCTSCMCVK + 2 Carbamidomethyl (C); Oxidation (M)** |
|  | 479 | 387.9558 | 773.8968 | 772.8932 | 1.0036 | 1 | 14 | 1.5e+02 | 1 | LRTEVR |
|  | 629 | **400.2180** | **1197.6319** | **1196.4628** | **1.1691** | **2** | **14** | **87** | **1** | **TYGTIVKMKR** |
|  | 1999 | **495.8062** | **989.5976** | **989.1508** | **0.4469** | **1** | **14** | **1e+02** | **1** | **GRPKGSEMK** |
|  | 3273 | **743.6469** | **1485.2790** | **1485.5786** | **-0.2996** | **2** | **14** | **92** | **1** | **YNGESSGKQGRSLC** |
|  | 159 | **369.1825** | **1104.5252** | **1105.2229** | **-0.6976** | **1** | **14** | **1.1e+02** | **1** | **EKSPQMGANK + Oxidation (M)** |
|  | 1383 | **436.2274** | **1305.6599** | **1305.6134** | **0.0464** | **2** | **14** | **1.1e+02** | **1** | **MERLRGMTLAK** |
|  | 1799 | **472.4532** | **942.8915** | **943.1022** | **-0.2107** | **2** | **14** | **1.2e+02** | **1** | **ATKATKAPR** |
|  | 2588 | **592.2502** | **1773.7286** | **1773.1508** | **0.5777** | **2** | **14** | **1.1e+02** | **1** | **FVIIHKRCVYYFK + Carbamidomethyl (C)** |
|  | 176 | **369.2539** | **1104.7396** | **1105.2443** | **-0.5047** | **1** | **13** | **1e+02** | **1** | **TIHTGGKTYK** |
|  | 489 | **388.1307** | **774.2467** | **773.7919** | **0.4548** | **0** | **13** | **1.4e+02** | **1** | **GSQPETR** |
|  | 1950 | **488.2101** | **1461.6081** | **1461.6798** | **-0.0718** | **1** | **13** | **1.3e+02** | **1** | **TCILVLGEKNDEK** |
|  | 2721 | **612.0608** | **1222.1068** | **1222.4370** | **-0.3302** | **1** | **13** | **1.2e+02** | **1** | **RTNPPGSCFLM** |
|  | 2792 | **629.6003** | **1257.1858** | **1256.5214** | **0.6644** | **1** | **13** | **1e+02** | **1** | **MAAQVGAVRVVR** |
|  | 3057 | **685.9801** | **2054.9181** | **2055.2693** | **-0.3512** | **2** | **13** | **95** | **1** | **QYYLGDKCQVSSFFKNQ** |
|  | 242 | **373.1140** | **744.2132** | **744.9211** | **-0.7079** | **0** | **13** | **1.4e+02** | **1** | **LAPFLGK** |
|  | 3076 | **687.1720** | **2058.4938** | **2058.2254** | **0.2685** | **0** | **13** | **1.2e+02** | **1** | **SPELIMFIYSNGDVEDGR + Oxidation (M)** |
|  | 382 | 385.0221 | 1152.0440 | 1152.2976 | -0.2535 | 1 | 13 | 1e+02 | 1 | KVSTSFGDAIK |
|  | 1001 | **413.5569** | **825.0991** | **823.9599** | **1.1392** | **0** | **13** | **1e+02** | **1** | **NVSFCVR** |
|  | 1188 | **423.2176** | **1266.6307** | **1267.4975** | **-0.8668** | **1** | **13** | **1.2e+02** | **1** | **FRLLTQMSAGK + Oxidation (M)** |
|  | 1505 | **449.4355** | **896.8563** | **897.1381** | **-0.2818** | **0** | **13** | **1.1e+02** | **1** | **MCNVCISK** |
|  | 1759 | **468.2506** | **1401.7297** | **1400.5836** | **1.1461** | **2** | **13** | **1.2e+02** | **1** | **SGNLPSKRVSISR** |
|  | 153 | **369.1185** | **1104.3332** | **1103.2897** | **1.0435** | **0** | **13** | **1.3e+02** | **1** | **ATVIGLPTNMS** |
|  | 173 | **369.2356** | **1104.6845** | **1105.3089** | **-0.6243** | **0** | **13** | **1e+02** | **1** | **AKPWAVCFPS** |
|  | 368 | **384.5734** | **1150.6980** | **1150.2453** | **0.4528** | **1** | **13** | **87** | **1** | **YGVARSDQVR** |
|  | 580 | **396.1698** | **1185.4871** | **1185.3340** | **0.1532** | **1** | **13** | **1.3e+02** | **1** | **CELSCDRGFR** |
|  | 1784 | **470.6774** | **1409.0101** | **1408.6452** | **0.3649** | **2** | **13** | **89** | **1** | **GLNLAPSPANKAKK** |
|  | 1823 | **474.6296** | **1420.8667** | **1421.5366** | **-0.6699** | **2** | **13** | **1.3e+02** | **1** | **DGMRNKETLEGR + Oxidation (M)** |
|  | 2373 | **541.4775** | **1080.9403** | **1080.3409** | **0.5994** | **2** | **13** | **95** | **1** | **KMLKSFLPT + Oxidation (M)** |
|  | 2905 | **666.8362** | **1997.4864** | **1998.2049** | **-0.7186** | **2** | **13** | **1.3e+02** | **1** | **QQELKDPHCRDEMAAAR** |
|  | 179 | **369.2634** | **1104.7680** | **1105.3520** | **-0.5839** | **2** | **13** | **1e+02** | **1** | **KIMSKIQNK + Oxidation (M)** |
|  | 444 | **386.9313** | **1157.7718** | **1158.2672** | **-0.4954** | **2** | **13** | **1.3e+02** | **1** | **AYRSNYTRK** |
|  | 1737 | **466.1312** | **930.2475** | **929.1566** | **1.0909** | **1** | **13** | **1.3e+02** | **1** | **LPKYALPK** |
|  | 1877 | **478.8361** | **955.6573** | **955.1129** | **0.5445** | **2** | **13** | **1.1e+02** | **1** | **NARKLPEK** |
|  | 2381 | **543.0477** | **1626.1210** | **1626.8528** | **-0.7318** | **2** | **13** | **1.1e+02** | **1** | **ETQVRTPVTGVSPKK** |
|  | 1064 | **418.7903** | **1253.3487** | **1253.5768** | **-0.2282** | **1** | **13** | **1.4e+02** | **1** | **AGAILVVTKLLR** |
|  | 3059 | **686.0289** | **2055.0646** | **2054.3475** | **0.7171** | **1** | **13** | **1e+02** | **1** | **YLLIDCGDTEACLAELRR** |
|  | 3235 | **741.5751** | **2221.7032** | **2220.6094** | **1.0938** | **1** | **13** | **1e+02** | **1** | **QLALEVIVTCSETAAAMLRK + Carbamidomethyl (C); Oxidation (M)** |
|  | 3534 | **996.8904** | **2987.6490** | **2987.1768** | **0.4722** | **2** | **13** | **94** | **1** | **DSSYKNGSRDTGSMDPDVQLCHCIER + Carbamidomethyl (C); Oxidation (M)** |
|  | 177 | **369.2593** | **1104.7558** | **1105.3089** | **-0.5530** | **0** | **13** | **1e+02** | **1** | **AKPWAVCFPS** |
|  | 1283 | **431.0033** | **1289.9878** | **1290.3205** | **-0.3327** | **0** | **13** | **1.5e+02** | **1** | **MSGGGPSGGGPGGSGR + Oxidation (M)** |
|  | 2044 | **503.4084** | **1507.2031** | **1507.5444** | **-0.3414** | **0** | **13** | **1.1e+02** | **1** | **AFGDNSSCTQHQR + Carbamidomethyl (C)** |
|  | 728 | **404.0142** | **806.0135** | **804.9550** | **1.0586** | **1** | **13** | **1.2e+02** | **1** | **MAAEAGKK** |
|  | 1415 | **440.3561** | **1318.0461** | **1317.4933** | **0.5527** | **2** | **13** | **1.1e+02** | **1** | **DKTLTGVKYHR** |
|  | 3131 | **708.8045** | **1415.5942** | **1416.6358** | **-1.0416** | **2** | **13** | **1.4e+02** | **1** | **THPHCCCRSRC + 2 Carbamidomethyl (C)** |
|  | 778 | **405.1671** | **1212.4790** | **1212.4389** | **0.0401** | **0** | **13** | **1.1e+02** | **1** | **INFVMCEVNK + Oxidation (M)** |
|  | 2030 | **502.2119** | **1503.6136** | **1504.6465** | **-1.0329** | **0** | **13** | **1.3e+02** | **1** | **NHCEGMGLANEWK + Oxidation (M)** |
|  | 2124 | **515.6605** | **1543.9594** | **1542.7824** | **1.1770** | **1** | **13** | **1.4e+02** | **1** | **GPMIDQRGLPMDGR** |
|  | 3044 | **685.0349** | **1368.0550** | **1367.6498** | **0.4053** | **2** | **13** | **1.1e+02** | **1** | **RVCPCAPRRPR + Carbamidomethyl (C)** |
|  | 683 | **402.4007** | **802.7867** | **802.8729** | **-0.0862** | **1** | **13** | **1.5e+02** | **1** | **DPTAGSKK** |
|  | 1763 | **469.1163** | **1404.3266** | **1403.5625** | **0.7642** | **0** | **13** | **1.2e+02** | **1** | **AGSSFAFCYHWK** |
|  | 1970 | **490.0465** | **1467.1173** | **1466.6613** | **0.4559** | **1** | **13** | **1.3e+02** | **1** | **LCKSGFSLDNGGLR** |
|  | 2192 | **520.1816** | **1557.5228** | **1557.7440** | **-0.2213** | **0** | **13** | **1.2e+02** | **1** | **FTAAYPTQSSIPFK** |
|  | 358 | **384.2079** | **766.4011** | **766.9283** | **-0.5272** | **0** | **13** | **94** | **1** | **TLALPPR** |
|  | 1285 | **431.0146** | **860.0145** | **858.8565** | **1.1581** | **0** | **13** | **1.5e+02** | **1** | **AGSSGGGGGPR** |
|  | 1754 | **467.9283** | **1400.7627** | **1399.5757** | **1.1870** | **0** | **13** | **1.3e+02** | **1** | **GAAHYEMMGTCR + Carbamidomethyl (C); Oxidation (M)** |
|  | 230 | **371.4283** | **1111.2628** | **1110.1781** | **1.0847** | **1** | **13** | **1.2e+02** | **1** | **TYNADSVKGR** |
|  | 373 | **384.9348** | **1151.7821** | **1151.3822** | **0.3998** | **1** | **13** | **1.1e+02** | **1** | **YRQLLGMVR + Oxidation (M)** |
|  | 907 | **407.8453** | **813.6758** | **813.8623** | **-0.1864** | **1** | **13** | **1.2e+02** | **1** | **GRSQGPGR** |
|  | 1968 | **489.9842** | **1466.9305** | **1466.6431** | **0.2874** | **1** | **13** | **1.3e+02** | **1** | **WPGPGNKGSNALLR** |
|  | 1992 | **494.2863** | **1479.8366** | **1478.6920** | **1.1446** | **0** | **13** | **1.2e+02** | **1** | **GEVTAALALLHGQAK** |
|  | 695 | **403.0200** | **1206.0379** | **1205.3421** | **0.6958** | **1** | **13** | **1.4e+02** | **1** | **VERQVQDMGK + Oxidation (M)** |
|  | 847 | **407.0271** | **1218.0591** | **1217.3990** | **0.6601** | **1** | **13** | **1.1e+02** | **1** | **MQASPTASRLR** |
|  | 948 | **409.0399** | **816.0649** | **814.9711** | **1.0938** | **0** | **13** | **1.4e+02** | **1** | **GHLTLFK** |
|  | 195 | **369.3037** | **1104.8888** | **1105.3089** | **-0.4201** | **0** | **13** | **1.1e+02** | **1** | **AKPWAVCFPS** |
|  | 3090 | **690.4996** | **2068.4767** | **2068.3362** | **0.1406** | **1** | **13** | **1.2e+02** | **1** | **KVSWTPARPSQSALLSPSR** |
|  | 302 | 377.5815 | 753.1481 | 752.7727 | 0.3755 | 0 | 13 | 90 | 1 | SGYSSPR |
|  | 764 | **405.0982** | **1212.2724** | **1211.4078** | **0.8646** | **0** | **13** | **1.2e+02** | **1** | **IYMETPGCPGK + Oxidation (M)** |
|  | 789 | **405.6941** | **1214.0601** | **1214.2794** | **-0.2193** | **0** | **13** | **92** | **1** | **DVVPSPDTQEK** |
|  | 842 | **406.9594** | **1217.8561** | **1217.4451** | **0.4109** | **1** | **13** | **1.1e+02** | **1** | **WICFHSLRR** |
|  | 1056 | **418.1228** | **1251.3462** | **1252.4398** | **-1.0936** | **0** | **13** | **1.3e+02** | **1** | **ETKPCFLATSR** |
|  | 1563 | **453.3131** | **1356.9171** | **1356.5292** | **0.3878** | **1** | **13** | **1.1e+02** | **1** | **IIHARDIFESR** |
|  | 2628 | **594.9453** | **1187.8758** | **1187.3033** | **0.5725** | **0** | **13** | **1.2e+02** | **1** | **QGLTANSLLDR** |
|  | 3007 | **681.7406** | **1361.4664** | **1362.5288** | **-1.0624** | **0** | **13** | **1.4e+02** | **1** | **QTPHSEIIFYK** |
|  | 104 | **366.0848** | **1095.2323** | **1094.3493** | **0.8831** | **1** | **13** | **1.4e+02** | **1** | **MGSACIKVTK + Carbamidomethyl (C)** |
|  | 324 | **379.9586** | **1136.8537** | **1136.2585** | **0.5952** | **1** | **13** | **1.4e+02** | **1** | **GKYSPTVQTR** |
|  | 1735 | **466.1061** | **1395.2961** | **1395.6464** | **-0.3504** | **1** | **13** | **1.3e+02** | **1** | **VLRGLLDQGIPSK** |
|  | 2860 | **661.5344** | **1981.5811** | **1982.3312** | **-0.7501** | **1** | **13** | **1e+02** | **1** | **QLISQIGNAMGYVRMIR + 2 Oxidation (M)** |
|  | 167 | **369.2239** | **1104.6495** | **1104.2614** | **0.3881** | **0** | **13** | **1.1e+02** | **1** | **AVAMMHQER + 2 Oxidation (M)** |
|  | 1104 | **420.1730** | **1257.4967** | **1258.4111** | **-0.9144** | **2** | **13** | **1.2e+02** | **1** | **NPARTCRDLR + Carbamidomethyl (C)** |
|  | 1198 | **424.0523** | **846.0898** | **844.9542** | **1.1357** | **0** | **13** | **1.5e+02** | **1** | **GNFSPVPK** |
|  | 2879 | **665.7721** | **1994.2941** | **1993.1554** | **1.1387** | **2** | **13** | **1.5e+02** | **1** | **QSKSTASFEELCSEYRK** |
|  | 3066 | **686.1592** | **2055.4554** | **2054.3841** | **1.0712** | **0** | **13** | **1.2e+02** | **1** | **IELIQDFEMPTVCTTIK + Carbamidomethyl (C); Oxidation (M)** |
|  | 1416 | **440.7270** | **1319.1588** | **1318.5029** | **0.6559** | **1** | **13** | **1.1e+02** | **1** | **SGLSSSLPRCGVR** |
|  | 1640 | **459.3259** | **916.6370** | **915.9906** | **0.6464** | **0** | **13** | **1.1e+02** | **1** | **SPGAAGLTSR** |
|  | 3415 | **829.2162** | **1656.4176** | **1655.7614** | **0.6562** | **0** | **13** | **1e+02** | **1** | **SSTTVSSFANSKPGSAK** |
|  | 3417 | 830.2797 | 2487.8168 | 2487.9019 | -0.0851 | 2 | 13 | 1.1e+02 | 1 | APLRLHSEKPECRIPAICSPR + 2 Carbamidomethyl (C) |
|  | 2061 | **505.2742** | **1512.8005** | **1513.6353** | **-0.8347** | **1** | **13** | **1.2e+02** | **1** | **RMEAGEAAPPAGAGGR + Oxidation (M)** |
|  | 2258 | **525.9796** | **1049.9443** | **1049.2507** | **0.6937** | **1** | **13** | **1.3e+02** | **1** | **HMVRQPPGK** |
|  | 3512 | **909.6377** | **1817.2606** | **1817.9976** | **-0.7370** | **1** | **13** | **1.2e+02** | **1** | **GFDNHSPIAKYTLQAR** |
|  | 891 | **407.7043** | **813.3939** | **812.9571** | **0.4368** | **2** | **13** | **1e+02** | **1** | **DRGPLKK** |
|  | 2793 | **629.7125** | **1257.4103** | **1256.3193** | **1.0910** | **0** | **13** | **1.5e+02** | **1** | **NTDVAQSPEAPK** |
|  | 2881 | **666.0391** | **1995.0950** | **1994.4017** | **0.6933** | **2** | **13** | **1.1e+02** | **1** | **CTIEEAPPTKKPLILKR + Carbamidomethyl (C)** |
|  | 1150 | **422.1120** | **842.2092** | **841.9949** | **0.2143** | **0** | **13** | **1.4e+02** | **1** | **GITLSVRP** |
|  | 1962 | **489.2693** | **1464.7857** | **1463.7071** | **1.0785** | **2** | **13** | **1.3e+02** | **1** | **ARKNCSQIALFR + Carbamidomethyl (C)** |
|  | 2114 | **513.8453** | **1538.5137** | **1538.7241** | **-0.2105** | **0** | **13** | **1.1e+02** | **1** | **LCYAASSLQSGVPSR** |
|  | 2202 | **521.1252** | **1560.3534** | **1559.7269** | **0.6265** | **2** | **13** | **1.2e+02** | **1** | **DCSELRSRHCTPV + Carbamidomethyl (C)** |
|  | 2384 | **544.9802** | **1631.9185** | **1630.8859** | **1.0326** | **2** | **13** | **1.4e+02** | **1** | **MEKFGMNFGGGPSKK + Oxidation (M)** |
|  | 2993 | **680.4455** | **2038.3143** | **2038.3497** | **-0.0354** | **1** | **13** | **1.2e+02** | **1** | **LMRYNDFLHDPLSLCK + Carbamidomethyl (C); Oxidation (M)** |
|  | 1300 | **431.4504** | **1291.3291** | **1292.4389** | **-1.1098** | **0** | **13** | **1.7e+02** | **1** | **ALHFAISEYNK** |
|  | 1397 | **437.1228** | **872.2309** | **873.0090** | **-0.7782** | **1** | **13** | **1.4e+02** | **1** | **REIVSAAK** |
|  | 1677 | **461.4826** | **1381.4255** | **1381.6218** | **-0.1962** | **0** | **13** | **1.4e+02** | **1** | **GPAAPPPTPVKPPR** |
|  | 2296 | **532.8561** | **1595.5462** | **1594.8372** | **0.7091** | **2** | **13** | **1.1e+02** | **1** | **MIDRIFSGAVTRGR + Oxidation (M)** |
|  | 288 | **377.0286** | **752.0423** | **751.9801** | **0.0623** | **2** | **13** | **1.2e+02** | **1** | **KVFKCK** |
|  | 312 | **378.3987** | **1132.1739** | **1132.3987** | **-0.2249** | **2** | **13** | **1.3e+02** | **1** | **KSAKLGFLLR** |
|  | 577 | **395.3134** | **1182.9179** | **1182.2836** | **0.6343** | **0** | **13** | **1.2e+02** | **1** | **QQEALSEHLK** |
|  | 1070 | **419.1185** | **1254.3333** | **1253.3647** | **0.9686** | **0** | **13** | **1.5e+02** | **1** | **AQLSNSAQGLHK** |
|  | 1210 | **426.1658** | **1275.4753** | **1274.4571** | **1.0183** | **2** | **13** | **1.3e+02** | **1** | **RPMASSARRAR + Oxidation (M)** |
|  | 1623 | **458.9018** | **1373.6832** | **1374.6755** | **-0.9923** | **2** | **13** | **1.4e+02** | **1** | **MMSLIPGPRRGK + 2 Oxidation (M)** |
|  | 2499 | **571.4855** | **1140.9562** | **1141.3875** | **-0.4313** | **1** | **13** | **99** | **1** | **QKPMAKGLPR + Oxidation (M)** |
|  | 2627 | **594.6682** | **1780.9823** | **1779.9661** | **1.0161** | **0** | **13** | **1.6e+02** | **1** | **GLEWMGIIYPGDGDTR** |
|  | 61 | **363.3175** | **1086.9303** | **1086.2260** | **0.7043** | **1** | **13** | **1.1e+02** | **1** | **CRHGAASLGSK** |
|  | 2408 | **551.8840** | **1101.7532** | **1101.3617** | **0.3915** | **2** | **13** | **1.1e+02** | **1** | **MSKLGKFFK + Oxidation (M)** |
|  | 1215 | **427.1100** | **1278.3080** | **1278.5249** | **-0.2170** | **1** | **13** | **1.2e+02** | **1** | **SPWGRLYLMR** |
|  | 431 | **386.4493** | **770.8838** | **771.8455** | **-0.9616** | **0** | **13** | **1.5e+02** | **1** | **MQGSGHR** |
|  | 1352 | **435.0341** | **1302.0800** | **1302.5283** | **-0.4483** | **1** | **13** | **1.1e+02** | **1** | **GWQAAAHMMRK + Oxidation (M)** |
|  | 2047 | **504.1985** | **1509.5734** | **1509.8371** | **-0.2637** | **1** | **13** | **1.4e+02** | **1** | **CCNPSVPKQCVMK + Carbamidomethyl (C); Oxidation (M)** |
|  | 2409 | **552.0349** | **1102.0549** | **1101.2176** | **0.8373** | **1** | **13** | **1.3e+02** | **1** | **AQGAAVARTTR** |
|  | 20 | **361.1276** | **1080.3606** | **1080.3274** | **0.0332** | **2** | **13** | **1.2e+02** | **1** | **RKLGIAPIGR** |
|  | 55 | **363.1967** | **1086.5678** | **1087.1861** | **-0.6183** | **0** | **13** | **1e+02** | **1** | **MSSSCSGLSR + Carbamidomethyl (C); Oxidation (M)** |
|  | 2022 | **500.9026** | **1499.6857** | **1500.8055** | **-1.1198** | **1** | **13** | **1.3e+02** | **1** | **VKVVLNMAEIQTR** |
|  | 518 | **389.1334** | **1164.3780** | **1163.4543** | **0.9237** | **1** | **13** | **1.4e+02** | **1** | **CIAMKQGVIK + Carbamidomethyl (C); Oxidation (M)** |
|  | 932 | 408.2015 | 814.3883 | 814.9066 | -0.5184 | 0 | 13 | 1.2e+02 | 1 | IHTGEMQ |
|  | 982 | **411.5802** | **821.1456** | **821.8994** | **-0.7538** | **0** | **13** | **1.2e+02** | **1** | **TSPMTNR + Oxidation (M)** |
|  | 1106 | **420.2054** | **838.3959** | **837.8804** | **0.5155** | **1** | **13** | **1.1e+02** | **1** | **RYTDQR** |
|  | 1510 | **450.0599** | **1347.1575** | **1346.5445** | **0.6130** | **2** | **13** | **1.2e+02** | **1** | **ARAPGRVPGGRPR** |
|  | 998 | **413.2608** | **1236.7603** | **1236.3740** | **0.3863** | **0** | **13** | **88** | **1** | **SELTNLAGLYR** |
|  | 2433 | **557.9451** | **1113.8754** | **1114.2510** | **-0.3757** | **0** | **13** | **1.3e+02** | **1** | **SFLAASSYIR** |
|  | 509 | **389.0588** | **1164.1542** | **1164.4008** | **-0.2467** | **0** | **13** | **1.4e+02** | **1** | **IMQFCHTLR + Oxidation (M)** |
|  | 1074 | **419.1689** | **1254.4846** | **1255.3412** | **-0.8566** | **2** | **13** | **1.5e+02** | **1** | **AGTPEREPRSR** |
|  | 1226 | **428.1153** | **854.2159** | **853.0175** | **1.1983** | **0** | **13** | **1.3e+02** | **1** | **AELAPFCC** |
|  | 2940 | **668.7384** | **2003.1930** | **2003.4069** | **-0.2138** | **1** | **13** | **1.5e+02** | **1** | **IKCFGFMMDYTLAVYK + Carbamidomethyl (C); Oxidation (M)** |
|  | 919 | **408.0747** | **814.1346** | **813.9251** | **0.2094** | **0** | **13** | **1.4e+02** | **1** | **CLATHGR + Carbamidomethyl (C)** |
|  | 2014 | **498.4382** | **1492.2923** | **1492.5035** | **-0.2111** | **1** | **13** | **1e+02** | **1** | **DQPRDYSGENPSK** |
|  | 2213 | **521.6119** | **1561.8136** | **1560.7113** | **1.1023** | **1** | **13** | **1.5e+02** | **1** | **WNKQNHFTSISAK** |
|  | 2412 | **552.6247** | **1654.8519** | **1653.8992** | **0.9527** | **1** | **13** | **1.6e+02** | **1** | **DVGPYGIRIEYCIR** |
|  | 3071 | **686.2037** | **2055.5890** | **2056.3934** | **-0.8043** | **1** | **13** | **1.2e+02** | **1** | **GHLIPSCVSSRAPAMCSSPR** |
|  | 1217 | **427.2663** | **852.5179** | **852.0329** | **0.4850** | **1** | **13** | **94** | **1** | **APQLAPKK** |
|  | 3182 | **737.1964** | **1472.3779** | **1472.7489** | **-0.3710** | **0** | **13** | **1.2e+02** | **1** | **MAIVQLVQSGAEVK** |
|  | 181 | **369.2646** | **1104.7717** | **1104.2812** | **0.4905** | **1** | **13** | **1.1e+02** | **1** | **VNLAMEGRAK + Oxidation (M)** |
|  | 194 | **369.2979** | **1104.8715** | **1105.3089** | **-0.4374** | **0** | **13** | **1.1e+02** | **1** | **AKPWAVCFPS** |
|  | 530 | **389.2563** | **1164.7467** | **1164.2868** | **0.4599** | **0** | **13** | **1.1e+02** | **1** | **MDVMDGCQFS + 2 Oxidation (M)** |
|  | 568 | **393.5013** | **1177.4817** | **1176.3042** | **1.1775** | **1** | **13** | **1.5e+02** | **1** | **MTSPSPSRAAR + Oxidation (M)** |
|  | 2577 | **590.2806** | **1767.8197** | **1768.9671** | **-1.1473** | **1** | **13** | **1.4e+02** | **1** | **QRSSTAMTVMADLGER + Oxidation (M)** |
|  | 3145 | **717.7611** | **2150.2612** | **2150.5379** | **-0.2768** | **2** | **13** | **1.4e+02** | **1** | **KPPASVKVSCKASGYTLVTTI** |
|  | 684 | **402.4082** | **802.8016** | **803.9271** | **-1.1254** | **0** | **13** | **1.7e+02** | **1** | **LPGAMGSR + Oxidation (M)** |
|  | 879 | **407.3539** | **1219.0397** | **1219.3320** | **-0.2924** | **1** | **13** | **1e+02** | **1** | **NGTAVCATNRR + Carbamidomethyl (C)** |
|  | 2507 | **573.2777** | **1716.8110** | **1717.9183** | **-1.1074** | **0** | **13** | **1.3e+02** | **1** | **TGQPPELLVYWASTR** |
|  | 2641 | **596.8909** | **1191.7670** | **1192.3645** | **-0.5976** | **0** | **13** | **1.2e+02** | **1** | **GLSPAPGLPDLR** |
|  | 739 | **404.0894** | **1209.2461** | **1210.4064** | **-1.1604** | **2** | **13** | **1.3e+02** | **1** | **QMYLEKRSR** |
|  | 1297 | **431.1711** | **860.3274** | **859.9670** | **0.3604** | **0** | **13** | **1.5e+02** | **1** | **ASALSLGNK** |
|  | 2828 | **648.0315** | **1941.0723** | **1940.2186** | **0.8537** | **1** | **13** | **1.2e+02** | **1** | **DFILLTMRVSTEEELK + Oxidation (M)** |
|  | 1751 | **467.7297** | **1400.1669** | **1399.5524** | **0.6145** | **0** | **13** | **1.1e+02** | **1** | **NVFPPPSQTWAR** |
|  | 2842 | **653.8571** | **1305.6995** | **1304.5194** | **1.1801** | **0** | **13** | **1.2e+02** | **1** | **CPAGGNPMPTMR + Carbamidomethyl (C); Oxidation (M)** |
|  | 2861 | **661.6573** | **1981.9499** | **1981.2805** | **0.6694** | **2** | **13** | **1.2e+02** | **1** | **HMSNKGMEHLYSMKQK + 2 Oxidation (M)** |
|  | 2931 | **668.5629** | **2002.6664** | **2003.3244** | **-0.6579** | **2** | **13** | **1.1e+02** | **1** | **SQKQVKVEMSGPVTVLTR + Oxidation (M)** |
|  | 2976 | **676.7302** | **2027.1685** | **2026.2764** | **0.8921** | **0** | **13** | **1.6e+02** | **1** | **MAACTMSVCSSACSDSWR + 2 Carbamidomethyl (C); Oxidation (M)** |
|  | 334 | **380.2155** | **758.4163** | **758.8235** | **-0.4072** | **0** | **13** | **1.2e+02** | **1** | **GTAAAAAAR** |
|  | 1870 | **478.1311** | **1431.3711** | **1430.6060** | **0.7651** | **1** | **13** | **1.2e+02** | **1** | **WAGFHTIKDDIK** |
|  | 1121 | **421.1390** | **1260.3948** | **1260.3940** | **0.0007** | **0** | **13** | **1.3e+02** | **1** | **VTSEVAASLQQK** |
|  | 1736 | **466.1098** | **1395.3073** | **1394.4633** | **0.8441** | **0** | **13** | **1.4e+02** | **1** | **MEEFATEGTDHK** |
|  | 2573 | **588.1602** | **1174.3057** | **1173.3647** | **0.9410** | **1** | **13** | **1.4e+02** | **1** | **LLPTHPPDRK** |
|  | 2623 | **593.8683** | **1185.7219** | **1185.3704** | **0.3515** | **1** | **13** | **1.1e+02** | **1** | **LLLKTDPTER** |
|  | 3112 | **699.5599** | **2095.6576** | **2096.3660** | **-0.7084** | **2** | **13** | **1e+02** | **1** | **CSTELIKNMKSASCGGSHL + 2 Carbamidomethyl (C); Oxidation (M)** |
|  | 770 | **405.1155** | **1212.3243** | **1211.4939** | **0.8304** | **1** | **13** | **1.2e+02** | **1** | **KVIVGGVDLLAK** |
|  | 1194 | **423.4342** | **844.8537** | **843.8817** | **0.9720** | **0** | **13** | **1.7e+02** | **1** | **GASLEDPR** |
|  | 1804 | **473.1018** | **944.1888** | **945.0750** | **-0.8862** | **1** | **13** | **1.4e+02** | **1** | **QSRVLSQK** |
|  | 1986 | **493.1698** | **1476.4872** | **1477.5815** | **-1.0942** | **2** | **13** | **1.3e+02** | **1** | **LGDDFNRELSRR** |
|  | 2250 | **524.9414** | **1571.8020** | **1570.7228** | **1.0792** | **0** | **13** | **1.4e+02** | **1** | **VCTELDNHLGINDK** |
|  | 2864 | **663.4542** | **1324.8937** | **1325.3829** | **-0.4892** | **1** | **13** | **1.3e+02** | **1** | **WSAYRDTPDSK** |
|  | 2805 | **635.6796** | **1904.0165** | **1903.1221** | **0.8944** | **1** | **13** | **1.5e+02** | **1** | **SPATLSLSPGERATLSCR + Carbamidomethyl (C)** |
|  | 1882 | **479.5238** | **1435.5491** | **1434.5785** | **0.9707** | **2** | **13** | **1.6e+02** | **1** | **MTPSEGARAGTGRK + Oxidation (M)** |
|  | 3544 | **1062.0989** | **2122.1830** | **2122.4019** | **-0.2189** | **1** | **13** | **1.1e+02** | **1** | **APKLMIYDVTDRPSGISSR + Oxidation (M)** |
|  | 706 | **403.5205** | **1207.5394** | **1206.3497** | **1.1896** | **0** | **13** | **1.6e+02** | **1** | **HIDGAYIYVR** |
|  | 1304 | 432.1916 | 862.3685 | 862.9282 | -0.5596 | 2 | 13 | 1.4e+02 | 1 | KKSSEER |
|  | 2866 | **663.6008** | **1325.1869** | **1325.4936** | **-0.3068** | **0** | **13** | **1e+02** | **1** | **ISHCPNILTDR + Carbamidomethyl (C)** |
|  | 263 | **375.0076** | **1122.0006** | **1121.2223** | **0.7784** | **0** | **13** | **1.5e+02** | **1** | **EATGVDINMR + Oxidation (M)** |
|  | 341 | **381.2225** | **760.4302** | **760.9024** | **-0.4722** | **0** | **13** | **1.3e+02** | **1** | **MVDAGLR** |
|  | 2912 | **667.1963** | **1998.5667** | **1999.2941** | **-0.7274** | **1** | **13** | **1.3e+02** | **1** | **VVSQHPSWVICKSGTSVK + Carbamidomethyl (C)** |
|  | 82 | **364.2979** | **1089.8715** | **1089.1604** | **0.7110** | **1** | **13** | **97** | **1** | **EKSQLQNSR** |
|  | 686 | **402.5582** | **803.1016** | **803.8244** | **-0.7227** | **1** | **13** | **1.4e+02** | **1** | **TRSGGGGGR** |
|  | 775 | **405.1424** | **808.2699** | **809.0530** | **-0.7830** | **1** | **13** | **1.2e+02** | **1** | **KMMLVR + 2 Oxidation (M)** |
|  | 2585 | **591.5861** | **1181.1575** | **1182.2623** | **-1.1048** | **1** | **13** | **1.2e+02** | **1** | **KEQCSSTQSK + Carbamidomethyl (C)** |
|  | 2941 | **668.7937** | **2003.3589** | **2002.3347** | **1.0243** | **2** | **13** | **1.6e+02** | **1** | **MNKMVTSEEQMKLTSTK + Oxidation (M)** |
|  | 1282 | **430.9349** | **859.8551** | **859.0949** | **0.7602** | **2** | **13** | **1.6e+02** | **1** | **ACLRIRK** |
|  | 2145 | **518.8182** | **1553.4325** | **1554.6241** | **-1.1916** | **1** | **13** | **1.1e+02** | **1** | **AGAGTEARAGPGAAGAGGR** |
|  | 2727 | **612.2649** | **1222.5150** | **1221.4489** | **1.0661** | **1** | **13** | **1.3e+02** | **1** | **APALASTPRLPK** |
|  | 760 | 405.0511 | 808.0873 | 808.8756 | -0.7883 | 0 | 13 | 1.2e+02 | 1 | DEAALYK |
|  | 1364 | **435.6632** | **1303.9675** | **1304.5573** | **-0.5898** | **0** | **13** | **1e+02** | **1** | **QPIQYCVVINK** |
|  | 1448 | **443.8258** | **1328.4552** | **1327.5528** | **0.9025** | **0** | **13** | **1.4e+02** | **1** | **AHELHMDPCMK + Oxidation (M)** |
|  | 1606 | **458.4174** | **914.8201** | **914.1073** | **0.7127** | **2** | **13** | **1.2e+02** | **1** | **RVKTALAR** |
|  | 2465 | **564.5243** | **1127.0338** | **1126.2917** | **0.7421** | **1** | **13** | **1.2e+02** | **1** | **SCGRCVCER + 2 Carbamidomethyl (C)** |
|  | 2629 | **594.9498** | **1781.8273** | **1781.9607** | **-0.1334** | **0** | **13** | **1.3e+02** | **1** | **LTSVSAADTAMYYCGGR + Oxidation (M)** |
|  | 3151 | **721.3767** | **1440.7386** | **1440.5546** | **0.1840** | **0** | **13** | **1.3e+02** | **1** | **TQNGLLSPPQEEK** |
|  | 3335 | **776.9147** | **2327.7218** | **2328.7224** | **-1.0006** | **1** | **13** | **1.5e+02** | **1** | **LAEILYYKILETVMVQETR + Oxidation (M)** |
|  | 3447 | **849.9669** | **2546.8784** | **2547.8859** | **-1.0075** | **2** | **13** | **1.5e+02** | **1** | **RLVFIDCPGKLAEHIEHEQQK + Carbamidomethyl (C)** |
|  | 3378 | **805.2783** | **2412.8126** | **2413.6495** | **-0.8369** | **1** | **13** | **1.3e+02** | **1** | **QAPGHRLEWMGWMNTGSGEPR + Oxidation (M)** |
|  | 408 | **386.0636** | **770.1124** | **770.8741** | **-0.7617** | **0** | **13** | **1.2e+02** | **1** | **AVSVNPGK** |
|  | 1220 | **427.3453** | **1279.0138** | **1279.4206** | **-0.4067** | **2** | **13** | **97** | **1** | **KCSSEVEAKNK + Carbamidomethyl (C)** |
|  | 2372 | **541.3718** | **1080.7289** | **1081.1585** | **-0.4296** | **1** | **13** | **1.1e+02** | **1** | **MESSDVNKR + Oxidation (M)** |
|  | 2471 | **565.4594** | **1693.3559** | **1693.9400** | **-0.5842** | **1** | **13** | **1.2e+02** | **1** | **LCDIDGAKVNTLMER + Oxidation (M)** |
|  | 3204 | **740.5060** | **1478.9972** | **1478.6954** | **0.3018** | **0** | **13** | **1.3e+02** | **1** | **GPCGPDCAPAAPGLPR** |
|  | 202 | **369.3443** | **1105.0108** | **1105.1567** | **-0.1458** | **1** | **13** | **1.4e+02** | **1** | **DPNKASASTSK** |
|  | 1030 | **415.6262** | **829.2375** | **828.8704** | **0.3672** | **0** | **13** | **1.3e+02** | **1** | **GSQPSTPR** |
|  | 1752 | **467.8053** | **1400.3938** | **1400.6743** | **-0.2805** | **2** | **13** | **1.3e+02** | **1** | **ALWPLRRFWR** |
|  | 3114 | **700.6354** | **2098.8840** | **2098.3604** | **0.5236** | **0** | **13** | **1e+02** | **1** | **LSCAASGFAFSTYGMQWVR + Oxidation (M)** |
|  | 3128 | **707.1195** | **1412.2242** | **1411.5799** | **0.6444** | **1** | **13** | **1.3e+02** | **1** | **FVSRSEMEADIK** |
|  | 13 | **360.4972** | **1078.4694** | **1078.3497** | **0.1197** | **2** | **13** | **1.5e+02** | **1** | **IIDKAKCCGK** |
|  | 366 | **384.5414** | **1150.6019** | **1151.2927** | **-0.6909** | **0** | **13** | **1.1e+02** | **1** | **GAQLCFEANAK** |
|  | 564 | **393.0568** | **1176.1481** | **1175.3987** | **0.7495** | **0** | **13** | **1.3e+02** | **1** | **LFIFETFCR** |
|  | 2011 | **498.2072** | **1491.5993** | **1490.7276** | **0.8717** | **1** | **13** | **1.3e+02** | **1** | **VIITCRASQSISR + Carbamidomethyl (C)** |
|  | 2374 | **541.5074** | **1621.5002** | **1621.8028** | **-0.3027** | **2** | **13** | **1.1e+02** | **1** | **ALQARTGASRVHAAGR** |
|  | 2446 | **562.1508** | **1683.4301** | **1682.8956** | **0.5345** | **0** | **13** | **1.4e+02** | **1** | **LLQAFCFPDSEWAR** |
|  | 554 | **390.1188** | **1167.3342** | **1168.1678** | **-0.8335** | **0** | **13** | **1.3e+02** | **1** | **DFALDSEESR** |
|  | 931 | **408.1729** | **814.3310** | **813.9019** | **0.4290** | **0** | **13** | **1.4e+02** | **1** | **GAQIQAAR** |
|  | 1591 | **457.4928** | **1369.4563** | **1368.5767** | **0.8797** | **0** | **13** | **1.6e+02** | **1** | **TGAVVIDMGTGTCK + Oxidation (M)** |
|  | 3395 | **816.4424** | **1630.8700** | **1630.8626** | **0.0073** | **1** | **13** | **1.3e+02** | **1** | **LAQHKSEIECFTPK** |
|  | 116 | **366.2339** | **730.4531** | **730.8531** | **-0.4000** | **0** | **13** | **1.2e+02** | **1** | **WFPGPK** |
|  | 148 | **368.3332** | **1101.9774** | **1102.1561** | **-0.1787** | **0** | **13** | **1.5e+02** | **1** | **EARPSNTAEK** |
|  | 374 | 384.9563 | 1151.8467 | 1152.3438 | -0.4971 | 1 | 13 | 1.2e+02 | 1 | HLLGAVGTKEK |
|  | 777 | **405.1638** | **1212.4691** | **1213.4699** | **-1.0008** | **2** | **13** | **1.2e+02** | **1** | **EKQRLLVLSK** |
|  | 973 | **411.0958** | **1230.2651** | **1230.3332** | **-0.0680** | **2** | **13** | **1.5e+02** | **1** | **FVNSAKHSGGRA** |
|  | 3095 | **694.5361** | **1387.0575** | **1387.5633** | **-0.5058** | **1** | **13** | **1.1e+02** | **1** | **MSKENSSCCLR + 2 Carbamidomethyl (C); Oxidation (M)** |
|  | 34 | **362.2232** | **722.4316** | **722.7964** | **-0.3647** | **1** | **13** | **1e+02** | **1** | **SAPHRR** |
|  | 2509 | **573.5392** | **1145.0636** | **1145.3101** | **-0.2465** | **2** | **13** | **1.2e+02** | **1** | **KVSAVSGDVRK** |
|  | 3453 | **852.5358** | **2554.5851** | **2555.6431** | **-1.0580** | **0** | **13** | **1.3e+02** | **1** | **DSAVYFCASSSAGSGGYGYTFGSGTR** |
|  | 114 | **366.2301** | **730.4455** | **730.8102** | **-0.3647** | **1** | **13** | **1.2e+02** | **1** | **ELVGRAS** |
|  | 1041 | **416.1766** | **830.3385** | **830.8863** | **-0.5478** | **1** | **13** | **1.6e+02** | **1** | **QSDTPRK** |
|  | 2045 | **504.0466** | **1006.0784** | **1007.1660** | **-1.0876** | **2** | **13** | **1.4e+02** | **1** | **KNSQKQMK + Oxidation (M)** |
|  | 297 | **377.1908** | **1128.5503** | **1127.3560** | **1.1943** | **0** | **13** | **1.1e+02** | **1** | **LCMDLMTER + Oxidation (M)** |
|  | 320 | **379.5311** | **757.0474** | **756.8276** | **0.2198** | **0** | **13** | **1.4e+02** | **1** | **QMGYSR + Oxidation (M)** |
|  | 972 | **411.0698** | **1230.1873** | **1231.3857** | **-1.1984** | **0** | **13** | **1.5e+02** | **1** | **HCAGPGECACAR + Carbamidomethyl (C)** |
|  | 1470 | **445.1763** | **1332.5067** | **1332.5855** | **-0.0788** | **0** | **13** | **1.6e+02** | **1** | **EPSLAYLLLWK** |
|  | 1851 | **476.2462** | **1425.7166** | **1424.5552** | **1.1614** | **0** | **13** | **1.3e+02** | **1** | **EWEGVFLSSGWK** |
|  | 2583 | **591.4508** | **1771.3302** | **1770.9694** | **0.3609** | **2** | **13** | **1.1e+02** | **1** | **NSSRFINRHNMPGPK + Oxidation (M)** |
|  | 3350 | **785.4507** | **2353.3299** | **2353.8017** | **-0.4718** | **2** | **13** | **1.3e+02** | **1** | **LPAAGMGDMMMATVKKGTPELR + 3 Oxidation (M)** |
|  | 3489 | **887.6459** | **2659.9154** | **2659.0045** | **0.9109** | **2** | **13** | **1.3e+02** | **1** | **ELTNITFGFRTRDANVIILHAEK** |
|  | 3490 | **887.6554** | **2659.9440** | **2659.1108** | **0.8332** | **1** | **13** | **1.2e+02** | **1** | **KVMCFHPWSDVTPPLMSVPEIR + Carbamidomethyl (C); 2 Oxidation (M)** |
|  | 3523 | **934.4562** | **2800.3464** | **2801.2067** | **-0.8603** | **1** | **13** | **1.2e+02** | **1** | **ALTPSSTWMLCASESPRWAWMCR + 2 Carbamidomethyl (C); Oxidation (M)** |
|  | 3545 | **1065.2444** | **3192.7110** | **3191.5342** | **1.1768** | **2** | **13** | **1.4e+02** | **1** | **AGYTEKQRVDFLGEAGIMGQFSHHNIIR + Oxidation (M)** |
|  | 3553 | **1135.8076** | **3404.4007** | **3404.6922** | **-0.2915** | **1** | **13** | **1.1e+02** | **1** | **CMQGKYAGAMESEPCVCTEADFDCDYGYER + 2 Oxidation (M)** |
|  | 3559 | **1187.3406** | **3558.9996** | **3558.7543** | **0.2452** | **2** | **13** | **1.3e+02** | **1** | **QRESSITSCCSTSSCDADDEGVRGTCEDASLCK + 2 Carbamidomethyl (C)** |
|  | 2356 | **539.9382** | **1616.7923** | **1616.8328** | **-0.0404** | **0** | **13** | **1.4e+02** | **1** | **DINDNPPIFPMTVK + Oxidation (M)** |
|  | 2656 | **599.9083** | **1197.8017** | **1198.2367** | **-0.4350** | **0** | **13** | **1.2e+02** | **1** | **YSQSDLEQTK** |
|  | 665 | **401.8747** | **801.7345** | **802.8762** | **-1.1416** | **1** | **13** | **1.6e+02** | **1** | **KSQGEVR** |
|  | 716 | **403.9388** | **1208.7942** | **1209.3389** | **-0.5447** | **1** | **13** | **1.4e+02** | **1** | **RTGATPPGCHR + Carbamidomethyl (C)** |
|  | 908 | **407.8473** | **1220.5198** | **1221.3592** | **-0.8394** | **0** | **13** | **1.4e+02** | **1** | **NNLFSSNILSL** |
|  | 2498 | **571.4302** | **1711.2684** | **1711.9967** | **-0.7283** | **0** | **13** | **1.1e+02** | **1** | **CFGFPMDYTLAVYK + Carbamidomethyl (C)** |
|  | 3231 | **741.2678** | **1480.5207** | **1479.6206** | **0.9002** | **2** | **13** | **1.3e+02** | **1** | **KRYNSHQMGQSK + Oxidation (M)** |
|  | 986 | **412.0958** | **1233.2653** | **1232.4500** | **0.8153** | **1** | **13** | **1.4e+02** | **1** | **RCVSLLLDADK** |
|  | 2777 | **625.0729** | **1872.1965** | **1873.0299** | **-0.8335** | **2** | **13** | **1.4e+02** | **1** | **GRTIPDKIGSTSGAEAANK** |
|  | 143 | **368.1588** | **1101.4541** | **1101.2177** | **0.2364** | **2** | **13** | **1.3e+02** | **1** | **VLTDEGRRR** |
|  | 771 | **405.1175** | **1212.3303** | **1211.3745** | **0.9558** | **2** | **13** | **1.3e+02** | **1** | **ANPRLANATKR** |
|  | 2110 | **512.3647** | **1022.7146** | **1023.0975** | **-0.3830** | **1** | **13** | **1.1e+02** | **1** | **KEAEVEYR** |
|  | 3363 | **790.1334** | **2367.3781** | **2366.6066** | **0.7715** | **1** | **13** | **1.1e+02** | **1** | **AQKEMSPSPPAAQDPGGTALVSAR** |
|  | 424 | **386.1880** | **770.3611** | **769.9355** | **0.4256** | **1** | **13** | **1.1e+02** | **1** | **AALRALR** |
|  | 1107 | **420.2177** | **1257.6310** | **1257.4364** | **0.1946** | **0** | **13** | **1.2e+02** | **1** | **MICAGFAASGEK + Carbamidomethyl (C); Oxidation (M)** |
|  | 2120 | **515.3795** | **1028.7441** | **1028.1636** | **0.5806** | **1** | **13** | **1.1e+02** | **1** | **LGKASGVPSGR** |
|  | 2660 | **600.3071** | **1797.8990** | **1797.9930** | **-0.0940** | **2** | **13** | **1.5e+02** | **1** | **TRMHGLNDALDNLRR + Oxidation (M)** |
|  | 2687 | **608.3573** | **1822.0497** | **1821.1357** | **0.9140** | **2** | **13** | **1.4e+02** | **1** | **MRAGQQLASMLRWTR + Oxidation (M)** |
|  | 899 | **407.7734** | **1220.2980** | **1221.4689** | **-1.1708** | **1** | **13** | **1.4e+02** | **1** | **APKLMIYDVR + Oxidation (M)** |
|  | 2075 | **506.0942** | **1010.1736** | **1009.1106** | **1.0629** | **0** | **13** | **1.3e+02** | **1** | **AVEEGLTYK** |
|  | 2569 | **586.6379** | **1171.2610** | **1171.2180** | **0.0430** | **0** | **13** | **1.6e+02** | **1** | **VELQDPNSNR** |
|  | 3197 | **740.3779** | **1478.7411** | **1479.6356** | **-0.8945** | **1** | **13** | **1.3e+02** | **1** | **KYWTSKPEPSTR** |
|  | 105 | **366.1053** | **1095.2939** | **1094.2896** | **1.0043** | **0** | **13** | **1.5e+02** | **1** | **CHTIMNCTR + Oxidation (M)** |
|  | 314 | **379.0419** | **756.0691** | **755.9025** | **0.1666** | **1** | **13** | **1.4e+02** | **1** | **LKGPDVK** |
|  | 347 | **382.2834** | **762.5519** | **761.8641** | **0.6879** | **1** | **13** | **1.3e+02** | **1** | **ATEKSVK** |
|  | 1411 | **438.1730** | **1311.4968** | **1311.3612** | **0.1356** | **1** | **13** | **1.5e+02** | **1** | **RAEDLSAEQHR** |
|  | 1578 | **456.2609** | **910.5069** | **910.0723** | **0.4347** | **1** | **13** | **1.1e+02** | **1** | **GLQTVHKK** |
|  | 2872 | **664.1359** | **1326.2569** | **1326.5282** | **-0.2713** | **2** | **13** | **1.3e+02** | **1** | **ILEARHREMR + Oxidation (M)** |
|  | 3058 | 686.0137 | 2055.0188 | 2056.2014 | -1.1826 | 2 | 13 | 1.1e+02 | 1 | TAAGAVSPDSRPETRRQTR |
|  | 133 | **367.2656** | **1098.7745** | **1099.2381** | **-0.4636** | **1** | **13** | **1.2e+02** | **1** | **NIPLEKETR** |
|  | 970 | **411.0121** | **1230.0143** | **1230.5237** | **-0.5094** | **0** | **13** | **1.5e+02** | **1** | **MASVCCLPPPR + Carbamidomethyl (C)** |
|  | 1319 | **432.9574** | **1295.8500** | **1296.4445** | **-0.5944** | **1** | **13** | **1.4e+02** | **1** | **EEIDTLKMSSK + Oxidation (M)** |
|  | 2334 | **537.5159** | **1073.0171** | **1072.1714** | **0.8457** | **0** | **13** | **1.4e+02** | **1** | **IHDGASPFTK** |
|  | 3158 | **725.6109** | **1449.2070** | **1448.5850** | **0.6221** | **1** | **13** | **1.1e+02** | **1** | **ARGGSVPHAGPWEK** |
|  | 3287 | **752.7332** | **2255.1775** | **2255.8305** | **-0.6531** | **2** | **13** | **1.2e+02** | **1** | **SLLNIMMLVPHKRYLVSIK** |
|  | 3410 | **827.5956** | **2479.7648** | **2480.9459** | **-1.1812** | **2** | **13** | **1.4e+02** | **1** | **LSCPITALGRTMATFPVAPRYAK + Oxidation (M)** |
|  | 95 | **365.2744** | **728.5340** | **728.8372** | **-0.3032** | **0** | **13** | **1.2e+02** | **1** | **EGAALLR** |
|  | 337 | **380.2560** | **758.4972** | **757.8586** | **0.6386** | **0** | **13** | **1.3e+02** | **1** | **HNGSCLK** |
|  | 651 | **401.3338** | **800.6528** | **800.8835** | **-0.2306** | **0** | **13** | **1.3e+02** | **1** | **DGVMAHR + Oxidation (M)** |
|  | 721 | **403.9795** | **805.9442** | **806.9260** | **-0.9818** | **0** | **13** | **1.4e+02** | **1** | **SMPAWLS + Oxidation (M)** |
|  | 1621 | **458.8721** | **1373.5941** | **1373.6012** | **-0.0070** | **2** | **13** | **1.5e+02** | **1** | **FLFSISKGYRR** |
|  | 2510 | **574.1020** | **1719.2838** | **1718.9693** | **0.3145** | **1** | **13** | **1.4e+02** | **1** | **NLCLLKTSESGLPSTR** |
|  | 573 | **394.1626** | **786.3104** | **785.8456** | **0.4648** | **0** | **13** | **1.4e+02** | **1** | **ALGSSHSK** |
|  | 2095 | **508.4089** | **1014.8031** | **1014.2000** | **0.6031** | **0** | **13** | **1.2e+02** | **1** | **VPTPLCTAR + Carbamidomethyl (C)** |
|  | 2564 | **585.6686** | **1753.9836** | **1753.9060** | **0.0776** | **1** | **13** | **1.6e+02** | **1** | **SEDPPSTPHFGKVDIK** |
|  | 2610 | **593.1445** | **1776.4114** | **1776.9276** | **-0.5162** | **2** | **13** | **1.4e+02** | **1** | **QGSRGAMGHYVLAERE + Oxidation (M)** |
|  | 2809 | 637.0549 | 1908.1426 | 1908.1404 | 0.0023 | 2 | 13 | 1.4e+02 | 1 | VDVGCLDNRGSVKAFAEK |
|  | 419 | **386.1675** | **1155.4805** | **1155.4771** | **0.0034** | **1** | **13** | **1.2e+02** | **1** | **MPPVPRCIVK + Oxidation (M)** |
|  | 1467 | **445.0885** | **888.1622** | **888.0635** | **0.0988** | **2** | **13** | **1.6e+02** | **1** | **KIGEKVSK** |
|  | 1601 | **458.1370** | **914.2593** | **915.0488** | **-0.7896** | **1** | **13** | **1.5e+02** | **1** | **GSAGIKGLGR** |
|  | 2096 | **508.4638** | **1014.9128** | **1015.2491** | **-0.3364** | **1** | **13** | **1.3e+02** | **1** | **IVGTKWLAK** |
|  | 2787 | **628.5688** | **1255.1229** | **1255.4207** | **-0.2978** | **2** | **13** | **1.1e+02** | **1** | **VKSPSKQAPGEK** |
|  | 3011 | **682.5758** | **2044.7052** | **2044.3313** | **0.3740** | **2** | **13** | **1.1e+02** | **1** | **MFQGQKGEPGDIKDIVGPK** |
|  | 547 | **389.9969** | **1166.9686** | **1166.2877** | **0.6809** | **1** | **13** | **1.4e+02** | **1** | **IVVADKDNHR** |
|  | 1212 | **426.1887** | **850.3625** | **850.9190** | **-0.5564** | **1** | **13** | **1.4e+02** | **1** | **KGGEAFSR** |
|  | 1345 | **434.7484** | **1301.2229** | **1301.4771** | **-0.2543** | **1** | **13** | **1.1e+02** | **1** | **TGWCPGRAGAIR + Carbamidomethyl (C)** |
|  | 1443 | **443.7431** | **1328.2070** | **1328.4714** | **-0.2643** | **1** | **13** | **1.2e+02** | **1** | **KGPGEVAGTVTGQK** |
|  | 1447 | **443.8107** | **1328.4100** | **1327.5327** | **0.8773** | **0** | **13** | **1.4e+02** | **1** | **NCAHPAIDAMLR + Oxidation (M)** |
|  | 2697 | **609.9541** | **1826.8401** | **1827.1091** | **-0.2689** | **2** | **13** | **1.3e+02** | **1** | **LQKELSMCEMEREK + Carbamidomethyl (C); Oxidation (M)** |
|  | 3108 | **698.5643** | **1395.1139** | **1394.5343** | **0.5795** | **1** | **13** | **1.1e+02** | **1** | **ETEAQRAGAACCK + Carbamidomethyl (C)** |
|  | 369 | **384.8280** | **1151.4618** | **1150.3280** | **1.1339** | **0** | **13** | **1.2e+02** | **1** | **GPLGSPASVPLR** |
|  | 376 | **384.9667** | **767.9186** | **767.9133** | **0.0053** | **0** | **13** | **1.2e+02** | **1** | **KPTPPTK** |
|  | 849 | **407.0539** | **1218.1394** | **1218.4466** | **-0.3072** | **0** | **13** | **1.3e+02** | **1** | **VCALLSCTSHK + Carbamidomethyl (C)** |
|  | 924 | **408.1140** | **1221.3197** | **1222.4004** | **-1.0808** | **2** | **13** | **1.5e+02** | **1** | **ARKSANLGHLR** |
|  | 557 | **391.2377** | **780.4606** | **780.9535** | **-0.4929** | **0** | **13** | **1e+02** | **1** | **ECMVVK + Carbamidomethyl (C); Oxidation (M)** |
|  | 1473 | **446.1500** | **890.2853** | **891.0078** | **-0.7225** | **1** | **13** | **1.6e+02** | **1** | **ARAEASMR** |
|  | 751 | **404.7698** | **807.5249** | **807.9572** | **-0.4323** | **0** | **13** | **1.3e+02** | **1** | **MFQPAAK + Oxidation (M)** |
|  | 937 | **408.3400** | **814.6652** | **815.8285** | **-1.1633** | **0** | **13** | **1.2e+02** | **1** | **GGGSGGPEAK** |
|  | 1395 | **437.1090** | **872.2032** | **873.0089** | **-0.8057** | **1** | **13** | **1.6e+02** | **1** | **EKGLGITR** |
|  | 2189 | **520.1224** | **1038.2301** | **1039.1796** | **-0.9496** | **0** | **13** | **1.3e+02** | **1** | **VTISIDTYK** |
|  | 2230 | **523.1718** | **1044.3288** | **1045.1940** | **-0.8652** | **0** | **13** | **1.5e+02** | **1** | **HCTPACATK + 2 Carbamidomethyl (C)** |
|  | 1830 | **475.7305** | **949.4462** | **949.1665** | **0.2798** | **1** | **13** | **1.2e+02** | **1** | **METIKTVK** |
|  | 3469 | **859.6213** | **2575.8418** | **2574.8665** | **0.9753** | **1** | **13** | **1.4e+02** | **1** | **QVSPLLASQSWSSRSMSPNLLNR + Oxidation (M)** |
|  | 97 | **365.7135** | **729.4122** | **728.7081** | **0.7041** | **0** | **13** | **1.5e+02** | **1** | **DSSGNHL** |
|  | 1129 | **421.7291** | **1262.1650** | **1261.2595** | **0.9056** | **1** | **13** | **1.2e+02** | **1** | **RQNGSSSSGPER** |
|  | 1482 | **447.1875** | **892.3602** | **892.0105** | **0.3496** | **0** | **13** | **1.5e+02** | **1** | **DIYAAAIR** |
|  | 1512 | **450.1553** | **898.2959** | **898.9171** | **-0.6212** | **0** | **13** | **1.5e+02** | **1** | **NNPNDPTK** |
|  | 2733 | **612.3546** | **1834.0417** | **1835.1308** | **-1.0892** | **1** | **13** | **1.4e+02** | **1** | **CYSSIGKVQIAFISYR** |
|  | 1401 | **437.1981** | **1308.5722** | **1307.3875** | **1.1847** | **0** | **13** | **1.6e+02** | **1** | **CASSSGPGGLTNEK** |
|  | 1656 | **460.1435** | **918.2723** | **917.9618** | **0.3105** | **0** | **13** | **1.6e+02** | **1** | **AWGSGVDGAV** |
|  | 2313 | **534.4294** | **1066.8441** | **1066.1620** | **0.6821** | **0** | **13** | **1.2e+02** | **1** | **VDTDFTQLK** |
|  | 2597 | **592.8025** | **1775.3853** | **1775.9395** | **-0.5542** | **1** | **13** | **1.3e+02** | **1** | **QMRIIHGAGYSEEER** |
|  | 3465 | **857.7893** | **2570.3457** | **2570.8398** | **-0.4941** | **2** | **13** | **1.1e+02** | **1** | **CGHEDLQLRTGCRSVDECNLHK + Carbamidomethyl (C)** |
|  | 392 | **385.2079** | **1152.6017** | **1153.1629** | **-0.5612** | **0** | **13** | **1e+02** | **1** | **HGGGGGGSSLDPR** |
|  | 816 | **406.2273** | **1215.6598** | **1214.5857** | **1.0741** | **2** | **13** | **1.1e+02** | **1** | **MELMCKKMK + Carbamidomethyl (C); Oxidation (M)** |
|  | 1757 | **468.2040** | **1401.5900** | **1400.6895** | **0.9005** | **2** | **13** | **1.5e+02** | **1** | **CKECGKAFICGK + 2 Carbamidomethyl (C)** |
|  | 2679 | **607.0688** | **1818.1842** | **1818.1054** | **0.0788** | **2** | **13** | **1.4e+02** | **1** | **GSFICHCDMGYSGKKGK** |
|  | 2766 | **622.6230** | **1243.2312** | **1242.5111** | **0.7201** | **2** | **13** | **1.4e+02** | **1** | **KSTNIIVRALK** |
|  | 2840 | 653.3883 | 1957.1427 | 1958.0929 | -0.9502 | 1 | 13 | 1.5e+02 | 1 | ISTYNANTNYTQKVQGR |
|  | 286 | 376.9611 | 751.9075 | 752.8554 | -0.9480 | 0 | 13 | 1.3e+02 | 1 | LAATSYK |
|  | 405 | **386.0190** | **1155.0350** | **1155.2882** | **-0.2532** | **1** | **13** | **1.2e+02** | **1** | **RMEGAPAGPGGR** |
|  | 871 | **407.2932** | **1218.8576** | **1219.3288** | **-0.4712** | **2** | **13** | **1.1e+02** | **1** | **KEKGCGNPGSSR** |
|  | 910 | 407.8531 | 1220.5371 | 1221.3844 | -0.8473 | 2 | 13 | 1.5e+02 | 1 | TDGCTGKNKIGK |
|  | 1556 | **452.3509** | **902.6871** | **903.0318** | **-0.3447** | **1** | **13** | **1.3e+02** | **1** | **EKVLETGK** |
|  | 1833 | **475.9448** | **949.8747** | **950.0251** | **-0.1504** | **0** | **13** | **1.5e+02** | **1** | **CIQTEDGK + Carbamidomethyl (C)** |
|  | 643 | **401.0887** | **800.1625** | **798.9702** | **1.1924** | **1** | **13** | **1.6e+02** | **1** | **SPLGLKGK** |
|  | 675 | **402.1186** | **802.2223** | **801.8913** | **0.3310** | **1** | **13** | **1.6e+02** | **1** | **DALTRAR** |
|  | 960 | **410.0036** | **1226.9887** | **1226.4440** | **0.5448** | **0** | **13** | **1.6e+02** | **1** | **CMVADEMGLGK + Carbamidomethyl (C); Oxidation (M)** |
|  | 994 | 413.0560 | 824.0972 | 822.9272 | 1.1700 | 0 | 13 | 1.3e+02 | 1 | GSVSVCSK + Carbamidomethyl (C) |
|  | 3286 | **752.7098** | **1503.4048** | **1503.6370** | **-0.2323** | **0** | **13** | **1.2e+02** | **1** | **TQSPGGCSAEAVLAR + Carbamidomethyl (C)** |
|  | 1846 | **476.2078** | **1425.6013** | **1424.7124** | **0.8889** | **1** | **13** | **1.4e+02** | **1** | **GMGYLHAKGILHK** |
|  | 3149 | **719.1755** | **2154.5044** | **2153.5268** | **0.9777** | **2** | **13** | **1.4e+02** | **1** | **MDLAESAKRLGPGCGMMAGGK + Carbamidomethyl (C); Oxidation (M)** |
|  | 352 | **382.8916** | **1145.6527** | **1146.3643** | **-0.7115** | **2** | **13** | **1.5e+02** | **1** | **MLSARAKTPR + Oxidation (M)** |
|  | 1762 | **469.0588** | **936.1029** | **934.9445** | **1.1584** | **1** | **13** | **1.5e+02** | **1** | **EDSKEAEK** |
|  | 1887 | **480.1497** | **958.2847** | **958.0257** | **0.2590** | **1** | **13** | **1.5e+02** | **1** | **YGGKGYEGK** |
|  | 1985 | **492.5137** | **1474.5188** | **1474.7682** | **-0.2493** | **2** | **13** | **1.6e+02** | **1** | **KNKVVAMCDYCK + Carbamidomethyl (C); Oxidation (M)** |
|  | 2963 | **671.0558** | **2010.1452** | **2010.2304** | **-0.0853** | **1** | **13** | **1.3e+02** | **1** | **GTFAQLSELHCDKLHVDP** |
|  | 3018 | **683.9541** | **1365.8934** | **1365.5807** | **0.3127** | **1** | **13** | **1.2e+02** | **1** | **GALVLGSSLKQHR** |
|  | 519 | **389.1350** | **1164.3829** | **1163.4543** | **0.9286** | **1** | **13** | **1.6e+02** | **1** | **CIAMKQGVIK + Carbamidomethyl (C); Oxidation (M)** |
|  | 2737 | **613.0515** | **1224.0882** | **1223.3387** | **0.7495** | **0** | **13** | **1.5e+02** | **1** | **QLNQQHSQIK** |
|  | 653 | **401.3568** | **1201.0483** | **1200.4499** | **0.5984** | **0** | **13** | **1.5e+02** | **1** | **TAVTTMMNMGK + Oxidation (M)** |
|  | 1673 | **460.7168** | **919.4189** | **919.9993** | **-0.5804** | **0** | **13** | **1.2e+02** | **1** | **LEQDMER** |
|  | 3423 | **833.6958** | **1665.3768** | **1665.8687** | **-0.4918** | **2** | **13** | **1.2e+02** | **1** | **IMRDVIDGGDQYRK** |
|  | 1235 | **428.5256** | **1282.5545** | **1281.4199** | **1.1347** | **1** | **13** | **1.5e+02** | **1** | **MMGQRSPASER + 2 Oxidation (M)** |
|  | 2097 | **508.5168** | **1015.0188** | **1015.1267** | **-0.1079** | **1** | **13** | **1.6e+02** | **1** | **SFGAAGPPRR** |
|  | 2863 | **662.5892** | **1984.7455** | **1984.1934** | **0.5522** | **2** | **13** | **1.1e+02** | **1** | **KGFTEVKSQNGEFMTHK + Oxidation (M)** |
|  | 3344 | **784.2078** | **2349.6011** | **2349.7382** | **-0.1371** | **1** | **13** | **1.4e+02** | **1** | **ELSVLLLEMKEAQEEIAFLK + Oxidation (M)** |
|  | 355 | **383.3436** | **1147.0085** | **1146.3592** | **0.6494** | **0** | **13** | **1.3e+02** | **1** | **MAAQPSWLVK + Oxidation (M)** |
|  | 1337 | **434.1528** | **1299.4361** | **1298.5131** | **0.9231** | **0** | **13** | **1.4e+02** | **1** | **MPHSFANLPLR + Oxidation (M)** |
|  | 1625 | **458.9636** | **915.9125** | **915.9510** | **-0.0385** | **1** | **13** | **1.6e+02** | **1** | **DSAPGSARR** |
|  | 1765 | **469.2309** | **936.4471** | **936.0897** | **0.3574** | **1** | **13** | **1.4e+02** | **1** | **KCYGPGVGR** |
|  | 2666 | **603.2541** | **1204.4934** | **1203.3891** | **1.1043** | **2** | **13** | **1.5e+02** | **1** | **TLKIRDVSSGK** |
|  | 106 | **366.1232** | **730.2316** | **729.7758** | **0.4559** | **0** | **13** | **1.6e+02** | **1** | **EEPLDK** |
|  | 262 | **374.4427** | **746.8707** | **745.8911** | **0.9796** | **0** | **13** | **2.1e+02** | **1** | **MGQGVVR** |
|  | 316 | **379.1204** | **1134.3391** | **1133.2543** | **1.0847** | **1** | **13** | **1.5e+02** | **1** | **GTLQKDGWTK** |
|  | 853 | **407.1084** | **1218.3030** | **1219.4727** | **-1.1697** | **0** | **13** | **1.4e+02** | **1** | **ELLPVLISAHK** |
|  | 866 | **407.2516** | **812.4885** | **811.9689** | **0.5196** | **1** | **13** | **1.1e+02** | **1** | **QKAPQIK** |
|  | 1035 | **415.9931** | **1244.9570** | **1245.4489** | **-0.4918** | **1** | **13** | **1.7e+02** | **1** | **QAKELMSGLPR + Oxidation (M)** |
|  | 1302 | **432.1506** | **1293.4296** | **1292.3762** | **1.0535** | **0** | **13** | **1.6e+02** | **1** | **STNSHLGTSNMK + Oxidation (M)** |
|  | 2331 | **537.1709** | **1608.4905** | **1607.8423** | **0.6482** | **2** | **13** | **1.6e+02** | **1** | **CGSLKNIRHRPGGGR** |
|  | 2121 | **515.4127** | **1543.2160** | **1542.7807** | **0.4352** | **1** | **13** | **1.2e+02** | **1** | **AIAPTPSALFRVGSR** |
|  | 3150 | **719.6667** | **1437.3187** | **1437.5540** | **-0.2353** | **0** | **13** | **1.2e+02** | **1** | **DEEVHAGLGELLR** |
|  | 365 | **384.3809** | **1150.1206** | **1149.2836** | **0.8370** | **0** | **13** | **1.5e+02** | **1** | **GHSHLCPSVR + Carbamidomethyl (C)** |
|  | 703 | **403.1693** | **1206.4859** | **1205.3884** | **1.0975** | **2** | **13** | **1.6e+02** | **1** | **MSEIQARAKR + Oxidation (M)** |
|  | 2655 | **598.3336** | **1791.9787** | **1792.8426** | **-0.8639** | **1** | **13** | **1.4e+02** | **1** | **MADGGGGGSGGAGPASTRASR + Oxidation (M)** |
|  | 2835 | **651.5581** | **1301.1014** | **1301.4044** | **-0.3030** | **1** | **13** | **1.2e+02** | **1** | **GALTGGYYDTRK** |
|  | 3152 | **723.1337** | **2166.3790** | **2165.4809** | **0.8981** | **2** | **13** | **1.4e+02** | **1** | **HCRVRYSQLLGLHEQLR + Carbamidomethyl (C)** |
|  | 3519 | **926.7063** | **2777.0967** | **2777.0943** | **0.0025** | **2** | **13** | **1.3e+02** | **1** | **FIGNGYCKFPSSTHPVNTDFTGKCV + Carbamidomethyl (C)** |
|  | 321 | **379.7120** | **1136.1138** | **1135.2273** | **0.8865** | **1** | **13** | **1.5e+02** | **1** | **DPDSFLKSAR** |
|  | 1011 | **414.0894** | **1239.2459** | **1239.4473** | **-0.2014** | **0** | **13** | **1.3e+02** | **1** | **LLHLGLGSGGCR + Carbamidomethyl (C)** |
|  | 1061 | **418.2697** | **1251.7871** | **1252.3405** | **-0.5534** | **2** | **13** | **1.2e+02** | **1** | **NPTSHRAGTGRV** |
|  | 1249 | **429.0812** | **856.1477** | **854.9705** | **1.1772** | **0** | **13** | **1.4e+02** | **1** | **AIECYTR** |
|  | 1949 | **488.2091** | **1461.6051** | **1462.6284** | **-1.0232** | **1** | **13** | **1.6e+02** | **1** | **RLTSAEVPMATDR + Oxidation (M)** |
|  | 2161 | **519.0159** | **1036.0171** | **1035.1066** | **0.9105** | **1** | **13** | **1.4e+02** | **1** | **SSENKLETK** |
|  | 2933 | **668.6680** | **2002.9817** | **2002.2761** | **0.7056** | **0** | **13** | **1.4e+02** | **1** | **QHLSNSDCVLLCLSISR + 2 Carbamidomethyl (C)** |
|  | 3454 | **852.8026** | **2555.3857** | **2554.8721** | **0.5135** | **2** | **13** | **1.1e+02** | **1** | **EWTTYPESTVGTLMSKLRELGR** |
|  | 833 | **406.7706** | **1217.2897** | **1218.3836** | **-1.0939** | **1** | **13** | **1.4e+02** | **1** | **NIESLCLDRR** |
|  | 1113 | **420.4692** | **1258.3855** | **1259.4804** | **-1.0948** | **1** | **13** | **1.6e+02** | **1** | **CLHFSGVGKVR + Carbamidomethyl (C)** |
|  | 1425 | **441.2903** | **1320.8489** | **1320.3896** | **0.4593** | **1** | **13** | **1.2e+02** | **1** | **LGRQMSDGDGER** |
|  | 1462 | **444.9706** | **1331.8896** | **1332.5742** | **-0.6846** | **2** | **13** | **1.7e+02** | **1** | **YMKLQHVNRK + Oxidation (M)** |
|  | 1843 | **476.1743** | **1425.5009** | **1426.6856** | **-1.1847** | **2** | **13** | **1.5e+02** | **1** | **AMKVDMDICRR + Carbamidomethyl (C); 2 Oxidation (M)** |
|  | 1969 | **490.0327** | **1467.0760** | **1467.6909** | **-0.6149** | **0** | **13** | **1.5e+02** | **1** | **GLPGFPGLHGMPGSK + Oxidation (M)** |
|  | 2757 | **616.6940** | **1847.0599** | **1848.0421** | **-0.9822** | **0** | **13** | **1.8e+02** | **1** | **YTPEQVDMATVTALHR + Oxidation (M)** |
|  | 3266 | **742.6614** | **2224.9621** | **2224.5813** | **0.3809** | **1** | **13** | **1.1e+02** | **1** | **MNCSRVFNLFCLYGNIEK + Carbamidomethyl (C); Oxidation (M)** |
|  | 3305 | **760.0924** | **1518.1700** | **1518.7776** | **-0.6076** | **2** | **13** | **1.2e+02** | **1** | **GAKNSETAAKVALMK** |
|  | 2491 | **569.4914** | **1136.9680** | **1136.4505** | **0.5175** | **2** | **13** | **1.2e+02** | **1** | **GKFGMVLLKK + Oxidation (M)** |
|  | 2552 | **583.0277** | **1746.0610** | **1747.0955** | **-1.0345** | **0** | **13** | **1.5e+02** | **1** | **MMVHCAGCERPILDR + Oxidation (M)** |
|  | 1711 | **463.2621** | **924.5095** | **924.0573** | **0.4522** | **0** | **13** | **1.2e+02** | **1** | **AASSCGLCGR** |
|  | 2067 | **505.5065** | **1513.4974** | **1512.7117** | **0.7857** | **2** | **13** | **1.4e+02** | **1** | **GHATIRKNPVYEK** |
|  | 2492 | **569.9632** | **1137.9116** | **1138.2247** | **-0.3130** | **0** | **13** | **1.5e+02** | **1** | **TPLFTSETDK** |
|  | 811 | 406.1271 | 810.2395 | 809.8455 | 0.3940 | 0 | 13 | 1.4e+02 | 1 | ACSSGSNK + Carbamidomethyl (C) |
|  | 834 | 406.7885 | 1217.3434 | 1217.4369 | -0.0936 | 0 | 13 | 1.4e+02 | 1 | LIITCHEGFK + Carbamidomethyl (C) |
|  | 2244 | **524.1523** | **1569.4349** | **1568.7039** | **0.7310** | **0** | **13** | **1.6e+02** | **1** | **SAESMSDGVGSFLPGK** |
|  | 3164 | **731.1125** | **2190.3155** | **2189.4247** | **0.8907** | **1** | **13** | **1.4e+02** | **1** | **SKTAAAAADVNGFKPLSGNELK** |
|  | 558 | **391.2544** | **780.4940** | **779.8163** | **0.6778** | **0** | **13** | **1.1e+02** | **1** | **SCSQPSTA** |
|  | 700 | **403.1487** | **804.2825** | **804.8522** | **-0.5697** | **1** | **13** | **1.6e+02** | **1** | **RGTSAASR** |
|  | 888 | 407.6146 | 813.2143 | 813.9650 | -0.7506 | 0 | 13 | 1.2e+02 | 1 | GAPPLCTR |
|  | 1079 | **419.2253** | **836.4359** | **836.9785** | **-0.5426** | **0** | **13** | **1.5e+02** | **1** | **GAVPLQPR** |
|  | 1684 | **461.8481** | **921.6814** | **922.0120** | **-0.3306** | **0** | **13** | **1.5e+02** | **1** | **ESAEEMVK** |
|  | 2911 | **667.0917** | **1998.2530** | **1998.2696** | **-0.0165** | **2** | **13** | **1.5e+02** | **1** | **VEKVLSNSTAHPSMRWR** |
|  | 2985 | 679.2632 | 1356.5116 | 1357.5951 | -1.0835 | 1 | 13 | 1.5e+02 | 1 | LLLQLEATKNSK |
|  | 3495 | **890.9636** | **1779.9125** | **1780.1882** | **-0.2758** | **2** | **13** | **1.6e+02** | **1** | **LMGRLASQCMALKSVR + Oxidation (M)** |
|  | 639 | **401.0739** | **1200.1994** | **1199.2694** | **0.9300** | **0** | **13** | **1.7e+02** | **1** | **WSNPEPNDLK** |
|  | 1047 | **417.1501** | **1248.4281** | **1248.4494** | **-0.0214** | **1** | **13** | **1.6e+02** | **1** | **LCAKSANAIETK** |
|  | 3473 | **861.6243** | **2581.8508** | **2581.7484** | **0.1025** | **1** | **13** | **1.5e+02** | **1** | **SPVIGSEVFLPNSNHVASGAGEAGRE** |
|  | 1267 | **430.0751** | **858.1354** | **857.0361** | **1.0993** | **0** | **13** | **1.7e+02** | **1** | **ANIMRPR** |
|  | 2635 | **595.8597** | **1784.5570** | **1784.0325** | **0.5245** | **2** | **13** | **1.3e+02** | **1** | **GYIGGSRGLVHGRLWR** |
|  | 988 | **412.2092** | **1233.6053** | **1233.4131** | **0.1922** | **0** | **13** | **1.4e+02** | **1** | **DFLAGGIAAAISK** |
|  | 1095 | **419.4437** | **1255.3088** | **1255.3346** | **-0.0257** | **1** | **13** | **2e+02** | **1** | **YVASSSKDGSVR** |
|  | 2329 | 536.2662 | 1070.5176 | 1070.3692 | 0.1484 | 1 | 13 | 1.5e+02 | 1 | FMKICALTK + Oxidation (M) |
|  | 2429 | **556.8196** | **1667.4366** | **1667.7953** | **-0.3587** | **0** | **13** | **1.2e+02** | **1** | **ASSVTTFTGEPNTCPR** |
|  | 2536 | **579.4254** | **1735.2539** | **1734.9471** | **0.3067** | **0** | **13** | **1.3e+02** | **1** | **DIKPSNLLVGEDGHIK** |
|  | 2728 | **612.2656** | **1222.5165** | **1222.5395** | **-0.0231** | **1** | **13** | **1.5e+02** | **1** | **YCKISALALLK** |
|  | 69 | **363.8991** | **1088.6751** | **1089.2482** | **-0.5731** | **0** | **12** | **1.3e+02** | **1** | **CPPWCAEAR + Carbamidomethyl (C)** |
|  | 290 | **377.0971** | **1128.2692** | **1128.2627** | **0.0065** | **1** | **12** | **1.4e+02** | **1** | **GERSLAHCAGK** |
|  | 630 | **400.2256** | **798.4365** | **797.9854** | **0.4511** | **0** | **12** | **1.1e+02** | **1** | **IGIAAVVR** |
|  | 886 | **407.5855** | **1219.7344** | **1220.4193** | **-0.6850** | **0** | **12** | **1.3e+02** | **1** | **ALFLYTSHLR** |
|  | 953 | **409.1981** | **1224.5720** | **1224.3421** | **0.2299** | **1** | **12** | **1.5e+02** | **1** | **MKVASGSTGDQK + Oxidation (M)** |
|  | 2651 | **597.9484** | **1790.8231** | **1790.9093** | **-0.0862** | **1** | **12** | **1.3e+02** | **1** | **QYWDIPDGTDCHRK + Carbamidomethyl (C)** |
|  | 984 | **411.6212** | **1231.8414** | **1231.3857** | **0.4556** | **0** | **12** | **1.3e+02** | **1** | **HCAGPGECACAR + Carbamidomethyl (C)** |
|  | 1546 | **452.0172** | **1353.0296** | **1353.3514** | **-0.3219** | **0** | **12** | **1.8e+02** | **1** | **NQGQLYSEGDSR** |
|  | 1983 | **492.3204** | **1473.9389** | **1473.5117** | **0.4273** | **2** | **12** | **1.2e+02** | **1** | **RSGQVGGGEGGGRGSR** |
|  | 2622 | **593.8508** | **1778.5301** | **1777.9173** | **0.6129** | **1** | **12** | **1.3e+02** | **1** | **SGQCQGRDQCPEPCR + 2 Carbamidomethyl (C)** |
|  | 1610 | **458.6344** | **1372.8811** | **1372.6117** | **0.2695** | **0** | **12** | **1.5e+02** | **1** | **AEVPTKPPLPPAR** |
|  | 1743 | **466.2339** | **930.4531** | **931.0319** | **-0.5787** | **1** | **12** | **1.6e+02** | **1** | **MSGHRSTR** |
|  | 852 | **407.1023** | **1218.2847** | **1218.4005** | **-0.1157** | **1** | **12** | **1.5e+02** | **1** | **SRVVISLDTTK** |
|  | 1308 | **432.3195** | **1293.9363** | **1294.6700** | **-0.7337** | **1** | **12** | **1.3e+02** | **1** | **LAKLVHFLLLK** |
|  | 1491 | **448.2460** | **1341.7158** | **1342.4546** | **-0.7388** | **0** | **12** | **1.4e+02** | **1** | **DDAQLSGLPSALR** |
|  | 2286 | **531.2866** | **1060.5585** | **1061.2165** | **-0.6581** | **2** | **12** | **1.6e+02** | **1** | **IEIRGCRSQ** |
|  | 2560 | **584.3566** | **1166.6984** | **1167.3981** | **-0.6998** | **0** | **12** | **1.5e+02** | **1** | **CAYCAAPILDK** |
|  | 3383 | **808.7452** | **2423.2135** | **2423.8065** | **-0.5930** | **1** | **12** | **1.2e+02** | **1** | **HIDGSNLVRLDPILCDCILEK + Carbamidomethyl (C)** |
|  | 2453 | **563.5646** | **1687.6716** | **1687.9158** | **-0.2442** | **0** | **12** | **1.5e+02** | **1** | **QVMAVLVVSGAAEQGGR + Oxidation (M)** |
|  | 256 | **374.2292** | **1119.6656** | **1119.3354** | **0.3301** | **1** | **12** | **1.5e+02** | **1** | **KLLQDIMSR + Oxidation (M)** |
|  | 804 | **406.1131** | **1215.3172** | **1214.4431** | **0.8742** | **1** | **12** | **1.5e+02** | **1** | **VLRAGMGAHFR** |
|  | 1536 | **451.5589** | **901.1030** | **900.9993** | **0.1037** | **0** | **12** | **2.1e+02** | **1** | **CPGSPSPTR** |
|  | 2910 | **667.0487** | **1998.1239** | **1998.3244** | **-0.2004** | **1** | **12** | **1.5e+02** | **1** | **MKSCAVSLTVAAVAFGDEAK** |
|  | 3121 | **702.2380** | **2103.6919** | **2104.3434** | **-0.6515** | **2** | **12** | **1.5e+02** | **1** | **FTISRDNSRNTLYLEMK + Oxidation (M)** |
|  | 2300 | **533.3931** | **1597.1570** | **1597.8543** | **-0.6972** | **1** | **12** | **1.2e+02** | **1** | **KYNINPVSMDICK + Carbamidomethyl (C); Oxidation (M)** |
|  | 856 | **407.1198** | **1218.3371** | **1218.3559** | **-0.0188** | **1** | **12** | **1.5e+02** | **1** | **KDASTSMTMSK + 2 Oxidation (M)** |
|  | 1248 | **429.0374** | **856.0599** | **854.9738** | **1.0861** | **1** | **12** | **1.4e+02** | **1** | **CGKAYTR + Carbamidomethyl (C)** |
|  | 1780 | **470.3683** | **1408.0826** | **1407.7021** | **0.3805** | **2** | **12** | **1.1e+02** | **1** | **MGRNSMKLPTLK + 2 Oxidation (M)** |
|  | 1899 | **481.4642** | **960.9137** | **960.0729** | **0.8407** | **1** | **12** | **1.5e+02** | **1** | **RAAAQQCR + Carbamidomethyl (C)** |
|  | 2587 | **592.2193** | **1182.4238** | **1183.4921** | **-1.0682** | **1** | **12** | **1.5e+02** | **1** | **GMRMTCPLCR + Oxidation (M)** |
|  | 2818 | **643.1625** | **1284.3102** | **1284.3343** | **-0.0241** | **0** | **12** | **1.4e+02** | **1** | **MEGNGTENSCSR** |
|  | 370 | **384.8438** | **1151.5091** | **1152.2595** | **-0.7504** | **0** | **12** | **1.3e+02** | **1** | **SCTSPTPCSR + 2 Carbamidomethyl (C)** |
|  | 615 | **399.8474** | **1196.5202** | **1195.4978** | **1.0224** | **2** | **12** | **1.3e+02** | **1** | **KSKWMAMLGK + Oxidation (M)** |
|  | 1355 | **435.1498** | **1302.4273** | **1303.4883** | **-1.0609** | **1** | **12** | **1.5e+02** | **1** | **RLVCLDVSENR** |
|  | 1724 | **464.2883** | **926.5618** | **927.0199** | **-0.4580** | **1** | **12** | **1.2e+02** | **1** | **RLNPNASR** |
|  | 2130 | **516.2058** | **1545.5953** | **1544.8547** | **0.7406** | **1** | **12** | **1.6e+02** | **1** | **VEVLLGMKVQGDIK + Oxidation (M)** |
|  | 2157 | **518.9699** | **1035.9250** | **1035.2191** | **0.7059** | **2** | **12** | **1.5e+02** | **1** | **TCKKELTR + Carbamidomethyl (C)** |
|  | 2159 | **518.9841** | **1553.9300** | **1553.6761** | **0.2540** | **1** | **12** | **1.5e+02** | **1** | **ERPGGSPRGPVTSEK** |
|  | 2162 | **519.0171** | **1036.0194** | **1035.2854** | **0.7340** | **2** | **12** | **1.5e+02** | **1** | **RVCECLKK + Carbamidomethyl (C)** |
|  | 2340 | **538.0654** | **1611.1741** | **1611.7567** | **-0.5825** | **1** | **12** | **1.6e+02** | **1** | **TNRFTASFQGIVDR** |
|  | 2724 | **612.1300** | **1833.3678** | **1833.9756** | **-0.6077** | **0** | **12** | **1.6e+02** | **1** | **QGPLDSIHPSPCPGEGSR** |
|  | 2119 | **515.2636** | **1542.7685** | **1541.8869** | **0.8815** | **2** | **12** | **1.5e+02** | **1** | **GLLRGGLPCARAMAR** |
|  | 2813 | **638.2930** | **1911.8567** | **1911.2948** | **0.5619** | **1** | **12** | **1.6e+02** | **1** | **KIYLCESVLCSFPRPR** |
|  | 425 | **386.2344** | **1155.6810** | **1155.3281** | **0.3530** | **2** | **12** | **1.1e+02** | **1** | **VRARYSMEK + Oxidation (M)** |
|  | 2059 | **505.0937** | **1512.2589** | **1512.6703** | **-0.4114** | **2** | **12** | **1.5e+02** | **1** | **ADVEKARQQAQIR** |
|  | 1274 | **430.2161** | **1287.6262** | **1287.5285** | **0.0977** | **0** | **12** | **1.7e+02** | **1** | **VMLLLDTAGGAAR** |
|  | 2216 | **522.1731** | **1563.4971** | **1563.7118** | **-0.2147** | **0** | **12** | **1.6e+02** | **1** | **NNWDAALISLQYR** |
|  | 3115 | **700.8696** | **1399.7245** | **1398.6307** | **1.0938** | **1** | **12** | **1.6e+02** | **1** | **VLKTPGCQSPGVGR** |
|  | 3188 | **739.9575** | **1477.9003** | **1478.6672** | **-0.7670** | **0** | **12** | **1.3e+02** | **1** | **QSLMLIATSNEGSK** |
|  | 3556 | **1159.6643** | **3475.9707** | **3476.6684** | **-0.6977** | **1** | **12** | **1.2e+02** | **1** | **GTPGPDSSGSLGSGEFTGVKELDDISQEIAQLQR** |
|  | 445 | **386.9460** | **1157.8158** | **1158.4594** | **-0.6436** | **0** | **12** | **1.7e+02** | **1** | **HTMLCMCCK + Carbamidomethyl (C); 2 Oxidation (M)** |
|  | 1232 | **428.3138** | **1281.9192** | **1281.3103** | **0.6088** | **0** | **12** | **1.1e+02** | **1** | **EEDSGHYWCR** |
|  | 1508 | **449.6501** | **1345.9281** | **1346.4467** | **-0.5187** | **2** | **12** | **1.3e+02** | **1** | **NQDESRKLTQK** |
|  | 1629 | 459.0482 | 916.0817 | 916.9325 | -0.8508 | 1 | 12 | 1.8e+02 | 1 | ESDKDAPR |
|  | 2367 | **541.0229** | **1620.0467** | **1619.8386** | **0.2081** | **2** | **12** | **1.5e+02** | **1** | **QMVPREEIKSTSAK + Oxidation (M)** |
|  | 2540 | **580.3340** | **1737.9800** | **1736.9648** | **1.0152** | **0** | **12** | **1.6e+02** | **1** | **MVLAWYMDDAPGDPR** |
|  | 212 | 371.0298 | 1110.0673 | 1110.2872 | -0.2200 | 1 | 12 | 1.2e+02 | 1 | TGSRCLAFQK |
|  | 301 | **377.3694** | **1129.0859** | **1128.3057** | **0.7801** | **1** | **12** | **1.5e+02** | **1** | **RLASSGLHCGK** |
|  | 934 | **408.2280** | **1221.6617** | **1221.3811** | **0.2807** | **1** | **12** | **1.3e+02** | **1** | **DTKLNMLNEK + Oxidation (M)** |
|  | 949 | **409.0407** | **1224.1001** | **1223.4499** | **0.6502** | **1** | **12** | **1.7e+02** | **1** | **AMHWVRAAPGK** |
|  | 999 | **413.4534** | **824.8921** | **824.9678** | **-0.0757** | **1** | **12** | **1.6e+02** | **1** | **RLPPSQK** |
|  | 958 | **409.3812** | **1225.1213** | **1224.3635** | **0.7579** | **0** | **12** | **1.6e+02** | **1** | **SAEGSNPPKPLK** |
|  | 971 | **411.0368** | **1230.0883** | **1229.4776** | **0.6108** | **1** | **12** | **1.6e+02** | **1** | **LRCMCTCNR + 2 Carbamidomethyl (C); Oxidation (M)** |
|  | 2438 | **559.1851** | **1674.5332** | **1673.8459** | **0.6872** | **1** | **12** | **1.6e+02** | **1** | **MLSLPGNELGEERGR + Oxidation (M)** |
|  | 1367 | **435.8096** | **1304.4065** | **1303.6008** | **0.8057** | **2** | **12** | **1.5e+02** | **1** | **CRMQLVREIR** |
|  | 2190 | **520.1472** | **1038.2795** | **1038.0921** | **0.1875** | **0** | **12** | **1.5e+02** | **1** | **SMNQNFSPN** |
|  | 323 | **379.9482** | **1136.8225** | **1136.3397** | **0.4828** | **0** | **12** | **1.8e+02** | **1** | **SPPTSELMMK + Oxidation (M)** |
|  | 1537 | **451.7802** | **1352.3185** | **1351.6106** | **0.7079** | **0** | **12** | **1.6e+02** | **1** | **LFQMVTIDQLK + Oxidation (M)** |
|  | 1557 | **452.6221** | **903.2295** | **903.1013** | **0.1282** | **1** | **12** | **1.6e+02** | **1** | **LVEQRMK** |
|  | 3279 | **745.2812** | **2232.8214** | **2232.3434** | **0.4779** | **0** | **12** | **1.5e+02** | **1** | **SSANAISTSWAGGSSTMWSSTR** |
|  | 80 | **364.2449** | **1089.7126** | **1089.1556** | **0.5571** | **0** | **12** | **1.1e+02** | **1** | **DDDKPFQPK** |
|  | 712 | **403.9234** | **1208.7480** | **1209.4548** | **-0.7068** | **0** | **12** | **1.6e+02** | **1** | **FMAAIEVITSK** |
|  | 1500 | **448.6362** | **1342.8864** | **1342.5688** | **0.3176** | **1** | **12** | **1.2e+02** | **1** | **LLRWTCSPGPR + Carbamidomethyl (C)** |
|  | 3134 | **711.9342** | **2132.7804** | **2133.3699** | **-0.5894** | **2** | **12** | **1.4e+02** | **1** | **QMDSSKPTGAPRGRLCEAR + Carbamidomethyl (C); Oxidation (M)** |
|  | 1002 | **413.5751** | **1237.7032** | **1237.4898** | **0.2135** | **1** | **12** | **1.2e+02** | **1** | **KIMNSLQEMK + Oxidation (M)** |
|  | 1053 | **418.0050** | **1250.9929** | **1250.4471** | **0.5458** | **0** | **12** | **1.7e+02** | **1** | **LVAAPVATANPAR** |
|  | 2274 | **529.1950** | **1056.3752** | **1056.1751** | **0.2001** | **0** | **12** | **1.6e+02** | **1** | **AGAAGLPTGWR** |
|  | 83 | **364.6903** | **1091.0488** | **1090.2281** | **0.8207** | **0** | **12** | **1.4e+02** | **1** | **DYCSQMLSK + Oxidation (M)** |
|  | 1900 | **481.4643** | **1441.3708** | **1441.5892** | **-0.2184** | **1** | **12** | **1.6e+02** | **1** | **EVRSTPLTATPGGR** |
|  | 2083 | **507.0674** | **1518.1801** | **1517.8557** | **0.3244** | **0** | **12** | **1.5e+02** | **1** | **LWCCTGNSIMVMK + 2 Oxidation (M)** |
|  | 2195 | **520.3237** | **1557.9490** | **1557.7058** | **0.2432** | **1** | **12** | **1.4e+02** | **1** | **ADEAWLANLAREAK** |
|  | 2297 | **533.0544** | **1064.0940** | **1063.2027** | **0.8912** | **1** | **12** | **1.6e+02** | **1** | **GTITIDTSKK** |
|  | 1788 | **471.3654** | **1411.0741** | **1411.6723** | **-0.5983** | **0** | **12** | **1.1e+02** | **1** | **AMSHMLCIGYGR + Carbamidomethyl (C); Oxidation (M)** |
|  | 2318 | **536.0010** | **1604.9809** | **1603.8159** | **1.1651** | **1** | **12** | **1.6e+02** | **1** | **SNSLASKSMEQFMK + Oxidation (M)** |
|  | 3225 | **740.8616** | **2219.5627** | **2218.4162** | **1.1465** | **1** | **12** | **1.8e+02** | **1** | **SHRPLADAHRSVQAAACQSR + Carbamidomethyl (C)** |
|  | 1429 | **442.2274** | **1323.6600** | **1322.4931** | **1.1668** | **2** | **12** | **1.4e+02** | **1** | **KCDELQFARR + Carbamidomethyl (C)** |
|  | 3154 | **724.2843** | **2169.8307** | **2169.4252** | **0.4056** | **2** | **12** | **1.6e+02** | **1** | **GSPPPPQRRYGHTMVAFDR** |
|  | 3198 | **740.4197** | **1478.8246** | **1479.6935** | **-0.8690** | **1** | **12** | **1.5e+02** | **1** | **EEKYLPELMAEK** |
|  | 773 | **405.1236** | **1212.3487** | **1211.4971** | **0.8516** | **2** | **12** | **1.5e+02** | **1** | **SPRVLIKTLGK** |
|  | 2788 | **628.5900** | **1255.1652** | **1254.4803** | **0.6848** | **2** | **12** | **1.3e+02** | **1** | **KSGLGHLKWTK** |
|  | 3549 | **1101.8728** | **3302.5962** | **3303.6309** | **-1.0347** | **0** | **12** | **1.4e+02** | **1** | **CLISGWGNTASSGADVPDELQCLDAPVLSQAK + Carbamidomethyl (C)** |
|  | 289 | **377.0507** | **1128.1298** | **1129.2674** | **-1.1376** | **2** | **12** | **1.5e+02** | **1** | **EKRQEQIAK** |
|  | 2605 | **593.0244** | **1776.0509** | **1776.2822** | **-0.2313** | **1** | **12** | **1.6e+02** | **1** | **TRCALLLLMVLMLGR + Carbamidomethyl (C); Oxidation (M)** |
|  | 131 | **367.2385** | **732.4623** | **732.7863** | **-0.3240** | **1** | **12** | **1.4e+02** | **1** | **RLDSSR** |
|  | 1636 | **459.1729** | **916.3310** | **917.0184** | **-0.6874** | **1** | **12** | **1.8e+02** | **1** | **LTKNAQDK** |
|  | 1712 | **463.4199** | **1387.2376** | **1387.7139** | **-0.4763** | **1** | **12** | **1.2e+02** | **1** | **IKSVALCVAACPR + Carbamidomethyl (C)** |
|  | 2459 | **564.2318** | **1126.4488** | **1126.2917** | **0.1572** | **1** | **12** | **1.7e+02** | **1** | **SCGRCVCER + 2 Carbamidomethyl (C)** |
|  | 3535 | **1008.1114** | **2014.2081** | **2015.3297** | **-1.1216** | **0** | **12** | **1.6e+02** | **1** | **ANLTIVTEFILMGFSTNK + Oxidation (M)** |
|  | 1016 | **414.6698** | **1240.9871** | **1241.3808** | **-0.3936** | **2** | **12** | **1.2e+02** | **1** | **MRRYAASGSSR** |
|  | 1185 | **423.1652** | **1266.4735** | **1266.3603** | **0.1132** | **0** | **12** | **1.8e+02** | **1** | **EHNIDPTAITR** |
|  | 1775 | **470.2031** | **1407.5872** | **1408.7100** | **-1.1228** | **0** | **12** | **1.5e+02** | **1** | **LVFFVNVGGVAMR** |
|  | 1923 | **486.3525** | **970.6902** | **971.1934** | **-0.5032** | **0** | **12** | **1.3e+02** | **1** | **VPTGLPFIK** |
|  | 2375 | **541.7767** | **1622.3080** | **1622.9334** | **-0.6253** | **2** | **12** | **1.3e+02** | **1** | **MGLSHSKTHLRVIK + Oxidation (M)** |
|  | 2719 | **611.9188** | **1221.8229** | **1221.4091** | **0.4137** | **1** | **12** | **1.3e+02** | **1** | **KGGLLQDVHVR** |
|  | 2962 | **670.9836** | **1339.9525** | **1340.5052** | **-0.5527** | **0** | **12** | **1.2e+02** | **1** | **VSSLHGASPTCPK + Carbamidomethyl (C)** |
|  | 1812 | **474.1540** | **1419.4398** | **1418.5788** | **0.8610** | **1** | **12** | **1.7e+02** | **1** | **NAQSEQLMGIRR + Oxidation (M)** |
|  | 2886 | **666.5001** | **1330.9855** | **1330.4839** | **0.5016** | **1** | **12** | **1.4e+02** | **1** | **LVSSDPEINTKK** |
|  | 3056 | **685.9357** | **2054.7848** | **2055.3588** | **-0.5740** | **2** | **12** | **1.3e+02** | **1** | **GQDKAIAEKPSCPSLNIRK** |
|  | 33 | **362.1937** | **1083.5590** | **1084.2930** | **-0.7340** | **0** | **12** | **1.2e+02** | **1** | **VLCAPAAGAVR + Carbamidomethyl (C)** |
|  | 1250 | **429.0921** | **1284.2541** | **1283.3047** | **0.9494** | **0** | **12** | **1.6e+02** | **1** | **DPQSPEGQAQAR** |
|  | 1379 | **436.1912** | **1305.5514** | **1304.4151** | **1.1364** | **2** | **12** | **1.6e+02** | **1** | **TPPRSYNASRR** |
|  | 1496 | 448.3535 | 894.6921 | 894.0694 | 0.6227 | 0 | 12 | 1.3e+02 | 1 | HLVNVLLS |
|  | 2708 | **610.1725** | **1827.4953** | **1827.1967** | **0.2986** | **0** | **12** | **1.7e+02** | **1** | **CPPPRPPTIPYPPLPK + Carbamidomethyl (C)** |
|  | 882 | **407.5302** | **1219.5683** | **1218.3689** | **1.1994** | **2** | **12** | **1.7e+02** | **1** | **RVAQHKGASHK** |
|  | 563 | **392.4116** | **782.8084** | **781.9017** | **0.9067** | **0** | **12** | **1.8e+02** | **1** | **MGGVSCGR + Oxidation (M)** |
|  | 1025 | **415.1391** | **828.2634** | **827.9252** | **0.3382** | **0** | **12** | **1.7e+02** | **1** | **ANASAIPGK** |
|  | 1715 | **464.0282** | **1389.0624** | **1389.6405** | **-0.5780** | **0** | **12** | **1.5e+02** | **1** | **VSCVEMTGHILK + Carbamidomethyl (C); Oxidation (M)** |
|  | 2407 | **551.5856** | **1101.1565** | **1100.2031** | **0.9534** | **1** | **12** | **2e+02** | **1** | **MKFENSSNK + Oxidation (M)** |
|  | 1006 | **414.0005** | **1238.9795** | **1238.4100** | **0.5695** | **0** | **12** | **1.4e+02** | **1** | **MPGEVQASYLK + Oxidation (M)** |
|  | 1565 | **453.6092** | **1357.8053** | **1357.5968** | **0.2086** | **1** | **12** | **1.7e+02** | **1** | **VFNKLIPDNIGK** |
|  | 1874 | **478.2250** | **954.4352** | **954.2505** | **0.1846** | **0** | **12** | **1.5e+02** | **1** | **LIYCLMAK** |
|  | 2056 | **505.0348** | **1512.0823** | **1511.5963** | **0.4860** | **1** | **12** | **1.6e+02** | **1** | **DADATEPRPDRIR** |
|  | 1020 | **414.9420** | **827.8693** | **826.9009** | **0.9684** | **1** | **12** | **1.6e+02** | **1** | **SGGPPRTR** |
|  | 1852 | 476.2814 | 1425.8221 | 1426.5994 | -0.7773 | 0 | 12 | 1.4e+02 | 1 | SPSTHVMPNWVR + Oxidation (M) |
|  | 2166 | **519.0398** | **1036.0648** | **1035.1346** | **0.9302** | **0** | **12** | **1.6e+02** | **1** | **MGGAAFGPDGR** |
|  | 2185 | **520.0997** | **1038.1847** | **1037.3410** | **0.8437** | **2** | **12** | **1.6e+02** | **1** | **KLTCGKLMK + Oxidation (M)** |
|  | 2443 | **561.0704** | **1120.1261** | **1119.2344** | **0.8917** | **1** | **12** | **1.7e+02** | **1** | **AKIHQTEHR** |
|  | 165 | **369.2090** | **736.4032** | **736.7982** | **-0.3950** | **1** | **12** | **1.4e+02** | **1** | **MRSGDR + Oxidation (M)** |
|  | 234 | **372.3290** | **742.6433** | **743.8056** | **-1.1623** | **0** | **12** | **1.4e+02** | **1** | **NGSPEIK** |
|  | 402 | **386.0057** | **769.9966** | **768.8185** | **1.1781** | **0** | **12** | **1.4e+02** | **1** | **HETQVR** |
|  | 1856 | **476.5283** | **951.0419** | **950.0253** | **1.0166** | **0** | **12** | **1.9e+02** | **1** | **MGAGASAEEK** |
|  | 2739 | **613.2419** | **1836.7037** | **1836.1239** | **0.5797** | **2** | **12** | **1.6e+02** | **1** | **QRSKLSLSHSMIPAHK + Oxidation (M)** |
|  | 3259 | **742.3837** | **2224.1290** | **2224.4704** | **-0.3414** | **0** | **12** | **1.6e+02** | **1** | **MASNSLFSTVTPCQQNFFW + Oxidation (M)** |
|  | 423 | **386.1800** | **770.3452** | **770.9139** | **-0.5687** | **0** | **12** | **1.3e+02** | **1** | **IVDVPTK** |
|  | 709 | **403.9109** | **805.8069** | **804.8954** | **0.9116** | **2** | **12** | **1.7e+02** | **1** | **ASRSKTR** |
|  | 2023 | **501.0835** | **1500.2283** | **1500.7406** | **-0.5123** | **1** | **12** | **1.7e+02** | **1** | **LDPVNAASFGKLIR** |
|  | 2630 | **594.9775** | **1781.9103** | **1781.0900** | **0.8202** | **2** | **12** | **1.7e+02** | **1** | **VLDKHLHLCIYDRR** |
|  | 3124 | **703.2720** | **1404.5293** | **1405.5968** | **-1.0675** | **0** | **12** | **1.7e+02** | **1** | **GSHMASLDMAEIK + Oxidation (M)** |
|  | 3244 | **741.8711** | **1481.7274** | **1481.7424** | **-0.0149** | **1** | **12** | **1.8e+02** | **1** | **QIPPATNKMCHNK** |
|  | 130 | **367.1899** | **1098.5476** | **1098.2963** | **0.2513** | **0** | **12** | **1.5e+02** | **1** | **CCIAAAYDLR** |
|  | 511 | **389.0742** | **1164.2004** | **1163.3680** | **0.8323** | **0** | **12** | **1.8e+02** | **1** | **LAISAVWTFR** |
|  | 572 | 394.1502 | 786.2857 | 785.8887 | 0.3969 | 1 | 12 | 1.7e+02 | 1 | RTAPAVAT |
|  | 1373 | **436.1060** | **1305.2960** | **1304.4580** | **0.8379** | **1** | **12** | **1.6e+02** | **1** | **VGRTGSHHAAALK** |
|  | 2603 | **592.9983** | **1775.9727** | **1777.0020** | **-1.0293** | **0** | **12** | **1.7e+02** | **1** | **GLEYMGLIYPGDYGTK** |
|  | 793 | **405.8857** | **1214.6348** | **1214.4797** | **0.1551** | **2** | **12** | **1.5e+02** | **1** | **RNIMKDMMK + 3 Oxidation (M)** |
|  | 1059 | **418.1863** | **1251.5367** | **1250.4536** | **1.0831** | **1** | **12** | **1.6e+02** | **1** | **GQFCGPCLRNR** |
|  | 3092 | **692.7261** | **2075.1560** | **2074.4117** | **0.7443** | **1** | **12** | **1.7e+02** | **1** | **DMIARPRQPVAQWHQLK** |
|  | 3230 | **741.0899** | **1480.1650** | **1479.6571** | **0.5080** | **1** | **12** | **1.3e+02** | **1** | **KQNAPMTLEEFR + Oxidation (M)** |
|  | 129 | **367.1660** | **1098.4759** | **1099.2398** | **-0.7639** | **0** | **12** | **1.6e+02** | **1** | **SAPFLTPGPGR** |
|  | 512 | **389.0754** | **776.1360** | **775.8971** | **0.2390** | **0** | **12** | **1.8e+02** | **1** | **GHMCNAK + Oxidation (M)** |
|  | 854 | **407.1140** | **1218.3200** | **1219.4826** | **-1.1627** | **2** | **12** | **1.6e+02** | **1** | **GVLGHLKARIR** |
|  | 1474 | **446.1879** | **890.3610** | **890.0195** | **0.3415** | **0** | **12** | **1.9e+02** | **1** | **GAQGMGLTR** |
|  | 1660 | **460.2976** | **1377.8707** | **1378.5713** | **-0.7006** | **0** | **12** | **1.5e+02** | **1** | **CSACEGLALTPDAK** |
|  | 2098 | 509.1266 | 1016.2385 | 1017.1772 | -0.9388 | 0 | 12 | 1.8e+02 | 1 | NLVSLGISSK |
|  | 2553 | **583.1794** | **1746.5162** | **1745.9068** | **0.6093** | **0** | **12** | **1.7e+02** | **1** | **GLEWMGLIDPADGEAR + Oxidation (M)** |
|  | 3550 | **1105.6672** | **2209.3197** | **2209.4773** | **-0.1577** | **1** | **12** | **1.3e+02** | **1** | **NYLELMVELPWNKSTTDR** |
|  | 625 | **400.1191** | **798.2235** | **797.9655** | **0.2580** | **0** | **12** | **1.5e+02** | **1** | **MHLELR** |
|  | 1535 | **451.4696** | **900.9244** | **900.1389** | **0.7855** | **1** | **12** | **2.3e+02** | **1** | **MMAKMEK + 2 Oxidation (M)** |
|  | 1721 | 464.2229 | 1389.6466 | 1388.6126 | 1.0340 | 0 | 12 | 1.6e+02 | 1 | DPGMGAMGGMGGGMF + Oxidation (M) |
|  | 2707 | **610.1442** | **1218.2737** | **1219.4545** | **-1.1809** | **1** | **12** | **1.7e+02** | **1** | **RSVIGSSCLIK + Carbamidomethyl (C)** |
|  | 746 | **404.4417** | **1210.3030** | **1210.3370** | **-0.0340** | **2** | **12** | **1.9e+02** | **1** | **SDNTVSKKGFK** |
|  | 1361 | **435.3691** | **868.7235** | **867.9959** | **0.7276** | **2** | **12** | **1.3e+02** | **1** | **APRSKGPR** |
|  | 1386 | **436.7892** | **871.5636** | **871.8951** | **-0.3315** | **0** | **12** | **1.7e+02** | **1** | **ENGVNSPR** |
|  | 2028 | **502.1050** | **1002.1953** | **1003.1707** | **-0.9754** | **0** | **12** | **1.8e+02** | **1** | **MPSLSPIDK + Oxidation (M)** |
|  | 239 | **372.5906** | **1114.7496** | **1115.2806** | **-0.5311** | **1** | **12** | **1.5e+02** | **1** | **QAQVEATKLK** |
|  | 1090 | **419.3541** | **836.6935** | **836.0134** | **0.6800** | **0** | **12** | **1.6e+02** | **1** | **LGCQLFR** |
|  | 1349 | **434.9352** | **1301.7835** | **1301.4890** | **0.2945** | **1** | **12** | **1.5e+02** | **1** | **TKGLQSGVDIGVK** |
|  | 1665 | **460.3499** | **1378.0274** | **1377.5567** | **0.4706** | **1** | **12** | **1.5e+02** | **1** | **VTARPAHGRAWR** |
|  | 2132 | 517.0354 | 1548.0840 | 1547.7077 | 0.3763 | 0 | 12 | 1.8e+02 | 1 | DNNMAPYYEALCK + Oxidation (M) |
|  | 2284 | **530.3685** | **1588.0834** | **1588.8245** | **-0.7411** | **0** | **12** | **1.6e+02** | **1** | **VMPNAIVQSVGVSSGK + Oxidation (M)** |
|  | 2483 | **568.0185** | **1701.0333** | **1700.8929** | **0.1405** | **1** | **12** | **1.7e+02** | **1** | **IPPAPSNEAPRLPADR** |
|  | 3435 | **845.3284** | **1688.6421** | **1688.9895** | **-0.3475** | **0** | **12** | **1.6e+02** | **1** | **GSLPILPQGCVWHPK + Carbamidomethyl (C)** |
|  | 16 | **360.5070** | **718.9992** | **718.7580** | **0.2412** | **0** | **12** | **1.8e+02** | **1** | **GSVWDR** |
|  | 1008 | **414.0508** | **1239.1303** | **1239.4310** | **-0.3007** | **2** | **12** | **1.4e+02** | **1** | **NRCQQCRFK + Carbamidomethyl (C)** |
|  | 2085 | **507.1805** | **1012.3462** | **1012.1179** | **0.2283** | **0** | **12** | **1.6e+02** | **1** | **ASLESVPPGR** |
|  | 3533 | **991.5953** | **1981.1759** | **1981.3185** | **-0.1426** | **1** | **12** | **1.5e+02** | **1** | **TDCPCATMGLGQKTLAVQK + Oxidation (M)** |
|  | 1825 | **475.0936** | **1422.2587** | **1421.5943** | **0.6644** | **0** | **12** | **1.9e+02** | **1** | **LLEIISQDSFTR** |
|  | 2544 | **581.4294** | **1160.8441** | **1161.3092** | **-0.4651** | **0** | **12** | **1.5e+02** | **1** | **LSFPHVNGYK** |
|  | 307 | **378.1889** | **754.3630** | **754.8382** | **-0.4751** | **1** | **12** | **1.2e+02** | **1** | **GRGVGPGR** |
|  | 1336 | **434.1335** | **866.2522** | **866.9645** | **-0.7123** | **0** | **12** | **1.6e+02** | **1** | **GLGHSALGR** |
|  | 1773 | **470.1769** | **1407.5085** | **1406.6079** | **0.9005** | **1** | **12** | **1.6e+02** | **1** | **VSITCRASQSINK** |
|  | 42 | **363.0709** | **1086.1904** | **1087.3581** | **-1.1677** | **1** | **12** | **1.7e+02** | **1** | **AVLLQRFIK** |
|  | 325 | **380.0004** | **1136.9789** | **1137.2630** | **-0.2840** | **0** | **12** | **1.9e+02** | **1** | **ESMPSWGISK + Oxidation (M)** |
|  | 1432 | **442.4685** | **1324.3833** | **1323.4748** | **0.9086** | **1** | **12** | **1.8e+02** | **1** | **DPAGMPHPETKK + Oxidation (M)** |
|  | 1927 | **487.0027** | **1457.9861** | **1458.7903** | **-0.8042** | **2** | **12** | **1.7e+02** | **1** | **GPKLKMPEMHFK + Oxidation (M)** |
|  | 1727 | **465.1305** | **928.2462** | **928.0013** | **0.2450** | **0** | **12** | **1.7e+02** | **1** | **GNLAPQGSGK** |
|  | 2243 | **524.1095** | **1046.2042** | **1046.1327** | **0.0716** | **2** | **12** | **1.8e+02** | **1** | **KEREVEEK** |
|  | 2273 | **529.1461** | **1056.2775** | **1057.1800** | **-0.9025** | **0** | **12** | **1.7e+02** | **1** | **MDFPGPHEK** |
|  | 2556 | **583.7582** | **1748.2524** | **1749.0417** | **-0.7894** | **1** | **12** | **1.7e+02** | **1** | **AGAPRCTYTFVLPPQK** |
|  | 975 | **411.1364** | **1230.3871** | **1230.3929** | **-0.0058** | **1** | **12** | **1.8e+02** | **1** | **GKECTCTSCK + 3 Carbamidomethyl (C)** |
|  | 2398 | **550.0261** | **1647.0562** | **1647.8499** | **-0.7937** | **0** | **12** | **1.6e+02** | **1** | **MSQWNQVQQLEIK + Oxidation (M)** |
|  | 2875 | **665.2397** | **1992.6969** | **1992.3180** | **0.3789** | **1** | **12** | **1.7e+02** | **1** | **QPEMKSGFMASFLDFLK + Oxidation (M)** |
|  | 825 | **406.3259** | **810.6370** | **809.9366** | **0.7005** | **1** | **12** | **1.3e+02** | **1** | **RFCASAR** |
|  | 1196 | **423.7903** | **1268.3488** | **1269.5348** | **-1.1860** | **0** | **12** | **1.9e+02** | **1** | **LLFSFHMCAK + Carbamidomethyl (C); Oxidation (M)** |
|  | 1974 | **490.2961** | **978.5774** | **978.2523** | **0.3251** | **1** | **12** | **1.5e+02** | **1** | **MLTVRFVL** |
|  | 3033 | 684.1815 | 1366.3483 | 1367.4891 | -1.1408 | 0 | 12 | 1.6e+02 | 1 | QGGSVCGCDPCER + Carbamidomethyl (C) |
|  | 10 | **360.4580** | **1078.3518** | **1078.2406** | **0.1112** | **1** | **12** | **2.3e+02** | **1** | **RSLLEMEGK + Oxidation (M)** |
|  | 1571 | **456.0961** | **1365.2662** | **1364.6127** | **0.6535** | **1** | **12** | **1.6e+02** | **1** | **MVPDGINPKLHK + Oxidation (M)** |
|  | 1902 | **482.0569** | **1443.1484** | **1443.7126** | **-0.5642** | **0** | **12** | **1.8e+02** | **1** | **NFMCHSMLGPFK + 2 Oxidation (M)** |
|  | 2885 | **666.4351** | **1996.2830** | **1995.1696** | **1.1134** | **1** | **12** | **1.7e+02** | **1** | **AAEEFVNIYYETMDKR + Oxidation (M)** |
|  | 3372 | **799.9279** | **1597.8409** | **1597.7795** | **0.0614** | **2** | **12** | **1.8e+02** | **1** | **AGGSLLPGGARGWRSR** |
|  | 3393 | **815.8551** | **2444.5431** | **2444.6389** | **-0.0957** | **2** | **12** | **1.8e+02** | **1** | **TNPNSGGTNYAQKFQGRVTMTR + Oxidation (M)** |
|  | 467 | **387.7805** | **773.5463** | **772.8469** | **0.6995** | **0** | **12** | **2e+02** | **1** | **SANPAVSK** |
|  | 1931 | **487.2929** | **1458.8566** | **1457.7838** | **1.0727** | **2** | **12** | **1.7e+02** | **1** | **KNSRTMLLPLLR + Oxidation (M)** |
|  | 2768 | **622.8464** | **1243.6781** | **1244.5253** | **-0.8472** | **0** | **12** | **1.5e+02** | **1** | **LWHMMAPLLT + 2 Oxidation (M)** |
|  | 1089 | **419.3413** | **836.6678** | **836.8709** | **-0.2030** | **0** | **12** | **1.7e+02** | **1** | **GGSMSGADR** |
|  | 2652 | **598.0507** | **1791.1298** | **1790.1117** | **1.0181** | **2** | **12** | **1.7e+02** | **1** | **AKLSIKGLIESALSFGR** |
|  | 3402 | **821.0889** | **1640.1630** | **1639.7915** | **0.3714** | **1** | **12** | **1.3e+02** | **1** | **AAPCPSTPGAAGRANNK + Carbamidomethyl (C)** |
|  | 1260 | **429.2341** | **1284.6800** | **1284.4683** | **0.2117** | **2** | **12** | **1.5e+02** | **1** | **ALSRTSGALPRR** |
|  | 2127 | **515.9810** | **1029.9471** | **1029.2543** | **0.6929** | **0** | **12** | **1.8e+02** | **1** | **MLLPSLSPR + Oxidation (M)** |
|  | 3157 | **724.9302** | **2171.7685** | **2172.4376** | **-0.6690** | **1** | **12** | **1.6e+02** | **1** | **KPYVLGHEASGTVEKVGSSVK** |
|  | 3232 | **741.4079** | **1480.8010** | **1481.6545** | **-0.8535** | **1** | **12** | **1.6e+02** | **1** | **KQLCSCDGGLSTR + 2 Carbamidomethyl (C)** |
|  | 2255 | **525.2897** | **1572.8468** | **1572.7372** | **0.1096** | **0** | **12** | **1.8e+02** | **1** | **SVIPEDSAVYYCAR** |
|  | 2315 | **534.7814** | **1067.5481** | **1068.2043** | **-0.6562** | **1** | **12** | **1.5e+02** | **1** | **KEFMSQGNK** |
|  | 2537 | **579.5817** | **1157.1487** | **1156.4152** | **0.7334** | **1** | **12** | **1.8e+02** | **1** | **ASIIVGDKLLK** |
|  | 2750 | **614.1594** | **1839.4561** | **1839.8461** | **-0.3900** | **0** | **12** | **1.7e+02** | **1** | **QEMEDDFDYYGQQR + Oxidation (M)** |
|  | 2856 | **659.0510** | **1316.0873** | **1315.5437** | **0.5436** | **1** | **12** | **1.7e+02** | **1** | **IHTGERPFKCK** |
|  | 3054 | **685.5547** | **2053.6421** | **2053.3429** | **0.2991** | **2** | **12** | **1.5e+02** | **1** | **EIGQSPSRKIMLNLEGHK + Oxidation (M)** |
|  | 669 | 401.9709 | 1202.8905 | 1203.3243 | -0.4338 | 0 | 12 | 2e+02 | 1 | YICYAEQTR + Carbamidomethyl (C) |
|  | 2353 | **539.3683** | **1615.0829** | **1615.8463** | **-0.7635** | **0** | **12** | **1.5e+02** | **1** | **VALQEEPASGINMIK + Oxidation (M)** |
|  | 65 | **363.3880** | **724.7612** | **723.9054** | **0.8558** | **0** | **12** | **2e+02** | **1** | **TLMGMR + Oxidation (M)** |
|  | 472 | **387.8665** | **773.7181** | **772.8932** | **0.8250** | **1** | **12** | **2.1e+02** | **1** | **IVRGSNK** |
|  | 3315 | **762.0708** | **2283.1902** | **2283.4367** | **-0.2464** | **0** | **12** | **1.4e+02** | **1** | **MSEHVEPAAPGPGPNGGGGGPAPAR + Oxidation (M)** |
|  | 3481 | **873.7236** | **2618.1487** | **2618.0323** | **0.1164** | **2** | **12** | **1.5e+02** | **1** | **ATMLISGQCQEVKVILEKTDEPGK** |
|  | 108 | **366.1754** | **1095.5040** | **1095.0839** | **0.4201** | **1** | **12** | **1.7e+02** | **1** | **NPRDGHDER** |
|  | 1230 | **428.2736** | **1281.7987** | **1281.4214** | **0.3772** | **0** | **12** | **1.2e+02** | **1** | **RPVAAAAAGSASPR** |
|  | 2355 | **539.8898** | **1077.7649** | **1078.2208** | **-0.4559** | **0** | **12** | **1.6e+02** | **1** | **AEKPDCMER** |
|  | 2523 | **576.1780** | **1150.3412** | **1150.1742** | **0.1670** | **0** | **12** | **1.7e+02** | **1** | **EEEGMTSDPR** |
|  | 2686 | **608.2882** | **1214.5616** | **1215.4660** | **-0.9043** | **2** | **12** | **1.8e+02** | **1** | **KMADKILPQR + Oxidation (M)** |
|  | 2989 | **680.0208** | **2037.0401** | **2036.1769** | **0.8632** | **1** | **12** | **1.5e+02** | **1** | **TPDTSTYCYETAEKITR + Carbamidomethyl (C)** |
|  | 27 | **362.1634** | **722.3121** | **722.7683** | **-0.4562** | **0** | **12** | **1.4e+02** | **1** | **MASNER + Oxidation (M)** |
|  | 473 | **387.8697** | **1160.5868** | **1160.3461** | **0.2407** | **2** | **12** | **2.1e+02** | **1** | **MKYHPDKNK** |
|  | 245 | **373.1682** | **744.3216** | **744.8799** | **-0.5583** | **2** | **12** | **1.9e+02** | **1** | **GDKAKVK** |
|  | 254 | **374.1851** | **746.3554** | **745.7832** | **0.5722** | **0** | **12** | **1.8e+02** | **1** | **QNGGWGK** |
|  | 534 | 389.3109 | 776.6071 | 775.8905 | 0.7166 | 0 | 12 | 1.6e+02 | 1 | LVSATTGK |
|  | 1081 | **419.2705** | **1254.7893** | **1254.5037** | **0.2856** | **2** | **12** | **1.7e+02** | **1** | **RMQICDKCGK + Carbamidomethyl (C); Oxidation (M)** |
|  | 53 | **363.1845** | **1086.5314** | **1087.1860** | **-0.6545** | **0** | **12** | **1.4e+02** | **1** | **SSGHFLPTGGK** |
|  | 378 | **384.9809** | **1151.9205** | **1152.3734** | **-0.4529** | **1** | **12** | **1.5e+02** | **1** | **HGILGACGRLR** |
|  | 2015 | **498.4945** | **994.9742** | **994.2334** | **0.7409** | **2** | **12** | **1.7e+02** | **1** | **LKVGFFKR** |
|  | 2357 | **539.9618** | **1616.8632** | **1615.8764** | **0.9868** | **2** | **12** | **1.8e+02** | **1** | **EKMKPERCGDAVPR** |
|  | 2555 | **583.5591** | **1747.6551** | **1747.9694** | **-0.3143** | **2** | **12** | **1.6e+02** | **1** | **SGASVKVSCKASGYTFR** |
|  | 3008 | **682.2041** | **2043.5901** | **2044.4158** | **-0.8257** | **0** | **12** | **1.7e+02** | **1** | **VALGITTVLTMTTISTHLR + Oxidation (M)** |
|  | 1783 | **470.6187** | **1408.8338** | **1409.5472** | **-0.7135** | **0** | **12** | **1.6e+02** | **1** | **CTCAQGYAPAPDGR** |
|  | 2182 | **520.0816** | **1557.2226** | **1557.8003** | **-0.5776** | **1** | **12** | **1.7e+02** | **1** | **RWCGMTGGACSCPR + Carbamidomethyl (C); Oxidation (M)** |
|  | 3153 | **723.4062** | **2167.1966** | **2167.4903** | **-0.2938** | **2** | **12** | **1.7e+02** | **1** | **CQECNNVIKTCSVLTKNR + 2 Carbamidomethyl (C)** |
|  | 3470 | **859.7419** | **2576.2037** | **2575.8483** | **0.3554** | **1** | **12** | **1.4e+02** | **1** | **LDLGLFSTKTGVEANNEHMVEVR + Oxidation (M)** |
|  | 2505 | **572.2007** | **1142.3866** | **1142.3061** | **0.0805** | **1** | **12** | **1.8e+02** | **1** | **QLSVKDTVPR** |
|  | 229 | 371.3508 | 740.6868 | 741.7931 | -1.1062 | 0 | 12 | 1.4e+02 | 1 | AGPGSPTR |
|  | 496 | **388.3431** | **774.6714** | **773.8315** | **0.8398** | **0** | **12** | **1.9e+02** | **1** | **QLEGEAK** |
|  | 1911 | **483.4660** | **1447.3758** | **1447.5541** | **-0.1782** | **2** | **12** | **1.8e+02** | **1** | **TSSRVSSRSAPQGK** |
|  | 3130 | **708.2874** | **2121.8399** | **2122.3158** | **-0.4759** | **1** | **12** | **1.7e+02** | **1** | **SSSGPVWSGASSACTSPAVGKAK** |
|  | 2838 | **652.4207** | **1954.2398** | **1954.2812** | **-0.0415** | **2** | **12** | **1.7e+02** | **1** | **KGNMSLICWYNKGHFR** |
|  | 3330 | **774.7155** | **2321.1242** | **2320.8360** | **0.2882** | **2** | **12** | **1.4e+02** | **1** | **KLLMMASVNDCYTLIRAALPP** |
|  | 394 | **385.2452** | **1152.7135** | **1152.3423** | **0.3712** | **0** | **12** | **1.2e+02** | **1** | **MSLSLSPDMR + Oxidation (M)** |
|  | 868 | **407.2724** | **1218.7951** | **1218.3371** | **0.4580** | **0** | **12** | **1.3e+02** | **1** | **FPYICYQNGG + Carbamidomethyl (C)** |
|  | 1486 | **447.3012** | **892.5876** | **893.0435** | **-0.4558** | **0** | **12** | **1.5e+02** | **1** | **AMAGEGMAR** |
|  | 3003 | **681.2421** | **2040.7040** | **2040.4949** | **0.2091** | **2** | **12** | **1.8e+02** | **1** | **VIIRFLTLMMKHGYTGK + 2 Oxidation (M)** |
|  | 59 | **363.2623** | **724.5099** | **723.8392** | **0.6707** | **1** | **12** | **1.4e+02** | **1** | **GTGMSKK + Oxidation (M)** |
|  | 1989 | **493.7187** | **1478.1338** | **1478.6574** | **-0.5236** | **2** | **12** | **1.5e+02** | **1** | **GSDCSPIMRRSGR + Carbamidomethyl (C)** |
|  | 2927 | **668.1841** | **2001.5301** | **2002.2716** | **-0.7415** | **0** | **12** | **1.8e+02** | **1** | **EGVYVMVGADVPFSSCLR + Carbamidomethyl (C); Oxidation (M)** |
|  | 3239 | **741.6810** | **1481.3472** | **1480.7511** | **0.5961** | **0** | **12** | **1.4e+02** | **1** | **AGCQVASTMLVWSK** |
|  | 3461 | **855.6604** | **1709.3060** | **1708.9960** | **0.3100** | **1** | **12** | **1.6e+02** | **1** | **LVPSQAEIEARLAALK** |
|  | 555 | **391.1779** | **780.3409** | **780.9534** | **-0.6124** | **0** | **12** | **1.5e+02** | **1** | **AMGIMDK + Oxidation (M)** |
|  | 1044 | **416.3094** | **830.6041** | **829.9645** | **0.6397** | **0** | **12** | **1.7e+02** | **1** | **TMAAAPPR + Oxidation (M)** |
|  | 1071 | **419.1334** | **1254.3779** | **1253.4692** | **0.9088** | **0** | **12** | **2.1e+02** | **1** | **DVMWIGFLTR + Oxidation (M)** |
|  | 1438 | **443.3086** | **1326.9035** | **1326.4521** | **0.4514** | **2** | **12** | **1.4e+02** | **1** | **KEGSKYEALSSK** |
|  | 2617 | **593.6409** | **1185.2670** | **1185.3307** | **-0.0637** | **2** | **12** | **2.2e+02** | **1** | **GKAITPKDNNK** |
|  | 2670 | **604.3527** | **1810.0358** | **1808.9679** | **1.0679** | **1** | **12** | **1.8e+02** | **1** | **NALSVTTTSQERAAMGR + Oxidation (M)** |
|  | 539 | **389.8077** | **1166.4010** | **1167.3567** | **-0.9558** | **1** | **12** | **1.8e+02** | **1** | **GAFGKVYLGQK** |
|  | 877 | **407.3465** | **1219.0172** | **1219.4547** | **-0.4374** | **1** | **12** | **1.4e+02** | **1** | **SAMQTLKGIVR + Oxidation (M)** |
|  | 1586 | **456.7434** | **911.4720** | **911.9987** | **-0.5266** | **0** | **12** | **1.3e+02** | **1** | **ASPGPGELGK** |
|  | 1697 | **463.0045** | **1385.9912** | **1386.5719** | **-0.5807** | **0** | **12** | **1.6e+02** | **1** | **VPPGMGPSSDWIK + Oxidation (M)** |
|  | 2058 | **505.0653** | **1512.1738** | **1512.7052** | **-0.5313** | **0** | **12** | **1.7e+02** | **1** | **MIYEMFSGDFTR + Oxidation (M)** |
|  | 1922 | **486.1365** | **1455.3873** | **1455.6574** | **-0.2700** | **2** | **12** | **1.7e+02** | **1** | **SSVDSTPCRTKMK + Oxidation (M)** |
|  | 3065 | **686.0817** | **2055.2228** | **2056.3190** | **-1.0961** | **1** | **12** | **1.7e+02** | **1** | **QEDAHEFLMFTVDAMKK + Oxidation (M)** |
|  | 3278 | **744.9023** | **2231.6847** | **2230.5462** | **1.1384** | **1** | **12** | **1.9e+02** | **1** | **ALYTKMVPAAVSHSEFWHR** |
|  | 18 | **360.5321** | **1078.5740** | **1078.2454** | **0.3286** | **0** | **12** | **1.7e+02** | **1** | **ECYRPLAAR** |
|  | 1192 | **423.3161** | **1266.9260** | **1267.3912** | **-0.4652** | **0** | **12** | **1.7e+02** | **1** | **LGDGLGAALGPGNR** |
|  | 2063 | **505.2982** | **1512.8723** | **1512.7266** | **0.1457** | **1** | **12** | **1.6e+02** | **1** | **NKTYVGTLLDCTK + Carbamidomethyl (C)** |
|  | 2515 | **574.9747** | **1147.9347** | **1147.3490** | **0.5857** | **2** | **12** | **1.9e+02** | **1** | **KCVRGLGSEAK** |
|  | 1069 | **419.0948** | **836.1748** | **834.9843** | **1.1905** | **1** | **12** | **2.1e+02** | **1** | **ASKMATAR** |
|  | 2626 | **594.5734** | **1187.1321** | **1186.3452** | **0.7869** | **2** | **12** | **1.7e+02** | **1** | **RNPCNRLGEK** |
|  | 3306 | **760.2009** | **2277.5806** | **2276.6113** | **0.9694** | **2** | **12** | **1.7e+02** | **1** | **LQGGKDFNMPLTISRITPGSK + Oxidation (M)** |
|  | 333 | **380.1993** | **1137.5758** | **1137.2218** | **0.3540** | **0** | **12** | **1.7e+02** | **1** | **MATTGTPTADR + Oxidation (M)** |
|  | 1108 | **420.2473** | **1257.7198** | **1258.4029** | **-0.6831** | **0** | **12** | **1.4e+02** | **1** | **TNMSWSFLTR + Oxidation (M)** |
|  | 1726 | **465.0636** | **928.1125** | **927.1391** | **0.9733** | **0** | **12** | **1.8e+02** | **1** | **APSILSVLK** |
|  | 1883 | **479.6101** | **1435.8081** | **1434.6197** | **1.1884** | **0** | **12** | **2.1e+02** | **1** | **NFCFMNPGMER + Carbamidomethyl (C); 2 Oxidation (M)** |
|  | 1919 | **485.6791** | **969.3434** | **970.2102** | **-0.8669** | **2** | **12** | **1.4e+02** | **1** | **EIIKAIKR** |
|  | 2402 | **550.6340** | **1099.2533** | **1100.2890** | **-1.0358** | **0** | **12** | **2.2e+02** | **1** | **GQCILSTPPK + Carbamidomethyl (C)** |
|  | 601 | **399.1953** | **1194.5639** | **1195.5625** | **-0.9986** | **2** | **12** | **1.4e+02** | **1** | **KKMMEIMIR + Oxidation (M)** |
|  | 946 | **408.9653** | **1223.8737** | **1224.3834** | **-0.5096** | **0** | **12** | **2e+02** | **1** | **TAIPGMYDNVK + Oxidation (M)** |
|  | 1084 | **419.3038** | **1254.8891** | **1255.4272** | **-0.5381** | **2** | **12** | **1.7e+02** | **1** | **TRTPPRASLTR** |
|  | 1644 | **459.7783** | **1376.3128** | **1376.4711** | **-0.1583** | **0** | **12** | **1.7e+02** | **1** | **DIQHAGVPGEEPK** |
|  | 659 | **401.7061** | **1202.0963** | **1201.3269** | **0.7694** | **0** | **12** | **1.7e+02** | **1** | **SIQASVDVSAPK** |
|  | 714 | **403.9355** | **1208.7843** | **1208.3722** | **0.4120** | **1** | **12** | **1.9e+02** | **1** | **ATAGLAFHRHK** |
|  | 1600 | **458.1349** | **914.2549** | **915.0060** | **-0.7510** | **0** | **12** | **1.9e+02** | **1** | **SVAQAGVQR** |
|  | 400 | **385.9860** | **1154.9358** | **1155.2337** | **-0.2979** | **0** | **12** | **1.6e+02** | **1** | **AASSSEISEMK + Oxidation (M)** |
|  | 817 | **406.2361** | **1215.6860** | **1216.4058** | **-0.7199** | **0** | **12** | **1.4e+02** | **1** | **CSYNFLTAIK + Carbamidomethyl (C)** |
|  | 889 | **407.6164** | **1219.8269** | **1220.4194** | **-0.5925** | **0** | **12** | **1.5e+02** | **1** | **TLCATDALCPR + Carbamidomethyl (C)** |
|  | 2519 | **575.4346** | **1723.2815** | **1722.9831** | **0.2985** | **1** | **12** | **1.5e+02** | **1** | **MDPMTTPENLVRYR** |
|  | 2578 | **590.3163** | **1178.6179** | **1178.3860** | **0.2319** | **1** | **12** | **1.9e+02** | **1** | **MSLCGARANAK + Carbamidomethyl (C)** |
|  | 2806 | **635.7256** | **1904.1546** | **1905.0487** | **-0.8941** | **0** | **12** | **2.1e+02** | **1** | **VNPTTANSDLNPGTTQMK + Oxidation (M)** |
|  | 3444 | **849.6821** | **2546.0242** | **2545.8537** | **0.1705** | **2** | **12** | **1.6e+02** | **1** | **VGHVFRKQHPYTFPGGSGTVFAR** |
|  | 3516 | 915.3739 | 1828.7330 | 1829.0650 | -0.3320 | 1 | 12 | 1.6e+02 | 1 | FLACGEFAQCVKNER + 2 Carbamidomethyl (C) |
|  | 488 | **388.1285** | **1161.3634** | **1160.2153** | **1.1482** | **0** | **12** | **2.2e+02** | **1** | **MSQAYSSSQR + Oxidation (M)** |
|  | 1195 | **423.7200** | **845.4252** | **844.9127** | **0.5125** | **0** | **12** | **1.8e+02** | **1** | **LPAGTSGSR** |
|  | 2251 | **524.9873** | **1571.9397** | **1571.7076** | **0.2321** | **0** | **12** | **1.9e+02** | **1** | **INNDGSSTIYACSVK** |
|  | 3125 | **703.8589** | **2108.5545** | **2108.3354** | **0.2190** | **2** | **12** | **2e+02** | **1** | **SIGDGQYKRCGVTSVPDIR + Carbamidomethyl (C)** |
|  | 3148 | **718.6808** | **2153.0204** | **2153.5268** | **-0.5064** | **2** | **12** | **1.5e+02** | **1** | **MDLAESAKRLGPGCGMMAGGK + Carbamidomethyl (C); Oxidation (M)** |
|  | 500 | **388.3977** | **774.7806** | **773.8580** | **0.9227** | **0** | **12** | **2.5e+02** | **1** | **CLGNPDR** |
|  | 1755 | **467.9930** | **933.9712** | **933.0840** | **0.8871** | **0** | **12** | **2e+02** | **1** | **NLTEGMLR** |
|  | 2026 | **501.9515** | **1502.8323** | **1503.6799** | **-0.8477** | **0** | **12** | **2e+02** | **1** | **NQLQVNNTQLTCK** |
|  | 2038 | **503.0121** | **1004.0093** | **1005.1550** | **-1.1456** | **0** | **12** | **2e+02** | **1** | **MGSHAHPLR** |
|  | 2551 | **582.7327** | **1163.4507** | **1162.3353** | **1.1153** | **0** | **12** | **2e+02** | **1** | **LAIVGGGYTPSK** |
|  | 2934 | **668.6757** | **1335.3365** | **1335.4473** | **-0.1107** | **2** | **12** | **1.8e+02** | **1** | **EAGGGGNGSRKMAK + Oxidation (M)** |
|  | 3196 | **740.3575** | **1478.7003** | **1478.7981** | **-0.0978** | **1** | **12** | **1.8e+02** | **1** | **KVIIVYVESICR + Carbamidomethyl (C)** |
|  | 1109 | **420.2632** | **838.5116** | **838.8835** | **-0.3719** | **1** | **12** | **1.4e+02** | **1** | **DSDCSGKK** |
|  | 3529 | **974.0221** | **1946.0294** | **1945.3078** | **0.7216** | **2** | **12** | **1.7e+02** | **1** | **LRAADELMCIDGIPVKGK + Oxidation (M)** |
|  | 1320 | **432.9954** | **863.9759** | **865.0515** | **-1.0755** | **0** | **12** | **1.9e+02** | **1** | **LLCDVFR** |
|  | 2279 | **529.9271** | **1586.7592** | **1585.6516** | **1.1076** | **0** | **12** | **2e+02** | **1** | **CPQEAGASDQPEPTR** |
|  | 26 | **362.1223** | **1083.3448** | **1084.2883** | **-0.9435** | **0** | **12** | **1.7e+02** | **1** | **NMEVAMMDK + Oxidation (M)** |
|  | 1683 | **461.8301** | **1382.4681** | **1381.7494** | **0.7187** | **1** | **12** | **1.8e+02** | **1** | **VVRYLQVMVMK + Oxidation (M)** |
|  | 1995 | **495.0378** | **1482.0913** | **1482.5978** | **-0.5066** | **0** | **12** | **2e+02** | **1** | **AHSCYNGCLASGDK + Carbamidomethyl (C)** |
|  | 2261 | **527.1658** | **1578.4753** | **1578.8972** | **-0.4219** | **0** | **12** | **1.8e+02** | **1** | **FGCRPVMLAGGLLASA + Oxidation (M)** |
|  | 306 | **378.1206** | **1131.3396** | **1130.2537** | **1.0858** | **0** | **12** | **1.7e+02** | **1** | **VNYLTQHQK** |
|  | 633 | **400.2588** | **798.5029** | **798.9734** | **-0.4705** | **1** | **12** | **1.3e+02** | **1** | **RLLLER** |
|  | 331 | **380.1555** | **758.2963** | **757.8304** | **0.4659** | **0** | **12** | **2e+02** | **1** | **IGYDYK** |
|  | 861 | **407.1901** | **1218.5480** | **1217.4022** | **1.1458** | **2** | **12** | **1.7e+02** | **1** | **LRTAGRDGLCR** |
|  | 2732 | **612.2913** | **1222.5677** | **1221.4339** | **1.1338** | **1** | **12** | **1.8e+02** | **1** | **CLRTHCCSGK + 2 Carbamidomethyl (C)** |
|  | 2217 | **522.2496** | **1042.4845** | **1042.1967** | **0.2877** | **2** | **12** | **1.9e+02** | **1** | **ALGGRAVRSR** |
|  | 2218 | **522.2541** | **1563.7403** | **1564.6768** | **-0.9366** | **0** | **12** | **1.9e+02** | **1** | **QQLWSEECYHGK + Carbamidomethyl (C)** |
|  | 1568 | **455.3577** | **1363.0510** | **1362.5570** | **0.4940** | **1** | **12** | **1.4e+02** | **1** | **ITRMLEHYQR + Oxidation (M)** |
|  | 1676 | **460.9290** | **1379.7647** | **1378.5977** | **1.1669** | **0** | **12** | **1.9e+02** | **1** | **LECGGMISAHCK + 2 Carbamidomethyl (C); Oxidation (M)** |
|  | 1916 | **485.2061** | **1452.5961** | **1452.6149** | **-0.0188** | **1** | **12** | **1.7e+02** | **1** | **EANIGSIHLSAGRK** |
|  | 1050 | **417.8794** | **833.7440** | **832.8988** | **0.8452** | **0** | **12** | **2e+02** | **1** | **TISVDGNK** |
|  | 1713 | **463.4942** | **1387.4604** | **1386.7027** | **0.7577** | **1** | **12** | **2e+02** | **1** | **AVANAAAMLVLKAK + Oxidation (M)** |
|  | 1945 | **488.1105** | **974.2063** | **975.1026** | **-0.8963** | **2** | **12** | **2.1e+02** | **1** | **KKASAWER** |
|  | 648 | **401.1761** | **1200.5060** | **1200.2758** | **0.2301** | **0** | **12** | **2e+02** | **1** | **QQYQTEMEK + Oxidation (M)** |
|  | 1341 | **434.2891** | **1299.8450** | **1299.4599** | **0.3851** | **2** | **12** | **1.4e+02** | **1** | **GQKGSSAMPHKR + Oxidation (M)** |
|  | 2172 | **519.0968** | **1036.1788** | **1035.1578** | **1.0211** | **1** | **12** | **1.8e+02** | **1** | **GGSVSFRGLR** |
|  | 2545 | **581.7143** | **1742.1207** | **1740.9600** | **1.1607** | **1** | **12** | **2.3e+02** | **1** | **TDIAPTPGALRGYRPR** |
|  | 2971 | **674.2384** | **2019.6930** | **2018.4994** | **1.1937** | **2** | **12** | **1.9e+02** | **1** | **GAAPRLAAPRPRPLGMMKK** |
|  | 98 | **365.8715** | **1094.5924** | **1095.2263** | **-0.6339** | **0** | **12** | **2.1e+02** | **1** | **ASLWTNMEK + Oxidation (M)** |
|  | 550 | **390.0259** | **778.0370** | **777.8649** | **0.1721** | **0** | **12** | **1.9e+02** | **1** | **LADAFNK** |
|  | 896 | **407.7588** | **813.5027** | **813.9003** | **-0.3975** | **0** | **12** | **1.9e+02** | **1** | **AAGAPGGWK** |
|  | 1057 | **418.1633** | **834.3119** | **833.8867** | **0.4251** | **0** | **12** | **1.9e+02** | **1** | **TNNAISSK** |
|  | 1391 | **437.0735** | **1308.1985** | **1307.5366** | **0.6619** | **0** | **12** | **2.1e+02** | **1** | **MQPDMSLNVIK + 2 Oxidation (M)** |
|  | 1709 | **463.1716** | **924.3284** | **925.0604** | **-0.7320** | **0** | **12** | **1.8e+02** | **1** | **GVGCLDFSK** |
|  | 2006 | **497.1411** | **992.2673** | **993.2289** | **-0.9616** | **1** | **12** | **2e+02** | **1** | **KMSMMGHR + Oxidation (M)** |
|  | 2142 | **518.7487** | **1035.4825** | **1035.1729** | **0.3097** | **0** | **12** | **1.5e+02** | **1** | **VMTSGTGAPAK + Oxidation (M)** |
|  | 2361 | **540.1912** | **1617.5515** | **1617.8222** | **-0.2707** | **0** | **12** | **1.9e+02** | **1** | **LLSDSGFYLCAWSR** |
|  | 2568 | **586.5674** | **1171.1200** | **1171.2612** | **-0.1412** | **1** | **12** | **1.7e+02** | **1** | **VQKGPTSPSDR** |
|  | 2827 | **646.8357** | **1291.6566** | **1291.4973** | **0.1594** | **0** | **12** | **1.7e+02** | **1** | **VGAFGFLALPGSR** |
|  | 867 | 407.2631 | 812.5114 | 811.7537 | 0.7577 | 0 | 12 | 1.4e+02 | 1 | SGDQDHPG |
|  | 1973 | **490.1470** | **1467.4189** | **1467.7556** | **-0.3366** | **1** | **12** | **1.9e+02** | **1** | **REVIMLACSFGNK** |
|  | 2729 | **612.2781** | **1222.5414** | **1221.4507** | **1.0907** | **1** | **12** | **1.8e+02** | **1** | **LVEVAGCKCSR + Carbamidomethyl (C)** |
|  | 443 | **386.9241** | **771.8335** | **772.8900** | **-1.0565** | **1** | **12** | **2.1e+02** | **1** | **EEVIRK** |
|  | 2135 | **517.5932** | **1549.7574** | **1550.6754** | **-0.9179** | **2** | **12** | **2.4e+02** | **1** | **GREPAVTKDPGPGGGR** |
|  | 2821 | **643.8633** | **1285.7118** | **1284.5678** | **1.1440** | **0** | **12** | **1.6e+02** | **1** | **KPGQAPLLVMSK + Oxidation (M)** |
|  | 2907 | **666.8578** | **1997.5512** | **1998.3059** | **-0.7547** | **2** | **12** | **1.8e+02** | **1** | **TITKMCEQALGKGCGADSK + Carbamidomethyl (C)** |
|  | 145 | **368.1791** | **734.3435** | **733.7263** | **0.6172** | **0** | **11** | **1.7e+02** | **1** | **DDHGYK** |
|  | 1099 | **419.7035** | **1256.0884** | **1255.3544** | **0.7340** | **0** | **11** | **1.4e+02** | **1** | **FQDSDMLEVR + Oxidation (M)** |
|  | 1255 | **429.1268** | **856.2389** | **856.0281** | **0.2107** | **2** | **11** | **1.9e+02** | **1** | **LAAKRAAR** |
|  | 1868 | **478.0045** | **953.9943** | **954.1464** | **-0.1521** | **0** | **11** | **1.7e+02** | **1** | **VCCSCSPQK** |
|  | 2672 | **606.0953** | **1815.2637** | **1814.9937** | **0.2699** | **0** | **11** | **1.7e+02** | **1** | **HGSTEPLVLAWSSQFR** |
|  | 2764 | **621.5511** | **1241.0875** | **1241.2715** | **-0.1839** | **1** | **11** | **1.4e+02** | **1** | **QEVGSHGRDTR** |
|  | 957 | **409.3275** | **816.6401** | **816.9259** | **-0.2857** | **1** | **11** | **1.7e+02** | **1** | **MSKHNGK + Oxidation (M)** |
|  | 1097 | 419.4839 | 1255.4296 | 1254.5055 | 0.9241 | 2 | 11 | 2.6e+02 | 1 | APKASRPPKMR + Oxidation (M) |
|  | 1905 | **482.1205** | **1443.3394** | **1443.5172** | **-0.1778** | **0** | **11** | **2e+02** | **1** | **FDLPETHSLDNR** |
|  | 91 | **365.2141** | **1092.6203** | **1093.1937** | **-0.5735** | **0** | **11** | **1.6e+02** | **1** | **DQPAHSGILR** |
|  | 3024 | **684.0289** | **2049.0644** | **2049.2187** | **-0.1542** | **1** | **11** | **1.6e+02** | **1** | **TQQSFEMVKGGYTLDSNK + Oxidation (M)** |
|  | 304 | **377.6735** | **753.3322** | **752.9267** | **0.4055** | **0** | **11** | **1.4e+02** | **1** | **MCCPSR + Carbamidomethyl (C)** |
|  | 1548 | **452.0688** | **1353.1842** | **1353.4377** | **-0.2536** | **1** | **11** | **2.3e+02** | **1** | **DTLFTAESGTRR** |
|  | 527 | **389.1656** | **1164.4746** | **1163.3450** | **1.1296** | **1** | **11** | **2e+02** | **1** | **ANSDIKMLQK + Oxidation (M)** |
|  | 1222 | **427.4287** | **852.8426** | **852.9812** | **-0.1386** | **1** | **11** | **2e+02** | **1** | **APGLSPRR** |
|  | 1867 | **477.9110** | **1430.7107** | **1430.5679** | **0.1427** | **0** | **11** | **1.8e+02** | **1** | **VAHEINHGIGQAGK** |
|  | 2107 | **511.5950** | **1531.7628** | **1530.7235** | **1.0393** | **2** | **11** | **2.3e+02** | **1** | **ELSWKDLLKDQR** |
|  | 2542 | **581.0861** | **1740.2362** | **1741.0645** | **-0.8283** | **0** | **11** | **2e+02** | **1** | **MQCVATCPTPPLPPR + 2 Carbamidomethyl (C); Oxidation (M)** |
|  | 2664 | **601.8152** | **1802.4236** | **1803.0294** | **-0.6058** | **1** | **11** | **1.6e+02** | **1** | **LCRDWDQMFAFAQR + Oxidation (M)** |
|  | 2904 | **666.8114** | **1331.6080** | **1332.4980** | **-0.8899** | **0** | **11** | **2.2e+02** | **1** | **EDLDVLGLSFPK** |
|  | 447 | **386.9574** | **771.9000** | **772.8533** | **-0.9534** | **1** | **11** | **2.1e+02** | **1** | **GSLGQRR** |
|  | 1213 | **426.2861** | **1275.8362** | **1275.5145** | **0.3217** | **1** | **11** | **1.5e+02** | **1** | **LCDSGELVAIKK** |
|  | 928 | **408.1418** | **1221.4031** | **1222.5395** | **-1.1364** | **1** | **11** | **2e+02** | **1** | **YCKISALALLK** |
|  | 1935 | **487.5074** | **1459.5001** | **1459.9060** | **-0.4059** | **1** | **11** | **2.4e+02** | **1** | **MYRMAVMAMVIK + Oxidation (M)** |
|  | 1940 | **488.0161** | **1461.0260** | **1460.6571** | **0.3689** | **1** | **11** | **2.2e+02** | **1** | **VHTGEKPYKCNK + Carbamidomethyl (C)** |
|  | 2079 | **506.2935** | **1515.8584** | **1515.7125** | **0.1459** | **0** | **11** | **1.7e+02** | **1** | **APPQASVQVPTPPAR** |
|  | 2487 | **568.9922** | **1703.9544** | **1704.8187** | **-0.8643** | **0** | **11** | **2e+02** | **1** | **TTGPPSGQMPDNPPHR + Oxidation (M)** |
|  | 2891 | **666.5394** | **1331.0641** | **1330.6178** | **0.4462** | **2** | **11** | **1.6e+02** | **1** | **ILIPKGSYGRVK** |
|  | 12 | **360.4928** | **1078.4563** | **1077.3651** | **1.0913** | **1** | **11** | **2.2e+02** | **1** | **MVCGRIALAK + Oxidation (M)** |
|  | 283 | **376.2744** | **750.5341** | **749.8118** | **0.7224** | **0** | **11** | **1.5e+02** | **1** | **YDPSLR** |
|  | 1718 | **464.1118** | **926.2088** | **926.0320** | **0.1768** | **0** | **11** | **1.8e+02** | **1** | **AGTPTRPAR** |
|  | 2069 | **505.6673** | **1513.9798** | **1513.8671** | **0.1128** | **2** | **11** | **1.7e+02** | **1** | **HMCKEIAIIPSKK + Oxidation (M)** |
|  | 2116 | **513.9196** | **1538.7365** | **1538.7672** | **-0.0307** | **0** | **11** | **1.9e+02** | **1** | **QVPAPLLPSCDATAR** |
|  | 2169 | **519.0533** | **1036.0919** | **1035.0701** | **1.0219** | **1** | **11** | **1.9e+02** | **1** | **SGATGGSRSQK** |
|  | 2819 | **643.4373** | **1927.2896** | **1928.2195** | **-0.9299** | **1** | **11** | **1.8e+02** | **1** | **ARRPSVTPLVWDDMLR + Oxidation (M)** |
|  | 1516 | **450.2170** | **898.4192** | **899.0065** | **-0.5874** | **1** | **11** | **1.9e+02** | **1** | **RATGIPER** |
|  | 1907 | **482.2731** | **1443.7971** | **1444.5882** | **-0.7910** | **0** | **11** | **1.9e+02** | **1** | **FAEAVGVQLEPER** |
|  | 2214 | **521.6440** | **1561.9100** | **1562.8320** | **-0.9220** | **0** | **11** | **2.1e+02** | **1** | **MVPPVVVGSPPGSPSR** |
|  | 2295 | **532.5466** | **1594.6177** | **1594.8587** | **-0.2409** | **2** | **11** | **2.1e+02** | **1** | **HWKVLGTTGVGVKGR** |
|  | 3541 | **1049.5525** | **3145.6353** | **3145.7094** | **-0.0741** | **0** | **11** | **1.6e+02** | **1** | **MLVVLNSVLMEQQDCALPLLTDVIQTNK + Oxidation (M)** |
|  | 1758 | **468.2194** | **934.4241** | **935.0784** | **-0.6544** | **1** | **11** | **2.1e+02** | **1** | **VEQKYLR** |
|  | 3491 | **887.8156** | **2660.4245** | **2659.9234** | **0.5011** | **2** | **11** | **1.4e+02** | **1** | **QEELGAVVDKEMAATSAAIEDAVRR** |
|  | 1120 | **421.1084** | **1260.3029** | **1261.4961** | **-1.1932** | **1** | **11** | **2e+02** | **1** | **LIGCAGPFATRR** |
|  | 1134 | **421.8514** | **1262.5320** | **1262.4397** | **0.0923** | **2** | **11** | **2e+02** | **1** | **SEMLQKAARGR + Oxidation (M)** |
|  | 1368 | **435.8597** | **1304.5569** | **1305.5074** | **-0.9505** | **1** | **11** | **1.9e+02** | **1** | **YAFKAAHPNMR** |
|  | 1530 | **451.1566** | **900.2985** | **900.1007** | **0.1978** | **2** | **11** | **2.1e+02** | **1** | **QTKHMKK** |
|  | 1699 | **463.0421** | **924.0694** | **924.0940** | **-0.0245** | **0** | **11** | **1.8e+02** | **1** | **VTPFYAVK** |
|  | 2853 | **655.9270** | **1309.8392** | **1310.5435** | **-0.7043** | **0** | **11** | **1.5e+02** | **1** | **LLPQVSAWAGLR** |
|  | 797 | **406.0121** | **1215.0141** | **1214.4614** | **0.5527** | **2** | **11** | **1.8e+02** | **1** | **MPCIKDSHRK** |
|  | 885 | **407.5805** | **813.1462** | **812.9521** | **0.1941** | **0** | **11** | **1.7e+02** | **1** | **VFSGLYK** |
|  | 2177 | **519.2227** | **1554.6458** | **1554.8131** | **-0.1673** | **2** | **11** | **1.9e+02** | **1** | **VMAAKKGPGPGGGVSGGK** |
|  | 2377 | **542.1703** | **1623.4887** | **1622.9267** | **0.5620** | **1** | **11** | **1.9e+02** | **1** | **MTFYLFGIRSFPK + Oxidation (M)** |
|  | 790 | **405.7666** | **1214.2776** | **1213.4218** | **0.8558** | **1** | **11** | **1.8e+02** | **1** | **YLELLSGYKK** |
|  | 2743 | **613.4297** | **1224.8446** | **1225.5235** | **-0.6789** | **1** | **11** | **1.7e+02** | **1** | **ELLLRSLLLR** |
|  | 238 | **372.4228** | **742.8308** | **743.8090** | **-0.9782** | **1** | **11** | **2.4e+02** | **1** | **SKGTSHK** |
|  | 1263 | **429.3572** | **856.6997** | **856.9832** | **-0.2835** | **0** | **11** | **1.6e+02** | **1** | **LYDTAMK + Oxidation (M)** |
|  | 1695 | **462.2672** | **922.5196** | **922.1014** | **0.4182** | **1** | **11** | **1.6e+02** | **1** | **TKVCSVTK + Carbamidomethyl (C)** |
|  | 3333 | **776.5454** | **2326.6141** | **2327.5353** | **-0.9213** | **1** | **11** | **1.9e+02** | **1** | **GNFQTIGLSAAARFNQCNTTR + Carbamidomethyl (C)** |
|  | 631 | **400.2345** | **1197.6813** | **1197.3711** | **0.3102** | **2** | **11** | **1.4e+02** | **1** | **LRAHRECQK + Carbamidomethyl (C)** |
|  | 1599 | **458.1247** | **914.2346** | **913.1193** | **1.1153** | **2** | **11** | **2e+02** | **1** | **SLGRPKKK** |
|  | 1746 | **466.4515** | **1396.3323** | **1396.5683** | **-0.2361** | **0** | **11** | **2e+02** | **1** | **MLYSLVGGQGSER** |
|  | 1956 | **488.3252** | **974.6357** | **974.0070** | **0.6287** | **1** | **11** | **1.7e+02** | **1** | **AMREEHSD** |
|  | 2751 | **614.2029** | **1226.3910** | **1225.3333** | **1.0577** | **0** | **11** | **1.9e+02** | **1** | **DPSGCCVGNDCR** |
|  | 2800 | **633.2029** | **1896.5867** | **1896.0602** | **0.5265** | **1** | **11** | **2.1e+02** | **1** | **EACNVPEPEEKFNMDK + Oxidation (M)** |
|  | 2983 | **679.0431** | **2034.1071** | **2034.4208** | **-0.3137** | **2** | **11** | **1.7e+02** | **1** | **LLLLGTSNSGKSTIVKQMK + Oxidation (M)** |
|  | 3111 | **699.3792** | **2095.1155** | **2095.3964** | **-0.2810** | **2** | **11** | **1.9e+02** | **1** | **LSLLSSTSKYKVTVGEVQR** |
|  | 354 | **383.2725** | **1146.7952** | **1146.3607** | **0.4345** | **0** | **11** | **1.6e+02** | **1** | **TLIQCLGSVGR** |
|  | 2337 | **537.9029** | **1610.6865** | **1609.6812** | **1.0053** | **2** | **11** | **1.9e+02** | **1** | **SSATGRSCRGASEQR + Carbamidomethyl (C)** |
|  | 2550 | **582.4644** | **1744.3709** | **1745.0034** | **-0.6325** | **2** | **11** | **1.6e+02** | **1** | **SLDIAPGDMLDKVIKN + Oxidation (M)** |
|  | 2379 | **542.3611** | **1082.7074** | **1083.1992** | **-0.4918** | **2** | **11** | **1.7e+02** | **1** | **EPRKVPDSR** |
|  | 3041 | **684.8865** | **1367.7582** | **1367.5572** | **0.2010** | **2** | **11** | **1.9e+02** | **1** | **TAAPSVRPEKRR** |
|  | 353 | **383.1566** | **1146.4478** | **1146.3378** | **0.1100** | **1** | **11** | **2e+02** | **1** | **HKVEPVYFK** |
|  | 694 | **402.9915** | **1205.9524** | **1205.3702** | **0.5822** | **1** | **11** | **2.2e+02** | **1** | **THPVRTNKPR** |
|  | 749 | **404.6737** | **1210.9990** | **1211.3745** | **-0.3755** | **2** | **11** | **1.5e+02** | **1** | **ANPRLANATKR** |
|  | 950 | **409.1534** | **1224.4381** | **1223.3574** | **1.0807** | **2** | **11** | **2.1e+02** | **1** | **AKRASEMASEK + Oxidation (M)** |
|  | 2508 | **573.2862** | **1716.8364** | **1715.9061** | **0.9303** | **2** | **11** | **2.1e+02** | **1** | **SGVVTKRSSLPVSNER** |
|  | 985 | 411.9931 | 1232.9572 | 1232.2150 | 0.7423 | 0 | 11 | 2e+02 | 1 | DSEDGAAGAVGGAR |
|  | 2094 | **508.3396** | **1521.9965** | **1521.6852** | **0.3113** | **2** | **11** | **1.7e+02** | **1** | **AFGQKSQLRGHHR** |
|  | 744 | **404.2879** | **1209.8416** | **1209.2956** | **0.5460** | **0** | **11** | **1.6e+02** | **1** | **CNSHLASHQR + Carbamidomethyl (C)** |
|  | 1454 | **444.0350** | **886.0552** | **886.9924** | **-0.9373** | **1** | **11** | **2.2e+02** | **1** | **LQGGKEQK** |
|  | 2101 | **509.2264** | **1524.6570** | **1524.6379** | **0.0190** | **1** | **11** | **2.2e+02** | **1** | **TRGYCSGGSCYTGR + Carbamidomethyl (C)** |
|  | 2948 | **668.9219** | **2003.7435** | **2004.1631** | **-0.4196** | **1** | **11** | **1.6e+02** | **1** | **KTSNSCIMENGHQPGAEK + Carbamidomethyl (C); Oxidation (M)** |
|  | 3127 | **706.5407** | **1411.0666** | **1411.6062** | **-0.5395** | **2** | **11** | **1.7e+02** | **1** | **RANGNLVSDKIPK** |
|  | 5 | **360.3848** | **718.7549** | **719.8106** | **-1.0557** | **0** | **11** | **2.7e+02** | **1** | **GGLGGGMR + Oxidation (M)** |
|  | 553 | **390.1022** | **1167.2844** | **1168.4724** | **-1.1879** | **2** | **11** | **1.9e+02** | **1** | **LFKCKECLK + Carbamidomethyl (C)** |
|  | 2868 | **663.6392** | **1987.8953** | **1988.2200** | **-0.3247** | **0** | **11** | **1.7e+02** | **1** | **QCSDAPVSVLQEDIVGSLK** |
|  | 696 | **403.0663** | **1206.1767** | **1206.2836** | **-0.1069** | **1** | **11** | **2.2e+02** | **1** | **LRLCDDSGPSSG** |
|  | 803 | **406.1074** | **1215.2999** | **1214.2410** | **1.0589** | **0** | **11** | **1.9e+02** | **1** | **EYEAQQQYR** |
|  | 930 | **408.1718** | **1221.4931** | **1220.3134** | **1.1796** | **1** | **11** | **2e+02** | **1** | **ANLGSGCEEKR + Carbamidomethyl (C)** |
|  | 1330 | **433.4552** | **864.8956** | **864.0486** | **0.8470** | **1** | **11** | **2.3e+02** | **1** | **MARGGAACK** |
|  | 1669 | **460.5358** | **1378.5853** | **1377.4776** | **1.1077** | **1** | **11** | **2.6e+02** | **1** | **APEDTVAEMKDR + Oxidation (M)** |
|  | 2316 | **534.8641** | **1067.7134** | **1067.1830** | **0.5303** | **1** | **11** | **1.9e+02** | **1** | **HQTGRTCHK** |
|  | 2688 | **608.3770** | **1214.7392** | **1214.4348** | **0.3044** | **0** | **11** | **2.1e+02** | **1** | **VPLVDQATCLR** |
|  | 3557 | **1177.6953** | **3530.0638** | **3529.9944** | **0.0693** | **2** | **11** | **1.5e+02** | **1** | **FLCSKATTPVNIDSQAQLADDVLRAPHPDMFK** |
|  | 481 | **387.9756** | **1160.9048** | **1160.2317** | **0.6730** | **0** | **11** | **2.5e+02** | **1** | **LGDIDAATEQK** |
|  | 1484 | **447.2080** | **1338.6018** | **1338.4264** | **0.1754** | **2** | **11** | **2.1e+02** | **1** | **FTISRDDSKNR** |
|  | 1040 | **416.1687** | **830.3226** | **829.9843** | **0.3383** | **1** | **11** | **2.4e+02** | **1** | **SDLVRIK** |
|  | 1871 | 478.1414 | 1431.4019 | 1431.4913 | -0.0895 | 0 | 11 | 1.9e+02 | 1 | ICNQNSSNPNQR + Carbamidomethyl (C) |
|  | 2767 | **622.6532** | **1864.9374** | **1864.1508** | **0.7867** | **2** | **11** | **2.3e+02** | **1** | **DMKVLYTNRAQAYMK + 2 Oxidation (M)** |
|  | 514 | **389.0921** | **1164.2541** | **1165.2863** | **-1.0321** | **1** | **11** | **2.3e+02** | **1** | **REMHGHGIGR + Oxidation (M)** |
|  | 1637 | **459.1764** | **1374.5069** | **1374.6871** | **-0.1801** | **0** | **11** | **2.2e+02** | **1** | **ELTPAMPVIFIK + Oxidation (M)** |
|  | 1335 | **434.1103** | **1299.3087** | **1300.5076** | **-1.1989** | **1** | **11** | **2e+02** | **1** | **CSPKMPPAPSGR + Carbamidomethyl (C); Oxidation (M)** |
|  | 2596 | **592.7754** | **1775.3040** | **1775.0176** | **0.2864** | **0** | **11** | **1.9e+02** | **1** | **VHTLAWSDTCNVLCR + Carbamidomethyl (C)** |
|  | 2846 | **654.3396** | **1959.9966** | **1960.1205** | **-0.1238** | **1** | **11** | **2e+02** | **1** | **VAQHRPPSYHPSQRSGR** |
|  | 2932 | **668.5693** | **2002.6858** | **2002.3458** | **0.3400** | **2** | **11** | **1.7e+02** | **1** | **CCIHRDLAARNCLVTEK + Carbamidomethyl (C)** |
|  | 788 | **405.2849** | **1212.8327** | **1212.3081** | **0.5246** | **0** | **11** | **1.5e+02** | **1** | **SECLQDADMK + Carbamidomethyl (C); Oxidation (M)** |
|  | 1360 | **435.3406** | **1302.9997** | **1303.4965** | **-0.4967** | **2** | **11** | **1.5e+02** | **1** | **MAGGVWGRSRAR** |
|  | 1543 | **451.9535** | **1352.8382** | **1353.5303** | **-0.6921** | **2** | **11** | **2.4e+02** | **1** | **RRASLPGGLSPSR** |
|  | 2225 | **522.9651** | **1043.9155** | **1044.1595** | **-0.2440** | **0** | **11** | **2.1e+02** | **1** | **LSNVNLQEK** |
|  | 2604 | **593.0095** | **1184.0041** | **1184.2516** | **-0.2474** | **0** | **11** | **2.1e+02** | **1** | **LDESFYGPEK** |
|  | 1436 | **443.1423** | **1326.4048** | **1325.5816** | **0.8233** | **1** | **11** | **2e+02** | **1** | **CNDMMTLGRLR + Oxidation (M)** |
|  | 1476 | **446.2482** | **1335.7225** | **1335.5748** | **0.1477** | **0** | **11** | **2e+02** | **1** | **MVAGANFYMCGR + Oxidation (M)** |
|  | 1504 | **449.3874** | **1345.1400** | **1345.5049** | **-0.3649** | **0** | **11** | **1.5e+02** | **1** | **GAGACPECGTPLR + 2 Carbamidomethyl (C)** |
|  | 1667 | **460.4606** | **1378.3595** | **1378.6393** | **-0.2797** | **0** | **11** | **2.3e+02** | **1** | **SAAVFVMLTGINR** |
|  | 1719 | **464.1213** | **1389.3417** | **1389.6203** | **-0.2786** | **0** | **11** | **1.9e+02** | **1** | **LMVHNWEYLGK** |
|  | 2987 | **679.8372** | **1357.6597** | **1358.4957** | **-0.8360** | **0** | **11** | **2.2e+02** | **1** | **VPVTIAPDDFER** |
|  | 676 | **402.1194** | **1203.3360** | **1202.3578** | **0.9781** | **1** | **11** | **2.3e+02** | **1** | **LPSQSLSSKQK** |
|  | 1333 | **434.0437** | **866.0726** | **864.9422** | **1.1303** | **0** | **11** | **1.9e+02** | **1** | **AFTLEER** |
|  | 1558 | **452.9459** | **1355.8156** | **1355.3754** | **0.4402** | **0** | **11** | **2.2e+02** | **1** | **QGAGGGGGGGAGWPGGR** |
|  | 1647 | **459.8617** | **1376.5629** | **1376.6151** | **-0.0522** | **0** | **11** | **2.2e+02** | **1** | **LAEMLPSVSSILT + Oxidation (M)** |
|  | 1347 | **434.8887** | **867.7626** | **867.9063** | **-0.1438** | **0** | **11** | **1.9e+02** | **1** | **HQGELER** |
|  | 1822 | **474.6027** | **1420.7858** | **1421.7718** | **-0.9859** | **1** | **11** | **2.5e+02** | **1** | **LRLLTSMVVMSR + Oxidation (M)** |
|  | 1865 | **477.7809** | **1430.3205** | **1430.6756** | **-0.3551** | **0** | **11** | **1.5e+02** | **1** | **APWPLSLPGCPHR** |
|  | 2019 | **500.0727** | **1497.1959** | **1496.6858** | **0.5101** | **0** | **11** | **2e+02** | **1** | **AQDFVQWLMNTK + Oxidation (M)** |
|  | 2033 | **502.4297** | **1002.8447** | **1002.3185** | **0.5262** | **2** | **11** | **1.8e+02** | **1** | **KVVKMLLR + Oxidation (M)** |
|  | 2206 | **521.2638** | **1040.5128** | **1041.1194** | **-0.6065** | **2** | **11** | **2e+02** | **1** | **KHGSEEAKR** |
|  | 3202 | **740.4896** | **2218.4467** | **2219.4906** | **-1.0439** | **2** | **11** | **2e+02** | **1** | **VDIDVPDVNIEGPEGKLKGPK** |
|  | 3362 | **790.1014** | **2367.2821** | **2366.7204** | **0.5617** | **1** | **11** | **1.6e+02** | **1** | **IGCLHARVSGPLWDAGLCPASSR** |
[truncated: 3,359,337 more chars]
