# Supplementary material for: Tobacco-induced hyperglycemia promotes lung cancer progression via cancer cell-macrophage interaction through paracrine IGF2/IR/NPM1-driven PD-L1 expression
Source: Nat Commun. 2024 Jun 8;15:4909. doi: 10.1038/s41467-024-49199-9 (PMC11162468; doi:10.1038/s41467-024-49199-9)
Supplement: Supplementary file 3 — Supplementary Data 1 [file 41467_2024_49199_MOESM3_ESM.zip › Supplementary Data 1/4.htm]

Peptide Summary Report (../data/20120627/F011180.dat)


# Mascot Search Results

```
User            : yprc
Email           : info
Search title    : 
MS data file    : 4.xml
Database        : NCBInr 110704 (14481393 sequences; 4958963357 residues)
Taxonomy        : Homo sapiens (human) (217342 sequences)
Timestamp       : 27 Jun 2012 at 05:12:14 GMT

|  |  |  |
| --- | --- | --- |
| Protein hits    : | gi|47115317 | VIM [Homo sapiens] |
|  | gi|193787214 | unnamed protein product [Homo sapiens] |
|  | gi|73760405 | thymopoietin isoform beta [Homo sapiens] |
|  | gi|13129104 | coiled-coil domain-containing protein 86 [Homo sapiens] |
|  | gi|62088174 | Lamina-associated polypeptide 2, isoforms beta/gamma variant [Homo sapiens] |
|  | gi|225131084 | titin [Homo sapiens] |
|  | gi|119631418 | titin, isoform CRA_a [Homo sapiens] |
|  | gi|825671 | B23 nucleophosmin (280 AA) [Homo sapiens] |
|  | gi|17066105 | Titin [Homo sapiens] |
|  | gi|1212992 | titin [Homo sapiens] |
|  | gi|119631424 | titin, isoform CRA_e [Homo sapiens] |
|  | gi|1790878 | microtubule-associated protein 1a [Homo sapiens] |
|  | gi|18157651 | bullous pemphigoid antigen 1 eA [Homo sapiens] |
|  | gi|119631904 | nebulin, isoform CRA_a [Homo sapiens] |
|  | gi|415819 | antigen of the monoclonal antibody Ki-67 [Homo sapiens] |
|  | gi|119629468 | hCG2011852 [Homo sapiens] |
|  | gi|806562 | nebulin [Homo sapiens] |
|  | gi|14790190 | msx2-interacting protein [Homo sapiens] |
|  | gi|119631421 | titin, isoform CRA_c [Homo sapiens] |
|  | gi|189306 | nucleolin [Homo sapiens] |
```

### Probability Based Mowse Score

Ions score is -10\*Log(P), where P is the
probability that the observed match is a random event.  
Individual ions scores
> 47 indicate identity or extensive homology (p<0.05).  
Protein scores
are derived from ions scores as a non-probabilistic basis for ranking protein
hits.

### Peptide Summary Report

|  |  |  |  |
| --- | --- | --- | --- |
|  | Peptide Summary Select Summary (protein hits) Select Summary (unassigned) Export Search Results |  | Help |
|  | Significance threshold p< | Max. number of hits |  |
|  | Standard scoring  MudPIT scoring | Ions score or expect cut-off | Show sub-sets |
|  | Show pop-ups  Suppress pop-ups | Sort unassigned  Decreasing Score Increasing query / Mr Decreasing Intensity | Require bold red |

  
 
                
             


  


  
     **Error tolerant**    

|  |  |
| --- | --- |
| **1.** | gi|47115317    **Mass:** 53579    **Score:** 779    **Queries matched:** 68   **emPAI:** 2.10 |
|  | VIM [Homo sapiens] |

|  |  |
| --- | --- |
|  | Check to include this hit in error tolerant search or archive report |
|  |  |

|  |  |  |  |  |  |  |  |  |  |  |
| --- | --- | --- | --- | --- | --- | --- | --- | --- | --- | --- |
|  | **Query** | **Observed** | **Mr(expt)** | **Mr(calc)** | **Delta** | **Miss** | **Score** | **Expect** | **Rank** | **Peptide** |
|  | 1725 | **458.0789** | **914.1431** | **913.9716** | **0.1715** | **0** | **25** | **7.6** | **1** | **R.SYVTTSTR.T** |
|  | 1731 | **458.2334** | **914.4520** | **913.9716** | **0.4803** | **0** | **(6)** | **6.4e+02** | **4** | **R.SYVTTSTR.T** |
|  | 2049 | **486.2525** | **970.4903** | **970.1276** | **0.3627** | **1** | **28** | **4.5** | **1** | **R.LRSSVPGVR.L** |
|  | 2050 | **486.2829** | **970.5510** | **970.1276** | **0.4235** | **1** | **(10)** | **2.2e+02** | **4** | **R.LRSSVPGVR.L** |
|  | 2538 | **547.5720** | **1093.1291** | **1093.1474** | **-0.0183** | **0** | **70** | **0.0003** | **1** | **K.FADLSEAANR.N** |
|  | 2728 | **587.5143** | **1173.0139** | **1173.4028** | **-0.3889** | **1** | **59** | **0.0029** | **1** | **R.TLLIKTVETR.D** |
|  | 2729 | **587.5765** | **1173.1383** | **1173.4028** | **-0.2645** | **1** | **(52)** | **0.015** | **1** | **R.TLLIKTVETR.D** |
|  | 618 | **392.4475** | **1174.3204** | **1173.4028** | **0.9175** | **1** | **(15)** | **91** | **4** | **R.TLLIKTVETR.D** |
|  | 619 | **392.4648** | **1174.3724** | **1173.4028** | **0.9695** | **1** | **(35)** | **1.1** | **1** | **R.TLLIKTVETR.D** |
|  | 1840 | **465.2312** | **1392.6714** | **1393.5016** | **-0.8302** | **1** | **7** | **5.6e+02** | **6** | **R.DVRQQYESVAAK.N** |
|  | 3191 | **714.9349** | **1427.8550** | **1428.5456** | **-0.6907** | **0** | **(61)** | **0.002** | **1** | **R.SLYASSPGGVYATR.S** |
|  | 3192 | **715.1526** | **1428.2904** | **1428.5456** | **-0.2552** | **0** | **(36)** | **0.72** | **1** | **R.SLYASSPGGVYATR.S** |
|  | 3193 | **715.1653** | **1428.3158** | **1428.5456** | **-0.2299** | **0** | **(55)** | **0.0076** | **1** | **R.SLYASSPGGVYATR.S** |
|  | 3194 | **715.1945** | **1428.3741** | **1428.5456** | **-0.1715** | **0** | **(70)** | **0.00029** | **1** | **R.SLYASSPGGVYATR.S** |
|  | 3196 | **715.3862** | **1428.7577** | **1428.5456** | **0.2120** | **0** | **(66)** | **0.00066** | **1** | **R.SLYASSPGGVYATR.S** |
|  | 3198 | **715.7172** | **1429.4197** | **1428.5456** | **0.8740** | **0** | **83** | **1.2e-05** | **1** | **R.SLYASSPGGVYATR.S** |
|  | 3222 | **736.3070** | **1470.5992** | **1470.6769** | **-0.0776** | **2** | **(22)** | **15** | **1** | **R.SSAVRLRSSVPGVR.L** |
|  | 2123 | **491.2927** | **1470.8559** | **1470.6769** | **0.1790** | **2** | **(9)** | **2.8e+02** | **7** | **R.SSAVRLRSSVPGVR.L** |
|  | 2124 | **491.4342** | **1471.2803** | **1470.6769** | **0.6035** | **2** | **(26)** | **5.2** | **1** | **R.SSAVRLRSSVPGVR.L** |
|  | 2125 | **491.4907** | **1471.4500** | **1470.6769** | **0.7731** | **2** | **(16)** | **67** | **2** | **R.SSAVRLRSSVPGVR.L** |
|  | 2126 | **491.4940** | **1471.4597** | **1470.6769** | **0.7828** | **2** | **26** | **6.2** | **2** | **R.SSAVRLRSSVPGVR.L** |
|  | 2127 | **491.5064** | **1471.4970** | **1470.6769** | **0.8201** | **2** | **(23)** | **14** | **1** | **R.SSAVRLRSSVPGVR.L** |
|  | 2180 | **499.1256** | **1494.3547** | **1494.6320** | **-0.2773** | **0** | **(10)** | **2.8e+02** | **2** | **R.MFGGPGTASRPSSSR.S** |
|  | 3307 | **748.1947** | **1494.3746** | **1494.6320** | **-0.2574** | **0** | **(25)** | **8.5** | **1** | **R.MFGGPGTASRPSSSR.S** |
|  | 3308 | **748.2500** | **1494.4852** | **1494.6320** | **-0.1468** | **0** | **(38)** | **0.38** | **1** | **R.MFGGPGTASRPSSSR.S** |
|  | 3309 | **748.3790** | **1494.7433** | **1495.6365** | **-0.8933** | **0** | **(19)** | **35** | **1** | **R.TYSLGSALRPSTSR.S** |
|  | 3310 | **748.4634** | **1494.9120** | **1495.6365** | **-0.7246** | **0** | **(38)** | **0.43** | **1** | **R.TYSLGSALRPSTSR.S** |
|  | 3311 | **748.7261** | **1495.4374** | **1495.6365** | **-0.1992** | **0** | **(40)** | **0.2** | **1** | **R.TYSLGSALRPSTSR.S** |
|  | 2188 | **499.6074** | **1495.8000** | **1495.6365** | **0.1635** | **0** | **(19)** | **38** | **1** | **R.TYSLGSALRPSTSR.S** |
|  | 2190 | **499.6129** | **1495.8164** | **1494.6320** | **1.1844** | **0** | **(26)** | **7.3** | **1** | **R.MFGGPGTASRPSSSR.S** |
|  | 2192 | **499.6666** | **1495.9775** | **1495.6365** | **0.3410** | **0** | **(14)** | **1.1e+02** | **2** | **R.TYSLGSALRPSTSR.S** |
|  | 2193 | **499.6929** | **1496.0564** | **1495.6365** | **0.4199** | **0** | **(14)** | **88** | **2** | **R.TYSLGSALRPSTSR.S** |
|  | 2194 | **499.7063** | **1496.0967** | **1495.6365** | **0.4602** | **0** | **(33)** | **0.99** | **1** | **R.TYSLGSALRPSTSR.S** |
|  | 3314 | **749.1981** | **1496.3813** | **1495.6365** | **0.7448** | **0** | **(16)** | **61** | **1** | **R.TYSLGSALRPSTSR.S** |
|  | 2197 | **499.8580** | **1496.5519** | **1495.6365** | **0.9154** | **0** | **44** | **0.096** | **1** | **R.TYSLGSALRPSTSR.S** |
|  | 2198 | **499.9446** | **1496.8116** | **1495.6365** | **1.1750** | **0** | **(6)** | **7e+02** | **7** | **R.TYSLGSALRPSTSR.S** |
|  | 3328 | **756.2330** | **1510.4513** | **1510.6314** | **-0.1801** | **0** | **(32)** | **1.5** | **1** | **R.MFGGPGTASRPSSSR.S + Oxidation (M)** |
|  | 2223 | **504.6824** | **1511.0249** | **1510.6314** | **0.3936** | **0** | **(38)** | **0.37** | **1** | **R.MFGGPGTASRPSSSR.S + Oxidation (M)** |
|  | 2224 | **504.7071** | **1511.0990** | **1510.6314** | **0.4676** | **0** | **(43)** | **0.12** | **1** | **R.MFGGPGTASRPSSSR.S + Oxidation (M)** |
|  | 3330 | **756.7134** | **1511.4120** | **1510.6314** | **0.7806** | **0** | **(30)** | **2** | **1** | **R.MFGGPGTASRPSSSR.S + Oxidation (M)** |
|  | 2225 | **504.8880** | **1511.6417** | **1510.6314** | **1.0104** | **0** | **48** | **0.04** | **1** | **R.MFGGPGTASRPSSSR.S + Oxidation (M)** |
|  | 3350 | **763.1797** | **1524.3446** | **1524.6296** | **-0.2850** | **1** | **53** | **0.013** | **1** | **K.NLQEAEEWYKSK.F** |
|  | 2257 | **509.4908** | **1525.4501** | **1524.6296** | **0.8204** | **1** | **(19)** | **39** | **1** | **K.NLQEAEEWYKSK.F** |
|  | 2567 | **551.6007** | **1651.7800** | **1650.8177** | **0.9623** | **1** | **50** | **0.034** | **1** | **R.RMFGGPGTASRPSSSR.S** |
|  | 3443 | **834.2712** | **1666.5276** | **1666.8171** | **-0.2895** | **1** | **(8)** | **4.3e+02** | **2** | **R.RMFGGPGTASRPSSSR.S + Oxidation (M)** |
|  | 2606 | **557.6163** | **1669.8268** | **1668.7997** | **1.0271** | **0** | **9** | **3.8e+02** | **3** | **R.ETNLDSLPLVDTHSK.R** |
|  | 3482 | **889.2980** | **1776.5812** | **1776.8612** | **-0.2801** | **1** | **(44)** | **0.1** | **1** | **K.FADLSEAANRNNDALR.Q** |
|  | 2776 | **593.4658** | **1777.3753** | **1776.8612** | **0.5141** | **1** | **49** | **0.03** | **1** | **K.FADLSEAANRNNDALR.Q** |
|  | 2858 | **609.2557** | **1824.7450** | **1824.9854** | **-0.2404** | **1** | **25** | **8.3** | **1** | **R.ETNLDSLPLVDTHSKR.T** |
|  | 2917 | **613.1045** | **1836.2913** | **1836.8238** | **-0.5325** | **0** | **(25)** | **8** | **1** | **R.DGQVINETSQHHDDLE.-** |
|  | 3495 | **919.1843** | **1836.3539** | **1836.8238** | **-0.4700** | **0** | **56** | **0.006** | **1** | **R.DGQVINETSQHHDDLE.-** |
|  | 3496 | **919.2162** | **1836.4176** | **1836.8238** | **-0.4062** | **0** | **(44)** | **0.084** | **1** | **R.DGQVINETSQHHDDLE.-** |
|  | 2918 | **613.2061** | **1836.5960** | **1836.8238** | **-0.2278** | **0** | **(11)** | **2.1e+02** | **1** | **R.DGQVINETSQHHDDLE.-** |
|  | 2920 | **613.5418** | **1837.6033** | **1836.8238** | **0.7794** | **0** | **(30)** | **2.2** | **1** | **R.DGQVINETSQHHDDLE.-** |
|  | 2921 | **613.6615** | **1837.9623** | **1836.8238** | **1.1385** | **0** | **(18)** | **51** | **1** | **R.DGQVINETSQHHDDLE.-** |
|  | 3503 | **965.3608** | **1928.7069** | **1929.0949** | **-0.3880** | **1** | **33** | **0.99** | **1** | **R.SLYASSPGGVYATRSSAVR.L** |
|  | 3000 | **644.2660** | **1929.7758** | **1929.0949** | **0.6809** | **1** | **(22)** | **18** | **1** | **R.SLYASSPGGVYATRSSAVR.L** |
|  | 3001 | **644.2709** | **1929.7904** | **1929.0949** | **0.6955** | **1** | **(24)** | **10** | **1** | **R.SLYASSPGGVYATRSSAVR.L** |
|  | 3504 | **965.9659** | **1929.9171** | **1929.0949** | **0.8222** | **1** | **(33)** | **1.1** | **1** | **R.SLYASSPGGVYATRSSAVR.L** |
|  | 3217 | **733.6558** | **2197.9451** | **2198.4382** | **-0.4931** | **2** | **(17)** | **43** | **1** | **R.SLYASSPGGVYATRSSAVRLR.S** |
|  | 3218 | **734.0574** | **2199.1499** | **2198.4382** | **0.7117** | **2** | **18** | **36** | **1** | **R.SLYASSPGGVYATRSSAVRLR.S** |
|  | 3397 | **798.0340** | **2391.0798** | **2391.5929** | **-0.5131** | **1** | **(36)** | **0.6** | **1** | **R.SYVTTSTRTYSLGSALRPSTSR.S** |
|  | 3399 | **798.1106** | **2391.3096** | **2390.5884** | **0.7213** | **1** | **(12)** | **1.2e+02** | **1** | **R.MFGGPGTASRPSSSRSYVTTSTR.T** |
|  | 3401 | **798.2310** | **2391.6707** | **2390.5884** | **1.0823** | **1** | **(14)** | **90** | **1** | **R.MFGGPGTASRPSSSRSYVTTSTR.T** |
|  | 3402 | **798.4841** | **2392.4302** | **2391.5929** | **0.8373** | **1** | **38** | **0.44** | **1** | **R.SYVTTSTRTYSLGSALRPSTSR.S** |
|  | 3407 | **803.2020** | **2406.5839** | **2406.5878** | **-0.0039** | **1** | **(24)** | **9.8** | **1** | **R.MFGGPGTASRPSSSRSYVTTSTR.T + Oxidation (M)** |
|  | 3408 | **803.2465** | **2406.7172** | **2406.5878** | **0.1294** | **1** | **26** | **6.7** | **1** | **R.MFGGPGTASRPSSSRSYVTTSTR.T + Oxidation (M)** |
|  | 3415 | **809.0010** | **2423.9809** | **2423.4624** | **0.5185** | **1** | **43** | **0.13** | **1** | **K.TVETRDGQVINETSQHHDDLE.-** |

  

|  |  |
| --- | --- |
|  | |
|  | **Proteins matching the same set of peptides:** |

|  |  |
| --- | --- |
|  | gi|62414289    **Mass:** 53651    **Score:** 777    **Queries matched:** 68 |
|  | vimentin [Homo sapiens] |

|  |  |
| --- | --- |
|  | gi|62896523    **Mass:** 53683    **Score:** 777    **Queries matched:** 68 |
|  | vimentin variant [Homo sapiens] |

---

|  |  |
| --- | --- |
| **2.** | gi|193787214    **Mass:** 46976    **Score:** 562    **Queries matched:** 54   **emPAI:** 1.42 |
|  | unnamed protein product [Homo sapiens] |

|  |  |
| --- | --- |
|  | Check to include this hit in error tolerant search or archive report |
|  |  |

|  |  |  |  |  |  |  |  |  |  |  |
| --- | --- | --- | --- | --- | --- | --- | --- | --- | --- | --- |
|  | **Query** | **Observed** | **Mr(expt)** | **Mr(calc)** | **Delta** | **Miss** | **Score** | **Expect** | **Rank** | **Peptide** |
|  | 1725 | 458.0789 | 914.1431 | 913.9716 | 0.1715 | 0 | 25 | 7.6 | 1 | R.SYVTTSTR.T |
|  | 1731 | 458.2334 | 914.4520 | 913.9716 | 0.4803 | 0 | (6) | 6.4e+02 | 4 | R.SYVTTSTR.T |
|  | 2049 | 486.2525 | 970.4903 | 970.1276 | 0.3627 | 1 | 28 | 4.5 | 1 | R.LRSSVPGVR.L |
|  | 2050 | 486.2829 | 970.5510 | 970.1276 | 0.4235 | 1 | (10) | 2.2e+02 | 4 | R.LRSSVPGVR.L |
|  | 2538 | 547.5720 | 1093.1291 | 1093.1474 | -0.0183 | 0 | 70 | 0.0003 | 1 | K.FADLSEAANR.N |
|  | 1689 | **455.1808** | **1362.5204** | **1363.4359** | **-0.9155** | **0** | **23** | **13** | **1** | **M.FGGPGTASRPSSSR.S** |
|  | 1840 | 465.2312 | 1392.6714 | 1393.5016 | -0.8302 | 1 | 7 | 5.6e+02 | 6 | R.DVRQQYESVAAK.N |
|  | 3191 | 714.9349 | 1427.8550 | 1428.5456 | -0.6907 | 0 | (61) | 0.002 | 1 | R.SLYASSPGGVYATR.S |
|  | 3192 | 715.1526 | 1428.2904 | 1428.5456 | -0.2552 | 0 | (36) | 0.72 | 1 | R.SLYASSPGGVYATR.S |
|  | 3193 | 715.1653 | 1428.3158 | 1428.5456 | -0.2299 | 0 | (55) | 0.0076 | 1 | R.SLYASSPGGVYATR.S |
|  | 3194 | 715.1945 | 1428.3741 | 1428.5456 | -0.1715 | 0 | (70) | 0.00029 | 1 | R.SLYASSPGGVYATR.S |
|  | 3196 | 715.3862 | 1428.7577 | 1428.5456 | 0.2120 | 0 | (66) | 0.00066 | 1 | R.SLYASSPGGVYATR.S |
|  | 3198 | 715.7172 | 1429.4197 | 1428.5456 | 0.8740 | 0 | 83 | 1.2e-05 | 1 | R.SLYASSPGGVYATR.S |
|  | 3222 | 736.3070 | 1470.5992 | 1470.6769 | -0.0776 | 2 | (22) | 15 | 1 | R.SSAVRLRSSVPGVR.L |
|  | 2123 | 491.2927 | 1470.8559 | 1470.6769 | 0.1790 | 2 | (9) | 2.8e+02 | 7 | R.SSAVRLRSSVPGVR.L |
|  | 2124 | 491.4342 | 1471.2803 | 1470.6769 | 0.6035 | 2 | (26) | 5.2 | 1 | R.SSAVRLRSSVPGVR.L |
|  | 2125 | 491.4907 | 1471.4500 | 1470.6769 | 0.7731 | 2 | (16) | 67 | 2 | R.SSAVRLRSSVPGVR.L |
|  | 2126 | 491.4940 | 1471.4597 | 1470.6769 | 0.7828 | 2 | 26 | 6.2 | 2 | R.SSAVRLRSSVPGVR.L |
|  | 2127 | 491.5064 | 1471.4970 | 1470.6769 | 0.8201 | 2 | (23) | 14 | 1 | R.SSAVRLRSSVPGVR.L |
|  | 2180 | 499.1256 | 1494.3547 | 1494.6320 | -0.2773 | 0 | (10) | 2.8e+02 | 2 | -.MFGGPGTASRPSSSR.S |
|  | 3307 | 748.1947 | 1494.3746 | 1494.6320 | -0.2574 | 0 | (25) | 8.5 | 1 | -.MFGGPGTASRPSSSR.S |
|  | 3308 | 748.2500 | 1494.4852 | 1494.6320 | -0.1468 | 0 | (38) | 0.38 | 1 | -.MFGGPGTASRPSSSR.S |
|  | 3309 | 748.3790 | 1494.7433 | 1495.6365 | -0.8933 | 0 | (19) | 35 | 1 | R.TYSLGSALRPSTSR.S |
|  | 3310 | 748.4634 | 1494.9120 | 1495.6365 | -0.7246 | 0 | (38) | 0.43 | 1 | R.TYSLGSALRPSTSR.S |
|  | 3311 | 748.7261 | 1495.4374 | 1495.6365 | -0.1992 | 0 | (40) | 0.2 | 1 | R.TYSLGSALRPSTSR.S |
|  | 2188 | 499.6074 | 1495.8000 | 1495.6365 | 0.1635 | 0 | (19) | 38 | 1 | R.TYSLGSALRPSTSR.S |
|  | 2190 | 499.6129 | 1495.8164 | 1494.6320 | 1.1844 | 0 | (26) | 7.3 | 1 | -.MFGGPGTASRPSSSR.S |
|  | 2192 | 499.6666 | 1495.9775 | 1495.6365 | 0.3410 | 0 | (14) | 1.1e+02 | 2 | R.TYSLGSALRPSTSR.S |
|  | 2193 | 499.6929 | 1496.0564 | 1495.6365 | 0.4199 | 0 | (14) | 88 | 2 | R.TYSLGSALRPSTSR.S |
|  | 2194 | 499.7063 | 1496.0967 | 1495.6365 | 0.4602 | 0 | (33) | 0.99 | 1 | R.TYSLGSALRPSTSR.S |
|  | 3314 | 749.1981 | 1496.3813 | 1495.6365 | 0.7448 | 0 | (16) | 61 | 1 | R.TYSLGSALRPSTSR.S |
|  | 2197 | 499.8580 | 1496.5519 | 1495.6365 | 0.9154 | 0 | 44 | 0.096 | 1 | R.TYSLGSALRPSTSR.S |
|  | 2198 | 499.9446 | 1496.8116 | 1495.6365 | 1.1750 | 0 | (6) | 7e+02 | 7 | R.TYSLGSALRPSTSR.S |
|  | 3328 | 756.2330 | 1510.4513 | 1510.6314 | -0.1801 | 0 | (32) | 1.5 | 1 | -.MFGGPGTASRPSSSR.S + Oxidation (M) |
|  | 2223 | 504.6824 | 1511.0249 | 1510.6314 | 0.3936 | 0 | (38) | 0.37 | 1 | -.MFGGPGTASRPSSSR.S + Oxidation (M) |
|  | 2224 | 504.7071 | 1511.0990 | 1510.6314 | 0.4676 | 0 | (43) | 0.12 | 1 | -.MFGGPGTASRPSSSR.S + Oxidation (M) |
|  | 3330 | 756.7134 | 1511.4120 | 1510.6314 | 0.7806 | 0 | (30) | 2 | 1 | -.MFGGPGTASRPSSSR.S + Oxidation (M) |
|  | 2225 | 504.8880 | 1511.6417 | 1510.6314 | 1.0104 | 0 | 48 | 0.04 | 1 | -.MFGGPGTASRPSSSR.S + Oxidation (M) |
|  | 3350 | 763.1797 | 1524.3446 | 1524.6296 | -0.2850 | 1 | 53 | 0.013 | 1 | K.NLQEAEEWYKSK.F |
|  | 2257 | 509.4908 | 1525.4501 | 1524.6296 | 0.8204 | 1 | (19) | 39 | 1 | K.NLQEAEEWYKSK.F |
|  | 3482 | 889.2980 | 1776.5812 | 1776.8612 | -0.2801 | 1 | (44) | 0.1 | 1 | K.FADLSEAANRNNDALR.Q |
|  | 2776 | 593.4658 | 1777.3753 | 1776.8612 | 0.5141 | 1 | 49 | 0.03 | 1 | K.FADLSEAANRNNDALR.Q |
|  | 3503 | 965.3608 | 1928.7069 | 1929.0949 | -0.3880 | 1 | 33 | 0.99 | 1 | R.SLYASSPGGVYATRSSAVR.L |
|  | 3000 | 644.2660 | 1929.7758 | 1929.0949 | 0.6809 | 1 | (22) | 18 | 1 | R.SLYASSPGGVYATRSSAVR.L |
|  | 3001 | 644.2709 | 1929.7904 | 1929.0949 | 0.6955 | 1 | (24) | 10 | 1 | R.SLYASSPGGVYATRSSAVR.L |
|  | 3504 | 965.9659 | 1929.9171 | 1929.0949 | 0.8222 | 1 | (33) | 1.1 | 1 | R.SLYASSPGGVYATRSSAVR.L |
|  | 3217 | 733.6558 | 2197.9451 | 2198.4382 | -0.4931 | 2 | (17) | 43 | 1 | R.SLYASSPGGVYATRSSAVRLR.S |
|  | 3218 | 734.0574 | 2199.1499 | 2198.4382 | 0.7117 | 2 | 18 | 36 | 1 | R.SLYASSPGGVYATRSSAVRLR.S |
|  | 3397 | 798.0340 | 2391.0798 | 2391.5929 | -0.5131 | 1 | (36) | 0.6 | 1 | R.SYVTTSTRTYSLGSALRPSTSR.S |
|  | 3399 | 798.1106 | 2391.3096 | 2390.5884 | 0.7213 | 1 | (12) | 1.2e+02 | 1 | -.MFGGPGTASRPSSSRSYVTTSTR.T |
|  | 3401 | 798.2310 | 2391.6707 | 2390.5884 | 1.0823 | 1 | (14) | 90 | 1 | -.MFGGPGTASRPSSSRSYVTTSTR.T |
|  | 3402 | 798.4841 | 2392.4302 | 2391.5929 | 0.8373 | 1 | 38 | 0.44 | 1 | R.SYVTTSTRTYSLGSALRPSTSR.S |
|  | 3407 | 803.2020 | 2406.5839 | 2406.5878 | -0.0039 | 1 | (24) | 9.8 | 1 | -.MFGGPGTASRPSSSRSYVTTSTR.T + Oxidation (M) |
|  | 3408 | 803.2465 | 2406.7172 | 2406.5878 | 0.1294 | 1 | 26 | 6.7 | 1 | -.MFGGPGTASRPSSSRSYVTTSTR.T + Oxidation (M) |

  


---

|  |  |
| --- | --- |
| **3.** | gi|73760405    **Mass:** 50670    **Score:** 176    **Queries matched:** 5   **emPAI:** 0.21 |
|  | thymopoietin isoform beta [Homo sapiens] |

|  |  |
| --- | --- |
|  | Check to include this hit in error tolerant search or archive report |
|  |  |

|  |  |  |  |  |  |  |  |  |  |  |
| --- | --- | --- | --- | --- | --- | --- | --- | --- | --- | --- |
|  | **Query** | **Observed** | **Mr(expt)** | **Mr(calc)** | **Delta** | **Miss** | **Score** | **Expect** | **Rank** | **Peptide** |
|  | 1714 | **457.6195** | **913.2242** | **912.0449** | **1.1792** | **0** | **(20)** | **23** | **1** | **K.GGPLQALTR.E** |
|  | 1715 | **457.6262** | **913.2377** | **912.0449** | **1.1927** | **0** | **32** | **1.6** | **1** | **K.GGPLQALTR.E** |
|  | 371 | **381.8171** | **1142.4292** | **1141.3643** | **1.0648** | **2** | **31** | **2.2** | **1** | **R.AKTPVTLKQR.R** |
|  | 3033 | **666.2634** | **1330.5120** | **1330.4888** | **0.0232** | **0** | **39** | **0.37** | **1** | **K.YGVNPGPIVGTTR.K** |
|  | 3472 | **857.8241** | **2570.4501** | **2570.6795** | **-0.2293** | **1** | **75** | **7e-05** | **1** | **K.GPPDFSSDEEREPTPVLGSGAAAAGR.S** |

  


---

|  |  |
| --- | --- |
| **4.** | gi|13129104    **Mass:** 40235    **Score:** 152    **Queries matched:** 7   **emPAI:** 0.17 |
|  | coiled-coil domain-containing protein 86 [Homo sapiens] |

|  |  |
| --- | --- |
|  | Check to include this hit in error tolerant search or archive report |
|  |  |

|  |  |  |  |  |  |  |  |  |  |  |
| --- | --- | --- | --- | --- | --- | --- | --- | --- | --- | --- |
|  | **Query** | **Observed** | **Mr(expt)** | **Mr(calc)** | **Delta** | **Miss** | **Score** | **Expect** | **Rank** | **Peptide** |
|  | 1719 | **457.8621** | **913.7095** | **913.0730** | **0.6365** | **0** | **15** | **77** | **1** | **K.AEVVQVIR.N** |
|  | 2501 | **539.7114** | **1077.4079** | **1077.2341** | **0.1738** | **1** | **26** | **7.2** | **1** | **K.QPPQQPAAKI.-** |
|  | 1803 | **460.8688** | **1379.5842** | **1378.5962** | **0.9881** | **0** | **15** | **98** | **1** | **R.FSQMLQDKPLR.T + Oxidation (M)** |
|  | 1892 | **470.3967** | **1408.1680** | **1407.6971** | **0.4710** | **2** | **(30)** | **1.8** | **1** | **K.KLNKEELPVIPK.G** |
|  | 1893 | **470.5886** | **1408.7437** | **1407.6971** | **1.0466** | **2** | **33** | **1.3** | **1** | **K.KLNKEELPVIPK.G** |
|  | 2035 | **484.5490** | **1450.6248** | **1451.7133** | **-1.0885** | **2** | **20** | **30** | **1** | **R.KAEVVQVIRNPAK.L** |
|  | 3014 | **654.3522** | **1960.0343** | **1960.2776** | **-0.2433** | **2** | **43** | **0.13** | **1** | **R.DTLALLQKQPPQQPAAKI.-** |

  


---

|  |  |
| --- | --- |
| **5.** | gi|62088174    **Mass:** 41806    **Score:** 151    **Queries matched:** 4   **emPAI:** 0.26 |
|  | Lamina-associated polypeptide 2, isoforms beta/gamma variant [Homo sapiens] |

|  |  |
| --- | --- |
|  | Check to include this hit in error tolerant search or archive report |
|  |  |

|  |  |  |  |  |  |  |  |  |  |  |
| --- | --- | --- | --- | --- | --- | --- | --- | --- | --- | --- |
|  | **Query** | **Observed** | **Mr(expt)** | **Mr(calc)** | **Delta** | **Miss** | **Score** | **Expect** | **Rank** | **Peptide** |
|  | 2206 | **502.0759** | **1002.1370** | **1003.1144** | **-0.9774** | **2** | **6** | **6.7e+02** | **10** | **R.SKRAGVSGGGK.G** |
|  | 371 | 381.8171 | 1142.4292 | 1141.3643 | 1.0648 | 2 | 31 | 2.2 | 1 | R.AKTPVTLKQR.R |
|  | 3033 | 666.2634 | 1330.5120 | 1330.4888 | 0.0232 | 0 | 39 | 0.37 | 1 | K.YGVNPGPIVGTTR.K |
|  | 3472 | 857.8241 | 2570.4501 | 2570.6795 | -0.2293 | 1 | 75 | 7e-05 | 1 | K.GPPDFSSDEEREPTPVLGSGAAAAGR.S |

  


---

|  |  |
| --- | --- |
| **6.** | gi|225131084    **Mass:** 3713667  **Score:** 137    **Queries matched:** 68 |
|  | titin [Homo sapiens] |

|  |  |
| --- | --- |
|  | Check to include this hit in error tolerant search or archive report |
|  |  |

|  |  |  |  |  |  |  |  |  |  |  |
| --- | --- | --- | --- | --- | --- | --- | --- | --- | --- | --- |
|  | **Query** | **Observed** | **Mr(expt)** | **Mr(calc)** | **Delta** | **Miss** | **Score** | **Expect** | **Rank** | **Peptide** |
|  | 83 | **365.1657** | **728.3167** | **728.9203** | **-0.6036** | **0** | **(7)** | **4.9e+02** | **3** | **K.VLVAVTK.K** |
|  | 86 | **365.4347** | **728.8546** | **728.9203** | **-0.0657** | **0** | **11** | **2.5e+02** | **3** | **K.VLVAVTK.K** |
|  | 227 | **371.2714** | **740.5279** | **739.8601** | **0.6679** | **1** | **13** | **93** | **2** | **K.KEAPPAK.V** |
|  | 349 | **380.0574** | **758.1000** | **757.9198** | **0.1802** | **0** | **14** | **1.3e+02** | **3** | **R.FLTLHK.V** |
|  | 363 | **380.4083** | **758.8017** | **757.9198** | **0.8819** | **0** | **(3)** | **1.9e+03** | **2** | **R.FLTLHK.V** |
|  | 714 | **401.1246** | **800.2344** | **800.9429** | **-0.7086** | **1** | **11** | **2.1e+02** | **5** | **K.KGDQILK.Q** |
|  | 716 | **401.2014** | **800.3881** | **800.9067** | **-0.5185** | **2** | **13** | **1.2e+02** | **2** | **K.DRKQVR.N** |
|  | 1059 | **410.9979** | **819.9811** | **818.9817** | **0.9994** | **0** | **6** | **7.6e+02** | **2** | **K.VPVTMTR.Y + Oxidation (M)** |
|  | 1110 | **414.0691** | **826.1233** | **824.9646** | **1.1588** | **0** | **7** | **4.9e+02** | **4** | **R.LHVETVK.I** |
|  | 1672 | **452.6298** | **903.2449** | **903.0614** | **0.1834** | **1** | **9** | **3.4e+02** | **1** | **R.QRIMAER.E** |
|  | 1724 | **458.0725** | **914.1303** | **913.0696** | **1.0607** | **0** | **10** | **2.9e+02** | **5** | **R.ITIENVPK.K** |
|  | 1933 | **474.4376** | **946.8605** | **948.0342** | **-1.1737** | **1** | **16** | **71** | **1** | **K.VSWAKDSR.E** |
|  | 1960 | **476.2941** | **950.5734** | **950.0468** | **0.5266** | **0** | **7** | **4.5e+02** | **4** | **K.AAEPPSPPGK.V** |
|  | 2031 | **484.1978** | **966.3809** | **967.1005** | **-0.7196** | **0** | **1** | **2.2e+03** | **6** | **K.VSECFVAR.D + Carbamidomethyl (C)** |
|  | 2306 | **517.6474** | **1033.2800** | **1034.1879** | **-0.9079** | **1** | **17** | **71** | **1** | **K.KGDATQLACK.V** |
|  | 2349 | **520.0552** | **1038.0956** | **1037.1074** | **0.9882** | **0** | **5** | **8.7e+02** | **9** | **K.TAGPDCNFR.V + Carbamidomethyl (C)** |
|  | 2360 | **520.9778** | **1039.9408** | **1040.1909** | **-0.2501** | **0** | **8** | **3.7e+02** | **3** | **K.AEFVCSISK.E + Carbamidomethyl (C)** |
|  | 2375 | **522.0974** | **1042.1800** | **1043.1320** | **-0.9519** | **0** | **5** | **9e+02** | **5** | **R.KPVSEVGDGR.W** |
|  | 2377 | **522.1971** | **1042.3794** | **1042.1670** | **0.2124** | **1** | **7** | **5.7e+02** | **8** | **K.INKMYSDR.A + Oxidation (M)** |
|  | 2473 | **536.1332** | **1070.2516** | **1070.1770** | **0.0745** | **1** | **4** | **1e+03** | **4** | **K.LDDGTYRCK.V** |
|  | 40 | **363.0770** | **1086.2087** | **1087.2077** | **-0.9990** | **2** | **8** | **3.8e+02** | **8** | **R.KYKMSSDGR.T + Oxidation (M)** |
|  | 50 | **363.1646** | **1086.4718** | **1086.2012** | **0.2705** | **1** | **14** | **86** | **2** | **K.KIEAHFDAR.S** |
|  | 2533 | **547.4041** | **1092.7933** | **1093.2732** | **-0.4799** | **0** | **2** | **1.2e+03** | **6** | **K.YISSLEILR.T** |
|  | 96 | **366.0843** | **1095.2307** | **1095.2761** | **-0.0454** | **2** | **9** | **3.5e+02** | **7** | **K.REAAMRAFK.T + Oxidation (M)** |
|  | 100 | **366.1522** | **1095.4344** | **1095.2761** | **0.1583** | **2** | **(9)** | **3.6e+02** | **8** | **K.REAAMRAFK.T + Oxidation (M)** |
|  | 2565 | **551.1006** | **1100.1864** | **1101.3170** | **-1.1306** | **0** | **8** | **4.3e+02** | **5** | **K.DVLPGSAVCLK.S** |
|  | 129 | **367.7991** | **1100.3752** | **1101.3170** | **-0.9417** | **0** | **(7)** | **6.1e+02** | **4** | **K.DVLPGSAVCLK.S** |
|  | 190 | **369.3158** | **1104.9252** | **1105.2925** | **-0.3673** | **1** | **6** | **5.9e+02** | **3** | **R.MSPARMSPGR.R + Oxidation (M)** |
|  | 314 | **377.1657** | **1128.4750** | **1128.2793** | **0.1956** | **1** | **10** | **2.6e+02** | **1** | **K.LTSGEAPGIRK.E** |
|  | 2656 | **566.2069** | **1130.3990** | **1130.3831** | **0.0160** | **1** | **15** | **87** | **5** | **K.TPPRIPPKPK.S** |
|  | 2683 | **574.3709** | **1146.7270** | **1147.3240** | **-0.5970** | **1** | **9** | **3.4e+02** | **5** | **R.YPAKLTLGER.E** |
|  | 434 | **386.0396** | **1155.0967** | **1155.3000** | **-0.2033** | **0** | **14** | **99** | **2** | **R.VEAMGMSSEAK.L + Oxidation (M)** |
|  | 544 | **388.8141** | **1163.4201** | **1163.2789** | **0.1412** | **2** | **6** | **8.4e+02** | **6** | **K.ISETKKSDQK.T** |
|  | 611 | **391.1789** | **1170.5145** | **1170.3658** | **0.1488** | **2** | **6** | **5.2e+02** | **6** | **R.KGIVVRAGGSAR.I** |
|  | 2760 | **592.7612** | **1183.5076** | **1183.3563** | **0.1513** | **1** | **5** | **9.1e+02** | **5** | **R.EKLYPPSPPR.W** |
|  | 683 | **399.8672** | **1196.5795** | **1197.4010** | **-0.8216** | **0** | **3** | **1.1e+03** | **9** | **K.VTGYLIEMQK.V + Oxidation (M)** |
|  | 733 | **401.9340** | **1202.7799** | **1203.3757** | **-0.5959** | **2** | **8** | **4.6e+02** | **3** | **K.QRQRIMAER.E + Oxidation (M)** |
|  | 865 | **406.0075** | **1215.0003** | **1215.3568** | **-0.3565** | **1** | **6** | **5.9e+02** | **4** | **K.DRVVIDNVGTK.S** |
|  | 911 | **406.9037** | **1217.6891** | **1217.3892** | **0.2999** | **0** | **11** | **1.9e+02** | **7** | **K.IELSPSMEAPK.I + Oxidation (M)** |
|  | 959 | **407.5059** | **1219.4954** | **1219.4315** | **0.0639** | **0** | **10** | **2.8e+02** | **4** | **K.VLGSSIHMECK.V + Oxidation (M)** |
|  | 1082 | **412.4707** | **1234.3898** | **1233.4382** | **0.9517** | **2** | **2** | **2.1e+03** | **5** | **K.ADMILKQDKR.I + Oxidation (M)** |
|  | 2946 | **623.2170** | **1244.4193** | **1243.3717** | **1.0476** | **2** | **13** | **1.3e+02** | **2** | **R.RDLPDGRWTK.A** |
|  | 2989 | **637.4632** | **1272.9116** | **1273.4738** | **-0.5622** | **0** | **25** | **7.1** | **3** | **K.EIELDFAVPLK.D** |
|  | 1409 | **433.4536** | **1297.3386** | **1297.5466** | **-0.2080** | **2** | **8** | **4.5e+02** | **4** | **K.GKTFVYLKWR.R** |
|  | 1414 | **434.1722** | **1299.4944** | **1300.3765** | **-0.8822** | **0** | **9** | **3.4e+02** | **6** | **K.GEQTWSHAGISK.T** |
|  | 1415 | **434.1731** | **1299.4971** | **1300.3765** | **-0.8794** | **0** | **(4)** | **9.4e+02** | **10** | **K.GEQTWSHAGISK.T** |
|  | 1531 | **441.1255** | **1320.3543** | **1321.4357** | **-1.0814** | **0** | **5** | **8.1e+02** | **8** | **R.DTIVVNAGETFR.L** |
|  | 1618 | **448.2582** | **1341.7524** | **1342.5227** | **-0.7703** | **1** | **3** | **1.2e+03** | **8** | **K.DAGFYVVCAKNR.F** |
|  | 3109 | **678.5944** | **1355.1739** | **1354.5995** | **0.5744** | **1** | **4** | **9e+02** | **7** | **K.YVHRLLIPSTR.M** |
|  | 1685 | **454.5326** | **1360.5757** | **1359.5764** | **0.9993** | **2** | **8** | **5.5e+02** | **8** | **K.LFVEGRDVRIR.S** |
|  | 2051 | **486.3709** | **1456.0905** | **1456.5624** | **-0.4719** | **1** | **12** | **1.3e+02** | **2** | **R.EVNSTHWSRVNK.S** |
|  | 2239 | **505.8279** | **1514.4615** | **1514.7243** | **-0.2628** | **1** | **6** | **4.8e+02** | **5** | **K.AADPIDPPGPPAKIR.I** |
|  | 3338 | **759.0032** | **1515.9916** | **1516.7635** | **-0.7719** | **2** | **7** | **4.7e+02** | **4** | **K.LRMPYDVPEPRK.Y + Oxidation (M)** |
|  | 3371 | **777.9435** | **1553.8722** | **1553.8002** | **0.0720** | **0** | **0** | **2.7e+03** | **5** | **K.QKPDIVLYPEPVR.V** |
|  | 2433 | **530.4683** | **1588.3826** | **1588.8543** | **-0.4717** | **2** | **14** | **1.1e+02** | **4** | **R.SPIRMSPARMSPAR.M + 2 Oxidation (M)** |
|  | 2451 | **534.0782** | **1599.2126** | **1598.7995** | **0.4130** | **1** | **3** | **1.3e+03** | **8** | **K.KAEAVATVVAAVDQAR.V** |
|  | 2568 | **551.6765** | **1652.0074** | **1650.9386** | **1.0688** | **1** | **6** | **9e+02** | **6** | **K.MDKVLGSSIHMECK.V + Carbamidomethyl (C); Oxidation (M)** |
|  | 2592 | **555.6692** | **1663.9854** | **1663.8230** | **0.1623** | **1** | **4** | **1.2e+03** | **6** | **K.LEDAGEVQLTAKDFK.T** |
|  | 2720 | **584.4496** | **1750.3268** | **1750.9699** | **-0.6431** | **0** | **5** | **7e+02** | **5** | **K.VSNVAGGVECSANLFVK.E + Carbamidomethyl (C)** |
|  | 2815 | **598.0847** | **1791.2318** | **1790.0688** | **1.1629** | **1** | **7** | **5.3e+02** | **7** | **R.LKVTSLMEGCDYQFR.V** |
|  | 2889 | **611.8750** | **1832.6028** | **1831.9980** | **0.6048** | **1** | **11** | **1.9e+02** | **1** | **R.MAHEGALTGVTTDQKEK.Q + Oxidation (M)** |
|  | 2891 | **611.9609** | **1832.8605** | **1832.0228** | **0.8377** | **2** | **8** | **4.3e+02** | **6** | **R.RDVASAQWSPLSATSKK.K** |
|  | 2904 | **612.2870** | **1833.8390** | **1834.0172** | **-0.1782** | **1** | **2** | **1.5e+03** | **10** | **K.NAAGVISKGSESTGPVTCR.D** |
|  | 2912 | **612.7262** | **1835.1564** | **1834.1644** | **0.9921** | **1** | **8** | **5e+02** | **4** | **R.AGSNLKVDIPISGKPLPK.V** |
|  | 3077 | **668.4992** | **2002.4754** | **2003.3074** | **-0.8320** | **2** | **8** | **3.9e+02** | **4** | **K.YRFRVLAENLAGPGKPSK.S** |
|  | 3332 | **757.9138** | **2270.7193** | **2269.6172** | **1.1020** | **1** | **20** | **28** | **1** | **R.ESRPVIVKEQTMLPELDLR.G + Oxidation (M)** |
|  | 3346 | **761.1796** | **2280.5165** | **2279.5757** | **0.9409** | **2** | **7** | **5.2e+02** | **2** | **R.VFAENETGLSRPRRTAMSIK.T + Oxidation (M)** |
|  | 3357 | **766.8203** | **2297.4386** | **2297.5807** | **-0.1421** | **0** | **6** | **6.9e+02** | **7** | **K.AENIVGLGLPDTTIPIECQEK.L + Carbamidomethyl (C)** |

  


---

|  |  |
| --- | --- |
| **7.** | gi|119631418    **Mass:** 3881215  **Score:** 135    **Queries matched:** 69 |
|  | titin, isoform CRA\_a [Homo sapiens] |

|  |  |
| --- | --- |
|  | Check to include this hit in error tolerant search or archive report |
|  |  |

|  |  |  |  |  |  |  |  |  |  |  |
| --- | --- | --- | --- | --- | --- | --- | --- | --- | --- | --- |
|  | **Query** | **Observed** | **Mr(expt)** | **Mr(calc)** | **Delta** | **Miss** | **Score** | **Expect** | **Rank** | **Peptide** |
|  | 83 | 365.1657 | 728.3167 | 728.9203 | -0.6036 | 0 | (7) | 4.9e+02 | 3 | K.VLVAVTK.K |
|  | 86 | 365.4347 | 728.8546 | 728.9203 | -0.0657 | 0 | 11 | 2.5e+02 | 3 | K.VLVAVTK.K |
|  | 227 | 371.2714 | 740.5279 | 739.8601 | 0.6679 | 1 | 13 | 93 | 2 | K.KEAPPAK.V |
|  | 349 | 380.0574 | 758.1000 | 757.9198 | 0.1802 | 0 | 14 | 1.3e+02 | 3 | R.FLTLHK.V |
|  | 363 | 380.4083 | 758.8017 | 757.9198 | 0.8819 | 0 | (3) | 1.9e+03 | 2 | R.FLTLHK.V |
|  | 714 | 401.1246 | 800.2344 | 800.9429 | -0.7086 | 1 | 11 | 2.1e+02 | 5 | K.KGDQILK.Q |
|  | 716 | 401.2014 | 800.3881 | 800.9067 | -0.5185 | 2 | 13 | 1.2e+02 | 2 | K.DRKQVR.N |
|  | 1059 | 410.9979 | 819.9811 | 818.9817 | 0.9994 | 0 | 6 | 7.6e+02 | 2 | K.VPVTMTR.Y + Oxidation (M) |
|  | 1110 | 414.0691 | 826.1233 | 824.9646 | 1.1588 | 0 | 7 | 4.9e+02 | 4 | R.LHVETVK.I |
|  | 1620 | **448.5572** | **895.0996** | **896.0458** | **-0.9462** | **1** | **3** | **1.4e+03** | **10** | **K.KPEAPRAK.V** |
|  | 1672 | 452.6298 | 903.2449 | 903.0614 | 0.1834 | 1 | 9 | 3.4e+02 | 1 | R.QRIMAER.E |
|  | 1724 | 458.0725 | 914.1303 | 913.0696 | 1.0607 | 0 | 10 | 2.9e+02 | 5 | R.ITIENVPK.K |
|  | 1933 | 474.4376 | 946.8605 | 948.0342 | -1.1737 | 1 | 16 | 71 | 1 | K.VSWAKDSR.E |
|  | 1960 | 476.2941 | 950.5734 | 950.0468 | 0.5266 | 0 | 7 | 4.5e+02 | 4 | K.AAEPPSPPGK.V |
|  | 2031 | 484.1978 | 966.3809 | 967.1005 | -0.7196 | 0 | 1 | 2.2e+03 | 6 | K.VSECFVAR.D + Carbamidomethyl (C) |
|  | 2306 | 517.6474 | 1033.2800 | 1034.1879 | -0.9079 | 1 | 17 | 71 | 1 | K.KGDATQLACK.V |
|  | 2349 | 520.0552 | 1038.0956 | 1037.1074 | 0.9882 | 0 | 5 | 8.7e+02 | 9 | K.TAGPDCNFR.V + Carbamidomethyl (C) |
|  | 2360 | 520.9778 | 1039.9408 | 1040.1909 | -0.2501 | 0 | 8 | 3.7e+02 | 3 | K.AEFVCSISK.E + Carbamidomethyl (C) |
|  | 2375 | 522.0974 | 1042.1800 | 1043.1320 | -0.9519 | 0 | 5 | 9e+02 | 5 | R.KPVSEVGDGR.W |
|  | 2377 | 522.1971 | 1042.3794 | 1042.1670 | 0.2124 | 1 | 7 | 5.7e+02 | 8 | K.INKMYSDR.A + Oxidation (M) |
|  | 2473 | 536.1332 | 1070.2516 | 1070.1770 | 0.0745 | 1 | 4 | 1e+03 | 4 | K.LDDGTYRCK.V |
|  | 40 | 363.0770 | 1086.2087 | 1087.2077 | -0.9990 | 2 | 8 | 3.8e+02 | 8 | R.KYKMSSDGR.T + Oxidation (M) |
|  | 50 | 363.1646 | 1086.4718 | 1086.2012 | 0.2705 | 1 | 14 | 86 | 2 | K.KIEAHFDAR.S |
|  | 2533 | 547.4041 | 1092.7933 | 1093.2732 | -0.4799 | 0 | 2 | 1.2e+03 | 6 | K.YISSLEILR.T |
|  | 96 | 366.0843 | 1095.2307 | 1095.2761 | -0.0454 | 2 | 9 | 3.5e+02 | 7 | K.REAAMRAFK.T + Oxidation (M) |
|  | 100 | 366.1522 | 1095.4344 | 1095.2761 | 0.1583 | 2 | (9) | 3.6e+02 | 8 | K.REAAMRAFK.T + Oxidation (M) |
|  | 2565 | 551.1006 | 1100.1864 | 1101.3170 | -1.1306 | 0 | 8 | 4.3e+02 | 5 | K.DVLPGSAVCLK.S |
|  | 129 | 367.7991 | 1100.3752 | 1101.3170 | -0.9417 | 0 | (7) | 6.1e+02 | 4 | K.DVLPGSAVCLK.S |
|  | 190 | 369.3158 | 1104.9252 | 1105.2925 | -0.3673 | 1 | 6 | 5.9e+02 | 3 | R.MSPARMSPGR.R + Oxidation (M) |
|  | 314 | 377.1657 | 1128.4750 | 1128.2793 | 0.1956 | 1 | 10 | 2.6e+02 | 1 | K.LTSGEAPGIRK.E |
|  | 2656 | 566.2069 | 1130.3990 | 1130.3831 | 0.0160 | 1 | 15 | 87 | 5 | K.TPPRIPPKPK.S |
|  | 2683 | 574.3709 | 1146.7270 | 1147.3240 | -0.5970 | 1 | 9 | 3.4e+02 | 5 | R.YPAKLTLGER.E |
|  | 434 | 386.0396 | 1155.0967 | 1155.3000 | -0.2033 | 0 | 14 | 99 | 2 | R.VEAMGMSSEAK.L + Oxidation (M) |
|  | 544 | 388.8141 | 1163.4201 | 1163.2789 | 0.1412 | 2 | 6 | 8.4e+02 | 6 | K.ISETKKSDQK.T |
|  | 611 | 391.1789 | 1170.5145 | 1170.3658 | 0.1488 | 2 | 6 | 5.2e+02 | 6 | R.KGIVVRAGGSAR.I |
|  | 2760 | 592.7612 | 1183.5076 | 1183.3563 | 0.1513 | 1 | 5 | 9.1e+02 | 5 | R.EKLYPPSPPR.W |
|  | 683 | 399.8672 | 1196.5795 | 1197.4010 | -0.8216 | 0 | 3 | 1.1e+03 | 9 | K.VTGYLIEMQK.V + Oxidation (M) |
|  | 733 | 401.9340 | 1202.7799 | 1203.3757 | -0.5959 | 2 | 8 | 4.6e+02 | 3 | K.QRQRIMAER.E + Oxidation (M) |
|  | 865 | 406.0075 | 1215.0003 | 1215.3568 | -0.3565 | 1 | 6 | 5.9e+02 | 4 | K.DRVVIDNVGTK.S |
|  | 911 | 406.9037 | 1217.6891 | 1217.3892 | 0.2999 | 0 | 11 | 1.9e+02 | 7 | K.IELSPSMEAPK.I + Oxidation (M) |
|  | 959 | 407.5059 | 1219.4954 | 1219.4315 | 0.0639 | 0 | 10 | 2.8e+02 | 4 | K.VLGSSIHMECK.V + Oxidation (M) |
|  | 1082 | 412.4707 | 1234.3898 | 1233.4382 | 0.9517 | 2 | 2 | 2.1e+03 | 5 | K.ADMILKQDKR.I + Oxidation (M) |
|  | 2946 | 623.2170 | 1244.4193 | 1243.3717 | 1.0476 | 2 | 13 | 1.3e+02 | 2 | R.RDLPDGRWTK.A |
|  | 2989 | 637.4632 | 1272.9116 | 1273.4738 | -0.5622 | 0 | 25 | 7.1 | 3 | K.EIELDFAVPLK.D |
|  | 1409 | 433.4536 | 1297.3386 | 1297.5466 | -0.2080 | 2 | 8 | 4.5e+02 | 4 | K.GKTFVYLKWR.R |
|  | 1414 | 434.1722 | 1299.4944 | 1300.3765 | -0.8822 | 0 | 9 | 3.4e+02 | 6 | K.GEQTWSHAGISK.T |
|  | 1415 | 434.1731 | 1299.4971 | 1300.3765 | -0.8794 | 0 | (4) | 9.4e+02 | 10 | K.GEQTWSHAGISK.T |
|  | 1531 | 441.1255 | 1320.3543 | 1321.4357 | -1.0814 | 0 | 5 | 8.1e+02 | 8 | R.DTIVVNAGETFR.L |
|  | 1618 | 448.2582 | 1341.7524 | 1342.5227 | -0.7703 | 1 | 3 | 1.2e+03 | 8 | K.DAGFYVVCAKNR.F |
|  | 3109 | 678.5944 | 1355.1739 | 1354.5995 | 0.5744 | 1 | 4 | 9e+02 | 7 | K.YVHRLLIPSTR.M |
|  | 1685 | 454.5326 | 1360.5757 | 1359.5764 | 0.9993 | 2 | 8 | 5.5e+02 | 8 | K.LFVEGRDVRIR.S |
|  | 2051 | 486.3709 | 1456.0905 | 1456.5624 | -0.4719 | 1 | 12 | 1.3e+02 | 2 | R.EVNSTHWSRVNK.S |
|  | 2239 | 505.8279 | 1514.4615 | 1514.7243 | -0.2628 | 1 | 6 | 4.8e+02 | 5 | K.AADPIDPPGPPAKIR.I |
|  | 3338 | 759.0032 | 1515.9916 | 1516.7635 | -0.7719 | 2 | 7 | 4.7e+02 | 4 | K.LRMPYDVPEPRK.Y + Oxidation (M) |
|  | 3371 | 777.9435 | 1553.8722 | 1553.8002 | 0.0720 | 0 | 0 | 2.7e+03 | 5 | K.QKPDIVLYPEPVR.V |
|  | 2433 | 530.4683 | 1588.3826 | 1588.8543 | -0.4717 | 2 | 14 | 1.1e+02 | 4 | R.SPIRMSPARMSPAR.M + 2 Oxidation (M) |
|  | 2451 | 534.0782 | 1599.2126 | 1598.7995 | 0.4130 | 1 | 3 | 1.3e+03 | 8 | K.KAEAVATVVAAVDQAR.V |
|  | 2568 | 551.6765 | 1652.0074 | 1650.9386 | 1.0688 | 1 | 6 | 9e+02 | 6 | K.MDKVLGSSIHMECK.V + Carbamidomethyl (C); Oxidation (M) |
|  | 2592 | 555.6692 | 1663.9854 | 1663.8230 | 0.1623 | 1 | 4 | 1.2e+03 | 6 | K.LEDAGEVQLTAKDFK.T |
|  | 2720 | 584.4496 | 1750.3268 | 1750.9699 | -0.6431 | 0 | 5 | 7e+02 | 5 | K.VSNVAGGVECSANLFVK.E + Carbamidomethyl (C) |
|  | 2815 | 598.0847 | 1791.2318 | 1790.0688 | 1.1629 | 1 | 7 | 5.3e+02 | 7 | R.LKVTSLMEGCDYQFR.V |
|  | 2889 | 611.8750 | 1832.6028 | 1831.9980 | 0.6048 | 1 | 11 | 1.9e+02 | 1 | R.MAHEGALTGVTTDQKEK.Q + Oxidation (M) |
|  | 2891 | 611.9609 | 1832.8605 | 1832.0228 | 0.8377 | 2 | 8 | 4.3e+02 | 6 | R.RDVASAQWSPLSATSKK.K |
|  | 2904 | 612.2870 | 1833.8390 | 1834.0172 | -0.1782 | 1 | 2 | 1.5e+03 | 10 | K.NAAGVISKGSESTGPVTCR.D |
|  | 2912 | 612.7262 | 1835.1564 | 1834.1644 | 0.9921 | 1 | 8 | 5e+02 | 4 | R.AGSNLKVDIPISGKPLPK.V |
|  | 3077 | 668.4992 | 2002.4754 | 2003.3074 | -0.8320 | 2 | 8 | 3.9e+02 | 4 | K.YRFRVLAENLAGPGKPSK.S |
|  | 3332 | 757.9138 | 2270.7193 | 2269.6172 | 1.1020 | 1 | 20 | 28 | 1 | R.ESRPVIVKEQTMLPELDLR.G + Oxidation (M) |
|  | 3346 | 761.1796 | 2280.5165 | 2279.5757 | 0.9409 | 2 | 7 | 5.2e+02 | 2 | R.VFAENETGLSRPRRTAMSIK.T + Oxidation (M) |
|  | 3357 | 766.8203 | 2297.4386 | 2297.5807 | -0.1421 | 0 | 6 | 6.9e+02 | 7 | K.AENIVGLGLPDTTIPIECQEK.L + Carbamidomethyl (C) |

  


---

|  |  |
| --- | --- |
| **8.** | gi|825671    **Mass:** 30938    **Score:** 128    **Queries matched:** 6   **emPAI:** 0.36 |
|  | B23 nucleophosmin (280 AA) [Homo sapiens] |

|  |  |
| --- | --- |
|  | Check to include this hit in error tolerant search or archive report |
|  |  |

|  |  |  |  |  |  |  |  |  |  |  |
| --- | --- | --- | --- | --- | --- | --- | --- | --- | --- | --- |
|  | **Query** | **Observed** | **Mr(expt)** | **Mr(calc)** | **Delta** | **Miss** | **Score** | **Expect** | **Rank** | **Peptide** |
|  | 1490 | **437.3590** | **872.7032** | **873.0519** | **-0.3487** | **1** | **5** | **7e+02** | **4** | **K.LLSISGKR.S** |
|  | 1855 | **466.4974** | **930.9801** | **930.9988** | **-0.0187** | **0** | **43** | **0.15** | **1** | **K.GPSSVEDIK.A** |
|  | 2655 | **566.1166** | **1130.2185** | **1130.2490** | **-0.0305** | **1** | **63** | **0.0013** | **1** | **K.GPSSVEDIKAK.M** |
|  | 326 | **377.9738** | **1130.8994** | **1130.2490** | **0.6504** | **1** | **(41)** | **0.17** | **1** | **K.GPSSVEDIKAK.M** |
|  | 327 | **377.9848** | **1130.9322** | **1130.2490** | **0.6833** | **1** | **(13)** | **1.1e+02** | **4** | **K.GPSSVEDIKAK.M** |
|  | 1226 | **420.2867** | **1257.8378** | **1257.3902** | **0.4476** | **1** | **16** | **50** | **3** | **K.TPKGPSSVEDIK.A** |

  

|  |  |
| --- | --- |
|  | |
|  | **Proteins matching the same set of peptides:** |

|  |  |
| --- | --- |
|  | gi|7020584    **Mass:** 19372    **Score:** 128    **Queries matched:** 6 |
|  | unnamed protein product [Homo sapiens] |

|  |  |
| --- | --- |
|  | gi|10835063    **Mass:** 32575    **Score:** 128    **Queries matched:** 6 |
|  | nucleophosmin isoform 1 [Homo sapiens] |

|  |  |
| --- | --- |
|  | gi|15214852    **Mass:** 32609    **Score:** 128    **Queries matched:** 6 |
|  | Nucleophosmin (nucleolar phosphoprotein B23, numatrin) [Homo sapiens] |

|  |  |
| --- | --- |
|  | gi|16876992    **Mass:** 32457    **Score:** 128    **Queries matched:** 6 |
|  | Nucleophosmin (nucleolar phosphoprotein B23, numatrin) [Homo sapiens] |

|  |  |
| --- | --- |
|  | gi|18314408    **Mass:** 32576    **Score:** 128    **Queries matched:** 6 |
|  | Nucleophosmin (nucleolar phosphoprotein B23, numatrin) [Homo sapiens] |

|  |  |
| --- | --- |
|  | gi|33694244    **Mass:** 32603    **Score:** 128    **Queries matched:** 6 |
|  | nucleophosmin [Homo sapiens] |

|  |  |
| --- | --- |
|  | gi|40353734    **Mass:** 29464    **Score:** 128    **Queries matched:** 6 |
|  | nucleophosmin isoform 2 [Homo sapiens] |

|  |  |
| --- | --- |
|  | gi|58220457    **Mass:** 32818    **Score:** 128    **Queries matched:** 6 |
|  | nucleophosmin [Homo sapiens] |

|  |  |
| --- | --- |
|  | gi|58220459    **Mass:** 32836    **Score:** 128    **Queries matched:** 6 |
|  | nucleophosmin [Homo sapiens] |

|  |  |
| --- | --- |
|  | gi|58220461    **Mass:** 32804    **Score:** 128    **Queries matched:** 6 |
|  | nucleophosmin [Homo sapiens] |

|  |  |
| --- | --- |
|  | gi|58220465    **Mass:** 32887    **Score:** 128    **Queries matched:** 6 |
|  | nucleophosmin [Homo sapiens] |

|  |  |
| --- | --- |
|  | gi|58220467    **Mass:** 32945    **Score:** 128    **Queries matched:** 6 |
|  | nucleophosmin [Homo sapiens] |

|  |  |
| --- | --- |
|  | gi|58220469    **Mass:** 32460    **Score:** 128    **Queries matched:** 6 |
|  | nucleophosmin [Homo sapiens] |

|  |  |
| --- | --- |
|  | gi|62913985    **Mass:** 25048    **Score:** 128    **Queries matched:** 6 |
|  | NPM1 protein [Homo sapiens] |

|  |  |
| --- | --- |
|  | gi|83641870    **Mass:** 28400    **Score:** 128    **Queries matched:** 6 |
|  | nucleophosmin isoform 3 [Homo sapiens] |

|  |  |
| --- | --- |
|  | gi|119581852    **Mass:** 31842    **Score:** 128    **Queries matched:** 6 |
|  | nucleophosmin (nucleolar phosphoprotein B23, numatrin), isoform CRA\_e [Homo sapiens] |

|  |  |
| --- | --- |
|  | gi|145904604    **Mass:** 29978    **Score:** 128    **Queries matched:** 6 |
|  | truncated nucleolar phosphoprotein B23 [Homo sapiens] |

|  |  |
| --- | --- |
|  | gi|197692173    **Mass:** 32574    **Score:** 128    **Queries matched:** 6 |
|  | nucleophosmin 1 isoform 1 [Homo sapiens] |

---

|  |  |
| --- | --- |
| **9.** | gi|17066105    **Mass:** 3816172  **Score:** 125    **Queries matched:** 66 |
|  | Titin [Homo sapiens] |

|  |  |
| --- | --- |
|  | Check to include this hit in error tolerant search or archive report |
|  |  |

|  |  |  |  |  |  |  |  |  |  |  |
| --- | --- | --- | --- | --- | --- | --- | --- | --- | --- | --- |
|  | **Query** | **Observed** | **Mr(expt)** | **Mr(calc)** | **Delta** | **Miss** | **Score** | **Expect** | **Rank** | **Peptide** |
|  | 83 | 365.1657 | 728.3167 | 728.9203 | -0.6036 | 0 | (7) | 4.9e+02 | 3 | K.VLVAVTK.K |
|  | 86 | 365.4347 | 728.8546 | 728.9203 | -0.0657 | 0 | 11 | 2.5e+02 | 3 | K.VLVAVTK.K |
|  | 227 | 371.2714 | 740.5279 | 739.8601 | 0.6679 | 1 | 13 | 93 | 2 | K.KEAPPAK.V |
|  | 714 | 401.1246 | 800.2344 | 800.9429 | -0.7086 | 1 | 11 | 2.1e+02 | 5 | K.KGDQILK.Q |
|  | 716 | 401.2014 | 800.3881 | 800.9067 | -0.5185 | 2 | 13 | 1.2e+02 | 2 | K.DRKQVR.N |
|  | 1059 | 410.9979 | 819.9811 | 818.9817 | 0.9994 | 0 | 6 | 7.6e+02 | 2 | K.VPVTMTR.Y + Oxidation (M) |
|  | 1110 | 414.0691 | 826.1233 | 824.9646 | 1.1588 | 0 | 7 | 4.9e+02 | 4 | R.LHVETVK.I |
|  | 1672 | 452.6298 | 903.2449 | 903.0614 | 0.1834 | 1 | 9 | 3.4e+02 | 1 | R.QRIMAER.E |
|  | 1724 | 458.0725 | 914.1303 | 913.0696 | 1.0607 | 0 | 10 | 2.9e+02 | 5 | R.ITIENVPK.K |
|  | 1933 | 474.4376 | 946.8605 | 948.0342 | -1.1737 | 1 | 16 | 71 | 1 | K.VSWAKDSR.E |
|  | 1960 | 476.2941 | 950.5734 | 950.0468 | 0.5266 | 0 | 7 | 4.5e+02 | 4 | K.AAEPPSPPGK.V |
|  | 2031 | 484.1978 | 966.3809 | 967.1005 | -0.7196 | 0 | 1 | 2.2e+03 | 6 | K.VSECFVAR.D + Carbamidomethyl (C) |
|  | 2306 | 517.6474 | 1033.2800 | 1034.1879 | -0.9079 | 1 | 17 | 71 | 1 | K.KGDATQLACK.V |
|  | 2349 | 520.0552 | 1038.0956 | 1037.1074 | 0.9882 | 0 | 5 | 8.7e+02 | 9 | K.TAGPDCNFR.V + Carbamidomethyl (C) |
|  | 2360 | 520.9778 | 1039.9408 | 1040.1909 | -0.2501 | 0 | 8 | 3.7e+02 | 3 | K.AEFVCSISK.E + Carbamidomethyl (C) |
|  | 2375 | 522.0974 | 1042.1800 | 1043.1320 | -0.9519 | 0 | 5 | 9e+02 | 5 | R.KPVSEVGDGR.W |
|  | 2377 | 522.1971 | 1042.3794 | 1042.1670 | 0.2124 | 1 | 7 | 5.7e+02 | 8 | K.INKMYSDR.A + Oxidation (M) |
|  | 2473 | 536.1332 | 1070.2516 | 1070.1770 | 0.0745 | 1 | 4 | 1e+03 | 4 | K.LDDGTYRCK.V |
|  | 40 | 363.0770 | 1086.2087 | 1087.2077 | -0.9990 | 2 | 8 | 3.8e+02 | 8 | R.KYKMSSDGR.T + Oxidation (M) |
|  | 50 | 363.1646 | 1086.4718 | 1086.2012 | 0.2705 | 1 | 14 | 86 | 2 | K.KIEAHFDAR.S |
|  | 2533 | 547.4041 | 1092.7933 | 1093.2732 | -0.4799 | 0 | 2 | 1.2e+03 | 6 | K.YISSLEILR.T |
|  | 96 | 366.0843 | 1095.2307 | 1095.2761 | -0.0454 | 2 | 9 | 3.5e+02 | 7 | K.REAAMRAFK.T + Oxidation (M) |
|  | 100 | 366.1522 | 1095.4344 | 1095.2761 | 0.1583 | 2 | (9) | 3.6e+02 | 8 | K.REAAMRAFK.T + Oxidation (M) |
|  | 2565 | 551.1006 | 1100.1864 | 1101.3170 | -1.1306 | 0 | 8 | 4.3e+02 | 5 | K.DVLPGSAVCLK.S |
|  | 129 | 367.7991 | 1100.3752 | 1101.3170 | -0.9417 | 0 | (7) | 6.1e+02 | 4 | K.DVLPGSAVCLK.S |
|  | 190 | 369.3158 | 1104.9252 | 1105.2925 | -0.3673 | 1 | 6 | 5.9e+02 | 3 | R.MSPARMSPGR.R + Oxidation (M) |
|  | 314 | 377.1657 | 1128.4750 | 1128.2793 | 0.1956 | 1 | 10 | 2.6e+02 | 1 | K.LTSGEAPGIRK.E |
|  | 2683 | 574.3709 | 1146.7270 | 1147.3240 | -0.5970 | 1 | 9 | 3.4e+02 | 5 | R.YPAKLTLGER.E |
|  | 434 | 386.0396 | 1155.0967 | 1155.3000 | -0.2033 | 0 | 14 | 99 | 2 | R.VEAMGMSSEAK.L + Oxidation (M) |
|  | 544 | 388.8141 | 1163.4201 | 1163.2789 | 0.1412 | 2 | 6 | 8.4e+02 | 6 | K.ISETKKSDQK.T |
|  | 611 | 391.1789 | 1170.5145 | 1170.3658 | 0.1488 | 2 | 6 | 5.2e+02 | 6 | R.KGIVVRAGGSAR.I |
|  | 2760 | 592.7612 | 1183.5076 | 1183.3563 | 0.1513 | 1 | 5 | 9.1e+02 | 5 | R.EKLYPPSPPR.W |
|  | 683 | 399.8672 | 1196.5795 | 1197.4010 | -0.8216 | 0 | 3 | 1.1e+03 | 9 | K.VTGYLIEMQK.V + Oxidation (M) |
|  | 733 | 401.9340 | 1202.7799 | 1203.3757 | -0.5959 | 2 | 8 | 4.6e+02 | 3 | K.QRQRIMAER.E + Oxidation (M) |
|  | 865 | 406.0075 | 1215.0003 | 1215.3568 | -0.3565 | 1 | 6 | 5.9e+02 | 4 | K.DRVVIDNVGTK.S |
|  | 911 | 406.9037 | 1217.6891 | 1217.3892 | 0.2999 | 0 | 11 | 1.9e+02 | 7 | K.IELSPSMEAPK.I + Oxidation (M) |
|  | 959 | 407.5059 | 1219.4954 | 1219.4315 | 0.0639 | 0 | 10 | 2.8e+02 | 4 | K.VLGSSIHMECK.V + Oxidation (M) |
|  | 1082 | 412.4707 | 1234.3898 | 1233.4382 | 0.9517 | 2 | 2 | 2.1e+03 | 5 | K.ADMILKQDKR.I + Oxidation (M) |
|  | 2946 | 623.2170 | 1244.4193 | 1243.3717 | 1.0476 | 2 | 13 | 1.3e+02 | 2 | R.RDLPDGRWTK.A |
|  | 2989 | 637.4632 | 1272.9116 | 1273.4738 | -0.5622 | 0 | 25 | 7.1 | 3 | K.EIELDFAVPLK.D |
|  | 1409 | 433.4536 | 1297.3386 | 1297.5466 | -0.2080 | 2 | 8 | 4.5e+02 | 4 | K.GKTFVYLKWR.R |
|  | 1414 | 434.1722 | 1299.4944 | 1300.3765 | -0.8822 | 0 | 9 | 3.4e+02 | 6 | K.GEQTWSHAGISK.T |
|  | 1415 | 434.1731 | 1299.4971 | 1300.3765 | -0.8794 | 0 | (4) | 9.4e+02 | 10 | K.GEQTWSHAGISK.T |
|  | 1531 | 441.1255 | 1320.3543 | 1321.4357 | -1.0814 | 0 | 5 | 8.1e+02 | 8 | R.DTIVVNAGETFR.L |
|  | 1618 | 448.2582 | 1341.7524 | 1342.5227 | -0.7703 | 1 | 3 | 1.2e+03 | 8 | K.DAGFYVVCAKNR.F |
|  | 3109 | 678.5944 | 1355.1739 | 1354.5995 | 0.5744 | 1 | 4 | 9e+02 | 7 | K.YVHRLLIPSTR.M |
|  | 1685 | 454.5326 | 1360.5757 | 1359.5764 | 0.9993 | 2 | 8 | 5.5e+02 | 8 | K.LFVEGRDVRIR.S |
|  | 2051 | 486.3709 | 1456.0905 | 1456.5624 | -0.4719 | 1 | 12 | 1.3e+02 | 2 | R.EVNSTHWSRVNK.S |
|  | 2239 | 505.8279 | 1514.4615 | 1514.7243 | -0.2628 | 1 | 6 | 4.8e+02 | 5 | K.AADPIDPPGPPAKIR.I |
|  | 3338 | 759.0032 | 1515.9916 | 1516.7635 | -0.7719 | 2 | 7 | 4.7e+02 | 4 | K.LRMPYDVPEPRK.Y + Oxidation (M) |
|  | 3371 | 777.9435 | 1553.8722 | 1553.8002 | 0.0720 | 0 | 0 | 2.7e+03 | 5 | K.QKPDIVLYPEPVR.V |
|  | 2433 | 530.4683 | 1588.3826 | 1588.8543 | -0.4717 | 2 | 14 | 1.1e+02 | 4 | R.SPIRMSPARMSPAR.M + 2 Oxidation (M) |
|  | 2451 | 534.0782 | 1599.2126 | 1598.7995 | 0.4130 | 1 | 3 | 1.3e+03 | 8 | K.KAEAVATVVAAVDQAR.V |
|  | 2568 | 551.6765 | 1652.0074 | 1650.9386 | 1.0688 | 1 | 6 | 9e+02 | 6 | K.MDKVLGSSIHMECK.V + Carbamidomethyl (C); Oxidation (M) |
|  | 2592 | 555.6692 | 1663.9854 | 1663.8230 | 0.1623 | 1 | 4 | 1.2e+03 | 6 | K.LEDAGEVQLTAKDFK.T |
|  | 2720 | 584.4496 | 1750.3268 | 1750.9699 | -0.6431 | 0 | 5 | 7e+02 | 5 | K.VSNVAGGVECSANLFVK.E + Carbamidomethyl (C) |
|  | 2815 | 598.0847 | 1791.2318 | 1790.0688 | 1.1629 | 1 | 7 | 5.3e+02 | 7 | R.LKVTSLMEGCDYQFR.V |
|  | 2889 | 611.8750 | 1832.6028 | 1831.9980 | 0.6048 | 1 | 11 | 1.9e+02 | 1 | R.MAHEGALTGVTTDQKEK.Q + Oxidation (M) |
|  | 2891 | 611.9609 | 1832.8605 | 1832.0228 | 0.8377 | 2 | 8 | 4.3e+02 | 6 | R.RDVASAQWSPLSATSKK.K |
|  | 2904 | 612.2870 | 1833.8390 | 1834.0172 | -0.1782 | 1 | 2 | 1.5e+03 | 10 | K.NAAGVISKGSESTGPVTCR.D |
|  | 2912 | 612.7262 | 1835.1564 | 1834.1644 | 0.9921 | 1 | 8 | 5e+02 | 4 | R.AGSNLKVDIPISGKPLPK.V |
|  | 3077 | 668.4992 | 2002.4754 | 2003.3074 | -0.8320 | 2 | 8 | 3.9e+02 | 4 | K.YRFRVLAENLAGPGKPSK.S |
|  | 3332 | 757.9138 | 2270.7193 | 2269.6172 | 1.1020 | 1 | 20 | 28 | 1 | R.ESRPVIVKEQTMLPELDLR.G + Oxidation (M) |
|  | 3346 | 761.1796 | 2280.5165 | 2279.5757 | 0.9409 | 2 | 7 | 5.2e+02 | 2 | R.VFAENETGLSRPRRTAMSIK.T + Oxidation (M) |
|  | 3357 | 766.8203 | 2297.4386 | 2297.5807 | -0.1421 | 0 | 6 | 6.9e+02 | 7 | K.AENIVGLGLPDTTIPIECQEK.L + Carbamidomethyl (C) |
|  | 3469 | **850.6427** | **2548.9059** | **2547.8780** | **1.0280** | **1** | **1** | **1.9e+03** | **10** | **K.FRVSAVNIAGIGEPGEVTDVIEMK.D + Oxidation (M)** |

  

|  |  |
| --- | --- |
|  | |
|  | **Proteins matching the same set of peptides:** |

|  |  |
| --- | --- |
|  | gi|108861911    **Mass:** 3816142  **Score:** 125    **Queries matched:** 66 |
|  | RecName: Full=Titin; AltName: Full=Connectin; AltName: Full=Rhabdomyosarcoma antigen MU-RMS-40.14 |

---

|  |  |
| --- | --- |
| **10.** | gi|1212992    **Mass:** 2993415  **Score:** 123    **Queries matched:** 60 |
|  | titin [Homo sapiens] |

|  |  |
| --- | --- |
|  | Check to include this hit in error tolerant search or archive report |
|  |  |

|  |  |  |  |  |  |  |  |  |  |  |
| --- | --- | --- | --- | --- | --- | --- | --- | --- | --- | --- |
|  | **Query** | **Observed** | **Mr(expt)** | **Mr(calc)** | **Delta** | **Miss** | **Score** | **Expect** | **Rank** | **Peptide** |
|  | 349 | 380.0574 | 758.1000 | 757.9198 | 0.1802 | 0 | 14 | 1.3e+02 | 3 | R.FLTLHK.V |
|  | 353 | **380.1512** | **758.2877** | **758.8633** | **-0.5755** | **0** | **3** | **1.4e+03** | **5** | **K.QLVGTNK.W** |
|  | 363 | 380.4083 | 758.8017 | 757.9198 | 0.8819 | 0 | (3) | 1.9e+03 | 2 | R.FLTLHK.V |
|  | 714 | 401.1246 | 800.2344 | 800.9429 | -0.7086 | 1 | 11 | 2.1e+02 | 5 | K.KGDQILK.Q |
|  | 1059 | 410.9979 | 819.9811 | 818.9817 | 0.9994 | 0 | 6 | 7.6e+02 | 2 | K.VPVTMTR.Y + Oxidation (M) |
|  | 1110 | 414.0691 | 826.1233 | 824.9646 | 1.1588 | 0 | 7 | 4.9e+02 | 4 | R.LHVETVK.I |
|  | 1672 | 452.6298 | 903.2449 | 903.0614 | 0.1834 | 1 | 9 | 3.4e+02 | 1 | R.QRIMAER.E |
|  | 1724 | 458.0725 | 914.1303 | 913.0696 | 1.0607 | 0 | 10 | 2.9e+02 | 5 | R.ITIENVPK.K |
|  | 1960 | 476.2941 | 950.5734 | 950.0468 | 0.5266 | 0 | 7 | 4.5e+02 | 4 | K.AAEPPSPPGK.V |
|  | 2031 | 484.1978 | 966.3809 | 967.1005 | -0.7196 | 0 | 1 | 2.2e+03 | 6 | K.VSECFVAR.D + Carbamidomethyl (C) |
|  | 2349 | 520.0552 | 1038.0956 | 1037.1074 | 0.9882 | 0 | 5 | 8.7e+02 | 9 | K.TAGPDCNFR.V + Carbamidomethyl (C) |
|  | 2360 | 520.9778 | 1039.9408 | 1040.1909 | -0.2501 | 0 | 8 | 3.7e+02 | 3 | K.AEFVCSISK.E + Carbamidomethyl (C) |
|  | 2375 | 522.0974 | 1042.1800 | 1043.1320 | -0.9519 | 0 | 5 | 9e+02 | 5 | R.KPVSEVGDGR.W |
|  | 2377 | 522.1971 | 1042.3794 | 1042.1670 | 0.2124 | 1 | 7 | 5.7e+02 | 8 | K.INKMYSDR.A + Oxidation (M) |
|  | 2473 | 536.1332 | 1070.2516 | 1070.1770 | 0.0745 | 1 | 4 | 1e+03 | 4 | K.LDDGTYRCK.V |
|  | 40 | 363.0770 | 1086.2087 | 1087.2077 | -0.9990 | 2 | 8 | 3.8e+02 | 8 | R.KYKMSSDGR.T + Oxidation (M) |
|  | 50 | 363.1646 | 1086.4718 | 1086.2012 | 0.2705 | 1 | 14 | 86 | 2 | K.KIEAHFDAR.S |
|  | 2527 | **546.7433** | **1091.4718** | **1092.2273** | **-0.7556** | **0** | **3** | **1.3e+03** | **9** | **R.NAMASASATIR.V** |
|  | 2533 | 547.4041 | 1092.7933 | 1093.2732 | -0.4799 | 0 | 2 | 1.2e+03 | 6 | K.YISSLEILR.T |
|  | 96 | 366.0843 | 1095.2307 | 1095.2761 | -0.0454 | 2 | 9 | 3.5e+02 | 7 | K.REAAMRAFK.T + Oxidation (M) |
|  | 100 | 366.1522 | 1095.4344 | 1095.2761 | 0.1583 | 2 | (9) | 3.6e+02 | 8 | K.REAAMRAFK.T + Oxidation (M) |
|  | 190 | 369.3158 | 1104.9252 | 1105.2925 | -0.3673 | 1 | 6 | 5.9e+02 | 3 | R.MSPARMSPGR.R + Oxidation (M) |
|  | 314 | 377.1657 | 1128.4750 | 1128.2793 | 0.1956 | 1 | 10 | 2.6e+02 | 1 | K.LTSGEAPGIRK.E |
|  | 2656 | 566.2069 | 1130.3990 | 1130.3831 | 0.0160 | 1 | 15 | 87 | 5 | K.TPPRIPPKPK.S |
|  | 2683 | 574.3709 | 1146.7270 | 1147.3240 | -0.5970 | 1 | 9 | 3.4e+02 | 5 | R.YPAKLTLGER.E |
|  | 434 | 386.0396 | 1155.0967 | 1155.3000 | -0.2033 | 0 | 14 | 99 | 2 | R.VEAMGMSSEAK.L + Oxidation (M) |
|  | 544 | 388.8141 | 1163.4201 | 1163.2789 | 0.1412 | 2 | 6 | 8.4e+02 | 6 | K.ISETKKSDQK.T |
|  | 611 | 391.1789 | 1170.5145 | 1170.3658 | 0.1488 | 2 | 6 | 5.2e+02 | 6 | R.KGIVVRAGGSAR.I |
|  | 2760 | 592.7612 | 1183.5076 | 1183.3563 | 0.1513 | 1 | 5 | 9.1e+02 | 5 | R.EKLYPPSPPR.W |
|  | 683 | 399.8672 | 1196.5795 | 1197.4010 | -0.8216 | 0 | 3 | 1.1e+03 | 9 | K.VTGYLIEMQK.V + Oxidation (M) |
|  | 733 | 401.9340 | 1202.7799 | 1203.3757 | -0.5959 | 2 | 8 | 4.6e+02 | 3 | K.QRQRIMAER.E + Oxidation (M) |
|  | 865 | 406.0075 | 1215.0003 | 1215.3568 | -0.3565 | 1 | 6 | 5.9e+02 | 4 | K.DRVVIDNVGTK.S |
|  | 911 | 406.9037 | 1217.6891 | 1217.3892 | 0.2999 | 0 | 11 | 1.9e+02 | 7 | K.IELSPSMEAPK.I + Oxidation (M) |
|  | 1082 | 412.4707 | 1234.3898 | 1233.4382 | 0.9517 | 2 | 2 | 2.1e+03 | 5 | K.ADMILKQDKR.I + Oxidation (M) |
|  | 2946 | 623.2170 | 1244.4193 | 1243.3717 | 1.0476 | 2 | 13 | 1.3e+02 | 2 | R.RDLPDGRWTK.A |
|  | 2989 | 637.4632 | 1272.9116 | 1273.4738 | -0.5622 | 0 | 25 | 7.1 | 3 | K.EIELDFAVPLK.D |
|  | 1409 | 433.4536 | 1297.3386 | 1297.5466 | -0.2080 | 2 | 8 | 4.5e+02 | 4 | K.GKTFVYLKWR.R |
|  | 1414 | 434.1722 | 1299.4944 | 1300.3765 | -0.8822 | 0 | 9 | 3.4e+02 | 6 | K.GEQTWSHAGISK.T |
|  | 1415 | 434.1731 | 1299.4971 | 1300.3765 | -0.8794 | 0 | (4) | 9.4e+02 | 10 | K.GEQTWSHAGISK.T |
|  | 1531 | 441.1255 | 1320.3543 | 1321.4357 | -1.0814 | 0 | 5 | 8.1e+02 | 8 | R.DTIVVNAGETFR.L |
|  | 1618 | 448.2582 | 1341.7524 | 1342.5227 | -0.7703 | 1 | 3 | 1.2e+03 | 8 | K.DAGFYVVCAKNR.F |
|  | 3109 | 678.5944 | 1355.1739 | 1354.5995 | 0.5744 | 1 | 4 | 9e+02 | 7 | K.YVHRLLIPSTR.M |
|  | 1685 | 454.5326 | 1360.5757 | 1359.5764 | 0.9993 | 2 | 8 | 5.5e+02 | 8 | K.LFVEGRDVRIR.S |
|  | 2051 | 486.3709 | 1456.0905 | 1456.5624 | -0.4719 | 1 | 12 | 1.3e+02 | 2 | R.EVNSTHWSRVNK.S |
|  | 2239 | 505.8279 | 1514.4615 | 1514.7243 | -0.2628 | 1 | 6 | 4.8e+02 | 5 | K.AADPIDPPGPPAKIR.I |
|  | 3338 | 759.0032 | 1515.9916 | 1516.7635 | -0.7719 | 2 | 7 | 4.7e+02 | 4 | K.LRMPYDVPEPRK.Y + Oxidation (M) |
|  | 3371 | 777.9435 | 1553.8722 | 1553.8002 | 0.0720 | 0 | 0 | 2.7e+03 | 5 | K.QKPDIVLYPEPVR.V |
|  | 2433 | 530.4683 | 1588.3826 | 1588.8543 | -0.4717 | 2 | 14 | 1.1e+02 | 4 | R.SPIRMSPARMSPAR.M + 2 Oxidation (M) |
|  | 2451 | 534.0782 | 1599.2126 | 1598.7995 | 0.4130 | 1 | 3 | 1.3e+03 | 8 | K.KAEAVATVVAAVDQAR.V |
|  | 2592 | 555.6692 | 1663.9854 | 1663.8230 | 0.1623 | 1 | 4 | 1.2e+03 | 6 | K.LEDAGEVQLTAKDFK.T |
|  | 2815 | 598.0847 | 1791.2318 | 1790.0688 | 1.1629 | 1 | 7 | 5.3e+02 | 7 | R.LKVTSLMEGCDYQFR.V |
|  | 2889 | 611.8750 | 1832.6028 | 1831.9980 | 0.6048 | 1 | 11 | 1.9e+02 | 1 | R.MAHEGALTGVTTDQKEK.Q + Oxidation (M) |
|  | 2891 | 611.9609 | 1832.8605 | 1832.0228 | 0.8377 | 2 | 8 | 4.3e+02 | 6 | R.RDVASAQWSPLSATSKK.K |
|  | 2904 | 612.2870 | 1833.8390 | 1834.0172 | -0.1782 | 1 | 2 | 1.5e+03 | 10 | K.NAAGVISKGSESTGPVTCR.D |
|  | 2912 | 612.7262 | 1835.1564 | 1834.1644 | 0.9921 | 1 | 8 | 5e+02 | 4 | R.AGSNLKVDIPISGKPLPK.V |
|  | 3077 | 668.4992 | 2002.4754 | 2003.3074 | -0.8320 | 2 | 8 | 3.9e+02 | 4 | K.YRFRVLAENLAGPGKPSK.S |
|  | 3332 | 757.9138 | 2270.7193 | 2269.6172 | 1.1020 | 1 | 20 | 28 | 1 | R.ESRPVIVKEQTMLPELDLR.G + Oxidation (M) |
|  | 3346 | 761.1796 | 2280.5165 | 2279.5757 | 0.9409 | 2 | 7 | 5.2e+02 | 2 | R.VFAENETGLSRPRRTAMSIK.T + Oxidation (M) |
|  | 3357 | 766.8203 | 2297.4386 | 2297.5807 | -0.1421 | 0 | 6 | 6.9e+02 | 7 | K.AENIVGLGLPDTTIPIECQEK.L + Carbamidomethyl (C) |
|  | 3469 | 850.6427 | 2548.9059 | 2547.8780 | 1.0280 | 1 | 1 | 1.9e+03 | 10 | K.FRVSAVNIAGIGEPGEVTDVIEMK.D + Oxidation (M) |

  


---

|  |  |
| --- | --- |
| **11.** | gi|119631424    **Mass:** 3014062  **Score:** 120    **Queries matched:** 58 |
|  | titin, isoform CRA\_e [Homo sapiens] |

|  |  |
| --- | --- |
|  | Check to include this hit in error tolerant search or archive report |
|  |  |

|  |  |  |  |  |  |  |  |  |  |  |
| --- | --- | --- | --- | --- | --- | --- | --- | --- | --- | --- |
|  | **Query** | **Observed** | **Mr(expt)** | **Mr(calc)** | **Delta** | **Miss** | **Score** | **Expect** | **Rank** | **Peptide** |
|  | 349 | 380.0574 | 758.1000 | 757.9198 | 0.1802 | 0 | 14 | 1.3e+02 | 3 | R.FLTLHK.V |
|  | 363 | 380.4083 | 758.8017 | 757.9198 | 0.8819 | 0 | (3) | 1.9e+03 | 2 | R.FLTLHK.V |
|  | 714 | 401.1246 | 800.2344 | 800.9429 | -0.7086 | 1 | 11 | 2.1e+02 | 5 | K.KGDQILK.Q |
|  | 1059 | 410.9979 | 819.9811 | 818.9817 | 0.9994 | 0 | 6 | 7.6e+02 | 2 | K.VPVTMTR.Y + Oxidation (M) |
|  | 1110 | 414.0691 | 826.1233 | 824.9646 | 1.1588 | 0 | 7 | 4.9e+02 | 4 | R.LHVETVK.I |
|  | 1672 | 452.6298 | 903.2449 | 903.0614 | 0.1834 | 1 | 9 | 3.4e+02 | 1 | R.QRIMAER.E |
|  | 1724 | 458.0725 | 914.1303 | 913.0696 | 1.0607 | 0 | 10 | 2.9e+02 | 5 | R.ITIENVPK.K |
|  | 1960 | 476.2941 | 950.5734 | 950.0468 | 0.5266 | 0 | 7 | 4.5e+02 | 4 | K.AAEPPSPPGK.V |
|  | 2031 | 484.1978 | 966.3809 | 967.1005 | -0.7196 | 0 | 1 | 2.2e+03 | 6 | K.VSECFVAR.D + Carbamidomethyl (C) |
|  | 2209 | **502.3960** | **1002.7772** | **1003.1077** | **-0.3306** | **1** | **6** | **6e+02** | **9** | **K.IEESERLK.Q** |
|  | 2349 | 520.0552 | 1038.0956 | 1037.1074 | 0.9882 | 0 | 5 | 8.7e+02 | 9 | K.TAGPDCNFR.V + Carbamidomethyl (C) |
|  | 2360 | 520.9778 | 1039.9408 | 1040.1909 | -0.2501 | 0 | 8 | 3.7e+02 | 3 | K.AEFVCSISK.E + Carbamidomethyl (C) |
|  | 2375 | 522.0974 | 1042.1800 | 1043.1320 | -0.9519 | 0 | 5 | 9e+02 | 5 | R.KPVSEVGDGR.W |
|  | 2377 | 522.1971 | 1042.3794 | 1042.1670 | 0.2124 | 1 | 7 | 5.7e+02 | 8 | K.INKMYSDR.A + Oxidation (M) |
|  | 2473 | 536.1332 | 1070.2516 | 1070.1770 | 0.0745 | 1 | 4 | 1e+03 | 4 | K.LDDGTYRCK.V |
|  | 40 | 363.0770 | 1086.2087 | 1087.2077 | -0.9990 | 2 | 8 | 3.8e+02 | 8 | R.KYKMSSDGR.T + Oxidation (M) |
|  | 50 | 363.1646 | 1086.4718 | 1086.2012 | 0.2705 | 1 | 14 | 86 | 2 | K.KIEAHFDAR.S |
|  | 2533 | 547.4041 | 1092.7933 | 1093.2732 | -0.4799 | 0 | 2 | 1.2e+03 | 6 | K.YISSLEILR.T |
|  | 96 | 366.0843 | 1095.2307 | 1095.2761 | -0.0454 | 2 | 9 | 3.5e+02 | 7 | K.REAAMRAFK.T + Oxidation (M) |
|  | 100 | 366.1522 | 1095.4344 | 1095.2761 | 0.1583 | 2 | (9) | 3.6e+02 | 8 | K.REAAMRAFK.T + Oxidation (M) |
|  | 190 | 369.3158 | 1104.9252 | 1105.2925 | -0.3673 | 1 | 6 | 5.9e+02 | 3 | R.MSPARMSPGR.R + Oxidation (M) |
|  | 314 | 377.1657 | 1128.4750 | 1128.2793 | 0.1956 | 1 | 10 | 2.6e+02 | 1 | K.LTSGEAPGIRK.E |
|  | 2656 | 566.2069 | 1130.3990 | 1130.3831 | 0.0160 | 1 | 15 | 87 | 5 | K.TPPRIPPKPK.S |
|  | 2683 | 574.3709 | 1146.7270 | 1147.3240 | -0.5970 | 1 | 9 | 3.4e+02 | 5 | R.YPAKLTLGER.E |
|  | 434 | 386.0396 | 1155.0967 | 1155.3000 | -0.2033 | 0 | 14 | 99 | 2 | R.VEAMGMSSEAK.L + Oxidation (M) |
|  | 544 | 388.8141 | 1163.4201 | 1163.2789 | 0.1412 | 2 | 6 | 8.4e+02 | 6 | K.ISETKKSDQK.T |
|  | 611 | 391.1789 | 1170.5145 | 1170.3658 | 0.1488 | 2 | 6 | 5.2e+02 | 6 | R.KGIVVRAGGSAR.I |
|  | 2760 | 592.7612 | 1183.5076 | 1183.3563 | 0.1513 | 1 | 5 | 9.1e+02 | 5 | R.EKLYPPSPPR.W |
|  | 683 | 399.8672 | 1196.5795 | 1197.4010 | -0.8216 | 0 | 3 | 1.1e+03 | 9 | K.VTGYLIEMQK.V + Oxidation (M) |
|  | 733 | 401.9340 | 1202.7799 | 1203.3757 | -0.5959 | 2 | 8 | 4.6e+02 | 3 | K.QRQRIMAER.E + Oxidation (M) |
|  | 865 | 406.0075 | 1215.0003 | 1215.3568 | -0.3565 | 1 | 6 | 5.9e+02 | 4 | K.DRVVIDNVGTK.S |
|  | 911 | 406.9037 | 1217.6891 | 1217.3892 | 0.2999 | 0 | 11 | 1.9e+02 | 7 | K.IELSPSMEAPK.I + Oxidation (M) |
|  | 1082 | 412.4707 | 1234.3898 | 1233.4382 | 0.9517 | 2 | 2 | 2.1e+03 | 5 | K.ADMILKQDKR.I + Oxidation (M) |
|  | 2946 | 623.2170 | 1244.4193 | 1243.3717 | 1.0476 | 2 | 13 | 1.3e+02 | 2 | R.RDLPDGRWTK.A |
|  | 2989 | 637.4632 | 1272.9116 | 1273.4738 | -0.5622 | 0 | 25 | 7.1 | 3 | K.EIELDFAVPLK.D |
|  | 1409 | 433.4536 | 1297.3386 | 1297.5466 | -0.2080 | 2 | 8 | 4.5e+02 | 4 | K.GKTFVYLKWR.R |
|  | 1414 | 434.1722 | 1299.4944 | 1300.3765 | -0.8822 | 0 | 9 | 3.4e+02 | 6 | K.GEQTWSHAGISK.T |
|  | 1415 | 434.1731 | 1299.4971 | 1300.3765 | -0.8794 | 0 | (4) | 9.4e+02 | 10 | K.GEQTWSHAGISK.T |
|  | 1531 | 441.1255 | 1320.3543 | 1321.4357 | -1.0814 | 0 | 5 | 8.1e+02 | 8 | R.DTIVVNAGETFR.L |
|  | 1618 | 448.2582 | 1341.7524 | 1342.5227 | -0.7703 | 1 | 3 | 1.2e+03 | 8 | K.DAGFYVVCAKNR.F |
|  | 3109 | 678.5944 | 1355.1739 | 1354.5995 | 0.5744 | 1 | 4 | 9e+02 | 7 | K.YVHRLLIPSTR.M |
|  | 1685 | 454.5326 | 1360.5757 | 1359.5764 | 0.9993 | 2 | 8 | 5.5e+02 | 8 | K.LFVEGRDVRIR.S |
|  | 2051 | 486.3709 | 1456.0905 | 1456.5624 | -0.4719 | 1 | 12 | 1.3e+02 | 2 | R.EVNSTHWSRVNK.S |
|  | 2239 | 505.8279 | 1514.4615 | 1514.7243 | -0.2628 | 1 | 6 | 4.8e+02 | 5 | K.AADPIDPPGPPAKIR.I |
|  | 3338 | 759.0032 | 1515.9916 | 1516.7635 | -0.7719 | 2 | 7 | 4.7e+02 | 4 | K.LRMPYDVPEPRK.Y + Oxidation (M) |
|  | 3371 | 777.9435 | 1553.8722 | 1553.8002 | 0.0720 | 0 | 0 | 2.7e+03 | 5 | K.QKPDIVLYPEPVR.V |
|  | 2433 | 530.4683 | 1588.3826 | 1588.8543 | -0.4717 | 2 | 14 | 1.1e+02 | 4 | R.SPIRMSPARMSPAR.M + 2 Oxidation (M) |
|  | 2451 | 534.0782 | 1599.2126 | 1598.7995 | 0.4130 | 1 | 3 | 1.3e+03 | 8 | K.KAEAVATVVAAVDQAR.V |
|  | 2592 | 555.6692 | 1663.9854 | 1663.8230 | 0.1623 | 1 | 4 | 1.2e+03 | 6 | K.LEDAGEVQLTAKDFK.T |
|  | 2815 | 598.0847 | 1791.2318 | 1790.0688 | 1.1629 | 1 | 7 | 5.3e+02 | 7 | R.LKVTSLMEGCDYQFR.V |
|  | 2889 | 611.8750 | 1832.6028 | 1831.9980 | 0.6048 | 1 | 11 | 1.9e+02 | 1 | R.MAHEGALTGVTTDQKEK.Q + Oxidation (M) |
|  | 2891 | 611.9609 | 1832.8605 | 1832.0228 | 0.8377 | 2 | 8 | 4.3e+02 | 6 | R.RDVASAQWSPLSATSKK.K |
|  | 2904 | 612.2870 | 1833.8390 | 1834.0172 | -0.1782 | 1 | 2 | 1.5e+03 | 10 | K.NAAGVISKGSESTGPVTCR.D |
|  | 2912 | 612.7262 | 1835.1564 | 1834.1644 | 0.9921 | 1 | 8 | 5e+02 | 4 | R.AGSNLKVDIPISGKPLPK.V |
|  | 3077 | 668.4992 | 2002.4754 | 2003.3074 | -0.8320 | 2 | 8 | 3.9e+02 | 4 | K.YRFRVLAENLAGPGKPSK.S |
|  | 3332 | 757.9138 | 2270.7193 | 2269.6172 | 1.1020 | 1 | 20 | 28 | 1 | R.ESRPVIVKEQTMLPELDLR.G + Oxidation (M) |
|  | 3346 | 761.1796 | 2280.5165 | 2279.5757 | 0.9409 | 2 | 7 | 5.2e+02 | 2 | R.VFAENETGLSRPRRTAMSIK.T + Oxidation (M) |
|  | 3357 | 766.8203 | 2297.4386 | 2297.5807 | -0.1421 | 0 | 6 | 6.9e+02 | 7 | K.AENIVGLGLPDTTIPIECQEK.L + Carbamidomethyl (C) |

  


---

|  |  |
| --- | --- |
| **12.** | gi|1790878    **Mass:** 306224   **Score:** 113    **Queries matched:** 25 |
|  | microtubule-associated protein 1a [Homo sapiens] |

|  |  |
| --- | --- |
|  | Check to include this hit in error tolerant search or archive report |
|  |  |

|  |  |  |  |  |  |  |  |  |  |  |
| --- | --- | --- | --- | --- | --- | --- | --- | --- | --- | --- |
|  | **Query** | **Observed** | **Mr(expt)** | **Mr(calc)** | **Delta** | **Miss** | **Score** | **Expect** | **Rank** | **Peptide** |
|  | 1038 | **409.0540** | **816.0933** | **816.8994** | **-0.8060** | **1** | **15** | **83** | **3** | **K.DKALDQK.V** |
|  | 1147 | **416.2507** | **830.4866** | **830.9259** | **-0.4394** | **1** | **14** | **1.1e+02** | **2** | **K.DKALEQK.D** |
|  | 1152 | **416.5774** | **831.1399** | **830.9259** | **0.2140** | **0** | **4** | **1.3e+03** | **4** | **K.AGPTALSSK.G** |
|  | 1648 | **450.2594** | **898.5040** | **899.0034** | **-0.4994** | **0** | **8** | **3.5e+02** | **2** | **K.DKPVSPTR.R** |
|  | 1720 | **457.9860** | **913.9571** | **915.0491** | **-1.0919** | **2** | **9** | **3e+02** | **8** | **R.AKEKVQGR.V** |
|  | 1833 | **464.0349** | **926.0549** | **927.0598** | **-1.0048** | **2** | **10** | **2.3e+02** | **1** | **K.EIPRERK.E** |
|  | 2004 | **480.0843** | **958.1538** | **957.0791** | **1.0747** | **0** | **8** | **4.9e+02** | **10** | **K.ELSSPISPK.S** |
|  | 2481 | **537.1235** | **1072.2323** | **1071.2679** | **0.9644** | **0** | **6** | **7.7e+02** | **5** | **K.EMQFLMEK.W + Oxidation (M)** |
|  | 2482 | **537.2155** | **1072.4161** | **1071.2679** | **1.1483** | **0** | **(1)** | **2.4e+03** | **8** | **K.EMQFLMEK.W + Oxidation (M)** |
|  | 13 | **360.4811** | **1078.4212** | **1079.1674** | **-0.7461** | **2** | **9** | **4.2e+02** | **4** | **K.DYRKVGGER.E** |
|  | 2522 | **544.2035** | **1086.3922** | **1086.1995** | **0.1927** | **1** | **6** | **7e+02** | **10** | **K.QQNKALEQK.G** |
|  | 168 | **369.2296** | **1104.6667** | **1105.3272** | **-0.6605** | **0** | **10** | **2.2e+02** | **2** | **K.EMQFLMYK.W + Oxidation (M)** |
|  | 192 | **369.3389** | **1104.9944** | **1105.3272** | **-0.3327** | **0** | **(10)** | **2.5e+02** | **4** | **K.EMQFLMYK.W + Oxidation (M)** |
|  | 2589 | **554.5231** | **1107.0314** | **1106.2475** | **0.7839** | **1** | **9** | **3.1e+02** | **1** | **K.MLEEKSPEK.V + Oxidation (M)** |
|  | 864 | **406.0013** | **1214.9819** | **1214.2892** | **0.6927** | **2** | **11** | **2e+02** | **3** | **K.RSPTPGKGSGDR.V** |
|  | 2989 | 637.4632 | 1272.9116 | 1272.5141 | 0.3976 | 2 | 25 | 7.3 | 4 | K.EIPMERKELK.K |
|  | 1340 | **429.0416** | **1284.1027** | **1283.3926** | **0.7101** | **1** | **9** | **3e+02** | **5** | **K.SSHWSKVPEAR.K** |
|  | 1776 | **459.8517** | **1376.5330** | **1375.6338** | **0.8992** | **1** | **10** | **2.7e+02** | **5** | **K.DSKEMQFLMCK.W + Oxidation (M)** |
|  | 1958 | **476.2142** | **1425.6205** | **1425.6296** | **-0.0091** | **1** | **11** | **2.3e+02** | **2** | **K.DSKEMQFLMHK.W + 2 Oxidation (M)** |
|  | 2012 | **481.0995** | **1440.2765** | **1440.5615** | **-0.2850** | **2** | **22** | **18** | **1** | **R.SPTPGKGSPDRVSR.X** |
|  | 2023 | **482.2937** | **1443.8588** | **1444.5502** | **-0.6913** | **2** | **9** | **3.2e+02** | **1** | **R.SPTPGKGSTDRVSR.X** |
|  | 2304 | **517.6087** | **1549.8039** | **1550.6905** | **-0.8865** | **1** | **7** | **7.5e+02** | **9** | **R.SRSMTSQVTPAEEK.D** |
|  | 2476 | **536.2349** | **1605.6824** | **1606.6474** | **-0.9650** | **0** | **4** | **1e+03** | **8** | **K.AENEEAAAYPAWER.G** |
|  | 2578 | **553.6078** | **1657.8012** | **1656.9231** | **0.8781** | **1** | **7** | **6.1e+02** | **4** | **K.EMQFLMAKWAGNSK.A + Oxidation (M)** |
|  | 2986 | **635.3240** | **1902.9498** | **1902.1987** | **0.7511** | **2** | **7** | **5.2e+02** | **5** | **K.EMQFLMTKWAGNSKAK.T + 2 Oxidation (M)** |

  


---

|  |  |
| --- | --- |
| **13.** | gi|18157651    **Mass:** 590870   **Score:** 104    **Queries matched:** 15 |
|  | bullous pemphigoid antigen 1 eA [Homo sapiens] |

|  |  |
| --- | --- |
|  | Check to include this hit in error tolerant search or archive report |
|  |  |

|  |  |  |  |  |  |  |  |  |  |  |
| --- | --- | --- | --- | --- | --- | --- | --- | --- | --- | --- |
|  | **Query** | **Observed** | **Mr(expt)** | **Mr(calc)** | **Delta** | **Miss** | **Score** | **Expect** | **Rank** | **Peptide** |
|  | 1389 | **432.4314** | **862.8480** | **861.8971** | **0.9509** | **1** | **11** | **2.2e+02** | **1** | **R.SEVDEKR.Q** |
|  | 1783 | **460.1273** | **918.2398** | **917.0582** | **1.1816** | **1** | **8** | **5.1e+02** | **7** | **K.ESLEKALK.Y** |
|  | 2519 | **543.9044** | **1085.7941** | **1085.2148** | **0.5792** | **1** | **9** | **3.6e+02** | **4** | **R.SARELIPGSR.D** |
|  | 2620 | **560.8689** | **1119.7230** | **1119.2957** | **0.4273** | **1** | **5** | **6.9e+02** | **4** | **R.SARELIMGSR.D** |
|  | 308 | **377.1355** | **1128.3844** | **1128.1967** | **0.1878** | **2** | **15** | **73** | **1** | **K.DSERAGTKHK.Q** |
|  | 357 | **380.2455** | **1137.7143** | **1137.2216** | **0.4927** | **0** | **(7)** | **5.5e+02** | **8** | **R.QSSINAMNEK.V + Oxidation (M)** |
|  | 359 | **380.2886** | **1137.8437** | **1137.2216** | **0.6222** | **0** | **14** | **94** | **4** | **R.QSSINAMNEK.V + Oxidation (M)** |
|  | 531 | **388.2324** | **1161.6750** | **1162.3188** | **-0.6438** | **1** | **5** | **8.1e+02** | **9** | **K.KDYHAELMR.E** |
|  | 715 | **401.1284** | **1200.3632** | **1201.3981** | **-1.0349** | **2** | **13** | **1.4e+02** | **3** | **K.KSRVMDFFR.R + Oxidation (M)** |
|  | 810 | **404.1814** | **1209.5221** | **1208.4038** | **1.1183** | **2** | **7** | **4.7e+02** | **8** | **K.ESLEKALKYK.E** |
|  | 3072 | **667.7573** | **1333.4999** | **1332.4168** | **1.0831** | **1** | **15** | **1e+02** | **1** | **R.DLDFDWHKEK.A** |
|  | 1627 | **449.3769** | **1345.1084** | **1344.5966** | **0.5118** | **2** | **13** | **1.1e+02** | **2** | **K.KELDKVVTTAIK.E** |
|  | 1923 | **473.6333** | **1417.8777** | **1417.6122** | **0.2655** | **0** | **6** | **7.5e+02** | **5** | **K.HLHQAVSIGQSLK.V** |
|  | 2073 | **487.8854** | **1460.6341** | **1459.5548** | **1.0793** | **1** | **9** | **3.4e+02** | **6** | **R.EGEKIATTAEPADK.V** |
|  | 2439 | **532.0931** | **1593.2571** | **1592.9222** | **0.3349** | **0** | **14** | **1.1e+02** | **1** | **K.FWCDHMSLIVTIK.D** |

  


---

|  |  |
| --- | --- |
| **14.** | gi|119631904    **Mass:** 740442   **Score:** 100    **Queries matched:** 24 |
|  | nebulin, isoform CRA\_a [Homo sapiens] |

|  |  |
| --- | --- |
|  | Check to include this hit in error tolerant search or archive report |
|  |  |

|  |  |  |  |  |  |  |  |  |  |  |
| --- | --- | --- | --- | --- | --- | --- | --- | --- | --- | --- |
|  | **Query** | **Observed** | **Mr(expt)** | **Mr(calc)** | **Delta** | **Miss** | **Score** | **Expect** | **Rank** | **Peptide** |
|  | 241 | **372.5738** | **743.1328** | **743.8918** | **-0.7590** | **1** | **24** | **10** | **7** | **R.KVQELK.T** |
|  | 242 | **372.6068** | **743.1989** | **743.8918** | **-0.6929** | **1** | **(24)** | **10** | **6** | **R.KVQELK.T** |
|  | 755 | **402.8230** | **803.6313** | **803.9039** | **-0.2726** | **1** | **14** | **1.1e+02** | **6** | **K.NLSSQKK.Y** |
|  | 1335 | **428.8970** | **855.7793** | **856.0480** | **-0.2688** | **1** | **(10)** | **2.2e+02** | **6** | **K.AHMLKTR.N** |
|  | 1337 | **428.9666** | **855.9184** | **856.0480** | **-0.1296** | **1** | **13** | **1.3e+02** | **2** | **K.AHMLKTR.N** |
|  | 1339 | **429.0055** | **855.9962** | **856.0480** | **-0.0518** | **1** | **(13)** | **1.4e+02** | **1** | **K.AHMLKTR.N** |
|  | 1344 | **429.0984** | **856.1820** | **856.0480** | **0.1339** | **1** | **(13)** | **1.4e+02** | **1** | **K.AHMLKTR.N** |
|  | 1345 | **429.1078** | **856.2008** | **856.0480** | **0.1527** | **1** | **(13)** | **1.4e+02** | **1** | **K.AHMLKTR.N** |
|  | 1452 | **436.0777** | **870.1406** | **870.9930** | **-0.8524** | **0** | **(9)** | **3.4e+02** | **5** | **R.QGLTLSPR.L** |
|  | 1468 | **436.2771** | **870.5395** | **870.9930** | **-0.4536** | **0** | **16** | **55** | **5** | **R.QGLTLSPR.L** |
|  | 2385 | **523.2018** | **1044.3889** | **1043.2179** | **1.1710** | **2** | **9** | **3.8e+02** | **5** | **K.LGLEEAKRK.G** |
|  | 2505 | **540.1440** | **1078.2733** | **1077.2160** | **1.0573** | **1** | **6** | **6.9e+02** | **7** | **K.NNAITMNKR.L + Oxidation (M)** |
|  | 1273 | **423.2770** | **1266.8089** | **1267.3452** | **-0.5363** | **1** | **10** | **2.2e+02** | **6** | **K.VTAQNSDKNYK.A** |
|  | 1412 | **434.1408** | **1299.4003** | **1299.4747** | **-0.0744** | **2** | **7** | **5.3e+02** | **8** | **R.LYRSVYEKNK.M** |
|  | 1473 | **437.0063** | **1307.9969** | **1307.4123** | **0.5845** | **1** | **(4)** | **1e+03** | **7** | **K.AARQAASDVQYK.K** |
|  | 1482 | **437.1614** | **1308.4621** | **1307.4123** | **1.0498** | **1** | **6** | **8.1e+02** | **8** | **K.AARQAASDVQYK.K** |
|  | 1487 | **437.2598** | **1308.7571** | **1308.4434** | **0.3137** | **1** | **3** | **1.2e+03** | **8** | **K.HAKTTELPQQR.S** |
|  | 1619 | **448.2787** | **1341.8140** | **1342.5179** | **-0.7039** | **0** | **6** | **5.9e+02** | **4** | **K.GTPLPVTPEMER.V + Oxidation (M)** |
|  | 1664 | **452.1415** | **1353.4025** | **1353.5469** | **-0.1445** | **1** | **6** | **8.6e+02** | **7** | **R.IARTTENLPCHV.-** |
|  | 1711 | **457.1163** | **1368.3268** | **1367.6317** | **0.6952** | **1** | **6** | **6.1e+02** | **5** | **K.KLTDSMDMVLAK.Q + Oxidation (M)** |
|  | 2312 | **518.3828** | **1552.1263** | **1552.6879** | **-0.5616** | **1** | **3** | **1e+03** | **4** | **R.EGWDEMKAGCDVR.L + Carbamidomethyl (C)** |
|  | 2424 | **528.3538** | **1582.0391** | **1582.7569** | **-0.7178** | **0** | **4** | **9.5e+02** | **7** | **R.GLNAMANETPDFMR.A + Oxidation (M)** |
|  | 2646 | **565.5944** | **1693.7609** | **1692.9073** | **0.8536** | **2** | **16** | **87** | **2** | **K.QVSDILYKAKGEDVK.H** |
|  | 3040 | **666.5009** | **1996.4804** | **1995.3038** | **1.1766** | **0** | **13** | **1.1e+02** | **2** | **K.FTSVPDSMGMMLAQHNTK.Q** |

  


---

|  |  |
| --- | --- |
| **15.** | gi|415819    **Mass:** 358741   **Score:** 98     **Queries matched:** 18 |
|  | antigen of the monoclonal antibody Ki-67 [Homo sapiens] |

|  |  |
| --- | --- |
|  | Check to include this hit in error tolerant search or archive report |
|  |  |

|  |  |  |  |  |  |  |  |  |  |  |
| --- | --- | --- | --- | --- | --- | --- | --- | --- | --- | --- |
|  | **Query** | **Observed** | **Mr(expt)** | **Mr(calc)** | **Delta** | **Miss** | **Score** | **Expect** | **Rank** | **Peptide** |
|  | 253 | **373.3954** | **744.7761** | **744.7474** | **0.0288** | **0** | **7** | **7e+02** | **10** | **K.EPAGEDK.G** |
|  | 362 | **380.3613** | **758.7078** | **758.8171** | **-0.1093** | **0** | **8** | **4.7e+02** | **10** | **K.EEPSAVK.F** |
|  | 1305 | **427.0841** | **852.1534** | **851.9965** | **0.1570** | **2** | **5** | **7.5e+02** | **8** | **R.RQPRAPK.E** |
|  | 1312 | **427.5075** | **853.0002** | **851.9965** | **1.0037** | **2** | **(5)** | **1.1e+03** | **8** | **R.RQPRAPK.E** |
|  | 2380 | **522.9301** | **1043.8454** | **1044.2457** | **-0.4003** | **2** | **13** | **1.3e+02** | **1** | **K.GIKALKESAK.Q** |
|  | 295 | **376.2114** | **1125.6119** | **1126.1377** | **-0.5258** | **0** | **14** | **78** | **2** | **R.TSGETTHTHR.E** |
|  | 2728 | 587.5143 | 1173.0139 | 1172.3783 | 0.6356 | 2 | 18 | 32 | 3 | K.TLTPRKLSTR.N |
|  | 2818 | **598.2502** | **1194.4856** | **1193.3909** | **1.0947** | **0** | **0** | **2.4e+03** | **4** | **K.CAPMSDLTDLK.S** |
|  | 2847 | **607.6393** | **1213.2639** | **1213.3808** | **-0.1169** | **1** | **13** | **1.4e+02** | **2** | **K.VQVKEEPSAVK.F** |
|  | 915 | **406.9736** | **1217.8985** | **1218.3837** | **-0.4852** | **1** | **8** | **3.7e+02** | **8** | **R.GGERVATCLQK.R + Carbamidomethyl (C)** |
|  | 2919 | **613.2952** | **1224.5756** | **1224.4064** | **0.1691** | **1** | **6** | **5.8e+02** | **4** | **K.IIKEQPQPSGK.Q** |
|  | 1517 | **439.3701** | **1315.0883** | **1315.4757** | **-0.3875** | **1** | **9** | **3.2e+02** | **10** | **R.RSGASEANLIVAK.S** |
|  | 3066 | **667.4209** | **1332.8270** | **1332.4369** | **0.3901** | **0** | **5** | **7.8e+02** | **3** | **K.AMDTPKPAGGDEK.D + Oxidation (M)** |
|  | 3107 | **676.6936** | **1351.3724** | **1350.5829** | **0.7896** | **1** | **16** | **81** | **2** | **K.DIKAFMGTPVQK.L + Oxidation (M)** |
|  | 2001 | **479.9791** | **1436.9152** | **1437.5576** | **-0.6424** | **1** | **3** | **1.3e+03** | **4** | **K.KPNPVEEEPSRR.R** |
|  | 2022 | **482.1763** | **1443.5068** | **1442.5245** | **0.9823** | **0** | **3** | **1.5e+03** | **8** | **K.SPPPESVDTPTSTK.Q** |
|  | 2543 | **548.8571** | **1643.5490** | **1642.8385** | **0.7104** | **2** | **6** | **4.8e+02** | **10** | **R.QCIRENGNVAKTPR.N + Carbamidomethyl (C)** |
|  | 2615 | **559.5201** | **1675.5383** | **1674.7880** | **0.7503** | **1** | **7** | **4.7e+02** | **6** | **K.SPPPESMDTPTSTRR.R + Oxidation (M)** |

  


---

|  |  |
| --- | --- |
| **16.** | gi|119629468    **Mass:** 784994   **Score:** 96     **Queries matched:** 23 |
|  | hCG2011852 [Homo sapiens] |

|  |  |
| --- | --- |
|  | Check to include this hit in error tolerant search or archive report |
|  |  |

|  |  |  |  |  |  |  |  |  |  |  |
| --- | --- | --- | --- | --- | --- | --- | --- | --- | --- | --- |
|  | **Query** | **Observed** | **Mr(expt)** | **Mr(calc)** | **Delta** | **Miss** | **Score** | **Expect** | **Rank** | **Peptide** |
|  | 674 | **399.1988** | **796.3828** | **795.8867** | **0.4961** | **1** | **(9)** | **2.6e+02** | **8** | **K.GAKLHDR.E** |
|  | 677 | **399.3655** | **796.7163** | **795.8867** | **0.8295** | **1** | **16** | **59** | **1** | **K.GAKLHDR.E** |
|  | 1171 | **418.0398** | **834.0648** | **835.0057** | **-0.9409** | **2** | **5** | **9.6e+02** | **6** | **K.GKKSLFR.H** |
|  | 1603 | **446.4411** | **890.8674** | **889.9933** | **0.8741** | **2** | **10** | **3.3e+02** | **8** | **R.KTPDSKSK.L** |
|  | 1641 | **450.1085** | **898.2021** | **899.0232** | **-0.8210** | **1** | **7** | **5.4e+02** | **8** | **R.QKMDFSK.K + Oxidation (M)** |
|  | 1754 | **459.0175** | **916.0203** | **917.0235** | **-1.0032** | **1** | **19** | **38** | **2** | **R.QFTTKHR.K** |
|  | 2178 | **499.0974** | **996.1800** | **996.0507** | **0.1293** | **0** | **3** | **1.2e+03** | **9** | **K.SPNTSEMSK.R + Oxidation (M)** |
|  | 199 | **369.4913** | **1105.4518** | **1106.1910** | **-0.7391** | **0** | **7** | **5.9e+02** | **6** | **K.CMGEQHNSGK.G + Oxidation (M)** |
|  | 232 | **372.1566** | **1113.4476** | **1112.2782** | **1.1693** | **0** | **10** | **2.4e+02** | **7** | **R.WTIPPETLR.K** |
|  | 820 | **404.9723** | **1211.8947** | **1212.4653** | **-0.5706** | **2** | **10** | **2.7e+02** | **5** | **K.ALEKIMHSKR.I** |
|  | 881 | **406.1362** | **1215.3865** | **1214.2428** | **1.1438** | **1** | **7** | **5.6e+02** | **9** | **K.NSISHDREEK.L** |
|  | 969 | **407.6347** | **1219.8820** | **1219.3686** | **0.5135** | **2** | **16** | **58** | **4** | **K.QEGKMQEGKGK.S** |
|  | 1054 | **410.1710** | **1227.4908** | **1226.4454** | **1.0453** | **0** | **5** | **8.2e+02** | **6** | **R.ISPMSHILNAK.E + Oxidation (M)** |
|  | 1346 | **429.1306** | **1284.3695** | **1283.5152** | **0.8543** | **1** | **9** | **3.6e+02** | **6** | **K.ELSCNLTTKMK.E + Oxidation (M)** |
|  | 1535 | **441.1893** | **1320.5458** | **1320.6215** | **-0.0756** | **2** | **16** | **71** | **1** | **K.MLPKCTDLKAK.Q + Carbamidomethyl (C); Oxidation (M)** |
|  | 3212 | **732.5955** | **1463.1761** | **1462.6529** | **0.5232** | **1** | **17** | **39** | **1** | **K.DPLHLKQAVNTAR.K** |
|  | 2134 | **492.1740** | **1473.4998** | **1473.7802** | **-0.2804** | **2** | **8** | **4.2e+02** | **3** | **K.VQKVKSGPGVMLSK.S + Oxidation (M)** |
|  | 2136 | **492.4354** | **1474.2841** | **1473.7802** | **0.5039** | **2** | **(8)** | **3.8e+02** | **6** | **K.VQKVKSGPGVMLSK.S + Oxidation (M)** |
|  | 2197 | 499.8580 | 1496.5519 | 1497.7368 | -1.1849 | 1 | 11 | 1.8e+02 | 4 | R.SLSGCTDMSSILRK.Q |
|  | 2199 | **500.0775** | **1497.2104** | **1497.7368** | **-0.5265** | **1** | **(9)** | **3.1e+02** | **1** | **R.SLSGCTDMSSILRK.Q** |
|  | 2859 | **609.3066** | **1824.8978** | **1825.1545** | **-0.2567** | **1** | **4** | **1.2e+03** | **6** | **K.VADMTSVLDPNKMYLK.A** |
|  | 2892 | **611.9626** | **1832.8658** | **1832.1304** | **0.7354** | **1** | **7** | **4.9e+02** | **9** | **K.QSPHMQEGIKCMEGLK.T + Oxidation (M)** |
|  | 3177 | **701.9554** | **2102.8440** | **2102.3014** | **0.5426** | **2** | **4** | **8.4e+02** | **7** | **R.KKDTQVLSESEFHVTPEK.N** |

  


---

|  |  |
| --- | --- |
| **17.** | gi|806562    **Mass:** 773204   **Score:** 93     **Queries matched:** 23 |
|  | nebulin [Homo sapiens] |

|  |  |
| --- | --- |
|  | Check to include this hit in error tolerant search or archive report |
|  |  |

|  |  |  |  |  |  |  |  |  |  |  |
| --- | --- | --- | --- | --- | --- | --- | --- | --- | --- | --- |
|  | **Query** | **Observed** | **Mr(expt)** | **Mr(calc)** | **Delta** | **Miss** | **Score** | **Expect** | **Rank** | **Peptide** |
|  | 241 | 372.5738 | 743.1328 | 743.8918 | -0.7590 | 1 | 24 | 10 | 7 | R.KVQELK.T |
|  | 242 | 372.6068 | 743.1989 | 743.8918 | -0.6929 | 1 | (24) | 10 | 6 | R.KVQELK.T |
|  | 705 | **400.2863** | **798.5579** | **797.9193** | **0.6386** | **0** | **4** | **8.2e+02** | **5** | **K.NTAMFSK.K** |
|  | 712 | **401.0640** | **800.1133** | **799.9185** | **0.1947** | **1** | **8** | **4.2e+02** | **3** | **R.RVAQAQK.A** |
|  | 755 | 402.8230 | 803.6313 | 803.9039 | -0.2726 | 1 | 14 | 1.1e+02 | 6 | K.NLSSQKK.Y |
|  | 1335 | 428.8970 | 855.7793 | 856.0480 | -0.2688 | 1 | (10) | 2.2e+02 | 6 | K.AHMLKTR.N |
|  | 1337 | 428.9666 | 855.9184 | 856.0480 | -0.1296 | 1 | 13 | 1.3e+02 | 2 | K.AHMLKTR.N |
|  | 1339 | 429.0055 | 855.9962 | 856.0480 | -0.0518 | 1 | (13) | 1.4e+02 | 1 | K.AHMLKTR.N |
|  | 1344 | 429.0984 | 856.1820 | 856.0480 | 0.1339 | 1 | (13) | 1.4e+02 | 1 | K.AHMLKTR.N |
|  | 1345 | 429.1078 | 856.2008 | 856.0480 | 0.1527 | 1 | (13) | 1.4e+02 | 1 | K.AHMLKTR.N |
|  | 2385 | 523.2018 | 1044.3889 | 1043.2179 | 1.1710 | 2 | 9 | 3.8e+02 | 5 | K.LGLEEAKRK.G |
|  | 96 | 366.0843 | 1095.2307 | 1095.2263 | 0.0044 | 0 | 9 | 3.8e+02 | 9 | K.MQDLFSPNK.Y + Oxidation (M) |
|  | 1273 | 423.2770 | 1266.8089 | 1267.3452 | -0.5363 | 1 | 10 | 2.2e+02 | 6 | K.VTAQNSDKNYK.A |
|  | 1412 | 434.1408 | 1299.4003 | 1299.4747 | -0.0744 | 2 | 7 | 5.3e+02 | 8 | R.LYRSVYEKNK.M |
|  | 1473 | 437.0063 | 1307.9969 | 1307.4123 | 0.5845 | 1 | (4) | 1e+03 | 7 | K.AARQAASDVQYK.K |
|  | 1482 | 437.1614 | 1308.4621 | 1307.4123 | 1.0498 | 1 | 6 | 8.1e+02 | 8 | K.AARQAASDVQYK.K |
|  | 1487 | 437.2598 | 1308.7571 | 1308.4434 | 0.3137 | 1 | 3 | 1.2e+03 | 8 | K.HAKTTELPQQR.S |
|  | 1619 | 448.2787 | 1341.8140 | 1342.5179 | -0.7039 | 0 | 6 | 5.9e+02 | 4 | K.GTPLPVTPEMER.V + Oxidation (M) |
|  | 1711 | 457.1163 | 1368.3268 | 1367.6317 | 0.6952 | 1 | 6 | 6.1e+02 | 5 | K.KLTDSMDMVLAK.Q + Oxidation (M) |
|  | 2312 | 518.3828 | 1552.1263 | 1552.6879 | -0.5616 | 1 | 3 | 1e+03 | 4 | R.EGWDEMKAGCDVR.L + Carbamidomethyl (C) |
|  | 2424 | 528.3538 | 1582.0391 | 1582.7569 | -0.7178 | 0 | 4 | 9.5e+02 | 7 | R.GLNAMANETPDFMR.A + Oxidation (M) |
|  | 2646 | 565.5944 | 1693.7609 | 1692.9073 | 0.8536 | 2 | 16 | 87 | 2 | K.QVSDILYKAKGEDVK.H |
|  | 3040 | 666.5009 | 1996.4804 | 1995.3038 | 1.1766 | 0 | 13 | 1.1e+02 | 2 | K.FTSVPDSMGMMLAQHNTK.Q |

  


---

|  |  |
| --- | --- |
| **18.** | gi|14790190    **Mass:** 402243   **Score:** 92     **Queries matched:** 14 |
|  | msx2-interacting protein [Homo sapiens] |

|  |  |
| --- | --- |
|  | Check to include this hit in error tolerant search or archive report |
|  |  |

|  |  |  |  |  |  |  |  |  |  |  |
| --- | --- | --- | --- | --- | --- | --- | --- | --- | --- | --- |
|  | **Query** | **Observed** | **Mr(expt)** | **Mr(calc)** | **Delta** | **Miss** | **Score** | **Expect** | **Rank** | **Peptide** |
|  | 363 | 380.4083 | 758.8017 | 759.8547 | -1.0529 | 2 | 3 | 1.9e+03 | 2 | R.KRNTNK.K |
|  | 543 | **388.6043** | **775.1939** | **775.8938** | **-0.6999** | **1** | **13** | **1.1e+02** | **5** | **K.ITRTASK.N** |
|  | 922 | **407.1080** | **812.2012** | **811.9459** | **0.2553** | **0** | **8** | **4.6e+02** | **9** | **R.FMELTR.M + Oxidation (M)** |
|  | 57 | **363.2500** | **1086.7278** | **1086.2029** | **0.5249** | **0** | **13** | **1.1e+02** | **2** | **R.LASQASRPTR.S** |
|  | 2628 | **562.3625** | **1122.7103** | **1123.3011** | **-0.5907** | **0** | **6** | **6.2e+02** | **4** | **K.QQMEMEIAK.S + Oxidation (M)** |
|  | 551 | **389.0629** | **1164.1666** | **1165.2319** | **-1.0653** | **1** | **(11)** | **2.3e+02** | **6** | **K.KSSPEMEDAR.V + Oxidation (M)** |
|  | 567 | **389.1444** | **1164.4110** | **1165.2319** | **-0.8209** | **1** | **16** | **67** | **4** | **K.KSSPEMEDAR.V + Oxidation (M)** |
|  | 831 | **405.0988** | **1212.2742** | **1213.3838** | **-1.1097** | **1** | **15** | **73** | **8** | **K.LDRLNTVASPK.D** |
|  | 876 | **406.0970** | **1215.2690** | **1216.3051** | **-1.0361** | **2** | **16** | **67** | **1** | **R.DDITREVRGR.R** |
|  | 892 | **406.2224** | **1215.6450** | **1215.3502** | **0.2947** | **0** | **11** | **1.5e+02** | **2** | **K.TVEAPLVTEEK.T** |
|  | 2314 | **518.4554** | **1552.3442** | **1552.7310** | **-0.3868** | **1** | **4** | **8.2e+02** | **6** | **K.RMDHVDFDICTK.R + Carbamidomethyl (C); Oxidation (M)** |
|  | 2330 | **518.9354** | **1553.7841** | **1553.7388** | **0.0453** | **2** | **7** | **4.8e+02** | **1** | **K.KMDGEYLGNNRLK.L + Oxidation (M)** |
|  | 2742 | **591.4896** | **1771.4467** | **1771.1998** | **0.2469** | **2** | **7** | **4.4e+02** | **9** | **K.NRLELMPCVVLTRVK.E** |
|  | 3496 | 919.2162 | 1836.4176 | 1837.0043 | -0.5867 | 2 | 9 | 2.4e+02 | 7 | R.ERTLQHGLYYASRSR.S |

  

|  |  |
| --- | --- |
|  | |
|  | **Proteins matching the same set of peptides:** |

|  |  |
| --- | --- |
|  | gi|119572141    **Mass:** 402244   **Score:** 92     **Queries matched:** 14 |
|  | spen homolog, transcriptional regulator (Drosophila) [Homo sapiens] |

---

|  |  |
| --- | --- |
| **19.** | gi|119631421    **Mass:** 631679   **Score:** 92     **Queries matched:** 16 |
|  | titin, isoform CRA\_c [Homo sapiens] |

|  |  |
| --- | --- |
|  | Check to include this hit in error tolerant search or archive report |
|  |  |

|  |  |  |  |  |  |  |  |  |  |  |
| --- | --- | --- | --- | --- | --- | --- | --- | --- | --- | --- |
|  | **Query** | **Observed** | **Mr(expt)** | **Mr(calc)** | **Delta** | **Miss** | **Score** | **Expect** | **Rank** | **Peptide** |
|  | 1110 | 414.0691 | 826.1233 | 824.9646 | 1.1588 | 0 | 7 | 4.9e+02 | 4 | R.LHVETVK.I |
|  | 50 | 363.1646 | 1086.4718 | 1086.2012 | 0.2705 | 1 | 14 | 86 | 2 | K.KIEAHFDAR.S |
|  | 190 | 369.3158 | 1104.9252 | 1105.2925 | -0.3673 | 1 | 6 | 5.9e+02 | 3 | R.MSPARMSPGR.R + Oxidation (M) |
|  | 222 | **371.2485** | **1110.7233** | **1111.2920** | **-0.5686** | **1** | **17** | **35** | **3** | **R.TSIVNPPQKK.I** |
|  | 226 | **371.2701** | **1110.7881** | **1111.2920** | **-0.5039** | **1** | **(17)** | **35** | **6** | **R.TSIVNPPQKK.I** |
|  | 2656 | 566.2069 | 1130.3990 | 1130.3831 | 0.0160 | 1 | 15 | 87 | 5 | K.TPPRIPPKPK.S |
|  | 911 | 406.9037 | 1217.6891 | 1217.3892 | 0.2999 | 0 | 11 | 1.9e+02 | 7 | K.IELSPSMEAPK.I + Oxidation (M) |
|  | 1163 | **417.8428** | **1250.5062** | **1249.3266** | **1.1796** | **0** | **11** | **2.2e+02** | **2** | **R.YSTPPGETLER.Y** |
|  | 3109 | 678.5944 | 1355.1739 | 1354.5995 | 0.5744 | 1 | 4 | 9e+02 | 7 | K.YVHRLLIPSTR.M |
|  | 1685 | 454.5326 | 1360.5757 | 1359.5764 | 0.9993 | 2 | 8 | 5.5e+02 | 8 | K.LFVEGRDVRIR.S |
|  | 1729 | **458.1549** | **1371.4425** | **1370.5477** | **0.8948** | **0** | **6** | **6.5e+02** | **7** | **K.EYMCIEPDNIK.Y + Oxidation (M)** |
|  | 3371 | 777.9435 | 1553.8722 | 1553.8002 | 0.0720 | 0 | 0 | 2.7e+03 | 5 | K.QKPDIVLYPEPVR.V |
|  | 2433 | 530.4683 | 1588.3826 | 1588.8543 | -0.4717 | 2 | 14 | 1.1e+02 | 4 | R.SPIRMSPARMSPAR.M + 2 Oxidation (M) |
|  | 2451 | 534.0782 | 1599.2126 | 1598.7995 | 0.4130 | 1 | 3 | 1.3e+03 | 8 | K.KAEAVATVVAAVDQAR.V |
|  | 2513 | **542.0307** | **1623.0699** | **1622.8043** | **0.2657** | **1** | **3** | **1.3e+03** | **9** | **K.AMPQDQVTQSPKHR.F** |
|  | 2889 | 611.8750 | 1832.6028 | 1831.9980 | 0.6048 | 1 | 11 | 1.9e+02 | 1 | R.MAHEGALTGVTTDQKEK.Q + Oxidation (M) |

  

|  |  |
| --- | --- |
|  | |
|  | **Proteins matching the same set of peptides:** |

|  |  |
| --- | --- |
|  | gi|110349721    **Mass:** 631560   **Score:** 91     **Queries matched:** 16 |
|  | titin isoform novex-3 [Homo sapiens] |

---

|  |  |
| --- | --- |
| **20.** | gi|189306    **Mass:** 76343    **Score:** 92     **Queries matched:** 7 |
|  | nucleolin [Homo sapiens] |

|  |  |
| --- | --- |
|  | Check to include this hit in error tolerant search or archive report |
|  |  |

|  |  |  |  |  |  |  |  |  |  |  |
| --- | --- | --- | --- | --- | --- | --- | --- | --- | --- | --- |
|  | **Query** | **Observed** | **Mr(expt)** | **Mr(calc)** | **Delta** | **Miss** | **Score** | **Expect** | **Rank** | **Peptide** |
|  | 424 | **385.3678** | **1153.0813** | **1152.3470** | **0.7343** | **1** | **30** | **2.5** | **1** | **R.AIRLELQGPR.G** |
|  | 570 | **389.1619** | **1164.4634** | **1165.2598** | **-0.7963** | **1** | **12** | **1.6e+02** | **3** | **R.GGGGDHKPQGKK.T** |
|  | 590 | **389.5620** | **1165.6638** | **1165.2598** | **0.4040** | **1** | **(12)** | **1.5e+02** | **1** | **R.GGGGDHKPQGKK.T** |
|  | 1550 | **442.4751** | **1324.4030** | **1323.3406** | **1.0625** | **0** | **12** | **2.1e+02** | **4** | **K.EAMEDGEIDGNK.V + Oxidation (M)** |
|  | 1807 | **461.6932** | **1382.0574** | **1381.6201** | **0.4373** | **2** | **5** | **7.1e+02** | **5** | **K.AAVTPGKKAAATPAK.K** |
|  | 1844 | **465.9627** | **1394.8660** | **1394.5358** | **0.3301** | **1** | **15** | **81** | **3** | **R.LELQGPRGSPNAR.S** |
|  | 2694 | **579.3253** | **1734.9538** | **1734.9570** | **-0.0033** | **2** | **22** | **18** | **1** | **R.AIRLELQGPRGSPNAR.S** |

  

|  |  |
| --- | --- |
|  | |
|  | **Proteins matching the same set of peptides:** |

|  |  |
| --- | --- |
|  | gi|31455187    **Mass:** 50950    **Score:** 92     **Queries matched:** 7 |
|  | NCL protein [Homo sapiens] |

|  |  |
| --- | --- |
|  | gi|55956788    **Mass:** 76613    **Score:** 92     **Queries matched:** 7 |
|  | nucleolin [Homo sapiens] |

|  |  |
| --- | --- |
|  | gi|119591366    **Mass:** 52027    **Score:** 92     **Queries matched:** 7 |
|  | nucleolin, isoform CRA\_a [Homo sapiens] |

---

**Peptide matches not assigned to protein hits:** (no details means no
match)  
  

|  |  |  |  |  |  |  |  |  |  |  |
| --- | --- | --- | --- | --- | --- | --- | --- | --- | --- | --- |
|  | **Query** | **Observed** | **Mr(expt)** | **Mr(calc)** | **Delta** | **Miss** | **Score** | **Expect** | **Rank** | **Peptide** |
|  | 2622 | **561.5932** | **1121.1716** | **1121.1577** | **0.0140** | **0** | **60** | **0.0028** | **1** | **AFEEDQVAGR** |
|  | 2981 | **634.6759** | **1267.3370** | **1267.5140** | **-0.1770** | **1** | **55** | **0.0082** | **1** | **ALPAPIEKTISK** |
|  | 1408 | **433.4066** | **1297.1975** | **1296.4326** | **0.7649** | **1** | **53** | **0.012** | **1** | **AIESSRDLLHR** |
|  | 1394 | **433.0323** | **1296.0748** | **1296.4326** | **-0.3579** | **1** | **49** | **0.032** | **1** | **AIESSRDLLHR** |
|  | 278 | **374.8306** | **1121.4697** | **1120.3880** | **1.0817** | **1** | **48** | **0.051** | **1** | **VGLKAPGIIPR** |
|  | 1246 | **421.8661** | **841.7174** | **841.9949** | **-0.2776** | **0** | **42** | **0.16** | **1** | **GITLSVRP** |
|  | 1245 | **421.8553** | **841.6958** | **841.9949** | **-0.2991** | **0** | **40** | **0.3** | **1** | **GITLSVRP** |
|  | 1244 | **421.8313** | **841.6478** | **841.9949** | **-0.3471** | **0** | **39** | **0.32** | **1** | **GITLSVRP** |
|  | 2217 | **504.1241** | **1006.2335** | **1007.1411** | **-0.9075** | **0** | **39** | **0.35** | **1** | **VSIVNQYGK** |
|  | 2924 | **615.1027** | **1228.1905** | **1228.3571** | **-0.1665** | **1** | **39** | **0.33** | **1** | **AAPGAEFAPNKR** |
|  | 2984 | **634.8794** | **1267.7440** | **1267.5140** | **0.2300** | **1** | **38** | **0.32** | **1** | **ALPAPIEKTISK** |
|  | 1264 | **422.2444** | **842.4740** | **841.9949** | **0.4790** | **0** | **37** | **0.48** | **1** | **GITLSVRP** |
|  | 1253 | **422.1202** | **842.2257** | **841.9949** | **0.2307** | **0** | **37** | **0.61** | **1** | **GITLSVRP** |
|  | 2408 | **524.5936** | **1047.1725** | **1046.1291** | **1.0433** | **0** | **36** | **0.79** | **1** | **LSSPADITDK** |
|  | 1004 | **408.1493** | **814.2837** | **813.9847** | **0.2990** | **0** | **35** | **0.81** | **1** | **QLLATLR** |
|  | 1838 | **464.8552** | **1391.5435** | **1390.6303** | **0.9132** | **1** | **35** | **0.76** | **1** | **RAPSSPVAKPGPVK** |
|  | 2516 | **543.1519** | **1084.2889** | **1083.2817** | **1.0072** | **1** | **35** | **0.85** | **1** | **ILKSPEIQR** |
|  | 1251 | **422.0760** | **842.1371** | **841.9949** | **0.1422** | **0** | **34** | **1.1** | **1** | **GITLSVRP** |
|  | 3377 | **783.8903** | **1565.7657** | **1566.7957** | **-1.0299** | **1** | **34** | **1.3** | **1** | **FKGPFTDVVTTNLK** |
|  | 1257 | **422.1456** | **842.2764** | **841.9949** | **0.2814** | **0** | **34** | **1.3** | **1** | **GITLSVRP** |
|  | 247 | **372.9965** | **743.9783** | **743.8915** | **0.0867** | **0** | **33** | **1.4** | **1** | **SGNLLLK** |
|  | 484 | **387.0974** | **772.1800** | **772.8932** | **-0.7131** | **1** | **33** | **1.5** | **1** | **KGISGGVR** |
|  | 1334 | **428.8937** | **855.7727** | **855.9785** | **-0.2058** | **0** | **33** | **1.3** | **1** | **SPSSLLPR** |
|  | 3378 | **784.1382** | **1566.2616** | **1566.7957** | **-0.5341** | **1** | **32** | **1.3** | **1** | **FKGPFTDVVTTNLK** |
|  | 244 | **372.7075** | **743.4003** | **743.8486** | **-0.4484** | **0** | **32** | **1.7** | **1** | **QAGVELK** |
|  | 3120 | **682.0800** | **2043.2177** | **2042.1695** | **1.0482** | **0** | **31** | **1.9** | **1** | **HAPINSAQHLDNVDQTGPK** |
|  | 3301 | **744.6049** | **1487.1949** | **1486.6316** | **0.5634** | **2** | **31** | **1.7** | **1** | **MQKSSSTRAGDMR + 2 Oxidation (M)** |
|  | 1313 | **427.5498** | **1279.6271** | **1280.4962** | **-0.8691** | **2** | **31** | **2.3** | **1** | **QRLKGDACVYK** |
|  | 2654 | **566.0508** | **1130.0868** | **1129.3517** | **0.7350** | **1** | **31** | **2.3** | **1** | **KITVLGANWK** |
|  | 1539 | **441.8452** | **881.6755** | **881.0706** | **0.6049** | **0** | **31** | **2** | **1** | **IGPLGLSPK** |
|  | 2827 | **600.6270** | **1199.2391** | **1198.4173** | **0.8219** | **0** | **31** | **2.5** | **1** | **AVFPSIVGRPR** |
|  | 567 | 389.1444 | 1164.4110 | 1165.3840 | -0.9731 | 0 | 31 | 2.5 | 1 | TLMLMAQEGR + Oxidation (M) |
|  | 240 | **372.4212** | **742.8277** | **742.9102** | **-0.0826** | **2** | **30** | **3** | **1** | **KARQIK** |
|  | 1613 | **448.0508** | **1341.1302** | **1341.7335** | **-0.6033** | **2** | **30** | **2.4** | **1** | **MMMLVGMGKRR + 2 Oxidation (M)** |
|  | 241 | 372.5738 | 743.1328 | 742.9035 | 0.2293 | 0 | 30 | 2.6 | 1 | AGLLELK |
|  | 3223 | **736.4252** | **2206.2533** | **2206.5414** | **-0.2880** | **1** | **29** | **3.4** | **1** | **MLYFAPDLVFNEYRMHK + 2 Oxidation (M)** |
|  | 1262 | **422.1990** | **842.3832** | **841.9949** | **0.3882** | **0** | **28** | **4** | **1** | **GITLSVRP** |
|  | 242 | 372.6068 | 743.1989 | 742.9466 | 0.2523 | 1 | 28 | 3.5 | 1 | KELILK |
|  | 946 | **407.2807** | **1218.8199** | **1218.4402** | **0.3797** | **1** | **28** | **3** | **1** | **LEVTSKVLTTK** |
|  | 1654 | **450.6399** | **899.2650** | **899.0464** | **0.2186** | **0** | **28** | **3.6** | **1** | **VQTVPLSR** |
|  | 1493 | **437.5493** | **873.0838** | **873.0719** | **0.0119** | **1** | **28** | **5.4** | **1** | **DICLPGKK** |
|  | 599 | **389.9913** | **777.9679** | **776.9431** | **1.0248** | **0** | **28** | **4.4** | **1** | **MSPIWK + Oxidation (M)** |
|  | 458 | **386.1685** | **770.3221** | **770.8771** | **-0.5550** | **0** | **28** | **3.9** | **1** | **GAQGGLLR** |
|  | 237 | **372.3408** | **1114.0002** | **1113.2251** | **0.7751** | **1** | **28** | **4.4** | **1** | **VNKTQHASTK** |
|  | 3204 | **719.0892** | **2154.2453** | **2154.6125** | **-0.3671** | **2** | **27** | **4.5** | **1** | **FNAIPIKLPMTFFTKLEK + Oxidation (M)** |
|  | 1911 | **472.6117** | **1414.8129** | **1414.6929** | **0.1200** | **0** | **27** | **5.5** | **1** | **QLGLVPFPPPPPR** |
|  | 2989 | 637.4632 | 1272.9116 | 1273.4771 | -0.5654 | 2 | 27 | 4.9 | 1 | DKIDKWDLIK |
|  | 272 | **374.3108** | **1119.9103** | **1120.3880** | **-0.4778** | **1** | **27** | **5.5** | **1** | **VGLKAPGIIPR** |
|  | 1363 | **430.3674** | **1288.0799** | **1287.4458** | **0.6342** | **1** | **27** | **5.1** | **1** | **RLLPDSSSGCPR** |
|  | 2690 | **576.7996** | **1151.5845** | **1151.2695** | **0.3149** | **0** | **27** | **4.4** | **1** | **SDSNYIIAIR** |
|  | 1478 | **437.0710** | **872.1272** | **872.0639** | **0.0633** | **1** | **27** | **6.3** | **1** | **LLLTKER** |
|  | 1267 | **422.3159** | **842.6171** | **841.9949** | **0.6221** | **0** | **27** | **5** | **1** | **GITLSVRP** |
|  | 1260 | **422.1897** | **842.3647** | **842.0379** | **0.3267** | **0** | **27** | **6.3** | **1** | **AVTLLGLR** |
|  | 2126 | 491.4940 | 1471.4597 | 1472.5468 | -1.0871 | 2 | 27 | 5.7 | 1 | RGGGCAEAGGDGPRR + Carbamidomethyl (C) |
|  | 1341 | **429.0541** | **856.0934** | **855.9785** | **0.1149** | **0** | **26** | **5.6** | **1** | **SPSSLLPR** |
|  | 430 | **385.9967** | **1154.9680** | **1154.3185** | **0.6496** | **1** | **26** | **5.9** | **1** | **SRVGMGGMEAK + 2 Oxidation (M)** |
|  | 882 | **406.1418** | **810.2688** | **809.9116** | **0.3572** | **0** | **26** | **6.7** | **1** | **GFFNGLR** |
|  | 1653 | **450.6075** | **899.2002** | **899.0099** | **0.1904** | **2** | **26** | **7** | **1** | **RDRSLPR** |
|  | 2635 | **563.6307** | **1687.8700** | **1687.8742** | **-0.0041** | **1** | **26** | **8.1** | **1** | **QAPGKGLEWVANMDR + Oxidation (M)** |
|  | 2350 | **520.0779** | **1557.2115** | **1556.6568** | **0.5547** | **2** | **26** | **7** | **1** | **MKEDGGAEFSARSR + Oxidation (M)** |
|  | 2795 | **595.6427** | **1783.9059** | **1783.0285** | **0.8775** | **1** | **26** | **9.3** | **1** | **GKTVPEELVKPEELSK** |
|  | 652 | **396.3463** | **1186.0168** | **1186.3206** | **-0.3038** | **2** | **26** | **7.4** | **1** | **DSMSMRSGRK + 2 Oxidation (M)** |
|  | 440 | **386.0802** | **1155.2184** | **1154.3464** | **0.8720** | **1** | **26** | **6.9** | **1** | **CIAATVAHRR + Carbamidomethyl (C)** |
|  | 1252 | **422.0955** | **842.1762** | **841.9949** | **0.1812** | **0** | **25** | **8.2** | **1** | **GITLSVRP** |
|  | 1343 | **429.0944** | **856.1741** | **856.9664** | **-0.7924** | **0** | **25** | **7.4** | **1** | **GAGLSSIPR** |
|  | 993 | **407.8957** | **813.7766** | **813.9847** | **-0.2081** | **0** | **25** | **7.7** | **1** | **QLLATLR** |
|  | 1696 | **456.2446** | **1365.7115** | **1365.5594** | **0.1522** | **1** | **25** | **7** | **1** | **KGACENCGAMTHK + Oxidation (M)** |
|  | 1912 | **472.7252** | **1415.1533** | **1414.5621** | **0.5912** | **1** | **25** | **6.5** | **1** | **KNSFSLSSFLER** |
|  | 1290 | **425.7770** | **1274.3089** | **1274.3378** | **-0.0290** | **1** | **25** | **8.1** | **1** | **RQVTAEQGEEK** |
|  | 1503 | **438.0699** | **874.1250** | **873.9937** | **0.1314** | **0** | **25** | **9.2** | **1** | **SLVNLGGSK** |
|  | 1608 | **447.0918** | **1338.2533** | **1338.5291** | **-0.2758** | **1** | **25** | **8.7** | **1** | **APKISMPDVDLH + Oxidation (M)** |
|  | 955 | **407.4147** | **1219.2218** | **1218.4052** | **0.8167** | **1** | **25** | **8.8** | **1** | **IGSKGNQIFVR** |
|  | 1007 | **408.2089** | **1221.6044** | **1222.4351** | **-0.8307** | **0** | **25** | **7.8** | **1** | **FGIAVLGYLNR** |
|  | 1468 | 436.2771 | 870.5395 | 870.0283 | 0.5112 | 0 | 25 | 6.6 | 1 | TPCPSLPR |
|  | 543 | 388.6043 | 775.1939 | 774.9024 | 0.2915 | 0 | 25 | 8.1 | 1 | IVYFSF |
|  | 1351 | **429.2855** | **1284.8344** | **1285.4497** | **-0.6153** | **1** | **25** | **7** | **1** | **NRSLASPLQATK** |
|  | 1069 | **411.2165** | **1230.6274** | **1229.4892** | **1.1381** | **1** | **25** | **8.1** | **1** | **TLTEHLLKMK + Oxidation (M)** |
|  | 985 | **407.8242** | **813.6337** | **813.8988** | **-0.2650** | **1** | **25** | **9.1** | **1** | **IVRGPASD** |
|  | 972 | **407.7127** | **1220.1160** | **1219.3436** | **0.7724** | **0** | **25** | **7.5** | **1** | **AWGQGTLVTVST** |
|  | 873 | **406.0816** | **1215.2227** | **1214.4350** | **0.7878** | **0** | **25** | **8.8** | **1** | **MIVAAEAVAAPR + Oxidation (M)** |
|  | 3159 | **686.2133** | **2055.6176** | **2055.2693** | **0.3483** | **2** | **25** | **9.2** | **1** | **QYYLGDKCQVSSFFKNQ** |
|  | 396 | **384.9377** | **767.8606** | **768.8335** | **-0.9728** | **0** | **24** | **8.3** | **1** | **SMSATEK + Oxidation (M)** |
|  | 2209 | 502.3960 | 1002.7772 | 1002.2288 | 0.5483 | 0 | 24 | 8.4 | 1 | AVIDCAGILK |
|  | 534 | **388.2480** | **774.4813** | **774.9024** | **-0.4211** | **0** | **24** | **9.3** | **1** | **IVYFSF** |
|  | 831 | 405.0988 | 1212.2742 | 1211.4111 | 0.8631 | 0 | 24 | 9.3 | 1 | LVEVCADFCR + Carbamidomethyl (C) |
|  | 3016 | **655.7045** | **1309.3942** | **1309.4466** | **-0.0524** | **2** | **24** | **11** | **1** | **SCQKDSEVGTKK** |
|  | 1734 | **458.2834** | **914.5521** | **914.0178** | **0.5343** | **0** | **24** | **8.6** | **1** | **ADLQAAAVR** |
|  | 1836 | **464.7000** | **927.3852** | **928.1140** | **-0.7288** | **1** | **24** | **8** | **1** | **AGAMRLGPR** |
|  | 981 | **407.7863** | **813.5579** | **812.9156** | **0.6424** | **0** | **24** | **10** | **1** | **FSGPLHR** |
|  | 3050 | **666.6744** | **1997.0011** | **1997.1937** | **-0.1925** | **2** | **24** | **10** | **1** | **VRNQIGSMQRLGSFSSDV + Oxidation (M)** |
|  | 2486 | **537.9554** | **1610.8442** | **1611.8015** | **-0.9573** | **0** | **24** | **11** | **1** | **RPQGAYSMPMSSGAR + Oxidation (M)** |
|  | 3296 | **743.5922** | **1485.1695** | **1484.5293** | **0.6403** | **0** | **24** | **8.8** | **1** | **AWGAGPPEGGGGGSATR** |
|  | 2893 | **612.0229** | **1222.0310** | **1221.4276** | **0.6034** | **1** | **24** | **11** | **1** | **KIGNVMVTTSR + Oxidation (M)** |
|  | 1291 | **425.8882** | **1274.6425** | **1274.3378** | **0.3046** | **1** | **24** | **11** | **1** | **RQVTAEQGEEK** |
|  | 2894 | **612.0516** | **1833.1327** | **1831.9763** | **1.1564** | **0** | **24** | **11** | **1** | **QDLSAPPGYTLTENVAR** |
|  | 709 | **400.9957** | **1199.9648** | **1200.3882** | **-0.4234** | **0** | **24** | **11** | **1** | **PAPHFLYSLR** |
|  | 703 | **400.1991** | **1197.5750** | **1196.4645** | **1.1106** | **1** | **24** | **8.6** | **1** | **TQMAEVRCMK** |
|  | 400 | **384.9993** | **1151.9757** | **1151.3144** | **0.6614** | **1** | **24** | **9.5** | **1** | **KDFTQLFPR** |
|  | 2242 | **506.0099** | **1010.0050** | **1009.1537** | **0.8513** | **1** | **24** | **10** | **1** | **ETKDPPPLL** |
|  | 43 | **363.0821** | **1086.2240** | **1085.2778** | **0.9463** | **1** | **24** | **12** | **1** | **KLHIGMENK + Oxidation (M)** |
|  | 61 | **363.2901** | **1086.8482** | **1087.9816** | **-1.1333** | **1** | **24** | **9.5** | **1** | **RSGGXCQXCR** |
|  | 293 | **376.1896** | **1125.5466** | **1126.1526** | **-0.6060** | **0** | **24** | **9.1** | **1** | **MTTLDSNNNT + Oxidation (M)** |
|  | 969 | 407.6347 | 1219.8820 | 1219.4297 | 0.4523 | 1 | 24 | 9.3 | 1 | FLQTIVKADGK |
|  | 3039 | **666.4562** | **1996.3464** | **1997.1257** | **-0.7794** | **1** | **24** | **11** | **1** | **RFSDQAAGPAIPTSNSYSK** |
|  | 1015 | **408.2680** | **1221.7819** | **1222.3095** | **-0.5275** | **1** | **24** | **9.7** | **1** | **GARCSGPSCSSP + 2 Carbamidomethyl (C)** |
|  | 2287 | **513.8025** | **1538.3853** | **1537.8104** | **0.5749** | **2** | **24** | **9.1** | **1** | **HQRKLLAAINAFR** |
|  | 1526 | **440.4392** | **1318.2955** | **1317.4900** | **0.8055** | **1** | **23** | **14** | **1** | **NPQVADLKAFSK** |
|  | 1399 | **433.1046** | **1296.2917** | **1297.4574** | **-1.1657** | **0** | **23** | **12** | **1** | **TCDPVEMSYPR** |
|  | 451 | **386.1087** | **1155.3038** | **1155.3247** | **-0.0208** | **2** | **23** | **11** | **1** | **EKYGDKMLR + Oxidation (M)** |
|  | 883 | **406.1420** | **1215.4039** | **1216.3033** | **-0.8993** | **1** | **23** | **12** | **1** | **HRDFLTNDAK** |
|  | 2026 | **483.3558** | **1447.0452** | **1447.6782** | **-0.6329** | **1** | **23** | **10** | **1** | **AKYFCAMGGGVTDK** |
|  | 1375 | **431.0636** | **1290.1685** | **1290.5358** | **-0.3673** | **2** | **23** | **15** | **1** | **KMASATRLIQR + Oxidation (M)** |
|  | 899 | **406.2734** | **1215.7981** | **1216.3000** | **-0.5019** | **0** | **23** | **9.5** | **1** | **ATHDQAVEAFK** |
|  | 1651 | **450.5142** | **1348.5203** | **1347.5624** | **0.9579** | **2** | **23** | **15** | **1** | **GVASSRFLPKGTK** |
|  | 2648 | **565.8311** | **1694.4712** | **1694.7606** | **-0.2895** | **1** | **23** | **11** | **1** | **DGADKGQHIGNQQAQK** |
|  | 846 | **405.2262** | **808.4377** | **807.9192** | **0.5185** | **1** | **23** | **10** | **1** | **RCVSTSR** |
|  | 2656 | 566.2069 | 1130.3990 | 1131.3246 | -0.9256 | 1 | 23 | 14 | 1 | AHLIKSSTFK |
|  | 1773 | **459.5696** | **1375.6867** | **1374.5397** | **1.1469** | **1** | **23** | **17** | **1** | **LQDKATVLTTER** |
|  | 3021 | **658.3064** | **1314.5980** | **1315.4823** | **-0.8843** | **2** | **23** | **13** | **1** | **LSGQTRTIARGR** |
|  | 1272 | **423.2590** | **1266.7547** | **1266.3621** | **0.3927** | **0** | **23** | **12** | **1** | **AHTFGHPPSSTK** |
|  | 1675 | **452.8045** | **1355.3913** | **1354.5085** | **0.8828** | **1** | **23** | **14** | **1** | **ALEEPANDIKVR** |
|  | 3418 | **809.6301** | **1617.2455** | **1616.7398** | **0.5057** | **1** | **23** | **12** | **1** | **HGQRGHGQQLLETR** |
|  | 926 | **407.1430** | **812.2713** | **811.9426** | **0.3287** | **0** | **23** | **13** | **1** | **EFIMEK + Oxidation (M)** |
|  | 2172 | **498.4286** | **994.8425** | **995.2363** | **-0.3939** | **1** | **23** | **11** | **1** | **GTVLYAMKL** |
|  | 129 | 367.7991 | 1100.3752 | 1099.2414 | 1.1338 | 0 | 23 | 15 | 1 | IIRPSETAGR |
|  | 3035 | **666.4125** | **1996.2153** | **1995.2389** | **0.9763** | **0** | **23** | **14** | **1** | **CSCCQAQLGDIGTSCYTK + 2 Carbamidomethyl (C)** |
|  | 3085 | **668.8153** | **2003.4238** | **2003.2680** | **0.1558** | **2** | **23** | **16** | **1** | **AVVMTQSPGPCRASVRDR + Carbamidomethyl (C); Oxidation (M)** |
|  | 939 | **407.2173** | **1218.6296** | **1219.4727** | **-0.8431** | **0** | **23** | **12** | **1** | **ELLPVLISAHK** |
|  | 1802 | **460.8298** | **919.6448** | **920.0042** | **-0.3594** | **0** | **23** | **15** | **1** | **GGSYMPHR + Oxidation (M)** |
|  | 1950 | **476.0224** | **1425.0451** | **1425.6743** | **-0.6291** | **2** | **23** | **15** | **1** | **VCSRLLGKGSSTMA + Oxidation (M)** |
|  | 2966 | **630.0604** | **1258.1061** | **1258.3781** | **-0.2721** | **0** | **23** | **14** | **1** | **TDQEVLGELVR** |
|  | 874 | **406.0855** | **1215.2343** | **1216.3050** | **-1.0708** | **2** | **23** | **14** | **1** | **EELARSEARR** |
|  | 84 | **365.1901** | **728.3654** | **728.7545** | **-0.3891** | **0** | **23** | **12** | **1** | **STTQHR** |
|  | 760 | **403.0562** | **1206.1463** | **1206.3300** | **-0.1837** | **0** | **23** | **16** | **1** | **GAMGGQSGLGVTR + Oxidation (M)** |
|  | 1102 | **413.4780** | **1237.4119** | **1236.3561** | **1.0558** | **1** | **23** | **15** | **1** | **SVMNTSNPRSK + Oxidation (M)** |
|  | 932 | **407.1604** | **1218.4589** | **1217.4354** | **1.0235** | **0** | **23** | **14** | **1** | **SEIIMSPATLR** |
|  | 3097 | **669.6713** | **2005.9916** | **2005.2603** | **0.7314** | **2** | **23** | **14** | **1** | **MWNFLSRNGELTKHQK + Oxidation (M)** |
|  | 455 | **386.1441** | **1155.4100** | **1154.2538** | **1.1563** | **1** | **23** | **13** | **1** | **EFGSGCAEAKR** |
|  | 94 | **366.0135** | **1095.0184** | **1094.3076** | **0.7108** | **0** | **23** | **17** | **1** | **LGGPVQALIAR** |
|  | 383 | **384.2008** | **1149.5803** | **1148.4198** | **1.1606** | **2** | **23** | **12** | **1** | **ILRSLGSKMK + Oxidation (M)** |
|  | 2415 | **525.5325** | **1573.5752** | **1573.7085** | **-0.1333** | **1** | **23** | **16** | **1** | **HSLNIGDYNRTVGK** |
|  | 604 | **390.0052** | **1166.9936** | **1166.4119** | **0.5817** | **0** | **22** | **15** | **1** | **MVNILMANTK + 2 Oxidation (M)** |
|  | 710 | **401.0114** | **1200.0119** | **1199.4464** | **0.5655** | **0** | **22** | **16** | **1** | **QCIHLGLGACK + Carbamidomethyl (C)** |
|  | 793 | **404.0421** | **1209.1042** | **1209.4798** | **-0.3756** | **1** | **22** | **15** | **1** | **MISTRVMDIK + Oxidation (M)** |
|  | 1122 | **415.1332** | **1242.3774** | **1242.3987** | **-0.0213** | **0** | **22** | **16** | **1** | **GSGSVVGELMYK + Oxidation (M)** |
|  | 443 | **386.0879** | **1155.2415** | **1155.3212** | **-0.0797** | **0** | **22** | **14** | **1** | **DFSAISLACTK** |
|  | 2651 | **565.8579** | **1694.5516** | **1694.9066** | **-0.3550** | **1** | **22** | **13** | **1** | **FVKINNSTNEGMNVK** |
|  | 412 | **385.0757** | **768.1366** | **767.8766** | **0.2599** | **1** | **22** | **14** | **1** | **LGSKAHR** |
|  | 2046 | **486.1162** | **970.2176** | **971.1157** | **-0.8980** | **2** | **22** | **14** | **1** | **RVGEGAVKR** |
|  | 3259 | **740.8045** | **1479.5942** | **1480.6618** | **-1.0676** | **0** | **22** | **17** | **1** | **MNVSFFASSMEAK + 2 Oxidation (M)** |
|  | 1263 | **422.2028** | **842.3909** | **841.9949** | **0.3959** | **0** | **22** | **16** | **1** | **GITLSVRP** |
|  | 1385 | **431.6772** | **1292.0096** | **1291.5023** | **0.5072** | **1** | **22** | **14** | **1** | **SAGVPSRVIHIR** |
|  | 1765 | **459.2141** | **916.4134** | **917.0847** | **-0.6712** | **1** | **22** | **18** | **1** | **GSQKGICPK** |
|  | 2283 | **513.6868** | **1538.0383** | **1538.7425** | **-0.7041** | **0** | **22** | **14** | **1** | **QPLMLMATSNEGSK + 2 Oxidation (M)** |
|  | 852 | **405.6026** | **1213.7855** | **1214.3918** | **-0.6062** | **0** | **22** | **12** | **1** | **MEGAPPGSLALR + Oxidation (M)** |
|  | 2248 | **506.5948** | **1516.7623** | **1516.7465** | **0.0157** | **1** | **22** | **19** | **1** | **WTKHICAICGDR + 2 Carbamidomethyl (C)** |
|  | 1621 | **448.7836** | **1343.3285** | **1343.5520** | **-0.2235** | **0** | **22** | **14** | **1** | **QLGCPTAVTAIGR + Carbamidomethyl (C)** |
|  | 1879 | **469.3301** | **936.6454** | **936.0267** | **0.6187** | **1** | **22** | **13** | **1** | **LEEPRHR** |
|  | 1973 | **477.7606** | **1430.2596** | **1429.5553** | **0.7043** | **2** | **22** | **12** | **1** | **WLSKSTTRSSSSC** |
|  | 81 | **364.6283** | **1090.8627** | **1090.2760** | **0.5868** | **0** | **22** | **13** | **1** | **GNLPPLVPAGR** |
|  | 729 | **401.8787** | **801.7425** | **802.8761** | **-1.1335** | **0** | **22** | **19** | **1** | **GGSNTLVR** |
|  | 1862 | **467.4302** | **932.8456** | **933.0379** | **-0.1923** | **0** | **22** | **15** | **1** | **SAPTTVMNP + Oxidation (M)** |
|  | 2583 | **554.0322** | **1106.0497** | **1106.2769** | **-0.2273** | **0** | **22** | **17** | **1** | **CLDNLCVNR + Carbamidomethyl (C)** |
|  | 1416 | **434.2080** | **1299.6019** | **1298.4353** | **1.1666** | **1** | **22** | **16** | **1** | **RCGRPGAQPSGR + Carbamidomethyl (C)** |
|  | 1488 | **437.2640** | **872.5133** | **873.0139** | **-0.5006** | **1** | **22** | **15** | **1** | **RLPAFNR** |
|  | 2892 | 611.9626 | 1832.8658 | 1832.0011 | 0.8646 | 1 | 22 | 15 | 1 | LQLNNRPNMTADEKTA + Oxidation (M) |
|  | 246 | **372.8572** | **743.6996** | **743.8486** | **-0.1491** | **0** | **22** | **20** | **1** | **QVQELK** |
|  | 2767 | **592.8878** | **1775.6413** | **1775.0345** | **0.6068** | **0** | **22** | **14** | **1** | **LTPPTMPPPPTTQGAPR + Oxidation (M)** |
|  | 1034 | **408.9687** | **1223.8838** | **1223.3504** | **0.5334** | **2** | **22** | **19** | **1** | **HRRHHLPDR** |
|  | 1269 | **423.0883** | **844.1618** | **843.0262** | **1.1356** | **2** | **22** | **20** | **1** | **VATVIRKG** |
|  | 379 | **382.4869** | **762.9590** | **762.8387** | **0.1204** | **1** | **22** | **24** | **1** | **GGCAGSRR** |
|  | 1273 | 423.2770 | 1266.8089 | 1266.4498 | 0.3590 | 0 | 22 | 17 | 1 | VARPAQLSAPTR |
|  | 1418 | **434.2182** | **1299.6324** | **1298.4519** | **1.1806** | **0** | **22** | **17** | **1** | **MDGMVPGNHQGR** |
|  | 3049 | **666.6399** | **1996.8975** | **1996.3730** | **0.5245** | **0** | **22** | **16** | **1** | **AATVTLMLAMLSTPMAEGR + 2 Oxidation (M)** |
|  | 1036 | **408.9813** | **1223.9218** | **1224.2856** | **-0.3638** | **1** | **22** | **21** | **1** | **RCGASGDECGR + 2 Carbamidomethyl (C)** |
|  | 39 | **363.0750** | **724.1352** | **723.8805** | **0.2547** | **0** | **22** | **19** | **1** | **FLAVMQ + Oxidation (M)** |
|  | 1323 | **428.2035** | **854.3921** | **854.9507** | **-0.5585** | **0** | **22** | **18** | **1** | **QSLPASPR** |
|  | 1216 | **419.7835** | **1256.3284** | **1255.3791** | **0.9493** | **0** | **21** | **18** | **1** | **SAAGPFAPPGDLR** |
|  | 1426 | **434.6857** | **867.3567** | **867.9445** | **-0.5878** | **0** | **21** | **14** | **1** | **EGPPPGFAP** |
|  | 3229 | **739.8044** | **1477.5940** | **1476.7227** | **0.8713** | **0** | **21** | **22** | **1** | **ATLGDVPKPGLRPR** |
|  | 2445 | **532.8403** | **1595.4988** | **1594.7906** | **0.7082** | **0** | **21** | **16** | **1** | **NGSIENCLHKPTPK + Carbamidomethyl (C)** |
|  | 167 | **369.2203** | **1104.6388** | **1104.2991** | **0.3397** | **0** | **21** | **17** | **1** | **QQLLIGAYAK** |
|  | 976 | **407.7250** | **1220.1529** | **1219.3901** | **0.7628** | **0** | **21** | **17** | **1** | **MDPFHNMAPK + 2 Oxidation (M)** |
|  | 1979 | **478.1108** | **954.2068** | **953.0589** | **1.1479** | **1** | **21** | **18** | **1** | **CCRSQSR + 2 Carbamidomethyl (C)** |
|  | 410 | **385.0502** | **1152.1283** | **1151.3178** | **0.8106** | **0** | **21** | **17** | **1** | **MAAMAVGGAGGSR + Oxidation (M)** |
|  | 877 | **406.1007** | **1215.2799** | **1215.4660** | **-0.1861** | **0** | **21** | **19** | **1** | **AAWLMMMGTR + 3 Oxidation (M)** |
|  | 1274 | **423.3342** | **1266.9804** | **1266.4711** | **0.5093** | **0** | **21** | **18** | **1** | **SPLCLNCGSCR + 2 Carbamidomethyl (C)** |
|  | 920 | **407.0921** | **1218.2541** | **1217.3327** | **0.9214** | **1** | **21** | **19** | **1** | **RLTASDIGTQR** |
|  | 2341 | **519.1926** | **1554.5555** | **1553.7602** | **0.7953** | **1** | **21** | **19** | **1** | **WLEVEIAPDIRGR** |
|  | 2897 | **612.1732** | **1833.4975** | **1834.2123** | **-0.7148** | **2** | **21** | **20** | **1** | **QQVLVFYTKLLGRIR** |
|  | 3052 | **666.6815** | **1997.0222** | **1997.1277** | **-0.1055** | **2** | **21** | **20** | **1** | **RPQPVKSELDEEEERR** |
|  | 618 | 392.4475 | 1174.3204 | 1173.4227 | 0.8976 | 0 | 21 | 22 | 1 | TLTMPTTAIPK |
|  | 2607 | **558.0845** | **1114.1542** | **1113.1883** | **0.9658** | **1** | **21** | **20** | **1** | **RGGGGGGGQIAAR** |
|  | 2959 | **628.6353** | **1255.2557** | **1256.3655** | **-1.1098** | **1** | **21** | **20** | **1** | **HTTDLDASKIR** |
|  | 341 | **379.2074** | **1134.5999** | **1134.2010** | **0.3989** | **1** | **21** | **16** | **1** | **RQQGSSLSGSK** |
|  | 210 | **370.9135** | **1109.7182** | **1109.2778** | **0.4403** | **0** | **21** | **15** | **1** | **ATEATAMAMGR** |
|  | 3139 | **684.3075** | **1366.6002** | **1367.6363** | **-1.0361** | **1** | **21** | **20** | **1** | **LGGLSISPAGIVKR** |
|  | 33 | **362.5938** | **1084.7592** | **1085.2545** | **-0.4954** | **0** | **21** | **16** | **1** | **QVQSLLELR** |
|  | 348 | **379.8121** | **1136.4142** | **1135.2339** | **1.1804** | **1** | **21** | **22** | **1** | **SPARHPGAGTGK** |
|  | 1104 | **413.5634** | **1237.6681** | **1238.3105** | **-0.6425** | **1** | **21** | **17** | **1** | **AQGPEPAARGER** |
|  | 546 | **388.9359** | **775.8570** | **775.8707** | **-0.0137** | **0** | **21** | **24** | **1** | **ACETQPK** |
|  | 3337 | **758.3327** | **1514.6506** | **1514.8536** | **-0.2029** | **0** | **21** | **20** | **1** | **CMSALSMPMLATSR + Oxidation (M)** |
|  | 1381 | **431.2561** | **1290.7460** | **1290.4528** | **0.2932** | **1** | **21** | **20** | **1** | **RWWCAAPSGTR** |
|  | 3375 | **780.1838** | **2337.5293** | **2336.6916** | **0.8378** | **2** | **21** | **19** | **1** | **GSEVTAMLEKGERMGCPAGCPR + Carbamidomethyl (C)** |
|  | 1634 | **449.8708** | **1346.5902** | **1346.3837** | **0.2065** | **0** | **21** | **21** | **1** | **AGGSCQAAQPEDR + Carbamidomethyl (C)** |
|  | 3514 | **1160.3964** | **3478.1669** | **3477.8685** | **0.2984** | **2** | **21** | **17** | **1** | **MGKCCHHCFPCCRGSGTSNVGTSGDHDNSFMK + Carbamidomethyl (C); Oxidation (M)** |
|  | 565 | **389.1380** | **1164.3919** | **1164.3331** | **0.0589** | **1** | **21** | **23** | **1** | **MSDRLGQITK + Oxidation (M)** |
|  | 968 | **407.6170** | **1219.8287** | **1219.4083** | **0.4205** | **2** | **21** | **17** | **1** | **GKKEGGSSMLPL + Oxidation (M)** |
|  | 1064 | **411.0679** | **1230.1815** | **1230.4989** | **-0.3173** | **0** | **21** | **23** | **1** | **MEAMVIGGGCCK + 2 Oxidation (M)** |
|  | 238 | **372.3439** | **1114.0096** | **1114.3388** | **-0.3292** | **2** | **21** | **20** | **1** | **KRIAIEGISK** |
|  | 324 | **377.3519** | **752.6891** | **752.9864** | **-0.2973** | **1** | **21** | **18** | **1** | **LSMKMK + Oxidation (M)** |
|  | 1285 | **424.3496** | **1270.0267** | **1270.4585** | **-0.4318** | **2** | **21** | **20** | **1** | **LKETSSFRMR + Oxidation (M)** |
|  | 1221 | **420.1574** | **1257.4500** | **1256.4614** | **0.9885** | **1** | **21** | **20** | **1** | **QGLRCVACGHR + Carbamidomethyl (C)** |
|  | 1365 | **430.7491** | **1289.2251** | **1289.3724** | **-0.1473** | **1** | **21** | **22** | **1** | **ERMGEPSQDPK + Oxidation (M)** |
|  | 2065 | **487.1868** | **972.3588** | **972.0968** | **0.2619** | **1** | **21** | **23** | **1** | **LNLAGRDLT** |
|  | 2674 | **571.4552** | **1140.8956** | **1140.2915** | **0.6041** | **0** | **21** | **17** | **1** | **NGGLEVWLPR** |
|  | 3292 | **742.7919** | **1483.5691** | **1482.7453** | **0.8237** | **1** | **21** | **24** | **1** | **SELAGFYPRLMAK** |
|  | 87 | **365.4724** | **728.9299** | **727.8262** | **1.1037** | **0** | **21** | **26** | **1** | **MAESFK + Oxidation (M)** |
|  | 487 | **387.3409** | **772.6670** | **773.7952** | **-1.1282** | **1** | **21** | **23** | **1** | **EAQDRR** |
|  | 136 | **368.0820** | **1101.2238** | **1101.1646** | **0.0592** | **0** | **21** | **23** | **1** | **TYSLQSTSSK** |
|  | 2987 | **635.7371** | **1904.1890** | **1903.1305** | **1.0585** | **2** | **21** | **25** | **1** | **ATASSCNSPFPQRPRKR** |
|  | 711 | **401.0636** | **1200.1686** | **1200.3719** | **-0.2032** | **1** | **21** | **24** | **1** | **RMLSHSSGAVR** |
|  | 511 | **387.9517** | **773.8886** | **773.8778** | **0.0108** | **0** | **21** | **27** | **1** | **LGLSTQR** |
|  | 2932 | **616.9712** | **1231.9276** | **1231.4025** | **0.5251** | **0** | **21** | **20** | **1** | **CPSVSGPGCPATR** |
|  | 222 | 371.2485 | 1110.7233 | 1110.3502 | 0.3731 | 2 | 21 | 14 | 1 | KEPRGALLVK |
|  | 489 | **387.5325** | **1159.5754** | **1160.3842** | **-0.8087** | **1** | **21** | **27** | **1** | **AMEDGGVKLLK** |
|  | 130 | **367.9214** | **1100.7422** | **1100.1866** | **0.5556** | **1** | **21** | **25** | **1** | **VRQPSPSSSR** |
|  | 2384 | **523.1869** | **1566.5385** | **1565.6947** | **0.8438** | **1** | **21** | **24** | **1** | **AFNRASHLTQHQR** |
|  | 2390 | **523.3923** | **1044.7698** | **1045.1923** | **-0.4226** | **0** | **21** | **19** | **1** | **LSSAHVYLR** |
|  | 2647 | **565.8066** | **1694.3978** | **1694.7606** | **-0.3629** | **1** | **21** | **20** | **1** | **DGADKGQHIGNQQAQK** |
|  | 226 | 371.2701 | 1110.7881 | 1110.3502 | 0.4378 | 2 | 21 | 15 | 1 | KEPRGALLVK |
|  | 313 | **377.1616** | **752.3085** | **751.8508** | **0.4577** | **0** | **21** | **21** | **1** | **QMWSGK + Oxidation (M)** |
|  | 1157 | **417.0752** | **832.1357** | **830.9724** | **1.1633** | **2** | **21** | **26** | **1** | **AKAASAGKK** |
|  | 1786 | **460.3317** | **918.6487** | **919.0577** | **-0.4090** | **1** | **21** | **21** | **1** | **AEVDKVCR** |
|  | 2051 | 486.3709 | 1456.0905 | 1456.6848 | -0.5944 | 1 | 21 | 18 | 1 | KVWALAESGAISPK |
|  | 2930 | **616.3795** | **1230.7443** | **1230.4556** | **0.2886** | **1** | **21** | **24** | **1** | **AADKFLSLIPR** |
|  | 2391 | **523.4474** | **1567.3202** | **1566.6435** | **0.6767** | **2** | **20** | **20** | **1** | **ESEDKDEAVKECGK** |
|  | 990 | **407.8479** | **813.6810** | **812.8247** | **0.8564** | **0** | **20** | **24** | **1** | **SHPETDK** |
|  | 717 | **401.2312** | **1200.6714** | **1200.3766** | **0.2948** | **1** | **20** | **22** | **1** | **GQHLCPHPRR** |
|  | 2730 | **588.2816** | **1174.5483** | **1175.2332** | **-0.6848** | **1** | **20** | **25** | **1** | **EPNGSGRMDGR** |
|  | 2236 | **505.3693** | **1008.7239** | **1008.2999** | **0.4240** | **2** | **20** | **19** | **1** | **MVDMKKLK + Oxidation (M)** |
|  | 1147 | 416.2507 | 830.4866 | 830.8896 | -0.4030 | 1 | 20 | 24 | 1 | RSAGDVAR |
|  | 1794 | **460.5146** | **1378.5217** | **1378.5713** | **-0.0497** | **0** | **20** | **31** | **1** | **CSACEGLALTPDAK** |
|  | 811 | **404.1848** | **1209.5322** | **1210.3418** | **-0.8096** | **0** | **20** | **23** | **1** | **FLQVHASSGHK** |
|  | 35 | **362.9286** | **723.8423** | **723.7778** | **0.0646** | **0** | **20** | **24** | **1** | **GPSPGPGR** |
|  | 2601 | **557.0238** | **1668.0492** | **1667.9026** | **0.1466** | **1** | **20** | **25** | **1** | **FLIYKASSLASGAPSR** |
|  | 289 | **375.2119** | **748.4091** | **748.9330** | **-0.5239** | **0** | **20** | **22** | **1** | **MGGFLPK** |
|  | 1934 | **474.4391** | **1420.2950** | **1420.5054** | **-0.2104** | **1** | **20** | **24** | **1** | **CSAGSQKAGSGEGPGK** |
|  | 2208 | **502.3418** | **1002.6688** | **1003.0631** | **-0.3943** | **0** | **20** | **22** | **1** | **VSGYSFTDK** |
|  | 2450 | **533.7610** | **1065.5073** | **1066.2961** | **-0.7887** | **0** | **20** | **20** | **1** | **MITAMNTIR + Oxidation (M)** |
|  | 2760 | 592.7612 | 1183.5076 | 1183.3530 | 0.1546 | 1 | 20 | 26 | 1 | KYFPLTASEK |
|  | 1949 | **475.9766** | **1424.9077** | **1424.5950** | **0.3127** | **0** | **20** | **27** | **1** | **EIVDLGGTLPGPEK** |
|  | 1359 | **430.1097** | **858.2046** | **857.9115** | **0.2932** | **1** | **20** | **31** | **1** | **NSANSPRL** |
|  | 1413 | **434.1664** | **1299.4770** | **1300.5504** | **-1.0735** | **0** | **20** | **25** | **1** | **HLCGMATDLALR** |
|  | 2064 | **487.1703** | **1458.4887** | **1459.6476** | **-1.1590** | **1** | **20** | **29** | **1** | **VVVTTTSQARELR** |
|  | 2216 | **504.0384** | **1509.0930** | **1509.7925** | **-0.6995** | **2** | **20** | **28** | **1** | **QKFVSMKVDHMK + 2 Oxidation (M)** |
|  | 1132 | **415.9516** | **829.8884** | **828.8686** | **1.0198** | **0** | **20** | **32** | **1** | **YYAQER** |
|  | 50 | 363.1646 | 1086.4718 | 1087.1911 | -0.7193 | 2 | 20 | 24 | 1 | GNRATKVDAR |
|  | 1853 | **466.2456** | **1395.7146** | **1394.6898** | **1.0247** | **2** | **20** | **27** | **1** | **RFFQRVLCLR + Carbamidomethyl (C)** |
|  | 1650 | **450.3594** | **898.7041** | **899.0497** | **-0.3456** | **1** | **20** | **22** | **1** | **VATISPRR** |
|  | 3073 | **667.7620** | **2000.2637** | **2001.3775** | **-1.1138** | **1** | **20** | **34** | **1** | **IHRSPLCEYMINFIHK** |
|  | 2128 | **491.5427** | **1471.6058** | **1472.6858** | **-1.0800** | **2** | **20** | **31** | **1** | **NKILTGADGKNLTK** |
|  | 1790 | **460.3900** | **918.7652** | **918.1554** | **0.6098** | **1** | **20** | **25** | **1** | **KSNICILK** |
|  | 18 | **361.1335** | **1080.3784** | **1081.2211** | **-0.8428** | **0** | **20** | **27** | **1** | **GYVPANYLGK** |
|  | 555 | **389.0813** | **776.1478** | **775.8740** | **0.2739** | **1** | **20** | **31** | **1** | **SPEGCRK** |
|  | 2735 | **589.6622** | **1177.3097** | **1176.3486** | **0.9610** | **1** | **20** | **36** | **1** | **GSPPRAYLCGR** |
|  | 351 | **380.1275** | **1137.3602** | **1136.3195** | **1.0407** | **0** | **20** | **32** | **1** | **SSGMSQLIGLK + Oxidation (M)** |
|  | 629 | **393.5198** | **785.0248** | **784.9402** | **0.0845** | **0** | **20** | **30** | **1** | **SPLIIDK** |
|  | 1462 | **436.2421** | **870.4693** | **871.0579** | **-0.5885** | **0** | **20** | **24** | **1** | **MVTMCDR + Oxidation (M)** |
|  | 2783 | **593.8618** | **1778.5633** | **1777.9122** | **0.6510** | **0** | **20** | **24** | **1** | **AFSESGSNLHALMNQR + Oxidation (M)** |
|  | 2616 | **559.9107** | **1676.7099** | **1676.7670** | **-0.0570** | **1** | **20** | **27** | **1** | **DWEKHHHICSGPSSG** |
|  | 2742 | 591.4896 | 1771.4467 | 1772.0369 | -0.5902 | 1 | 20 | 22 | 1 | LCIYTDHAVSPRQLR |
|  | 1754 | 459.0175 | 916.0203 | 917.0451 | -1.0248 | 1 | 20 | 32 | 1 | QMVDPRR + Oxidation (M) |
|  | 1328 | **428.3061** | **1281.8960** | **1281.4411** | **0.4549** | **2** | **20** | **20** | **1** | **RSLAGGSDCFRL** |
|  | 109 | **367.0454** | **732.0761** | **732.8325** | **-0.7564** | **0** | **20** | **31** | **1** | **CHSACR + Carbamidomethyl (C)** |
|  | 563 | **389.1099** | **776.2050** | **776.8786** | **-0.6735** | **1** | **20** | **32** | **1** | **NTKLSSK** |
|  | 235 | **372.2883** | **1113.8427** | **1113.3111** | **0.5317** | **2** | **20** | **22** | **1** | **CDKCGKAFK + 2 Carbamidomethyl (C)** |
|  | 1144 | **416.1760** | **1245.5059** | **1244.4411** | **1.0648** | **1** | **20** | **34** | **1** | **ASTVVKGVASGLR** |
|  | 1261 | **422.1930** | **842.3711** | **841.9949** | **0.3762** | **0** | **20** | **31** | **1** | **GITLSVRP** |
|  | 2017 | **481.9819** | **1442.9236** | **1443.5667** | **-0.6431** | **1** | **20** | **31** | **1** | **AFSAKSNLNAHQR** |
|  | 2079 | **488.0582** | **974.1017** | **973.0088** | **1.0929** | **2** | **20** | **33** | **1** | **GGGSGGGRRGR** |
|  | 2629 | **562.3659** | **1684.0755** | **1682.8759** | **1.1996** | **1** | **20** | **27** | **1** | **SGQKTTIPASAPAGLQR** |
|  | 706 | **400.3445** | **798.6742** | **798.8941** | **-0.2199** | **2** | **20** | **22** | **1** | **RGPARSR** |
|  | 2057 | **486.7901** | **971.5655** | **971.1518** | **0.4137** | **0** | **20** | **25** | **1** | **LLQLSLER** |
|  | 893 | **406.2289** | **1215.6647** | **1216.3496** | **-0.6850** | **1** | **20** | **23** | **1** | **YSSALVRHQR** |
|  | 2093 | **488.4238** | **974.8329** | **974.1427** | **0.6902** | **2** | **20** | **26** | **1** | **AVRCQGRK + Carbamidomethyl (C)** |
|  | 123 | **367.2775** | **1098.8102** | **1099.2428** | **-0.4326** | **0** | **20** | **27** | **1** | **GAAWAGGLLQR** |
|  | 3135 | **684.0978** | **1366.1808** | **1365.4700** | **0.7108** | **1** | **20** | **29** | **1** | **AAMDSRASDAINK + Oxidation (M)** |
|  | 1914 | **472.7883** | **1415.3426** | **1414.5621** | **0.7806** | **1** | **19** | **26** | **1** | **KNSFSLSSFLER** |
|  | 755 | 402.8230 | 803.6313 | 803.9269 | -0.2957 | 0 | 19 | 34 | 1 | ICEGLGR + Carbamidomethyl (C) |
|  | 1658 | **451.6873** | **901.3599** | **901.0159** | **0.3441** | **1** | **19** | **29** | **1** | **KAELESPK** |
|  | 807 | **404.1572** | **1209.4493** | **1208.3706** | **1.0787** | **2** | **19** | **30** | **1** | **AQKIAEHQRK** |
|  | 1704 | **456.8995** | **1367.6763** | **1366.5854** | **1.0908** | **0** | **19** | **30** | **1** | **CYCQAMQVYK + 2 Carbamidomethyl (C); Oxidation (M)** |
|  | 276 | **374.7124** | **747.4101** | **746.8527** | **0.5574** | **2** | **19** | **33** | **1** | **KKTENK** |
|  | 1075 | **411.6607** | **1231.9599** | **1232.3441** | **-0.3842** | **1** | **19** | **26** | **1** | **SLNVQKASTER** |
|  | 2060 | **486.8869** | **1457.6384** | **1458.7321** | **-1.0937** | **2** | **19** | **31** | **1** | **MRNVGISSILRGR** |
|  | 457 | **386.1649** | **1155.4724** | **1156.2697** | **-0.7973** | **1** | **19** | **27** | **1** | **FRTMESNQK + Oxidation (M)** |
|  | 3150 | **686.0234** | **2055.0480** | **2054.3510** | **0.6969** | **2** | **19** | **25** | **1** | **LDVPEDLRQMCAKEAAHK** |
|  | 3249 | **740.6086** | **1479.2024** | **1478.6127** | **0.5897** | **2** | **19** | **25** | **1** | **HLSSASRGPEKGPR** |
|  | 3233 | **740.4210** | **1478.8273** | **1477.6692** | **1.1580** | **2** | **19** | **30** | **1** | **FSHLTRHKTIHT** |
|  | 1149 | **416.3708** | **1246.0901** | **1245.5098** | **0.5803** | **0** | **19** | **34** | **1** | **IIGNSAFLLIGK** |
|  | 197 | **369.3641** | **1105.0701** | **1104.2879** | **0.7823** | **2** | **19** | **35** | **1** | **RSAVVMGRGR + Oxidation (M)** |
|  | 671 | **399.1494** | **796.2840** | **796.9577** | **-0.6736** | **1** | **19** | **28** | **1** | **KGLVPQR** |
|  | 2755 | **592.7064** | **1775.0969** | **1776.1269** | **-1.0300** | **2** | **19** | **38** | **1** | **IEAVKLQMEPKMQSK + Oxidation (M)** |
|  | 3383 | **787.9478** | **2360.8213** | **2360.7295** | **0.0918** | **1** | **19** | **34** | **1** | **ERLHFSTFLVDVMSPVGIGQK** |
|  | 1391 | **432.9471** | **1295.8191** | **1296.5319** | **-0.7128** | **1** | **19** | **32** | **1** | **QYESLKILICS** |
|  | 2442 | **532.4185** | **1062.8221** | **1062.2412** | **0.5810** | **1** | **19** | **27** | **1** | **MGTIQEAGKK** |
|  | 436 | **386.0601** | **770.1055** | **769.8032** | **0.3023** | **0** | **19** | **29** | **1** | **SEPANPR** |
|  | 1226 | 420.2867 | 1257.8378 | 1258.5056 | -0.6678 | 0 | 19 | 24 | 1 | SLVNLEPVFLK |
|  | 2426 | **528.8340** | **1055.6532** | **1055.1808** | **0.4724** | **0** | **19** | **25** | **1** | **FEVSPSYVK** |
|  | 630 | **393.9831** | **785.9513** | **784.9005** | **1.0508** | **0** | **19** | **34** | **1** | **LTEIGPR** |
|  | 967 | **407.6042** | **1219.7905** | **1219.3470** | **0.4435** | **1** | **19** | **27** | **1** | **QGKGQSVQPYK** |
|  | 2344 | **519.4017** | **1555.1830** | **1554.7204** | **0.4627** | **0** | **19** | **25** | **1** | **YDVVLDAIDTMQR + Oxidation (M)** |
|  | 3302 | **744.7056** | **2231.0945** | **2230.4981** | **0.5964** | **2** | **19** | **27** | **1** | **MIISSNADRLLDPQKLEDR + Oxidation (M)** |
|  | 754 | **402.6452** | **803.2756** | **802.8810** | **0.3946** | **1** | **19** | **30** | **1** | **WAGSARR** |
|  | 1673 | **452.7115** | **1355.1124** | **1354.6210** | **0.4913** | **2** | **19** | **29** | **1** | **KEGKLIMGIGHR + Oxidation (M)** |
|  | 2787 | **594.1602** | **1779.4583** | **1779.0694** | **0.3889** | **1** | **19** | **36** | **1** | **VLTPDCPKAVHTGTAIR** |
|  | 3283 | **742.5352** | **1483.0557** | **1482.5783** | **0.4774** | **2** | **19** | **30** | **1** | **GRRYPDMDTESR** |
|  | 504 | **387.8941** | **1160.6601** | **1160.3611** | **0.2991** | **0** | **19** | **40** | **1** | **TLLSGSASVVVK** |
|  | 1916 | **472.8205** | **1415.4393** | **1414.6779** | **0.7614** | **2** | **19** | **31** | **1** | **IICPSPRRYPR + Carbamidomethyl (C)** |
|  | 230 | **372.0844** | **1113.2312** | **1112.3693** | **0.8618** | **2** | **19** | **32** | **1** | **LRLRGSAVIK** |
|  | 1466 | **436.2685** | **870.5221** | **870.9932** | **-0.4710** | **1** | **19** | **26** | **1** | **KAVPGNSAK** |
|  | 1844 | 465.9627 | 1394.8660 | 1394.6585 | 0.2074 | 2 | 19 | 34 | 1 | LEIKRTVAAPSPL |
|  | 2750 | **592.6106** | **1774.8096** | **1774.9483** | **-0.1386** | **2** | **19** | **35** | **1** | **MNISETRYKESWSK + Oxidation (M)** |
|  | 2235 | **505.3498** | **1513.0273** | **1512.7316** | **0.2957** | **1** | **19** | **26** | **1** | **QAPGKGPEWVANMK** |
|  | 577 | **389.1904** | **776.3660** | **775.9601** | **0.4060** | **1** | **19** | **32** | **1** | **LAGTMRK** |
|  | 95 | **366.0433** | **1095.1078** | **1095.2065** | **-0.0987** | **1** | **19** | **39** | **1** | **GPSSKSGGPPPK** |
|  | 538 | **388.3928** | **1162.1563** | **1161.2928** | **0.8635** | **1** | **19** | **44** | **1** | **ARVPGGCTSGTR** |
|  | 875 | **406.0914** | **1215.2519** | **1215.3801** | **-0.1281** | **1** | **19** | **32** | **1** | **EEERVAMVPR** |
|  | 1923 | 473.6333 | 1417.8777 | 1418.6237 | -0.7459 | 1 | 19 | 36 | 1 | CPPGVVPARHNSK + Carbamidomethyl (C) |
|  | 1002 | **408.0511** | **1221.1313** | **1220.3167** | **0.8145** | **1** | **19** | **35** | **1** | **DCGGGLQTRTR + Carbamidomethyl (C)** |
|  | 250 | **373.1409** | **1116.4005** | **1115.2377** | **1.1629** | **2** | **19** | **40** | **1** | **GKASPTSPDKK** |
|  | 164 | **369.2014** | **736.3879** | **736.8347** | **-0.4467** | **0** | **19** | **30** | **1** | **MASSPTK + Oxidation (M)** |
|  | 2246 | **506.4035** | **1516.1883** | **1515.6611** | **0.5272** | **0** | **19** | **26** | **1** | **VSIQPLDETAVTDK** |
|  | 1549 | **442.3463** | **882.6777** | **882.0124** | **0.6653** | **0** | **19** | **26** | **1** | **ALIYSTSK** |
|  | 1699 | **456.4096** | **910.8044** | **911.0568** | **-0.2524** | **0** | **19** | **27** | **1** | **IIPGGIADR** |
|  | 74 | **364.2225** | **726.4303** | **725.7505** | **0.6797** | **0** | **19** | **25** | **1** | **EHLGDR** |
|  | 2159 | **495.1453** | **1482.4138** | **1482.7354** | **-0.3216** | **2** | **19** | **36** | **1** | **LRFPGGSRPLRAR** |
|  | 3063 | **667.1633** | **1998.4678** | **1997.9879** | **0.4800** | **2** | **19** | **35** | **1** | **NQGGYGGSSSSSSYGSGRRF** |
|  | 1843 | **465.9570** | **929.8992** | **929.1169** | **0.7823** | **1** | **19** | **37** | **1** | **IHSKGIFK** |
|  | 3424 | **816.4468** | **2446.3182** | **2446.6449** | **-0.3268** | **1** | **19** | **35** | **1** | **CYEATDTETGSAYAVKVIPQSR + Carbamidomethyl (C)** |
|  | 1708 | **457.0275** | **1368.0602** | **1367.5537** | **0.5066** | **1** | **19** | **35** | **1** | **EKPFRCGECGK + 2 Carbamidomethyl (C)** |
|  | 3469 | **850.6427** | **2548.9059** | **2549.8522** | **-0.9463** | **0** | **19** | **32** | **1** | **QQQEDMTILLSEVSAACLHCEK + Carbamidomethyl (C); Oxidation (M)** |
|  | 495 | **387.8411** | **1160.5012** | **1160.3227** | **0.1785** | **1** | **19** | **43** | **1** | **LVIAGNPAYRS** |
|  | 1449 | **435.7142** | **1304.1203** | **1304.5210** | **-0.4006** | **1** | **19** | **27** | **1** | **ELHLGSRMPHK** |
|  | 1599 | **446.1641** | **890.3135** | **889.9998** | **0.3137** | **2** | **19** | **40** | **1** | **RKSGLSSR** |
|  | 2066 | **487.2012** | **1458.5813** | **1458.7440** | **-0.1626** | **2** | **19** | **38** | **1** | **KAKTIPVQAFEVK** |
|  | 3137 | **684.1113** | **1366.2079** | **1365.4948** | **0.7131** | **1** | **19** | **35** | **1** | **RPSGISSRFSGSK** |
|  | 1784 | **460.1757** | **1377.5050** | **1376.6449** | **0.8600** | **0** | **19** | **39** | **1** | **AVCVEAGMIALAR + Carbamidomethyl (C); Oxidation (M)** |
|  | 1635 | **449.9520** | **1346.8338** | **1346.4501** | **0.3838** | **1** | **19** | **33** | **1** | **GRYPEVHGEFR** |
|  | 3423 | **812.6647** | **1623.3146** | **1623.7705** | **-0.4560** | **2** | **19** | **30** | **1** | **EHLERGRGNLEWK** |
|  | 356 | **380.2365** | **1137.6875** | **1137.4436** | **0.2439** | **2** | **19** | **33** | **1** | **MKCPMLRSR + Oxidation (M)** |
|  | 1512 | **438.4818** | **1312.4233** | **1312.4704** | **-0.0471** | **0** | **19** | **48** | **1** | **YKPTGEYVTVR** |
|  | 147 | **368.2947** | **1101.8619** | **1102.2720** | **-0.4101** | **2** | **19** | **32** | **1** | **EVRGVRAGCR** |
|  | 1547 | **442.2991** | **1323.8751** | **1324.3633** | **-0.4881** | **1** | **19** | **27** | **1** | **DKPRGSGSGGGGHR** |
|  | 1791 | **460.4260** | **918.8373** | **919.9346** | **-1.0973** | **1** | **19** | **35** | **1** | **KSDAGEWQ** |
|  | 3486 | **898.0237** | **1794.0326** | **1793.0543** | **0.9782** | **1** | **19** | **40** | **1** | **QGISLCPYLKSHSFR + Carbamidomethyl (C)** |
|  | 231 | **372.1539** | **742.2930** | **742.8225** | **-0.5294** | **0** | **19** | **33** | **1** | **WPAVDR** |
|  | 1090 | **413.2036** | **1236.5886** | **1236.3560** | **0.2325** | **1** | **19** | **30** | **1** | **QKTENVSTCAR** |
|  | 2797 | **595.8652** | **1784.5733** | **1785.1301** | **-0.5567** | **2** | **19** | **31** | **1** | **RCWRSCLSCCMSHR + Carbamidomethyl (C)** |
|  | 107 | **366.4788** | **1096.4142** | **1097.2455** | **-0.8313** | **0** | **19** | **47** | **1** | **MGLPSGPGPER** |
|  | 1346 | 429.1306 | 1284.3695 | 1284.3771 | -0.0076 | 0 | 19 | 37 | 1 | WDESSILAAHR |
|  | 1417 | **434.2114** | **866.4081** | **866.9844** | **-0.5764** | **0** | **19** | **34** | **1** | **CYQAGIR + Carbamidomethyl (C)** |
|  | 1017 | **408.2929** | **1221.8566** | **1222.4123** | **-0.5556** | **2** | **19** | **31** | **1** | **MKNASESKLSK** |
|  | 1624 | **448.9466** | **1343.8176** | **1343.5704** | **0.2473** | **1** | **19** | **33** | **1** | **QDFPLVKAAVQK** |
|  | 745 | **402.1901** | **1203.5480** | **1203.3243** | **0.2237** | **0** | **19** | **40** | **1** | **YICYAEQTR + Carbamidomethyl (C)** |
|  | 2668 | **570.3689** | **1708.0845** | **1707.9185** | **0.1660** | **1** | **19** | **36** | **1** | **SILEILSKSPDSYQK** |
|  | 3006 | **648.9714** | **1295.9281** | **1296.4326** | **-0.5045** | **1** | **19** | **29** | **1** | **AIESSRDLLHR** |
|  | 291 | **376.1151** | **1125.3230** | **1126.3909** | **-1.0680** | **1** | **19** | **35** | **1** | **EHFVLLKIK** |
|  | 716 | 401.2014 | 800.3881 | 800.8172 | -0.4290 | 0 | 18 | 36 | 1 | GTDGAAGPR |
|  | 2170 | **496.5655** | **1486.6743** | **1485.6880** | **0.9863** | **2** | **18** | **49** | **1** | **NVTTPISKRISNR** |
|  | 977 | **407.7526** | **1220.2356** | **1221.2982** | **-1.0626** | **1** | **18** | **37** | **1** | **RMGSGYFDSW + Oxidation (M)** |
|  | 1338 | **428.9844** | **855.9541** | **854.9739** | **0.9802** | **0** | **18** | **36** | **1** | **APQHQMK + Oxidation (M)** |
|  | 3411 | **805.1174** | **2412.3301** | **2412.6135** | **-0.2834** | **1** | **18** | **30** | **1** | **AFVCDQCGAQFSKEDALETHR + Carbamidomethyl (C)** |
|  | 715 | 401.1284 | 1200.3632 | 1201.3699 | -1.0068 | 2 | 18 | 42 | 1 | KQEIVAEKEK |
|  | 888 | **406.1995** | **1215.5762** | **1215.4198** | **0.1564** | **0** | **18** | **33** | **1** | **VVDLMVHTASK + Oxidation (M)** |
|  | 2008 | **480.1925** | **1437.5553** | **1437.6385** | **-0.0832** | **0** | **18** | **40** | **1** | **LMEQCASLEELR + Oxidation (M)** |
|  | 1926 | **473.8546** | **1418.5415** | **1417.7169** | **0.8246** | **1** | **18** | **42** | **1** | **VLEMSMKSACFR + Oxidation (M)** |
|  | 259 | **374.0724** | **1119.1949** | **1118.1950** | **0.9999** | **0** | **18** | **46** | **1** | **NNTVGILETAS** |
|  | 1028 | **408.8389** | **1223.4946** | **1224.4328** | **-0.9382** | **1** | **18** | **42** | **1** | **ICSGLSYGRIR** |
|  | 1629 | **449.4452** | **896.8757** | **897.9987** | **-1.1230** | **1** | **18** | **38** | **1** | **RDSAMYR** |
|  | 851 | **405.5145** | **1213.5212** | **1214.3917** | **-0.8705** | **0** | **18** | **42** | **1** | **TPPEAIALCSR + Carbamidomethyl (C)** |
|  | 1131 | **415.8627** | **1244.5658** | **1243.3933** | **1.1726** | **0** | **18** | **45** | **1** | **MDPASSNRPLR** |
|  | 229 | **371.7576** | **1112.2507** | **1111.3382** | **0.9125** | **1** | **18** | **34** | **1** | **RVNAALVIQK** |
|  | 256 | **373.9403** | **1118.7988** | **1119.3141** | **-0.5153** | **1** | **18** | **50** | **1** | **EYLRVNVVK** |
|  | 471 | **386.8933** | **1157.6577** | **1158.3730** | **-0.7154** | **0** | **18** | **43** | **1** | **GAVCLSIPGWR** |
|  | 2433 | 530.4683 | 1588.3826 | 1587.8031 | 0.5796 | 1 | 18 | 37 | 1 | NYAMHWVRQTPGK |
|  | 92 | **366.0062** | **1094.9965** | **1094.1124** | **0.8841** | **0** | **18** | **44** | **1** | **FHGDMSGPSSG + Oxidation (M)** |
|  | 1373 | **431.0278** | **1290.0611** | **1290.5358** | **-0.4747** | **2** | **18** | **48** | **1** | **KMASATRLIQR + Oxidation (M)** |
|  | 1160 | **417.2855** | **832.5562** | **832.9452** | **-0.3890** | **2** | **18** | **35** | **1** | **KSLSKDR** |
|  | 2870 | **610.0921** | **1827.2541** | **1826.0564** | **1.1978** | **0** | **18** | **40** | **1** | **ENSSEVVQPFLMGCGTK** |
|  | 908 | **406.8141** | **811.6134** | **810.9593** | **0.6541** | **0** | **18** | **38** | **1** | **GSFIMAW** |
|  | 2715 | **584.0445** | **1749.1113** | **1749.9468** | **-0.8355** | **2** | **18** | **42** | **1** | **WSKYKHDLAASCQGR** |
|  | 3416 | **809.2639** | **1616.5131** | **1616.7398** | **-0.2268** | **1** | **18** | **37** | **1** | **HGQRGHGQQLLETR** |
|  | 662 | **399.0637** | **1194.1690** | **1195.3520** | **-1.1830** | **1** | **18** | **36** | **1** | **MFRGSGGLQAR + Oxidation (M)** |
|  | 903 | **406.3167** | **1215.9281** | **1215.4463** | **0.4818** | **2** | **18** | **30** | **1** | **TVVVGTKSRLR** |
|  | 488 | **387.5148** | **773.0148** | **772.9164** | **0.0984** | **1** | **18** | **52** | **1** | **ECLPRR** |
|  | 695 | **400.1237** | **1197.3490** | **1196.3367** | **1.0122** | **1** | **18** | **36** | **1** | **RADGCIYGVSR** |
|  | 753 | **402.5413** | **1204.6016** | **1203.4553** | **1.1463** | **1** | **18** | **49** | **1** | **VMNIRTVLNK + Oxidation (M)** |
|  | 378 | **382.3262** | **1143.9563** | **1144.2342** | **-0.2778** | **0** | **18** | **40** | **1** | **ETDVSPFPPR** |
|  | 2307 | **517.7211** | **1033.4274** | **1034.0902** | **-0.6628** | **2** | **18** | **37** | **1** | **GRGGDRGGFR** |
|  | 2965 | **629.9316** | **1886.7728** | **1886.1528** | **0.6199** | **1** | **18** | **33** | **1** | **LGCQDAFPEVYDKICK + Carbamidomethyl (C)** |
|  | 300 | **377.0115** | **1128.0123** | **1128.3224** | **-0.3101** | **2** | **18** | **37** | **1** | **SPLERLGKTK** |
|  | 513 | **387.9998** | **1160.9772** | **1160.4916** | **0.4856** | **2** | **18** | **51** | **1** | **ALKKALLLYK** |
|  | 1215 | **419.7476** | **837.4803** | **838.0525** | **-0.5722** | **1** | **18** | **35** | **1** | **LALPRIR** |
|  | 1527 | **440.5383** | **879.0618** | **878.0901** | **0.9717** | **0** | **18** | **54** | **1** | **QPIMVFK + Oxidation (M)** |
|  | 3032 | **666.2609** | **1330.5069** | **1331.5813** | **-1.0743** | **2** | **18** | **41** | **1** | **KEPITREAMLK + Oxidation (M)** |
|  | 3056 | **666.7217** | **1997.1430** | **1997.2168** | **-0.0738** | **2** | **18** | **48** | **1** | **RELALECDYQREAACAR** |
|  | 1625 | **449.3450** | **1345.0129** | **1344.5815** | **0.4315** | **0** | **18** | **31** | **1** | **FYCCPIEGCPR + Carbamidomethyl (C)** |
|  | 3034 | **666.3291** | **1995.9651** | **1996.3697** | **-0.4045** | **0** | **18** | **41** | **1** | **ILSEVLGMDAGEMVIQMK + 2 Oxidation (M)** |
|  | 3054 | **666.6927** | **1997.0561** | **1996.2023** | **0.8538** | **1** | **18** | **44** | **1** | **CQEETTQKLVLEYQAR + Carbamidomethyl (C)** |
|  | 1929 | **474.1091** | **946.2035** | **947.0016** | **-0.7981** | **1** | **18** | **46** | **1** | **AEKVETDR** |
|  | 185 | **369.2928** | **1104.8563** | **1104.2879** | **0.5684** | **2** | **18** | **37** | **1** | **RSAVVMGRGR + Oxidation (M)** |
|  | 1759 | **459.1144** | **916.2140** | **916.9358** | **-0.7217** | **1** | **18** | **47** | **1** | **GPSEDTRR** |
|  | 1603 | 446.4411 | 890.8674 | 892.0105 | -1.1432 | 0 | 18 | 48 | 1 | QTLDFLR |
|  | 1834 | **464.0827** | **926.1506** | **925.0472** | **1.1034** | **2** | **18** | **40** | **1** | **RKGPSAGPR** |
|  | 1175 | **418.1389** | **834.2630** | **833.8504** | **0.4126** | **1** | **18** | **43** | **1** | **ANSSGRSR** |
|  | 2518 | **543.8274** | **1628.4600** | **1628.7424** | **-0.2823** | **0** | **18** | **36** | **1** | **SSGPYGGGGQYFAKPR** |
|  | 3044 | **666.5736** | **1331.1324** | **1330.4276** | **0.7049** | **0** | **18** | **34** | **1** | **EGNMTTATNHVR** |
|  | 2853 | **608.6344** | **1822.8810** | **1821.9849** | **0.8962** | **0** | **18** | **48** | **1** | **HNSTGQGNVSVSLVPPTK** |
|  | 30 | **362.3268** | **1083.9584** | **1084.2499** | **-0.2916** | **0** | **18** | **37** | **1** | **QILEAMSHR** |
|  | 1517 | **439.3701** | **1315.0883** | **1315.4297** | **-0.3414** | **1** | **18** | **38** | **1** | **TVKEEAEKPER** |
|  | 890 | **406.2132** | **1215.6175** | **1215.3996** | **0.2179** | **2** | **18** | **35** | **1** | **LKSKLEDNIR** |
|  | 113 | **367.1414** | **1098.4022** | **1099.2215** | **-0.8194** | **0** | **18** | **44** | **1** | **THMLNAAADR** |
|  | 1753 | **458.9796** | **915.9445** | **917.0202** | **-1.0756** | **0** | **18** | **48** | **1** | **GAPTVPGYR** |
|  | 2047 | **486.1278** | **1455.3612** | **1455.6124** | **-0.2513** | **0** | **18** | **40** | **1** | **VTNEVASLAIPADR** |
|  | 2599 | **556.6710** | **1666.9909** | **1667.0719** | **-0.0811** | **2** | **18** | **51** | **1** | **LMKLLQRLPNSVVR** |
|  | 2267 | **511.6144** | **1531.8210** | **1532.6599** | **-0.8390** | **1** | **18** | **52** | **1** | **SELHRHIEEGLGR** |
|  | 128 | **367.4771** | **1099.4092** | **1099.2811** | **0.1280** | **0** | **18** | **55** | **1** | **IDGVSLLVQR** |
|  | 347 | **379.6947** | **757.3746** | **757.8354** | **-0.4609** | **0** | **18** | **41** | **1** | **GTGLSPAR** |
|  | 2675 | **571.9553** | **1712.8436** | **1712.0842** | **0.7594** | **0** | **18** | **42** | **1** | **IIAMGLLLPFSDCFR + Oxidation (M)** |
|  | 1266 | **422.3081** | **842.6015** | **841.9949** | **0.6065** | **0** | **18** | **38** | **1** | **GITLSVRP** |
|  | 2701 | **580.7775** | **1739.3102** | **1740.1838** | **-0.8736** | **2** | **18** | **41** | **1** | **GLVLKVIPPIKHIAGGK** |
|  | 3037 | **666.4406** | **1330.8663** | **1331.4271** | **-0.5608** | **0** | **18** | **42** | **1** | **LAQYEPPQEEK** |
|  | 3053 | **666.6912** | **1331.3675** | **1330.5500** | **0.8175** | **1** | **18** | **46** | **1** | **AQIPKMEQELK + Oxidation (M)** |
|  | 808 | **404.1719** | **1209.4935** | **1209.2727** | **0.2209** | **2** | **18** | **43** | **1** | **RGSSKGHDTHK** |
|  | 4 | **360.3365** | **718.6583** | **718.7796** | **-0.1213** | **0** | **18** | **52** | **1** | **MNDSPR** |
|  | 2725 | **586.1782** | **1755.5125** | **1756.1023** | **-0.5898** | **2** | **18** | **45** | **1** | **KSVAHNMTMPNKLLR + Oxidation (M)** |
|  | 2817 | **598.1468** | **1791.4182** | **1791.0550** | **0.3632** | **0** | **18** | **43** | **1** | **TPLDWKPSLLEGHLGK** |
|  | 2 | **360.3217** | **718.6287** | **718.7133** | **-0.0846** | **0** | **18** | **47** | **1** | **GDEATAR** |
|  | 747 | **402.2923** | **1203.8548** | **1204.4019** | **-0.5470** | **1** | **18** | **41** | **1** | **HALVNKMYGR + Oxidation (M)** |
|  | 3165 | **688.7655** | **2063.2743** | **2063.1245** | **0.1499** | **2** | **18** | **53** | **1** | **CSQHGSSSEESTKRTSHSK** |
|  | 724 | **401.8335** | **1202.4784** | **1203.4370** | **-0.9585** | **1** | **18** | **51** | **1** | **QPRVGILAPPR** |
|  | 120 | **367.2104** | **732.4061** | **731.7585** | **0.6476** | **1** | **18** | **39** | **1** | **GRATDGR** |
|  | 2523 | **544.9170** | **1087.8192** | **1087.2935** | **0.5257** | **0** | **18** | **47** | **1** | **CSLALSALPR + Carbamidomethyl (C)** |
|  | 391 | **384.7831** | **1151.3271** | **1151.3575** | **-0.0304** | **1** | **18** | **40** | **1** | **MSQALCQKDK** |
|  | 3454 | **846.5271** | **2536.5591** | **2537.0076** | **-0.4484** | **1** | **18** | **46** | **1** | **RLFCTMEPEPVQPGMLIDVCK + 2 Carbamidomethyl (C); Oxidation (M)** |
|  | 605 | **390.0259** | **1167.0556** | **1166.3258** | **0.7299** | **0** | **18** | **45** | **1** | **AVFVPDIYSR** |
|  | 728 | **401.8660** | **801.7173** | **800.8570** | **0.8603** | **0** | **18** | **52** | **1** | **GSSPGAAVGV** |
|  | 1320 | **428.0158** | **1281.0253** | **1280.5362** | **0.4891** | **2** | **18** | **44** | **1** | **MRDVVLFEKK + Oxidation (M)** |
|  | 3142 | **685.1069** | **1368.1991** | **1367.5572** | **0.6419** | **2** | **18** | **46** | **1** | **TAAPSVRPEKRR** |
|  | 3256 | **740.7516** | **2219.2326** | **2219.5187** | **-0.2861** | **0** | **18** | **42** | **1** | **GSMNLMLQVNMTSGHSSAPPK + 2 Oxidation (M)** |
|  | 1479 | **437.0755** | **872.1363** | **873.0504** | **-0.9141** | **0** | **18** | **50** | **1** | **EIVPLFR** |
|  | 2423 | **527.4365** | **1052.8581** | **1052.2697** | **0.5885** | **0** | **18** | **34** | **1** | **TMAVVASVCR + Oxidation (M)** |
|  | 284 | **375.1506** | **1122.4295** | **1122.2501** | **0.1795** | **2** | **18** | **48** | **1** | **DMKKLSGPSSG + Oxidation (M)** |
|  | 398 | **384.9755** | **767.9363** | **766.8887** | **1.0476** | **1** | **18** | **39** | **1** | **AAPPGAKR** |
|  | 681 | **399.7761** | **1196.3062** | **1195.3884** | **0.9178** | **0** | **18** | **39** | **1** | **MTSLFAQEIR** |
|  | 2864 | **609.9273** | **1217.8398** | **1218.4268** | **-0.5869** | **0** | **18** | **38** | **1** | **VWPWVAMASR + Oxidation (M)** |
|  | 3293 | **742.8383** | **2225.4926** | **2224.6230** | **0.8696** | **2** | **18** | **53** | **1** | **DVMQETIRNLDCVVMKWK + Oxidation (M)** |
|  | 288 | **375.2044** | **748.3940** | **748.8885** | **-0.4945** | **1** | **18** | **41** | **1** | **MTPKEK + Oxidation (M)** |
|  | 591 | **389.6052** | **1165.7935** | **1166.4352** | **-0.6417** | **1** | **18** | **38** | **1** | **KMGGAMAPPMK + 3 Oxidation (M)** |
|  | 85 | **365.3493** | **1093.0258** | **1093.2354** | **-0.2095** | **1** | **18** | **51** | **1** | **RSSPQAMPCT + Oxidation (M)** |
|  | 104 | **366.2358** | **1095.6853** | **1095.1701** | **0.5152** | **2** | **18** | **41** | **1** | **SPYSRSRSR** |
|  | 580 | **389.2086** | **776.4025** | **776.9449** | **-0.5424** | **1** | **18** | **42** | **1** | **IKTEMR** |
|  | 983 | **407.8041** | **813.5935** | **812.8875** | **0.7060** | **0** | **18** | **47** | **1** | **GCFASSEL** |
|  | 3130 | **684.0032** | **1365.9916** | **1365.5807** | **0.4108** | **1** | **18** | **37** | **1** | **GALVLGSSLKQHR** |
|  | 3254 | **740.7277** | **2219.1608** | **2220.2762** | **-1.1154** | **1** | **18** | **39** | **1** | **HGENEHNLQGRIGGDSGLSSR** |
|  | 472 | **386.8933** | **1157.6577** | **1157.2809** | **0.3768** | **2** | **18** | **50** | **1** | **KVIGEDRQGR** |
|  | 1459 | **436.1857** | **870.3567** | **871.0394** | **-0.6828** | **2** | **18** | **46** | **1** | **LLDARRK** |
|  | 2355 | **520.2667** | **1557.7780** | **1557.6875** | **0.0905** | **0** | **18** | **44** | **1** | **LNSNLHACDSGASIR** |
|  | 1317 | **427.8130** | **1280.4170** | **1280.4398** | **-0.0228** | **2** | **18** | **47** | **1** | **CWNRSGGCRK + 2 Carbamidomethyl (C)** |
|  | 2100 | **488.5267** | **975.0386** | **974.1129** | **0.9257** | **0** | **18** | **59** | **1** | **LDGYGMMR + 2 Oxidation (M)** |
|  | 3070 | **667.6834** | **1333.3519** | **1332.4417** | **0.9103** | **0** | **18** | **49** | **1** | **AAPDSPAPFGSCR + Carbamidomethyl (C)** |
|  | 3076 | **667.8481** | **1333.6815** | **1332.5708** | **1.1107** | **0** | **18** | **47** | **1** | **GIKPGMPSIFNR + Oxidation (M)** |
|  | 3082 | **668.7631** | **1335.5113** | **1335.5467** | **-0.0353** | **1** | **18** | **56** | **1** | **MKEPLLGGECDK + Oxidation (M)** |
|  | 295 | 376.2114 | 1125.6119 | 1126.2635 | -0.6516 | 1 | 18 | 35 | 1 | AKSPGLTPEAR |
|  | 1957 | **476.2058** | **1425.5954** | **1425.5930** | **0.0023** | **1** | **18** | **46** | **1** | **GGTGSVGLAAAPVRGR** |
|  | 3125 | **683.8636** | **1365.7125** | **1365.5611** | **0.1514** | **2** | **18** | **46** | **1** | **GCKSVGEHKVHK + Carbamidomethyl (C)** |
|  | 157 | **369.1648** | **1104.4721** | **1104.2680** | **0.2041** | **2** | **18** | **45** | **1** | **HRSDRMMR + Oxidation (M)** |
|  | 1377 | **431.0759** | **1290.2055** | **1290.5374** | **-0.3318** | **2** | **18** | **57** | **1** | **NMSWSRLIRK** |
|  | 2768 | **592.9677** | **1775.8810** | **1775.0162** | **0.8648** | **0** | **18** | **46** | **1** | **CSASMSLHNSLVKPER + Oxidation (M)** |
|  | 3081 | **668.7585** | **1335.5023** | **1336.5430** | **-1.0406** | **2** | **18** | **57** | **1** | **VLATQPGPGRGRK** |
|  | 359 | 380.2886 | 1137.8437 | 1137.1588 | 0.6849 | 1 | 18 | 43 | 1 | TAETETSRSR |
|  | 780 | **403.9292** | **1208.7653** | **1209.4151** | **-0.6497** | **0** | **18** | **48** | **1** | **VAVIGGGYCVSK + Carbamidomethyl (C)** |
|  | 1016 | **408.2814** | **1221.8220** | **1222.4819** | **-0.6598** | **2** | **18** | **39** | **1** | **TRAPMRIMSK + 2 Oxidation (M)** |
|  | 395 | **384.9275** | **767.8401** | **767.8766** | **-0.0365** | **1** | **18** | **40** | **1** | **AKGHSLR** |
|  | 75 | **364.2239** | **1089.6496** | **1089.2432** | **0.4064** | **1** | **18** | **34** | **1** | **AKLSTSGAINK** |
|  | 1866 | **468.2088** | **1401.6042** | **1400.7490** | **0.8552** | **0** | **18** | **51** | **1** | **MLPQVLGCELLK + Carbamidomethyl (C)** |
|  | 2212 | **502.5588** | **1003.1028** | **1002.2753** | **0.8276** | **1** | **18** | **61** | **1** | **MNLIVKLR + Oxidation (M)** |
|  | 2382 | **523.0898** | **1566.2474** | **1566.5786** | **-0.3313** | **0** | **18** | **50** | **1** | **ETYYYDSSGYYR** |
|  | 3036 | **666.4308** | **1996.2702** | **1997.1656** | **-0.8955** | **1** | **18** | **46** | **1** | **KPAELDEDSESSKCCSIR** |
|  | 1162 | **417.7477** | **1250.2208** | **1249.4558** | **0.7649** | **0** | **17** | **46** | **1** | **TYVSTITVPLR** |
|  | 1863 | **467.7704** | **1400.2891** | **1399.6038** | **0.6853** | **2** | **17** | **42** | **1** | **VPRGGIPPRAHSR** |
|  | 163 | **369.1911** | **1104.5510** | **1104.3011** | **0.2500** | **0** | **17** | **42** | **1** | **MHPSLATMGK + 2 Oxidation (M)** |
|  | 712 | 401.0640 | 800.1133 | 798.9736 | 1.1397 | 1 | 17 | 52 | 1 | LRGGAVVK |
|  | 338 | **378.3712** | **1132.0915** | **1131.4106** | **0.6808** | **2** | **17** | **43** | **1** | **ALNLIGKKFK** |
|  | 1357 | **429.6046** | **857.1945** | **858.0209** | **-0.8264** | **1** | **17** | **47** | **1** | **LMGKTHR + Oxidation (M)** |
|  | 135 | **368.0801** | **1101.2182** | **1101.2111** | **0.0071** | **0** | **17** | **52** | **1** | **VESSPQVLSR** |
|  | 1225 | **420.2262** | **838.4377** | **837.9682** | **0.4695** | **1** | **17** | **39** | **1** | **MKCNGGR + Carbamidomethyl (C); Oxidation (M)** |
|  | 3288 | **742.7092** | **1483.4037** | **1483.5995** | **-0.1958** | **0** | **17** | **37** | **1** | **ACDTVPTGGVYTDK + Carbamidomethyl (C)** |
|  | 2860 | **609.5340** | **1825.5798** | **1825.0946** | **0.4852** | **0** | **17** | **40** | **1** | **LSLPMPASGTSLSAAIHR + Oxidation (M)** |
|  | 1367 | **430.8983** | **859.7818** | **858.8565** | **0.9254** | **0** | **17** | **58** | **1** | **AGSSGGGGGPR** |
|  | 863 | **405.9955** | **1214.9642** | **1214.4150** | **0.5492** | **1** | **17** | **44** | **1** | **FCRMAASLDK + Carbamidomethyl (C); Oxidation (M)** |
|  | 1851 | **466.2262** | **930.4376** | **931.0896** | **-0.6520** | **0** | **17** | **50** | **1** | **APSIALGFR** |
|  | 233 | **372.1812** | **1113.5215** | **1113.2515** | **0.2700** | **1** | **17** | **39** | **1** | **QRCPGGVSGPR** |
|  | 2016 | **481.9350** | **961.8552** | **962.1320** | **-0.2768** | **2** | **17** | **51** | **1** | **LMRQSRR + Oxidation (M)** |
|  | 1043 | **409.0907** | **1224.2498** | **1225.4345** | **-1.1846** | **0** | **17** | **54** | **1** | **PVQLVVSGAEVK** |
|  | 2185 | **499.3147** | **996.6146** | **997.1493** | **-0.5348** | **0** | **17** | **40** | **1** | **LLALSGPGGGR** |
|  | 3209 | **729.6050** | **1457.1952** | **1456.6947** | **0.5004** | **1** | **17** | **39** | **1** | **RPYGRNKPLISR** |
|  | 3215 | **733.2386** | **1464.4624** | **1463.4805** | **0.9819** | **0** | **17** | **46** | **1** | **EPSPQCGGSLSEES + Carbamidomethyl (C)** |
|  | 3238 | **740.5125** | **1479.0102** | **1478.7517** | **0.2585** | **1** | **17** | **46** | **1** | **LYEEKLGMLGTPK** |
|  | 3279 | **742.0406** | **1482.0665** | **1482.6393** | **-0.5728** | **1** | **17** | **36** | **1** | **APQGGYYKTALGTR** |
|  | 799 | **404.0816** | **1209.2225** | **1210.3831** | **-1.1606** | **1** | **17** | **50** | **1** | **SAHSIAQLQKK** |
|  | 1242 | **421.8080** | **841.6013** | **840.8779** | **0.7235** | **0** | **17** | **51** | **1** | **SSTYEVR** |
|  | 243 | **372.6499** | **1114.9276** | **1114.3620** | **0.5656** | **1** | **17** | **43** | **1** | **RHITMLSIK + Oxidation (M)** |
|  | 503 | **387.8929** | **773.7711** | **772.8568** | **0.9144** | **2** | **17** | **59** | **1** | **RRGTAGR** |
|  | 1717 | **457.6819** | **913.3490** | **913.0962** | **0.2528** | **0** | **17** | **38** | **1** | **PPMVNVTR** |
|  | 2286 | **513.7158** | **1538.1253** | **1538.7490** | **-0.6237** | **1** | **17** | **40** | **1** | **SMSLNIGGAKGSMNR + Oxidation (M)** |
|  | 3261 | **740.8978** | **1479.7809** | **1480.6847** | **-0.9039** | **1** | **17** | **52** | **1** | **KIENCNYAVELGK** |
|  | 528 | **388.1856** | **1161.5347** | **1161.3058** | **0.2288** | **1** | **17** | **52** | **1** | **DKLNSSLIGSK** |
|  | 505 | **387.9045** | **773.7942** | **772.9761** | **0.8182** | **2** | **17** | **60** | **1** | **AKLKSVK** |
|  | 1038 | 409.0540 | 816.0933 | 816.0007 | 0.0926 | 2 | 17 | 54 | 1 | KITAKQK |
|  | 756 | **402.9628** | **803.9108** | **802.9158** | **0.9950** | **1** | **17** | **56** | **1** | **ATNIEKK** |
|  | 792 | **404.0320** | **1209.0737** | **1209.3538** | **-0.2801** | **0** | **17** | **50** | **1** | **RPVGASFSFGGK** |
|  | 2201 | **501.0340** | **1000.0532** | **999.0760** | **0.9773** | **0** | **17** | **51** | **1** | **AIPSGGDPSAK** |
|  | 704 | **400.2812** | **1197.8216** | **1197.3415** | **0.4801** | **1** | **17** | **35** | **1** | **VPKGAAPGSQTGK** |
|  | 2070 | **487.4313** | **972.8479** | **972.0573** | **0.7906** | **2** | **17** | **46** | **1** | **GDKGQKSPR** |
|  | 2089 | **488.2863** | **1461.8368** | **1462.7542** | **-0.9174** | **0** | **17** | **49** | **1** | **GMFGLMTVSSCVSK + Oxidation (M)** |
|  | 1271 | **423.2560** | **1266.7459** | **1266.4711** | **0.2747** | **0** | **17** | **47** | **1** | **SPLCLNCGSCR + 2 Carbamidomethyl (C)** |
|  | 36 | **363.0313** | **1086.0719** | **1086.2063** | **-0.1344** | **2** | **17** | **50** | **1** | **SPSALRGSRR** |
|  | 1616 | **448.2090** | **1341.6047** | **1340.6573** | **0.9473** | **0** | **17** | **49** | **1** | **HVINMCLNVIK + Carbamidomethyl (C)** |
|  | 2763 | **592.7867** | **1775.3381** | **1775.8535** | **-0.5155** | **1** | **17** | **46** | **1** | **QFMDEQAAEREHER** |
|  | 3306 | **748.1288** | **1494.2429** | **1494.5194** | **-0.2765** | **0** | **17** | **45** | **1** | **VEGTPSDPNPNDPR** |
|  | 1163 | 417.8428 | 1250.5062 | 1250.3794 | 0.1269 | 0 | 17 | 54 | 1 | GTDGAVSEMVLR + Oxidation (M) |
|  | 2438 | **532.0552** | **1062.0956** | **1061.2963** | **0.7993** | **1** | **17** | **53** | **1** | **TPALKMSVSK** |
|  | 2520 | **544.0070** | **1628.9989** | **1627.9265** | **1.0723** | **0** | **17** | **56** | **1** | **LMPGSGLALAPSTCPR + Carbamidomethyl (C)** |
|  | 93 | **366.0102** | **1095.0084** | **1095.2082** | **-0.1998** | **0** | **17** | **57** | **1** | **DCMGEVVNR + Carbamidomethyl (C); Oxidation (M)** |
|  | 439 | **386.0788** | **770.1428** | **769.7998** | **0.3430** | **0** | **17** | **47** | **1** | **ESPAQAPA** |
|  | 3065 | **667.3892** | **1332.7635** | **1331.6507** | **1.1129** | **1** | **17** | **52** | **1** | **MPRYCAAICCK + Carbamidomethyl (C); Oxidation (M)** |
|  | 3376 | **780.9511** | **1559.8874** | **1558.9025** | **0.9849** | **0** | **17** | **53** | **1** | **YGEGLIFLMMLQK + Oxidation (M)** |
|  | 1054 | 410.1710 | 1227.4908 | 1227.3674 | 0.1234 | 0 | 17 | 55 | 1 | GKPYNCEECGK |
|  | 1229 | **420.3439** | **838.6731** | **838.9977** | **-0.3246** | **2** | **17** | **39** | **1** | **AIHSKRK** |
|  | 1560 | **443.4658** | **884.9167** | **886.0476** | **-1.1308** | **1** | **17** | **58** | **1** | **LDNVVAKK** |
|  | 121 | **367.2429** | **1098.7064** | **1099.3012** | **-0.5948** | **0** | **17** | **46** | **1** | **EIPVPLMER + Oxidation (M)** |
|  | 1529 | **440.8524** | **1319.5350** | **1318.4349** | **1.1001** | **0** | **17** | **59** | **1** | **MYGCDLGSDWR + Oxidation (M)** |
|  | 1919 | **472.9661** | **1415.8760** | **1415.6379** | **0.2381** | **1** | **17** | **54** | **1** | **SMYLCASSLERR** |
|  | 2144 | **493.9675** | **985.9202** | **985.2697** | **0.6505** | **0** | **17** | **53** | **1** | **MAMGLMCGR + Oxidation (M)** |
|  | 3240 | **740.5286** | **2218.5637** | **2217.7049** | **0.8588** | **2** | **17** | **49** | **1** | **EMMDMCFIICTMARHRR + Carbamidomethyl (C); Oxidation (M)** |
|  | 1231 | **420.4108** | **838.8068** | **838.0080** | **0.7988** | **1** | **17** | **49** | **1** | **GMMKGGNK + Oxidation (M)** |
|  | 1354 | **429.4219** | **856.8291** | **857.9149** | **-1.0858** | **1** | **17** | **56** | **1** | **AARSGPSGR** |
|  | 158 | **369.1654** | **1104.4741** | **1105.3089** | **-0.8347** | **0** | **17** | **51** | **1** | **AKPWAVCFPS** |
|  | 1707 | **457.0164** | **1368.0269** | **1367.5351** | **0.4918** | **0** | **17** | **51** | **1** | **ITCHLGNNPWR + Carbamidomethyl (C)** |
|  | 2908 | **612.4404** | **1834.2989** | **1835.0233** | **-0.7243** | **1** | **17** | **45** | **1** | **ENMKLPQPPEGQCYSN** |
|  | 156 | **369.1616** | **1104.4626** | **1105.3089** | **-0.8463** | **0** | **17** | **52** | **1** | **AKPWAVCFPS** |
|  | 1481 | **437.0974** | **1308.2700** | **1308.4617** | **-0.1917** | **1** | **17** | **59** | **1** | **MDDSIRSLISR + Oxidation (M)** |
|  | 1540 | **442.0428** | **882.0708** | **880.9912** | **1.0795** | **1** | **17** | **46** | **1** | **LAQKEHR** |
|  | 159 | **369.1701** | **1104.4882** | **1103.3395** | **1.1488** | **2** | **17** | **51** | **1** | **INVAAKRGMK + Oxidation (M)** |
|  | 174 | **369.2533** | **1104.7376** | **1105.3089** | **-0.5712** | **0** | **17** | **45** | **1** | **AKPWAVCFPS** |
|  | 177 | **369.2692** | **1104.7855** | **1105.3089** | **-0.5234** | **0** | **17** | **45** | **1** | **AKPWAVCFPS** |
|  | 426 | **385.8437** | **1154.5088** | **1155.3479** | **-0.8391** | **2** | **17** | **44** | **1** | **LVDDGKKKPR** |
|  | 1309 | **427.3593** | **1279.0559** | **1278.5035** | **0.5523** | **0** | **17** | **38** | **1** | **SLPQIAAVARPR** |
|  | 1026 | **408.7285** | **1223.1632** | **1222.2200** | **0.9432** | **1** | **17** | **48** | **1** | **TDRAGWEDSW** |
|  | 1457 | **436.1603** | **870.3059** | **870.0945** | **0.2114** | **2** | **17** | **53** | **1** | **KCFKCK + 2 Carbamidomethyl (C)** |
|  | 2968 | **630.1935** | **1258.3722** | **1258.3781** | **-0.0060** | **0** | **17** | **52** | **1** | **TDQEVLGELVR** |
|  | 2972 | **631.4408** | **1260.8668** | **1260.4021** | **0.4647** | **1** | **17** | **53** | **1** | **APLSYGGQVGRR** |
|  | 3108 | **678.0016** | **1353.9884** | **1353.5703** | **0.4181** | **0** | **17** | **43** | **1** | **RPGSAATTKPIVR** |
|  | 215 | **371.1100** | **740.2052** | **740.8051** | **-0.5999** | **1** | **17** | **43** | **1** | **EEKAHK** |
|  | 633 | **394.6029** | **1180.7865** | **1180.2976** | **0.4890** | **1** | **17** | **47** | **1** | **VSNQNRFCR + Carbamidomethyl (C)** |
|  | 1874 | **469.1338** | **1404.3792** | **1403.6024** | **0.7768** | **1** | **17** | **53** | **1** | **YFGTNSVICSKK + Carbamidomethyl (C)** |
|  | 2115 | **490.1967** | **978.3785** | **977.1798** | **1.1988** | **2** | **17** | **53** | **1** | **MDKGGKTLK** |
|  | 178 | **369.2719** | **1104.7934** | **1105.3089** | **-0.5155** | **0** | **17** | **45** | **1** | **AKPWAVCFPS** |
|  | 2129 | **491.7248** | **1472.1522** | **1471.6547** | **0.4975** | **1** | **17** | **38** | **1** | **INGEKLAELLSER** |
|  | 402 | **385.0038** | **767.9927** | **768.8183** | **-0.8256** | **0** | **17** | **45** | **1** | **QTHLDR** |
|  | 1414 | 434.1722 | 1299.4944 | 1299.3966 | 0.0977 | 1 | 17 | 50 | 1 | GQGSGRGLGLGQGR |
|  | 2009 | **480.1949** | **958.3750** | **959.1431** | **-0.7681** | **0** | **17** | **55** | **1** | **TWVTVVVR** |
|  | 266 | **374.2183** | **746.4219** | **745.9339** | **0.4879** | **1** | **17** | **51** | **1** | **RCGGLLK** |
|  | 2542 | **548.5780** | **1642.7118** | **1643.9094** | **-1.1976** | **2** | **17** | **57** | **1** | **AAKALLAMARVNENR + Oxidation (M)** |
|  | 3088 | **668.8730** | **1335.7312** | **1335.5101** | **0.2211** | **2** | **17** | **48** | **1** | **QLQDFWKKSR** |
|  | 1546 | **442.2108** | **1323.6102** | **1322.5990** | **1.0112** | **2** | **17** | **49** | **1** | **TILNRIISKHK** |
|  | 2055 | **486.7014** | **971.3881** | **971.0295** | **0.3586** | **1** | **17** | **45** | **1** | **NRASGVPDR** |
|  | 2603 | **557.1068** | **1668.2983** | **1668.7897** | **-0.4915** | **2** | **17** | **51** | **1** | **RTYEGGNALDGGRMR + Oxidation (M)** |
|  | 3447 | **841.8478** | **1681.6808** | **1681.8018** | **-0.1211** | **0** | **17** | **46** | **1** | **GHTGEKPYECSECGK + Carbamidomethyl (C)** |
|  | 3468 | **850.3313** | **1698.6478** | **1697.9733** | **0.6745** | **2** | **17** | **49** | **1** | **RVLQLEKQNSLIEK** |
|  | 3478 | **874.5349** | **1747.0550** | **1746.0046** | **1.0504** | **2** | **17** | **54** | **1** | **CEKEAVLQRCHCR + 3 Carbamidomethyl (C)** |
|  | 150 | **368.5057** | **734.9966** | **735.7918** | **-0.7952** | **1** | **17** | **61** | **1** | **GGRGSFR** |
|  | 2080 | **488.1111** | **974.2074** | **973.1281** | **1.0792** | **2** | **17** | **59** | **1** | **ESKLKAAAR** |
|  | 110 | **367.0563** | **1098.1468** | **1097.2522** | **0.8947** | **2** | **17** | **58** | **1** | **KHMKTHSGR + Oxidation (M)** |
|  | 3258 | **740.8016** | **2219.3827** | **2220.3478** | **-0.9651** | **0** | **17** | **59** | **1** | **SGTELSAVGSVCSDADCQEYAK** |
|  | 2058 | **486.8275** | **971.6402** | **972.1235** | **-0.4833** | **0** | **17** | **50** | **1** | **SSPRPCIR + Carbamidomethyl (C)** |
|  | 1947 | **475.7834** | **1424.3282** | **1424.5555** | **-0.2273** | **1** | **17** | **47** | **1** | **SLTEKTPTGTFSR** |
|  | 2696 | **579.7538** | **1736.2392** | **1736.0464** | **0.1928** | **2** | **17** | **54** | **1** | **MHCSGAMKVLGSKGEK + Carbamidomethyl (C); Oxidation (M)** |
|  | 188 | **369.3036** | **1104.8886** | **1105.3089** | **-0.4203** | **0** | **17** | **48** | **1** | **AKPWAVCFPS** |
|  | 189 | **369.3123** | **1104.9146** | **1104.3011** | **0.6135** | **0** | **17** | **49** | **1** | **MHPSLATMGK + 2 Oxidation (M)** |
|  | 1597 | **446.0650** | **1335.1729** | **1335.4025** | **-0.2295** | **0** | **17** | **62** | **1** | **GGGCVWEAEAGGSR** |
|  | 126 | **367.2891** | **1098.8450** | **1099.3110** | **-0.4659** | **1** | **17** | **49** | **1** | **CLKGGRPVGR + Carbamidomethyl (C)** |
|  | 161 | **369.1843** | **1104.5306** | **1105.3089** | **-0.7782** | **0** | **17** | **50** | **1** | **AKPWAVCFPS** |
|  | 1112 | **414.1903** | **826.3659** | **825.9525** | **0.4134** | **0** | **17** | **46** | **1** | **LLGPPSSR** |
|  | 2136 | 492.4354 | 1474.2841 | 1473.8229 | 0.4612 | 2 | 17 | 43 | 1 | IKKITAIITQGCK + Carbamidomethyl (C) |
|  | 2251 | **507.2872** | **1518.8393** | **1518.7362** | **0.1032** | **1** | **17** | **52** | **1** | **EAMPLFLSPDSRR** |
|  | 885 | **406.1879** | **1215.5415** | **1214.4563** | **1.0853** | **0** | **17** | **49** | **1** | **MDCPALPPGWK** |
|  | 1308 | **427.2986** | **1278.8736** | **1279.3624** | **-0.4889** | **0** | **17** | **39** | **1** | **APAGAAAVSASGHGR** |
|  | 2823 | **599.0353** | **1196.0559** | **1195.3452** | **0.7107** | **0** | **17** | **53** | **1** | **ALLGYADNQCK** |
|  | 2896 | **612.1501** | **1833.4283** | **1834.0287** | **-0.6005** | **0** | **17** | **55** | **1** | **EQIELPEVPSEPLPEK** |
|  | 3002 | **644.4860** | **1286.9571** | **1286.5619** | **0.3952** | **1** | **17** | **46** | **1** | **AKAIAINTFLPK** |
|  | 186 | **369.2960** | **1104.8657** | **1104.2778** | **0.5879** | **0** | **17** | **48** | **1** | **VLMDLQNQK + Oxidation (M)** |
|  | 1944 | **475.6378** | **1423.8914** | **1423.6201** | **0.2712** | **1** | **17** | **56** | **1** | **WQDVCSRASLCR** |
|  | 1181 | **418.8746** | **835.7344** | **835.9657** | **-0.2313** | **0** | **17** | **64** | **1** | **MEAASALK + Oxidation (M)** |
|  | 165 | **369.2157** | **1104.6248** | **1105.3089** | **-0.6840** | **0** | **17** | **47** | **1** | **AKPWAVCFPS** |
|  | 3491 | **901.6152** | **1801.2157** | **1800.9721** | **0.2435** | **2** | **17** | **51** | **1** | **IGQYLSSNRDHKAVGR** |
|  | 3497 | **922.3500** | **1842.6852** | **1842.0592** | **0.6260** | **0** | **17** | **47** | **1** | **GVPSSPDHMLAMVEEAR + Oxidation (M)** |
|  | 8 | **360.3788** | **1078.1143** | **1079.2252** | **-1.1110** | **0** | **17** | **74** | **1** | **EGLLANTMSK + Oxidation (M)** |
|  | 3144 | **685.1127** | **2052.3158** | **2053.3250** | **-1.0091** | **2** | **17** | **57** | **1** | **RISVQPSSSLSARMMSGSR + Oxidation (M)** |
|  | 96 | 366.0843 | 1095.2307 | 1094.1588 | 1.0719 | 0 | 17 | 60 | 1 | SPTHYSSCR + Carbamidomethyl (C) |
|  | 603 | **389.9998** | **1166.9774** | **1167.3187** | **-0.3414** | **0** | **17** | **56** | **1** | **GATQTPRPALR** |
|  | 661 | **399.0338** | **1194.0792** | **1194.2548** | **-0.1756** | **2** | **17** | **48** | **1** | **DPKGSREYSR** |
|  | 680 | **399.7509** | **1196.2307** | **1197.4259** | **-1.1952** | **1** | **17** | **45** | **1** | **LPVKYPDLPR** |
|  | 1769 | **459.2825** | **1374.8252** | **1375.5908** | **-0.7655** | **2** | **17** | **51** | **1** | **SKKNEIMVAPDK + Oxidation (M)** |
|  | 2547 | **549.5119** | **1645.5135** | **1644.8262** | **0.6873** | **1** | **17** | **45** | **1** | **ALIGDDVGLTSYKHR** |
|  | 20 | **361.9975** | **1082.9702** | **1083.3247** | **-0.3545** | **0** | **17** | **53** | **1** | **LGGGLLVLVSR** |
|  | 248 | **373.0857** | **1116.2349** | **1117.3629** | **-1.1280** | **2** | **17** | **67** | **1** | **MSAPAAKVSKK** |
|  | 499 | **387.8770** | **773.7393** | **772.9993** | **0.7400** | **2** | **17** | **66** | **1** | **AAPKMKK** |
|  | 2967 | **630.1589** | **1258.3030** | **1258.3781** | **-0.0752** | **0** | **17** | **55** | **1** | **TDQEVLGELVR** |
|  | 3321 | **751.2725** | **1500.5301** | **1499.7296** | **0.8006** | **1** | **17** | **51** | **1** | **AEEFFLVQNKMK + Oxidation (M)** |
|  | 1288 | **425.1764** | **1272.5069** | **1271.4017** | **1.1053** | **0** | **17** | **59** | **1** | **HCSAPEDPIFR** |
|  | 1425 | **434.5935** | **1300.7584** | **1300.4795** | **0.2790** | **0** | **17** | **49** | **1** | **SMADEQEIMCK + Oxidation (M)** |
|  | 414 | **385.0992** | **1152.2753** | **1152.1750** | **0.1002** | **1** | **17** | **49** | **1** | **HDETHTREK** |
|  | 921 | **407.1048** | **1218.2922** | **1218.3804** | **-0.0882** | **0** | **17** | **55** | **1** | **FYWSVMGSNK** |
|  | 2775 | **593.3710** | **1777.0909** | **1777.1328** | **-0.0419** | **1** | **17** | **57** | **1** | **SKYLPELLGNTGLLMK** |
|  | 191 | **369.3304** | **1104.9689** | **1105.3089** | **-0.3400** | **0** | **17** | **54** | **1** | **AKPWAVCFPS** |
|  | 404 | **385.0221** | **1152.0442** | **1152.2147** | **-0.1705** | **0** | **17** | **49** | **1** | **GTNNSFQVASK** |
|  | 2982 | **634.8188** | **1901.4344** | **1901.2190** | **0.2154** | **2** | **17** | **52** | **1** | **MSDNLERCFHRAMMK + 2 Oxidation (M)** |
|  | 1492 | **437.4446** | **872.8744** | **872.9625** | **-0.0882** | **0** | **17** | **70** | **1** | **EAIDLGQK** |
|  | 2958 | **628.2847** | **1881.8320** | **1881.9987** | **-0.1666** | **1** | **17** | **55** | **1** | **FLSESSSGPAPHGEPARR** |
|  | 71 | **364.1451** | **1089.4133** | **1090.2612** | **-0.8479** | **2** | **17** | **48** | **1** | **IRMGSSQRR** |
|  | 501 | **387.8850** | **1160.6329** | **1160.4272** | **0.2058** | **1** | **17** | **68** | **1** | **KLEMGNLVLK + Oxidation (M)** |
|  | 658 | **398.2125** | **1191.6153** | **1191.3584** | **0.2570** | **2** | **17** | **49** | **1** | **IKKADNSACNK** |
|  | 1218 | **420.1106** | **1257.3097** | **1257.5409** | **-0.2312** | **1** | **17** | **54** | **1** | **FVEKMTIFVK + Oxidation (M)** |
|  | 1726 | **458.0901** | **1371.2483** | **1370.4679** | **0.7803** | **0** | **17** | **57** | **1** | **GTSNPNLYWYR** |
|  | 3502 | **961.2211** | **2880.6410** | **2881.6196** | **-0.9785** | **1** | **17** | **44** | **1** | **QRMCMMLSMCACVYVCMCVCVR + 5 Carbamidomethyl (C); 2 Oxidation (M)** |
|  | 536 | **388.3244** | **1161.9510** | **1162.2541** | **-0.3031** | **1** | **17** | **57** | **1** | **YNFSNYKAR** |
|  | 598 | **389.9912** | **1166.9513** | **1167.1647** | **-0.2135** | **0** | **17** | **57** | **1** | **GDSAQSTACDR + Carbamidomethyl (C)** |
|  | 770 | **403.7473** | **805.4798** | **805.9180** | **-0.4383** | **0** | **17** | **55** | **1** | **AYPAGSIK** |
|  | 2444 | **532.8139** | **1063.6130** | **1063.2308** | **0.3823** | **0** | **17** | **48** | **1** | **VCFIGGPNTR** |
|  | 3048 | **666.6145** | **1996.8213** | **1997.2699** | **-0.4486** | **2** | **17** | **46** | **1** | **MATELSDAILEGYPKSKK + Oxidation (M)** |
|  | 2071 | **487.6570** | **973.2993** | **972.1200** | **1.1793** | **0** | **17** | **61** | **1** | **DLLQPGACR** |
|  | 1520 | **440.1537** | **1317.4390** | **1316.5514** | **0.8875** | **0** | **17** | **67** | **1** | **GSGSACSLLCCCGR** |
|  | 3438 | **828.1722** | **1654.3297** | **1654.8657** | **-0.5360** | **2** | **17** | **44** | **1** | **QENQVLKLANQKNK** |
|  | 790 | **404.0285** | **1209.0632** | **1208.3723** | **0.6910** | **1** | **17** | **58** | **1** | **MNNSRCAALR + Carbamidomethyl (C); Oxidation (M)** |
|  | 872 | **406.0573** | **810.0997** | **809.9530** | **0.1468** | **0** | **17** | **54** | **1** | **CGLSMWN** |
|  | 1808 | **461.7127** | **921.4106** | **921.0303** | **0.3803** | **0** | **17** | **45** | **1** | **ENVTLSCR** |
|  | 2335 | **519.0811** | **1036.1473** | **1037.2533** | **-1.1059** | **2** | **17** | **56** | **1** | **ISTKSVFKK** |
|  | 3200 | **716.3115** | **1430.6083** | **1429.4709** | **1.1374** | **1** | **17** | **56** | **1** | **SCSSASSRSQQSSK** |
|  | 494 | **387.8334** | **773.6520** | **773.8779** | **-0.2259** | **1** | **17** | **69** | **1** | **QGSNKIK** |
|  | 1089 | **413.2006** | **1236.5797** | **1236.5017** | **0.0780** | **0** | **17** | **48** | **1** | **MPLAGMSLGSLK + 2 Oxidation (M)** |
|  | 2568 | 551.6765 | 1652.0074 | 1651.8868 | 0.1206 | 1 | 17 | 71 | 1 | GMDQGLLGMCPGERR + 2 Oxidation (M) |
|  | 1780 | **459.9924** | **917.9701** | **917.0416** | **0.9285** | **0** | **17** | **64** | **1** | **SQPQGCLGK** |
|  | 2432 | **530.3865** | **1058.7582** | **1058.1002** | **0.6580** | **1** | **17** | **54** | **1** | **GEAEGPGGKEK** |
|  | 1499 | **437.8166** | **1310.4276** | **1311.4653** | **-1.0377** | **0** | **17** | **65** | **1** | **WQLCYDISAR + Carbamidomethyl (C)** |
|  | 2193 | 499.6929 | 1496.0564 | 1495.7273 | 0.3291 | 0 | 17 | 47 | 1 | HQLSHPGLPGALLR |
|  | 2138 | **492.6646** | **1474.9715** | **1475.6701** | **-0.6986** | **2** | **17** | **53** | **1** | **RDSAAAGMPGALSKK + Oxidation (M)** |
|  | 1483 | **437.1713** | **1308.4916** | **1307.4752** | **1.0164** | **0** | **17** | **64** | **1** | **EAAQAIFPSMAR + Oxidation (M)** |
|  | 2164 | **495.9218** | **989.8287** | **991.0143** | **-1.1855** | **0** | **17** | **62** | **1** | **ESQGSGTAVR** |
|  | 3080 | **668.6874** | **2003.0400** | **2002.2646** | **0.7754** | **0** | **17** | **61** | **1** | **ELLEQVDEQTFLQIIAI** |
|  | 3107 | 676.6936 | 1351.3724 | 1351.5344 | -0.1619 | 0 | 17 | 65 | 1 | SRPSAPGLPCPGR + Carbamidomethyl (C) |
|  | 3166 | **694.3635** | **2080.0684** | **2079.3722** | **0.6962** | **0** | **17** | **58** | **1** | **EMGDVSSGMSSSIMQLYLK + Oxidation (M)** |
|  | 687 | **400.0082** | **798.0016** | **797.9424** | **0.0593** | **1** | **17** | **49** | **1** | **KGLLPDR** |
|  | 3071 | **667.7407** | **1333.4667** | **1334.4557** | **-0.9890** | **0** | **17** | **73** | **1** | **GNSPCWSSWIPT** |
|  | 835 | **405.1174** | **1212.3301** | **1211.3912** | **0.9390** | **0** | **17** | **54** | **1** | **GPLTACSGPPVR + Carbamidomethyl (C)** |
|  | 880 | **406.1242** | **1215.3504** | **1216.4690** | **-1.1185** | **0** | **17** | **56** | **1** | **MFPFIMEGTK + Oxidation (M)** |
|  | 1787 | **460.3320** | **918.6493** | **919.0558** | **-0.4065** | **0** | **17** | **51** | **1** | **LWDMNPK + Oxidation (M)** |
|  | 2145 | **494.0924** | **986.1700** | **985.0892** | **1.0808** | **0** | **17** | **60** | **1** | **LEPETAPTK** |
|  | 764 | **403.1469** | **1206.4186** | **1207.4273** | **-1.0087** | **1** | **17** | **64** | **1** | **GPWVLRAPVGR** |
|  | 1141 | **416.1419** | **1245.4036** | **1245.4489** | **-0.0452** | **1** | **17** | **69** | **1** | **QAKELMSGLPR + Oxidation (M)** |
|  | 2040 | **485.8053** | **1454.3938** | **1454.6541** | **-0.2602** | **1** | **17** | **45** | **1** | **GARMGNLPQPELR + Oxidation (M)** |
|  | 79 | **364.3462** | **1090.0165** | **1091.1996** | **-1.1831** | **1** | **17** | **54** | **1** | **MQGDNSGVRK** |
|  | 553 | **389.0743** | **1164.2008** | **1164.3196** | **-0.1188** | **1** | **17** | **66** | **1** | **CQLCAGSGRR + 2 Carbamidomethyl (C)** |
|  | 2125 | 491.4907 | 1471.4500 | 1470.6320 | 0.8180 | 0 | 17 | 57 | 1 | SQPGTWLSVSRPR |
|  | 2494 | **538.3552** | **1074.6957** | **1075.2631** | **-0.5674** | **0** | **17** | **54** | **1** | **MSQPPQCLR + Oxidation (M)** |
|  | 1970 | **477.5593** | **953.1039** | **951.9399** | **1.1640** | **0** | **17** | **65** | **1** | **SGSGGGGGGGFR** |
|  | 1994 | **479.1548** | **1434.4423** | **1433.6168** | **0.8255** | **1** | **17** | **60** | **1** | **VWQVGPHVARER** |
|  | 1189 | **419.2026** | **1254.5857** | **1253.4313** | **1.1545** | **1** | **17** | **65** | **1** | **VEVTHCGQPKR** |
|  | 1687 | **455.1003** | **1362.2789** | **1361.5904** | **0.6885** | **0** | **17** | **58** | **1** | **VPAWGCCPASWK + Carbamidomethyl (C)** |
|  | 2638 | **564.3126** | **1126.6105** | **1126.3449** | **0.2656** | **0** | **17** | **63** | **1** | **VTTIMEMASK + Oxidation (M)** |
|  | 906 | **406.6711** | **1216.9911** | **1216.3878** | **0.6033** | **2** | **17** | **44** | **1** | **CEKCGKGYNSK** |
|  | 2118 | **490.3500** | **1468.0278** | **1467.5881** | **0.4397** | **0** | **17** | **48** | **1** | **AFGQSSSLIHHQR** |
|  | 558 | **389.0925** | **1164.2554** | **1164.3298** | **-0.0743** | **0** | **16** | **67** | **1** | **MASVLNSLEGK + Oxidation (M)** |
|  | 1330 | **428.3799** | **1282.1174** | **1281.5275** | **0.5899** | **1** | **16** | **45** | **1** | **SPKAQRPVACPK** |
|  | 1403 | **433.1738** | **1296.4991** | **1297.4574** | **-0.9582** | **0** | **16** | **59** | **1** | **TCDPVEMSYPR** |
|  | 2173 | **498.6069** | **1492.7984** | **1493.7496** | **-0.9512** | **0** | **16** | **69** | **1** | **MTINWAELLSGCR** |
|  | 833 | **405.1004** | **1212.2791** | **1213.4268** | **-1.1477** | **2** | **16** | **57** | **1** | **GPLATGGIKKSGK** |
|  | 3490 | **901.4845** | **1800.9542** | **1800.0155** | **0.9387** | **0** | **16** | **55** | **1** | **TLEENLGITIQDIGVGK** |
|  | 922 | 407.1080 | 812.2012 | 812.9139 | -0.7128 | 0 | 16 | 59 | 1 | QPAGALTR |
|  | 1125 | **415.2300** | **828.4452** | **828.9563** | **-0.5112** | **1** | **16** | **54** | **1** | **RGIALDGK** |
|  | 1645 | **450.1770** | **1347.5087** | **1347.4512** | **0.0575** | **0** | **16** | **62** | **1** | **LCLPGSSNSPASAS + Carbamidomethyl (C)** |
|  | 2692 | **578.6493** | **1732.9257** | **1732.8981** | **0.0276** | **2** | **16** | **72** | **1** | **LIQPPGDPRGGREGQR** |
|  | 1848 | **466.0401** | **930.0654** | **931.0916** | **-1.0262** | **2** | **16** | **64** | **1** | **VAASRTKAK** |
|  | 320 | **377.2363** | **752.4578** | **751.8939** | **0.5640** | **0** | **16** | **45** | **1** | **MLPGYR + Oxidation (M)** |
|  | 1948 | **475.8359** | **1424.4856** | **1425.5187** | **-1.0330** | **1** | **16** | **62** | **1** | **DMELDSSSIEKR + Oxidation (M)** |
|  | 595 | **389.9744** | **777.9341** | **777.7374** | **0.1967** | **0** | **16** | **62** | **1** | **GNSSGNDK** |
|  | 1222 | **420.1669** | **1257.4786** | **1257.3370** | **0.1417** | **1** | **16** | **58** | **1** | **PGGCSRGPAAGDGR** |
|  | 1232 | **420.4276** | **1258.2608** | **1258.4229** | **-0.1621** | **1** | **16** | **62** | **1** | **KEDFLKPPER** |
|  | 2269 | **511.8654** | **1532.5741** | **1532.7794** | **-0.2053** | **0** | **16** | **57** | **1** | **NMVFLFTDTQVCV + Oxidation (M)** |
|  | 980 | **407.7861** | **1220.3361** | **1219.3106** | **1.0256** | **0** | **16** | **62** | **1** | **DHQRPSGVPAR** |
|  | 2084 | **488.2100** | **974.4051** | **974.0699** | **0.3353** | **1** | **16** | **67** | **1** | **KTVNDELR** |
|  | 956 | **407.4163** | **812.8179** | **812.9968** | **-0.1789** | **1** | **16** | **66** | **1** | **AGKLSPLK** |
|  | 1755 | **459.0189** | **916.0231** | **915.9476** | **0.0755** | **0** | **16** | **69** | **1** | **ANSLGPSDR** |
|  | 3327 | **755.2309** | **2262.6705** | **2261.6827** | **0.9878** | **2** | **16** | **61** | **1** | **DMFKLLLGLLQADRTVVNSK** |
|  | 122 | **367.2682** | **1098.7823** | **1099.2398** | **-0.4575** | **0** | **16** | **54** | **1** | **SAPFLTPGPGR** |
|  | 1123 | **415.1711** | **828.3274** | **828.9563** | **-0.6289** | **0** | **16** | **63** | **1** | **LLAASQAR** |
|  | 1406 | **433.2526** | **1296.7357** | **1297.4574** | **-0.7217** | **0** | **16** | **50** | **1** | **TCDPVEMSYPR** |
|  | 13 | 360.4811 | 1078.4212 | 1079.1890 | -0.7677 | 1 | 16 | 75 | 1 | MAPTSASSRR + Oxidation (M) |
|  | 933 | **407.1837** | **1218.5289** | **1218.3192** | **0.2097** | **0** | **16** | **57** | **1** | **GPHTSPSHTLGK** |
|  | 1885 | **470.1099** | **1407.3076** | **1407.5034** | **-0.1958** | **1** | **16** | **55** | **1** | **EMEKLAQGDQDK + Oxidation (M)** |
|  | 1772 | **459.3998** | **916.7848** | **916.0303** | **0.7544** | **0** | **16** | **57** | **1** | **SSSAPLGLGK** |
|  | 3492 | **902.8000** | **2705.3780** | **2705.0895** | **0.2885** | **2** | **16** | **48** | **1** | **SIDLSALNLTELVNGMLSRALKDSK + Oxidation (M)** |
|  | 3403 | **802.5475** | **1603.0803** | **1601.8909** | **1.1894** | **2** | **16** | **60** | **1** | **ADARLAIARLVSAFK** |
|  | 234 | **372.2799** | **1113.8175** | **1113.2681** | **0.5495** | **0** | **16** | **47** | **1** | **SSCAMTWVR + Carbamidomethyl (C); Oxidation (M)** |
|  | 257 | **374.0068** | **745.9988** | **744.8797** | **1.1190** | **1** | **16** | **77** | **1** | **KGTAALGK** |
|  | 1900 | **472.1595** | **942.3042** | **942.0711** | **0.2331** | **1** | **16** | **62** | **1** | **SKDPVQLR** |
|  | 2293 | **515.2032** | **1028.3917** | **1027.2186** | **1.1732** | **1** | **16** | **64** | **1** | **IRGISAVSPK** |
|  | 2646 | 565.5944 | 1693.7609 | 1693.7694 | -0.0085 | 1 | 16 | 74 | 1 | SKADGGTSDYAAPLQGR |
|  | 1504 | **438.0866** | **1311.2376** | **1310.4991** | **0.7385** | **0** | **16** | **72** | **1** | **LPEVQVLNVSGR** |
|  | 3007 | **649.6262** | **1297.2377** | **1296.5502** | **0.6875** | **2** | **16** | **51** | **1** | **CPWRPRLGRR** |
|  | 1524 | **440.3996** | **878.7844** | **877.9229** | **0.8616** | **1** | **16** | **63** | **1** | **AECRDER** |
|  | 2680 | **573.7379** | **1145.4610** | **1144.3715** | **1.0896** | **1** | **16** | **66** | **1** | **IRNQMVCHK + Oxidation (M)** |
|  | 517 | **388.0722** | **1161.1943** | **1160.2649** | **0.9295** | **1** | **16** | **75** | **1** | **AGMGRAGAGSPGR + Oxidation (M)** |
|  | 2067 | **487.2061** | **972.3975** | **972.1369** | **0.2607** | **1** | **16** | **68** | **1** | **TSPAEVLKK** |
|  | 2564 | **551.0029** | **1649.9866** | **1649.8875** | **0.0992** | **1** | **16** | **64** | **1** | **VRQPWSIPVLPDDK** |
|  | 3003 | **646.7937** | **1937.3589** | **1938.2245** | **-0.8655** | **2** | **16** | **72** | **1** | **EEKMSVLSPEIKCETSK** |
|  | 3246 | **740.5914** | **2218.7521** | **2218.5354** | **0.2167** | **1** | **16** | **52** | **1** | **APRAQAVVGDLLELHCEALR + Carbamidomethyl (C)** |
|  | 1602 | **446.2182** | **1335.6323** | **1334.5056** | **1.1267** | **2** | **16** | **68** | **1** | **TECISSRAGRVR** |
|  | 2401 | **524.2202** | **1569.6383** | **1568.8825** | **0.7558** | **1** | **16** | **68** | **1** | **TGQVQGLLRMQPLK** |
|  | 3498 | **934.7268** | **2801.1582** | **2802.1483** | **-0.9901** | **2** | **16** | **54** | **1** | **AEIASCSYEARQLGIKNGMFFGHAK + Carbamidomethyl (C); Oxidation (M)** |
|  | 991 | **407.8637** | **1220.5690** | **1221.4108** | **-0.8418** | **1** | **16** | **62** | **1** | **MECGLNNRIR + Oxidation (M)** |
|  | 1797 | **460.7066** | **1379.0977** | **1378.6029** | **0.4949** | **2** | **16** | **54** | **1** | **KGGHPPAVKAGGMR + Oxidation (M)** |
|  | 3051 | **666.6805** | **1997.0195** | **1996.3347** | **0.6847** | **1** | **16** | **64** | **1** | **TVSSTLNIPINRITCHVK** |
|  | 125 | **367.2804** | **1098.8191** | **1099.2647** | **-0.4456** | **1** | **16** | **58** | **1** | **CPKGTQVPGR + Carbamidomethyl (C)** |
|  | 456 | **386.1494** | **770.2840** | **769.8463** | **0.4378** | **0** | **16** | **57** | **1** | **SAPSGPVR** |
|  | 1350 | **429.2470** | **856.4792** | **857.0889** | **-0.6098** | **0** | **16** | **54** | **1** | **IIIISVSL** |
|  | 1614 | **448.1161** | **1341.3261** | **1341.5130** | **-0.1869** | **0** | **16** | **62** | **1** | **QMSCNPSFGGIGK + Oxidation (M)** |
|  | 1733 | **458.2572** | **914.4996** | **914.9164** | **-0.4167** | **0** | **16** | **60** | **1** | **GNGIGSNEAP** |
|  | 682 | **399.8116** | **797.6085** | **796.8716** | **0.7370** | **1** | **16** | **55** | **1** | **QKHDAAK** |
|  | 114 | **367.1450** | **1098.4128** | **1099.2051** | **-0.7923** | **2** | **16** | **66** | **1** | **RGVGSSPGARR** |
|  | 596 | **389.9875** | **777.9603** | **776.8372** | **1.1232** | **0** | **16** | **65** | **1** | **AHSFTSK** |
|  | 938 | **407.2122** | **1218.6144** | **1217.4189** | **1.1955** | **0** | **16** | **53** | **1** | **MGPLPGAGVSCR + Carbamidomethyl (C); Oxidation (M)** |
|  | 214 | **371.0689** | **1110.1846** | **1110.3502** | **-0.1657** | **2** | **16** | **54** | **1** | **IKTPKGTLPR** |
|  | 3136 | **684.1005** | **2049.2792** | **2049.2616** | **0.0176** | **0** | **16** | **63** | **1** | **LPTMAEVNGEQDFIDLTR** |
|  | 491 | **387.5906** | **773.1664** | **772.8898** | **0.2765** | **0** | **16** | **64** | **1** | **SLPTSLR** |
|  | 493 | **387.8303** | **773.6457** | **773.8779** | **-0.2322** | **1** | **16** | **78** | **1** | **RETLQK** |
|  | 2363 | **521.1013** | **1040.1879** | **1040.2389** | **-0.0510** | **0** | **16** | **62** | **1** | **CVDMGCAGLR + Oxidation (M)** |
|  | 124 | **367.2794** | **732.5441** | **732.8491** | **-0.3050** | **0** | **16** | **59** | **1** | **ALAEACR** |
|  | 559 | **389.0936** | **776.1724** | **775.8740** | **0.2984** | **1** | **16** | **72** | **1** | **MGKGPDR + Oxidation (M)** |
|  | 1048 | **409.4322** | **816.8496** | **817.8908** | **-1.0412** | **1** | **16** | **85** | **1** | **NRVGASSK** |
|  | 2793 | **595.1747** | **1782.5021** | **1782.0698** | **0.4322** | **1** | **16** | **72** | **1** | **LLNSPAKTLPGACGSPQK** |
|  | 842 | **405.1841** | **1212.5303** | **1213.3208** | **-0.7906** | **0** | **16** | **58** | **1** | **EGEQICLHER** |
|  | 1276 | **423.4196** | **1267.2367** | **1267.5006** | **-0.2639** | **1** | **16** | **80** | **1** | **LAAENHCLRIK** |
|  | 1348 | **429.2133** | **1284.6176** | **1283.4985** | **1.1191** | **1** | **16** | **62** | **1** | **VCDFGFAKQLR** |
|  | 3335 | **758.1704** | **1514.3260** | **1513.8470** | **0.4790** | **1** | **16** | **60** | **1** | **IQRLMDVGLIAIR + Oxidation (M)** |
|  | 26 | **362.1346** | **1083.3815** | **1083.1096** | **0.2719** | **1** | **16** | **59** | **1** | **NKTNSNYDK** |
|  | 1508 | **438.2034** | **1311.5880** | **1311.3763** | **0.2118** | **0** | **16** | **71** | **1** | **MAGSSTGGGGVGETK + Oxidation (M)** |
|  | 2081 | **488.1253** | **1461.3537** | **1461.7113** | **-0.3575** | **1** | **16** | **73** | **1** | **AEAGMGMWRLAPR + Oxidation (M)** |
|  | 750 | **402.3920** | **1204.1539** | **1205.3454** | **-1.1915** | **2** | **16** | **79** | **1** | **DEKMRQQVR + Oxidation (M)** |
|  | 1915 | **472.7952** | **1415.3634** | **1414.6564** | **0.7070** | **2** | **16** | **57** | **1** | **QPIACPKCERR + 2 Carbamidomethyl (C)** |
|  | 3084 | **668.7675** | **2003.2804** | **2003.2197** | **0.0607** | **0** | **16** | **80** | **1** | **LSHSDEKPFECPICNQR** |
|  | 273 | **374.4359** | **746.8570** | **745.8281** | **1.0289** | **2** | **16** | **94** | **1** | **NSKGGRK** |
|  | 1124 | **415.1718** | **828.3288** | **828.9563** | **-0.6276** | **1** | **16** | **68** | **1** | **GQDLLKR** |
|  | 1631 | **449.6682** | **1345.9826** | **1346.4680** | **-0.4855** | **0** | **16** | **52** | **1** | **CGWDIAGDPGSIR** |
|  | 3132 | **684.0298** | **2049.0672** | **2048.1708** | **0.8964** | **0** | **16** | **56** | **1** | **VGNGFGGYCSSTSCYHFDF** |
|  | 2142 | **493.3155** | **984.6161** | **984.1541** | **0.4620** | **1** | **16** | **58** | **1** | **TLATPAVRR** |
|  | 2285 | **513.7035** | **1025.3922** | **1025.1166** | **0.2756** | **0** | **16** | **56** | **1** | **ENPVLGSGPR** |
|  | 467 | **386.4466** | **1156.3176** | **1156.2347** | **0.0830** | **0** | **16** | **77** | **1** | **GAFGAGHCSGHR** |
|  | 478 | **387.0312** | **1158.0714** | **1157.3452** | **0.7261** | **0** | **16** | **76** | **1** | **FCIGLHSAPR + Carbamidomethyl (C)** |
|  | 1411 | **434.0768** | **1299.2084** | **1298.5312** | **0.6771** | **1** | **16** | **62** | **1** | **ELLLSELLGRR** |
|  | 1454 | **436.1104** | **870.2060** | **871.0807** | **-0.8747** | **1** | **16** | **67** | **1** | **KIVLWGR** |
|  | 1605 | **446.9756** | **891.9365** | **891.9925** | **-0.0560** | **0** | **16** | **72** | **1** | **MAVQGSQR + Oxidation (M)** |
|  | 339 | **378.5024** | **1132.4851** | **1133.3419** | **-0.8568** | **0** | **16** | **63** | **1** | **ALSIFWLGAR** |
|  | 823 | **405.0052** | **1211.9936** | **1211.3315** | **0.6620** | **1** | **16** | **62** | **1** | **QRSSLTVHQR** |
|  | 1830 | **463.8009** | **1388.3805** | **1388.5961** | **-0.2155** | **2** | **16** | **57** | **1** | **RLKMSGGASATGPR** |
|  | 2007 | **480.1522** | **958.2895** | **959.1430** | **-0.8534** | **0** | **16** | **70** | **1** | **WVVVSTLR** |
|  | 2790 | **594.8384** | **1187.6620** | **1187.3483** | **0.3137** | **2** | **16** | **58** | **1** | **EPLERRYPK** |
|  | 2886 | **611.1879** | **1830.5414** | **1830.1758** | **0.3657** | **1** | **16** | **70** | **1** | **AEFIATRSMDFIGMIK** |
|  | 5 | **360.3528** | **1078.0362** | **1077.3204** | **0.7158** | **0** | **16** | **84** | **1** | **GLQPPVVVLR** |
|  | 1710 | **457.0981** | **912.1814** | **913.1127** | **-0.9312** | **1** | **16** | **66** | **1** | **IVPELKSK** |
|  | 1993 | **479.0858** | **1434.2354** | **1433.5025** | **0.7329** | **1** | **16** | **66** | **1** | **FGEYCSSENGKGR** |
|  | 349 | 380.0574 | 758.1000 | 757.9250 | 0.1751 | 2 | 16 | 77 | 1 | LRKVSR |
|  | 551 | 389.0629 | 1164.1666 | 1165.2748 | -1.1082 | 0 | 16 | 75 | 1 | LGSGGGAGVMESK + Oxidation (M) |
|  | 647 | **396.1869** | **790.3591** | **789.8757** | **0.4834** | **0** | **16** | **70** | **1** | **WVQETK** |
|  | 651 | **396.3420** | **790.6693** | **791.8038** | **-1.1345** | **0** | **16** | **65** | **1** | **LEDGGSSK** |
|  | 1747 | **458.8453** | **1373.5137** | **1372.5056** | **1.0082** | **1** | **16** | **74** | **1** | **DKCGVCGGDNSSCK** |
|  | 1804 | **460.9612** | **919.9077** | **919.9397** | **-0.0320** | **1** | **16** | **69** | **1** | **SPGSRSSSR** |
|  | 2123 | 491.2927 | 1470.8559 | 1471.6582 | -0.8022 | 0 | 16 | 59 | 1 | GLCVCSSFDQTVR + Carbamidomethyl (C) |
|  | 3184 | **708.1898** | **2121.5473** | **2121.3506** | **0.1966** | **1** | **16** | **65** | **1** | **TNPDLLAVGYGHFGFKEQK** |
|  | 645 | **396.1545** | **1185.4415** | **1185.3475** | **0.0940** | **0** | **16** | **75** | **1** | **VETTDCPVPPK** |
|  | 1019 | **408.2978** | **814.5807** | **814.8438** | **-0.2630** | **0** | **16** | **56** | **1** | **GSSGPTGPR** |
|  | 1475 | **437.0368** | **872.0588** | **871.0330** | **1.0258** | **1** | **16** | **75** | **1** | **AKEVLPSK** |
|  | 3458 | **847.4070** | **1692.7993** | **1691.9292** | **0.8701** | **1** | **16** | **66** | **1** | **AFDRHCNMVLENVK + Oxidation (M)** |
|  | 2496 | **538.4070** | **1074.7992** | **1074.2089** | **0.5903** | **0** | **16** | **56** | **1** | **MATEIGSPPR + Oxidation (M)** |
|  | 2708 | **582.2792** | **1743.8155** | **1743.9839** | **-0.1683** | **1** | **16** | **72** | **1** | **LIYMVSNRDSGVPHR** |
|  | 265 | **374.2155** | **1119.6244** | **1120.3416** | **-0.7173** | **0** | **16** | **66** | **1** | **SAILHEPLLK** |
|  | 1379 | **431.1009** | **1290.2805** | **1289.3920** | **0.8885** | **0** | **16** | **80** | **1** | **SSGLSSSLQPAGAK** |
|  | 2004 | **480.0843** | **958.1538** | **959.0983** | **-0.9445** | **2** | **16** | **71** | **1** | **LKDKAADAK** |
|  | 819 | **404.9539** | **1211.8397** | **1211.3680** | **0.4717** | **0** | **16** | **63** | **1** | **SCCDSPLPFSR** |
|  | 140 | **368.1793** | **1101.5158** | **1102.2853** | **-0.7695** | **0** | **16** | **61** | **1** | **FQGMVTMTR + 2 Oxidation (M)** |
|  | 476 | **386.9944** | **1157.9612** | **1158.4114** | **-0.4502** | **0** | **16** | **76** | **1** | **KPNIAIVEMK + Oxidation (M)** |
|  | 2437 | **531.2833** | **1590.8276** | **1591.7652** | **-0.9376** | **2** | **16** | **72** | **1** | **WRILEEEKFEGR** |
|  | 2640 | **564.4061** | **1690.1960** | **1689.8636** | **0.3324** | **1** | **16** | **61** | **1** | **AEVHKSLDSYAASLAK** |
|  | 1743 | **458.7447** | **1373.2119** | **1373.4738** | **-0.2619** | **1** | **16** | **61** | **1** | **GSSGSSGHFPARVK** |
|  | 2294 | **515.2274** | **1028.4399** | **1028.1835** | **0.2565** | **0** | **16** | **70** | **1** | **EGQLTPMPR** |
|  | 2456 | **534.4601** | **1066.9054** | **1067.2162** | **-0.3108** | **1** | **16** | **61** | **1** | **KQQFCISDV** |
|  | 3140 | **684.7008** | **2051.0802** | **2050.2397** | **0.8406** | **2** | **16** | **72** | **1** | **AFHHPASFLGKDSSQHRK** |
|  | 1325 | **428.2431** | **1281.7071** | **1282.5538** | **-0.8467** | **1** | **16** | **51** | **1** | **MVEAAMKSPMR + 2 Oxidation (M)** |
|  | 1884 | **470.0698** | **938.1248** | **938.0856** | **0.0392** | **2** | **16** | **62** | **1** | **RIAKEHGK** |
|  | 2876 | **610.1532** | **1827.4374** | **1827.8667** | **-0.4292** | **1** | **16** | **71** | **1** | **EGPAGGTGGSGGPGGSLGSRGR** |
|  | 1402 | **433.1362** | **864.2576** | **864.0238** | **0.2338** | **0** | **16** | **69** | **1** | **FQSMPVR** |
|  | 2295 | **515.3236** | **1542.9487** | **1542.7358** | **0.2128** | **0** | **16** | **63** | **1** | **NLQASGLTTLGQALR** |
|  | 2829 | **601.7244** | **1201.4341** | **1200.2989** | **1.1351** | **0** | **16** | **82** | **1** | **EGLGASSHITTK** |
|  | 429 | **385.9852** | **769.9556** | **768.8150** | **1.1405** | **0** | **16** | **60** | **1** | **AEGDHLK** |
|  | 1873 | **469.0844** | **936.1539** | **935.0354** | **1.1186** | **0** | **16** | **69** | **1** | **ISYSNPVR** |
|  | 3128 | **683.9537** | **2048.8390** | **2048.5004** | **0.3386** | **2** | **16** | **56** | **1** | **MRCLTTPMLLRALAQAAR + 2 Oxidation (M)** |
|  | 803 | **404.1170** | **806.2193** | **806.9245** | **-0.7052** | **0** | **16** | **70** | **1** | **MEDGVLK + Oxidation (M)** |
|  | 1315 | **427.6728** | **1279.9962** | **1279.5332** | **0.4630** | **2** | **16** | **57** | **1** | **MIMAKTAADRR + Oxidation (M)** |
|  | 1380 | **431.1444** | **1290.4111** | **1291.5142** | **-1.1030** | **2** | **16** | **82** | **1** | **KEKEAVLAMEK + Oxidation (M)** |
|  | 2593 | **555.7958** | **1109.5768** | **1109.0674** | **0.5094** | **0** | **16** | **56** | **1** | **DSSEHGAGGHR** |
|  | 2964 | **629.8324** | **1257.6500** | **1257.3900** | **0.2600** | **0** | **16** | **62** | **1** | **EEEALLGEIVR** |
|  | 441 | **386.0824** | **1155.2250** | **1155.2337** | **-0.0086** | **0** | **16** | **64** | **1** | **AASSSEISEMK + Oxidation (M)** |
|  | 996 | **407.9359** | **1220.7857** | **1221.4656** | **-0.6799** | **1** | **16** | **70** | **1** | **SMPKLEIFEK** |
|  | 674 | 399.1988 | 796.3828 | 795.8869 | 0.4959 | 0 | 16 | 54 | 1 | VVTANHR |
|  | 723 | **401.7251** | **1202.1532** | **1201.3947** | **0.7586** | **1** | **16** | **72** | **1** | **YFTDKQMLR** |
|  | 1611 | **447.5246** | **893.0344** | **892.9507** | **0.0836** | **0** | **16** | **86** | **1** | **TINTETSK** |
|  | 3340 | **759.3424** | **1516.6700** | **1515.6676** | **1.0024** | **2** | **16** | **69** | **1** | **AALTDELLKDRDR** |
|  | 2237 | **505.3877** | **1513.1408** | **1513.5656** | **-0.4247** | **0** | **16** | **53** | **1** | **GDWETQYFGPGTR** |
|  | 3126 | **683.9016** | **2048.6827** | **2049.3776** | **-0.6949** | **1** | **16** | **61** | **1** | **MSRSTWSALACMWEMSR + Oxidation (M)** |
|  | 943 | **407.2644** | **812.5141** | **811.9689** | **0.5451** | **0** | **16** | **53** | **1** | **SVLAGPLR** |
|  | 1392 | **432.9697** | **863.9245** | **863.9758** | **-0.0512** | **0** | **16** | **71** | **1** | **MTISPDGK + Oxidation (M)** |
|  | 732 | **401.9026** | **1202.6855** | **1202.4540** | **0.2315** | **2** | **16** | **81** | **1** | **MAGAMPARGGKR** |
|  | 1185 | **419.1202** | **1254.3384** | **1253.4277** | **0.9106** | **0** | **16** | **82** | **1** | **YLCASSLVGQGR** |
|  | 1983 | **478.2158** | **954.4169** | **955.0913** | **-0.6744** | **0** | **16** | **67** | **1** | **MFQTAWR + Oxidation (M)** |
|  | 2110 | **490.1113** | **978.2079** | **978.0818** | **0.1261** | **0** | **16** | **72** | **1** | **AASAMDAVSR** |
|  | 1074 | **411.6202** | **1231.8384** | **1231.4903** | **0.3481** | **2** | **16** | **60** | **1** | **KRPIVPEHKK** |
|  | 3388 | **789.2292** | **1576.4436** | **1576.8848** | **-0.4412** | **0** | **16** | **65** | **1** | **MSCCSCCPVGCAK + 5 Carbamidomethyl (C)** |
|  | 1358 | **429.7043** | **857.3938** | **857.0957** | **0.2980** | **2** | **16** | **63** | **1** | **KKTIVLR** |
|  | 417 | **385.1351** | **768.2555** | **768.8151** | **-0.5596** | **0** | **16** | **62** | **1** | **GPGGAEGPK** |
|  | 1083 | **412.8773** | **1235.6096** | **1234.4875** | **1.1221** | **1** | **16** | **64** | **1** | **TTFMLRFSCL + Oxidation (M)** |
|  | 854 | **405.7637** | **1214.2688** | **1215.3830** | **-1.1141** | **0** | **16** | **67** | **1** | **VSPCWPGWAR + Carbamidomethyl (C)** |
|  | 1371 | **431.0170** | **1290.0287** | **1290.3800** | **-0.3513** | **0** | **16** | **87** | **1** | **EYQWLHTGEK** |
|  | 361 | **380.3297** | **1137.9669** | **1137.2464** | **0.7204** | **0** | **16** | **71** | **1** | **EAHAQAGVQVK** |
|  | 771 | **403.8112** | **1208.4115** | **1207.3778** | **1.0337** | **0** | **16** | **73** | **1** | **AAPMLDMSEAR + Oxidation (M)** |
|  | 1882 | **470.0233** | **938.0318** | **937.0578** | **0.9740** | **1** | **16** | **65** | **1** | **RLSATPHR** |
|  | 2372 | **521.5811** | **1561.7210** | **1562.7026** | **-0.9816** | **1** | **16** | **83** | **1** | **MKSDNHSFLGDSPK** |
|  | 3167 | **695.3683** | **2083.0827** | **2082.3279** | **0.7547** | **1** | **16** | **72** | **1** | **AESRPGMAAHACNPSTLGRR** |
|  | 927 | **407.1430** | **1218.4069** | **1218.4482** | **-0.0413** | **1** | **16** | **70** | **1** | **CKVLGYYCR + 2 Carbamidomethyl (C)** |
|  | 1865 | **468.0639** | **1401.1695** | **1401.5269** | **-0.3573** | **0** | **16** | **78** | **1** | **SDHQLVAGPPQPR** |
|  | 384 | **384.2426** | **1149.7056** | **1149.3878** | **0.3178** | **0** | **16** | **54** | **1** | **WHGCVFLCK + Carbamidomethyl (C)** |
|  | 1955 | **476.1784** | **1425.5130** | **1425.6511** | **-0.1381** | **2** | **16** | **72** | **1** | **KDDVCPGMKCISG + Carbamidomethyl (C); Oxidation (M)** |
|  | 3147 | **685.8213** | **1369.6278** | **1368.6194** | **1.0084** | **2** | **16** | **88** | **1** | **INLSKIDKYFK** |
|  | 719 | **401.6267** | **1201.8580** | **1202.4921** | **-0.6341** | **1** | **16** | **68** | **1** | **YMVFRSMLR** |
|  | 2091 | **488.3176** | **1461.9307** | **1462.5238** | **-0.5930** | **1** | **16** | **65** | **1** | **NQTSNGFPGAEGRK** |
|  | 1731 | 458.2334 | 914.4520 | 914.0410 | 0.4110 | 0 | 16 | 73 | 1 | MINDLHR + Oxidation (M) |
|  | 3460 | **847.8134** | **1693.6119** | **1692.8924** | **0.7196** | **2** | **16** | **59** | **1** | **GNSRASSSSMKIPLNK + Oxidation (M)** |
|  | 694 | **400.1157** | **798.2166** | **798.9736** | **-0.7570** | **2** | **16** | **65** | **1** | **TPLGKKR** |
|  | 1685 | 454.5326 | 1360.5757 | 1360.4238 | 0.1519 | 1 | 16 | 91 | 1 | DKLPSTEVSGPSSG |
|  | 3325 | **754.8152** | **2261.4236** | **2260.4894** | **0.9342** | **1** | **16** | **85** | **1** | **ASGFTFDHHAMTWVRQAPGK + Oxidation (M)** |
|  | 1088 | **413.1879** | **1236.5414** | **1235.3497** | **1.1918** | **1** | **16** | **64** | **1** | **SHDGKTPQLPR** |
|  | 1398 | **433.1030** | **1296.2867** | **1297.4574** | **-1.1706** | **0** | **16** | **74** | **1** | **TCDPVEMSYPR** |
|  | 195 | **369.3504** | **1105.0289** | **1104.2680** | **0.7610** | **2** | **16** | **76** | **1** | **HRSDRMMR + Oxidation (M)** |
|  | 776 | **403.9220** | **805.8293** | **804.8490** | **0.9803** | **1** | **16** | **75** | **1** | **EKGSTGAR** |
|  | 2276 | **512.4459** | **1534.3156** | **1534.6263** | **-0.3107** | **1** | **16** | **59** | **1** | **KGDDGTPSQPGPPGPK** |
|  | 1282 | **423.7718** | **1268.2932** | **1268.4640** | **-0.1708** | **0** | **16** | **81** | **1** | **WPCECPPSPPR** |
|  | 1887 | **470.2137** | **1407.6189** | **1408.7281** | **-1.1093** | **2** | **16** | **68** | **1** | **DLVRKLIVLDPK** |
|  | 1774 | **459.7132** | **917.4115** | **916.9325** | **0.4791** | **1** | **16** | **66** | **1** | **DEPGSERK** |
|  | 1910 | **472.5174** | **943.0201** | **943.1219** | **-0.1019** | **0** | **16** | **89** | **1** | **TSLPCIPR + Carbamidomethyl (C)** |
|  | 1610 | **447.3342** | **892.6536** | **892.9738** | **-0.3203** | **0** | **16** | **63** | **1** | **CSLSVAQE + Carbamidomethyl (C)** |
|  | 2884 | **610.3440** | **1218.6732** | **1218.4700** | **0.2033** | **2** | **16** | **77** | **1** | **LKLRVAEMSR + Oxidation (M)** |
|  | 261 | **374.0902** | **746.1657** | **745.7816** | **0.3841** | **0** | **16** | **90** | **1** | **ASGLQDR** |
|  | 540 | **388.4569** | **1162.3486** | **1163.3849** | **-1.0362** | **2** | **16** | **1.1e+02** | **1** | **KKILEEMEK + Oxidation (M)** |
|  | 1121 | **415.1033** | **1242.2877** | **1242.3853** | **-0.0975** | **2** | **16** | **78** | **1** | **GRISISRDNPK** |
|  | 393 | **384.8946** | **767.7743** | **766.7991** | **0.9752** | **0** | **15** | **63** | **1** | **DSYGGLR** |
|  | 838 | **405.1555** | **1212.4443** | **1212.5051** | **-0.0608** | **0** | **15** | **69** | **1** | **LATPGLMIRPK + Oxidation (M)** |
|  | 1180 | **418.5406** | **835.0663** | **834.9194** | **0.1469** | **0** | **15** | **99** | **1** | **GLQTNFR** |
|  | 1532 | **441.1453** | **1320.4138** | **1320.5020** | **-0.0882** | **1** | **15** | **76** | **1** | **CTAQCGGGIQARR** |
|  | 2281 | **513.5584** | **1537.6531** | **1536.6900** | **0.9631** | **1** | **15** | **86** | **1** | **GFDGLPGLPGEKGHR** |
|  | 236 | **372.2973** | **742.5798** | **742.9035** | **-0.3238** | **0** | **15** | **58** | **1** | **LLEALGK** |
|  | 49 | **363.1603** | **724.3059** | **723.8209** | **0.4850** | **1** | **15** | **67** | **1** | **KHPGTGK** |
|  | 1156 | **417.0609** | **832.1070** | **830.9294** | **1.1777** | **2** | **15** | **85** | **1** | **KSKSPER** |
|  | 2061 | **486.9299** | **1457.7674** | **1456.6436** | **1.1239** | **1** | **15** | **79** | **1** | **ANVIVTGGDDKVIR** |
|  | 432 | **386.0125** | **770.0102** | **769.8892** | **0.1211** | **0** | **15** | **66** | **1** | **KPGGNLGK** |
|  | 525 | **388.1565** | **1161.4474** | **1161.3754** | **0.0720** | **1** | **15** | **87** | **1** | **QVSARLCGLSK** |
|  | 2405 | **524.3254** | **1046.6361** | **1046.2200** | **0.4160** | **0** | **15** | **75** | **1** | **SSLNPILFR** |
|  | 2461 | **535.6845** | **1604.0314** | **1602.8507** | **1.1806** | **0** | **15** | **77** | **1** | **FEMYSSLAWIAIR + Oxidation (M)** |
|  | 78 | **364.2921** | **1089.8541** | **1089.2848** | **0.5693** | **0** | **15** | **55** | **1** | **EAVVNAVFIK** |
|  | 116 | **367.1575** | **1098.4504** | **1098.1673** | **0.2831** | **1** | **15** | **74** | **1** | **HETAQKGEAK** |
|  | 691 | **400.0583** | **798.1018** | **796.9575** | **1.1442** | **1** | **15** | **65** | **1** | **GNLPLRK** |
|  | 1788 | **460.3491** | **918.6835** | **918.0266** | **0.6569** | **1** | **15** | **66** | **1** | **EKMTHEK + Oxidation (M)** |
|  | 523 | **388.1349** | **1161.3825** | **1162.3039** | **-0.9214** | **2** | **15** | **90** | **1** | **SFRRAPSWR** |
|  | 1812 | **461.9578** | **921.9007** | **921.0735** | **0.8273** | **0** | **15** | **72** | **1** | **SLVSVCTR + Carbamidomethyl (C)** |
|  | 1470 | **436.3564** | **870.6980** | **870.0712** | **0.6267** | **0** | **15** | **59** | **1** | **LAHASIMK** |
|  | 1835 | **464.2617** | **1389.7631** | **1388.7154** | **1.0477** | **1** | **15** | **68** | **1** | **IIKKPMDLSTVK + Oxidation (M)** |
|  | 2653 | **565.9707** | **1694.8899** | **1695.9344** | **-1.0444** | **0** | **15** | **80** | **1** | **CITFCPTPDQADPCK + Carbamidomethyl (C)** |
|  | 2883 | **610.3434** | **1218.6720** | **1219.4050** | **-0.7330** | **0** | **15** | **79** | **1** | **LIGEVMIGEDK + Oxidation (M)** |
|  | 579 | **389.1963** | **776.3778** | **776.9200** | **-0.5422** | **0** | **15** | **72** | **1** | **LGVTYPK** |
|  | 1561 | **443.4658** | **1327.3753** | **1326.5942** | **0.7811** | **1** | **15** | **87** | **1** | **CLAHRGGGLCALR** |
|  | 263 | **374.1473** | **746.2799** | **746.8923** | **-0.6125** | **0** | **15** | **88** | **1** | **LSLVSTK** |
|  | 2643 | **565.2582** | **1692.7524** | **1692.8727** | **-0.1203** | **1** | **15** | **83** | **1** | **MAQEVDTAQGAEMRR** |
|  | 1067 | **411.1266** | **1230.3577** | **1229.4874** | **0.8703** | **0** | **15** | **82** | **1** | **LTFCTLALYK + Carbamidomethyl (C)** |
|  | 3087 | **668.8427** | **2003.5058** | **2004.0703** | **-0.5645** | **1** | **15** | **79** | **1** | **MRGSHHHHHHGSENSEK** |
|  | 1718 | **457.7644** | **913.5140** | **912.9917** | **0.5224** | **1** | **15** | **61** | **1** | **GRFSSGFR** |
|  | 3206 | **723.1045** | **1444.1942** | **1443.7093** | **0.4849** | **0** | **15** | **70** | **1** | **VFTSMSLFNILR + Oxidation (M)** |
|  | 1039 | **409.0648** | **1224.1721** | **1223.3373** | **0.8348** | **1** | **15** | **86** | **1** | **QDYGPKVNFR** |
|  | 1567 | **443.8214** | **1328.4421** | **1328.4944** | **-0.0523** | **1** | **15** | **79** | **1** | **SCGSSSSLRLFK + Carbamidomethyl (C)** |
|  | 881 | 406.1362 | 1215.3865 | 1215.3598 | 0.0267 | 0 | 15 | 75 | 1 | GDAPTCGICHK + 2 Carbamidomethyl (C) |
|  | 406 | **385.0254** | **1152.0539** | **1151.3093** | **0.7446** | **0** | **15** | **68** | **1** | **DIYINLVSSK** |
|  | 911 | **406.9037** | **1217.6891** | **1218.3789** | **-0.6898** | **1** | **15** | **68** | **1** | **TFQGKFMDTK + Oxidation (M)** |
|  | 1052 | **410.0909** | **818.1670** | **817.0518** | **1.1152** | **1** | **15** | **86** | **1** | **LCVVGKAK** |
|  | 1096 | **413.2846** | **1236.8317** | **1236.3560** | **0.4757** | **1** | **15** | **53** | **1** | **QKTENVSTCAR** |
|  | 1374 | **431.0510** | **1290.1307** | **1290.3404** | **-0.2097** | **1** | **15** | **96** | **1** | **GNTSSERAALER** |
|  | 1376 | **431.0666** | **860.1184** | **859.0949** | **1.0235** | **2** | **15** | **95** | **1** | **ACLRIRK** |
|  | 2948 | **624.4763** | **1870.4068** | **1871.0125** | **-0.6057** | **1** | **15** | **66** | **1** | **KCQEQSSNVEISSNSCK** |
|  | 668 | **399.1194** | **796.2240** | **796.9741** | **-0.7501** | **0** | **15** | **70** | **1** | **YLCSIAK** |
|  | 786 | **404.0036** | **805.9924** | **805.8536** | **0.1388** | **0** | **15** | **80** | **1** | **AMPSGPSSG + Oxidation (M)** |
|  | 2555 | **550.1855** | **1647.5343** | **1646.8639** | **0.6704** | **2** | **15** | **78** | **1** | **MPGGRDALKSSVDAVK + Oxidation (M)** |
|  | 2606 | 557.6163 | 1669.8268 | 1670.0512 | -0.2243 | 2 | 15 | 91 | 1 | VMDLICEKCMKQR + Carbamidomethyl (C); Oxidation (M) |
|  | 539 | **388.4176** | **774.8204** | **774.9025** | **-0.0821** | **0** | **15** | **1.1e+02** | **1** | **GTLVIVSS** |
|  | 1241 | **421.5919** | **1261.7534** | **1261.3869** | **0.3665** | **0** | **15** | **70** | **1** | **LSVHVIEGDHR** |
|  | 2880 | **610.2697** | **1827.7870** | **1826.9200** | **0.8670** | **0** | **15** | **81** | **1** | **DSAWGSGGGQQSVNHLVK** |
|  | 3303 | **745.3201** | **1488.6254** | **1487.7718** | **0.8536** | **2** | **15** | **75** | **1** | **APFLGIPRGTMRR + Oxidation (M)** |
|  | 1596 | **445.6659** | **889.3170** | **890.0595** | **-0.7425** | **1** | **15** | **73** | **1** | **VLDMEKR** |
|  | 1762 | **459.1576** | **916.3004** | **917.0582** | **-0.7578** | **1** | **15** | **87** | **1** | **KSLEAELK** |
|  | 1981 | **478.1810** | **954.3472** | **954.1247** | **0.2225** | **1** | **15** | **75** | **1** | **GALPARELK** |
|  | 3152 | **686.0420** | **1370.0692** | **1370.6421** | **-0.5729** | **2** | **15** | **68** | **1** | **ENRLPLFVKVR** |
|  | 2782 | **593.7565** | **1778.2474** | **1778.0148** | **0.2326** | **0** | **15** | **86** | **1** | **INCGPALTWMEIDNK + Carbamidomethyl (C); Oxidation (M)** |
|  | 2919 | 613.2952 | 1224.5756 | 1225.4607 | -0.8852 | 0 | 15 | 77 | 1 | ALAGCVVQLSHK |
|  | 1360 | **430.1099** | **1287.3075** | **1286.5521** | **0.7554** | **2** | **15** | **94** | **1** | **RSWRPRGMLK** |
|  | 1366 | **430.8889** | **1289.6445** | **1289.2697** | **0.3748** | **2** | **15** | **95** | **1** | **DQDERDRAER** |
|  | 1566 | **443.8175** | **1328.4303** | **1329.4791** | **-1.0488** | **0** | **15** | **80** | **1** | **EQAHNLTIEMK + Oxidation (M)** |
|  | 1278 | **423.6556** | **1267.9446** | **1268.4707** | **-0.5261** | **2** | **15** | **76** | **1** | **KGVRPVRDWR** |
|  | 583 | **389.2250** | **776.4352** | **776.8587** | **-0.4234** | **0** | **15** | **71** | **1** | **GSCTQPK + Carbamidomethyl (C)** |
|  | 2931 | **616.5803** | **1231.1459** | **1230.3680** | **0.7779** | **1** | **15** | **70** | **1** | **IIQDSDKVNAK** |
|  | 3396 | **797.5303** | **2389.5686** | **2390.6528** | **-1.0842** | **2** | **15** | **76** | **1** | **SESQCRVVVLRGSTSDLGHCEK** |
|  | 522 | **388.1291** | **774.2435** | **773.9176** | **0.3259** | **1** | **15** | **94** | **1** | **ILDAKSK** |
|  | 2358 | **520.6696** | **1039.3243** | **1038.1999** | **1.1245** | **0** | **15** | **80** | **1** | **QGNSCMVATK** |
|  | 29 | **362.3116** | **1083.9127** | **1084.3541** | **-0.4415** | **1** | **15** | **65** | **1** | **IKYLGIHLK** |
|  | 785 | **404.0028** | **1208.9863** | **1209.4151** | **-0.4288** | **0** | **15** | **81** | **1** | **VAVIGGGYCVSK + Carbamidomethyl (C)** |
|  | 2168 | **496.3868** | **990.7588** | **990.1323** | **0.6266** | **1** | **15** | **70** | **1** | **EEKTPGCVK** |
|  | 2613 | **558.5266** | **1115.0384** | **1115.2608** | **-0.2223** | **0** | **15** | **74** | **1** | **ASPSSCPLTPR** |
|  | 3484 | **894.4924** | **1786.9701** | **1788.1028** | **-1.1327** | **2** | **15** | **78** | **1** | **KKSSVVNSSCMGGMCR + 2 Carbamidomethyl (C)** |
|  | 714 | 401.1246 | 800.2344 | 800.9034 | -0.6690 | 1 | 15 | 88 | 1 | KASPGSVR |
|  | 1451 | **435.7753** | **1304.3038** | **1303.6605** | **0.6434** | **2** | **15** | **72** | **1** | **AMKILMKVGHC + Carbamidomethyl (C); Oxidation (M)** |
|  | 1975 | **477.7851** | **953.5553** | **953.1997** | **0.3557** | **1** | **15** | **60** | **1** | **ILGFMKTK + Oxidation (M)** |
|  | 1510 | **438.3521** | **1312.0340** | **1312.4305** | **-0.3965** | **2** | **15** | **74** | **1** | **GFGGRYGVEKDK** |
|  | 1671 | **452.5646** | **903.1144** | **902.9887** | **0.1257** | **0** | **15** | **1e+02** | **1** | **EDFCMDK + Oxidation (M)** |
|  | 2232 | **505.1360** | **1008.2572** | **1008.1077** | **0.1495** | **0** | **15** | **77** | **1** | **SANQMGVSAK + Oxidation (M)** |
|  | 3143 | **685.1104** | **2052.3089** | **2053.4307** | **-1.1219** | **0** | **15** | **83** | **1** | **YMCHVQHEGLPEPLMLR** |
|  | 117 | **367.1833** | **1098.5278** | **1098.2070** | **0.3208** | **0** | **15** | **73** | **1** | **AIAEELAPER** |
|  | 275 | **374.6781** | **1121.0120** | **1121.2684** | **-0.2563** | **0** | **15** | **78** | **1** | **GAPWLFCDR + Carbamidomethyl (C)** |
|  | 915 | 406.9736 | 1217.8985 | 1218.3408 | -0.4422 | 1 | 15 | 72 | 1 | EDGAMPSARLR + Oxidation (M) |
|  | 2422 | **527.3228** | **1578.9463** | **1578.8114** | **0.1349** | **1** | **15** | **72** | **1** | **CVKYTEIHPEMR + Carbamidomethyl (C); Oxidation (M)** |
|  | 532 | **388.2346** | **1161.6818** | **1161.4153** | **0.2665** | **1** | **15** | **77** | **1** | **CVLQSVSGLKK** |
|  | 3160 | **686.3000** | **2055.8780** | **2055.3573** | **0.5207** | **2** | **15** | **80** | **1** | **SRVTISVDMWNKQFSLK + Oxidation (M)** |
|  | 1058 | **410.5742** | **1228.7004** | **1228.3157** | **0.3846** | **1** | **15** | **81** | **1** | **AAPGRSSTPTQR** |
|  | 2737 | **590.4543** | **1768.3409** | **1767.9571** | **0.3837** | **0** | **15** | **66** | **1** | **LALSMLGQSGAYGAADAR + Oxidation (M)** |
|  | 232 | 372.1566 | 1113.4476 | 1113.2681 | 0.1795 | 1 | 15 | 72 | 1 | NTPIVKATNR |
|  | 2056 | **486.7358** | **1457.1853** | **1457.6151** | **-0.4297** | **1** | **15** | **68** | **1** | **GVMAAQAAPSRQDR** |
|  | 137 | **368.1589** | **1101.4545** | **1102.3250** | **-0.8705** | **2** | **15** | **80** | **1** | **AVSIISKEKK** |
|  | 1428 | **434.8205** | **867.6262** | **866.8967** | **0.7296** | **0** | **15** | **77** | **1** | **SHCDYWG** |
|  | 2059 | **486.8777** | **1457.6109** | **1456.7164** | **0.8945** | **2** | **15** | **84** | **1** | **LLMQRGRPKSDR** |
|  | 3096 | **669.6231** | **2005.8472** | **2006.3327** | **-0.4856** | **2** | **15** | **67** | **1** | **LQKCFLSRGCGSYCAGAK + 2 Carbamidomethyl (C)** |
|  | 569 | **389.1608** | **776.3068** | **776.8356** | **-0.5288** | **2** | **15** | **87** | **1** | **DSKGKDK** |
|  | 1745 | **458.8085** | **1373.4033** | **1373.6626** | **-0.2592** | **1** | **15** | **85** | **1** | **ELVLMKFVNHK + Oxidation (M)** |
|  | 1857 | **467.2052** | **932.3956** | **933.0855** | **-0.6900** | **0** | **15** | **85** | **1** | **WELLCGR + Carbamidomethyl (C)** |
|  | 64 | **363.4639** | **1087.3696** | **1088.2452** | **-0.8756** | **2** | **15** | **92** | **1** | **ARLECRGGAR** |
|  | 621 | **392.8776** | **1175.6107** | **1176.3438** | **-0.7331** | **0** | **15** | **72** | **1** | **MAAAAEGVLATR + Oxidation (M)** |
|  | 1068 | **411.1968** | **1230.5683** | **1231.3760** | **-0.8076** | **0** | **15** | **81** | **1** | **DGPGPASLSAMTK** |
|  | 1326 | **428.2440** | **1281.7100** | **1282.4077** | **-0.6977** | **1** | **15** | **62** | **1** | **RGPGPGGFGAQGPK** |
|  | 3404 | **802.6400** | **2404.8979** | **2403.7143** | **1.1836** | **2** | **15** | **72** | **1** | **MCQWLPDAESKASCSGRFWK + Carbamidomethyl (C); Oxidation (M)** |
|  | 958 | **407.4763** | **1219.4069** | **1219.3288** | **0.0781** | **2** | **15** | **96** | **1** | **KEKGCGNPGSSR** |
|  | 2524 | **545.0535** | **1088.0922** | **1087.2475** | **0.8447** | **1** | **15** | **90** | **1** | **MTFTKTSQK + Oxidation (M)** |
|  | 2039 | **485.3314** | **968.6481** | **968.0222** | **0.6258** | **0** | **15** | **61** | **1** | **VANYTSTGR** |
|  | 3273 | **741.7733** | **2222.2978** | **2221.4483** | **0.8494** | **1** | **15** | **81** | **1** | **AQYGHPSPLGMAAREELYSK + Oxidation (M)** |
|  | 99 | **366.1470** | **730.2792** | **729.7360** | **0.5432** | **0** | **15** | **86** | **1** | **GGGEEPGK** |
|  | 1570 | **443.8547** | **1328.5420** | **1328.6203** | **-0.0783** | **1** | **15** | **85** | **1** | **LAPAQVLKMTEK** |
|  | 2914 | **612.8219** | **1223.6290** | **1224.4759** | **-0.8469** | **1** | **15** | **72** | **1** | **HLCISGTPKIR** |
|  | 844 | **405.2020** | **1212.5838** | **1213.3886** | **-0.8048** | **0** | **15** | **70** | **1** | **LNSCHFAYCR** |
|  | 847 | **405.2647** | **1212.7720** | **1213.3591** | **-0.5871** | **0** | **15** | **62** | **1** | **QGDFYVPEMK** |
|  | 1903 | **472.2558** | **942.4968** | **942.0711** | **0.4257** | **2** | **15** | **78** | **1** | **PPKGKSGSGK** |
|  | 3 | **360.3342** | **718.6536** | **719.8289** | **-1.1752** | **0** | **15** | **96** | **1** | **FVADLR** |
|  | 809 | **404.1769** | **1209.5085** | **1208.3211** | **1.1874** | **1** | **15** | **81** | **1** | **KGSFLEGSEVR** |
|  | 3224 | **736.5635** | **1471.1123** | **1471.4843** | **-0.3720** | **0** | **15** | **74** | **1** | **DGGTDTQYFGPGTR** |
|  | 3380 | **784.6985** | **1567.3822** | **1567.6779** | **-0.2957** | **1** | **15** | **65** | **1** | **MAVSAGSARTSPSSDK + Oxidation (M)** |
|  | 65 | **363.5708** | **1087.6902** | **1088.2136** | **-0.5234** | **0** | **15** | **64** | **1** | **EGLTIWNQK** |
|  | 60 | **363.2806** | **724.5465** | **723.9022** | **0.6443** | **0** | **15** | **68** | **1** | **ECMVVK + Oxidation (M)** |
|  | 2871 | **610.1064** | **1827.2970** | **1828.0556** | **-0.7586** | **2** | **15** | **85** | **1** | **VKGSASFGSKSLSCLGGSR** |
|  | 678 | **399.4262** | **796.8376** | **796.8732** | **-0.0356** | **0** | **15** | **85** | **1** | **SGGSCCAR + Carbamidomethyl (C)** |
|  | 734 | **401.9485** | **1202.8233** | **1202.2801** | **0.5432** | **1** | **15** | **98** | **1** | **SSFPAHSSRAR** |
|  | 1170 | **417.9936** | **833.9724** | **833.9530** | **0.0194** | **0** | **15** | **89** | **1** | **IVTCGADR** |
|  | 2718 | **584.2324** | **1749.6751** | **1749.1263** | **0.5488** | **0** | **15** | **82** | **1** | **MVLPTCPMAEFALPR + Carbamidomethyl (C); Oxidation (M)** |
|  | 19 | **361.6536** | **1081.9385** | **1082.2588** | **-0.3203** | **2** | **15** | **65** | **1** | **FLHIRRSGP** |
|  | 82 | **364.9934** | **1091.9581** | **1091.2822** | **0.6759** | **1** | **15** | **85** | **1** | **TLKLCNSQK + Carbamidomethyl (C)** |
|  | 1148 | **416.2680** | **830.5212** | **829.9379** | **0.5833** | **0** | **15** | **81** | **1** | **GLDIEGVK** |
|  | 597 | **389.9901** | **777.9653** | **776.9450** | **1.0203** | **0** | **15** | **85** | **1** | **MVVVTGR + Oxidation (M)** |
|  | 929 | **407.1534** | **1218.4379** | **1219.3886** | **-0.9507** | **2** | **15** | **82** | **1** | **SRVTISVDKSK** |
|  | 1045 | **409.1418** | **1224.4032** | **1224.4329** | **-0.0298** | **1** | **15** | **91** | **1** | **RHSALCIPEAK** |
|  | 1976 | **477.8305** | **1430.4693** | **1431.6324** | **-1.1631** | **1** | **15** | **74** | **1** | **TPEAEVAFQALKK** |
|  | 388 | **384.3539** | **1150.0395** | **1150.3942** | **-0.3547** | **2** | **15** | **73** | **1** | **KAIFDRLCK + Carbamidomethyl (C)** |
|  | 1065 | **411.0975** | **1230.2702** | **1230.4377** | **-0.1674** | **1** | **15** | **93** | **1** | **AASQMPVVKGAR + Oxidation (M)** |
|  | 1238 | **421.2121** | **1260.6142** | **1261.4911** | **-0.8769** | **1** | **15** | **78** | **1** | **TGGQLSLGKCLK + Carbamidomethyl (C)** |
|  | 346 | **379.5956** | **757.1764** | **756.8936** | **0.2828** | **1** | **15** | **75** | **1** | **RGNGLIK** |
|  | 401 | **385.0002** | **767.9856** | **768.9443** | **-0.9587** | **1** | **15** | **73** | **1** | **GLVQPKK** |
|  | 496 | **387.8433** | **773.6717** | **772.8038** | **0.8680** | **0** | **15** | **1e+02** | **1** | **SLGEDPR** |
|  | 1044 | **409.1356** | **816.2565** | **815.9545** | **0.3020** | **0** | **15** | **91** | **1** | **YMMVDSA** |
|  | 1628 | **449.4400** | **896.8652** | **897.9722** | **-1.1069** | **0** | **15** | **80** | **1** | **VDDLPPSR** |
|  | 2712 | **583.2034** | **1746.5879** | **1746.8567** | **-0.2688** | **1** | **15** | **87** | **1** | **NSLGAEQGNEKGLGGCR + Carbamidomethyl (C)** |
|  | 366 | **381.2329** | **1140.6766** | **1140.3580** | **0.3186** | **1** | **15** | **81** | **1** | **MAACGGTCKNK + Carbamidomethyl (C)** |
|  | 1627 | 449.3769 | 1345.1084 | 1345.4126 | -0.3041 | 1 | 15 | 64 | 1 | EEAEVKVEQER |
|  | 1678 | **453.4185** | **904.8223** | **904.1322** | **0.6900** | **2** | **15** | **83** | **1** | **RSLIKCK + Carbamidomethyl (C)** |
|  | 1930 | **474.1367** | **1419.3879** | **1418.5755** | **0.8124** | **0** | **15** | **92** | **1** | **CVASNAAGADSLAIR** |
|  | 788 | **404.0103** | **1209.0086** | **1208.3427** | **0.6659** | **2** | **15** | **87** | **1** | **GQKKADSMEAK + Oxidation (M)** |
|  | 855 | **405.8276** | **1214.4607** | **1214.3554** | **0.1054** | **1** | **15** | **82** | **1** | **SCAPGSRGAPVR + Carbamidomethyl (C)** |
|  | 1059 | 410.9979 | 819.9811 | 818.8922 | 1.0889 | 1 | 15 | 91 | 1 | DDPTKMP + Oxidation (M) |
|  | 1752 | **458.9515** | **915.8882** | **916.0305** | **-0.1424** | **0** | **15** | **96** | **1** | **VPATATQTK** |
|  | 2723 | **584.5900** | **1167.1653** | **1166.5410** | **0.6243** | **0** | **15** | **85** | **1** | **VLLFVMWMK** |
|  | 3046 | **666.5945** | **1996.7613** | **1996.2701** | **0.4911** | **1** | **15** | **69** | **1** | **YRVETCCVIGNYYSLR + Carbamidomethyl (C)** |
|  | 2134 | 492.1740 | 1473.4998 | 1473.6924 | -0.1926 | 1 | 15 | 82 | 1 | ECNSATMMEGLKK + 2 Oxidation (M) |
|  | 1370 | **431.0058** | **859.9969** | **859.0949** | **0.9019** | **2** | **15** | **1.1e+02** | **1** | **ACLRIRK** |
|  | 3276 | **741.9219** | **2222.7435** | **2221.6452** | **1.0982** | **2** | **15** | **81** | **1** | **ATLESLLVGNCQRIFCLRK + Carbamidomethyl (C)** |
|  | 1347 | **429.1434** | **1284.4081** | **1283.4769** | **0.9312** | **1** | **15** | **87** | **1** | **LNIAVEQVNKR** |
|  | 1998 | **479.5503** | **957.0857** | **957.1718** | **-0.0860** | **0** | **15** | **1.1e+02** | **1** | **CALTPCPPR** |
|  | 262 | **374.1380** | **1119.3917** | **1118.2250** | **1.1668** | **1** | **15** | **1e+02** | **1** | **SSSCHVGSARK** |
|  | 2561 | **550.8714** | **1099.7280** | **1099.2878** | **0.4402** | **2** | **15** | **74** | **1** | **SLLTPRRTR** |
|  | 69 | **364.1199** | **1089.3374** | **1088.2800** | **1.0575** | **0** | **15** | **80** | **1** | **AGAGGPFPACLK** |
|  | 428 | **385.9483** | **769.8819** | **770.9635** | **-1.0816** | **2** | **15** | **75** | **1** | **KKAGVLR** |
|  | 86 | 365.4347 | 728.8546 | 728.7081 | 0.1465 | 0 | 15 | 1.1e+02 | 1 | DSSGNHL |
|  | 971 | **407.7093** | **1220.1057** | **1219.4133** | **0.6924** | **1** | **15** | **72** | **1** | **AVFAKEQPACR** |
|  | 139 | **368.1760** | **1101.5057** | **1100.3141** | **1.1916** | **1** | **15** | **79** | **1** | **KPKYPSRPK** |
|  | 1924 | **473.6355** | **945.2562** | **944.1100** | **1.1461** | **1** | **15** | **93** | **1** | **ACHSKITK + Carbamidomethyl (C)** |
|  | 1985 | **478.2279** | **1431.6616** | **1430.6459** | **1.0157** | **1** | **15** | **83** | **1** | **YYSVLYPLERK** |
|  | 3104 | **673.4775** | **2017.4104** | **2017.4350** | **-0.0246** | **1** | **15** | **84** | **1** | **VLVFCLLPSKDVQSLSLR** |
|  | 90 | **365.9260** | **1094.7559** | **1094.3145** | **0.4414** | **2** | **15** | **1e+02** | **1** | **RALGVPVARR** |
|  | 245 | **372.8252** | **1115.4535** | **1116.1378** | **-0.6843** | **0** | **15** | **99** | **1** | **EEPGEQWNK** |
|  | 912 | **406.9122** | **811.8096** | **810.9413** | **0.8684** | **1** | **15** | **78** | **1** | **GEPKPRK** |
|  | 1024 | **408.3710** | **1222.0908** | **1221.4339** | **0.6570** | **1** | **15** | **86** | **1** | **HVIWKNCHK + Carbamidomethyl (C)** |
|  | 1528 | **440.6234** | **1318.8481** | **1318.5706** | **0.2774** | **2** | **15** | **85** | **1** | **ILPKLRATNHR** |
|  | 1537 | **441.5032** | **880.9916** | **881.1587** | **-0.1671** | **2** | **15** | **1e+02** | **1** | **LSMKMKK + Oxidation (M)** |
|  | 1578 | **444.6664** | **887.3180** | **886.9926** | **0.3253** | **1** | **15** | **81** | **1** | **TRAPAVATT** |
|  | 211 | **370.9930** | **739.9712** | **740.8051** | **-0.8338** | **0** | **15** | **66** | **1** | **SPAVPDR** |
|  | 224 | **371.2569** | **740.4990** | **740.8051** | **-0.3060** | **1** | **15** | **56** | **1** | **EEKAHK** |
|  | 775 | **403.8990** | **805.7833** | **805.8305** | **-0.0471** | **1** | **15** | **88** | **1** | **DGTEKEK** |
|  | 1331 | **428.4072** | **854.7995** | **854.0320** | **0.7675** | **0** | **15** | **73** | **1** | **CGIIHVR + Carbamidomethyl (C)** |
|  | 1421 | **434.2767** | **1299.8078** | **1300.5702** | **-0.7624** | **0** | **15** | **66** | **1** | **VLLNIGQQMLR + Oxidation (M)** |
|  | 2186 | **499.3440** | **996.6733** | **996.0970** | **0.5763** | **1** | **15** | **68** | **1** | **DLSKSMSGR + Oxidation (M)** |
|  | 2470 | **536.0339** | **1605.0796** | **1604.7441** | **0.3355** | **1** | **15** | **86** | **1** | **DRLGQSSMLGEQGAR** |
|  | 1820 | **462.5348** | **1384.5821** | **1384.5560** | **0.0261** | **0** | **15** | **98** | **1** | **LRPEDTAVYFCA** |
|  | 1917 | **472.8485** | **943.6822** | **943.0193** | **0.6628** | **1** | **15** | **89** | **1** | **AANRTSAPR** |
|  | 187 | **369.2988** | **736.5828** | **736.8793** | **-0.2965** | **0** | **15** | **79** | **1** | **AFQMPK + Oxidation (M)** |
|  | 2302 | **517.4376** | **1032.8605** | **1032.1456** | **0.7148** | **1** | **15** | **78** | **1** | **EIDLKETGK** |
|  | 2430 | **529.8603** | **1057.7058** | **1057.1616** | **0.5442** | **0** | **15** | **83** | **1** | **NGAVQTIAQR** |
|  | 2759 | **592.7178** | **1775.1311** | **1774.0315** | **1.0997** | **0** | **15** | **1e+02** | **1** | **TCVSSMMFPQCGHTR + Carbamidomethyl (C); 2 Oxidation (M)** |
|  | 184 | **369.2926** | **736.5704** | **736.8793** | **-0.3088** | **0** | **15** | **79** | **1** | **AFQMPK + Oxidation (M)** |
|  | 1661 | **452.0782** | **1353.2126** | **1352.4959** | **0.7166** | **1** | **15** | **1.1e+02** | **1** | **DPASAKHSLLSAR** |
|  | 3068 | **667.5483** | **1999.6228** | **2000.3866** | **-0.7637** | **2** | **15** | **75** | **1** | **TNSRFVKVVLMSATISCK + Oxidation (M)** |
|  | 3417 | **809.2883** | **1616.5619** | **1616.7398** | **-0.1779** | **1** | **15** | **84** | **1** | **HGQRGHGQQLLETR** |
|  | 325 | **377.5972** | **753.1796** | **753.8899** | **-0.7103** | **1** | **15** | **65** | **1** | **SLPGPKR** |
|  | 1471 | **436.4049** | **870.7950** | **871.0327** | **-0.2377** | **0** | **15** | **80** | **1** | **QALELLGK** |
|  | 1473 | 437.0063 | 1307.9969 | 1308.6767 | -0.6799 | 0 | 15 | 1e+02 | 1 | LIGMLLACCLSR + Oxidation (M) |
|  | 2842 | **606.6309** | **1816.8706** | **1815.9804** | **0.8902** | **0** | **15** | **93** | **1** | **GSVGDPGMEGPMCSSCR + 2 Carbamidomethyl (C); 2 Oxidation (M)** |
|  | 45 | **363.1301** | **1086.3681** | **1086.3255** | **0.0427** | **0** | **15** | **89** | **1** | **FIACLMSTK + Carbamidomethyl (C); Oxidation (M)** |
|  | 2254 | **508.0317** | **1521.0729** | **1521.8047** | **-0.7318** | **2** | **15** | **92** | **1** | **ARMNALDLNMKTK + Oxidation (M)** |
|  | 2905 | **612.3128** | **1833.9162** | **1834.0582** | **-0.1420** | **1** | **15** | **92** | **1** | **LPGGLEPKGELDCHQLA + Carbamidomethyl (C)** |
|  | 102 | **366.2059** | **730.3970** | **730.8102** | **-0.4132** | **1** | **15** | **82** | **1** | **ALKEDR** |
|  | 227 | 371.2714 | 740.5279 | 740.8051 | -0.2771 | 1 | 15 | 58 | 1 | EEKAHK |
|  | 321 | **377.2527** | **752.4905** | **751.8924** | **0.5982** | **1** | **15** | **68** | **1** | **STKVSCK** |
|  | 514 | **388.0025** | **773.9902** | **772.9131** | **1.0771** | **0** | **15** | **1.1e+02** | **1** | **SIHAMAK + Oxidation (M)** |
|  | 3245 | **740.5881** | **1479.1615** | **1478.6540** | **0.5075** | **0** | **15** | **76** | **1** | **FPPHCFPSGCSGSR** |
|  | 2951 | **626.8857** | **1877.6351** | **1877.1958** | **0.4392** | **1** | **15** | **71** | **1** | **VRDMYATHLASGMLGVR** |
|  | 492 | **387.8186** | **773.6225** | **773.7951** | **-0.1726** | **0** | **15** | **1.1e+02** | **1** | **QGGNSGVR** |
|  | 588 | **389.2711** | **776.5273** | **775.8475** | **0.6798** | **0** | **15** | **81** | **1** | **ATPTSTAK** |
|  | 826 | **405.0161** | **1212.0262** | **1212.3859** | **-0.3597** | **2** | **15** | **84** | **1** | **MTPGGGPGARRR** |
|  | 1202 | **419.2875** | **1254.8403** | **1254.4955** | **0.3449** | **2** | **15** | **87** | **1** | **EMIDFSKLKK + Oxidation (M)** |
|  | 1329 | **428.3792** | **1282.1155** | **1281.3981** | **0.7173** | **0** | **15** | **68** | **1** | **SHMQGSEHQIK** |
|  | 3304 | **746.8771** | **1491.7394** | **1491.6926** | **0.0468** | **0** | **15** | **1e+02** | **1** | **RPCSDMEGAALLR + Carbamidomethyl (C); Oxidation (M)** |
|  | 3382 | **787.4042** | **2359.1904** | **2358.6969** | **0.4935** | **1** | **15** | **90** | **1** | **FLNGCVPLSHQVAGHMYGKDK + Carbamidomethyl (C)** |
|  | 1227 | **420.2877** | **838.5606** | **837.8557** | **0.7049** | **0** | **15** | **68** | **1** | **SQADDMR + Oxidation (M)** |
|  | 1809 | **461.7278** | **1382.1614** | **1382.5584** | **-0.3971** | **0** | **15** | **72** | **1** | **IQPTTPSEPTAIK** |
|  | 2866 | **609.9900** | **1826.9478** | **1826.1308** | **0.8170** | **2** | **15** | **89** | **1** | **YRQIFSVMVNQRIR + Oxidation (M)** |
|  | 3211 | **730.7028** | **1459.3907** | **1459.6441** | **-0.2534** | **1** | **15** | **77** | **1** | **LLREVTGGAISSEK** |
|  | 2689 | **575.9805** | **1149.9463** | **1149.3150** | **0.6312** | **0** | **15** | **92** | **1** | **GEIGMASIDLK + Oxidation (M)** |
|  | 2947 | **623.2535** | **1866.7384** | **1866.1400** | **0.5984** | **0** | **15** | **94** | **1** | **VVCVYGGTGISEQIAELK** |
|  | 673 | **399.1681** | **1194.4820** | **1194.4435** | **0.0386** | **1** | **15** | **79** | **1** | **KAVIFCLSADK** |
|  | 1220 | **420.1460** | **1257.4158** | **1258.3598** | **-0.9440** | **0** | **15** | **89** | **1** | **SAYDMYHLSR + Oxidation (M)** |
|  | 820 | 404.9723 | 1211.8947 | 1212.4190 | -0.5243 | 1 | 15 | 85 | 1 | SPVAKAGCGLGVPG |
|  | 1268 | **422.4565** | **1264.3474** | **1265.3905** | **-1.0432** | **0** | **15** | **1.2e+02** | **1** | **ISADETLCQTK + Carbamidomethyl (C)** |
|  | 2544 | **548.9326** | **1643.7755** | **1642.8996** | **0.8759** | **1** | **15** | **88** | **1** | **LRAAFAGLQELQGLR** |
|  | 1438 | **435.1316** | **868.2483** | **869.0005** | **-0.7521** | **2** | **15** | **88** | **1** | **KRYDCK + Carbamidomethyl (C)** |
|  | 1796 | **460.6723** | **919.3298** | **919.9760** | **-0.6462** | **0** | **15** | **80** | **1** | **GENFYYK** |
|  | 752 | **402.5190** | **803.0232** | **802.8729** | **0.1503** | **1** | **15** | **1.2e+02** | **1** | **DPTAGSKK** |
|  | 2537 | **547.5548** | **1093.0948** | **1092.2885** | **0.8063** | **1** | **15** | **94** | **1** | **YSLAEKILR** |
|  | 2796 | **595.8389** | **1784.4946** | **1785.0937** | **-0.5991** | **1** | **15** | **79** | **1** | **STLMNILAGYRETGMK** |
|  | 634 | **394.7218** | **1181.1432** | **1182.3266** | **-1.1833** | **0** | **15** | **90** | **1** | **GGFDWNLVFK** |
|  | 840 | **405.1824** | **1212.5250** | **1213.4267** | **-0.9017** | **1** | **15** | **83** | **1** | **LLTNLGLGERK** |
|  | 1588 | **445.1500** | **1332.4277** | **1332.3740** | **0.0538** | **1** | **15** | **1.1e+02** | **1** | **RASEDTTSGSPPK** |
|  | 2282 | **513.6818** | **1538.0231** | **1537.7659** | **0.2572** | **2** | **15** | **85** | **1** | **LQPRISAASSVPRR** |
|  | 568 | **389.1546** | **776.2944** | **776.9232** | **-0.6288** | **1** | **15** | **99** | **1** | **KGIFGQK** |
|  | 2042 | **486.0325** | **970.0503** | **969.0799** | **0.9704** | **1** | **15** | **87** | **1** | **HMKTHSGR + Oxidation (M)** |
|  | 2677 | **572.5532** | **1714.6375** | **1715.0405** | **-0.4030** | **1** | **15** | **89** | **1** | **MVSKMIIENFETLK + 2 Oxidation (M)** |
|  | 1433 | **435.0249** | **1302.0524** | **1301.5123** | **0.5402** | **1** | **15** | **82** | **1** | **RAVAELDMATPK** |
|  | 2099 | **488.5240** | **975.0332** | **975.0146** | **0.0185** | **0** | **15** | **1.2e+02** | **1** | **TLTGGGGGGGGGK** |
|  | 2113 | **490.1689** | **978.3230** | **977.1432** | **1.1798** | **2** | **15** | **95** | **1** | **SGRKACSIR** |
|  | 3278 | **742.0284** | **1482.0421** | **1481.8120** | **0.2301** | **1** | **15** | **70** | **1** | **MAVRRPGCFLCR + Carbamidomethyl (C); Oxidation (M)** |
|  | 1430 | **434.8720** | **867.7292** | **867.0062** | **0.7231** | **0** | **15** | **87** | **1** | **CMAGVQSR + Oxidation (M)** |
|  | 1594 | **445.2672** | **888.5196** | **887.9806** | **0.5391** | **1** | **15** | **91** | **1** | **RSSEGCCF** |
|  | 423 | **385.3392** | **1152.9955** | **1153.3734** | **-0.3779** | **0** | **15** | **69** | **1** | **VVSTTCTLACR** |
|  | 521 | **388.1172** | **1161.3294** | **1161.3492** | **-0.0198** | **1** | **15** | **1.1e+02** | **1** | **FMETTMNKK + 2 Oxidation (M)** |
|  | 607 | **390.1097** | **1167.3070** | **1166.2662** | **1.0409** | **2** | **15** | **91** | **1** | **QDMSKSNGKR + Oxidation (M)** |
|  | 2260 | **510.0640** | **1018.1131** | **1018.1671** | **-0.0539** | **1** | **15** | **1e+02** | **1** | **THTGLKSFK** |
|  | 473 | **386.9047** | **771.7945** | **770.8840** | **0.9106** | **2** | **15** | **1e+02** | **1** | **GGRVARR** |
|  | 1142 | **416.1428** | **1245.4061** | **1245.4339** | **-0.0278** | **1** | **15** | **1.1e+02** | **1** | **VVKAHASLHGAR** |
|  | 2799 | **596.1024** | **1785.2851** | **1785.0725** | **0.2126** | **1** | **15** | **99** | **1** | **KCLMPASTTMESTGVR + Carbamidomethyl (C); Oxidation (M)** |
|  | 63 | **363.3190** | **724.6232** | **724.8901** | **-0.2669** | **0** | **15** | **82** | **1** | **MLTCNK + Oxidation (M)** |
|  | 510 | **387.9442** | **773.8736** | **772.9330** | **0.9406** | **2** | **15** | **1.2e+02** | **1** | **DIKKAAK** |
|  | 730 | **401.8917** | **801.7685** | **800.9862** | **0.7824** | **2** | **15** | **1.1e+02** | **1** | **KKTSPLK** |
|  | 1644 | **450.1735** | **1347.4984** | **1346.6173** | **0.8811** | **2** | **15** | **97** | **1** | **IRYVLRVSEVI** |
|  | 2101 | **489.2047** | **976.3946** | **976.1487** | **0.2460** | **0** | **15** | **1e+02** | **1** | **AVSCAEIVK + Carbamidomethyl (C)** |
|  | 685 | **400.0048** | **1196.9923** | **1196.2937** | **0.6986** | **0** | **15** | **79** | **1** | **NPSLPGECHSR** |
|  | 3243 | **740.5657** | **2218.6748** | **2218.3799** | **0.2950** | **0** | **15** | **84** | **1** | **EPHSPCSEIGSACSQEQPFK + Carbamidomethyl (C)** |
|  | 1209 | **419.3869** | **1255.1384** | **1255.2548** | **-0.1163** | **1** | **14** | **99** | **1** | **HGKGGGGGGGGGESGK** |
|  | 2946 | 623.2170 | 1244.4193 | 1245.2997 | -0.8804 | 0 | 14 | 98 | 1 | ASGGTGAGSQSLPR |
|  | 821 | **404.9868** | **807.9587** | **808.9021** | **-0.9433** | **0** | **14** | **88** | **1** | **CAGTSWK + Carbamidomethyl (C)** |
|  | 978 | **407.7659** | **1220.2754** | **1219.3900** | **0.8855** | **0** | **14** | **95** | **1** | **NNCTFFSCVK + Carbamidomethyl (C)** |
|  | 1084 | **413.0061** | **1235.9961** | **1235.3067** | **0.6895** | **1** | **14** | **84** | **1** | **REPQPDPSPGR** |
|  | 999 | **408.0051** | **813.9954** | **812.9371** | **1.0583** | **1** | **14** | **96** | **1** | **GPRGPCLG + Carbamidomethyl (C)** |
|  | 1484 | **437.1764** | **1308.5070** | **1307.4569** | **1.0501** | **0** | **14** | **1e+02** | **1** | **GLPGFPGPQGPAGR** |
|  | 3134 | **684.0814** | **1366.1481** | **1366.5920** | **-0.4440** | **2** | **14** | **92** | **1** | **ALRSYALCTRR + Carbamidomethyl (C)** |
|  | 772 | **403.8339** | **1208.4794** | **1207.3562** | **1.1233** | **0** | **14** | **98** | **1** | **MELFPAEAQR + Oxidation (M)** |
|  | 2953 | **627.0917** | **1252.1686** | **1251.4765** | **0.6920** | **0** | **14** | **94** | **1** | **MLQSLAGSSCVR** |
|  | 3362 | **774.9611** | **1547.9073** | **1547.6679** | **0.2394** | **0** | **14** | **94** | **1** | **AAGGDWGTLGITTTAR** |
|  | 143 | **368.2342** | **1101.6803** | **1102.2024** | **-0.5221** | **2** | **14** | **82** | **1** | **ENRGKGGAVSK** |
|  | 1480 | **437.0800** | **1308.2180** | **1307.3677** | **0.8502** | **1** | **14** | **1.1e+02** | **1** | **ASASTRESTLER** |
|  | 368 | **381.2858** | **1140.8351** | **1141.3840** | **-0.5489** | **1** | **14** | **92** | **1** | **SKEALIHCLK** |
|  | 1335 | 428.8970 | 855.7793 | 855.9985 | -0.2192 | 1 | 14 | 86 | 1 | GVDYMKK + Oxidation (M) |
|  | 1444 | **435.2628** | **1302.7663** | **1302.5004** | **0.2659** | **1** | **14** | **72** | **1** | **KAVGTPMAASTPR + Oxidation (M)** |
|  | 2814 | **597.9738** | **1790.8991** | **1792.0700** | **-1.1709** | **2** | **14** | **91** | **1** | **SPKCSGKAVAMMHQER + 2 Oxidation (M)** |
|  | 312 | **377.1451** | **1128.4132** | **1129.3467** | **-0.9336** | **0** | **14** | **89** | **1** | **LYLSGAIVFF** |
|  | 434 | 386.0396 | 1155.0967 | 1156.2893 | -1.1927 | 0 | 14 | 85 | 1 | NLIDAAVDGLR |
|  | 664 | **399.0825** | **796.1502** | **795.9694** | **0.1808** | **0** | **14** | **87** | **1** | **GALGLLPR** |
|  | 2512 | **541.9025** | **1622.6852** | **1621.8987** | **0.7865** | **0** | **14** | **89** | **1** | **LQGAGLPMESAILHGK** |
|  | 2753 | **592.6776** | **1775.0107** | **1774.9363** | **0.0743** | **1** | **14** | **1.2e+02** | **1** | **QWLNSGHINDVRHAK** |
|  | 781 | **403.9600** | **1208.8577** | **1209.4877** | **-0.6300** | **2** | **14** | **99** | **1** | **VLILGRRLNR** |
|  | 2582 | **554.0045** | **1105.9943** | **1105.0738** | **0.9205** | **0** | **14** | **1e+02** | **1** | **STPGSNSADGGR** |
|  | 1256 | **422.1281** | **842.2414** | **842.8936** | **-0.6522** | **0** | **14** | **1.1e+02** | **1** | **ENAPLNVS** |
|  | 1434 | **435.0377** | **1302.0909** | **1302.4637** | **-0.3728** | **1** | **14** | **85** | **1** | **VGGGLSMGRAQGGR** |
|  | 3163 | **687.1915** | **1372.3683** | **1373.4290** | **-1.0608** | **1** | **14** | **94** | **1** | **GVEGSAGAGKEAQGR** |
|  | 160 | **369.1726** | **736.3305** | **736.8793** | **-0.5488** | **0** | **14** | **92** | **1** | **AFQMPK + Oxidation (M)** |
|  | 1143 | **416.1489** | **1245.4246** | **1245.3361** | **0.0885** | **0** | **14** | **1.1e+02** | **1** | **LNDSIAEELNK** |
|  | 1564 | **443.7903** | **885.5658** | **886.0310** | **-0.4652** | **1** | **14** | **94** | **1** | **ADMKHLR + Oxidation (M)** |
|  | 2413 | **525.2266** | **1048.4383** | **1048.2610** | **0.1774** | **0** | **14** | **1e+02** | **1** | **AMCCVPDPGR** |
|  | 3338 | 759.0032 | 1515.9916 | 1515.8165 | 0.1750 | 1 | 14 | 79 | 1 | TLGLKQSTCLGLPK + Carbamidomethyl (C) |
|  | 609 | **391.0887** | **1170.2440** | **1170.3160** | **-0.0721** | **0** | **14** | **93** | **1** | **QEGAGLELVVR** |
|  | 22 | **362.0288** | **722.0428** | **720.8617** | **1.1812** | **0** | **14** | **91** | **1** | **MAPCAR + Carbamidomethyl (C); Oxidation (M)** |
|  | 670 | **399.1370** | **1194.3889** | **1194.4317** | **-0.0428** | **1** | **14** | **87** | **1** | **KHLQRPIFR** |
|  | 183 | **369.2882** | **736.5616** | **736.8793** | **-0.3177** | **0** | **14** | **86** | **1** | **AFQMPK + Oxidation (M)** |
|  | 2572 | **552.4183** | **1654.2328** | **1654.0334** | **0.1994** | **2** | **14** | **85** | **1** | **GEVKPRILLMGLRR + Oxidation (M)** |
|  | 979 | **407.7715** | **1220.2924** | **1220.4163** | **-0.1239** | **0** | **14** | **99** | **1** | **FVSFPTQVPAK** |
|  | 1942 | **475.4623** | **948.9098** | **949.1266** | **-0.2168** | **2** | **14** | **1e+02** | **1** | **CSGLDKKAK** |
|  | 2847 | 607.6393 | 1213.2639 | 1214.3688 | -1.1049 | 1 | 14 | 1.1e+02 | 1 | VEVEVGKEGLR |
|  | 58 | **363.2569** | **1086.7484** | **1086.2822** | **0.4662** | **0** | **14** | **79** | **1** | **GDILLSSLIR** |
|  | 304 | **377.1174** | **1128.3301** | **1127.1590** | **1.1712** | **0** | **14** | **93** | **1** | **GVDASGETTYK** |
|  | 474 | **386.9352** | **1157.7834** | **1158.3947** | **-0.6113** | **0** | **14** | **1.1e+02** | **1** | **MNLPQCPALR + Oxidation (M)** |
|  | 1758 | **459.1099** | **1374.3076** | **1374.6755** | **-0.3679** | **2** | **14** | **1.1e+02** | **1** | **MMSLIPGPRRGK + 2 Oxidation (M)** |
|  | 700 | **400.1824** | **1197.5249** | **1197.2950** | **0.2299** | **0** | **14** | **82** | **1** | **ASTAASSALGFSK** |
|  | 1732 | **458.2375** | **914.4603** | **914.0211** | **0.4392** | **1** | **14** | **98** | **1** | **EGGLGRAVR** |
|  | 373 | **382.1262** | **1143.3565** | **1142.3242** | **1.0323** | **0** | **14** | **1.1e+02** | **1** | **EAFMPFSIGK + Oxidation (M)** |
|  | 1304 | **427.0812** | **1278.2214** | **1278.5451** | **-0.3237** | **1** | **14** | **92** | **1** | **ACRMEFCSTCK** |
|  | 1467 | **436.2754** | **1305.8039** | **1306.4856** | **-0.6816** | **1** | **14** | **76** | **1** | **EKQDLMQSLAK + Oxidation (M)** |
|  | 2534 | **547.4727** | **1639.3958** | **1639.9575** | **-0.5617** | **1** | **14** | **79** | **1** | **EVRLSVPPLVEVMR + Oxidation (M)** |
|  | 2291 | **514.5009** | **1026.9869** | **1027.2615** | **-0.2746** | **1** | **14** | **90** | **1** | **SQVALKIIR** |
|  | 3083 | **668.7659** | **2003.2754** | **2003.3868** | **-0.1114** | **1** | **14** | **1.2e+02** | **1** | **NTAVTGKIYIYILHIVGK** |
|  | 907 | **406.7764** | **1217.3071** | **1218.2794** | **-0.9723** | **1** | **14** | **93** | **1** | **HERTHNAEKP** |
|  | 1073 | **411.4593** | **820.9038** | **820.8683** | **0.0355** | **0** | **14** | **1.2e+02** | **1** | **SSMEDPR** |
|  | 2754 | **592.6947** | **1183.3746** | **1182.3681** | **1.0065** | **0** | **14** | **1.2e+02** | **1** | **LPTTGYLVYR** |
|  | 34 | **362.7073** | **1085.0998** | **1086.1103** | **-1.0104** | **1** | **14** | **95** | **1** | **DERASLPPSD** |
|  | 787 | **404.0088** | **1209.0042** | **1208.3658** | **0.6384** | **2** | **14** | **1e+02** | **1** | **KTHAGEKFYK** |
|  | 1681 | **454.1911** | **1359.5510** | **1360.6488** | **-1.0977** | **1** | **14** | **1.1e+02** | **1** | **LPPKPQFLHRK** |
|  | 2910 | **612.4740** | **1834.3998** | **1834.0400** | **0.3598** | **2** | **14** | **81** | **1** | **RSVRLIDAFDIWGQGT** |
|  | 119 | **367.2060** | **1098.5958** | **1098.2699** | **0.3259** | **0** | **14** | **89** | **1** | **NYAELTIMK + Oxidation (M)** |
|  | 718 | **401.2407** | **1200.7000** | **1201.3980** | **-0.6980** | **0** | **14** | **88** | **1** | **ELHPQPPPMR** |
|  | 1400 | **433.1194** | **1296.3362** | **1296.5220** | **-0.1859** | **2** | **14** | **1e+02** | **1** | **IRKQQQVAGIR** |
|  | 1789 | **460.3897** | **918.7646** | **917.9669** | **0.7977** | **2** | **14** | **88** | **1** | **SKRGGEER** |
|  | 1870 | **468.5743** | **1402.7007** | **1403.4931** | **-0.7925** | **0** | **14** | **1.2e+02** | **1** | **VHYSIDTENPTK** |
|  | 3511 | **1078.8801** | **2155.7455** | **2154.5478** | **1.1977** | **0** | **14** | **86** | **1** | **ALMIAMEYAPGGTLAEFIQK** |
|  | 450 | **386.1069** | **770.1991** | **769.8893** | **0.3098** | **1** | **14** | **91** | **1** | **AKLGEPR** |
|  | 1880 | **469.4547** | **936.8947** | **936.1479** | **0.7468** | **0** | **14** | **93** | **1** | **SVVMPMEK + Oxidation (M)** |
|  | 2901 | **612.2514** | **1833.7320** | **1834.0900** | **-0.3579** | **2** | **14** | **1e+02** | **1** | **SCLPRVSGAMARNTAAR + Carbamidomethyl (C); Oxidation (M)** |
|  | 3145 | **685.2601** | **2052.7582** | **2053.5336** | **-0.7753** | **2** | **14** | **1e+02** | **1** | **LHMLLLKGMATMNKEMK + 4 Oxidation (M)** |
|  | 336 | **378.2841** | **1131.8300** | **1131.4155** | **0.4145** | **2** | **14** | **70** | **1** | **WLLIRFKR** |
|  | 1538 | **441.7726** | **881.5304** | **882.0124** | **-0.4821** | **0** | **14** | **80** | **1** | **ALIYSTSK** |
|  | 1099 | **413.3771** | **1237.1091** | **1236.2037** | **0.9055** | **1** | **14** | **77** | **1** | **AGSSGSGEVGASRD** |
|  | 1420 | **434.2751** | **1299.8033** | **1299.4102** | **0.3931** | **1** | **14** | **75** | **1** | **YCNASVTNSVKG + Carbamidomethyl (C)** |
|  | 1952 | **476.1460** | **1425.4158** | **1425.6377** | **-0.2219** | **2** | **14** | **1e+02** | **1** | **RVLTAYAHRNPK** |
|  | 2691 | **578.1970** | **1154.3793** | **1153.3088** | **1.0705** | **0** | **14** | **1e+02** | **1** | **VFSENSCLVR** |
|  | 2872 | **610.1095** | **1218.2042** | **1218.4021** | **-0.1979** | **2** | **14** | **1e+02** | **1** | **NVPEAFKGTKK** |
|  | 1008 | **408.2201** | **1221.6380** | **1222.2433** | **-0.6052** | **1** | **14** | **89** | **1** | **ANEEDKNCSR + Carbamidomethyl (C)** |
|  | 11 | **360.4428** | **718.8708** | **719.7676** | **-0.8968** | **0** | **14** | **1.4e+02** | **1** | **GGSCPSR + Carbamidomethyl (C)** |
|  | 374 | **382.1919** | **762.3690** | **762.8322** | **-0.4632** | **0** | **14** | **1e+02** | **1** | **MASEGPR + Oxidation (M)** |
|  | 527 | **388.1688** | **1161.4843** | **1160.3842** | **1.1001** | **0** | **14** | **1.1e+02** | **1** | **AQQDVIMVLK + Oxidation (M)** |
|  | 815 | **404.3825** | **1210.1253** | **1210.2973** | **-0.1719** | **1** | **14** | **98** | **1** | **DAPPPTRAETR** |
|  | 435 | **386.0552** | **1155.1434** | **1155.2419** | **-0.0984** | **1** | **14** | **93** | **1** | **YSAVGDRCER** |
|  | 693 | **400.1145** | **1197.3213** | **1196.3366** | **0.9847** | **1** | **14** | **90** | **1** | **RNAGLTCGGYK + Carbamidomethyl (C)** |
|  | 1355 | **429.4556** | **856.8963** | **855.9388** | **0.9576** | **1** | **14** | **1.2e+02** | **1** | **ATQGHSKK** |
|  | 2611 | **558.4197** | **1114.8246** | **1114.3654** | **0.4591** | **1** | **14** | **86** | **1** | **RVMAAQVALR** |
|  | 343 | **379.3434** | **1135.0082** | **1135.3614** | **-0.3532** | **1** | **14** | **89** | **1** | **IIGRMCVGDR + Oxidation (M)** |
|  | 2076 | **487.9844** | **973.9540** | **973.0819** | **0.8721** | **2** | **14** | **1.1e+02** | **1** | **KKEEPASGK** |
|  | 2298 | **516.1093** | **1030.2037** | **1030.2425** | **-0.0387** | **1** | **14** | **1.1e+02** | **1** | **KASMLEVPR** |
|  | 323 | **377.3182** | **1128.9325** | **1129.3136** | **-0.3811** | **0** | **14** | **79** | **1** | **HCDGGMILQR** |
|  | 1046 | **409.1714** | **1224.4922** | **1223.4199** | **1.0722** | **1** | **14** | **1.1e+02** | **1** | **KINPALYNYK** |
|  | 3164 | **688.4813** | **2062.4218** | **2061.4529** | **0.9689** | **1** | **14** | **99** | **1** | **SSMILMRHLLMDAQVQR + 2 Oxidation (M)** |
|  | 319 | **377.2337** | **1128.6789** | **1129.1847** | **-0.5057** | **2** | **14** | **76** | **1** | **DSRDIRDPR** |
|  | 3271 | **741.7211** | **2222.1412** | **2222.7577** | **-0.6164** | **1** | **14** | **82** | **1** | **RPDQLAMLMNKLILMGCVK + 3 Oxidation (M)** |
|  | 181 | **369.2786** | **1104.8136** | **1105.3371** | **-0.5235** | **1** | **14** | **88** | **1** | **RVAFCPCPR + Carbamidomethyl (C)** |
|  | 502 | **387.8854** | **1160.6340** | **1161.3193** | **-0.6853** | **2** | **14** | **1.2e+02** | **1** | **MRHGEGRMR + 2 Oxidation (M)** |
|  | 418 | **385.1361** | **768.2575** | **768.8812** | **-0.6237** | **0** | **14** | **89** | **1** | **AICYSR + Carbamidomethyl (C)** |
|  | 537 | **388.3543** | **1162.0408** | **1161.2446** | **0.7962** | **0** | **14** | **1.1e+02** | **1** | **IHDPYDCNK + Carbamidomethyl (C)** |
|  | 1214 | **419.4878** | **1255.4412** | **1254.5055** | **0.9357** | **2** | **14** | **1.4e+02** | **1** | **APKASRPPKMR + Oxidation (M)** |
|  | 3356 | **766.1750** | **2295.5030** | **2296.6226** | **-1.1196** | **2** | **14** | **94** | **1** | **GCSLGAPGTMVKLGNNFAEKGTK + Oxidation (M)** |
|  | 3489 | **900.7390** | **2699.1949** | **2699.0287** | **0.1662** | **2** | **14** | **86** | **1** | **KINSQNQPTGIHREPPPLPFSVNK** |
|  | 337 | **378.3390** | **1131.9947** | **1131.3064** | **0.6883** | **1** | **14** | **79** | **1** | **QRQQMELAK** |
|  | 778 | **403.9271** | **805.8393** | **807.0120** | **-1.1727** | **0** | **14** | **1e+02** | **1** | **ACIPYLK** |
|  | 1234 | **420.4584** | **1258.3529** | **1259.3943** | **-1.0413** | **1** | **14** | **1.1e+02** | **1** | **RAPPYCGADPR + Carbamidomethyl (C)** |
|  | 1494 | **437.5786** | **873.1423** | **874.0601** | **-0.9177** | **0** | **14** | **1.2e+02** | **1** | **VAMTPALR + Oxidation (M)** |
|  | 2855 | **609.0714** | **1216.1279** | **1215.4032** | **0.7248** | **2** | **14** | **1.1e+02** | **1** | **RVQESTKVLR** |
|  | 1771 | **459.3821** | **916.7494** | **915.9940** | **0.7554** | **2** | **14** | **92** | **1** | **KQNDQRK** |
|  | 578 | **389.1946** | **1164.5618** | **1165.3179** | **-0.7561** | **1** | **14** | **98** | **1** | **TQDKMLAQSK + Oxidation (M)** |
|  | 171 | **369.2505** | **736.4862** | **736.8793** | **-0.3931** | **0** | **14** | **89** | **1** | **AFQMPK + Oxidation (M)** |
|  | 486 | **387.2011** | **1158.5811** | **1159.3215** | **-0.7404** | **1** | **14** | **1e+02** | **1** | **VFHDGCLRR + Carbamidomethyl (C)** |
|  | 622 | **393.0012** | **783.9876** | **783.9126** | **0.0750** | **0** | **14** | **93** | **1** | **VIPGAAEK** |
|  | 824 | **405.0142** | **808.0135** | **807.9556** | **0.0580** | **1** | **14** | **95** | **1** | **SLEGMKK + Oxidation (M)** |
|  | 1301 | **426.3506** | **1276.0298** | **1275.3207** | **0.7090** | **0** | **14** | **84** | **1** | **GSAYGLSGADSYK** |
|  | 2178 | 499.0974 | 996.1800 | 995.1090 | 1.0710 | 2 | 14 | 98 | 1 | KNKMDESK + Oxidation (M) |
|  | 1713 | **457.5629** | **913.1110** | **913.0730** | **0.0381** | **1** | **14** | **1.2e+02** | **1** | **RTVAAPSVI** |
|  | 1867 | **468.2468** | **934.4787** | **935.0818** | **-0.6030** | **2** | **14** | **1.1e+02** | **1** | **LSRERFK** |
|  | 2628 | 562.3625 | 1122.7103 | 1122.4025 | 0.3079 | 2 | 14 | 96 | 1 | KEMAMLQKK + Oxidation (M) |
|  | 2956 | **627.6671** | **1253.3194** | **1252.4398** | **0.8797** | **0** | **14** | **1.1e+02** | **1** | **GMPGLPGPAGTPGK + Oxidation (M)** |
|  | 665 | **399.1003** | **1194.2787** | **1193.2898** | **0.9888** | **1** | **14** | **93** | **1** | **CAERHYDTAK** |
|  | 1793 | **460.5115** | **919.0082** | **917.9238** | **1.0844** | **1** | **14** | **1.3e+02** | **1** | **EAGSGGRER** |
|  | 1861 | **467.3904** | **1399.1491** | **1399.6449** | **-0.4959** | **1** | **14** | **88** | **1** | **QLSHLCLRCNR + Carbamidomethyl (C)** |
|  | 2050 | 486.2829 | 970.5510 | 971.0692 | -0.5181 | 0 | 14 | 94 | 1 | NRPGSALEK |
|  | 2165 | **496.2167** | **990.4186** | **991.2328** | **-0.8142** | **1** | **14** | **1.1e+02** | **1** | **ALVRCNMK + Carbamidomethyl (C)** |
|  | 444 | **386.0880** | **770.1612** | **769.8891** | **0.2720** | **0** | **14** | **96** | **1** | **LGLSGPAR** |
|  | 497 | **387.8660** | **773.7172** | **774.8627** | **-1.1456** | **1** | **14** | **1.2e+02** | **1** | **IEKTER** |
|  | 576 | **389.1853** | **1164.5337** | **1164.2950** | **0.2388** | **0** | **14** | **1e+02** | **1** | **MGPPHSGPGGVR + Oxidation (M)** |
|  | 1063 | **411.0491** | **1230.1250** | **1230.3729** | **-0.2478** | **0** | **14** | **1.1e+02** | **1** | **NVSGHLPPGAPGK** |
|  | 3391 | **791.6211** | **2371.8411** | **2370.7720** | **1.0691** | **1** | **14** | **92** | **1** | **RICNPTSCWLPLDMELLHR + Carbamidomethyl (C); Oxidation (M)** |
|  | 271 | **374.2907** | **746.5666** | **745.7798** | **0.7867** | **0** | **14** | **1e+02** | **1** | **DLAGWGQ** |
|  | 369 | **381.2990** | **760.5833** | **760.9420** | **-0.3587** | **0** | **14** | **1e+02** | **1** | **GVIECLK** |
|  | 1571 | **443.9154** | **1328.7240** | **1329.5916** | **-0.8676** | **1** | **14** | **1.1e+02** | **1** | **ITKHICAICGDR** |
|  | 2385 | 523.2018 | 1044.3889 | 1044.2291 | 0.1598 | 0 | 14 | 1.1e+02 | 1 | MIQAAVQQR |
|  | 574 | **389.1812** | **1164.5216** | **1165.2748** | **-0.7532** | **0** | **14** | **1e+02** | **1** | **LGSGGGAGVMESK + Oxidation (M)** |
|  | 2428 | **529.5239** | **1585.5494** | **1585.6844** | **-0.1350** | **2** | **14** | **1.1e+02** | **1** | **SRGGLGAGAAGGGGAGRTR** |
|  | 2077 | **487.9966** | **973.9783** | **973.1693** | **0.8090** | **0** | **14** | **1.2e+02** | **1** | **LLIFGASPR** |
|  | 2558 | **550.7534** | **1649.2381** | **1649.8244** | **-0.5863** | **0** | **14** | **93** | **1** | **DFWMSSNLHQLQK + Oxidation (M)** |
|  | 951 | **407.3640** | **1219.0697** | **1218.5327** | **0.5369** | **2** | **14** | **86** | **1** | **LKKAGASCPICK** |
|  | 1031 | **408.8668** | **1223.5781** | **1224.4494** | **-0.8713** | **0** | **14** | **1.1e+02** | **1** | **GTAQVLPGILQK** |
|  | 1250 | **422.0664** | **1263.1770** | **1263.4407** | **-0.2638** | **0** | **14** | **1.1e+02** | **1** | **LLFNPSGPYQK** |
|  | 1729 | 458.1549 | 1371.4425 | 1371.5788 | -0.1363 | 0 | 14 | 1.1e+02 | 1 | MNELEAFNMLK + 2 Oxidation (M) |
|  | 1920 | **473.3170** | **1416.9290** | **1417.4353** | **-0.5064** | **1** | **14** | **94** | **1** | **DEKGTEAGASTHSK** |
|  | 2231 | **505.0952** | **1008.1755** | **1008.1705** | **0.0051** | **0** | **14** | **1e+02** | **1** | **MAGDLAGMGGI + Oxidation (M)** |
|  | 3266 | **741.5835** | **2221.7283** | **2221.4896** | **0.2387** | **0** | **14** | **87** | **1** | **YEGLWMSCVSQSTGQIQCK + Carbamidomethyl (C); Oxidation (M)** |
|  | 112 | **367.1414** | **1098.4019** | **1099.3044** | **-0.9025** | **1** | **14** | **1.1e+02** | **1** | **MSIDHLKQK** |
|  | 627 | **393.4875** | **784.9603** | **783.9556** | **1.0048** | **0** | **14** | **1.2e+02** | **1** | **VTPILNK** |
|  | 987 | **407.8265** | **1220.4574** | **1221.4241** | **-0.9667** | **0** | **14** | **1.1e+02** | **1** | **CSITVSQSLVK + Carbamidomethyl (C)** |
|  | 892 | 406.2224 | 1215.6450 | 1215.2707 | 0.3743 | 2 | 14 | 84 | 1 | DRAPGEKGEEK |
|  | 975 | **407.7240** | **813.4332** | **813.9434** | **-0.5102** | **0** | **14** | **93** | **1** | **GPPIQFR** |
|  | 1071 | **411.2879** | **1230.8416** | **1230.3929** | **0.4488** | **1** | **14** | **89** | **1** | **NKECTCTSCK + 2 Carbamidomethyl (C)** |
|  | 1228 | **420.2881** | **1257.8423** | **1257.3571** | **0.4852** | **2** | **14** | **79** | **1** | **TRSPGRAEVER** |
|  | 1382 | **431.2687** | **1290.7840** | **1291.5022** | **-0.7182** | **2** | **14** | **1e+02** | **1** | **QRKIGGHGPTLK** |
|  | 2021 | **482.1277** | **962.2407** | **962.9611** | **-0.7203** | **1** | **14** | **1.1e+02** | **1** | **DASSGKENR** |
|  | 2785 | **594.0355** | **1779.0844** | **1778.1262** | **0.9582** | **2** | **14** | **1.1e+02** | **1** | **KHGVVPLATYMRVYK + Oxidation (M)** |
|  | 155 | **369.1471** | **1104.4192** | **1103.3096** | **1.1096** | **0** | **14** | **1.1e+02** | **1** | **YLTFGIPPPV** |
|  | 1018 | **408.2960** | **1221.8658** | **1222.3477** | **-0.4819** | **1** | **14** | **88** | **1** | **SFATKNEAVQK** |
|  | 2253 | **507.9624** | **1520.8650** | **1521.8212** | **-0.9562** | **2** | **14** | **1e+02** | **1** | **ASMASLKKTISQIK + Oxidation (M)** |
|  | 2297 | **515.9739** | **1029.9330** | **1031.0813** | **-1.1484** | **1** | **14** | **1.1e+02** | **1** | **DTGDAGLRAR** |
|  | 722 | **401.7177** | **1202.1310** | **1202.4273** | **-0.2964** | **1** | **14** | **1.1e+02** | **1** | **AALKALVSGCGR + Carbamidomethyl (C)** |
|  | 1212 | **419.4732** | **836.9316** | **837.9633** | **-1.0316** | **2** | **14** | **1.4e+02** | **1** | **FSNSKKK** |
|  | 2121 | **490.6198** | **979.2247** | **978.0420** | **1.1827** | **0** | **14** | **1.2e+02** | **1** | **RPSGMDSGR + Oxidation (M)** |
|  | 655 | **397.2373** | **1188.6898** | **1189.4038** | **-0.7139** | **0** | **14** | **91** | **1** | **CSEIVFFCGK + Carbamidomethyl (C)** |
|  | 1601 | **446.2134** | **890.4121** | **891.0043** | **-0.5923** | **1** | **14** | **1.1e+02** | **1** | **RGLCGEEK** |
|  | 3040 | 666.5009 | 1996.4804 | 1996.1114 | 0.3690 | 0 | 14 | 92 | 1 | TEDTAVYYCTTETSFHK |
|  | 134 | **368.0540** | **1101.1399** | **1101.3234** | **-0.1835** | **1** | **14** | **1.2e+02** | **1** | **IIDAALRACR** |
|  | 1800 | **460.7916** | **919.5685** | **920.0853** | **-0.5168** | **0** | **14** | **99** | **1** | **QMLISNAK + Oxidation (M)** |
|  | 1937 | **474.9824** | **947.9499** | **947.9863** | **-0.0363** | **0** | **14** | **1.2e+02** | **1** | **VSEDTELR** |
|  | 154 | **369.1436** | **1104.4085** | **1105.0072** | **-0.5987** | **1** | **14** | **1.1e+02** | **1** | **XDRAGLVXPR** |
|  | 2644 | **565.5085** | **1693.5033** | **1692.8096** | **0.6937** | **2** | **14** | **93** | **1** | **CPSQGSGGQTKSSARGGK** |
|  | 2747 | **592.4434** | **1774.3079** | **1775.0409** | **-0.7330** | **1** | **14** | **86** | **1** | **VIVPATGGCHRLEHTK + Carbamidomethyl (C)** |
|  | 1450 | **435.7471** | **1304.2190** | **1303.5049** | **0.7142** | **0** | **14** | **87** | **1** | **MYSTALMAGASGK + Oxidation (M)** |
|  | 606 | **390.0562** | **1167.1465** | **1168.2755** | **-1.1290** | **1** | **14** | **1.1e+02** | **1** | **ASMLEDSGSKK + Oxidation (M)** |
|  | 1716 | **457.6756** | **913.3364** | **914.1006** | **-0.7642** | **1** | **14** | **83** | **1** | **AALAKAIEK** |
|  | 1964 | **476.5981** | **951.1815** | **950.0683** | **1.1133** | **0** | **14** | **1.2e+02** | **1** | **SDLLEMAR + Oxidation (M)** |
|  | 1333 | **428.6329** | **855.2510** | **855.0798** | **0.1711** | **2** | **14** | **78** | **1** | **KKTIIPR** |
|  | 2786 | **594.0634** | **1186.1119** | **1185.4168** | **0.6952** | **0** | **14** | **1.2e+02** | **1** | **VSCLSFMLGR + Carbamidomethyl (C); Oxidation (M)** |
|  | 252 | **373.2009** | **744.3870** | **744.8831** | **-0.4960** | **2** | **14** | **1.1e+02** | **1** | **KSRGLGK** |
|  | 415 | **385.1210** | **1152.3407** | **1153.3751** | **-1.0343** | **1** | **14** | **94** | **1** | **MWKMSVEAR + Oxidation (M)** |
|  | 725 | **401.8417** | **1202.5029** | **1203.4187** | **-0.9158** | **2** | **14** | **1.2e+02** | **1** | **RRMNLQTLR + Oxidation (M)** |
|  | 1009 | **408.2323** | **1221.6747** | **1221.4456** | **0.2291** | **0** | **14** | **93** | **1** | **LTPPLVPTLDR** |
|  | 1353 | **429.3582** | **1285.0525** | **1284.3623** | **0.6902** | **1** | **14** | **88** | **1** | **NGDAGPGPCRQGR** |
|  | 2602 | **557.0923** | **1112.1698** | **1111.2076** | **0.9622** | **0** | **14** | **1e+02** | **1** | **CVDVCSDASR + Carbamidomethyl (C)** |
|  | 3141 | **685.0688** | **1368.1228** | **1368.5847** | **-0.4620** | **1** | **14** | **1.1e+02** | **1** | **LMPSRCNTQYR** |
|  | 586 | **389.2661** | **776.5173** | **776.9879** | **-0.4705** | **1** | **14** | **96** | **1** | **ITKIMR + Oxidation (M)** |
|  | 739 | **402.0215** | **1203.0423** | **1203.4322** | **-0.3899** | **2** | **14** | **1.3e+02** | **1** | **MGDMGDPPKKK** |
|  | 1184 | **419.1100** | **836.2052** | **835.7736** | **0.4316** | **0** | **14** | **1.3e+02** | **1** | **ADDTSGDR** |
|  | 1555 | **443.2205** | **1326.6392** | **1327.5525** | **-0.9133** | **1** | **14** | **1e+02** | **1** | **ILPRNQCEGIK + Carbamidomethyl (C)** |
|  | 2971 | **631.3550** | **1891.0428** | **1892.2054** | **-1.1627** | **2** | **14** | **1.2e+02** | **1** | **LDAQGRCAPMKSISSSLK** |
|  | 644 | **396.1352** | **1185.3834** | **1185.3292** | **0.0542** | **0** | **14** | **1.2e+02** | **1** | **METASTGMGIR + 2 Oxidation (M)** |
|  | 974 | **407.7196** | **1220.1367** | **1220.4627** | **-0.3259** | **1** | **14** | **95** | **1** | **CGMISPRVDVK + Oxidation (M)** |
|  | 1485 | **437.1789** | **1308.5146** | **1309.4677** | **-0.9531** | **0** | **14** | **1.2e+02** | **1** | **DHYNLFVAFAL** |
|  | 1690 | **455.3337** | **1362.9790** | **1362.4893** | **0.4897** | **1** | **14** | **83** | **1** | **TAGEPMERSQPC + Carbamidomethyl (C)** |
|  | 2266 | **511.6103** | **1531.8088** | **1531.6904** | **0.1184** | **1** | **14** | **1.3e+02** | **1** | **TKVCTTPSSGHAASGK** |
|  | 176 | **369.2690** | **736.5232** | **736.8131** | **-0.2899** | **0** | **14** | **94** | **1** | **SASFTPK** |
|  | 1223 | **420.1895** | **1257.5462** | **1256.3903** | **1.1559** | **0** | **14** | **1e+02** | **1** | **QAPGQGPEWMR** |
|  | 1989 | **478.3328** | **954.6507** | **955.0650** | **-0.4142** | **0** | **14** | **83** | **1** | **DCMASSPTK + Oxidation (M)** |
|  | 3129 | **683.9773** | **1365.9398** | **1365.5411** | **0.3987** | **0** | **14** | **87** | **1** | **VVGVRPNGALGGNR** |
|  | 3381 | **785.2939** | **1568.5731** | **1567.7040** | **0.8691** | **2** | **14** | **1e+02** | **1** | **NKFGEIREYQQR** |
|  | 89 | **365.8773** | **729.7397** | **728.7513** | **0.9885** | **0** | **14** | **1.2e+02** | **1** | **GDGVPER** |
|  | 526 | **388.1663** | **774.3178** | **774.0268** | **0.2910** | **0** | **14** | **1.2e+02** | **1** | **LLIMIR + Oxidation (M)** |
|  | 758 | **402.9728** | **1205.8963** | **1206.3665** | **-0.4702** | **0** | **14** | **1.2e+02** | **1** | **VANSEAMILDK + Oxidation (M)** |
|  | 1649 | **450.2615** | **1347.7623** | **1347.4810** | **0.2812** | **1** | **14** | **95** | **1** | **WEAVQARGGAFR** |
|  | 2005 | **480.0970** | **1437.2690** | **1436.5447** | **0.7243** | **0** | **14** | **1.1e+02** | **1** | **VTVGDTSCTGQGPSK** |
|  | 264 | **374.1479** | **746.2810** | **745.8262** | **0.4547** | **0** | **14** | **1.3e+02** | **1** | **LWGTGGR** |
|  | 466 | **386.3764** | **1156.1071** | **1156.3177** | **-0.2105** | **1** | **14** | **1.1e+02** | **1** | **EKHFMVGHR + Oxidation (M)** |
|  | 659 | **398.9106** | **795.8064** | **796.8749** | **-1.0684** | **0** | **14** | **95** | **1** | **GPGSRPAR** |
|  | 1419 | **434.2211** | **1299.6412** | **1300.4182** | **-0.7770** | **0** | **14** | **99** | **1** | **AASVAAATTSPTPR** |
|  | 545 | **388.9301** | **775.8453** | **776.7528** | **-0.9074** | **1** | **14** | **1.3e+02** | **1** | **EDRDSR** |
|  | 1119 | **415.0679** | **828.1211** | **827.0036** | **1.1175** | **0** | **14** | **1.1e+02** | **1** | **MPPDVIR** |
|  | 1845 | **466.0083** | **930.0019** | **930.0834** | **-0.0815** | **0** | **14** | **1.2e+02** | **1** | **YVFCGWR** |
|  | 2791 | **594.8530** | **1187.6912** | **1188.2850** | **-0.5938** | **1** | **14** | **94** | **1** | **LDIKNEDDVK** |
|  | 2835 | **605.6482** | **1209.2816** | **1210.3864** | **-1.1048** | **2** | **14** | **1.3e+02** | **1** | **SLLGSKQGHKR** |
|  | 587 | **389.2693** | **1164.7858** | **1164.3976** | **0.3881** | **0** | **14** | **98** | **1** | **APQVLVLAPTR** |
|  | 656 | **397.6491** | **1189.9251** | **1190.3488** | **-0.4237** | **0** | **14** | **89** | **1** | **FNNYVDCMK + Carbamidomethyl (C)** |
|  | 1372 | **431.0233** | **860.0318** | **858.8565** | **1.1754** | **0** | **14** | **1.3e+02** | **1** | **AGSSGGGGGPR** |
|  | 1423 | **434.4509** | **1300.3304** | **1300.5719** | **-0.2415** | **0** | **14** | **1.2e+02** | **1** | **MHIAGFILVQR + Oxidation (M)** |
|  | 701 | **400.1830** | **798.3512** | **798.9306** | **-0.5793** | **2** | **14** | **92** | **1** | **AKKARPE** |
|  | 1114 | **414.2923** | **1239.8547** | **1240.4921** | **-0.6373** | **0** | **14** | **77** | **1** | **LMVCYETLPR + Oxidation (M)** |
|  | 1727 | **458.0982** | **914.1816** | **915.0457** | **-0.8641** | **1** | **14** | **1.1e+02** | **1** | **RTVAAPSTL** |
|  | 2326 | **518.8936** | **1553.6587** | **1554.7636** | **-1.1049** | **1** | **14** | **1.1e+02** | **1** | **DKEGPECPPTVVVK + Carbamidomethyl (C)** |
|  | 2933 | **618.5222** | **1852.5445** | **1853.1066** | **-0.5621** | **0** | **14** | **91** | **1** | **VACMAQVSSCSPSLPSR + 2 Carbamidomethyl (C); Oxidation (M)** |
|  | 3158 | **686.1724** | **2055.4949** | **2055.5146** | **-0.0196** | **2** | **14** | **1.1e+02** | **1** | **CTSCKKCCCSCCPVGCAK + 4 Carbamidomethyl (C)** |
|  | 1669 | **452.4256** | **1354.2547** | **1353.6778** | **0.5770** | **1** | **14** | **1.2e+02** | **1** | **MMRTQCLLGLR + 2 Oxidation (M)** |
|  | 2264 | **511.5958** | **1531.7653** | **1532.8275** | **-1.0622** | **1** | **14** | **1.3e+02** | **1** | **KNCVPVTLAMVER + Carbamidomethyl (C); Oxidation (M)** |
|  | 170 | **369.2446** | **1104.7115** | **1105.3089** | **-0.5973** | **0** | **14** | **96** | **1** | **AKPWAVCFPS** |
|  | 849 | **405.3460** | **808.6773** | **807.8908** | **0.7865** | **0** | **14** | **86** | **1** | **ANAEYIK** |
|  | 1859 | **467.2364** | **932.4580** | **933.0693** | **-0.6113** | **1** | **14** | **1.1e+02** | **1** | **GHTRVHVK** |
|  | 1901 | **472.1652** | **942.3156** | **941.1493** | **1.1663** | **2** | **14** | **1.1e+02** | **1** | **IMKSKYR + Oxidation (M)** |
|  | 3326 | **755.1983** | **1508.3818** | **1507.5280** | **0.8539** | **2** | **14** | **1.1e+02** | **1** | **RHQQGRGDDSSHK** |
|  | 566 | **389.1392** | **1164.3953** | **1165.3228** | **-0.9275** | **1** | **14** | **1.2e+02** | **1** | **MSWVRQASGK + Oxidation (M)** |
|  | 2229 | **505.0605** | **1008.1061** | **1009.1802** | **-1.0740** | **0** | **14** | **1.1e+02** | **1** | **GMTVYGLPR + Oxidation (M)** |
|  | 2473 | 536.1332 | 1070.2516 | 1071.0988 | -0.8473 | 0 | 14 | 1.1e+02 | 1 | HLDAASGDTGK |
|  | 3162 | **687.0219** | **1372.0289** | **1371.4991** | **0.5298** | **1** | **14** | **90** | **1** | **NLADKGNQTAAIR** |
|  | 182 | **369.2867** | **1104.8380** | **1105.3089** | **-0.4709** | **0** | **14** | **97** | **1** | **AKPWAVCFPS** |
|  | 327 | 377.9848 | 1130.9322 | 1131.2402 | -0.3080 | 1 | 14 | 94 | 1 | EIQSTLRER |
|  | 547 | **389.0140** | **1164.0198** | **1163.4974** | **0.5224** | **1** | **14** | **1.3e+02** | **1** | **MVIKQMLIR + 2 Oxidation (M)** |
|  | 612 | **391.1995** | **1170.5762** | **1170.3360** | **0.2403** | **0** | **14** | **86** | **1** | **VMNATAYGISK + Oxidation (M)** |
|  | 1436 | **435.0881** | **868.1614** | **868.1433** | **0.0181** | **2** | **14** | **1e+02** | **1** | **MCAKCKK + Carbamidomethyl (C)** |
|  | 1805 | **461.0069** | **919.9990** | **920.0422** | **-0.0432** | **0** | **14** | **1.2e+02** | **1** | **NGLLDMNK + Oxidation (M)** |
|  | 3041 | **666.5094** | **1996.5060** | **1997.2384** | **-0.7323** | **0** | **14** | **94** | **1** | **ATVCDCHGHVETCPGPWK + Carbamidomethyl (C)** |
|  | 297 | **376.2542** | **750.4937** | **750.8214** | **-0.3276** | **0** | **14** | **85** | **1** | **SAAGAGCSK** |
|  | 762 | **403.0694** | **804.1240** | **804.9581** | **-0.8342** | **2** | **14** | **1.2e+02** | **1** | **LRAKNTC** |
|  | 834 | **405.1141** | **1212.3202** | **1211.3714** | **0.9489** | **1** | **14** | **1e+02** | **1** | **SLPSVSGPVRGR** |
|  | 1876 | **469.2628** | **1404.7662** | **1405.5338** | **-0.7676** | **0** | **14** | **1e+02** | **1** | **TQTHATLCSTSAK + Carbamidomethyl (C)** |
|  | 2135 | **492.1828** | **1473.5261** | **1472.7060** | **0.8202** | **0** | **14** | **1.1e+02** | **1** | **IDISAVMSVHSAVK + Oxidation (M)** |
|  | 1254 | **422.1247** | **842.2345** | **841.9122** | **0.3224** | **1** | **14** | **1.2e+02** | **1** | **GAAGEPGKR** |
|  | 2435 | **530.8295** | **1589.4662** | **1588.8142** | **0.6520** | **2** | **14** | **1e+02** | **1** | **AFQWAVQRAISRR** |
|  | 2598 | **556.4341** | **1666.2803** | **1666.8750** | **-0.5947** | **1** | **14** | **94** | **1** | **FDKCMVCGGDGSGCSK + Carbamidomethyl (C); Oxidation (M)** |
|  | 3244 | **740.5814** | **2218.7219** | **2217.5443** | **1.1776** | **1** | **14** | **96** | **1** | **VVPLVQMGETDANVAKFLNR + Oxidation (M)** |
|  | 442 | **386.0853** | **1155.2338** | **1154.3151** | **0.9187** | **1** | **14** | **1e+02** | **1** | **KEMAINDCSK + Oxidation (M)** |
|  | 742 | **402.1232** | **1203.3474** | **1203.3756** | **-0.0282** | **2** | **14** | **1.3e+02** | **1** | **CQLNAARSKR + Carbamidomethyl (C)** |
|  | 1505 | **438.1028** | **1311.2862** | **1310.5222** | **0.7639** | **1** | **14** | **1.3e+02** | **1** | **CTLGSKVGYQVR** |
|  | 68 | **364.0846** | **1089.2316** | **1089.2848** | **-0.0531** | **0** | **14** | **1.1e+02** | **1** | **EAVVNAVFIK** |
|  | 828 | **405.0316** | **808.0485** | **807.9590** | **0.0895** | **1** | **14** | **1e+02** | **1** | **STAVKMR + Oxidation (M)** |
|  | 1140 | **416.1344** | **1245.3811** | **1245.4291** | **-0.0480** | **1** | **14** | **1.3e+02** | **1** | **SQAKMSGMFAR + 2 Oxidation (M)** |
|  | 1940 | **475.3419** | **948.6690** | **949.1051** | **-0.4361** | **1** | **14** | **97** | **1** | **SKIVAEFR** |
|  | 2119 | **490.4521** | **978.8895** | **978.1942** | **0.6953** | **1** | **14** | **98** | **1** | **ALSRAMACR** |
|  | 2252 | **507.7589** | **1520.2546** | **1520.8382** | **-0.5836** | **2** | **14** | **91** | **1** | **MAKSAEVKLAIFGR** |
|  | 721 | **401.6806** | **1202.0197** | **1201.3797** | **0.6401** | **1** | **14** | **1.1e+02** | **1** | **LGSPHSCKCGR + Carbamidomethyl (C)** |
|  | 152 | **369.0448** | **1104.1123** | **1105.2440** | **-1.1317** | **0** | **14** | **1.3e+02** | **1** | **LNILNNNYK** |
|  | 344 | **379.3630** | **1135.0668** | **1135.3183** | **-0.2515** | **1** | **14** | **1.2e+02** | **1** | **QRLATFFPR** |
|  | 3057 | **666.7660** | **1997.2758** | **1998.2249** | **-0.9491** | **2** | **14** | **1.4e+02** | **1** | **APSPSGLMSPSRLPGSRER + Oxidation (M)** |
|  | 1642 | **450.1360** | **898.2573** | **899.0480** | **-0.7907** | **2** | **14** | **1.2e+02** | **1** | **SSKKFFR** |
|  | 2868 | **610.0475** | **1218.0802** | **1218.3389** | **-0.2588** | **0** | **14** | **1.1e+02** | **1** | **EDEHFICIR + Carbamidomethyl (C)** |
|  | 3210 | **729.7789** | **1457.5430** | **1456.6717** | **0.8713** | **2** | **14** | **1.2e+02** | **1** | **SPRRMSFSGIFR + Oxidation (M)** |
|  | 350 | **380.1191** | **1137.3352** | **1137.2433** | **0.0919** | **1** | **14** | **1.3e+02** | **1** | **EPPGEEPVRK** |
|  | 646 | **396.1803** | **1185.5188** | **1185.3042** | **0.2146** | **0** | **14** | **1.2e+02** | **1** | **SSEQYLEAMK** |
|  | 676 | **399.2757** | **796.5367** | **796.8748** | **-0.3382** | **1** | **14** | **79** | **1** | **SHRLER** |
|  | 2804 | **596.7380** | **1787.1918** | **1786.0834** | **1.1084** | **0** | **14** | **1.4e+02** | **1** | **LVHLCSNFLHSSPMK + Carbamidomethyl (C); Oxidation (M)** |
|  | 3452 | **845.5904** | **1689.1660** | **1689.8703** | **-0.7043** | **0** | **14** | **1.1e+02** | **1** | **AHVQLVESGGGVVQPGR** |
|  | 3483 | **890.9294** | **1779.8441** | **1780.1270** | **-0.2828** | **1** | **14** | **1.1e+02** | **1** | **INGKVAERPQHMLMR** |
|  | 169 | **369.2405** | **1104.6994** | **1104.2991** | **0.4003** | **0** | **14** | **98** | **1** | **QQLLIGAYAK** |
|  | 1908 | **472.3984** | **1414.1730** | **1414.6911** | **-0.5182** | **1** | **14** | **91** | **1** | **AFPAIQILSQAKK** |
|  | 3127 | **683.9175** | **2048.7303** | **2048.5004** | **0.2298** | **2** | **14** | **95** | **1** | **MRCLTTPMLLRALAQAAR + 2 Oxidation (M)** |
|  | 97 | **366.1350** | **1095.3829** | **1096.1681** | **-0.7852** | **0** | **14** | **1.2e+02** | **1** | **MAEDGDPFAK + Oxidation (M)** |
|  | 131 | **367.9931** | **1100.9571** | **1100.2892** | **0.6680** | **0** | **14** | **1.3e+02** | **1** | **SLPASITMHK + Oxidation (M)** |
|  | 1542 | **442.0663** | **1323.1766** | **1322.4918** | **0.6848** | **2** | **14** | **1e+02** | **1** | **KMAASTRVEASR + Oxidation (M)** |
|  | 2130 | **491.7723** | **981.5298** | **981.1037** | **0.4260** | **0** | **14** | **83** | **1** | **FSSASLLTR** |
|  | 2493 | **538.2238** | **1611.6491** | **1612.7412** | **-1.0921** | **0** | **14** | **1.2e+02** | **1** | **DNGSLALPADAHLYR** |
|  | 307 | **377.1213** | **752.2278** | **752.0017** | **0.2261** | **2** | **14** | **1.1e+02** | **1** | **MKKMSK** |
|  | 3269 | **741.6672** | **2221.9794** | **2221.6389** | **0.3405** | **2** | **14** | **87** | **1** | **LDSLPPVIKFCTSAADMKIR + Oxidation (M)** |
|  | 549 | **389.0364** | **1164.0870** | **1164.3544** | **-0.2674** | **0** | **14** | **1.3e+02** | **1** | **LSRPLPLPASN** |
|  | 561 | **389.1063** | **776.1979** | **775.8922** | **0.3057** | **1** | **14** | **1.3e+02** | **1** | **SKEVWK** |
|  | 1041 | **409.0713** | **1224.1917** | **1224.4296** | **-0.2379** | **1** | **14** | **1.3e+02** | **1** | **AKGCTIIFTDR** |
|  | 2533 | 547.4041 | 1092.7933 | 1093.2766 | -0.4832 | 0 | 14 | 92 | 1 | CWMDIQPK + Carbamidomethyl (C); Oxidation (M) |
|  | 2619 | **560.5930** | **1678.7569** | **1677.7322** | **1.0247** | **2** | **14** | **1.4e+02** | **1** | **NDSSRSEASRPSTRK** |
|  | 3121 | **682.2577** | **2043.7509** | **2044.3679** | **-0.6170** | **2** | **14** | **1.1e+02** | **1** | **TSLDPSLEIYKKMFEVK + Oxidation (M)** |
|  | 173 | **369.2527** | **1104.7360** | **1105.3089** | **-0.5729** | **0** | **14** | **99** | **1** | **AKPWAVCFPS** |
|  | 699 | **400.1683** | **1197.4827** | **1197.4108** | **0.0719** | **1** | **14** | **99** | **1** | **ATHARIGQICK** |
|  | 905 | **406.4248** | **1216.2521** | **1215.5040** | **0.7482** | **0** | **14** | **1.2e+02** | **1** | **YQIMIPLPPK + Oxidation (M)** |
|  | 1161 | **417.4525** | **832.8901** | **832.9021** | **-0.0119** | **1** | **14** | **1.5e+02** | **1** | **SSDKQLR** |
|  | 1243 | **421.8134** | **841.6120** | **840.9440** | **0.6680** | **0** | **14** | **1.2e+02** | **1** | **MGQSQFK + Oxidation (M)** |
|  | 1971 | **477.5921** | **1429.7542** | **1428.6368** | **1.1175** | **2** | **14** | **1.2e+02** | **1** | **CNECGKTFSVKR + Carbamidomethyl (C)** |
|  | 2511 | **541.2797** | **1620.8170** | **1621.8557** | **-1.0387** | **1** | **14** | **1.1e+02** | **1** | **SGKGPILCELQTYR + Carbamidomethyl (C)** |
|  | 2641 | **564.4513** | **1690.3317** | **1689.8766** | **0.4551** | **1** | **14** | **99** | **1** | **QGCAQAMQWQPRGR + Carbamidomethyl (C); Oxidation (M)** |
|  | 2738 | **590.6172** | **1179.2196** | **1180.3556** | **-1.1360** | **1** | **14** | **1.3e+02** | **1** | **MQCDNNIKSK** |
|  | 199 | 369.4913 | 1105.4518 | 1105.3089 | 0.1430 | 0 | 14 | 1.3e+02 | 1 | AKPWAVCFPS |
|  | 2580 | **553.7373** | **1105.4598** | **1104.3242** | **1.1357** | **2** | **14** | **1.1e+02** | **1** | **KQGKAGLQMK + Oxidation (M)** |
|  | 2963 | **629.8166** | **1257.6185** | **1258.4905** | **-0.8720** | **1** | **14** | **1.1e+02** | **1** | **ITPNCLIKGSR + Carbamidomethyl (C)** |
|  | 385 | **384.2500** | **1149.7279** | **1149.3598** | **0.3682** | **0** | **14** | **87** | **1** | **ILFVGPCESGK** |
|  | 408 | **385.0462** | **768.0776** | **768.7753** | **-0.6978** | **0** | **14** | **1e+02** | **1** | **DAEAAHR** |
|  | 481 | **387.0629** | **1158.1666** | **1157.3172** | **0.8494** | **0** | **14** | **1.4e+02** | **1** | **LGQSVVADQLK** |
|  | 1643 | **450.1539** | **1347.4394** | **1346.4899** | **0.9496** | **1** | **14** | **1.2e+02** | **1** | **MEKEPGPEGGCR + Carbamidomethyl (C)** |
|  | 1730 | **458.2142** | **1371.6204** | **1371.6234** | **-0.0030** | **0** | **14** | **1.2e+02** | **1** | **LFAVLEQLSPVR** |
|  | 1895 | **470.8818** | **1409.6232** | **1408.5196** | **1.1036** | **2** | **14** | **1.1e+02** | **1** | **RTAQEVETYRR** |
|  | 727 | **401.8431** | **801.6714** | **801.9577** | **-0.2863** | **1** | **14** | **1.3e+02** | **1** | **MTAPARR** |
|  | 269 | **374.2635** | **746.5121** | **745.8015** | **0.7107** | **0** | **14** | **1.1e+02** | **1** | **EGIGNPC + Carbamidomethyl (C)** |
|  | 639 | **395.2619** | **1182.7634** | **1183.4623** | **-0.6988** | **0** | **14** | **1e+02** | **1** | **MVLMEGLCSK + Carbamidomethyl (C); Oxidation (M)** |
|  | 1395 | **433.0520** | **1296.1337** | **1295.5490** | **0.5848** | **0** | **14** | **1.2e+02** | **1** | **MSTLFPSLFPR** |
|  | 3345 | **760.4724** | **2278.3951** | **2277.4424** | **0.9527** | **1** | **14** | **1.1e+02** | **1** | **DPSEDTPAVDGSTDMDRMPLK** |
|  | 3372 | **777.9727** | **2330.8960** | **2329.7601** | **1.1358** | **1** | **14** | **1.1e+02** | **1** | **EIHCPGYVLPVKVVAECGCQK + Carbamidomethyl (C)** |
|  | 1208 | **419.3839** | **1255.1297** | **1254.3082** | **0.8215** | **0** | **14** | **1.2e+02** | **1** | **SGWSNAYAGSVR** |
|  | 1378 | **431.0871** | **1290.2391** | **1290.5143** | **-0.2751** | **2** | **14** | **1.4e+02** | **1** | **ASVFSNLRIRK** |
|  | 2196 | **499.7378** | **1496.1912** | **1495.7275** | **0.4637** | **1** | **14** | **90** | **1** | **MMITRGWEGWGR + Oxidation (M)** |
|  | 2392 | **523.5500** | **1567.6280** | **1566.8786** | **0.7494** | **0** | **14** | **1.4e+02** | **1** | **MVTMVSVLEDLIGK + 2 Oxidation (M)** |
|  | 2993 | **640.1836** | **1917.5286** | **1918.1485** | **-0.6198** | **1** | **14** | **1.2e+02** | **1** | **SIEVENDFLPVEKTIGK** |
|  | 461 | **386.2173** | **1155.6299** | **1156.5079** | **-0.8781** | **0** | **14** | **84** | **1** | **LCCCFLCPVR** |
|  | 1748 | **458.8533** | **915.6918** | **915.0026** | **0.6891** | **0** | **14** | **1.3e+02** | **1** | **ALETQPTR** |
|  | 1992 | **478.7939** | **1433.3595** | **1433.5705** | **-0.2110** | **1** | **14** | **96** | **1** | **GSDDMELHRMSR** |
|  | 2321 | **518.7476** | **1035.4803** | **1035.1346** | **0.3458** | **0** | **14** | **93** | **1** | **MGGAAFGPDGR** |
|  | 763 | **403.1115** | **1206.3124** | **1205.4281** | **0.8843** | **2** | **14** | **1.3e+02** | **1** | **IMLQRESGKK + Oxidation (M)** |
|  | 1361 | **430.1872** | **858.3597** | **857.9776** | **0.3820** | **0** | **14** | **1.3e+02** | **1** | **GGPGCAALR + Carbamidomethyl (C)** |
|  | 1648 | 450.2594 | 898.5040 | 899.0016 | -0.4975 | 0 | 14 | 1e+02 | 1 | DSVYIFR |
|  | 965 | **407.6015** | **813.1882** | **814.0096** | **-0.8214** | **1** | **14** | **97** | **1** | **CWHLKK** |
|  | 1062 | **411.0390** | **820.0633** | **820.0325** | **0.0308** | **0** | **14** | **1.2e+02** | **1** | **GPMMQIK + Oxidation (M)** |
|  | 1217 | **420.0704** | **838.1260** | **838.9975** | **-0.8716** | **1** | **14** | **1.1e+02** | **1** | **GGARLPLR** |
|  | 2371 | **521.5125** | **1561.5152** | **1560.8224** | **0.6927** | **2** | **14** | **1.1e+02** | **1** | **QMYVALNGKGAPRR** |
|  | 194 | **369.3456** | **1105.0147** | **1105.3089** | **-0.2942** | **0** | **14** | **1.2e+02** | **1** | **AKPWAVCFPS** |
|  | 464 | **386.2702** | **1155.7884** | **1155.3727** | **0.4158** | **1** | **14** | **84** | **1** | **AFVTRQFMR** |
|  | 509 | **387.9167** | **773.8186** | **773.9178** | **-0.0992** | **1** | **14** | **1.4e+02** | **1** | **KDVTALK** |
|  | 2192 | 499.6666 | 1495.9775 | 1495.7273 | 0.2502 | 0 | 14 | 1.1e+02 | 1 | HQLSHPGLPGALLR |
|  | 362 | **380.3613** | **758.7078** | **757.8718** | **0.8359** | **0** | **14** | **1.4e+02** | **1** | **LGIIVDE** |
|  | 1169 | **417.9932** | **1250.9575** | **1251.4750** | **-0.5175** | **1** | **14** | **1.2e+02** | **1** | **RLEEMMWEK** |
|  | 2074 | **487.9239** | **973.8330** | **973.1297** | **0.7033** | **1** | **14** | **1.4e+02** | **1** | **AIPRFNQK** |
|  | 3227 | **739.2654** | **1476.5160** | **1475.5674** | **0.9486** | **2** | **14** | **1.2e+02** | **1** | **RDSGVGASLTRSNR** |
|  | 1332 | **428.4536** | **1282.3385** | **1282.4077** | **-0.0692** | **1** | **14** | **1.2e+02** | **1** | **RGPGPGGFGAQGPK** |
|  | 2899 | **612.2192** | **1222.4236** | **1223.3456** | **-0.9220** | **2** | **14** | **1.2e+02** | **1** | **ARAGRGAASGPPR** |
|  | 909 | **406.8639** | **1217.5695** | **1218.3375** | **-0.7680** | **0** | **14** | **1.1e+02** | **1** | **VVDELDNQMR** |
|  | 1196 | **419.2511** | **1254.7310** | **1254.5055** | **0.2256** | **2** | **14** | **1.2e+02** | **1** | **APKASRPPKMR + Oxidation (M)** |
|  | 1211 | **419.4573** | **1255.3497** | **1255.4485** | **-0.0988** | **1** | **14** | **1.6e+02** | **1** | **LCGGGGPWAGPKR** |
|  | 2400 | **524.1921** | **1569.5542** | **1568.9437** | **0.6105** | **0** | **14** | **1.3e+02** | **1** | **ICICQQESMLLCK + Carbamidomethyl (C)** |
|  | 2826 | **600.5000** | **1798.4778** | **1799.0822** | **-0.6043** | **0** | **14** | **96** | **1** | **AGQSPACGCWTPVLPVR + Carbamidomethyl (C)** |
|  | 3181 | **705.7675** | **1409.5201** | **1409.5241** | **-0.0039** | **0** | **14** | **1.3e+02** | **1** | **SFSNSLSQSPCPR** |
|  | 111 | **367.0634** | **1098.1682** | **1099.3276** | **-1.1594** | **0** | **14** | **1.3e+02** | **1** | **IHMGEKPCK + Carbamidomethyl (C)** |
|  | 375 | **382.2125** | **762.4102** | **761.8242** | **0.5860** | **1** | **14** | **1.1e+02** | **1** | **ARAETSK** |
|  | 2820 | **598.2728** | **1194.5309** | **1194.3409** | **0.1900** | **1** | **14** | **1.2e+02** | **1** | **KPSSRSGGPPPK** |
|  | 57 | 363.2500 | 1086.7278 | 1087.1461 | -0.4183 | 0 | 14 | 96 | 1 | EANAAAAQWR |
|  | 192 | 369.3389 | 1104.9944 | 1105.3089 | -0.3144 | 0 | 14 | 1.2e+02 | 1 | AKPWAVCFPS |
|  | 1129 | **415.8359** | **829.6571** | **828.9167** | **0.7404** | **1** | **14** | **1.4e+02** | **1** | **IVGGRDGR** |
|  | 1164 | **417.8519** | **1250.5334** | **1250.4456** | **0.0879** | **0** | **14** | **1.3e+02** | **1** | **AVDLECMQAVR + Oxidation (M)** |
|  | 1453 | **436.1074** | **1305.3001** | **1304.4564** | **0.8437** | **1** | **14** | **1.2e+02** | **1** | **VSQHDFRLFR** |
|  | 2122 | **491.2823** | **1470.8249** | **1470.6586** | **0.1663** | **2** | **14** | **1.1e+02** | **1** | **CPRASSMSGSCGRR + Oxidation (M)** |
|  | 1921 | **473.3532** | **944.6916** | **945.1561** | **-0.4644** | **1** | **13** | **1.1e+02** | **1** | **EFPLAIKK** |
|  | 409 | **385.0500** | **1152.1277** | **1151.3577** | **0.7700** | **1** | **13** | **1e+02** | **1** | **VEAAMKSPMR + 2 Oxidation (M)** |
|  | 649 | **396.2065** | **1185.5973** | **1185.3274** | **0.2698** | **0** | **13** | **1.2e+02** | **1** | **LSETSAHLTVK** |
|  | 797 | **404.0751** | **1209.2032** | **1209.4380** | **-0.2348** | **1** | **13** | **1.2e+02** | **1** | **LLLRGANPDLK** |
|  | 850 | **405.3581** | **1213.0522** | **1213.3655** | **-0.3133** | **0** | **13** | **94** | **1** | **GGWEPGMGLGPR** |
|  | 1756 | **459.0483** | **1374.1228** | **1373.5481** | **0.5746** | **0** | **13** | **1.3e+02** | **1** | **LEDILESINSIK** |
|  | 2027 | **483.7814** | **1448.3221** | **1447.5771** | **0.7451** | **2** | **13** | **1e+02** | **1** | **SQMNGKRGNAAGEK** |
|  | 1932 | **474.3775** | **946.7402** | **947.1323** | **-0.3920** | **0** | **13** | **1e+02** | **1** | **KPIVPEHK** |
|  | 2758 | **592.7147** | **1183.4147** | **1182.2390** | **1.1757** | **0** | **13** | **1.4e+02** | **1** | **YNTAPPTFGGGT** |
|  | 1204 | **419.3229** | **1254.9465** | **1255.5051** | **-0.5585** | **1** | **13** | **1.2e+02** | **1** | **AKLSMINTMSK + 2 Oxidation (M)** |
|  | 1531 | 441.1255 | 1320.3543 | 1320.5616 | -0.2074 | 0 | 13 | 1.2e+02 | 1 | GTHMLQCLCGK + 2 Carbamidomethyl (C); Oxidation (M) |
|  | 144 | **368.2343** | **1101.6807** | **1102.2620** | **-0.5813** | **0** | **13** | **1e+02** | **1** | **CDECPFTCK + Carbamidomethyl (C)** |
|  | 153 | **369.1147** | **1104.3219** | **1105.3089** | **-0.9870** | **0** | **13** | **1.3e+02** | **1** | **AKPWAVCFPS** |
|  | 1113 | **414.2441** | **826.4734** | **826.9637** | **-0.4902** | **0** | **13** | **86** | **1** | **LGQPPCR + Carbamidomethyl (C)** |
|  | 1569 | **443.8335** | **885.6522** | **884.9735** | **0.6787** | **1** | **13** | **1.2e+02** | **1** | **APEKSEPK** |
|  | 1775 | **459.7424** | **1376.2049** | **1376.5356** | **-0.3307** | **0** | **13** | **1.1e+02** | **1** | **EMDITQLVQQR + Oxidation (M)** |
|  | 2233 | **505.1520** | **1512.4339** | **1511.6392** | **0.7948** | **0** | **13** | **1.2e+02** | **1** | **PTCQVDQFSCGNGR** |
|  | 2838 | **606.3030** | **1815.8868** | **1816.0264** | **-0.1397** | **0** | **13** | **1.2e+02** | **1** | **ITLVLQQPQSGGPQGHR** |
|  | 963 | **407.5647** | **813.1147** | **811.9525** | **1.1622** | **1** | **13** | **1.1e+02** | **1** | **AKGMPHR + Oxidation (M)** |
|  | 998 | **407.9612** | **813.9076** | **812.9124** | **0.9953** | **0** | **13** | **1.2e+02** | **1** | **GATYVFR** |
|  | 1928 | **474.0955** | **1419.2644** | **1419.7738** | **-0.5094** | **0** | **13** | **1.3e+02** | **1** | **VDLLAPMIIHGIK** |
|  | 24 | **362.1057** | **1083.2948** | **1083.2851** | **0.0096** | **0** | **13** | **1.1e+02** | **1** | **TLVTGALRPR** |
|  | 380 | **383.2277** | **1146.6609** | **1146.3163** | **0.3446** | **0** | **13** | **1e+02** | **1** | **VERPPSPFSM** |
|  | 427 | **385.9322** | **769.8495** | **768.8615** | **0.9881** | **0** | **13** | **1e+02** | **1** | **GPTGRPGK** |
|  | 859 | **405.9094** | **809.8040** | **809.0133** | **0.7907** | **1** | **13** | **1.1e+02** | **1** | **RMTVMR + Oxidation (M)** |
|  | 937 | **407.2098** | **812.4049** | **811.8184** | **0.5865** | **0** | **13** | **1e+02** | **1** | **MSGDSSGR + Oxidation (M)** |
|  | 1292 | **425.9130** | **1274.7169** | **1275.4334** | **-0.7164** | **0** | **13** | **1.2e+02** | **1** | **FCISIHDEVR + Carbamidomethyl (C)** |
|  | 1117 | **414.4277** | **1240.2608** | **1240.3232** | **-0.0623** | **1** | **13** | **1.2e+02** | **1** | **SQGPSPSPAREK** |
|  | 1133 | **415.9820** | **1244.9237** | **1244.5469** | **0.3768** | **1** | **13** | **1.4e+02** | **1** | **VLLAAAVCTKAGK** |
|  | 1832 | **464.0052** | **1388.9934** | **1389.4716** | **-0.4782** | **0** | **13** | **1.1e+02** | **1** | **MDSSEEHAGCPAR** |
|  | 198 | **369.3654** | **1105.0740** | **1105.3089** | **-0.2349** | **0** | **13** | **1.4e+02** | **1** | **AKPWAVCFPS** |
|  | 193 | **369.3394** | **1104.9961** | **1105.3089** | **-0.3128** | **0** | **13** | **1.2e+02** | **1** | **AKPWAVCFPS** |
|  | 954 | **407.4120** | **1219.2137** | **1218.4218** | **0.7919** | **0** | **13** | **1.3e+02** | **1** | **FLQCPPDLTGK** |
|  | 1210 | **419.4311** | **1255.2710** | **1256.3259** | **-1.0548** | **1** | **13** | **1.6e+02** | **1** | **APQTGPGTSRER** |
|  | 1749 | **458.8778** | **1373.6113** | **1374.3773** | **-0.7661** | **1** | **13** | **1.3e+02** | **1** | **HSSGRSNTSTANR** |
|  | 1936 | **474.9071** | **947.7994** | **947.0874** | **0.7119** | **1** | **13** | **1.3e+02** | **1** | **KSLTNSGIK** |
|  | 2034 | **484.4363** | **966.8579** | **966.1770** | **0.6809** | **1** | **13** | **97** | **1** | **RMALSMIE + Oxidation (M)** |
|  | 625 | **393.4458** | **784.8769** | **783.9124** | **0.9644** | **0** | **13** | **1.4e+02** | **1** | **EPALNIK** |
|  | 1201 | **419.2853** | **836.5558** | **835.8629** | **0.6930** | **0** | **13** | **1.2e+02** | **1** | **DHFFDR** |
|  | 2167 | **496.3232** | **1485.9476** | **1485.8304** | **0.1171** | **1** | **13** | **1.1e+02** | **1** | **LASIMSKLPLATPK + Oxidation (M)** |
|  | 542 | **388.4839** | **774.9531** | **774.8858** | **0.0673** | **0** | **13** | **1.7e+02** | **1** | **GSLGCPGK + Carbamidomethyl (C)** |
|  | 779 | **403.9290** | **805.8431** | **805.9213** | **-0.0782** | **0** | **13** | **1.2e+02** | **1** | **AAYNLVR** |
|  | 1110 | 414.0691 | 826.1233 | 824.9446 | 1.1788 | 0 | 13 | 1.1e+02 | 1 | SQAFNMK |
|  | 2595 | **555.9747** | **1664.9018** | **1665.9251** | **-1.0233** | **2** | **13** | **1.2e+02** | **1** | **KYIAKVSLAVTDTEK** |
|  | 166 | **369.2161** | **1104.6262** | **1105.3089** | **-0.6827** | **0** | **13** | **1.1e+02** | **1** | **AKPWAVCFPS** |
|  | 1598 | **446.1424** | **1335.4050** | **1335.5335** | **-0.1285** | **2** | **13** | **1.4e+02** | **1** | **MASVALGRSKGSR + Oxidation (M)** |
|  | 162 | **369.1897** | **1104.5470** | **1105.3089** | **-0.7619** | **0** | **13** | **1.1e+02** | **1** | **AKPWAVCFPS** |
|  | 686 | **400.0049** | **1196.9926** | **1196.3797** | **0.6128** | **0** | **13** | **1e+02** | **1** | **VGHNEIIGVCR** |
|  | 1259 | **422.1867** | **842.3586** | **841.9951** | **0.3636** | **0** | **13** | **1.3e+02** | **1** | **TSIVVAPR** |
|  | 1356 | **429.4890** | **1285.4449** | **1285.4497** | **-0.0049** | **1** | **13** | **1.5e+02** | **1** | **NRSLASPLQATK** |
|  | 2174 | **498.7054** | **995.3960** | **995.0725** | **0.3235** | **1** | **13** | **99** | **1** | **TMSSQGRGR + Oxidation (M)** |
|  | 2528 | **546.8652** | **1091.7157** | **1091.1962** | **0.5194** | **0** | **13** | **1.1e+02** | **1** | **EVAQGCTAQK + Carbamidomethyl (C)** |
|  | 315 | **377.1667** | **1128.4781** | **1127.3775** | **1.1006** | **2** | **13** | **1.1e+02** | **1** | **KALVGLSKSPK** |
|  | 1577 | **444.2797** | **886.5445** | **886.9894** | **-0.4448** | **1** | **13** | **1.1e+02** | **1** | **TDPEAKVK** |
|  | 2309 | **517.8363** | **1033.6578** | **1033.0939** | **0.5639** | **1** | **13** | **1.1e+02** | **1** | **LDDTQASRK** |
|  | 2485 | **537.9409** | **1610.8006** | **1610.8828** | **-0.0822** | **1** | **13** | **1.3e+02** | **1** | **MHHLSLFFTGHRK** |
|  | 52 | **363.1964** | **1086.5671** | **1087.2077** | **-0.6406** | **1** | **13** | **1e+02** | **1** | **SQMSRFSTK + Oxidation (M)** |
|  | 1693 | **456.1088** | **1365.3041** | **1364.7417** | **0.5624** | **1** | **13** | **1.1e+02** | **1** | **KLLVPGLPCLVR + Carbamidomethyl (C)** |
|  | 200 | **369.5065** | **1105.4974** | **1106.3369** | **-0.8394** | **1** | **13** | **1.3e+02** | **1** | **LGNMTVTVKK + Oxidation (M)** |
|  | 251 | **373.1992** | **1116.5755** | **1117.2104** | **-0.6349** | **1** | **13** | **1.3e+02** | **1** | **GEKGDVGSAGLK** |
|  | 743 | **402.1299** | **802.2450** | **802.8762** | **-0.6311** | **0** | **13** | **1.4e+02** | **1** | **GSGTTRPK** |
|  | 751 | **402.4899** | **1204.4476** | **1204.3325** | **0.1151** | **0** | **13** | **1.7e+02** | **1** | **VDTSAFSPRPK** |
|  | 1130 | **415.8592** | **1244.5555** | **1243.3933** | **1.1622** | **0** | **13** | **1.4e+02** | **1** | **MDPASSNRPLR** |
|  | 1778 | **459.9586** | **1376.8537** | **1377.5965** | **-0.7429** | **2** | **13** | **1.4e+02** | **1** | **ELQAACRARGMR + Oxidation (M)** |
|  | 101 | **366.1991** | **1095.5750** | **1096.2575** | **-0.6825** | **2** | **13** | **1.1e+02** | **1** | **WKSGKDMTK + Oxidation (M)** |
|  | 832 | **405.0997** | **1212.2768** | **1211.5368** | **0.7400** | **0** | **13** | **1.2e+02** | **1** | **YLVLANMLMK + Oxidation (M)** |
|  | 1761 | **459.1432** | **916.2716** | **917.0816** | **-0.8100** | **0** | **13** | **1.4e+02** | **1** | **TPMTGVSPK** |
|  | 2833 | **603.0909** | **1806.2507** | **1807.1624** | **-0.9117** | **1** | **13** | **1.3e+02** | **1** | **FEKMISGMYMGELVR + Oxidation (M)** |
|  | 40 | 363.0770 | 1086.2087 | 1086.1982 | 0.0105 | 0 | 13 | 1.3e+02 | 1 | MPSSMTSGTR + 2 Oxidation (M) |
|  | 1397 | **433.0869** | **864.1589** | **862.9759** | **1.1830** | **0** | **13** | **1.2e+02** | **1** | **AAHPAGALR** |
|  | 37 | **363.0400** | **1086.0978** | **1085.1853** | **0.9125** | **0** | **13** | **1.3e+02** | **1** | **SEEVFDMTK** |
|  | 657 | **398.2124** | **1191.6150** | **1191.3367** | **0.2784** | **0** | **13** | **1.1e+02** | **1** | **WCGSINPSPCA + Carbamidomethyl (C)** |
|  | 1902 | **472.2298** | **1413.6673** | **1412.6126** | **1.0547** | **0** | **13** | **1.2e+02** | **1** | **EIVEHMVQHFK + Oxidation (M)** |
|  | 2777 | **593.6104** | **1185.2059** | **1185.3737** | **-0.1678** | **1** | **13** | **1.4e+02** | **1** | **TRQIAQEIVK** |
|  | 3091 | **669.4334** | **2005.2779** | **2006.1308** | **-0.8530** | **1** | **13** | **1.2e+02** | **1** | **ILAPSERDEGSYYCACD + 2 Carbamidomethyl (C)** |
|  | 108 | **366.9055** | **1097.6942** | **1097.3084** | **0.3858** | **0** | **13** | **1.4e+02** | **1** | **ALLCEDMFR** |
|  | 2073 | 487.8854 | 1460.6341 | 1459.6044 | 1.0298 | 0 | 13 | 1.4e+02 | 1 | SPEECPPLCSQR + 2 Carbamidomethyl (C) |
|  | 2200 | **500.5155** | **999.0162** | **999.0760** | **-0.0597** | **0** | **13** | **1.3e+02** | **1** | **AIPSGGDPSAK** |
|  | 2562 | **550.8768** | **1099.7388** | **1100.1714** | **-0.4327** | **0** | **13** | **1.1e+02** | **1** | **HCPGEHAHR + Carbamidomethyl (C)** |
|  | 2986 | 635.3240 | 1902.9498 | 1903.0210 | -0.0713 | 2 | 13 | 1.2e+02 | 1 | DAQAGKEPGGGRAHSSHLK |
|  | 1095 | **413.2588** | **1236.7543** | **1236.4405** | **0.3138** | **0** | **13** | **86** | **1** | **AHKPCEIDPVK** |
|  | 1369 | **431.0011** | **859.9874** | **858.9395** | **1.0479** | **1** | **13** | **1.5e+02** | **1** | **KSSGPVER** |
|  | 1701 | **456.5406** | **911.0664** | **912.1047** | **-1.0383** | **0** | **13** | **1.4e+02** | **1** | **LLTMGSFK + Oxidation (M)** |
|  | 1721 | **458.0411** | **914.0674** | **915.0058** | **-0.9385** | **1** | **13** | **1.3e+02** | **1** | **ILDRENR** |
|  | 1035 | **408.9753** | **815.9359** | **815.9178** | **0.0181** | **1** | **13** | **1.4e+02** | **1** | **KQLGAGSR** |
|  | 1136 | **416.0260** | **1245.0559** | **1245.4291** | **-0.3732** | **1** | **13** | **1.5e+02** | **1** | **SQAKMSGMFAR + 2 Oxidation (M)** |
|  | 1684 | **454.3253** | **1359.9538** | **1359.5564** | **0.3974** | **1** | **13** | **1.1e+02** | **1** | **LFDNAMLRAHR + Oxidation (M)** |
|  | 1746 | **458.8192** | **915.6237** | **915.0422** | **0.5815** | **0** | **13** | **1.3e+02** | **1** | **ILNADLEK** |
|  | 2925 | **615.4282** | **1228.8415** | **1229.3433** | **-0.5018** | **1** | **13** | **1.2e+02** | **1** | **NNDIRLELSR** |
|  | 533 | **388.2350** | **1161.6828** | **1161.2928** | **0.3900** | **1** | **13** | **1.2e+02** | **1** | **ARVPGGCTSGTR** |
|  | 1640 | **450.1060** | **1347.2958** | **1348.4825** | **-1.1867** | **0** | **13** | **1.2e+02** | **1** | **TQTHATLCSTSAK** |
|  | 1237 | **421.1186** | **840.2224** | **841.0069** | **-0.7845** | **0** | **13** | **1.3e+02** | **1** | **NITLPGVK** |
|  | 1692 | **456.1033** | **1365.2876** | **1364.6128** | **0.6749** | **1** | **13** | **1.2e+02** | **1** | **GLERDMPKPPPK** |
|  | 3430 | **817.6918** | **2450.0533** | **2450.7061** | **-0.6528** | **1** | **13** | **1e+02** | **1** | **GGCNEVNQCLSTTQNKIFQTHK** |
|  | 541 | **388.4782** | **774.9416** | **775.8954** | **-0.9537** | **0** | **13** | **1.8e+02** | **1** | **QLHPPGK** |
|  | 1183 | **419.1067** | **1254.2979** | **1255.4884** | **-1.1906** | **1** | **13** | **1.5e+02** | **1** | **VKAGTCCATCK + 3 Carbamidomethyl (C)** |
|  | 1412 | 434.1408 | 1299.4003 | 1300.4164 | -1.0161 | 0 | 13 | 1.2e+02 | 1 | CCTEGGISSTTK + 2 Carbamidomethyl (C) |
|  | 2594 | **555.9244** | **1664.7511** | **1665.8836** | **-1.1324** | **0** | **13** | **1.2e+02** | **1** | **NVQFVFDAVTDGIIK** |
|  | 298 | **376.2815** | **750.5483** | **750.8646** | **-0.3163** | **0** | **13** | **97** | **1** | **MTGSTVR** |
|  | 309 | **377.1358** | **1128.3852** | **1129.3087** | **-0.9235** | **1** | **13** | **1.2e+02** | **1** | **KFHILSQEK** |
|  | 871 | **406.0535** | **1215.1382** | **1215.3418** | **-0.2035** | **1** | **13** | **1.2e+02** | **1** | **SEGCNRMSCR + Carbamidomethyl (C); Oxidation (M)** |
|  | 901 | **406.2912** | **1215.8515** | **1216.3399** | **-0.4883** | **1** | **13** | **96** | **1** | **SGYVFGTGTKVT** |
|  | 283 | **375.1303** | **1122.3688** | **1123.1720** | **-0.8032** | **0** | **13** | **1.4e+02** | **1** | **ASSLESTVSSR** |
|  | 2891 | 611.9609 | 1832.8605 | 1833.9956 | -1.1351 | 1 | 13 | 1.1e+02 | 1 | VIPEDGPAAQNPENVKR |
|  | 285 | **375.1547** | **1122.4420** | **1121.3513** | **1.0906** | **0** | **13** | **1.3e+02** | **1** | **YHAIVYPMK** |
|  | 841 | **405.1830** | **1212.5270** | **1211.3699** | **1.1571** | **2** | **13** | **1.1e+02** | **1** | **KEREMAEMR + 2 Oxidation (M)** |
|  | 1814 | **462.0245** | **922.0342** | **923.0280** | **-0.9938** | **1** | **13** | **1.2e+02** | **1** | **LKSSHHSK** |
|  | 175 | **369.2688** | **1104.7843** | **1105.3089** | **-0.5246** | **0** | **13** | **1.1e+02** | **1** | **AKPWAVCFPS** |
|  | 333 | **378.1920** | **1131.5537** | **1131.3147** | **0.2390** | **2** | **13** | **93** | **1** | **ARWQQRMR** |
|  | 397 | **384.9727** | **1151.8958** | **1151.3575** | **0.5383** | **0** | **13** | **1.1e+02** | **1** | **IGTNQMAVCAK + Oxidation (M)** |
|  | 477 | **387.0059** | **1157.9954** | **1157.3438** | **0.6517** | **0** | **13** | **1.4e+02** | **1** | **ILNHSTSVMR** |
|  | 1410 | **433.8062** | **1298.3965** | **1298.4057** | **-0.0092** | **1** | **13** | **1.2e+02** | **1** | **VSEEGPAARAGVR** |
|  | 1486 | **437.2111** | **1308.6111** | **1309.5108** | **-0.8997** | **0** | **13** | **1.4e+02** | **1** | **LTLIALDGGSPPR** |
|  | 789 | **404.0249** | **1209.0525** | **1209.4631** | **-0.4105** | **0** | **13** | **1.3e+02** | **1** | **ALPCPAHVVFR** |
|  | 1476 | **437.0369** | **872.0589** | **872.0244** | **0.0346** | **2** | **13** | **1.4e+02** | **1** | **KAEARVAK** |
|  | 179 | **369.2726** | **1104.7958** | **1105.3089** | **-0.5131** | **0** | **13** | **1.1e+02** | **1** | **AKPWAVCFPS** |
|  | 1270 | **423.1483** | **844.2818** | **844.0341** | **0.2478** | **1** | **13** | **1.5e+02** | **1** | **LPPSMRK + Oxidation (M)** |
|  | 3281 | **742.0635** | **1482.1123** | **1482.5550** | **-0.4427** | **1** | **13** | **98** | **1** | **GESGEPGPKGQQGVR** |
|  | 886 | **406.1905** | **1215.5493** | **1215.3136** | **0.2357** | **1** | **13** | **1.2e+02** | **1** | **RLDGIEEVER** |
|  | 1362 | **430.2205** | **1287.6393** | **1287.4458** | **0.1935** | **1** | **13** | **1.3e+02** | **1** | **RLLPDSSSGCPR** |
|  | 1519 | **440.1080** | **1317.3017** | **1317.5347** | **-0.2330** | **0** | **13** | **1.5e+02** | **1** | **HIMAEMIATER + Oxidation (M)** |
|  | 448 | **386.0998** | **1155.2772** | **1155.3247** | **-0.0475** | **2** | **13** | **1.2e+02** | **1** | **KSGMAFKSGDK** |
|  | 1918 | **472.8979** | **1415.6714** | **1416.6028** | **-0.9314** | **1** | **13** | **1.3e+02** | **1** | **NAIPAPTTTKSCR + Carbamidomethyl (C)** |
|  | 2033 | **484.4193** | **1450.2358** | **1449.6261** | **0.6096** | **0** | **13** | **1e+02** | **1** | **VISALAEVDNCTSK** |
|  | 2275 | **512.3314** | **1533.9719** | **1532.7827** | **1.1893** | **0** | **13** | **1.1e+02** | **1** | **AEMTLMTNFFATR** |
|  | 2988 | **636.2570** | **1905.7487** | **1906.3232** | **-0.5745** | **2** | **13** | **1.3e+02** | **1** | **ISFVCQIVRVGRMELR** |
|  | 3487 | **898.1267** | **2691.3580** | **2691.0846** | **0.2733** | **1** | **13** | **1.1e+02** | **1** | **AASLMNIPSTSSCLTESPFKVHLEK** |
|  | 438 | **386.0697** | **1155.1868** | **1154.4026** | **0.7842** | **1** | **13** | **1.2e+02** | **1** | **CAGMKQIFSL + Carbamidomethyl (C)** |
|  | 2452 | **534.1016** | **1066.1883** | **1066.3192** | **-0.1308** | **0** | **13** | **1.4e+02** | **1** | **MVHVPGLWK** |
|  | 2477 | **536.2448** | **1070.4747** | **1070.1987** | **0.2761** | **0** | **13** | **1.3e+02** | **1** | **MSSQCLETR + Oxidation (M)** |
|  | 310 | **377.1374** | **1128.3901** | **1129.3732** | **-0.9831** | **0** | **13** | **1.2e+02** | **1** | **LSPLSLCGLAR** |
|  | 2342 | **519.2588** | **1554.7542** | **1553.7157** | **1.0385** | **1** | **13** | **1.3e+02** | **1** | **IVEVGPRDGLQNEK** |
|  | 3280 | **742.0468** | **1482.0788** | **1482.6874** | **-0.6085** | **1** | **13** | **98** | **1** | **SCRGDPAGLPPCPR + Carbamidomethyl (C)** |
|  | 1842 | **465.7951** | **1394.3631** | **1394.5990** | **-0.2358** | **2** | **13** | **1.2e+02** | **1** | **QFKVRQAECTK + Carbamidomethyl (C)** |
|  | 31 | **362.3542** | **722.6935** | **722.8114** | **-0.1178** | **0** | **13** | **1.3e+02** | **1** | **MSVQSR + Oxidation (M)** |
|  | 1695 | **456.1851** | **1365.5332** | **1366.6549** | **-1.1218** | **2** | **13** | **1.2e+02** | **1** | **CALCAEIFKRR + Carbamidomethyl (C)** |
|  | 2386 | **523.2510** | **1044.4873** | **1045.1476** | **-0.6603** | **0** | **13** | **1.4e+02** | **1** | **SLAAAGELSAR** |
|  | 731 | **401.8987** | **801.7827** | **800.8140** | **0.9687** | **0** | **13** | **1.5e+02** | **1** | **APVDEDR** |
|  | 1536 | **441.3122** | **880.6096** | **880.9666** | **-0.3570** | **1** | **13** | **1e+02** | **1** | **EAGSSMRK + Oxidation (M)** |
|  | 2300 | **517.2037** | **1548.5890** | **1549.7055** | **-1.1164** | **0** | **13** | **1.4e+02** | **1** | **GFTVSSNYMSWVR + Oxidation (M)** |
|  | 3268 | **741.6592** | **2221.9554** | **2222.6303** | **-0.6749** | **2** | **13** | **1e+02** | **1** | **HVKDKNIIELVHQVSMGMK + Oxidation (M)** |
|  | 2205 | **501.5337** | **1501.5790** | **1502.7034** | **-1.1245** | **2** | **13** | **1.6e+02** | **1** | **GWRTGVQRGCGLR + Carbamidomethyl (C)** |
|  | 2830 | **602.5334** | **1203.0520** | **1203.3955** | **-0.3435** | **1** | **13** | **1.1e+02** | **1** | **ACCHSTGAGKLR** |
|  | 897 | **406.2392** | **1215.6954** | **1215.5486** | **0.1468** | **2** | **13** | **1e+02** | **1** | **LLALCKKEIK + Carbamidomethyl (C)** |
|  | 2792 | **594.9403** | **1781.7988** | **1781.8682** | **-0.0694** | **0** | **13** | **1.3e+02** | **1** | **EEFTDEEMQYDMAK + Oxidation (M)** |
|  | 239 | **372.3509** | **1114.0305** | **1114.2280** | **-0.1975** | **0** | **13** | **1.3e+02** | **1** | **QWMEVATFS + Oxidation (M)** |
|  | 1297 | **426.1615** | **850.3083** | **851.0416** | **-0.7333** | **0** | **13** | **1.3e+02** | **1** | **DVPLPVVI** |
|  | 1025 | **408.4179** | **1222.2316** | **1222.4818** | **-0.2502** | **2** | **13** | **1.6e+02** | **1** | **GCKVMASGKLR + Carbamidomethyl (C); Oxidation (M)** |
|  | 1307 | **427.2179** | **1278.6314** | **1278.4374** | **0.1940** | **1** | **13** | **1.1e+02** | **1** | **FPMNSRQPGTK + Oxidation (M)** |
|  | 2873 | **610.1119** | **1827.3135** | **1826.2316** | **1.0818** | **1** | **13** | **1.3e+02** | **1** | **LIKPYTRIHIPFISK** |
|  | 3252 | **740.6485** | **1479.2822** | **1478.6505** | **0.6317** | **0** | **13** | **1e+02** | **1** | **FICDCTGTGYWGR** |
|  | 1945 | **475.7073** | **1424.0998** | **1423.4879** | **0.6120** | **2** | **13** | **1.1e+02** | **1** | **RSDPGTSGEAYKR** |
|  | 1961 | **476.3317** | **950.6485** | **950.1179** | **0.5306** | **2** | **13** | **1e+02** | **1** | **SLTSCRKR** |
|  | 2540 | **547.9809** | **1093.9470** | **1093.2303** | **0.7167** | **1** | **13** | **1.3e+02** | **1** | **EAFATKGELK** |
|  | 767 | **403.1880** | **1206.5419** | **1207.3793** | **-0.8375** | **1** | **13** | **1.4e+02** | **1** | **RLEIEHSVPK** |
|  | 1001 | **408.0453** | **1221.1138** | **1221.3147** | **-0.2010** | **0** | **13** | **1.3e+02** | **1** | **IWAEYDPEAK** |
|  | 2531 | **547.2534** | **1638.7379** | **1639.9327** | **-1.1948** | **1** | **13** | **1.4e+02** | **1** | **LSAASVGDMVMATVKK + 2 Oxidation (M)** |
|  | 2553 | **550.0248** | **1647.0522** | **1645.8958** | **1.1564** | **2** | **13** | **1.3e+02** | **1** | **MKEMYQSAEAKVSK + Oxidation (M)** |
|  | 1739 | **458.5547** | **1372.6420** | **1371.5440** | **1.0980** | **0** | **13** | **1.7e+02** | **1** | **LGSVTQGFRPPGR** |
|  | 2453 | **534.1779** | **1599.5116** | **1599.0541** | **0.4575** | **0** | **13** | **1.4e+02** | **1** | **LGGILPLVTILQCMK** |
|  | 3250 | **740.6196** | **2218.8367** | **2218.4894** | **0.3473** | **2** | **13** | **1.1e+02** | **1** | **QGPPPSEKECAPTPAPVTRAK + Carbamidomethyl (C)** |
|  | 216 | **371.1349** | **1110.3825** | **1111.2505** | **-0.8680** | **1** | **13** | **1.1e+02** | **1** | **ITFARDNFK** |
|  | 1324 | **428.2186** | **1281.6335** | **1282.5170** | **-0.8834** | **2** | **13** | **1.1e+02** | **1** | **CLRFFQQRK + Carbamidomethyl (C)** |
|  | 55 | **363.2039** | **1086.5896** | **1087.2308** | **-0.6413** | **2** | **13** | **1.1e+02** | **1** | **SKATPAARASK** |
|  | 316 | **377.2059** | **752.3970** | **752.9052** | **-0.5082** | **0** | **13** | **1e+02** | **1** | **KPAKPGR** |
|  | 420 | **385.2137** | **1152.6189** | **1152.4118** | **0.2071** | **1** | **13** | **93** | **1** | **KSVSCLCCPR + Carbamidomethyl (C)** |
|  | 1134 | **415.9845** | **1244.9312** | **1245.4754** | **-0.5441** | **1** | **13** | **1.6e+02** | **1** | **LSHNTMMKQR** |
|  | 1337 | 428.9666 | 855.9184 | 854.9077 | 1.0108 | 0 | 13 | 1.3e+02 | 1 | TNEAVGHK |
|  | 1854 | **466.3945** | **1396.1612** | **1395.6269** | **0.5344** | **2** | **13** | **1.2e+02** | **1** | **GQEVYVKKTMGR** |
|  | 2716 | **584.1112** | **1749.3114** | **1750.1356** | **-0.8242** | **2** | **13** | **1.3e+02** | **1** | **FKRPDELLHLLKLK** |
|  | 3508 | **1062.0319** | **3183.0734** | **3182.5736** | **0.4998** | **1** | **13** | **96** | **1** | **CSCNNGEMCDRFQGCLCSPGWQGLQCER + Carbamidomethyl (C)** |
|  | 449 | **386.1061** | **1155.2962** | **1154.3365** | **0.9597** | **0** | **13** | **1.2e+02** | **1** | **MTYALVSNQK** |
|  | 1336 | **428.9160** | **855.8172** | **854.8678** | **0.9494** | **0** | **13** | **1.2e+02** | **1** | **NAAGHTER** |
|  | 1652 | **450.5892** | **899.1637** | **899.0477** | **0.1159** | **0** | **13** | **1.4e+02** | **1** | **WPLGLASR** |
|  | 2578 | 553.6078 | 1657.8012 | 1656.9448 | 0.8564 | 2 | 13 | 1.7e+02 | 1 | MNPRTKLLTDVGPAK + Oxidation (M) |
|  | 1182 | **419.0564** | **1254.1469** | **1253.5537** | **0.5933** | **2** | **13** | **1.6e+02** | **1** | **LKMLSYLKNK + Oxidation (M)** |
|  | 1841 | **465.3063** | **1392.8966** | **1392.5168** | **0.3798** | **1** | **13** | **1.1e+02** | **1** | **EKEIHQSHVASK** |
|  | 3018 | **656.0885** | **1965.2433** | **1964.3162** | **0.9272** | **2** | **13** | **1.3e+02** | **1** | **DKNVMMSARTLIHLFR + 2 Oxidation (M)** |
|  | 372 | **382.0669** | **1143.1785** | **1142.2582** | **0.9203** | **0** | **13** | **1.6e+02** | **1** | **MTAEPMSESK + 2 Oxidation (M)** |
|  | 620 | **392.5954** | **1174.7641** | **1175.5049** | **-0.7408** | **0** | **13** | **97** | **1** | **MSPAMLSVLVK** |
|  | 648 | **396.2007** | **1185.5798** | **1185.3292** | **0.2506** | **0** | **13** | **1.3e+02** | **1** | **METASTGMGIR + 2 Oxidation (M)** |
|  | 961 | **407.5351** | **1219.5830** | **1219.3685** | **0.2145** | **0** | **13** | **1.4e+02** | **1** | **MAAADTAAAASIR** |
|  | 1850 | **466.1230** | **1395.3470** | **1394.5096** | **0.8374** | **0** | **13** | **1.4e+02** | **1** | **NSMFVHQDSVSK + Oxidation (M)** |
|  | 3253 | **740.6505** | **1479.2861** | **1478.7949** | **0.4913** | **1** | **13** | **1.1e+02** | **1** | **APSDLLVLTKMFK + Oxidation (M)** |
|  | 593 | **389.7500** | **1166.2278** | **1167.3586** | **-1.1307** | **0** | **13** | **1.3e+02** | **1** | **HSISVSAVIVR** |
|  | 879 | **406.1128** | **1215.3163** | **1216.4506** | **-1.1343** | **0** | **13** | **1.3e+02** | **1** | **LLEQMGALAVR + Oxidation (M)** |
|  | 1858 | **467.2177** | **1398.6309** | **1399.6121** | **-0.9812** | **1** | **13** | **1.4e+02** | **1** | **MYYKFSGFTQK** |
|  | 2526 | **546.4124** | **1636.2149** | **1635.8888** | **0.3261** | **2** | **13** | **1.1e+02** | **1** | **SLLQCQYRNINRK** |
|  | 105 | **366.2806** | **730.5464** | **730.7702** | **-0.2239** | **0** | **13** | **1.2e+02** | **1** | **GLSGNAGR** |
|  | 617 | **392.3447** | **1174.0119** | **1173.4224** | **0.5895** | **0** | **13** | **1.1e+02** | **1** | **IIGMETLIGIN** |
|  | 1047 | **409.2573** | **1224.7498** | **1225.4825** | **-0.7327** | **2** | **13** | **1.2e+02** | **1** | **MSTIRMKASGK + Oxidation (M)** |
|  | 1427 | **434.7490** | **1301.2249** | **1300.4179** | **0.8070** | **0** | **13** | **1e+02** | **1** | **IVGNGSEQQLQK** |
|  | 1883 | **470.0557** | **1407.1449** | **1406.6676** | **0.4773** | **0** | **13** | **1.2e+02** | **1** | **QLEVLVSPTCSCK** |
|  | 2011 | **480.6996** | **1439.0768** | **1438.6281** | **0.4486** | **0** | **13** | **1.2e+02** | **1** | **QNQSSLSPVLLPR** |
|  | 2630 | **562.4357** | **1684.2848** | **1683.9220** | **0.3628** | **1** | **13** | **1.1e+02** | **1** | **TYPTVKICNYEGPAK** |
|  | 942 | **407.2532** | **1218.7374** | **1218.2679** | **0.4696** | **0** | **13** | **1e+02** | **1** | **SDEGLPDGLSTK** |
|  | 570 | 389.1619 | 1164.4634 | 1165.3180 | -0.8545 | 2 | 13 | 1.4e+02 | 1 | MEKKSEELR + Oxidation (M) |
|  | 1550 | 442.4751 | 1324.4030 | 1323.5226 | 0.8805 | 2 | 13 | 1.5e+02 | 1 | KSPQMKNCCNG + 2 Carbamidomethyl (C) |
|  | 1815 | **462.0361** | **1383.0860** | **1383.6358** | **-0.5498** | **1** | **13** | **1.3e+02** | **1** | **KSQVGAIIITPTR** |
|  | 2757 | **592.7087** | **1183.4027** | **1184.3063** | **-0.9036** | **2** | **13** | **1.6e+02** | **1** | **KSKQHQSSVR** |
|  | 572 | **389.1752** | **1164.5034** | **1163.3235** | **1.1800** | **0** | **13** | **1.4e+02** | **1** | **LRPSSLSVAFS** |
|  | 857 | **405.8978** | **809.7808** | **808.9286** | **0.8521** | **1** | **13** | **1.2e+02** | **1** | **HREVLR** |
|  | 1314 | **427.6496** | **1279.9266** | **1279.4451** | **0.4815** | **1** | **13** | **1.1e+02** | **1** | **LKALGDELHQR** |
|  | 1386 | **431.9974** | **861.9800** | **861.9368** | **0.0432** | **0** | **13** | **1.5e+02** | **1** | **TELSSTPK** |
|  | 3384 | **788.3964** | **2362.1669** | **2361.7639** | **0.4031** | **0** | **13** | **1.3e+02** | **1** | **MPSCSCALMAPCGPAAGPAAVER + 3 Carbamidomethyl (C)** |
|  | 3425 | **816.5750** | **2446.7029** | **2446.8483** | **-0.1455** | **2** | **13** | **1.3e+02** | **1** | **IEQCTRGSLFMCSIVQGCKR + 3 Carbamidomethyl (C); Oxidation (M)** |
|  | 3509 | **1068.3157** | **2134.6166** | **2134.4771** | **0.1395** | **1** | **13** | **1.1e+02** | **1** | **AMGKLGTSLSSGHVLMNGTLK + 2 Oxidation (M)** |
|  | 54 | **363.2028** | **1086.5863** | **1086.2228** | **0.3635** | **1** | **13** | **1.1e+02** | **1** | **DMNGKSLHGK** |
|  | 1623 | **448.9305** | **1343.7695** | **1344.5151** | **-0.7457** | **1** | **13** | **1.2e+02** | **1** | **QLQSGKALADWK** |
|  | 1811 | **461.8656** | **921.7164** | **921.0337** | **0.6827** | **2** | **13** | **1.3e+02** | **1** | **NRDGKMGK + Oxidation (M)** |
|  | 2733 | **589.4077** | **1765.2010** | **1764.0599** | **1.1411** | **1** | **13** | **1.3e+02** | **1** | **CVVMTQSPGPCRASVR + Carbamidomethyl (C); Oxidation (M)** |
|  | 41 | **363.0791** | **724.1435** | **723.9022** | **0.2413** | **0** | **13** | **1.4e+02** | **1** | **ECMVVK + Oxidation (M)** |
|  | 360 | **380.3162** | **1137.9264** | **1138.2543** | **-0.3279** | **0** | **13** | **1.4e+02** | **1** | **NMNGSYSLPR** |
|  | 1150 | **416.4188** | **830.8227** | **831.0122** | **-0.1894** | **1** | **13** | **1.8e+02** | **1** | **KSGVLTVK** |
|  | 2087 | **488.2529** | **974.4911** | **974.1326** | **0.3584** | **0** | **13** | **1.5e+02** | **1** | **EACSIPGIGK** |
|  | 2406 | **524.3573** | **1570.0497** | **1570.7196** | **-0.6698** | **0** | **13** | **1.2e+02** | **1** | **GFSGDMGILGDTGISK + Oxidation (M)** |
|  | 2969 | **630.3744** | **1258.7340** | **1258.4015** | **0.3325** | **2** | **13** | **1.4e+02** | **1** | **METKDTKYAR + Oxidation (M)** |
|  | 1953 | **476.1487** | **1425.4239** | **1425.5664** | **-0.1426** | **1** | **13** | **1.4e+02** | **1** | **ALCNGDYDRTIK + Carbamidomethyl (C)** |
|  | 2801 | **596.4545** | **1190.8942** | **1190.2924** | **0.6017** | **0** | **13** | **1.2e+02** | **1** | **CPSAHTAPGHR + Carbamidomethyl (C)** |
|  | 91 | **365.9480** | **1094.8218** | **1094.2646** | **0.5572** | **1** | **13** | **1.6e+02** | **1** | **EQNLPLPKR** |
|  | 759 | **403.0157** | **804.0167** | **803.9056** | **0.1111** | **1** | **13** | **1.6e+02** | **1** | **VATRNFP** |
|  | 782 | **403.9666** | **1208.8776** | **1208.1967** | **0.6809** | **0** | **13** | **1.4e+02** | **1** | **ANDHGYDNFR** |
|  | 2377 | 522.1971 | 1042.3794 | 1042.2761 | 0.1032 | 2 | 13 | 1.4e+02 | 1 | GKLKLQSIR |
|  | 23 | **362.0594** | **722.1041** | **722.8327** | **-0.7286** | **0** | **13** | **1.3e+02** | **1** | **HQPTLK** |
|  | 1105 | **413.5815** | **1237.7222** | **1238.3717** | **-0.6495** | **0** | **13** | **1e+02** | **1** | **FMAGEGGLDWR** |
|  | 1482 | 437.1614 | 1308.4621 | 1309.4795 | -1.0173 | 2 | 13 | 1.5e+02 | 1 | RGCGSRISGGMGR + Oxidation (M) |
|  | 1574 | **443.9342** | **1328.7804** | **1328.5112** | **0.2693** | **0** | **13** | **1.5e+02** | **1** | **MTNMGPVDTVTY** |
|  | 2368 | **521.3067** | **1560.8979** | **1559.9586** | **0.9394** | **0** | **13** | **1.3e+02** | **1** | **MMGICPSCALWGMK + 2 Oxidation (M)** |
|  | 2398 | **524.1746** | **1569.5017** | **1569.8693** | **-0.3676** | **2** | **13** | **1.5e+02** | **1** | **MIMSRMKVEQER + 2 Oxidation (M)** |
|  | 196 | **369.3581** | **736.7013** | **735.9160** | **0.7854** | **0** | **13** | **1.5e+02** | **1** | **VNICCK + Carbamidomethyl (C)** |
|  | 560 | **389.1063** | **1164.2966** | **1165.3690** | **-1.0724** | **1** | **13** | **1.5e+02** | **1** | **SRSPHPLCIR** |
|  | 1166 | **417.8861** | **833.7574** | **832.9235** | **0.8340** | **0** | **13** | **1.4e+02** | **1** | **GGPAWCDK** |
|  | 2210 | **502.5121** | **1504.5141** | **1503.7050** | **0.8091** | **0** | **13** | **1.6e+02** | **1** | **TPWAERPMCGPSR + Oxidation (M)** |
|  | 2637 | **564.3054** | **1689.8939** | **1689.8853** | **0.0086** | **0** | **13** | **1.5e+02** | **1** | **VMTQSPATLSLSPGER + Oxidation (M)** |
|  | 1568 | **443.8235** | **1328.4482** | **1328.5326** | **-0.0844** | **1** | **13** | **1.4e+02** | **1** | **EYGEMSKFLPK** |
|  | 601 | **389.9973** | **777.9799** | **778.8100** | **-0.8300** | **0** | **13** | **1.4e+02** | **1** | **AYPGDTR** |
|  | 2441 | **532.2029** | **1062.3910** | **1061.2529** | **1.1380** | **0** | **13** | **1.5e+02** | **1** | **SVLGGDCLLK + Carbamidomethyl (C)** |
|  | 3161 | **686.9381** | **2057.7922** | **2057.3498** | **0.4423** | **2** | **13** | **1.1e+02** | **1** | **HYEKKMAGSTLNFCLEI + Carbamidomethyl (C); Oxidation (M)** |
|  | 2289 | **514.1041** | **1539.2902** | **1538.7227** | **0.5675** | **2** | **13** | **1.3e+02** | **1** | **AYETSKMYRDFK** |
|  | 2705 | **581.3872** | **1741.1394** | **1741.8772** | **-0.7377** | **0** | **13** | **1.4e+02** | **1** | **RPTPMVPGAQEEEER + Oxidation (M)** |
|  | 3255 | **740.7401** | **2219.1982** | **2218.3304** | **0.8678** | **0** | **13** | **1.2e+02** | **1** | **ESHTPVSIQEEIVGDFSSEK** |
|  | 1168 | **417.9811** | **1250.9212** | **1250.3609** | **0.5603** | **0** | **13** | **1.5e+02** | **1** | **YMTGECNYGGR** |
|  | 2244 | **506.2081** | **1515.6021** | **1516.7004** | **-1.0983** | **2** | **13** | **1.3e+02** | **1** | **SHRCEEYCKAYK** |
|  | 2706 | **582.1195** | **1743.3363** | **1743.9573** | **-0.6209** | **2** | **13** | **1.5e+02** | **1** | **GVWQINLSKVDDNKK** |
|  | 3090 | **668.9709** | **2003.8907** | **2004.4014** | **-0.5108** | **1** | **13** | **1.1e+02** | **1** | **TALKQQGVLGLNMAPCMR + Carbamidomethyl (C); Oxidation (M)** |
|  | 3148 | **685.9654** | **2054.8740** | **2054.4817** | **0.3923** | **2** | **13** | **1.1e+02** | **1** | **LLLTRFIFCSATMRTHK + Oxidation (M)** |
|  | 204 | **370.1425** | **1107.4052** | **1107.2601** | **0.1450** | **0** | **13** | **1.2e+02** | **1** | **LLQFVTGSSR** |
|  | 640 | **395.2700** | **788.5251** | **787.8616** | **0.6636** | **1** | **13** | **1.3e+02** | **1** | **AEGKVER** |
|  | 2305 | **517.6406** | **1549.8995** | **1549.7055** | **0.1941** | **0** | **13** | **1.7e+02** | **1** | **ECDMCFSQASSLR + Carbamidomethyl (C); Oxidation (M)** |
|  | 2778 | **593.6141** | **1185.2134** | **1185.3904** | **-0.1770** | **1** | **13** | **1.6e+02** | **1** | **SFSLKESIMK + Oxidation (M)** |
|  | 3061 | **667.1165** | **1998.3274** | **1998.2333** | **0.0941** | **0** | **13** | **1.4e+02** | **1** | **TIDYEEFVAMMTGESFK** |
|  | 254 | **373.4444** | **1117.3109** | **1117.3593** | **-0.0484** | **1** | **13** | **2.1e+02** | **1** | **LIKLQDMEK** |
|  | 258 | **374.0615** | **1119.1623** | **1119.2496** | **-0.0873** | **0** | **13** | **1.7e+02** | **1** | **MDAPTSAAVTR** |
|  | 2359 | **520.6939** | **1039.3730** | **1039.1896** | **0.1835** | **1** | **13** | **1.3e+02** | **1** | **VPGVQGQARK** |
|  | 2929 | **616.3396** | **1230.6644** | **1231.4008** | **-0.7363** | **0** | **13** | **1.5e+02** | **1** | **FLASVPGTGVQR** |
|  | 666 | **399.1044** | **796.1940** | **796.8948** | **-0.7008** | **0** | **13** | **1.3e+02** | **1** | **HSPPMGR + Oxidation (M)** |
|  | 1495 | **437.6392** | **873.2636** | **873.0123** | **0.2513** | **2** | **13** | **1.3e+02** | **1** | **KLNQSRK** |
|  | 3060 | **667.0448** | **1998.1122** | **1997.1475** | **0.9648** | **2** | **13** | **1.3e+02** | **1** | **RMSKSVPAFLQDESDDR + Oxidation (M)** |
|  | 3476 | **871.8134** | **2612.4179** | **2613.0208** | **-0.6029** | **1** | **13** | **1.1e+02** | **1** | **GICPKQENIAGSQPHTKPSVFVMK + Oxidation (M)** |
|  | 1682 | **454.2068** | **1359.5981** | **1358.5850** | **1.0131** | **2** | **13** | **1.5e+02** | **1** | **EDFPKCKGCFK + Carbamidomethyl (C)** |
|  | 1813 | **462.0176** | **922.0204** | **923.0679** | **-1.0475** | **1** | **13** | **1.4e+02** | **1** | **YAVVTRSK** |
|  | 3155 | **686.0989** | **2055.2745** | **2055.2000** | **0.0744** | **1** | **13** | **1.4e+02** | **1** | **KSAAAEAPTDDQPTDLSLPK** |
|  | 217 | **371.1856** | **1110.5347** | **1110.3254** | **0.2094** | **0** | **13** | **95** | **1** | **AMLSYVWPK + Oxidation (M)** |
|  | 1000 | **408.0251** | **814.0354** | **814.9332** | **-0.8977** | **1** | **13** | **1.4e+02** | **1** | **SVARIGGR** |
|  | 1764 | **459.2068** | **1374.5983** | **1375.6817** | **-1.0834** | **1** | **13** | **1.6e+02** | **1** | **DLKCCMGMCGK + 3 Carbamidomethyl (C); Oxidation (M)** |
|  | 2096 | **488.4520** | **974.8891** | **974.0666** | **0.8226** | **0** | **13** | **1.4e+02** | **1** | **DSSVAAPSLK** |
|  | 405 | **385.0248** | **1152.0522** | **1152.3734** | **-0.3213** | **1** | **13** | **1.2e+02** | **1** | **HGILGACGRLR** |
|  | 884 | **406.1772** | **1215.5093** | **1214.3984** | **1.1109** | **1** | **13** | **1.3e+02** | **1** | **MAAARAAPAAAAR + Oxidation (M)** |
|  | 843 | **405.1985** | **1212.5734** | **1211.4176** | **1.1557** | **2** | **13** | **1.2e+02** | **1** | **KVGAAGGARGVIR** |
|  | 878 | **406.1008** | **1215.2803** | **1216.4127** | **-1.1323** | **1** | **13** | **1.4e+02** | **1** | **RMHAGEKPYK** |
|  | 931 | **407.1594** | **1218.4559** | **1217.3327** | **1.1232** | **1** | **13** | **1.4e+02** | **1** | **GATRDIGSALTR** |
|  | 2455 | **534.3445** | **1066.6742** | **1067.2594** | **-0.5852** | **0** | **13** | **1.4e+02** | **1** | **MVPQVKPGPD** |
|  | 1327 | **428.3007** | **1281.8800** | **1282.3896** | **-0.5096** | **1** | **13** | **1e+02** | **1** | **GMRADSAPGGHAR** |
|  | 1587 | **445.1208** | **888.2267** | **888.0436** | **0.1832** | **1** | **13** | **1.7e+02** | **1** | **QSMKGPPK + Oxidation (M)** |
|  | 305 | **377.1197** | **1128.3370** | **1128.2365** | **0.1005** | **1** | **13** | **1.4e+02** | **1** | **VPSAGDVEKAR** |
|  | 2487 | **537.9896** | **1610.9467** | **1611.6889** | **-0.7423** | **1** | **13** | **1.5e+02** | **1** | **MAREAEFEAEQER + Oxidation (M)** |
|  | 624 | **393.4354** | **1177.2841** | **1176.3671** | **0.9170** | **1** | **13** | **1.7e+02** | **1** | **TMDVCPKGPR + Carbamidomethyl (C); Oxidation (M)** |
|  | 982 | **407.7998** | **1220.3772** | **1219.3205** | **1.0567** | **1** | **13** | **1.5e+02** | **1** | **AEDTAKYFCAT** |
|  | 1128 | **415.5773** | **1243.7098** | **1243.4114** | **0.2984** | **2** | **13** | **1.5e+02** | **1** | **KSKNYATIYR** |
|  | 1300 | **426.2704** | **850.5260** | **850.0201** | **0.5059** | **0** | **13** | **1.2e+02** | **1** | **AQLIIHR** |
|  | 1638 | **450.0679** | **1347.1815** | **1346.3705** | **0.8111** | **0** | **13** | **1.4e+02** | **1** | **HAPEHHPGHGDR** |
|  | 1751 | **458.9391** | **915.8634** | **916.0736** | **-0.2102** | **0** | **13** | **1.6e+02** | **1** | **MAQEYMK + Oxidation (M)** |
|  | 2072 | **487.7388** | **973.4628** | **973.0883** | **0.3744** | **1** | **13** | **1.3e+02** | **1** | **SSGVGAARLR** |
|  | 2570 | **552.0959** | **1102.1771** | **1101.2541** | **0.9230** | **1** | **13** | **1.6e+02** | **1** | **ESITVKTAPR** |
|  | 2618 | **560.3997** | **1118.7845** | **1118.2200** | **0.5646** | **0** | **13** | **1.3e+02** | **1** | **DSAVYFCASR** |
|  | 3031 | **666.2448** | **1995.7121** | **1995.3101** | **0.4019** | **2** | **13** | **1.4e+02** | **1** | **LLVQRLNMGTQGDLHRK + Oxidation (M)** |
|  | 994 | **407.9263** | **813.8378** | **812.9090** | **0.9288** | **0** | **13** | **1.4e+02** | **1** | **FQIDYK** |
|  | 1219 | **420.1372** | **1257.3895** | **1256.4715** | **0.9181** | **0** | **13** | **1.4e+02** | **1** | **MLQLGPPLSER + Oxidation (M)** |
|  | 3427 | **816.8895** | **2447.6464** | **2447.8514** | **-0.2050** | **1** | **13** | **1.6e+02** | **1** | **MDYLRCAAGSCIPSAIVSFTVSR** |
|  | 910 | **406.8846** | **1217.6318** | **1217.3477** | **0.2840** | **0** | **13** | **1.3e+02** | **1** | **AEDTAVFYCAK** |
|  | 407 | **385.0321** | **1152.0742** | **1151.4668** | **0.6075** | **0** | **13** | **1.2e+02** | **1** | **TNVALMCMLR** |
|  | 2898 | **612.2137** | **1222.4127** | **1222.3775** | **0.0352** | **1** | **13** | **1.5e+02** | **1** | **MHRAVDPPGAR + Oxidation (M)** |
|  | 2943 | **622.2567** | **1242.4986** | **1241.4352** | **1.0634** | **1** | **13** | **1.5e+02** | **1** | **YGTDLLLYRK** |
|  | 2954 | **627.2900** | **1878.8479** | **1878.2683** | **0.5797** | **1** | **13** | **1.4e+02** | **1** | **AVVACFRMAPLYNLPR + Carbamidomethyl (C)** |
|  | 3094 | **669.5345** | **2005.5813** | **2005.2770** | **0.3043** | **1** | **13** | **1.2e+02** | **1** | **EEISSPDMCPRPWKWK + Oxidation (M)** |
|  | 1340 | 429.0416 | 1284.1027 | 1284.4849 | -0.3822 | 0 | 13 | 1.4e+02 | 1 | LNIPMSQVNPR + Oxidation (M) |
|  | 2094 | **488.4260** | **1462.2557** | **1461.5574** | **0.6983** | **1** | **13** | **1.3e+02** | **1** | **GDSGSTPMPRDGLR + Oxidation (M)** |
|  | 2296 | **515.7935** | **1544.3582** | **1543.7190** | **0.6392** | **1** | **13** | **1.2e+02** | **1** | **TLSSKISYNDFLR** |
|  | 2446 | **532.9520** | **1595.8337** | **1595.7803** | **0.0535** | **1** | **13** | **1.5e+02** | **1** | **GFPGCNKNATLGFGR + Carbamidomethyl (C)** |
|  | 988 | **407.8371** | **1220.4892** | **1221.4093** | **-0.9200** | **1** | **13** | **1.5e+02** | **1** | **GRAPLDAPAVVR** |
|  | 2327 | **518.9028** | **1035.7909** | **1036.2239** | **-0.4330** | **1** | **13** | **1.4e+02** | **1** | **GMDPVDKMK + Oxidation (M)** |
|  | 3077 | 668.4992 | 2002.4754 | 2001.2880 | 1.1874 | 1 | 13 | 1.3e+02 | 1 | ACPNPGKNGLSNSCILLDK + Carbamidomethyl (C) |
|  | 355 | **380.2244** | **1137.6509** | **1138.1848** | **-0.5338** | **0** | **13** | **1.3e+02** | **1** | **FNTENLGESK** |
|  | 602 | **389.9978** | **1166.9711** | **1167.3221** | **-0.3509** | **2** | **13** | **1.5e+02** | **1** | **KRQSVSGLHR** |
|  | 1311 | **427.3977** | **1279.1709** | **1279.4435** | **-0.2726** | **0** | **13** | **1.2e+02** | **1** | **LLSDCANVCER + Carbamidomethyl (C)** |
|  | 3208 | **725.7393** | **1449.4637** | **1448.6630** | **0.8008** | **0** | **13** | **1.4e+02** | **1** | **LPGAEVGVPQVSAPK** |
|  | 1593 | **445.2076** | **888.4004** | **888.1263** | **0.2741** | **0** | **13** | **1.6e+02** | **1** | **MLPSLLSK** |
|  | 934 | **407.1922** | **1218.5544** | **1218.3176** | **0.2369** | **1** | **13** | **1.3e+02** | **1** | **TLVEQRSGGSGK** |
|  | 2535 | **547.4854** | **1639.4339** | **1639.7632** | **-0.3294** | **0** | **13** | **1.2e+02** | **1** | **GLDWLAVISHDGDNK** |
|  | 966 | **407.6028** | **1219.7864** | **1219.4812** | **0.3052** | **2** | **13** | **1.2e+02** | **1** | **MMKLSNRAPR + Oxidation (M)** |
|  | 387 | **384.3082** | **766.6016** | **765.9039** | **0.6977** | **1** | **13** | **1.1e+02** | **1** | **RLPAPGR** |
|  | 1442 | **435.1971** | **1302.5693** | **1303.5146** | **-0.9454** | **1** | **13** | **1.4e+02** | **1** | **SCSRCLHTALR + Carbamidomethyl (C)** |
|  | 2317 | **518.7144** | **1553.1209** | **1552.6863** | **0.4346** | **0** | **13** | **1.3e+02** | **1** | **DNLMRPPGMTSSSQ + 2 Oxidation (M)** |
|  | 889 | **406.2131** | **1215.6171** | **1215.4444** | **0.1728** | **0** | **13** | **1.2e+02** | **1** | **LCPAMGYTFR + Carbamidomethyl (C)** |
|  | 2874 | **610.1373** | **1218.2598** | **1219.4082** | **-1.1485** | **1** | **13** | **1.5e+02** | **1** | **RIEILESECK** |
|  | 2163 | **495.4784** | **1483.4130** | **1483.5776** | **-0.1646** | **0** | **13** | **1.5e+02** | **1** | **WIDATSGIYNSEK** |
|  | 2581 | **553.8872** | **1658.6394** | **1658.7517** | **-0.1123** | **0** | **13** | **1.3e+02** | **1** | **QLAGGGAGTSSHQACTGR** |
|  | 357 | 380.2455 | 1137.7143 | 1137.3476 | 0.3667 | 1 | 13 | 1.4e+02 | 1 | ITMSVDLSKK + Oxidation (M) |
|  | 1289 | **425.3093** | **848.6039** | **848.9064** | **-0.3025** | **0** | **13** | **1.3e+02** | **1** | **AHSPGAGPR** |
|  | 1898 | **471.2645** | **1410.7714** | **1410.5387** | **0.2328** | **2** | **13** | **1.3e+02** | **1** | **IEKVANRGHSSGR** |
|  | 1956 | **476.2008** | **950.3868** | **950.0070** | **0.3799** | **0** | **13** | **1.5e+02** | **1** | **GPSNTAFTR** |
|  | 822 | **405.0041** | **1211.9901** | **1212.4256** | **-0.4355** | **1** | **13** | **1.4e+02** | **1** | **RLVAGQGCVGPR** |
|  | 1030 | **408.8590** | **815.7032** | **814.8438** | **0.8595** | **0** | **13** | **1.6e+02** | **1** | **GPSQVGDR** |
|  | 2097 | **488.5030** | **974.9912** | **975.0978** | **-0.1066** | **1** | **13** | **1.8e+02** | **1** | **KVVDNTTAK** |
|  | 2153 | **494.6365** | **1480.8873** | **1479.8094** | **1.0779** | **1** | **13** | **1.7e+02** | **1** | **MALRYPMAVGLNK + Oxidation (M)** |
|  | 2388 | **523.3387** | **1044.6626** | **1044.1662** | **0.4964** | **2** | **13** | **1.4e+02** | **1** | **LKGNNKSQR** |
|  | 3098 | **669.6956** | **2006.0645** | **2007.2084** | **-1.1439** | **2** | **13** | **1.6e+02** | **1** | **DHKSVTADTGKCGLSMWN + Carbamidomethyl (C)** |
|  | 1349 | **429.2469** | **1284.7186** | **1285.4480** | **-0.7294** | **0** | **13** | **1.3e+02** | **1** | **WEAALVAEQLR** |
|  | 1680 | **454.1796** | **1359.5167** | **1360.4484** | **-0.9317** | **0** | **13** | **1.6e+02** | **1** | **EQDVYICDYR + Carbamidomethyl (C)** |
|  | 2697 | **579.9424** | **1157.8701** | **1158.3055** | **-0.4354** | **2** | **13** | **1.5e+02** | **1** | **KSTPPSSAKQK** |
|  | 1534 | **441.1736** | **880.3324** | **880.0862** | **0.2462** | **0** | **13** | **1.5e+02** | **1** | **KPPKPVSK** |
|  | 1657 | **451.2185** | **1350.6333** | **1351.5345** | **-0.9011** | **1** | **13** | **1.6e+02** | **1** | **AASPPRMPGSQPR** |
|  | 1977 | **477.8528** | **953.6908** | **952.9743** | **0.7165** | **1** | **13** | **1.4e+02** | **1** | **GGHRGGGGGGGK** |
|  | 2092 | **488.3479** | **1462.0216** | **1461.6830** | **0.3386** | **1** | **13** | **1.3e+02** | **1** | **IRNTDINLLMDK + Oxidation (M)** |
|  | 2902 | **612.2546** | **1833.7417** | **1834.0900** | **-0.3482** | **2** | **13** | **1.5e+02** | **1** | **SCLPRVSGAMARNTAAR + Carbamidomethyl (C); Oxidation (M)** |
|  | 746 | **402.2068** | **1203.5982** | **1203.3891** | **0.2091** | **2** | **13** | **1.5e+02** | **1** | **EYKCTSCKK + 2 Carbamidomethyl (C)** |
|  | 1554 | **443.1732** | **1326.4973** | **1326.4140** | **0.0833** | **1** | **13** | **1.4e+02** | **1** | **GDTCGNCTKGEK + 2 Carbamidomethyl (C)** |
|  | 1847 | **466.0324** | **1395.0751** | **1394.6421** | **0.4330** | **2** | **13** | **1.6e+02** | **1** | **GAVETFMKLRAR + Oxidation (M)** |
|  | 306 | **377.1213** | **1128.3417** | **1127.3808** | **0.9609** | **1** | **12** | **1.4e+02** | **1** | **FKAMMASIGR + Oxidation (M)** |
|  | 433 | **386.0245** | **770.0343** | **770.9851** | **-0.9508** | **1** | **12** | **1.3e+02** | **1** | **CMSMRK + Oxidation (M)** |
|  | 1070 | **411.2631** | **1230.7671** | **1230.4324** | **0.3347** | **0** | **12** | **1.3e+02** | **1** | **LSCAASGFIFSK** |
|  | 1667 | **452.3403** | **1353.9987** | **1354.5103** | **-0.5115** | **1** | **12** | **1.4e+02** | **1** | **SLMNNSKEEMR + Oxidation (M)** |
|  | 3219 | **734.2305** | **1466.4462** | **1467.5205** | **-1.0743** | **1** | **12** | **1.4e+02** | **1** | **SDRYSGHDDLMR + Oxidation (M)** |
|  | 720 | **401.6319** | **801.2491** | **800.8569** | **0.3921** | **0** | **12** | **1.4e+02** | **1** | **ATIPEDR** |
|  | 869 | **406.0471** | **1215.1192** | **1215.3417** | **-0.2225** | **1** | **12** | **1.4e+02** | **1** | **RAAGSGSFFCR + Carbamidomethyl (C)** |
|  | 952 | **407.3832** | **1219.1275** | **1218.4252** | **0.7024** | **0** | **12** | **1.4e+02** | **1** | **LPQGTMFPATR** |
|  | 2424 | 528.3538 | 1582.0391 | 1582.7139 | -0.6748 | 0 | 12 | 1.2e+02 | 1 | MFGSATTDNHNPMK + 2 Oxidation (M) |
|  | 3138 | **684.1934** | **1366.3721** | **1365.5709** | **0.8011** | **0** | **12** | **1.4e+02** | **1** | **VEQPIIEEPALK** |
|  | 735 | **401.9571** | **801.8995** | **800.8835** | **1.0160** | **0** | **12** | **1.8e+02** | **1** | **DGVMAHR + Oxidation (M)** |
|  | 1477 | **437.0381** | **872.0614** | **871.0758** | **0.9856** | **0** | **12** | **1.7e+02** | **1** | **GLALTVGLK** |
|  | 2063 | **487.1359** | **1458.3856** | **1457.6580** | **0.7276** | **0** | **12** | **1.6e+02** | **1** | **ARPGAAAVCTLGGTR + Carbamidomethyl (C)** |
|  | 389 | **384.6456** | **767.2764** | **766.8853** | **0.3911** | **0** | **12** | **1.1e+02** | **1** | **GAAGLPGPK** |
|  | 777 | **403.9231** | **1208.7472** | **1209.4380** | **-0.6908** | **1** | **12** | **1.5e+02** | **1** | **LLLRGANPDLK** |
|  | 898 | **406.2572** | **810.4996** | **809.9318** | **0.5678** | **1** | **12** | **1.2e+02** | **1** | **KTSAMTR + Oxidation (M)** |
|  | 2086 | **488.2303** | **1461.6686** | **1461.6006** | **0.0680** | **2** | **12** | **1.6e+02** | **1** | **ARSESVGRMDEPK** |
|  | 2762 | **592.7858** | **1183.5569** | **1184.3659** | **-0.8090** | **2** | **12** | **1.4e+02** | **1** | **SFCDKSTLRK** |
|  | 914 | **406.9496** | **811.8843** | **812.9570** | **-1.0727** | **1** | **12** | **1.4e+02** | **1** | **KSPIAAAR** |
|  | 1404 | **433.1853** | **1296.5336** | **1296.4924** | **0.0413** | **0** | **12** | **1.5e+02** | **1** | **DVVYLQMSGLR + Oxidation (M)** |
|  | 2404 | **524.3167** | **1569.9280** | **1568.8960** | **1.0320** | **1** | **12** | **1.5e+02** | **1** | **VLEFLEKTCQVML + Oxidation (M)** |
|  | 3086 | **668.8169** | **2003.4285** | **2004.2575** | **-0.8289** | **2** | **12** | **1.7e+02** | **1** | **GWGSPAAVCSRNCTVSPRR** |
|  | 1258 | **422.1553** | **842.2959** | **841.9949** | **0.3009** | **0** | **12** | **1.7e+02** | **1** | **GITLSVRP** |
|  | 1891 | **470.3546** | **1408.0417** | **1407.7464** | **0.2953** | **1** | **12** | **1.1e+02** | **1** | **GARGALLLALLLAR** |
|  | 1966 | **476.9221** | **951.8295** | **951.0166** | **0.8130** | **1** | **12** | **1.5e+02** | **1** | **ACDTGGSGRK** |
|  | 2621 | **561.4139** | **1681.2195** | **1681.9145** | **-0.6950** | **2** | **12** | **1.3e+02** | **1** | **LDMPRGSLPSTAHRK + Oxidation (M)** |
|  | 3456 | **846.8043** | **2537.3906** | **2537.0472** | **0.3434** | **1** | **12** | **1.3e+02** | **1** | **SIGGAPTFNVIVTMTAKTLGLLMGK + Oxidation (M)** |
|  | 2082 | **488.1632** | **974.3117** | **975.0147** | **-0.7030** | **1** | **12** | **1.7e+02** | **1** | **TAIQREGNS** |
|  | 2821 | **598.3324** | **1791.9750** | **1792.1295** | **-0.1545** | **2** | **12** | **1.5e+02** | **1** | **SKIAVFDKMWTYMR + Oxidation (M)** |
|  | 3011 | **652.4316** | **1954.2728** | **1954.0597** | **0.2130** | **2** | **12** | **1.5e+02** | **1** | **EEEQEHSNKAPRALTSK** |
|  | 3221 | **736.0022** | **1469.9896** | **1470.7183** | **-0.7287** | **0** | **12** | **1.2e+02** | **1** | **AMGNRPMEMMDR + 2 Oxidation (M)** |
|  | 3290 | **742.7117** | **2225.1130** | **2224.5018** | **0.6112** | **0** | **12** | **1.2e+02** | **1** | **GQVPSHLHGSTPIHLGATAGMR** |
|  | 16 | **360.5424** | **1078.6049** | **1078.2406** | **0.3643** | **1** | **12** | **1.4e+02** | **1** | **RSLLEMEGK + Oxidation (M)** |
|  | 3207 | **723.8781** | **1445.7413** | **1446.6469** | **-0.9056** | **0** | **12** | **1.7e+02** | **1** | **GEKPYECYICGK + Carbamidomethyl (C)** |
|  | 3412 | **805.2447** | **1608.4746** | **1608.8008** | **-0.3262** | **2** | **12** | **1.5e+02** | **1** | **QTPSCWCAGKESRR** |
|  | 142 | **368.2253** | **734.4358** | **734.7559** | **-0.3200** | **1** | **12** | **1.3e+02** | **1** | **SSEEKR** |
|  | 512 | **387.9934** | **1160.9581** | **1161.3291** | **-0.3711** | **0** | **12** | **1.9e+02** | **1** | **LEAGAMVLADR + Oxidation (M)** |
|  | 796 | **404.0658** | **806.1168** | **806.8880** | **-0.7711** | **1** | **12** | **1.5e+02** | **1** | **CKEGTGR + Carbamidomethyl (C)** |
|  | 945 | **407.2694** | **812.5240** | **812.0152** | **0.5088** | **1** | **12** | **1.2e+02** | **1** | **ALARLLR** |
|  | 2036 | **485.1172** | **1452.3294** | **1452.5704** | **-0.2410** | **0** | **12** | **1.4e+02** | **1** | **AQGTGSVCPSCGGSK + 2 Carbamidomethyl (C)** |
|  | 2045 | **486.1159** | **970.2171** | **971.1089** | **-0.8918** | **0** | **12** | **1.4e+02** | **1** | **TLTLHTSAK** |
|  | 2206 | **502.0759** | **1002.1370** | **1001.1017** | **1.0353** | **2** | **12** | **1.7e+02** | **1** | **NKLSGGGGRR** |
|  | 2976 | **632.5496** | **1894.6265** | **1895.2724** | **-0.6458** | **0** | **12** | **1.3e+02** | **1** | **GTLCSMGMVQQLVALVR + Carbamidomethyl (C); 2 Oxidation (M)** |
|  | 575 | **389.1843** | **776.3538** | **776.9231** | **-0.5693** | **0** | **12** | **1.5e+02** | **1** | **LAAFISR** |
|  | 1037 | **409.0229** | **1224.0466** | **1223.3439** | **0.7027** | **2** | **12** | **1.7e+02** | **1** | **ERCSCGNGKR + 2 Carbamidomethyl (C)** |
|  | 1553 | **443.1544** | **884.2940** | **885.0593** | **-0.7653** | **0** | **12** | **1.5e+02** | **1** | **EQAIALLK** |
|  | 1724 | 458.0725 | 914.1303 | 913.0098 | 1.1205 | 0 | 12 | 1.5e+02 | 1 | LCNSTYR + Carbamidomethyl (C) |
|  | 1750 | **458.8931** | **915.7713** | **915.9873** | **-0.2160** | **0** | **12** | **1.7e+02** | **1** | **IAEVGAGGDK** |
|  | 453 | **386.1152** | **770.2156** | **770.9021** | **-0.6864** | **0** | **12** | **1.4e+02** | **1** | **AMHWAR** |
|  | 1686 | **454.8223** | **1361.4448** | **1361.6517** | **-0.2069** | **0** | **12** | **1.6e+02** | **1** | **IMHYMYQLCK + 2 Oxidation (M)** |
|  | 679 | **399.7481** | **1196.2222** | **1195.4549** | **0.7673** | **2** | **12** | **1.3e+02** | **1** | **KPKKVAGAATPK** |
|  | 1081 | **412.2561** | **822.4974** | **821.9176** | **0.5799** | **0** | **12** | **1.3e+02** | **1** | **GFEAAVTK** |
|  | 1213 | **419.4751** | **1255.4030** | **1256.4979** | **-1.0949** | **2** | **12** | **2.1e+02** | **1** | **LLSNRSKALVR** |
|  | 1443 | **435.2176** | **1302.6306** | **1303.4635** | **-0.8329** | **1** | **12** | **1.4e+02** | **1** | **VSFHSIKQSTAV** |
|  | 2182 | **499.1742** | **996.3336** | **996.1250** | **0.2087** | **1** | **12** | **1.5e+02** | **1** | **GRAVLGPNGR** |
|  | 2458 | **534.7008** | **1601.0802** | **1600.4639** | **0.6164** | **0** | **12** | **1.6e+02** | **1** | **TMDYXEVSNTFXR + Oxidation (M)** |
|  | 2657 | **566.2838** | **1695.8293** | **1694.8385** | **0.9907** | **0** | **12** | **1.6e+02** | **1** | **SNLCALCIGDEEGENK** |
|  | 3284 | **742.5356** | **2224.5848** | **2225.4617** | **-0.8770** | **1** | **12** | **1.4e+02** | **1** | **LRLPGSSNSSASVSQAAGIIGPR** |
|  | 1087 | **413.1643** | **1236.4708** | **1237.4119** | **-0.9410** | **2** | **12** | **1.4e+02** | **1** | **KMRCEWDGGR** |
|  | 1383 | **431.2969** | **1290.8686** | **1290.3852** | **0.4835** | **2** | **12** | **1.5e+02** | **1** | **GERSVKEWSGR** |
|  | 1637 | **450.0098** | **1347.0073** | **1346.4867** | **0.5206** | **1** | **12** | **1.5e+02** | **1** | **EAEEMCSKFTR + Oxidation (M)** |
|  | 2454 | **534.2526** | **1599.7357** | **1599.7673** | **-0.0316** | **0** | **12** | **1.7e+02** | **1** | **CQQSDCGMLGNWK + 2 Carbamidomethyl (C); Oxidation (M)** |
|  | 3010 | **651.4244** | **1951.2511** | **1950.1837** | **1.0675** | **2** | **12** | **1.5e+02** | **1** | **SSRGRGSFGMQVVSVGGPGK** |
|  | 3092 | **669.4580** | **2005.3519** | **2006.3460** | **-0.9942** | **1** | **12** | **1.5e+02** | **1** | **GDGTGALLLGLVVPAAGKGQLV** |
|  | 3346 | 761.1796 | 2280.5165 | 2280.7093 | -0.1927 | 1 | 12 | 1.5e+02 | 1 | VAMNILNSGRFSMGSVVAGLLK + Oxidation (M) |
|  | 1108 | **414.0497** | **1239.1271** | **1238.3916** | **0.7354** | **0** | **12** | **1.3e+02** | **1** | **DLMACAQTGSGK + Carbamidomethyl (C)** |
|  | 1205 | **419.3365** | **1254.9874** | **1255.4886** | **-0.5012** | **1** | **12** | **1.5e+02** | **1** | **DAMMAMNGKVR + 2 Oxidation (M)** |
|  | 2739 | **590.6805** | **1179.3463** | **1179.2597** | **0.0866** | **0** | **12** | **1.8e+02** | **1** | **GNESALWDCK + Carbamidomethyl (C)** |
|  | 3358 | **769.6377** | **1537.2606** | **1536.7745** | **0.4861** | **1** | **12** | **1.2e+02** | **1** | **LQHAAELIKTVASR** |
|  | 168 | 369.2296 | 1104.6667 | 1104.2879 | 0.3788 | 2 | 12 | 1.3e+02 | 1 | RSAVVMGRGR + Oxidation (M) |
|  | 2000 | **479.9258** | **957.8368** | **958.1399** | **-0.3032** | **1** | **12** | **1.6e+02** | **1** | **TRAAPCALR** |
|  | 2586 | **554.1325** | **1106.2502** | **1105.3091** | **0.9412** | **2** | **12** | **1.6e+02** | **1** | **GKVKGTQEMK** |
|  | 416 | **385.1250** | **768.2353** | **767.8304** | **0.4049** | **1** | **12** | **1.4e+02** | **1** | **SYSKQR** |
|  | 1066 | **411.1101** | **1230.3081** | **1229.3404** | **0.9678** | **1** | **12** | **1.7e+02** | **1** | **GSPVTTTKADPR** |
|  | 1432 | **435.0137** | **1302.0189** | **1301.4294** | **0.5896** | **1** | **12** | **1.4e+02** | **1** | **ADVLRGSGHSAMT** |
|  | 1487 | 437.2598 | 1308.7571 | 1308.6767 | 0.0804 | 0 | 12 | 1.5e+02 | 1 | LIGMLLACCLSR + Oxidation (M) |
|  | 554 | **389.0778** | **1164.2111** | **1163.4342** | **0.7769** | **0** | **12** | **1.8e+02** | **1** | **CCLFCFWK + 2 Carbamidomethyl (C)** |
|  | 761 | **403.0683** | **1206.1827** | **1205.3421** | **0.8407** | **1** | **12** | **1.7e+02** | **1** | **ERTTMADPLR + Oxidation (M)** |
|  | 959 | 407.5059 | 1219.4954 | 1219.3670 | 0.1284 | 0 | 12 | 1.8e+02 | 1 | TSVYFCASVSR |
|  | 1996 | **479.1906** | **1434.5495** | **1434.6877** | **-0.1382** | **2** | **12** | **1.6e+02** | **1** | **RQQMRQEALMK + Oxidation (M)** |
|  | 2131 | **491.9915** | **981.9681** | **983.0998** | **-1.1316** | **0** | **12** | **1.4e+02** | **1** | **NTGTVGFCK + Carbamidomethyl (C)** |
|  | 2703 | **581.0730** | **1160.1312** | **1159.3297** | **0.8015** | **0** | **12** | **1.6e+02** | **1** | **LESLTDIIQK** |
|  | 3117 | **680.0767** | **2037.2080** | **2037.2297** | **-0.0217** | **0** | **12** | **1.5e+02** | **1** | **STCPSAAPSASAPAMTTVENK + Oxidation (M)** |
|  | 459 | **386.1704** | **770.3260** | **770.7912** | **-0.4652** | **0** | **12** | **1.3e+02** | **1** | **EGAAGNPR** |
|  | 2713 | **583.2701** | **1746.7883** | **1747.0491** | **-0.2608** | **1** | **12** | **1.7e+02** | **1** | **TGPIVSGALQVNCKMR + Carbamidomethyl (C); Oxidation (M)** |
|  | 2732 | **589.1069** | **1176.1991** | **1176.3470** | **-0.1480** | **1** | **12** | **1.7e+02** | **1** | **RNVSLELSCR** |
|  | 1283 | **424.1504** | **1269.4290** | **1269.3378** | **0.0912** | **0** | **12** | **1.7e+02** | **1** | **EGCLAFGTDDGK + Carbamidomethyl (C)** |
|  | 2566 | **551.4403** | **1651.2988** | **1650.8077** | **0.4911** | **0** | **12** | **1.3e+02** | **1** | **FIQVPENMSIDEGR + Oxidation (M)** |
|  | 27 | **362.2211** | **1083.6411** | **1083.2620** | **0.3792** | **2** | **12** | **1.2e+02** | **1** | **RGMKGGFTIT + Oxidation (M)** |
|  | 550 | **389.0462** | **1164.1163** | **1163.4115** | **0.7049** | **0** | **12** | **1.7e+02** | **1** | **PMTVVTMASAR** |
|  | 1151 | **416.5172** | **1246.5295** | **1245.5098** | **1.0196** | **0** | **12** | **2.2e+02** | **1** | **IIGNSAFLLIGK** |
|  | 1188 | **419.1829** | **1254.5265** | **1254.4525** | **0.0740** | **0** | **12** | **1.8e+02** | **1** | **NETFVTVMLGK + Oxidation (M)** |
|  | 1728 | **458.1492** | **1371.4255** | **1372.6132** | **-1.1876** | **0** | **12** | **1.6e+02** | **1** | **TPLHMAANMLNK + 2 Oxidation (M)** |
|  | 1819 | **462.1905** | **1383.5494** | **1382.5900** | **0.9594** | **1** | **12** | **1.5e+02** | **1** | **QMMTMHNTKGR + 3 Oxidation (M)** |
|  | 1935 | **474.5842** | **1420.7305** | **1419.7094** | **1.0212** | **0** | **12** | **2e+02** | **1** | **VLFPGIEAHPVLK** |
|  | 1279 | **423.6750** | **845.3352** | **845.9407** | **-0.6055** | **1** | **12** | **1.5e+02** | **1** | **KQPSGTTK** |
|  | 2265 | **511.6003** | **1531.7786** | **1530.7022** | **1.0764** | **0** | **12** | **1.9e+02** | **1** | **KPGMPNVSNDLSQK + Oxidation (M)** |
|  | 2085 | **488.2144** | **974.4140** | **975.0131** | **-0.5991** | **0** | **12** | **1.7e+02** | **1** | **YYTGTQSR** |
|  | 2378 | **522.2263** | **1563.6568** | **1564.7615** | **-1.1047** | **1** | **12** | **1.6e+02** | **1** | **EDFKQMSPGIIQR + Oxidation (M)** |
|  | 2849 | **608.0435** | **1214.0721** | **1214.3123** | **-0.2401** | **1** | **12** | **1.6e+02** | **1** | **NDHLRQMER + Oxidation (M)** |
|  | 2937 | **620.9414** | **1859.8020** | **1859.2814** | **0.5206** | **2** | **12** | **1.3e+02** | **1** | **KLSFYYLIMAKGGIVR** |
|  | 3262 | **740.9202** | **2219.7385** | **2219.7301** | **0.0084** | **0** | **12** | **1.6e+02** | **1** | **YLFILGIQILACALAASILR + Carbamidomethyl (C)** |
|  | 274 | **374.4644** | **1120.3712** | **1120.2556** | **0.1156** | **0** | **12** | **2.3e+02** | **1** | **FAYWSGYVK** |
|  | 891 | **406.2222** | **810.4296** | **810.0161** | **0.4135** | **1** | **12** | **1.3e+02** | **1** | **FGLAKMK + Oxidation (M)** |
|  | 1770 | **459.2912** | **1374.8514** | **1375.4813** | **-0.6299** | **0** | **12** | **1.4e+02** | **1** | **LGGVSSTEELDIR** |
|  | 132 | **368.0145** | **1101.0212** | **1101.1712** | **-0.1499** | **1** | **12** | **1.8e+02** | **1** | **GNKGSTGPAGQK** |
|  | 1922 | **473.4950** | **944.9752** | **944.9889** | **-0.0137** | **1** | **12** | **2e+02** | **1** | **ATGDGSPGRK** |
|  | 2137 | **492.4398** | **1474.2973** | **1473.7619** | **0.5354** | **2** | **12** | **1.3e+02** | **1** | **RIYSVKRPDVLK** |
|  | 1316 | **427.7281** | **1280.1621** | **1280.5212** | **-0.3591** | **1** | **12** | **1.3e+02** | **1** | **MTFCCCRSTR + Carbamidomethyl (C); Oxidation (M)** |
|  | 3336 | **758.3262** | **1514.6376** | **1513.7394** | **0.8982** | **0** | **12** | **1.5e+02** | **1** | **GQMLMPNFGYGGNK** |
|  | 1760 | **459.1204** | **916.2259** | **917.1710** | **-0.9450** | **2** | **12** | **1.8e+02** | **1** | **SKPKKCVK** |
|  | 2497 | **538.4152** | **1612.2233** | **1612.6904** | **-0.4670** | **1** | **12** | **1.3e+02** | **1** | **YTDEKETEGSKPTK** |
|  | 3334 | **758.0461** | **1514.0775** | **1514.6895** | **-0.6120** | **2** | **12** | **1.2e+02** | **1** | **VFHQFSNSKRHK** |
|  | 431 | **385.9988** | **1154.9743** | **1155.3429** | **-0.3686** | **0** | **12** | **1.4e+02** | **1** | **MSLGGSDTMLK + Oxidation (M)** |
|  | 519 | **388.1021** | **774.1894** | **774.8593** | **-0.6699** | **0** | **12** | **1.9e+02** | **1** | **EGSIEIK** |
|  | 757 | **402.9648** | **803.9148** | **802.8795** | **1.0353** | **1** | **12** | **1.8e+02** | **1** | **SRGTAGVR** |
|  | 1011 | **408.2550** | **1221.7429** | **1222.4371** | **-0.6941** | **1** | **12** | **1.3e+02** | **1** | **VPGRAAPGVALSK** |
|  | 1573 | **443.9189** | **1328.7347** | **1328.4712** | **0.2634** | **1** | **12** | **1.7e+02** | **1** | **DLEIEVEGLRR** |
|  | 2615 | 559.5201 | 1675.5383 | 1674.8291 | 0.7092 | 0 | 12 | 1.4e+02 | 1 | MPDDVWLVDLDSNR |
|  | 12 | **360.4665** | **1078.3772** | **1079.1210** | **-0.7437** | **0** | **12** | **2.1e+02** | **1** | **PTFAGSQESR** |
|  | 62 | **363.3183** | **724.6219** | **724.8504** | **-0.2285** | **0** | **12** | **1.4e+02** | **1** | **NCVMSR + Oxidation (M)** |
|  | 77 | **364.2869** | **726.5591** | **726.8611** | **-0.3021** | **0** | **12** | **1.1e+02** | **1** | **ELPALGK** |
|  | 1694 | **456.1813** | **910.3479** | **911.0621** | **-0.7142** | **1** | **12** | **1.5e+02** | **1** | **MNRTAMR + 2 Oxidation (M)** |
|  | 581 | **389.2119** | **776.4090** | **775.8509** | **0.5581** | **0** | **12** | **1.5e+02** | **1** | **SGVVTASR** |
|  | 3420 | **810.8991** | **2429.6752** | **2430.7859** | **-1.1108** | **1** | **12** | **1.8e+02** | **1** | **ASGCQSCPACLMERMNWLSR + 3 Carbamidomethyl (C); Oxidation (M)** |
|  | 138 | **368.1700** | **1101.4880** | **1101.1546** | **0.3334** | **1** | **12** | **1.5e+02** | **1** | **KGNCSQHDR + Carbamidomethyl (C)** |
|  | 935 | **407.1926** | **1218.5555** | **1219.4380** | **-0.8825** | **1** | **12** | **1.4e+02** | **1** | **AICVVCDRAR + 2 Carbamidomethyl (C)** |
|  | 2536 | **547.5391** | **1639.5952** | **1638.9660** | **0.6292** | **2** | **12** | **1.5e+02** | **1** | **MLSVFKKEDTIIAK + Oxidation (M)** |
|  | 1032 | **408.8951** | **1223.6633** | **1223.2925** | **0.3707** | **0** | **12** | **1.8e+02** | **1** | **EAENQAGVLHVG** |
|  | 1051 | **409.7827** | **1226.3260** | **1227.3508** | **-1.0248** | **0** | **12** | **1.8e+02** | **1** | **TECGDCHFCR + Carbamidomethyl (C)** |
|  | 1511 | **438.3750** | **1312.1027** | **1312.4305** | **-0.3277** | **0** | **12** | **1.5e+02** | **1** | **SGCSSQSISPMR + Carbamidomethyl (C); Oxidation (M)** |
|  | 2234 | **505.2433** | **1512.7076** | **1513.6353** | **-0.9277** | **1** | **12** | **1.6e+02** | **1** | **RMEAGEAAPPAGAGGR + Oxidation (M)** |
|  | 133 | **368.0172** | **734.0197** | **732.8277** | **1.1921** | **0** | **12** | **1.8e+02** | **1** | **AVPAYGR** |
|  | 301 | **377.0727** | **1128.1958** | **1128.3223** | **-0.1265** | **1** | **12** | **1.6e+02** | **1** | **SSGLASKLPLR** |
|  | 769 | **403.7382** | **805.4615** | **805.9430** | **-0.4814** | **1** | **12** | **1.5e+02** | **1** | **LGAMDKR + Oxidation (M)** |
|  | 287 | **375.1798** | **748.3448** | **747.7546** | **0.5903** | **2** | **12** | **1.6e+02** | **1** | **RGSGKED** |
|  | 1159 | **417.2449** | **1248.7125** | **1249.3367** | **-0.6241** | **1** | **12** | **1.5e+02** | **1** | **EHSVHVERTR** |
|  | 1856 | **467.1943** | **1398.5607** | **1398.5493** | **0.0113** | **1** | **12** | **1.7e+02** | **1** | **RWAPPAAADCQR + Carbamidomethyl (C)** |
|  | 663 | **399.0716** | **1194.1926** | **1195.3669** | **-1.1742** | **0** | **12** | **1.5e+02** | **1** | **TFFISPGSLAR** |
|  | 1779 | **459.9591** | **1376.8551** | **1377.4160** | **-0.5609** | **0** | **12** | **1.8e+02** | **1** | **LEFQSDHSTTGR** |
|  | 1927 | **474.0355** | **946.0562** | **945.0287** | **1.0275** | **0** | **12** | **1.8e+02** | **1** | **LQDVVSER** |
|  | 2207 | **502.2199** | **1503.6376** | **1503.6818** | **-0.0442** | **0** | **12** | **1.7e+02** | **1** | **VGECGQKPFTQPR + Carbamidomethyl (C)** |
|  | 2810 | **597.6923** | **1193.3699** | **1192.3713** | **0.9986** | **0** | **12** | **2e+02** | **1** | **MAQHGAMGAFR + Oxidation (M)** |
|  | 741 | **402.0752** | **1203.2035** | **1203.2616** | **-0.0580** | **1** | **12** | **1.9e+02** | **1** | **REAEYPGPER** |
|  | 1502 | **437.8673** | **1310.5798** | **1309.5158** | **1.0640** | **2** | **12** | **1.9e+02** | **1** | **LLYKQDFARR** |
|  | 2498 | **538.9249** | **1613.7524** | **1612.8477** | **0.9048** | **2** | **12** | **1.7e+02** | **1** | **MMEQKMKEEQER + Oxidation (M)** |
|  | 3017 | **655.9098** | **1309.8048** | **1310.6115** | **-0.8067** | **2** | **12** | **1.3e+02** | **1** | **LMGSLIQKHRK** |
|  | 3277 | **741.9371** | **1481.8594** | **1480.8205** | **1.0388** | **2** | **12** | **1.5e+02** | **1** | **GALCKPLPIREKR** |
|  | 643 | **396.0193** | **790.0239** | **790.9283** | **-0.9044** | **0** | **12** | **1.9e+02** | **1** | **TALEVCR** |
|  | 1173 | **418.1259** | **1251.3556** | **1251.4948** | **-0.1392** | **1** | **12** | **1.7e+02** | **1** | **IFKLEQQMAK + Oxidation (M)** |
|  | 3235 | **740.4636** | **2218.3687** | **2219.4128** | **-1.0441** | **1** | **12** | **1.6e+02** | **1** | **EVEPAPVGGEHPSAAAPGPGKHK** |
|  | 3236 | **740.4640** | **2218.3698** | **2218.6496** | **-0.2798** | **2** | **12** | **1.6e+02** | **1** | **TLRRFRPMCWAASPLLSR + Carbamidomethyl (C)** |
|  | 3291 | **742.7656** | **1483.5165** | **1482.7256** | **0.7909** | **2** | **12** | **1.5e+02** | **1** | **VCSRLLGKGSSTMA + Carbamidomethyl (C); Oxidation (M)** |
|  | 1154 | **416.8297** | **1247.4669** | **1246.4766** | **0.9902** | **0** | **12** | **1.9e+02** | **1** | **MALDLASLASVR** |
|  | 1249 | **422.0327** | **1263.0760** | **1262.3734** | **0.7026** | **1** | **12** | **1.6e+02** | **1** | **FSGQSYVRYR** |
|  | 1506 | **438.1099** | **1311.3074** | **1310.4113** | **0.8962** | **0** | **12** | **1.9e+02** | **1** | **TGQWVDGATFTK** |
|  | 1665 | **452.3062** | **1353.8965** | **1353.4377** | **0.4588** | **1** | **12** | **1.5e+02** | **1** | **ELGPGPEAENGRK** |
|  | 1798 | **460.7394** | **1379.1961** | **1378.5795** | **0.6167** | **0** | **12** | **1.4e+02** | **1** | **VSQNWIVGCCR + 2 Carbamidomethyl (C)** |
|  | 2394 | **523.8431** | **1568.5072** | **1567.8547** | **0.6525** | **2** | **12** | **1.5e+02** | **1** | **VGTHGGCLKRAIDLK** |
|  | 3027 | **662.9199** | **1985.7376** | **1985.1846** | **0.5529** | **2** | **12** | **1.3e+02** | **1** | **TRSDDTAVYFCARGPGLR** |
|  | 3099 | **670.2606** | **2007.7595** | **2007.4879** | **0.2716** | **1** | **12** | **1.6e+02** | **1** | **DMRVPAQLLGLLLLWLR** |
|  | 209 | **370.8137** | **1109.4189** | **1109.2397** | **0.1792** | **1** | **12** | **1.3e+02** | **1** | **GSPGVGAGRVPR** |
|  | 970 | **407.6709** | **1219.9904** | **1219.4166** | **0.5739** | **2** | **12** | **1.3e+02** | **1** | **VKKGGSYMGHR** |
|  | 1507 | **438.1450** | **1311.4128** | **1311.5665** | **-0.1537** | **1** | **12** | **1.8e+02** | **1** | **IIPTLPSDKLSK** |
|  | 59 | **363.2615** | **1086.7623** | **1086.3471** | **0.4153** | **1** | **12** | **1.3e+02** | **1** | **LFSYCVKVK** |
|  | 556 | **389.0852** | **776.1557** | **776.9017** | **-0.7461** | **0** | **12** | **1.9e+02** | **1** | **TCVELR + Carbamidomethyl (C)** |
|  | 1897 | **471.2625** | **1410.7654** | **1409.6517** | **1.1136** | **2** | **12** | **1.4e+02** | **1** | **LTTLKSSSSAMRK** |
|  | 1501 | **437.8507** | **1310.5299** | **1310.5006** | **0.0292** | **0** | **12** | **1.9e+02** | **1** | **YEQVHILPVGR** |
|  | 100 | 366.1522 | 1095.4344 | 1096.2624 | -0.8280 | 0 | 12 | 1.7e+02 | 1 | ECMNGCVQR + Carbamidomethyl (C) |
|  | 480 | **387.0451** | **1158.1132** | **1158.3287** | **-0.2154** | **1** | **12** | **1.9e+02** | **1** | **IKEVEHMTR + Oxidation (M)** |
|  | 1305 | 427.0841 | 852.1534 | 853.0823 | -0.9288 | 0 | 12 | 1.6e+02 | 1 | LPDMCMK + Oxidation (M) |
|  | 1663 | **452.1408** | **1353.4003** | **1353.5869** | **-0.1866** | **2** | **12** | **2e+02** | **1** | **YIEMKRVAEAK + Oxidation (M)** |
|  | 616 | **392.1915** | **1173.5522** | **1174.3745** | **-0.8222** | **1** | **12** | **1.4e+02** | **1** | **KPAMTTPTRR + Oxidation (M)** |
|  | 1056 | **410.1966** | **1227.5675** | **1227.3938** | **0.1738** | **0** | **12** | **1.7e+02** | **1** | **VQCGGGLVQPGR + Carbamidomethyl (C)** |
|  | 1939 | **475.2945** | **1422.8614** | **1421.7334** | **1.1280** | **2** | **12** | **1.5e+02** | **1** | **LMKTSRCGLWAR** |
|  | 1968 | **477.2982** | **1428.8724** | **1429.6445** | **-0.7722** | **1** | **12** | **1.3e+02** | **1** | **RIPLGGLSSMENR** |
|  | 571 | **389.1628** | **1164.4664** | **1165.3029** | **-0.8365** | **1** | **12** | **1.8e+02** | **1** | **GRNPLVEQPR** |
|  | 1255 | **422.1266** | **842.2385** | **842.9136** | **-0.6751** | **0** | **12** | **1.8e+02** | **1** | **AMVSDASF + Oxidation (M)** |
|  | 3045 | **666.5945** | **1331.1742** | **1330.6178** | **0.5563** | **2** | **12** | **1.3e+02** | **1** | **ILIPKGSYGRVK** |
|  | 1197 | **419.2521** | **836.4895** | **836.0352** | **0.4543** | **1** | **12** | **1.6e+02** | **1** | **AAGCCGVKK** |
|  | 1239 | **421.2293** | **1260.6657** | **1259.5233** | **1.1423** | **1** | **12** | **1.4e+02** | **1** | **LLFLSMHRAR + Oxidation (M)** |
|  | 1518 | **439.4162** | **1315.2265** | **1315.4991** | **-0.2726** | **1** | **12** | **1.7e+02** | **1** | **MKVTSLDGGHVR + Oxidation (M)** |
|  | 1662 | **452.1122** | **902.2096** | **901.0192** | **1.1905** | **2** | **12** | **2e+02** | **1** | **TKEPGGKGK** |
|  | 2983 | **634.8643** | **1901.5708** | **1902.2071** | **-0.6363** | **2** | **12** | **1.3e+02** | **1** | **CQTPAAKPAKMRQVSNR + Oxidation (M)** |
|  | 637 | **395.1992** | **1182.5755** | **1182.3684** | **0.2071** | **0** | **12** | **1.6e+02** | **1** | **VTETVMNGGMK + Oxidation (M)** |
|  | 1545 | **442.1860** | **1323.5359** | **1322.4850** | **1.0510** | **1** | **12** | **1.6e+02** | **1** | **KTVIDCSISNDK** |
|  | 2597 | **556.3842** | **1110.7535** | **1110.3090** | **0.4446** | **2** | **12** | **1.5e+02** | **1** | **KGASKMDAMR + Oxidation (M)** |
|  | 3124 | **683.7814** | **1365.5480** | **1364.5696** | **0.9784** | **0** | **12** | **2e+02** | **1** | **MAWSNMGNMFK + 3 Oxidation (M)** |
|  | 802 | **404.1076** | **806.2004** | **805.0014** | **1.1991** | **2** | **12** | **1.7e+02** | **1** | **KRTVCAK** |
|  | 1094 | **413.2583** | **1236.7528** | **1236.3858** | **0.3671** | **2** | **12** | **1.2e+02** | **1** | **EAGAGCMGRRR + Carbamidomethyl (C); Oxidation (M)** |
|  | 2447 | **532.9625** | **1595.8654** | **1594.7827** | **1.0827** | **2** | **12** | **1.7e+02** | **1** | **MEKKESNLETELK + Oxidation (M)** |
|  | 3361 | **773.6978** | **1545.3807** | **1544.8135** | **0.5672** | **1** | **12** | **1.3e+02** | **1** | **MEMTEMTGVSLKR + 2 Oxidation (M)** |
|  | 1322 | **428.1976** | **1281.5706** | **1280.4036** | **1.1671** | **0** | **12** | **1.7e+02** | **1** | **ASPDMDGYPALK + Oxidation (M)** |
|  | 1768 | **459.2542** | **1374.7405** | **1375.5507** | **-0.8103** | **1** | **12** | **1.7e+02** | **1** | **SLADCIKQDIGR + Carbamidomethyl (C)** |
|  | 2068 | **487.2325** | **1458.6754** | **1457.7010** | **0.9743** | **2** | **12** | **1.8e+02** | **1** | **CEECGKGFICRR + Carbamidomethyl (C)** |
|  | 3067 | **667.5449** | **1999.6126** | **1999.2361** | **0.3765** | **1** | **12** | **1.4e+02** | **1** | **LNQARSMSGHPEAAQMVR + Oxidation (M)** |
|  | 172 | **369.2520** | **1104.7339** | **1105.2444** | **-0.5105** | **1** | **12** | **1.5e+02** | **1** | **SGYEGRVPLK** |
|  | 529 | **388.1878** | **1161.5412** | **1162.1682** | **-0.6270** | **1** | **12** | **1.8e+02** | **1** | **DSDARAAAETR** |
|  | 1021 | **408.3202** | **1221.9384** | **1222.4321** | **-0.4937** | **0** | **12** | **1.4e+02** | **1** | **NEVMEAGLCLK + Oxidation (M)** |
|  | 1792 | **460.4375** | **918.8602** | **918.9914** | **-0.1311** | **2** | **12** | **1.7e+02** | **1** | **TDKGGEKGK** |
|  | 2375 | 522.0974 | 1042.1800 | 1041.3081 | 0.8720 | 0 | 12 | 1.7e+02 | 1 | AIMLGAKPPK + Oxidation (M) |
|  | 3287 | **742.6581** | **2224.9523** | **2225.4586** | **-0.5063** | **0** | **12** | **1.3e+02** | **1** | **LSCTASGFTFSTYDMHWVR + Oxidation (M)** |
|  | 1943 | **475.5874** | **949.1600** | **950.0914** | **-0.9314** | **0** | **12** | **2.1e+02** | **1** | **ICEEPTCR** |
|  | 2625 | **562.1699** | **1122.3251** | **1121.1362** | **1.1889** | **0** | **12** | **1.7e+02** | **1** | **MDPASGEGEGR + Oxidation (M)** |
|  | 66 | **363.9426** | **1088.8055** | **1089.3062** | **-0.5006** | **0** | **12** | **1.5e+02** | **1** | **IVGQLMDGLK + Oxidation (M)** |
|  | 253 | **373.3954** | **744.7761** | **743.8486** | **0.9275** | **1** | **12** | **2.5e+02** | **1** | **TPLKTAN** |
|  | 3323 | **753.2933** | **1504.5719** | **1503.6306** | **0.9413** | **1** | **12** | **1.6e+02** | **1** | **LDTGNSMTKYTEK + Oxidation (M)** |
|  | 465 | **386.2707** | **1155.7898** | **1155.3245** | **0.4653** | **0** | **12** | **1.2e+02** | **1** | **SSIYLCSVAR + Carbamidomethyl (C)** |
|  | 3175 | **700.2705** | **1398.5262** | **1397.5399** | **0.9863** | **2** | **12** | **1.6e+02** | **1** | **VAGAGESLEPRRR** |
|  | 3459 | **847.7352** | **1693.4556** | **1692.8722** | **0.5833** | **1** | **12** | **1.4e+02** | **1** | **QSPGKGLEWIGDIHR** |
|  | 868 | **406.0399** | **1215.0976** | **1214.4231** | **0.6744** | **1** | **12** | **1.5e+02** | **1** | **HLRGAGHVLVR** |
|  | 1633 | **449.8305** | **1346.4693** | **1347.4780** | **-1.0086** | **1** | **12** | **1.7e+02** | **1** | **AVREGMCDSDHK** |
|  | 2184 | **499.2161** | **1494.6262** | **1494.6054** | **0.0208** | **0** | **12** | **1.7e+02** | **1** | **QSPPASGEVNLGPNK** |
|  | 3451 | **845.2505** | **2532.7295** | **2533.8809** | **-1.1515** | **2** | **12** | **1.5e+02** | **1** | **HQLRQLESQFVLMQQDLTRM + 2 Oxidation (M)** |
|  | 632 | **394.2427** | **1179.7059** | **1179.4154** | **0.2904** | **1** | **12** | **1.4e+02** | **1** | **LAVALLGGGPRR** |
|  | 1647 | **450.2367** | **898.4587** | **899.0098** | **-0.5512** | **1** | **12** | **1.6e+02** | **1** | **RAAAAAGGVR** |
|  | 1846 | **466.0265** | **930.0381** | **929.0358** | **1.0024** | **1** | **12** | **1.8e+02** | **1** | **GRGLVAGGSR** |
|  | 2176 | **498.8041** | **1493.3901** | **1492.8295** | **0.5605** | **1** | **12** | **1.4e+02** | **1** | **MCKGLAGLPASCLR + Carbamidomethyl (C); Oxidation (M)** |
|  | 2337 | **519.0865** | **1036.1582** | **1036.1822** | **-0.0240** | **1** | **12** | **1.7e+02** | **1** | **ENLKTLYR** |
|  | 2609 | **558.3605** | **1672.0592** | **1671.9376** | **0.1216** | **1** | **12** | **1.7e+02** | **1** | **QLFGGGTRLTVLGQPK** |
|  | 468 | **386.7627** | **1157.2658** | **1158.1515** | **-0.8857** | **0** | **12** | **1.8e+02** | **1** | **MDNGTDSSTSK + Oxidation (M)** |
|  | 1364 | **430.4212** | **1288.2414** | **1288.4936** | **-0.2521** | **0** | **12** | **2e+02** | **1** | **VVCPELDCASPR** |
|  | 2974 | **631.9863** | **1261.9578** | **1262.2838** | **-0.3261** | **0** | **12** | **1.6e+02** | **1** | **SLLEGEGSSGGGGR** |
|  | 1152 | 416.5774 | 831.1399 | 831.9156 | -0.7757 | 0 | 12 | 2e+02 | 1 | TPGPAAYR |
|  | 1676 | **453.0825** | **904.1502** | **903.0814** | **1.0688** | **0** | **12** | **1.8e+02** | **1** | **LSHMMER** |
|  | 3173 | **697.6805** | **1393.3463** | **1394.5080** | **-1.1617** | **1** | **12** | **1.4e+02** | **1** | **DAKAVMAGSSGEQK + Oxidation (M)** |
|  | 340 | **379.1646** | **1134.4717** | **1134.2888** | **0.1828** | **1** | **12** | **1.6e+02** | **1** | **KGVEHGPAAIR** |
|  | 1576 | **444.1233** | **1329.3477** | **1328.5822** | **0.7655** | **1** | **12** | **1.9e+02** | **1** | **FRTGIMGVIYR + Oxidation (M)** |
|  | 2695 | **579.4272** | **1735.2594** | **1736.0181** | **-0.7588** | **0** | **12** | **1.4e+02** | **1** | **AEEFILAMHLTDMAK + Oxidation (M)** |
|  | 3131 | **684.0093** | **2049.0057** | **2048.2551** | **0.7505** | **0** | **12** | **1.4e+02** | **1** | **LEVLNFDFQANAQLSNPK** |
|  | 267 | **374.2343** | **746.4537** | **746.7731** | **-0.3193** | **1** | **12** | **1.7e+02** | **1** | **AGSGRGSR** |
|  | 936 | **407.2078** | **1218.6011** | **1218.3806** | **0.2206** | **1** | **12** | **1.4e+02** | **1** | **EGRVLQEEMK** |
|  | 2322 | **518.8094** | **1035.6040** | **1035.1364** | **0.4676** | **2** | **12** | **1.4e+02** | **1** | **ECREAGKSR** |
|  | 2425 | **528.5109** | **1582.5104** | **1582.7336** | **-0.2232** | **0** | **12** | **1.5e+02** | **1** | **VEVGLSSCQGDYIR + Carbamidomethyl (C)** |
|  | 2507 | **540.2296** | **1078.4443** | **1079.2070** | **-0.7627** | **2** | **12** | **1.8e+02** | **1** | **DYELRQKK** |
|  | 482 | **387.0766** | **1158.2075** | **1158.3485** | **-0.1409** | **1** | **12** | **2e+02** | **1** | **CSECGKAFSVK** |
|  | 1306 | **427.1823** | **852.3498** | **853.0806** | **-0.7307** | **0** | **12** | **1.6e+02** | **1** | **LMVIFSK + Oxidation (M)** |
|  | 1321 | **428.0957** | **1281.2650** | **1280.4666** | **0.7985** | **0** | **12** | **1.7e+02** | **1** | **FLASVSTVLESK** |
|  | 1875 | **469.2087** | **1404.6039** | **1404.5325** | **0.0714** | **1** | **12** | **1.7e+02** | **1** | **MPGGCSRGPAAGDGR + Oxidation (M)** |
|  | 2746 | **592.3577** | **1774.0508** | **1775.1187** | **-1.0679** | **0** | **12** | **1.7e+02** | **1** | **LLMMASVNDCYTLIR + 2 Oxidation (M)** |
|  | 801 | **404.1053** | **1209.2937** | **1210.3335** | **-1.0398** | **0** | **12** | **1.8e+02** | **1** | **TSYLTELIDR** |
|  | 1116 | **414.4151** | **1240.2232** | **1239.4310** | **0.7923** | **2** | **12** | **1.7e+02** | **1** | **NRCQQCRFK + Carbamidomethyl (C)** |
|  | 2054 | **486.5895** | **1456.7463** | **1455.7633** | **0.9830** | **1** | **12** | **2.1e+02** | **1** | **MTMKVDMSGLQAK + Oxidation (M)** |
|  | 2684 | **575.1191** | **1722.3351** | **1721.8674** | **0.4677** | **0** | **12** | **1.8e+02** | **1** | **CVCDPGYTGDDCGMR + 2 Carbamidomethyl (C); Oxidation (M)** |
|  | 280 | **375.0162** | **1122.0265** | **1122.1407** | **-0.1142** | **0** | **12** | **2e+02** | **1** | **ADDGSLTSQTK** |
|  | 1050 | **409.7413** | **817.4679** | **816.8994** | **0.5685** | **0** | **12** | **1.7e+02** | **1** | **LQPASSSK** |
|  | 1233 | **420.4541** | **1258.3401** | **1258.3351** | **0.0051** | **1** | **12** | **1.9e+02** | **1** | **SHVDKFSEYF** |
|  | 1559 | **443.4344** | **884.8541** | **885.0166** | **-0.1625** | **1** | **12** | **1.8e+02** | **1** | **TPPEVSKK** |
|  | 2788 | **594.7695** | **1781.2864** | **1780.1252** | **1.1613** | **2** | **12** | **1.8e+02** | **1** | **MGVFCLGPWGSGRKLR + Oxidation (M)** |
|  | 2822 | **598.7360** | **1195.4571** | **1194.4451** | **1.0121** | **0** | **12** | **1.9e+02** | **1** | **FYICSPVPLR** |
|  | 3156 | **686.0996** | **1370.1844** | **1369.6357** | **0.5487** | **1** | **12** | **1.7e+02** | **1** | **QQSPLMAVLRAR** |
|  | 707 | **400.7955** | **1199.3642** | **1198.3807** | **0.9835** | **2** | **12** | **1.8e+02** | **1** | **AVWAARRAAAR** |
|  | 1060 | **411.0292** | **1230.0654** | **1229.2144** | **0.8511** | **1** | **12** | **1.8e+02** | **1** | **AAPDDDREGGAR** |
|  | 2211 | **502.5538** | **1504.6391** | **1503.5859** | **1.0532** | **0** | **12** | **2.3e+02** | **1** | **LTTTSSSGTSATTAAM + Oxidation (M)** |
|  | 2241 | **506.0078** | **1010.0009** | **1009.2412** | **0.7597** | **0** | **12** | **1.6e+02** | **1** | **WLTPVIPAL** |
|  | 2869 | **610.0677** | **1218.1206** | **1217.4588** | **0.6618** | **0** | **12** | **1.8e+02** | **1** | **AAMMTEVLAHK + Oxidation (M)** |
|  | 672 | **399.1514** | **1194.4319** | **1193.3082** | **1.1238** | **0** | **12** | **1.6e+02** | **1** | **DAASCMTVHDK + Oxidation (M)** |
|  | 950 | **407.3538** | **1219.0393** | **1219.4547** | **-0.4154** | **2** | **12** | **1.4e+02** | **1** | **NTQVLKKMNK + Oxidation (M)** |
|  | 2500 | **539.1896** | **1076.3645** | **1075.3043** | **1.0602** | **1** | **12** | **1.9e+02** | **1** | **SLKLLAQFR** |
|  | 2379 | **522.8749** | **1043.7350** | **1043.1815** | **0.5535** | **0** | **12** | **1.7e+02** | **1** | **CGASGQHPMR** |
|  | 2554 | **550.1841** | **1098.3534** | **1097.2670** | **1.0864** | **1** | **12** | **1.7e+02** | **1** | **NNSCLSKMK + Carbamidomethyl (C); Oxidation (M)** |
|  | 260 | **374.0808** | **1119.2204** | **1119.2728** | **-0.0524** | **2** | **12** | **2.1e+02** | **1** | **KQVRSSVTSK** |
|  | 611 | 391.1789 | 1170.5145 | 1171.4513 | -0.9368 | 0 | 12 | 1.5e+02 | 1 | LPPTLCTWLK |
|  | 697 | **400.1412** | **798.2676** | **798.8477** | **-0.5800** | **1** | **12** | **1.6e+02** | **1** | **GRGGAEPR** |
|  | 1076 | **412.0765** | **1233.2074** | **1233.4580** | **-0.2506** | **2** | **12** | **1.8e+02** | **1** | **SSLKTRLTISK** |
|  | 2032 | **484.3899** | **1450.1475** | **1449.6541** | **0.4934** | **2** | **12** | **1.4e+02** | **1** | **LSLKNDAPQAKHK** |
|  | 2840 | **606.5662** | **1211.1175** | **1210.2475** | **0.8701** | **0** | **12** | **1.4e+02** | **1** | **EEQSSDAGLFK** |
|  | 684 | **399.8678** | **797.7208** | **797.8961** | **-0.1753** | **0** | **12** | **1.4e+02** | **1** | **QPPESLK** |
|  | 737 | **402.0082** | **802.0016** | **802.8729** | **-0.8712** | **1** | **12** | **2.1e+02** | **1** | **NVADKEK** |
|  | 1176 | **418.1886** | **834.3624** | **833.8438** | **0.5187** | **1** | **12** | **1.8e+02** | **1** | **GGTRDELS** |
|  | 2745 | **592.3553** | **1774.0437** | **1772.9488** | **1.0948** | **0** | **12** | **1.7e+02** | **1** | **YSQLTSLDVGFNTISK** |
|  | 3154 | **686.0923** | **2055.2547** | **2054.4753** | **0.7794** | **2** | **12** | **1.7e+02** | **1** | **VIPKKPPPSPQPTGKIEIK** |
|  | 3364 | **776.2020** | **1550.3893** | **1549.7055** | **0.6838** | **1** | **12** | **1.7e+02** | **1** | **NLDTGGNKSVLMER + Oxidation (M)** |
|  | 713 | **401.0804** | **1200.2189** | **1201.3517** | **-1.1328** | **0** | **12** | **2e+02** | **1** | **LMSSAEECCR + Carbamidomethyl (C); Oxidation (M)** |
|  | 2671 | **570.7113** | **1709.1117** | **1707.9269** | **1.1849** | **1** | **12** | **1.9e+02** | **1** | **ILKSQNNSSVDPCMR + Oxidation (M)** |
|  | 3400 | **798.1196** | **2391.3367** | **2391.7184** | **-0.3817** | **1** | **12** | **1.4e+02** | **1** | **FIISRDDSSNMLYLQMNSLK + Oxidation (M)** |
|  | 557 | **389.0856** | **776.1564** | **776.9016** | **-0.7452** | **1** | **12** | **2e+02** | **1** | **DRIASCL** |
|  | 1590 | **445.1576** | **1332.4505** | **1333.6203** | **-1.1698** | **1** | **12** | **2e+02** | **1** | **TPMRTINPLMK + 2 Oxidation (M)** |
|  | 1776 | 459.8517 | 1376.5330 | 1375.6369 | 0.8960 | 2 | 12 | 1.9e+02 | 1 | SKMLLEINRQK + Oxidation (M) |
|  | 2261 | **510.6453** | **1528.9138** | **1527.8174** | **1.0964** | **2** | **12** | **2.1e+02** | **1** | **RVCSNPGPRCLLR + Carbamidomethyl (C)** |
|  | 392 | **384.8648** | **1151.5722** | **1151.2697** | **0.3024** | **1** | **12** | **1.5e+02** | **1** | **KLTVEENYR** |
|  | 1100 | **413.4103** | **1237.2088** | **1237.3423** | **-0.1335** | **1** | **12** | **1.6e+02** | **1** | **RAGCPYQADEK** |
|  | 2029 | **484.0601** | **966.1054** | **965.1505** | **0.9549** | **0** | **12** | **1.8e+02** | **1** | **ILIISHNR** |
|  | 2399 | **524.1819** | **1046.3490** | **1047.2727** | **-0.9237** | **1** | **12** | **1.9e+02** | **1** | **SKLALSLCR + Carbamidomethyl (C)** |
|  | 3264 | **741.1830** | **1480.3512** | **1479.6522** | **0.6990** | **0** | **12** | **1.7e+02** | **1** | **ADVIITSGGVSMGEK + Oxidation (M)** |
|  | 1005 | **408.1557** | **1221.4450** | **1220.3137** | **1.1313** | **1** | **12** | **1.9e+02** | **1** | **EMSQRDVSPR + Oxidation (M)** |
|  | 2370 | **521.4777** | **1561.4108** | **1561.7342** | **-0.3234** | **2** | **12** | **1.4e+02** | **1** | **YDKLKQNLESNLP** |
|  | 3153 | **686.0786** | **2055.2137** | **2054.4817** | **0.7319** | **2** | **12** | **1.7e+02** | **1** | **LLLTRFIFCSATMRTHK + Oxidation (M)** |
|  | 1562 | **443.7565** | **885.4982** | **886.0326** | **-0.5344** | **1** | **12** | **1.5e+02** | **1** | **YGGFMRR** |
|  | 1988 | **478.3017** | **954.5886** | **954.9406** | **-0.3519** | **0** | **12** | **1.4e+02** | **1** | **GQEGQDHGK** |
|  | 2329 | **518.9178** | **1553.7312** | **1553.8666** | **-0.1354** | **2** | **12** | **1.7e+02** | **1** | **CVMKMVAANKEEK + Carbamidomethyl (C); Oxidation (M)** |
|  | 2545 | **548.9672** | **1095.9197** | **1095.3355** | **0.5841** | **1** | **12** | **1.7e+02** | **1** | **RLELLVQPK** |
|  | 2652 | **565.8697** | **1129.7246** | **1129.1749** | **0.5497** | **0** | **12** | **1.6e+02** | **1** | **DEAPVADGVEK** |
|  | 148 | **368.3018** | **1101.8834** | **1101.1713** | **0.7120** | **1** | **12** | **1.6e+02** | **1** | **EAEAARAVER** |
|  | 1085 | **413.0747** | **1236.2019** | **1236.3344** | **-0.1324** | **0** | **12** | **1.6e+02** | **1** | **DFPAYHGVPHP** |
|  | 1174 | **418.1278** | **1251.3613** | **1251.4548** | **-0.0935** | **0** | **12** | **1.9e+02** | **1** | **ALLASNSCFIR + Carbamidomethyl (C)** |
|  | 2798 | **596.0175** | **1785.0304** | **1785.0274** | **0.0029** | **0** | **12** | **1.9e+02** | **1** | **AQPPMYYDILTGYPR** |
|  | 2912 | 612.7262 | 1835.1564 | 1835.1308 | 0.0256 | 1 | 12 | 2.1e+02 | 1 | CYSSIGKVQIAFISYR |
|  | 2098 | **488.5031** | **974.9913** | **974.0930** | **0.8984** | **0** | **12** | **2.2e+02** | **1** | **MLSDHSIR + Oxidation (M)** |
|  | 1589 | **445.1567** | **1332.4480** | **1332.5855** | **-0.1375** | **0** | **12** | **2.1e+02** | **1** | **EPSLAYLLLWK** |
|  | 2030 | **484.1664** | **1449.4769** | **1450.5529** | **-1.0761** | **1** | **12** | **1.7e+02** | **1** | **TDQFLRDAVETR** |
|  | 2204 | **501.3450** | **1501.0129** | **1500.7604** | **0.2525** | **0** | **12** | **1.5e+02** | **1** | **MQLLDENHLFIK** |
|  | 2230 | **505.0934** | **1512.2580** | **1511.6423** | **0.6156** | **1** | **12** | **1.7e+02** | **1** | **YNHKTDAALWHR** |
|  | 3185 | **708.2233** | **2121.6476** | **2121.3690** | **0.2786** | **2** | **12** | **1.7e+02** | **1** | **MGESDDSILRLAKADGIVSK + Oxidation (M)** |
|  | 270 | **374.2778** | **1119.8112** | **1120.3666** | **-0.5553** | **0** | **12** | **1.8e+02** | **1** | **VVWIFCPTR** |
|  | 2255 | **508.1443** | **1521.4106** | **1520.6888** | **0.7218** | **0** | **12** | **1.8e+02** | **1** | **ELHLSFTQGFWR** |
|  | 2488 | **538.0128** | **1074.0107** | **1074.1691** | **-0.1583** | **0** | **12** | **1.8e+02** | **1** | **CVVGSPGAGDR + Carbamidomethyl (C)** |
|  | 1027 | **408.8331** | **1223.4770** | **1224.4973** | **-1.0203** | **2** | **12** | **1.9e+02** | **1** | **LPIGKRNWLK** |
|  | 1452 | 436.0777 | 870.1406 | 871.0759 | -0.9353 | 0 | 12 | 1.7e+02 | 1 | AGAILVVTK |
|  | 1516 | **439.2893** | **1314.8457** | **1315.5203** | **-0.6746** | **1** | **12** | **1.6e+02** | **1** | **GLWKGVSLTAQR** |
|  | 2258 | **509.5305** | **1017.0461** | **1018.2133** | **-1.1672** | **1** | **12** | **2.2e+02** | **1** | **GYRLAVIAR** |
|  | 2365 | **521.1758** | **1040.3368** | **1039.1499** | **1.1869** | **2** | **12** | **1.7e+02** | **1** | **SRSPRSPPR** |
|  | 2419 | **527.0477** | **1052.0807** | **1051.2235** | **0.8572** | **1** | **12** | **1.7e+02** | **1** | **HKMPAPAQR + Oxidation (M)** |
|  | 2744 | **592.3433** | **1774.0076** | **1775.0343** | **-1.0267** | **2** | **12** | **1.8e+02** | **1** | **ELIMGEDPAQPRKYK** |
|  | 592 | **389.7216** | **777.4284** | **777.9294** | **-0.5010** | **0** | **12** | **1.7e+02** | **1** | **LLQSCSK** |
|  | 690 | **400.0438** | **1197.1093** | **1196.2935** | **0.8157** | **0** | **12** | **1.5e+02** | **1** | **LNGFCAASGGSR + Carbamidomethyl (C)** |
|  | 1818 | **462.1778** | **1383.5112** | **1383.5481** | **-0.0369** | **0** | **12** | **1.7e+02** | **1** | **YTCFAENFMGK + Carbamidomethyl (C); Oxidation (M)** |
|  | 2133 | **492.1447** | **982.2745** | **983.0817** | **-0.8071** | **1** | **12** | **1.7e+02** | **1** | **MTCERDR + Carbamidomethyl (C); Oxidation (M)** |
|  | 370 | **381.3636** | **760.7123** | **759.9640** | **0.7484** | **2** | **12** | **2.1e+02** | **1** | **KRLGMR** |
|  | 1783 | 460.1273 | 918.2398 | 917.0416 | 1.1982 | 0 | 12 | 2e+02 | 1 | SQPQGCLGK |
|  | 2774 | **593.2957** | **1776.8648** | **1777.9689** | **-1.1041** | **2** | **12** | **2e+02** | **1** | **QFEKVSEEKENALVK** |
|  | 2909 | **612.4530** | **1834.3368** | **1833.1381** | **1.1987** | **1** | **12** | **1.5e+02** | **1** | **RSIYVLLYGSALPAPGR** |
|  | 1496 | **437.6929** | **873.3710** | **872.9295** | **0.4414** | **2** | **12** | **1.7e+02** | **1** | **AEREGRR** |
|  | 1575 | **444.0827** | **1329.2260** | **1328.4150** | **0.8111** | **0** | **12** | **2e+02** | **1** | **NGHASEGMTRPR + Oxidation (M)** |
|  | 2262 | **510.7560** | **1529.2459** | **1529.7671** | **-0.5213** | **2** | **12** | **1.6e+02** | **1** | **LTIMGTRTHRTAR + Oxidation (M)** |
|  | 2345 | **519.4971** | **1036.9794** | **1038.0443** | **-1.0649** | **0** | **12** | **1.6e+02** | **1** | **EEMDEAGNK + Oxidation (M)** |
|  | 2885 | **611.0413** | **1830.1018** | **1831.1141** | **-1.0123** | **0** | **12** | **1.9e+02** | **1** | **DYMSIILMWLGDDVK + 2 Oxidation (M)** |
|  | 1431 | **434.9008** | **867.7867** | **866.9613** | **0.8254** | **0** | **12** | **1.6e+02** | **1** | **LDQVGAHK** |
|  | 861 | **405.9271** | **809.8395** | **808.9684** | **0.8711** | **1** | **12** | **1.7e+02** | **1** | **GPPAKIAR** |
|  | 1042 | **409.0875** | **816.1602** | **817.0287** | **-0.8685** | **2** | **12** | **2e+02** | **1** | **TKVKTIK** |
|  | 2381 | **522.9384** | **1565.7931** | **1565.7977** | **-0.0046** | **1** | **12** | **1.9e+02** | **1** | **TTCKCHGVSGSCAVR + Carbamidomethyl (C)** |
|  | 322 | **377.2780** | **1128.8118** | **1129.1780** | **-0.3663** | **0** | **12** | **1.4e+02** | **1** | **EIATEGQEPR** |
|  | 446 | **386.0914** | **1155.2521** | **1155.2599** | **-0.0078** | **0** | **12** | **1.7e+02** | **1** | **EHHISELYK** |
|  | 530 | **388.1878** | **774.3608** | **774.8229** | **-0.4620** | **1** | **12** | **1.9e+02** | **1** | **NSNQKGK** |
|  | 864 | 406.0013 | 1214.9819 | 1215.2258 | -0.2439 | 0 | 12 | 1.7e+02 | 1 | QTFGYGASGPSSG |
|  | 924 | **407.1288** | **1218.3643** | **1219.4528** | **-1.0886** | **0** | **12** | **1.8e+02** | **1** | **VQWCSLSVIK + Carbamidomethyl (C)** |
|  | 2083 | **488.1817** | **974.3487** | **974.2056** | **0.1431** | **1** | **12** | **2e+02** | **1** | **CLPVARCR + Carbamidomethyl (C)** |
|  | 2280 | **513.4672** | **1537.3793** | **1536.7148** | **0.6645** | **1** | **12** | **1.5e+02** | **1** | **NHIENTLKAHMGR + Oxidation (M)** |
|  | 2915 | **612.8856** | **1835.6347** | **1835.0466** | **0.5881** | **1** | **12** | **1.5e+02** | **1** | **VSPGQTARFTCSGDALPK** |
|  | 1396 | **433.0562** | **1296.1464** | **1296.5618** | **-0.4155** | **2** | **12** | **1.8e+02** | **1** | **LVAALQGKKVNR** |
|  | 1583 | **445.0322** | **888.0497** | **887.0321** | **1.0175** | **0** | **12** | **2.2e+02** | **1** | **LAENLISK** |
|  | 2215 | **503.4933** | **1004.9717** | **1004.1372** | **0.8346** | **0** | **12** | **2e+02** | **1** | **IGFPSTSPAK** |
|  | 2410 | **524.9325** | **1571.7753** | **1571.8816** | **-0.1062** | **1** | **12** | **1.9e+02** | **1** | **KMGFPEIILPGDVR** |
|  | 2522 | **544.2035** | **1086.3922** | **1086.1566** | **0.2356** | **1** | **12** | **2.1e+02** | **1** | **LTRQPSGPSSG** |
|  | 2532 | **547.2879** | **1638.8415** | **1638.9109** | **-0.0694** | **0** | **12** | **1.8e+02** | **1** | **MDWGGPALGIPHLCR + Oxidation (M)** |
|  | 3028 | **663.1380** | **1324.2612** | **1325.3812** | **-1.1200** | **0** | **12** | **1.8e+02** | **1** | **LQDVHAEAEGEK** |
|  | 296 | **376.2433** | **750.4717** | **749.8367** | **0.6351** | **0** | **12** | **1.4e+02** | **1** | **NVSGSMR** |
|  | 1111 | **414.1561** | **826.2974** | **825.9525** | **0.3448** | **1** | **12** | **1.6e+02** | **1** | **AKTLHEK** |
|  | 3151 | **686.0295** | **2055.0664** | **2054.2034** | **0.8630** | **1** | **12** | **1.5e+02** | **1** | **VSGDAQKQGCDCECLGGGR + 3 Carbamidomethyl (C)** |
|  | 28 | **362.2348** | **1083.6822** | **1083.3033** | **0.3789** | **1** | **12** | **1.4e+02** | **1** | **LCKCEECAK + Carbamidomethyl (C)** |
|  | 860 | **405.9245** | **809.8342** | **810.8584** | **-1.0242** | **1** | **12** | **1.7e+02** | **1** | **KAADGGHR** |
|  | 2214 | **502.8013** | **1003.5878** | **1004.1801** | **-0.5924** | **0** | **12** | **1.6e+02** | **1** | **EILKPNYK** |
|  | 311 | **377.1436** | **1128.4088** | **1127.3776** | **1.0312** | **1** | **12** | **1.7e+02** | **1** | **EKMVGAAFMK + Oxidation (M)** |
|  | 1155 | **416.9616** | **1247.8627** | **1248.4362** | **-0.5734** | **2** | **12** | **2.1e+02** | **1** | **FRVHGTPGKHI** |
|  | 1941 | **475.3608** | **1423.0602** | **1422.5612** | **0.4990** | **2** | **12** | **1.6e+02** | **1** | **DTKEEMGELARK + Oxidation (M)** |
|  | 3179 | **702.5255** | **2104.5544** | **2105.4326** | **-0.8782** | **1** | **12** | **1.6e+02** | **1** | **DWYDVKAPAMFSIITMGK + 2 Oxidation (M)** |
|  | 485 | **387.0987** | **772.1827** | **771.9083** | **0.2743** | **1** | **12** | **2.1e+02** | **1** | **RTGLGLR** |
|  | 1600 | **446.1706** | **1335.4896** | **1336.4687** | **-0.9791** | **0** | **12** | **2.1e+02** | **1** | **EEVAQNVVSMSK + Oxidation (M)** |
|  | 1705 | **456.9748** | **1367.9021** | **1368.4589** | **-0.5567** | **1** | **12** | **1.8e+02** | **1** | **DPSRDCHHCGK + 2 Carbamidomethyl (C)** |
|  | 2429 | **529.5928** | **1585.7561** | **1585.6516** | **0.1046** | **1** | **12** | **2.3e+02** | **1** | **MASATSSSQRDWDK + Oxidation (M)** |
|  | 151 | **368.6255** | **735.2363** | **735.8498** | **-0.6135** | **0** | **12** | **1.7e+02** | **1** | **LTGNMGK + Oxidation (M)** |
|  | 1077 | **412.0977** | **1233.2709** | **1234.3833** | **-1.1124** | **0** | **12** | **1.9e+02** | **1** | **MESRPSTSALR** |
|  | 1509 | **438.2475** | **1311.7204** | **1312.4731** | **-0.7527** | **0** | **12** | **1.8e+02** | **1** | **LGWYFDLWGR** |
|  | 1580 | **444.9983** | **1331.9728** | **1332.5743** | **-0.6015** | **2** | **12** | **2.2e+02** | **1** | **MGPVPTSFGKRR** |
|  | 2037 | **485.1565** | **1452.4473** | **1452.5058** | **-0.0585** | **1** | **12** | **1.7e+02** | **1** | **FDHDGSNSKGNMK + Oxidation (M)** |
|  | 2605 | **557.4612** | **1669.3616** | **1669.9900** | **-0.6284** | **2** | **12** | **1.4e+02** | **1** | **TMRPLPGRIEVRTK + Oxidation (M)** |
|  | 3299 | **744.2130** | **1486.4112** | **1487.5748** | **-1.1635** | **1** | **12** | **1.8e+02** | **1** | **TPGNSSKKPSQGSGR** |
|  | 1448 | **435.6725** | **869.3302** | **869.0219** | **0.3083** | **0** | **12** | **1.4e+02** | **1** | **SPIHLFR** |
|  | 2844 | **607.2158** | **1818.6253** | **1818.9810** | **-0.3558** | **2** | **12** | **1.8e+02** | **1** | **SVYEKRVPQEQADAAK** |
|  | 3260 | **740.8690** | **2219.5849** | **2220.6060** | **-1.0211** | **1** | **12** | **2.1e+02** | **1** | **AVYQAVLSLKNIPVLETAYK** |
|  | 631 | **394.2210** | **786.4272** | **785.8192** | **0.6081** | **0** | **12** | **1.6e+02** | **1** | **DFMDDK + Oxidation (M)** |
|  | 1626 | **449.3481** | **1345.0221** | **1344.4309** | **0.5911** | **0** | **12** | **1.4e+02** | **1** | **ERPTLSAEAAGSR** |
|  | 190 | 369.3158 | 1104.9252 | 1104.2991 | 0.6261 | 1 | 12 | 1.7e+02 | 1 | GKLAIGITANF |
|  | 317 | **377.2083** | **1128.6027** | **1128.2861** | **0.3166** | **2** | **12** | **1.4e+02** | **1** | **DSRPRKLTR** |
|  | 669 | **399.1264** | **1194.3569** | **1194.4435** | **-0.0865** | **1** | **12** | **1.7e+02** | **1** | **MLAKISTTWK + Oxidation (M)** |
|  | 989 | **407.8425** | **1220.5052** | **1219.4530** | **1.0523** | **2** | **12** | **1.9e+02** | **1** | **HKILMFKTEG + Oxidation (M)** |
|  | 995 | **407.9283** | **813.8418** | **814.8437** | **-1.0019** | **0** | **12** | **1.9e+02** | **1** | **QNSPAGNK** |
|  | 2025 | **483.2932** | **1446.8575** | **1445.7054** | **1.1521** | **1** | **12** | **1.7e+02** | **1** | **ARLTVNVLPSFTK** |
|  | 2748 | **592.5133** | **1774.5177** | **1774.8902** | **-0.3725** | **2** | **12** | **1.5e+02** | **1** | **RAGAVQNTNDSSALSKR** |
|  | 973 | **407.7170** | **1220.1290** | **1219.3455** | **0.7835** | **2** | **12** | **1.6e+02** | **1** | **KNKQSVSATEK** |
|  | 2376 | **522.1732** | **1563.4975** | **1562.7289** | **0.7686** | **0** | **12** | **1.9e+02** | **1** | **CQCEPGFQLGPNNR** |
|  | 2521 | **544.0210** | **1629.0408** | **1628.9147** | **0.1261** | **1** | **12** | **2e+02** | **1** | **LMDEVAGIVAARHCK + Oxidation (M)** |
|  | 44 | **363.1120** | **724.2092** | **724.8902** | **-0.6810** | **0** | **12** | **1.9e+02** | **1** | **MMNVSK + Oxidation (M)** |
|  | 475 | **386.9483** | **1157.8228** | **1157.2443** | **0.5785** | **2** | **12** | **2.1e+02** | **1** | **RTAGAGAAGGRGR** |
|  | 708 | **400.9880** | **1199.9419** | **1199.3971** | **0.5449** | **0** | **12** | **2e+02** | **1** | **CTSPPGLSYMK + Oxidation (M)** |
|  | 827 | **405.0170** | **1212.0289** | **1212.2699** | **-0.2409** | **0** | **12** | **1.7e+02** | **1** | **IGGGPGDAADVQR** |
|  | 1029 | **408.8508** | **1223.5302** | **1222.4172** | **1.1131** | **1** | **12** | **2e+02** | **1** | **RVYMASLPNR + Oxidation (M)** |
|  | 2274 | **512.2645** | **1533.7714** | **1532.8057** | **0.9657** | **1** | **12** | **1.9e+02** | **1** | **CKYLTKPEPQIR + Carbamidomethyl (C)** |
|  | 3101 | **671.0790** | **2010.2148** | **2011.1458** | **-0.9311** | **2** | **12** | **1.7e+02** | **1** | **VEAEEEKAKSGYDEWIK** |
|  | 3429 | **817.5182** | **1633.0216** | **1633.8034** | **-0.7818** | **1** | **12** | **1.8e+02** | **1** | **DYDLCINCYNRK + 2 Carbamidomethyl (C)** |
|  | 390 | **384.7684** | **1151.2829** | **1150.2816** | **1.0014** | **0** | **12** | **1.7e+02** | **1** | **VALISFDGSNK** |
|  | 498 | **387.8717** | **1160.5929** | **1160.3510** | **0.2419** | **2** | **12** | **2.2e+02** | **1** | **RRMLAAAAER + Oxidation (M)** |
|  | 1139 | **416.1053** | **830.1958** | **831.0386** | **-0.8428** | **2** | **12** | **2.3e+02** | **1** | **KRLSCPK** |
|  | 1280 | **423.6961** | **1268.0662** | **1268.4228** | **-0.3565** | **1** | **12** | **1.8e+02** | **1** | **ATSGPQTPRVVR** |
|  | 1491 | **437.3838** | **872.7529** | **873.0754** | **-0.3225** | **1** | **12** | **1.9e+02** | **1** | **KPKTMPR + Oxidation (M)** |
|  | 1691 | **455.7934** | **909.5721** | **909.1290** | **0.4431** | **1** | **12** | **1.7e+02** | **1** | **RQLMSMK + Oxidation (M)** |
|  | 2389 | **523.3644** | **1044.7141** | **1044.2092** | **0.5049** | **2** | **12** | **1.6e+02** | **1** | **GKSRNITLR** |
|  | 2734 | **589.6146** | **1177.2145** | **1176.2576** | **0.9568** | **0** | **12** | **2.3e+02** | **1** | **GPLGSCADEATR** |
|  | 1091 | **413.2493** | **1236.7256** | **1236.4404** | **0.2853** | **0** | **12** | **1.3e+02** | **1** | **MLVGITSSWSR** |
|  | 1552 | **443.1462** | **1326.4165** | **1326.6077** | **-0.1912** | **0** | **12** | **1.8e+02** | **1** | **ILRPSIGDKPCK** |
|  | 1958 | 476.2142 | 1425.6205 | 1426.5298 | -0.9093 | 1 | 12 | 1.9e+02 | 1 | YSEDLPYEVRR |
|  | 2339 | **519.1010** | **1036.1872** | **1037.2397** | **-1.0525** | **1** | **12** | **1.8e+02** | **1** | **RGNPLHLCK** |
|  | 2509 | **540.5524** | **1618.6349** | **1617.9813** | **0.6536** | **2** | **12** | **2e+02** | **1** | **MRRALMNGISSLIR** |
|  | 2721 | **584.5181** | **1167.0213** | **1168.2141** | **-1.1927** | **0** | **12** | **1.5e+02** | **1** | **ASPDDPSGLPGR** |
|  | 290 | **376.0993** | **1125.2756** | **1124.2426** | **1.0330** | **0** | **11** | **1.8e+02** | **1** | **QIAPEYFEK** |
|  | 1612 | **448.0288** | **1341.0643** | **1340.5531** | **0.5113** | **1** | **11** | **1.8e+02** | **1** | **SFKGCAHAPLPR + Carbamidomethyl (C)** |
|  | 6 | **360.3772** | **718.7397** | **718.9056** | **-0.1659** | **1** | **11** | **2.6e+02** | **1** | **DVKMVK** |
|  | 286 | **375.1611** | **1122.4612** | **1121.3331** | **1.1282** | **0** | **11** | **2e+02** | **1** | **NVLIPRPGQK** |
|  | 1138 | **416.0699** | **1245.1877** | **1244.4643** | **0.7234** | **1** | **11** | **2.2e+02** | **1** | **RMSDVPVGVIR + Oxidation (M)** |
|  | 1664 | 452.1415 | 1353.4025 | 1353.5468 | -0.1444 | 0 | 11 | 2.3e+02 | 1 | LHVGNISPACTNK |
|  | 2292 | **514.9679** | **1027.9210** | **1027.2617** | **0.6593** | **1** | **11** | **1.8e+02** | **1** | **CPPKCPTPK + Carbamidomethyl (C)** |
|  | 334 | **378.1943** | **1131.5608** | **1130.4047** | **1.1562** | **2** | **11** | **1.4e+02** | **1** | **MKVLAVEGKR** |
|  | 531 | 388.2324 | 1161.6750 | 1162.2957 | -0.6207 | 1 | 11 | 1.8e+02 | 1 | SWGRGTLVTVS |
|  | 2075 | **487.9446** | **1460.8117** | **1461.5820** | **-0.7704** | **0** | **11** | **2.1e+02** | **1** | **GASIHLVGDSVNHR** |
|  | 2250 | **507.2105** | **1012.4062** | **1012.1872** | **0.2190** | **0** | **11** | **1.9e+02** | **1** | **GLLMAHSQR** |
|  | 2476 | 536.2349 | 1605.6824 | 1604.5853 | 1.0971 | 1 | 11 | 2e+02 | 1 | EARAGGDEDGEELEK |
|  | 2854 | **608.7299** | **1823.1674** | **1822.9315** | **0.2359** | **0** | **11** | **2.3e+02** | **1** | **TSGGGAGGPGAGGVMGCTEAR + Carbamidomethyl (C); Oxidation (M)** |
|  | 3177 | 701.9554 | 2102.8440 | 2103.2942 | -0.4502 | 1 | 11 | 1.5e+02 | 1 | GAAGAVTQSLSRTPTATTSGIR |
|  | 853 | **405.6795** | **809.3443** | **808.9205** | **0.4238** | **0** | **11** | **1.4e+02** | **1** | **AETMGPMG + Oxidation (M)** |
|  | 1849 | **466.0435** | **930.0722** | **929.0921** | **0.9801** | **0** | **11** | **2e+02** | **1** | **ILDSPCPK + Carbamidomethyl (C)** |
|  | 2483 | **537.2661** | **1608.7760** | **1609.8501** | **-1.0741** | **2** | **11** | **2.1e+02** | **1** | **GWMQARTLFKESR** |
|  | 2543 | **548.8571** | **1643.5490** | **1642.7905** | **0.7585** | **1** | **11** | **1.5e+02** | **1** | **SQWYNDIAASMKGR + Oxidation (M)** |
|  | 282 | **375.0836** | **1122.2286** | **1122.2733** | **-0.0447** | **0** | **11** | **2.1e+02** | **1** | **ETNCCVTGPAK** |
|  | 817 | **404.8734** | **1211.5979** | **1210.4661** | **1.1318** | **0** | **11** | **1.8e+02** | **1** | **IPQTMAAFGMK + Oxidation (M)** |
|  | 862 | **405.9488** | **809.8828** | **810.9444** | **-1.0616** | **1** | **11** | **1.7e+02** | **1** | **ALPAQRR** |
|  | 1103 | **413.5241** | **1237.5502** | **1238.4118** | **-0.8615** | **1** | **11** | **1.9e+02** | **1** | **SRMTITVDTSK** |
|  | 1187 | **419.1413** | **1254.4016** | **1255.3393** | **-0.9376** | **0** | **11** | **2.2e+02** | **1** | **QGNGVWSSPAPR** |
|  | 1606 | **447.0386** | **892.0625** | **893.0432** | **-0.9808** | **0** | **11** | **2.1e+02** | **1** | **ACLNGCAK + 2 Carbamidomethyl (C)** |
|  | 2111 | **490.1252** | **978.2357** | **977.1201** | **1.1157** | **1** | **11** | **1.9e+02** | **1** | **CEKACNPR + Carbamidomethyl (C)** |
|  | 3239 | **740.5282** | **2218.5624** | **2219.6310** | **-1.0686** | **2** | **11** | **1.8e+02** | **1** | **DNHLLKYRALLLERPVLR** |
|  | 1523 | **440.3734** | **1318.0979** | **1318.4764** | **-0.3786** | **1** | **11** | **1.8e+02** | **1** | **QKADSLSTLVTR** |
|  | 3157 | **686.1598** | **2055.4572** | **2054.3841** | **1.0731** | **0** | **11** | **1.9e+02** | **1** | **IELIQDFEMPTVCTTIK + Carbamidomethyl (C); Oxidation (M)** |
|  | 425 | **385.3898** | **1153.1471** | **1152.2180** | **0.9292** | **0** | **11** | **1.8e+02** | **1** | **SPSQGAQGPAPR** |
|  | 2141 | **493.2729** | **984.5310** | **984.0250** | **0.5060** | **0** | **11** | **1.9e+02** | **1** | **DSQRPEPR** |
|  | 636 | **395.1564** | **1182.4470** | **1181.3404** | **1.1067** | **0** | **11** | **2.1e+02** | **1** | **MGSVLQEGCGK + Carbamidomethyl (C); Oxidation (M)** |
|  | 1248 | **422.0208** | **1263.0401** | **1263.4029** | **-0.3628** | **2** | **11** | **1.9e+02** | **1** | **RRGASVDDFLK** |
|  | 2273 | **512.2334** | **1533.6780** | **1533.6398** | **0.0382** | **1** | **11** | **1.9e+02** | **1** | **EELSASSARTQNLK** |
|  | 2475 | **536.2039** | **1605.5894** | **1604.7028** | **0.8866** | **1** | **11** | **2e+02** | **1** | **NEGSPSRACSPASWR** |
|  | 2529 | **547.1929** | **1638.5564** | **1638.8185** | **-0.2621** | **1** | **11** | **2e+02** | **1** | **LEEESAQLKEMCR + Carbamidomethyl (C); Oxidation (M)** |
|  | 2766 | **592.8336** | **1775.4787** | **1774.9930** | **0.4857** | **0** | **11** | **1.6e+02** | **1** | **CFSQESQIAMVCQER + Oxidation (M)** |
|  | 2904 | **612.2870** | **1833.8390** | **1834.9804** | **-1.1414** | **1** | **11** | **1.9e+02** | **1** | **GPAAKVSGGGDTEHPVIDK** |
|  | 3012 | **653.0636** | **1956.1686** | **1955.1337** | **1.0349** | **1** | **11** | **1.9e+02** | **1** | **DPCYLAGPGSRSLSCSER + Carbamidomethyl (C)** |
|  | 146 | **368.2686** | **1101.7836** | **1101.3201** | **0.4634** | **0** | **11** | **1.7e+02** | **1** | **QLLMVGGLDR** |
|  | 294 | **376.2034** | **1125.5879** | **1125.3815** | **0.2065** | **2** | **11** | **1.5e+02** | **1** | **FMDKKLSLK + Oxidation (M)** |
|  | 1619 | 448.2787 | 1341.8140 | 1342.5076 | -0.6937 | 1 | 11 | 1.5e+02 | 1 | ETHLGCGCRGPR + Carbamidomethyl (C) |
|  | 1962 | **476.3824** | **950.7500** | **950.9471** | **-0.1971** | **0** | **11** | **1.5e+02** | **1** | **TISEDSGSR** |
|  | 2420 | **527.0704** | **1052.1261** | **1051.1936** | **0.9324** | **1** | **11** | **1.9e+02** | **1** | **AAGDYSLVKK** |
|  | 2935 | **620.2402** | **1857.6985** | **1857.1199** | **0.5786** | **1** | **11** | **1.8e+02** | **1** | **LPVATRENPVINDCCR + Carbamidomethyl (C)** |
|  | 1055 | **410.1726** | **1227.4956** | **1228.4880** | **-0.9923** | **0** | **11** | **2.1e+02** | **1** | **VLVHCAMGVSR + Carbamidomethyl (C)** |
|  | 1405 | **433.2154** | **864.4160** | **863.8698** | **0.5463** | **0** | **11** | **1.8e+02** | **1** | **SQESAAGSK** |
|  | 1177 | **418.1912** | **834.3677** | **833.8835** | **0.4841** | **0** | **11** | **1.9e+02** | **1** | **AIESSAEK** |
|  | 2464 | **535.8296** | **1069.6444** | **1069.1675** | **0.4769** | **0** | **11** | **1.5e+02** | **1** | **DVFGVFNSGK** |
|  | 1101 | **413.4120** | **1237.2137** | **1237.4898** | **-0.2761** | **0** | **11** | **1.8e+02** | **1** | **VMMTAQLGNLK + 2 Oxidation (M)** |
|  | 255 | **373.8735** | **745.7323** | **745.8495** | **-0.1172** | **0** | **11** | **2.5e+02** | **1** | **WPAGCR + Carbamidomethyl (C)** |
|  | 302 | **377.0912** | **1128.2516** | **1129.2640** | **-1.0125** | **0** | **11** | **1.9e+02** | **1** | **GGILDTAIVDR** |
|  | 552 | **389.0704** | **776.1260** | **774.9471** | **1.1789** | **0** | **11** | **2.2e+02** | **1** | **LFAPSIK** |
|  | 1674 | **452.7403** | **903.4659** | **904.0496** | **-0.5837** | **2** | **11** | **1.7e+02** | **1** | **MPRNKSR + Oxidation (M)** |
|  | 1785 | **460.2496** | **1377.7265** | **1378.5053** | **-0.7787** | **1** | **11** | **2e+02** | **1** | **VKLTTDMDPSQQ + Oxidation (M)** |
|  | 3214 | **733.2033** | **2196.5878** | **2197.5927** | **-1.0049** | **0** | **11** | **1.9e+02** | **1** | **MYICPFMGAVSGTLTVTDFK + Oxidation (M)** |
|  | 1679 | **454.1592** | **906.3036** | **907.1098** | **-0.8062** | **0** | **11** | **2.1e+02** | **1** | **CCLDKPTK** |
|  | 1969 | **477.4981** | **1429.4722** | **1430.5384** | **-1.0662** | **0** | **11** | **2e+02** | **1** | **SEDFSLPAYMDR** |
|  | 46 | **363.1418** | **1086.4034** | **1086.1550** | **0.2484** | **1** | **11** | **1.9e+02** | **1** | **YDTYGSPRK** |
|  | 866 | **406.0255** | **810.0363** | **808.9669** | **1.0694** | **0** | **11** | **1.8e+02** | **1** | **MQEMVR + Oxidation (M)** |
|  | 2669 | **570.4247** | **1138.8347** | **1139.2606** | **-0.4259** | **0** | **11** | **1.6e+02** | **1** | **APSSSLGAFFR** |
|  | 2862 | **609.8661** | **1826.5761** | **1827.1918** | **-0.6157** | **2** | **11** | **1.6e+02** | **1** | **DIMKDECSMLKLQLK + 2 Oxidation (M)** |
|  | 1737 | **458.3502** | **1372.0285** | **1371.4925** | **0.5360** | **1** | **11** | **1.6e+02** | **1** | **IEAEEAIKGLNGQ** |
|  | 2062 | **487.1294** | **972.2440** | **971.1783** | **1.0657** | **1** | **11** | **2.1e+02** | **1** | **APLGCKGGLR** |
|  | 2106 | **489.6990** | **977.3833** | **977.1548** | **0.2284** | **0** | **11** | **1.7e+02** | **1** | **SITVYIPGK** |
|  | 2672 | **570.8474** | **1709.5199** | **1710.0520** | **-0.5321** | **2** | **11** | **1.5e+02** | **1** | **AMSLVLPGPPGKWRGK + Oxidation (M)** |
|  | 3029 | **663.6718** | **1987.9931** | **1988.2466** | **-0.2535** | **0** | **11** | **1.9e+02** | **1** | **MDLSCSCATGGSCTCASSCK + Carbamidomethyl (C); Oxidation (M)** |
|  | 1287 | **424.7657** | **1271.2750** | **1270.4567** | **0.8183** | **1** | **11** | **2e+02** | **1** | **EKPYGFNKCGK** |
|  | 1293 | **425.9833** | **1274.9278** | **1275.2994** | **-0.3715** | **0** | **11** | **2e+02** | **1** | **MDEDGDESIHK** |
|  | 1530 | **441.1135** | **880.2123** | **879.9785** | **0.2338** | **0** | **11** | **2e+02** | **1** | **LNMETTR + Oxidation (M)** |
|  | 1806 | **461.6475** | **921.2801** | **921.0502** | **0.2300** | **0** | **11** | **1.7e+02** | **1** | **CECDPLK + 2 Carbamidomethyl (C)** |
|  | 2484 | **537.3536** | **1072.6925** | **1073.2438** | **-0.5513** | **1** | **11** | **2e+02** | **1** | **NLATIGTKQK** |
|  | 1995 | **479.1846** | **1434.5317** | **1433.5440** | **0.9878** | **2** | **11** | **2e+02** | **1** | **RDEGPTDLSMKGGA** |
|  | 2541 | **548.4847** | **1094.9547** | **1095.3375** | **-0.3828** | **1** | **11** | **1.6e+02** | **1** | **AMRSTGMVVK + Oxidation (M)** |
|  | 2805 | **596.8666** | **1787.5777** | **1786.7717** | **0.8060** | **1** | **11** | **1.8e+02** | **1** | **DNSRHSASQDGQDTIR** |
|  | 3069 | **667.6014** | **1999.7821** | **2000.2823** | **-0.5001** | **2** | **11** | **1.7e+02** | **1** | **QKDPTATSTLMRSCMTR + Carbamidomethyl (C); Oxidation (M)** |
|  | 422 | **385.2882** | **1152.8425** | **1152.3734** | **0.4690** | **1** | **11** | **1.4e+02** | **1** | **HGILGACGRLR** |
|  | 696 | **400.1283** | **1197.3626** | **1197.4259** | **-0.0632** | **1** | **11** | **1.8e+02** | **1** | **LPVKYPDLPR** |
|  | 1203 | **419.3034** | **1254.8880** | **1254.3927** | **0.4953** | **1** | **11** | **1.9e+02** | **1** | **LNQPGTPTRTAV** |
|  | 118 | **367.1861** | **732.3575** | **732.7830** | **-0.4255** | **0** | **11** | **1.8e+02** | **1** | **GLSDSVR** |
|  | 399 | **384.9822** | **1151.9245** | **1152.3734** | **-0.4490** | **1** | **11** | **1.7e+02** | **1** | **HGILGACGRLR** |
|  | 2147 | **494.1724** | **1479.4951** | **1479.7233** | **-0.2282** | **1** | **11** | **2.1e+02** | **1** | **MIPPGECTYAGRK + Carbamidomethyl (C)** |
|  | 2569 | **551.7255** | **1101.4361** | **1101.2556** | **0.1806** | **0** | **11** | **2e+02** | **1** | **ILQDVPFNR** |
|  | 2913 | **612.7803** | **1835.3186** | **1836.2928** | **-0.9742** | **2** | **11** | **2e+02** | **1** | **KPIDLRAIGKLPIVMR + Oxidation (M)** |
|  | 1126 | **415.3619** | **1243.0635** | **1243.2838** | **-0.2204** | **0** | **11** | **1.8e+02** | **1** | **DHSGSLGSSQLR** |
|  | 2191 | **499.6383** | **1495.8928** | **1496.8198** | **-0.9270** | **1** | **11** | **2e+02** | **1** | **VLAKLACGLNKPNR** |
|  | 2772 | **593.1647** | **1184.3146** | **1183.4835** | **0.8310** | **1** | **11** | **2.1e+02** | **1** | **IKQQLLLISK** |
|  | 208 | **370.7526** | **739.4904** | **739.9095** | **-0.4191** | **0** | **11** | **1.6e+02** | **1** | **CHICHK** |
|  | 524 | **388.1531** | **774.2914** | **773.9045** | **0.3869** | **1** | **11** | **2.3e+02** | **1** | **RPDRCK** |
|  | 3248 | **740.6085** | **1479.2023** | **1479.7185** | **-0.5162** | **0** | **11** | **1.6e+02** | **1** | **SIVTAEVSSMPACK + Carbamidomethyl (C)** |
|  | 894 | **406.2292** | **1215.6653** | **1216.4672** | **-0.8019** | **1** | **11** | **1.6e+02** | **1** | **LLLTTESAIKK** |
|  | 925 | **407.1307** | **1218.3699** | **1219.4182** | **-1.0482** | **1** | **11** | **1.9e+02** | **1** | **NCRACECHVK + Carbamidomethyl (C)** |
|  | 1795 | **460.6603** | **919.3059** | **919.0543** | **0.2516** | **0** | **11** | **1.8e+02** | **1** | **VSGPSCLEK** |
|  | 2020 | **482.0875** | **962.1603** | **963.0023** | **-0.8421** | **0** | **11** | **2.1e+02** | **1** | **GGTWSQAEK** |
|  | 2154 | **494.6675** | **987.3202** | **988.0965** | **-0.7763** | **2** | **11** | **2.1e+02** | **1** | **ESPKAGKSGK** |
|  | 2409 | **524.7454** | **1571.2139** | **1571.8169** | **-0.6030** | **2** | **11** | **1.7e+02** | **1** | **IESTKNEQLRLLK** |
|  | 1439 | **435.1599** | **868.3050** | **868.9972** | **-0.6921** | **1** | **11** | **1.9e+02** | **1** | **ECGKAFSK** |
|  | 1831 | **463.9707** | **1388.8898** | **1388.4453** | **0.4445** | **1** | **11** | **1.8e+02** | **1** | **GHAESTSYRQPR** |
|  | 2053 | **486.5016** | **970.9885** | **972.0969** | **-1.1084** | **0** | **11** | **2.2e+02** | **1** | **TSLLASGAPR** |
|  | 2514 | **542.5005** | **1624.4793** | **1623.8616** | **0.6176** | **2** | **11** | **1.7e+02** | **1** | **WAWPVARAGGAVARR** |
|  | 1107 | **413.9950** | **1238.9629** | **1238.2825** | **0.6804** | **1** | **11** | **1.7e+02** | **1** | **ESSKTPCGNAASS** |
|  | 1609 | **447.0935** | **892.1722** | **891.9494** | **0.2229** | **0** | **11** | **2.2e+02** | **1** | **MQNAESGR** |
|  | 2499 | **539.1078** | **1076.2008** | **1075.2383** | **0.9625** | **0** | **11** | **2.1e+02** | **1** | **SHMLAEPFK + Oxidation (M)** |
|  | 3274 | **741.7891** | **2222.3450** | **2222.6036** | **-0.2586** | **1** | **11** | **2.1e+02** | **1** | **AGCKEPCIITACEDVVSLWK + Carbamidomethyl (C)** |
|  | 918 | **407.0232** | **1218.0475** | **1217.4388** | **0.6087** | **1** | **11** | **1.8e+02** | **1** | **LGQMDTKGLVR** |
|  | 2539 | **547.8459** | **1093.6770** | **1093.0632** | **0.6138** | **1** | **11** | **1.5e+02** | **1** | **NTSRSEEDR** |
|  | 1429 | **434.8546** | **1301.5417** | **1301.5335** | **0.0081** | **1** | **11** | **1.9e+02** | **1** | **VLIKYGNEPLR** |
|  | 1615 | **448.1690** | **1341.4848** | **1341.5759** | **-0.0911** | **0** | **11** | **2e+02** | **1** | **SQLLHLASPMTK + Oxidation (M)** |
|  | 2624 | **561.8861** | **1682.6362** | **1681.6513** | **0.9849** | **1** | **11** | **1.7e+02** | **1** | **GRCSSYSDSSSSSSER** |
|  | 3242 | **740.5490** | **2218.6249** | **2219.6247** | **-0.9998** | **2** | **11** | **1.9e+02** | **1** | **VSIYKLTGAVMHYGNMKFK + 2 Oxidation (M)** |
|  | 80 | **364.5424** | **1090.6051** | **1091.3502** | **-0.7451** | **1** | **11** | **1.6e+02** | **1** | **RMPGLGWMK + Oxidation (M)** |
|  | 600 | **389.9924** | **1166.9549** | **1166.3869** | **0.5680** | **0** | **11** | **2e+02** | **1** | **CVEEILGYLK** |
|  | 2279 | **513.2220** | **1536.6440** | **1536.7099** | **-0.0659** | **0** | **11** | **2e+02** | **1** | **FLNDTSLPHSCFR** |
|  | 2041 | **485.9890** | **1454.9449** | **1454.6508** | **0.2941** | **1** | **11** | **1.9e+02** | **1** | **QRSYFCMSSGLC + Carbamidomethyl (C); Oxidation (M)** |
|  | 2361 | **521.0699** | **1040.1251** | **1041.1193** | **-0.9942** | **1** | **11** | **1.9e+02** | **1** | **SGVPAQVRGGN** |
|  | 51 | **363.1738** | **724.3329** | **724.8686** | **-0.5357** | **1** | **11** | **1.7e+02** | **1** | **MFKSNV** |
|  | 1828 | **463.2938** | **1386.8592** | **1387.5784** | **-0.7192** | **1** | **11** | **1.5e+02** | **1** | **EMATMKEEFQK + Oxidation (M)** |
|  | 2013 | **481.1064** | **1440.2971** | **1440.6059** | **-0.3089** | **1** | **11** | **2.2e+02** | **1** | **HLFTSHSTGIGRK** |
|  | 2395 | **523.9363** | **1568.7867** | **1567.8100** | **0.9766** | **1** | **11** | **2.2e+02** | **1** | **LHLYASMLYERR + Oxidation (M)** |
|  | 1886 | **470.1324** | **938.2501** | **937.0942** | **1.1559** | **0** | **11** | **1.9e+02** | **1** | **IHAEVQLK** |
|  | 3294 | **742.8492** | **2225.5256** | **2226.5592** | **-1.0336** | **1** | **11** | **2.4e+02** | **1** | **VSCRVSGHIFPPQSITWVR + Carbamidomethyl (C)** |
|  | 1020 | **408.3073** | **814.5999** | **813.8589** | **0.7410** | **0** | **11** | **1.7e+02** | **1** | **SSGGTLHR** |
|  | 1890 | **470.3030** | **938.5912** | **938.2080** | **0.3831** | **1** | **11** | **1.5e+02** | **1** | **GLGLKIPLK** |
|  | 1907 | **472.3783** | **1414.1128** | **1413.6818** | **0.4310** | **0** | **11** | **1.6e+02** | **1** | **MADPEVCCFITK + Carbamidomethyl (C)** |
|  | 2219 | **504.3165** | **1509.9274** | **1508.8438** | **1.0836** | **0** | **11** | **1.9e+02** | **1** | **TLTVALFTLICCP + 2 Carbamidomethyl (C)** |
|  | 2417 | **526.3875** | **1576.1404** | **1576.6212** | **-0.4809** | **0** | **11** | **1.7e+02** | **1** | **GYSYGPHFDYWGQ** |
|  | 1118 | **415.0078** | **1242.0012** | **1241.3094** | **0.6917** | **0** | **11** | **2.1e+02** | **1** | **GATEADYHLHK** |
|  | 180 | **369.2766** | **1104.8077** | **1105.2229** | **-0.4152** | **1** | **11** | **1.8e+02** | **1** | **EKSPQMGANK + Oxidation (M)** |
|  | 299 | **376.2823** | **750.5499** | **750.7998** | **-0.2499** | **0** | **11** | **1.6e+02** | **1** | **GFNSQAK** |
|  | 1868 | **468.2728** | **1401.7963** | **1400.6859** | **1.1104** | **0** | **11** | **2e+02** | **1** | **GGLIGLAACSIALGK + Carbamidomethyl (C)** |
|  | 2090 | **488.3154** | **1461.9239** | **1462.5653** | **-0.6413** | **0** | **11** | **1.9e+02** | **1** | **MSCGPAAAPQDGEGR + Oxidation (M)** |
|  | 479 | **387.0342** | **1158.0805** | **1158.3302** | **-0.2497** | **0** | **11** | **2.3e+02** | **1** | **MSCATSGLCR + 2 Carbamidomethyl (C); Oxidation (M)** |
[truncated: 3,307,119 more chars]
