# Supplementary material for: Tobacco-induced hyperglycemia promotes lung cancer progression via cancer cell-macrophage interaction through paracrine IGF2/IR/NPM1-driven PD-L1 expression
Source: Nat Commun. 2024 Jun 8;15:4909. doi: 10.1038/s41467-024-49199-9 (PMC11162468; doi:10.1038/s41467-024-49199-9)
Supplement: Supplementary file 3 — Supplementary Data 1 [file 41467_2024_49199_MOESM3_ESM.zip › Supplementary Data 1/5.htm]

Peptide Summary Report (../data/20120627/F011179.dat)


# Mascot Search Results

```
User            : yprc
Email           : info
Search title    : 
MS data file    : 5.xml
Database        : NCBInr 110704 (14481393 sequences; 4958963357 residues)
Taxonomy        : Homo sapiens (human) (217342 sequences)
Timestamp       : 27 Jun 2012 at 05:12:23 GMT

|  |  |  |
| --- | --- | --- |
| Protein hits    : | gi|14043072 | heterogeneous nuclear ribonucleoproteins A2/B1 isoform B1 [Homo sapiens] |
|  | gi|825671 | B23 nucleophosmin (280 AA) [Homo sapiens] |
|  | gi|8923427 | OCIA domain-containing protein 1 isoform 1 [Homo sapiens] |
|  | gi|36102 | unnamed protein product [Homo sapiens] |
|  | gi|36138 | ribosomal protein L6 [Homo sapiens] |
|  | gi|194380042 | unnamed protein product [Homo sapiens] |
|  | gi|2780748 | hnRNP JKTBP [Homo sapiens] |
|  | gi|1911429 | A0=heterogeneous nuclear ribonucleoprotein [human, placenta, Peptide, 305 aa] |
|  | gi|17066105 | Titin [Homo sapiens] |
|  | gi|225131084 | titin [Homo sapiens] |
|  | gi|119631418 | titin, isoform CRA_a [Homo sapiens] |
|  | gi|2606094 | Cyr61 protein [Homo sapiens] |
|  | gi|21397154 | BX1 [Homo sapiens] |
|  | gi|1790878 | microtubule-associated protein 1a [Homo sapiens] |
|  | gi|119590550 | hCG37498, isoform CRA_b [Homo sapiens] |
|  | gi|17066104 | N2B-Titin Isoform [Homo sapiens] |
|  | gi|119631420 | titin, isoform CRA_b [Homo sapiens] |
|  | gi|21930287 | ninein-Lm isoform [Homo sapiens] |
|  | gi|1212992 | titin [Homo sapiens] |
|  | gi|46107962 | inositol 1,4,5-trisphosphate receptor type 1 [Homo sapiens] |
```

### Probability Based Mowse Score

Ions score is -10\*Log(P), where P is the
probability that the observed match is a random event.  
Individual ions scores
> 47 indicate identity or extensive homology (p<0.05).  
Protein scores
are derived from ions scores as a non-probabilistic basis for ranking protein
hits.

### Peptide Summary Report

|  |  |  |  |
| --- | --- | --- | --- |
|  | Peptide Summary Select Summary (protein hits) Select Summary (unassigned) Export Search Results |  | Help |
|  | Significance threshold p< | Max. number of hits |  |
|  | Standard scoring  MudPIT scoring | Ions score or expect cut-off | Show sub-sets |
|  | Show pop-ups  Suppress pop-ups | Sort unassigned  Decreasing Score Increasing query / Mr Decreasing Intensity | Require bold red |

  
 
                
             


  


  
     **Error tolerant**    

|  |  |
| --- | --- |
| **1.** | gi|14043072    **Mass:** 37429    **Score:** 794    **Queries matched:** 57   **emPAI:** 4.01 |
|  | heterogeneous nuclear ribonucleoproteins A2/B1 isoform B1 [Homo sapiens] |

|  |  |
| --- | --- |
|  | Check to include this hit in error tolerant search or archive report |
|  |  |

|  |  |  |  |  |  |  |  |  |  |  |
| --- | --- | --- | --- | --- | --- | --- | --- | --- | --- | --- |
|  | **Query** | **Observed** | **Mr(expt)** | **Mr(calc)** | **Delta** | **Miss** | **Score** | **Expect** | **Rank** | **Peptide** |
|  | 2117 | **507.2463** | **1012.4779** | **1013.0213** | **-0.5434** | **0** | **(41)** | **0.19** | **1** | **R.GGNFGFGDSR.G** |
|  | 2118 | **507.2508** | **1012.4868** | **1013.0213** | **-0.5345** | **0** | **(51)** | **0.023** | **1** | **R.GGNFGFGDSR.G** |
|  | 2119 | **507.2771** | **1012.5394** | **1013.0213** | **-0.4819** | **0** | **53** | **0.013** | **1** | **R.GGNFGFGDSR.G** |
|  | 2283 | **530.1606** | **1058.3064** | **1057.1982** | **1.1081** | **0** | **54** | **0.013** | **1** | **K.TLETVPLER.K** |
|  | 2284 | **530.1671** | **1058.3194** | **1057.1982** | **1.1212** | **0** | **(35)** | **0.85** | **1** | **K.TLETVPLER.K** |
|  | 1338 | **438.4319** | **1312.2737** | **1313.3356** | **-1.0619** | **1** | **(6)** | **7.4e+02** | **3** | **R.SGRGGNFGFGDSR.G** |
|  | 1340 | **438.4864** | **1312.4371** | **1313.3356** | **-0.8985** | **1** | **(8)** | **5.6e+02** | **3** | **R.SGRGGNFGFGDSR.G** |
|  | 1341 | **438.4883** | **1312.4427** | **1313.3356** | **-0.8929** | **1** | **(21)** | **28** | **1** | **R.SGRGGNFGFGDSR.G** |
|  | 2932 | **657.3751** | **1312.7355** | **1313.3356** | **-0.6001** | **1** | **(56)** | **0.0065** | **1** | **R.SGRGGNFGFGDSR.G** |
|  | 2933 | **657.4470** | **1312.8791** | **1313.3356** | **-0.4564** | **1** | **(37)** | **0.54** | **1** | **R.SGRGGNFGFGDSR.G** |
|  | 1342 | **438.6354** | **1312.8840** | **1313.3356** | **-0.4515** | **1** | **(14)** | **97** | **1** | **R.SGRGGNFGFGDSR.G** |
|  | 1343 | **438.6799** | **1313.0174** | **1313.3356** | **-0.3181** | **1** | **(14)** | **84** | **1** | **R.SGRGGNFGFGDSR.G** |
|  | 2934 | **657.6188** | **1313.2228** | **1313.3356** | **-0.1128** | **1** | **68** | **0.00033** | **1** | **R.SGRGGNFGFGDSR.G** |
|  | 1344 | **438.7497** | **1313.2269** | **1313.3356** | **-0.1087** | **1** | **(23)** | **13** | **1** | **R.SGRGGNFGFGDSR.G** |
|  | 2935 | **657.6522** | **1313.2897** | **1313.3356** | **-0.0459** | **1** | **(19)** | **27** | **2** | **R.SGRGGNFGFGDSR.G** |
|  | 2936 | **657.8130** | **1313.6113** | **1313.3356** | **0.2757** | **1** | **(47)** | **0.06** | **1** | **R.SGRGGNFGFGDSR.G** |
|  | 1345 | **439.0098** | **1314.0071** | **1313.3356** | **0.6716** | **1** | **(14)** | **1e+02** | **1** | **R.SGRGGNFGFGDSR.G** |
|  | 1347 | **439.0369** | **1314.0884** | **1313.3356** | **0.7529** | **1** | **(34)** | **1.2** | **1** | **R.SGRGGNFGFGDSR.G** |
|  | 1349 | **439.0734** | **1314.1979** | **1313.3356** | **0.8623** | **1** | **(15)** | **89** | **1** | **R.SGRGGNFGFGDSR.G** |
|  | 1350 | **439.0896** | **1314.2467** | **1313.3356** | **0.9111** | **1** | **(22)** | **19** | **1** | **R.SGRGGNFGFGDSR.G** |
|  | 2939 | **658.1794** | **1314.3441** | **1313.3356** | **1.0085** | **1** | **(21)** | **22** | **1** | **R.SGRGGNFGFGDSR.G** |
|  | 1351 | **439.1394** | **1314.3959** | **1313.3356** | **1.0604** | **1** | **(15)** | **1e+02** | **1** | **R.SGRGGNFGFGDSR.G** |
|  | 1352 | **439.1542** | **1314.4405** | **1313.3356** | **1.1050** | **1** | **(11)** | **2.4e+02** | **3** | **R.SGRGGNFGFGDSR.G** |
|  | 3147 | **689.3374** | **1376.6600** | **1377.4208** | **-0.7608** | **0** | **(58)** | **0.0044** | **1** | **R.GGGGNFGPGPGSNFR.G** |
|  | 3148 | **689.6422** | **1377.2696** | **1377.4208** | **-0.1511** | **0** | **(23)** | **10** | **1** | **R.GGGGNFGPGPGSNFR.G** |
|  | 3149 | **689.6897** | **1377.3646** | **1377.4208** | **-0.0562** | **0** | **74** | **9.1e-05** | **1** | **R.GGGGNFGPGPGSNFR.G** |
|  | 3152 | **690.1207** | **1378.2266** | **1377.4208** | **0.8058** | **0** | **(46)** | **0.059** | **1** | **R.GGGGNFGPGPGSNFR.G** |
|  | 3154 | **690.2966** | **1378.5785** | **1377.4208** | **1.1577** | **0** | **(37)** | **0.51** | **1** | **R.GGGGNFGPGPGSNFR.G** |
|  | 3499 | **824.8953** | **1647.7757** | **1648.7968** | **-1.0211** | **1** | **59** | **0.0033** | **1** | **K.ALSRQEMQEVQSSR.S** |
|  | 2421 | **550.4805** | **1648.4194** | **1648.7968** | **-0.3774** | **1** | **(42)** | **0.15** | **1** | **K.ALSRQEMQEVQSSR.S** |
|  | 3500 | **825.2527** | **1648.4907** | **1648.7968** | **-0.3061** | **1** | **(53)** | **0.012** | **1** | **K.ALSRQEMQEVQSSR.S** |
|  | 2422 | **550.6982** | **1649.0726** | **1648.7968** | **0.2757** | **1** | **(18)** | **42** | **1** | **K.ALSRQEMQEVQSSR.S** |
|  | 2425 | **550.8389** | **1649.4944** | **1648.7968** | **0.6976** | **1** | **(57)** | **0.0045** | **1** | **K.ALSRQEMQEVQSSR.S** |
|  | 2456 | **556.0515** | **1665.1324** | **1664.7962** | **0.3361** | **1** | **(30)** | **2.4** | **1** | **K.ALSRQEMQEVQSSR.S + Oxidation (M)** |
|  | 2460 | **556.1133** | **1665.3177** | **1664.7962** | **0.5214** | **1** | **(47)** | **0.049** | **1** | **K.ALSRQEMQEVQSSR.S + Oxidation (M)** |
|  | 3509 | **833.7169** | **1665.4191** | **1664.7962** | **0.6228** | **1** | **(32)** | **1.5** | **1** | **K.ALSRQEMQEVQSSR.S + Oxidation (M)** |
|  | 3510 | **833.7171** | **1665.4194** | **1664.7962** | **0.6232** | **1** | **(40)** | **0.22** | **1** | **K.ALSRQEMQEVQSSR.S + Oxidation (M)** |
|  | 2681 | **593.5059** | **1777.4954** | **1776.9691** | **0.5263** | **2** | **(41)** | **0.17** | **1** | **R.KALSRQEMQEVQSSR.S** |
|  | 2731 | **598.3676** | **1792.0805** | **1792.9686** | **-0.8881** | **2** | **53** | **0.012** | **1** | **R.KALSRQEMQEVQSSR.S + Oxidation (M)** |
|  | 3552 | **897.8074** | **1793.6000** | **1792.9686** | **0.6314** | **2** | **(27)** | **4.2** | **1** | **R.KALSRQEMQEVQSSR.S + Oxidation (M)** |
|  | 2928 | **656.2645** | **1965.7714** | **1965.1106** | **0.6609** | **2** | **14** | **1.1e+02** | **1** | **K.ALSRQEMQEVQSSRSGR.G + Oxidation (M)** |
|  | 3576 | **1095.7946** | **2189.5743** | **2190.2254** | **-0.6510** | **0** | **(76)** | **5.3e-05** | **1** | **R.NMGGPYGGGNYGPGGSGGSGGYGGR.S** |
|  | 3242 | **731.3827** | **2191.1259** | **2190.2254** | **0.9005** | **0** | **(74)** | **0.00011** | **1** | **R.NMGGPYGGGNYGPGGSGGSGGYGGR.S** |
|  | 3577 | **1103.7709** | **2205.5270** | **2206.2248** | **-0.6978** | **0** | **(81)** | **1.7e-05** | **1** | **R.NMGGPYGGGNYGPGGSGGSGGYGGR.S + Oxidation (M)** |
|  | 3255 | **736.3891** | **2206.1451** | **2206.2248** | **-0.0796** | **0** | **(70)** | **0.00025** | **1** | **R.NMGGPYGGGNYGPGGSGGSGGYGGR.S + Oxidation (M)** |
|  | 3578 | **1104.1960** | **2206.3773** | **2206.2248** | **0.1525** | **0** | **105** | **6.9e-08** | **1** | **R.NMGGPYGGGNYGPGGSGGSGGYGGR.S + Oxidation (M)** |
|  | 3256 | **736.5948** | **2206.7622** | **2206.2248** | **0.5374** | **0** | **(14)** | **91** | **1** | **R.NMGGPYGGGNYGPGGSGGSGGYGGR.S + Oxidation (M)** |
|  | 3257 | **736.6233** | **2206.8477** | **2206.2248** | **0.6229** | **0** | **(100)** | **2.1e-07** | **1** | **R.NMGGPYGGGNYGPGGSGGSGGYGGR.S + Oxidation (M)** |
|  | 3379 | **757.8483** | **2270.5226** | **2271.2783** | **-0.7556** | **1** | **82** | **1.9e-05** | **1** | **R.GGGGNFGPGPGSNFRGGSDGYGSGR.G** |
|  | 3382 | **758.0688** | **2271.1842** | **2271.2783** | **-0.0941** | **1** | **(4)** | **8.7e+02** | **6** | **R.GGGGNFGPGPGSNFRGGSDGYGSGR.G** |
|  | 3384 | **758.1085** | **2271.3034** | **2271.2783** | **0.0251** | **1** | **(54)** | **0.0083** | **1** | **R.GGGGNFGPGPGSNFRGGSDGYGSGR.G** |
|  | 3385 | **758.1166** | **2271.3277** | **2271.2783** | **0.0495** | **1** | **(56)** | **0.0052** | **1** | **R.GGGGNFGPGPGSNFRGGSDGYGSGR.G** |
|  | 3386 | **758.1556** | **2271.4447** | **2271.2783** | **0.1665** | **1** | **(21)** | **20** | **1** | **R.GGGGNFGPGPGSNFRGGSDGYGSGR.G** |
|  | 3388 | **758.2168** | **2271.6282** | **2271.2783** | **0.3499** | **1** | **(54)** | **0.0091** | **1** | **R.GGGGNFGPGPGSNFRGGSDGYGSGR.G** |
|  | 3389 | **758.3091** | **2271.9051** | **2271.2783** | **0.6268** | **1** | **(63)** | **0.0014** | **1** | **R.GGGGNFGPGPGSNFRGGSDGYGSGR.G** |
|  | 3547 | **872.0658** | **2613.1752** | **2612.6611** | **0.5142** | **2** | **127** | **5.4e-10** | **1** | **R.NMGGPYGGGNYGPGGSGGSGGYGGRSRY.- + Oxidation (M)** |
|  | 3570 | **990.5245** | **2968.5514** | **2968.9955** | **-0.4440** | **1** | **111** | **2e-08** | **1** | **K.SGNFGGSRNMGGPYGGGNYGPGGSGGSGGYGGR.S + Oxidation (M)** |

  


---

|  |  |
| --- | --- |
| **2.** | gi|825671    **Mass:** 30938    **Score:** 467    **Queries matched:** 34   **emPAI:** 1.77 |
|  | B23 nucleophosmin (280 AA) [Homo sapiens] |

|  |  |
| --- | --- |
|  | Check to include this hit in error tolerant search or archive report |
|  |  |

|  |  |  |  |  |  |  |  |  |  |  |
| --- | --- | --- | --- | --- | --- | --- | --- | --- | --- | --- |
|  | **Query** | **Observed** | **Mr(expt)** | **Mr(calc)** | **Delta** | **Miss** | **Score** | **Expect** | **Rank** | **Peptide** |
|  | 535 | **392.3037** | **782.5926** | **782.9261** | **-0.3335** | **0** | **36** | **0.48** | **1** | **K.FINYVK.N** |
|  | 540 | **392.4283** | **782.8418** | **782.9261** | **-0.0842** | **0** | **(20)** | **29** | **6** | **K.FINYVK.N** |
|  | 546 | **392.8993** | **783.7838** | **782.9261** | **0.8577** | **0** | **(14)** | **87** | **3** | **K.FINYVK.N** |
|  | 663 | **402.1827** | **802.3507** | **802.9158** | **-0.5652** | **0** | **13** | **1.5e+02** | **9** | **R.TVSLGAGAK.D** |
|  | 1321 | **437.3789** | **872.7430** | **873.0519** | **-0.3090** | **1** | **(25)** | **8.3** | **1** | **K.LLSISGKR.S** |
|  | 1326 | **437.6862** | **873.3575** | **873.0519** | **0.3056** | **1** | **43** | **0.11** | **1** | **K.LLSISGKR.S** |
|  | 1726 | **466.3013** | **930.5878** | **930.9988** | **-0.4109** | **0** | **62** | **0.0014** | **1** | **K.GPSSVEDIK.A** |
|  | 2004 | **493.3562** | **984.6976** | **985.1356** | **-0.4379** | **1** | **30** | **2.4** | **1** | **K.GGSLPKVEAK.F** |
|  | 2006 | **493.6165** | **985.2181** | **985.1356** | **0.0826** | **1** | **(21)** | **22** | **1** | **K.GGSLPKVEAK.F** |
|  | 2509 | **565.8708** | **1129.7268** | **1130.2490** | **-0.5222** | **1** | **44** | **0.085** | **1** | **K.GPSSVEDIKAK.M** |
|  | 272 | **377.9616** | **1130.8626** | **1130.2490** | **0.6136** | **1** | **(40)** | **0.22** | **1** | **K.GPSSVEDIKAK.M** |
|  | 273 | **377.9851** | **1130.9332** | **1130.2490** | **0.6843** | **1** | **(43)** | **0.11** | **1** | **K.GPSSVEDIKAK.M** |
|  | 276 | **378.0894** | **1131.2459** | **1130.2490** | **0.9969** | **1** | **(15)** | **72** | **1** | **K.GPSSVEDIKAK.M** |
|  | 2757 | **606.0721** | **1210.1295** | **1210.4214** | **-0.2918** | **1** | **15** | **71** | **1** | **K.VEAKFINYVK.N** |
|  | 1050 | **420.0242** | **1257.0503** | **1257.3902** | **-0.3398** | **1** | **33** | **1.3** | **1** | **K.TPKGPSSVEDIK.A** |
|  | 2873 | **629.6028** | **1257.1908** | **1257.3902** | **-0.1994** | **1** | **(15)** | **75** | **2** | **K.TPKGPSSVEDIK.A** |
|  | 3064 | **681.2502** | **1360.4857** | **1360.5825** | **-0.0968** | **1** | **42** | **0.18** | **1** | **K.FINYVKNCFR.M + Carbamidomethyl (C)** |
|  | 1537 | **454.5298** | **1360.5674** | **1360.5825** | **-0.0151** | **1** | **(34)** | **1.3** | **2** | **K.FINYVKNCFR.M + Carbamidomethyl (C)** |
|  | 3067 | **681.5954** | **1361.1760** | **1360.5825** | **0.5936** | **1** | **(38)** | **0.33** | **1** | **K.FINYVKNCFR.M + Carbamidomethyl (C)** |
|  | 1541 | **454.8203** | **1361.4387** | **1360.5825** | **0.8562** | **1** | **(28)** | **4.5** | **2** | **K.FINYVKNCFR.M + Carbamidomethyl (C)** |
|  | 1542 | **454.8269** | **1361.4586** | **1361.5641** | **-0.1054** | **1** | **(18)** | **45** | **1** | **K.MQASIEKGGSLPK.V + Oxidation (M)** |
|  | 3069 | **681.7735** | **1361.5322** | **1361.5641** | **-0.0318** | **1** | **43** | **0.14** | **2** | **K.MQASIEKGGSLPK.V + Oxidation (M)** |
|  | 1545 | **454.8988** | **1361.6741** | **1360.5825** | **1.0917** | **1** | **(21)** | **23** | **1** | **K.FINYVKNCFR.M + Carbamidomethyl (C)** |
|  | 1546 | **455.0782** | **1362.2126** | **1361.5641** | **0.6485** | **1** | **(23)** | **13** | **2** | **K.MQASIEKGGSLPK.V + Oxidation (M)** |
|  | 1924 | **486.3779** | **1456.1116** | **1456.6404** | **-0.5287** | **2** | **(45)** | **0.06** | **1** | **K.TPKGPSSVEDIKAK.M** |
|  | 3238 | **729.2542** | **1456.4936** | **1456.6404** | **-0.1467** | **2** | **(32)** | **1.5** | **1** | **K.TPKGPSSVEDIKAK.M** |
|  | 1929 | **486.7809** | **1457.3206** | **1456.6404** | **0.6802** | **2** | **55** | **0.0066** | **1** | **K.TPKGPSSVEDIKAK.M** |
|  | 3240 | **729.7126** | **1457.4104** | **1456.6404** | **0.7700** | **2** | **(44)** | **0.099** | **1** | **K.TPKGPSSVEDIKAK.M** |
|  | 1931 | **486.9054** | **1457.6940** | **1456.6404** | **1.0536** | **2** | **(15)** | **88** | **1** | **K.TPKGPSSVEDIKAK.M** |
|  | 2275 | **528.9927** | **1583.9560** | **1583.7816** | **0.1745** | **2** | **8** | **4.5e+02** | **7** | **K.TPKTPKGPSSVEDIK.A** |
|  | 2657 | **591.7463** | **1772.2167** | **1773.0600** | **-0.8433** | **2** | **46** | **0.067** | **2** | **K.MQASIEKGGSLPKVEAK.F** |
|  | 2718 | **597.3652** | **1789.0733** | **1789.0594** | **0.0140** | **2** | **(36)** | **0.71** | **1** | **K.MQASIEKGGSLPKVEAK.F + Oxidation (M)** |
|  | 2723 | **597.5167** | **1789.5278** | **1789.0594** | **0.4685** | **2** | **(34)** | **1** | **2** | **K.MQASIEKGGSLPKVEAK.F + Oxidation (M)** |
|  | 2725 | **597.5521** | **1789.6342** | **1789.0594** | **0.5748** | **2** | **(4)** | **8.9e+02** | **10** | **K.MQASIEKGGSLPKVEAK.F + Oxidation (M)** |

  

|  |  |
| --- | --- |
|  | |
|  | **Proteins matching the same set of peptides:** |

|  |  |
| --- | --- |
|  | gi|10835063    **Mass:** 32575    **Score:** 467    **Queries matched:** 34 |
|  | nucleophosmin isoform 1 [Homo sapiens] |

|  |  |
| --- | --- |
|  | gi|15214852    **Mass:** 32609    **Score:** 467    **Queries matched:** 34 |
|  | Nucleophosmin (nucleolar phosphoprotein B23, numatrin) [Homo sapiens] |

|  |  |
| --- | --- |
|  | gi|18314408    **Mass:** 32576    **Score:** 467    **Queries matched:** 34 |
|  | Nucleophosmin (nucleolar phosphoprotein B23, numatrin) [Homo sapiens] |

|  |  |
| --- | --- |
|  | gi|33694244    **Mass:** 32603    **Score:** 467    **Queries matched:** 34 |
|  | nucleophosmin [Homo sapiens] |

|  |  |
| --- | --- |
|  | gi|40353734    **Mass:** 29464    **Score:** 467    **Queries matched:** 34 |
|  | nucleophosmin isoform 2 [Homo sapiens] |

|  |  |
| --- | --- |
|  | gi|58220457    **Mass:** 32818    **Score:** 467    **Queries matched:** 34 |
|  | nucleophosmin [Homo sapiens] |

|  |  |
| --- | --- |
|  | gi|58220459    **Mass:** 32836    **Score:** 467    **Queries matched:** 34 |
|  | nucleophosmin [Homo sapiens] |

|  |  |
| --- | --- |
|  | gi|58220461    **Mass:** 32804    **Score:** 467    **Queries matched:** 34 |
|  | nucleophosmin [Homo sapiens] |

|  |  |
| --- | --- |
|  | gi|58220465    **Mass:** 32887    **Score:** 467    **Queries matched:** 34 |
|  | nucleophosmin [Homo sapiens] |

|  |  |
| --- | --- |
|  | gi|58220467    **Mass:** 32945    **Score:** 467    **Queries matched:** 34 |
|  | nucleophosmin [Homo sapiens] |

|  |  |
| --- | --- |
|  | gi|58220469    **Mass:** 32460    **Score:** 467    **Queries matched:** 34 |
|  | nucleophosmin [Homo sapiens] |

|  |  |
| --- | --- |
|  | gi|119581852    **Mass:** 31842    **Score:** 467    **Queries matched:** 34 |
|  | nucleophosmin (nucleolar phosphoprotein B23, numatrin), isoform CRA\_e [Homo sapiens] |

|  |  |
| --- | --- |
|  | gi|197692173    **Mass:** 32574    **Score:** 467    **Queries matched:** 34 |
|  | nucleophosmin 1 isoform 1 [Homo sapiens] |

---

|  |  |
| --- | --- |
| **3.** | gi|8923427    **Mass:** 27626    **Score:** 265    **Queries matched:** 13   **emPAI:** 0.77 |
|  | OCIA domain-containing protein 1 isoform 1 [Homo sapiens] |

|  |  |
| --- | --- |
|  | Check to include this hit in error tolerant search or archive report |
|  |  |

|  |  |  |  |  |  |  |  |  |  |  |
| --- | --- | --- | --- | --- | --- | --- | --- | --- | --- | --- |
|  | **Query** | **Observed** | **Mr(expt)** | **Mr(calc)** | **Delta** | **Miss** | **Score** | **Expect** | **Rank** | **Peptide** |
|  | 2213 | **520.0671** | **1038.1195** | **1037.1239** | **0.9956** | **0** | **28** | **4.1** | **1** | **K.NITYEELR.N** |
|  | 2216 | **520.0979** | **1038.1810** | **1037.1239** | **1.0571** | **0** | **(27)** | **5.5** | **1** | **K.NITYEELR.N** |
|  | 2739 | **600.1332** | **1198.2516** | **1198.3260** | **-0.0745** | **0** | **55** | **0.0087** | **1** | **K.LENSPLGEALR.S** |
|  | 2740 | **600.1467** | **1198.2787** | **1198.3260** | **-0.0474** | **0** | **(36)** | **0.79** | **1** | **K.LENSPLGEALR.S** |
|  | 2823 | **614.3654** | **1226.7161** | **1226.2485** | **0.4676** | **1** | **55** | **0.0089** | **1** | **K.VNKYGDTWDE.-** |
|  | 2825 | **614.5704** | **1227.1261** | **1226.2485** | **0.8776** | **1** | **(44)** | **0.097** | **1** | **K.VNKYGDTWDE.-** |
|  | 1138 | **427.8427** | **1280.5061** | **1279.3988** | **1.1072** | **1** | **(17)** | **58** | **2** | **K.NITYEELRNK.N** |
|  | 2897 | **641.2783** | **1280.5417** | **1279.3988** | **1.1429** | **1** | **35** | **0.88** | **1** | **K.NITYEELRNK.N** |
|  | 2954 | **664.1727** | **1326.3307** | **1326.4983** | **-0.1677** | **1** | **57** | **0.0055** | **1** | **K.KLENSPLGEALR.S** |
|  | 1384 | **443.2706** | **1326.7895** | **1326.4983** | **0.2912** | **1** | **(55)** | **0.0074** | **1** | **K.KLENSPLGEALR.S** |
|  | 2177 | **517.8835** | **1550.6285** | **1549.6871** | **0.9413** | **2** | **14** | **1.2e+02** | **3** | **K.NITYEELRNKNR.E** |
|  | 2514 | **567.0273** | **1698.0599** | **1697.8474** | **0.2124** | **1** | **14** | **1e+02** | **2** | **K.LENSPLGEALRSGQAR.R** |
|  | 2772 | **609.3624** | **1825.0651** | **1826.0197** | **-0.9546** | **2** | **10** | **2.8e+02** | **4** | **K.KLENSPLGEALRSGQAR.R** |

  


---

|  |  |
| --- | --- |
| **4.** | gi|36102    **Mass:** 34152    **Score:** 256    **Queries matched:** 22   **emPAI:** 0.59 |
|  | unnamed protein product [Homo sapiens] |

|  |  |
| --- | --- |
|  | Check to include this hit in error tolerant search or archive report |
|  |  |

|  |  |  |  |  |  |  |  |  |  |  |
| --- | --- | --- | --- | --- | --- | --- | --- | --- | --- | --- |
|  | **Query** | **Observed** | **Mr(expt)** | **Mr(calc)** | **Delta** | **Miss** | **Score** | **Expect** | **Rank** | **Peptide** |
|  | 819 | **407.1963** | **1218.5667** | **1218.4202** | **0.1465** | **0** | **7** | **4.2e+02** | **6** | **K.IEVIEIMTDR.G** |
|  | 1334 | **438.3320** | **1311.9739** | **1312.3508** | **-0.3768** | **1** | **(11)** | **2.1e+02** | **10** | **R.GGNFSGRGGFGGSR.G** |
|  | 1341 | 438.4883 | 1312.4427 | 1312.3508 | 0.0919 | 1 | (14) | 1.5e+02 | 2 | R.GGNFSGRGGFGGSR.G |
|  | 2932 | 657.3751 | 1312.7355 | 1312.3508 | 0.3847 | 1 | 23 | 14 | 3 | R.GGNFSGRGGFGGSR.G |
|  | 1342 | 438.6354 | 1312.8840 | 1312.3508 | 0.5333 | 1 | (8) | 4.1e+02 | 5 | R.GGNFSGRGGFGGSR.G |
|  | 1344 | 438.7497 | 1313.2269 | 1312.3508 | 0.8761 | 1 | (16) | 56 | 3 | R.GGNFSGRGGFGGSR.G |
|  | 2384 | **543.7966** | **1628.3677** | **1628.7424** | **-0.3746** | **0** | **(19)** | **30** | **1** | **R.SSGPYGGGGQYFAKPR.N** |
|  | 3484 | **815.2007** | **1628.3866** | **1628.7424** | **-0.3558** | **0** | **(80)** | **2.2e-05** | **1** | **R.SSGPYGGGGQYFAKPR.N** |
|  | 2385 | **544.0020** | **1628.9837** | **1628.7424** | **0.2413** | **0** | **(38)** | **0.46** | **1** | **R.SSGPYGGGGQYFAKPR.N** |
|  | 3485 | **815.6315** | **1629.2482** | **1628.7424** | **0.5058** | **0** | **92** | **1.5e-06** | **1** | **R.SSGPYGGGGQYFAKPR.N** |
|  | 2387 | **544.1498** | **1629.4272** | **1628.7424** | **0.6848** | **0** | **(5)** | **9.3e+02** | **9** | **R.SSGPYGGGGQYFAKPR.N** |
|  | 2388 | **544.1760** | **1629.5059** | **1628.7424** | **0.7635** | **0** | **(70)** | **0.00033** | **1** | **R.SSGPYGGGGQYFAKPR.N** |
|  | 2391 | **544.2142** | **1629.6203** | **1628.7424** | **0.8780** | **0** | **(38)** | **0.47** | **1** | **R.SSGPYGGGGQYFAKPR.N** |
|  | 3526 | **848.1669** | **1694.3191** | **1694.6282** | **-0.3092** | **0** | **98** | **3.6e-07** | **1** | **R.NQGGYGGSSSSSSYGSGR.R** |
|  | 2978 | **666.7511** | **1997.2311** | **1997.9879** | **-0.7567** | **2** | **(3)** | **1.5e+03** | **5** | **R.NQGGYGGSSSSSSYGSGRRF.-** |
|  | 3571 | **999.7671** | **1997.5194** | **1997.9879** | **-0.4685** | **2** | **36** | **0.58** | **1** | **R.NQGGYGGSSSSSSYGSGRRF.-** |
|  | 3572 | **999.7892** | **1997.5636** | **1997.9879** | **-0.4243** | **2** | **(23)** | **12** | **1** | **R.NQGGYGGSSSSSSYGSGRRF.-** |
|  | 2984 | **666.8665** | **1997.5772** | **1997.9879** | **-0.4107** | **2** | **(35)** | **0.73** | **1** | **R.NQGGYGGSSSSSSYGSGRRF.-** |
|  | 2985 | **666.9254** | **1997.7539** | **1997.9879** | **-0.2340** | **2** | **(13)** | **1e+02** | **1** | **R.NQGGYGGSSSSSSYGSGRRF.-** |
|  | 2988 | **667.2573** | **1998.7496** | **1997.9879** | **0.7618** | **2** | **(22)** | **17** | **1** | **R.NQGGYGGSSSSSSYGSGRRF.-** |
|  | 2989 | **667.3077** | **1998.9010** | **1997.9879** | **0.9132** | **2** | **(29)** | **3.2** | **1** | **R.NQGGYGGSSSSSSYGSGRRF.-** |
|  | 2991 | **667.4020** | **1999.1838** | **1997.9879** | **1.1959** | **2** | **(23)** | **13** | **1** | **R.NQGGYGGSSSSSSYGSGRRF.-** |

  

|  |  |
| --- | --- |
|  | |
|  | **Proteins matching the same set of peptides:** |

|  |  |
| --- | --- |
|  | gi|4504445    **Mass:** 34196    **Score:** 256    **Queries matched:** 22 |
|  | heterogeneous nuclear ribonucleoprotein A1 isoform a [Homo sapiens] |

|  |  |
| --- | --- |
|  | gi|13436308    **Mass:** 31752    **Score:** 256    **Queries matched:** 22 |
|  | Unknown (protein for IMAGE:3615335) [Homo sapiens] |

|  |  |
| --- | --- |
|  | gi|14043070    **Mass:** 38746    **Score:** 256    **Queries matched:** 22 |
|  | heterogeneous nuclear ribonucleoprotein A1 isoform b [Homo sapiens] |

|  |  |
| --- | --- |
|  | gi|47939618    **Mass:** 34180    **Score:** 256    **Queries matched:** 22 |
|  | Heterogeneous nuclear ribonucleoprotein A1 [Homo sapiens] |

---

|  |  |
| --- | --- |
| **5.** | gi|36138    **Mass:** 32861    **Score:** 228    **Queries matched:** 10   **emPAI:** 0.33 |
|  | ribosomal protein L6 [Homo sapiens] |

|  |  |
| --- | --- |
|  | Check to include this hit in error tolerant search or archive report |
|  |  |

|  |  |  |  |  |  |  |  |  |  |  |
| --- | --- | --- | --- | --- | --- | --- | --- | --- | --- | --- |
|  | **Query** | **Observed** | **Mr(expt)** | **Mr(calc)** | **Delta** | **Miss** | **Score** | **Expect** | **Rank** | **Peptide** |
|  | 1920 | **486.1636** | **970.3124** | **970.1209** | **0.1915** | **0** | **29** | **3.1** | **2** | **K.AVDSQILPK.I** |
|  | 2532 | **571.0667** | **1140.1186** | **1139.2144** | **0.9043** | **0** | **52** | **0.014** | **1** | **R.YYPTEDVPR.K** |
|  | 2585 | **580.5323** | **1159.0499** | **1158.3498** | **0.7001** | **0** | **44** | **0.095** | **1** | **K.AIPQLQGYLR.S** |
|  | 2587 | **580.6172** | **1159.2197** | **1158.3498** | **0.8699** | **0** | **(37)** | **0.63** | **1** | **K.AIPQLQGYLR.S** |
|  | 732 | **405.0878** | **1212.2413** | **1211.4507** | **0.7906** | **1** | **(10)** | **2.3e+02** | **3** | **K.AVDSQILPKIK.A** |
|  | 740 | **405.1414** | **1212.4022** | **1211.4507** | **0.9514** | **1** | **21** | **20** | **1** | **K.AVDSQILPKIK.A** |
|  | 1111 | **423.7955** | **1268.3642** | **1267.3867** | **0.9776** | **1** | **24** | **12** | **1** | **R.YYPTEDVPRK.L** |
|  | 1163 | **429.0829** | **1284.2267** | **1284.5017** | **-0.2750** | **0** | **10** | **2.4e+02** | **5** | **K.VLATVTKPVGGDK.N** |
|  | 1733 | **467.5903** | **1399.7488** | **1399.6797** | **0.0691** | **1** | **15** | **97** | **1** | **K.IKAIPQLQGYLR.S** |
|  | 2272 | **528.8292** | **1583.4653** | **1582.8396** | **0.6257** | **2** | **32** | **1.2** | **1** | **R.KIDQKAVDSQILPK.I** |

  

|  |  |
| --- | --- |
|  | |
|  | **Proteins matching the same set of peptides:** |

|  |  |
| --- | --- |
|  | gi|21410970    **Mass:** 32726    **Score:** 228    **Queries matched:** 10 |
|  | Ribosomal protein L6 [Homo sapiens] |

|  |  |
| --- | --- |
|  | gi|9802306    **Mass:** 32891    **Score:** 226    **Queries matched:** 10 |
|  | DNA-binding protein TAXREB107 [Homo sapiens] |

|  |  |
| --- | --- |
|  | gi|16753227    **Mass:** 32728    **Score:** 226    **Queries matched:** 10 |
|  | 60S ribosomal protein L6 [Homo sapiens] |

|  |  |
| --- | --- |
|  | gi|18088374    **Mass:** 32747    **Score:** 226    **Queries matched:** 10 |
|  | Ribosomal protein L6 [Homo sapiens] |

|  |  |
| --- | --- |
|  | gi|18490263    **Mass:** 32742    **Score:** 226    **Queries matched:** 10 |
|  | Ribosomal protein L6 [Homo sapiens] |

|  |  |
| --- | --- |
|  | gi|189053182    **Mass:** 32701    **Score:** 226    **Queries matched:** 10 |
|  | unnamed protein product [Homo sapiens] |

---

|  |  |
| --- | --- |
| **6.** | gi|194380042    **Mass:** 39680    **Score:** 164    **Queries matched:** 10   **emPAI:** 0.27 |
|  | unnamed protein product [Homo sapiens] |

|  |  |
| --- | --- |
|  | Check to include this hit in error tolerant search or archive report |
|  |  |

|  |  |  |  |  |  |  |  |  |  |  |
| --- | --- | --- | --- | --- | --- | --- | --- | --- | --- | --- |
|  | **Query** | **Observed** | **Mr(expt)** | **Mr(calc)** | **Delta** | **Miss** | **Score** | **Expect** | **Rank** | **Peptide** |
|  | 1838 | **477.3827** | **952.7505** | **952.0443** | **0.7062** | **0** | **7** | **4.3e+02** | **4** | **K.TCGTGISTR.V + Carbamidomethyl (C)** |
|  | 2184 | **518.7803** | **1035.5458** | **1035.1991** | **0.3466** | **0** | **48** | **0.035** | **1** | **R.CCTPQLTR.T + 2 Carbamidomethyl (C)** |
|  | 2290 | **531.1835** | **1060.3522** | **1061.2548** | **-0.9026** | **0** | **9** | **3.3e+02** | **4** | **R.LPVFGMEPR.I + Oxidation (M)** |
|  | 2631 | **587.6198** | **1173.2248** | **1173.3611** | **-0.1363** | **0** | **34** | **1.2** | **1** | **R.ILYNPLQGQK.C** |
|  | 2633 | **587.6935** | **1173.3723** | **1173.3611** | **0.0112** | **0** | **(10)** | **3.1e+02** | **3** | **R.ILYNPLQGQK.C** |
|  | 2634 | **587.9193** | **1173.8237** | **1173.3611** | **0.4626** | **0** | **(24)** | **8.8** | **1** | **R.ILYNPLQGQK.C** |
|  | 2636 | **588.1608** | **1174.3068** | **1173.3611** | **0.9456** | **0** | **(32)** | **1.8** | **1** | **R.ILYNPLQGQK.C** |
|  | 801 | **406.8445** | **1217.5114** | **1217.4405** | **0.0709** | **1** | **20** | **26** | **1** | **K.RLPVFGMEPR.I + Oxidation (M)** |
|  | 1931 | 486.9054 | 1457.6940 | 1456.8173 | 0.8767 | 1 | 6 | 6.9e+02 | 7 | R.LALSTCGCCKVCAK.Q + Carbamidomethyl (C) |
|  | 2791 | **611.7431** | **1832.2071** | **1831.0166** | **1.1906** | **2** | **44** | **0.11** | **1** | **R.VTNDNPECRLVKETR.I + Carbamidomethyl (C)** |

  


---

|  |  |
| --- | --- |
| **7.** | gi|2780748    **Mass:** 33588    **Score:** 139    **Queries matched:** 6   **emPAI:** 0.33 |
|  | hnRNP JKTBP [Homo sapiens] |

|  |  |
| --- | --- |
|  | Check to include this hit in error tolerant search or archive report |
|  |  |

|  |  |  |  |  |  |  |  |  |  |  |
| --- | --- | --- | --- | --- | --- | --- | --- | --- | --- | --- |
|  | **Query** | **Observed** | **Mr(expt)** | **Mr(calc)** | **Delta** | **Miss** | **Score** | **Expect** | **Rank** | **Peptide** |
|  | 2040 | **499.0577** | **996.1006** | **996.0720** | **0.0285** | **0** | **13** | **1.2e+02** | **1** | **K.DLTEYLSR.F** |
|  | 2396 | **545.3458** | **1088.6769** | **1089.2451** | **-0.5682** | **1** | **59** | **0.0032** | **1** | **K.VAQPKEVYR.Q** |
|  | 69 | **364.2359** | **1089.6856** | **1089.2451** | **0.4406** | **1** | **(34)** | **0.8** | **1** | **K.VAQPKEVYR.Q** |
|  | 416 | **387.1507** | **1158.4298** | **1157.3636** | **1.0663** | **1** | **7** | **5.8e+02** | **8** | **R.SRGFGFVLFK.D** |
|  | 1800 | **474.4251** | **1420.2531** | **1419.6049** | **0.6481** | **1** | **27** | **5.1** | **1** | **R.YHQIGSGKCEIK.V + Carbamidomethyl (C)** |
|  | 2949 | **663.3253** | **1986.9538** | **1986.1925** | **0.7612** | **2** | **35** | **0.83** | **1** | **K.VAQPKEVYRQQQQQQK.G** |

  

|  |  |
| --- | --- |
|  | |
|  | **Proteins matching the same set of peptides:** |

|  |  |
| --- | --- |
|  | gi|15010818    **Mass:** 27191    **Score:** 139    **Queries matched:** 6 |
|  | JKTBP1delta6 [Homo sapiens] |

|  |  |
| --- | --- |
|  | gi|38327502    **Mass:** 33213    **Score:** 139    **Queries matched:** 6 |
|  | CArG binding factor [Homo sapiens] |

|  |  |
| --- | --- |
|  | gi|149521754    **Mass:** 30184    **Score:** 139    **Queries matched:** 6 |
|  | PREDICTED: similar to heterogeneous nuclear ribonucleoprotein D-like, isoform 1 [Ornithorhynchus anatinus] |

|  |  |
| --- | --- |
|  | gi|194390304    **Mass:** 30214    **Score:** 139    **Queries matched:** 6 |
|  | unnamed protein product [Homo sapiens] |

|  |  |
| --- | --- |
|  | gi|14110407    **Mass:** 46437    **Score:** 138    **Queries matched:** 6 |
|  | heterogeneous nuclear ribonucleoprotein D-like isoform a [Homo sapiens] |

|  |  |
| --- | --- |
|  | gi|39644771    **Mass:** 45883    **Score:** 138    **Queries matched:** 6 |
|  | HNRPDL protein [Homo sapiens] |

|  |  |
| --- | --- |
|  | gi|332801090    **Mass:** 40040    **Score:** 138    **Queries matched:** 6 |
|  | heterogeneous nuclear ribonucleoprotein D-like isoform b [Homo sapiens] |

---

|  |  |
| --- | --- |
| **8.** | gi|1911429    **Mass:** 30900    **Score:** 136    **Queries matched:** 4   **emPAI:** 0.23 |
|  | A0=heterogeneous nuclear ribonucleoprotein [human, placenta, Peptide, 305 aa] |

|  |  |
| --- | --- |
|  | Check to include this hit in error tolerant search or archive report |
|  |  |

|  |  |  |  |  |  |  |  |  |  |  |
| --- | --- | --- | --- | --- | --- | --- | --- | --- | --- | --- |
|  | **Query** | **Observed** | **Mr(expt)** | **Mr(calc)** | **Delta** | **Miss** | **Score** | **Expect** | **Rank** | **Peptide** |
|  | 3084 | **684.0952** | **1366.1756** | **1365.4949** | **0.6808** | **1** | **5** | **7.9e+02** | **10** | **R.EDSARPGAHAKVK.K** |
|  | 2336 | **536.2499** | **1605.7276** | **1606.6922** | **-0.9645** | **1** | **(12)** | **1.6e+02** | **1** | **K.AVPKEDIYSGGGGGGSR.S** |
|  | 3470 | **804.1877** | **1606.3607** | **1606.6922** | **-0.3314** | **1** | **74** | **9.8e-05** | **1** | **K.AVPKEDIYSGGGGGGSR.S** |
|  | 2579 | **579.6553** | **1735.9438** | **1734.8645** | **1.0794** | **2** | **57** | **0.0061** | **1** | **K.KAVPKEDIYSGGGGGGSR.S** |

  

|  |  |
| --- | --- |
|  | |
|  | **Proteins matching the same set of peptides:** |

|  |  |
| --- | --- |
|  | gi|5803036    **Mass:** 30840    **Score:** 136    **Queries matched:** 4 |
|  | heterogeneous nuclear ribonucleoprotein A0 [Homo sapiens] |

---

|  |  |
| --- | --- |
| **9.** | gi|17066105    **Mass:** 3816172  **Score:** 130    **Queries matched:** 57 |
|  | Titin [Homo sapiens] |

|  |  |
| --- | --- |
|  | Check to include this hit in error tolerant search or archive report |
|  |  |

|  |  |  |  |  |  |  |  |  |  |  |
| --- | --- | --- | --- | --- | --- | --- | --- | --- | --- | --- |
|  | **Query** | **Observed** | **Mr(expt)** | **Mr(calc)** | **Delta** | **Miss** | **Score** | **Expect** | **Rank** | **Peptide** |
|  | 184 | **371.0591** | **740.1034** | **738.9612** | **1.1422** | **1** | **4** | **7.8e+02** | **6** | **R.KLIIPR.G** |
|  | 190 | **371.1832** | **740.3516** | **739.8601** | **0.4915** | **1** | **(11)** | **1.5e+02** | **4** | **K.KEAPPAK.V** |
|  | 192 | **371.2608** | **740.5068** | **739.8601** | **0.6467** | **1** | **14** | **72** | **3** | **K.KEAPPAK.V** |
|  | 240 | **374.9745** | **747.9342** | **748.7791** | **-0.8449** | **0** | **13** | **1.7e+02** | **2** | **R.ELSADSK.H** |
|  | 296 | **380.0652** | **758.1157** | **758.9064** | **-0.7906** | **1** | **10** | **3.2e+02** | **2** | **R.VKGLTNK.K** |
|  | 347 | **385.0210** | **768.0272** | **767.9133** | **0.1139** | **0** | **8** | **3.4e+02** | **6** | **R.GVPVPTAK.W** |
|  | 370 | **386.0157** | **770.0165** | **768.9875** | **1.0291** | **1** | **7** | **4.4e+02** | **3** | **K.KVVAKPK.E** |
|  | 372 | **386.0449** | **770.0750** | **769.9292** | **0.1458** | **1** | **20** | **24** | **2** | **K.VAVPEKK.V** |
|  | 445 | **388.1121** | **774.2093** | **774.9041** | **-0.6947** | **0** | **7** | **7e+02** | **8** | **K.EPAAFLK.R** |
|  | 839 | **407.5888** | **813.1628** | **812.0089** | **1.1540** | **1** | **9** | **3.2e+02** | **3** | **R.VPEVIKK.A** |
|  | 1357 | **439.9832** | **877.9516** | **876.9777** | **0.9739** | **0** | **8** | **5.2e+02** | **6** | **K.CLEGQTAR.F** |
|  | 1470 | **448.2874** | **894.5599** | **895.0545** | **-0.4945** | **0** | **7** | **3.9e+02** | **4** | **K.KPEVPPTK.V** |
|  | 1511 | **451.2696** | **900.5244** | **900.0375** | **0.4869** | **1** | **2** | **1.5e+03** | **6** | **R.LGRLGASAR.L** |
|  | 1531 | **453.4450** | **904.8752** | **904.0197** | **0.8556** | **1** | **9** | **3.9e+02** | **8** | **R.LSIDNSKK.G** |
|  | 1603 | **458.9693** | **915.9238** | **914.9199** | **1.0040** | **1** | **20** | **30** | **1** | **R.SESDGHKR.R** |
|  | 1667 | **462.1082** | **922.2016** | **922.0763** | **0.1253** | **0** | **4** | **1.1e+03** | **10** | **K.ITGYIVEK.K** |
|  | 1759 | **470.0428** | **938.0709** | **938.1321** | **-0.0611** | **2** | **(3)** | **1.1e+03** | **10** | **K.RAAPLVRR.R** |
|  | 1768 | **470.4817** | **938.9487** | **938.1321** | **0.8167** | **2** | **17** | **52** | **2** | **K.RAAPLVRR.R** |
|  | 2312 | **534.3156** | **1066.6164** | **1067.2161** | **-0.5997** | **0** | **4** | **1.2e+03** | **3** | **K.YMIAEDLGR.G** |
|  | 40 | **363.1039** | **1086.2896** | **1087.2938** | **-1.0042** | **1** | **5** | **8.8e+02** | **9** | **R.DGVPLKATMR.F** |
|  | 91 | **366.2093** | **1095.6058** | **1095.2761** | **0.3297** | **2** | **6** | **6.2e+02** | **7** | **K.REAAMRAFK.T + Oxidation (M)** |
|  | 2414 | **549.4994** | **1096.9840** | **1097.3514** | **-0.3674** | **1** | **8** | **3.2e+02** | **3** | **R.KPIIERTLK.A** |
|  | 2479 | **562.0752** | **1122.1356** | **1121.2919** | **0.8437** | **1** | **12** | **1.8e+02** | **1** | **R.MSPARMSPGR.R + 2 Oxidation (M)** |
|  | 257 | **376.2853** | **1125.8337** | **1125.3187** | **0.5150** | **1** | **6** | **5.1e+02** | **7** | **K.DKPAVAPATKK.A** |
|  | 404 | **386.9664** | **1157.8769** | **1157.3620** | **0.5150** | **0** | **5** | **1e+03** | **9** | **R.VSVFNLHITK.C** |
|  | 524 | **391.2444** | **1170.7112** | **1170.3658** | **0.3454** | **2** | **7** | **4.3e+02** | **3** | **R.KGIVVRAGGSAR.I** |
|  | 678 | **403.2116** | **1206.6126** | **1205.4909** | **1.1217** | **2** | **9** | **3.1e+02** | **4** | **K.KSLGWFKVLK.E** |
|  | 820 | **407.2014** | **1218.5822** | **1219.4315** | **-0.8493** | **0** | **13** | **1e+02** | **1** | **K.VLGSSIHMECK.V + Oxidation (M)** |
|  | 861 | **407.8979** | **1220.6715** | **1221.3595** | **-0.6880** | **0** | **12** | **1.8e+02** | **2** | **K.IADFSTNLVNK.D** |
|  | 928 | **412.1732** | **1233.4973** | **1234.2938** | **-0.7965** | **1** | **17** | **58** | **2** | **K.MPDDDGGDRIK.G + Oxidation (M)** |
|  | 1073 | **422.0147** | **1263.0219** | **1263.4658** | **-0.4439** | **2** | **12** | **1.8e+02** | **2** | **K.RDLPSKSWMK.A + Oxidation (M)** |
|  | 2885 | **637.6091** | **1273.2035** | **1273.4738** | **-0.2704** | **0** | **23** | **12** | **4** | **K.EIELDFAVPLK.D** |
|  | 2897 | 641.2783 | 1280.5417 | 1279.4174 | 1.1244 | 1 | 9 | 3.5e+02 | 10 | K.EVTIMEEKER.A + Oxidation (M) |
|  | 1171 | **430.0873** | **1287.2398** | **1286.5073** | **0.7324** | **2** | **9** | **3.6e+02** | **4** | **K.RCNAAAQLVRK.E + Carbamidomethyl (C)** |
|  | 1254 | **434.2907** | **1299.8500** | **1300.3765** | **-0.5266** | **0** | **4** | **7.6e+02** | **5** | **K.GEQTWSHAGISK.T** |
|  | 2935 | 657.6522 | 1313.2897 | 1312.4058 | 0.8839 | 1 | 15 | 84 | 5 | K.DEAKFECEVSR.E |
|  | 1533 | **454.0694** | **1359.1860** | **1358.5819** | **0.6041** | **1** | **6** | **7.8e+02** | **6** | **K.EPAAPPKVPEVPK.K** |
|  | 3147 | 689.3374 | 1376.6600 | 1376.6449 | 0.0152 | 1 | 10 | 2.7e+02 | 4 | R.LDCKIAGSLPMR.V + Carbamidomethyl (C); Oxidation (M) |
|  | 1697 | **464.0195** | **1389.0364** | **1388.5862** | **0.4503** | **0** | **3** | **1.4e+03** | **8** | **K.VCEGDIVQLEVK.V + Carbamidomethyl (C)** |
|  | 1924 | 486.3779 | 1456.1116 | 1455.7020 | 0.4097 | 2 | 6 | 5.2e+02 | 9 | R.CNKMPVKDTTYR.V |
|  | 3285 | **740.6399** | **1479.2650** | **1479.7467** | **-0.4817** | **2** | **4** | **9e+02** | **7** | **R.VNKVPVTMTRYR.S + Oxidation (M)** |
|  | 2096 | **505.7566** | **1514.2477** | **1514.6847** | **-0.4370** | **2** | **1** | **1.4e+03** | **7** | **R.DATKRTWSVVSHK.C** |
|  | 2104 | **506.0691** | **1515.1851** | **1514.6566** | **0.5285** | **1** | **3** | **1.1e+03** | **6** | **R.DEVDPPRISMDPK.Y + Oxidation (M)** |
|  | 2130 | **508.4141** | **1522.2200** | **1522.6849** | **-0.4649** | **1** | **5** | **6.5e+02** | **9** | **K.YVCQAKNDAGIQR.C + Carbamidomethyl (C)** |
|  | 2176 | **517.6781** | **1550.0121** | **1549.7285** | **0.2836** | **1** | **4** | **1.1e+03** | **10** | **R.VPGLIEGNEYRFR.I** |
|  | 2235 | **521.1406** | **1560.3997** | **1560.6255** | **-0.2258** | **2** | **5** | **7.2e+02** | **7** | **K.DRGEVIRESENTR.I** |
|  | 2273 | **528.9186** | **1583.7336** | **1584.8935** | **-1.1600** | **0** | **15** | **86** | **1** | **K.ELPLIFITPLSDVK.V** |
|  | 2311 | **534.1686** | **1599.4837** | **1598.7995** | **0.6842** | **1** | **8** | **4.6e+02** | **5** | **K.KAEAVATVVAAVDQAR.V** |
|  | 2315 | **534.5234** | **1600.5481** | **1600.8120** | **-0.2638** | **0** | **6** | **6.3e+02** | **5** | **K.ADNSVGAVASSAVLVIK.E** |
|  | 2326 | **536.0461** | **1605.1163** | **1605.8500** | **-0.7337** | **0** | **1** | **1.9e+03** | **9** | **K.YGVGEPLESAPVLMK.N + Oxidation (M)** |
|  | 2785 | **610.2767** | **1827.8080** | **1828.1384** | **-0.3304** | **2** | **5** | **8.1e+02** | **10** | **K.KGDATQLACKVTGTPPIK.I** |
|  | 2796 | **612.1584** | **1833.4530** | **1834.1644** | **-0.7114** | **1** | **3** | **1.2e+03** | **8** | **R.AGSNLKVDIPISGKPLPK.V** |
|  | 2945 | **662.0758** | **1983.2052** | **1983.2683** | **-0.0630** | **2** | **11** | **1.9e+02** | **3** | **R.KLKETNGLSGSSVVMECK.V + Carbamidomethyl (C); Oxidation (M)** |
|  | 3031 | **670.3690** | **2008.0849** | **2008.3275** | **-0.2426** | **2** | **7** | **5.5e+02** | **4** | **K.RPRTASPHFTVSKISVPK.T** |
|  | 3155 | **690.5618** | **2068.6631** | **2069.5163** | **-0.8531** | **2** | **4** | **9.6e+02** | **5** | **K.ACRPIKPPGPPINPKLKDK.S** |
|  | 3255 | 736.3891 | 2206.1451 | 2205.5122 | 0.6330 | 2 | 6 | 6.1e+02 | 6 | R.FVAEEKLSFAVPQRVEVTR.H |
|  | 3566 | **972.0663** | **2913.1767** | **2912.2278** | **0.9488** | **1** | **11** | **2e+02** | **1** | **R.TSVSLAWSVPEDEGGSKVTGYLIEMQK.V** |

  

|  |  |
| --- | --- |
|  | |
|  | **Proteins matching the same set of peptides:** |

|  |  |
| --- | --- |
|  | gi|108861911    **Mass:** 3816142  **Score:** 130    **Queries matched:** 57 |
|  | RecName: Full=Titin; AltName: Full=Connectin; AltName: Full=Rhabdomyosarcoma antigen MU-RMS-40.14 |

---

|  |  |
| --- | --- |
| **10.** | gi|225131084    **Mass:** 3713667  **Score:** 130    **Queries matched:** 58 |
|  | titin [Homo sapiens] |

|  |  |
| --- | --- |
|  | Check to include this hit in error tolerant search or archive report |
|  |  |

|  |  |  |  |  |  |  |  |  |  |  |
| --- | --- | --- | --- | --- | --- | --- | --- | --- | --- | --- |
|  | **Query** | **Observed** | **Mr(expt)** | **Mr(calc)** | **Delta** | **Miss** | **Score** | **Expect** | **Rank** | **Peptide** |
|  | 184 | 371.0591 | 740.1034 | 738.9612 | 1.1422 | 1 | 4 | 7.8e+02 | 6 | R.KLIIPR.G |
|  | 190 | 371.1832 | 740.3516 | 739.8601 | 0.4915 | 1 | (11) | 1.5e+02 | 4 | K.KEAPPAK.V |
|  | 192 | 371.2608 | 740.5068 | 739.8601 | 0.6467 | 1 | 14 | 72 | 3 | K.KEAPPAK.V |
|  | 240 | 374.9745 | 747.9342 | 748.7791 | -0.8449 | 0 | 13 | 1.7e+02 | 2 | R.ELSADSK.H |
|  | 296 | 380.0652 | 758.1157 | 758.9064 | -0.7906 | 1 | 10 | 3.2e+02 | 2 | R.VKGLTNK.K |
|  | 300 | **380.1822** | **758.3496** | **757.9198** | **0.4298** | **0** | **0** | **2.4e+03** | **8** | **R.FLTLHK.V** |
|  | 347 | 385.0210 | 768.0272 | 767.9133 | 0.1139 | 0 | 8 | 3.4e+02 | 6 | R.GVPVPTAK.W |
|  | 370 | 386.0157 | 770.0165 | 768.9875 | 1.0291 | 1 | 7 | 4.4e+02 | 3 | K.KVVAKPK.E |
|  | 372 | 386.0449 | 770.0750 | 769.9292 | 0.1458 | 1 | 20 | 24 | 2 | K.VAVPEKK.V |
|  | 445 | 388.1121 | 774.2093 | 774.9041 | -0.6947 | 0 | 7 | 7e+02 | 8 | K.EPAAFLK.R |
|  | 839 | 407.5888 | 813.1628 | 812.0089 | 1.1540 | 1 | 9 | 3.2e+02 | 3 | R.VPEVIKK.A |
|  | 1357 | 439.9832 | 877.9516 | 876.9777 | 0.9739 | 0 | 8 | 5.2e+02 | 6 | K.CLEGQTAR.F |
|  | 1470 | 448.2874 | 894.5599 | 895.0545 | -0.4945 | 0 | 7 | 3.9e+02 | 4 | K.KPEVPPTK.V |
|  | 1511 | 451.2696 | 900.5244 | 900.0375 | 0.4869 | 1 | 2 | 1.5e+03 | 6 | R.LGRLGASAR.L |
|  | 1531 | 453.4450 | 904.8752 | 904.0197 | 0.8556 | 1 | 9 | 3.9e+02 | 8 | R.LSIDNSKK.G |
|  | 1603 | 458.9693 | 915.9238 | 914.9199 | 1.0040 | 1 | 20 | 30 | 1 | R.SESDGHKR.R |
|  | 1667 | 462.1082 | 922.2016 | 922.0763 | 0.1253 | 0 | 4 | 1.1e+03 | 10 | K.ITGYIVEK.K |
|  | 1759 | 470.0428 | 938.0709 | 938.1321 | -0.0611 | 2 | (3) | 1.1e+03 | 10 | K.RAAPLVRR.R |
|  | 1768 | 470.4817 | 938.9487 | 938.1321 | 0.8167 | 2 | 17 | 52 | 2 | K.RAAPLVRR.R |
|  | 2312 | 534.3156 | 1066.6164 | 1067.2161 | -0.5997 | 0 | 4 | 1.2e+03 | 3 | K.YMIAEDLGR.G |
|  | 40 | 363.1039 | 1086.2896 | 1087.2938 | -1.0042 | 1 | 5 | 8.8e+02 | 9 | R.DGVPLKATMR.F |
|  | 91 | 366.2093 | 1095.6058 | 1095.2761 | 0.3297 | 2 | 6 | 6.2e+02 | 7 | K.REAAMRAFK.T + Oxidation (M) |
|  | 2414 | 549.4994 | 1096.9840 | 1097.3514 | -0.3674 | 1 | 8 | 3.2e+02 | 3 | R.KPIIERTLK.A |
|  | 2479 | 562.0752 | 1122.1356 | 1121.2919 | 0.8437 | 1 | 12 | 1.8e+02 | 1 | R.MSPARMSPGR.R + 2 Oxidation (M) |
|  | 257 | 376.2853 | 1125.8337 | 1125.3187 | 0.5150 | 1 | 6 | 5.1e+02 | 7 | K.DKPAVAPATKK.A |
|  | 404 | 386.9664 | 1157.8769 | 1157.3620 | 0.5150 | 0 | 5 | 1e+03 | 9 | R.VSVFNLHITK.C |
|  | 524 | 391.2444 | 1170.7112 | 1170.3658 | 0.3454 | 2 | 7 | 4.3e+02 | 3 | R.KGIVVRAGGSAR.I |
|  | 678 | 403.2116 | 1206.6126 | 1205.4909 | 1.1217 | 2 | 9 | 3.1e+02 | 4 | K.KSLGWFKVLK.E |
|  | 820 | 407.2014 | 1218.5822 | 1219.4315 | -0.8493 | 0 | 13 | 1e+02 | 1 | K.VLGSSIHMECK.V + Oxidation (M) |
|  | 861 | 407.8979 | 1220.6715 | 1221.3595 | -0.6880 | 0 | 12 | 1.8e+02 | 2 | K.IADFSTNLVNK.D |
|  | 928 | 412.1732 | 1233.4973 | 1234.2938 | -0.7965 | 1 | 17 | 58 | 2 | K.MPDDDGGDRIK.G + Oxidation (M) |
|  | 1073 | 422.0147 | 1263.0219 | 1263.4658 | -0.4439 | 2 | 12 | 1.8e+02 | 2 | K.RDLPSKSWMK.A + Oxidation (M) |
|  | 2885 | 637.6091 | 1273.2035 | 1273.4738 | -0.2704 | 0 | 23 | 12 | 4 | K.EIELDFAVPLK.D |
|  | 2897 | 641.2783 | 1280.5417 | 1279.4174 | 1.1244 | 1 | 9 | 3.5e+02 | 10 | K.EVTIMEEKER.A + Oxidation (M) |
|  | 1171 | 430.0873 | 1287.2398 | 1286.5073 | 0.7324 | 2 | 9 | 3.6e+02 | 4 | K.RCNAAAQLVRK.E + Carbamidomethyl (C) |
|  | 1254 | 434.2907 | 1299.8500 | 1300.3765 | -0.5266 | 0 | 4 | 7.6e+02 | 5 | K.GEQTWSHAGISK.T |
|  | 2935 | 657.6522 | 1313.2897 | 1312.4058 | 0.8839 | 1 | 15 | 84 | 5 | K.DEAKFECEVSR.E |
|  | 1533 | 454.0694 | 1359.1860 | 1358.5819 | 0.6041 | 1 | 6 | 7.8e+02 | 6 | K.EPAAPPKVPEVPK.K |
|  | 3147 | 689.3374 | 1376.6600 | 1376.6449 | 0.0152 | 1 | 10 | 2.7e+02 | 4 | R.LDCKIAGSLPMR.V + Carbamidomethyl (C); Oxidation (M) |
|  | 1697 | 464.0195 | 1389.0364 | 1388.5862 | 0.4503 | 0 | 3 | 1.4e+03 | 8 | K.VCEGDIVQLEVK.V + Carbamidomethyl (C) |
|  | 1924 | 486.3779 | 1456.1116 | 1455.7020 | 0.4097 | 2 | 6 | 5.2e+02 | 9 | R.CNKMPVKDTTYR.V |
|  | 3285 | 740.6399 | 1479.2650 | 1479.7467 | -0.4817 | 2 | 4 | 9e+02 | 7 | R.VNKVPVTMTRYR.S + Oxidation (M) |
|  | 2096 | 505.7566 | 1514.2477 | 1514.6847 | -0.4370 | 2 | 1 | 1.4e+03 | 7 | R.DATKRTWSVVSHK.C |
|  | 2104 | 506.0691 | 1515.1851 | 1514.6566 | 0.5285 | 1 | 3 | 1.1e+03 | 6 | R.DEVDPPRISMDPK.Y + Oxidation (M) |
|  | 2130 | 508.4141 | 1522.2200 | 1522.6849 | -0.4649 | 1 | 5 | 6.5e+02 | 9 | K.YVCQAKNDAGIQR.C + Carbamidomethyl (C) |
|  | 2176 | 517.6781 | 1550.0121 | 1549.7285 | 0.2836 | 1 | 4 | 1.1e+03 | 10 | R.VPGLIEGNEYRFR.I |
|  | 2235 | 521.1406 | 1560.3997 | 1560.6255 | -0.2258 | 2 | 5 | 7.2e+02 | 7 | K.DRGEVIRESENTR.I |
|  | 2273 | 528.9186 | 1583.7336 | 1584.8935 | -1.1600 | 0 | 15 | 86 | 1 | K.ELPLIFITPLSDVK.V |
|  | 2311 | 534.1686 | 1599.4837 | 1598.7995 | 0.6842 | 1 | 8 | 4.6e+02 | 5 | K.KAEAVATVVAAVDQAR.V |
|  | 2315 | 534.5234 | 1600.5481 | 1600.8120 | -0.2638 | 0 | 6 | 6.3e+02 | 5 | K.ADNSVGAVASSAVLVIK.A |
|  | 2326 | 536.0461 | 1605.1163 | 1605.8500 | -0.7337 | 0 | 1 | 1.9e+03 | 9 | K.YGVGEPLESAPVLMK.N + Oxidation (M) |
|  | 2785 | 610.2767 | 1827.8080 | 1828.1384 | -0.3304 | 2 | 5 | 8.1e+02 | 10 | K.KGDATQLACKVTGTPPIK.I |
|  | 2796 | 612.1584 | 1833.4530 | 1834.1644 | -0.7114 | 1 | 3 | 1.2e+03 | 8 | R.AGSNLKVDIPISGKPLPK.V |
|  | 2945 | 662.0758 | 1983.2052 | 1983.2683 | -0.0630 | 2 | 11 | 1.9e+02 | 3 | R.KLKETNGLSGSSVVMECK.V + Carbamidomethyl (C); Oxidation (M) |
|  | 3031 | 670.3690 | 2008.0849 | 2008.3275 | -0.2426 | 2 | 7 | 5.5e+02 | 4 | K.RPRTASPHFTVSKISVPK.T |
|  | 3155 | 690.5618 | 2068.6631 | 2069.5163 | -0.8531 | 2 | 4 | 9.6e+02 | 5 | K.ACRPIKPPGPPINPKLKDK.S |
|  | 3255 | 736.3891 | 2206.1451 | 2205.5122 | 0.6330 | 2 | 6 | 6.1e+02 | 6 | R.FVAEEKLSFAVPQRVEVTR.H |
|  | 3566 | 972.0663 | 2913.1767 | 2912.2278 | 0.9488 | 1 | 11 | 2e+02 | 1 | R.TSVSLAWSVPEDEGGSKVTGYLIEMQK.V |

  


---

|  |  |
| --- | --- |
| **11.** | gi|119631418    **Mass:** 3881215  **Score:** 126    **Queries matched:** 60 |
|  | titin, isoform CRA\_a [Homo sapiens] |

|  |  |
| --- | --- |
|  | Check to include this hit in error tolerant search or archive report |
|  |  |

|  |  |  |  |  |  |  |  |  |  |  |
| --- | --- | --- | --- | --- | --- | --- | --- | --- | --- | --- |
|  | **Query** | **Observed** | **Mr(expt)** | **Mr(calc)** | **Delta** | **Miss** | **Score** | **Expect** | **Rank** | **Peptide** |
|  | 184 | 371.0591 | 740.1034 | 738.9612 | 1.1422 | 1 | 4 | 7.8e+02 | 6 | R.KLIIPR.G |
|  | 190 | 371.1832 | 740.3516 | 739.8601 | 0.4915 | 1 | (11) | 1.5e+02 | 4 | K.KEAPPAK.V |
|  | 192 | 371.2608 | 740.5068 | 739.8601 | 0.6467 | 1 | 14 | 72 | 3 | K.KEAPPAK.V |
|  | 240 | 374.9745 | 747.9342 | 748.7791 | -0.8449 | 0 | 13 | 1.7e+02 | 2 | R.ELSADSK.H |
|  | 296 | 380.0652 | 758.1157 | 758.9064 | -0.7906 | 1 | 10 | 3.2e+02 | 2 | R.VKGLTNK.K |
|  | 300 | 380.1822 | 758.3496 | 757.9198 | 0.4298 | 0 | 0 | 2.4e+03 | 8 | R.FLTLHK.V |
|  | 347 | 385.0210 | 768.0272 | 767.9133 | 0.1139 | 0 | 8 | 3.4e+02 | 6 | R.GVPVPTAK.W |
|  | 370 | 386.0157 | 770.0165 | 768.9875 | 1.0291 | 1 | 7 | 4.4e+02 | 3 | K.KVVAKPK.E |
|  | 372 | 386.0449 | 770.0750 | 769.9292 | 0.1458 | 1 | 20 | 24 | 2 | K.VAVPEKK.V |
|  | 445 | 388.1121 | 774.2093 | 774.9041 | -0.6947 | 0 | 7 | 7e+02 | 8 | K.EPAAFLK.R |
|  | 839 | 407.5888 | 813.1628 | 812.0089 | 1.1540 | 1 | 9 | 3.2e+02 | 3 | R.VPEVIKK.A |
|  | 1357 | 439.9832 | 877.9516 | 876.9777 | 0.9739 | 0 | 8 | 5.2e+02 | 6 | K.CLEGQTAR.F |
|  | 1470 | 448.2874 | 894.5599 | 895.0545 | -0.4945 | 0 | 7 | 3.9e+02 | 4 | K.KPEVPPTK.V |
|  | 1474 | **448.4339** | **894.8530** | **896.0458** | **-1.1927** | **1** | **(1)** | **1.9e+03** | **6** | **K.KPEAPRAK.V** |
|  | 1475 | **448.4523** | **894.8898** | **896.0458** | **-1.1559** | **1** | **4** | **1.2e+03** | **5** | **K.KPEAPRAK.V** |
|  | 1511 | 451.2696 | 900.5244 | 900.0375 | 0.4869 | 1 | 2 | 1.5e+03 | 6 | R.LGRLGASAR.L |
|  | 1531 | 453.4450 | 904.8752 | 904.0197 | 0.8556 | 1 | 9 | 3.9e+02 | 8 | R.LSIDNSKK.G |
|  | 1603 | 458.9693 | 915.9238 | 914.9199 | 1.0040 | 1 | 20 | 30 | 1 | R.SESDGHKR.R |
|  | 1667 | 462.1082 | 922.2016 | 922.0763 | 0.1253 | 0 | 4 | 1.1e+03 | 10 | K.ITGYIVEK.K |
|  | 1759 | 470.0428 | 938.0709 | 938.1321 | -0.0611 | 2 | (3) | 1.1e+03 | 10 | K.RAAPLVRR.R |
|  | 1768 | 470.4817 | 938.9487 | 938.1321 | 0.8167 | 2 | 17 | 52 | 2 | K.RAAPLVRR.R |
|  | 2312 | 534.3156 | 1066.6164 | 1067.2161 | -0.5997 | 0 | 4 | 1.2e+03 | 3 | K.YMIAEDLGR.G |
|  | 40 | 363.1039 | 1086.2896 | 1087.2938 | -1.0042 | 1 | 5 | 8.8e+02 | 9 | R.DGVPLKATMR.F |
|  | 91 | 366.2093 | 1095.6058 | 1095.2761 | 0.3297 | 2 | 6 | 6.2e+02 | 7 | K.REAAMRAFK.T + Oxidation (M) |
|  | 2414 | 549.4994 | 1096.9840 | 1097.3514 | -0.3674 | 1 | 8 | 3.2e+02 | 3 | R.KPIIERTLK.A |
|  | 2479 | 562.0752 | 1122.1356 | 1121.2919 | 0.8437 | 1 | 12 | 1.8e+02 | 1 | R.MSPARMSPGR.R + 2 Oxidation (M) |
|  | 257 | 376.2853 | 1125.8337 | 1125.3187 | 0.5150 | 1 | 6 | 5.1e+02 | 7 | K.DKPAVAPATKK.A |
|  | 404 | 386.9664 | 1157.8769 | 1157.3620 | 0.5150 | 0 | 5 | 1e+03 | 9 | R.VSVFNLHITK.C |
|  | 524 | 391.2444 | 1170.7112 | 1170.3658 | 0.3454 | 2 | 7 | 4.3e+02 | 3 | R.KGIVVRAGGSAR.I |
|  | 678 | 403.2116 | 1206.6126 | 1205.4909 | 1.1217 | 2 | 9 | 3.1e+02 | 4 | K.KSLGWFKVLK.E |
|  | 820 | 407.2014 | 1218.5822 | 1219.4315 | -0.8493 | 0 | 13 | 1e+02 | 1 | K.VLGSSIHMECK.V + Oxidation (M) |
|  | 861 | 407.8979 | 1220.6715 | 1221.3595 | -0.6880 | 0 | 12 | 1.8e+02 | 2 | K.IADFSTNLVNK.D |
|  | 928 | 412.1732 | 1233.4973 | 1234.2938 | -0.7965 | 1 | 17 | 58 | 2 | K.MPDDDGGDRIK.G + Oxidation (M) |
|  | 1073 | 422.0147 | 1263.0219 | 1263.4658 | -0.4439 | 2 | 12 | 1.8e+02 | 2 | K.RDLPSKSWMK.A + Oxidation (M) |
|  | 2885 | 637.6091 | 1273.2035 | 1273.4738 | -0.2704 | 0 | 23 | 12 | 4 | K.EIELDFAVPLK.D |
|  | 2897 | 641.2783 | 1280.5417 | 1279.4174 | 1.1244 | 1 | 9 | 3.5e+02 | 10 | K.EVTIMEEKER.A + Oxidation (M) |
|  | 1171 | 430.0873 | 1287.2398 | 1286.5073 | 0.7324 | 2 | 9 | 3.6e+02 | 4 | K.RCNAAAQLVRK.E + Carbamidomethyl (C) |
|  | 1254 | 434.2907 | 1299.8500 | 1300.3765 | -0.5266 | 0 | 4 | 7.6e+02 | 5 | K.GEQTWSHAGISK.T |
|  | 2935 | 657.6522 | 1313.2897 | 1312.4058 | 0.8839 | 1 | 15 | 84 | 5 | K.DEAKFECEVSR.E |
|  | 1533 | 454.0694 | 1359.1860 | 1358.5819 | 0.6041 | 1 | 6 | 7.8e+02 | 6 | K.EPAAPPKVPEVPK.K |
|  | 3147 | 689.3374 | 1376.6600 | 1376.6449 | 0.0152 | 1 | 10 | 2.7e+02 | 4 | R.LDCKIAGSLPMR.V + Carbamidomethyl (C); Oxidation (M) |
|  | 1697 | 464.0195 | 1389.0364 | 1388.5862 | 0.4503 | 0 | 3 | 1.4e+03 | 8 | K.VCEGDIVQLEVK.V + Carbamidomethyl (C) |
|  | 1924 | 486.3779 | 1456.1116 | 1455.7020 | 0.4097 | 2 | 6 | 5.2e+02 | 9 | R.CNKMPVKDTTYR.V |
|  | 3285 | 740.6399 | 1479.2650 | 1479.7467 | -0.4817 | 2 | 4 | 9e+02 | 7 | R.VNKVPVTMTRYR.S + Oxidation (M) |
|  | 2096 | 505.7566 | 1514.2477 | 1514.6847 | -0.4370 | 2 | 1 | 1.4e+03 | 7 | R.DATKRTWSVVSHK.C |
|  | 2104 | 506.0691 | 1515.1851 | 1514.6566 | 0.5285 | 1 | 3 | 1.1e+03 | 6 | R.DEVDPPRISMDPK.Y + Oxidation (M) |
|  | 2130 | 508.4141 | 1522.2200 | 1522.6849 | -0.4649 | 1 | 5 | 6.5e+02 | 9 | K.YVCQAKNDAGIQR.C + Carbamidomethyl (C) |
|  | 2176 | 517.6781 | 1550.0121 | 1549.7285 | 0.2836 | 1 | 4 | 1.1e+03 | 10 | R.VPGLIEGNEYRFR.I |
|  | 2235 | 521.1406 | 1560.3997 | 1560.6255 | -0.2258 | 2 | 5 | 7.2e+02 | 7 | K.DRGEVIRESENTR.I |
|  | 2273 | 528.9186 | 1583.7336 | 1584.8935 | -1.1600 | 0 | 15 | 86 | 1 | K.ELPLIFITPLSDVK.V |
|  | 2311 | 534.1686 | 1599.4837 | 1598.7995 | 0.6842 | 1 | 8 | 4.6e+02 | 5 | K.KAEAVATVVAAVDQAR.V |
|  | 2315 | 534.5234 | 1600.5481 | 1600.8120 | -0.2638 | 0 | 6 | 6.3e+02 | 5 | K.ADNSVGAVASSAVLVIK.A |
|  | 2326 | 536.0461 | 1605.1163 | 1605.8500 | -0.7337 | 0 | 1 | 1.9e+03 | 9 | K.YGVGEPLESAPVLMK.N + Oxidation (M) |
|  | 2785 | 610.2767 | 1827.8080 | 1828.1384 | -0.3304 | 2 | 5 | 8.1e+02 | 10 | K.KGDATQLACKVTGTPPIK.I |
|  | 2796 | 612.1584 | 1833.4530 | 1834.1644 | -0.7114 | 1 | 3 | 1.2e+03 | 8 | R.AGSNLKVDIPISGKPLPK.V |
|  | 2945 | 662.0758 | 1983.2052 | 1983.2683 | -0.0630 | 2 | 11 | 1.9e+02 | 3 | R.KLKETNGLSGSSVVMECK.V + Carbamidomethyl (C); Oxidation (M) |
|  | 3031 | 670.3690 | 2008.0849 | 2008.3275 | -0.2426 | 2 | 7 | 5.5e+02 | 4 | K.RPRTASPHFTVSKISVPK.T |
|  | 3155 | 690.5618 | 2068.6631 | 2069.5163 | -0.8531 | 2 | 4 | 9.6e+02 | 5 | K.ACRPIKPPGPPINPKLKDK.S |
|  | 3255 | 736.3891 | 2206.1451 | 2205.5122 | 0.6330 | 2 | 6 | 6.1e+02 | 6 | R.FVAEEKLSFAVPQRVEVTR.H |
|  | 3566 | 972.0663 | 2913.1767 | 2912.2278 | 0.9488 | 1 | 11 | 2e+02 | 1 | R.TSVSLAWSVPEDEGGSKVTGYLIEMQK.V |

  


---

|  |  |
| --- | --- |
| **12.** | gi|2606094    **Mass:** 42069    **Score:** 123    **Queries matched:** 5   **emPAI:** 0.16 |
|  | Cyr61 protein [Homo sapiens] |

|  |  |
| --- | --- |
|  | Check to include this hit in error tolerant search or archive report |
|  |  |

|  |  |  |  |  |  |  |  |  |  |  |
| --- | --- | --- | --- | --- | --- | --- | --- | --- | --- | --- |
|  | **Query** | **Observed** | **Mr(expt)** | **Mr(calc)** | **Delta** | **Miss** | **Score** | **Expect** | **Rank** | **Peptide** |
|  | 1838 | 477.3827 | 952.7505 | 952.0443 | 0.7062 | 0 | 7 | 4.3e+02 | 4 | K.TCGTGISTR.V + Carbamidomethyl (C) |
|  | 2184 | 518.7803 | 1035.5458 | 1035.1991 | 0.3466 | 0 | 48 | 0.035 | 1 | R.CCTPQLTR.T + 2 Carbamidomethyl (C) |
|  | 2290 | 531.1835 | 1060.3522 | 1061.2548 | -0.9026 | 0 | 9 | 3.3e+02 | 4 | R.IPVFGMEPR.I + Oxidation (M) |
|  | 801 | 406.8445 | 1217.5114 | 1217.4405 | 0.0709 | 1 | 20 | 26 | 1 | K.RIPVFGMEPR.I + Oxidation (M) |
|  | 2791 | 611.7431 | 1832.2071 | 1831.0166 | 1.1906 | 2 | 44 | 0.11 | 1 | R.VTNDNPECRLVKETR.I + Carbamidomethyl (C) |

  

|  |  |
| --- | --- |
|  | |
|  | **Proteins matching the same set of peptides:** |

|  |  |
| --- | --- |
|  | gi|6649849    **Mass:** 37246    **Score:** 122    **Queries matched:** 5 |
|  | tumor RMS cell line RD specific product [Homo sapiens] |

---

|  |  |
| --- | --- |
| **13.** | gi|21397154    **Mass:** 6122     **Score:** 122    **Queries matched:** 2   **emPAI:** 1.53 |
|  | BX1 [Homo sapiens] |

|  |  |
| --- | --- |
|  | Check to include this hit in error tolerant search or archive report |
|  |  |

|  |  |  |  |  |  |  |  |  |  |  |
| --- | --- | --- | --- | --- | --- | --- | --- | --- | --- | --- |
|  | **Query** | **Observed** | **Mr(expt)** | **Mr(calc)** | **Delta** | **Miss** | **Score** | **Expect** | **Rank** | **Peptide** |
|  | 3563 | **956.2227** | **1910.4305** | **1910.8642** | **-0.4336** | **0** | **89** | **2.9e-06** | **1** | **R.SSGSPYGGGYGSGGGSGGYGSR.R** |
|  | 3266 | **739.3077** | **2214.9009** | **2214.2238** | **0.6771** | **2** | **33** | **1.2** | **1** | **R.SSGSPYGGGYGSGGGSGGYGSRRF.-** |

  

|  |  |
| --- | --- |
|  | |
|  | **Proteins matching the same set of peptides:** |

|  |  |
| --- | --- |
|  | gi|34740329    **Mass:** 39594    **Score:** 122    **Queries matched:** 2 |
|  | heterogeneous nuclear ribonucleoprotein A3 [Homo sapiens] |

|  |  |
| --- | --- |
|  | gi|51873980    **Mass:** 17868    **Score:** 122    **Queries matched:** 2 |
|  | HNRPA3 protein [Homo sapiens] |

|  |  |
| --- | --- |
|  | gi|119631468    **Mass:** 37029    **Score:** 122    **Queries matched:** 2 |
|  | heterogeneous nuclear ribonucleoprotein A3, isoform CRA\_a [Homo sapiens] |

|  |  |
| --- | --- |
|  | gi|194381858    **Mass:** 14040    **Score:** 122    **Queries matched:** 2 |
|  | unnamed protein product [Homo sapiens] |

|  |  |
| --- | --- |
|  | gi|194388148    **Mass:** 34189    **Score:** 122    **Queries matched:** 2 |
|  | unnamed protein product [Homo sapiens] |

---

|  |  |
| --- | --- |
| **14.** | gi|1790878    **Mass:** 306224   **Score:** 115    **Queries matched:** 22 |
|  | microtubule-associated protein 1a [Homo sapiens] |

|  |  |
| --- | --- |
|  | Check to include this hit in error tolerant search or archive report |
|  |  |

|  |  |  |  |  |  |  |  |  |  |  |
| --- | --- | --- | --- | --- | --- | --- | --- | --- | --- | --- |
|  | **Query** | **Observed** | **Mr(expt)** | **Mr(calc)** | **Delta** | **Miss** | **Score** | **Expect** | **Rank** | **Peptide** |
|  | 1184 | **430.8930** | **859.7712** | **858.9361** | **0.8352** | **0** | **13** | **1.6e+02** | **9** | **K.AGPEALSSK.G** |
|  | 1354 | **439.5455** | **877.0763** | **876.9150** | **0.1613** | **1** | **7** | **7e+02** | **7** | **K.GSTDRVSR.X** |
|  | 1463 | **447.5187** | **893.0226** | **892.0092** | **1.0134** | **1** | **17** | **61** | **3** | **K.ATTKTGVSK.L** |
|  | 1506 | **450.5448** | **899.0748** | **899.9881** | **-0.9132** | **1** | **7** | **6.7e+02** | **5** | **K.EIPEERK.E** |
|  | 2342 | **537.0525** | **1072.0902** | **1071.2679** | **0.8223** | **0** | **4** | **1.2e+03** | **4** | **K.EMQFLMEK.W + Oxidation (M)** |
|  | 2553 | **575.1180** | **1148.2213** | **1148.2261** | **-0.0048** | **1** | **8** | **4.6e+02** | **4** | **R.SPTPGKGSFDR.V** |
|  | 583 | **398.1838** | **1191.5293** | **1191.2725** | **0.2569** | **1** | **14** | **1.1e+02** | **5** | **K.VMGEREEEGR.A** |
|  | 707 | **404.1296** | **1209.3667** | **1209.4417** | **-0.0749** | **2** | **14** | **1.1e+02** | **2** | **K.AKVPGRVVIDR.S** |
|  | 1039 | **419.3567** | **1255.0481** | **1255.4205** | **-0.3725** | **2** | **9** | **3.6e+02** | **9** | **K.EIPNERKELK.K** |
|  | 1155 | **428.6439** | **1282.9096** | **1283.4374** | **-0.5278** | **2** | **16** | **52** | **1** | **K.VPGRVEIDRSR.A** |
|  | 1156 | **428.6975** | **1283.0703** | **1283.4374** | **-0.3671** | **2** | **(5)** | **5.7e+02** | **5** | **K.VPGRVEIDRSR.A** |
|  | 1168 | **429.5185** | **1285.5332** | **1286.4378** | **-0.9046** | **2** | **9** | **4.5e+02** | **8** | **K.QQTKALEQKGR.D** |
|  | 1292 | **436.1372** | **1305.3894** | **1304.4334** | **0.9561** | **2** | **7** | **5.4e+02** | **7** | **K.RSPTPGKGSMDR.V + Oxidation (M)** |
|  | 1435 | **445.2054** | **1332.5940** | **1331.4720** | **1.1220** | **2** | **8** | **4.9e+02** | **4** | **K.VSEKKDQALEGK.Y** |
|  | 3016 | **668.9286** | **1335.8424** | **1335.3745** | **0.4679** | **1** | **7** | **4.6e+02** | **5** | **K.TESSEALDAEKR.K** |
|  | 3040 | **673.1771** | **1344.3395** | **1343.4478** | **0.8917** | **2** | **13** | **1.3e+02** | **2** | **K.RSPTPGKGSWDR.V** |
|  | 3182 | **700.1757** | **1398.3365** | **1397.6162** | **0.7204** | **2** | **13** | **1.1e+02** | **1** | **K.IPVEKDKALEQK.D** |
|  | 1735 | **467.8481** | **1400.5221** | **1400.6632** | **-0.1411** | **2** | **12** | **1.9e+02** | **8** | **K.DSKEMQFLMKK.W + Oxidation (M)** |
|  | 2234 | **521.0015** | **1559.9824** | **1558.8197** | **1.1627** | **1** | **12** | **1.6e+02** | **1** | **K.GLVNGLKAGPFALSSK.G** |
|  | 2742 | **600.8131** | **1799.4172** | **1799.9792** | **-0.5621** | **1** | **9** | **3.1e+02** | **3** | **K.TAALGLEESLVQEGRAR.E** |
|  | 2972 | **666.5201** | **1996.5383** | **1996.0517** | **0.4866** | **2** | **12** | **1.3e+02** | **3** | **K.SEPQDFKEADSWGDTKR.T** |
|  | 2980 | **666.7712** | **1997.2915** | **1997.1906** | **0.1010** | **0** | **11** | **2.5e+02** | **2** | **K.AQESSEKPPEKPCKPER.V + Carbamidomethyl (C)** |

  


---

|  |  |
| --- | --- |
| **15.** | gi|119590550    **Mass:** 10980    **Score:** 113    **Queries matched:** 6   **emPAI:** 1.27 |
|  | hCG37498, isoform CRA\_b [Homo sapiens] |

|  |  |
| --- | --- |
|  | Check to include this hit in error tolerant search or archive report |
|  |  |

|  |  |  |  |  |  |  |  |  |  |  |
| --- | --- | --- | --- | --- | --- | --- | --- | --- | --- | --- |
|  | **Query** | **Observed** | **Mr(expt)** | **Mr(calc)** | **Delta** | **Miss** | **Score** | **Expect** | **Rank** | **Peptide** |
|  | 1726 | 466.3013 | 930.5878 | 930.9988 | -0.4109 | 0 | 62 | 0.0014 | 1 | K.GPSSVEDIK.A |
|  | 2509 | 565.8708 | 1129.7268 | 1130.2490 | -0.5222 | 1 | 44 | 0.085 | 1 | K.GPSSVEDIKAK.M |
|  | 272 | 377.9616 | 1130.8626 | 1130.2490 | 0.6136 | 1 | (40) | 0.22 | 1 | K.GPSSVEDIKAK.M |
|  | 273 | 377.9851 | 1130.9332 | 1130.2490 | 0.6843 | 1 | (43) | 0.11 | 1 | K.GPSSVEDIKAK.M |
|  | 276 | 378.0894 | 1131.2459 | 1130.2490 | 0.9969 | 1 | (15) | 72 | 1 | K.GPSSVEDIKAK.M |
|  | 1709 | **465.0312** | **1392.0714** | **1391.6562** | **0.4151** | **1** | **7** | **5.7e+02** | **7** | **K.MQASIEKCGSLPK.V** |

  


---

|  |  |
| --- | --- |
| **16.** | gi|17066104    **Mass:** 2993008  **Score:** 110    **Queries matched:** 38 |
|  | N2B-Titin Isoform [Homo sapiens] |

|  |  |
| --- | --- |
|  | Check to include this hit in error tolerant search or archive report |
|  |  |

|  |  |  |  |  |  |  |  |  |  |  |
| --- | --- | --- | --- | --- | --- | --- | --- | --- | --- | --- |
|  | **Query** | **Observed** | **Mr(expt)** | **Mr(calc)** | **Delta** | **Miss** | **Score** | **Expect** | **Rank** | **Peptide** |
|  | 184 | 371.0591 | 740.1034 | 738.9612 | 1.1422 | 1 | 4 | 7.8e+02 | 6 | R.KLIIPR.G |
|  | 296 | 380.0652 | 758.1157 | 758.9064 | -0.7906 | 1 | 10 | 3.2e+02 | 2 | R.VKGLTNK.K |
|  | 347 | 385.0210 | 768.0272 | 767.9133 | 0.1139 | 0 | 8 | 3.4e+02 | 6 | R.GVPVPTAK.W |
|  | 1357 | 439.9832 | 877.9516 | 876.9777 | 0.9739 | 0 | 8 | 5.2e+02 | 6 | K.CLEGQTAR.F |
|  | 1511 | 451.2696 | 900.5244 | 900.0375 | 0.4869 | 1 | 2 | 1.5e+03 | 6 | R.LGRLGASAR.L |
|  | 1531 | 453.4450 | 904.8752 | 904.0197 | 0.8556 | 1 | 9 | 3.9e+02 | 8 | R.LSIDNSKK.G |
|  | 1603 | 458.9693 | 915.9238 | 914.9199 | 1.0040 | 1 | 20 | 30 | 1 | R.SESDGHKR.R |
|  | 1667 | 462.1082 | 922.2016 | 922.0763 | 0.1253 | 0 | 4 | 1.1e+03 | 10 | K.ITGYIVEK.K |
|  | 1759 | 470.0428 | 938.0709 | 938.1321 | -0.0611 | 2 | (3) | 1.1e+03 | 10 | K.RAAPLVRR.R |
|  | 1768 | 470.4817 | 938.9487 | 938.1321 | 0.8167 | 2 | 17 | 52 | 2 | K.RAAPLVRR.R |
|  | 2312 | 534.3156 | 1066.6164 | 1067.2161 | -0.5997 | 0 | 4 | 1.2e+03 | 3 | K.YMIAEDLGR.G |
|  | 40 | 363.1039 | 1086.2896 | 1087.2938 | -1.0042 | 1 | 5 | 8.8e+02 | 9 | R.DGVPLKATMR.F |
|  | 91 | 366.2093 | 1095.6058 | 1095.2761 | 0.3297 | 2 | 6 | 6.2e+02 | 7 | K.REAAMRAFK.T + Oxidation (M) |
|  | 2414 | 549.4994 | 1096.9840 | 1097.3514 | -0.3674 | 1 | 8 | 3.2e+02 | 3 | R.KPIIERTLK.A |
|  | 2479 | 562.0752 | 1122.1356 | 1121.2919 | 0.8437 | 1 | 12 | 1.8e+02 | 1 | R.MSPARMSPGR.R + 2 Oxidation (M) |
|  | 524 | 391.2444 | 1170.7112 | 1170.3658 | 0.3454 | 2 | 7 | 4.3e+02 | 3 | R.KGIVVRAGGSAR.I |
|  | 678 | 403.2116 | 1206.6126 | 1205.4909 | 1.1217 | 2 | 9 | 3.1e+02 | 4 | K.KSLGWFKVLK.E |
|  | 861 | 407.8979 | 1220.6715 | 1221.3595 | -0.6880 | 0 | 12 | 1.8e+02 | 2 | K.IADFSTNLVNK.D |
|  | 928 | 412.1732 | 1233.4973 | 1234.2938 | -0.7965 | 1 | 17 | 58 | 2 | K.MPDDDGGDRIK.G + Oxidation (M) |
|  | 1073 | 422.0147 | 1263.0219 | 1263.4658 | -0.4439 | 2 | 12 | 1.8e+02 | 2 | K.RDLPSKSWMK.A + Oxidation (M) |
|  | 2885 | 637.6091 | 1273.2035 | 1273.4738 | -0.2704 | 0 | 23 | 12 | 4 | K.EIELDFAVPLK.D |
|  | 1171 | 430.0873 | 1287.2398 | 1286.5073 | 0.7324 | 2 | 9 | 3.6e+02 | 4 | K.RCNAAAQLVRK.E + Carbamidomethyl (C) |
|  | 1254 | 434.2907 | 1299.8500 | 1300.3765 | -0.5266 | 0 | 4 | 7.6e+02 | 5 | K.GEQTWSHAGISK.T |
|  | 2935 | 657.6522 | 1313.2897 | 1312.4058 | 0.8839 | 1 | 15 | 84 | 5 | K.DEAKFECEVSR.E |
|  | 1697 | 464.0195 | 1389.0364 | 1388.5862 | 0.4503 | 0 | 3 | 1.4e+03 | 8 | K.VCEGDIVQLEVK.V + Carbamidomethyl (C) |
|  | 1902 | **483.9167** | **1448.7279** | **1449.6295** | **-0.9017** | **1** | **11** | **2.2e+02** | **1** | **K.QVTQEAIMKETR.K + Oxidation (M)** |
|  | 1924 | 486.3779 | 1456.1116 | 1455.7020 | 0.4097 | 2 | 6 | 5.2e+02 | 9 | R.CNKMPVKDTTYR.V |
|  | 3285 | 740.6399 | 1479.2650 | 1479.7467 | -0.4817 | 2 | 4 | 9e+02 | 7 | R.VNKVPVTMTRYR.S + Oxidation (M) |
|  | 2096 | 505.7566 | 1514.2477 | 1514.6847 | -0.4370 | 2 | 1 | 1.4e+03 | 7 | R.DATKRTWSVVSHK.C |
|  | 2104 | 506.0691 | 1515.1851 | 1514.6566 | 0.5285 | 1 | 3 | 1.1e+03 | 6 | R.DEVDPPRISMDPK.Y + Oxidation (M) |
|  | 2176 | 517.6781 | 1550.0121 | 1549.7285 | 0.2836 | 1 | 4 | 1.1e+03 | 10 | R.VPGLIEGNEYRFR.I |
|  | 2273 | 528.9186 | 1583.7336 | 1584.8935 | -1.1600 | 0 | 15 | 86 | 1 | K.ELPLIFITPLSDVK.V |
|  | 2311 | 534.1686 | 1599.4837 | 1598.7995 | 0.6842 | 1 | 8 | 4.6e+02 | 5 | K.KAEAVATVVAAVDQAR.V |
|  | 2326 | 536.0461 | 1605.1163 | 1605.8500 | -0.7337 | 0 | 1 | 1.9e+03 | 9 | K.YGVGEPLESAPVLMK.N + Oxidation (M) |
|  | 2796 | 612.1584 | 1833.4530 | 1834.1644 | -0.7114 | 1 | 3 | 1.2e+03 | 8 | R.AGSNLKVDIPISGKPLPK.V |
|  | 3031 | 670.3690 | 2008.0849 | 2008.3275 | -0.2426 | 2 | 7 | 5.5e+02 | 4 | K.RPRTASPHFTVSKISVPK.T |
|  | 3155 | 690.5618 | 2068.6631 | 2069.5163 | -0.8531 | 2 | 4 | 9.6e+02 | 5 | K.ACRPIKPPGPPINPKLKDK.S |
|  | 3566 | 972.0663 | 2913.1767 | 2912.2278 | 0.9488 | 1 | 11 | 2e+02 | 1 | R.TSVSLAWSVPEDEGGSKVTGYLIEMQK.V |

  


---

|  |  |
| --- | --- |
| **17.** | gi|119631420    **Mass:** 2993014  **Score:** 109    **Queries matched:** 39 |
|  | titin, isoform CRA\_b [Homo sapiens] |

|  |  |
| --- | --- |
|  | Check to include this hit in error tolerant search or archive report |
|  |  |

|  |  |  |  |  |  |  |  |  |  |  |
| --- | --- | --- | --- | --- | --- | --- | --- | --- | --- | --- |
|  | **Query** | **Observed** | **Mr(expt)** | **Mr(calc)** | **Delta** | **Miss** | **Score** | **Expect** | **Rank** | **Peptide** |
|  | 184 | 371.0591 | 740.1034 | 738.9612 | 1.1422 | 1 | 4 | 7.8e+02 | 6 | R.KLIIPR.G |
|  | 296 | 380.0652 | 758.1157 | 758.9064 | -0.7906 | 1 | 10 | 3.2e+02 | 2 | R.VKGLTNK.K |
|  | 300 | 380.1822 | 758.3496 | 757.9198 | 0.4298 | 0 | 0 | 2.4e+03 | 8 | R.FLTLHK.V |
|  | 347 | 385.0210 | 768.0272 | 767.9133 | 0.1139 | 0 | 8 | 3.4e+02 | 6 | R.GVPVPTAK.W |
|  | 1357 | 439.9832 | 877.9516 | 876.9777 | 0.9739 | 0 | 8 | 5.2e+02 | 6 | K.CLEGQTAR.F |
|  | 1511 | 451.2696 | 900.5244 | 900.0375 | 0.4869 | 1 | 2 | 1.5e+03 | 6 | R.LGRLGASAR.L |
|  | 1531 | 453.4450 | 904.8752 | 904.0197 | 0.8556 | 1 | 9 | 3.9e+02 | 8 | R.LSIDNSKK.G |
|  | 1603 | 458.9693 | 915.9238 | 914.9199 | 1.0040 | 1 | 20 | 30 | 1 | R.SESDGHKR.R |
|  | 1667 | 462.1082 | 922.2016 | 922.0763 | 0.1253 | 0 | 4 | 1.1e+03 | 10 | K.ITGYIVEK.K |
|  | 1759 | 470.0428 | 938.0709 | 938.1321 | -0.0611 | 2 | (3) | 1.1e+03 | 10 | K.RAAPLVRR.R |
|  | 1768 | 470.4817 | 938.9487 | 938.1321 | 0.8167 | 2 | 17 | 52 | 2 | K.RAAPLVRR.R |
|  | 2312 | 534.3156 | 1066.6164 | 1067.2161 | -0.5997 | 0 | 4 | 1.2e+03 | 3 | K.YMIAEDLGR.G |
|  | 40 | 363.1039 | 1086.2896 | 1087.2938 | -1.0042 | 1 | 5 | 8.8e+02 | 9 | R.DGVPLKATMR.F |
|  | 91 | 366.2093 | 1095.6058 | 1095.2761 | 0.3297 | 2 | 6 | 6.2e+02 | 7 | K.REAAMRAFK.T + Oxidation (M) |
|  | 2414 | 549.4994 | 1096.9840 | 1097.3514 | -0.3674 | 1 | 8 | 3.2e+02 | 3 | R.KPIIERTLK.A |
|  | 2479 | 562.0752 | 1122.1356 | 1121.2919 | 0.8437 | 1 | 12 | 1.8e+02 | 1 | R.MSPARMSPGR.R + 2 Oxidation (M) |
|  | 524 | 391.2444 | 1170.7112 | 1170.3658 | 0.3454 | 2 | 7 | 4.3e+02 | 3 | R.KGIVVRAGGSAR.I |
|  | 678 | 403.2116 | 1206.6126 | 1205.4909 | 1.1217 | 2 | 9 | 3.1e+02 | 4 | K.KSLGWFKVLK.E |
|  | 861 | 407.8979 | 1220.6715 | 1221.3595 | -0.6880 | 0 | 12 | 1.8e+02 | 2 | K.IADFSTNLVNK.D |
|  | 928 | 412.1732 | 1233.4973 | 1234.2938 | -0.7965 | 1 | 17 | 58 | 2 | K.MPDDDGGDRIK.G + Oxidation (M) |
|  | 1073 | 422.0147 | 1263.0219 | 1263.4658 | -0.4439 | 2 | 12 | 1.8e+02 | 2 | K.RDLPSKSWMK.A + Oxidation (M) |
|  | 2885 | 637.6091 | 1273.2035 | 1273.4738 | -0.2704 | 0 | 23 | 12 | 4 | K.EIELDFAVPLK.D |
|  | 1171 | 430.0873 | 1287.2398 | 1286.5073 | 0.7324 | 2 | 9 | 3.6e+02 | 4 | K.RCNAAAQLVRK.E + Carbamidomethyl (C) |
|  | 1254 | 434.2907 | 1299.8500 | 1300.3765 | -0.5266 | 0 | 4 | 7.6e+02 | 5 | K.GEQTWSHAGISK.T |
|  | 2935 | 657.6522 | 1313.2897 | 1312.4058 | 0.8839 | 1 | 15 | 84 | 5 | K.DEAKFECEVSR.E |
|  | 1697 | 464.0195 | 1389.0364 | 1388.5862 | 0.4503 | 0 | 3 | 1.4e+03 | 8 | K.VCEGDIVQLEVK.V + Carbamidomethyl (C) |
|  | 1902 | 483.9167 | 1448.7279 | 1449.6295 | -0.9017 | 1 | 11 | 2.2e+02 | 1 | K.QVTQEAIMKETR.K + Oxidation (M) |
|  | 1924 | 486.3779 | 1456.1116 | 1455.7020 | 0.4097 | 2 | 6 | 5.2e+02 | 9 | R.CNKMPVKDTTYR.V |
|  | 3285 | 740.6399 | 1479.2650 | 1479.7467 | -0.4817 | 2 | 4 | 9e+02 | 7 | R.VNKVPVTMTRYR.S + Oxidation (M) |
|  | 2096 | 505.7566 | 1514.2477 | 1514.6847 | -0.4370 | 2 | 1 | 1.4e+03 | 7 | R.DATKRTWSVVSHK.C |
|  | 2104 | 506.0691 | 1515.1851 | 1514.6566 | 0.5285 | 1 | 3 | 1.1e+03 | 6 | R.DEVDPPRISMDPK.Y + Oxidation (M) |
|  | 2176 | 517.6781 | 1550.0121 | 1549.7285 | 0.2836 | 1 | 4 | 1.1e+03 | 10 | R.VPGLIEGNEYRFR.I |
|  | 2273 | 528.9186 | 1583.7336 | 1584.8935 | -1.1600 | 0 | 15 | 86 | 1 | K.ELPLIFITPLSDVK.V |
|  | 2311 | 534.1686 | 1599.4837 | 1598.7995 | 0.6842 | 1 | 8 | 4.6e+02 | 5 | K.KAEAVATVVAAVDQAR.V |
|  | 2326 | 536.0461 | 1605.1163 | 1605.8500 | -0.7337 | 0 | 1 | 1.9e+03 | 9 | K.YGVGEPLESAPVLMK.N + Oxidation (M) |
|  | 2796 | 612.1584 | 1833.4530 | 1834.1644 | -0.7114 | 1 | 3 | 1.2e+03 | 8 | R.AGSNLKVDIPISGKPLPK.V |
|  | 3031 | 670.3690 | 2008.0849 | 2008.3275 | -0.2426 | 2 | 7 | 5.5e+02 | 4 | K.RPRTASPHFTVSKISVPK.T |
|  | 3155 | 690.5618 | 2068.6631 | 2069.5163 | -0.8531 | 2 | 4 | 9.6e+02 | 5 | K.ACRPIKPPGPPINPKLKDK.S |
|  | 3566 | 972.0663 | 2913.1767 | 2912.2278 | 0.9488 | 1 | 11 | 2e+02 | 1 | R.TSVSLAWSVPEDEGGSKVTGYLIEMQK.V |

  

|  |  |
| --- | --- |
|  | |
|  | **Proteins matching the same set of peptides:** |

|  |  |
| --- | --- |
|  | gi|119631423    **Mass:** 3006829  **Score:** 109    **Queries matched:** 39 |
|  | titin, isoform CRA\_d [Homo sapiens] |

|  |  |
| --- | --- |
|  | gi|119631424    **Mass:** 3014062  **Score:** 109    **Queries matched:** 39 |
|  | titin, isoform CRA\_e [Homo sapiens] |

---

|  |  |
| --- | --- |
| **18.** | gi|21930287    **Mass:** 243254   **Score:** 109    **Queries matched:** 10 |
|  | ninein-Lm isoform [Homo sapiens] |

|  |  |
| --- | --- |
|  | Check to include this hit in error tolerant search or archive report |
|  |  |

|  |  |  |  |  |  |  |  |  |  |  |
| --- | --- | --- | --- | --- | --- | --- | --- | --- | --- | --- |
|  | **Query** | **Observed** | **Mr(expt)** | **Mr(calc)** | **Delta** | **Miss** | **Score** | **Expect** | **Rank** | **Peptide** |
|  | 215 | **373.4192** | **744.8235** | **744.8333** | **-0.0098** | **0** | **15** | **1.4e+02** | **2** | **R.LQDELK.K** |
|  | 364 | **385.4182** | **768.8216** | **768.8997** | **-0.0780** | **0** | **14** | **97** | **1** | **R.QEMMSK.L + Oxidation (M)** |
|  | 2180 | **518.7057** | **1035.3966** | **1035.1512** | **0.2454** | **1** | **10** | **2.3e+02** | **2** | **R.EQEKFNLK.E** |
|  | 1292 | 436.1372 | 1305.3894 | 1306.4857 | -1.0962 | 1 | 8 | 4.1e+02 | 3 | K.QLDETVVSCKK.A + Carbamidomethyl (C) |
|  | 1834 | **476.7546** | **1427.2416** | **1426.6174** | **0.6241** | **1** | **15** | **69** | **1** | **K.LQRNLENVLAEK.F** |
|  | 2045 | **499.8594** | **1496.5560** | **1495.6548** | **0.9012** | **1** | **11** | **1.9e+02** | **1** | **R.AEMSTEISRLQSK.I + Oxidation (M)** |
|  | 2219 | **520.1560** | **1557.4458** | **1556.6997** | **0.7461** | **1** | **6** | **6.2e+02** | **6** | **R.LERLEMEHAQER.Q + Oxidation (M)** |
|  | 2483 | **562.8944** | **1685.6611** | **1685.7920** | **-0.1310** | **1** | **21** | **19** | **4** | **K.QEQKSWEHQSASLK.S** |
|  | 2691 | **594.3549** | **1780.0424** | **1780.9545** | **-0.9121** | **2** | **13** | **1.5e+02** | **2** | **R.ERAEMSTEISRLQSK.I + Oxidation (M)** |
|  | 2945 | 662.0758 | 1983.2052 | 1983.2715 | -0.0662 | 0 | 4 | 9.7e+02 | 8 | K.AMMHDLQITCSEMQQK.V + Carbamidomethyl (C); 2 Oxidation (M) |

  

|  |  |
| --- | --- |
|  | |
|  | **Proteins matching the same set of peptides:** |

|  |  |
| --- | --- |
|  | gi|52699551    **Mass:** 231103   **Score:** 109    **Queries matched:** 10 |
|  | ninein isoform 6 [Homo sapiens] |

---

|  |  |
| --- | --- |
| **19.** | gi|1212992    **Mass:** 2993415  **Score:** 108    **Queries matched:** 39 |
|  | titin [Homo sapiens] |

|  |  |
| --- | --- |
|  | Check to include this hit in error tolerant search or archive report |
|  |  |

|  |  |  |  |  |  |  |  |  |  |  |
| --- | --- | --- | --- | --- | --- | --- | --- | --- | --- | --- |
|  | **Query** | **Observed** | **Mr(expt)** | **Mr(calc)** | **Delta** | **Miss** | **Score** | **Expect** | **Rank** | **Peptide** |
|  | 184 | 371.0591 | 740.1034 | 738.9612 | 1.1422 | 1 | 4 | 7.8e+02 | 6 | R.KLIIPR.G |
|  | 296 | 380.0652 | 758.1157 | 758.9064 | -0.7906 | 1 | 10 | 3.2e+02 | 2 | R.VKGLTNK.K |
|  | 300 | 380.1822 | 758.3496 | 757.9198 | 0.4298 | 0 | 0 | 2.4e+03 | 8 | R.FLTLHK.V |
|  | 347 | 385.0210 | 768.0272 | 767.9133 | 0.1139 | 0 | 8 | 3.4e+02 | 6 | R.GVPVPTAK.W |
|  | 1511 | 451.2696 | 900.5244 | 900.0375 | 0.4869 | 1 | 2 | 1.5e+03 | 6 | R.LGRLGASAR.L |
|  | 1531 | 453.4450 | 904.8752 | 904.0197 | 0.8556 | 1 | 9 | 3.9e+02 | 8 | R.LSIDNSKK.G |
|  | 1603 | 458.9693 | 915.9238 | 914.9199 | 1.0040 | 1 | 20 | 30 | 1 | R.SESDGHKR.R |
|  | 1667 | 462.1082 | 922.2016 | 922.0763 | 0.1253 | 0 | 4 | 1.1e+03 | 10 | K.ITGYIVEK.K |
|  | 1759 | 470.0428 | 938.0709 | 938.1321 | -0.0611 | 2 | (3) | 1.1e+03 | 10 | K.RAAPLVRR.R |
|  | 1768 | 470.4817 | 938.9487 | 938.1321 | 0.8167 | 2 | 17 | 52 | 2 | K.RAAPLVRR.R |
|  | 2312 | 534.3156 | 1066.6164 | 1067.2161 | -0.5997 | 0 | 4 | 1.2e+03 | 3 | K.YMIAEDLGR.G |
|  | 40 | 363.1039 | 1086.2896 | 1087.2938 | -1.0042 | 1 | 5 | 8.8e+02 | 9 | R.DGVPLKATMR.F |
|  | 91 | 366.2093 | 1095.6058 | 1095.2761 | 0.3297 | 2 | 6 | 6.2e+02 | 7 | K.REAAMRAFK.T + Oxidation (M) |
|  | 2414 | 549.4994 | 1096.9840 | 1097.3514 | -0.3674 | 1 | 8 | 3.2e+02 | 3 | R.KPIIERTLK.A |
|  | 2479 | 562.0752 | 1122.1356 | 1121.2919 | 0.8437 | 1 | 12 | 1.8e+02 | 1 | R.MSPARMSPGR.R + 2 Oxidation (M) |
|  | 524 | 391.2444 | 1170.7112 | 1170.3658 | 0.3454 | 2 | 7 | 4.3e+02 | 3 | R.KGIVVRAGGSAR.I |
|  | 678 | 403.2116 | 1206.6126 | 1205.4909 | 1.1217 | 2 | 9 | 3.1e+02 | 4 | K.KSLGWFKVLK.E |
|  | 861 | 407.8979 | 1220.6715 | 1221.3595 | -0.6880 | 0 | 12 | 1.8e+02 | 2 | K.IADFSTNLVNK.D |
|  | 928 | 412.1732 | 1233.4973 | 1234.2938 | -0.7965 | 1 | 17 | 58 | 2 | K.MPDDDGGDRIK.G + Oxidation (M) |
|  | 1073 | 422.0147 | 1263.0219 | 1263.4658 | -0.4439 | 2 | 12 | 1.8e+02 | 2 | K.RDLPSKSWMK.A + Oxidation (M) |
|  | 2885 | 637.6091 | 1273.2035 | 1273.4738 | -0.2704 | 0 | 23 | 12 | 4 | K.EIELDFAVPLK.D |
|  | 1171 | 430.0873 | 1287.2398 | 1286.5073 | 0.7324 | 2 | 9 | 3.6e+02 | 4 | K.RCNAAAQLVRK.E + Carbamidomethyl (C) |
|  | 1254 | 434.2907 | 1299.8500 | 1300.3765 | -0.5266 | 0 | 4 | 7.6e+02 | 5 | K.GEQTWSHAGISK.T |
|  | 2935 | 657.6522 | 1313.2897 | 1312.4058 | 0.8839 | 1 | 15 | 84 | 5 | K.DEAKFECEVSR.E |
|  | 1697 | 464.0195 | 1389.0364 | 1388.5862 | 0.4503 | 0 | 3 | 1.4e+03 | 8 | K.VCEGDIVQLEVK.V + Carbamidomethyl (C) |
|  | 1902 | 483.9167 | 1448.7279 | 1449.6295 | -0.9017 | 1 | 11 | 2.2e+02 | 1 | K.QVTQEAIMKETR.K + Oxidation (M) |
|  | 1924 | 486.3779 | 1456.1116 | 1455.7020 | 0.4097 | 2 | 6 | 5.2e+02 | 9 | R.CNKMPVKDTTYR.V |
|  | 3285 | 740.6399 | 1479.2650 | 1479.7467 | -0.4817 | 2 | 4 | 9e+02 | 7 | R.VNKVPVTMTRYR.S + Oxidation (M) |
|  | 2096 | 505.7566 | 1514.2477 | 1514.6847 | -0.4370 | 2 | 1 | 1.4e+03 | 7 | R.DATKRTWSVVSHK.C |
|  | 2104 | 506.0691 | 1515.1851 | 1514.6566 | 0.5285 | 1 | 3 | 1.1e+03 | 6 | R.DEVDPPRISMDPK.Y + Oxidation (M) |
|  | 2176 | 517.6781 | 1550.0121 | 1549.7285 | 0.2836 | 1 | 4 | 1.1e+03 | 10 | R.VPGLIEGNEYRFR.I |
|  | 2273 | 528.9186 | 1583.7336 | 1584.8935 | -1.1600 | 0 | 15 | 86 | 1 | K.ELPLIFITPLSDVK.V |
|  | 2311 | 534.1686 | 1599.4837 | 1598.7995 | 0.6842 | 1 | 8 | 4.6e+02 | 5 | K.KAEAVATVVAAVDQAR.V |
|  | 2326 | 536.0461 | 1605.1163 | 1605.8500 | -0.7337 | 0 | 1 | 1.9e+03 | 9 | K.YGVGEPLESAPVLMK.N + Oxidation (M) |
|  | 2796 | 612.1584 | 1833.4530 | 1834.1644 | -0.7114 | 1 | 3 | 1.2e+03 | 8 | R.AGSNLKVDIPISGKPLPK.V |
|  | 3031 | 670.3690 | 2008.0849 | 2008.3275 | -0.2426 | 2 | 7 | 5.5e+02 | 4 | K.RPRTASPHFTVSKISVPK.T |
|  | 3137 | **687.4576** | **2059.3508** | **2059.3475** | **0.0032** | **1** | **3** | **1.2e+03** | **7** | **K.TLYRISGLVEGTMHYFR.V + Oxidation (M)** |
|  | 3155 | 690.5618 | 2068.6631 | 2069.5163 | -0.8531 | 2 | 4 | 9.6e+02 | 5 | K.ACRPIKPPGPPINPKLKDK.S |
|  | 3566 | 972.0663 | 2913.1767 | 2912.2278 | 0.9488 | 1 | 11 | 2e+02 | 1 | R.TSVSLAWSVPEDEGGSKVTGYLIEMQK.V |

  


---

|  |  |
| --- | --- |
| **20.** | gi|46107962    **Mass:** 308488   **Score:** 107    **Queries matched:** 18 |
|  | inositol 1,4,5-trisphosphate receptor type 1 [Homo sapiens] |

|  |  |
| --- | --- |
|  | Check to include this hit in error tolerant search or archive report |
|  |  |

|  |  |  |  |  |  |  |  |  |  |  |
| --- | --- | --- | --- | --- | --- | --- | --- | --- | --- | --- |
|  | **Query** | **Observed** | **Mr(expt)** | **Mr(calc)** | **Delta** | **Miss** | **Score** | **Expect** | **Rank** | **Peptide** |
|  | 470 | **389.0421** | **776.0694** | **775.8740** | **0.1954** | **0** | **15** | **94** | **3** | **R.DVVCQR.F + Carbamidomethyl (C)** |
|  | 535 | 392.3037 | 782.5926 | 781.9199 | 0.6727 | 1 | 22 | 13 | 7 | R.MKFDNK.T |
|  | 1608 | **459.0829** | **916.1511** | **915.0009** | **1.1501** | **0** | **7** | **6.5e+02** | **9** | **R.DVLSYYR.Y** |
|  | 1665 | **462.0286** | **922.0425** | **920.9906** | **1.0519** | **1** | **5** | **8e+02** | **6** | **R.ACNNTKDR.K** |
|  | 2091 | **505.2357** | **1008.4565** | **1008.0679** | **0.3887** | **1** | **12** | **1.5e+02** | **3** | **R.ACNNTSDRK.H** |
|  | 2179 | **518.3987** | **1034.7826** | **1034.1481** | **0.6344** | **1** | **8** | **3.5e+02** | **8** | **R.ACNNTIDRK.H** |
|  | 319 | **382.2053** | **1143.5938** | **1144.1927** | **-0.5989** | **0** | **5** | **7.3e+02** | **8** | **K.TEEGNNKPQK.H** |
|  | 767 | **406.0598** | **1215.1573** | **1216.2983** | **-1.1411** | **1** | **13** | **1.3e+02** | **2** | **R.ELAQDAKEGQK.E** |
|  | 784 | **406.2736** | **1215.7987** | **1216.2983** | **-0.4996** | **1** | **(3)** | **9.4e+02** | **4** | **R.ELAQDAKEGQK.E** |
|  | 1054 | **420.1806** | **1257.5196** | **1258.3780** | **-0.8584** | **0** | **10** | **2.6e+02** | **4** | **R.LQDIVSALEDR.L** |
|  | 1259 | **435.0409** | **1302.1004** | **1302.4339** | **-0.3335** | **1** | **5** | **6.7e+02** | **9** | **R.DQLLEASAATRK.A** |
|  | 1379 | **442.6682** | **1324.9823** | **1324.4610** | **0.5213** | **0** | **10** | **1.9e+02** | **2** | **K.EIYTSNHMWK.L + Oxidation (M)** |
|  | 3078 | **683.9784** | **1365.9420** | **1365.6622** | **0.2799** | **1** | **5** | **7.4e+02** | **4** | **K.VLQTLREMMTK.D + Oxidation (M)** |
|  | 1659 | **461.8153** | **1382.4238** | **1383.5483** | **-1.1244** | **1** | **1** | **1.9e+03** | **7** | **K.AEDTKMQEIMR.L + 2 Oxidation (M)** |
|  | 3380 | **757.8884** | **1513.7621** | **1513.7378** | **0.0242** | **1** | **13** | **1.3e+02** | **2** | **K.TTCFICGLEREK.F + 2 Carbamidomethyl (C)** |
|  | 3381 | **758.0023** | **1513.9899** | **1513.7378** | **0.2520** | **1** | **(4)** | **8e+02** | **10** | **K.TTCFICGLEREK.F + 2 Carbamidomethyl (C)** |
|  | 3437 | **780.4032** | **1558.7916** | **1557.7755** | **1.0162** | **1** | **5** | **8.1e+02** | **5** | **K.SHSIVQKTAMNWR.L** |
|  | 3054 | **680.1223** | **2037.3448** | **2036.3556** | **0.9892** | **2** | **13** | **1.3e+02** | **1** | **K.TTCFICGLERMKFDNK.T + 2 Carbamidomethyl (C); Oxidation (M)** |

  


---

**Peptide matches not assigned to protein hits:** (no details means no
match)  
  

|  |  |  |  |  |  |  |  |  |  |  |
| --- | --- | --- | --- | --- | --- | --- | --- | --- | --- | --- |
|  | **Query** | **Observed** | **Mr(expt)** | **Mr(calc)** | **Delta** | **Miss** | **Score** | **Expect** | **Rank** | **Peptide** |
|  | 2884 | **636.6591** | **1271.3033** | **1271.3753** | **-0.0719** | **0** | **57** | **0.0061** | **1** | **STGEAFVQFASK** |
|  | 3551 | **886.2092** | **1770.4037** | **1769.9102** | **0.4935** | **0** | **54** | **0.0081** | **1** | **SGASAAPAASAAAALAPSATR** |
|  | 2657 | 591.7463 | 1772.2167 | 1773.0168 | -0.8002 | 1 | 47 | 0.052 | 1 | MQASIEQGGSLPKVEAK |
|  | 3069 | 681.7735 | 1361.5322 | 1361.5210 | 0.0113 | 0 | 44 | 0.14 | 1 | MQASIEQGGSLPK + Oxidation (M) |
|  | 1066 | **421.6475** | **841.2803** | **841.9949** | **-0.7147** | **0** | **42** | **0.13** | **1** | **GITLSVRP** |
|  | 2860 | **626.7341** | **1251.4535** | **1250.2717** | **1.1818** | **0** | **40** | **0.31** | **1** | **TYGEPESAGPSR** |
|  | 2955 | **664.2658** | **1326.5168** | **1326.5383** | **-0.0214** | **1** | **40** | **0.27** | **1** | **KASGPPVSELITK** |
|  | 1065 | **421.6052** | **841.1955** | **841.9949** | **-0.7994** | **0** | **40** | **0.25** | **1** | **GITLSVRP** |
|  | 1075 | **422.0446** | **842.0745** | **841.9949** | **0.0795** | **0** | **37** | **0.49** | **1** | **GITLSVRP** |
|  | 1069 | **421.9155** | **841.8163** | **841.9949** | **-0.1787** | **0** | **37** | **0.48** | **1** | **GITLSVRP** |
|  | 1104 | **423.0588** | **844.1029** | **843.0262** | **1.0767** | **2** | **37** | **0.61** | **1** | **VATVIRKG** |
|  | 2741 | **600.1536** | **1198.2925** | **1198.3660** | **-0.0735** | **0** | **37** | **0.58** | **1** | **ASGPPVSELITK** |
|  | 1098 | **422.2534** | **842.4921** | **841.9949** | **0.4972** | **0** | **37** | **0.5** | **1** | **GITLSVRP** |
|  | 1085 | **422.1570** | **842.2993** | **841.9949** | **0.3044** | **0** | **36** | **0.68** | **1** | **GITLSVRP** |
|  | 1080 | **422.1196** | **842.2245** | **841.9949** | **0.2295** | **0** | **36** | **0.72** | **1** | **GITLSVRP** |
|  | 1084 | **422.1561** | **842.2974** | **841.9949** | **0.3024** | **0** | **36** | **0.75** | **1** | **GITLSVRP** |
|  | 2942 | **658.8185** | **1315.6222** | **1315.3035** | **0.3187** | **0** | **36** | **0.75** | **1** | **DQDGDQQLGPSR** |
|  | 1537 | 454.5298 | 1360.5674 | 1360.5363 | 0.0311 | 0 | 35 | 0.99 | 1 | SCSSQGSWEMMK |
|  | 326 | **382.4201** | **1144.2380** | **1144.3666** | **-0.1285** | **1** | **35** | **1.2** | **1** | **TNVPVKLFAR** |
|  | 2723 | 597.5167 | 1789.5278 | 1789.0163 | 0.5116 | 1 | 34 | 1 | 1 | MQASIEQGGSLPKVEAK + Oxidation (M) |
|  | 1920 | 486.1636 | 970.3124 | 969.1360 | 1.1763 | 0 | 33 | 1.4 | 1 | VISGQQLPK |
|  | 2057 | **501.4158** | **1501.2251** | **1500.7177** | **0.5075** | **0** | **32** | **1.3** | **1** | **AEMTLVTNFFATR** |
|  | 2056 | **501.3158** | **1500.9251** | **1500.7177** | **0.2075** | **0** | **32** | **1.5** | **1** | **AEMTLVTNFFATR** |
|  | 2170 | **516.7256** | **1547.1546** | **1546.5990** | **0.5556** | **2** | **32** | **1.6** | **1** | **NVADDTRSEDLRR** |
|  | 3081 | **684.0820** | **1366.1493** | **1365.5807** | **0.5685** | **1** | **32** | **1.8** | **1** | **GALVLGSSLKQHR** |
|  | 2872 | **629.3367** | **1256.6587** | **1257.4810** | **-0.8224** | **1** | **31** | **2.1** | **1** | **LEFAPKAVLNR** |
|  | 1003 | **417.2656** | **832.5163** | **831.9570** | **0.5593** | **0** | **31** | **1.8** | **1** | **SISISVAR** |
|  | 1541 | 454.8203 | 1361.4387 | 1360.4236 | 1.0151 | 0 | 31 | 2.2 | 1 | FLDNFDSPYDK |
|  | 1110 | **423.6176** | **845.2203** | **844.9590** | **0.2613** | **1** | **31** | **2.2** | **1** | **SSRGGLLR** |
|  | 1708 | **465.0188** | **928.0228** | **927.9815** | **0.0413** | **0** | **30** | **2.5** | **1** | **SSGFSCAGR + Carbamidomethyl (C)** |
|  | 3443 | **784.7422** | **1567.4696** | **1566.7957** | **0.6739** | **1** | **30** | **2** | **1** | **FKGPFTDVVTTNLK** |
|  | 2935 | 657.6522 | 1313.2897 | 1312.6273 | 0.6623 | 0 | 29 | 2.8 | 1 | CASPIPLCLCHR |
|  | 1901 | **483.7598** | **1448.2571** | **1447.5921** | **0.6651** | **1** | **29** | **2.7** | **1** | **NLVPGESVYGEKR** |
|  | 636 | **401.6486** | **1201.9235** | **1202.3165** | **-0.3929** | **0** | **29** | **3** | **1** | **GTHLEVFGGGTK** |
|  | 371 | **386.0170** | **770.0192** | **770.8327** | **-0.8134** | **0** | **29** | **3** | **1** | **DCSCTSR** |
|  | 1500 | **450.3137** | **898.6125** | **899.0099** | **-0.3973** | **2** | **29** | **3** | **1** | **RDRSLPR** |
|  | 2270 | **527.0103** | **1578.0088** | **1578.6886** | **-0.6799** | **1** | **28** | **3.7** | **1** | **LADAHDNQGRPRTK** |
|  | 540 | 392.4283 | 782.8418 | 782.9694 | -0.1275 | 1 | 28 | 4.5 | 1 | EMTKMK + Oxidation (M) |
|  | 2490 | **564.3094** | **1126.6041** | **1126.3943** | **0.2098** | **0** | **28** | **4.3** | **1** | **MPFNFLVCR** |
|  | 1280 | **435.8610** | **869.7073** | **870.0283** | **-0.3210** | **0** | **28** | **4.1** | **1** | **TPCPSLPR** |
|  | 2226 | **520.2020** | **1557.5837** | **1556.7593** | **0.8244** | **2** | **28** | **4.1** | **1** | **ASEEKIKQLGAGTPK** |
|  | 1867 | **479.1936** | **956.3725** | **956.1010** | **0.2715** | **1** | **28** | **4.4** | **1** | **ARTVLSPGR** |
|  | 3346 | **744.6967** | **1487.3785** | **1486.6694** | **0.7091** | **1** | **28** | **3.6** | **1** | **SLALETVQNDLRK** |
|  | 2885 | 637.6091 | 1273.2035 | 1273.4771 | -0.2736 | 2 | 28 | 4.2 | 1 | DKIDKWDLIK |
|  | 1074 | **422.0420** | **842.0692** | **841.9949** | **0.0743** | **0** | **27** | **4.9** | **1** | **GITLSVRP** |
|  | 2926 | **655.5599** | **1309.1051** | **1309.3387** | **-0.2336** | **0** | **27** | **4** | **1** | **ESTNLANFNDGK** |
|  | 681 | **403.2876** | **1206.8406** | **1207.3859** | **-0.5452** | **2** | **27** | **4.5** | **1** | **DCKFNCHKR + Carbamidomethyl (C)** |
|  | 1728 | **466.6066** | **931.1985** | **930.1266** | **1.0718** | **0** | **27** | **5.8** | **1** | **MAAAVAAVAR** |
|  | 3373 | **755.7363** | **1509.4578** | **1510.6046** | **-1.1469** | **0** | **27** | **4.6** | **1** | **YYWQENHLETK** |
|  | 3439 | **784.2195** | **1566.4243** | **1566.7957** | **-0.3714** | **1** | **27** | **5** | **1** | **FKGPFTDVVTTNLK** |
|  | 2476 | **561.1247** | **1120.2346** | **1120.2111** | **0.0235** | **1** | **27** | **5.6** | **1** | **VDSLEKSNTK** |
|  | 2729 | **598.1064** | **1791.2972** | **1791.8677** | **-0.5706** | **0** | **27** | **5.1** | **1** | **ECIDDETEEICGGHGK + Carbamidomethyl (C)** |
|  | 58 | **364.0582** | **1089.1525** | **1089.2267** | **-0.0742** | **1** | **27** | **5** | **1** | **GEAAAGAAGMKR** |
|  | 1083 | **422.1552** | **842.2957** | **841.9949** | **0.3008** | **0** | **27** | **5.9** | **1** | **GITLSVRP** |
|  | 631 | **401.1414** | **1200.4019** | **1201.4129** | **-1.0111** | **1** | **27** | **5.9** | **1** | **VTAKELSLVNK** |
|  | 1093 | **422.2094** | **842.4039** | **841.9949** | **0.4090** | **0** | **27** | **5.7** | **1** | **GITLSVRP** |
|  | 2478 | **561.7878** | **1121.5609** | **1121.3266** | **0.2343** | **1** | **27** | **4.6** | **1** | **SIDVKYIGVK** |
|  | 2483 | 562.8944 | 1685.6611 | 1684.8456 | 0.8155 | 2 | 27 | 4.7 | 1 | TENPTKNELAQSPKK |
|  | 3127 | **686.2892** | **2055.8454** | **2056.4528** | **-0.6075** | **2** | **26** | **6.2** | **1** | **MGHSKQIRILLLNEMEK + Oxidation (M)** |
|  | 3474 | **806.2714** | **1610.5281** | **1609.7458** | **0.7823** | **2** | **26** | **6** | **1** | **GSRGSRGPSPLAGPSAR** |
|  | 903 | **409.0612** | **816.1077** | **815.9178** | **0.1899** | **0** | **26** | **7.2** | **1** | **SACYCR + 2 Carbamidomethyl (C)** |
|  | 1092 | **422.2026** | **842.3904** | **841.9949** | **0.3954** | **0** | **26** | **6.8** | **1** | **GITLSVRP** |
|  | 1546 | 455.0782 | 1362.2126 | 1361.5210 | 0.6916 | 0 | 26 | 6.5 | 1 | MQASIEQGGSLPK + Oxidation (M) |
|  | 429 | **387.8948** | **1160.6623** | **1161.3538** | **-0.6915** | **0** | **26** | **8.1** | **1** | **VLIPIHEANR** |
|  | 432 | **387.9015** | **773.7882** | **772.8931** | **0.8951** | **1** | **26** | **8.1** | **1** | **KIQQTR** |
|  | 1158 | **428.7969** | **855.5790** | **855.9389** | **-0.3599** | **1** | **26** | **6.1** | **1** | **ERVAEPR** |
|  | 255 | **376.2614** | **750.5080** | **749.8367** | **0.6713** | **0** | **26** | **5.3** | **1** | **NVSGSMR** |
|  | 1089 | **422.1858** | **842.3567** | **841.9949** | **0.3618** | **0** | **26** | **7.5** | **1** | **GITLSVRP** |
|  | 3008 | **668.7117** | **2003.1128** | **2003.2214** | **-0.1085** | **0** | **26** | **8.2** | **1** | **QPPGGQGFPASAAPAQVPAVR** |
|  | 1318 | **437.2863** | **872.5577** | **872.0175** | **0.5402** | **0** | **26** | **6.5** | **1** | **LPLLDSSK** |
|  | 355 | **385.1291** | **1152.3650** | **1151.3591** | **1.0059** | **1** | **26** | **6.3** | **1** | **THKLTVLPSR** |
|  | 1504 | **450.4020** | **898.7893** | **899.0099** | **-0.2206** | **2** | **26** | **6** | **1** | **RDRSLPR** |
|  | 3120 | **686.1542** | **1370.2936** | **1370.3391** | **-0.0455** | **1** | **26** | **7** | **1** | **SSGSFDGGERESR** |
|  | 3072 | **682.4038** | **2044.1893** | **2043.3468** | **0.8425** | **1** | **26** | **7.1** | **1** | **LERCMSIVTSMTAGVSER + Carbamidomethyl (C); Oxidation (M)** |
|  | 1091 | **422.2020** | **842.3893** | **841.9949** | **0.3943** | **0** | **26** | **7.7** | **1** | **GITLSVRP** |
|  | 51 | **363.2187** | **1086.6339** | **1086.1582** | **0.4756** | **1** | **25** | **6.2** | **1** | **YYADSVRGR** |
|  | 3440 | **784.2556** | **1566.4963** | **1566.7078** | **-0.2115** | **1** | **25** | **7.3** | **1** | **FQESQEEIKSLTK** |
|  | 980 | **416.0378** | **830.0607** | **830.8399** | **-0.7791** | **0** | **25** | **9.1** | **1** | **SQEQPDK** |
|  | 976 | **415.9396** | **829.8644** | **831.0352** | **-1.1708** | **1** | **25** | **9.1** | **1** | **TPKAACLK** |
|  | 3219 | **718.8036** | **1435.5924** | **1434.5518** | **1.0406** | **0** | **25** | **9.1** | **1** | **VEGNFNPFASPQK** |
|  | 661 | **402.1106** | **1203.3095** | **1203.3043** | **0.0052** | **0** | **25** | **9** | **1** | **NWTITDANIR** |
|  | 2977 | **666.6864** | **1997.0370** | **1996.3264** | **0.7106** | **0** | **25** | **8.2** | **1** | **GLYLTEMEWMSLVMYN + Oxidation (M)** |
|  | 1572 | **457.9700** | **1370.8878** | **1370.3391** | **0.5488** | **1** | **25** | **7.8** | **1** | **SSGSFDGGERESR** |
|  | 1077 | **422.0915** | **842.1683** | **841.9949** | **0.1733** | **0** | **25** | **8.7** | **1** | **GITLSVRP** |
|  | 1959 | **488.7759** | **1463.3056** | **1462.6944** | **0.6112** | **0** | **25** | **6.8** | **1** | **YLRPPNTSLFVR** |
|  | 1801 | **474.4446** | **946.8745** | **945.9769** | **0.8976** | **1** | **25** | **7.9** | **1** | **AARAEDSAR** |
|  | 1955 | **488.5968** | **975.1789** | **974.0914** | **1.0875** | **0** | **25** | **10** | **1** | **ASPMSYFR + Oxidation (M)** |
|  | 555 | **394.0748** | **786.1348** | **785.8887** | **0.2462** | **1** | **25** | **9.2** | **1** | **GDTKLPR** |
|  | 3058 | **680.5546** | **2038.6415** | **2038.3501** | **0.2915** | **2** | **25** | **6.7** | **1** | **SSSGSVLVLAMRVCSDKNK + Carbamidomethyl (C)** |
|  | 1577 | **458.1274** | **1371.3600** | **1370.5758** | **0.7842** | **1** | **25** | **8.6** | **1** | **NCTDGLCMQNKK + Oxidation (M)** |
|  | 2037 | **498.0886** | **994.1625** | **994.1886** | **-0.0262** | **0** | **25** | **8.5** | **1** | **PLPVALQTR** |
|  | 1078 | **422.1036** | **842.1924** | **841.9949** | **0.1974** | **0** | **25** | **9.6** | **1** | **GITLSVRP** |
|  | 1442 | **445.9583** | **1334.8526** | **1334.5882** | **0.2643** | **0** | **25** | **10** | **1** | **YQHIGLVAMFR** |
|  | 1711 | **465.1726** | **1392.4956** | **1393.5497** | **-1.0540** | **2** | **25** | **9** | **1** | **KCKTHDSMSGAGR + Oxidation (M)** |
|  | 480 | **389.1410** | **1164.4009** | **1165.3295** | **-0.9286** | **2** | **25** | **9.6** | **1** | **HMRRHTAEK** |
|  | 3347 | **745.4657** | **2233.3749** | **2232.4942** | **0.8808** | **2** | **25** | **8.8** | **1** | **ESDSFCTACPIFEKQWRK + Carbamidomethyl (C)** |
|  | 1768 | 470.4817 | 938.9487 | 939.1567 | -0.2079 | 1 | 25 | 8.9 | 1 | VTVPLVRR |
|  | 3441 | **784.2866** | **2349.8375** | **2348.7003** | **1.1372** | **1** | **25** | **8.6** | **1** | **MSTCCWCTPGGASTIDFLKR + 3 Carbamidomethyl (C)** |
|  | 989 | **416.2222** | **1245.6445** | **1245.4108** | **0.2338** | **1** | **25** | **9.5** | **1** | **GPCHTYKVQR + Carbamidomethyl (C)** |
|  | 2533 | **571.3922** | **1140.7696** | **1141.3875** | **-0.6178** | **1** | **25** | **8.1** | **1** | **QKPMAKGLPR + Oxidation (M)** |
|  | 293 | **379.7109** | **757.4070** | **757.8322** | **-0.4252** | **0** | **24** | **9.4** | **1** | **DPIQGTK** |
|  | 3206 | **710.7493** | **2129.2256** | **2128.3646** | **0.8610** | **0** | **24** | **10** | **1** | **HFQLALIDCNPNTLSNAEK** |
|  | 2963 | **665.2850** | **1328.5553** | **1328.5175** | **0.0378** | **1** | **24** | **9.7** | **1** | **EQLTTRLQALR** |
|  | 1606 | **459.0398** | **916.0648** | **917.1127** | **-1.0479** | **2** | **24** | **11** | **1** | **LFGRLRR** |
|  | 1017 | **419.0409** | **1254.1005** | **1253.3648** | **0.7357** | **0** | **24** | **11** | **1** | **TLQNTPSLHSR** |
|  | 1269 | **435.2672** | **1302.7793** | **1303.4618** | **-0.6826** | **1** | **24** | **7.4** | **1** | **ESAIASTEVKLR** |
|  | 1574 | **458.0002** | **1370.9784** | **1370.3391** | **0.6393** | **1** | **24** | **10** | **1** | **SSGSFDGGERESR** |
|  | 803 | **406.8892** | **1217.6453** | **1218.4467** | **-0.8013** | **0** | **24** | **8.9** | **1** | **RPSAASLMLPC + Carbamidomethyl (C); Oxidation (M)** |
|  | 431 | **387.8989** | **1160.6744** | **1161.3969** | **-0.7225** | **2** | **24** | **12** | **1** | **LKQVRNYIK** |
|  | 1779 | **471.7752** | **1412.3035** | **1412.5479** | **-0.2444** | **0** | **24** | **7.9** | **1** | **TLSDNVNLPQGVR** |
|  | 3129 | **686.3564** | **1370.6981** | **1371.6682** | **-0.9700** | **0** | **24** | **10** | **1** | **HCLMSPSVAILGK + Oxidation (M)** |
|  | 1964 | **489.3872** | **976.7596** | **976.1519** | **0.6078** | **1** | **24** | **9** | **1** | **MLNISRDK** |
|  | 2214 | **520.0771** | **1557.2091** | **1557.7473** | **-0.5382** | **1** | **24** | **10** | **1** | **LAQLSEEKNSAVLR** |
|  | 3397 | **759.9702** | **1517.9256** | **1518.6947** | **-0.7691** | **1** | **24** | **9.6** | **1** | **DGRVSENTVCLIR + Carbamidomethyl (C)** |
|  | 2908 | **647.0403** | **1292.0658** | **1291.4064** | **0.6594** | **0** | **24** | **10** | **1** | **NLVPGESVYGEK** |
|  | 1827 | **476.3279** | **1425.9615** | **1425.5946** | **0.3669** | **0** | **24** | **8.6** | **1** | **IFHAGDCGMHHK + Carbamidomethyl (C); Oxidation (M)** |
|  | 3074 | **683.7740** | **1365.5333** | **1365.5429** | **-0.0095** | **2** | **24** | **13** | **1** | **MEGASRVCRQGR + Oxidation (M)** |
|  | 368 | **386.0023** | **1154.9846** | **1155.3114** | **-0.3268** | **2** | **24** | **9.6** | **1** | **RVVRDGQGIR** |
|  | 1019 | **419.0479** | **836.0810** | **834.9196** | **1.1614** | **1** | **24** | **13** | **1** | **KSWTSAR** |
|  | 2558 | **576.0868** | **1725.2382** | **1725.9750** | **-0.7368** | **2** | **24** | **11** | **1** | **MRSWLLRNGQATHR** |
|  | 1258 | **434.9269** | **1301.7586** | **1302.4637** | **-0.7050** | **1** | **24** | **9.8** | **1** | **VGGGLSMGRAQGGR** |
|  | 1102 | **422.7148** | **843.4148** | **843.0262** | **0.3886** | **2** | **24** | **10** | **1** | **VATVIRKG** |
|  | 3197 | **705.7609** | **2114.2604** | **2115.3912** | **-1.1308** | **2** | **24** | **13** | **1** | **AKEVSPMSAPNMPSIERDR** |
|  | 2892 | **639.4565** | **1276.8983** | **1276.3983** | **0.5000** | **1** | **24** | **11** | **1** | **LRVTEGGEPYR** |
|  | 20 | **361.9556** | **1082.8446** | **1082.2903** | **0.5543** | **0** | **24** | **11** | **1** | **DPPSIILLSK** |
|  | 749 | **405.1943** | **1212.5607** | **1213.3641** | **-0.8033** | **0** | **24** | **9.9** | **1** | **GSTPLHMAVER + Oxidation (M)** |
|  | 1358 | **439.9940** | **1316.9599** | **1317.5114** | **-0.5515** | **0** | **24** | **13** | **1** | **TQLQLQEMAQK** |
|  | 2835 | **618.0249** | **1234.0350** | **1234.3616** | **-0.3265** | **1** | **24** | **12** | **1** | **NFSGRIEGVQK** |
|  | 1189 | **431.0260** | **1290.0558** | **1290.5358** | **-0.4800** | **2** | **24** | **14** | **1** | **KMASATRLIQR + Oxidation (M)** |
|  | 2435 | **552.3341** | **1653.9801** | **1654.7336** | **-0.7534** | **2** | **24** | **13** | **1** | **YVTDRKSAEQNESK** |
|  | 479 | **389.1104** | **1164.3092** | **1163.2623** | **1.0469** | **1** | **24** | **13** | **1** | **ELNEEMARR + Oxidation (M)** |
|  | 1107 | **423.4052** | **1267.1934** | **1267.5157** | **-0.3223** | **1** | **23** | **14** | **1** | **SPVEAKLPWLK** |
|  | 373 | **386.0708** | **1155.1903** | **1156.3525** | **-1.1622** | **1** | **23** | **11** | **1** | **SFSKNSMVIK + Oxidation (M)** |
|  | 1583 | **458.2973** | **1371.8696** | **1371.5721** | **0.2976** | **2** | **23** | **10** | **1** | **CEQCGRGFRCR + Carbamidomethyl (C)** |
|  | 3412 | **766.1580** | **1530.3013** | **1530.7054** | **-0.4041** | **0** | **23** | **11** | **1** | **CSCGQGYSLMPDGR + Carbamidomethyl (C)** |
|  | 136 | **369.2433** | **1104.7077** | **1104.2991** | **0.4086** | **0** | **23** | **10** | **1** | **QQLLIGAYAK** |
|  | 2705 | **596.2800** | **1190.5453** | **1189.3625** | **1.1827** | **1** | **23** | **13** | **1** | **MTAEYACTRK + Oxidation (M)** |
|  | 2440 | **552.9228** | **1655.7462** | **1654.8642** | **0.8820** | **0** | **23** | **13** | **1** | **LSCAASGFTFGGYAMR + Oxidation (M)** |
|  | 626 | **401.0212** | **800.0276** | **798.8873** | **1.1403** | **0** | **23** | **13** | **1** | **AGANITPR** |
|  | 105 | **367.4052** | **1099.1933** | **1099.3674** | **-0.1741** | **1** | **23** | **16** | **1** | **MICFKSVAK + Carbamidomethyl (C); Oxidation (M)** |
|  | 488 | **389.2094** | **1164.6061** | **1165.3395** | **-0.7334** | **0** | **23** | **11** | **1** | **VPVLESHSAVK** |
|  | 767 | 406.0598 | 1215.1573 | 1215.3402 | -0.1829 | 1 | 23 | 12 | 1 | KSPQAMSHASR + Oxidation (M) |
|  | 1205 | **431.4650** | **1291.3728** | **1292.5466** | **-1.1738** | **0** | **23** | **17** | **1** | **VIEALQGMFIR + Oxidation (M)** |
|  | 1406 | **443.9005** | **1328.6792** | **1328.5358** | **0.1434** | **0** | **23** | **13** | **1** | **QSLNQFMAVYK** |
|  | 102 | **367.3171** | **1098.9292** | **1099.2810** | **-0.3519** | **1** | **23** | **12** | **1** | **YIAWYQKK** |
|  | 690 | **403.8070** | **805.5993** | **804.8887** | **0.7106** | **1** | **23** | **13** | **1** | **GSKGLSEK** |
|  | 553 | **393.5591** | **785.1035** | **784.8775** | **0.2260** | **0** | **23** | **12** | **1** | **MNDYVK + Oxidation (M)** |
|  | 3124 | **686.2034** | **1370.3921** | **1369.6390** | **0.7530** | **2** | **23** | **13** | **1** | **VALAALPCRRGSR** |
|  | 423 | **387.7313** | **773.4478** | **773.9176** | **-0.4698** | **1** | **23** | **15** | **1** | **LDSKALK** |
|  | 3288 | **740.6882** | **2219.0425** | **2218.5786** | **0.4640** | **1** | **23** | **10** | **1** | **GHMENSSAWVVEIQMLKCR** |
|  | 2086 | **504.7645** | **1511.2714** | **1511.7023** | **-0.4309** | **1** | **23** | **10** | **1** | **VHIEMGPDGRVTGK + Oxidation (M)** |
|  | 2322 | **535.6687** | **1069.3226** | **1070.1589** | **-0.8362** | **1** | **23** | **15** | **1** | **CSCKNTDSR + Carbamidomethyl (C)** |
|  | 463 | **388.9747** | **1163.9018** | **1164.3398** | **-0.4379** | **2** | **23** | **15** | **1** | **LMSSRRGVSR + Oxidation (M)** |
|  | 2232 | **520.5750** | **1558.7029** | **1558.8481** | **-0.1452** | **2** | **23** | **15** | **1** | **KACKACDCHPVGAAGK** |
|  | 2347 | **537.4713** | **1609.3916** | **1609.7424** | **-0.3508** | **1** | **23** | **12** | **1** | **ERELQHAALGGTATR** |
|  | 2093 | **505.5275** | **1009.0403** | **1008.2351** | **0.8052** | **0** | **23** | **14** | **1** | **MVIIGNSFK** |
|  | 2668 | **592.6520** | **1183.2892** | **1184.2631** | **-0.9739** | **1** | **23** | **16** | **1** | **NSQQGPNVGKR** |
|  | 1409 | **443.9155** | **885.8163** | **886.0509** | **-0.2346** | **0** | **23** | **15** | **1** | **MFQASMR + Oxidation (M)** |
|  | 2390 | **544.2028** | **1629.5861** | **1628.6084** | **0.9778** | **1** | **23** | **16** | **1** | **SSGGSYRDSYDSYGK** |
|  | 2734 | **599.6328** | **1795.8763** | **1796.2106** | **-0.3344** | **1** | **23** | **16** | **1** | **LRCGQMMLAQALICR + Carbamidomethyl (C); 2 Oxidation (M)** |
|  | 2095 | **505.6566** | **1513.9476** | **1514.8500** | **-0.9025** | **1** | **23** | **13** | **1** | **VALLKLVSLTGSWK** |
|  | 1593 | **458.7874** | **1373.3400** | **1372.5470** | **0.7930** | **0** | **23** | **14** | **1** | **DCCYSIHQMEK + Oxidation (M)** |
|  | 139 | **369.2492** | **736.4836** | **736.9224** | **-0.4387** | **1** | **23** | **12** | **1** | **FAKMPK + Oxidation (M)** |
|  | 2909 | **648.0735** | **1294.1322** | **1294.5493** | **-0.4171** | **2** | **23** | **14** | **1** | **RNFCKSCLPAR** |
|  | 1037 | **419.2822** | **1254.8243** | **1254.2587** | **0.5657** | **0** | **23** | **14** | **1** | **DGEEEFPFER** |
|  | 676 | **403.0574** | **1206.1500** | **1205.3005** | **0.8496** | **0** | **23** | **16** | **1** | **ATNSSGHMWAK + Oxidation (M)** |
|  | 422 | **387.5754** | **1159.7041** | **1160.3410** | **-0.6369** | **0** | **23** | **15** | **1** | **LEEEVCLLR + Carbamidomethyl (C)** |
|  | 3007 | **668.7054** | **2003.0942** | **2004.1681** | **-1.0739** | **2** | **23** | **17** | **1** | **LGGPRQPPSPPATSSAGDRR** |
|  | 295 | **379.9626** | **1136.8655** | **1136.2553** | **0.6102** | **1** | **23** | **17** | **1** | **VYDRVSVEAV** |
|  | 3015 | **668.9076** | **2003.7006** | **2003.1849** | **0.5157** | **2** | **23** | **12** | **1** | **GSRSSCGAGSCRLGPGPGPSR + Carbamidomethyl (C)** |
|  | 3239 | **729.3527** | **2185.0358** | **2185.3466** | **-0.3108** | **0** | **23** | **15** | **1** | **YAGSALQYEDVSTAVQNLQK** |
|  | 663 | 402.1827 | 802.3507 | 802.8728 | -0.5221 | 0 | 23 | 16 | 1 | GVNADSIK |
|  | 3212 | **715.1265** | **1428.2381** | **1428.5456** | **-0.3075** | **0** | **23** | **15** | **1** | **SLYASSPGGVYATR** |
|  | 2694 | **594.7045** | **1187.3943** | **1186.4015** | **0.9927** | **2** | **22** | **19** | **1** | **LLAKASEQKAK** |
|  | 2404 | **548.2647** | **1094.5146** | **1094.1605** | **0.3542** | **1** | **22** | **15** | **1** | **QMQSTRDGR + Oxidation (M)** |
|  | 9 | **360.4431** | **1078.3070** | **1079.2088** | **-0.9018** | **0** | **22** | **21** | **1** | **CHGVSGSCTTK** |
|  | 2547 | **573.2338** | **1144.4529** | **1143.3336** | **1.1192** | **0** | **22** | **16** | **1** | **LTQVQNSLLK** |
|  | 2924 | **655.5020** | **1963.4837** | **1964.2067** | **-0.7230** | **1** | **22** | **13** | **1** | **LKHEHCEELLTQALSGR** |
|  | 544 | **392.7813** | **783.5478** | **782.9710** | **0.5769** | **0** | **22** | **15** | **1** | **VTVPLVR** |
|  | 3229 | **724.8766** | **1447.7384** | **1446.7413** | **0.9970** | **2** | **22** | **18** | **1** | **LIGMERMNQRAK** |
|  | 262 | **377.1408** | **1128.4003** | **1128.3026** | **0.0978** | **1** | **22** | **15** | **1** | **MRLSYLSSR + Oxidation (M)** |
|  | 99 | **367.1707** | **1098.4898** | **1099.2795** | **-0.7898** | **2** | **22** | **15** | **1** | **YLKQYKEK** |
|  | 1231 | **433.0935** | **1296.2583** | **1295.4168** | **0.8416** | **0** | **22** | **16** | **1** | **MSSSATAVETPAK + Oxidation (M)** |
|  | 1509 | **451.2220** | **900.4292** | **899.9879** | **0.4413** | **0** | **22** | **17** | **1** | **ALAAGADSPK** |
|  | 1184 | 430.8930 | 859.7712 | 859.9706 | -0.1993 | 2 | 22 | 19 | 1 | ATEAGKRK |
|  | 817 | **407.1389** | **1218.3946** | **1217.3955** | **0.9990** | **0** | **22** | **16** | **1** | **GDLCAISLVGNR** |
|  | 1101 | **422.5567** | **843.0986** | **842.9401** | **0.1586** | **1** | **22** | **19** | **1** | **GESAPVRK** |
|  | 847 | **407.7915** | **1220.3522** | **1220.2888** | **0.0635** | **0** | **22** | **17** | **1** | **ATAASPSTDFPR** |
|  | 1164 | **429.1849** | **856.3550** | **856.0215** | **0.3334** | **0** | **22** | **16** | **1** | **VTIASLPR** |
|  | 2888 | **638.5939** | **1275.1730** | **1274.4504** | **0.7226** | **0** | **22** | **14** | **1** | **VCVEHHTFFR** |
|  | 1648 | **460.7212** | **1379.1413** | **1378.4025** | **0.7389** | **0** | **22** | **14** | **1** | **EGGDCSSTSCYR + 2 Carbamidomethyl (C)** |
|  | 2979 | **666.7517** | **1997.2330** | **1997.2267** | **0.0062** | **0** | **22** | **20** | **1** | **CIELLYAALTSSSTDQPK + Carbamidomethyl (C)** |
|  | 233 | **374.2719** | **746.5290** | **746.9189** | **-0.3899** | **1** | **22** | **16** | **1** | **MIKEAR** |
|  | 1934 | **487.0464** | **972.0779** | **973.0834** | **-1.0054** | **0** | **22** | **18** | **1** | **TPPFEINR** |
|  | 1226 | **432.8228** | **1295.4461** | **1296.4990** | **-1.0529** | **1** | **22** | **17** | **1** | **MIPGQPPASARR + Oxidation (M)** |
|  | 2287 | **530.6456** | **1588.9145** | **1587.9918** | **0.9227** | **1** | **22** | **23** | **1** | **LQSLLRVLVQMCK + Carbamidomethyl (C)** |
|  | 208 | **372.6413** | **743.2677** | **743.8949** | **-0.6272** | **1** | **22** | **15** | **1** | **ASGRLIK** |
|  | 304 | **380.2300** | **1137.6678** | **1137.3739** | **0.2939** | **0** | **22** | **16** | **1** | **MMSLLGGLQR + 2 Oxidation (M)** |
|  | 1953 | **488.5194** | **975.0240** | **974.0302** | **0.9939** | **1** | **22** | **22** | **1** | **RAVDSAEAR** |
|  | 46 | **363.1837** | **1086.5288** | **1087.1877** | **-0.6590** | **1** | **22** | **14** | **1** | **VAQTRLDER** |
|  | 1035 | **419.2673** | **1254.7798** | **1255.3346** | **-0.5547** | **1** | **22** | **17** | **1** | **YVASSSKDGSVR** |
|  | 2127 | **508.2201** | **1521.6381** | **1522.6453** | **-1.0072** | **1** | **22** | **19** | **1** | **GQRGKPFQCSDSR + Carbamidomethyl (C)** |
|  | 2863 | **627.3027** | **1252.5907** | **1251.4350** | **1.1557** | **1** | **22** | **17** | **1** | **GYWGGPAFLRK** |
|  | 434 | **387.9092** | **1160.7055** | **1160.3475** | **0.3580** | **1** | **22** | **22** | **1** | **ITCGGIDIGRR** |
|  | 3011 | **668.7627** | **2003.2659** | **2004.1680** | **-0.9021** | **2** | **22** | **22** | **1** | **WFREFGHGPVSEAKSNR** |
|  | 441 | **388.0095** | **1161.0063** | **1160.4138** | **0.5925** | **1** | **22** | **22** | **1** | **MALGPRCGAIR + Oxidation (M)** |
|  | 8 | **360.4212** | **1078.2415** | **1079.1905** | **-0.9489** | **1** | **22** | **25** | **1** | **MGAHAGKHSSP** |
|  | 1179 | **430.3823** | **858.7498** | **858.9858** | **-0.2360** | **2** | **22** | **18** | **1** | **GQRSGVKK** |
|  | 700 | **404.0050** | **1208.9928** | **1209.4184** | **-0.4255** | **0** | **22** | **19** | **1** | **YMFCSMAQR + Carbamidomethyl (C); Oxidation (M)** |
|  | 1943 | **488.0834** | **1461.2280** | **1460.6571** | **0.5710** | **2** | **22** | **21** | **1** | **RSPSYPPPGCGKSK** |
|  | 2464 | **557.1514** | **1668.4321** | **1667.7305** | **0.7017** | **0** | **22** | **18** | **1** | **DGFNPADVEAGLYGSR** |
|  | 2946 | **662.8489** | **1985.5245** | **1985.0521** | **0.4724** | **0** | **22** | **18** | **1** | **ATFPEDQFPNSSQNGSCR** |
|  | 282 | **378.2664** | **1131.7771** | **1132.1024** | **-0.3253** | **1** | **22** | **13** | **1** | **GNRGDAGGSDAR** |
|  | 643 | **401.9059** | **1202.6955** | **1203.2632** | **-0.5678** | **1** | **22** | **22** | **1** | **EAAQRVAESSR** |
|  | 1146 | **428.2606** | **854.5065** | **854.9474** | **-0.4409** | **0** | **21** | **14** | **1** | **DLPGSAPAK** |
|  | 1540 | **454.8071** | **1361.3992** | **1360.4751** | **0.9241** | **0** | **21** | **19** | **1** | **SGRPPGTGVVYDR** |
|  | 3189 | **702.7230** | **1403.4311** | **1403.4998** | **-0.0686** | **1** | **21** | **19** | **1** | **QPRHPSAEGAPEK** |
|  | 3399 | **760.1418** | **2277.4032** | **2277.5329** | **-0.1298** | **0** | **21** | **17** | **1** | **LSCAASGFTFSNYYMSWIR + Carbamidomethyl (C); Oxidation (M)** |
|  | 1434 | **445.1480** | **888.2812** | **887.9575** | **0.3238** | **0** | **21** | **22** | **1** | **DPTMGSHK + Oxidation (M)** |
|  | 2020 | **495.4128** | **988.8109** | **988.1395** | **0.6714** | **1** | **21** | **17** | **1** | **QGMKDCYK + Oxidation (M)** |
|  | 1131 | **426.9206** | **1277.7395** | **1278.4357** | **-0.6961** | **1** | **21** | **18** | **1** | **QYFFETRCK + Carbamidomethyl (C)** |
|  | 474 | **389.0673** | **776.1197** | **776.8768** | **-0.7570** | **0** | **21** | **22** | **1** | **FSPINSL** |
|  | 269 | **377.3048** | **1128.8923** | **1128.2264** | **0.6659** | **2** | **21** | **15** | **1** | **GAHMDRGRGR + Oxidation (M)** |
|  | 3523 | **846.2155** | **1690.4161** | **1690.0345** | **0.3816** | **1** | **21** | **18** | **1** | **KMEMEMEQVFEMK** |
|  | 3171 | **697.5093** | **2089.5057** | **2089.5687** | **-0.0631** | **1** | **21** | **18** | **1** | **CCVCGHLILEKILQAMGK + 2 Carbamidomethyl (C); Oxidation (M)** |
|  | 3336 | **743.4917** | **2227.4529** | **2228.3813** | **-0.9283** | **0** | **21** | **21** | **1** | **HDLSLMSHGSQYGMHPDQR + 2 Oxidation (M)** |
|  | 660 | **402.0966** | **802.1783** | **802.8759** | **-0.6976** | **0** | **21** | **23** | **1** | **GNSGSLIR** |
|  | 2348 | **538.0017** | **1610.9830** | **1609.8847** | **1.0982** | **0** | **21** | **21** | **1** | **DMLLANPHELSLLK + Oxidation (M)** |
|  | 1981 | **490.8191** | **979.6235** | **979.1311** | **0.4924** | **2** | **21** | **17** | **1** | **KRVFLSTE** |
|  | 597 | **399.0641** | **1194.1702** | **1193.4406** | **0.7296** | **1** | **21** | **18** | **1** | **MMVNLRAQSK + Oxidation (M)** |
|  | 3370 | **753.7837** | **1505.5526** | **1505.6961** | **-0.1435** | **1** | **21** | **22** | **1** | **LRSELEMVSGNVR + Oxidation (M)** |
|  | 764 | **405.9918** | **1214.9533** | **1215.3168** | **-0.3635** | **1** | **21** | **19** | **1** | **LKQQQQESAR** |
|  | 2054 | **501.1899** | **1000.3651** | **1001.0787** | **-0.7136** | **1** | **21** | **22** | **1** | **DRETMGHR** |
|  | 468 | **389.0391** | **776.0633** | **775.9568** | **0.1066** | **0** | **21** | **23** | **1** | **LAVSQMK** |
|  | 3345 | **744.6503** | **1487.2858** | **1486.7422** | **0.5435** | **2** | **21** | **17** | **1** | **QLGCGRAVLTQKR + Carbamidomethyl (C)** |
|  | 2081 | **504.2212** | **1509.6415** | **1509.7539** | **-0.1124** | **0** | **21** | **22** | **1** | **CGICEQCGPGCGIGGR** |
|  | 342 | **384.9988** | **1151.9743** | **1153.1381** | **-1.1638** | **0** | **21** | **18** | **1** | **SCNGGSGGGDGTGK** |
|  | 421 | **387.3919** | **772.7691** | **771.9250** | **0.8441** | **0** | **21** | **28** | **1** | **LCPQPSK** |
|  | 3068 | **681.6646** | **1361.3144** | **1360.6075** | **0.7070** | **0** | **21** | **19** | **1** | **MRPAAGPCPTFR + Carbamidomethyl (C)** |
|  | 1450 | **446.3696** | **1336.0867** | **1336.4934** | **-0.4066** | **1** | **21** | **19** | **1** | **SSVGPVRSSLGYK** |
|  | 2864 | **627.7935** | **1880.3582** | **1881.0784** | **-0.7202** | **0** | **21** | **21** | **1** | **LYVHPDSPNTGAHWMR** |
|  | 3132 | **686.7229** | **1371.4310** | **1370.5627** | **0.8683** | **2** | **21** | **23** | **1** | **VRRVSVAHFGSR** |
|  | 1225 | **432.8098** | **1295.4071** | **1294.5626** | **0.8445** | **2** | **21** | **22** | **1** | **QKGELMIGFKK + Oxidation (M)** |
|  | 1549 | **455.2505** | **1362.7295** | **1361.5954** | **1.1340** | **2** | **21** | **19** | **1** | **RRAPAAGLPLPSR** |
|  | 2560 | **576.4820** | **1726.4238** | **1727.0558** | **-0.6320** | **1** | **21** | **17** | **1** | **LALASRLLPDFLLER** |
|  | 901 | **409.0064** | **1223.9971** | **1224.5192** | **-0.5220** | **2** | **21** | **24** | **1** | **KHREMVALLK** |
|  | 3420 | **770.6659** | **2308.9755** | **2309.6429** | **-0.6674** | **2** | **21** | **17** | **1** | **NAVHRVLKAVYDSGYCVSSIK** |
|  | 1138 | 427.8427 | 1280.5061 | 1280.3853 | 0.1208 | 0 | 21 | 22 | 1 | NDVFYLQPER |
|  | 2499 | **565.0903** | **1128.1658** | **1127.2085** | **0.9573** | **0** | **21** | **23** | **1** | **QVQLQDSGPR** |
|  | 2975 | **666.6547** | **1996.9418** | **1997.2746** | **-0.3328** | **1** | **21** | **21** | **1** | **ADIKEMGLSLQWLYSAR + Oxidation (M)** |
|  | 3066 | **681.3502** | **2041.0285** | **2040.2863** | **0.7422** | **2** | **21** | **23** | **1** | **DCKFHHHKLTLADMDR + Carbamidomethyl (C); Oxidation (M)** |
|  | 3281 | **740.5244** | **2218.5509** | **2218.6017** | **-0.0508** | **2** | **21** | **21** | **1** | **GPLGLRAALQQVTLTATPRVR** |
|  | 783 | **406.2611** | **1215.7612** | **1216.4456** | **-0.6845** | **0** | **21** | **17** | **1** | **MYFIISDLSK** |
|  | 3403 | **760.9382** | **2279.7925** | **2279.6089** | **0.1836** | **1** | **21** | **23** | **1** | **MAGVEDKNMVFLFTDTQVCV + 2 Oxidation (M)** |
|  | 3099 | **685.0417** | **1368.0687** | **1367.6498** | **0.4189** | **2** | **21** | **21** | **1** | **RVCPCAPRRPR + Carbamidomethyl (C)** |
|  | 2043 | **499.4102** | **1495.2083** | **1495.6167** | **-0.4084** | **1** | **21** | **17** | **1** | **DRAEMTWGGLSTR + Oxidation (M)** |
|  | 687 | **403.7898** | **805.5649** | **804.9780** | **0.5869** | **0** | **21** | **24** | **1** | **LPCNCGAK** |
|  | 758 | **405.2993** | **1212.8757** | **1212.4387** | **0.4369** | **1** | **21** | **17** | **1** | **QLLQVNKLEK** |
|  | 1046 | **419.5361** | **1255.5862** | **1254.4835** | **1.1027** | **1** | **21** | **30** | **1** | **VALGLGLGRGWR** |
|  | 2689 | **594.0734** | **1779.1979** | **1778.1327** | **1.0652** | **2** | **21** | **25** | **1** | **KRKPYRPGIGGFMVR + Oxidation (M)** |
|  | 2307 | **533.9287** | **1598.7640** | **1599.7907** | **-1.0268** | **2** | **21** | **23** | **1** | **RHLSRTISVSGSTAK** |
|  | 2174 | **517.6060** | **1549.7957** | **1549.7103** | **0.0854** | **1** | **21** | **30** | **1** | **AFSCSSSIRIHER + Carbamidomethyl (C)** |
|  | 213 | **373.1832** | **1116.5275** | **1117.2104** | **-0.6828** | **1** | **21** | **24** | **1** | **GEKGDVGSAGLK** |
|  | 28 | **362.2435** | **722.4722** | **722.8312** | **-0.3589** | **0** | **21** | **18** | **1** | **VCCPSAS + Carbamidomethyl (C)** |
|  | 1153 | **428.5129** | **855.0110** | **855.0334** | **-0.0225** | **0** | **21** | **25** | **1** | **VTLLGQPK** |
|  | 736 | **405.1066** | **1212.2977** | **1211.4063** | **0.8914** | **1** | **20** | **22** | **1** | **KESMPSLMEK + 2 Oxidation (M)** |
|  | 207 | **372.6182** | **743.2215** | **742.8638** | **0.3577** | **0** | **20** | **21** | **1** | **QNAIVAK** |
|  | 2253 | **523.6677** | **1045.3205** | **1045.1923** | **0.1282** | **0** | **20** | **27** | **1** | **LSSAHVYLR** |
|  | 372 | 386.0449 | 770.0750 | 770.8708 | -0.7958 | 0 | 20 | 21 | 1 | LGVPEEK |
|  | 2449 | **554.2858** | **1106.5568** | **1107.2189** | **-0.6621** | **0** | **20** | **25** | **1** | **ACATSCPSDPR** |
|  | 1718 | **466.1024** | **1395.2850** | **1394.6582** | **0.6268** | **0** | **20** | **25** | **1** | **GNIGTGLLGLPLAAK** |
|  | 2769 | **608.4934** | **1214.9720** | **1215.3550** | **-0.3830** | **1** | **20** | **20** | **1** | **TYYTPSLKSR** |
|  | 199 | **372.1636** | **742.3124** | **742.8607** | **-0.5482** | **0** | **20** | **21** | **1** | **GITVPEK** |
|  | 1731 | **467.2064** | **932.3979** | **933.1055** | **-0.7076** | **1** | **20** | **25** | **1** | **ILAFREGK** |
|  | 3021 | **669.4454** | **2005.3139** | **2004.2442** | **1.0697** | **2** | **20** | **24** | **1** | **RYLSTYTATSSTSRALIL** |
|  | 1644 | **460.6666** | **1378.9775** | **1379.6308** | **-0.6532** | **1** | **20** | **22** | **1** | **VPVHVIRMEQR + Oxidation (M)** |
|  | 190 | 371.1832 | 740.3516 | 739.8203 | 0.5313 | 0 | 20 | 17 | 1 | QPGSVPR |
|  | 1214 | **432.2164** | **1293.6269** | **1294.4763** | **-0.8494** | **0** | **20** | **25** | **1** | **LQSCAEIDLFR** |
|  | 1438 | **445.2621** | **1332.7640** | **1333.5192** | **-0.7552** | **1** | **20** | **25** | **1** | **TLVSCARSFHR + Carbamidomethyl (C)** |
|  | 2517 | **567.6675** | **1133.3202** | **1132.3293** | **0.9908** | **1** | **20** | **31** | **1** | **MAEIYKYAK + Oxidation (M)** |
|  | 209 | **372.7206** | **743.4265** | **742.8640** | **0.5625** | **0** | **20** | **27** | **1** | **DAVIAVR** |
|  | 3494 | **820.4451** | **2458.3130** | **2458.7681** | **-0.4550** | **0** | **20** | **24** | **1** | **GQCSPSLQECIRPNPFSPSSPVK** |
|  | 728 | **405.0310** | **1212.0709** | **1211.2869** | **0.7841** | **1** | **20** | **23** | **1** | **MERDGCAGGGSR + Oxidation (M)** |
|  | 2267 | **525.3752** | **1048.7356** | **1049.2209** | **-0.4853** | **1** | **20** | **22** | **1** | **KSAVQLFEK** |
|  | 280 | **378.2345** | **754.4543** | **754.7853** | **-0.3310** | **0** | **20** | **18** | **1** | **AFSESSK** |
|  | 2398 | **547.4308** | **1092.8468** | **1093.1874** | **-0.3406** | **1** | **20** | **20** | **1** | **DFPSTAKEAK** |
|  | 246 | **375.1941** | **748.3734** | **747.8639** | **0.5095** | **1** | **20** | **24** | **1** | **CVDARK + Carbamidomethyl (C)** |
|  | 684 | **403.3860** | **1207.1360** | **1206.3665** | **0.7695** | **0** | **20** | **29** | **1** | **VANSEAMILDK + Oxidation (M)** |
|  | 1760 | **470.0668** | **1407.1783** | **1407.6359** | **-0.4575** | **1** | **20** | **23** | **1** | **LSMSRVVLSQGSK + Oxidation (M)** |
|  | 217 | **373.8781** | **745.7414** | **745.8495** | **-0.1080** | **0** | **20** | **33** | **1** | **WPAGCR + Carbamidomethyl (C)** |
|  | 2867 | **628.3003** | **1254.5858** | **1254.3512** | **0.2346** | **0** | **20** | **25** | **1** | **SGAELCTGCGSR + 2 Carbamidomethyl (C)** |
|  | 3088 | **684.1173** | **2049.3298** | **2049.3345** | **-0.0048** | **2** | **20** | **25** | **1** | **KKPSDTAKPAAPGGGRAAQLK** |
|  | 2590 | **581.0463** | **1740.1166** | **1739.0901** | **1.0266** | **2** | **20** | **28** | **1** | **EKQRMLIEMQSAMK + Oxidation (M)** |
|  | 2335 | **536.2279** | **1070.4410** | **1070.2202** | **0.2209** | **0** | **20** | **27** | **1** | **EMPGAPSPLR + Oxidation (M)** |
|  | 3237 | **728.3032** | **2181.8873** | **2181.5600** | **0.3273** | **2** | **20** | **25** | **1** | **RKDTPALHMSPFAAGVTLLR** |
|  | 3075 | **683.8375** | **1365.6603** | **1364.5945** | **1.0658** | **2** | **20** | **29** | **1** | **AFYKGTVPRLGR** |
|  | 229 | **374.2270** | **1119.6588** | **1120.2952** | **-0.6364** | **0** | **20** | **26** | **1** | **ALLLYDLAQT** |
|  | 831 | **407.4076** | **1219.2006** | **1218.3573** | **0.8433** | **2** | **20** | **28** | **1** | **EKLAQASKESK** |
|  | 926 | **412.1019** | **1233.2836** | **1232.3871** | **0.8965** | **1** | **20** | **28** | **1** | **TKGLQGTTALSR** |
|  | 1437 | **445.2469** | **888.4791** | **888.0469** | **0.4323** | **1** | **20** | **28** | **1** | **MAPERLR + Oxidation (M)** |
|  | 789 | **406.3259** | **1215.9556** | **1215.4046** | **0.5510** | **1** | **20** | **21** | **1** | **HKAIAPTPGAPR** |
|  | 2696 | **595.0669** | **1782.1785** | **1781.9241** | **0.2544** | **1** | **20** | **30** | **1** | **WGQASSDRAAVCGECGK + Carbamidomethyl (C)** |
|  | 2269 | **526.6278** | **1051.2408** | **1051.1970** | **0.0439** | **0** | **20** | **33** | **1** | **SVVLPASNHK** |
|  | 3535 | **856.4203** | **2566.2387** | **2567.0350** | **-0.7963** | **2** | **20** | **26** | **1** | **IYPQKRTVAVTIIPSIVNANQIK** |
|  | 403 | **386.8816** | **771.7485** | **772.8103** | **-1.0619** | **1** | **20** | **31** | **1** | **GAEGARGR** |
|  | 1079 | **422.1080** | **842.2013** | **841.9949** | **0.2063** | **0** | **20** | **31** | **1** | **GITLSVRP** |
|  | 1371 | **441.4250** | **880.8352** | **881.0772** | **-0.2420** | **1** | **20** | **26** | **1** | **LGLRGPLR** |
|  | 3332 | **742.9238** | **2225.7493** | **2226.5711** | **-0.8218** | **0** | **20** | **29** | **1** | **MTEVMMNTQPMEEIGLSPR + 2 Oxidation (M)** |
|  | 3445 | **785.2461** | **2352.7161** | **2352.4193** | **0.2968** | **0** | **20** | **27** | **1** | **LSPDFYEESETDPGAEELPAR** |
|  | 520 | **391.1786** | **780.3423** | **780.9535** | **-0.6111** | **0** | **20** | **24** | **1** | **ECMVVK + Carbamidomethyl (C); Oxidation (M)** |
|  | 1670 | **462.1407** | **922.2665** | **922.0464** | **0.2201** | **1** | **20** | **28** | **1** | **RQGHGVLR** |
|  | 1977 | **490.2648** | **1467.7723** | **1467.6479** | **0.1244** | **1** | **20** | **28** | **1** | **CPCERGLTCEGDK + Carbamidomethyl (C)** |
|  | 3481 | **812.4523** | **1622.8898** | **1622.9053** | **-0.0155** | **1** | **20** | **28** | **1** | **MGALDQNLTSAKVMK + Oxidation (M)** |
|  | 1566 | **457.2857** | **1368.8349** | **1367.6365** | **1.1985** | **0** | **20** | **25** | **1** | **LDVALFMSNAMR** |
|  | 1463 | 447.5187 | 893.0226 | 893.0634 | -0.0408 | 1 | 20 | 37 | 1 | MSVIGSRK + Oxidation (M) |
|  | 1403 | **443.8399** | **1328.4974** | **1329.6497** | **-1.1522** | **1** | **20** | **31** | **1** | **FIPDPKAGIMIK** |
|  | 206 | **372.3478** | **742.6808** | **742.8242** | **-0.1433** | **1** | **19** | **29** | **1** | **QPTRNK** |
|  | 2153 | **514.1049** | **1026.1949** | **1026.2323** | **-0.0373** | **1** | **19** | **29** | **1** | **TTGMLKTCR + Oxidation (M)** |
|  | 2640 | **589.6312** | **1765.8713** | **1767.0403** | **-1.1690** | **1** | **19** | **38** | **1** | **IRTASPVLWDLRPSR** |
|  | 1117 | **425.2325** | **1272.6753** | **1273.3481** | **-0.6728** | **0** | **19** | **28** | **1** | **GDLDFTYVTSR** |
|  | 458 | **388.3956** | **1162.1647** | **1162.3833** | **-0.2186** | **0** | **19** | **40** | **1** | **SSGPILCQVCR** |
|  | 757 | **405.2914** | **1212.8521** | **1212.3328** | **0.5192** | **0** | **19** | **23** | **1** | **AGHCAPSEAIEK** |
|  | 896 | **408.8985** | **1223.6733** | **1223.5028** | **0.1705** | **0** | **19** | **33** | **1** | **VALLYLAAVYK** |
|  | 3378 | **757.8414** | **1513.6681** | **1514.6796** | **-1.0115** | **1** | **19** | **34** | **1** | **ESDNNVKLIVLDR** |
|  | 444 | **388.0424** | **1161.1052** | **1161.2695** | **-0.1644** | **2** | **19** | **37** | **1** | **EATSISRNRK** |
|  | 2555 | **575.2527** | **1722.7359** | **1722.9151** | **-0.1792** | **1** | **19** | **32** | **1** | **EISMSVGLGRSQLDSK + Oxidation (M)** |
|  | 2052 | **501.0857** | **1500.2349** | **1499.6665** | **0.5684** | **0** | **19** | **31** | **1** | **TCNAYSSSMICCS + 2 Carbamidomethyl (C); Oxidation (M)** |
|  | 2374 | **540.2936** | **1617.8586** | **1616.7698** | **1.0888** | **0** | **19** | **31** | **1** | **WDDFSCSCPALTSGK** |
|  | 1222 | **432.6170** | **1294.8289** | **1294.4814** | **0.3476** | **0** | **19** | **28** | **1** | **MTLNTFHFAGR** |
|  | 605 | **399.2082** | **1194.6025** | **1194.4104** | **0.1921** | **2** | **19** | **23** | **1** | **KRLDAAPHMR** |
|  | 3275 | **740.3686** | **2218.0836** | **2218.5370** | **-0.4534** | **2** | **19** | **30** | **1** | **YAAALCAGTLWPEAAELRRR** |
|  | 3525 | **848.0203** | **1694.0257** | **1693.8111** | **0.2147** | **0** | **19** | **33** | **1** | **TMSTEQAHSGEGPMSK + Oxidation (M)** |
|  | 1173 | **430.2007** | **858.3867** | **859.0452** | **-0.6585** | **0** | **19** | **35** | **1** | **LTCLGQPK** |
|  | 3310 | **741.7247** | **1481.4346** | **1480.6218** | **0.8127** | **1** | **19** | **25** | **1** | **QLSENFKTSSLAR** |
|  | 1677 | **462.7648** | **1385.2723** | **1384.5809** | **0.6914** | **1** | **19** | **24** | **1** | **DLTVITREGKPR** |
|  | 541 | **392.4555** | **1174.3443** | **1174.3096** | **0.0348** | **1** | **19** | **36** | **1** | **KNNIPANFTR** |
|  | 928 | 412.1732 | 1233.4973 | 1234.3799 | -0.8826 | 0 | 19 | 33 | 1 | CYMDAEACSK + 2 Carbamidomethyl (C) |
|  | 325 | **382.3777** | **1144.1109** | **1145.1443** | **-1.0334** | **2** | **19** | **40** | **1** | **RDQERDGNR** |
|  | 2475 | **560.7099** | **1119.4050** | **1119.1832** | **0.2218** | **0** | **19** | **36** | **1** | **EATEGATATLR** |
|  | 1132 | **427.0601** | **1278.1582** | **1278.5815** | **-0.4233** | **0** | **19** | **30** | **1** | **LTPSLLCSMVAK + Oxidation (M)** |
|  | 667 | **402.4355** | **1204.2844** | **1203.3459** | **0.9384** | **1** | **19** | **45** | **1** | **TDKGATASAIIR** |
|  | 759 | **405.4176** | **1213.2305** | **1213.4268** | **-0.1963** | **1** | **19** | **34** | **1** | **KEFSACAIGCK + Carbamidomethyl (C)** |
|  | 3217 | **718.2994** | **2151.8761** | **2151.3619** | **0.5142** | **0** | **19** | **32** | **1** | **MTTTSAAAYGTHLSPHVPHR + Oxidation (M)** |
|  | 1792 | **472.8955** | **1415.6644** | **1414.5503** | **1.1140** | **1** | **19** | **34** | **1** | **SAAGGAGGCRAGLGPR + Carbamidomethyl (C)** |
|  | 1462 | **447.4916** | **1339.4527** | **1339.4176** | **0.0351** | **1** | **19** | **41** | **1** | **QPAGPQRGATGGSR** |
|  | 424 | **387.7577** | **773.5005** | **772.9331** | **0.5675** | **1** | **19** | **39** | **1** | **LVRSVTV** |
|  | 1251 | **434.1878** | **1299.5412** | **1298.4751** | **1.0661** | **2** | **19** | **32** | **1** | **MHKAGTNLRDR** |
|  | 271 | **377.7405** | **1130.1994** | **1131.3212** | **-1.1219** | **0** | **19** | **31** | **1** | **LEPQGLLYAK** |
|  | 2167 | **516.2604** | **1545.7591** | **1546.8339** | **-1.0748** | **1** | **19** | **35** | **1** | **CTYLQNKMSLCSR** |
|  | 2743 | **600.9890** | **1799.9447** | **1800.9606** | **-1.0159** | **1** | **19** | **33** | **1** | **DEEINQQSQLVEKLK** |
|  | 2645 | **589.9293** | **1177.8439** | **1178.4655** | **-0.6217** | **0** | **19** | **31** | **1** | **MCLLLGATGVGK + Oxidation (M)** |
|  | 2125 | **508.1701** | **1521.4880** | **1522.6453** | **-1.1572** | **1** | **19** | **35** | **1** | **GQRGKPFQCSDSR + Carbamidomethyl (C)** |
|  | 2328 | **536.1053** | **1070.1958** | **1069.1742** | **1.0216** | **1** | **19** | **34** | **1** | **TCDGGMRTR + Carbamidomethyl (C); Oxidation (M)** |
|  | 56 | **363.2963** | **1086.8668** | **1086.2626** | **0.6042** | **0** | **19** | **28** | **1** | **MHISDIGAVK + Oxidation (M)** |
|  | 3111 | **685.9913** | **1369.9678** | **1370.5096** | **-0.5418** | **0** | **19** | **27** | **1** | **SQAAVGAVPEGAWK** |
|  | 3512 | **839.6044** | **2515.7911** | **2515.8047** | **-0.0136** | **2** | **19** | **32** | **1** | **SGSPDPEVPSRASPPVWHAVRMR** |
|  | 842 | **407.6744** | **1220.0010** | **1219.3685** | **0.6324** | **0** | **19** | **27** | **1** | **SASATALSAGPMR** |
|  | 1962 | **489.2933** | **1464.8577** | **1465.6802** | **-0.8224** | **2** | **19** | **33** | **1** | **MSEGRGLPPPPRR + Oxidation (M)** |
|  | 850 | **407.8422** | **1220.5044** | **1220.3846** | **0.1199** | **1** | **19** | **35** | **1** | **ACQGRGFHCK + 2 Carbamidomethyl (C)** |
|  | 2838 | **619.8264** | **1856.4571** | **1855.9596** | **0.4974** | **1** | **19** | **29** | **1** | **SNVALPANSEDGGRAIER** |
|  | 1420 | **444.8464** | **887.6780** | **886.9924** | **0.6856** | **1** | **19** | **40** | **1** | **INAENKAK** |
|  | 1694 | **464.0043** | **1388.9908** | **1388.6076** | **0.3833** | **1** | **19** | **33** | **1** | **SIFVLPNDDLKK** |
|  | 2970 | **666.3559** | **1330.6970** | **1330.4887** | **0.2083** | **0** | **19** | **35** | **1** | **GPPGPQGATGPLGPK** |
|  | 1159 | **428.9975** | **1283.9704** | **1283.3511** | **0.6193** | **1** | **19** | **33** | **1** | **TDSHEARAGALR** |
|  | 3518 | **844.3817** | **2530.1230** | **2529.8225** | **0.3005** | **0** | **19** | **33** | **1** | **SSLPAISDAHSDLLSAICQGFQLR** |
|  | 35 | **363.0794** | **1086.2160** | **1085.2546** | **0.9614** | **0** | **19** | **35** | **1** | **EMGNSLGCFK** |
|  | 98 | **367.1560** | **1098.4458** | **1097.2853** | **1.1606** | **0** | **19** | **35** | **1** | **STVLFSGGCVK** |
|  | 1932 | **486.9378** | **1457.7912** | **1456.6436** | **1.1477** | **1** | **19** | **37** | **1** | **ANVIVTGGDDKVIR** |
|  | 670 | **402.7697** | **803.5246** | **802.8727** | **0.6518** | **0** | **19** | **39** | **1** | **ASEQLAGK** |
|  | 2427 | **551.5861** | **1101.1573** | **1100.2693** | **0.8880** | **2** | **19** | **43** | **1** | **VRKQLEAEK** |
|  | 97 | **366.3949** | **1096.1625** | **1095.1701** | **0.9925** | **2** | **19** | **48** | **1** | **SRRSPSYSR** |
|  | 2467 | **558.1706** | **1114.3264** | **1115.2805** | **-0.9541** | **0** | **19** | **36** | **1** | **SSVEQALLLR** |
|  | 2285 | **530.2001** | **1058.3854** | **1057.2015** | **1.1839** | **1** | **19** | **41** | **1** | **ECDCGKAFK + Carbamidomethyl (C)** |
|  | 3116 | **686.0847** | **2055.2320** | **2054.4205** | **0.8115** | **1** | **19** | **34** | **1** | **MMKNRPFMGSISQQNIR + Oxidation (M)** |
|  | 509 | **390.2628** | **1167.7662** | **1168.4295** | **-0.6633** | **1** | **19** | **28** | **1** | **MEFPKMLTR + Oxidation (M)** |
|  | 598 | **399.0823** | **796.1498** | **795.9283** | **0.2216** | **0** | **19** | **33** | **1** | **QAMSGMR + Oxidation (M)** |
|  | 2418 | **549.8136** | **1646.4186** | **1646.8884** | **-0.4698** | **1** | **19** | **28** | **1** | **MELCGATRLGYFGR + Carbamidomethyl (C); Oxidation (M)** |
|  | 302 | **380.2154** | **1137.6239** | **1138.3601** | **-0.7362** | **0** | **19** | **34** | **1** | **CGALALWSCSK** |
|  | 2956 | **664.4313** | **1990.2716** | **1990.1728** | **0.0988** | **0** | **19** | **35** | **1** | **DLKPENLLYYHPGTDSK** |
|  | 673 | **403.0026** | **1205.9855** | **1205.4065** | **0.5791** | **0** | **19** | **41** | **1** | **LSCAASGFMFR + Oxidation (M)** |
|  | 1353 | **439.2913** | **1314.8516** | **1314.5308** | **0.3208** | **0** | **19** | **32** | **1** | **GPGVLASGPGVMCP + Carbamidomethyl (C); Oxidation (M)** |
|  | 625 | **400.9446** | **1199.8116** | **1200.3652** | **-0.5536** | **0** | **19** | **39** | **1** | **MLVNELNHSK + Oxidation (M)** |
|  | 3262 | **737.8746** | **2210.6017** | **2211.3842** | **-0.7824** | **1** | **19** | **42** | **1** | **MDESKEGSIQGLEEMQVER + Oxidation (M)** |
|  | 1316 | **437.2173** | **1308.6299** | **1308.5065** | **0.1234** | **1** | **19** | **39** | **1** | **KEPAPPPGVCTR + Carbamidomethyl (C)** |
|  | 1199 | **431.1761** | **1290.5060** | **1291.4826** | **-0.9766** | **2** | **19** | **43** | **1** | **VFRMGPREQR + Oxidation (M)** |
|  | 2537 | **571.7947** | **1141.5747** | **1141.1986** | **0.3761** | **2** | **19** | **31** | **1** | **AGGKDGGRNPGR** |
|  | 3247 | **733.3410** | **2197.0008** | **2196.5766** | **0.4243** | **2** | **18** | **36** | **1** | **MARMNRPAPVEVTYKNMR + 2 Oxidation (M)** |
|  | 3319 | **742.1094** | **2223.3060** | **2223.5386** | **-0.2326** | **1** | **18** | **32** | **1** | **CSPWNRPGPLAPRSHMSWK + Oxidation (M)** |
|  | 2138 | **511.1650** | **1530.4729** | **1529.6479** | **0.8250** | **1** | **18** | **39** | **1** | **TLADVNGVIEEDRV** |
|  | 86 | **366.0841** | **1095.2302** | **1095.2033** | **0.0270** | **1** | **18** | **41** | **1** | **KDVDAAYVSK** |
|  | 1211 | **432.1635** | **1293.4682** | **1292.5019** | **0.9662** | **0** | **18** | **40** | **1** | **SSMNLLSLAAAAK + Oxidation (M)** |
|  | 2371 | **540.1784** | **1617.5130** | **1617.8457** | **-0.3327** | **0** | **18** | **39** | **1** | **QHLVTVCESGMQTK + Carbamidomethyl (C)** |
|  | 3574 | **1004.2200** | **3009.6377** | **3009.1620** | **0.4757** | **0** | **18** | **34** | **1** | **WSSWYNGHRPEPGLGGGDFETFENLR** |
|  | 2995 | **667.8586** | **2000.5538** | **2001.2849** | **-0.7311** | **1** | **18** | **38** | **1** | **SHNSFLLKIAPSLDSFPK** |
|  | 1201 | **431.1875** | **1290.5403** | **1289.5063** | **1.0341** | **1** | **18** | **44** | **1** | **MPWSSRGALLR + Oxidation (M)** |
|  | 695 | **403.9517** | **1208.8330** | **1208.3211** | **0.5119** | **2** | **18** | **39** | **1** | **QGYSKGPSEKK** |
|  | 1166 | **429.2315** | **856.4483** | **857.0094** | **-0.5611** | **0** | **18** | **34** | **1** | **NATGLILR** |
|  | 89 | **366.1549** | **1095.4426** | **1095.2063** | **0.2363** | **0** | **18** | **38** | **1** | **SFTQSSGLIR** |
|  | 651 | **401.9702** | **801.9255** | **800.9430** | **0.9825** | **0** | **18** | **45** | **1** | **IAAQAVTK** |
|  | 1188 | **431.0241** | **1290.0502** | **1289.4599** | **0.5902** | **0** | **18** | **47** | **1** | **TCASWLTTPPR + Carbamidomethyl (C)** |
|  | 721 | **404.9281** | **1211.7620** | **1211.5189** | **0.2432** | **1** | **18** | **36** | **1** | **LAVMSMEMRK + Oxidation (M)** |
|  | 3391 | **758.5170** | **2272.5287** | **2273.1983** | **-0.6695** | **0** | **18** | **37** | **1** | **NNWSEEDPDYPDYSGSQNR** |
|  | 400 | **386.8432** | **771.6717** | **770.8807** | **0.7910** | **1** | **18** | **41** | **1** | **NVVGARR** |
|  | 719 | **404.8847** | **807.7546** | **806.9062** | **0.8485** | **1** | **18** | **35** | **1** | **KFNSPSK** |
|  | 1866 | **479.0292** | **1434.0655** | **1433.5190** | **0.5465** | **0** | **18** | **38** | **1** | **GTFATLSELHDDK** |
|  | 1198 | **431.1700** | **1290.4880** | **1290.3867** | **0.1012** | **2** | **18** | **46** | **1** | **LQSSGRSQSKGR** |
|  | 352 | **385.1140** | **1152.3198** | **1151.3176** | **1.0022** | **0** | **18** | **34** | **1** | **HSPGGFLALPR** |
|  | 3322 | **742.4611** | **2224.3612** | **2225.3706** | **-1.0094** | **1** | **18** | **37** | **1** | **YQGVNLYVKNLDDGINDER** |
|  | 401 | **386.8557** | **771.6966** | **772.8103** | **-1.1137** | **1** | **18** | **43** | **1** | **QAEGGRR** |
|  | 602 | **399.1333** | **1194.3776** | **1195.3091** | **-0.9314** | **2** | **18** | **35** | **1** | **KSRHDSMYR + Oxidation (M)** |
|  | 3414 | **768.2697** | **2301.7870** | **2302.5675** | **-0.7805** | **2** | **18** | **39** | **1** | **VQSAHSPSPLNKKACFPGDYR** |
|  | 1185 | **431.0078** | **1290.0013** | **1290.5358** | **-0.5346** | **2** | **18** | **48** | **1** | **KMASATRLIQR + Oxidation (M)** |
|  | 404 | 386.9664 | 1157.8769 | 1158.2872 | -0.4102 | 1 | 18 | 44 | 1 | QADMRNSSMC + Oxidation (M) |
|  | 1789 | **472.5548** | **1414.6422** | **1414.5837** | **0.0585** | **0** | **18** | **48** | **1** | **SQMDGLIPGVEPR + Oxidation (M)** |
|  | 3502 | **828.3542** | **1654.6937** | **1654.8888** | **-0.1951** | **0** | **18** | **36** | **1** | **NQNGTHALSILLMSR** |
|  | 3278 | **740.4359** | **2218.2856** | **2217.4151** | **0.8704** | **2** | **18** | **39** | **1** | **KGEEALFTTRESVVDYCNR** |
|  | 1382 | **443.1700** | **1326.4880** | **1326.6310** | **-0.1430** | **2** | **18** | **39** | **1** | **VCRGYLVKMGGK + Oxidation (M)** |
|  | 3094 | **684.4226** | **1366.8304** | **1367.5572** | **-0.7267** | **2** | **18** | **39** | **1** | **TAAPSVRPEKRR** |
|  | 2869 | **628.9659** | **1883.8757** | **1884.1586** | **-0.2829** | **1** | **18** | **35** | **1** | **ICEVWACNLDEEMKK + Carbamidomethyl (C); Oxidation (M)** |
|  | 2394 | **544.6291** | **1630.8653** | **1629.8545** | **1.0108** | **0** | **18** | **54** | **1** | **LATIANFSALGLEPGR** |
|  | 1443 | **445.9618** | **1334.8632** | **1334.4528** | **0.4104** | **0** | **18** | **46** | **1** | **MVTTDGHTLSEK + Oxidation (M)** |
|  | 3097 | **684.9362** | **1367.8575** | **1367.5935** | **0.2641** | **1** | **18** | **35** | **1** | **MYNVRIMSPQP + 2 Oxidation (M)** |
|  | 2943 | **659.3673** | **1975.0798** | **1975.1400** | **-0.0603** | **1** | **18** | **41** | **1** | **GPELLMYTYSSGNKEGGR + Oxidation (M)** |
|  | 3151 | **690.0956** | **2067.2646** | **2067.2785** | **-0.0140** | **1** | **18** | **38** | **1** | **LWAESSLRATEDMEVWK + Oxidation (M)** |
|  | 234 | **374.2800** | **1119.8177** | **1119.2776** | **0.5402** | **2** | **18** | **39** | **1** | **VRGGAGRGVYK** |
|  | 1331 | **438.1732** | **1311.4976** | **1311.4673** | **0.0303** | **0** | **18** | **44** | **1** | **SHGLSPASPVCTR** |
|  | 1560 | **456.4444** | **1366.3111** | **1366.5210** | **-0.2099** | **1** | **18** | **36** | **1** | **DGPSSKVFGFVAR** |
|  | 1666 | **462.0687** | **1383.1838** | **1382.6031** | **0.5808** | **0** | **18** | **39** | **1** | **SPLSEKPPLTWK** |
|  | 693 | **403.9363** | **1208.7868** | **1209.3322** | **-0.5453** | **0** | **18** | **42** | **1** | **SAQGCPLGHSVP + Carbamidomethyl (C)** |
|  | 2951 | **663.9396** | **1988.7966** | **1988.2662** | **0.5303** | **0** | **18** | **32** | **1** | **QQMETQIGALILATDISR** |
|  | 18 | **361.8671** | **721.7195** | **722.7898** | **-1.0703** | **0** | **18** | **39** | **1** | **GPSTHPK** |
|  | 753 | **405.2280** | **1212.6617** | **1212.4256** | **0.2362** | **1** | **18** | **32** | **1** | **RLVAGQGCVGPR** |
|  | 1238 | **433.2563** | **1296.7469** | **1296.5319** | **0.2150** | **1** | **18** | **34** | **1** | **QYESLKILICS** |
|  | 467 | **389.0343** | **776.0538** | **776.8356** | **-0.7817** | **2** | **18** | **47** | **1** | **DSKGKDK** |
|  | 546 | 392.8993 | 783.7838 | 783.8761 | -0.0923 | 1 | 18 | 36 | 1 | SRGHSIK |
|  | 413 | **387.0819** | **1158.2236** | **1158.1829** | **0.0407** | **1** | **18** | **48** | **1** | **RPSGDEDRAR** |
|  | 2658 | **591.9331** | **1772.7771** | **1772.9339** | **-0.1568** | **1** | **18** | **35** | **1** | **FSGSKSGTSASLAISGCR + Carbamidomethyl (C)** |
|  | 3082 | **684.0890** | **1366.1632** | **1365.5709** | **0.5923** | **0** | **18** | **40** | **1** | **VEQPIIEEPALK** |
|  | 3102 | **685.1273** | **2052.3598** | **2053.3780** | **-1.0182** | **0** | **18** | **43** | **1** | **EPGGSCMAALTVTLMVLSSP + Carbamidomethyl (C); 2 Oxidation (M)** |
|  | 570 | **396.1598** | **790.3048** | **789.9187** | **0.3861** | **0** | **18** | **46** | **1** | **QIFPSAK** |
|  | 1951 | **488.3950** | **974.7751** | **974.1145** | **0.6607** | **0** | **18** | **36** | **1** | **LQVGASPFR** |
|  | 1041 | **419.4327** | **1255.2759** | **1256.3672** | **-1.0913** | **1** | **18** | **54** | **1** | **NHAPGATDAFKK** |
|  | 815 | **407.0982** | **812.1816** | **811.9241** | **0.2574** | **0** | **18** | **41** | **1** | **GFINYAK** |
|  | 1709 | 465.0312 | 1392.0714 | 1392.5531 | -0.4817 | 0 | 18 | 42 | 1 | QVYEGLDIITNK |
|  | 619 | **400.1926** | **1197.5556** | **1198.3724** | **-0.8168** | **1** | **18** | **34** | **1** | **QLGVAQGELKR** |
|  | 1620 | **459.7745** | **1376.3013** | **1375.4897** | **0.8116** | **2** | **18** | **39** | **1** | **WKSGGREDVSVR** |
|  | 3311 | **741.7422** | **1481.4696** | **1480.5988** | **0.8708** | **0** | **18** | **36** | **1** | **EDAHADPEIQPMK** |
|  | 339 | **384.9247** | **1151.7520** | **1151.2483** | **0.5037** | **0** | **18** | **36** | **1** | **QTGPATTMNSK + Oxidation (M)** |
|  | 1011 | **418.0856** | **834.1564** | **833.8439** | **0.3126** | **0** | **18** | **44** | **1** | **STEASPSR** |
|  | 1445 | **446.1194** | **1335.3359** | **1334.5387** | **0.7972** | **1** | **18** | **47** | **1** | **EIGNIISDAMKK + Oxidation (M)** |
|  | 2364 | **538.4772** | **1612.4095** | **1612.7166** | **-0.3071** | **0** | **18** | **36** | **1** | **EPGTTSESIFTCQR + Carbamidomethyl (C)** |
|  | 3000 | **668.5247** | **2002.5518** | **2002.2947** | **0.2571** | **0** | **18** | **36** | **1** | **KPSAFKPAIEMQNSVPNK + Oxidation (M)** |
|  | 677 | **403.1304** | **804.2461** | **804.8522** | **-0.6062** | **1** | **18** | **47** | **1** | **RGTSAASR** |
|  | 344 | **385.0046** | **767.9945** | **769.0090** | **-1.0145** | **0** | **18** | **37** | **1** | **MVMAACK + Oxidation (M)** |
|  | 1334 | **438.3320** | **1311.9739** | **1311.5268** | **0.4472** | **0** | **18** | **39** | **1** | **GLGMCVDFLNSV + Carbamidomethyl (C)** |
|  | 3143 | **688.4207** | **2062.2398** | **2061.3453** | **0.8945** | **2** | **18** | **43** | **1** | **DMLDHGKFLDCVVRAGER** |
|  | 3153 | **690.2642** | **2067.7703** | **2068.1591** | **-0.3888** | **1** | **18** | **42** | **1** | **ESGVPDRISSSGSGTDFTLR** |
|  | 3479 | **810.8408** | **2429.5001** | **2428.7555** | **0.7446** | **2** | **18** | **41** | **1** | **ELVLALYDYQEKSPREVTMK + Oxidation (M)** |
|  | 840 | **407.5915** | **1219.7524** | **1220.4193** | **-0.6669** | **0** | **18** | **37** | **1** | **ALFLYTSHLR** |
|  | 906 | **410.0390** | **1227.0948** | **1226.4905** | **0.6044** | **1** | **18** | **47** | **1** | **MVADMCRAAVK + 2 Oxidation (M)** |
|  | 1377 | **442.4096** | **1324.2066** | **1323.5575** | **0.6492** | **1** | **18** | **35** | **1** | **NTKVIDLDMFK** |
|  | 1149 | **428.3526** | **1282.0356** | **1281.4115** | **0.6242** | **1** | **18** | **30** | **1** | **SAKDDFTSIIGK** |
|  | 861 | 407.8979 | 1220.6715 | 1220.5137 | 0.1577 | 2 | 18 | 44 | 1 | MWRSCLRLR |
|  | 2473 | **559.8254** | **1676.4541** | **1676.0854** | **0.3687** | **2** | **18** | **37** | **1** | **IVRSKARPLLHVMR** |
|  | 2338 | **536.5417** | **1606.6029** | **1606.7847** | **-0.1819** | **2** | **18** | **48** | **1** | **RLPGIGDERGGPLGGR** |
|  | 3208 | **712.0544** | **2133.1410** | **2133.5584** | **-0.4175** | **2** | **18** | **36** | **1** | **AGNCYILAEPKLAFVIRVR** |
|  | 192 | 371.2608 | 740.5068 | 740.8051 | -0.2983 | 1 | 18 | 28 | 1 | KEAHEK |
|  | 1447 | **446.1427** | **1335.4059** | **1334.4560** | **0.9500** | **0** | **18** | **51** | **1** | **NPSMDSLGAVSTR** |
|  | 2810 | **613.2506** | **1836.7297** | **1837.1023** | **-0.3726** | **2** | **18** | **43** | **1** | **VKEEEQKIFGLMVDR + Oxidation (M)** |
|  | 274 | **378.0517** | **754.0886** | **752.9017** | **1.1869** | **0** | **18** | **40** | **1** | **LASPLPR** |
|  | 393 | **386.2567** | **1155.7481** | **1156.2431** | **-0.4950** | **0** | **18** | **32** | **1** | **EGGGPDGLDVLK** |
|  | 2506 | **565.6188** | **1693.8341** | **1694.8436** | **-1.0094** | **1** | **18** | **56** | **1** | **KYGGPPPGWDAAPPER** |
|  | 3316 | **741.9553** | **1481.8957** | **1481.7194** | **0.1764** | **0** | **18** | **37** | **1** | **MHVAQPAVVLASSR + Oxidation (M)** |
|  | 150 | **369.3177** | **1104.9308** | **1104.2680** | **0.6628** | **2** | **18** | **41** | **1** | **HRSDRMMR + Oxidation (M)** |
|  | 3458 | **793.9081** | **2378.7023** | **2378.6203** | **0.0819** | **2** | **18** | **51** | **1** | **STSLRRAGGQGPSCLDGLFPSSGK** |
|  | 2874 | **630.4049** | **1888.1925** | **1889.1121** | **-0.9195** | **0** | **18** | **44** | **1** | **MLENQNSLLSSSSGMFK + Oxidation (M)** |
|  | 1130 | **426.5113** | **851.0078** | **851.9037** | **-0.8959** | **1** | **18** | **54** | **1** | **YQEEKR** |
|  | 2687 | **593.7814** | **1185.5480** | **1185.3737** | **0.1742** | **2** | **18** | **44** | **1** | **NKEVKDALLR** |
|  | 216 | **373.6029** | **745.1910** | **744.8400** | **0.3510** | **1** | **18** | **46** | **1** | **LSGGQKR** |
|  | 797 | **406.7429** | **1217.2064** | **1218.2745** | **-1.0681** | **1** | **18** | **39** | **1** | **STQAELERER** |
|  | 1569 | **457.8220** | **1370.4437** | **1370.5461** | **-0.1024** | **0** | **18** | **43** | **1** | **VPIALVGPDDVEF** |
|  | 3087 | **684.1129** | **1366.2110** | **1365.5096** | **0.7015** | **0** | **18** | **44** | **1** | **NIISLMDTSGNGK + Oxidation (M)** |
|  | 2669 | **592.6984** | **1183.3819** | **1183.3994** | **-0.0174** | **1** | **18** | **54** | **1** | **MMGIGKNTTSK + Oxidation (M)** |
|  | 31 | **363.0226** | **1086.0455** | **1085.2546** | **0.7908** | **0** | **18** | **45** | **1** | **LLVAGATVDAR** |
|  | 2507 | **565.7098** | **1694.1072** | **1694.6699** | **-0.5627** | **1** | **18** | **53** | **1** | **DSGRGDSVSESGSDALR** |
|  | 2110 | **506.4081** | **1516.2022** | **1515.7852** | **0.4169** | **2** | **18** | **35** | **1** | **AFTVRCGLTRHVR** |
|  | 3043 | **677.0785** | **2028.2133** | **2028.2671** | **-0.0538** | **0** | **18** | **47** | **1** | **YSHLITSHQFQPSELIK** |
|  | 36 | **363.0796** | **1086.2165** | **1086.1982** | **0.0183** | **0** | **18** | **46** | **1** | **MPSSMTSGTR + 2 Oxidation (M)** |
|  | 1030 | **419.1928** | **1254.5564** | **1255.4453** | **-0.8889** | **1** | **18** | **52** | **1** | **MRALGYSQWK + Oxidation (M)** |
|  | 391 | **386.1906** | **770.3663** | **769.8893** | **0.4771** | **1** | **18** | **36** | **1** | **APGLEKR** |
|  | 2164 | **516.1637** | **1030.3126** | **1029.1516** | **1.1610** | **2** | **18** | **49** | **1** | **LAREQREK** |
|  | 3216 | **717.6685** | **1433.3223** | **1433.6779** | **-0.3557** | **1** | **18** | **37** | **1** | **IRLQMQTQPFR + Oxidation (M)** |
|  | 1006 | **417.9117** | **833.8086** | **832.9418** | **0.8667** | **1** | **18** | **49** | **1** | **KLSAESAK** |
|  | 471 | **389.0455** | **1164.1144** | **1163.4143** | **0.7001** | **0** | **18** | **51** | **1** | **GWVALVLHLR** |
|  | 2207 | **519.1855** | **1554.5343** | **1555.7131** | **-1.1788** | **0** | **18** | **45** | **1** | **TSEGADGCNLLCCGR + Carbamidomethyl (C)** |
|  | 3031 | 670.3690 | 2008.0849 | 2009.0934 | -1.0085 | 2 | 18 | 47 | 1 | ETPREGTAFNISSWDKNG |
|  | 1368 | **441.1849** | **1320.5326** | **1321.5297** | **-0.9971** | **1** | **18** | **46** | **1** | **ALAALWRIDHR** |
|  | 1520 | **452.1441** | **1353.4101** | **1354.4886** | **-1.0784** | **1** | **18** | **56** | **1** | **DFCSGKDGLLSR + Carbamidomethyl (C)** |
|  | 2024 | **495.5972** | **1483.7693** | **1483.6970** | **0.0724** | **2** | **18** | **59** | **1** | **RFQLYRSMNPR + Oxidation (M)** |
|  | 2541 | **572.2759** | **1142.5370** | **1141.3675** | **1.1694** | **2** | **18** | **49** | **1** | **LRARGQVLTK** |
|  | 2850 | **624.0291** | **1246.0433** | **1246.3688** | **-0.3255** | **0** | **18** | **46** | **1** | **SGHILSYDNIK** |
|  | 2952 | **664.0370** | **1989.0888** | **1989.2742** | **-0.1854** | **2** | **18** | **42** | **1** | **RLAFDITYTLEYSRLK** |
|  | 1150 | **428.4218** | **854.8287** | **855.9753** | **-1.1465** | **0** | **18** | **43** | **1** | **DIGPEVVK** |
|  | 2472 | **558.6813** | **1115.3478** | **1114.2512** | **1.0966** | **0** | **18** | **57** | **1** | **GPTPLFPETR** |
|  | 2972 | 666.5201 | 1996.5383 | 1997.2746 | -0.7364 | 1 | 18 | 39 | 1 | ADIKEMGLSLQWLYSAR + Oxidation (M) |
|  | 450 | **388.2072** | **1161.5993** | **1162.3638** | **-0.7645** | **2** | **18** | **46** | **1** | **TTVSMPRSRK** |
|  | 3027 | **669.8916** | **2006.6526** | **2006.2272** | **0.4255** | **2** | **18** | **41** | **1** | **SCTSMADGRMSTARDMGR + Carbamidomethyl (C); Oxidation (M)** |
|  | 26 | **362.2002** | **1083.5785** | **1084.2730** | **-0.6945** | **1** | **18** | **36** | **1** | **QLRLAGTIGR** |
|  | 1391 | **443.4482** | **1327.3225** | **1327.5097** | **-0.1872** | **1** | **18** | **51** | **1** | **MLSLRGPDTHGK + Oxidation (M)** |
|  | 451 | **388.2190** | **774.4233** | **774.8595** | **-0.4362** | **0** | **18** | **45** | **1** | **VSEAIEK** |
|  | 1484 | **449.4745** | **896.9343** | **895.9414** | **0.9930** | **1** | **18** | **52** | **1** | **SCSRGSSR + Carbamidomethyl (C)** |
|  | 3259 | **737.2643** | **2208.7707** | **2208.4249** | **0.3457** | **2** | **18** | **44** | **1** | **LERTSSVSPSTAERELSIVF** |
|  | 3314 | **741.8503** | **2222.5289** | **2223.5257** | **-0.9968** | **2** | **18** | **53** | **1** | **KGMMIDEADEFVAGPQNKVK + Oxidation (M)** |
|  | 671 | **402.8938** | **1205.6592** | **1206.3729** | **-0.7137** | **1** | **18** | **52** | **1** | **RITCGGNTIGSK** |
|  | 1626 | **460.1071** | **918.1993** | **918.1192** | **0.0802** | **2** | **17** | **52** | **1** | **MRRAGLAK + Oxidation (M)** |
|  | 2039 | **498.8504** | **995.6860** | **995.0921** | **0.5940** | **0** | **17** | **43** | **1** | **FGQGGGLGFR** |
|  | 2488 | **564.2323** | **1689.6747** | **1688.9304** | **0.7444** | **2** | **17** | **50** | **1** | **TSRMAPPASRAPQMR + 2 Oxidation (M)** |
|  | 2840 | **620.0063** | **1237.9979** | **1237.2945** | **0.7035** | **0** | **17** | **43** | **1** | **CSENGTVDVADK** |
|  | 1717 | **465.9760** | **929.9373** | **929.0125** | **0.9248** | **0** | **17** | **50** | **1** | **DGTHACLGR** |
|  | 1729 | **467.1888** | **1398.5444** | **1399.7247** | **-1.1803** | **2** | **17** | **50** | **1** | **HSIKGMNMPKLK + Oxidation (M)** |
|  | 2594 | **581.2493** | **1160.4838** | **1161.2744** | **-0.7906** | **0** | **17** | **51** | **1** | **HNPSQRPGLR** |
|  | 1740 | **468.3355** | **1401.9844** | **1401.5023** | **0.4822** | **0** | **17** | **42** | **1** | **VTPTCHSSTSEPR** |
|  | 1129 | **426.4560** | **850.8973** | **849.8910** | **1.0062** | **0** | **17** | **57** | **1** | **YQQQQR** |
|  | 517 | **391.1519** | **1170.4334** | **1170.2399** | **0.1935** | **2** | **17** | **45** | **1** | **GGREAGPGSRAR** |
|  | 697 | **403.9800** | **1208.9179** | **1209.4812** | **-0.5633** | **1** | **17** | **49** | **1** | **IAMLWGSGKCK + Oxidation (M)** |
|  | 814 | **407.0938** | **1218.2591** | **1217.5051** | **0.7540** | **2** | **17** | **47** | **1** | **KTCIVHKMNK + Oxidation (M)** |
|  | 1308 | **437.1127** | **872.2107** | **871.9614** | **0.2493** | **1** | **17** | **55** | **1** | **EMGHRVSG** |
|  | 3513 | **840.4973** | **1678.9797** | **1678.0530** | **0.9267** | **2** | **17** | **46** | **1** | **IILARKQNSCCAIM + 2 Carbamidomethyl (C)** |
|  | 1763 | **470.1210** | **1407.3409** | **1407.5744** | **-0.2335** | **2** | **17** | **44** | **1** | **SRRASLSEIGFGK** |
|  | 2429 | **551.7594** | **1652.2560** | **1651.9713** | **0.2847** | **1** | **17** | **44** | **1** | **MVRGVIMGGMDVWGQ + Oxidation (M)** |
|  | 3104 | **685.2693** | **2052.7857** | **2052.2871** | **0.4985** | **2** | **17** | **51** | **1** | **KKALLAQEQVSFFEEER** |
|  | 3236 | **728.2722** | **1454.5297** | **1455.6523** | **-1.1226** | **1** | **17** | **46** | **1** | **AVGPASILKEVEDK** |
|  | 3531 | **850.6906** | **2549.0495** | **2547.8844** | **1.1650** | **1** | **17** | **41** | **1** | **SHVIWTLKMECSETHVQGSCAK + Carbamidomethyl (C); Oxidation (M)** |
|  | 3103 | **685.2234** | **1368.4320** | **1367.5572** | **0.8748** | **2** | **17** | **50** | **1** | **TAAPSVRPEKRR** |
|  | 768 | **406.0705** | **1215.1892** | **1215.3781** | **-0.1889** | **0** | **17** | **46** | **1** | **IGDTAVYFCAR** |
|  | 1813 | **475.6132** | **1423.8175** | **1424.6480** | **-0.8305** | **1** | **17** | **56** | **1** | **ALTARAGVPAAALSR** |
|  | 2821 | **614.3495** | **1840.0263** | **1839.0169** | **1.0094** | **1** | **17** | **49** | **1** | **QPIRGYVQPADTGHTAK** |
|  | 834 | **407.4598** | **1219.3572** | **1219.3900** | **-0.0329** | **1** | **17** | **57** | **1** | **EFAVLSELRR** |
|  | 2112 | **506.6908** | **1517.0502** | **1517.5957** | **-0.5454** | **0** | **17** | **43** | **1** | **DFVAGAIGGQDPDGAK** |
|  | 2356 | **538.1157** | **1611.3250** | **1611.6723** | **-0.3473** | **0** | **17** | **51** | **1** | **GARPGGGDAGGTPGETVR** |
|  | 3098 | **685.0243** | **1368.0338** | **1367.4690** | **0.5648** | **0** | **17** | **44** | **1** | **WPGSHLATSQQR** |
|  | 215 | 373.4192 | 744.8235 | 745.7832 | -0.9596 | 0 | 17 | 73 | 1 | GAGGGGWGK |
|  | 584 | **398.1980** | **1191.5718** | **1192.2421** | **-0.6702** | **1** | **17** | **45** | **1** | **DGARGAHGAVGAPG** |
|  | 723 | **404.9924** | **1211.9551** | **1211.2404** | **0.7147** | **0** | **17** | **45** | **1** | **EENNTGECCGR** |
|  | 1994 | **492.1879** | **1473.5416** | **1472.6031** | **0.9385** | **0** | **17** | **48** | **1** | **MGANINMQDAYGR + 2 Oxidation (M)** |
|  | 1147 | **428.2631** | **1281.7670** | **1282.5121** | **-0.7451** | **0** | **17** | **35** | **1** | **MLKPSAHLTER** |
|  | 2484 | **563.1934** | **1686.5579** | **1685.9246** | **0.6333** | **2** | **17** | **46** | **1** | **SCKKAVCDFPGFGGGR + Carbamidomethyl (C)** |
|  | 120 | **369.0861** | **1104.2361** | **1105.3089** | **-1.0728** | **0** | **17** | **56** | **1** | **AKPWAVCFPS** |
|  | 2072 | **503.0007** | **1505.9800** | **1506.7518** | **-0.7718** | **1** | **17** | **54** | **1** | **SSIHRPRLLTAQK** |
|  | 2878 | **632.2695** | **1262.5243** | **1261.4667** | **1.0576** | **1** | **17** | **54** | **1** | **TFSVPVKQEVK** |
|  | 3114 | **686.0181** | **2055.0320** | **2054.2599** | **0.7722** | **0** | **17** | **40** | **1** | **DVGWGLGGPPSSSSTVAAPLAK** |
|  | 470 | 389.0421 | 776.0694 | 775.8740 | 0.1954 | 1 | 17 | 55 | 1 | SPEGCRK |
|  | 775 | **406.1353** | **1215.3836** | **1214.4431** | **0.9405** | **1** | **17** | **48** | **1** | **VLRAGMGAHFR** |
|  | 3350 | **746.9912** | **1491.9676** | **1490.8136** | **1.1540** | **0** | **17** | **40** | **1** | **CELCLGIMGGKPR + 2 Carbamidomethyl (C)** |
|  | 230 | **374.2408** | **746.4669** | **745.9075** | **0.5594** | **1** | **17** | **48** | **1** | **ASSKILK** |
|  | 999 | **417.0172** | **832.0196** | **831.9157** | **0.1039** | **0** | **17** | **57** | **1** | **FGVPAEGR** |
|  | 2000 | **492.6419** | **983.2690** | **982.1151** | **1.1539** | **1** | **17** | **50** | **1** | **ECGKVFSR + Carbamidomethyl (C)** |
|  | 791 | **406.4477** | **810.8806** | **809.8604** | **1.0202** | **0** | **17** | **56** | **1** | **EEELYK** |
|  | 891 | **408.5862** | **1222.7364** | **1223.4201** | **-0.6837** | **1** | **17** | **47** | **1** | **CMELLDGKNGK + Oxidation (M)** |
|  | 982 | **416.0746** | **830.1344** | **830.0272** | **0.1072** | **1** | **17** | **60** | **1** | **TLSLIRK** |
|  | 3100 | **685.0864** | **1368.1579** | **1367.5572** | **0.6008** | **2** | **17** | **51** | **1** | **TAAPSVRPEKRR** |
|  | 165 | **370.0377** | **738.0605** | **738.9168** | **-0.8562** | **0** | **17** | **46** | **1** | **SQMVMK + Oxidation (M)** |
|  | 669 | **402.4809** | **802.9470** | **802.9424** | **0.0047** | **0** | **17** | **70** | **1** | **KPGTCAAR** |
|  | 1289 | **436.1004** | **1305.2791** | **1304.5243** | **0.7549** | **2** | **17** | **50** | **1** | **MWSRRGLGVSR** |
|  | 653 | **401.9994** | **1202.9761** | **1202.4455** | **0.5305** | **0** | **17** | **59** | **1** | **EICCCSISCK + 2 Carbamidomethyl (C)** |
|  | 1482 | **449.2871** | **896.5595** | **897.1349** | **-0.5754** | **0** | **17** | **38** | **1** | **MEMMLDK** |
|  | 2922 | **655.1561** | **1962.4462** | **1962.1976** | **0.2487** | **1** | **17** | **49** | **1** | **EPRVQAPGLGPCGRPASGR + Carbamidomethyl (C)** |
|  | 1257 | **434.6973** | **1301.0698** | **1300.4611** | **0.6087** | **0** | **17** | **37** | **1** | **SEVLVQQTLQR** |
|  | 3425 | **775.1458** | **2322.4151** | **2321.7873** | **0.6278** | **0** | **17** | **44** | **1** | **LCADCHVLIWLCGVLWPSHR** |
|  | 428 | **387.8779** | **773.7410** | **772.8517** | **0.8894** | **0** | **17** | **61** | **1** | **FSNIHR** |
|  | 500 | **389.9110** | **777.8072** | **776.8372** | **0.9700** | **0** | **17** | **51** | **1** | **AHSFTSK** |
|  | 2238 | **521.2545** | **1560.7412** | **1559.8046** | **0.9366** | **2** | **17** | **49** | **1** | **LKEIAFPRTDELK** |
|  | 3433 | **778.4142** | **1554.8136** | **1555.7449** | **-0.9313** | **2** | **17** | **48** | **1** | **HRRMHGMTPGSTR + 2 Oxidation (M)** |
|  | 3498 | **823.1994** | **1644.3840** | **1644.9190** | **-0.5350** | **2** | **17** | **45** | **1** | **ALRATYQLTKARPR** |
|  | 1388 | **443.3845** | **1327.1312** | **1326.5479** | **0.5833** | **0** | **17** | **39** | **1** | **GPWCCPPAPWR + Carbamidomethyl (C)** |
|  | 1275 | **435.7159** | **1304.1255** | **1303.4618** | **0.6636** | **1** | **17** | **39** | **1** | **ESAIASTEVKLR** |
|  | 47 | **363.1889** | **724.3631** | **723.8192** | **0.5439** | **0** | **17** | **42** | **1** | **CEACGK + 2 Carbamidomethyl (C)** |
|  | 2852 | **624.2496** | **1869.7267** | **1870.1289** | **-0.4021** | **0** | **17** | **53** | **1** | **ALYDYMGSEMMAVTQK + 2 Oxidation (M)** |
|  | 3164 | **695.4344** | **2083.2812** | **2083.3741** | **-0.0929** | **1** | **17** | **52** | **1** | **SRVPVPGPGAAAAPCPAPASPR + Carbamidomethyl (C)** |
|  | 642 | **401.8730** | **1202.5967** | **1203.2632** | **-0.6665** | **1** | **17** | **59** | **1** | **QSSVLERDNR** |
|  | 2007 | **493.7572** | **1478.2494** | **1478.7633** | **-0.5138** | **1** | **17** | **42** | **1** | **HCCPVCRWPSYK** |
|  | 3505 | **830.8894** | **1659.7640** | **1658.9193** | **0.8448** | **2** | **17** | **54** | **1** | **CSCQTKDMSVRLSGK + Oxidation (M)** |
|  | 131 | **369.2246** | **1104.6516** | **1105.3089** | **-0.6573** | **0** | **17** | **44** | **1** | **AKPWAVCFPS** |
|  | 729 | **405.0373** | **808.0599** | **808.8607** | **-0.8009** | **0** | **17** | **47** | **1** | **ASSGTCQR** |
|  | 2937 | **657.8398** | **1970.4974** | **1971.2360** | **-0.7386** | **1** | **17** | **48** | **1** | **EKANVNVPADTPLMLQSK + Oxidation (M)** |
|  | 699 | **403.9960** | **1208.9657** | **1208.3871** | **0.5786** | **0** | **17** | **53** | **1** | **MAWGTGTLLSR + Oxidation (M)** |
|  | 157 | **369.3633** | **1105.0677** | **1104.3011** | **0.7666** | **0** | **17** | **58** | **1** | **MHPSLATMGK + 2 Oxidation (M)** |
|  | 1915 | **485.4249** | **968.8350** | **968.0238** | **0.8112** | **1** | **17** | **38** | **1** | **SFASSGRWA** |
|  | 2584 | **580.3824** | **1738.1252** | **1739.0056** | **-0.8804** | **0** | **17** | **50** | **1** | **IHTGERPYVCMECGK + Oxidation (M)** |
|  | 1016 | **419.0215** | **1254.0422** | **1253.4443** | **0.5979** | **0** | **17** | **60** | **1** | **LPPITGGASELAK** |
|  | 2941 | **658.6267** | **1315.2386** | **1315.3035** | **-0.0648** | **0** | **17** | **44** | **1** | **DQDGDQQLGPSR** |
|  | 363 | **385.3862** | **1153.1364** | **1152.3821** | **0.7544** | **0** | **17** | **49** | **1** | **LDSVIEMCVK + Oxidation (M)** |
|  | 127 | **369.1897** | **1104.5469** | **1104.3011** | **0.2458** | **0** | **17** | **46** | **1** | **MHPSLATMGK + 2 Oxidation (M)** |
|  | 129 | **369.2027** | **1104.5860** | **1104.3011** | **0.2849** | **0** | **17** | **46** | **1** | **MHPSLATMGK + 2 Oxidation (M)** |
|  | 2161 | **515.7560** | **1029.4972** | **1029.1898** | **0.3074** | **1** | **17** | **45** | **1** | **KHLSYVVSP** |
|  | 1407 | **443.9032** | **1328.6873** | **1327.5079** | **1.1794** | **1** | **17** | **54** | **1** | **YWDLKSGVCTR** |
|  | 2227 | **520.2262** | **1557.6564** | **1557.7508** | **-0.0944** | **1** | **17** | **51** | **1** | **GLMHVPPASESRMGG + 2 Oxidation (M)** |
|  | 91 | 366.2093 | 1095.6058 | 1095.2512 | 0.3546 | 1 | 17 | 48 | 1 | KYGSVTVWR |
|  | 595 | **398.9446** | **1193.8116** | **1193.2948** | **0.5168** | **2** | **17** | **46** | **1** | **IRDGSSSCRR + Carbamidomethyl (C)** |
|  | 3165 | **696.0861** | **1390.1573** | **1389.7265** | **0.4308** | **1** | **17** | **53** | **1** | **DVCIVLAKEMIR** |
|  | 3533 | **852.5275** | **1703.0402** | **1702.9039** | **0.1363** | **0** | **17** | **50** | **1** | **EQSMSSVGFCVINSAK + Oxidation (M)** |
|  | 148 | **369.2893** | **1104.8458** | **1104.3011** | **0.5448** | **0** | **17** | **47** | **1** | **MHPSLATMGK + 2 Oxidation (M)** |
|  | 859 | **407.8802** | **1220.6185** | **1220.2489** | **0.3695** | **1** | **17** | **52** | **1** | **AADEGPEGRYR** |
|  | 1020 | **419.0857** | **1254.2350** | **1253.3664** | **0.8686** | **1** | **17** | **62** | **1** | **SWTYGITRGGR** |
|  | 707 | 404.1296 | 1209.3667 | 1209.3520 | 0.0147 | 0 | 17 | 53 | 1 | ALAALSAPPNER |
|  | 1174 | **430.2067** | **858.3985** | **858.8963** | **-0.4977** | **0** | **17** | **58** | **1** | **QDGSLSPR** |
|  | 2632 | **587.6787** | **1760.0140** | **1760.9910** | **-0.9771** | **0** | **17** | **66** | **1** | **VQLVHSGGGLVQPGGSLR** |
|  | 2695 | **594.8040** | **1781.3897** | **1782.0037** | **-0.6140** | **1** | **17** | **48** | **1** | **DLDVAILLGSVPREER** |
|  | 154 | **369.3376** | **1104.9906** | **1104.3011** | **0.6895** | **0** | **17** | **53** | **1** | **MHPSLATMGK + 2 Oxidation (M)** |
|  | 258 | **376.9744** | **751.9339** | **751.7830** | **0.1510** | **0** | **17** | **49** | **1** | **SSSSLSGK** |
|  | 389 | **386.1709** | **770.3270** | **770.9403** | **-0.6132** | **0** | **17** | **45** | **1** | **VLQHMK + Oxidation (M)** |
|  | 1469 | **448.2727** | **1341.7960** | **1341.5957** | **0.2003** | **0** | **17** | **43** | **1** | **YGIICMEDLIR + Oxidation (M)** |
|  | 1632 | **460.2422** | **1377.7043** | **1377.5517** | **0.1526** | **2** | **17** | **56** | **1** | **RSSQHPRVQGLL** |
|  | 1390 | **443.4453** | **1327.3136** | **1327.4171** | **-0.1035** | **0** | **17** | **56** | **1** | **FSDEGMEVIER + Oxidation (M)** |
|  | 2010 | **494.1459** | **1479.4154** | **1479.7630** | **-0.3475** | **1** | **17** | **56** | **1** | **TFEALKFFVHLK** |
|  | 2063 | **502.1349** | **1002.2551** | **1003.0217** | **-0.7665** | **0** | **17** | **58** | **1** | **TEAGGSPEQK** |
|  | 3203 | **708.2089** | **1414.4031** | **1414.6266** | **-0.2236** | **0** | **17** | **52** | **1** | **LMSPENTLLQPR + Oxidation (M)** |
|  | 126 | **369.1834** | **1104.5280** | **1103.3395** | **1.1885** | **2** | **17** | **50** | **1** | **INVAAKRGMK + Oxidation (M)** |
|  | 865 | **407.9536** | **1220.8388** | **1221.4093** | **-0.5705** | **1** | **17** | **54** | **1** | **RSAPLGPVAPTR** |
|  | 412 | **387.0765** | **1158.2074** | **1157.3024** | **0.9051** | **0** | **17** | **63** | **1** | **AASGACMTCNR + Carbamidomethyl (C); Oxidation (M)** |
|  | 1669 | **462.1163** | **922.2178** | **923.0430** | **-0.8252** | **0** | **17** | **51** | **1** | **DITMQSTK** |
|  | 121 | **369.1304** | **1104.3691** | **1105.3089** | **-0.9397** | **0** | **17** | **59** | **1** | **AKPWAVCFPS** |
|  | 634 | **401.4783** | **1201.4126** | **1200.3022** | **1.1104** | **1** | **17** | **73** | **1** | **EVGNGAKQEIR** |
|  | 21 | **362.1188** | **1083.3342** | **1082.2957** | **1.0386** | **0** | **17** | **51** | **1** | **MTVVTMASAR + Oxidation (M)** |
|  | 1125 | **426.1995** | **1275.5764** | **1276.3768** | **-0.8004** | **0** | **17** | **54** | **1** | **AQNSSGVMGGPQK + Oxidation (M)** |
|  | 2625 | **586.5232** | **1756.5474** | **1756.9543** | **-0.4069** | **1** | **17** | **44** | **1** | **GQKGESCLICDIDGYR** |
|  | 2514 | 567.0273 | 1698.0599 | 1697.1343 | 0.9255 | 1 | 17 | 55 | 1 | IQNPMILKVVATLLK + Oxidation (M) |
|  | 3300 | **741.0992** | **1480.1837** | **1480.5326** | **-0.3489** | **1** | **17** | **46** | **1** | **SSFKDSSEHSLEK** |
|  | 49 | **363.2151** | **724.4155** | **723.8392** | **0.5763** | **1** | **17** | **44** | **1** | **MSKTNK + Oxidation (M)** |
|  | 981 | **416.0601** | **830.1053** | **830.9691** | **-0.8637** | **0** | **17** | **66** | **1** | **GSVTGAVLK** |
|  | 2746 | **602.5040** | **1202.9933** | **1202.3595** | **0.6338** | **0** | **17** | **46** | **1** | **SLEVLWNVSR** |
|  | 142 | **369.2585** | **1104.7534** | **1105.3089** | **-0.5555** | **0** | **17** | **47** | **1** | **AKPWAVCFPS** |
|  | 2526 | **569.3999** | **1136.7850** | **1136.2648** | **0.5202** | **1** | **17** | **49** | **1** | **DLRGAALAHGR** |
|  | 449 | **388.1816** | **774.3484** | **774.8262** | **-0.4779** | **1** | **17** | **59** | **1** | **TRAGSQR** |
|  | 3313 | **741.8375** | **2222.4902** | **2221.4467** | **1.0435** | **1** | **17** | **61** | **1** | **RVAEVWMDEYAEYIYQR** |
|  | 583 | 398.1838 | 1191.5293 | 1191.3565 | 0.1728 | 0 | 17 | 53 | 1 | IWDAASGQCLK |
|  | 1869 | **479.6571** | **1435.9490** | **1435.5797** | **0.3693** | **0** | **17** | **52** | **1** | **ITLGASTVSSVSSAR** |
|  | 146 | **369.2690** | **1104.7850** | **1105.3089** | **-0.5239** | **0** | **17** | **48** | **1** | **AKPWAVCFPS** |
|  | 2107 | **506.2296** | **1515.6665** | **1515.8000** | **-0.1336** | **1** | **17** | **53** | **1** | **ASCLPAMLLDPRR + Carbamidomethyl (C); Oxidation (M)** |
|  | 2549 | **573.4182** | **1144.8216** | **1144.1927** | **0.6290** | **1** | **17** | **48** | **1** | **NLDPDDTARK** |
|  | 849 | **407.8109** | **1220.4105** | **1219.3685** | **1.0420** | **2** | **17** | **57** | **1** | **NAKKCLEDNK + Carbamidomethyl (C)** |
|  | 2295 | **532.6313** | **1063.2478** | **1063.2077** | **0.0401** | **0** | **17** | **69** | **1** | **AELPPGPAVGR** |
|  | 3363 | **751.3454** | **1500.6760** | **1499.6317** | **1.0443** | **2** | **17** | **52** | **1** | **RSTNLAANTPGKGGR** |
|  | 135 | **369.2414** | **1104.7019** | **1105.3089** | **-0.6069** | **0** | **17** | **48** | **1** | **AKPWAVCFPS** |
|  | 751 | **405.2142** | **808.4136** | **808.8358** | **-0.4222** | **0** | **17** | **44** | **1** | **YLEDNR** |
|  | 1936 | **487.1722** | **972.3297** | **973.2342** | **-0.9045** | **1** | **17** | **61** | **1** | **KMILVVDR** |
|  | 143 | **369.2635** | **1104.7684** | **1105.2443** | **-0.4760** | **1** | **17** | **48** | **1** | **TIHTGGKTYK** |
|  | 114 | **368.2962** | **734.5776** | **734.8434** | **-0.2659** | **0** | **17** | **49** | **1** | **GLVGYAR** |
|  | 356 | **385.1380** | **1152.3917** | **1151.4220** | **0.9698** | **1** | **17** | **48** | **1** | **LLFGRGTMLK + Oxidation (M)** |
|  | 386 | **386.1507** | **770.2866** | **769.8891** | **0.3975** | **0** | **17** | **50** | **1** | **GASLGPIR** |
|  | 1966 | **489.6904** | **977.3660** | **977.0308** | **0.3353** | **1** | **17** | **48** | **1** | **QVKESSGSR** |
|  | 2853 | **625.1060** | **1248.1971** | **1247.3571** | **0.8401** | **1** | **17** | **57** | **1** | **EKGWSSLPTSR** |
|  | 3139 | **687.9363** | **2060.7867** | **2060.5210** | **0.2656** | **0** | **17** | **46** | **1** | **MSVCLDMFLTIICEGSIK + Carbamidomethyl (C)** |
|  | 3140 | **688.2411** | **1374.4675** | **1375.4465** | **-0.9790** | **2** | **17** | **56** | **1** | **WRAGESEEQRK** |
|  | 1853 | **478.0067** | **953.9985** | **953.0110** | **0.9876** | **0** | **17** | **51** | **1** | **SSSVPHSPR** |
|  | 2800 | **612.2228** | **1833.6462** | **1834.2355** | **-0.5894** | **2** | **17** | **57** | **1** | **VFPIRLMLRLGAEYR** |
|  | 1658 | **461.8004** | **1382.3792** | **1382.4390** | **-0.0599** | **1** | **17** | **50** | **1** | **ESRLENGHGLDR** |
|  | 2280 | **530.0131** | **1058.0113** | **1058.2308** | **-0.2195** | **1** | **17** | **61** | **1** | **WIEVKANAK** |
|  | 1440 | **445.5211** | **889.0275** | **887.9804** | **1.0470** | **0** | **17** | **79** | **1** | **SAAGNSILR** |
|  | 2132 | **509.0367** | **1016.0587** | **1017.0979** | **-1.0392** | **1** | **17** | **61** | **1** | **VTRTAQGER** |
|  | 34 | **363.0594** | **1086.1560** | **1086.2064** | **-0.0504** | **2** | **17** | **57** | **1** | **RQDSRVVAR** |
|  | 760 | **405.5417** | **1213.6028** | **1214.4216** | **-0.8188** | **1** | **17** | **55** | **1** | **ASHMPALGCRR + Oxidation (M)** |
|  | 806 | **406.9290** | **811.8432** | **810.8767** | **0.9665** | **1** | **17** | **50** | **1** | **SGSCSSRK** |
|  | 1366 | **441.1612** | **1320.4614** | **1320.5583** | **-0.0969** | **1** | **17** | **56** | **1** | **GLSSLLKSLSCR + Carbamidomethyl (C)** |
|  | 1634 | **460.2903** | **1377.8487** | **1377.5023** | **0.3464** | **0** | **17** | **51** | **1** | **NPEPGPVSGTAVPR** |
|  | 2789 | **611.2855** | **1220.5563** | **1221.2784** | **-0.7221** | **0** | **17** | **58** | **1** | **AYCSSSSCNSR + Carbamidomethyl (C)** |
|  | 704 | **404.1005** | **1209.2792** | **1208.4119** | **0.8673** | **1** | **17** | **57** | **1** | **RITCGGNCIGSK** |
|  | 202 | **372.2124** | **742.4101** | **742.8607** | **-0.4506** | **0** | **17** | **44** | **1** | **SAVIPEK** |
|  | 311 | **380.6336** | **1138.8786** | **1139.2191** | **-0.3406** | **0** | **17** | **54** | **1** | **HSQEELLQR** |
|  | 477 | **389.0966** | **776.1784** | **775.8491** | **0.3293** | **0** | **17** | **62** | **1** | **GQEFAPK** |
|  | 1051 | **420.0250** | **838.0353** | **836.8909** | **1.1444** | **1** | **17** | **52** | **1** | **KSSTSATR** |
|  | 1854 | **478.0133** | **1431.0177** | **1430.5434** | **0.4744** | **0** | **17** | **52** | **1** | **PGSSGAGMEAAVAPGR + Oxidation (M)** |
|  | 3254 | **735.8285** | **1469.6422** | **1470.6071** | **-0.9649** | **2** | **17** | **64** | **1** | **KQSGGKTQQYDCK** |
|  | 3349 | **746.6614** | **2236.9620** | **2236.5624** | **0.3995** | **1** | **17** | **44** | **1** | **ILDVIDDTIEMETGLKAMGR + Oxidation (M)** |
|  | 664 | **402.2136** | **1203.6185** | **1202.4259** | **1.1926** | **1** | **17** | **56** | **1** | **SGMSMASRLCK + 2 Oxidation (M)** |
|  | 3477 | **809.1030** | **1616.1912** | **1616.8110** | **-0.6199** | **1** | **17** | **42** | **1** | **KLISSDYYIWNSK** |
|  | 2419 | **549.9575** | **1097.9003** | **1097.1841** | **0.7161** | **1** | **17** | **57** | **1** | **EYQAGRFAR** |
|  | 3017 | **669.1426** | **1336.2705** | **1335.4192** | **0.8513** | **0** | **17** | **57** | **1** | **LFEVGESPSNTR** |
|  | 503 | **389.9737** | **1166.8989** | **1166.2874** | **0.6115** | **0** | **17** | **58** | **1** | **CDGCGLNYHK + Carbamidomethyl (C)** |
|  | 1476 | **448.5228** | **1342.5463** | **1341.5528** | **0.9935** | **0** | **17** | **65** | **1** | **QFAVGLFFPSTK** |
|  | 1851 | **477.9380** | **1430.7920** | **1430.4803** | **0.3116** | **1** | **17** | **53** | **1** | **IRDAGNDSASTAPR** |
|  | 639 | **401.8387** | **1202.4938** | **1203.3028** | **-0.8091** | **0** | **17** | **66** | **1** | **SGTSASLAISGPR** |
|  | 1294 | **436.2119** | **870.4090** | **869.9653** | **0.4438** | **1** | **17** | **56** | **1** | **KGGPLGEGR** |
|  | 1417 | **444.1661** | **886.3174** | **885.1455** | **1.1719** | **1** | **17** | **65** | **1** | **KAILTLVK** |
|  | 3520 | **845.9012** | **2534.6816** | **2535.7892** | **-1.1077** | **2** | **17** | **64** | **1** | **YTFGSTVHYSCTGKRSLLGQSSR** |
|  | 727 | **405.0226** | **1212.0457** | **1212.3560** | **-0.3104** | **0** | **17** | **53** | **1** | **GGTGSVGLAAAPVR** |
|  | 87 | **366.1194** | **1095.3362** | **1095.3805** | **-0.0443** | **2** | **17** | **63** | **1** | **SAIMVKKMR + 2 Oxidation (M)** |
|  | 2306 | **533.8537** | **1598.5389** | **1598.8210** | **-0.2820** | **0** | **17** | **47** | **1** | **VTEGAGCMSELAVCR + Carbamidomethyl (C); Oxidation (M)** |
|  | 3167 | **696.3365** | **2085.9873** | **2085.2870** | **0.7002** | **1** | **17** | **60** | **1** | **EGYPCQDGMCGYCHHRR + Carbamidomethyl (C); Oxidation (M)** |
|  | 185 | **371.0745** | **1110.2013** | **1110.3553** | **-0.1540** | **0** | **17** | **49** | **1** | **MHCASQMMR + Oxidation (M)** |
|  | 475 | **389.0682** | **1164.1824** | **1165.3494** | **-1.1669** | **2** | **17** | **65** | **1** | **TPRAGVPGVRR** |
|  | 1531 | 453.4450 | 904.8752 | 905.0709 | -0.1956 | 1 | 17 | 65 | 1 | AEVEVCKK |
|  | 2485 | **563.8382** | **1688.4924** | **1687.8726** | **0.6198** | **2** | **17** | **46** | **1** | **DLERSHTACATLDKK** |
|  | 1181 | **430.7198** | **1289.1372** | **1288.4917** | **0.6454** | **1** | **17** | **55** | **1** | **KGAYAGGLVLDPK** |
|  | 2079 | **504.1775** | **1509.5103** | **1509.5354** | **-0.0251** | **0** | **17** | **62** | **1** | **YNTPGQNYSNHSK** |
|  | 3108 | **685.9172** | **2054.7295** | **2054.4817** | **0.2478** | **2** | **17** | **49** | **1** | **LLLTRFIFCSATMRTHK + Oxidation (M)** |
|  | 1192 | **431.0895** | **860.1642** | **861.0216** | **-0.8574** | **1** | **17** | **70** | **1** | **QMRSPVK + Oxidation (M)** |
|  | 473 | **389.0564** | **1164.1471** | **1164.3164** | **-0.1693** | **1** | **17** | **67** | **1** | **AYRSYVLHR** |
|  | 912 | **411.0860** | **1230.2359** | **1230.4389** | **-0.2030** | **0** | **17** | **65** | **1** | **ALLHFNSICR + Carbamidomethyl (C)** |
|  | 1555 | **456.1783** | **1365.5129** | **1366.5159** | **-1.0031** | **2** | **17** | **57** | **1** | **KTLDGELDGKYK** |
|  | 2098 | **505.8766** | **1514.6078** | **1513.6353** | **0.9725** | **1** | **17** | **54** | **1** | **RMEAGEAAPPAGAGGR + Oxidation (M)** |
|  | 511 | **391.0222** | **780.0297** | **780.8657** | **-0.8360** | **0** | **17** | **56** | **1** | **VYADSVK** |
|  | 2462 | **556.3715** | **1110.7283** | **1111.3348** | **-0.6066** | **0** | **17** | **57** | **1** | **ELLIIGGVAAR** |
|  | 160 | **369.3893** | **1105.1457** | **1104.2991** | **0.8465** | **1** | **16** | **73** | **1** | **GKLAIGITANF** |
|  | 171 | **370.2057** | **1107.5950** | **1108.2465** | **-0.6515** | **0** | **16** | **42** | **1** | **AFGNPISFQK** |
|  | 2974 | **666.6112** | **1331.2076** | **1330.5915** | **0.6162** | **0** | **16** | **49** | **1** | **AAPSVTLFPPSLM** |
|  | 1171 | 430.0873 | 1287.2398 | 1286.3470 | 0.8928 | 0 | 16 | 70 | 1 | EMESCTSAAER + Carbamidomethyl (C); Oxidation (M) |
|  | 2730 | **598.1738** | **1194.3328** | **1193.2914** | **1.0413** | **1** | **16** | **58** | **1** | **RCSNDSTLAAR** |
|  | 3434 | **778.6409** | **1555.2671** | **1554.7930** | **0.4741** | **2** | **16** | **47** | **1** | **IKLQASREAGAAALR** |
|  | 38 | **363.0899** | **724.1650** | **724.8901** | **-0.7251** | **1** | **16** | **61** | **1** | **GKCTLM + Carbamidomethyl (C); Oxidation (M)** |
|  | 365 | **385.9190** | **1154.7348** | **1154.3149** | **0.4199** | **0** | **16** | **52** | **1** | **TFSPTYGLLR** |
|  | 2438 | **552.8838** | **1655.6292** | **1655.8985** | **-0.2693** | **1** | **16** | **56** | **1** | **LSGFENQMRLCWR + Oxidation (M)** |
|  | 2683 | **593.5261** | **1777.5562** | **1777.7122** | **-0.1560** | **0** | **16** | **50** | **1** | **VNGDDHHEEDMDMSD + 2 Oxidation (M)** |
|  | 3447 | **786.9962** | **2357.9665** | **2358.7104** | **-0.7439** | **2** | **16** | **56** | **1** | **MEELKGQMLKFSSMAPDLDR + 2 Oxidation (M)** |
|  | 369 | **386.0071** | **1154.9993** | **1154.3199** | **0.6794** | **1** | **16** | **52** | **1** | **NGKLNVSPIGR** |
|  | 3522 | **846.2081** | **2535.6022** | **2535.9384** | **-0.3362** | **1** | **16** | **54** | **1** | **CVPACGEGFYPEEMPGLPHKVCR + Oxidation (M)** |
|  | 61 | **364.1220** | **1089.3439** | **1088.2536** | **1.0904** | **0** | **16** | **56** | **1** | **DFTLTLPPGK** |
|  | 641 | **401.8584** | **1202.5529** | **1202.3578** | **0.1951** | **1** | **16** | **71** | **1** | **DAESTLGRIIK** |
|  | 84 | **365.9478** | **729.8807** | **730.9195** | **-1.0387** | **1** | **16** | **70** | **1** | **MSKLPR** |
|  | 609 | **399.4896** | **796.9643** | **796.8749** | **0.0895** | **0** | **16** | **62** | **1** | **AGSGPRPR** |
|  | 1278 | **435.8433** | **869.6717** | **870.1341** | **-0.4624** | **2** | **16** | **59** | **1** | **KIQKLIK** |
|  | 702 | **404.0819** | **1209.2236** | **1208.2811** | **0.9424** | **0** | **16** | **63** | **1** | **DAGLAQHSGPQK** |
|  | 1265 | **435.1769** | **1302.5085** | **1301.5088** | **0.9996** | **0** | **16** | **57** | **1** | **SAEPAEALVLACK** |
|  | 614 | **400.0973** | **798.1797** | **797.0008** | **1.1790** | **1** | **16** | **55** | **1** | **LRVGKPK** |
|  | 2914 | **651.2357** | **1300.4567** | **1300.4394** | **0.0172** | **0** | **16** | **60** | **1** | **YFCALGEGGAQK + Carbamidomethyl (C)** |
|  | 2168 | **516.2816** | **1545.8227** | **1545.7863** | **0.0363** | **1** | **16** | **64** | **1** | **VCADAAALSVCGPRR + Carbamidomethyl (C)** |
|  | 3457 | **793.1260** | **2376.3558** | **2376.5895** | **-0.2338** | **2** | **16** | **47** | **1** | **WCRSETWVCISNREHQER + Carbamidomethyl (C)** |
|  | 2414 | 549.4994 | 1096.9840 | 1097.2056 | -0.2216 | 0 | 16 | 49 | 1 | QCSHLTSHK + Carbamidomethyl (C) |
|  | 2672 | **592.7720** | **1183.5293** | **1182.3469** | **1.1824** | **1** | **16** | **60** | **1** | **YMPTEAVRVT + Oxidation (M)** |
|  | 3493 | **819.7830** | **2456.3267** | **2455.6352** | **0.6915** | **1** | **16** | **48** | **1** | **IMSCSPDTQCSRDHSMEDPDK + Carbamidomethyl (C); Oxidation (M)** |
|  | 1423 | **444.8847** | **1331.6318** | **1332.4368** | **-0.8050** | **0** | **16** | **71** | **1** | **MYTASSSAETLR + Oxidation (M)** |
|  | 1640 | **460.5598** | **1378.6571** | **1378.4272** | **0.2299** | **0** | **16** | **78** | **1** | **AHYSSNPSGGGCGGK** |
|  | 3250 | **734.2341** | **1466.4535** | **1465.6918** | **0.7617** | **0** | **16** | **58** | **1** | **AGYQVSKPEVIFK** |
|  | 493 | **389.3044** | **776.5940** | **775.8045** | **0.7896** | **0** | **16** | **56** | **1** | **DEVAQSK** |
|  | 1263 | **435.1306** | **868.2464** | **868.0323** | **0.2142** | **0** | **16** | **59** | **1** | **LSPVISPR** |
|  | 1887 | **481.0280** | **960.0412** | **959.0534** | **0.9878** | **0** | **16** | **67** | **1** | **GYSYTLQK** |
|  | 2623 | **586.2351** | **1755.6831** | **1756.0393** | **-0.3561** | **1** | **16** | **63** | **1** | **MCRSSNSMAFLAHIR + 2 Oxidation (M)** |
|  | 2626 | **586.6584** | **1756.9530** | **1756.8517** | **0.1013** | **1** | **16** | **75** | **1** | **KAGQLSQGAAEEDHGCR** |
|  | 3475 | **807.7850** | **1613.5553** | **1613.7310** | **-0.1758** | **1** | **16** | **48** | **1** | **FFSRSSDYIAHQR** |
|  | 1012 | **418.1417** | **1251.4030** | **1252.4429** | **-1.0399** | **0** | **16** | **64** | **1** | **ICPENHSALLR** |
|  | 1748 | **469.2560** | **1404.7460** | **1403.7334** | **1.0126** | **2** | **16** | **57** | **1** | **RALMMMKFAEK + 3 Oxidation (M)** |
|  | 266 | **377.1573** | **1128.4499** | **1127.3244** | **1.1255** | **1** | **16** | **56** | **1** | **IGMGRPGQRR** |
|  | 506 | **389.9954** | **1166.9640** | **1167.2047** | **-0.2406** | **0** | **16** | **63** | **1** | **SMETAEEGSAR** |
|  | 2386 | **544.0219** | **1629.0434** | **1628.9590** | **0.0843** | **0** | **16** | **69** | **1** | **CCALCSWLLCWDR + Carbamidomethyl (C)** |
|  | 1310 | **437.1572** | **1308.4493** | **1308.3738** | **0.0755** | **0** | **16** | **69** | **1** | **LYHEDSQCSQV** |
|  | 694 | **403.9487** | **805.8826** | **806.8664** | **-0.9838** | **1** | **16** | **64** | **1** | **RGDGFQK** |
|  | 1361 | **440.3958** | **1318.1653** | **1317.5381** | **0.6272** | **1** | **16** | **63** | **1** | **AGVPNPCAMGTKR + Oxidation (M)** |
|  | 1010 | **418.0370** | **1251.0887** | **1251.4584** | **-0.3697** | **0** | **16** | **65** | **1** | **GPVVVHCSAGIGR** |
|  | 1135 | **427.2729** | **1278.7965** | **1279.4816** | **-0.6852** | **1** | **16** | **46** | **1** | **TKTLLFISNDK** |
|  | 390 | **386.1763** | **1155.5068** | **1155.3212** | **0.1856** | **0** | **16** | **52** | **1** | **DFSAISLACTK** |
|  | 1223 | **432.6171** | **863.2193** | **862.9742** | **0.2451** | **0** | **16** | **58** | **1** | **ALGYFHR** |
|  | 1421 | **444.8531** | **1331.5370** | **1332.5757** | **-1.0387** | **2** | **16** | **73** | **1** | **TLLRGGMSLRGR + Oxidation (M)** |
|  | 2286 | **530.2276** | **1587.6606** | **1588.7418** | **-1.0811** | **1** | **16** | **73** | **1** | **DRSAASPVVSSMPER** |
|  | 970 | **415.2167** | **828.4187** | **828.8703** | **-0.4516** | **0** | **16** | **59** | **1** | **QDGPGLSR** |
|  | 141 | **369.2573** | **1104.7497** | **1104.3011** | **0.4486** | **0** | **16** | **55** | **1** | **MHPSLATMGK + 2 Oxidation (M)** |
|  | 1177 | **430.3370** | **858.6592** | **857.9578** | **0.7013** | **1** | **16** | **58** | **1** | **RGQSLAAR** |
|  | 1282 | **435.9229** | **1304.7465** | **1304.4580** | **0.2884** | **1** | **16** | **58** | **1** | **VGRTGSHHAAALK** |
|  | 2611 | **584.0084** | **1166.0019** | **1166.2843** | **-0.2823** | **1** | **16** | **67** | **1** | **GTGASGSFKLNK** |
|  | 543 | **392.7126** | **783.4105** | **782.8832** | **0.5273** | **0** | **16** | **50** | **1** | **MMEDNK + Oxidation (M)** |
|  | 1940 | **487.8582** | **973.7016** | **973.1249** | **0.5768** | **0** | **16** | **70** | **1** | **MAYGADMAK + Oxidation (M)** |
|  | 2053 | **501.1813** | **1500.5216** | **1500.7177** | **-0.1960** | **0** | **16** | **67** | **1** | **AEMTLVTNFFATR** |
|  | 2202 | **519.0765** | **1554.2073** | **1553.8413** | **0.3659** | **1** | **16** | **62** | **1** | **IVILNNLEELKQK** |
|  | 3407 | **761.7318** | **1521.4488** | **1520.7770** | **0.6719** | **2** | **16** | **51** | **1** | **VMDMIRAQEQKR + Oxidation (M)** |
|  | 3042 | **674.0312** | **1346.0477** | **1346.3607** | **-0.3130** | **1** | **16** | **58** | **1** | **SDSDQNKGSGVPR** |
|  | 3406 | **761.2676** | **1520.5204** | **1519.8317** | **0.6886** | **2** | **16** | **63** | **1** | **ACSGAGGGAAMKLKLK + Carbamidomethyl (C)** |
|  | 973 | **415.3709** | **1243.0906** | **1242.3388** | **0.7517** | **0** | **16** | **60** | **1** | **GQLEALQVDGGR** |
|  | 1573 | **458.0002** | **1370.9784** | **1370.3391** | **0.6393** | **1** | **16** | **64** | **1** | **SSGSFDGGERESR** |
|  | 1985 | **491.3288** | **1470.9643** | **1470.6073** | **0.3570** | **1** | **16** | **51** | **1** | **HLMKVDSDGSHTK + Oxidation (M)** |
|  | 947 | **413.8948** | **1238.6623** | **1238.3934** | **0.2690** | **2** | **16** | **53** | **1** | **APLDPKANREK** |
|  | 1206 | **431.5585** | **1291.6532** | **1291.4329** | **0.2204** | **0** | **16** | **79** | **1** | **MATFPGPGSSPAR + Oxidation (M)** |
|  | 2960 | **664.9395** | **1991.7962** | **1991.2720** | **0.5242** | **2** | **16** | **54** | **1** | **KAREVGMGSGAELGFPLQK + Oxidation (M)** |
|  | 63 | **364.1444** | **1089.4111** | **1090.3407** | **-0.9296** | **2** | **16** | **56** | **1** | **LSAKMALRGK + Oxidation (M)** |
|  | 852 | **407.8593** | **1220.5558** | **1219.3999** | **1.1559** | **0** | **16** | **64** | **1** | **HCHNMGVLHR + Oxidation (M)** |
|  | 1881 | **480.2806** | **958.5465** | **959.1845** | **-0.6380** | **2** | **16** | **61** | **1** | **STVVKGKIK** |
|  | 2290 | 531.1835 | 1060.3522 | 1061.1870 | -0.8348 | 2 | 16 | 69 | 1 | SKLKEEAEK |
|  | 2808 | **613.1227** | **1836.3459** | **1836.0494** | **0.2964** | **0** | **16** | **62** | **1** | **VLTNWEDGLTVAYGAVK** |
|  | 1293 | **436.1892** | **870.3636** | **869.9654** | **0.3982** | **1** | **16** | **65** | **1** | **ASPAPGSRK** |
|  | 1398 | **443.7050** | **885.3951** | **886.0706** | **-0.6755** | **0** | **16** | **53** | **1** | **CLDAPVLR** |
|  | 3427 | **777.0397** | **2328.0968** | **2327.6812** | **0.4156** | **2** | **16** | **53** | **1** | **GEGINVRCRVTLYSCFPIDK + Carbamidomethyl (C)** |
|  | 897 | **408.9083** | **1223.7028** | **1224.5156** | **-0.8128** | **0** | **16** | **71** | **1** | **NLCLPISLVPR** |
|  | 212 | **373.1732** | **1116.4974** | **1115.3304** | **1.1670** | **0** | **16** | **70** | **1** | **MPRPGAAMPR + 2 Oxidation (M)** |
|  | 218 | **373.9980** | **1118.9719** | **1119.3354** | **-0.3636** | **1** | **16** | **84** | **1** | **MLKLSGEGLR + Oxidation (M)** |
|  | 417 | **387.2131** | **1158.6172** | **1158.2473** | **0.3700** | **0** | **16** | **63** | **1** | **GGSSFPPCGHR + Carbamidomethyl (C)** |
|  | 1654 | **461.5802** | **1381.7183** | **1380.7297** | **0.9887** | **2** | **16** | **74** | **1** | **GMRMTCPLCRR + Carbamidomethyl (C)** |
|  | 2368 | **539.9715** | **1616.8923** | **1617.8522** | **-0.9599** | **2** | **16** | **68** | **1** | **DLISFYGVRHVRR** |
|  | 1190 | **431.0437** | **860.0726** | **859.0056** | **1.0671** | **1** | **16** | **80** | **1** | **CRGLADPK** |
|  | 1850 | **477.9124** | **953.8099** | **953.1566** | **0.6533** | **1** | **16** | **61** | **1** | **AYEMALKK** |
|  | 2771 | **608.6199** | **1215.2250** | **1214.4779** | **0.7471** | **0** | **16** | **70** | **1** | **MATFPCQLCGK + Oxidation (M)** |
|  | 220 | **374.0704** | **1119.1889** | **1120.1727** | **-0.9838** | **0** | **16** | **79** | **1** | **GYCSGGSCYSH** |
|  | 1954 | **488.5892** | **1462.7454** | **1463.7074** | **-0.9619** | **2** | **16** | **84** | **1** | **MKISQDPHPRVR** |
|  | 2471 | **558.4852** | **1114.9556** | **1115.2773** | **-0.3218** | **0** | **16** | **56** | **1** | **ASVGLEVLAEK** |
|  | 2938 | **658.0027** | **1313.9906** | **1313.6353** | **0.3552** | **1** | **16** | **54** | **1** | **LLRVQLCHMK + Carbamidomethyl (C); Oxidation (M)** |
|  | 3071 | **682.2017** | **1362.3885** | **1361.4895** | **0.8991** | **2** | **16** | **64** | **1** | **RSGGWGRDPAMR + Oxidation (M)** |
|  | 866 | **408.0018** | **1220.9832** | **1220.5137** | **0.4695** | **2** | **16** | **66** | **1** | **MWRSCLRLR** |
|  | 756 | **405.2570** | **1212.7490** | **1212.4022** | **0.3467** | **2** | **16** | **49** | **1** | **GDALNLRGLKR** |
|  | 1628 | **460.1725** | **1377.4952** | **1377.4623** | **0.0329** | **0** | **16** | **72** | **1** | **EDITHSAQHALR** |
|  | 2177 | 517.8835 | 1550.6285 | 1550.7149 | -0.0865 | 1 | 16 | 68 | 1 | DLKEQQPQALAPGR |
|  | 960 | **414.6951** | **1241.0631** | **1240.3232** | **0.7400** | **1** | **16** | **49** | **1** | **SQGPSPSPAREK** |
|  | 2559 | **576.1331** | **1150.2515** | **1149.2059** | **1.0455** | **0** | **16** | **66** | **1** | **NTETEESLVK** |
|  | 1414 | **443.9990** | **885.9833** | **885.0246** | **0.9587** | **1** | **16** | **72** | **1** | **RLAWSPR** |
|  | 924 | **411.5395** | **1231.5963** | **1232.4536** | **-0.8573** | **2** | **16** | **76** | **1** | **MRVLVDAREK + Oxidation (M)** |
|  | 1785 | **472.3736** | **1414.0986** | **1414.4976** | **-0.3989** | **1** | **16** | **53** | **1** | **TYDRDNSGMIDK** |
|  | 2820 | **614.2011** | **1839.5810** | **1840.1906** | **-0.6096** | **1** | **16** | **64** | **1** | **AMASLETIGPLMNGMKK + 3 Oxidation (M)** |
|  | 3079 | **684.0172** | **2049.0293** | **2050.1933** | **-1.1640** | **1** | **16** | **55** | **1** | **HSEGPSNFCSICNREGQK + Carbamidomethyl (C)** |
|  | 1917 | **485.9058** | **969.7969** | **969.0932** | **0.7037** | **1** | **16** | **62** | **1** | **DIKETVHK** |
|  | 1957 | **488.7145** | **1463.1213** | **1462.6495** | **0.4717** | **1** | **16** | **58** | **1** | **TYLFKGTQYWR** |
|  | 2546 | **573.0703** | **1144.1258** | **1144.3633** | **-0.2374** | **1** | **16** | **68** | **1** | **AAEPPPPKIPK** |
|  | 1945 | **488.1240** | **974.2331** | **973.1281** | **1.1050** | **1** | **16** | **75** | **1** | **KASVTIQAR** |
|  | 2947 | **663.1412** | **1986.4015** | **1986.2255** | **0.1760** | **1** | **16** | **65** | **1** | **QGPAITDEILLSKAEWSK** |
|  | 361 | **385.2570** | **1152.7488** | **1153.3734** | **-0.6246** | **1** | **16** | **46** | **1** | **TMSCSDKILR** |
|  | 478 | **389.0970** | **776.1793** | **775.8987** | **0.2807** | **1** | **16** | **74** | **1** | **FINARR** |
|  | 3268 | **739.7225** | **2216.1454** | **2216.4932** | **-0.3478** | **1** | **16** | **58** | **1** | **VLQTEQAVKEYNALVAQGVR** |
|  | 460 | **388.4627** | **774.9106** | **774.9503** | **-0.0396** | **0** | **16** | **95** | **1** | **CPLCWP + Carbamidomethyl (C)** |
|  | 567 | **396.0665** | **1185.1774** | **1184.4319** | **0.7455** | **2** | **16** | **77** | **1** | **NLAITLRKAGK** |
|  | 1127 | **426.3781** | **1276.1120** | **1276.5025** | **-0.3906** | **0** | **16** | **58** | **1** | **SDMICGYACLK + Carbamidomethyl (C); Oxidation (M)** |
|  | 2252 | **523.6337** | **1567.8790** | **1567.8067** | **0.0723** | **1** | **16** | **85** | **1** | **SLMARGTYLEWPK + Oxidation (M)** |
|  | 252 | **376.1448** | **1125.4122** | **1125.2787** | **0.1334** | **1** | **16** | **62** | **1** | **VKLAGDAAAGPR** |
|  | 495 | **389.5270** | **777.0392** | **776.9846** | **0.0547** | **0** | **16** | **74** | **1** | **MIVGTLK + Oxidation (M)** |
|  | 1919 | **486.0786** | **970.1423** | **971.1519** | **-1.0096** | **1** | **16** | **63** | **1** | **ENIKQIVK** |
|  | 2971 | **666.4267** | **1996.2579** | **1996.2883** | **-0.0304** | **1** | **16** | **67** | **1** | **TIRDLSTFSQNMTQIIK** |
|  | 37 | **363.0801** | **1086.2182** | **1087.1810** | **-0.9628** | **0** | **16** | **69** | **1** | **SSPEEQLGIK** |
|  | 1893 | **482.2552** | **962.4955** | **962.2081** | **0.2875** | **1** | **16** | **70** | **1** | **IKIMSNLK + Oxidation (M)** |
|  | 3145 | **689.0294** | **1376.0441** | **1376.5207** | **-0.4766** | **1** | **16** | **58** | **1** | **ASSWHVCPRSCS + Carbamidomethyl (C)** |
|  | 1973 | **490.1552** | **978.2956** | **977.1663** | **1.1293** | **1** | **16** | **70** | **1** | **ACGLCARAR + Carbamidomethyl (C)** |
|  | 2005 | **493.5143** | **1477.5206** | **1478.6192** | **-1.0987** | **2** | **16** | **79** | **1** | **AAADRGRPGPAAGRR** |
|  | 195 | **371.3336** | **1110.9788** | **1111.3182** | **-0.3395** | **1** | **16** | **49** | **1** | **HCILQNKQK** |
|  | 730 | **405.0750** | **1212.2027** | **1211.3661** | **0.8366** | **0** | **16** | **63** | **1** | **YIYIGGIQER** |
|  | 1044 | **419.5165** | **837.0182** | **837.8987** | **-0.8804** | **0** | **16** | **91** | **1** | **GSMGSLDR + Oxidation (M)** |
|  | 2843 | **621.3147** | **1240.6146** | **1240.4290** | **0.1856** | **0** | **16** | **67** | **1** | **LSCAVSGLTFSR** |
|  | 2873 | 629.6028 | 1257.1908 | 1257.4760 | -0.2853 | 0 | 16 | 61 | 1 | FLYEVFAQLK |
|  | 3095 | **684.8639** | **1367.7130** | **1367.5935** | **0.1195** | **1** | **16** | **71** | **1** | **MYNVRIMSPQP + 2 Oxidation (M)** |
|  | 3115 | **686.0829** | **2055.2265** | **2054.2879** | **0.9386** | **1** | **16** | **65** | **1** | **SAYAAPKHATQAFFDCLR + Carbamidomethyl (C)** |
|  | 261 | **377.1266** | **752.2385** | **752.8374** | **-0.5989** | **0** | **16** | **65** | **1** | **MASVSSR + Oxidation (M)** |
|  | 1585 | **458.4230** | **1372.2468** | **1372.5289** | **-0.2820** | **2** | **16** | **64** | **1** | **KEPVAGSPFERR** |
|  | 1586 | **458.4463** | **914.8777** | **914.9630** | **-0.0852** | **1** | **16** | **74** | **1** | **HSSVDRSK** |
|  | 1678 | **462.8709** | **923.7270** | **923.0246** | **0.7024** | **1** | **16** | **65** | **1** | **GETGKYLR** |
|  | 1121 | **425.9912** | **849.9676** | **850.9438** | **-0.9762** | **1** | **16** | **70** | **1** | **TSRATGCR** |
|  | 2256 | **524.1357** | **1046.2567** | **1045.1923** | **1.0644** | **0** | **16** | **74** | **1** | **LSSAHVYLR** |
|  | 211 | **373.1121** | **1116.3141** | **1116.3531** | **-0.0390** | **1** | **16** | **84** | **1** | **VSGKVLLSWK** |
|  | 1601 | **458.9542** | **915.8935** | **915.0522** | **0.8413** | **2** | **16** | **77** | **1** | **IGGKGTARR** |
|  | 1635 | **460.3028** | **918.5907** | **918.9483** | **-0.3575** | **0** | **16** | **61** | **1** | **SVSEDGGLR** |
|  | 2649 | **590.4707** | **1178.9266** | **1178.4075** | **0.5191** | **1** | **16** | **56** | **1** | **SRAAGFMGLLR** |
|  | 4 | **360.3710** | **1078.0908** | **1079.1890** | **-1.0981** | **1** | **16** | **92** | **1** | **MAPTSASSRR + Oxidation (M)** |
|  | 283 | **378.2879** | **1131.8416** | **1132.1024** | **-0.2607** | **1** | **16** | **48** | **1** | **GANDGRNGESR** |
|  | 1256 | **434.3383** | **1299.9928** | **1299.5163** | **0.4765** | **0** | **16** | **52** | **1** | **CLMSEVEAVFR + Oxidation (M)** |
|  | 1983 | **491.1200** | **980.2253** | **979.0449** | **1.1804** | **0** | **16** | **67** | **1** | **AFSDPSSLR** |
|  | 2826 | **615.4208** | **1843.2402** | **1843.9689** | **-0.7288** | **1** | **16** | **66** | **1** | **TLCPAAAAEESRDAEPR + Carbamidomethyl (C)** |
|  | 452 | **388.2517** | **1161.7330** | **1161.2247** | **0.5083** | **1** | **16** | **66** | **1** | **WEAAQASKDR** |
|  | 385 | **386.1467** | **770.2787** | **769.9324** | **0.3463** | **1** | **16** | **63** | **1** | **LGPVTRK** |
|  | 735 | **405.0950** | **1212.2628** | **1211.4109** | **0.8519** | **0** | **16** | **65** | **1** | **LQELQVNLVR** |
|  | 1025 | **419.1598** | **1254.4572** | **1255.4272** | **-0.9700** | **2** | **16** | **81** | **1** | **TRTPPRASLTR** |
|  | 1764 | **470.1655** | **1407.4743** | **1406.4522** | **1.0221** | **0** | **16** | **65** | **1** | **CSLAEGGASSDCYS + Carbamidomethyl (C)** |
|  | 790 | **406.3445** | **810.6743** | **810.9644** | **-0.2901** | **0** | **16** | **54** | **1** | **MHGPLTR** |
|  | 1186 | **431.0127** | **860.0107** | **859.0289** | **0.9818** | **0** | **16** | **86** | **1** | **MATPMHR + Oxidation (M)** |
|  | 2748 | **602.9179** | **1805.7316** | **1806.0946** | **-0.3631** | **2** | **16** | **60** | **1** | **MATSSRLALWEQKIR + Oxidation (M)** |
|  | 2910 | **648.3159** | **1941.9254** | **1941.3390** | **0.5864** | **0** | **16** | **69** | **1** | **MLAAVTGQLALVQLLVER + Oxidation (M)** |
|  | 95 | **366.3313** | **1095.9716** | **1095.3189** | **0.6527** | **1** | **16** | **77** | **1** | **AHLLPSKACR** |
|  | 1372 | **442.1344** | **882.2540** | **881.1568** | **1.0972** | **1** | **16** | **67** | **1** | **VLLPAIKK** |
|  | 3575 | **1050.2476** | **2098.4803** | **2098.3177** | **0.1627** | **1** | **16** | **64** | **1** | **QLQAESVSEVVVNRVDVAR** |
|  | 1613 | **459.3773** | **1375.1098** | **1375.3986** | **-0.2889** | **1** | **16** | **63** | **1** | **AKEEAGATAADEGR** |
|  | 1809 | **475.2498** | **1422.7273** | **1422.7347** | **-0.0075** | **1** | **16** | **75** | **1** | **MQILGSTLKLFR + Oxidation (M)** |
|  | 44 | **363.1641** | **1086.4702** | **1086.2426** | **0.2276** | **1** | **16** | **62** | **1** | **VDNAASRLLK** |
|  | 402 | **386.8808** | **771.7469** | **771.9084** | **-0.1616** | **1** | **16** | **77** | **1** | **AIRVSAR** |
|  | 1140 | **428.0753** | **854.1359** | **854.0056** | **0.1302** | **0** | **16** | **69** | **1** | **GLLVPEAR** |
|  | 2871 | **629.1677** | **1884.4810** | **1883.3242** | **1.1568** | **0** | **16** | **70** | **1** | **FCLLSCGILTFLAVLGR + Carbamidomethyl (C)** |
|  | 3264 | **738.1335** | **2211.3785** | **2211.4998** | **-0.1214** | **1** | **16** | **68** | **1** | **YLQRIVNHPTMLQDPDVR + Oxidation (M)** |
|  | 350 | **385.0780** | **1152.2118** | **1151.4668** | **0.7451** | **0** | **16** | **62** | **1** | **TNVALMCMLR** |
|  | 2439 | **552.8977** | **1103.7806** | **1104.2877** | **-0.5071** | **2** | **16** | **68** | **1** | **RRIGSMTGAR** |
|  | 265 | **377.1542** | **1128.4405** | **1127.3773** | **1.0632** | **1** | **16** | **64** | **1** | **QIAVKTLGIGK** |
|  | 1059 | **420.4650** | **838.9153** | **839.9426** | **-1.0274** | **1** | **16** | **78** | **1** | **SLTARHR** |
|  | 589 | **398.2778** | **794.5409** | **794.8557** | **-0.3148** | **0** | **16** | **58** | **1** | **DPSIAHR** |
|  | 596 | **398.9486** | **1193.8237** | **1194.2578** | **-0.4340** | **0** | **16** | **62** | **1** | **WHQGSGYFGR** |
|  | 741 | **405.1428** | **808.2709** | **809.0530** | **-0.7821** | **1** | **16** | **66** | **1** | **KMMLVR + 2 Oxidation (M)** |
|  | 1960 | **488.8050** | **1463.3927** | **1463.5780** | **-0.1853** | **1** | **16** | **63** | **1** | **APGTCGWSASARSR + Carbamidomethyl (C)** |
|  | 3161 | **693.4451** | **1384.8754** | **1383.6771** | **1.1982** | **0** | **16** | **69** | **1** | **KPGQAPLLVLYGK** |
|  | 221 | **374.1100** | **1119.3079** | **1119.2561** | **0.0518** | **1** | **16** | **85** | **1** | **RGACSVGTNVR** |
|  | 1700 | **464.0986** | **1389.2737** | **1388.6374** | **0.6364** | **0** | **16** | **67** | **1** | **GSCCSCCPVGCAK + 3 Carbamidomethyl (C)** |
|  | 2704 | **596.0792** | **1190.1437** | **1189.4287** | **0.7150** | **1** | **16** | **76** | **1** | **ALSKVVSLCNR** |
|  | 3227 | **724.2720** | **1446.5292** | **1447.6617** | **-1.1325** | **2** | **16** | **71** | **1** | **YNARHPSTKVMK + Oxidation (M)** |
|  | 613 | **400.0952** | **1197.2635** | **1196.4165** | **0.8470** | **0** | **16** | **65** | **1** | **VVVTEISYMR** |
|  | 1401 | **443.7979** | **1328.3714** | **1328.5327** | **-0.1613** | **0** | **16** | **71** | **1** | **EMCDMEQVLSK + Oxidation (M)** |
|  | 2282 | **530.1309** | **1058.2469** | **1057.2314** | **1.0156** | **2** | **16** | **80** | **1** | **MKVHSAGRR + Oxidation (M)** |
|  | 291 | **379.5858** | **757.1568** | **757.7892** | **-0.6324** | **0** | **16** | **64** | **1** | **EQAGEPK** |
|  | 1916 | **485.6529** | **969.2911** | **970.0412** | **-0.7502** | **0** | **16** | **63** | **1** | **GNLTGAPGQR** |
|  | 2818 | **613.8317** | **1838.4730** | **1839.0567** | **-0.5837** | **1** | **16** | **60** | **1** | **LQSLMADSMDTLEGRR + Oxidation (M)** |
|  | 1062 | **421.1526** | **1260.4356** | **1259.4092** | **1.0264** | **2** | **16** | **73** | **1** | **AAEISKQEQKK** |
|  | 1043 | **419.4951** | **836.9755** | **835.9906** | **0.9849** | **1** | **16** | **97** | **1** | **TVKAYVR** |
|  | 1262 | **435.1011** | **1302.2810** | **1303.4037** | **-1.1227** | **1** | **16** | **66** | **1** | **SQGYARSECFR** |
|  | 2346 | **537.4146** | **1609.2215** | **1608.6716** | **0.5499** | **1** | **16** | **65** | **1** | **HNGPNDASDGTVRLR** |
|  | 844 | **407.7340** | **1220.1799** | **1220.3780** | **-0.1982** | **1** | **16** | **66** | **1** | **VESQLIGRYR** |
|  | 2087 | **504.8895** | **1511.6464** | **1511.7023** | **-0.0559** | **1** | **16** | **71** | **1** | **VHIEMGPDGRVTGK + Oxidation (M)** |
|  | 1473 | **448.4018** | **1342.1833** | **1342.5657** | **-0.3824** | **1** | **16** | **59** | **1** | **QVRITFGFSCK + Carbamidomethyl (C)** |
|  | 1547 | **455.1426** | **1362.4057** | **1361.6501** | **0.7556** | **2** | **16** | **72** | **1** | **MDILKSEILRK + Oxidation (M)** |
|  | 580 | **397.3245** | **1188.9513** | **1188.3083** | **0.6430** | **1** | **16** | **64** | **1** | **MQSSSKETYK** |
|  | 656 | **402.0709** | **1203.1906** | **1203.3924** | **-0.2018** | **0** | **16** | **84** | **1** | **SHISCMPGTVR + Oxidation (M)** |
|  | 748 | **405.1906** | **808.3664** | **808.9850** | **-0.6185** | **1** | **16** | **64** | **1** | **FIPKAMS + Oxidation (M)** |
|  | 2027 | **495.9752** | **1484.9033** | **1485.6897** | **-0.7864** | **1** | **16** | **76** | **1** | **KMDSGCCPQCQR + 2 Carbamidomethyl (C); Oxidation (M)** |
|  | 110 | **368.1081** | **1101.3020** | **1102.1990** | **-0.8970** | **0** | **16** | **79** | **1** | **SLVQAAETQR** |
|  | 492 | **389.3001** | **776.5855** | **775.8922** | **0.6933** | **1** | **16** | **66** | **1** | **SKEVWK** |
|  | 969 | **415.1588** | **1242.4541** | **1242.2973** | **0.1567** | **0** | **16** | **76** | **1** | **ALGEEGNPGGWR** |
|  | 1311 | **437.1572** | **872.2995** | **872.0175** | **0.2820** | **0** | **16** | **81** | **1** | **LPLLDSSK** |
|  | 2760 | **606.7206** | **1817.1396** | **1818.1438** | **-1.0042** | **2** | **16** | **82** | **1** | **TDSPEFLTMMARKMK + 2 Oxidation (M)** |
|  | 964 | **415.0845** | **828.1542** | **828.9563** | **-0.8022** | **0** | **16** | **76** | **1** | **ALAGTLQR** |
|  | 1783 | **472.1934** | **942.3719** | **943.0128** | **-0.6409** | **0** | **16** | **73** | **1** | **TPATTPEAR** |
|  | 2925 | **655.5204** | **1309.0260** | **1308.5028** | **0.5232** | **0** | **16** | **61** | **1** | **LLAAGFDNSCIK + Carbamidomethyl (C)** |
|  | 1040 | **419.3762** | **1255.1063** | **1255.3178** | **-0.2115** | **1** | **16** | **76** | **1** | **QFGSKCNSGDR + Carbamidomethyl (C)** |
|  | 2982 | **666.7866** | **1997.3377** | **1996.2437** | **1.0940** | **0** | **16** | **88** | **1** | **SPSDFMSLLISTVPGSWR + Oxidation (M)** |
|  | 3380 | 757.8884 | 1513.7621 | 1513.6600 | 0.1021 | 2 | 16 | 81 | 1 | TPGGLFRSHNGKSR |
|  | 889 | **408.3496** | **1222.0267** | **1222.4370** | **-0.4103** | **2** | **16** | **65** | **1** | **LEKAKVELHR** |
|  | 2674 | **592.8094** | **1183.6040** | **1183.3629** | **0.2411** | **0** | **16** | **64** | **1** | **THLFGRPTVR** |
|  | 2900 | **642.5533** | **1283.0919** | **1283.3941** | **-0.3022** | **1** | **16** | **56** | **1** | **EAAAGGAQLRSPR** |
|  | 979 | **416.0327** | **1245.0761** | **1245.2567** | **-0.1807** | **0** | **16** | **88** | **1** | **GHLGSDSGSTATR** |
|  | 1094 | **422.2115** | **842.4083** | **841.9752** | **0.4331** | **0** | **16** | **75** | **1** | **MPASAPPR + Oxidation (M)** |
|  | 2997 | **667.9794** | **2000.9159** | **2000.2556** | **0.6604** | **0** | **16** | **63** | **1** | **LNSSDDGTQGCMGLPCVVM + Carbamidomethyl (C); Oxidation (M)** |
|  | 101 | **367.2918** | **1098.8533** | **1098.1211** | **0.7323** | **1** | **16** | **68** | **1** | **ERYSVDSDK** |
|  | 1821 | **476.1916** | **1425.5526** | **1426.5347** | **-0.9821** | **1** | **16** | **74** | **1** | **EVAPITRGQGDQR** |
|  | 2013 | **494.1881** | **1479.5421** | **1479.6819** | **-0.1398** | **1** | **16** | **78** | **1** | **IVRDDMLCAGNTR + Oxidation (M)** |
|  | 827 | **407.3119** | **1218.9135** | **1218.4233** | **0.4902** | **1** | **16** | **56** | **1** | **LNTLCERLLS + Carbamidomethyl (C)** |
|  | 1623 | **459.8653** | **1376.5737** | **1375.5921** | **0.9816** | **0** | **16** | **81** | **1** | **CLSQILHTEFK + Carbamidomethyl (C)** |
|  | 1464 | **447.5368** | **893.0589** | **893.0201** | **0.0387** | **0** | **16** | **91** | **1** | **SICSSLAR + Carbamidomethyl (C)** |
|  | 1042 | **419.4364** | **1255.2869** | **1255.3346** | **-0.0476** | **1** | **16** | **97** | **1** | **YVASSSKDGSVR** |
|  | 1707 | **464.2881** | **926.5614** | **927.0166** | **-0.4552** | **0** | **16** | **60** | **1** | **NQLSNVPR** |
|  | 2646 | **590.0670** | **1767.1787** | **1765.9843** | **1.1944** | **1** | **16** | **78** | **1** | **LTNSEGKDFCGGVIIR + Carbamidomethyl (C)** |
|  | 3437 | 780.4032 | 1558.7916 | 1559.6904 | -0.8987 | 2 | 16 | 73 | 1 | DHDRQCGRQSCVR |
|  | 1383 | **443.2647** | **884.5146** | **885.0463** | **-0.5317** | **0** | **16** | **60** | **1** | **CSRPPAVR** |
|  | 2319 | **534.7054** | **1067.3961** | **1066.2746** | **1.1216** | **1** | **16** | **77** | **1** | **LEAMAKTFR** |
|  | 2785 | **610.2767** | **1827.8080** | **1827.1568** | **0.6512** | **2** | **16** | **76** | **1** | **AGRRGLDPTGTVILLCK + Carbamidomethyl (C)** |
|  | 3076 | **683.9443** | **1365.8739** | **1364.6741** | **1.1998** | **1** | **16** | **61** | **1** | **MPCSVLTLAKTK + Carbamidomethyl (C); Oxidation (M)** |
|  | 3298 | **740.8552** | **1479.6957** | **1479.7215** | **-0.0259** | **1** | **15** | **86** | **1** | **KCGCSEGLASLVQK + Carbamidomethyl (C)** |
|  | 3309 | **741.7125** | **2222.1154** | **2221.3790** | **0.7364** | **1** | **15** | **60** | **1** | **EPNLEPMVEKQESENSCNK + Oxidation (M)** |
|  | 3390 | **758.5025** | **1514.9902** | **1514.6994** | **0.2908** | **1** | **15** | **72** | **1** | **EDKEEILMLHNK + Oxidation (M)** |
|  | 62 | **364.1353** | **1089.3836** | **1090.3375** | **-0.9539** | **1** | **15** | **66** | **1** | **TSVLQVMGKK** |
|  | 294 | **379.7620** | **1136.2638** | **1137.3310** | **-1.0672** | **0** | **15** | **82** | **1** | **QTMGDCVVLR + Oxidation (M)** |
|  | 894 | **408.8284** | **815.6420** | **814.8902** | **0.7519** | **1** | **15** | **79** | **1** | **TPGQRTR** |
|  | 1568 | **457.6735** | **913.3321** | **912.9949** | **0.3373** | **1** | **15** | **59** | **1** | **GPGWRGAGR** |
|  | 299 | **380.1085** | **1137.3032** | **1137.2433** | **0.0599** | **0** | **15** | **85** | **1** | **APPDSPTTPVR** |
|  | 792 | **406.5361** | **811.0575** | **809.9134** | **1.1441** | **1** | **15** | **75** | **1** | **VQHKGNK** |
|  | 1797 | **474.0906** | **946.1664** | **945.0964** | **1.0701** | **0** | **15** | **84** | **1** | **WVCQAPGK + Carbamidomethyl (C)** |
|  | 1235 | **433.1710** | **1296.4908** | **1296.5319** | **-0.0411** | **1** | **15** | **75** | **1** | **QYESLKILICS** |
|  | 2965 | **665.3755** | **1993.1043** | **1992.2846** | **0.8197** | **0** | **15** | **77** | **1** | **SPHLAAACPASPGWASCLPR** |
|  | 375 | **386.0849** | **1155.2326** | **1155.3015** | **-0.0688** | **0** | **15** | **70** | **1** | **IPSSTTLSPPR** |
|  | 2289 | **531.1716** | **1060.3284** | **1060.2021** | **0.1262** | **1** | **15** | **82** | **1** | **AAATKLASAEK** |
|  | 1375 | **442.2683** | **1323.7828** | **1324.4395** | **-0.6567** | **0** | **15** | **59** | **1** | **GGSPLAAPQGGSPTK** |
|  | 2016 | **494.2834** | **1479.8282** | **1479.8045** | **0.0237** | **0** | **15** | **75** | **1** | **AMFFSMGFIVAVK + 2 Oxidation (M)** |
|  | 787 | **406.2842** | **1215.8304** | **1215.2938** | **0.5366** | **1** | **15** | **58** | **1** | **GRFTMSGDNSK + Oxidation (M)** |
|  | 975 | **415.9282** | **829.8415** | **830.9359** | **-1.0943** | **2** | **15** | **90** | **1** | **GRLSSRR** |
|  | 1871 | **479.9840** | **957.9532** | **959.0618** | **-1.1085** | **2** | **15** | **80** | **1** | **KQKNATGGR** |
|  | 2120 | **507.5360** | **1519.5857** | **1520.6263** | **-1.0406** | **1** | **15** | **88** | **1** | **YEEDMHRPGSRK + Oxidation (M)** |
|  | 1592 | **458.7160** | **915.4172** | **914.9629** | **0.4543** | **1** | **15** | **69** | **1** | **AAGDGSPGRK** |
|  | 2067 | **502.5067** | **1002.9985** | **1003.1558** | **-0.1573** | **1** | **15** | **88** | **1** | **GFPSVVRGGK** |
|  | 435 | **387.9093** | **773.8039** | **772.8701** | **0.9338** | **0** | **15** | **95** | **1** | **AMHEASK** |
|  | 1961 | **489.2251** | **1464.6532** | **1465.7382** | **-1.0849** | **1** | **15** | **82** | **1** | **MLRAMVASGSELGK + Oxidation (M)** |
|  | 2837 | **619.7126** | **1856.1158** | **1855.0775** | **1.0382** | **2** | **15** | **89** | **1** | **QKSKISLYCLSQDEGR** |
|  | 3409 | **762.5399** | **2284.5976** | **2284.6348** | **-0.0373** | **0** | **15** | **75** | **1** | **WLHAGSSNMAVLEVPLLSGFR** |
|  | 3422 | **771.9334** | **2312.7781** | **2311.6803** | **1.0977** | **2** | **15** | **82** | **1** | **MSASAATGILDPCIYRVSVRK + Carbamidomethyl (C); Oxidation (M)** |
|  | 1266 | **435.1908** | **1302.5502** | **1303.4632** | **-0.9130** | **0** | **15** | **73** | **1** | **ALFQIEQGIER** |
|  | 2656 | **591.5764** | **1771.7071** | **1770.9843** | **0.7227** | **0** | **15** | **70** | **1** | **CICGTAHSVLDEGPVR + 2 Carbamidomethyl (C)** |
|  | 2675 | **592.8940** | **1775.6600** | **1775.0424** | **0.6175** | **1** | **15** | **66** | **1** | **RGHGCVLLCECLDSR + 2 Carbamidomethyl (C)** |
|  | 763 | **405.9290** | **809.8431** | **809.8704** | **-0.0272** | **1** | **15** | **71** | **1** | **GTSSFRR** |
|  | 2904 | **644.6555** | **1287.2963** | **1287.5103** | **-0.2141** | **2** | **15** | **79** | **1** | **GQTLTVAKWRK** |
|  | 1254 | 434.2907 | 1299.8500 | 1299.5426 | 0.3073 | 0 | 15 | 59 | 1 | RPGVTAGILEMR |
|  | 3078 | 683.9784 | 1365.9420 | 1365.5807 | 0.3613 | 1 | 15 | 63 | 1 | GALVLGSSLKQHR |
|  | 3091 | **684.2872** | **1366.5597** | **1365.5807** | **0.9789** | **1** | **15** | **77** | **1** | **GALVLGSSLKQHR** |
|  | 3315 | **741.9329** | **2222.7764** | **2223.5387** | **-0.7623** | **2** | **15** | **71** | **1** | **SGFVFRSCGMHWVRQAPGK + Carbamidomethyl (C); Oxidation (M)** |
|  | 514 | **391.1127** | **780.2106** | **780.9567** | **-0.7461** | **1** | **15** | **75** | **1** | **AGCGSMKK** |
|  | 575 | **396.4524** | **1186.3350** | **1185.3291** | **1.0059** | **1** | **15** | **1.1e+02** | **1** | **YEKLVSSGFR** |
|  | 1307 | **437.0864** | **872.1579** | **870.9897** | **1.1682** | **0** | **15** | **89** | **1** | **NELPSAIK** |
|  | 1418 | **444.2077** | **1329.6009** | **1330.4443** | **-0.8433** | **2** | **15** | **87** | **1** | **ESRSPAKEEVAK** |
|  | 1742 | **468.4560** | **1402.3459** | **1401.5799** | **0.7660** | **0** | **15** | **83** | **1** | **GRPGAAGPRPRPGR** |
|  | 462 | **388.9308** | **775.8469** | **774.8230** | **1.0239** | **0** | **15** | **91** | **1** | **KPSGGSSR** |
|  | 1157 | **428.7288** | **1283.1642** | **1282.4689** | **0.6952** | **0** | **15** | **58** | **1** | **MNGHQSLSALPK** |
|  | 2080 | **504.1930** | **1006.3712** | **1006.1746** | **0.1966** | **0** | **15** | **85** | **1** | **MLVSGAGDIK + Oxidation (M)** |
|  | 2180 | 518.7057 | 1035.3966 | 1035.1296 | 0.2670 | 0 | 15 | 70 | 1 | DSLAACAAEGK |
|  | 3312 | **741.7744** | **1481.5340** | **1482.7221** | **-1.1881** | **2** | **15** | **78** | **1** | **NAKYLLKGEYVGK** |
|  | 408 | **387.0267** | **772.0386** | **771.8820** | **0.1566** | **0** | **15** | **90** | **1** | **MDELHK** |
|  | 1097 | **422.2520** | **842.4891** | **842.1008** | **0.3883** | **0** | **15** | **71** | **1** | **GIAIIPMK** |
|  | 1103 | **422.7341** | **843.4535** | **843.0262** | **0.4273** | **2** | **15** | **75** | **1** | **VATVIRKG** |
|  | 1193 | **431.0945** | **860.1742** | **859.0949** | **1.0793** | **2** | **15** | **97** | **1** | **ACLRIRK** |
|  | 2406 | **549.0707** | **1096.1266** | **1095.1682** | **0.9583** | **0** | **15** | **75** | **1** | **NCECQTQGR + Carbamidomethyl (C)** |
|  | 196 | **371.3807** | **1111.1199** | **1111.2952** | **-0.1753** | **0** | **15** | **74** | **1** | **VFAADCLCR + 2 Carbamidomethyl (C)** |
|  | 333 | **384.2512** | **1149.7314** | **1149.1695** | **0.5619** | **1** | **15** | **60** | **1** | **DGSGTGGSKVER** |
|  | 2994 | **667.6791** | **2000.0151** | **1999.1750** | **0.8400** | **0** | **15** | **83** | **1** | **VLSTTKPFEYETPETEK** |
|  | 668 | **402.4565** | **1204.3475** | **1205.2342** | **-0.8868** | **0** | **15** | **1.1e+02** | **1** | **SDSYLAEHQR** |
|  | 3272 | **740.2250** | **2217.6529** | **2216.5197** | **1.1333** | **1** | **15** | **76** | **1** | **RPDALRMISNADPSIPPPPR + Oxidation (M)** |
|  | 1819 | **476.1434** | **950.2720** | **951.0779** | **-0.8059** | **0** | **15** | **79** | **1** | **LVVYGASSR** |
|  | 3317 | **742.0199** | **2223.0375** | **2223.2889** | **-0.2514** | **0** | **15** | **60** | **1** | **VYCDMNTENGGMDSDSEPSR + Oxidation (M)** |
|  | 3329 | **742.7306** | **1483.4464** | **1482.7551** | **0.6913** | **2** | **15** | **65** | **1** | **CHKRILVASWGR + Carbamidomethyl (C)** |
|  | 2131 | **508.5850** | **1522.7329** | **1523.7776** | **-1.0447** | **2** | **15** | **1e+02** | **1** | **GITAERKAMMQQK + 2 Oxidation (M)** |
|  | 2344 | **537.1814** | **1608.5220** | **1609.7144** | **-1.1924** | **0** | **15** | **90** | **1** | **CATVTENATGDLATSR** |
|  | 198 | **371.9876** | **741.9605** | **740.9343** | **1.0262** | **1** | **15** | **69** | **1** | **KPKAVAK** |
|  | 485 | **389.1796** | **1164.5165** | **1165.3840** | **-0.8675** | **0** | **15** | **81** | **1** | **TLMLMAQEGR + Oxidation (M)** |
|  | 2586 | **580.5595** | **1738.6564** | **1738.9459** | **-0.2895** | **2** | **15** | **76** | **1** | **ARRMGLPYDQGDMGR + Oxidation (M)** |
|  | 52 | **363.2198** | **1086.6372** | **1087.1677** | **-0.5305** | **0** | **15** | **65** | **1** | **QCGAEAAPQR + Carbamidomethyl (C)** |
|  | 530 | **391.3353** | **1170.9838** | **1170.3359** | **0.6479** | **0** | **15** | **64** | **1** | **LTGTAYMELR + Oxidation (M)** |
|  | 1651 | **460.8265** | **1379.4574** | **1380.5476** | **-1.0902** | **0** | **15** | **83** | **1** | **VPDMCSAGSASLSR** |
|  | 615 | **400.1089** | **798.2030** | **797.9424** | **0.2606** | **1** | **15** | **73** | **1** | **KGLLPDR** |
|  | 616 | **400.1154** | **1197.3242** | **1197.4906** | **-0.1664** | **1** | **15** | **73** | **1** | **VHVTLKIEMK** |
|  | 1369 | **441.3142** | **880.6136** | **880.9600** | **-0.3463** | **0** | **15** | **63** | **1** | **EDTLEMK + Oxidation (M)** |
|  | 1682 | **463.0471** | **1386.1190** | **1385.5658** | **0.5532** | **2** | **15** | **74** | **1** | **VAGKPEGKATDKGK** |
|  | 1672 | **462.2293** | **922.4438** | **923.0213** | **-0.5775** | **0** | **15** | **75** | **1** | **ITEYLER** |
|  | 2369 | **540.0190** | **1617.0348** | **1616.7698** | **0.2650** | **0** | **15** | **83** | **1** | **WDDFSCSCPALTSGK** |
|  | 3006 | **668.6922** | **2003.0544** | **2003.3044** | **-0.2499** | **2** | **15** | **87** | **1** | **VKHLEVSSASMAEDLCRK** |
|  | 1018 | **419.0459** | **1254.1154** | **1254.4389** | **-0.3235** | **1** | **15** | **93** | **1** | **LPLNLASQSRR** |
|  | 1167 | **429.3064** | **1284.8969** | **1284.3721** | **0.5248** | **0** | **15** | **66** | **1** | **LGENGLPISGPSSG** |
|  | 2116 | **507.1279** | **1518.3614** | **1517.7480** | **0.6134** | **1** | **15** | **77** | **1** | **SLRLSCTASGFTFK** |
|  | 2710 | **596.7726** | **1191.5305** | **1190.3356** | **1.1949** | **1** | **15** | **88** | **1** | **CSSAFVRHQR** |
|  | 566 | **396.0471** | **1185.1190** | **1186.2693** | **-1.1503** | **0** | **15** | **92** | **1** | **SSAPTTPPSVDK** |
|  | 838 | **407.5787** | **1219.7140** | **1219.5209** | **0.1932** | **1** | **15** | **71** | **1** | **SLGNVIMVCRK** |
|  | 886 | **408.2674** | **1221.7801** | **1221.4457** | **0.3344** | **2** | **15** | **68** | **1** | **KTAKAEAIVYK** |
|  | 937 | **412.7889** | **1235.3446** | **1234.4296** | **0.9150** | **2** | **15** | **76** | **1** | **RMVTGNGSLKR + Oxidation (M)** |
|  | 977 | **415.9761** | **829.9374** | **828.9183** | **1.0191** | **1** | **15** | **96** | **1** | **WTGGPRR** |
|  | 1196 | **431.1468** | **860.2788** | **859.0949** | **1.1839** | **2** | **15** | **96** | **1** | **ACLRIRK** |
|  | 1297 | **436.2793** | **1305.8157** | **1305.4576** | **0.3581** | **0** | **15** | **63** | **1** | **GVSLDLSNCELR** |
|  | 1491 | **450.1498** | **1347.4273** | **1348.4578** | **-1.0304** | **0** | **15** | **84** | **1** | **EPEPPGVVGGPGEK** |
|  | 3223 | **721.9020** | **1441.7892** | **1440.7088** | **1.0804** | **0** | **15** | **78** | **1** | **MVLDGGMGTMIQR + 2 Oxidation (M)** |
|  | 2773 | **609.5156** | **1217.0163** | **1216.3000** | **0.7163** | **0** | **15** | **69** | **1** | **ATHDQAVEAFK** |
|  | 242 | **375.0486** | **748.0823** | **748.9313** | **-0.8490** | **1** | **15** | **94** | **1** | **LCTKTAL** |
|  | 3029 | **670.2318** | **2007.6731** | **2007.3902** | **0.2828** | **1** | **15** | **82** | **1** | **ARPCWRPLGWVQKCIH + Carbamidomethyl (C)** |
|  | 675 | **403.0462** | **1206.1164** | **1206.4325** | **-0.3161** | **0** | **15** | **89** | **1** | **QCLPSLDLSCK** |
|  | 113 | **368.2922** | **1101.8545** | **1101.3267** | **0.5278** | **2** | **15** | **72** | **1** | **CLIQQKRR + Carbamidomethyl (C)** |
|  | 2725 | **597.5521** | **1789.6342** | **1789.8965** | **-0.2623** | **1** | **15** | **73** | **1** | **EFLQDQNPDRESALK** |
|  | 1595 | **458.8043** | **1373.3908** | **1374.5728** | **-1.1820** | **1** | **15** | **85** | **1** | **NMMAACDPRHGR + Oxidation (M)** |
|  | 2208 | **519.9053** | **1037.7959** | **1037.2152** | **0.5807** | **1** | **15** | **79** | **1** | **SKVTSCPGCR** |
|  | 2353 | **538.0916** | **1611.2527** | **1611.8013** | **-0.5487** | **1** | **15** | **87** | **1** | **ECCINRFQQVESR** |
|  | 1049 | **419.7552** | **1256.2433** | **1256.3657** | **-0.1224** | **1** | **15** | **73** | **1** | **GESRPETPKQK** |
|  | 2693 | **594.7045** | **1187.3942** | **1188.3710** | **-0.9769** | **1** | **15** | **1e+02** | **1** | **SGLLSVDKIEK** |
|  | 1766 | **470.3434** | **938.6719** | **937.9995** | **0.6725** | **0** | **15** | **59** | **1** | **QAGGGAAPGPR** |
|  | 3092 | **684.4045** | **2050.1913** | **2050.3027** | **-0.1114** | **2** | **15** | **82** | **1** | **CPQCNNRKEFPQEMPR + Carbamidomethyl (C); Oxidation (M)** |
|  | 124 | **369.1525** | **736.2902** | **736.8777** | **-0.5875** | **1** | **15** | **85** | **1** | **KIDSMK + Oxidation (M)** |
|  | 2474 | **560.1548** | **1677.4422** | **1677.9209** | **-0.4787** | **1** | **15** | **92** | **1** | **RNNMTASMFDLSMK + 2 Oxidation (M)** |
|  | 3231 | **725.8528** | **2174.5362** | **2175.3767** | **-0.8406** | **1** | **15** | **98** | **1** | **PVLAMAEAASGAGGTSLEGERGK + Oxidation (M)** |
|  | 3307 | **741.5967** | **2221.7681** | **2221.4267** | **0.3413** | **0** | **15** | **67** | **1** | **VQLVQHAIQAASSIDAEDGLR** |
|  | 3519 | **845.1364** | **2532.3871** | **2531.8203** | **0.5667** | **2** | **15** | **65** | **1** | **FPKCAELQGYCPVTYKDGNQR + 2 Carbamidomethyl (C)** |
|  | 665 | **402.2756** | **1203.8047** | **1204.4285** | **-0.6237** | **2** | **15** | **78** | **1** | **VRTPALVHRR** |
|  | 983 | **416.0945** | **1245.2614** | **1244.3945** | **0.8668** | **0** | **15** | **1e+02** | **1** | **DLGLSVLDVSAR** |
|  | 1995 | **492.2206** | **1473.6396** | **1474.6600** | **-1.0205** | **0** | **15** | **81** | **1** | **DFLPLLWNWDR** |
|  | 1788 | **472.4250** | **1414.2530** | **1414.6911** | **-0.4382** | **1** | **15** | **70** | **1** | **AFPAIQILSQAKK** |
|  | 407 | **387.0015** | **1157.9822** | **1157.3074** | **0.6749** | **1** | **15** | **93** | **1** | **EGARPVCRGR + Carbamidomethyl (C)** |
|  | 3348 | **746.6084** | **1491.2020** | **1490.7245** | **0.4776** | **2** | **15** | **68** | **1** | **SSGKLMSTDKAPLR** |
|  | 2557 | **575.5248** | **1149.0348** | **1148.2874** | **0.7474** | **0** | **15** | **72** | **1** | **MIPIGEESTR + Oxidation (M)** |
|  | 3020 | **669.4270** | **2005.2588** | **2006.3048** | **-1.0459** | **1** | **15** | **82** | **1** | **CFPPLISEHEFKENMK + Carbamidomethyl (C)** |
|  | 1639 | **460.5468** | **919.0788** | **918.0266** | **1.0523** | **1** | **15** | **1.1e+02** | **1** | **ESSRVPMP + Oxidation (M)** |
|  | 2123 | **507.9565** | **1520.8473** | **1521.7170** | **-0.8698** | **1** | **15** | **85** | **1** | **TETIRPASVYTKR** |
|  | 3118 | **686.0992** | **2055.2756** | **2056.3583** | **-1.0828** | **2** | **15** | **82** | **1** | **HHLAEAISRCCMWGRNR + Oxidation (M)** |
|  | 392 | **386.2368** | **1155.6883** | **1155.2849** | **0.4034** | **0** | **15** | **61** | **1** | **HELQVQDMR** |
|  | 902 | **409.0187** | **816.0226** | **815.8716** | **0.1510** | **0** | **15** | **95** | **1** | **VLEGAGDR** |
|  | 24 | **362.1836** | **1083.5287** | **1084.2485** | **-0.7198** | **0** | **15** | **66** | **1** | **MDPAASSCMR + Oxidation (M)** |
|  | 2607 | **583.7964** | **1748.3670** | **1748.9541** | **-0.5871** | **1** | **15** | **76** | **1** | **EMRQAPAASPSSAVFSL** |
|  | 984 | **416.1038** | **1245.2893** | **1246.3938** | **-1.1045** | **0** | **15** | **1e+02** | **1** | **ASCITPGAATAAR + Carbamidomethyl (C)** |
|  | 2845 | **623.1255** | **1244.2362** | **1244.4409** | **-0.2047** | **2** | **15** | **87** | **1** | **TGARTLADIKAK** |
|  | 107 | **367.7157** | **1100.1249** | **1101.2109** | **-1.0860** | **1** | **15** | **90** | **1** | **EIIEAEKNR** |
|  | 1831 | **476.3905** | **1426.1493** | **1426.6058** | **-0.4565** | **0** | **15** | **68** | **1** | **LPAHPRPHDCAR + Carbamidomethyl (C)** |
|  | 877 | **408.1490** | **1221.4249** | **1220.2473** | **1.1776** | **0** | **15** | **89** | **1** | **LPGSSNSSASASR** |
|  | 1656 | **461.7468** | **1382.2181** | **1383.3561** | **-1.1379** | **0** | **15** | **68** | **1** | **MASASQGADDDGSR + Oxidation (M)** |
|  | 3205 | **708.9479** | **1415.8811** | **1415.6413** | **0.2398** | **2** | **15** | **71** | **1** | **SSMNFGMKGRLR + 2 Oxidation (M)** |
|  | 809 | **407.0162** | **1218.0264** | **1218.3773** | **-0.3508** | **1** | **15** | **77** | **1** | **MIPTDEEKQK** |
|  | 1348 | **439.0464** | **876.0781** | **874.9819** | **1.0963** | **0** | **15** | **93** | **1** | **ETCMYR + Carbamidomethyl (C); Oxidation (M)** |
|  | 1795 | **473.8320** | **945.6492** | **945.0731** | **0.5761** | **0** | **15** | **90** | **1** | **ETWLQLR** |
|  | 10 | **360.4565** | **1078.3472** | **1079.2900** | **-0.9428** | **0** | **15** | **1.2e+02** | **1** | **MFTFASMTK + Oxidation (M)** |
|  | 151 | **369.3282** | **1104.9623** | **1104.1224** | **0.8399** | **0** | **15** | **82** | **1** | **ESEEEVGPTK** |
|  | 251 | **376.1316** | **750.2485** | **749.8365** | **0.4119** | **0** | **15** | **80** | **1** | **TSGLGCR + Carbamidomethyl (C)** |
|  | 995 | **416.5193** | **1246.5357** | **1245.3810** | **1.1547** | **1** | **15** | **1.2e+02** | **1** | **KWDELAVQEK** |
|  | 1972 | **490.1461** | **1467.4162** | **1467.6282** | **-0.2120** | **1** | **15** | **88** | **1** | **KMQMDGNVSGTQR + Oxidation (M)** |
|  | 1055 | **420.2083** | **1257.6028** | **1257.3270** | **0.2757** | **0** | **15** | **78** | **1** | **CALSDYQGGSEK** |
|  | 270 | **377.3778** | **752.7408** | **751.8278** | **0.9130** | **0** | **15** | **87** | **1** | **FTVETR** |
|  | 289 | **379.4763** | **756.9379** | **755.8794** | **1.0585** | **0** | **15** | **1.1e+02** | **1** | **VDMTFK + Oxidation (M)** |
|  | 1937 | **487.1884** | **972.3620** | **972.0540** | **0.3080** | **1** | **15** | **94** | **1** | **KNTPEDLR** |
|  | 2752 | **604.1852** | **1809.5336** | **1808.9348** | **0.5987** | **0** | **15** | **91** | **1** | **LASVLGSESSLDSEVTSK** |
|  | 2861 | **626.7531** | **1877.2370** | **1876.1482** | **1.0888** | **0** | **15** | **98** | **1** | **LPASSVASGPRPCAPRPR + Carbamidomethyl (C)** |
|  | 1175 | **430.2216** | **1287.6427** | **1287.4458** | **0.1969** | **1** | **15** | **89** | **1** | **RLLPDSSSGCPR** |
|  | 1988 | **491.7735** | **981.5322** | **981.0641** | **0.4681** | **1** | **15** | **64** | **1** | **EPAGHSQKK** |
|  | 2465 | **557.1820** | **1668.5238** | **1668.0091** | **0.5148** | **1** | **15** | **84** | **1** | **KQVEAGVCVTMEMVK + Oxidation (M)** |
|  | 2667 | **592.6454** | **1774.9140** | **1773.9439** | **0.9701** | **2** | **15** | **1e+02** | **1** | **RRSSSVVSAEMSGCSSK + Oxidation (M)** |
|  | 1233 | **433.1436** | **1296.4086** | **1296.5319** | **-0.1233** | **1** | **15** | **87** | **1** | **QYESLKILICS** |
|  | 2345 | **537.2057** | **1608.5949** | **1608.7711** | **-0.1762** | **1** | **15** | **99** | **1** | **SCVSVEWAEGGATKGK** |
|  | 3144 | **688.6809** | **1375.3470** | **1374.5430** | **0.8041** | **0** | **15** | **81** | **1** | **LEVSSSCGPQCHK** |
|  | 2633 | 587.6935 | 1173.3723 | 1172.3303 | 1.0420 | 0 | 15 | 1.1e+02 | 1 | TFPQNPVELK |
|  | 2957 | **664.4408** | **1990.3002** | **1991.2934** | **-0.9931** | **2** | **15** | **84** | **1** | **KGPPLDGTECAPGKWCFK + Carbamidomethyl (C)** |
|  | 781 | **406.1916** | **1215.5528** | **1215.4196** | **0.1331** | **1** | **15** | **79** | **1** | **LPETREQVLM** |
|  | 76 | **365.1080** | **1092.3018** | **1091.3040** | **0.9979** | **2** | **15** | **93** | **1** | **LKDVVRAYK** |
|  | 565 | **395.8871** | **789.7595** | **789.7880** | **-0.0285** | **0** | **15** | **99** | **1** | **SPPGTSGST** |
|  | 1378 | **442.6571** | **883.2995** | **882.9442** | **0.3553** | **0** | **15** | **69** | **1** | **GPDAHCQR** |
|  | 1736 | **468.0626** | **934.1105** | **933.9677** | **0.1427** | **0** | **15** | **96** | **1** | **DLAADHHR** |
|  | 3453 | **789.8306** | **2366.4695** | **2366.7787** | **-0.3091** | **0** | **15** | **86** | **1** | **MQSCCLFAYDGCVPLEAVMGR + Carbamidomethyl (C); Oxidation (M)** |
|  | 1756 | **469.9846** | **1406.9318** | **1406.6757** | **0.2560** | **2** | **15** | **79** | **1** | **KLLNKVSLGHAAR** |
|  | 3012 | **668.7827** | **2003.3260** | **2003.3474** | **-0.0214** | **2** | **15** | **1.1e+02** | **1** | **HIKKQGLVTVCESGMQTK + Oxidation (M)** |
|  | 3284 | **740.6305** | **1479.2462** | **1479.7611** | **-0.5149** | **0** | **15** | **69** | **1** | **LILTPATFSGLPHL** |
|  | 875 | **408.1243** | **1221.3507** | **1222.3477** | **-0.9969** | **1** | **15** | **93** | **1** | **SFATKNEAVQK** |
|  | 1100 | **422.4098** | **842.8047** | **841.9534** | **0.8513** | **0** | **15** | **97** | **1** | **AEALWPR** |
|  | 1790 | **472.5686** | **943.1224** | **944.0009** | **-0.8785** | **0** | **15** | **1.1e+02** | **1** | **PTTSSAQPR** |
|  | 2361 | **538.2874** | **1074.5600** | **1074.1922** | **0.3678** | **2** | **15** | **91** | **1** | **ATKAKNTGQR** |
|  | 3215 | **715.9518** | **1429.8888** | **1430.7519** | **-0.8632** | **1** | **15** | **75** | **1** | **EITALAPSIMKIK + Oxidation (M)** |
|  | 6 | **360.3774** | **718.7400** | **719.8289** | **-1.0888** | **0** | **15** | **1.2e+02** | **1** | **FVADLR** |
|  | 585 | **398.1993** | **794.3839** | **794.9370** | **-0.5531** | **0** | **15** | **80** | **1** | **MTSPCEK** |
|  | 3195 | **704.1989** | **2109.5744** | **2109.5173** | **0.0571** | **1** | **15** | **84** | **1** | **AALAACPSSPFPPAMPRVLR + Carbamidomethyl (C)** |
|  | 100 | **367.2756** | **1098.8047** | **1098.2102** | **0.5945** | **0** | **15** | **80** | **1** | **IPAAGASQLDR** |
|  | 1565 | **457.2715** | **1368.7923** | **1368.5800** | **0.2123** | **2** | **15** | **79** | **1** | **QSEIAMDRMKK + 2 Oxidation (M)** |
|  | 773 | **406.1181** | **810.2215** | **809.9102** | **0.3113** | **1** | **15** | **86** | **1** | **YGTKSVR** |
|  | 1197 | **431.1683** | **860.3218** | **860.8690** | **-0.5473** | **0** | **15** | **1e+02** | **1** | **DLNSQER** |
|  | 1747 | **469.0812** | **1404.2214** | **1403.4783** | **0.7431** | **1** | **15** | **89** | **1** | **MERAGPAGEEGGAR + Oxidation (M)** |
|  | 3487 | **816.6326** | **2446.8755** | **2447.6606** | **-0.7851** | **1** | **15** | **82** | **1** | **NLDLSYNQLHSLGSEQFRGLR** |
|  | 123 | **369.1503** | **1104.4286** | **1105.2692** | **-0.8405** | **0** | **15** | **92** | **1** | **CMMAQYNR + Carbamidomethyl (C); 2 Oxidation (M)** |
|  | 418 | **387.2672** | **772.5197** | **771.7760** | **0.7437** | **0** | **15** | **85** | **1** | **AEPADNR** |
|  | 2576 | **579.0988** | **1734.2741** | **1733.0836** | **1.1905** | **1** | **15** | **88** | **1** | **RAVMTILLWAQSSLK + Oxidation (M)** |
|  | 2641 | **589.7052** | **1177.3956** | **1176.2776** | **1.1180** | **2** | **15** | **1.1e+02** | **1** | **KGAAAATSSKTPS** |
|  | 285 | **379.2165** | **756.4183** | **755.8196** | **0.5987** | **0** | **15** | **70** | **1** | **AALDGGPR** |
|  | 387 | **386.1534** | **1155.4382** | **1154.3165** | **1.1216** | **0** | **15** | **79** | **1** | **DDILINRPAK** |
|  | 835 | **407.4754** | **1219.4039** | **1218.3620** | **1.0419** | **1** | **15** | **1e+02** | **1** | **GLLRDNDLFR** |
|  | 507 | **389.9991** | **1166.9751** | **1167.2046** | **-0.2295** | **1** | **15** | **91** | **1** | **QDEDNGMKSK + Oxidation (M)** |
|  | 1105 | **423.1241** | **1266.3503** | **1266.4299** | **-0.0797** | **1** | **15** | **1e+02** | **1** | **HELSVSCAKHR** |
|  | 3177 | **698.3735** | **1394.7323** | **1395.6280** | **-0.8957** | **0** | **15** | **87** | **1** | **QCGGLQGFLIFR + Carbamidomethyl (C)** |
|  | 545 | **392.8651** | **783.7153** | **782.9046** | **0.8107** | **0** | **15** | **79** | **1** | **MIFDNK + Oxidation (M)** |
|  | 1501 | **450.3345** | **1347.9812** | **1347.4580** | **0.5233** | **2** | **15** | **72** | **1** | **MEGAGGANDKKNR** |
|  | 439 | **387.9760** | **773.9371** | **773.8812** | **0.0559** | **1** | **15** | **1.1e+02** | **1** | **RITSAAR** |
|  | 1195 | **431.0967** | **860.1786** | **859.0949** | **1.0836** | **2** | **15** | **1.1e+02** | **1** | **ACLRIRK** |
|  | 59 | **364.1007** | **1089.2799** | **1088.2519** | **1.0279** | **0** | **15** | **84** | **1** | **TLISDIEAVK** |
|  | 440 | **387.9887** | **773.9626** | **773.9176** | **0.0450** | **1** | **15** | **1.1e+02** | **1** | **LDSALKK** |
|  | 456 | **388.3281** | **774.6413** | **773.8781** | **0.7633** | **1** | **15** | **91** | **1** | **SSPVTKR** |
|  | 3047 | **678.7831** | **2033.3271** | **2033.3136** | **0.0135** | **2** | **15** | **1.1e+02** | **1** | **IPFRGAPCSDAAGRQVPYK** |
|  | 227 | **374.2039** | **1119.5896** | **1119.2279** | **0.3617** | **0** | **15** | **89** | **1** | **LSFQVPGSER** |
|  | 988 | **416.1844** | **1245.5312** | **1246.3293** | **-0.7981** | **2** | **15** | **1e+02** | **1** | **AEDNEAKWKR** |
|  | 1172 | **430.1827** | **858.3507** | **857.9910** | **0.3597** | **0** | **15** | **1e+02** | **1** | **EALDVLAK** |
|  | 1227 | **433.0322** | **1296.0744** | **1296.5319** | **-0.4575** | **1** | **15** | **91** | **1** | **QYESLKILICS** |
|  | 1306 | **437.0363** | **872.0579** | **870.9932** | **1.0647** | **0** | **15** | **1e+02** | **1** | **KPLPSSSR** |
|  | 2553 | 575.1180 | 1148.2213 | 1149.2954 | -1.0741 | 0 | 15 | 95 | 1 | NSSMTTAYMK + Oxidation (M) |
|  | 1698 | **464.0247** | **926.0347** | **925.0819** | **0.9529** | **0** | **15** | **85** | **1** | **FLVGFTNK** |
|  | 3002 | **668.5969** | **2002.7686** | **2003.2776** | **-0.5091** | **2** | **15** | **76** | **1** | **LEDSAKYFCALGEKFPK + Carbamidomethyl (C)** |
|  | 228 | **374.2221** | **746.4294** | **746.8758** | **-0.4464** | **0** | **15** | **88** | **1** | **MGPLSAR + Oxidation (M)** |
|  | 305 | **380.2404** | **758.4659** | **758.8268** | **-0.3608** | **1** | **15** | **84** | **1** | **LSGGGGRR** |
|  | 777 | **406.1699** | **1215.4874** | **1215.3797** | **0.1077** | **0** | **15** | **86** | **1** | **SEAACLAAGPGIR** |
|  | 1063 | **421.1974** | **1260.5702** | **1260.4933** | **0.0768** | **2** | **15** | **87** | **1** | **CCVPAGRAATRR** |
|  | 1594 | **458.7952** | **1373.3633** | **1374.3244** | **-0.9610** | **0** | **15** | **90** | **1** | **DSGDENDPIQER** |
|  | 3265 | **739.1973** | **1476.3798** | **1476.7392** | **-0.3594** | **0** | **15** | **91** | **1** | **CYEQFMAPLCAK + Carbamidomethyl (C); Oxidation (M)** |
|  | 1567 | **457.5375** | **913.0602** | **912.0449** | **1.0152** | **0** | **15** | **1.1e+02** | **1** | **GGPLQALTR** |
|  | 446 | **388.1136** | **774.2124** | **774.9124** | **-0.7000** | **1** | **15** | **1.1e+02** | **1** | **CRECHK** |
|  | 720 | **404.9092** | **1211.7055** | **1212.3528** | **-0.6472** | **0** | **15** | **83** | **1** | **KPSESAPLDLR** |
|  | 1252 | **434.1971** | **1299.5693** | **1298.4832** | **1.0860** | **0** | **15** | **86** | **1** | **SALYYVDLIGGK** |
|  | 3280 | **740.5016** | **1478.9884** | **1479.7399** | **-0.7515** | **1** | **15** | **88** | **1** | **MGQKIAPYSVEIK + Oxidation (M)** |
|  | 1137 | **427.7817** | **1280.3229** | **1279.4649** | **0.8579** | **0** | **15** | **90** | **1** | **LITAANFCQNGK** |
|  | 2480 | **562.2599** | **1683.7575** | **1684.7925** | **-1.0350** | **2** | **15** | **93** | **1** | **TRSRECNNPPPSGGGR** |
|  | 2682 | **593.5074** | **1777.5000** | **1777.8612** | **-0.3612** | **0** | **15** | **76** | **1** | **APSDTGSLSSPVEQDCK + Carbamidomethyl (C)** |
|  | 2751 | **603.8448** | **1808.5122** | **1808.1981** | **0.3141** | **2** | **15** | **79** | **1** | **RLYIITLGCLAPYRR** |
|  | 349 | **385.0494** | **1152.1261** | **1152.3622** | **-0.2360** | **1** | **15** | **79** | **1** | **SLLTTGSAMKK + Oxidation (M)** |
|  | 1826 | **476.2884** | **1425.8429** | **1425.4622** | **0.3808** | **0** | **15** | **80** | **1** | **APDQPQHFGDGTR** |
|  | 68 | **364.2083** | **1089.6026** | **1089.1573** | **0.4453** | **0** | **15** | **65** | **1** | **PSTLSASVGDR** |
|  | 734 | **405.0934** | **808.1720** | **807.9157** | **0.2563** | **0** | **15** | **86** | **1** | **TALTSCR + Carbamidomethyl (C)** |
|  | 1631 | **460.2165** | **1377.6272** | **1376.4297** | **1.1975** | **1** | **15** | **99** | **1** | **KSPETGGTAASQSR** |
|  | 1106 | **423.1962** | **1266.5664** | **1266.5544** | **0.0120** | **1** | **15** | **1e+02** | **1** | **MVEAAMKSPMR + Oxidation (M)** |
|  | 800 | **406.8387** | **1217.4940** | **1217.5016** | **-0.0077** | **0** | **15** | **88** | **1** | **SLPMPACLLTR + Oxidation (M)** |
|  | 22 | **362.1430** | **1083.4068** | **1084.2499** | **-0.8431** | **1** | **15** | **83** | **1** | **CLREEIHK + Carbamidomethyl (C)** |
|  | 1036 | **419.2788** | **1254.8143** | **1255.3393** | **-0.5250** | **0** | **15** | **89** | **1** | **GSPGGPGAAGFPGAR** |
|  | 1906 | **484.1823** | **966.3499** | **966.0460** | **0.3039** | **0** | **15** | **92** | **1** | **ATYLDDLR** |
|  | 178 | **370.5211** | **1108.5412** | **1109.1502** | **-0.6090** | **0** | **15** | **72** | **1** | **APPSGQGGPEGR** |
|  | 1589 | **458.6021** | **915.1894** | **914.0608** | **1.1286** | **0** | **15** | **1e+02** | **1** | **GQLAASVLR** |
|  | 2759 | **606.6870** | **1817.0387** | **1816.0415** | **0.9972** | **0** | **15** | **1.1e+02** | **1** | **ALMGHFQDGLSYSVFK + Oxidation (M)** |
|  | 323 | **382.3306** | **1143.9697** | **1144.2357** | **-0.2660** | **1** | **15** | **94** | **1** | **DGLPATEKSAR** |
|  | 1458 | **447.1601** | **1338.4581** | **1339.3214** | **-0.8633** | **0** | **15** | **97** | **1** | **GTGYSGYGGGDYW** |
|  | 3168 | **696.5844** | **2086.7309** | **2086.2404** | **0.4904** | **1** | **15** | **76** | **1** | **SADTAVYFCARLDYYDR + Carbamidomethyl (C)** |
|  | 381 | **386.1243** | **1155.3507** | **1154.4025** | **0.9482** | **0** | **15** | **85** | **1** | **GLSQAALLLLR** |
|  | 436 | **387.9139** | **1160.7196** | **1160.4138** | **0.3058** | **1** | **15** | **1.1e+02** | **1** | **MALGPRCGAIR + Oxidation (M)** |
|  | 491 | **389.2474** | **1164.7200** | **1165.1887** | **-0.4687** | **0** | **15** | **83** | **1** | **CSAEPDSSQNK** |
|  | 430 | **387.8965** | **1160.6673** | **1161.3956** | **-0.7283** | **2** | **15** | **1.1e+02** | **1** | **MEKMTHEKK** |
|  | 481 | **389.1520** | **1164.4338** | **1163.3898** | **1.0440** | **0** | **15** | **1e+02** | **1** | **QFIMPVVSAR + Oxidation (M)** |
|  | 501 | **389.9209** | **777.8271** | **777.7805** | **0.0465** | **0** | **15** | **95** | **1** | **SGTEAASR** |
|  | 1073 | 422.0147 | 1263.0219 | 1263.3781 | -0.3562 | 0 | 15 | 94 | 1 | SMTSAHGSASALK + Oxidation (M) |
|  | 1191 | **431.0818** | **860.1489** | **859.9274** | **0.2216** | **1** | **15** | **1.1e+02** | **1** | **NLGESRGK** |
|  | 204 | **372.2888** | **742.5627** | **742.9698** | **-0.4071** | **0** | **15** | **72** | **1** | **MAAPILK** |
|  | 1395 | **443.6223** | **885.2297** | **886.0127** | **-0.7829** | **0** | **15** | **85** | **1** | **VAHHGLPR** |
|  | 3014 | **668.8588** | **1335.7029** | **1336.5014** | **-0.7986** | **2** | **15** | **89** | **1** | **EKGRGCSICNR + 2 Carbamidomethyl (C)** |
|  | 93 | **366.2437** | **1095.7090** | **1095.2526** | **0.4563** | **0** | **15** | **85** | **1** | **ALQSIHGTLR** |
|  | 497 | **389.8653** | **1166.5737** | **1167.3618** | **-0.7880** | **0** | **15** | **93** | **1** | **CHAEGIPMPR + Carbamidomethyl (C)** |
|  | 1243 | **433.3451** | **864.6754** | **864.9868** | **-0.3114** | **0** | **15** | **75** | **1** | **IHAFSYK** |
|  | 2502 | **565.2452** | **1692.7135** | **1691.9736** | **0.7399** | **1** | **14** | **1e+02** | **1** | **GFPSGMAAAGRHLCFL + Carbamidomethyl (C)** |
|  | 705 | **404.1105** | **1209.3094** | **1209.3919** | **-0.0824** | **0** | **14** | **95** | **1** | **MEEWPCIASK + Oxidation (M)** |
|  | 967 | **415.1324** | **1242.3750** | **1241.4138** | **0.9611** | **0** | **14** | **99** | **1** | **NTAYVQMSSLK** |
|  | 1133 | **427.1041** | **1278.2900** | **1279.4253** | **-1.1353** | **1** | **14** | **91** | **1** | **NLTTHRCSYK + Carbamidomethyl (C)** |
|  | 137 | **369.2447** | **736.4747** | **736.8793** | **-0.4046** | **0** | **14** | **82** | **1** | **AFQMPK + Oxidation (M)** |
|  | 3368 | **752.7316** | **1503.4485** | **1502.7617** | **0.6868** | **1** | **14** | **79** | **1** | **AVMQSQKPPKNCR + Oxidation (M)** |
|  | 710 | **404.1761** | **806.3375** | **805.9033** | **0.4342** | **1** | **14** | **92** | **1** | **RSSMGPR + Oxidation (M)** |
|  | 2691 | 594.3549 | 1780.0424 | 1780.0078 | 0.0346 | 0 | 14 | 1e+02 | 1 | SCAVSLTTAAVAFGPEAK + Carbamidomethyl (C) |
|  | 3291 | **740.7105** | **2219.1094** | **2218.4262** | **0.6831** | **2** | **14** | **78** | **1** | **TLFDCDEQQRDYLMERR** |
|  | 2278 | **529.1266** | **1584.3576** | **1583.7018** | **0.6557** | **2** | **14** | **95** | **1** | **DSLKGGGALEKESHR** |
|  | 2048 | **500.1649** | **998.3151** | **997.1959** | **1.1192** | **1** | **14** | **93** | **1** | **CMRFDLR + Carbamidomethyl (C)** |
|  | 3451 | **789.5564** | **2365.6470** | **2366.4754** | **-0.8284** | **0** | **14** | **88** | **1** | **TLDSLMQFGNHGEGAEPSAGGQF + Oxidation (M)** |
|  | 718 | **404.8772** | **1211.6093** | **1212.3793** | **-0.7699** | **2** | **14** | **88** | **1** | **DRSKYGMLAR + Oxidation (M)** |
|  | 726 | **405.0108** | **1212.0101** | **1211.2867** | **0.7235** | **0** | **14** | **88** | **1** | **NNPNGFQVHGK** |
|  | 1139 | **427.8956** | **853.7765** | **854.8829** | **-1.1064** | **0** | **14** | **93** | **1** | **AGGMSDSSK + Oxidation (M)** |
|  | 1357 | 439.9832 | 877.9516 | 878.0058 | -0.0541 | 0 | 14 | 1.1e+02 | 1 | AEGTVVMR + Oxidation (M) |
|  | 1479 | **449.1808** | **1344.5202** | **1343.5255** | **0.9946** | **1** | **14** | **90** | **1** | **DEISQVLGKLNK** |
|  | 2258 | **524.2493** | **1046.4838** | **1046.2202** | **0.2636** | **0** | **14** | **1e+02** | **1** | **LFSVPSQLR** |
|  | 2552 | **574.7784** | **1721.3132** | **1720.9090** | **0.4041** | **2** | **14** | **87** | **1** | **MERRAAGPGWAAYER** |
|  | 3246 | **732.6735** | **1463.3321** | **1463.5499** | **-0.2177** | **0** | **14** | **75** | **1** | **NEEVFNYGNHLK** |
|  | 1145 | **428.2089** | **1281.6046** | **1282.5521** | **-0.9475** | **2** | **14** | **81** | **1** | **VKGKGYSVSVMK** |
|  | 1926 | **486.5244** | **971.0339** | **971.1088** | **-0.0749** | **0** | **14** | **1.1e+02** | **1** | **GLGQLGEAVK** |
|  | 2299 | **532.9059** | **1595.6957** | **1596.8495** | **-1.1539** | **1** | **14** | **96** | **1** | **CSPGPCDRCIFSAQL** |
|  | 2962 | **665.2067** | **1328.3985** | **1327.5113** | **0.8873** | **1** | **14** | **97** | **1** | **SQFCGARVFGAK + Carbamidomethyl (C)** |
|  | 3090 | **684.1825** | **2049.5253** | **2049.2849** | **0.2404** | **2** | **14** | **92** | **1** | **LGVASTEAQRGVSFKLEEK** |
|  | 159 | **369.3691** | **1105.0851** | **1105.3089** | **-0.2238** | **1** | **14** | **1.1e+02** | **1** | **MDLGIGLSKR + Oxidation (M)** |
|  | 601 | **399.1219** | **1194.3434** | **1195.3736** | **-1.0302** | **2** | **14** | **86** | **1** | **KQNGSCGARMK + Oxidation (M)** |
|  | 862 | **407.9146** | **1220.7215** | **1220.5039** | **0.2176** | **0** | **14** | **96** | **1** | **CMQIPISFPK + Carbamidomethyl (C)** |
|  | 515 | **391.1389** | **1170.3944** | **1169.3710** | **1.0234** | **0** | **14** | **91** | **1** | **TLVQILTEPR** |
|  | 2827 | **615.4653** | **1843.3738** | **1844.0514** | **-0.6776** | **0** | **14** | **82** | **1** | **LWNCDITSDGCCDLTK + Carbamidomethyl (C)** |
|  | 3323 | **742.5461** | **1483.0774** | **1483.6474** | **-0.5700** | **1** | **14** | **88** | **1** | **YGADVHTQSKFCK** |
|  | 3338 | **743.7596** | **2228.2566** | **2227.3900** | **0.8665** | **2** | **14** | **91** | **1** | **NTDGGTTDYAAPVKGRFTISR** |
|  | 149 | **369.2908** | **736.5668** | **736.8793** | **-0.3125** | **0** | **14** | **86** | **1** | **AFQMPK + Oxidation (M)** |
|  | 1161 | **429.0516** | **856.0884** | **854.9508** | **1.1376** | **1** | **14** | **91** | **1** | **SSSPPPRK** |
|  | 2310 | **534.1648** | **1066.3148** | **1066.1519** | **0.1629** | **1** | **14** | **1e+02** | **1** | **GMDRGGFGGGR** |
|  | 588 | **398.2700** | **794.5253** | **793.7369** | **0.7884** | **0** | **14** | **80** | **1** | **SSGSEGDR** |
|  | 1216 | **432.2704** | **862.5261** | **862.0527** | **0.4735** | **2** | **14** | **84** | **1** | **TKAMRQK** |
|  | 1510 | **451.2509** | **900.4870** | **900.0345** | **0.4525** | **1** | **14** | **96** | **1** | **VTSAAAPKR** |
|  | 1615 | **459.3981** | **916.7815** | **916.0435** | **0.7379** | **1** | **14** | **90** | **1** | **CCHARGR + 2 Carbamidomethyl (C)** |
|  | 3327 | **742.6605** | **1483.3063** | **1482.6825** | **0.6238** | **0** | **14** | **73** | **1** | **VNFSLTPGAGPVPAR** |
|  | 1230 | **433.0859** | **1296.2355** | **1296.5319** | **-0.2965** | **1** | **14** | **96** | **1** | **QYESLKILICS** |
|  | 1536 | **454.3412** | **1360.0013** | **1359.5319** | **0.4694** | **2** | **14** | **83** | **1** | **KPRATTEVSSRK** |
|  | 1597 | **458.8649** | **915.7150** | **916.0155** | **-0.3005** | **0** | **14** | **1.1e+02** | **1** | **HFCQEPR** |
|  | 2650 | **590.5044** | **1178.9940** | **1179.1524** | **-0.1584** | **1** | **14** | **78** | **1** | **EQGSSSEKDGR** |
|  | 1114 | **424.2311** | **1269.6712** | **1269.4704** | **0.2009** | **2** | **14** | **95** | **1** | **KDGVCGPPPSKK + Carbamidomethyl (C)** |
|  | 2501 | **565.1193** | **1128.2239** | **1128.3223** | **-0.0985** | **1** | **14** | **1e+02** | **1** | **RAQLLLESAK** |
|  | 2618 | **584.6357** | **1167.2567** | **1166.3474** | **0.9094** | **0** | **14** | **1.1e+02** | **1** | **MIEGVAYEVR** |
|  | 2799 | **612.1804** | **1222.3461** | **1223.3838** | **-1.0377** | **1** | **14** | **99** | **1** | **AQRMATDMQR + Oxidation (M)** |
|  | 1805 | **475.0660** | **1422.1758** | **1421.6605** | **0.5152** | **0** | **14** | **1.1e+02** | **1** | **NAAEAMLLQLGYK** |
|  | 2876 | **631.1154** | **1260.2159** | **1261.3387** | **-1.1228** | **0** | **14** | **1e+02** | **1** | **QPPDYWGQGTL** |
|  | 2930 | **656.8817** | **1311.7486** | **1312.4702** | **-0.7215** | **0** | **14** | **79** | **1** | **LLPQPPDTEFR** |
|  | 696 | **403.9576** | **805.9005** | **804.9383** | **0.9622** | **0** | **14** | **1e+02** | **1** | **VPGCCNR + Carbamidomethyl (C)** |
|  | 662 | **402.1178** | **802.2208** | **801.8848** | **0.3360** | **0** | **14** | **1.1e+02** | **1** | **LDSGVPSK** |
|  | 1684 | **463.0741** | **924.1334** | **923.0645** | **1.0690** | **0** | **14** | **90** | **1** | **ASLGFTVTK** |
|  | 3130 | **686.3584** | **2056.0530** | **2055.4398** | **0.6132** | **0** | **14** | **97** | **1** | **VGWQCLPSILQTGEIVALK** |
|  | 232 | **374.2713** | **746.5278** | **745.7834** | **0.7445** | **0** | **14** | **96** | **1** | **HAFDTR** |
|  | 629 | **401.0948** | **1200.2621** | **1199.2695** | **0.9926** | **0** | **14** | **1.1e+02** | **1** | **TGELFFGEGSR** |
|  | 1086 | **422.1678** | **842.3209** | **841.9535** | **0.3675** | **0** | **14** | **1.1e+02** | **1** | **DWIGVPR** |
|  | 3160 | **692.2538** | **2073.7394** | **2074.4117** | **-0.6724** | **1** | **14** | **94** | **1** | **DMIARPRQPVAQWHQLK** |
|  | 3185 | **701.5144** | **2101.5210** | **2100.4208** | **1.1002** | **2** | **14** | **91** | **1** | **YLDKELAGRVQQIQLLGR** |
|  | 3269 | **740.1903** | **2217.5488** | **2218.4656** | **-0.9169** | **0** | **14** | **94** | **1** | **LFLTGWDSNIVGHAALNFDK** |
|  | 201 | **372.2073** | **1113.5998** | **1112.4091** | **1.1907** | **2** | **14** | **76** | **1** | **KIGCYAAMKK** |
|  | 2142 | **512.4354** | **1022.8559** | **1023.1038** | **-0.2479** | **0** | **14** | **79** | **1** | **HNEALNGLR** |
|  | 3117 | **686.0933** | **2055.2576** | **2055.3294** | **-0.0718** | **0** | **14** | **95** | **1** | **MEIDFLPSTMTTPTPVSR + 2 Oxidation (M)** |
|  | 3480 | **811.4122** | **1620.8096** | **1621.8805** | **-1.0710** | **2** | **14** | **97** | **1** | **GKDIFHQLHLSKAK** |
|  | 425 | **387.8486** | **1160.5236** | **1159.4423** | **1.0813** | **0** | **14** | **1.2e+02** | **1** | **AITTLMAGILR** |
|  | 971 | **415.2597** | **1242.7568** | **1243.5258** | **-0.7689** | **2** | **14** | **84** | **1** | **FMMRKNNMR + Oxidation (M)** |
|  | 70 | **364.2695** | **726.5242** | **727.7202** | **-1.1960** | **0** | **14** | **71** | **1** | **TSDSYR** |
|  | 1290 | **436.1075** | **1305.3002** | **1306.3811** | **-1.0810** | **0** | **14** | **99** | **1** | **GLEWVSATSGSGR** |
|  | 2409 | **549.2084** | **1644.6029** | **1643.8401** | **0.7629** | **2** | **14** | **95** | **1** | **KQSQPVVTASDISKR** |
|  | 2822 | **614.3546** | **1226.6944** | **1227.4782** | **-0.7838** | **1** | **14** | **99** | **1** | **RTVAAPFCLHL** |
|  | 3077 | **683.9551** | **1365.8954** | **1365.5807** | **0.3146** | **1** | **14** | **80** | **1** | **GALVLGSSLKQHR** |
|  | 1468 | **448.2039** | **1341.5895** | **1341.5576** | **0.0318** | **0** | **14** | **98** | **1** | **GNPVPILIPEHR** |
|  | 1735 | 467.8481 | 1400.5221 | 1399.5740 | 0.9481 | 1 | 14 | 1.1e+02 | 1 | VDASSMWLYRR + Oxidation (M) |
|  | 464 | **388.9765** | **775.9382** | **776.9630** | **-1.0247** | **1** | **14** | **1.1e+02** | **1** | **EIKFIK** |
|  | 600 | **399.1191** | **796.2234** | **796.9577** | **-0.7343** | **1** | **14** | **90** | **1** | **KGLVPQR** |
|  | 1392 | **443.5306** | **1327.5696** | **1326.6077** | **0.9619** | **1** | **14** | **1.2e+02** | **1** | **GTPINRIPIMAK + Oxidation (M)** |
|  | 2726 | **597.6810** | **1790.0209** | **1789.1286** | **0.8923** | **2** | **14** | **1.3e+02** | **1** | **CGLTSLHSLSNCKKLK + Carbamidomethyl (C)** |
|  | 505 | **389.9825** | **777.9501** | **778.8084** | **-0.8582** | **1** | **14** | **1e+02** | **1** | **TSKGSGDK** |
|  | 2317 | **534.5988** | **1600.7741** | **1600.7953** | **-0.0212** | **1** | **14** | **1.3e+02** | **1** | **LIPGGDNVTVCKDNR** |
|  | 2376 | **540.4586** | **1618.3537** | **1617.8491** | **0.5046** | **0** | **14** | **81** | **1** | **MNCSAGNPNVVVTVR + Carbamidomethyl (C)** |
|  | 3136 | **687.0186** | **1372.0223** | **1371.5656** | **0.4568** | **0** | **14** | **81** | **1** | **LMNSRPEKPSGR** |
|  | 12 | **360.4866** | **1078.4375** | **1079.1857** | **-0.7481** | **0** | **14** | **1.2e+02** | **1** | **TPTVTSNTCR** |
|  | 1058 | **420.3041** | **838.5934** | **837.9649** | **0.6286** | **0** | **14** | **76** | **1** | **MCADLNR + Oxidation (M)** |
|  | 3176 | **698.3495** | **2092.0263** | **2092.2451** | **-0.2188** | **2** | **14** | **97** | **1** | **VQQKEGGSDLGMSGNSEPKK + Oxidation (M)** |
|  | 3536 | **856.5589** | **2566.6545** | **2565.8320** | **0.8225** | **1** | **14** | **94** | **1** | **VRVMGFGTDAFDTWGQGTMVTVSS + Oxidation (M)** |
|  | 108 | **367.9564** | **733.8981** | **732.8492** | **1.0489** | **0** | **14** | **1.1e+02** | **1** | **AGEMLGR** |
|  | 447 | **388.1371** | **1161.3891** | **1162.3203** | **-0.9313** | **1** | **14** | **1.2e+02** | **1** | **RITCGGNGIGSK** |
|  | 1430 | **445.0952** | **888.1756** | **886.9924** | **1.1831** | **0** | **14** | **1.2e+02** | **1** | **NLLSVGER** |
|  | 2964 | **665.3157** | **1328.6166** | **1327.5295** | **1.0870** | **1** | **14** | **1e+02** | **1** | **KQLAIGVNEVTR** |
|  | 2999 | **668.3473** | **2002.0197** | **2002.2283** | **-0.2086** | **0** | **14** | **1.1e+02** | **1** | **WWCEMEPCLEGEECK + 2 Carbamidomethyl (C); Oxidation (M)** |
|  | 3123 | **686.1870** | **2055.5387** | **2056.3303** | **-0.7916** | **2** | **14** | **98** | **1** | **WMASAWKGSSRTVWCQR + Oxidation (M)** |
|  | 1411 | **443.9283** | **1328.7628** | **1329.5206** | **-0.7578** | **1** | **14** | **1.1e+02** | **1** | **EYKCGDLVFAK + Carbamidomethyl (C)** |
|  | 2470 | **558.4555** | **1114.8962** | **1114.2956** | **0.6006** | **0** | **14** | **86** | **1** | **LAALNGLSLSR** |
|  | 828 | **407.3549** | **1219.0427** | **1219.4163** | **-0.3737** | **1** | **14** | **80** | **1** | **CPNAYLGIRR + Carbamidomethyl (C)** |
|  | 1194 | **431.0966** | **1290.2677** | **1290.3852** | **-0.1175** | **2** | **14** | **1.2e+02** | **1** | **GERSVKEWSGR** |
|  | 1303 | **437.0276** | **1308.0608** | **1308.3970** | **-0.3363** | **0** | **14** | **1.1e+02** | **1** | **VAAGSLSADAGFSR** |
|  | 1875 | **480.0975** | **1437.2704** | **1437.6831** | **-0.4127** | **1** | **14** | **1.1e+02** | **1** | **LLSHGKEVGSIIGK** |
|  | 2090 | **505.1039** | **1512.2897** | **1511.8095** | **0.4801** | **1** | **14** | **98** | **1** | **VLQGAAWLLGRSLK** |
|  | 2352 | **538.0906** | **1611.2497** | **1610.8381** | **0.4117** | **2** | **14** | **1.1e+02** | **1** | **DKGNCGVGKSCLCNR + Carbamidomethyl (C)** |
|  | 2365 | **539.0743** | **1614.2007** | **1613.7941** | **0.4066** | **1** | **14** | **1.1e+02** | **1** | **VVNCSDPGIPANSKR + Carbamidomethyl (C)** |
|  | 3324 | **742.5601** | **2224.6580** | **2224.5072** | **0.1508** | **0** | **14** | **90** | **1** | **TTQVVSADSTIQMLFYMEK + 2 Oxidation (M)** |
|  | 1543 | **454.8310** | **1361.4708** | **1361.5457** | **-0.0749** | **1** | **14** | **1.1e+02** | **1** | **FDLIGLEGRVSR** |
|  | 1913 | **485.1581** | **1452.4523** | **1452.7805** | **-0.3282** | **0** | **14** | **95** | **1** | **LETILALLVALQR** |
|  | 3383 | **758.0870** | **1514.1592** | **1513.6981** | **0.4611** | **1** | **14** | **79** | **1** | **RLACMFGSAVQSNN + Oxidation (M)** |
|  | 562 | **395.2803** | **1182.8188** | **1183.4258** | **-0.6069** | **1** | **14** | **93** | **1** | **WLMVHVDKR** |
|  | 1802 | **474.5353** | **1420.5838** | **1419.6299** | **0.9539** | **1** | **14** | **1.3e+02** | **1** | **TATPCSLPSACRR + Carbamidomethyl (C)** |
|  | 1982 | **490.9382** | **979.8617** | **980.0992** | **-0.2375** | **1** | **14** | **1e+02** | **1** | **TMWSRGDK** |
|  | 2548 | **573.2524** | **1716.7351** | **1717.9234** | **-1.1882** | **2** | **14** | **1.1e+02** | **1** | **SYECPQCGKAFSRK + 2 Carbamidomethyl (C)** |
|  | 1118 | **425.3425** | **848.6703** | **848.9494** | **-0.2791** | **0** | **14** | **91** | **1** | **HLGVPNGR** |
|  | 2778 | **610.1237** | **1218.2325** | **1219.3670** | **-1.1344** | **1** | **14** | **1e+02** | **1** | **MEAKEEFAHK** |
|  | 3178 | **698.6058** | **2092.7953** | **2093.3506** | **-0.5552** | **0** | **14** | **80** | **1** | **CHPETGACVCPPGHSGAPCR + 2 Carbamidomethyl (C)** |
|  | 405 | **386.9694** | **771.9240** | **772.8038** | **-0.8798** | **0** | **14** | **1.1e+02** | **1** | **LEDGSPR** |
|  | 426 | **387.8589** | **773.7031** | **773.9012** | **-0.1980** | **0** | **14** | **1.2e+02** | **1** | **APAMSAAR** |
|  | 1467 | **448.1778** | **1341.5112** | **1340.5531** | **0.9582** | **1** | **14** | **1e+02** | **1** | **SFKGCAHAPLPR + Carbamidomethyl (C)** |
|  | 2160 | **515.5884** | **1029.1620** | **1030.2471** | **-1.0852** | **1** | **14** | **1.3e+02** | **1** | **IRGIGHFCK** |
|  | 2186 | **518.8197** | **1553.4369** | **1552.7359** | **0.7011** | **2** | **14** | **82** | **1** | **NHFHKRSTTLSPK** |
|  | 1048 | **419.7299** | **837.4450** | **836.9801** | **0.4649** | **1** | **14** | **82** | **1** | **AGGNCCKK + Carbamidomethyl (C)** |
|  | 1363 | **440.5461** | **1318.6161** | **1318.5011** | **0.1150** | **0** | **14** | **1.3e+02** | **1** | **CAAPVGNNGLAFGK** |
|  | 2237 | **521.2245** | **1040.4342** | **1041.2667** | **-0.8325** | **0** | **14** | **1e+02** | **1** | **CVIMCQGSK + Carbamidomethyl (C); Oxidation (M)** |
|  | 948 | **413.9891** | **1238.9451** | **1238.4961** | **0.4490** | **1** | **14** | **88** | **1** | **EQMVSFLLKK + Oxidation (M)** |
|  | 1633 | **460.2571** | **1377.7493** | **1377.5020** | **0.2472** | **0** | **14** | **1e+02** | **1** | **LFQVQGTGANNTK** |
|  | 3286 | **740.6438** | **1479.2728** | **1478.6492** | **0.6236** | **2** | **14** | **81** | **1** | **KKDGSSSVPLSFAR** |
|  | 654 | **402.0072** | **801.9996** | **802.0402** | **-0.0407** | **1** | **14** | **1.2e+02** | **1** | **RILCGIK** |
|  | 1229 | **433.0533** | **1296.1377** | **1295.4495** | **0.6881** | **2** | **14** | **1e+02** | **1** | **RRATNSCQISC + Carbamidomethyl (C)** |
|  | 1260 | **435.0938** | **868.1728** | **867.8999** | **0.2730** | **0** | **14** | **94** | **1** | **ATFAEDSK** |
|  | 1852 | **477.9703** | **953.9259** | **953.0293** | **0.8966** | **1** | **14** | **93** | **1** | **EEMAKSSR + Oxidation (M)** |
|  | 2028 | **496.4454** | **1486.3140** | **1486.7076** | **-0.3936** | **1** | **14** | **94** | **1** | **EGTVSYVISYLKK** |
|  | 2288 | **530.9600** | **1589.8577** | **1588.8427** | **1.0151** | **1** | **14** | **1.1e+02** | **1** | **VEFVQELPKTITGK** |
|  | 2530 | **570.8712** | **1709.5915** | **1708.7395** | **0.8520** | **2** | **14** | **81** | **1** | **SSSRNLGSSGGEKEEGK** |
|  | 2593 | **581.1904** | **1740.5489** | **1741.0661** | **-0.5171** | **0** | **14** | **1.1e+02** | **1** | **MDCVPPIQCSCSMGGR + Carbamidomethyl (C)** |
|  | 2907 | **646.9052** | **1291.7955** | **1292.5498** | **-0.7543** | **1** | **14** | **83** | **1** | **KAIACLLFGGSR + Carbamidomethyl (C)** |
|  | 516 | **391.1499** | **1170.4275** | **1170.2930** | **0.1345** | **0** | **14** | **97** | **1** | **MSFVGENSGVK + Oxidation (M)** |
|  | 3209 | **713.4509** | **1424.8870** | **1425.5864** | **-0.6994** | **2** | **14** | **1e+02** | **1** | **GSPGLKGDKGIPGDK** |
|  | 2857 | **625.9193** | **1249.8238** | **1249.3779** | **0.4460** | **1** | **14** | **84** | **1** | **VFHQFSNSKR** |
|  | 1365 | **441.0668** | **880.1189** | **879.8261** | **0.2928** | **0** | **14** | **1.1e+02** | **1** | **SDSENSNK** |
|  | 1427 | **445.0274** | **888.0400** | **887.0820** | **0.9580** | **0** | **14** | **1.2e+02** | **1** | **MAAPGPGMR** |
|  | 2450 | **554.3205** | **1659.9393** | **1660.0331** | **-0.0937** | **1** | **14** | **1.1e+02** | **1** | **VVIIQTPINMKTFR** |
|  | 3253 | **734.7172** | **1467.4195** | **1466.6796** | **0.7399** | **0** | **14** | **88** | **1** | **DLTGLSCLLGMDR + Carbamidomethyl (C); Oxidation (M)** |
|  | 579 | **397.1942** | **1188.5606** | **1189.4054** | **-0.8449** | **0** | **14** | **1e+02** | **1** | **TALMHACLEK + Carbamidomethyl (C); Oxidation (M)** |
|  | 1428 | **445.0291** | **888.0434** | **886.9959** | **1.0476** | **2** | **14** | **1.2e+02** | **1** | **LRGTRVAD** |
|  | 2179 | 518.3987 | 1034.7826 | 1034.1664 | 0.6162 | 1 | 14 | 87 | 1 | SLSTKWANK |
|  | 2304 | **533.3469** | **1064.6791** | **1064.1526** | **0.5265** | **0** | **14** | **93** | **1** | **NPLQPGPGER** |
|  | 45 | **363.1719** | **1086.4935** | **1086.3255** | **0.1680** | **0** | **14** | **90** | **1** | **FIACLMSTK + Carbamidomethyl (C); Oxidation (M)** |
|  | 621 | **400.2155** | **1197.6243** | **1198.4270** | **-0.8028** | **2** | **14** | **77** | **1** | **CCRGACRCR + 3 Carbamidomethyl (C)** |
|  | 1005 | **417.5774** | **833.1399** | **833.0112** | **0.1287** | **1** | **14** | **1.1e+02** | **1** | **NSCLIKR** |
|  | 2606 | **583.7713** | **1748.2917** | **1748.0771** | **0.2147** | **1** | **14** | **1e+02** | **1** | **CVGVMLVGPTGGGKTTVR + Oxidation (M)** |
|  | 316 | **381.2812** | **1140.8216** | **1140.2487** | **0.5729** | **0** | **14** | **1e+02** | **1** | **GGMNGGSSSVCK + Carbamidomethyl (C)** |
|  | 3473 | **805.9082** | **2414.7024** | **2415.6984** | **-0.9960** | **0** | **14** | **1.2e+02** | **1** | **CAFQFPGSPPGGGGTYLCLATEK + 2 Carbamidomethyl (C)** |
|  | 659 | **402.0957** | **802.1766** | **802.9158** | **-0.7393** | **0** | **14** | **1.2e+02** | **1** | **SVITIDR** |
|  | 2671 | **592.7540** | **1183.4933** | **1183.3181** | **0.1751** | **1** | **14** | **1.1e+02** | **1** | **ALRGSAPEQVR** |
|  | 3138 | **687.4644** | **2059.3709** | **2059.3275** | **0.0433** | **0** | **14** | **1e+02** | **1** | **ICELSAMTCADGPCFNGGR + 2 Carbamidomethyl (C)** |
|  | 284 | **379.0961** | **1134.2662** | **1134.2593** | **0.0069** | **0** | **14** | **1.1e+02** | **1** | **MTMETQMSQ + 3 Oxidation (M)** |
|  | 1270 | **435.3176** | **1302.9307** | **1302.4338** | **0.4969** | **0** | **14** | **78** | **1** | **QQTLQLSDTLR** |
|  | 1737 | **468.1738** | **1401.4991** | **1401.6526** | **-0.1535** | **1** | **14** | **1.1e+02** | **1** | **EPSRFSLLLAIR** |
|  | 374 | **386.0756** | **1155.2046** | **1154.2769** | **0.9277** | **0** | **14** | **98** | **1** | **MDGPGFGGMNR + Oxidation (M)** |
|  | 1339 | **438.4437** | **874.8726** | **874.8989** | **-0.0263** | **0** | **14** | **1.3e+02** | **1** | **GAAGAAGASSR** |
|  | 1978 | **490.3397** | **1467.9968** | **1468.6279** | **-0.6311** | **0** | **14** | **86** | **1** | **MDVWGLGTTVTVSS + Oxidation (M)** |
|  | 1879 | **480.1918** | **1437.5531** | **1436.7415** | **0.8116** | **2** | **14** | **1.1e+02** | **1** | **LREVVLGPGKQLK** |
|  | 2145 | **512.6364** | **1023.2579** | **1023.1404** | **0.1175** | **0** | **14** | **1.2e+02** | **1** | **GAGVQLYSTK** |
|  | 57 | **364.0200** | **1089.0379** | **1088.1726** | **0.8653** | **0** | **14** | **95** | **1** | **SATSVDQRPK** |
|  | 564 | **395.4114** | **1183.2120** | **1182.4361** | **0.7760** | **2** | **14** | **1.4e+02** | **1** | **SKLTFSCLRK** |
|  | 2367 | **539.8049** | **1077.5950** | **1078.1824** | **-0.5875** | **1** | **14** | **89** | **1** | **LKGGAHPGEGR** |
|  | 2877 | **631.6367** | **1891.8878** | **1891.0518** | **0.8360** | **2** | **14** | **1.1e+02** | **1** | **RAAAEAGDPKEAGAGCMGR + Carbamidomethyl (C); Oxidation (M)** |
|  | 337 | **384.8493** | **767.6838** | **766.8885** | **0.7953** | **0** | **14** | **92** | **1** | **HSAAILR** |
|  | 1268 | **435.2048** | **1302.5921** | **1303.4734** | **-0.8812** | **2** | **14** | **98** | **1** | **HAKPGARGPEKR** |
|  | 1465 | **447.9710** | **893.9273** | **892.9191** | **1.0082** | **0** | **14** | **1e+02** | **1** | **AHGAGGGGPGR** |
|  | 769 | **406.0884** | **1215.2430** | **1214.2874** | **0.9556** | **1** | **14** | **1e+02** | **1** | **RGSSFLAGEHPG** |
|  | 883 | **408.1996** | **1221.5765** | **1220.4395** | **1.1370** | **1** | **14** | **1e+02** | **1** | **QMESMKYCK + Carbamidomethyl (C); Oxidation (M)** |
|  | 1234 | **433.1702** | **1296.4885** | **1297.5050** | **-1.0165** | **1** | **14** | **1.1e+02** | **1** | **AHLFGNLEKLR** |
|  | 2508 | **565.7512** | **1129.4877** | **1130.1726** | **-0.6850** | **1** | **14** | **1.1e+02** | **1** | **AGTGARGAGAEGR** |
|  | 313 | **381.1436** | **1140.4086** | **1140.2885** | **0.1201** | **1** | **14** | **1.2e+02** | **1** | **LFRFDVESK** |
|  | 334 | **384.2757** | **1149.8049** | **1149.3680** | **0.4370** | **0** | **14** | **81** | **1** | **CVCNPAGCLR + 2 Carbamidomethyl (C)** |
|  | 798 | **406.8043** | **811.5939** | **811.9061** | **-0.3122** | **0** | **14** | **1e+02** | **1** | **GPAPCDRP** |
|  | 1444 | **446.0662** | **890.1176** | **888.9652** | **1.1524** | **0** | **14** | **1.2e+02** | **1** | **GLSGELGTR** |
|  | 2230 | **520.2655** | **1557.7743** | **1558.7998** | **-1.0255** | **0** | **14** | **1e+02** | **1** | **GAAPSGPAILEGLFCR** |
|  | 3105 | **685.8319** | **2054.4736** | **2055.3790** | **-0.9054** | **2** | **14** | **1.3e+02** | **1** | **VAEEMKCTLGSKVGYQVR + Carbamidomethyl (C)** |
|  | 3141 | **688.3597** | **1374.7047** | **1375.4813** | **-0.7766** | **0** | **14** | **1.1e+02** | **1** | **TLTSDVANLANEK** |
|  | 357 | **385.1655** | **1152.4742** | **1153.5258** | **-1.0515** | **2** | **14** | **88** | **1** | **LAIMMMKKR + 2 Oxidation (M)** |
|  | 799 | **406.8378** | **1217.4911** | **1218.4664** | **-0.9753** | **0** | **14** | **1e+02** | **1** | **TNMSLGLILTR** |
|  | 1319 | **437.3639** | **872.7131** | **873.9126** | **-1.1995** | **0** | **14** | **1e+02** | **1** | **HEASGFAR** |
|  | 1993 | **492.1835** | **1473.5284** | **1474.7133** | **-1.1849** | **2** | **14** | **1e+02** | **1** | **CSRTHLHKFSCR** |
|  | 2652 | **590.5183** | **1179.0218** | **1178.4077** | **0.6142** | **1** | **14** | **87** | **1** | **MHIVRQPPGK + Oxidation (M)** |
|  | 189 | **371.1733** | **1110.4978** | **1110.1781** | **0.3198** | **1** | **14** | **75** | **1** | **TYNADSVKGR** |
|  | 837 | **407.5676** | **1219.6806** | **1219.2990** | **0.3816** | **1** | **14** | **98** | **1** | **EELESLRSEK** |
|  | 1220 | **432.4408** | **1294.3001** | **1294.5229** | **-0.2227** | **2** | **14** | **1.3e+02** | **1** | **KKVGLTGFECR + Carbamidomethyl (C)** |
|  | 1688 | **463.4136** | **1387.2185** | **1387.6443** | **-0.4258** | **1** | **14** | **82** | **1** | **CGDILGVTSKLPK + Carbamidomethyl (C)** |
|  | 343 | **385.0001** | **1151.9781** | **1151.2980** | **0.6801** | **2** | **14** | **93** | **1** | **MRKAASAASSR + Oxidation (M)** |
|  | 1277 | **435.7998** | **869.5848** | **870.0482** | **-0.4633** | **0** | **14** | **1e+02** | **1** | **NYMMPAK + Oxidation (M)** |
|  | 1523 | **452.4182** | **1354.2325** | **1354.4456** | **-0.2131** | **0** | **14** | **1.1e+02** | **1** | **GISQEQMNEFR + Oxidation (M)** |
|  | 1914 | **485.2102** | **1452.6084** | **1453.5999** | **-0.9915** | **0** | **14** | **1e+02** | **1** | **QSTMFNPCPSER + Carbamidomethyl (C)** |
|  | 1969 | **490.0280** | **978.0413** | **977.1333** | **0.9080** | **0** | **14** | **1.1e+02** | **1** | **AELEALGMK + Oxidation (M)** |
|  | 140 | **369.2556** | **1104.7447** | **1104.2614** | **0.4833** | **0** | **14** | **94** | **1** | **AVAMMHQER + 2 Oxidation (M)** |
|  | 839 | 407.5888 | 813.1628 | 813.9449 | -0.7821 | 1 | 14 | 93 | 1 | RLGGGLGGK |
|  | 996 | **416.5249** | **831.0351** | **829.9660** | **1.0691** | **0** | **14** | **1.5e+02** | **1** | **QWMPPR + Oxidation (M)** |
|  | 3287 | **740.6647** | **1479.3146** | **1478.7800** | **0.5346** | **2** | **14** | **85** | **1** | **GPKFKMPDMHFK + Oxidation (M)** |
|  | 376 | **386.0882** | **1155.2425** | **1156.4038** | **-1.1613** | **2** | **14** | **1e+02** | **1** | **RLGCKSSMCR + Oxidation (M)** |
|  | 340 | **384.9598** | **767.9048** | **768.9245** | **-1.0197** | **1** | **14** | **94** | **1** | **FMAKTR + Oxidation (M)** |
|  | 863 | **407.9229** | **1220.7466** | **1221.4277** | **-0.6811** | **2** | **14** | **1.1e+02** | **1** | **KSTKTAMEVAR** |
|  | 1515 | **451.6295** | **901.2441** | **900.0544** | **1.1898** | **0** | **14** | **1.2e+02** | **1** | **EPTMVAPR** |
|  | 2624 | **586.2872** | **1170.5596** | **1171.3671** | **-0.8075** | **0** | **14** | **1.1e+02** | **1** | **MLVNELTHSK** |
|  | 2677 | **592.9238** | **1183.8329** | **1184.3873** | **-0.5544** | **1** | **14** | **95** | **1** | **HNVLEKGFLK** |
|  | 2768 | **608.1412** | **1821.4015** | **1821.0464** | **0.3552** | **0** | **14** | **1.1e+02** | **1** | **MPSAQHVHGPSANICSK + Carbamidomethyl (C)** |
|  | 3267 | **739.4103** | **2215.2088** | **2215.4438** | **-0.2350** | **2** | **14** | **1.1e+02** | **1** | **CTNSEKKYGPPQTCEQCK + 3 Carbamidomethyl (C)** |
|  | 1453 | **447.0491** | **1338.1250** | **1337.4615** | **0.6636** | **2** | **14** | **1.2e+02** | **1** | **KMRDDGQGWTK + Oxidation (M)** |
|  | 1949 | **488.2086** | **1461.6037** | **1460.5892** | **1.0145** | **1** | **14** | **1.2e+02** | **1** | **WYYDSAVSVKSR** |
|  | 2945 | 662.0758 | 1983.2052 | 1984.3023 | -1.0971 | 1 | 14 | 1.1e+02 | 1 | NAGLTCGGYKVIHCSGYLK |
|  | 2978 | 666.7511 | 1997.2311 | 1998.3756 | -1.1444 | 1 | 14 | 1.3e+02 | 1 | MSSAQCPALVCVMSRLR + 2 Carbamidomethyl (C); 2 Oxidation (M) |
|  | 235 | **374.2986** | **746.5824** | **747.7512** | **-1.1688** | **0** | **14** | **1.1e+02** | **1** | **LSGSGPSSG** |
|  | 298 | **380.0929** | **1137.2566** | **1136.2121** | **1.0446** | **0** | **14** | **1.2e+02** | **1** | **LHEEDAPPTK** |
|  | 1938 | **487.2182** | **972.4216** | **972.1399** | **0.2817** | **1** | **14** | **1.2e+02** | **1** | **IGLSDARLK** |
|  | 2647 | **590.1398** | **1178.2648** | **1178.3595** | **-0.0948** | **0** | **14** | **1.1e+02** | **1** | **SQSTLSCALLR** |
|  | 3040 | 673.1771 | 1344.3395 | 1343.5523 | 0.7872 | 2 | 14 | 1.1e+02 | 1 | EAKLEMVHKSR + Oxidation (M) |
|  | 461 | **388.8246** | **1163.4517** | **1163.3068** | **0.1448** | **1** | **14** | **1.2e+02** | **1** | **GTHKGYCLER** |
|  | 119 | **368.9793** | **1103.9158** | **1103.2234** | **0.6923** | **0** | **14** | **1.2e+02** | **1** | **SELIELGQSK** |
|  | 559 | **394.5236** | **1180.5486** | **1181.3451** | **-0.7965** | **1** | **14** | **1.3e+02** | **1** | **IGAGPGLKGQQR** |
|  | 701 | **404.0695** | **1209.1864** | **1208.1967** | **0.9897** | **0** | **14** | **1.1e+02** | **1** | **ANDHGYDNFR** |
|  | 3055 | **680.4286** | **2038.2638** | **2037.0670** | **1.1967** | **2** | **14** | **1.1e+02** | **1** | **YDQHGNSRQGEPRDYSK** |
|  | 152 | **369.3301** | **1104.9682** | **1104.2991** | **0.6690** | **0** | **14** | **1.1e+02** | **1** | **QQLLIGAYAK** |
|  | 1322 | **437.5211** | **1309.5410** | **1310.5022** | **-0.9612** | **2** | **14** | **1.5e+02** | **1** | **KQLSNSAKGLHK** |
|  | 3146 | **689.3269** | **2064.9585** | **2065.3355** | **-0.3770** | **2** | **14** | **1.1e+02** | **1** | **AMVCGRQLSGAGSETLKQR + Carbamidomethyl (C); Oxidation (M)** |
|  | 550 | **393.4830** | **1177.4269** | **1178.3395** | **-0.9126** | **1** | **14** | **1.3e+02** | **1** | **KNGQCCIADL + 2 Carbamidomethyl (C)** |
|  | 2130 | 508.4141 | 1522.2200 | 1521.6373 | 0.5827 | 0 | 14 | 93 | 1 | AAAAAAGERPNAQPAR |
|  | 3135 | **686.9498** | **2057.8273** | **2058.3149** | **-0.4876** | **2** | **14** | **87** | **1** | **SKAKSCENDLEMGMLNSK + Carbamidomethyl (C); Oxidation (M)** |
|  | 3179 | **698.9004** | **1395.7860** | **1394.6203** | **1.1657** | **0** | **14** | **1e+02** | **1** | **AVVNAAIPAYHLR** |
|  | 939 | **413.1387** | **1236.3938** | **1236.3278** | **0.0660** | **0** | **14** | **1e+02** | **1** | **DAYKPITDADK** |
|  | 961 | **414.9543** | **827.8939** | **827.0484** | **0.8455** | **1** | **14** | **1.1e+02** | **1** | **MMDKMR + Oxidation (M)** |
|  | 1047 | **419.6386** | **1255.8936** | **1256.3259** | **-0.4323** | **1** | **14** | **84** | **1** | **APQTGPGTSRER** |
|  | 1381 | **442.8940** | **1325.6597** | **1325.4506** | **0.2090** | **1** | **14** | **1e+02** | **1** | **RAYSLQQDNCK** |
|  | 2457 | **556.0567** | **1110.0986** | **1109.3588** | **0.7398** | **0** | **14** | **1.1e+02** | **1** | **TLLPIPTVAGK** |
|  | 2832 | **616.7000** | **1231.3852** | **1230.3714** | **1.0139** | **0** | **14** | **1.4e+02** | **1** | **VVCSCTEGYR + 2 Carbamidomethyl (C)** |
|  | 3330 | **742.8250** | **2225.4527** | **2225.3741** | **0.0786** | **0** | **14** | **1.3e+02** | **1** | **DCVSNNSLSSNASLPSVQSCR + Carbamidomethyl (C)** |
|  | 1182 | **430.8323** | **1289.4748** | **1288.5347** | **0.9401** | **0** | **14** | **1.3e+02** | **1** | **VFIGNLNTAIVK** |
|  | 1525 | **452.4645** | **902.9142** | **902.9886** | **-0.0745** | **0** | **14** | **1.4e+02** | **1** | **LVTQDAEK** |
|  | 1815 | **476.0517** | **1425.1329** | **1425.5666** | **-0.4337** | **1** | **14** | **1.2e+02** | **1** | **ERMEPTYQLSR + Oxidation (M)** |
|  | 2076 | **503.3262** | **1506.9564** | **1506.7868** | **0.1697** | **2** | **14** | **1.1e+02** | **1** | **KLEMCSEIKGPQK + Oxidation (M)** |
|  | 3306 | **741.5837** | **1481.1527** | **1480.7924** | **0.3602** | **1** | **14** | **92** | **1** | **RLLQTMDMIISK + 2 Oxidation (M)** |
|  | 395 | **386.4825** | **1156.4253** | **1157.2545** | **-0.8292** | **1** | **14** | **1.3e+02** | **1** | **EKYDSAAAMR + Oxidation (M)** |
|  | 1187 | **431.0159** | **1290.0255** | **1289.4351** | **0.5904** | **1** | **14** | **1.4e+02** | **1** | **LASASLLDTDKR** |
|  | 1645 | **460.6949** | **1379.0627** | **1379.5909** | **-0.5282** | **1** | **14** | **95** | **1** | **RCPAFHEMCR + 2 Carbamidomethyl (C); Oxidation (M)** |
|  | 2896 | **640.7159** | **1919.1255** | **1920.1113** | **-0.9858** | **0** | **14** | **1.4e+02** | **1** | **TARPFGIQAPGGTSQMER + Oxidation (M)** |
|  | 3360 | **750.4884** | **1498.9620** | **1498.7479** | **0.2141** | **0** | **14** | **1.1e+02** | **1** | **ALWPAQMSAAPAIR + Oxidation (M)** |
|  | 96 | **366.3942** | **1096.1605** | **1095.0956** | **1.0649** | **0** | **14** | **1.5e+02** | **1** | **MEGEQGEDGK + Oxidation (M)** |
|  | 244 | **375.1021** | **1122.2841** | **1122.2285** | **0.0556** | **1** | **14** | **1.2e+02** | **1** | **FADSNLKAEK** |
|  | 263 | **377.1436** | **1128.4086** | **1128.3058** | **0.1028** | **0** | **14** | **1e+02** | **1** | **FWPPHSVCR** |
|  | 577 | **396.9910** | **1187.9508** | **1188.2451** | **-0.2943** | **0** | **14** | **1.2e+02** | **1** | **SSQGTPALGGGEK** |
|  | 737 | **405.1284** | **1212.3631** | **1211.2173** | **1.1457** | **0** | **14** | **1e+02** | **1** | **SQSCSDTAQER** |
|  | 1169 | **429.5598** | **857.1048** | **856.0249** | **1.0800** | **1** | **14** | **1.3e+02** | **1** | **TKKPGGLR** |
|  | 2105 | **506.1059** | **1515.2955** | **1514.6564** | **0.6391** | **0** | **14** | **1e+02** | **1** | **ESSQEAAITIYMR + Oxidation (M)** |
|  | 2137 | **510.9240** | **1019.8333** | **1019.1402** | **0.6931** | **2** | **14** | **1.2e+02** | **1** | **GMGRRSGNGK** |
|  | 2511 | **566.1791** | **1695.5151** | **1694.9496** | **0.5654** | **2** | **14** | **1.2e+02** | **1** | **AVRCYESLIAKAEGK + Carbamidomethyl (C)** |
|  | 2772 | 609.3624 | 1825.0651 | 1824.0452 | 1.0199 | 0 | 14 | 1.2e+02 | 1 | QLGAASVGPGPLGFPATQR |
|  | 2868 | **628.4053** | **1882.1936** | **1882.1988** | **-0.0052** | **1** | **14** | **1.1e+02** | **1** | **CRSAAHVCLCLGHLER + 2 Carbamidomethyl (C)** |
|  | 2923 | **655.4205** | **1308.8263** | **1309.4661** | **-0.6399** | **0** | **14** | **1.1e+02** | **1** | **YDVYLINPQGK** |
|  | 1115 | **425.1975** | **1272.5704** | **1271.5755** | **0.9949** | **1** | **14** | **1.2e+02** | **1** | **TLMLKGLRPSR** |
|  | 1610 | **459.2640** | **916.5133** | **916.0752** | **0.4381** | **0** | **14** | **1.1e+02** | **1** | **FVVLADPR** |
|  | 1643 | **460.6588** | **919.3029** | **919.0576** | **0.2454** | **0** | **14** | **1e+02** | **1** | **LMAGASPTR + Oxidation (M)** |
|  | 1855 | **478.0220** | **1431.0439** | **1430.5251** | **0.5189** | **1** | **14** | **1e+02** | **1** | **GPGAGPGAAVDYRSR** |
|  | 2617 | **584.5251** | **1750.5531** | **1750.0958** | **0.4572** | **1** | **14** | **89** | **1** | **HRQIVKPGFNISILK** |
|  | 3026 | **669.8691** | **2006.5853** | **2006.0829** | **0.5023** | **0** | **14** | **1.1e+02** | **1** | **FSYISSSSSYTNYADSVK** |
|  | 3334 | **743.3190** | **1484.6232** | **1483.6872** | **0.9360** | **1** | **14** | **1.1e+02** | **1** | **HAYVKDDTMFLK + Oxidation (M)** |
|  | 802 | **406.8603** | **1217.5586** | **1216.4538** | **1.1048** | **1** | **14** | **1e+02** | **1** | **LQCLARGSAGIK** |
|  | 1300 | **436.3886** | **1306.1435** | **1306.5582** | **-0.4147** | **1** | **14** | **92** | **1** | **QWHLGCFKCK + Carbamidomethyl (C)** |
|  | 1304 | **437.0316** | **1308.0727** | **1308.5510** | **-0.4783** | **0** | **14** | **1.2e+02** | **1** | **WMGPIPPAVNAR** |
|  | 1519 | **451.9161** | **1352.7261** | **1351.5309** | **1.1951** | **0** | **14** | **1.3e+02** | **1** | **QAASQCFAGLSLR** |
|  | 279 | **378.1927** | **1131.5558** | **1132.2944** | **-0.7387** | **1** | **14** | **82** | **1** | **VGGAKINTNCR** |
|  | 416 | 387.1507 | 1158.4298 | 1158.3697 | 0.0601 | 0 | 14 | 1.3e+02 | 1 | LHLDYIGPCK |
|  | 927 | **412.1677** | **1233.4809** | **1233.2394** | **0.2415** | **0** | **14** | **1.2e+02** | **1** | **ELSDLEEENR** |
|  | 1151 | **428.4257** | **1282.2548** | **1281.5820** | **0.6728** | **0** | **14** | **1e+02** | **1** | **TSQMMVLLLTL + 2 Oxidation (M)** |
|  | 1630 | **460.2043** | **1377.5909** | **1376.4066** | **1.1843** | **0** | **14** | **1.2e+02** | **1** | **STNHEPSEMSNK + Oxidation (M)** |
|  | 2556 | **575.3792** | **1723.1155** | **1721.9931** | **1.1224** | **0** | **14** | **1.1e+02** | **1** | **GMWASLDALWEMPAK + Oxidation (M)** |
|  | 3274 | **740.3554** | **2218.0441** | **2218.4314** | **-0.3873** | **2** | **14** | **1.1e+02** | **1** | **GHESEDSMSTLAGRRGMRPK + Oxidation (M)** |
|  | 14 | **360.8882** | **1079.6426** | **1080.1672** | **-0.5246** | **0** | **14** | **1.2e+02** | **1** | **EDVAVASMDK + Oxidation (M)** |
|  | 1208 | **431.7443** | **861.4739** | **861.0612** | **0.4126** | **0** | **14** | **1e+02** | **1** | **LAAVVSACK** |
|  | 3200 | **707.2177** | **1412.4206** | **1413.5328** | **-1.1121** | **1** | **14** | **1.1e+02** | **1** | **RPKLPQTSDDEK** |
|  | 1221 | **432.4838** | **1294.4293** | **1295.3304** | **-0.9011** | **0** | **14** | **1.4e+02** | **1** | **SCNEQIEESEK** |
|  | 1516 | **451.6575** | **1351.9504** | **1352.6682** | **-0.7177** | **2** | **14** | **1.1e+02** | **1** | **MWLSMGKKLSR + Oxidation (M)** |
|  | 1930 | **486.8990** | **1457.6750** | **1456.5376** | **1.1374** | **0** | **14** | **1.2e+02** | **1** | **EPAGACGGPGSSVDPR** |
|  | 1739 | **468.2167** | **1401.6279** | **1401.5916** | **0.0364** | **1** | **14** | **1.2e+02** | **1** | **MEARSGVGPGGVIR + Oxidation (M)** |
|  | 752 | **405.2255** | **1212.6542** | **1212.3328** | **0.3214** | **0** | **14** | **88** | **1** | **AGHCAPSEAIEK** |
|  | 1415 | **443.9999** | **885.9850** | **886.0474** | **-0.0625** | **0** | **14** | **1.2e+02** | **1** | **LSEIVGLR** |
|  | 1897 | **483.2151** | **1446.6231** | **1446.7100** | **-0.0869** | **0** | **14** | **1.2e+02** | **1** | **SIPLAMAPVFEQK + Oxidation (M)** |
|  | 2399 | **547.5032** | **1639.4875** | **1639.8510** | **-0.3635** | **1** | **14** | **95** | **1** | **QIALGTSKLNNLDPR** |
|  | 2431 | **551.9053** | **1101.7958** | **1101.2540** | **0.5418** | **1** | **14** | **1.1e+02** | **1** | **YVSWYQKK** |
|  | 2958 | **664.5370** | **1990.5888** | **1991.3546** | **-0.7658** | **1** | **14** | **90** | **1** | **IIYNVLHGSTMKGITSLK + Oxidation (M)** |
|  | 54 | **363.2425** | **724.4701** | **724.8521** | **-0.3819** | **1** | **14** | **92** | **1** | **HSRVVK** |
|  | 267 | **377.2088** | **1128.6041** | **1129.0952** | **-0.4910** | **0** | **14** | **86** | **1** | **GSGSSQSSGYGR** |
|  | 415 | **387.1325** | **1158.3753** | **1158.4794** | **-0.1040** | **1** | **14** | **1.3e+02** | **1** | **CVMKMVCANK + 2 Oxidation (M)** |
|  | 992 | **416.3289** | **830.6431** | **830.9259** | **-0.2828** | **1** | **14** | **1.1e+02** | **1** | **GLEAADKK** |
|  | 1212 | **432.1683** | **1293.4828** | **1294.4566** | **-0.9738** | **2** | **14** | **1.2e+02** | **1** | **TLKAGYTEKQR** |
|  | 2720 | **597.4000** | **1192.7853** | **1192.4740** | **0.3113** | **2** | **14** | **1.2e+02** | **1** | **FMKNKIGQVK** |
|  | 2764 | **607.8702** | **1213.7257** | **1213.2548** | **0.4709** | **1** | **14** | **93** | **1** | **ADSPPKEEQGR** |
|  | 103 | **367.3263** | **1098.9567** | **1099.2614** | **-0.3046** | **0** | **14** | **1.1e+02** | **1** | **HSPTNTIVCK** |
|  | 804 | **406.8892** | **1217.6455** | **1218.2759** | **-0.6304** | **0** | **14** | **1e+02** | **1** | **WSLGSGAEQQR** |
|  | 1529 | **453.0562** | **904.0976** | **902.9026** | **1.1951** | **0** | **14** | **1.2e+02** | **1** | **SEEANEPK** |
|  | 2516 | **567.5152** | **1133.0156** | **1132.3308** | **0.6848** | **0** | **14** | **96** | **1** | **ISQEIGNLMK** |
|  | 2858 | **626.3210** | **1250.6273** | **1249.4624** | **1.1650** | **0** | **14** | **1.1e+02** | **1** | **ACQFPAPMAAAR + Oxidation (M)** |
|  | 2920 | **653.7572** | **1305.4996** | **1304.5557** | **0.9439** | **1** | **14** | **1.4e+02** | **1** | **LDSICQTVLKGK** |
|  | 132 | **369.2281** | **1104.6623** | **1104.2991** | **0.3632** | **0** | **14** | **99** | **1** | **QQLLIGAYAK** |
|  | 1611 | **459.2674** | **916.5200** | **916.1431** | **0.3770** | **2** | **14** | **1.1e+02** | **1** | **KLKACTPR** |
|  | 1661 | **461.8949** | **921.7750** | **921.0618** | **0.7133** | **2** | **14** | **1.1e+02** | **1** | **RSPHLRR** |
|  | 1971 | **490.1140** | **1467.3199** | **1467.6494** | **-0.3296** | **2** | **14** | **1.2e+02** | **1** | **DLSKGGCKNGYLR + Carbamidomethyl (C)** |
|  | 2655 | **591.5071** | **1771.4991** | **1771.0274** | **0.4717** | **2** | **14** | **91** | **1** | **EFLRFSFSRVINAGK** |
|  | 2987 | **667.2531** | **1998.7370** | **1999.3555** | **-0.6185** | **2** | **14** | **1.2e+02** | **1** | **MKSCAVSLTTAAVAFGDKAK** |
|  | 1835 | **476.8690** | **951.7233** | **951.0134** | **0.7100** | **1** | **14** | **1.1e+02** | **1** | **CEEETKR + Carbamidomethyl (C)** |
|  | 1910 | **484.4266** | **1450.2576** | **1450.6257** | **-0.3682** | **2** | **14** | **91** | **1** | **QRVRCVNEGYAR** |
|  | 3010 | **668.7596** | **1335.5045** | **1336.6273** | **-1.1228** | **2** | **14** | **1.4e+02** | **1** | **AKGLAPKLHVFR** |
|  | 256 | **376.2808** | **1125.8202** | **1125.3183** | **0.5019** | **0** | **14** | **88** | **1** | **SLPLLEGQIR** |
|  | 409 | **387.0396** | **772.0644** | **770.9202** | **1.1442** | **2** | **14** | **1.3e+02** | **1** | **KLAGLRN** |
|  | 1683 | **463.0637** | **1386.1688** | **1385.5688** | **0.6000** | **1** | **14** | **1.1e+02** | **1** | **QAKVAQNSGLTLR** |
|  | 354 | **385.1263** | **1152.3569** | **1153.3353** | **-0.9784** | **1** | **14** | **1e+02** | **1** | **RSGMYVHCK + Carbamidomethyl (C); Oxidation (M)** |
|  | 1008 | **417.9992** | **833.9836** | **835.0206** | **-1.0370** | **0** | **14** | **1.2e+02** | **1** | **LMDLLSK + Oxidation (M)** |
|  | 1691 | **463.8229** | **1388.4466** | **1387.7322** | **0.7145** | **1** | **14** | **1.1e+02** | **1** | **LCPFKLVSQKPK** |
|  | 2571 | **578.7579** | **1155.5011** | **1155.2583** | **0.2427** | **0** | **14** | **1.1e+02** | **1** | **ESPPEILSGAR** |
|  | 1090 | **422.1896** | **842.3645** | **841.9949** | **0.3695** | **0** | **14** | **1.2e+02** | **1** | **GITLSVRP** |
|  | 1276 | **435.7428** | **1304.2063** | **1303.6605** | **0.5458** | **2** | **14** | **93** | **1** | **AMKILMKVGHC + Carbamidomethyl (C); Oxidation (M)** |
|  | 1487 | **450.0219** | **1347.0435** | **1346.5542** | **0.4892** | **0** | **14** | **1.1e+02** | **1** | **AAPIPQGFSCLSR** |
|  | 2104 | 506.0691 | 1515.1851 | 1515.6280 | -0.4429 | 0 | 14 | 1.1e+02 | 1 | MTRPDCSSDFNSR |
|  | 71 | **364.3088** | **1089.9043** | **1090.2283** | **-0.3239** | **2** | **14** | **87** | **1** | **AKEAVTKSEK** |
|  | 870 | **408.0800** | **1221.2177** | **1220.3965** | **0.8212** | **1** | **14** | **1.2e+02** | **1** | **SEQMVKEITR** |
|  | 1072 | **422.0114** | **1263.0119** | **1263.4658** | **-0.4539** | **0** | **14** | **1.2e+02** | **1** | **SPLHPMSEIPR** |
|  | 1386 | **443.3533** | **884.6918** | **885.0463** | **-0.3544** | **0** | **14** | **89** | **1** | **CSRPPAVR** |
|  | 2030 | **496.4818** | **1486.4233** | **1486.7804** | **-0.3571** | **1** | **14** | **1.2e+02** | **1** | **FLVGPDGIPIMRR + Oxidation (M)** |
|  | 3193 | **703.8158** | **2108.4252** | **2109.4029** | **-0.9777** | **1** | **14** | **1.4e+02** | **1** | **VDGLPQQIESVSDKCLLHK** |
|  | 1596 | **458.8214** | **915.6280** | **915.0292** | **0.5988** | **1** | **14** | **1.2e+02** | **1** | **MAEGKAHR + Oxidation (M)** |
|  | 2983 | **666.7869** | **1997.3386** | **1997.4011** | **-0.0625** | **2** | **14** | **1.4e+02** | **1** | **MKKMEMEMEQVFEMK + 3 Oxidation (M)** |
|  | 40 | 363.1039 | 1086.2896 | 1086.2295 | 0.0601 | 2 | 14 | 1.2e+02 | 1 | MERGPGARGR |
|  | 1655 | **461.6319** | **921.2490** | **921.0272** | **0.2219** | **0** | **14** | **1.1e+02** | **1** | **SATTTVMNP** |
|  | 3374 | **756.4213** | **1510.8279** | **1511.6293** | **-0.8015** | **2** | **14** | **1.1e+02** | **1** | **EKEKILSSDDYGK** |
|  | 747 | **405.1901** | **1212.5481** | **1213.3705** | **-0.8224** | **0** | **14** | **1e+02** | **1** | **GGGRPAAGMAAAAR** |
|  | 2492 | **564.4538** | **1690.3392** | **1689.8901** | **0.4492** | **1** | **14** | **1e+02** | **1** | **FQLLEGPPESMGRGR + Oxidation (M)** |
|  | 1323 | **437.5255** | **873.0363** | **873.0089** | **0.0274** | **0** | **14** | **1.6e+02** | **1** | **LLVQTGSR** |
|  | 1710 | **465.0788** | **928.1429** | **928.0477** | **0.0951** | **1** | **14** | **1.2e+02** | **1** | **LQEGRVAR** |
|  | 1968 | **490.0119** | **1467.0135** | **1466.5787** | **0.4349** | **1** | **14** | **1.2e+02** | **1** | **MNPNRGNTGYAQK + Oxidation (M)** |
|  | 7 | **360.3910** | **1078.1507** | **1078.3248** | **-0.1741** | **0** | **14** | **1.6e+02** | **1** | **CLIFEGLVK + Carbamidomethyl (C)** |
|  | 2588 | **580.7054** | **1159.3960** | **1160.3842** | **-0.9882** | **1** | **14** | **1.4e+02** | **1** | **ELMKAQSLPK + Oxidation (M)** |
|  | 3252 | **734.4826** | **2200.4256** | **2199.5288** | **0.8969** | **1** | **14** | **1.2e+02** | **1** | **MWATGSLPDFPAAAKFLGFR + Oxidation (M)** |
|  | 335 | **384.6000** | **767.1852** | **766.9283** | **0.2568** | **0** | **14** | **83** | **1** | **ALPTLPR** |
|  | 1590 | **458.6066** | **1372.7976** | **1371.6913** | **1.1063** | **1** | **14** | **1.3e+02** | **1** | **VQGLVISQMLRK** |
|  | 3188 | **702.5924** | **2104.7550** | **2105.1280** | **-0.3730** | **2** | **14** | **93** | **1** | **KEREEDENEELEEGLEK** |
|  | 144 | **369.2677** | **1104.7810** | **1105.3520** | **-0.5709** | **2** | **14** | **1e+02** | **1** | **KIMSKIQNK + Oxidation (M)** |
|  | 147 | **369.2780** | **1104.8119** | **1105.3089** | **-0.4970** | **0** | **14** | **1e+02** | **1** | **AKPWAVCFPS** |
|  | 205 | **372.3362** | **742.6576** | **741.9188** | **0.7387** | **0** | **14** | **1.1e+02** | **1** | **ALVAQLK** |
|  | 319 | 382.2053 | 1143.5938 | 1142.4101 | 1.1837 | 0 | 14 | 1.2e+02 | 1 | AFYLGSMLIK |
|  | 685 | **403.4297** | **804.8447** | **804.8490** | **-0.0042** | **0** | **14** | **1.5e+02** | **1** | **SQVSSAAR** |
|  | 1400 | **443.7897** | **885.5646** | **886.0904** | **-0.5259** | **1** | **14** | **1.2e+02** | **1** | **QSLLGKLK** |
|  | 1460 | **447.1895** | **1338.5465** | **1339.5172** | **-0.9707** | **1** | **14** | **1.2e+02** | **1** | **NSMFTAGKGVAEK** |
|  | 3263 | **737.9638** | **1473.9128** | **1473.7603** | **0.1525** | **2** | **14** | **1e+02** | **1** | **MKHMAIPKSSSPK + 2 Oxidation (M)** |
|  | 1773 | **471.1095** | **1410.3062** | **1410.6579** | **-0.3516** | **0** | **13** | **1.1e+02** | **1** | **GTHLSLFFVVYK** |
|  | 17 | **361.3398** | **1080.9972** | **1080.2365** | **0.7607** | **0** | **13** | **1.2e+02** | **1** | **VQPAFISYR** |
|  | 94 | **366.2700** | **730.5253** | **730.8168** | **-0.2915** | **2** | **13** | **1.1e+02** | **1** | **GGARRSK** |
|  | 611 | **400.0136** | **798.0125** | **799.0134** | **-1.0009** | **1** | **13** | **1e+02** | **1** | **KIGVVVGK** |
|  | 974 | **415.4197** | **828.8247** | **828.9596** | **-0.1349** | **1** | **13** | **1.5e+02** | **1** | **LGQSIRR** |
|  | 1719 | **466.1278** | **930.2409** | **929.0721** | **1.1687** | **1** | **13** | **1.3e+02** | **1** | **AIKDGANIK** |
|  | 2254 | **523.8676** | **1568.5807** | **1569.7136** | **-1.1329** | **0** | **13** | **1.2e+02** | **1** | **EMGQVEISSCTVDR + Oxidation (M)** |
|  | 191 | **371.2599** | **1110.7575** | **1110.1781** | **0.5794** | **1** | **13** | **77** | **1** | **TYNADSVKGR** |
|  | 1029 | **419.1901** | **1254.5480** | **1253.4760** | **1.0720** | **1** | **13** | **1.4e+02** | **1** | **MRGCPMAGGSVR + 2 Oxidation (M)** |
|  | 1582 | **458.2944** | **1371.8609** | **1372.6532** | **-0.7922** | **2** | **13** | **1e+02** | **1** | **KTVQMMFKEAK + 2 Oxidation (M)** |
|  | 1816 | **476.1022** | **950.1897** | **950.0285** | **0.1612** | **0** | **13** | **1.3e+02** | **1** | **CPSSLSNSR** |
|  | 2091 | 505.2357 | 1008.4565 | 1008.1507 | 0.3059 | 1 | 13 | 1.2e+02 | 1 | ISICSSDKR |
|  | 2562 | **577.0128** | **1152.0107** | **1151.2946** | **0.7162** | **1** | **13** | **1.2e+02** | **1** | **ISCKASGGSVSR** |
|  | 2592 | **581.1444** | **1160.2740** | **1161.2595** | **-0.9855** | **0** | **13** | **1.3e+02** | **1** | **AITDIIEGDSK** |
|  | 2851 | **624.1595** | **1246.3043** | **1245.3228** | **0.9815** | **0** | **13** | **1.2e+02** | **1** | **CALGGHGSSNTGK + Carbamidomethyl (C)** |
|  | 351 | **385.0899** | **1152.2474** | **1152.3672** | **-0.1197** | **1** | **13** | **1.1e+02** | **1** | **LERTMTVFR** |
|  | 739 | **405.1349** | **808.2550** | **807.9141** | **0.3409** | **0** | **13** | **1.1e+02** | **1** | **AMWGEAK + Oxidation (M)** |
|  | 1404 | **443.8680** | **1328.5818** | **1327.4832** | **1.0985** | **1** | **13** | **1.2e+02** | **1** | **SLSGVHEEKTLK** |
|  | 1422 | **444.8625** | **1331.5655** | **1331.4353** | **0.1302** | **0** | **13** | **1.4e+02** | **1** | **SCSSLGCGSNGFR + Carbamidomethyl (C)** |
|  | 1925 | **486.4142** | **970.8136** | **971.1121** | **-0.2985** | **0** | **13** | **95** | **1** | **LQQAGSVLR** |
|  | 746 | **405.1886** | **808.3624** | **807.9556** | **0.4068** | **2** | **13** | **1.1e+02** | **1** | **SKGLKME + Oxidation (M)** |
|  | 1842 | **477.5891** | **1429.7451** | **1430.5682** | **-0.8231** | **0** | **13** | **1.3e+02** | **1** | **TPPPARPAAPGEGGR** |
|  | 138 | **369.2448** | **1104.7123** | **1105.3089** | **-0.5966** | **0** | **13** | **1e+02** | **1** | **AKPWAVCFPS** |
|  | 1026 | **419.1725** | **1254.4954** | **1254.4111** | **0.0843** | **1** | **13** | **1.4e+02** | **1** | **SRITMSIDTSK + Oxidation (M)** |
|  | 1163 | 429.0829 | 1284.2267 | 1284.5281 | -0.3014 | 2 | 13 | 1.1e+02 | 1 | KVQKVPCSPNK + Carbamidomethyl (C) |
|  | 1228 | **433.0376** | **1296.0905** | **1295.3586** | **0.7320** | **1** | **13** | **1.2e+02** | **1** | **NETRSGQQFTK** |
|  | 1367 | **441.1736** | **1320.4988** | **1320.4641** | **0.0347** | **2** | **13** | **1.2e+02** | **1** | **AGPPGGRRPRSGR** |
|  | 3342 | **744.3643** | **1486.7137** | **1486.6958** | **0.0179** | **0** | **13** | **1.2e+02** | **1** | **CVCTGGAIQCGDFR + Carbamidomethyl (C)** |
|  | 353 | **385.1152** | **1152.3234** | **1151.3576** | **0.9659** | **1** | **13** | **1.1e+02** | **1** | **EKELCQMVR + Oxidation (M)** |
|  | 848 | **407.7943** | **1220.3607** | **1219.3222** | **1.0385** | **0** | **13** | **1.2e+02** | **1** | **VDIDQGADMQK** |
|  | 882 | **408.1983** | **1221.5728** | **1222.4355** | **-0.8626** | **2** | **13** | **1.2e+02** | **1** | **TCPDLMRDKK + Oxidation (M)** |
|  | 1087 | **422.1798** | **1263.5172** | **1264.4720** | **-0.9548** | **1** | **13** | **1.3e+02** | **1** | **NFKEPLLTFR** |
|  | 1533 | 454.0694 | 1359.1860 | 1358.6761 | 0.5099 | 2 | 13 | 1.3e+02 | 1 | MCPECKRCFK + 2 Carbamidomethyl (C) |
|  | 904 | **409.0701** | **1224.1882** | **1223.4382** | **0.7500** | **0** | **13** | **1.4e+02** | **1** | **ESPPLLPLGTPC** |
|  | 1039 | 419.3567 | 1255.0481 | 1254.4342 | 0.6139 | 0 | 13 | 1.2e+02 | 1 | TYSKPIFGSVR |
|  | 1207 | **431.6998** | **861.3848** | **862.0494** | **-0.6645** | **1** | **13** | **1.1e+02** | **1** | **IVEAMKR + Oxidation (M)** |
|  | 1302 | **436.7838** | **871.5527** | **870.9932** | **0.5596** | **0** | **13** | **1.2e+02** | **1** | **KPLPSSSR** |
|  | 145 | **369.2679** | **1104.7815** | **1105.3089** | **-0.5274** | **0** | **13** | **1.1e+02** | **1** | **AKPWAVCFPS** |
|  | 341 | **384.9987** | **1151.9740** | **1151.3358** | **0.6381** | **0** | **13** | **1e+02** | **1** | **SIFAMALQDR** |
|  | 857 | **407.8681** | **813.7215** | **813.8591** | **-0.1376** | **1** | **13** | **1.2e+02** | **1** | **GEHTSRK** |
|  | 1237 | **433.2339** | **864.4530** | **864.0038** | **0.4491** | **2** | **13** | **1.1e+02** | **1** | **RYLREK** |
|  | 2178 | **518.2736** | **1551.7987** | **1551.7275** | **0.0712** | **0** | **13** | **1.3e+02** | **1** | **LCCLGGGCGGGGGGGQK + 3 Carbamidomethyl (C)** |
|  | 2980 | 666.7712 | 1997.2915 | 1998.3290 | -1.0375 | 2 | 13 | 1.5e+02 | 1 | FVVLADPRQAGIDSLLRK |
|  | 1387 | **443.3655** | **884.7162** | **885.0429** | **-0.3267** | **0** | **13** | **93** | **1** | **MQPSLPGR** |
|  | 3119 | **686.1105** | **2055.3092** | **2056.3190** | **-1.0097** | **1** | **13** | **1.2e+02** | **1** | **QEDAHEFLMFTVDAMKK + Oxidation (M)** |
|  | 3126 | **686.2745** | **1370.5343** | **1369.5480** | **0.9863** | **0** | **13** | **1.2e+02** | **1** | **CKPSPTWHQTK + Carbamidomethyl (C)** |
|  | 689 | **403.8064** | **1208.3970** | **1207.3562** | **1.0408** | **0** | **13** | **1.3e+02** | **1** | **MEGPLSVFGDR** |
|  | 1859 | **478.1354** | **954.2561** | **954.0421** | **0.2140** | **2** | **13** | **1.1e+02** | **1** | **KHEKQER** |
|  | 2206 | **519.1196** | **1036.2245** | **1035.2208** | **1.0037** | **1** | **13** | **1.2e+02** | **1** | **MRGGFPQVK + Oxidation (M)** |
|  | 522 | **391.2048** | **1170.5922** | **1170.3410** | **0.2513** | **1** | **13** | **94** | **1** | **MPDCTSKCR + 2 Carbamidomethyl (C); Oxidation (M)** |
|  | 3466 | **800.5801** | **2398.7181** | **2399.7176** | **-0.9995** | **1** | **13** | **1.1e+02** | **1** | **LNEVNLSMQGKNVTVFTVFDK + Oxidation (M)** |
|  | 180 | **370.6508** | **1108.9304** | **1109.1568** | **-0.2264** | **1** | **13** | **80** | **1** | **GGRSPAGASGHR** |
|  | 288 | **379.4031** | **1135.1872** | **1135.3731** | **-0.1858** | **0** | **13** | **1.5e+02** | **1** | **DVLVPSVLMF + Oxidation (M)** |
|  | 1152 | **428.4866** | **854.9584** | **854.9110** | **0.0474** | **1** | **13** | **1.3e+02** | **1** | **EPRSPGGR** |
|  | 1496 | **450.2150** | **1347.6229** | **1346.4302** | **1.1927** | **1** | **13** | **1.3e+02** | **1** | **NMTPGQNREQR + Oxidation (M)** |
|  | 224 | **374.1728** | **1119.4962** | **1118.3276** | **1.1686** | **0** | **13** | **1.3e+02** | **1** | **ECMLPGVQGK + Carbamidomethyl (C)** |
|  | 868 | **408.0204** | **814.0260** | **813.0233** | **1.0027** | **1** | **13** | **1.2e+02** | **1** | **HIRIMK + Oxidation (M)** |
|  | 1752 | **469.4806** | **1405.4196** | **1404.5029** | **0.9167** | **1** | **13** | **1.3e+02** | **1** | **TPDCSSGPVKEER** |
|  | 2094 | **505.5625** | **1009.1102** | **1008.2999** | **0.8103** | **2** | **13** | **1.4e+02** | **1** | **MVDMKKLK + Oxidation (M)** |
|  | 1119 | **425.5026** | **848.9905** | **849.9738** | **-0.9834** | **0** | **13** | **1.6e+02** | **1** | **IIFGSGTR** |
|  | 1218 | **432.4175** | **1294.2303** | **1294.4782** | **-0.2479** | **1** | **13** | **1.3e+02** | **1** | **FGNMTRVYYK + Oxidation (M)** |
|  | 1495 | **450.1906** | **1347.5497** | **1347.6038** | **-0.0541** | **0** | **13** | **1.3e+02** | **1** | **APISGAMSMPLTR + Oxidation (M)** |
|  | 1775 | **471.2026** | **940.3904** | **940.1148** | **0.2756** | **0** | **13** | **1.1e+02** | **1** | **MGFTIDIK + Oxidation (M)** |
|  | 2155 | **515.0056** | **1027.9963** | **1027.1953** | **0.8010** | **1** | **13** | **1.2e+02** | **1** | **AYSCALKSGK** |
|  | 2366 | **539.5096** | **1615.5066** | **1615.7603** | **-0.2538** | **0** | **13** | **1.2e+02** | **1** | **DTSASTAYMPLSSLR + Oxidation (M)** |
|  | 187 | **371.1020** | **1110.2839** | **1111.3352** | **-1.0513** | **1** | **13** | **1e+02** | **1** | **VGVTVVGPQKK** |
|  | 1649 | **460.7594** | **919.5040** | **918.9515** | **0.5524** | **1** | **13** | **1.1e+02** | **1** | **SRQAAGTGTA** |
|  | 448 | **388.1619** | **1161.4634** | **1161.3290** | **0.1344** | **0** | **13** | **1.4e+02** | **1** | **ANLAVTLCNDK** |
|  | 1817 | **476.1181** | **950.2214** | **951.1225** | **-0.9010** | **1** | **13** | **1.3e+02** | **1** | **CIKGDQCK + Carbamidomethyl (C)** |
|  | 3201 | **707.6982** | **1413.3817** | **1413.6404** | **-0.2587** | **1** | **13** | **1.1e+02** | **1** | **VKMQTFPDLYR + Oxidation (M)** |
|  | 1554 | **456.1771** | **1365.5090** | **1365.5574** | **-0.0484** | **0** | **13** | **1.2e+02** | **1** | **NFLNLQMNSLR + Oxidation (M)** |
|  | 2042 | **499.1990** | **1494.5749** | **1494.5045** | **0.0705** | **2** | **13** | **1.2e+02** | **1** | **GCSDREDNAESRR** |
|  | 2529 | **570.2986** | **1707.8738** | **1708.8718** | **-0.9980** | **0** | **13** | **1.3e+02** | **1** | **INMNGINNSSGMVDAR + Oxidation (M)** |
|  | 599 | **399.0951** | **1194.2632** | **1195.3685** | **-1.1054** | **0** | **13** | **1.1e+02** | **1** | **IQGVIAQPVDR** |
|  | 706 | **404.1198** | **806.2248** | **806.8895** | **-0.6647** | **0** | **13** | **1.3e+02** | **1** | **GAWACGSR** |
|  | 923 | **411.4347** | **1231.2818** | **1232.3706** | **-1.0887** | **1** | **13** | **1.5e+02** | **1** | **VQGMEGLNRGR + Oxidation (M)** |
|  | 1553 | **455.9308** | **1364.7704** | **1364.4784** | **0.2919** | **0** | **13** | **1.2e+02** | **1** | **CSSLPYAEGFAY + Carbamidomethyl (C)** |
|  | 2711 | **596.9071** | **1191.7994** | **1191.3153** | **0.4841** | **0** | **13** | **1.1e+02** | **1** | **AAGQTAQMLER + Oxidation (M)** |
|  | 106 | **367.5260** | **1099.5557** | **1100.1634** | **-0.6076** | **1** | **13** | **1.3e+02** | **1** | **EGYSEGRMR + Oxidation (M)** |
|  | 910 | **410.3972** | **1228.1693** | **1229.3418** | **-1.1725** | **1** | **13** | **1.4e+02** | **1** | **RYQSTVNAYK** |
|  | 161 | **369.3956** | **1105.1646** | **1105.3089** | **-0.1442** | **0** | **13** | **1.6e+02** | **1** | **AKPWAVCFPS** |
|  | 1591 | **458.6883** | **915.3618** | **915.0126** | **0.3492** | **2** | **13** | **1.1e+02** | **1** | **SGGRVQRR** |
|  | 557 | **394.2303** | **786.4459** | **786.8370** | **-0.3910** | **1** | **13** | **1.1e+02** | **1** | **GPGRASSR** |
|  | 1379 | 442.6682 | 1324.9823 | 1325.4325 | -0.4502 | 1 | 13 | 99 | 1 | AGPPRGDWGVASR |
|  | 2525 | **569.3378** | **1136.6608** | **1136.3031** | **0.3577** | **0** | **13** | **1.3e+02** | **1** | **GSCGSCPVTLR + Carbamidomethyl (C)** |
|  | 1 | **360.3308** | **718.6469** | **718.7133** | **-0.0664** | **0** | **13** | **1.4e+02** | **1** | **GDEATAR** |
|  | 944 | **413.4089** | **1237.2045** | **1237.3457** | **-0.1412** | **1** | **13** | **1.1e+02** | **1** | **RGSEMYPGGAGR** |
|  | 1108 | **423.4712** | **1267.3913** | **1268.4225** | **-1.0312** | **1** | **13** | **1.8e+02** | **1** | **ASLPGGLSPSRAR** |
|  | 666 | **402.3959** | **802.7771** | **802.9193** | **-0.1422** | **2** | **13** | **1.6e+02** | **1** | **REVKGSK** |
|  | 2003 | **493.2880** | **1476.8418** | **1476.5967** | **0.2451** | **1** | **13** | **1.2e+02** | **1** | **ISHRSVHAASVQQG** |
|  | 125 | **369.1616** | **1104.4626** | **1105.3089** | **-0.8463** | **0** | **13** | **1.3e+02** | **1** | **AKPWAVCFPS** |
|  | 1562 | **456.5893** | **1366.7458** | **1366.6069** | **0.1389** | **0** | **13** | **1.2e+02** | **1** | **LHAVVQGLGPTFK** |
|  | 1618 | **459.6259** | **1375.8554** | **1376.6250** | **-0.7697** | **2** | **13** | **1.3e+02** | **1** | **ILRRMITSNEK + Oxidation (M)** |
|  | 1804 | **475.0189** | **948.0229** | **948.1387** | **-0.1157** | **0** | **13** | **1.4e+02** | **1** | **TVTMAGKPK + Oxidation (M)** |
|  | 2100 | **505.8955** | **1514.6644** | **1513.7348** | **0.9296** | **1** | **13** | **1.2e+02** | **1** | **EVERVVVDALSGLK** |
|  | 2776 | **610.0698** | **1827.1873** | **1826.1539** | **1.0334** | **2** | **13** | **1.3e+02** | **1** | **CLKNENCSIMRMNR + 2 Carbamidomethyl (C)** |
|  | 193 | **371.2970** | **740.5792** | **740.7620** | **-0.1828** | **1** | **13** | **83** | **1** | **KHSSADP** |
|  | 1652 | **461.1681** | **1380.4822** | **1381.5372** | **-1.0551** | **1** | **13** | **1.3e+02** | **1** | **SRAAPSPQEALVR** |
|  | 2848 | **623.7159** | **1868.1255** | **1869.0828** | **-0.9573** | **1** | **13** | **1.6e+02** | **1** | **AAVSRLGNMSQIDMSEK + 2 Oxidation (M)** |
|  | 3478 | **810.7531** | **1619.4913** | **1618.7923** | **0.6990** | **1** | **13** | **99** | **1** | **QPAQDTAPTPAPRLR** |
|  | 454 | **388.3027** | **1161.8860** | **1162.3006** | **-0.4146** | **1** | **13** | **1.2e+02** | **1** | **EAAPCECARGR** |
|  | 755 | **405.2549** | **1212.7424** | **1213.3655** | **-0.6231** | **0** | **13** | **96** | **1** | **GGWEPGMGLGPR** |
|  | 155 | **369.3522** | **1105.0345** | **1105.3089** | **-0.2743** | **0** | **13** | **1.4e+02** | **1** | **AKPWAVCFPS** |
|  | 1142 | **428.0822** | **1281.2243** | **1280.3672** | **0.8571** | **0** | **13** | **1.3e+02** | **1** | **HPPSPVSECGDR** |
|  | 2302 | **533.2133** | **1596.6176** | **1597.6392** | **-1.0216** | **1** | **13** | **1.3e+02** | **1** | **NDVSRENSTVDFSK** |
|  | 2788 | **611.1940** | **1830.5599** | **1831.2335** | **-0.6736** | **2** | **13** | **1.4e+02** | **1** | **FPSVYLRCKMVVCR + 2 Carbamidomethyl (C); Oxidation (M)** |
|  | 2990 | **667.3473** | **1999.0197** | **1999.3338** | **-0.3141** | **1** | **13** | **1.3e+02** | **1** | **MLENKVIETLPGFQHVK + Oxidation (M)** |
|  | 1183 | **430.8432** | **859.6717** | **859.9872** | **-0.3155** | **0** | **13** | **1.5e+02** | **1** | **CTESVPPK** |
|  | 2463 | **556.4688** | **1666.3843** | **1666.9644** | **-0.5802** | **0** | **13** | **1.1e+02** | **1** | **RPPCCCACQEVQTTK** |
|  | 2554 | **575.1815** | **1722.5224** | **1722.8735** | **-0.3512** | **0** | **13** | **1.4e+02** | **1** | **YLQGMLEAGEGGAPSSR** |
|  | 243 | **375.0694** | **1122.1859** | **1123.2350** | **-1.0490** | **1** | **13** | **1.5e+02** | **1** | **MADVSAEEKK + Oxidation (M)** |
|  | 268 | **377.2914** | **752.5680** | **752.8621** | **-0.2941** | **1** | **13** | **98** | **1** | **APPQGRK** |
|  | 1689 | **463.5311** | **925.0474** | **925.0372** | **0.0101** | **1** | **13** | **1.4e+02** | **1** | **KLSAESYK** |
|  | 990 | **416.2590** | **1245.7549** | **1245.3412** | **0.4138** | **0** | **13** | **1.3e+02** | **1** | **TWNAGPAVSTNK** |
|  | 1064 | **421.3474** | **840.6800** | **839.8897** | **0.7904** | **0** | **13** | **1.1e+02** | **1** | **DLSSGFSK** |
|  | 3376 | **757.7841** | **2270.3300** | **2270.6730** | **-0.3430** | **0** | **13** | **1.2e+02** | **1** | **QVAAINPNHPLAQMPLPPSMK + Oxidation (M)** |
|  | 3580 | **1124.1201** | **2246.2255** | **2246.5010** | **-0.2755** | **1** | **13** | **88** | **1** | **TLSGCTFGPKASGQTGCMSPSR + Carbamidomethyl (C); Oxidation (M)** |
|  | 130 | **369.2198** | **1104.6373** | **1105.3089** | **-0.6716** | **0** | **13** | **1.1e+02** | **1** | **AKPWAVCFPS** |
|  | 2036 | **498.0321** | **994.0495** | **993.0500** | **0.9995** | **0** | **13** | **1.3e+02** | **1** | **SAPAQSDSCK** |
|  | 2309 | **534.1098** | **1066.2048** | **1066.2281** | **-0.0232** | **0** | **13** | **1.4e+02** | **1** | **SMQLFDNVI** |
|  | 260 | **377.1184** | **752.2220** | **752.9864** | **-0.7643** | **1** | **13** | **1.2e+02** | **1** | **LKMMAK + 2 Oxidation (M)** |
|  | 635 | **401.6007** | **801.1866** | **800.8835** | **0.3032** | **0** | **13** | **1.2e+02** | **1** | **DGVMAHR + Oxidation (M)** |
|  | 1286 | **436.0671** | **1305.1792** | **1304.4778** | **0.7014** | **2** | **13** | **1.2e+02** | **1** | **CKRGLGYHGEK + Carbamidomethyl (C)** |
|  | 2169 | **516.5585** | **1031.1023** | **1030.0900** | **1.0123** | **0** | **13** | **1.6e+02** | **1** | **GNDSSVPINK** |
|  | 2187 | **518.8365** | **1553.4875** | **1553.7171** | **-0.2297** | **1** | **13** | **1.1e+02** | **1** | **KLSDGQVGGFINYR** |
|  | 2543 | **572.6948** | **1143.3749** | **1143.1315** | **0.2434** | **1** | **13** | **1.6e+02** | **1** | **GGGGGRGGGGGGGQR** |
|  | 122 | **369.1393** | **1104.3957** | **1105.3089** | **-0.9132** | **0** | **13** | **1.4e+02** | **1** | **AKPWAVCFPS** |
|  | 1209 | **431.9978** | **1292.9713** | **1292.4440** | **0.5274** | **0** | **13** | **1.4e+02** | **1** | **CQCPAGGAFEGPR** |
|  | 2663 | **592.5751** | **1183.1354** | **1182.3022** | **0.8332** | **2** | **13** | **1.2e+02** | **1** | **MEKSASKEEK + Oxidation (M)** |
|  | 2736 | **599.8286** | **1197.6424** | **1198.3593** | **-0.7168** | **2** | **13** | **1.1e+02** | **1** | **AAHRSGMKNAR** |
|  | 240 | 374.9745 | 747.9342 | 747.9035 | 0.0306 | 0 | 13 | 1.5e+02 | 1 | ALATNMK |
|  | 253 | **376.2054** | **750.3960** | **750.7784** | **-0.3824** | **0** | **13** | **1e+02** | **1** | **CSEAER + Carbamidomethyl (C)** |
|  | 360 | **385.2455** | **1152.7143** | **1152.2775** | **0.4367** | **0** | **13** | **90** | **1** | **DCEYPLISR + Carbamidomethyl (C)** |
|  | 794 | **406.7041** | **811.3934** | **810.8567** | **0.5367** | **0** | **13** | **98** | **1** | **GSCDCNR + Carbamidomethyl (C)** |
|  | 933 | **412.3314** | **1233.9719** | **1233.4813** | **0.4906** | **1** | **13** | **1.1e+02** | **1** | **SMFRMGELCK + 2 Oxidation (M)** |
|  | 2209 | **519.9057** | **1037.7966** | **1038.2858** | **-0.4892** | **2** | **13** | **1.3e+02** | **1** | **IKKFAIYR** |
|  | 2841 | **620.4323** | **1238.8497** | **1239.3747** | **-0.5250** | **0** | **13** | **1.2e+02** | **1** | **IIHLDVDSEAK** |
|  | 3070 | **682.1106** | **1362.2064** | **1362.4661** | **-0.2597** | **0** | **13** | **1.3e+02** | **1** | **TMEDQDLKPNR + Oxidation (M)** |
|  | 1374 | **442.2389** | **1323.6944** | **1323.5010** | **0.1934** | **1** | **13** | **1.1e+02** | **1** | **ARTLLGAESHLR** |
|  | 1466 | **448.0389** | **1341.0945** | **1341.3823** | **-0.2878** | **0** | **13** | **1.3e+02** | **1** | **DFVSYPSAGNER** |
|  | 406 | **386.9847** | **1157.9320** | **1157.3008** | **0.6312** | **1** | **13** | **1.5e+02** | **1** | **YTSMTGRLGR + Oxidation (M)** |
|  | 1459 | **447.1843** | **892.3537** | **892.5161** | **-0.1623** | **0** | **13** | **1.4e+02** | **1** | **HPVPXXXX** |
|  | 1807 | **475.2137** | **1422.6191** | **1422.6306** | **-0.0115** | **2** | **13** | **1.4e+02** | **1** | **EGKLGCKDCSAVR + Carbamidomethyl (C)** |
|  | 778 | **406.1771** | **1215.5092** | **1215.3996** | **0.1096** | **1** | **13** | **1.2e+02** | **1** | **LYECKQCSK + 2 Carbamidomethyl (C)** |
|  | 1641 | **460.5655** | **1378.6744** | **1378.5748** | **0.0996** | **1** | **13** | **1.7e+02** | **1** | **DSPDLEIMMRR + Oxidation (M)** |
|  | 2382 | **542.5276** | **1624.5606** | **1623.7691** | **0.7915** | **0** | **13** | **1.2e+02** | **1** | **THTGTKPFGCSDCR + 2 Carbamidomethyl (C)** |
|  | 568 | **396.0849** | **1185.2325** | **1186.2493** | **-1.0167** | **1** | **13** | **1.5e+02** | **1** | **DDYCEAGTKK + Carbamidomethyl (C)** |
|  | 1244 | **433.4848** | **1297.4322** | **1297.5750** | **-0.1427** | **2** | **13** | **1.6e+02** | **1** | **TMSMGRMRGVR + Oxidation (M)** |
|  | 1267 | **435.1972** | **1302.5694** | **1302.4507** | **0.1188** | **0** | **13** | **1.2e+02** | **1** | **AAAMTPPEEELK + Oxidation (M)** |
|  | 2577 | **579.5280** | **1735.5619** | **1735.0828** | **0.4790** | **0** | **13** | **1.1e+02** | **1** | **LAPLHQLLQPMAGCAR + Oxidation (M)** |
|  | 2781 | **610.1671** | **1218.3194** | **1219.4727** | **-1.1533** | **0** | **13** | **1.4e+02** | **1** | **ELLPVLISAHK** |
|  | 330 | **384.0992** | **1149.2756** | **1148.2722** | **1.0033** | **1** | **13** | **1.3e+02** | **1** | **LRGGAYIGEGR** |
|  | 688 | **403.7973** | **1208.3698** | **1208.3442** | **0.0256** | **0** | **13** | **1.4e+02** | **1** | **VACGYTHTSAAK** |
|  | 956 | **414.3592** | **826.7036** | **826.0815** | **0.6221** | **1** | **13** | **97** | **1** | **ALKLIIR** |
|  | 1757 | **469.9885** | **1406.9433** | **1406.6661** | **0.2773** | **1** | **13** | **1.2e+02** | **1** | **IPVADTPEVPIKK** |
|  | 2062 | **502.0503** | **1503.1288** | **1502.6922** | **0.4366** | **1** | **13** | **1.4e+02** | **1** | **MGREEQIQTSVPK** |
|  | 2188 | **518.8601** | **1035.7054** | **1035.3036** | **0.4019** | **2** | **13** | **1.2e+02** | **1** | **LCGVYPKKK** |
|  | 2387 | 544.1498 | 1629.4272 | 1628.7807 | 0.6465 | 2 | 13 | 1.5e+02 | 1 | LSKSKDFYPGTTER |
|  | 3196 | **705.6981** | **2114.0722** | **2113.3369** | **0.7353** | **2** | **13** | **1.2e+02** | **1** | **ARLPVTTWRISAGSGGQAER** |
|  | 890 | **408.3505** | **1222.0294** | **1222.3576** | **-0.3282** | **2** | **13** | **1.2e+02** | **1** | **DPVRRGPVGGGR** |
|  | 2089 | **504.9984** | **1511.9729** | **1512.7052** | **-0.7323** | **0** | **13** | **1.3e+02** | **1** | **APEAQVSVQPLFVQ** |
|  | 3170 | **696.7574** | **2087.2502** | **2087.3989** | **-0.1488** | **1** | **13** | **1.5e+02** | **1** | **QKANDVLLSHMITFLNDK** |
|  | 533 | **391.6991** | **1172.0751** | **1173.2636** | **-1.1885** | **1** | **13** | **1.1e+02** | **1** | **GTHTCTRQGR + Carbamidomethyl (C)** |
|  | 586 | **398.1999** | **1191.5776** | **1190.5192** | **1.0584** | **0** | **13** | **1.2e+02** | **1** | **LLATVCLGGCLK** |
|  | 1767 | **470.4159** | **1408.2255** | **1407.5976** | **0.6279** | **0** | **13** | **1e+02** | **1** | **MHPAGLAAAAAGTPR + Oxidation (M)** |
|  | 569 | **396.1597** | **1185.4570** | **1184.3458** | **1.1112** | **1** | **13** | **1.5e+02** | **1** | **AAIQELQQKR** |
|  | 1309 | **437.1227** | **872.2307** | **873.0056** | **-0.7749** | **0** | **13** | **1.5e+02** | **1** | **IIEVSGQK** |
|  | 1200 | **431.1795** | **1290.5163** | **1290.3800** | **0.1363** | **0** | **13** | **1.6e+02** | **1** | **EYQWLHTGEK** |
|  | 1741 | **468.3669** | **934.7190** | **935.1198** | **-0.4008** | **0** | **13** | **1.2e+02** | **1** | **NPLSACSIM** |
|  | 2531 | **570.9451** | **1139.8754** | **1140.3331** | **-0.4577** | **0** | **13** | **1.2e+02** | **1** | **SQPGLCNMYK** |
|  | 321 | **382.2903** | **1143.8489** | **1143.2077** | **0.6411** | **0** | **13** | **1.3e+02** | **1** | **IEAADLNGANR** |
|  | 608 | **399.4362** | **796.8575** | **797.9852** | **-1.1277** | **0** | **13** | **1.4e+02** | **1** | **GLNLLIR** |
|  | 922 | **411.4339** | **1231.2795** | **1231.3857** | **-0.1062** | **0** | **13** | **1.7e+02** | **1** | **HCAGPGECACAR + Carbamidomethyl (C)** |
|  | 943 | **413.2973** | **1236.8697** | **1237.3160** | **-0.4462** | **0** | **13** | **92** | **1** | **DDAAPAPPVADAK** |
|  | 1830 | **476.3886** | **1426.1437** | **1425.6161** | **0.5275** | **0** | **13** | **1.1e+02** | **1** | **QYCGAACTCHVR + 2 Carbamidomethyl (C)** |
|  | 2644 | **589.8395** | **1766.4963** | **1765.9001** | **0.5962** | **1** | **13** | **1.2e+02** | **1** | **AMSPENQEYFEKHR** |
|  | 286 | **379.2540** | **1134.7399** | **1135.2091** | **-0.4692** | **1** | **13** | **1.1e+02** | **1** | **GRDPQCTSSGK** |
|  | 617 | **400.1276** | **798.2403** | **797.9226** | **0.3178** | **0** | **13** | **1.2e+02** | **1** | **MPGGPSPR** |
|  | 622 | **400.2894** | **1197.8461** | **1198.2862** | **-0.4401** | **0** | **13** | **95** | **1** | **SCCSYISHQN + Carbamidomethyl (C)** |
|  | 678 | 403.2116 | 1206.6126 | 1205.4449 | 1.1677 | 1 | 13 | 1.3e+02 | 1 | MTVPKEMPEK + Oxidation (M) |
|  | 725 | **405.0097** | **808.0046** | **806.9542** | **1.0504** | **1** | **13** | **1.2e+02** | **1** | **CGSPKCR + Carbamidomethyl (C)** |
|  | 48 | **363.2040** | **724.3933** | **723.8591** | **0.5342** | **0** | **13** | **1.1e+02** | **1** | **TPMCEK + Oxidation (M)** |
|  | 637 | **401.6717** | **1201.9928** | **1201.3582** | **0.6347** | **1** | **13** | **1.3e+02** | **1** | **SPPTGACWRAR** |
|  | 898 | **408.9104** | **1223.7090** | **1222.5232** | **1.1859** | **0** | **13** | **1.5e+02** | **1** | **SCCSCCPMSCAK** |
|  | 2360 | **538.2668** | **1611.7784** | **1610.7521** | **1.0263** | **1** | **13** | **1.4e+02** | **1** | **SRQGGSVCGCDPCER + Carbamidomethyl (C)** |
|  | 1176 | **430.2499** | **858.4850** | **858.0176** | **0.4675** | **0** | **13** | **1.3e+02** | **1** | **MLPSASPR** |
|  | 2305 | **533.7609** | **1065.5069** | **1065.2648** | **0.2421** | **0** | **13** | **1.1e+02** | **1** | **AGQTGVCLIM + Carbamidomethyl (C); Oxidation (M)** |
|  | 3410 | **765.0150** | **2292.0227** | **2291.7799** | **0.2428** | **2** | **13** | **1.1e+02** | **1** | **MAAFHGAGLKRYLLTVMAAAAK** |
|  | 379 | **386.1154** | **1155.3242** | **1154.2538** | **1.0704** | **1** | **13** | **1.2e+02** | **1** | **EFGSGCAEAKR** |
|  | 872 | **408.0952** | **1221.2635** | **1222.4321** | **-1.1686** | **1** | **13** | **1.5e+02** | **1** | **EKDALCLDMK + Carbamidomethyl (C)** |
|  | 895 | **408.8712** | **1223.5915** | **1224.2160** | **-0.6246** | **1** | **13** | **1.5e+02** | **1** | **AGSRASEGQDSC + Carbamidomethyl (C)** |
|  | 1786 | **472.3770** | **1414.1089** | **1413.6883** | **0.4205** | **2** | **13** | **1.1e+02** | **1** | **TAHKAARLGITMK + Oxidation (M)** |
|  | 2608 | **583.8397** | **1165.6647** | **1166.4563** | **-0.7916** | **0** | **13** | **1.1e+02** | **1** | **LLALAAAALLAR** |
|  | 1617 | **459.5964** | **917.1780** | **916.0536** | **1.1243** | **0** | **13** | **1.6e+02** | **1** | **ELCEAPVR** |
|  | 2293 | **532.0604** | **1062.1061** | **1062.2014** | **-0.0953** | **1** | **13** | **1.4e+02** | **1** | **GQNMTKDIR** |
|  | 2915 | **652.6823** | **1955.0248** | **1956.0524** | **-1.0277** | **2** | **13** | **1.5e+02** | **1** | **CAKDTDSEEEIREAFR + Carbamidomethyl (C)** |
|  | 3122 | **686.1757** | **2055.5048** | **2056.1713** | **-0.6665** | **1** | **13** | **1.3e+02** | **1** | **GQDGAGKSNPTHLGSALDMEA** |
|  | 3218 | **718.3363** | **2151.9867** | **2152.5400** | **-0.5533** | **0** | **13** | **1.3e+02** | **1** | **MGLWQQISNCMPDLALCR + Carbamidomethyl (C); Oxidation (M)** |
|  | 3489 | **817.1821** | **2448.5240** | **2447.7039** | **0.8201** | **0** | **13** | **1.2e+02** | **1** | **MCPHPGYSPHLASYTLTSGNTR + Carbamidomethyl (C)** |
|  | 3503 | **828.4727** | **2482.3958** | **2481.7733** | **0.6225** | **1** | **13** | **1.3e+02** | **1** | **FTGHCTCRPGVSGVRCDQCAR + 4 Carbamidomethyl (C)** |
|  | 3530 | **849.7772** | **2546.3093** | **2545.6038** | **0.7055** | **0** | **13** | **1e+02** | **1** | **SCVTDCEEEAGTESQQGTESSHCK** |
|  | 3537 | **859.8687** | **2576.5838** | **2575.9788** | **0.6050** | **0** | **13** | **1.2e+02** | **1** | **CELQICGLAMADAGEYLCVCGQER** |
|  | 3559 | **939.4414** | **2815.3020** | **2816.1418** | **-0.8398** | **1** | **13** | **1.2e+02** | **1** | **CGECHHTCGTCVGPGREECIHCAK + 5 Carbamidomethyl (C)** |
|  | 3560 | **944.4043** | **2830.1907** | **2831.3124** | **-1.1216** | **1** | **13** | **1.2e+02** | **1** | **AKLMHLLLMSFSVCLMTSGDPSSSAR + 3 Oxidation (M)** |
|  | 380 | **386.1187** | **1155.3340** | **1154.3368** | **0.9972** | **1** | **13** | **1.2e+02** | **1** | **RTVMVEEFK + Oxidation (M)** |
|  | 1113 | **424.1314** | **1269.3720** | **1268.4424** | **0.9295** | **0** | **13** | **1.5e+02** | **1** | **TSCMYGANCYR** |
|  | 1396 | **443.6340** | **1327.8799** | **1328.6632** | **-0.7833** | **1** | **13** | **1.2e+02** | **1** | **LPLLMDTLRLK + Oxidation (M)** |
|  | 1839 | **477.4536** | **952.8924** | **953.1035** | **-0.2112** | **2** | **13** | **1.2e+02** | **1** | **RIPGAGARR** |
|  | 2437 | **552.4171** | **1654.2292** | **1653.8132** | **0.4160** | **0** | **13** | **1.2e+02** | **1** | **FQLSIHEMDDPHGK** |
|  | 2573 | **578.8835** | **1733.6285** | **1732.8951** | **0.7334** | **2** | **13** | **1.1e+02** | **1** | **RGGELRTEEPPPPAAR** |
|  | 935 | **412.4139** | **1234.2196** | **1233.3555** | **0.8642** | **0** | **13** | **1.5e+02** | **1** | **MRPGGETVASGR + Oxidation (M)** |
|  | 1699 | **464.0322** | **926.0495** | **925.0821** | **0.9675** | **0** | **13** | **1.3e+02** | **1** | **TVSGMDGMK** |
|  | 2595 | **581.2826** | **1160.5504** | **1160.3310** | **0.2194** | **1** | **13** | **1.4e+02** | **1** | **ACLHCRGTSR + Carbamidomethyl (C)** |
|  | 2780 | **610.1447** | **1218.2746** | **1217.4819** | **0.7928** | **2** | **13** | **1.4e+02** | **1** | **KLLGPSSMTRK** |
|  | 3411 | **765.2000** | **2292.5779** | **2292.5410** | **0.0368** | **1** | **13** | **1.3e+02** | **1** | **IGLLDRVTNDTESDINYLLK** |
|  | 3438 | **784.2081** | **2349.6022** | **2349.6868** | **-0.0845** | **0** | **13** | **1.3e+02** | **1** | **ATVLQQLGWMHHNMDLVPDK + Oxidation (M)** |
|  | 346 | **385.0201** | **1152.0383** | **1151.3822** | **0.6560** | **1** | **13** | **1.2e+02** | **1** | **YRQLLGMVR + Oxidation (M)** |
|  | 257 | 376.2853 | 1125.8337 | 1126.2038 | -0.3702 | 0 | 13 | 1.1e+02 | 1 | AGQATEGHCPR |
|  | 378 | **386.1114** | **1155.3121** | **1156.3755** | **-1.0635** | **0** | **13** | **1.3e+02** | **1** | **MQQAGMALYK + Oxidation (M)** |
|  | 811 | **407.0472** | **1218.1195** | **1217.3510** | **0.7686** | **0** | **13** | **1.2e+02** | **1** | **VPNMAEWDQK** |
|  | 836 | **407.4804** | **1219.4191** | **1220.3715** | **-0.9524** | **0** | **13** | **1.6e+02** | **1** | **FDYMDCELK + Carbamidomethyl (C)** |
|  | 987 | **416.1647** | **830.3146** | **830.9029** | **-0.5882** | **1** | **13** | **1.6e+02** | **1** | **DTKSYSM** |
|  | 1877 | **480.1412** | **958.2677** | **958.1334** | **0.1343** | **1** | **13** | **1.4e+02** | **1** | **TKGLPDCPK** |
|  | 1829 | **476.3525** | **1426.0354** | **1425.6710** | **0.3645** | **1** | **13** | **1.1e+02** | **1** | **VKVLGSGAFGTVYK** |
|  | 2610 | **584.0033** | **1748.9877** | **1750.0796** | **-1.0918** | **2** | **13** | **1.4e+02** | **1** | **MSAVCGGAARMLRTPGR + Oxidation (M)** |
|  | 3371 | **755.1593** | **2262.4557** | **2262.5432** | **-0.0875** | **1** | **13** | **1.3e+02** | **1** | **ANQVVIHEDFIQSCFDRLK** |
|  | 383 | **386.1381** | **1155.3921** | **1154.4241** | **0.9680** | **0** | **13** | **1.2e+02** | **1** | **CSLILFFVR + Carbamidomethyl (C)** |
|  | 1697 | 464.0195 | 1389.0364 | 1389.5743 | -0.5378 | 1 | 13 | 1.3e+02 | 1 | KEVMLQNGETPK + Oxidation (M) |
|  | 972 | **415.2811** | **828.5475** | **827.9650** | **0.5824** | **0** | **13** | **1.2e+02** | **1** | **EPLGATIK** |
|  | 1213 | **432.1704** | **862.3260** | **862.9249** | **-0.5989** | **1** | **13** | **1.5e+02** | **1** | **DSEVGTKK** |
|  | 1236 | **433.2231** | **1296.6473** | **1297.4870** | **-0.8397** | **1** | **13** | **1.3e+02** | **1** | **IPQGQRVSCPR + Carbamidomethyl (C)** |
|  | 1393 | **443.6064** | **885.1980** | **884.9801** | **0.2179** | **1** | **13** | **1.3e+02** | **1** | **RATDVPAR** |
|  | 1575 | **458.0489** | **914.0830** | **912.9901** | **1.0929** | **0** | **13** | **1.4e+02** | **1** | **AGPATNVQR** |
|  | 2085 | **504.4250** | **1510.2527** | **1509.7724** | **0.4803** | **1** | **13** | **1.2e+02** | **1** | **SFTVCSRFFFIR** |
|  | 1486 | **449.7485** | **1346.2232** | **1346.6157** | **-0.3924** | **1** | **13** | **1.1e+02** | **1** | **KMDPAPSLGCSLK** |
|  | 1891 | **481.5392** | **961.0636** | **961.1339** | **-0.0704** | **0** | **13** | **1.7e+02** | **1** | **AELEALGMK** |
|  | 3449 | **789.0897** | **2364.2470** | **2364.7392** | **-0.4922** | **0** | **13** | **1e+02** | **1** | **YGNLGHVNIGAIQEPLAFILPK** |
|  | 2961 | **665.1108** | **1992.3103** | **1993.2729** | **-0.9625** | **1** | **13** | **1.4e+02** | **1** | **GEPGMHGAPGPMGPKGPPGHK** |
|  | 3004 | **668.6736** | **2002.9988** | **2003.2214** | **-0.2226** | **0** | **13** | **1.4e+02** | **1** | **QPPGGQGFPASAAPAQVPAVR** |
|  | 1551 | **455.4650** | **1363.3727** | **1362.5703** | **0.8025** | **0** | **13** | **1.5e+02** | **1** | **LSSFIGAGSPSLVK** |
|  | 1653 | **461.5063** | **920.9979** | **920.1087** | **0.8892** | **0** | **13** | **1.6e+02** | **1** | **MTPPATMR + Oxidation (M)** |
|  | 2415 | **549.5082** | **1097.0016** | **1096.2012** | **0.8004** | **2** | **13** | **1.1e+02** | **1** | **ADRTGPAPRR** |
|  | 845 | **407.7418** | **1220.2031** | **1220.3317** | **-0.1286** | **2** | **13** | **1.3e+02** | **1** | **EAELGARAKEF** |
|  | 2133 | **509.4429** | **1016.8710** | **1016.2341** | **0.6369** | **0** | **13** | **1.3e+02** | **1** | **MAPFFEMK + Oxidation (M)** |
|  | 2172 | **517.5509** | **1549.6305** | **1548.6957** | **0.9348** | **1** | **13** | **1.7e+02** | **1** | **EIQKLGADYGLDAR** |
|  | 2737 | **600.0079** | **1797.0016** | **1798.0509** | **-1.0493** | **1** | **13** | **1.5e+02** | **1** | **SWAPCSKACGGGIQFTK + Carbamidomethyl (C)** |
|  | 3009 | **668.7312** | **2003.1714** | **2004.2988** | **-1.1273** | **2** | **13** | **1.7e+02** | **1** | **RGLFAPTGCRWSCPAPER** |
|  | 807 | **406.9295** | **1217.7662** | **1218.4002** | **-0.6340** | **1** | **13** | **1.3e+02** | **1** | **LGGSKISSLSAAK** |
|  | 1679 | **462.9808** | **923.9468** | **922.9783** | **0.9685** | **0** | **13** | **1.3e+02** | **1** | **DADAAYAVK** |
|  | 2793 | **612.1003** | **1833.2787** | **1833.9969** | **-0.7183** | **0** | **13** | **1.4e+02** | **1** | **ASGYSFNAYYIHWVR** |
|  | 640 | **401.8566** | **801.6984** | **800.8636** | **0.8348** | **1** | **13** | **1.6e+02** | **1** | **AGRVGGER** |
|  | 1241 | **433.3073** | **864.5999** | **864.9456** | **-0.3457** | **1** | **13** | **1.1e+02** | **1** | **FRAGEASK** |
|  | 1271 | **435.3252** | **1302.9534** | **1302.5049** | **0.4485** | **0** | **13** | **1e+02** | **1** | **LCEGPIHHLQR** |
|  | 2271 | **528.5390** | **1055.0632** | **1055.2301** | **-0.1669** | **0** | **13** | **1.5e+02** | **1** | **ATLQLAWPR** |
|  | 3085 | **684.0959** | **1366.1771** | **1365.5793** | **0.5979** | **2** | **13** | **1.4e+02** | **1** | **EGKLGCKDCSAVR** |
|  | 674 | **403.0099** | **1206.0074** | **1206.4426** | **-0.4352** | **2** | **13** | **1.6e+02** | **1** | **VLRCKTNCR + 2 Carbamidomethyl (C)** |
|  | 1232 | **433.1069** | **1296.2985** | **1297.4837** | **-1.1851** | **1** | **13** | **1.4e+02** | **1** | **MLTSRNGSLFR + Oxidation (M)** |
|  | 1636 | **460.3106** | **1377.9097** | **1378.5584** | **-0.6487** | **2** | **13** | **1.2e+02** | **1** | **MDVVARGTTTRR + Oxidation (M)** |
|  | 1892 | **482.0556** | **1443.1445** | **1442.5110** | **0.6335** | **1** | **13** | **1.5e+02** | **1** | **SDDTAVYACARDR** |
|  | 1948 | **488.1711** | **1461.4913** | **1462.6313** | **-1.1401** | **1** | **13** | **1.6e+02** | **1** | **AQNISSNANMLRK + Oxidation (M)** |
|  | 3455 | **791.1643** | **1580.3138** | **1580.9543** | **-0.6405** | **1** | **13** | **1.3e+02** | **1** | **CELLCLAKIWFK + 2 Carbamidomethyl (C)** |
|  | 42 | **363.1429** | **1086.4066** | **1086.3287** | **0.0778** | **2** | **13** | **1.4e+02** | **1** | **GRLALKTLSK** |
|  | 1571 | **457.9550** | **1370.8429** | **1370.5542** | **0.2886** | **2** | **13** | **1.4e+02** | **1** | **KTLDTGAIPGKNR** |
|  | 3211 | **714.7891** | **1427.5635** | **1427.6453** | **-0.0819** | **0** | **13** | **1.7e+02** | **1** | **CDTYQLIMEVR + Carbamidomethyl (C)** |
|  | 3468 | **802.6895** | **2405.0462** | **2405.4866** | **-0.4405** | **0** | **13** | **1.1e+02** | **1** | **DEGGFGAGGGGFDYWGQGTLVTVSA** |
|  | 307 | **380.2837** | **1137.8290** | **1138.2708** | **-0.4418** | **0** | **13** | **1.3e+02** | **1** | **TLGSNATYIAK** |
|  | 843 | **407.6958** | **1220.0652** | **1220.3433** | **-0.2780** | **1** | **13** | **1.1e+02** | **1** | **RHFNAPSHVR** |
|  | 314 | **381.1516** | **1140.4327** | **1141.3824** | **-0.9497** | **0** | **13** | **1.6e+02** | **1** | **SFILDFMLR** |
|  | 2315 | 534.5234 | 1600.5481 | 1599.7673 | 0.7808 | 1 | 13 | 1.5e+02 | 1 | GHNAMYWYKQSAK + Oxidation (M) |
|  | 1836 | **477.0828** | **952.1508** | **951.1059** | **1.0450** | **0** | **13** | **1.4e+02** | **1** | **CPCGCGQAR + Carbamidomethyl (C)** |
|  | 936 | **412.4423** | **1234.3046** | **1235.4226** | **-1.1180** | **2** | **13** | **1.7e+02** | **1** | **RMGPPVGGHRR + Oxidation (M)** |
|  | 1724 | **466.2415** | **1395.7022** | **1396.5981** | **-0.8960** | **2** | **13** | **1.5e+02** | **1** | **ILTRNEKNRPR** |
|  | 2064 | **502.1716** | **1002.3284** | **1001.1399** | **1.1885** | **1** | **13** | **1.6e+02** | **1** | **FPRLPESR** |
|  | 2498 | **564.9071** | **1127.7994** | **1128.3887** | **-0.5892** | **1** | **13** | **1.4e+02** | **1** | **TAWMNCMKK + Oxidation (M)** |
|  | 754 | **405.2420** | **1212.7039** | **1212.3360** | **0.3679** | **0** | **13** | **1.1e+02** | **1** | **CGYCSGGSCYR + Carbamidomethyl (C)** |
|  | 1958 | **488.7565** | **975.4983** | **975.1639** | **0.3344** | **0** | **13** | **1.2e+02** | **1** | **ASIMNVNVK** |
|  | 652 | **401.9728** | **1202.8963** | **1202.3826** | **0.5136** | **1** | **13** | **1.7e+02** | **1** | **LYCKGAQTYR** |
|  | 1203 | **431.3255** | **1290.9543** | **1290.5724** | **0.3820** | **2** | **13** | **1.4e+02** | **1** | **KTISPQMKTLK + Oxidation (M)** |
|  | 2173 | **517.5900** | **1549.7477** | **1549.8098** | **-0.0620** | **0** | **13** | **1.9e+02** | **1** | **DLLPNVCAFPMEK + Carbamidomethyl (C); Oxidation (M)** |
|  | 2212 | **520.0431** | **1038.0714** | **1039.1665** | **-1.0951** | **1** | **13** | **1.4e+02** | **1** | **MPYETRSR** |
|  | 1178 | **430.3475** | **1288.0202** | **1288.3909** | **-0.3706** | **0** | **13** | **1.3e+02** | **1** | **MGSRPTNDASPR** |
|  | 1478 | **449.0451** | **896.0754** | **896.1319** | **-0.0564** | **2** | **13** | **1.3e+02** | **1** | **VLVPGRKK** |
|  | 1861 | **478.1631** | **1431.4673** | **1431.7696** | **-0.3024** | **1** | **13** | **1.4e+02** | **1** | **GALGALMACLRIAR + Oxidation (M)** |
|  | 2241 | **521.6612** | **1041.3076** | **1042.3013** | **-0.9937** | **2** | **13** | **1.5e+02** | **1** | **KRMAMAMR + 3 Oxidation (M)** |
|  | 2849 | **623.8188** | **1245.6228** | **1245.3693** | **0.2535** | **1** | **13** | **1.3e+02** | **1** | **TQHTSISGCRR** |
|  | 197 | **371.7942** | **741.5737** | **740.7619** | **0.8118** | **0** | **13** | **1.3e+02** | **1** | **YTSNTR** |
|  | 2359 | **538.2607** | **1074.5066** | **1075.2399** | **-0.7333** | **0** | **13** | **1.5e+02** | **1** | **VAASQGGGMLGK** |
|  | 672 | **402.9504** | **1205.8291** | **1205.4263** | **0.4028** | **1** | **13** | **1.6e+02** | **1** | **EKFGPIGLNCK** |
|  | 913 | **411.1354** | **1230.3840** | **1230.4640** | **-0.0799** | **2** | **13** | **1.5e+02** | **1** | **MCLGARNHKK + Carbamidomethyl (C); Oxidation (M)** |
|  | 1001 | **417.1591** | **832.3035** | **831.8710** | **0.4325** | **0** | **13** | **1.6e+02** | **1** | **NSPNVSSK** |
|  | 1812 | **475.5410** | **1423.6009** | **1423.5076** | **0.0933** | **0** | **13** | **1.8e+02** | **1** | **ANQNFDEMEGIR** |
|  | 2816 | **613.7377** | **1838.1908** | **1837.0126** | **1.1782** | **0** | **13** | **1.6e+02** | **1** | **CFPNSSVIEEDGEGLLK** |
|  | 954 | **414.3022** | **1239.8844** | **1239.3318** | **0.5525** | **0** | **13** | **1e+02** | **1** | **TFTVTESGEIR** |
|  | 2275 | 528.9927 | 1583.9560 | 1584.6633 | -0.7073 | 0 | 13 | 1.4e+02 | 1 | ETSISACPNNSYNK + Carbamidomethyl (C) |
|  | 2690 | **594.1567** | **1779.4480** | **1780.0410** | **-0.5929** | **1** | **13** | **1.6e+02** | **1** | **MAAPGPGMREPPGRPAR + 2 Oxidation (M)** |
|  | 398 | **386.7750** | **1157.3029** | **1157.4548** | **-0.1519** | **2** | **13** | **1.5e+02** | **1** | **VIRLVRVFR** |
|  | 3418 | **769.2016** | **2304.5826** | **2304.2341** | **0.3486** | **0** | **13** | **1.4e+02** | **1** | **MEEGGSTGSAGSDSSTSGSGGAQQR + Oxidation (M)** |
|  | 3524 | **846.7355** | **1691.4562** | **1691.9653** | **-0.5091** | **0** | **13** | **1.2e+02** | **1** | **EILNLIHLEELSLR** |
|  | 377 | **386.0953** | **770.1759** | **769.7817** | **0.3942** | **0** | **13** | **1.3e+02** | **1** | **SSCSSSGR** |
|  | 692 | **403.8591** | **1208.5552** | **1208.5565** | **-0.0013** | **1** | **13** | **1.5e+02** | **1** | **MEKMTMMMK + 3 Oxidation (M)** |
|  | 2887 | **637.9349** | **1273.8551** | **1273.4174** | **0.4376** | **0** | **13** | **1.3e+02** | **1** | **LSCAGAGFTFSR + Carbamidomethyl (C)** |
|  | 186 | **371.0789** | **1110.2146** | **1111.2025** | **-0.9880** | **0** | **13** | **1.2e+02** | **1** | **YGVSEQTSLK** |
|  | 1168 | 429.5185 | 1285.5332 | 1286.4346 | -0.9013 | 2 | 13 | 1.8e+02 | 1 | KKTPGLSEQNGK |
|  | 2224 | **520.1747** | **1038.3347** | **1037.1969** | **1.1378** | **0** | **13** | **1.4e+02** | **1** | **NVPCSHRPK** |
|  | 893 | **408.7940** | **1223.3599** | **1222.3709** | **0.9891** | **1** | **13** | **1.5e+02** | **1** | **DVPEGYKVCR + Carbamidomethyl (C)** |
|  | 1405 | **443.8932** | **1328.6573** | **1328.5408** | **0.1165** | **2** | **13** | **1.5e+02** | **1** | **KMGDHLTNLRK + Oxidation (M)** |
|  | 1622 | **459.8636** | **1376.5687** | **1375.5478** | **1.0209** | **1** | **13** | **1.6e+02** | **1** | **EDDKPEMVIKR + Oxidation (M)** |
|  | 2402 | **548.0889** | **1641.2444** | **1640.9220** | **0.3224** | **2** | **13** | **1.4e+02** | **1** | **AREVKLLLLGAGESGK** |
|  | 3421 | **771.0873** | **1540.1598** | **1539.6494** | **0.5104** | **1** | **13** | **1.1e+02** | **1** | **LSGGGRPAEREAPDK** |
|  | 1242 | **433.3089** | **1296.9046** | **1296.4925** | **0.4121** | **2** | **13** | **1.2e+02** | **1** | **MFGQGTKVEVKG + Oxidation (M)** |
|  | 2389 | **544.1777** | **1629.5110** | **1628.7605** | **0.7505** | **0** | **13** | **1.7e+02** | **1** | **DSYPSFLICEQNR + Carbamidomethyl (C)** |
|  | 2792 | **611.8922** | **1221.7696** | **1221.4521** | **0.3175** | **2** | **13** | **1.2e+02** | **1** | **LIKQLGEHKR** |
|  | 1054 | 420.1806 | 1257.5196 | 1258.5306 | -1.0109 | 2 | 13 | 1.4e+02 | 1 | MEERLKVIPK + Oxidation (M) |
|  | 1327 | **437.8347** | **1310.4818** | **1309.4233** | **1.0585** | **1** | **13** | **1.7e+02** | **1** | **GDVFFPKVNETG** |
|  | 3377 | **757.8340** | **1513.6532** | **1513.7327** | **-0.0795** | **0** | **13** | **1.6e+02** | **1** | **ALPDLWEAGISTLK** |
|  | 359 | **385.1955** | **1152.5643** | **1153.2889** | **-0.7246** | **1** | **13** | **1.1e+02** | **1** | **KAEEATVHLR** |
|  | 1272 | **435.3732** | **1303.0974** | **1303.5546** | **-0.4571** | **1** | **13** | **1.1e+02** | **1** | **QERMPVLQMR + Oxidation (M)** |
|  | 1557 | **456.2569** | **910.4991** | **909.9446** | **0.5545** | **0** | **13** | **1.2e+02** | **1** | **YGQWSGGR** |
|  | 1814 | **475.7091** | **949.4035** | **949.8761** | **-0.4726** | **0** | **13** | **1.3e+02** | **1** | **DSNNNSDGK** |
|  | 2245 | **523.0165** | **1566.0275** | **1566.8238** | **-0.7963** | **1** | **13** | **1.5e+02** | **1** | **KIGCVEAGGVSHKPK + Carbamidomethyl (C)** |
|  | 2331 | **536.1469** | **1070.2789** | **1070.1985** | **0.0804** | **1** | **13** | **1.5e+02** | **1** | **YFSKQELR** |
|  | 2803 | **612.2622** | **1222.5096** | **1221.3594** | **1.1502** | **0** | **13** | **1.5e+02** | **1** | **LNEIVLDYSR** |
|  | 3107 | **685.8780** | **2054.6118** | **2053.5336** | **1.0782** | **2** | **13** | **1.5e+02** | **1** | **LHMLLLKGMATMNKEMK + 4 Oxidation (M)** |
|  | 438 | **387.9599** | **1160.8575** | **1160.2782** | **0.5794** | **0** | **13** | **1.8e+02** | **1** | **FFSQFTEVR** |
|  | 472 | **389.0504** | **776.0860** | **775.8939** | **0.1922** | **2** | **13** | **1.7e+02** | **1** | **TAKSGGKK** |
|  | 655 | **402.0378** | **802.0608** | **802.8778** | **-0.8170** | **1** | **13** | **1.7e+02** | **1** | **REPPHPA** |
|  | 994 | **416.5063** | **1246.4966** | **1245.2999** | **1.1967** | **1** | **13** | **2.1e+02** | **1** | **GASVDRDLQER** |
|  | 2023 | **495.5792** | **1483.7155** | **1484.8079** | **-1.0924** | **2** | **13** | **1.9e+02** | **1** | **TRLAVMSMEMRK + 2 Oxidation (M)** |
|  | 50 | **363.2187** | **724.4225** | **723.9022** | **0.5204** | **0** | **13** | **1.2e+02** | **1** | **ECMVVK + Oxidation (M)** |
|  | 1704 | **464.1804** | **926.3459** | **927.0565** | **-0.7105** | **1** | **13** | **1.5e+02** | **1** | **KESTLPPR** |
|  | 2619 | **585.4167** | **1753.2281** | **1752.9441** | **0.2839** | **1** | **13** | **1.3e+02** | **1** | **SIKYSIWCSTEHGNK** |
|  | 2698 | **595.2183** | **1188.4219** | **1189.4254** | **-1.0035** | **1** | **13** | **1.7e+02** | **1** | **MLLVGKDGNVK + Oxidation (M)** |
|  | 2707 | **596.4005** | **1190.7861** | **1190.4116** | **0.3745** | **0** | **13** | **1.5e+02** | **1** | **LLGLYHTMDK** |
|  | 3545 | **871.6632** | **1741.3116** | **1742.1187** | **-0.8071** | **2** | **13** | **1.4e+02** | **1** | **LRTMLVRTHMQDLK** |
|  | 239 | **374.7053** | **747.3959** | **747.9037** | **-0.5078** | **1** | **13** | **1.6e+02** | **1** | **KVAGMDK** |
|  | 1333 | **438.2833** | **874.5517** | **874.0186** | **0.5332** | **1** | **13** | **1.3e+02** | **1** | **GMGRYFK + Oxidation (M)** |
|  | 1883 | **480.4701** | **1438.3881** | **1437.5111** | **0.8770** | **0** | **13** | **1.5e+02** | **1** | **ASQSSPGDFSISVR** |
|  | 1895 | **483.0437** | **964.0726** | **964.0948** | **-0.0221** | **0** | **13** | **1.6e+02** | **1** | **NQIDMSLK + Oxidation (M)** |
|  | 2240 | **521.4253** | **1561.2537** | **1561.8501** | **-0.5964** | **1** | **13** | **1.1e+02** | **1** | **ALCRIPAPNPLPGSR** |
|  | 2407 | **549.0847** | **1096.1547** | **1095.2545** | **0.9002** | **2** | **13** | **1.4e+02** | **1** | **CCRKESGQK + Carbamidomethyl (C)** |
|  | 2604 | **583.1351** | **1746.3832** | **1745.8194** | **0.5638** | **0** | **13** | **1.5e+02** | **1** | **TTPANPVGPSGGMSDDDK** |
|  | 3041 | **673.8602** | **2018.5585** | **2019.3516** | **-0.7930** | **2** | **13** | **1.5e+02** | **1** | **DMHVVAKIKSGNCLFQR + Carbamidomethyl (C); Oxidation (M)** |
|  | 134 | **369.2382** | **1104.6923** | **1105.0072** | **-0.3149** | **1** | **13** | **1.3e+02** | **1** | **XDRAGLVXPR** |
|  | 722 | **404.9919** | **807.9691** | **807.9803** | **-0.0112** | **0** | **13** | **1.4e+02** | **1** | **LPPNVLR** |
|  | 1240 | **433.2933** | **864.5719** | **864.9672** | **-0.3953** | **1** | **13** | **1.2e+02** | **1** | **MASAKDAR + Oxidation (M)** |
|  | 1832 | **476.4684** | **950.9221** | **950.0467** | **0.8754** | **0** | **13** | **1.5e+02** | **1** | **AEYEAIVR** |
|  | 223 | **374.1490** | **746.2832** | **745.9309** | **0.3523** | **1** | **12** | **1.7e+02** | **1** | **VAKGPMK + Oxidation (M)** |
|  | 310 | **380.3491** | **1138.0252** | **1137.4022** | **0.6230** | **2** | **12** | **1.7e+02** | **1** | **RTRHVMLPK** |
|  | 658 | **402.0919** | **802.1691** | **802.9591** | **-0.7899** | **2** | **12** | **1.7e+02** | **1** | **ATVEKKK** |
|  | 1045 | **419.5287** | **1255.5640** | **1256.4317** | **-0.8678** | **1** | **12** | **2e+02** | **1** | **EPSSKHISLCR** |
|  | 1217 | **432.2787** | **1293.8139** | **1294.3920** | **-0.5781** | **0** | **12** | **1.3e+02** | **1** | **MSEASGNLNSLR + Oxidation (M)** |
|  | 1397 | **443.6464** | **1327.9171** | **1328.4297** | **-0.5127** | **0** | **12** | **1.3e+02** | **1** | **SEQPGLAADVWR** |
|  | 1604 | **458.9716** | **1373.8927** | **1373.6474** | **0.2453** | **1** | **12** | **1.7e+02** | **1** | **SLQAPCLCRGGLR** |
|  | 3 | **360.3499** | **1078.0274** | **1079.1870** | **-1.1597** | **0** | **12** | **1.9e+02** | **1** | **CINTHGSYK + Carbamidomethyl (C)** |
|  | 399 | **386.8340** | **1157.4797** | **1156.3591** | **1.1206** | **2** | **12** | **1.6e+02** | **1** | **KSMHRGSPIK + Oxidation (M)** |
|  | 433 | **387.9043** | **1160.6907** | **1160.3443** | **0.3464** | **0** | **12** | **1.8e+02** | **1** | **LAALMSSPQSR** |
|  | 2363 | **538.4636** | **1074.9125** | **1075.3475** | **-0.4351** | **2** | **12** | **1.3e+02** | **1** | **VLYKGVLKR** |
|  | 275 | **378.0729** | **1131.1965** | **1130.2191** | **0.9775** | **2** | **12** | **1.4e+02** | **1** | **ARRGSGVGAGSR** |
|  | 536 | **392.3343** | **1173.9808** | **1173.3465** | **0.6343** | **1** | **12** | **1.2e+02** | **1** | **MAAANKGNKPR + Oxidation (M)** |
|  | 732 | 405.0878 | 1212.2413 | 1211.4937 | 0.7476 | 0 | 12 | 1.4e+02 | 1 | LGCIYPSSLMK |
|  | 776 | **406.1579** | **1215.4514** | **1215.3767** | **0.0747** | **0** | **12** | **1.4e+02** | **1** | **VTPQMGTPSPGK + Oxidation (M)** |
|  | 841 | **407.6360** | **1219.8860** | **1220.4126** | **-0.5267** | **2** | **12** | **1.2e+02** | **1** | **RGWHHCRLR** |
|  | 2684 | **593.5941** | **1777.7602** | **1776.9890** | **0.7712** | **0** | **12** | **1.6e+02** | **1** | **SVSSWASSITVPRPFR** |
|  | 1431 | **445.1064** | **888.1980** | **888.0684** | **0.1296** | **1** | **12** | **1.7e+02** | **1** | **FVITPRR** |
|  | 1743 | **468.4599** | **934.9050** | **935.2491** | **-0.3441** | **1** | **12** | **1.6e+02** | **1** | **MMGLKLVK + Oxidation (M)** |
|  | 410 | **387.0689** | **1158.1845** | **1158.2838** | **-0.0993** | **0** | **12** | **1.8e+02** | **1** | **SLCACSDDCK + 2 Carbamidomethyl (C)** |
|  | 1143 | **428.0979** | **1281.2715** | **1281.4214** | **-0.1499** | **0** | **12** | **1.5e+02** | **1** | **RPVAAAAAGSASPR** |
|  | 1455 | **447.0973** | **892.1799** | **892.0985** | **0.0814** | **1** | **12** | **1.6e+02** | **1** | **GAMGKCPTK** |
|  | 1616 | **459.5766** | **917.1385** | **917.0153** | **0.1232** | **0** | **12** | **1.9e+02** | **1** | **DSVTGTLPK** |
|  | 3183 | **700.6810** | **1399.3473** | **1398.5364** | **0.8109** | **0** | **12** | **1.3e+02** | **1** | **SSTLSSSSMSLSPK** |
|  | 3199 | **707.1669** | **2118.4784** | **2118.3980** | **0.0804** | **1** | **12** | **1.4e+02** | **1** | **NPLVEQPRACACGEAFAWR** |
|  | 502 | **389.9211** | **777.8274** | **776.8621** | **0.9654** | **1** | **12** | **1.5e+02** | **1** | **RMEDAR** |
|  | 682 | **403.3235** | **1206.9482** | **1206.2702** | **0.6779** | **1** | **12** | **1.4e+02** | **1** | **SWRHGSGTYR** |
|  | 822 | **407.2457** | **1218.7150** | **1218.3837** | **0.3314** | **2** | **12** | **1.1e+02** | **1** | **KGQRDLMSGLN** |
|  | 1446 | **446.1202** | **1335.3385** | **1334.5023** | **0.8362** | **2** | **12** | **1.7e+02** | **1** | **TTKDLEGVRGCR** |
|  | 1614 | **459.3853** | **916.7558** | **917.0184** | **-0.2626** | **0** | **12** | **1.4e+02** | **1** | **AASAAAAGSLK** |
|  | 1905 | **484.1209** | **966.2270** | **965.0615** | **1.1656** | **0** | **12** | **1.5e+02** | **1** | **VSTAFVADR** |
|  | 2084 | **504.3459** | **1510.0156** | **1510.7955** | **-0.7799** | **0** | **12** | **1.3e+02** | **1** | **TMCMSTPVLEPTK + Carbamidomethyl (C); Oxidation (M)** |
|  | 2534 | **571.4310** | **1140.8473** | **1140.3795** | **0.4678** | **1** | **12** | **1.2e+02** | **1** | **ACFKAAMGIR + Carbamidomethyl (C); Oxidation (M)** |
|  | 3343 | **744.4871** | **2230.4390** | **2231.3422** | **-0.9032** | **2** | **12** | **1.5e+02** | **1** | **VGGRWVSAEKVNEGGSGGGGSQR** |
|  | 2839 | **619.8650** | **1237.7152** | **1236.5267** | **1.1885** | **2** | **12** | **1.2e+02** | **1** | **KTPPAPMAPAKK** |
|  | 3464 | **799.0312** | **2394.0714** | **2394.7260** | **-0.6546** | **1** | **12** | **1.3e+02** | **1** | **RCPPTSSPASTCPALPPTTPLGSR** |
|  | 248 | **375.3930** | **1123.1569** | **1123.2861** | **-0.1291** | **1** | **12** | **1.9e+02** | **1** | **LSRFMGEGAR** |
|  | 603 | **399.1842** | **1194.5303** | **1195.2791** | **-0.7487** | **0** | **12** | **1.3e+02** | **1** | **FGTNIDLSSNK** |
|  | 1556 | **456.2189** | **1365.6344** | **1365.6239** | **0.0105** | **1** | **12** | **1.4e+02** | **1** | **KPEAGGVIRAIVR** |
|  | 2171 | **517.5184** | **1549.5331** | **1548.7406** | **0.7926** | **1** | **12** | **1.7e+02** | **1** | **LRTQLPSMPQSDC + Carbamidomethyl (C); Oxidation (M)** |
|  | 2889 | **639.0778** | **1276.1407** | **1276.4627** | **-0.3220** | **0** | **12** | **1.5e+02** | **1** | **STLNLQMNSLR** |
|  | 818 | **407.1584** | **1218.4531** | **1217.3327** | **1.1203** | **1** | **12** | **1.5e+02** | **1** | **RLTASDIGTQR** |
|  | 2151 | **513.9290** | **1538.7649** | **1539.7140** | **-0.9491** | **1** | **12** | **1.5e+02** | **1** | **ESMGHRYIEVFR + Oxidation (M)** |
|  | 2433 | **552.0553** | **1102.0958** | **1101.2375** | **0.8584** | **1** | **12** | **1.6e+02** | **1** | **KMNSEISHR** |
|  | 2535 | **571.4814** | **1711.4222** | **1711.9817** | **-0.5596** | **1** | **12** | **1.2e+02** | **1** | **EQLLDRMCMTLGGR + Carbamidomethyl (C); 2 Oxidation (M)** |
|  | 2813 | **613.3302** | **1836.9684** | **1837.1087** | **-0.1403** | **2** | **12** | **1.5e+02** | **1** | **LRSRVQIGVVGGSDYCK** |
|  | 1154 | **428.5905** | **1282.7495** | **1282.5570** | **0.1925** | **2** | **12** | **1.2e+02** | **1** | **KCSTGCPRGMVK + Oxidation (M)** |
|  | 1317 | **437.2713** | **872.5279** | **872.9659** | **-0.4381** | **1** | **12** | **1.4e+02** | **1** | **QAQAAKEK** |
|  | 1410 | **443.9221** | **885.8295** | **887.0157** | **-1.1862** | **0** | **12** | **1.6e+02** | **1** | **LMDLSHR + Oxidation (M)** |
|  | 1489 | **450.0798** | **1347.2172** | **1346.5494** | **0.6679** | **0** | **12** | **1.5e+02** | **1** | **GILVNMDDNIVK + Oxidation (M)** |
|  | 1638 | **460.4160** | **918.8172** | **918.0082** | **0.8090** | **0** | **12** | **1.4e+02** | **1** | **SGVFAAGGPR** |
|  | 1975 | **490.2097** | **1467.6070** | **1467.6928** | **-0.0858** | **2** | **12** | **1.5e+02** | **1** | **MSGRGVGSVSQFKK** |
|  | 3057 | **680.5353** | **2038.5837** | **2039.3564** | **-0.7727** | **2** | **12** | **1.3e+02** | **1** | **DSGLTTFFKMVSMRDCK + Carbamidomethyl (C); Oxidation (M)** |
|  | 3416 | **768.8947** | **2303.6618** | **2304.5370** | **-0.8752** | **0** | **12** | **1.7e+02** | **1** | **MSGECQSPHCPGTSAEFFFK + 2 Carbamidomethyl (C)** |
|  | 561 | **395.0383** | **1182.0927** | **1181.2776** | **0.8152** | **0** | **12** | **1.8e+02** | **1** | **NMGVGVSSTASR + Oxidation (M)** |
|  | 1605 | **458.9721** | **1373.8941** | **1374.6705** | **-0.7764** | **2** | **12** | **1.7e+02** | **1** | **AKFENLCKFMK + Oxidation (M)** |
|  | 1878 | **480.1760** | **1437.5058** | **1438.5853** | **-1.0795** | **0** | **12** | **1.6e+02** | **1** | **AANVTGPGGVPVQGSK** |
|  | 2424 | **550.7910** | **1649.3509** | **1648.8994** | **0.4514** | **0** | **12** | **1.3e+02** | **1** | **MIAVCQNVALSAEDK + Carbamidomethyl (C)** |
|  | 3125 | **686.2700** | **1370.5253** | **1370.5972** | **-0.0720** | **0** | **12** | **1.5e+02** | **1** | **ADPECMLGHLLR + Oxidation (M)** |
|  | 3190 | **702.8381** | **2105.4922** | **2105.4360** | **0.0562** | **1** | **12** | **1.8e+02** | **1** | **TLHSAIVVTFRDVAVTFTK** |
|  | 3282 | **740.5425** | **1479.0702** | **1479.6555** | **-0.5853** | **1** | **12** | **1.4e+02** | **1** | **LSRDDISTAAGMVK + Oxidation (M)** |
|  | 1833 | **476.5016** | **1426.4825** | **1425.6710** | **0.8115** | **1** | **12** | **1.7e+02** | **1** | **VKVLGSGAFGTVYK** |
|  | 2574 | **578.9207** | **1733.7400** | **1733.8533** | **-0.1133** | **2** | **12** | **1.4e+02** | **1** | **FKKLADMYGGDDSDR + Oxidation (M)** |
|  | 2642 | **589.8010** | **1766.3807** | **1767.1199** | **-0.7392** | **2** | **12** | **1.4e+02** | **1** | **KMGYYKILDVMYSR** |
|  | 1305 | **437.0349** | **872.0550** | **870.9700** | **1.0851** | **0** | **12** | **1.7e+02** | **1** | **TCFTAGGSK** |
|  | 2198 | **519.0233** | **1036.0318** | **1035.1346** | **0.8973** | **0** | **12** | **1.5e+02** | **1** | **MGGAAFGPDGR** |
|  | 2522 | **569.1174** | **1704.3301** | **1704.9228** | **-0.5927** | **0** | **12** | **1.6e+02** | **1** | **MDLQQPAANLSELCR + Oxidation (M)** |
|  | 2566 | **577.6339** | **1153.2530** | **1154.2536** | **-1.0006** | **0** | **12** | **1.8e+02** | **1** | **DAGMQLQGYR + Oxidation (M)** |
|  | 2862 | **627.2778** | **1252.5409** | **1251.4782** | **1.0626** | **1** | **12** | **1.5e+02** | **1** | **RTLVLSPAAAPR** |
|  | 3541 | **864.9595** | **2591.8564** | **2592.9398** | **-1.0834** | **0** | **12** | **1.7e+02** | **1** | **NTLYLQMLSLEPENTAVYYCAR** |
|  | 2754 | **605.6315** | **1209.2482** | **1208.3210** | **0.9271** | **1** | **12** | **1.7e+02** | **1** | **VYRSQGELEK** |
|  | 3046 | **678.6841** | **2033.0301** | **2034.1213** | **-1.0912** | **1** | **12** | **1.5e+02** | **1** | **YDAFGEDSSSAMGVENRAK** |
|  | 3202 | **707.8302** | **2120.4684** | **2121.3494** | **-0.8810** | **1** | **12** | **1.8e+02** | **1** | **GRVTMTTDTSTSTAYMELR** |
|  | 3290 | **740.7042** | **1479.3937** | **1479.8127** | **-0.4190** | **1** | **12** | **1.3e+02** | **1** | **KTHMISMHPIIR + Oxidation (M)** |
|  | 231 | **374.2471** | **1119.7192** | **1119.3288** | **0.3904** | **0** | **12** | **1.5e+02** | **1** | **LLEVLSGEML + Oxidation (M)** |
|  | 784 | 406.2736 | 1215.7987 | 1216.4507 | -0.6521 | 1 | 12 | 1.2e+02 | 1 | QEIIRVAMEK |
|  | 1761 | **470.0794** | **938.1440** | **937.0942** | **1.0498** | **0** | **12** | **1.4e+02** | **1** | **ELLPAAAPR** |
|  | 1840 | **477.5078** | **1429.5012** | **1428.8222** | **0.6789** | **2** | **12** | **1.7e+02** | **1** | **VVKMILKLLEDK** |
|  | 2762 | **607.0885** | **1818.2433** | **1817.9001** | **0.3432** | **1** | **12** | **1.5e+02** | **1** | **DYLSKQEFSSDEEIK** |
|  | 3001 | **668.5962** | **1335.1776** | **1335.4853** | **-0.3077** | **0** | **12** | **1.3e+02** | **1** | **AGAHSVYACELSK** |
|  | 552 | **393.5231** | **1177.5470** | **1177.3733** | **0.1738** | **0** | **12** | **1.6e+02** | **1** | **VQAMQPAFASK** |
|  | 1576 | **458.1028** | **1371.2863** | **1371.5656** | **-0.2793** | **2** | **12** | **1.6e+02** | **1** | **AKMRNLETQHK + Oxidation (M)** |
|  | 2032 | **497.0016** | **991.9884** | **992.1048** | **-0.1165** | **0** | **12** | **1.6e+02** | **1** | **ALCGLDESK + Carbamidomethyl (C)** |
|  | 2139 | **511.5038** | **1531.4894** | **1531.8557** | **-0.3664** | **1** | **12** | **1.6e+02** | **1** | **SIDPALSMLIKSIK + Oxidation (M)** |
|  | 2542 | **572.6021** | **1714.7842** | **1715.8613** | **-1.0771** | **2** | **12** | **1.8e+02** | **1** | **IDYADSVKGRFTTSR** |
|  | 3261 | **737.6420** | **1473.2692** | **1472.6264** | **0.6428** | **1** | **12** | **1.2e+02** | **1** | **HGMALNDTKQVSR + Oxidation (M)** |
|  | 2190 | **518.9246** | **1553.7515** | **1554.7038** | **-0.9522** | **2** | **12** | **1.5e+02** | **1** | **SIPAGAEPGEKGSARK** |
|  | 2219 | 520.1560 | 1557.4458 | 1557.7690 | -0.3231 | 1 | 12 | 1.5e+02 | 1 | DMQMDKSELGCLR + 2 Oxidation (M) |
|  | 2750 | **603.5632** | **1205.1116** | **1204.3972** | **0.7144** | **1** | **12** | **1.4e+02** | **1** | **SRVTMPVDTAK** |
|  | 1313 | **437.1845** | **1308.5314** | **1308.4384** | **0.0930** | **0** | **12** | **1.7e+02** | **1** | **YNSAPPLFGGGTK** |
|  | 1771 | **470.9888** | **939.9629** | **939.0275** | **0.9354** | **1** | **12** | **1.4e+02** | **1** | **HKVSEPSR** |
|  | 2670 | **592.7136** | **1183.4125** | **1182.3997** | **1.0128** | **2** | **12** | **1.9e+02** | **1** | **KHSVRLCSPR** |
|  | 3289 | **740.6913** | **1479.3678** | **1479.6224** | **-0.2546** | **2** | **12** | **1.2e+02** | **1** | **NGRAVMTTSSRQR + Oxidation (M)** |
|  | 1141 | **428.0801** | **1281.2181** | **1281.4811** | **-0.2630** | **1** | **12** | **1.5e+02** | **1** | **LRDAFSAQMVK + Oxidation (M)** |
|  | 1312 | **437.1813** | **1308.5217** | **1307.3295** | **1.1923** | **1** | **12** | **1.7e+02** | **1** | **ERNQASWSSSR** |
|  | 2071 | **502.7693** | **1003.5238** | **1003.2218** | **0.3019** | **1** | **12** | **1.4e+02** | **1** | **QLVCRWAK** |
|  | 225 | **374.1768** | **746.3387** | **745.8680** | **0.4708** | **1** | **12** | **1.6e+02** | **1** | **KTSGVVR** |
|  | 1991 | **492.0925** | **982.1703** | **981.1667** | **1.0035** | **0** | **12** | **1.5e+02** | **1** | **YICSTVPAK** |
|  | 2489 | **564.2501** | **1689.7280** | **1688.8557** | **0.8724** | **0** | **12** | **1.7e+02** | **1** | **QDCDTFDLHEKPLK** |
|  | 2899 | **641.9435** | **1281.8722** | **1281.4380** | **0.4342** | **2** | **12** | **1.2e+02** | **1** | **AGGMSDSSKWKK** |
|  | 2330 | **536.1407** | **1070.2667** | **1070.3127** | **-0.0460** | **2** | **12** | **1.6e+02** | **1** | **KLGIAPCGRR** |
|  | 3556 | **920.4410** | **1838.8673** | **1838.0936** | **0.7737** | **1** | **12** | **1.4e+02** | **1** | **ALPTGASLSSMARVSFAR + Oxidation (M)** |
|  | 1412 | **443.9423** | **885.8698** | **886.0276** | **-0.1578** | **0** | **12** | **1.7e+02** | **1** | **EMTHLQK** |
|  | 1435 | 445.2054 | 1332.5940 | 1332.5708 | 0.0232 | 0 | 12 | 1.8e+02 | 1 | GIKPGMPSIFNR + Oxidation (M) |
|  | 2165 | **516.2192** | **1545.6355** | **1544.6624** | **0.9731** | **1** | **12** | **1.7e+02** | **1** | **KNLQEEIDALESR** |
|  | 539 | **392.4122** | **1174.2145** | **1173.4261** | **0.7884** | **0** | **12** | **1.7e+02** | **1** | **QPAMMMFSSK + Oxidation (M)** |
|  | 627 | **401.0509** | **800.0871** | **798.8907** | **1.1964** | **1** | **12** | **1.7e+02** | **1** | **RNPGSLR** |
|  | 419 | **387.3460** | **1159.0159** | **1158.3484** | **0.6675** | **1** | **12** | **1.7e+02** | **1** | **CDTCGKSFGLK** |
|  | 592 | **398.8733** | **795.7319** | **796.8650** | **-1.1331** | **0** | **12** | **1.4e+02** | **1** | **FTISSDK** |
|  | 985 | **416.1565** | **1245.4474** | **1244.3764** | **1.0710** | **0** | **12** | **1.9e+02** | **1** | **MSTQYFGPGTR** |
|  | 3271 | **740.2250** | **1478.4352** | **1478.6954** | **-0.2603** | **2** | **12** | **1.5e+02** | **1** | **RLAPGKDEQVPIR** |
|  | 824 | **407.2546** | **1218.7417** | **1218.3771** | **0.3645** | **0** | **12** | **1.2e+02** | **1** | **ALQEMLANTVE** |
|  | 1360 | **440.2784** | **1317.8132** | **1318.5874** | **-0.7742** | **0** | **12** | **1.5e+02** | **1** | **TSCCSCCPVGCAK + Carbamidomethyl (C)** |
|  | 1600 | **458.9036** | **915.7925** | **915.9940** | **-0.2015** | **1** | **12** | **1.8e+02** | **1** | **INSDGRVR** |
|  | 3297 | **740.8396** | **1479.6644** | **1478.6457** | **1.0188** | **0** | **12** | **1.8e+02** | **1** | **ALIFSTDDFFFR** |
|  | 1096 | **422.2223** | **1263.6448** | **1264.4275** | **-0.7827** | **1** | **12** | **1.6e+02** | **1** | **SGSPDVKGPPPVK** |
|  | 711 | **404.1862** | **1209.5363** | **1209.3058** | **0.2305** | **0** | **12** | **1.5e+02** | **1** | **VAPHALSEEEK** |
|  | 1015 | **418.6533** | **1252.9378** | **1253.2987** | **-0.3609** | **0** | **12** | **1.6e+02** | **1** | **GPDNSMGFGAER + Oxidation (M)** |
|  | 1362 | **440.5016** | **1318.4825** | **1318.4351** | **0.0474** | **2** | **12** | **2.1e+02** | **1** | **SPREPGYKAEGK** |
|  | 2135 | **510.1857** | **1527.5350** | **1526.5644** | **0.9707** | **0** | **12** | **1.7e+02** | **1** | **FHGYGPEENSFSR** |
|  | 2540 | **572.1627** | **1142.3107** | **1142.2431** | **0.0676** | **0** | **12** | **1.6e+02** | **1** | **EQAPNMAEPR** |
|  | 3482 | **813.6432** | **1625.2717** | **1624.6810** | **0.5907** | **0** | **12** | **1.4e+02** | **1** | **MEIDDGTSAWGDPSK + Oxidation (M)** |
|  | 1481 | **449.2794** | **896.5441** | **896.9873** | **-0.4432** | **1** | **12** | **1.2e+02** | **1** | **SLSSYRGK** |
|  | 1843 | **477.6062** | **1429.7963** | **1428.6299** | **1.1664** | **0** | **12** | **1.6e+02** | **1** | **ALLLTGSNEIEIR** |
|  | 2092 | **505.2637** | **1512.7689** | **1511.5945** | **1.1745** | **0** | **12** | **1.5e+02** | **1** | **VGNTNTQYFGPGTR** |
|  | 2372 | **540.2709** | **1078.5271** | **1078.3250** | **0.2021** | **0** | **12** | **1.6e+02** | **1** | **EIFMVALQK** |
|  | 2981 | **666.7762** | **1997.3066** | **1998.3691** | **-1.0625** | **2** | **12** | **1.9e+02** | **1** | **EEMVSAAFMKKYIHVAK + Oxidation (M)** |
|  | 3096 | **684.8678** | **2051.5812** | **2051.3466** | **0.2346** | **0** | **12** | **1.6e+02** | **1** | **HNNGQPIWFTLGILEALK** |
|  | 67 | **364.2018** | **1089.5833** | **1089.2682** | **0.3151** | **0** | **12** | **1.2e+02** | **1** | **CHTGVKPYK + Carbamidomethyl (C)** |
|  | 109 | **368.1039** | **1101.2895** | **1101.1745** | **0.1150** | **1** | **12** | **1.7e+02** | **1** | **RLGPGSSGGSAR** |
|  | 2393 | **544.4189** | **1630.2347** | **1631.0000** | **-0.7654** | **1** | **12** | **1.5e+02** | **1** | **CLCGGRAKPCVPLGTR** |
|  | 3113 | **686.0172** | **1370.0196** | **1370.6370** | **-0.6174** | **2** | **12** | **1.3e+02** | **1** | **DLISKLLVRDAK** |
|  | 3387 | **758.2058** | **2271.5953** | **2272.5398** | **-0.9446** | **2** | **12** | **1.5e+02** | **1** | **CLSEQIADAYSSFRSVRAIR** |
|  | 115 | **368.3421** | **1102.0042** | **1101.1630** | **0.8413** | **0** | **12** | **1.8e+02** | **1** | **FDLYDVDSK** |
|  | 560 | **394.6527** | **1180.9360** | **1181.4050** | **-0.4690** | **1** | **12** | **1.4e+02** | **1** | **LQYVKVGSCK + Carbamidomethyl (C)** |
|  | 1295 | **436.2186** | **870.4225** | **870.9883** | **-0.5658** | **0** | **12** | **1.5e+02** | **1** | **MIMDDSK + 2 Oxidation (M)** |
|  | 1324 | **437.5661** | **873.1175** | **873.0520** | **0.0654** | **1** | **12** | **2e+02** | **1** | **ITATIAKR** |
|  | 2260 | **524.5566** | **1047.0985** | **1046.2003** | **0.8982** | **0** | **12** | **2e+02** | **1** | **VPEGSWLCR** |
|  | 3131 | **686.4098** | **2056.2072** | **2057.2308** | **-1.0236** | **1** | **12** | **1.6e+02** | **1** | **ENGGAADVAAVGRARPPSPHK** |
|  | 3490 | **817.8593** | **2450.5556** | **2449.9286** | **0.6269** | **2** | **12** | **1.6e+02** | **1** | **MLSSIKCVLVGDSAVGKTSLLVR + Carbamidomethyl (C); Oxidation (M)** |
|  | 1822 | **476.1954** | **1425.5639** | **1424.6846** | **0.8794** | **1** | **12** | **1.6e+02** | **1** | **SMMSPKISTPWK + 2 Oxidation (M)** |
|  | 2247 | **523.2253** | **1044.4359** | **1045.1280** | **-0.6921** | **1** | **12** | **1.7e+02** | **1** | **GHVERSVMD + Oxidation (M)** |
|  | 2316 | **534.5736** | **1067.1324** | **1067.1700** | **-0.0375** | **0** | **12** | **2e+02** | **1** | **MFSPTPDEK + Oxidation (M)** |
|  | 2413 | **549.4476** | **1645.3207** | **1644.9802** | **0.3405** | **1** | **12** | **1.3e+02** | **1** | **MCKGLAALPHSCLER + Oxidation (M)** |
|  | 2774 | **610.0099** | **1827.0075** | **1827.9563** | **-0.9488** | **2** | **12** | **1.6e+02** | **1** | **ARGSPSSQGTGGVGAAGVRR** |
|  | 876 | **408.1255** | **1221.3543** | **1220.2951** | **1.0592** | **0** | **12** | **1.7e+02** | **1** | **GAAAHLDPGQQR** |
|  | 907 | **410.0639** | **818.1130** | **818.9848** | **-0.8717** | **0** | **12** | **1.7e+02** | **1** | **MGCCFSR + Oxidation (M)** |
|  | 2140 | **511.5314** | **1531.5720** | **1532.6916** | **-1.1196** | **0** | **12** | **1.9e+02** | **1** | **TEVALLASEAVGSASK** |
|  | 2894 | **639.9236** | **1916.7486** | **1916.1674** | **0.5812** | **2** | **12** | **1.3e+02** | **1** | **MKRNEQNTVAISQRPK + Oxidation (M)** |
|  | 3515 | **841.4652** | **2521.3735** | **2521.8986** | **-0.5251** | **2** | **12** | **1.6e+02** | **1** | **ACPEAGCPAGMEVVTCANRCPRR + 2 Carbamidomethyl (C); Oxidation (M)** |
|  | 85 | **366.0675** | **1095.1803** | **1094.2482** | **0.9322** | **1** | **12** | **1.8e+02** | **1** | **RPFKGSSCR + Carbamidomethyl (C)** |
|  | 796 | **406.7427** | **1217.2059** | **1216.4277** | **0.7783** | **2** | **12** | **1.4e+02** | **1** | **VKLTPKAASSSK** |
|  | 2802 | **612.2522** | **1222.4896** | **1221.4125** | **1.0772** | **1** | **12** | **1.6e+02** | **1** | **MHSLGCFRDR** |
|  | 3549 | **873.0083** | **2616.0027** | **2616.0378** | **-0.0351** | **1** | **12** | **1.8e+02** | **1** | **MENFDYSNEEHMTLLKMILIK + Oxidation (M)** |
|  | 1261 | **435.1002** | **868.1855** | **867.0275** | **1.1580** | **0** | **12** | **1.5e+02** | **1** | **AALQHCPK** |
|  | 1909 | **484.3619** | **966.7090** | **967.1037** | **-0.3947** | **0** | **12** | **1.3e+02** | **1** | **CHLEPNVR** |
|  | 2713 | **596.9423** | **1787.8046** | **1786.9822** | **0.8224** | **2** | **12** | **1.6e+02** | **1** | **DWSNKMPDMAYERK + Oxidation (M)** |
|  | 542 | **392.4941** | **782.9734** | **782.9694** | **0.0040** | **0** | **12** | **1.8e+02** | **1** | **MVSGMVK + 2 Oxidation (M)** |
|  | 966 | **415.1304** | **1242.3689** | **1242.4184** | **-0.0495** | **1** | **12** | **1.7e+02** | **1** | **LKETIEDLPGK** |
|  | 3106 | **685.8514** | **1369.6880** | **1370.5740** | **-0.8861** | **0** | **12** | **1.8e+02** | **1** | **HTIASGVDCGLLK + Carbamidomethyl (C)** |
|  | 823 | **407.2506** | **1218.7296** | **1219.4528** | **-0.7233** | **0** | **12** | **1.2e+02** | **1** | **SNPQLMAAFIK** |
|  | 2702 | **595.6897** | **1784.0469** | **1783.0564** | **0.9906** | **2** | **12** | **2.1e+02** | **1** | **ESKNMWIFDRIVTK + Oxidation (M)** |
|  | 3101 | **685.1240** | **1368.2333** | **1368.4907** | **-0.2574** | **0** | **12** | **1.7e+02** | **1** | **ESMEASPEAMVR + 2 Oxidation (M)** |
|  | 3357 | **749.7910** | **1497.5673** | **1497.5894** | **-0.0222** | **1** | **12** | **1.7e+02** | **1** | **ESCFELASQDRR + Carbamidomethyl (C)** |
|  | 2051 | **501.0109** | **1500.0104** | **1500.6347** | **-0.6243** | **1** | **12** | **1.7e+02** | **1** | **EDCEAFRGLFTR + Carbamidomethyl (C)** |
|  | 2311 | 534.1686 | 1599.4837 | 1598.8008 | 0.6829 | 1 | 12 | 1.8e+02 | 1 | HRPDLIDYSKLNK |
|  | 2742 | 600.8131 | 1799.4172 | 1799.0344 | 0.3827 | 1 | 12 | 1.4e+02 | 1 | TAASLRVTGEPEVTIVR |
|  | 3446 | **786.2645** | **2355.7714** | **2355.7111** | **0.0603** | **2** | **12** | **1.6e+02** | **1** | **QNNWEEAAKLLKEAINKPMK** |
|  | 459 | **388.4409** | **1162.3006** | **1161.3275** | **0.9731** | **0** | **12** | **2.3e+02** | **1** | **MSTIAAFYGGK + Oxidation (M)** |
|  | 1668 | **462.1140** | **922.2132** | **921.1345** | **1.0787** | **1** | **12** | **1.6e+02** | **1** | **ILSYKIGK** |
|  | 2703 | **595.6927** | **1784.0559** | **1782.9685** | **1.0874** | **1** | **12** | **2.1e+02** | **1** | **DDAKNTLYLEMNSLR** |
|  | 1837 | **477.3253** | **1428.9539** | **1429.7572** | **-0.8034** | **2** | **12** | **1.3e+02** | **1** | **MRAPGALLARMSR** |
|  | 1067 | **421.7570** | **1262.2489** | **1263.4030** | **-1.1541** | **1** | **12** | **1.6e+02** | **1** | **TPPTVPNPRER** |
|  | 1796 | **474.0594** | **1419.1561** | **1418.6468** | **0.5093** | **2** | **12** | **1.8e+02** | **1** | **QPGRACWVDKCR** |
|  | 1965 | **489.4827** | **976.9507** | **977.1616** | **-0.2109** | **1** | **12** | **1.8e+02** | **1** | **AKRPPSPPK** |
|  | 2968 | **666.3372** | **1995.9893** | **1995.2818** | **0.7075** | **0** | **12** | **1.6e+02** | **1** | **HCLSNISDPFIFNLCSK + Carbamidomethyl (C)** |
|  | 3073 | **683.6750** | **2048.0028** | **2048.4158** | **-0.4130** | **2** | **12** | **1.5e+02** | **1** | **RALGVLGQPGGLQMRLPER** |
|  | 245 | **375.1777** | **1122.5108** | **1121.3496** | **1.1612** | **0** | **12** | **1.6e+02** | **1** | **AMIPLQAYAK + Oxidation (M)** |
|  | 2441 | **553.0211** | **1656.0410** | **1656.9050** | **-0.8640** | **2** | **12** | **1.8e+02** | **1** | **DLNMSSSRGPIPVKR** |
|  | 3367 | **752.6123** | **1503.2098** | **1503.6834** | **-0.4736** | **1** | **12** | **1.4e+02** | **1** | **TMSQGRTISQAPAR** |
|  | 855 | **407.8652** | **1220.5735** | **1219.3965** | **1.1770** | **2** | **12** | **1.6e+02** | **1** | **LRFNGTSIRR** |
|  | 2758 | **606.5052** | **1816.4934** | **1816.9899** | **-0.4965** | **1** | **12** | **1.3e+02** | **1** | **CSWGMEEKAAASASCR + 2 Carbamidomethyl (C); Oxidation (M)** |
|  | 712 | **404.1998** | **1209.5773** | **1210.3385** | **-0.7611** | **1** | **12** | **1.5e+02** | **1** | **KCGNCGEISDK + Carbamidomethyl (C)** |
|  | 1004 | **417.3505** | **832.6861** | **831.8709** | **0.8152** | **0** | **12** | **1.5e+02** | **1** | **LSQAEER** |
|  | 2011 | **494.1745** | **1479.5014** | **1479.5678** | **-0.0663** | **0** | **12** | **1.8e+02** | **1** | **AVEHMFETEDGSK** |
|  | 2664 | **592.5875** | **1774.7404** | **1774.0495** | **0.6909** | **1** | **12** | **1.6e+02** | **1** | **DIFHVATLACVNKTNK** |
|  | 2836 | **619.2618** | **1236.5089** | **1237.3160** | **-0.8071** | **0** | **12** | **1.6e+02** | **1** | **DDAAPAPPVADAK** |
|  | 2859 | **626.3680** | **1876.0820** | **1875.0475** | **1.0345** | **1** | **12** | **1.7e+02** | **1** | **ALGVAVGGGVDGSRDELFR** |
|  | 941 | **413.2313** | **1236.6717** | **1237.3839** | **-0.7122** | **1** | **12** | **1.2e+02** | **1** | **SRVNMSIDTSK** |
|  | 3084 | **684.0952** | **1366.1756** | **1365.4948** | **0.6809** | **1** | **12** | **1.6e+02** | **1** | **RPSGISSRFSGSK** |
|  | 1448 | **446.1528** | **1335.4363** | **1334.4593** | **0.9771** | **2** | **12** | **1.9e+02** | **1** | **RLKNEMENER + Oxidation (M)** |
|  | 1587 | **458.4491** | **914.8834** | **915.0722** | **-0.1887** | **1** | **12** | **1.8e+02** | **1** | **CAAPGGVRK + Carbamidomethyl (C)** |
|  | 1874 | **480.0665** | **958.1183** | **958.1136** | **0.0047** | **2** | **12** | **1.7e+02** | **1** | **VENKRTVL** |
|  | 2354 | **538.1000** | **1611.2778** | **1611.8015** | **-0.5237** | **0** | **12** | **1.8e+02** | **1** | **RPQGAYSMPMSSGAR + Oxidation (M)** |
|  | 2568 | **578.2719** | **1154.5290** | **1155.3097** | **-0.7806** | **2** | **12** | **1.7e+02** | **1** | **FSSRLFSRR** |
|  | 469 | **389.0409** | **1164.1005** | **1164.3148** | **-0.2143** | **2** | **12** | **1.9e+02** | **1** | **EVRNKIYSR** |
|  | 826 | **407.3006** | **1218.8797** | **1218.1851** | **0.6947** | **0** | **12** | **1.3e+02** | **1** | **NSDIEHSSDSK** |
|  | 1947 | **488.1710** | **974.3273** | **973.2093** | **1.1180** | **1** | **12** | **1.8e+02** | **1** | **EKLVPFLK** |
|  | 2580 | **579.7513** | **1736.2319** | **1735.8427** | **0.3892** | **1** | **12** | **1.8e+02** | **1** | **LGINFKDEEEETSPK** |
|  | 3016 | 668.9286 | 1335.8424 | 1334.6926 | 1.1498 | 1 | 12 | 1.4e+02 | 1 | MPAFLGLKCLGK + Carbamidomethyl (C) |
|  | 3039 | **672.6780** | **2015.0118** | **2015.1871** | **-0.1754** | **1** | **12** | **1.6e+02** | **1** | **SLLNKDLSLENGAHAYNR** |
|  | 770 | **406.0902** | **1215.2485** | **1214.3306** | **0.9179** | **1** | **12** | **1.6e+02** | **1** | **ATFYASSVRGR** |
|  | 604 | **399.1942** | **1194.5603** | **1194.4253** | **0.1350** | **1** | **12** | **1.3e+02** | **1** | **RGSPCSMLSLK + Oxidation (M)** |
|  | 1642 | **460.5843** | **1378.7307** | **1377.5683** | **1.1624** | **0** | **12** | **2e+02** | **1** | **CSVLAAANPVYGR + Carbamidomethyl (C)** |
|  | 2523 | **569.1862** | **1704.5363** | **1704.9939** | **-0.4576** | **1** | **12** | **1.8e+02** | **1** | **YVWRFCIGLHSAPR** |
|  | 1667 | **462.1082** | **922.2016** | **922.0797** | **0.1219** | **0** | **12** | **1.6e+02** | **1** | **YVATLTVR** |
|  | 2228 | **520.2345** | **1557.6813** | **1556.6780** | **1.0033** | **0** | **12** | **1.7e+02** | **1** | **LHDHGHIQEETLK** |
|  | 2798 | **612.1697** | **1833.4869** | **1834.0602** | **-0.5733** | **1** | **12** | **1.7e+02** | **1** | **TEWSACSKTCGMGISTR + Oxidation (M)** |
|  | 236 | **374.3142** | **746.6136** | **745.7850** | **0.8285** | **1** | **12** | **1.8e+02** | **1** | **ERASAGR** |
|  | 743 | **405.1750** | **1212.5030** | **1211.4741** | **1.0289** | **0** | **12** | **1.5e+02** | **1** | **AQVPMVLTAGPK** |
|  | 885 | **408.2189** | **1221.6345** | **1220.4808** | **1.1537** | **0** | **12** | **1.5e+02** | **1** | **QCLPYAVVTVK** |
|  | 2121 | **507.7744** | **1520.3009** | **1519.4810** | **0.8199** | **1** | **12** | **1.4e+02** | **1** | **DTPAGTGEREDDEK** |
|  | 2420 | **550.1624** | **1647.4649** | **1646.8820** | **0.5829** | **2** | **12** | **1.7e+02** | **1** | **KKVIDEQAFLSPGSK** |
|  | 2763 | **607.7709** | **1820.2906** | **1820.9619** | **-0.6713** | **2** | **12** | **1.7e+02** | **1** | **ERCAQDACQAQAREK + 2 Carbamidomethyl (C)** |
|  | 2903 | **644.1697** | **1286.3246** | **1285.4532** | **0.8714** | **2** | **12** | **1.7e+02** | **1** | **LRGTVVGGRQDK** |
|  | 795 | **406.7348** | **1217.1821** | **1216.4126** | **0.7696** | **0** | **12** | **1.4e+02** | **1** | **HTAGVLFCVGGR** |
|  | 959 | **414.6224** | **1240.8450** | **1240.4258** | **0.4192** | **1** | **12** | **1.3e+02** | **1** | **ESGLLMFKGDK + Oxidation (M)** |
|  | 1933 | **487.0098** | **1458.0073** | **1458.5731** | **-0.5658** | **0** | **12** | **1.8e+02** | **1** | **SQGQEIETILANR** |
|  | 2735 | **599.7009** | **1796.0806** | **1796.0101** | **0.0705** | **0** | **12** | **2.1e+02** | **1** | **GLEWMGWINPYSGGTK** |
|  | 2770 | **608.5204** | **1822.5390** | **1822.0261** | **0.5129** | **0** | **12** | **1.4e+02** | **1** | **LEEALQGSLAQMESCR + Carbamidomethyl (C)** |
|  | 805 | **406.8949** | **811.7750** | **810.9611** | **0.8140** | **0** | **12** | **1.5e+02** | **1** | **MGSLTFR** |
|  | 3511 | **838.6101** | **2512.8081** | **2511.8869** | **0.9212** | **1** | **12** | **1.6e+02** | **1** | **DGDCSKGFFLVSLLVEIAAASAIK + Carbamidomethyl (C)** |
|  | 1028 | **419.1890** | **836.3633** | **836.0121** | **0.3512** | **2** | **12** | **1.9e+02** | **1** | **SNMTKKK** |
|  | 1825 | **476.2560** | **950.4972** | **951.1392** | **-0.6420** | **1** | **12** | **1.6e+02** | **1** | **SIMTDIKK + Oxidation (M)** |
|  | 686 | **403.7344** | **1208.1811** | **1209.3787** | **-1.1975** | **2** | **12** | **1.6e+02** | **1** | **GGNMKRVFER + Oxidation (M)** |
|  | 3024 | **669.7811** | **2006.3212** | **2006.2486** | **0.0726** | **2** | **12** | **2.1e+02** | **1** | **MSEPAGDVRQNPCGSKACR** |
|  | 112 | **368.2439** | **1101.7096** | **1102.2621** | **-0.5524** | **1** | **12** | **1.5e+02** | **1** | **LEKHMTAEK + Oxidation (M)** |
|  | 1329 | **437.9982** | **1310.9723** | **1311.5333** | **-0.5610** | **2** | **12** | **2e+02** | **1** | **NVLQRDLRIGK** |
|  | 2539 | **572.1162** | **1142.2176** | **1143.1913** | **-0.9736** | **1** | **12** | **1.7e+02** | **1** | **YGCGSSRGGSR + Carbamidomethyl (C)** |
|  | 1162 | **429.0587** | **1284.1541** | **1283.4802** | **0.6738** | **2** | **12** | **1.6e+02** | **1** | **LARRLQELER** |
|  | 2596 | **581.5693** | **1161.1238** | **1160.2830** | **0.8407** | **2** | **12** | **1.7e+02** | **1** | **KRSHAGYQTI** |
|  | 3465 | **799.0579** | **2394.1514** | **2394.6779** | **-0.5265** | **1** | **12** | **1.4e+02** | **1** | **TALDDTVLQVMEMINENAKSR + Oxidation (M)** |
|  | 2 | **360.3403** | **718.6658** | **719.7858** | **-1.1200** | **0** | **12** | **2e+02** | **1** | **IPYGDR** |
|  | 1544 | **454.8312** | **1361.4715** | **1361.5027** | **-0.0312** | **1** | **12** | **1.8e+02** | **1** | **TNKGPAFTLQER** |
|  | 1650 | **460.8231** | **1379.4470** | **1379.5015** | **-0.0545** | **1** | **12** | **1.8e+02** | **1** | **MERAGPSFGQQR + Oxidation (M)** |
|  | 2162 | **515.8906** | **1029.7665** | **1029.0689** | **0.6976** | **2** | **12** | **1.8e+02** | **1** | **EPDGRGRSR** |
|  | 2801 | **612.2301** | **1833.6681** | **1832.9214** | **0.7467** | **1** | **12** | **1.8e+02** | **1** | **LGADPRVYAEDGSTPER** |
|  | 3035 | **671.0460** | **2010.1159** | **2011.3013** | **-1.1854** | **1** | **12** | **1.5e+02** | **1** | **EACSILAEALCDMDIRNK + Oxidation (M)** |
|  | 3220 | **719.6171** | **2155.8290** | **2155.5180** | **0.3111** | **2** | **12** | **1.4e+02** | **1** | **AKKAMGYQPLVTMDDAMER** |
|  | 153 | **369.3316** | **1104.9726** | **1104.2117** | **0.7610** | **1** | **12** | **1.7e+02** | **1** | **KELTSSGGPTK** |
|  | 226 | **374.1992** | **1119.5754** | **1120.3402** | **-0.7648** | **0** | **12** | **1.7e+02** | **1** | **MDQPEMILK + Oxidation (M)** |
|  | 813 | **407.0912** | **812.1677** | **812.9538** | **-0.7860** | **0** | **12** | **1.7e+02** | **1** | **ADIAAKPK** |
|  | 1621 | **459.8264** | **1376.4569** | **1377.5020** | **-1.0451** | **0** | **12** | **1.9e+02** | **1** | **HTLTTIAAAGIDHG** |
|  | 1900 | **483.7513** | **1448.2318** | **1447.6996** | **0.5321** | **1** | **12** | **1.5e+02** | **1** | **VSADAMLRALLGSK + Oxidation (M)** |
|  | 1921 | **486.2039** | **1455.5895** | **1454.7818** | **0.8077** | **2** | **12** | **1.7e+02** | **1** | **MIRDGCVMKITR + 2 Oxidation (M)** |
|  | 2201 | **519.0599** | **1036.1050** | **1035.1794** | **0.9256** | **2** | **12** | **1.7e+02** | **1** | **AKGGSATCRK + Carbamidomethyl (C)** |
|  | 297 | **380.0654** | **758.1160** | **757.8586** | **0.2573** | **0** | **12** | **2e+02** | **1** | **IEHTCR** |
|  | 856 | **407.8675** | **1220.5803** | **1221.3828** | **-0.8025** | **0** | **12** | **1.7e+02** | **1** | **EMPFTNPLTR + Oxidation (M)** |
|  | 858 | **407.8710** | **1220.5909** | **1221.3645** | **-0.7735** | **0** | **12** | **1.7e+02** | **1** | **EPFAHSLPPAR** |
|  | 1528 | **452.9810** | **903.9473** | **903.9370** | **0.0103** | **0** | **12** | **1.9e+02** | **1** | **VAGNETSAR** |
|  | 1559 | **456.4243** | **910.8338** | **911.0240** | **-0.1902** | **2** | **12** | **1.4e+02** | **1** | **RTHSRVR** |
|  | 1769 | **470.8243** | **1409.4508** | **1409.7378** | **-0.2869** | **2** | **12** | **1.5e+02** | **1** | **FQMVKQKIPFK + Oxidation (M)** |
|  | 2159 | **515.5345** | **1029.0542** | **1028.1587** | **0.8955** | **0** | **12** | **2e+02** | **1** | **MSSCGLETK + Carbamidomethyl (C); Oxidation (M)** |
|  | 3150 | **689.8764** | **2066.6070** | **2067.2390** | **-0.6319** | **2** | **12** | **1.6e+02** | **1** | **FKKEHEEFETMENVNR** |
|  | 23 | **362.1713** | **722.3278** | **721.8664** | **0.4614** | **0** | **12** | **1.4e+02** | **1** | **SITMVR + Oxidation (M)** |
|  | 3137 | 687.4576 | 2059.3508 | 2059.3275 | 0.0232 | 0 | 12 | 1.7e+02 | 1 | ICELSAMTCADGPCFNGGR + 2 Carbamidomethyl (C) |
|  | 249 | **375.4989** | **1123.4745** | **1122.2948** | **1.1798** | **0** | **12** | **2e+02** | **1** | **YLDVCPVSAR** |
|  | 1219 | **432.4251** | **1294.2531** | **1293.4041** | **0.8490** | **1** | **12** | **1.9e+02** | **1** | **KMNELETEQR + Oxidation (M)** |
|  | 1376 | **442.3460** | **1324.0159** | **1324.5208** | **-0.5049** | **0** | **12** | **1.3e+02** | **1** | **VTQEAAVIFTFV** |
|  | 1721 | **466.1787** | **1395.5139** | **1394.5741** | **0.9398** | **1** | **12** | **1.8e+02** | **1** | **DCPSRAMGLASGLT + Oxidation (M)** |
|  | 1998 | **492.4425** | **1474.3054** | **1473.5463** | **0.7591** | **0** | **12** | **1.4e+02** | **1** | **GAAGSGDAAAAAEWIR** |
|  | 3244 | **732.1477** | **2193.4209** | **2194.4393** | **-1.0184** | **0** | **12** | **1.7e+02** | **1** | **NSLDFPSFINLSNVLLGESK** |
|  | 713 | **404.2312** | **1209.6715** | **1209.4364** | **0.2351** | **0** | **12** | **1.4e+02** | **1** | **NLLPVLHFEK** |
|  | 484 | **389.1775** | **776.3403** | **776.8355** | **-0.4953** | **0** | **12** | **1.8e+02** | **1** | **ATTASQAK** |
|  | 1426 | **444.9889** | **887.9630** | **886.9924** | **0.9706** | **0** | **12** | **2.1e+02** | **1** | **QQGNTVLK** |
|  | 1810 | **475.3613** | **948.7078** | **948.1219** | **0.5860** | **0** | **12** | **1.5e+02** | **1** | **NCVLGPCSR** |
|  | 2688 | **593.8761** | **1778.6061** | **1777.9173** | **0.6889** | **1** | **12** | **1.5e+02** | **1** | **SGQCQGRDQCPEPCR + 2 Carbamidomethyl (C)** |
|  | 2805 | **612.3345** | **1833.9812** | **1835.0081** | **-1.0269** | **0** | **12** | **1.8e+02** | **1** | **EPGAGAQGALWPGPGPCNR** |
|  | 3321 | **742.3231** | **1482.6315** | **1481.5620** | **1.0695** | **1** | **12** | **1.7e+02** | **1** | **APYWTNTEKDEK** |
|  | 322 | **382.3232** | **1143.9475** | **1143.2740** | **0.6734** | **0** | **12** | **1.7e+02** | **1** | **HASNMLGELR + Oxidation (M)** |
|  | 1288 | **436.0813** | **870.1479** | **869.0155** | **1.1324** | **1** | **12** | **1.7e+02** | **1** | **AIEKDMM + 2 Oxidation (M)** |
|  | 1534 | **454.1519** | **906.2891** | **907.0071** | **-0.7179** | **1** | **12** | **1.9e+02** | **1** | **AKGGSATCR + Carbamidomethyl (C)** |
|  | 1847 | **477.7120** | **953.4093** | **954.1282** | **-0.7189** | **2** | **12** | **1.3e+02** | **1** | **RVDLPRAK** |
|  | 2444 | **553.3835** | **1104.7522** | **1104.2116** | **0.5405** | **1** | **12** | **1.6e+02** | **1** | **DEAAKLQTTK** |
|  | 72 | **364.3089** | **726.6030** | **725.7937** | **0.8093** | **0** | **12** | **1.3e+02** | **1** | **VISHDR** |
|  | 1457 | **447.1595** | **892.3042** | **893.0432** | **-0.7391** | **0** | **12** | **1.8e+02** | **1** | **ACLNGCAK + 2 Carbamidomethyl (C)** |
|  | 2154 | **514.7048** | **1541.0923** | **1540.6754** | **0.4169** | **0** | **12** | **1.5e+02** | **1** | **ALDTEAAPAPTAAWR** |
|  | 2220 | **520.1650** | **1038.3153** | **1037.3425** | **0.9728** | **2** | **12** | **1.7e+02** | **1** | **KVPKALLIR** |
|  | 3065 | **681.2754** | **1360.5360** | **1359.4919** | **1.0441** | **2** | **12** | **1.8e+02** | **1** | **RTSLEGGQVRTR** |
|  | 527 | **391.2681** | **1170.7822** | **1171.3834** | **-0.6012** | **0** | **12** | **1.3e+02** | **1** | **LLEAIISLDGK** |
|  | 1264 | **435.1433** | **1302.4077** | **1302.3015** | **0.1061** | **0** | **12** | **1.7e+02** | **1** | **DPQISEAEEER** |
|  | 1385 | **443.3074** | **884.6000** | **884.9800** | **-0.3799** | **0** | **12** | **1.3e+02** | **1** | **RPAAGTGQK** |
|  | 2012 | **494.1835** | **986.3522** | **986.1667** | **0.1855** | **0** | **12** | **1.9e+02** | **1** | **MYCVLADR + Oxidation (M)** |
|  | 482 | **389.1596** | **776.3045** | **775.8078** | **0.4967** | **0** | **12** | **1.9e+02** | **1** | **STDQAVR** |
|  | 931 | **412.2516** | **1233.7328** | **1234.3367** | **-0.6040** | **0** | **12** | **1.5e+02** | **1** | **EVDALDGLCSR + Carbamidomethyl (C)** |
|  | 940 | **413.1697** | **824.3246** | **823.9398** | **0.3847** | **0** | **12** | **1.6e+02** | **1** | **ISGHIAAR** |
|  | 958 | **414.6039** | **1240.7897** | **1240.5384** | **0.2513** | **1** | **12** | **1.4e+02** | **1** | **FLSCMSVLRGK** |
|  | 1123 | **426.1224** | **1275.3450** | **1275.3770** | **-0.0321** | **1** | **12** | **1.8e+02** | **1** | **RQAAAHTDHLR** |
|  | 2505 | **565.5347** | **1129.0547** | **1128.4086** | **0.6461** | **2** | **12** | **1.7e+02** | **1** | **KIGCYAAMKK + Oxidation (M)** |
|  | 3157 | **690.7028** | **1379.3907** | **1378.5930** | **0.7978** | **1** | **12** | **1.8e+02** | **1** | **MDPFTEKLLER** |
|  | 317 | **381.3523** | **1141.0346** | **1140.1674** | **0.8672** | **0** | **12** | **2e+02** | **1** | **SSGGGAGAVHGAGR** |
|  | 2157 | **515.1763** | **1542.5066** | **1542.8220** | **-0.3154** | **2** | **12** | **1.8e+02** | **1** | **LGFKAKQGYVIYR** |
|  | 39 | **363.0922** | **1086.2543** | **1085.2546** | **0.9997** | **0** | **12** | **1.8e+02** | **1** | **LLVAGATVDAR** |
|  | 203 | **372.2740** | **742.5333** | **741.9190** | **0.6144** | **1** | **12** | **1.4e+02** | **1** | **KPKEIK** |
|  | 1027 | **419.1817** | **1254.5228** | **1254.3893** | **0.1335** | **0** | **12** | **2e+02** | **1** | **QLLEQPQAEAK** |
|  | 2341 | **536.9874** | **1071.9599** | **1071.2941** | **0.6658** | **0** | **12** | **2e+02** | **1** | **NSVCIIPLR + Carbamidomethyl (C)** |
|  | 3048 | **678.8406** | **2033.4996** | **2032.5356** | **0.9639** | **1** | **12** | **1.9e+02** | **1** | **MLLSIFCLHLNDLPMKK + Oxidation (M)** |
|  | 2182 | **518.7533** | **1035.4918** | **1036.1657** | **-0.6739** | **1** | **12** | **1.4e+02** | **1** | **CQPGFSGKR + Carbamidomethyl (C)** |
|  | 2340 | **536.7124** | **1607.1150** | **1606.7417** | **0.3733** | **1** | **12** | **1.8e+02** | **1** | **QRLTEPQHGLGSQR** |
|  | 175 | **370.2242** | **1107.6506** | **1107.1293** | **0.5212** | **0** | **12** | **1.2e+02** | **1** | **GSSGSSGGEAIAK** |
|  | 899 | **408.9145** | **1223.7213** | **1224.2160** | **-0.4948** | **1** | **12** | **2e+02** | **1** | **AGSRASEGQDSC + Carbamidomethyl (C)** |
|  | 1000 | **417.1069** | **1248.2986** | **1247.4034** | **0.8952** | **0** | **12** | **2e+02** | **1** | **MDGMGPPQWGR + Oxidation (M)** |
|  | 1425 | **444.9505** | **887.8862** | **887.0354** | **0.8508** | **1** | **12** | **2.1e+02** | **1** | **ALKNSLGGK** |
|  | 1759 | **470.0428** | **938.0709** | **937.1340** | **0.9369** | **1** | **12** | **1.6e+02** | **1** | **KSLLTTFK** |
|  | 2017 | **494.4527** | **1480.3360** | **1479.7199** | **0.6161** | **0** | **12** | **1.6e+02** | **1** | **EMMAIWPNLSQK + 2 Oxidation (M)** |
|  | 2856 | **625.7645** | **1874.2714** | **1874.0827** | **0.1887** | **1** | **12** | **2e+02** | **1** | **VSSVTAADTAVYFCARR + Carbamidomethyl (C)** |
|  | 437 | **387.9302** | **1160.7684** | **1160.3013** | **0.4671** | **1** | **12** | **2.2e+02** | **1** | **NKMDIQGNPK + Oxidation (M)** |
|  | 881 | **408.1963** | **1221.5667** | **1221.3447** | **0.2220** | **1** | **12** | **1.7e+02** | **1** | **RDTISQMTGGR** |
|  | 1451 | **446.9673** | **891.9198** | **891.0012** | **0.9186** | **0** | **12** | **1.9e+02** | **1** | **SAATTVMNP** |
|  | 1820 | **476.1841** | **950.3534** | **950.0962** | **0.2572** | **0** | **12** | **1.8e+02** | **1** | **HQVQLLGR** |
|  | 556 | **394.1613** | **786.3079** | **786.8768** | **-0.5689** | **1** | **12** | **1.8e+02** | **1** | **RAAASPSK** |
|  | 951 | **414.2099** | **1239.6074** | **1240.4357** | **-0.8283** | **2** | **12** | **1.5e+02** | **1** | **ERVCAEHLRK** |
|  | 1432 | **445.1084** | **1332.3031** | **1332.4169** | **-0.1138** | **1** | **12** | **2e+02** | **1** | **TGGKEAASGTTPQK** |
|  | 306 | **380.2763** | **758.5378** | **758.8186** | **-0.2807** | **0** | **12** | **1.7e+02** | **1** | **ASGYSFK** |
|  | 1076 | **422.0910** | **1263.2507** | **1264.4506** | **-1.1998** | **1** | **12** | **2e+02** | **1** | **TLVKDMWDTR** |
|  | 1967 | **489.7972** | **1466.3695** | **1465.6801** | **0.6893** | **1** | **12** | **1.5e+02** | **1** | **RCAASGAPPAAVAPAR** |
|  | 1974 | **490.1575** | **978.3002** | **978.0619** | **0.2383** | **2** | **12** | **1.8e+02** | **1** | **SKKGQSTSR** |
|  | 1007 | **417.9409** | **1250.8004** | **1251.4583** | **-0.6579** | **1** | **12** | **1.9e+02** | **1** | **GGAIRMGIFSSR** |
|  | 1424 | **444.8928** | **887.7709** | **886.9959** | **0.7750** | **2** | **12** | **2.1e+02** | **1** | **VRAGEIRS** |
|  | 2659 | **592.3932** | **1182.7716** | **1182.1546** | **0.6170** | **0** | **12** | **1.7e+02** | **1** | **SYNPEGESSGR** |
|  | 2329 | **536.1183** | **1605.3329** | **1605.7504** | **-0.4175** | **0** | **12** | **1.8e+02** | **1** | **ECCFTFTLNGNSR + 2 Carbamidomethyl (C)** |
|  | 679 | **403.2596** | **804.5045** | **803.9007** | **0.6038** | **1** | **12** | **1.6e+02** | **1** | **VDLSKDK** |
|  | 930 | **412.2245** | **1233.6515** | **1234.4661** | **-0.8146** | **2** | **12** | **1.6e+02** | **1** | **DMVSKKLELR + Oxidation (M)** |
|  | 1870 | **479.7975** | **1436.3702** | **1436.5926** | **-0.2224** | **1** | **12** | **1.6e+02** | **1** | **HDLMRSSQPGVPP + Oxidation (M)** |
|  | 27 | **362.2361** | **1083.6862** | **1083.2620** | **0.4242** | **2** | **12** | **1.4e+02** | **1** | **MRGDYGKLK + Oxidation (M)** |
|  | 73 | **364.3362** | **1089.9863** | **1089.2482** | **0.7380** | **1** | **12** | **1.6e+02** | **1** | **VIDAAKHAHK** |
|  | 2811 | **613.2633** | **1836.7677** | **1836.0047** | **0.7630** | **0** | **12** | **1.8e+02** | **1** | **EGLGEYTCTCLEGFEGK** |
|  | 3032 | **670.8074** | **2009.3999** | **2010.2721** | **-0.8721** | **1** | **12** | **2.1e+02** | **1** | **MMDETEIGSCFGRYGSVK** |
|  | 860 | **407.8878** | **1220.6414** | **1220.3349** | **0.3064** | **1** | **12** | **1.8e+02** | **1** | **DKLSHIHDQK** |
|  | 554 | **393.5779** | **1177.7115** | **1177.2622** | **0.4492** | **0** | **12** | **1.6e+02** | **1** | **SQLTINADTSK** |
|  | 921 | **411.3869** | **1231.1384** | **1231.4108** | **-0.2723** | **2** | **12** | **1.8e+02** | **1** | **RPPPEAPRRR** |
|  | 1352 | 439.1542 | 1314.4405 | 1314.5704 | -0.1299 | 2 | 12 | 2e+02 | 1 | LDELQKLISKK |
|  | 2250 | **523.5486** | **1045.0825** | **1044.1993** | **0.8832** | **0** | **12** | **2.2e+02** | **1** | **LVDITDIQK** |
|  | 3109 | **685.9281** | **2054.7621** | **2054.3974** | **0.3647** | **2** | **12** | **1.5e+02** | **1** | **GPDGKMHGNKCAMCASVFK + Carbamidomethyl (C); Oxidation (M)** |
|  | 623 | **400.3789** | **798.7430** | **798.9471** | **-0.2040** | **0** | **12** | **1.6e+02** | **1** | **ISAAMYK + Oxidation (M)** |
|  | 851 | **407.8511** | **1220.5311** | **1221.3662** | **-0.8350** | **2** | **12** | **1.9e+02** | **1** | **VAYLSGGRDKR** |
|  | 1706 | **464.2783** | **926.5417** | **926.0252** | **0.5165** | **0** | **12** | **1.5e+02** | **1** | **ELGIETHK** |
|  | 2055 | **501.3145** | **1000.6142** | **1000.1135** | **0.5007** | **1** | **12** | **1.7e+02** | **1** | **QRNGLSGLR** |
|  | 2616 | **584.3290** | **1166.6432** | **1166.2199** | **0.4233** | **1** | **12** | **1.8e+02** | **1** | **ADQDSEAMKR + Oxidation (M)** |
|  | 3504 | **829.6888** | **1657.3629** | **1656.8849** | **0.4780** | **0** | **12** | **1.5e+02** | **1** | **NFINPPVPLQGPHAR** |
|  | 510 | **390.6977** | **1169.0709** | **1169.3266** | **-0.2557** | **0** | **12** | **1.5e+02** | **1** | **MEKPSSMDTK + Oxidation (M)** |
|  | 581 | **397.3945** | **1189.1612** | **1188.3147** | **0.8465** | **0** | **12** | **2e+02** | **1** | **MAAAAGAAAAPGSR + Oxidation (M)** |
|  | 978 | **416.0308** | **1245.0702** | **1245.4274** | **-0.3572** | **0** | **12** | **2.2e+02** | **1** | **DEGCAQMMCK + 2 Carbamidomethyl (C); Oxidation (M)** |
|  | 1122 | **426.0197** | **850.0246** | **848.9694** | **1.0553** | **1** | **12** | **1.9e+02** | **1** | **KYDHMR** |
|  | 2678 | **592.9516** | **1183.8884** | **1183.2551** | **0.6334** | **1** | **12** | **1.7e+02** | **1** | **GNNSYGQCGRK** |
|  | 3210 | **713.5114** | **2137.5119** | **2136.3207** | **1.1912** | **1** | **12** | **1.7e+02** | **1** | **FMAGAEETNNKSCFWAEK + Carbamidomethyl (C); Oxidation (M)** |
|  | 19 | **361.9394** | **721.8640** | **722.9206** | **-1.0566** | **1** | **12** | **1.8e+02** | **1** | **CRMLK + Carbamidomethyl (C); Oxidation (M)** |
|  | 1124 | **426.1547** | **1275.4419** | **1275.4350** | **0.0069** | **1** | **12** | **1.9e+02** | **1** | **DAAAALAAMNGRK + Oxidation (M)** |
|  | 1165 | **429.2222** | **1284.6444** | **1284.5082** | **0.1362** | **1** | **12** | **1.7e+02** | **1** | **CSPKMPPAPSGR + Carbamidomethyl (C)** |
|  | 1858 | **478.1096** | **1431.3067** | **1430.5880** | **0.7187** | **1** | **12** | **1.7e+02** | **1** | **YQPGGRYLSMSR + Oxidation (M)** |
|  | 2156 | **515.0790** | **1028.1432** | **1027.0959** | **1.0472** | **1** | **12** | **1.9e+02** | **1** | **GTPGAGGAGRAR** |
|  | 628 | **401.0887** | **800.1626** | **798.9702** | **1.1924** | **0** | **12** | **2.1e+02** | **1** | **LIGKPSGK** |
|  | 703 | **404.0893** | **1209.2457** | **1209.4382** | **-0.1925** | **1** | **12** | **1.9e+02** | **1** | **RTLSLQAPPVK** |
|  | 1841 | **477.5450** | **1429.6128** | **1428.5473** | **1.0655** | **0** | **12** | **2e+02** | **1** | **DSGACFSGAAASCPK + Carbamidomethyl (C)** |
|  | 2065 | **502.2220** | **1002.4292** | **1002.0798** | **0.3494** | **0** | **12** | **2e+02** | **1** | **TQDLEGALR** |
|  | 2279 | **529.5819** | **1057.1490** | **1056.1075** | **1.0416** | **0** | **12** | **2.3e+02** | **1** | **FSEMQNER + Oxidation (M)** |
|  | 548 | **393.2964** | **1176.8672** | **1176.3008** | **0.5664** | **0** | **12** | **1.4e+02** | **1** | **THGTGIMNTTK + Oxidation (M)** |
|  | 934 | **412.3459** | **822.6769** | **821.7437** | **0.9333** | **0** | **12** | **1.6e+02** | **1** | **DSDGEDGK** |
|  | 1354 | 439.5455 | 877.0763 | 875.9069 | 1.1694 | 0 | 12 | 2.3e+02 | 1 | GGSEGGCPR + Carbamidomethyl (C) |
|  | 2442 | **553.2026** | **1656.5856** | **1656.9065** | **-0.3210** | **2** | **12** | **2e+02** | **1** | **YCQKFQGRVTITR + Carbamidomethyl (C)** |
|  | 2582 | **579.8546** | **1736.5417** | **1736.0214** | **0.5203** | **0** | **12** | **1.6e+02** | **1** | **TPVGFIGLGNMGNPMAK + 2 Oxidation (M)** |
|  | 1031 | **419.1935** | **1254.5583** | **1253.4046** | **1.1536** | **0** | **12** | **2.1e+02** | **1** | **EQAAALPPTLSR** |
|  | 1095 | **422.2216** | **1263.6425** | **1264.4489** | **-0.8064** | **1** | **12** | **1.8e+02** | **1** | **KAEEMALSLTR + Oxidation (M)** |
|  | 1627 | **460.1594** | **918.3039** | **918.0067** | **0.2973** | **2** | **12** | **2.1e+02** | **1** | **KEAEASRK** |
|  | 1676 | **462.6717** | **1384.9928** | **1385.5641** | **-0.5713** | **1** | **12** | **1.4e+02** | **1** | **SLMKMDDASISR + 2 Oxidation (M)** |
|  | 3251 | **734.4463** | **2200.3167** | **2200.7404** | **-0.4238** | **2** | **12** | **1.8e+02** | **1** | **GKEAPPCMLCRGPCRPLCLR** |
|  | 874 | **408.1210** | **1221.3407** | **1221.2751** | **0.0657** | **2** | **12** | **2e+02** | **1** | **KQRSDYFDY** |
|  | 1454 | **447.0654** | **1338.1741** | **1337.5409** | **0.6332** | **1** | **12** | **2e+02** | **1** | **QKVIIADCGEYV** |
|  | 1485 | **449.4788** | **1345.4143** | **1344.5632** | **0.8511** | **1** | **12** | **2.1e+02** | **1** | **ATKHICAICGDR + Carbamidomethyl (C)** |
|  | 1057 | **420.2675** | **1257.7805** | **1257.3701** | **0.4103** | **1** | **12** | **1.4e+02** | **1** | **CVTSNSLAKDGY** |
|  | 3400 | **760.1981** | **2277.5720** | **2276.7650** | **0.8070** | **1** | **12** | **1.8e+02** | **1** | **WNIKSQTFICMLLCPFCR + Carbamidomethyl (C); Oxidation (M)** |
|  | 1727 | **466.3857** | **1396.1351** | **1396.7207** | **-0.5857** | **2** | **12** | **1.6e+02** | **1** | **KIVRTEIGVLLR** |
|  | 2373 | **540.2764** | **1617.8069** | **1618.7643** | **-0.9573** | **0** | **12** | **1.9e+02** | **1** | **VWFGDVEAETMYR + Oxidation (M)** |
|  | 3156 | **690.6913** | **1379.3679** | **1378.4471** | **0.9209** | **1** | **12** | **1.8e+02** | **1** | **KESQNLENNFR** |
|  | 1647 | **460.7185** | **1379.1333** | **1379.6242** | **-0.4908** | **0** | **12** | **1.6e+02** | **1** | **MFFVEMCVDAR + 2 Oxidation (M)** |
|  | 2412 | **549.4350** | **1645.2828** | **1645.9800** | **-0.6971** | **2** | **12** | **1.5e+02** | **1** | **FKDELDIMKFICK + Oxidation (M)** |
|  | 3134 | **686.8795** | **2057.6164** | **2057.3218** | **0.2946** | **0** | **12** | **1.7e+02** | **1** | **TTSDLFLEVTSATSLQICK** |
|  | 571 | **396.1937** | **790.3725** | **789.8558** | **0.5168** | **0** | **12** | **1.9e+02** | **1** | **HAWETM + Oxidation (M)** |
|  | 1751 | **469.4671** | **1405.3791** | **1404.5887** | **0.7904** | **0** | **12** | **1.8e+02** | **1** | **ELSIQCLGVETR + Carbamidomethyl (C)** |
|  | 2083 | **504.2933** | **1509.8578** | **1509.6434** | **0.2145** | **0** | **12** | **1.9e+02** | **1** | **VTPAHSPADAEMGAR** |
|  | 2326 | 536.0461 | 1605.1163 | 1604.8487 | 0.2676 | 0 | 12 | 1.9e+02 | 1 | MPPGGGGEVVFSCPVR + Oxidation (M) |
|  | 2377 | **540.5437** | **1079.0726** | **1078.1976** | **0.8751** | **0** | **12** | **1.9e+02** | **1** | **LHTSTMSSSK** |
|  | 3192 | **703.7430** | **1405.4712** | **1404.5290** | **0.9421** | **1** | **12** | **2.1e+02** | **1** | **WDTHYAQSLRK** |
|  | 74 | **364.4153** | **1090.2237** | **1091.2639** | **-1.0403** | **0** | **11** | **2.1e+02** | **1** | **CLAWCEPR + 2 Carbamidomethyl (C)** |
|  | 264 | **377.1452** | **752.2756** | **751.8907** | **0.3850** | **0** | **11** | **1.7e+02** | **1** | **MTPFEK** |
|  | 761 | **405.8333** | **1214.4777** | **1214.4333** | **0.0444** | **1** | **11** | **1.8e+02** | **1** | **MDCCVDIKSK + Carbamidomethyl (C); Oxidation (M)** |
|  | 1619 | **459.6437** | **917.2726** | **917.0863** | **0.1863** | **0** | **11** | **1.8e+02** | **1** | **CIPSVWR + Carbamidomethyl (C)** |
|  | 1997 | **492.3879** | **1474.1416** | **1473.6689** | **0.4726** | **0** | **11** | **1.5e+02** | **1** | **ALTTVTIQDGLGVW** |
|  | 2886 | **637.7476** | **1910.2207** | **1911.1941** | **-0.9734** | **2** | **11** | **2.4e+02** | **1** | **MTGPRPRQKAAVAEGAVR + Oxidation (M)** |
|  | 396 | **386.6844** | **1157.0310** | **1157.4068** | **-0.3758** | **2** | **11** | **1.6e+02** | **1** | **LRFSKASCVM + Oxidation (M)** |
|  | 2875 | **630.7848** | **1259.5548** | **1259.4391** | **0.1157** | **2** | **11** | **2.1e+02** | **1** | **LPGSCVRSERR** |
|  | 16 | **361.2212** | **1080.6413** | **1080.1057** | **0.5356** | **0** | **11** | **1.5e+02** | **1** | **LGFSSSDPDR** |
|  | 900 | **408.9680** | **1223.8817** | **1223.3622** | **0.5195** | **1** | **11** | **2.1e+02** | **1** | **CARVPSSGFSR + Carbamidomethyl (C)** |
|  | 1770 | **470.9866** | **939.9584** | **940.0584** | **-0.1001** | **1** | **11** | **1.7e+02** | **1** | **RAAGAELPR** |
|  | 3005 | **668.6826** | **2003.0257** | **2002.2548** | **0.7709** | **1** | **11** | **2e+02** | **1** | **DCGAPCEPGRANGLMYFK + Carbamidomethyl (C); Oxidation (M)** |
|  | 715 | **404.7627** | **1211.2659** | **1212.3642** | **-1.0983** | **2** | **11** | **1.8e+02** | **1** | **RLREAFHAGR** |
|  | 1722 | **466.1814** | **930.3480** | **931.0468** | **-0.6988** | **1** | **11** | **2e+02** | **1** | **KFHADSVK** |
|  | 2114 | **506.9282** | **1517.7623** | **1517.7877** | **-0.0254** | **0** | **11** | **1.9e+02** | **1** | **VMAGALEGDIFIGPK** |
|  | 2986 | **666.9495** | **1331.8841** | **1332.5757** | **-0.6916** | **2** | **11** | **1.6e+02** | **1** | **TLLARLSSCRGR** |
|  | 3180 | **698.9579** | **1395.9010** | **1395.6450** | **0.2560** | **1** | **11** | **1.5e+02** | **1** | **QLEGKSCSLVGMK + Oxidation (M)** |
|  | 819 | 407.1963 | 1218.5667 | 1218.3375 | 0.2292 | 0 | 11 | 1.7e+02 | 1 | EGVGMQDPTLR + Oxidation (M) |
|  | 909 | **410.2108** | **1227.6104** | **1227.4120** | **0.1983** | **1** | **11** | **1.8e+02** | **1** | **IANFVEHKAAK** |
|  | 717 | **404.7845** | **1211.3314** | **1212.4387** | **-1.1073** | **0** | **11** | **1.8e+02** | **1** | **LDVLLALASAAR** |
|  | 1521 | **452.2378** | **1353.6911** | **1353.5239** | **0.1672** | **1** | **11** | **2.2e+02** | **1** | **GPQDAVLVRVGDK** |
|  | 3337 | **743.5504** | **2227.6291** | **2228.4641** | **-0.8350** | **0** | **11** | **1.9e+02** | **1** | **SLPAPVAQRPDSPGGGLQAPGQK** |
|  | 649 | **401.9348** | **801.8547** | **802.8795** | **-1.0247** | **2** | **11** | **2.2e+02** | **1** | **KGRGETR** |
|  | 916 | **411.2039** | **820.3931** | **819.9511** | **0.4420** | **0** | **11** | **1.8e+02** | **1** | **LGIAPHGR** |
|  | 1024 | **419.1397** | **1254.3969** | **1253.4478** | **0.9491** | **0** | **11** | **2.2e+02** | **1** | **KPGLGVPGSSGAVK** |
|  | 1082 | **422.1454** | **842.2760** | **841.9752** | **0.3008** | **0** | **11** | **2.1e+02** | **1** | **SPSPAPMR** |
|  | 1734 | **467.7954** | **1400.3640** | **1401.5634** | **-1.1994** | **0** | **11** | **1.8e+02** | **1** | **YFCAAMYSSASK + Carbamidomethyl (C); Oxidation (M)** |
|  | 3061 | **681.1766** | **2040.5077** | **2039.5498** | **0.9579** | **1** | **11** | **1.9e+02** | **1** | **KPTLISVLVIIFILRGTR** |
|  | 529 | **391.3264** | **1170.9572** | **1170.2698** | **0.6874** | **1** | **11** | **1.4e+02** | **1** | **ELSPEGPGKEK** |
|  | 582 | **398.1494** | **1191.4262** | **1191.3187** | **0.1075** | **1** | **11** | **2e+02** | **1** | **MGKVGAGGGSQAR + Oxidation (M)** |
|  | 1598 | **458.8725** | **1373.5953** | **1372.5257** | **1.0697** | **0** | **11** | **2.1e+02** | **1** | **MATTATMATSGSAR + Oxidation (M)** |
|  | 1780 | **472.1616** | **1413.4625** | **1414.5903** | **-1.1278** | **1** | **11** | **1.9e+02** | **1** | **RAPGPATASAAAAMR + Oxidation (M)** |
|  | 2239 | **521.4170** | **1561.2288** | **1561.7807** | **-0.5519** | **1** | **11** | **1.5e+02** | **1** | **EVQSLGQAKVYALR** |
|  | 955 | **414.3088** | **1239.9042** | **1239.5172** | **0.3870** | **2** | **11** | **1.4e+02** | **1** | **HRCPKLAMAR + Carbamidomethyl (C)** |
|  | 2189 | **518.9155** | **1553.7244** | **1554.7052** | **-0.9807** | **0** | **11** | **1.9e+02** | **1** | **QWMYGCDLGPDGR + Carbamidomethyl (C)** |
|  | 2194 | **518.9893** | **1035.9637** | **1036.2237** | **-0.2599** | **0** | **11** | **1.9e+02** | **1** | **PELPVPAWK** |
|  | 133 | **369.2297** | **1104.6670** | **1105.2031** | **-0.5360** | **2** | **11** | **1.7e+02** | **1** | **KTPSSSSRQK** |
|  | 1579 | **458.1706** | **914.3265** | **914.0608** | **0.2656** | **0** | **11** | **2e+02** | **1** | **GQLAASVLR** |
|  | 2765 | **607.9363** | **1820.7867** | **1820.0070** | **0.7797** | **1** | **11** | **1.7e+02** | **1** | **AQSKDIFTQPSGSPIPF** |
|  | 3089 | **684.1755** | **2049.5044** | **2050.4665** | **-0.9621** | **2** | **11** | **1.9e+02** | **1** | **MYGKIIFVLLLSDTHKR + Oxidation (M)** |
|  | 3276 | **740.3907** | **2218.1499** | **2217.5692** | **0.5807** | **2** | **11** | **1.9e+02** | **1** | **ELRQSTMFNPCPSERMMK + 2 Oxidation (M)** |
|  | 644 | **401.9089** | **1202.7046** | **1203.3079** | **-0.6032** | **1** | **11** | **2.2e+02** | **1** | **HPSSRSGGPPPK** |
|  | 2294 | **532.4028** | **1594.1863** | **1593.7963** | **0.3901** | **0** | **11** | **1.6e+02** | **1** | **VLQEYEMEVVPNK + Oxidation (M)** |
|  | 2370 | **540.1255** | **1617.3543** | **1616.9418** | **0.4124** | **0** | **11** | **2e+02** | **1** | **LMPGWCAPISDLLK + Carbamidomethyl (C); Oxidation (M)** |
|  | 3448 | **788.7834** | **1575.5521** | **1576.7325** | **-1.1803** | **2** | **11** | **1.6e+02** | **1** | **CEGEVNTRFSLKH + Carbamidomethyl (C)** |
|  | 3508 | **833.2148** | **2496.6224** | **2495.6742** | **0.9482** | **1** | **11** | **1.7e+02** | **1** | **DSSAGRPSMSVSASSEEWKSIPAI + Oxidation (M)** |
|  | 1908 | **484.3065** | **1449.8974** | **1449.6541** | **0.2432** | **2** | **11** | **1.6e+02** | **1** | **LSLKNDAPQAKHK** |
|  | 1935 | **487.1500** | **1458.4278** | **1459.6028** | **-1.1750** | **0** | **11** | **2.1e+02** | **1** | **SDCGVDCCCDPTK + 2 Carbamidomethyl (C)** |
|  | 816 | **407.1115** | **1218.3124** | **1218.4899** | **-0.1775** | **2** | **11** | **1.9e+02** | **1** | **MDKLPPSMRK + Oxidation (M)** |
|  | 846 | **407.7695** | **1220.2862** | **1221.3859** | **-1.0997** | **1** | **11** | **2e+02** | **1** | **WQEATRCLSK** |
|  | 168 | **370.1941** | **738.3733** | **737.8243** | **0.5491** | **0** | **11** | **1.4e+02** | **1** | **EPQMHP** |
|  | 2685 | **593.6230** | **1185.2313** | **1184.4485** | **0.7828** | **0** | **11** | **2.3e+02** | **1** | **ALACIPVQELK** |
|  | 1419 | **444.8289** | **1331.4647** | **1331.4985** | **-0.0338** | **1** | **11** | **2.3e+02** | **1** | **SPPGSSRELVMR + Oxidation (M)** |
|  | 1806 | **475.0999** | **948.1851** | **947.1287** | **1.0564** | **0** | **11** | **2.2e+02** | **1** | **SGGLLYPLK** |
|  | 2026 | **495.8503** | **1484.5289** | **1485.5109** | **-0.9820** | **0** | **11** | **2e+02** | **1** | **SYDPADPPGEAGPGR** |
|  | 892 | **408.7929** | **1223.3566** | **1224.4132** | **-1.0566** | **2** | **11** | **2.1e+02** | **1** | **EHKCPHCDKK** |
|  | 3158 | **690.7338** | **1379.4527** | **1379.6473** | **-0.1945** | **1** | **11** | **2.2e+02** | **1** | **MVNKDMNGFPVK** |
|  | 2058 | **501.5964** | **1501.7670** | **1500.5685** | **1.1984** | **0** | **11** | **2.5e+02** | **1** | **QEYDEAGPSIVHR** |
|  | 2332 | **536.1505** | **1070.2862** | **1069.1891** | **1.0972** | **0** | **11** | **2e+02** | **1** | **GMSYLEDVR** |
|  | 2944 | **661.0104** | **1980.0090** | **1980.1202** | **-0.1112** | **1** | **11** | **1.8e+02** | **1** | **MSSKVQQQEYNTNGPPR + Oxidation (M)** |
|  | 607 | **399.3495** | **796.6842** | **796.8732** | **-0.1890** | **0** | **11** | **1.5e+02** | **1** | **GFSPPHR** |
|  | 1296 | **436.2617** | **1305.7628** | **1306.4639** | **-0.7011** | **0** | **11** | **1.6e+02** | **1** | **LSTELQYPSLR** |
|  | 1911 | **484.5347** | **1450.5818** | **1451.6434** | **-1.0616** | **1** | **11** | **2.3e+02** | **1** | **KILQSNDCIEAAF** |
|  | 2068 | **502.6521** | **1504.9341** | **1505.6695** | **-0.7354** | **1** | **11** | **2.2e+02** | **1** | **TLASKTISISEEAR** |
|  | 2205 | **519.1125** | **1554.3153** | **1554.8115** | **-0.4962** | **1** | **11** | **1.9e+02** | **1** | **LTSKQMMGMHNTK + 3 Oxidation (M)** |
|  | 2787 | **610.3798** | **1218.7449** | **1218.4270** | **0.3179** | **2** | **11** | **2e+02** | **1** | **MRVKELAESR** |
|  | 3245 | **732.1916** | **2193.5528** | **2194.3071** | **-0.7544** | **0** | **11** | **1.9e+02** | **1** | **LWEPADQEAPSTTEYSEIK** |
|  | 55 | **363.2748** | **724.5348** | **723.7745** | **0.7604** | **0** | **11** | **1.6e+02** | **1** | **GTFGGGTK** |
|  | 1776 | **471.4048** | **940.7947** | **941.0599** | **-0.2651** | **1** | **11** | **1.5e+02** | **1** | **VTKGAFGDM + Oxidation (M)** |
|  | 2443 | **553.2662** | **1656.7765** | **1656.9814** | **-0.2048** | **2** | **11** | **2.1e+02** | **1** | **EPEVPIKKLETMVK + Oxidation (M)** |
|  | 2881 | **634.4664** | **1900.3771** | **1901.1078** | **-0.7307** | **1** | **11** | **1.7e+02** | **1** | **LHLQGQTMQDPFGEKR + Oxidation (M)** |
|  | 1052 | **420.1039** | **838.1931** | **838.9547** | **-0.7616** | **2** | **11** | **1.9e+02** | **1** | **RGPEPRK** |
|  | 1259 | 435.0409 | 1302.1004 | 1302.3909 | -0.2905 | 0 | 11 | 1.8e+02 | 1 | ASLQASTTAPEAR |
|  | 3341 | **744.2279** | **1486.4410** | **1485.7048** | **0.7363** | **0** | **11** | **1.9e+02** | **1** | **MDPSQPVVAALVSR + Oxidation (M)** |
|  | 918 | **411.2321** | **820.4495** | **819.9033** | **0.5463** | **0** | **11** | **1.7e+02** | **1** | **FYHPEK** |
|  | 1904 | **484.0974** | **1449.2702** | **1449.5665** | **-0.2963** | **0** | **11** | **1.9e+02** | **1** | **QCGDVSSEHSCLK + Carbamidomethyl (C)** |
|  | 75 | **365.0130** | **728.0113** | **728.7115** | **-0.7002** | **0** | **11** | **2.1e+02** | **1** | **NSSEHR** |
|  | 1753 | **469.5288** | **1405.5643** | **1405.4925** | **0.0718** | **1** | **11** | **2.3e+02** | **1** | **HPRDYSGEMEGK** |
|  | 2103 | **506.0681** | **1010.1213** | **1011.0900** | **-0.9686** | **0** | **11** | **1.8e+02** | **1** | **HVNTNLGEK** |
|  | 2898 | **641.3918** | **1280.7689** | **1281.4611** | **-0.6922** | **1** | **11** | **2.2e+02** | **1** | **LPERGTALPTAR** |
|  | 2966 | **665.4000** | **1328.7853** | **1329.6118** | **-0.8265** | **2** | **11** | **2e+02** | **1** | **ARPASMPLKSKK + Oxidation (M)** |
|  | 3198 | **706.2535** | **2115.7384** | **2115.4140** | **0.3244** | **2** | **11** | **1.9e+02** | **1** | **CEVKLLESGGGLVQRGGSLR + Carbamidomethyl (C)** |
|  | 587 | **398.2343** | **1191.6809** | **1191.3203** | **0.3606** | **1** | **11** | **1.7e+02** | **1** | **METARNWGAR** |
|  | 1506 | 450.5448 | 899.0748 | 899.0695 | 0.0054 | 1 | 11 | 2.4e+02 | 1 | DHAKMGIK |
|  | 1524 | **452.4390** | **1354.2948** | **1353.5221** | **0.7727** | **0** | **11** | **2.2e+02** | **1** | **DSSIHGPGPIFVK** |
|  | 1923 | **486.2502** | **1455.7286** | **1456.6433** | **-0.9148** | **1** | **11** | **1.9e+02** | **1** | **KELNALIGLAGDSR** |
|  | 2784 | **610.2721** | **1218.5294** | **1218.3607** | **0.1688** | **0** | **11** | **2.1e+02** | **1** | **EKPTHCSECGK** |
|  | 2919 | **653.5676** | **1957.6807** | **1958.1561** | **-0.4754** | **1** | **11** | **1.7e+02** | **1** | **AQVLRGTVTDFMGFDER + Oxidation (M)** |
|  | 3013 | **668.8430** | **2003.5067** | **2003.3490** | **0.1577** | **2** | **11** | **2e+02** | **1** | **NSMKVFLKQGECASVHPK** |
|  | 13 | **360.5146** | **1078.5215** | **1079.2683** | **-0.7468** | **0** | **11** | **2.1e+02** | **1** | **FSACYFTLK** |
|  | 2301 | **533.1280** | **1596.3618** | **1595.8451** | **0.5167** | **0** | **11** | **2e+02** | **1** | **MGTQVPVCPGHSGLR + Carbamidomethyl (C)** |
|  | 2428 | **551.5978** | **1101.1808** | **1100.4182** | **0.7626** | **0** | **11** | **2.5e+02** | **1** | **LFMCLLCGK + Carbamidomethyl (C); Oxidation (M)** |
|  | 397 | **386.7497** | **1157.2269** | **1157.2792** | **-0.0523** | **2** | **11** | **2.1e+02** | **1** | **FFKGGGSSKSR** |
|  | 2804 | **612.3033** | **1833.8879** | **1834.2093** | **-0.3214** | **0** | **11** | **2e+02** | **1** | **LMAVVASTVLGLVQNMR + 2 Oxidation (M)** |
|  | 3194 | **703.8415** | **1405.6682** | **1404.5921** | **1.0761** | **1** | **11** | **2.3e+02** | **1** | **ARMSSPALTAGASGK** |
|  | 3557 | **923.7113** | **2768.1117** | **2768.0645** | **0.0473** | **0** | **11** | **1.8e+02** | **1** | **VQFAPEKPGPQPSAETTPHFLMSDR** |
|  | 347 | 385.0210 | 768.0272 | 767.9230 | 0.1041 | 2 | 11 | 1.7e+02 | 1 | RRAPLR |
|  | 254 | **376.2396** | **750.4645** | **750.8182** | **-0.3537** | **0** | **11** | **1.5e+02** | **1** | **MDGDVAK + Oxidation (M)** |
|  | 1033 | **419.2214** | **1254.6420** | **1253.4509** | **1.1911** | **1** | **11** | **2.1e+02** | **1** | **LALVNNTQPKR** |
|  | 2545 | **573.0169** | **1716.0285** | **1714.9377** | **1.0909** | **0** | **11** | **2.1e+02** | **1** | **NSEGEPVCNACGLYMK** |
|  | 2648 | **590.2859** | **1178.5570** | **1178.3564** | **0.2006** | **0** | **11** | **2.1e+02** | **1** | **LDQTTMNVIK + Oxidation (M)** |
|  | 3299 | **740.9749** | **2219.9026** | **2220.5464** | **-0.6438** | **1** | **11** | **1.7e+02** | **1** | **MTTAILERLSTLSVSGQQLR + Oxidation (M)** |
|  | 80 | **365.4062** | **728.7976** | **727.8527** | **0.9449** | **2** | **11** | **2.6e+02** | **1** | **KTSKHK** |
|  | 320 | **382.2896** | **1143.8468** | **1144.2986** | **-0.4518** | **0** | **11** | **1.9e+02** | **1** | **IDCQGIPPSSK** |
|  | 1629 | **460.1806** | **918.3465** | **918.0728** | **0.2736** | **0** | **11** | **2.2e+02** | **1** | **MAASVAAAAR** |
|  | 1744 | **468.4825** | **1402.4254** | **1403.4981** | **-1.0727** | **0** | **11** | **2.4e+02** | **1** | **DPMATGDDCGHIR + Oxidation (M)** |
|  | 1021 | **419.0910** | **1254.2509** | **1255.4455** | **-1.1945** | **1** | **11** | **2.3e+02** | **1** | **VCKSCGGEEMR + Carbamidomethyl (C)** |
|  | 3328 | **742.7101** | **2225.1081** | **2225.5498** | **-0.4417** | **2** | **11** | **1.6e+02** | **1** | **VHVERNAMDGYRTLCVAFK + Oxidation (M)** |
|  | 1210 | **432.1043** | **862.1939** | **861.0878** | **1.1061** | **1** | **11** | **2.2e+02** | **1** | **KVMACPR + Carbamidomethyl (C)** |
|  | 1399 | **443.7309** | **1328.1704** | **1327.6206** | **0.5499** | **1** | **11** | **1.7e+02** | **1** | **KAPHFCPVCLR + Carbamidomethyl (C)** |
|  | 1992 | **492.1140** | **982.2132** | **982.2193** | **-0.0060** | **0** | **11** | **1.9e+02** | **1** | **IQVVWPIK** |
|  | 2761 | **606.8479** | **1817.5215** | **1817.1800** | **0.3415** | **0** | **11** | **1.6e+02** | **1** | **LLGCAALCLLAADSFHAK** |
|  | 176 | **370.2874** | **1107.8402** | **1107.2388** | **0.6014** | **1** | **11** | **1.4e+02** | **1** | **LRGMDSSTPK + Oxidation (M)** |
|  | 1180 | **430.5269** | **1288.5586** | **1288.3611** | **0.1974** | **0** | **11** | **2.8e+02** | **1** | **LVESDAEAEAVR** |
|  | 1782 | **472.1733** | **1413.4977** | **1413.5626** | **-0.0649** | **2** | **11** | **2e+02** | **1** | **KSGHPAPSSMRSR + Oxidation (M)** |
|  | 3028 | **670.1519** | **2007.4334** | **2006.5020** | **0.9314** | **2** | **11** | **2e+02** | **1** | **SKTPPMFLCIKVGKPMR + Carbamidomethyl (C); Oxidation (M)** |
|  | 3381 | **758.0023** | **1513.9899** | **1514.7956** | **-0.8057** | **2** | **11** | **1.6e+02** | **1** | **ARLRVLMVVDGNR + Oxidation (M)** |
|  | 88 | **366.1494** | **1095.4260** | **1094.2415** | **1.1844** | **0** | **11** | **2.1e+02** | **1** | **CSGITFHTTK** |
|  | 318 | **382.0900** | **762.1652** | **761.7778** | **0.3874** | **0** | **11** | **2.4e+02** | **1** | **LGDNTDK** |
|  | 884 | **408.2112** | **1221.6115** | **1221.4108** | **0.2007** | **1** | **11** | **1.9e+02** | **1** | **MECGLNNRIR + Oxidation (M)** |
|  | 2263 | **524.6782** | **1571.0123** | **1571.7359** | **-0.7236** | **0** | **11** | **2.2e+02** | **1** | **QPRPPPAPGDLDVGR** |
|  | 2834 | **617.9475** | **1850.8204** | **1850.0079** | **0.8124** | **2** | **11** | **1.9e+02** | **1** | **ANRLVVAPARGGGGGGGGGGGR** |
|  | 158 | **369.3651** | **1105.0733** | **1106.2540** | **-1.1807** | **1** | **11** | **2.3e+02** | **1** | **RLGMGEGGVSK + Oxidation (M)** |
|  | 486 | **389.1970** | **776.3791** | **776.9200** | **-0.5408** | **0** | **11** | **1.9e+02** | **1** | **LGVTYPK** |
|  | 537 | **392.3813** | **1174.1217** | **1173.4261** | **0.6956** | **0** | **11** | **2e+02** | **1** | **QPAMMMFSSK + Oxidation (M)** |
|  | 1828 | **476.3412** | **1426.0014** | **1426.6837** | **-0.6824** | **0** | **11** | **1.6e+02** | **1** | **GRPSLILDTPAMR** |
|  | 2417 | **549.7413** | **1097.4678** | **1098.2518** | **-0.7840** | **0** | **11** | **1.8e+02** | **1** | **DLSTSCLMR + Carbamidomethyl (C); Oxidation (M)** |
|  | 2477 | **561.5856** | **1681.7345** | **1682.8679** | **-1.1333** | **1** | **11** | **2.3e+02** | **1** | **DEEKMEIQEMQLK + 2 Oxidation (M)** |
|  | 513 | **391.1005** | **1170.2794** | **1170.2930** | **-0.0136** | **1** | **11** | **2e+02** | **1** | **KEDMEYALR + Oxidation (M)** |
|  | 591 | **398.8241** | **1193.4502** | **1192.3432** | **1.1071** | **0** | **11** | **2e+02** | **1** | **ALEVNMSLSGR + Oxidation (M)** |
|  | 3214 | **715.7018** | **2144.0834** | **2144.5350** | **-0.4516** | **2** | **11** | **1.8e+02** | **1** | **KADMSVLEISGMIMNRVSF + Oxidation (M)** |
|  | 292 | **379.6170** | **1135.8287** | **1136.3676** | **-0.5389** | **1** | **11** | **1.8e+02** | **1** | **QCVFAIATKR** |
|  | 367 | **385.9726** | **769.9305** | **768.8119** | **1.1186** | **0** | **11** | **1.8e+02** | **1** | **FEGTSTK** |
|  | 1023 | **419.1209** | **1254.3406** | **1255.4650** | **-1.1245** | **0** | **11** | **2.4e+02** | **1** | **WPTAASALGLLR** |
|  | 1475 | 448.4523 | 894.8898 | 896.0689 | -1.1790 | 2 | 11 | 2.1e+02 | 1 | KFRCSAK + Carbamidomethyl (C) |
|  | 1680 | **462.9991** | **1385.9753** | **1385.5658** | **0.4095** | **2** | **11** | **1.9e+02** | **1** | **KTSATVGPKAPSGGK** |
|  | 2122 | **507.7979** | **1013.5810** | **1014.1172** | **-0.5361** | **0** | **11** | **1.7e+02** | **1** | **DRPPCSPSR** |
|  | 2448 | **553.9233** | **1658.7477** | **1658.8480** | **-0.1004** | **1** | **11** | **2.1e+02** | **1** | **TEVLSEDLLQVEKR** |
|  | 2786 | **610.3757** | **1828.1050** | **1828.1879** | **-0.0829** | **2** | **11** | **2.1e+02** | **1** | **CQYCRYQKCLAMGM + 2 Carbamidomethyl (C); Oxidation (M)** |
|  | 3476 | **809.0898** | **1616.1649** | **1616.6823** | **-0.5173** | **1** | **11** | **1.6e+02** | **1** | **QQDVQTPSAKEEEK** |
|  | 169 | **370.1945** | **1107.5615** | **1107.3082** | **0.2533** | **1** | **11** | **1.5e+02** | **1** | **RNLTCMQGGK** |
|  | 574 | **396.3867** | **1186.1380** | **1186.3221** | **-0.1841** | **0** | **11** | **2.5e+02** | **1** | **METHHGICSR + Oxidation (M)** |
|  | 2665 | **592.6121** | **1183.2093** | **1183.3960** | **-0.1866** | **0** | **11** | **2.2e+02** | **1** | **FTVNIISVYK** |
|  | 1416 | **444.1342** | **1329.3804** | **1328.5143** | **0.8661** | **1** | **11** | **2.4e+02** | **1** | **NILDSKPTANKK** |
|  | 2143 | **512.4590** | **1534.3548** | **1533.7292** | **0.6255** | **0** | **11** | **1.7e+02** | **1** | **GSGCSCTAPALSGPVAR** |
|  | 2567 | **577.7881** | **1153.5614** | **1152.3704** | **1.1910** | **2** | **11** | **1.8e+02** | **1** | **LMASRGRSFK** |
|  | 2973 | **666.6108** | **1996.8103** | **1997.2863** | **-0.4760** | **2** | **11** | **1.7e+02** | **1** | **CPDVDECRLGLARCHPR + Carbamidomethyl (C)** |
|  | 79 | **365.3049** | **1092.8924** | **1092.2422** | **0.6502** | **0** | **11** | **1.9e+02** | **1** | **QTTLYLEPK** |
|  | 1799 | **474.3478** | **1420.0213** | **1419.6927** | **0.3285** | **0** | **11** | **1.8e+02** | **1** | **LLHDCQQMLMR + 2 Oxidation (M)** |
|  | 2313 | **534.3596** | **1066.7045** | **1066.2992** | **0.4052** | **2** | **11** | **2e+02** | **1** | **KRLLAFYR** |
|  | 2378 | **541.1605** | **1620.4594** | **1619.8167** | **0.6427** | **0** | **11** | **2.1e+02** | **1** | **QPSNLPGTVAVHILSS** |
|  | 2493 | **564.4733** | **1126.9318** | **1127.2133** | **-0.2816** | **2** | **11** | **1.8e+02** | **1** | **YGRLGPRDHG** |
|  | 278 | **378.1440** | **1131.4098** | **1131.2401** | **0.1696** | **0** | **11** | **1.8e+02** | **1** | **YSEFHALHK** |
[truncated: 3,378,132 more chars]
