# Supplementary material for: Tobacco-induced hyperglycemia promotes lung cancer progression via cancer cell-macrophage interaction through paracrine IGF2/IR/NPM1-driven PD-L1 expression
Source: Nat Commun. 2024 Jun 8;15:4909. doi: 10.1038/s41467-024-49199-9 (PMC11162468; doi:10.1038/s41467-024-49199-9)
Supplement: Supplementary file 3 — Supplementary Data 1 [file 41467_2024_49199_MOESM3_ESM.zip › Supplementary Data 1/6.htm]

Peptide Summary Report (../data/20120627/F011167.dat)


# Mascot Search Results

```
User            : yprc
Email           : info
Search title    : 
MS data file    : 6-1.xml
Database        : NCBInr 110704 (14481393 sequences; 4958963357 residues)
Taxonomy        : Homo sapiens (human) (217342 sequences)
Timestamp       : 27 Jun 2012 at 01:46:53 GMT

|  |  |  |
| --- | --- | --- |
| Protein hits    : | gi|4501887 | actin, cytoplasmic 2 [Homo sapiens] |
|  | gi|14250401 | actin, beta [Homo sapiens] |
|  | gi|62897625 | beta actin variant [Homo sapiens] |
|  | gi|4501881 | actin, alpha skeletal muscle [Homo sapiens] |
|  | gi|178045 | gamma-actin [Homo sapiens] |
|  | gi|145309046 | POTE-2 alpha-actin [Homo sapiens] |
|  | gi|134133226 | POTE ankyrin domain family member E [Homo sapiens] |
|  | gi|542850 | heterogeneous nuclear ribonucleoprotein G - human |
|  | gi|63055057 | beta-actin-like protein 2 [Homo sapiens] |
|  | gi|532313 | NF45 protein [Homo sapiens] |
|  | gi|1835786 | nucleolar protein p40 [Homo sapiens] |
|  | gi|194388816 | unnamed protein product [Homo sapiens] |
|  | gi|158260881 | unnamed protein product [Homo sapiens] |
|  | gi|74739412 | RecName: Full=Putative beta-actin-like protein 3; AltName: Full=Kappa-actin; AltName: Full=POTE ankyrin domain family member K |
|  | gi|119626277 | heterogeneous nuclear ribonucleoprotein D (AU-rich element RNA binding protein 1, 37kDa), isoform CRA_a [Homo sapiens] |
|  | gi|13027602 | DDRGK domain-containing protein 1 precursor [Homo sapiens] |
|  | gi|14198113 | POLR1C protein [Homo sapiens] |
|  | gi|34364637 | hypothetical protein [Homo sapiens] |
|  | gi|34526063 | unnamed protein product [Homo sapiens] |
|  | gi|16553682 | unnamed protein product [Homo sapiens] |
```

### Probability Based Mowse Score

Ions score is -10\*Log(P), where P is the
probability that the observed match is a random event.  
Individual ions scores
> 47 indicate identity or extensive homology (p<0.05).  
Protein scores
are derived from ions scores as a non-probabilistic basis for ranking protein
hits.

### Peptide Summary Report

|  |  |  |  |
| --- | --- | --- | --- |
|  | Peptide Summary Select Summary (protein hits) Select Summary (unassigned) Export Search Results |  | Help |
|  | Significance threshold p< | Max. number of hits |  |
|  | Standard scoring  MudPIT scoring | Ions score or expect cut-off | Show sub-sets |
|  | Show pop-ups  Suppress pop-ups | Sort unassigned  Decreasing Score Increasing query / Mr Decreasing Intensity | Require bold red |

  
 
             
  
             


  


  
     **Error tolerant**    

|  |  |
| --- | --- |
| **1.** | gi|4501887    **Mass:** 41792    **Score:** 1025   **Queries matched:** 94   **emPAI:** 5.20 |
|  | actin, cytoplasmic 2 [Homo sapiens] |

|  |  |
| --- | --- |
|  | Check to include this hit in error tolerant search or archive report |
|  |  |

|  |  |  |  |  |  |  |  |  |  |  |
| --- | --- | --- | --- | --- | --- | --- | --- | --- | --- | --- |
|  | **Query** | **Observed** | **Mr(expt)** | **Mr(calc)** | **Delta** | **Miss** | **Score** | **Expect** | **Rank** | **Peptide** |
|  | 590 | **398.5465** | **795.0782** | **794.9384** | **0.1398** | **0** | **26** | **6.6** | **1** | **K.IIAPPER.K** |
|  | 1481 | **489.0091** | **976.0033** | **976.0012** | **0.0022** | **0** | **87** | **5.6e-06** | **1** | **K.AGFAGDDAPR.A** |
|  | 1482 | **489.0325** | **976.0502** | **976.0012** | **0.0490** | **0** | **(76)** | **7.3e-05** | **1** | **K.AGFAGDDAPR.A** |
|  | 1483 | **489.0872** | **976.1597** | **976.0012** | **0.1585** | **0** | **(62)** | **0.0016** | **1** | **K.AGFAGDDAPR.A** |
|  | 1484 | **489.1371** | **976.2593** | **976.0012** | **0.2582** | **0** | **(43)** | **0.13** | **1** | **K.AGFAGDDAPR.A** |
|  | 1614 | **518.7917** | **1035.5687** | **1036.2683** | **-0.6996** | **1** | **19** | **26** | **1** | **K.IKIIAPPER.K** |
|  | 1996 | **581.5563** | **1161.0978** | **1161.3689** | **-0.2712** | **0** | **(37)** | **0.55** | **1** | **K.EITALAPSTMK.I** |
|  | 444 | **388.9354** | **1163.7841** | **1164.4406** | **-0.6565** | **2** | **(19)** | **40** | **1** | **K.IKIIAPPERK.Y** |
|  | 446 | **388.9509** | **1163.8305** | **1164.4406** | **-0.6101** | **2** | **(5)** | **8.9e+02** | **8** | **K.IKIIAPPERK.Y** |
|  | 488 | **389.4004** | **1165.1789** | **1164.4406** | **0.7383** | **2** | **45** | **0.096** | **1** | **K.IKIIAPPERK.Y** |
|  | 560 | **391.4145** | **1171.2214** | **1171.3936** | **-0.1722** | **0** | **31** | **2.3** | **1** | **R.HQGVMVGMGQK.D** |
|  | 2025 | **586.6212** | **1171.2275** | **1171.3936** | **-0.1660** | **0** | **(25)** | **10** | **1** | **R.HQGVMVGMGQK.D** |
|  | 2026 | **587.2489** | **1172.4830** | **1171.3936** | **1.0895** | **0** | **(10)** | **2.6e+02** | **1** | **R.HQGVMVGMGQK.D** |
|  | 2036 | **589.6276** | **1177.2403** | **1177.3683** | **-0.1280** | **0** | **52** | **0.022** | **1** | **K.EITALAPSTMK.I + Oxidation (M)** |
|  | 2037 | **589.6486** | **1177.2823** | **1177.3683** | **-0.0860** | **0** | **(33)** | **1.6** | **2** | **K.EITALAPSTMK.I + Oxidation (M)** |
|  | 2129 | **599.6302** | **1197.2456** | **1198.4173** | **-1.1717** | **0** | **(5)** | **8.7e+02** | **3** | **R.AVFPSIVGRPR.H** |
|  | 2130 | **599.7516** | **1197.4884** | **1198.4173** | **-0.9289** | **0** | **(44)** | **0.12** | **1** | **R.AVFPSIVGRPR.H** |
|  | 2131 | **599.7771** | **1197.5394** | **1198.4173** | **-0.8778** | **0** | **(39)** | **0.32** | **1** | **R.AVFPSIVGRPR.H** |
|  | 2132 | **599.7792** | **1197.5436** | **1198.4173** | **-0.8737** | **0** | **(36)** | **0.63** | **1** | **R.AVFPSIVGRPR.H** |
|  | 2133 | **599.8466** | **1197.6785** | **1198.4173** | **-0.7388** | **0** | **(53)** | **0.012** | **1** | **R.AVFPSIVGRPR.H** |
|  | 2135 | **600.0215** | **1198.0283** | **1198.4173** | **-0.3890** | **0** | **(12)** | **1.8e+02** | **1** | **R.AVFPSIVGRPR.H** |
|  | 2137 | **600.2160** | **1198.4172** | **1198.4173** | **-0.0000** | **0** | **(71)** | **0.00024** | **1** | **R.AVFPSIVGRPR.H** |
|  | 2138 | **600.2161** | **1198.4173** | **1198.4173** | **0.0001** | **0** | **(37)** | **0.6** | **1** | **R.AVFPSIVGRPR.H** |
|  | 2139 | **600.2168** | **1198.4188** | **1198.4173** | **0.0015** | **0** | **(34)** | **1.1** | **1** | **R.AVFPSIVGRPR.H** |
|  | 2140 | **600.2443** | **1198.4737** | **1198.4173** | **0.0565** | **0** | **83** | **1.4e-05** | **1** | **R.AVFPSIVGRPR.H** |
|  | 2141 | **600.2688** | **1198.5228** | **1198.4173** | **0.1055** | **0** | **(54)** | **0.012** | **1** | **R.AVFPSIVGRPR.H** |
|  | 2142 | **600.3383** | **1198.6617** | **1198.4173** | **0.2445** | **0** | **(64)** | **0.0011** | **1** | **R.AVFPSIVGRPR.H** |
|  | 619 | **400.6896** | **1199.0467** | **1198.4173** | **0.6295** | **0** | **(28)** | **3.8** | **1** | **R.AVFPSIVGRPR.H** |
|  | 620 | **400.7009** | **1199.0806** | **1198.4173** | **0.6633** | **0** | **(18)** | **34** | **1** | **R.AVFPSIVGRPR.H** |
|  | 621 | **400.7180** | **1199.1318** | **1198.4173** | **0.7145** | **0** | **(22)** | **13** | **1** | **R.AVFPSIVGRPR.H** |
|  | 622 | **400.7871** | **1199.3392** | **1198.4173** | **0.9220** | **0** | **(15)** | **90** | **5** | **R.AVFPSIVGRPR.H** |
|  | 2143 | **600.7864** | **1199.5580** | **1198.4173** | **1.1407** | **0** | **(30)** | **2.4** | **1** | **R.AVFPSIVGRPR.H** |
|  | 1225 | **452.7126** | **1355.1157** | **1354.3795** | **0.7362** | **1** | **(32)** | **1.5** | **1** | **K.DSYVGDEAQSKR.G** |
|  | 2687 | **678.6069** | **1355.1991** | **1354.3795** | **0.8196** | **1** | **50** | **0.022** | **1** | **K.DSYVGDEAQSKR.G** |
|  | 2841 | **709.9313** | **1417.8479** | **1418.6982** | **-0.8504** | **1** | **(17)** | **44** | **2** | **K.EITALAPSTMKIK.I + Oxidation (M)** |
|  | 2843 | **710.1817** | **1418.3486** | **1418.6982** | **-0.3496** | **1** | **(20)** | **29** | **2** | **K.EITALAPSTMKIK.I + Oxidation (M)** |
|  | 1367 | **474.2588** | **1419.7542** | **1418.6982** | **1.0560** | **1** | **34** | **0.99** | **1** | **K.EITALAPSTMKIK.I + Oxidation (M)** |
|  | 1558 | **506.8081** | **1517.4021** | **1516.5679** | **0.8342** | **0** | **10** | **2.2e+02** | **2** | **K.QEYDESGPSIVHR.K** |
|  | 3118 | **775.4117** | **1548.8086** | **1548.8665** | **-0.0580** | **1** | **(6)** | **6.8e+02** | **4** | **R.MQKEITALAPSTMK.I** |
|  | 1651 | **522.7468** | **1565.2181** | **1564.8660** | **0.3522** | **1** | **(30)** | **2.1** | **1** | **R.MQKEITALAPSTMK.I + Oxidation (M)** |
|  | 1653 | **522.8632** | **1565.5673** | **1564.8660** | **0.7014** | **1** | **32** | **1.5** | **1** | **R.MQKEITALAPSTMK.I + Oxidation (M)** |
|  | 3161 | **791.5933** | **1581.1717** | **1580.8654** | **0.3064** | **1** | **(32)** | **1.7** | **1** | **R.MQKEITALAPSTMK.I + 2 Oxidation (M)** |
|  | 1819 | **542.3186** | **1623.9336** | **1623.8683** | **0.0653** | **1** | **23** | **14** | **1** | **R.LDLAGRDLTDYLMK.I** |
|  | 1850 | **549.4825** | **1645.4255** | **1644.7403** | **0.6852** | **1** | **10** | **2.3e+02** | **2** | **K.QEYDESGPSIVHRK.C** |
|  | 3324 | **895.8816** | **1789.7484** | **1790.9243** | **-1.1759** | **0** | **(14)** | **76** | **1** | **K.SYELPDGQVITIGNER.F** |
|  | 3325 | **895.8936** | **1789.7723** | **1790.9243** | **-1.1520** | **0** | **(31)** | **1.6** | **1** | **K.SYELPDGQVITIGNER.F** |
|  | 3326 | **896.1632** | **1790.3116** | **1790.9243** | **-0.6127** | **0** | **(35)** | **0.6** | **1** | **K.SYELPDGQVITIGNER.F** |
|  | 3327 | **896.1948** | **1790.3749** | **1790.9243** | **-0.5495** | **0** | **(11)** | **1.6e+02** | **2** | **K.SYELPDGQVITIGNER.F** |
|  | 3328 | **896.2452** | **1790.4757** | **1790.9243** | **-0.4486** | **0** | **(36)** | **0.52** | **1** | **K.SYELPDGQVITIGNER.F** |
|  | 3329 | **896.3129** | **1790.6110** | **1790.9243** | **-0.3134** | **0** | **(48)** | **0.039** | **1** | **K.SYELPDGQVITIGNER.F** |
|  | 2118 | **597.9269** | **1790.7586** | **1790.9243** | **-0.1657** | **0** | **(4)** | **7.9e+02** | **5** | **K.SYELPDGQVITIGNER.F** |
|  | 3331 | **896.4506** | **1790.8863** | **1790.9243** | **-0.0380** | **0** | **(43)** | **0.12** | **1** | **K.SYELPDGQVITIGNER.F** |
|  | 3332 | **896.5997** | **1791.1847** | **1790.9243** | **0.2603** | **0** | **(64)** | **0.0011** | **1** | **K.SYELPDGQVITIGNER.F** |
|  | 3333 | **896.7216** | **1791.4283** | **1790.9243** | **0.5040** | **0** | **(79)** | **3.2e-05** | **1** | **K.SYELPDGQVITIGNER.F** |
|  | 2121 | **598.1779** | **1791.5116** | **1790.9243** | **0.5872** | **0** | **(48)** | **0.046** | **1** | **K.SYELPDGQVITIGNER.F** |
|  | 2122 | **598.2000** | **1791.5779** | **1790.9243** | **0.6535** | **0** | **(55)** | **0.0075** | **1** | **K.SYELPDGQVITIGNER.F** |
|  | 3334 | **896.9148** | **1791.8148** | **1790.9243** | **0.8905** | **0** | **(70)** | **0.00024** | **1** | **K.SYELPDGQVITIGNER.F** |
|  | 3335 | **896.9431** | **1791.8714** | **1790.9243** | **0.9471** | **0** | **(83)** | **1.2e-05** | **1** | **K.SYELPDGQVITIGNER.F** |
|  | 3336 | **897.0674** | **1792.1200** | **1790.9243** | **1.1957** | **0** | **87** | **5.3e-06** | **1** | **K.SYELPDGQVITIGNER.F** |
|  | 3344 | **904.0286** | **1806.0423** | **1806.1958** | **-0.1535** | **2** | **(28)** | **5.1** | **1** | **R.MQKEITALAPSTMKIK.I + Oxidation (M)** |
|  | 2156 | **603.3624** | **1807.0651** | **1806.1958** | **0.8693** | **2** | **(24)** | **10** | **1** | **R.MQKEITALAPSTMKIK.I + Oxidation (M)** |
|  | 2194 | **608.2214** | **1821.6421** | **1822.1953** | **-0.5531** | **2** | **(3)** | **1.5e+03** | **7** | **R.MQKEITALAPSTMKIK.I + 2 Oxidation (M)** |
|  | 2195 | **608.2873** | **1821.8397** | **1822.1953** | **-0.3555** | **2** | **(13)** | **1.4e+02** | **1** | **R.MQKEITALAPSTMKIK.I + 2 Oxidation (M)** |
|  | 2198 | **608.5206** | **1822.5395** | **1822.1953** | **0.3443** | **2** | **33** | **1.2** | **1** | **R.MQKEITALAPSTMKIK.I + 2 Oxidation (M)** |
|  | 3383 | **927.5835** | **1853.1522** | **1852.0488** | **1.1034** | **0** | **11** | **1.9e+02** | **2** | **M.EEEIAALVIDNGSGMCK.A + Carbamidomethyl (C); Oxidation (M)** |
|  | 2498 | **652.1713** | **1953.4916** | **1954.2269** | **-0.7353** | **0** | **(16)** | **69** | **1** | **R.VAPEEHPVLLTEAPLNPK.A** |
|  | 3535 | **977.9032** | **1953.7916** | **1954.2269** | **-0.4353** | **0** | **(44)** | **0.077** | **2** | **R.VAPEEHPVLLTEAPLNPK.A** |
|  | 3536 | **978.2371** | **1954.4593** | **1954.2269** | **0.2324** | **0** | **69** | **0.00025** | **1** | **R.VAPEEHPVLLTEAPLNPK.A** |
|  | 2501 | **652.5559** | **1954.6456** | **1954.2269** | **0.4186** | **0** | **(47)** | **0.043** | **1** | **R.VAPEEHPVLLTEAPLNPK.A** |
|  | 2502 | **652.6416** | **1954.9026** | **1954.2269** | **0.6757** | **0** | **(43)** | **0.12** | **1** | **R.VAPEEHPVLLTEAPLNPK.A** |
|  | 2503 | **652.7772** | **1955.3095** | **1954.2269** | **1.0825** | **0** | **(35)** | **1.1** | **1** | **R.VAPEEHPVLLTEAPLNPK.A** |
|  | 3556 | **1047.9919** | **2093.9691** | **2094.2839** | **-0.3148** | **1** | **42** | **0.13** | **1** | **K.SYELPDGQVITIGNERFR.C** |
|  | 3557 | **1047.9972** | **2093.9796** | **2094.2839** | **-0.3043** | **1** | **(39)** | **0.26** | **1** | **K.SYELPDGQVITIGNERFR.C** |
|  | 2809 | **699.1260** | **2094.3559** | **2094.2839** | **0.0720** | **1** | **(13)** | **1.4e+02** | **1** | **K.SYELPDGQVITIGNERFR.C** |
|  | 2813 | **699.4652** | **2095.3735** | **2094.2839** | **1.0895** | **1** | **(29)** | **2.9** | **1** | **K.SYELPDGQVITIGNERFR.C** |
|  | 2866 | **719.4661** | **2155.3760** | **2156.4032** | **-1.0271** | **1** | **(43)** | **0.12** | **1** | **K.AGFAGDDAPRAVFPSIVGRPR.H** |
|  | 2867 | **719.5731** | **2155.6972** | **2156.4032** | **-0.7060** | **1** | **(19)** | **26** | **1** | **K.AGFAGDDAPRAVFPSIVGRPR.H** |
|  | 2868 | **719.7181** | **2156.1322** | **2156.4032** | **-0.2709** | **1** | **(37)** | **0.44** | **1** | **K.AGFAGDDAPRAVFPSIVGRPR.H** |
|  | 2869 | **719.7473** | **2156.2196** | **2156.4032** | **-0.1836** | **1** | **(34)** | **0.97** | **1** | **K.AGFAGDDAPRAVFPSIVGRPR.H** |
|  | 3566 | **1079.3804** | **2156.7460** | **2156.4032** | **0.3428** | **1** | **(27)** | **4** | **1** | **K.AGFAGDDAPRAVFPSIVGRPR.H** |
|  | 2870 | **719.9481** | **2156.8220** | **2156.4032** | **0.4188** | **1** | **(26)** | **4.9** | **1** | **K.AGFAGDDAPRAVFPSIVGRPR.H** |
|  | 2871 | **720.0771** | **2157.2093** | **2156.4032** | **0.8061** | **1** | **(49)** | **0.025** | **1** | **K.AGFAGDDAPRAVFPSIVGRPR.H** |
|  | 2872 | **720.1254** | **2157.3539** | **2156.4032** | **0.9508** | **1** | **(51)** | **0.02** | **1** | **K.AGFAGDDAPRAVFPSIVGRPR.H** |
|  | 2873 | **720.2019** | **2157.5835** | **2156.4032** | **1.1804** | **1** | **57** | **0.0055** | **1** | **K.AGFAGDDAPRAVFPSIVGRPR.H** |
|  | 2899 | **733.1710** | **2196.4909** | **2195.6214** | **0.8695** | **2** | **9** | **3.1e+02** | **1** | **K.EITALAPSTMKIKIIAPPER.K + Oxidation (M)** |
|  | 3572 | **1116.2848** | **2230.5548** | **2231.4382** | **-0.8834** | **0** | **(18)** | **36** | **1** | **K.DLYANTVLSGGTTMYPGIADR.M + Oxidation (M)** |
|  | 3006 | **744.5515** | **2230.6322** | **2231.4382** | **-0.8060** | **0** | **21** | **21** | **1** | **K.DLYANTVLSGGTTMYPGIADR.M + Oxidation (M)** |
|  | 3103 | **766.0936** | **2295.2587** | **2295.5931** | **-0.3344** | **1** | **52** | **0.012** | **2** | **R.VAPEEHPVLLTEAPLNPKANR.E** |
|  | 3104 | **766.4434** | **2296.3079** | **2295.5931** | **0.7148** | **1** | **(24)** | **8.9** | **1** | **R.VAPEEHPVLLTEAPLNPKANR.E** |
|  | 3147 | **787.4149** | **2359.2226** | **2359.6105** | **-0.3880** | **1** | **40** | **0.24** | **1** | **R.KDLYANTVLSGGTTMYPGIADR.M + Oxidation (M)** |
|  | 3258 | **836.7892** | **2507.3454** | **2507.7578** | **-0.4124** | **2** | **58** | **0.0029** | **1** | **R.HQGVMVGMGQKDSYVGDEAQSKR.G** |
|  | 3282 | **851.8923** | **2552.6548** | **2552.8794** | **-0.2246** | **2** | **15** | **78** | **1** | **R.VAPEEHPVLLTEAPLNPKANREK.M** |
|  | 3313 | **879.0190** | **2634.0350** | **2634.9352** | **-0.9003** | **1** | **21** | **23** | **1** | **K.DLYANTVLSGGTTMYPGIADRMQK.E + 2 Oxidation (M)** |
|  | 3474 | **957.2186** | **2868.6337** | **2868.1801** | **0.4536** | **1** | **9** | **2.5e+02** | **1** | **-.MEEEIAALVIDNGSGMCKAGFAGDDAPR.A** |

  

|  |  |
| --- | --- |
|  | |
|  | **Proteins matching the same set of peptides:** |

|  |  |
| --- | --- |
|  | gi|297702014    **Mass:** 39799    **Score:** 1025   **Queries matched:** 94 |
|  | PREDICTED: actin, cytoplasmic 2-like isoform 3 [Pongo abelii] |

---

|  |  |
| --- | --- |
| **2.** | gi|14250401    **Mass:** 41005    **Score:** 1011   **Queries matched:** 93   **emPAI:** 5.44 |
|  | actin, beta [Homo sapiens] |

|  |  |
| --- | --- |
|  | Check to include this hit in error tolerant search or archive report |
|  |  |

|  |  |  |  |  |  |  |  |  |  |  |
| --- | --- | --- | --- | --- | --- | --- | --- | --- | --- | --- |
|  | **Query** | **Observed** | **Mr(expt)** | **Mr(calc)** | **Delta** | **Miss** | **Score** | **Expect** | **Rank** | **Peptide** |
|  | 590 | 398.5465 | 795.0782 | 794.9384 | 0.1398 | 0 | 26 | 6.6 | 1 | K.IIAPPER.K |
|  | 1481 | 489.0091 | 976.0033 | 976.0012 | 0.0022 | 0 | 87 | 5.6e-06 | 1 | K.AGFAGDDAPR.A |
|  | 1482 | 489.0325 | 976.0502 | 976.0012 | 0.0490 | 0 | (76) | 7.3e-05 | 1 | K.AGFAGDDAPR.A |
|  | 1483 | 489.0872 | 976.1597 | 976.0012 | 0.1585 | 0 | (62) | 0.0016 | 1 | K.AGFAGDDAPR.A |
|  | 1484 | 489.1371 | 976.2593 | 976.0012 | 0.2582 | 0 | (43) | 0.13 | 1 | K.AGFAGDDAPR.A |
|  | 1614 | 518.7917 | 1035.5687 | 1036.2683 | -0.6996 | 1 | 19 | 26 | 1 | K.IKIIAPPER.K |
|  | 194 | **374.9718** | **1121.8933** | **1122.3163** | **-0.4229** | **0** | **6** | **7.2e+02** | **3** | **-.LVVDNGSGMCK.A** |
|  | 1996 | 581.5563 | 1161.0978 | 1161.3689 | -0.2712 | 0 | (37) | 0.55 | 1 | K.EITALAPSTMK.I |
|  | 444 | 388.9354 | 1163.7841 | 1164.4406 | -0.6565 | 2 | (19) | 40 | 1 | K.IKIIAPPERK.Y |
|  | 446 | 388.9509 | 1163.8305 | 1164.4406 | -0.6101 | 2 | (5) | 8.9e+02 | 8 | K.IKIIAPPERK.Y |
|  | 488 | 389.4004 | 1165.1789 | 1164.4406 | 0.7383 | 2 | 45 | 0.096 | 1 | K.IKIIAPPERK.Y |
|  | 560 | 391.4145 | 1171.2214 | 1171.3936 | -0.1722 | 0 | 31 | 2.3 | 1 | R.HQGVMVGMGQK.D |
|  | 2025 | 586.6212 | 1171.2275 | 1171.3936 | -0.1660 | 0 | (25) | 10 | 1 | R.HQGVMVGMGQK.D |
|  | 2026 | 587.2489 | 1172.4830 | 1171.3936 | 1.0895 | 0 | (10) | 2.6e+02 | 1 | R.HQGVMVGMGQK.D |
|  | 2036 | 589.6276 | 1177.2403 | 1177.3683 | -0.1280 | 0 | 52 | 0.022 | 1 | K.EITALAPSTMK.I + Oxidation (M) |
|  | 2037 | 589.6486 | 1177.2823 | 1177.3683 | -0.0860 | 0 | (33) | 1.6 | 2 | K.EITALAPSTMK.I + Oxidation (M) |
|  | 2129 | 599.6302 | 1197.2456 | 1198.4173 | -1.1717 | 0 | (5) | 8.7e+02 | 3 | R.AVFPSIVGRPR.H |
|  | 2130 | 599.7516 | 1197.4884 | 1198.4173 | -0.9289 | 0 | (44) | 0.12 | 1 | R.AVFPSIVGRPR.H |
|  | 2131 | 599.7771 | 1197.5394 | 1198.4173 | -0.8778 | 0 | (39) | 0.32 | 1 | R.AVFPSIVGRPR.H |
|  | 2132 | 599.7792 | 1197.5436 | 1198.4173 | -0.8737 | 0 | (36) | 0.63 | 1 | R.AVFPSIVGRPR.H |
|  | 2133 | 599.8466 | 1197.6785 | 1198.4173 | -0.7388 | 0 | (53) | 0.012 | 1 | R.AVFPSIVGRPR.H |
|  | 2135 | 600.0215 | 1198.0283 | 1198.4173 | -0.3890 | 0 | (12) | 1.8e+02 | 1 | R.AVFPSIVGRPR.H |
|  | 2137 | 600.2160 | 1198.4172 | 1198.4173 | -0.0000 | 0 | (71) | 0.00024 | 1 | R.AVFPSIVGRPR.H |
|  | 2138 | 600.2161 | 1198.4173 | 1198.4173 | 0.0001 | 0 | (37) | 0.6 | 1 | R.AVFPSIVGRPR.H |
|  | 2139 | 600.2168 | 1198.4188 | 1198.4173 | 0.0015 | 0 | (34) | 1.1 | 1 | R.AVFPSIVGRPR.H |
|  | 2140 | 600.2443 | 1198.4737 | 1198.4173 | 0.0565 | 0 | 83 | 1.4e-05 | 1 | R.AVFPSIVGRPR.H |
|  | 2141 | 600.2688 | 1198.5228 | 1198.4173 | 0.1055 | 0 | (54) | 0.012 | 1 | R.AVFPSIVGRPR.H |
|  | 2142 | 600.3383 | 1198.6617 | 1198.4173 | 0.2445 | 0 | (64) | 0.0011 | 1 | R.AVFPSIVGRPR.H |
|  | 619 | 400.6896 | 1199.0467 | 1198.4173 | 0.6295 | 0 | (28) | 3.8 | 1 | R.AVFPSIVGRPR.H |
|  | 620 | 400.7009 | 1199.0806 | 1198.4173 | 0.6633 | 0 | (18) | 34 | 1 | R.AVFPSIVGRPR.H |
|  | 621 | 400.7180 | 1199.1318 | 1198.4173 | 0.7145 | 0 | (22) | 13 | 1 | R.AVFPSIVGRPR.H |
|  | 622 | 400.7871 | 1199.3392 | 1198.4173 | 0.9220 | 0 | (15) | 90 | 5 | R.AVFPSIVGRPR.H |
|  | 2143 | 600.7864 | 1199.5580 | 1198.4173 | 1.1407 | 0 | (30) | 2.4 | 1 | R.AVFPSIVGRPR.H |
|  | 1225 | 452.7126 | 1355.1157 | 1354.3795 | 0.7362 | 1 | (32) | 1.5 | 1 | K.DSYVGDEAQSKR.G |
|  | 2687 | 678.6069 | 1355.1991 | 1354.3795 | 0.8196 | 1 | 50 | 0.022 | 1 | K.DSYVGDEAQSKR.G |
|  | 2841 | 709.9313 | 1417.8479 | 1418.6982 | -0.8504 | 1 | (17) | 44 | 2 | K.EITALAPSTMKIK.I + Oxidation (M) |
|  | 2843 | 710.1817 | 1418.3486 | 1418.6982 | -0.3496 | 1 | (20) | 29 | 2 | K.EITALAPSTMKIK.I + Oxidation (M) |
|  | 1367 | 474.2588 | 1419.7542 | 1418.6982 | 1.0560 | 1 | 34 | 0.99 | 1 | K.EITALAPSTMKIK.I + Oxidation (M) |
|  | 1558 | 506.8081 | 1517.4021 | 1516.5679 | 0.8342 | 0 | 10 | 2.2e+02 | 2 | K.QEYDESGPSIVHR.K |
|  | 3118 | 775.4117 | 1548.8086 | 1548.8665 | -0.0580 | 1 | (6) | 6.8e+02 | 4 | R.MQKEITALAPSTMK.I |
|  | 1651 | 522.7468 | 1565.2181 | 1564.8660 | 0.3522 | 1 | (30) | 2.1 | 1 | R.MQKEITALAPSTMK.I + Oxidation (M) |
|  | 1653 | 522.8632 | 1565.5673 | 1564.8660 | 0.7014 | 1 | 32 | 1.5 | 1 | R.MQKEITALAPSTMK.I + Oxidation (M) |
|  | 3161 | 791.5933 | 1581.1717 | 1580.8654 | 0.3064 | 1 | (32) | 1.7 | 1 | R.MQKEITALAPSTMK.I + 2 Oxidation (M) |
|  | 1819 | 542.3186 | 1623.9336 | 1623.8683 | 0.0653 | 1 | 23 | 14 | 1 | R.LDLAGRDLTDYLMK.I |
|  | 1850 | 549.4825 | 1645.4255 | 1644.7403 | 0.6852 | 1 | 10 | 2.3e+02 | 2 | K.QEYDESGPSIVHRK.C |
|  | 3324 | 895.8816 | 1789.7484 | 1790.9243 | -1.1759 | 0 | (14) | 76 | 1 | K.SYELPDGQVITIGNER.F |
|  | 3325 | 895.8936 | 1789.7723 | 1790.9243 | -1.1520 | 0 | (31) | 1.6 | 1 | K.SYELPDGQVITIGNER.F |
|  | 3326 | 896.1632 | 1790.3116 | 1790.9243 | -0.6127 | 0 | (35) | 0.6 | 1 | K.SYELPDGQVITIGNER.F |
|  | 3327 | 896.1948 | 1790.3749 | 1790.9243 | -0.5495 | 0 | (11) | 1.6e+02 | 2 | K.SYELPDGQVITIGNER.F |
|  | 3328 | 896.2452 | 1790.4757 | 1790.9243 | -0.4486 | 0 | (36) | 0.52 | 1 | K.SYELPDGQVITIGNER.F |
|  | 3329 | 896.3129 | 1790.6110 | 1790.9243 | -0.3134 | 0 | (48) | 0.039 | 1 | K.SYELPDGQVITIGNER.F |
|  | 2118 | 597.9269 | 1790.7586 | 1790.9243 | -0.1657 | 0 | (4) | 7.9e+02 | 5 | K.SYELPDGQVITIGNER.F |
|  | 3331 | 896.4506 | 1790.8863 | 1790.9243 | -0.0380 | 0 | (43) | 0.12 | 1 | K.SYELPDGQVITIGNER.F |
|  | 3332 | 896.5997 | 1791.1847 | 1790.9243 | 0.2603 | 0 | (64) | 0.0011 | 1 | K.SYELPDGQVITIGNER.F |
|  | 3333 | 896.7216 | 1791.4283 | 1790.9243 | 0.5040 | 0 | (79) | 3.2e-05 | 1 | K.SYELPDGQVITIGNER.F |
|  | 2121 | 598.1779 | 1791.5116 | 1790.9243 | 0.5872 | 0 | (48) | 0.046 | 1 | K.SYELPDGQVITIGNER.F |
|  | 2122 | 598.2000 | 1791.5779 | 1790.9243 | 0.6535 | 0 | (55) | 0.0075 | 1 | K.SYELPDGQVITIGNER.F |
|  | 3334 | 896.9148 | 1791.8148 | 1790.9243 | 0.8905 | 0 | (70) | 0.00024 | 1 | K.SYELPDGQVITIGNER.F |
|  | 3335 | 896.9431 | 1791.8714 | 1790.9243 | 0.9471 | 0 | (83) | 1.2e-05 | 1 | K.SYELPDGQVITIGNER.F |
|  | 3336 | 897.0674 | 1792.1200 | 1790.9243 | 1.1957 | 0 | 87 | 5.3e-06 | 1 | K.SYELPDGQVITIGNER.F |
|  | 3344 | 904.0286 | 1806.0423 | 1806.1958 | -0.1535 | 2 | (28) | 5.1 | 1 | R.MQKEITALAPSTMKIK.I + Oxidation (M) |
|  | 2156 | 603.3624 | 1807.0651 | 1806.1958 | 0.8693 | 2 | (24) | 10 | 1 | R.MQKEITALAPSTMKIK.I + Oxidation (M) |
|  | 2194 | 608.2214 | 1821.6421 | 1822.1953 | -0.5531 | 2 | (3) | 1.5e+03 | 7 | R.MQKEITALAPSTMKIK.I + 2 Oxidation (M) |
|  | 2195 | 608.2873 | 1821.8397 | 1822.1953 | -0.3555 | 2 | (13) | 1.4e+02 | 1 | R.MQKEITALAPSTMKIK.I + 2 Oxidation (M) |
|  | 2198 | 608.5206 | 1822.5395 | 1822.1953 | 0.3443 | 2 | 33 | 1.2 | 1 | R.MQKEITALAPSTMKIK.I + 2 Oxidation (M) |
|  | 2498 | 652.1713 | 1953.4916 | 1954.2269 | -0.7353 | 0 | (16) | 69 | 1 | R.VAPEEHPVLLTEAPLNPK.A |
|  | 3535 | 977.9032 | 1953.7916 | 1954.2269 | -0.4353 | 0 | (44) | 0.077 | 2 | R.VAPEEHPVLLTEAPLNPK.A |
|  | 3536 | 978.2371 | 1954.4593 | 1954.2269 | 0.2324 | 0 | 69 | 0.00025 | 1 | R.VAPEEHPVLLTEAPLNPK.A |
|  | 2501 | 652.5559 | 1954.6456 | 1954.2269 | 0.4186 | 0 | (47) | 0.043 | 1 | R.VAPEEHPVLLTEAPLNPK.A |
|  | 2502 | 652.6416 | 1954.9026 | 1954.2269 | 0.6757 | 0 | (43) | 0.12 | 1 | R.VAPEEHPVLLTEAPLNPK.A |
|  | 2503 | 652.7772 | 1955.3095 | 1954.2269 | 1.0825 | 0 | (35) | 1.1 | 1 | R.VAPEEHPVLLTEAPLNPK.A |
|  | 3556 | 1047.9919 | 2093.9691 | 2094.2839 | -0.3148 | 1 | 42 | 0.13 | 1 | K.SYELPDGQVITIGNERFR.C |
|  | 3557 | 1047.9972 | 2093.9796 | 2094.2839 | -0.3043 | 1 | (39) | 0.26 | 1 | K.SYELPDGQVITIGNERFR.C |
|  | 2809 | 699.1260 | 2094.3559 | 2094.2839 | 0.0720 | 1 | (13) | 1.4e+02 | 1 | K.SYELPDGQVITIGNERFR.C |
|  | 2813 | 699.4652 | 2095.3735 | 2094.2839 | 1.0895 | 1 | (29) | 2.9 | 1 | K.SYELPDGQVITIGNERFR.C |
|  | 2866 | 719.4661 | 2155.3760 | 2156.4032 | -1.0271 | 1 | (43) | 0.12 | 1 | K.AGFAGDDAPRAVFPSIVGRPR.H |
|  | 2867 | 719.5731 | 2155.6972 | 2156.4032 | -0.7060 | 1 | (19) | 26 | 1 | K.AGFAGDDAPRAVFPSIVGRPR.H |
|  | 2868 | 719.7181 | 2156.1322 | 2156.4032 | -0.2709 | 1 | (37) | 0.44 | 1 | K.AGFAGDDAPRAVFPSIVGRPR.H |
|  | 2869 | 719.7473 | 2156.2196 | 2156.4032 | -0.1836 | 1 | (34) | 0.97 | 1 | K.AGFAGDDAPRAVFPSIVGRPR.H |
|  | 3566 | 1079.3804 | 2156.7460 | 2156.4032 | 0.3428 | 1 | (27) | 4 | 1 | K.AGFAGDDAPRAVFPSIVGRPR.H |
|  | 2870 | 719.9481 | 2156.8220 | 2156.4032 | 0.4188 | 1 | (26) | 4.9 | 1 | K.AGFAGDDAPRAVFPSIVGRPR.H |
|  | 2871 | 720.0771 | 2157.2093 | 2156.4032 | 0.8061 | 1 | (49) | 0.025 | 1 | K.AGFAGDDAPRAVFPSIVGRPR.H |
|  | 2872 | 720.1254 | 2157.3539 | 2156.4032 | 0.9508 | 1 | (51) | 0.02 | 1 | K.AGFAGDDAPRAVFPSIVGRPR.H |
|  | 2873 | 720.2019 | 2157.5835 | 2156.4032 | 1.1804 | 1 | 57 | 0.0055 | 1 | K.AGFAGDDAPRAVFPSIVGRPR.H |
|  | 2899 | 733.1710 | 2196.4909 | 2195.6214 | 0.8695 | 2 | 9 | 3.1e+02 | 1 | K.EITALAPSTMKIKIIAPPER.K + Oxidation (M) |
|  | 3572 | 1116.2848 | 2230.5548 | 2231.4382 | -0.8834 | 0 | (18) | 36 | 1 | K.DLYANTVLSGGTTMYPGIADR.M + Oxidation (M) |
|  | 3006 | 744.5515 | 2230.6322 | 2231.4382 | -0.8060 | 0 | 21 | 21 | 1 | K.DLYANTVLSGGTTMYPGIADR.M + Oxidation (M) |
|  | 3103 | 766.0936 | 2295.2587 | 2295.5931 | -0.3344 | 1 | 52 | 0.012 | 2 | R.VAPEEHPVLLTEAPLNPKANR.E |
|  | 3104 | 766.4434 | 2296.3079 | 2295.5931 | 0.7148 | 1 | (24) | 8.9 | 1 | R.VAPEEHPVLLTEAPLNPKANR.E |
|  | 3147 | 787.4149 | 2359.2226 | 2359.6105 | -0.3880 | 1 | 40 | 0.24 | 1 | R.KDLYANTVLSGGTTMYPGIADR.M + Oxidation (M) |
|  | 3258 | 836.7892 | 2507.3454 | 2507.7578 | -0.4124 | 2 | 58 | 0.0029 | 1 | R.HQGVMVGMGQKDSYVGDEAQSKR.G |
|  | 3282 | 851.8923 | 2552.6548 | 2552.8794 | -0.2246 | 2 | 15 | 78 | 1 | R.VAPEEHPVLLTEAPLNPKANREK.M |
|  | 3313 | 879.0190 | 2634.0350 | 2634.9352 | -0.9003 | 1 | 21 | 23 | 1 | K.DLYANTVLSGGTTMYPGIADRMQK.E + 2 Oxidation (M) |

  


---

|  |  |
| --- | --- |
| **3.** | gi|62897625    **Mass:** 41764    **Score:** 933    **Queries matched:** 83   **emPAI:** 3.96 |
|  | beta actin variant [Homo sapiens] |

|  |  |
| --- | --- |
|  | Check to include this hit in error tolerant search or archive report |
|  |  |

|  |  |  |  |  |  |  |  |  |  |  |
| --- | --- | --- | --- | --- | --- | --- | --- | --- | --- | --- |
|  | **Query** | **Observed** | **Mr(expt)** | **Mr(calc)** | **Delta** | **Miss** | **Score** | **Expect** | **Rank** | **Peptide** |
|  | 590 | 398.5465 | 795.0782 | 794.9384 | 0.1398 | 0 | 26 | 6.6 | 1 | K.IIAPPER.K |
|  | 1481 | 489.0091 | 976.0033 | 976.0012 | 0.0022 | 0 | 87 | 5.6e-06 | 1 | K.AGFAGDDAPR.A |
|  | 1482 | 489.0325 | 976.0502 | 976.0012 | 0.0490 | 0 | (76) | 7.3e-05 | 1 | K.AGFAGDDAPR.A |
|  | 1483 | 489.0872 | 976.1597 | 976.0012 | 0.1585 | 0 | (62) | 0.0016 | 1 | K.AGFAGDDAPR.A |
|  | 1484 | 489.1371 | 976.2593 | 976.0012 | 0.2582 | 0 | (43) | 0.13 | 1 | K.AGFAGDDAPR.A |
|  | 1614 | 518.7917 | 1035.5687 | 1036.2683 | -0.6996 | 1 | 19 | 26 | 1 | K.IKIIAPPER.K |
|  | 1996 | 581.5563 | 1161.0978 | 1161.3689 | -0.2712 | 0 | (37) | 0.55 | 1 | K.EITALAPSTMK.I |
|  | 444 | 388.9354 | 1163.7841 | 1164.4406 | -0.6565 | 2 | (19) | 40 | 1 | K.IKIIAPPERK.Y |
|  | 446 | 388.9509 | 1163.8305 | 1164.4406 | -0.6101 | 2 | (5) | 8.9e+02 | 8 | K.IKIIAPPERK.Y |
|  | 488 | 389.4004 | 1165.1789 | 1164.4406 | 0.7383 | 2 | 45 | 0.096 | 1 | K.IKIIAPPERK.Y |
|  | 560 | 391.4145 | 1171.2214 | 1171.3936 | -0.1722 | 0 | 31 | 2.3 | 1 | R.HQGVMVGMGQK.D |
|  | 2025 | 586.6212 | 1171.2275 | 1171.3936 | -0.1660 | 0 | (25) | 10 | 1 | R.HQGVMVGMGQK.D |
|  | 2026 | 587.2489 | 1172.4830 | 1171.3936 | 1.0895 | 0 | (10) | 2.6e+02 | 1 | R.HQGVMVGMGQK.D |
|  | 2036 | 589.6276 | 1177.2403 | 1177.3683 | -0.1280 | 0 | 52 | 0.022 | 1 | K.EITALAPSTMK.I + Oxidation (M) |
|  | 2037 | 589.6486 | 1177.2823 | 1177.3683 | -0.0860 | 0 | (33) | 1.6 | 2 | K.EITALAPSTMK.I + Oxidation (M) |
|  | 2129 | 599.6302 | 1197.2456 | 1198.4173 | -1.1717 | 0 | (5) | 8.7e+02 | 3 | R.AVFPSIVGRPR.H |
|  | 2130 | 599.7516 | 1197.4884 | 1198.4173 | -0.9289 | 0 | (44) | 0.12 | 1 | R.AVFPSIVGRPR.H |
|  | 2131 | 599.7771 | 1197.5394 | 1198.4173 | -0.8778 | 0 | (39) | 0.32 | 1 | R.AVFPSIVGRPR.H |
|  | 2132 | 599.7792 | 1197.5436 | 1198.4173 | -0.8737 | 0 | (36) | 0.63 | 1 | R.AVFPSIVGRPR.H |
|  | 2133 | 599.8466 | 1197.6785 | 1198.4173 | -0.7388 | 0 | (53) | 0.012 | 1 | R.AVFPSIVGRPR.H |
|  | 2135 | 600.0215 | 1198.0283 | 1198.4173 | -0.3890 | 0 | (12) | 1.8e+02 | 1 | R.AVFPSIVGRPR.H |
|  | 2137 | 600.2160 | 1198.4172 | 1198.4173 | -0.0000 | 0 | (71) | 0.00024 | 1 | R.AVFPSIVGRPR.H |
|  | 2138 | 600.2161 | 1198.4173 | 1198.4173 | 0.0001 | 0 | (37) | 0.6 | 1 | R.AVFPSIVGRPR.H |
|  | 2139 | 600.2168 | 1198.4188 | 1198.4173 | 0.0015 | 0 | (34) | 1.1 | 1 | R.AVFPSIVGRPR.H |
|  | 2140 | 600.2443 | 1198.4737 | 1198.4173 | 0.0565 | 0 | 83 | 1.4e-05 | 1 | R.AVFPSIVGRPR.H |
|  | 2141 | 600.2688 | 1198.5228 | 1198.4173 | 0.1055 | 0 | (54) | 0.012 | 1 | R.AVFPSIVGRPR.H |
|  | 2142 | 600.3383 | 1198.6617 | 1198.4173 | 0.2445 | 0 | (64) | 0.0011 | 1 | R.AVFPSIVGRPR.H |
|  | 619 | 400.6896 | 1199.0467 | 1198.4173 | 0.6295 | 0 | (28) | 3.8 | 1 | R.AVFPSIVGRPR.H |
|  | 620 | 400.7009 | 1199.0806 | 1198.4173 | 0.6633 | 0 | (18) | 34 | 1 | R.AVFPSIVGRPR.H |
|  | 621 | 400.7180 | 1199.1318 | 1198.4173 | 0.7145 | 0 | (22) | 13 | 1 | R.AVFPSIVGRPR.H |
|  | 622 | 400.7871 | 1199.3392 | 1198.4173 | 0.9220 | 0 | (15) | 90 | 5 | R.AVFPSIVGRPR.H |
|  | 2143 | 600.7864 | 1199.5580 | 1198.4173 | 1.1407 | 0 | (30) | 2.4 | 1 | R.AVFPSIVGRPR.H |
|  | 1225 | 452.7126 | 1355.1157 | 1354.3795 | 0.7362 | 1 | (32) | 1.5 | 1 | K.DSYVGDEAQSKR.G |
|  | 2687 | 678.6069 | 1355.1991 | 1354.3795 | 0.8196 | 1 | 50 | 0.022 | 1 | K.DSYVGDEAQSKR.G |
|  | 2841 | 709.9313 | 1417.8479 | 1418.6982 | -0.8504 | 1 | (17) | 44 | 2 | K.EITALAPSTMKIK.I + Oxidation (M) |
|  | 2843 | 710.1817 | 1418.3486 | 1418.6982 | -0.3496 | 1 | (20) | 29 | 2 | K.EITALAPSTMKIK.I + Oxidation (M) |
|  | 1367 | 474.2588 | 1419.7542 | 1418.6982 | 1.0560 | 1 | 34 | 0.99 | 1 | K.EITALAPSTMKIK.I + Oxidation (M) |
|  | 1558 | 506.8081 | 1517.4021 | 1516.5679 | 0.8342 | 0 | 10 | 2.2e+02 | 2 | K.QEYDESGPSIVHR.K |
|  | 1759 | **537.2552** | **1608.7436** | **1608.9219** | **-0.1783** | **2** | **14** | **1.1e+02** | **1** | **R.MRKEITALAPSTMK.I + 2 Oxidation (M)** |
|  | 1819 | 542.3186 | 1623.9336 | 1623.8683 | 0.0653 | 1 | 23 | 14 | 1 | R.LDLAGRDLTDYLMK.I |
|  | 1850 | 549.4825 | 1645.4255 | 1644.7403 | 0.6852 | 1 | 10 | 2.3e+02 | 2 | K.QEYDESGPSIVHRK.C |
|  | 3324 | 895.8816 | 1789.7484 | 1790.9243 | -1.1759 | 0 | (14) | 76 | 1 | K.SYELPDGQVITIGNER.F |
|  | 3325 | 895.8936 | 1789.7723 | 1790.9243 | -1.1520 | 0 | (31) | 1.6 | 1 | K.SYELPDGQVITIGNER.F |
|  | 3326 | 896.1632 | 1790.3116 | 1790.9243 | -0.6127 | 0 | (35) | 0.6 | 1 | K.SYELPDGQVITIGNER.F |
|  | 3327 | 896.1948 | 1790.3749 | 1790.9243 | -0.5495 | 0 | (11) | 1.6e+02 | 2 | K.SYELPDGQVITIGNER.F |
|  | 3328 | 896.2452 | 1790.4757 | 1790.9243 | -0.4486 | 0 | (36) | 0.52 | 1 | K.SYELPDGQVITIGNER.F |
|  | 3329 | 896.3129 | 1790.6110 | 1790.9243 | -0.3134 | 0 | (48) | 0.039 | 1 | K.SYELPDGQVITIGNER.F |
|  | 2118 | 597.9269 | 1790.7586 | 1790.9243 | -0.1657 | 0 | (4) | 7.9e+02 | 5 | K.SYELPDGQVITIGNER.F |
|  | 3331 | 896.4506 | 1790.8863 | 1790.9243 | -0.0380 | 0 | (43) | 0.12 | 1 | K.SYELPDGQVITIGNER.F |
|  | 3332 | 896.5997 | 1791.1847 | 1790.9243 | 0.2603 | 0 | (64) | 0.0011 | 1 | K.SYELPDGQVITIGNER.F |
|  | 3333 | 896.7216 | 1791.4283 | 1790.9243 | 0.5040 | 0 | (79) | 3.2e-05 | 1 | K.SYELPDGQVITIGNER.F |
|  | 2121 | 598.1779 | 1791.5116 | 1790.9243 | 0.5872 | 0 | (48) | 0.046 | 1 | K.SYELPDGQVITIGNER.F |
|  | 2122 | 598.2000 | 1791.5779 | 1790.9243 | 0.6535 | 0 | (55) | 0.0075 | 1 | K.SYELPDGQVITIGNER.F |
|  | 3334 | 896.9148 | 1791.8148 | 1790.9243 | 0.8905 | 0 | (70) | 0.00024 | 1 | K.SYELPDGQVITIGNER.F |
|  | 3335 | 896.9431 | 1791.8714 | 1790.9243 | 0.9471 | 0 | (83) | 1.2e-05 | 1 | K.SYELPDGQVITIGNER.F |
|  | 3336 | 897.0674 | 1792.1200 | 1790.9243 | 1.1957 | 0 | 87 | 5.3e-06 | 1 | K.SYELPDGQVITIGNER.F |
|  | 2498 | 652.1713 | 1953.4916 | 1954.2269 | -0.7353 | 0 | (16) | 69 | 1 | R.VAPEEHPVLLTEAPLNPK.A |
|  | 3535 | 977.9032 | 1953.7916 | 1954.2269 | -0.4353 | 0 | (44) | 0.077 | 2 | R.VAPEEHPVLLTEAPLNPK.A |
|  | 3536 | 978.2371 | 1954.4593 | 1954.2269 | 0.2324 | 0 | 69 | 0.00025 | 1 | R.VAPEEHPVLLTEAPLNPK.A |
|  | 2501 | 652.5559 | 1954.6456 | 1954.2269 | 0.4186 | 0 | (47) | 0.043 | 1 | R.VAPEEHPVLLTEAPLNPK.A |
|  | 2502 | 652.6416 | 1954.9026 | 1954.2269 | 0.6757 | 0 | (43) | 0.12 | 1 | R.VAPEEHPVLLTEAPLNPK.A |
|  | 2503 | 652.7772 | 1955.3095 | 1954.2269 | 1.0825 | 0 | (35) | 1.1 | 1 | R.VAPEEHPVLLTEAPLNPK.A |
|  | 3556 | 1047.9919 | 2093.9691 | 2094.2839 | -0.3148 | 1 | 42 | 0.13 | 1 | K.SYELPDGQVITIGNERFR.C |
|  | 3557 | 1047.9972 | 2093.9796 | 2094.2839 | -0.3043 | 1 | (39) | 0.26 | 1 | K.SYELPDGQVITIGNERFR.C |
|  | 2809 | 699.1260 | 2094.3559 | 2094.2839 | 0.0720 | 1 | (13) | 1.4e+02 | 1 | K.SYELPDGQVITIGNERFR.C |
|  | 2813 | 699.4652 | 2095.3735 | 2094.2839 | 1.0895 | 1 | (29) | 2.9 | 1 | K.SYELPDGQVITIGNERFR.C |
|  | 2866 | 719.4661 | 2155.3760 | 2156.4032 | -1.0271 | 1 | (43) | 0.12 | 1 | K.AGFAGDDAPRAVFPSIVGRPR.H |
|  | 2867 | 719.5731 | 2155.6972 | 2156.4032 | -0.7060 | 1 | (19) | 26 | 1 | K.AGFAGDDAPRAVFPSIVGRPR.H |
|  | 2868 | 719.7181 | 2156.1322 | 2156.4032 | -0.2709 | 1 | (37) | 0.44 | 1 | K.AGFAGDDAPRAVFPSIVGRPR.H |
|  | 2869 | 719.7473 | 2156.2196 | 2156.4032 | -0.1836 | 1 | (34) | 0.97 | 1 | K.AGFAGDDAPRAVFPSIVGRPR.H |
|  | 3566 | 1079.3804 | 2156.7460 | 2156.4032 | 0.3428 | 1 | (27) | 4 | 1 | K.AGFAGDDAPRAVFPSIVGRPR.H |
|  | 2870 | 719.9481 | 2156.8220 | 2156.4032 | 0.4188 | 1 | (26) | 4.9 | 1 | K.AGFAGDDAPRAVFPSIVGRPR.H |
|  | 2871 | 720.0771 | 2157.2093 | 2156.4032 | 0.8061 | 1 | (49) | 0.025 | 1 | K.AGFAGDDAPRAVFPSIVGRPR.H |
|  | 2872 | 720.1254 | 2157.3539 | 2156.4032 | 0.9508 | 1 | (51) | 0.02 | 1 | K.AGFAGDDAPRAVFPSIVGRPR.H |
|  | 2873 | 720.2019 | 2157.5835 | 2156.4032 | 1.1804 | 1 | 57 | 0.0055 | 1 | K.AGFAGDDAPRAVFPSIVGRPR.H |
|  | 2899 | 733.1710 | 2196.4909 | 2195.6214 | 0.8695 | 2 | 9 | 3.1e+02 | 1 | K.EITALAPSTMKIKIIAPPER.K + Oxidation (M) |
|  | 3572 | 1116.2848 | 2230.5548 | 2231.4382 | -0.8834 | 0 | (18) | 36 | 1 | K.DLYANTVLSGGTTMYPGIADR.M + Oxidation (M) |
|  | 3006 | 744.5515 | 2230.6322 | 2231.4382 | -0.8060 | 0 | 21 | 21 | 1 | K.DLYANTVLSGGTTMYPGIADR.M + Oxidation (M) |
|  | 3103 | 766.0936 | 2295.2587 | 2295.5931 | -0.3344 | 1 | 52 | 0.012 | 2 | R.VAPEEHPVLLTEAPLNPKANR.E |
|  | 3104 | 766.4434 | 2296.3079 | 2295.5931 | 0.7148 | 1 | (24) | 8.9 | 1 | R.VAPEEHPVLLTEAPLNPKANR.E |
|  | 3147 | 787.4149 | 2359.2226 | 2359.6105 | -0.3880 | 1 | 40 | 0.24 | 1 | R.KDLYANTVLSGGTTMYPGIADR.M + Oxidation (M) |
|  | 3258 | 836.7892 | 2507.3454 | 2507.7578 | -0.4124 | 2 | 58 | 0.0029 | 1 | R.HQGVMVGMGQKDSYVGDEAQSKR.G |
|  | 3282 | 851.8923 | 2552.6548 | 2552.8794 | -0.2246 | 2 | 15 | 78 | 1 | R.VAPEEHPVLLTEAPLNPKANREK.M |

  


---

|  |  |
| --- | --- |
| **4.** | gi|4501881    **Mass:** 42051    **Score:** 781    **Queries matched:** 79   **emPAI:** 3.21 |
|  | actin, alpha skeletal muscle [Homo sapiens] |

|  |  |
| --- | --- |
|  | Check to include this hit in error tolerant search or archive report |
|  |  |

|  |  |  |  |  |  |  |  |  |  |  |
| --- | --- | --- | --- | --- | --- | --- | --- | --- | --- | --- |
|  | **Query** | **Observed** | **Mr(expt)** | **Mr(calc)** | **Delta** | **Miss** | **Score** | **Expect** | **Rank** | **Peptide** |
|  | 590 | 398.5465 | 795.0782 | 794.9384 | 0.1398 | 0 | 26 | 6.6 | 1 | K.IIAPPER.K |
|  | 1481 | 489.0091 | 976.0033 | 976.0012 | 0.0022 | 0 | 87 | 5.6e-06 | 1 | K.AGFAGDDAPR.A |
|  | 1482 | 489.0325 | 976.0502 | 976.0012 | 0.0490 | 0 | (76) | 7.3e-05 | 1 | K.AGFAGDDAPR.A |
|  | 1483 | 489.0872 | 976.1597 | 976.0012 | 0.1585 | 0 | (62) | 0.0016 | 1 | K.AGFAGDDAPR.A |
|  | 1484 | 489.1371 | 976.2593 | 976.0012 | 0.2582 | 0 | (43) | 0.13 | 1 | K.AGFAGDDAPR.A |
|  | 1614 | 518.7917 | 1035.5687 | 1036.2683 | -0.6996 | 1 | 19 | 26 | 1 | K.IKIIAPPER.K |
|  | 1996 | 581.5563 | 1161.0978 | 1161.3689 | -0.2712 | 0 | (37) | 0.55 | 1 | K.EITALAPSTMK.I |
|  | 444 | 388.9354 | 1163.7841 | 1164.4406 | -0.6565 | 2 | (19) | 40 | 1 | K.IKIIAPPERK.Y |
|  | 446 | 388.9509 | 1163.8305 | 1164.4406 | -0.6101 | 2 | (5) | 8.9e+02 | 8 | K.IKIIAPPERK.Y |
|  | 488 | 389.4004 | 1165.1789 | 1164.4406 | 0.7383 | 2 | 45 | 0.096 | 1 | K.IKIIAPPERK.Y |
|  | 560 | 391.4145 | 1171.2214 | 1171.3936 | -0.1722 | 0 | 31 | 2.3 | 1 | R.HQGVMVGMGQK.D |
|  | 2025 | 586.6212 | 1171.2275 | 1171.3936 | -0.1660 | 0 | (25) | 10 | 1 | R.HQGVMVGMGQK.D |
|  | 2026 | 587.2489 | 1172.4830 | 1171.3936 | 1.0895 | 0 | (10) | 2.6e+02 | 1 | R.HQGVMVGMGQK.D |
|  | 2036 | 589.6276 | 1177.2403 | 1177.3683 | -0.1280 | 0 | 52 | 0.022 | 1 | K.EITALAPSTMK.I + Oxidation (M) |
|  | 2037 | 589.6486 | 1177.2823 | 1177.3683 | -0.0860 | 0 | (33) | 1.6 | 2 | K.EITALAPSTMK.I + Oxidation (M) |
|  | 2129 | 599.6302 | 1197.2456 | 1198.4173 | -1.1717 | 0 | (5) | 8.7e+02 | 3 | R.AVFPSIVGRPR.H |
|  | 2130 | 599.7516 | 1197.4884 | 1198.4173 | -0.9289 | 0 | (44) | 0.12 | 1 | R.AVFPSIVGRPR.H |
|  | 2131 | 599.7771 | 1197.5394 | 1198.4173 | -0.8778 | 0 | (39) | 0.32 | 1 | R.AVFPSIVGRPR.H |
|  | 2132 | 599.7792 | 1197.5436 | 1198.4173 | -0.8737 | 0 | (36) | 0.63 | 1 | R.AVFPSIVGRPR.H |
|  | 2133 | 599.8466 | 1197.6785 | 1198.4173 | -0.7388 | 0 | (53) | 0.012 | 1 | R.AVFPSIVGRPR.H |
|  | 2135 | 600.0215 | 1198.0283 | 1198.4173 | -0.3890 | 0 | (12) | 1.8e+02 | 1 | R.AVFPSIVGRPR.H |
|  | 2137 | 600.2160 | 1198.4172 | 1198.4173 | -0.0000 | 0 | (71) | 0.00024 | 1 | R.AVFPSIVGRPR.H |
|  | 2138 | 600.2161 | 1198.4173 | 1198.4173 | 0.0001 | 0 | (37) | 0.6 | 1 | R.AVFPSIVGRPR.H |
|  | 2139 | 600.2168 | 1198.4188 | 1198.4173 | 0.0015 | 0 | (34) | 1.1 | 1 | R.AVFPSIVGRPR.H |
|  | 2140 | 600.2443 | 1198.4737 | 1198.4173 | 0.0565 | 0 | 83 | 1.4e-05 | 1 | R.AVFPSIVGRPR.H |
|  | 2141 | 600.2688 | 1198.5228 | 1198.4173 | 0.1055 | 0 | (54) | 0.012 | 1 | R.AVFPSIVGRPR.H |
|  | 2142 | 600.3383 | 1198.6617 | 1198.4173 | 0.2445 | 0 | (64) | 0.0011 | 1 | R.AVFPSIVGRPR.H |
|  | 619 | 400.6896 | 1199.0467 | 1198.4173 | 0.6295 | 0 | (28) | 3.8 | 1 | R.AVFPSIVGRPR.H |
|  | 620 | 400.7009 | 1199.0806 | 1198.4173 | 0.6633 | 0 | (18) | 34 | 1 | R.AVFPSIVGRPR.H |
|  | 621 | 400.7180 | 1199.1318 | 1198.4173 | 0.7145 | 0 | (22) | 13 | 1 | R.AVFPSIVGRPR.H |
|  | 622 | 400.7871 | 1199.3392 | 1198.4173 | 0.9220 | 0 | (15) | 90 | 5 | R.AVFPSIVGRPR.H |
|  | 2143 | 600.7864 | 1199.5580 | 1198.4173 | 1.1407 | 0 | (30) | 2.4 | 1 | R.AVFPSIVGRPR.H |
|  | 1225 | 452.7126 | 1355.1157 | 1354.3795 | 0.7362 | 1 | (32) | 1.5 | 1 | K.DSYVGDEAQSKR.G |
|  | 2687 | 678.6069 | 1355.1991 | 1354.3795 | 0.8196 | 1 | 50 | 0.022 | 1 | K.DSYVGDEAQSKR.G |
|  | 2841 | 709.9313 | 1417.8479 | 1418.6982 | -0.8504 | 1 | (17) | 44 | 2 | K.EITALAPSTMKIK.I + Oxidation (M) |
|  | 2843 | 710.1817 | 1418.3486 | 1418.6982 | -0.3496 | 1 | (20) | 29 | 2 | K.EITALAPSTMKIK.I + Oxidation (M) |
|  | 1367 | 474.2588 | 1419.7542 | 1418.6982 | 1.0560 | 1 | 34 | 0.99 | 1 | K.EITALAPSTMKIK.I + Oxidation (M) |
|  | 3118 | 775.4117 | 1548.8086 | 1548.8665 | -0.0580 | 1 | (6) | 6.8e+02 | 4 | R.MQKEITALAPSTMK.I |
|  | 1651 | 522.7468 | 1565.2181 | 1564.8660 | 0.3522 | 1 | (30) | 2.1 | 1 | R.MQKEITALAPSTMK.I + Oxidation (M) |
|  | 1653 | 522.8632 | 1565.5673 | 1564.8660 | 0.7014 | 1 | 32 | 1.5 | 1 | R.MQKEITALAPSTMK.I + Oxidation (M) |
|  | 3161 | 791.5933 | 1581.1717 | 1580.8654 | 0.3064 | 1 | (32) | 1.7 | 1 | R.MQKEITALAPSTMK.I + 2 Oxidation (M) |
|  | 1819 | 542.3186 | 1623.9336 | 1623.8683 | 0.0653 | 1 | 23 | 14 | 1 | R.LDLAGRDLTDYLMK.I |
|  | 3324 | 895.8816 | 1789.7484 | 1790.9243 | -1.1759 | 0 | (14) | 76 | 1 | K.SYELPDGQVITIGNER.F |
|  | 3325 | 895.8936 | 1789.7723 | 1790.9243 | -1.1520 | 0 | (31) | 1.6 | 1 | K.SYELPDGQVITIGNER.F |
|  | 3326 | 896.1632 | 1790.3116 | 1790.9243 | -0.6127 | 0 | (35) | 0.6 | 1 | K.SYELPDGQVITIGNER.F |
|  | 3327 | 896.1948 | 1790.3749 | 1790.9243 | -0.5495 | 0 | (11) | 1.6e+02 | 2 | K.SYELPDGQVITIGNER.F |
|  | 3328 | 896.2452 | 1790.4757 | 1790.9243 | -0.4486 | 0 | (36) | 0.52 | 1 | K.SYELPDGQVITIGNER.F |
|  | 3329 | 896.3129 | 1790.6110 | 1790.9243 | -0.3134 | 0 | (48) | 0.039 | 1 | K.SYELPDGQVITIGNER.F |
|  | 2118 | 597.9269 | 1790.7586 | 1790.9243 | -0.1657 | 0 | (4) | 7.9e+02 | 5 | K.SYELPDGQVITIGNER.F |
|  | 3331 | 896.4506 | 1790.8863 | 1790.9243 | -0.0380 | 0 | (43) | 0.12 | 1 | K.SYELPDGQVITIGNER.F |
|  | 3332 | 896.5997 | 1791.1847 | 1790.9243 | 0.2603 | 0 | (64) | 0.0011 | 1 | K.SYELPDGQVITIGNER.F |
|  | 3333 | 896.7216 | 1791.4283 | 1790.9243 | 0.5040 | 0 | (79) | 3.2e-05 | 1 | K.SYELPDGQVITIGNER.F |
|  | 2121 | 598.1779 | 1791.5116 | 1790.9243 | 0.5872 | 0 | (48) | 0.046 | 1 | K.SYELPDGQVITIGNER.F |
|  | 2122 | 598.2000 | 1791.5779 | 1790.9243 | 0.6535 | 0 | (55) | 0.0075 | 1 | K.SYELPDGQVITIGNER.F |
|  | 3334 | 896.9148 | 1791.8148 | 1790.9243 | 0.8905 | 0 | (70) | 0.00024 | 1 | K.SYELPDGQVITIGNER.F |
|  | 3335 | 896.9431 | 1791.8714 | 1790.9243 | 0.9471 | 0 | (83) | 1.2e-05 | 1 | K.SYELPDGQVITIGNER.F |
|  | 3336 | 897.0674 | 1792.1200 | 1790.9243 | 1.1957 | 0 | 87 | 5.3e-06 | 1 | K.SYELPDGQVITIGNER.F |
|  | 3344 | 904.0286 | 1806.0423 | 1806.1958 | -0.1535 | 2 | (28) | 5.1 | 1 | R.MQKEITALAPSTMKIK.I + Oxidation (M) |
|  | 2156 | 603.3624 | 1807.0651 | 1806.1958 | 0.8693 | 2 | (24) | 10 | 1 | R.MQKEITALAPSTMKIK.I + Oxidation (M) |
|  | 2194 | 608.2214 | 1821.6421 | 1822.1953 | -0.5531 | 2 | (3) | 1.5e+03 | 7 | R.MQKEITALAPSTMKIK.I + 2 Oxidation (M) |
|  | 2195 | 608.2873 | 1821.8397 | 1822.1953 | -0.3555 | 2 | (13) | 1.4e+02 | 1 | R.MQKEITALAPSTMKIK.I + 2 Oxidation (M) |
|  | 2198 | 608.5206 | 1822.5395 | 1822.1953 | 0.3443 | 2 | 33 | 1.2 | 1 | R.MQKEITALAPSTMKIK.I + 2 Oxidation (M) |
|  | 2503 | 652.7772 | 1955.3095 | 1956.1997 | -0.8902 | 0 | 11 | 2.2e+02 | 3 | R.VAPEEHPTLLTEAPLNPK.A |
|  | 3556 | 1047.9919 | 2093.9691 | 2094.2839 | -0.3148 | 1 | 42 | 0.13 | 1 | K.SYELPDGQVITIGNERFR.C |
|  | 3557 | 1047.9972 | 2093.9796 | 2094.2839 | -0.3043 | 1 | (39) | 0.26 | 1 | K.SYELPDGQVITIGNERFR.C |
|  | 2809 | 699.1260 | 2094.3559 | 2094.2839 | 0.0720 | 1 | (13) | 1.4e+02 | 1 | K.SYELPDGQVITIGNERFR.C |
|  | 2813 | 699.4652 | 2095.3735 | 2094.2839 | 1.0895 | 1 | (29) | 2.9 | 1 | K.SYELPDGQVITIGNERFR.C |
|  | 2866 | 719.4661 | 2155.3760 | 2156.4032 | -1.0271 | 1 | (43) | 0.12 | 1 | K.AGFAGDDAPRAVFPSIVGRPR.H |
|  | 2867 | 719.5731 | 2155.6972 | 2156.4032 | -0.7060 | 1 | (19) | 26 | 1 | K.AGFAGDDAPRAVFPSIVGRPR.H |
|  | 2868 | 719.7181 | 2156.1322 | 2156.4032 | -0.2709 | 1 | (37) | 0.44 | 1 | K.AGFAGDDAPRAVFPSIVGRPR.H |
|  | 2869 | 719.7473 | 2156.2196 | 2156.4032 | -0.1836 | 1 | (34) | 0.97 | 1 | K.AGFAGDDAPRAVFPSIVGRPR.H |
|  | 3566 | 1079.3804 | 2156.7460 | 2156.4032 | 0.3428 | 1 | (27) | 4 | 1 | K.AGFAGDDAPRAVFPSIVGRPR.H |
|  | 2870 | 719.9481 | 2156.8220 | 2156.4032 | 0.4188 | 1 | (26) | 4.9 | 1 | K.AGFAGDDAPRAVFPSIVGRPR.H |
|  | 2871 | 720.0771 | 2157.2093 | 2156.4032 | 0.8061 | 1 | (49) | 0.025 | 1 | K.AGFAGDDAPRAVFPSIVGRPR.H |
|  | 2872 | 720.1254 | 2157.3539 | 2156.4032 | 0.9508 | 1 | (51) | 0.02 | 1 | K.AGFAGDDAPRAVFPSIVGRPR.H |
|  | 2873 | 720.2019 | 2157.5835 | 2156.4032 | 1.1804 | 1 | 57 | 0.0055 | 1 | K.AGFAGDDAPRAVFPSIVGRPR.H |
|  | 2899 | 733.1710 | 2196.4909 | 2195.6214 | 0.8695 | 2 | 9 | 3.1e+02 | 1 | K.EITALAPSTMKIKIIAPPER.K + Oxidation (M) |
|  | 3258 | 836.7892 | 2507.3454 | 2507.7578 | -0.4124 | 2 | 58 | 0.0029 | 1 | R.HQGVMVGMGQKDSYVGDEAQSKR.G |
|  | 3313 | 879.0190 | 2634.0350 | 2633.9736 | 0.0613 | 1 | 7 | 6.2e+02 | 3 | K.DLYANNVMSGGTTMYPGIADRMQK.E |

  


---

|  |  |
| --- | --- |
| **5.** | gi|178045    **Mass:** 25878    **Score:** 435    **Queries matched:** 37   **emPAI:** 1.34 |
|  | gamma-actin [Homo sapiens] |

|  |  |
| --- | --- |
|  | Check to include this hit in error tolerant search or archive report |
|  |  |

|  |  |  |  |  |  |  |  |  |  |  |
| --- | --- | --- | --- | --- | --- | --- | --- | --- | --- | --- |
|  | **Query** | **Observed** | **Mr(expt)** | **Mr(calc)** | **Delta** | **Miss** | **Score** | **Expect** | **Rank** | **Peptide** |
|  | 590 | 398.5465 | 795.0782 | 794.9384 | 0.1398 | 0 | 26 | 6.6 | 1 | K.IIAPPER.K |
|  | 1614 | 518.7917 | 1035.5687 | 1036.2683 | -0.6996 | 1 | 19 | 26 | 1 | K.IKIIAPPER.K |
|  | 1996 | 581.5563 | 1161.0978 | 1160.4272 | 0.6705 | 1 | (31) | 1.8 | 2 | K.KITALAPSTMK.I |
|  | 444 | 388.9354 | 1163.7841 | 1164.4406 | -0.6565 | 2 | (19) | 40 | 1 | K.IKIIAPPERK.Y |
|  | 446 | 388.9509 | 1163.8305 | 1164.4406 | -0.6101 | 2 | (5) | 8.9e+02 | 8 | K.IKIIAPPERK.Y |
|  | 488 | 389.4004 | 1165.1789 | 1164.4406 | 0.7383 | 2 | 45 | 0.096 | 1 | K.IKIIAPPERK.Y |
|  | 2036 | 589.6276 | 1177.2403 | 1176.4267 | 0.8137 | 1 | 45 | 0.11 | 2 | K.KITALAPSTMK.I + Oxidation (M) |
|  | 2037 | 589.6486 | 1177.2823 | 1176.4267 | 0.8557 | 1 | (38) | 0.59 | 1 | K.KITALAPSTMK.I + Oxidation (M) |
|  | 2841 | 709.9313 | 1417.8479 | 1417.7565 | 0.0913 | 2 | (18) | 36 | 1 | K.KITALAPSTMKIK.I + Oxidation (M) |
|  | 2843 | 710.1817 | 1418.3486 | 1417.7565 | 0.5921 | 2 | 29 | 3.6 | 1 | K.KITALAPSTMKIK.I + Oxidation (M) |
|  | 1558 | 506.8081 | 1517.4021 | 1516.5679 | 0.8342 | 0 | 10 | 2.2e+02 | 2 | K.QEYDESGPSIVHR.K |
|  | 3118 | 775.4117 | 1548.8086 | 1547.9249 | 0.8837 | 2 | 18 | 46 | 1 | R.MQKKITALAPSTMK.I |
|  | 1819 | 542.3186 | 1623.9336 | 1623.8683 | 0.0653 | 1 | 23 | 14 | 1 | R.LDLAGRDLTDYLMK.I |
|  | 1850 | 549.4825 | 1645.4255 | 1644.7403 | 0.6852 | 1 | 10 | 2.3e+02 | 2 | K.QEYDESGPSIVHRK.C |
|  | 3324 | 895.8816 | 1789.7484 | 1790.9243 | -1.1759 | 0 | (14) | 76 | 1 | K.SYELPDGQVITIGNER.F |
|  | 3325 | 895.8936 | 1789.7723 | 1790.9243 | -1.1520 | 0 | (31) | 1.6 | 1 | K.SYELPDGQVITIGNER.F |
|  | 3326 | 896.1632 | 1790.3116 | 1790.9243 | -0.6127 | 0 | (35) | 0.6 | 1 | K.SYELPDGQVITIGNER.F |
|  | 3327 | 896.1948 | 1790.3749 | 1790.9243 | -0.5495 | 0 | (11) | 1.6e+02 | 2 | K.SYELPDGQVITIGNER.F |
|  | 3328 | 896.2452 | 1790.4757 | 1790.9243 | -0.4486 | 0 | (36) | 0.52 | 1 | K.SYELPDGQVITIGNER.F |
|  | 3329 | 896.3129 | 1790.6110 | 1790.9243 | -0.3134 | 0 | (48) | 0.039 | 1 | K.SYELPDGQVITIGNER.F |
|  | 2118 | 597.9269 | 1790.7586 | 1790.9243 | -0.1657 | 0 | (4) | 7.9e+02 | 5 | K.SYELPDGQVITIGNER.F |
|  | 3331 | 896.4506 | 1790.8863 | 1790.9243 | -0.0380 | 0 | (43) | 0.12 | 1 | K.SYELPDGQVITIGNER.F |
|  | 3332 | 896.5997 | 1791.1847 | 1790.9243 | 0.2603 | 0 | (64) | 0.0011 | 1 | K.SYELPDGQVITIGNER.F |
|  | 3333 | 896.7216 | 1791.4283 | 1790.9243 | 0.5040 | 0 | (79) | 3.2e-05 | 1 | K.SYELPDGQVITIGNER.F |
|  | 2121 | 598.1779 | 1791.5116 | 1790.9243 | 0.5872 | 0 | (48) | 0.046 | 1 | K.SYELPDGQVITIGNER.F |
|  | 2122 | 598.2000 | 1791.5779 | 1790.9243 | 0.6535 | 0 | (55) | 0.0075 | 1 | K.SYELPDGQVITIGNER.F |
|  | 3334 | 896.9148 | 1791.8148 | 1790.9243 | 0.8905 | 0 | (70) | 0.00024 | 1 | K.SYELPDGQVITIGNER.F |
|  | 3335 | 896.9431 | 1791.8714 | 1790.9243 | 0.9471 | 0 | (83) | 1.2e-05 | 1 | K.SYELPDGQVITIGNER.F |
|  | 3336 | 897.0674 | 1792.1200 | 1790.9243 | 1.1957 | 0 | 87 | 5.3e-06 | 1 | K.SYELPDGQVITIGNER.F |
|  | 3556 | 1047.9919 | 2093.9691 | 2094.2839 | -0.3148 | 1 | 42 | 0.13 | 1 | K.SYELPDGQVITIGNERFR.C |
|  | 3557 | 1047.9972 | 2093.9796 | 2094.2839 | -0.3043 | 1 | (39) | 0.26 | 1 | K.SYELPDGQVITIGNERFR.C |
|  | 2809 | 699.1260 | 2094.3559 | 2094.2839 | 0.0720 | 1 | (13) | 1.4e+02 | 1 | K.SYELPDGQVITIGNERFR.C |
|  | 2813 | 699.4652 | 2095.3735 | 2094.2839 | 1.0895 | 1 | (29) | 2.9 | 1 | K.SYELPDGQVITIGNERFR.C |
|  | 3572 | 1116.2848 | 2230.5548 | 2231.4382 | -0.8834 | 0 | (18) | 36 | 1 | K.DLYANTVLSGGTTMYPGIADR.M + Oxidation (M) |
|  | 3006 | 744.5515 | 2230.6322 | 2231.4382 | -0.8060 | 0 | 21 | 21 | 1 | K.DLYANTVLSGGTTMYPGIADR.M + Oxidation (M) |
|  | 3147 | 787.4149 | 2359.2226 | 2359.6105 | -0.3880 | 1 | 40 | 0.24 | 1 | R.KDLYANTVLSGGTTMYPGIADR.M + Oxidation (M) |
|  | 3313 | 879.0190 | 2634.0350 | 2634.9352 | -0.9003 | 1 | 21 | 23 | 1 | K.DLYANTVLSGGTTMYPGIADRMQK.K + 2 Oxidation (M) |

  


---

|  |  |
| --- | --- |
| **6.** | gi|145309046    **Mass:** 121369   **Score:** 404    **Queries matched:** 58   **emPAI:** 0.27 |
|  | POTE-2 alpha-actin [Homo sapiens] |

|  |  |
| --- | --- |
|  | Check to include this hit in error tolerant search or archive report |
|  |  |

|  |  |  |  |  |  |  |  |  |  |  |
| --- | --- | --- | --- | --- | --- | --- | --- | --- | --- | --- |
|  | **Query** | **Observed** | **Mr(expt)** | **Mr(calc)** | **Delta** | **Miss** | **Score** | **Expect** | **Rank** | **Peptide** |
|  | 1481 | 489.0091 | 976.0033 | 976.0012 | 0.0022 | 0 | 87 | 5.6e-06 | 1 | K.AGFAGDDAPR.A |
|  | 1482 | 489.0325 | 976.0502 | 976.0012 | 0.0490 | 0 | (76) | 7.3e-05 | 1 | K.AGFAGDDAPR.A |
|  | 1483 | 489.0872 | 976.1597 | 976.0012 | 0.1585 | 0 | (62) | 0.0016 | 1 | K.AGFAGDDAPR.A |
|  | 1484 | 489.1371 | 976.2593 | 976.0012 | 0.2582 | 0 | (43) | 0.13 | 1 | K.AGFAGDDAPR.A |
|  | 1865 | **550.2676** | **1098.5205** | **1098.3660** | **0.1545** | **2** | **11** | **2e+02** | **3** | **R.SKMGKWCCR.C** |
|  | 2129 | 599.6302 | 1197.2456 | 1198.4173 | -1.1717 | 0 | (5) | 8.7e+02 | 3 | R.AVFPSIVGRPR.Q |
|  | 2130 | 599.7516 | 1197.4884 | 1198.4173 | -0.9289 | 0 | (44) | 0.12 | 1 | R.AVFPSIVGRPR.Q |
|  | 2131 | 599.7771 | 1197.5394 | 1198.4173 | -0.8778 | 0 | (39) | 0.32 | 1 | R.AVFPSIVGRPR.Q |
|  | 2132 | 599.7792 | 1197.5436 | 1198.4173 | -0.8737 | 0 | (36) | 0.63 | 1 | R.AVFPSIVGRPR.Q |
|  | 2133 | 599.8466 | 1197.6785 | 1198.4173 | -0.7388 | 0 | (53) | 0.012 | 1 | R.AVFPSIVGRPR.Q |
|  | 2135 | 600.0215 | 1198.0283 | 1198.4173 | -0.3890 | 0 | (12) | 1.8e+02 | 1 | R.AVFPSIVGRPR.Q |
|  | 2137 | 600.2160 | 1198.4172 | 1198.4173 | -0.0000 | 0 | (71) | 0.00024 | 1 | R.AVFPSIVGRPR.Q |
|  | 2138 | 600.2161 | 1198.4173 | 1198.4173 | 0.0001 | 0 | (37) | 0.6 | 1 | R.AVFPSIVGRPR.Q |
|  | 2139 | 600.2168 | 1198.4188 | 1198.4173 | 0.0015 | 0 | (34) | 1.1 | 1 | R.AVFPSIVGRPR.Q |
|  | 2140 | 600.2443 | 1198.4737 | 1198.4173 | 0.0565 | 0 | 83 | 1.4e-05 | 1 | R.AVFPSIVGRPR.Q |
|  | 2141 | 600.2688 | 1198.5228 | 1198.4173 | 0.1055 | 0 | (54) | 0.012 | 1 | R.AVFPSIVGRPR.Q |
|  | 2142 | 600.3383 | 1198.6617 | 1198.4173 | 0.2445 | 0 | (64) | 0.0011 | 1 | R.AVFPSIVGRPR.Q |
|  | 619 | 400.6896 | 1199.0467 | 1198.4173 | 0.6295 | 0 | (28) | 3.8 | 1 | R.AVFPSIVGRPR.Q |
|  | 620 | 400.7009 | 1199.0806 | 1198.4173 | 0.6633 | 0 | (18) | 34 | 1 | R.AVFPSIVGRPR.Q |
|  | 621 | 400.7180 | 1199.1318 | 1198.4173 | 0.7145 | 0 | (22) | 13 | 1 | R.AVFPSIVGRPR.Q |
|  | 622 | 400.7871 | 1199.3392 | 1198.4173 | 0.9220 | 0 | (15) | 90 | 5 | R.AVFPSIVGRPR.Q |
|  | 2143 | 600.7864 | 1199.5580 | 1198.4173 | 1.1407 | 0 | (30) | 2.4 | 1 | R.AVFPSIVGRPR.Q |
|  | 720 | **404.7637** | **1211.2688** | **1212.4686** | **-1.1997** | **2** | **(11)** | **1.9e+02** | **3** | **R.SKMGKWCCR.C + 2 Carbamidomethyl (C)** |
|  | 1558 | 506.8081 | 1517.4021 | 1516.5679 | 0.8342 | 0 | 10 | 2.2e+02 | 2 | K.QEYDESGPSIVHR.K |
|  | 3161 | 791.5933 | 1581.1717 | 1580.9316 | 0.2402 | 1 | 5 | 8.4e+02 | 9 | R.MQKEIAALAPSMMK.I + 2 Oxidation (M) |
|  | 1850 | 549.4825 | 1645.4255 | 1644.7403 | 0.6852 | 1 | 10 | 2.3e+02 | 2 | K.QEYDESGPSIVHRK.C |
|  | 1854 | **549.8562** | **1646.5464** | **1647.0242** | **-0.4777** | **1** | **4** | **7.7e+02** | **7** | **K.MGKWCCHCFPCCR.G + Carbamidomethyl (C); Oxidation (M)** |
|  | 3324 | 895.8816 | 1789.7484 | 1790.9243 | -1.1759 | 0 | (14) | 76 | 1 | K.SYELPDGQVITIGNER.F |
|  | 3325 | 895.8936 | 1789.7723 | 1790.9243 | -1.1520 | 0 | (31) | 1.6 | 1 | K.SYELPDGQVITIGNER.F |
|  | 3326 | 896.1632 | 1790.3116 | 1790.9243 | -0.6127 | 0 | (35) | 0.6 | 1 | K.SYELPDGQVITIGNER.F |
|  | 3327 | 896.1948 | 1790.3749 | 1790.9243 | -0.5495 | 0 | (11) | 1.6e+02 | 2 | K.SYELPDGQVITIGNER.F |
|  | 3328 | 896.2452 | 1790.4757 | 1790.9243 | -0.4486 | 0 | (36) | 0.52 | 1 | K.SYELPDGQVITIGNER.F |
|  | 3329 | 896.3129 | 1790.6110 | 1790.9243 | -0.3134 | 0 | (48) | 0.039 | 1 | K.SYELPDGQVITIGNER.F |
|  | 2118 | 597.9269 | 1790.7586 | 1790.9243 | -0.1657 | 0 | (4) | 7.9e+02 | 5 | K.SYELPDGQVITIGNER.F |
|  | 3331 | 896.4506 | 1790.8863 | 1790.9243 | -0.0380 | 0 | (43) | 0.12 | 1 | K.SYELPDGQVITIGNER.F |
|  | 3332 | 896.5997 | 1791.1847 | 1790.9243 | 0.2603 | 0 | (64) | 0.0011 | 1 | K.SYELPDGQVITIGNER.F |
|  | 3333 | 896.7216 | 1791.4283 | 1790.9243 | 0.5040 | 0 | (79) | 3.2e-05 | 1 | K.SYELPDGQVITIGNER.F |
|  | 2121 | 598.1779 | 1791.5116 | 1790.9243 | 0.5872 | 0 | (48) | 0.046 | 1 | K.SYELPDGQVITIGNER.F |
|  | 2122 | 598.2000 | 1791.5779 | 1790.9243 | 0.6535 | 0 | (55) | 0.0075 | 1 | K.SYELPDGQVITIGNER.F |
|  | 3334 | 896.9148 | 1791.8148 | 1790.9243 | 0.8905 | 0 | (70) | 0.00024 | 1 | K.SYELPDGQVITIGNER.F |
|  | 3335 | 896.9431 | 1791.8714 | 1790.9243 | 0.9471 | 0 | (83) | 1.2e-05 | 1 | K.SYELPDGQVITIGNER.F |
|  | 3336 | 897.0674 | 1792.1200 | 1790.9243 | 1.1957 | 0 | 87 | 5.3e-06 | 1 | K.SYELPDGQVITIGNER.F |
|  | 3556 | 1047.9919 | 2093.9691 | 2094.2839 | -0.3148 | 1 | 42 | 0.13 | 1 | K.SYELPDGQVITIGNERFR.C |
|  | 3557 | 1047.9972 | 2093.9796 | 2094.2839 | -0.3043 | 1 | (39) | 0.26 | 1 | K.SYELPDGQVITIGNERFR.C |
|  | 2809 | 699.1260 | 2094.3559 | 2094.2839 | 0.0720 | 1 | (13) | 1.4e+02 | 1 | K.SYELPDGQVITIGNERFR.C |
|  | 2813 | 699.4652 | 2095.3735 | 2094.2839 | 1.0895 | 1 | (29) | 2.9 | 1 | K.SYELPDGQVITIGNERFR.C |
|  | 2866 | 719.4661 | 2155.3760 | 2156.4032 | -1.0271 | 1 | (43) | 0.12 | 1 | K.AGFAGDDAPRAVFPSIVGRPR.Q |
|  | 2867 | 719.5731 | 2155.6972 | 2156.4032 | -0.7060 | 1 | (19) | 26 | 1 | K.AGFAGDDAPRAVFPSIVGRPR.Q |
|  | 2868 | 719.7181 | 2156.1322 | 2156.4032 | -0.2709 | 1 | (37) | 0.44 | 1 | K.AGFAGDDAPRAVFPSIVGRPR.Q |
|  | 2869 | 719.7473 | 2156.2196 | 2156.4032 | -0.1836 | 1 | (34) | 0.97 | 1 | K.AGFAGDDAPRAVFPSIVGRPR.Q |
|  | 3566 | 1079.3804 | 2156.7460 | 2156.4032 | 0.3428 | 1 | (27) | 4 | 1 | K.AGFAGDDAPRAVFPSIVGRPR.Q |
|  | 2870 | 719.9481 | 2156.8220 | 2156.4032 | 0.4188 | 1 | (26) | 4.9 | 1 | K.AGFAGDDAPRAVFPSIVGRPR.Q |
|  | 2871 | 720.0771 | 2157.2093 | 2156.4032 | 0.8061 | 1 | (49) | 0.025 | 1 | K.AGFAGDDAPRAVFPSIVGRPR.Q |
|  | 2872 | 720.1254 | 2157.3539 | 2156.4032 | 0.9508 | 1 | (51) | 0.02 | 1 | K.AGFAGDDAPRAVFPSIVGRPR.Q |
|  | 2873 | 720.2019 | 2157.5835 | 2156.4032 | 1.1804 | 1 | 57 | 0.0055 | 1 | K.AGFAGDDAPRAVFPSIVGRPR.Q |
|  | 3395 | **940.0254** | **2817.0540** | **2817.2245** | **-0.1705** | **2** | **4** | **1e+03** | **5** | **R.KDLYTNTVLSGGTTMYPGMAHRMQK.E + Oxidation (M)** |
|  | 3441 | **948.0242** | **2841.0503** | **2840.1650** | **0.8853** | **0** | **(11)** | **2.3e+02** | **1** | **K.ALQLNELTMDDDTAVLVIDNGSGMCK.A + Carbamidomethyl (C); Oxidation (M)** |
|  | 3442 | **948.0761** | **2841.2060** | **2840.1650** | **1.0409** | **0** | **11** | **2.2e+02** | **1** | **K.ALQLNELTMDDDTAVLVIDNGSGMCK.A + Carbamidomethyl (C); Oxidation (M)** |

  

|  |  |
| --- | --- |
|  | |
|  | **Proteins matching the same set of peptides:** |

|  |  |
| --- | --- |
|  | gi|153791352    **Mass:** 121443   **Score:** 402    **Queries matched:** 58 |
|  | POTE ankyrin domain family member F [Homo sapiens] |

---

|  |  |
| --- | --- |
| **7.** | gi|134133226    **Mass:** 121362   **Score:** 403    **Queries matched:** 59   **emPAI:** 0.27 |
|  | POTE ankyrin domain family member E [Homo sapiens] |

|  |  |
| --- | --- |
|  | Check to include this hit in error tolerant search or archive report |
|  |  |

|  |  |  |  |  |  |  |  |  |  |  |
| --- | --- | --- | --- | --- | --- | --- | --- | --- | --- | --- |
|  | **Query** | **Observed** | **Mr(expt)** | **Mr(calc)** | **Delta** | **Miss** | **Score** | **Expect** | **Rank** | **Peptide** |
|  | 1481 | 489.0091 | 976.0033 | 976.0012 | 0.0022 | 0 | 87 | 5.6e-06 | 1 | K.AGFAGDDAPR.A |
|  | 1482 | 489.0325 | 976.0502 | 976.0012 | 0.0490 | 0 | (76) | 7.3e-05 | 1 | K.AGFAGDDAPR.A |
|  | 1483 | 489.0872 | 976.1597 | 976.0012 | 0.1585 | 0 | (62) | 0.0016 | 1 | K.AGFAGDDAPR.A |
|  | 1484 | 489.1371 | 976.2593 | 976.0012 | 0.2582 | 0 | (43) | 0.13 | 1 | K.AGFAGDDAPR.A |
|  | 1865 | 550.2676 | 1098.5205 | 1098.3660 | 0.1545 | 2 | 11 | 2e+02 | 3 | R.SKMGKWCCR.C |
|  | 2129 | 599.6302 | 1197.2456 | 1198.4173 | -1.1717 | 0 | (5) | 8.7e+02 | 3 | R.AVFPSIVGRPR.Q |
|  | 2130 | 599.7516 | 1197.4884 | 1198.4173 | -0.9289 | 0 | (44) | 0.12 | 1 | R.AVFPSIVGRPR.Q |
|  | 2131 | 599.7771 | 1197.5394 | 1198.4173 | -0.8778 | 0 | (39) | 0.32 | 1 | R.AVFPSIVGRPR.Q |
|  | 2132 | 599.7792 | 1197.5436 | 1198.4173 | -0.8737 | 0 | (36) | 0.63 | 1 | R.AVFPSIVGRPR.Q |
|  | 2133 | 599.8466 | 1197.6785 | 1198.4173 | -0.7388 | 0 | (53) | 0.012 | 1 | R.AVFPSIVGRPR.Q |
|  | 2135 | 600.0215 | 1198.0283 | 1198.4173 | -0.3890 | 0 | (12) | 1.8e+02 | 1 | R.AVFPSIVGRPR.Q |
|  | 2137 | 600.2160 | 1198.4172 | 1198.4173 | -0.0000 | 0 | (71) | 0.00024 | 1 | R.AVFPSIVGRPR.Q |
|  | 2138 | 600.2161 | 1198.4173 | 1198.4173 | 0.0001 | 0 | (37) | 0.6 | 1 | R.AVFPSIVGRPR.Q |
|  | 2139 | 600.2168 | 1198.4188 | 1198.4173 | 0.0015 | 0 | (34) | 1.1 | 1 | R.AVFPSIVGRPR.Q |
|  | 2140 | 600.2443 | 1198.4737 | 1198.4173 | 0.0565 | 0 | 83 | 1.4e-05 | 1 | R.AVFPSIVGRPR.Q |
|  | 2141 | 600.2688 | 1198.5228 | 1198.4173 | 0.1055 | 0 | (54) | 0.012 | 1 | R.AVFPSIVGRPR.Q |
|  | 2142 | 600.3383 | 1198.6617 | 1198.4173 | 0.2445 | 0 | (64) | 0.0011 | 1 | R.AVFPSIVGRPR.Q |
|  | 619 | 400.6896 | 1199.0467 | 1198.4173 | 0.6295 | 0 | (28) | 3.8 | 1 | R.AVFPSIVGRPR.Q |
|  | 620 | 400.7009 | 1199.0806 | 1198.4173 | 0.6633 | 0 | (18) | 34 | 1 | R.AVFPSIVGRPR.Q |
|  | 621 | 400.7180 | 1199.1318 | 1198.4173 | 0.7145 | 0 | (22) | 13 | 1 | R.AVFPSIVGRPR.Q |
|  | 622 | 400.7871 | 1199.3392 | 1198.4173 | 0.9220 | 0 | (15) | 90 | 5 | R.AVFPSIVGRPR.Q |
|  | 2143 | 600.7864 | 1199.5580 | 1198.4173 | 1.1407 | 0 | (30) | 2.4 | 1 | R.AVFPSIVGRPR.Q |
|  | 720 | 404.7637 | 1211.2688 | 1212.4686 | -1.1997 | 2 | (11) | 1.9e+02 | 3 | R.SKMGKWCCR.C + 2 Carbamidomethyl (C) |
|  | 1558 | 506.8081 | 1517.4021 | 1516.5679 | 0.8342 | 0 | 10 | 2.2e+02 | 2 | K.QEYDESGPSIVHR.K |
|  | 3161 | 791.5933 | 1581.1717 | 1580.9316 | 0.2402 | 1 | 5 | 8.4e+02 | 9 | R.MQKEIAALAPSMMK.I + 2 Oxidation (M) |
|  | 1850 | 549.4825 | 1645.4255 | 1644.7403 | 0.6852 | 1 | 10 | 2.3e+02 | 2 | K.QEYDESGPSIVHRK.C |
|  | 1854 | 549.8562 | 1646.5464 | 1647.0242 | -0.4777 | 1 | 4 | 7.7e+02 | 7 | K.MGKWCCHCFPCCR.G + Carbamidomethyl (C); Oxidation (M) |
|  | 3324 | 895.8816 | 1789.7484 | 1790.9243 | -1.1759 | 0 | (14) | 76 | 1 | K.SYELPDGQVITIGNER.F |
|  | 3325 | 895.8936 | 1789.7723 | 1790.9243 | -1.1520 | 0 | (31) | 1.6 | 1 | K.SYELPDGQVITIGNER.F |
|  | 3326 | 896.1632 | 1790.3116 | 1790.9243 | -0.6127 | 0 | (35) | 0.6 | 1 | K.SYELPDGQVITIGNER.F |
|  | 3327 | 896.1948 | 1790.3749 | 1790.9243 | -0.5495 | 0 | (11) | 1.6e+02 | 2 | K.SYELPDGQVITIGNER.F |
|  | 3328 | 896.2452 | 1790.4757 | 1790.9243 | -0.4486 | 0 | (36) | 0.52 | 1 | K.SYELPDGQVITIGNER.F |
|  | 3329 | 896.3129 | 1790.6110 | 1790.9243 | -0.3134 | 0 | (48) | 0.039 | 1 | K.SYELPDGQVITIGNER.F |
|  | 2118 | 597.9269 | 1790.7586 | 1790.9243 | -0.1657 | 0 | (4) | 7.9e+02 | 5 | K.SYELPDGQVITIGNER.F |
|  | 3331 | 896.4506 | 1790.8863 | 1790.9243 | -0.0380 | 0 | (43) | 0.12 | 1 | K.SYELPDGQVITIGNER.F |
|  | 3332 | 896.5997 | 1791.1847 | 1790.9243 | 0.2603 | 0 | (64) | 0.0011 | 1 | K.SYELPDGQVITIGNER.F |
|  | 3333 | 896.7216 | 1791.4283 | 1790.9243 | 0.5040 | 0 | (79) | 3.2e-05 | 1 | K.SYELPDGQVITIGNER.F |
|  | 2121 | 598.1779 | 1791.5116 | 1790.9243 | 0.5872 | 0 | (48) | 0.046 | 1 | K.SYELPDGQVITIGNER.F |
|  | 2122 | 598.2000 | 1791.5779 | 1790.9243 | 0.6535 | 0 | (55) | 0.0075 | 1 | K.SYELPDGQVITIGNER.F |
|  | 3334 | 896.9148 | 1791.8148 | 1790.9243 | 0.8905 | 0 | (70) | 0.00024 | 1 | K.SYELPDGQVITIGNER.F |
|  | 3335 | 896.9431 | 1791.8714 | 1790.9243 | 0.9471 | 0 | (83) | 1.2e-05 | 1 | K.SYELPDGQVITIGNER.F |
|  | 3336 | 897.0674 | 1792.1200 | 1790.9243 | 1.1957 | 0 | 87 | 5.3e-06 | 1 | K.SYELPDGQVITIGNER.F |
|  | 3556 | 1047.9919 | 2093.9691 | 2094.2839 | -0.3148 | 1 | 42 | 0.13 | 1 | K.SYELPDGQVITIGNERFR.C |
|  | 3557 | 1047.9972 | 2093.9796 | 2094.2839 | -0.3043 | 1 | (39) | 0.26 | 1 | K.SYELPDGQVITIGNERFR.C |
|  | 2809 | 699.1260 | 2094.3559 | 2094.2839 | 0.0720 | 1 | (13) | 1.4e+02 | 1 | K.SYELPDGQVITIGNERFR.C |
|  | 2813 | 699.4652 | 2095.3735 | 2094.2839 | 1.0895 | 1 | (29) | 2.9 | 1 | K.SYELPDGQVITIGNERFR.C |
|  | 2866 | 719.4661 | 2155.3760 | 2156.4032 | -1.0271 | 1 | (43) | 0.12 | 1 | K.AGFAGDDAPRAVFPSIVGRPR.Q |
|  | 2867 | 719.5731 | 2155.6972 | 2156.4032 | -0.7060 | 1 | (19) | 26 | 1 | K.AGFAGDDAPRAVFPSIVGRPR.Q |
|  | 2868 | 719.7181 | 2156.1322 | 2156.4032 | -0.2709 | 1 | (37) | 0.44 | 1 | K.AGFAGDDAPRAVFPSIVGRPR.Q |
|  | 2869 | 719.7473 | 2156.2196 | 2156.4032 | -0.1836 | 1 | (34) | 0.97 | 1 | K.AGFAGDDAPRAVFPSIVGRPR.Q |
|  | 3566 | 1079.3804 | 2156.7460 | 2156.4032 | 0.3428 | 1 | (27) | 4 | 1 | K.AGFAGDDAPRAVFPSIVGRPR.Q |
|  | 2870 | 719.9481 | 2156.8220 | 2156.4032 | 0.4188 | 1 | (26) | 4.9 | 1 | K.AGFAGDDAPRAVFPSIVGRPR.Q |
|  | 2871 | 720.0771 | 2157.2093 | 2156.4032 | 0.8061 | 1 | (49) | 0.025 | 1 | K.AGFAGDDAPRAVFPSIVGRPR.Q |
|  | 2872 | 720.1254 | 2157.3539 | 2156.4032 | 0.9508 | 1 | (51) | 0.02 | 1 | K.AGFAGDDAPRAVFPSIVGRPR.Q |
|  | 2873 | 720.2019 | 2157.5835 | 2156.4032 | 1.1804 | 1 | 57 | 0.0055 | 1 | K.AGFAGDDAPRAVFPSIVGRPR.Q |
|  | 3025 | **750.1422** | **2247.4045** | **2248.4671** | **-1.0627** | **2** | **3** | **1.1e+03** | **2** | **K.EKQMLKISSENSNPEQELK.L + Oxidation (M)** |
|  | 3395 | 940.0254 | 2817.0540 | 2817.2245 | -0.1705 | 2 | 4 | 1e+03 | 5 | R.KDLYTNTVLSGGTTMYPGMAHRMQK.E + Oxidation (M) |
|  | 3441 | 948.0242 | 2841.0503 | 2840.1650 | 0.8853 | 0 | (11) | 2.3e+02 | 1 | K.ALQLNELTMDDDTAVLVIDNGSGMCK.A + Carbamidomethyl (C); Oxidation (M) |
|  | 3442 | 948.0761 | 2841.2060 | 2840.1650 | 1.0409 | 0 | 11 | 2.2e+02 | 1 | K.ALQLNELTMDDDTAVLVIDNGSGMCK.A + Carbamidomethyl (C); Oxidation (M) |

  


---

|  |  |
| --- | --- |
| **8.** | gi|542850    **Mass:** 47447    **Score:** 394    **Queries matched:** 25   **emPAI:** 0.83 |
|  | heterogeneous nuclear ribonucleoprotein G - human |

|  |  |
| --- | --- |
|  | Check to include this hit in error tolerant search or archive report |
|  |  |

|  |  |  |  |  |  |  |  |  |  |  |
| --- | --- | --- | --- | --- | --- | --- | --- | --- | --- | --- |
|  | **Query** | **Observed** | **Mr(expt)** | **Mr(calc)** | **Delta** | **Miss** | **Score** | **Expect** | **Rank** | **Peptide** |
|  | 1044 | **425.3841** | **848.7533** | **848.9429** | **-0.1895** | **0** | **41** | **0.18** | **1** | **R.DVYLSPR.D** |
|  | 1215 | **451.9318** | **901.8489** | **902.0271** | **-0.1782** | **0** | **7** | **6.4e+02** | **6** | **R.GLPPSMER.G + Oxidation (M)** |
|  | 1040 | **424.4781** | **1270.4120** | **1270.5263** | **-0.1143** | **2** | **8** | **5.6e+02** | **6** | **R.GPLPVKRGPPPR.S** |
|  | 1369 | **474.3261** | **1419.9562** | **1419.4098** | **0.5465** | **1** | **34** | **0.96** | **1** | **K.DSYSSRDYPSSR.D** |
|  | 3003 | **743.8450** | **1485.6752** | **1486.6232** | **-0.9480** | **0** | **28** | **4.4** | **1** | **R.GFAFVTFESPADAK.D** |
|  | 3039 | **753.6146** | **1505.2145** | **1505.6133** | **-0.3988** | **1** | **6** | **4.9e+02** | **2** | **R.SAPSGPVRSSSGMGGR.A + Oxidation (M)** |
|  | 1603 | **516.7375** | **1547.1903** | **1546.7298** | **0.4605** | **2** | **39** | **0.26** | **1** | **R.SGGPPPKRSAPSGPVR.S** |
|  | 1777 | **539.4774** | **1615.4099** | **1615.6958** | **-0.2859** | **1** | **8** | **3.7e+02** | **1** | **R.DVYLSPRDDGYSTK.D** |
|  | 1900 | **557.9742** | **1670.9006** | **1670.7809** | **0.1197** | **1** | **(12)** | **1.8e+02** | **2** | **R.SAPPTRGPPPSYGGSSR.Y** |
|  | 1901 | **558.1505** | **1671.4292** | **1670.7809** | **0.6483** | **1** | **(18)** | **42** | **1** | **R.SAPPTRGPPPSYGGSSR.Y** |
|  | 1902 | **558.1992** | **1671.5753** | **1670.7809** | **0.7944** | **1** | **21** | **20** | **1** | **R.SAPPTRGPPPSYGGSSR.Y** |
|  | 2007 | **583.7521** | **1748.2341** | **1747.9462** | **0.2879** | **1** | **47** | **0.053** | **1** | **K.AIKVEQATKPSFESGR.R** |
|  | 2009 | **583.9014** | **1748.6819** | **1747.9462** | **0.7358** | **1** | **(17)** | **43** | **1** | **K.AIKVEQATKPSFESGR.R** |
|  | 2010 | **583.9164** | **1748.7270** | **1747.9462** | **0.7808** | **1** | **(44)** | **0.096** | **1** | **K.AIKVEQATKPSFESGR.R** |
|  | 3394 | **940.0071** | **1877.9994** | **1878.8656** | **-0.8662** | **2** | **0** | **2.5e+03** | **10** | **R.DDYPSRGYSDRDGYGR.D** |
|  | 2416 | **634.5275** | **1900.5602** | **1900.0521** | **0.5082** | **1** | **(64)** | **0.00079** | **1** | **R.GFAFVTFESPADAKDAAR.D** |
|  | 2418 | **634.5968** | **1900.7682** | **1900.0521** | **0.7162** | **1** | **(64)** | **0.00081** | **1** | **R.GFAFVTFESPADAKDAAR.D** |
|  | 2420 | **634.6261** | **1900.8561** | **1900.0521** | **0.8041** | **1** | **(21)** | **18** | **1** | **R.GFAFVTFESPADAKDAAR.D** |
|  | 3458 | **951.4393** | **1900.8639** | **1900.0521** | **0.8118** | **1** | **(70)** | **0.00024** | **1** | **R.GFAFVTFESPADAKDAAR.D** |
|  | 2422 | **634.6667** | **1900.9781** | **1900.0521** | **0.9260** | **1** | **70** | **0.00025** | **1** | **R.GFAFVTFESPADAKDAAR.D** |
|  | 2423 | **634.6943** | **1901.0607** | **1900.0521** | **1.0086** | **1** | **(69)** | **0.00037** | **1** | **R.GFAFVTFESPADAKDAAR.D** |
|  | 2428 | **635.7421** | **1904.2042** | **1904.1319** | **0.0724** | **2** | **12** | **1.8e+02** | **1** | **K.AIKVEQATKPSFESGRR.G** |
|  | 3028 | **750.5337** | **2248.5789** | **2248.4920** | **0.0868** | **2** | **43** | **0.12** | **1** | **K.SLDGKAIKVEQATKPSFESGR.R** |
|  | 3029 | **750.5612** | **2248.6613** | **2248.4920** | **0.1692** | **2** | **(14)** | **90** | **1** | **K.SLDGKAIKVEQATKPSFESGR.R** |
|  | 3114 | **771.5081** | **2311.5020** | **2311.3741** | **0.1279** | **2** | **29** | **3.4** | **1** | **R.DVYLSPRDDGYSTKDSYSSR.D** |

  

|  |  |
| --- | --- |
|  | |
|  | **Proteins matching the same set of peptides:** |

|  |  |
| --- | --- |
|  | gi|56699409    **Mass:** 42331    **Score:** 392    **Queries matched:** 25 |
|  | heterogeneous nuclear ribonucleoprotein G isoform 1 [Homo sapiens] |

|  |  |
| --- | --- |
|  | gi|297711163    **Mass:** 38070    **Score:** 392    **Queries matched:** 25 |
|  | PREDICTED: heterogeneous nuclear ribonucleoprotein G-like isoform 4 [Pongo abelii] |

---

|  |  |
| --- | --- |
| **9.** | gi|63055057    **Mass:** 42003    **Score:** 300    **Queries matched:** 34   **emPAI:** 0.70 |
|  | beta-actin-like protein 2 [Homo sapiens] |

|  |  |
| --- | --- |
|  | Check to include this hit in error tolerant search or archive report |
|  |  |

|  |  |  |  |  |  |  |  |  |  |  |
| --- | --- | --- | --- | --- | --- | --- | --- | --- | --- | --- |
|  | **Query** | **Observed** | **Mr(expt)** | **Mr(calc)** | **Delta** | **Miss** | **Score** | **Expect** | **Rank** | **Peptide** |
|  | 590 | 398.5465 | 795.0782 | 794.9384 | 0.1398 | 0 | 26 | 6.6 | 1 | K.IIAPPER.K |
|  | 1614 | 518.7917 | 1035.5687 | 1036.2683 | -0.6996 | 1 | 19 | 26 | 1 | K.IKIIAPPER.K |
|  | 444 | 388.9354 | 1163.7841 | 1164.4406 | -0.6565 | 2 | (19) | 40 | 1 | K.IKIIAPPERK.Y |
|  | 446 | 388.9509 | 1163.8305 | 1164.4406 | -0.6101 | 2 | (5) | 8.9e+02 | 8 | K.IKIIAPPERK.Y |
|  | 488 | 389.4004 | 1165.1789 | 1164.4406 | 0.7383 | 2 | 45 | 0.096 | 1 | K.IKIIAPPERK.Y |
|  | 560 | 391.4145 | 1171.2214 | 1171.3936 | -0.1722 | 0 | 31 | 2.3 | 1 | R.HQGVMVGMGQK.D |
|  | 2025 | 586.6212 | 1171.2275 | 1171.3936 | -0.1660 | 0 | (25) | 10 | 1 | R.HQGVMVGMGQK.D |
|  | 2026 | 587.2489 | 1172.4830 | 1171.3936 | 1.0895 | 0 | (10) | 2.6e+02 | 1 | R.HQGVMVGMGQK.D |
|  | 1819 | 542.3186 | 1623.9336 | 1623.8683 | 0.0653 | 1 | 23 | 14 | 1 | R.LDLAGRDLTDYLMK.I |
|  | 3324 | 895.8816 | 1789.7484 | 1790.9243 | -1.1759 | 0 | (14) | 76 | 1 | R.SYELPDGQVITIGNER.F |
|  | 3325 | 895.8936 | 1789.7723 | 1790.9243 | -1.1520 | 0 | (31) | 1.6 | 1 | R.SYELPDGQVITIGNER.F |
|  | 3326 | 896.1632 | 1790.3116 | 1790.9243 | -0.6127 | 0 | (35) | 0.6 | 1 | R.SYELPDGQVITIGNER.F |
|  | 3327 | 896.1948 | 1790.3749 | 1790.9243 | -0.5495 | 0 | (11) | 1.6e+02 | 2 | R.SYELPDGQVITIGNER.F |
|  | 3328 | 896.2452 | 1790.4757 | 1790.9243 | -0.4486 | 0 | (36) | 0.52 | 1 | R.SYELPDGQVITIGNER.F |
|  | 3329 | 896.3129 | 1790.6110 | 1790.9243 | -0.3134 | 0 | (48) | 0.039 | 1 | R.SYELPDGQVITIGNER.F |
|  | 2118 | 597.9269 | 1790.7586 | 1790.9243 | -0.1657 | 0 | (4) | 7.9e+02 | 5 | R.SYELPDGQVITIGNER.F |
|  | 3331 | 896.4506 | 1790.8863 | 1790.9243 | -0.0380 | 0 | (43) | 0.12 | 1 | R.SYELPDGQVITIGNER.F |
|  | 3332 | 896.5997 | 1791.1847 | 1790.9243 | 0.2603 | 0 | (64) | 0.0011 | 1 | R.SYELPDGQVITIGNER.F |
|  | 3333 | 896.7216 | 1791.4283 | 1790.9243 | 0.5040 | 0 | (79) | 3.2e-05 | 1 | R.SYELPDGQVITIGNER.F |
|  | 2121 | 598.1779 | 1791.5116 | 1790.9243 | 0.5872 | 0 | (48) | 0.046 | 1 | R.SYELPDGQVITIGNER.F |
|  | 2122 | 598.2000 | 1791.5779 | 1790.9243 | 0.6535 | 0 | (55) | 0.0075 | 1 | R.SYELPDGQVITIGNER.F |
|  | 3334 | 896.9148 | 1791.8148 | 1790.9243 | 0.8905 | 0 | (70) | 0.00024 | 1 | R.SYELPDGQVITIGNER.F |
|  | 3335 | 896.9431 | 1791.8714 | 1790.9243 | 0.9471 | 0 | (83) | 1.2e-05 | 1 | R.SYELPDGQVITIGNER.F |
|  | 3336 | 897.0674 | 1792.1200 | 1790.9243 | 1.1957 | 0 | 87 | 5.3e-06 | 1 | R.SYELPDGQVITIGNER.F |
|  | 2498 | 652.1713 | 1953.4916 | 1954.2268 | -0.7352 | 0 | (11) | 2e+02 | 3 | R.VAPDEHPILLTEAPLNPK.I |
|  | 3535 | 977.9032 | 1953.7916 | 1954.2268 | -0.4352 | 0 | (13) | 90 | 3 | R.VAPDEHPILLTEAPLNPK.I |
|  | 3536 | 978.2371 | 1954.4593 | 1954.2268 | 0.2325 | 0 | (27) | 4.3 | 2 | R.VAPDEHPILLTEAPLNPK.I |
|  | 2501 | 652.5559 | 1954.6456 | 1954.2268 | 0.4187 | 0 | (28) | 3.1 | 2 | R.VAPDEHPILLTEAPLNPK.I |
|  | 2502 | 652.6416 | 1954.9026 | 1954.2268 | 0.6758 | 0 | 29 | 3.1 | 2 | R.VAPDEHPILLTEAPLNPK.I |
|  | 2503 | 652.7772 | 1955.3095 | 1954.2268 | 1.0827 | 0 | (24) | 13 | 2 | R.VAPDEHPILLTEAPLNPK.I |
|  | 3556 | 1047.9919 | 2093.9691 | 2094.2839 | -0.3148 | 1 | 42 | 0.13 | 1 | R.SYELPDGQVITIGNERFR.C |
|  | 3557 | 1047.9972 | 2093.9796 | 2094.2839 | -0.3043 | 1 | (39) | 0.26 | 1 | R.SYELPDGQVITIGNERFR.C |
|  | 2809 | 699.1260 | 2094.3559 | 2094.2839 | 0.0720 | 1 | (13) | 1.4e+02 | 1 | R.SYELPDGQVITIGNERFR.C |
|  | 2813 | 699.4652 | 2095.3735 | 2094.2839 | 1.0895 | 1 | (29) | 2.9 | 1 | R.SYELPDGQVITIGNERFR.C |

  


---

|  |  |
| --- | --- |
| **10.** | gi|532313    **Mass:** 44697    **Score:** 244    **Queries matched:** 12   **emPAI:** 0.43 |
|  | NF45 protein [Homo sapiens] |

|  |  |
| --- | --- |
|  | Check to include this hit in error tolerant search or archive report |
|  |  |

|  |  |  |  |  |  |  |  |  |  |  |
| --- | --- | --- | --- | --- | --- | --- | --- | --- | --- | --- |
|  | **Query** | **Observed** | **Mr(expt)** | **Mr(calc)** | **Delta** | **Miss** | **Score** | **Expect** | **Rank** | **Peptide** |
|  | 2301 | **619.1886** | **1236.3624** | **1236.5032** | **-0.1408** | **0** | **20** | **25** | **2** | **K.ILITTVPPNLR.K** |
|  | 2829 | **705.3199** | **1408.6250** | **1409.6697** | **-1.0447** | **0** | **31** | **2.2** | **1** | **K.ILPTLEAVAALGNK.V** |
|  | 2833 | **706.1121** | **1410.2093** | **1409.6697** | **0.5396** | **0** | **(2)** | **1.5e+03** | **8** | **K.ILPTLEAVAALGNK.V** |
|  | 1979 | **578.5441** | **1732.6100** | **1731.9402** | **0.6699** | **0** | **50** | **0.021** | **1** | **R.VKPAPDETSFSEALLK.R** |
|  | 2400 | **630.3583** | **1888.0527** | **1888.1259** | **-0.0732** | **1** | **(14)** | **1e+02** | **1** | **R.VKPAPDETSFSEALLKR.N** |
|  | 2401 | **630.6438** | **1888.9092** | **1888.1259** | **0.7834** | **1** | **(22)** | **19** | **1** | **R.VKPAPDETSFSEALLKR.N** |
|  | 2402 | **630.6711** | **1888.9913** | **1888.1259** | **0.8654** | **1** | **33** | **1.4** | **1** | **R.VKPAPDETSFSEALLKR.N** |
|  | 3558 | **1050.3284** | **2098.6420** | **2099.2987** | **-0.6568** | **0** | **(34)** | **0.79** | **1** | **R.NQDLAPNSAEQASILSLVTK.I** |
|  | 3559 | **1050.3817** | **2098.7486** | **2099.2987** | **-0.5501** | **0** | **(26)** | **5** | **1** | **R.NQDLAPNSAEQASILSLVTK.I** |
|  | 2818 | **700.6626** | **2098.9656** | **2099.2987** | **-0.3331** | **0** | **44** | **0.091** | **1** | **R.NQDLAPNSAEQASILSLVTK.I** |
|  | 2819 | **700.8467** | **2099.5179** | **2099.2987** | **0.2191** | **0** | **(37)** | **0.58** | **1** | **R.NQDLAPNSAEQASILSLVTK.I** |
|  | 3037 | **752.6464** | **2254.9169** | **2255.4844** | **-0.5675** | **1** | **66** | **0.00057** | **1** | **K.RNQDLAPNSAEQASILSLVTK.I** |

  

|  |  |
| --- | --- |
|  | |
|  | **Proteins matching the same set of peptides:** |

|  |  |
| --- | --- |
|  | gi|13385872    **Mass:** 43062    **Score:** 244    **Queries matched:** 12 |
|  | interleukin enhancer-binding factor 2 [Mus musculus] |

|  |  |
| --- | --- |
|  | gi|62898213    **Mass:** 43048    **Score:** 244    **Queries matched:** 12 |
|  | interleukin enhancer binding factor 2 variant [Homo sapiens] |

|  |  |
| --- | --- |
|  | gi|197101499    **Mass:** 38910    **Score:** 244    **Queries matched:** 12 |
|  | interleukin enhancer-binding factor 2 [Pongo abelii] |

---

|  |  |
| --- | --- |
| **11.** | gi|1835786    **Mass:** 34819    **Score:** 194    **Queries matched:** 8   **emPAI:** 0.31 |
|  | nucleolar protein p40 [Homo sapiens] |

|  |  |
| --- | --- |
|  | Check to include this hit in error tolerant search or archive report |
|  |  |

|  |  |  |  |  |  |  |  |  |  |  |
| --- | --- | --- | --- | --- | --- | --- | --- | --- | --- | --- |
|  | **Query** | **Observed** | **Mr(expt)** | **Mr(calc)** | **Delta** | **Miss** | **Score** | **Expect** | **Rank** | **Peptide** |
|  | 290 | **385.9564** | **769.8979** | **770.8807** | **-0.9827** | **2** | **8** | **3.3e+02** | **2** | **K.GPSAKRR.Y** |
|  | 1870 | **551.1417** | **1100.2685** | **1100.2693** | **-0.0008** | **1** | **35** | **0.93** | **1** | **K.VQTEVLQKR.Q** |
|  | 2301 | 619.1886 | 1236.3624 | 1236.4635 | -0.1011 | 0 | 30 | 2.8 | 1 | R.QAQAAVLAVLPR.L |
|  | 2947 | **740.6990** | **1479.3833** | **1478.7816** | **0.6017** | **2** | **9** | **2.8e+02** | **7** | **K.AHMMNAIKKYQK.G + Oxidation (M)** |
|  | 1583 | **512.0073** | **1532.9998** | **1533.8533** | **-0.8535** | **0** | **40** | **0.28** | **1** | **R.GLLKPGLNVVLEGPK.K** |
|  | 2511 | **654.6926** | **1961.0557** | **1962.2339** | **-1.1782** | **2** | **10** | **3e+02** | **3** | **K.AVNDVNGLKQCLAEFKR.D + Carbamidomethyl (C)** |
|  | 2876 | **722.5995** | **2164.7765** | **2164.3737** | **0.4027** | **1** | **67** | **0.00044** | **1** | **K.GFSDKLDFLEGDQKPLAQR.K** |
|  | 2877 | **722.7763** | **2165.3067** | **2164.3737** | **0.9330** | **1** | **(52)** | **0.017** | **1** | **K.GFSDKLDFLEGDQKPLAQR.K** |

  

|  |  |
| --- | --- |
|  | |
|  | **Proteins matching the same set of peptides:** |

|  |  |
| --- | --- |
|  | gi|237649015    **Mass:** 34852    **Score:** 194    **Queries matched:** 8 |
|  | probable rRNA-processing protein EBP2 isoform 2 [Homo sapiens] |

|  |  |
| --- | --- |
|  | gi|237649012    **Mass:** 40684    **Score:** 192    **Queries matched:** 8 |
|  | probable rRNA-processing protein EBP2 isoform 1 [Homo sapiens] |

---

|  |  |
| --- | --- |
| **12.** | gi|194388816    **Mass:** 35390    **Score:** 168    **Queries matched:** 4   **emPAI:** 0.20 |
|  | unnamed protein product [Homo sapiens] |

|  |  |
| --- | --- |
|  | Check to include this hit in error tolerant search or archive report |
|  |  |

|  |  |  |  |  |  |  |  |  |  |  |
| --- | --- | --- | --- | --- | --- | --- | --- | --- | --- | --- |
|  | **Query** | **Observed** | **Mr(expt)** | **Mr(calc)** | **Delta** | **Miss** | **Score** | **Expect** | **Rank** | **Peptide** |
|  | 831 | **407.4111** | **1219.2112** | **1220.3400** | **-1.1287** | **1** | **8** | **4.3e+02** | **5** | **K.TGHSRGFGFVR.F** |
|  | 3295 | **864.1780** | **1726.3412** | **1726.7626** | **-0.4214** | **0** | **86** | **5.4e-06** | **1** | **R.FGGNPGGFGNQGGFGNSR.G** |
|  | 2681 | **676.5164** | **2026.5269** | **2027.0769** | **-0.5500** | **1** | **(51)** | **0.018** | **1** | **R.SGRFGGNPGGFGNQGGFGNSR.G** |
|  | 2683 | **676.6510** | **2026.9308** | **2027.0769** | **-0.1460** | **1** | **74** | **9.8e-05** | **1** | **R.SGRFGGNPGGFGNQGGFGNSR.G** |

  


---

|  |  |
| --- | --- |
| **13.** | gi|158260881    **Mass:** 45322    **Score:** 167    **Queries matched:** 10   **emPAI:** 0.24 |
|  | unnamed protein product [Homo sapiens] |

|  |  |
| --- | --- |
|  | Check to include this hit in error tolerant search or archive report |
|  |  |

|  |  |  |  |  |  |  |  |  |  |  |
| --- | --- | --- | --- | --- | --- | --- | --- | --- | --- | --- |
|  | **Query** | **Observed** | **Mr(expt)** | **Mr(calc)** | **Delta** | **Miss** | **Score** | **Expect** | **Rank** | **Peptide** |
|  | 687 | **403.9472** | **805.8797** | **807.0120** | **-1.1324** | **0** | **10** | **2.7e+02** | **6** | **K.ACIPYLK.K** |
|  | 1439 | **486.7882** | **971.5616** | **971.1088** | **0.4528** | **0** | **35** | **0.71** | **1** | **K.DGANIVIAAK.T** |
|  | 1519 | **498.4687** | **994.9226** | **995.0444** | **-0.1218** | **0** | **34** | **0.98** | **1** | **R.SGAVEETFR.I** |
|  | 1764 | **537.6209** | **1073.2270** | **1074.3378** | **-1.1108** | **1** | **10** | **3.5e+02** | **1** | **K.IKGNMALAIK.L + Oxidation (M)** |
|  | 460 | **389.0084** | **1164.0031** | **1163.2773** | **0.7258** | **0** | **10** | **3.2e+02** | **4** | **K.VESTGAVPEFK.E** |
|  | 2261 | **612.6327** | **1223.2506** | **1222.4354** | **0.8152** | **0** | **1** | **2.1e+03** | **8** | **R.LDLMMNVNTR.G + Oxidation (M)** |
|  | 2618 | **668.8165** | **1335.6183** | **1335.5054** | **0.1129** | **1** | **13** | **1.6e+02** | **2** | **R.SGAVEETFRIVK.D** |
|  | 2685 | **677.4164** | **1352.8180** | **1352.5588** | **0.2591** | **0** | **40** | **0.28** | **1** | **R.LAGCTVFITGASR.G + Carbamidomethyl (C)** |
|  | 1278 | **460.7346** | **1379.1815** | **1379.6058** | **-0.4243** | **1** | **12** | **1.6e+02** | **3** | **K.LEKLMNQMNAR.L + 2 Oxidation (M)** |
|  | 3463 | **954.5056** | **2860.4947** | **2861.2952** | **-0.8006** | **1** | **6** | **5.3e+02** | **5** | **K.QHCAYTIAKYGMSMYVLGMAEEFK.G + Carbamidomethyl (C); 2 Oxidation (M)** |

  

|  |  |
| --- | --- |
|  | |
|  | **Proteins matching the same set of peptides:** |

|  |  |
| --- | --- |
|  | gi|40254986    **Mass:** 45394    **Score:** 166    **Queries matched:** 10 |
|  | hydroxysteroid dehydrogenase-like protein 2 isoform 1 [Homo sapiens] |

---

|  |  |
| --- | --- |
| **14.** | gi|74739412    **Mass:** 42016    **Score:** 161    **Queries matched:** 23   **emPAI:** 0.35 |
|  | RecName: Full=Putative beta-actin-like protein 3; AltName: Full=Kappa-actin; AltName: Full=POTE ankyrin domain family member K |

|  |  |
| --- | --- |
|  | Check to include this hit in error tolerant search or archive report |
|  |  |

|  |  |  |  |  |  |  |  |  |  |  |
| --- | --- | --- | --- | --- | --- | --- | --- | --- | --- | --- |
|  | **Query** | **Observed** | **Mr(expt)** | **Mr(calc)** | **Delta** | **Miss** | **Score** | **Expect** | **Rank** | **Peptide** |
|  | 1614 | 518.7917 | 1035.5687 | 1035.3266 | 0.2421 | 2 | 6 | 5.4e+02 | 4 | K.IKIIAPPKR.K |
|  | 1558 | 506.8081 | 1517.4021 | 1516.5679 | 0.8342 | 0 | 10 | 2.2e+02 | 2 | K.QEYDESGPSIVHR.K |
|  | 1850 | 549.4825 | 1645.4255 | 1644.7403 | 0.6852 | 1 | 10 | 2.3e+02 | 2 | K.QEYDESGPSIVHRK.C |
|  | 2021 | **585.6941** | **1754.0601** | **1752.9179** | **1.1422** | **0** | **7** | **6.3e+02** | **4** | **M.DDDTAVLVIDNGSGMCK.A** |
|  | 3324 | 895.8816 | 1789.7484 | 1790.9243 | -1.1759 | 0 | (14) | 76 | 1 | K.SYELPDGQVITIGNER.F |
|  | 3325 | 895.8936 | 1789.7723 | 1790.9243 | -1.1520 | 0 | (31) | 1.6 | 1 | K.SYELPDGQVITIGNER.F |
|  | 3326 | 896.1632 | 1790.3116 | 1790.9243 | -0.6127 | 0 | (35) | 0.6 | 1 | K.SYELPDGQVITIGNER.F |
|  | 3327 | 896.1948 | 1790.3749 | 1790.9243 | -0.5495 | 0 | (11) | 1.6e+02 | 2 | K.SYELPDGQVITIGNER.F |
|  | 3328 | 896.2452 | 1790.4757 | 1790.9243 | -0.4486 | 0 | (36) | 0.52 | 1 | K.SYELPDGQVITIGNER.F |
|  | 3329 | 896.3129 | 1790.6110 | 1790.9243 | -0.3134 | 0 | (48) | 0.039 | 1 | K.SYELPDGQVITIGNER.F |
|  | 2118 | 597.9269 | 1790.7586 | 1790.9243 | -0.1657 | 0 | (4) | 7.9e+02 | 5 | K.SYELPDGQVITIGNER.F |
|  | 3331 | 896.4506 | 1790.8863 | 1790.9243 | -0.0380 | 0 | (43) | 0.12 | 1 | K.SYELPDGQVITIGNER.F |
|  | 3332 | 896.5997 | 1791.1847 | 1790.9243 | 0.2603 | 0 | (64) | 0.0011 | 1 | K.SYELPDGQVITIGNER.F |
|  | 3333 | 896.7216 | 1791.4283 | 1790.9243 | 0.5040 | 0 | (79) | 3.2e-05 | 1 | K.SYELPDGQVITIGNER.F |
|  | 2121 | 598.1779 | 1791.5116 | 1790.9243 | 0.5872 | 0 | (48) | 0.046 | 1 | K.SYELPDGQVITIGNER.F |
|  | 2122 | 598.2000 | 1791.5779 | 1790.9243 | 0.6535 | 0 | (55) | 0.0075 | 1 | K.SYELPDGQVITIGNER.F |
|  | 3334 | 896.9148 | 1791.8148 | 1790.9243 | 0.8905 | 0 | (70) | 0.00024 | 1 | K.SYELPDGQVITIGNER.F |
|  | 3335 | 896.9431 | 1791.8714 | 1790.9243 | 0.9471 | 0 | (83) | 1.2e-05 | 1 | K.SYELPDGQVITIGNER.F |
|  | 3336 | 897.0674 | 1792.1200 | 1790.9243 | 1.1957 | 0 | 87 | 5.3e-06 | 1 | K.SYELPDGQVITIGNER.F |
|  | 3556 | 1047.9919 | 2093.9691 | 2094.2839 | -0.3148 | 1 | 42 | 0.13 | 1 | K.SYELPDGQVITIGNERFR.C |
|  | 3557 | 1047.9972 | 2093.9796 | 2094.2839 | -0.3043 | 1 | (39) | 0.26 | 1 | K.SYELPDGQVITIGNERFR.C |
|  | 2809 | 699.1260 | 2094.3559 | 2094.2839 | 0.0720 | 1 | (13) | 1.4e+02 | 1 | K.SYELPDGQVITIGNERFR.C |
|  | 2813 | 699.4652 | 2095.3735 | 2094.2839 | 1.0895 | 1 | (29) | 2.9 | 1 | K.SYELPDGQVITIGNERFR.C |

  


---

|  |  |
| --- | --- |
| **15.** | gi|119626277    **Mass:** 40800    **Score:** 159    **Queries matched:** 7   **emPAI:** 0.26 |
|  | heterogeneous nuclear ribonucleoprotein D (AU-rich element RNA binding protein 1, 37kDa), isoform CRA\_a [Homo sapiens] |

|  |  |
| --- | --- |
|  | Check to include this hit in error tolerant search or archive report |
|  |  |

|  |  |  |  |  |  |  |  |  |  |  |
| --- | --- | --- | --- | --- | --- | --- | --- | --- | --- | --- |
|  | **Query** | **Observed** | **Mr(expt)** | **Mr(calc)** | **Delta** | **Miss** | **Score** | **Expect** | **Rank** | **Peptide** |
|  | 2800 | **696.5590** | **1391.1031** | **1390.6068** | **0.4963** | **1** | **10** | **2.4e+02** | **1** | **K.YHNVGLSKCEIK.V** |
|  | 3010 | **745.0671** | **1488.1195** | **1488.6374** | **-0.5179** | **0** | **(54)** | **0.0087** | **1** | **K.IFVGGLSPDTPEEK.I** |
|  | 3012 | **745.5574** | **1489.1000** | **1488.6374** | **0.4626** | **0** | **56** | **0.0057** | **1** | **K.IFVGGLSPDTPEEK.I** |
|  | 1552 | **505.5680** | **1513.6817** | **1513.7842** | **-0.1025** | **1** | **9** | **3.5e+02** | **9** | **R.DAHALECCRLVVK.Q + Carbamidomethyl (C)** |
|  | 2307 | **620.7620** | **1859.2637** | **1859.1492** | **0.1145** | **2** | **(20)** | **27** | **1** | **R.GFCFITFKEEEPVKK.I + Carbamidomethyl (C)** |
|  | 2309 | **621.0110** | **1860.0108** | **1859.1492** | **0.8616** | **2** | **37** | **0.53** | **1** | **R.GFCFITFKEEEPVKK.I + Carbamidomethyl (C)** |
|  | 2446 | **641.1971** | **1920.5693** | **1920.1907** | **0.3785** | **1** | **47** | **0.065** | **1** | **K.FGEVVDCTLKLDPITGR.S + Carbamidomethyl (C)** |

  


---

|  |  |
| --- | --- |
| **16.** | gi|13027602    **Mass:** 35610    **Score:** 147    **Queries matched:** 7   **emPAI:** 0.31 |
|  | DDRGK domain-containing protein 1 precursor [Homo sapiens] |

|  |  |
| --- | --- |
|  | Check to include this hit in error tolerant search or archive report |
|  |  |

|  |  |  |  |  |  |  |  |  |  |  |
| --- | --- | --- | --- | --- | --- | --- | --- | --- | --- | --- |
|  | **Query** | **Observed** | **Mr(expt)** | **Mr(calc)** | **Delta** | **Miss** | **Score** | **Expect** | **Rank** | **Peptide** |
|  | 310 | **386.0516** | **770.0884** | **769.7998** | **0.2885** | **0** | **(6)** | **5.4e+02** | **5** | **R.ESPAQAPA.-** |
|  | 312 | **386.0527** | **770.0907** | **769.7998** | **0.2908** | **0** | **(4)** | **9.9e+02** | **8** | **R.ESPAQAPA.-** |
|  | 315 | **386.0775** | **770.1403** | **769.7998** | **0.3405** | **0** | **7** | **4.9e+02** | **8** | **R.ESPAQAPA.-** |
|  | 2842 | **710.1138** | **1418.2128** | **1418.5509** | **-0.3381** | **0** | **46** | **0.07** | **1** | **R.VAQPGPLEPEEPR.A** |
|  | 2363 | **626.9685** | **1877.8833** | **1877.9651** | **-0.0818** | **0** | **61** | **0.0018** | **1** | **R.AASAGQEPLHNEELAGAGR.V** |
|  | 2657 | **672.0131** | **2013.0170** | **2013.2180** | **-0.2009** | **1** | **36** | **0.6** | **1** | **R.VAQPGPLEPEEPRAGGRPR.R** |
|  | 2658 | **672.0798** | **2013.2173** | **2013.2180** | **-0.0006** | **1** | **(33)** | **1.3** | **1** | **R.VAQPGPLEPEEPRAGGRPR.R** |

  

|  |  |
| --- | --- |
|  | |
|  | **Proteins matching the same set of peptides:** |

|  |  |
| --- | --- |
|  | gi|14044070    **Mass:** 35640    **Score:** 147    **Queries matched:** 7 |
|  | DDRGK domain containing 1 [Homo sapiens] |

|  |  |
| --- | --- |
|  | gi|119630948    **Mass:** 35253    **Score:** 147    **Queries matched:** 7 |
|  | chromosome 20 open reading frame 116, isoform CRA\_a [Homo sapiens] |

---

|  |  |
| --- | --- |
| **17.** | gi|14198113    **Mass:** 38461    **Score:** 135    **Queries matched:** 5   **emPAI:** 0.18 |
|  | POLR1C protein [Homo sapiens] |

|  |  |
| --- | --- |
|  | Check to include this hit in error tolerant search or archive report |
|  |  |

|  |  |  |  |  |  |  |  |  |  |  |
| --- | --- | --- | --- | --- | --- | --- | --- | --- | --- | --- |
|  | **Query** | **Observed** | **Mr(expt)** | **Mr(calc)** | **Delta** | **Miss** | **Score** | **Expect** | **Rank** | **Peptide** |
|  | 1476 | **488.5407** | **975.0666** | **975.1424** | **-0.0757** | **0** | **59** | **0.004** | **1** | **R.VVLGEFGVR.N** |
|  | 1862 | **550.2067** | **1098.3987** | **1098.2088** | **0.1899** | **0** | **54** | **0.0095** | **1** | **K.FSPVATASYR.L** |
|  | 2147 | **601.7899** | **1201.5649** | **1201.4176** | **0.1473** | **0** | **5** | **7.3e+02** | **2** | **R.LGLIPIHADPR.L** |
|  | 823 | **407.3037** | **1218.8888** | **1218.4054** | **0.4834** | **1** | **16** | **54** | **1** | **R.SRVVLGEFGVR.N** |
|  | 830 | **407.3971** | **1219.1691** | **1218.4054** | **0.7637** | **1** | **(15)** | **81** | **4** | **R.SRVVLGEFGVR.N** |

  

|  |  |
| --- | --- |
|  | |
|  | **Proteins matching the same set of peptides:** |

|  |  |
| --- | --- |
|  | gi|42560246    **Mass:** 39249    **Score:** 135    **Queries matched:** 5 |
|  | DNA-directed RNA polymerases I and III subunit RPAC1 [Homo sapiens] |

|  |  |
| --- | --- |
|  | gi|57209068    **Mass:** 35837    **Score:** 135    **Queries matched:** 5 |
|  | polymerase (RNA) I polypeptide C, 30kDa [Homo sapiens] |

|  |  |
| --- | --- |
|  | gi|3335138    **Mass:** 38646    **Score:** 133    **Queries matched:** 5 |
|  | RNA polymerase I 40kD subunit [Homo sapiens] |

---

|  |  |
| --- | --- |
| **18.** | gi|34364637    **Mass:** 50926    **Score:** 134    **Queries matched:** 6   **emPAI:** 0.06 |
|  | hypothetical protein [Homo sapiens] |

|  |  |
| --- | --- |
|  | Check to include this hit in error tolerant search or archive report |
|  |  |

|  |  |  |  |  |  |  |  |  |  |  |
| --- | --- | --- | --- | --- | --- | --- | --- | --- | --- | --- |
|  | **Query** | **Observed** | **Mr(expt)** | **Mr(calc)** | **Delta** | **Miss** | **Score** | **Expect** | **Rank** | **Peptide** |
|  | 999 | **419.7696** | **837.5245** | **838.0029** | **-0.4785** | **0** | **18** | **37** | **2** | **K.ALPAPIEK.T** |
|  | 1047 | **426.5892** | **851.1637** | **850.9803** | **0.1834** | **0** | **38** | **0.36** | **1** | **K.DTLMISR.T + Oxidation (M)** |
|  | 2048 | **592.6963** | **1183.3778** | **1183.3381** | **0.0397** | **2** | **15** | **99** | **3** | **K.EYKCRVSNK.A + Carbamidomethyl (C)** |
|  | 2414 | **634.2466** | **1266.4784** | **1267.5140** | **-1.0357** | **1** | **(10)** | **2.6e+02** | **2** | **K.ALPAPIEKTISK.A** |
|  | 2425 | **635.0229** | **1268.0311** | **1267.5140** | **0.5171** | **1** | **(35)** | **0.8** | **1** | **K.ALPAPIEKTISK.A** |
|  | 2426 | **635.2228** | **1268.4308** | **1267.5140** | **0.9167** | **1** | **63** | **0.0014** | **1** | **K.ALPAPIEKTISK.A** |

  


---

|  |  |
| --- | --- |
| **19.** | gi|34526063    **Mass:** 51348    **Score:** 133    **Queries matched:** 6   **emPAI:** 0.06 |
|  | unnamed protein product [Homo sapiens] |

|  |  |
| --- | --- |
|  | Check to include this hit in error tolerant search or archive report |
|  |  |

|  |  |  |  |  |  |  |  |  |  |  |
| --- | --- | --- | --- | --- | --- | --- | --- | --- | --- | --- |
|  | **Query** | **Observed** | **Mr(expt)** | **Mr(calc)** | **Delta** | **Miss** | **Score** | **Expect** | **Rank** | **Peptide** |
|  | 999 | 419.7696 | 837.5245 | 838.0029 | -0.4785 | 0 | 18 | 37 | 2 | K.ALPAPIEK.T |
|  | 1047 | 426.5892 | 851.1637 | 850.9803 | 0.1834 | 0 | 38 | 0.36 | 1 | K.DTLMISR.T + Oxidation (M) |
|  | 2414 | 634.2466 | 1266.4784 | 1267.5140 | -1.0357 | 1 | (10) | 2.6e+02 | 2 | K.ALPAPIEKTISK.A |
|  | 2425 | 635.0229 | 1268.0311 | 1267.5140 | 0.5171 | 1 | (35) | 0.8 | 1 | K.ALPAPIEKTISK.A |
|  | 2426 | 635.2228 | 1268.4308 | 1267.5140 | 0.9167 | 1 | 63 | 0.0014 | 1 | K.ALPAPIEKTISK.A |
|  | 2616 | **668.7710** | **1335.5272** | **1335.4390** | **0.0882** | **0** | **14** | **1.2e+02** | **3** | **K.TEDTAIYYCTR.E** |

  


---

|  |  |
| --- | --- |
| **20.** | gi|16553682    **Mass:** 52843    **Score:** 128    **Queries matched:** 6   **emPAI:** 0.06 |
|  | unnamed protein product [Homo sapiens] |

|  |  |
| --- | --- |
|  | Check to include this hit in error tolerant search or archive report |
|  |  |

|  |  |  |  |  |  |  |  |  |  |  |
| --- | --- | --- | --- | --- | --- | --- | --- | --- | --- | --- |
|  | **Query** | **Observed** | **Mr(expt)** | **Mr(calc)** | **Delta** | **Miss** | **Score** | **Expect** | **Rank** | **Peptide** |
|  | 999 | 419.7696 | 837.5245 | 838.0029 | -0.4785 | 0 | 18 | 37 | 2 | K.ALPAPIEK.T |
|  | 1047 | 426.5892 | 851.1637 | 850.9803 | 0.1834 | 0 | 38 | 0.36 | 1 | K.DTLMISR.T + Oxidation (M) |
|  | 426 | **388.4026** | **1162.1856** | **1161.4801** | **0.7055** | **2** | **11** | **3.1e+02** | **8** | **-.MDVMCKKMK.H + 3 Oxidation (M)** |
|  | 2414 | 634.2466 | 1266.4784 | 1267.5140 | -1.0357 | 1 | (10) | 2.6e+02 | 2 | K.ALPAPIEKTISK.A |
|  | 2425 | 635.0229 | 1268.0311 | 1267.5140 | 0.5171 | 1 | (35) | 0.8 | 1 | K.ALPAPIEKTISK.A |
|  | 2426 | 635.2228 | 1268.4308 | 1267.5140 | 0.9167 | 1 | 63 | 0.0014 | 1 | K.ALPAPIEKTISK.A |

  


---

**Peptide matches not assigned to protein hits:** (no details means no
match)  
  

|  |  |  |  |  |  |  |  |  |  |  |
| --- | --- | --- | --- | --- | --- | --- | --- | --- | --- | --- |
|  | **Query** | **Observed** | **Mr(expt)** | **Mr(calc)** | **Delta** | **Miss** | **Score** | **Expect** | **Rank** | **Peptide** |
|  | 2592 | **667.7041** | **1333.3934** | **1334.4740** | **-1.0806** | **0** | **69** | **0.00038** | **1** | **ISEIEDAAFLAR** |
|  | 2618 | 668.8165 | 1335.6183 | 1334.4740 | 1.1442 | 0 | 69 | 0.00038 | 1 | ISEIEDAAFLAR |
|  | 3103 | 766.0936 | 2295.2587 | 2294.6083 | 0.6504 | 1 | 57 | 0.0039 | 1 | VAPEEHPVLLTQAPLNPKANR |
|  | 1586 | **513.5487** | **1025.0826** | **1025.2027** | **-0.1200** | **0** | **55** | **0.0087** | **1** | **IGGIGTVPVGR** |
|  | 3535 | 977.9032 | 1953.7916 | 1953.2421 | 0.5495 | 0 | 52 | 0.011 | 1 | VAPEEHPVLLTQAPLNPK |
|  | 2808 | **698.8281** | **1395.6415** | **1395.5208** | **0.1207** | **2** | **45** | **0.087** | **1** | **RNEIDAEPPAKR** |
|  | 3137 | **784.1548** | **2349.4422** | **2350.5903** | **-1.1481** | **0** | **44** | **0.099** | **1** | **AVAFQNPQTHVIENLHAAAYR** |
|  | 2541 | **662.6748** | **1323.3348** | **1323.5374** | **-0.2026** | **1** | **42** | **0.16** | **1** | **ILPQDLERAGLV** |
|  | 3310 | **878.3164** | **2631.9270** | **2630.7694** | **1.1576** | **2** | **42** | **0.14** | **1** | **ESSLPSKEALEPSGENVIQNKESTG** |
|  | 1020 | **421.8640** | **841.7131** | **841.9949** | **-0.2818** | **0** | **40** | **0.26** | **1** | **GITLSVRP** |
|  | 491 | **389.8602** | **777.7056** | **776.9018** | **0.8038** | **0** | **39** | **0.36** | **1** | **AGVDMLR + Oxidation (M)** |
|  | 1500 | **492.8413** | **983.6678** | **983.1245** | **0.5433** | **0** | **38** | **0.42** | **1** | **HGSLGFLPR** |
|  | 1025 | **422.1163** | **842.2179** | **841.9949** | **0.2229** | **0** | **38** | **0.52** | **1** | **GITLSVRP** |
|  | 1029 | **422.1875** | **842.3602** | **841.9949** | **0.3653** | **0** | **37** | **0.57** | **1** | **GITLSVRP** |
|  | 1925 | **563.6075** | **1687.8005** | **1686.8878** | **0.9126** | **1** | **36** | **0.75** | **1** | **QLVRGEPNVSEICSR** |
|  | 1232 | **454.8475** | **1361.5202** | **1360.5825** | **0.9378** | **1** | **35** | **0.79** | **1** | **FINYVKNCFR + Carbamidomethyl (C)** |
|  | 2895 | **730.6120** | **2188.8138** | **2189.3802** | **-0.5664** | **2** | **35** | **0.68** | **1** | **ATVLESEGTRESAINVAEGKK** |
|  | 2698 | **681.0837** | **1360.1527** | **1360.5825** | **-0.4298** | **1** | **35** | **0.89** | **1** | **FINYVKNCFR + Carbamidomethyl (C)** |
|  | 1977 | **576.9958** | **1727.9652** | **1727.9167** | **0.0484** | **2** | **34** | **1.1** | **1** | **VRVTELEDEVRNLR** |
|  | 2663 | **672.7581** | **1343.5013** | **1343.4858** | **0.0155** | **0** | **33** | **1.4** | **1** | **TAAAVAAQSGILDR** |
|  | 3146 | **786.4497** | **1570.8846** | **1569.7796** | **1.1051** | **0** | **31** | **2.2** | **1** | **QTFSPFGQIMEIR + Oxidation (M)** |
|  | 3141 | **784.7363** | **1567.4578** | **1566.7957** | **0.6621** | **1** | **30** | **1.9** | **1** | **FKGPFTDVVTTNLK** |
|  | 719 | **404.7549** | **807.4950** | **806.8598** | **0.6352** | **0** | **30** | **2.3** | **1** | **APFALSSD** |
|  | 1598 | **514.9982** | **1027.9817** | **1028.2067** | **-0.2250** | **1** | **30** | **2.6** | **1** | **RSSIPITVR** |
|  | 1606 | **517.2952** | **1032.5756** | **1033.1784** | **-0.6028** | **1** | **30** | **3.1** | **1** | **KGFADIPTGK** |
|  | 212 | **377.1292** | **1128.3656** | **1129.1350** | **-0.7694** | **0** | **29** | **3.1** | **1** | **GPGAEGGSGSPEK** |
|  | 928 | **411.9472** | **821.8797** | **821.8994** | **-0.0197** | **0** | **28** | **4.2** | **1** | **MQSPSTR + Oxidation (M)** |
|  | 1131 | **435.8130** | **869.6112** | **870.0283** | **-0.4171** | **0** | **28** | **4** | **1** | **TPCPSLPR** |
|  | 167 | **372.4192** | **742.8235** | **742.9466** | **-0.1231** | **0** | **28** | **5.5** | **1** | **VLGTLLK** |
|  | 653 | **402.0732** | **1203.1975** | **1204.2593** | **-1.0618** | **2** | **28** | **5.2** | **1** | **GGRGGGRGGGGGFR** |
|  | 271 | **384.9922** | **1151.9545** | **1151.2927** | **0.6617** | **0** | **28** | **3.8** | **1** | **GAQLCFEANAK** |
|  | 235 | **379.4824** | **1135.4249** | **1136.1937** | **-0.7688** | **0** | **28** | **5.7** | **1** | **GATGECNISER** |
|  | 1858 | **550.0800** | **1647.2177** | **1646.7992** | **0.4185** | **1** | **27** | **4.9** | **1** | **QLRFEDVVNQSSPK** |
|  | 614 | **400.2990** | **798.5832** | **797.9823** | **0.6009** | **0** | **26** | **4.3** | **1** | **AVPLTVAK** |
|  | 1487 | **489.3497** | **1465.0268** | **1465.6603** | **-0.6334** | **1** | **26** | **5.6** | **1** | **RPMSSRGWVCDR + Oxidation (M)** |
|  | 3 | **360.4430** | **718.8712** | **718.7101** | **0.1611** | **0** | **26** | **9.1** | **1** | **SPSADDK** |
|  | 687 | 403.9472 | 805.8797 | 806.9508 | -1.0711 | 0 | 26 | 7.2 | 1 | FFQPLR |
|  | 2906 | **738.1948** | **2211.5621** | **2210.5548** | **1.0073** | **1** | **26** | **7.2** | **1** | **QCQCTSVGAQNTVICSKLAAK + Carbamidomethyl (C)** |
|  | 1600 | **515.3380** | **1028.6612** | **1028.2067** | **0.4546** | **1** | **26** | **6.3** | **1** | **RSSIPITVR** |
|  | 1607 | **517.3007** | **1032.5867** | **1033.0907** | **-0.5040** | **1** | **26** | **7.6** | **1** | **KLSEGDGAEK** |
|  | 413 | **388.0282** | **774.0416** | **772.9331** | **1.1085** | **1** | **26** | **9.5** | **1** | **KAVASVAK** |
|  | 2900 | **733.3376** | **1464.6605** | **1464.6655** | **-0.0049** | **0** | **25** | **7.5** | **1** | **CFLGCELPPEGSR + Carbamidomethyl (C)** |
|  | 1725 | **534.0229** | **1066.0310** | **1065.2301** | **0.8008** | **1** | **25** | **8.4** | **1** | **AGVIRAVSHR** |
|  | 163 | **372.3206** | **742.6265** | **742.9466** | **-0.3202** | **1** | **25** | **6.5** | **1** | **KELILK** |
|  | 2053 | **592.7731** | **1183.5314** | **1184.3857** | **-0.8544** | **1** | **25** | **7.7** | **1** | **EEVGALAKVLR** |
|  | 2777 | **688.2310** | **1374.4473** | **1373.3793** | **1.0679** | **0** | **25** | **8.1** | **1** | **GEEVNGDATAGSIPG** |
|  | 174 | **373.2284** | **1116.6629** | **1117.2584** | **-0.5954** | **1** | **25** | **8.2** | **1** | **LPEVNRSFR** |
|  | 3140 | **784.7126** | **1567.4105** | **1566.7957** | **0.6148** | **1** | **25** | **6.6** | **1** | **FKGPFTDVVTTNLK** |
|  | 209 | **377.0703** | **1128.1889** | **1127.3343** | **0.8545** | **1** | **25** | **8.2** | **1** | **ALDEKIVALR** |
|  | 1795 | **540.3447** | **1618.0120** | **1618.7924** | **-0.7803** | **0** | **25** | **8.2** | **1** | **QVHLVESGGGVVQPGR** |
|  | 281 | **385.2096** | **1152.6066** | **1153.2854** | **-0.6788** | **0** | **25** | **6.2** | **1** | **SLLAAAPGPTGAQ** |
|  | 1195 | **448.1245** | **894.2341** | **893.0419** | **1.1923** | **1** | **25** | **8.9** | **1** | **YTVKVQR** |
|  | 382 | **387.8152** | **773.6156** | **772.9990** | **0.6166** | **0** | **25** | **11** | **1** | **LIGCLVR** |
|  | 143 | **370.9902** | **1109.9485** | **1109.2363** | **0.7122** | **1** | **25** | **7** | **1** | **RLTSSYGAVR** |
|  | 481 | **389.1414** | **776.2679** | **775.9568** | **0.3112** | **1** | **25** | **10** | **1** | **MKLGSPK + Oxidation (M)** |
|  | 2065 | **593.0580** | **1776.1518** | **1775.2079** | **0.9438** | **1** | **24** | **10** | **1** | **IRNMLNIYLVWLVK** |
|  | 480 | **389.1296** | **776.2444** | **775.8044** | **0.4400** | **0** | **24** | **11** | **1** | **ELGQSDK** |
|  | 2572 | **666.6678** | **1331.3209** | **1330.5715** | **0.7494** | **0** | **24** | **9.6** | **1** | **MFLLSGDPCFK + Carbamidomethyl (C); Oxidation (M)** |
|  | 1406 | **480.3379** | **958.6609** | **959.0585** | **-0.3976** | **2** | **24** | **8.5** | **1** | **EEEKRLR** |
|  | 23 | **363.1425** | **1086.4055** | **1086.1765** | **0.2290** | **0** | **24** | **10** | **1** | **GENPPTSCPK + Carbamidomethyl (C)** |
|  | 1288 | **461.6855** | **1382.0345** | **1382.6279** | **-0.5935** | **0** | **24** | **8.4** | **1** | **LLIMAVHPESTR + Oxidation (M)** |
|  | 1926 | **563.9864** | **1688.9372** | **1688.9700** | **-0.0328** | **1** | **24** | **11** | **1** | **QLVRGEPNVSMICSR** |
|  | 159 | **372.1764** | **742.3380** | **742.9499** | **-0.6119** | **1** | **24** | **9** | **1** | **TRLILK** |
|  | 1478 | **488.6296** | **1462.8667** | **1462.8236** | **0.0432** | **2** | **24** | **12** | **1** | **KNCLMGFSCKGMK + Oxidation (M)** |
|  | 1136 | **436.1027** | **870.1905** | **870.0283** | **0.1623** | **0** | **24** | **11** | **1** | **TPCPSLPR** |
|  | 2563 | **666.4574** | **1330.9000** | **1331.4985** | **-0.5985** | **1** | **24** | **11** | **1** | **CNRPSVKVEDK + Carbamidomethyl (C)** |
|  | 2647 | **670.7792** | **1339.5436** | **1339.5402** | **0.0033** | **1** | **24** | **13** | **1** | **VPEQQRQITLK** |
|  | 1141 | **436.3015** | **870.5882** | **870.0049** | **0.5833** | **0** | **24** | **8.9** | **1** | **ASGGPALLGK** |
|  | 835 | **407.4363** | **1219.2867** | **1218.3010** | **0.9857** | **1** | **24** | **13** | **1** | **RSTPGCSPGGSR + Carbamidomethyl (C)** |
|  | 613 | **400.2686** | **798.5225** | **798.8443** | **-0.3217** | **0** | **23** | **8.5** | **1** | **LGDPQNR** |
|  | 981 | **419.1617** | **1254.4628** | **1253.4759** | **0.9869** | **2** | **23** | **14** | **1** | **MRRVYAGSWK** |
|  | 170 | **372.5732** | **743.1315** | **742.9697** | **0.1618** | **0** | **23** | **11** | **1** | **CLPLLK + Carbamidomethyl (C)** |
|  | 1365 | **474.1656** | **1419.4746** | **1420.6180** | **-1.1434** | **1** | **23** | **13** | **1** | **MNERVNTGLGACR** |
|  | 1229 | **454.3058** | **1359.8951** | **1359.6376** | **0.2575** | **1** | **23** | **11** | **1** | **MRCGTIFDCLK + Carbamidomethyl (C); Oxidation (M)** |
|  | 668 | **402.9551** | **803.8954** | **802.9391** | **0.9563** | **0** | **23** | **14** | **1** | **MAAQSAPK** |
|  | 276 | **385.0774** | **1152.2100** | **1151.3576** | **0.8524** | **1** | **23** | **11** | **1** | **EKELCQMVR + Oxidation (M)** |
|  | 1115 | **434.5908** | **1300.7501** | **1300.3351** | **0.4150** | **0** | **23** | **11** | **1** | **DLQSSQGGQQPR** |
|  | 1465 | **488.1523** | **1461.4349** | **1461.6219** | **-0.1871** | **1** | **23** | **14** | **1** | **FRGKPTEASIEAR** |
|  | 999 | 419.7696 | 837.5245 | 836.9419 | 0.5825 | 1 | 23 | 12 | 1 | ALGARGHR |
|  | 1405 | **480.3351** | **958.6555** | **959.0155** | **-0.3600** | **0** | **23** | **11** | **1** | **TGAGVSQSPR** |
|  | 1165 | **441.6051** | **881.1954** | **880.0464** | **1.1491** | **1** | **23** | **11** | **1** | **NKAVVPPR** |
|  | 91 | **369.2444** | **1104.7111** | **1104.2680** | **0.4431** | **2** | **23** | **11** | **1** | **HRSDRMMR + Oxidation (M)** |
|  | 531 | **390.0553** | **1167.1438** | **1166.3719** | **0.7719** | **0** | **23** | **13** | **1** | **AYYLACGFCR** |
|  | 641 | **401.8733** | **1202.5976** | **1201.4660** | **1.1316** | **2** | **23** | **15** | **1** | **RLPREMMPR + Oxidation (M)** |
|  | 2903 | **734.4902** | **1466.9657** | **1467.4295** | **-0.4638** | **0** | **23** | **13** | **1** | **EQAPDCSSSDGSER** |
|  | 1567 | **508.0566** | **1014.0985** | **1015.1616** | **-1.0631** | **0** | **23** | **13** | **1** | **YDDMAAAMK** |
|  | 245 | **380.0594** | **1137.1560** | **1138.3421** | **-1.1861** | **0** | **23** | **16** | **1** | **CVDMGCAGLR + 2 Carbamidomethyl (C)** |
|  | 528 | **390.0404** | **1167.0990** | **1167.3354** | **-0.2363** | **0** | **23** | **14** | **1** | **VMLTQFAEGR + Oxidation (M)** |
|  | 162 | **372.2578** | **742.5008** | **742.9466** | **-0.4459** | **1** | **23** | **10** | **1** | **KELILK** |
|  | 706 | **404.1225** | **1209.3453** | **1210.3419** | **-0.9965** | **1** | **23** | **14** | **1** | **QQGAGMTDKCR + Oxidation (M)** |
|  | 407 | **387.9278** | **1160.7612** | **1160.3213** | **0.4400** | **1** | **23** | **17** | **1** | **CSECGKAFTSK** |
|  | 1676 | **525.0673** | **1572.1798** | **1572.8499** | **-0.6701** | **0** | **23** | **15** | **1** | **CVRPVGTAYYQMK + Carbamidomethyl (C)** |
|  | 1133 | **435.8403** | **869.6659** | **870.0102** | **-0.3443** | **1** | **23** | **14** | **1** | **MASRSMR + 2 Oxidation (M)** |
|  | 1085 | **431.1218** | **860.2289** | **860.9520** | **-0.7231** | **0** | **23** | **17** | **1** | **TNESALVK** |
|  | 46 | **364.5131** | **1090.5172** | **1091.2872** | **-0.7701** | **1** | **23** | **13** | **1** | **VYCINPARR** |
|  | 861 | **407.8226** | **1220.4455** | **1219.3106** | **1.1350** | **0** | **23** | **15** | **1** | **DHQRPSGVPAR** |
|  | 3251 | **832.5630** | **1663.1112** | **1662.9143** | **0.1969** | **2** | **22** | **15** | **1** | **AGQAPRLLMYGASRR + Oxidation (M)** |
|  | 287 | **385.9321** | **1154.7741** | **1154.1445** | **0.6297** | **0** | **22** | **13** | **1** | **EDQQSFTGSR** |
|  | 1492 | **490.0551** | **978.0953** | **977.0753** | **1.0200** | **0** | **22** | **16** | **1** | **AFQQVQTR** |
|  | 2212 | **608.9021** | **1823.6841** | **1823.0820** | **0.6021** | **0** | **22** | **13** | **1** | **VQCEVHLLASGGGLVQR + Carbamidomethyl (C)** |
|  | 865 | **407.8395** | **1220.4964** | **1221.3213** | **-0.8250** | **0** | **22** | **16** | **1** | **SIPFSNTHYR** |
|  | 758 | **405.8554** | **1214.5440** | **1215.4627** | **-0.9187** | **1** | **22** | **15** | **1** | **GTGIISAPMPKK + Oxidation (M)** |
|  | 606 | **399.9038** | **1196.6892** | **1196.4226** | **0.2665** | **1** | **22** | **13** | **1** | **LRCIGSIQHL + Carbamidomethyl (C)** |
|  | 752 | **405.5410** | **1213.6009** | **1214.3304** | **-0.7296** | **0** | **22** | **15** | **1** | **ALEERPWASR** |
|  | 2950 | **740.7886** | **1479.5624** | **1480.7591** | **-1.1968** | **1** | **22** | **17** | **1** | **AGSGRLVLRPWIR** |
|  | 1094 | **432.8955** | **1295.6644** | **1296.5319** | **-0.8676** | **1** | **22** | **16** | **1** | **QYESLKILICS** |
|  | 154 | **371.2678** | **1110.7813** | **1111.2490** | **-0.4677** | **1** | **22** | **10** | **1** | **KEPEKPIDR** |
|  | 2383 | **628.4951** | **1254.9755** | **1254.4805** | **0.4950** | **2** | **22** | **13** | **1** | **WKKIAEAVPGR** |
|  | 160 | **372.2241** | **742.4335** | **742.8607** | **-0.4272** | **0** | **22** | **12** | **1** | **TGVLEPK** |
|  | 649 | **401.9867** | **1202.9380** | **1202.3196** | **0.6184** | **1** | **22** | **19** | **1** | **EDARLGSWLR** |
|  | 909 | **409.1119** | **1224.3135** | **1223.3837** | **0.9298** | **1** | **22** | **18** | **1** | **MCNVDNSRIR + Oxidation (M)** |
|  | 145 | **371.0016** | **739.9885** | **740.8479** | **-0.8594** | **0** | **22** | **12** | **1** | **GLALDPR** |
|  | 753 | **405.5686** | **1213.6836** | **1214.3456** | **-0.6619** | **0** | **22** | **14** | **1** | **FTASSSSGMVPK + Oxidation (M)** |
|  | 607 | **399.9189** | **1196.7347** | **1196.3103** | **0.4243** | **1** | **22** | **14** | **1** | **KGIVEHEEQK** |
|  | 314 | **386.0565** | **1155.1472** | **1155.3281** | **-0.1809** | **2** | **22** | **15** | **1** | **VRARYSMEK + Oxidation (M)** |
|  | 1061 | **429.1595** | **856.3041** | **856.9664** | **-0.6623** | **0** | **22** | **16** | **1** | **LVGDGIQR** |
|  | 906 | **409.0468** | **1224.1183** | **1224.3898** | **-0.2716** | **0** | **22** | **19** | **1** | **CHGLSLPVENR** |
|  | 1542 | **504.9044** | **1511.6909** | **1511.7387** | **-0.0478** | **2** | **22** | **16** | **1** | **DKMDAFILSAEKK + Oxidation (M)** |
|  | 423 | **388.3314** | **1161.9721** | **1161.3293** | **0.6428** | **1** | **22** | **17** | **1** | **GKLEAEEVMR** |
|  | 432 | **388.6524** | **775.2900** | **774.9024** | **0.3877** | **0** | **22** | **16** | **1** | **IVYFSF** |
|  | 147 | **371.0793** | **1110.2158** | **1111.3333** | **-1.1175** | **1** | **22** | **14** | **1** | **FSFITLGAKK** |
|  | 655 | **402.1089** | **1203.3045** | **1203.3361** | **-0.0316** | **2** | **22** | **20** | **1** | **RSRCSGRPER** |
|  | 3219 | **817.3243** | **2448.9509** | **2447.7523** | **1.1986** | **0** | **22** | **16** | **1** | **TLEPLFDETFEFFVPMEEVK** |
|  | 1167 | **442.2247** | **1323.6518** | **1324.3633** | **-0.7114** | **1** | **22** | **16** | **1** | **DKPRGSGSGGGGHR** |
|  | 60 | **366.2162** | **730.4176** | **729.8519** | **0.5657** | **1** | **22** | **16** | **1** | **ARCQPR** |
|  | 2920 | **740.2549** | **2217.7425** | **2218.5900** | **-0.8476** | **0** | **22** | **17** | **1** | **MAQSINITELNLPQLEMLK + 2 Oxidation (M)** |
|  | 409 | **387.9524** | **773.8900** | **772.9331** | **0.9569** | **1** | **22** | **22** | **1** | **LVRSVTV** |
|  | 960 | **416.0681** | **830.1213** | **829.0027** | **1.1186** | **2** | **22** | **21** | **1** | **RLGSKIR** |
|  | 3115 | **771.6710** | **2311.9907** | **2311.5859** | **0.4048** | **1** | **22** | **14** | **1** | **LLYYDVSTVRDVLESGLSPGK** |
|  | 720 | 404.7637 | 1211.2688 | 1212.3112 | -1.0424 | 1 | 22 | 17 | 1 | EYNFLDQRK |
|  | 1372 | **475.5108** | **949.0068** | **950.1593** | **-1.1524** | **1** | **22** | **23** | **1** | **KAFCNVLR** |
|  | 1284 | **460.8243** | **1379.4507** | **1379.6058** | **-0.1552** | **2** | **22** | **19** | **1** | **MLCEEAAQKRK + Carbamidomethyl (C); Oxidation (M)** |
|  | 133 | **369.4945** | **1105.4613** | **1106.2556** | **-0.7942** | **1** | **22** | **21** | **1** | **RGPEVGGFCK + Carbamidomethyl (C)** |
|  | 377 | **387.8043** | **1160.3908** | **1160.3048** | **0.0860** | **2** | **22** | **22** | **1** | **HKSQTRMEK + Oxidation (M)** |
|  | 3237 | **823.2871** | **2466.8392** | **2467.8562** | **-1.0170** | **1** | **22** | **18** | **1** | **AEILCMGNSFGVSPTMDKEYMK + Oxidation (M)** |
|  | 176 | **373.3691** | **744.7235** | **745.7784** | **-1.0549** | **1** | **22** | **25** | **1** | **KNLDEAG** |
|  | 85 | **369.1965** | **1104.5672** | **1104.2879** | **0.2794** | **2** | **21** | **17** | **1** | **RSAVVMGRGR + Oxidation (M)** |
|  | 2331 | **623.7875** | **1245.5603** | **1245.3842** | **0.1760** | **1** | **21** | **20** | **1** | **QVKGTGAFLTHS** |
|  | 1474 | **488.4868** | **1462.4384** | **1461.6499** | **0.7884** | **1** | **21** | **22** | **1** | **GAHGMAVWYTRGR** |
|  | 2713 | **684.0209** | **1366.0270** | **1365.5807** | **0.4462** | **1** | **21** | **16** | **1** | **GALVLGSSLKQHR** |
|  | 416 | **388.0854** | **774.1559** | **774.8262** | **-0.6703** | **1** | **21** | **23** | **1** | **GSAAARSR** |
|  | 1250 | **458.7925** | **1373.3553** | **1372.5716** | **0.7836** | **1** | **21** | **19** | **1** | **AGLPRDLTFNLR** |
|  | 1467 | **488.2211** | **1461.6412** | **1460.6106** | **1.0306** | **1** | **21** | **21** | **1** | **AEDTAIYYCAKGR** |
|  | 946 | **414.0049** | **1238.9925** | **1239.4061** | **-0.4137** | **1** | **21** | **16** | **1** | **QRGACCEQCK + 2 Carbamidomethyl (C)** |
|  | 186 | **374.1984** | **746.3820** | **745.8281** | **0.5539** | **2** | **21** | **19** | **1** | **GNKGRSK** |
|  | 2089 | **595.1683** | **1188.3219** | **1187.3862** | **0.9356** | **1** | **21** | **22** | **1** | **KLSLTSPLNSK** |
|  | 2325 | **623.6667** | **1245.3187** | **1245.3428** | **-0.0241** | **1** | **21** | **23** | **1** | **GKSLTQNSAPSR** |
|  | 1490 | **489.9383** | **977.8618** | **978.1646** | **-0.3028** | **1** | **21** | **20** | **1** | **LSPAKMSTK + Oxidation (M)** |
|  | 1638 | **521.5364** | **1561.5870** | **1560.6471** | **0.9399** | **2** | **21** | **21** | **1** | **DPMYRREGSYDR + Oxidation (M)** |
|  | 1462 | **488.1424** | **1461.4049** | **1462.5357** | **-1.1307** | **0** | **21** | **22** | **1** | **SMTEGSTVNTEYK + Oxidation (M)** |
|  | 1510 | **495.8682** | **989.7217** | **989.0844** | **0.6372** | **1** | **21** | **21** | **1** | **ESLRIESR** |
|  | 2754 | **686.0906** | **2055.2496** | **2054.3443** | **0.9052** | **0** | **21** | **19** | **1** | **STAALSGEAASCSPIIMPYK + Carbamidomethyl (C)** |
|  | 2729 | **684.7758** | **1367.5367** | **1368.6015** | **-1.0647** | **1** | **21** | **25** | **1** | **LSPNTMVTPHKK + Oxidation (M)** |
|  | 982 | **419.1835** | **1254.5284** | **1253.4478** | **1.0807** | **0** | **21** | **23** | **1** | **KPGLGVPGSSGAVK** |
|  | 1407 | **480.3774** | **1438.1101** | **1438.6085** | **-0.4983** | **2** | **21** | **17** | **1** | **DRLEGMAAFREK + Oxidation (M)** |
|  | 2338 | **624.0167** | **1869.0280** | **1870.1388** | **-1.1108** | **1** | **21** | **20** | **1** | **KDAMTGAHSASMCRPYK + Oxidation (M)** |
|  | 2631 | **669.6724** | **2005.9949** | **2005.1711** | **0.8238** | **1** | **21** | **19** | **1** | **TSVVTQSAPICSQEEGRGR** |
|  | 350 | **387.3266** | **772.6384** | **772.8898** | **-0.2514** | **0** | **21** | **21** | **1** | **IVGTDIR** |
|  | 384 | **387.8215** | **1160.4423** | **1159.3165** | **1.1258** | **0** | **21** | **26** | **1** | **SMAAAAASLGGPR** |
|  | 366 | **387.7530** | **1160.2368** | **1160.3443** | **-0.1075** | **1** | **21** | **25** | **1** | **RITCSGDALPK** |
|  | 1373 | **475.5579** | **949.1011** | **948.0327** | **1.0684** | **1** | **21** | **27** | **1** | **SSTPKTATR** |
|  | 2184 | **607.5929** | **1819.7565** | **1819.8317** | **-0.0751** | **0** | **21** | **19** | **1** | **SQEGENEEGSEGELVVK** |
|  | 591 | **398.7334** | **1193.1779** | **1192.3679** | **0.8100** | **1** | **21** | **19** | **1** | **RGFASITITAR** |
|  | 664 | **402.4578** | **1204.3512** | **1205.3817** | **-1.0305** | **1** | **21** | **30** | **1** | **DLLMKAETER** |
|  | 479 | **389.1240** | **1164.3498** | **1163.3484** | **1.0014** | **1** | **21** | **23** | **1** | **MLASTQKVNR + Oxidation (M)** |
|  | 722 | **404.8264** | **1211.4569** | **1212.4256** | **-0.9687** | **1** | **21** | **21** | **1** | **RLVAGQGCVGPR** |
|  | 3262 | **839.2848** | **2514.8322** | **2514.7254** | **0.1068** | **2** | **21** | **20** | **1** | **GAQGPAGPKGEPGQDGEMGPKGPPGPK + Oxidation (M)** |
|  | 755 | **405.7336** | **1214.1785** | **1214.3701** | **-0.1917** | **1** | **21** | **19** | **1** | **LTGNSKYIYR** |
|  | 385 | **387.8268** | **1160.4584** | **1161.2928** | **-0.8344** | **1** | **21** | **27** | **1** | **ARVPGGCTSGTR** |
|  | 2568 | **666.5959** | **1996.7657** | **1996.3135** | **0.4522** | **1** | **21** | **18** | **1** | **SRMVGTPYWMAPEVVTR + Oxidation (M)** |
|  | 221 | **377.9838** | **1130.9293** | **1130.2057** | **0.7236** | **0** | **21** | **19** | **1** | **LQELEQENK** |
|  | 297 | **385.9945** | **769.9742** | **769.8462** | **0.1280** | **0** | **21** | **19** | **1** | **SLSPNPR** |
|  | 568 | **392.4185** | **782.8223** | **782.9046** | **-0.0823** | **0** | **21** | **25** | **1** | **YNDLMK** |
|  | 40 | **363.5521** | **725.0893** | **723.9022** | **1.1872** | **0** | **21** | **18** | **1** | **ECMVVK + Oxidation (M)** |
|  | 1285 | **461.1400** | **1380.3978** | **1380.4847** | **-0.0869** | **2** | **21** | **24** | **1** | **SKMAKSGGSDQER** |
|  | 3045 | **757.0773** | **2268.2096** | **2268.6573** | **-0.4476** | **1** | **21** | **17** | **1** | **MARCGEGSAAPMVLLGSAGVCSK + Carbamidomethyl (C); Oxidation (M)** |
|  | 1459 | **488.1186** | **974.2224** | **973.1116** | **1.1108** | **1** | **21** | **26** | **1** | **GRGVAGPMGR + Oxidation (M)** |
|  | 1480 | **488.8950** | **975.7751** | **975.1839** | **0.5913** | **2** | **21** | **24** | **1** | **VSLTKTAKK** |
|  | 3347 | **910.7787** | **2729.3139** | **2728.8096** | **0.5043** | **0** | **21** | **18** | **1** | **PMILGYWQVXGLAXAIXLLLEYTD + Oxidation (M)** |
|  | 1832 | **544.2947** | **1629.8620** | **1629.8365** | **0.0255** | **1** | **21** | **26** | **1** | **DEAQVCAIIERVQR** |
|  | 1470 | **488.3228** | **974.6307** | **975.0578** | **-0.4271** | **1** | **21** | **21** | **1** | **ARELSSGQK** |
|  | 223 | **378.0661** | **1131.1761** | **1131.3230** | **-0.1469** | **1** | **21** | **21** | **1** | **SVSERLSILK** |
|  | 24 | **363.1427** | **1086.4058** | **1087.2921** | **-0.8862** | **0** | **21** | **23** | **1** | **MSFIVFSTR** |
|  | 1090 | **432.2372** | **862.4596** | **861.8537** | **0.6059** | **0** | **21** | **22** | **1** | **SGQASGEGGL** |
|  | 2749 | **685.9309** | **1369.8470** | **1369.5246** | **0.3224** | **1** | **21** | **19** | **1** | **KESLGHWSQGLK** |
|  | 3488 | **965.9731** | **2894.8973** | **2895.2518** | **-0.3546** | **2** | **21** | **18** | **1** | **AAVAGEDGRMIAGQVLDINLAAEPKVNR + Oxidation (M)** |
|  | 47 | **365.1478** | **728.2808** | **727.8527** | **0.4282** | **1** | **20** | **23** | **1** | **KAERPK** |
|  | 1375 | **475.6147** | **949.2146** | **949.0405** | **0.1741** | **0** | **20** | **27** | **1** | **MGLSSPDAR + Oxidation (M)** |
|  | 1170 | **442.5685** | **883.1223** | **882.9642** | **0.1581** | **1** | **20** | **25** | **1** | **SVERQHK** |
|  | 1276 | **460.6696** | **1378.9866** | **1378.6575** | **0.3291** | **0** | **20** | **21** | **1** | **VDLAPPALVVAWK** |
|  | 978 | **419.0842** | **1254.2303** | **1255.3393** | **-1.1089** | **0** | **20** | **28** | **1** | **GSPGGPGAAGFPGAR** |
|  | 1839 | **546.9305** | **1091.8463** | **1092.3765** | **-0.5302** | **1** | **20** | **25** | **1** | **MLMADKVLR + Oxidation (M)** |
|  | 1013 | **420.7903** | **1259.3487** | **1258.3417** | **1.0070** | **1** | **20** | **23** | **1** | **SRGTSGGSSLPPR** |
|  | 736 | **405.1810** | **808.3472** | **807.8728** | **0.4745** | **0** | **20** | **22** | **1** | **MESQAAR + Oxidation (M)** |
|  | 1976 | **576.8469** | **1727.5186** | **1728.1118** | **-0.5932** | **2** | **20** | **19** | **1** | **MSAKILNFSCLKCR + 2 Carbamidomethyl (C)** |
|  | 2392 | **628.9589** | **1255.9031** | **1255.4271** | **0.4759** | **2** | **20** | **21** | **1** | **RAAGTPGLQTRK** |
|  | 618 | **400.5068** | **798.9989** | **797.8994** | **1.0995** | **0** | **20** | **27** | **1** | **AVGGLEPR** |
|  | 1952 | **571.4319** | **1711.2735** | **1711.9254** | **-0.6520** | **2** | **20** | **20** | **1** | **GSLPHAPRRAPAPSAAR** |
|  | 723 | **404.9481** | **1211.8221** | **1212.4606** | **-0.6385** | **1** | **20** | **23** | **1** | **SVAMCEMEKK + Carbamidomethyl (C)** |
|  | 419 | **388.1989** | **1161.5747** | **1161.2247** | **0.3499** | **1** | **20** | **26** | **1** | **WEAAQASKDR** |
|  | 3136 | **780.9208** | **2339.7402** | **2340.7001** | **-0.9599** | **1** | **20** | **28** | **1** | **KVAERPLGEMGSPPAAGFVTLGR** |
|  | 633 | **401.5619** | **1201.6634** | **1201.3333** | **0.3301** | **1** | **20** | **28** | **1** | **TSRGALSPSLGR** |
|  | 67 | **368.1180** | **1101.3318** | **1102.2422** | **-0.9104** | **1** | **20** | **28** | **1** | **RLVGVDQSTK** |
|  | 769 | **406.1481** | **1215.4220** | **1216.5170** | **-1.0949** | **0** | **20** | **25** | **1** | **MMKPSCSWCK + Oxidation (M)** |
|  | 2327 | **623.7203** | **1868.1388** | **1867.1377** | **1.0011** | **1** | **20** | **32** | **1** | **LWPCSNARACCSNLSK + 2 Carbamidomethyl (C)** |
|  | 953 | **415.9392** | **829.8636** | **830.8863** | **-1.0226** | **0** | **20** | **31** | **1** | **QTPSVSGR** |
|  | 2932 | **740.5245** | **2218.5514** | **2218.3551** | **0.1963** | **1** | **20** | **25** | **1** | **LMELHGEGSSSPKATGDETGAK + Oxidation (M)** |
|  | 169 | **372.4791** | **742.9435** | **743.8519** | **-0.9084** | **1** | **20** | **31** | **1** | **KNINQK** |
|  | 352 | **387.4968** | **1159.4682** | **1159.3348** | **0.1335** | **0** | **20** | **36** | **1** | **FTCACPDQFK** |
|  | 2787 | **690.8981** | **2069.6720** | **2068.5463** | **1.1257** | **1** | **20** | **24** | **1** | **LLILADMADVMRLLSHLK + Oxidation (M)** |
|  | 2971 | **741.8159** | **2222.4254** | **2222.5885** | **-0.1631** | **2** | **20** | **29** | **1** | **GFWKQPPLIWDVNPKQIR** |
|  | 2451 | **641.4749** | **1280.9349** | **1280.3639** | **0.5711** | **1** | **20** | **25** | **1** | **CRAPEGFEEDK** |
|  | 165 | **372.3850** | **742.7552** | **742.9466** | **-0.1915** | **1** | **20** | **32** | **1** | **KELILK** |
|  | 1055 | **428.7858** | **855.5568** | **855.9785** | **-0.4217** | **0** | **20** | **24** | **1** | **SPSSLLPR** |
|  | 1174 | **443.5887** | **1327.7439** | **1327.4435** | **0.3004** | **1** | **20** | **28** | **1** | **NEGSQAKPGSPKK** |
|  | 1380 | **475.7776** | **949.5404** | **950.0022** | **-0.4618** | **1** | **20** | **24** | **1** | **QSTKTEEK** |
|  | 1666 | **524.0107** | **1569.0101** | **1568.8099** | **0.2002** | **1** | **20** | **28** | **1** | **EMCKEFLVLGEAPS + Oxidation (M)** |
|  | 660 | **402.1834** | **1203.5281** | **1204.4170** | **-0.8889** | **0** | **20** | **29** | **1** | **MLEHTEAVMK + Oxidation (M)** |
|  | 400 | **387.8923** | **1160.6547** | **1161.2896** | **-0.6348** | **1** | **20** | **33** | **1** | **SETSHMSVKR** |
|  | 1012 | **420.5249** | **1258.5525** | **1258.4460** | **0.1066** | **0** | **20** | **29** | **1** | **MLYQAGVFASR + Oxidation (M)** |
|  | 1324 | **466.7833** | **931.5517** | **931.1509** | **0.4008** | **0** | **20** | **24** | **1** | **LLCGADVLK** |
|  | 635 | **401.6534** | **801.2920** | **800.9066** | **0.3853** | **2** | **20** | **25** | **1** | **KAEARAR** |
|  | 3038 | **753.5779** | **1505.1411** | **1504.8141** | **0.3270** | **1** | **20** | **25** | **1** | **NLDVMKEAMVQVK** |
|  | 222 | **378.0325** | **754.0503** | **752.8787** | **1.1716** | **0** | **20** | **24** | **1** | **MGPGFTK + Oxidation (M)** |
|  | 2715 | **684.0372** | **1366.0596** | **1365.6621** | **0.3975** | **1** | **20** | **24** | **1** | **VKLSCMANSVIK + Carbamidomethyl (C); Oxidation (M)** |
|  | 1703 | **532.7465** | **1595.2174** | **1595.8664** | **-0.6491** | **2** | **20** | **24** | **1** | **IDRATCPQHLKWK** |
|  | 2518 | **656.5076** | **1966.5005** | **1966.2043** | **0.2962** | **2** | **20** | **23** | **1** | **CNECGKVFRYNSYLGR + Carbamidomethyl (C)** |
|  | 2772 | **687.2924** | **1372.5701** | **1371.6252** | **0.9449** | **1** | **20** | **28** | **1** | **KAGSFEMIMLGR + 2 Oxidation (M)** |
|  | 42 | **364.1688** | **1089.4841** | **1090.2793** | **-0.7952** | **1** | **20** | **21** | **1** | **RHVISIHTK** |
|  | 2960 | **741.4691** | **2221.3850** | **2221.3638** | **0.0211** | **1** | **20** | **27** | **1** | **QSLSSATAQGQPMGREENWK + Oxidation (M)** |
|  | 1088 | **431.4907** | **860.9667** | **860.0732** | **0.8935** | **0** | **20** | **39** | **1** | **MLDAAVIK** |
|  | 801 | **406.8249** | **1217.4526** | **1218.3854** | **-0.9328** | **1** | **20** | **27** | **1** | **MRGFPLGGPDR + Oxidation (M)** |
|  | 2450 | **641.4265** | **1280.8382** | **1280.4334** | **0.4049** | **0** | **20** | **31** | **1** | **FQSNHMDMVR + Oxidation (M)** |
|  | 2707 | **683.8379** | **2048.4915** | **2049.1374** | **-0.6459** | **0** | **20** | **31** | **1** | **IQVSSGENMAGTAEGEGQQR** |
|  | 2840 | **709.7932** | **2126.3575** | **2125.3611** | **0.9964** | **1** | **20** | **34** | **1** | **EEKDPGMGAMGGMGGGMGGGMF + 4 Oxidation (M)** |
|  | 155 | **371.2776** | **1110.8108** | **1111.3381** | **-0.5273** | **1** | **20** | **18** | **1** | **QLLQAALRAK** |
|  | 1384 | **475.8855** | **949.7562** | **949.0007** | **0.7555** | **0** | **20** | **30** | **1** | **QTPGGSSCR + Carbamidomethyl (C)** |
|  | 766 | **406.0210** | **1215.0407** | **1215.5720** | **-0.5312** | **0** | **20** | **26** | **1** | **LVMALMPVGLR + Oxidation (M)** |
|  | 2092 | **595.2739** | **1188.5331** | **1189.4254** | **-0.8923** | **1** | **20** | **32** | **1** | **MLLVGKDGNVK + Oxidation (M)** |
|  | 478 | **389.1183** | **1164.3328** | **1163.3532** | **0.9796** | **2** | **20** | **32** | **1** | **GWLSRGMKGR + Oxidation (M)** |
|  | 393 | **387.8546** | **1160.5415** | **1159.3562** | **1.1852** | **0** | **20** | **35** | **1** | **LILSPDMQAR + Oxidation (M)** |
|  | 733 | **405.1439** | **1212.4094** | **1213.2613** | **-0.8519** | **2** | **20** | **27** | **1** | **GENGKNARDPR** |
|  | 1075 | **430.9833** | **1289.9278** | **1289.3588** | **0.5691** | **1** | **20** | **36** | **1** | **VWGSNWNDRR** |
|  | 1252 | **458.8615** | **1373.5623** | **1374.4567** | **-0.8944** | **1** | **20** | **32** | **1** | **WPSRDALSEWQ** |
|  | 1923 | **563.4113** | **1687.2116** | **1687.0151** | **0.1965** | **0** | **20** | **23** | **1** | **AQLIGPLVFGGMNLTR** |
|  | 1605 | **517.2627** | **1548.7659** | **1548.8515** | **-0.0856** | **1** | **20** | **32** | **1** | **YWVVWVRQMPGK** |
|  | 270 | **384.9659** | **767.9170** | **766.9086** | **1.0085** | **0** | **20** | **25** | **1** | **KPGMYR + Oxidation (M)** |
|  | 466 | **389.0303** | **1164.0686** | **1164.4159** | **-0.3473** | **1** | **20** | **33** | **1** | **TTMLKIATAAK + Oxidation (M)** |
|  | 1956 | **572.6496** | **1714.9266** | **1714.8152** | **0.1115** | **1** | **19** | **37** | **1** | **SQSMHASSNGGSHATKK** |
|  | 2356 | **625.9829** | **1249.9510** | **1249.4343** | **0.5168** | **1** | **19** | **27** | **1** | **ASLDKELMAQK + Oxidation (M)** |
|  | 1546 | **505.0957** | **1008.1766** | **1007.0979** | **1.0787** | **0** | **19** | **29** | **1** | **DLDFASLAR** |
|  | 446 | 388.9509 | 1163.8305 | 1164.2287 | -0.3981 | 2 | 19 | 35 | 1 | KNGAGPYSRNT |
|  | 149 | **371.1538** | **1110.4392** | **1110.3269** | **0.1122** | **1** | **19** | **23** | **1** | **SFGIRMILAS + Oxidation (M)** |
|  | 1270 | **459.9291** | **1376.7652** | **1376.6234** | **0.1419** | **1** | **19** | **33** | **1** | **DGWIGVRSVMLK + Oxidation (M)** |
|  | 69 | **368.1645** | **1101.4714** | **1101.1779** | **0.2935** | **2** | **19** | **29** | **1** | **GRDGRASAGVR** |
|  | 2087 | **595.0129** | **1188.0111** | **1188.3742** | **-0.3631** | **0** | **19** | **34** | **1** | **ILYSFATAFR** |
|  | 3083 | **760.0349** | **1518.0550** | **1518.6731** | **-0.6181** | **2** | **19** | **25** | **1** | **LRELFGKDEQQR** |
|  | 386 | **387.8275** | **773.6401** | **774.7336** | **-1.0934** | **0** | **19** | **37** | **1** | **DEGSDPR** |
|  | 1293 | **462.0461** | **1383.1161** | **1382.6311** | **0.4849** | **2** | **19** | **30** | **1** | **LVHIKGNCGKEK + Carbamidomethyl (C)** |
|  | 530 | **390.0456** | **778.0765** | **776.9018** | **1.1747** | **0** | **19** | **32** | **1** | **MLSSPAR + Oxidation (M)** |
|  | 232 | **379.4313** | **1135.2716** | **1134.3037** | **0.9679** | **0** | **19** | **40** | **1** | **AGQEMSLAALK + Oxidation (M)** |
|  | 506 | **389.9753** | **777.9358** | **777.9938** | **-0.0580** | **0** | **19** | **33** | **1** | **LLLLGLH** |
|  | 993 | **419.3142** | **1254.9204** | **1254.3082** | **0.6122** | **0** | **19** | **31** | **1** | **SGWSNAYAGSVR** |
|  | 3560 | **1051.4939** | **2100.9730** | **2100.4804** | **0.4926** | **0** | **19** | **26** | **1** | **NEGNKPLSLPTIFCNIVLK** |
|  | 1312 | **464.4246** | **1390.2516** | **1391.4095** | **-1.1579** | **1** | **19** | **26** | **1** | **HPGGDRTGNHTSR** |
|  | 1160 | **440.1661** | **1317.4762** | **1316.5863** | **0.8899** | **1** | **19** | **38** | **1** | **LLLVSQLSTSKK** |
|  | 2952 | **740.8099** | **2219.4076** | **2218.5006** | **0.9070** | **2** | **19** | **36** | **1** | **MAGHRIIQCSDTSCGGNGVRR** |
|  | 1690 | **528.6135** | **1055.2123** | **1054.1776** | **1.0346** | **0** | **19** | **38** | **1** | **EAYIAMGQR + Oxidation (M)** |
|  | 868 | **407.8496** | **1220.5267** | **1220.4031** | **0.1236** | **2** | **19** | **32** | **1** | **RRPSGSKSMAK + Oxidation (M)** |
|  | 372 | **387.7833** | **1160.3278** | **1160.1902** | **0.1376** | **0** | **19** | **39** | **1** | **DWYGVDYWG** |
|  | 427 | **388.4232** | **774.8315** | **773.8779** | **0.9537** | **1** | **19** | **45** | **1** | **NQKSGLK** |
|  | 734 | **405.1548** | **1212.4424** | **1211.3233** | **1.1190** | **0** | **19** | **30** | **1** | **TESCSVAQAGMQ** |
|  | 1221 | **452.3553** | **1354.0437** | **1354.3825** | **-0.3389** | **0** | **19** | **30** | **1** | **NHEISNDAGELR** |
|  | 962 | **416.1170** | **1245.3287** | **1245.3395** | **-0.0108** | **1** | **19** | **39** | **1** | **ELEEGKAGSGLR** |
|  | 549 | **391.1344** | **1170.3810** | **1169.3941** | **0.9869** | **1** | **19** | **31** | **1** | **SLILKCSSYR** |
|  | 2397 | **629.7968** | **1257.5787** | **1256.4317** | **1.1470** | **1** | **19** | **34** | **1** | **AQAMLPNRVDAA** |
|  | 1648 | **522.4944** | **1564.4610** | **1564.7466** | **-0.2856** | **2** | **19** | **30** | **1** | **AFTRSSTLFNHKR** |
|  | 460 | 389.0084 | 1164.0031 | 1163.3284 | 0.6747 | 1 | 19 | 37 | 1 | QLRFPPSYR |
|  | 2575 | **666.6906** | **1997.0495** | **1997.9864** | **-0.9369** | **2** | **19** | **35** | **1** | **SPELSSSRDRYNSDNDR** |
|  | 142 | **370.9766** | **739.9385** | **740.8911** | **-0.9526** | **0** | **19** | **25** | **1** | **SPLGVLR** |
|  | 1461 | **488.1241** | **974.2333** | **974.0748** | **0.1586** | **1** | **19** | **37** | **1** | **KFNSSHVR** |
|  | 2743 | **685.8046** | **2054.3917** | **2054.3443** | **0.0474** | **1** | **19** | **42** | **1** | **NDISQKAEILLSSSKPVPK** |
|  | 130 | **369.4250** | **1105.2530** | **1105.2892** | **-0.0362** | **1** | **19** | **42** | **1** | **CAEPMRTPK + Carbamidomethyl (C); Oxidation (M)** |
|  | 1965 | **574.5163** | **1147.0178** | **1146.2482** | **0.7696** | **0** | **19** | **29** | **1** | **IDVSIEAASGGK** |
|  | 3316 | **881.7417** | **1761.4686** | **1761.9064** | **-0.4377** | **0** | **19** | **28** | **1** | **SMAASGNLGHTPFVDEL + Oxidation (M)** |
|  | 640 | **401.8654** | **1202.5739** | **1201.4443** | **1.1296** | **2** | **19** | **39** | **1** | **MRLSKWQPR** |
|  | 2702 | **682.2006** | **1362.3863** | **1361.4975** | **0.8888** | **0** | **19** | **33** | **1** | **IGPLTGDAFDIWG** |
|  | 168 | **372.4760** | **742.9372** | **743.8950** | **-0.9578** | **2** | **19** | **41** | **1** | **KLERAK** |
|  | 601 | **399.2039** | **1194.5895** | **1194.3822** | **0.2073** | **0** | **19** | **26** | **1** | **MIDMLAANSGR + Oxidation (M)** |
|  | 391 | **387.8391** | **1160.4952** | **1160.3227** | **0.1724** | **1** | **19** | **41** | **1** | **LVIAGNPAYRS** |
|  | 1213 | **450.9770** | **899.9392** | **900.0328** | **-0.0936** | **1** | **19** | **37** | **1** | **KTCSSCSK + Carbamidomethyl (C)** |
|  | 2782 | **689.0237** | **1376.0326** | **1375.5292** | **0.5033** | **1** | **19** | **29** | **1** | **REIQGLFDELR** |
|  | 380 | **387.8109** | **1160.4105** | **1161.2200** | **-0.8095** | **1** | **19** | **41** | **1** | **TASEGSEQPKK** |
|  | 1729 | **534.5979** | **1067.1810** | **1068.1878** | **-1.0067** | **2** | **19** | **44** | **1** | **EPPQSARRK** |
|  | 144 | **370.9947** | **1109.9619** | **1109.2728** | **0.6891** | **0** | **19** | **26** | **1** | **TITSAATAFVK** |
|  | 730 | **405.1237** | **1212.3490** | **1213.3042** | **-0.9553** | **1** | **19** | **33** | **1** | **ERGVGAAINNGR** |
|  | 2030 | **587.5513** | **1759.6316** | **1758.7845** | **0.8471** | **0** | **19** | **31** | **1** | **CGAELGSPGGGGGGGGGGGAGGR + Carbamidomethyl (C)** |
|  | 1052 | **428.0620** | **1281.1640** | **1280.4285** | **0.7355** | **0** | **19** | **34** | **1** | **EMDAGGDMIAVR + Oxidation (M)** |
|  | 31 | **363.2043** | **724.3938** | **723.7961** | **0.5977** | **0** | **19** | **29** | **1** | **SSGNTMK** |
|  | 367 | **387.7608** | **773.5068** | **773.9440** | **-0.4373** | **1** | **19** | **42** | **1** | **LRGGMQI** |
|  | 2517 | **656.0742** | **1965.2005** | **1966.1232** | **-0.9227** | **2** | **19** | **34** | **1** | **GHQRASTLVGLGNSRADAR** |
|  | 1829 | **543.6044** | **1627.7909** | **1627.7347** | **0.0563** | **1** | **19** | **43** | **1** | **QEVDYMTQTRGQR + Oxidation (M)** |
|  | 2593 | **667.7711** | **2000.2912** | **2001.3531** | **-1.0619** | **2** | **19** | **44** | **1** | **VQWGAEVKKPGASVKVSCK** |
|  | 669 | **402.9763** | **1205.9068** | **1206.3266** | **-0.4199** | **0** | **19** | **40** | **1** | **MLSGIGAEGEAR + Oxidation (M)** |
|  | 210 | **377.0899** | **752.1651** | **752.7943** | **-0.6292** | **0** | **19** | **34** | **1** | **SCESATR** |
|  | 2388 | **628.7346** | **1255.4545** | **1255.5098** | **-0.0554** | **2** | **19** | **43** | **1** | **RALLEKLGSLR** |
|  | 513 | **389.9964** | **1166.9669** | **1166.4517** | **0.5153** | **1** | **19** | **36** | **1** | **LEMMIKELK + 2 Oxidation (M)** |
|  | 961 | **416.0820** | **830.1493** | **829.9875** | **0.1617** | **2** | **19** | **44** | **1** | **KKSAGLAR** |
|  | 1286 | **461.3436** | **1381.0087** | **1380.5676** | **0.4411** | **2** | **19** | **29** | **1** | **DLVKSKMSETSR** |
|  | 3124 | **776.8447** | **1551.6747** | **1550.6685** | **1.0061** | **0** | **19** | **40** | **1** | **DIPGYCSGGSCYSK + 2 Carbamidomethyl (C)** |
|  | 1148 | **437.1336** | **1308.3786** | **1308.4632** | **-0.0847** | **1** | **19** | **40** | **1** | **TYGPECQRNLK** |
|  | 1402 | **479.8682** | **957.7216** | **957.0791** | **0.6426** | **0** | **19** | **37** | **1** | **SLPSTPDIK** |
|  | 476 | **389.1088** | **1164.3043** | **1163.3018** | **1.0025** | **0** | **19** | **40** | **1** | **MGGSLEANQLK + Oxidation (M)** |
|  | 2149 | **602.1295** | **1202.2441** | **1201.3731** | **0.8710** | **1** | **19** | **37** | **1** | **LSVEIDTLRR** |
|  | 806 | **406.8872** | **1217.6394** | **1218.4236** | **-0.7841** | **1** | **19** | **32** | **1** | **SMQPGTSLAKAK** |
|  | 1054 | **428.2913** | **1281.8518** | **1281.5206** | **0.3311** | **1** | **19** | **25** | **1** | **LSDFGLCTGLKK** |
|  | 2784 | **690.2683** | **2067.7828** | **2067.2370** | **0.5457** | **1** | **19** | **36** | **1** | **EQIDNLAMRSFGENDALK + Oxidation (M)** |
|  | 59 | **366.1658** | **1095.4751** | **1096.3071** | **-0.8319** | **1** | **19** | **35** | **1** | **MNHQALVRK** |
|  | 840 | **407.5410** | **1219.6008** | **1220.3712** | **-0.7704** | **0** | **19** | **37** | **1** | **SLNESLNALFI** |
|  | 2637 | **669.8399** | **1337.6650** | **1336.5165** | **1.1486** | **1** | **19** | **39** | **1** | **EPNQKAFLMSR + Oxidation (M)** |
|  | 925 | **411.5096** | **1231.5066** | **1231.2780** | **0.2285** | **1** | **19** | **46** | **1** | **GAASWEGAGGRGR** |
|  | 2954 | **740.9341** | **1479.8534** | **1479.7002** | **0.1532** | **2** | **19** | **34** | **1** | **KQRVDMYLPADK + Oxidation (M)** |
|  | 1194 | **447.4684** | **892.9220** | **893.9868** | **-1.0647** | **1** | **19** | **43** | **1** | **GPKGEPGPR** |
|  | 94 | **369.2627** | **736.5106** | **735.9343** | **0.5763** | **0** | **19** | **32** | **1** | **FMITPK** |
|  | 1083 | **431.0809** | **860.1470** | **859.9655** | **0.1816** | **0** | **19** | **44** | **1** | **DWEALVK** |
|  | 1982 | **579.0157** | **1156.0167** | **1156.3326** | **-0.3159** | **0** | **19** | **37** | **1** | **GSMFDELMAR** |
|  | 440 | **388.8837** | **775.7526** | **774.8196** | **0.9330** | **0** | **19** | **42** | **1** | **IEADATR** |
|  | 791 | **406.6865** | **1217.0373** | **1217.2434** | **-0.2061** | **1** | **19** | **27** | **1** | **EGEKGVNNGGEK** |
|  | 589 | **398.3275** | **1191.9604** | **1192.2140** | **-0.2536** | **0** | **19** | **31** | **1** | **QNQDMTPSQGA + Oxidation (M)** |
|  | 2113 | **597.3948** | **1789.1623** | **1789.0659** | **0.0964** | **2** | **19** | **39** | **1** | **QLAVELQSRTTMQKR** |
|  | 2616 | 668.7710 | 1335.5272 | 1335.5514 | -0.0242 | 1 | 19 | 46 | 1 | KQQQELHLALK |
|  | 158 | **371.4249** | **740.8351** | **739.9495** | **0.8856** | **1** | **19** | **37** | **1** | **KPKVIR** |
|  | 2705 | **682.7872** | **1363.5597** | **1362.5338** | **1.0259** | **0** | **19** | **44** | **1** | **LCHMDTIDWR + Carbamidomethyl (C); Oxidation (M)** |
|  | 3311 | **878.5095** | **2632.5064** | **2632.0439** | **0.4624** | **1** | **19** | **35** | **1** | **LMEDYELRQLVLEVMHNPMDR** |
|  | 2998 | **742.8922** | **1483.7695** | **1484.6138** | **-0.8443** | **1** | **19** | **42** | **1** | **QLAAERAALEQER** |
|  | 1152 | **438.0811** | **874.1475** | **873.9557** | **0.1918** | **0** | **18** | **43** | **1** | **HVVYSGGR** |
|  | 1597 | **514.5692** | **1540.6855** | **1540.7202** | **-0.0348** | **1** | **18** | **43** | **1** | **ENEPSVTQLVRLR** |
|  | 1073 | **430.7315** | **1289.1724** | **1288.4041** | **0.7683** | **1** | **18** | **36** | **1** | **VELDEARLSEK** |
|  | 53 | **366.0306** | **730.0464** | **728.9200** | **1.1263** | **0** | **18** | **43** | **1** | **SLGLVLK** |
|  | 979 | **419.1354** | **1254.3840** | **1255.4638** | **-1.0798** | **2** | **18** | **44** | **1** | **KPEVKKVDAGGK** |
|  | 54 | **366.0377** | **1095.0910** | **1096.2575** | **-1.1665** | **2** | **18** | **44** | **1** | **WKSGKDMTK + Oxidation (M)** |
|  | 2609 | **668.6736** | **1335.3324** | **1335.3810** | **-0.0486** | **0** | **18** | **37** | **1** | **VYFQEQGHGDR** |
|  | 2048 | 592.6963 | 1183.3778 | 1183.2784 | 0.0994 | 1 | 18 | 46 | 1 | RRPNSGPAGGSK |
|  | 1449 | **487.9504** | **1460.8290** | **1460.6552** | **0.1737** | **1** | **18** | **43** | **1** | **DPCNSSLASLRLK + Carbamidomethyl (C)** |
|  | 1512 | **496.0519** | **1485.1335** | **1484.6087** | **0.5247** | **0** | **18** | **41** | **1** | **LHLLQWEEDSSK** |
|  | 2861 | **717.5287** | **2149.5639** | **2148.4806** | **1.0833** | **0** | **18** | **35** | **1** | **INVVAAMFSAPPFPAAVSSQK + Oxidation (M)** |
|  | 70 | **368.1782** | **1101.5125** | **1101.1713** | **0.3412** | **1** | **18** | **34** | **1** | **DQKGVPQSSR** |
|  | 1568 | **508.0754** | **1521.2041** | **1520.7735** | **0.4306** | **1** | **18** | **39** | **1** | **CLIQMGAAVEAKDR + Oxidation (M)** |
|  | 2175 | **606.6860** | **1211.3573** | **1211.3713** | **-0.0140** | **1** | **18** | **44** | **1** | **GVVRWEYFR** |
|  | 285 | **385.8907** | **769.7665** | **768.8613** | **0.9052** | **0** | **18** | **33** | **1** | **QLAGQPR** |
|  | 373 | **387.7919** | **1160.3536** | **1161.1801** | **-0.8265** | **1** | **18** | **46** | **1** | **EEGASDLRER** |
|  | 1552 | 505.5680 | 1513.6817 | 1512.6055 | 1.0762 | 0 | 18 | 44 | 1 | EGNCSGCIQDCNR + 2 Carbamidomethyl (C) |
|  | 1689 | **528.5646** | **1582.6716** | **1583.8063** | **-1.1347** | **0** | **18** | **44** | **1** | **VCPPLSHSESFGVPK** |
|  | 1294 | **462.1061** | **1383.2962** | **1382.3928** | **0.9035** | **1** | **18** | **38** | **1** | **DCEDGRDEFHC + Carbamidomethyl (C)** |
|  | 246 | **380.0609** | **1137.1607** | **1137.3590** | **-0.1983** | **0** | **18** | **46** | **1** | **NAMVCFHCR + Carbamidomethyl (C)** |
|  | 1169 | **442.4612** | **1324.3613** | **1325.3878** | **-1.0266** | **1** | **18** | **42** | **1** | **LHAASEAEERGR** |
|  | 628 | **401.0242** | **1200.0505** | **1201.2457** | **-1.1951** | **2** | **18** | **43** | **1** | **RKDDSYFDR** |
|  | 1175 | **443.7982** | **1328.3725** | **1328.5192** | **-0.1467** | **2** | **18** | **39** | **1** | **RHGKELINFSK** |
|  | 533 | **390.0602** | **1167.1584** | **1166.3507** | **0.8077** | **1** | **18** | **39** | **1** | **MAVSSPRYQK** |
|  | 1011 | **420.4980** | **1258.4719** | **1258.4080** | **0.0639** | **2** | **18** | **44** | **1** | **ARARGPCPTDSK** |
|  | 2692 | **680.5347** | **1359.0546** | **1358.4377** | **0.6169** | **0** | **18** | **33** | **1** | **AQEDMAAHVGASR + Oxidation (M)** |
|  | 622 | 400.7871 | 1199.3392 | 1199.3538 | -0.0146 | 0 | 18 | 41 | 1 | ALSQDLPLNTK |
|  | 754 | **405.7008** | **1214.0803** | **1214.2411** | **-0.1607** | **1** | **18** | **30** | **1** | **EFERYGGNDK** |
|  | 677 | **403.1075** | **1206.3003** | **1205.3454** | **0.9549** | **2** | **18** | **45** | **1** | **DEKMRQQVR + Oxidation (M)** |
|  | 876 | **407.9086** | **1220.7035** | **1221.3447** | **-0.6411** | **1** | **18** | **40** | **1** | **RDTISQMTGGR** |
|  | 1345 | **471.3320** | **1410.9738** | **1410.4874** | **0.4865** | **0** | **18** | **29** | **1** | **LHHVSPADSGEYV** |
|  | 1394 | **477.2927** | **952.5705** | **952.0229** | **0.5477** | **0** | **18** | **33** | **1** | **AEAGPSAPPR** |
|  | 1990 | **579.9418** | **1736.8033** | **1736.9648** | **-0.1615** | **1** | **18** | **40** | **1** | **VVYQPGYPKSIASTAR** |
|  | 183 | **374.1739** | **1119.4994** | **1119.2775** | **0.2220** | **2** | **18** | **44** | **1** | **RAAGFLRSNK** |
|  | 1408 | **481.0196** | **960.0245** | **960.0831** | **-0.0586** | **0** | **18** | **45** | **1** | **NVTIVESAK** |
|  | 1030 | **422.2391** | **842.4635** | **841.9321** | **0.5314** | **0** | **18** | **38** | **1** | **DCPSAPPR** |
|  | 908 | **409.1025** | **1224.2854** | **1224.3634** | **-0.0780** | **1** | **18** | **46** | **1** | **ESPGELISKHK** |
|  | 120 | **369.3462** | **1105.0165** | **1104.2879** | **0.7286** | **2** | **18** | **43** | **1** | **RSAVVMGRGR + Oxidation (M)** |
|  | 743 | **405.2685** | **808.5222** | **807.9638** | **0.5584** | **0** | **18** | **31** | **1** | **APRPCHK** |
|  | 153 | **371.2622** | **1110.7644** | **1110.3502** | **0.4142** | **2** | **18** | **27** | **1** | **KEPRGALLVK** |
|  | 171 | **372.6044** | **1114.7911** | **1114.2527** | **0.5384** | **1** | **18** | **37** | **1** | **KLLADQAEAR** |
|  | 2282 | **615.5327** | **1229.0505** | **1228.4848** | **0.5658** | **1** | **18** | **35** | **1** | **KCKPEEMHVK** |
|  | 874 | **407.8916** | **1220.6527** | **1221.4077** | **-0.7549** | **1** | **18** | **41** | **1** | **TMNPSEMQRK** |
|  | 1434 | **485.9110** | **1454.7107** | **1453.5751** | **1.1356** | **0** | **18** | **39** | **1** | **FSYSHMSASVPPQ + Oxidation (M)** |
|  | 3046 | **757.1948** | **2268.5623** | **2267.6061** | **0.9562** | **1** | **18** | **39** | **1** | **QCQCTSVGAQNTVICSKLAAK + 2 Carbamidomethyl (C)** |
|  | 257 | **384.4981** | **1150.4722** | **1151.2696** | **-0.7975** | **0** | **18** | **43** | **1** | **ALQATVGNSYK** |
|  | 84 | **369.1898** | **1104.5471** | **1104.2779** | **0.2692** | **1** | **18** | **38** | **1** | **NGKLMESTPK** |
|  | 121 | **369.3471** | **1105.0191** | **1104.2680** | **0.7511** | **2** | **18** | **44** | **1** | **HRSDRMMR + Oxidation (M)** |
|  | 472 | **389.0538** | **1164.1393** | **1165.2232** | **-1.0839** | **1** | **18** | **48** | **1** | **AGRAGAQAGGGHR** |
|  | 1223 | **452.6185** | **1354.8334** | **1355.6470** | **-0.8136** | **0** | **18** | **43** | **1** | **ALGIHLLWGTMK + Oxidation (M)** |
|  | 1062 | **429.1662** | **1284.4764** | **1283.4937** | **0.9828** | **0** | **18** | **43** | **1** | **MAEDPAALKPPK + Oxidation (M)** |
|  | 1214 | **451.8586** | **1352.5537** | **1351.4252** | **1.1285** | **2** | **18** | **50** | **1** | **AEFSANKDSARR** |
|  | 1198 | **448.5442** | **895.0737** | **894.0530** | **1.0208** | **1** | **18** | **48** | **1** | **GRFACGVGK** |
|  | 1228 | **453.8497** | **1358.5268** | **1358.5967** | **-0.0698** | **2** | **18** | **46** | **1** | **VQRRAAHSMMR + Oxidation (M)** |
|  | 631 | **401.3203** | **1200.9388** | **1200.3438** | **0.5950** | **0** | **18** | **38** | **1** | **SCAATSGLSTMR + Oxidation (M)** |
|  | 1211 | **450.6470** | **899.2793** | **899.0098** | **0.2694** | **1** | **18** | **38** | **1** | **RAAAAAGGVR** |
|  | 3214 | **815.8704** | **2444.5891** | **2444.6587** | **-0.0696** | **2** | **18** | **47** | **1** | **SCPPSPDLCSHSPSSGSRWKNSK** |
|  | 1261 | **459.2028** | **1374.5861** | **1374.4935** | **0.0926** | **2** | **18** | **48** | **1** | **EAEKKLPSDTEK** |
|  | 1206 | **449.9811** | **897.9474** | **898.0466** | **-0.0992** | **1** | **18** | **41** | **1** | **GCRCSGCR + Carbamidomethyl (C)** |
|  | 1967 | **574.7371** | **1147.4593** | **1148.4859** | **-1.0266** | **1** | **18** | **46** | **1** | **LMIMLRSIR + Oxidation (M)** |
|  | 2908 | **738.2826** | **2211.8256** | **2210.6608** | **1.1648** | **0** | **18** | **43** | **1** | **MALAGLAMGCIDTVANMQLVR + 2 Oxidation (M)** |
|  | 362 | **387.7374** | **1160.1901** | **1159.2339** | **0.9563** | **1** | **18** | **50** | **1** | **HERAMQSER + Oxidation (M)** |
|  | 1336 | **468.2715** | **1401.7924** | **1400.6069** | **1.1855** | **2** | **18** | **42** | **1** | **NPRVERPCKSSK** |
|  | 2085 | **594.9252** | **1781.7533** | **1780.9361** | **0.8172** | **1** | **18** | **39** | **1** | **SHSSGSVLPLGELEGRR** |
|  | 2178 | **606.7291** | **1817.1650** | **1817.2629** | **-0.0979** | **1** | **18** | **49** | **1** | **MLLYLMGLSRLISYK + Oxidation (M)** |
|  | 795 | **406.7663** | **1217.2768** | **1216.3383** | **0.9386** | **1** | **18** | **41** | **1** | **SPEEKLTPTSK** |
|  | 1105 | **433.0862** | **1296.2366** | **1296.5220** | **-0.2855** | **2** | **18** | **43** | **1** | **IRKQQQVAGIR** |
|  | 1663 | **523.6954** | **1045.3761** | **1046.1291** | **-0.7531** | **0** | **18** | **45** | **1** | **LSSPADITDK** |
|  | 207 | **377.0659** | **1128.1754** | **1129.0952** | **-0.9198** | **0** | **18** | **42** | **1** | **GSGSSQSSGYGR** |
|  | 602 | **399.2258** | **796.4369** | **795.8852** | **0.5517** | **0** | **18** | **31** | **1** | **QACMDR + Carbamidomethyl (C); Oxidation (M)** |
|  | 2205 | **608.7596** | **1215.5045** | **1216.4970** | **-0.9925** | **1** | **18** | **50** | **1** | **IAASMRALVLR + Oxidation (M)** |
|  | 693 | **403.9856** | **1208.9347** | **1209.3322** | **-0.3975** | **0** | **18** | **45** | **1** | **GAEAFQTCVQR** |
|  | 627 | **401.0116** | **800.0083** | **800.9032** | **-0.8949** | **0** | **18** | **47** | **1** | **AVATNGLR** |
|  | 520 | **390.0168** | **1167.0283** | **1168.1711** | **-1.1428** | **1** | **18** | **45** | **1** | **EASGTQSERFG** |
|  | 951 | **415.8921** | **829.7694** | **828.8737** | **0.8957** | **1** | **18** | **52** | **1** | **KSAGHSSR** |
|  | 1329 | **467.5925** | **1399.7554** | **1399.5524** | **0.2030** | **0** | **18** | **53** | **1** | **NVFPPPSQTWAR** |
|  | 1661 | **523.3379** | **1566.9915** | **1565.9169** | **1.0746** | **0** | **18** | **42** | **1** | **LMDLMLVCTNVPK + Carbamidomethyl (C); 2 Oxidation (M)** |
|  | 2577 | **666.7202** | **1997.1385** | **1998.2975** | **-1.1590** | **2** | **18** | **52** | **1** | **CGKAFMHNFQLQKHHR + Oxidation (M)** |
|  | 3186 | **803.8853** | **2408.6336** | **2408.6595** | **-0.0259** | **0** | **18** | **52** | **1** | **FIMQQNLGEEEIEQMLVNDQ** |
|  | 1632 | **520.4576** | **1558.3508** | **1558.7668** | **-0.4160** | **2** | **18** | **35** | **1** | **GGRGARIVWVCDGR + Carbamidomethyl (C)** |
|  | 1888 | **555.7860** | **1664.3359** | **1663.7070** | **0.6288** | **0** | **18** | **36** | **1** | **EAAANTNRPSPGGHER** |
|  | 18 | **363.0793** | **1086.2157** | **1085.2546** | **0.9611** | **0** | **18** | **45** | **1** | **EMGNSLGCFK** |
|  | 2027 | **587.2775** | **1758.8104** | **1757.9395** | **0.8710** | **1** | **18** | **48** | **1** | **LEEITVSRPDSKEVR** |
|  | 329 | **386.1474** | **1155.4201** | **1154.3199** | **1.1002** | **2** | **18** | **41** | **1** | **AQKGQKAPAQK** |
|  | 1431 | **485.2801** | **968.5454** | **968.1482** | **0.3972** | **2** | **18** | **39** | **1** | **IPKEPKEK** |
|  | 1507 | **494.7513** | **987.4877** | **987.1844** | **0.3033** | **2** | **18** | **39** | **1** | **RLVCGARR + Carbamidomethyl (C)** |
|  | 1454 | **488.0587** | **974.1026** | **975.2070** | **-1.1044** | **2** | **18** | **51** | **1** | **KCIEKVAK + Carbamidomethyl (C)** |
|  | 904 | **409.0041** | **1223.9902** | **1224.3766** | **-0.3864** | **2** | **18** | **51** | **1** | **CSWRRCSGR + 2 Carbamidomethyl (C)** |
|  | 114 | **369.3286** | **1104.9635** | **1104.2680** | **0.6955** | **2** | **18** | **43** | **1** | **HRSDRMMR + Oxidation (M)** |
|  | 600 | **399.1687** | **1194.4839** | **1195.4185** | **-0.9345** | **2** | **18** | **39** | **1** | **VRAAATVPRVR** |
|  | 1867 | **550.6114** | **1648.8122** | **1647.7392** | **1.0730** | **1** | **18** | **56** | **1** | **YQEGVDDPDPAKWK** |
|  | 862 | **407.8362** | **1220.4864** | **1221.3678** | **-0.8814** | **1** | **18** | **47** | **1** | **QSCATGPRNCK + Carbamidomethyl (C)** |
|  | 1404 | **480.1422** | **958.2695** | **959.1017** | **-0.8321** | **2** | **18** | **48** | **1** | **SEAVAKKAR** |
|  | 1559 | **507.0167** | **1518.0280** | **1517.6835** | **0.3445** | **2** | **18** | **43** | **1** | **LSTDLKQKVSNER** |
|  | 1761 | **537.4595** | **1609.3564** | **1609.9943** | **-0.6378** | **2** | **18** | **42** | **1** | **LPVTKMKYSGNLMK** |
|  | 625 | **400.9844** | **799.9541** | **800.9032** | **-0.9492** | **0** | **18** | **48** | **1** | **AVATNGLR** |
|  | 426 | 388.4026 | 1162.1856 | 1162.4214 | -0.2358 | 2 | 18 | 62 | 1 | LKEYLQIKK |
|  | 421 | **388.2192** | **774.4236** | **774.8627** | **-0.4390** | **0** | **18** | **44** | **1** | **INSTVNK** |
|  | 2170 | **606.4327** | **1816.2760** | **1815.8096** | **0.4665** | **1** | **18** | **39** | **1** | **KEPGGGGGGGGGGGGGGGGVSSEK** |
|  | 1043 | **425.0971** | **1272.2691** | **1271.4216** | **0.8476** | **0** | **18** | **50** | **1** | **GPITAVAFAPDGR** |
|  | 2290 | **616.9935** | **1847.9584** | **1847.1481** | **0.8103** | **1** | **18** | **46** | **1** | **CSGTISAHCKLCLPGSR + 2 Carbamidomethyl (C)** |
|  | 2737 | **685.2548** | **1368.4949** | **1367.5572** | **0.9377** | **2** | **18** | **49** | **1** | **TAAPSVRPEKRR** |
|  | 1253 | **458.8677** | **1373.5809** | **1373.5764** | **0.0044** | **0** | **18** | **51** | **1** | **THTGEKPYLCPK** |
|  | 1989 | **579.8367** | **1736.4878** | **1735.9569** | **0.5310** | **1** | **18** | **40** | **1** | **KLNPPDESGPGCMSCK + Carbamidomethyl (C); Oxidation (M)** |
|  | 2083 | **594.7949** | **1781.3624** | **1782.0069** | **-0.6445** | **2** | **18** | **43** | **1** | **ELEGILLPSDRDRLR** |
|  | 2378 | **628.3073** | **1254.5997** | **1254.4125** | **0.1872** | **0** | **18** | **47** | **1** | **INMLTAGYAER + Oxidation (M)** |
|  | 1268 | **459.8799** | **1376.6175** | **1375.4432** | **1.1743** | **0** | **18** | **51** | **1** | **GYQGNSGAPGSPGVK** |
|  | 1972 | **575.7300** | **1149.4453** | **1148.2923** | **1.1530** | **1** | **18** | **50** | **1** | **ENKMTWAPR + Oxidation (M)** |
|  | 1502 | **494.1488** | **1479.4242** | **1478.5680** | **0.8563** | **1** | **18** | **49** | **1** | **MSAQGSKDEGHCR + Carbamidomethyl (C); Oxidation (M)** |
|  | 1551 | **505.4044** | **1008.7941** | **1008.2332** | **0.5608** | **0** | **18** | **36** | **1** | **WCFLDVLL** |
|  | 1048 | **427.7037** | **1280.0888** | **1280.4749** | **-0.3860** | **1** | **18** | **39** | **1** | **MSACASAVREAGK** |
|  | 1455 | **488.0829** | **1461.2264** | **1461.7443** | **-0.5179** | **1** | **18** | **53** | **1** | **KGSLAALYDLAVLK** |
|  | 626 | **400.9867** | **799.9585** | **798.8907** | **1.0679** | **1** | **17** | **50** | **1** | **RGLGGSPR** |
|  | 3105 | **766.4969** | **1530.9791** | **1530.7269** | **0.2522** | **1** | **17** | **45** | **1** | **DQQLHIEKAPPVR** |
|  | 650 | **401.9989** | **1202.9745** | **1202.3645** | **0.6101** | **1** | **17** | **56** | **1** | **HGKSCMNPQK + Carbamidomethyl (C); Oxidation (M)** |
|  | 1608 | **517.5073** | **1549.4998** | **1548.7606** | **0.7392** | **1** | **17** | **50** | **1** | **ITESPRASMGVLSGK + Oxidation (M)** |
|  | 1938 | **567.6577** | **1133.3005** | **1132.1356** | **1.1650** | **0** | **17** | **59** | **1** | **SPLSQGDSSAPS** |
|  | 586 | **397.0988** | **792.1829** | **793.0103** | **-0.8274** | **0** | **17** | **52** | **1** | **ALAIMCR + Oxidation (M)** |
|  | 624 | **400.9838** | **799.9529** | **800.9032** | **-0.9504** | **0** | **17** | **50** | **1** | **AVATNGLR** |
|  | 662 | **402.3710** | **1204.0907** | **1205.2838** | **-1.1931** | **1** | **17** | **52** | **1** | **DAGHGQISHKR** |
|  | 1071 | **430.0712** | **858.1276** | **857.0062** | **1.1213** | **0** | **17** | **55** | **1** | **QVLADIAK** |
|  | 1543 | **505.0343** | **1512.0807** | **1512.7399** | **-0.6591** | **1** | **17** | **46** | **1** | **RCFPHVHAVSFR + Carbamidomethyl (C)** |
|  | 484 | **389.1883** | **776.3618** | **775.9418** | **0.4200** | **2** | **17** | **47** | **1** | **RGRFLK** |
|  | 1668 | **524.2277** | **1046.4405** | **1046.0497** | **0.3909** | **1** | **17** | **52** | **1** | **NSPSQKDDR** |
|  | 1718 | **533.6576** | **1597.9506** | **1598.8723** | **-0.9217** | **2** | **17** | **54** | **1** | **MHKSRMYSQCVR + Carbamidomethyl (C); Oxidation (M)** |
|  | 610 | **400.0540** | **798.0933** | **796.9577** | **1.1357** | **1** | **17** | **41** | **1** | **KGLVPQR** |
|  | 2714 | **684.0363** | **1366.0577** | **1365.6025** | **0.4552** | **2** | **17** | **42** | **1** | **HNKMMKDCSAGK + Oxidation (M)** |
|  | 2719 | **684.0948** | **1366.1749** | **1365.6621** | **0.5129** | **2** | **17** | **47** | **1** | **GMLEKIDMIRK + 2 Oxidation (M)** |
|  | 111 | **369.3259** | **1104.9554** | **1104.3277** | **0.6278** | **2** | **17** | **46** | **1** | **RVAAMSVAKR + Oxidation (M)** |
|  | 401 | **387.8971** | **773.7794** | **772.9760** | **0.8035** | **2** | **17** | **59** | **1** | **IIGSKKK** |
|  | 1121 | **434.9247** | **867.8345** | **868.9805** | **-1.1459** | **0** | **17** | **42** | **1** | **KPGGNIQR** |
|  | 1100 | **433.0276** | **1296.0605** | **1295.4168** | **0.6437** | **0** | **17** | **49** | **1** | **MSSSATAVETPAK + Oxidation (M)** |
|  | 1988 | **579.7856** | **1736.3348** | **1737.0559** | **-0.7211** | **2** | **17** | **45** | **1** | **KMPNVFQNLVSCKR + Carbamidomethyl (C); Oxidation (M)** |
|  | 2731 | **685.0253** | **1368.0358** | **1367.5572** | **0.4786** | **2** | **17** | **44** | **1** | **TAAPSVRPEKRR** |
|  | 3293 | **862.8241** | **2585.4501** | **2584.9276** | **0.5226** | **2** | **17** | **38** | **1** | **NNFLFGSRCWMTRFSAENIFK + Oxidation (M)** |
|  | 20 | **363.1073** | **1086.2997** | **1085.2563** | **1.0435** | **1** | **17** | **49** | **1** | **PRAAGIPEFK** |
|  | 1166 | **442.1993** | **1323.5757** | **1323.4748** | **0.1009** | **2** | **17** | **46** | **1** | **KAKMTDSDHFK + Oxidation (M)** |
|  | 1610 | **517.5922** | **1033.1697** | **1032.1307** | **1.0390** | **0** | **17** | **63** | **1** | **AFSSCQSFR** |
|  | 632 | **401.3460** | **800.6773** | **800.9034** | **-0.2261** | **1** | **17** | **46** | **1** | **RELEVR** |
|  | 1429 | **484.3304** | **1449.9692** | **1450.6686** | **-0.6995** | **2** | **17** | **38** | **1** | **EKHNALRCLPGR + Carbamidomethyl (C)** |
|  | 1452 | **488.0069** | **1460.9985** | **1460.6340** | **0.3645** | **0** | **17** | **56** | **1** | **EMVGGCCVCSDER + Carbamidomethyl (C); Oxidation (M)** |
|  | 2580 | **666.8323** | **1997.4747** | **1998.3470** | **-0.8724** | **0** | **17** | **52** | **1** | **MLENLALISSLGCWCGSK + Carbamidomethyl (C); Oxidation (M)** |
|  | 1081 | **431.0464** | **860.0780** | **860.0732** | **0.0048** | **0** | **17** | **61** | **1** | **MLDAAVIK** |
|  | 2648 | **670.8441** | **1339.6735** | **1338.5589** | **1.1146** | **2** | **17** | **50** | **1** | **VRLPRSAGDLVR** |
|  | 124 | **369.3713** | **1105.0917** | **1105.3089** | **-0.2171** | **0** | **17** | **58** | **1** | **AKPWAVCFPS** |
|  | 1063 | **429.1691** | **1284.4852** | **1285.4864** | **-1.0011** | **1** | **17** | **50** | **1** | **TKEGSIVDPLVK** |
|  | 757 | **405.8481** | **1214.5223** | **1213.4268** | **1.0954** | **1** | **17** | **48** | **1** | **KEFSACAIGCK + Carbamidomethyl (C)** |
|  | 2001 | **582.2113** | **1743.6117** | **1744.0202** | **-0.4085** | **0** | **17** | **53** | **1** | **AEPLGLLAVMEWTAAR + Oxidation (M)** |
|  | 104 | **369.2957** | **1104.8650** | **1105.3089** | **-0.4439** | **0** | **17** | **45** | **1** | **AKPWAVCFPS** |
|  | 1550 | **505.2578** | **1512.7512** | **1513.6353** | **-0.8841** | **1** | **17** | **48** | **1** | **RMEAGEAAPPAGAGGR + Oxidation (M)** |
|  | 3188 | **804.3769** | **2410.1085** | **2410.6307** | **-0.5222** | **2** | **17** | **49** | **1** | **ALNGEQAAGHARQFHAMATRTR + Oxidation (M)** |
|  | 88 | **369.2118** | **1104.6131** | **1105.2626** | **-0.6494** | **0** | **17** | **44** | **1** | **SNSMLELAPK + Oxidation (M)** |
|  | 748 | **405.5073** | **808.9999** | **808.8756** | **0.1242** | **0** | **17** | **55** | **1** | **DEAALYK** |
|  | 7 | **360.4879** | **718.9610** | **717.7715** | **1.1894** | **0** | **17** | **61** | **1** | **AALSSNR** |
|  | 125 | **369.3735** | **1105.0984** | **1105.3089** | **-0.2104** | **0** | **17** | **60** | **1** | **AKPWAVCFPS** |
|  | 665 | **402.6670** | **1204.9788** | **1205.3418** | **-0.3630** | **0** | **17** | **46** | **1** | **YFNPCYATAR** |
|  | 1298 | **462.9849** | **1385.9325** | **1386.5802** | **-0.6476** | **1** | **17** | **46** | **1** | **QLASGVAAPGMRGR + Oxidation (M)** |
|  | 1848 | **548.6631** | **1095.3114** | **1094.3491** | **0.9623** | **1** | **17** | **57** | **1** | **SSAIACAKLCK** |
|  | 112 | **369.3270** | **1104.9587** | **1105.3089** | **-0.3501** | **0** | **17** | **48** | **1** | **AKPWAVCFPS** |
|  | 468 | **389.0411** | **1164.1012** | **1164.3778** | **-0.2765** | **0** | **17** | **56** | **1** | **TFQCEMCFR** |
|  | 2075 | **593.7396** | **1778.1967** | **1777.9507** | **0.2460** | **2** | **17** | **58** | **1** | **DSVPNKAGGMKDVQTSK + Oxidation (M)** |
|  | 2972 | **741.8337** | **1481.6526** | **1481.6231** | **0.0294** | **2** | **17** | **57** | **1** | **AGAGAGAGARRPRGTR** |
|  | 1049 | **427.7771** | **853.5394** | **852.9149** | **0.6246** | **0** | **17** | **50** | **1** | **SGSSCAWR** |
|  | 2905 | **737.5638** | **1473.1129** | **1472.7721** | **0.3408** | **1** | **17** | **45** | **1** | **VVLSGHPFKIFTK** |
|  | 924 | **411.4805** | **1231.4194** | **1231.5050** | **-0.0856** | **1** | **17** | **65** | **1** | **LIIAMLEREK + Oxidation (M)** |
|  | 2330 | **623.7875** | **1868.3403** | **1868.1643** | **0.1760** | **1** | **17** | **53** | **1** | **WVGVAMASRVLSAYVSR + Oxidation (M)** |
|  | 298 | **385.9954** | **769.9761** | **769.8662** | **0.1099** | **0** | **17** | **44** | **1** | **EYGVMR + Oxidation (M)** |
|  | 2934 | **740.5563** | **2218.6466** | **2218.5156** | **0.1311** | **2** | **17** | **46** | **1** | **ISGGNDKCGFPMKQGVLTHGR + Oxidation (M)** |
|  | 302 | **386.0165** | **1155.0273** | **1155.3013** | **-0.2741** | **0** | **17** | **44** | **1** | **GEACDIPYCK + Carbamidomethyl (C)** |
|  | 357 | **387.6927** | **1160.0559** | **1159.2901** | **0.7657** | **1** | **17** | **52** | **1** | **GAVTGGEEAKLK** |
|  | 418 | **388.1745** | **1161.5014** | **1161.2910** | **0.2105** | **0** | **17** | **57** | **1** | **CFDLVTHNR + Carbamidomethyl (C)** |
|  | 741 | **405.2274** | **1212.6601** | **1213.4285** | **-0.7684** | **2** | **17** | **40** | **1** | **LFDHANKLKK** |
|  | 2280 | **614.9486** | **1227.8824** | **1227.3672** | **0.5152** | **0** | **17** | **44** | **1** | **QLVEADINGLR** |
|  | 797 | **406.7822** | **1217.3243** | **1216.2536** | **1.0708** | **0** | **17** | **50** | **1** | **DGAYSLFDNSK** |
|  | 83 | **369.1897** | **1104.5469** | **1104.3011** | **0.2458** | **0** | **17** | **46** | **1** | **MHPSLATMGK + 2 Oxidation (M)** |
|  | 90 | **369.2374** | **1104.6899** | **1105.3752** | **-0.6852** | **1** | **17** | **46** | **1** | **IRGGMLAMPK + 2 Oxidation (M)** |
|  | 1558 | 506.8081 | 1517.4021 | 1517.6403 | -0.2382 | 1 | 17 | 42 | 1 | ENLYFQGSREFK |
|  | 3106 | **767.4083** | **2299.2026** | **2299.6812** | **-0.4786** | **0** | **17** | **51** | **1** | **MIIDASGESGLTQLLMTEVMK + 2 Oxidation (M)** |
|  | 148 | **371.0818** | **1110.2232** | **1110.2839** | **-0.0607** | **0** | **17** | **44** | **1** | **NSGIAGGMFLK + Oxidation (M)** |
|  | 1056 | **428.9162** | **1283.7264** | **1284.4866** | **-0.7601** | **1** | **17** | **48** | **1** | **MEPRAPWIER** |
|  | 1986 | **579.5967** | **1735.7679** | **1735.0600** | **0.7079** | **1** | **17** | **59** | **1** | **FCRMDVAVLSYCVR + Carbamidomethyl (C); Oxidation (M)** |
|  | 3315 | **880.5610** | **1759.1073** | **1758.9059** | **0.2014** | **0** | **17** | **51** | **1** | **ESEMNAIAADMCTNAR + 2 Oxidation (M)** |
|  | 505 | **389.9751** | **777.9354** | **777.8435** | **0.0919** | **0** | **17** | **54** | **1** | **CGVEADK + Carbamidomethyl (C)** |
|  | 1450 | **487.9636** | **973.9125** | **974.1095** | **-0.1971** | **1** | **17** | **59** | **1** | **AKLDELSAK** |
|  | 2620 | **668.8605** | **2003.5592** | **2004.1960** | **-0.6368** | **2** | **17** | **50** | **1** | **HTAFQNQGNHPCKHKTR** |
|  | 434 | **388.7977** | **1163.3708** | **1164.2650** | **-0.8942** | **0** | **17** | **61** | **1** | **LAQENSDLFK** |
|  | 369 | **387.7754** | **1160.3040** | **1159.3995** | **0.9045** | **1** | **17** | **63** | **1** | **MKATQVLPGSK** |
|  | 940 | **413.4051** | **1237.1932** | **1236.2880** | **0.9052** | **0** | **17** | **47** | **1** | **GPLGSYADEATR** |
|  | 964 | **416.2123** | **1245.6147** | **1245.3661** | **0.2486** | **1** | **17** | **57** | **1** | **KSQAAPGSSPCR + Carbamidomethyl (C)** |
|  | 1203 | **449.7256** | **897.4365** | **897.1150** | **0.3215** | **1** | **17** | **42** | **1** | **MQKMLSK + 2 Oxidation (M)** |
|  | 1933 | **565.3564** | **1693.0472** | **1692.7052** | **0.3420** | **2** | **17** | **55** | **1** | **DGNNSEASGPYRRGGR** |
|  | 96 | **369.2630** | **1104.7669** | **1105.3089** | **-0.5419** | **0** | **17** | **46** | **1** | **AKPWAVCFPS** |
|  | 462 | **389.0118** | **776.0088** | **775.9168** | **0.0919** | **0** | **17** | **61** | **1** | **LSGLCAR + Carbamidomethyl (C)** |
|  | 1878 | **552.2043** | **1653.5909** | **1654.7797** | **-1.1889** | **1** | **17** | **59** | **1** | **FSASRSGNTASLTISR** |
|  | 954 | **415.9655** | **1244.8743** | **1244.3549** | **0.5194** | **1** | **17** | **63** | **1** | **EKSRPDLGGASK** |
|  | 2623 | **669.0095** | **2004.0064** | **2003.3011** | **0.7053** | **2** | **17** | **46** | **1** | **LPRLSCEPVMEEKAQEK + Oxidation (M)** |
|  | 2722 | **684.1202** | **1366.2257** | **1366.6549** | **-0.4292** | **2** | **17** | **53** | **1** | **IAGIRGIQGVVRK** |
|  | 2795 | **694.8540** | **2081.5398** | **2080.4084** | **1.1315** | **1** | **17** | **58** | **1** | **SQRMVITAPPVTNQPVTPK + Oxidation (M)** |
|  | 2017 | **584.3863** | **1750.1367** | **1750.9101** | **-0.7734** | **1** | **17** | **50** | **1** | **WVSENIAFFGGDPRR** |
|  | 474 | **389.0680** | **776.1212** | **776.9231** | **-0.8019** | **0** | **17** | **61** | **1** | **ALSALFR** |
|  | 2217 | **609.9524** | **1826.8350** | **1828.0076** | **-1.1725** | **0** | **17** | **48** | **1** | **SSDSEMAIFGEAAPFLR** |
|  | 623 | **400.9597** | **799.9046** | **800.9032** | **-0.9987** | **0** | **17** | **57** | **1** | **AVATNGLR** |
|  | 914 | **410.2293** | **1227.6658** | **1228.2197** | **-0.5539** | **0** | **17** | **50** | **1** | **EDNSYNVTSSL** |
|  | 2557 | **666.2054** | **1995.5942** | **1996.2189** | **-0.6247** | **0** | **17** | **53** | **1** | **FYLPPTPGSEFIGDVTQK** |
|  | 2959 | **741.4082** | **2221.2024** | **2220.5362** | **0.6662** | **2** | **17** | **52** | **1** | **NQLFHAVQRLQRVQNQLK** |
|  | 537 | **390.0758** | **1167.2052** | **1167.3900** | **-0.1848** | **2** | **17** | **54** | **1** | **RCMARSAGCR + Carbamidomethyl (C)** |
|  | 363 | **387.7375** | **1160.1903** | **1161.3291** | **-1.1389** | **0** | **17** | **63** | **1** | **LEAGAMVLADR + Oxidation (M)** |
|  | 508 | **389.9883** | **1166.9427** | **1166.4119** | **0.5308** | **0** | **17** | **55** | **1** | **MVNILMANTK + 2 Oxidation (M)** |
|  | 310 | 386.0516 | 770.0884 | 770.7879 | -0.6996 | 0 | 17 | 49 | 1 | QHTEEK |
|  | 1027 | **422.1798** | **1263.5173** | **1263.3198** | **0.1974** | **0** | **17** | **59** | **1** | **DHLQQQGGQPR** |
|  | 1310 | **464.2026** | **1389.5856** | **1390.6267** | **-1.0412** | **1** | **17** | **54** | **1** | **VYGKSENILVLR** |
|  | 3345 | **904.7629** | **1807.5111** | **1807.9979** | **-0.4868** | **0** | **17** | **44** | **1** | **SEINLPGNCVAMDWDK + Oxidation (M)** |
|  | 1414 | **481.7882** | **1442.3425** | **1441.5692** | **0.7732** | **0** | **17** | **47** | **1** | **CTPASGGTSLHTPR + Carbamidomethyl (C)** |
|  | 1099 | **433.0257** | **864.0366** | **864.9901** | **-0.9535** | **0** | **17** | **56** | **1** | **CCEAGLR + 2 Carbamidomethyl (C)** |
|  | 1997 | **582.0272** | **1743.0595** | **1742.0292** | **1.0303** | **2** | **17** | **57** | **1** | **SAMEALKQKSLYNCR** |
|  | 2144 | **601.0214** | **1200.0279** | **1199.3621** | **0.6658** | **1** | **17** | **56** | **1** | **RALSWAAATPR** |
|  | 512 | **389.9950** | **1166.9629** | **1166.3507** | **0.6122** | **1** | **17** | **56** | **1** | **MSKEGVFVNR** |
|  | 1060 | **429.1371** | **856.2594** | **856.1274** | **0.1319** | **0** | **17** | **56** | **1** | **CLIVALPK** |
|  | 431 | **388.5939** | **1162.7595** | **1162.2047** | **0.5549** | **0** | **17** | **53** | **1** | **DSEDSQAVIAK** |
|  | 1701 | **532.6022** | **1594.7843** | **1593.8886** | **0.8957** | **1** | **17** | **70** | **1** | **DLLEPGCSILLRHK** |
|  | 2611 | **668.6768** | **2003.0081** | **2002.2997** | **0.7084** | **2** | **17** | **56** | **1** | **ATQEAFMKRAMANCQAAK + 2 Oxidation (M)** |
|  | 2697 | **680.8649** | **2039.5726** | **2040.3657** | **-0.7931** | **2** | **17** | **55** | **1** | **QIWVRGLAGVENVTELKK** |
|  | 398 | **387.8758** | **773.7368** | **772.8932** | **0.8436** | **2** | **17** | **67** | **1** | **KRGDGLK** |
|  | 2979 | **742.2332** | **2223.6773** | **2224.6676** | **-0.9903** | **2** | **17** | **52** | **1** | **MSTNICSFKDRCVSILCCK + Carbamidomethyl (C); Oxidation (M)** |
|  | 1695 | **530.3717** | **1588.0929** | **1588.8291** | **-0.7362** | **1** | **17** | **53** | **1** | **RLQCPFEQELLR + Carbamidomethyl (C)** |
|  | 4 | **360.4670** | **1078.3788** | **1077.2308** | **1.1479** | **0** | **17** | **75** | **1** | **GNVFLLDATK** |
|  | 2508 | **653.4629** | **1304.9110** | **1304.5375** | **0.3735** | **2** | **17** | **54** | **1** | **YGKELVQIARK** |
|  | 598 | **399.1356** | **1194.3847** | **1193.3526** | **1.0320** | **1** | **17** | **50** | **1** | **QDGPLASKLHK** |
|  | 942 | **413.6658** | **1237.9753** | **1238.4131** | **-0.4378** | **1** | **17** | **38** | **1** | **LDFQSCKEIR** |
|  | 430 | **388.5677** | **1162.6808** | **1163.2357** | **-0.5550** | **0** | **17** | **56** | **1** | **CTDCDYTTNK** |
|  | 551 | **391.1438** | **780.2728** | **779.8163** | **0.4565** | **0** | **17** | **53** | **1** | **MGEGGEGK + Oxidation (M)** |
|  | 916 | **410.2643** | **818.5138** | **817.8941** | **0.6197** | **2** | **17** | **50** | **1** | **KSRNASR** |
|  | 1042 | **424.8954** | **1271.6640** | **1270.5642** | **1.0997** | **0** | **17** | **61** | **1** | **PGITGTIHLCMK** |
|  | 2253 | **612.1843** | **1833.5308** | **1833.0522** | **0.4786** | **1** | **17** | **57** | **1** | **AAAAAETPEVLRECGCK + 2 Carbamidomethyl (C)** |
|  | 2755 | **686.1259** | **2055.3556** | **2054.2879** | **1.0676** | **1** | **17** | **55** | **1** | **SAYAAPKHATQAFFDCLR + Carbamidomethyl (C)** |
|  | 920 | **411.1346** | **1230.3815** | **1230.4194** | **-0.0379** | **1** | **17** | **60** | **1** | **MCGECEACRR + Carbamidomethyl (C); Oxidation (M)** |
|  | 1273 | **460.1896** | **1377.5467** | **1377.6129** | **-0.0662** | **0** | **17** | **62** | **1** | **GCQLCVQPACGK + 3 Carbamidomethyl (C)** |
|  | 2126 | **599.4495** | **1196.8841** | **1197.3813** | **-0.4971** | **0** | **17** | **45** | **1** | **EKPQTLPSAVK** |
|  | 1132 | **435.8249** | **1304.4524** | **1305.6134** | **-1.1610** | **2** | **17** | **55** | **1** | **MERLRGMTLAK** |
|  | 173 | **373.1189** | **744.2231** | **743.8089** | **0.4142** | **0** | **17** | **69** | **1** | **GEGINVR** |
|  | 912 | **409.1713** | **1224.4916** | **1225.3548** | **-0.8632** | **0** | **17** | **60** | **1** | **ASDTAMYHCAR** |
|  | 1180 | **444.0322** | **886.0497** | **884.8905** | **1.1592** | **0** | **17** | **62** | **1** | **GGVPGGDGAGAA** |
|  | 1762 | **537.5457** | **1609.6150** | **1610.7074** | **-1.0924** | **1** | **17** | **66** | **1** | **GRSSSMGLQEYHSR + Oxidation (M)** |
|  | 1535 | **504.1938** | **1006.3728** | **1006.0753** | **0.2975** | **2** | **17** | **61** | **1** | **RTSSAGSRGK** |
|  | 346 | **386.4576** | **770.9003** | **769.8496** | **1.0508** | **1** | **17** | **66** | **1** | **SARSPPR** |
|  | 501 | **389.9599** | **777.9050** | **777.9079** | **-0.0029** | **0** | **17** | **58** | **1** | **SINGFLK** |
|  | 676 | **403.0641** | **1206.1700** | **1206.3085** | **-0.1385** | **1** | **17** | **64** | **1** | **KRPSEGNYQK** |
|  | 902 | **408.9187** | **1223.7340** | **1223.4035** | **0.3305** | **0** | **17** | **63** | **1** | **QCVAACCSEPR + Carbamidomethyl (C)** |
|  | 3277 | **847.9343** | **1693.8537** | **1694.9960** | **-1.1422** | **2** | **17** | **65** | **1** | **LAGQGNMAPKKSLHVK + Oxidation (M)** |
|  | 538 | **390.0796** | **778.1444** | **777.8898** | **0.2546** | **0** | **17** | **58** | **1** | **GTGMQIR + Oxidation (M)** |
|  | 1845 | **547.4301** | **1639.2682** | **1639.7632** | **-0.4951** | **0** | **17** | **46** | **1** | **GLDWLAVISHDGDNK** |
|  | 2927 | **740.4467** | **2218.3178** | **2217.5837** | **0.7341** | **0** | **17** | **57** | **1** | **MAGEQKPSSNLLEQFILLAK** |
|  | 1110 | **433.2004** | **1296.5790** | **1297.4574** | **-0.8784** | **0** | **17** | **56** | **1** | **TCDPVEMSYPR** |
|  | 1347 | **471.5743** | **941.1338** | **941.0632** | **0.0706** | **0** | **17** | **62** | **1** | **GFMVSTGSR** |
|  | 2717 | **684.0815** | **2049.2223** | **2050.3425** | **-1.1202** | **1** | **17** | **56** | **1** | **SRNPLMVNSNYYMMAAR + 2 Oxidation (M)** |
|  | 682 | **403.7824** | **1208.3251** | **1209.3787** | **-1.0536** | **1** | **17** | **60** | **1** | **RQMEAPGAPPR** |
|  | 2944 | **740.6428** | **2218.9063** | **2219.4953** | **-0.5891** | **0** | **17** | **45** | **1** | **YLRPSCTGYIDHDISMFK + Carbamidomethyl (C); Oxidation (M)** |
|  | 1346 | **471.5308** | **1411.5703** | **1410.6017** | **0.9686** | **1** | **17** | **64** | **1** | **RQDAPKPTPAACR** |
|  | 179 | **374.1140** | **1119.3199** | **1119.2279** | **0.0920** | **1** | **17** | **69** | **1** | **DAKHFISSSK** |
|  | 336 | **386.2232** | **1155.6474** | **1156.4153** | **-0.7680** | **2** | **17** | **42** | **1** | **KSLLPTLEKK** |
|  | 770 | **406.1577** | **1215.4509** | **1216.4357** | **-0.9849** | **1** | **17** | **56** | **1** | **IRHLVHSVQK** |
|  | 2576 | **666.7202** | **1997.1385** | **1997.3427** | **-0.2042** | **0** | **17** | **68** | **1** | **MGLNPPGLTSALKPQMEGR** |
|  | 1453 | **488.0199** | **1461.0375** | **1460.6422** | **0.3954** | **1** | **17** | **66** | **1** | **GRMASGCARPPEGR + Oxidation (M)** |
|  | 182 | **374.1730** | **746.3312** | **746.8094** | **-0.4782** | **0** | **17** | **62** | **1** | **SLDGLSR** |
|  | 1706 | **532.8011** | **1063.5875** | **1064.1525** | **-0.5650** | **0** | **17** | **49** | **1** | **QFIAAQGSSR** |
|  | 2858 | **715.5181** | **2143.5320** | **2143.3976** | **0.1344** | **1** | **17** | **58** | **1** | **SLMDLANDACQLLSGERYK + Oxidation (M)** |
|  | 543 | **391.0915** | **780.1682** | **781.0627** | **-0.8945** | **0** | **17** | **56** | **1** | **QMIMMK** |
|  | 1458 | **488.1017** | **1461.2829** | **1460.7034** | **0.5795** | **2** | **17** | **66** | **1** | **WMTSLAPNRRTK** |
|  | 1903 | **558.3075** | **1671.9003** | **1672.9308** | **-1.0305** | **2** | **17** | **61** | **1** | **RVAACQADRHSLMAK + Oxidation (M)** |
|  | 2266 | **613.6045** | **1837.7913** | **1837.1882** | **0.6031** | **0** | **17** | **53** | **1** | **VLDVLDLCVVVLQSHK + Carbamidomethyl (C)** |
|  | 731 | **405.1300** | **1212.3679** | **1213.4071** | **-1.0391** | **0** | **17** | **55** | **1** | **QPAMAPGVTGLR + Oxidation (M)** |
|  | 762 | **405.9346** | **1214.7815** | **1214.3968** | **0.3848** | **1** | **17** | **54** | **1** | **GTMGNGMCSRK + Carbamidomethyl (C); Oxidation (M)** |
|  | 834 | **407.4363** | **1219.2867** | **1219.4082** | **-0.1216** | **1** | **17** | **67** | **1** | **GCDVLAKSLEK + Carbamidomethyl (C)** |
|  | 1536 | **504.5302** | **1007.0457** | **1007.2321** | **-0.1864** | **1** | **17** | **69** | **1** | **VAKLCTCR + 2 Carbamidomethyl (C)** |
|  | 1611 | **518.3368** | **1551.9882** | **1550.7995** | **1.1887** | **0** | **17** | **53** | **1** | **ALGEMLAVSGCVGATR + Oxidation (M)** |
|  | 2284 | **615.7771** | **1844.3091** | **1843.1975** | **1.1116** | **1** | **17** | **64** | **1** | **FSLGAVGGLSLHVCIKGK + Carbamidomethyl (C)** |
|  | 201 | **375.9642** | **749.9136** | **748.8486** | **1.0650** | **0** | **17** | **59** | **1** | **MAAANGSK** |
|  | 1112 | **434.0698** | **1299.1873** | **1299.4828** | **-0.2955** | **2** | **17** | **55** | **1** | **SIRGNLNTRLR** |
|  | 278 | **385.1261** | **1152.3562** | **1152.3867** | **-0.0305** | **0** | **17** | **52** | **1** | **VGILSGLGALPR** |
|  | 3379 | **925.7992** | **1849.5836** | **1849.1575** | **0.4261** | **0** | **17** | **48** | **1** | **MCSGPTLLVQESLDCPR** |
|  | 3472 | **956.9178** | **2867.7314** | **2868.1617** | **-0.4303** | **2** | **17** | **42** | **1** | **DTQDLNLTLQQADLRDIYRTLHPK** |
|  | 39 | **363.4071** | **1087.1990** | **1086.2856** | **0.9134** | **1** | **16** | **69** | **1** | **KSTALALGGLR** |
|  | 1710 | **532.8785** | **1595.6134** | **1594.6350** | **0.9784** | **0** | **16** | **56** | **1** | **QPQSQSGTSDQVLYG** |
|  | 3178 | **801.1481** | **2400.4220** | **2400.0432** | **0.3788** | **1** | **16** | **47** | **1** | **PLFLKLFFGLLNMLMFIWR** |
|  | 2120 | **598.1266** | **1791.3576** | **1792.0864** | **-0.7289** | **0** | **16** | **58** | **1** | **MAIVEAASCGLQVVSTR + Carbamidomethyl (C)** |
|  | 2989 | **742.6122** | **2224.8144** | **2223.6778** | **1.1365** | **2** | **16** | **46** | **1** | **RTVYTMVKYFSILIFLGR + Oxidation (M)** |
|  | 1031 | **422.2769** | **1263.8084** | **1264.2137** | **-0.4053** | **0** | **16** | **53** | **1** | **ENSAGGGGDSAQSK** |
|  | 318 | **386.1004** | **1155.2790** | **1156.3294** | **-1.0503** | **1** | **16** | **55** | **1** | **VTDPVGALKEK** |
|  | 637 | **401.7609** | **801.5071** | **800.9065** | **0.6005** | **1** | **16** | **68** | **1** | **QALSRAR** |
|  | 990 | **419.2999** | **836.5850** | **836.0353** | **0.5497** | **0** | **16** | **58** | **1** | **SCMKPVR + Oxidation (M)** |
|  | 1617 | **518.8754** | **1035.7360** | **1035.3069** | **0.4291** | **2** | **16** | **55** | **1** | **FAKRMAALK** |
|  | 1667 | **524.1602** | **1569.4583** | **1568.8163** | **0.6420** | **0** | **16** | **64** | **1** | **AEFIQAAAAACLSMR + Oxidation (M)** |
|  | 1241 | **458.1924** | **1371.5552** | **1370.6803** | **0.8749** | **1** | **16** | **62** | **1** | **MVKISMDIFVR + 2 Oxidation (M)** |
|  | 1246 | **458.7662** | **1373.2763** | **1373.5763** | **-0.3000** | **0** | **16** | **55** | **1** | **EPLVWLCAASER** |
|  | 1350 | **472.1327** | **1413.3759** | **1412.5246** | **0.8513** | **0** | **16** | **58** | **1** | **SSSNPASLCLYDR** |
|  | 348 | **386.8706** | **771.7265** | **772.8568** | **-1.1303** | **2** | **16** | **66** | **1** | **GAGRTRR** |
|  | 2650 | **671.1421** | **2010.4041** | **2009.3253** | **1.0788** | **1** | **16** | **57** | **1** | **SMTVEVSGKWIPASLAIGF + Oxidation (M)** |
|  | 1335 | **468.1028** | **1401.2864** | **1400.4048** | **0.8815** | **1** | **16** | **65** | **1** | **KHSDLEEEEER** |
|  | 1874 | **551.8378** | **1101.6609** | **1101.2985** | **0.3623** | **0** | **16** | **52** | **1** | **GGLPITQLFR** |
|  | 2000 | **582.1828** | **1743.5262** | **1742.9046** | **0.6216** | **1** | **16** | **64** | **1** | **HINESLKELNCEEK + Carbamidomethyl (C)** |
|  | 2968 | **741.7723** | **2222.2948** | **2221.4299** | **0.8649** | **1** | **16** | **60** | **1** | **KDELQYCALSNGHACLENR + Carbamidomethyl (C)** |
|  | 1183 | **445.0556** | **888.0964** | **888.8393** | **-0.7428** | **0** | **16** | **73** | **1** | **DGNGGGGGGGGK** |
|  | 615 | **400.3156** | **1197.9247** | **1198.3278** | **-0.4031** | **1** | **16** | **44** | **1** | **GIVDSKYFNR** |
|  | 1045 | **425.7159** | **849.4170** | **849.0324** | **0.3846** | **0** | **16** | **53** | **1** | **CMCMSSR + 2 Oxidation (M)** |
|  | 2218 | **610.0143** | **1827.0209** | **1828.1370** | **-1.1161** | **1** | **16** | **62** | **1** | **TVPGSTMKMGSLEMQSK + Oxidation (M)** |
|  | 217 | **377.3104** | **752.6061** | **751.8755** | **0.7305** | **0** | **16** | **48** | **1** | **SCLCNR + Carbamidomethyl (C)** |
|  | 839 | **407.5107** | **1219.5099** | **1220.3995** | **-0.8897** | **1** | **16** | **70** | **1** | **CSNTSGLAARIK** |
|  | 3229 | **819.7943** | **1637.5737** | **1636.8637** | **0.7100** | **0** | **16** | **49** | **1** | **NFEALQIDDIMAIK + Oxidation (M)** |
|  | 49 | **365.4404** | **728.8661** | **729.8286** | **-0.9626** | **1** | **16** | **78** | **1** | **GTALRGR** |
|  | 1235 | **456.2933** | **1365.8578** | **1366.6914** | **-0.8336** | **2** | **16** | **47** | **1** | **KAPLGKLTVAQIK** |
|  | 1271 | **460.0659** | **918.1171** | **917.9603** | **0.1568** | **1** | **16** | **69** | **1** | **KDPGSTEGK** |
|  | 64 | **367.2463** | **1098.7168** | **1098.3577** | **0.3592** | **0** | **16** | **55** | **1** | **CIQMAISLM + Carbamidomethyl (C); 2 Oxidation (M)** |
|  | 236 | **379.6263** | **1135.8567** | **1136.3012** | **-0.4446** | **0** | **16** | **54** | **1** | **IQQQILEHK** |
|  | 1017 | **421.6047** | **841.1946** | **842.0216** | **-0.8270** | **1** | **16** | **54** | **1** | **MPPAVRR + Oxidation (M)** |
|  | 1057 | **428.9315** | **855.8482** | **855.9321** | **-0.0839** | **0** | **16** | **56** | **1** | **ASSLSTYK** |
|  | 375 | **387.8007** | **1160.3800** | **1161.2928** | **-0.9128** | **1** | **16** | **75** | **1** | **ARVPGGCTSGTR** |
|  | 454 | **388.9850** | **775.9551** | **775.8508** | **0.1044** | **0** | **16** | **71** | **1** | **SGLVSTGR** |
|  | 1157 | **439.9898** | **1316.9473** | **1317.5149** | **-0.5675** | **1** | **16** | **70** | **1** | **VGVNTSPSCRLK + Carbamidomethyl (C)** |
|  | 1422 | **483.9401** | **1448.7980** | **1448.5883** | **0.2097** | **1** | **16** | **64** | **1** | **CSVARSAGSCHGTR + Carbamidomethyl (C)** |
|  | 2234 | **610.3475** | **1218.6802** | **1218.4749** | **0.2053** | **2** | **16** | **64** | **1** | **KPKQVFCRR + Carbamidomethyl (C)** |
|  | 2820 | **701.1216** | **1400.2284** | **1399.7412** | **0.4872** | **1** | **16** | **60** | **1** | **GELLLKMNKPLK + Oxidation (M)** |
|  | 963 | **416.2109** | **1245.6105** | **1244.5040** | **1.1066** | **0** | **16** | **67** | **1** | **VLVSGASMVLPR + Oxidation (M)** |
|  | 81 | **369.1873** | **1104.5399** | **1104.2680** | **0.2719** | **2** | **16** | **56** | **1** | **HRSDRMMR + Oxidation (M)** |
|  | 1499 | **492.4811** | **982.9474** | **982.0918** | **0.8556** | **1** | **16** | **58** | **1** | **SKLQTYSR** |
|  | 2510 | **653.8712** | **1305.7277** | **1305.3699** | **0.3578** | **0** | **16** | **53** | **1** | **NFLSYDCGSDK + Carbamidomethyl (C)** |
|  | 2271 | **614.0770** | **1839.2089** | **1839.0632** | **0.1457** | **2** | **16** | **61** | **1** | **LGRDAEAAHRAGLAFGVK** |
|  | 2565 | **666.5194** | **1996.5361** | **1997.3661** | **-0.8300** | **2** | **16** | **52** | **1** | **TKVVMKGQNVSMFCSHK + Carbamidomethyl (C); Oxidation (M)** |
|  | 3371 | **922.6005** | **1843.1861** | **1842.0988** | **1.0873** | **1** | **16** | **57** | **1** | **ESELKPLVGESRSIAVK** |
|  | 3405 | **940.6204** | **2818.8389** | **2819.5547** | **-0.7158** | **0** | **16** | **56** | **1** | **PMANLCLLIVPILIAMAFLMLTEQK + 2 Oxidation (M)** |
|  | 843 | **407.6267** | **1219.8580** | **1220.5670** | **-0.7089** | **0** | **16** | **51** | **1** | **MMYFVIAAMK + Oxidation (M)** |
|  | 295 | **385.9901** | **769.9654** | **769.9507** | **0.0148** | **0** | **16** | **54** | **1** | **SMMSPMA + Oxidation (M)** |
|  | 612 | **400.2582** | **1197.7525** | **1197.3912** | **0.3613** | **2** | **16** | **44** | **1** | **RVGSSRALVPR** |
|  | 2270 | **613.9712** | **1838.8914** | **1838.0073** | **0.8841** | **0** | **16** | **57** | **1** | **MDGYSCDGVQGICFGGR + Carbamidomethyl (C); Oxidation (M)** |
|  | 922 | **411.2107** | **1230.6099** | **1230.4194** | **0.1905** | **1** | **16** | **59** | **1** | **MCGECEACRR + Carbamidomethyl (C); Oxidation (M)** |
|  | 493 | **389.8957** | **777.7765** | **777.8881** | **-0.1115** | **0** | **16** | **63** | **1** | **MPGSWGK + Oxidation (M)** |
|  | 1028 | **422.1833** | **1263.5277** | **1262.4332** | **1.0945** | **1** | **16** | **68** | **1** | **ETSPRLGVMEK + Oxidation (M)** |
|  | 1264 | **459.2799** | **1374.8175** | **1374.5231** | **0.2945** | **0** | **16** | **60** | **1** | **GAVPAAGSSALNGMR + Oxidation (M)** |
|  | 652 | **402.0384** | **802.0621** | **802.9159** | **-0.8538** | **1** | **16** | **74** | **1** | **VDGKGSIK** |
|  | 1634 | **521.3867** | **1040.7587** | **1040.1974** | **0.5612** | **0** | **16** | **48** | **1** | **LCVAGEVHR + Carbamidomethyl (C)** |
|  | 3054 | **758.0875** | **2271.2404** | **2270.6760** | **0.5644** | **2** | **16** | **49** | **1** | **ELLLKPHSYGRFIRWLNK** |
|  | 161 | **372.2289** | **742.4429** | **742.9302** | **-0.4872** | **1** | **16** | **49** | **1** | **KMLSHK** |
|  | 2432 | **637.6953** | **1910.0638** | **1909.9722** | **0.0915** | **1** | **16** | **79** | **1** | **HEHSPDQAEPIHSRNR** |
|  | 1509 | **495.8355** | **1484.4843** | **1484.6536** | **-0.1694** | **1** | **16** | **61** | **1** | **CYKCGSTDHEITK** |
|  | 1865 | 550.2676 | 1098.5205 | 1098.2966 | 0.2239 | 0 | 16 | 66 | 1 | TLKPKPATSR |
|  | 1111 | **433.2570** | **1296.7487** | **1297.4574** | **-0.7087** | **0** | **16** | **53** | **1** | **TCDPVEMSYPR** |
|  | 1696 | **530.6273** | **1588.8598** | **1588.7299** | **0.1298** | **2** | **16** | **87** | **1** | **ADALRVHQETRHR** |
|  | 1826 | **543.0867** | **1084.1586** | **1083.2057** | **0.9529** | **2** | **16** | **62** | **1** | **RRAAGGGSPVR** |
|  | 1513 | **496.2560** | **990.4973** | **990.2065** | **0.2908** | **2** | **16** | **68** | **1** | **AHKLPLRR** |
|  | 2894 | **730.5331** | **2188.5773** | **2189.5189** | **-0.9417** | **0** | **16** | **62** | **1** | **SHAPWSIMCVTAHGLSHLNK** |
|  | 3040 | **754.1167** | **2259.3279** | **2260.4215** | **-1.0936** | **1** | **16** | **59** | **1** | **QAAAREAEGTAGAWGVFPEVSR** |
|  | 3205 | **809.8101** | **2426.4080** | **2426.6401** | **-0.2321** | **2** | **16** | **56** | **1** | **EFEELHRRLDEEQQVLLSR** |
|  | 1244 | **458.7478** | **915.4809** | **915.0458** | **0.4351** | **1** | **16** | **58** | **1** | **LESRVSPK** |
|  | 1420 | **483.5461** | **965.0773** | **966.1354** | **-1.0581** | **0** | **16** | **81** | **1** | **LLVTIHDR** |
|  | 1557 | **506.7836** | **1517.3285** | **1516.6870** | **0.6416** | **1** | **16** | **51** | **1** | **SCAGNRPRLSGWR + Carbamidomethyl (C)** |
|  | 56 | **366.1210** | **1095.3408** | **1096.2608** | **-0.9199** | **1** | **16** | **71** | **1** | **FKTCTGGAVR + Carbamidomethyl (C)** |
|  | 663 | **402.4116** | **1204.2125** | **1205.3023** | **-1.0898** | **1** | **16** | **86** | **1** | **RCNVSDEVQR** |
|  | 10 | **362.0841** | **1083.2302** | **1082.2160** | **1.0143** | **1** | **16** | **62** | **1** | **VGMCSGSGRR + Carbamidomethyl (C); Oxidation (M)** |
|  | 1395 | **477.8373** | **1430.4897** | **1430.6893** | **-0.1996** | **2** | **16** | **59** | **1** | **KEEMVSAAFMKK + 2 Oxidation (M)** |
|  | 3459 | **951.6359** | **2851.8854** | **2852.2269** | **-0.3415** | **2** | **16** | **58** | **1** | **SHALEELTSANDGRTLSPGILGRLCLK** |
|  | 150 | **371.1613** | **1110.4616** | **1109.2794** | **1.1821** | **2** | **16** | **48** | **1** | **EEAIKHVRK** |
|  | 1763 | **537.5886** | **1073.1625** | **1072.2193** | **0.9431** | **1** | **16** | **85** | **1** | **AARASLGLASR** |
|  | 3327 | 896.1948 | 1790.3749 | 1790.0275 | 0.3474 | 1 | 16 | 50 | 1 | ESYLPVPGRFLTSAPR |
|  | 896 | **408.1983** | **1221.5726** | **1221.3428** | **0.2298** | **1** | **16** | **64** | **1** | **ENRCYISHLS** |
|  | 883 | **407.9840** | **813.9532** | **814.8437** | **-0.8904** | **0** | **16** | **67** | **1** | **QNSPAGNK** |
|  | 1724 | **534.0093** | **1066.0038** | **1067.1599** | **-1.1561** | **1** | **16** | **72** | **1** | **SPAPQRQQR** |
|  | 319 | **386.1054** | **1155.2941** | **1155.4571** | **-0.1630** | **2** | **16** | **61** | **1** | **GPLVKMGGRIK** |
|  | 1009 | **420.4411** | **1258.3010** | **1257.4629** | **0.8382** | **1** | **16** | **71** | **1** | **VKCSGPGLGAGVR + Carbamidomethyl (C)** |
|  | 435 | **388.7981** | **775.5814** | **774.8197** | **0.7618** | **0** | **16** | **76** | **1** | **TSPDSLR** |
|  | 470 | **389.0509** | **776.0870** | **775.9169** | **0.1702** | **1** | **16** | **74** | **1** | **NCLNKK + Carbamidomethyl (C)** |
|  | 1296 | **462.1442** | **1383.4104** | **1382.5418** | **0.8687** | **1** | **16** | **64** | **1** | **GKMELGGDSGFLR + Oxidation (M)** |
|  | 2189 | **607.8416** | **1820.5025** | **1820.9837** | **-0.4812** | **0** | **16** | **55** | **1** | **MATRPPDRPEGPHTSR + Oxidation (M)** |
|  | 445 | **388.9400** | **1163.7979** | **1164.3562** | **-0.5582** | **0** | **16** | **77** | **1** | **AFFALVANGVR** |
|  | 1015 | **421.1758** | **1260.5052** | **1259.4092** | **1.0959** | **2** | **16** | **67** | **1** | **AAEISKQEQKK** |
|  | 2865 | **719.1765** | **2154.5074** | **2155.5210** | **-1.0136** | **1** | **16** | **67** | **1** | **MVGPGTGIAPFIGFLQHREK** |
|  | 698 | **404.0286** | **1209.0637** | **1209.2758** | **-0.2121** | **2** | **16** | **68** | **1** | **GIRDGAHGRDR** |
|  | 2904 | **735.3424** | **1468.6700** | **1467.6908** | **0.9792** | **0** | **16** | **65** | **1** | **AMTYNQWLLVGR + Oxidation (M)** |
|  | 923 | **411.3380** | **1230.9919** | **1231.4453** | **-0.4534** | **0** | **16** | **58** | **1** | **IFSMSWCWR + Oxidation (M)** |
|  | 1381 | **475.7817** | **1424.3229** | **1423.6218** | **0.7011** | **1** | **16** | **59** | **1** | **RAPGQGKPWTGLR** |
|  | 2276 | **614.3825** | **1840.1253** | **1840.1292** | **-0.0039** | **1** | **16** | **66** | **1** | **TALATSPITVGDLGIVRR** |
|  | 1961 | **573.3916** | **1717.1526** | **1718.0874** | **-0.9348** | **1** | **16** | **62** | **1** | **VATDPKEVLLLMSACK** |
|  | 2874 | **720.2444** | **2157.7112** | **2157.4245** | **0.2867** | **2** | **16** | **65** | **1** | **VFTPERGLEVPKSDTAPWK** |
|  | 790 | **406.6721** | **811.3293** | **811.8829** | **-0.5535** | **0** | **16** | **50** | **1** | **GSLPPGER** |
|  | 1719 | **533.7123** | **1598.1147** | **1597.7285** | **0.3862** | **0** | **16** | **61** | **1** | **MGFESLSHCPGSSSR + Oxidation (M)** |
|  | 1163 | **440.8965** | **879.7782** | **878.9753** | **0.8028** | **1** | **16** | **72** | **1** | **LGQYRSR** |
|  | 2035 | **589.5541** | **1177.0934** | **1176.1916** | **0.9018** | **1** | **16** | **63** | **1** | **DRGETGVGEEK** |
|  | 3370 | **921.5032** | **1840.9916** | **1840.1509** | **0.8407** | **2** | **16** | **61** | **1** | **LCPSMGLKAETRDSMGK + Oxidation (M)** |
|  | 370 | **387.7777** | **1160.3110** | **1159.2902** | **1.0208** | **0** | **16** | **81** | **1** | **VYCNMTEDK + Carbamidomethyl (C)** |
|  | 648 | **401.9517** | **801.8885** | **802.8762** | **-0.9876** | **1** | **16** | **79** | **1** | **KSQGEVR** |
|  | 1134 | **435.8781** | **869.7414** | **870.8839** | **-1.1424** | **0** | **16** | **65** | **1** | **SCPEHPSD** |
|  | 2101 | **596.0956** | **1190.1764** | **1189.4254** | **0.7510** | **1** | **16** | **71** | **1** | **MLLVGKDGNVK + Oxidation (M)** |
|  | 1272 | **460.1643** | **918.3138** | **918.0082** | **0.3057** | **0** | **16** | **75** | **1** | **NSAVSWVR** |
|  | 1040 | 424.4781 | 1270.4120 | 1270.4153 | -0.0033 | 0 | 16 | 91 | 1 | GPLGSMPGPAAGSR + Oxidation (M) |
|  | 2910 | **738.3357** | **2211.9849** | **2211.5000** | **0.4850** | **2** | **16** | **69** | **1** | **LQPNPTCCSMLKSASSSRER + Oxidation (M)** |
|  | 128 | **369.3871** | **1105.1390** | **1104.2680** | **0.8710** | **2** | **16** | **84** | **1** | **HRSDRMMR + Oxidation (M)** |
|  | 1410 | **481.1075** | **960.2003** | **959.1642** | **1.0361** | **0** | **16** | **74** | **1** | **IIAQCLNK + Carbamidomethyl (C)** |
|  | 1844 | **547.4050** | **1639.1929** | **1639.6839** | **-0.4909** | **0** | **16** | **55** | **1** | **EGVCCGGGGSGSAGGGGSAR + Carbamidomethyl (C)** |
|  | 2034 | **588.5848** | **1175.1549** | **1174.4751** | **0.6798** | **1** | **16** | **70** | **1** | **INLLVKSFIK** |
|  | 2974 | **741.8884** | **1481.7621** | **1480.5987** | **1.1634** | **1** | **16** | **74** | **1** | **AYRTYDPFDMW + Oxidation (M)** |
|  | 1079 | **431.0295** | **860.0442** | **859.0949** | **0.9493** | **2** | **16** | **85** | **1** | **ACLRIRK** |
|  | 1192 | **447.2808** | **892.5469** | **892.1182** | **0.4287** | **0** | **16** | **60** | **1** | **AMIFPWK** |
|  | 796 | **406.7672** | **811.5195** | **811.8151** | **-0.2956** | **0** | **16** | **65** | **1** | **MDSGSGDK + Oxidation (M)** |
|  | 1817 | **542.2870** | **1623.8388** | **1624.8199** | **-0.9812** | **1** | **16** | **69** | **1** | **TGNNGCLVSPHIREK** |
|  | 497 | **389.9182** | **777.8215** | **776.9018** | **0.9198** | **1** | **16** | **70** | **1** | **CEGVGGKK** |
|  | 516 | **390.0074** | **778.0000** | **776.8470** | **1.1529** | **1** | **16** | **69** | **1** | **RHHTAR** |
|  | 1392 | **476.3120** | **950.6092** | **951.1194** | **-0.5101** | **0** | **16** | **56** | **1** | **MTPEQMAK + Oxidation (M)** |
|  | 3255 | **835.4497** | **1668.8846** | **1669.9021** | **-1.0174** | **1** | **16** | **65** | **1** | **NVAGRSIPAPFEHMK + Oxidation (M)** |
|  | 2645 | **670.6886** | **1339.3624** | **1339.5634** | **-0.2010** | **1** | **16** | **70** | **1** | **ELCLVRAHDVK + Carbamidomethyl (C)** |
|  | 349 | **387.2842** | **772.5536** | **772.8932** | **-0.3396** | **1** | **16** | **66** | **1** | **IVRGSNK** |
|  | 405 | **387.9167** | **1160.7281** | **1161.2863** | **-0.5582** | **0** | **16** | **86** | **1** | **MVASSPPSVDR + Oxidation (M)** |
|  | 1647 | **522.1213** | **1042.2279** | **1042.1420** | **0.0858** | **1** | **16** | **70** | **1** | **RIDFDLYT** |
|  | 344 | **386.3076** | **1155.9007** | **1155.3032** | **0.5975** | **1** | **16** | **50** | **1** | **KDSVFASFVR** |
|  | 2079 | **594.1920** | **1779.5539** | **1779.9529** | **-0.3991** | **2** | **16** | **76** | **1** | **GAKGLADPSHTQAGWRK** |
|  | 3166 | **793.9691** | **1585.9235** | **1585.7790** | **0.1445** | **1** | **16** | **70** | **1** | **ECGKTFSWGSSLVK + Carbamidomethyl (C)** |
|  | 830 | 407.3971 | 1219.1691 | 1219.3022 | -0.1331 | 1 | 16 | 67 | 1 | QKSEGSGGTQLK |
|  | 1122 | **435.0851** | **868.1555** | **869.0866** | **-0.9311** | **0** | **16** | **64** | **1** | **KPPAGMLR** |
|  | 1058 | **429.0270** | **1284.0589** | **1284.5264** | **-0.4674** | **1** | **16** | **66** | **1** | **IKVDCNTCTCK + Carbamidomethyl (C)** |
|  | 2760 | **686.2001** | **1370.3855** | **1370.4184** | **-0.0330** | **0** | **16** | **68** | **1** | **IEDTGGSGGSYSIK** |
|  | 22 | **363.1394** | **1086.3959** | **1085.2412** | **1.1547** | **1** | **16** | **68** | **1** | **HRCGGSLLSR** |
|  | 392 | **387.8509** | **1160.5306** | **1160.3013** | **0.2293** | **1** | **16** | **84** | **1** | **NKMDIQGNPK + Oxidation (M)** |
|  | 1619 | **518.9348** | **1553.7821** | **1553.6958** | **0.0863** | **0** | **16** | **69** | **1** | **QFEAQNLSMQSVR + Oxidation (M)** |
|  | 2111 | **597.1383** | **1192.2618** | **1191.4642** | **0.7976** | **1** | **16** | **77** | **1** | **LLKQLECCK + 2 Carbamidomethyl (C)** |
|  | 2601 | **668.1094** | **2001.3060** | **2002.2997** | **-0.9937** | **2** | **16** | **73** | **1** | **ATQEAFMKRAMANCQAAK + 2 Oxidation (M)** |
|  | 536 | **390.0691** | **1167.1852** | **1166.2181** | **0.9671** | **0** | **16** | **70** | **1** | **GDMGDPGFGGEK** |
|  | 1306 | **463.9567** | **1388.8480** | **1388.5345** | **0.3135** | **1** | **16** | **64** | **1** | **GPIGHRGNTGPLGR** |
|  | 1353 | **472.3499** | **942.6850** | **943.0987** | **-0.4137** | **1** | **16** | **56** | **1** | **LEIDGKLR** |
|  | 1479 | **488.6809** | **1463.0206** | **1462.5817** | **0.4389** | **0** | **16** | **65** | **1** | **DLDCSLEAAAELR + Carbamidomethyl (C)** |
|  | 2015 | **584.3661** | **1166.7174** | **1166.3240** | **0.3934** | **0** | **16** | **68** | **1** | **LLDIEEAVHK** |
|  | 2182 | **607.0132** | **1818.0174** | **1817.0314** | **0.9860** | **1** | **16** | **67** | **1** | **FKTSVSFTVTMGANGNR** |
|  | 708 | **404.1356** | **1209.3845** | **1208.4104** | **0.9741** | **1** | **16** | **71** | **1** | **EPTGRLPALVR** |
|  | 1265 | **459.7847** | **917.5547** | **917.1279** | **0.4268** | **0** | **16** | **67** | **1** | **NIMVVVSR** |
|  | 2038 | **589.8564** | **1766.5472** | **1766.8599** | **-0.3128** | **0** | **16** | **62** | **1** | **QVSAGQTSVYALDENGK** |
|  | 867 | **407.8413** | **1220.5018** | **1220.4395** | **0.0624** | **1** | **16** | **72** | **1** | **QMESMKYCK + Carbamidomethyl (C); Oxidation (M)** |
|  | 2735 | **685.1784** | **2052.5130** | **2053.4042** | **-0.8911** | **1** | **16** | **72** | **1** | **MQNKLCFSLDPFQLPAK + Carbamidomethyl (C); Oxidation (M)** |
|  | 134 | **369.5453** | **1105.6139** | **1106.3249** | **-0.7111** | **2** | **16** | **59** | **1** | **INLIGHRKR** |
|  | 690 | **403.9651** | **1208.8730** | **1209.3969** | **-0.5239** | **1** | **16** | **73** | **1** | **RDLMACAQTGK + Oxidation (M)** |
|  | 739 | **405.2124** | **808.4100** | **807.9374** | **0.4727** | **0** | **16** | **57** | **1** | **VPAHASVK** |
|  | 234 | **379.4441** | **1135.3100** | **1134.4165** | **0.8935** | **0** | **16** | **89** | **1** | **SMMCPPGMHK + Oxidation (M)** |
|  | 1082 | **431.0704** | **1290.1890** | **1289.5013** | **0.6877** | **0** | **16** | **88** | **1** | **TLAMDTILANAR** |
|  | 1159 | **440.1495** | **1317.4263** | **1317.4702** | **-0.0439** | **0** | **16** | **84** | **1** | **QHMEVELPYR + Oxidation (M)** |
|  | 110 | **369.3217** | **1104.9428** | **1105.3536** | **-0.4108** | **1** | **16** | **67** | **1** | **RGFAMLPGLK + Oxidation (M)** |
|  | 248 | **380.1486** | **1137.4235** | **1137.1754** | **0.2482** | **0** | **16** | **78** | **1** | **EGEAGAAMEEK + Oxidation (M)** |
|  | 359 | **387.7218** | **1160.1432** | **1160.2336** | **-0.0903** | **0** | **16** | **79** | **1** | **DAVTYTEHPK** |
|  | 2941 | **740.6082** | **2218.8023** | **2219.5316** | **-0.7294** | **1** | **16** | **58** | **1** | **GCLGASSRPRWRPLGAQPPR + Carbamidomethyl (C)** |
|  | 1158 | **440.0490** | **878.0832** | **877.9443** | **0.1390** | **0** | **16** | **80** | **1** | **QGQHPPSK** |
|  | 1966 | **574.5209** | **1720.5404** | **1719.9178** | **0.6227** | **1** | **16** | **63** | **1** | **YVFRTENGGQVMYR** |
|  | 1224 | **452.6233** | **1354.8477** | **1355.4336** | **-0.5859** | **1** | **16** | **72** | **1** | **AGYQQKGDTCER** |
|  | 402 | **387.8988** | **773.7828** | **772.8468** | **0.9359** | **0** | **16** | **88** | **1** | **AEADVIR** |
|  | 1387 | **476.0502** | **950.0857** | **951.0746** | **-0.9889** | **0** | **16** | **76** | **1** | **ISFAVSEAK** |
|  | 303 | **386.0186** | **1155.0335** | **1154.3002** | **0.7333** | **2** | **16** | **61** | **1** | **RAAGFMRSDK + Oxidation (M)** |
|  | 1173 | **443.5118** | **885.0089** | **886.0476** | **-1.0387** | **1** | **16** | **86** | **1** | **DLVNVAKK** |
|  | 225 | **378.1172** | **1131.3294** | **1130.2905** | **1.0389** | **0** | **16** | **65** | **1** | **MDLTTTMSSK + Oxidation (M)** |
|  | 540 | **390.0863** | **1167.2368** | **1167.3817** | **-0.1448** | **1** | **16** | **72** | **1** | **MELHIPQKR + Oxidation (M)** |
|  | 1601 | **515.5095** | **1029.0041** | **1028.1140** | **0.8902** | **0** | **16** | **75** | **1** | **DDPLGEVGVK** |
|  | 1973 | **575.7955** | **1724.3642** | **1724.9956** | **-0.6313** | **2** | **16** | **61** | **1** | **KNSTGSSKLTPLVPAPK** |
|  | 2704 | **682.6362** | **1363.2577** | **1364.4422** | **-1.1845** | **2** | **16** | **58** | **1** | **QNRVNSKETEC + Carbamidomethyl (C)** |
|  | 231 | **379.3802** | **1135.1185** | **1134.3682** | **0.7502** | **1** | **16** | **84** | **1** | **SAQKFSLILK** |
|  | 2209 | **608.8197** | **1215.6246** | **1216.4506** | **-0.8260** | **0** | **16** | **65** | **1** | **LMGITLPATQR + Oxidation (M)** |
|  | 3148 | **788.0383** | **2361.0928** | **2360.7160** | **0.3768** | **2** | **16** | **59** | **1** | **CPCCPDKCCCPEARYAAGK + 6 Carbamidomethyl (C)** |
|  | 564 | **392.3481** | **782.6813** | **782.8848** | **-0.2034** | **0** | **16** | **61** | **1** | **KPAPGADK** |
|  | 2057 | **592.8578** | **1183.7008** | **1184.2764** | **-0.5756** | **0** | **16** | **61** | **1** | **NGADFDITGMK + Oxidation (M)** |
|  | 14 | **362.4116** | **1084.2126** | **1085.2580** | **-1.0454** | **0** | **16** | **82** | **1** | **MGPPSAPPCR + Carbamidomethyl (C); Oxidation (M)** |
|  | 102 | **369.2909** | **1104.8504** | **1104.2381** | **0.6124** | **1** | **16** | **65** | **1** | **SCLAKGSPER + Carbamidomethyl (C)** |
|  | 820 | **407.2319** | **1218.6736** | **1219.4730** | **-0.7993** | **2** | **16** | **57** | **1** | **KVFEAAVKSIK** |
|  | 921 | **411.1664** | **1230.4771** | **1230.4624** | **0.0147** | **1** | **16** | **78** | **1** | **AAKCSCCMGAR + 2 Carbamidomethyl (C); Oxidation (M)** |
|  | 2247 | **612.1297** | **1222.2446** | **1222.3741** | **-0.1294** | **1** | **16** | **74** | **1** | **ERGPGLCTSFR** |
|  | 1722 | **533.9664** | **1065.9181** | **1066.1471** | **-0.2290** | **0** | **16** | **72** | **1** | **SSQQGSSVMR** |
|  | 2745 | **685.8696** | **1369.7245** | **1369.5728** | **0.1516** | **1** | **16** | **77** | **1** | **RPRTILTTQQR** |
|  | 3226 | **819.1663** | **1636.3177** | **1635.9271** | **0.3907** | **0** | **16** | **57** | **1** | **ALAFNGVMFGDRPLK** |
|  | 312 | 386.0527 | 770.0907 | 770.8725 | -0.7818 | 0 | 16 | 67 | 1 | SCMTDAK + Oxidation (M) |
|  | 559 | **391.2687** | **1170.7840** | **1170.4285** | **0.3555** | **1** | **16** | **54** | **1** | **ACSPALRALLR** |
|  | 2415 | **634.3697** | **1900.0869** | **1898.9995** | **1.0874** | **0** | **16** | **71** | **1** | **DIQMTQSSSTLSASVGDR + Oxidation (M)** |
|  | 325 | **386.1255** | **1155.3543** | **1154.3597** | **0.9946** | **0** | **16** | **67** | **1** | **LFCMGDVNAK + Carbamidomethyl (C)** |
|  | 328 | **386.1460** | **1155.4157** | **1155.4554** | **-0.0396** | **1** | **16** | **67** | **1** | **HIAVFPCKIK** |
|  | 475 | **389.0802** | **1164.2185** | **1164.1640** | **0.0545** | **0** | **16** | **83** | **1** | **SNNADCQAER + Carbamidomethyl (C)** |
|  | 1351 | **472.1933** | **1413.5578** | **1412.6637** | **0.8941** | **2** | **16** | **73** | **1** | **RAPAGGSLGRALMR** |
|  | 2032 | **588.1725** | **1761.4953** | **1761.0953** | **0.4000** | **2** | **16** | **79** | **1** | **EILRLGFKSCLYYR** |
|  | 189 | **374.2659** | **746.5170** | **746.8097** | **-0.2927** | **1** | **16** | **72** | **1** | **KVEDTR** |
|  | 2055 | **592.8364** | **1775.4871** | **1775.0129** | **0.4742** | **1** | **16** | **62** | **1** | **EEKDPGMGAMGGMGGGMF + Oxidation (M)** |
|  | 3365 | **920.4297** | **2758.2669** | **2759.1021** | **-0.8352** | **1** | **16** | **67** | **1** | **WEAAHVVEQLRAYLEGTCVEWLR** |
|  | 192 | **374.4028** | **1120.1861** | **1119.3357** | **0.8505** | **0** | **16** | **1.1e+02** | **1** | **IMATSGVVAVR + Oxidation (M)** |
|  | 371 | **387.7805** | **773.5462** | **772.8038** | **0.7424** | **0** | **16** | **88** | **1** | **SGEPLDR** |
|  | 2264 | **613.2236** | **1836.6487** | **1835.9866** | **0.6621** | **0** | **16** | **73** | **1** | **SLRPSAEMIETTNDSGK** |
|  | 339 | **386.2361** | **770.4573** | **769.8031** | **0.6542** | **0** | **16** | **53** | **1** | **AHSAEQK** |
|  | 1101 | **433.0442** | **1296.1104** | **1296.5319** | **-0.4215** | **1** | **16** | **75** | **1** | **QYESLKILICS** |
|  | 2936 | **740.5807** | **2218.7201** | **2218.3351** | **0.3849** | **1** | **16** | **64** | **1** | **EYQEPEVPESNQKQWQAK** |
|  | 3017 | **748.7527** | **1495.4907** | **1496.6741** | **-1.1834** | **2** | **16** | **69** | **1** | **AQRARQLGLEGAAR** |
|  | 68 | **368.1495** | **1101.4263** | **1100.2495** | **1.1769** | **1** | **16** | **76** | **1** | **ADKSPCPQVR** |
|  | 1328 | **467.3007** | **932.5867** | **933.1669** | **-0.5803** | **1** | **16** | **64** | **1** | **LAKLMTEK** |
|  | 1570 | **508.6285** | **1522.8633** | **1522.8522** | **0.0110** | **0** | **16** | **91** | **1** | **LFTLVSACIPVFR + Carbamidomethyl (C)** |
|  | 1919 | **562.5115** | **1123.0082** | **1122.3177** | **0.6904** | **0** | **16** | **62** | **1** | **LPSPQQLLAR** |
|  | 277 | **385.1228** | **768.2308** | **767.9563** | **0.2746** | **1** | **16** | **66** | **1** | **KIPSPVK** |
|  | 1645 | **522.0867** | **1042.1587** | **1041.1623** | **0.9964** | **2** | **16** | **75** | **1** | **GRNGDPGKIK** |
|  | 2963 | **741.5596** | **2221.6567** | **2221.6207** | **0.0361** | **1** | **16** | **67** | **1** | **RVALGMAMDQVNALCEQLVK + 2 Oxidation (M)** |
|  | 686 | **403.9453** | **1208.8138** | **1208.3458** | **0.4680** | **0** | **15** | **77** | **1** | **WPTWMSSSAR** |
|  | 809 | **406.9595** | **1217.8563** | **1217.3707** | **0.4856** | **1** | **15** | **68** | **1** | **IAQESKDIWK** |
|  | 2472 | **647.3131** | **1938.9172** | **1938.3663** | **0.5509** | **2** | **15** | **74** | **1** | **RKLIQQQLVLLLHAHK** |
|  | 62 | **366.2870** | **1095.8387** | **1096.3022** | **-0.4635** | **0** | **15** | **71** | **1** | **LCANEEMMR** |
|  | 1150 | **437.9297** | **1310.7668** | **1311.6395** | **-0.8727** | **1** | **15** | **85** | **1** | **LWMMKMATQR + Oxidation (M)** |
|  | 3078 | **759.1221** | **2274.3440** | **2273.1983** | **1.1458** | **0** | **15** | **65** | **1** | **NNWSEEDPDYPDYSGSQNR** |
|  | 2630 | **669.6187** | **1337.2225** | **1337.3984** | **-0.1759** | **0** | **15** | **61** | **1** | **QHAEALQQEQR** |
|  | 157 | **371.3955** | **740.7763** | **739.8601** | **0.9162** | **1** | **15** | **73** | **1** | **KEAPPAK** |
|  | 1960 | **573.3032** | **1716.8873** | **1715.8262** | **1.0611** | **2** | **15** | **79** | **1** | **RGDLLSGANGGTRGTQR** |
|  | 2734 | **685.1195** | **1368.2242** | **1367.6748** | **0.5495** | **2** | **15** | **78** | **1** | **SKIVEEMLKMK + 2 Oxidation (M)** |
|  | 583 | **396.1035** | **1185.2884** | **1186.4661** | **-1.1777** | **0** | **15** | **87** | **1** | **LIPFMLSPPR + Oxidation (M)** |
|  | 2078 | **594.1827** | **1186.3507** | **1187.3266** | **-0.9759** | **0** | **15** | **83** | **1** | **QLGCTSPTPAR + Carbamidomethyl (C)** |
|  | 1275 | **460.2460** | **1377.7157** | **1378.6192** | **-0.9035** | **1** | **15** | **79** | **1** | **KDLGPKPALIGNR** |
|  | 306 | **386.0314** | **770.0480** | **769.9324** | **0.1156** | **1** | **15** | **66** | **1** | **APLSKVR** |
|  | 509 | **389.9885** | **777.9621** | **777.7773** | **0.1849** | **1** | **15** | **77** | **1** | **QKDATTD** |
|  | 1376 | **475.6665** | **1423.9773** | **1423.6120** | **0.3654** | **0** | **15** | **71** | **1** | **QLICEEMGSVNK + Carbamidomethyl (C); Oxidation (M)** |
|  | 2880 | **724.8134** | **2171.4181** | **2170.4230** | **0.9951** | **2** | **15** | **91** | **1** | **LTQGLQEALDRADLLKTER** |
|  | 2980 | **742.3162** | **2223.9263** | **2223.3766** | **0.5497** | **1** | **15** | **73** | **1** | **GKPSPWQSSMSTGEGSKEAQK + Oxidation (M)** |
|  | 1222 | **452.5994** | **903.1839** | **902.9554** | **0.2285** | **1** | **15** | **87** | **1** | **TRSGGAAQR** |
|  | 3305 | **873.6882** | **2618.0425** | **2618.9558** | **-0.9133** | **1** | **15** | **70** | **1** | **HYPQTEGMTTLAVRETLVDLLSK + Oxidation (M)** |
|  | 471 | **389.0514** | **1164.1320** | **1164.2287** | **-0.0966** | **2** | **15** | **86** | **1** | **KNGAGPYSRNT** |
|  | 1428 | **484.2538** | **966.4929** | **967.0375** | **-0.5446** | **0** | **15** | **73** | **1** | **DGAQGPVAPR** |
|  | 857 | **407.7733** | **1220.2977** | **1220.3317** | **-0.0340** | **1** | **15** | **78** | **1** | **SIFPGGGDKTNK** |
|  | 1877 | **551.9216** | **1101.8285** | **1101.2159** | **0.6126** | **0** | **15** | **77** | **1** | **SVAQAGVQWR** |
|  | 2192 | **607.9734** | **1213.9320** | **1214.4846** | **-0.5526** | **2** | **15** | **74** | **1** | **GRQAVLGMVRK** |
|  | 1515 | **496.5828** | **1486.7261** | **1485.7093** | **1.0168** | **1** | **15** | **1e+02** | **1** | **QALQASIGPRCWGV** |
|  | 2594 | **667.7719** | **2000.2934** | **2000.4709** | **-0.1775** | **2** | **15** | **97** | **1** | **MVASAGSLFGGMVLKKFLK + Oxidation (M)** |
|  | 2682 | **676.5250** | **2026.5529** | **2027.3257** | **-0.7728** | **2** | **15** | **67** | **1** | **VLYSQGVKLFRFDAEVR** |
|  | 2220 | **610.0458** | **1827.1152** | **1826.2701** | **0.8451** | **0** | **15** | **79** | **1** | **KPELVFSPMVPLPMPK + Oxidation (M)** |
|  | 1362 | **474.0372** | **946.0596** | **947.1320** | **-1.0724** | **0** | **15** | **86** | **1** | **FTLLINAR** |
|  | 1778 | **539.5414** | **1615.6020** | **1616.7714** | **-1.1695** | **0** | **15** | **83** | **1** | **ASGGTFTSYAITWVR** |
|  | 105 | **369.3029** | **1104.8866** | **1104.2547** | **0.6319** | **0** | **15** | **70** | **1** | **LDTYCMSAK + Carbamidomethyl (C); Oxidation (M)** |
|  | 681 | **403.7718** | **1208.2932** | **1209.4828** | **-1.1897** | **2** | **15** | **79** | **1** | **KQALKFHPLK** |
|  | 989 | **419.2791** | **1254.8152** | **1254.4788** | **0.3364** | **1** | **15** | **75** | **1** | **CDICGKVFNQK** |
|  | 1130 | **435.7783** | **869.5419** | **869.0202** | **0.5216** | **0** | **15** | **71** | **1** | **AGSPLVGLR** |
|  | 3079 | **759.1931** | **2274.5570** | **2275.5684** | **-1.0114** | **2** | **15** | **74** | **1** | **CRMLIQENQELGRQLSQGR + Oxidation (M)** |
|  | 2812 | **699.4299** | **1396.8451** | **1395.6897** | **1.1554** | **2** | **15** | **77** | **1** | **AKVVGKGPLATGGIK** |
|  | 580 | **394.6802** | **1181.0184** | **1181.3884** | **-0.3700** | **1** | **15** | **70** | **1** | **MMGSWGVGRGK + Oxidation (M)** |
|  | 751 | **405.5227** | **1213.5459** | **1214.3719** | **-0.8260** | **2** | **15** | **83** | **1** | **DLAAADVKKQR** |
|  | 1836 | **546.2941** | **1635.8600** | **1635.8839** | **-0.0239** | **0** | **15** | **81** | **1** | **QASGNIVYGVFCLHK** |
|  | 1370 | **474.3549** | **1420.0425** | **1420.6824** | **-0.6399** | **2** | **15** | **68** | **1** | **TCNRFRLLLER** |
|  | 2054 | **592.7813** | **1775.3218** | **1774.2183** | **1.1034** | **0** | **15** | **74** | **1** | **QMLISLAPPVLTLHLK** |
|  | 2210 | **608.8241** | **1215.6334** | **1216.3232** | **-0.6898** | **0** | **15** | **70** | **1** | **QFFSNCTVDR** |
|  | 1149 | **437.1630** | **872.3111** | **872.9263** | **-0.6151** | **0** | **15** | **87** | **1** | **RPSGVSDR** |
|  | 675 | **403.0515** | **1206.1322** | **1205.4530** | **0.6792** | **1** | **15** | **85** | **1** | **LVGAGHVVAKVR** |
|  | 2193 | **608.0172** | **1214.0196** | **1214.4597** | **-0.4400** | **1** | **15** | **80** | **1** | **IYMHCFKTR + Oxidation (M)** |
|  | 2511 | 654.6926 | 1961.0557 | 1960.1351 | 0.9206 | 2 | 15 | 87 | 1 | CSSGGFGSRSLYNLRGNK + Carbamidomethyl (C) |
|  | 1077 | **431.0112** | **860.0076** | **859.0949** | **0.9126** | **2** | **15** | **97** | **1** | **ACLRIRK** |
|  | 1809 | **541.5270** | **1081.0393** | **1080.2778** | **0.7614** | **0** | **15** | **72** | **1** | **AVLLAGPPGTGK** |
|  | 61 | **366.2304** | **1095.6691** | **1096.2375** | **-0.5685** | **0** | **15** | **72** | **1** | **VHYECTGCK + Carbamidomethyl (C)** |
|  | 2554 | **666.0706** | **1330.1263** | **1330.5568** | **-0.4304** | **2** | **15** | **77** | **1** | **DVQALRDMVRK** |
|  | 2746 | **685.8836** | **1369.7524** | **1370.5508** | **-0.7983** | **0** | **15** | **80** | **1** | **NFSYPLNFLQK** |
|  | 3201 | **807.4330** | **1612.8512** | **1611.9470** | **0.9041** | **1** | **15** | **77** | **1** | **LQCVASKLQVLPQK + Carbamidomethyl (C)** |
|  | 3408 | **941.0159** | **2820.0254** | **2819.2371** | **0.7884** | **2** | **15** | **78** | **1** | **EMPGKGGVWKVLFKPPTSDAEFLER** |
|  | 247 | **380.1064** | **1137.2972** | **1136.1740** | **1.1231** | **1** | **15** | **90** | **1** | **TAATASDSTRR** |
|  | 1051 | **428.0491** | **1281.1251** | **1280.4398** | **0.6854** | **2** | **15** | **78** | **1** | **CWNRSGGCRK + 2 Carbamidomethyl (C)** |
|  | 2614 | **668.7387** | **1335.4626** | **1336.5959** | **-1.1333** | **1** | **15** | **97** | **1** | **MINLKTEEFVL** |
|  | 3561 | **1051.9945** | **2101.9742** | **2102.2894** | **-0.3151** | **0** | **15** | **55** | **1** | **QAPGQRPEWMGWVNTGSGK + Oxidation (M)** |
|  | 2292 | **617.4520** | **1849.3339** | **1850.0347** | **-0.7008** | **1** | **15** | **76** | **1** | **EETSPKVPHLIQESQK** |
|  | 866 | **407.8406** | **1220.4996** | **1219.4314** | **1.0682** | **0** | **15** | **82** | **1** | **RPPFSIDLFK** |
|  | 1473 | **488.4690** | **974.9232** | **974.1574** | **0.7658** | **1** | **15** | **82** | **1** | **KLAPYNIR** |
|  | 1970 | **575.1233** | **1722.3477** | **1721.9002** | **0.4475** | **2** | **15** | **85** | **1** | **GLHSWQKAAHCDRR + Carbamidomethyl (C)** |
|  | 3448 | **948.6179** | **1895.2211** | **1894.3918** | **0.8292** | **1** | **15** | **74** | **1** | **VLCVLKDLLVAAIVHCK + Carbamidomethyl (C)** |
|  | 166 | **372.3907** | **742.7665** | **742.8871** | **-0.1205** | **0** | **15** | **97** | **1** | **CLVHSK + Carbamidomethyl (C)** |
|  | 288 | **385.9496** | **1154.8265** | **1154.3034** | **0.5231** | **0** | **15** | **70** | **1** | **NRPMGLHGTR + Oxidation (M)** |
|  | 2600 | **668.0969** | **2001.2684** | **2002.2897** | **-1.0213** | **0** | **15** | **83** | **1** | **IPAMTTATNAGVEGSLIVEK** |
|  | 3107 | **767.6083** | **2299.8027** | **2299.6812** | **0.1214** | **0** | **15** | **71** | **1** | **MIIDASGESGLTQLLMTEVMK + 2 Oxidation (M)** |
|  | 2437 | **638.5537** | **1275.0926** | **1275.4930** | **-0.4004** | **1** | **15** | **66** | **1** | **APKLINYEVTK** |
|  | 2522 | **657.0363** | **1968.0866** | **1969.1607** | **-1.0741** | **1** | **15** | **74** | **1** | **EPGRPGSSVTVSVKGQEVR** |
|  | 2744 | **685.8582** | **1369.7015** | **1370.5161** | **-0.8146** | **2** | **15** | **89** | **1** | **GARRAGAEVFSHL** |
|  | 3021 | **749.1356** | **1496.2565** | **1495.5601** | **0.6964** | **0** | **15** | **74** | **1** | **RPGGQHGGDWSWR** |
|  | 721 | **404.7950** | **807.5753** | **807.8728** | **-0.2975** | **0** | **15** | **78** | **1** | **ASMNTER** |
|  | 854 | **407.7626** | **1220.2657** | **1219.4117** | **0.8541** | **2** | **15** | **81** | **1** | **AKIMSEQERK** |
|  | 901 | **408.8958** | **815.7768** | **814.9946** | **0.7823** | **0** | **15** | **89** | **1** | **MMMGSAR + 2 Oxidation (M)** |
|  | 2467 | **646.3828** | **1290.7508** | **1289.5842** | **1.1667** | **1** | **15** | **85** | **1** | **LIKSVINMDIK + Oxidation (M)** |
|  | 595 | **399.0805** | **796.1463** | **794.9801** | **1.1662** | **1** | **15** | **74** | **1** | **KMSMPAV + 2 Oxidation (M)** |
|  | 903 | **408.9819** | **1223.9236** | **1223.3788** | **0.5449** | **2** | **15** | **91** | **1** | **AITTGKSRFVSG** |
|  | 1838 | **546.8323** | **1091.6498** | **1092.2903** | **-0.6405** | **0** | **15** | **69** | **1** | **LCPSGIEMAR + Oxidation (M)** |
|  | 2180 | **606.8026** | **1211.5903** | **1210.4212** | **1.1691** | **0** | **15** | **70** | **1** | **NTGMNMSITLI + Oxidation (M)** |
|  | 2465 | **646.1894** | **1935.5460** | **1936.3241** | **-0.7781** | **2** | **15** | **86** | **1** | **LWLQFVYMRFNFKK + Oxidation (M)** |
|  | 1440 | **486.9684** | **971.9220** | **972.0554** | **-0.1335** | **0** | **15** | **84** | **1** | **LSEGWQPR** |
|  | 1443 | **487.2904** | **972.5660** | **972.0969** | **0.4691** | **1** | **15** | **82** | **1** | **NKLLEAER** |
|  | 1702 | **532.6413** | **1594.9017** | **1595.9891** | **-1.0874** | **2** | **15** | **1e+02** | **1** | **YYLTPVRMAIIKK** |
|  | 725 | **404.9768** | **1211.9082** | **1212.4403** | **-0.5322** | **1** | **15** | **76** | **1** | **AFIHDLKLQK** |
|  | 2771 | **687.1765** | **2058.5074** | **2058.2585** | **0.2488** | **2** | **15** | **81** | **1** | **QEEACQCQCACRDSAKER** |
|  | 636 | **401.6640** | **801.3132** | **801.9310** | **-0.6178** | **1** | **15** | **77** | **1** | **KSLADIR** |
|  | 3502 | **968.5743** | **2902.7009** | **2903.4429** | **-0.7421** | **1** | **15** | **74** | **1** | **MLFRTVLHLLSVDVSTAEMMPENLR** |
|  | 773 | **406.2132** | **1215.6173** | **1216.4079** | **-0.7905** | **1** | **15** | **68** | **1** | **MAPEVAAVERK + Oxidation (M)** |
|  | 1035 | **423.7146** | **1268.1217** | **1267.4330** | **0.6888** | **1** | **15** | **80** | **1** | **ANCQKSTEQMK** |
|  | 1765 | **537.6254** | **1609.8541** | **1609.7770** | **0.0771** | **0** | **15** | **1.1e+02** | **1** | **DLLAYAFALAGNQDK** |
|  | 604 | **399.2947** | **796.5746** | **795.8867** | **0.6878** | **1** | **15** | **58** | **1** | **GAKLHDR** |
|  | 2254 | **612.1868** | **1222.3588** | **1223.5342** | **-1.1755** | **1** | **15** | **85** | **1** | **HMTILIRIAR** |
|  | 3412 | **941.4938** | **1880.9728** | **1881.1995** | **-0.2267** | **0** | **15** | **73** | **1** | **MATHTFLQESYFMMK + Oxidation (M)** |
|  | 188 | **374.2307** | **1119.6699** | **1120.3237** | **-0.6538** | **2** | **15** | **81** | **1** | **MNKKAGVAASK + Oxidation (M)** |
|  | 1091 | **432.2552** | **1293.7434** | **1294.4350** | **-0.6916** | **0** | **15** | **74** | **1** | **GQTITISCTGTR + Carbamidomethyl (C)** |
|  | 197 | **375.4238** | **1123.2491** | **1122.2416** | **1.0075** | **2** | **15** | **1.1e+02** | **1** | **HQKRGAAAGAR** |
|  | 656 | **402.1220** | **1203.3439** | **1204.3538** | **-1.0100** | **0** | **15** | **96** | **1** | **IICADTQGVER** |
|  | 772 | **406.1939** | **1215.5596** | **1215.3334** | **0.2263** | **0** | **15** | **75** | **1** | **SNWPPMYTFG + Oxidation (M)** |
|  | 859 | **407.8002** | **1220.3785** | **1219.3701** | **1.0085** | **1** | **15** | **86** | **1** | **WRSSGQAPMLA + Oxidation (M)** |
|  | 2790 | **691.0952** | **2070.2635** | **2069.3821** | **0.8813** | **1** | **15** | **85** | **1** | **DINAVSIDMLLIVHSEKR + Oxidation (M)** |
|  | 642 | **401.8811** | **1202.6211** | **1203.2433** | **-0.6221** | **0** | **15** | **97** | **1** | **ASVCSSGGGEHR + Carbamidomethyl (C)** |
|  | 1963 | **573.8766** | **1145.7385** | **1145.2634** | **0.4751** | **1** | **15** | **71** | **1** | **ISQENEIGKK** |
|  | 2776 | **687.9193** | **2060.7358** | **2060.4581** | **0.2776** | **0** | **15** | **71** | **1** | **YLSTMLMLGNILGTTMER + Oxidation (M)** |
|  | 358 | **387.7063** | **773.3979** | **772.8467** | **0.5511** | **0** | **15** | **88** | **1** | **EAGLDLR** |
|  | 2086 | **594.9752** | **1781.9033** | **1782.9936** | **-1.0903** | **1** | **15** | **90** | **1** | **VMATTGGTNLRDDIMR + 2 Oxidation (M)** |
|  | 2188 | **607.7598** | **1820.2571** | **1819.7520** | **0.5051** | **2** | **15** | **91** | **1** | **NASKGQGGAGARDDEEEE** |
|  | 3275 | **845.5961** | **2533.7662** | **2532.6729** | **1.0934** | **1** | **15** | **81** | **1** | **DPFPNSSTAAKSFEDLTDHPVTR** |
|  | 1869 | **551.0061** | **1099.9974** | **1100.1351** | **-0.1377** | **0** | **15** | **86** | **1** | **YGEQPASYVS** |
|  | 1835 | **546.2154** | **1635.6240** | **1634.7885** | **0.8355** | **1** | **15** | **89** | **1** | **NSPSFQGRVTISVDK** |
|  | 672 | **403.0193** | **804.0239** | **803.8177** | **0.2062** | **0** | **15** | **96** | **1** | **GESELGGR** |
|  | 1788 | **540.2231** | **1078.4314** | **1079.3380** | **-0.9066** | **1** | **15** | **88** | **1** | **LMMLVRER + 2 Oxidation (M)** |
|  | 1942 | **568.6977** | **1703.0709** | **1702.9152** | **0.1557** | **2** | **15** | **1e+02** | **1** | **HRLVGGNRLHLDTSK** |
|  | 832 | **407.4117** | **1219.2130** | **1218.3689** | **0.8441** | **2** | **15** | **90** | **1** | **RVAQHKGASHK** |
|  | 397 | **387.8666** | **773.7184** | **772.8899** | **0.8286** | **1** | **15** | **1e+02** | **1** | **KLGDVNK** |
|  | 1717 | **533.5997** | **1597.7768** | **1598.7927** | **-1.0159** | **1** | **15** | **99** | **1** | **EGLPTEEIKNVLEK** |
|  | 1199 | **448.6013** | **1342.7816** | **1342.4214** | **0.3602** | **1** | **15** | **77** | **1** | **GGLGAGAAGGGGAGRTR** |
|  | 1914 | **561.1746** | **1680.5017** | **1679.9964** | **0.5053** | **2** | **15** | **93** | **1** | **KDLIVMLKDTDMNK + Oxidation (M)** |
|  | 605 | **399.7397** | **797.4647** | **796.8716** | **0.5932** | **1** | **15** | **68** | **1** | **QKHDAAK** |
|  | 654 | **402.0826** | **802.1505** | **801.0093** | **1.1412** | **1** | **15** | **98** | **1** | **GPLKCVK + Carbamidomethyl (C)** |
|  | 910 | **409.1277** | **816.2405** | **816.9690** | **-0.7284** | **0** | **15** | **93** | **1** | **EMLRPR + Oxidation (M)** |
|  | 608 | **399.9882** | **797.9615** | **796.9377** | **1.0238** | **0** | **15** | **73** | **1** | **ACHTLPR** |
|  | 771 | **406.1853** | **1215.5338** | **1216.5766** | **-1.0427** | **0** | **15** | **78** | **1** | **MLYSLMMIAK + Oxidation (M)** |
|  | 816 | **407.1475** | **812.2801** | **812.9801** | **-0.7000** | **0** | **15** | **84** | **1** | **ACLTLHR** |
|  | 1538 | **504.6307** | **1007.2466** | **1007.2321** | **0.0145** | **1** | **15** | **97** | **1** | **VAKLCTCR + 2 Carbamidomethyl (C)** |
|  | 262 | **384.8340** | **1151.4798** | **1152.3456** | **-0.8658** | **0** | **15** | **75** | **1** | **VMATSGCAAIR + Carbamidomethyl (C); Oxidation (M)** |
|  | 1191 | **447.1846** | **892.3545** | **891.9642** | **0.3902** | **0** | **15** | **90** | **1** | **TNTFLEAP** |
|  | 1656 | **523.0334** | **1044.0521** | **1045.1841** | **-1.1320** | **0** | **15** | **88** | **1** | **IELLEATEK** |
|  | 2633 | **669.7151** | **2006.1231** | **2005.2419** | **0.8811** | **2** | **15** | **99** | **1** | **VWRNPLNLFRGAEYNR** |
|  | 2019 | **584.8212** | **1751.4413** | **1750.9664** | **0.4749** | **0** | **15** | **70** | **1** | **VQQVELICEYNDLK + Carbamidomethyl (C)** |
|  | 1237 | **457.1944** | **1368.5610** | **1368.5537** | **0.0074** | **1** | **15** | **88** | **1** | **MPGTTTTTTSVKK + Oxidation (M)** |
|  | 107 | **369.3151** | **1104.9232** | **1104.2991** | **0.6241** | **0** | **15** | **81** | **1** | **QQLLIGAYAK** |
|  | 309 | **386.0503** | **1155.1286** | **1155.3032** | **-0.1745** | **1** | **15** | **77** | **1** | **MQQSSECKSK** |
|  | 644 | **401.8872** | **801.7596** | **800.9429** | **0.8166** | **0** | **15** | **1e+02** | **1** | **EIGTIIR** |
|  | 997 | **419.7629** | **1256.2665** | **1255.5297** | **0.7367** | **1** | **15** | **78** | **1** | **ILERHLMISK + Oxidation (M)** |
|  | 2982 | **742.4260** | **2224.2557** | **2223.5352** | **0.7205** | **0** | **15** | **83** | **1** | **LIHNWVMAQHMQSHAPYK + 2 Oxidation (M)** |
|  | 659 | **402.1802** | **802.3456** | **802.8346** | **-0.4890** | **0** | **15** | **95** | **1** | **GYAEAHR** |
|  | 2724 | **684.3469** | **2050.0186** | **2050.4100** | **-0.3914** | **2** | **15** | **85** | **1** | **LWQRLCQLKGPTCQYR + Carbamidomethyl (C)** |
|  | 2846 | **711.1859** | **1420.3570** | **1421.4274** | **-1.0703** | **1** | **15** | **87** | **1** | **RVSEAGDSSTEGAR** |
|  | 594 | **399.0254** | **796.0361** | **794.9386** | **1.0975** | **0** | **15** | **76** | **1** | **TPTVIHK** |
|  | 855 | **407.7697** | **1220.2870** | **1219.3436** | **0.9433** | **0** | **15** | **88** | **1** | **AWGQGTLVTVST** |
|  | 1053 | **428.2628** | **1281.7664** | **1282.3150** | **-0.5486** | **0** | **15** | **62** | **1** | **DGEYGINFDPR** |
|  | 1086 | **431.2034** | **1290.5881** | **1290.3835** | **0.2047** | **0** | **15** | **1e+02** | **1** | **AEAASPGCDCPGR + Carbamidomethyl (C)** |
|  | 1954 | **572.1549** | **1713.4425** | **1714.0638** | **-0.6212** | **1** | **15** | **89** | **1** | **MMCWPWPLSSKCR + Carbamidomethyl (C); 2 Oxidation (M)** |
|  | 1257 | **459.0140** | **916.0132** | **917.0448** | **-1.0317** | **0** | **15** | **99** | **1** | **AVCALGNNR** |
|  | 1881 | **552.5012** | **1654.4815** | **1653.8399** | **0.6416** | **1** | **15** | **79** | **1** | **MAAMAVGGAGGSRVSSGR + 2 Oxidation (M)** |
|  | 3395 | 940.0254 | 2817.0540 | 2816.1966 | 0.8574 | 0 | 15 | 88 | 1 | GSPQLVCSLPGPQGPPGPPGAPGPSGMMGR + 2 Oxidation (M) |
|  | 1498 | **492.3160** | **982.6172** | **983.1396** | **-0.5224** | **0** | **15** | **73** | **1** | **DVFMSGLSK** |
|  | 2701 | **681.6975** | **1361.3802** | **1361.6106** | **-0.2303** | **2** | **15** | **89** | **1** | **METIERKISVR** |
|  | 3517 | **971.2472** | **1940.4796** | **1940.3029** | **0.1767** | **0** | **15** | **66** | **1** | **LMPGTYTLEITSIPLYK** |
|  | 746 | **405.4847** | **808.9547** | **808.9254** | **0.0293** | **2** | **15** | **98** | **1** | **GYKGKTR** |
|  | 1340 | **469.7133** | **1406.1177** | **1405.5538** | **0.5639** | **0** | **15** | **67** | **1** | **CYTCQVSNSVSSK** |
|  | 3015 | **747.3658** | **1492.7169** | **1493.6371** | **-0.9202** | **0** | **15** | **87** | **1** | **GNEQLFGTMDTALP** |
|  | 16 | **363.0351** | **724.0555** | **724.8056** | **-0.7501** | **0** | **15** | **88** | **1** | **IPPADGR** |
|  | 1537 | **504.5623** | **1007.1098** | **1007.2321** | **-0.1224** | **1** | **15** | **1.1e+02** | **1** | **VAKLCTCR + 2 Carbamidomethyl (C)** |
|  | 2249 | **612.1398** | **1222.2648** | **1221.4489** | **0.8158** | **1** | **15** | **89** | **1** | **APALASTPRLPK** |
|  | 3525 | **975.2377** | **2922.6908** | **2923.4559** | **-0.7651** | **2** | **15** | **69** | **1** | **TGQLAAIKVMDVTGAVECVNNVTCKVCR** |
|  | 1004 | **420.1678** | **1257.4811** | **1258.3831** | **-0.9020** | **0** | **15** | **86** | **1** | **SGTRPGPFSQPK** |
|  | 3449 | **948.7001** | **1895.3854** | **1894.3918** | **0.9935** | **1** | **15** | **80** | **1** | **VLCVLKDLLVAAIVHCK + Carbamidomethyl (C)** |
|  | 11 | **362.1638** | **722.3128** | **721.8232** | **0.4896** | **0** | **15** | **72** | **1** | **NSLMNK + Oxidation (M)** |
|  | 783 | **406.4007** | **1216.1798** | **1217.2878** | **-1.1080** | **1** | **15** | **87** | **1** | **RDGDIWGQGTL** |
|  | 1317 | **466.0554** | **1395.1440** | **1395.5174** | **-0.3734** | **0** | **15** | **95** | **1** | **GMFDHDNLMQST** |
|  | 1339 | **469.4836** | **936.9525** | **936.0846** | **0.8679** | **0** | **15** | **95** | **1** | **TASLLNSCK** |
|  | 1430 | **484.8876** | **967.7604** | **967.0638** | **0.6966** | **0** | **15** | **84** | **1** | **SSLQCHHR** |
|  | 2316 | **621.7538** | **1862.2392** | **1862.0517** | **0.1874** | **1** | **15** | **1e+02** | **1** | **NQELPLRTTWDFWR** |
|  | 122 | **369.3474** | **736.6799** | **736.8793** | **-0.1994** | **0** | **15** | **92** | **1** | **AFQMPK + Oxidation (M)** |
|  | 443 | **388.9305** | **775.8463** | **776.9663** | **-1.1200** | **2** | **15** | **1e+02** | **1** | **KLKNFK** |
|  | 1096 | **432.9433** | **863.8718** | **865.0332** | **-1.1613** | **0** | **15** | **89** | **1** | **KPGHIWK** |
|  | 1909 | **560.6735** | **1678.9982** | **1678.8443** | **0.1539** | **1** | **15** | **1.1e+02** | **1** | **AYSRAGATAGTISTPVR** |
|  | 3469 | **956.0802** | **1910.1456** | **1909.1712** | **0.9744** | **0** | **15** | **99** | **1** | **HVQLMQSGGGLVQPGGSLK + Oxidation (M)** |
|  | 36 | **363.3562** | **1087.0463** | **1086.1933** | **0.8531** | **0** | **15** | **93** | **1** | **TVDVTPETPK** |
|  | 1137 | **436.1105** | **870.2062** | **869.0235** | **1.1827** | **1** | **15** | **90** | **1** | **ALSRGLPR** |
|  | 1388 | **476.0630** | **950.1113** | **948.9974** | **1.1139** | **0** | **15** | **95** | **1** | **GDDLGMEGR** |
|  | 2106 | **596.7582** | **1191.5017** | **1192.4108** | **-0.9091** | **0** | **15** | **1e+02** | **1** | **LADALCACWAR** |
|  | 2856 | **714.0858** | **1426.1568** | **1426.5330** | **-0.3762** | **0** | **15** | **77** | **1** | **STCQGTDTAGINCR** |
|  | 1584 | **512.3401** | **1533.9983** | **1533.8118** | **0.1864** | **1** | **15** | **75** | **1** | **RILFSLQEIWTK** |
|  | 2028 | **587.3293** | **1172.6438** | **1172.2922** | **0.3516** | **2** | **15** | **93** | **1** | **RLNPKSSGADK** |
|  | 2747 | **685.9274** | **1369.8400** | **1369.4553** | **0.3846** | **0** | **15** | **74** | **1** | **SEDTYVYYCAR** |
|  | 51 | **365.9711** | **1094.8911** | **1095.1666** | **-0.2755** | **0** | **15** | **1e+02** | **1** | **VDGEAHGALAR** |
|  | 495 | **389.9083** | **777.8017** | **776.8836** | **0.9182** | **1** | **15** | **91** | **1** | **VRAEFR** |
|  | 1022 | **422.0216** | **842.0284** | **842.8572** | **-0.8287** | **1** | **15** | **91** | **1** | **GRGGDPER** |
|  | 726 | **405.0065** | **1211.9972** | **1211.4308** | **0.5664** | **1** | **15** | **84** | **1** | **NLVSLGSYKCK** |
|  | 2341 | **624.1418** | **1869.4034** | **1868.2269** | **1.1764** | **1** | **15** | **93** | **1** | **CYHQQLLMQLVKYK + Carbamidomethyl (C); Oxidation (M)** |
|  | 347 | **386.5988** | **1156.7742** | **1157.3255** | **-0.5513** | **1** | **15** | **75** | **1** | **LTVAYHARAR** |
|  | 822 | **407.2659** | **812.5171** | **812.8709** | **-0.3539** | **0** | **15** | **68** | **1** | **EGAAAPAAR** |
|  | 1501 | **493.2303** | **984.4458** | **984.0647** | **0.3811** | **0** | **15** | **93** | **1** | **KPGTPENNK** |
|  | 2043 | **591.6429** | **1181.2710** | **1180.4021** | **0.8689** | **2** | **15** | **1e+02** | **1** | **LEKMNAMRR + 2 Oxidation (M)** |
|  | 740 | **405.2214** | **1212.6420** | **1213.4303** | **-0.7884** | **2** | **15** | **72** | **1** | **EAARTAVLRVK** |
|  | 2532 | **658.7560** | **1973.2458** | **1972.1591** | **1.0866** | **1** | **15** | **1.1e+02** | **1** | **DDQNVLAGESLKWGQAIK** |
|  | 3481 | **962.8889** | **2885.6446** | **2886.2748** | **-0.6303** | **2** | **15** | **64** | **1** | **SHQRVACGVESSVAAIFGKHCHSHLPK** |
|  | 763 | **405.9681** | **1214.8822** | **1214.2428** | **0.6394** | **0** | **15** | **83** | **1** | **QHSSGQENTVK** |
|  | 25 | **363.1466** | **1086.4177** | **1086.1335** | **0.2843** | **0** | **15** | **86** | **1** | **MDTSAQDFR + Oxidation (M)** |
|  | 599 | **399.1401** | **1194.3981** | **1195.3669** | **-0.9687** | **0** | **15** | **82** | **1** | **TFFISPGSLAR** |
|  | 630 | **401.2274** | **1200.6601** | **1200.4512** | **0.2088** | **1** | **15** | **82** | **1** | **MIPDSQKLLR** |
|  | 709 | **404.1461** | **806.2775** | **806.9277** | **-0.6501** | **0** | **15** | **91** | **1** | **SCLVNSGK** |
|  | 1219 | **452.2353** | **1353.6837** | **1353.5220** | **0.1617** | **1** | **15** | **1.1e+02** | **1** | **FGTYPALASKNGK** |
|  | 1378 | **475.7540** | **949.4932** | **950.1115** | **-0.6183** | **1** | **15** | **79** | **1** | **KMSAAEAVK + Oxidation (M)** |
|  | 1334 | **468.0303** | **934.0458** | **935.0570** | **-1.0112** | **1** | **15** | **1e+02** | **1** | **NVSADAKCK** |
|  | 1669 | **524.2288** | **1569.6641** | **1569.8028** | **-0.1387** | **0** | **15** | **99** | **1** | **FSQCGHVMFAEIK + Carbamidomethyl (C); Oxidation (M)** |
|  | 507 | **389.9863** | **777.9578** | **776.9017** | **1.0560** | **0** | **15** | **93** | **1** | **AMEALAR + Oxidation (M)** |
|  | 1125 | **435.1348** | **1302.3822** | **1301.4276** | **0.9546** | **1** | **15** | **87** | **1** | **YWAKSCESATR** |
|  | 3050 | **757.8427** | **2270.5058** | **2270.5686** | **-0.0628** | **1** | **15** | **1e+02** | **1** | **CSWTHGAGYVCKCPPQFSGK + 2 Carbamidomethyl (C)** |
|  | 63 | **367.2164** | **732.4180** | **731.7121** | **0.7060** | **0** | **15** | **82** | **1** | **GAAADDGR** |
|  | 2634 | **669.7617** | **2006.2630** | **2006.2766** | **-0.0137** | **2** | **15** | **1.1e+02** | **1** | **LLQRERPSAASGRPVGRR** |
|  | 2973 | **741.8851** | **2222.6330** | **2222.4628** | **0.1703** | **0** | **15** | **99** | **1** | **GGGLLVRPSPPNSGPLAAPGDHR** |
|  | 521 | **390.0207** | **1167.0399** | **1167.2923** | **-0.2524** | **1** | **15** | **93** | **1** | **VCGKGAENFDK** |
|  | 688 | **403.9511** | **1208.8312** | **1209.4003** | **-0.5690** | **2** | **15** | **95** | **1** | **MRTKAAGCAER + Oxidation (M)** |
|  | 1108 | **433.1444** | **1296.4111** | **1296.5319** | **-0.1208** | **1** | **15** | **92** | **1** | **QYESLKILICS** |
|  | 486 | **389.2999** | **1164.8774** | **1164.4425** | **0.4350** | **1** | **15** | **83** | **1** | **SVVMTLCQKR** |
|  | 814 | **407.0813** | **1218.2217** | **1219.3288** | **-1.1070** | **2** | **15** | **89** | **1** | **KEKGCGNPGSSR** |
|  | 2366 | **627.0627** | **1878.1661** | **1877.1743** | **0.9918** | **1** | **15** | **90** | **1** | **DCCYSIHQMEKMCR + 2 Carbamidomethyl (C); Oxidation (M)** |
|  | 109 | **369.3191** | **736.6234** | **736.8793** | **-0.2559** | **0** | **15** | **87** | **1** | **AFQMPK + Oxidation (M)** |
|  | 1098 | **432.9915** | **863.9682** | **864.0486** | **-0.0804** | **1** | **15** | **94** | **1** | **MARGGAACK** |
|  | 2357 | **625.9965** | **1874.9672** | **1874.1039** | **0.8634** | **0** | **15** | **88** | **1** | **SYFGGLLCVCWSPDGR + 2 Carbamidomethyl (C)** |
|  | 2520 | **656.9825** | **1967.9255** | **1968.3184** | **-0.3929** | **0** | **15** | **73** | **1** | **MSSFVVVMGFLVEMTSR + 3 Oxidation (M)** |
|  | 1 | **360.3728** | **1078.0961** | **1078.1792** | **-0.0830** | **0** | **15** | **1.2e+02** | **1** | **TFNIQSVNR** |
|  | 695 | **404.0136** | **806.0124** | **805.8769** | **0.1356** | **1** | **15** | **94** | **1** | **SSEKVTR** |
|  | 1348 | **471.9369** | **1412.7885** | **1412.6986** | **0.0899** | **0** | **15** | **86** | **1** | **CMSTSASALLACVR** |
|  | 3053 | **758.0491** | **2271.1250** | **2271.4922** | **-0.3671** | **1** | **15** | **70** | **1** | **HQHDRVCGDAMFQLQENVK + Oxidation (M)** |
|  | 799 | **406.7936** | **1217.3586** | **1217.3345** | **0.0241** | **1** | **15** | **89** | **1** | **FSSSVRIHER** |
|  | 1640 | **521.8159** | **1562.4256** | **1563.5336** | **-1.1080** | **1** | **15** | **74** | **1** | **RSYSSSSTEEDTSK** |
|  | 645 | **401.9145** | **801.8142** | **800.8140** | **1.0002** | **0** | **15** | **1.1e+02** | **1** | **APVDEDR** |
|  | 1070 | **429.8112** | **1286.4115** | **1286.4759** | **-0.0644** | **0** | **15** | **1e+02** | **1** | **LKPGDPGGSAFLK** |
|  | 1260 | **459.1521** | **1374.4341** | **1374.6075** | **-0.1733** | **0** | **15** | **1e+02** | **1** | **MFSEVLHGLIGR + Oxidation (M)** |
|  | 2190 | **607.8431** | **1213.6715** | **1213.3954** | **0.2761** | **1** | **15** | **77** | **1** | **RPHPPGQLRR** |
|  | 1847 | **547.9435** | **1093.8723** | **1093.1907** | **0.6816** | **1** | **15** | **90** | **1** | **GAYGVVEKDR** |
|  | 2926 | **740.4453** | **2218.3138** | **2218.5338** | **-0.2201** | **1** | **15** | **92** | **1** | **SMDHATCESRIHTSLIGCIK + Oxidation (M)** |
|  | 692 | **403.9755** | **1208.9043** | **1209.2661** | **-0.3618** | **2** | **15** | **97** | **1** | **GGAFKKTDDDR** |
|  | 694 | **404.0027** | **1208.9858** | **1208.3642** | **0.6216** | **2** | **15** | **96** | **1** | **KRGTLVDYEK** |
|  | 884 | **407.9926** | **813.9703** | **814.9264** | **-0.9561** | **0** | **15** | **95** | **1** | **GIAQGLEK** |
|  | 338 | **386.2343** | **1155.6806** | **1156.2480** | **-0.5675** | **0** | **14** | **68** | **1** | **YSEPPHGLTR** |
|  | 2802 | **696.7995** | **1391.5842** | **1392.6457** | **-1.0615** | **0** | **14** | **1.1e+02** | **1** | **SWLCLIASSCPR + Carbamidomethyl (C)** |
|  | 998 | **419.7675** | **1256.2803** | **1255.3873** | **0.8929** | **2** | **14** | **86** | **1** | **RAGRNLGEGVAR** |
|  | 239 | **379.8510** | **1136.5310** | **1137.3755** | **-0.8445** | **0** | **14** | **1e+02** | **1** | **MSNMALLWR + Oxidation (M)** |
|  | 828 | **407.3916** | **1219.1526** | **1219.4163** | **-0.2637** | **1** | **14** | **89** | **1** | **CPNAYLGIRR + Carbamidomethyl (C)** |
|  | 1488 | **489.6125** | **977.2102** | **976.1949** | **1.0152** | **1** | **14** | **1.1e+02** | **1** | **LTKGMGLTR** |
|  | 2750 | **686.0231** | **2055.0470** | **2054.4239** | **0.6232** | **2** | **14** | **78** | **1** | **HPLLCPRRTMPVTSGCR + 2 Carbamidomethyl (C); Oxidation (M)** |
|  | 800 | **406.8242** | **1217.4506** | **1216.4505** | **1.0000** | **0** | **14** | **91** | **1** | **NILVNSNLVCK** |
|  | 1712 | **532.9160** | **1595.7257** | **1596.8048** | **-1.0791** | **1** | **14** | **96** | **1** | **DEQYMWIQKALR + Oxidation (M)** |
|  | 787 | **406.5676** | **811.1205** | **810.1039** | **1.0166** | **1** | **14** | **80** | **1** | **MMCLKK + Carbamidomethyl (C)** |
|  | 1302 | **463.0900** | **1386.2477** | **1385.6483** | **0.5994** | **1** | **14** | **87** | **1** | **LLSAAKILADATAK** |
|  | 1899 | **557.5183** | **1113.0218** | **1112.1873** | **0.8345** | **0** | **14** | **78** | **1** | **YSSVTEDAIK** |
|  | 2003 | **582.3273** | **1743.9596** | **1745.0118** | **-1.0521** | **2** | **14** | **99** | **1** | **MPQTFRDPATAPLRK + Oxidation (M)** |
|  | 2807 | **698.5747** | **2092.7019** | **2093.2961** | **-0.5941** | **1** | **14** | **75** | **1** | **ERMLSLTQEPGEGQDMQK + Oxidation (M)** |
|  | 396 | **387.8598** | **773.7048** | **772.8897** | **0.8151** | **0** | **14** | **1.1e+02** | **1** | **SALIQNK** |
|  | 578 | **394.0584** | **786.1020** | **786.9579** | **-0.8558** | **0** | **14** | **1e+02** | **1** | **SPLPVFK** |
|  | 794 | **406.7226** | **1217.1455** | **1218.3177** | **-1.1721** | **1** | **14** | **76** | **1** | **QTSGRSGVGVAVT** |
|  | 1893 | **556.2704** | **1665.7892** | **1664.9718** | **0.8174** | **2** | **14** | **1e+02** | **1** | **SVCERAKAGCAPLMR + Carbamidomethyl (C); Oxidation (M)** |
|  | 2621 | **668.8610** | **1335.7071** | **1336.4685** | **-0.7613** | **0** | **14** | **90** | **1** | **ADDTFVYFCAK + Carbamidomethyl (C)** |
|  | 1006 | **420.2029** | **1257.5865** | **1257.3104** | **0.2760** | **0** | **14** | **87** | **1** | **ADGAAAGAGGSPSLR** |
|  | 1102 | **433.0455** | **864.0763** | **862.9362** | **1.1400** | **1** | **14** | **96** | **1** | **AHNTKHR** |
|  | 3443 | **948.1858** | **2841.5352** | **2841.2906** | **0.2446** | **0** | **14** | **79** | **1** | **FMGHSPLERPQLLPSYTASAMFMGR + Oxidation (M)** |
|  | 238 | **379.8474** | **1136.5200** | **1136.3445** | **0.1755** | **1** | **14** | **1.1e+02** | **1** | **ILGGVPAPERK** |
|  | 208 | **377.0702** | **1128.1885** | **1129.3072** | **-1.1187** | **2** | **14** | **92** | **1** | **GDLKGVKGDLK** |
|  | 364 | **387.7473** | **1160.2199** | **1159.3086** | **0.9113** | **0** | **14** | **1.1e+02** | **1** | **MSEMEEEMK + Oxidation (M)** |
|  | 254 | **382.8604** | **1145.5592** | **1146.2782** | **-0.7190** | **1** | **14** | **1e+02** | **1** | **DVVERACQAR** |
|  | 410 | **387.9656** | **1160.8746** | **1160.4736** | **0.4011** | **2** | **14** | **1.2e+02** | **1** | **CSICGKMFKK + Oxidation (M)** |
|  | 885 | **408.0219** | **1221.0434** | **1221.3147** | **-0.2714** | **0** | **14** | **97** | **1** | **IWAEYDPEAK** |
|  | 1003 | **420.1596** | **1257.4566** | **1258.3798** | **-0.9231** | **0** | **14** | **93** | **1** | **DWIVVVAGEDR** |
|  | 2490 | **650.7579** | **1299.5011** | **1299.5244** | **-0.0233** | **2** | **14** | **1.1e+02** | **1** | **QQIPVFRQKR** |
|  | 439 | **388.8364** | **1163.4871** | **1164.3992** | **-0.9121** | **1** | **14** | **1.1e+02** | **1** | **EMCKLLNTR + Carbamidomethyl (C)** |
|  | 2171 | **606.4427** | **1210.8706** | **1210.4264** | **0.4442** | **1** | **14** | **78** | **1** | **VRPAKASPSLGK** |
|  | 356 | **387.6514** | **773.2880** | **773.9176** | **-0.6296** | **1** | **14** | **95** | **1** | **DGTIKIK** |
|  | 826 | **407.3533** | **1219.0378** | **1219.4083** | **-0.3705** | **1** | **14** | **76** | **1** | **EEALMELKTR** |
|  | 1007 | **420.2061** | **1257.5961** | **1257.4362** | **0.1598** | **0** | **14** | **87** | **1** | **SLIVAGLGDGSIR** |
|  | 684 | **403.9344** | **805.8540** | **805.8536** | **0.0004** | **0** | **14** | **99** | **1** | **AMPSGPSSG + Oxidation (M)** |
|  | 442 | **388.8927** | **775.7706** | **775.8772** | **-0.1067** | **1** | **14** | **1.1e+02** | **1** | **RAAACER** |
|  | 95 | **369.2628** | **1104.7664** | **1104.2746** | **0.4918** | **0** | **14** | **85** | **1** | **AIDPSLVDMK + Oxidation (M)** |
|  | 737 | **405.1815** | **1212.5224** | **1213.5791** | **-1.0567** | **2** | **14** | **88** | **1** | **KVLGACKLLLR** |
|  | 2098 | **595.8797** | **1784.6169** | **1783.9767** | **0.6403** | **1** | **14** | **83** | **1** | **AKMNTGMEESEHFIK + 2 Oxidation (M)** |
|  | 2145 | **601.3154** | **1800.9241** | **1799.7639** | **1.1602** | **0** | **14** | **1e+02** | **1** | **AEAGAEAGGGAGPGAEDEAGR** |
|  | 1254 | **458.8680** | **915.7212** | **915.0921** | **0.6292** | **1** | **14** | **1.1e+02** | **1** | **GCTKMFR + Carbamidomethyl (C); Oxidation (M)** |
|  | 2162 | **605.3583** | **1813.0527** | **1812.9778** | **0.0748** | **0** | **14** | **1e+02** | **1** | **QLQPHPYLEEAAFNR** |
|  | 1688 | **528.5197** | **1582.5368** | **1581.7243** | **0.8125** | **2** | **14** | **92** | **1** | **GEKGEMGEKGEMGDK** |
|  | 249 | **380.1708** | **1137.4902** | **1138.2411** | **-0.7509** | **2** | **14** | **1e+02** | **1** | **ARRGGGGSAPPR** |
|  | 853 | **407.7626** | **1220.2655** | **1219.4959** | **0.7696** | **0** | **14** | **98** | **1** | **AVFQCAVLALK + Carbamidomethyl (C)** |
|  | 29 | **363.1810** | **1086.5207** | **1087.2673** | **-0.7465** | **0** | **14** | **82** | **1** | **LSPTVVGLSSK** |
|  | 1033 | **423.0222** | **844.0297** | **843.0094** | **1.0202** | **0** | **14** | **1.1e+02** | **1** | **ACQRPLR** |
|  | 2125 | **599.4059** | **1196.7971** | **1197.4538** | **-0.6567** | **1** | **14** | **89** | **1** | **GALRCLLGPAAR** |
|  | 178 | **374.0883** | **746.1618** | **746.8097** | **-0.6479** | **0** | **14** | **1.2e+02** | **1** | **DGTVTVR** |
|  | 272 | **385.0147** | **1152.0220** | **1151.3326** | **0.6894** | **0** | **14** | **85** | **1** | **EFAAMEAAALK** |
|  | 849 | **407.6957** | **1220.0650** | **1221.2518** | **-1.1868** | **0** | **14** | **80** | **1** | **EASCSQDGNLPT** |
|  | 1065 | **429.2541** | **1284.7401** | **1283.5815** | **1.1586** | **2** | **14** | **82** | **1** | **CAIEADMKMKK + Oxidation (M)** |
|  | 2328 | **623.7424** | **1245.4701** | **1246.3922** | **-0.9221** | **1** | **14** | **1.2e+02** | **1** | **SPTDPKACNWK** |
|  | 585 | **396.3127** | **1185.9159** | **1186.3982** | **-0.4824** | **0** | **14** | **94** | **1** | **VLELVSITANK** |
|  | 697 | **404.0250** | **806.0352** | **805.8352** | **0.2000** | **0** | **14** | **1e+02** | **1** | **QGGSGWSK** |
|  | 749 | **405.5078** | **1213.5013** | **1214.4117** | **-0.9104** | **0** | **14** | **1.1e+02** | **1** | **SFQPITMSCK + Carbamidomethyl (C); Oxidation (M)** |
|  | 1767 | **538.2062** | **1074.3977** | **1074.2155** | **0.1822** | **1** | **14** | **1e+02** | **1** | **RAAGGVPSMGR + Oxidation (M)** |
|  | 311 | **386.0526** | **1155.1358** | **1154.2969** | **0.8389** | **2** | **14** | **90** | **1** | **KRAFQDMDK + Oxidation (M)** |
|  | 1109 | **433.1484** | **1296.4231** | **1296.5319** | **-0.1088** | **1** | **14** | **99** | **1** | **QYESLKILICS** |
|  | 3471 | **956.8662** | **2867.5765** | **2868.2212** | **-0.6448** | **0** | **14** | **71** | **1** | **GGCASGLYPDAFAPVAQFVNWIDSIIV + Carbamidomethyl (C)** |
|  | 1104 | **433.0847** | **1296.2318** | **1296.5319** | **-0.3001** | **1** | **14** | **99** | **1** | **QYESLKILICS** |
|  | 2058 | **592.8616** | **1775.5627** | **1775.0557** | **0.5070** | **1** | **14** | **83** | **1** | **LENITWARYPLAVTK** |
|  | 2107 | **596.7778** | **1191.5408** | **1190.3900** | **1.1507** | **1** | **14** | **1.1e+02** | **1** | **LWGKEGYLPK** |
|  | 2118 | 597.9269 | 1790.7586 | 1790.8401 | -0.0814 | 0 | 14 | 83 | 1 | EGDVGSSFDAMSEQCR + Carbamidomethyl (C); Oxidation (M) |
|  | 251 | **382.2670** | **1143.7788** | **1143.3319** | **0.4469** | **0** | **14** | **95** | **1** | **VEGDIWALLK** |
|  | 1046 | **426.1200** | **1275.3379** | **1274.5133** | **0.8246** | **1** | **14** | **1e+02** | **1** | **GLMCQGPEKVR + Carbamidomethyl (C)** |
|  | 1182 | **444.9080** | **887.8012** | **888.0005** | **-0.1993** | **0** | **14** | **1.2e+02** | **1** | **MELAPGDR** |
|  | 1491 | **490.0495** | **978.0842** | **977.0970** | **0.9873** | **0** | **14** | **1e+02** | **1** | **MAAQAAGVSR + Oxidation (M)** |
|  | 1549 | **505.1349** | **1512.3825** | **1512.7761** | **-0.3936** | **0** | **14** | **96** | **1** | **NMLNPWVPSWLR** |
|  | 2528 | **658.1814** | **1314.3480** | **1314.5076** | **-0.1595** | **0** | **14** | **96** | **1** | **IATENGAPMPLGK + Oxidation (M)** |
|  | 241 | **380.0050** | **757.9952** | **758.8468** | **-0.8515** | **0** | **14** | **1.1e+02** | **1** | **GMGHVSR + Oxidation (M)** |
|  | 456 | **388.9864** | **1163.9369** | **1163.2688** | **0.6681** | **2** | **14** | **1.1e+02** | **1** | **NQSMRGNGRK + Oxidation (M)** |
|  | 804 | **406.8355** | **811.6563** | **811.9243** | **-0.2680** | **0** | **14** | **95** | **1** | **TECATCK + Carbamidomethyl (C)** |
|  | 1107 | **433.1428** | **1296.4061** | **1296.5319** | **-0.1258** | **1** | **14** | **1e+02** | **1** | **QYESLKILICS** |
|  | 2559 | **666.3051** | **1330.5953** | **1330.4474** | **0.1479** | **1** | **14** | **99** | **1** | **VTEVRADTGGLGR** |
|  | 3211 | **815.7258** | **2444.1553** | **2443.8114** | **0.3439** | **1** | **14** | **83** | **1** | **SLGYMPSEVELAIIMQRLDMD + 2 Oxidation (M)** |
|  | 19 | **363.1037** | **1086.2890** | **1087.2706** | **-0.9816** | **0** | **14** | **1e+02** | **1** | **SNSGFMAMVK + Oxidation (M)** |
|  | 1290 | **461.8902** | **1382.6484** | **1382.5883** | **0.0602** | **1** | **14** | **99** | **1** | **LTQRYVSVMNR + Oxidation (M)** |
|  | 1371 | **475.2510** | **1422.7308** | **1423.6399** | **-0.9092** | **0** | **14** | **1.1e+02** | **1** | **CQQGPLQPLQVR + Carbamidomethyl (C)** |
|  | 211 | **377.0925** | **1128.2554** | **1129.3073** | **-1.0518** | **0** | **14** | **96** | **1** | **VLSQQAASVVK** |
|  | 1005 | **420.1839** | **1257.5296** | **1256.4614** | **1.0682** | **1** | **14** | **96** | **1** | **QGLRCVACGHR + Carbamidomethyl (C)** |
|  | 1344 | **471.2825** | **1410.8252** | **1410.5519** | **0.2733** | **1** | **14** | **80** | **1** | **THTGEKSYICSK + Carbamidomethyl (C)** |
|  | 1831 | **544.0638** | **1086.1129** | **1086.3123** | **-0.1994** | **1** | **14** | **1.1e+02** | **1** | **NAVIRVMQR** |
|  | 825 | **407.3281** | **1218.9620** | **1219.3999** | **-0.4378** | **0** | **14** | **77** | **1** | **HCHNMGVLHR + Oxidation (M)** |
|  | 206 | **377.0037** | **751.9925** | **750.8182** | **1.1744** | **0** | **14** | **94** | **1** | **ATMADDK** |
|  | 548 | **391.1303** | **1170.3689** | **1171.3306** | **-0.9617** | **1** | **14** | **96** | **1** | **VIRADPQGCR + Carbamidomethyl (C)** |
|  | 2834 | **707.0330** | **2118.0769** | **2118.4150** | **-0.3381** | **1** | **14** | **79** | **1** | **RGHVFEESQVAGTPMFVVK** |
|  | 3134 | **779.6331** | **2335.8770** | **2335.6019** | **0.2751** | **1** | **14** | **82** | **1** | **DGGVAIIDQWICAHARNPWR + Carbamidomethyl (C)** |
|  | 33 | **363.2502** | **1086.7283** | **1086.1949** | **0.5334** | **0** | **14** | **82** | **1** | **SMDAMSSPTK + 2 Oxidation (M)** |
|  | 833 | **407.4289** | **1219.2646** | **1219.2195** | **0.0451** | **2** | **14** | **1.1e+02** | **1** | **GSDKRGDNQDK** |
|  | 1287 | **461.4937** | **1381.4590** | **1382.6344** | **-1.1755** | **1** | **14** | **1.2e+02** | **1** | **CFEARICACPGR + Carbamidomethyl (C)** |
|  | 1413 | **481.4244** | **1441.2511** | **1440.6838** | **0.5673** | **0** | **14** | **89** | **1** | **VLVSGLQGLGAEVAK** |
|  | 2382 | **628.4315** | **1254.8483** | **1255.4668** | **-0.6186** | **2** | **14** | **95** | **1** | **IKAPSRNTIQK** |
|  | 2642 | **670.5841** | **2008.7301** | **2008.1301** | **0.6000** | **1** | **14** | **82** | **1** | **SNCITENPAGPYGQRTDK + Carbamidomethyl (C)** |
|  | 2986 | **742.5318** | **2224.5732** | **2223.5075** | **1.0657** | **2** | **14** | **94** | **1** | **SHGVMSKACTNDDQMKTPLK + 2 Oxidation (M)** |
|  | 774 | **406.2208** | **1215.6403** | **1216.3217** | **-0.6814** | **0** | **14** | **82** | **1** | **EGTVGSVDGHMK** |
|  | 1041 | **424.8307** | **847.6467** | **847.0346** | **0.6121** | **0** | **14** | **1.1e+02** | **1** | **MIVLNNK + Oxidation (M)** |
|  | 1875 | **551.8461** | **1101.6774** | **1101.3385** | **0.3389** | **1** | **14** | **88** | **1** | **KLPTAFLPSK** |
|  | 2636 | **669.8120** | **2006.4139** | **2007.1883** | **-0.7745** | **2** | **14** | **1.2e+02** | **1** | **LKWEDGLSSGGRGCSELR + Carbamidomethyl (C)** |
|  | 1493 | **490.2273** | **978.4398** | **979.0746** | **-0.6348** | **1** | **14** | **1e+02** | **1** | **TYHSCRR + Carbamidomethyl (C)** |
|  | 2711 | **683.9940** | **2048.9599** | **2048.2571** | **0.7028** | **0** | **14** | **83** | **1** | **GSPGAMEPQCQFPDKPSSK + Carbamidomethyl (C)** |
|  | 948 | **414.3110** | **1239.9107** | **1239.4526** | **0.4582** | **2** | **14** | **72** | **1** | **MFHRKDHLR** |
|  | 1604 | **517.1890** | **1548.5447** | **1548.6115** | **-0.0668** | **1** | **14** | **1.1e+02** | **1** | **DSAQTSVTQAQREK** |
|  | 2814 | **699.7628** | **2096.2663** | **2096.3415** | **-0.0752** | **0** | **14** | **1.1e+02** | **1** | **SYELMQPPSVSVSPGQMAR + 2 Oxidation (M)** |
|  | 1023 | **422.1005** | **842.1863** | **842.9830** | **-0.7968** | **1** | **14** | **1.1e+02** | **1** | **AGVLAERK** |
|  | 2191 | **607.8447** | **1213.6745** | **1212.5069** | **1.1677** | **2** | **14** | **85** | **1** | **LCTCRKDMVK + Oxidation (M)** |
|  | 3373 | **924.6998** | **2771.0773** | **2772.1624** | **-1.0850** | **1** | **14** | **92** | **1** | **GSNVALMLDVRSLGAVEPICSVNTPR + Carbamidomethyl (C); Oxidation (M)** |
|  | 3533 | **976.2872** | **1950.5597** | **1951.3371** | **-0.7774** | **0** | **14** | **74** | **1** | **AGLGMCALAAALVVHCYSK + Carbamidomethyl (C); Oxidation (M)** |
|  | 1415 | **481.8286** | **1442.4636** | **1442.6467** | **-0.1831** | **2** | **14** | **99** | **1** | **RGFCAPAGGRSGMC + Carbamidomethyl (C); Oxidation (M)** |
|  | 494 | **389.9082** | **1166.7025** | **1166.3272** | **0.3753** | **0** | **14** | **1e+02** | **1** | **QALSIPDPLGR** |
|  | 728 | **405.0121** | **808.0094** | **806.9725** | **1.0370** | **0** | **14** | **95** | **1** | **FAPAVMR + Oxidation (M)** |
|  | 2077 | **594.1729** | **1779.4964** | **1780.0110** | **-0.5146** | **1** | **14** | **1.1e+02** | **1** | **YSKDTGLMSCGWCQK + Carbamidomethyl (C); Oxidation (M)** |
|  | 1409 | **481.0214** | **960.0279** | **959.0983** | **0.9297** | **1** | **14** | **1.1e+02** | **1** | **VSQELKGAK** |
|  | 3272 | **843.5581** | **1685.1014** | **1686.0058** | **-0.9043** | **0** | **14** | **98** | **1** | **TQMAALCQGPSKPLPK + Oxidation (M)** |
|  | 1283 | **460.8165** | **1379.4272** | **1379.5827** | **-0.1554** | **0** | **14** | **1e+02** | **1** | **ACDHMPSLMSSK + Carbamidomethyl (C); Oxidation (M)** |
|  | 2444 | **640.5763** | **1279.1378** | **1279.4254** | **-0.2875** | **0** | **14** | **83** | **1** | **MAEHNPNLTPR** |
|  | 1630 | **520.2493** | **1038.4838** | **1038.1336** | **0.3502** | **0** | **14** | **1e+02** | **1** | **MDSIGSSGLR + Oxidation (M)** |
|  | 252 | **382.3493** | **762.6839** | **761.8472** | **0.8366** | **0** | **14** | **1.1e+02** | **1** | **CDLNAAR** |
|  | 264 | **384.9285** | **767.8422** | **767.8304** | **0.0118** | **0** | **14** | **89** | **1** | **GSPSPPAR** |
|  | 707 | **404.1318** | **1209.3732** | **1209.2727** | **0.1005** | **2** | **14** | **1e+02** | **1** | **RGSSKGHDTHK** |
|  | 482 | **389.1448** | **1164.4122** | **1164.2670** | **0.1452** | **0** | **14** | **1.1e+02** | **1** | **SMMSSYTADR + Oxidation (M)** |
|  | 307 | **386.0318** | **770.0488** | **769.8264** | **0.2225** | **0** | **14** | **91** | **1** | **CVHSSHT** |
|  | 503 | **389.9678** | **777.9208** | **776.9018** | **1.0190** | **0** | **14** | **1.1e+02** | **1** | **DLSGMVR** |
|  | 1162 | **440.5262** | **1318.5564** | **1318.5626** | **-0.0062** | **0** | **14** | **1.4e+02** | **1** | **IPLGPSEMSTMR** |
|  | 629 | **401.0576** | **800.1005** | **800.9464** | **-0.8459** | **1** | **14** | **1.2e+02** | **1** | **TVKANIR** |
|  | 2756 | **686.1340** | **2055.3799** | **2054.4205** | **0.9595** | **1** | **14** | **1e+02** | **1** | **MMKNRPFMGSISQQNIR + Oxidation (M)** |
|  | 268 | **384.9548** | **767.8948** | **766.7561** | **1.1386** | **0** | **14** | **90** | **1** | **AQDYDR** |
|  | 2337 | **623.9514** | **1868.8321** | **1869.1073** | **-0.2753** | **1** | **14** | **90** | **1** | **NHSNTITGAKQIPCSLK + Carbamidomethyl (C)** |
|  | 657 | **402.1330** | **1203.3768** | **1204.4367** | **-1.0598** | **0** | **14** | **1.2e+02** | **1** | **NGMLDLSVVLK + Oxidation (M)** |
|  | 1714 | **533.1250** | **1596.3528** | **1596.8297** | **-0.4768** | **1** | **14** | **1.1e+02** | **1** | **MFGCDLGQDGRLLR + Oxidation (M)** |
|  | 78 | **369.1738** | **1104.4992** | **1105.3089** | **-0.8096** | **0** | **14** | **1e+02** | **1** | **AKPWAVCFPS** |
|  | 851 | **407.7269** | **1220.1586** | **1220.2442** | **-0.0855** | **0** | **14** | **94** | **1** | **TSSAEESPTGVR** |
|  | 952 | **415.9098** | **829.8049** | **828.8933** | **0.9115** | **0** | **14** | **1.2e+02** | **1** | **TGACLHGN + Carbamidomethyl (C)** |
|  | 1599 | **515.2288** | **1542.6641** | **1542.6945** | **-0.0304** | **2** | **14** | **1.1e+02** | **1** | **DLDRRLFLDHDK** |
|  | 2859 | **716.4538** | **2146.3392** | **2145.3971** | **0.9421** | **0** | **14** | **1e+02** | **1** | **LCDLSSEHTTVCTTGMPHR + Carbamidomethyl (C)** |
|  | 907 | **409.0906** | **816.1664** | **815.9593** | **0.2071** | **1** | **14** | **1.2e+02** | **1** | **GAKVQWK** |
|  | 2355 | **625.8732** | **1874.5973** | **1875.2048** | **-0.6074** | **2** | **14** | **87** | **1** | **PEGRNALMLCAPRMSR + Carbamidomethyl (C); Oxidation (M)** |
|  | 3001 | **743.6689** | **1485.3230** | **1485.6616** | **-0.3386** | **0** | **14** | **83** | **1** | **DMAGASGGVAAPLPQK + Oxidation (M)** |
|  | 3241 | **824.9342** | **2471.7804** | **2472.7103** | **-0.9298** | **1** | **14** | **1.2e+02** | **1** | **QPGASPSQERKPTGVSVIYWER** |
|  | 691 | **403.9720** | **805.9293** | **804.8721** | **1.0573** | **0** | **14** | **1.1e+02** | **1** | **AAGQGDMR** |
|  | 704 | **404.1151** | **1209.3231** | **1209.2727** | **0.0504** | **2** | **14** | **1.1e+02** | **1** | **RGSSKGHDTHK** |
|  | 164 | **372.3221** | **1113.9441** | **1113.2897** | **0.6544** | **1** | **14** | **89** | **1** | **MPDCTSKCR + Carbamidomethyl (C); Oxidation (M)** |
|  | 316 | **386.0873** | **1155.2398** | **1154.2951** | **0.9447** | **0** | **14** | **99** | **1** | **SSMVNFLGFH + Oxidation (M)** |
|  | 945 | **413.8210** | **1238.4408** | **1237.4468** | **0.9940** | **0** | **14** | **93** | **1** | **NMDMKPGSTLK + Oxidation (M)** |
|  | 1119 | **434.8326** | **867.6503** | **867.9924** | **-0.3421** | **0** | **14** | **1e+02** | **1** | **HCSYACK + Carbamidomethyl (C)** |
|  | 1496 | **492.1639** | **982.3130** | **981.1668** | **1.1462** | **1** | **14** | **1e+02** | **1** | **SFQKELVM** |
|  | 2726 | **684.4318** | **1366.8489** | **1367.5572** | **-0.7083** | **2** | **14** | **1e+02** | **1** | **TAAPSVRPEKRR** |
|  | 408 | **387.9511** | **1160.8312** | **1161.3291** | **-0.4980** | **0** | **14** | **1.3e+02** | **1** | **LEAGAMVLADR + Oxidation (M)** |
|  | 467 | **389.0352** | **776.0556** | **774.9720** | **1.0836** | **0** | **14** | **1.2e+02** | **1** | **MLVLQR + Oxidation (M)** |
|  | 703 | **404.1124** | **1209.3151** | **1208.3624** | **0.9527** | **0** | **14** | **1.1e+02** | **1** | **IASGPTTAACME + Carbamidomethyl (C)** |
|  | 837 | **407.4760** | **1219.4058** | **1219.3983** | **0.0075** | **2** | **14** | **1.3e+02** | **1** | **QNPCGSKACRR** |
|  | 2573 | **666.6709** | **1996.9905** | **1997.3013** | **-0.3108** | **1** | **14** | **1e+02** | **1** | **CEMPRSLTLEVCQCDNR** |
|  | 3435 | **947.5980** | **2839.7719** | **2839.3556** | **0.4163** | **2** | **14** | **98** | **1** | **LLFAPNLLLDSNKGMEHLYSMKCK + Carbamidomethyl (C); Oxidation (M)** |
|  | 75 | **369.1086** | **1104.3036** | **1105.3089** | **-1.0053** | **0** | **14** | **1.2e+02** | **1** | **AKPWAVCFPS** |
|  | 412 | **387.9841** | **773.9535** | **774.8661** | **-0.9126** | **2** | **14** | **1.3e+02** | **1** | **KGESRAK** |
|  | 1815 | **542.2750** | **1623.8029** | **1624.7387** | **-0.9358** | **1** | **14** | **1.1e+02** | **1** | **GGMPNRGNYNQNFR** |
|  | 2011 | **583.9190** | **1748.7348** | **1748.9971** | **-0.2622** | **1** | **14** | **98** | **1** | **SAVSRAGSLLWMVATSP + Oxidation (M)** |
|  | 2654 | **671.6697** | **2011.9869** | **2011.2320** | **0.7548** | **0** | **14** | **1e+02** | **1** | **EVVYTDYIDISVAVATPR** |
|  | 103 | **369.2950** | **1104.8630** | **1105.3089** | **-0.4459** | **0** | **14** | **96** | **1** | **AKPWAVCFPS** |
|  | 977 | **419.0204** | **836.0261** | **835.8796** | **0.1466** | **0** | **14** | **1.3e+02** | **1** | **TPCDEGSK** |
|  | 2096 | **595.7407** | **1189.4665** | **1188.3944** | **1.0722** | **0** | **14** | **1.2e+02** | **1** | **IVPVVSQSECK** |
|  | 956 | **415.9907** | **829.9665** | **828.9599** | **1.0067** | **1** | **14** | **1.3e+02** | **1** | **AARVAVSR** |
|  | 1544 | **505.0418** | **1512.1033** | **1511.7253** | **0.3779** | **2** | **14** | **1e+02** | **1** | **RYPSGEERCIACK** |
|  | 2335 | **623.9039** | **1245.7931** | **1245.3444** | **0.4487** | **0** | **14** | **89** | **1** | **CTSCNWDFR + 2 Carbamidomethyl (C)** |
|  | 3446 | **948.5825** | **1895.1503** | **1894.9859** | **0.1644** | **0** | **14** | **1e+02** | **1** | **NVSIPSSEALSSDPSYNK** |
|  | 880 | **407.9496** | **1220.8266** | **1221.4556** | **-0.6290** | **2** | **14** | **1.1e+02** | **1** | **LSRRALPGPVR** |
|  | 1562 | **507.3467** | **1519.0180** | **1519.8069** | **-0.7889** | **0** | **14** | **88** | **1** | **IYISGMAPRPSLAK + Oxidation (M)** |
|  | 1900 | 557.9742 | 1670.9006 | 1669.9766 | 0.9240 | 2 | 14 | 1.1e+02 | 1 | MPRIGQGRRPVCAGR + Oxidation (M) |
|  | 2732 | **685.0349** | **2052.0826** | **2052.3783** | **-0.2957** | **2** | **14** | **1e+02** | **1** | **GCPDNITVMVVKFRNSSK + Carbamidomethyl (C)** |
|  | 3271 | **843.4116** | **1684.8085** | **1684.8469** | **-0.0385** | **0** | **14** | **1e+02** | **1** | **LNFDLIQELSHEAR** |
|  | 2604 | **668.4945** | **2002.4613** | **2003.1768** | **-0.7154** | **1** | **14** | **1e+02** | **1** | **KPGREESPSQASGFSLVAR** |
|  | 126 | **369.3777** | **1105.1111** | **1105.3089** | **-0.1978** | **0** | **14** | **1.3e+02** | **1** | **AKPWAVCFPS** |
|  | 290 | 385.9564 | 769.8979 | 769.9322 | -0.0343 | 0 | 14 | 95 | 1 | GALAALVR |
|  | 525 | **390.0352** | **1167.0833** | **1166.2695** | **0.8138** | **2** | **14** | **1.1e+02** | **1** | **ASCSGKRQSSR** |
|  | 781 | **406.3674** | **1216.0801** | **1216.3017** | **-0.2215** | **1** | **14** | **90** | **1** | **KEELNQSVNR** |
|  | 1425 | **484.0096** | **966.0045** | **967.1896** | **-1.1851** | **0** | **14** | **1.1e+02** | **1** | **LALCALHAR** |
|  | 1019 | **421.8316** | **1262.4726** | **1263.3814** | **-0.9087** | **1** | **14** | **1.2e+02** | **1** | **MESTPSRGLNR + Oxidation (M)** |
|  | 869 | **407.8597** | **813.7046** | **814.8437** | **-1.1391** | **0** | **14** | **1.1e+02** | **1** | **QNSPAGNK** |
|  | 2265 | **613.2690** | **1224.5233** | **1225.4607** | **-0.9374** | **0** | **14** | **1.1e+02** | **1** | **ALAGCVVQLSHK** |
|  | 2342 | **624.1536** | **1869.4385** | **1870.0707** | **-0.6322** | **1** | **14** | **1.1e+02** | **1** | **INRTLETANCMSSQTK + Carbamidomethyl (C); Oxidation (M)** |
|  | 2574 | **666.6901** | **1997.0480** | **1997.3260** | **-0.2780** | **2** | **14** | **1.2e+02** | **1** | **RLLRQIVIQNENTMPR + Oxidation (M)** |
|  | 79 | **369.1750** | **1104.5028** | **1103.3395** | **1.1633** | **2** | **14** | **1e+02** | **1** | **INVAAKRGMK + Oxidation (M)** |
|  | 390 | **387.8375** | **773.6603** | **774.7336** | **-1.0733** | **0** | **14** | **1.3e+02** | **1** | **DEGSDPR** |
|  | 570 | **392.4374** | **782.8600** | **782.8848** | **-0.0247** | **0** | **14** | **1.2e+02** | **1** | **KPAPGADK** |
|  | 673 | **403.0251** | **1206.0531** | **1206.4808** | **-0.4277** | **2** | **14** | **1.2e+02** | **1** | **GKKAAPVLPVAR** |
|  | 939 | **413.3514** | **824.6880** | **825.8698** | **-1.1817** | **0** | **14** | **79** | **1** | **AVHSQER** |
|  | 1087 | **431.2623** | **860.5099** | **861.0614** | **-0.5515** | **0** | **14** | **1.1e+02** | **1** | **TAVAAVAMK** |
|  | 1852 | **549.5787** | **1645.7140** | **1644.8132** | **0.9009** | **1** | **14** | **1.2e+02** | **1** | **MAPRTPHPSPAHSSR + Oxidation (M)** |
|  | 116 | **369.3316** | **1104.9725** | **1104.2547** | **0.7179** | **0** | **14** | **1.1e+02** | **1** | **LDTYCMSAK + Carbamidomethyl (C); Oxidation (M)** |
|  | 926 | **411.6114** | **1231.8122** | **1231.4801** | **0.3320** | **0** | **14** | **97** | **1** | **APLLLIYDVSK** |
|  | 986 | **419.2333** | **836.4519** | **835.9259** | **0.5260** | **1** | **14** | **1.1e+02** | **1** | **CKDAGQSK** |
|  | 2914 | **738.7473** | **1475.4797** | **1474.5710** | **0.9088** | **0** | **14** | **1.1e+02** | **1** | **PGGSGPGFPTLEGSSK** |
|  | 603 | **399.2408** | **1194.7003** | **1194.4435** | **0.2568** | **1** | **14** | **78** | **1** | **MLAKISTTWK + Oxidation (M)** |
|  | 785 | **406.5222** | **1216.5446** | **1217.4156** | **-0.8710** | **0** | **14** | **1.2e+02** | **1** | **CPANMTPALDK + Carbamidomethyl (C)** |
|  | 1993 | **580.6317** | **1738.8728** | **1738.9162** | **-0.0434** | **0** | **14** | **1.4e+02** | **1** | **MQHPASGPAEVLSSSPK + Oxidation (M)** |
|  | 185 | **374.1804** | **1119.5191** | **1120.3170** | **-0.7980** | **1** | **14** | **1.1e+02** | **1** | **KMIETEELK** |
|  | 204 | **376.2103** | **750.4058** | **750.8214** | **-0.4156** | **0** | **14** | **86** | **1** | **SAAGAGCSK** |
|  | 1282 | **460.7528** | **1379.2363** | **1379.5645** | **-0.3282** | **2** | **14** | **95** | **1** | **AQKDKGLHVEVR** |
|  | 1472 | **488.3642** | **974.7136** | **974.0698** | **0.6438** | **0** | **14** | **97** | **1** | **AQTAVSELR** |
|  | 2839 | **709.2634** | **1416.5121** | **1416.5748** | **-0.0627** | **0** | **14** | **1.1e+02** | **1** | **VSESSPPGTAFPLK** |
|  | 294 | **385.9830** | **1154.9269** | **1154.2769** | **0.6500** | **0** | **14** | **96** | **1** | **MDGPGFGGMNR + Oxidation (M)** |
|  | 1089 | **432.1115** | **1293.3123** | **1292.3762** | **0.9361** | **0** | **14** | **1.2e+02** | **1** | **STNSHLGTSNMK + Oxidation (M)** |
|  | 1680 | **525.4340** | **1048.8531** | **1049.2290** | **-0.3759** | **0** | **14** | **94** | **1** | **CTLSRPCR + 2 Carbamidomethyl (C)** |
|  | 2172 | **606.6235** | **1211.2323** | **1212.3760** | **-1.1437** | **1** | **14** | **1.1e+02** | **1** | **GAMDSKTFLSR** |
|  | 2354 | **625.8511** | **1874.5310** | **1874.2366** | **0.2944** | **2** | **14** | **94** | **1** | **HTGQTVLLTLMRRGMK + 2 Oxidation (M)** |
|  | 113 | **369.3279** | **1104.9614** | **1104.3011** | **0.6603** | **0** | **14** | **1.1e+02** | **1** | **MHPSLATMGK + 2 Oxidation (M)** |
|  | 404 | **387.9106** | **773.8065** | **772.8501** | **0.9564** | **1** | **14** | **1.4e+02** | **1** | **EAERLR** |
|  | 567 | **392.4094** | **782.8040** | **782.8848** | **-0.0808** | **0** | **14** | **1.2e+02** | **1** | **KPAPGADK** |
|  | 933 | **412.6989** | **1235.0745** | **1235.3217** | **-0.2472** | **0** | **14** | **84** | **1** | **ELPEMDATSSR** |
|  | 1278 | 460.7346 | 1379.1815 | 1378.6360 | 0.5455 | 1 | 14 | 95 | 1 | VDFLKGMLQAEK |
|  | 1873 | **551.8275** | **1652.4602** | **1652.9892** | **-0.5290** | **2** | **14** | **96** | **1** | **MPRGLRPGCPVRTGR** |
|  | 2350 | **625.1727** | **1248.3307** | **1247.4234** | **0.9073** | **1** | **14** | **1.2e+02** | **1** | **MFAELEARHK + Oxidation (M)** |
|  | 2739 | **685.2804** | **2052.8190** | **2052.2905** | **0.5285** | **0** | **14** | **1.2e+02** | **1** | **GWNACPEGRPEFSEVVMK + Oxidation (M)** |
|  | 2766 | **686.3610** | **2056.0607** | **2056.4992** | **-0.4384** | **1** | **14** | **1.1e+02** | **1** | **LHLRCPVFMSFLPLPNR + Oxidation (M)** |
|  | 230 | **379.3640** | **756.7132** | **756.8970** | **-0.1839** | **2** | **14** | **1.2e+02** | **1** | **LAGRGRK** |
|  | 438 | **388.8359** | **1163.4855** | **1163.2359** | **0.2497** | **1** | **14** | **1.3e+02** | **1** | **EESSSSKPGKK** |
|  | 1000 | **419.8161** | **1256.4262** | **1255.4222** | **1.0041** | **1** | **14** | **1.1e+02** | **1** | **EVIDGVPGKWR** |
|  | 1435 | **486.0254** | **1455.0540** | **1454.6077** | **0.4463** | **0** | **14** | **1e+02** | **1** | **GSMGDPGLPGPQGLR + Oxidation (M)** |
|  | 2613 | **668.7003** | **1335.3859** | **1334.5453** | **0.8405** | **2** | **14** | **1.3e+02** | **1** | **ETGIKALRSGMR + Oxidation (M)** |
|  | 65 | **367.3134** | **1098.9180** | **1098.3577** | **0.5603** | **0** | **14** | **1.1e+02** | **1** | **CIQMAISLM + Carbamidomethyl (C); 2 Oxidation (M)** |
|  | 82 | **369.1892** | **1104.5455** | **1105.3089** | **-0.7634** | **0** | **14** | **1e+02** | **1** | **AKPWAVCFPS** |
|  | 129 | **369.4064** | **1105.1969** | **1105.2443** | **-0.0474** | **1** | **14** | **1.4e+02** | **1** | **TIHTGGKTYK** |
|  | 428 | **388.4679** | **1162.3814** | **1163.4129** | **-1.0315** | **1** | **14** | **1.6e+02** | **1** | **VVAGVAKALAHK** |
|  | 667 | **402.8355** | **1205.4844** | **1205.2741** | **0.2104** | **0** | **14** | **1.3e+02** | **1** | **SVDEFPEINR** |
|  | 863 | **407.8372** | **813.6597** | **812.8709** | **0.7887** | **0** | **14** | **1.2e+02** | **1** | **EHASLTR** |
|  | 2958 | **741.2906** | **1480.5664** | **1479.6568** | **0.9095** | **0** | **14** | **1.1e+02** | **1** | **ACNLDVILGFDGSR** |
|  | 76 | **369.1641** | **1104.4700** | **1105.3089** | **-0.8389** | **0** | **14** | **1.1e+02** | **1** | **AKPWAVCFPS** |
|  | 968 | **418.0193** | **1251.0357** | **1251.4548** | **-0.4191** | **0** | **14** | **1.2e+02** | **1** | **ALLASNSCFIR + Carbamidomethyl (C)** |
|  | 1314 | **466.0143** | **930.0138** | **929.0291** | **0.9847** | **0** | **14** | **1.2e+02** | **1** | **ISPGQAGTAK** |
|  | 1389 | **476.1039** | **1425.2896** | **1425.5466** | **-0.2570** | **0** | **14** | **1.2e+02** | **1** | **HQFPHFLEENK** |
|  | 1975 | **576.6641** | **1726.9702** | **1727.7906** | **-0.8205** | **1** | **14** | **1.3e+02** | **1** | **CPGGSHEAGRCEADPAG + 2 Carbamidomethyl (C)** |
|  | 26 | **363.1660** | **1086.4760** | **1086.3255** | **0.1505** | **0** | **14** | **99** | **1** | **FIACLMSTK + Carbamidomethyl (C); Oxidation (M)** |
|  | 729 | **405.1041** | **1212.2902** | **1211.2867** | **1.0035** | **0** | **14** | **1.1e+02** | **1** | **NNPNGFQVHGK** |
|  | 1390 | **476.2178** | **1425.6313** | **1424.6877** | **0.9436** | **0** | **14** | **1.1e+02** | **1** | **TTCFICGLERPK + Carbamidomethyl (C)** |
|  | 1768 | **538.2386** | **1611.6938** | **1610.8547** | **0.8391** | **1** | **14** | **1.2e+02** | **1** | **QPCWSFPMGQKTK + Carbamidomethyl (C); Oxidation (M)** |
|  | 2716 | **684.0437** | **2049.1089** | **2049.3741** | **-0.2652** | **0** | **14** | **1e+02** | **1** | **AVLGCLCSASVLGSYAGPAPR + Carbamidomethyl (C)** |
|  | 510 | **389.9931** | **777.9715** | **778.9043** | **-0.9328** | **1** | **14** | **1.1e+02** | **1** | **GCQRCR + Carbamidomethyl (C)** |
|  | 3439 | **947.6975** | **2840.0704** | **2839.1008** | **0.9695** | **1** | **14** | **1e+02** | **1** | **LSSVTAADTAVYYCARGYCSGGSCYR + 2 Carbamidomethyl (C)** |
|  | 810 | **406.9779** | **1217.9115** | **1218.3108** | **-0.3993** | **0** | **14** | **1e+02** | **1** | **QLSSELGDLEK** |
|  | 2994 | **742.8365** | **2225.4875** | **2225.5612** | **-0.0737** | **1** | **14** | **1.3e+02** | **1** | **TLTPWGVFLENMGKEDFIK** |
|  | 293 | **385.9773** | **1154.9098** | **1154.2969** | **0.6129** | **0** | **14** | **98** | **1** | **FMAASVASTNR** |
|  | 1520 | **498.5748** | **1492.7023** | **1491.7107** | **0.9917** | **1** | **14** | **1.3e+02** | **1** | **GSSGSSGPIKMGWLK** |
|  | 1080 | **431.0319** | **1290.0734** | **1290.5075** | **-0.4341** | **1** | **14** | **1.4e+02** | **1** | **AIASLKGFAGLDK** |
|  | 218 | **377.3116** | **1128.9128** | **1129.3486** | **-0.4358** | **0** | **14** | **89** | **1** | **VAPFTLGPSLK** |
|  | 2263 | **612.8571** | **1835.5490** | **1836.0478** | **-0.4988** | **1** | **14** | **93** | **1** | **REAGISDYLTIEELVK** |
|  | 529 | **390.0454** | **1167.1140** | **1166.1537** | **0.9603** | **1** | **14** | **1.2e+02** | **1** | **SESSSPRSSGSV** |
|  | 1830 | **543.9967** | **1085.9786** | **1085.2363** | **0.7423** | **0** | **14** | **1.3e+02** | **1** | **CVHEYIHK + Carbamidomethyl (C)** |
|  | 100 | **369.2843** | **1104.8308** | **1105.3089** | **-0.4780** | **0** | **14** | **1e+02** | **1** | **AKPWAVCFPS** |
|  | 827 | **407.3861** | **1219.1362** | **1218.3141** | **0.8220** | **1** | **14** | **1.1e+02** | **1** | **LKLSSGNEENK** |
|  | 2004 | **582.5655** | **1744.6743** | **1744.8561** | **-0.1818** | **2** | **14** | **1.1e+02** | **1** | **DKDHPSDPSGKSLSFK** |
|  | 2041 | **591.2869** | **1770.8384** | **1771.0903** | **-0.2518** | **1** | **14** | **1.2e+02** | **1** | **LWPPTRAAPPGLPTCPP** |
|  | 2287 | **616.2900** | **1845.8479** | **1845.0197** | **0.8283** | **0** | **14** | **1.2e+02** | **1** | **DNSNNMLYLQMNSLR + 2 Oxidation (M)** |
|  | 2545 | **663.6871** | **1988.0392** | **1988.2549** | **-0.2156** | **1** | **14** | **1.2e+02** | **1** | **MCLSGSTCRAMRPESGR + 2 Carbamidomethyl (C); 2 Oxidation (M)** |
|  | 3569 | **1092.4883** | **3274.4427** | **3274.3998** | **0.0429** | **2** | **14** | **84** | **1** | **ARTEAQSLDSAVPLTNGDTEDDADKMHVDR + Oxidation (M)** |
|  | 1349 | **472.1163** | **942.2179** | **941.0830** | **1.1349** | **0** | **14** | **1.1e+02** | **1** | **NSLGVPAGVK** |
|  | 1468 | **488.2726** | **1461.7956** | **1461.6416** | **0.1539** | **1** | **14** | **1.2e+02** | **1** | **YEKCGHENLQLK** |
|  | 2656 | **671.7925** | **1341.5702** | **1340.4919** | **1.0783** | **2** | **14** | **1.3e+02** | **1** | **DSGRRLRPIDR** |
|  | 441 | **388.8914** | **1163.6520** | **1164.4424** | **-0.7904** | **1** | **14** | **1.3e+02** | **1** | **TLMLMAQKGR + Oxidation (M)** |
|  | 574 | **392.5464** | **1174.6169** | **1175.3591** | **-0.7422** | **2** | **14** | **97** | **1** | **KDDARAQLMK** |
|  | 705 | **404.1215** | **806.2281** | **805.8569** | **0.3713** | **0** | **14** | **1.2e+02** | **1** | **DTPACSR + Carbamidomethyl (C)** |
|  | 798 | **406.7824** | **1217.3251** | **1217.3278** | **-0.0028** | **0** | **14** | **1.1e+02** | **1** | **ETVPTWDTIR** |
|  | 661 | **402.2497** | **1203.7270** | **1203.4750** | **0.2520** | **0** | **14** | **1.1e+02** | **1** | **MACLLETPIR + Carbamidomethyl (C)** |
|  | 2072 | **593.6318** | **1185.2489** | **1185.4001** | **-0.1513** | **1** | **14** | **1.4e+02** | **1** | **GGRCVPSLAGLR** |
|  | 3287 | **857.8554** | **1713.6960** | **1712.9053** | **0.7908** | **1** | **14** | **94** | **1** | **HRNENTCPLPQEMK + Oxidation (M)** |
|  | 1177 | **443.8206** | **1328.4397** | **1329.4642** | **-1.0245** | **1** | **14** | **1.2e+02** | **1** | **QQFGPSRSLGPR** |
|  | 1245 | **458.7586** | **1373.2537** | **1372.3947** | **0.8590** | **0** | **14** | **1.1e+02** | **1** | **AEEEAATPGGGVER** |
|  | 1263 | **459.2195** | **1374.6364** | **1374.5446** | **0.0918** | **1** | **14** | **1.3e+02** | **1** | **DIFIDGVVARNR** |
|  | 2018 | **584.4368** | **1750.2881** | **1750.9912** | **-0.7031** | **1** | **14** | **93** | **1** | **GLLGVKSSFYPQDALR** |
|  | 2992 | **742.6597** | **1483.3046** | **1482.7072** | **0.5973** | **2** | **14** | **88** | **1** | **APHKGIIRDSTCK + Carbamidomethyl (C)** |
|  | 3570 | **1104.4542** | **2206.8937** | **2207.4943** | **-0.6007** | **2** | **14** | **77** | **1** | **ALPAWKSQNKVHWAHSHPL** |
|  | 768 | **406.0735** | **810.1322** | **808.9666** | **1.1655** | **0** | **14** | **1.1e+02** | **1** | **ITCANCK + Carbamidomethyl (C)** |
|  | 1126 | **435.1507** | **1302.4298** | **1303.4634** | **-1.0336** | **0** | **14** | **1.1e+02** | **1** | **NVYATTVGHLTK** |
|  | 3193 | **804.7333** | **2411.1777** | **2411.7344** | **-0.5568** | **1** | **14** | **89** | **1** | **WTQLGAFYPFMRNHNSLLSL + Oxidation (M)** |
|  | 573 | **392.5168** | **1174.5284** | **1173.4044** | **1.1240** | **2** | **14** | **1.2e+02** | **1** | **KLENGPKFLK** |
|  | 992 | **419.3123** | **836.6099** | **836.9353** | **-0.3255** | **1** | **14** | **1.1e+02** | **1** | **KYSLNGR** |
|  | 1860 | **550.1610** | **1647.4609** | **1646.7524** | **0.7084** | **0** | **14** | **1.2e+02** | **1** | **NGIGGADYYFGLDVW** |
|  | 32 | **363.2212** | **1086.6413** | **1086.3920** | **0.2493** | **1** | **14** | **95** | **1** | **MASVKKPVVK** |
|  | 450 | **388.9616** | **775.9084** | **775.9634** | **-0.0549** | **2** | **14** | **1.3e+02** | **1** | **GLMKRR + Oxidation (M)** |
|  | 457 | **388.9919** | **775.9689** | **774.9106** | **1.0583** | **1** | **14** | **1.3e+02** | **1** | **ALPYRR** |
|  | 596 | **399.1069** | **796.1990** | **796.9577** | **-0.7587** | **1** | **14** | **1.1e+02** | **1** | **KGLVPQR** |
|  | 864 | **407.8395** | **1220.4963** | **1219.3468** | **1.1494** | **1** | **14** | **1.2e+02** | **1** | **SKINYGGDIPR** |
|  | 89 | **369.2144** | **1104.6209** | **1105.2660** | **-0.6451** | **0** | **14** | **1e+02** | **1** | **DMVGQVAITR + Oxidation (M)** |
|  | 1868 | **550.7542** | **1649.2405** | **1649.9718** | **-0.7314** | **0** | **14** | **1e+02** | **1** | **IPTCPLFVTGLGSCK + 2 Carbamidomethyl (C)** |
|  | 2216 | **609.9343** | **1217.8539** | **1218.3389** | **-0.4851** | **0** | **14** | **1e+02** | **1** | **EDEHFICIR + Carbamidomethyl (C)** |
|  | 3286 | **857.6139** | **2569.8195** | **2568.9251** | **0.8944** | **1** | **14** | **1.1e+02** | **1** | **MAGIELERCQQQANEVMEIMR + Carbamidomethyl (C); 2 Oxidation (M)** |
|  | 41 | **364.1386** | **726.2623** | **726.8644** | **-0.6021** | **1** | **14** | **1e+02** | **1** | **LLRAPGT** |
|  | 1095 | **432.9106** | **1295.7095** | **1296.5319** | **-0.8224** | **1** | **14** | **1.2e+02** | **1** | **QYESLKILICS** |
|  | 2495 | **651.9156** | **1952.7246** | **1953.3298** | **-0.6052** | **2** | **14** | **94** | **1** | **CMKIPTGQGYAAKIINTK + Oxidation (M)** |
|  | 894 | **408.1831** | **1221.5271** | **1222.4371** | **-0.9099** | **1** | **14** | **1.2e+02** | **1** | **VPGRAAPGVALSK** |
|  | 2345 | **624.2445** | **1869.7113** | **1870.2231** | **-0.5117** | **2** | **14** | **1.2e+02** | **1** | **GLEKAKVIQPGWSAMVR** |
|  | 2607 | **668.5969** | **1335.1791** | **1334.6034** | **0.5757** | **1** | **14** | **99** | **1** | **LSAMKEFAYMK + Oxidation (M)** |
|  | 2901 | **733.5896** | **1465.1644** | **1464.6290** | **0.5354** | **1** | **14** | **97** | **1** | **RVGLSAELGAGAHAR** |
|  | 2928 | **740.4474** | **2218.3200** | **2217.6980** | **0.6219** | **1** | **14** | **1.2e+02** | **1** | **KHVFLQVILTVSQIMWCR + Oxidation (M)** |
|  | 99 | **369.2770** | **1104.8089** | **1105.3089** | **-0.5000** | **0** | **14** | **1e+02** | **1** | **AKPWAVCFPS** |
|  | 689 | **403.9517** | **805.8887** | **804.9087** | **0.9800** | **0** | **14** | **1.2e+02** | **1** | **APMVDEK + Oxidation (M)** |
|  | 1810 | **541.6638** | **1621.9693** | **1620.8280** | **1.1412** | **1** | **14** | **1.3e+02** | **1** | **LAVTAHSMAYGTNRI + Oxidation (M)** |
|  | 970 | **418.0438** | **1251.1093** | **1250.3644** | **0.7449** | **2** | **13** | **1.2e+02** | **1** | **DAPPGARGPEKR** |
|  | 1153 | **438.2443** | **874.4738** | **873.9904** | **0.4834** | **0** | **13** | **1.2e+02** | **1** | **LALEEATK** |
|  | 1274 | **460.2407** | **1377.6998** | **1377.5053** | **0.1945** | **0** | **13** | **1.2e+02** | **1** | **GTSLHLAGPGGTPGR** |
|  | 1705 | **532.7662** | **1595.2763** | **1594.8506** | **0.4258** | **2** | **13** | **1e+02** | **1** | **APLTKPSKKEAPAEK** |
|  | 3181 | **801.7868** | **1601.5588** | **1600.9013** | **0.6576** | **1** | **13** | **1.1e+02** | **1** | **NMPVQDLPRLSIAM + Oxidation (M)** |
|  | 106 | **369.3064** | **1104.8971** | **1105.3089** | **-0.4118** | **0** | **13** | **1.1e+02** | **1** | **AKPWAVCFPS** |
|  | 117 | **369.3351** | **1104.9830** | **1105.3089** | **-0.3259** | **0** | **13** | **1.2e+02** | **1** | **AKPWAVCFPS** |
|  | 463 | **389.0154** | **1164.0242** | **1163.3898** | **0.6344** | **0** | **13** | **1.4e+02** | **1** | **QFIMPVVSAR + Oxidation (M)** |
|  | 556 | **391.2547** | **780.4946** | **780.9534** | **-0.4588** | **0** | **13** | **87** | **1** | **AMGIMDK + Oxidation (M)** |
|  | 1857 | **549.9784** | **1646.9130** | **1647.7359** | **-0.8229** | **0** | **13** | **1.2e+02** | **1** | **VDAQSSAGEEDVLLSK** |
|  | 1922 | **563.0524** | **1124.0900** | **1123.2231** | **0.8669** | **1** | **13** | **1.1e+02** | **1** | **LGGVNAVGHSRG** |
|  | 2168 | **606.2426** | **1815.7055** | **1815.1860** | **0.5195** | **1** | **13** | **1.1e+02** | **1** | **IFVFEPPPGVKANMLR** |
|  | 2223 | **610.1300** | **1218.2452** | **1217.2897** | **0.9556** | **0** | **13** | **1.2e+02** | **1** | **CDTCSNSFGQR** |
|  | 119 | **369.3430** | **1105.0069** | **1105.2626** | **-0.2558** | **1** | **13** | **1.2e+02** | **1** | **EIEAKMEQK** |
|  | 127 | **369.3842** | **1105.1303** | **1105.3089** | **-0.1786** | **0** | **13** | **1.5e+02** | **1** | **AKPWAVCFPS** |
|  | 815 | **407.0986** | **1218.2735** | **1219.4514** | **-1.1778** | **2** | **13** | **1.2e+02** | **1** | **KGGEKMALLEK + Oxidation (M)** |
|  | 1672 | **524.3736** | **1570.0986** | **1570.8420** | **-0.7434** | **1** | **13** | **1.1e+02** | **1** | **GTLCPALRGGCRPGGR** |
|  | 1837 | **546.7745** | **1637.3014** | **1636.8308** | **0.4707** | **1** | **13** | **1e+02** | **1** | **SMHSSATLIQSRFR + Oxidation (M)** |
|  | 2244 | **611.2075** | **1830.6004** | **1831.0396** | **-0.4392** | **1** | **13** | **1.3e+02** | **1** | **TWNPNVPESPRIPAPR** |
|  | 2736 | **685.2509** | **1368.4869** | **1367.5572** | **0.9298** | **2** | **13** | **1.2e+02** | **1** | **TAAPSVRPEKRR** |
|  | 485 | **389.2264** | **776.4380** | **775.8475** | **0.5904** | **0** | **13** | **1.1e+02** | **1** | **ATTTPASK** |
|  | 1138 | **436.1756** | **1305.5046** | **1305.5323** | **-0.0277** | **1** | **13** | **1.2e+02** | **1** | **MARPGMERWR + Oxidation (M)** |
|  | 2752 | **686.0323** | **2055.0747** | **2054.2648** | **0.8099** | **1** | **13** | **1e+02** | **1** | **AECELHSSLDRYLMNTR + Oxidation (M)** |
|  | 1259 | **459.0750** | **1374.2030** | **1374.5891** | **-0.3862** | **1** | **13** | **1.4e+02** | **1** | **CCKILNGAGEPR + 2 Carbamidomethyl (C)** |
|  | 2183 | **607.1768** | **1212.3389** | **1211.4807** | **0.8582** | **2** | **13** | **1.2e+02** | **1** | **VRGPPVSCIKR** |
|  | 2753 | **686.0355** | **2055.0842** | **2055.3358** | **-0.2515** | **1** | **13** | **1e+02** | **1** | **TSDCLCLQGKEPPSPAGPVR** |
|  | 132 | **369.4730** | **1105.3969** | **1105.3089** | **0.0880** | **0** | **13** | **1.5e+02** | **1** | **AKPWAVCFPS** |
|  | 1179 | **443.8788** | **1328.6142** | **1329.5305** | **-0.9163** | **1** | **13** | **1.2e+02** | **1** | **HSVRAPSFCGIR** |
|  | 1564 | **507.5143** | **1519.5208** | **1519.7374** | **-0.2167** | **1** | **13** | **1.3e+02** | **1** | **EQKDLSLTPFTIK** |
|  | 3204 | **809.3691** | **1616.7235** | **1616.9205** | **-0.1970** | **2** | **13** | **1.2e+02** | **1** | **AIIDTGLKKTTQCPK** |
|  | 761 | **405.9213** | **1214.7418** | **1215.2970** | **-0.5551** | **0** | **13** | **1.1e+02** | **1** | **EMQNLSQHGR + Oxidation (M)** |
|  | 1770 | **538.3876** | **1612.1405** | **1611.9537** | **0.1869** | **2** | **13** | **1e+02** | **1** | **CCQAGMVLGGRKFK + 2 Carbamidomethyl (C)** |
|  | 2024 | **586.5367** | **1756.5881** | **1756.9892** | **-0.4012** | **2** | **13** | **1e+02** | **1** | **LEDYYKIDEKSLIK** |
|  | 3495 | **967.6903** | **1933.3658** | **1934.0685** | **-0.7026** | **0** | **13** | **1.1e+02** | **1** | **ASQDQTMCMDVDECER + Carbamidomethyl (C); Oxidation (M)** |
|  | 1727 | **534.5576** | **1067.1005** | **1066.1700** | **0.9304** | **0** | **13** | **1.4e+02** | **1** | **GHIQFEAHK** |
|  | 1779 | **539.9220** | **1616.7438** | **1617.7381** | **-0.9942** | **0** | **13** | **1.2e+02** | **1** | **NGSYRPSYEEMLR + Oxidation (M)** |
|  | 651 | **402.0372** | **802.0596** | **800.8835** | **1.1761** | **0** | **13** | **1.4e+02** | **1** | **DGVMAHR + Oxidation (M)** |
|  | 959 | **416.0670** | **1245.1788** | **1244.4427** | **0.7361** | **0** | **13** | **1.5e+02** | **1** | **QAPVPPPKPASR** |
|  | 2482 | **649.6737** | **1297.3326** | **1298.4885** | **-1.1559** | **1** | **13** | **1.3e+02** | **1** | **QRMFSPMEEK + Oxidation (M)** |
|  | 2548 | **664.8566** | **1327.6984** | **1326.5002** | **1.1982** | **1** | **13** | **1.2e+02** | **1** | **MGTLGQCSEKTR + Oxidation (M)** |
|  | 2730 | **685.0134** | **1368.0121** | **1367.5572** | **0.4549** | **2** | **13** | **1.1e+02** | **1** | **TAAPSVRPEKRR** |
|  | 376 | **387.8021** | **1160.3840** | **1159.3448** | **1.0393** | **2** | **13** | **1.5e+02** | **1** | **EVPPLRRHR** |
|  | 2942 | **740.6185** | **1479.2223** | **1478.5416** | **0.6807** | **1** | **13** | **97** | **1** | **AKQPSDVSSECDR + Carbamidomethyl (C)** |
|  | 517 | **390.0100** | **1167.0079** | **1166.3306** | **0.6773** | **1** | **13** | **1.2e+02** | **1** | **TALHEAAKLGR** |
|  | 2810 | **699.2880** | **1396.5611** | **1395.6765** | **0.8847** | **2** | **13** | **1.2e+02** | **1** | **TGHVIAVKQMRR** |
|  | 93 | **369.2591** | **1104.7550** | **1104.2991** | **0.4559** | **0** | **13** | **1.1e+02** | **1** | **QQLLIGAYAK** |
|  | 123 | **369.3652** | **1105.0734** | **1106.2540** | **-1.1805** | **1** | **13** | **1.4e+02** | **1** | **RLGMGEGGVSK + Oxidation (M)** |
|  | 852 | **407.7526** | **1220.2357** | **1219.3470** | **0.8887** | **1** | **13** | **1.2e+02** | **1** | **QGKGQSVQPYK** |
|  | 1145 | **436.9689** | **871.9230** | **872.9261** | **-1.0031** | **1** | **13** | **1.4e+02** | **1** | **NEALRDR** |
|  | 2237 | **610.4545** | **1828.3412** | **1827.1568** | **1.1844** | **2** | **13** | **1.1e+02** | **1** | **AGRRGLDPTGTVILLCK + Carbamidomethyl (C)** |
|  | 2461 | **643.3970** | **1927.1687** | **1926.2017** | **0.9670** | **0** | **13** | **1.2e+02** | **1** | **QVQLVCSGGALVQPGGSLR + Carbamidomethyl (C)** |
|  | 2599 | **668.0112** | **1334.0077** | **1333.6186** | **0.3891** | **2** | **13** | **1.1e+02** | **1** | **FGVLGSSKVLAKK** |
|  | 433 | **388.6618** | **1162.9631** | **1162.3373** | **0.6259** | **1** | **13** | **1.2e+02** | **1** | **MTSYMKVDR + 2 Oxidation (M)** |
|  | 727 | **405.0077** | **808.0005** | **809.0530** | **-1.0524** | **1** | **13** | **1.1e+02** | **1** | **MVIMKR + 2 Oxidation (M)** |
|  | 351 | **387.3500** | **1159.0278** | **1159.3101** | **-0.2822** | **0** | **13** | **1.4e+02** | **1** | **DEGPMVSAPIK + Oxidation (M)** |
|  | 1128 | **435.2217** | **1302.6430** | **1301.4724** | **1.1706** | **1** | **13** | **1.1e+02** | **1** | **NNTGDKVLGPMR** |
|  | 1277 | **460.6914** | **1379.0520** | **1379.5694** | **-0.5174** | **2** | **13** | **1.1e+02** | **1** | **SGAVQKCSACRGR + Carbamidomethyl (C)** |
|  | 2349 | **625.0911** | **1872.2512** | **1872.1710** | **0.0802** | **0** | **13** | **1.3e+02** | **1** | **ETIMVTLHGATNLPACK + Carbamidomethyl (C); Oxidation (M)** |
|  | 355 | **387.6101** | **1159.8081** | **1160.4121** | **-0.6040** | **2** | **13** | **1.2e+02** | **1** | **IIFGKGTRLR** |
|  | 1220 | **452.2571** | **1353.7491** | **1354.5085** | **-0.7595** | **1** | **13** | **1.4e+02** | **1** | **ALEEPANDIKVR** |
|  | 1486 | **489.2717** | **976.5286** | **977.1336** | **-0.6050** | **0** | **13** | **1.3e+02** | **1** | **VTMPVDTAK + Oxidation (M)** |
|  | 2588 | **667.4409** | **1999.3006** | **1999.2726** | **0.0280** | **0** | **13** | **1.2e+02** | **1** | **MCASSLTSPVLGAHEVPSR + Carbamidomethyl (C)** |
|  | 3383 | 927.5835 | 1853.1522 | 1852.1020 | 1.0503 | 2 | 13 | 1.1e+02 | 1 | GSRSCKDAAVGTLVQMR + Carbamidomethyl (C); Oxidation (M) |
|  | 3491 | **966.1272** | **1930.2396** | **1931.1536** | **-0.9140** | **1** | **13** | **1.2e+02** | **1** | **ATLSLDTSKNQFSLHLR** |
|  | 937 | **413.3002** | **1236.8785** | **1236.3825** | **0.4961** | **1** | **13** | **85** | **1** | **SRCPVSTCNR + 2 Carbamidomethyl (C)** |
|  | 1573 | **509.2878** | **1524.8412** | **1524.6165** | **0.2247** | **1** | **13** | **1.3e+02** | **1** | **CDKAFHSSSSYHR** |
|  | 299 | **386.0035** | **769.9923** | **768.9642** | **1.0281** | **1** | **13** | **1.1e+02** | **1** | **ISKFMK + Oxidation (M)** |
|  | 609 | **400.0435** | **798.0721** | **796.9561** | **1.1161** | **0** | **13** | **1e+02** | **1** | **MSQMLR + 2 Oxidation (M)** |
|  | 873 | **407.8872** | **813.7596** | **812.9139** | **0.8456** | **0** | **13** | **1.2e+02** | **1** | **LSTSHLR** |
|  | 1208 | **450.2787** | **1347.8138** | **1348.5769** | **-0.7631** | **1** | **13** | **1e+02** | **1** | **MAVLPPQARGHR + Oxidation (M)** |
|  | 1733 | **534.7526** | **1067.4903** | **1068.0983** | **-0.6079** | **0** | **13** | **1.1e+02** | **1** | **QSPSPNDPAR** |
|  | 2803 | **696.8573** | **2087.5497** | **2086.3763** | **1.1735** | **2** | **13** | **1.3e+02** | **1** | **HIMEVHKEKGEGCSICNR + Oxidation (M)** |
|  | 80 | **369.1780** | **1104.5119** | **1105.3089** | **-0.7969** | **0** | **13** | **1.2e+02** | **1** | **AKPWAVCFPS** |
|  | 2537 | **662.1803** | **1322.3458** | **1321.3480** | **0.9979** | **2** | **13** | **1.2e+02** | **1** | **KEQEKGEGSDSK** |
|  | 895 | **408.1858** | **1221.5352** | **1222.4783** | **-0.9431** | **0** | **13** | **1.3e+02** | **1** | **IMTTLNMLGGR + Oxidation (M)** |
|  | 1463 | **488.1428** | **974.2707** | **974.9684** | **-0.6977** | **0** | **13** | **1.4e+02** | **1** | **QLEDEEGR** |
|  | 2181 | **606.8204** | **1211.6261** | **1211.3250** | **0.3011** | **0** | **13** | **1e+02** | **1** | **GTQVPTLAPGDR** |
|  | 3110 | **768.8423** | **2303.5049** | **2303.6551** | **-0.1503** | **2** | **13** | **1.4e+02** | **1** | **VTMTRDTAISTAYMELKSLR + Oxidation (M)** |
|  | 527 | **390.0359** | **778.0570** | **776.9017** | **1.1552** | **1** | **13** | **1.3e+02** | **1** | **ETLQRM** |
|  | 3374 | **924.7794** | **1847.5441** | **1847.0207** | **0.5233** | **2** | **13** | **1e+02** | **1** | **TKFTQDNLCHAQRER** |
|  | 215 | **377.2859** | **1128.8354** | **1129.2673** | **-0.4319** | **1** | **13** | **95** | **1** | **LDIAGRDITR** |
|  | 452 | **388.9673** | **775.9198** | **775.8508** | **0.0691** | **0** | **13** | **1.4e+02** | **1** | **NSTSLVR** |
|  | 544 | **391.0938** | **780.1727** | **779.8393** | **0.3335** | **0** | **13** | **1.2e+02** | **1** | **FQGWDK** |
|  | 1846 | **547.6497** | **1093.2847** | **1093.2800** | **0.0047** | **0** | **13** | **1.5e+02** | **1** | **CMAWPSQVR + Oxidation (M)** |
|  | 576 | **392.6210** | **783.2272** | **783.0389** | **0.1883** | **1** | **13** | **90** | **1** | **MMMARK + Oxidation (M)** |
|  | 955 | **415.9817** | **1244.9231** | **1245.2800** | **-0.3569** | **1** | **13** | **1.5e+02** | **1** | **SPCDPDRDQR + Carbamidomethyl (C)** |
|  | 1780 | **539.9895** | **1616.9463** | **1617.9052** | **-0.9589** | **1** | **13** | **1.3e+02** | **1** | **NILETLLQMKAAEK + Oxidation (M)** |
|  | 35 | **363.3344** | **1086.9812** | **1086.2426** | **0.7385** | **1** | **13** | **1.2e+02** | **1** | **LRQQALETK** |
|  | 86 | **369.1993** | **1104.5758** | **1105.3089** | **-0.7331** | **0** | **13** | **1.1e+02** | **1** | **AKPWAVCFPS** |
|  | 2768 | **687.0972** | **2058.2695** | **2059.2880** | **-1.0185** | **0** | **13** | **1.2e+02** | **1** | **MDKPCGCPPGVCDHGTGDR + 2 Carbamidomethyl (C)** |
|  | 98 | **369.2763** | **1104.8068** | **1105.3089** | **-0.5020** | **0** | **13** | **1.1e+02** | **1** | **AKPWAVCFPS** |
|  | 712 | **404.1889** | **1209.5444** | **1209.5494** | **-0.0049** | **2** | **13** | **1.2e+02** | **1** | **MMRLMKEVR + Oxidation (M)** |
|  | 1068 | **429.4879** | **1285.4416** | **1286.4560** | **-1.0144** | **0** | **13** | **1.6e+02** | **1** | **LAGGAFPPCDGGPK** |
|  | 1442 | **487.2782** | **1458.8124** | **1459.5796** | **-0.7671** | **0** | **13** | **1.3e+02** | **1** | **MSGNPDVLEYYR + Oxidation (M)** |
|  | 579 | **394.2308** | **1179.6703** | **1179.2847** | **0.3857** | **0** | **13** | **1.1e+02** | **1** | **GQNFVFGPGTR** |
|  | 1377 | **475.6740** | **1423.9999** | **1424.3418** | **-0.3419** | **0** | **13** | **1.2e+02** | **1** | **TNQNSSSDSEAER** |
|  | 2970 | **741.7941** | **1481.5735** | **1482.5565** | **-0.9831** | **1** | **13** | **1.3e+02** | **1** | **KWEAAHEAEQQR** |
|  | 13 | **362.2864** | **722.5581** | **722.8081** | **-0.2500** | **0** | **13** | **99** | **1** | **MQTSEK** |
|  | 499 | **389.9492** | **777.8836** | **778.8316** | **-0.9480** | **0** | **13** | **1.3e+02** | **1** | **MDGVDAR + Oxidation (M)** |
|  | 1385 | **475.9915** | **1424.9523** | **1425.6114** | **-0.6591** | **0** | **13** | **1.3e+02** | **1** | **AVCSQEAMTGPCR + Carbamidomethyl (C); Oxidation (M)** |
|  | 3360 | **919.2686** | **2754.7835** | **2754.9890** | **-0.2055** | **0** | **13** | **1e+02** | **1** | **CECSQGWNGEHCTIEGCPGLCNSNGR** |
|  | 213 | **377.1825** | **1128.5252** | **1128.2626** | **0.2626** | **0** | **13** | **1e+02** | **1** | **TILNNGHTCR** |
|  | 2229 | **610.1934** | **1218.3721** | **1217.4025** | **0.9696** | **1** | **13** | **1.3e+02** | **1** | **TRRPGTQMVR + Oxidation (M)** |
|  | 3317 | **888.5536** | **2662.6386** | **2662.0217** | **0.6169** | **0** | **13** | **1.3e+02** | **1** | **MSSSTPFDLLSCSYFGYTCPLLR + Carbamidomethyl (C); Oxidation (M)** |
|  | 1069 | **429.5833** | **1285.7279** | **1286.4560** | **-0.7281** | **0** | **13** | **1.4e+02** | **1** | **LAGGAFPPCDGGPK** |
|  | 1383 | **475.8823** | **1424.6248** | **1424.6828** | **-0.0580** | **1** | **13** | **1.4e+02** | **1** | **LELTCDTKTICK + Carbamidomethyl (C)** |
|  | 666 | **402.7890** | **1205.3449** | **1204.4235** | **0.9215** | **1** | **13** | **1.4e+02** | **1** | **KMAAAAGGPCVR + Carbamidomethyl (C); Oxidation (M)** |
|  | 1849 | **549.2885** | **1096.5621** | **1096.2607** | **0.3015** | **1** | **13** | **1.3e+02** | **1** | **GKSMGALFGGR + Oxidation (M)** |
|  | 2930 | **740.4921** | **2218.4540** | **2217.4860** | **0.9681** | **1** | **13** | **1.3e+02** | **1** | **EPHFAHVLNAWGAFNPKGPK** |
|  | 2996 | **742.8560** | **2225.5459** | **2225.4338** | **0.1121** | **2** | **13** | **1.5e+02** | **1** | **WFDLKDADINMVTEEDKR** |
|  | 97 | **369.2656** | **1104.7747** | **1105.3304** | **-0.5557** | **2** | **13** | **1.1e+02** | **1** | **LTAFIREKK** |
|  | 919 | **411.0956** | **820.1764** | **820.9990** | **-0.8226** | **0** | **13** | **1.4e+02** | **1** | **LTPGMFR** |
|  | 490 | **389.8597** | **1166.5570** | **1165.4253** | **1.1316** | **0** | **13** | **1.3e+02** | **1** | **ILKPSIPLER** |
|  | 1613 | **518.7772** | **1035.5397** | **1035.2192** | **0.3205** | **2** | **13** | **1e+02** | **1** | **RKSEMLQK + Oxidation (M)** |
|  | 671 | **403.0178** | **804.0208** | **803.8577** | **0.1631** | **1** | **13** | **1.5e+02** | **1** | **PSEKDTK** |
|  | 756 | **405.8364** | **1214.4871** | **1213.3838** | **1.1033** | **0** | **13** | **1.3e+02** | **1** | **LEDVVGGCCYR** |
|  | 1894 | **556.6140** | **1666.8197** | **1665.8026** | **1.0171** | **1** | **13** | **1.6e+02** | **1** | **SDDTAVYYCARDMR** |
|  | 2177 | **606.7095** | **1817.1064** | **1816.0845** | **1.0219** | **0** | **13** | **1.5e+02** | **1** | **NCAECDIVLYLSEMR + Carbamidomethyl (C)** |
|  | 2238 | **610.4630** | **1218.9112** | **1219.4545** | **-0.5433** | **1** | **13** | **1.1e+02** | **1** | **RSVIGSSCLIK + Carbamidomethyl (C)** |
|  | 2409 | **632.3212** | **1893.9415** | **1894.9709** | **-1.0294** | **1** | **13** | **1.4e+02** | **1** | **SCGELSESPGAGKGASGSTR + Carbamidomethyl (C)** |
|  | 2632 | **669.7076** | **2006.1008** | **2007.1569** | **-1.0562** | **0** | **13** | **1.5e+02** | **1** | **SMYLCASSLGDYEQYFG + Carbamidomethyl (C); Oxidation (M)** |
|  | 6 | **360.4838** | **1078.4291** | **1079.1673** | **-0.7381** | **0** | **13** | **1.6e+02** | **1** | **GGQHSQAAPVK** |
|  | 152 | **371.2151** | **1110.6230** | **1110.2211** | **0.4019** | **1** | **13** | **84** | **1** | **DLRAPPEQGK** |
|  | 1014 | **421.0559** | **1260.1456** | **1259.4753** | **0.6703** | **0** | **13** | **1.2e+02** | **1** | **MGLIQTAEQLR** |
|  | 342 | **386.2875** | **1155.8403** | **1155.3247** | **0.5156** | **2** | **13** | **94** | **1** | **RKDADMLYK + Oxidation (M)** |
|  | 888 | **408.0568** | **1221.1483** | **1221.3678** | **-0.2195** | **1** | **13** | **1.4e+02** | **1** | **QSCATGPRNCK + Carbamidomethyl (C)** |
|  | 2947 | 740.6990 | 1479.3833 | 1478.6589 | 0.7244 | 2 | 13 | 1.1e+02 | 1 | HALSAKAVQRQNR |
|  | 1411 | **481.1527** | **960.2906** | **960.1079** | **0.1828** | **0** | **13** | **1.4e+02** | **1** | **MPPTHSYK** |
|  | 3228 | **819.7238** | **1637.4328** | **1637.7874** | **-0.3546** | **0** | **13** | **1e+02** | **1** | **EEAMCQEELPLSSR + Oxidation (M)** |
|  | 3542 | **996.4795** | **2986.4163** | **2986.4521** | **-0.0358** | **2** | **13** | **1.1e+02** | **1** | **LSCPPRPEPELPADVSRFAALMSCRSR** |
|  | 2186 | **607.6749** | **1213.3349** | **1213.3358** | **-0.0008** | **1** | **13** | **1.6e+02** | **1** | **SLELDQFKFS** |
|  | 597 | **399.1184** | **1194.3331** | **1194.2578** | **0.0753** | **0** | **13** | **1.2e+02** | **1** | **WHQGSGYFGR** |
|  | 890 | **408.0906** | **1221.2496** | **1221.4057** | **-0.1562** | **1** | **13** | **1.4e+02** | **1** | **LNNTLYRSLK** |
|  | 1147 | **437.0160** | **872.0172** | **871.9877** | **0.0295** | **2** | **13** | **1.5e+02** | **1** | **SGAIGRRR** |
|  | 572 | **392.4825** | **782.9502** | **782.8865** | **0.0638** | **1** | **13** | **1.5e+02** | **1** | **RAMENM + 2 Oxidation (M)** |
|  | 1050 | **427.8747** | **1280.6019** | **1281.4199** | **-0.8180** | **1** | **13** | **1.3e+02** | **1** | **RVEDMMNQAR + 2 Oxidation (M)** |
|  | 1412 | **481.1889** | **960.3630** | **961.1339** | **-0.7710** | **0** | **13** | **1.4e+02** | **1** | **CVEQIIEK** |
|  | 2945 | **740.6514** | **2218.9319** | **2218.5156** | **0.4164** | **2** | **13** | **1e+02** | **1** | **ISGGNDKCGFPMKQGVLTHGR + Oxidation (M)** |
|  | 735 | **405.1671** | **1212.4790** | **1212.3992** | **0.0798** | **2** | **13** | **1.2e+02** | **1** | **ETLPGARNKVK** |
|  | 1641 | **521.8654** | **1562.5739** | **1562.8120** | **-0.2381** | **2** | **13** | **1.2e+02** | **1** | **VPAQKATGQKAAPAPK** |
|  | 526 | **390.0356** | **778.0565** | **776.9018** | **1.1547** | **1** | **13** | **1.3e+02** | **1** | **CEGVGGKK** |
|  | 539 | **390.0806** | **778.1463** | **777.9312** | **0.2152** | **0** | **13** | **1.3e+02** | **1** | **AFMGPQK** |
|  | 683 | **403.8694** | **805.7240** | **805.8784** | **-0.1544** | **0** | **13** | **1.3e+02** | **1** | **AGAEGFVR** |
|  | 1529 | **501.8660** | **1502.5757** | **1501.7503** | **0.8254** | **2** | **13** | **1.4e+02** | **1** | **QQELCADLKAKVR** |
|  | 3153 | **789.6210** | **1577.2273** | **1576.7507** | **0.4766** | **2** | **13** | **1.1e+02** | **1** | **TLTSDRLTLARDSK** |
|  | 280 | **385.2015** | **1152.5824** | **1151.4041** | **1.1784** | **2** | **13** | **95** | **1** | **KMMAEVVRR + 2 Oxidation (M)** |
|  | 958 | **416.0378** | **1245.0914** | **1245.3661** | **-0.2747** | **1** | **13** | **1.6e+02** | **1** | **KSQAAPGSSPCR + Carbamidomethyl (C)** |
|  | 2764 | **686.2952** | **1370.5756** | **1371.6299** | **-1.0544** | **2** | **13** | **1.3e+02** | **1** | **ASIRIQWTRLK** |
|  | 2824 | **702.0146** | **2103.0218** | **2103.4070** | **-0.3852** | **2** | **13** | **1.1e+02** | **1** | **CRVRAEDPPAAAAAMPTMR + Carbamidomethyl (C); 2 Oxidation (M)** |
|  | 334 | **386.1763** | **1155.5068** | **1156.3308** | **-0.8239** | **0** | **13** | **1.1e+02** | **1** | **AIEALPPGNFK** |
|  | 575 | **392.5726** | **1174.6955** | **1174.3545** | **0.3411** | **1** | **13** | **1e+02** | **1** | **CHRTPMQTK + Carbamidomethyl (C); Oxidation (M)** |
|  | 786 | **406.5605** | **811.1063** | **809.9548** | **1.1515** | **0** | **13** | **1.2e+02** | **1** | **SMNCLAR + Oxidation (M)** |
|  | 2608 | **668.6024** | **2002.7851** | **2002.2546** | **0.5304** | **0** | **13** | **1.1e+02** | **1** | **CAVHGGSFTSFYITWIR + Carbamidomethyl (C)** |
|  | 550 | **391.1418** | **1170.4034** | **1170.3410** | **0.0624** | **1** | **13** | **1.3e+02** | **1** | **MPDCTSKCR + 2 Carbamidomethyl (C); Oxidation (M)** |
|  | 702 | **404.1034** | **806.1920** | **804.9980** | **1.1941** | **1** | **13** | **1.4e+02** | **1** | **MSLAQKK** |
|  | 1196 | **448.2527** | **894.4906** | **894.0034** | **0.4872** | **0** | **13** | **1.2e+02** | **1** | **SMDPGYPK** |
|  | 1699 | **532.2947** | **1062.5746** | **1062.1996** | **0.3749** | **0** | **13** | **1.4e+02** | **1** | **QLYNEHMK** |
|  | 821 | **407.2324** | **812.4500** | **812.9139** | **-0.4639** | **0** | **13** | **1e+02** | **1** | **QLTAPQR** |
|  | 1981 | **578.9348** | **1155.8548** | **1156.3159** | **-0.4610** | **1** | **13** | **1.3e+02** | **1** | **RMNDILNHK + Oxidation (M)** |
|  | 2509 | **653.7008** | **1305.3868** | **1306.4427** | **-1.0558** | **0** | **13** | **1.5e+02** | **1** | **MVVSADPLSSER + Oxidation (M)** |
|  | 87 | **369.2054** | **1104.5942** | **1105.2657** | **-0.6716** | **0** | **13** | **1.2e+02** | **1** | **SGDLSMNLLR** |
|  | 1631 | **520.2770** | **1557.8088** | **1558.7984** | **-0.9897** | **0** | **13** | **1.3e+02** | **1** | **NLQEQVMAVTAQVK** |
|  | 2040 | **590.3516** | **1768.0325** | **1767.0586** | **0.9739** | **2** | **13** | **1.4e+02** | **1** | **LTKLRNTIMEQYTR** |
|  | 2612 | **668.6865** | **2003.0374** | **2002.5924** | **0.4450** | **0** | **13** | **1.4e+02** | **1** | **MLPGCIFLMILLIPQVK + Carbamidomethyl (C); Oxidation (M)** |
|  | 184 | **374.1798** | **746.3449** | **746.7699** | **-0.4250** | **1** | **13** | **1.4e+02** | **1** | **ARESER** |
|  | 1386 | **476.0184** | **950.0220** | **950.0932** | **-0.0711** | **2** | **13** | **1.4e+02** | **1** | **VAYRKDAK** |
|  | 2082 | **594.7701** | **1781.2883** | **1782.1194** | **-0.8311** | **1** | **13** | **1.4e+02** | **1** | **LRNMLNPWVPSWLR** |
|  | 140 | **370.9413** | **1109.8016** | **1110.3501** | **-0.5486** | **1** | **13** | **1e+02** | **1** | **GAIIGLRVGGVV** |
|  | 764 | **405.9720** | **1214.8940** | **1214.3456** | **0.5484** | **0** | **13** | **1.2e+02** | **1** | **FTASSSSGMVPK + Oxidation (M)** |
|  | 1495 | **491.3747** | **1471.1020** | **1471.6086** | **-0.5065** | **1** | **13** | **1e+02** | **1** | **TDSKGIIEPPEGTK** |
|  | 451 | **388.9636** | **1163.8687** | **1163.4974** | **0.3713** | **1** | **13** | **1.6e+02** | **1** | **MVIKQMLIR + 2 Oxidation (M)** |
|  | 1330 | **467.7411** | **933.4675** | **934.1186** | **-0.6511** | **2** | **13** | **1.2e+02** | **1** | **LARKGSMR + Oxidation (M)** |
|  | 1525 | **500.1235** | **1497.3483** | **1496.7952** | **0.5532** | **2** | **13** | **1.3e+02** | **1** | **KIPVPANRYTPLK** |
|  | 131 | **369.4304** | **736.8459** | **735.8499** | **0.9961** | **0** | **13** | **1.7e+02** | **1** | **VAGTMNK + Oxidation (M)** |
|  | 532 | **390.0565** | **778.0981** | **777.8252** | **0.2730** | **1** | **13** | **1.4e+02** | **1** | **SDRSWK** |
|  | 553 | **391.1949** | **780.3750** | **779.8196** | **0.5555** | **0** | **13** | **1.1e+02** | **1** | **AGMDGSSR** |
|  | 2211 | **608.8530** | **1823.5369** | **1824.0488** | **-0.5118** | **2** | **13** | **1.2e+02** | **1** | **CRDTMRDSLSQVLQR + Oxidation (M)** |
|  | 2526 | **657.6316** | **1313.2484** | **1313.6104** | **-0.3620** | **0** | **13** | **1.1e+02** | **1** | **MGFQPPAALLLR** |
|  | 2922 | **740.2701** | **1478.5254** | **1477.6875** | **0.8379** | **1** | **13** | **1.3e+02** | **1** | **LRGNCPVDIFSTR** |
|  | 3519 | **971.2826** | **1940.5504** | **1940.3029** | **0.2475** | **0** | **13** | **98** | **1** | **LMPGTYTLEITSIPLYK** |
|  | 836 | **407.4555** | **812.8962** | **812.8909** | **0.0054** | **0** | **13** | **1.6e+02** | **1** | **MGPSSYR + Oxidation (M)** |
|  | 1953 | **571.5043** | **1711.4908** | **1711.9568** | **-0.4660** | **2** | **13** | **1.1e+02** | **1** | **HISTLNIQLSDSKKK** |
|  | 2046 | **592.5658** | **1774.6752** | **1774.9102** | **-0.2349** | **1** | **13** | **1.2e+02** | **1** | **QDSLARTSMVGNPENR** |
|  | 2332 | **623.8066** | **1868.3978** | **1869.1506** | **-0.7528** | **2** | **13** | **1.3e+02** | **1** | **GFPKSCCKLEDCTPQR + Carbamidomethyl (C)** |
|  | 2596 | **667.8569** | **1333.6991** | **1332.5492** | **1.1498** | **0** | **13** | **1.4e+02** | **1** | **SPAMAGGLCAIER + Carbamidomethyl (C)** |
|  | 3256 | **836.1185** | **1670.2223** | **1669.7464** | **0.4758** | **0** | **13** | **1e+02** | **1** | **ADYIDTEGTFRPER** |
|  | 3265 | **839.6395** | **2515.8964** | **2516.8441** | **-0.9476** | **2** | **13** | **1.3e+02** | **1** | **SCAVSLTTAAVAFGDEAKKMWEGK + Oxidation (M)** |
|  | 3401 | **940.4384** | **2818.2929** | **2817.1796** | **1.1132** | **1** | **13** | **1.2e+02** | **1** | **IDILVNNGGMSQRSLCMDTSLDVYR + Oxidation (M)** |
|  | 3565 | **1079.1565** | **2156.2982** | **2157.3665** | **-1.0683** | **2** | **13** | **1.3e+02** | **1** | **HARETTNMKTQTVASYFR + Oxidation (M)** |
|  | 453 | **388.9732** | **775.9316** | **776.8372** | **-0.9056** | **0** | **13** | **1.6e+02** | **1** | **FSGVPDR** |
|  | 917 | **410.9916** | **1229.9526** | **1230.4989** | **-0.5463** | **0** | **13** | **1.5e+02** | **1** | **MEAMVIGGGCCK + 2 Oxidation (M)** |
|  | 1074 | **430.8077** | **859.6006** | **858.9626** | **0.6380** | **0** | **13** | **1.6e+02** | **1** | **LHEGTMR + Oxidation (M)** |
|  | 1097 | **432.9577** | **1295.8508** | **1296.5319** | **-0.6811** | **1** | **13** | **1.4e+02** | **1** | **QYESLKILICS** |
|  | 1958 | **573.0112** | **1144.0076** | **1143.2061** | **0.8014** | **0** | **13** | **1.4e+02** | **1** | **NTDIAYYQR** |
|  | 2442 | **639.9708** | **1277.9269** | **1277.5321** | **0.3948** | **0** | **13** | **1.2e+02** | **1** | **GFLLAPCVSSVK + Carbamidomethyl (C)** |
|  | 3276 | **847.2382** | **2538.6923** | **2539.8889** | **-1.1966** | **2** | **13** | **1.2e+02** | **1** | **QCEDVFCKHPCLKTNMSTQNR + Carbamidomethyl (C)** |
|  | 616 | **400.3205** | **798.6262** | **798.8477** | **-0.2214** | **1** | **13** | **97** | **1** | **GRGGAEPR** |
|  | 1239 | **457.7524** | **913.4901** | **913.0928** | **0.3973** | **1** | **13** | **1.1e+02** | **1** | **SYMGKSLK** |
|  | 1307 | **464.0106** | **1389.0095** | **1389.5526** | **-0.5430** | **0** | **13** | **1.3e+02** | **1** | **LVEDIFQQNVGK** |
|  | 1333 | **467.9851** | **933.9555** | **933.0874** | **0.8681** | **1** | **13** | **1.5e+02** | **1** | **LSCGVKNR + Carbamidomethyl (C)** |
|  | 1843 | **547.2073** | **1638.5998** | **1638.7804** | **-0.1806** | **2** | **13** | **1.4e+02** | **1** | **SKANSGATAYAASVKGR** |
|  | 2708 | **683.9442** | **2048.8105** | **2048.5102** | **0.3002** | **0** | **13** | **1.1e+02** | **1** | **VACAVAGDLFTLLFPLIPM + Carbamidomethyl (C)** |
|  | 2835 | **707.1831** | **2118.5271** | **2119.5155** | **-0.9883** | **2** | **13** | **1.3e+02** | **1** | **SLGGHMTMMHSRNSCKTLK** |
|  | 643 | **401.8832** | **1202.6274** | **1202.4707** | **0.1567** | **2** | **13** | **1.6e+02** | **1** | **KPRASVKVSCK** |
|  | 935 | **412.8617** | **1235.5630** | **1236.5495** | **-0.9865** | **1** | **13** | **1.3e+02** | **1** | **KSCFLCLPLR + Carbamidomethyl (C)** |
|  | 2105 | **596.7574** | **1787.2502** | **1788.0150** | **-0.7649** | **2** | **13** | **1.6e+02** | **1** | **EYACTAVDMISRRTR + Oxidation (M)** |
|  | 151 | **371.1696** | **1110.4867** | **1109.3224** | **1.1642** | **1** | **13** | **98** | **1** | **SIVRLSVHAK** |
|  | 1187 | **446.1495** | **1335.4263** | **1335.5020** | **-0.0757** | **1** | **13** | **1.6e+02** | **1** | **KDELFTGPTLSK** |
|  | 1460 | **488.1220** | **974.2293** | **973.1712** | **1.0580** | **2** | **13** | **1.5e+02** | **1** | **VTITKAGRK** |
|  | 2102 | **596.2648** | **1190.5149** | **1190.2693** | **0.2456** | **1** | **13** | **1.5e+02** | **1** | **DPPPGRGPGAGGR** |
|  | 52 | **366.0262** | **1095.0566** | **1096.1050** | **-1.0485** | **0** | **13** | **1.6e+02** | **1** | **LAYNESEDR** |
|  | 871 | **407.8820** | **1220.6237** | **1221.4459** | **-0.8222** | **2** | **13** | **1.4e+02** | **1** | **VKASVTTPKYK** |
|  | 1560 | **507.0805** | **1012.1462** | **1011.1297** | **1.0165** | **0** | **13** | **1.3e+02** | **1** | **ASVHDIIEK** |
|  | 1789 | **540.2650** | **1617.7729** | **1618.8951** | **-1.1222** | **1** | **13** | **1.4e+02** | **1** | **MVFLGRINEVEPAK + Oxidation (M)** |
|  | 2358 | **626.0276** | **1875.0606** | **1875.1716** | **-0.1111** | **0** | **13** | **1.4e+02** | **1** | **GSALCAMDGIVPDIAVGTK + Carbamidomethyl (C)** |
|  | 2762 | **686.2118** | **1370.4088** | **1369.5031** | **0.9057** | **0** | **13** | **1.4e+02** | **1** | **GEANGCPGLEPLR + Carbamidomethyl (C)** |
|  | 406 | **387.9243** | **773.8338** | **774.8860** | **-1.0521** | **0** | **13** | **1.7e+02** | **1** | **SEMGVPR** |
|  | 742 | **405.2446** | **1212.7115** | **1213.4039** | **-0.6923** | **1** | **13** | **1e+02** | **1** | **MSGFSSVAATKK** |
|  | 718 | **404.7177** | **1211.1309** | **1211.4640** | **-0.3331** | **2** | **13** | **1.1e+02** | **1** | **RHMLGKCPNR** |
|  | 2723 | **684.2047** | **1366.3945** | **1365.5429** | **0.8517** | **2** | **13** | **1.3e+02** | **1** | **MEGASRVCRQGR + Oxidation (M)** |
|  | 1475 | **488.5244** | **975.0339** | **975.9964** | **-0.9624** | **1** | **13** | **1.8e+02** | **1** | **VKDDDQEK** |
|  | 2157 | **603.5659** | **1807.6754** | **1806.9967** | **0.6787** | **0** | **13** | **1.2e+02** | **1** | **NQVERPQMTFGSLQR + Oxidation (M)** |
|  | 2984 | **742.4741** | **2224.4002** | **2223.4444** | **0.9558** | **2** | **13** | **1.3e+02** | **1** | **FGGAFCRYADEMKEIQER + Carbamidomethyl (C); Oxidation (M)** |
|  | 322 | **386.1195** | **770.2242** | **769.8893** | **0.3349** | **0** | **13** | **1.3e+02** | **1** | **AKPAQGAK** |
|  | 1747 | **536.2751** | **1070.5354** | **1070.1838** | **0.3516** | **1** | **13** | **1.5e+02** | **1** | **DVRGPSMHR + Oxidation (M)** |
|  | 2179 | **606.7988** | **1211.5829** | **1211.3664** | **0.2165** | **1** | **13** | **1.2e+02** | **1** | **WSASFTVTKGK** |
|  | 180 | **374.1195** | **1119.3363** | **1119.2775** | **0.0588** | **0** | **13** | **1.7e+02** | **1** | **LQAPGPAGRPR** |
|  | 557 | **391.2575** | **1170.7504** | **1171.3006** | **-0.5502** | **0** | **13** | **1e+02** | **1** | **AEQLIGGLGGEK** |
|  | 918 | **411.0610** | **1230.1607** | **1230.2621** | **-0.1013** | **0** | **13** | **1.5e+02** | **1** | **TYSDEANQMR + Oxidation (M)** |
|  | 1737 | **535.4346** | **1603.2817** | **1603.7562** | **-0.4745** | **1** | **13** | **1.1e+02** | **1** | **TELMDHSRTTLQR + Oxidation (M)** |
|  | 1446 | **487.4902** | **972.9656** | **973.2604** | **-0.2949** | **1** | **13** | **1.7e+02** | **1** | **RLLCPLCR** |
|  | 1802 | **540.5920** | **1079.1692** | **1078.1579** | **1.0113** | **1** | **13** | **1.7e+02** | **1** | **PDMDSSRVR + Oxidation (M)** |
|  | 1643 | **521.9216** | **1562.7425** | **1562.7074** | **0.0351** | **1** | **13** | **1.4e+02** | **1** | **RSGSIQQMEQLNR + Oxidation (M)** |
|  | 320 | **386.1057** | **1155.2948** | **1155.2203** | **0.0745** | **0** | **13** | **1.3e+02** | **1** | **DGAVCSGSSCR + 2 Carbamidomethyl (C)** |
|  | 788 | **406.6033** | **811.1919** | **811.8151** | **-0.6232** | **0** | **13** | **1.1e+02** | **1** | **MDSGSGDK + Oxidation (M)** |
|  | 1240 | **457.9427** | **913.8705** | **913.0730** | **0.7976** | **2** | **13** | **1.4e+02** | **1** | **VPKNAKEK** |
|  | 1589 | **513.8347** | **1538.4820** | **1537.7675** | **0.7144** | **2** | **13** | **1.2e+02** | **1** | **VYGKTSHLRAHLR** |
|  | 279 | **385.1804** | **1152.5189** | **1152.2113** | **0.3076** | **0** | **13** | **1.1e+02** | **1** | **DETGAYLIDR** |
|  | 1629 | **520.1160** | **1557.3259** | **1557.8569** | **-0.5309** | **1** | **13** | **1.4e+02** | **1** | **RVATMTSPVLVDIR** |
|  | 803 | **406.8336** | **1217.4787** | **1218.4086** | **-0.9299** | **2** | **13** | **1.4e+02** | **1** | **SSRIFPSLRR** |
|  | 949 | **415.1017** | **1242.2830** | **1241.3508** | **0.9322** | **0** | **13** | **1.5e+02** | **1** | **VCDACFNDLQG + Carbamidomethyl (C)** |
|  | 966 | **416.3201** | **1245.9382** | **1245.4026** | **0.5356** | **0** | **13** | **1.4e+02** | **1** | **SVDVPSIPSCGK + Carbamidomethyl (C)** |
|  | 2646 | **670.7509** | **2009.2306** | **2010.2172** | **-0.9866** | **2** | **13** | **1.7e+02** | **1** | **NAIRVHHALATRASDYSK** |
|  | 233 | **379.4382** | **1135.2923** | **1134.3749** | **0.9174** | **1** | **13** | **1.8e+02** | **1** | **ALRLPVAAPAR** |
|  | 1477 | **488.5621** | **975.1094** | **974.1377** | **0.9718** | **0** | **13** | **1.9e+02** | **1** | **HMLPSAFR + Oxidation (M)** |
|  | 2827 | **703.5848** | **2107.7322** | **2107.4947** | **0.2374** | **1** | **13** | **1.2e+02** | **1** | **MLSRLECSDAIMAYCSLK + Carbamidomethyl (C); Oxidation (M)** |
|  | 2997 | **742.8691** | **2225.5853** | **2226.7481** | **-1.1628** | **1** | **13** | **1.7e+02** | **1** | **MLLSSTCMVVLLFRHQMK + Carbamidomethyl (C); 2 Oxidation (M)** |
|  | 308 | **386.0402** | **1155.0983** | **1155.2203** | **-0.1220** | **0** | **13** | **1.3e+02** | **1** | **DGAVCSGSSCR + 2 Carbamidomethyl (C)** |
|  | 425 | **388.3866** | **1162.1377** | **1163.2803** | **-1.1426** | **0** | **13** | **1.8e+02** | **1** | **ALDDFVLGSAR** |
|  | 779 | **406.3431** | **1216.0072** | **1216.5349** | **-0.5277** | **0** | **13** | **1.1e+02** | **1** | **ECLPLIIFLR** |
|  | 1530 | **502.2811** | **1503.8212** | **1503.6800** | **0.1412** | **1** | **13** | **1.5e+02** | **1** | **QTLCLQAQREEK + Carbamidomethyl (C)** |
|  | 2050 | **592.7130** | **1183.4111** | **1184.3458** | **-0.9347** | **0** | **13** | **1.7e+02** | **1** | **AALAGAGATVGLGR** |
|  | 2240 | **610.9203** | **1829.7389** | **1830.0100** | **-0.2712** | **2** | **13** | **1.2e+02** | **1** | **KDLHDANTDLIGRHPK** |
|  | 2275 | **614.3534** | **1840.0380** | **1841.1669** | **-1.1289** | **2** | **13** | **1.4e+02** | **1** | **GLEPPMLQRAAQRMAR + Oxidation (M)** |
|  | 48 | **365.1816** | **1092.5228** | **1092.3119** | **0.2109** | **1** | **13** | **1.2e+02** | **1** | **HYAMKVLSK + Oxidation (M)** |
|  | 1201 | **449.4840** | **896.9533** | **897.0104** | **-0.0571** | **0** | **13** | **1.6e+02** | **1** | **CLSAYQR + Carbamidomethyl (C)** |
|  | 1331 | **467.9415** | **933.8682** | **933.0874** | **0.7808** | **0** | **13** | **1.5e+02** | **1** | **CCNFSCTR** |
|  | 282 | **385.2863** | **768.5579** | **767.8056** | **0.7523** | **0** | **13** | **99** | **1** | **SSGGEGMK + Oxidation (M)** |
|  | 365 | **387.7530** | **1160.2368** | **1161.2233** | **-0.9865** | **2** | **13** | **1.7e+02** | **1** | **DGEVKSQRDK** |
|  | 2204 | **608.7172** | **1823.1293** | **1822.0477** | **1.0817** | **1** | **13** | **1.8e+02** | **1** | **IIKDFMIQGGDPTGTGR + Oxidation (M)** |
|  | 2250 | **612.1774** | **1833.5101** | **1834.0881** | **-0.5780** | **2** | **13** | **1.5e+02** | **1** | **ALVAHTQAIKADNGRLR** |
|  | 2561 | **666.3391** | **1995.9952** | **1995.3068** | **0.6883** | **1** | **13** | **1.4e+02** | **1** | **LMLPHGRLSLVTGQSGQGK + Oxidation (M)** |
|  | 776 | **406.2731** | **1215.7970** | **1215.3767** | **0.4204** | **0** | **13** | **1.1e+02** | **1** | **VTPQMGTPSPGK + Oxidation (M)** |
|  | 973 | **418.3640** | **834.7132** | **834.9676** | **-0.2544** | **1** | **13** | **1.2e+02** | **1** | **SCPNCRR** |
|  | 3063 | **758.4211** | **1514.8275** | **1514.6912** | **0.1363** | **2** | **13** | **1.4e+02** | **1** | **LRHPGPGDRGTVPR** |
|  | 17 | **363.0572** | **1086.1495** | **1086.2823** | **-0.1328** | **0** | **13** | **1.5e+02** | **1** | **ALITTDLLAR** |
|  | 115 | **369.3304** | **736.6460** | **736.8347** | **-0.1887** | **1** | **13** | **1.4e+02** | **1** | **EAKDMK + Oxidation (M)** |
|  | 2278 | **614.5719** | **1227.1290** | **1227.4368** | **-0.3078** | **0** | **13** | **1.2e+02** | **1** | **IGCQTLPSRPR** |
|  | 565 | **392.3849** | **1174.1325** | **1174.3942** | **-0.2617** | **1** | **13** | **1.4e+02** | **1** | **APKSACGVCPGK + Carbamidomethyl (C)** |
|  | 1266 | **459.8753** | **1376.6037** | **1375.5475** | **1.0562** | **0** | **13** | **1.6e+02** | **1** | **CPDGFFASGFSLK** |
|  | 1927 | **564.0085** | **1689.0035** | **1689.8821** | **-0.8786** | **1** | **13** | **1.4e+02** | **1** | **KDGAVESISVPDMVDK** |
|  | 500 | **389.9553** | **1166.8437** | **1166.2859** | **0.5578** | **0** | **13** | **1.5e+02** | **1** | **LWGASPPDPAR** |
|  | 1889 | **555.8307** | **1109.6467** | **1110.2229** | **-0.5762** | **1** | **13** | **1.1e+02** | **1** | **PDMDSSRMR + Oxidation (M)** |
|  | 2965 | **741.6011** | **1481.1874** | **1481.6761** | **-0.4888** | **1** | **13** | **1.2e+02** | **1** | **ECGKAFYCCSSAR + Carbamidomethyl (C)** |
|  | 387 | **387.8312** | **1160.4715** | **1160.2352** | **0.2364** | **0** | **13** | **1.7e+02** | **1** | **ESDSAKPASLR** |
|  | 818 | **407.1850** | **1218.5328** | **1217.3692** | **1.1636** | **0** | **13** | **1.3e+02** | **1** | **AELDSTVLLTR** |
|  | 1882 | **552.7297** | **1103.4447** | **1103.1807** | **0.2640** | **1** | **13** | **1.5e+02** | **1** | **EVSKAPEESK** |
|  | 2114 | **597.4543** | **1789.3409** | **1788.9270** | **0.4139** | **0** | **13** | **1.3e+02** | **1** | **SPPAENEVSTPMQALTT + Oxidation (M)** |
|  | 205 | **376.3791** | **750.7433** | **750.8877** | **-0.1443** | **0** | **13** | **1.5e+02** | **1** | **CVDVCR + Carbamidomethyl (C)** |
|  | 1969 | **575.0504** | **1722.1291** | **1721.9932** | **0.1359** | **0** | **13** | **1.5e+02** | **1** | **DLHPDQMLMEAFFK** |
|  | 2398 | **629.8374** | **1886.4900** | **1886.1163** | **0.3737** | **0** | **13** | **1.3e+02** | **1** | **MATQAHSLSYAGCNFLR + Oxidation (M)** |
|  | 2419 | **634.5995** | **1267.1842** | **1266.5063** | **0.6779** | **0** | **13** | **1.2e+02** | **1** | **LMPSPSPPTPVK + Oxidation (M)** |
|  | 327 | **386.1367** | **1155.3879** | **1156.3374** | **-0.9496** | **1** | **13** | **1.3e+02** | **1** | **HLTAQGFVRK** |
|  | 394 | **387.8581** | **1160.5521** | **1160.1954** | **0.3567** | **1** | **13** | **1.7e+02** | **1** | **GSSTSGSQGHKK** |
|  | 1248 | **458.7872** | **915.5596** | **915.1750** | **0.3846** | **2** | **13** | **1.4e+02** | **1** | **AMKKMYK + Oxidation (M)** |
|  | 1436 | **486.2932** | **970.5716** | **970.1658** | **0.4059** | **1** | **13** | **1.3e+02** | **1** | **GAAMTMKTK + 2 Oxidation (M)** |
|  | 1834 | **546.1334** | **1635.3779** | **1635.8839** | **-0.5060** | **0** | **13** | **1.6e+02** | **1** | **QASGNIVYGVFCLHK** |
|  | 304 | **386.0211** | **1155.0411** | **1154.2802** | **0.7608** | **2** | **13** | **1.2e+02** | **1** | **EHTRASGIRK** |
|  | 424 | **388.3390** | **774.6633** | **774.8826** | **-0.2193** | **0** | **13** | **1.6e+02** | **1** | **DEPLCAK** |
|  | 593 | **398.9925** | **795.9701** | **796.9113** | **-0.9411** | **1** | **13** | **1.3e+02** | **1** | **KGLPQAPS** |
|  | 1289 | **461.8133** | **1382.4176** | **1382.6909** | **-0.2732** | **1** | **13** | **1.4e+02** | **1** | **VCLKLPGGSYMAK + Oxidation (M)** |
|  | 1485 | **489.2389** | **976.4629** | **977.1333** | **-0.6704** | **0** | **13** | **1.5e+02** | **1** | **AELEALGMK + Oxidation (M)** |
|  | 1526 | **500.1845** | **1497.5314** | **1496.8595** | **0.6719** | **1** | **13** | **1.4e+02** | **1** | **SCLKAPLLLGNVLR** |
|  | 1114 | **434.5104** | **1300.5090** | **1300.4659** | **0.0430** | **1** | **13** | **1.6e+02** | **1** | **GSRGTWSPALLR** |
|  | 2206 | **608.7703** | **1823.2888** | **1824.0668** | **-0.7780** | **2** | **13** | **1.6e+02** | **1** | **RSGCLKNYCECYEAK + Carbamidomethyl (C)** |
|  | 1320 | **466.1398** | **1395.3971** | **1396.5486** | **-1.1515** | **1** | **13** | **1.6e+02** | **1** | **APEVAAAAAAAAKER** |
|  | 1853 | **549.6534** | **1645.9382** | **1645.8542** | **0.0840** | **1** | **13** | **1.7e+02** | **1** | **TQPQGQVVIFSEKGK** |
|  | 523 | **390.0269** | **1167.0585** | **1167.2478** | **-0.1893** | **0** | **13** | **1.5e+02** | **1** | **TAMSSETPTSR** |
|  | 1036 | **423.9893** | **845.9639** | **845.9422** | **0.0217** | **0** | **13** | **1.7e+02** | **1** | **SSIFHAGK** |
|  | 1713 | **533.0057** | **1595.9950** | **1595.7524** | **0.2426** | **0** | **13** | **1.5e+02** | **1** | **SMTAADTAVYYCAR + Carbamidomethyl (C); Oxidation (M)** |
|  | 57 | **366.1561** | **1095.4461** | **1095.2081** | **0.2381** | **0** | **13** | **1.5e+02** | **1** | **TNCVDCLDR + Carbamidomethyl (C)** |
|  | 469 | **389.0440** | **1164.1098** | **1164.2435** | **-0.1338** | **0** | **13** | **1.6e+02** | **1** | **QINGATCSIEE** |
|  | 658 | **402.1785** | **1203.5133** | **1202.4508** | **1.0625** | **1** | **13** | **1.6e+02** | **1** | **VKVGMTVHCR + Carbamidomethyl (C); Oxidation (M)** |
|  | 1106 | **433.1309** | **1296.3705** | **1297.4574** | **-1.0869** | **0** | **13** | **1.5e+02** | **1** | **TCDPVEMSYPR** |
|  | 1625 | **519.0938** | **1036.1727** | **1035.2207** | **0.9521** | **1** | **13** | **1.4e+02** | **1** | **QKWGMTIR + Oxidation (M)** |
|  | 2767 | **687.0482** | **1372.0817** | **1371.5242** | **0.5575** | **1** | **13** | **1.3e+02** | **1** | **CPRASSMSGSCGR + Carbamidomethyl (C); Oxidation (M)** |
|  | 3051 | **757.9221** | **2270.7442** | **2271.6645** | **-0.9203** | **1** | **13** | **1.5e+02** | **1** | **MPCAELVREPGCGCCSVCAR + 3 Carbamidomethyl (C); Oxidation (M)** |
|  | 156 | **371.2819** | **740.5491** | **739.8203** | **0.7288** | **0** | **13** | **95** | **1** | **RPNPEK** |
|  | 2169 | **606.4233** | **1816.2478** | **1817.0345** | **-0.7866** | **2** | **13** | **1.3e+02** | **1** | **RDVFLERVCGPNGNLA + Carbamidomethyl (C)** |
|  | 3246 | **828.6010** | **2482.7809** | **2481.7818** | **0.9991** | **1** | **13** | **1.4e+02** | **1** | **TTPSACTLVMCSSVESGLPGRDGR + Carbamidomethyl (C)** |
|  | 5 | **360.4675** | **1078.3802** | **1078.3068** | **0.0735** | **0** | **13** | **2e+02** | **1** | **ALMSPAGMLR + 2 Oxidation (M)** |
|  | 50 | **365.4535** | **1093.3383** | **1094.1853** | **-0.8470** | **1** | **13** | **1.9e+02** | **1** | **AQPSHVRGSR** |
|  | 1267 | **459.8784** | **1376.6129** | **1377.5285** | **-0.9157** | **1** | **13** | **1.6e+02** | **1** | **DSFCSLHSLRR + Carbamidomethyl (C)** |
|  | 1280 | **460.7424** | **919.4700** | **919.1669** | **0.3031** | **1** | **13** | **1.3e+02** | **1** | **SGRIPMMK** |
|  | 1854 | **549.8562** | **1646.5464** | **1645.8805** | **0.6659** | **0** | **13** | **1.2e+02** | **1** | **ATVDPAGLALHPACPR + Carbamidomethyl (C)** |
|  | 1813 | **541.9380** | **1081.8613** | **1081.1766** | **0.6848** | **1** | **13** | **1.5e+02** | **1** | **LSPSYGSDKK** |
|  | 34 | **363.2785** | **1086.8134** | **1086.2064** | **0.6071** | **2** | **13** | **1.2e+02** | **1** | **RQDSRVVAR** |
|  | 1156 | **438.6581** | **1312.9523** | **1313.5889** | **-0.6367** | **0** | **13** | **1.3e+02** | **1** | **MEIQLMHNLGK** |
|  | 1295 | **462.1363** | **1383.3867** | **1383.5333** | **-0.1466** | **1** | **13** | **1.4e+02** | **1** | **GSAARHPSSPCSVK** |
|  | 1366 | **474.2107** | **946.4066** | **945.2239** | **1.1827** | **1** | **13** | **1.6e+02** | **1** | **MALITLRK** |
|  | 1554 | **505.9182** | **1514.7324** | **1513.6748** | **1.0576** | **0** | **13** | **1.4e+02** | **1** | **LAGEAPAAQQAAQCK + Carbamidomethyl (C)** |
|  | 315 | 386.0775 | 770.1403 | 770.9600 | -0.8197 | 0 | 12 | 1.4e+02 | 1 | ILASVLR |
|  | 674 | **403.0327** | **1206.0760** | **1206.2702** | **-0.1943** | **1** | **12** | **1.7e+02** | **1** | **SWRHGSGTYR** |
|  | 1178 | **443.8515** | **1328.5323** | **1329.5869** | **-1.0545** | **0** | **12** | **1.5e+02** | **1** | **MCSSCLVCEPK + 2 Carbamidomethyl (C); Oxidation (M)** |
|  | 1741 | **535.9485** | **1069.8822** | **1069.1229** | **0.7593** | **0** | **12** | **1.5e+02** | **1** | **SESTSFQGVK** |
|  | 2323 | **623.6377** | **1867.8909** | **1868.2320** | **-0.3411** | **1** | **12** | **1.6e+02** | **1** | **CRQSLLCMVMWSHK + 2 Carbamidomethyl (C); 2 Oxidation (M)** |
|  | 317 | **386.0942** | **1155.2605** | **1156.3325** | **-1.0719** | **0** | **12** | **1.4e+02** | **1** | **GGFFVDLFVR** |
|  | 2174 | **606.6805** | **1211.3463** | **1211.2819** | **0.0644** | **1** | **12** | **1.7e+02** | **1** | **GRFTISSDNSK** |
|  | 3440 | **947.7021** | **2840.0843** | **2839.1008** | **0.9835** | **1** | **12** | **1.3e+02** | **1** | **LSSVTAADTAVYYCARGYCSGGSCYR + 2 Carbamidomethyl (C)** |
|  | 1321 | **466.1447** | **1395.4118** | **1394.5572** | **0.8546** | **1** | **12** | **1.6e+02** | **1** | **HHWQDLLKCTN** |
|  | 2665 | **672.8395** | **1343.6642** | **1343.5688** | **0.0954** | **1** | **12** | **1.6e+02** | **1** | **NVLSVGTAVKDLK** |
|  | 1026 | **422.1274** | **1263.3601** | **1264.3907** | **-1.0306** | **0** | **12** | **1.7e+02** | **1** | **AAAVAGGDHGGLLR** |
|  | 1528 | **500.6879** | **1499.0414** | **1498.7679** | **0.2735** | **1** | **12** | **1.3e+02** | **1** | **RSPIFTHLVTSLK** |
|  | 2406 | **631.9675** | **1261.9202** | **1261.4483** | **0.4718** | **1** | **12** | **1.4e+02** | **1** | **SATVCERGTPIK** |
|  | 259 | **384.6568** | **1150.9483** | **1151.3576** | **-0.4093** | **1** | **12** | **1.1e+02** | **1** | **NVSVKDVCCGK** |
|  | 898 | **408.3061** | **1221.8961** | **1222.2200** | **-0.3239** | **1** | **12** | **1.3e+02** | **1** | **TDRAGWEDSW** |
|  | 1185 | **445.1753** | **1332.5036** | **1333.6037** | **-1.1001** | **1** | **12** | **1.7e+02** | **1** | **HMMIRGENMSK** |
|  | 1659 | **523.3045** | **1044.5942** | **1044.2060** | **0.3883** | **0** | **12** | **1.5e+02** | **1** | **MAGCSLSFR + Carbamidomethyl (C); Oxidation (M)** |
|  | 534 | **390.0668** | **1167.1783** | **1168.3299** | **-1.1516** | **1** | **12** | **1.5e+02** | **1** | **CEHVQARLR + Carbamidomethyl (C)** |
|  | 974 | **418.7441** | **835.4734** | **836.0386** | **-0.5652** | **1** | **12** | **1.6e+02** | **1** | **RQMVMR + Oxidation (M)** |
|  | 1066 | **429.3259** | **1284.9554** | **1285.3703** | **-0.4149** | **2** | **12** | **1.2e+02** | **1** | **AHTRKSGATGGSR** |
|  | 571 | **392.4534** | **1174.3380** | **1173.3864** | **0.9517** | **1** | **12** | **1.7e+02** | **1** | **KPATDGMAVRK** |
|  | 714 | **404.1935** | **1209.5584** | **1210.3816** | **-0.8232** | **0** | **12** | **1.4e+02** | **1** | **DMLLVGCSSGR + Carbamidomethyl (C); Oxidation (M)** |
|  | 1279 | **460.7410** | **919.4672** | **919.0376** | **0.4295** | **1** | **12** | **1.3e+02** | **1** | **SLSRSTLR** |
|  | 2165 | **605.7778** | **1814.3113** | **1813.9893** | **0.3221** | **2** | **12** | **1.5e+02** | **1** | **DVSKRLGCHGGGSQEVK + Carbamidomethyl (C)** |
|  | 2203 | **608.6677** | **1822.9808** | **1822.9235** | **0.0574** | **1** | **12** | **1.9e+02** | **1** | **AEPEKNGEVVHTPETSV** |
|  | 3250 | **832.3942** | **2494.1603** | **2493.9631** | **0.1973** | **2** | **12** | **1.5e+02** | **1** | **GANPVEIRRGVMLVVDVVIAELK + Oxidation (M)** |
|  | 3266 | **840.8632** | **1679.7117** | **1679.8707** | **-0.1590** | **0** | **12** | **1.4e+02** | **1** | **MADTQTQVAPTPTMR + 2 Oxidation (M)** |
|  | 266 | **384.9316** | **1151.7727** | **1152.1286** | **-0.3559** | **0** | **12** | **1.3e+02** | **1** | **GSGFPDGEGSSR** |
|  | 335 | **386.1953** | **1155.5636** | **1156.2694** | **-0.7059** | **0** | **12** | **1.2e+02** | **1** | **IGSLGYSGCSR + Carbamidomethyl (C)** |
|  | 1469 | **488.2782** | **974.5416** | **973.9904** | **0.5513** | **1** | **12** | **1.6e+02** | **1** | **SRSTSSHGR** |
|  | 2653 | **671.5844** | **2011.7309** | **2011.3893** | **0.3416** | **1** | **12** | **1.2e+02** | **1** | **VKFIPGSALNGMVEMMDR + Oxidation (M)** |
|  | 2775 | **687.9185** | **1373.8221** | **1373.4290** | **0.3931** | **1** | **12** | **1.3e+02** | **1** | **GVEGSAGAGKEAQGR** |
|  | 2500 | **652.4740** | **1954.3998** | **1954.2119** | **0.1880** | **2** | **12** | **1.4e+02** | **1** | **TDDGKGLGMQLKGPLGPGGR** |
|  | 1316 | **466.0508** | **1395.1304** | **1394.6583** | **0.4720** | **0** | **12** | **1.6e+02** | **1** | **LNALAAIVQGSLPK** |
|  | 2061 | **592.9282** | **1775.7625** | **1774.7944** | **0.9680** | **0** | **12** | **1.4e+02** | **1** | **EVAEDAAEVQTGDVADR** |
|  | 2957 | **741.1512** | **1480.2876** | **1479.5873** | **0.7003** | **0** | **12** | **1.4e+02** | **1** | **AGTLGEQIFSIEGGT** |
|  | 984 | **419.2201** | **1254.6380** | **1255.4638** | **-0.8258** | **2** | **12** | **1.6e+02** | **1** | **KPEVKKVDAGGK** |
|  | 1566 | **507.8997** | **1013.7846** | **1013.1307** | **0.6539** | **0** | **12** | **1.6e+02** | **1** | **CGCVGSDCR + 2 Carbamidomethyl (C)** |
|  | 2199 | **608.5352** | **1822.5833** | **1822.0527** | **0.5306** | **1** | **12** | **1.3e+02** | **1** | **HRGLRPVSDTLFSSFC** |
|  | 2983 | **742.4647** | **2224.3718** | **2223.5569** | **0.8149** | **2** | **12** | **1.5e+02** | **1** | **GQQKAHVSFKPTVAQQRICP** |
|  | 378 | **387.8083** | **1160.4027** | **1160.4138** | **-0.0112** | **1** | **12** | **1.9e+02** | **1** | **MALGPRCGAIR + Oxidation (M)** |
|  | 972 | **418.2082** | **834.4017** | **834.9379** | **-0.5362** | **0** | **12** | **1.5e+02** | **1** | **MGVENSAK** |
|  | 2615 | **668.7632** | **2003.2674** | **2002.2535** | **1.0139** | **2** | **12** | **1.9e+02** | **1** | **KSMEVLSVTDGGSPVPARR + Oxidation (M)** |
|  | 379 | **387.8083** | **1160.4028** | **1159.3331** | **1.0696** | **1** | **12** | **1.9e+02** | **1** | **GGLEKTVSLQK** |
|  | 1895 | **556.8232** | **1667.4474** | **1666.8536** | **0.5938** | **2** | **12** | **1.3e+02** | **1** | **TPAQASFEKMQEKR + Oxidation (M)** |
|  | 2395 | **629.2952** | **1256.5756** | **1255.4668** | **1.1088** | **0** | **12** | **1.6e+02** | **1** | **CFYCCTGCGK + 3 Carbamidomethyl (C)** |
|  | 3414 | **941.6058** | **1881.1969** | **1880.0674** | **1.1295** | **0** | **12** | **1.4e+02** | **1** | **MSSPPHSGQTVHPPCSSK + Oxidation (M)** |
|  | 265 | **384.9309** | **767.8470** | **768.8151** | **-0.9681** | **0** | **12** | **1.3e+02** | **1** | **GPGGAEGPK** |
|  | 1715 | **533.3646** | **1064.7145** | **1064.1676** | **0.5468** | **0** | **12** | **1.3e+02** | **1** | **ALEEAMEQK + Oxidation (M)** |
|  | 2099 | **595.9700** | **1784.8877** | **1785.1200** | **-0.2323** | **1** | **12** | **1.6e+02** | **1** | **EALWQHPHLPGLKMK** |
|  | 2173 | **606.6665** | **1816.9773** | **1817.0993** | **-0.1219** | **2** | **12** | **1.7e+02** | **1** | **MQTPGGSSCRKEAVLPR** |
|  | 2773 | **687.5402** | **2059.5983** | **2059.2599** | **0.3385** | **0** | **12** | **1.3e+02** | **1** | **MDPNYSCTTGGSCTCAGSCK + Carbamidomethyl (C); Oxidation (M)** |
|  | 289 | **385.9540** | **1154.8399** | **1154.4025** | **0.4373** | **0** | **12** | **1.3e+02** | **1** | **GLSQAALLLLR** |
|  | 2431 | **637.5081** | **1909.5020** | **1909.1464** | **0.3556** | **1** | **12** | **1.4e+02** | **1** | **CNTGERSLSLLNCEEIK** |
|  | 2506 | **653.0747** | **1956.2019** | **1956.2112** | **-0.0092** | **2** | **12** | **1.6e+02** | **1** | **LFWRDVKLAGGHGVSASR** |
|  | 2564 | **666.4836** | **1330.9524** | **1330.4904** | **0.4619** | **2** | **12** | **1.4e+02** | **1** | **DIEKVLSAGSRR** |
|  | 3002 | **743.8288** | **2228.4642** | **2227.4083** | **1.0560** | **1** | **12** | **1.8e+02** | **1** | **VSCKTSGYTFSDYDVNWVR** |
|  | 1084 | **431.0956** | **860.1764** | **859.0949** | **1.0815** | **2** | **12** | **1.9e+02** | **1** | **ACLRIRK** |
|  | 1374 | **475.5603** | **1423.6586** | **1423.5986** | **0.0601** | **1** | **12** | **2e+02** | **1** | **SFVCAWNLDRR + Carbamidomethyl (C)** |
|  | 2064 | **593.0104** | **1184.0060** | **1183.3976** | **0.6084** | **0** | **12** | **1.6e+02** | **1** | **LGECLCPSFSK** |
|  | 2638 | **669.8627** | **2006.5658** | **2006.1922** | **0.3737** | **2** | **12** | **1.5e+02** | **1** | **SMWHSSHHSEGRKMHR + Oxidation (M)** |
|  | 639 | **401.8505** | **1202.5292** | **1201.4805** | **1.0487** | **0** | **12** | **1.9e+02** | **1** | **LWTLSPLLCR** |
|  | 1693 | **529.4025** | **1585.1852** | **1585.7410** | **-0.5557** | **1** | **12** | **1.3e+02** | **1** | **AIEAVHNTAMRDGGK + Oxidation (M)** |
|  | 2725 | **684.4159** | **2050.2255** | **2051.3486** | **-1.1231** | **1** | **12** | **1.5e+02** | **1** | **RGDVLAAWSGIRPLVTDPK** |
|  | 203 | **376.1494** | **1125.4262** | **1126.2668** | **-0.8406** | **0** | **12** | **1.4e+02** | **1** | **GCSVGGWMTGR + Oxidation (M)** |
|  | 2066 | **593.0931** | **1184.1714** | **1184.1888** | **-0.0174** | **0** | **12** | **1.6e+02** | **1** | **SDCENSETATK** |
|  | 2938 | **740.6021** | **2218.7840** | **2218.4692** | **0.3147** | **0** | **12** | **1.3e+02** | **1** | **ACMLTTAAAHDGDVNVISWSR** |
|  | 2969 | **741.7803** | **1481.5458** | **1480.4912** | **1.0546** | **0** | **12** | **1.6e+02** | **1** | **GSSGSSGGTVSATSPNK** |
|  | 807 | **406.9523** | **1217.8348** | **1217.3727** | **0.4622** | **2** | **12** | **1.4e+02** | **1** | **VRDALTAEKSK** |
|  | 1236 | **456.7870** | **911.5591** | **911.0172** | **0.5419** | **1** | **12** | **1.3e+02** | **1** | **ITDAARHK** |
|  | 1618 | **518.9019** | **1035.7889** | **1035.2010** | **0.5880** | **1** | **12** | **1.5e+02** | **1** | **EVARPAHKK** |
|  | 496 | **389.9140** | **777.8133** | **776.7908** | **1.0225** | **0** | **12** | **1.6e+02** | **1** | **AEDEWK** |
|  | 838 | **407.5087** | **1219.5039** | **1220.3994** | **-0.8955** | **1** | **12** | **1.8e+02** | **1** | **HWYECLKNK** |
|  | 1269 | **459.9218** | **1376.7434** | **1375.5493** | **1.1941** | **0** | **12** | **1.7e+02** | **1** | **PVYPGEPASISCR** |
|  | 3004 | **743.8943** | **2228.6609** | **2227.6714** | **0.9894** | **2** | **12** | **1.7e+02** | **1** | **VVKTNISRQTIVHIIQFCK** |
|  | 3296 | **866.7360** | **2597.1857** | **2596.0100** | **1.1757** | **1** | **12** | **1.3e+02** | **1** | **NTLYLQMNGLRAEDMAVYYCVK** |
|  | 487 | **389.3078** | **776.6008** | **775.8905** | **0.7103** | **0** | **12** | **1.4e+02** | **1** | **LVSATTGK** |
|  | 1704 | **532.7501** | **1595.2282** | **1595.8118** | **-0.5837** | **0** | **12** | **1.4e+02** | **1** | **GIDIGDDMVSGLIYK** |
|  | 1231 | **454.7944** | **1361.3610** | **1360.6026** | **0.7585** | **0** | **12** | **1.5e+02** | **1** | **VLLTHEVMCSR + Carbamidomethyl (C); Oxidation (M)** |
|  | 1417 | **482.1021** | **962.1894** | **963.1712** | **-0.9819** | **1** | **12** | **1.7e+02** | **1** | **IDAYKLLK** |
|  | 1934 | **565.4382** | **1693.2923** | **1693.9830** | **-0.6906** | **1** | **12** | **1.4e+02** | **1** | **SFCCPPLSPSKLGPF + 2 Carbamidomethyl (C)** |
|  | 2071 | **593.5879** | **1185.1610** | **1185.3340** | **-0.1730** | **1** | **12** | **1.6e+02** | **1** | **QQTVLSRTPR** |
|  | 3172 | **797.7710** | **2390.2908** | **2389.6432** | **0.6477** | **2** | **12** | **1.3e+02** | **1** | **RGSSAWPGSCSAPPASKVTSASLK + Carbamidomethyl (C)** |
|  | 1615 | **518.8481** | **1553.5223** | **1552.8635** | **0.6588** | **0** | **12** | **1.3e+02** | **1** | **MIVRPPSHVELCR + Oxidation (M)** |
|  | 2084 | **594.9235** | **1187.8321** | **1188.4852** | **-0.6530** | **1** | **12** | **1.4e+02** | **1** | **MWLKVGGLLR + Oxidation (M)** |
|  | 2348 | **624.4696** | **1870.3866** | **1870.1088** | **0.2778** | **1** | **12** | **1.4e+02** | **1** | **VENLLGISSLEKTDPVR** |
|  | 243 | **380.0486** | **1137.1235** | **1138.3006** | **-1.1772** | **1** | **12** | **1.9e+02** | **1** | **ACAAFLSSRR + Carbamidomethyl (C)** |
|  | 2153 | **602.9126** | **1805.7156** | **1805.0189** | **0.6968** | **1** | **12** | **1.4e+02** | **1** | **MNPCKNNSTCTDLYK + Carbamidomethyl (C); Oxidation (M)** |
|  | 3343 | **900.2618** | **1798.5089** | **1798.0925** | **0.4164** | **1** | **12** | **1.2e+02** | **1** | **FLNPSKPHFSPITKGK** |
|  | 842 | **407.6198** | **1219.8373** | **1219.2560** | **0.5812** | **0** | **12** | **1.3e+02** | **1** | **TIVEAASDEER** |
|  | 1816 | **542.2773** | **1623.8099** | **1622.9318** | **0.8781** | **1** | **12** | **1.6e+02** | **1** | **MRSVNDIMPMIGAR + 2 Oxidation (M)** |
|  | 2326 | **623.6669** | **1245.3190** | **1244.3978** | **0.9211** | **1** | **12** | **1.9e+02** | **1** | **SSNIRQAEVLK** |
|  | 2505 | **652.9020** | **1955.6838** | **1955.2415** | **0.4423** | **2** | **12** | **1.3e+02** | **1** | **YGNIDAVHLKAMVDKHK + Oxidation (M)** |
|  | 1032 | **422.9973** | **1265.9696** | **1265.4006** | **0.5690** | **2** | **12** | **1.8e+02** | **1** | **MSAGGSARKSTGR** |
|  | 1076 | **430.9843** | **1289.9307** | **1289.5479** | **0.3828** | **2** | **12** | **2e+02** | **1** | **VKLAGVSQMGRK + Oxidation (M)** |
|  | 1721 | **533.9548** | **1598.8423** | **1598.8241** | **0.0183** | **0** | **12** | **1.5e+02** | **1** | **NFDVGHVPMAGLLGR + Oxidation (M)** |
|  | 2334 | **623.8718** | **1868.5933** | **1868.0533** | **0.5400** | **2** | **12** | **1.3e+02** | **1** | **SVALEKSGSHAKETAPQK** |
|  | 3339 | **897.3417** | **2689.0029** | **2688.9228** | **0.0800** | **1** | **12** | **1.5e+02** | **1** | **TNLTSSAMEPWKNQLSNSTQGLHK + Oxidation (M)** |
|  | 717 | **404.6990** | **1211.0749** | **1210.4032** | **0.6718** | **1** | **12** | **1.3e+02** | **1** | **GSFGKVMLAER + Oxidation (M)** |
|  | 744 | **405.2706** | **808.5265** | **807.9174** | **0.6091** | **0** | **12** | **1.2e+02** | **1** | **CYGPGVGR** |
|  | 2381 | **628.3597** | **1882.0570** | **1881.1995** | **0.8576** | **1** | **12** | **1.6e+02** | **1** | **RVDFETFLPMLQAVAK + Oxidation (M)** |
|  | 1746 | **536.0961** | **1605.2662** | **1604.7659** | **0.5003** | **0** | **12** | **1.6e+02** | **1** | **RPAGPGSPATSPPASVR** |
|  | 2042 | **591.4235** | **1771.2482** | **1770.9597** | **0.2885** | **1** | **12** | **1.4e+02** | **1** | **FDTVYDQMVQPQKR + Oxidation (M)** |
|  | 1171 | **443.3323** | **1326.9747** | **1327.6389** | **-0.6641** | **1** | **12** | **1.2e+02** | **1** | **LCKVMPNMYR + Carbamidomethyl (C); Oxidation (M)** |
|  | 1886 | **554.5900** | **1660.7477** | **1661.8981** | **-1.1504** | **0** | **12** | **1.9e+02** | **1** | **AIAMSLGQDIPMDQR + Oxidation (M)** |
|  | 2543 | **663.3960** | **1987.1658** | **1986.0830** | **1.0828** | **0** | **12** | **1.6e+02** | **1** | **YFNIDPNATQASGNCGTR + Carbamidomethyl (C)** |
|  | 2544 | **663.5255** | **1987.5544** | **1988.2150** | **-0.6606** | **1** | **12** | **1.3e+02** | **1** | **RIHSGERPFVCQECGR + 2 Carbamidomethyl (C)** |
|  | 3520 | **972.6322** | **1943.2496** | **1942.2362** | **1.0134** | **2** | **12** | **1.4e+02** | **1** | **MDDFQLKGIVEEKFVK + Oxidation (M)** |
|  | 577 | **393.5342** | **1177.5804** | **1177.1811** | **0.3993** | **2** | **12** | **1.6e+02** | **1** | **KGGDAGDRSEW** |
|  | 1242 | **458.2956** | **1371.8646** | **1371.5390** | **0.3256** | **0** | **12** | **1.4e+02** | **1** | **GCSPIFCSISSDR** |
|  | 1548 | **505.1331** | **1512.3770** | **1511.7418** | **0.6352** | **2** | **12** | **1.6e+02** | **1** | **KYSGCLKDIEISR** |
|  | 2068 | **593.2402** | **1776.6985** | **1775.9761** | **0.7225** | **1** | **12** | **1.7e+02** | **1** | **IQSVMKQDSGQYTFK + Oxidation (M)** |
|  | 1155 | **438.2623** | **1311.7648** | **1312.4916** | **-0.7268** | **0** | **12** | **1.5e+02** | **1** | **DTLYLQMGSLR + Oxidation (M)** |
|  | 2023 | **586.4272** | **1756.2594** | **1755.1341** | **1.1253** | **2** | **12** | **1.4e+02** | **1** | **RLHLCTSFMDMLKK + 2 Oxidation (M)** |
|  | 2230 | **610.2086** | **1218.4023** | **1218.3756** | **0.0268** | **0** | **12** | **1.7e+02** | **1** | **YSMLFSPTEK + Oxidation (M)** |
|  | 1995 | **580.9012** | **1159.7877** | **1160.3409** | **-0.5532** | **0** | **12** | **1.4e+02** | **1** | **LELEIQQCK + Carbamidomethyl (C)** |
|  | 2257 | **612.3359** | **1222.6570** | **1223.4484** | **-0.7914** | **2** | **12** | **1.6e+02** | **1** | **MTPSPAKHRAK** |
|  | 2515 | **655.5266** | **1963.5577** | **1963.3080** | **0.2496** | **0** | **12** | **1.3e+02** | **1** | **NPTFMCLALHCIANVGSR + Oxidation (M)** |
|  | 1580 | **511.8790** | **1532.6149** | **1533.5934** | **-0.9785** | **0** | **12** | **1.6e+02** | **1** | **ASLASLDSNPSTNEK** |
|  | 587 | **397.9418** | **1190.8033** | **1191.3436** | **-0.5402** | **0** | **12** | **1.6e+02** | **1** | **QRPSPRPTPR** |
|  | 1238 | **457.6966** | **913.3784** | **914.0180** | **-0.6396** | **2** | **12** | **1.2e+02** | **1** | **THTGDKKK** |
|  | 1433 | **485.8857** | **1454.6348** | **1454.6044** | **0.0303** | **0** | **12** | **1.5e+02** | **1** | **MYSSALSQPGLER + Oxidation (M)** |
|  | 2581 | **666.8490** | **1997.5248** | **1998.1552** | **-0.6304** | **0** | **12** | **1.6e+02** | **1** | **CTSSTQEPLNASEAFCPR + Carbamidomethyl (C)** |
|  | 2987 | **742.5515** | **1483.0882** | **1483.6917** | **-0.6035** | **0** | **12** | **1.5e+02** | **1** | **ANQNLICDNLLPR** |
|  | 77 | **369.1704** | **736.3261** | **736.8793** | **-0.5532** | **0** | **12** | **1.6e+02** | **1** | **AFQMPK + Oxidation (M)** |
|  | 782 | **406.3697** | **1216.0869** | **1215.4428** | **0.6441** | **1** | **12** | **1.4e+02** | **1** | **FVSATFRLFK** |
|  | 1146 | **436.9884** | **871.9620** | **872.9659** | **-1.0040** | **0** | **12** | **1.8e+02** | **1** | **ATSSAALPR** |
|  | 1172 | **443.4586** | **1327.3538** | **1327.5478** | **-0.1940** | **0** | **12** | **1.8e+02** | **1** | **LLESHFMPPEK** |
|  | 2821 | **701.2609** | **1400.5071** | **1399.5540** | **0.9530** | **1** | **12** | **1.6e+02** | **1** | **AVHSWSRISTAGK** |
|  | 2857 | **715.4205** | **2143.2392** | **2143.2205** | **0.0187** | **0** | **12** | **1.7e+02** | **1** | **DEGSYYCACDPLGDAYTDK + Carbamidomethyl (C)** |
|  | 1322 | **466.1736** | **1395.4985** | **1395.5242** | **-0.0257** | **2** | **12** | **1.7e+02** | **1** | **RRTAAPTDPSAPR** |
|  | 403 | **387.9077** | **773.8005** | **773.9176** | **-0.1171** | **1** | **12** | **2e+02** | **1** | **DTIGKLK** |
|  | 1827 | **543.1316** | **1626.3726** | **1625.9586** | **0.4140** | **1** | **12** | **1.6e+02** | **1** | **LLPALAARLAGHVPAR** |
|  | 1400 | **479.7118** | **1436.1131** | **1435.7337** | **0.3795** | **2** | **12** | **1.4e+02** | **1** | **SMLEKFKLVNAR** |
|  | 1781 | **539.9941** | **1077.9735** | **1078.2404** | **-0.2669** | **0** | **12** | **1.7e+02** | **1** | **AQTMGNSLLK + Oxidation (M)** |
|  | 2578 | **666.7960** | **1997.3657** | **1998.3686** | **-1.0029** | **2** | **12** | **1.9e+02** | **1** | **LARDQLIYLLEQLPGKK** |
|  | 3093 | **763.0112** | **1524.0076** | **1523.7561** | **0.2515** | **2** | **12** | **1.3e+02** | **1** | **KVRQGVEEMLYR + Oxidation (M)** |
|  | 3553 | **1016.6586** | **2031.3024** | **2032.1501** | **-0.8477** | **1** | **12** | **1.5e+02** | **1** | **SGSEGPVGAGEGDRMEGIGAAK** |
|  | 1205 | **449.9178** | **897.8208** | **898.0615** | **-0.2406** | **0** | **12** | **1.5e+02** | **1** | **MWCLSSR + Oxidation (M)** |
|  | 1671 | **524.3127** | **1569.9161** | **1571.0094** | **-1.0933** | **1** | **12** | **1.7e+02** | **1** | **LHMILKPFMLRR + Oxidation (M)** |
|  | 2811 | **699.3496** | **2095.0267** | **2094.3934** | **0.6333** | **2** | **12** | **1.6e+02** | **1** | **GEKGDLGMMGLPGSRGPMGSK + 2 Oxidation (M)** |
|  | 824 | **407.3124** | **1218.9151** | **1219.5638** | **-0.6487** | **2** | **12** | **1.2e+02** | **1** | **AAKRIMIICK + Carbamidomethyl (C); Oxidation (M)** |
|  | 1932 | **565.0011** | **1691.9811** | **1692.8692** | **-0.8880** | **0** | **12** | **1.8e+02** | **1** | **TSQTPFHSSYCCGFK** |
|  | 253 | **382.3823** | **1144.1246** | **1144.3531** | **-0.2285** | **2** | **12** | **2.1e+02** | **1** | **KRLCQFHR + Carbamidomethyl (C)** |
|  | 647 | **401.9387** | **1202.7940** | **1202.3627** | **0.4312** | **1** | **12** | **1.9e+02** | **1** | **AFDLSGPIARR** |
|  | 332 | **386.1750** | **1155.5028** | **1155.3046** | **0.1981** | **1** | **12** | **1.4e+02** | **1** | **QPASQLQKQK** |
|  | 732 | **405.1416** | **1212.4026** | **1212.4158** | **-0.0131** | **1** | **12** | **1.5e+02** | **1** | **MEKLQYAVSK + Oxidation (M)** |
|  | 1016 | **421.3394** | **840.6641** | **841.0103** | **-0.3462** | **2** | **12** | **1.3e+02** | **1** | **ELKKAPR** |
|  | 1602 | **516.4830** | **1030.9512** | **1031.2123** | **-0.2611** | **0** | **12** | **1.6e+02** | **1** | **AVPPARPPAR** |
|  | 2306 | **620.7197** | **1859.1370** | **1858.9669** | **0.1701** | **2** | **12** | **1.9e+02** | **1** | **RDHVFEDSYRELHR** |
|  | 2385 | **628.6349** | **1255.2550** | **1254.5416** | **0.7134** | **1** | **12** | **1.7e+02** | **1** | **GLCPRVDLLLV + Carbamidomethyl (C)** |
|  | 2939 | **740.6038** | **1479.1929** | **1479.5462** | **-0.3533** | **0** | **12** | **1.3e+02** | **1** | **AGTNCFGSTMEASTS + Oxidation (M)** |
|  | 3571 | **1106.7366** | **2211.4584** | **2211.5014** | **-0.0431** | **2** | **12** | **1.5e+02** | **1** | **RGPQLVERIDGLVSQIDCGR** |
|  | 261 | **384.7696** | **1151.2867** | **1150.3775** | **0.9092** | **2** | **12** | **1.5e+02** | **1** | **KHRSLLLQR** |
|  | 1423 | **483.9465** | **1448.8174** | **1449.6474** | **-0.8300** | **0** | **12** | **1.7e+02** | **1** | **TNGDILVLYNLSK** |
|  | 1916 | **561.7011** | **1121.3873** | **1120.2373** | **1.1500** | **0** | **12** | **1.9e+02** | **1** | **CDNNATTIIR** |
|  | 2208 | **608.8027** | **1215.5907** | **1216.4124** | **-0.8218** | **1** | **12** | **1.6e+02** | **1** | **HICGRLFGEK + Carbamidomethyl (C)** |
|  | 969 | **418.0237** | **834.0325** | **834.0194** | **0.0132** | **0** | **12** | **1.8e+02** | **1** | **MVAAPCAR + Oxidation (M)** |
|  | 2273 | **614.2223** | **1839.6447** | **1839.0153** | **0.6293** | **2** | **12** | **1.6e+02** | **1** | **GSEAGPDARSLGEPLVKR** |
|  | 1010 | **420.4583** | **838.9017** | **838.0527** | **0.8490** | **1** | **12** | **1.8e+02** | **1** | **WMMARK + Oxidation (M)** |
|  | 1912 | **560.7993** | **1679.3756** | **1679.9380** | **-0.5625** | **1** | **12** | **1.4e+02** | **1** | **YNLPSWCDRVLWK** |
|  | 2281 | **615.5217** | **1843.5428** | **1843.1098** | **0.4330** | **1** | **12** | **1.4e+02** | **1** | **MDLLNSKNNLVIDTPR** |
|  | 2288 | **616.4496** | **1846.3266** | **1846.0474** | **0.2791** | **0** | **12** | **1.5e+02** | **1** | **HSVYCASCSLGLESFW + Carbamidomethyl (C)** |
|  | 2300 | **618.6804** | **1853.0191** | **1852.9543** | **0.0648** | **1** | **12** | **2e+02** | **1** | **AEDADMRNELEEMQR + Oxidation (M)** |
|  | 2339 | **624.0803** | **1869.2188** | **1870.0229** | **-0.8041** | **0** | **12** | **1.7e+02** | **1** | **DIVVTQSPDSLAVSPGER** |
|  | 3346 | **907.9543** | **2720.8409** | **2721.0426** | **-0.2018** | **2** | **12** | **1.7e+02** | **1** | **QEEMKKLSLLNGGLQEEELSTSLK + Oxidation (M)** |
|  | 455 | **388.9851** | **775.9554** | **776.8652** | **-0.9098** | **1** | **12** | **1.9e+02** | **1** | **ARGCGGTR** |
|  | 1556 | **506.3698** | **1010.7248** | **1010.1930** | **0.5319** | **2** | **12** | **1.3e+02** | **1** | **GFFKRSIR** |
|  | 2067 | **593.2244** | **1776.6509** | **1777.0717** | **-0.4207** | **1** | **12** | **1.8e+02** | **1** | **KICILGDCSGVVGDGGVK + Carbamidomethyl (C)** |
|  | 2533 | **659.0728** | **1974.1961** | **1974.3060** | **-0.1100** | **0** | **12** | **1.7e+02** | **1** | **TTCFQPTCVYSCCQPSCC** |
|  | 1396 | **477.9643** | **1430.8707** | **1430.4339** | **0.4368** | **0** | **12** | **1.5e+02** | **1** | **DPTQALNEQGDSR** |
|  | 1078 | **431.0218** | **860.0289** | **859.0949** | **0.9339** | **2** | **12** | **2.1e+02** | **1** | **ACLRIRK** |
|  | 1226 | **452.8547** | **1355.5419** | **1355.6058** | **-0.0640** | **1** | **12** | **1.8e+02** | **1** | **HIMSERNVLLK + Oxidation (M)** |
|  | 710 | **404.1572** | **1209.4495** | **1209.3506** | **0.0989** | **1** | **12** | **1.7e+02** | **1** | **DVPEGYKVFR** |
|  | 1039 | **424.3500** | **1270.0278** | **1270.4318** | **-0.4041** | **0** | **12** | **1.6e+02** | **1** | **SAIQVPTDLAQK** |
|  | 2375 | **628.0984** | **1881.2730** | **1881.0486** | **0.2244** | **1** | **12** | **1.6e+02** | **1** | **WNSKLYDLPDEPFTR** |
|  | 3209 | **810.9559** | **2429.8456** | **2429.5113** | **0.3343** | **1** | **12** | **1.9e+02** | **1** | **TNGYCSGGSCYTGRDYFDYW + 2 Carbamidomethyl (C)** |
|  | 860 | **407.8205** | **1220.4394** | **1221.3645** | **-0.9251** | **0** | **12** | **1.7e+02** | **1** | **EPFAHSLPPAR** |
|  | 1444 | **487.4276** | **972.8403** | **972.2228** | **0.6175** | **1** | **12** | **1.6e+02** | **1** | **LKSAVLTLK** |
|  | 1673 | **524.4925** | **1046.9702** | **1046.2020** | **0.7682** | **1** | **12** | **1.6e+02** | **1** | **AARAELEMR** |
|  | 1803 | **540.6218** | **1618.8431** | **1618.7872** | **0.0559** | **0** | **12** | **2e+02** | **1** | **NLANLEHAFYVAEK** |
|  | 2112 | **597.2512** | **1192.4875** | **1193.3098** | **-0.8222** | **2** | **12** | **1.9e+02** | **1** | **WVRSDSTKSK** |
|  | 936 | **412.9670** | **1235.8788** | **1235.6264** | **0.2524** | **2** | **12** | **1.5e+02** | **1** | **MMKIPKGMATK** |
|  | 2619 | **668.8378** | **2003.4911** | **2004.1998** | **-0.7086** | **1** | **12** | **1.8e+02** | **1** | **MEPSPEPPSLESMKGDTR + Oxidation (M)** |
|  | 2672 | **674.7075** | **1347.4003** | **1346.4436** | **0.9567** | **1** | **12** | **1.9e+02** | **1** | **GDIAEGVSVSREK** |
|  | 3061 | **758.2989** | **2271.8745** | **2270.8617** | **1.0128** | **2** | **12** | **1.6e+02** | **1** | **LCGSIMCKKCMELISLPLAK + Carbamidomethyl (C); 2 Oxidation (M)** |
|  | 3499 | **968.1122** | **2901.3144** | **2901.2082** | **0.1062** | **1** | **12** | **1.7e+02** | **1** | **HLYDPKLGTDQPLDQATISLQMGTNK + Oxidation (M)** |
|  | 1164 | **441.4986** | **1321.4735** | **1322.4946** | **-1.0211** | **0** | **12** | **1.9e+02** | **1** | **GSCPGQGCCQGLR + Carbamidomethyl (C)** |
|  | 1403 | **480.1252** | **1437.3534** | **1436.6158** | **0.7376** | **2** | **12** | **1.8e+02** | **1** | **VNGEHKEKVLQR** |
|  | 1545 | **505.0481** | **1008.0814** | **1006.9969** | **1.0846** | **0** | **12** | **1.6e+02** | **1** | **NGDGGCGTGGR + Carbamidomethyl (C)** |
|  | 1574 | **510.4276** | **1528.2605** | **1527.8969** | **0.3635** | **1** | **12** | **1.5e+02** | **1** | **TVPTMLRAPGCLLR** |
|  | 383 | **387.8192** | **1160.4354** | **1161.2697** | **-0.8343** | **2** | **12** | **2.1e+02** | **1** | **RSSRQVSDVK** |
|  | 808 | **406.9579** | **811.9011** | **810.9410** | **0.9600** | **0** | **12** | **1.5e+02** | **1** | **LQGPLQR** |
|  | 1983 | **579.0193** | **1156.0239** | **1155.3247** | **0.6992** | **1** | **12** | **1.7e+02** | **1** | **AGPGKMQEPPK + Oxidation (M)** |
|  | 2294 | **617.5093** | **1849.5057** | **1849.1095** | **0.3961** | **0** | **12** | **1.5e+02** | **1** | **YQPETSIPCTTLIPTK + Carbamidomethyl (C)** |
|  | 139 | **370.8679** | **1109.5816** | **1110.3072** | **-0.7256** | **0** | **12** | **1.3e+02** | **1** | **MFCTLNTHK + Oxidation (M)** |
|  | 260 | **384.7182** | **767.4216** | **766.8424** | **0.5793** | **0** | **12** | **1.4e+02** | **1** | **APPTEPR** |
|  | 1188 | **446.1981** | **1335.5721** | **1336.5232** | **-0.9511** | **1** | **12** | **1.9e+02** | **1** | **RMSGVRPQSFR + Oxidation (M)** |
|  | 2778 | **688.3136** | **1374.6124** | **1374.4138** | **0.1986** | **1** | **12** | **1.7e+02** | **1** | **SGKSAPGTAQGEER** |
|  | 1123 | **435.0938** | **1302.2591** | **1303.4005** | **-1.1414** | **0** | **12** | **1.5e+02** | **1** | **ESQAFSFCTAGR** |
|  | 1401 | **479.7200** | **1436.1379** | **1436.5447** | **-0.4067** | **0** | **12** | **1.4e+02** | **1** | **VTVGDTSCTGQGPSK** |
|  | 1592 | **513.8972** | **1538.6695** | **1538.6382** | **0.0313** | **0** | **12** | **1.6e+02** | **1** | **QGHTSSVEYDMLR + Oxidation (M)** |
|  | 1681 | **526.0151** | **1575.0231** | **1575.7825** | **-0.7595** | **1** | **12** | **1.8e+02** | **1** | **KGDVVTILEACENK + Carbamidomethyl (C)** |
|  | 2322 | **623.5708** | **1867.6902** | **1867.1813** | **0.5090** | **2** | **12** | **1.4e+02** | **1** | **RPLHSSAMEVQTKKVR** |
|  | 2664 | **672.8076** | **2015.4007** | **2014.2622** | **1.1384** | **2** | **12** | **1.9e+02** | **1** | **TNYENRIYSLKVECGPK** |
|  | 3005 | **743.9497** | **1485.8846** | **1484.6998** | **1.1848** | **1** | **12** | **1.5e+02** | **1** | **EIQRQSTLQLLR** |
|  | 1024 | **422.1137** | **1263.3190** | **1263.3565** | **-0.0374** | **1** | **12** | **1.9e+02** | **1** | **TDFDRSAPNIK** |
|  | 1034 | **423.2507** | **1266.7298** | **1267.5805** | **-0.8506** | **0** | **12** | **1.6e+02** | **1** | **VMPLSKPVPATK** |
|  | 3060 | **758.2821** | **2271.8241** | **2271.4922** | **0.3320** | **1** | **12** | **1.6e+02** | **1** | **HQHDRVCGDAMFQLQENVK + Oxidation (M)** |
|  | 563 | **392.2503** | **1173.7287** | **1174.4404** | **-0.7117** | **2** | **12** | **1.3e+02** | **1** | **RKIECPICR + Carbamidomethyl (C)** |
|  | 889 | **408.0694** | **1221.1860** | **1221.3879** | **-0.2019** | **2** | **12** | **1.8e+02** | **1** | **KDTRPMSRSK + Oxidation (M)** |
|  | 1154 | **438.2552** | **1311.7436** | **1312.5199** | **-0.7763** | **2** | **12** | **1.6e+02** | **1** | **RHVEQKIQFK** |
|  | 1616 | **518.8733** | **1553.5977** | **1553.8065** | **-0.2088** | **1** | **12** | **1.6e+02** | **1** | **VSGNAALQHLFIRK** |
|  | 1754 | **536.7607** | **1071.5066** | **1072.2195** | **-0.7129** | **1** | **12** | **1.6e+02** | **1** | **MQMRFDGR + 2 Oxidation (M)** |
|  | 1186 | **446.0763** | **890.1377** | **889.0083** | **1.1294** | **1** | **12** | **2e+02** | **1** | **TKSLLDGR** |
|  | 1249 | **458.7881** | **1373.3421** | **1373.6028** | **-0.2607** | **2** | **12** | **1.7e+02** | **1** | **WISFKSFFRR** |
|  | 1093 | **432.8332** | **863.6515** | **863.9758** | **-0.3242** | **0** | **12** | **1.8e+02** | **1** | **MTISPDGK + Oxidation (M)** |
|  | 1784 | **540.1438** | **1078.2728** | **1077.2141** | **1.0587** | **0** | **12** | **1.8e+02** | **1** | **DLANSCWLR** |
|  | 1866 | **550.4449** | **1648.3127** | **1647.9396** | **0.3731** | **0** | **12** | **1.4e+02** | **1** | **MNECCLCGTSVAMR + 2 Carbamidomethyl (C); Oxidation (M)** |
|  | 1935 | **565.9275** | **1129.8402** | **1129.2246** | **0.6156** | **1** | **12** | **1.7e+02** | **1** | **SSAPVEEVRR** |
|  | 1974 | **576.1611** | **1725.4612** | **1725.9256** | **-0.4643** | **2** | **12** | **1.7e+02** | **1** | **LHYFNARGKMESTR + Oxidation (M)** |
|  | 2590 | **667.6779** | **2000.0114** | **2000.2540** | **-0.2426** | **1** | **12** | **1.8e+02** | **1** | **FDEVQSSGGMILSVCKDK + Carbamidomethyl (C)** |
|  | 3187 | **804.1688** | **2409.4843** | **2408.6595** | **0.8248** | **0** | **12** | **1.5e+02** | **1** | **FIMQQNLGEEEIEQMLVNDQ** |
|  | 1652 | **522.8235** | **1565.4483** | **1564.8064** | **0.6419** | **1** | **12** | **1.5e+02** | **1** | **VECKCHGVSGSCEVK** |
|  | 3378 | **925.3080** | **2772.9018** | **2773.0877** | **-0.1859** | **2** | **12** | **1.4e+02** | **1** | **RGETGSTLVKMNRPIQVKPADSESR + Oxidation (M)** |
|  | 777 | **406.3029** | **1215.8864** | **1216.2621** | **-0.3757** | **2** | **12** | **1.3e+02** | **1** | **VRGEREEEGR** |
|  | 524 | **390.0284** | **1167.0632** | **1168.2605** | **-1.1973** | **0** | **12** | **1.7e+02** | **1** | **SGRPQQSSPPK** |
|  | 1281 | **460.7466** | **1379.2178** | **1379.5412** | **-0.3234** | **1** | **12** | **1.5e+02** | **1** | **CQEPFNSITKR + Carbamidomethyl (C)** |
|  | 313 | **386.0551** | **1155.1430** | **1154.3399** | **0.8031** | **1** | **12** | **1.6e+02** | **1** | **STLMFGQRAK + Oxidation (M)** |
|  | 870 | **407.8634** | **1220.5680** | **1220.3995** | **0.1685** | **1** | **12** | **1.7e+02** | **1** | **TQNTRSIICK + Carbamidomethyl (C)** |
|  | 872 | **407.8856** | **1220.6347** | **1221.4075** | **-0.7729** | **0** | **12** | **1.7e+02** | **1** | **DACSALCVVAR + 2 Carbamidomethyl (C)** |
|  | 274 | **385.0292** | **768.0437** | **766.9700** | **1.0737** | **0** | **12** | **1.5e+02** | **1** | **VATMMSK** |
|  | 1541 | **504.8683** | **1511.5829** | **1511.7023** | **-0.1194** | **1** | **12** | **1.7e+02** | **1** | **VHIEMGPDGRVTGK + Oxidation (M)** |
|  | 1907 | **560.5170** | **1678.5289** | **1679.0163** | **-0.4874** | **2** | **12** | **1.6e+02** | **1** | **CCWQKQLALTKQTK** |
|  | 2033 | **588.2592** | **1761.7555** | **1761.9262** | **-0.1708** | **1** | **12** | **1.8e+02** | **1** | **KDFLSAPQSLLEEER** |
|  | 2049 | **592.7085** | **1183.4022** | **1183.2785** | **0.1238** | **2** | **12** | **2.1e+02** | **1** | **TPRESAPGRGR** |
|  | 2491 | **651.1941** | **1950.5601** | **1951.2545** | **-0.6944** | **2** | **12** | **1.7e+02** | **1** | **GRVTMLVNTSKNQFSLR** |
|  | 2516 | **655.8614** | **1964.5620** | **1965.2761** | **-0.7141** | **2** | **12** | **1.5e+02** | **1** | **VSAMSPEEFGKLALWKR + Oxidation (M)** |
|  | 802 | **406.8250** | **1217.4530** | **1218.3176** | **-0.8646** | **0** | **12** | **1.7e+02** | **1** | **GDTCTMAGYAR + Carbamidomethyl (C); Oxidation (M)** |
|  | 975 | **418.9011** | **1253.6812** | **1254.3845** | **-0.7033** | **0** | **12** | **2e+02** | **1** | **GDDLLAMMDEL + 2 Oxidation (M)** |
|  | 1683 | **526.1609** | **1050.3070** | **1050.2537** | **0.0533** | **0** | **12** | **1.8e+02** | **1** | **VQVMDACLR + Oxidation (M)** |
|  | 1728 | **534.5883** | **1600.7426** | **1601.8297** | **-1.0871** | **1** | **12** | **2.2e+02** | **1** | **VCACHLCGGRQDPDK** |
|  | 1766 | **538.0959** | **1611.2657** | **1611.7466** | **-0.4810** | **0** | **12** | **1.8e+02** | **1** | **ELGFGATVIENIEGY** |
|  | 2387 | **628.7091** | **1255.4034** | **1255.4222** | **-0.0187** | **1** | **12** | **2.1e+02** | **1** | **WSTLPKSSPPR** |
|  | 845 | **407.6370** | **1219.8888** | **1219.4828** | **0.4060** | **1** | **12** | **1.4e+02** | **1** | **RPSKPRLIPR** |
|  | 3112 | **768.9548** | **2303.8421** | **2303.7390** | **0.1031** | **1** | **12** | **1.7e+02** | **1** | **IITLAGPTNAIFKAFAWIIDK** |
|  | 793 | **406.7149** | **1217.1224** | **1218.3161** | **-1.1936** | **1** | **12** | **1.4e+02** | **1** | **ASRMTTSSSQM + 2 Oxidation (M)** |
|  | 891 | **408.1298** | **1221.3672** | **1221.4025** | **-0.0353** | **2** | **12** | **1.8e+02** | **1** | **KDYLGKATVGGI** |
|  | 1709 | **532.8161** | **1063.6174** | **1064.1678** | **-0.5504** | **0** | **12** | **1.5e+02** | **1** | **EDVAVASMDK** |
|  | 1824 | **542.6323** | **1624.8746** | **1624.9076** | **-0.0330** | **1** | **12** | **2.1e+02** | **1** | **LLASRGTCLGTCCR + 3 Carbamidomethyl (C)** |
|  | 2242 | **611.1810** | **1220.3473** | **1220.2870** | **0.0602** | **1** | **12** | **1.8e+02** | **1** | **EPKYYYDSR** |
|  | 856 | **407.7723** | **1220.2948** | **1219.4330** | **0.8618** | **1** | **12** | **1.8e+02** | **1** | **LARIVEIYSR** |
|  | 2955 | **740.9512** | **1479.8876** | **1480.7145** | **-0.8270** | **2** | **12** | **1.6e+02** | **1** | **RNPVLAALRSAQGK** |
|  | 947 | **414.0079** | **1239.0015** | **1239.3998** | **-0.3982** | **1** | **12** | **1.5e+02** | **1** | **TVTISCTRSSGK** |
|  | 1139 | **436.1784** | **1305.5130** | **1304.4316** | **1.0815** | **0** | **12** | **1.8e+02** | **1** | **GSPAGTGPPFTCR + Carbamidomethyl (C)** |
|  | 1627 | **520.0325** | **1557.0752** | **1557.7555** | **-0.6803** | **2** | **12** | **1.7e+02** | **1** | **GTWAAALSARERLR** |
|  | 2351 | **625.2354** | **1872.6839** | **1872.1926** | **0.4912** | **0** | **12** | **1.8e+02** | **1** | **SCTQVAGCLVSLSMLSTR + Oxidation (M)** |
|  | 2628 | **669.4854** | **2005.4341** | **2006.3922** | **-0.9581** | **1** | **12** | **1.6e+02** | **1** | **DPSCLINACLNKLLLYR + Carbamidomethyl (C)** |
|  | 3052 | **758.0086** | **1514.0024** | **1513.7146** | **0.2878** | **1** | **12** | **1.4e+02** | **1** | **GSDHLDQLKEIMK** |
|  | 2718 | **684.0870** | **2049.2389** | **2048.1096** | **1.1293** | **0** | **12** | **1.7e+02** | **1** | **NNPNESVTANAATNSPSCTR** |
|  | 3522 | **975.0170** | **1948.0192** | **1947.2159** | **0.8032** | **0** | **12** | **1.6e+02** | **1** | **SSHTDSVFLQCIQLLQK** |
|  | 44 | **364.3401** | **1089.9982** | **1089.2268** | **0.7714** | **0** | **12** | **1.6e+02** | **1** | **RPGQAAGMSAK + Oxidation (M)** |
|  | 368 | **387.7644** | **1160.2709** | **1159.2007** | **1.0702** | **0** | **12** | **2.1e+02** | **1** | **LQAESPEEEK** |
|  | 1670 | **524.2435** | **1046.4723** | **1045.2999** | **1.1723** | **1** | **12** | **1.9e+02** | **1** | **YACKICCK + 2 Carbamidomethyl (C)** |
|  | 2163 | **605.6854** | **1814.0341** | **1814.1415** | **-0.1074** | **1** | **12** | **2.2e+02** | **1** | **CCLAVGARAGVVPSQLR + 2 Carbamidomethyl (C)** |
|  | 2641 | **670.4099** | **1338.8049** | **1338.4726** | **0.3323** | **2** | **12** | **1.8e+02** | **1** | **KNEDLSHALRR** |
|  | 1588 | **513.7812** | **1538.3216** | **1538.7076** | **-0.3860** | **1** | **12** | **1.4e+02** | **1** | **VQDGGGLVQPGRSLR** |
|  | 2097 | **595.7965** | **1784.3674** | **1784.0029** | **0.3644** | **1** | **12** | **1.6e+02** | **1** | **DNIKNTLHVEMNSLR** |
|  | 2146 | **601.7500** | **1802.2278** | **1801.1083** | **1.1196** | **0** | **12** | **2e+02** | **1** | **LPTSSIMTESAEMMIK + 2 Oxidation (M)** |
|  | 2786 | **690.7220** | **2069.1440** | **2068.3379** | **0.8061** | **2** | **12** | **1.9e+02** | **1** | **QKNKPDMNYETMGRALR + Oxidation (M)** |
|  | 3301 | **870.9254** | **2609.7541** | **2609.1393** | **0.6148** | **2** | **12** | **1.9e+02** | **1** | **LLEGLTLLLNLNVAGARKQLLSCR** |
|  | 74 | **369.0196** | **1104.0367** | **1103.2070** | **0.8297** | **1** | **12** | **2e+02** | **1** | **CEDELGPRK + Carbamidomethyl (C)** |
|  | 1251 | **458.8091** | **1373.4051** | **1374.5663** | **-1.1612** | **2** | **12** | **1.9e+02** | **1** | **KGGVDPTASCVRK + Carbamidomethyl (C)** |
|  | 3019 | **748.9609** | **2243.8606** | **2244.5531** | **-0.6924** | **2** | **12** | **1.6e+02** | **1** | **SMRYTVSNLSMQTHAARFK + Oxidation (M)** |
|  | 2761 | **686.2030** | **2055.5868** | **2055.2693** | **0.3175** | **2** | **12** | **1.7e+02** | **1** | **QYYLGDKCQVSSFFKNQ** |
|  | 3180 | **801.1855** | **2400.5343** | **2400.6445** | **-0.1103** | **1** | **12** | **1.7e+02** | **1** | **FQGRVTMTTDTSTTTAHMELR + Oxidation (M)** |
|  | 477 | **389.1147** | **776.2147** | **775.7646** | **0.4501** | **0** | **12** | **2e+02** | **1** | **AQNDNSK** |
|  | 1230 | **454.6092** | **1360.8054** | **1360.4964** | **0.3090** | **1** | **12** | **1.8e+02** | **1** | **APGQTARISCGGDK** |
|  | 1523 | **499.4759** | **1495.4056** | **1494.6964** | **0.7092** | **1** | **12** | **1.5e+02** | **1** | **LANSCRTEMQLR + Carbamidomethyl (C); Oxidation (M)** |
|  | 395 | **387.8581** | **1160.5521** | **1161.3324** | **-0.7803** | **1** | **12** | **2.1e+02** | **1** | **LMEQERALR + Oxidation (M)** |
|  | 813 | **407.0737** | **1218.1988** | **1217.3295** | **0.8693** | **1** | **12** | **1.7e+02** | **1** | **ELTRSLEDVR** |
|  | 1532 | **502.3763** | **1002.7378** | **1003.1343** | **-0.3965** | **1** | **12** | **1.5e+02** | **1** | **RTVAAPSGMN** |
|  | 2256 | **612.2795** | **1222.5443** | **1223.4447** | **-0.9004** | **1** | **12** | **1.8e+02** | **1** | **QILKQHLPCS + Carbamidomethyl (C)** |
|  | 2486 | **650.5839** | **1948.7296** | **1948.1890** | **0.5406** | **0** | **12** | **1.4e+02** | **1** | **ADHLAQPQPQGHLAPVLR** |
|  | 447 | **388.9525** | **1163.8352** | **1164.3315** | **-0.4963** | **0** | **12** | **2.1e+02** | **1** | **ATAVMPDGQFK** |
|  | 1103 | **433.0476** | **1296.1206** | **1296.6448** | **-0.5241** | **1** | **12** | **1.8e+02** | **1** | **MSFFKMCCVK + Carbamidomethyl (C); Oxidation (M)** |
|  | 1569 | **508.1940** | **1014.3733** | **1015.0952** | **-0.7220** | **0** | **12** | **1.9e+02** | **1** | **EYTIGNAMT + Oxidation (M)** |
|  | 2091 | **595.1915** | **1782.5524** | **1781.9857** | **0.5667** | **0** | **12** | **2e+02** | **1** | **SNGGSSTHACMPSTVAMK + Oxidation (M)** |
|  | 3453 | **949.1316** | **1896.2484** | **1895.2241** | **1.0243** | **1** | **12** | **1.7e+02** | **1** | **FSYGIELPFKAMHLLE** |
|  | 1585 | **513.4130** | **1537.2169** | **1537.7758** | **-0.5589** | **1** | **12** | **1.4e+02** | **1** | **LASSKSCLDPEFLK** |
|  | 1665 | **523.9821** | **1568.9240** | **1569.8441** | **-0.9202** | **2** | **12** | **1.9e+02** | **1** | **FKCCQISIEEGKGK** |
|  | 331 | **386.1680** | **1155.4817** | **1154.3398** | **1.1419** | **1** | **12** | **1.5e+02** | **1** | **GATSIVYRCK + Carbamidomethyl (C)** |
|  | 2291 | **617.0220** | **1232.0292** | **1231.4917** | **0.5374** | **1** | **12** | **1.9e+02** | **1** | **LPGARCAIQMR + Oxidation (M)** |
|  | 3165 | **792.9166** | **2375.7277** | **2376.6626** | **-0.9349** | **1** | **12** | **2.1e+02** | **1** | **AVSHKTFGISLEEVLVNEFTR** |
|  | 415 | **388.0780** | **1161.2119** | **1160.2764** | **0.9356** | **0** | **12** | **2.2e+02** | **1** | **DFNQLGPLEK** |
|  | 858 | **407.8000** | **1220.3780** | **1220.3168** | **0.0611** | **1** | **12** | **1.9e+02** | **1** | **AATPTSCSRNR + Carbamidomethyl (C)** |
|  | 1218 | **452.1408** | **1353.4003** | **1354.4886** | **-1.0882** | **1** | **12** | **2.2e+02** | **1** | **TDSGKQLGNCFK + Carbamidomethyl (C)** |
|  | 1856 | **549.9711** | **1646.8910** | **1647.9361** | **-1.0451** | **1** | **12** | **1.8e+02** | **1** | **CDVLRNPLYLQSVK** |
|  | 3142 | **784.9832** | **1567.9516** | **1566.7560** | **1.1957** | **2** | **12** | **1.7e+02** | **1** | **DKMCRDLAGEVEK + Carbamidomethyl (C); Oxidation (M)** |
|  | 1398 | **478.9515** | **1433.8323** | **1434.6395** | **-0.8072** | **1** | **12** | **1.8e+02** | **1** | **SFCSSGCLNKFK + 2 Carbamidomethyl (C)** |
|  | 1527 | **500.5181** | **999.0215** | **999.1686** | **-0.1472** | **1** | **12** | **2e+02** | **1** | **AQTRAALLR** |
|  | 1883 | **553.5233** | **1657.5478** | **1657.8632** | **-0.3154** | **0** | **12** | **1.7e+02** | **1** | **QLTFGMCLVGGDTDK + Carbamidomethyl (C); Oxidation (M)** |
|  | 2333 | **623.8405** | **1245.6661** | **1245.4092** | **0.2570** | **2** | **12** | **1.6e+02** | **1** | **KKMNSEISHR + Oxidation (M)** |
|  | 3018 | **748.7913** | **1495.5679** | **1496.6629** | **-1.0950** | **0** | **12** | **1.9e+02** | **1** | **ANPMDTESTIVMR + 2 Oxidation (M)** |
|  | 330 | **386.1582** | **1155.4525** | **1154.3199** | **1.1326** | **2** | **12** | **1.6e+02** | **1** | **AQKGQKAPAQK** |
|  | 3363 | **920.1693** | **2757.4858** | **2758.3742** | **-0.8884** | **2** | **12** | **1.5e+02** | **1** | **LPNGKLKCDVCGMVCIGPNVLMVHK + Carbamidomethyl (C); 2 Oxidation (M)** |
|  | 388 | **387.8330** | **1160.4769** | **1159.3399** | **1.1371** | **0** | **12** | **2.2e+02** | **1** | **CATTPMAVHR + Carbamidomethyl (C); Oxidation (M)** |
|  | 581 | **395.1295** | **1182.3664** | **1183.4392** | **-1.0728** | **1** | **12** | **2e+02** | **1** | **KLMSTALEMK + 2 Oxidation (M)** |
|  | 2167 | **606.1174** | **1210.2201** | **1209.4400** | **0.7801** | **1** | **12** | **1.7e+02** | **1** | **LQQATKMMSR + Oxidation (M)** |
|  | 2390 | **628.7794** | **1255.5441** | **1255.3806** | **0.1634** | **1** | **12** | **2e+02** | **1** | **NNQKSEPLIGR** |
|  | 3476 | **959.1249** | **2874.3525** | **2875.1278** | **-0.7754** | **0** | **12** | **1.8e+02** | **1** | **QPPGQGMEYIGSIYYSGSTHYNPALK + Oxidation (M)** |
|  | 198 | **375.5351** | **1123.5830** | **1123.1983** | **0.3847** | **1** | **12** | **1.7e+02** | **1** | **RGSEGGTACTK + Carbamidomethyl (C)** |
|  | 967 | **416.8284** | **1247.4631** | **1246.5246** | **0.9386** | **2** | **12** | **2.1e+02** | **1** | **CGRKFQGPILK** |
|  | 1379 | **475.7760** | **1424.3057** | **1424.6927** | **-0.3869** | **1** | **12** | **1.6e+02** | **1** | **WVPEIRCHCPK + Carbamidomethyl (C)** |
|  | 459 | **389.0048** | **775.9949** | **775.9601** | **0.0348** | **1** | **12** | **2.1e+02** | **1** | **ALMAAKR + Oxidation (M)** |
|  | 175 | **373.2931** | **744.5715** | **743.8055** | **0.7659** | **0** | **12** | **1.9e+02** | **1** | **IENQVAA** |
|  | 2546 | **663.7441** | **1988.2103** | **1988.2663** | **-0.0561** | **0** | **12** | **2.2e+02** | **1** | **EMAVVLLANLAQGDSLATR + Oxidation (M)** |
|  | 417 | **388.1076** | **1161.3006** | **1160.4306** | **0.8701** | **2** | **12** | **2.2e+02** | **1** | **DLKKLAAVMR + Oxidation (M)** |
|  | 436 | **388.8123** | **775.6098** | **774.9291** | **0.6807** | **0** | **12** | **2.1e+02** | **1** | **APMTTVR** |
|  | 448 | **388.9591** | **1163.8551** | **1163.3847** | **0.4703** | **0** | **12** | **2.1e+02** | **1** | **NLTMDTLVIK + Oxidation (M)** |
|  | 1067 | **429.4156** | **856.8165** | **856.9731** | **-0.1567** | **2** | **12** | **1.9e+02** | **1** | **RRAELGR** |
|  | 275 | **385.0708** | **1152.1901** | **1153.2937** | **-1.1036** | **1** | **12** | **1.6e+02** | **1** | **AFRASSHLHK** |
|  | 1001 | **420.0465** | **1257.1173** | **1256.4285** | **0.6888** | **1** | **12** | **1.7e+02** | **1** | **SAFNKLASSMGK + Oxidation (M)** |
|  | 1726 | **534.4449** | **1600.3125** | **1600.8531** | **-0.5407** | **1** | **12** | **1.6e+02** | **1** | **LIEDVDALKSWLAK** |
|  | 2104 | **596.6825** | **1787.0253** | **1786.9804** | **0.0449** | **0** | **12** | **2.5e+02** | **1** | **SPGLGDAAMYLCATSTR + Carbamidomethyl (C); Oxidation (M)** |
|  | 899 | **408.7421** | **1223.2041** | **1222.2829** | **0.9212** | **0** | **12** | **1.8e+02** | **1** | **LGLSESDENCR** |
|  | 1732 | **534.7155** | **1067.4161** | **1068.2044** | **-0.7883** | **1** | **12** | **1.8e+02** | **1** | **DRMVEQFK + Oxidation (M)** |
|  | 1800 | **540.4802** | **1078.9455** | **1078.1808** | **0.7647** | **0** | **12** | **1.5e+02** | **1** | **CNEQPCQTR** |
|  | 3047 | **757.6791** | **1513.3434** | **1512.7762** | **0.5671** | **2** | **12** | **1.4e+02** | **1** | **RCRDPINVEGLLK** |
|  | 177 | **373.9941** | **1118.9601** | **1119.2496** | **-0.2895** | **1** | **12** | **2.4e+02** | **1** | **MRDVSAAPEK + Oxidation (M)** |
|  | 194 | 374.9718 | 1121.8933 | 1121.1874 | 0.7060 | 1 | 12 | 2.2e+02 | 1 | CSSGFDRHR + Carbamidomethyl (C) |
|  | 784 | **406.5083** | **1216.5028** | **1216.3249** | **0.1780** | **0** | **12** | **2e+02** | **1** | **MHSAGTPGLSSR + Oxidation (M)** |
|  | 1311 | **464.2333** | **1389.6778** | **1389.6650** | **0.0128** | **0** | **12** | **1.8e+02** | **1** | **LHDPQLMGIIPR** |
|  | 1448 | **487.9143** | **1460.7207** | **1460.8509** | **-0.1301** | **2** | **12** | **2.1e+02** | **1** | **MLPVLKMLTSRR + Oxidation (M)** |
|  | 2459 | **642.8800** | **1925.6178** | **1926.2285** | **-0.6106** | **1** | **12** | **1.4e+02** | **1** | **HMMAHTGDGPHKCTVCGK + Oxidation (M)** |
|  | 3144 | **785.4364** | **1568.8580** | **1567.9175** | **0.9405** | **0** | **12** | **1.8e+02** | **1** | **LCCLHKPGIGLTGAK + Carbamidomethyl (C)** |
|  | 3385 | **928.4010** | **1854.7872** | **1855.1920** | **-0.4047** | **2** | **12** | **1.7e+02** | **1** | **KPPHVELKARPKLGER** |
|  | 760 | **405.9062** | **809.7977** | **809.9728** | **-0.1751** | **0** | **12** | **1.7e+02** | **1** | **ILCNYK + Carbamidomethyl (C)** |
|  | 1655 | **522.9633** | **1043.9117** | **1043.1087** | **0.8031** | **0** | **12** | **1.9e+02** | **1** | **GEMLSYGDR + Oxidation (M)** |
|  | 2845 | **711.1359** | **1420.2569** | **1420.5485** | **-0.2915** | **1** | **12** | **1.8e+02** | **1** | **RQGISSMQESGPK + Oxidation (M)** |
|  | 1072 | **430.5478** | **859.0808** | **857.9116** | **1.1692** | **1** | **12** | **2.5e+02** | **1** | **RGGAEGSPK** |
|  | 2006 | **583.0153** | **1746.0238** | **1745.8871** | **0.1367** | **0** | **12** | **1.9e+02** | **1** | **WGGCSEDIEFGGMVSR + Oxidation (M)** |
|  | 2374 | **628.0156** | **1881.0245** | **1881.2007** | **-0.1762** | **0** | **12** | **1.8e+02** | **1** | **NLTPMNYNIFFHLLK + Oxidation (M)** |
|  | 196 | **375.1513** | **1122.4317** | **1121.2652** | **1.1665** | **0** | **12** | **2e+02** | **1** | **LQSMLQTER + Oxidation (M)** |
|  | 2644 | **670.6527** | **1339.2906** | **1338.5141** | **0.7766** | **0** | **12** | **1.7e+02** | **1** | **DHYGIMMYHR + Oxidation (M)** |
|  | 420 | **388.2180** | **774.4211** | **774.8196** | **-0.3985** | **0** | **12** | **1.8e+02** | **1** | **DEAATLR** |
|  | 1851 | **549.4989** | **1096.9830** | **1096.3682** | **0.6148** | **1** | **12** | **1.5e+02** | **1** | **VHKFLALLR** |
|  | 1209 | **450.3244** | **1347.9511** | **1347.6681** | **0.2830** | **0** | **12** | **1.5e+02** | **1** | **MAIIGGFLSAVLR** |
|  | 1243 | **458.4538** | **1372.3391** | **1372.6331** | **-0.2939** | **0** | **12** | **2.1e+02** | **1** | **LHPFFPSTMAPK** |
|  | 2408 | **632.1310** | **1893.3708** | **1892.1838** | **1.1869** | **2** | **12** | **2e+02** | **1** | **SNVSLLEPYQKCGLKR + Carbamidomethyl (C)** |
|  | 200 | **375.7842** | **1124.3304** | **1125.2622** | **-0.9318** | **1** | **11** | **1.9e+02** | **1** | **NTHGCKVGPGR** |
|  | 286 | **385.8907** | **769.7667** | **768.9441** | **0.8225** | **0** | **11** | **1.6e+02** | **1** | **ASLLLPR** |
|  | 2158 | **604.2927** | **1809.8558** | **1809.9607** | **-0.1049** | **1** | **11** | **2e+02** | **1** | **KVSGSGNHHTGAACLASR + Carbamidomethyl (C)** |
|  | 2336 | **623.9478** | **1245.8807** | **1246.3788** | **-0.4981** | **1** | **11** | **1.6e+02** | **1** | **GRGAAGGPPTHLR** |
|  | 2617 | **668.7977** | **1335.5807** | **1334.5005** | **1.0801** | **1** | **11** | **2.3e+02** | **1** | **AGGHDAGKLYAMK + Oxidation (M)** |
|  | 502 | **389.9604** | **777.9061** | **776.9231** | **0.9829** | **0** | **11** | **1.9e+02** | **1** | **ALSALFR** |
|  | 1451 | **487.9817** | **973.9487** | **973.0486** | **0.9001** | **2** | **11** | **2.1e+02** | **1** | **ERAGRAASR** |
|  | 1658 | **523.1026** | **1044.1904** | **1044.1928** | **-0.0023** | **2** | **11** | **2e+02** | **1** | **MRGAGPRGAR + Oxidation (M)** |
|  | 2597 | **667.8740** | **2000.5999** | **2000.3878** | **0.2121** | **0** | **11** | **1.8e+02** | **1** | **AVGLMLLVWDTPLGQACR + Carbamidomethyl (C)** |
|  | 3092 | **762.4029** | **2284.1865** | **2283.5840** | **0.6026** | **1** | **11** | **1.9e+02** | **1** | **LSCAASKFTFNNYAMTWVR + Carbamidomethyl (C); Oxidation (M)** |
|  | 678 | **403.2664** | **804.5181** | **804.9581** | **-0.4400** | **1** | **11** | **1.7e+02** | **1** | **GATKGCLR** |
|  | 1511 | **495.9879** | **989.9609** | **989.0910** | **0.8699** | **2** | **11** | **2e+02** | **1** | **LRGGTSSRR** |
|  | 1804 | **541.0885** | **1620.2433** | **1619.9062** | **0.3371** | **1** | **11** | **1.9e+02** | **1** | **SVTVRLPGLHNSLVK** |
|  | 1825 | **542.6342** | **1083.2537** | **1083.2702** | **-0.0165** | **2** | **11** | **2.2e+02** | **1** | **KCHHFQKR** |
|  | 2295 | **618.1794** | **1851.5162** | **1851.1817** | **0.3344** | **2** | **11** | **2.1e+02** | **1** | **VTAVHKANIMLRGTPAR + Oxidation (M)** |
|  | 3008 | **745.0065** | **2231.9972** | **2232.5524** | **-0.5551** | **2** | **11** | **1.5e+02** | **1** | **EFYPVKEFEVYHKLMEK + Oxidation (M)** |
|  | 1662 | **523.4667** | **1044.9186** | **1044.1860** | **0.7325** | **0** | **11** | **1.7e+02** | **1** | **THLGLTCSGR** |
|  | 2389 | **628.7787** | **1883.3139** | **1883.2431** | **0.0708** | **0** | **11** | **2.1e+02** | **1** | **AAGCLLAGMHTLLQQALR + Oxidation (M)** |
|  | 987 | **419.2576** | **836.5005** | **835.9919** | **0.5085** | **0** | **11** | **1.9e+02** | **1** | **CIQCQK + 2 Carbamidomethyl (C)** |
|  | 3425 | **946.8138** | **2837.4194** | **2836.2989** | **1.1204** | **1** | **11** | **1.5e+02** | **1** | **VCHQVLGSAPARPDLAPRFSAMALVR + Carbamidomethyl (C); Oxidation (M)** |
|  | 337 | **386.2278** | **1155.6613** | **1155.2416** | **0.4196** | **0** | **11** | **1.4e+02** | **1** | **NGGYSCTQIR + Carbamidomethyl (C)** |
|  | 449 | **388.9594** | **775.9040** | **776.8588** | **-0.9547** | **0** | **11** | **2.2e+02** | **1** | **VEDCVR + Carbamidomethyl (C)** |
|  | 3555 | **1028.7274** | **3083.1601** | **3082.6571** | **0.5030** | **0** | **11** | **1.7e+02** | **1** | **MAHFEEMGMCVETDMELLVCTFCIK + 3 Carbamidomethyl (C)** |
|  | 181 | **374.1532** | **746.2916** | **746.9586** | **-0.6669** | **1** | **11** | **2.2e+02** | **1** | **MILKDK** |
|  | 483 | **389.1482** | **776.2817** | **775.9418** | **0.3399** | **2** | **11** | **2.1e+02** | **1** | **RGRFLK** |
|  | 1457 | **488.0928** | **974.1708** | **973.1248** | **1.0459** | **2** | **11** | **2.2e+02** | **1** | **KAQIEEKK** |
|  | 2373 | **628.0087** | **1881.0038** | **1880.0523** | **0.9516** | **2** | **11** | **1.8e+02** | **1** | **QQQPTREFSGGWRMR + Oxidation (M)** |
|  | 2051 | **592.7509** | **1775.2304** | **1774.7448** | **0.4856** | **0** | **11** | **2e+02** | **1** | **TDSSTTNLFSEEVETD** |
|  | 2088 | **595.0175** | **1188.0203** | **1187.3483** | **0.6720** | **0** | **11** | **2.1e+02** | **1** | **VPAPQATPPGPR** |
|  | 2115 | **597.7258** | **1790.1553** | **1790.0704** | **0.0849** | **0** | **11** | **2.3e+02** | **1** | **MCISSTGNAGQVPAVGGIK** |
|  | 2695 | **680.6807** | **2039.0198** | **2038.3069** | **0.7129** | **2** | **11** | **1.8e+02** | **1** | **GPRISAPNVDFNLEGPKVK** |
|  | 141 | **370.9503** | **1109.8286** | **1110.2410** | **-0.4124** | **0** | **11** | **1.5e+02** | **1** | **ELYAMDVNR** |
|  | 2740 | **685.2972** | **2052.8695** | **2052.2953** | **0.5743** | **1** | **11** | **2e+02** | **1** | **EAFQLQGMPNSSGLMNRR + Oxidation (M)** |
|  | 2931 | **740.5108** | **2218.5102** | **2219.4408** | **-0.9305** | **1** | **11** | **1.9e+02** | **1** | **MERGWPQGDSCPGERPAACR + Oxidation (M)** |
|  | 263 | **384.8627** | **1151.5658** | **1151.3757** | **0.1902** | **0** | **11** | **1.7e+02** | **1** | **QQMVEFLIK + Oxidation (M)** |
|  | 847 | **407.6915** | **1220.0524** | **1220.5071** | **-0.4547** | **2** | **11** | **1.6e+02** | **1** | **IREKHLAILK** |
|  | 1256 | **458.8995** | **1373.6764** | **1372.6793** | **0.9971** | **2** | **11** | **2.2e+02** | **1** | **IRELAINIRMK + Oxidation (M)** |
|  | 2246 | **612.1209** | **1222.2269** | **1221.3149** | **0.9121** | **0** | **11** | **2e+02** | **1** | **FYSVNVDYSK** |
|  | 2691 | **680.0781** | **2037.2120** | **2037.2822** | **-0.0702** | **2** | **11** | **1.9e+02** | **1** | **NNLKGRLHLESPSPGVYR** |
|  | 1955 | **572.3047** | **1142.5946** | **1142.3489** | **0.2457** | **1** | **11** | **2e+02** | **1** | **LRILVQESGK** |
|  | 2913 | **738.5918** | **2212.7532** | **2212.6135** | **0.1398** | **1** | **11** | **1.7e+02** | **1** | **FAWLWLQFVYMRFNFK + Oxidation (M)** |
|  | 3289 | **859.4082** | **2575.2024** | **2576.0270** | **-0.8245** | **1** | **11** | **1.8e+02** | **1** | **FMPAAEGTVCGLSMWCRQGQCVK + Carbamidomethyl (C); Oxidation (M)** |
|  | 1711 | **532.8923** | **1595.6548** | **1595.7176** | **-0.0627** | **2** | **11** | **1.9e+02** | **1** | **DSCGKGEMATGNGRR + Carbamidomethyl (C)** |
|  | 2008 | **583.8824** | **1748.6250** | **1748.1347** | **0.4902** | **0** | **11** | **1.7e+02** | **1** | **GMTLVTPLQLLLFASK + Oxidation (M)** |
|  | 2080 | **594.6903** | **1781.0488** | **1780.0755** | **0.9732** | **2** | **11** | **2.5e+02** | **1** | **NVPEHLAKLFKIEGGK** |
|  | 2200 | **608.5858** | **1215.1568** | **1215.2275** | **-0.0707** | **0** | **11** | **1.8e+02** | **1** | **EHEQTDITSR** |
|  | 2207 | **608.7783** | **1823.3126** | **1824.1581** | **-0.8455** | **1** | **11** | **2e+02** | **1** | **MMGRCMGAAQFVGGTCR + 3 Oxidation (M)** |
|  | 3242 | **825.5127** | **2473.5159** | **2472.8574** | **0.6585** | **2** | **11** | **1.9e+02** | **1** | **KIIPQCIARDFTDLIVINEDR** |
|  | 2757 | **686.1675** | **2055.4803** | **2056.3438** | **-0.8636** | **2** | **11** | **1.9e+02** | **1** | **DVTLTREMITLSFRTTR + Oxidation (M)** |
|  | 2883 | **725.8852** | **1449.7556** | **1450.7022** | **-0.9466** | **2** | **11** | **2.1e+02** | **1** | **VEMKRTVAAPSVF + Oxidation (M)** |
|  | 3421 | **944.6497** | **2830.9268** | **2830.3044** | **0.6224** | **1** | **11** | **1.7e+02** | **1** | **TLAAKMAALASMAHGMAASPAILEETQK + Oxidation (M)** |
|  | 1896 | **556.9481** | **1667.8220** | **1666.8701** | **0.9519** | **1** | **11** | **2e+02** | **1** | **VVISTDTSKNQFSLK** |
|  | 2677 | **674.9248** | **2021.7522** | **2022.2695** | **-0.5173** | **2** | **11** | **1.7e+02** | **1** | **TSYRHCSIETMEVPRR + Carbamidomethyl (C)** |
|  | 3506 | **969.0250** | **2904.0529** | **2903.2920** | **0.7609** | **0** | **11** | **1.8e+02** | **1** | **SMLSLESTTLAAQHCCYGDNMQLITR + Oxidation (M)** |
|  | 679 | **403.6254** | **1207.8540** | **1208.3259** | **-0.4718** | **0** | **11** | **1.7e+02** | **1** | **APWSQAAGAPPR** |
|  | 2624 | **669.1194** | **2004.3360** | **2005.3266** | **-0.9906** | **1** | **11** | **2e+02** | **1** | **HKLMANGVMGDGHPLFHK + Oxidation (M)** |
|  | 2977 | **741.9609** | **2222.8606** | **2223.4623** | **-0.6017** | **0** | **11** | **1.6e+02** | **1** | **LDAFLNQMFWSSSLTQYR + Oxidation (M)** |
|  | 3000 | **743.1593** | **2226.4557** | **2226.6369** | **-0.1812** | **1** | **11** | **2e+02** | **1** | **CWDYMCEPLCPASCYLKK + Carbamidomethyl (C); Oxidation (M)** |
|  | 2258 | **612.3422** | **1222.6695** | **1223.3819** | **-0.7123** | **1** | **11** | **2e+02** | **1** | **NNRLGGPSALPK** |
|  | 3043 | **754.8845** | **2261.6314** | **2260.8023** | **0.8291** | **2** | **11** | **2.4e+02** | **1** | **MALCLELLKQCSSCLVAYKK + Oxidation (M)** |
|  | 1124 | **435.1185** | **1302.3332** | **1302.5002** | **-0.1669** | **1** | **11** | **1.9e+02** | **1** | **SDVPINLKNMR + Oxidation (M)** |
|  | 2595 | **667.8555** | **2000.5442** | **1999.4696** | **1.0747** | **2** | **11** | **2e+02** | **1** | **RMGMIICKACSMLPAER + Carbamidomethyl (C); 2 Oxidation (M)** |
|  | 3223 | **818.4415** | **1634.8683** | **1634.8313** | **0.0369** | **0** | **11** | **1.8e+02** | **1** | **ELTSHQDLNIPVLR** |
|  | 216 | **377.2928** | **752.5708** | **752.8189** | **-0.2481** | **0** | **11** | **1.5e+02** | **1** | **IHAAEGR** |
|  | 699 | **404.0380** | **1209.0919** | **1209.4200** | **-0.3281** | **0** | **11** | **2e+02** | **1** | **MCGLGCAPSAGSR** |
|  | 1393 | **477.2362** | **1428.6864** | **1427.5428** | **1.1437** | **1** | **11** | **1.9e+02** | **1** | **EVMGSKEPNAGHR + Oxidation (M)** |
|  | 2187 | **607.7153** | **1820.1238** | **1820.8825** | **-0.7587** | **0** | **11** | **2.3e+02** | **1** | **SDDTAVYYCASGYDYK** |
|  | 3342 | **898.6830** | **1795.3512** | **1794.9828** | **0.3684** | **1** | **11** | **1.8e+02** | **1** | **LSTPSPMQPPPDEARR + Oxidation (M)** |
|  | 284 | **385.7435** | **769.4722** | **768.8184** | **0.6538** | **0** | **11** | **1.7e+02** | **1** | **GGQGPPTR** |
|  | 2550 | **665.6714** | **1329.3281** | **1329.5038** | **-0.1757** | **0** | **11** | **1.9e+02** | **1** | **NSVYLQLHSLR** |
|  | 3220 | **817.3748** | **1632.7347** | **1631.8078** | **0.9270** | **1** | **11** | **1.9e+02** | **1** | **EGVELHAGPKYEMR + Oxidation (M)** |
|  | 267 | **384.9469** | **1151.8185** | **1151.4452** | **0.3734** | **1** | **11** | **1.7e+02** | **1** | **LCMPKGLSFR** |
|  | 670 | **403.0134** | **804.0120** | **802.9175** | **1.0945** | **1** | **11** | **2.3e+02** | **1** | **QKVWDK** |
|  | 2412 | **633.9296** | **1265.8445** | **1265.4339** | **0.4106** | **1** | **11** | **1.6e+02** | **1** | **TDAVAEMKAVSK + Oxidation (M)** |
|  | 3450 | **948.7987** | **2843.3739** | **2844.1751** | **-0.8012** | **2** | **11** | **1.6e+02** | **1** | **AGGAAGGACAFLGMSRHERCTCSCSPGR + 2 Carbamidomethyl (C); Oxidation (M)** |
|  | 2991 | **742.6583** | **2224.9526** | **2225.7121** | **-0.7595** | **1** | **11** | **1.5e+02** | **1** | **MCLVYPASKMFPFIMEGTK + 2 Oxidation (M)** |
|  | 1576 | **510.7666** | **1529.2775** | **1528.7773** | **0.5003** | **2** | **11** | **1.7e+02** | **1** | **KAPRVWELGGCANK** |
|  | 2441 | **639.6315** | **1915.8722** | **1915.1329** | **0.7393** | **1** | **11** | **1.9e+02** | **1** | **QPSMTLQRAVSLEGEPR + Oxidation (M)** |
|  | 3084 | **760.0577** | **1518.1007** | **1518.6203** | **-0.5196** | **0** | **11** | **1.6e+02** | **1** | **EVTWEALEGEVEK** |
|  | 250 | **380.2057** | **1137.5949** | **1138.1715** | **-0.5766** | **1** | **11** | **1.9e+02** | **1** | **DGGWDCRSSR** |
|  | 985 | **419.2328** | **836.4509** | **836.9969** | **-0.5460** | **1** | **11** | **2e+02** | **1** | **MAASSVKK + Oxidation (M)** |
|  | 994 | **419.3243** | **836.6338** | **836.9802** | **-0.3463** | **0** | **11** | **1.9e+02** | **1** | **FRPYVR** |
|  | 1687 | **527.8348** | **1053.6548** | **1053.1301** | **0.5247** | **1** | **11** | **1.6e+02** | **1** | **SRDPAPASPR** |
|  | 1168 | **442.2873** | **1323.8397** | **1323.5825** | **0.2572** | **2** | **11** | **1.5e+02** | **1** | **GREEAIMKMVK + 2 Oxidation (M)** |
|  | 1391 | **476.2576** | **1425.7507** | **1425.6774** | **0.0733** | **1** | **11** | **1.9e+02** | **1** | **DRYLHIAVVIAR** |
|  | 2059 | **592.8833** | **1183.7518** | **1183.3777** | **0.3742** | **1** | **11** | **1.7e+02** | **1** | **SFSCKSNLIK + Carbamidomethyl (C)** |
|  | 2738 | **685.2725** | **1368.5301** | **1367.5572** | **0.9730** | **2** | **11** | **2.1e+02** | **1** | **TAAPSVRPEKRR** |
|  | 3036 | **752.5406** | **2254.5996** | **2253.4505** | **1.1491** | **1** | **11** | **1.9e+02** | **1** | **SCCGGCFYGETEKHNFSVER** |
|  | 3244 | **827.1006** | **1652.1864** | **1651.8915** | **0.2948** | **1** | **11** | **1.6e+02** | **1** | **CSFCLRICNEHGR + 2 Carbamidomethyl (C)** |
|  | 3466 | **955.3577** | **2863.0508** | **2862.1491** | **0.9017** | **0** | **11** | **1.6e+02** | **1** | **SPAELQGGQVDLTGATCPYTLSNMTYK + Oxidation (M)** |
|  | 360 | **387.7287** | **773.4426** | **772.8038** | **0.6389** | **0** | **11** | **2.3e+02** | **1** | **SLGEDPR** |
|  | 1760 | **537.3239** | **1608.9494** | **1607.8293** | **1.1200** | **2** | **11** | **2.2e+02** | **1** | **REYIKQNPMATEK** |
|  | 1929 | **564.5835** | **1127.1522** | **1127.2979** | **-0.1457** | **2** | **11** | **2.3e+02** | **1** | **AIAPKRSGTAR** |
|  | 2279 | **614.5875** | **1840.7402** | **1840.0909** | **0.6493** | **0** | **11** | **1.7e+02** | **1** | **VLWLAENPCCGTSPHR + Carbamidomethyl (C)** |
|  | 2823 | **701.6833** | **2102.0277** | **2102.4585** | **-0.4308** | **1** | **11** | **1.7e+02** | **1** | **MKVSLGNGEMGVSAHLQPCK + Oxidation (M)** |
|  | 256 | **384.4609** | **766.9071** | **765.8592** | **1.0479** | **0** | **11** | **2.2e+02** | **1** | **CSCSPR + 2 Carbamidomethyl (C)** |
|  | 775 | **406.2282** | **1215.6626** | **1216.3049** | **-0.6423** | **2** | **11** | **1.6e+02** | **1** | **KKAGAGNANSNGK** |
|  | 983 | **419.2002** | **1254.5783** | **1255.4601** | **-0.8818** | **0** | **11** | **2.2e+02** | **1** | **EIQSILPGISAK** |
|  | 66 | **367.8940** | **1100.6597** | **1101.3833** | **-0.7236** | **2** | **11** | **2.3e+02** | **1** | **MMKFLSGKK + 2 Oxidation (M)** |
|  | 3290 | **859.5189** | **2575.5346** | **2576.0869** | **-0.5523** | **2** | **11** | **1.9e+02** | **1** | **NTLAVANMKVSRPVMEKVPYALK + Oxidation (M)** |
|  | 38 | **363.3821** | **724.7494** | **723.9022** | **0.8472** | **0** | **11** | **2.3e+02** | **1** | **ECMVVK + Oxidation (M)** |
|  | 929 | **411.9490** | **821.8833** | **820.9974** | **0.8859** | **1** | **11** | **2.1e+02** | **1** | **LQMSKAK + Oxidation (M)** |
|  | 2283 | **615.7402** | **1844.1985** | **1843.0833** | **1.1152** | **1** | **11** | **2.4e+02** | **1** | **QLTADYFEKTWLSLK** |
|  | 8 | **360.5783** | **1078.7127** | **1079.2038** | **-0.4911** | **0** | **11** | **1.8e+02** | **1** | **EAVSFALSQK** |
|  | 1418 | **482.6700** | **1444.9879** | **1445.6456** | **-0.6577** | **2** | **11** | **1.9e+02** | **1** | **RQSLKCNPDAWK** |
|  | 2286 | **616.1196** | **1845.3367** | **1846.1219** | **-0.7852** | **2** | **11** | **2.1e+02** | **1** | **RFCGLSQPRNLAQLSR** |
|  | 886 | **408.0257** | **1221.0550** | **1221.4125** | **-0.3575** | **1** | **11** | **2.1e+02** | **1** | **MHSLGCFRDR** |
|  | 1161 | **440.3227** | **1317.9458** | **1317.5612** | **0.3846** | **2** | **11** | **1.9e+02** | **1** | **EVLRMRGTISR** |
|  | 3521 | **972.6886** | **1943.3624** | **1942.2362** | **1.1262** | **2** | **11** | **1.8e+02** | **1** | **MDDFQLKGIVEEKFVK + Oxidation (M)** |
|  | 137 | **370.2202** | **738.4255** | **737.8243** | **0.6013** | **0** | **11** | **1.4e+02** | **1** | **EPQMHP** |
|  | 831 | 407.4111 | 1219.2112 | 1220.3717 | -1.1604 | 1 | 11 | 2.2e+02 | 1 | VSSSYPVEPKK |
|  | 1905 | **558.7011** | **1115.3873** | **1114.3390** | **1.0484** | **2** | **11** | **2.4e+02** | **1** | **VRVIKLSGGVS** |
|  | 2817 | **700.5290** | **2098.5648** | **2097.3974** | **1.1674** | **2** | **11** | **1.8e+02** | **1** | **TPKGTQGVVTNFEIFRMR + Oxidation (M)** |
|  | 2674 | **674.7789** | **1347.5431** | **1347.7128** | **-0.1698** | **0** | **11** | **2.5e+02** | **1** | **TLCCFCCPCLK + 2 Carbamidomethyl (C)** |
|  | 3407 | **940.8235** | **2819.4483** | **2819.8573** | **-0.4091** | **2** | **11** | **1.5e+02** | **1** | **SASPDDDLGSSNWEAADLGNEERKQK** |
|  | 514 | **390.0019** | **1166.9834** | **1167.4246** | **-0.4412** | **0** | **11** | **2.1e+02** | **1** | **MVLLAQHLAR + Oxidation (M)** |
|  | 3323 | **893.3262** | **2676.9563** | **2676.8943** | **0.0621** | **2** | **11** | **1.9e+02** | **1** | **AARVSARVFVTDENDNAPVFASPSR** |
|  | 1959 | **573.2224** | **1716.6449** | **1717.0249** | **-0.3800** | **1** | **11** | **2.2e+02** | **1** | **IRVDAMHGVMGPYVR + Oxidation (M)** |
|  | 2252 | **612.1818** | **1222.3487** | **1223.3557** | **-1.0069** | **0** | **11** | **2.1e+02** | **1** | **EVGYSTHMVGK + Oxidation (M)** |
|  | 991 | **419.3082** | **1254.9024** | **1254.3927** | **0.5097** | **1** | **11** | **2e+02** | **1** | **LNQPGTPTRTAV** |
|  | 1227 | **453.0078** | **904.0009** | **903.0331** | **0.9678** | **0** | **11** | **2.3e+02** | **1** | **IAAPGQSLF** |
|  | 2014 | **584.2019** | **1166.3890** | **1165.2499** | **1.1391** | **0** | **11** | **2e+02** | **1** | **LAEAESEAAFK** |
|  | 3123 | **775.7904** | **2324.3490** | **2323.5885** | **0.7606** | **1** | **11** | **1.9e+02** | **1** | **AVAAAAAMGKTANSPGSGARPDPVR** |
|  | 199 | **375.6998** | **1124.0773** | **1123.2780** | **0.7994** | **1** | **11** | **1.9e+02** | **1** | **ELADTTAMKK + Oxidation (M)** |
|  | 811 | **407.0342** | **1218.0804** | **1217.3990** | **0.6813** | **1** | **11** | **1.9e+02** | **1** | **GSRAAPVCSLTR** |
|  | 2161 | **605.0218** | **1812.0432** | **1813.1286** | **-1.0854** | **2** | **11** | **2.2e+02** | **1** | **DLFTYTVKCIQIRR + Carbamidomethyl (C)** |
|  | 1008 | **420.4046** | **838.7944** | **838.9942** | **-0.1998** | **0** | **11** | **1.9e+02** | **1** | **LNPLVQR** |
|  | 2012 | **583.9786** | **1165.9425** | **1166.3123** | **-0.3699** | **0** | **11** | **2.2e+02** | **1** | **GQATGCCCRPG + 2 Carbamidomethyl (C)** |
|  | 700 | **404.0468** | **806.0788** | **805.9828** | **0.0961** | **1** | **11** | **2.1e+02** | **1** | **CLSVKEK** |
|  | 1660 | **523.3286** | **1566.9637** | **1566.6862** | **0.2774** | **0** | **11** | **2e+02** | **1** | **DDSGASQICSETLIK** |
|  | 3239 | **823.6243** | **2467.8506** | **2466.9821** | **0.8685** | **2** | **11** | **1.9e+02** | **1** | **SVLILPPRAGTPKLFNAALAMTGK** |
|  | 229 | **379.1109** | **756.2070** | **755.8628** | **0.3443** | **1** | **11** | **2.1e+02** | **1** | **TPSAPRK** |
|  | 724 | **404.9491** | **807.8833** | **806.9707** | **0.9126** | **1** | **11** | **1.9e+02** | **1** | **LCTASKGK** |
|  | 1190 | **446.9825** | **1337.9252** | **1338.5475** | **-0.6223** | **1** | **11** | **2.3e+02** | **1** | **EVDMMKEALEK + Oxidation (M)** |
|  | 2709 | **683.9535** | **2048.8383** | **2049.2517** | **-0.4134** | **1** | **11** | **1.7e+02** | **1** | **SGLAVQAVTQHTPRASVGNR** |
|  | 21 | **363.1286** | **1086.3635** | **1087.2556** | **-0.8920** | **0** | **11** | **2.1e+02** | **1** | **QFHTCAKPR** |
|  | 3248 | **832.1851** | **2493.5330** | **2493.9282** | **-0.3952** | **1** | **11** | **1.7e+02** | **1** | **SMKETVPVHPPCVSLWRPGCR + 2 Carbamidomethyl (C)** |
|  | 240 | **379.9582** | **1136.8523** | **1137.2448** | **-0.3924** | **0** | **11** | **2.4e+02** | **1** | **SQGACVTPASGC + Carbamidomethyl (C)** |
|  | 685 | **403.9399** | **805.8650** | **806.8697** | **-1.0046** | **1** | **11** | **2.2e+02** | **1** | **GFSNRAR** |
|  | 844 | **407.6270** | **813.2392** | **813.9850** | **-0.7458** | **0** | **11** | **1.7e+02** | **1** | **MFEMTR** |
|  | 1247 | **458.7690** | **1373.2850** | **1372.5253** | **0.7596** | **0** | **11** | **2e+02** | **1** | **SNYPPPLSSAALR** |
|  | 2990 | **742.6418** | **2224.9034** | **2225.4338** | **-0.5304** | **2** | **11** | **1.6e+02** | **1** | **WFDLKDADINMVTEEDKR** |
|  | 1581 | **511.9237** | **1532.7489** | **1532.8011** | **-0.0521** | **1** | **11** | **2.1e+02** | **1** | **VTGKPDAETLKVMK + Oxidation (M)** |
|  | 2324 | **623.6394** | **1245.2640** | **1244.4722** | **0.7918** | **2** | **11** | **2.2e+02** | **1** | **RRFLCHLSR + Carbamidomethyl (C)** |
|  | 2748 | **685.9285** | **2054.7632** | **2054.4372** | **0.3260** | **1** | **11** | **1.7e+02** | **1** | **ATVSAQYRGVMGTILTMVR** |
|  | 429 | **388.5650** | **775.1152** | **774.8892** | **0.2261** | **1** | **11** | **2.2e+02** | **1** | **GSRAPCK + Carbamidomethyl (C)** |
|  | 931 | **412.2586** | **1233.7536** | **1234.3999** | **-0.6462** | **0** | **11** | **1.8e+02** | **1** | **MAAVSPCPPTSAS + Oxidation (M)** |
|  | 1772 | **538.4706** | **1074.9264** | **1074.1724** | **0.7540** | **1** | **11** | **1.8e+02** | **1** | **GSSKASHMNR** |
|  | 1790 | **540.2653** | **1617.7736** | **1618.0358** | **-0.2622** | **0** | **11** | **2.2e+02** | **1** | **LIALTLLGMGLALFR + Oxidation (M)** |
|  | 1822 | **542.4692** | **1082.9237** | **1082.1481** | **0.7756** | **0** | **11** | **1.7e+02** | **1** | **SCATFSSSHR** |
|  | 269 | **384.9572** | **1151.8495** | **1151.2483** | **0.6012** | **0** | **11** | **1.8e+02** | **1** | **QTGPATTMNSK + Oxidation (M)** |
|  | 634 | **401.5863** | **1201.7367** | **1202.3925** | **-0.6558** | **2** | **11** | **2.2e+02** | **1** | **RYIRACGAHR** |
|  | 879 | **407.9484** | **1220.8231** | **1220.2507** | **0.5724** | **1** | **11** | **2.2e+02** | **1** | **SRGTPSGTQSSR** |
|  | 1850 | 549.4825 | 1645.4255 | 1644.8893 | 0.5362 | 2 | 11 | 1.7e+02 | 1 | FLAKTCSTKDQQFK |
|  | 2805 | **697.6895** | **1393.3641** | **1392.7087** | **0.6554** | **1** | **11** | **1.8e+02** | **1** | **RIFGLLMGTLQK + Oxidation (M)** |
|  | 1650 | **522.6890** | **1565.0447** | **1564.8658** | **0.1789** | **0** | **11** | **2.2e+02** | **1** | **MIAICQSVALSVADK + Oxidation (M)** |
|  | 759 | **405.8849** | **809.7551** | **808.9220** | **0.8331** | **0** | **11** | **2e+02** | **1** | **GLPGPPGSK** |
|  | 1313 | **464.8783** | **927.7418** | **927.0366** | **0.7053** | **0** | **11** | **2.1e+02** | **1** | **AVSTSFGCR** |
|  | 1059 | **429.1008** | **1284.2803** | **1283.6046** | **0.6757** | **1** | **11** | **2.1e+02** | **1** | **VPPPPRAVIPLK** |
|  | 227 | **378.1663** | **1131.4767** | **1130.2969** | **1.1798** | **0** | **11** | **1.7e+02** | **1** | **TPPQQPPPLR** |
|  | 237 | **379.7940** | **757.5733** | **756.8442** | **0.7291** | **0** | **11** | **2.3e+02** | **1** | **DPLAVDK** |
|  | 713 | **404.1905** | **1209.5494** | **1208.4949** | **1.0545** | **2** | **11** | **2e+02** | **1** | **MLGGGMKLKNK + 2 Oxidation (M)** |
|  | 2108 | **596.8620** | **1787.5638** | **1787.2800** | **0.2838** | **1** | **11** | **2e+02** | **1** | **VGLIALKLGMMPLWTK + Oxidation (M)** |
|  | 2487 | **650.6339** | **1299.2530** | **1298.4717** | **0.7813** | **1** | **11** | **1.9e+02** | **1** | **EHRGSVICLER** |
|  | 2606 | **668.5533** | **2002.6379** | **2002.3656** | **0.2722** | **2** | **11** | **1.8e+02** | **1** | **IQNCRNVTSLPCLSLRK + Carbamidomethyl (C)** |
|  | 3434 | **947.5312** | **2839.5716** | **2840.3007** | **-0.7291** | **2** | **11** | **1.9e+02** | **1** | **GHVECVDVLINQGASILVKDYILKR + Carbamidomethyl (C)** |
|  | 411 | **387.9733** | **773.9318** | **772.8900** | **1.0419** | **0** | **11** | **2.7e+02** | **1** | **VVTALDR** |
|  | 1037 | **424.0078** | **846.0007** | **844.8764** | **1.1244** | **2** | **11** | **2.6e+02** | **1** | **RDNRER** |
|  | 3222 | **817.8878** | **1633.7609** | **1632.7262** | **1.0346** | **0** | **11** | **2.4e+02** | **1** | **ETPPAEGEGHEAPIAK** |
|  | 1092 | **432.7323** | **1295.1748** | **1294.4798** | **0.6950** | **1** | **11** | **1.9e+02** | **1** | **HGPKGLAGPMGEK + Oxidation (M)** |
|  | 2134 | **599.9850** | **1197.9552** | **1198.2833** | **-0.3281** | **1** | **11** | **2.2e+02** | **1** | **STKEGPETPPR** |
|  | 2881 | **725.1620** | **1448.3093** | **1447.6764** | **0.6330** | **0** | **11** | **2.1e+02** | **1** | **CSYFMAWELPK + Carbamidomethyl (C); Oxidation (M)** |
|  | 2975 | **741.8944** | **2222.6611** | **2222.4131** | **0.2479** | **0** | **11** | **2.3e+02** | **1** | **LSCAASGETFSNYEMNWVR + Carbamidomethyl (C)** |
|  | 3176 | **800.2869** | **2397.8386** | **2397.6653** | **0.1733** | **2** | **11** | **2e+02** | **1** | **TKMSSCSQDHRSLVLQNDMK + Carbamidomethyl (C); 2 Oxidation (M)** |
|  | 146 | **371.0265** | **1110.0573** | **1110.3238** | **-0.2665** | **0** | **11** | **1.7e+02** | **1** | **QLEAPLPPMV + Oxidation (M)** |
|  | 1018 | **421.8102** | **841.6056** | **840.9670** | **0.6385** | **0** | **11** | **2.3e+02** | **1** | **LGPAGASLR** |
|  | 1785 | **540.1635** | **1078.3122** | **1078.1593** | **0.1530** | **1** | **11** | **2.2e+02** | **1** | **WNDRACGEK** |
|  | 592 | **398.9844** | **1193.9310** | **1193.3526** | **0.5783** | **0** | **11** | **1.9e+02** | **1** | **ASAPLPGLSAPGR** |
|  | 846 | **407.6774** | **813.3400** | **813.9667** | **-0.6266** | **0** | **11** | **1.8e+02** | **1** | **MCTGGCAR + Oxidation (M)** |
|  | 1808 | **541.4761** | **1621.4060** | **1621.7003** | **-0.2942** | **0** | **11** | **1.7e+02** | **1** | **VDSENSIEQPAAFSK** |
|  | 2480 | **649.6146** | **1945.8217** | **1946.2397** | **-0.4180** | **2** | **11** | **1.7e+02** | **1** | **RASCERVSPPPPLPHFR** |
|  | 2540 | **662.4160** | **1322.8173** | **1322.5544** | **0.2629** | **0** | **11** | **2.1e+02** | **1** | **HMFGDLVCSWK** |
|  | 2897 | **732.3691** | **1462.7234** | **1462.5553** | **0.1680** | **0** | **11** | **2.1e+02** | **1** | **IGSSSPDSEITELK** |
|  | 765 | **405.9836** | **1214.9285** | **1215.2970** | **-0.3685** | **0** | **11** | **2e+02** | **1** | **EMQNLSQHGR + Oxidation (M)** |
|  | 1202 | **449.5662** | **897.1177** | **897.0137** | **0.1039** | **0** | **11** | **2.4e+02** | **1** | **LHSQCPR + Carbamidomethyl (C)** |
|  | 2219 | **610.0162** | **1218.0177** | **1217.4352** | **0.5824** | **0** | **11** | **2.2e+02** | **1** | **IQLINNMLDK + Oxidation (M)** |
|  | 2781 | **689.0090** | **2064.0049** | **2063.3311** | **0.6738** | **0** | **11** | **1.8e+02** | **1** | **ILDAGDYDLQVGIVEALCR** |
|  | 3025 | 750.1422 | 2247.4045 | 2246.4531 | 0.9514 | 1 | 11 | 1.9e+02 | 1 | RASPPVSPIPVSEYCESENK + Carbamidomethyl (C) |
|  | 943 | **413.7090** | **1238.1050** | **1237.4468** | **0.6582** | **0** | **11** | **1.5e+02** | **1** | **NMDMKPGSTLK + Oxidation (M)** |
|  | 1622 | **519.0058** | **1035.9968** | **1035.1942** | **0.8027** | **0** | **11** | **2.1e+02** | **1** | **SLGVIFGGGTK** |
|  | 2728 | **684.4678** | **1366.9208** | **1367.4509** | **-0.5301** | **2** | **11** | **2.1e+02** | **1** | **RSGGRGGGGDCGFK + Carbamidomethyl (C)** |
|  | 3192 | **804.7168** | **2411.1282** | **2410.8177** | **0.3105** | **2** | **11** | **1.7e+02** | **1** | **RMPLNAYVLNFYKHNCLTR + Carbamidomethyl (C)** |
|  | 244 | **380.0562** | **1137.1463** | **1137.4402** | **-0.2939** | **1** | **11** | **2.5e+02** | **1** | **MMNCPKILR + 2 Oxidation (M)** |
|  | 1609 | **517.5886** | **1549.7437** | **1548.7441** | **0.9996** | **2** | **11** | **2.8e+02** | **1** | **DSSRTMDVCPKGPR** |
|  | 172 | **373.0887** | **744.1626** | **743.8520** | **0.3107** | **1** | **11** | **2.7e+02** | **1** | **VNAAKNK** |
|  | 1539 | **504.7282** | **1007.4416** | **1007.0549** | **0.3866** | **0** | **11** | **1.8e+02** | **1** | **DLAGEEAFR** |
|  | 2610 | **668.6744** | **2003.0011** | **2002.2034** | **0.7977** | **0** | **11** | **2.2e+02** | **1** | **VIQGLVAGETAQQICEDLD** |
|  | 1258 | **459.0250** | **916.0351** | **915.0125** | **1.0226** | **2** | **11** | **2.5e+02** | **1** | **GRTAGARAR** |
|  | 1807 | **541.3661** | **1621.0761** | **1620.8329** | **0.2432** | **2** | **11** | **1.9e+02** | **1** | **RGRVGGWEGAFLQGM** |
|  | 2427 | **635.3442** | **1903.0105** | **1902.0929** | **0.9177** | **1** | **11** | **2.2e+02** | **1** | **WVSEVMAPAEAPSRAER + Oxidation (M)** |
|  | 138 | **370.3580** | **738.7012** | **737.8228** | **0.8785** | **0** | **11** | **1.9e+02** | **1** | **MTEVSR + Oxidation (M)** |
|  | 326 | **386.1352** | **1155.3835** | **1154.3400** | **1.0435** | **1** | **11** | **2e+02** | **1** | **KMAASAAVFSR + Oxidation (M)** |
|  | 780 | **406.3548** | **810.6949** | **811.8863** | **-1.1914** | **0** | **11** | **1.8e+02** | **1** | **HQVSVSR** |
|  | 1234 | **456.1131** | **1365.3172** | **1366.4133** | **-1.0961** | **0** | **11** | **2.1e+02** | **1** | **VSSNCDFSEHNK** |
|  | 3208 | **810.7742** | **1619.5336** | **1619.9527** | **-0.4191** | **1** | **11** | **1.7e+02** | **1** | **MPHFKSCHCPVCSK + Oxidation (M)** |
|  | 45 | **364.4487** | **726.8826** | **725.7474** | **1.1352** | **0** | **11** | **2.5e+02** | **1** | **ETSSFR** |
|  | 1397 | **478.3643** | **1432.0708** | **1432.6418** | **-0.5710** | **0** | **11** | **1.7e+02** | **1** | **IASAQLLGMDIER + Oxidation (M)** |
|  | 3218 | **817.1325** | **1632.2502** | **1632.8835** | **-0.6332** | **0** | **11** | **1.8e+02** | **1** | **CASMVHLSQACQPK + 2 Carbamidomethyl (C); Oxidation (M)** |
|  | 12 | **362.2311** | **722.4474** | **722.7931** | **-0.3456** | **0** | **11** | **1.7e+02** | **1** | **VHPNTR** |
|  | 1354 | **473.1669** | **1416.4784** | **1416.5994** | **-0.1210** | **0** | **11** | **2.5e+02** | **1** | **HSLESSLIDIMR + Oxidation (M)** |
|  | 2155 | **603.3373** | **1806.9897** | **1806.9456** | **0.0441** | **1** | **11** | **2.3e+02** | **1** | **KALSSCVVDEEQDVER** |
|  | 2468 | **646.4125** | **1936.2153** | **1936.4120** | **-0.1967** | **2** | **11** | **2.3e+02** | **1** | **MIIPVRCFTCGKIVGNK + Carbamidomethyl (C)** |
|  | 2929 | **740.4910** | **1478.9672** | **1478.6821** | **0.2851** | **1** | **11** | **2.2e+02** | **1** | **GAIPARNRPGPGCR + Carbamidomethyl (C)** |
|  | 3259 | **837.4249** | **2509.2526** | **2509.8594** | **-0.6068** | **2** | **11** | **2.1e+02** | **1** | **MVGFIIGRGGEQISRIQAESGCK + Carbamidomethyl (C); Oxidation (M)** |
|  | 3261 | **839.2826** | **2514.8256** | **2515.8394** | **-1.0138** | **1** | **11** | **2.1e+02** | **1** | **QRLSSLNLTPDPEMEPPPKPPR + Oxidation (M)** |
|  | 3263 | **839.4365** | **1676.8583** | **1675.8454** | **1.0129** | **1** | **11** | **2.2e+02** | **1** | **RDAQCACTVGPSASPR + Carbamidomethyl (C)** |
|  | 1212 | **450.9034** | **1349.6880** | **1349.5947** | **0.0933** | **0** | **11** | **2.4e+02** | **1** | **VIIGLAFDCVDK + Carbamidomethyl (C)** |
|  | 1291 | **461.8956** | **921.7764** | **921.0702** | **0.7063** | **0** | **11** | **2.2e+02** | **1** | **LSVCVSEK + Carbamidomethyl (C)** |
|  | 1720 | **533.9243** | **1598.7508** | **1599.7691** | **-1.0184** | **1** | **11** | **2.2e+02** | **1** | **TFSQTYSLTCHRR** |
|  | 2022 | **586.1481** | **1170.2815** | **1171.3473** | **-1.0658** | **2** | **11** | **2.3e+02** | **1** | **GTPKVGDTIRK** |
|  | 957 | **416.0323** | **830.0499** | **829.9875** | **0.0623** | **2** | **11** | **2.7e+02** | **1** | **KKSAGLAR** |
|  | 1143 | **436.5932** | **871.1716** | **871.9614** | **-0.7897** | **1** | **11** | **2.2e+02** | **1** | **EMGHRVSG** |
|  | 2785 | **690.5414** | **2068.6022** | **2068.3593** | **0.2428** | **1** | **11** | **2e+02** | **1** | **VQALHTAYHVLRQMESGK** |
|  | 1814 | **542.2338** | **1082.4529** | **1083.2702** | **-0.8173** | **2** | **11** | **2.2e+02** | **1** | **KCHHFQKR** |
|  | 2031 | **587.5750** | **1759.7027** | **1759.8242** | **-0.1216** | **1** | **11** | **2.2e+02** | **1** | **YFYNQEEYVRFDS** |
|  | 2591 | **667.6798** | **2000.0173** | **1999.3766** | **0.6406** | **1** | **11** | **2.4e+02** | **1** | **CFTFTRYILQVLFYK + Carbamidomethyl (C)** |
|  | 3168 | **796.8657** | **2387.5748** | **2387.8141** | **-0.2393** | **0** | **11** | **2.6e+02** | **1** | **QILTLNVNLMLGQAQECLLEK + Oxidation (M)** |
|  | 324 | **386.1240** | **1155.3498** | **1154.2307** | **1.1191** | **0** | **11** | **2.1e+02** | **1** | **DNVEPVPTQR** |
|  | 3391 | **938.4319** | **2812.2735** | **2811.1469** | **1.1266** | **1** | **11** | **2e+02** | **1** | **TGLDIYEGYGQTETVLICGNFKGMK + Carbamidomethyl (C); Oxidation (M)** |
|  | 195 | **375.0173** | **1122.0297** | **1121.2686** | **0.7612** | **0** | **11** | **2.5e+02** | **1** | **MAPFTHDFR** |
|  | 2643 | **670.6080** | **2008.8019** | **2008.4317** | **0.3703** | **2** | **11** | **1.8e+02** | **1** | **LIKSEAVWENMARMCVK** |
|  | 2679 | **675.7733** | **2024.2978** | **2025.1759** | **-0.8781** | **1** | **11** | **2.8e+02** | **1** | **GDKGPSDAEVVDEISMMGR + 2 Oxidation (M)** |
|  | 2962 | **741.5529** | **1481.0909** | **1480.6418** | **0.4491** | **1** | **11** | **2e+02** | **1** | **ADIPAKYAETMDR** |
|  | 3322 | **892.6565** | **2674.9473** | **2675.0719** | **-0.1246** | **2** | **11** | **2.2e+02** | **1** | **AAPLLHTRLPGDAAASPSAVKMLGASR + Oxidation (M)** |
|  | 1363 | **474.0559** | **946.0970** | **946.0199** | **0.0771** | **1** | **11** | **2.5e+02** | **1** | **SLQNSGGRK** |
|  | 2455 | **642.3621** | **1924.0640** | **1924.1478** | **-0.0838** | **1** | **11** | **2.2e+02** | **1** | **HLAHIASFSSKTNMHAR + Oxidation (M)** |
|  | 1700 | **532.5207** | **1594.5399** | **1593.7663** | **0.7736** | **2** | **11** | **2.3e+02** | **1** | **QCGKTLSHSSSFRR** |
|  | 3496 | **967.7965** | **1933.5782** | **1934.1561** | **-0.5778** | **1** | **11** | **1.9e+02** | **1** | **QAQREAEMDSIPMGLNK + Oxidation (M)** |
|  | 300 | **386.0091** | **770.0034** | **769.9555** | **0.0479** | **0** | **11** | **2e+02** | **1** | **RPIPCK + Carbamidomethyl (C)** |
|  | 1456 | **488.0920** | **1461.2540** | **1461.7264** | **-0.4724** | **1** | **11** | **2.6e+02** | **1** | **KPKVMLTGTLSDR + Oxidation (M)** |
|  | 738 | **405.2075** | **1212.6002** | **1212.3360** | **0.2642** | **0** | **11** | **1.9e+02** | **1** | **LAAALCDHDQR** |
|  | 1327 | **466.9063** | **1397.6968** | **1396.6364** | **1.0604** | **2** | **11** | **2.4e+02** | **1** | **RNRDTMMSLLK + 2 Oxidation (M)** |
|  | 1664 | **523.8901** | **1568.6482** | **1568.6823** | **-0.0340** | **0** | **11** | **2.4e+02** | **1** | **LTEMAGTMNLNDGAD + Oxidation (M)** |
|  | 1184 | **445.0854** | **888.1561** | **887.0984** | **1.0577** | **1** | **11** | **2.7e+02** | **1** | **IICEGPKK** |
|  | 2123 | **598.2133** | **1194.4117** | **1194.3043** | **0.1075** | **2** | **11** | **2.2e+02** | **1** | **GGKGRGGPGPAQR** |
|  | 3094 | **763.3989** | **1524.7829** | **1523.6515** | **1.1314** | **1** | **11** | **2.3e+02** | **1** | **AFDSAAAKALEHHR** |
|  | 3280 | **851.5757** | **2551.7049** | **2550.9910** | **0.7139** | **0** | **11** | **2.2e+02** | **1** | **HEFFGVAMLEAVYCGCYPLCPK + Carbamidomethyl (C); Oxidation (M)** |
|  | 2076 | **593.9416** | **1778.8028** | **1778.1655** | **0.6372** | **1** | **11** | **2.2e+02** | **1** | **DLLLLCNSPCISKGCAK** |
|  | 2151 | **602.5015** | **1804.4824** | **1803.9713** | **0.5111** | **1** | **11** | **1.9e+02** | **1** | **GQKGSVGDPGMEGPMGQR + Oxidation (M)** |
|  | 3341 | **898.6534** | **1795.2921** | **1795.0753** | **0.2168** | **2** | **11** | **2.1e+02** | **1** | **LLNGPPRGIRASSPMGR + Oxidation (M)** |
|  | 1657 | **523.0462** | **1044.0776** | **1043.2179** | **0.8598** | **0** | **11** | **2.4e+02** | **1** | **MSQFGCGIK + Carbamidomethyl (C); Oxidation (M)** |
|  | 2411 | **633.6005** | **1265.1861** | **1264.5762** | **0.6099** | **1** | **11** | **2e+02** | **1** | **KLMENILLYK** |
|  | 3175 | **799.1722** | **2394.4945** | **2393.7395** | **0.7551** | **2** | **11** | **1.9e+02** | **1** | **NLRSPMVEALCENKEDCPCGK + Carbamidomethyl (C)** |
|  | 2081 | **594.6991** | **1781.0751** | **1779.9745** | **1.1007** | **1** | **11** | **2.9e+02** | **1** | **GGCGSCGCSKGACGSCGGSK + 2 Carbamidomethyl (C)** |
|  | 2566 | **666.5416** | **1996.6027** | **1996.2785** | **0.3242** | **2** | **11** | **1.9e+02** | **1** | **YACRGGGTCQMDAFMRR + Carbamidomethyl (C); Oxidation (M)** |
|  | 354 | **387.6031** | **1159.7873** | **1159.2915** | **0.4957** | **0** | **11** | **2.3e+02** | **1** | **TLWDQLDLR** |
|  | 646 | **401.9268** | **1202.7583** | **1202.4274** | **0.3308** | **2** | **11** | **2.7e+02** | **1** | **AGKKCEALAVGR** |
|  | 1674 | **524.9341** | **1571.7803** | **1570.8175** | **0.9628** | **2** | **11** | **2.4e+02** | **1** | **DNATITMCRMRAR + 2 Oxidation (M)** |
|  | 1744 | **536.0022** | **1069.9896** | **1071.1750** | **-1.1854** | **2** | **11** | **2.3e+02** | **1** | **HCRERASR + Carbamidomethyl (C)** |
|  | 437 | **388.8266** | **775.6384** | **774.8197** | **0.8188** | **1** | **11** | **2.6e+02** | **1** | **AAGQKDVS** |
|  | 552 | **391.1587** | **1170.4539** | **1169.4553** | **0.9986** | **0** | **11** | **2.1e+02** | **1** | **LLLSWLVPTK** |
|  | 1368 | **474.2624** | **1419.7650** | **1419.7044** | **0.0606** | **0** | **11** | **2.4e+02** | **1** | **MCYLSTLTVPYI + Oxidation (M)** |
[truncated: 3,422,293 more chars]
