# Supplementary material for: Tobacco-induced hyperglycemia promotes lung cancer progression via cancer cell-macrophage interaction through paracrine IGF2/IR/NPM1-driven PD-L1 expression
Source: Nat Commun. 2024 Jun 8;15:4909. doi: 10.1038/s41467-024-49199-9 (PMC11162468; doi:10.1038/s41467-024-49199-9)
Supplement: Supplementary file 4 — Reporting Summary [file 41467_2024_49199_MOESM4_ESM.pdf]

nature portfolio

Corresponding author(s): Ho-Young Lee

Last updated by author(s): Apr 30, 2024

Reporting Summary

Nature Portfolio wishes to improve the reproducibility of the work that we publish. This form provides structure for consistency and transparency in reporting. For further information on Nature Portfolio policies, see our Editorial Policies and the Editorial Policy Checklist.

Statistics

For all statistical analyses, confirm that the following items are present in the figure legend, table legend, main text, or Methods section.

n/a	Confirmed	
☐	☒	The exact sample size (n) for each experimental group/condition, given as a discrete number and unit of measurement
☐	☒	A statement on whether measurements were taken from distinct samples or whether the same sample was measured repeatedly
☐	☒	The statistical test(s) used AND whether they are one- or two-sided<br>*Only common tests should be described solely by name; describe more complex techniques in the Methods section.*
☐	☒	A description of all covariates tested
☐	☒	A description of any assumptions or corrections, such as tests of normality and adjustment for multiple comparisons
☐	☒	A full description of the statistical parameters including central tendency (e.g. means) or other basic estimates (e.g. regression coefficient) AND variation (e.g. standard deviation) or associated estimates of uncertainty (e.g. confidence intervals)
☐	☒	For null hypothesis testing, the test statistic (e.g. F, t, r) with confidence intervals, effect sizes, degrees of freedom and P value noted<br>*Give P values as exact values whenever suitable.*
☒	☐	For Bayesian analysis, information on the choice of priors and Markov chain Monte Carlo settings
☒	☐	For hierarchical and complex designs, identification of the appropriate level for tests and full reporting of outcomes
☐	☒	Estimates of effect sizes (e.g. Cohen's d, Pearson's r), indicating how they were calculated

Our web collection on statistics for biologists contains articles on many of the points above.

Software and code

Policy information about availability of computer code

Data collection	ImageJ (ver. 1.53k, NIH), LAS X Core (ver. 3.7.4, Leica), ZEN imaging software 2 (ver. 3.1, Carl Zeiss), IVIS-Spectrum microCT and Living Image software (ver. 4.2, PerkinElmer)
Data analysis	GraphPad Prism (ver. 10, GraphPad software), ELDA online software (http://bioinf.wehi.edu.au/software/elda/), Cytoscape (ver. 3.7.2, Cytoscape consortium), online venn-diagram drawing tool (https://bioinformatics.psb.ugent.be/webtools/Venn/), CalR software (https://calrapp.org/)

For manuscripts utilizing custom algorithms or software that are central to the research but not yet described in published literature, software must be made available to editors and reviewers. We strongly encourage code deposition in a community repository (e.g. GitHub). See the Nature Portfolio guidelines for submitting code & software for further information.

Data

Policy information about availability of data

All manuscripts must include a data availability statement. This statement should provide the following information, where applicable:

- Accession codes, unique identifiers, or web links for publicly available datasets
- A description of any restrictions on data availability
- For clinical datasets or third party data, please ensure that the statement adheres to our policy

A set of the mass spectrometry proteomics data (the IGF2-treated group) have been deposited in to the ProteomeXchange Consortium via the PRIDE85 partner repository with the dataset identifier PXD051519. Another set of the mass spectrometry proteomics data (the EGFP-IR overexpressed group) cannot be available in

a public repository due to the unavailability of raw LC-MS/MS data, and the processed data are available in Supplementary Data 1. The publicly available human lung cancer data used in this study are available GEO database under accession code GSE30219 (https://www.ncbi.nlm.nih.gov/geo/query/acc.cgi?acc=GSE30219), GSE37745 (https://www.ncbi.nlm.nih.gov/geo/query/acc.cgi?acc=GSE37745), GSE50081 ((https://www.ncbi.nlm.nih.gov/geo/query/acc.cgi?acc=GSE50081), and GSE77803 ((https://www.ncbi.nlm.nih.gov/geo/query/acc.cgi?acc=GSE77803). The remaining data are available within the Article, Supplementary Information, or Source Data file. Source data are provided with this paper.

Research involving human participants, their data, or biological material

Policy information about studies with human participants or human data. See also policy information about sex, gender (identity/presentation), and sexual orientation and race, ethnicity and racism.

Reporting on sex and gender	Sex was not considered in the study design. For the analysis, lung cancer tissues derived from both males and females were used.
Reporting on race, ethnicity, or other socially relevant groupings	We did not use the socially constructed or socially relevant categorization variables in this study.
Population characteristics	Patient-derived lung cancer tissues with a history of smoking were used for analysis. Other characteristics were not taken into account when collecting samples. For the analysis using a commercially available tissue microarray, sex, age, a smoking history (2015 WHO classification) and other clinicopathological features, including pathology grade, TNM, and clinical stage according to the 8th UICC/AJCC TNM edition for lung cancer staging, that were provided by the manufacturer were used for analysis.
Recruitment	Patient-derived lung cancer tissues from three non-smokers and five smokers were used for analysis. A commercially available tissue microarray used for this study contains lung cancer specimens derived from 35 smokers.
Ethics oversight	Experiments using patient-derived tissues were conducted using the protocol approved by Seoul National University Institutional review board (IRB; approval No. E1608/001-001).

Note that full information on the approval of the study protocol must also be provided in the manuscript.

Field-specific reporting

Please select the one below that is the best fit for your research. If you are not sure, read the appropriate sections before making your selection.

[x] Life sciences [ ] Behavioural & social sciences [ ] Ecological, evolutionary & environmental sciences

For a reference copy of the document with all sections, see nature.com/documents/nr-reporting-summary-flat.pdf

Life sciences study design

All studies must disclose on these points even when the disclosure is negative.

Sample size	The sample sizes for the in vitro and in vivo tests were not statistically calculated and were manually determined based on previous experiments and experience.
Data exclusions	No data were excluded from the analyses of in vitro and in vivo experimental results. For analysis of publicly available datasets, data not relevant to the current study, such as data from normal lung tissues, were excluded for analysis.
Replication	All in vitro experiments were performed at least twice independently, and a representative result from at least two independent experiments with similar results is shown. The experimental repeats and total animal numbers for each in vivo experiment are summarized in the supplementary table. For in vitro experiments, the nature of replicates is indicated in relevant figure legends.
Randomization	All the animals in the mouse experiments were randomly allocated to different groups. For cell line-based experiments, individual wells or dishes were assigned to treatment groups in a random manner.
Blinding	Measurement of tumor size and metastasis in the mouse experiments was performed in a blinded fashion. Because the same individual carried out the entire experimental procedure in the case of in vitro experiments, blinding allocation during experiments and result analysis was not possible.

Reporting for specific materials, systems and methods

We require information from authors about some types of materials, experimental systems and methods used in many studies. Here, indicate whether each material, system or method listed is relevant to your study. If you are not sure if a list item applies to your research, read the appropriate section before selecting a response.

Materials & experimental systems

n/a	Involved in the study	
	x	Antibodies
	x	Eukaryotic cell lines
x		Palaeontology and archaeology
	x	Animals and other organisms
x		Clinical data
x		Dual use research of concern
x		Plants

Methods

n/a	Involved in the study	
x		ChIP-seq
	x	Flow cytometry
x		MRI-based neuroimaging

Antibodies

Antibodies used

anti-CD45 antibody (BD Biosciences, cat # 550539, clone 30-F11)
anti-F4/80 antibody (Bio-Rad, cat # MCA497)
anti-MPO antibody (Abcam, cat # ab208670)
anti-alpha-SMA (Sigma-Aldrich, cat # A2547)
anti-CD4 antibody (Bio-Rad, cat # MCA1767T)
anti-CD8 antibody (Abcam, cat # ab22378)
anti-arginase 1 antibody (Cell Signaling, cat # 93668, clone D4E3M, lot #1)
anti-iNOS antibody (Abcam, cat # ab178945, lot #GR3240243-6)
anti-granzyme B antibody (R&D Systems, cat # AF-1885)
anti-Foxp3 antibody (Novus Biologicals, cat # MAB8214, clone 1054C. lot #CIKJ0215011)
anti-GLUT1 antibody (Novus Biologicals, cat # NB110-39113. lot #G-6)
anti-GLUT3 antibody (Proteintech, cat # 20413-1-AP)
anti-NA+K+ ATPase antibody (Cell Signaling, cat # 3010, lot #5)
anti-pIGF-1R (Y1131)/IR (Y1146) antibody (Cell Signaling, cat # 3021)
anti-pIGF-1R (Y1135/36)/pIR (Y1150/51) antibody (Cell Signaling, cat # 3024, clone 19H7, lot #15)
anti-IR antibody (Santa Cruz, cat # sc-711, clone C-19, lot #0216; Santa Cruz, cat # sc-57342, clone CT-3, lot #K3021; Cell Signaling, cat # 3020, clone L55B10, lot #6)
anti-IGF-1R antibody (Cell Signaling, cat # 3027, lot #16)
anti-IGF2 antibody (Abcam, cat # ab9574)
anti-NPM1 antibody (Abcam, cat # ab10530, lot #GR3324640-1; Santa Cruz, cat # sc-32256, lot #F0419)
anti-PD-L1 antibody (Cell Signaling, cat # 13684, clone E1L3N, lot #18)
anti-OCT-A antibody (Santa Cruz, cat # sc-166355, clone H-5, lot #B2522; Santa Cruz, cat # sc-807, clone D-8)
anti-human CD274 antibody (Biolegend, cat # 329402, clone 29E2A3, lot #B299336)
anti-mouse CD274 antibody (BioLegend, cat # 124302, clone 10F.9G2, lot #B220492)
anti-mouse PD-L1 antibody (BioXCell, cat # BE0101, clone 10F.9G2, lot #751220D1)
HRP-conjugated goat anti-mouse secondary antibody (GeneTex, cat # 213111-01)
HRP-conjugated goat anti-rabbit secondary antibody (GeneTex, cat # 213110-01)
APC/Cy7-conjugated anti-mouse CD45 antibody (BIoLegend, cat # 103116, clone 30-F11, lot # B279824)
PE/Cy7-conjugated anti-mouse F4/80 antibody (BioLegend, cat # 123114, clone BM8, lot # B371901)

Validation

All primary antibodies used in this study were validated by the manufacturer. Information about their validation data or citation can be found on the manufacturer's website by searching for the catalog number of antibodies provided in the Methods section.

Eukaryotic cell lines

Policy information about cell lines and Sex and Gender in Research

Cell line source(s)

A549 (cat. no. CCL-185) and LLC (cat. no. CRL-1642) cells were purchased from the American Type Culture Collection (ATCC).
H226Br cells were provided by Dr. John V. Heymach (University of Texas M. D. Anderson Cancer Center, Houston, TX, USA).
THP-1 cells were provided by Dr. Kyu-Won Kim (Seoul National University, Seoul, Republic of Korea).
L929 cells (cat. no. 10001) were acquired from the Korean Cell Line Bank (Seoul, Republic of Korea).

Authentication

Authentication and verification of human cancer cell lines were performed using the AmplFLSTR identifier PCR Amplification Kit (Applied Biosystems, Foster, CA; cat. no. 4322288). Cells cultured for fewer than two months after resuscitation of validated cells were used in this study.

Mycoplasma contamination

All cell lines used for this study were mycoplasma-free.

Commonly misidentified lines
(See ICLAC register)

No commonly misidentified cell lines were used.

Animals and other research organisms

Policy information about studies involving animals; ARRIVE guidelines recommended for reporting animal research, and Sex and Gender in Research

Laboratory animals	C57BL/6J (B6) mice were purchased from DBL (Chungcheongbuk-do, Republic of Korea) and FVB/N mice from Japan SLC, Inc. (Hamamatsu, Japan). Mice were maintained in a pathogen-free environment, freely accessed food and water, and housed at 22±2°C with a 12:12-h light:dark cycle. We used 2-month-old male mice for experiments.
Wild animals	This study did not involve wild animals.
Reporting on sex	The prevalence of smoking has been generally known to be higher in men than women (DOI: 10.1016/j.ypmed.2015.06.009). In addition, a previous report demonstrated that male smokers had a significantly greater risk of metabolic syndrome than female smokers did (DOI: 10.1371/journal.pone.0047791). Based on these findings, this study used male mice for in vivo experiments.
Field-collected samples	This study did not involve samples collected from the field.
Ethics oversight	All mouse experiments complied with the Seoul National University's Institutional Animal Care and Use Committee authorized protocols (approval no. SNU-190628, SNU-200721, and SNU-231212-2-2) and the Institutional Animal Care and Use Committee of Asan Institute for Life Sciences (No. 2023-40-308).

Note that full information on the approval of the study protocol must also be provided in the manuscript.

Plants

Seed stocks	*Report on the source of all seed stocks or other plant material used. If applicable, state the seed stock centre and catalogue number. If plant specimens were collected from the field, describe the collection location, date and sampling procedures.*
Novel plant genotypes	*Describe the methods by which all novel plant genotypes were produced. This includes those generated by transgenic approaches, gene editing, chemical/radiation-based mutagenesis and hybridization. For transgenic lines, describe the transformation method, the number of independent lines analyzed and the generation upon which experiments were performed. For gene-edited lines, describe the editor used, the endogenous sequence targeted for editing, the targeting guide RNA sequence (if applicable) and how the editor was applied.*
Authentication	*Describe any authentication procedures for each seed stock used or novel genotype generated. Describe any experiments used to assess the effect of a mutation and, where applicable, how potential secondary effects (e.g. second site T-DNA insertions, mosiacism, off-target gene editing) were examined.*

Flow Cytometry

Plots

Confirm that:

- [x] The axis labels state the marker and fluorochrome used (e.g. CD4-FITC).
- [x] The axis scales are clearly visible. Include numbers along axes only for bottom left plot of group (a 'group' is an analysis of identical markers).
- [x] All plots are contour plots with outliers or pseudocolor plots.
- [x] A numerical value for number of cells or percentage (with statistics) is provided.

Methodology

Sample preparation	Tumorous lungs were excised from C57BL/6J mice. After collecting lung tumor pieces, the remaining non-tumor regions of the lung were dissociated into single cells using the mouse lung dissociation kit (cat no. 130-095-927, Miltenyi Biotec) according to the manufacturer's instructions. RBCs were lysed by incubating cell pellets with RBC lysis buffer (Blogems). Isolated cells were incubated on ice for 15 min with TruStain fcX (cat. no. 101320, BioLegend, San Diego, CA, USA) diluted in FACS buffer (PBS containing 1% BSA, 2 mM EDTA, and 0.05% sodium azide) at a 1:50 ratio. The cells were then stained for 30 min on ice with allophycocyanin (APC)/cyanine7 (Cy7)-conjugated anti-mouse CD45 (cat. no. 103116, BioLegend, 1:100 ratio) and phycoerythrin (PE)/Cy7-conjugated anti-mouse F4/80 (cat. no. 123114, BioLegend, 1:100 ratio) antibodies. After being washed twice with FACS buffer, cells were sorted using a FACS Aria III flow cytometer (BD Biosciences, San Jose, CA, USA).
Instrument	FACS Aria III (BD Biosciences)
Software	FACSDiva (ver. 9.0.1, BD Biosciences) was used for acquisition and analysis
Cell population abundance	The abundance of the CD45+F4/80+ live cell population within post-sort fracitons was minimal at 25,000 cells/test. The purity (> 90%) was determined by flow cytometry.
Gating strategy	Initial cell populations were gated for exclusion of cell debris and dead cells using a FSC-A/SSC-A plot. Singlets were gated out using a FSC-A/FSC-H plot. Macrophages were gated out based on CD45 and F4/80 staining. Macrophages were defined as the

CD45+F4/80+ population (CD45-APC/Cy7-A > 1,000 and F4/80-PE/Cy7-A > 1,000).

☒ Tick this box to confirm that a figure exemplifying the gating strategy is provided in the Supplementary Information.
